# Revisiting inconsistency in large pharmacogenomic studies

**DOI:** 10.12688/f1000research.9611.3

**Published:** 2017-08-11

**Authors:** Zhaleh Safikhani, Petr Smirnov, Mark Freeman, Nehme El-Hachem, Adrian She, Quevedo Rene, Anna Goldenberg, Nicolai J. Birkbak, Christos Hatzis, Leming Shi, Andrew H. Beck, Hugo J.W.L. Aerts, John Quackenbush, Benjamin Haibe-Kains

**Affiliations:** 1 Department of Medical Biophysics, University of Toronto, Toronto, M5G 1L7, Canada; 2 Princess Margaret Cancer Centre, University Health Network, Toronto, M5G 1L7, Canada; 3 Institut de Recherches Cliniques de Montréal, Montréal, H2W 1R7, Canada; 4 Department of Computer Science, University of Toronto, Toronto, M5S 2E4, Canada; 5 Hospital for Sick Children, Toronto, M5G 1X8, Canada; 6 University College London, London, WC1E 6BT, UK; 7 Yale Cancer Center, Yale University, New Haven, CT, 06510, USA; 8 Section of Medical Oncology, Yale University School of Medicine, New Haven, CT, 06520, USA; 9 University of Arkansas for Medical Sciences, Little Rock, AR, 72205, USA; 10 Fudan University, Shanghai City, 200135, China; 11 Department of Pathology, Beth Israel Deaconess Medical Center and Harvard Medical School, Boston, MA, 02215, USA; 12 Department of Biostatistics and Computational Biology and Center for Cancer Computational Biology, Boston, MA, 02215, USA; 13 Department of Radiation Oncology and Radiology, Dana-Farber Cancer Institute, Harvard Medical School, Boston, MA, 02215, USA; 14 Department of Cancer Biology, Dana-Farber Cancer Institute, Boston, MA, 02215, USA; 15 Ontario Institute of Cancer Research, Toronto, M5G 1L7, Canada

**Keywords:** drug sensitivity, cancer, pharmacogenomics, consistency, pharmacogenomic agreement

## Abstract

In 2013, we published a comparative analysis of mutation and gene expression profiles and drug sensitivity measurements for 15 drugs characterized in the 471 cancer cell lines screened in the Genomics of Drug Sensitivity in Cancer (GDSC) and Cancer Cell Line Encyclopedia (CCLE). While we found good concordance in gene expression profiles, there was substantial inconsistency in the drug responses reported by the GDSC and CCLE projects. We received extensive feedback on the comparisons that we performed. This feedback, along with the release of new data, prompted us to revisit our initial analysis. We present a new analysis using these expanded data, where we address the most significant suggestions for improvements on our published analysis — that targeted therapies and broad cytotoxic drugs should have been treated differently in assessing consistency, that consistency of both molecular profiles and drug sensitivity measurements should be compared across cell lines, and that the software analysis tools provided should have been easier to run, particularly as the GDSC and CCLE released additional data.

Our re-analysis supports our previous finding that gene expression data are significantly more consistent than drug sensitivity measurements. Using new statistics to assess data consistency allowed identification of two broad effect drugs and three targeted drugs with moderate to good consistency in drug sensitivity data between GDSC and CCLE. For three other targeted drugs, there were not enough sensitive cell lines to assess the consistency of the pharmacological profiles. We found evidence of inconsistencies in pharmacological phenotypes for the remaining eight drugs.

Overall, our findings suggest that the drug sensitivity data in GDSC and CCLE continue to present challenges for robust biomarker discovery. This re-analysis provides additional support for the argument that experimental standardization and validation of pharmacogenomic response will be necessary to advance the broad use of large pharmacogenomic screens.

Box 1. **Summary box**

In 2013 we reported inconsistency in the drug sensitivity phenotypes measured by the Genomics of Drug Sensitivity in Cancer (GDSC) and the Cancer Cell Lines Encyclopedia (CCLE) studies. Here we revisit that analysis and address a number of potential concerns raised about our initial methodology:

- ***Different drugs should be compared based on the observed pattern of response.*** To address this concern, we considered drugs falling into three classes: (1) drugs with no observed activity in any of the cell lines; (2) drugs with sensitivity observed for only a small subset of cell lines; and (3) drugs producing a response in a large number of cell lines. For each class, we assessed the correlation in drug response between studies using a variety of metrics, selecting the metric that performed best in each individual comparison. While no metric identified any substantial consistency for the first class (sorafenib, erlotinib, and PHA−665752) due to no activity, judicious choice of metric found high consistency for three of eight highly targeted therapies in the second class (nilotinib, crizotinib, and PLX4720), but no metric found better than moderate correlation for two of four broad effect drugs in the third class (PD−0332901 and 17-AAG).
- ***Measure of consistency for targeted drugs.*** Beyond considering drug response profiles, targeted drugs should be treated differently when assessing consistency. We used six different statistics to test consistency, using both continuous and discretized drug sensitivity data. We confirmed that Spearman rank correlation, used in our 2013 study, does not detect consistency for the three targeted therapies profiled by GDSC and CCLE. Other statistics, such as Somers’ Dxy or Matthews correlation coefficient, yielded moderate to high consistency for specific drugs, but there was no single metric that found good consistency for each of the targeted drugs.
- ***Consistency of molecular profiles across cell lines.*** In our initial published analysis, we reported correlations based on comparing drug response “across cell lines” while gene expression levels were compared “between cell lines.” It has been suggested it would be more appropriate to compute correlations “across cell lines” for both molecular and pharmacological data. Here we report a number of statistical measures of consistency for both gene expression and drug response compared across cell lines and confirm our initial finding that gene expression is significantly more consistent than the reported drug phenotypes.
- ***Some published biomarkers are reproducible between studies.*** In our initial comparative study we found that the majority of known biomarkers predictive of drugs response are reproducible across studies. We extended the list of known biomarkers and found that seven out of 11 are significant in GDSC and CCLE. While one can find such anecdotal examples, they do not lead to a general process for discovering a new biomarker in one study that can be applied to another study.
- ***Research reproducibility.*** The code we provided with our original paper was incompatible with updated releases of the GDSC and CCLE datasets. We developed *PharmacoGx*, which is a flexible, open-source software package based on the statistical language R, and used it to derive the results reported here.

## Introduction

The goal of precision medicine is the identification of the best therapy for each patient and their own unique manifestation of a disease. This is particularly important in oncology where multiple cytotoxic and targeted drugs are available, but their therapeutic benefits are often insufficient or limited to a subset of cancer patients. Large-scale pharmacogenomics studies in which experimental and approved drugs are screened against panels of molecularly characterized cancer cell lines, have been proposed as a means for identifying drugs effective against specific cancers and for developing genomic biomarkers predictive of drug response. The Genomics of Drug Sensitivity in Cancer project (GDSC, referred to as the Cancer Genome Project [CGP] in our initial study) ^1^, and the Cancer Cell Line Encyclopedia (CCLE) ^2^ have each reported results of such screens, providing data on drug sensitivities and molecular profiles for collections of representative cancer cell lines.

Presented with these two large studies, our hope was that we could use the data to identify new molecular biomarkers of drug response in one study that would predict response in the second. We ^3^ and others ^4– 6^ reported difficulties in building and validating biomarkers of response using the GDSC and CCLE datasets, even when the analysis was limited to the drugs and cell lines screened in both studies. To understand the cause of this failure, we compared the gene expression profiles and the drug response data reported by the GDSC and CCLE ^7, 8^. We found that, although the gene expression data showed reasonable consistency between the two studies, the drug sensitivity measurements were surprisingly inconsistent. This inconsistency can be clearly seen by plotting drug response reported for each of the 15 drugs provided in both GDSC and CCLE for the 471 cell lines assayed by both studies ^7– 10^. Since the publication of our comparative analysis, we received a great deal of constructive feedback from the scientific community regarding multiple aspects of the analysis we reported, including suggestions for analytical methods that might uncover greater consistency between the studies. Moreover, both GDSC and CCLE have released new drug sensitivity and molecular profiling data, allowing us not only to revisit our initial analysis, but also to extend it using these new data.

To begin, we investigated alternative statistics to assess the inter-study consistency for drugs exhibiting different patterns of response across the collection of cell lines common to both studies. We then considered statistical methods for targeted drugs expected to be sensitive only in a subset of cell lines. We compared consistency estimates between continuous and discretized molecular features (gene expression, copy number variations and mutations) and drug sensitivity data, and importantly, assessed how potential discordance may affect the discovery of molecular features (biomarkers) predictive of drug response. We also revisited our analysis of consistency of molecular data between studies and evaluated “known biomarkers” of response expected to be predictive in these studies.

This extensive reanalysis found that by selecting specific statistical measures on a case-by-case basis, one can identify moderate to good consistency for two broad effect and three targeted therapies. However, overall, our results support our initial observations that drug sensitivity data in GDSC and CCLE are inconsistent for the majority of the drugs, even when considering metrics yielding the highest consistency for individual drugs. Our present analysis adds further evidence supporting the need for robust and standardized experimental pipelines to assure generation of comparable, biologically relevant measures of drug response as well as unbiased statistical and machine learning methods to better predict response. Failure to do so will continue to limit the potential for use of large-scale pharmacogenomic screens in reliable drug development and precision medicine applications.

## Results

**Figure 1.**
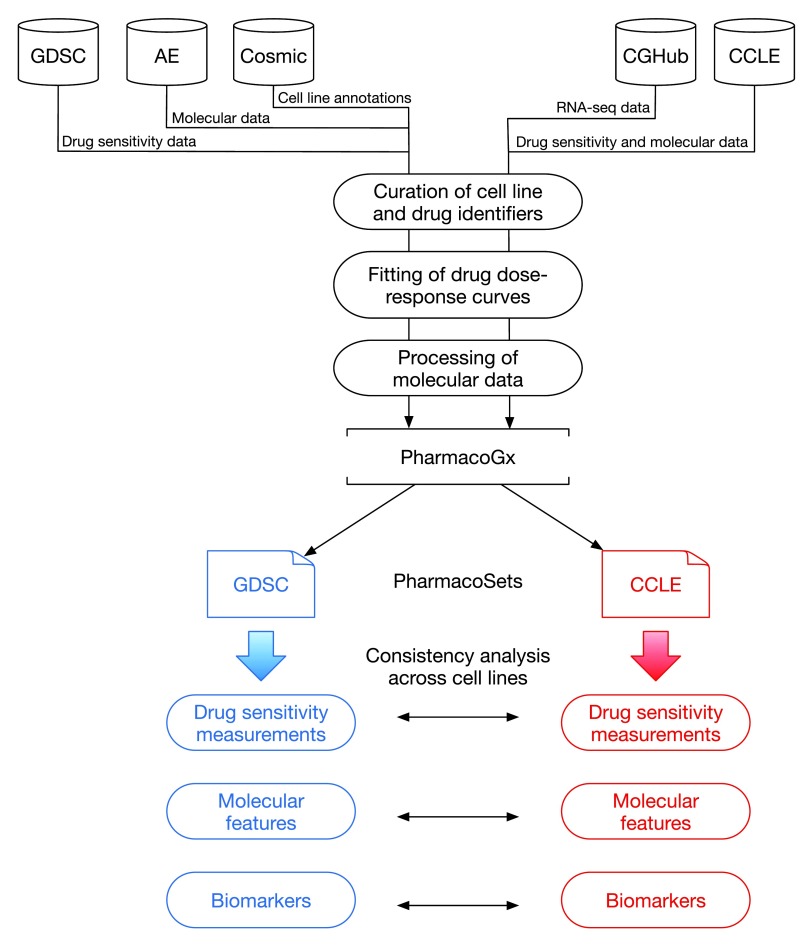
Analysis design. GDSC: Genomics of Drug Sensitivity in Cancer; AE: ArrayExpress; Cosmic: Catalogue of Somatic Mutations in Cancer; CGHub: Cancer Genomics Hub; CCLE: Cancer Cell Line Encyclopedia.

The overall analysis design of our study is represented in Figure 1.

### Intersection between GDSC and CCLE

**Figure 2.**
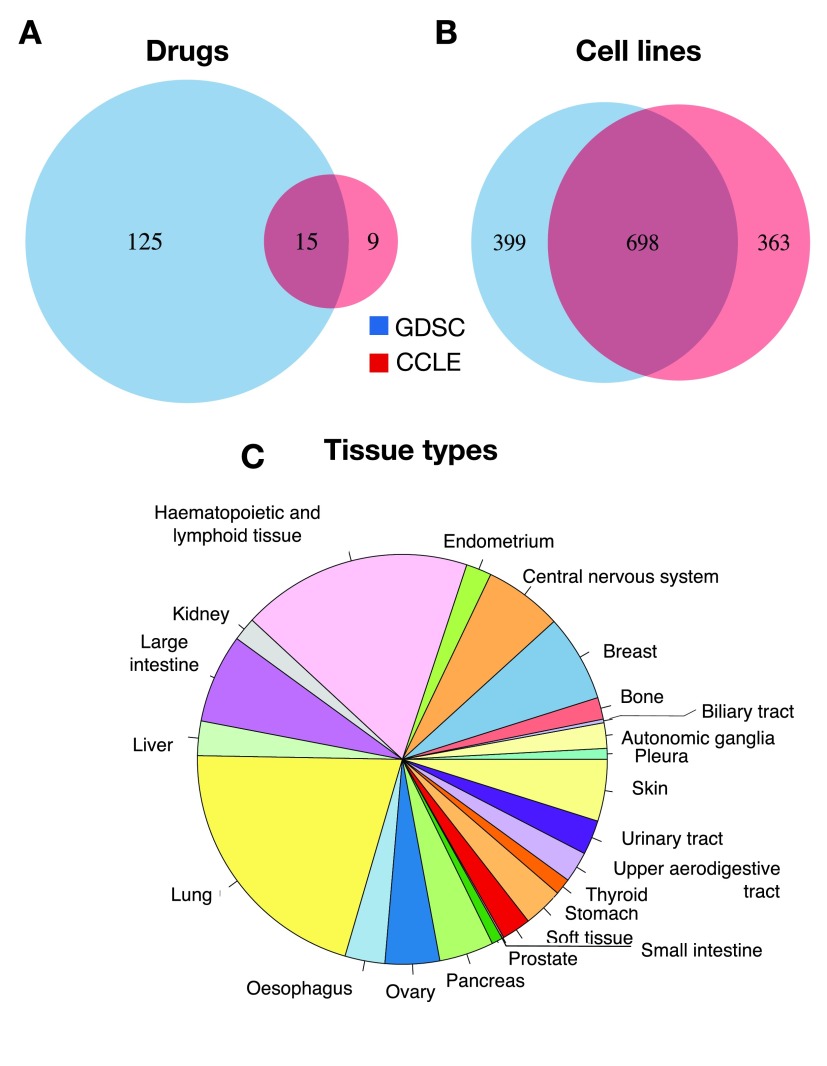
Intersection between GDSC and CCLE. Overlap of ( **A**) drugs, ( **B**) cell lines and ( **C**) tissue types.

To identify the largest set of cell lines and drugs profiled by both GDSC and CCLE, we used the *PharmacoGx* computational platform ^11^ that is able to store, analyze, and compare curated pharmacogenomic datasets. We created curated datasets for the new releases of the GDSC (July 2015) and CCLE (February 2015) projects. The improved curation of new data using *PharmacoGx* ^11^ identified 15 drugs in common between GDSC and CCLE as well 698 cell lines, originating from 23 tissue types ( Figure 2). This is the same number of shared drugs but the updated datasets contains a larger number of common cell lines than the 471 reported in our previous analysis ^7^.

### Comparing single nucleotide polymorphism (SNP) fingerprints

**Figure 3.**
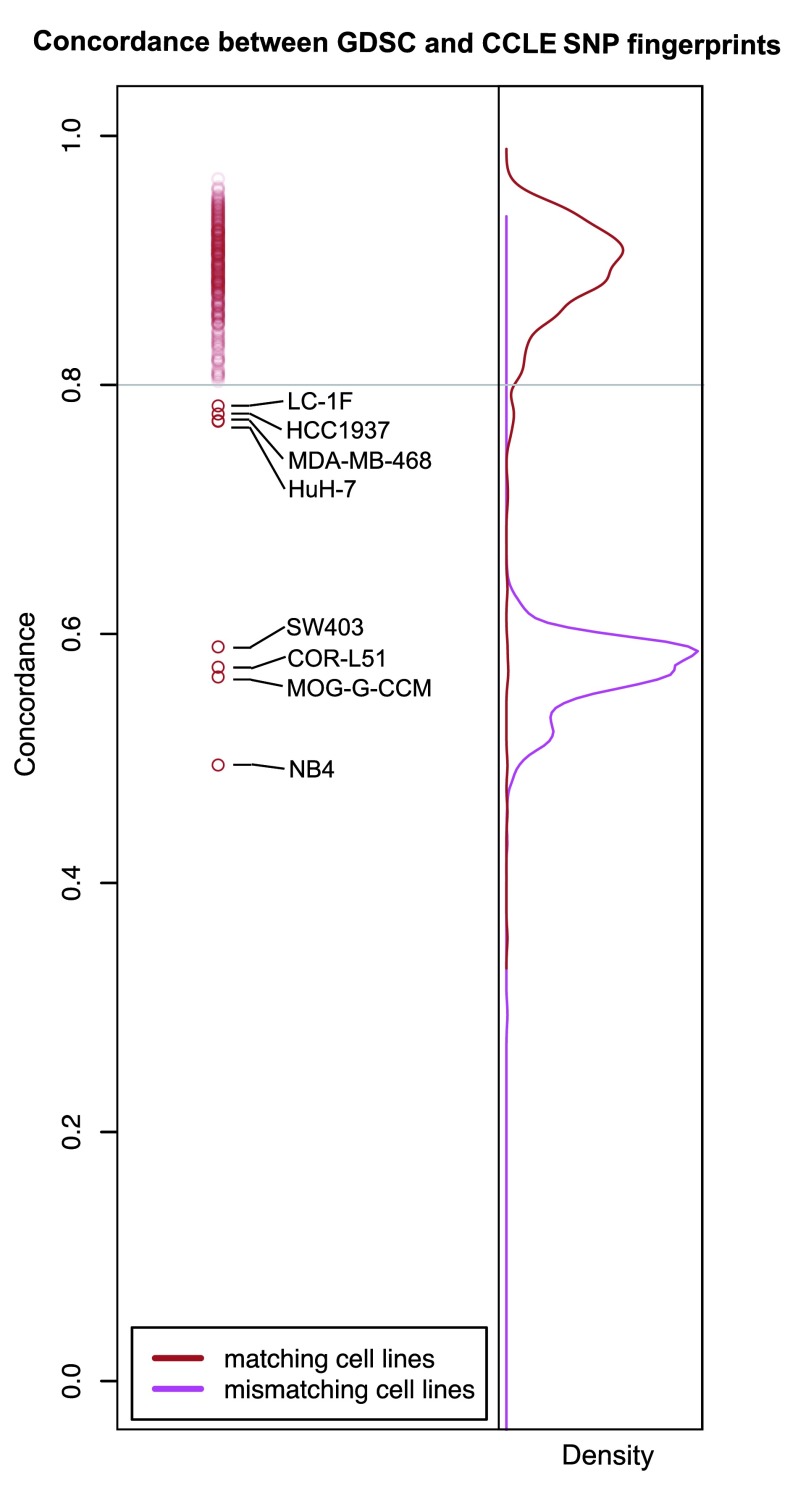
SNP fingerprinting between cancer cell lines screened in GDSC and CCLE.

To check the accuracy of cell line name matching, we compared single nucleotide polymorphism (SNP) fingerprints using data released in both studies. We first controlled for the quality of the SNP arrays and excluded 11 of 1,396 profiles due to low quality (see Methods). We then compared SNP fingerprints of cell lines with identical name using > 80% as threshold for concordance ^12, 13^. Consistent with the results reported by the CCLE ^2^, the vast majority of cell lines had highly concordant fingerprints (462 out of 470 cell lines with SNP profiles available in both GDSC and CCLE; Dataset 1). We found eight cell lines with same identifier but different SNP identity ( Figure 3); these were removed from our subsequent analyses to avoid discrepancies due to the use of possibly mislabeled or contaminated cell lines.

### Estimation and filtering of drug dose-response curves

We used the viability measures for each drug concentration in GDSC and CCLE to fit dose-response curves and assess their quality. An important factor influencing the fitting of drug dose-response curves is the range of concentration used for each cell line/drug combination. In CCLE, all dose-response curves were measured at eight concentrations: 2.5×10 ^-3^, 8×10 ^-3^, 2.5×10 ^-2^, 8×10 ^-2^, 2.5×10 ^-1^, 8×10 ^-1^, 2.5, and 8 μM. However, in GDSC response was measured at a different set of concentrations for each drug. The minimum concentrations for different drugs range from 3.125×10 ^-5^ to 15.625 μM. In each case, the concentrations tested by GDSC form a geometric sequence of nine terms with a common ratio of two between successive concentrations. Thus, the maximum concentration tested for each drug is 256 times the minimum concentration for that drug and ranges from 8×10 ^-3^ to 4000 μM.

**Figure 4.**
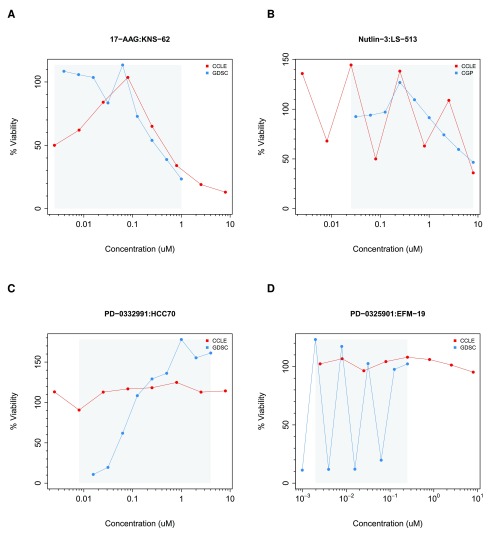
Examples of noisy drug dose-response curves identified during the filtering process in GDSC and CCLE. The grey area represents the common concentration range between studies. ( **A**) JNS-62 cell line treated with 17-AAG; ( **B**) LS-513 treated with nutlin-3; ( **C**) HCC70 cell lines treated with PD-0332991; and ( **D**) EFM-19 cell line treated with PD-0325901. Parameters have been set to ∈ = 25 and *ρ* = 0.80 ( Supplementary methods). Red curve in ( **A**) is the noisy due to violation of constraint 2, redcurve in ( **B**) due to violation of constraint 1, blue curve in ( **C**) is the noisy due to violation of constraint 2, blue curve in ( **B**) due to violation of constraint 1 ( Supplementary methods).

To properly fit drug dose-response curves, one must make multiple assumptions regarding the cell viability measurements generated by the pharmacological platform used in a given study. For instance, one assumes that viability ranges between 0% and 100% after data normalization and that consecutive viability measurements remain stable or decrease monotonically reflecting response to the drug being tested. Quality controls were implemented to flag dose-response curves that strongly violate these assumptions ( Supplementary Methods). We identified 2315 (2.9%) and 123 (1%) dose-response curves that failed to pass in GDSC and CCLE, respectively, as depicted in Figure 4 (all noisy curves are provided in Supplementary File 1. We excluded these cases to avoid erroneous curve fitting.

We used least squares optimization to fit a three-parameter sigmoid model (Methods) for the drug dose-response curves in GDSC and CCLE ( Supplementary File 2). For each fitted curve, we computed the most widely used drug activity metrics, that are the area under the curve (AUC) and the drug concentration required to inhibit 50% of cell viability (IC _50_).

### Consistency of drug sensitivity data

**Figure 5.**
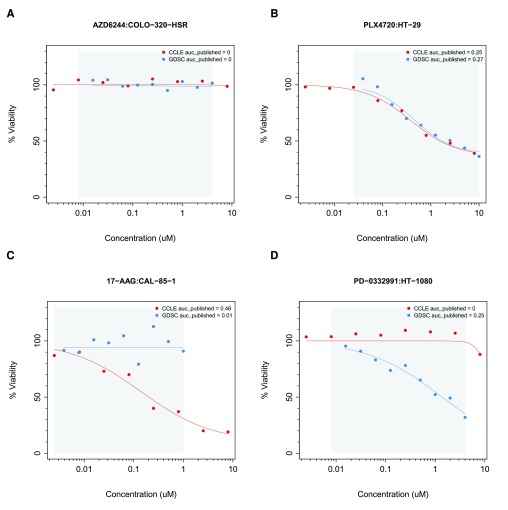
Examples of ( **A**, **B**) consistent and ( **C**, **D**) inconsistent drug dose-response curves in GDSC and CCLE. The grey area represents the common concentration range between studies. ( **A**) COLO-320-HSR cell line treated with AZD6244; ( **B**) HT-29 treated with PLX4720; ( **C**) CAL-85-1 cell lines treated with 17-AAG; and ( **D**) HT-1080 cell line treated with PD-0332991.

We began by computing the area between the two drug dose-response curves (ABC) to assess consistency of cell viability data for each cell line combination screened in both GDSC and CCLE using the common concentration range. ABC measures the difference between two drug-dose response curves by estimating the absolute area between these curves, which ranges from 0% (perfect consistency) to 100% (perfect inconsistency). The ABC statistic identified highly consistent ( Figure 5A, B) and highly inconsistent ( Figure 5C, D) dose-response curves between GDSC and CCLE. The mean of the ABC estimates for all drug-cell line combinations was 10% ( Supplementary Figure 1A), with paclitaxel yielding the highest discrepancies ( Supplementary Figure 1B).

We compared biological replicates in GDSC, which were performed independently at the Massachusetts General Hospital (MGH) and the Wellcome Trust Sanger Institute (WTSI). These experiments are comprised of 577 cell lines treated with AZD6482, a PI3Kβ inhibitor screened in GDSC ( Supplementary File 3). We computed the ABC of these biological replicates and observed both highly consistent and inconsistent cases ( Supplementary Figure 2). We then computed the median ABC values for each pair of drugs in GDSC and used these as a distance metric for complete linkage hierarchical clustering. We found that the MGH- and WTSI-administered AZD6482 experiments clustered together, suggesting that the differences between dose-response curves of biological replicates were smaller than the differences observed between different drugs ( Supplementary Figure 3A). We performed the same clustering analysis by computing the ABC-based distance between all the drugs in GDSC and CCLE and observed that only three out of the 15 common drugs clustered tightly (17-AAG, lapatinib, and PHA−665752; Supplementary Figure 3B). Despite the small number of cell lines exhibiting sensitivity to PHA−665752 and lapatinib, these drugs closely clustered between GDSC and CCLE; however this was not the case for other targeted therapies, such as AZD0530, nilotinib, crizotinib and TAE684 Supplementary Figure 3B).

**Figure 6.**
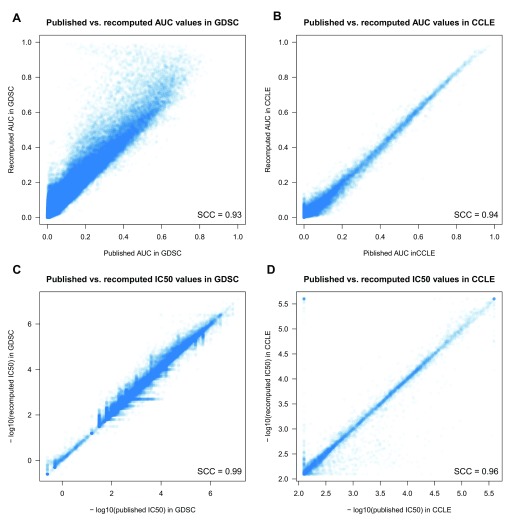
Comparison between published and recomputed drug sensitivity values between GDSC and CCLE. ( **A**) AUC in GDSC; ( **B**) AUC in CCLE; ( **C**) IC _50_ in GDSC; and ( **D**) IC _50_ in CCLE. SCC stands for Spearman correlation coefficient.

Although the ABC values provide a measure of the degree of consistency between studies, it is the AUC and IC _50_ estimates, and their correlation with molecular features (such as mutational status and gene expression) that are commonly used to assess drug response. Therefore we revisited our comparative analysis of the drug sensitivity data using the expanded data and the standardized methods implemented in our *PharmacoGx* platform. Using the same three-parameter sigmoid model to fit drug dose-response curves in GDSC and CCLE (see Methods), we recomputed AUC and IC _50_ values and observed very high correlation between published and recomputed drug sensitivity values for each study individually (Spearman > 0.93; Figure 6; Dataset 2).

It has been suggested that some of the observed inconsistencies between the GDSC and CCLE may be due to the nature of targeted therapies, which are expected to have selective activity against some cell lines ^10, 14, 15^. This is a reasonable assumption as the measured response in insensitive cell lines may represent random technical noise that one should not expect to be correlated between experiments. We therefore decided to clearly discriminate between targeted drugs with narrow growth inhibition effects and drugs with broader effects. We used the full GDSC and CCLE datasets to compare the variation of the drug sensitivity data of known targeted and cytotoxic therapies as classified in the original studies ( Supplementary Figure 4). We observed that drugs can be classified in these two categories based on median absolute deviation (MAD) of the estimated AUC values (Youden’s optimal cutoff ^16^ of AUC MAD > 0.13 for cytotoxic drugs). We then used this cutoff on the common drug-cell line combinations in GDSC and CCLE to define three classes of drugs ( Supplementary Figure 5):

- **No/little effect**: Drugs with minimal observed activity (typically active in less than five sensitive cell lines with AUC > 0.2 or IC _50_ < 1 µM in either study). This class includes sorafenib, erlotinib and PHA−665752.
- **Narrow effect**: Targeted drugs with activity observed for only a small subset of cell lines (AUC MAD ≤ 0.13). This group includes nilotinib, lapatinib, nutlin-3, PLX4720, crizotinib, PD-0332991, AZD0530, and TAE684.
- **Broad effect:** Drugs producing a response in a large number of cell lines (AUC MAD > 0.13). This includes AZD6244, PD-0325901, 17-AAG and paclitaxel.

**Figure 7.**
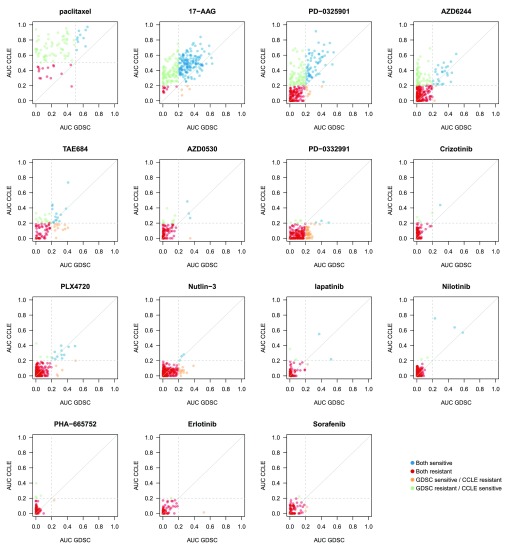
Comparison of AUC values as published in GDSC and CCLE. Cell lines with AUC >0.2 were considered as sensitive (AUC >0.4 for paclitaxel). In case of perfect consistency, all points would lie on the grey diagonal. The drugs are ranked based on their category: broad effect (AZD6244, PD–0325901, 17-AAG and paclitaxel), narrow effect (nilotinib, lapatinib, nutlin-3, PLX4720, crizotinib, PD-0332991, AZD0530, and TAE684) and no/little effect (sorafenib, erlotinib and PHA–665752).

**Figure 8.**
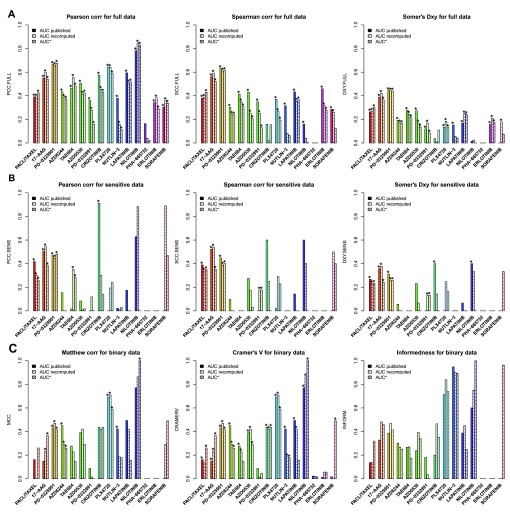
Consistency of AUC values as published and recomputed within *PharmacoGx*, with AUC* being computed using the common concentration range between GDSC and CCLE. The consistency is computed across cell lines, i.e., for each drug, a vector of drug sensitivity measures (AUC, IC _50_,...) is extracted from GDSC and CCLE and compared. ( **A**) Consistency assessed using the full set of cancer cell lines screened in both studies. ( **B**) Consistency assessed using only sensitive cell lines (AUC > 0.2 and AUC > 0.4 for targeted and cytotoxic drugs, respectively). ( **C**) Consistently assessed by discretizing the drug sensitivity data using the aforementioned cutoffs for AUC. PCC: Pearson correlation coefficient; SCC: Spearman rank-based correlation coefficient; DXY: Somers’ Dxy rank correlation; MCC: Matthews correlation coefficient; CRAMERV: Cramer’s V statistic; INFORM: Informedness. The symbol ’*’ indicates whether the consistency is statistically significant (p<0.05).

We then compared the AUC ( Figure 7, Supplementary Figure 6 and Supplementary Figure 7 for published AUC, recomputed AUC and AUC computed over the common concentration range, respectively) and IC _50_ ( Supplementary Figure 8 and Supplementary Figure 9) values and calculated the consistency of drug sensitivity data between studies using all common cases and only those that the data suggested were sensitive in at least one study ( Figure 8 and Supplementary Figure 10 for AUC and IC _50_, respectively, and Dataset 3). Given that no single metric can capture all forms of consistency, we extended our previous study by using the Pearson correlation ^17^, Spearman ^18^, and Somers’ Dxy ^19^ rank correlation coefficients to quantify the consistency of continuous drug sensitivity measurements across studies (see Methods).

As expected, no consistency was observed for drugs with “no effect” ( Figure 8A). For AUC of drugs with narrow and broad effects, Somers’ Dxy was the most stringent, with consistency estimated to be < 0.4 except for two drugs (PD-0325901 and 17-AAG), which were also the two drugs identified as the most consistent using Spearman correlation (ρ ~ 0.6; Figure 8A). However, these statistics did not capture potential consistency for the most targeted therapies, nilotinib, crizotinib, and PLX4720, for which the Pearson correlation coefficient gave the best evidence of concordance, as this statistics is strongly influenced by a small number of highly sensitive cell lines ( Figure 7). Our results concur with the recent comparative study published by the GDSC and CCLE investigators ^15^.

We then restricted our analysis to the cell lines identified as sensitive in at least one study and computed the same consistency measures ( Figure 8B). To our surprise, eliminating the insensitive cell lines resulted in decreased consistency for most drugs, which suggests a high level of inconsistency across sensitive cell lines, with the only exceptions of the targeted drugs nilotinib and crizotinib.

To test whether discretization of drug sensitivity data into binary calls (“insensitive” vs. “sensitive”; see Methods) improves consistency across studies, we used three association statistics, the Matthews correlation coefficient ^20^, Cramer’s V ^21^, and the informedness ^22^ statistics ( Figure 8C). These statistics are designed for use with imbalanced classes, which is particularly relevant in large pharmacogenomic datasets where, for targeted therapies, there are often many more insensitive cell lines than sensitive ones. As expected, some of the targeted therapies, nilotinib and PLX4720 (and nutlin-3 using informedness), yielded high level of consistency, but this was not the case for the other targeted therapies. We also found that the drug sensitivity calls for drugs with broader inhibitory effects were also poorly correlated between studies ( Figure 8C).

We performed the same analysis using IC _50_ values truncated to the maximum concentration used for each drug in each study separately. We observed similar patterns with nilotinib and crizotinib yielding moderate to high consistency across studies ( Supplementary Figure 10). Note that Somers’ Dxy rank correlation is biased in the presence of many repeated values in the datasets being analyzed, which is the case for truncated IC _50 _— pairs of cell line with identical IC _50_ values in one dataset but not in the other will not be taken into account as evidence of inconsistency — which explains the artifactual perfect consistency it suggests for both nilotinib and crizotinib.

### Consistency of molecular profiles across cell lines

Discovering new biomarkers predictive of drug response requires both robust pharmacological data and molecular profiles. In our original study, we showed that the gene expression profiles for each cell line profiled by both GDSC and CCLE were highly consistent. However, we found that mutation profiles were only moderately consistent, a result that was later confirmed by Hudson *et al.* ^23^.

There have been questions as to whether the measures of consistency we reported for drug response should be compared to those we reported for gene expression. Specifically, we reported correlations based on comparing drug response “across cell lines,” meaning that we examined the correlation of response of each cell line to a particular drug reported by the GDSC with the response of the same cell line to the same drug reported by the CCLE. In contrast we reported correlation of gene expression levels “between cell lines,” meaning that we compared the expression of all genes within each cell line in the GDSC to the expression of all genes in the same cell line in the CCLE (see Supplementary Methods). It has been suggested that a more valid comparison would be to compare both drug response and gene expression across cell lines. We report the results of such an “across cell lines” analysis of gene expression here, computed using techniques analogous to those we used to compare drug response.

**Figure 9.**
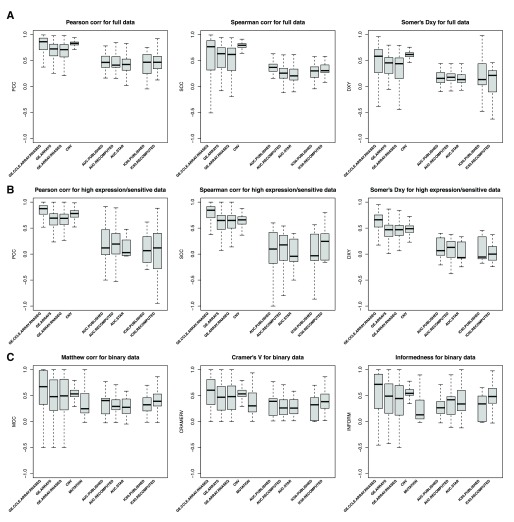
Consistency of molecular profiles (gene expression, copy number variation and mutation) and drug sensitivity data between GDSC and CCLE using multiple consistency measures. ( **A**) Consistency assessed using the full set of cancer cell lines screened in both studies. ( **B**) Consistency assessed using only sensitive cell lines (AUC >0.2 / IC _50_ <1 µM and AUC >0.4 / IC _50_ <10 µM for targeted and cytotoxic drugs, respectively). ( **C**) Consistently assessed by discretizing the molecular and drug sensitivity data. GE.CCLE.ARRAY.RNASEQ: Consistency between gene expression data generated using Affymetrix HG-U133PLUS2 microarray and Illumina RNA-seq platforms within CCLE; GE.ARRAYS: Consistency between gene expression data generated using Affymetrix HG-U133A and HG-U133PLUS2 microarray platforms in GDSC and CCLE, respectively; GE.ARRAY.RNASEQ: Consistency between gene expression data generated using Affymetrix HG-U133A microarray and Illumina RNA-seq platforms in GDSC and CCLE, respectively; CNV: Consistency of copy number variation data in CCLE and GDSC, respectively; MUTATION: Consistency of mutation profiles in CCLE and GDSC, respectively. PCC: Pearson correlation coefficient; SCC: Spearman rank-based correlation coefficient; DXY: Somers’ Dxy rank correlation; MCC: Matthews correlation coefficient; CRAMERV: Cramer’s V statistic; INFORM: Informedness.

We began by comparing the distribution of gene expression measurements generated using the microarray Affymetrix HG-U219 platform in GDSC, the microarray Affymetrix HG-U133PLUS2 platform and the new Illumina RNA-seq data in CCLE ( Supplementary Figure 11). We observed similar bimodal distributions, suggesting the presence of a natural cutoff to discriminate between lowly vs. highly expressed genes. We therefore fit a mixture of two gaussians and identified an expression cutoff for each platform separately ( Supplementary Figure 11). We then compared the consistency of continuous and discretized gene expression values between ( *i*) the microarray Affymetrix HG-U133PLUS2 and Illumina RNA-seq platforms within CCLE (intra-lab consistency); ( *ii*) the microarray Affymetrix HG-U219 and HG-U133PLUS2 platforms used in GDSC and CCLE, respectively (microarray, inter-lab consistency); and ( *iii*) the microarray Affymetrix HG-U219 and Illumina RNA-seq platforms used in GDSC and CCLE, respectively (inter-lab consistency). We performed a similar analysis for CNV log-ratios and observed high consistency across cell lines ( Figure 9A). Supporting our previous observations, we found that CNV and gene expression measurements are significantly more consistent than drug sensitivity values when using all cell lines (Wilcoxon rank sum test p-value < 0.05; Figure 9A; Supplementary Figure 12A).

Similarly to the filtering we performed for drug sensitivity data, we subsequently restricted our analysis to the cell lines showing high expression of a given gene/cell line combination in at least one study. Again, CNV and gene expression measurements were significantly more consistent than drug sensitivity values in this case (Wilcoxon rank sum test p-value < 0.05; Figure 9B; Supplementary Figure 12B). When dichotomizing data into lowly/highly expressing, amplifications/deletions, and wild type/mutated cell lines and insensitive/sensitive cell lines, the CNV and gene expression data were still more consistent ( Figure 9C) although the difference was not always significant ( Supplementary Figure 12C). Concurring with the report of Hudson *et al.* ^23^, we observed low consistency for mutation calls across cell lines ( Figure 9C).

### Consistency of gene-drug associations

The primary goal of the GDSC and CCLE studies was to identify new genomic predictors of drug response for both targeted and cytotoxic therapies. We therefore evaluated whether the good consistency in drug sensitivity data observed for nilotinib, PLX4720 and crizotinib, and the moderate consistency observed for 17-AAG and PD-0332901 would translate in reproducible biomarkers. We estimated gene–drug associations by fitting, for each gene and drug, a linear regression model including gene expression, CNV and mutations as predictors of drug sensitivity, adjusted for tissue source (see Methods). As illustrated in Figure 1, we used the molecular and pharmacological data generated independently in GDSC and CCLE to identify and compare gene-drug associations. This approach prevents any information leak between the two datasets, which may lead to overoptimistic consistency between the studies, as in the recent comparative study published by the GDSC and CCLE investigators ^9^. Given the high correlation between the published and recomputed AUC values in each study ( Figure 6) and their similar consistency ( Figure 9), all gene-drug associations were computed using published AUC for clarity.

We first computed the strength and significance of each gene in both datasets separately. Similarly to our initial study ^7^, the strength of a given gene-drug association is provided by the standardized coefficient associated to the corresponding gene profile in the linear model and its significance is provided by the p-value of this coefficient (see Methods). We then identified gene-drug associations that were reproducible in both datasets (same sign and False Discovery Rate [FDR] < 5%) or that were dataset-specific (different sign or significant in only one dataset) using continuous ( Supplementary Figure 13 and Supplementary Figure 14 for common and all cell lines, respectively) and discretized ( Supplementary Figure 15 and Supplementary Figure 15 for common and all cell lines, respectively) published AUC values as drug sensitivity data. We assessed the overlap of gene-drug associations discovered in both datasets using the Jaccard index ^24^. All Jaccard indices were low, with nilotinib yielded the largest overlap of gene-drug associations (32%), followed by PD-0325901 and erlotinib (almost 20%), while the other drugs yielded less than 15% overlap ( Supplementary Figure 17). Our results further indicate that larger overlap exists for gene-drug associations identified using the continuous drug sensitivity data compared with associations using discretized drug sensitivity calls (Wilcoxon signed rank test p-value of 4×10 ^-2^ and 2×10 ^-3^ for the common set and the full set of cell lines, respectively). We therefore focused our analyses on the gene-drug associations identified using continuous published AUC values. The number (and identity) of gene-drug associations computed using continuous published AUC values are provided in Supplementary Table 1 and Supplementary Table 2 ( Dataset 5 and Dataset 6) for common and all cell lines, respectively.

Given that simply intersecting significant gene-drug associations identified in each dataset separately yielded poor reproducibility for all drugs, we sought to more closely mimic the biomarker discovery and validation process. We therefore used one dataset to discover significant gene-drug associations and test whether this subset of markers validated in an independent dataset. Using the discovery dataset, gene-drug associations are first ranked by nominal p-values and their FDR is computed. An association is selected if it is part of the top 100 markers and its FDR is less than 5%. This procedure ensure to control for both significance and number of selected biomarkers, which can vary with respect to the cell line panel used for the analysis (larger panels enable the identification of more significant biomarkers due to increased statistical power). A gene-drug association is validated in an independent dataset if its nominal p-value is less than 0.05 and its “direction”, that is whether the marker is associated with sensitivity or resistance, is identical to the one estimated during the discovery process.

**Figure 10.**
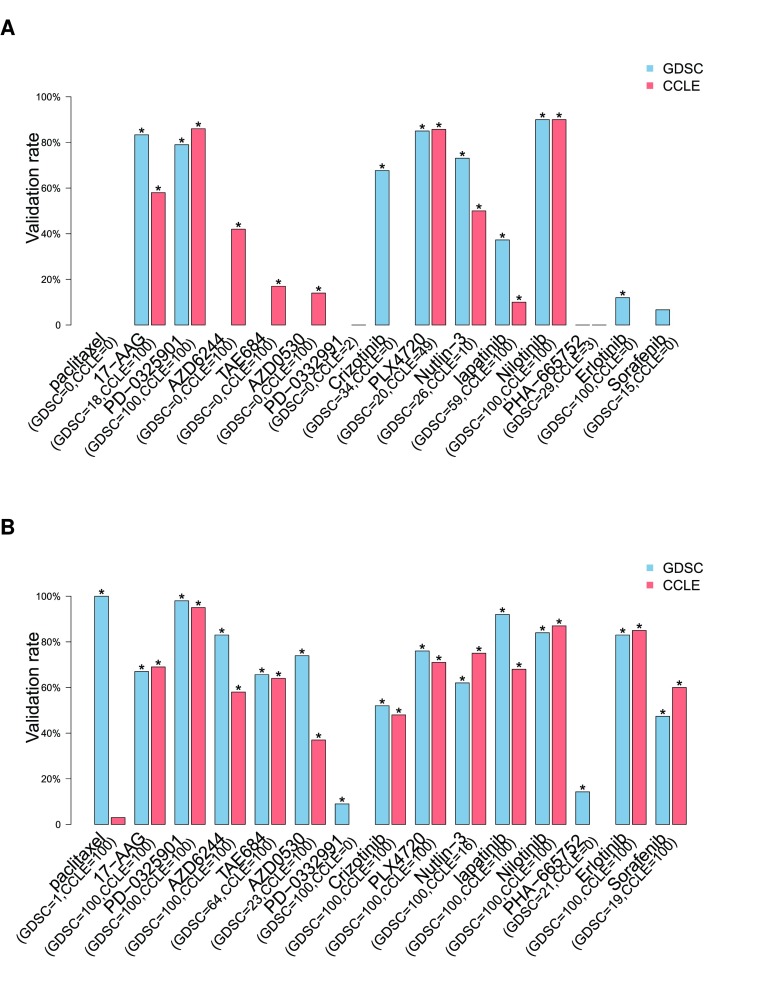
Proportion of gene-drug associations identified in a discovery set (top 100 gene-drug associations as ranked by p-values and FDR < 5%) and validated in an independent validation dataset. In blue and red are the gene-drug associations identified in GDSC and CCLE, respectively. Associations are identified using molecular profiles including gene expression, mutation and copy number variation data as input and ( **A**) continuous published AUC values as output in a linear model using only common cell lines or ( **B**) all cell lines. The number of selected gene-drugs associations in each datasets is provided in parentheses. The symbol ’*’ represents the significance of the proportion of validated gene-drug associations, computed as the frequency of 1000 random subsets of markers of the same size having equal or greater validation rate compared to the observed rate.

We computed the proportions of validated gene-drug associations for each drug using all available genomic molecular data profiles in GDSC as discovery set and CCLE as validation set, and *vice versa* ( Figure 10). Overall, we found that biomarkers for PD-0325901, PLX4720 and nilotinib yielded a high validation rate (> 80%) with either dataset as discovery set using the common cell lines screened in GDSC and CCLE ( Figure 10A). When using the entire cell line panels used in each study, two more drugs -- lapatinib and erlotinib -- yielded high validation rate ( Figure 10B). 17-AAG, and TAE684 yielded validation rate between 60% and 80%, while the other drugs yielded a validation rate around 50% or lower. For ten out of the fifteen drugs, using the entire panel of cell lines screened in each study ( Figure 10B) improved the validation rate compared to limiting the analysis to common cell lines ( Figure 10A). However, validation rate decreased for three drugs, suggesting that using large, but different panels of cell lines may increase statistical power but could also introduce biases in the biomarker discovery process.

We then investigated whether higher validation rates would be obtained by using more stringent significance threshold and relaxing the constraint on the number of significant associations in the discovery set ( Supplementary Figure 18 and Supplementary Figure 19). Using common cell lines, we found that proportion of validated gene-drug association monotonically increases with FDR stringency for six drugs, with very high validation rate for the most stringent FDR cutoff (validation rate > 80% for FDR < 0.1%) for 17-AAG, PD-0325901, PLX4720 and nilotinib using either dataset as discovery set ( Supplementary Figure 18). Using the entire panel of cell lines in each study actually improved validation rate for six drugs, AZD6244, TAE684, AZD0530, lapatinib — and erlotinib and sorafenib, for which insufficient number of sensitive cell lines were screened in both GDSC and CCLE ( Supplementary Figure 19). However, validation rate decreased for 17-AAG, crizotinib and PLX4720, which suggests again that large, but different panels of cell lines might introduce selection bias for some drugs.

### Known biomarkers

As reported in the original GDSC (1) and CCLE (2) publications and in recent reports ^10, 14, 15^, several known biomarkers for targeted therapies have been shown to be predictive in both GDSC and CCLE. In our initial comparative study we also found the following known gene-drug associations:

- BRAF mutations were significantly associated with sensitivity to MEK inhibitors (AZD6244 and PD-0325901) and BRAF ^V600E^ inhibitor (PLX4720) with nominal p-values < 0.01; see Supplementary File 10–Supplementary File 13 of our initial study.
- ERBB2 expression was significantly associated with sensitivity to lapatinib with nominal p-value = 0.04 and 8.4×10 ^-15 ^for GDSC and CCLE, respectively; see Supplementary File 4 and Supplementary File 5 of our initial study.
- NQ01 expression was significantly associated with sensitivity to 17-AAG with nominal p-value = 2.4×10 ^-13^ and 6.2×10 ^-14^ for GDSC and CCLE, respectively; see Supplementary File 4 and Supplementary File 5 of our initial study.
- MDM2 expression was significantly associated with sensitivity to Nutlin-3 with nominal p-value = 7.7×10 ^-18^ and 7×10 ^-8^ for GDSC and CCLE, respectively; see Supplementary File 4 and Supplementary File 5 of our initial study.
- ALK expression was significantly associated with sensitivity to TAE684 with nominal p-value = 1.6×10 ^-9^ and 1.7×10 ^-9^ or GDSC and CCLE, respectively; see Supplementary File 4 and Supplementary File 5 of our initial study.

We revisited our biomarker analysis using the new data released by GDSC and CCLE to test whether additional known biomarkers can be identified. We recomputed all gene-drug associations based on expression, mutation, gene-fusion and amplification data using the common cell lines between studies Dataset 5, and entire panel of cell lines in each study ( Dataset 6). We confirmed the reproducibility of the known associations reported in our initial study, but we were not able to find reproducible associations for EGFR mutations with response to AZD0530 and erlotinib, and HGF expression with response to crizotinib ( Table 1). The reproducibility of the majority of these previously known associations attests to the relevance of the GDSC and CCLE datasets although our results demonstrated that the noise and inconsistency in drug sensitivity data render discovery of new biomarkers difficult for the majority of the drugs.

**Table 1.** List of known gene-drug associations with their effect size and significance in GDSC and CCLE. Gene-drug associations were estimated using the full panel of cell lines and AUC as measure of drug sensitivity.

Drug	Gene	Type	GDSC effect size	GDSC pvalue	CCLE effect size	CCLE pvalue	Reproducible
Nilotinib	BCR_ABL	fusion	6.13	1.10E-51	5.84	2.60E-28	YES
17-AAG	NQO1	expression	0.55	5.30E-39	0.6	4.70E-29	YES
	HSP90AA1	expression	0	9.00E-01	0.02	6.40E-01	NS
	HSP90AB1	expression	0.01	7.40E-01	0	9.40E-01	NS
PD-0325901	BRAF	mutation	0.83	6.40E-09	0.82	8.10E-10	YES
	MAP2K1	expression	-0.07	7.10E-02	-0.02	6.70E-01	NS
	MAP2K2	expression	0.02	5.60E-01	0.03	5.10E-01	NS
AZD6244	BRAF	mutation	0.93	6.10E-10	0.86	3.70E-10	YES
	MAP2K1	expression	-0.04	2.80E-01	-0.06	1.90E-01	NS
	MAP2K2	expression	0.01	8.40E-01	0.01	7.70E-01	NS
TAE684	ALK	expression	0.28	2.20E-07	0.26	1.10E-08	YES
AZD0530	EGFR	mutation	0.03	9.50E-01	0.51	8.20E-03	NO
	BCR_ABL	fusion	3.87	2.60E-18	3.35	3.50E-09	YES
	SRC	expression	0.07	2.80E-01	0.07	1.60E-01	NS
PD-0332991	CDK4	expression	0.03	5.10E-01	0	9.50E-01	NS
	CDK6	expression	0.08	7.50E-02	-0.02	6.60E-01	NS
Crizotinib	HGF	expression	-0.03	6.50E-01	0.28	1.30E-09	NO
	MET	amplification	0.1	8.10E-02	0.29	3.80E-09	NO
	ALK	expression	0.58	3.90E-33	0.13	6.80E-03	YES
PLX4720	BRAF	mutation	1.75	8.60E-46	1.38	2.20E-27	YES
Nutlin-3	MDM2	expression	0.39	2.00E-25	0.31	8.40E-12	YES
lapatinib	ERBB2	expression	0.42	1.10E-12	0.53	3.40E-33	YES
		amplification	0.24	8.40E-06	0.39	4.20E-19	YES
	EGFR	expression	0.26	1.20E-04	0.16	7.20E-03	YES
PHA-665752	HGF	expression	0.04	4.90E-01	0.06	2.00E-01	NS
	MET	amplification	0.22	2.20E-04	0.02	6.80E-01	NO
Erlotinib	EGFR	mutation	0.71	1.90E-01	1.27	2.40E-12	NO
Sorafenib	PDGFRA	expression	0.06	3.90E-01	0.13	7.90E-03	NO
	KDR	expression	0.01	8.70E-01	-0.02	6.90E-01	NS
	KIT	mutation			0.11	6.30E-01	
		expression	0.06	3.50E-01	-0.01	7.70E-01	NS
	FLT1	expression	-0.04	5.20E-01	0.01	8.30E-01	NS
	FLT3	expression	0.32	4.80E-08	0.33	1.10E-13	YES
	FLT4	expression	0.12	4.30E-02	-0.02	6.40E-01	NO
	RAF1	expression	0.03	6.40E-01	0.06	2.30E-01	NS
	BRAF	mutation	-0.34	1.70E-01	0.14	3.80E-01	NS
	BRAF	expression	-0.11	9.30E-02	0.02	7.40E-01	NS

## Discussion

Our original motivation in analyzing the GDSC and CCLE data was to discover predictive genomic biomarkers of drug response. When we applied a number of methods using one study to select genomic features and to train a classifier, and then applied it to predict reported drug response in the second study, our predictive models failed to validate for half of the drugs tested ^3^. Indeed, out of nine predictors yielding concordance index ^25^ ≥0.65 in cross-validation in the training set (GDSC), only four were validated in identical cell lines treated with the same drugs in the validation set (CCLE) ^3^.

As we explored the reasons for this failure, we first checked whether cell lines could have drifted and consequently exhibited different transcriptional profiles between GDSC and CCLE. We found that any genome-wide molecular profile in one study would almost always identify “itself” (its purported biological replica) as being most similar among the cell lines in the other study. In a way this is not surprising. When gene expression studies were in their infancy, there were many reports that compared the results from studies and found that they were inconsistent and unreproducible in new studies — as demonstrated by the countless microarray signatures that fail to reproduce beyond their initial publication. As a result, scientists involved in gene expression studies “circled the wagons” and developed both much more standardized laboratory protocols and “best practices” for reproducible analysis, including data normalization and batch corrections, that now mean that independent measurements from different laboratories are far more often consistent and so can be used for signature development and validation ^26, 27^.

Unexpectedly, when we compared phenotypic measures of drug response that were released by the GDSC and CCLE projects, we found discrepancies in growth inhibition effects of multiple anticancer agents. What that means in practice is that, for some drugs, a molecular biomarker of drug response learned from one study would not likely be predictive of the reported response in the other. And consequently, neither of the studies might be useful in predicting response in patients as many had hoped when these large pharmacogenomic screens were published.

The feedback from the scientific community on our analysis, the availability of new data from the GDSC and CCLE, as well as improvements in the *PharmacoGx* software platform we developed to support this type of analyses ^11^, prompted us to revisit the question of consistency in these studies to see if we could find a principled way to identify correlated drug response phenotypes. By testing a variety of methods of classifying the data, and choosing the metric which gave the best consistency for each drug, we were able to find moderate to good consistency of sensitivity data for two broad effect and three targeted drugs. We also confirmed the overall lack of consistency between the studies for eight drugs, while there were not enough sensitive cell lines that had been screened by both GDSC and CCLE to properly assess consistency for the remaining three drugs. The summary box included with this paper briefly describes the most significant issues that people have raised in discussing our previous findings with us and summarizes what we have found in our reanalysis.

Some have suggested that one way to improve correlation would have been to compare the studies and throw out the most discordant data as noise and then compare the remaining concordant data. While this would certainly find concordance in the remaining data, the approach is equivalent to fitting data to a desired result, which is bad practice and certainly could not be extended to other data sets or to the classification of patient tumors as responsive or nonresponsive to a particular therapy. There is, however, merit in the suggestion that one would not expect to see correlation in noise. And noise is precisely what one would expect to see in drug response data from cell lines that are resistant to a particular drug or nonresponsive across the range of doses tested. As reported here, filtering the data in each study independently to classify cell lines in a binary fashion, and then comparing the binary classification between studies using a variety of metrics developed to handle the intricacies of this sort of response data, also failed to find simple correlations in the data, except for three of the targeted therapies, nilotinib, PLX4720 and crizotinib. What this ultimately means is that the most and the least sensitive cell lines would not appear to be the same when comparing the two studies.

There are many reasons for potential differences in measured phenotypes reported by the GDSC and CCLE, including substantial differences in doses used for each drug and in the methods used to both assay cell viability and to estimate drug response parameters. By comparing GDSC and CCLE with an independent pharmacogenomic dataset published by GlaxoSmithKline (GSK), we showed that higher consistency is achieved when the same pharmacological assay is used (GSK and CCLE used the CellTiter-Glo assay, while GDSC used Syto60) ^7, 8^. Genentech also used the CellTiter-Glo assay and observed higher consistency of drug sensitivity data with CCLE compared to GDSC ^10^. The authors elegantly evaluated the impact of cell viability readout, growth medium, and seeding density. They observed only weak impact of the choice of pharmacological assay as their follow-up screen with the Syto60 assay clustered closer to their own CellTiter-Glo screen than GDSC, suggesting that other parameters might have driven the inconsistency observed with GDSC ^10^. They further showed that increased fetal bovine serum and seeding cell density had a systematic effect on mean cell viability. Pozdeyev *et al.* showed that restricting the computation of AUC to the concentration range shared between GDSC and CCLE, the equivalent of our AUC* drug sensitivity measure, yielded a small, but statistically significant improvement in consistent of pharmacological profiles ^28^. Ultimately what our analysis and these recent reports suggest is that not only drug sensitivity measurements must be carefully and appropriately compared, but also that there is a pressing need for more robust pharmacological assays and standardized computational methods for modeling drug response. However, in the absence of a “gold standard” screening platform demonstrated to accurately recapitulate drug response *in vivo*, the use of multiple assays is critical to probe different biological aspects of growth inhibition. Given that GDSC and CCLE used different pharmacological assays, it makes the release of these pharmacogenomic data even more valuable.

The primary goal of the GDSC and CCLE studies was to link molecular features of a large panel of cancer cell lines to their sensitivity to cytotoxic and targeted drugs. The reproducibility of most of the known gene-drug associations provides evidence that these large pharmacogenomic datasets are biologically relevant. When we investigated whether we could find significant gene-drug associations discovered in one dataset that validate in the other independent dataset, we observed over 75% validation rate for the most significant molecular biomarkers for eight of 15 drugs, which is a major improvement over our initial comparative study. However, this does not suggest that one can use these studies to find new, reproducible gene-drug associations for the rest of the drugs, as the majority of associations can be found in only one dataset but not in both. However, GDSC and CCLE could be jointly analyzed to identify biomarkers that are robust to the use of different biological assays, and are therefore more likely to work in new biological contexts ^29^.

This study has several potential limitations. First, while the raw drug sensitivity data are publicly available for GDSC, these data have not been released within the CCLE study. We could not fit the drug dose-response curves using the technical triplicates but rather relied on the published median sensitivity values. The lack of technical replicates in CCLE also prevented us to assess the level of noise of the drug sensitivity measurements. Second, we discretized drug sensitivity values by selecting a common threshold to discriminate between insensitive (AUC ≤ 0.2 and IC _50_ ≥ 1 µM) and the rest of the cell lines for all the targeted agents. However, it is clear that such a threshold could be optimized for each drug, which might have an impact on the consistency of drug phenotypes and gene-drug associations based on binary sensitivity calls, as was done in breast cancer ^30^ and in our response to the critic of Geeleher *et al.* ^31, 32^. Lastly, the current set of mutations assessed in both study is small (64 mutations), which drastically limits the search for mutation-based and other genomic aberrations associated with drug response. The exome-sequencing data available within the new GDSC1000 dataset will enable to better explore the genomic space of biomarkers in cancer cell lines, and their reproducibility across studies.

## Conclusion

As is true of many scientists working in genomics and oncology, we were excited when the GDSC and CCLE released their initial data sets and were hopeful that these projects would help to accelerate drug discovery and further the development of precision medicine in oncology. However, what we found initially, and what the reanalysis presented here further indicates, is that there are inconsistencies between the measured phenotypic response to drugs in these studies. Even in our reanalysis, where we used methods specific to individual drugs and the response characteristics of the cell lines tested, we were only able to find new biomarkers consistently predictive of response for around half of the drugs screened in both studies. Consequently, it is challenging to use the data from these studies to develop general purpose classification rules for all drugs.

Our finding that molecular profiles are significantly more consistent than drug sensitivity data, indicates that the main barrier to biomarker development using these data is the discrepancy in the reported response phenotypes for many drugs. The experimental protocols and pharmacological assays used in the GDSC and CCLE studies are the state-of-the-art for high-throughput drug screening projects. Even though technical and biological replicates are necessary to assess and account for noise in drug sensitivity measurements, it is clear that the assays used in GDSC and CCLE probe different aspects of the biology underlying drug-induced growth inhibition. Without knowing which assay is more relevant for *in vivo* drug response, more research will be required to best leverage these complementary assays for robust biomarker discovery.

From having worked in large-scale genomic analyses, we recognize the challenges involved in planning and executing such studies and commend the GDSC and CCLE for their work and for making all the data available. However, we strongly encourage the GDSC, the CCLE, the pharmacogenomics and bioinformatics communities as a whole, to invest the necessary time and effort to account for the noise in drug response measurements and the complementary nature of different assays in order to assure that these studies are relevant for predicting response in patients. The recent report from Genentech is a significant step in this direction. Ultimately, that effort will help to assure that mammoth undertakings in drug characterization can deliver on their promise to identify better therapies and biomarkers predictive of response.

## Methods

### The PharmacoGx platform

The lack of standardization of cell line and drug identifiers hinders comparison of molecular and pharmacological data between large-scale pharmacogenomic studies, such as the GDSC and CCLE. To address this issue we developed *PharmacoGx*, a computational platform enabling users to download and interrogate large pharmacogenomic datasets that were extensively curated to ensure maximum overlap and consistency ^11^. *PharmacoGx* provides ( *i*) a new object class, called *PharmacoSet*, that acts as a container for the high-throughput pharmacological and molecular data generated in large pharmacogenomics studies (detailed structure provided in Supplementary Methods); and ( *ii*) a set of parallelized functions to assess the reproducibility of pharmacological and molecular data and to identify molecular features associated with drug effects. The *PharmacoGx* package is open-source and publicly available on Bioconductor.

### The GDSC (formerly CGP) dataset

***Drug sensitivity data.*** We used the data release 5 (June 2014) with 6,734 new IC _50_ values for a total of 79,903 drug dose-response curves for 139 different drugs tested on a panel of up to 672 unique cell lines. The data are accessible from ftp://ftp.sanger.ac.uk/pub4/cancerrxgene/releases/release-5.0/.

***Molecular profiles.*** Gene expression data were downloaded from ArrayExpress, accession number E-MTAB-3610. These new data were generated using Affymetrix HG-U219 microarray platform. We processed and normalized the CEL files using RMA ^33^ with BrainArray ^34^ chip description file based on Ensembl gene identifiers (version 19). This resulted in a matrix of normalized expression for 17,616 unique Ensembl gene ids. SNP array data for the Genome-Wide Human SNP Array 6.0 platform were downloaded from GEO with the accession number GSE36139. We processed the raw CEL data using Affymetrix Power Tools (APT) v1.16.1. Copy number segments were generated using HAPSEG v1.1.1 ^35^ based on RMA-normalized signal intensities and Birdseed v2-called genotypes. These segments were further refined using ABSOLUTE v1.0.6 ^36^ to identify allele-specificity within each segment. Mutation and gene fusion calls were downloaded from the GDSC website and processed as in our initial study ^7^.

### The CCLE dataset

***Drug sensitivity data.*** We used the drug sensitivity data available from the CCLE website ( https://portals.broadinstitute.org/ccle/data/browseData) and updated on February 2015 with a total number of 11,670 dose-response curves for 24 drugs tested in a panel of up to 504 cell lines.

***Molecular profiles.*** Gene expression data were downloaded from the CCLE website and CGHub ^37^ for the Affymetrix HG-U133PLUS2 and Illumina HiSeq 2500 platforms, respectively. SNP array data were downloaded from EMBL-EBI with the accession number EGAD00010000644. Normalization of microarray data (1036 cell lines) and SNP array data (1190 cell lines) was performed the same way than for GDSC. RNA-seq data (935 cell lines) were downloaded as BAM files previously aligned using TopHat ^38^ and the quantification of gene expression was performed using Cufflinks ^38^ based on Ensembl GrCh37 human reference genome. Mutation data were retrieved from the CCLE website and processed as in our initial study ^7^.

### Curation of drug and cell line identifiers

The lack of standardization for cell line names and drug identifiers represents a major barrier for performing comparative analyses of large pharmacogenomics studies, such as GDSC and CCLE. We therefore curated these datasets to maximize the overlap in cell lines and drugs by assigning a unique identifier to each cell line and drug. Entities with the same unique identifier were matched. Manual search was then applied to match any remaining cell lines or drugs which were not matched based on string similarity; annotations were consistently extracted from Cellosaurus ^39^. The cell line curation was validated by ensuring that the cell lines with matched name had a similar SNP fingerprint (see below). The drug curation was validated by examining the extended fingerprint of each of their SMILES strings ^40^ and ensuring that the Tanimoto similarity ^41^ between two drugs called as the same, as determined by this fingerprint, was above 0.95. 

### Cell line identity using SNP fingerprinting

To assess the identity of cell lines from GDSC and CCLE, data of low quality were first excluded from our analysis panel (detailed procedure described in Supplementary Methods). Of the 973 CEL files from GDSC, only 66 fell below the 0.4 threshold (6.88%) for contrast QC scores, indicating issues in resolving base calls. Additionally, five of the 1,190 CEL files from CCLE had an absolute difference between contrast QC scores for Nsp and Sty fragments greater than 2, thus indicating some issues with the efficacy of one enzyme set during sample preparation. CEL files with contrast QC scores indicative of some sort of issue with the assay that would affect the genotype call rate or birdseed accuracy were removed and genotype calling was conducted on the remaining CEL files using Birdseed version 2. The resulting files were then filtered to keep only the 1006 SNP fingerprints that originated from CEL files that had a common cell line annotation between GDSC and CCLE (503 CEL files from each). Finally, pairwise concordances of all SNP fingerprints were generated according to the method outlined by Hong *et al.* ^12^.

### Drug dose-response curves

To identify artefactual drug dose-response curves due to experimental or normalization issues, we developed simple quality controls (QC; details in Supplementary Methods). Briefly, we checked whether normalized viability measurements range between 0% and 100% and that drug-response curve is monotically non-increasing as expected. The drug dose-response curves which did not pass these simple QC were flagged and removed from subsequent analyses as the curve fitting would have yielded erroneous results.

All dose-response curves were fitted to the equation

$$y(x)=E_{\infty }+\frac{1-E_{\infty }}{\left(1+{\left(\frac{x}{EC_{50}}\right)}^{HS}\right)}$$

where *y* = 0 denotes death of all infected cells, *y* = *y*(0) = 1 denotes no effect of the drug dose, *EC*_inf_ is the viability observed in the presence of an arbitrarily large concentration of drug, *EC*_50_ is the concentration at which viability is reduced by half as much as it is in the presence of an arbitrarily large concentration of drug, and *HS* is a parameter describing the cooperativity of binding. *HS* < 1 denotes negative binding cooperativity, *HS* = 1 denotes noncooperative binding, and *HS* > 1 denotes positive binding cooperativity. The parameters of the curves were fitted using the least squares optimization framework. Comparison of our dose-response curve model with those used in the GDSC and CCLE publications is provided in Supplementary Methods.

### Discretization of pharmacogenomic data

***Drug sensitivity data.*** To discretize the drug sensitivity data, we used AUC ≤ 0.2 (IC _50_ ≥ 1 µM) and AUC ≤ 0.4 (IC _50_ ≥ 10 µM) to identify the “insensitive” cell lines for targeted and cytotoxic drugs, respectively, while the rest of the cell lines are classified as “sensitive”. These reasonable, although somewhat arbitrary, cutoffs enabled to explore the potential of such binary drug sensitivity calls as new drug phenotypic measures to find consistency in drug sensitivity data and gene-drug associations.

***Gene expression data.*** To discretize the drug sensitivity data into lowly vs. highly expressed genes, we fit a mixture of two Gaussians of unequal variance using the full distribution of expression values of the 17,401 genes in common between GDSC and CCLE datasets. We defined the expression threshold as the expression value for which the posterior probability of belonging to the left tail of the highly expression distribution is 10%.

***Mutation data.*** Similarly to the GDSC and CCLE publications, we transformed the original mutation data into binary values that represent the absence (0) or presence (1) of any missense mutations in a given gene in a given cell line.

### Gene-drug associations

We assessed the association, across cell lines, between a molecular feature and response to a given drug, referred to as gene-drug association, using a linear regression model adjusted for tissue source:

*Y = β _0_ + β _i_G _i_ + β _t_T*

where *Y* denotes the drug sensitivity variable, *G _i_* and *T* denote the expression of gene *i* and the tissue source respectively, and *β*s are the regression coefficients. The strength of gene-drug association is quantified by *β _i_*, above and beyond the relationship between drug sensitivity and tissue source. The variables *Y* and *G* are scaled (standard deviation equals to 1) to estimate standardized coefficients from the linear model. Significance of the gene-drug association is estimated by the statistical significance of *β _i_* (two-sided t test). When applicable, p-values were corrected for multiple testing using the FDR approach ^42^.

As we recognized that continuous drug sensitivity is not normally distributed, which violates one of the assumption of the linear regression model described above, we also assessed the consistency of gene-drug association using discretized (binary) drug sensitivity calls as the response variable in a logistic regression model adjusted for tissue source, similarly to the linear regression model.

### Measure of consistency

***Area between curves (ABC).*** To quantify the difference between two dose-response curves, we computed the area between curves (ABC). ABC is calculated by taking the unsigned area between the two curves over the intersection of the concentration range tested in the two experiments of interest, and normalizing that area by the length of the intersection interval. In the present study, we compared the curves fitted for the same drug-cell line combinations tested both in GDSC and CCLE. Further details are provided in Supplementary Methods.

***Pearson correlation coefficient (PCC).*** PCC is a measure of the linear correlation between two variables, giving a value between +1 and −1 inclusive, where 1 represents total positive correlation, 0 represents no correlation, and −1 represents total negative correlation ^17^. PCC is sensitive to the presence of outliers, like a few sensitive cell lines in the case of drug sensitivity data measured for targeted therapies or genes rarely expressed.

***Spearman rank correlation coefficient (SCC).*** SCC is a nonparametric measure of statistical dependence between two variables and is defined as the Pearson correlation coefficient between the ranked variables ^18^. It assesses how well the relationship between two variables can be described using a monotonic function. If there are no repeated data values, a perfect Spearman correlation of +1 or −1 occurs when each of the variables is a perfect monotone function of the other. Contrary to PCC, SCC can capture non linear relationship between variables but is insensitive to outliers, which is frequent for drug sensitivity data measured for targeted therapies or genes rarely expressed.

***Somers’ Dxy rank correlation (DXY).*** DXY is a non-parametric measure of association equivalent to ( *C* - 0.5) * 2 where *C* represents the concordance index ^25^ that is the probability that two variables will rank a random pair of samples the same way ^19^.

***Matthews correlation coefficient (MCC).*** MCC ^20^ is used in machine learning as a measure of the quality of classification predictions. It takes into account true and false positives and negatives, acting as a balanced measure which can be used when the classes are of different sizes. MCC is in essence a correlation coefficient between two binary classifications; it returns a value between −1 (perfect opposite classification) and +1 (identical classifications), with 0 representing association no better than random chance.

***Cramer’s V (CRAMERV).*** CRAMERV is a measure of association between two nominal variables, based on Pearson’s chi-squared statistic, giving a value between 0 (no association) and +1 (perfect association) ^21^. In the case of 2×2 contingency table, such as binary drug sensitivity or gene expression measurements, CRAMERV is equivalent to the Phi coefficient.

***Informedness (INFORM).*** For a 2×2 contingency table comparing two binary classifications, INFORM can be defined as Specificity + Sensitivity - 1, which is equivalent to true positive rate - false positive rate ^22^. The magnitude of INFORM gives the probability of an informed decision between the two classes, where INFORM > 0 represents appropriate use of information, 0 represents chance-level decision, < 0 represents perverse use of information.

## Data and software availability

Open Science Framework: Dataset: Revisiting inconsistency in large pharmacogenomics studies, doi 10.17605/osf.io/xxxx ^43^

Data: The list of all the pharmacogenomic datasets available through the *PharmacoGx* platform can be obtained from R using the *availablePSets()* function from the R/Bioconductor library *PharmacoGx*.

The GDSC and CCLE *PharmacoSets* used in this study are available from pmgenomics.ca/bhklab/sites/default/files/downloads/ using the *downloadPset()* function.

Code: The R code necessary to replicate all the results presented in this article is available from the cdrug2 GitHub repository.

## Supplementary material

**Supplementary file 1.**

All the noisy curves identified in GDSC and CCLE.

**Supplementary file 2.**

All drug dose-response curves in common between GDSC and CCLE.

**Supplementary file 3.**

All drug dose-response curves for replicated experiments using AZD6482 in GDSC.

**Supplementary figures and tables.**

**Supplementary Datasets**

**Dataset 1.** SNP fingerprints of all the cell lines profiled with SNP arrays in GDSC and CCLE. Data used to generate Figure 3.

**Dataset 2.** AUC and IC _50_ values as published and recomputed using PharmacoGx. Data used to generate Figure 6.

**Dataset 3.** Consistency measures for AUC, AUC* (STAR) and IC _50_ values as published and recomputed using PharmacoGx, across cell lines. Data used to generate Figure 8.

**Dataset 4.** Consistency measures for molecular profiles across cell lines. GE.CCLE.ARRAY.RNASEQ: Consistency between gene expression data generated using Affymetrix HG-U133PLUS2 microarray and Illumina RNA-seq platforms within CCLE; GE.ARRAYS: Consistency between gene expression data generated using Affymetrix HG-U133A and HG-U133PLUS2 microarray platforms in GDSC and CCLE, respectively; GE.ARRAY.RNASEQ: Consistency between gene expression data generated using Affymetrix HG-U133A microarray and Illumina RNA-seq platforms in GDSC and CCLE, respectively; CNV: Consistency of copy number variation data in CCLE and GDSC, respectively; MUTATION: Consistency of mutation profiles in CCLE and GDSC, respectively. Data used to generate Figure 12.

**Dataset 5.** Spreadsheets reporting the statistics (effect size and significance) for all expression-based, mutation-based and amplification-based gene-drug associations for each drug using the common cell lines screened both in GDSC and CCLE. Gene-drug associations were estimated the relevant molecular profile of genes as input and continuous published AUC as output in a linear regression model adjusted for tissue source. Data used to generate Supplementary Table 1 and Supplementary Figure 13.

**Dataset 6.** Spreadsheets reporting the statistics (effect size and significance) for all expression-based, mutation-based and amplification-based gene-drug associations for each drug using the entire panel of cell lines in GDSC and CCLE. Gene-drug associations were estimated using the relevant molecular profile of genes as input and continuous published AUC as output in a linear regression model adjusted for tissue source. Data used to generate Table 1, Figure 10, Supplementary Table 2 and Supplementary Figure 14.
