# Supplementary figures and images for: Revisiting inconsistency in large pharmacogenomic studies
Source: F1000Res. 2017 Aug 11;5:2333. Originally published 2016 Sep 16. [Version 3] doi: 10.12688/f1000research.9611.3 (PMC5580432; doi:10.12688/f1000research.9611.3)

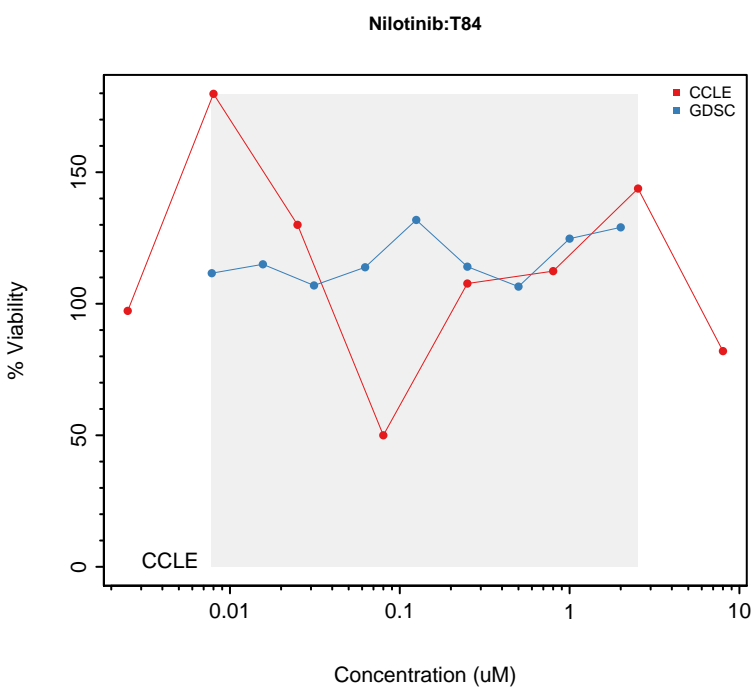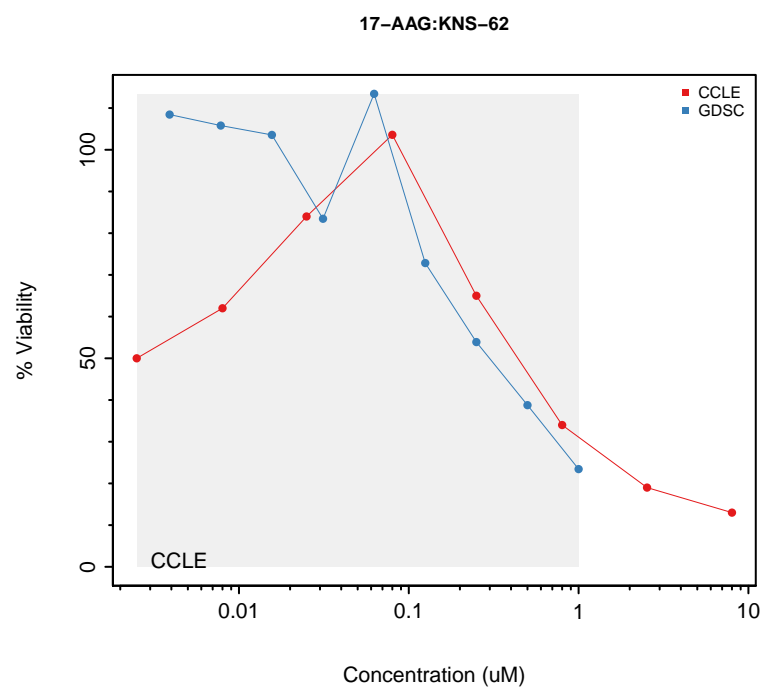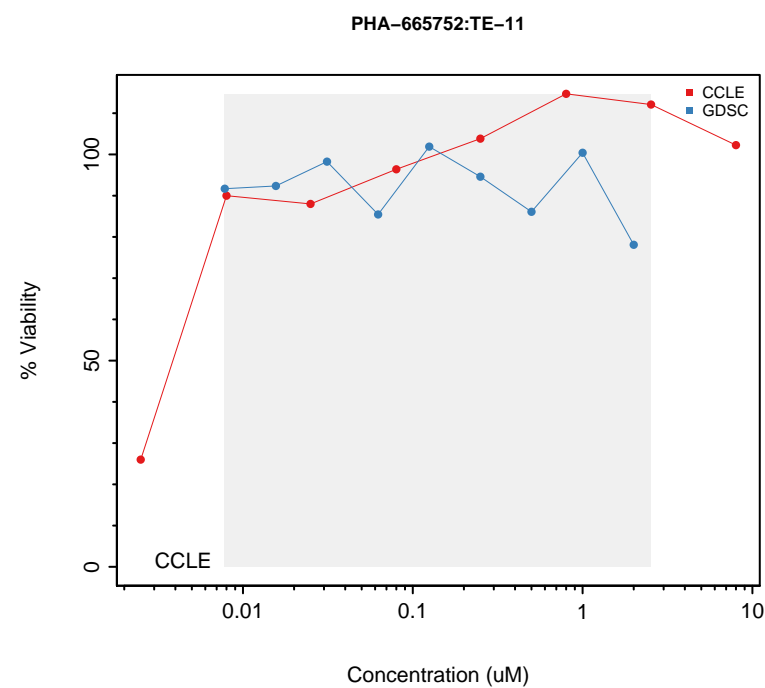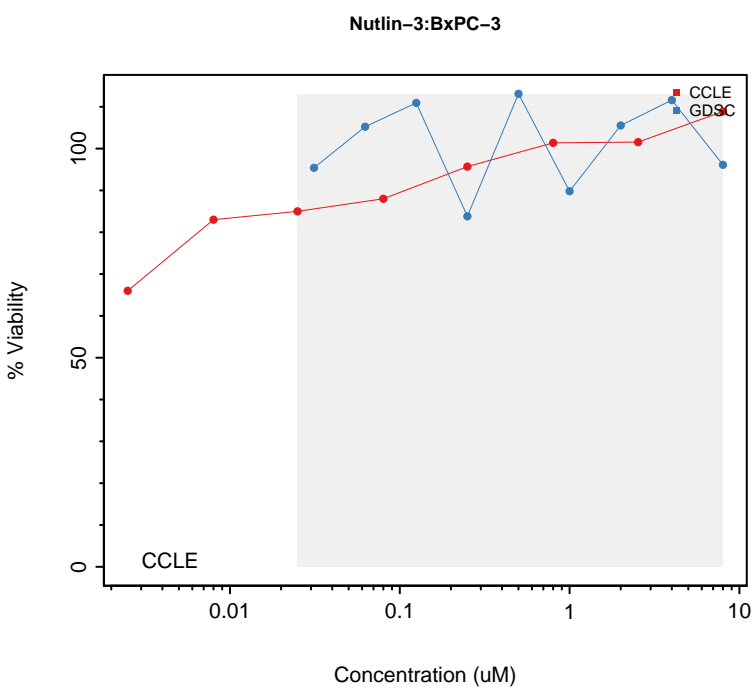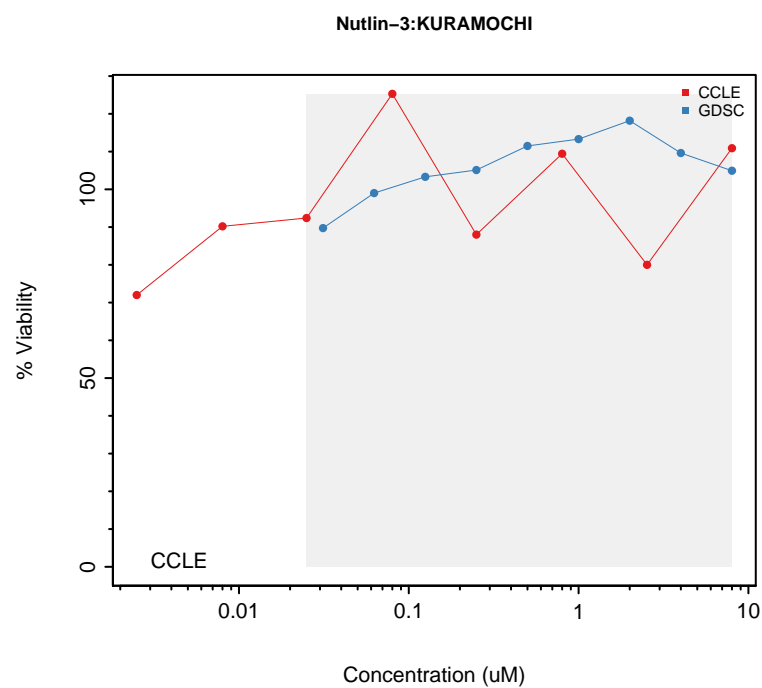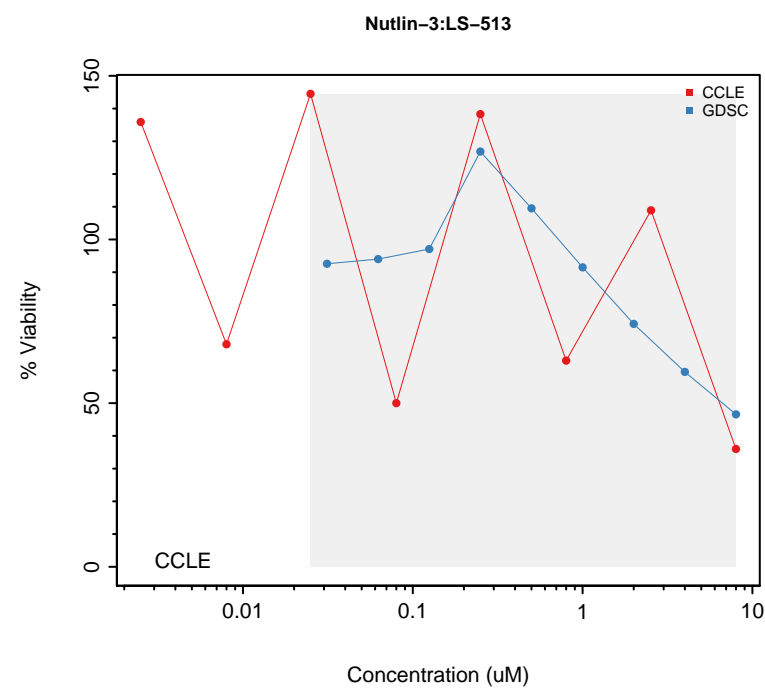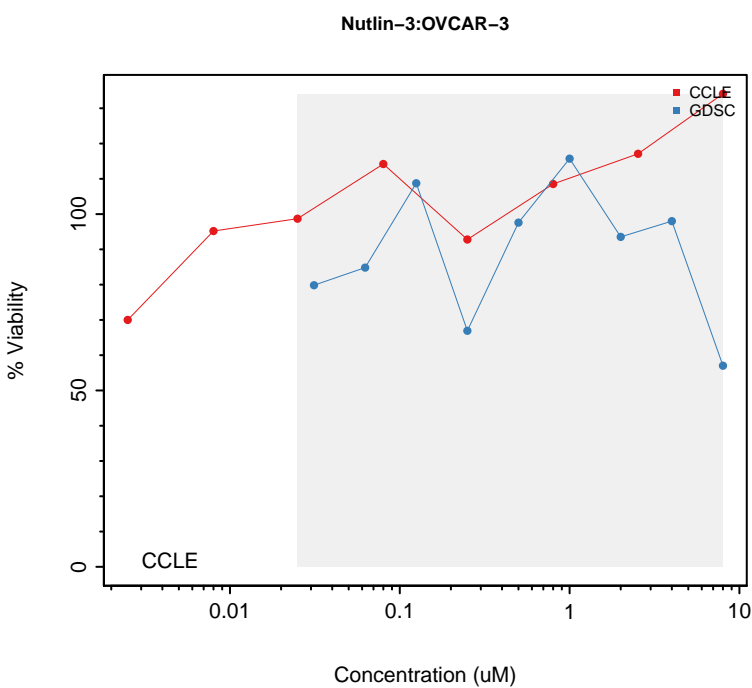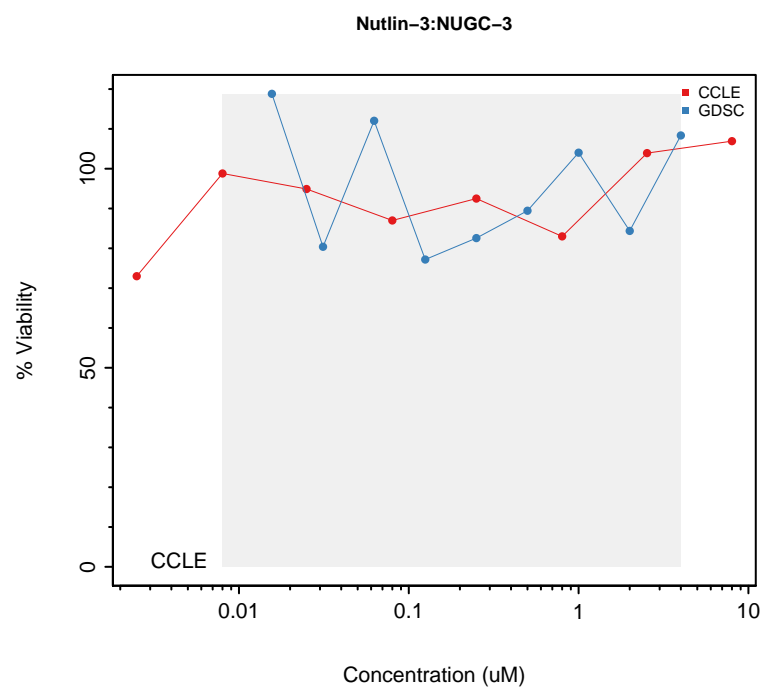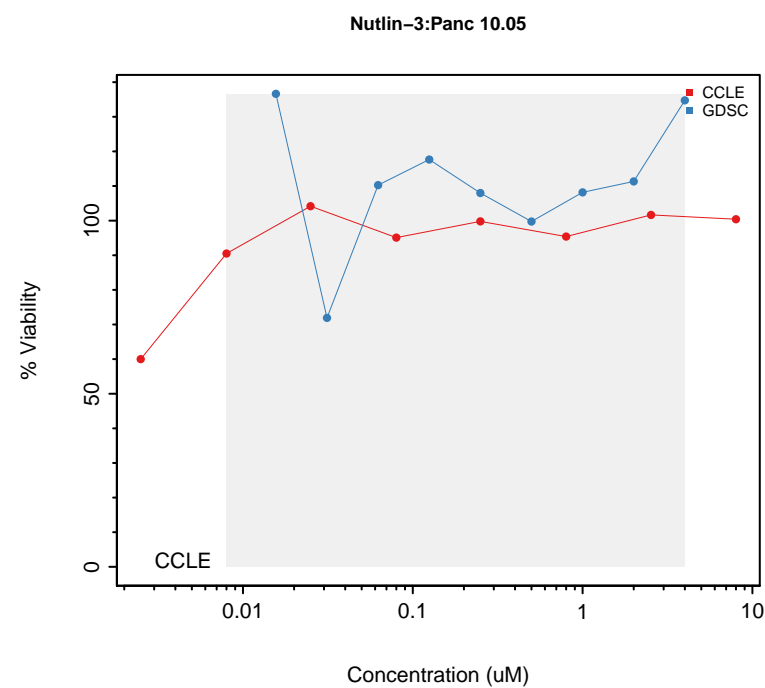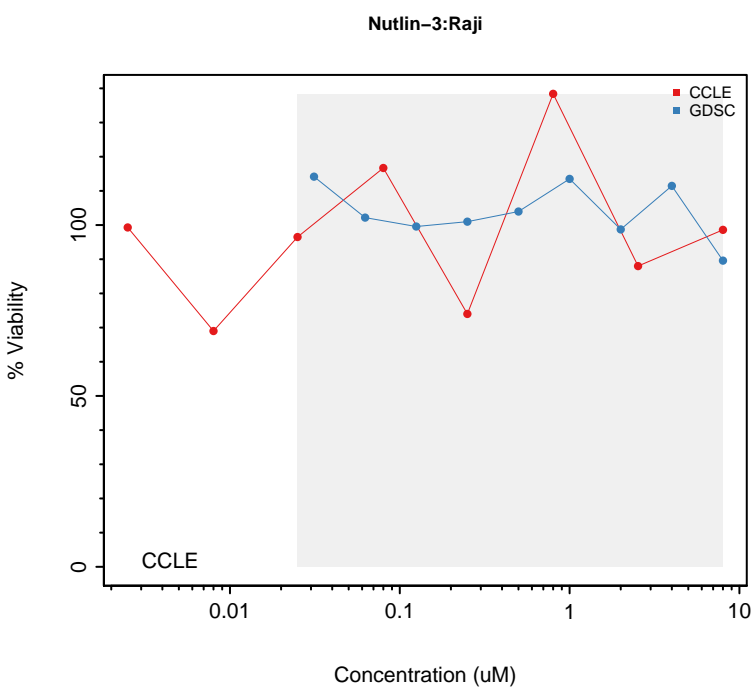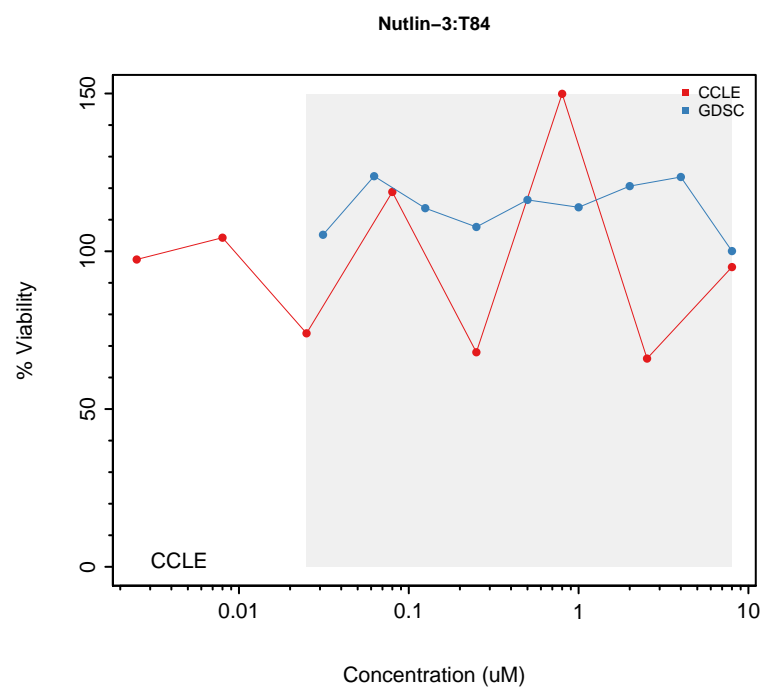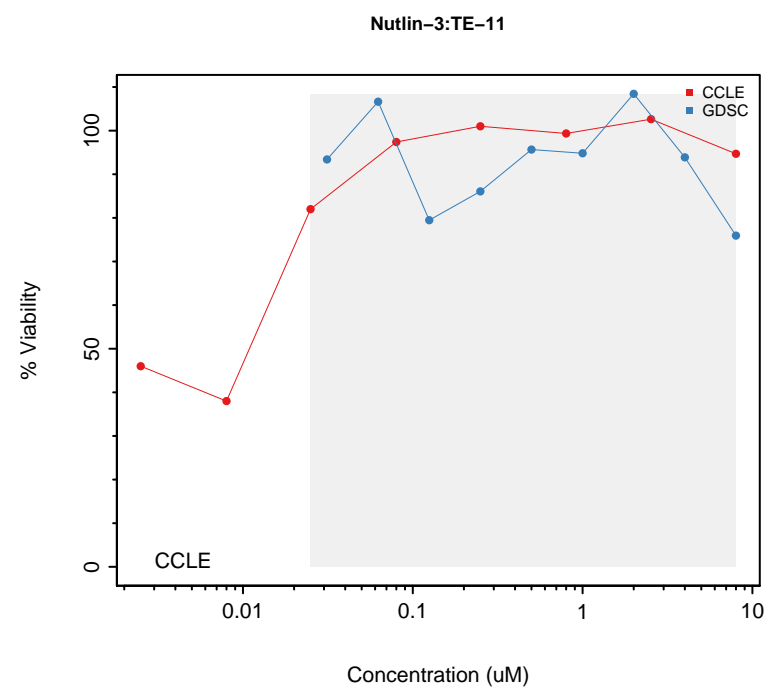

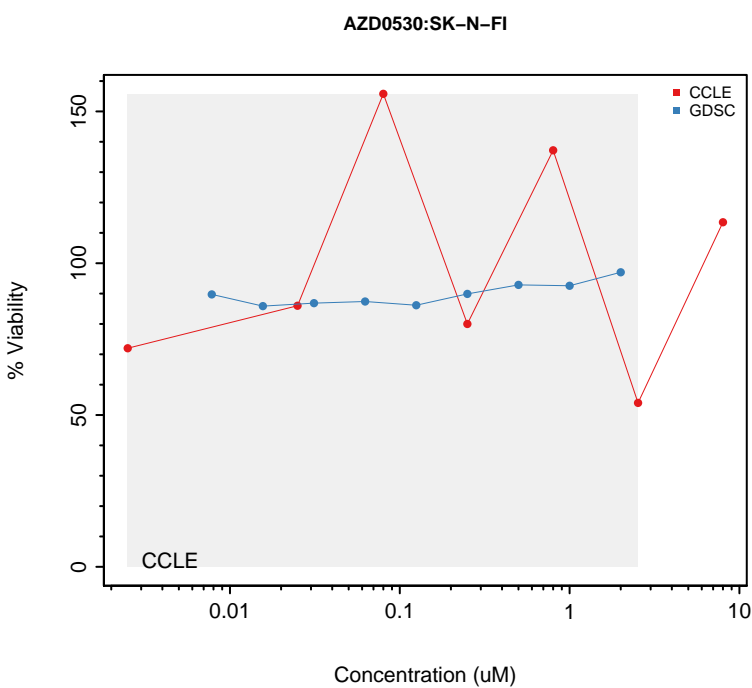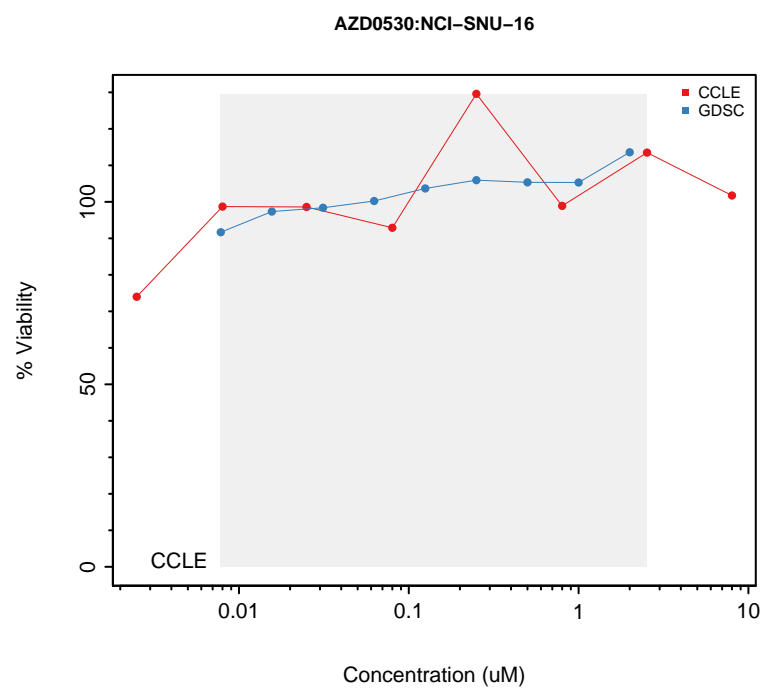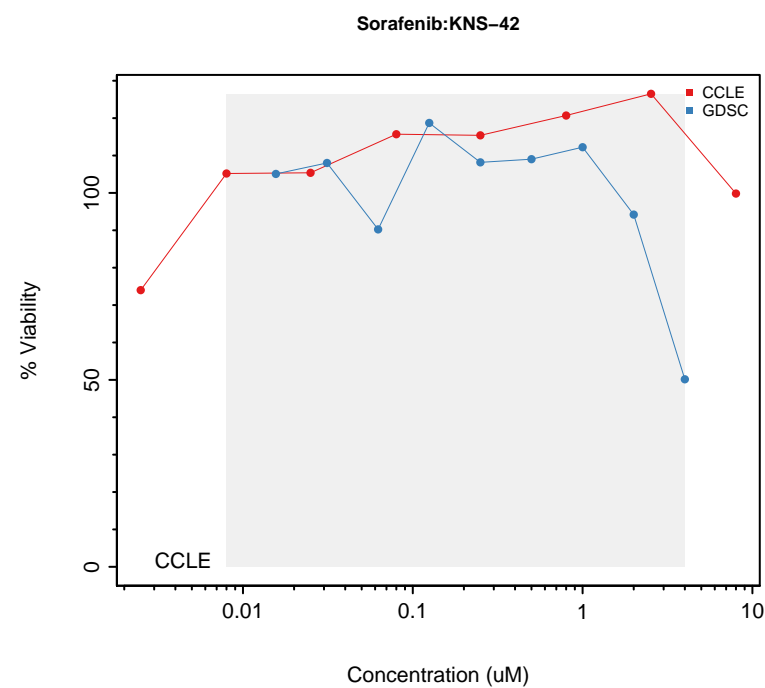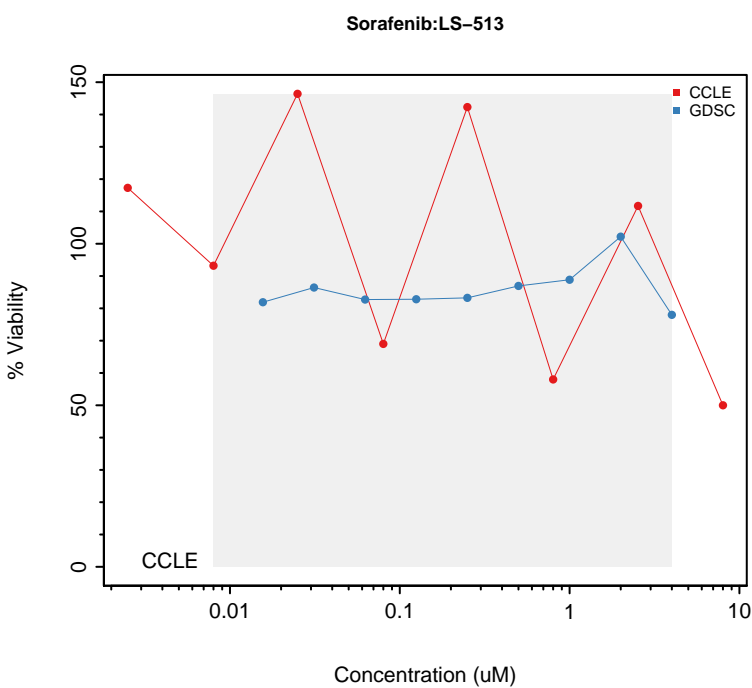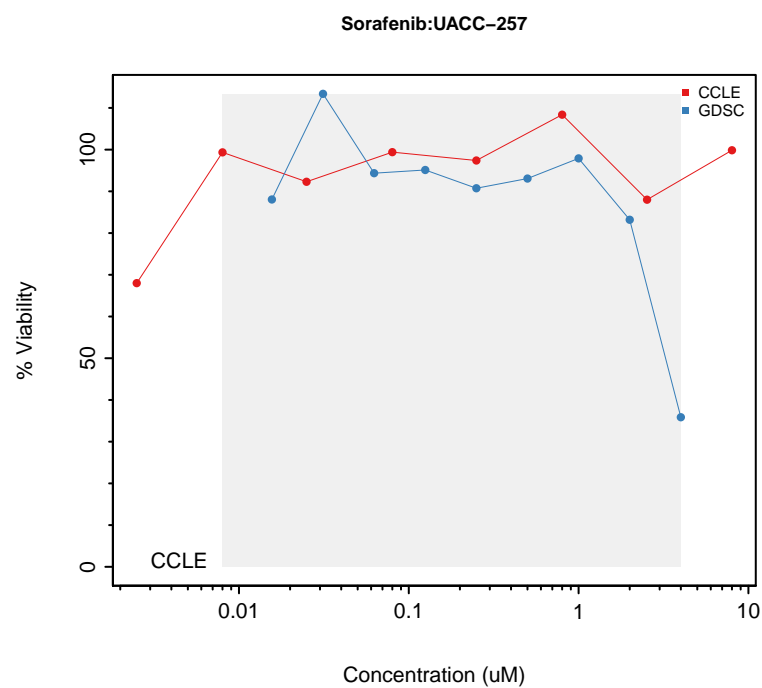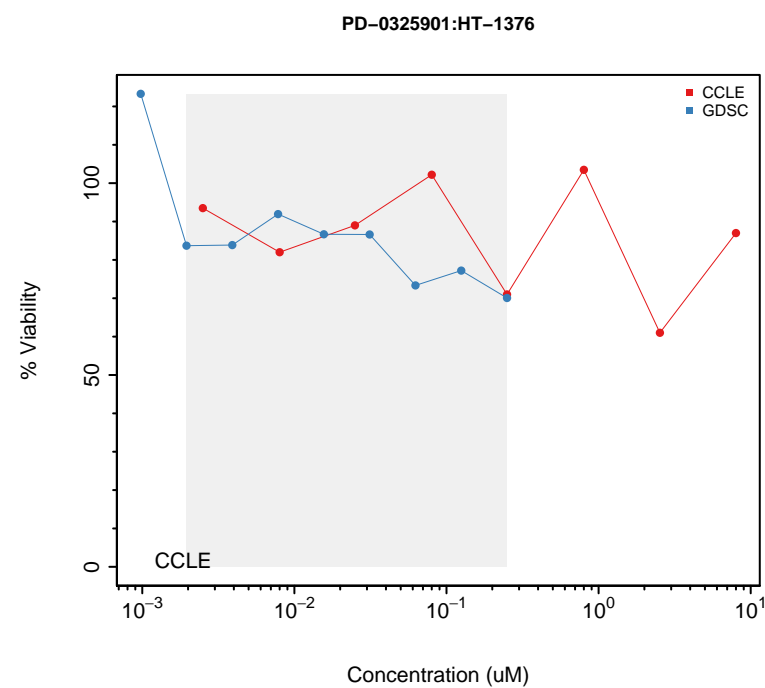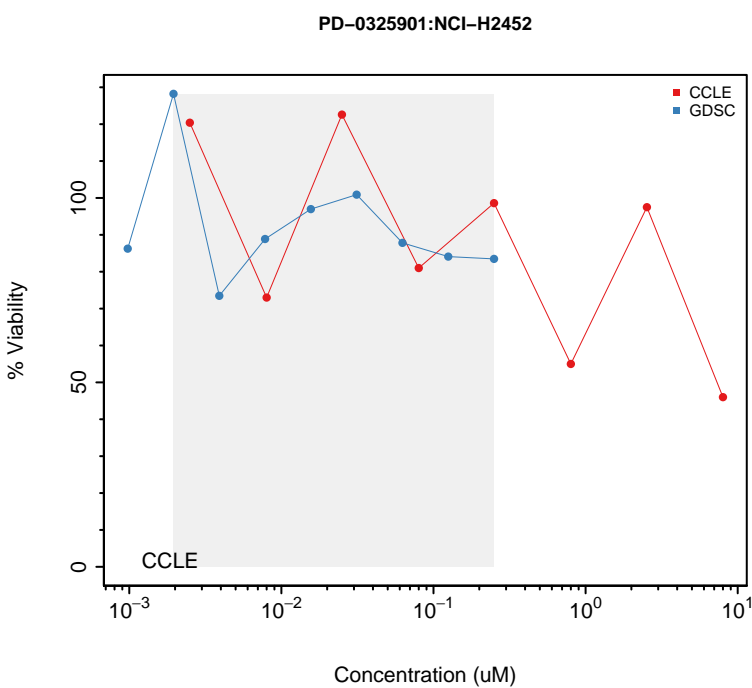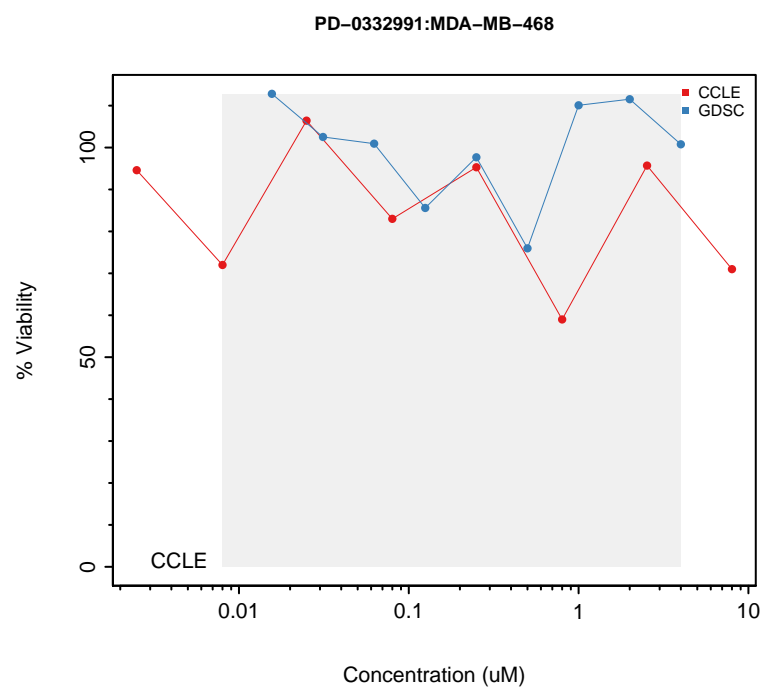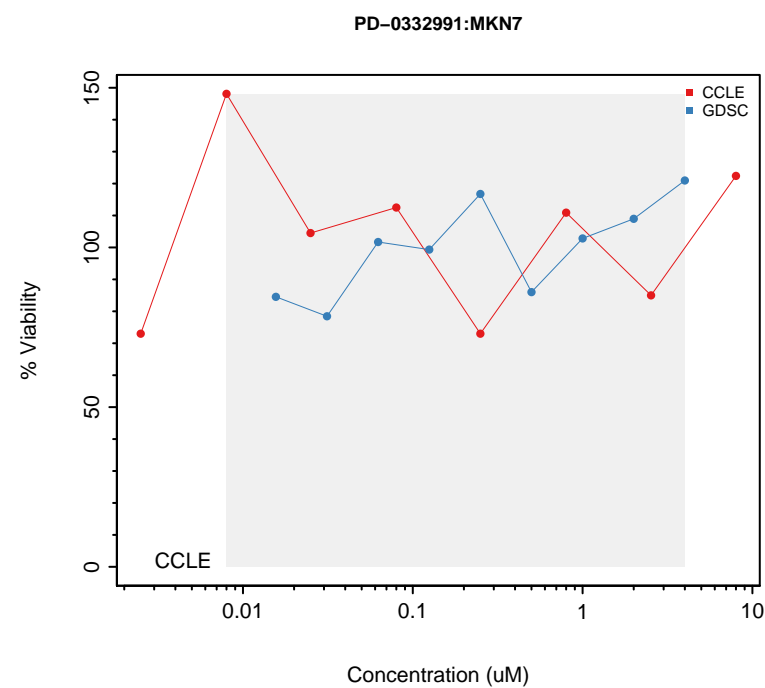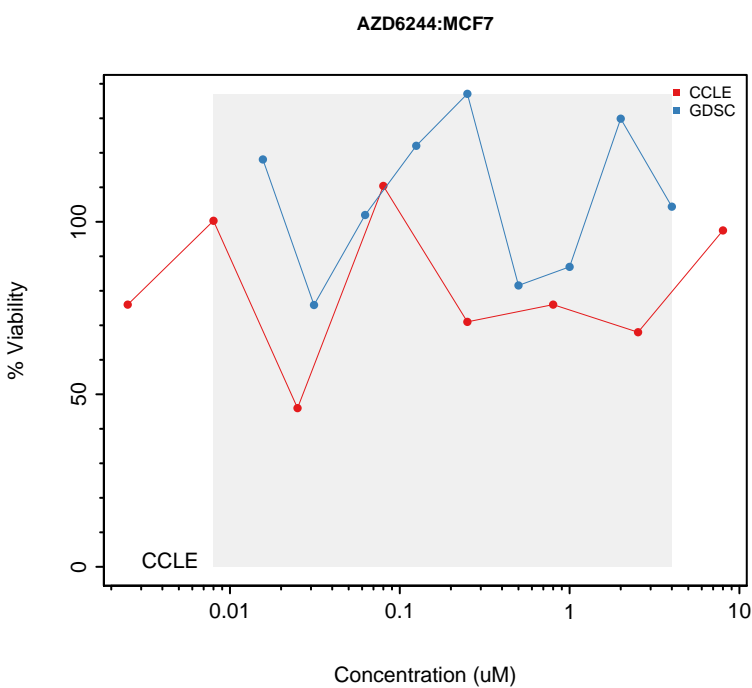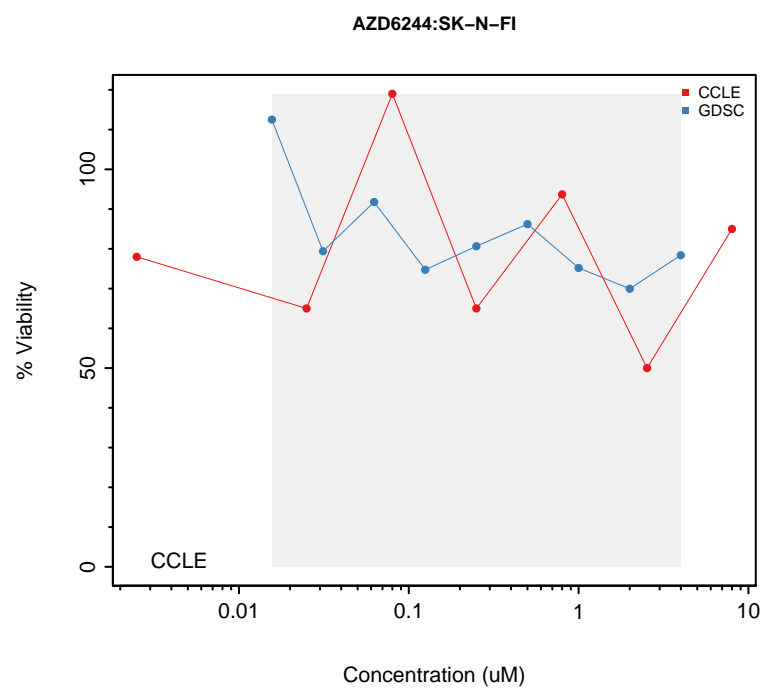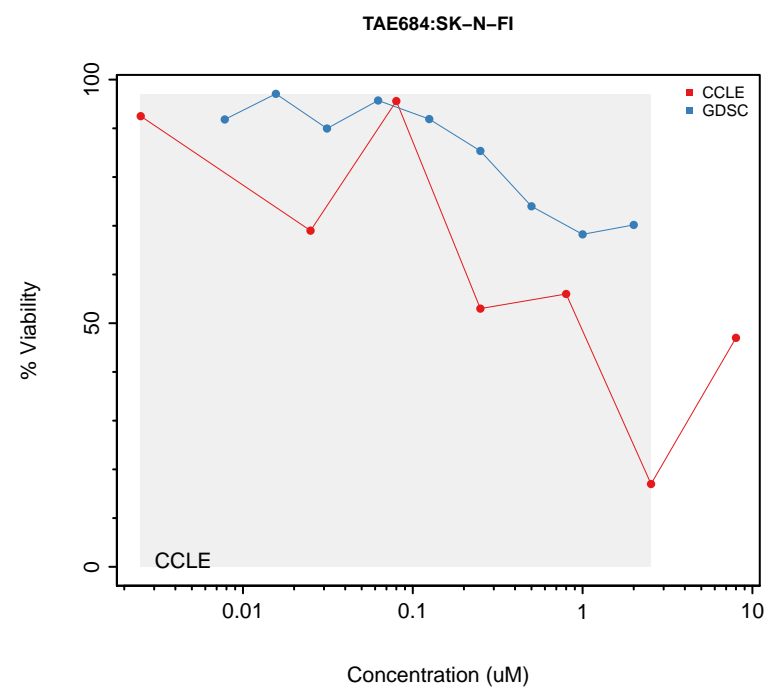

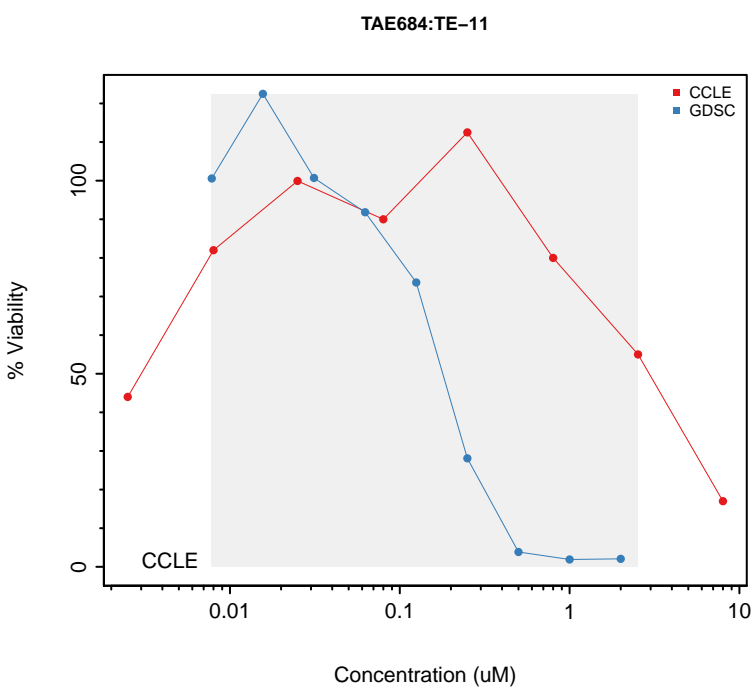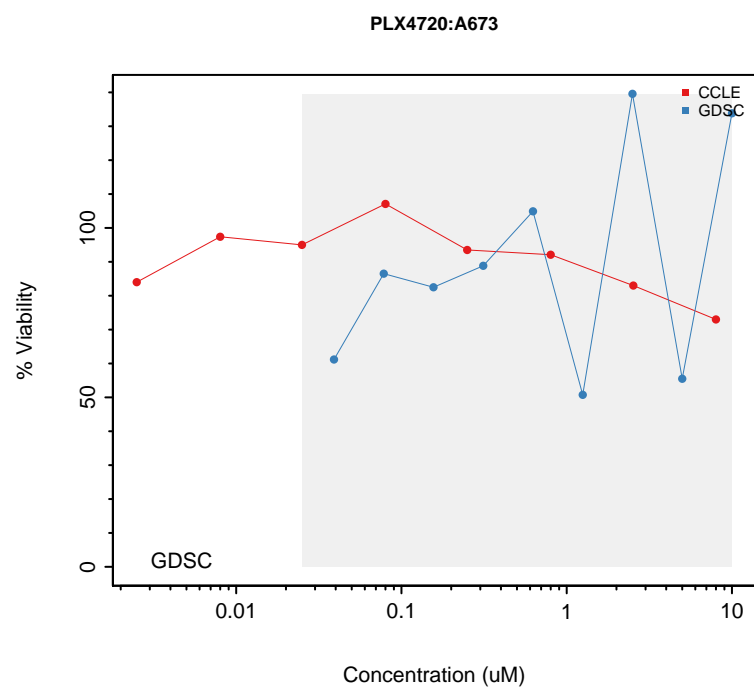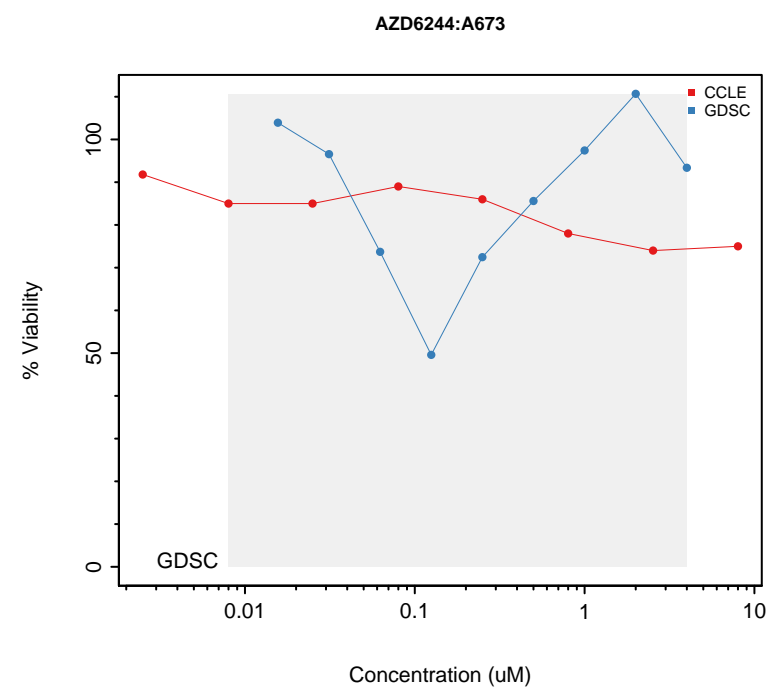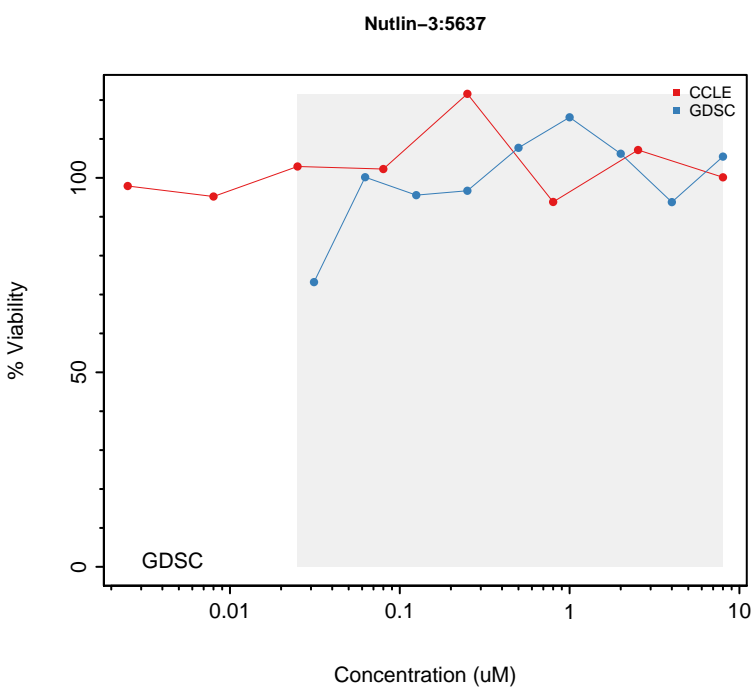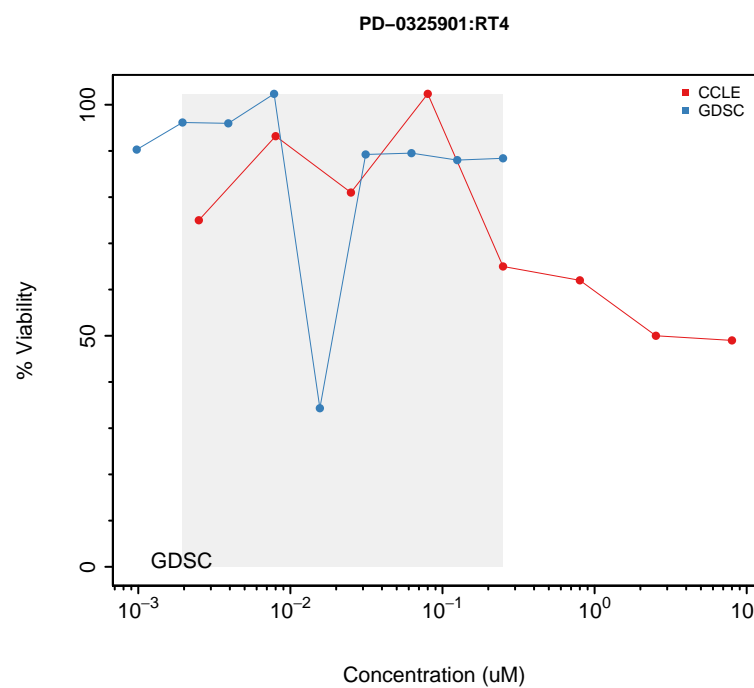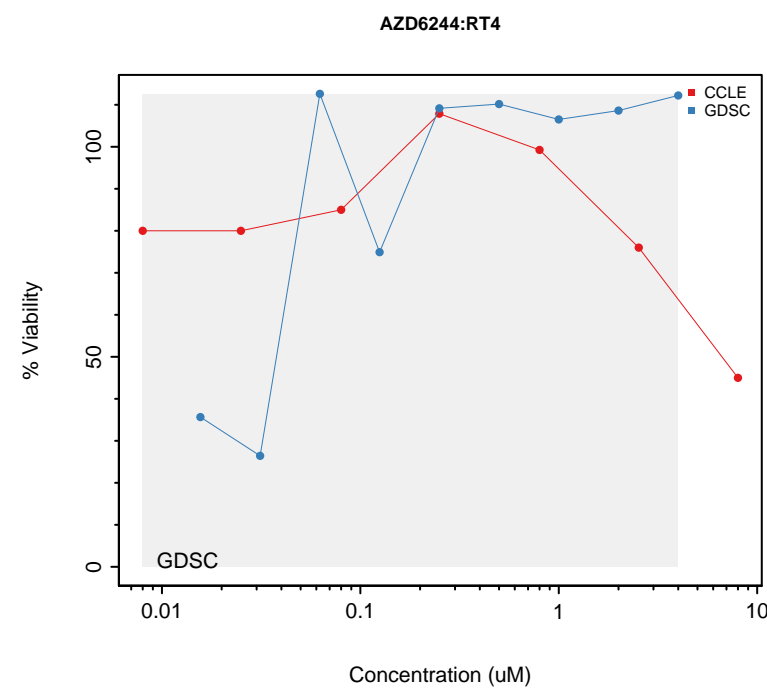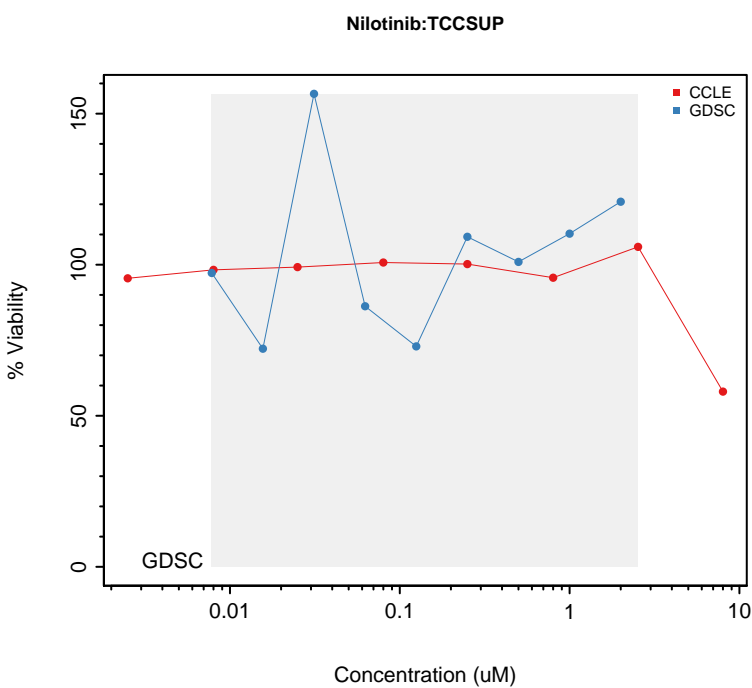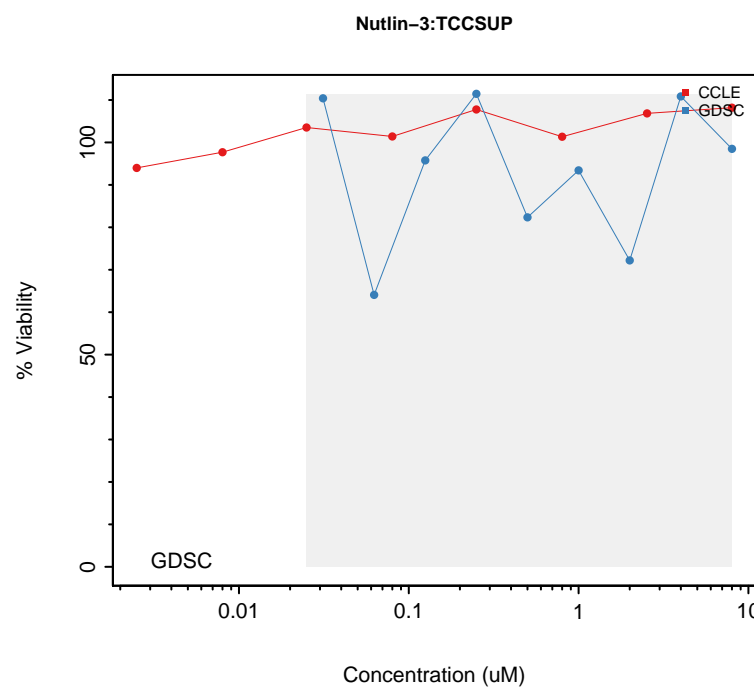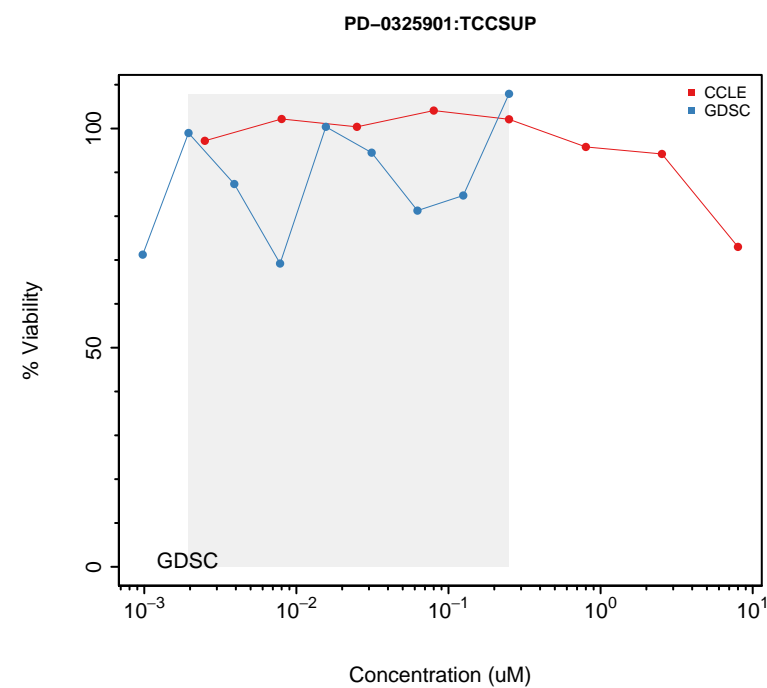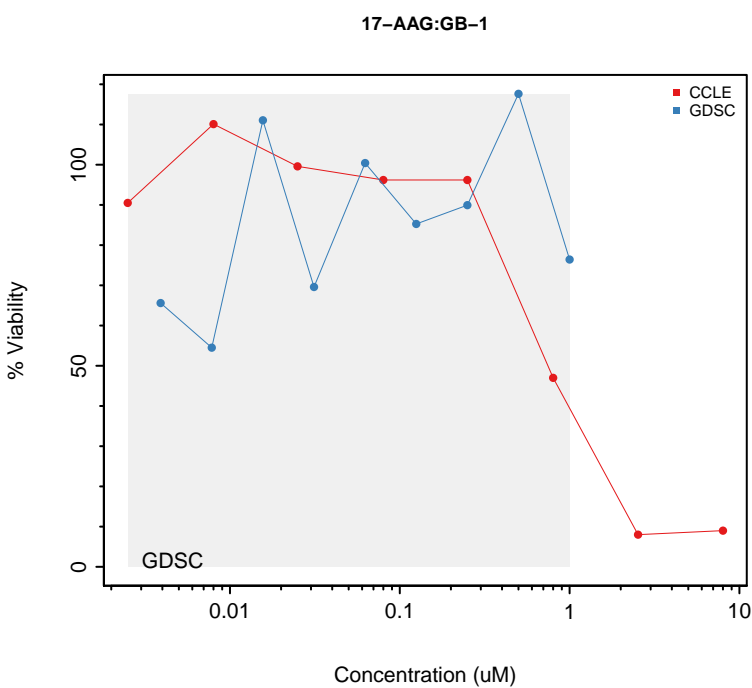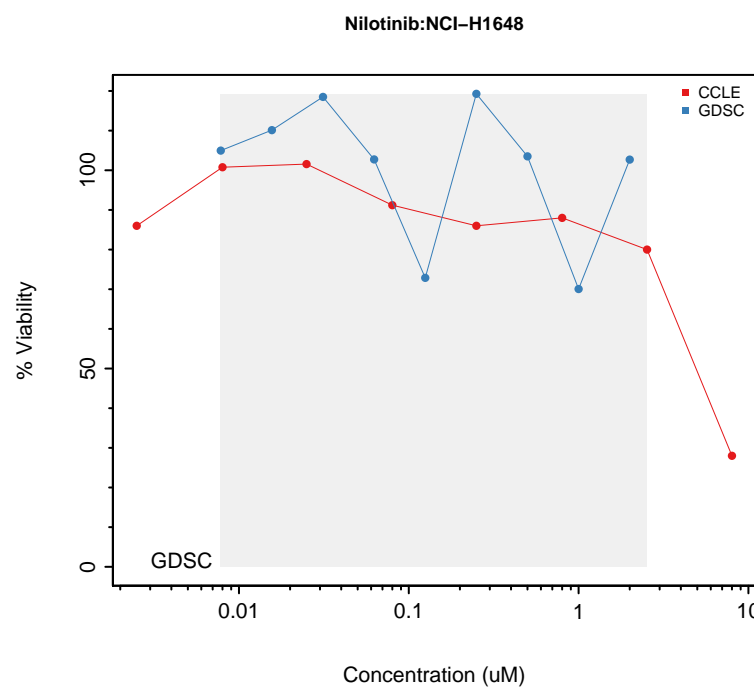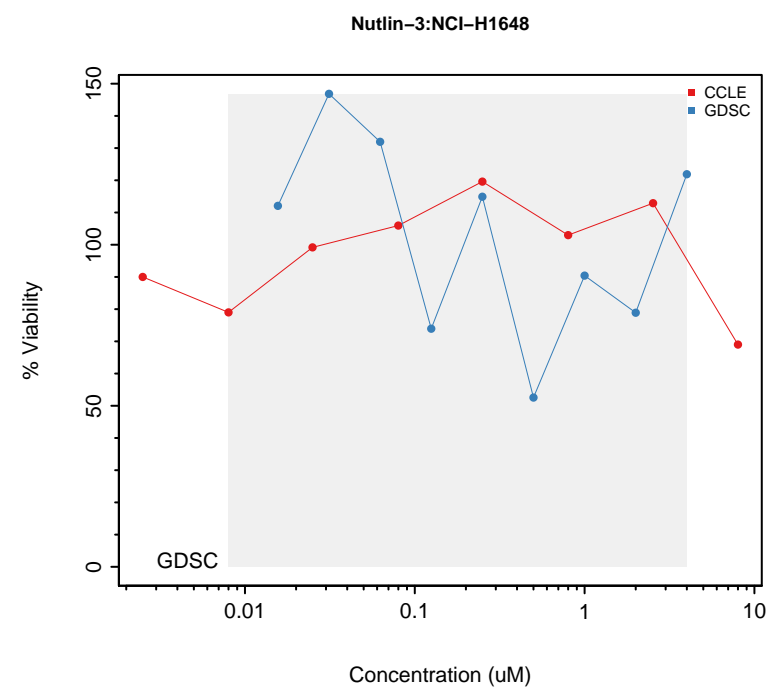

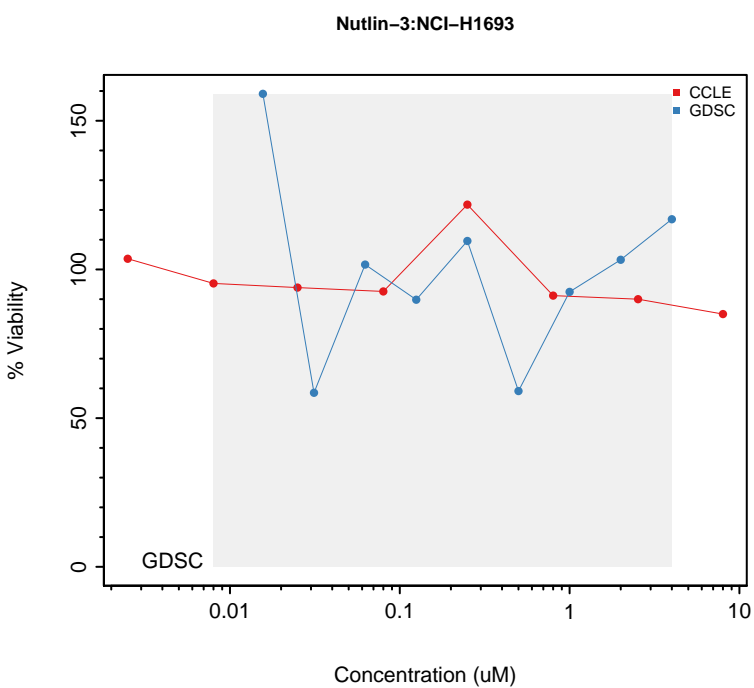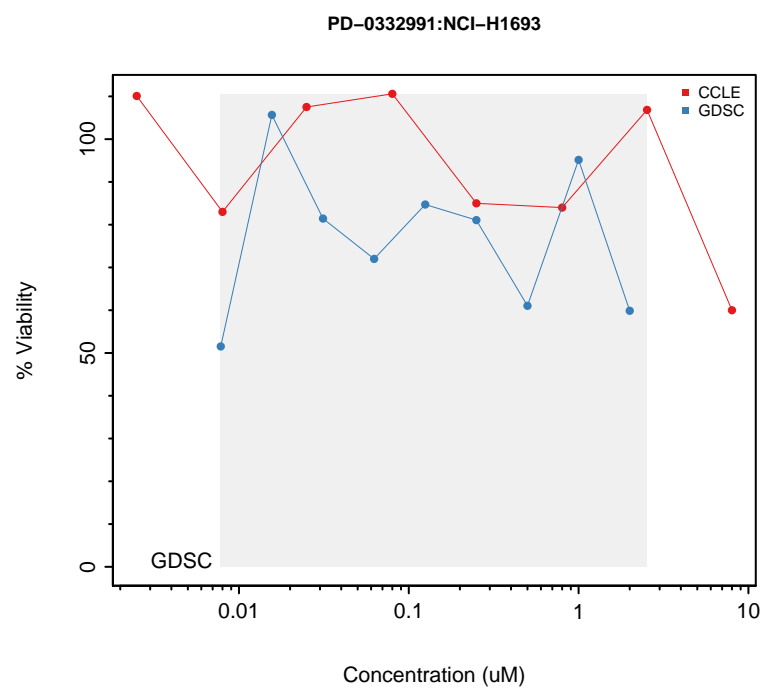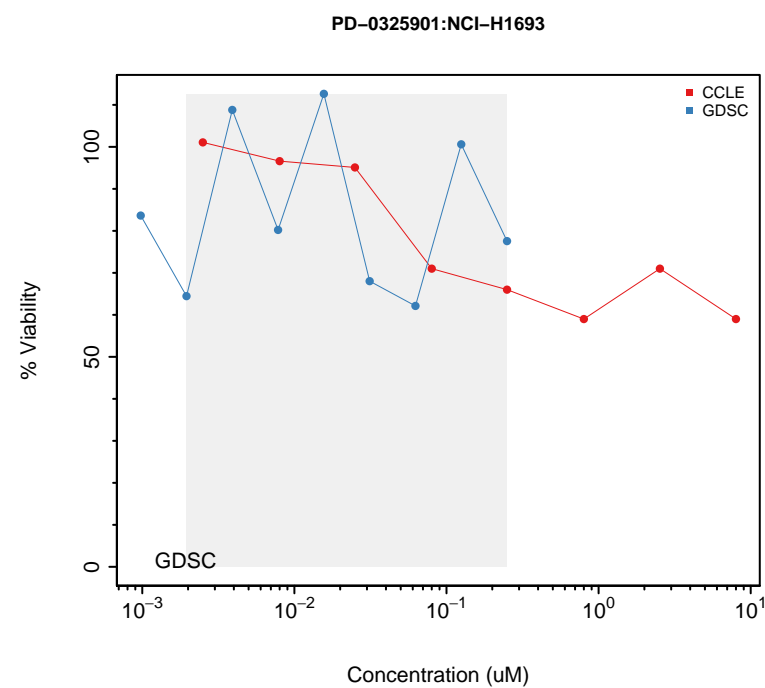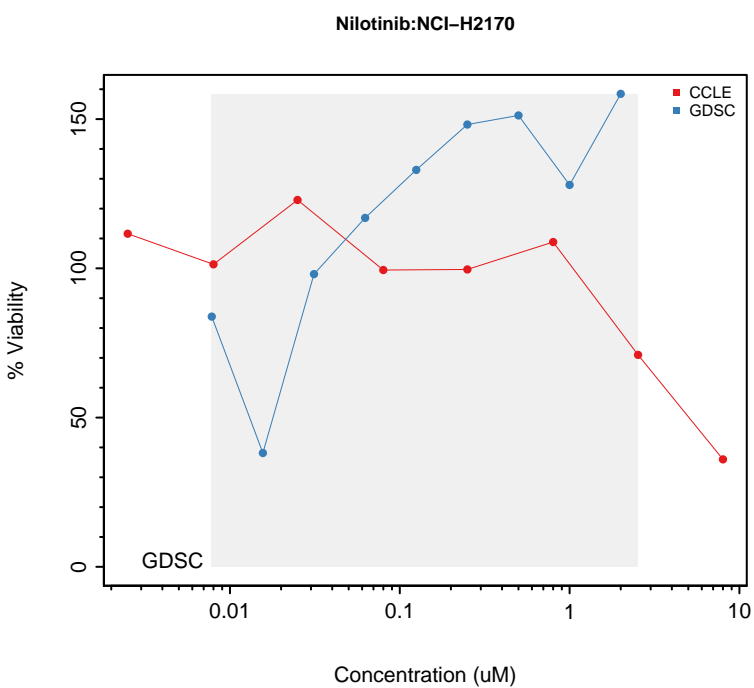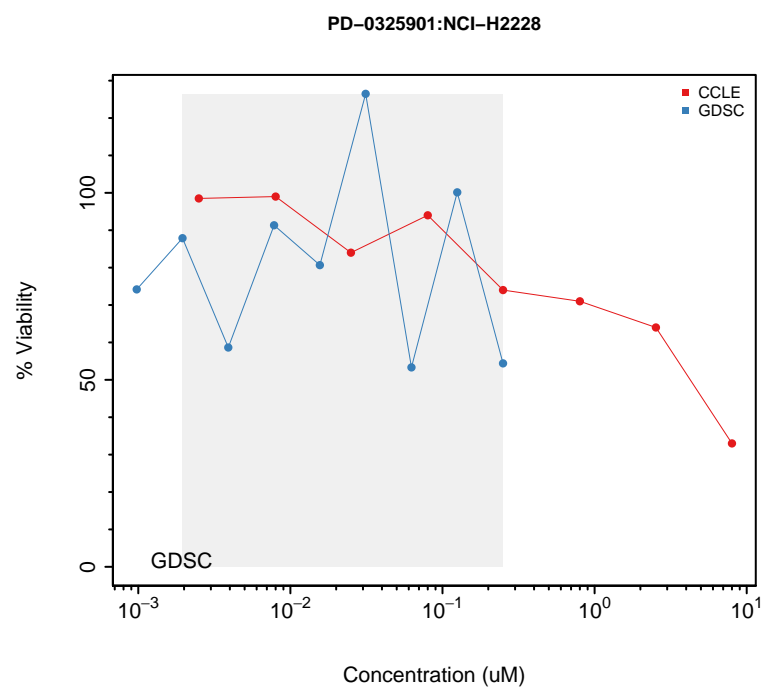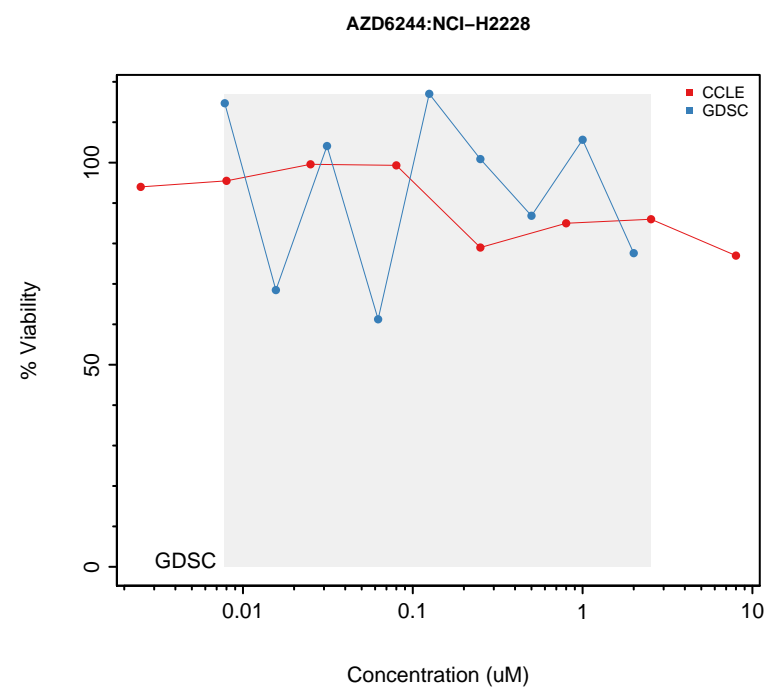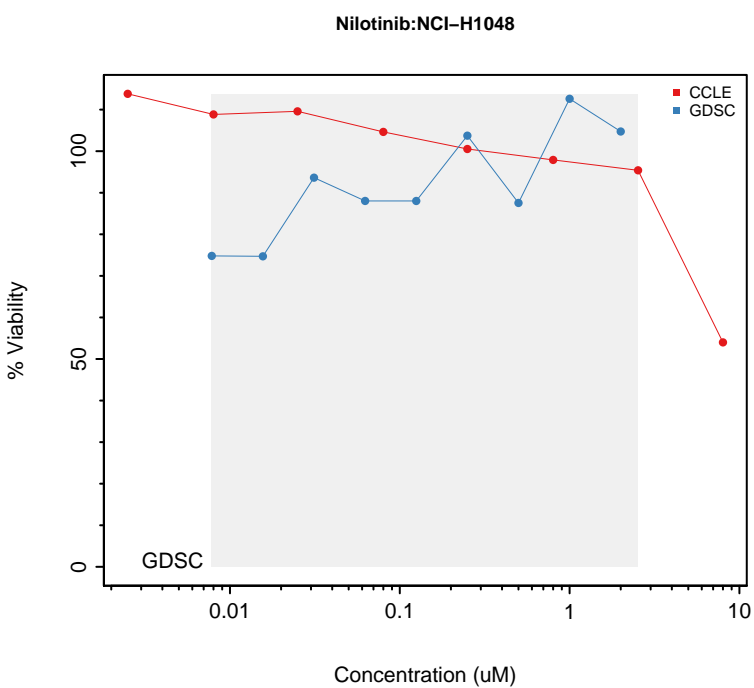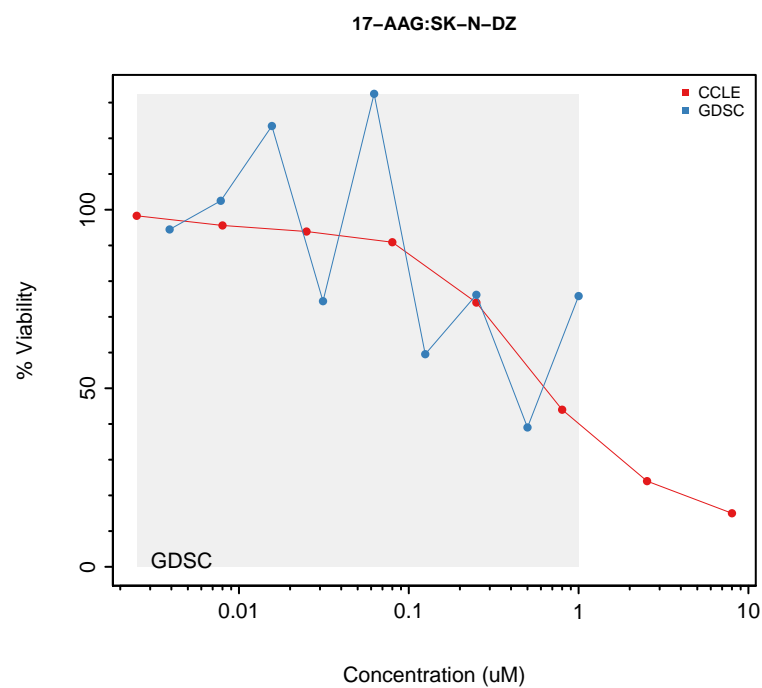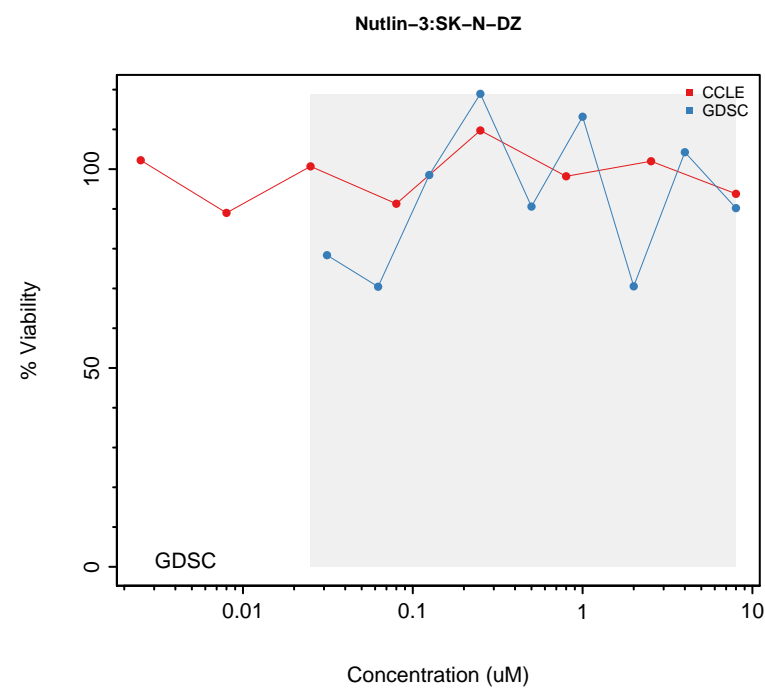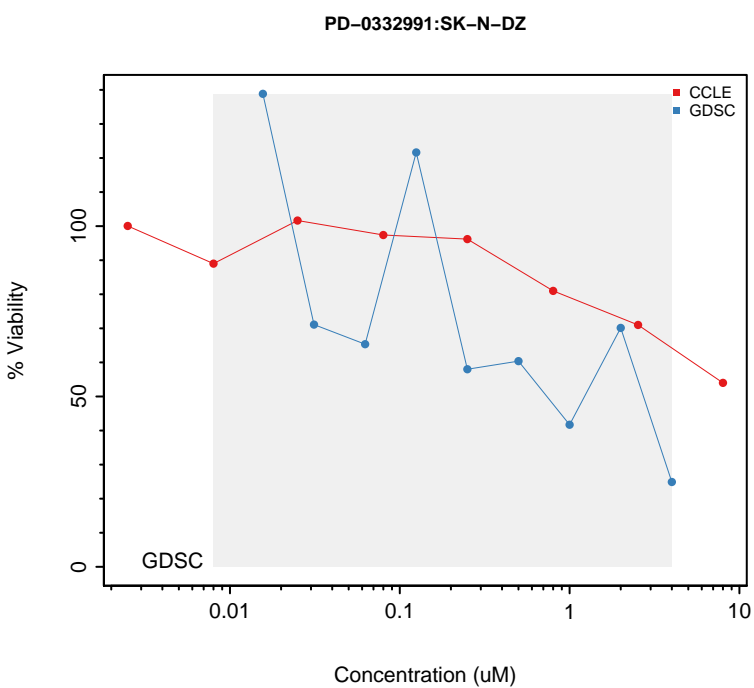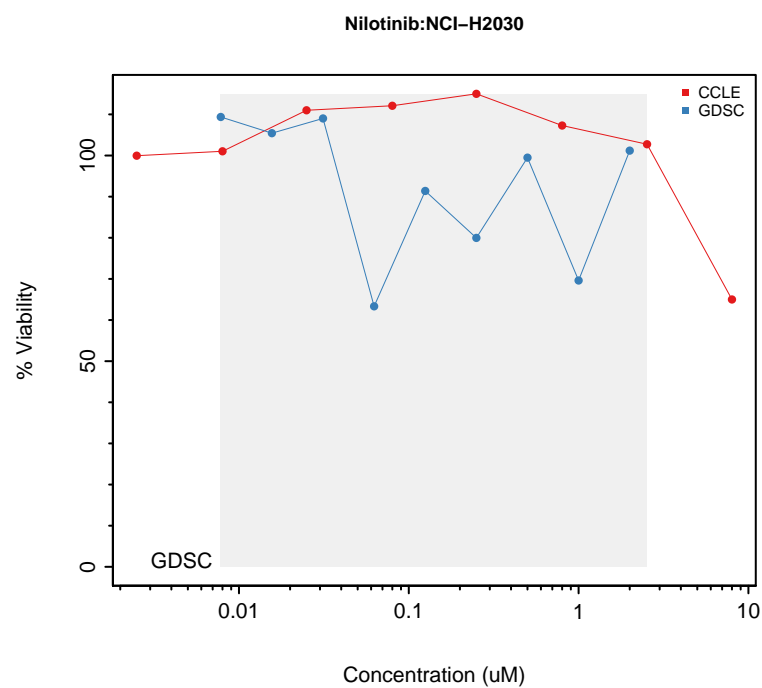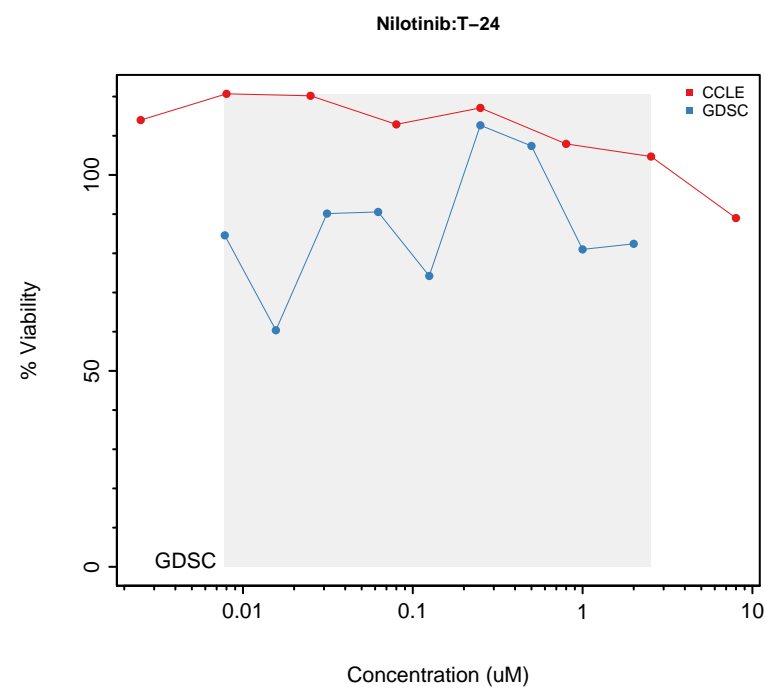

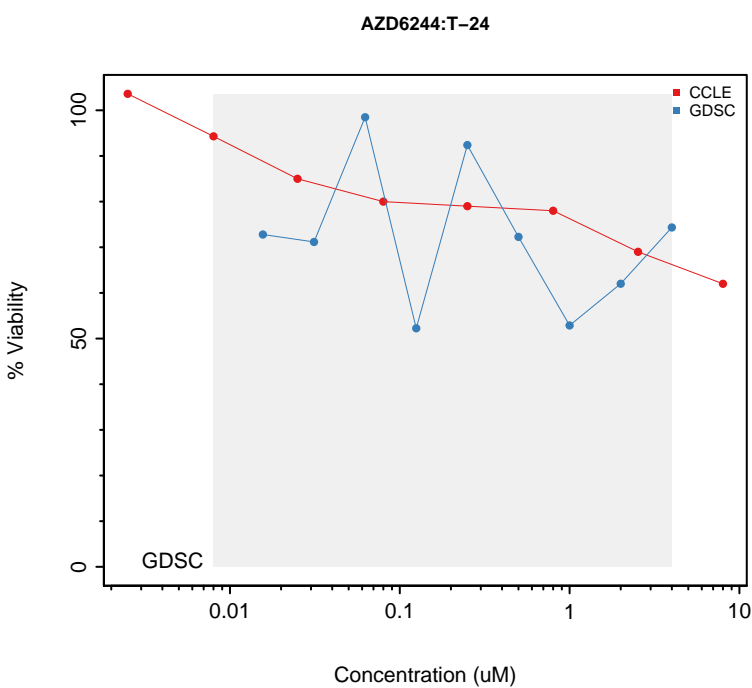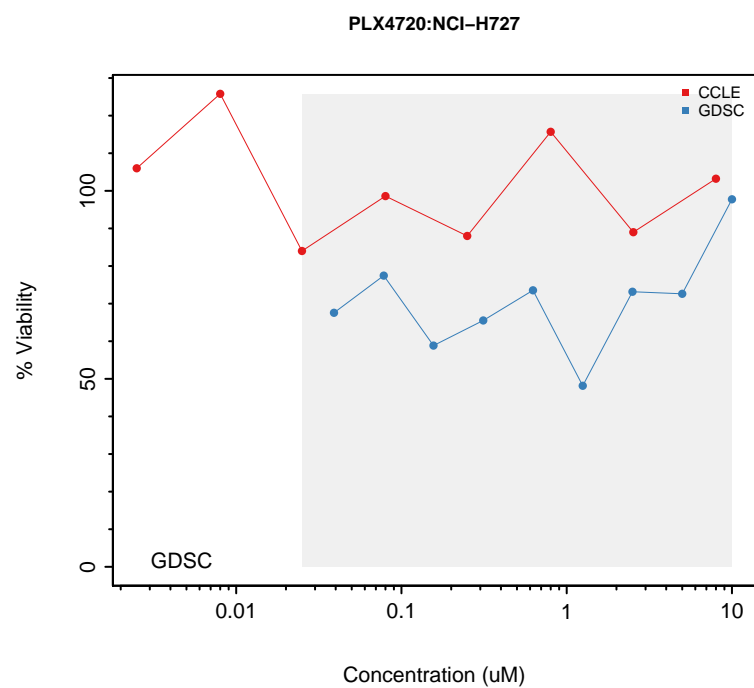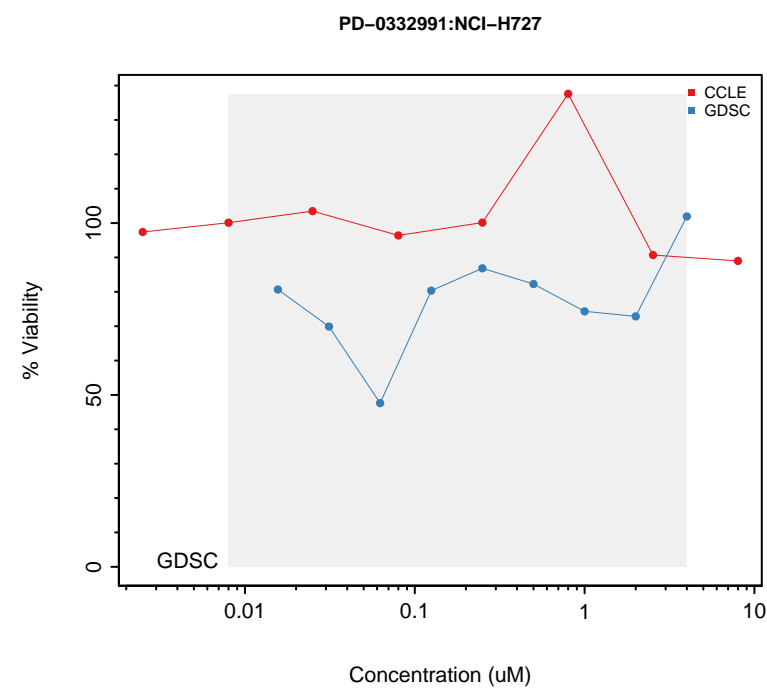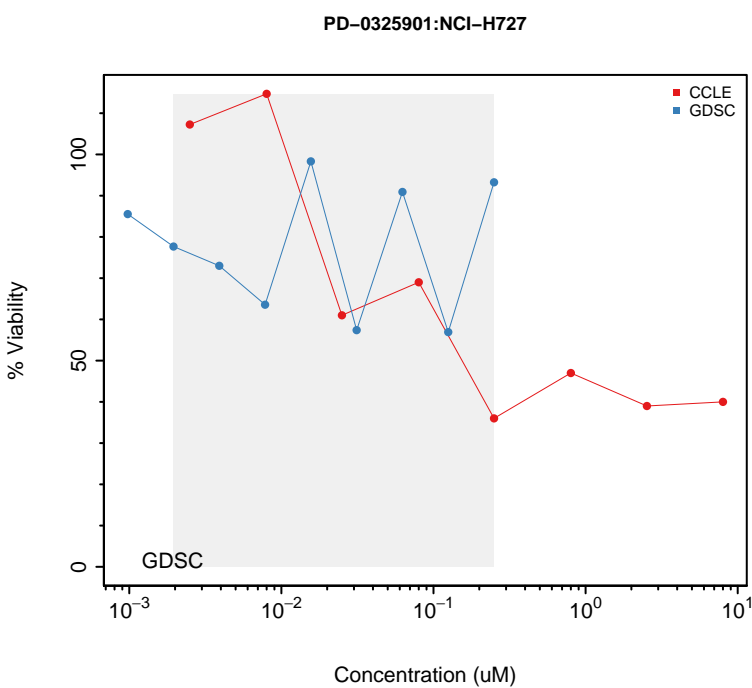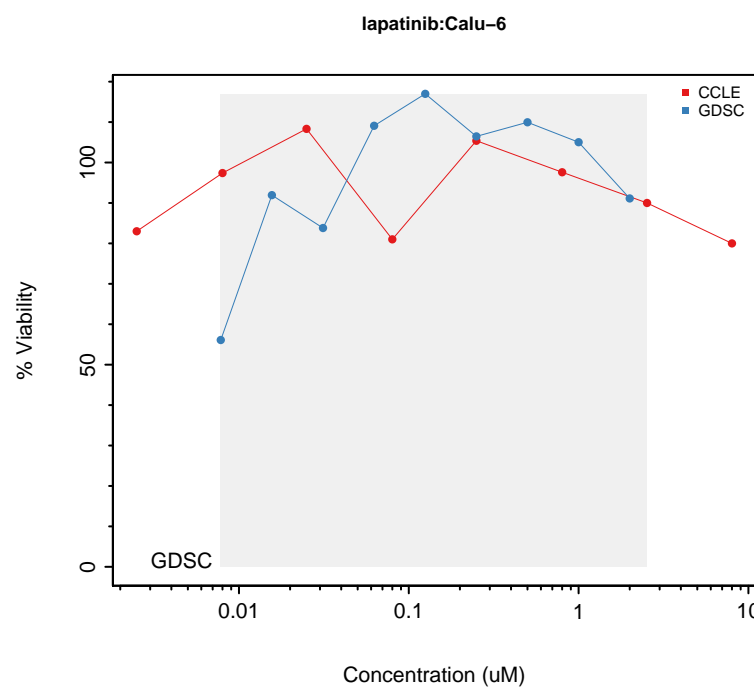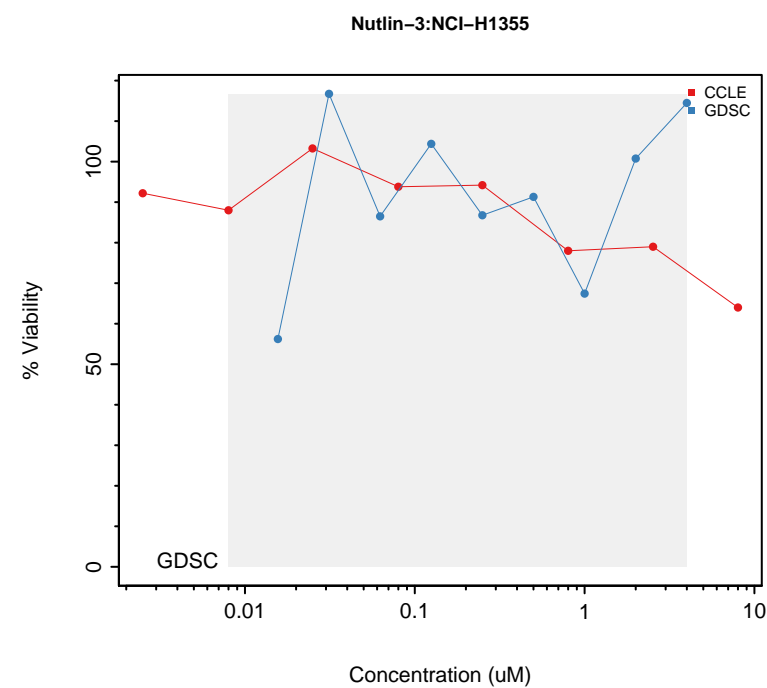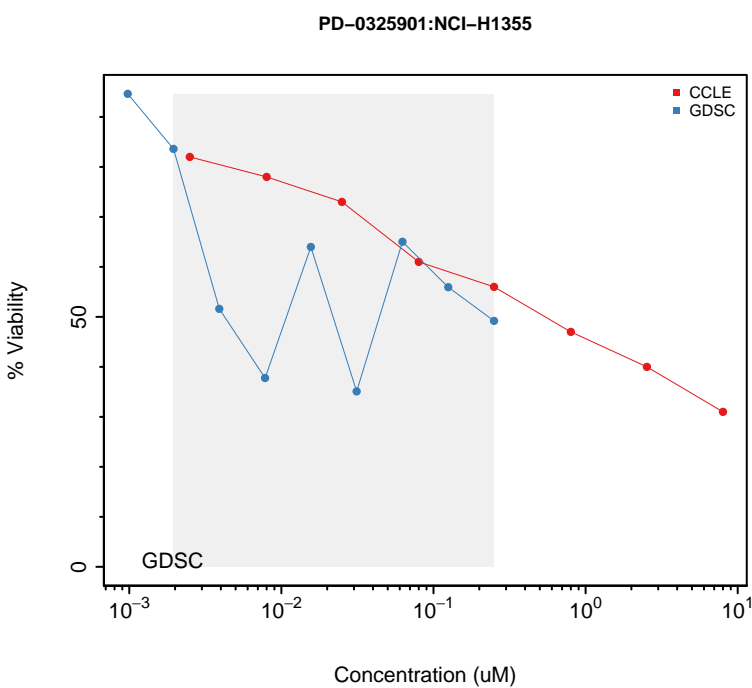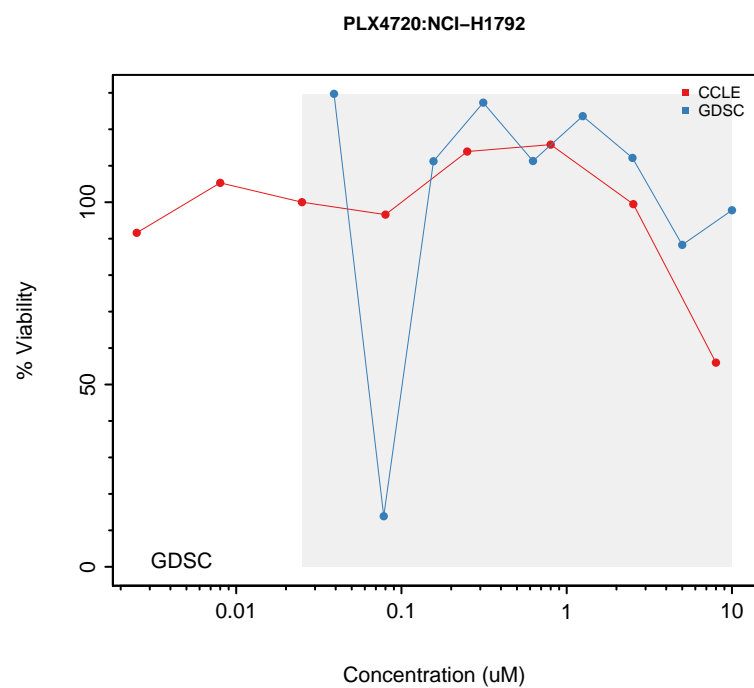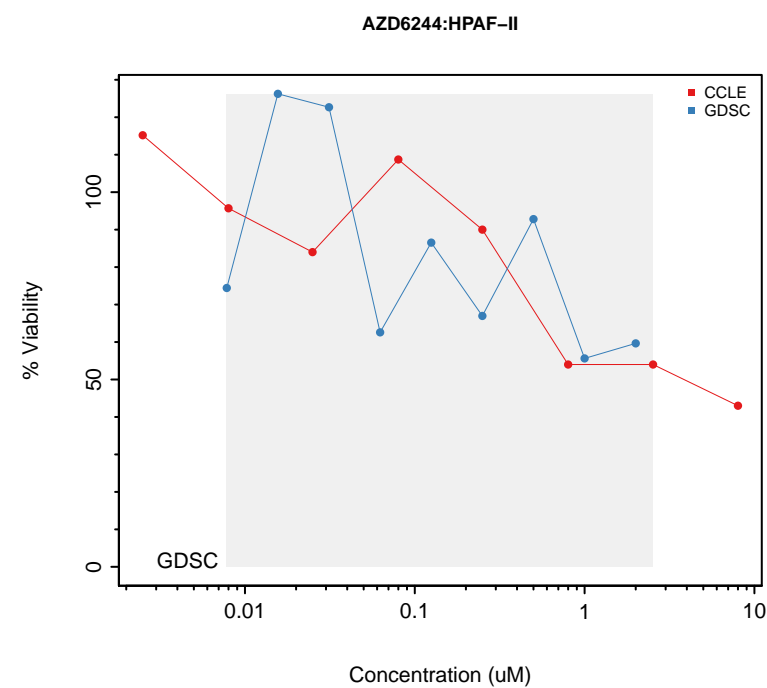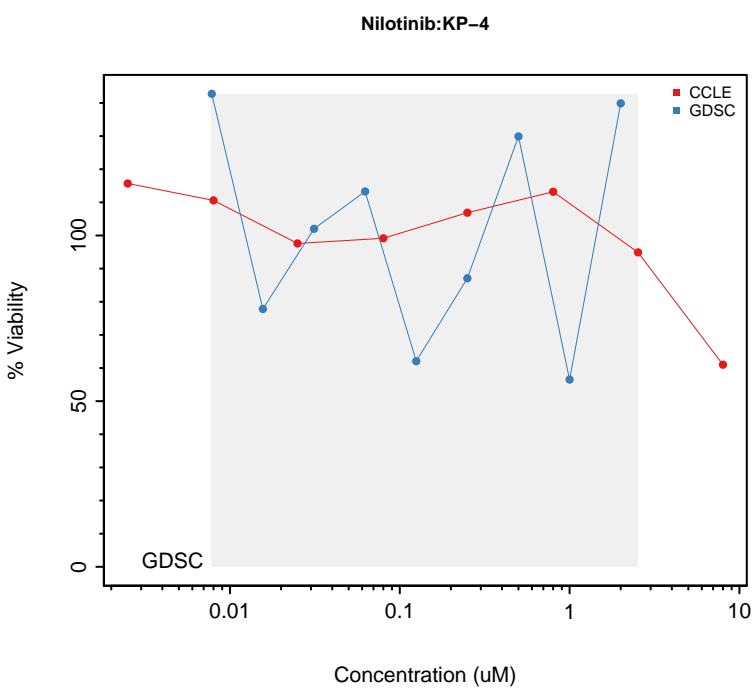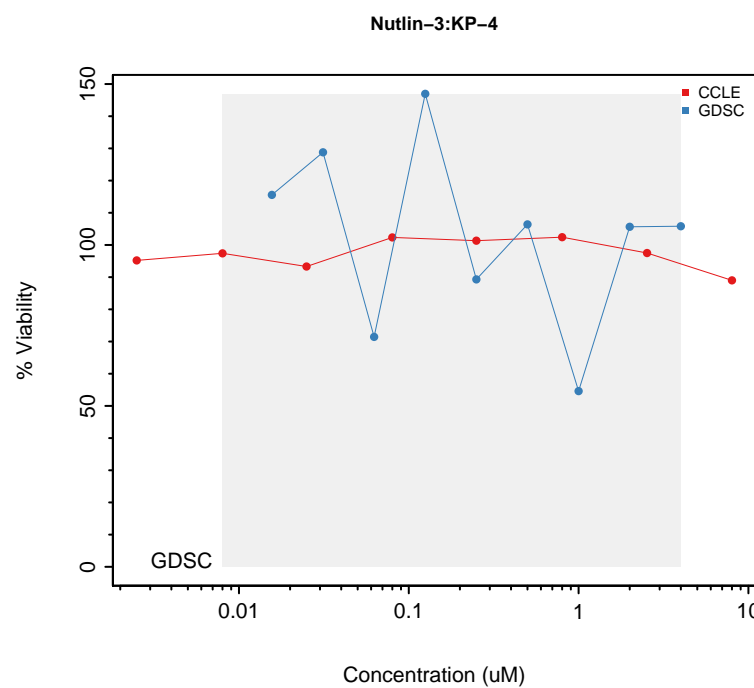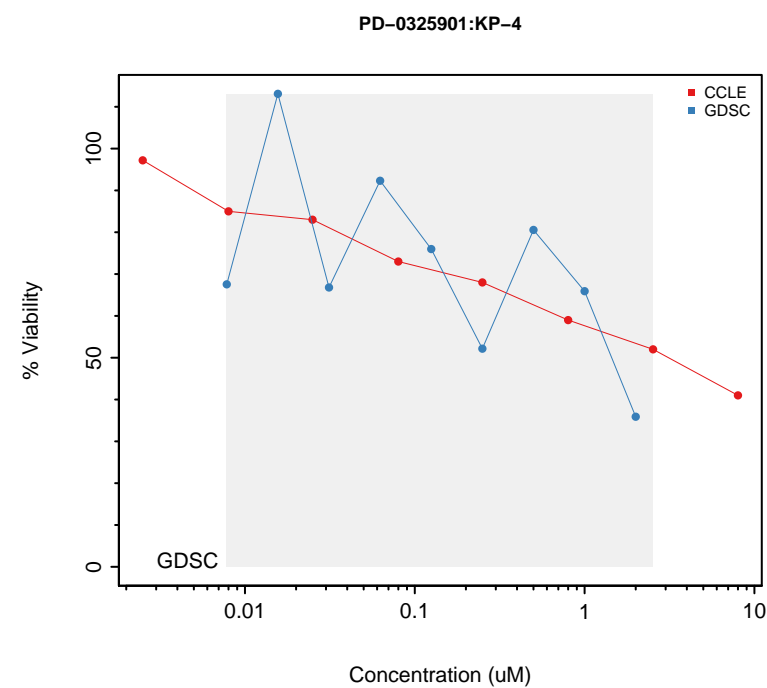

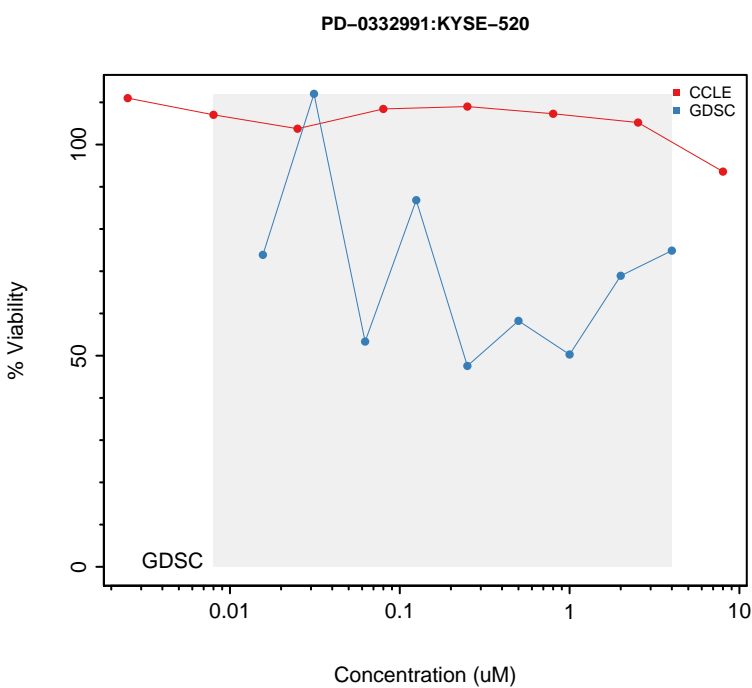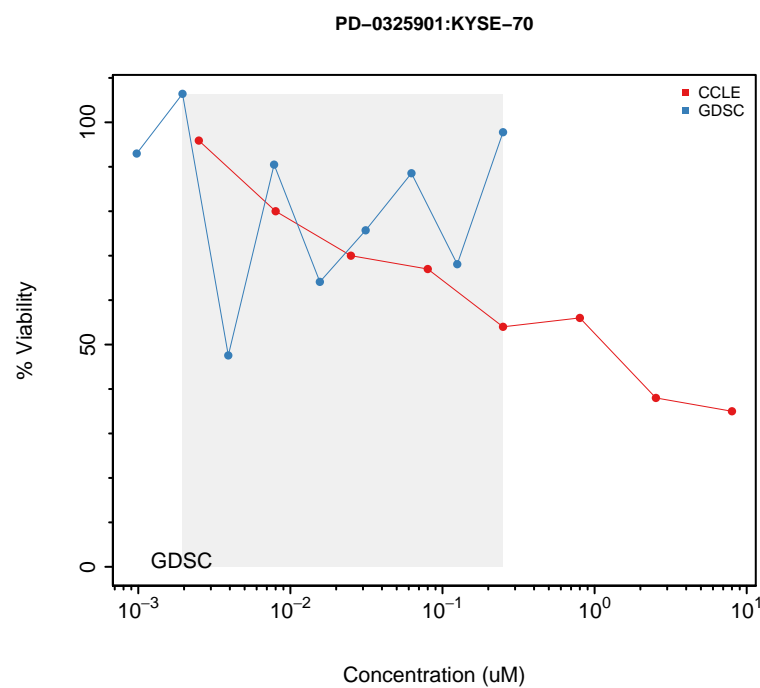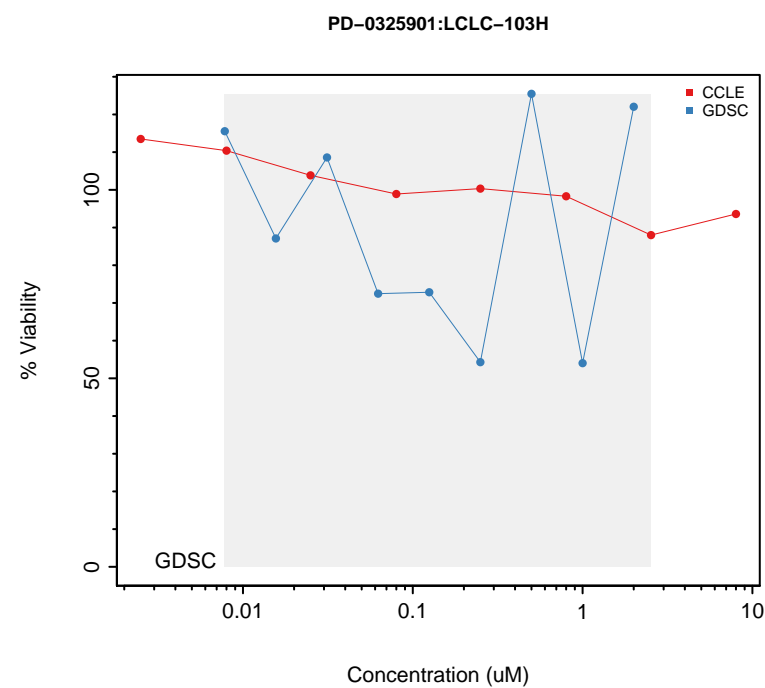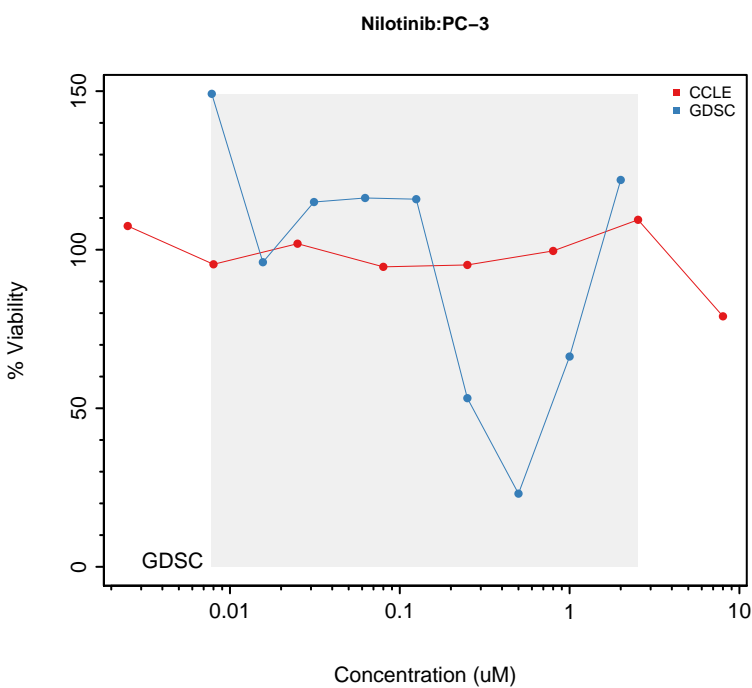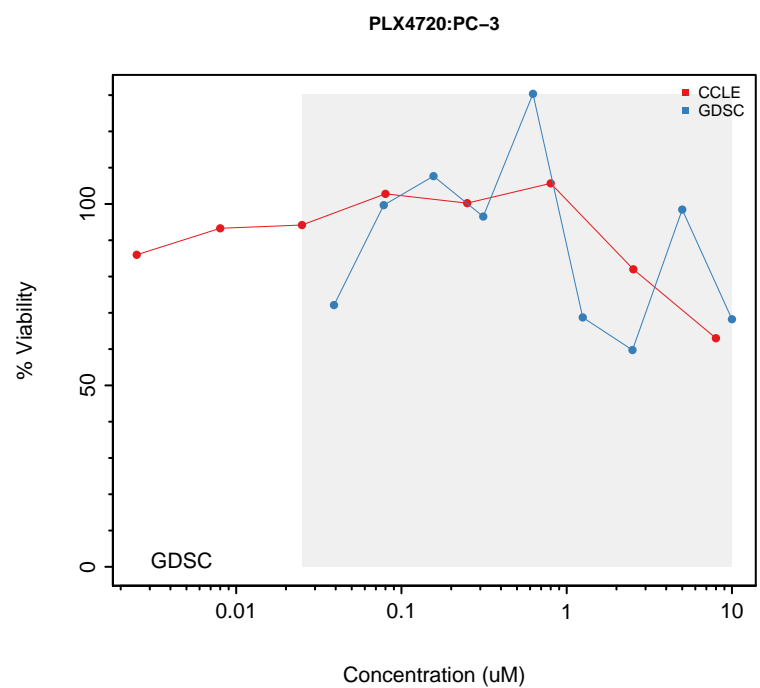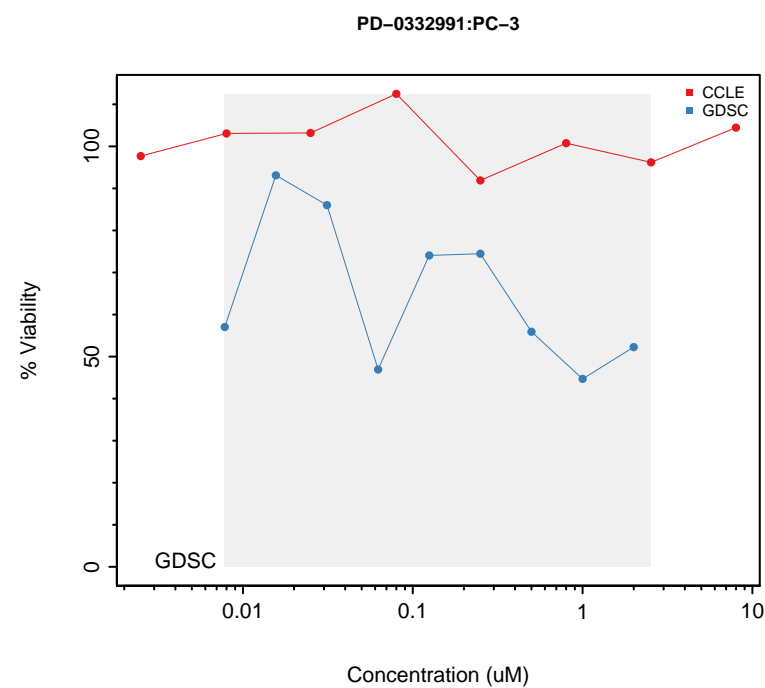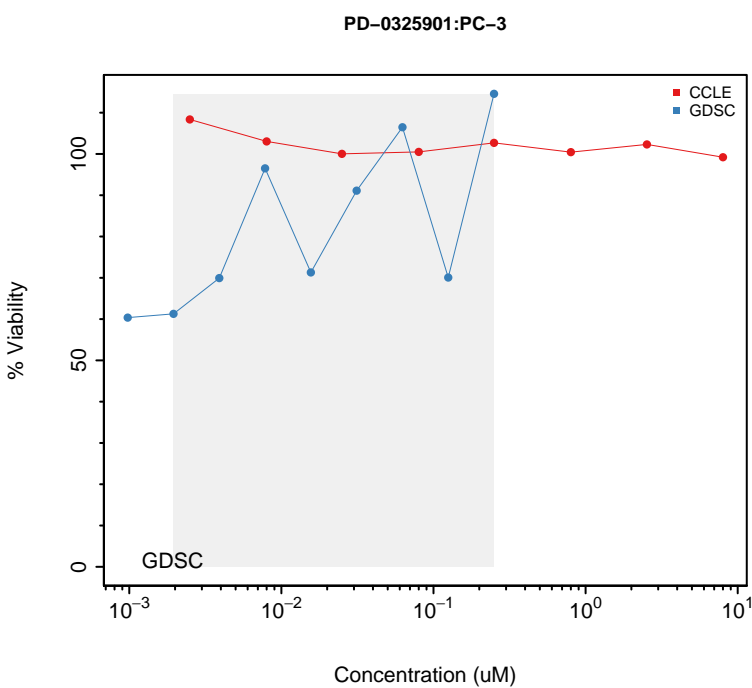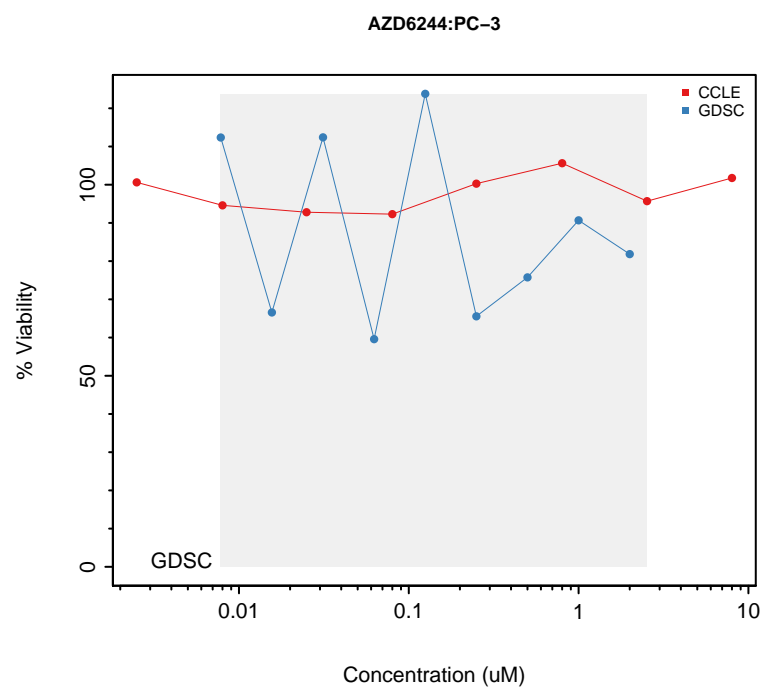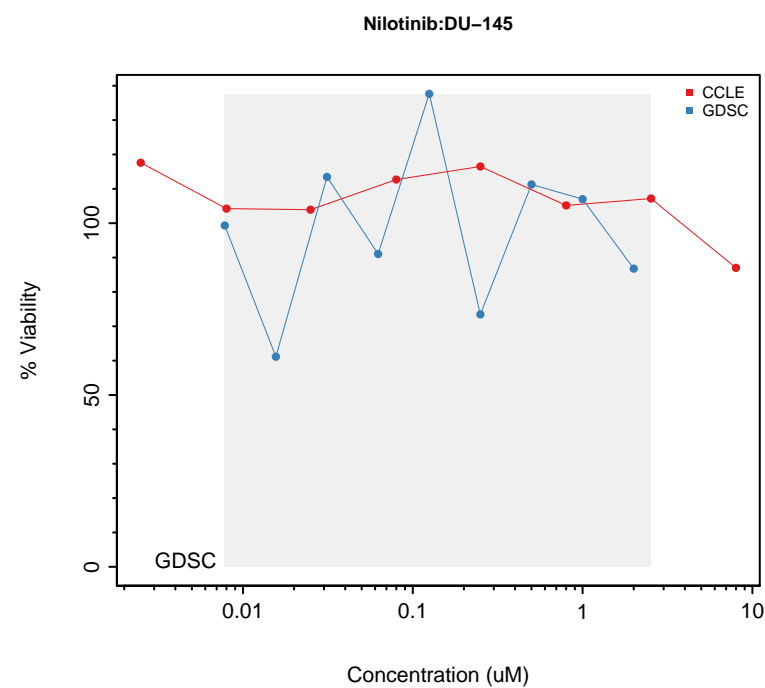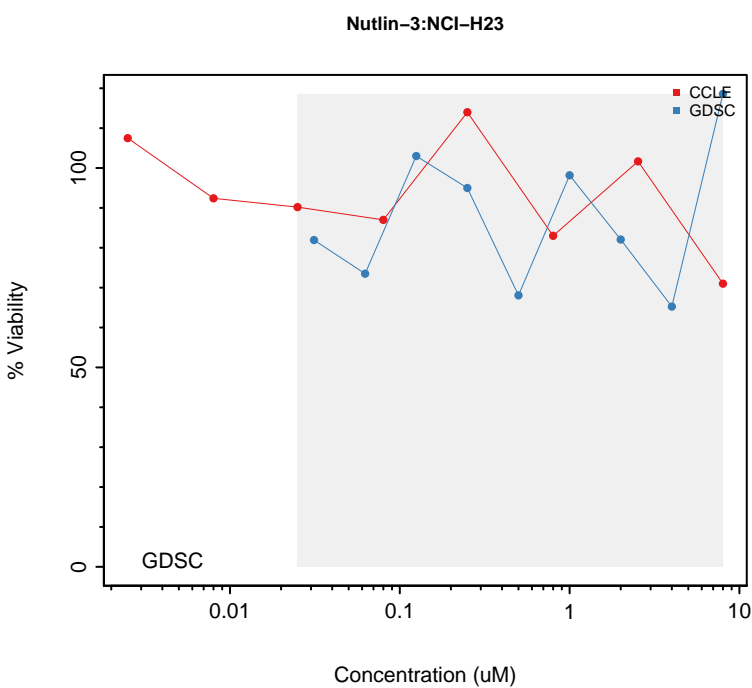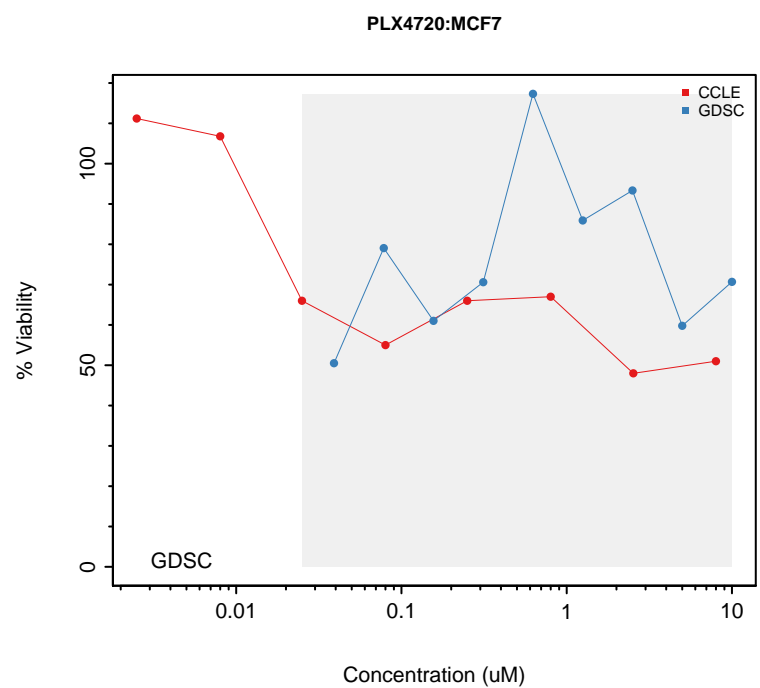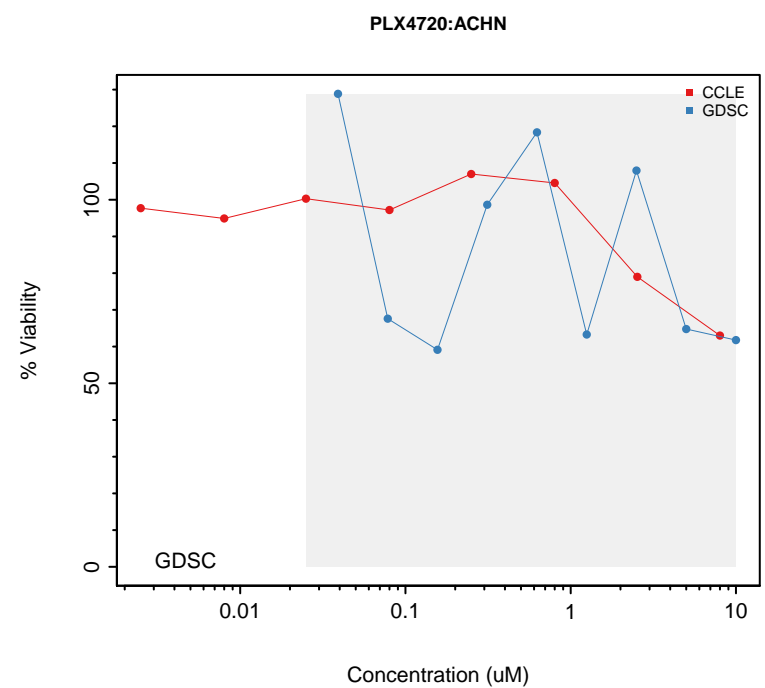

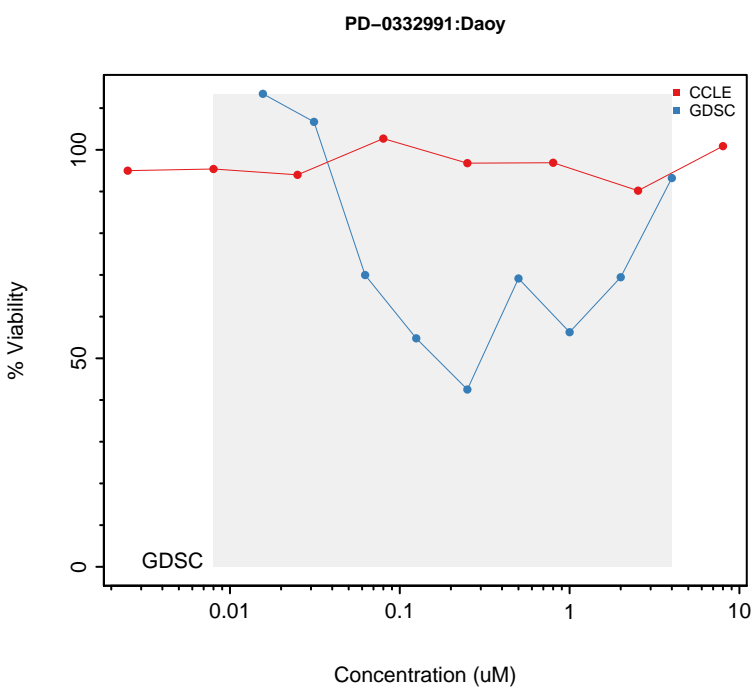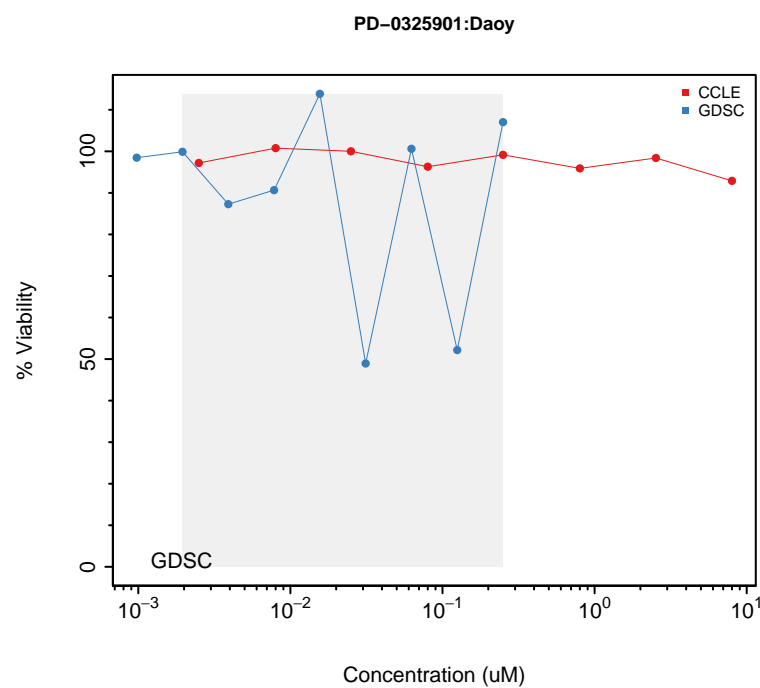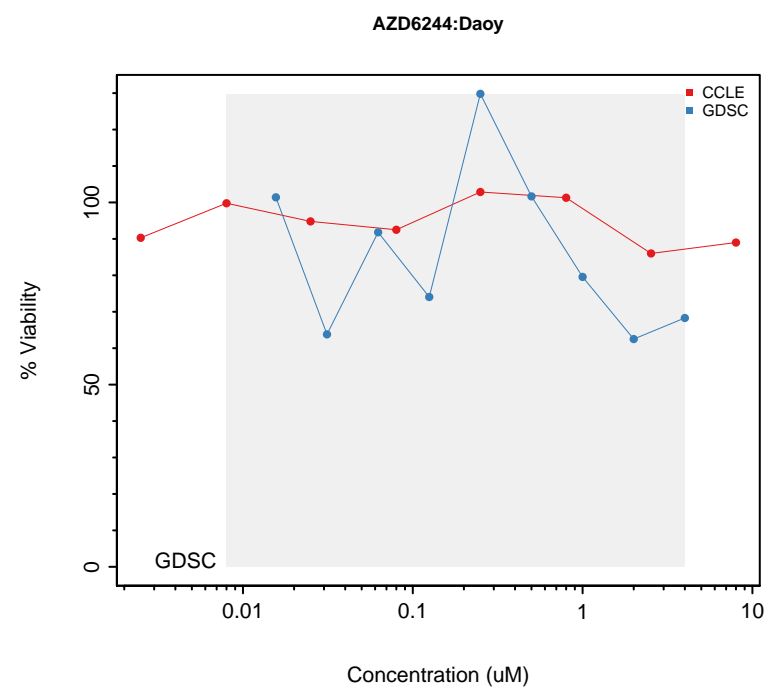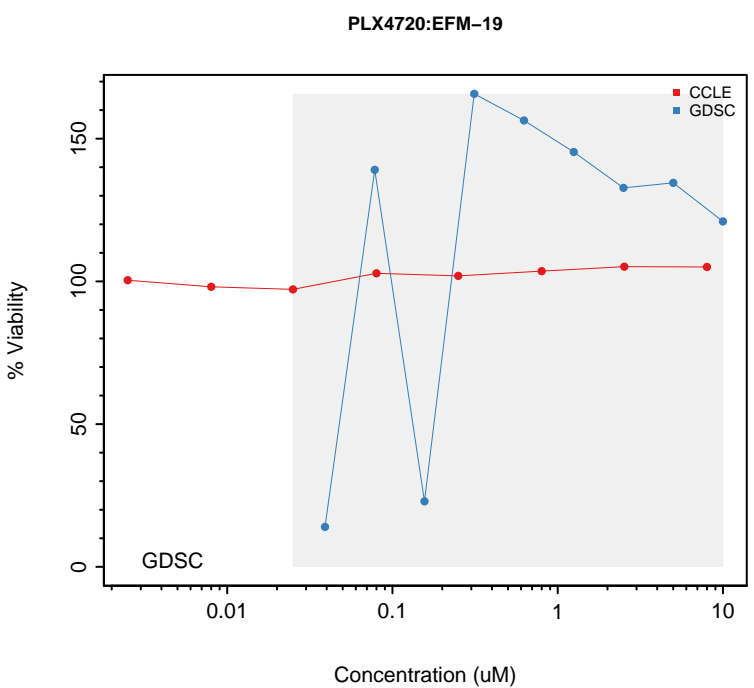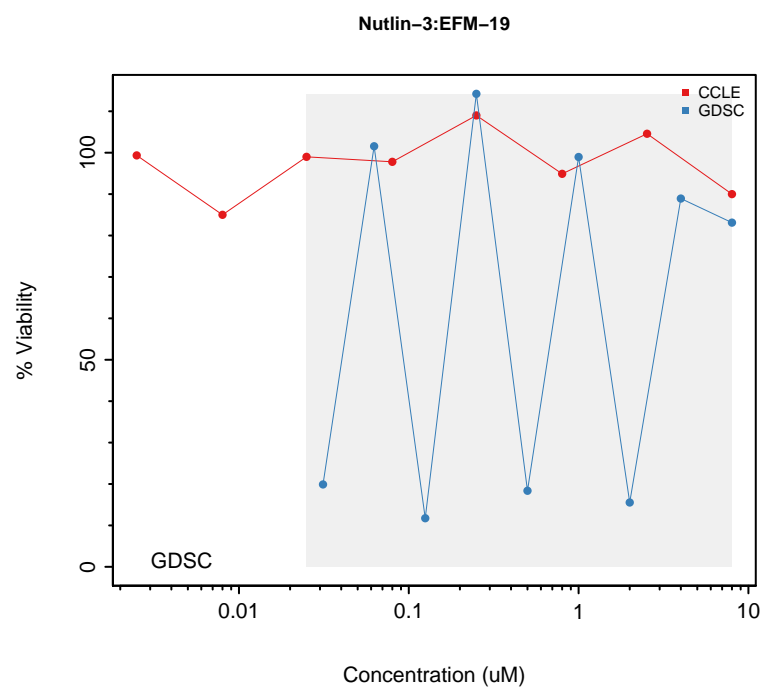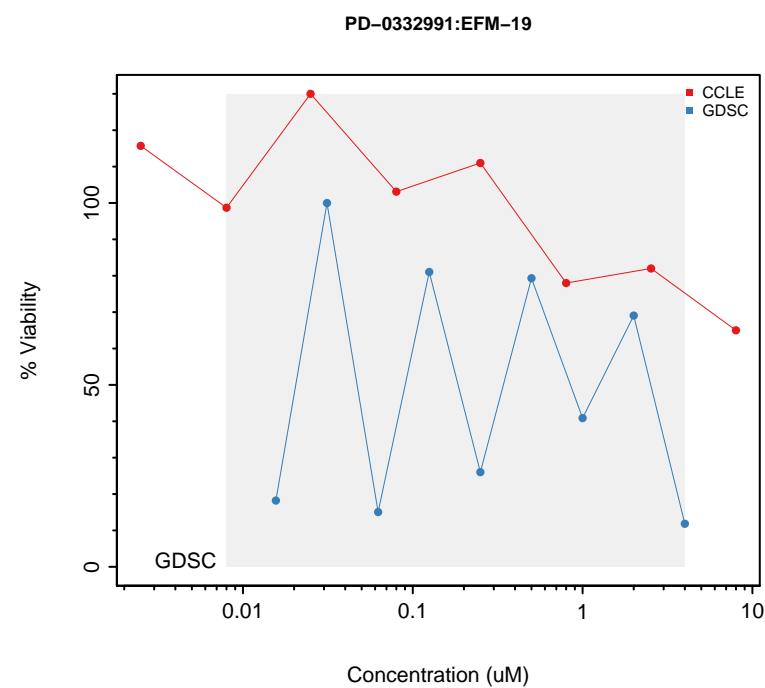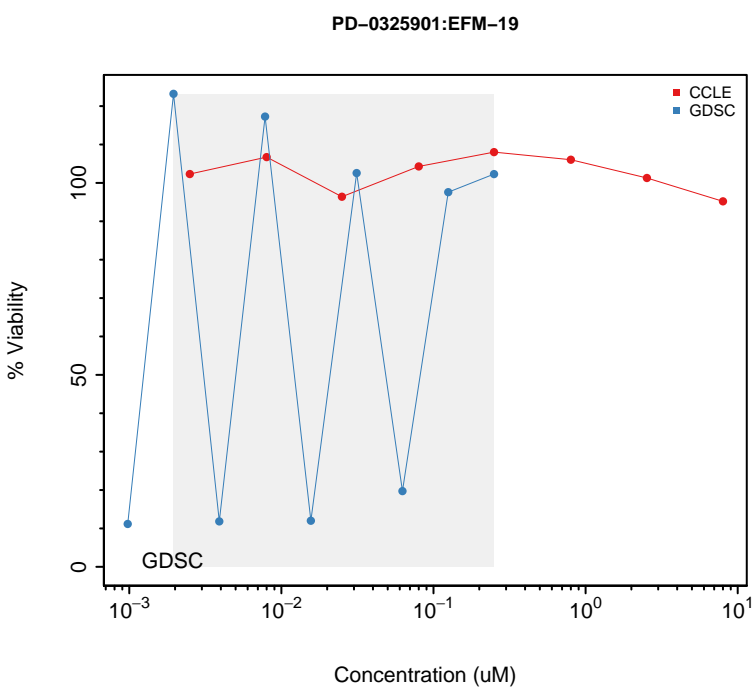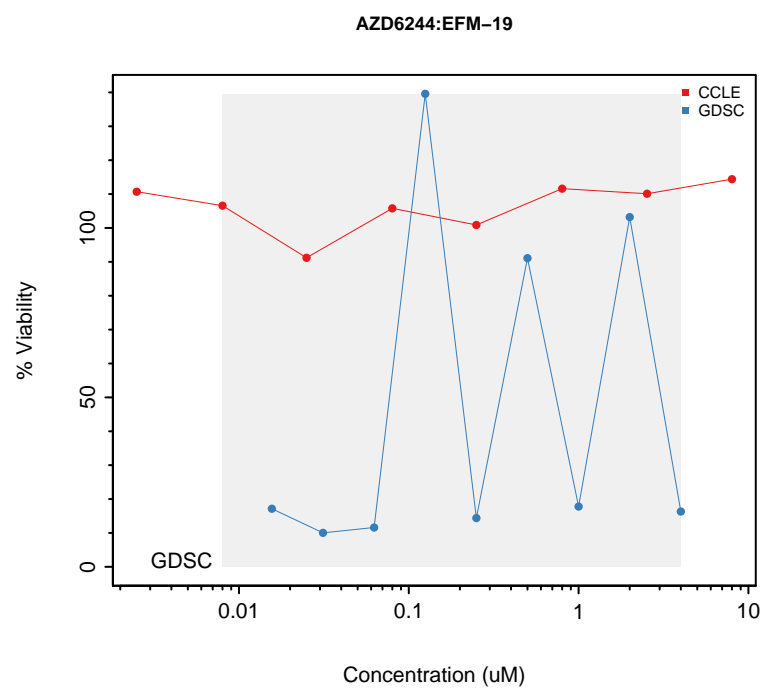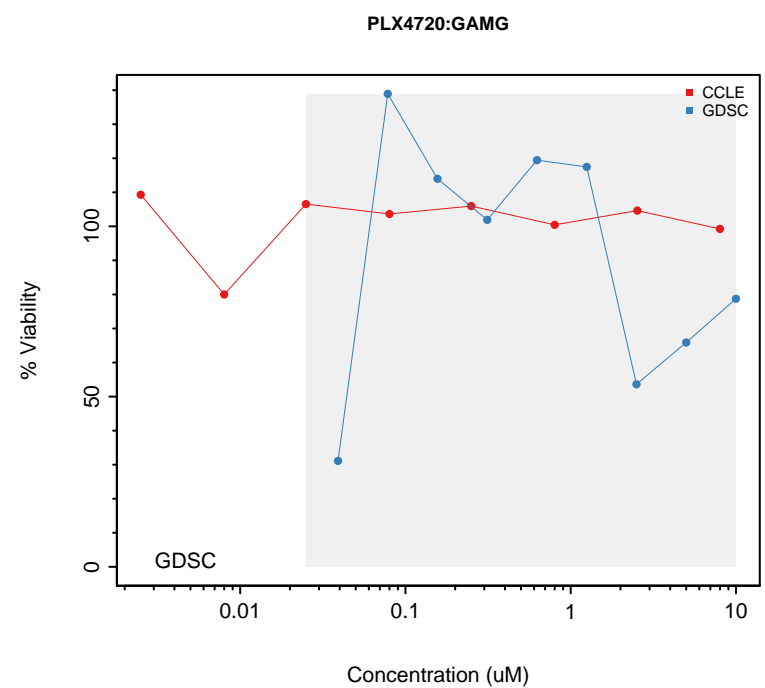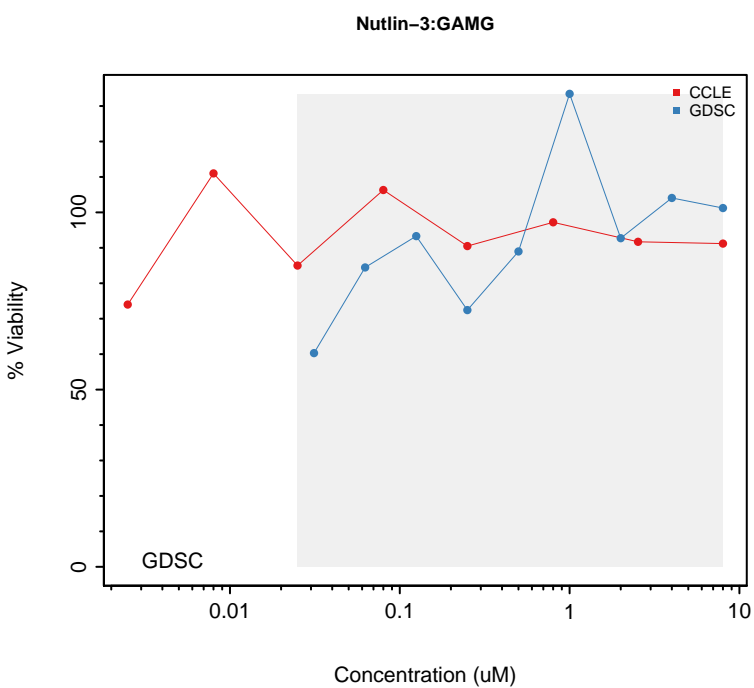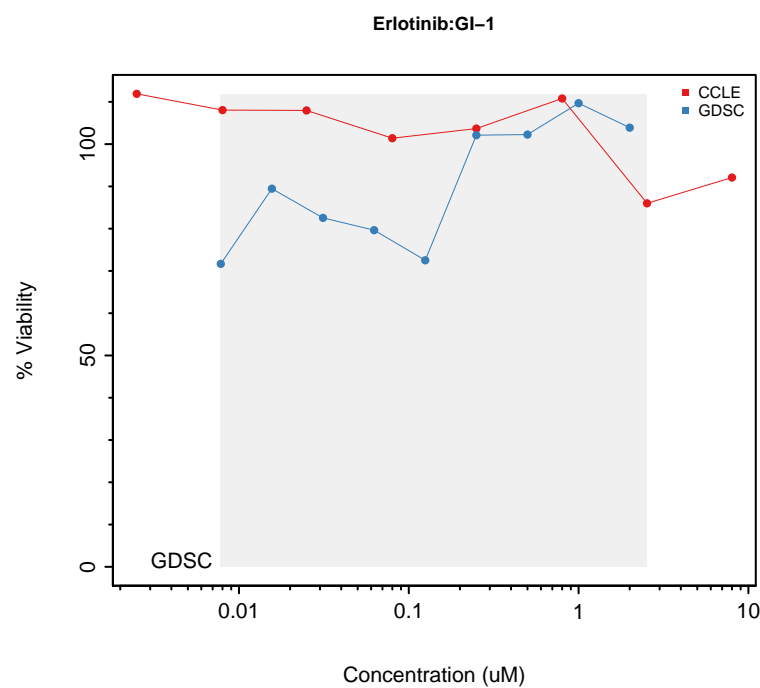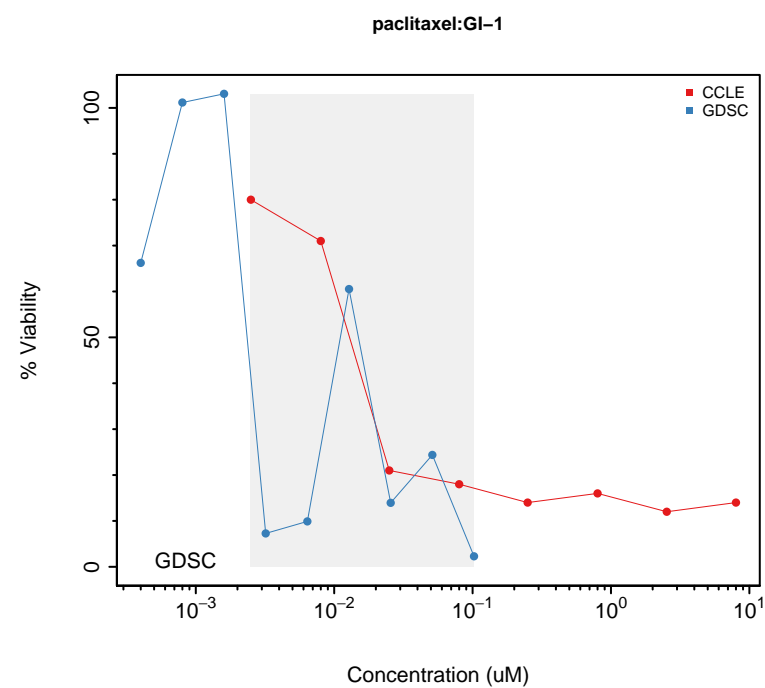

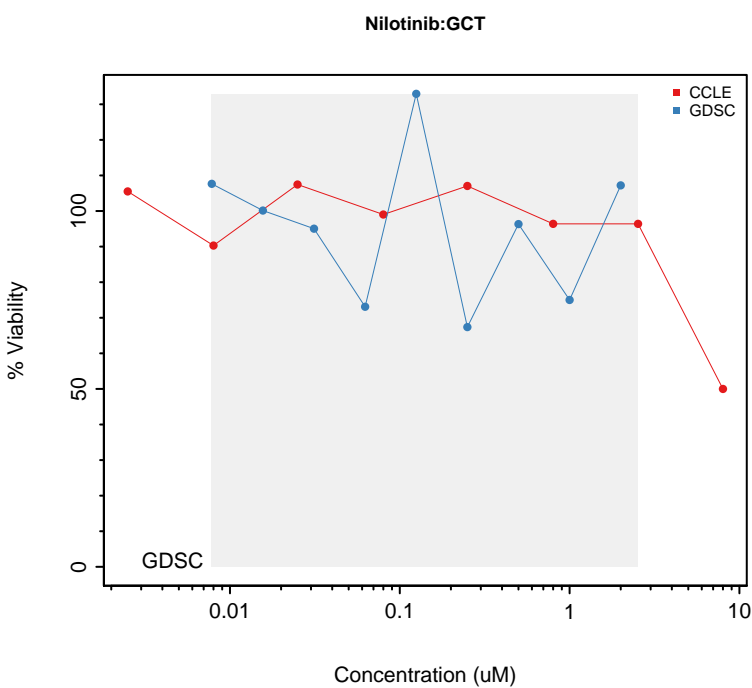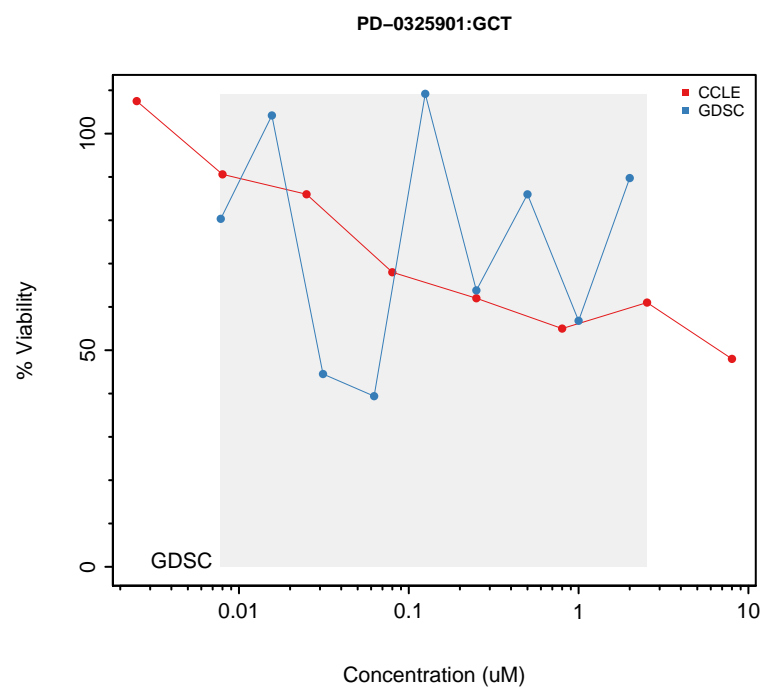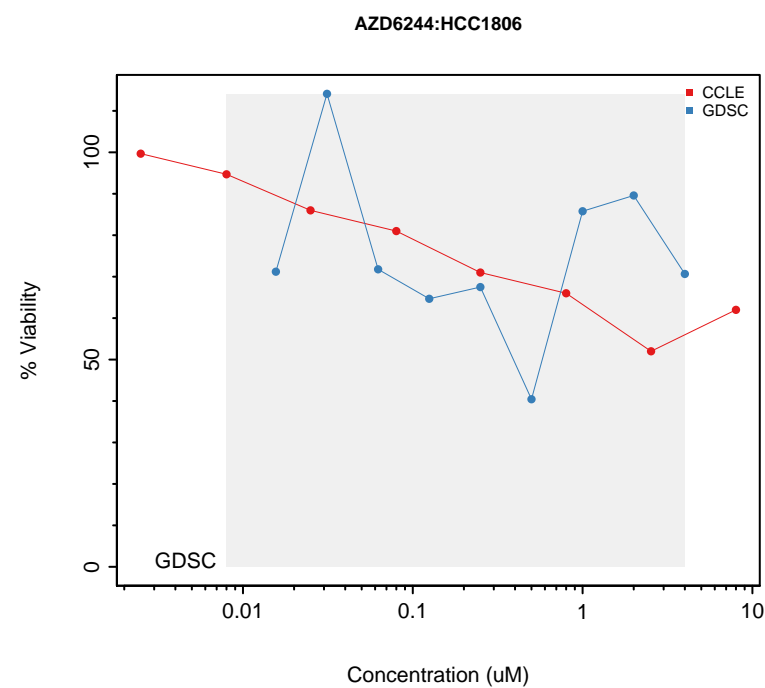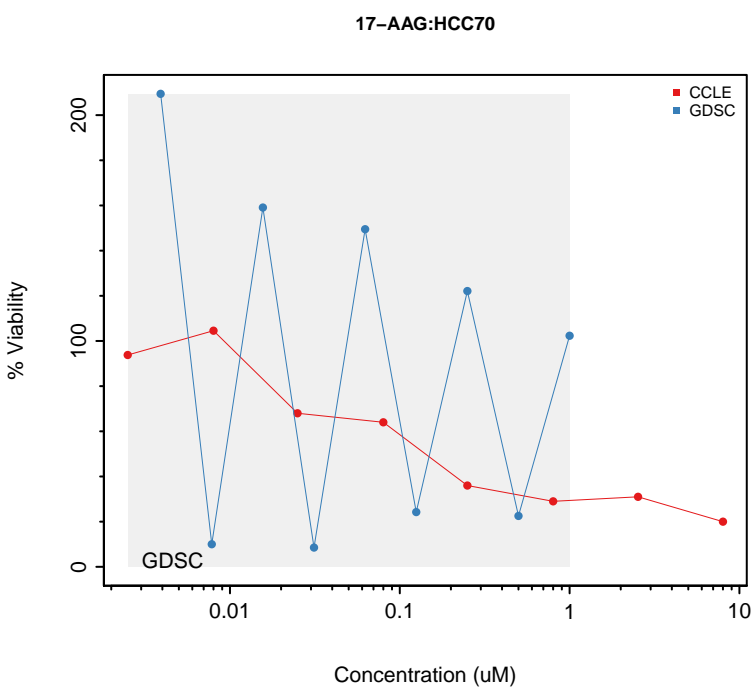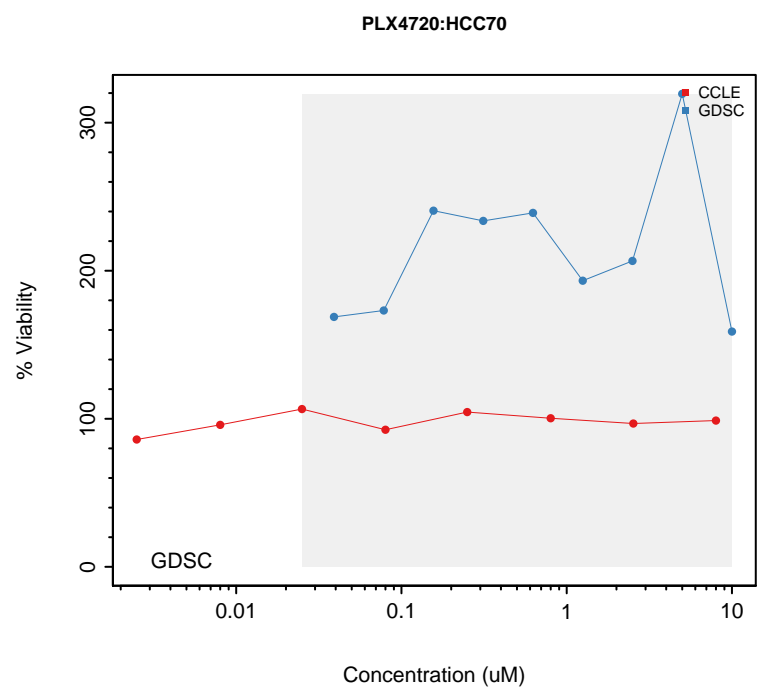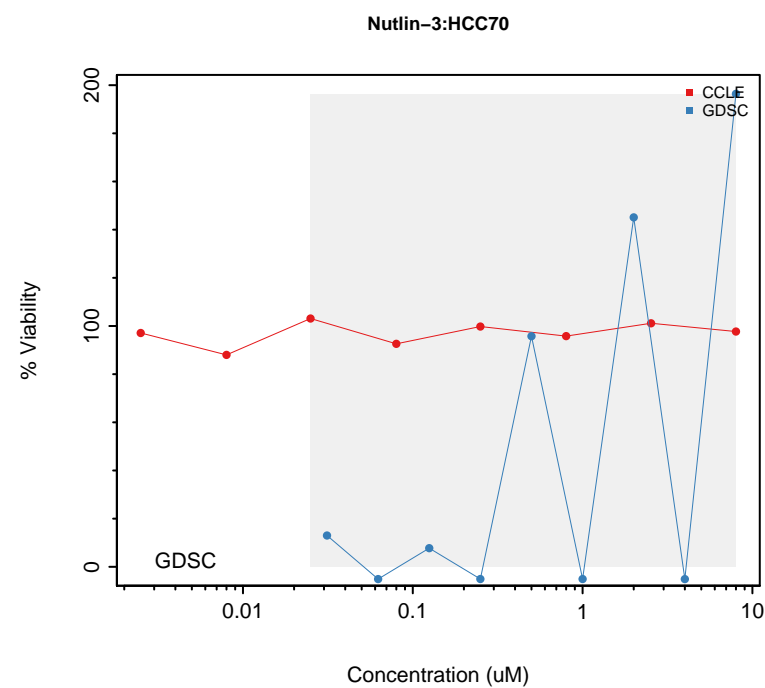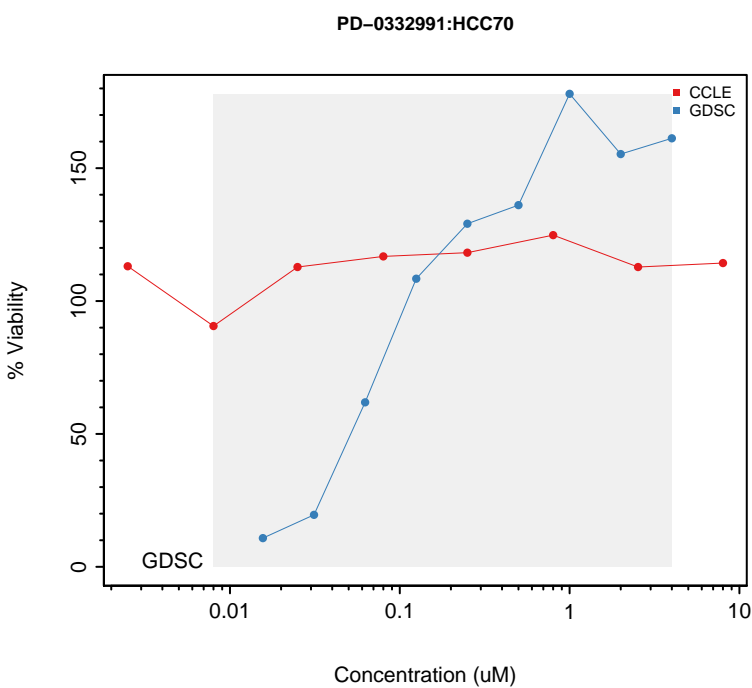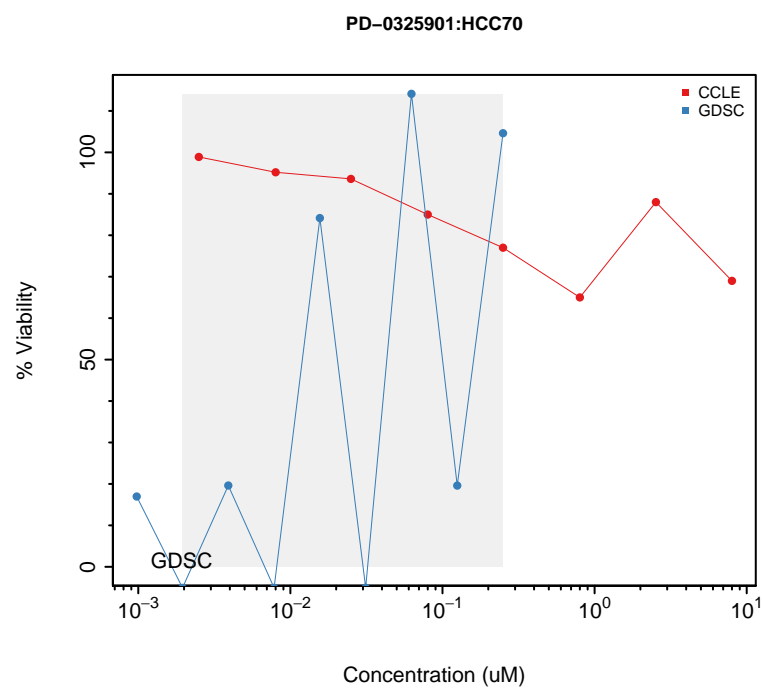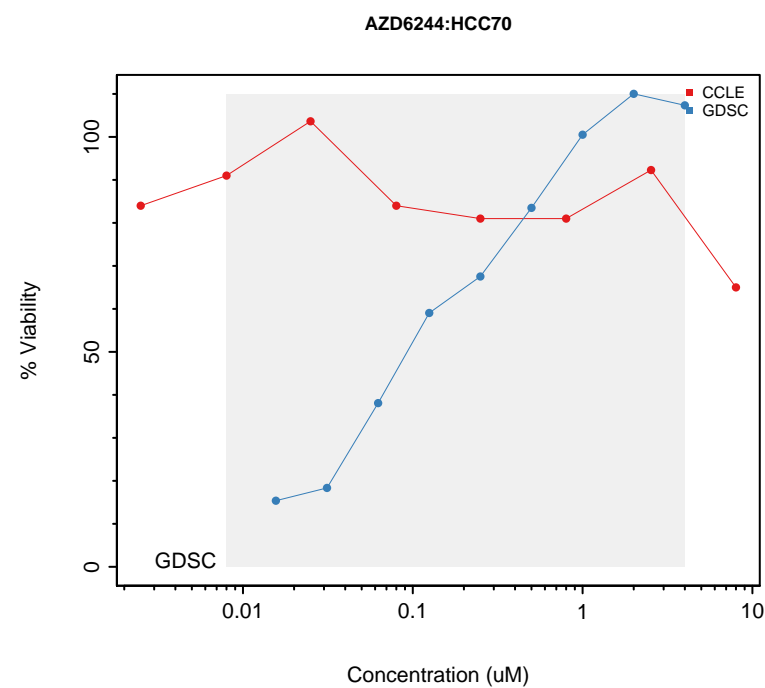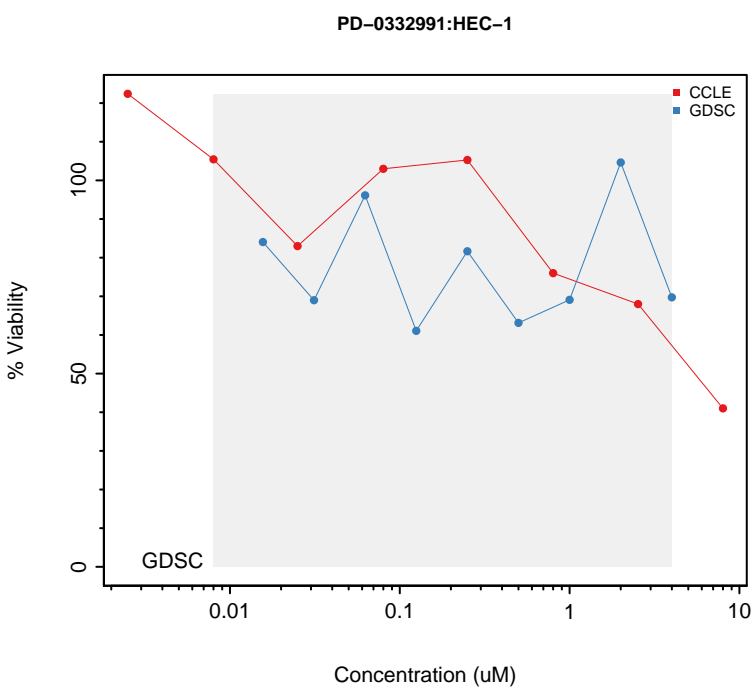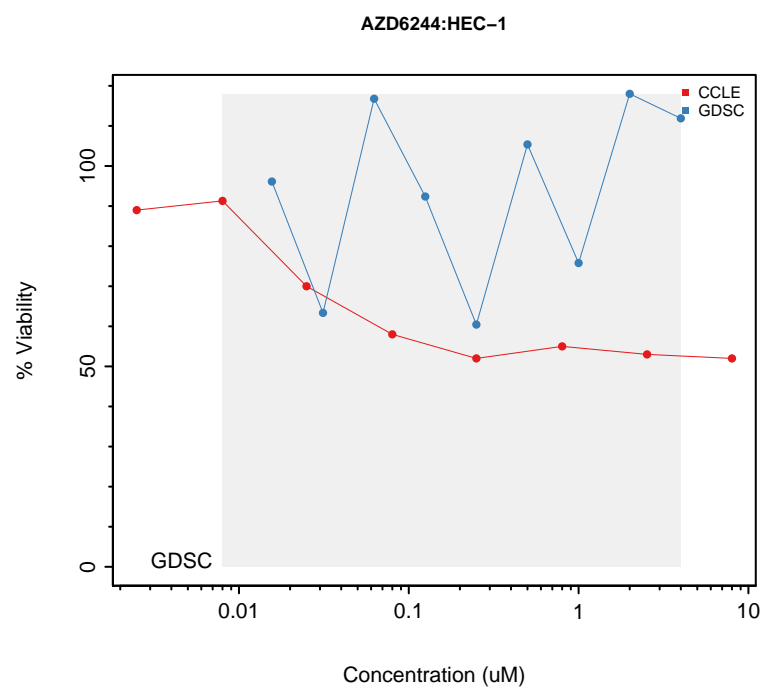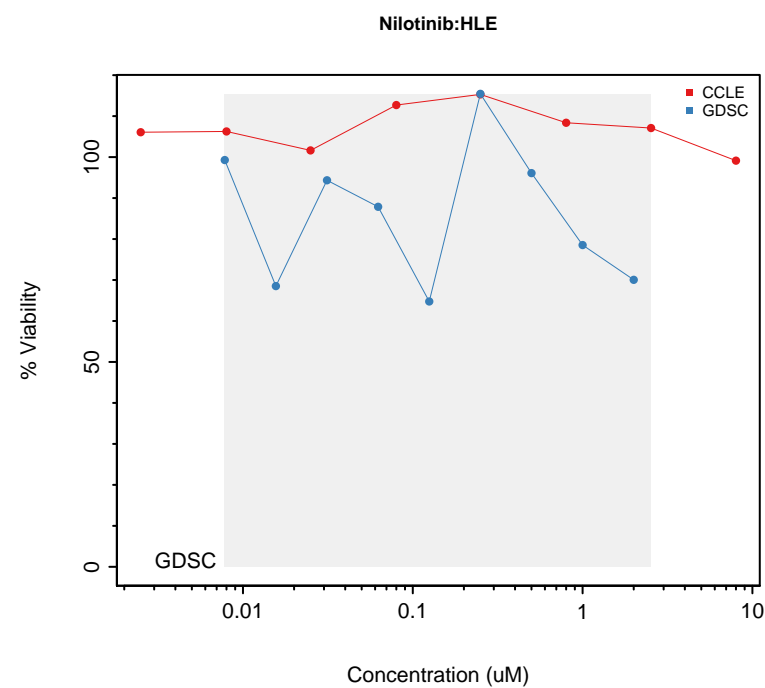

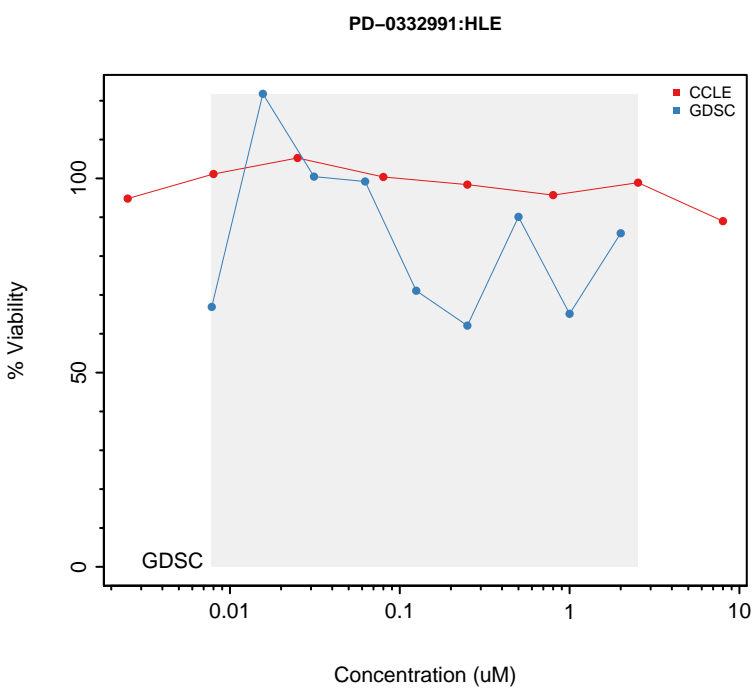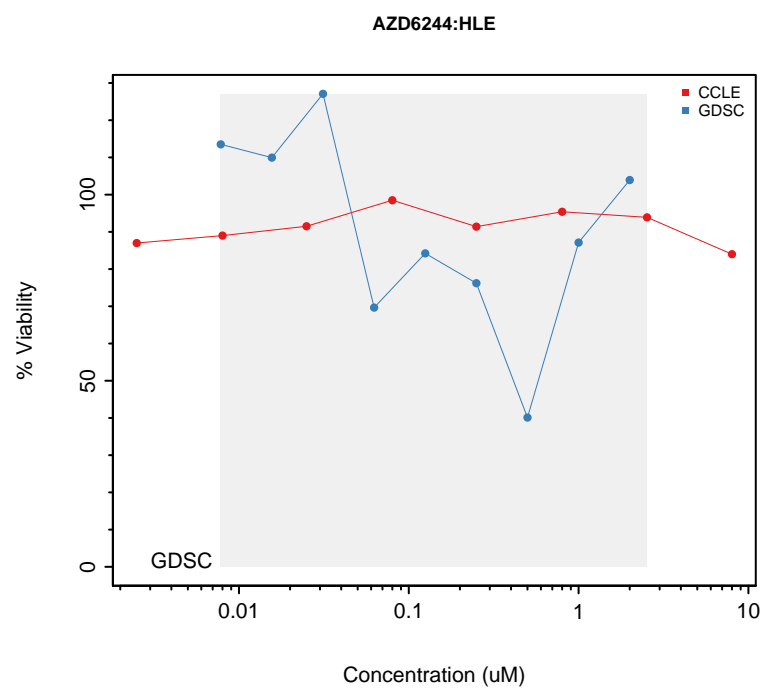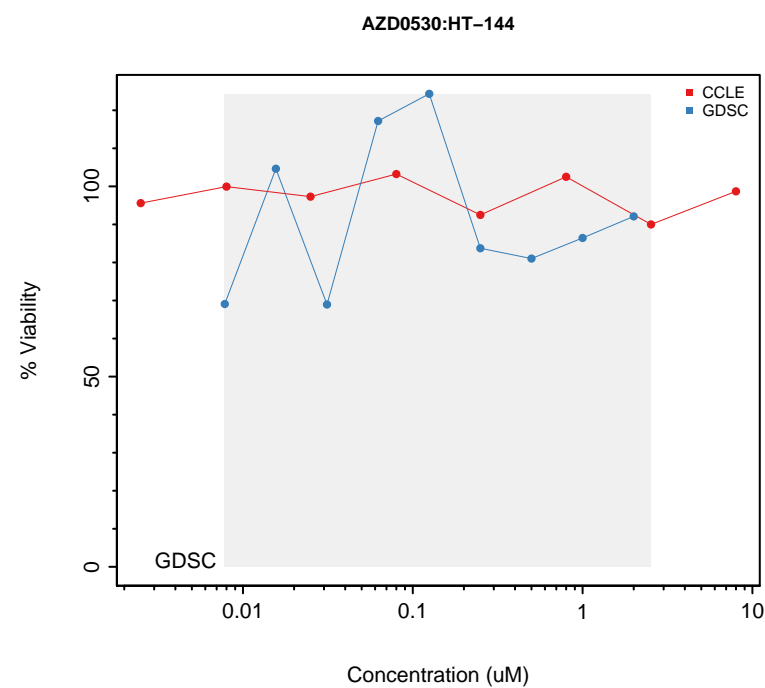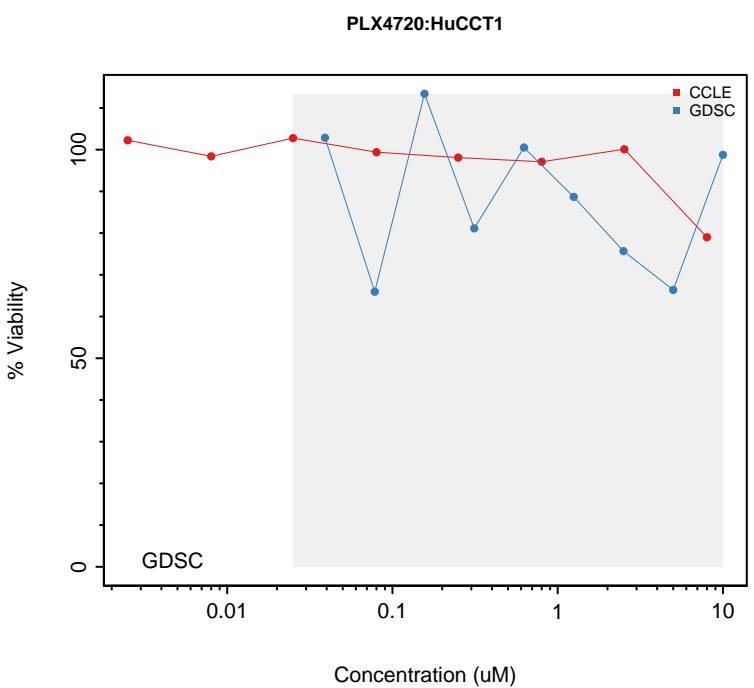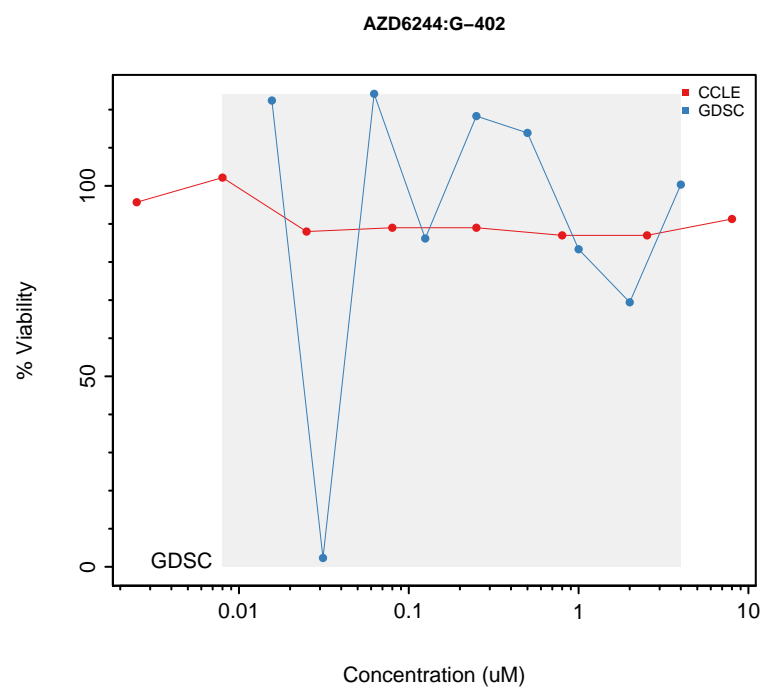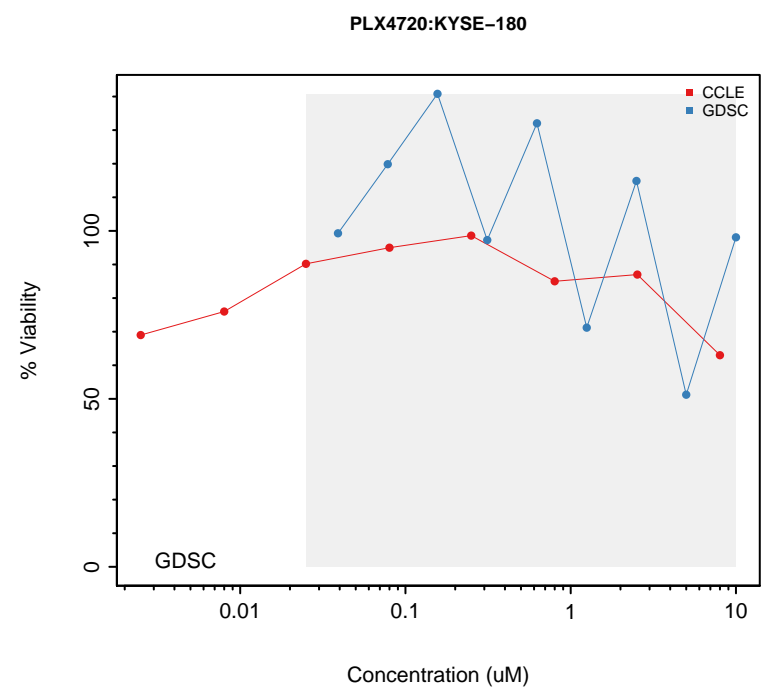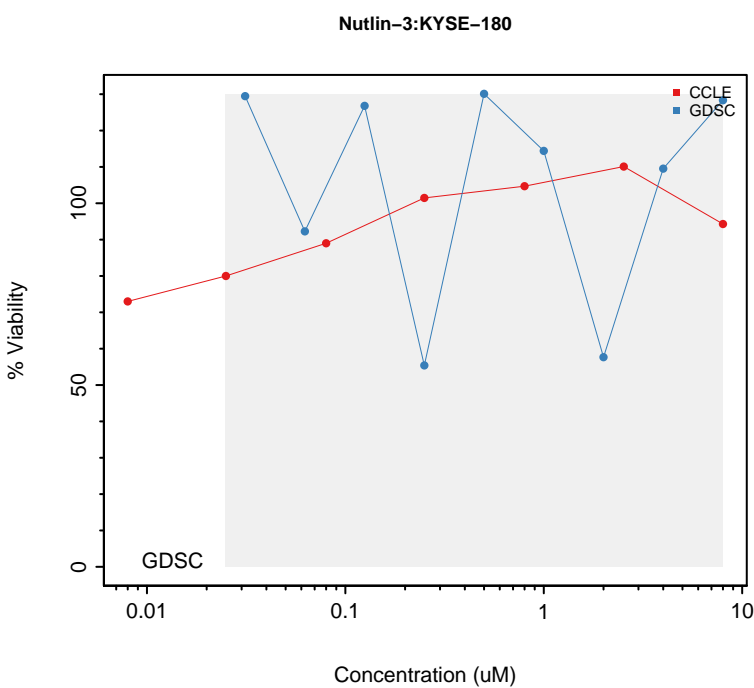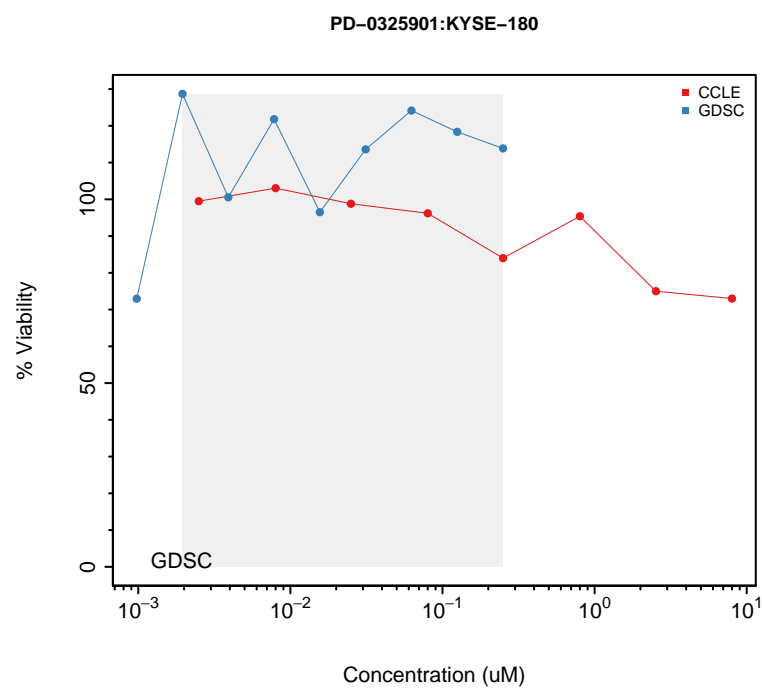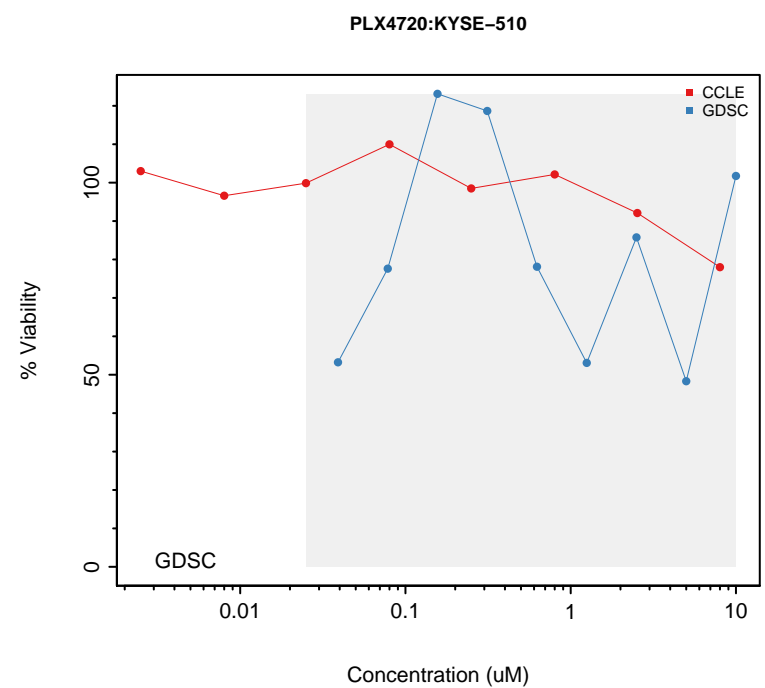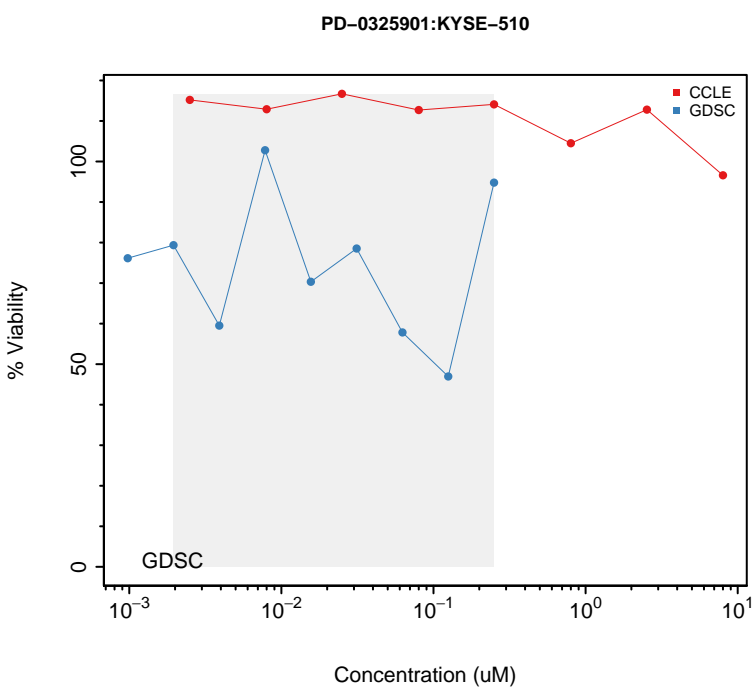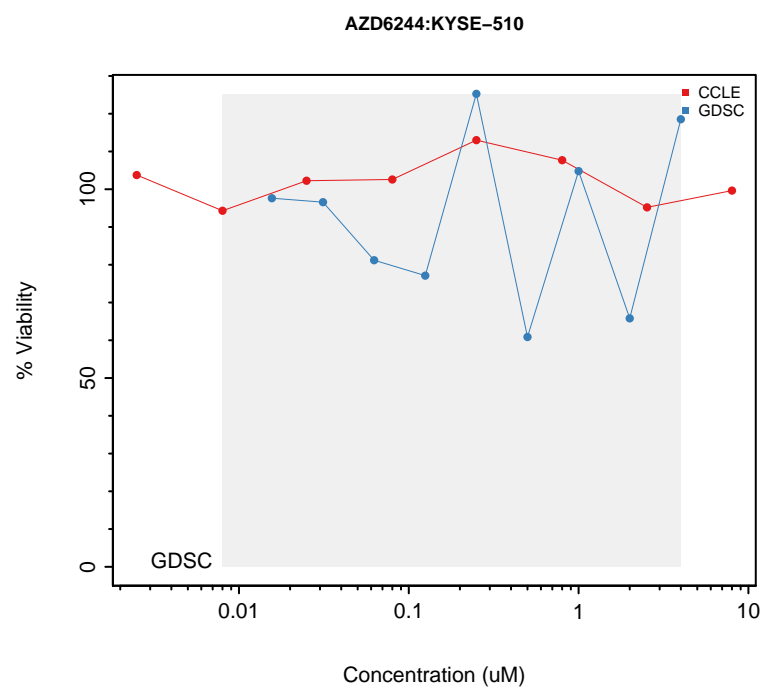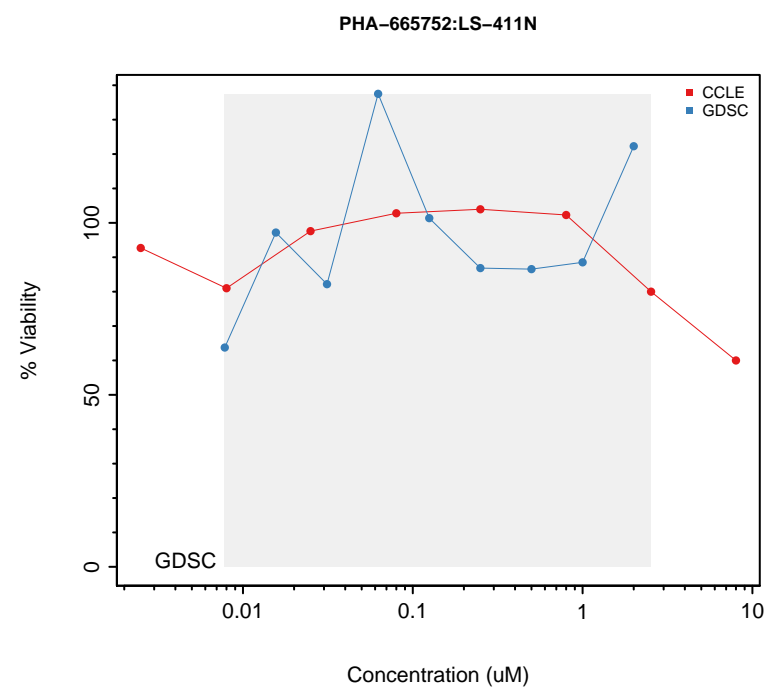

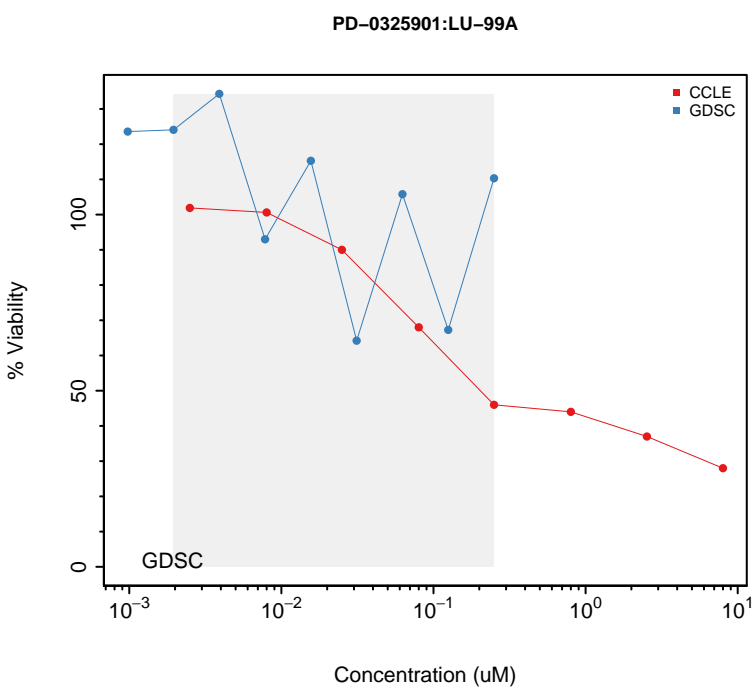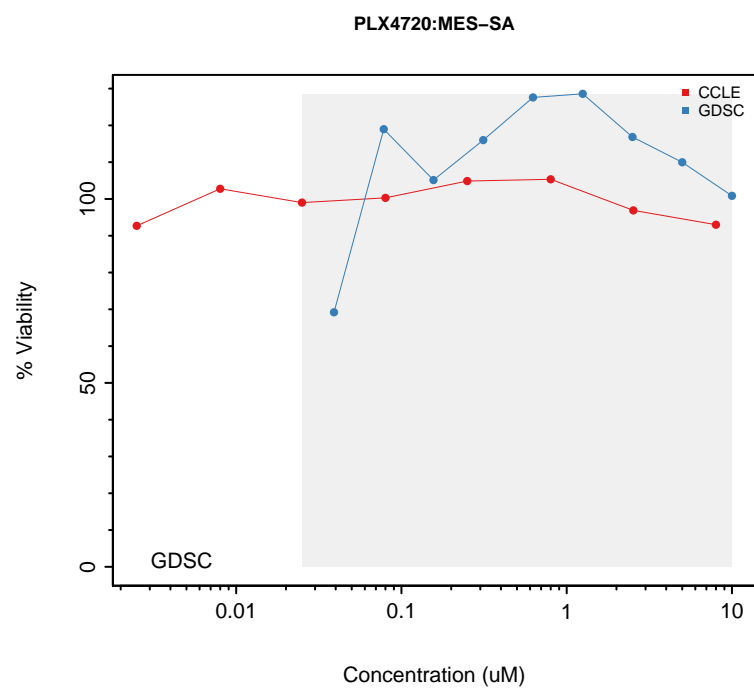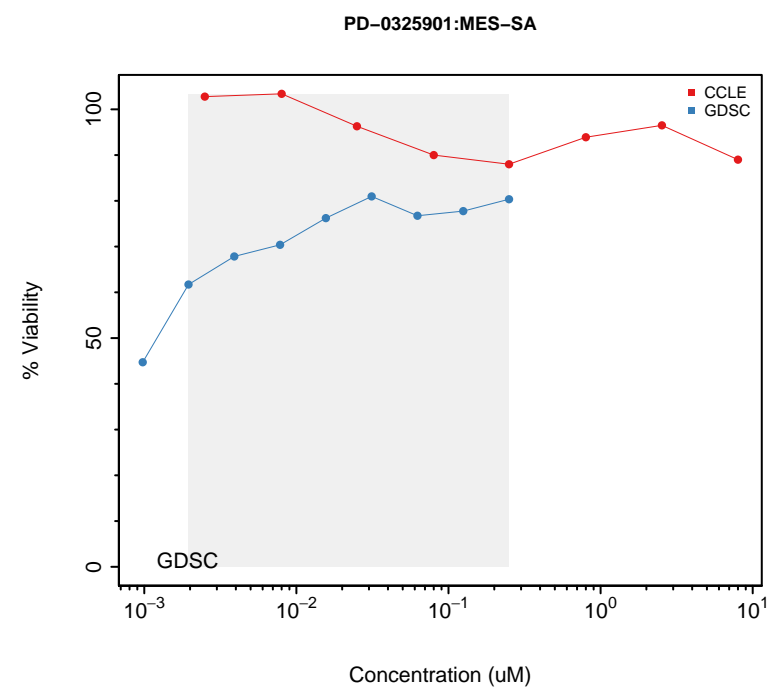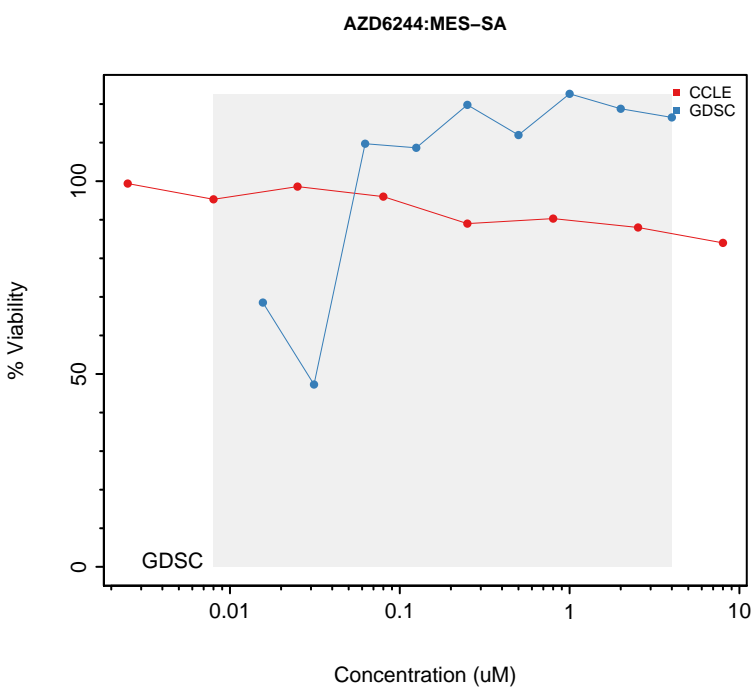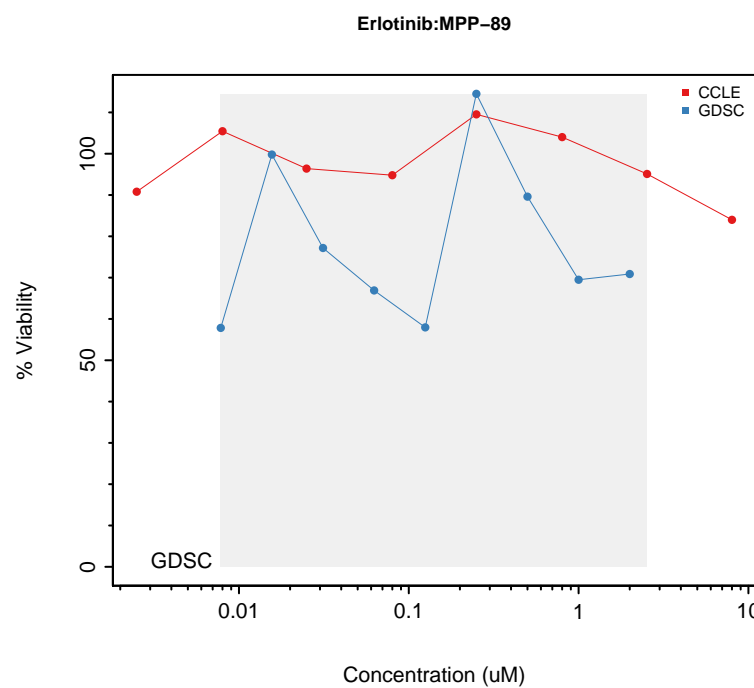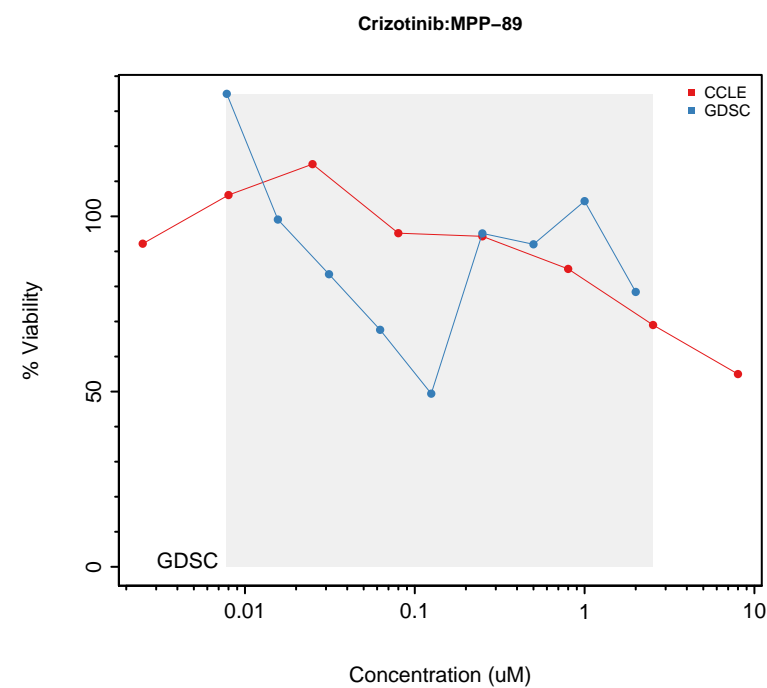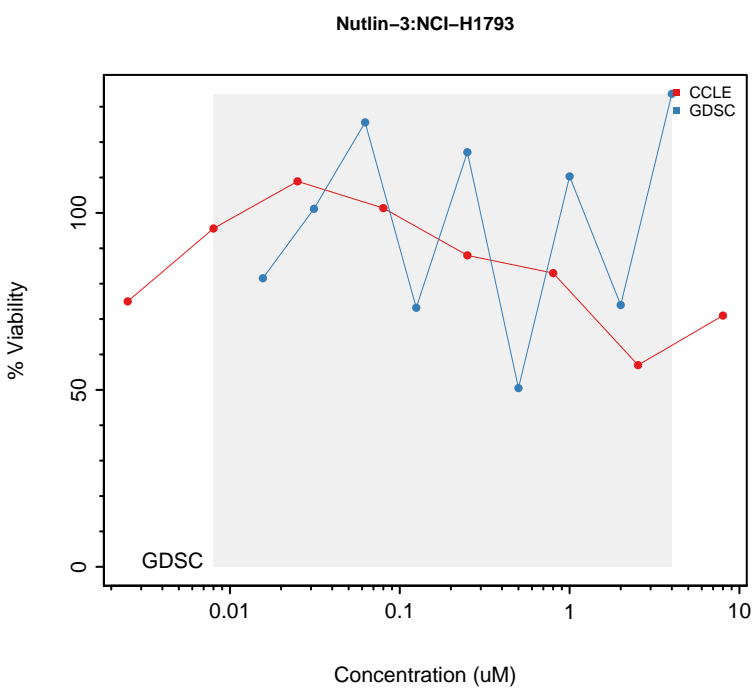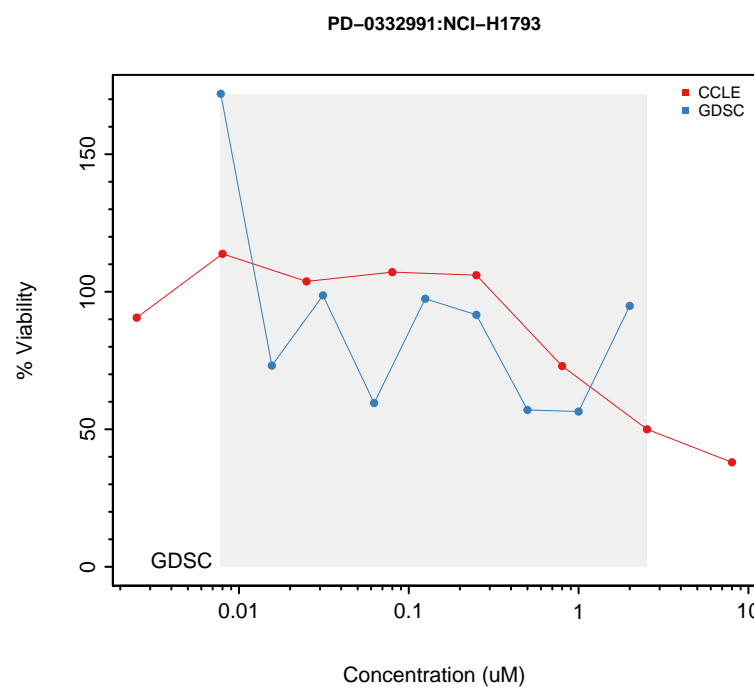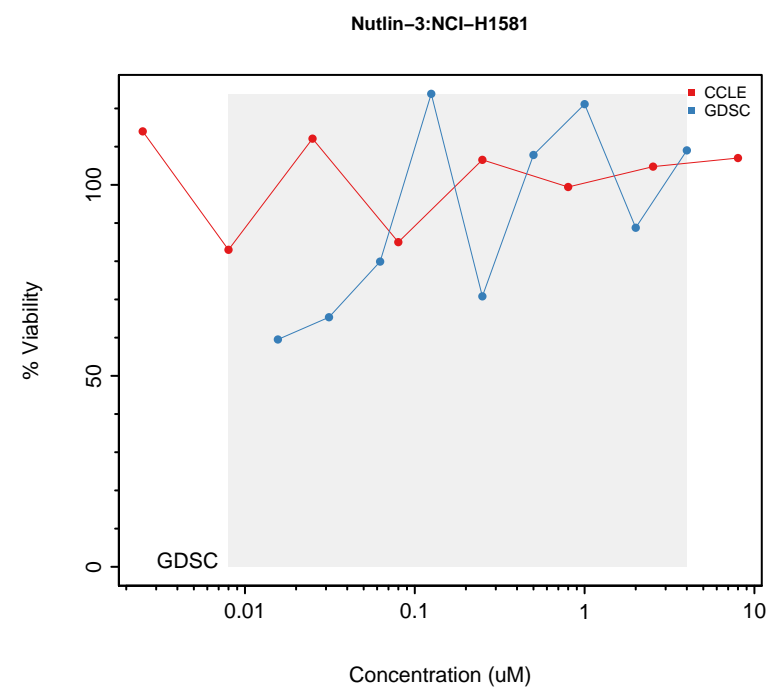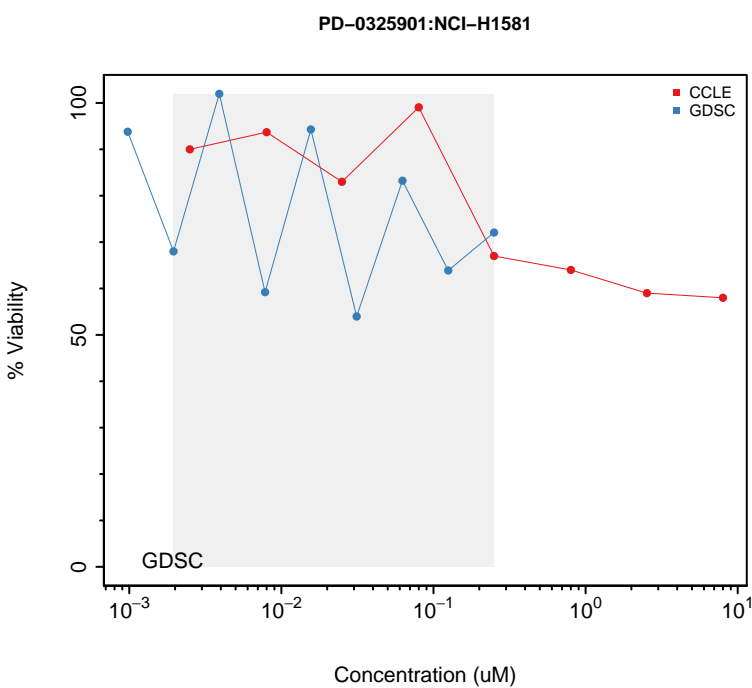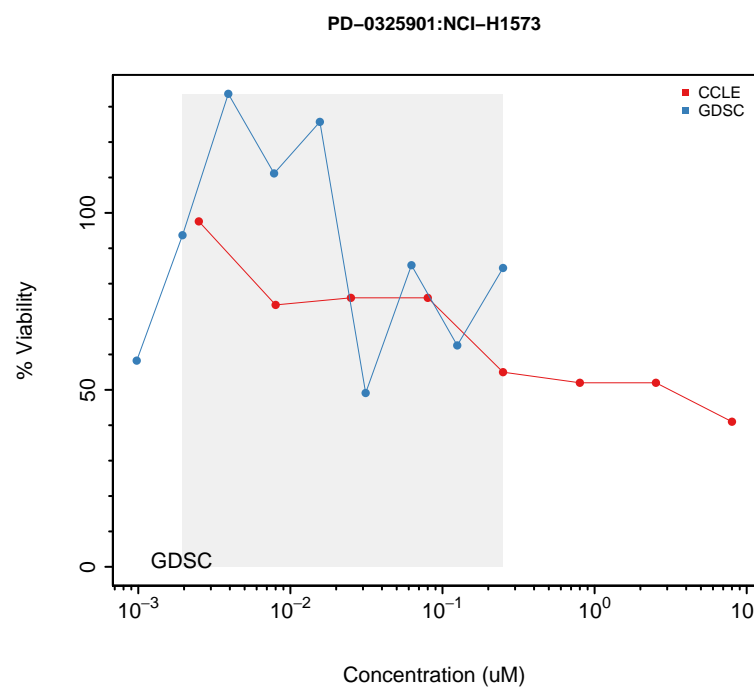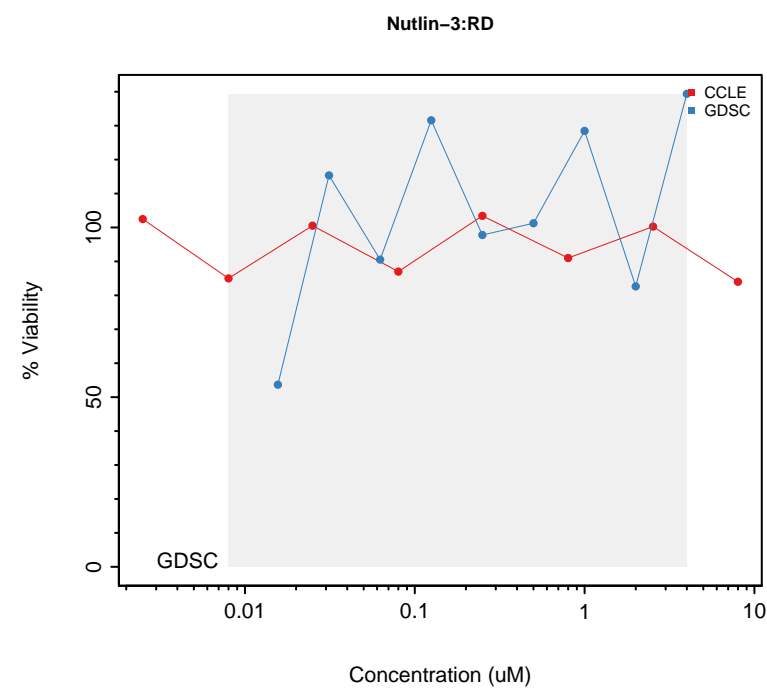

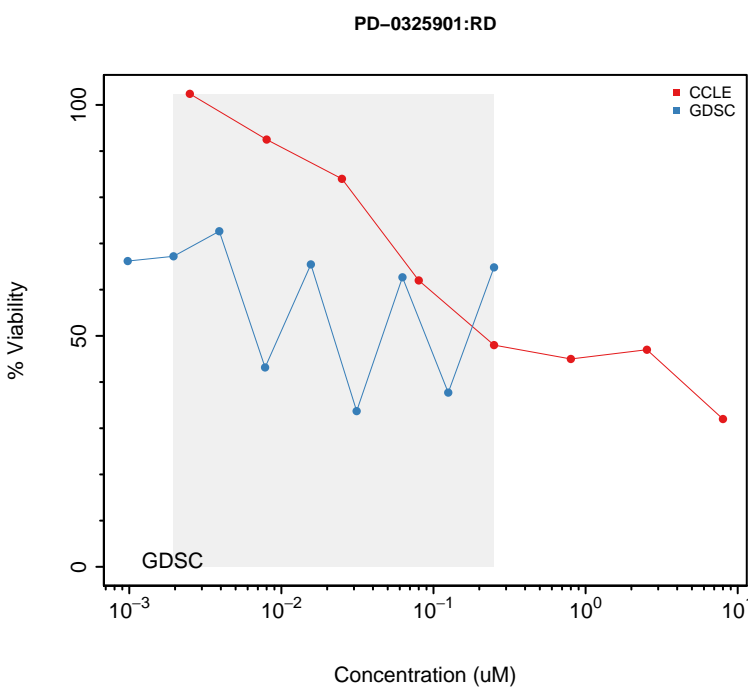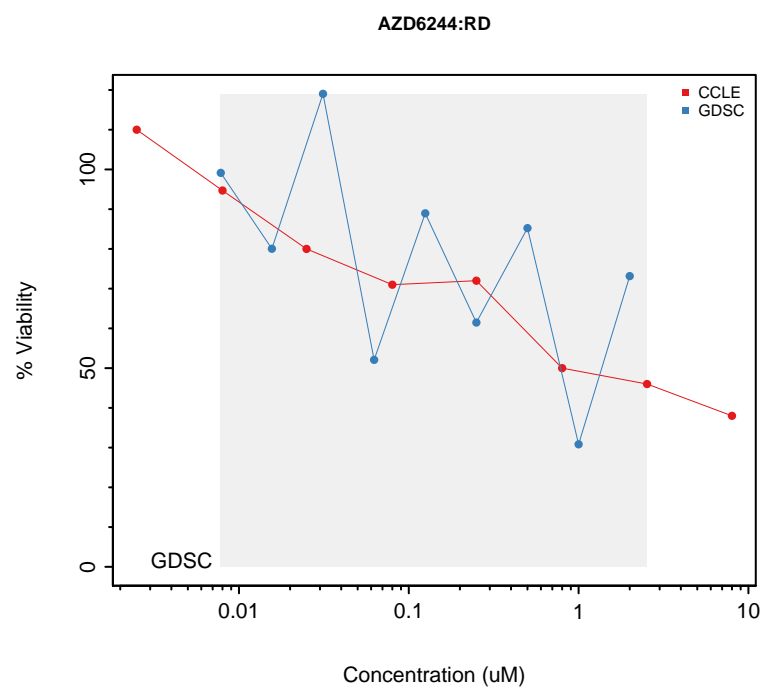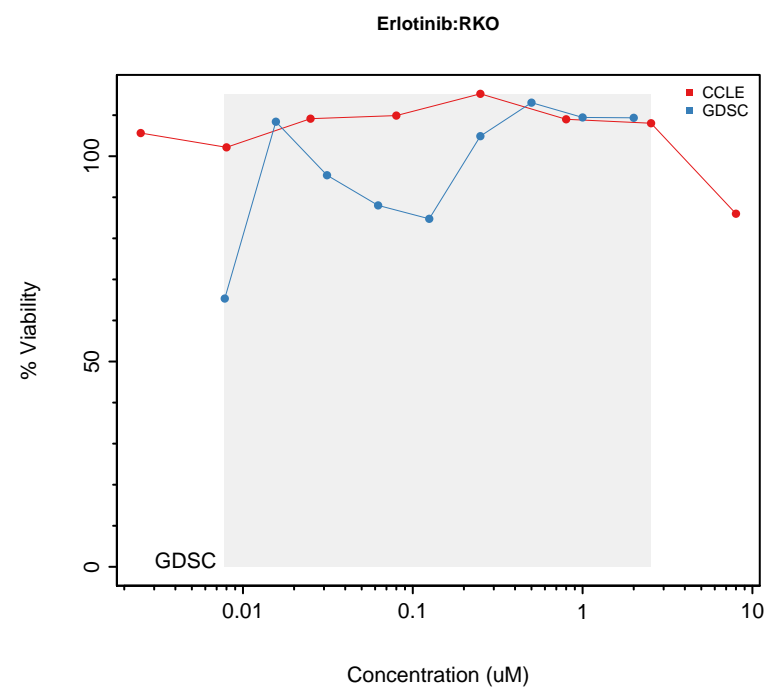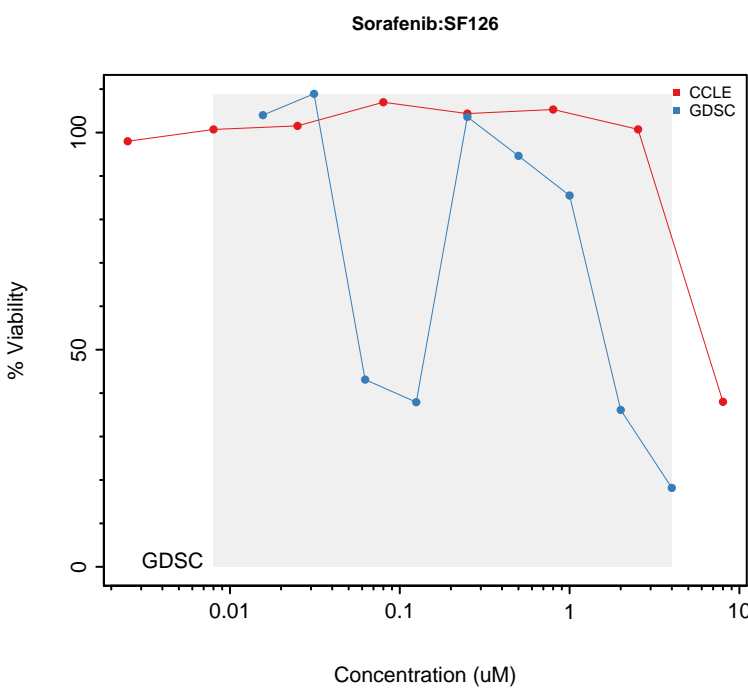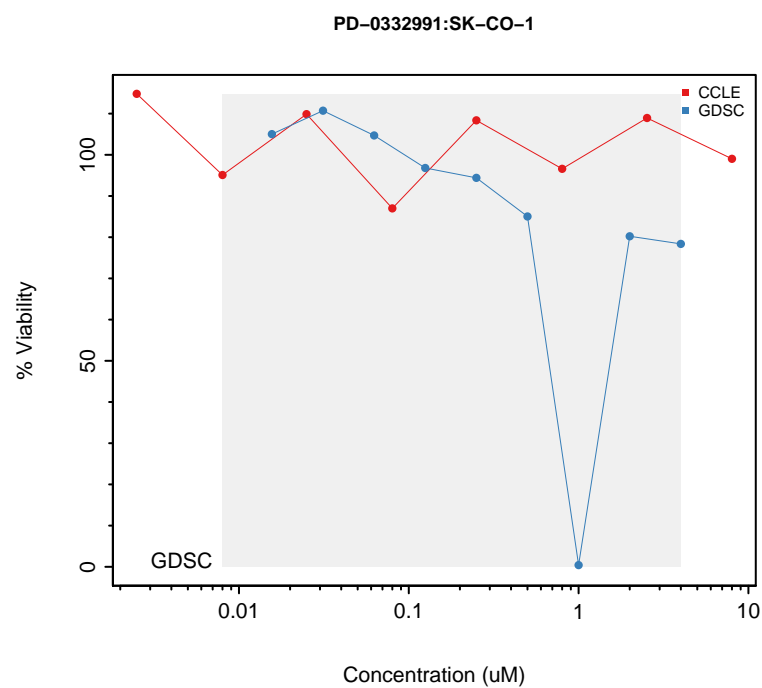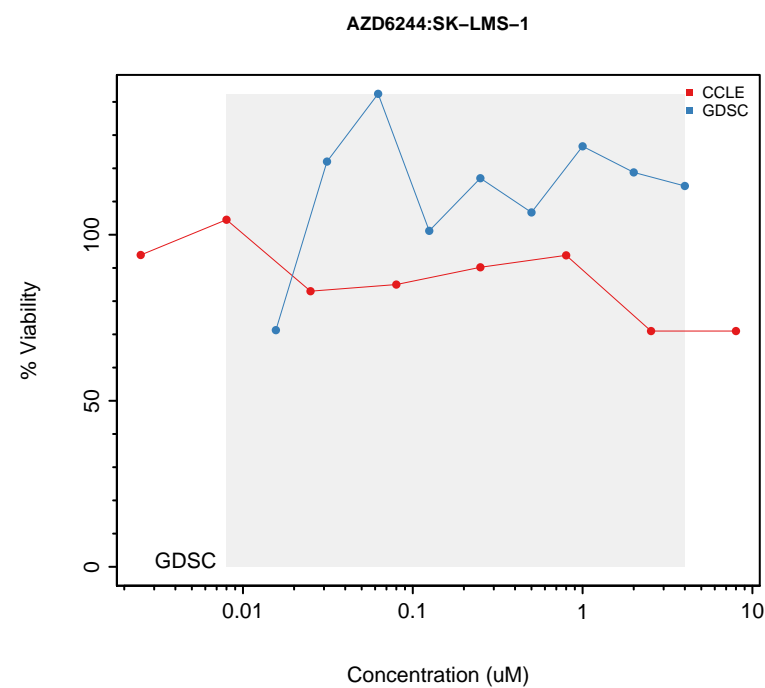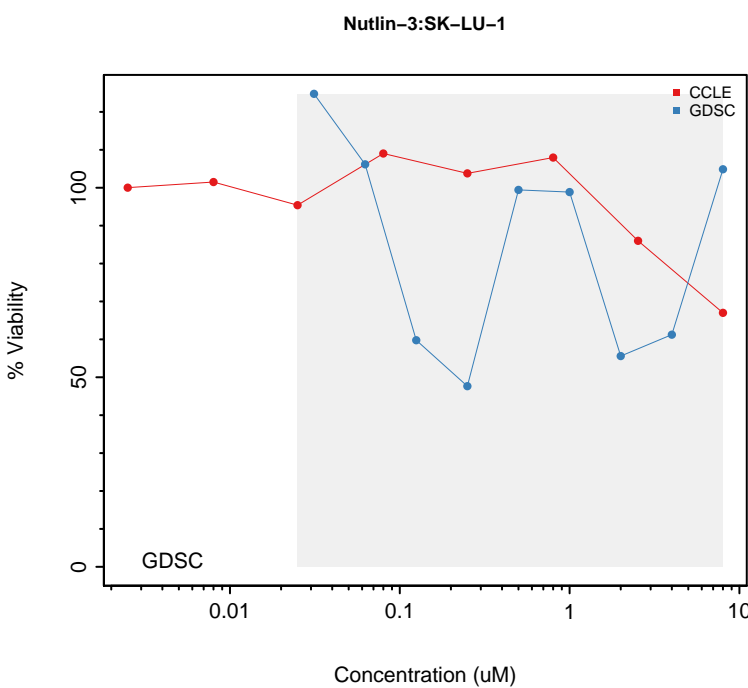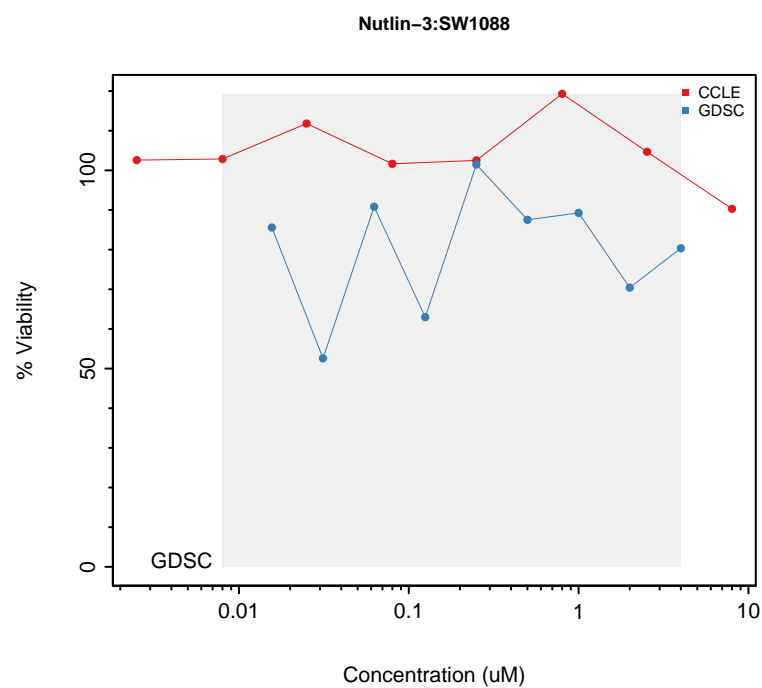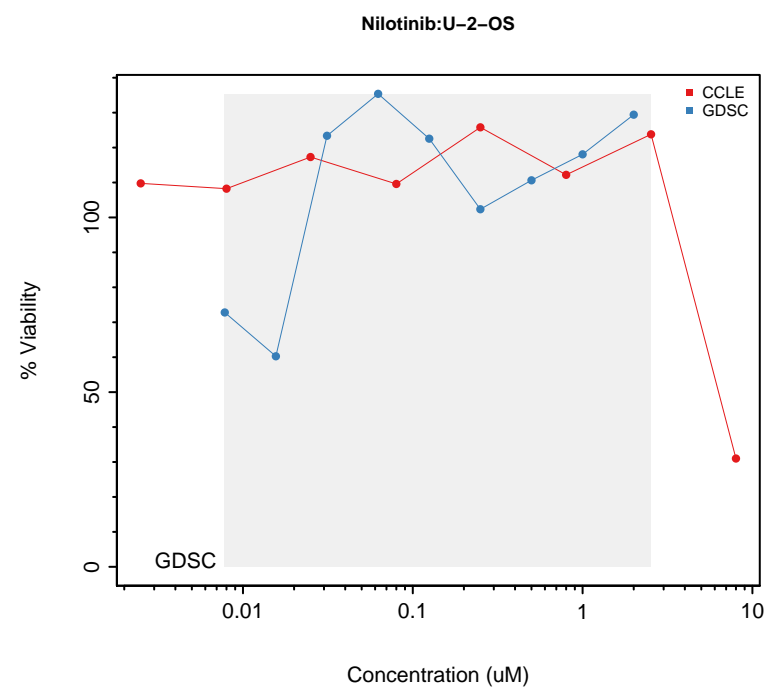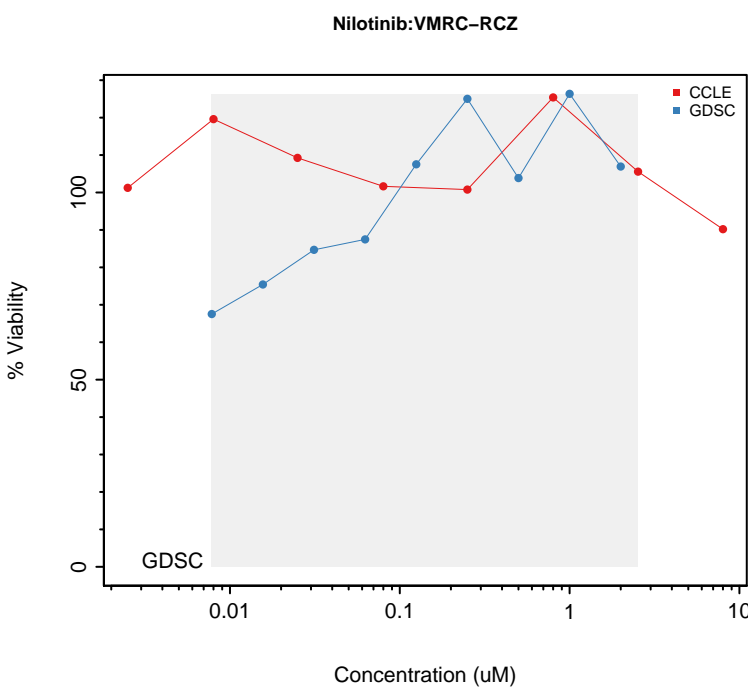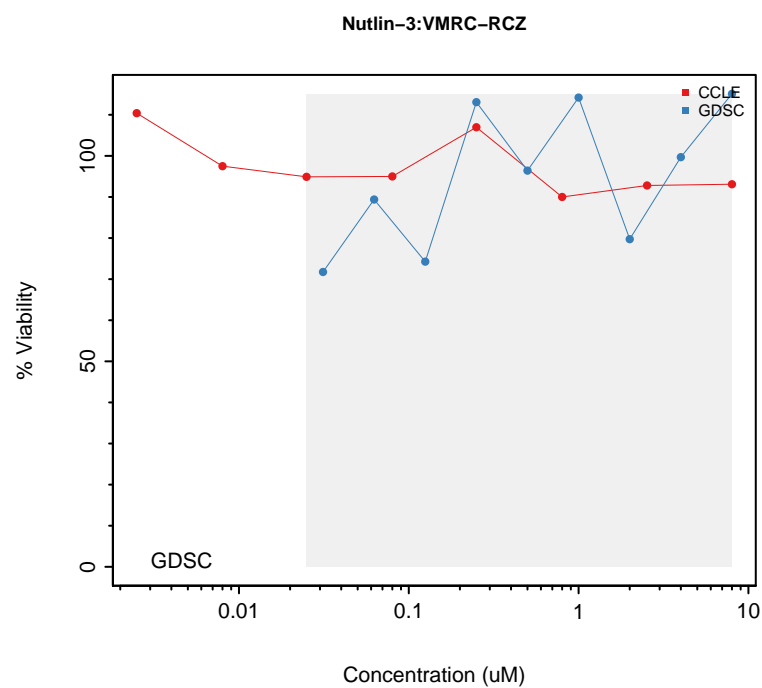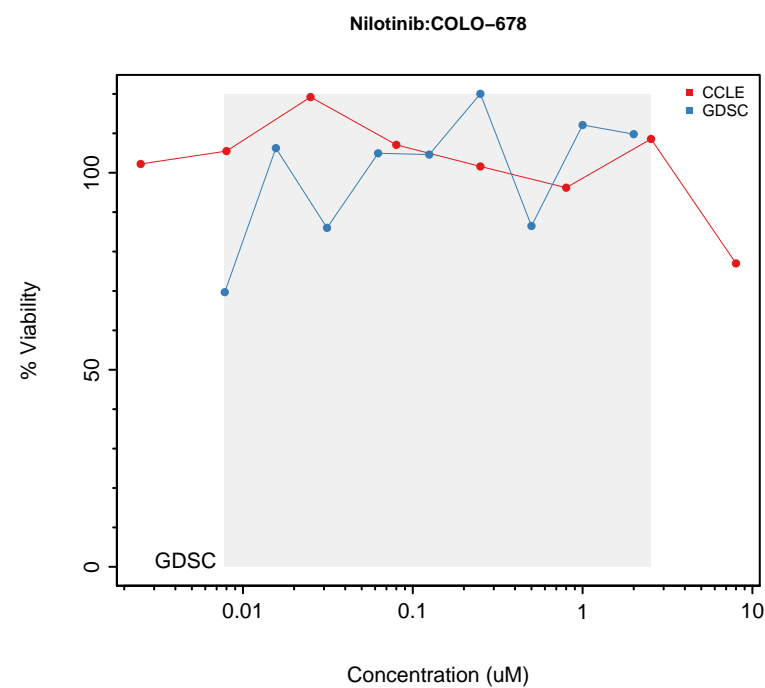

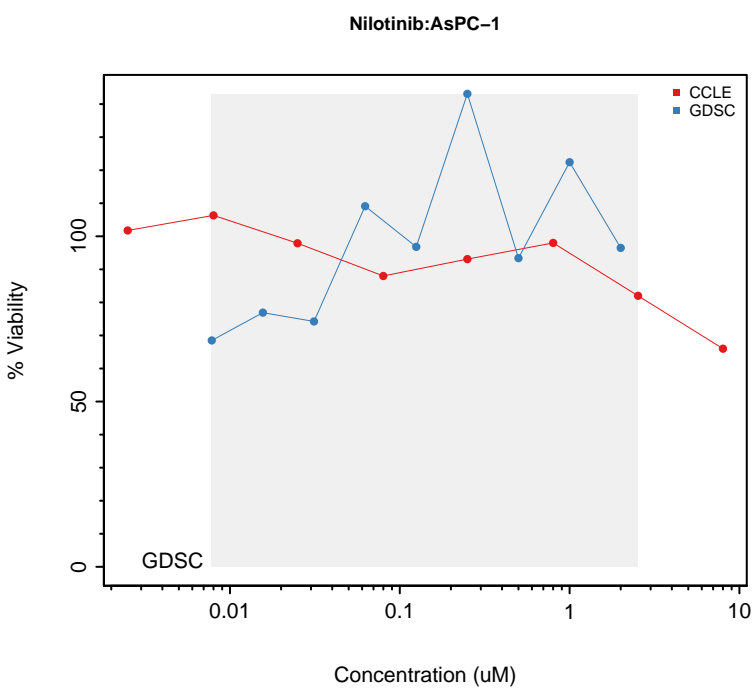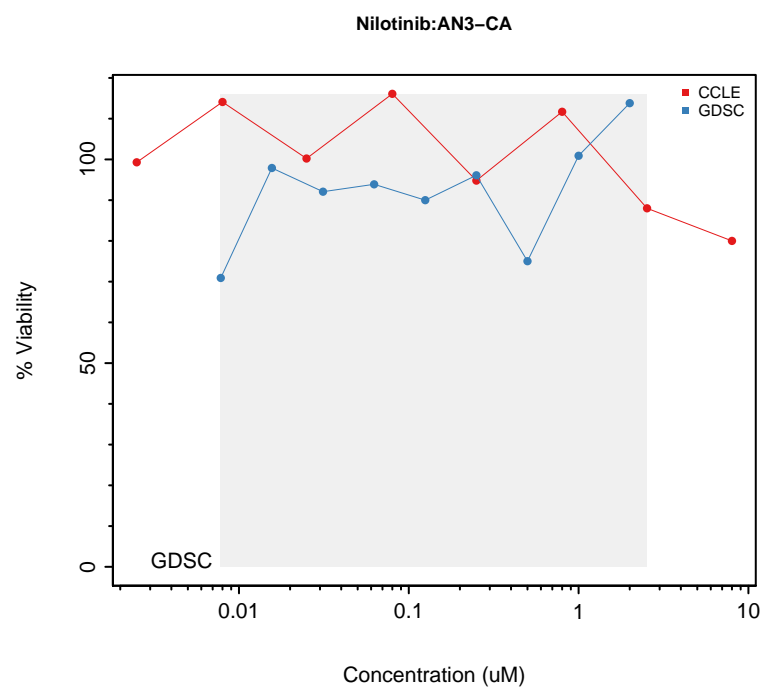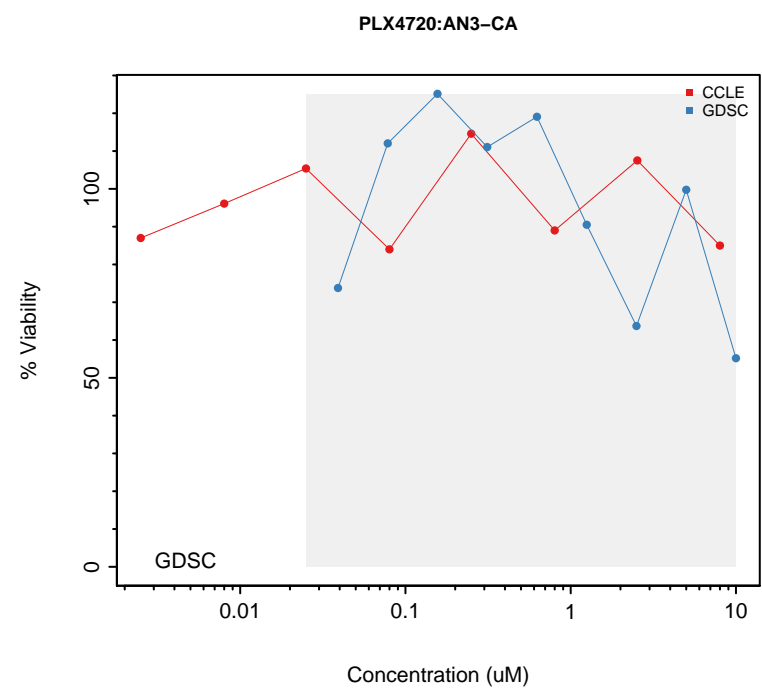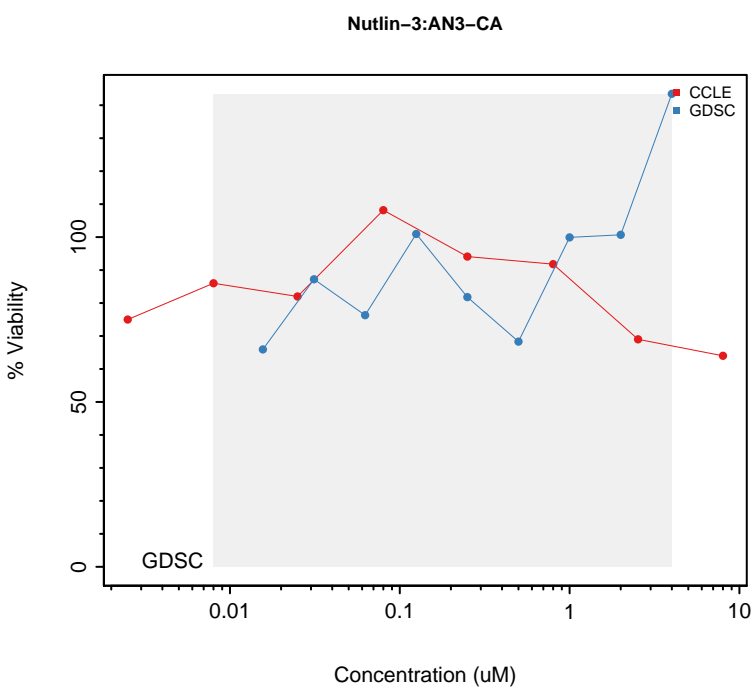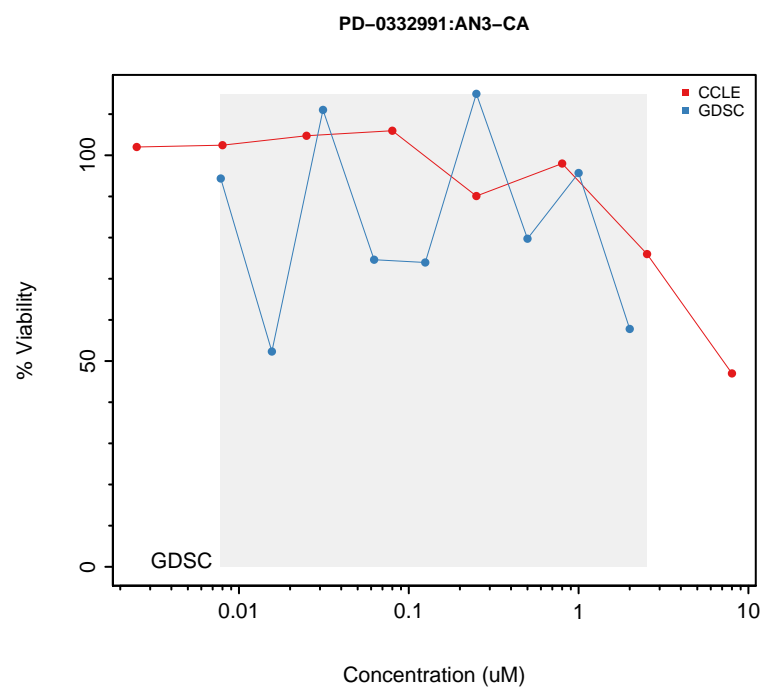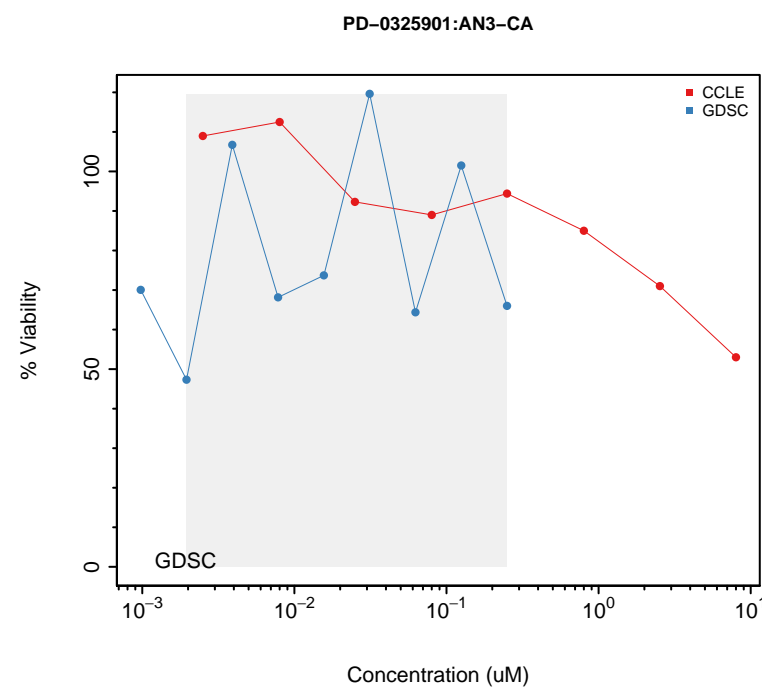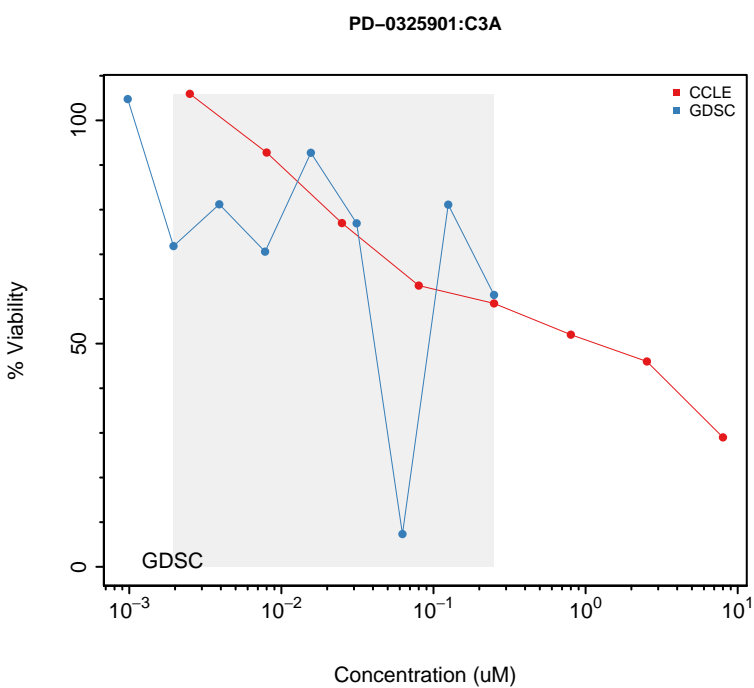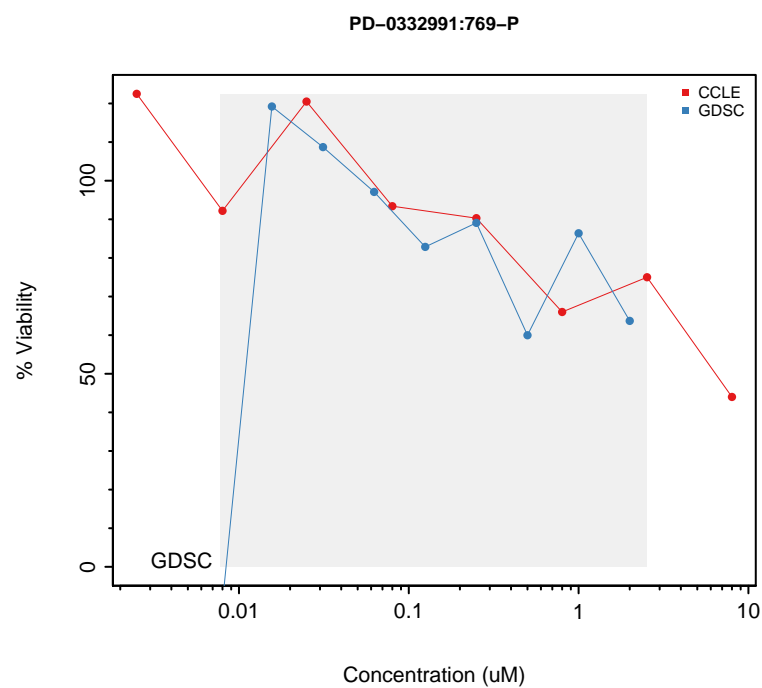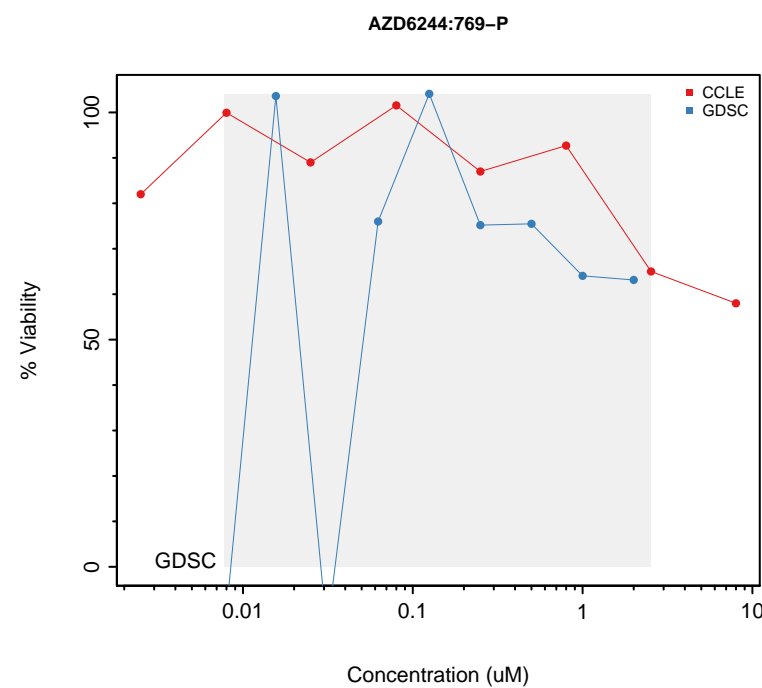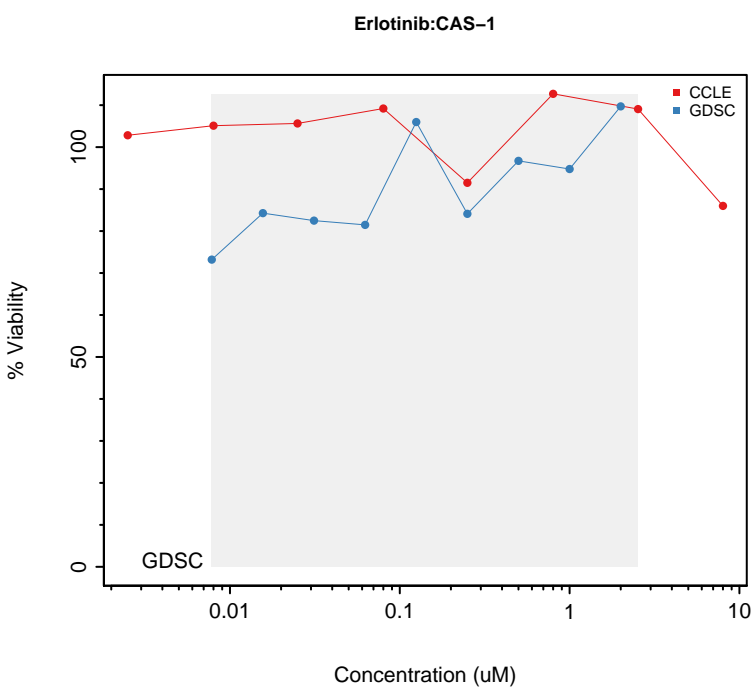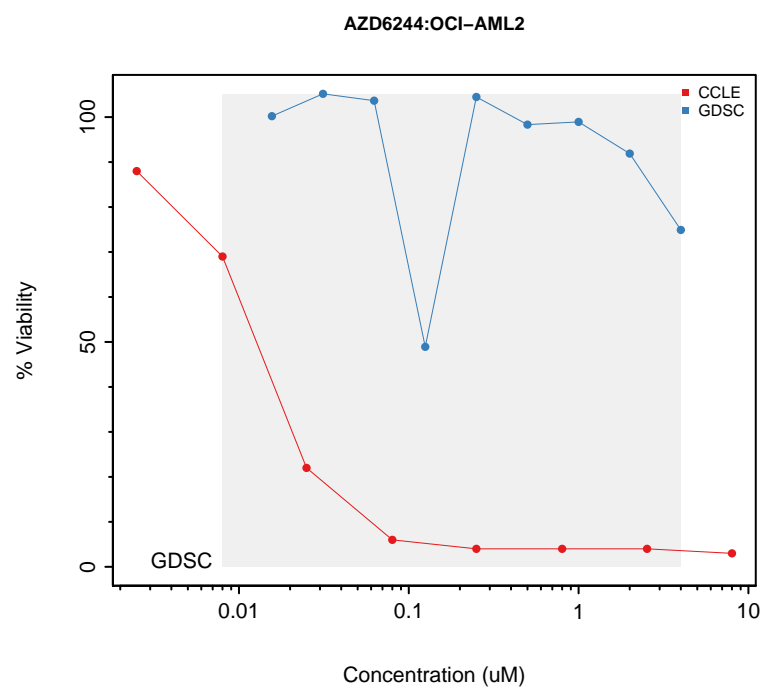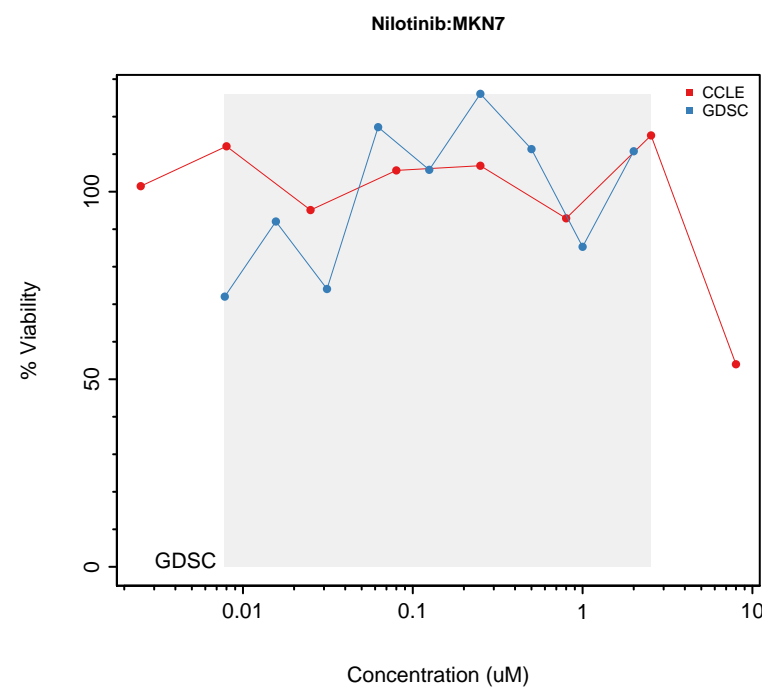

Nilotinib:MDA-MB-157

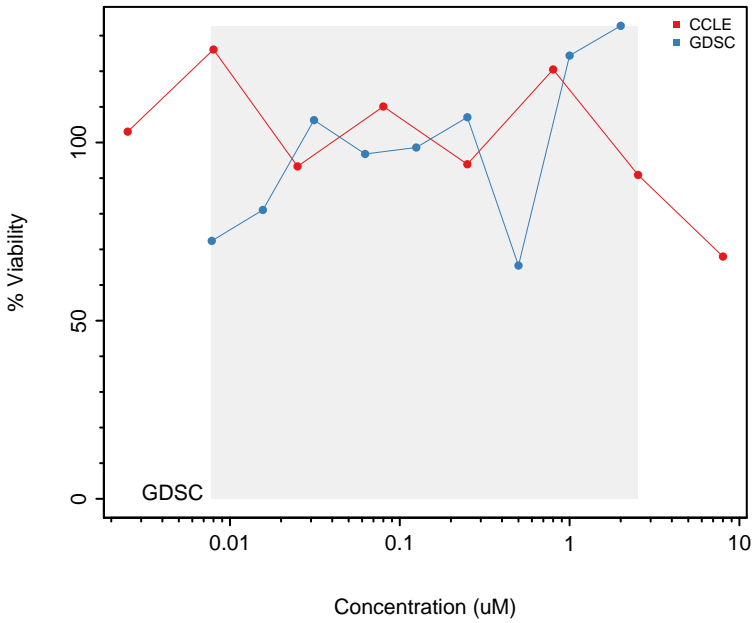

Nutlin-3:MDA-MB-157

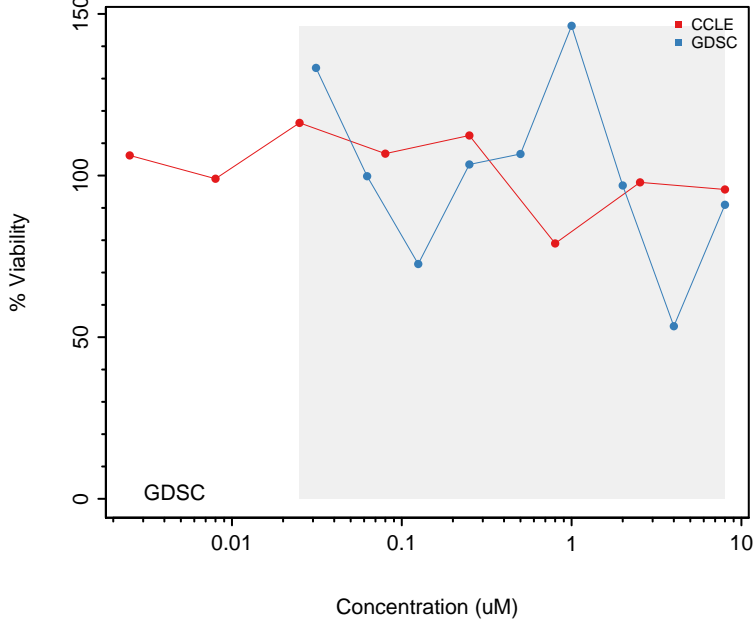

AZD6244:MDA-MB-157

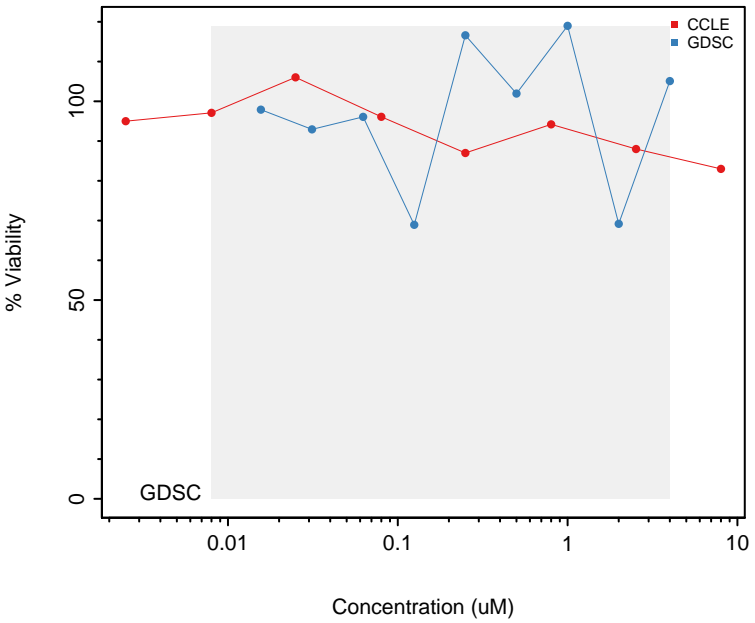

PD-0332991:Panc 10.05

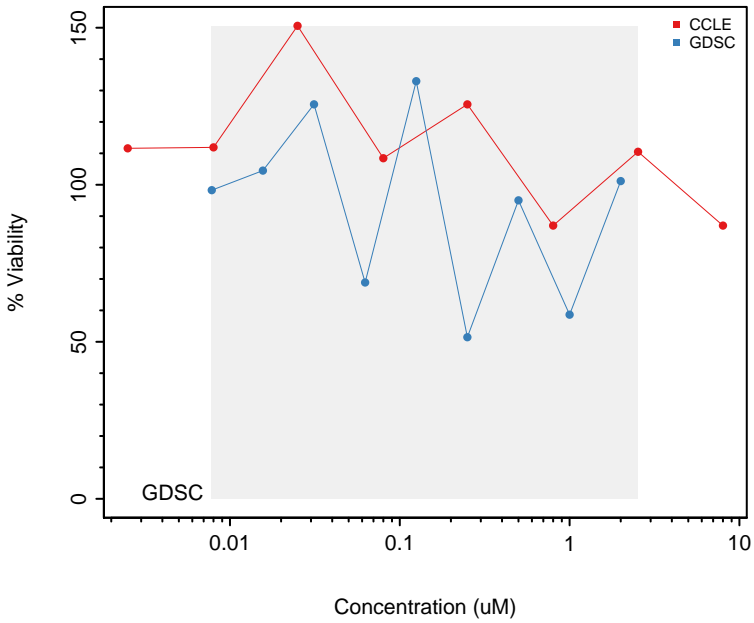

AZD6244:TE-9

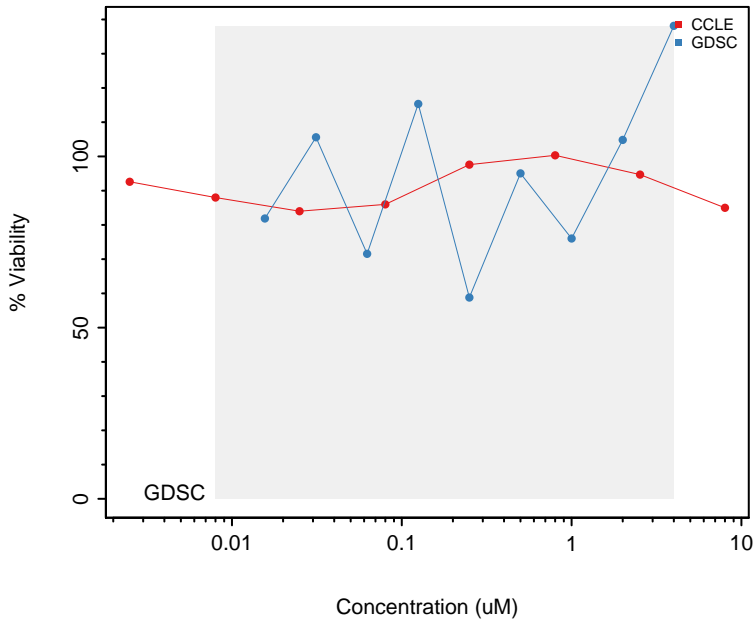

Nutlin-3:BT-474

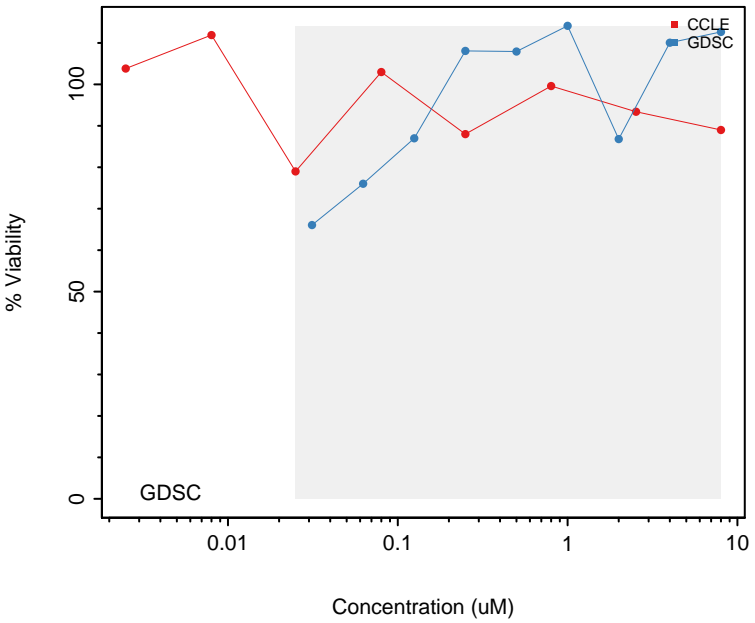

Supplement: Supplementary file 1 [file f1000research-5-13399-s0000.tgz › 394d66a3-b043-4008-8bed-cc7a9b955ae9.pdf]
