# Supplementary material for: Revisiting inconsistency in large pharmacogenomic studies
Source: F1000Res. 2017 Aug 11;5:2333. Originally published 2016 Sep 16. [Version 3] doi: 10.12688/f1000research.9611.3 (PMC5580432; doi:10.12688/f1000research.9611.3)
Supplement: Supplementary file 2 [file f1000research-5-13399-s0001.tgz › c5d8cf68-ad71-47a2-9275-03e5d8e36339.pdf]

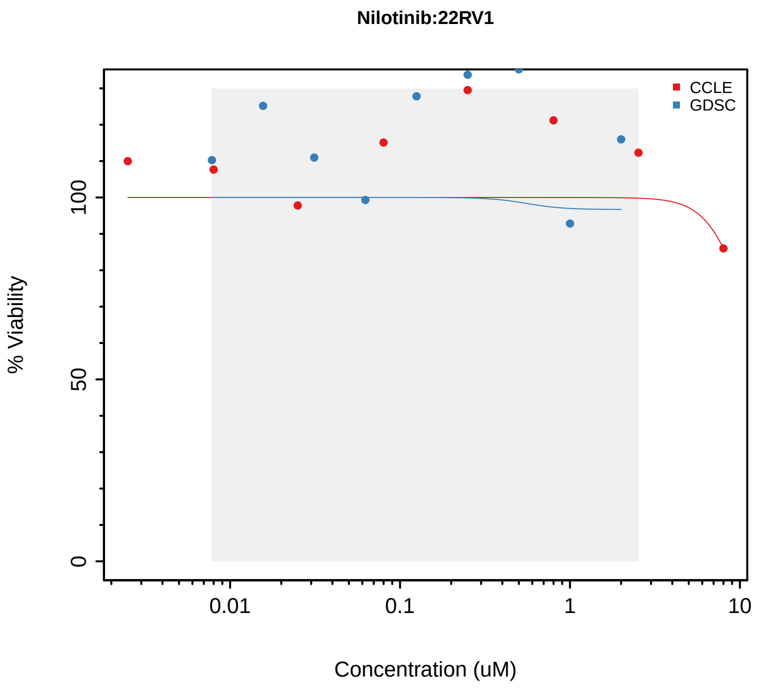
Nilotinib:22RV1
CCLE
GDSC
% Viability
0
50
100
0.01
0.1
1
10
Concentration (uM)

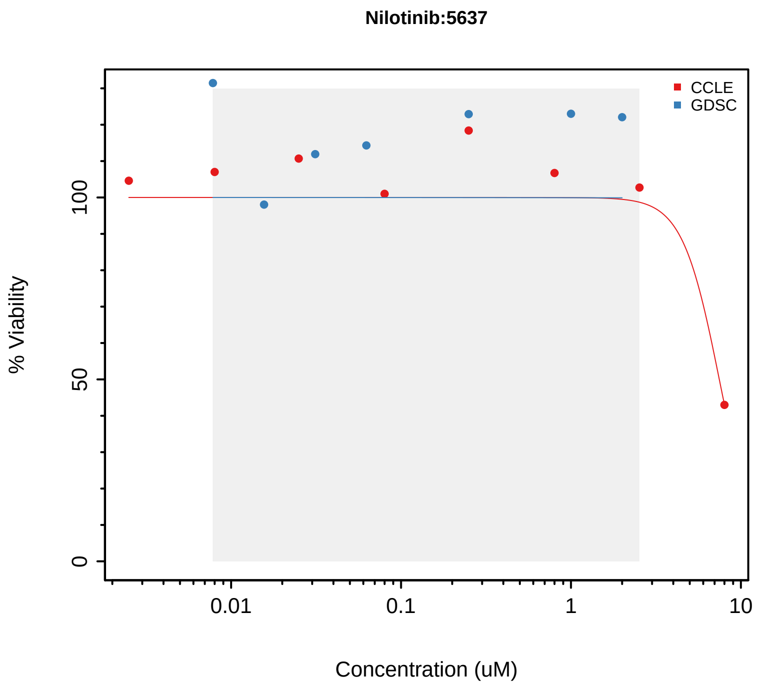
Nilotinib:5637
CCLE
GDSC
% Viability
0
50
100
0.01
0.1
1
10
Concentration (uM)

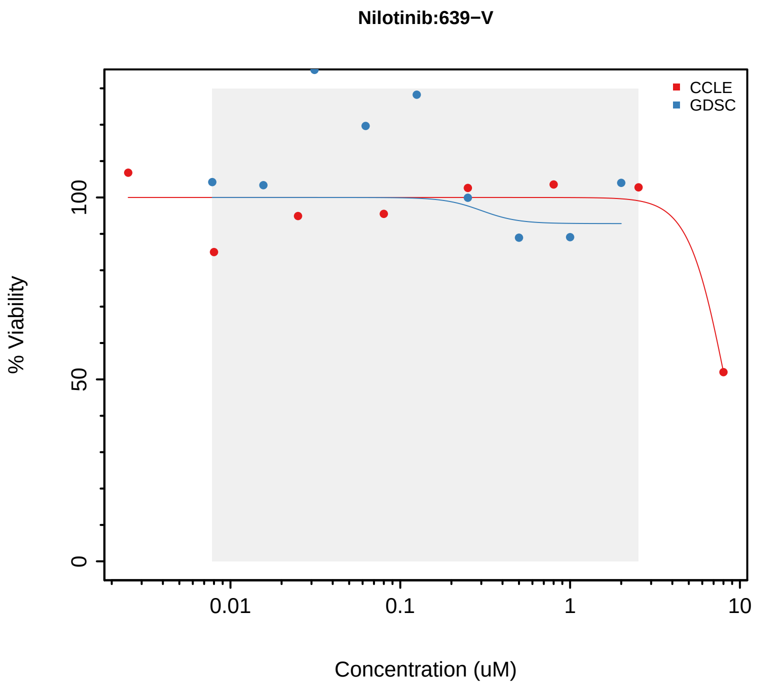
Nilotinib:639−V
CCLE
GDSC
% Viability
0
50
100
0.01
0.1
1
10
Concentration (uM)

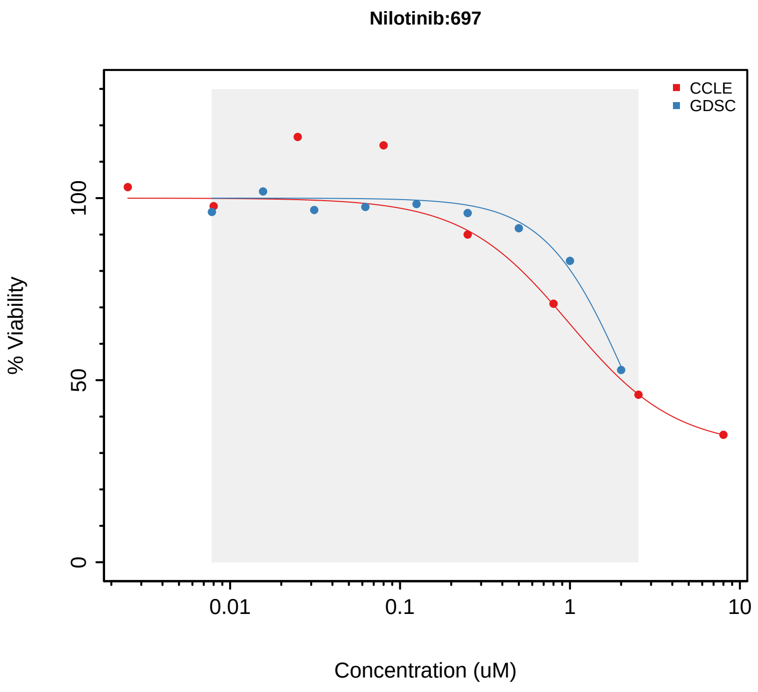
Nilotinib:697
CCLE
GDSC
% Viability
0
50
100
0.01
0.1
1
10
Concentration (uM)

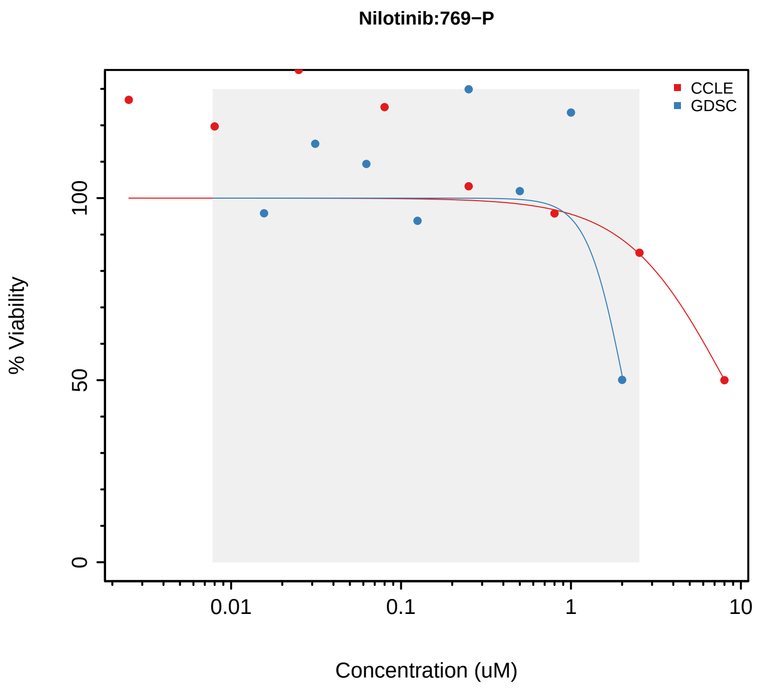
Nilotinib:769−P
CCLE
GDSC
% Viability
0
50
100
0.01
0.1
1
10
Concentration (uM)

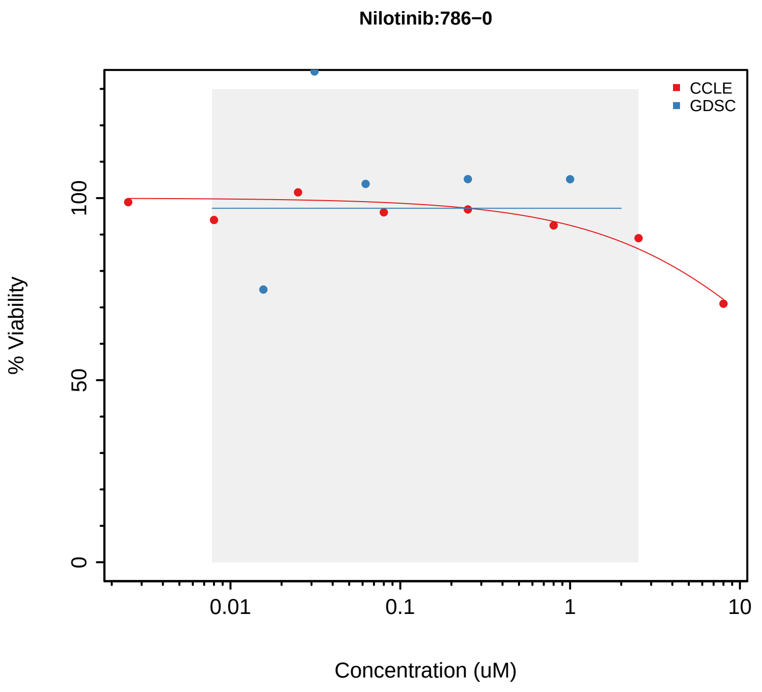
Nilotinib:786−0
CCLE
GDSC
% Viability
0
50
100
0.01
0.1
1
10
Concentration (uM)

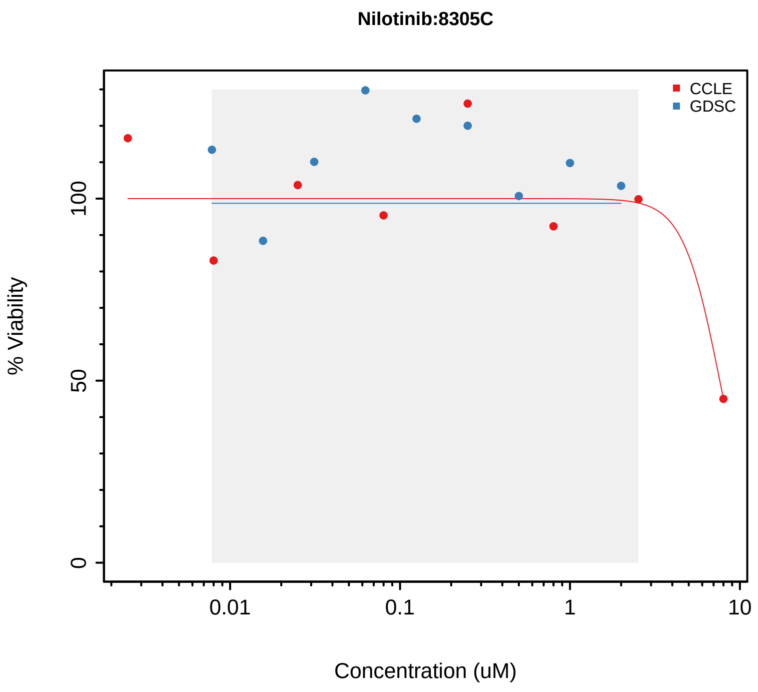
Nilotinib:8305C
CCLE
GDSC
% Viability
0
50
100
0.01
0.1
1
10
Concentration (uM)

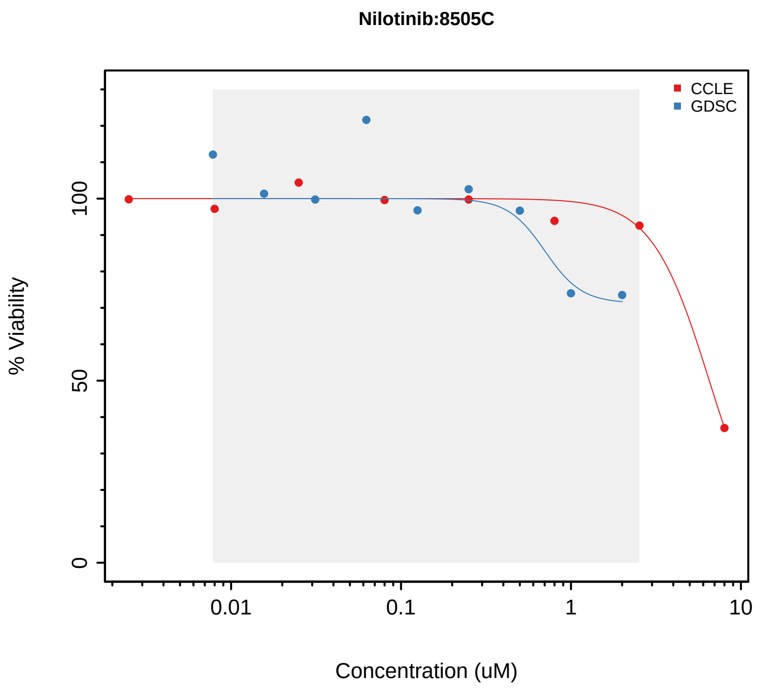
Nilotinib:8505C
CCLE
GDSC
% Viability
0
50
100
0.01
0.1
1
10
Concentration (uM)

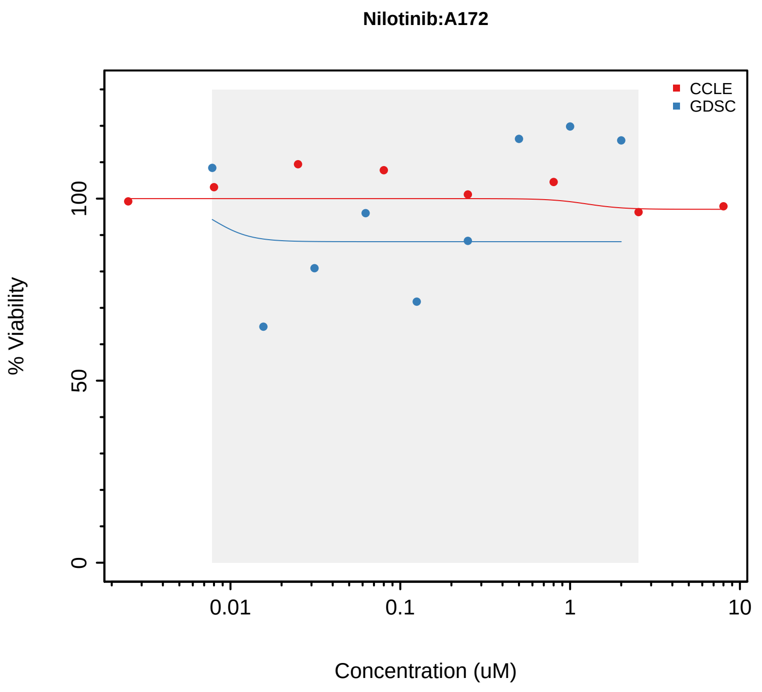
Nilotinib:A172
CCLE
GDSC
% Viability
0
50
100
0.01
0.1
1
10
Concentration (uM)

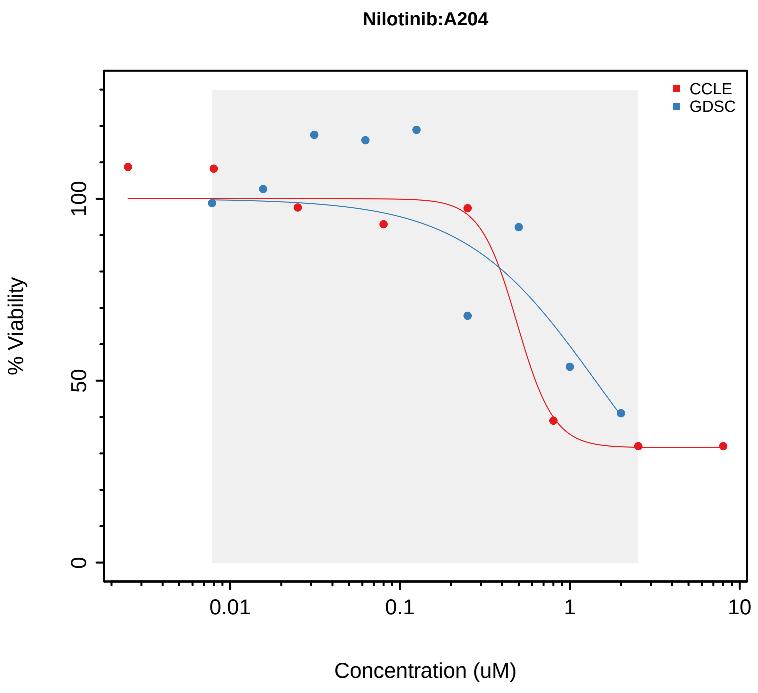
Nilotinib:A204
CCLE
GDSC
% Viability
0
50
100
0.01
0.1
1
10
Concentration (uM)

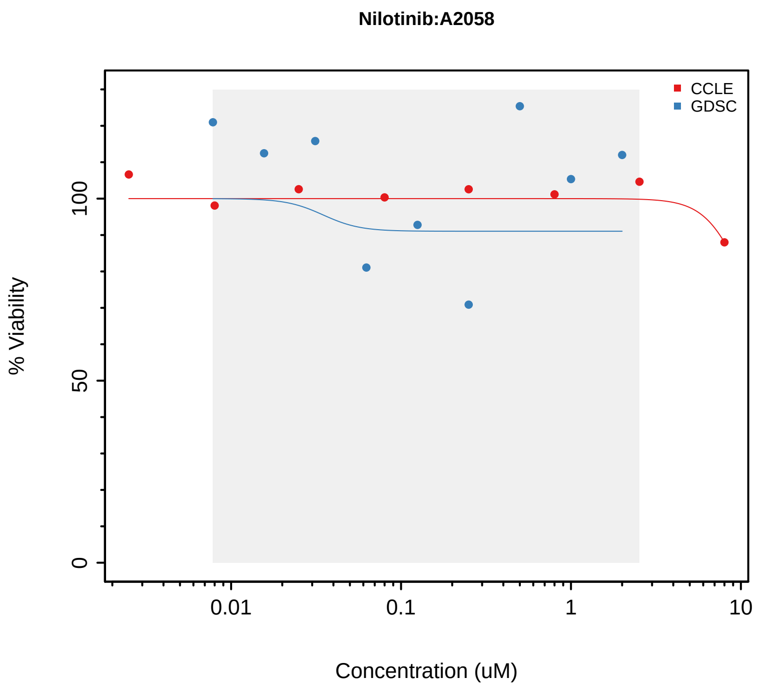
Nilotinib:A2058
CCLE
GDSC
% Viability
0
50
100
0.01
0.1
1
10
Concentration (uM)

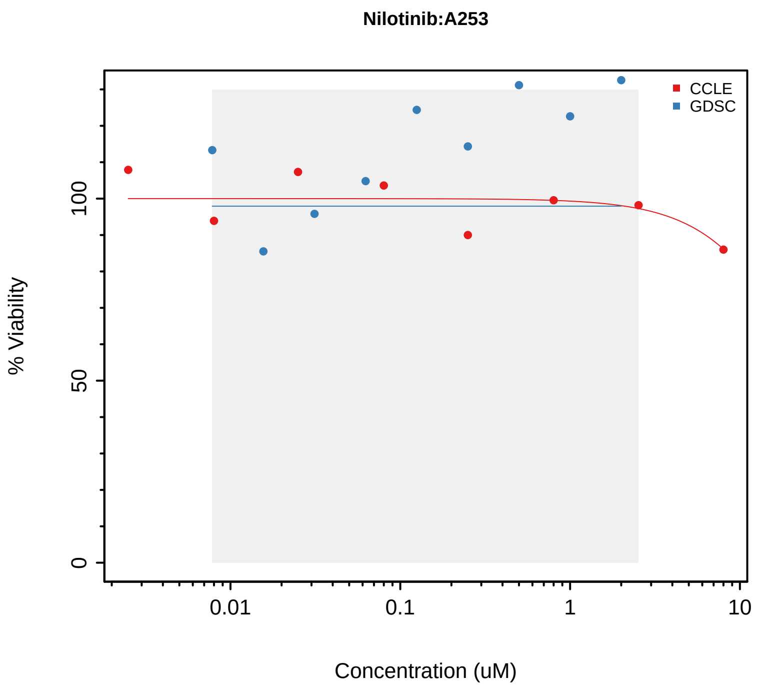
Nilotinib:A253
CCLE
GDSC
% Viability
0
50
100
0.01
0.1
1
10
Concentration (uM)

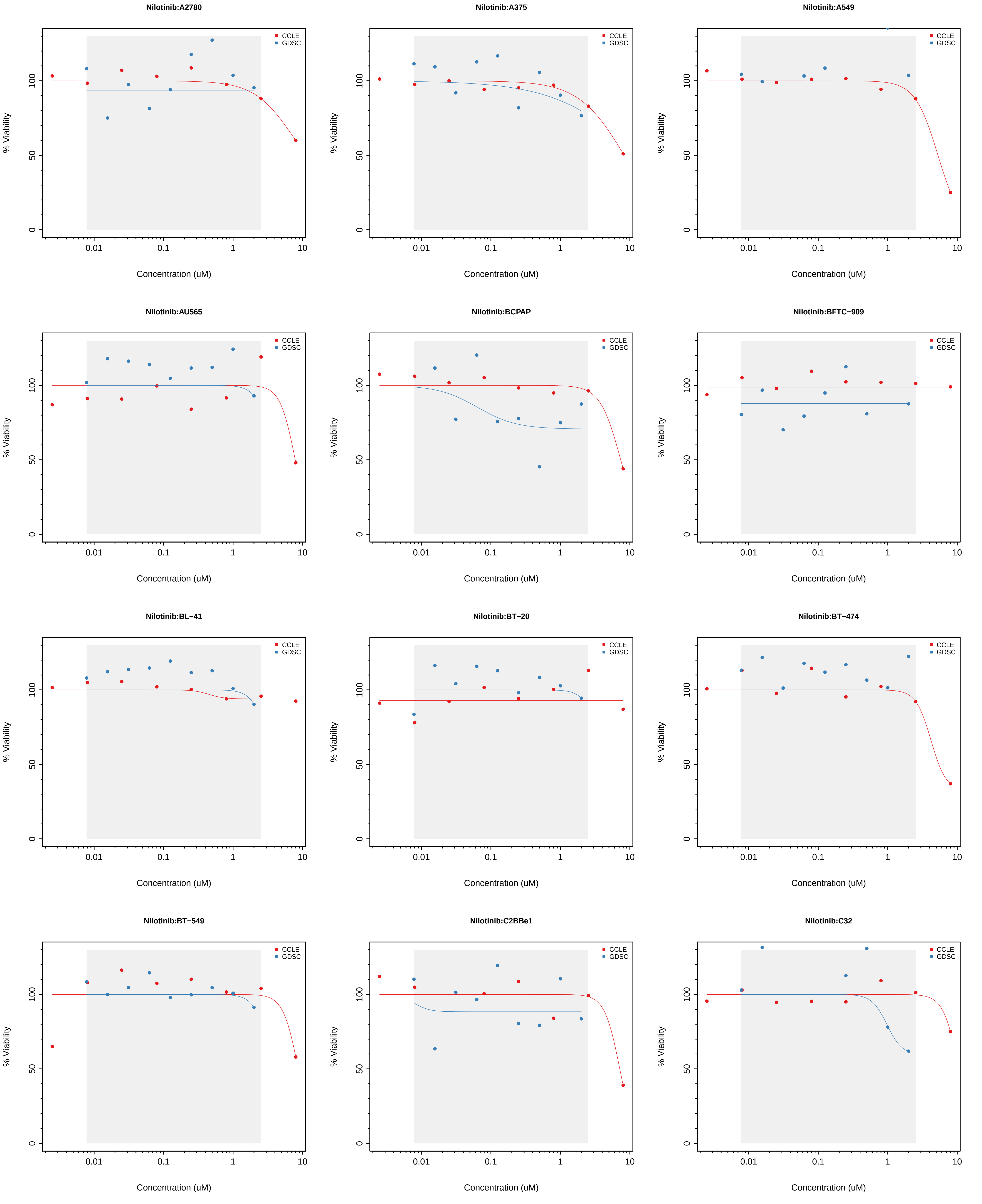

Nilotinib:A2780
Nilotinib:A375
Nilotinib:A549
Nilotinib:AU565
Nilotinib:BCPAP
Nilotinib:BFTC−909
Nilotinib:BL−41
Nilotinib:BT−20
Nilotinib:BT−474
Nilotinib:BT−549
Nilotinib:C2BBe1
Nilotinib:C32
CCLE
GDSC
% Viability
0
50
100
0.01
0.1
1
10
Concentration (uM)

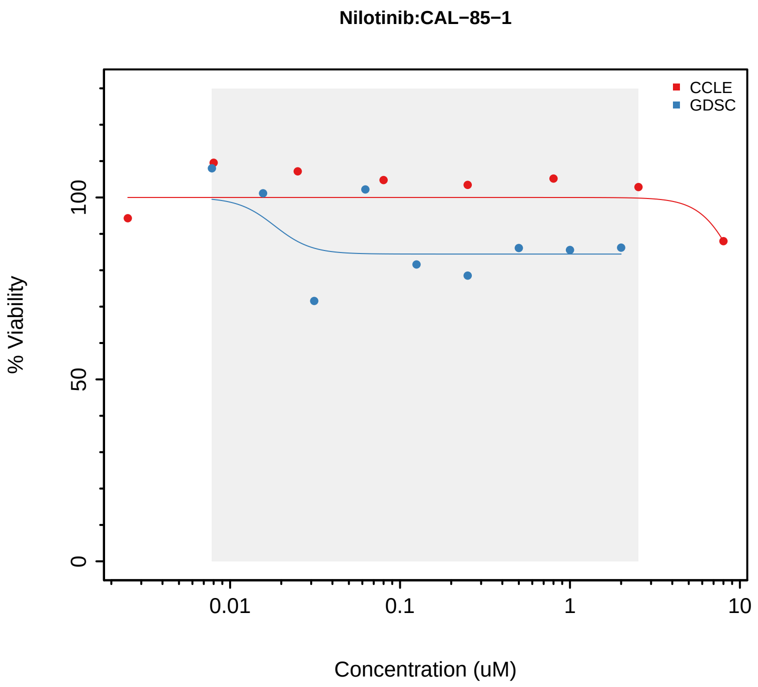
Nilotinib:CAL−85−1
CCLE
GDSC
% Viability
0
50
100
0.01
0.1
1
10
Concentration (uM)

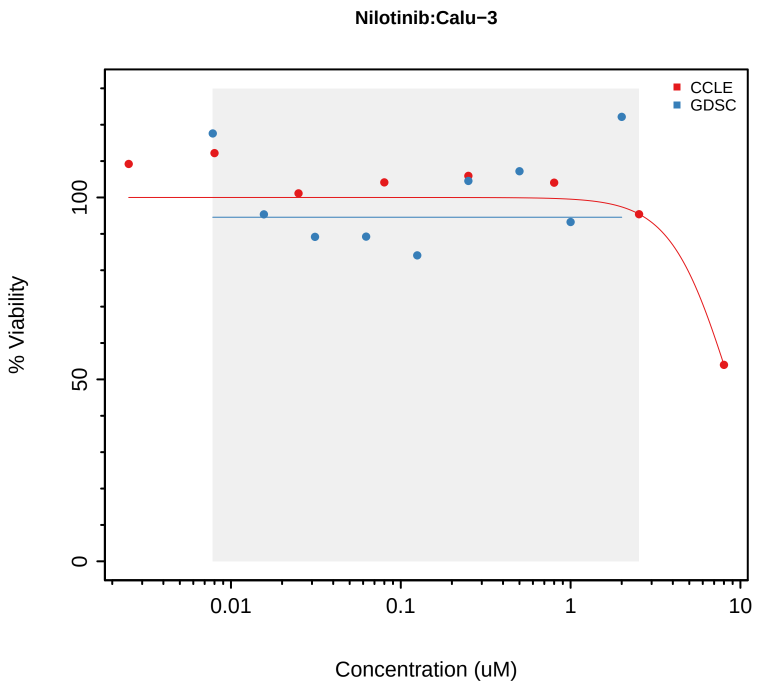
Nilotinib:Calu−3
CCLE
GDSC
% Viability
0
50
100
0.01
0.1
1
10
Concentration (uM)

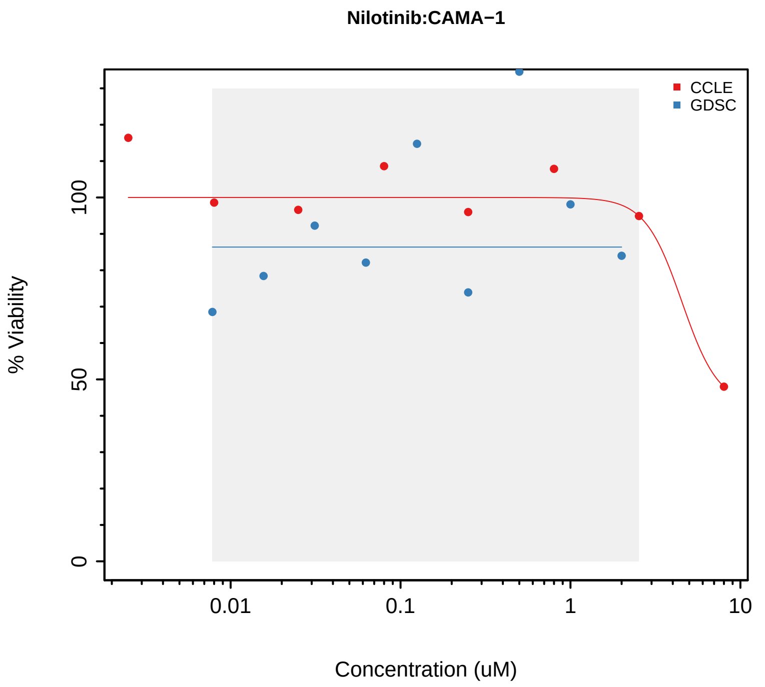
Nilotinib:CAMA−1
CCLE
GDSC
% Viability
0
50
100
0.01
0.1
1
10
Concentration (uM)

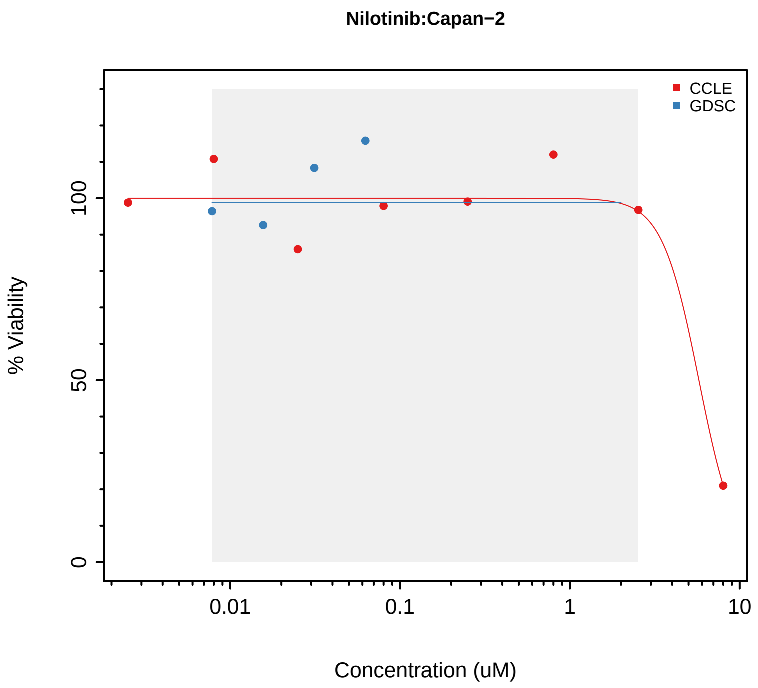
Nilotinib:Capan−2
CCLE
GDSC
% Viability
0
50
100
0.01
0.1
1
10
Concentration (uM)

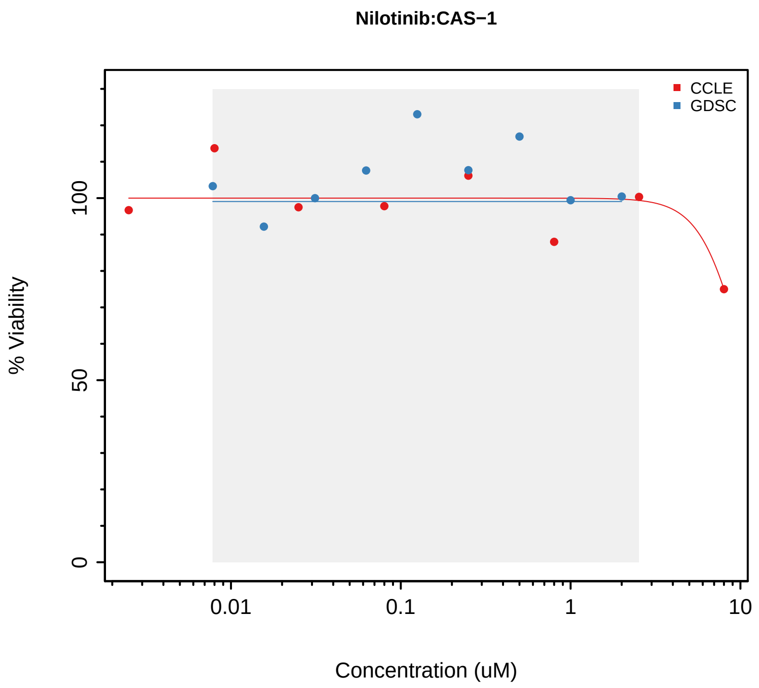
Nilotinib:CAS−1
CCLE
GDSC
% Viability
0
50
100
0.01
0.1
1
10
Concentration (uM)

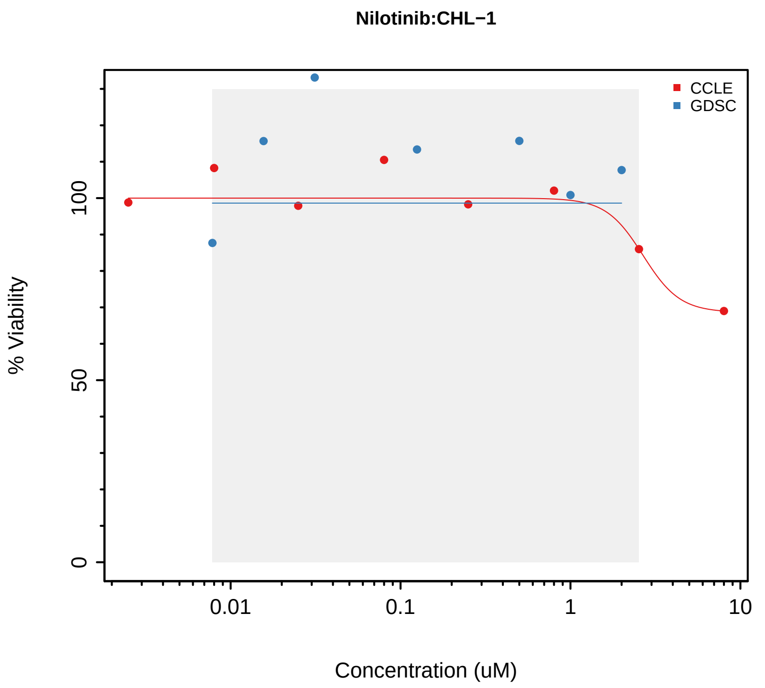
Nilotinib:CHL−1
CCLE
GDSC
% Viability
0
50
100
0.01
0.1
1
10
Concentration (uM)

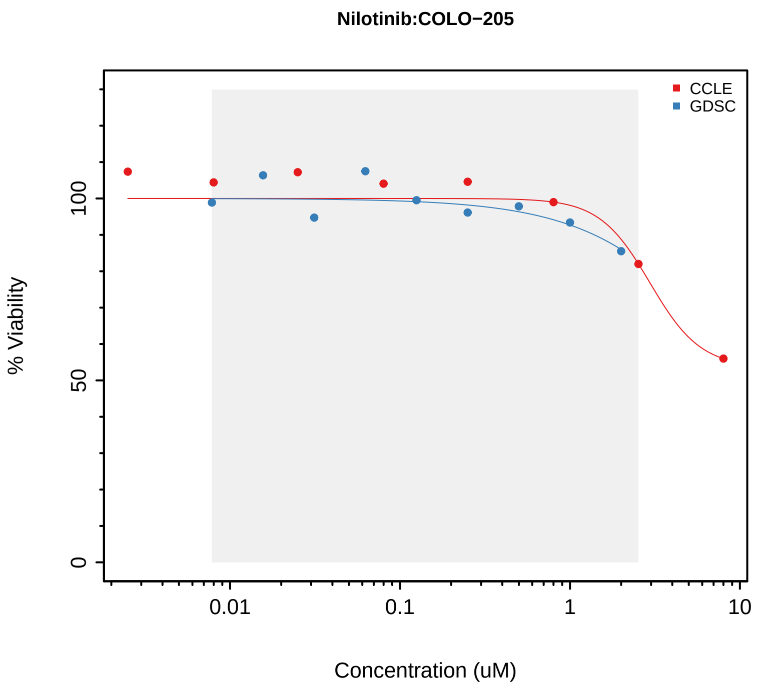
Nilotinib:COLO−205
CCLE
GDSC
% Viability
0
50
100
0.01
0.1
1
10
Concentration (uM)

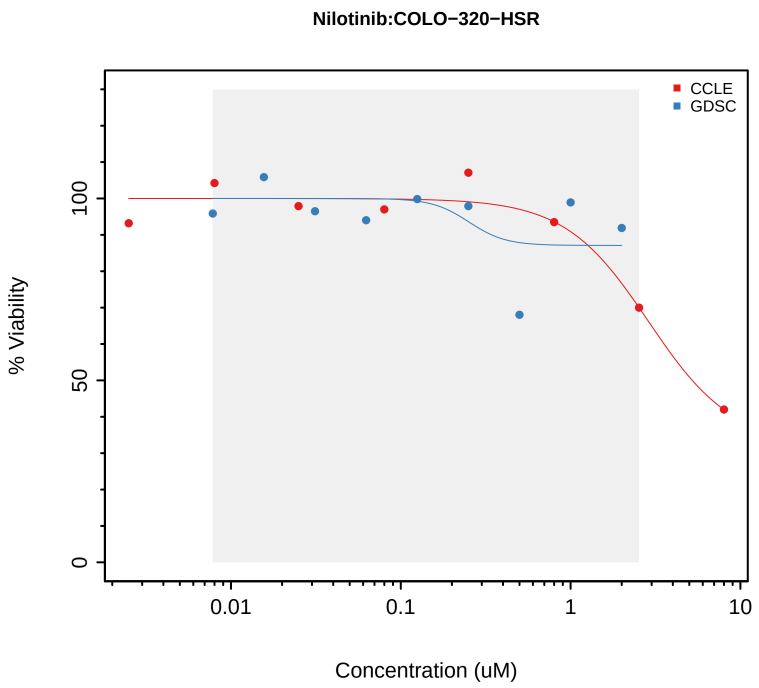
Nilotinib:COLO−320−HSR
CCLE
GDSC
% Viability
0
50
100
0.01
0.1
1
10
Concentration (uM)

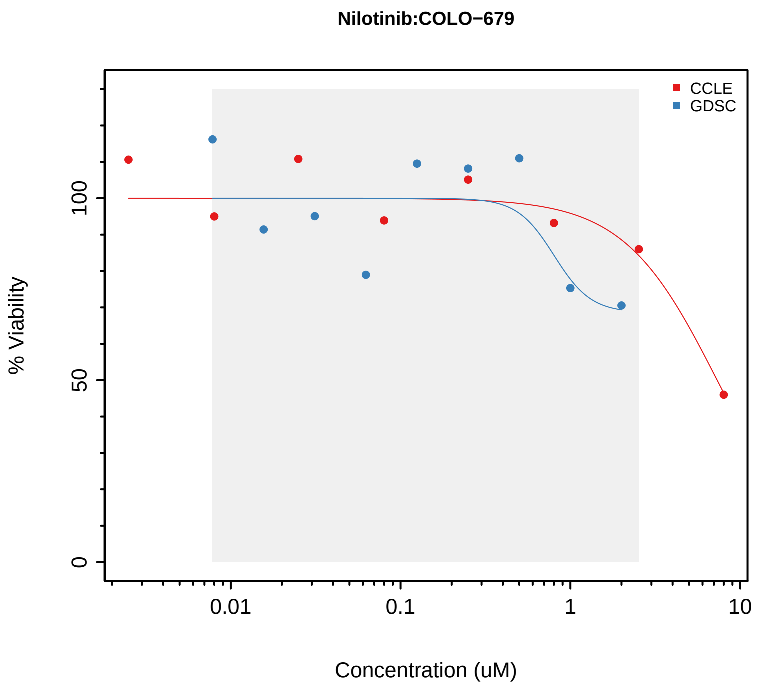
Nilotinib:COLO−679
CCLE
GDSC
% Viability
0
50
100
0.01
0.1
1
10
Concentration (uM)

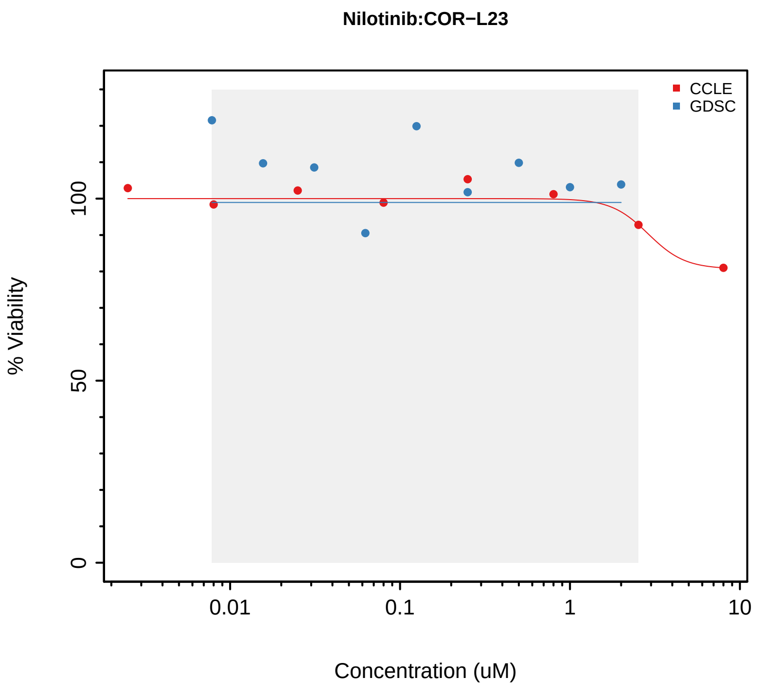
Nilotinib:COR−L23
CCLE
GDSC
% Viability
0
50
100
0.01
0.1
1
10
Concentration (uM)

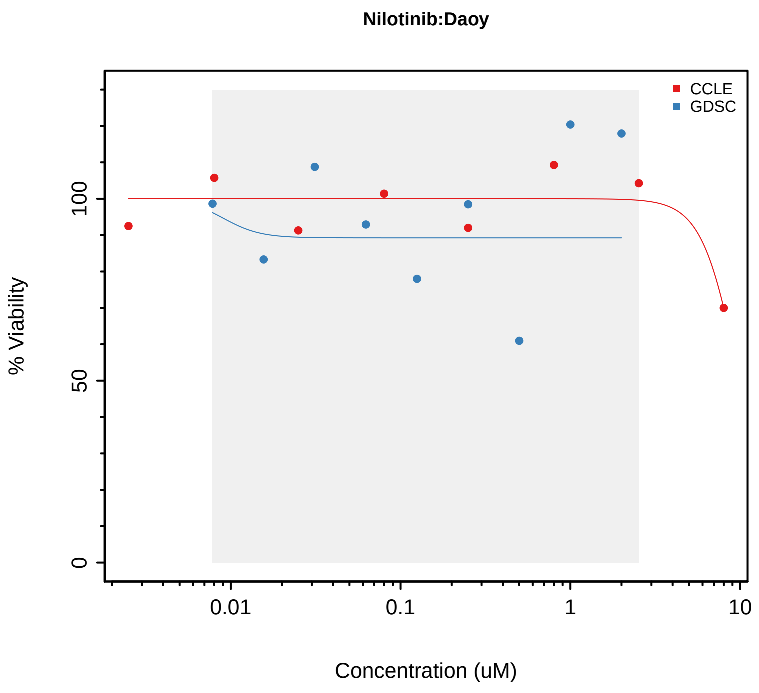
Nilotinib:Daoy
CCLE
GDSC
% Viability
0
50
100
0.01
0.1
1
10
Concentration (uM)

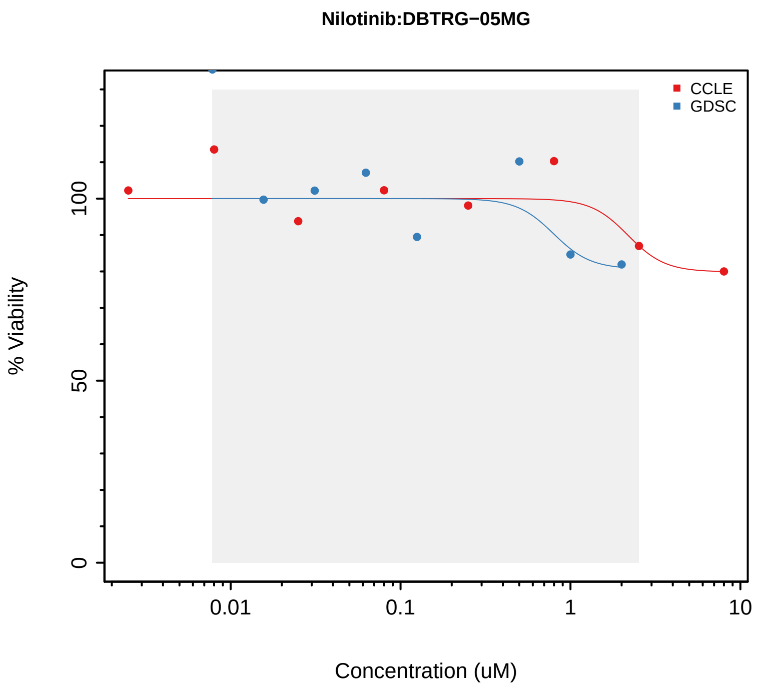
Nilotinib:DBTRG−05MG
CCLE
GDSC
% Viability
0
50
100
0.01
0.1
1
10
Concentration (uM)

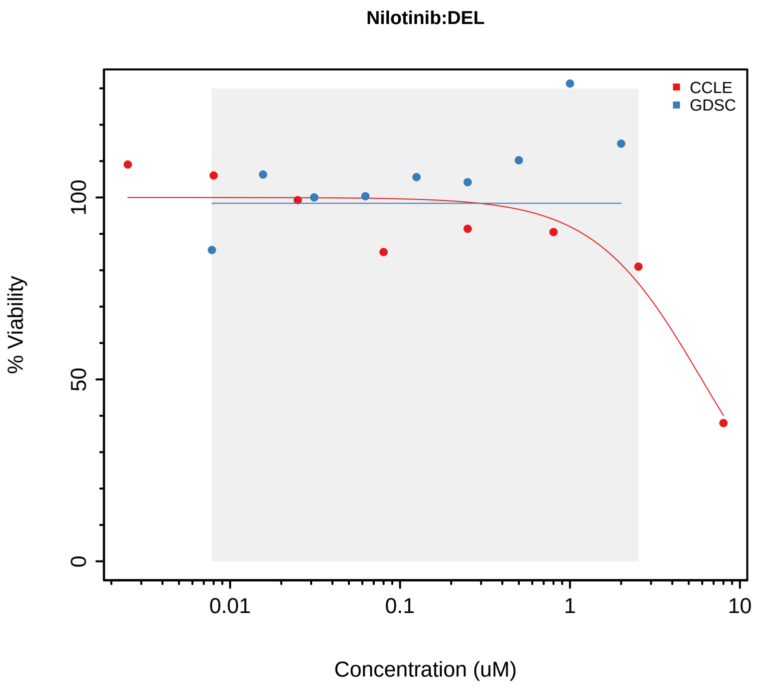
Nilotinib:DEL
CCLE
GDSC
% Viability
0
50
100
0.01
0.1
1
10
Concentration (uM)

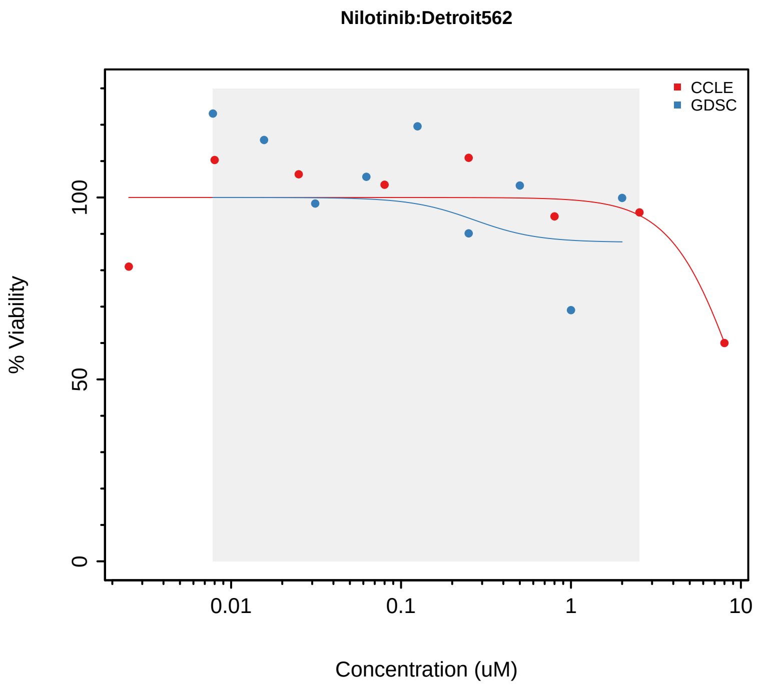
Nilotinib:Detroit562
CCLE
GDSC
% Viability
0
50
100
0.01
0.1
1
10
Concentration (uM)

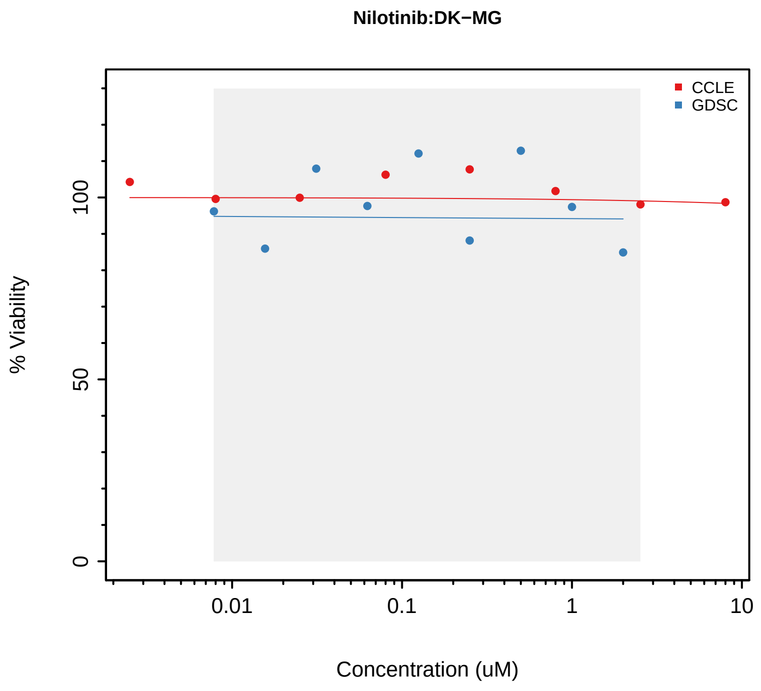
Nilotinib:DK−MG
CCLE
GDSC
% Viability
0
50
100
0.01
0.1
1
10
Concentration (uM)

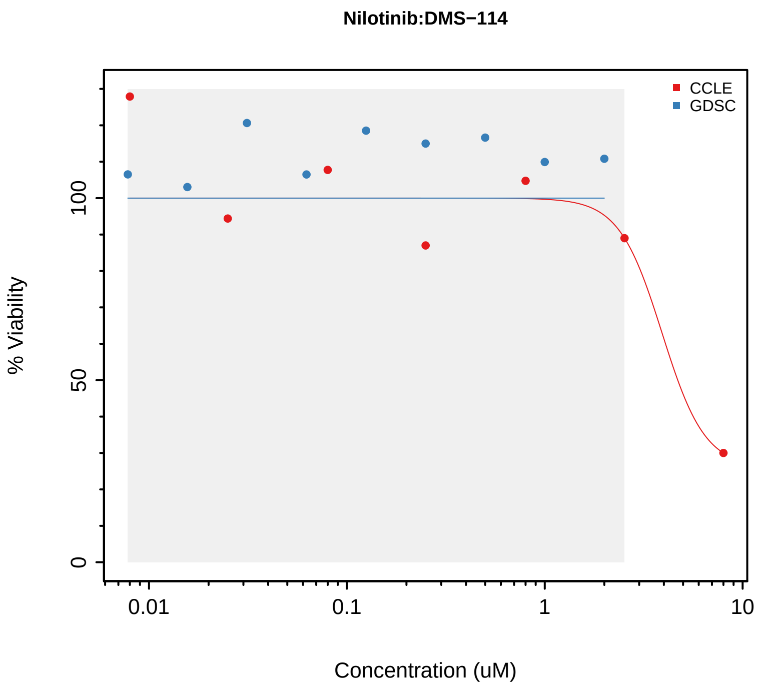
Nilotinib:DMS−114
CCLE
GDSC
% Viability
0
50
100
0.01
0.1
1
10
Concentration (uM)

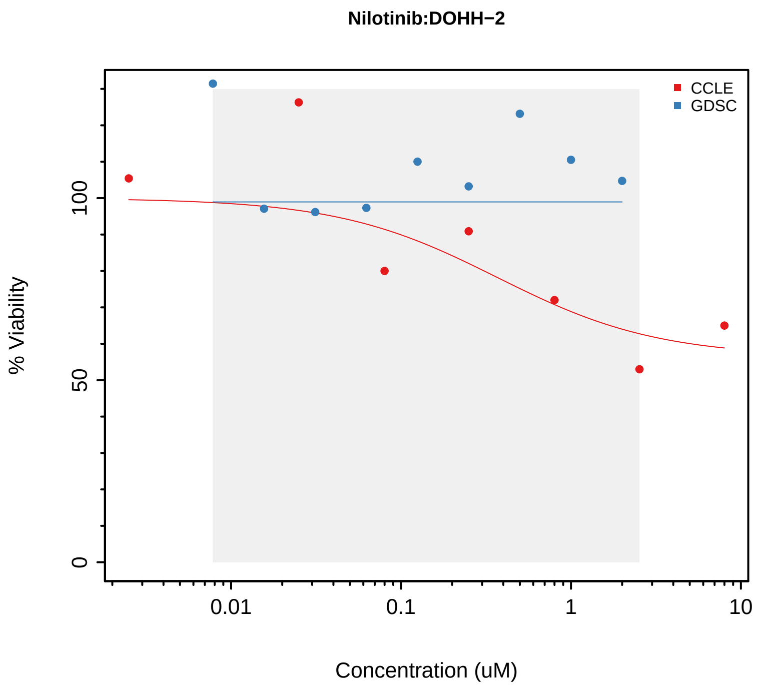
Nilotinib:DOHH−2
CCLE
GDSC
% Viability
0
50
100
0.01
0.1
1
10
Concentration (uM)

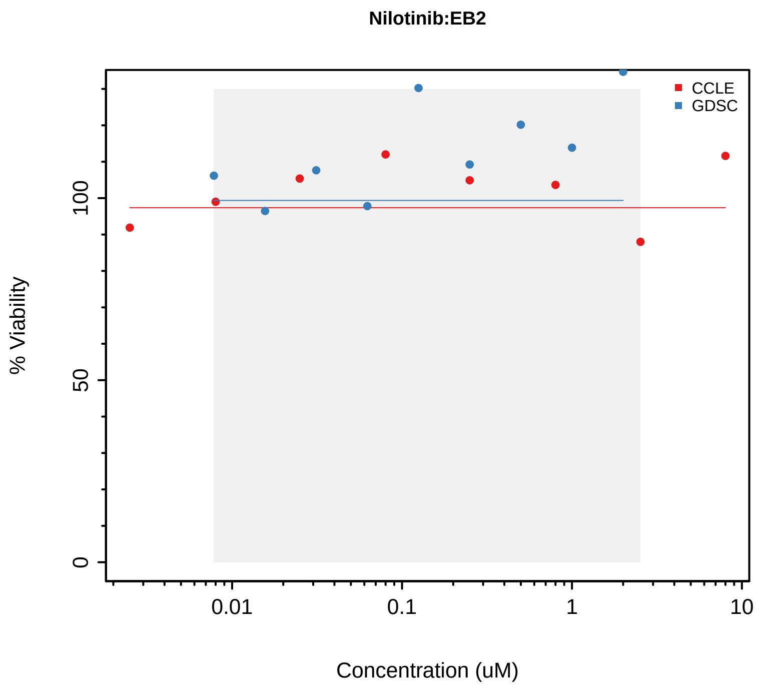
Nilotinib:EB2
CCLE
GDSC
% Viability
0
50
100
0.01
0.1
1
10
Concentration (uM)

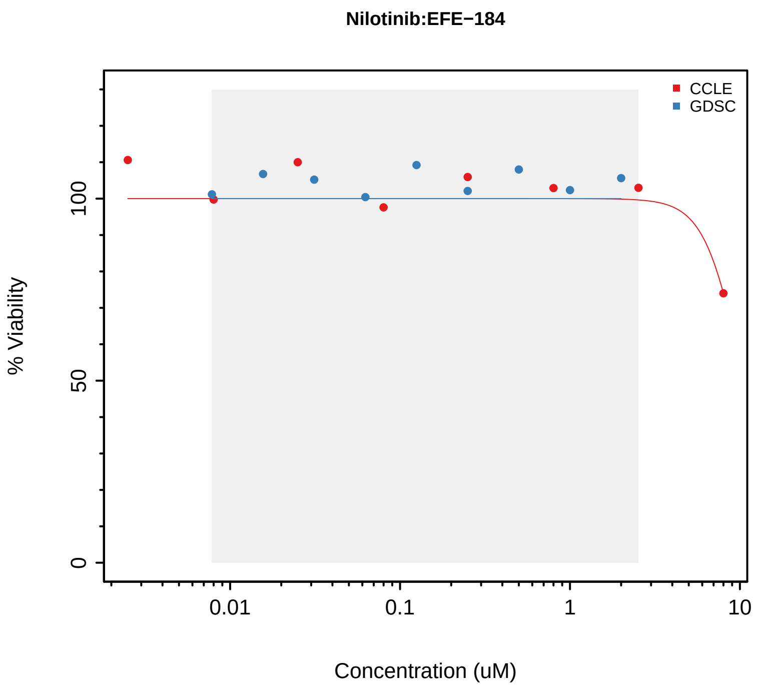
Nilotinib:EFE−184
CCLE
GDSC
% Viability
0
50
100
0.01
0.1
1
10
Concentration (uM)

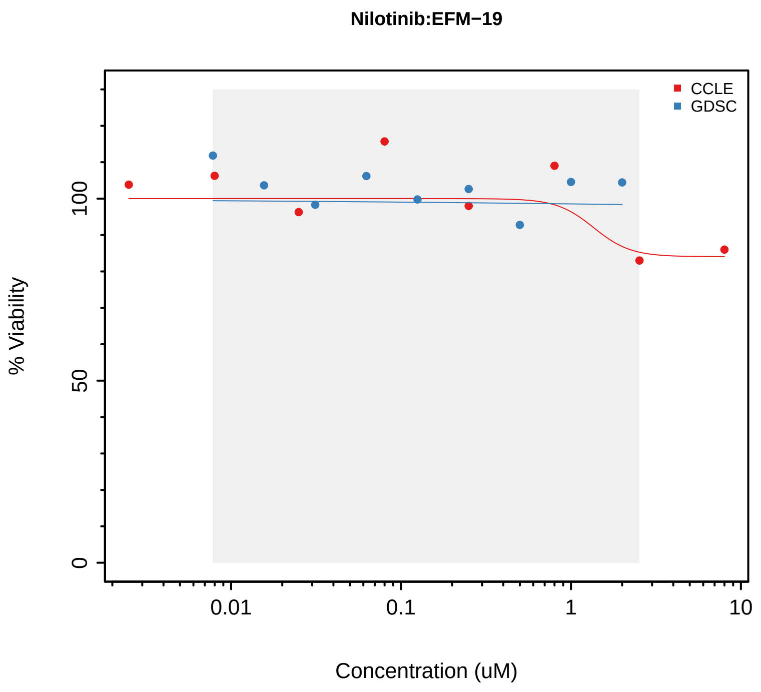
Nilotinib:EFM−19
CCLE
GDSC
% Viability
0
50
100
0.01
0.1
1
10
Concentration (uM)

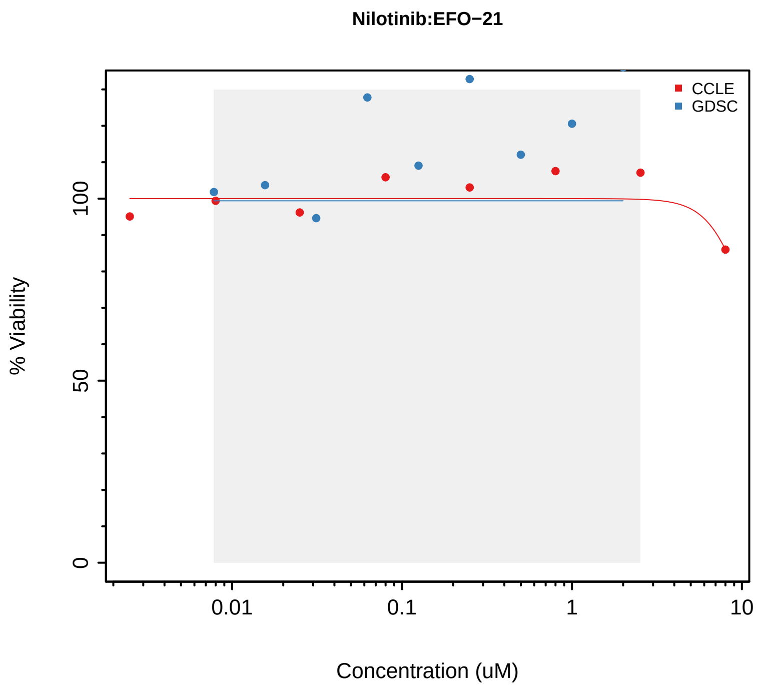
Nilotinib:EFO−21
CCLE
GDSC
% Viability
0
50
100
0.01
0.1
1
10
Concentration (uM)

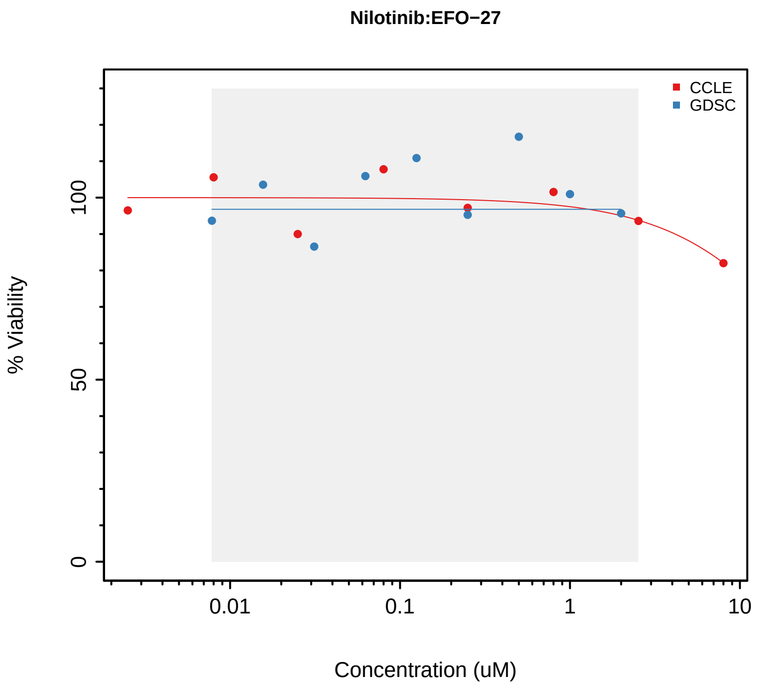
Nilotinib:EFO−27
CCLE
GDSC
% Viability
0
50
100
0.01
0.1
1
10
Concentration (uM)

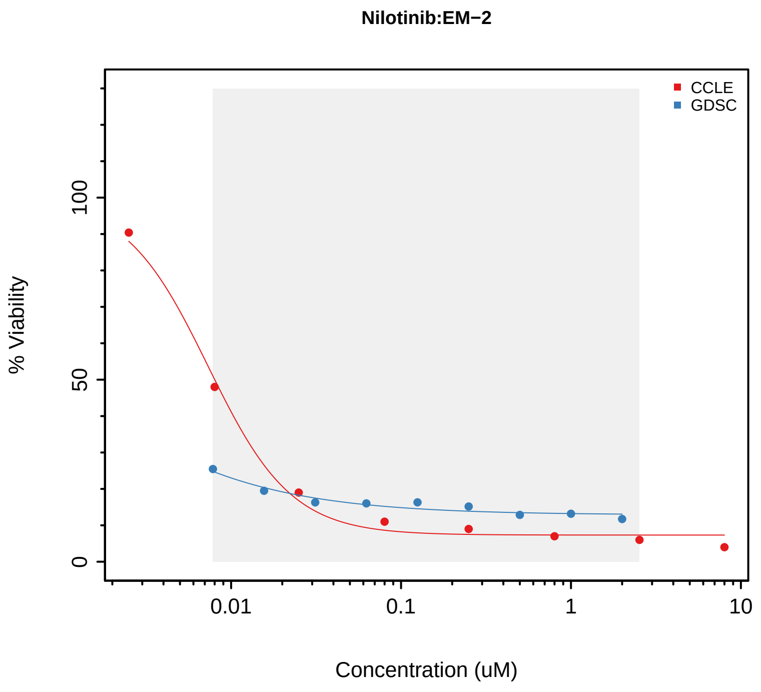
Nilotinib:EM−2
CCLE
GDSC
% Viability
0
50
100
0.01
0.1
1
10
Concentration (uM)

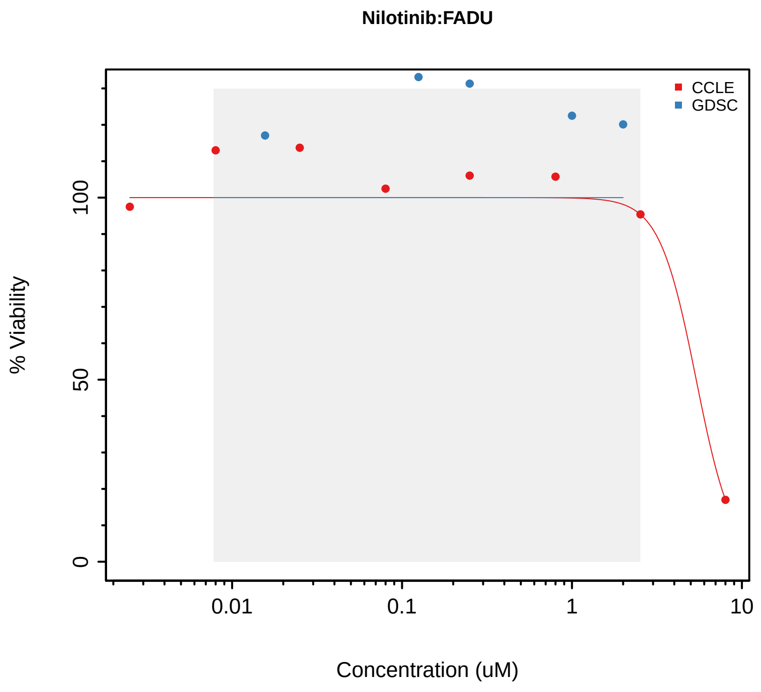
Nilotinib:FADU
CCLE
GDSC
% Viability
0
50
100
0.01
0.1
1
10
Concentration (uM)

Nilotinib:G−361

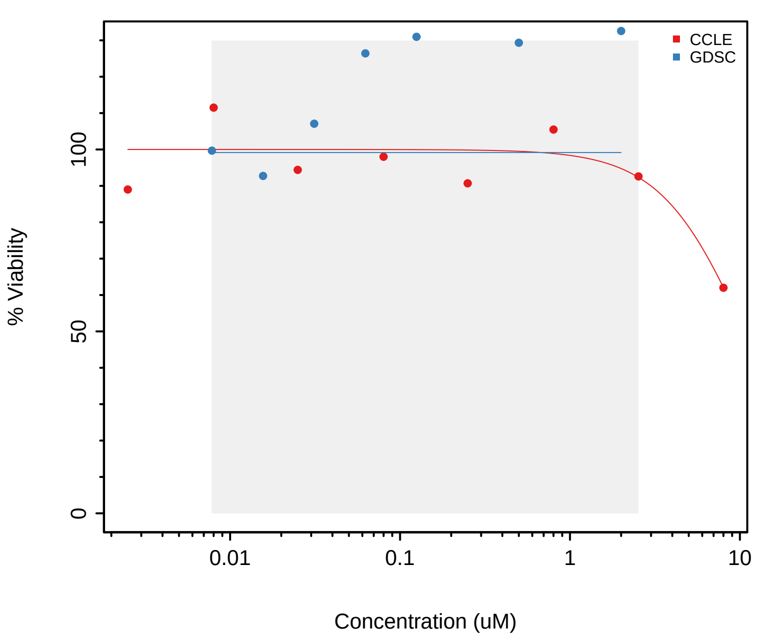

Nilotinib:G−401

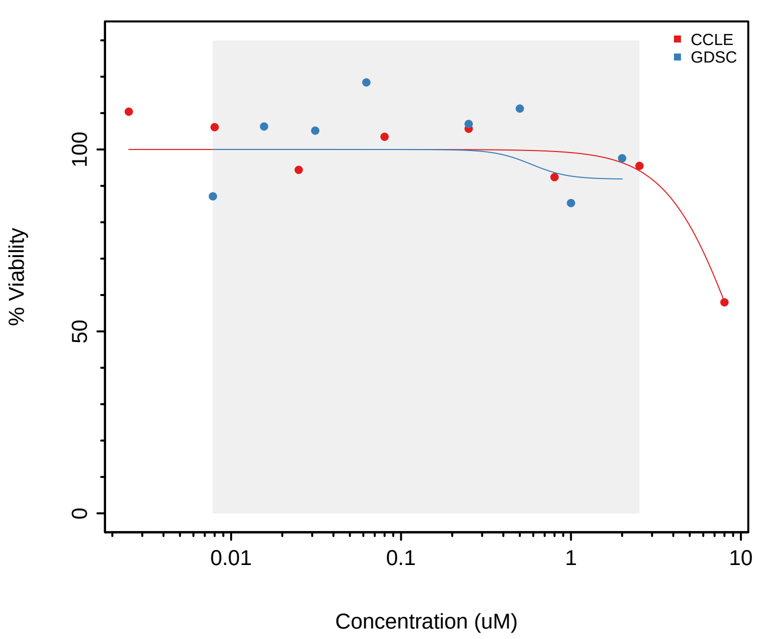

Nilotinib:GB−1

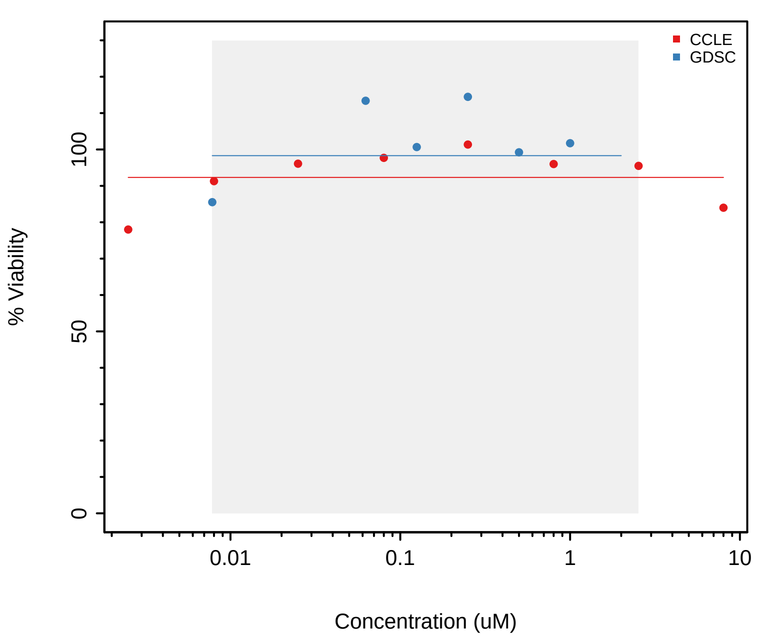

Nilotinib:GCIY

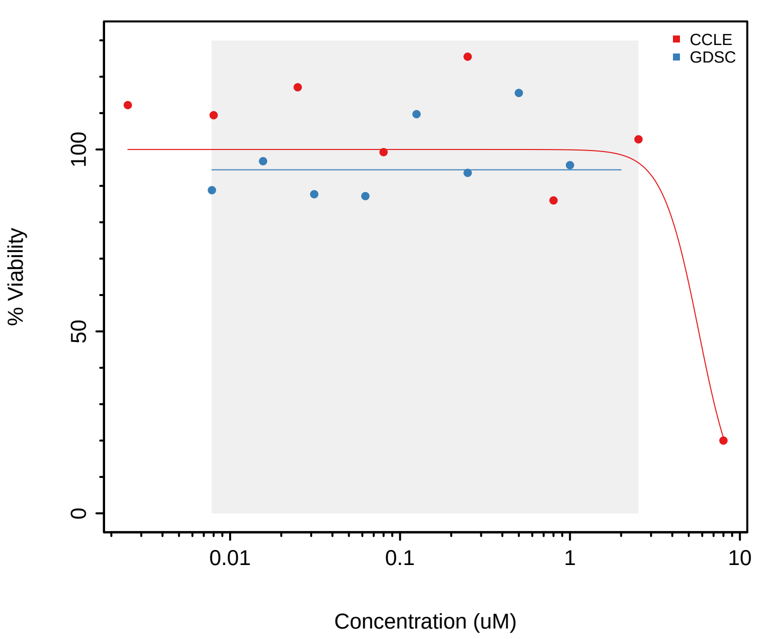

Nilotinib:GMS−10

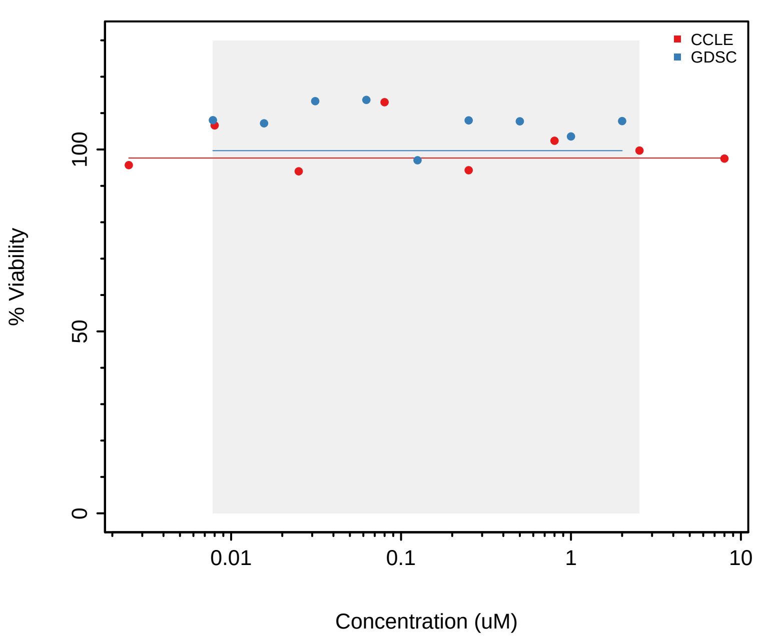

Nilotinib:H4

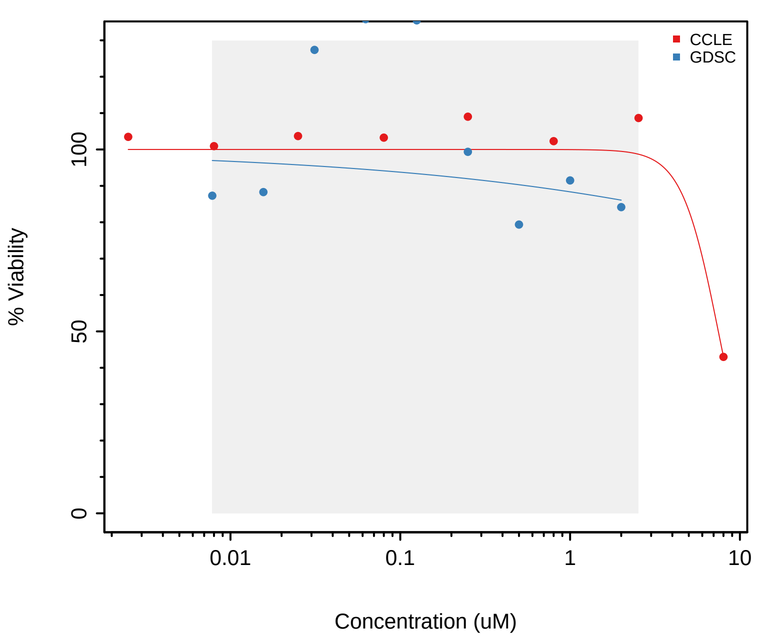

Nilotinib:HCC1187

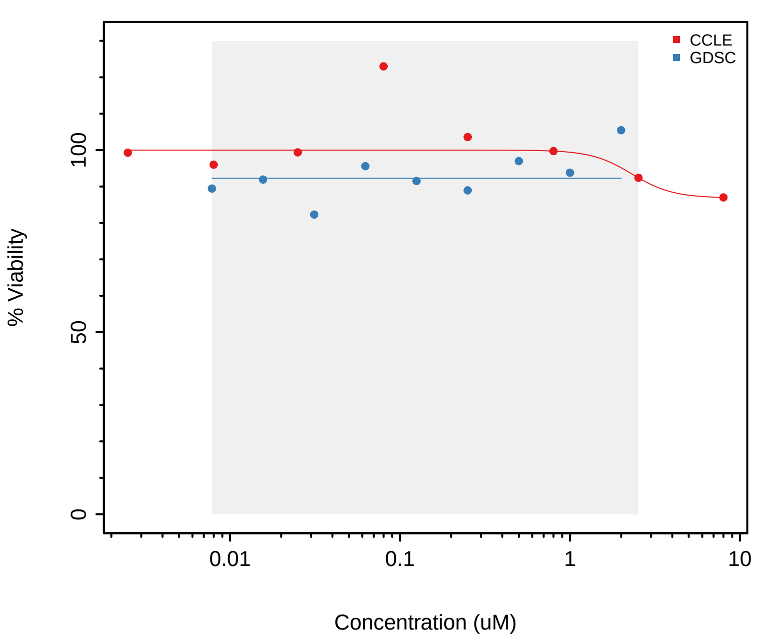

Nilotinib:HCC1569

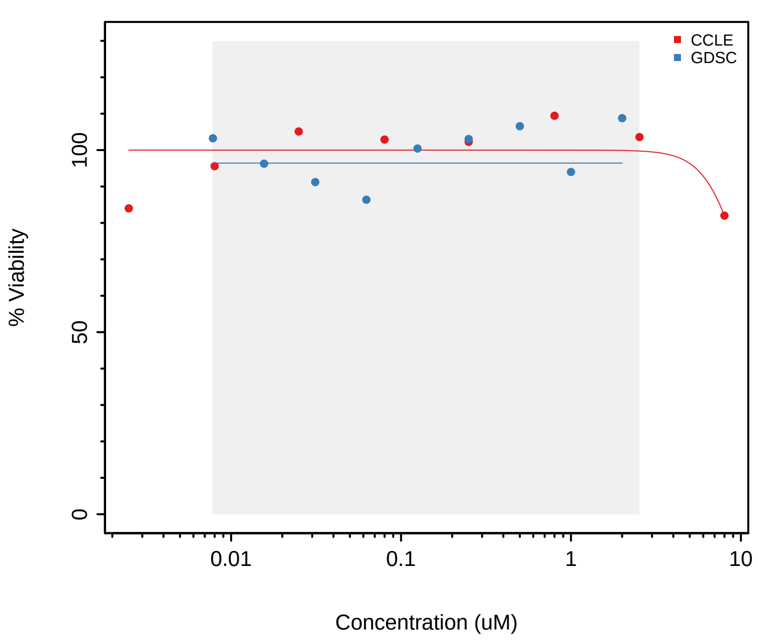

Nilotinib:HCC1806

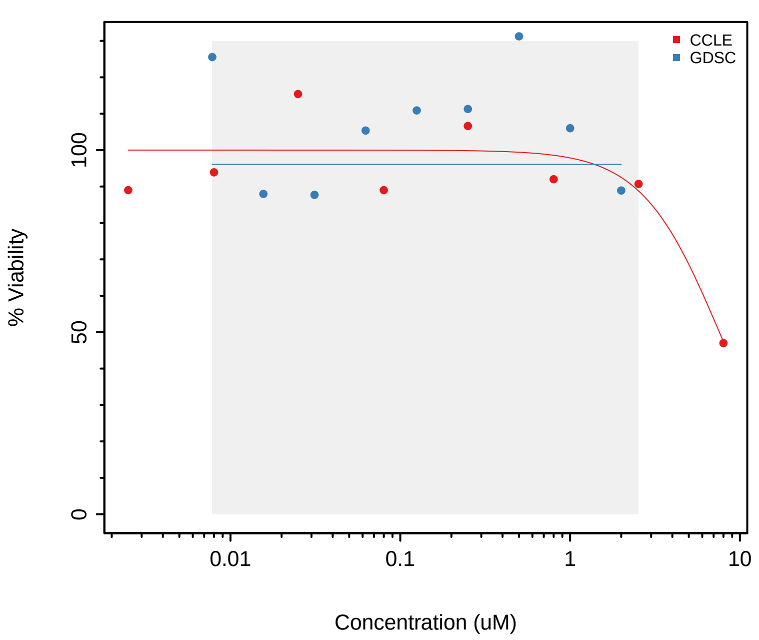

Nilotinib:HCC1954

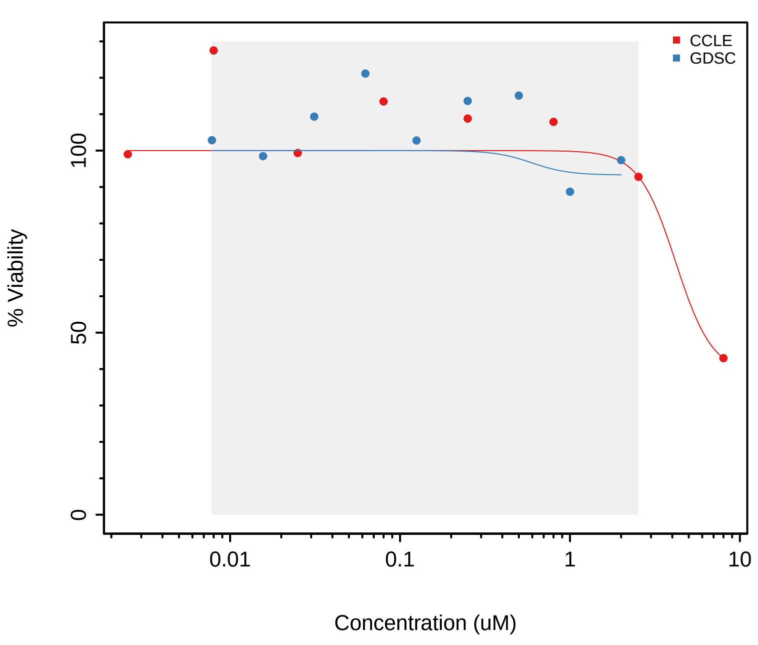

Nilotinib:HCC70

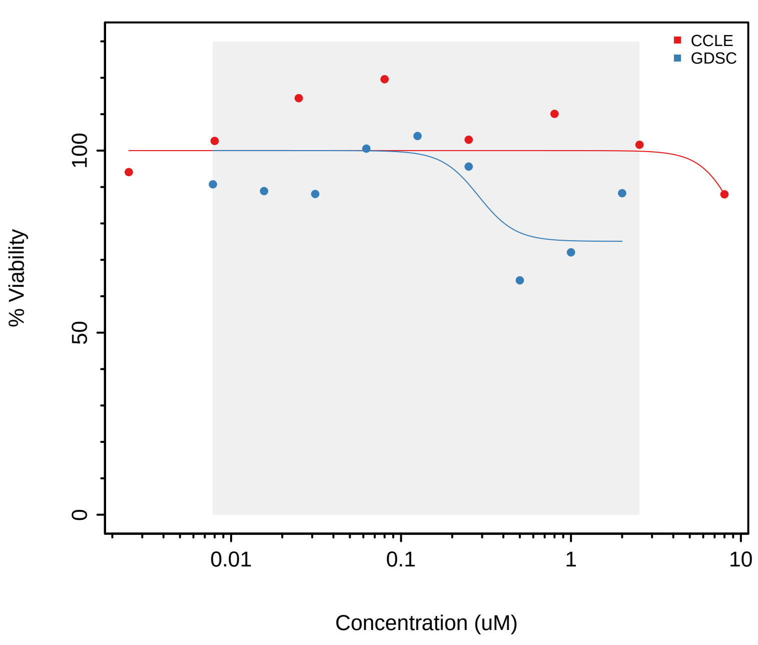

Nilotinib:HCT−116

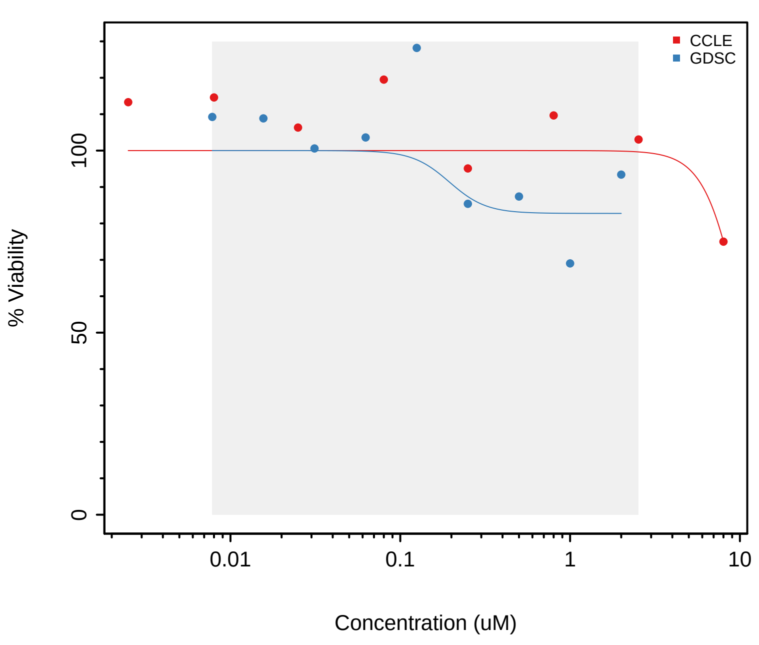

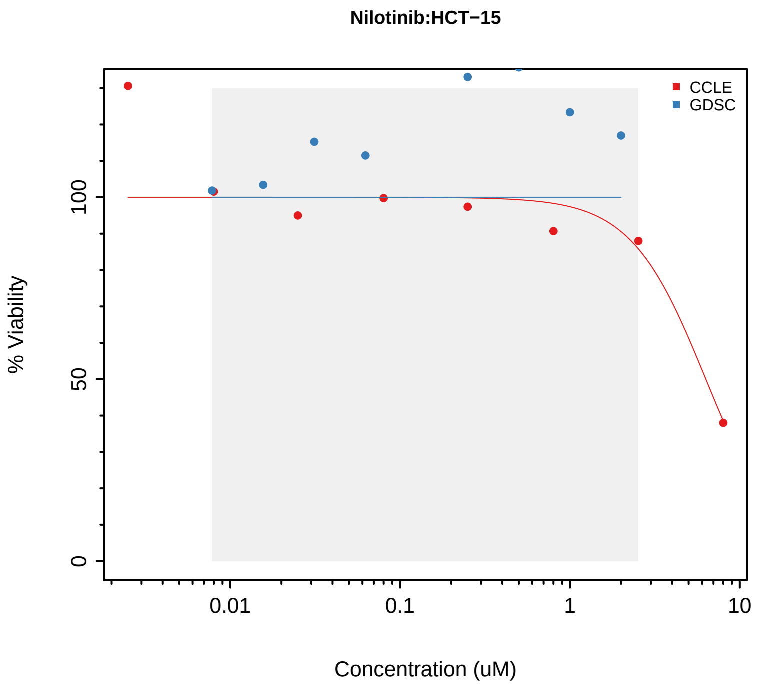
Nilotinib:HCT−15
CCLE
GDSC
% Viability
0 50 100
0.01 0.1 1 10
Concentration (uM)

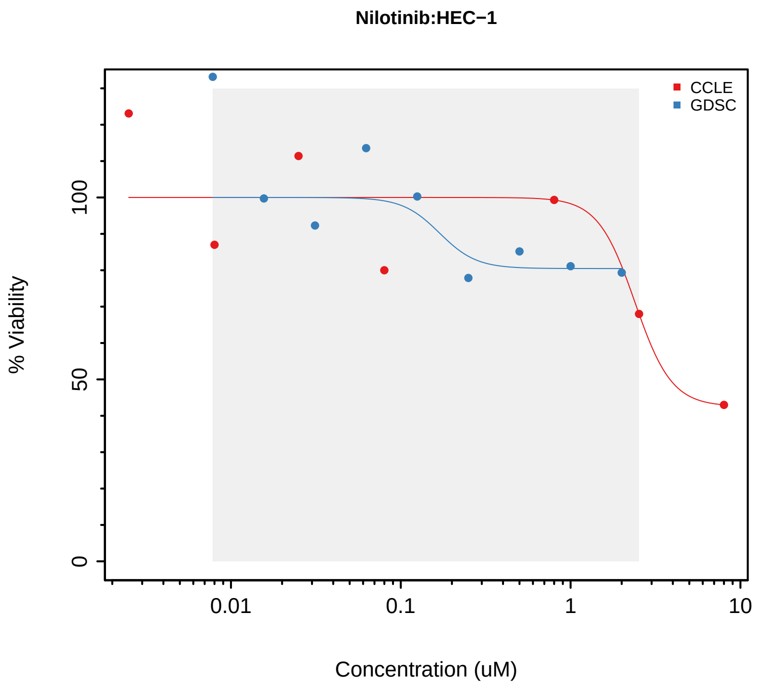
Nilotinib:HEC−1
CCLE
GDSC
% Viability
0 50 100
0.01 0.1 1 10
Concentration (uM)

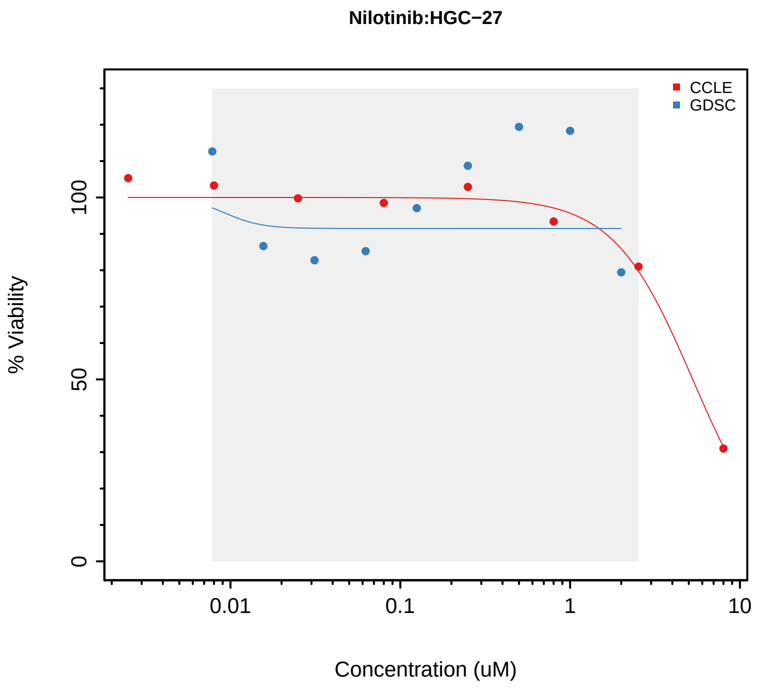
Nilotinib:HGC−27
CCLE
GDSC
% Viability
0 50 100
0.01 0.1 1 10
Concentration (uM)

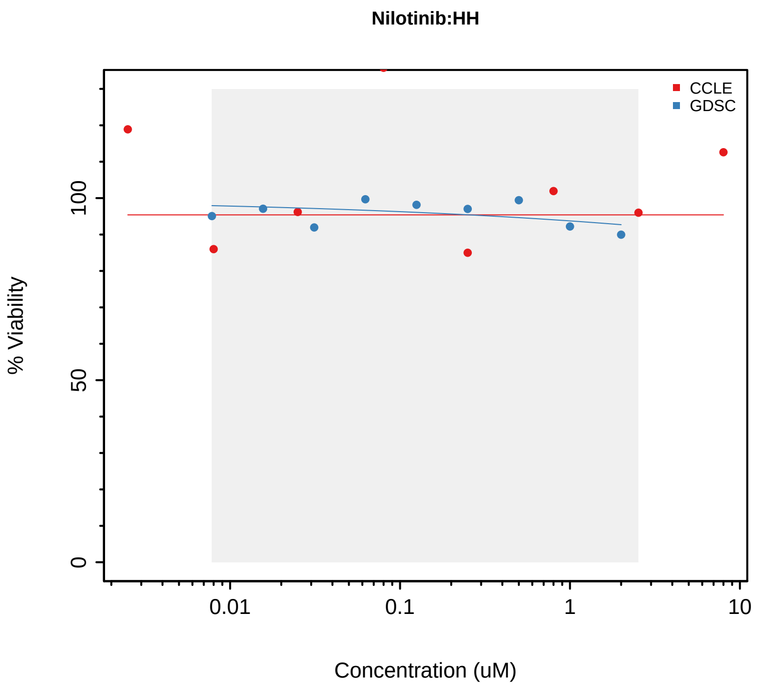
Nilotinib:HH
CCLE
GDSC
% Viability
0 50 100
0.01 0.1 1 10
Concentration (uM)

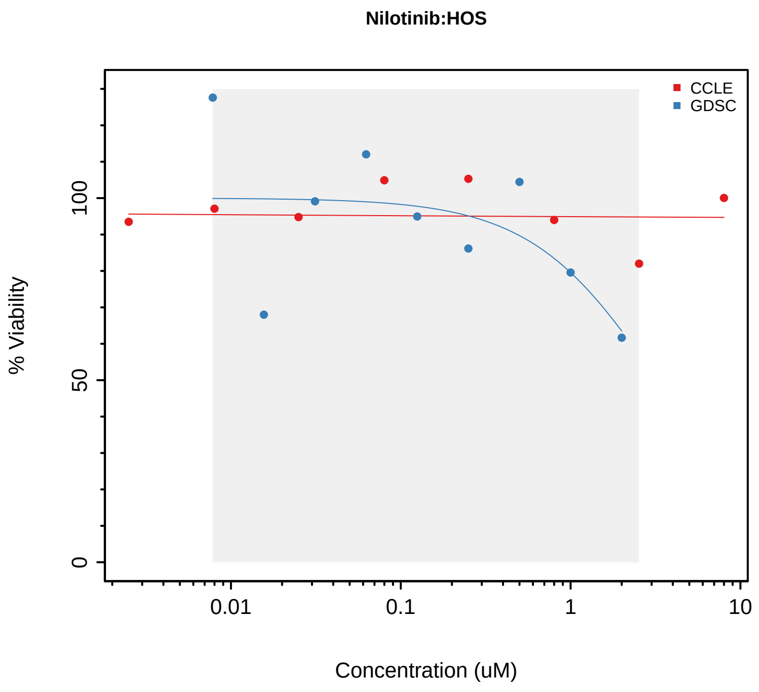
Nilotinib:HOS
CCLE
GDSC
% Viability
0 50 100
0.01 0.1 1 10
Concentration (uM)

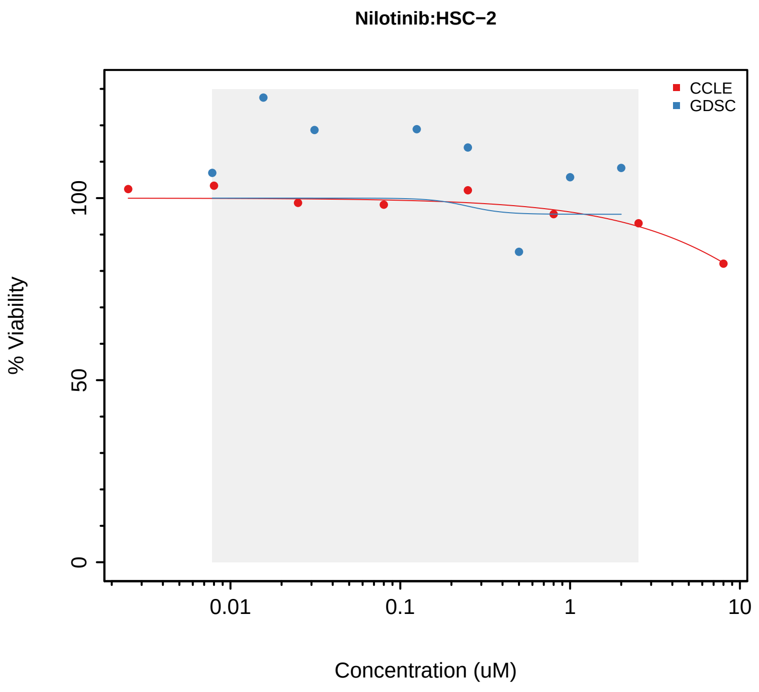
Nilotinib:HSC−2
CCLE
GDSC
% Viability
0 50 100
0.01 0.1 1 10
Concentration (uM)

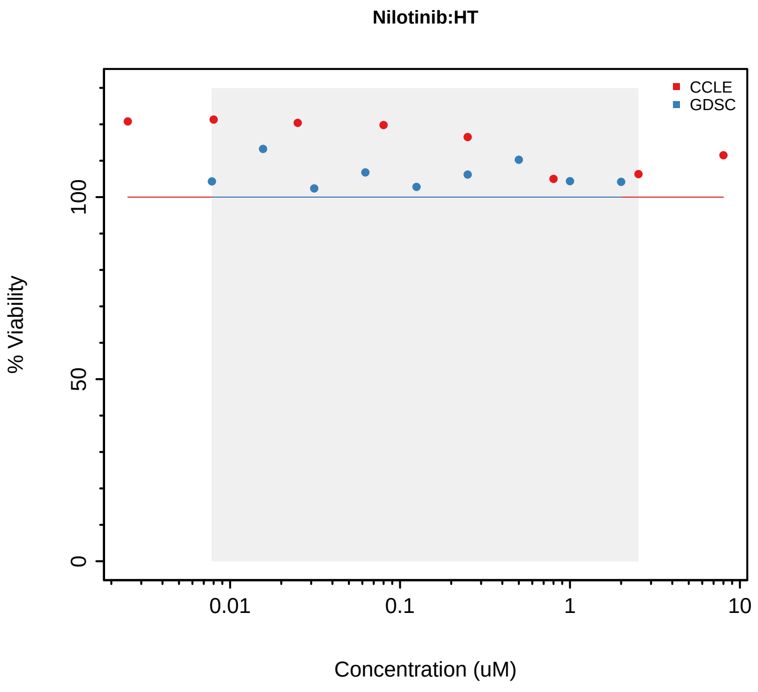
Nilotinib:HT
CCLE
GDSC
% Viability
0 50 100
0.01 0.1 1 10
Concentration (uM)

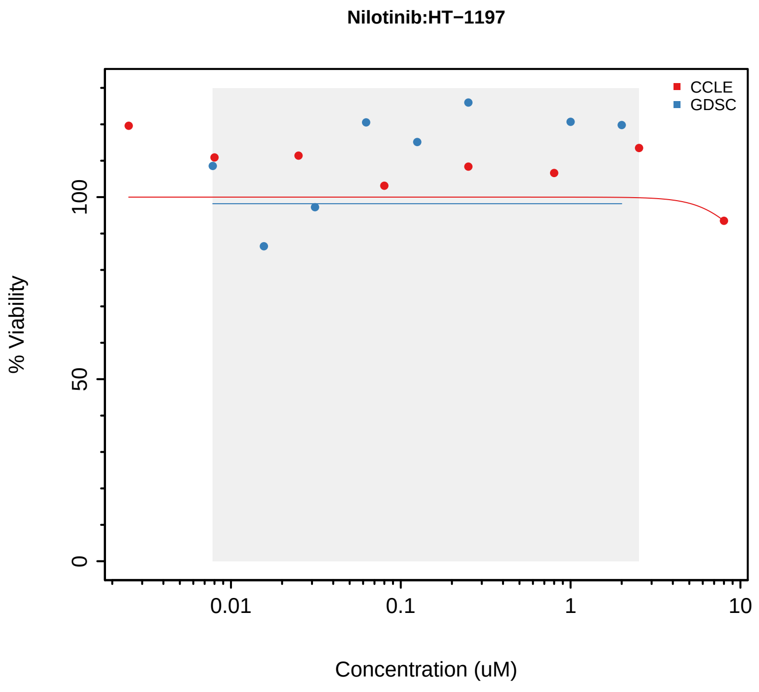
Nilotinib:HT−1197
CCLE
GDSC
% Viability
0 50 100
0.01 0.1 1 10
Concentration (uM)

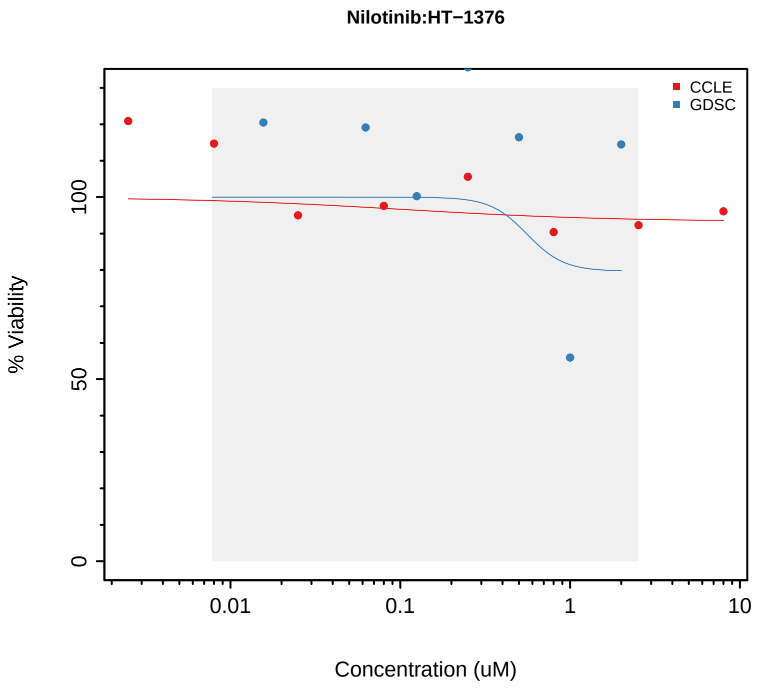
Nilotinib:HT−1376
CCLE
GDSC
% Viability
0 50 100
0.01 0.1 1 10
Concentration (uM)

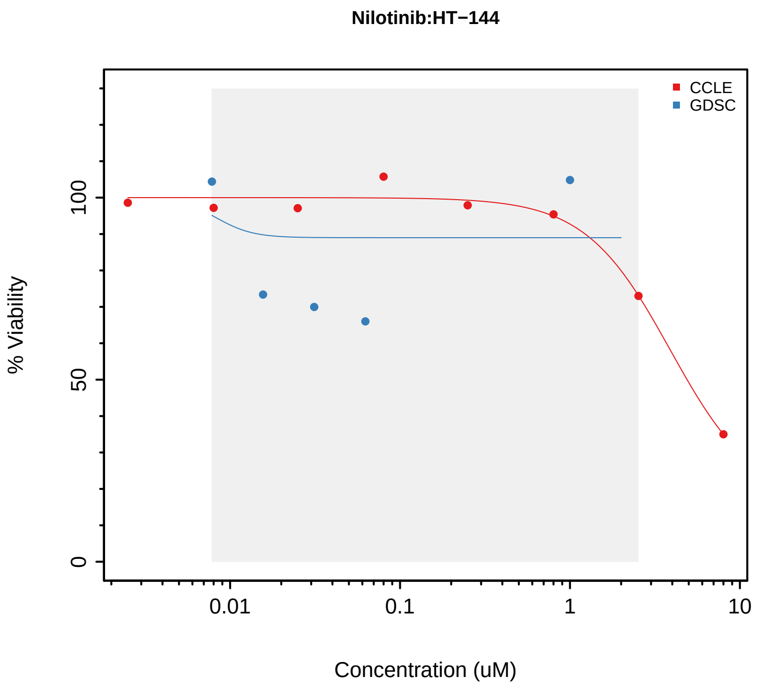
Nilotinib:HT−144
CCLE
GDSC
% Viability
0 50 100
0.01 0.1 1 10
Concentration (uM)

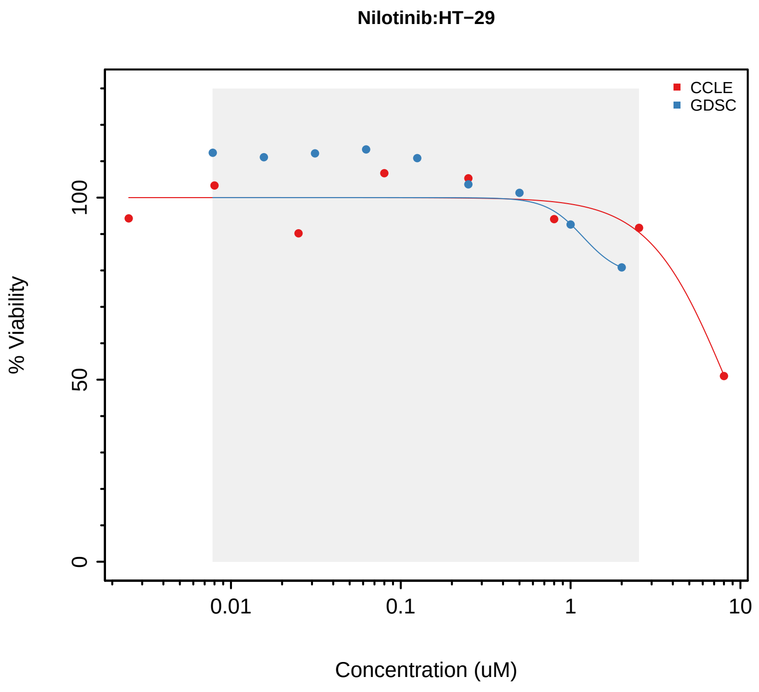
Nilotinib:HT−29
CCLE
GDSC
% Viability
0 50 100
0.01 0.1 1 10
Concentration (uM)

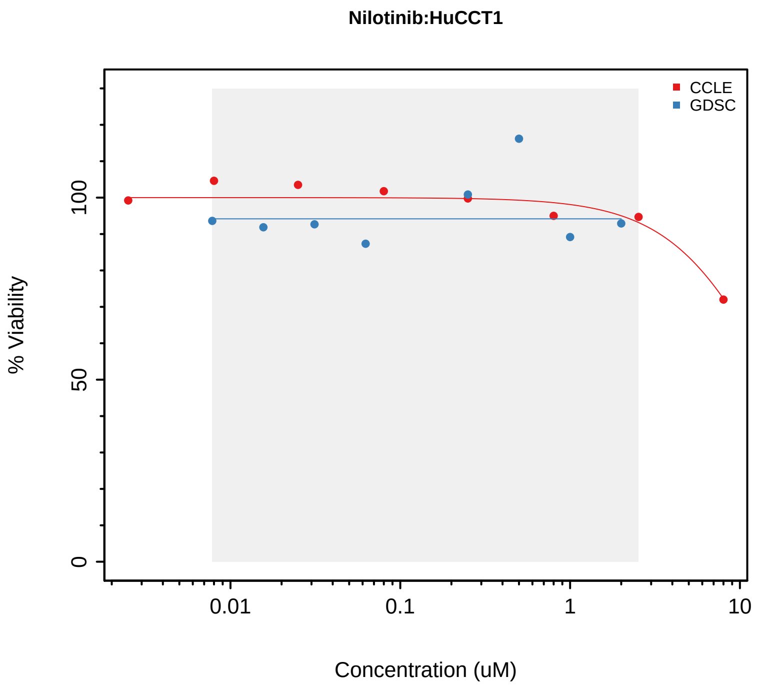
Nilotinib:HuCCT1
CCLE
GDSC
% Viability
0 50 100
0.01 0.1 1 10
Concentration (uM)

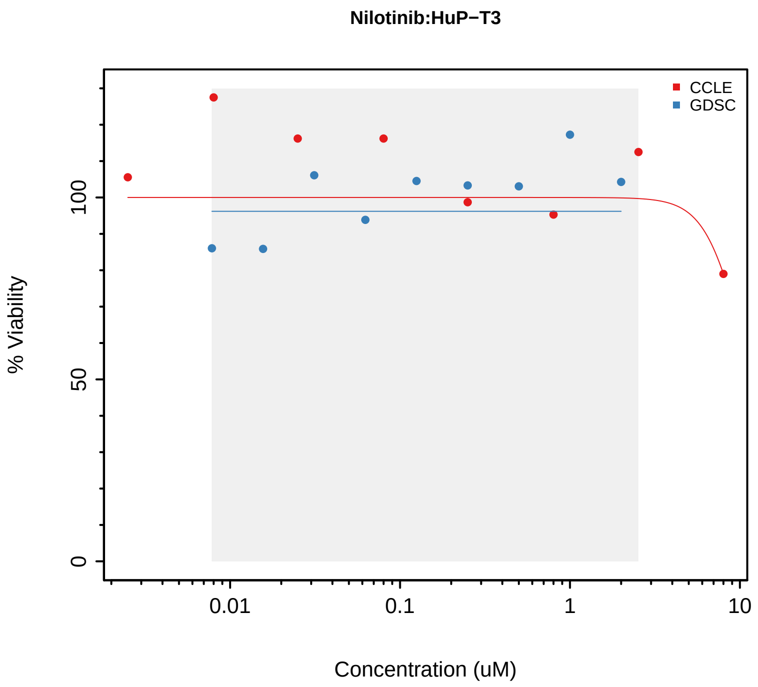
Nilotinib:HuP−T3
CCLE
GDSC
% Viability
0
50
100
0.01
0.1
1
10
Concentration (uM)

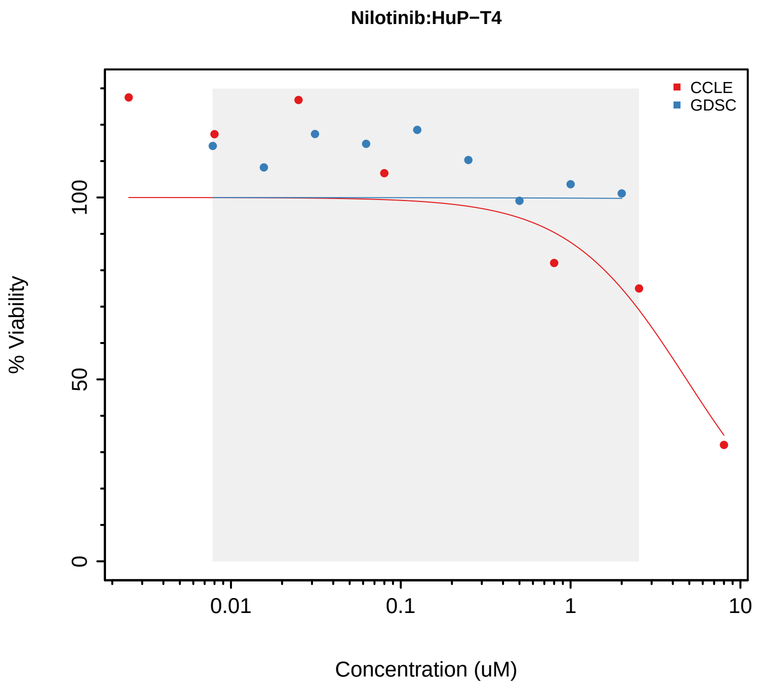
Nilotinib:HuP−T4
CCLE
GDSC
% Viability
0
50
100
0.01
0.1
1
10
Concentration (uM)

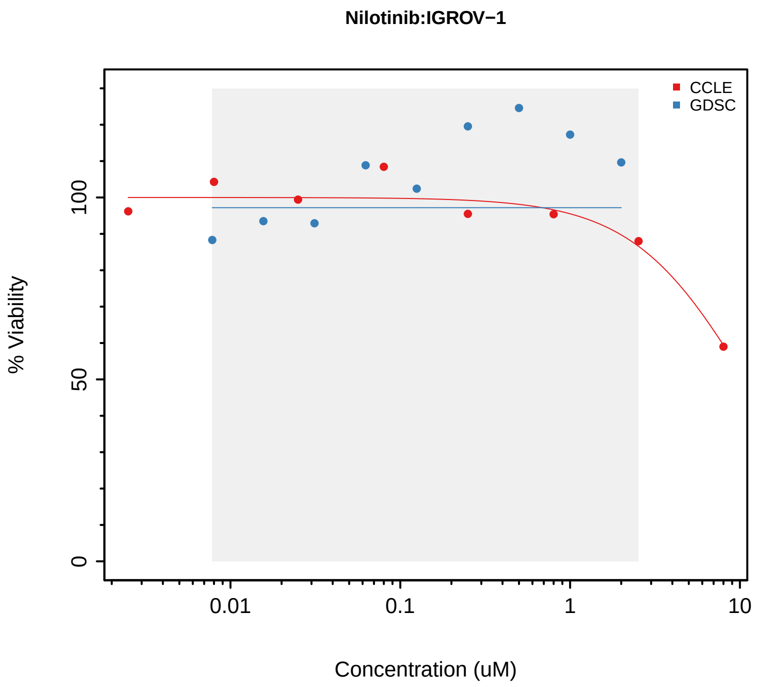
Nilotinib:IGROV−1
CCLE
GDSC
% Viability
0
50
100
0.01
0.1
1
10
Concentration (uM)

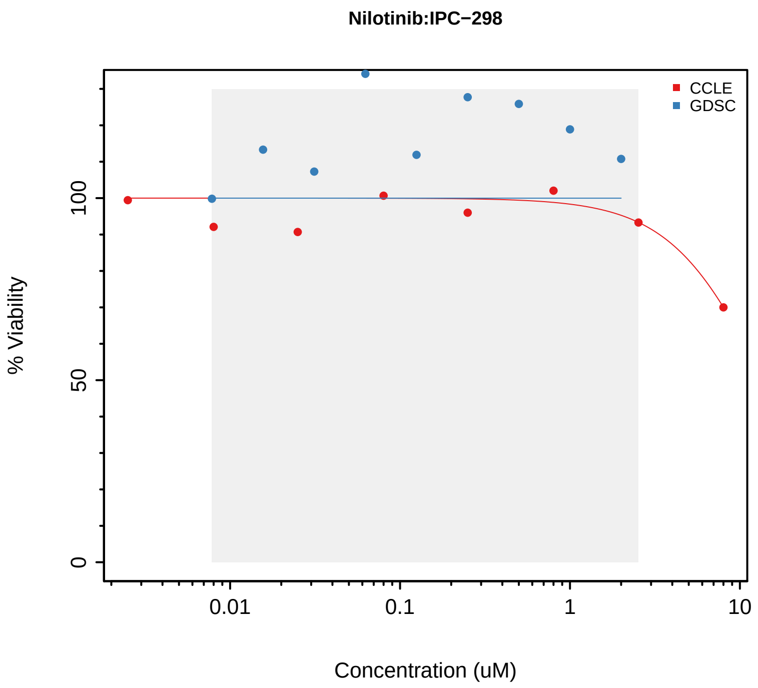
Nilotinib:IPC−298
CCLE
GDSC
% Viability
0
50
100
0.01
0.1
1
10
Concentration (uM)

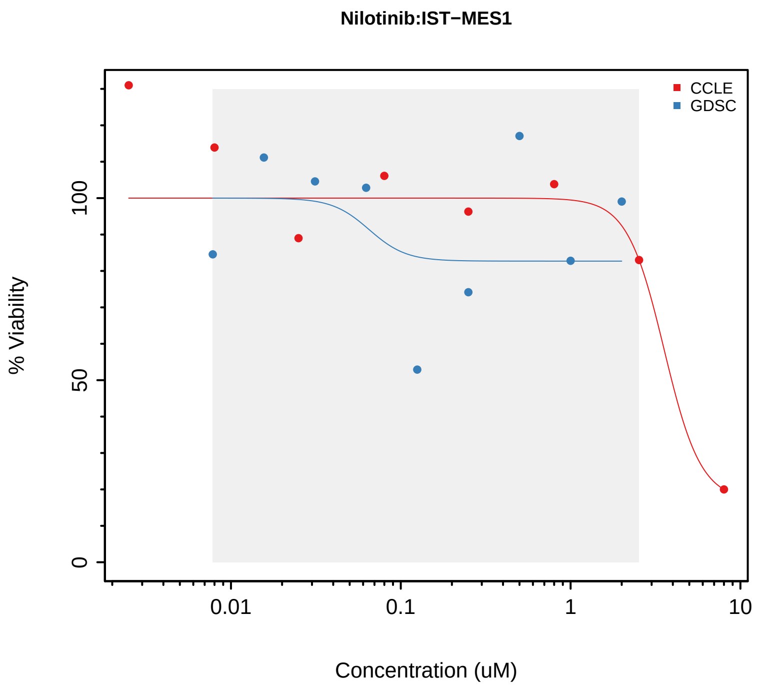
Nilotinib:IST−MES1
CCLE
GDSC
% Viability
0
50
100
0.01
0.1
1
10
Concentration (uM)

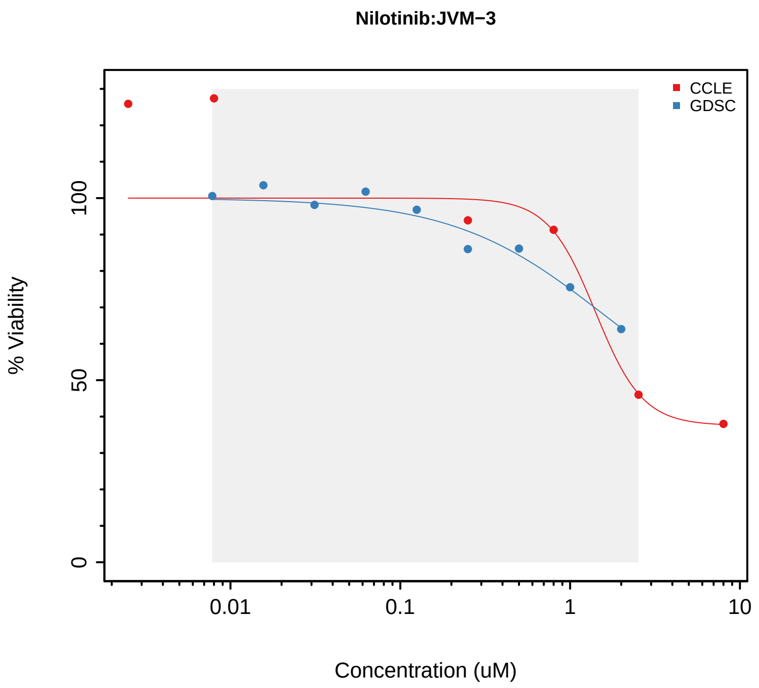
Nilotinib:JVM−3
CCLE
GDSC
% Viability
0
50
100
0.01
0.1
1
10
Concentration (uM)

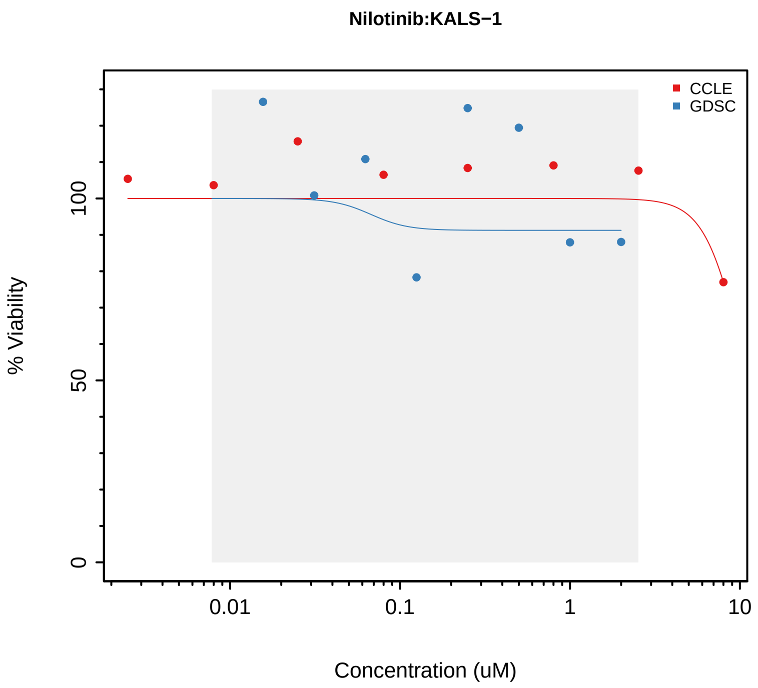
Nilotinib:KALS−1
CCLE
GDSC
% Viability
0
50
100
0.01
0.1
1
10
Concentration (uM)

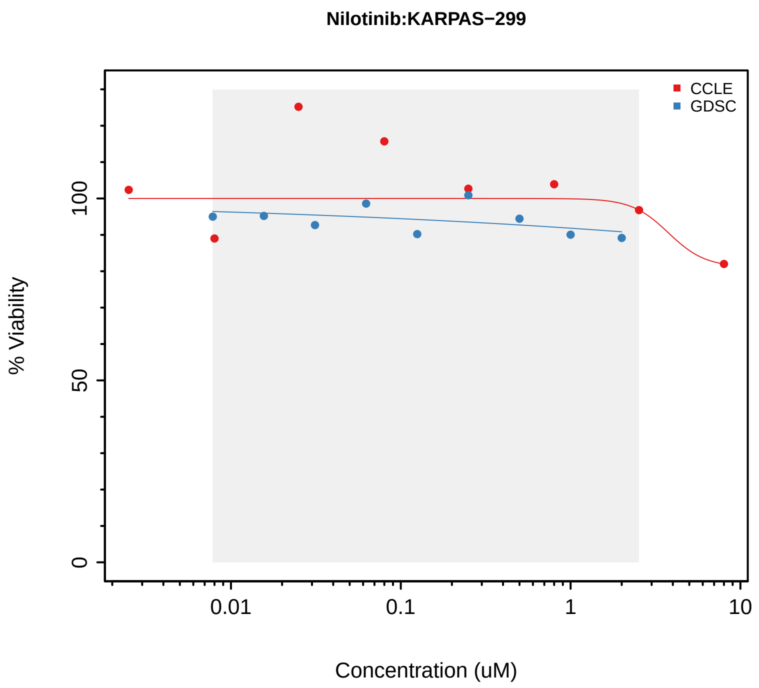
Nilotinib:KARPAS−299
CCLE
GDSC
% Viability
0
50
100
0.01
0.1
1
10
Concentration (uM)

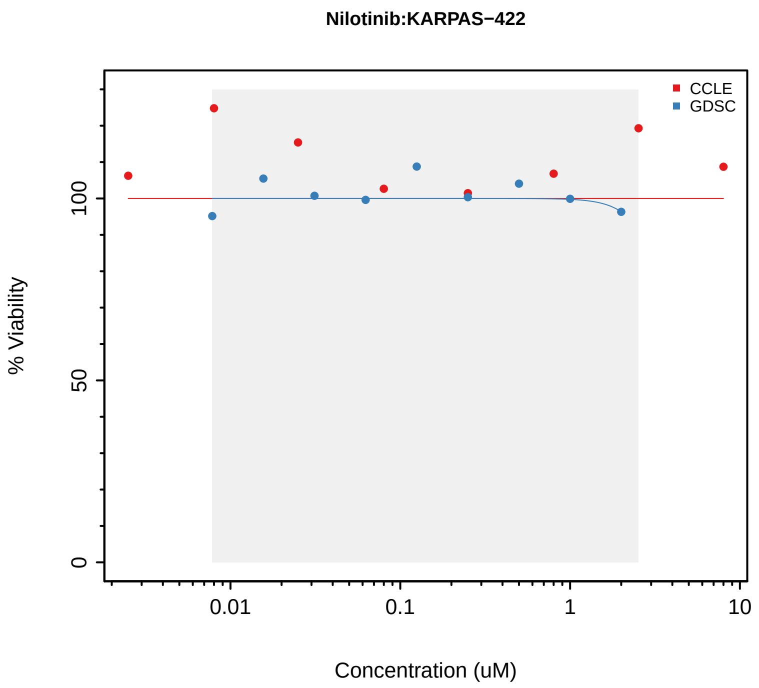
Nilotinib:KARPAS−422
CCLE
GDSC
% Viability
0
50
100
0.01
0.1
1
10
Concentration (uM)

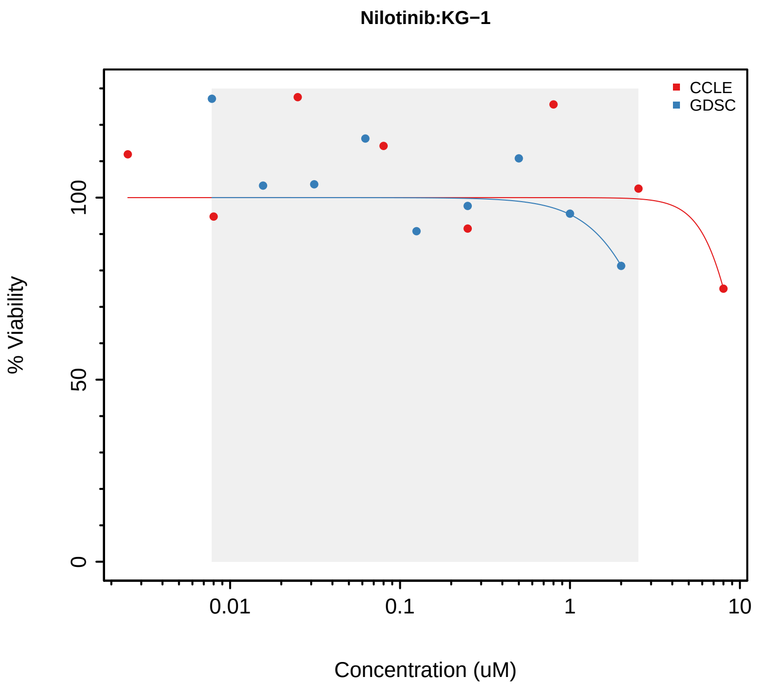
Nilotinib:KG−1
CCLE
GDSC
% Viability
0
50
100
0.01
0.1
1
10
Concentration (uM)

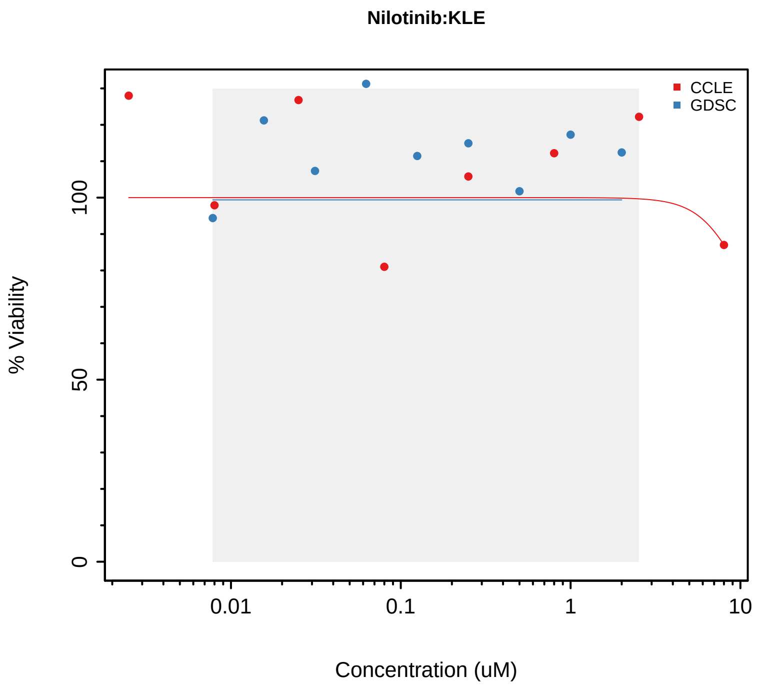
Nilotinib:KLE
CCLE
GDSC
% Viability
0
50
100
0.01
0.1
1
10
Concentration (uM)

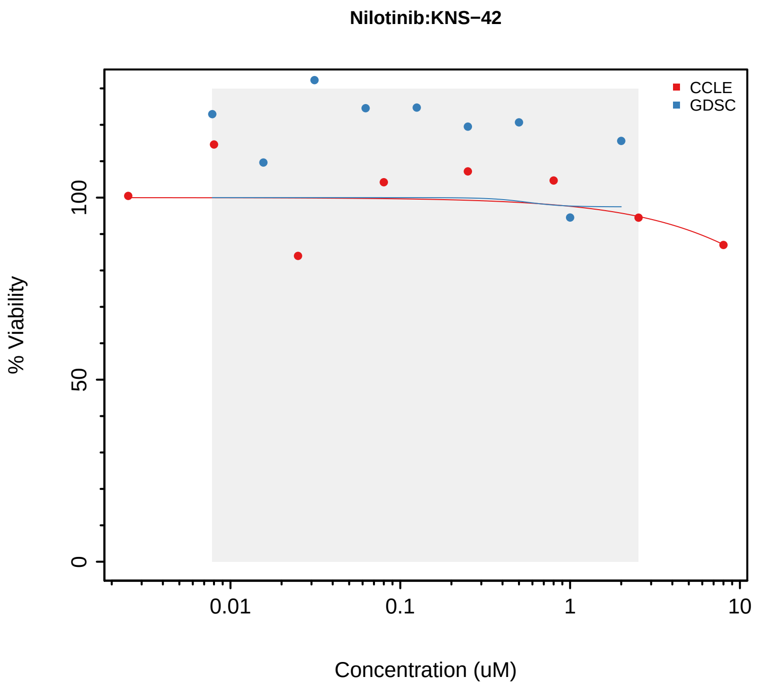
Nilotinib:KNS−42
CCLE
GDSC
% Viability
0
50
100
0.01
0.1
1
10
Concentration (uM)

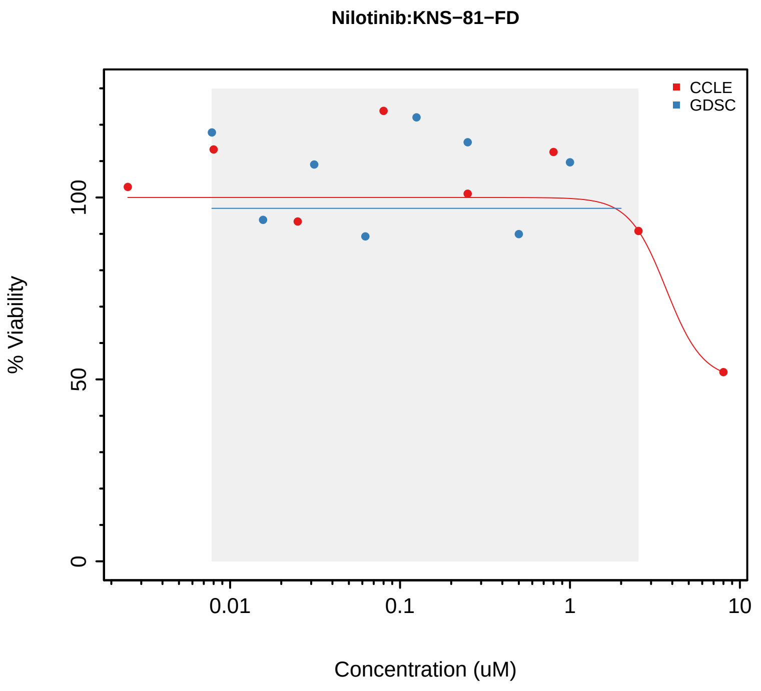
Nilotinib:KNS−81−FD
CCLE
GDSC
% Viability
0
50
100
0.01
0.1
1
10
Concentration (uM)

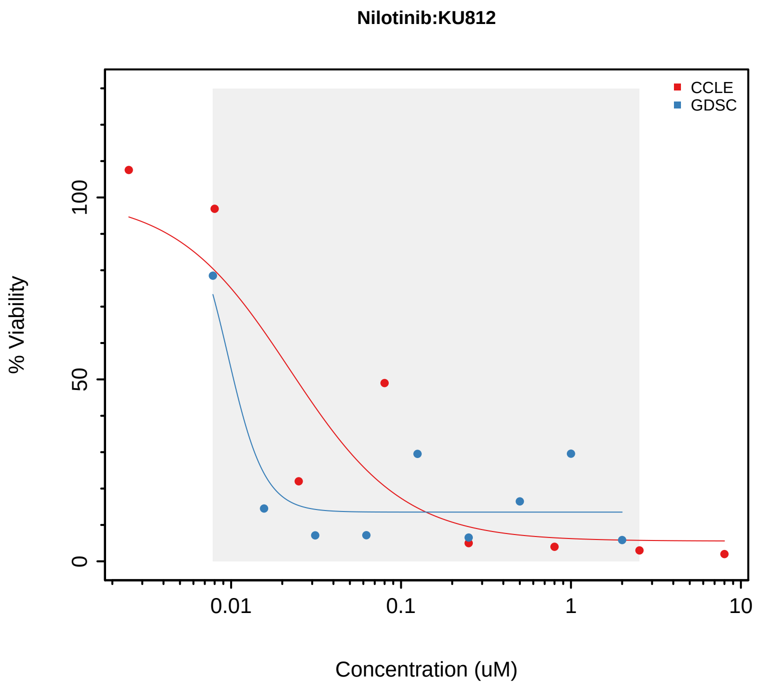
Nilotinib:KU812
CCLE
GDSC
% Viability
0
50
100
0.01
0.1
1
10
Concentration (uM)

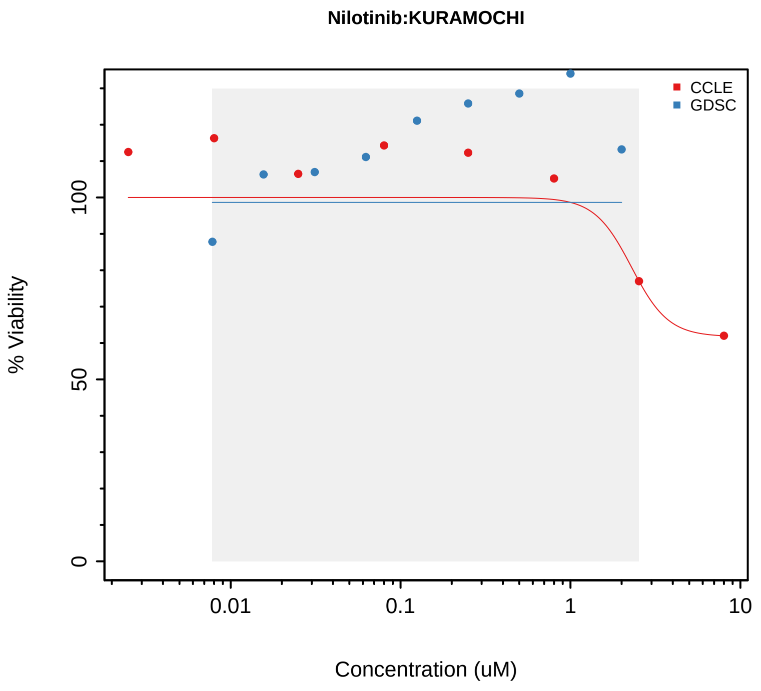
Nilotinib:KURAMOCHI
CCLE
GDSC
% Viability
0
50
100
0.01
0.1
1
10
Concentration (uM)

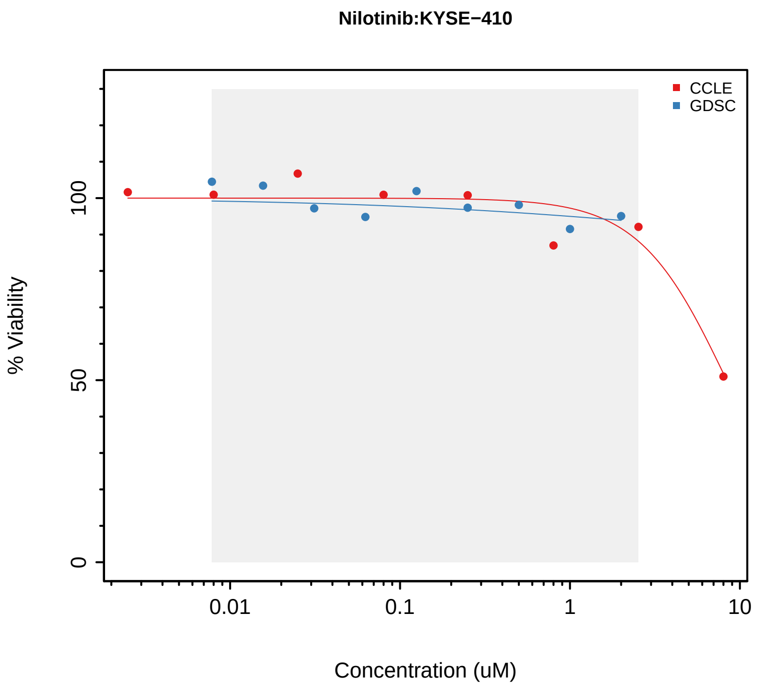
Nilotinib:KYSE−410
CCLE
GDSC
% Viability
0
50
100
0.01
0.1
1
10
Concentration (uM)

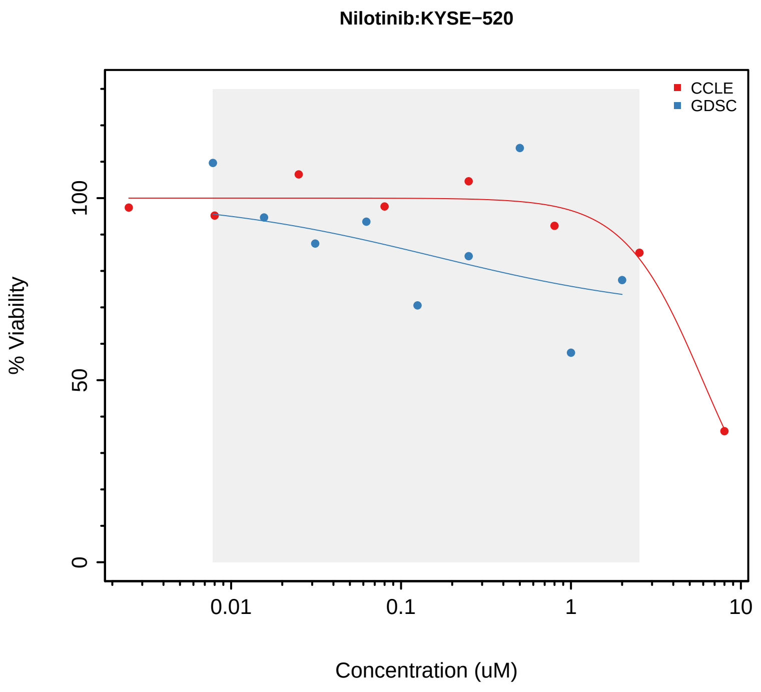
Nilotinib:KYSE−520
CCLE
GDSC
% Viability
0
50
100
0.01
0.1
1
10
Concentration (uM)

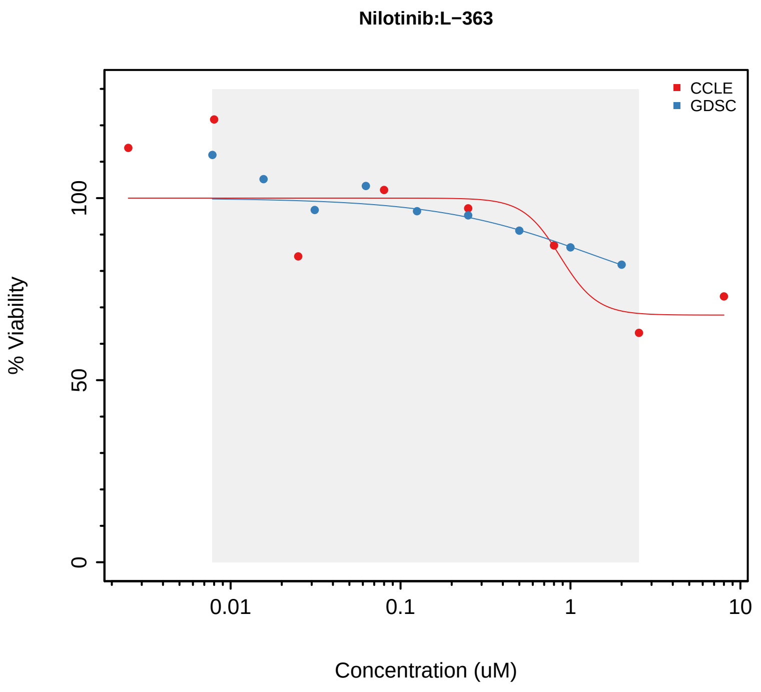
Nilotinib:L−363
CCLE
GDSC
% Viability
0
50
100
0.01
0.1
1
10
Concentration (uM)

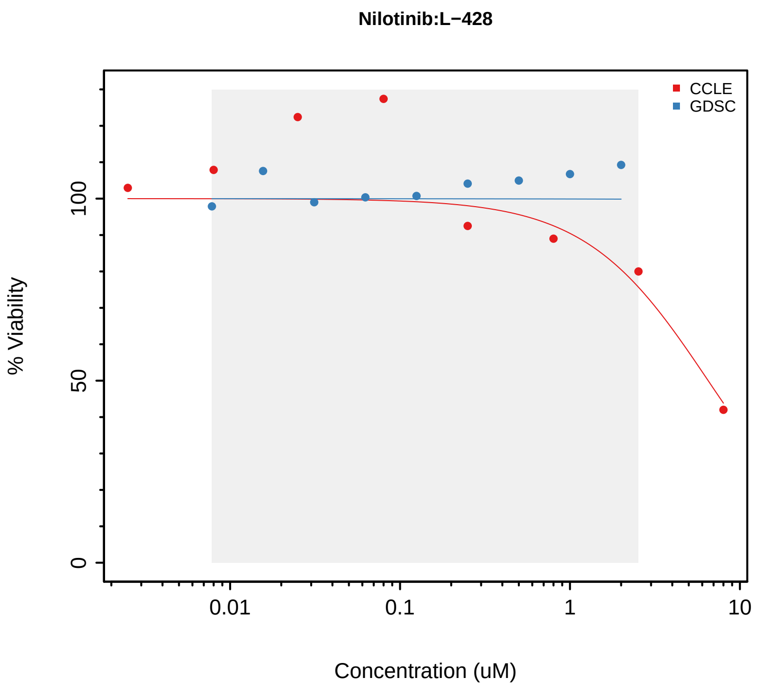
Nilotinib:L−428
CCLE
GDSC
% Viability
0
50
100
0.01
0.1
1
10
Concentration (uM)

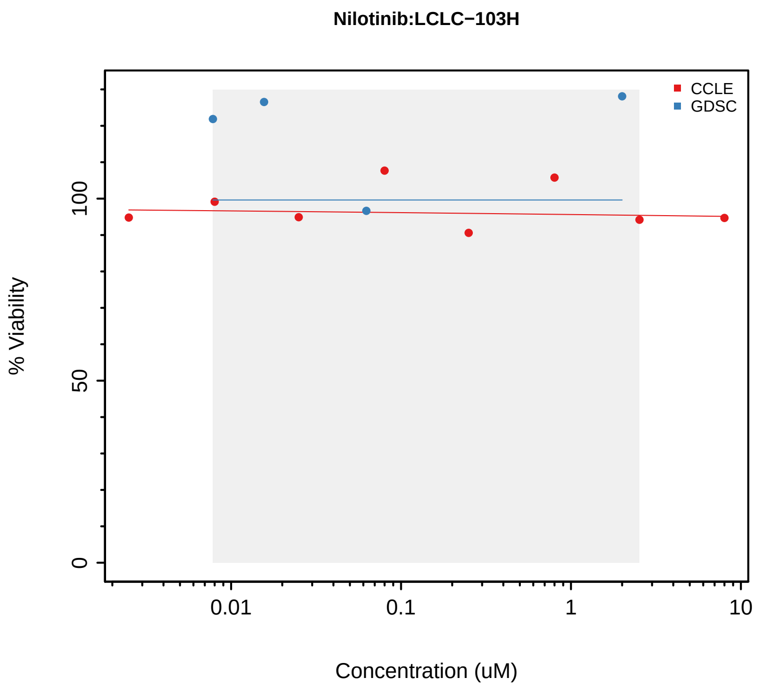
Nilotinib:LCLC−103H
CCLE
GDSC
% Viability
0
50
100
0.01
0.1
1
10
Concentration (uM)

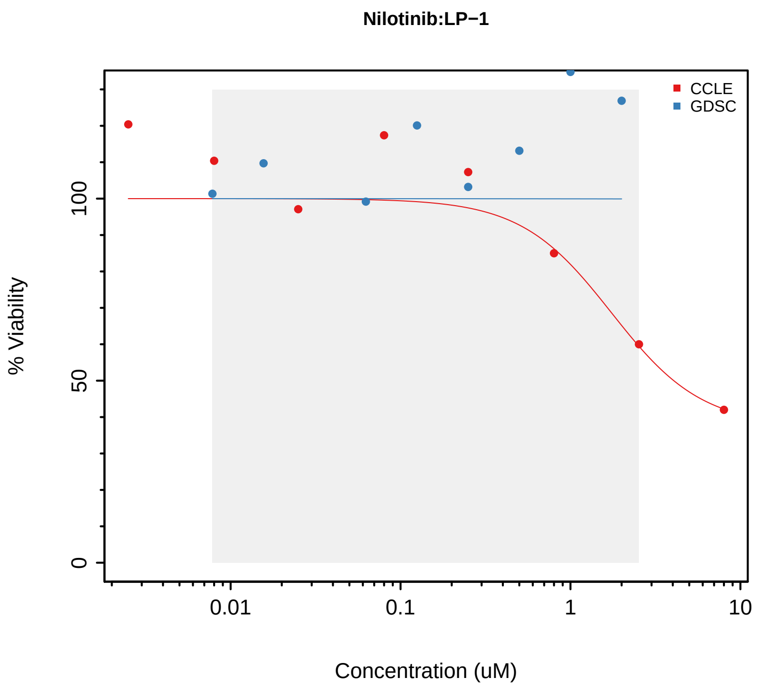
Nilotinib:LP−1
CCLE
GDSC
% Viability
0
50
100
0.01
0.1
1
10
Concentration (uM)

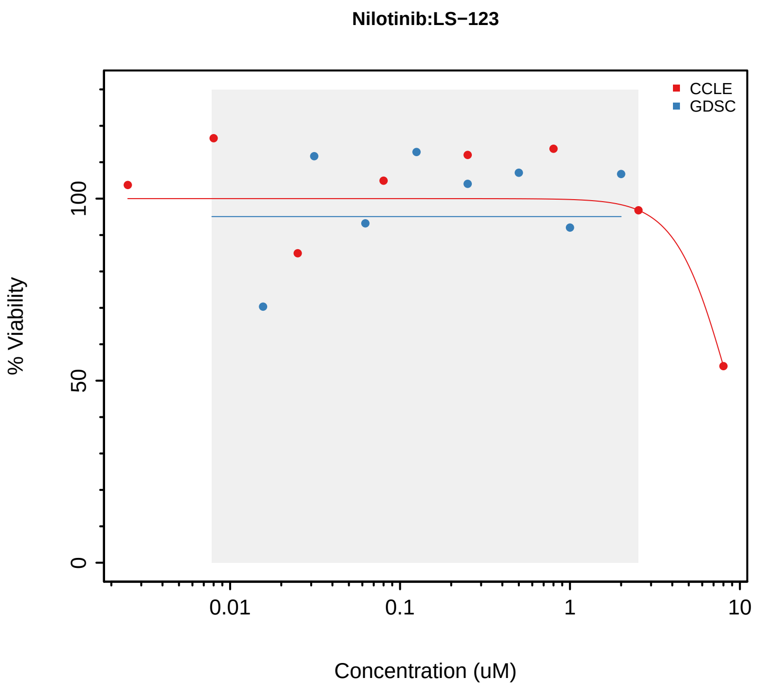
Nilotinib:LS−123
CCLE
GDSC
% Viability
0
50
100
0.01
0.1
1
10
Concentration (uM)

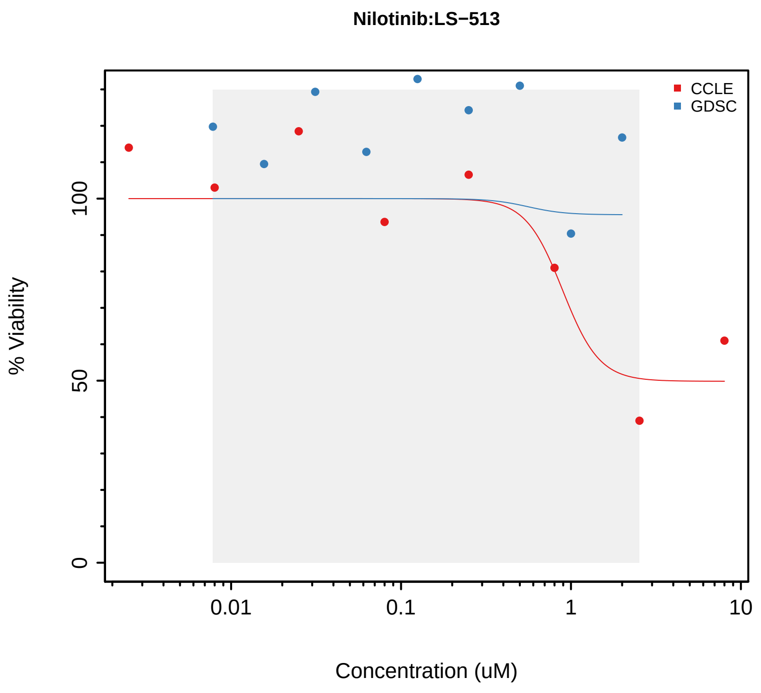
Nilotinib:LS−513
CCLE
GDSC
% Viability
0
50
100
0.01
0.1
1
10
Concentration (uM)

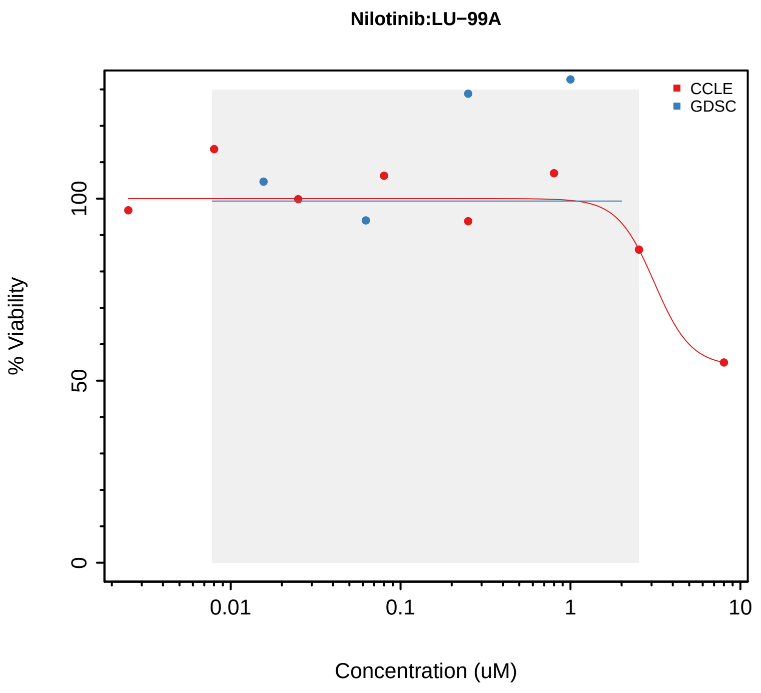
Nilotinib:LU−99A
CCLE
GDSC
% Viability
0
50
100
0.01
0.1
1
10
Concentration (uM)

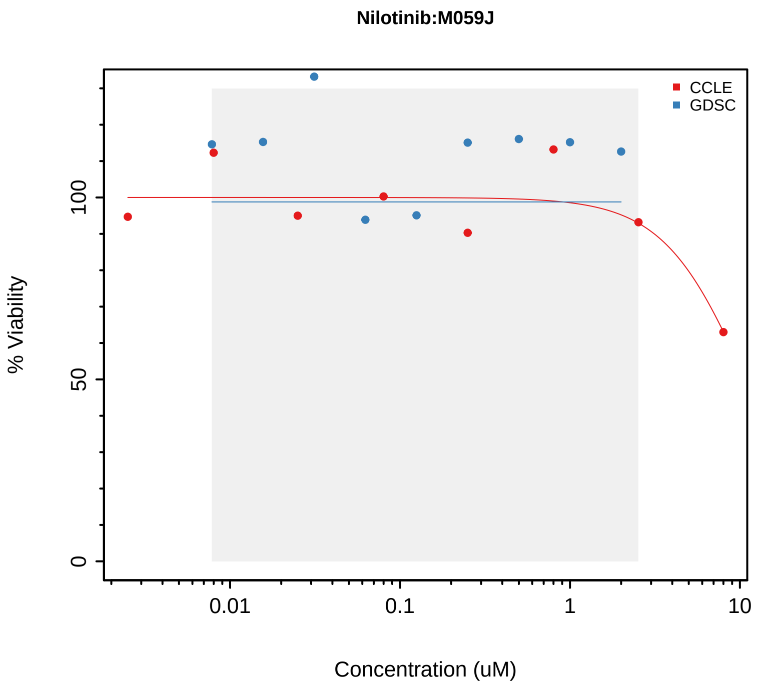
Nilotinib:M059J
CCLE
GDSC
% Viability
0
50
100
0.01
0.1
1
10
Concentration (uM)

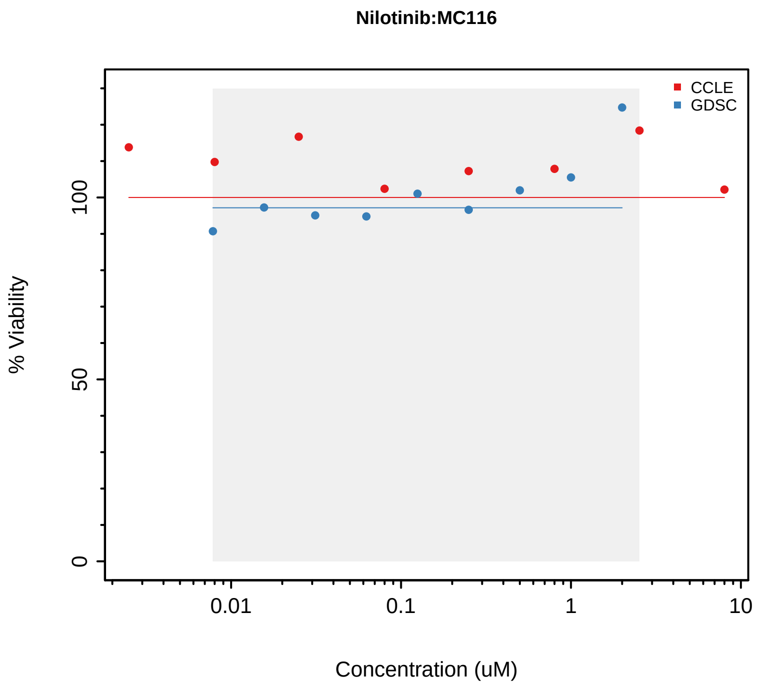
Nilotinib:MC116
CCLE
GDSC
% Viability
0
50
100
0.01
0.1
1
10
Concentration (uM)

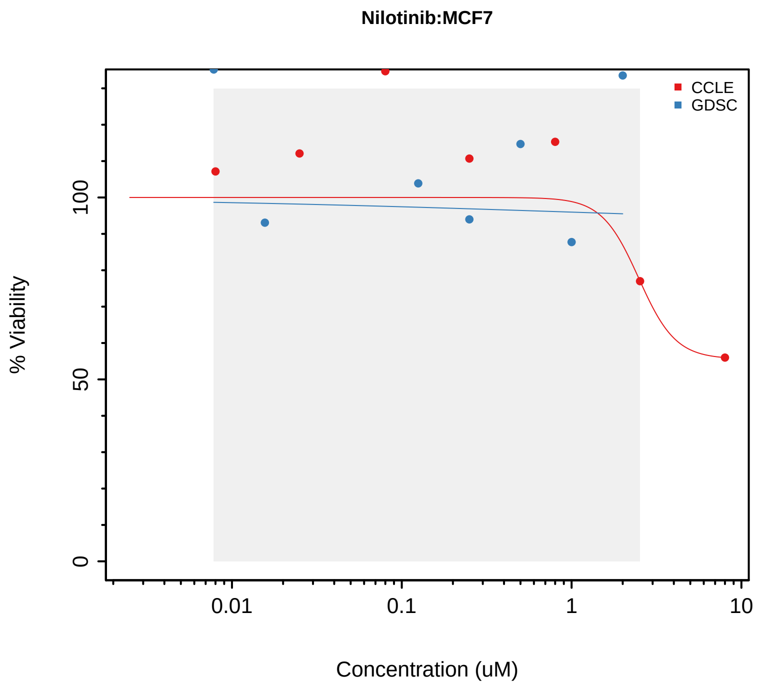
Nilotinib:MCF7
CCLE
GDSC
% Viability
0
50
100
0.01
0.1
1
10
Concentration (uM)

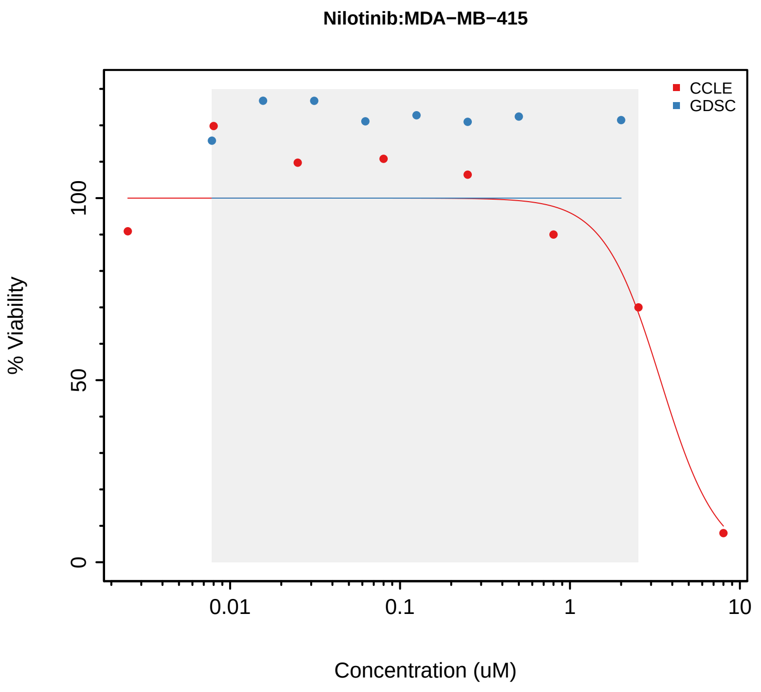
Nilotinib:MDA−MB−415
CCLE
GDSC
% Viability
0
50
100
0.01
0.1
1
10
Concentration (uM)

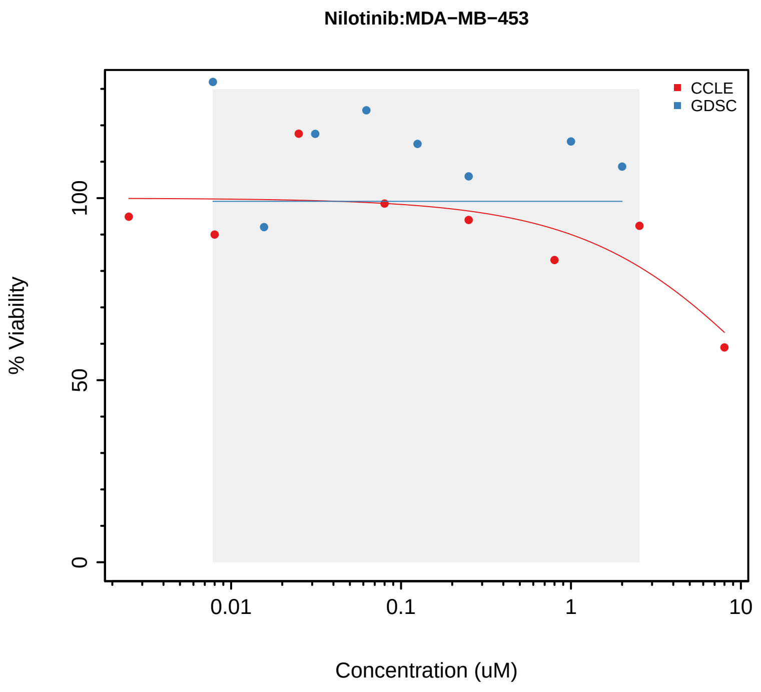
Nilotinib:MDA−MB−453
CCLE
GDSC
% Viability
0
50
100
0.01
0.1
1
10
Concentration (uM)

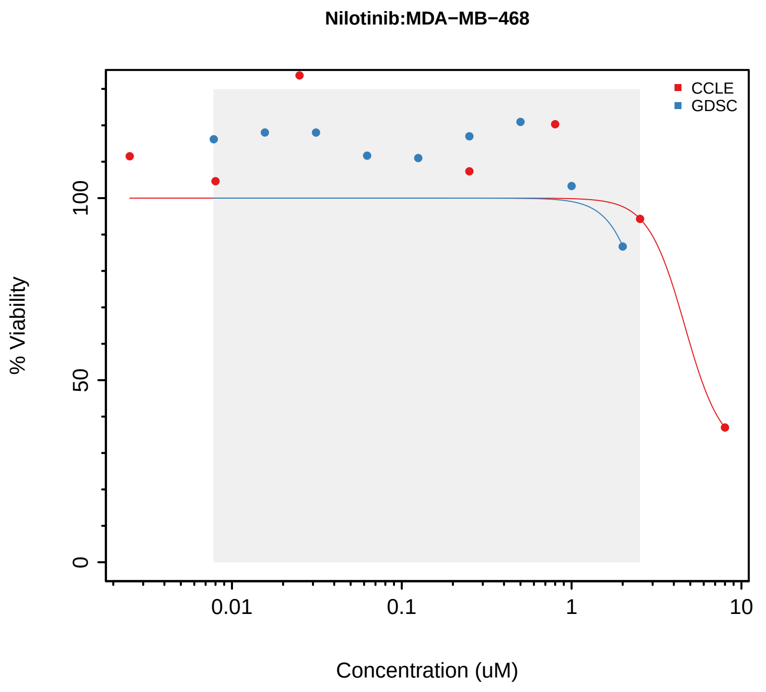
Nilotinib:MDA−MB−468
CCLE
GDSC
% Viability
0
50
100
0.01
0.1
1
10
Concentration (uM)

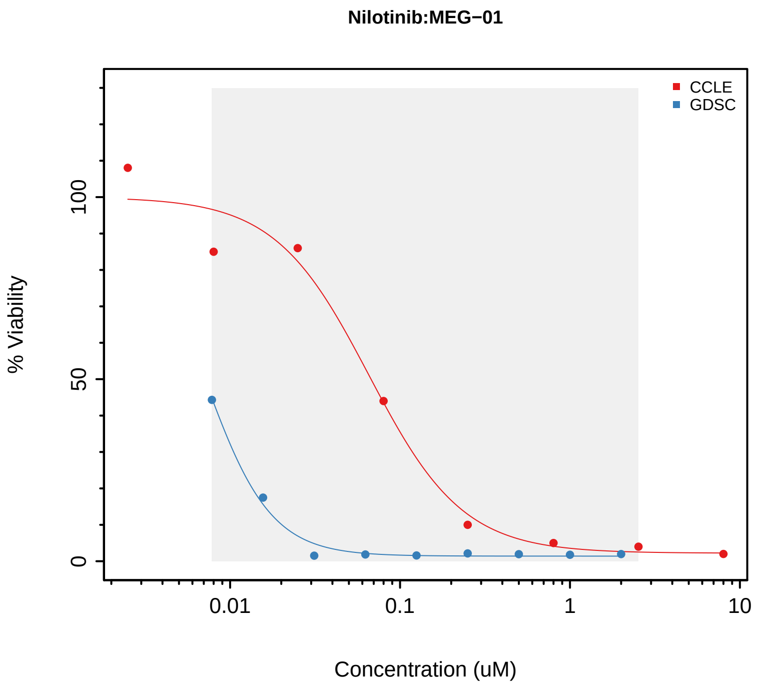
Nilotinib:MEG−01
CCLE
GDSC
% Viability
0
50
100
0.01
0.1
1
10
Concentration (uM)

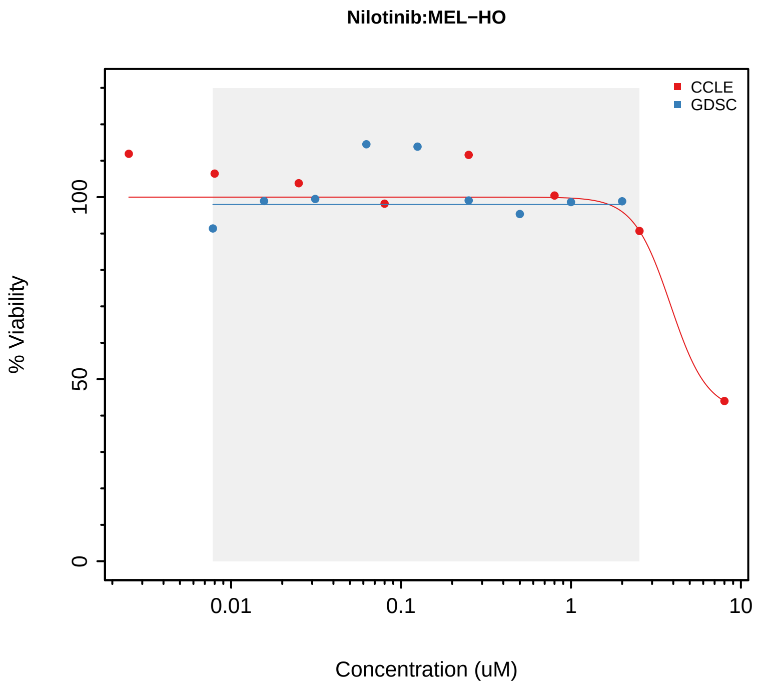
Nilotinib:MEL−HO
CCLE
GDSC
% Viability
0
50
100
0.01
0.1
1
10
Concentration (uM)

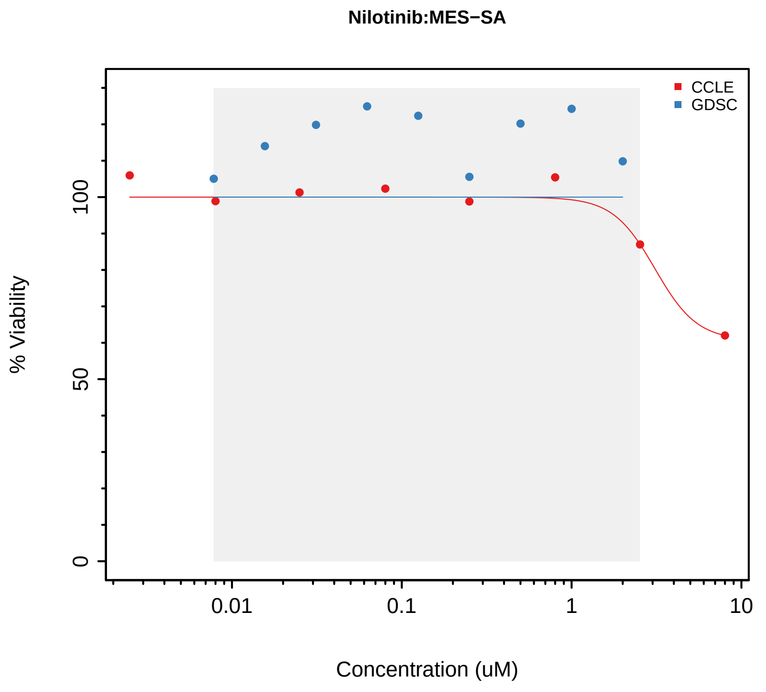
Nilotinib:MES−SA
CCLE
GDSC
% Viability
0
50
100
0.01
0.1
1
10
Concentration (uM)

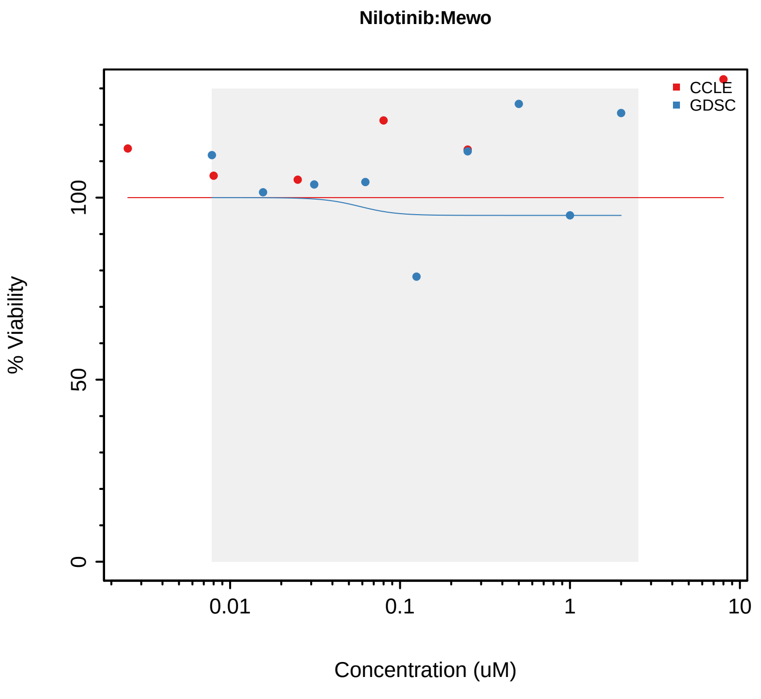
Nilotinib:Mewo
CCLE
GDSC
% Viability
0
50
100
0.01
0.1
1
10
Concentration (uM)

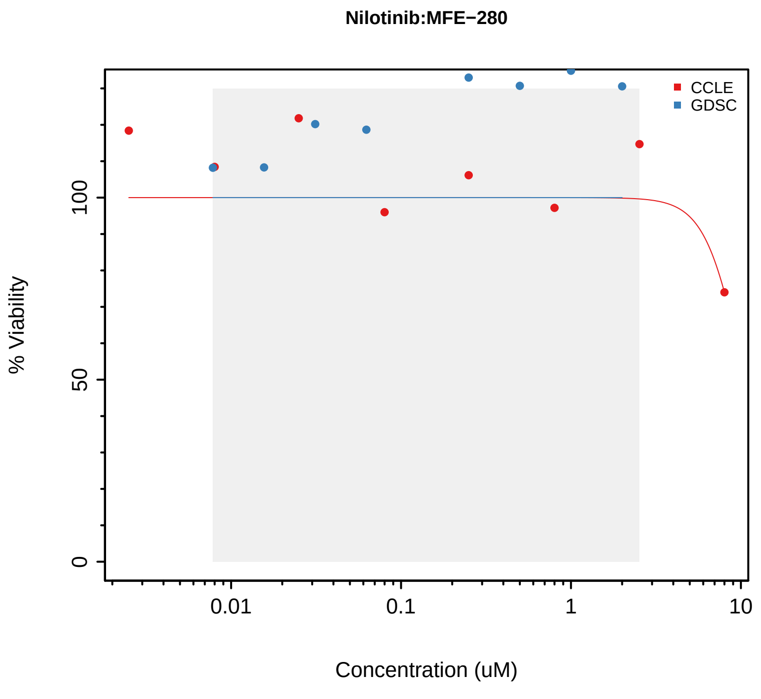
Nilotinib:MFE−280
CCLE
GDSC
% Viability
0
50
100
0.01
0.1
1
10
Concentration (uM)

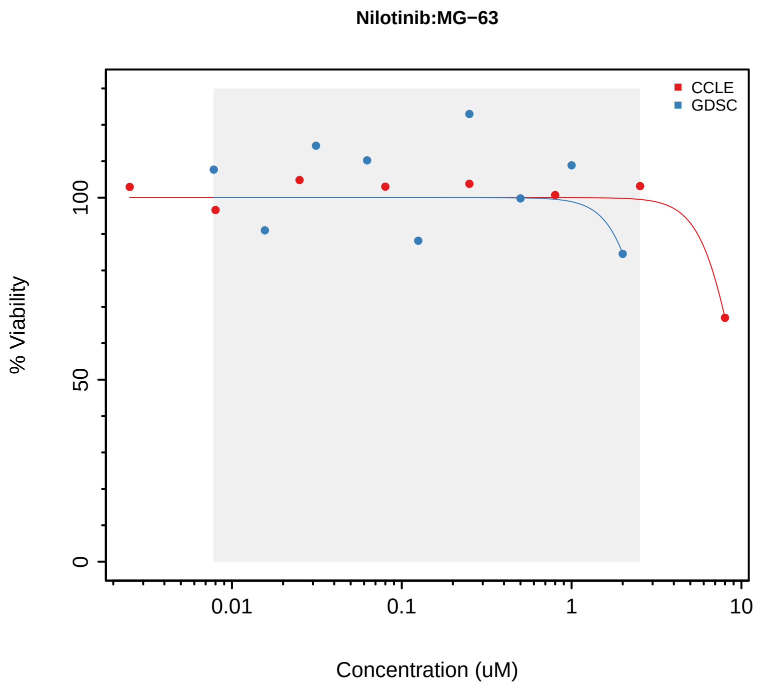
Nilotinib:MG−63
CCLE
GDSC
% Viability
0
50
100
0.01
0.1
1
10
Concentration (uM)

Nilotinib:MHH−ES−1

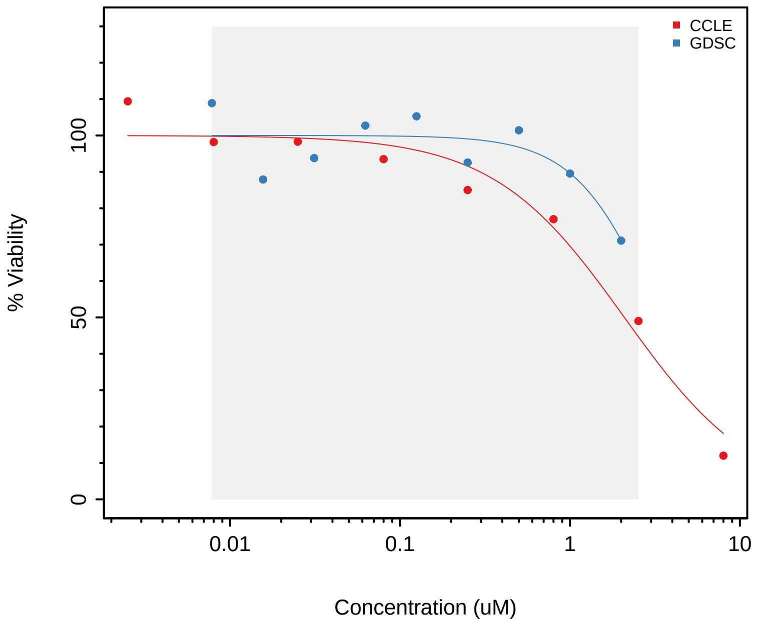

Nilotinib:MIA−PaCa−2

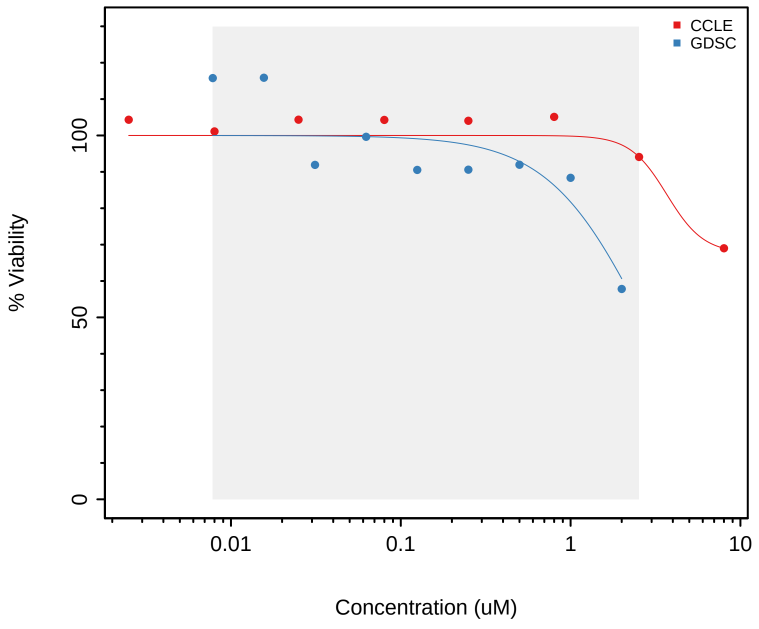

Nilotinib:MKN45

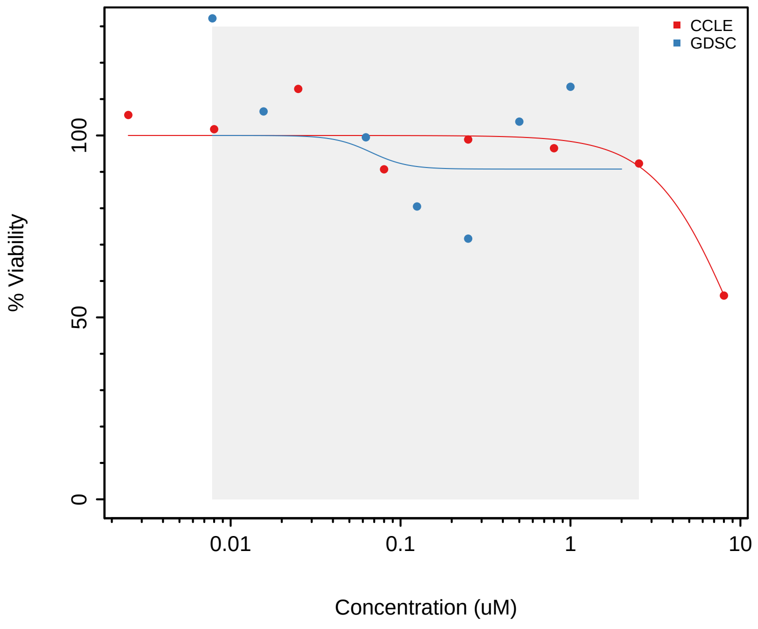

Nilotinib:MOLT−16

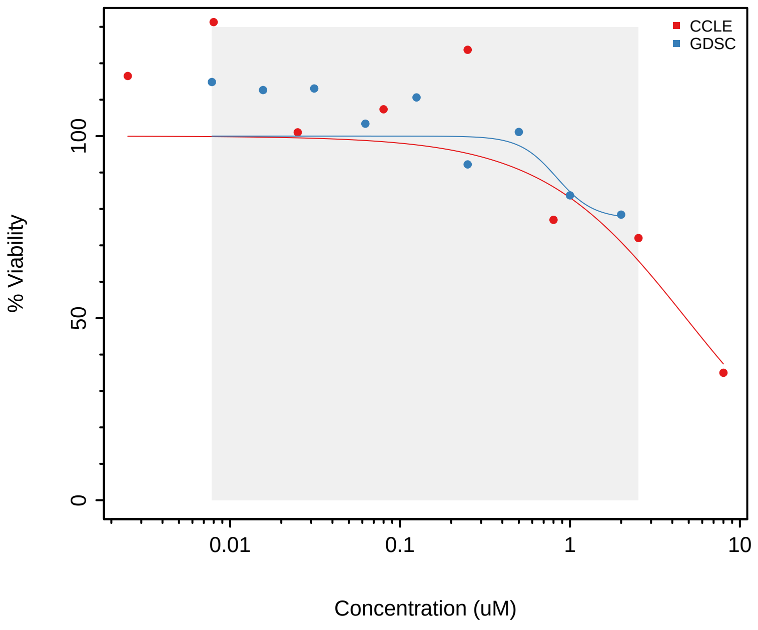

Nilotinib:MPP−89

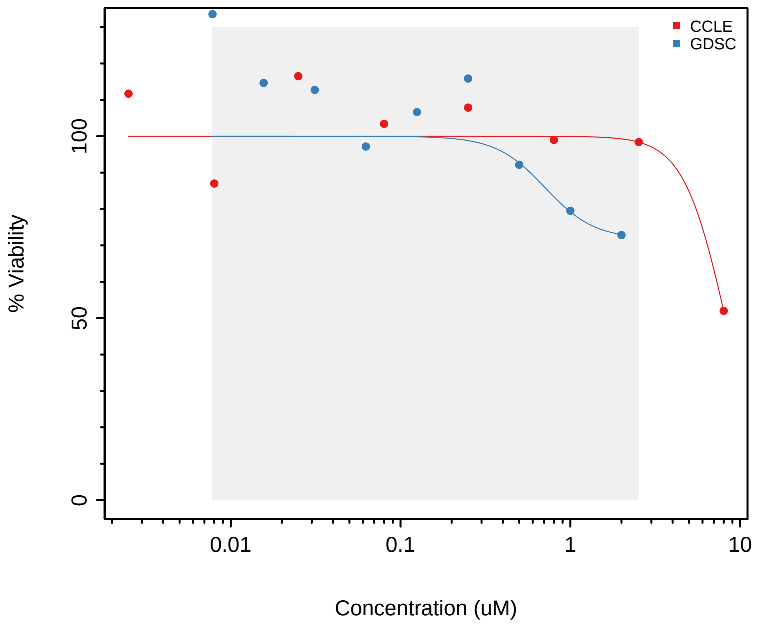

Nilotinib:MSTO−211H

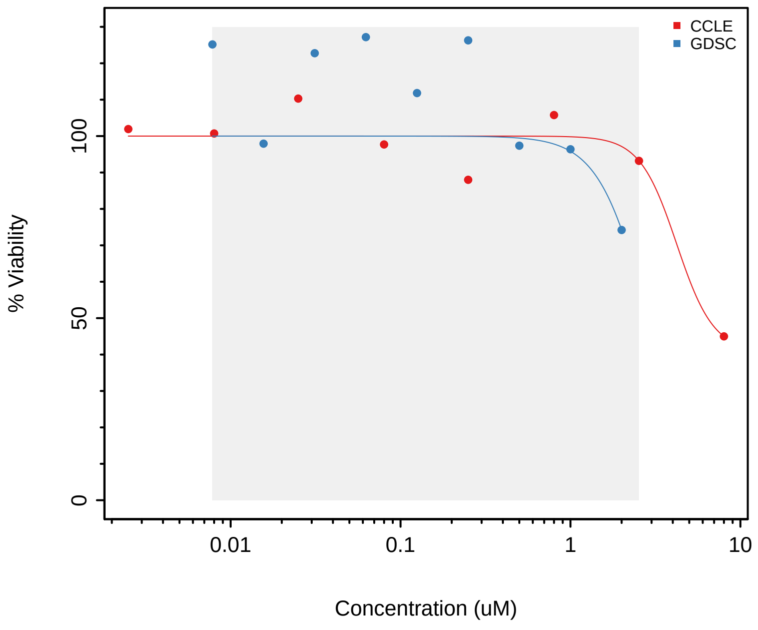

Nilotinib:NCI−H1092

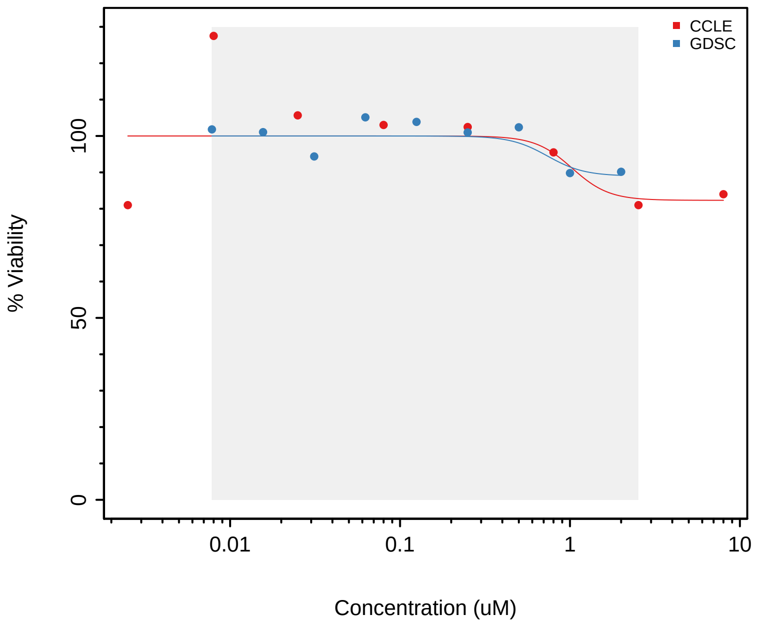

Nilotinib:NCI−H1355

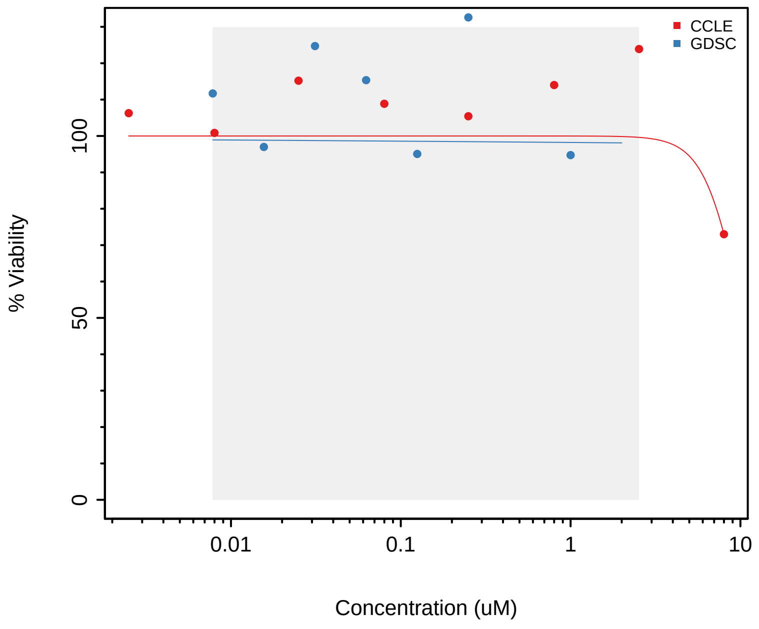

Nilotinib:NCI−H1563

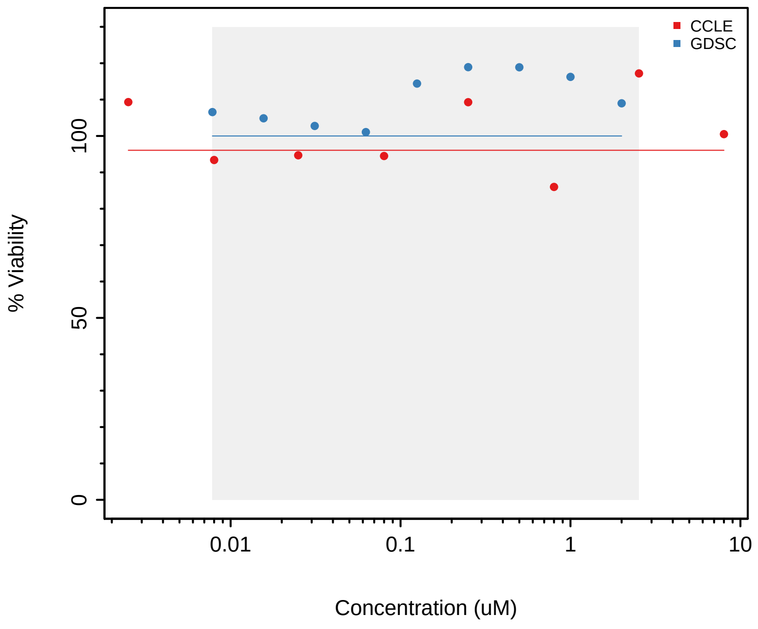

Nilotinib:NCI−H1573

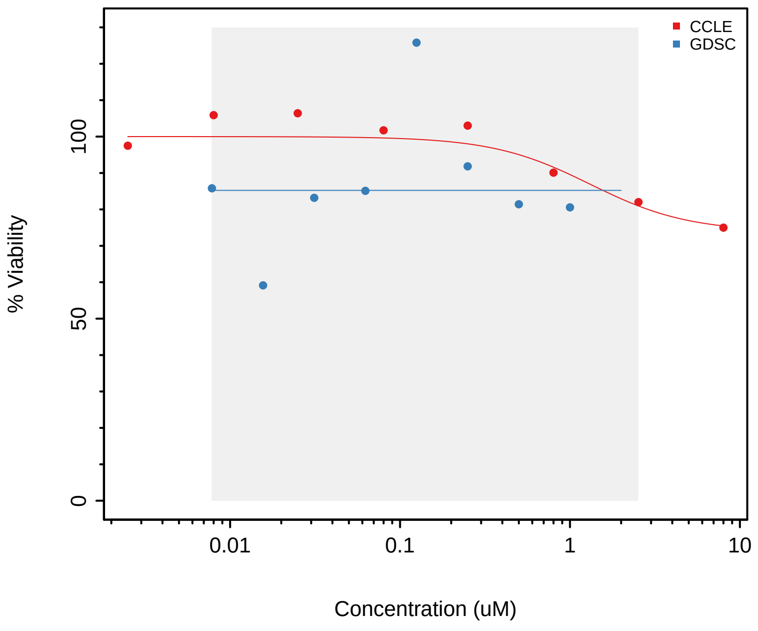

Nilotinib:NCI−H1581

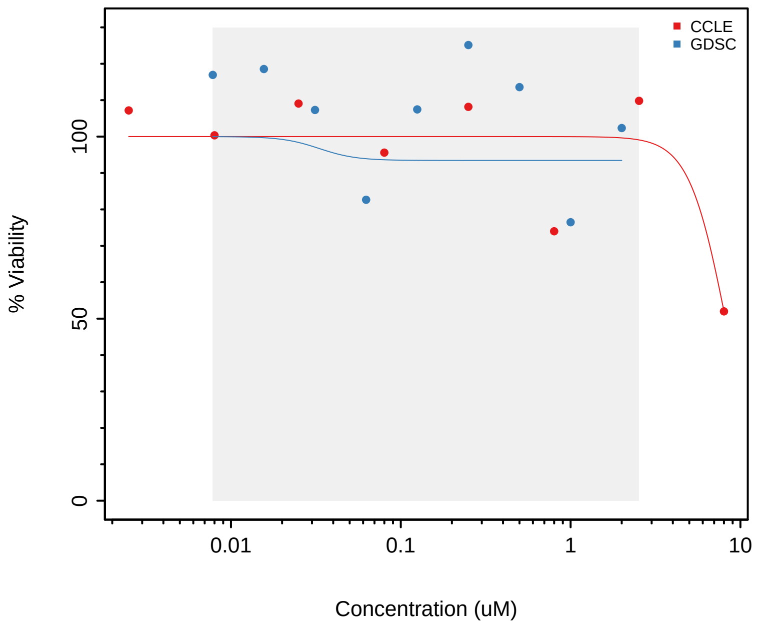

Nilotinib:NCI−H1650

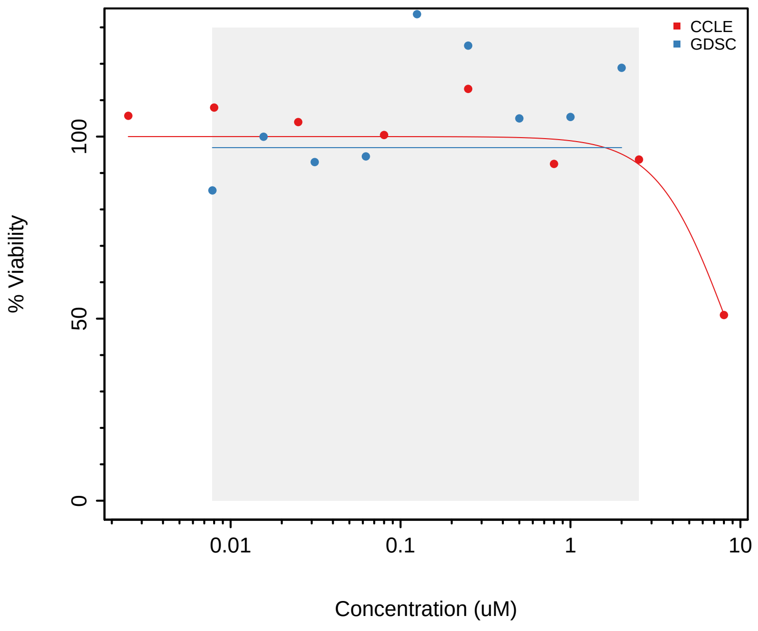

Nilotinib:NCI−H1651

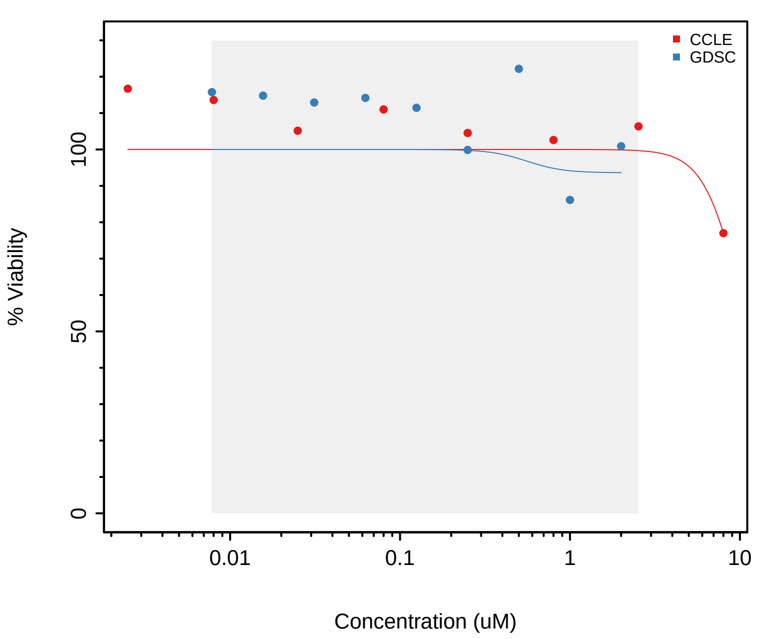

Nilotinib:NCI−H1666

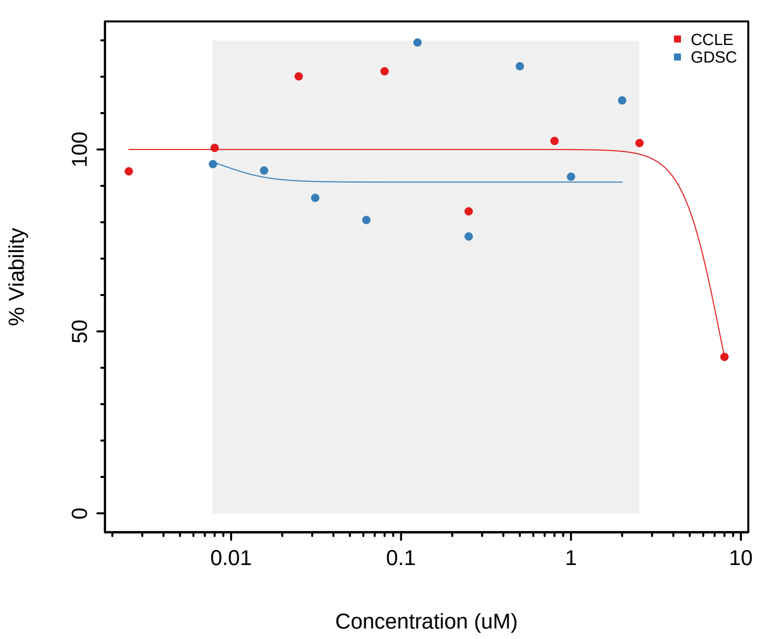

Nilotinib:NCI−H1693

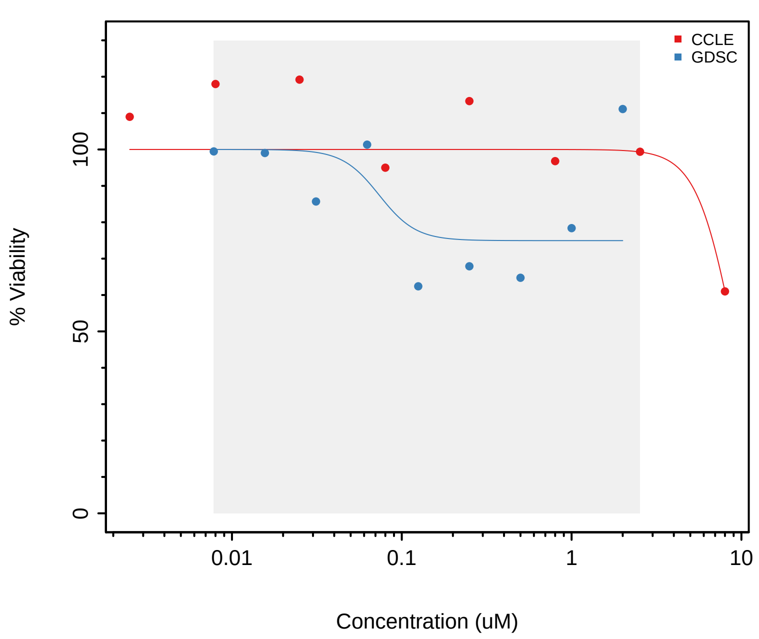

Nilotinib:NCI−H1694

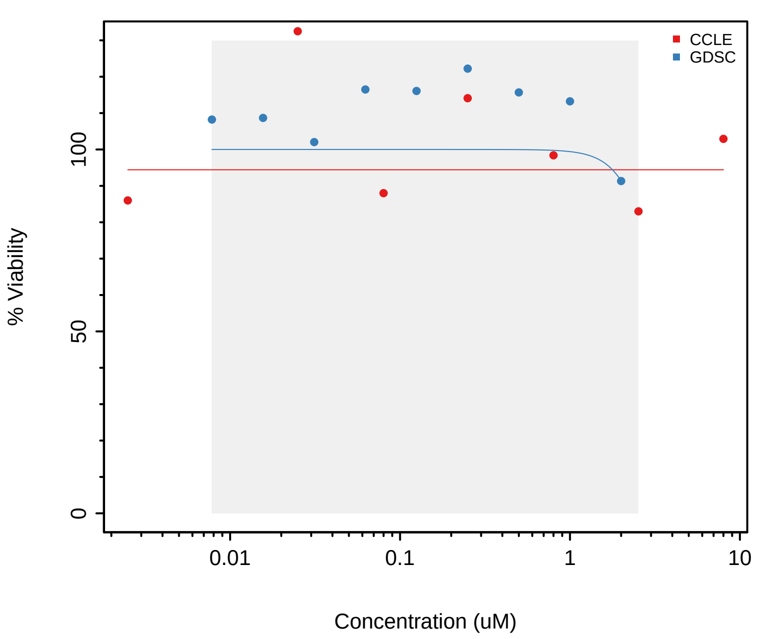

Nilotinib:NCI−H1792

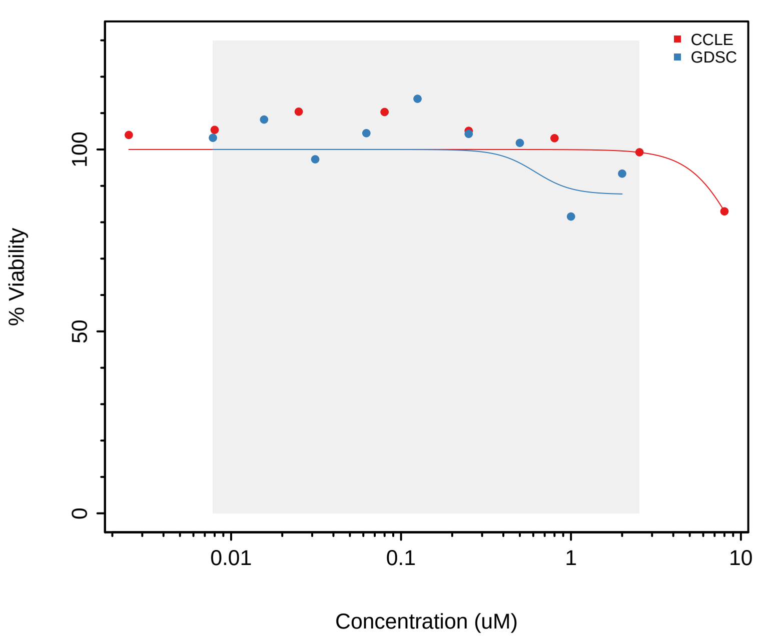

Nilotinib:NCI−H1793

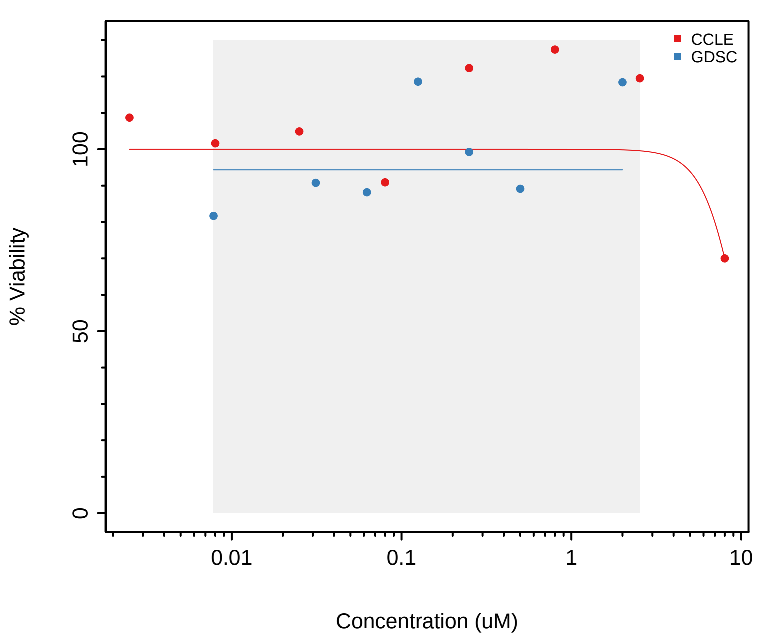

Nilotinib:NCI−H1975

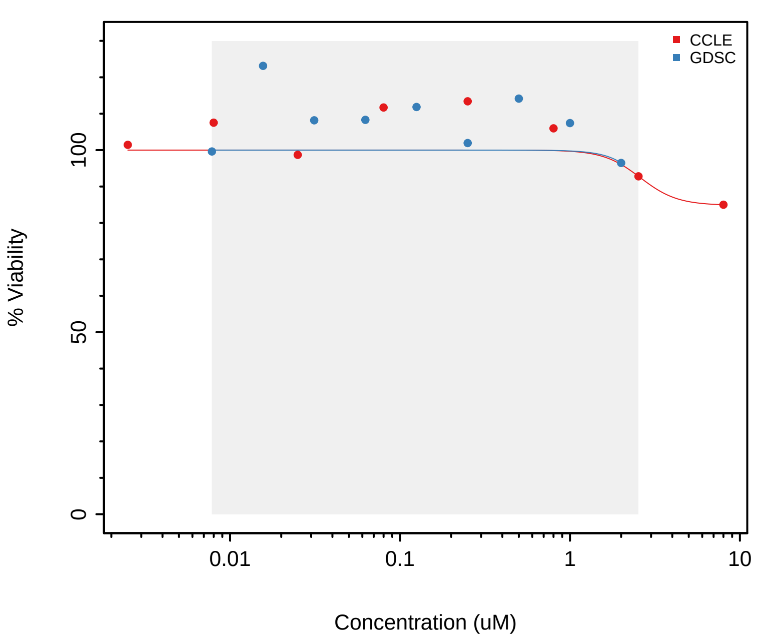

Nilotinib:NCI−H2009

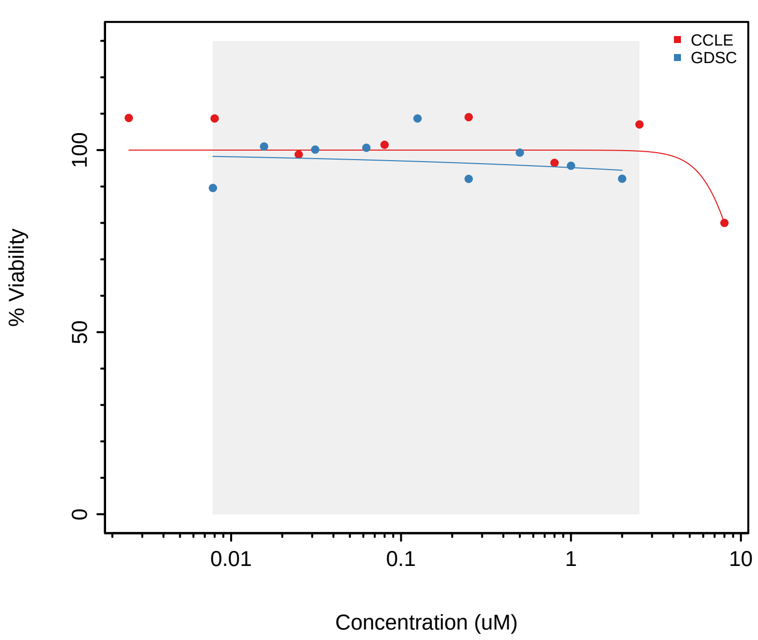

Nilotinib:NCI−H2052

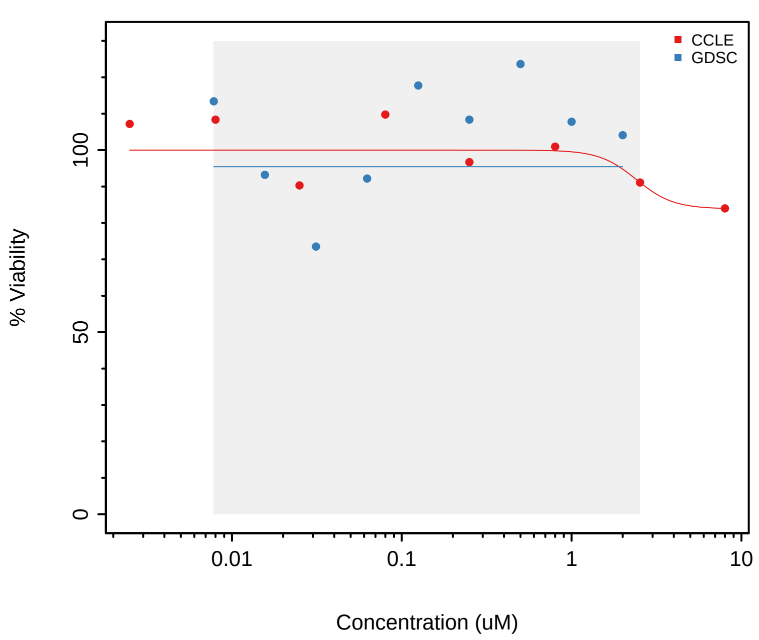

Nilotinib:NCI−H2087

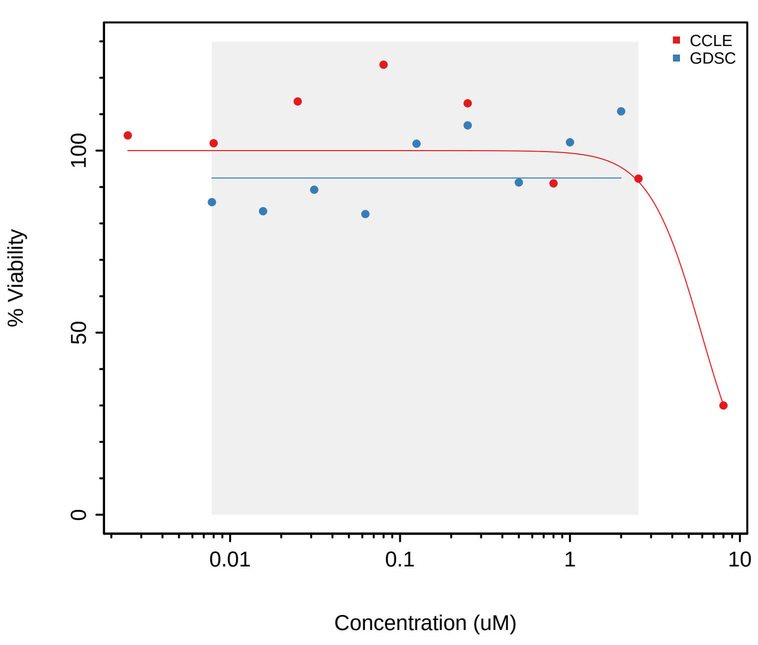

Nilotinib:NCI−H2122

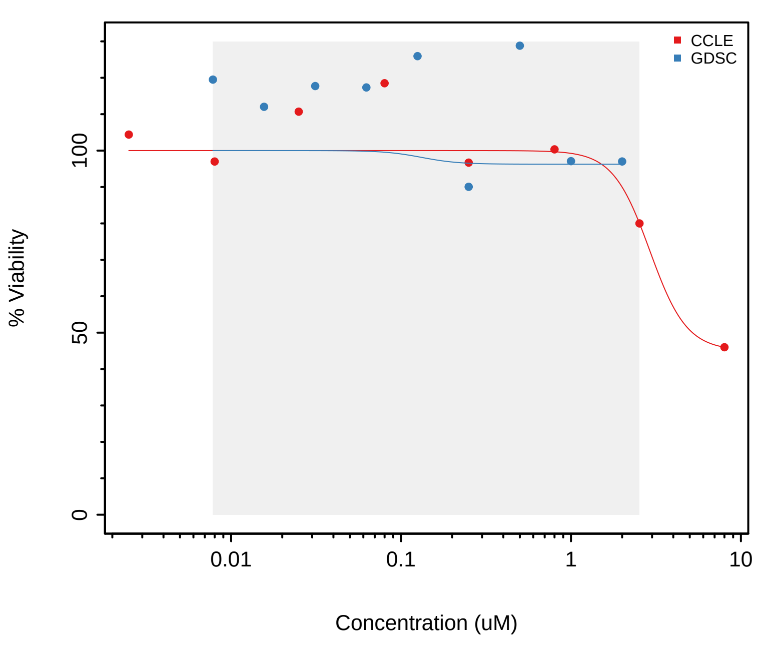

Nilotinib:NCI−H2228

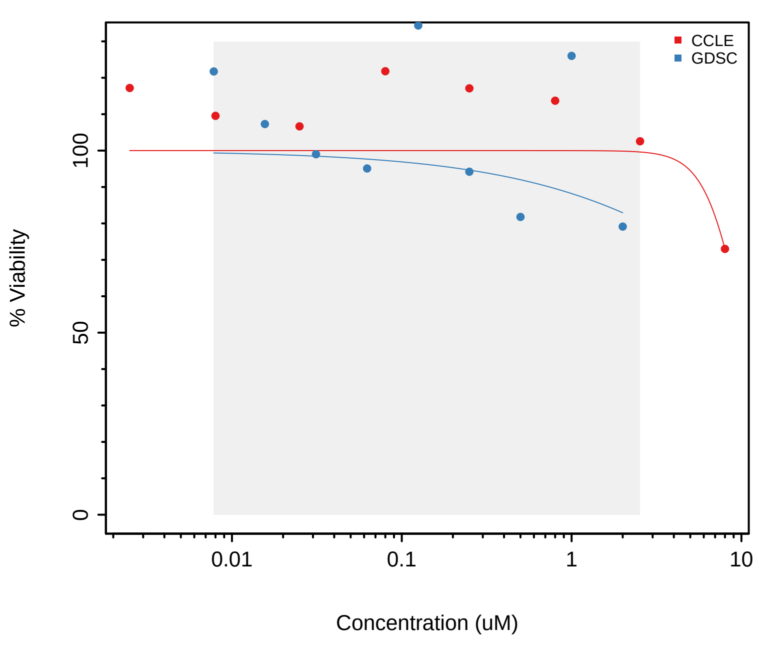

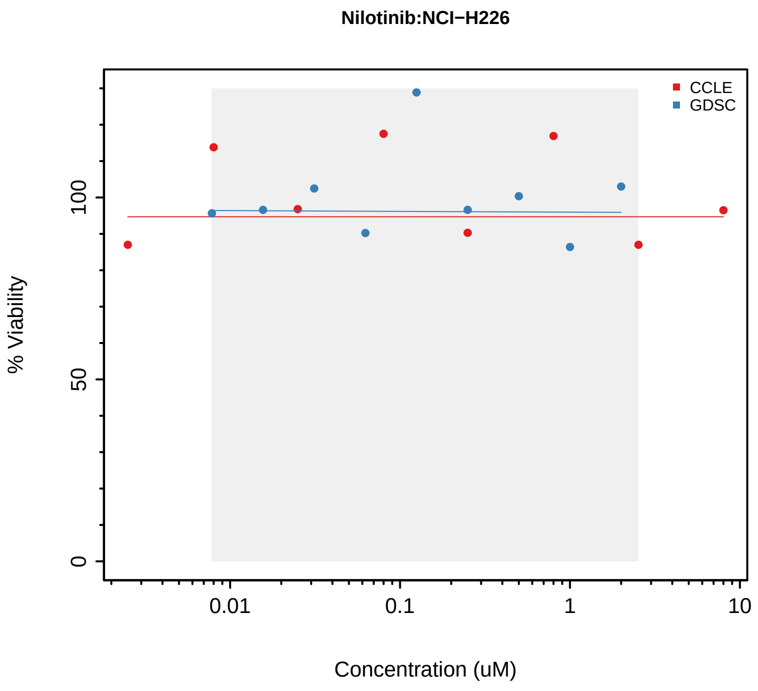
Nilotinib:NCI−H226
CCLE
GDSC
% Viability
Concentration (uM)

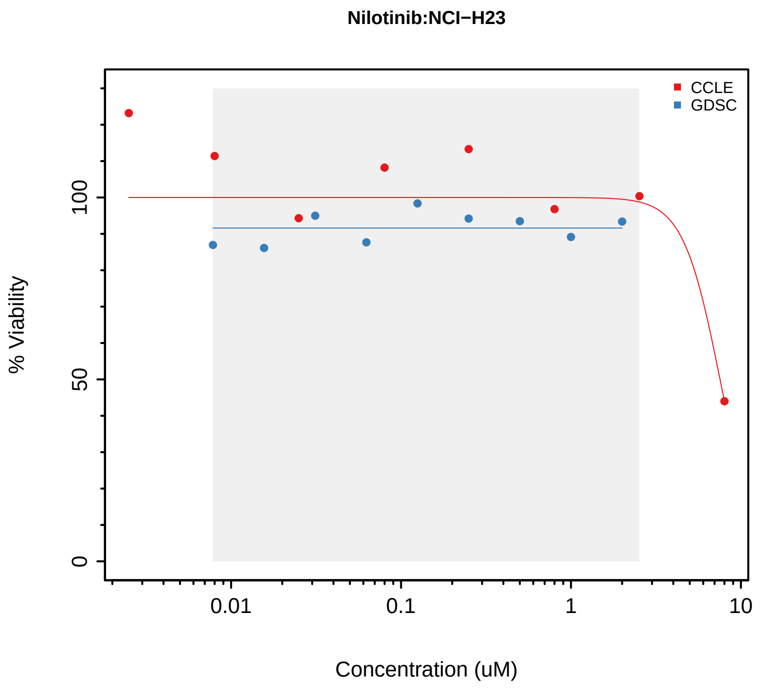
Nilotinib:NCI−H23
CCLE
GDSC
% Viability
Concentration (uM)

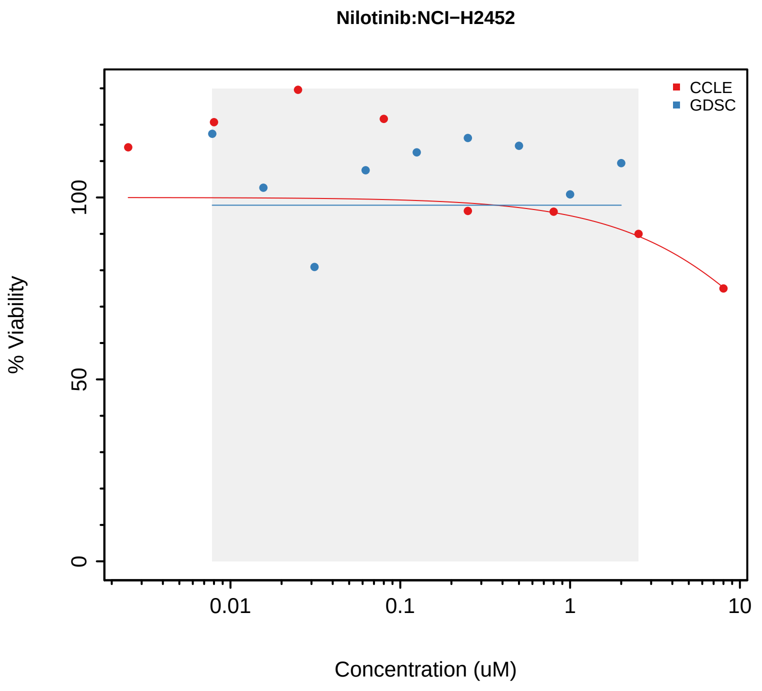
Nilotinib:NCI−H2452
CCLE
GDSC
% Viability
Concentration (uM)

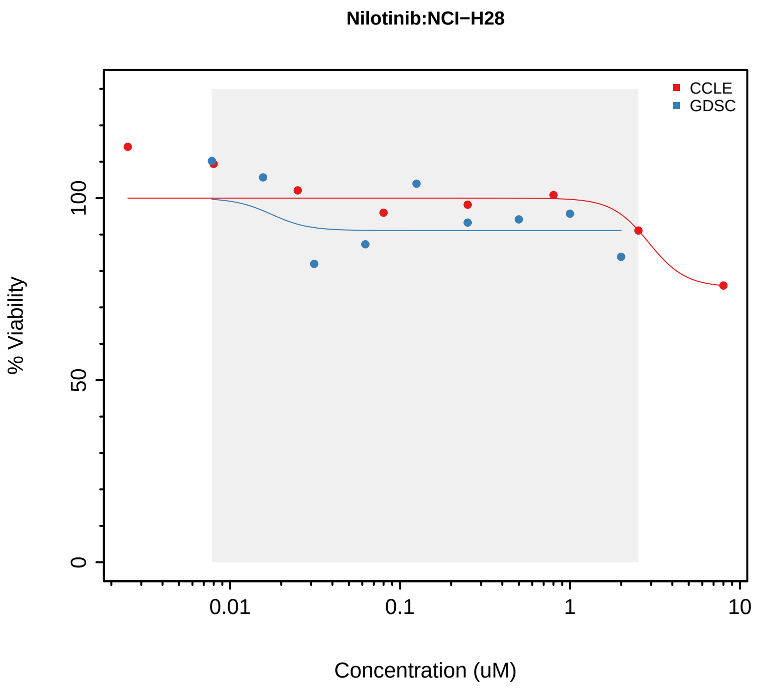
Nilotinib:NCI−H28
CCLE
GDSC
% Viability
Concentration (uM)

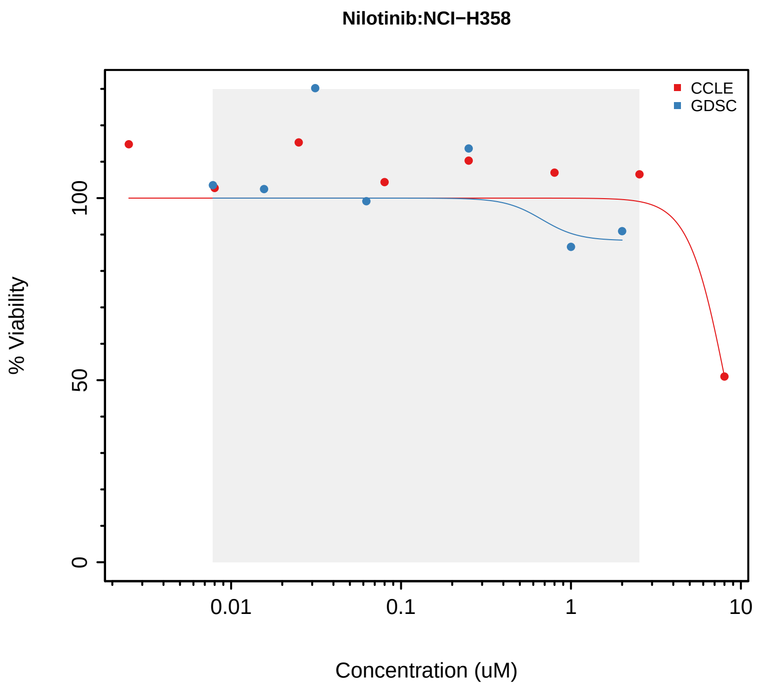
Nilotinib:NCI−H358
CCLE
GDSC
% Viability
Concentration (uM)

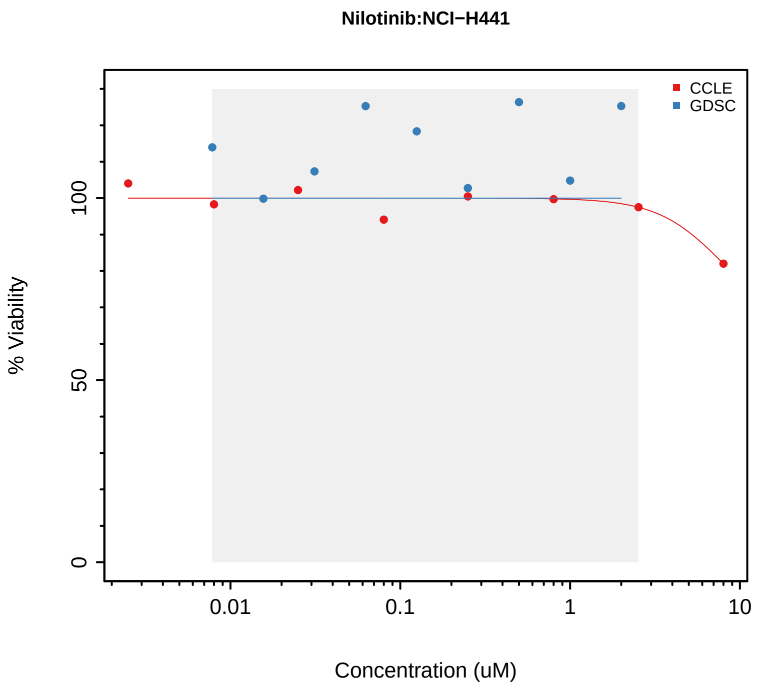
Nilotinib:NCI−H441
CCLE
GDSC
% Viability
Concentration (uM)

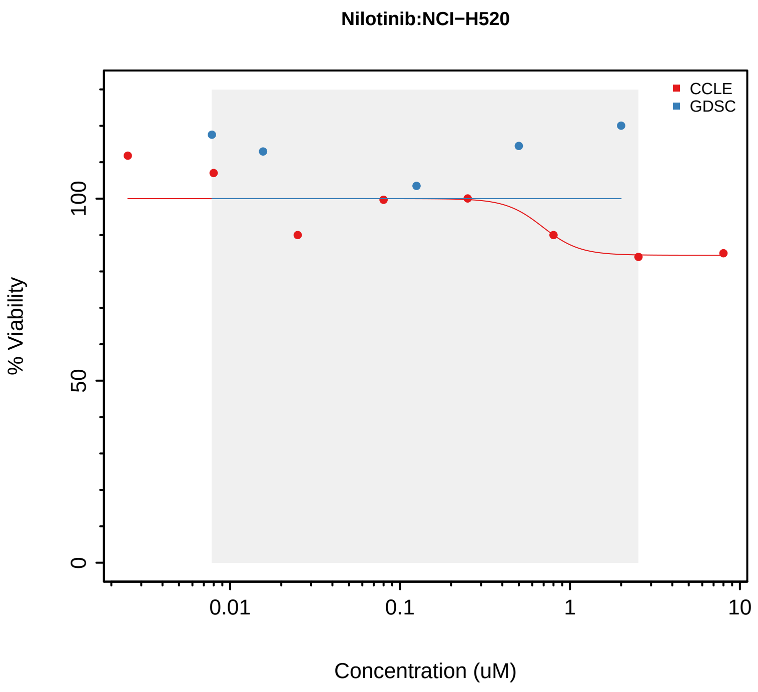
Nilotinib:NCI−H520
CCLE
GDSC
% Viability
Concentration (uM)

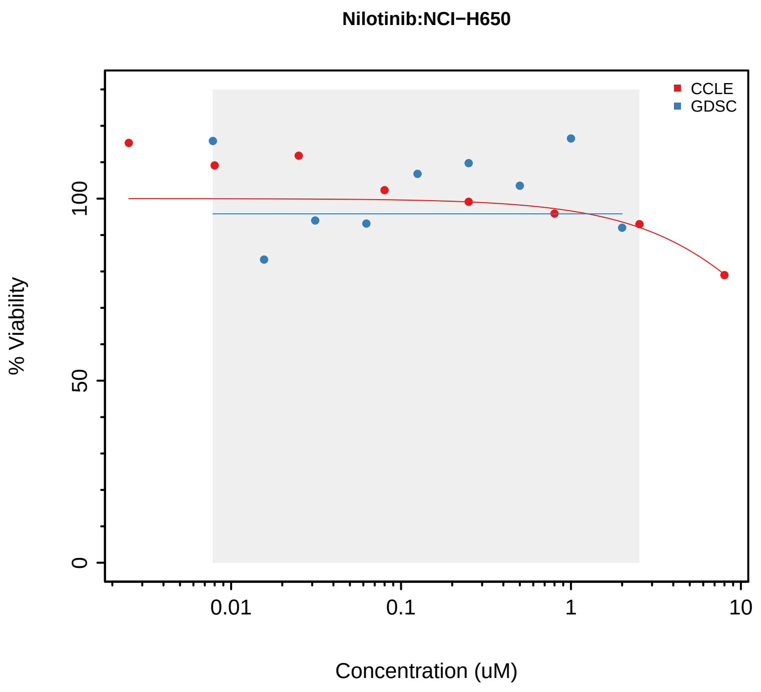
Nilotinib:NCI−H650
CCLE
GDSC
% Viability
Concentration (uM)

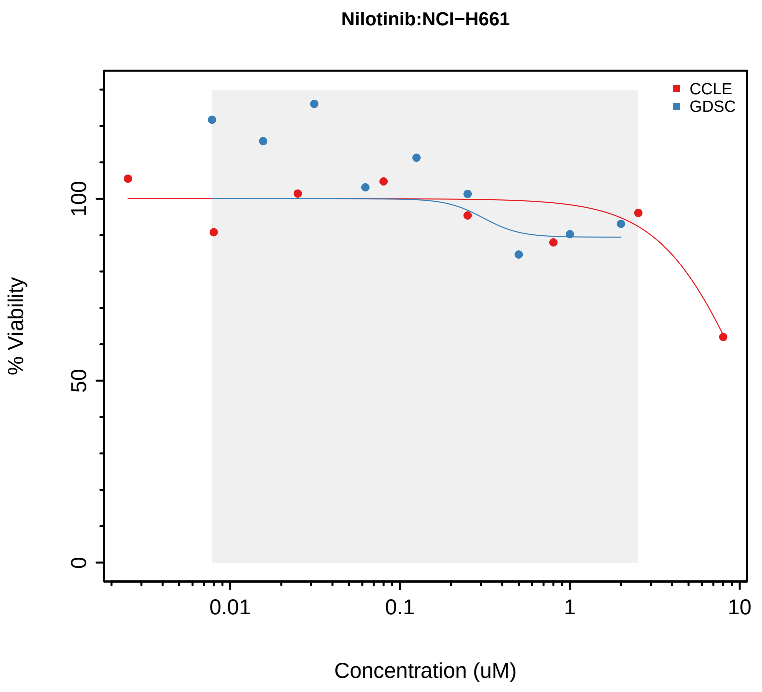
Nilotinib:NCI−H661
CCLE
GDSC
% Viability
Concentration (uM)

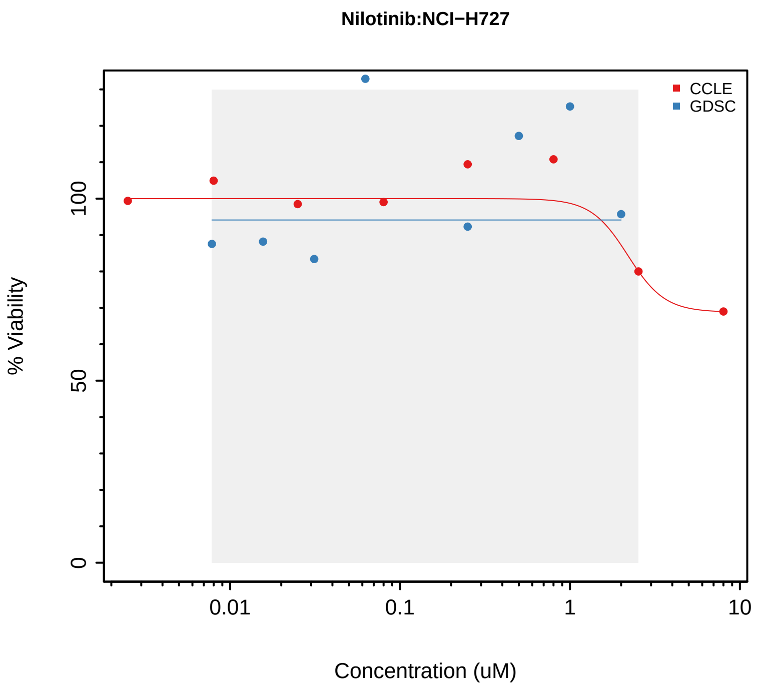
Nilotinib:NCI−H727
CCLE
GDSC
% Viability
Concentration (uM)

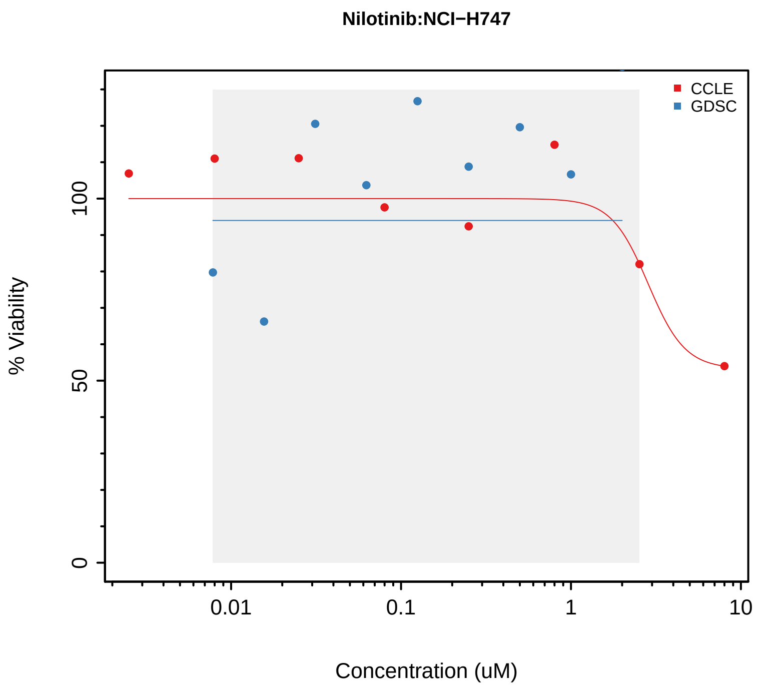
Nilotinib:NCI−H747
CCLE
GDSC
% Viability
Concentration (uM)

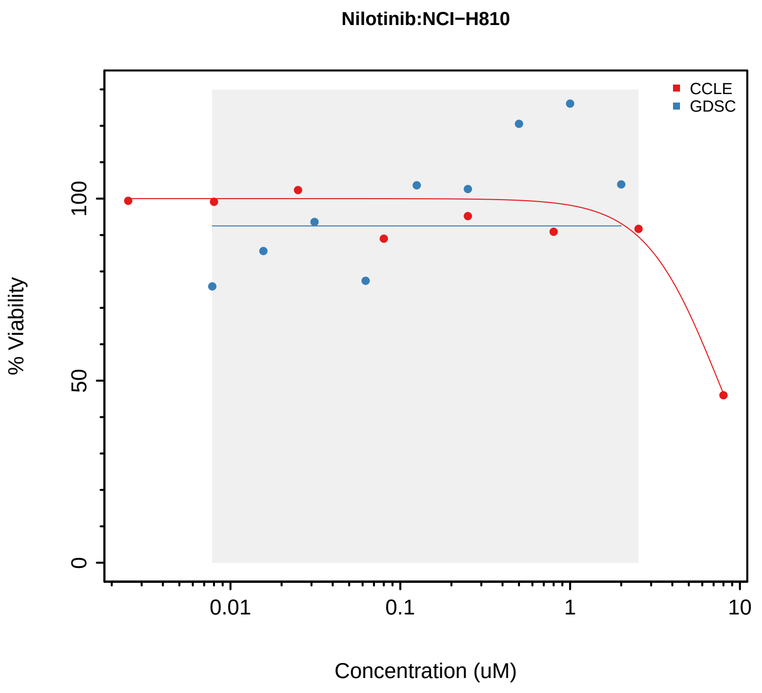
Nilotinib:NCI−H810
CCLE
GDSC
% Viability
Concentration (uM)

Nilotinib:NCI−N87

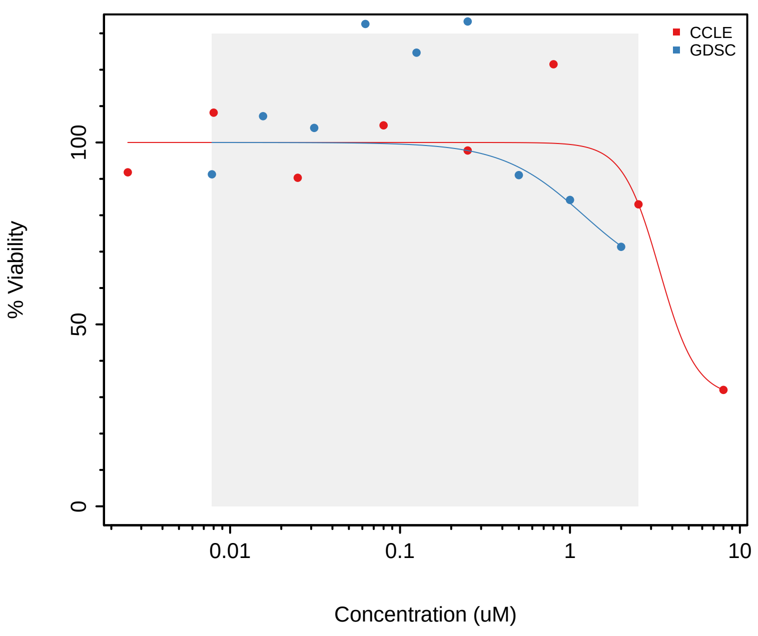

Nilotinib:OVCAR−3

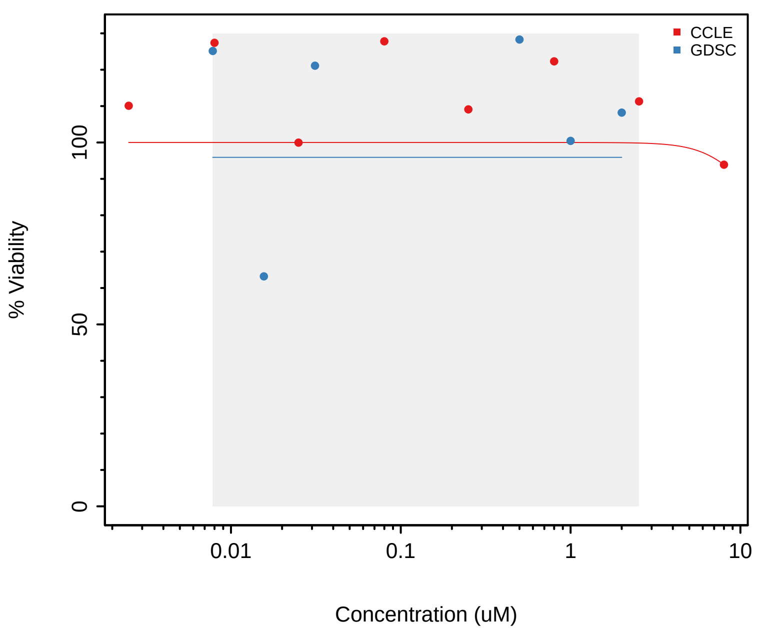

Nilotinib:NUGC−3

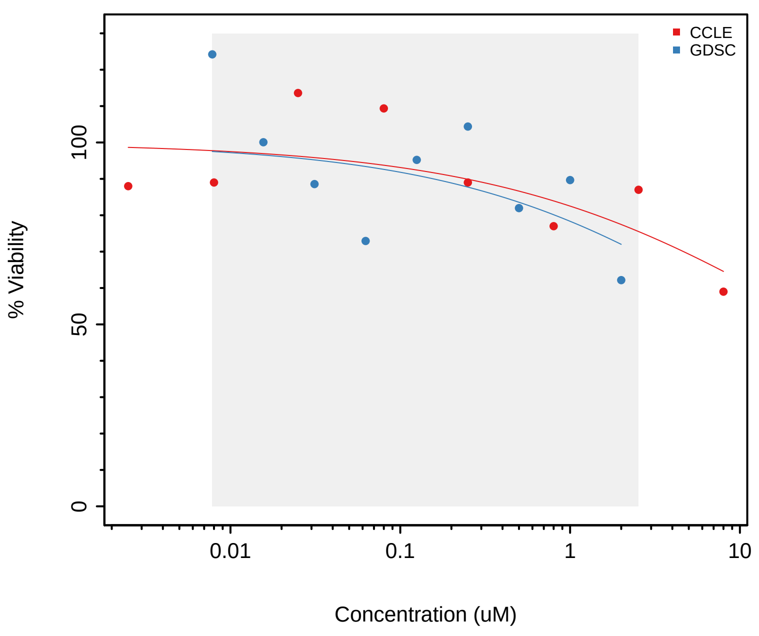

Nilotinib:OC−314

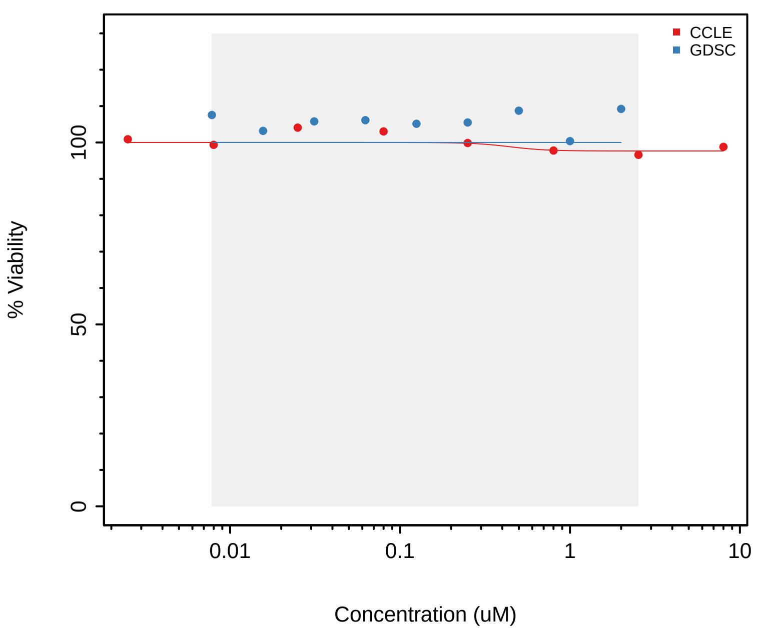

Nilotinib:OCI−AML2

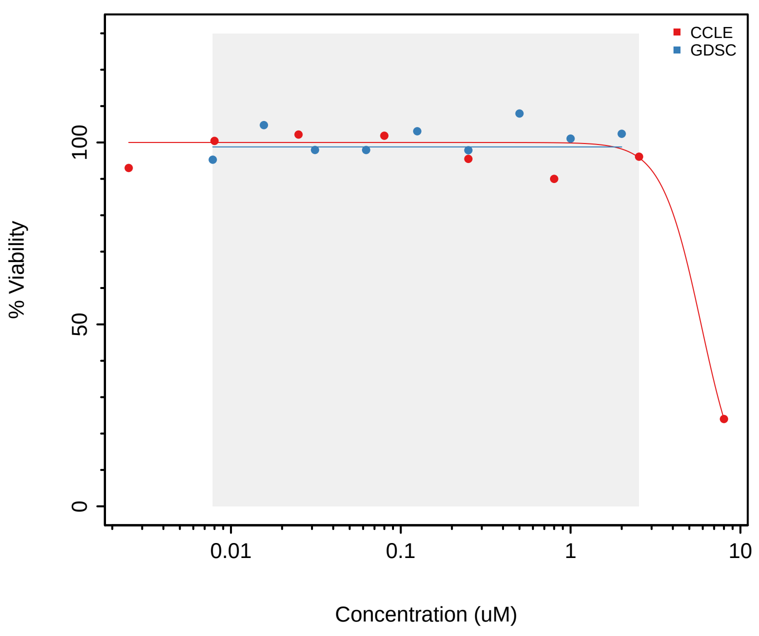

Nilotinib:OE33

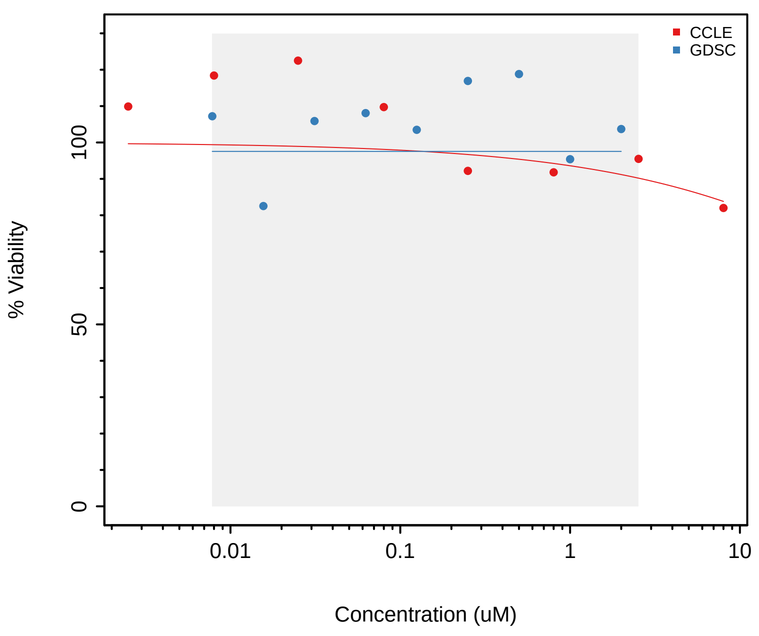

Nilotinib:ONS−76

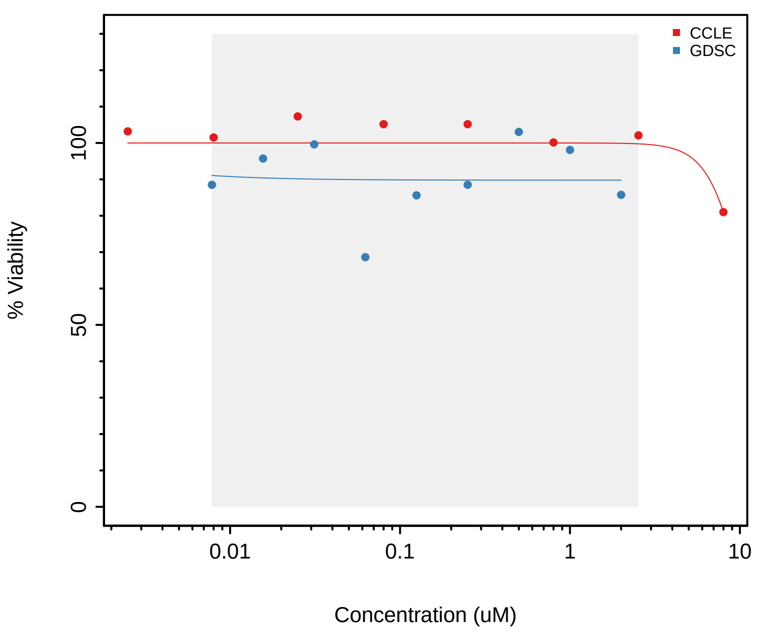

Nilotinib:OPM−2

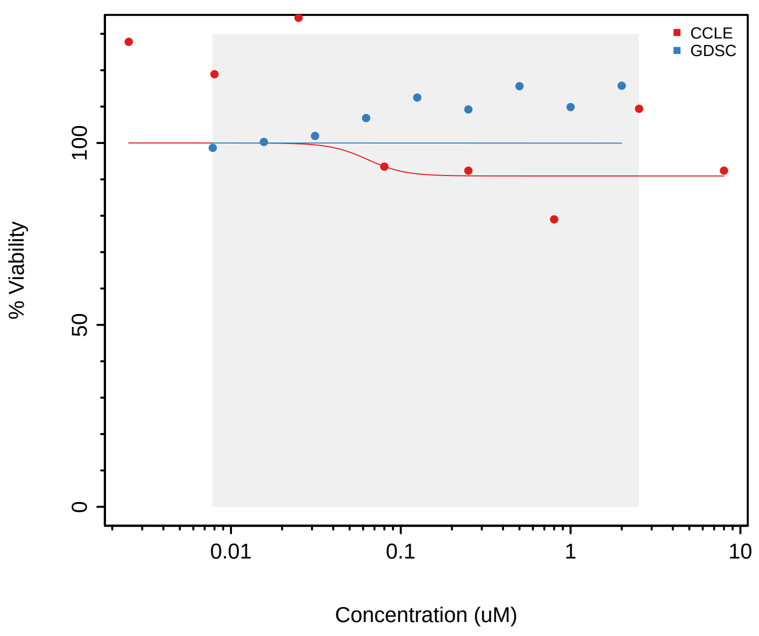

Nilotinib:OVCAR−4

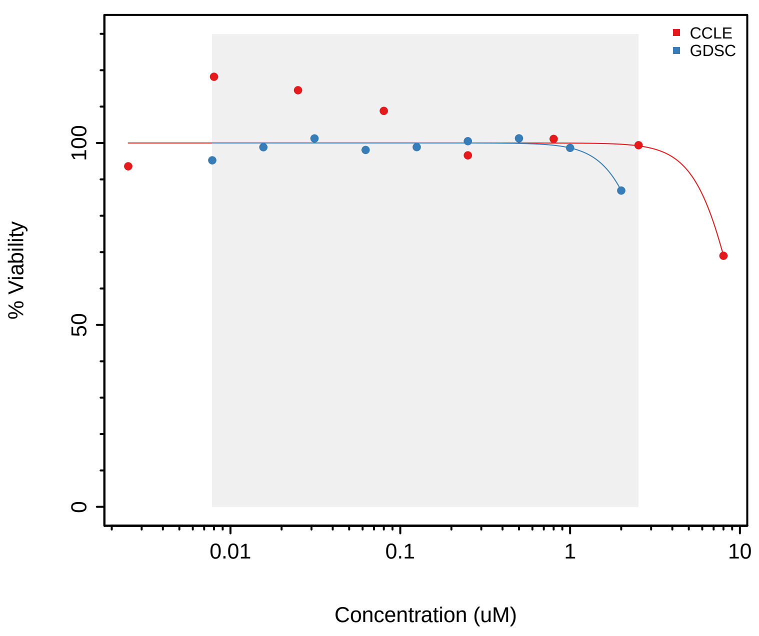

Nilotinib:OVCAR−8

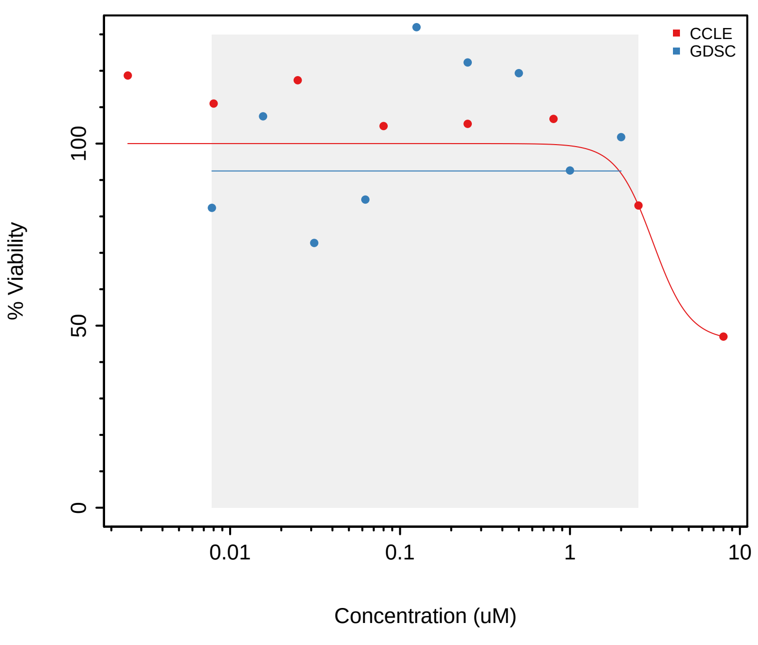

Nilotinib:P12−ICHIKAWA

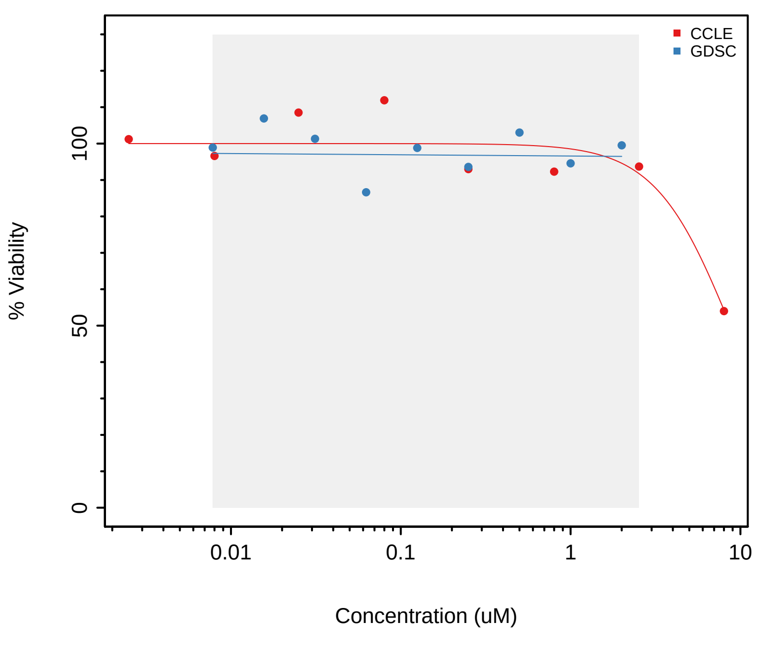

Nilotinib:P31−FUJ

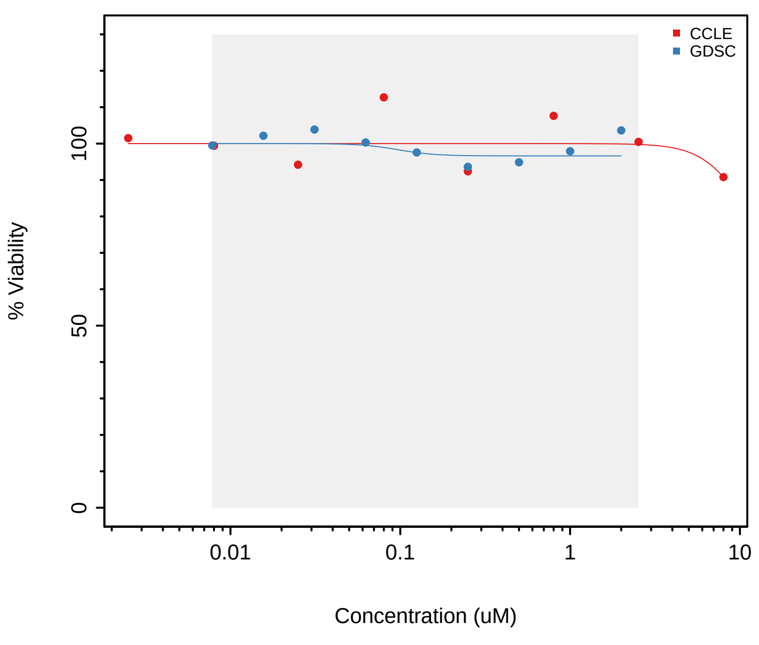

Nilotinib:Panc 03.27

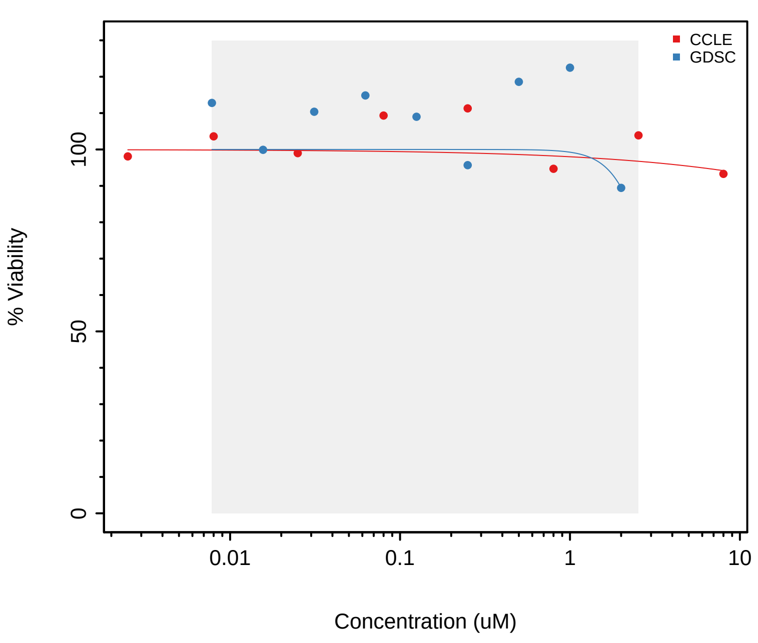

Nilotinib:Panc 10.05

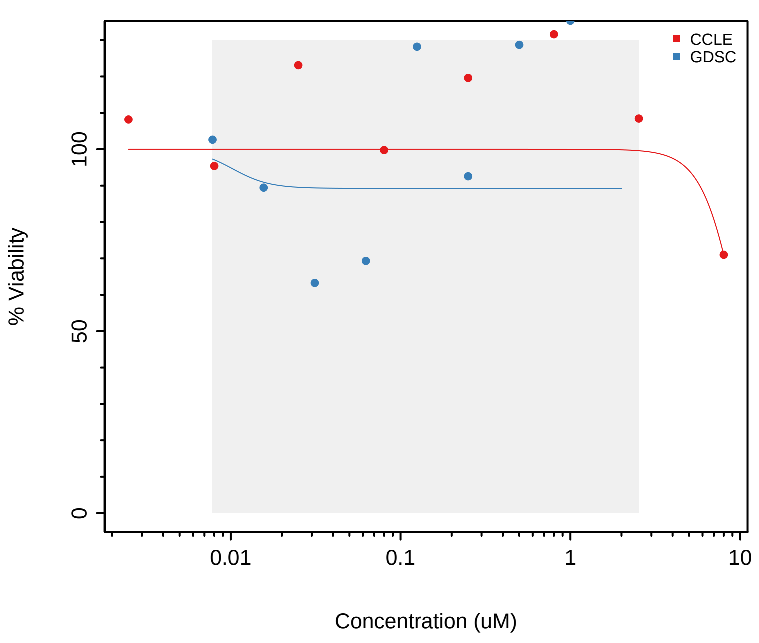

Nilotinib:Raji

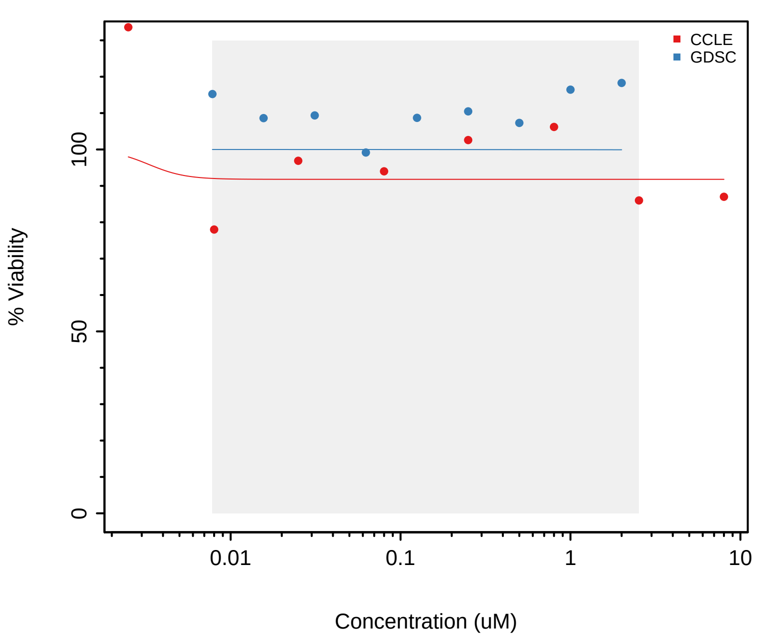

Nilotinib:REH

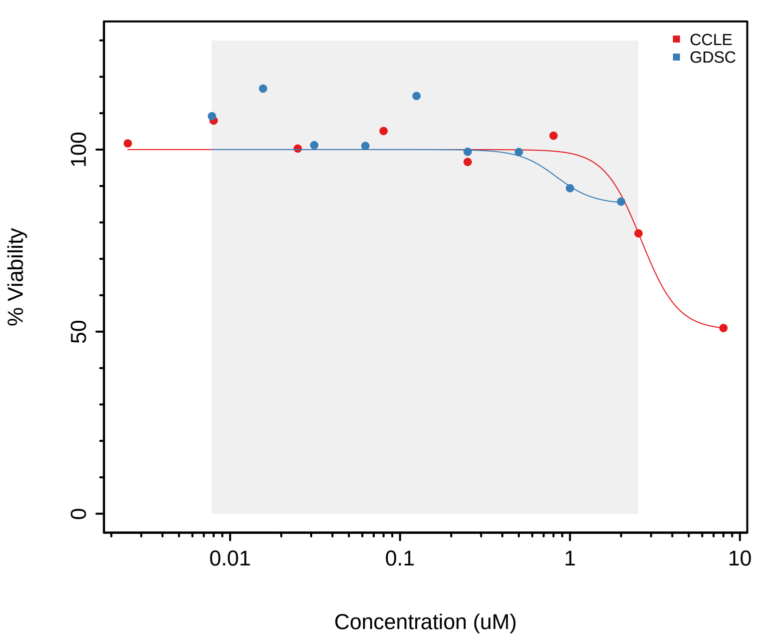

Nilotinib:RERF−LC−MS

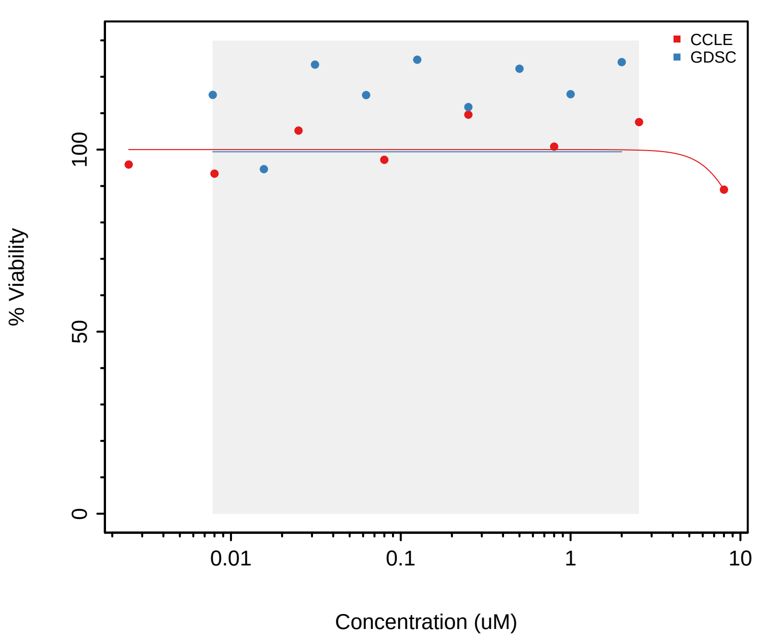

Nilotinib:RKO

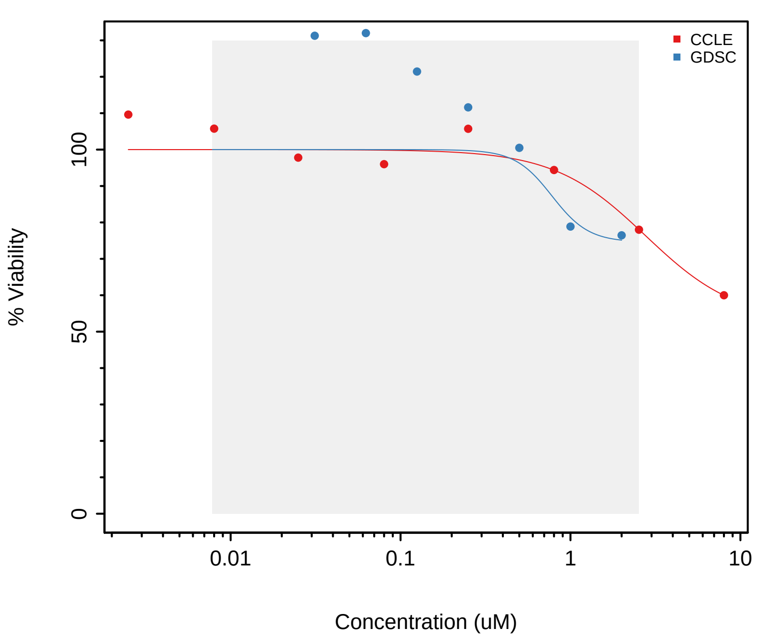

Nilotinib:RL95−2

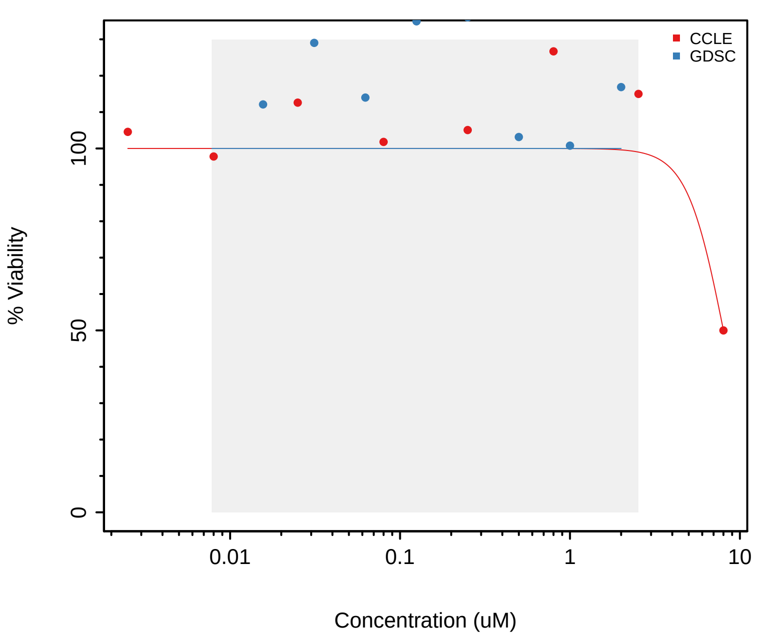

Nilotinib:RPMI−8402

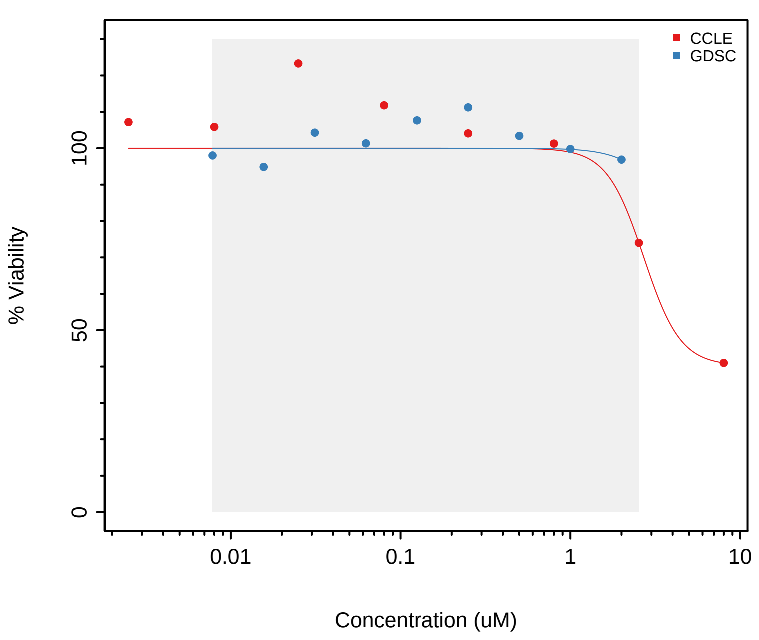

Nilotinib:RT−112

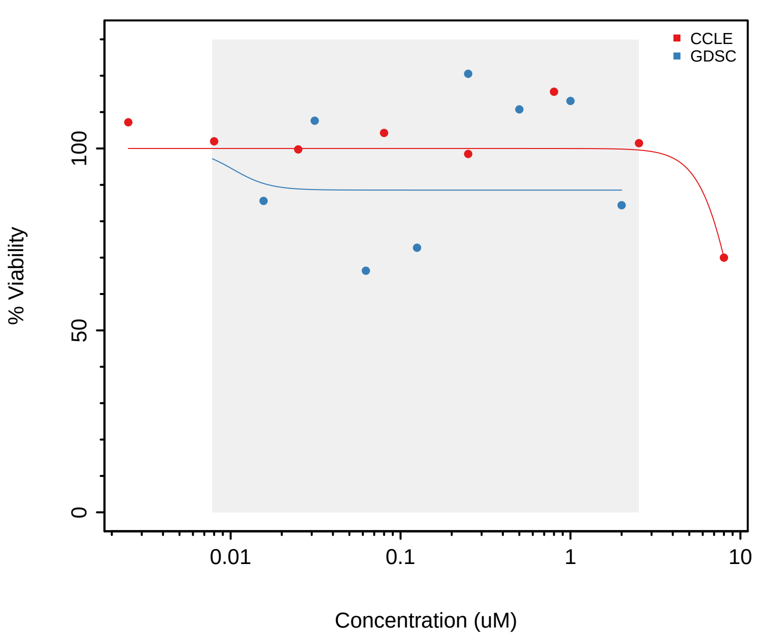

Nilotinib:RT4

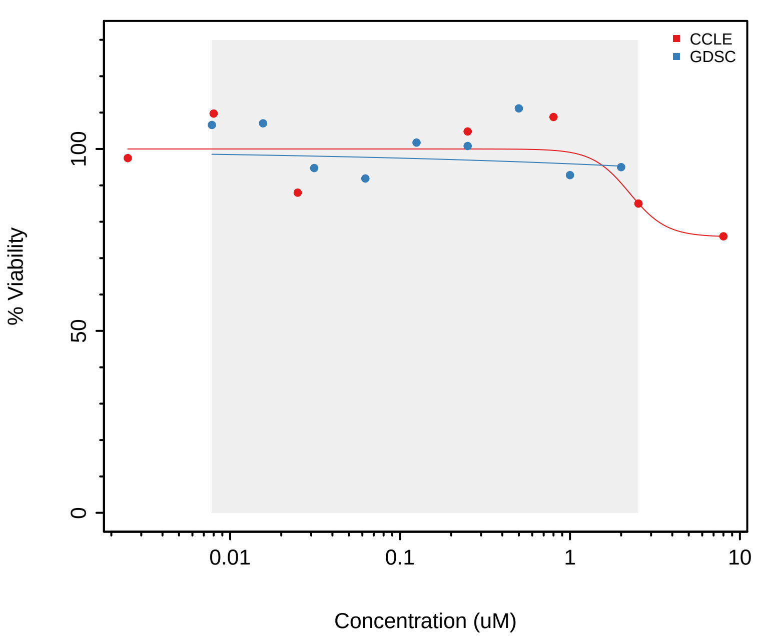

Nilotinib:RVH−421

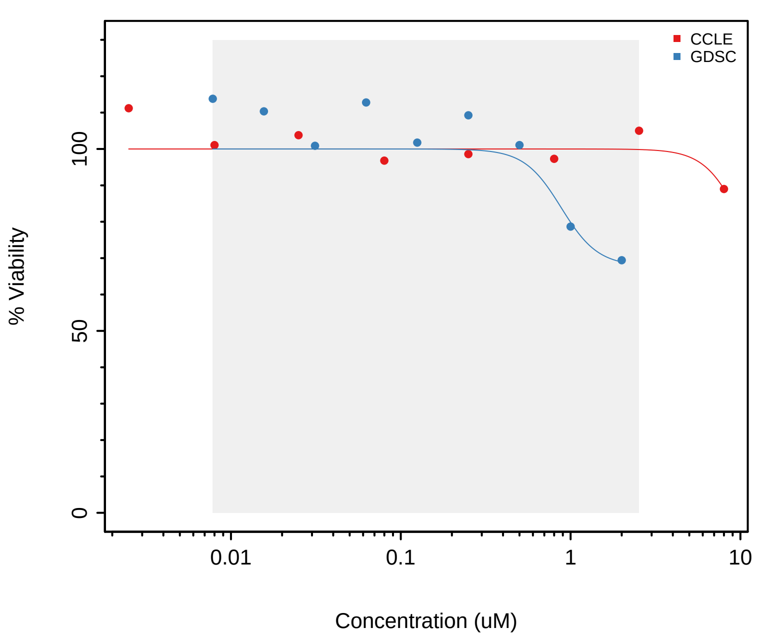

Nilotinib:Saos−2

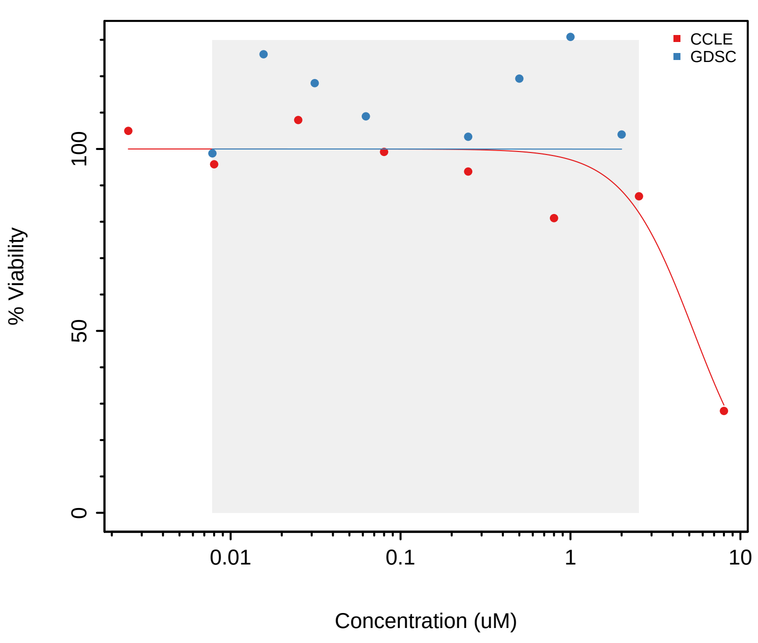

Nilotinib:SBC−5

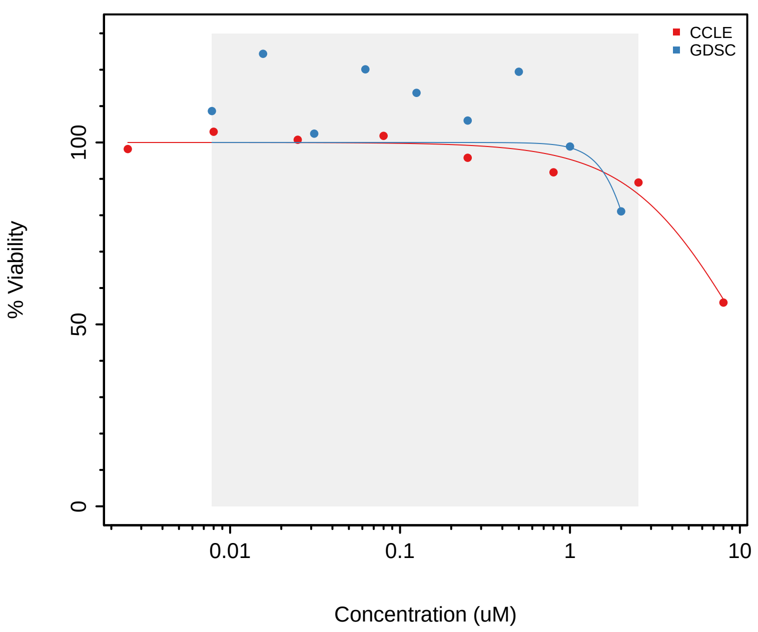

Nilotinib:SF126

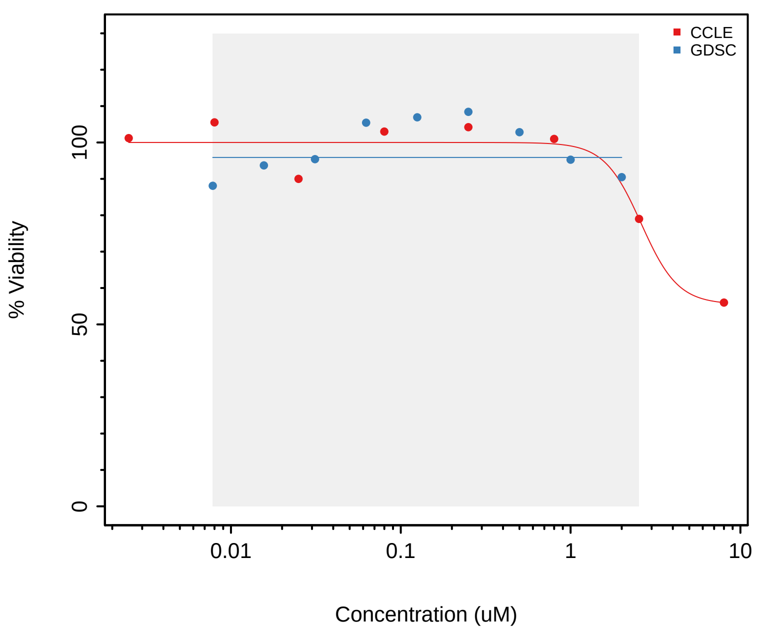

Nilotinib:SHP−77

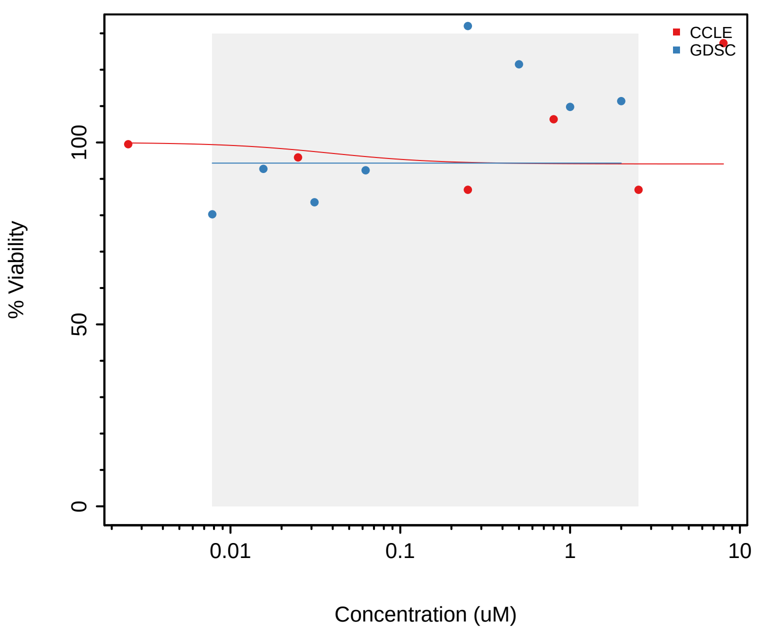

Nilotinib:SIMA

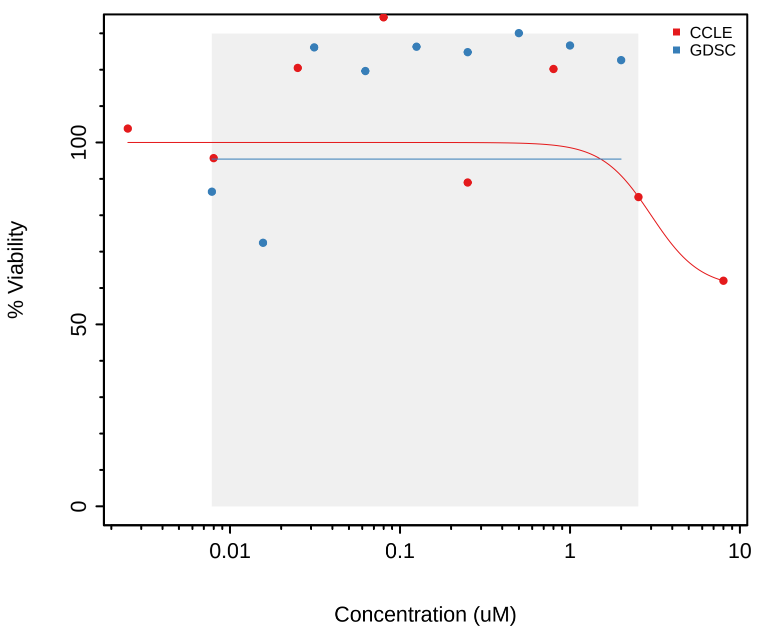

Nilotinib:SJRH30

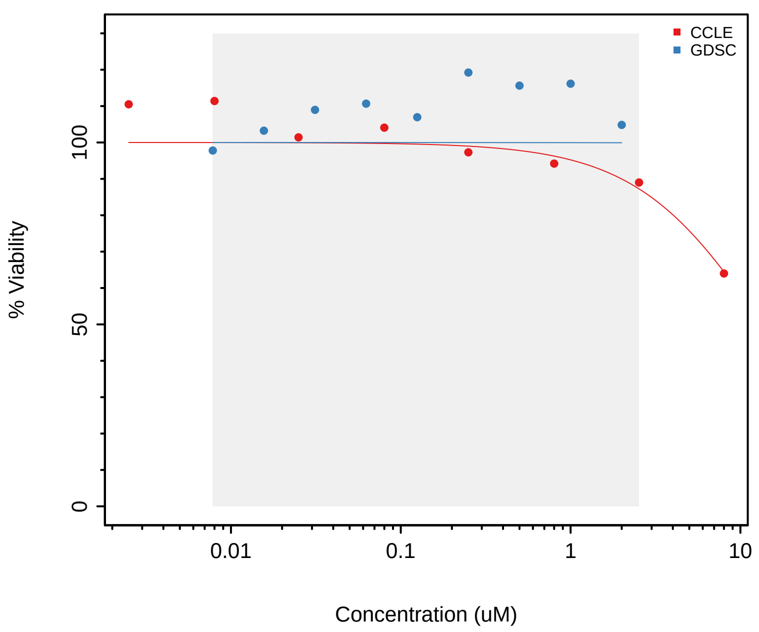

Nilotinib:SK−HEP−1

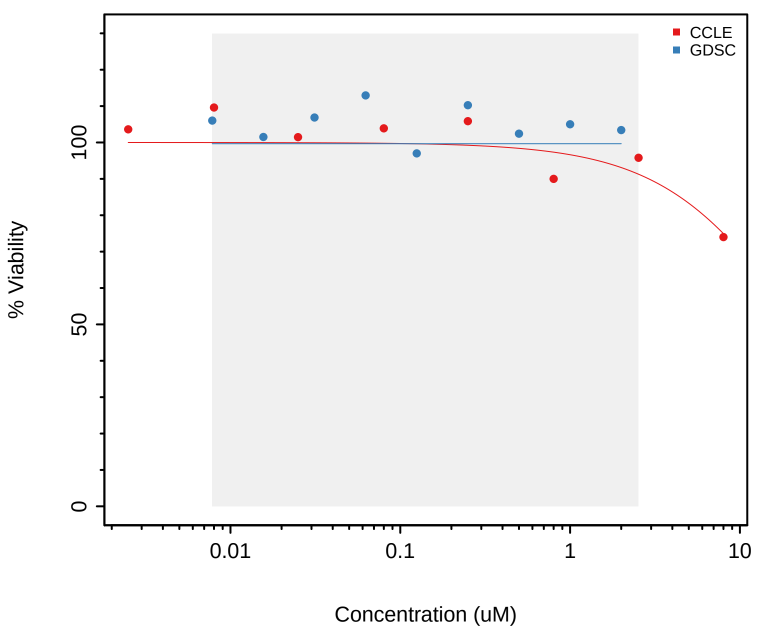

Nilotinib:SK−LU−1

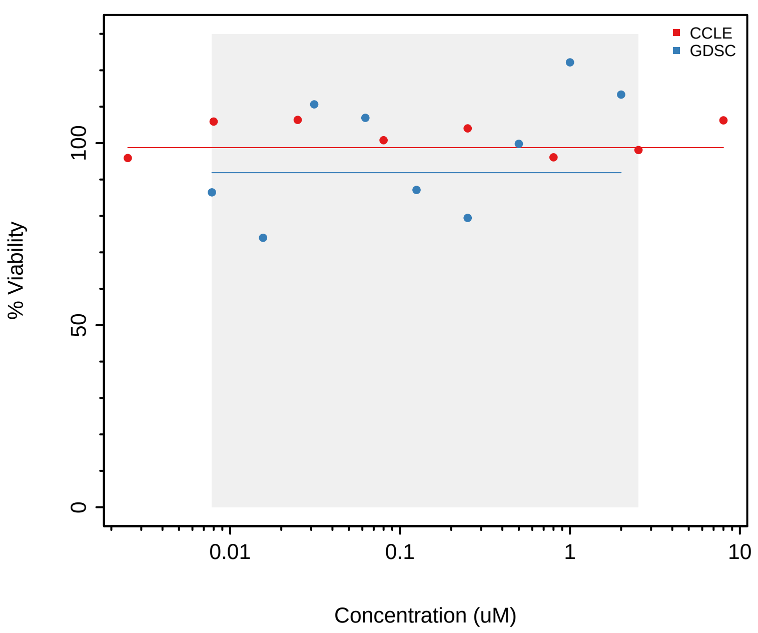

Nilotinib:SK−MEL−2

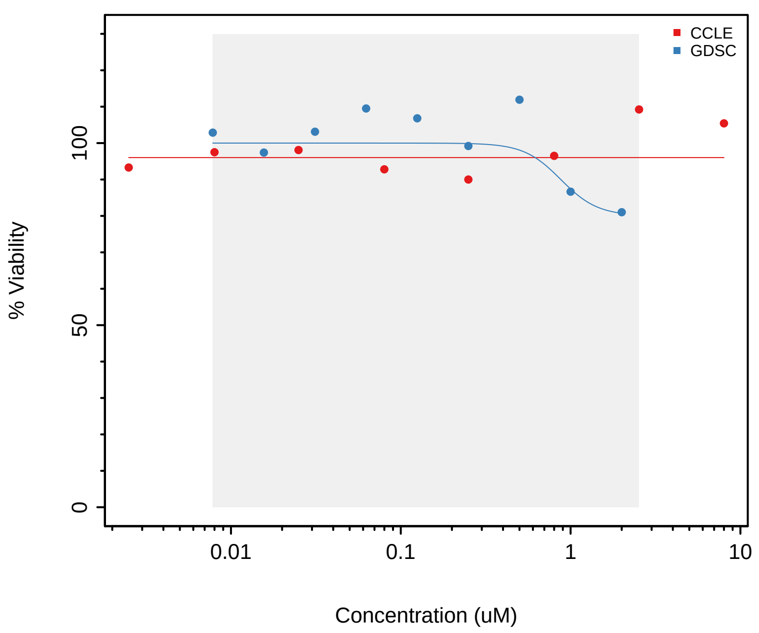

Nilotinib:SK−MEL−24

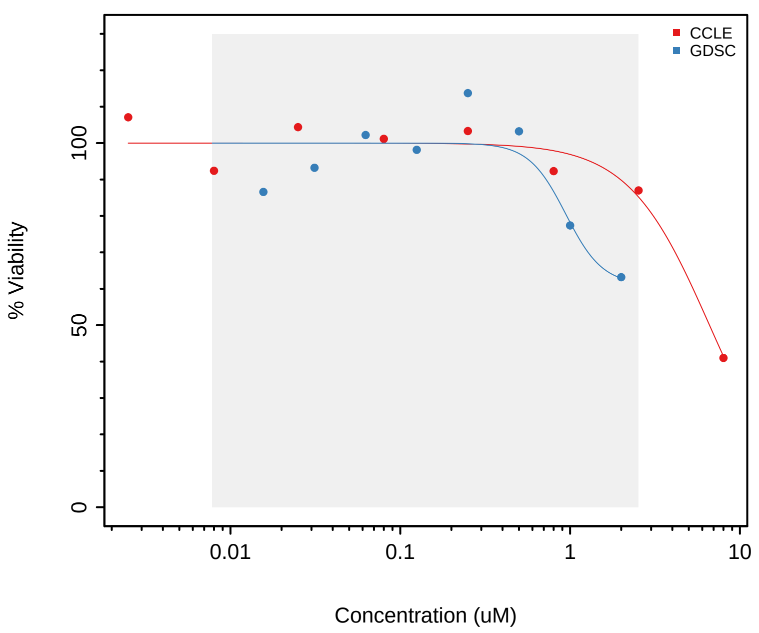

Nilotinib:SK−MEL−30

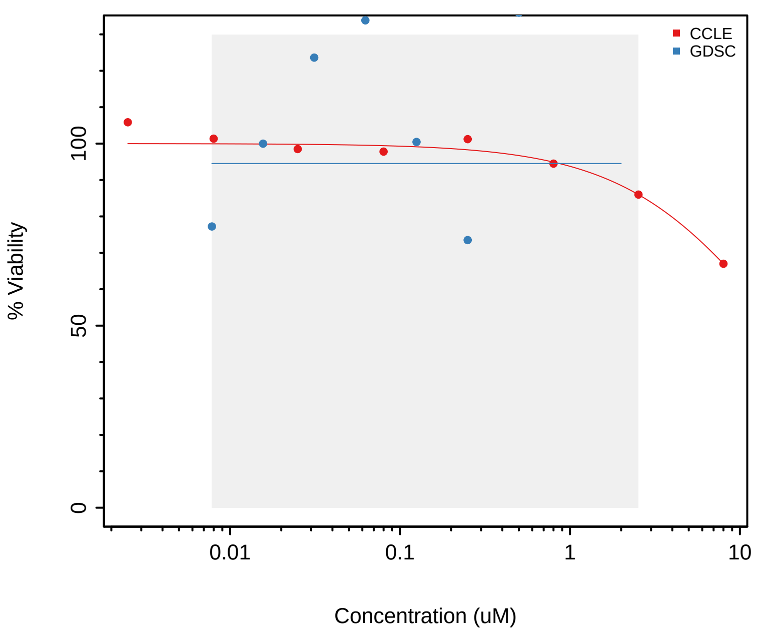

Nilotinib:SK−MEL−5

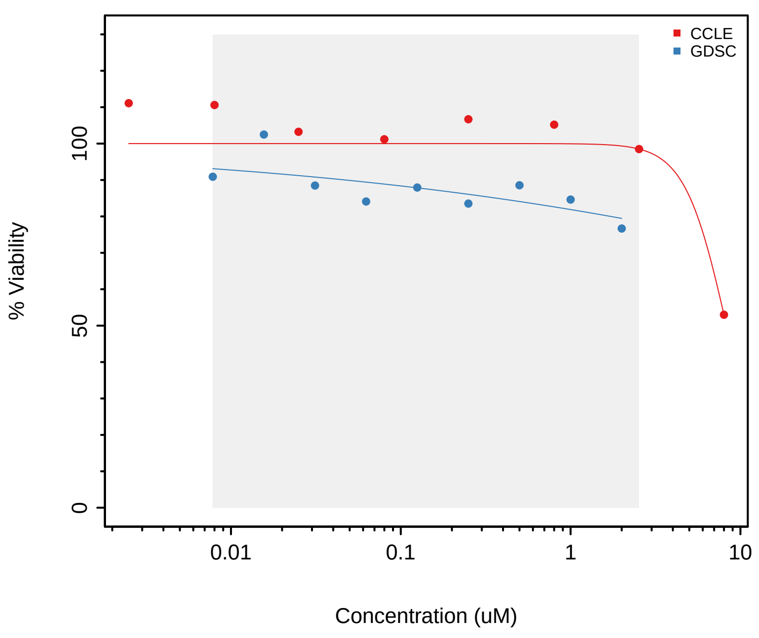

Nilotinib:SK−MM−2

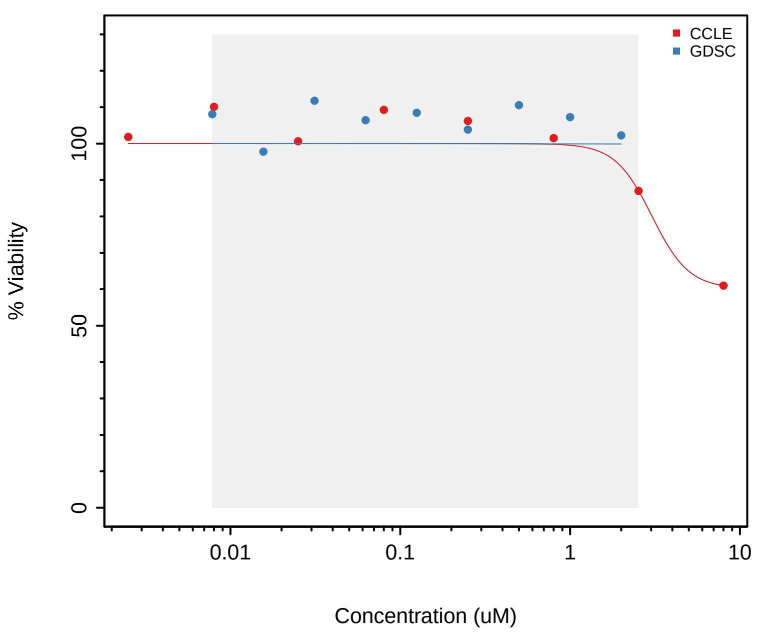

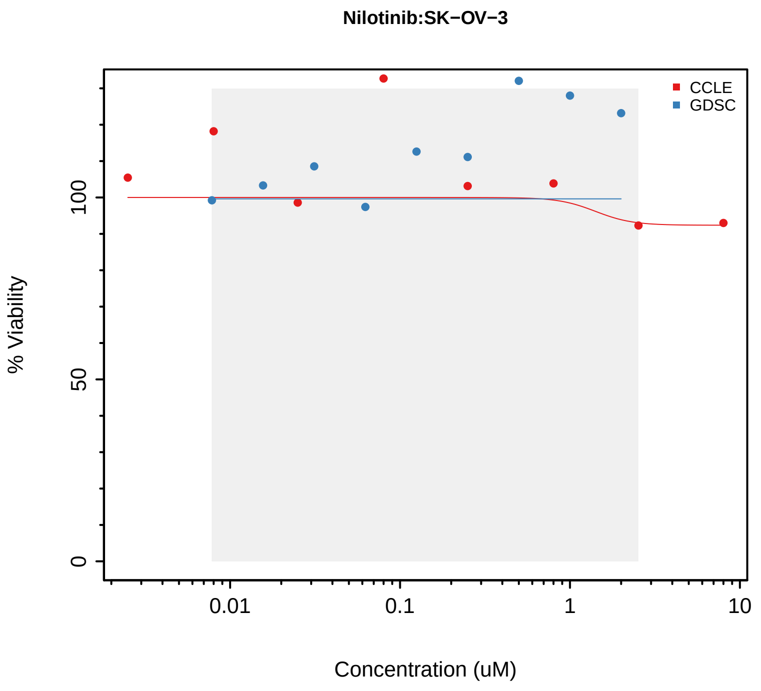
Nilotinib:SK−OV−3
CCLE
GDSC
% Viability
0
50
100
0.01
0.1
1
10
Concentration (uM)

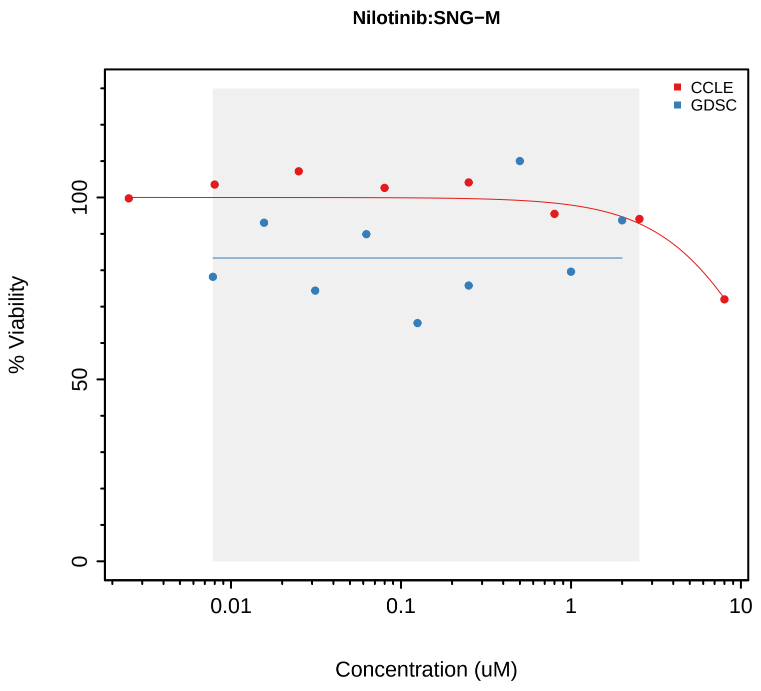
Nilotinib:SNG−M
CCLE
GDSC
% Viability
0
50
100
0.01
0.1
1
10
Concentration (uM)

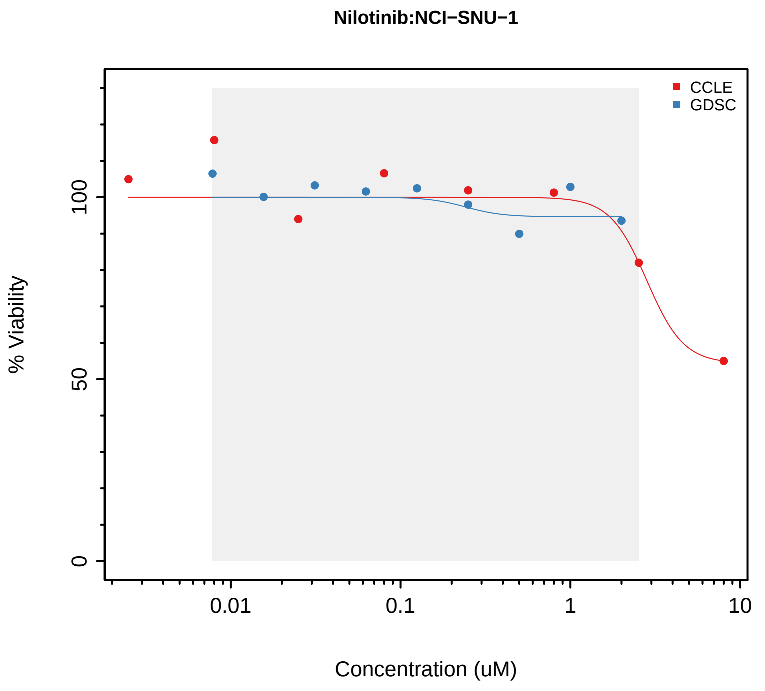
Nilotinib:NCI−SNU−1
CCLE
GDSC
% Viability
0
50
100
0.01
0.1
1
10
Concentration (uM)

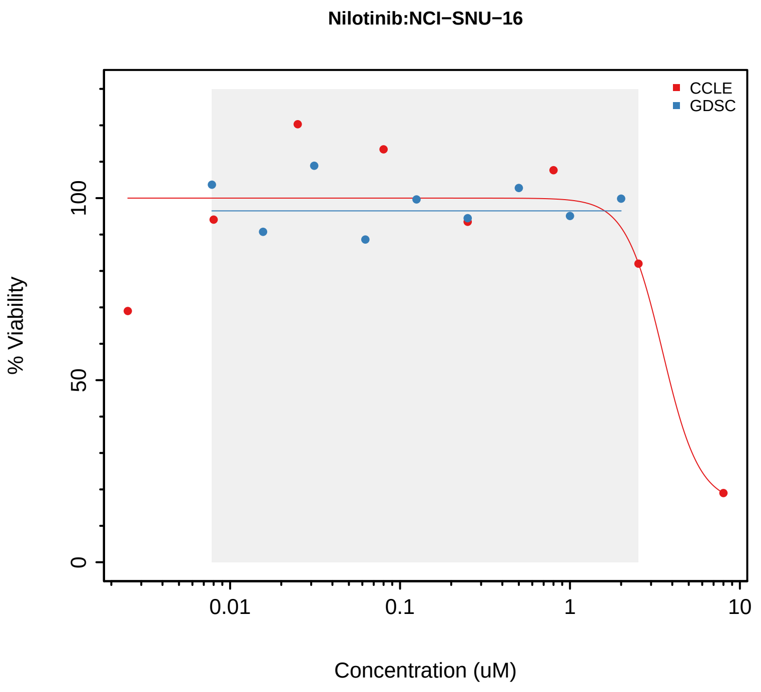
Nilotinib:NCI−SNU−16
CCLE
GDSC
% Viability
0
50
100
0.01
0.1
1
10
Concentration (uM)

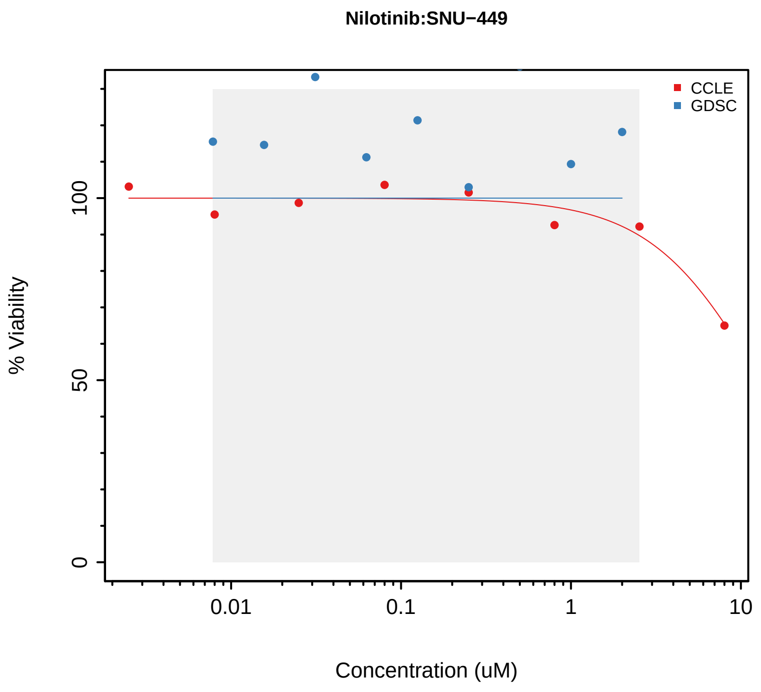
Nilotinib:SNU−449
CCLE
GDSC
% Viability
0
50
100
0.01
0.1
1
10
Concentration (uM)

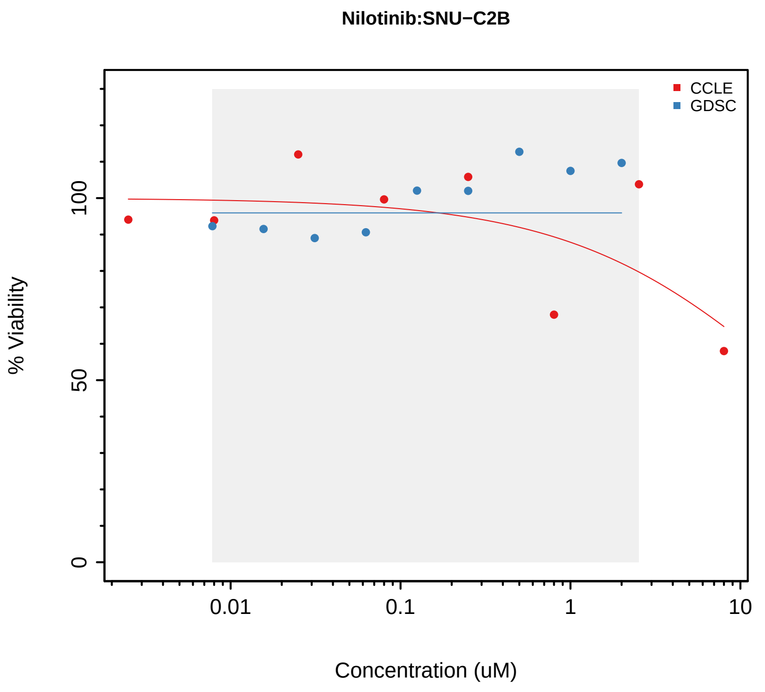
Nilotinib:SNU−C2B
CCLE
GDSC
% Viability
0
50
100
0.01
0.1
1
10
Concentration (uM)

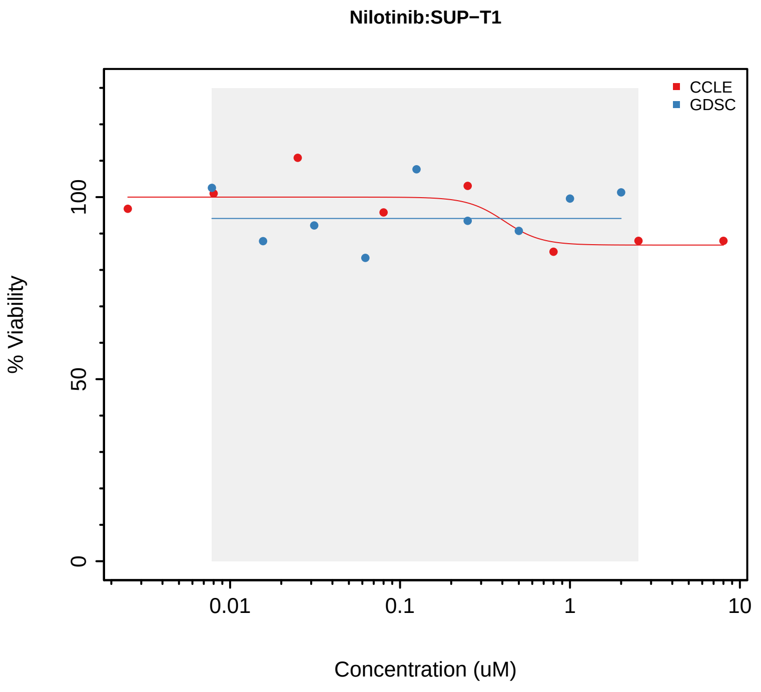
Nilotinib:SUP−T1
CCLE
GDSC
% Viability
0
50
100
0.01
0.1
1
10
Concentration (uM)

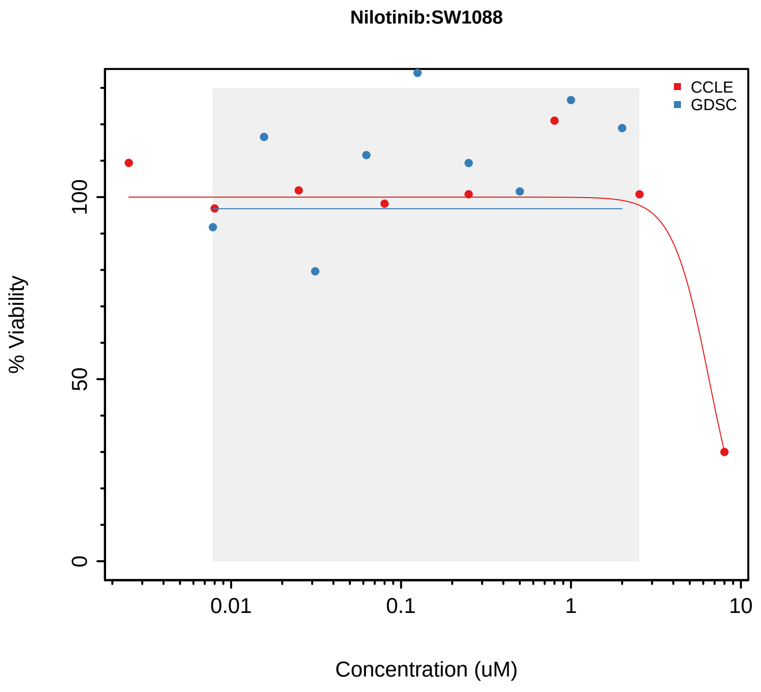
Nilotinib:SW1088
CCLE
GDSC
% Viability
0
50
100
0.01
0.1
1
10
Concentration (uM)

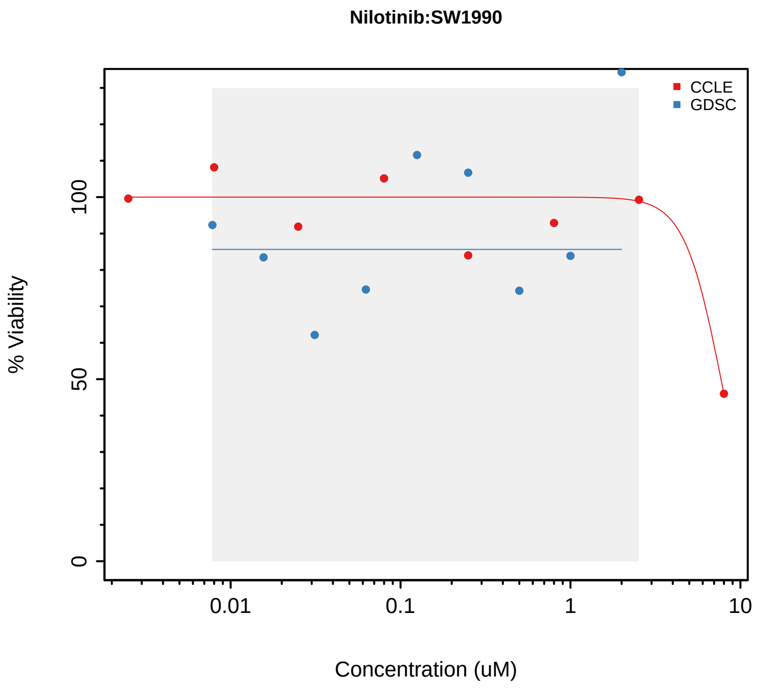
Nilotinib:SW1990
CCLE
GDSC
% Viability
0
50
100
0.01
0.1
1
10
Concentration (uM)

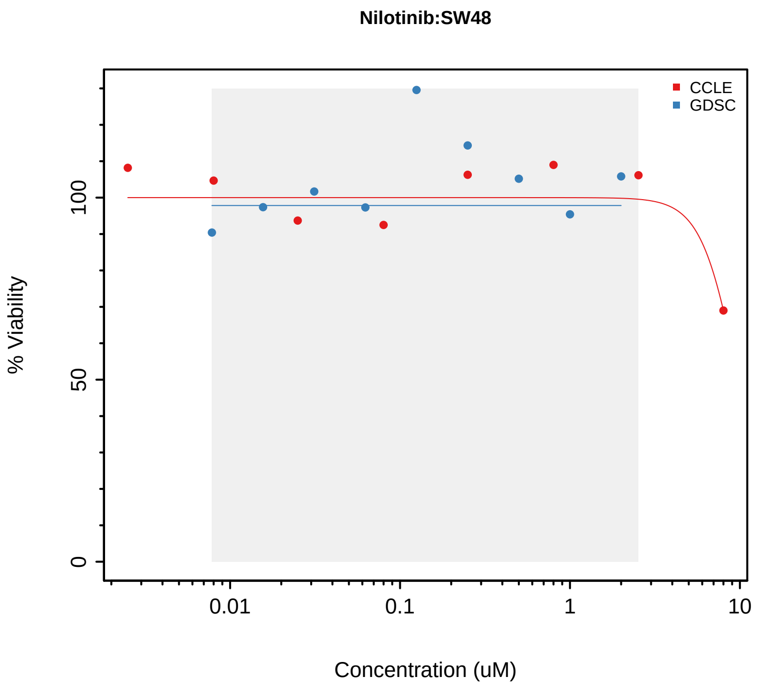
Nilotinib:SW48
CCLE
GDSC
% Viability
0
50
100
0.01
0.1
1
10
Concentration (uM)

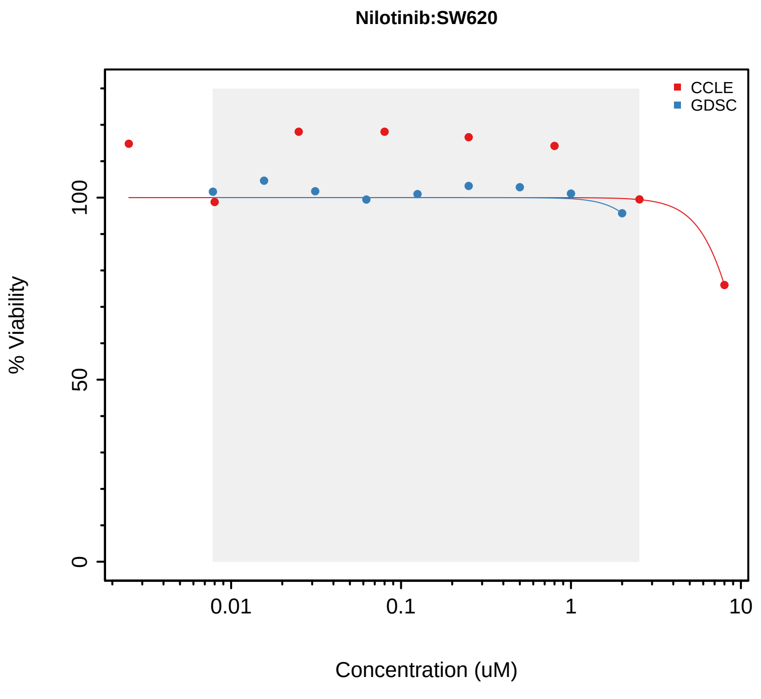
Nilotinib:SW620
CCLE
GDSC
% Viability
0
50
100
0.01
0.1
1
10
Concentration (uM)

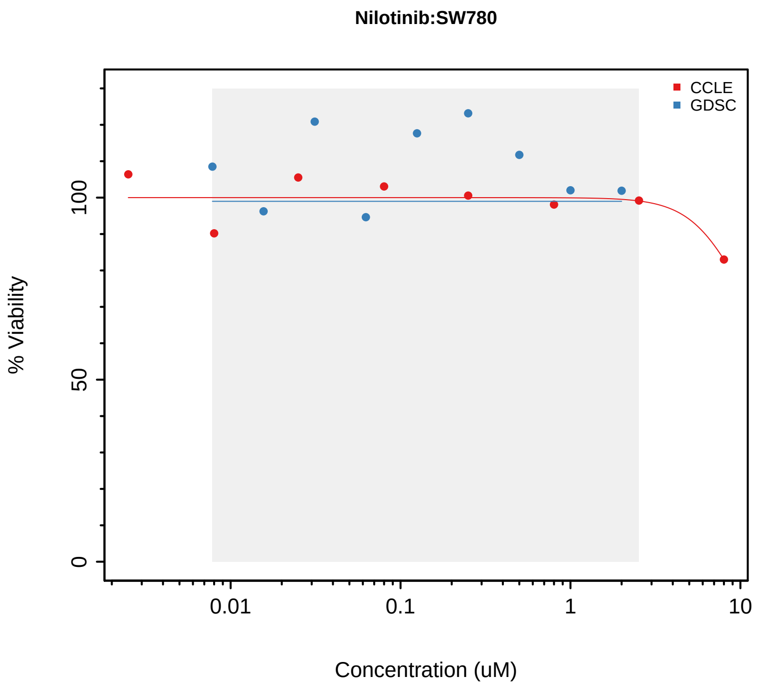
Nilotinib:SW780
CCLE
GDSC
% Viability
0
50
100
0.01
0.1
1
10
Concentration (uM)

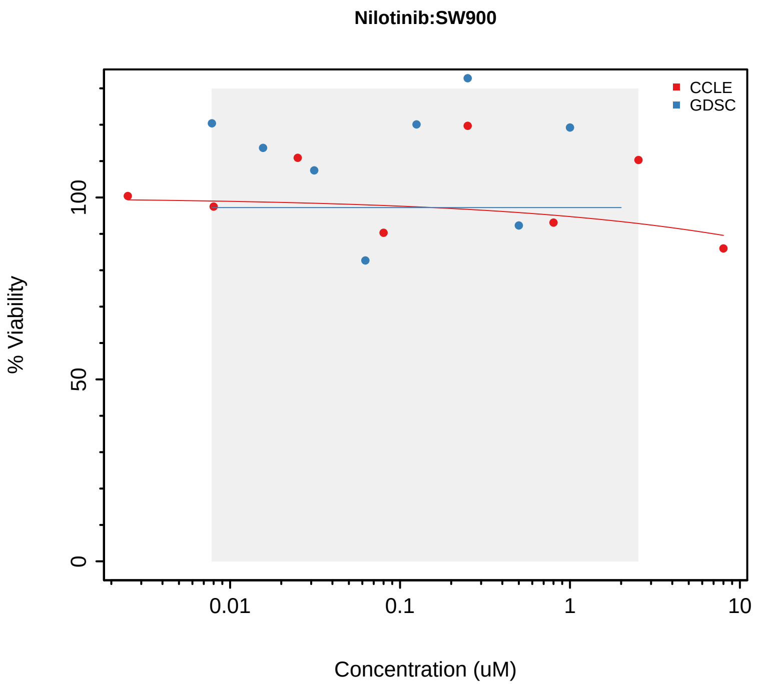
Nilotinib:SW900
CCLE
GDSC
% Viability
0
50
100
0.01
0.1
1
10
Concentration (uM)

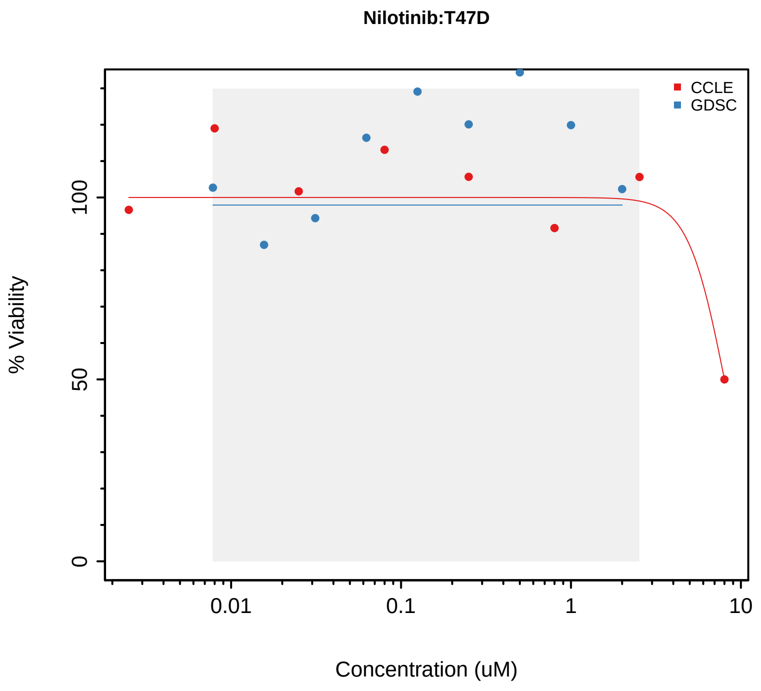
Nilotinib:T47D
CCLE
GDSC
% Viability
0
50
100
0.01
0.1
1
10
Concentration (uM)

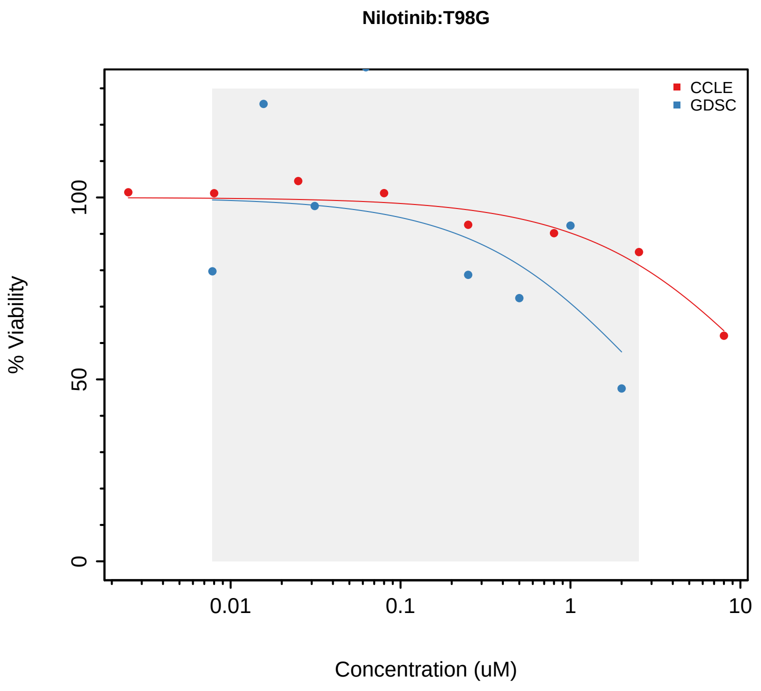
Nilotinib:T98G
CCLE
GDSC
% Viability
0
50
100
0.01
0.1
1
10
Concentration (uM)

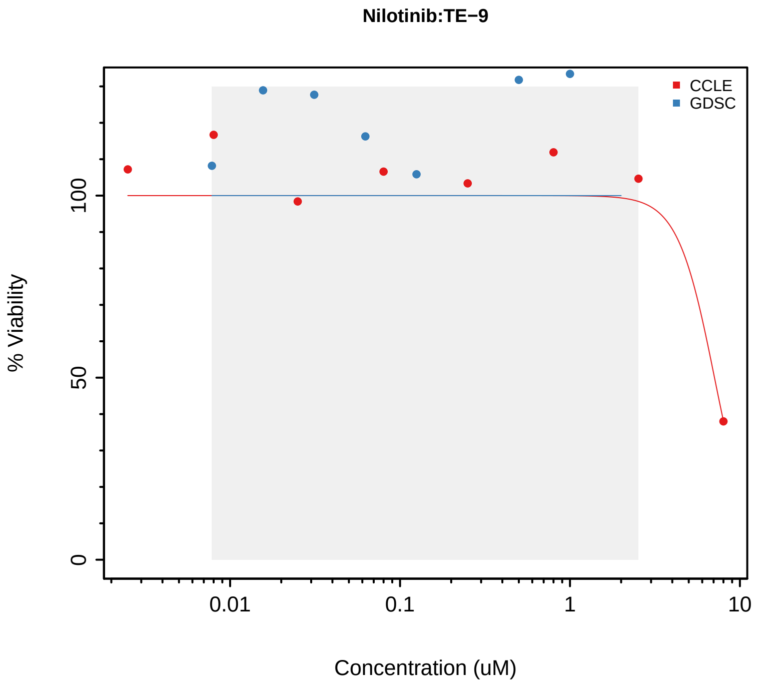
Nilotinib:TE−9
CCLE
GDSC
% Viability
0
50
100
0.01
0.1
1
10
Concentration (uM)

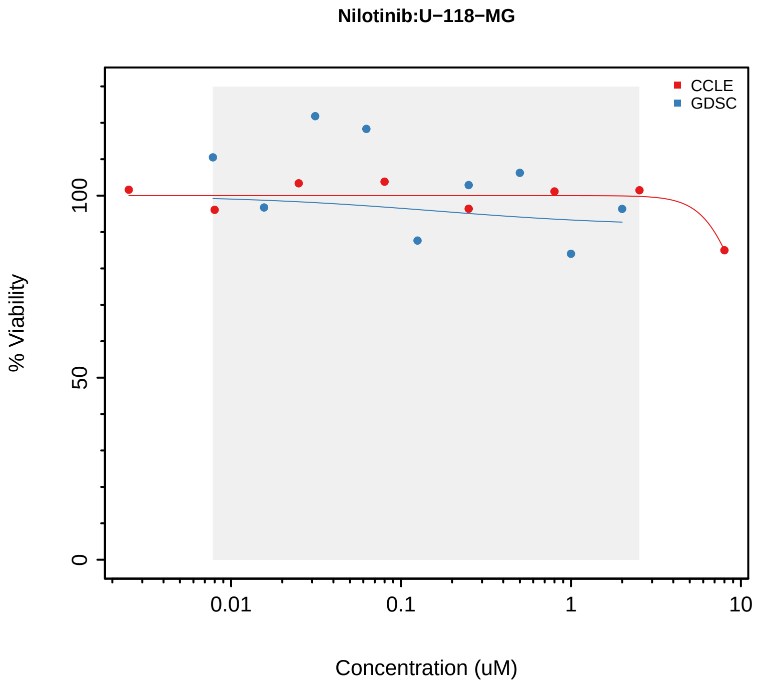
Nilotinib:U−118−MG
CCLE
GDSC
% Viability
0
50
100
0.01
0.1
1
10
Concentration (uM)

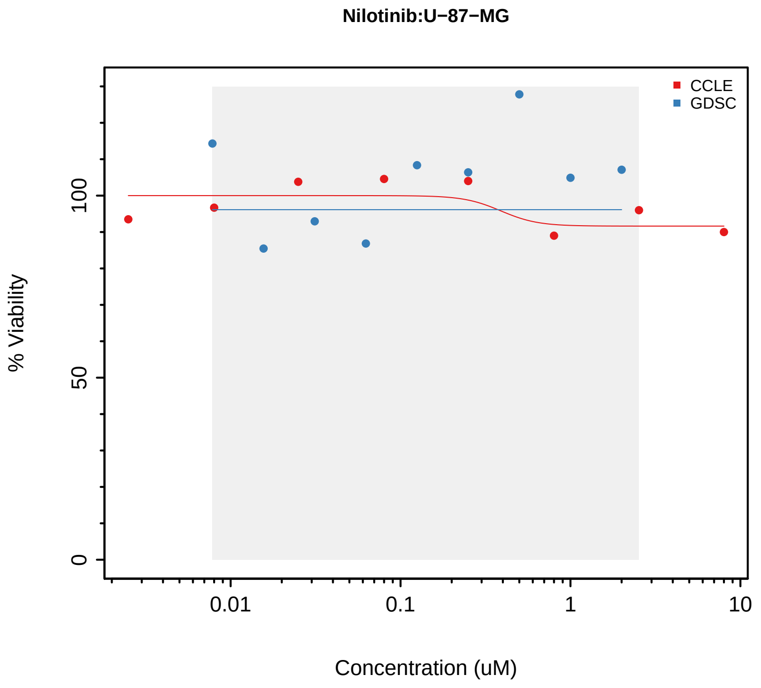
Nilotinib:U−87−MG
CCLE
GDSC
% Viability
0
50
100
0.01
0.1
1
10
Concentration (uM)

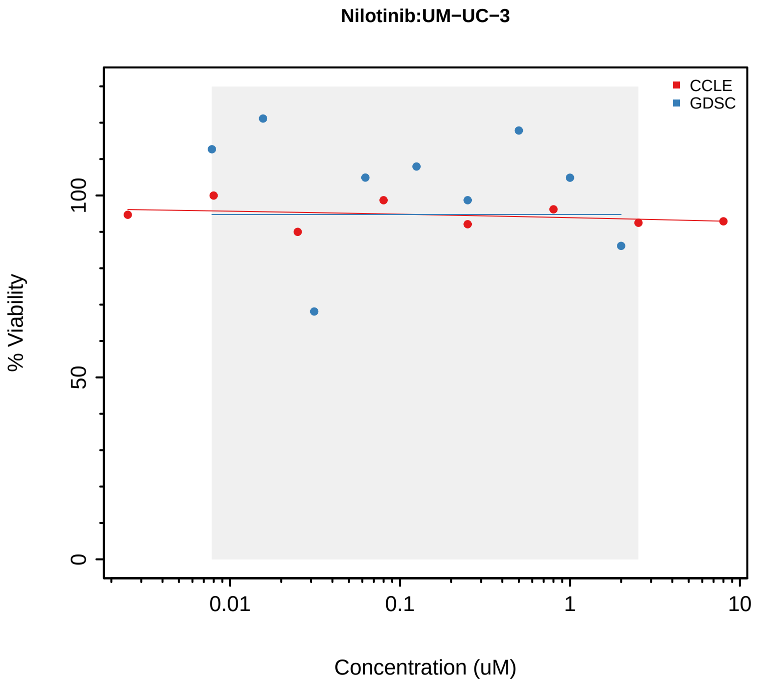
Nilotinib:UM−UC−3
CCLE
GDSC
% Viability
0
50
100
0.01
0.1
1
10
Concentration (uM)

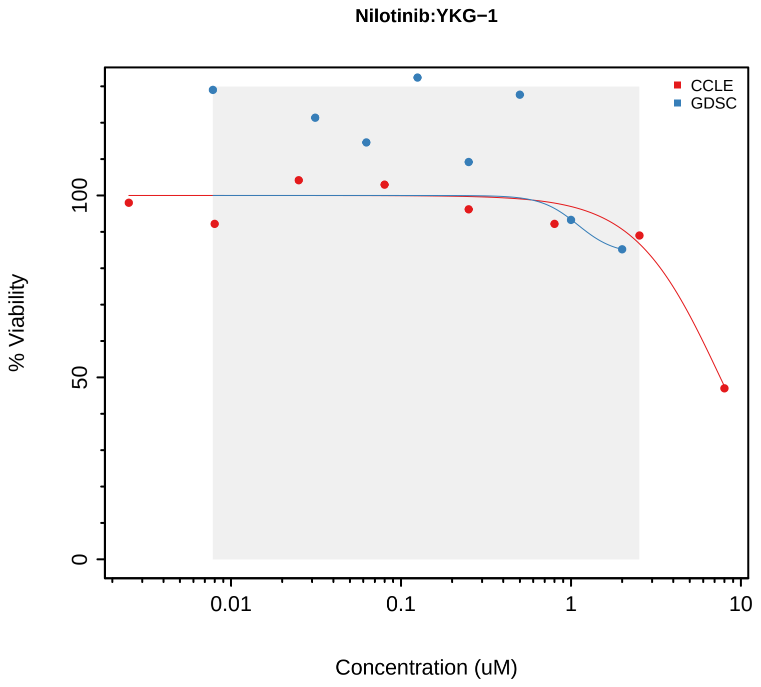
Nilotinib:YKG−1
CCLE
GDSC
% Viability
0
50
100
0.01
0.1
1
10
Concentration (uM)

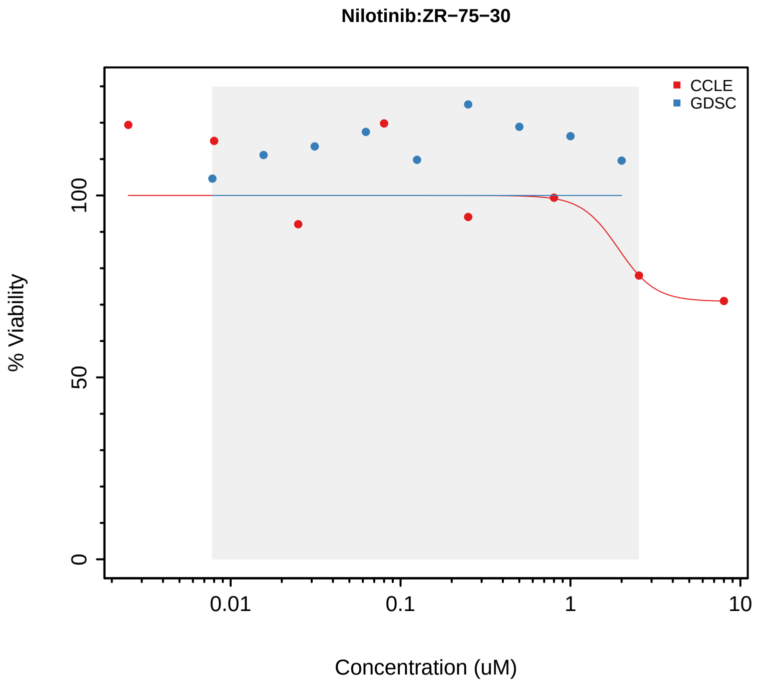
Nilotinib:ZR−75−30
CCLE
GDSC
% Viability
0
50
100
0.01
0.1
1
10
Concentration (uM)

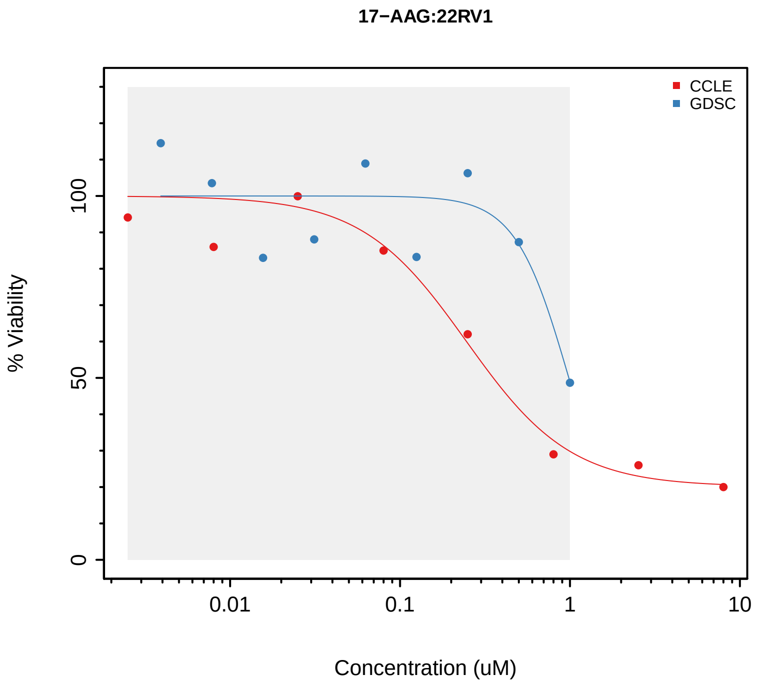
17−AAG:22RV1
CCLE
GDSC
% Viability
0
50
100
0.01
0.1
1
10
Concentration (uM)

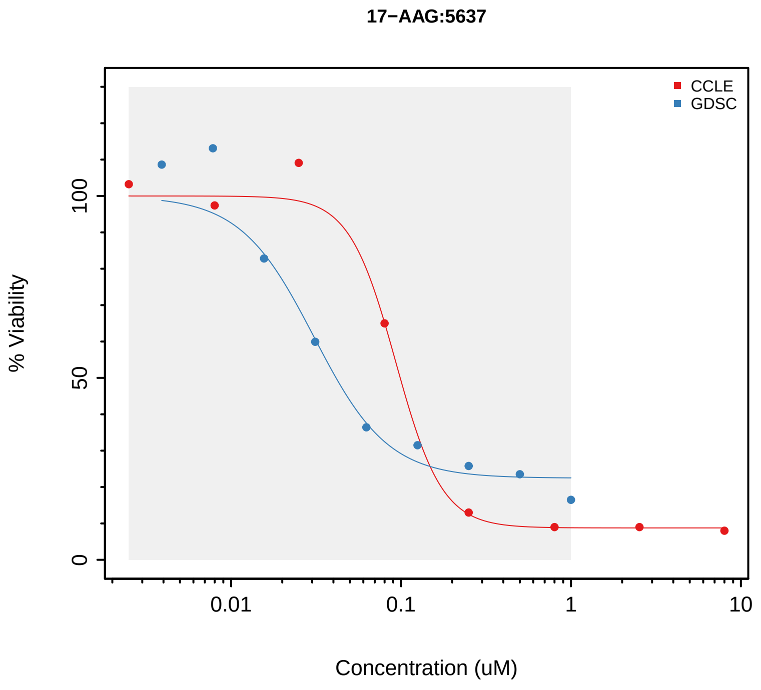
17−AAG:5637
CCLE
GDSC
% Viability
0
50
100
0.01
0.1
1
10
Concentration (uM)

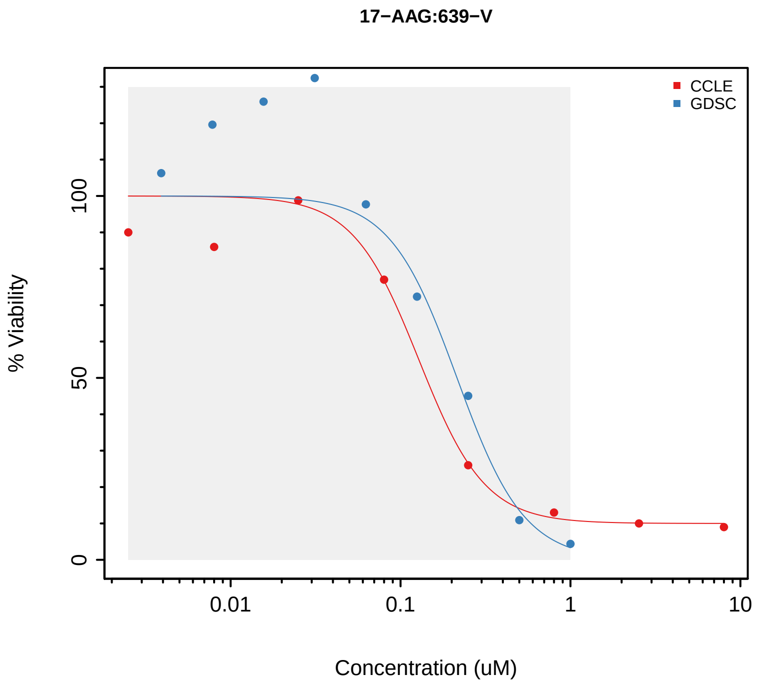
17−AAG:639−V
CCLE
GDSC
% Viability
0
50
100
0.01
0.1
1
10
Concentration (uM)

17−AAG:697

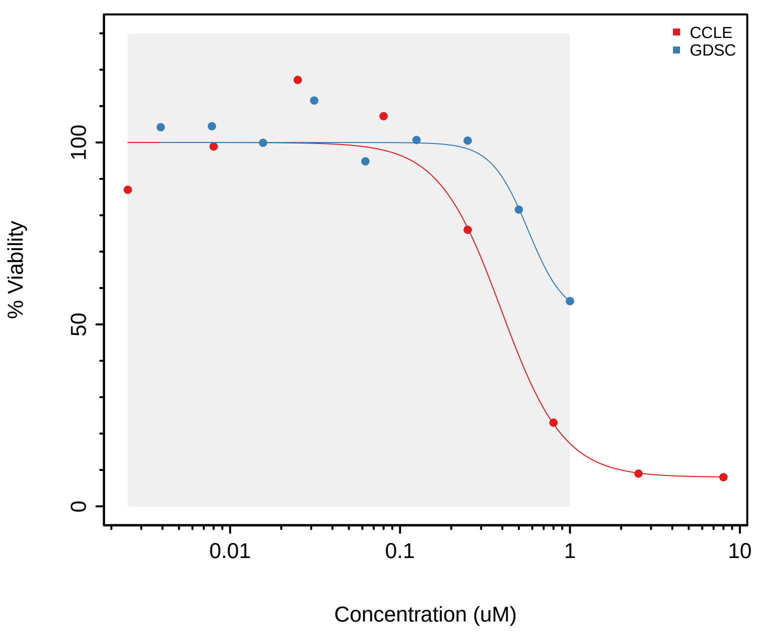

17−AAG:769−P

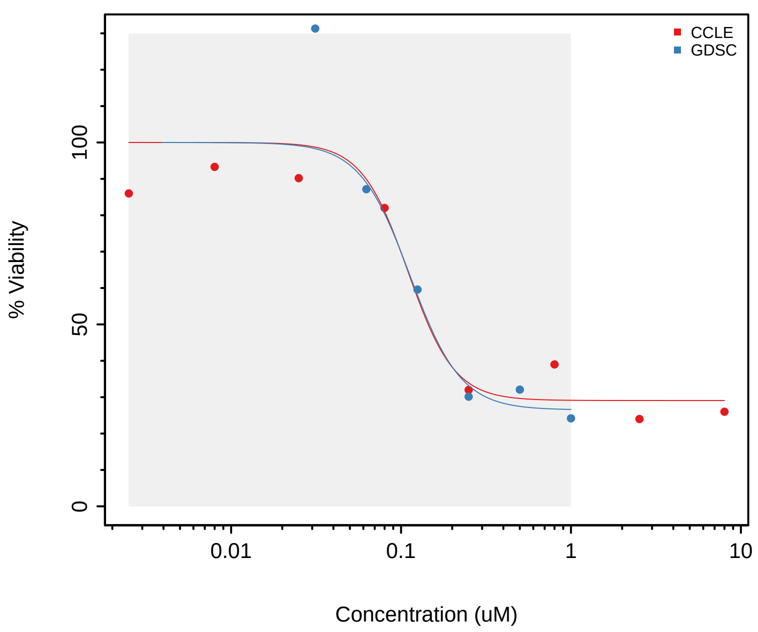

17−AAG:786−0

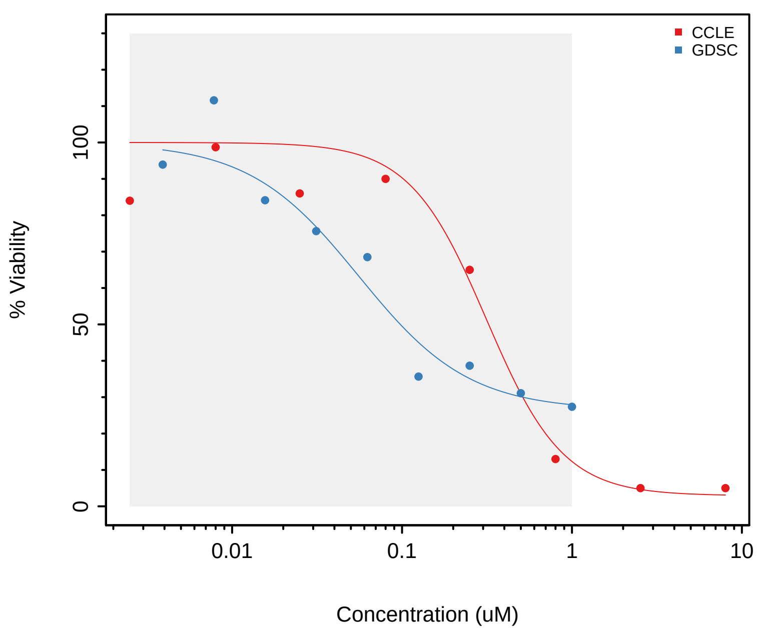

17−AAG:8305C

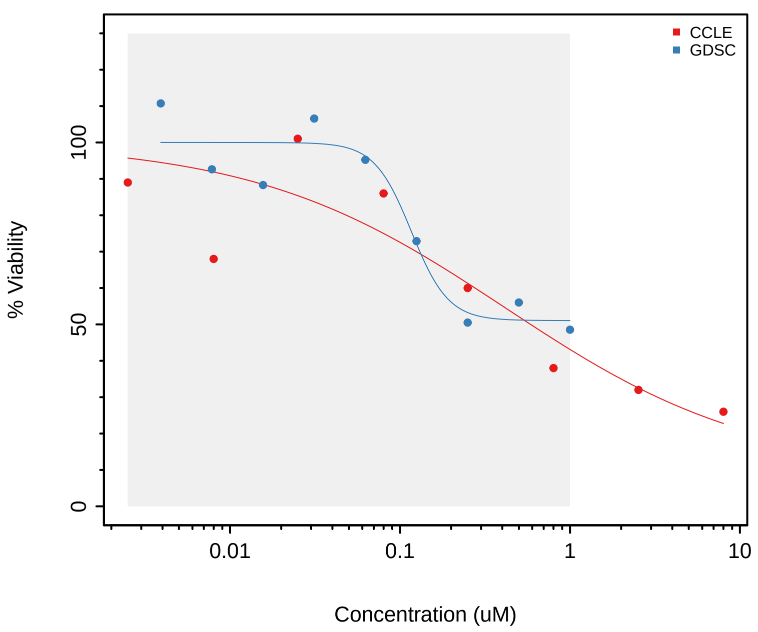

17−AAG:8505C

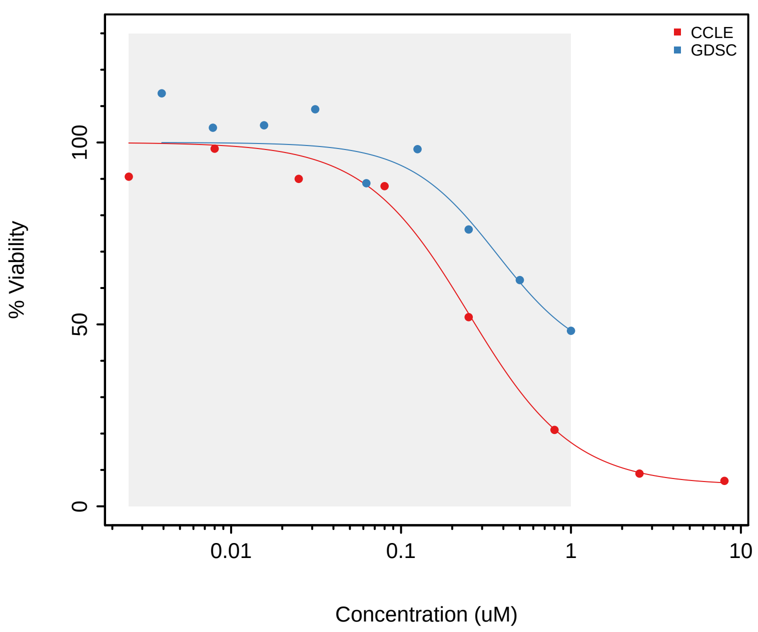

17−AAG:8−MG−BA

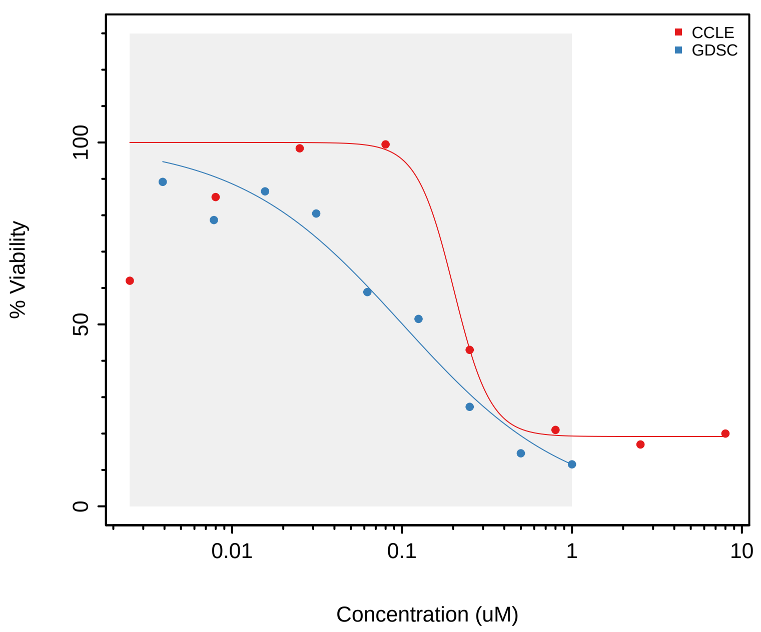

17−AAG:A172

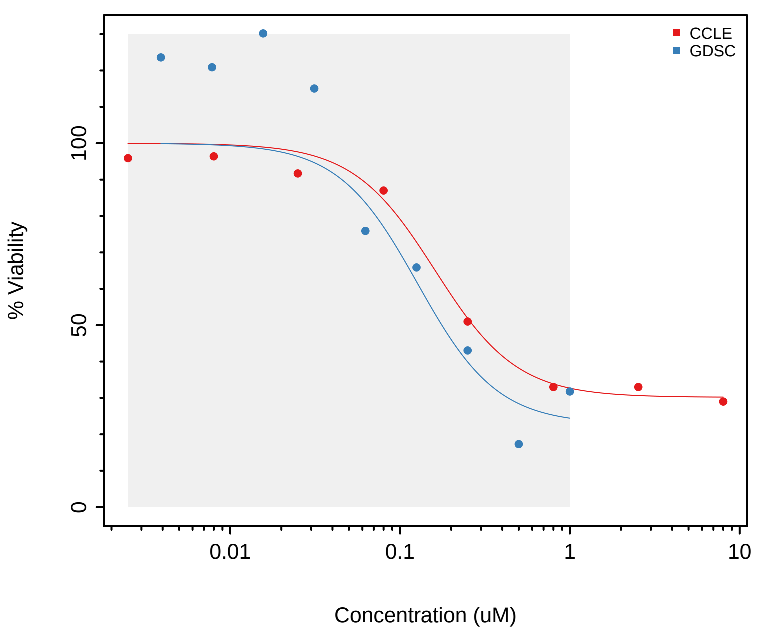

17−AAG:A204

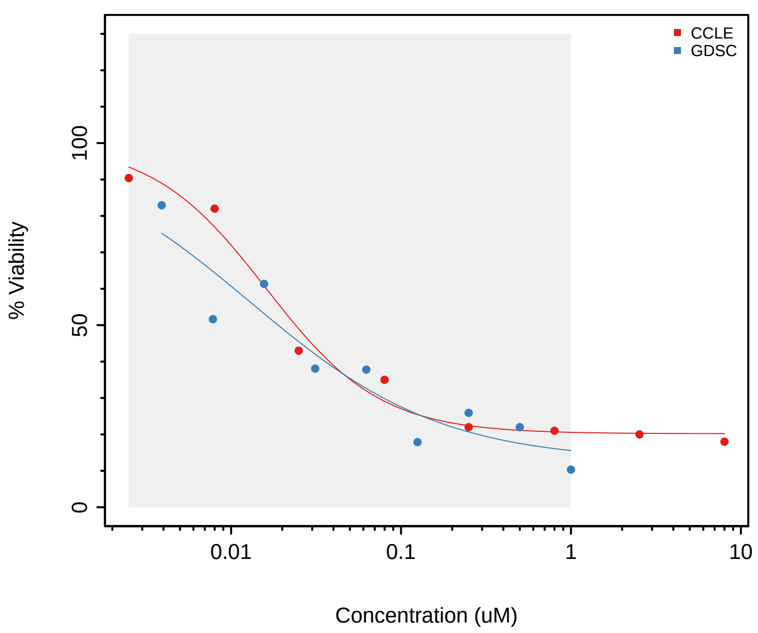

17−AAG:A2058

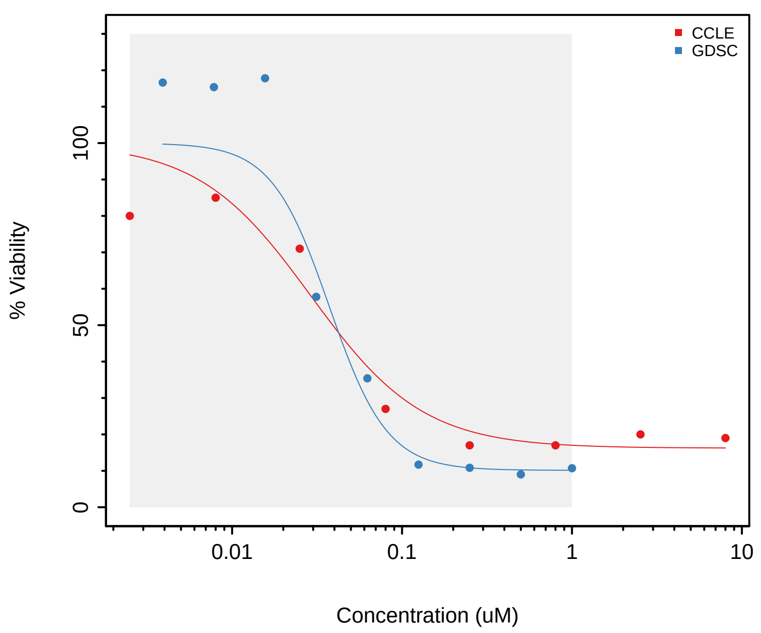

17−AAG:A253

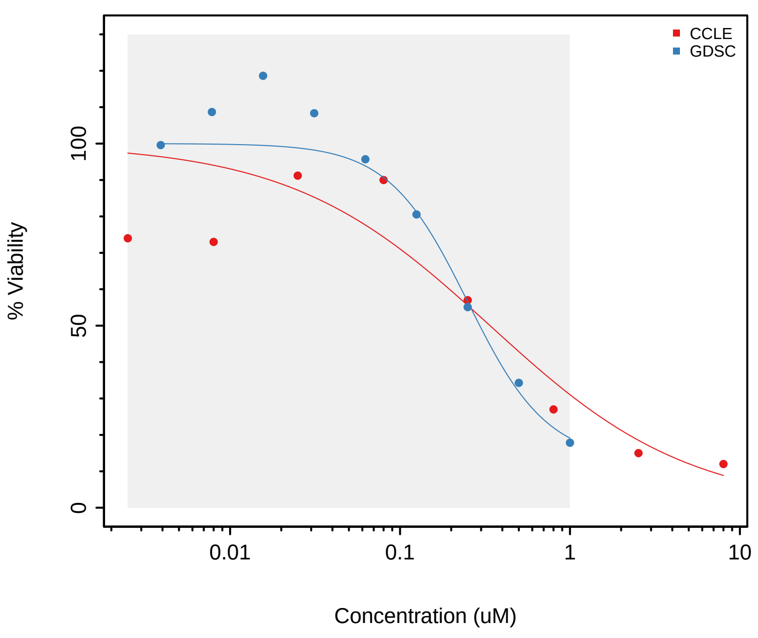

17−AAG:A2780

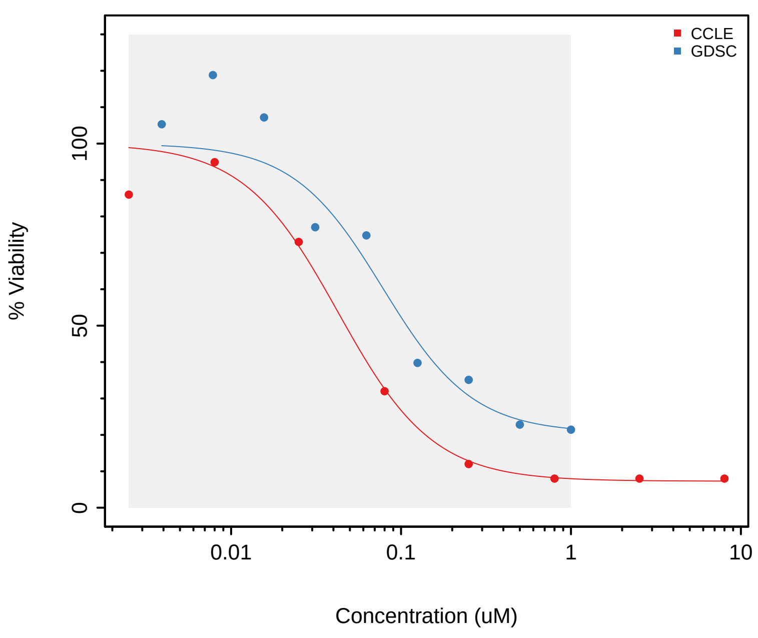

17−AAG:A375

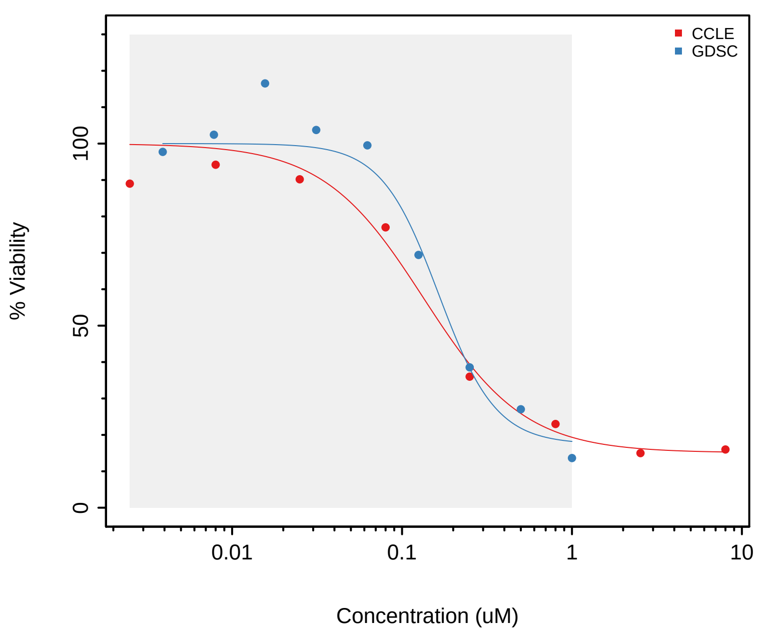

17−AAG:A549

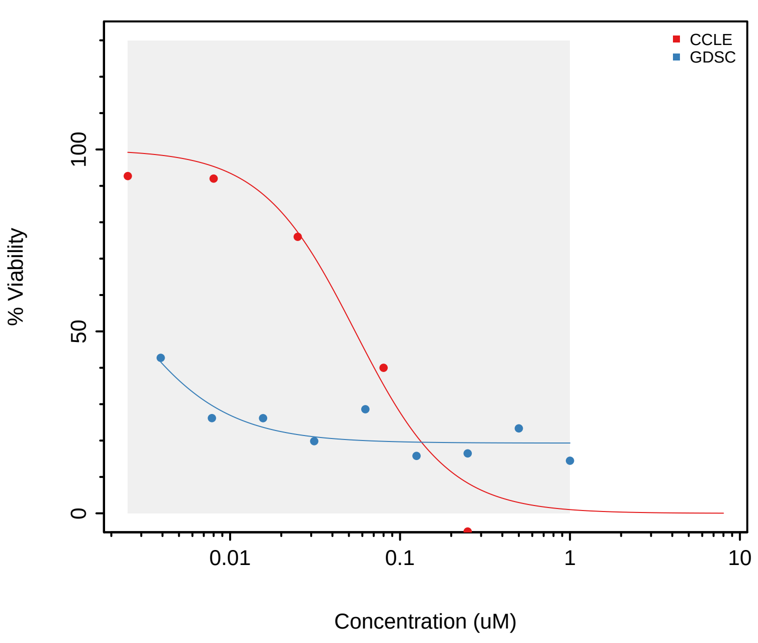

17−AAG:A673

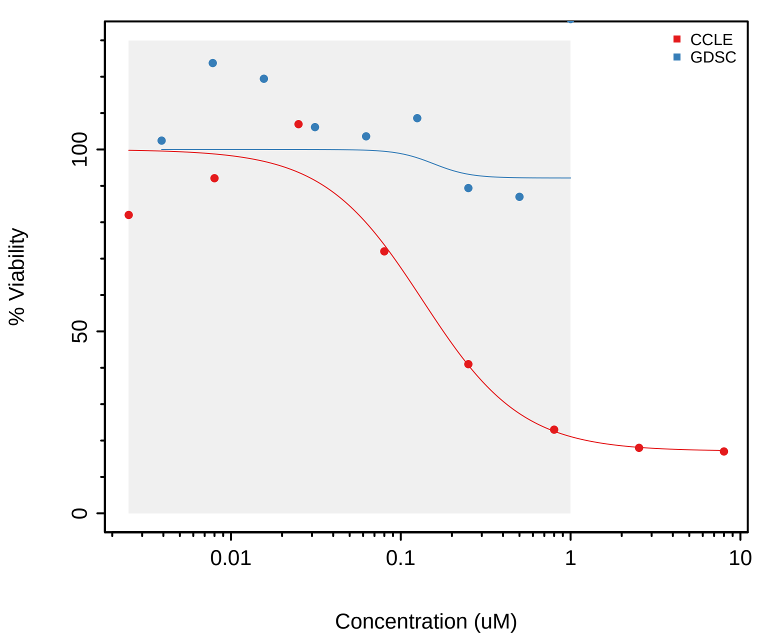

17−AAG:ACHN

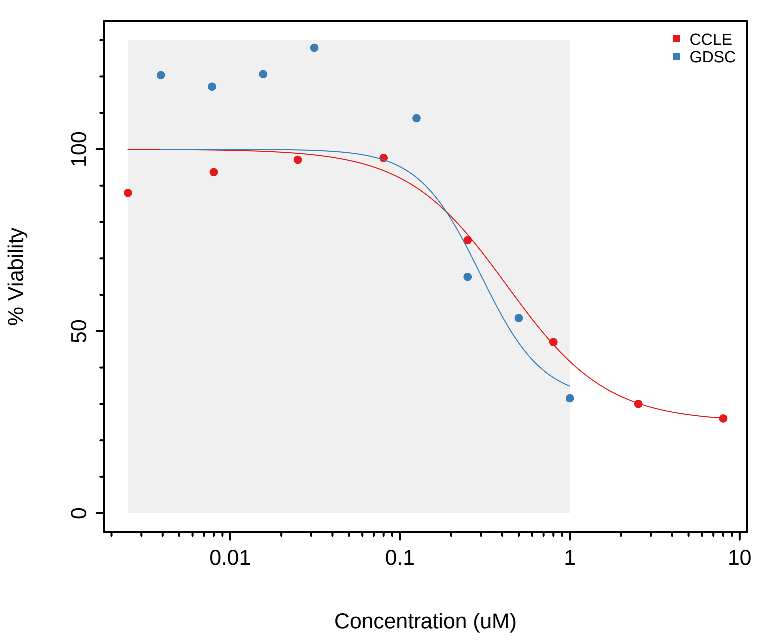

17−AAG:AN3−CA

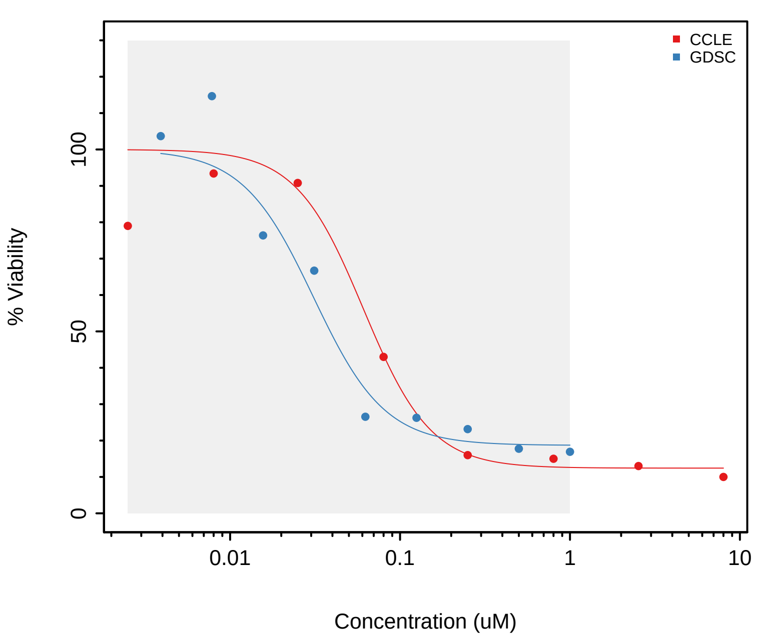

17−AAG:AsPC−1

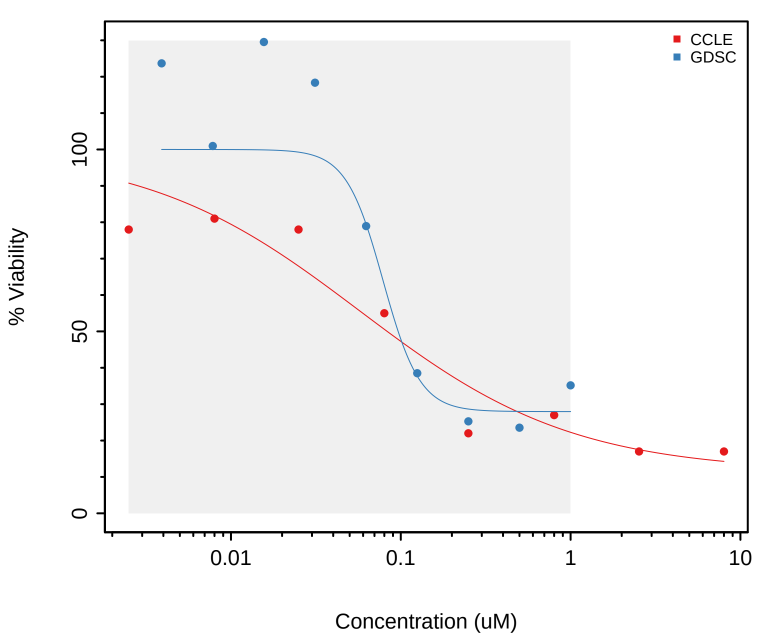

17−AAG:AU565

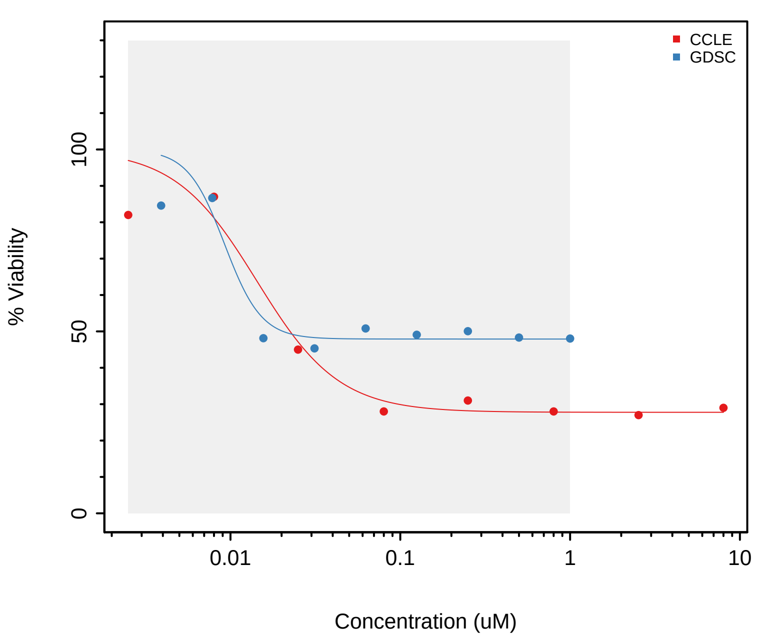

17−AAG:BCPAP

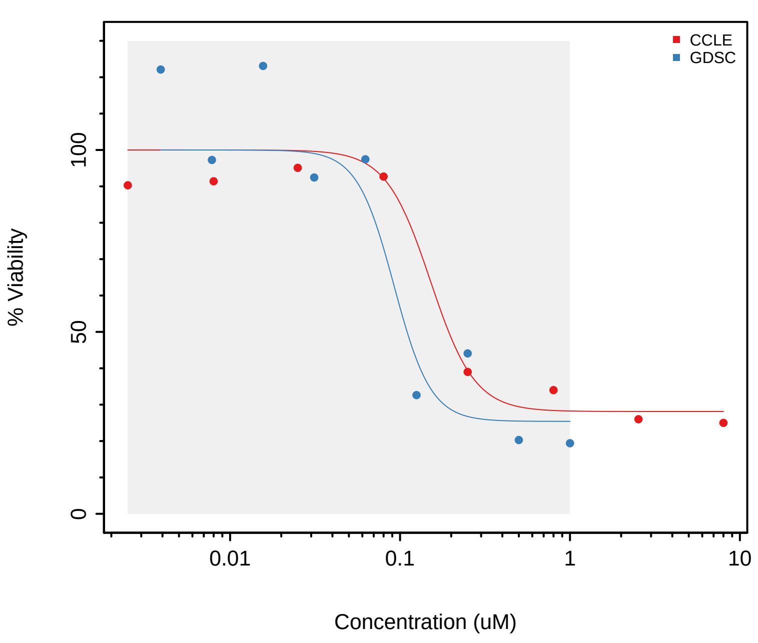

17−AAG:BFTC−909

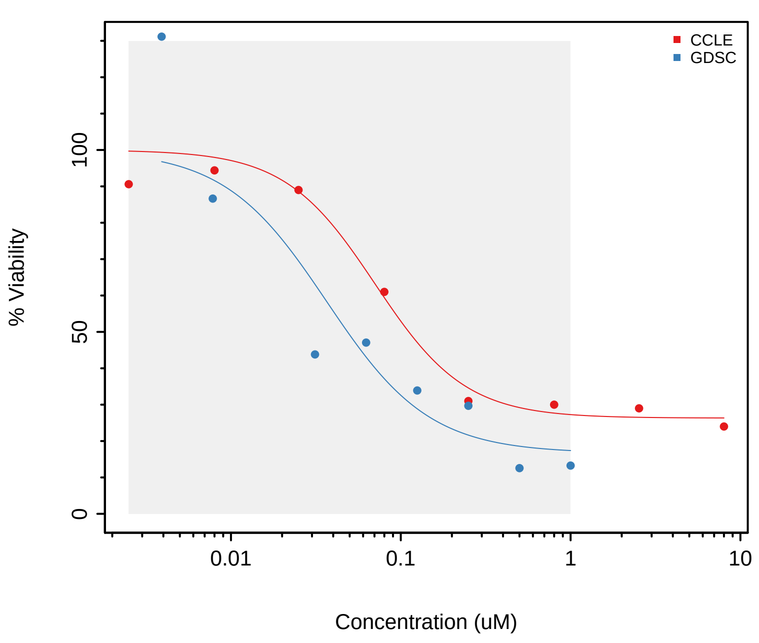

17−AAG:BL−41

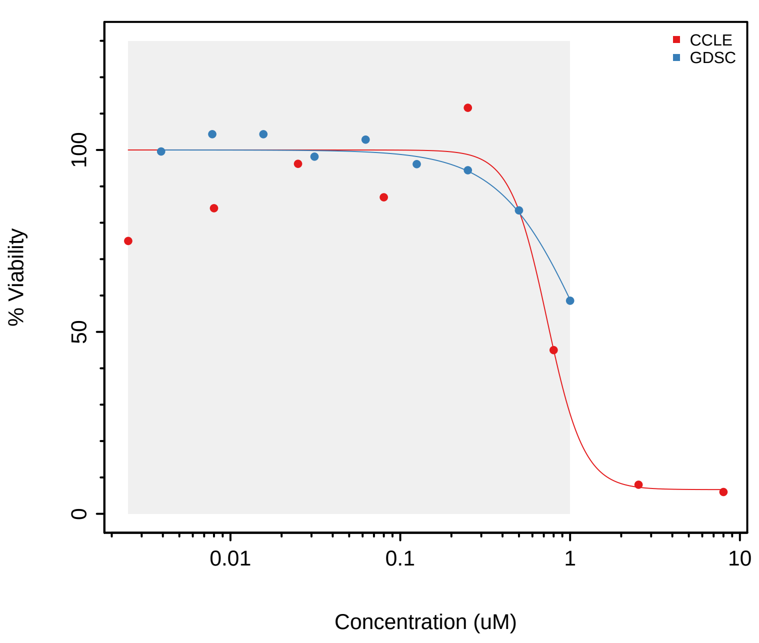

17−AAG:BT−20

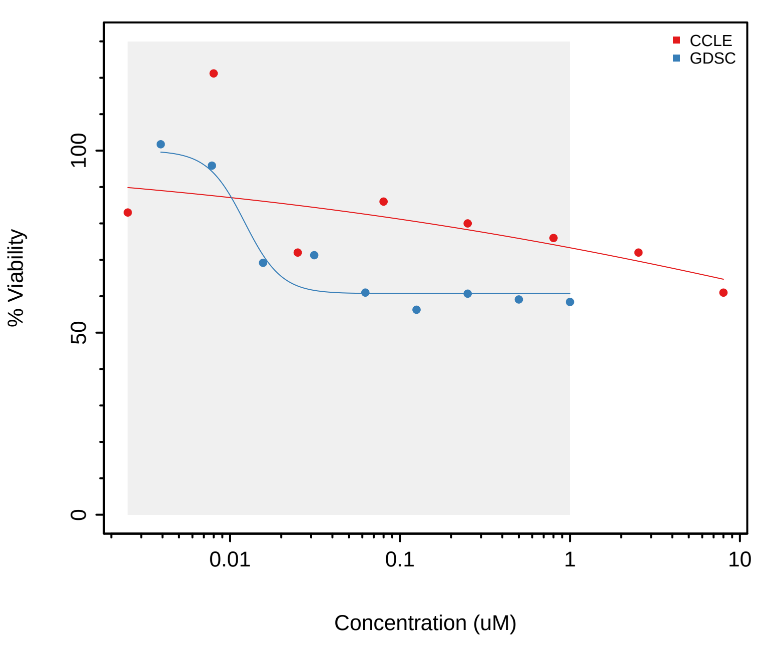

17−AAG:BT−474

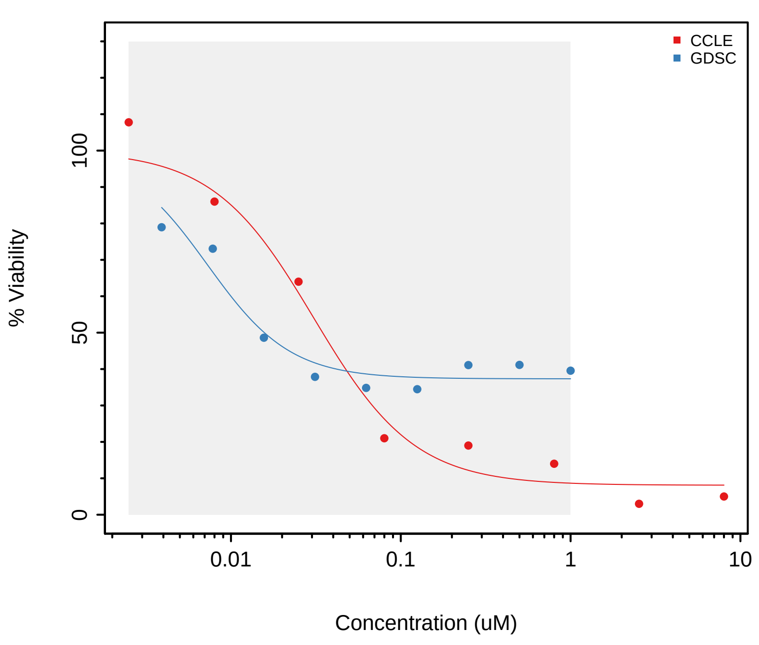

17−AAG:BT−549

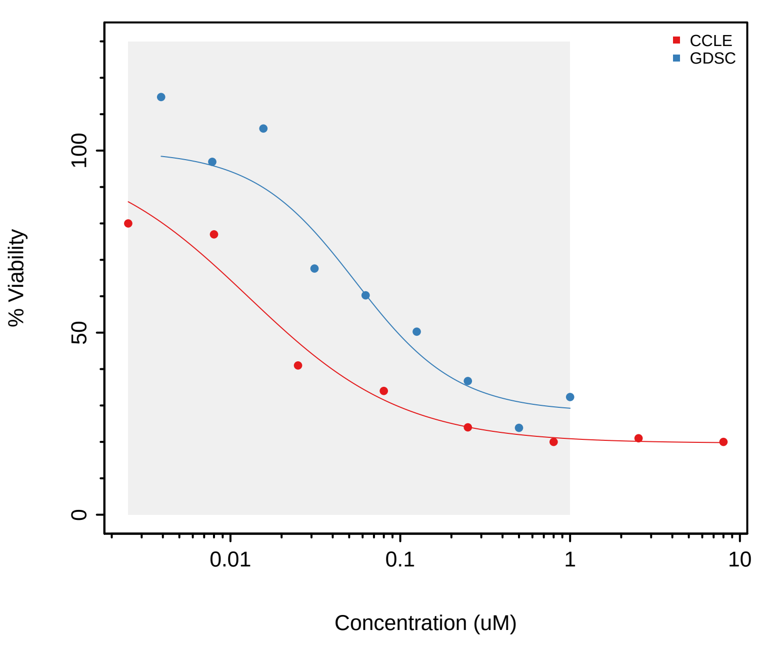

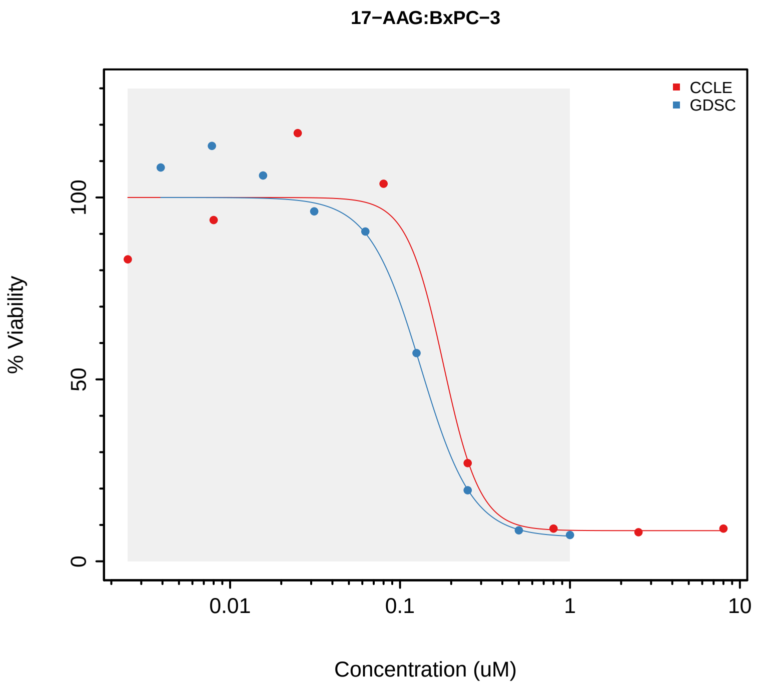
17−AAG:BxPC−3
CCLE
GDSC
% Viability
0
50
100
0.01
0.1
1
10
Concentration (uM)

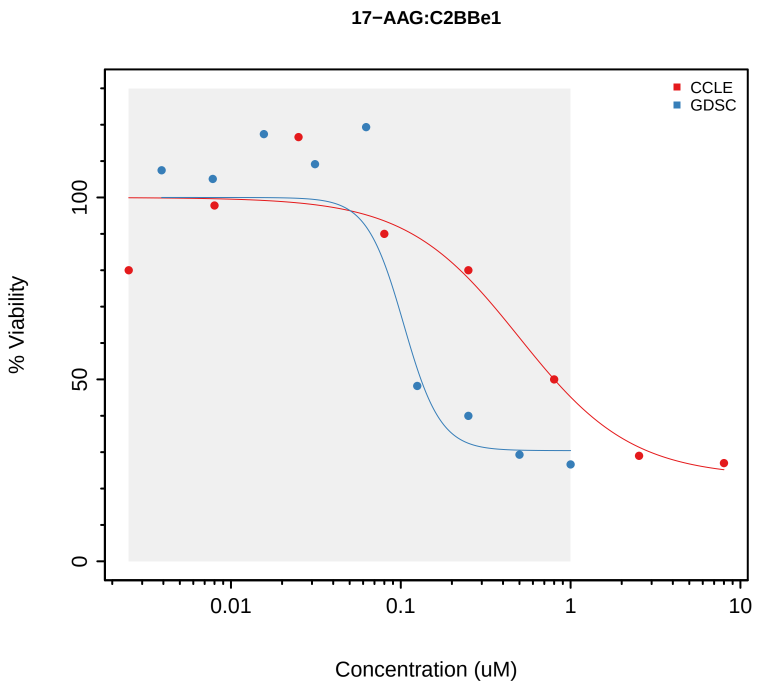
17−AAG:C2BBe1
CCLE
GDSC
% Viability
0
50
100
0.01
0.1
1
10
Concentration (uM)

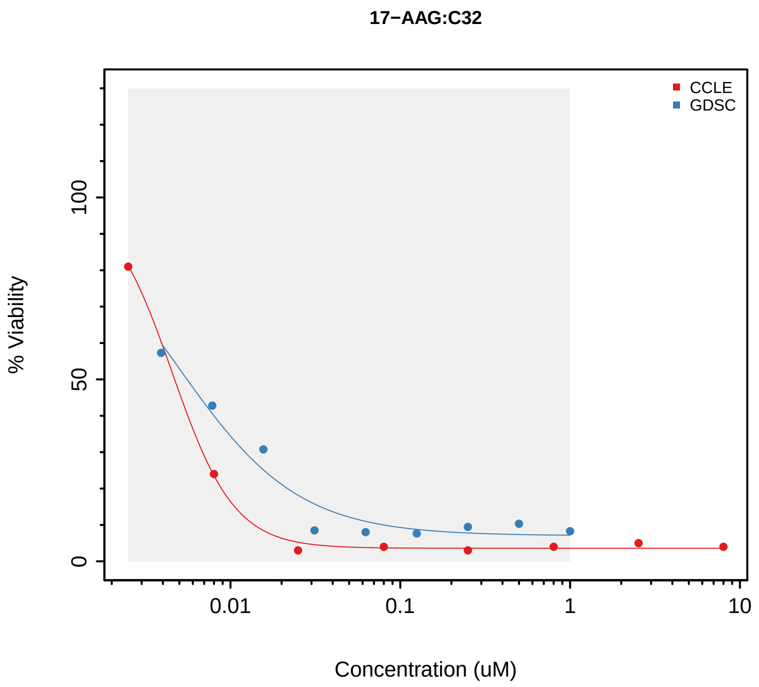
17−AAG:C32
CCLE
GDSC
% Viability
0
50
100
0.01
0.1
1
10
Concentration (uM)

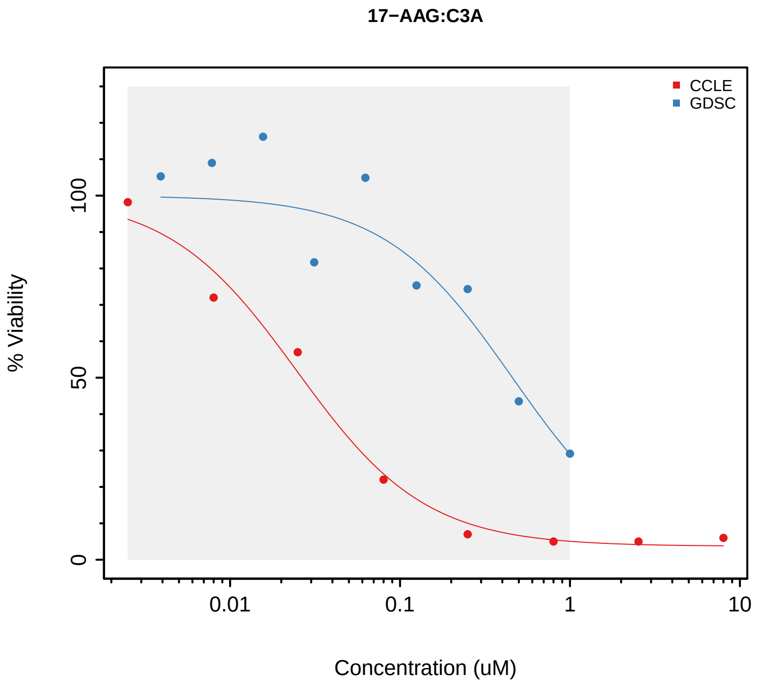
17−AAG:C3A
CCLE
GDSC
% Viability
0
50
100
0.01
0.1
1
10
Concentration (uM)

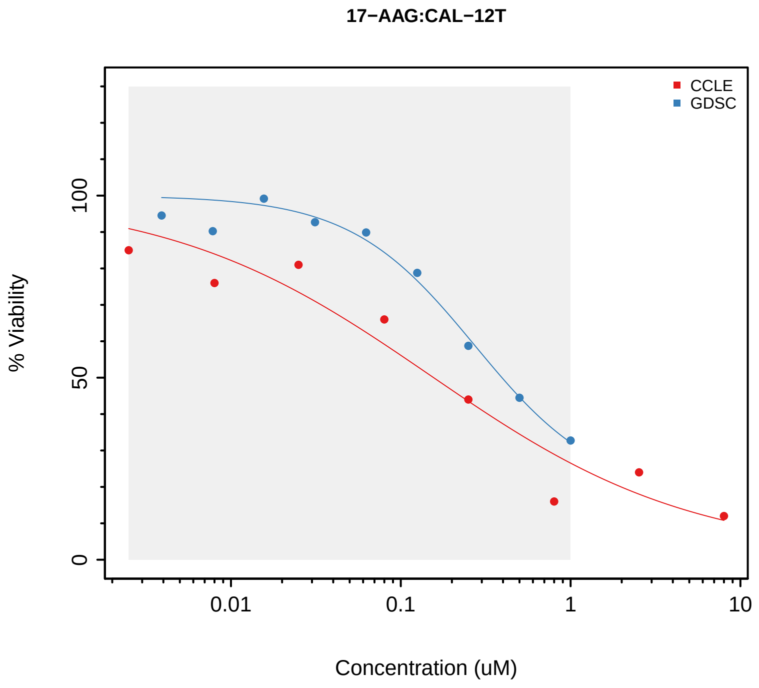
17−AAG:CAL−12T
CCLE
GDSC
% Viability
0
50
100
0.01
0.1
1
10
Concentration (uM)

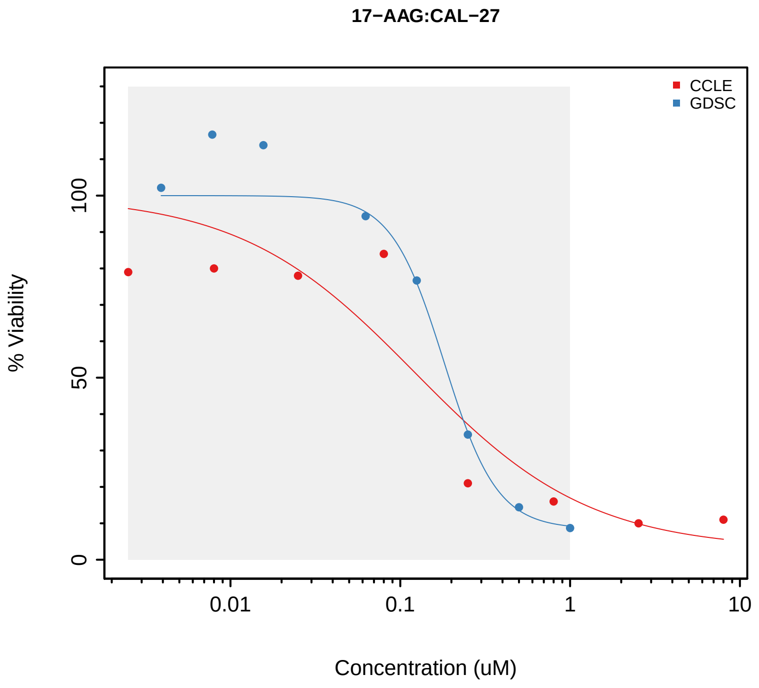
17−AAG:CAL−27
CCLE
GDSC
% Viability
0
50
100
0.01
0.1
1
10
Concentration (uM)

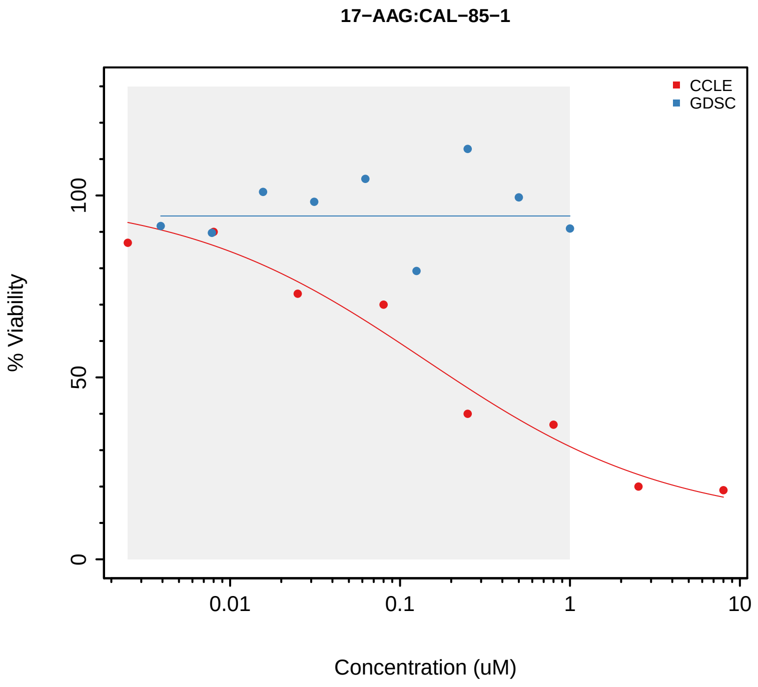
17−AAG:CAL−85−1
CCLE
GDSC
% Viability
0
50
100
0.01
0.1
1
10
Concentration (uM)

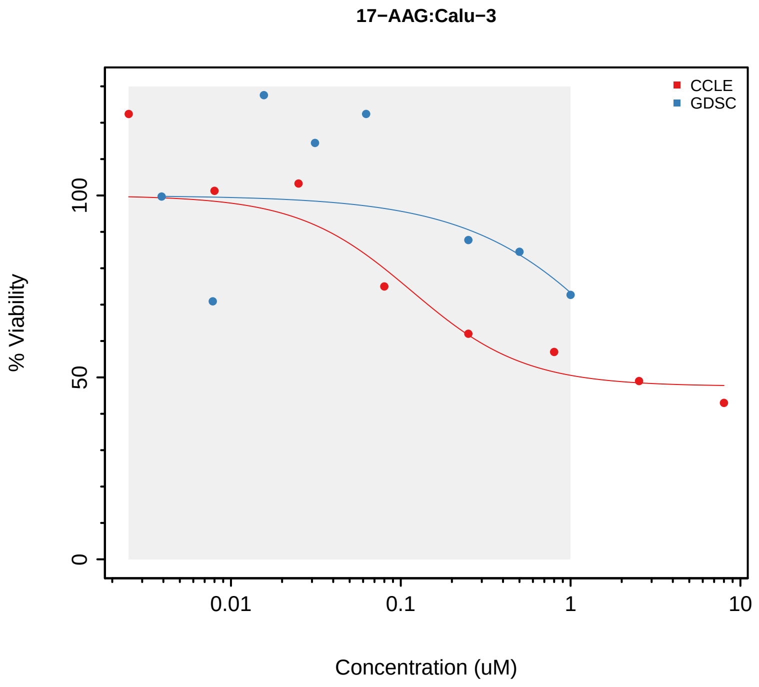
17−AAG:Calu−3
CCLE
GDSC
% Viability
0
50
100
0.01
0.1
1
10
Concentration (uM)

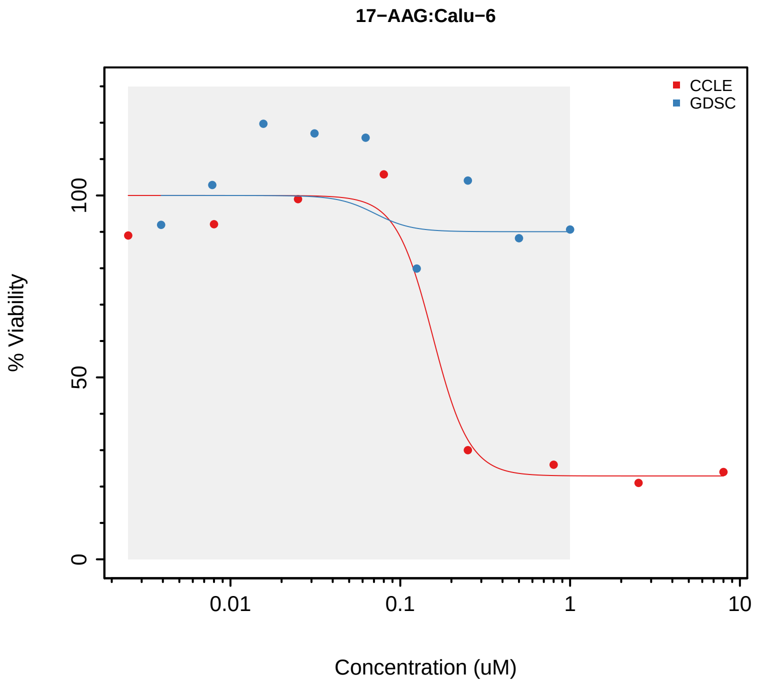
17−AAG:Calu−6
CCLE
GDSC
% Viability
0
50
100
0.01
0.1
1
10
Concentration (uM)

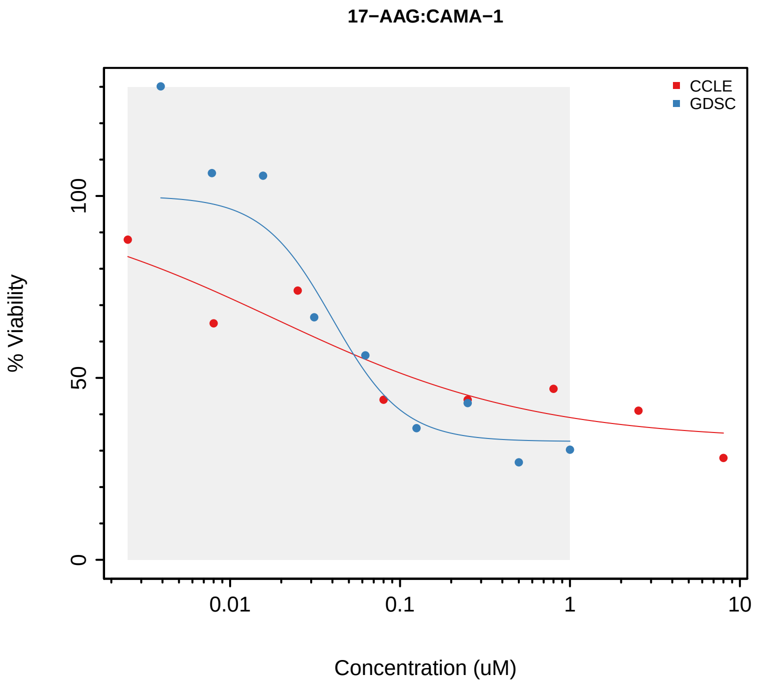
17−AAG:CAMA−1
CCLE
GDSC
% Viability
0
50
100
0.01
0.1
1
10
Concentration (uM)

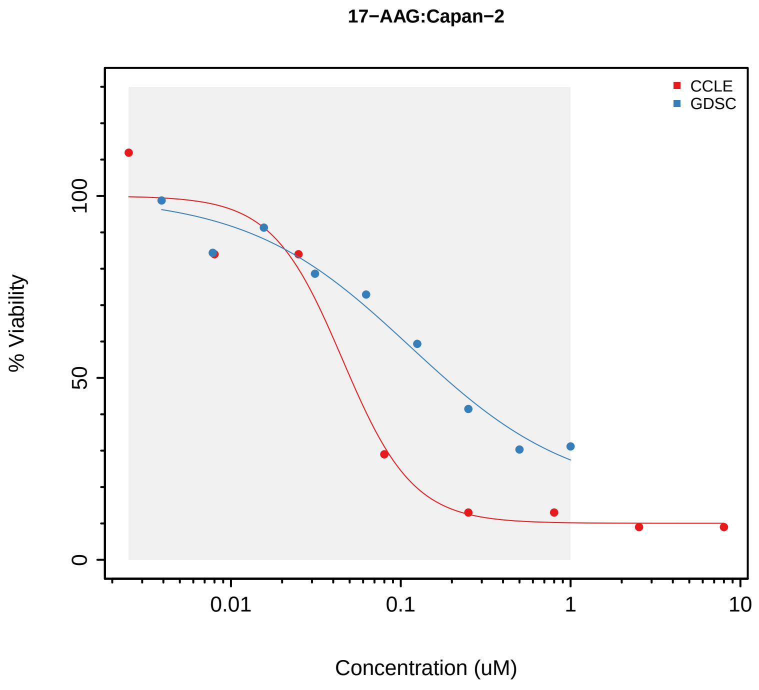
17−AAG:Capan−2
CCLE
GDSC
% Viability
0
50
100
0.01
0.1
1
10
Concentration (uM)

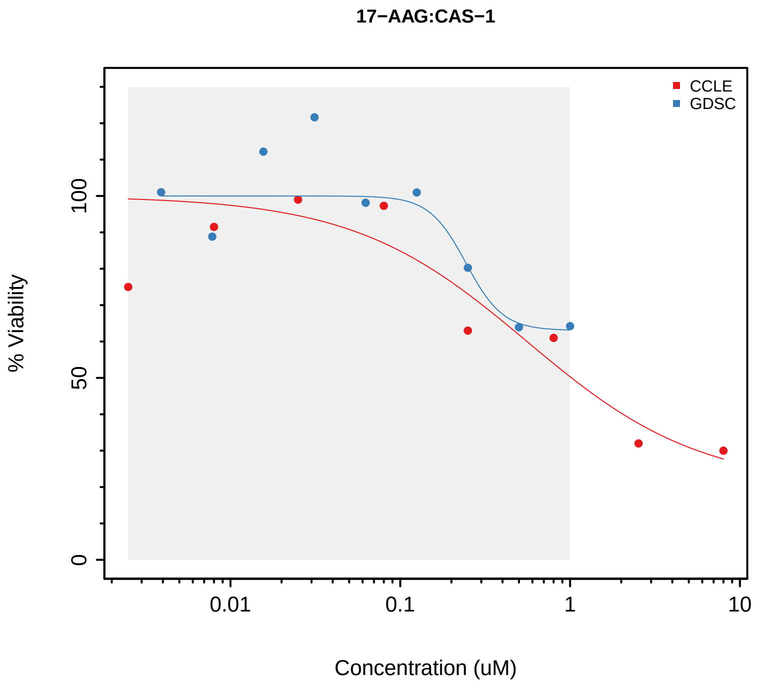
17−AAG:CAS−1
CCLE
GDSC
% Viability
0
50
100
0.01
0.1
1
10
Concentration (uM)

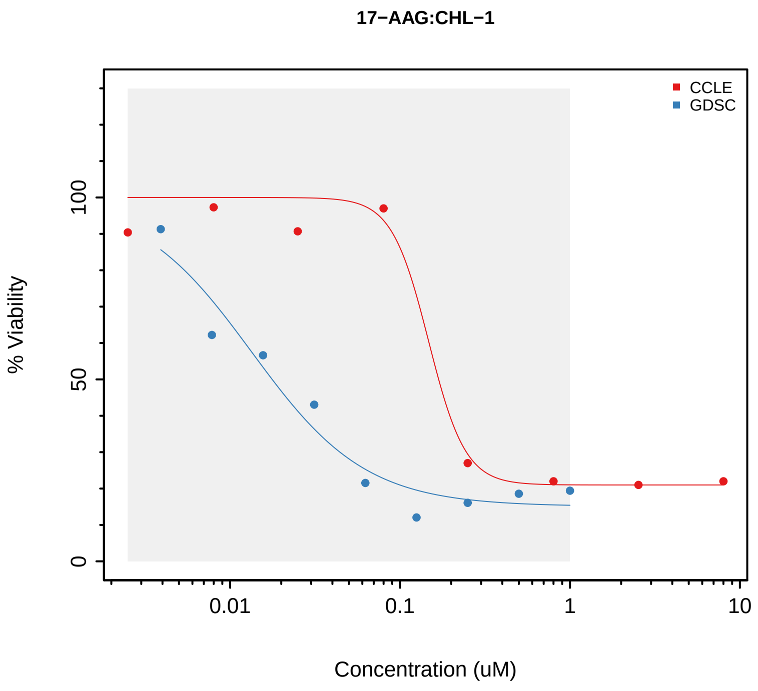
17−AAG:CHL−1
CCLE
GDSC
% Viability
0
50
100
0.01
0.1
1
10
Concentration (uM)

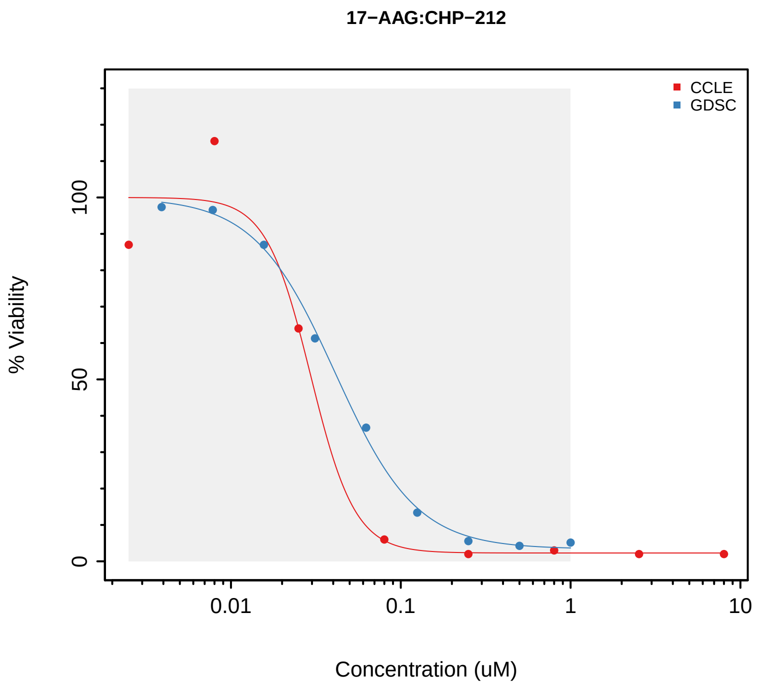
17−AAG:CHP−212
CCLE
GDSC
% Viability
0
50
100
0.01
0.1
1
10
Concentration (uM)

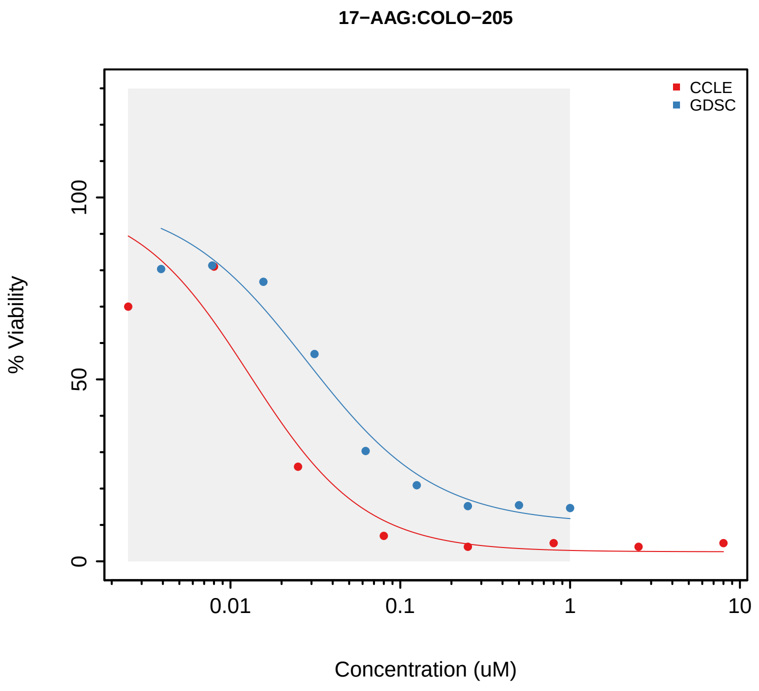
17−AAG:COLO−205
CCLE
GDSC
% Viability
0
50
100
0.01
0.1
1
10
Concentration (uM)

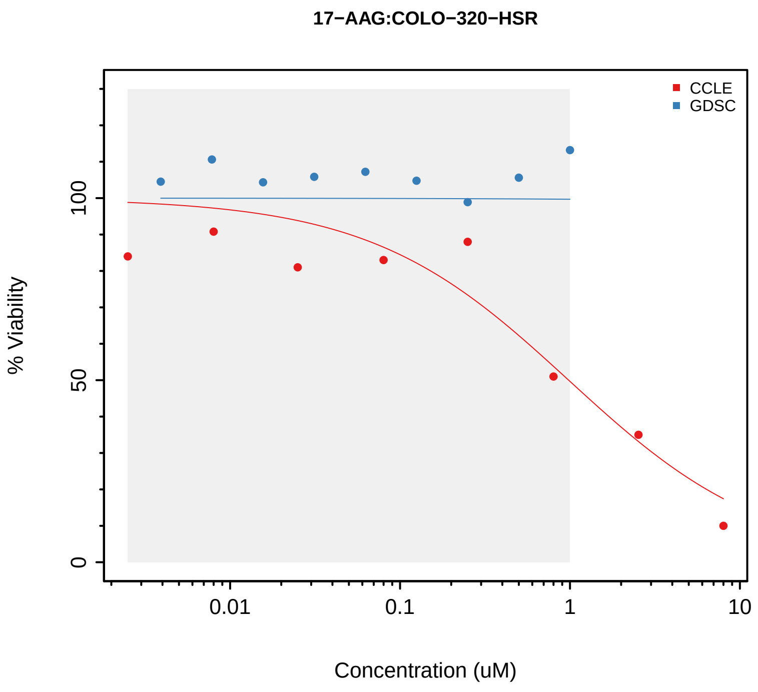
17−AAG:COLO−320−HSR
CCLE
GDSC
% Viability
0
50
100
0.01
0.1
1
10
Concentration (uM)

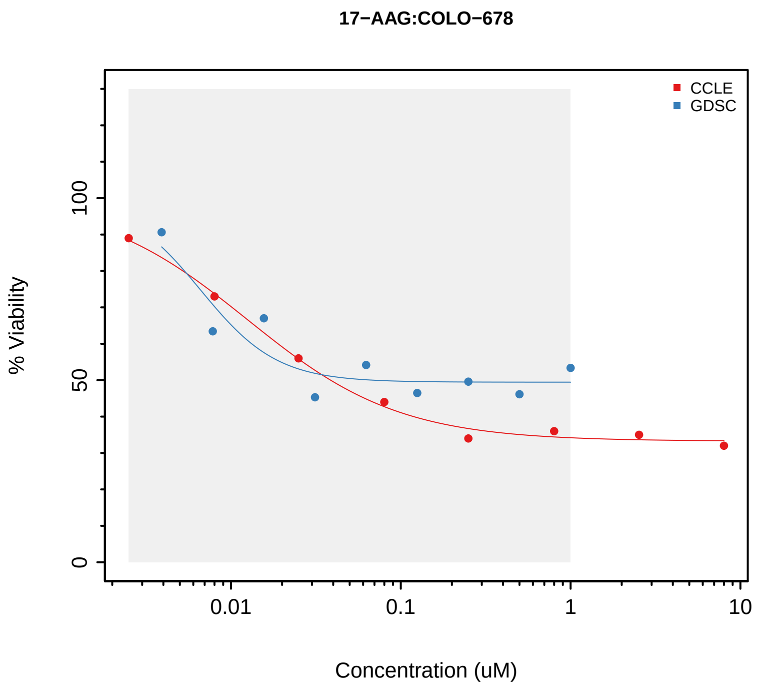
17−AAG:COLO−678
CCLE
GDSC
% Viability
0
50
100
0.01
0.1
1
10
Concentration (uM)

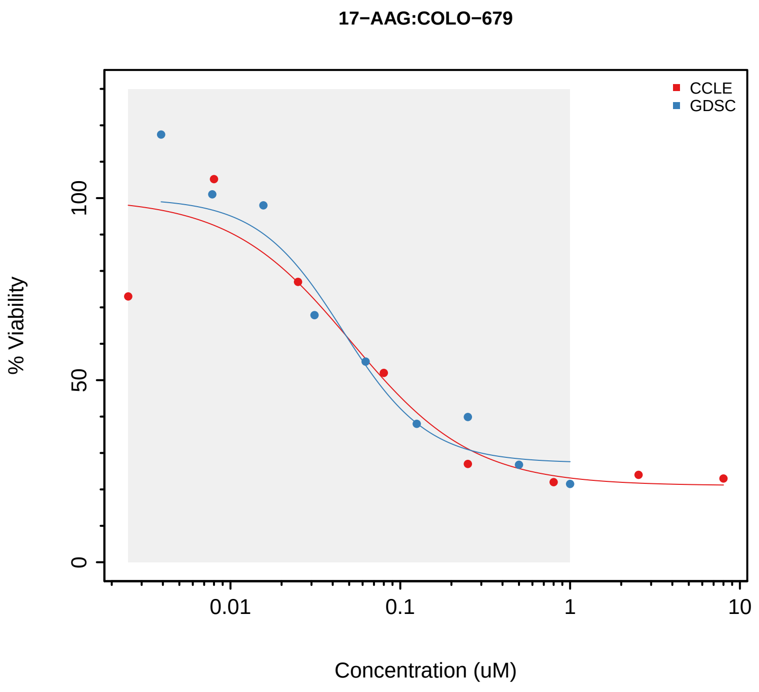
17−AAG:COLO−679
CCLE
GDSC
% Viability
0
50
100
0.01
0.1
1
10
Concentration (uM)

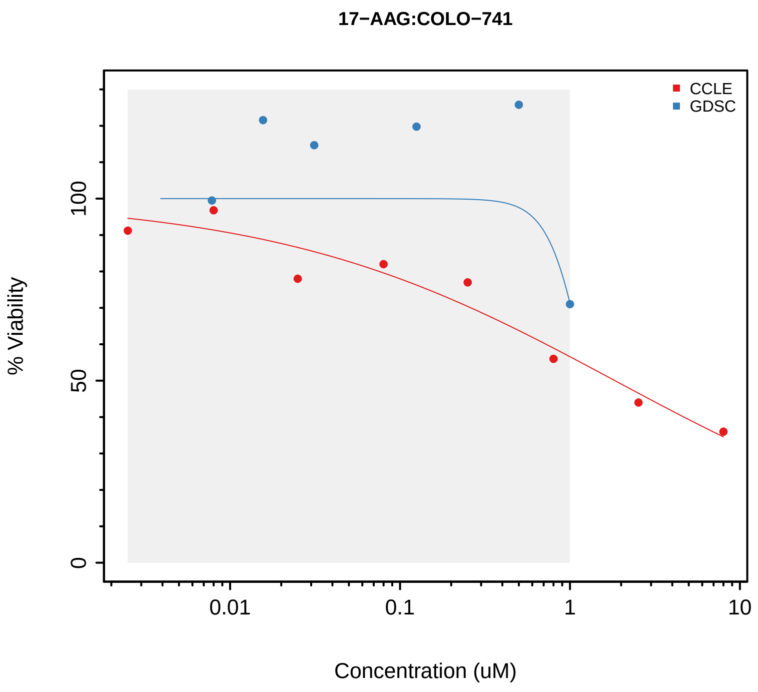
17−AAG:COLO−741
CCLE
GDSC
% Viability
0
50
100
0.01
0.1
1
10
Concentration (uM)

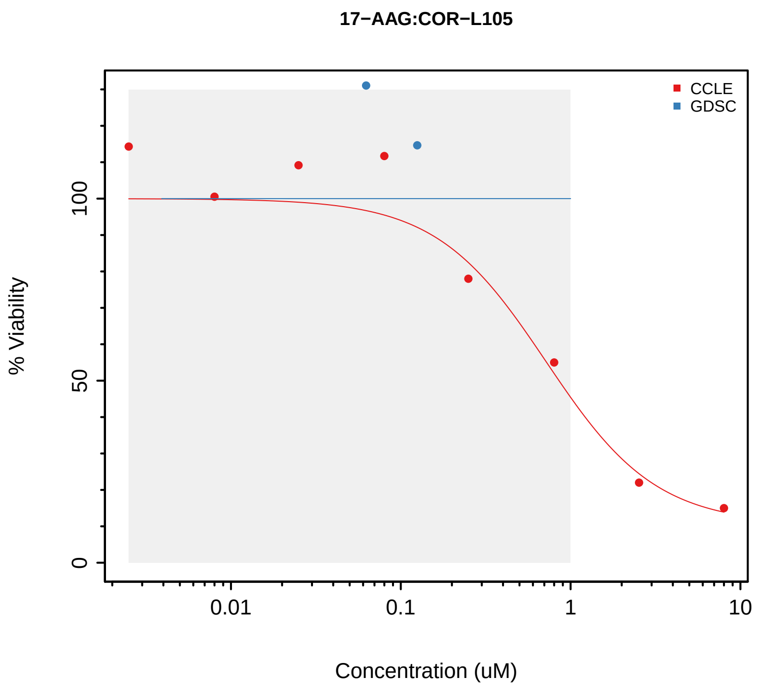
17−AAG:COR−L105
CCLE
GDSC
% Viability
0
50
100
0.01
0.1
1
10
Concentration (uM)

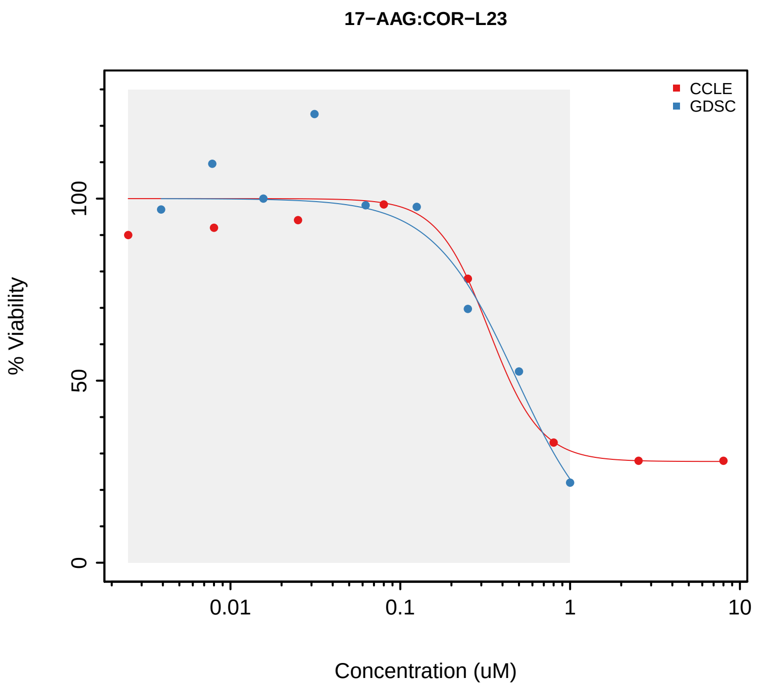
17−AAG:COR−L23
CCLE
GDSC
% Viability
0
50
100
0.01
0.1
1
10
Concentration (uM)

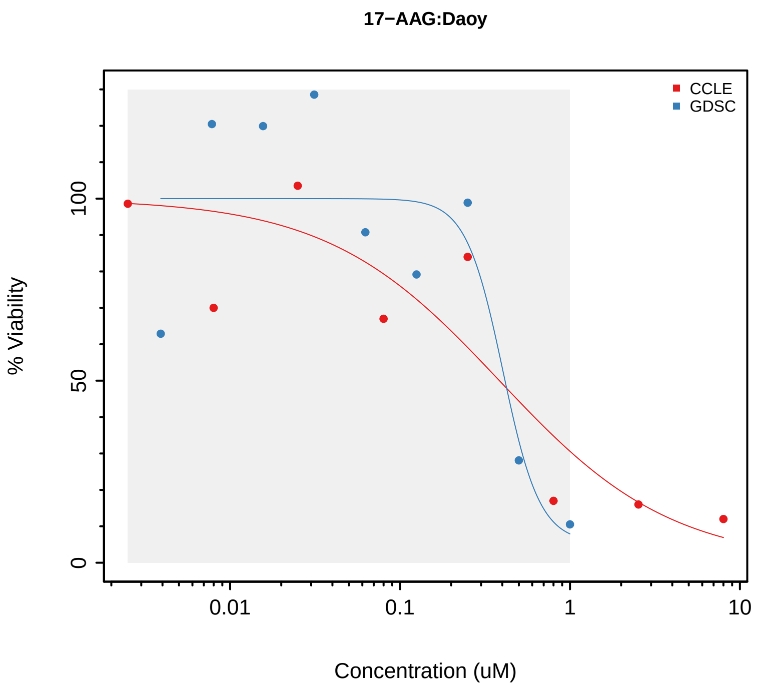
17−AAG:Daoy
CCLE
GDSC
% Viability
0
50
100
0.01
0.1
1
10
Concentration (uM)

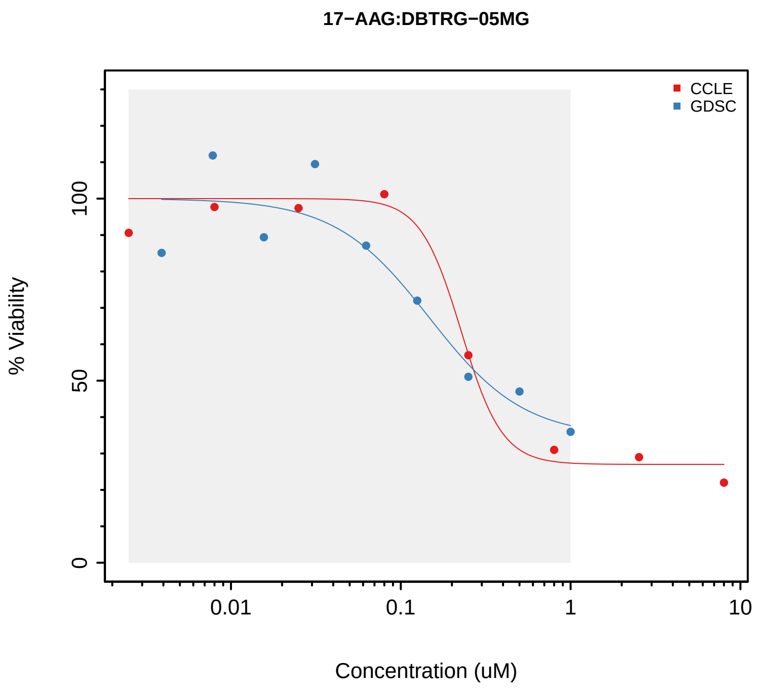
17−AAG:DBTRG−05MG
CCLE
GDSC
% Viability
0
50
100
0.01
0.1
1
10
Concentration (uM)

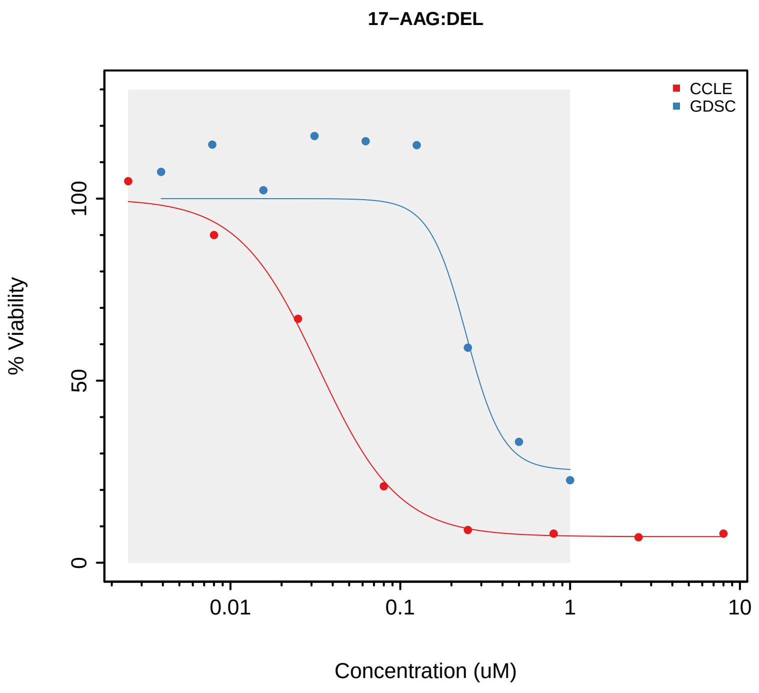
17−AAG:DEL
CCLE
GDSC
% Viability
0
50
100
0.01
0.1
1
10
Concentration (uM)

17−AAG:Detroit562

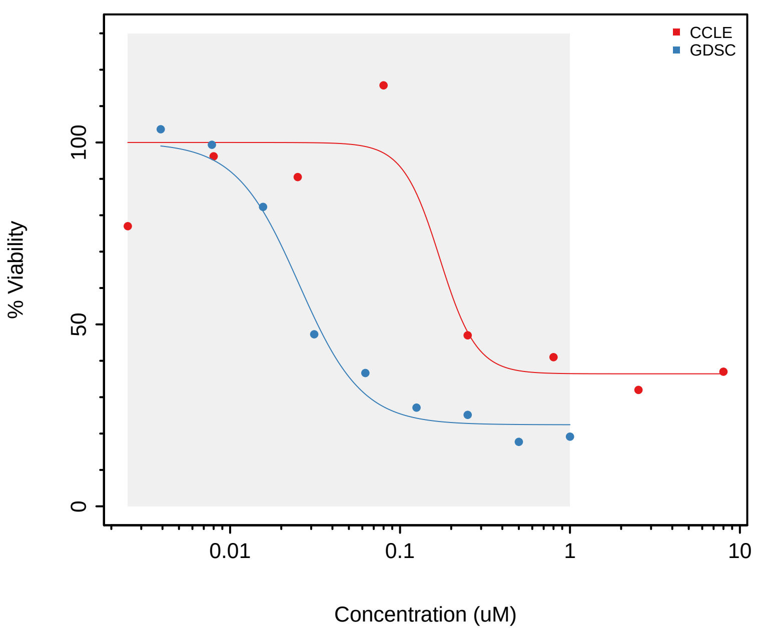

17−AAG:DK−MG

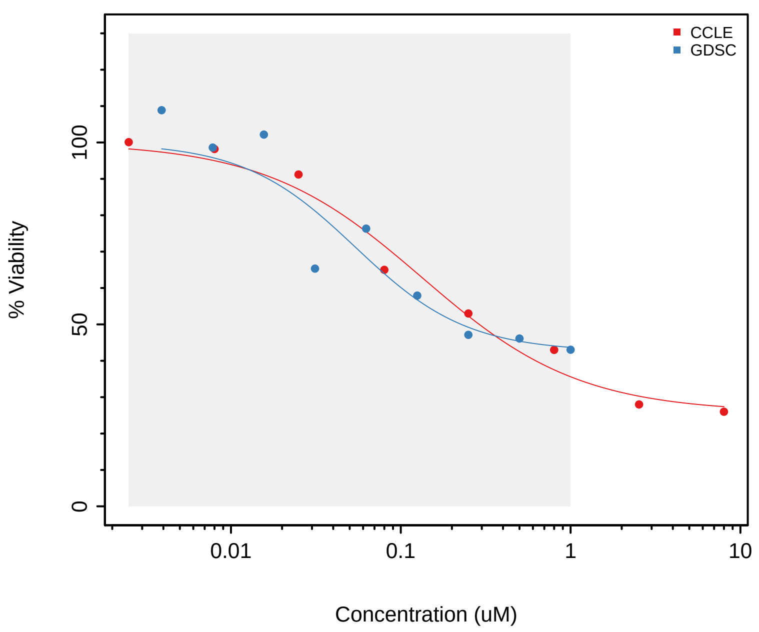

17−AAG:DMS−114

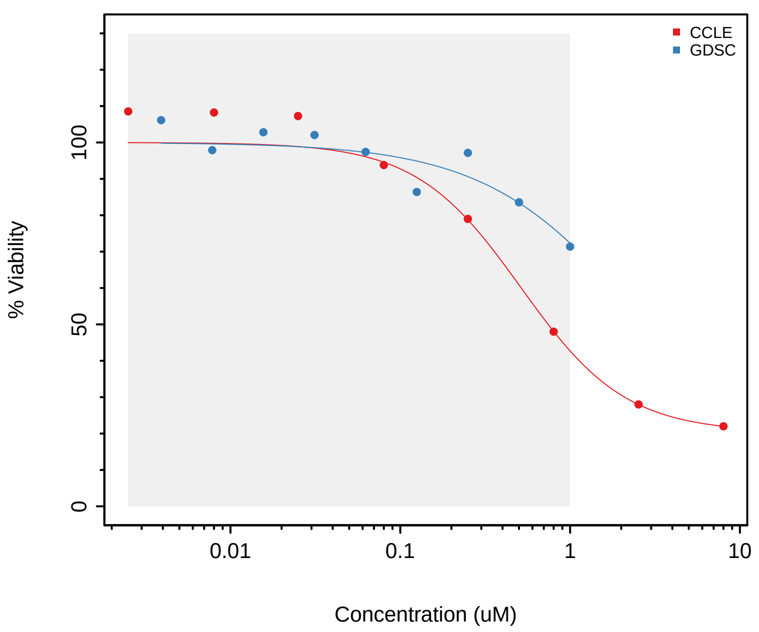

17−AAG:DOHH−2

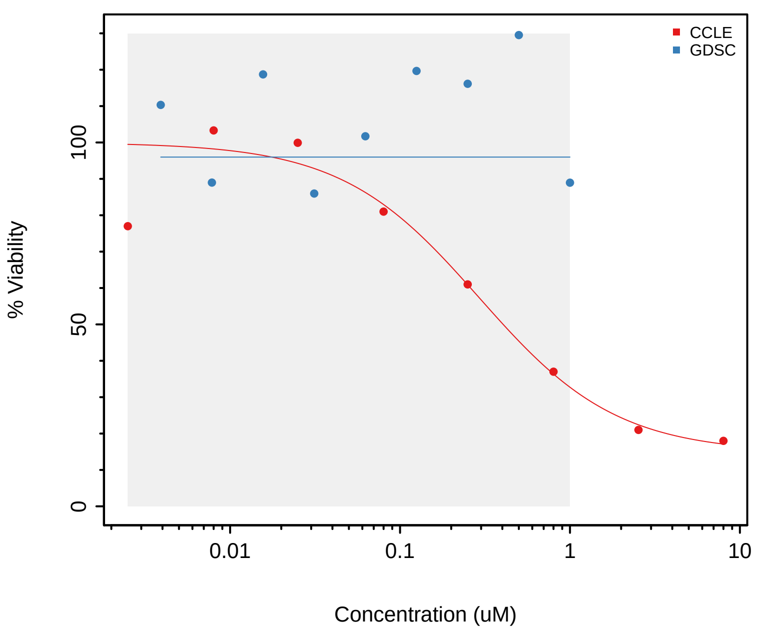

17−AAG:DU−145

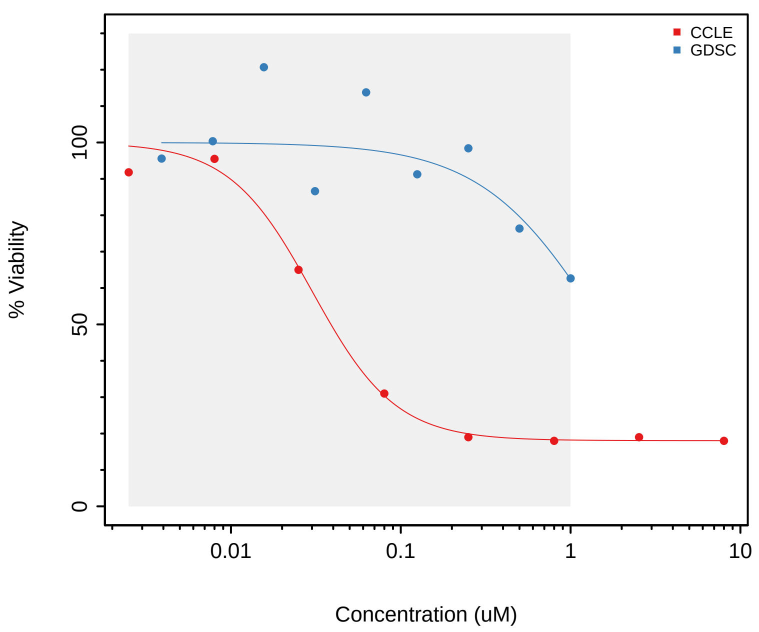

17−AAG:EB2

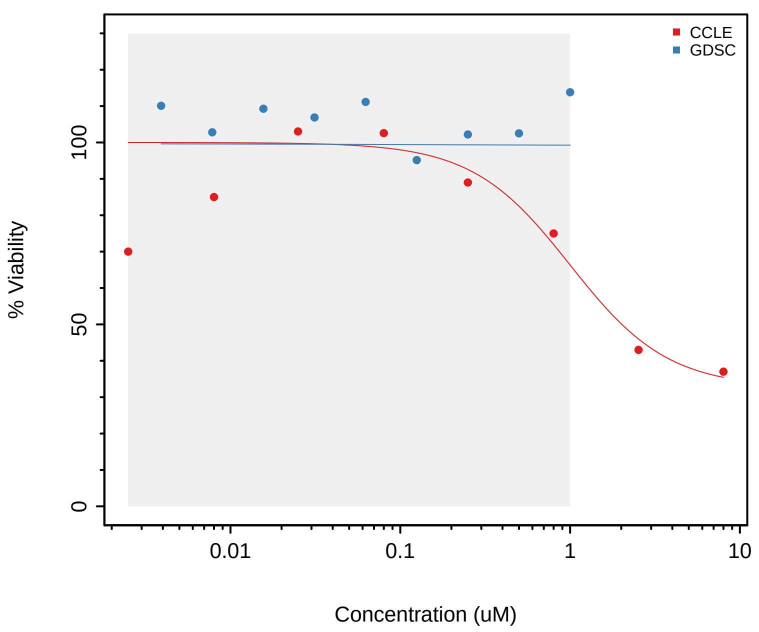

17−AAG:EFE−184

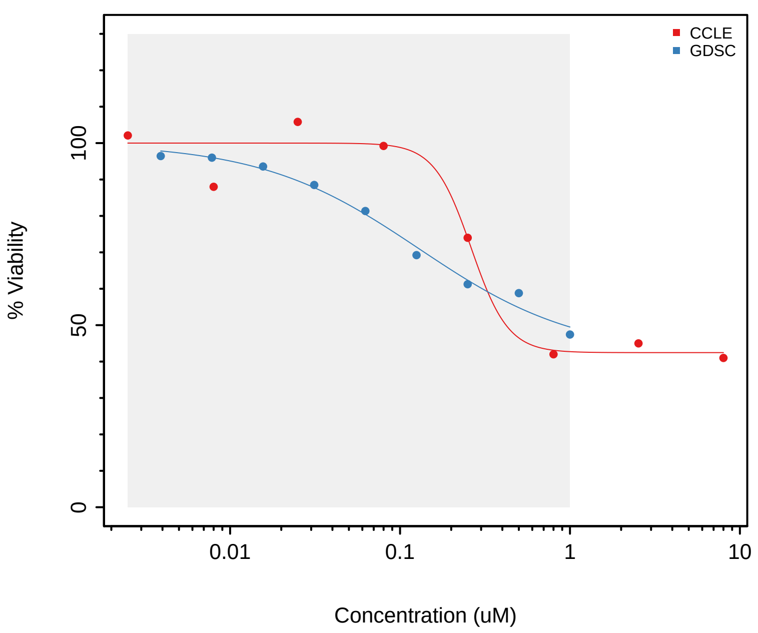

17−AAG:EFM−19

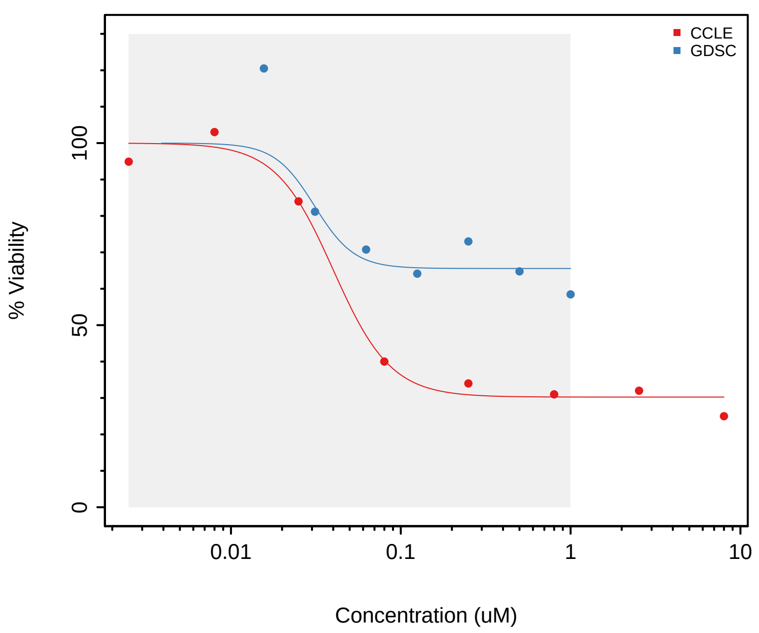

17−AAG:EFO−21

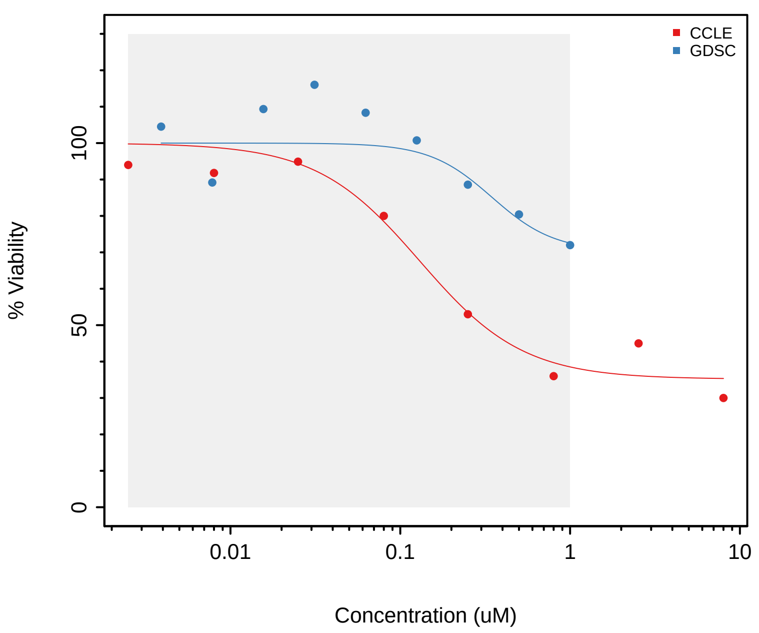

17−AAG:EFO−27

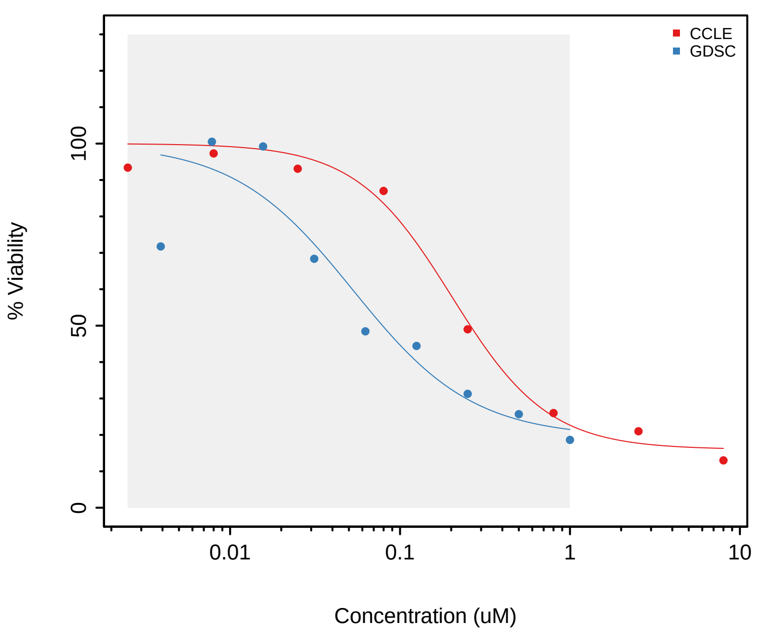

17−AAG:EM−2

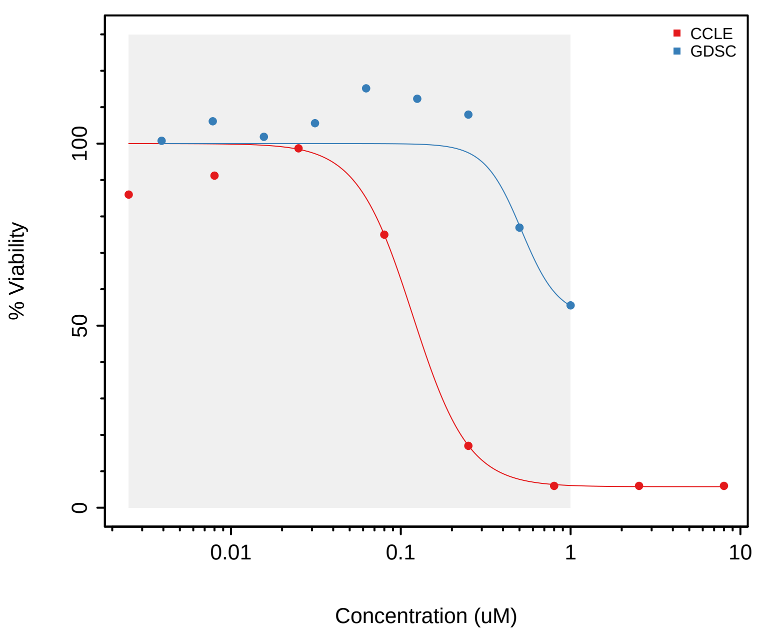

17−AAG:ESS−1

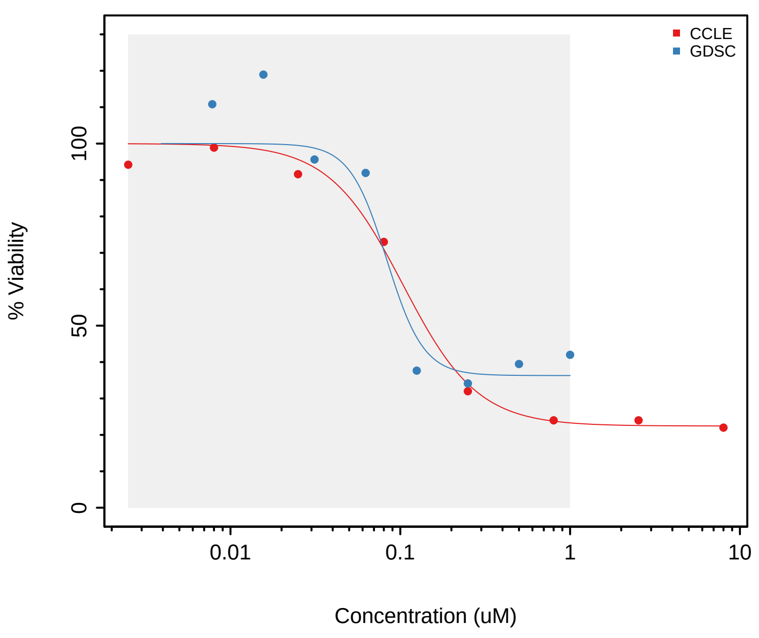

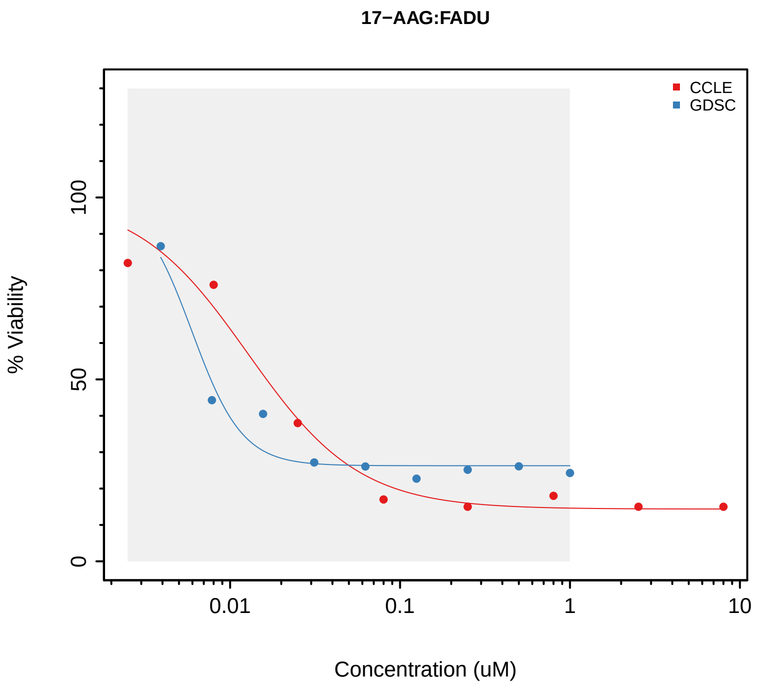
17−AAG:FADU
CCLE
GDSC
% Viability
0
50
100
0.01
0.1
1
10
Concentration (uM)

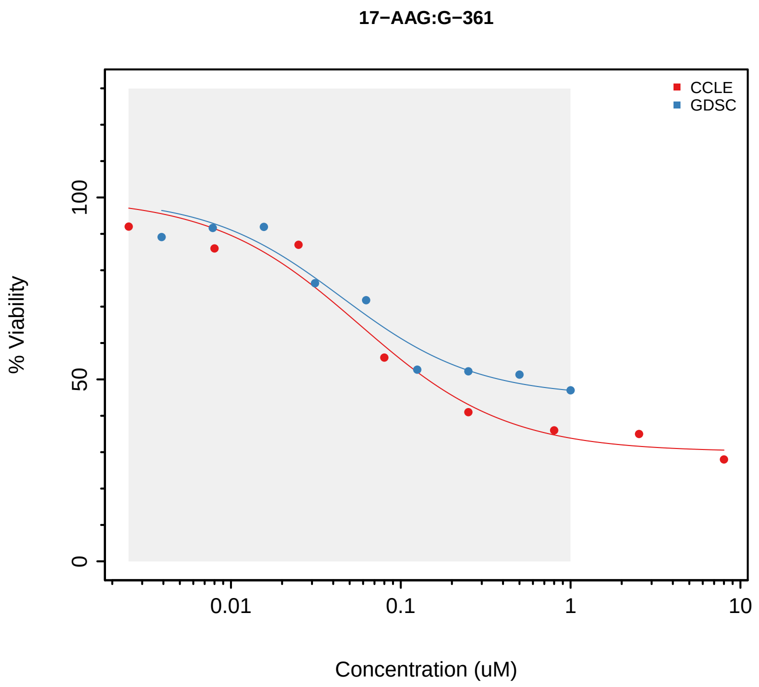
17−AAG:G−361
CCLE
GDSC
% Viability
0
50
100
0.01
0.1
1
10
Concentration (uM)

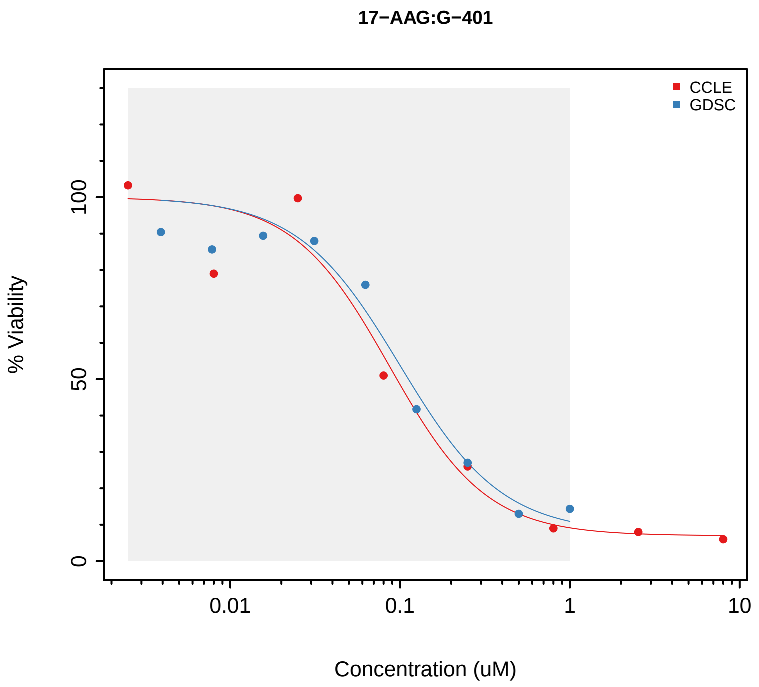
17−AAG:G−401
CCLE
GDSC
% Viability
0
50
100
0.01
0.1
1
10
Concentration (uM)

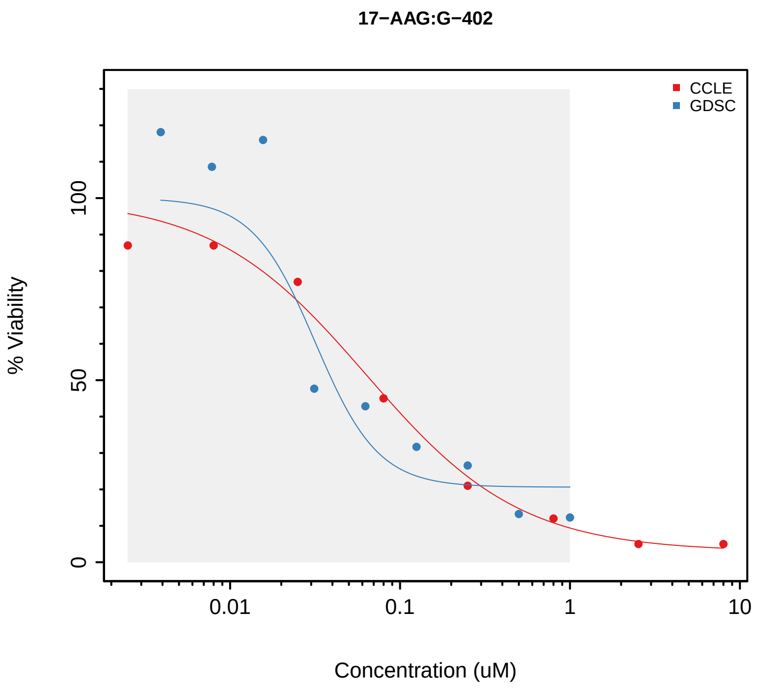
17−AAG:G−402
CCLE
GDSC
% Viability
0
50
100
0.01
0.1
1
10
Concentration (uM)

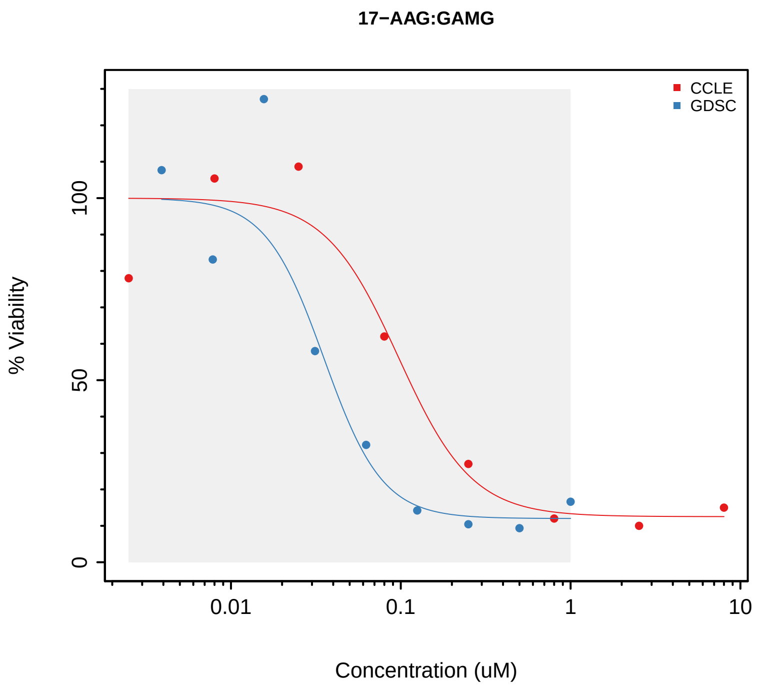
17−AAG:GAMG
CCLE
GDSC
% Viability
0
50
100
0.01
0.1
1
10
Concentration (uM)

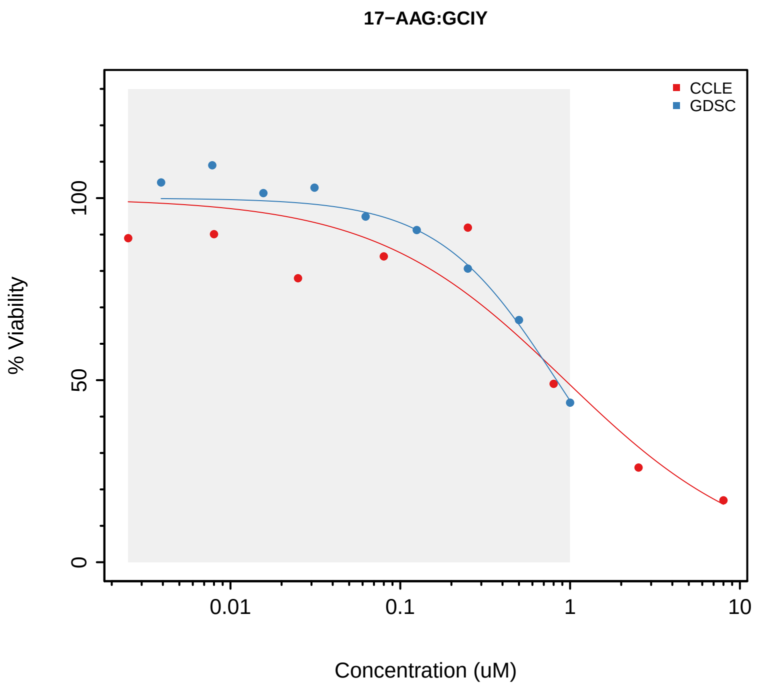
17−AAG:GCIY
CCLE
GDSC
% Viability
0
50
100
0.01
0.1
1
10
Concentration (uM)

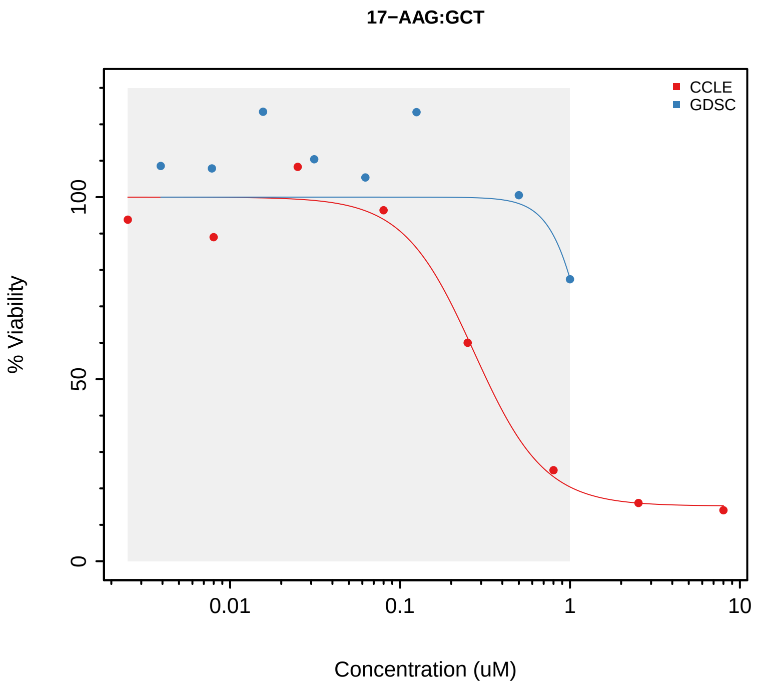
17−AAG:GCT
CCLE
GDSC
% Viability
0
50
100
0.01
0.1
1
10
Concentration (uM)

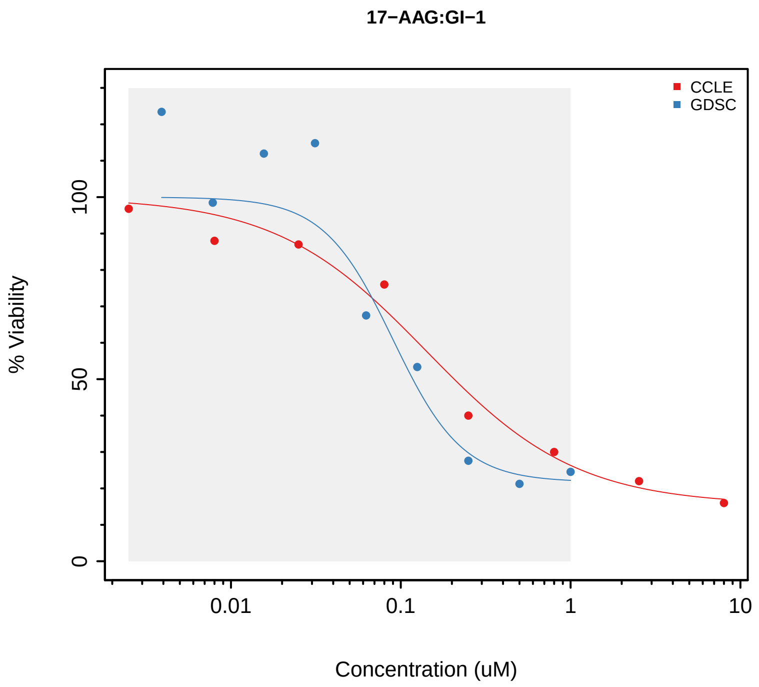
17−AAG:GI−1
CCLE
GDSC
% Viability
0
50
100
0.01
0.1
1
10
Concentration (uM)

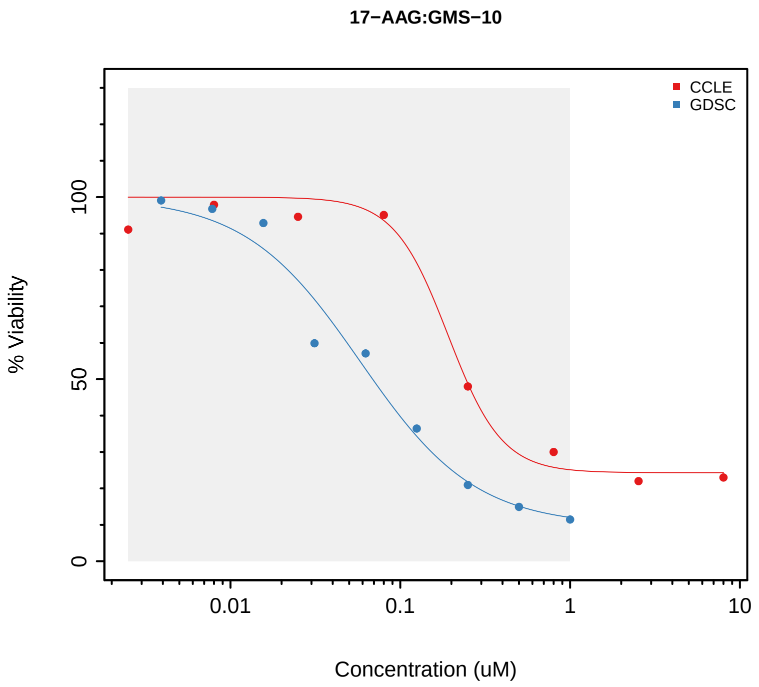
17−AAG:GMS−10
CCLE
GDSC
% Viability
0
50
100
0.01
0.1
1
10
Concentration (uM)

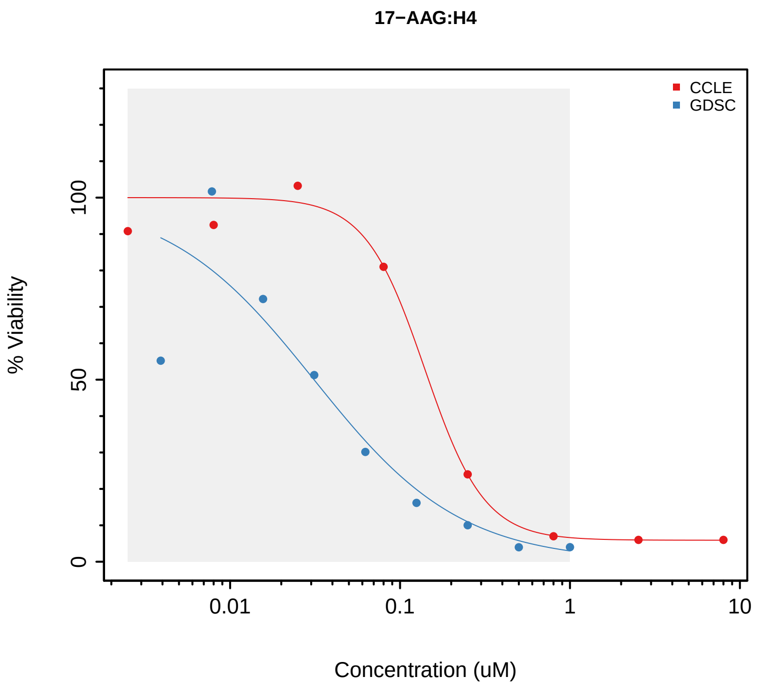
17−AAG:H4
CCLE
GDSC
% Viability
0
50
100
0.01
0.1
1
10
Concentration (uM)

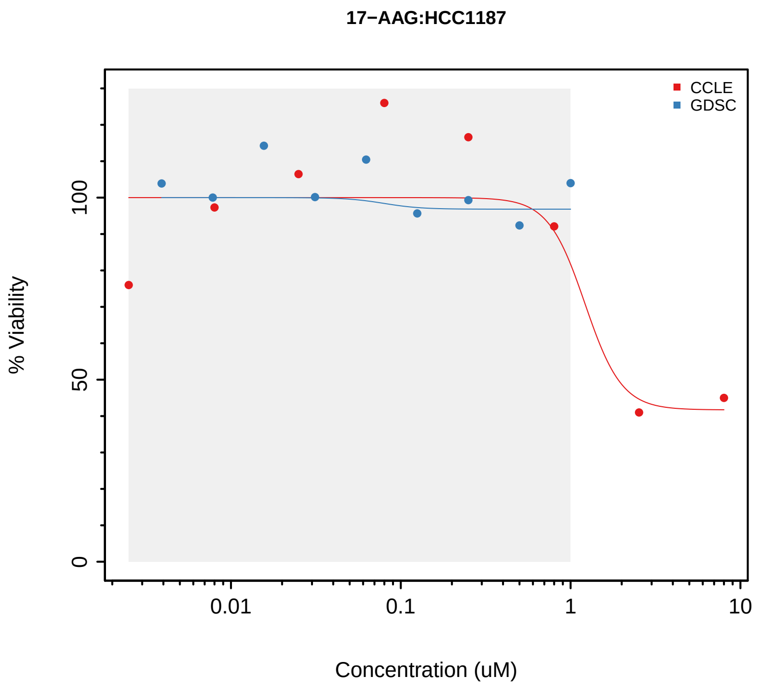
17−AAG:HCC1187
CCLE
GDSC
% Viability
0
50
100
0.01
0.1
1
10
Concentration (uM)

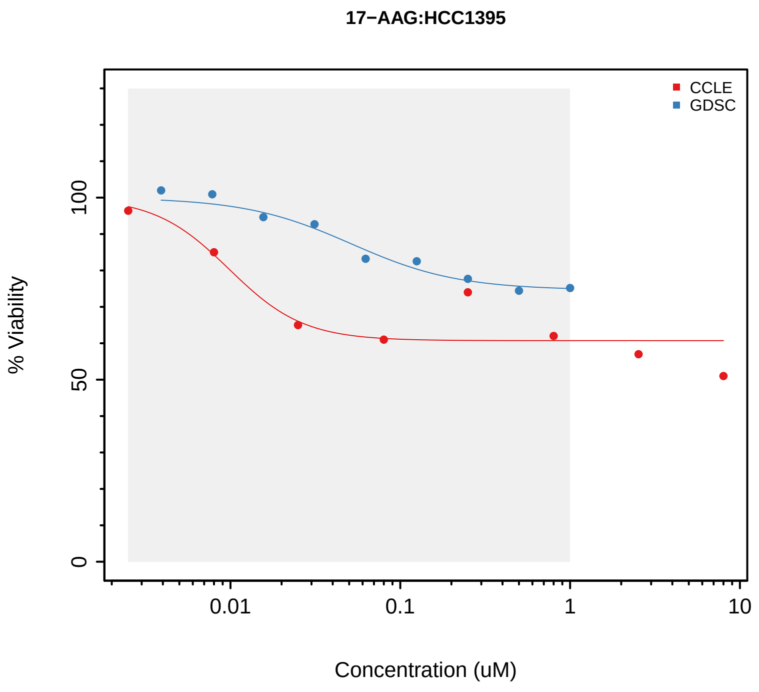
17−AAG:HCC1395
CCLE
GDSC
% Viability
0
50
100
0.01
0.1
1
10
Concentration (uM)

17−AAG:HCC1569

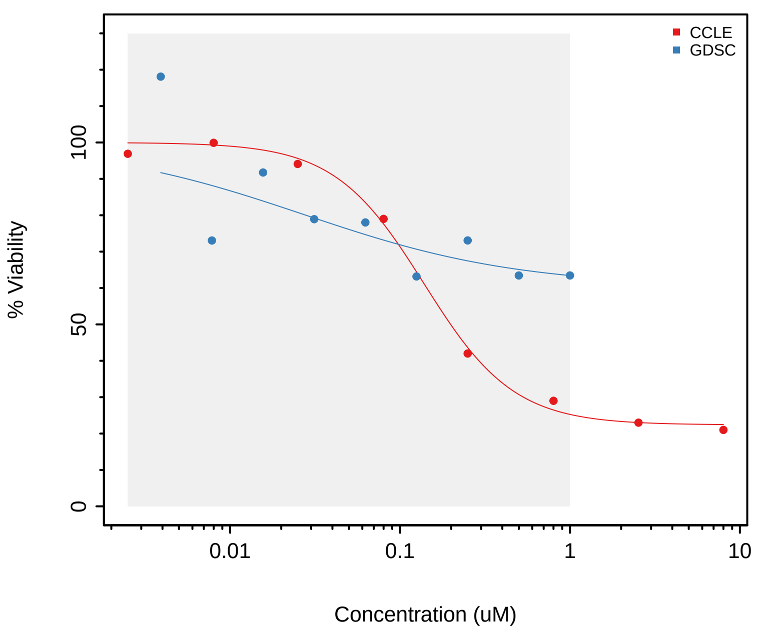

17−AAG:HCC1806

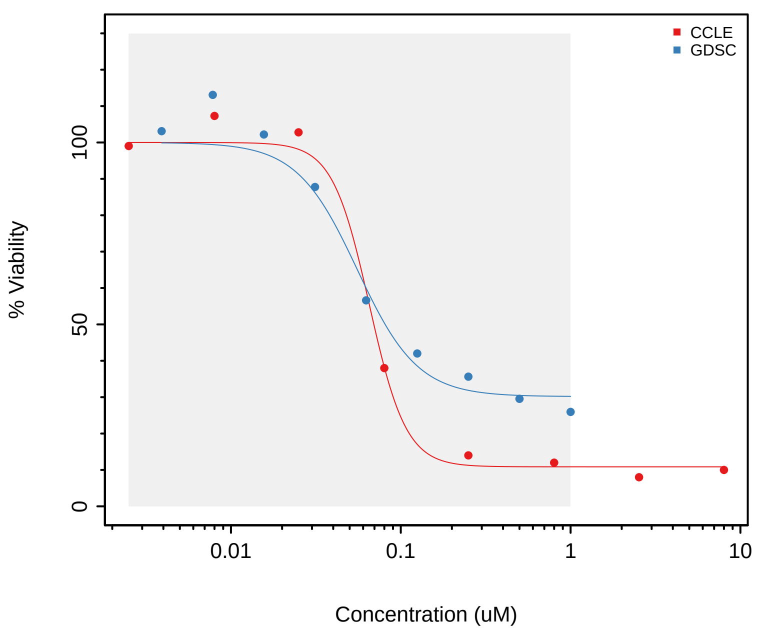

17−AAG:HCC1954

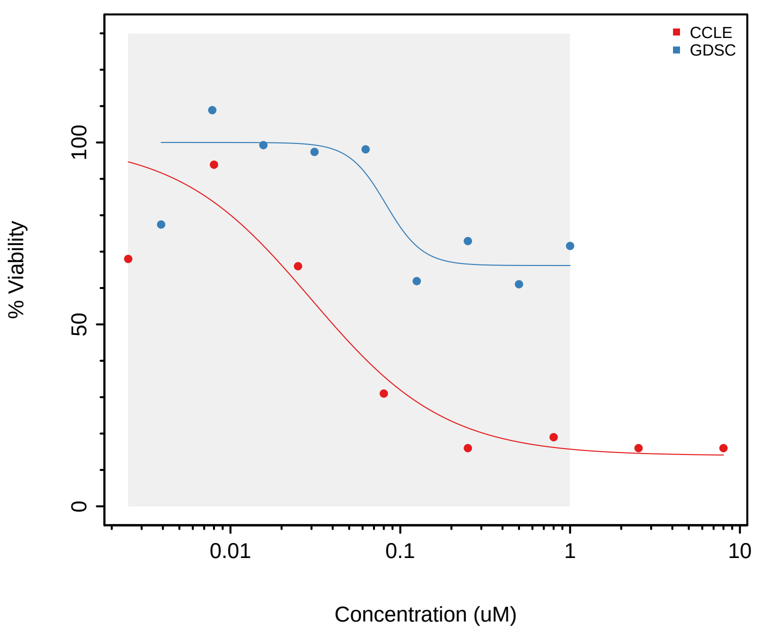

17−AAG:HCT−116

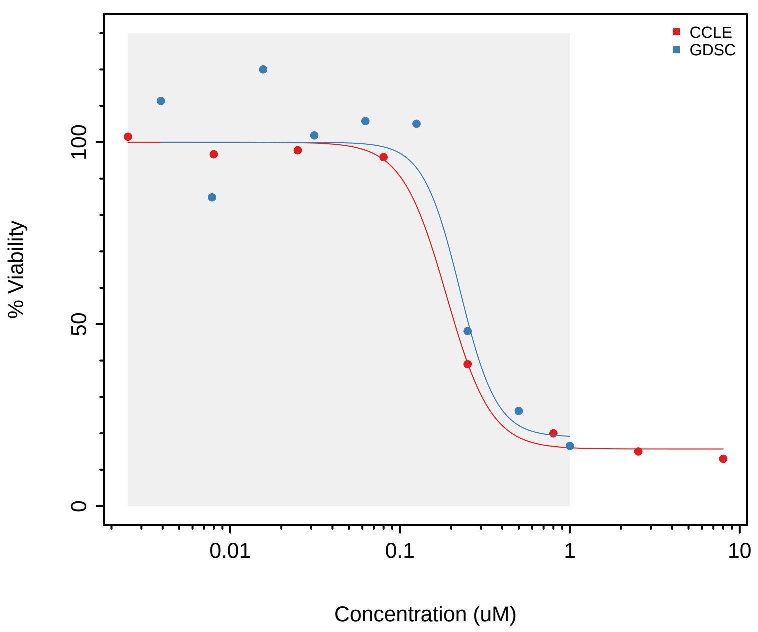

17−AAG:HCT−15

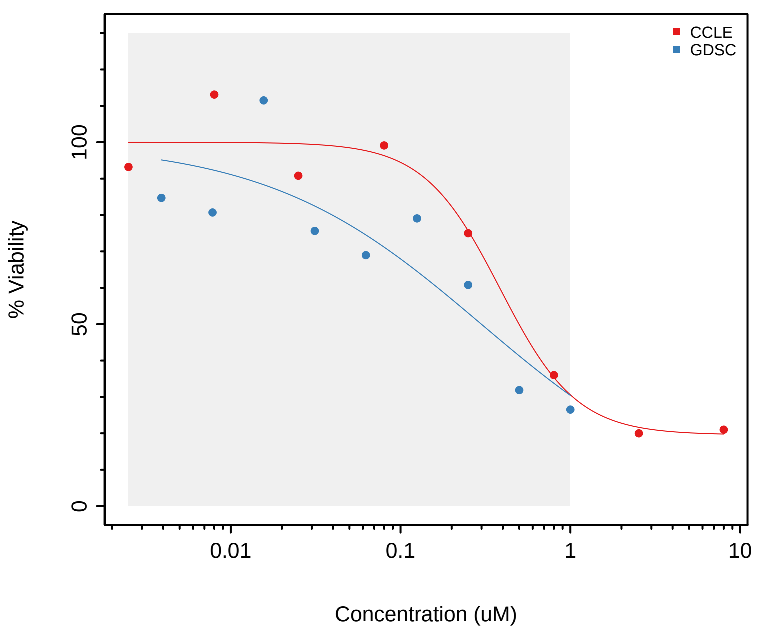

17−AAG:HD−MY−Z

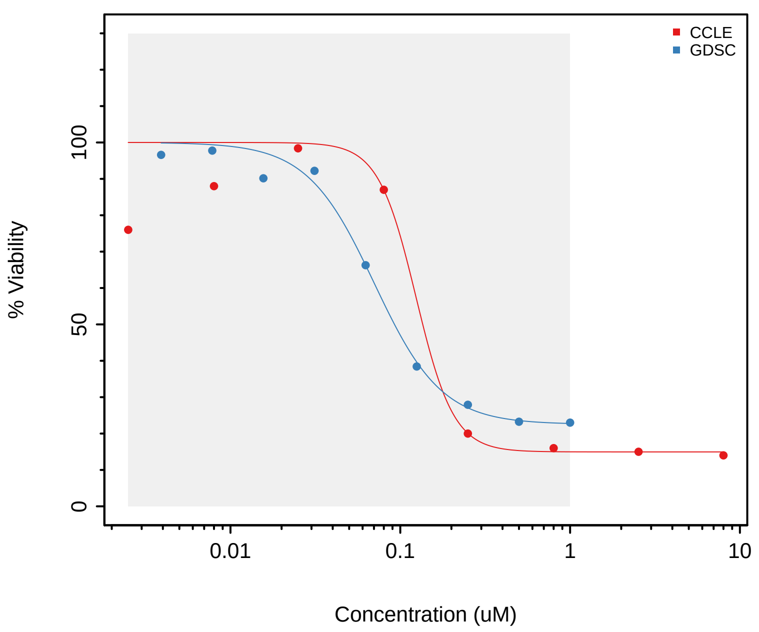

17−AAG:HEC−1

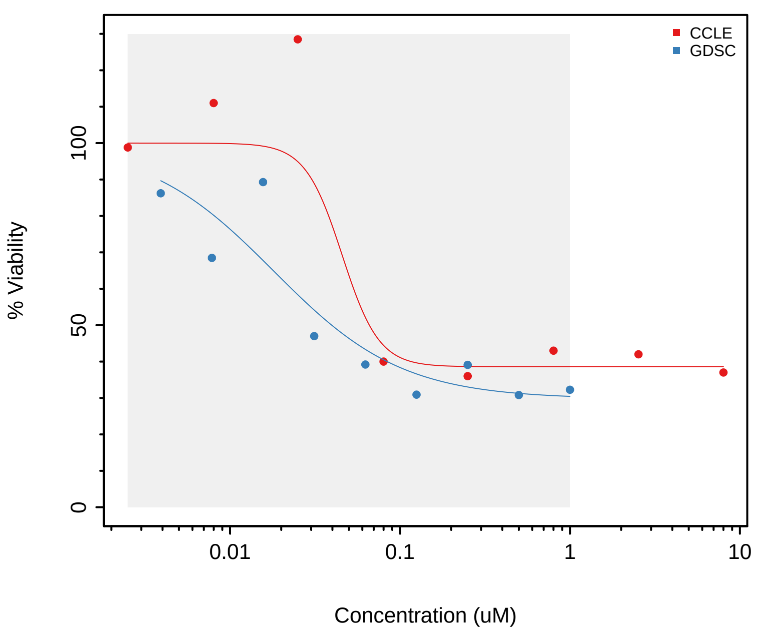

17−AAG:HGC−27

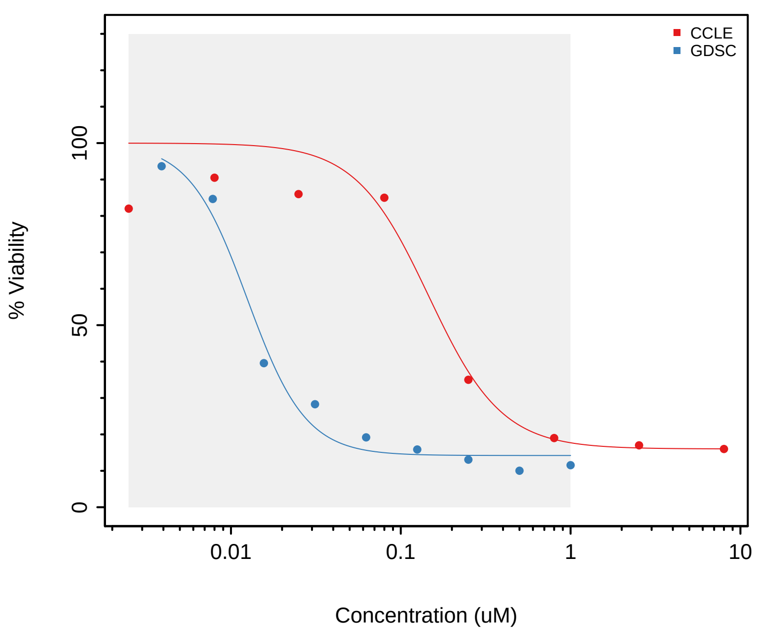

17−AAG:HH

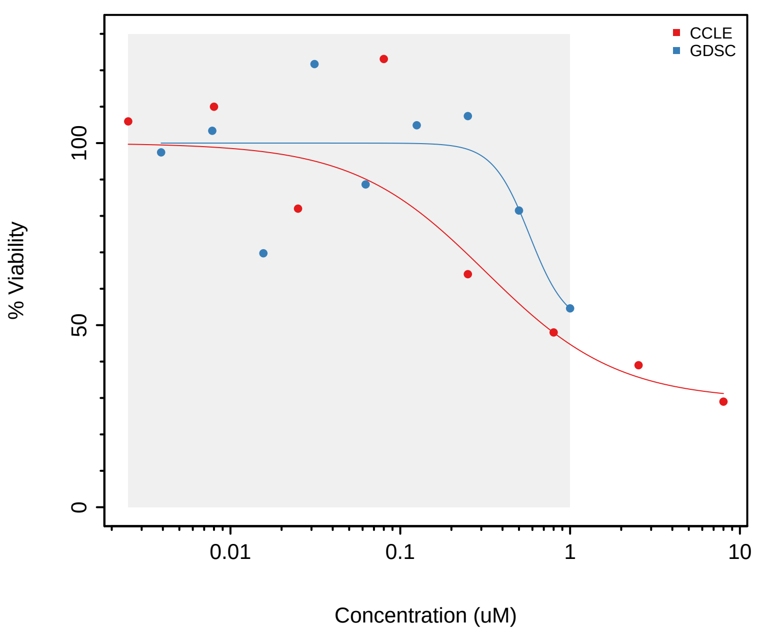

17−AAG:HLE

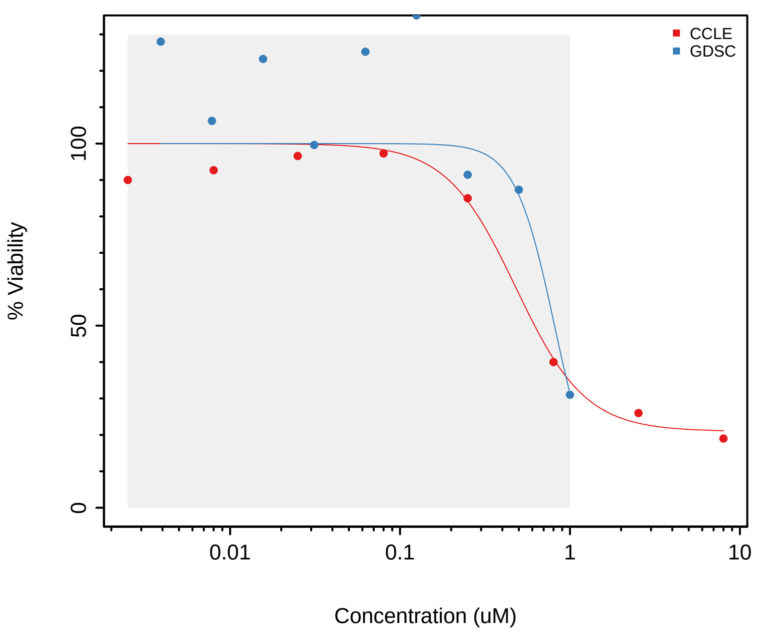

17−AAG:HOS

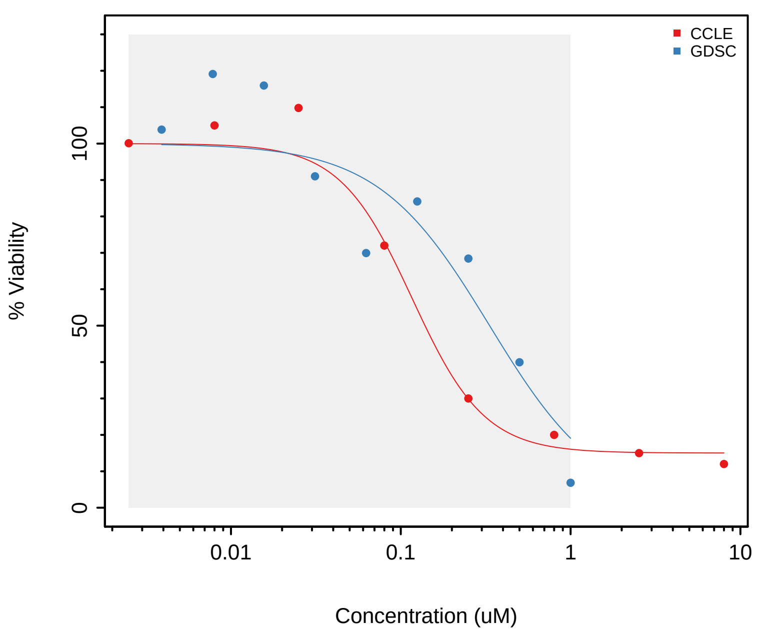

17−AAG:HPAF−II

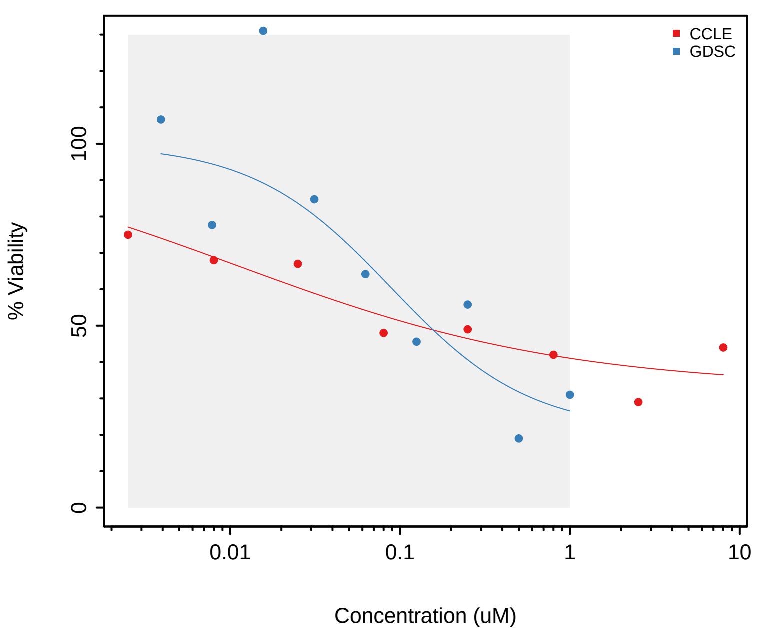

17−AAG:Hs−578−T

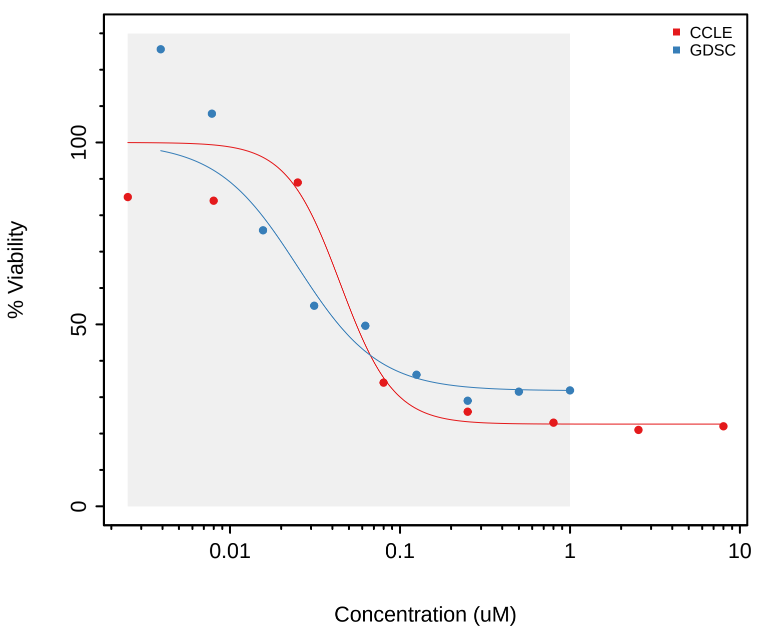

17−AAG:HSC−2

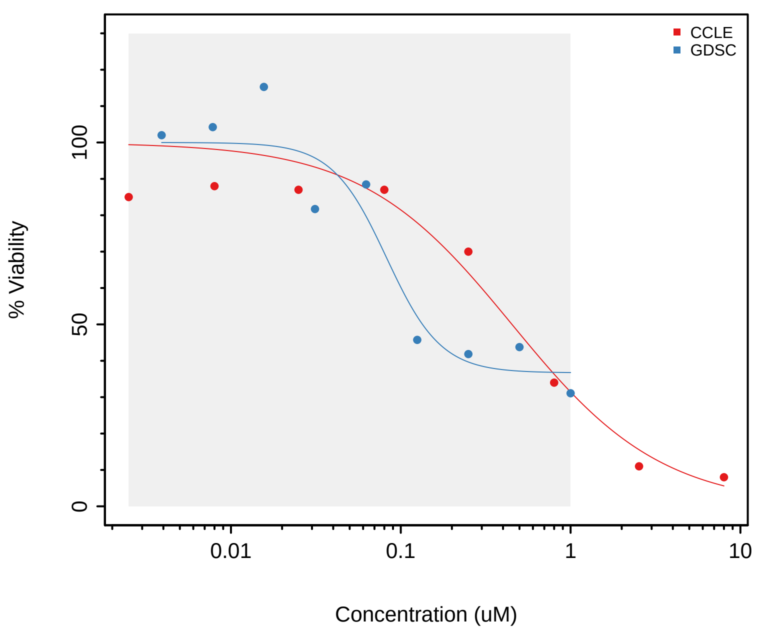

17−AAG:HT

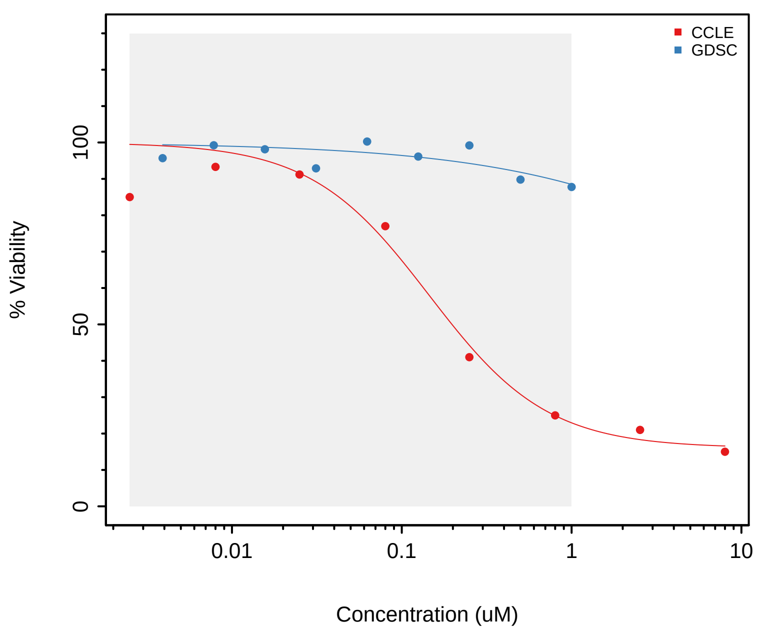

17−AAG:HT−1080

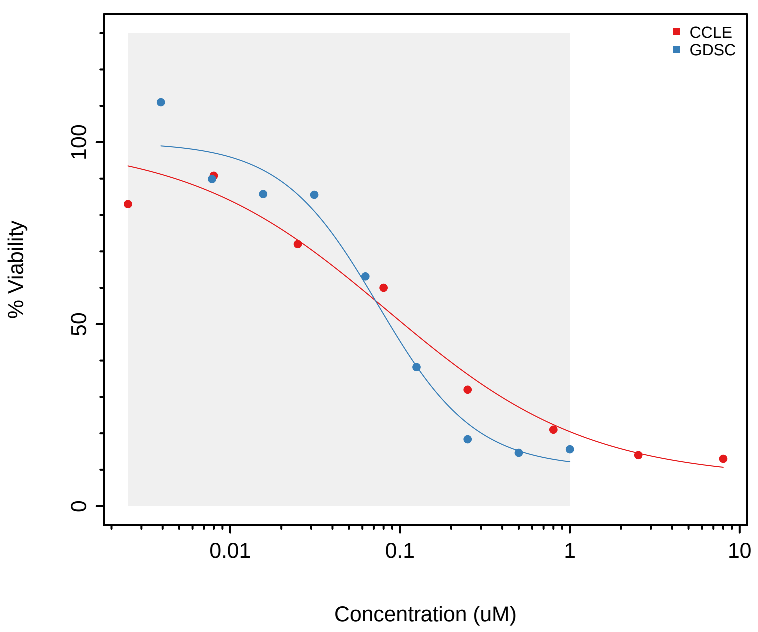

17−AAG:HT−1197

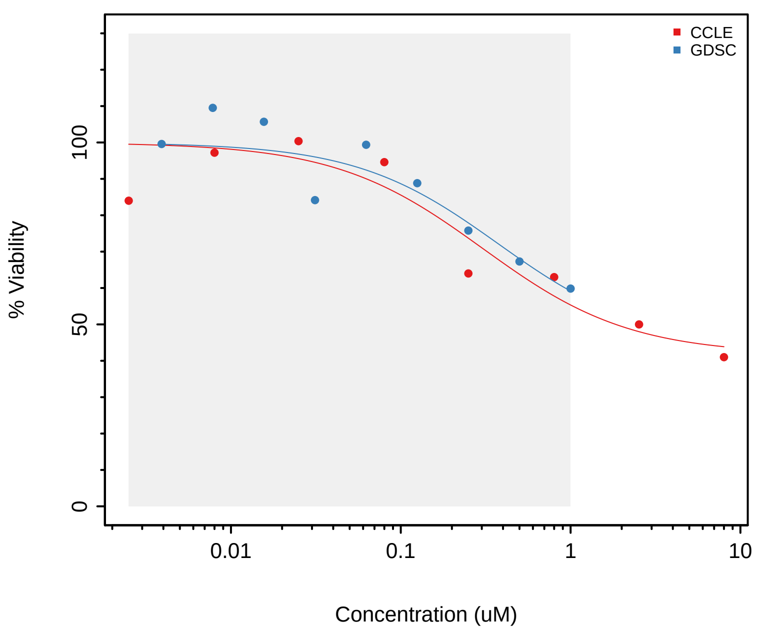

17−AAG:HT−1376

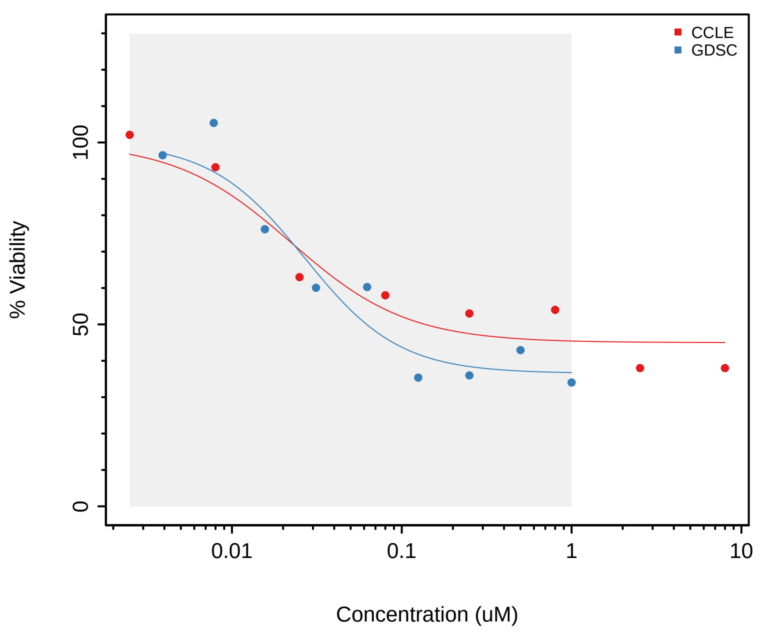

17−AAG:HT−144

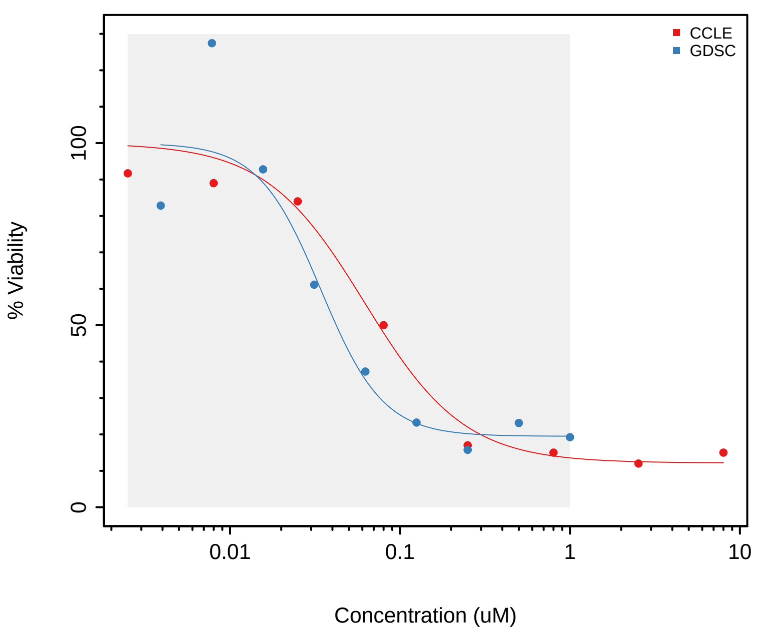

17−AAG:HT−29

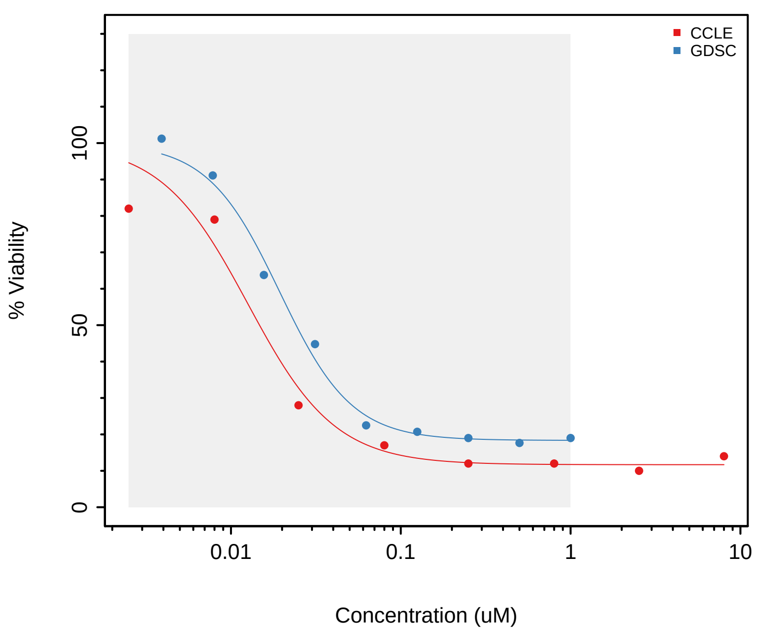

17−AAG:HuCCT1

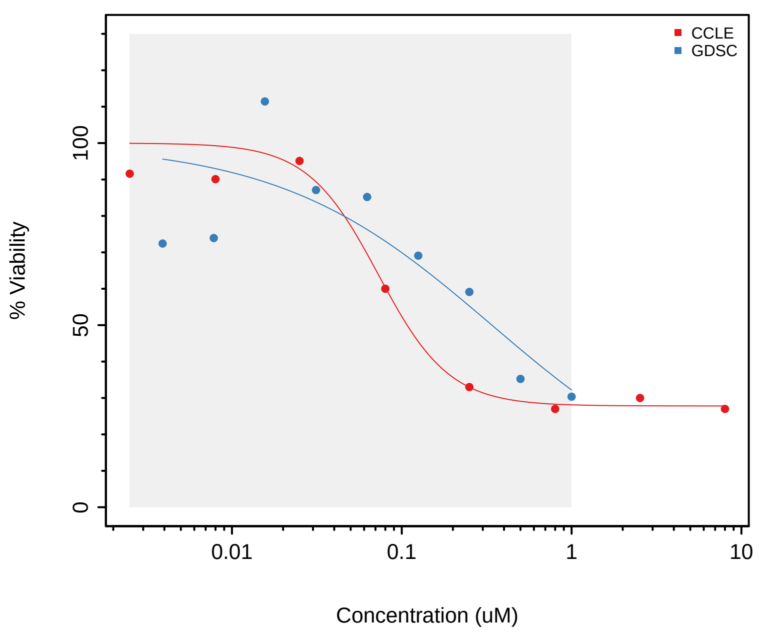

17−AAG:HuP−T3

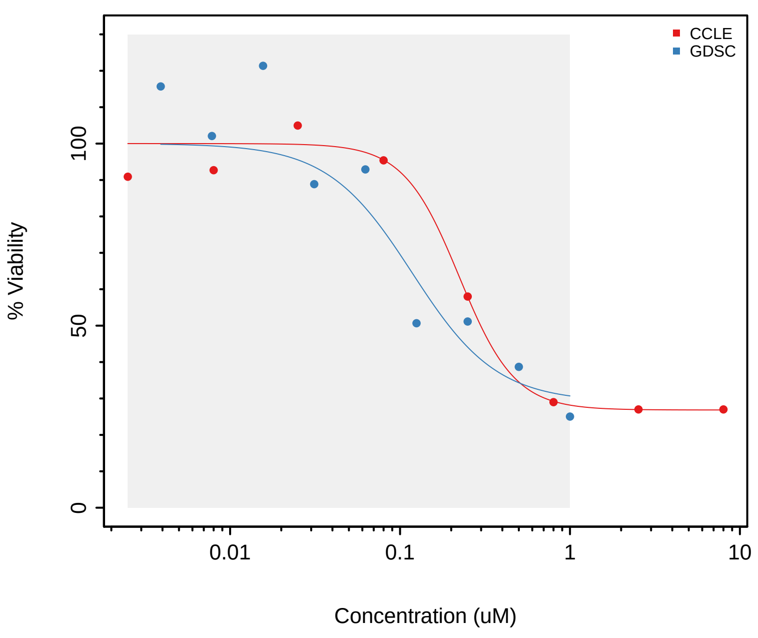

17−AAG:HuP−T4

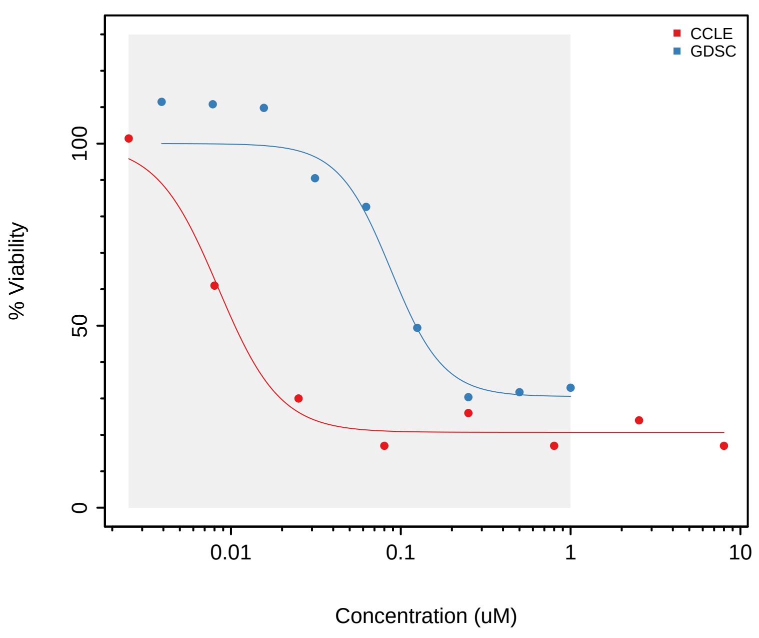

17−AAG:IA−LM

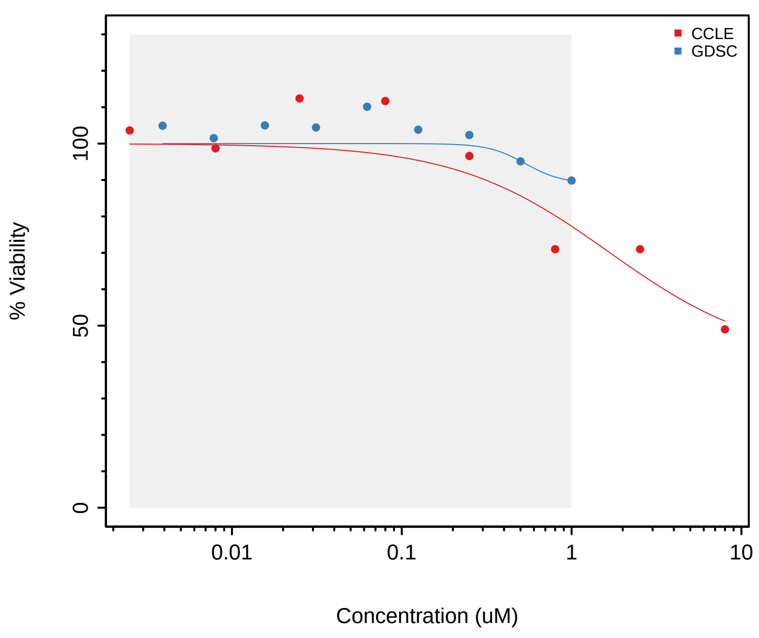

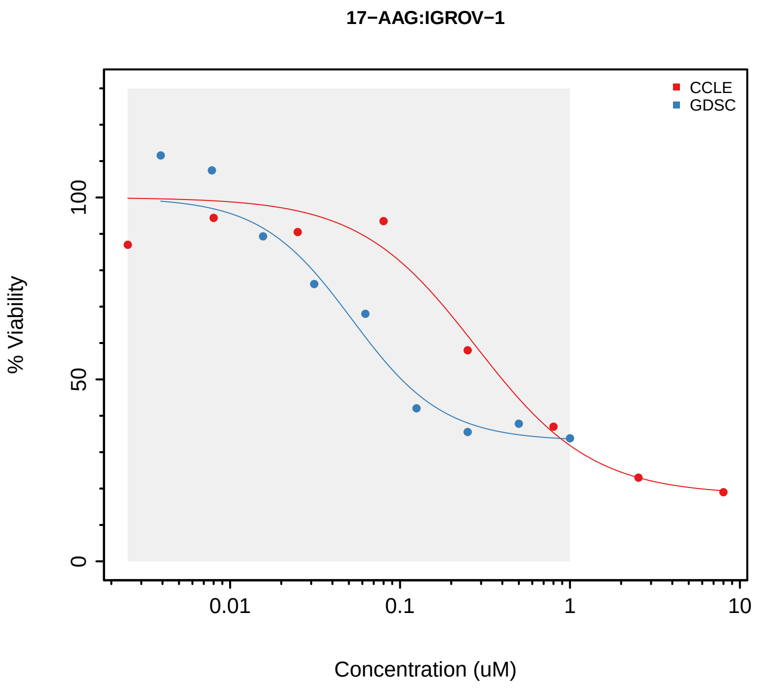
17−AAG:IGROV−1
CCLE
GDSC
% Viability
0
50
100
0.01
0.1
1
10
Concentration (uM)

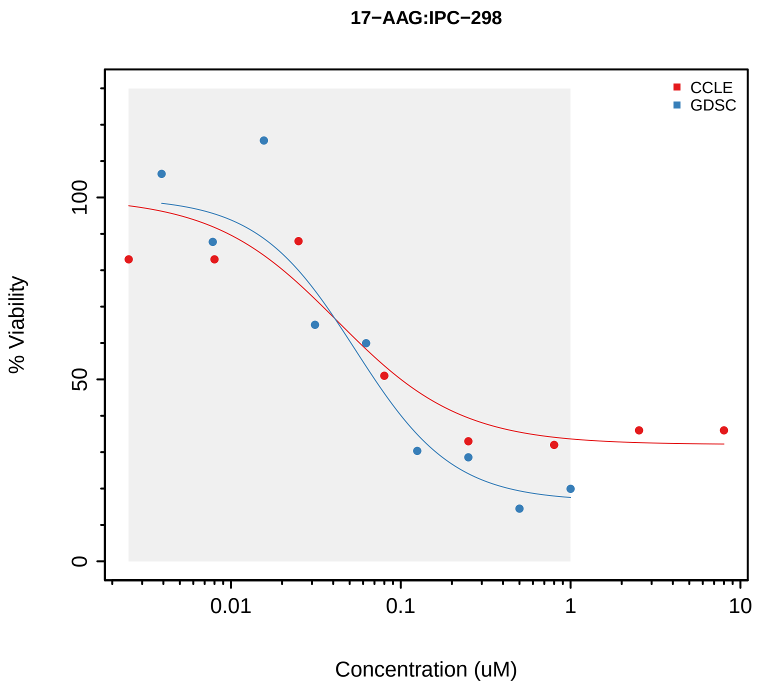
17−AAG:IPC−298
CCLE
GDSC
% Viability
0
50
100
0.01
0.1
1
10
Concentration (uM)

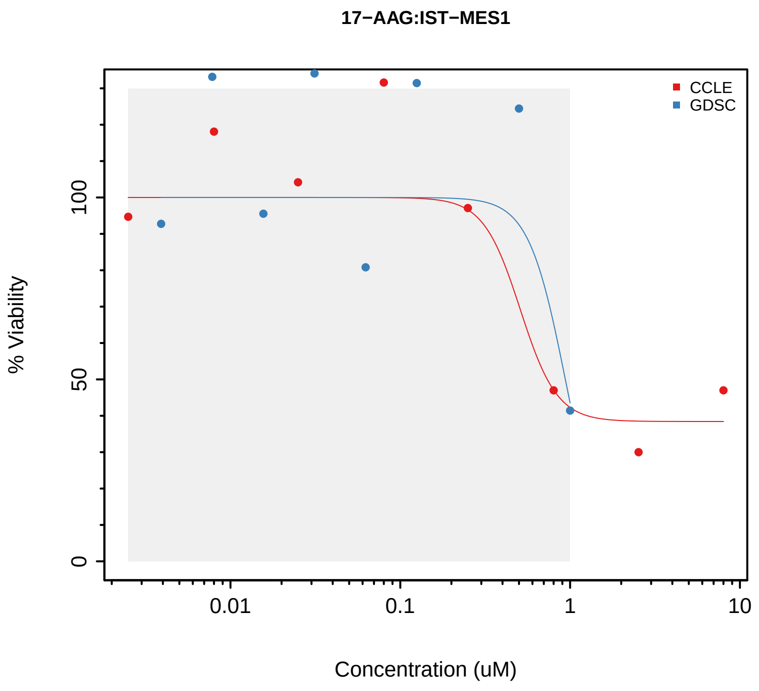
17−AAG:IST−MES1
CCLE
GDSC
% Viability
0
50
100
0.01
0.1
1
10
Concentration (uM)

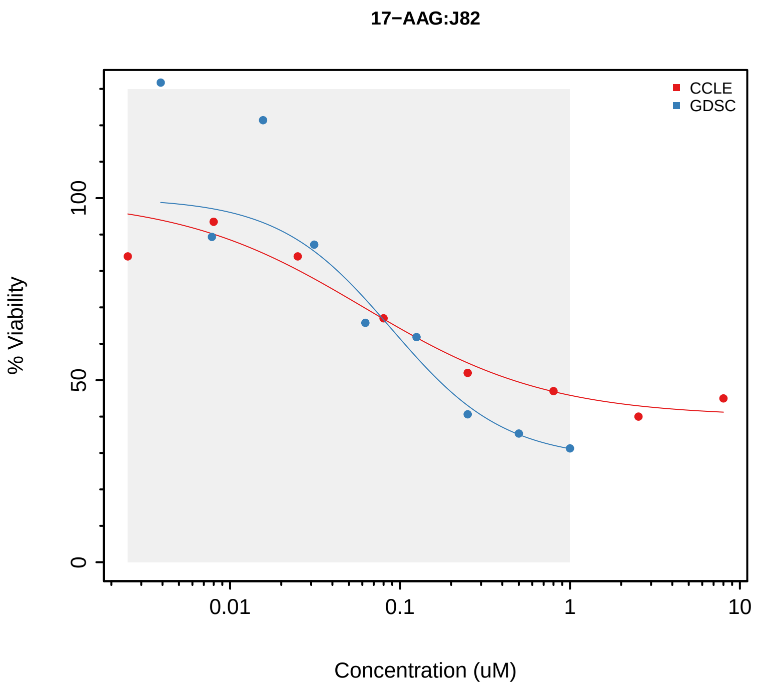
17−AAG:J82
CCLE
GDSC
% Viability
0
50
100
0.01
0.1
1
10
Concentration (uM)

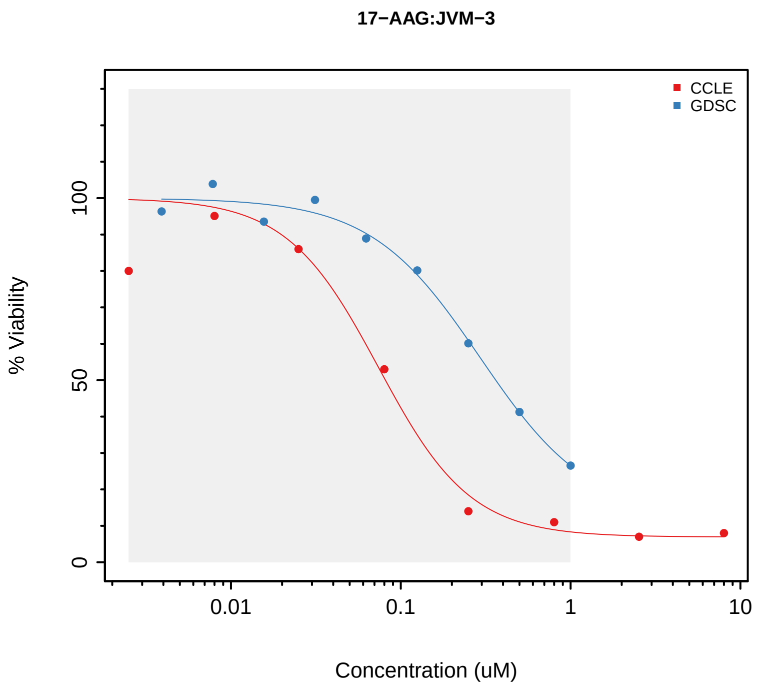
17−AAG:JVM−3
CCLE
GDSC
% Viability
0
50
100
0.01
0.1
1
10
Concentration (uM)

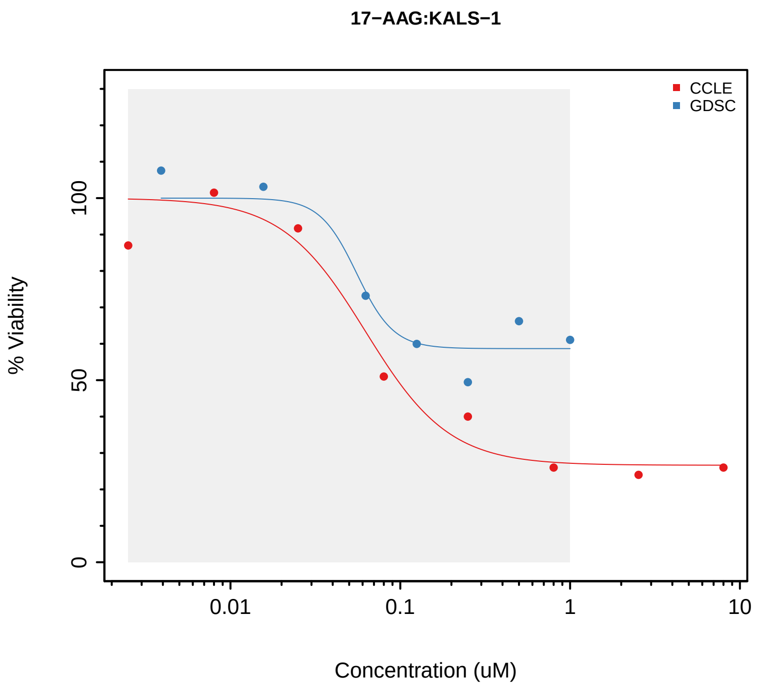
17−AAG:KALS−1
CCLE
GDSC
% Viability
0
50
100
0.01
0.1
1
10
Concentration (uM)

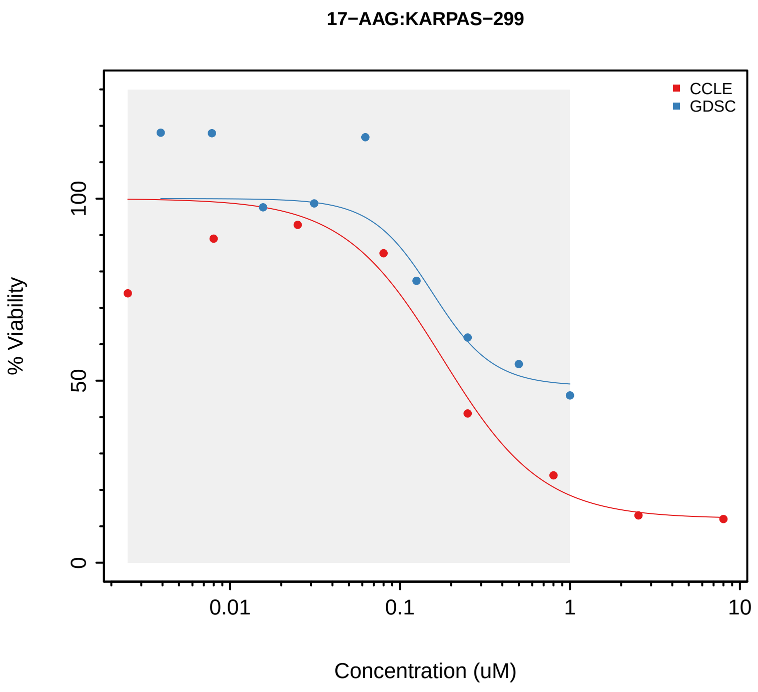
17−AAG:KARPAS−299
CCLE
GDSC
% Viability
0
50
100
0.01
0.1
1
10
Concentration (uM)

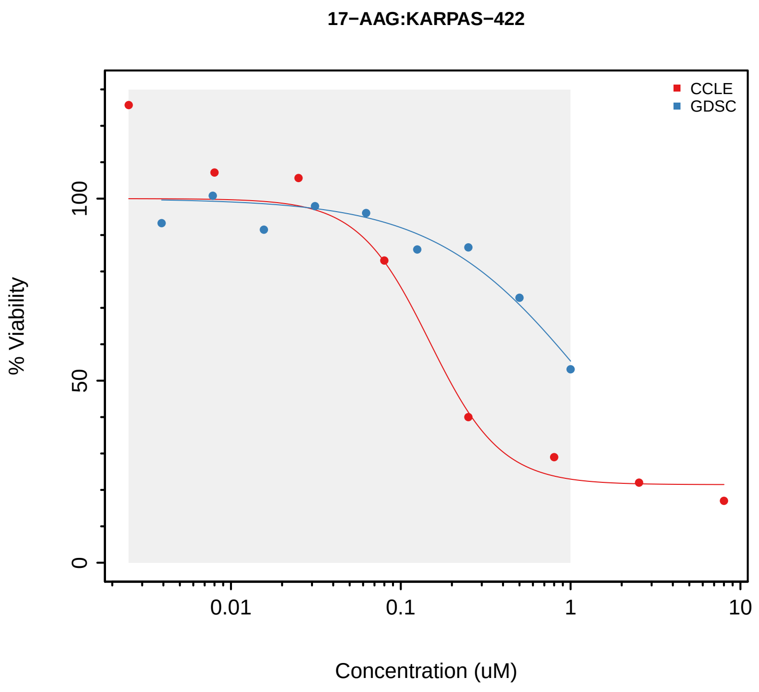
17−AAG:KARPAS−422
CCLE
GDSC
% Viability
0
50
100
0.01
0.1
1
10
Concentration (uM)

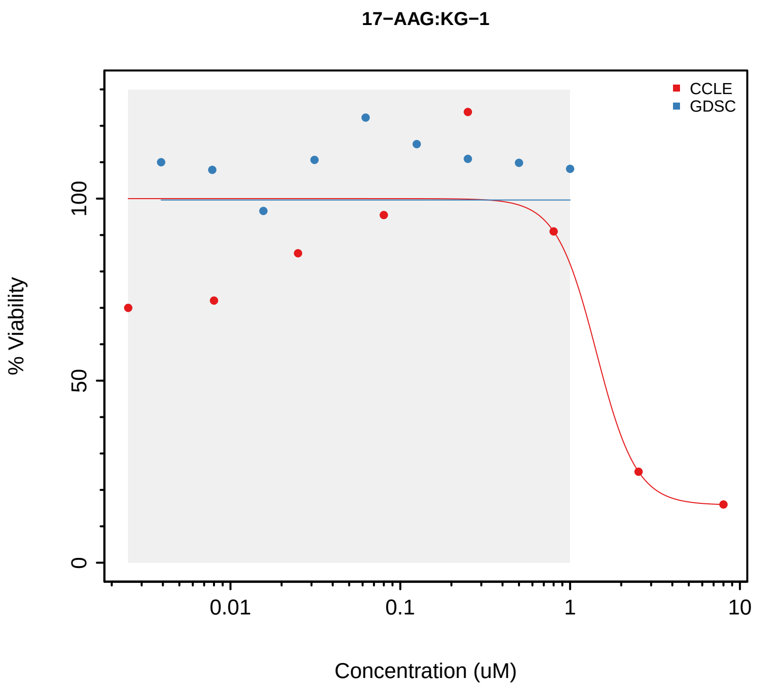
17−AAG:KG−1
CCLE
GDSC
% Viability
0
50
100
0.01
0.1
1
10
Concentration (uM)

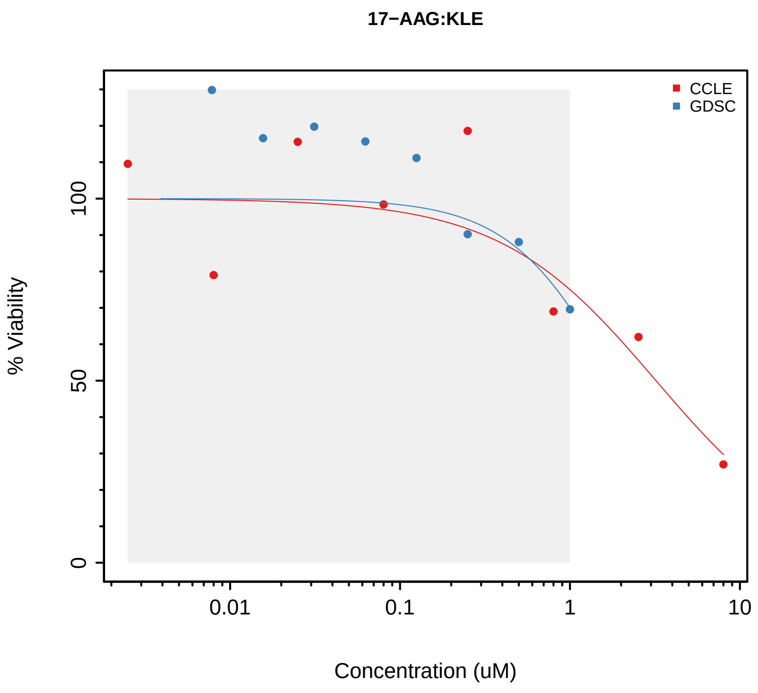
17−AAG:KLE
CCLE
GDSC
% Viability
0
50
100
0.01
0.1
1
10
Concentration (uM)

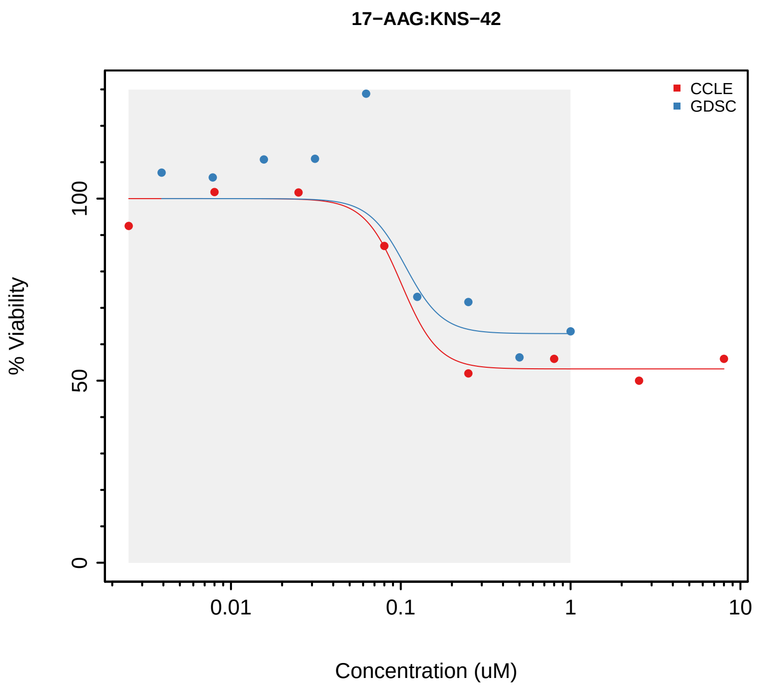
17−AAG:KNS−42
CCLE
GDSC
% Viability
0
50
100
0.01
0.1
1
10
Concentration (uM)

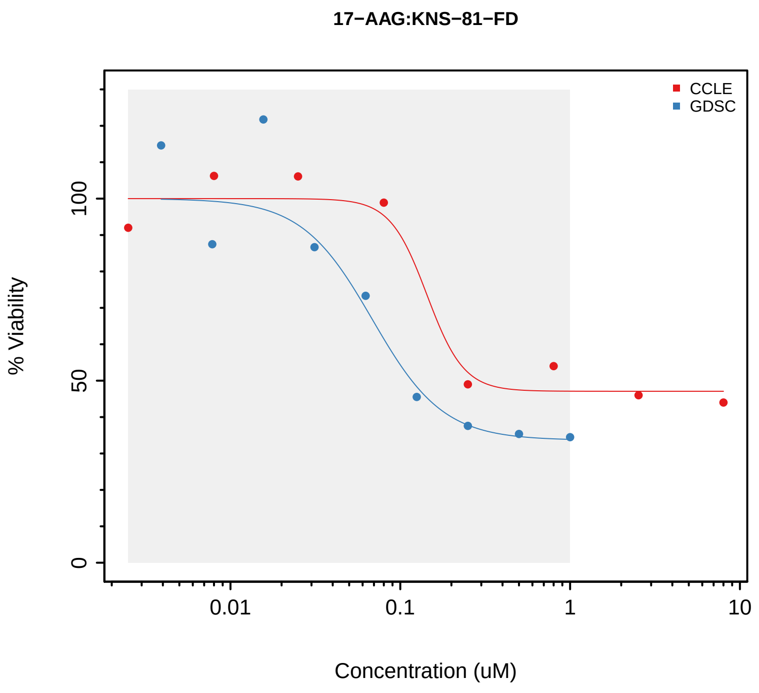
17−AAG:KNS−81−FD
CCLE
GDSC
% Viability
0
50
100
0.01
0.1
1
10
Concentration (uM)

17−AAG:KP−4

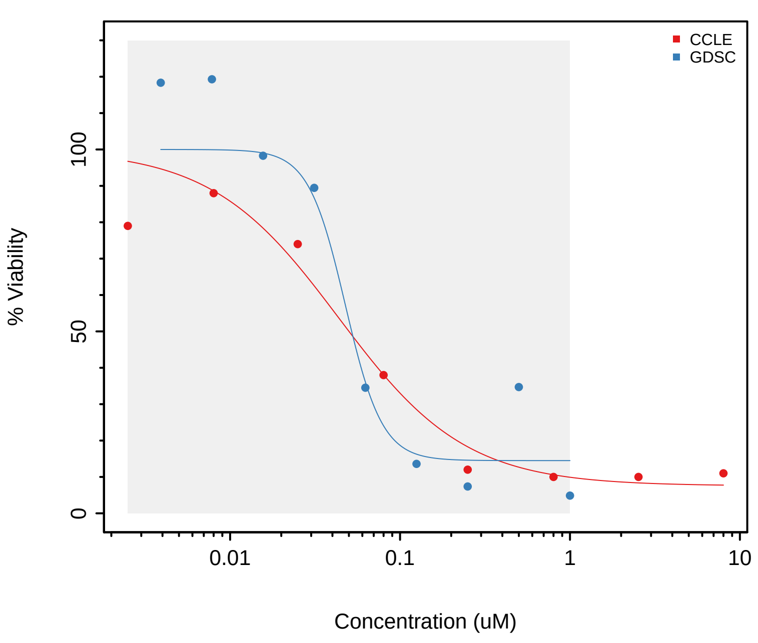

17−AAG:KU812

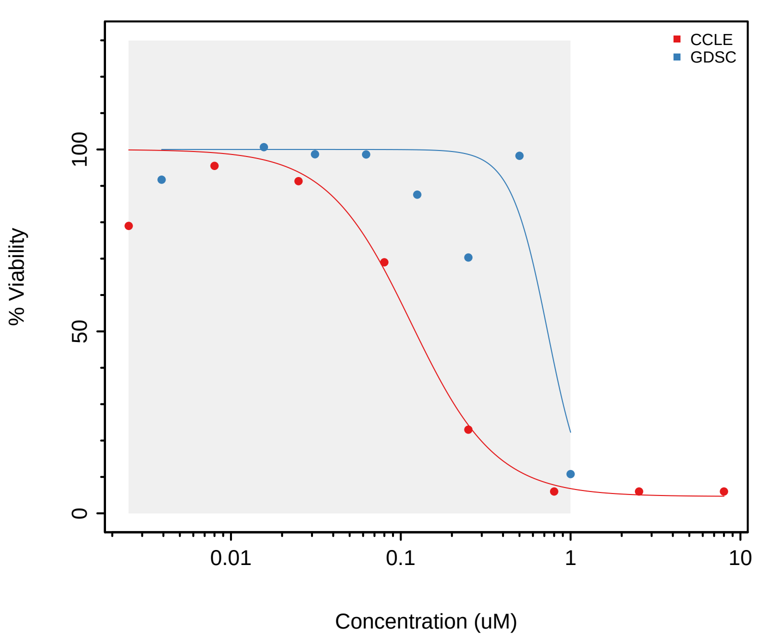

17−AAG:KURAMOCHI

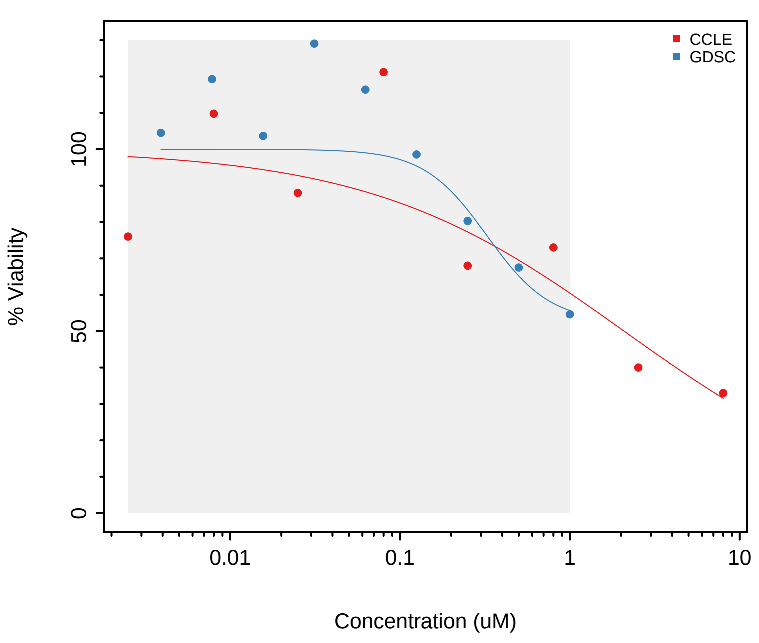

17−AAG:KYSE−140

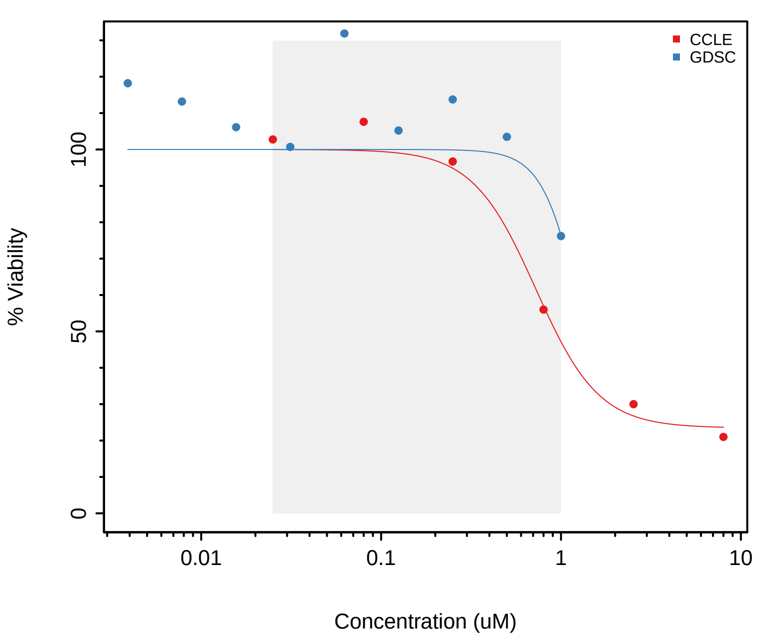

17−AAG:KYSE−150

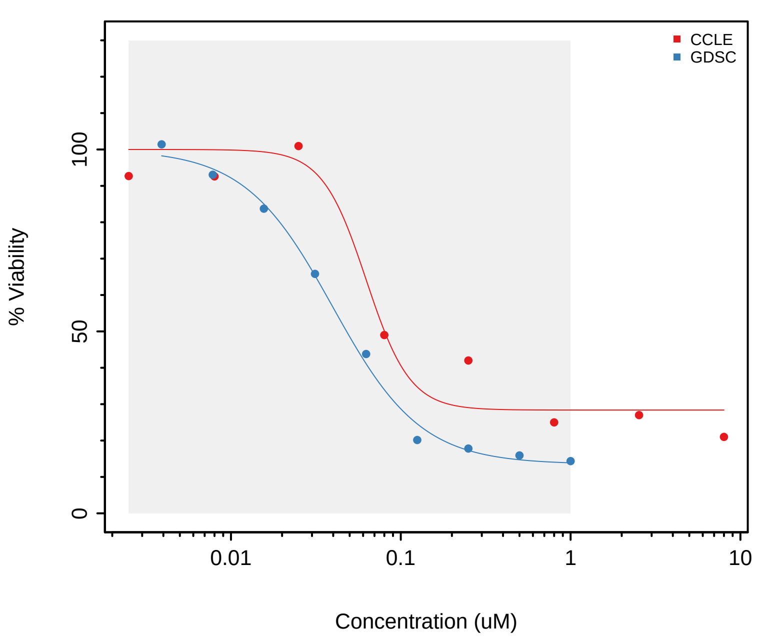

17−AAG:KYSE−180

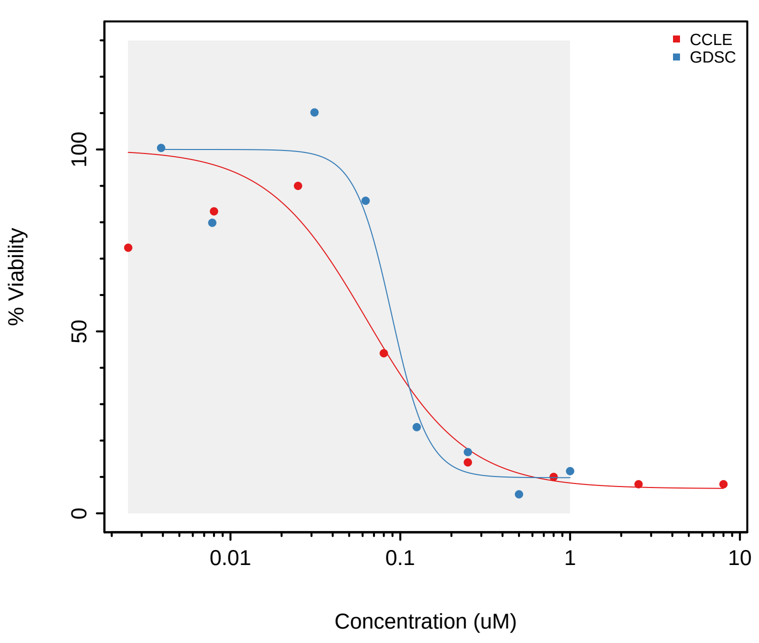

17−AAG:KYSE−410

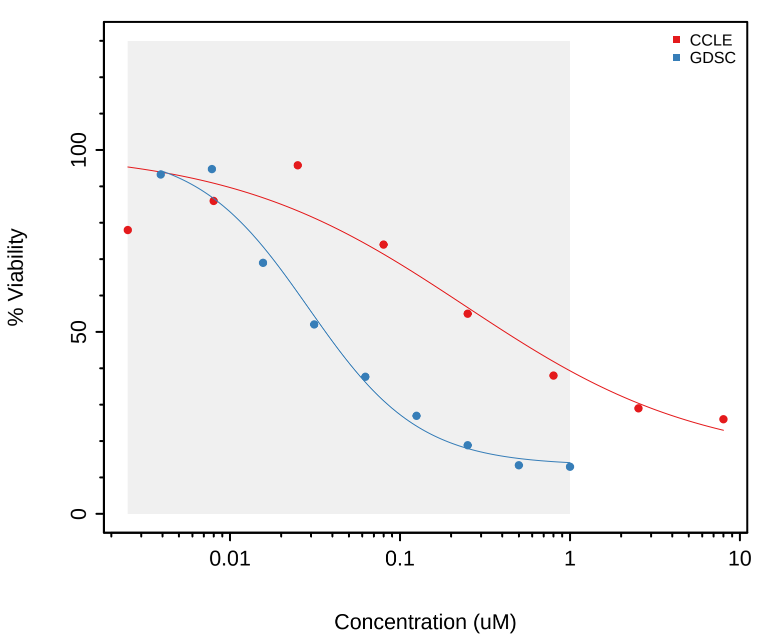

17−AAG:KYSE−450

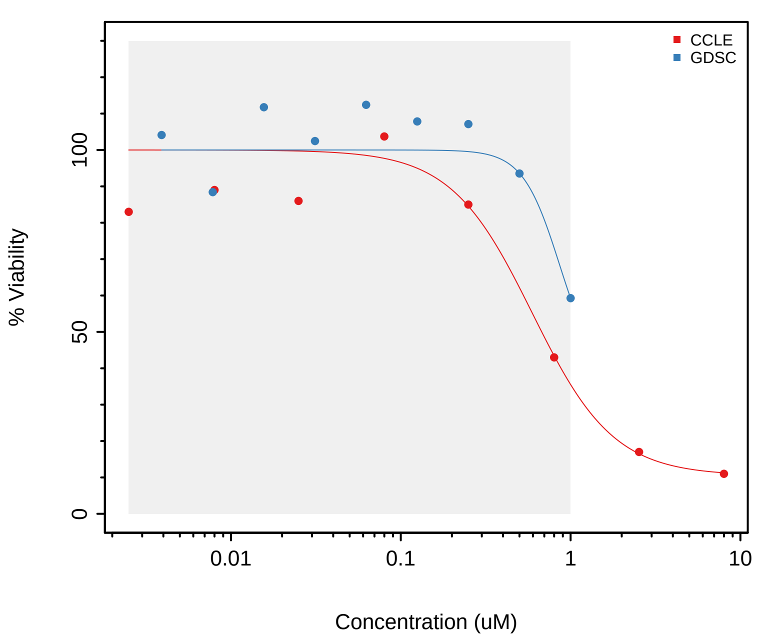

17−AAG:KYSE−510

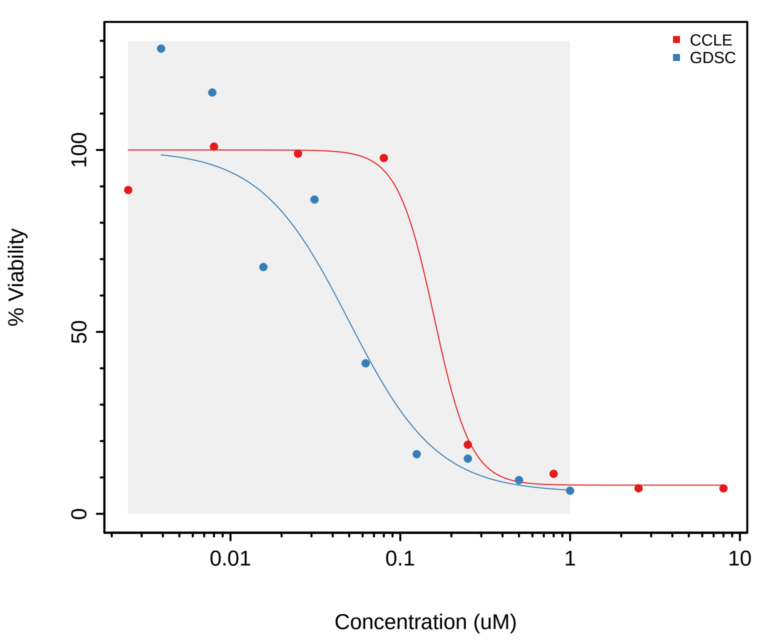

17−AAG:KYSE−520

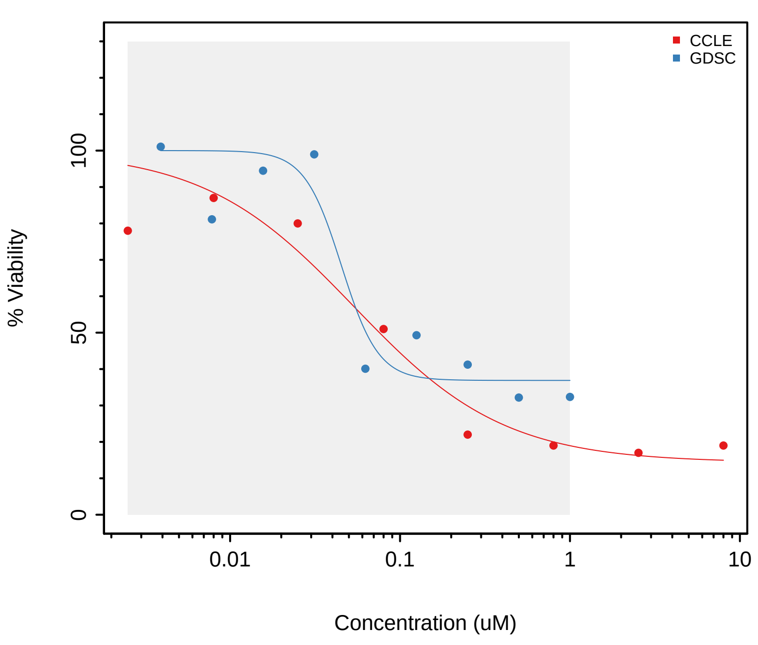

17−AAG:KYSE−70

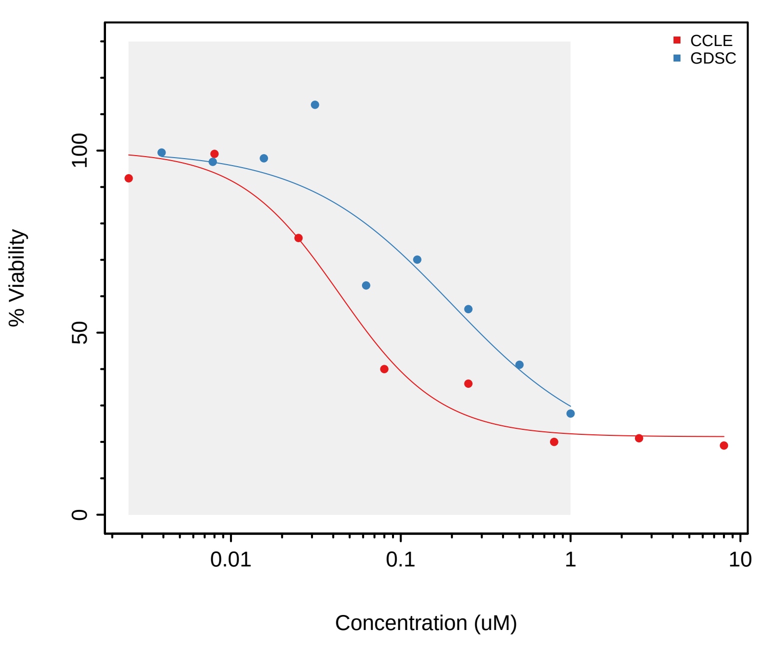

17−AAG:L−363

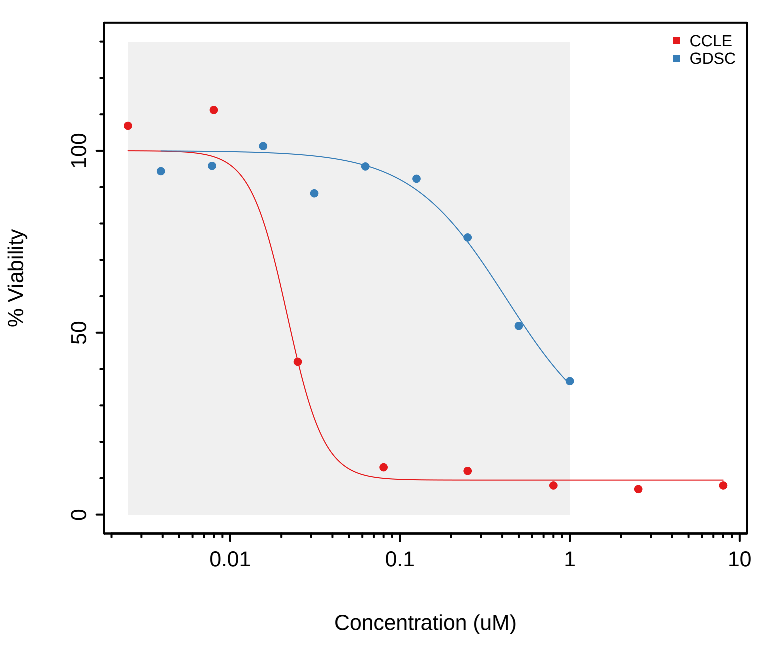

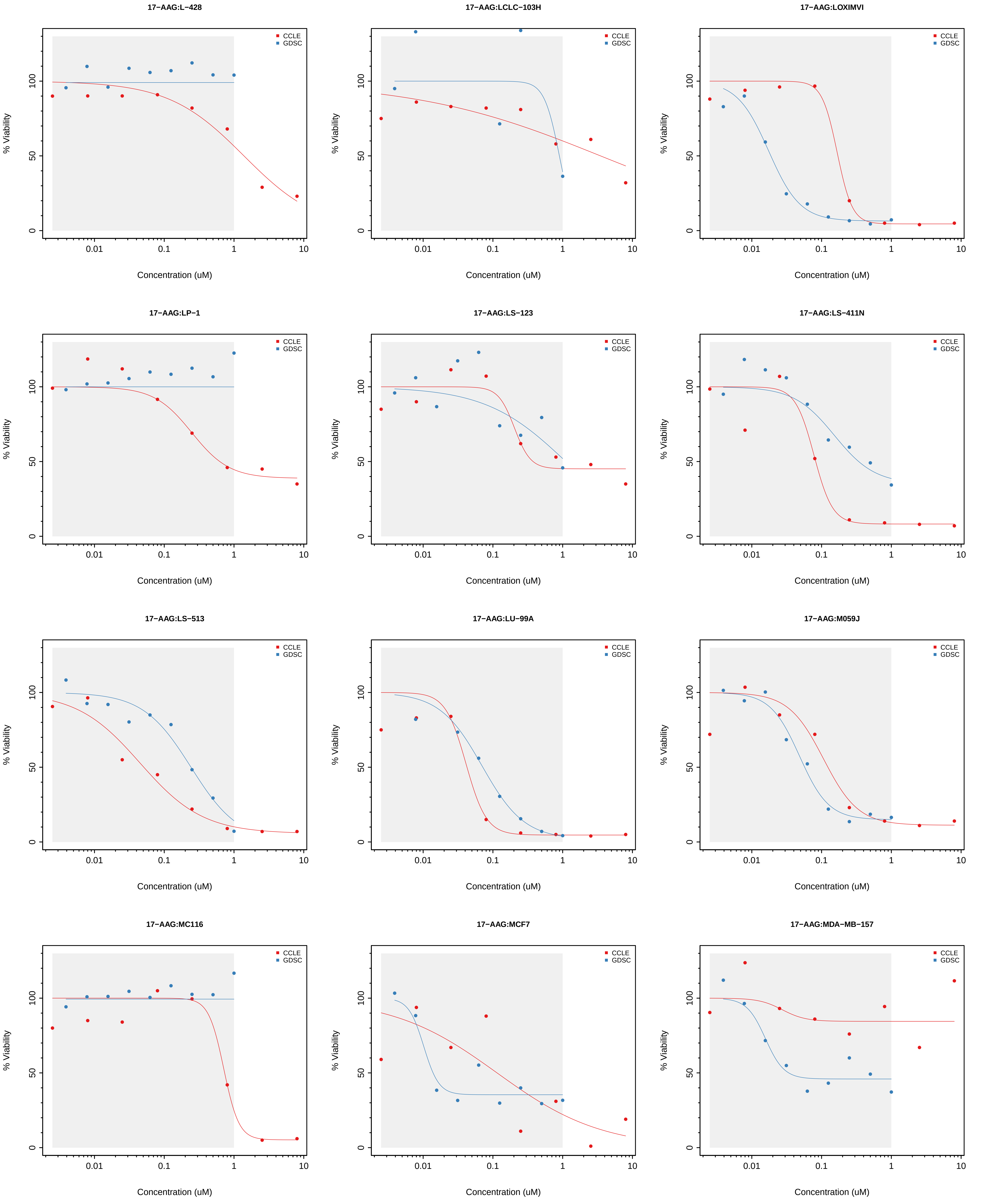
17−AAG:L−428
17−AAG:LCLC−103H
17−AAG:LOXIMVI
17−AAG:LP−1
17−AAG:LS−123
17−AAG:LS−411N
17−AAG:LS−513
17−AAG:LU−99A
17−AAG:M059J
17−AAG:MC116
17−AAG:MCF7
17−AAG:MDA−MB−157
CCLE
GDSC
% Viability
Concentration (uM)
0
50
100
0.01
0.1
1
10

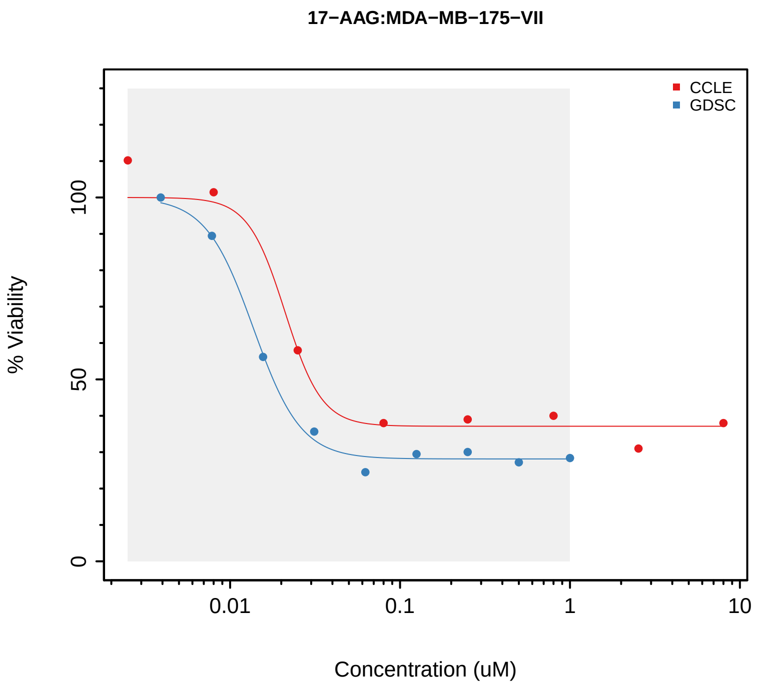
17−AAG:MDA−MB−175−VII
CCLE
GDSC
% Viability
0
50
100
0.01
0.1
1
10
Concentration (uM)

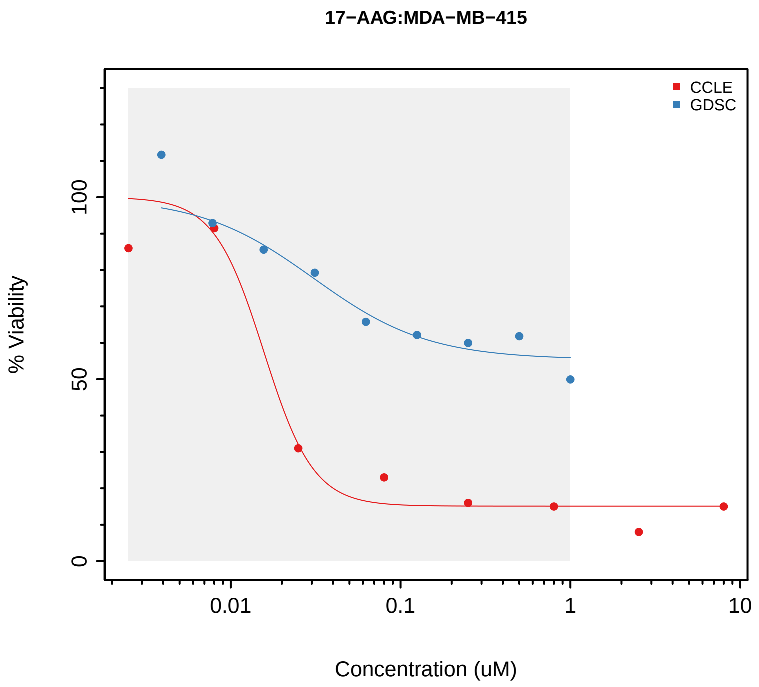
17−AAG:MDA−MB−415
CCLE
GDSC
% Viability
0
50
100
0.01
0.1
1
10
Concentration (uM)

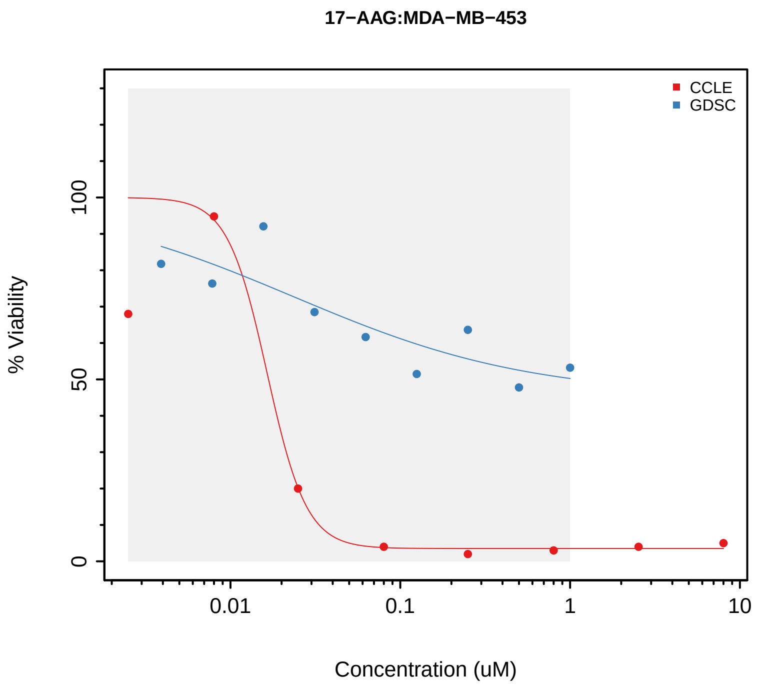
17−AAG:MDA−MB−453
CCLE
GDSC
% Viability
0
50
100
0.01
0.1
1
10
Concentration (uM)

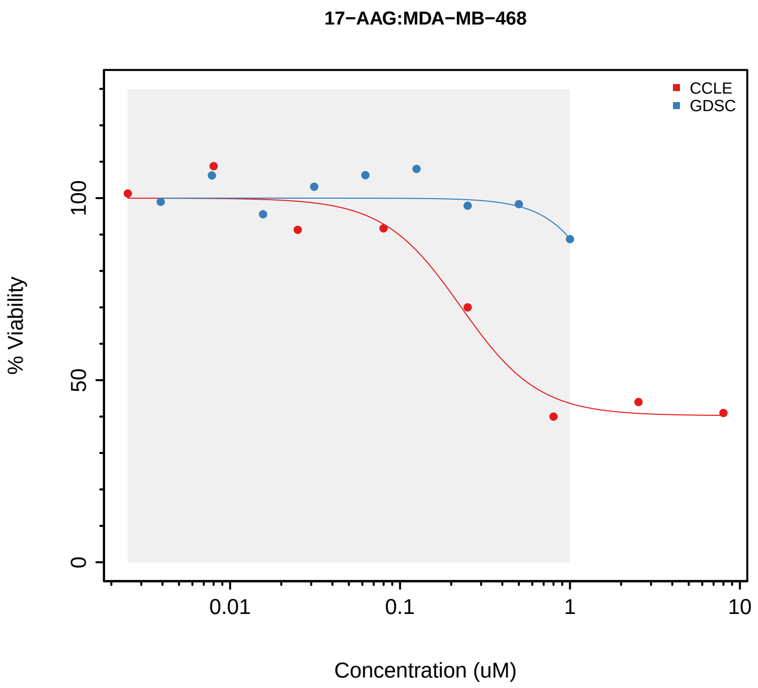
17−AAG:MDA−MB−468
CCLE
GDSC
% Viability
0
50
100
0.01
0.1
1
10
Concentration (uM)

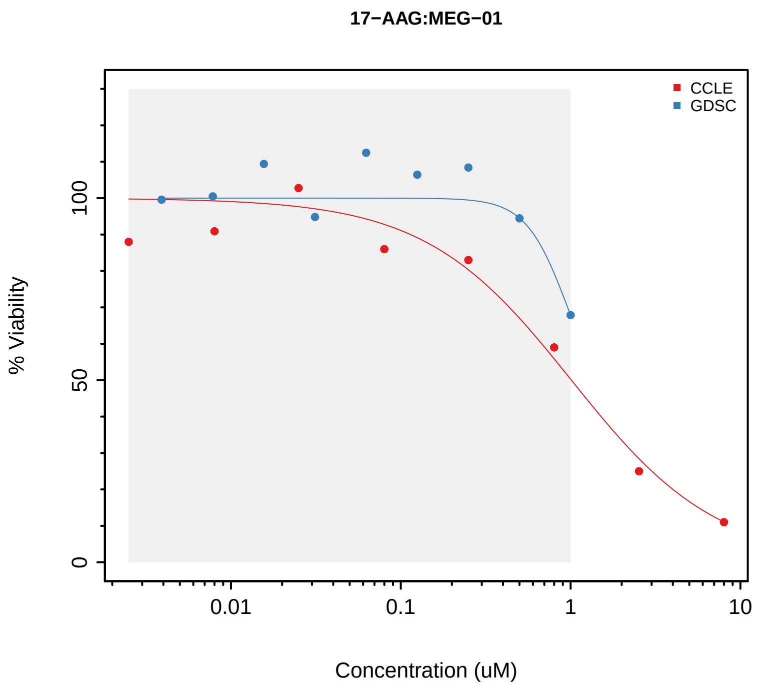
17−AAG:MEG−01
CCLE
GDSC
% Viability
0
50
100
0.01
0.1
1
10
Concentration (uM)

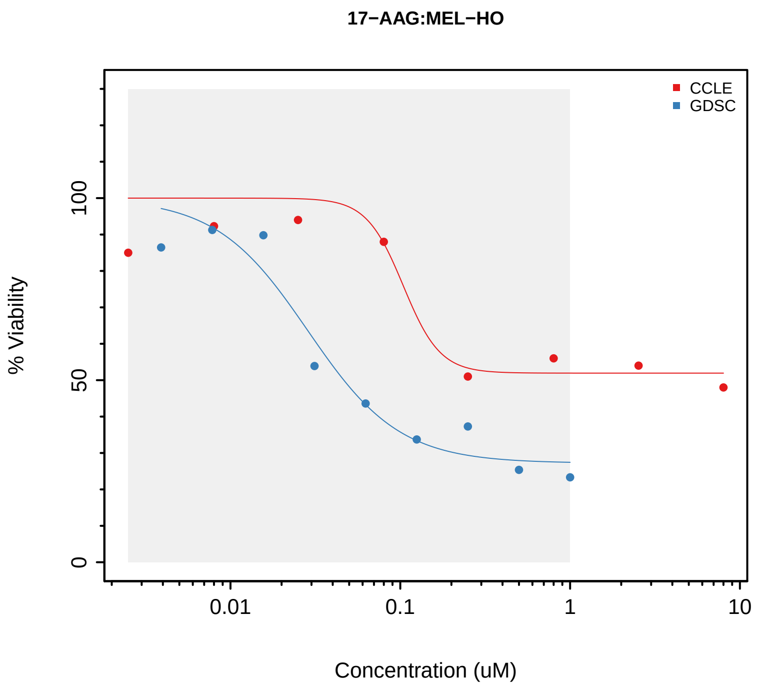
17−AAG:MEL−HO
CCLE
GDSC
% Viability
0
50
100
0.01
0.1
1
10
Concentration (uM)

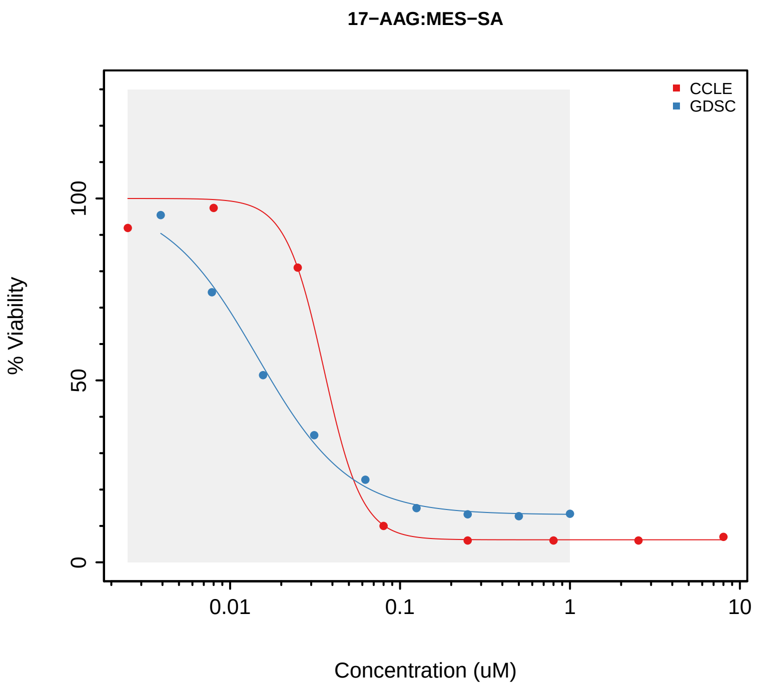
17−AAG:MES−SA
CCLE
GDSC
% Viability
0
50
100
0.01
0.1
1
10
Concentration (uM)

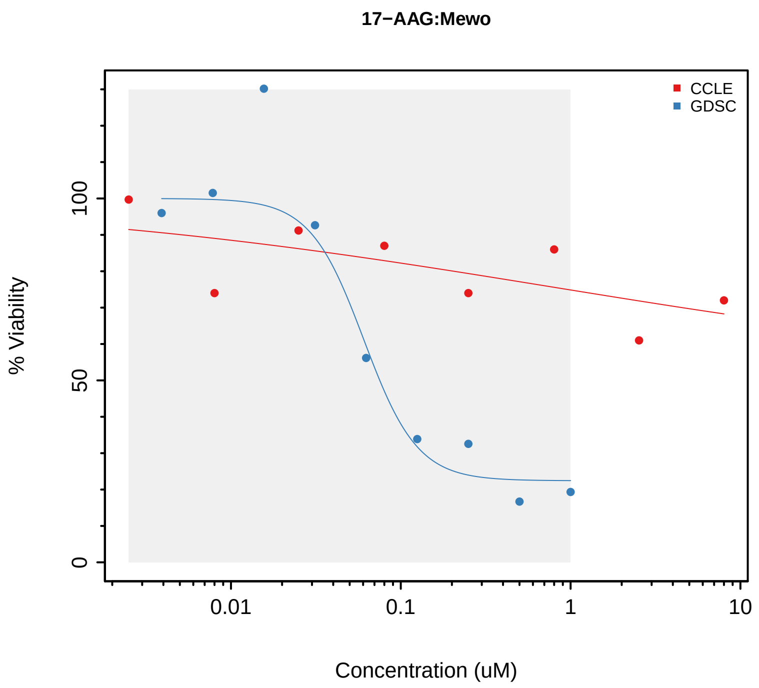
17−AAG:Mewo
CCLE
GDSC
% Viability
0
50
100
0.01
0.1
1
10
Concentration (uM)

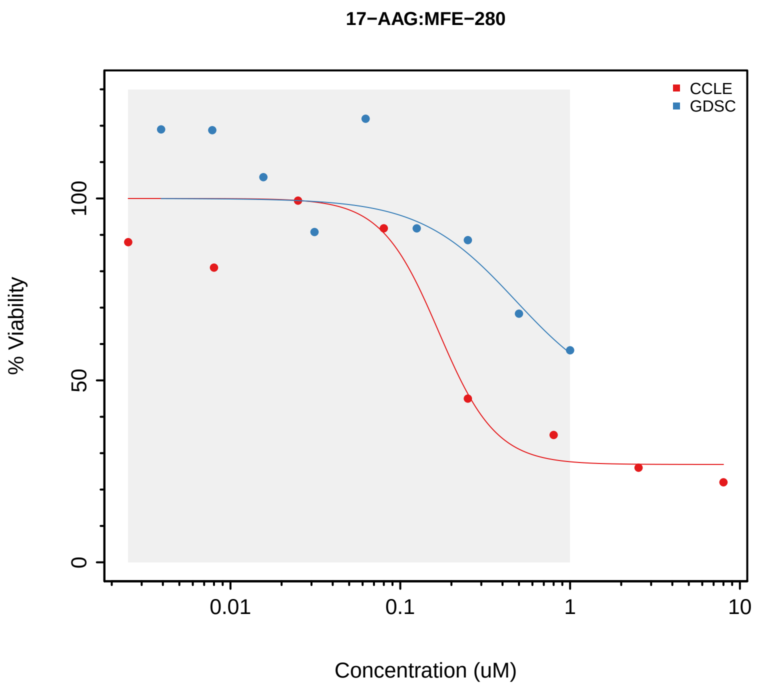
17−AAG:MFE−280
CCLE
GDSC
% Viability
0
50
100
0.01
0.1
1
10
Concentration (uM)

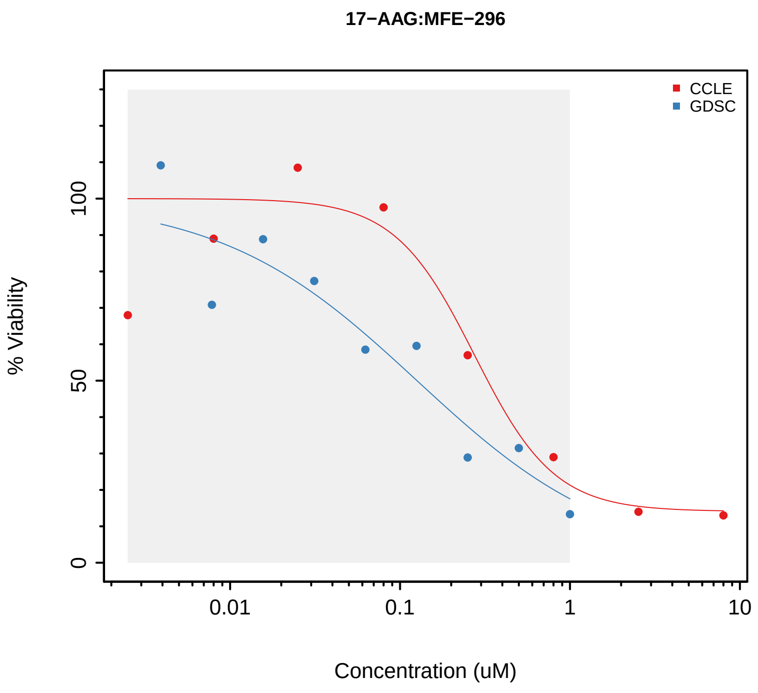
17−AAG:MFE−296
CCLE
GDSC
% Viability
0
50
100
0.01
0.1
1
10
Concentration (uM)

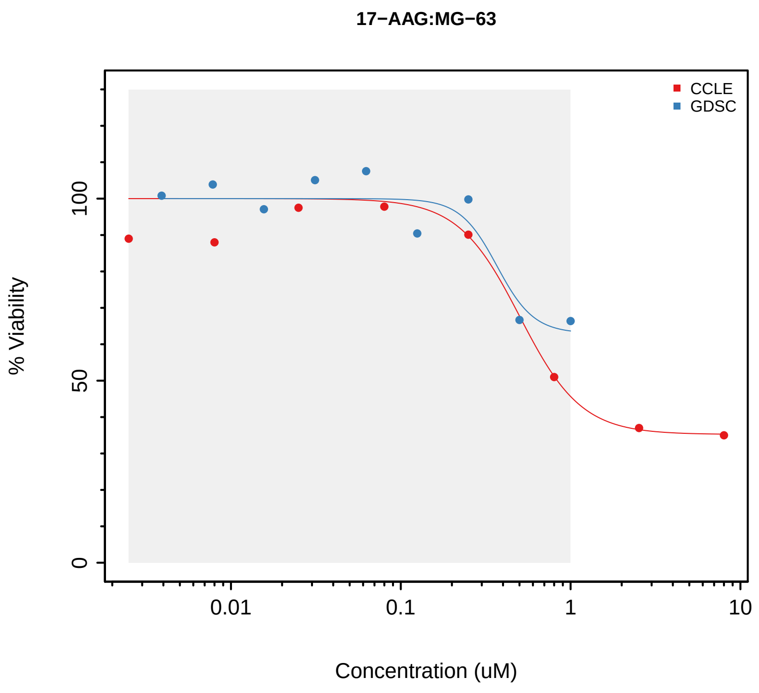
17−AAG:MG−63
CCLE
GDSC
% Viability
0
50
100
0.01
0.1
1
10
Concentration (uM)

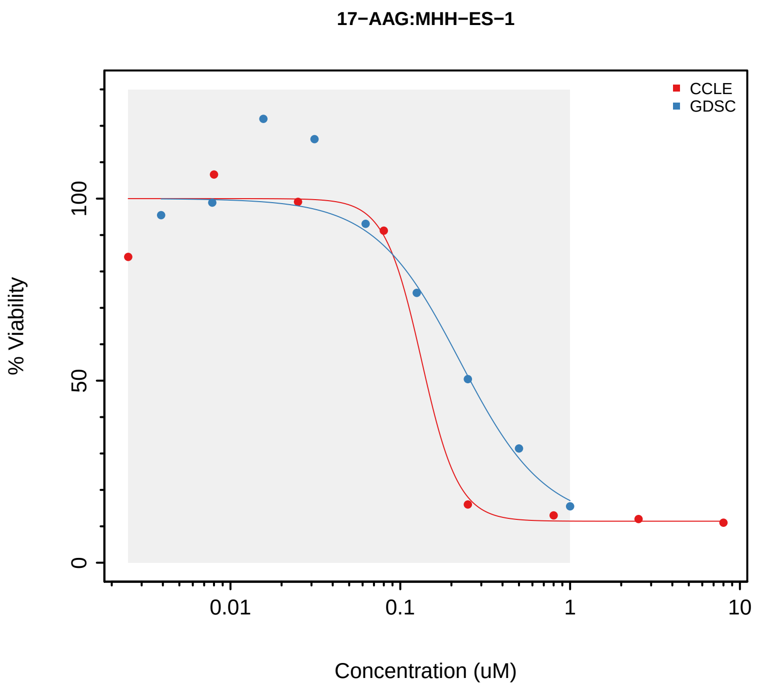
17−AAG:MHH−ES−1
CCLE
GDSC
% Viability
0
50
100
0.01
0.1
1
10
Concentration (uM)

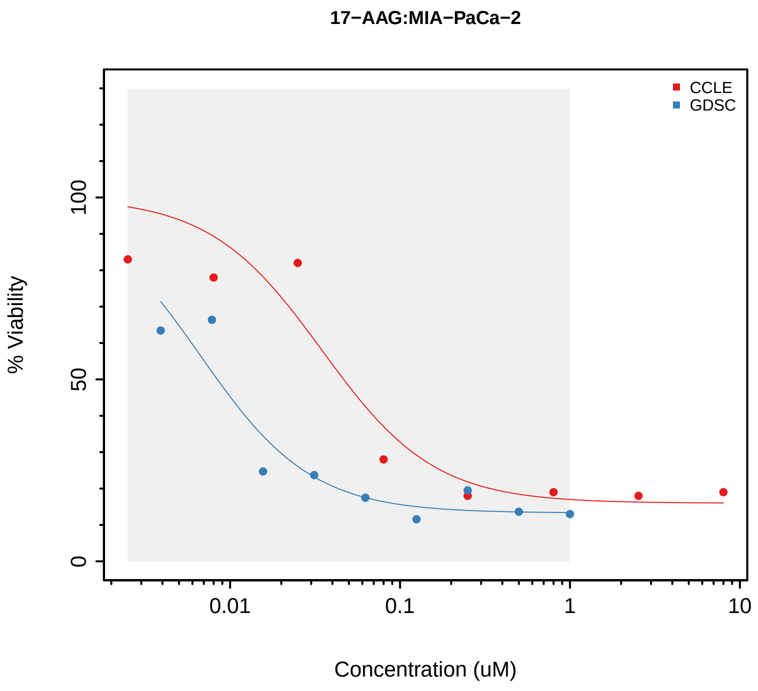
17−AAG:MIA−PaCa−2
CCLE
GDSC
% Viability
Concentration (uM)

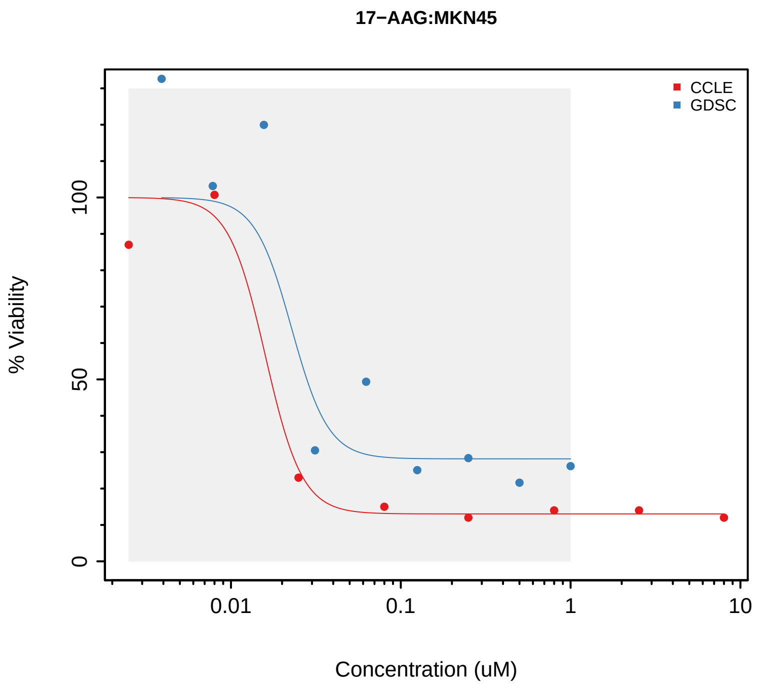
17−AAG:MKN45
CCLE
GDSC
% Viability
Concentration (uM)

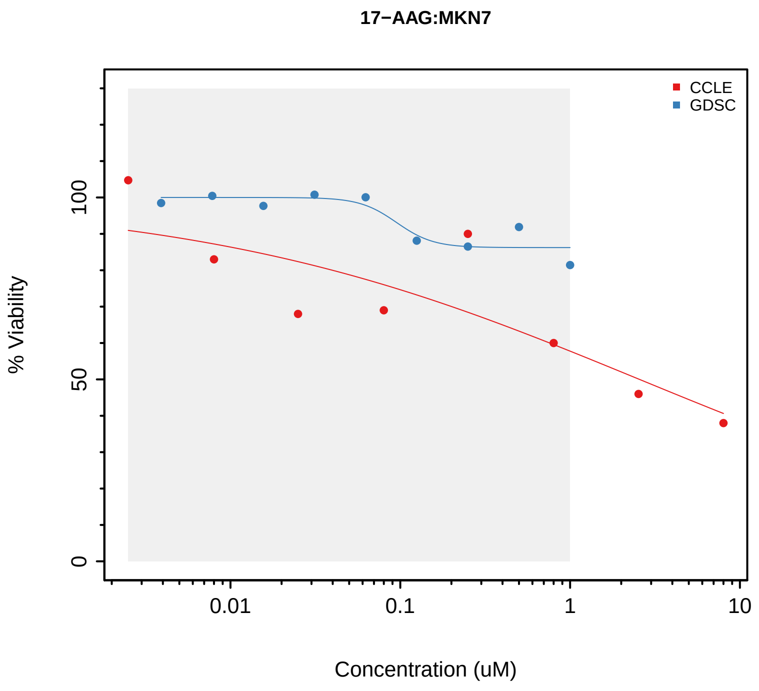
17−AAG:MKN7
CCLE
GDSC
% Viability
Concentration (uM)

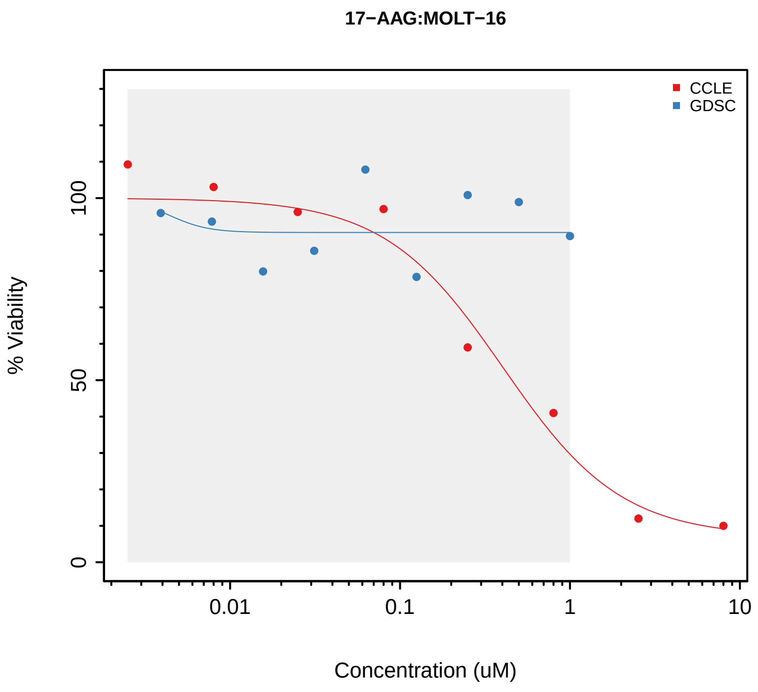
17−AAG:MOLT−16
CCLE
GDSC
% Viability
Concentration (uM)

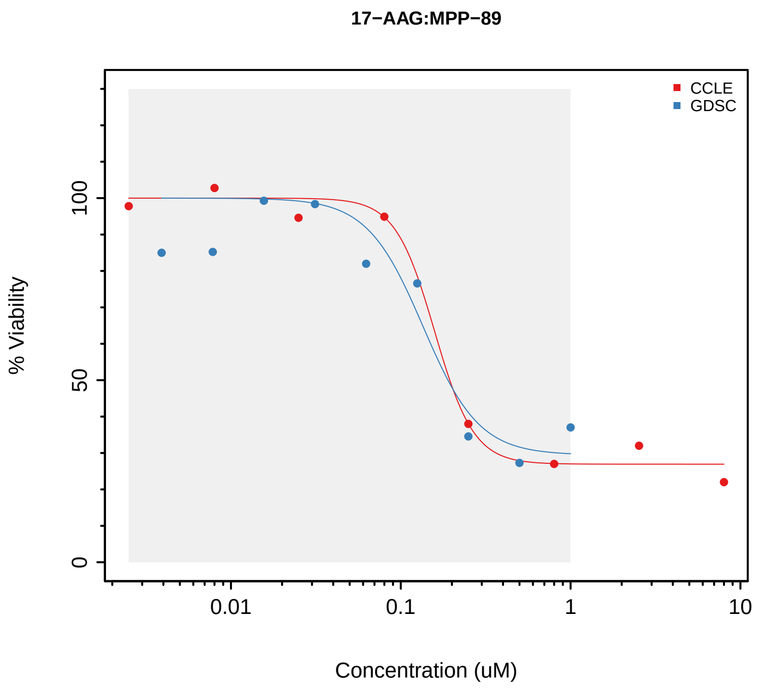
17−AAG:MPP−89
CCLE
GDSC
% Viability
Concentration (uM)

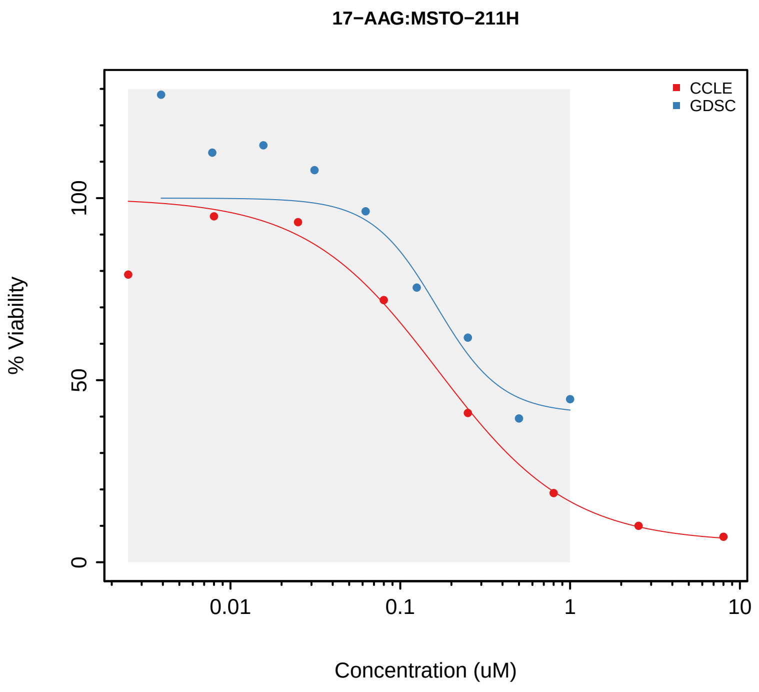
17−AAG:MSTO−211H
CCLE
GDSC
% Viability
Concentration (uM)

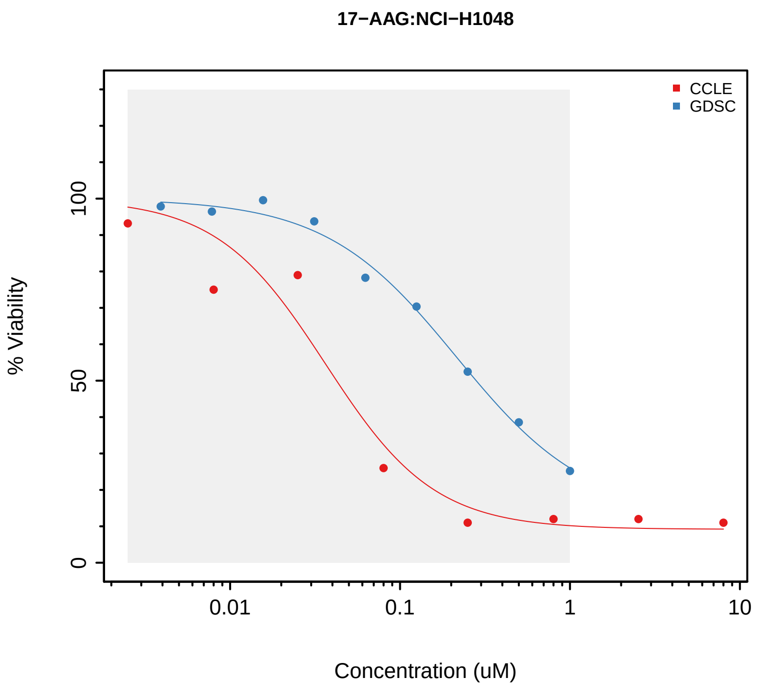
17−AAG:NCI−H1048
CCLE
GDSC
% Viability
Concentration (uM)

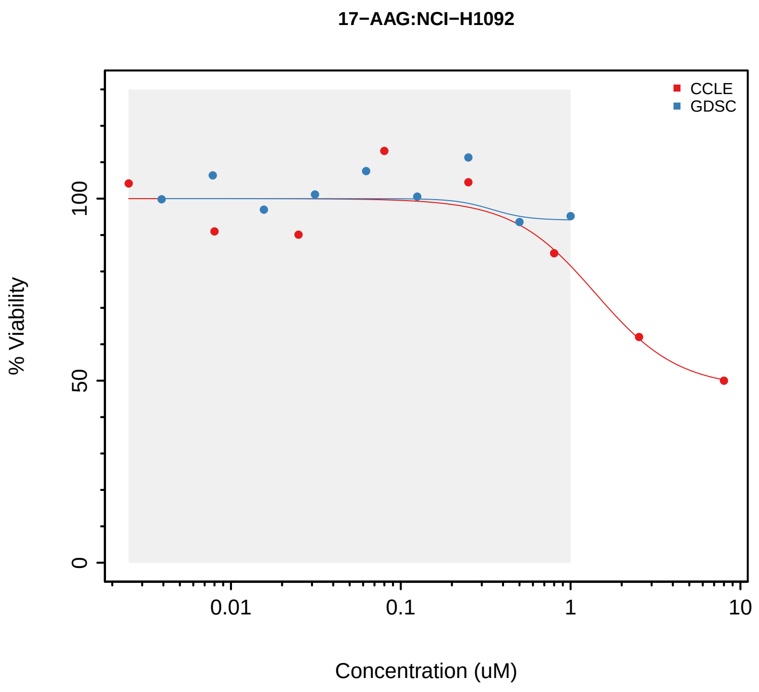
17−AAG:NCI−H1092
CCLE
GDSC
% Viability
Concentration (uM)

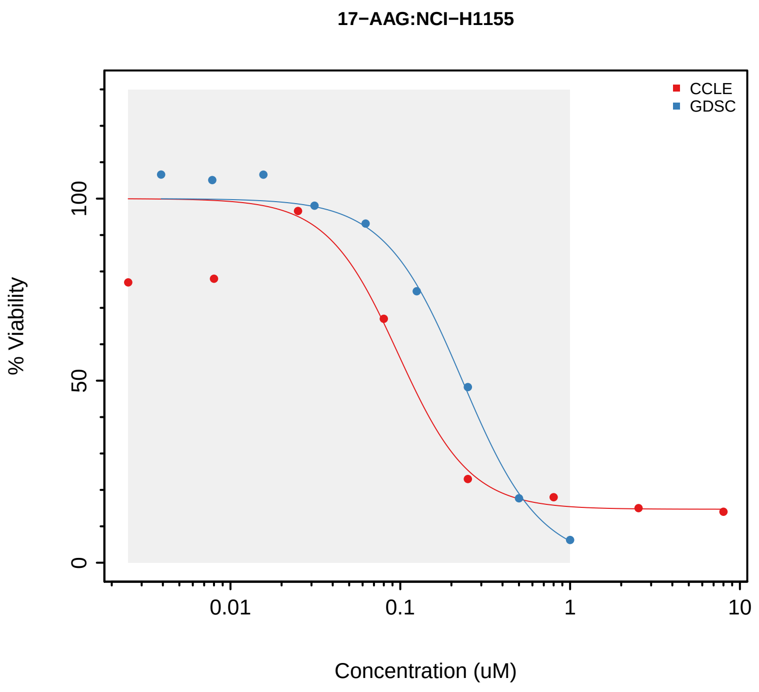
17−AAG:NCI−H1155
CCLE
GDSC
% Viability
Concentration (uM)

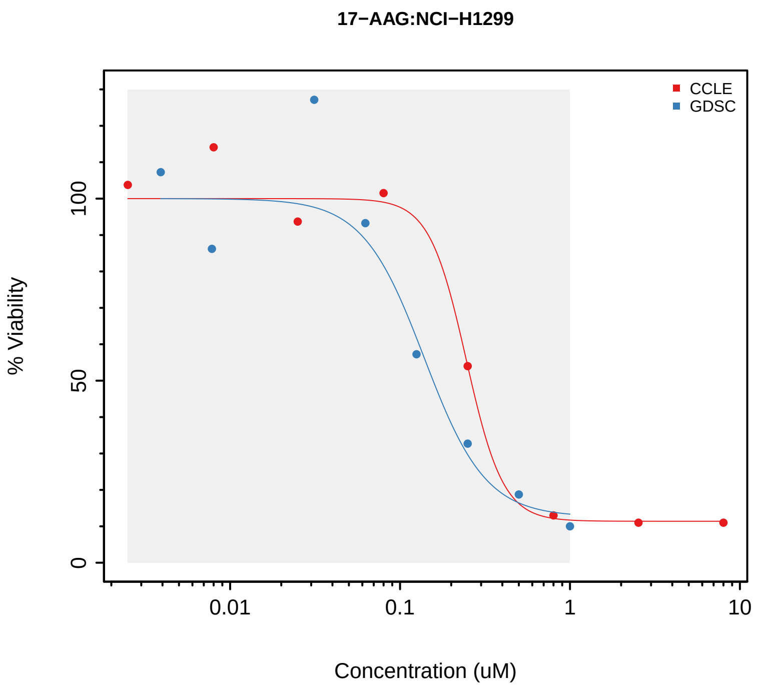
17−AAG:NCI−H1299
CCLE
GDSC
% Viability
Concentration (uM)

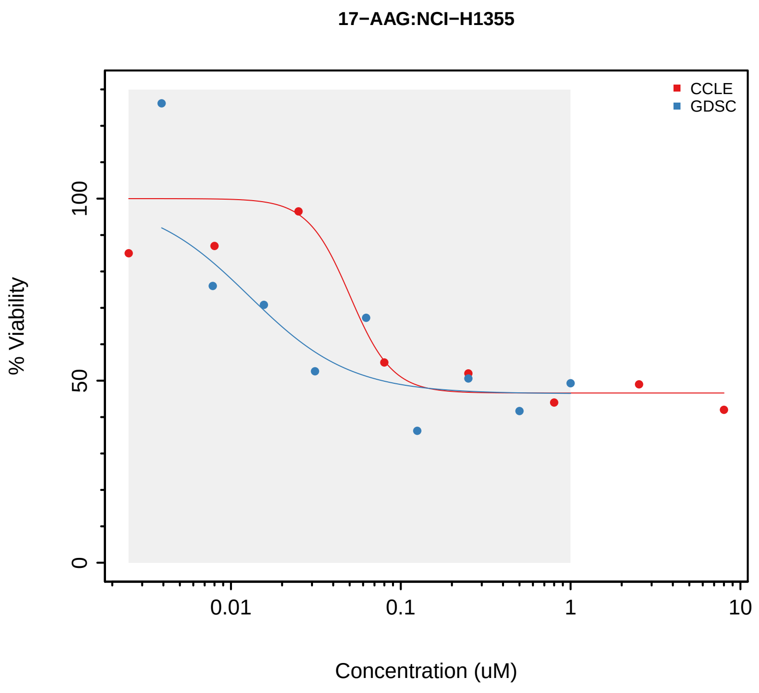
17−AAG:NCI−H1355
CCLE
GDSC
% Viability
Concentration (uM)

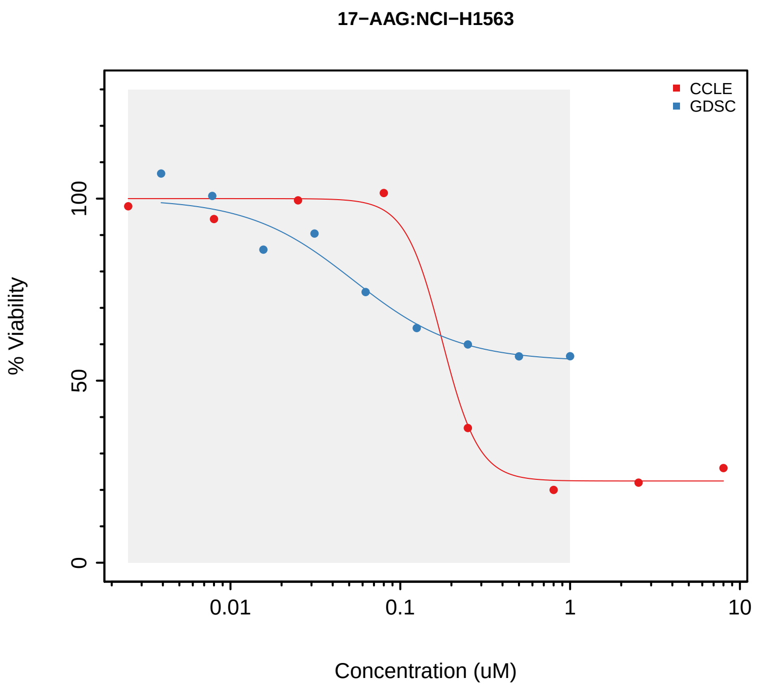
17−AAG:NCI−H1563
CCLE
GDSC
% Viability
Concentration (uM)

17−AAG:NCI−H1573

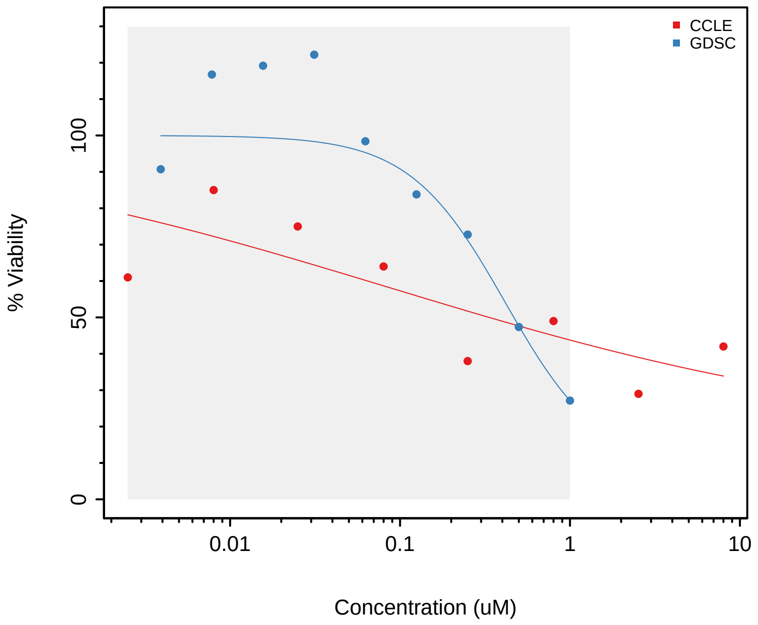

17−AAG:NCI−H1581

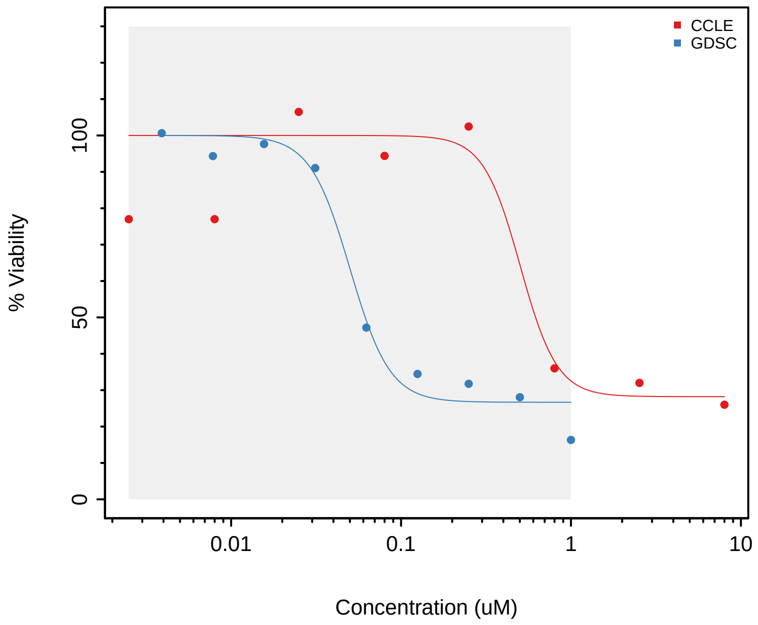

17−AAG:NCI−H1648

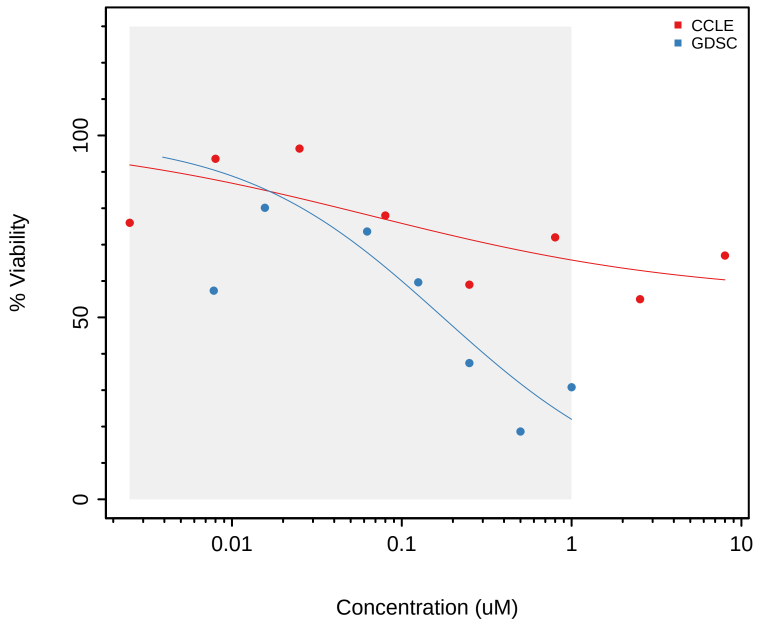

17−AAG:NCI−H1650

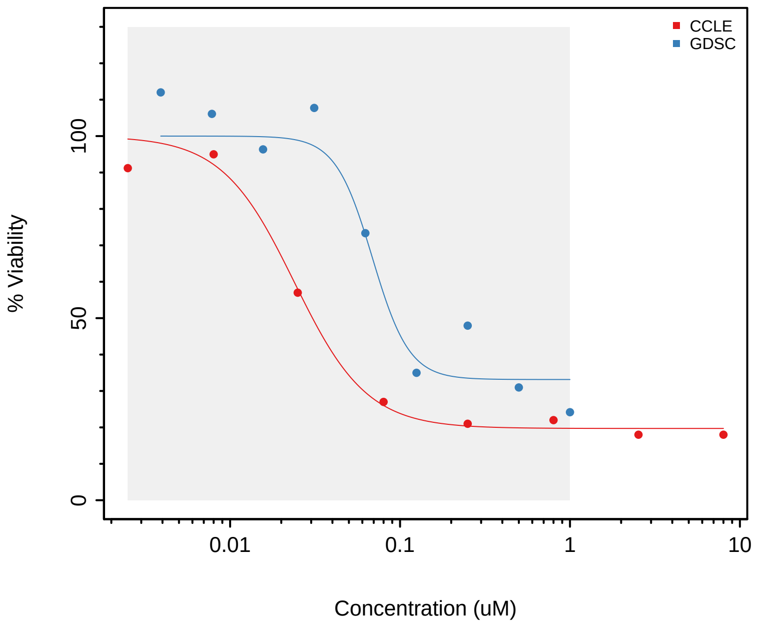

17−AAG:NCI−H1651

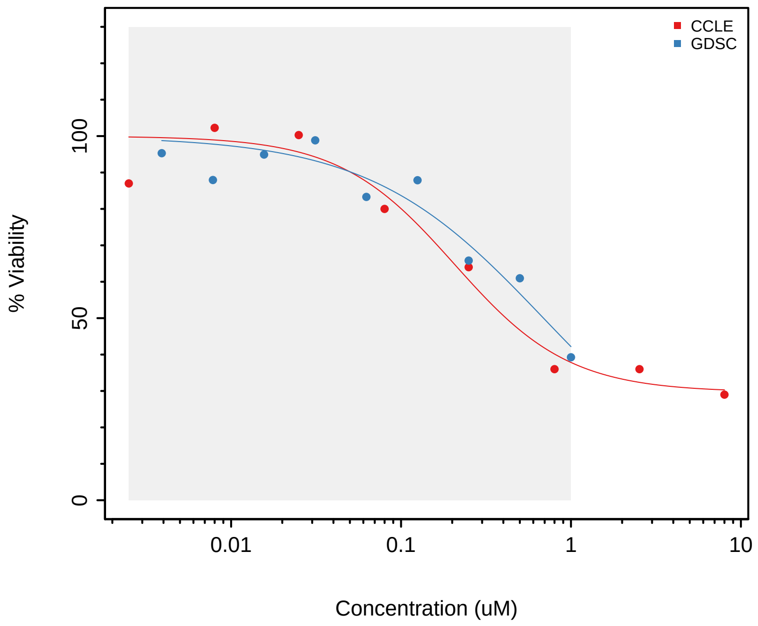

17−AAG:NCI−H1666

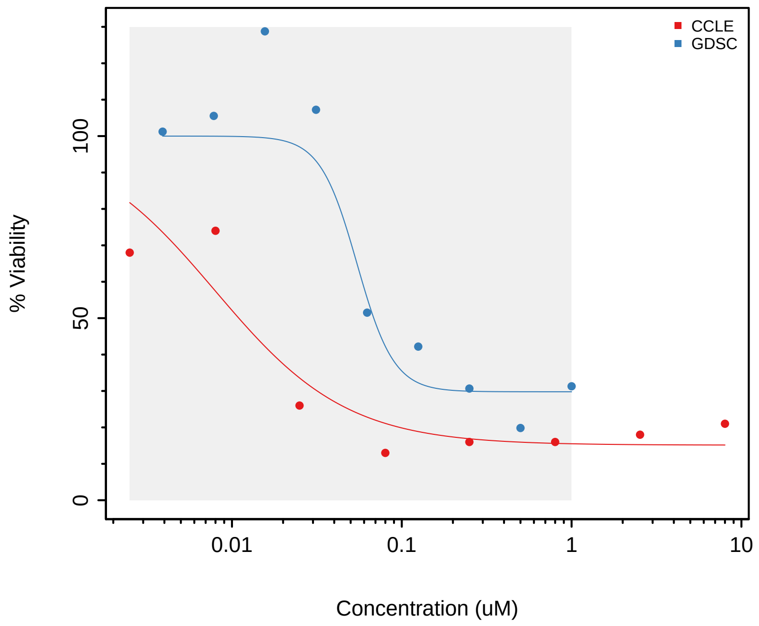

17−AAG:NCI−H1693

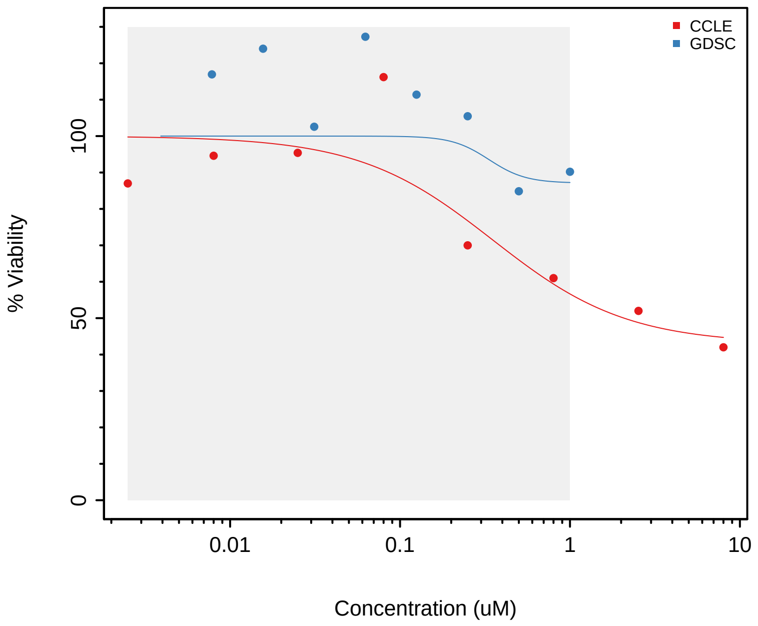

17−AAG:NCI−H1694

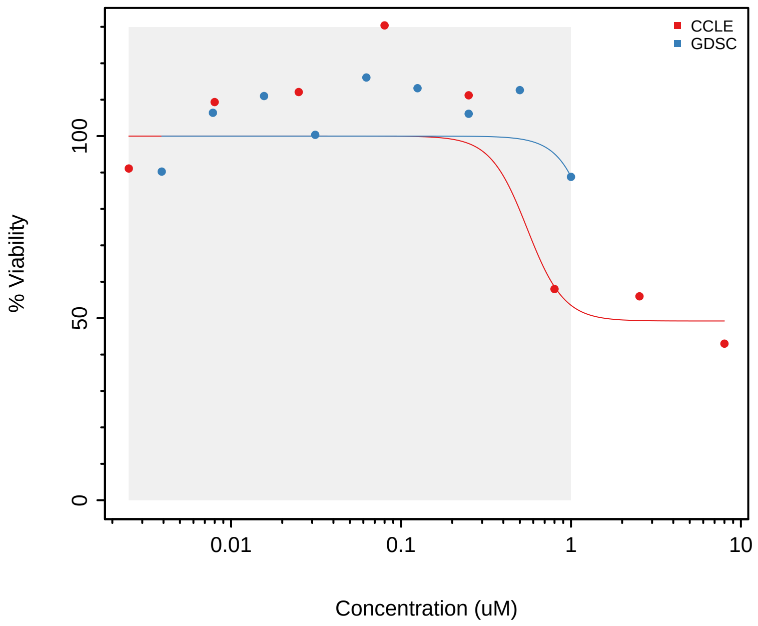

17−AAG:NCI−H1703

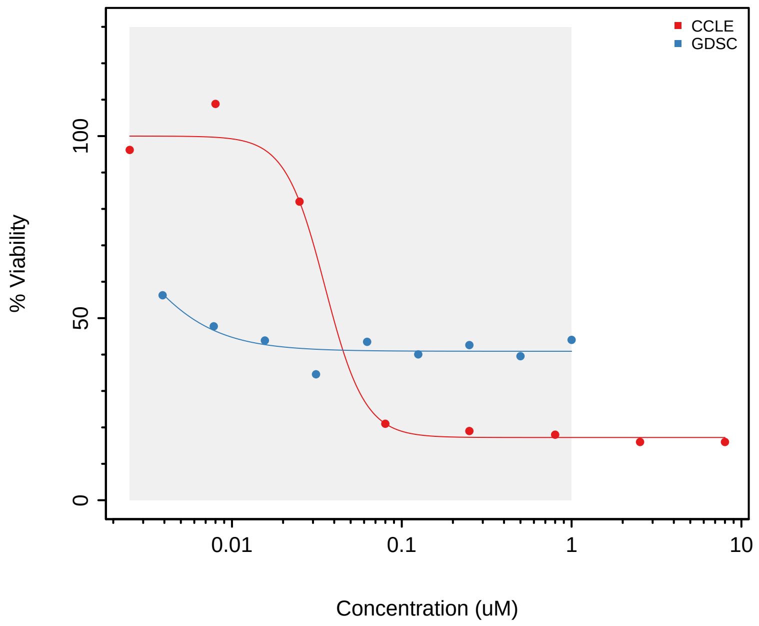

17−AAG:NCI−H1792

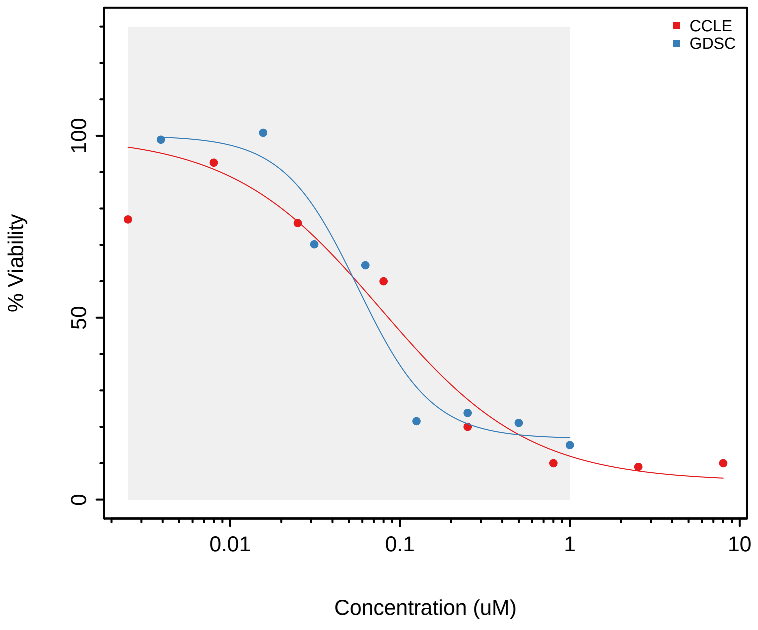

17−AAG:NCI−H1793

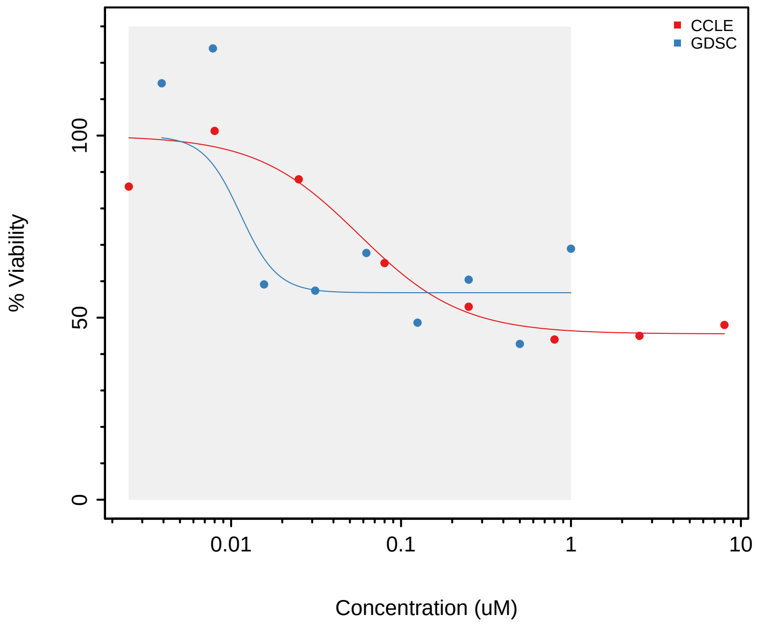

17−AAG:NCI−H1975

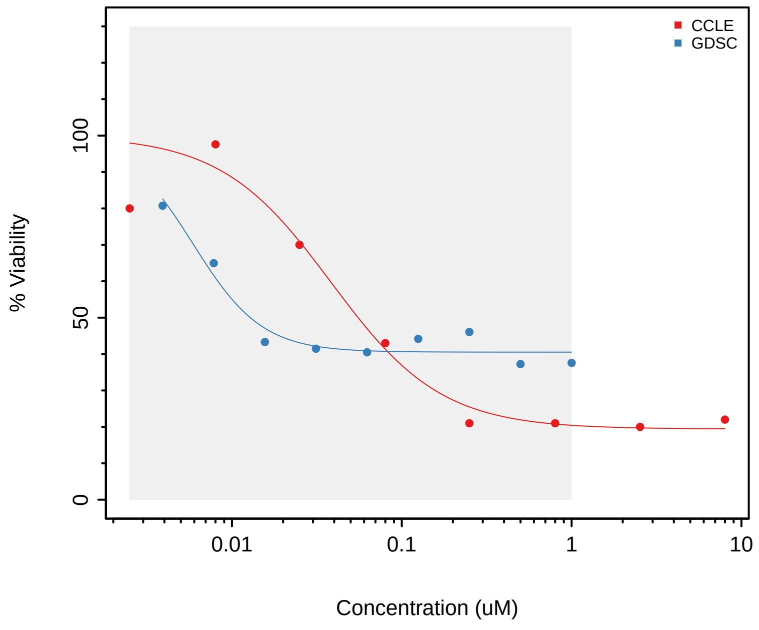

17−AAG:NCI−H2009

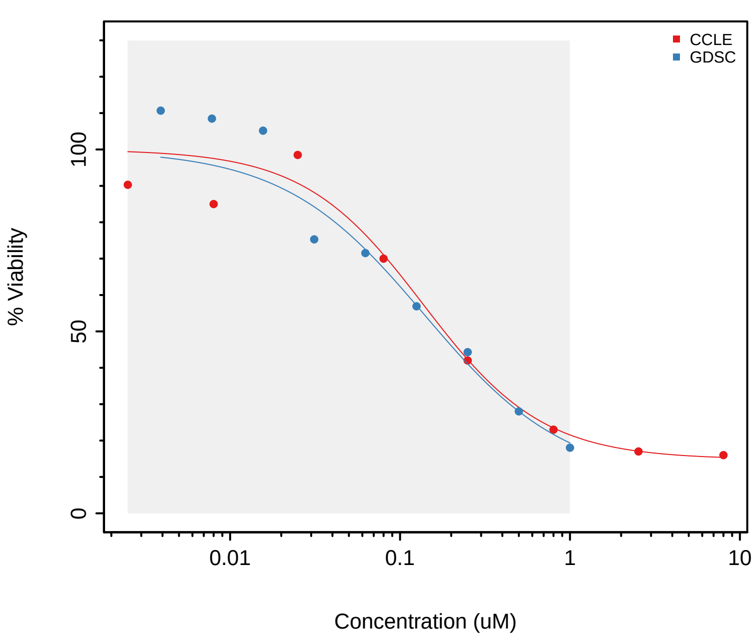

17−AAG:NCI−H2030

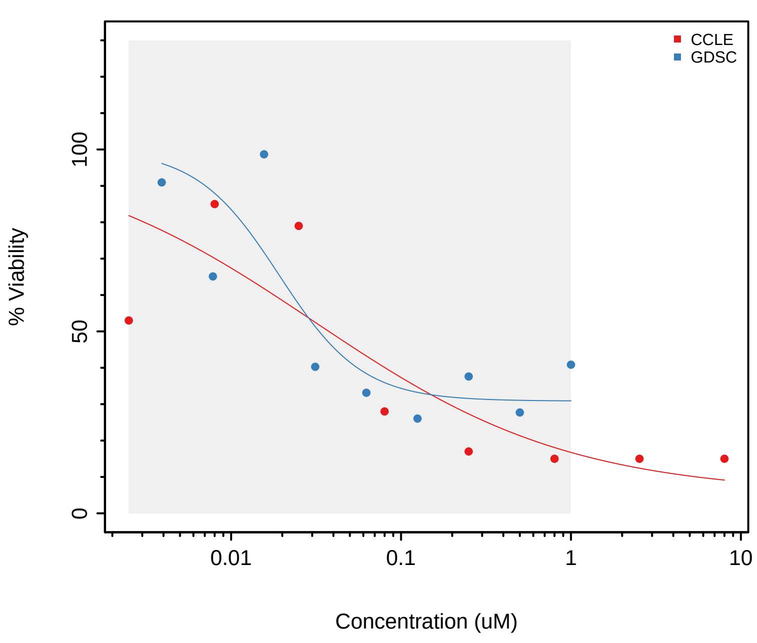

17−AAG:NCI−H2052

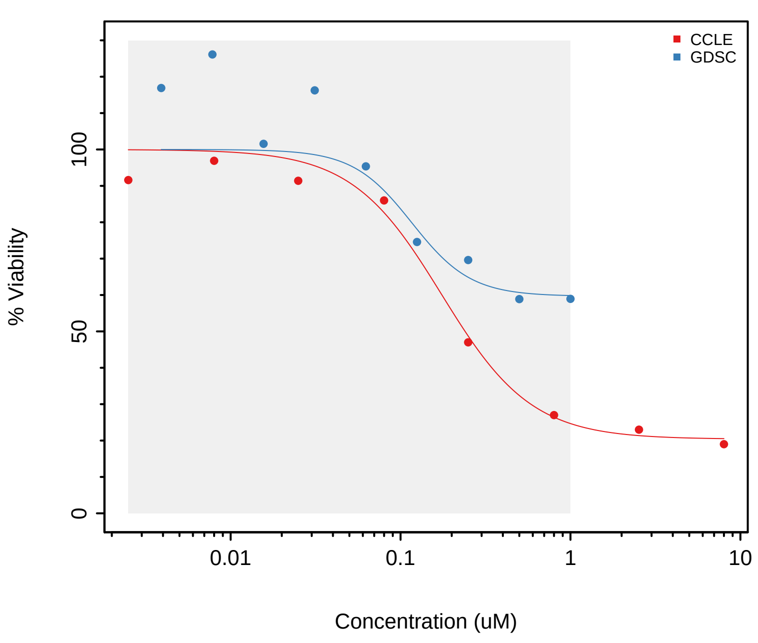

17−AAG:NCI−H2087

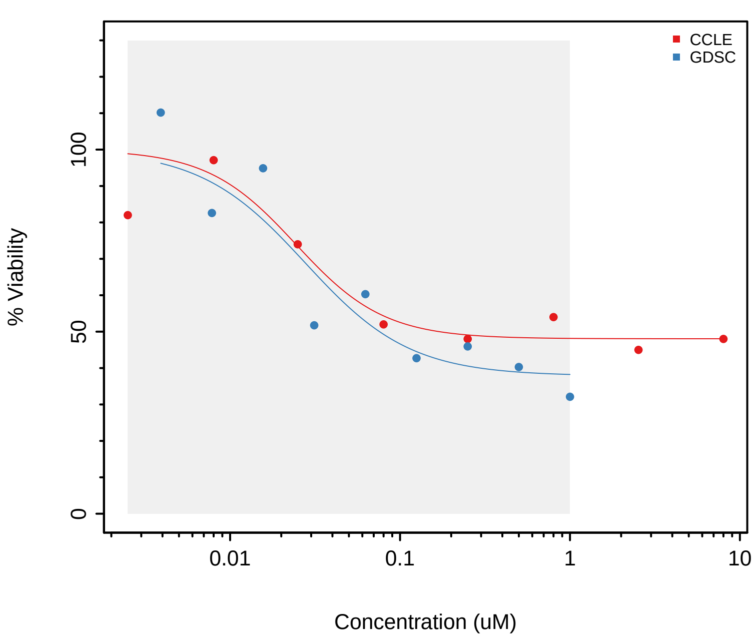

17−AAG:NCI−H2122

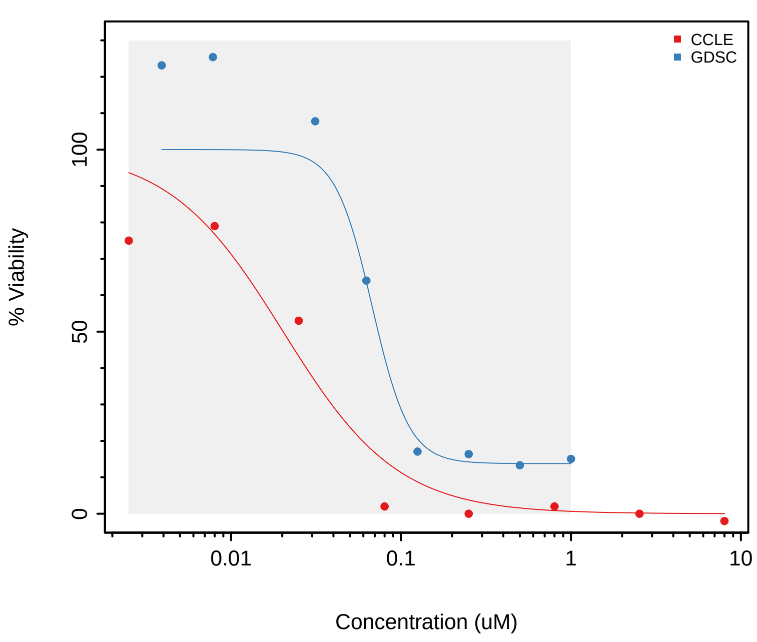

17−AAG:NCI−H2170

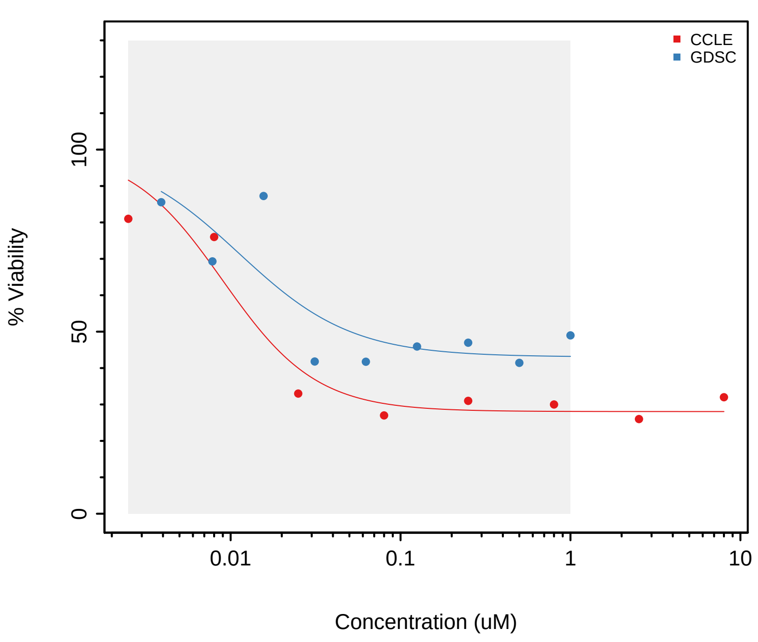

17−AAG:NCI−H2228

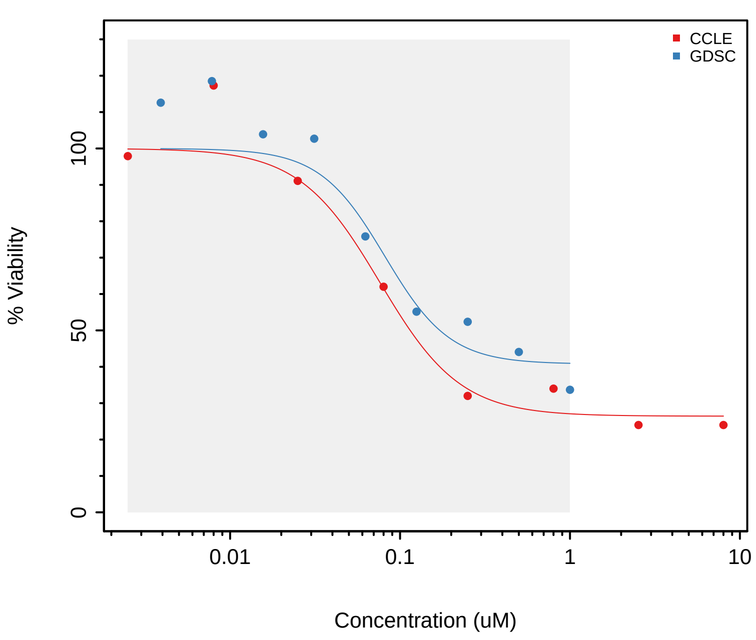

17−AAG:NCI−H226

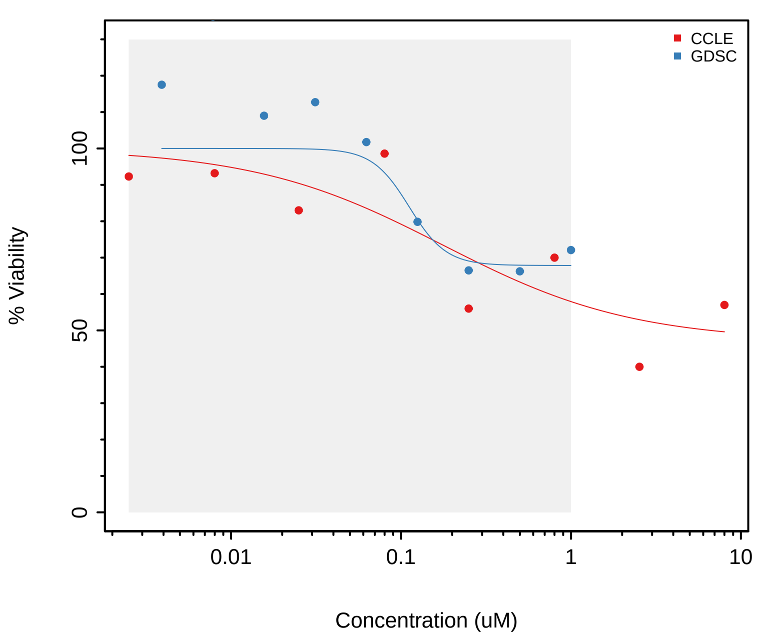

17−AAG:NCI−H23

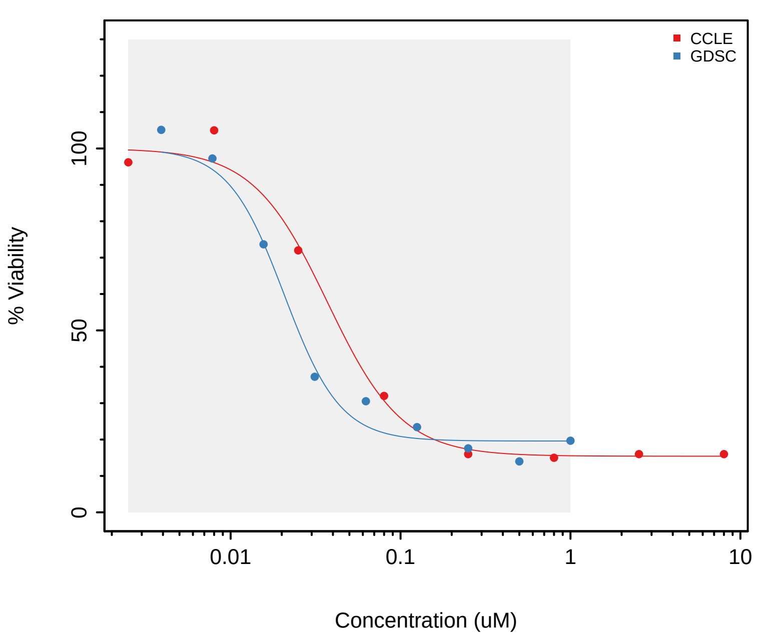

17−AAG:NCI−H2452

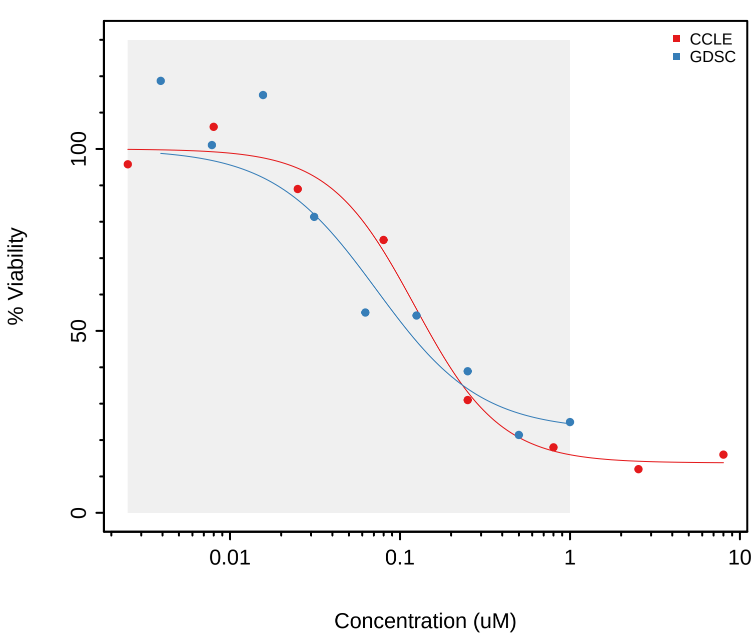

17−AAG:NCI−H28

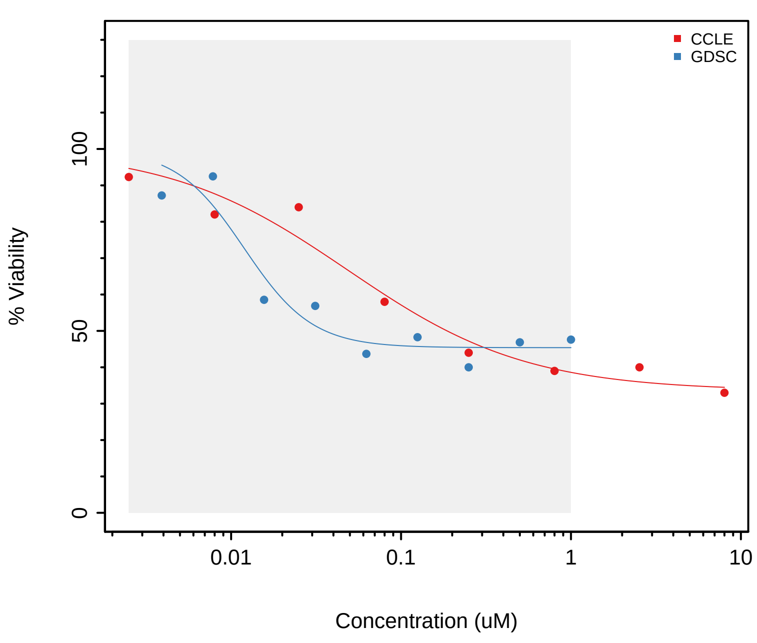

17−AAG:NCI−H358

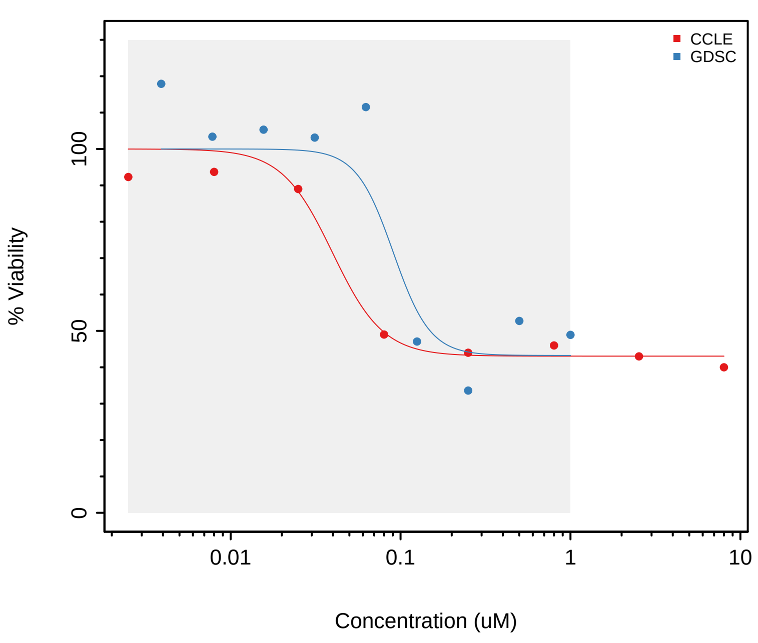

17−AAG:NCI−H441

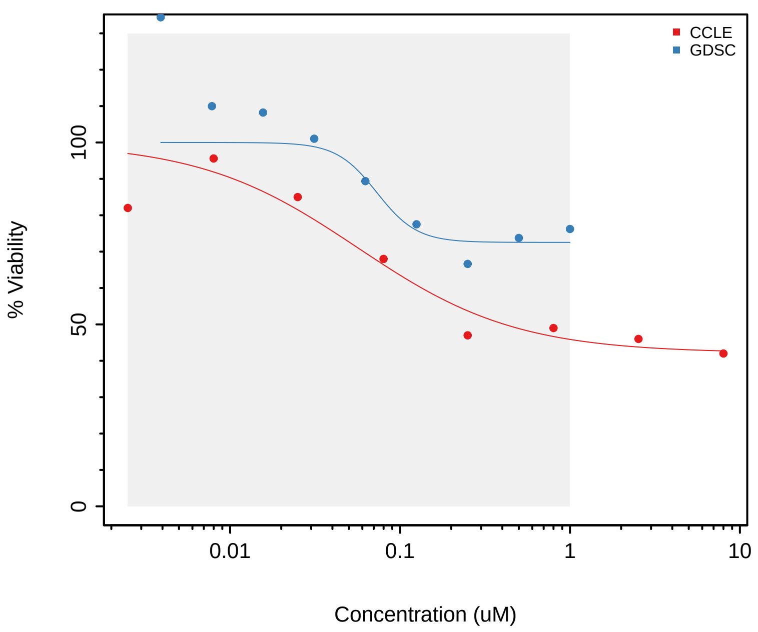

17−AAG:NCI−H460

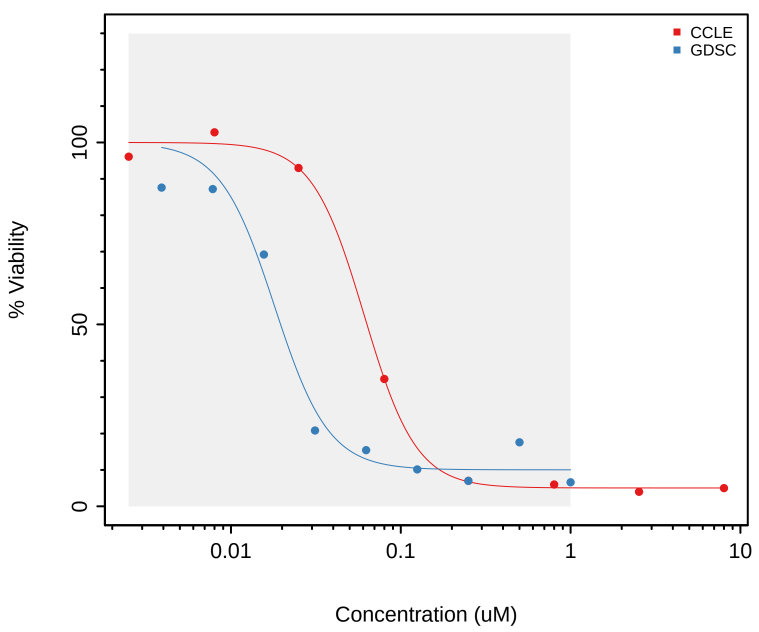

17−AAG:NCI−H520

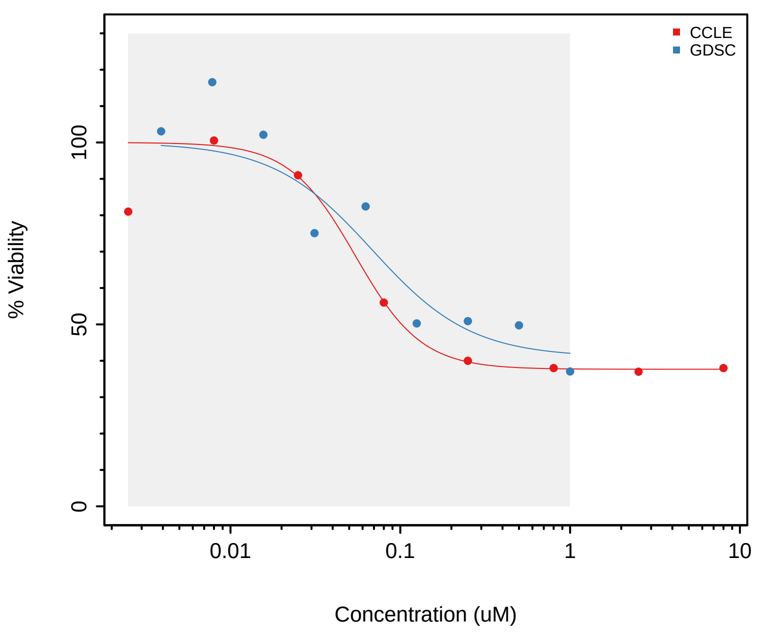

17−AAG:NCI−H522

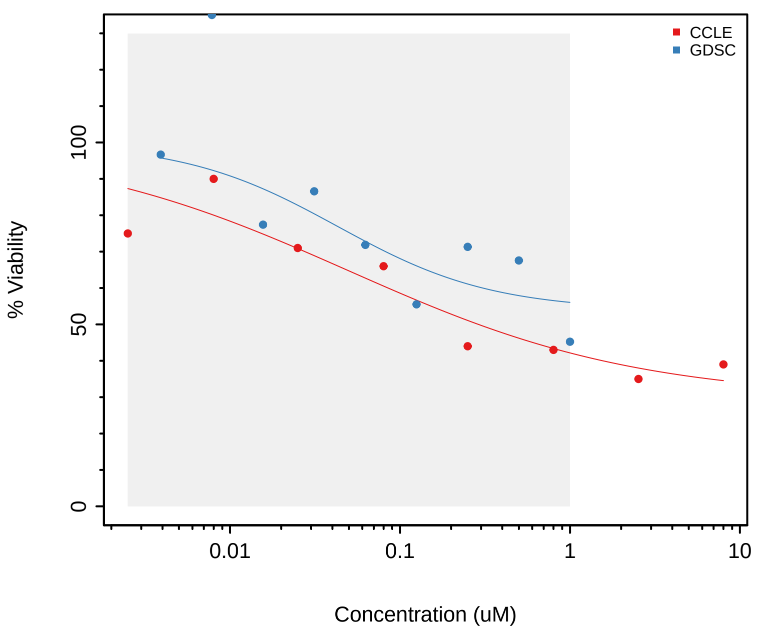

17−AAG:NCI−H650

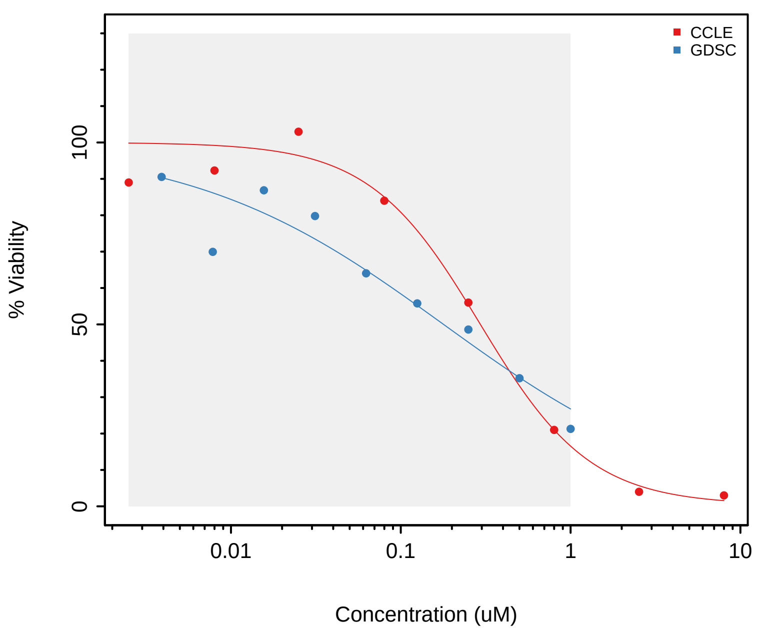

17−AAG:NCI−H661

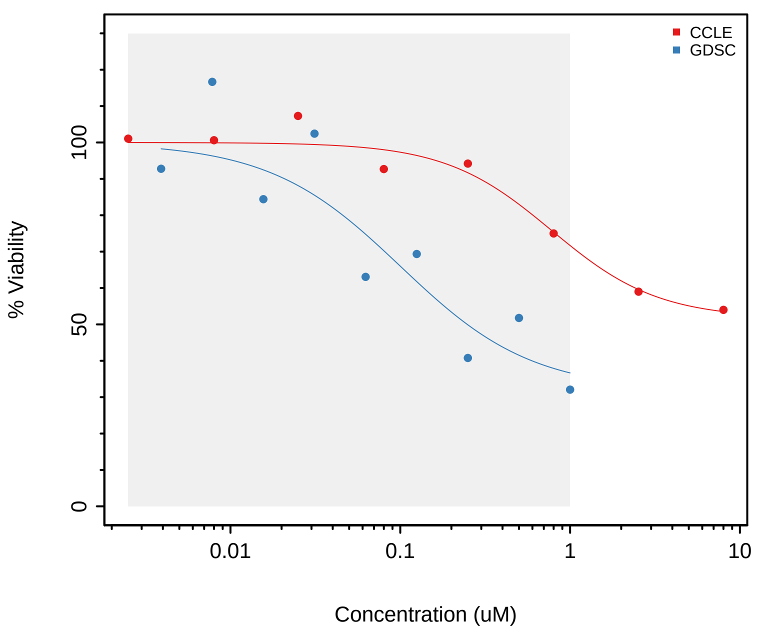

17−AAG:NCI−H727

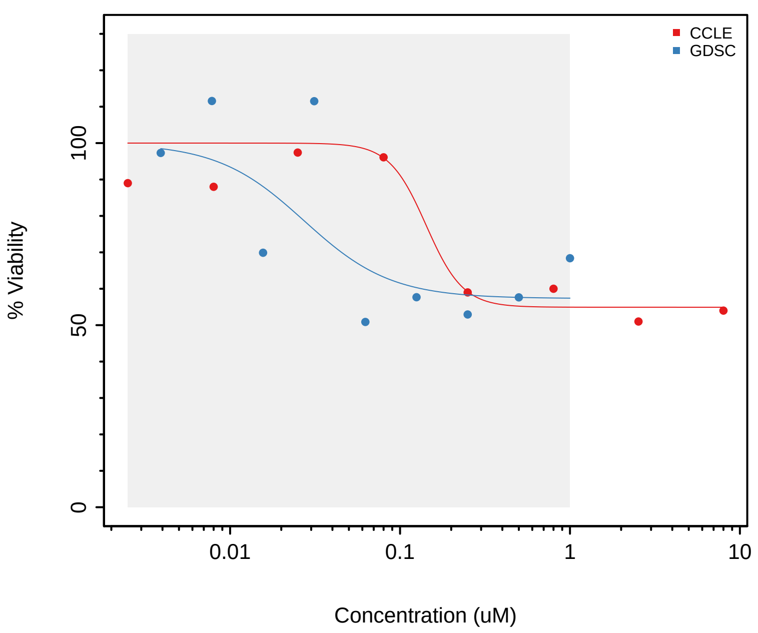

17−AAG:NCI−H747

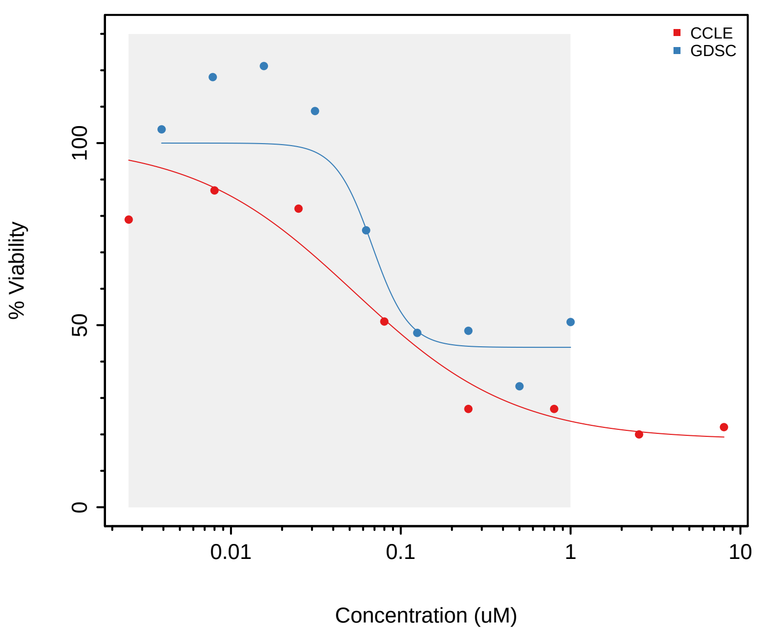

17−AAG:NCI−H810

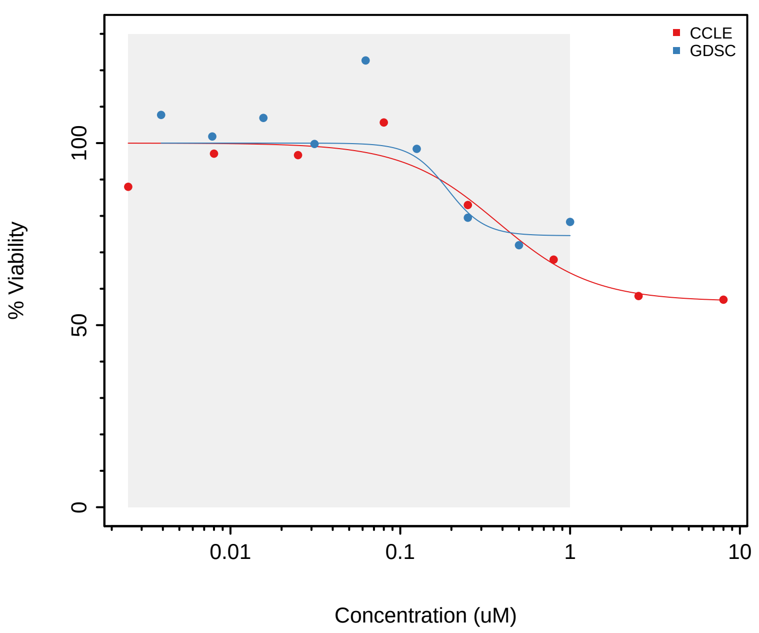

17−AAG:NCI−N87

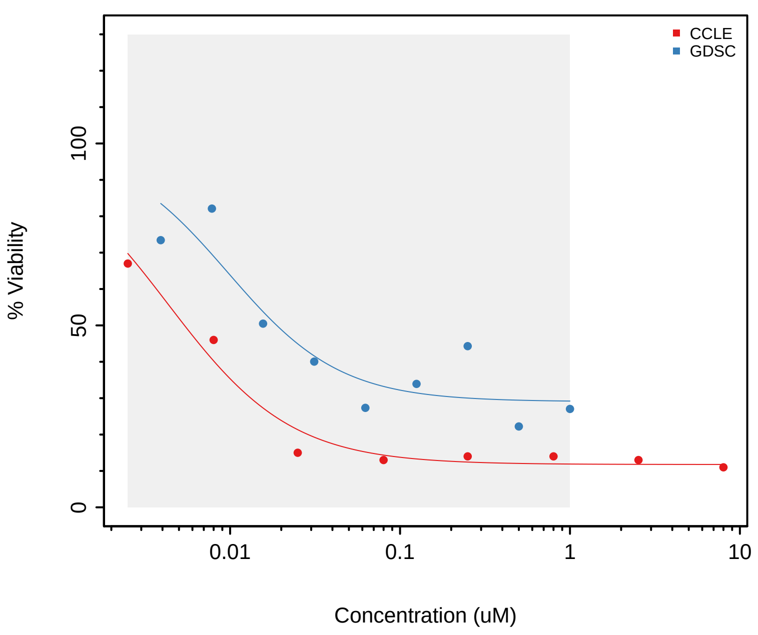

17−AAG:OVCAR−3

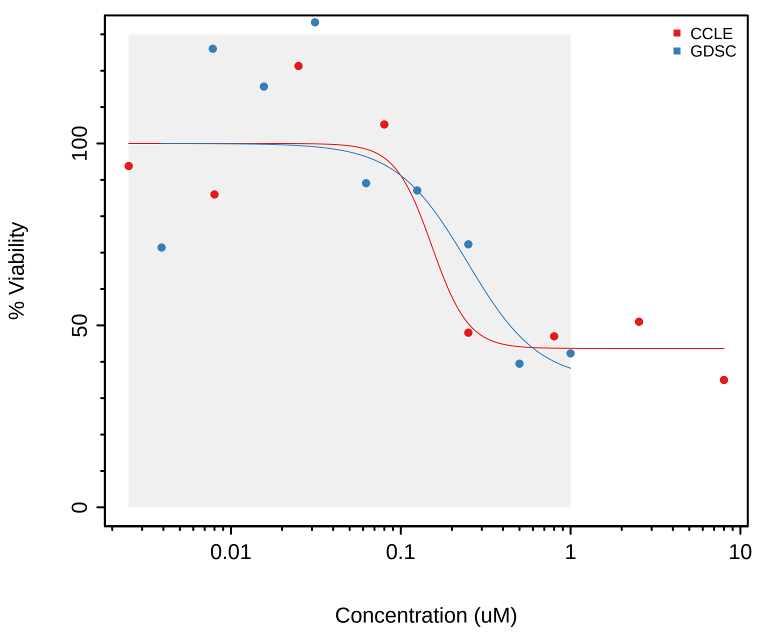

17−AAG:NUGC−3

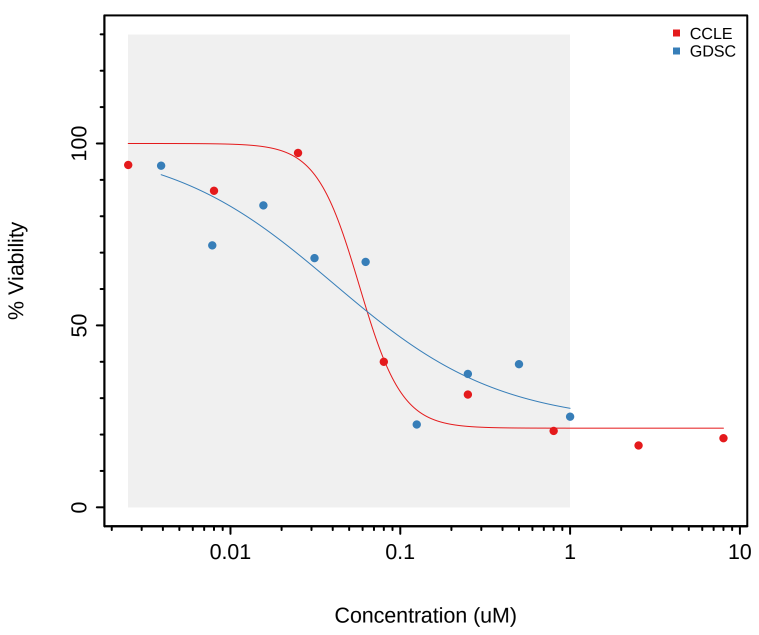

17−AAG:OC−314

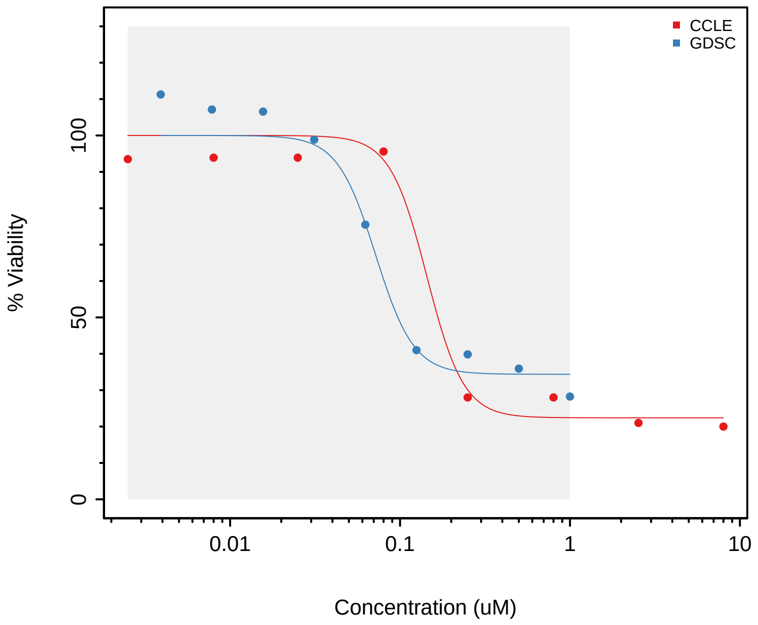

17−AAG:OCI−AML2

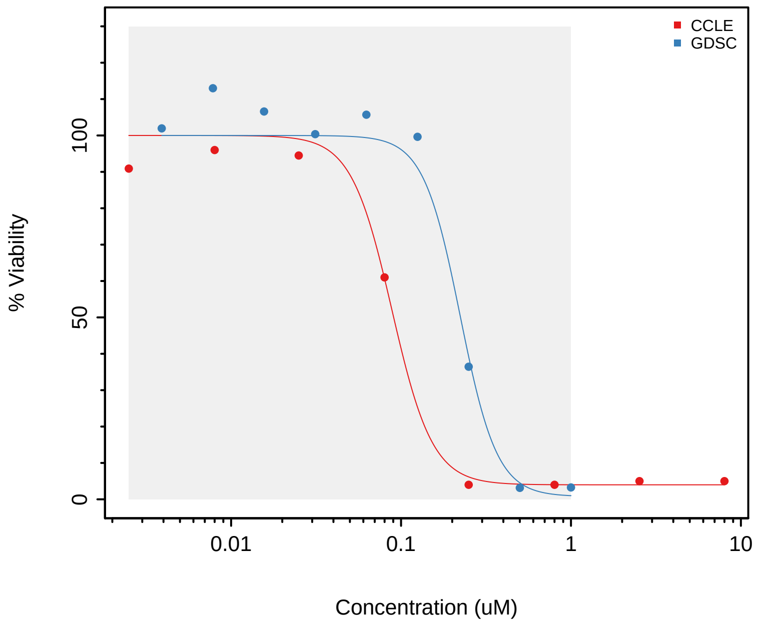

17−AAG:OE33

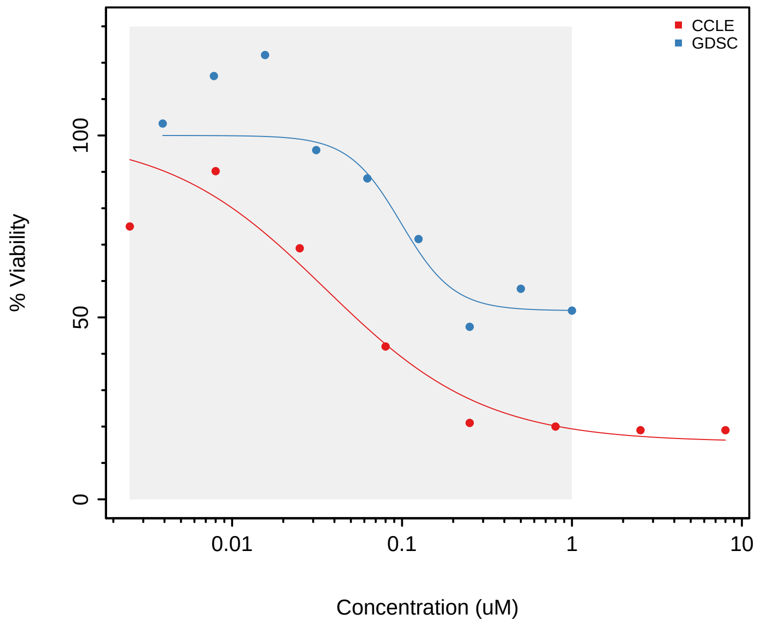

17−AAG:ONS−76

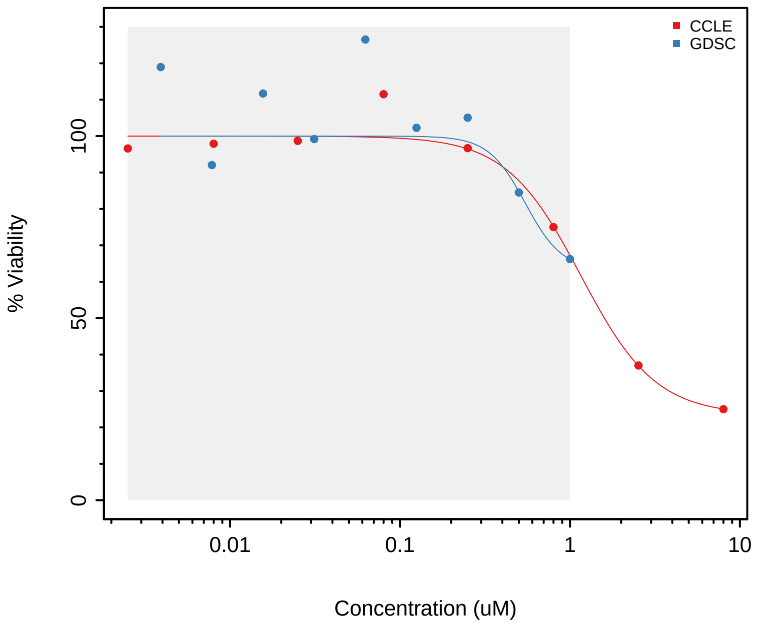

17−AAG:OPM−2

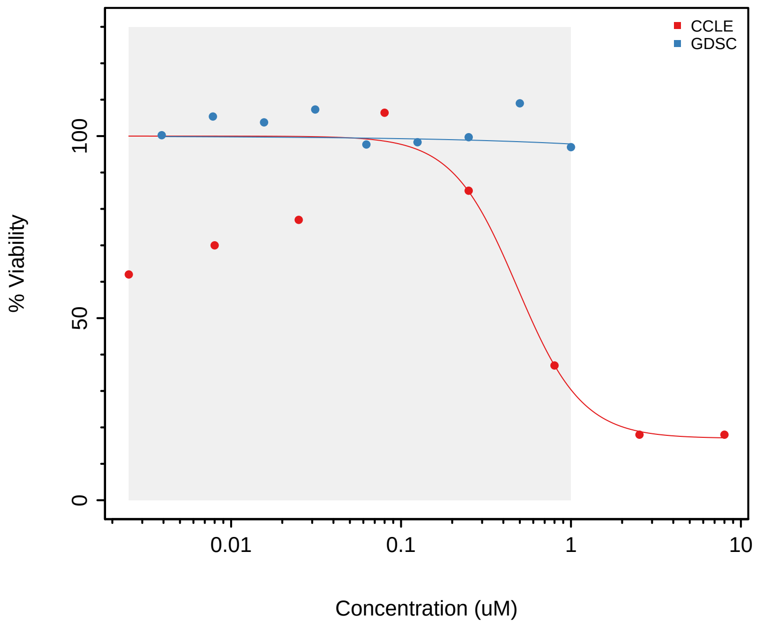

17−AAG:OVCAR−4

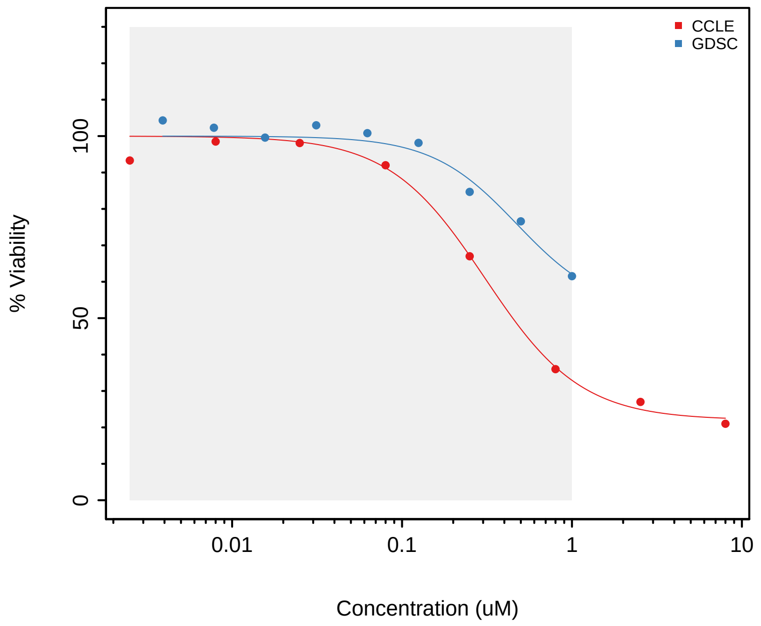

17−AAG:OVCAR−8

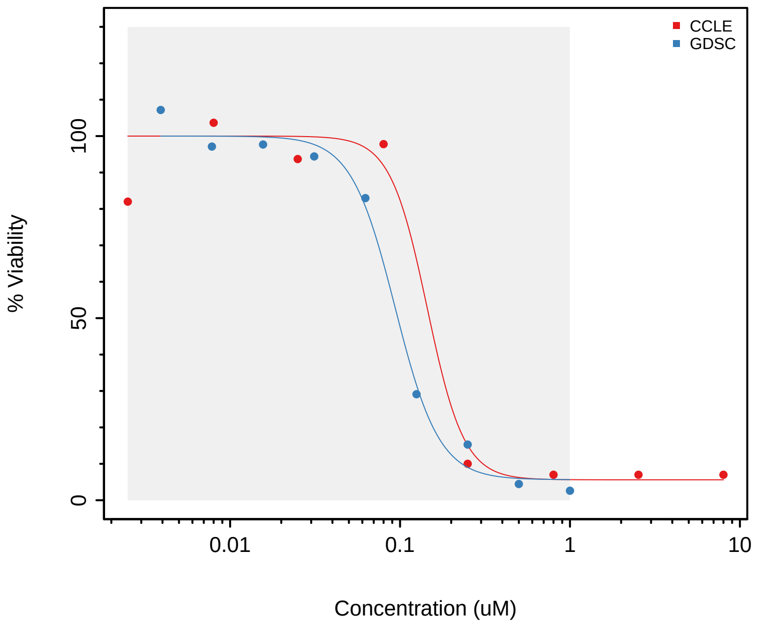

17−AAG:P12−ICHIKAWA

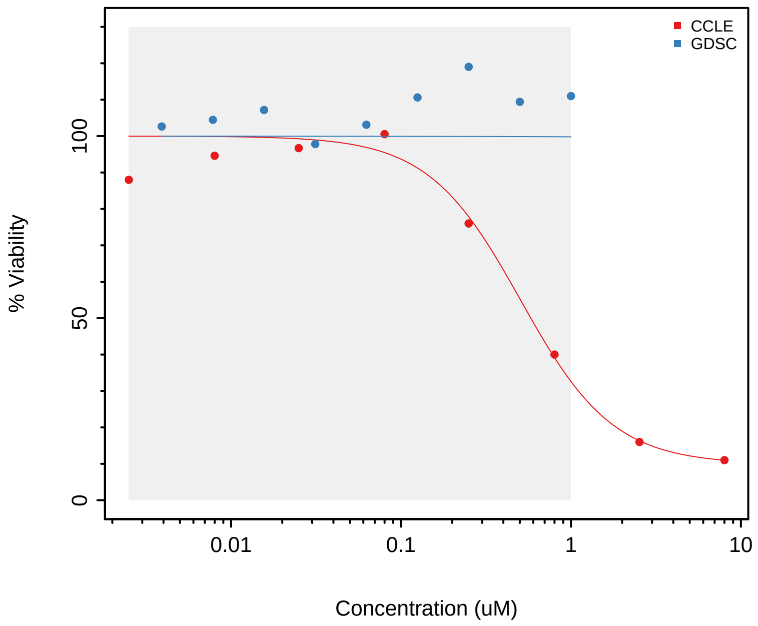

17−AAG:P31−FUJ

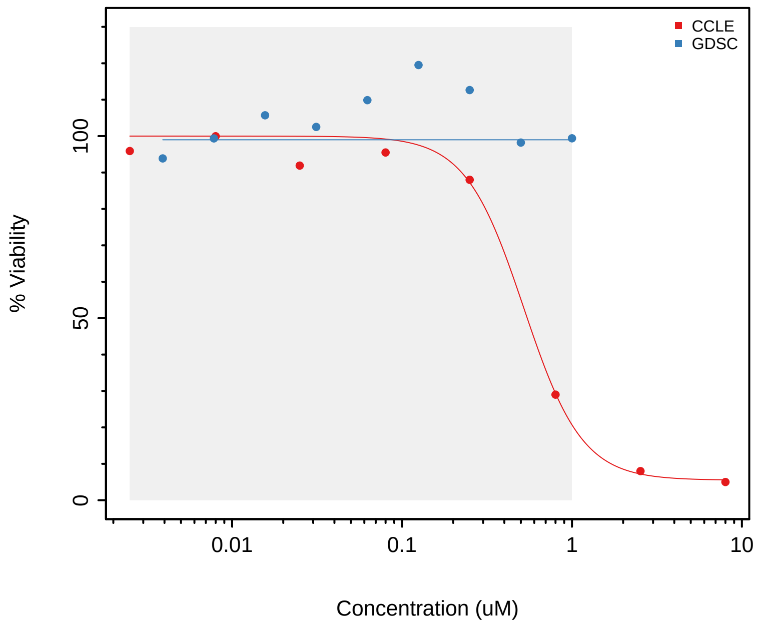

17−AAG:Panc 03.27

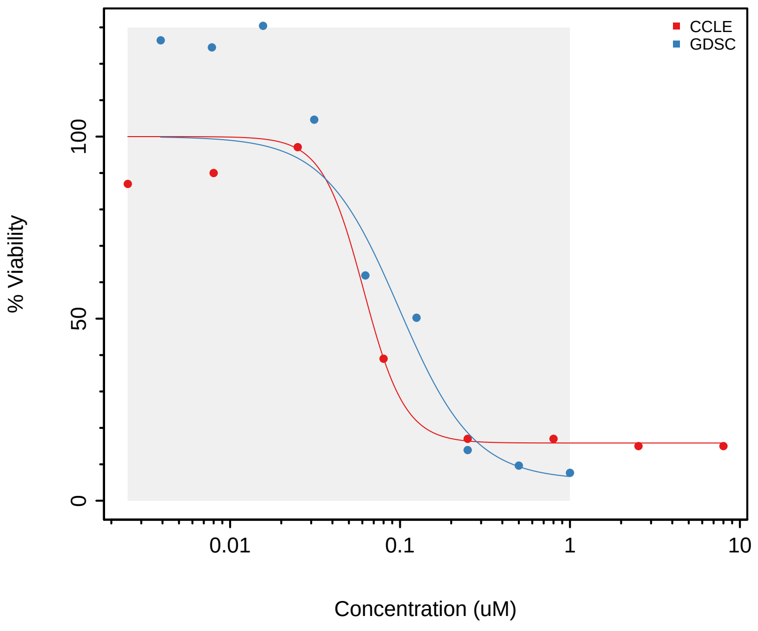

17−AAG:Panc 10.05

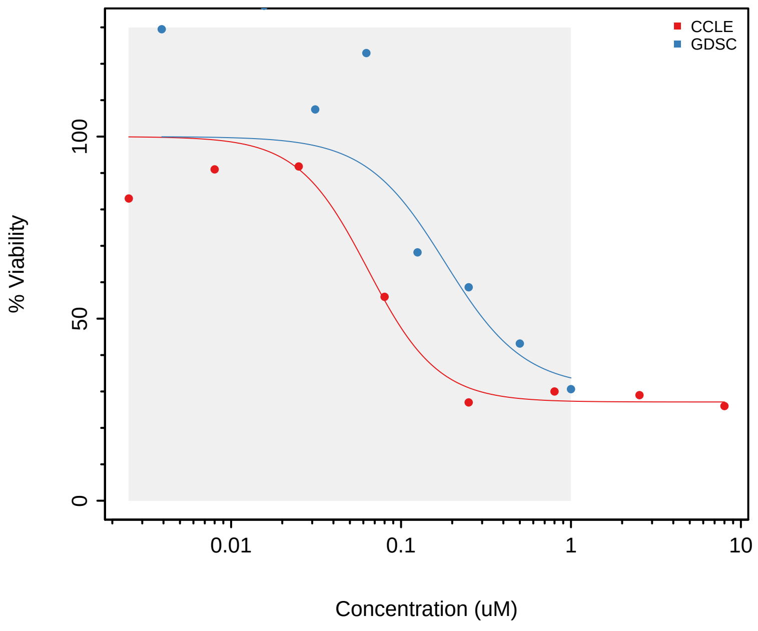

17−AAG:PC−14

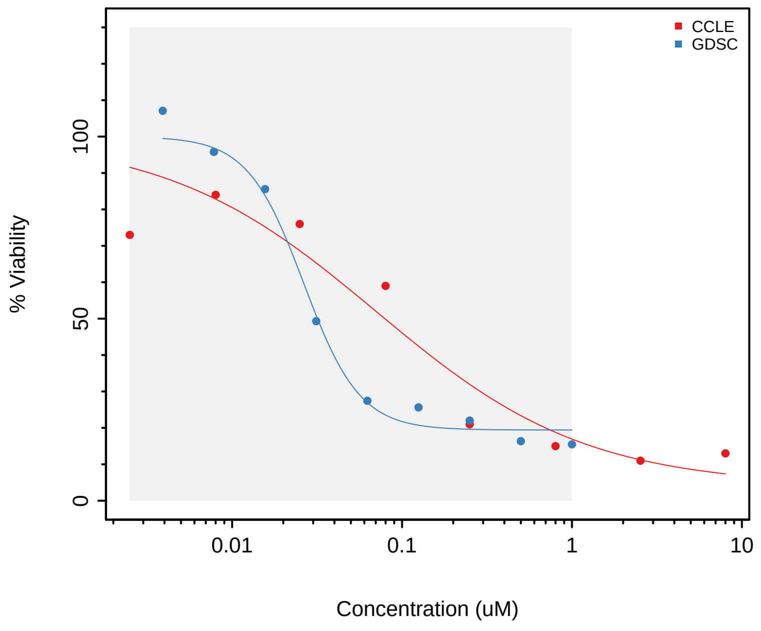

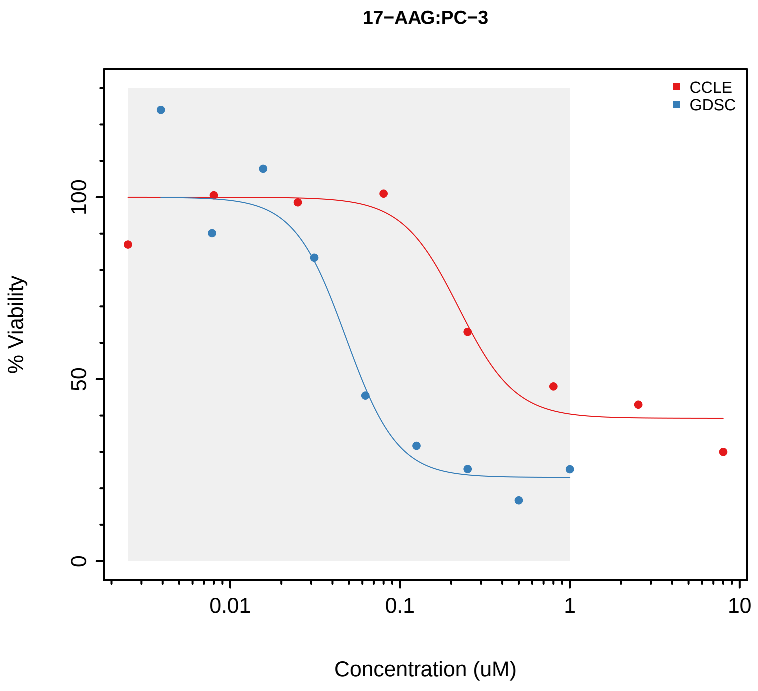
17−AAG:PC−3
CCLE
GDSC
% Viability
0
50
100
0.01
0.1
1
10
Concentration (uM)

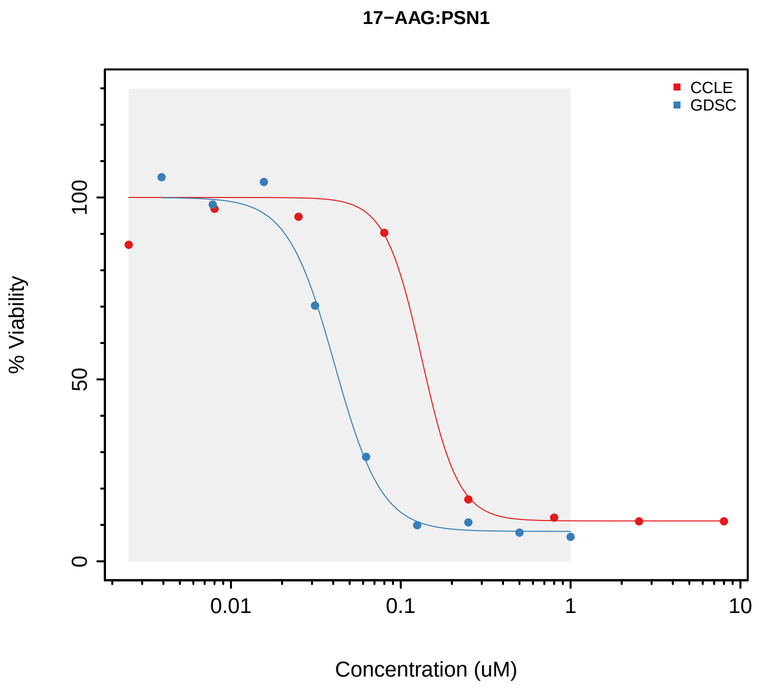
17−AAG:PSN1
CCLE
GDSC
% Viability
0
50
100
0.01
0.1
1
10
Concentration (uM)

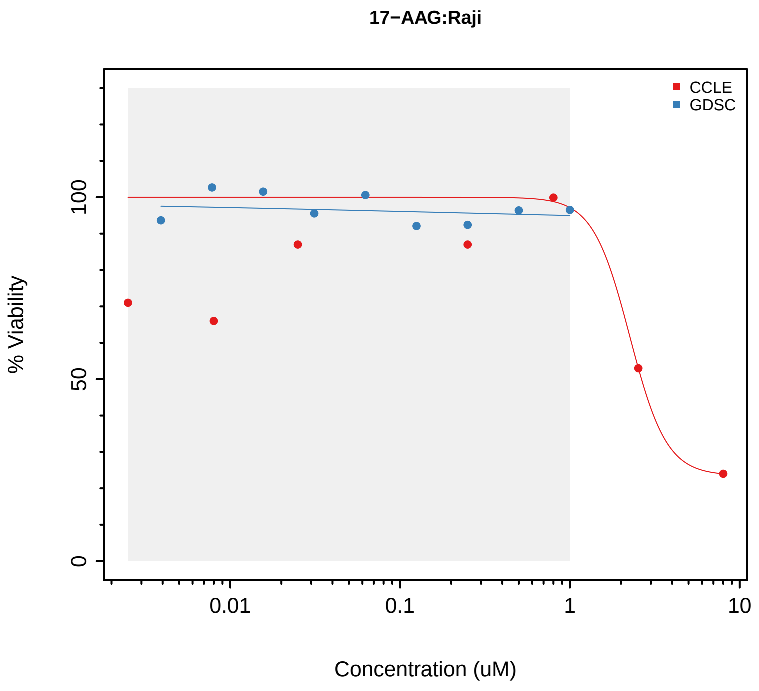
17−AAG:Raji
CCLE
GDSC
% Viability
0
50
100
0.01
0.1
1
10
Concentration (uM)

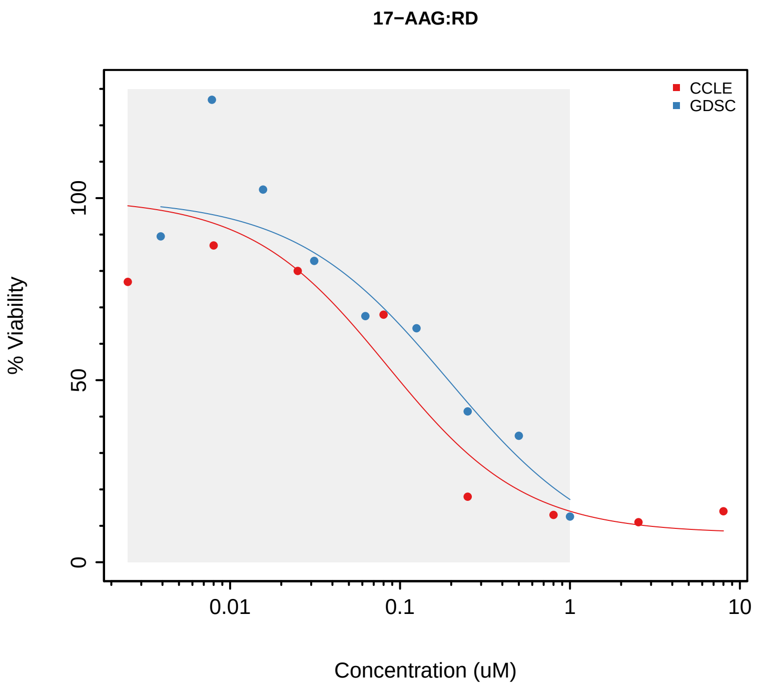
17−AAG:RD
CCLE
GDSC
% Viability
0
50
100
0.01
0.1
1
10
Concentration (uM)

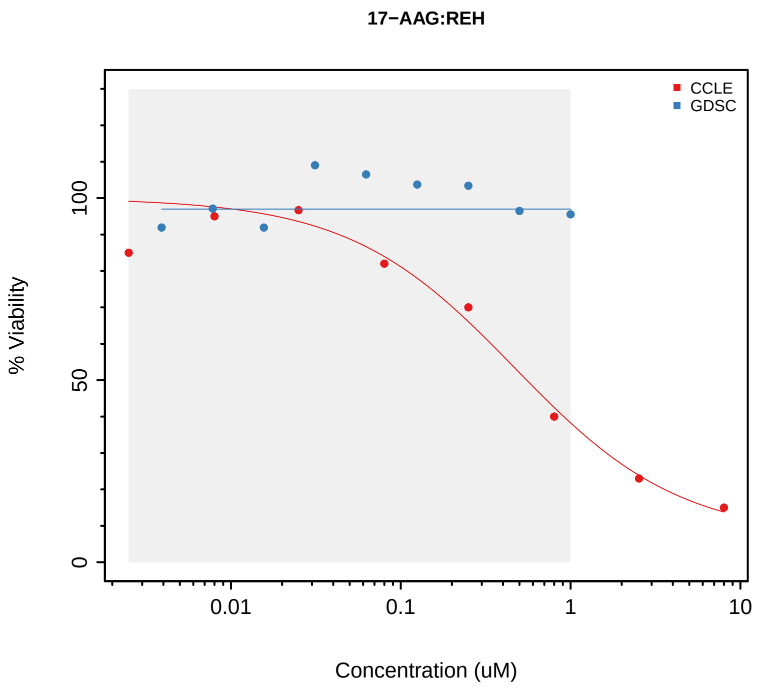
17−AAG:REH
CCLE
GDSC
% Viability
0
50
100
0.01
0.1
1
10
Concentration (uM)

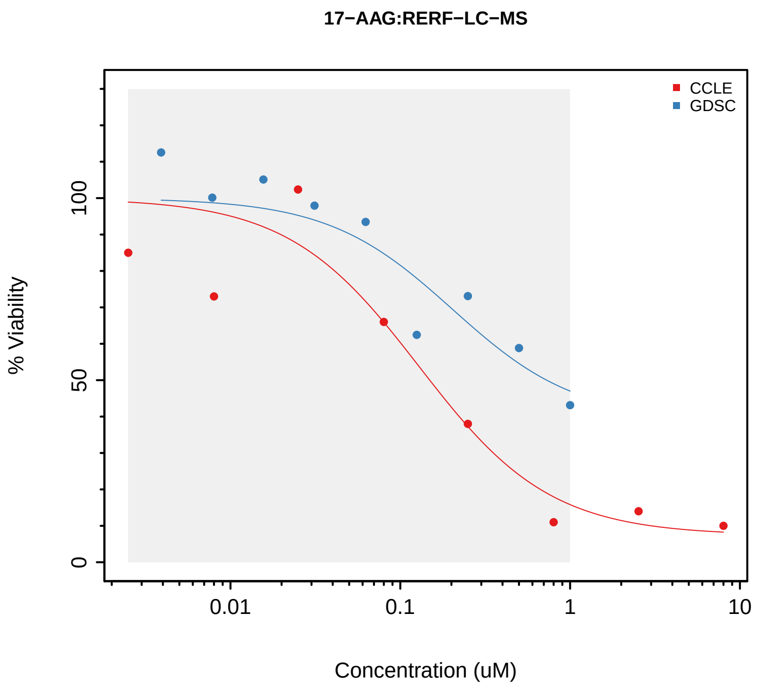
17−AAG:RERF−LC−MS
CCLE
GDSC
% Viability
0
50
100
0.01
0.1
1
10
Concentration (uM)

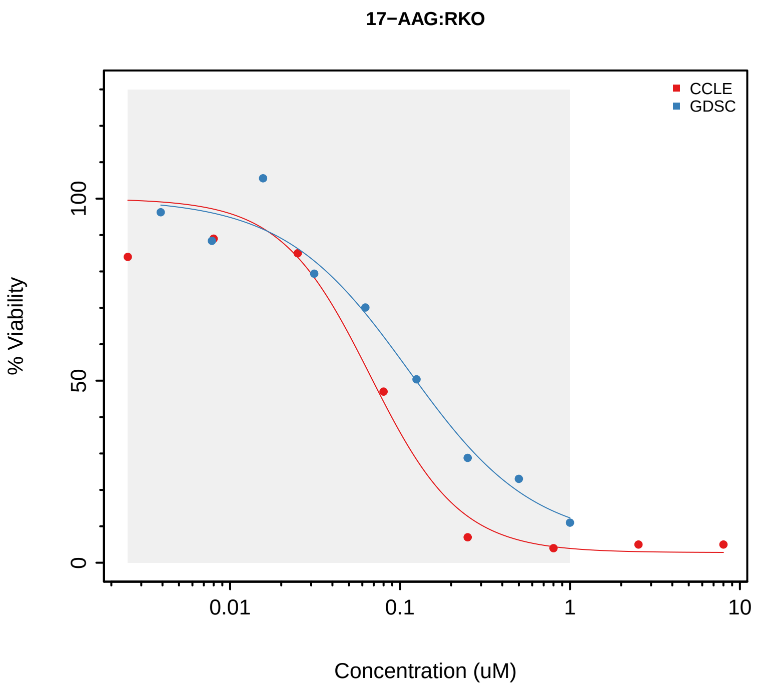
17−AAG:RKO
CCLE
GDSC
% Viability
0
50
100
0.01
0.1
1
10
Concentration (uM)

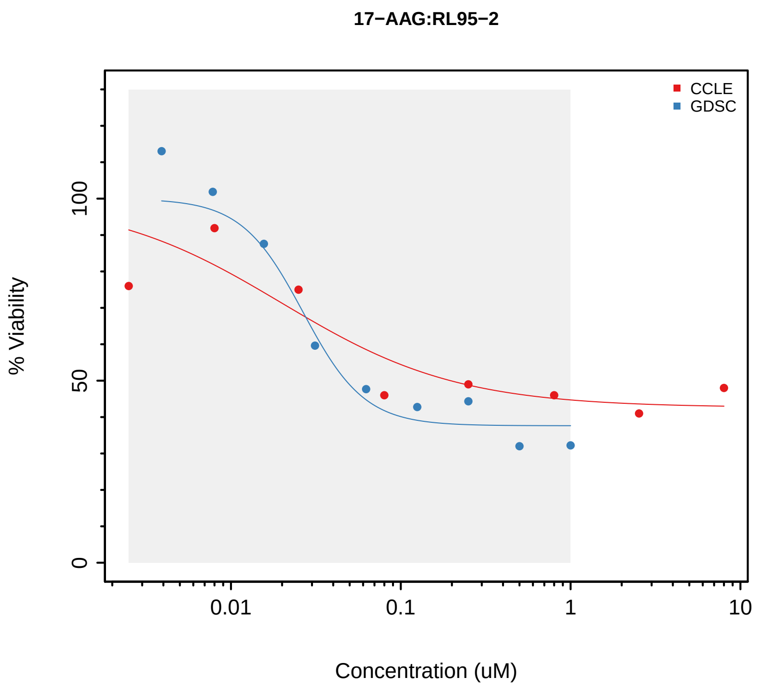
17−AAG:RL95−2
CCLE
GDSC
% Viability
0
50
100
0.01
0.1
1
10
Concentration (uM)

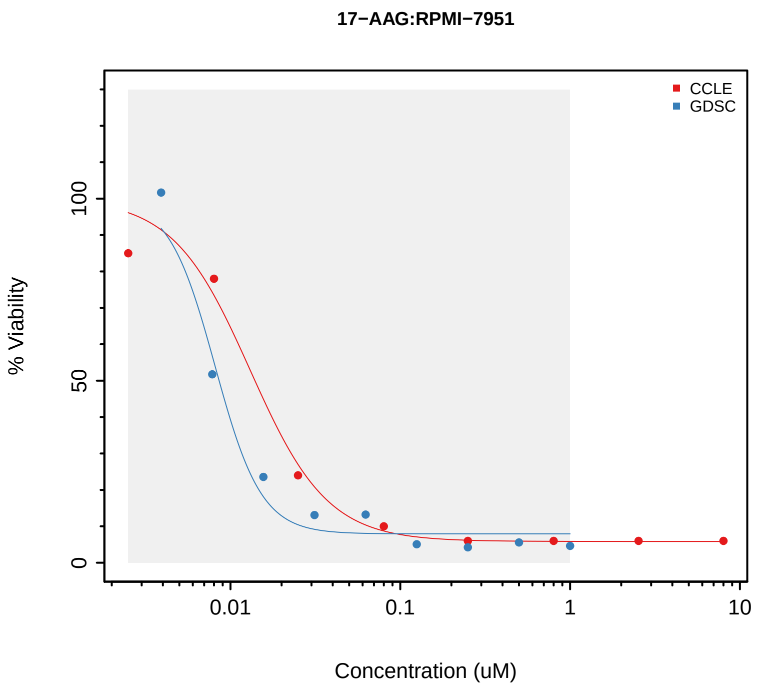
17−AAG:RPMI−7951
CCLE
GDSC
% Viability
0
50
100
0.01
0.1
1
10
Concentration (uM)

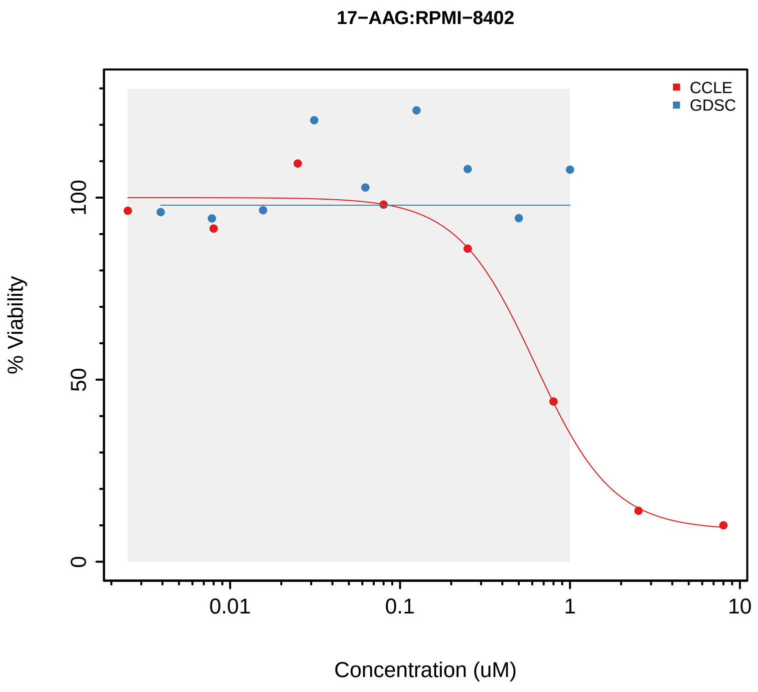
17−AAG:RPMI−8402
CCLE
GDSC
% Viability
0
50
100
0.01
0.1
1
10
Concentration (uM)

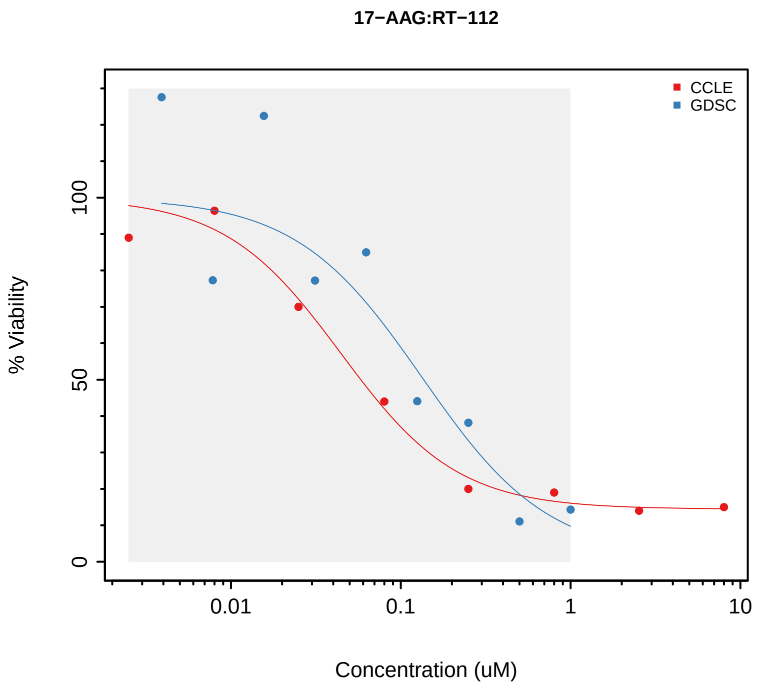
17−AAG:RT−112
CCLE
GDSC
% Viability
0
50
100
0.01
0.1
1
10
Concentration (uM)

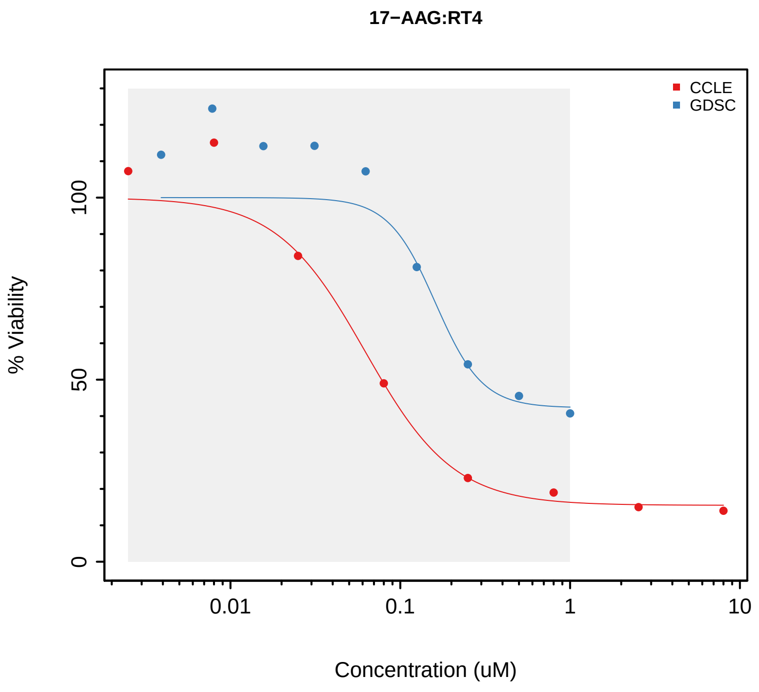
17−AAG:RT4
CCLE
GDSC
% Viability
0
50
100
0.01
0.1
1
10
Concentration (uM)

17−AAG:RVH−421

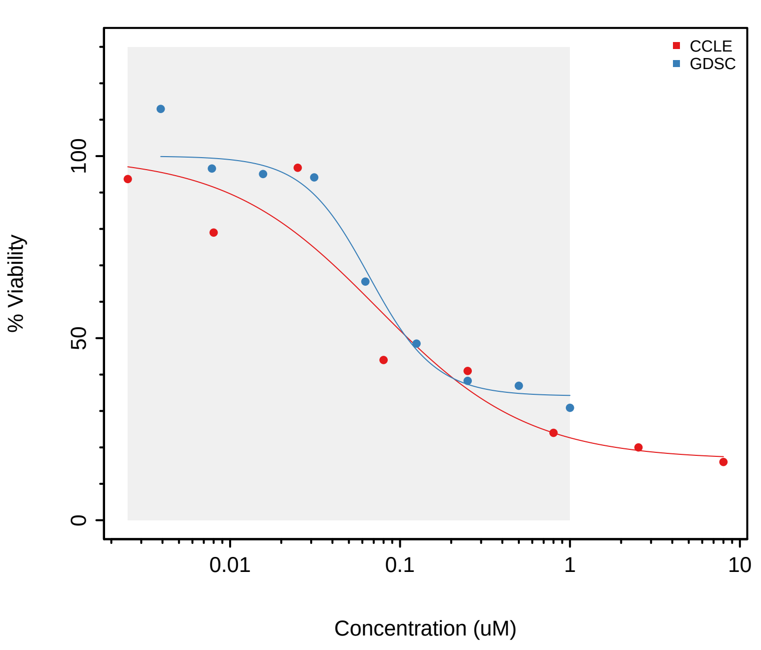

17−AAG:Saos−2

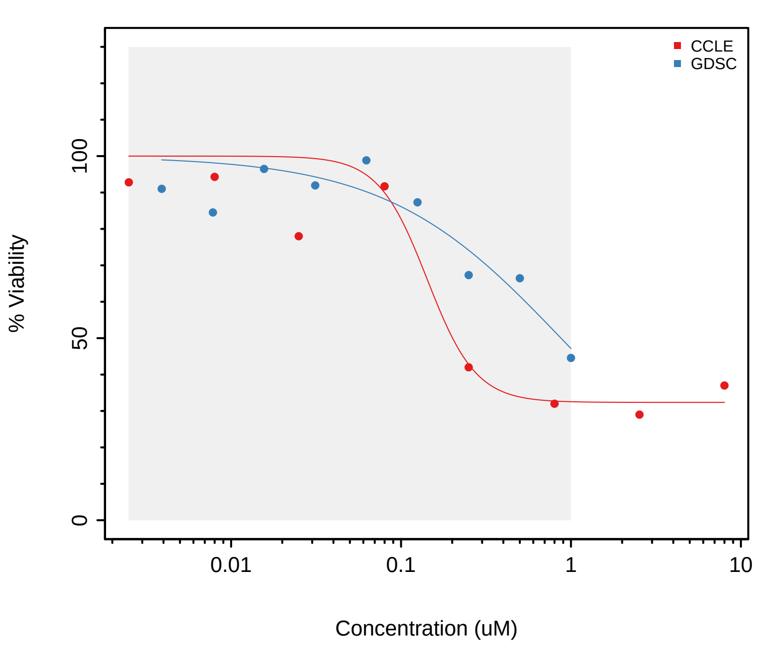

17−AAG:SBC−5

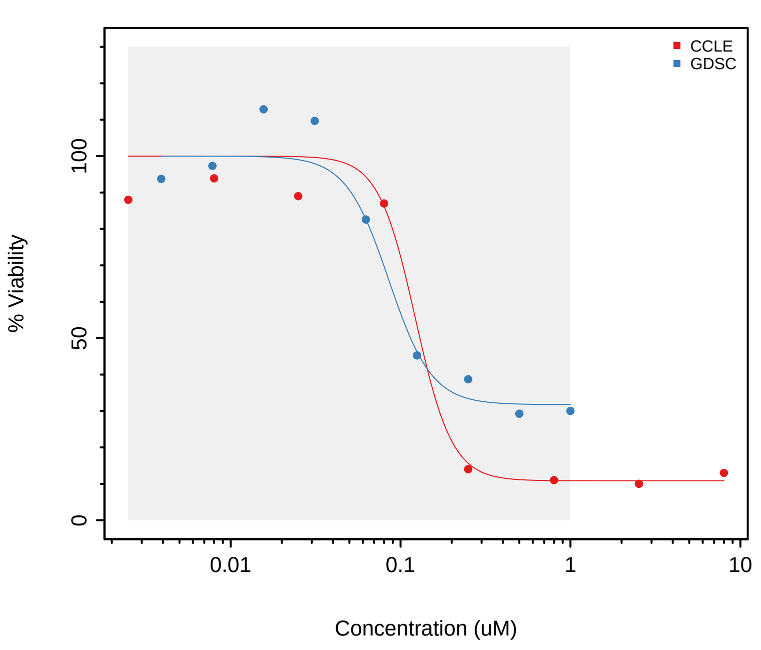

17−AAG:SCC−25

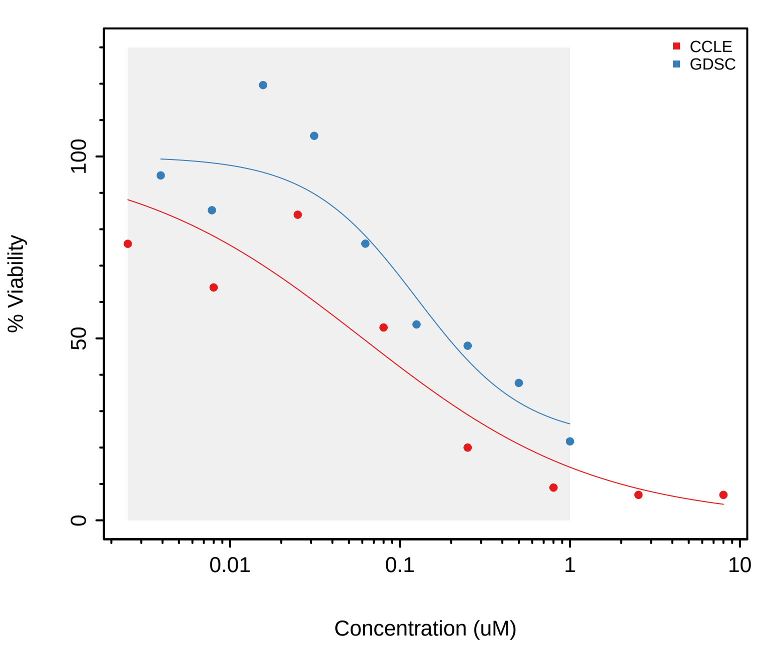

17−AAG:SCC−9

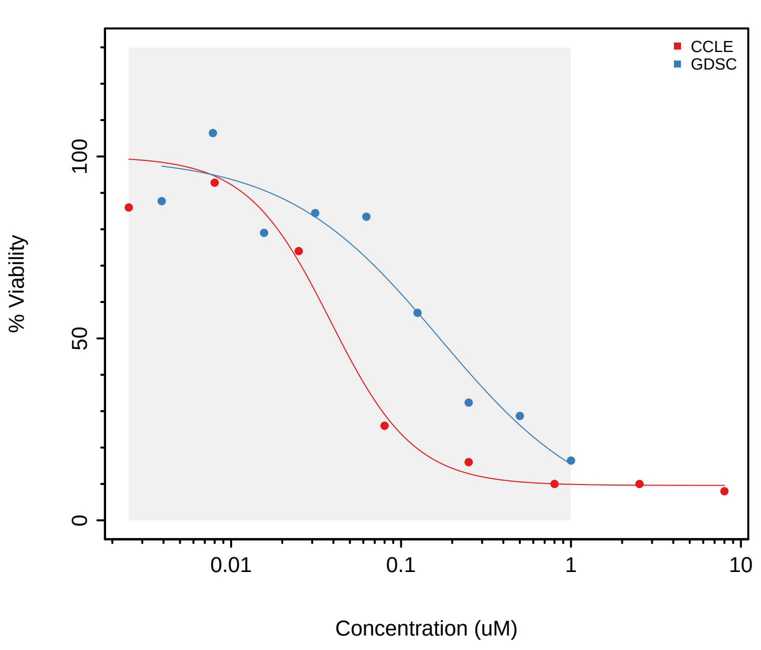

17−AAG:SF126

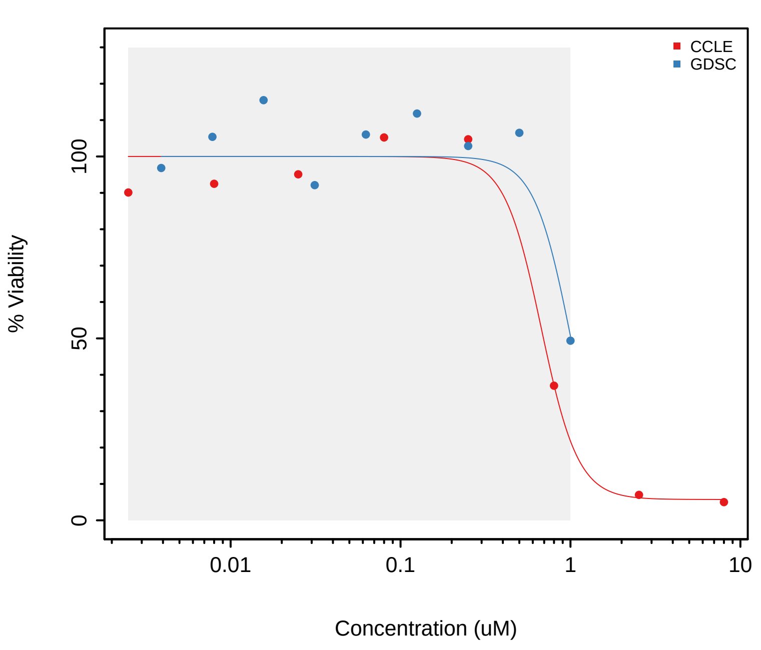

17−AAG:SF295

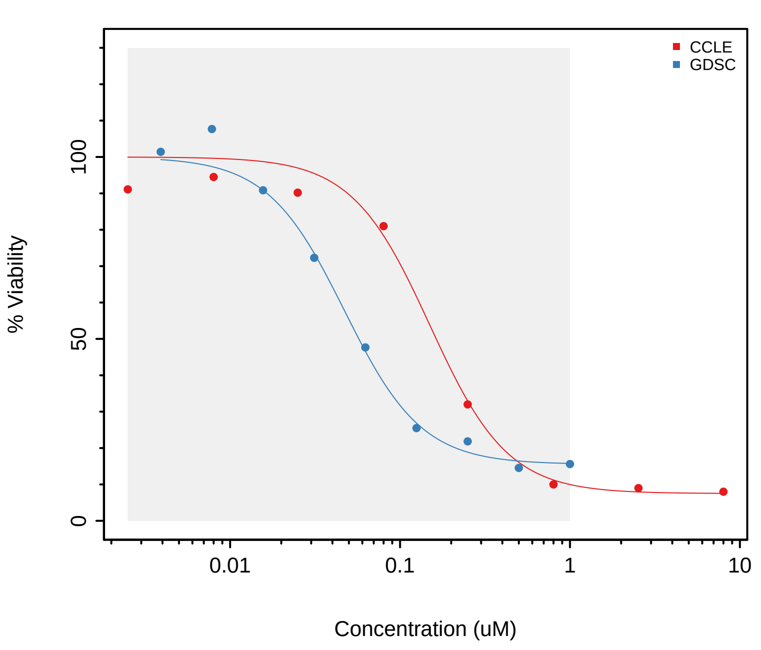

17−AAG:SHP−77

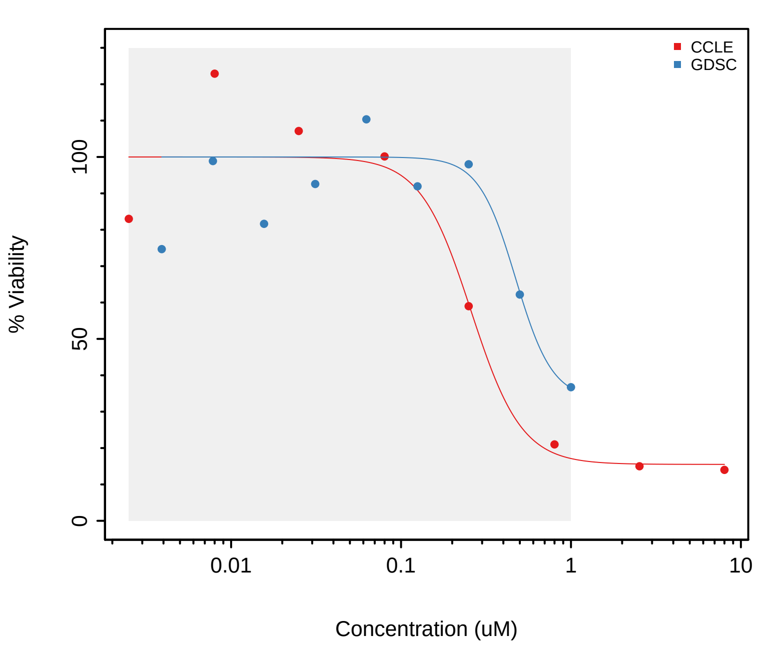

17−AAG:SIMA

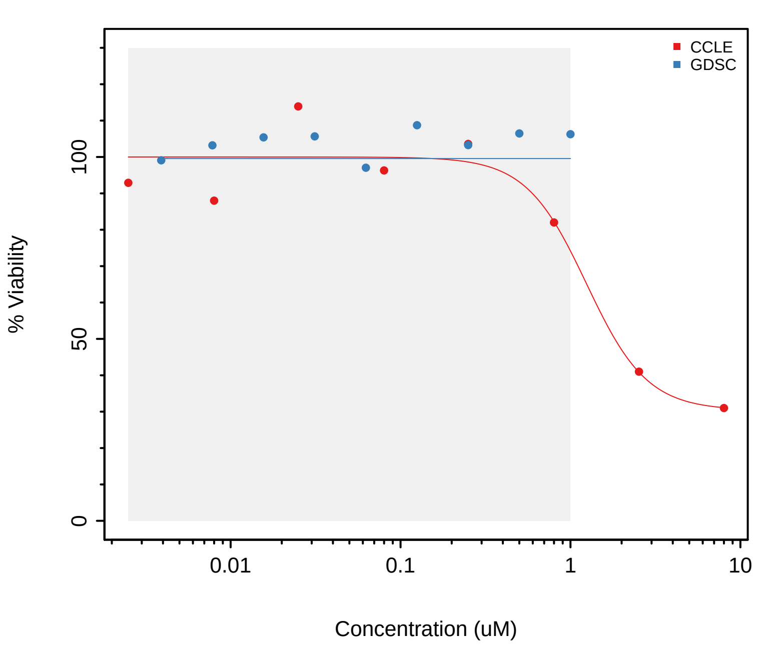

17−AAG:SJRH30

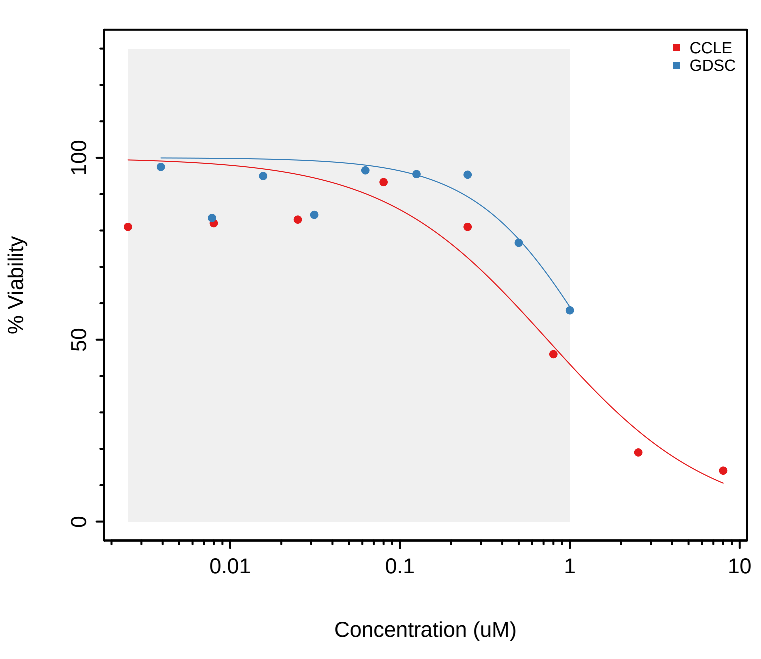

17−AAG:SJSA−1

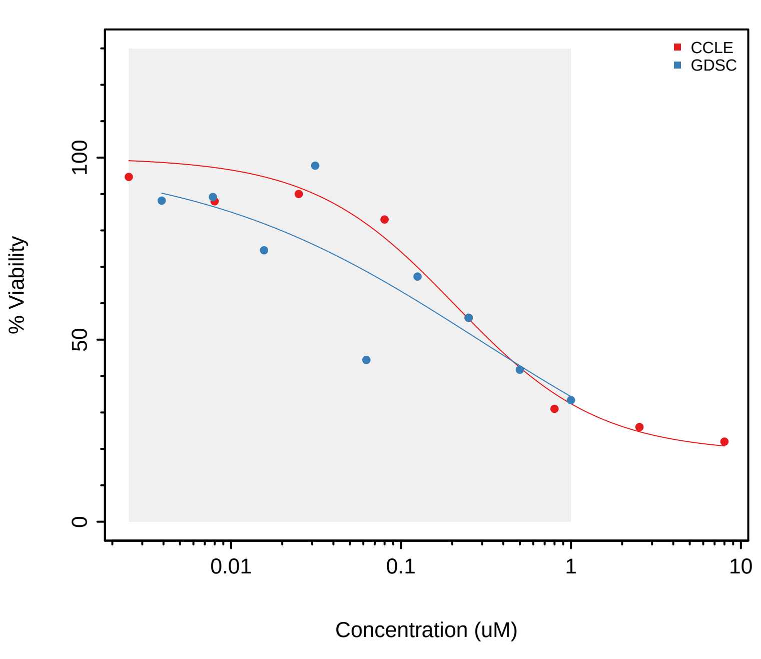

17−AAG:SK−CO−1

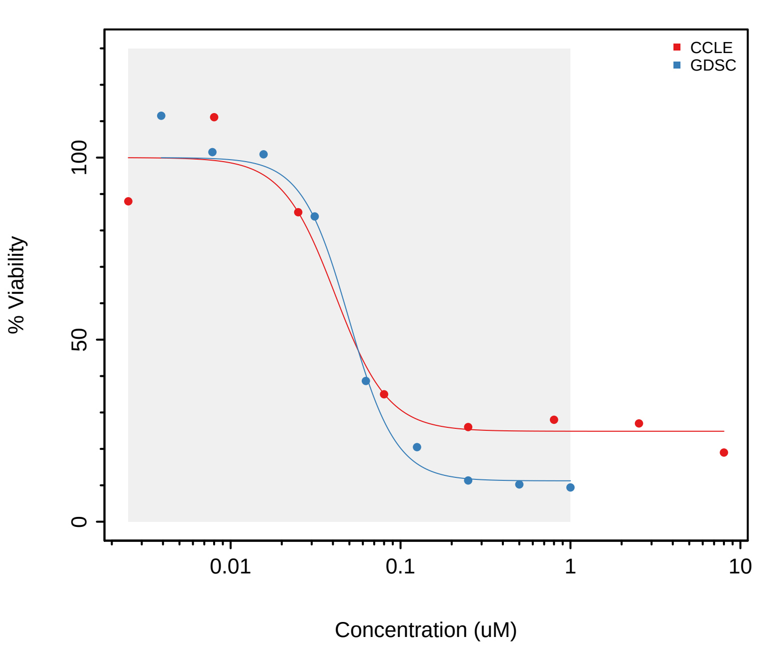

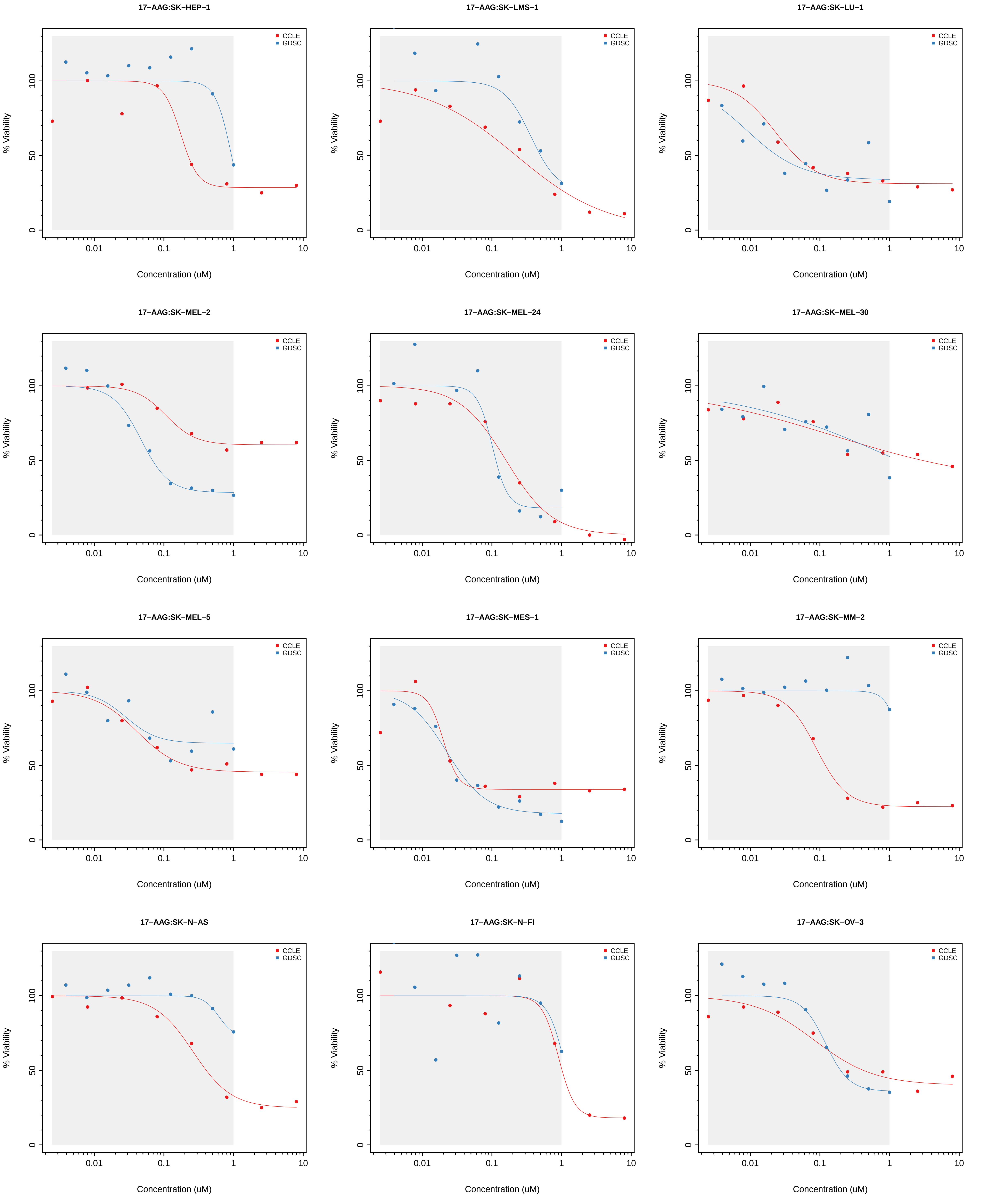
17−AAG:SK−HEP−1
17−AAG:SK−LMS−1
17−AAG:SK−LU−1
17−AAG:SK−MEL−2
17−AAG:SK−MEL−24
17−AAG:SK−MEL−30
17−AAG:SK−MEL−5
17−AAG:SK−MES−1
17−AAG:SK−MM−2
17−AAG:SK−N−AS
17−AAG:SK−N−FI
17−AAG:SK−OV−3
CCLE
GDSC
% Viability
0
50
100
Concentration (uM)
0.01
0.1
1
10

17−AAG:SNG−M

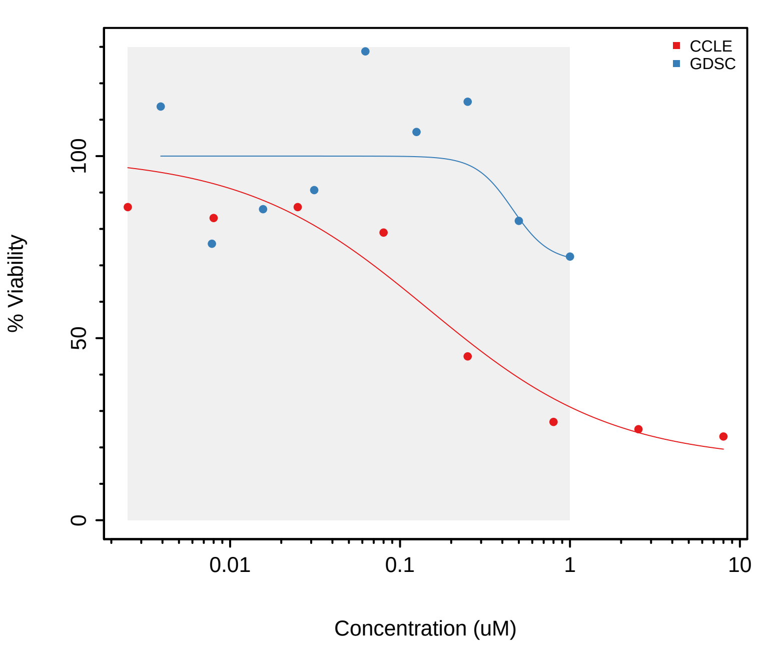

17−AAG:NCI−SNU−1

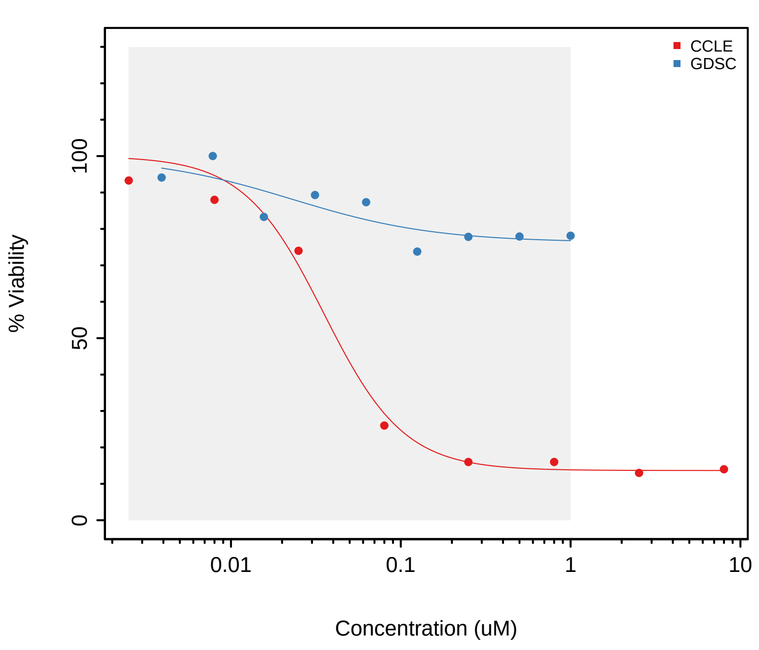

17−AAG:NCI−SNU−16

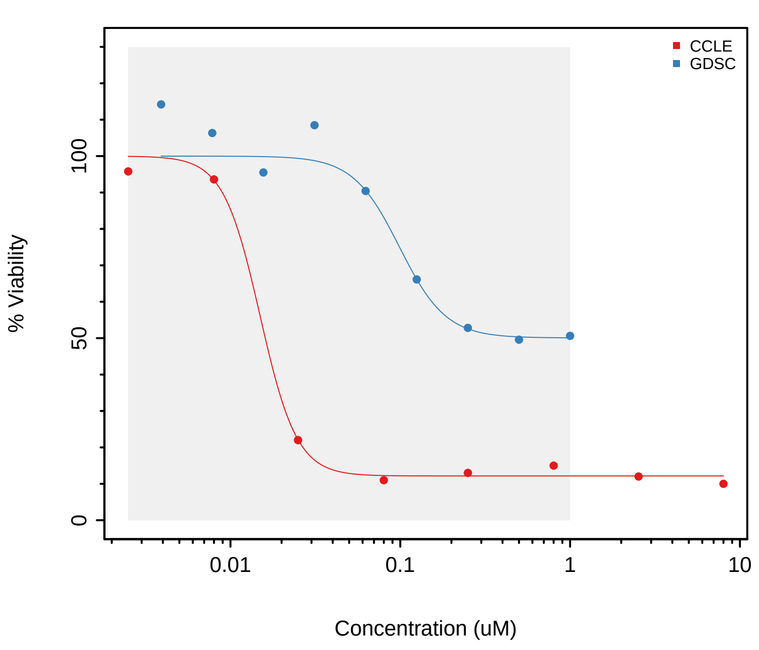

17−AAG:SNU−387

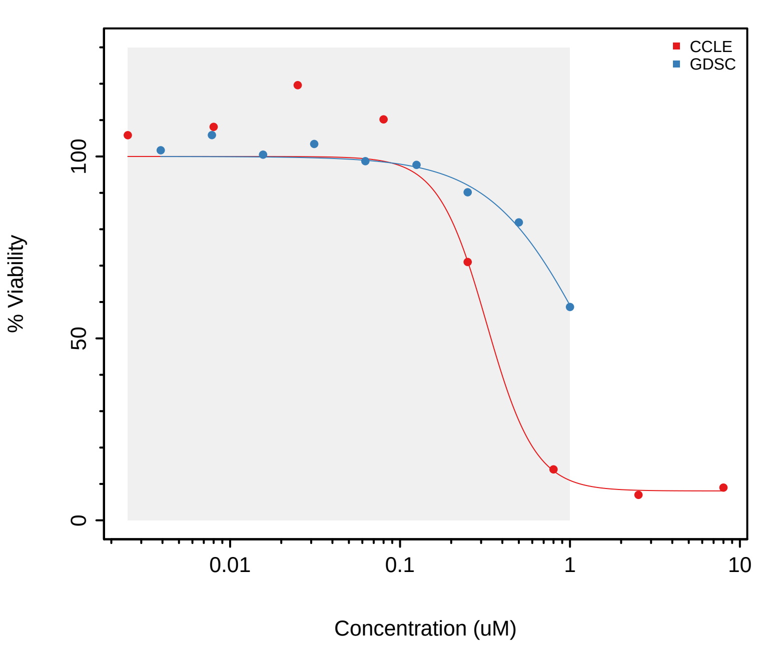

17−AAG:SNU−423

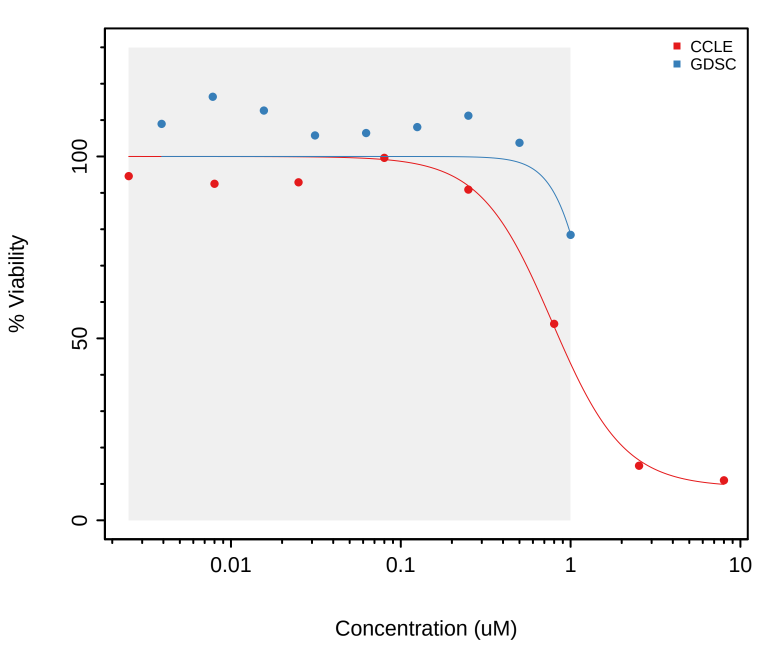

17−AAG:SNU−449

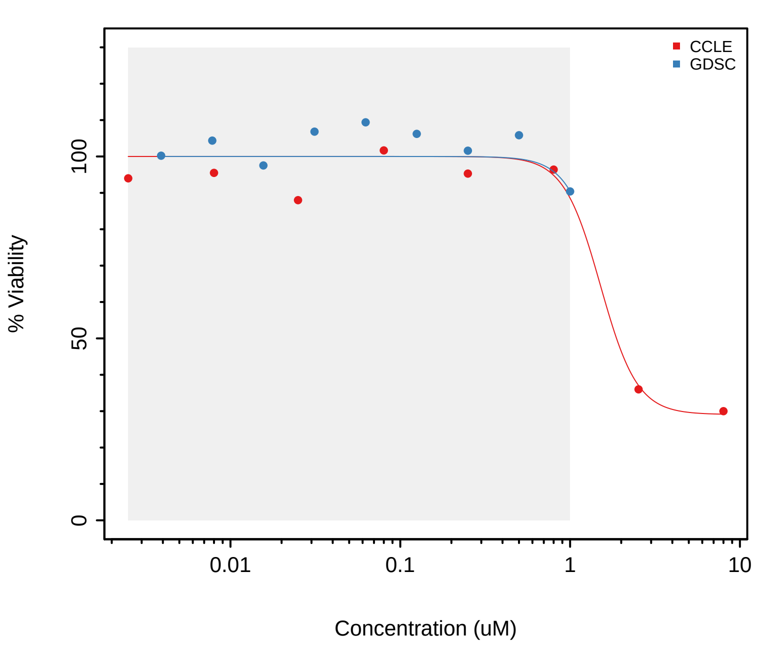

17−AAG:SNU−C2B

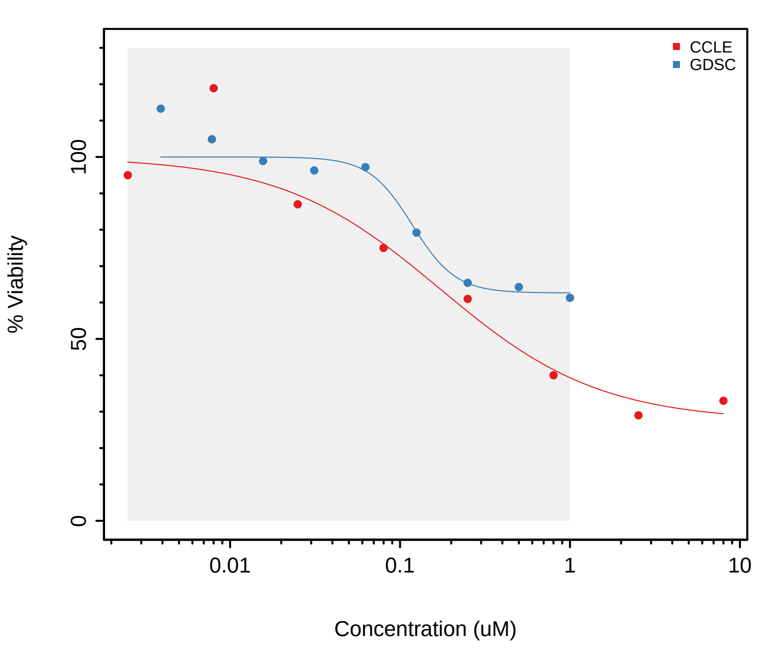

17−AAG:SUP−T1

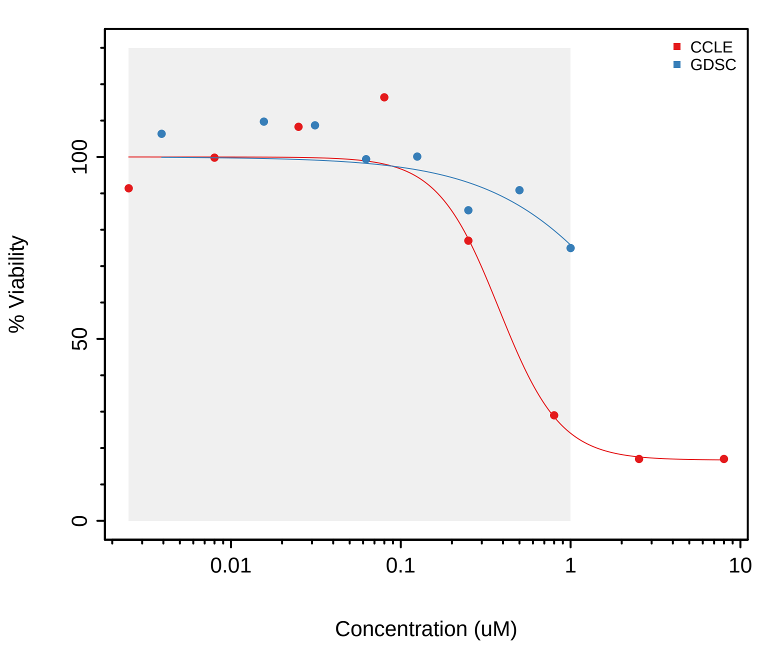

17−AAG:SW1088

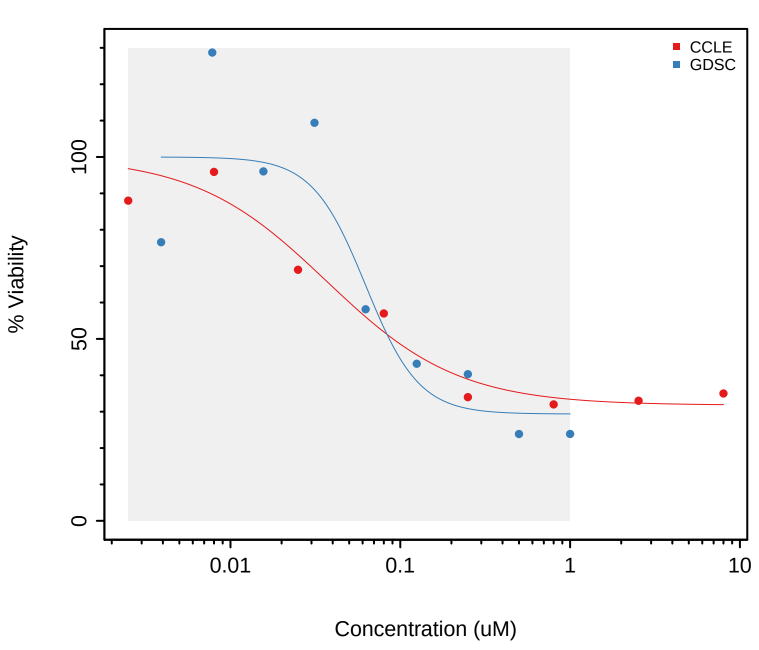

17−AAG:SW1417

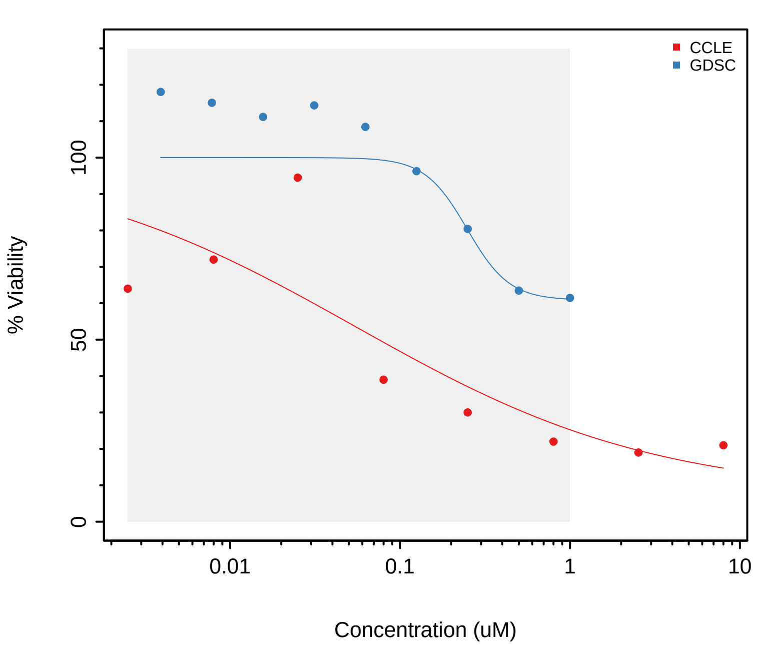

17−AAG:SW1573

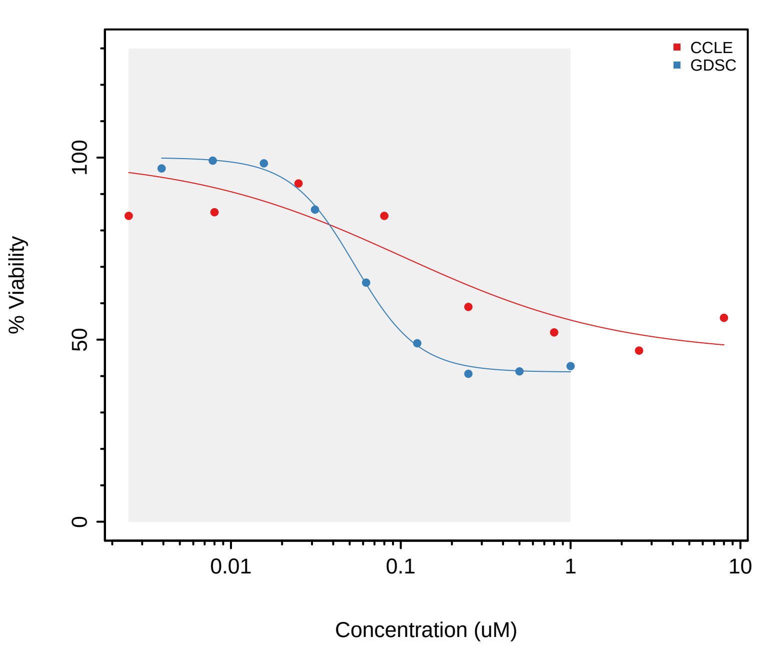

17−AAG:SW1990

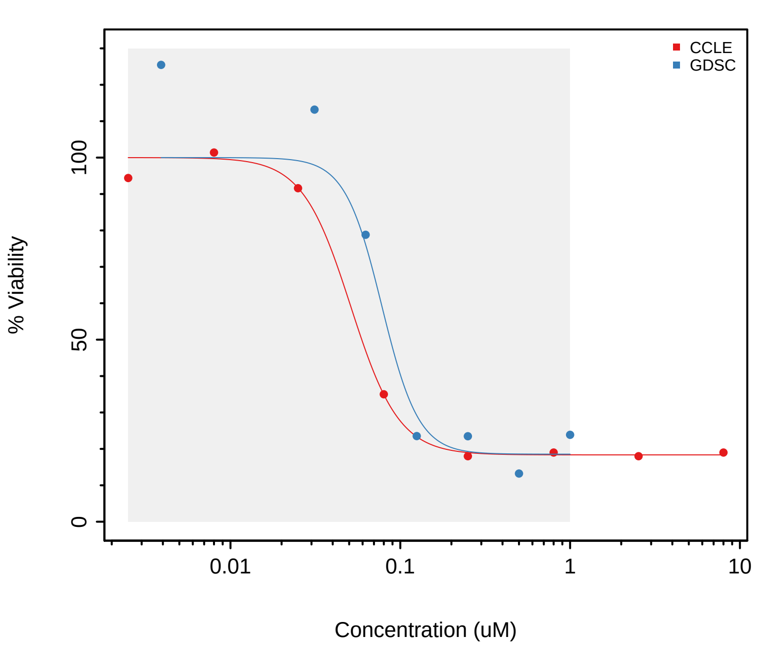

17−AAG:SW48

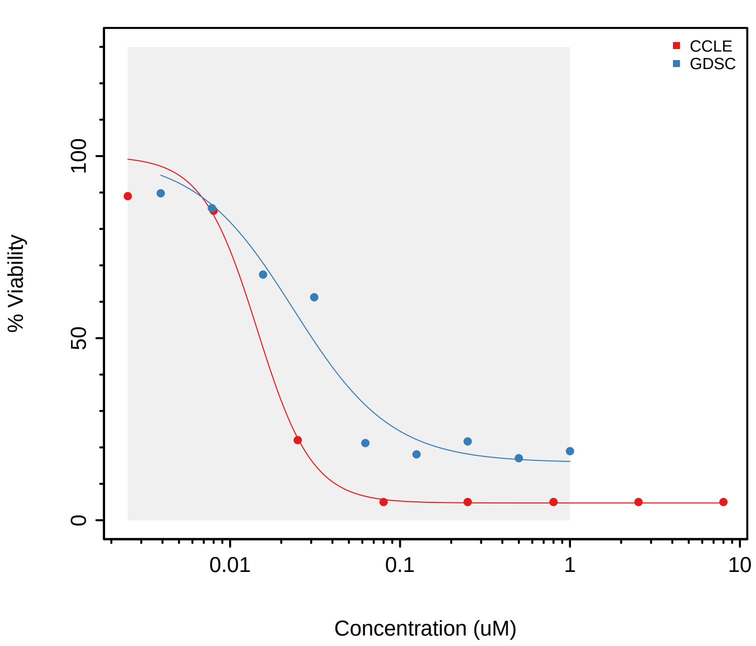

17−AAG:SW620

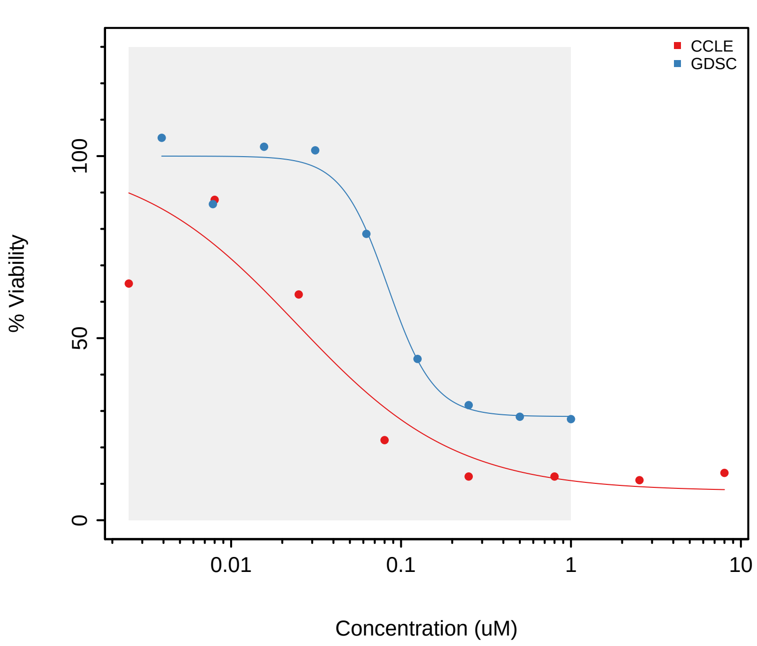

17−AAG:SW780

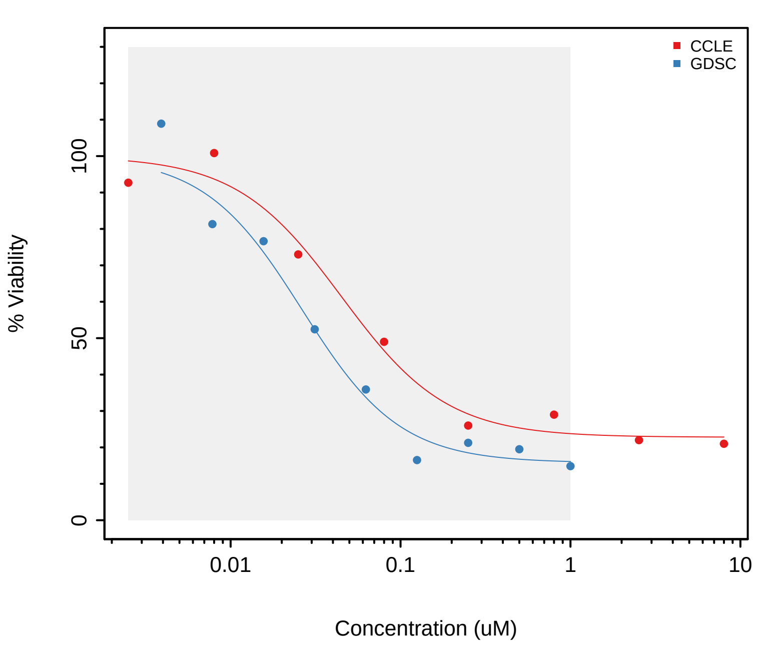

17−AAG:SW900

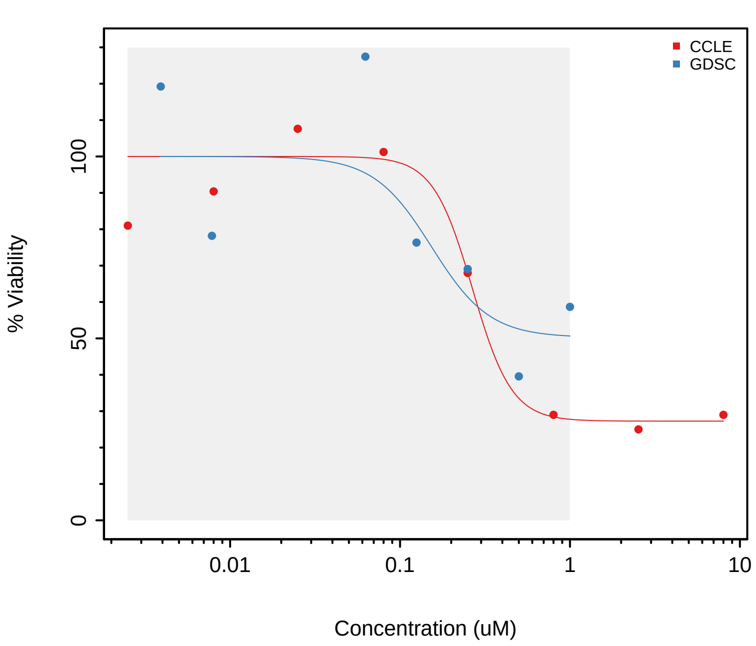

17−AAG:T−24

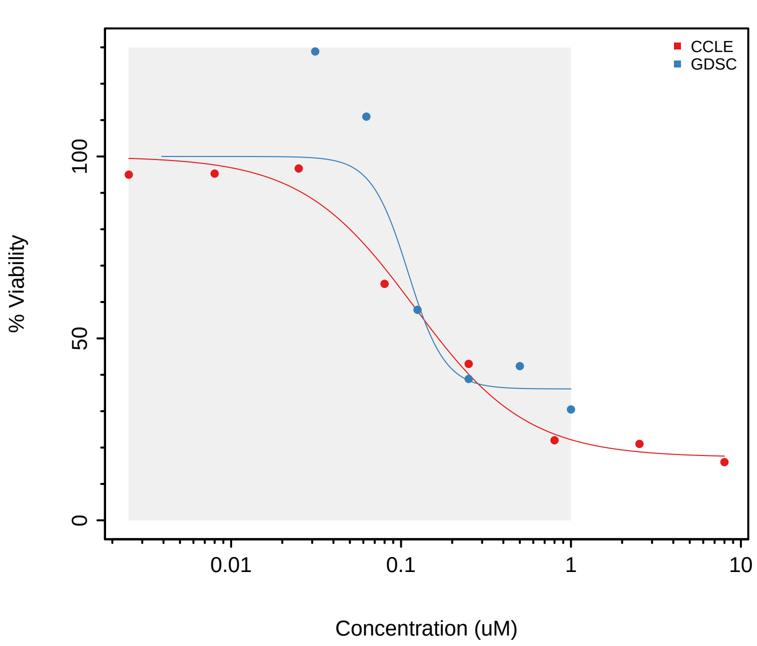

17−AAG:T47D

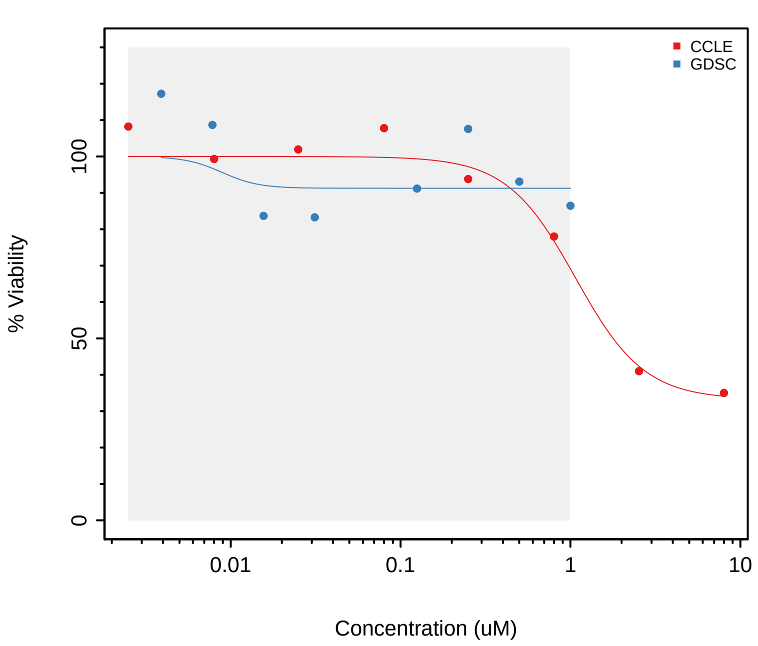

17−AAG:T84

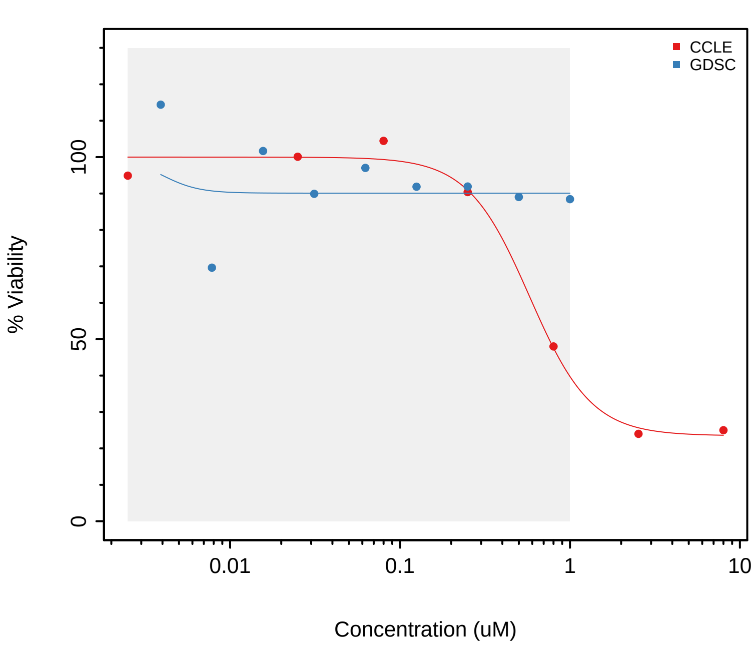

17−AAG:T98G

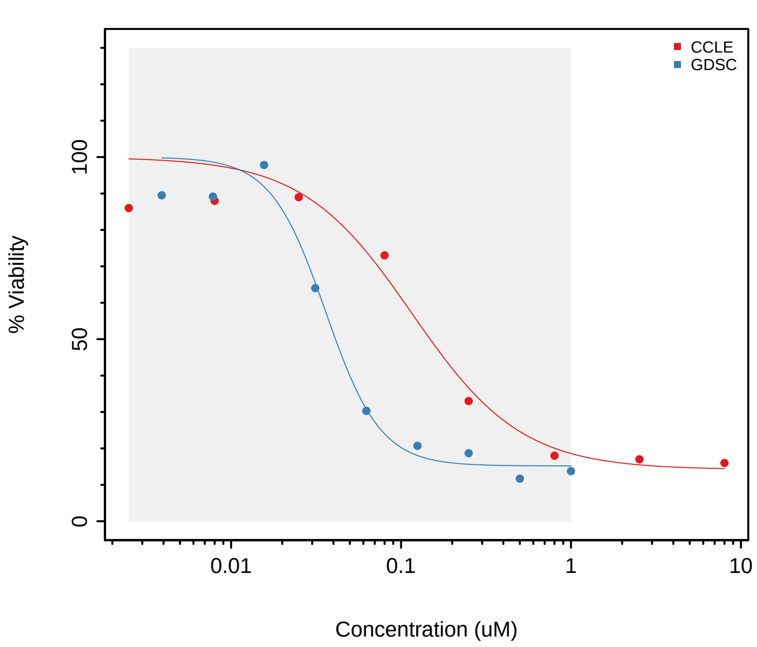

17−AAG:TCCSUP

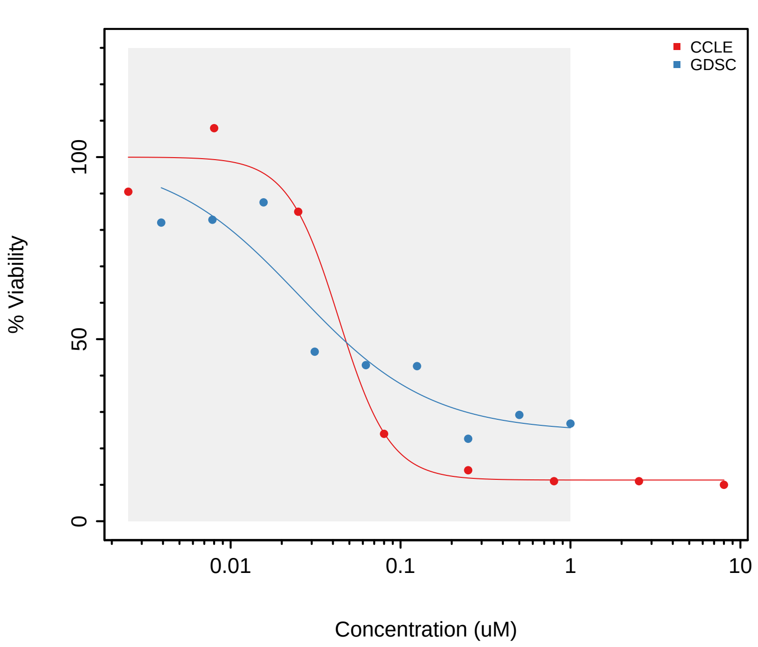

17−AAG:TE−1

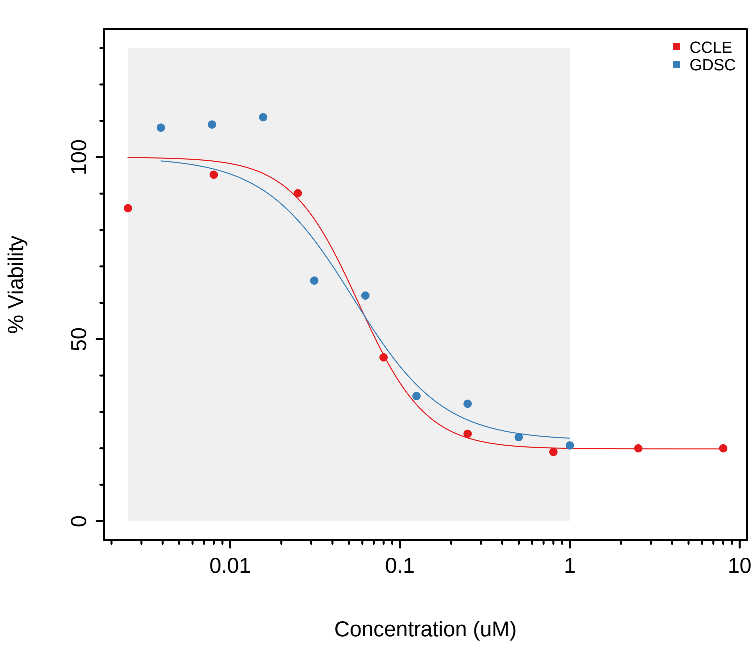

17−AAG:TE−11

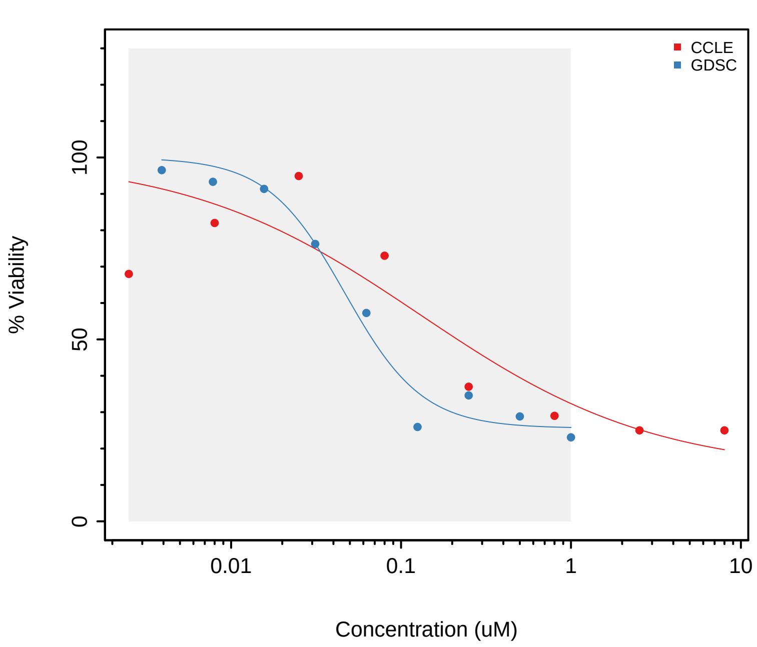

17−AAG:TE−15

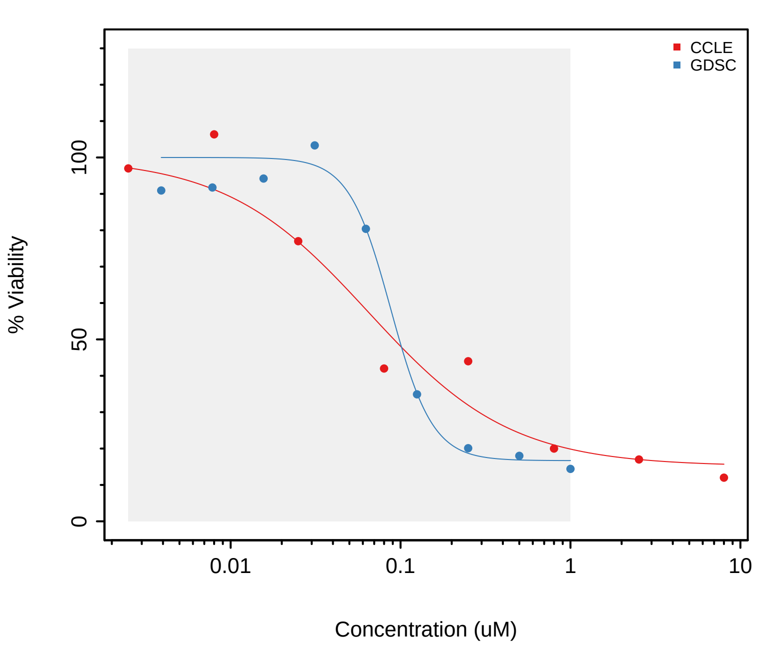

17−AAG:TE−5

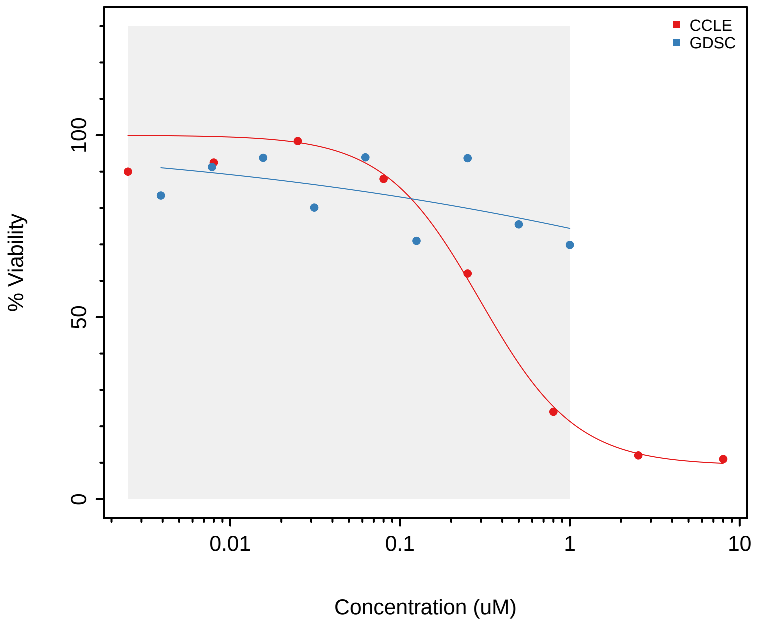

17−AAG:TE−9

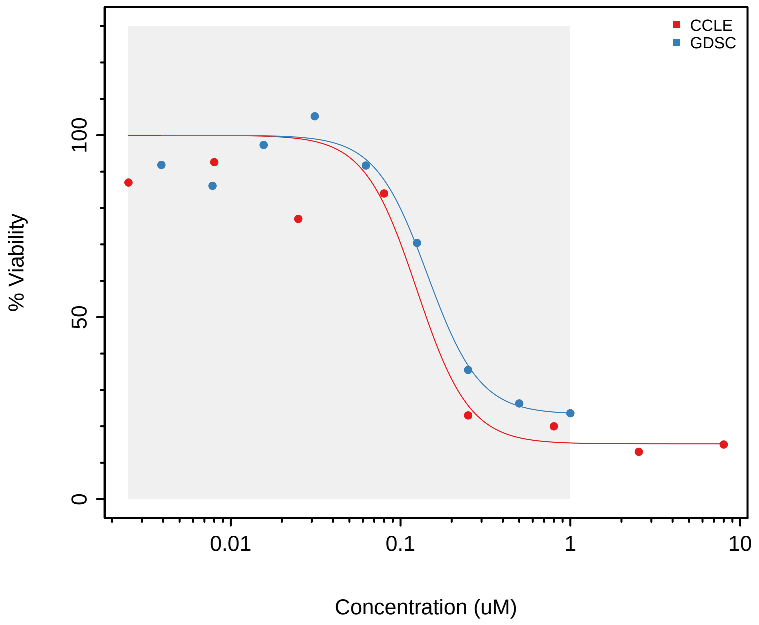

17−AAG:TYK−nu

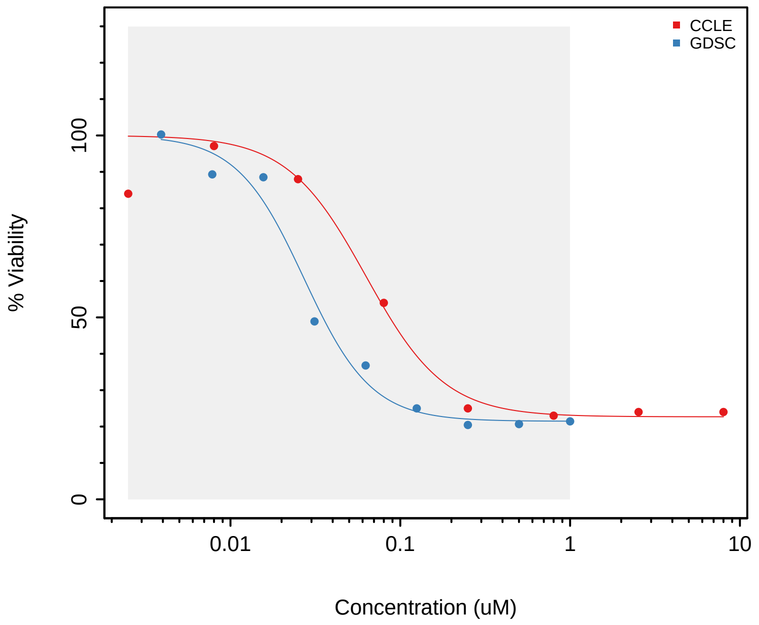

17−AAG:U−118−MG

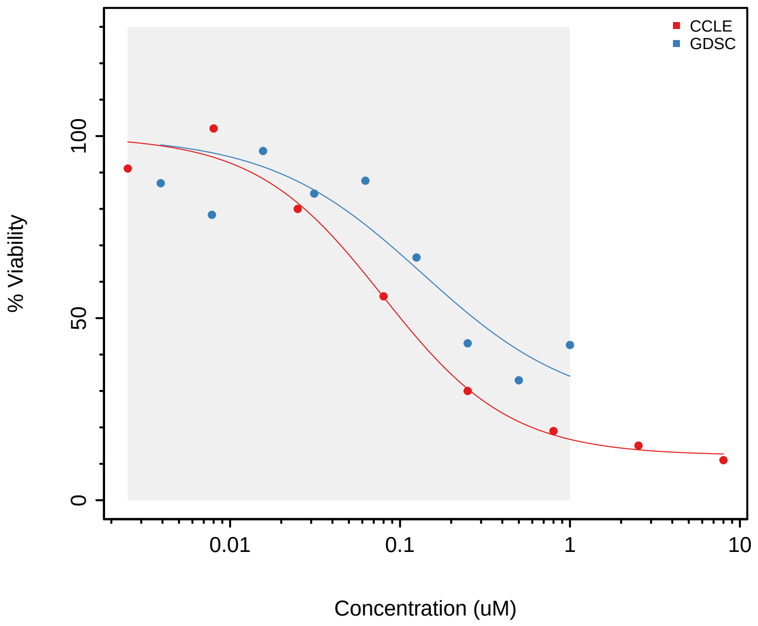

17−AAG:U−2−OS

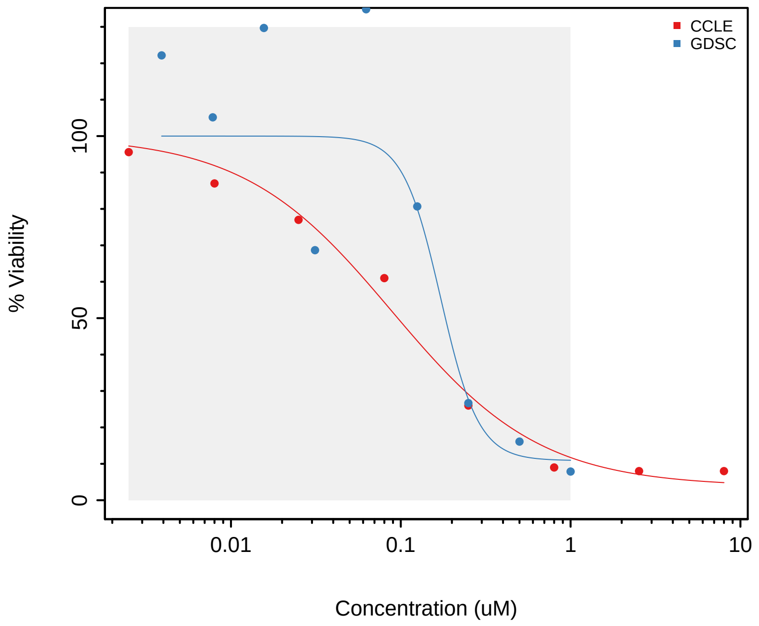

17−AAG:U−87−MG

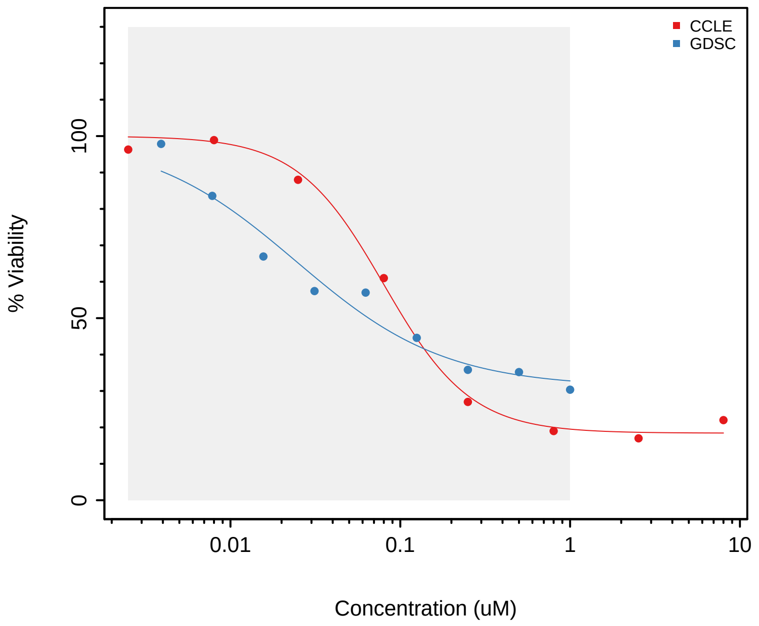

17−AAG:UACC−257

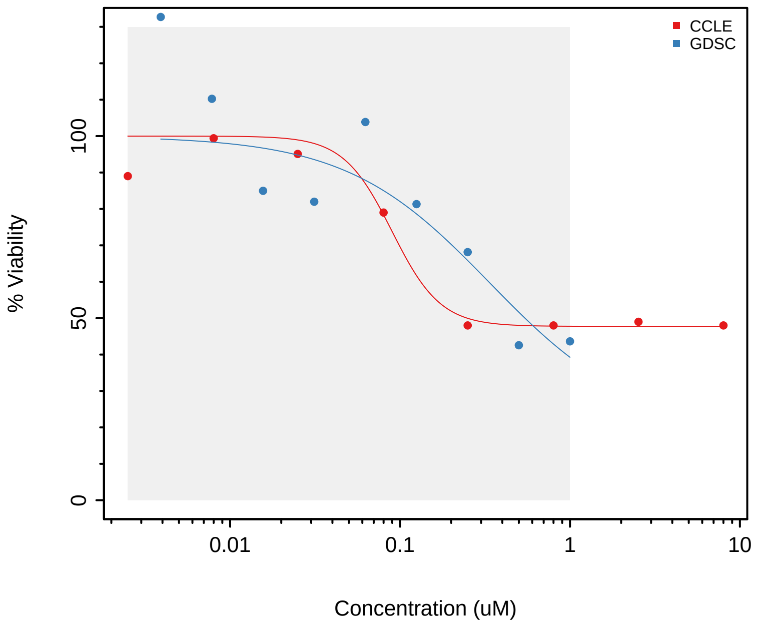

17−AAG:UACC−62

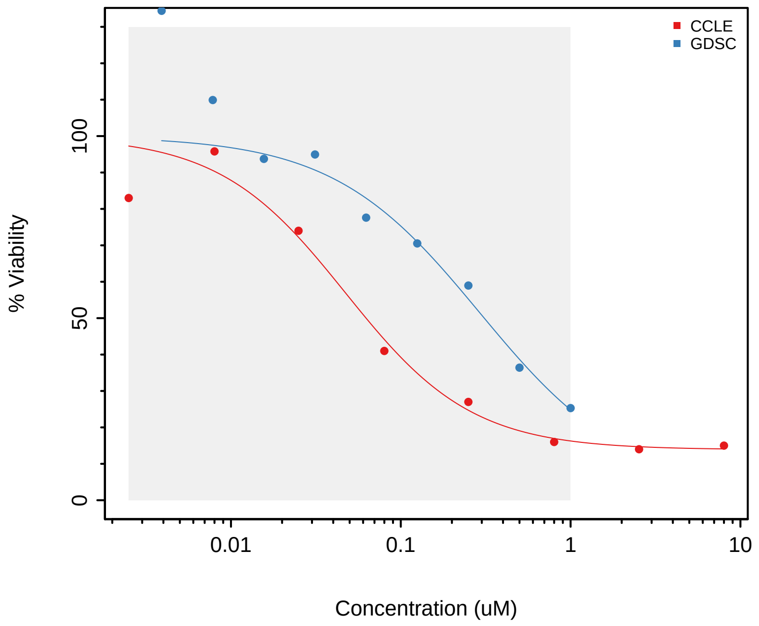

17−AAG:UM−UC−3

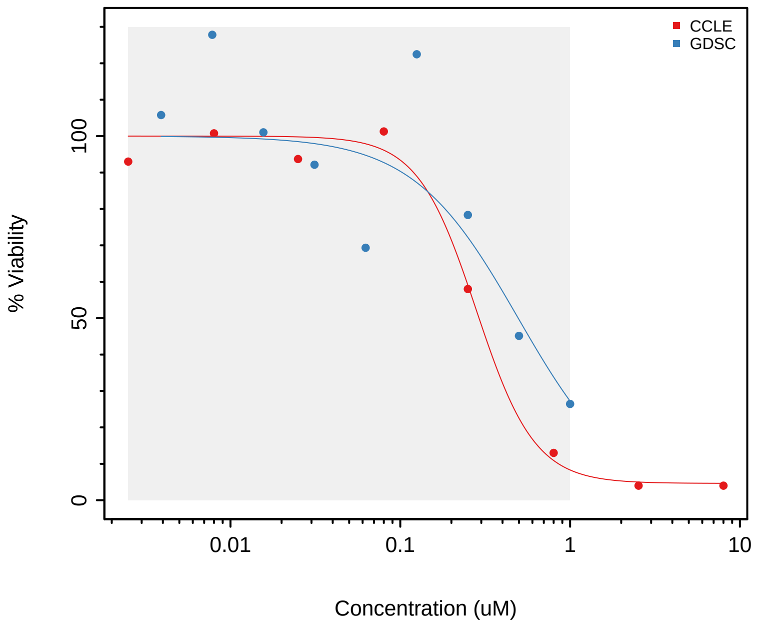

17−AAG:VMRC−RCZ

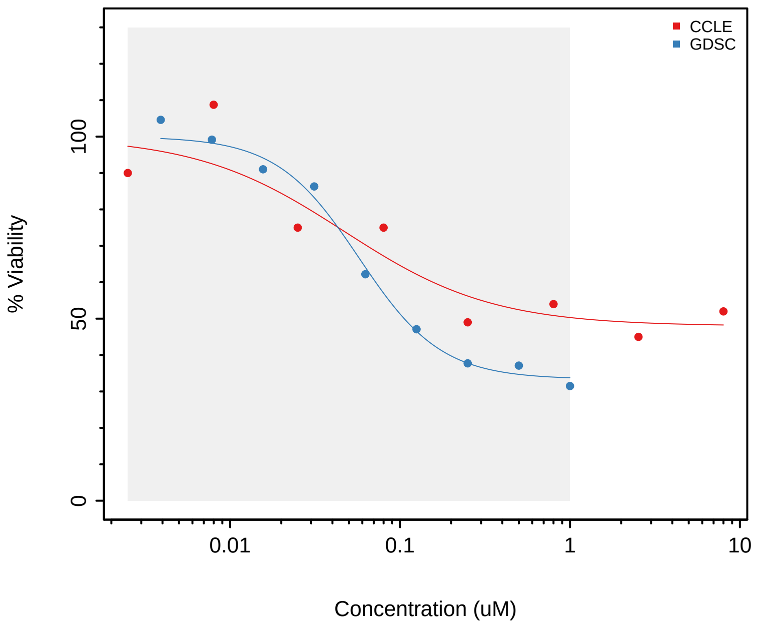

17−AAG:WM−115

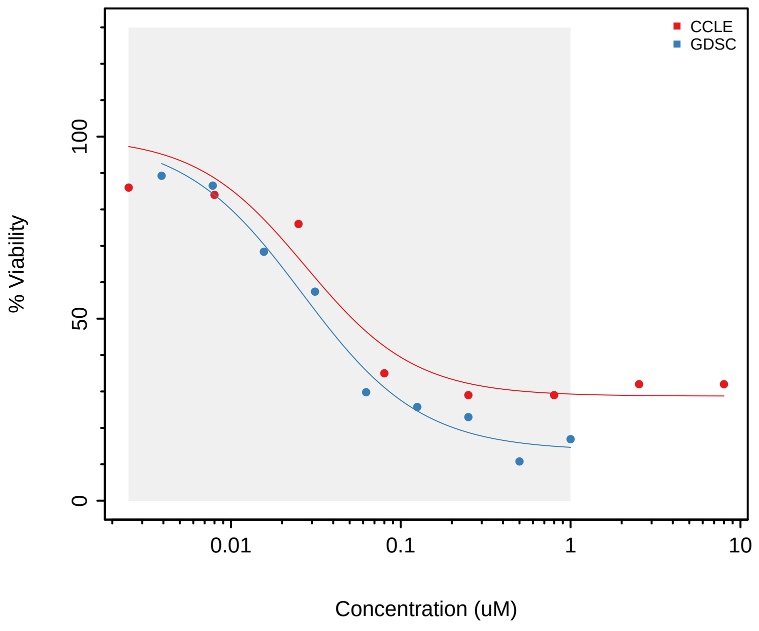

17−AAG:YKG−1

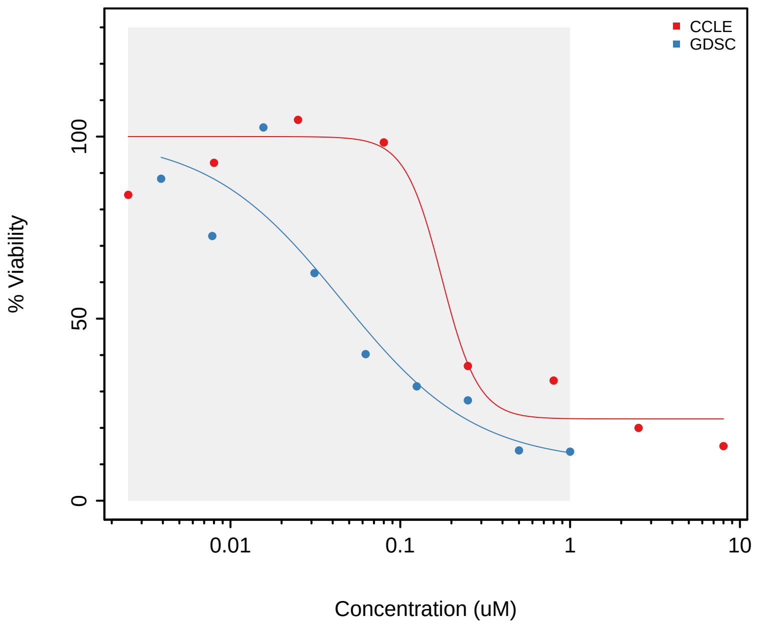

17−AAG:ZR−75−30

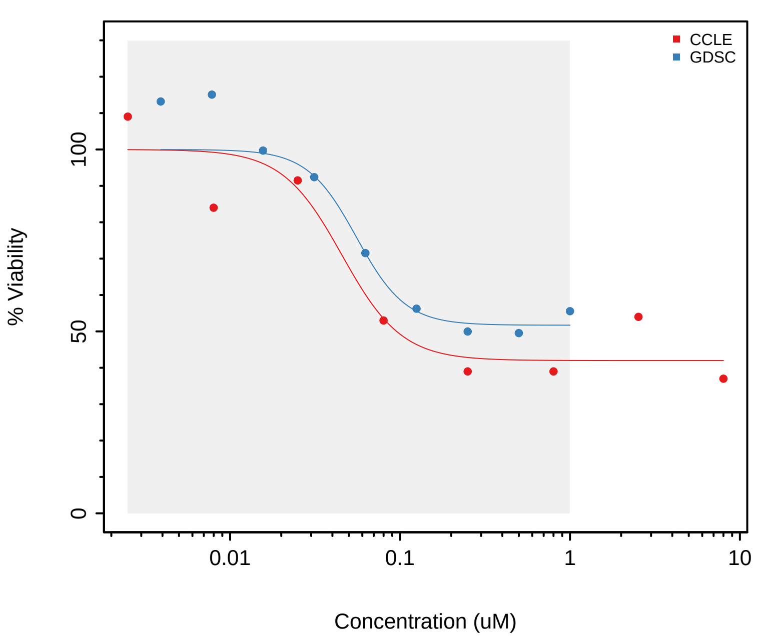

PHA−665752:697

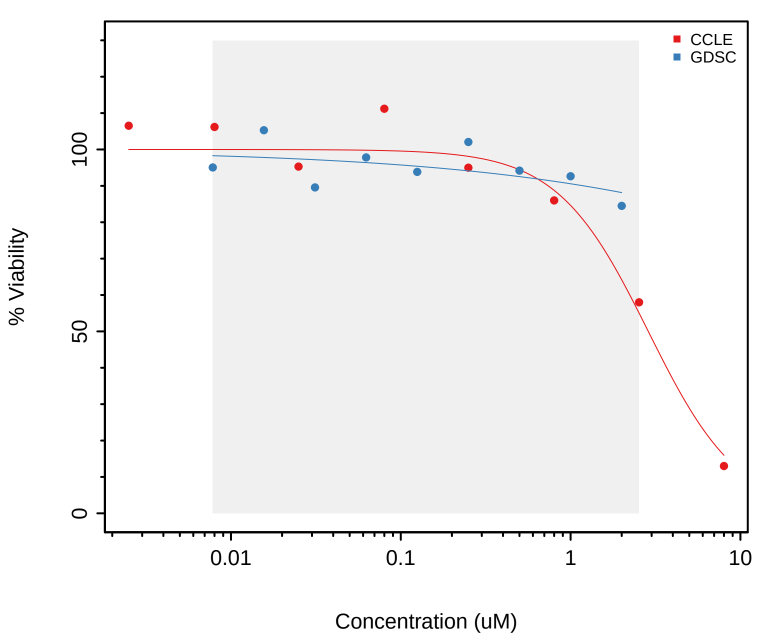

PHA−665752:8−MG−BA

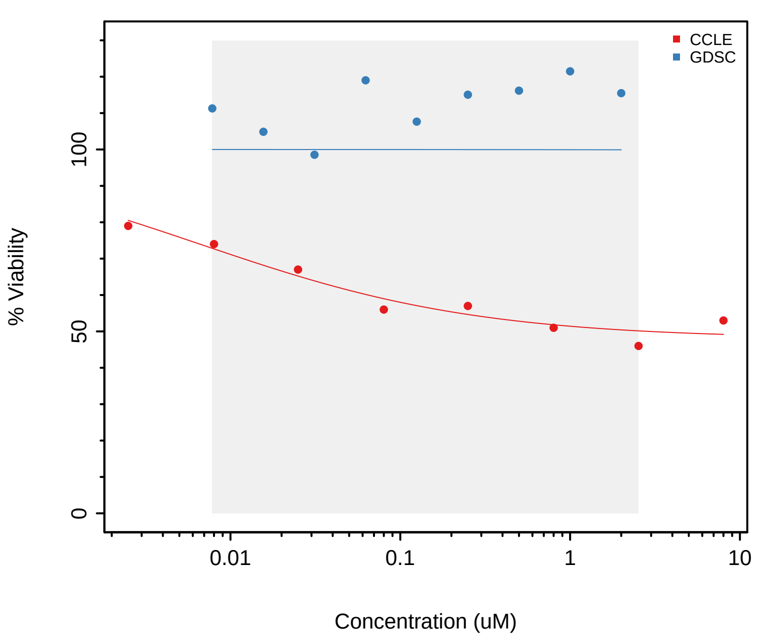

PHA−665752:A253

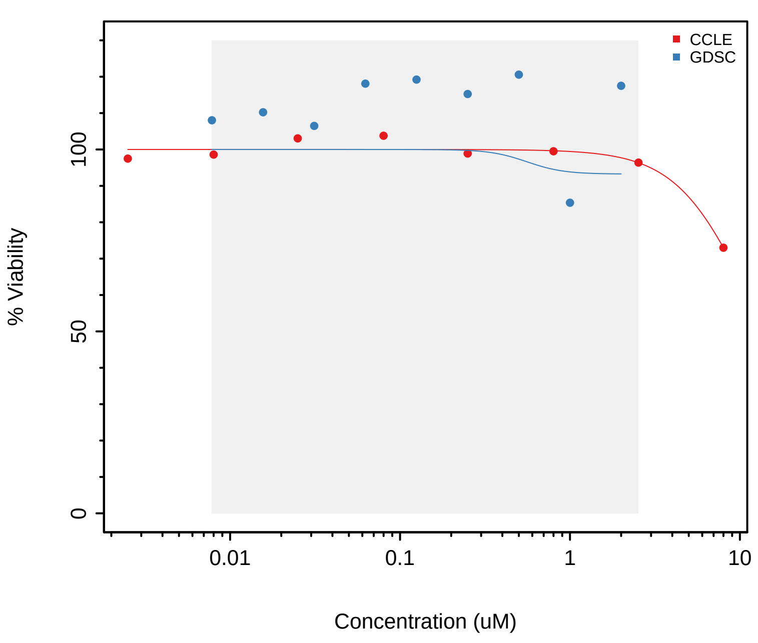

PHA−665752:BL−41

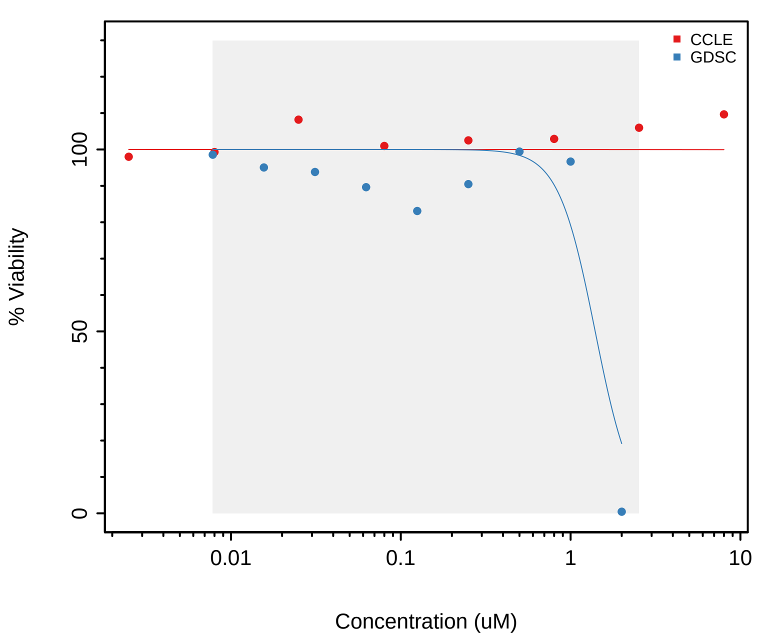

PHA−665752:BL−70

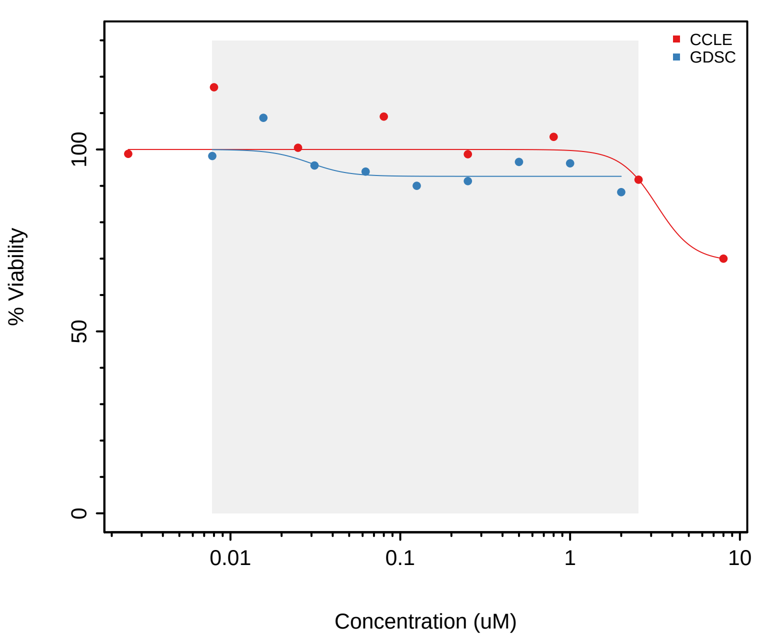

PHA−665752:BT−474

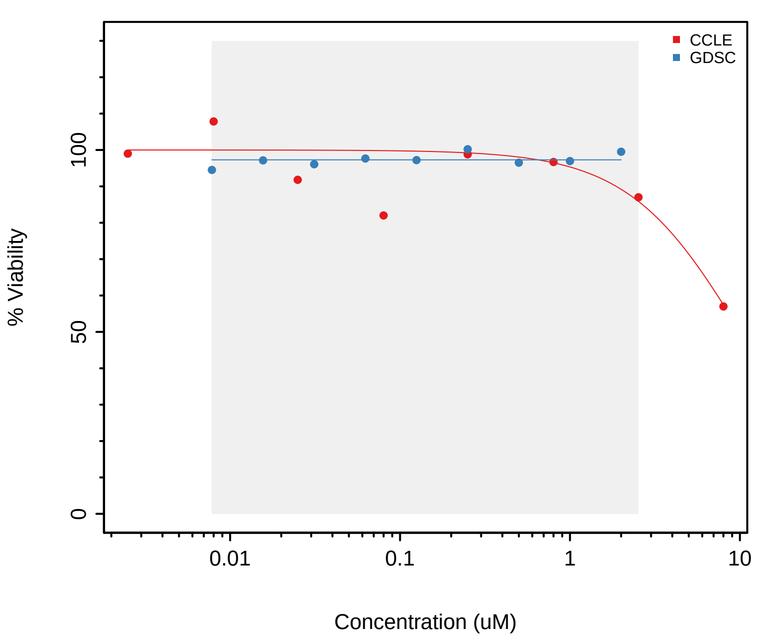

PHA−665752:C2BBe1

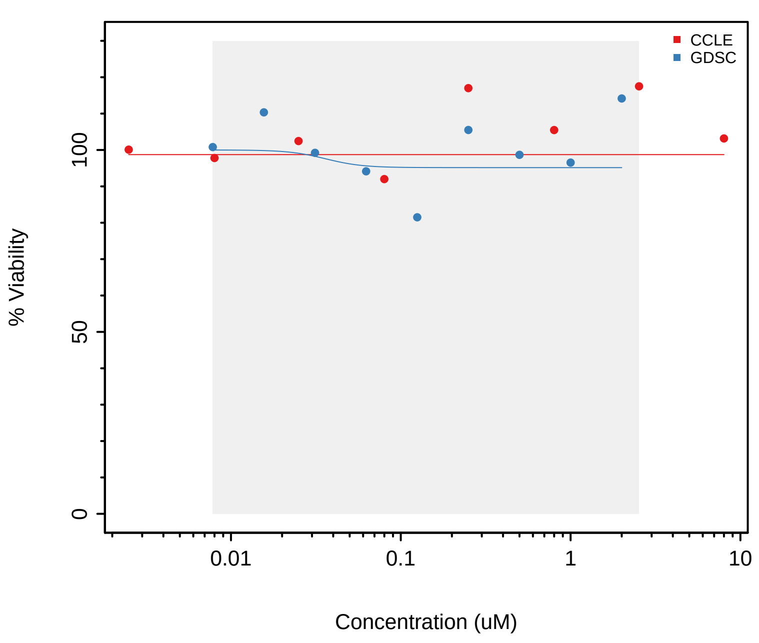

PHA−665752:Calu−6

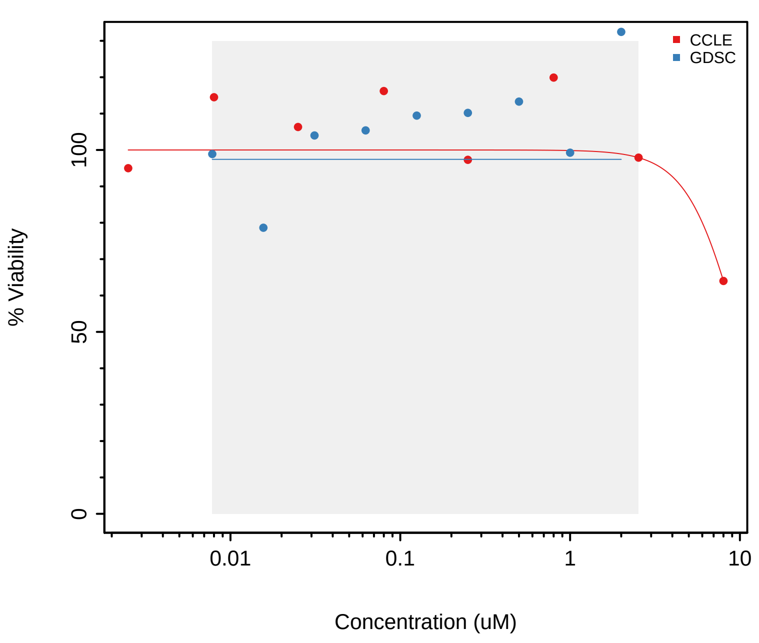

PHA−665752:CAS−1

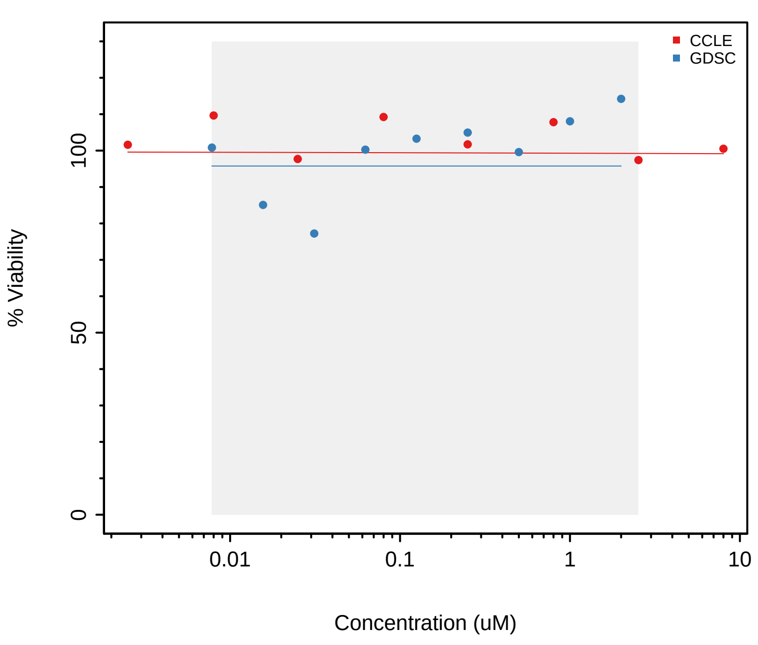

PHA−665752:COLO−320−HSR

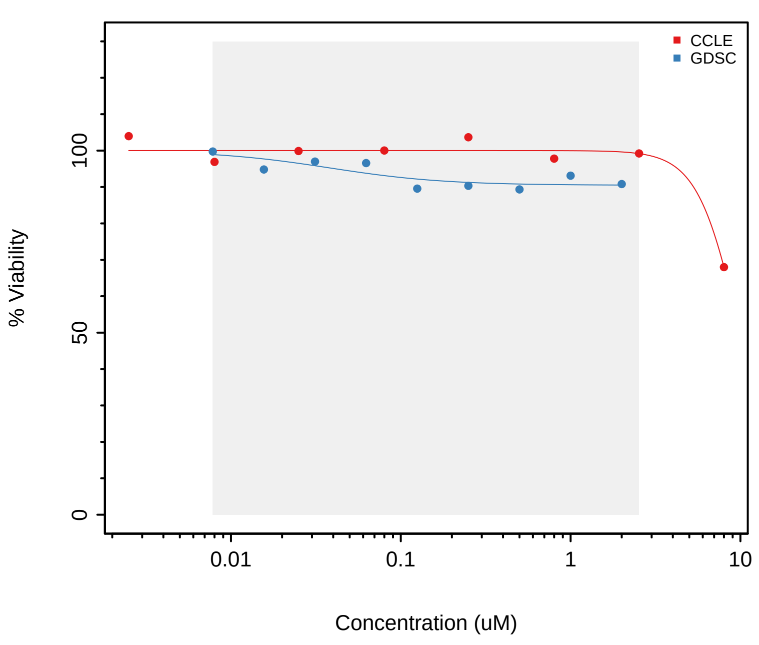

PHA−665752:DEL

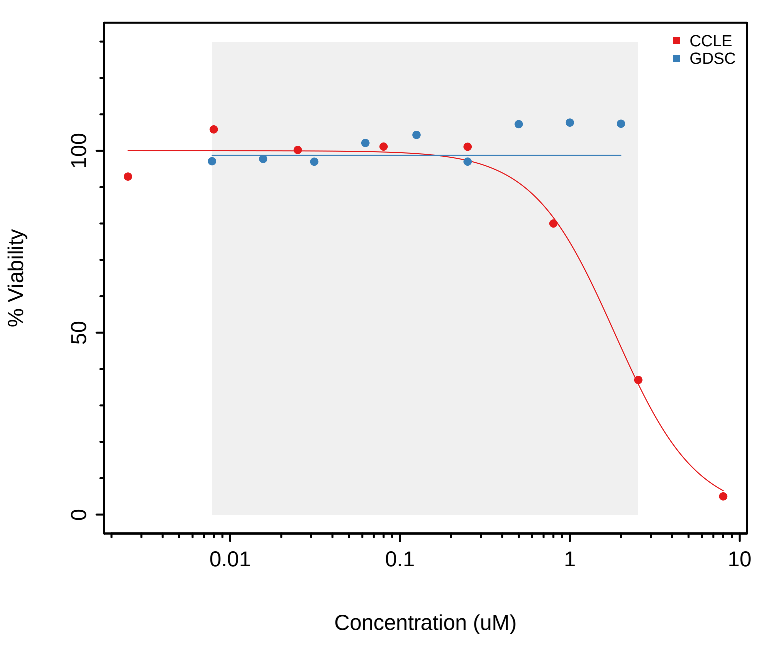

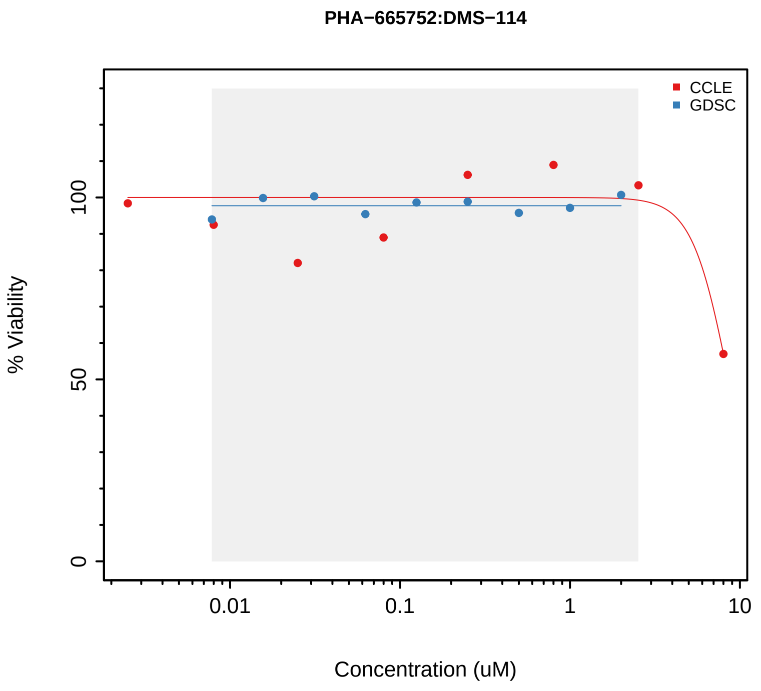
PHA−665752:DMS−114
CCLE
GDSC
% Viability
Concentration (uM)

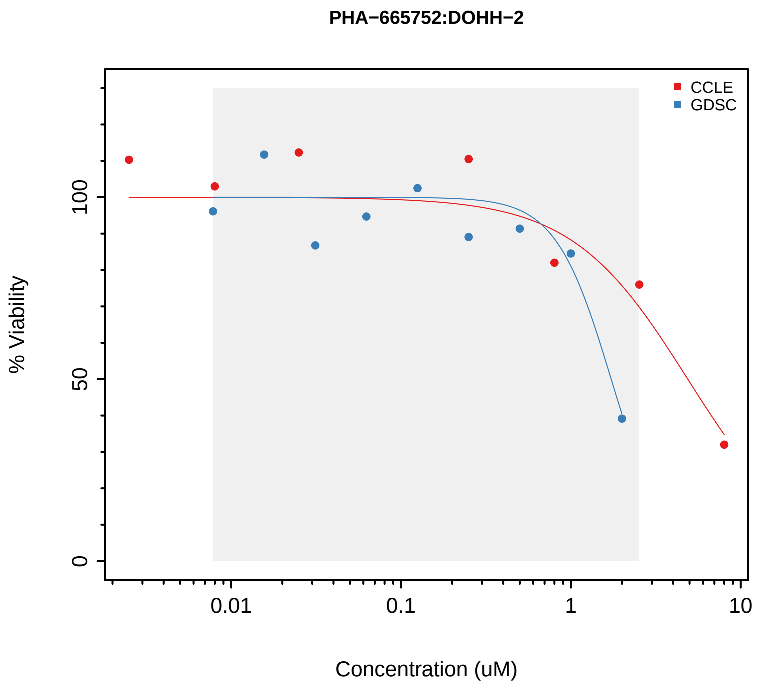
PHA−665752:DOHH−2
CCLE
GDSC
% Viability
Concentration (uM)

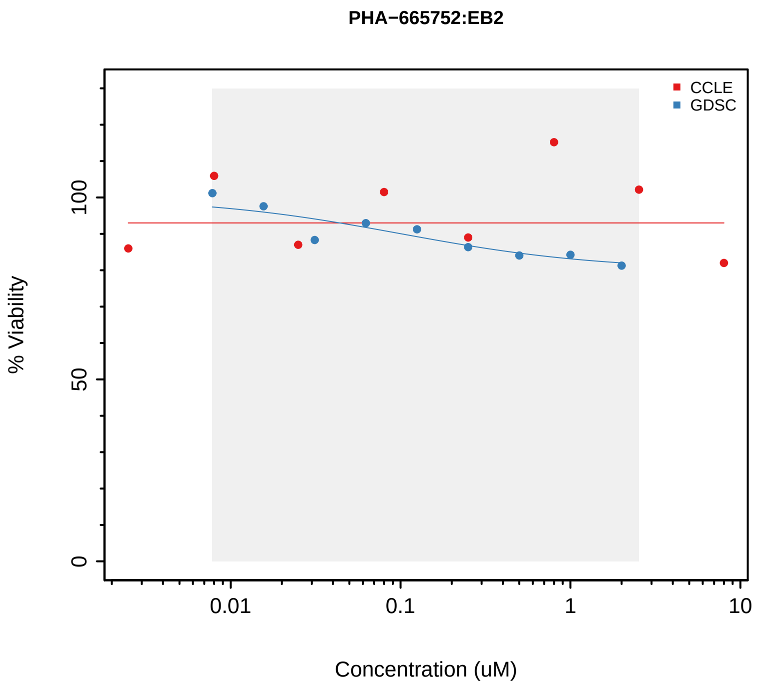
PHA−665752:EB2
CCLE
GDSC
% Viability
Concentration (uM)

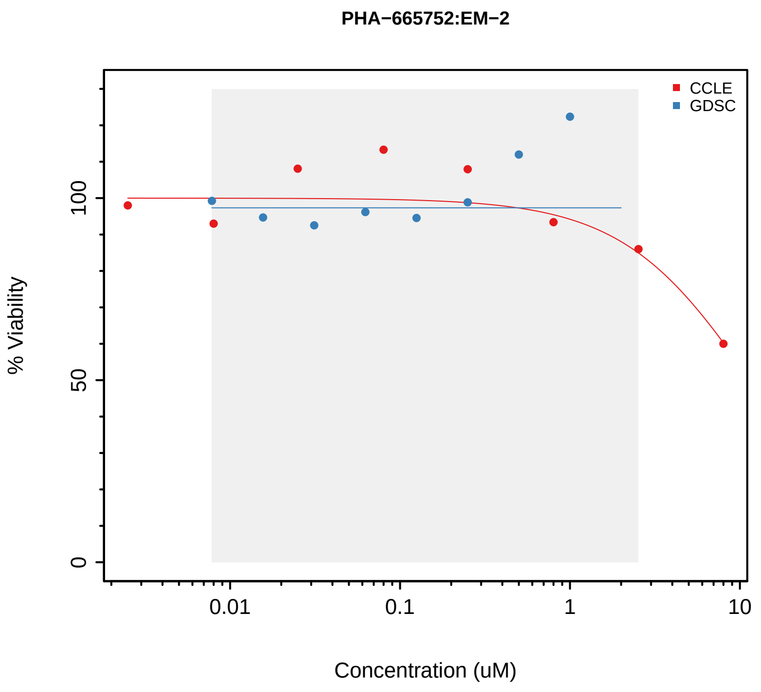
PHA−665752:EM−2
CCLE
GDSC
% Viability
Concentration (uM)

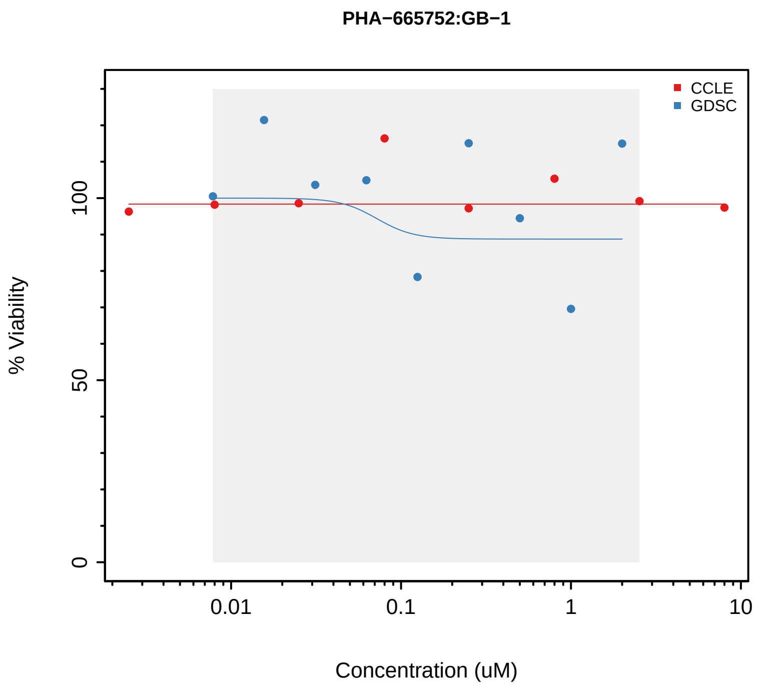
PHA−665752:GB−1
CCLE
GDSC
% Viability
Concentration (uM)

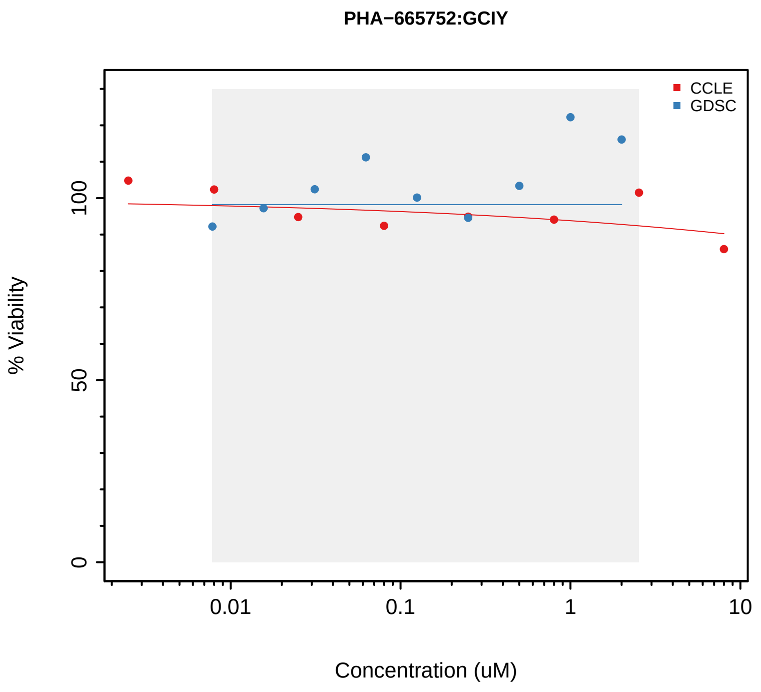
PHA−665752:GCIY
CCLE
GDSC
% Viability
Concentration (uM)

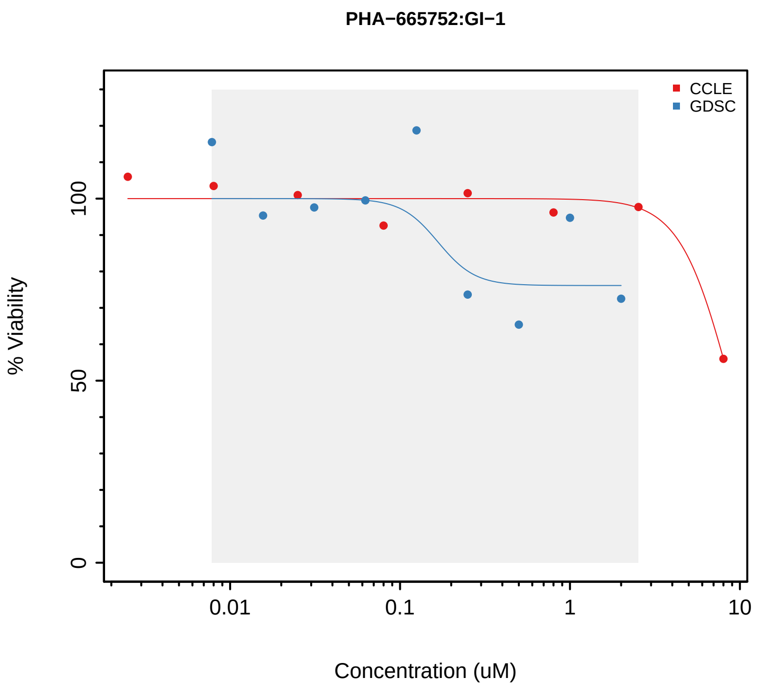
PHA−665752:GI−1
CCLE
GDSC
% Viability
Concentration (uM)

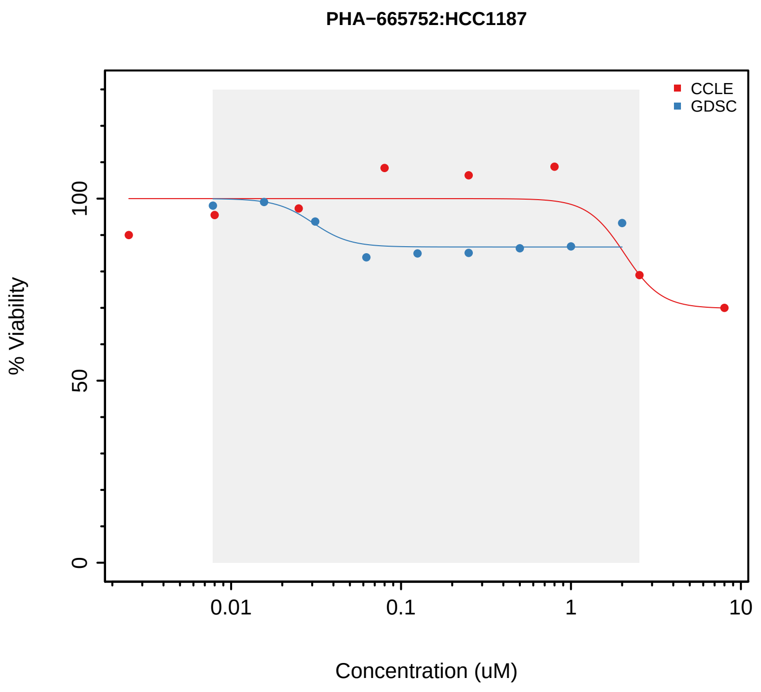
PHA−665752:HCC1187
CCLE
GDSC
% Viability
Concentration (uM)

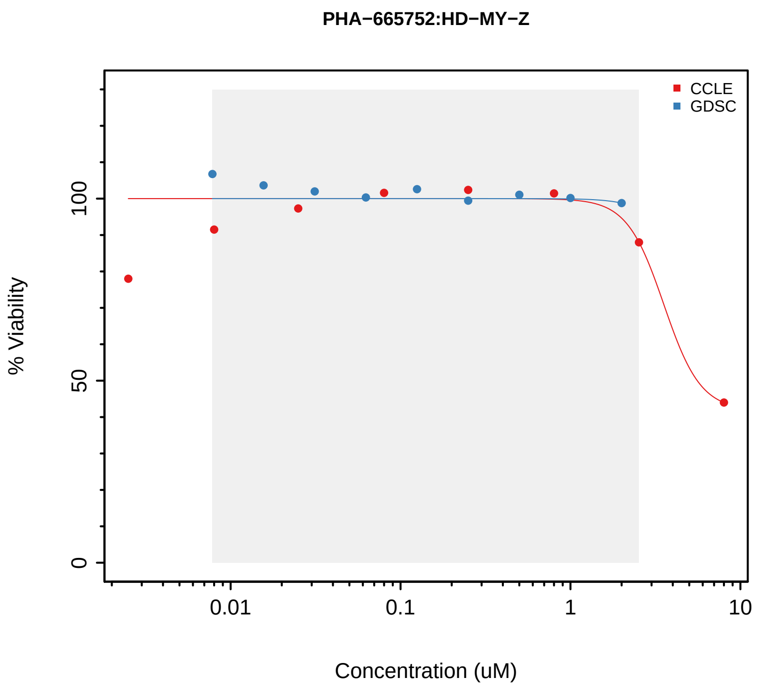
PHA−665752:HD−MY−Z
CCLE
GDSC
% Viability
Concentration (uM)

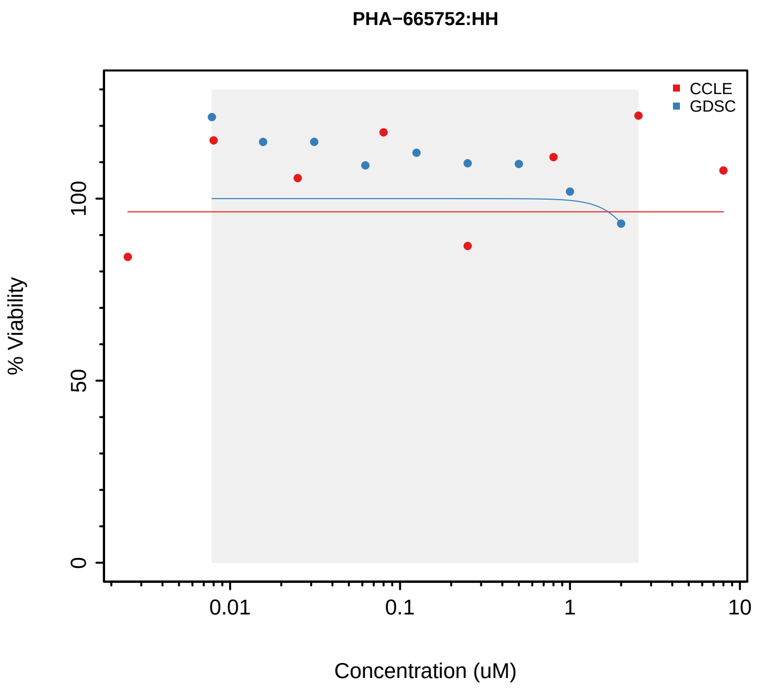
PHA−665752:HH
CCLE
GDSC
% Viability
Concentration (uM)

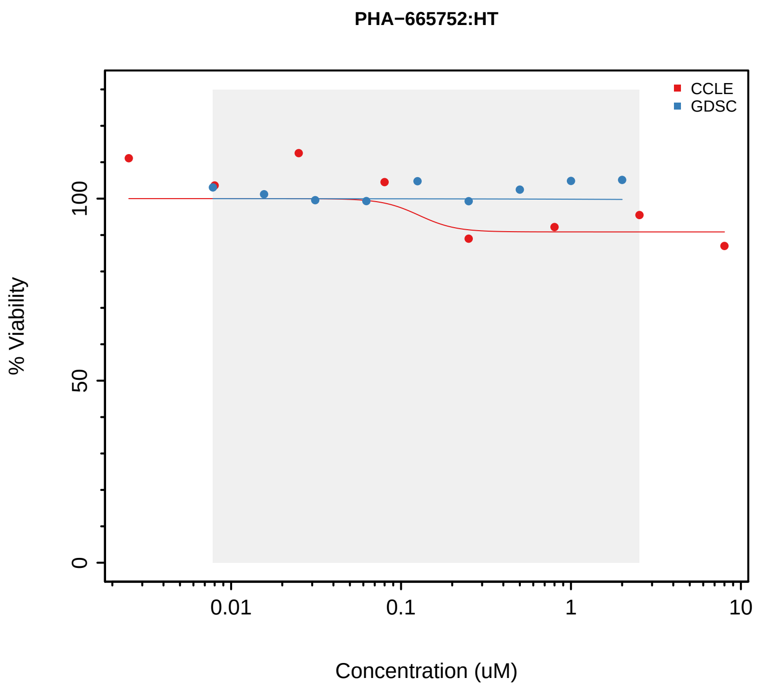
PHA−665752:HT
CCLE
GDSC
% Viability
Concentration (uM)

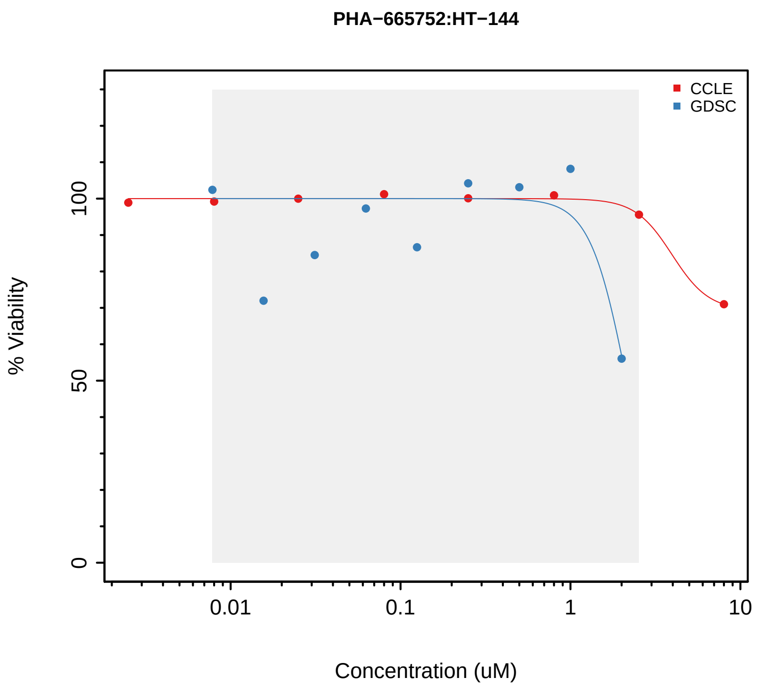
PHA−665752:HT−144
CCLE
GDSC
% Viability
Concentration (uM)

PHA−665752:IA−LM

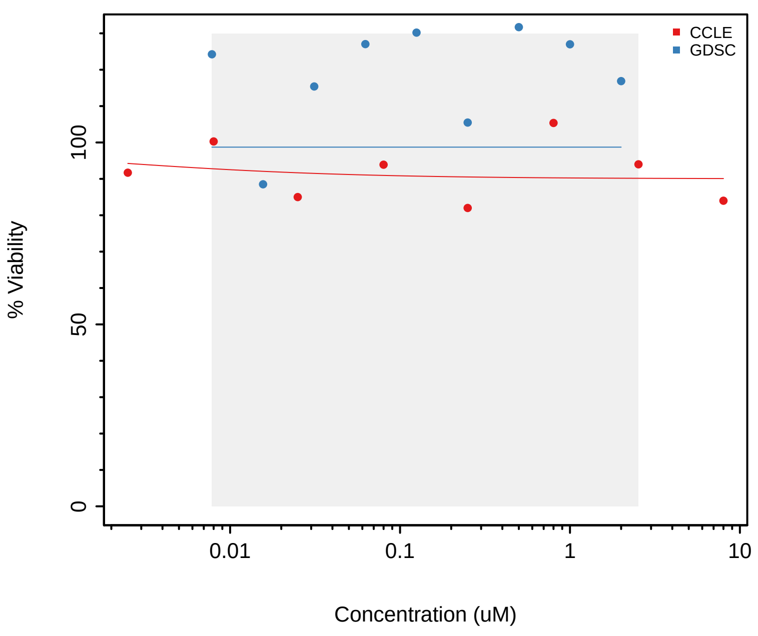

PHA−665752:IST−MES1

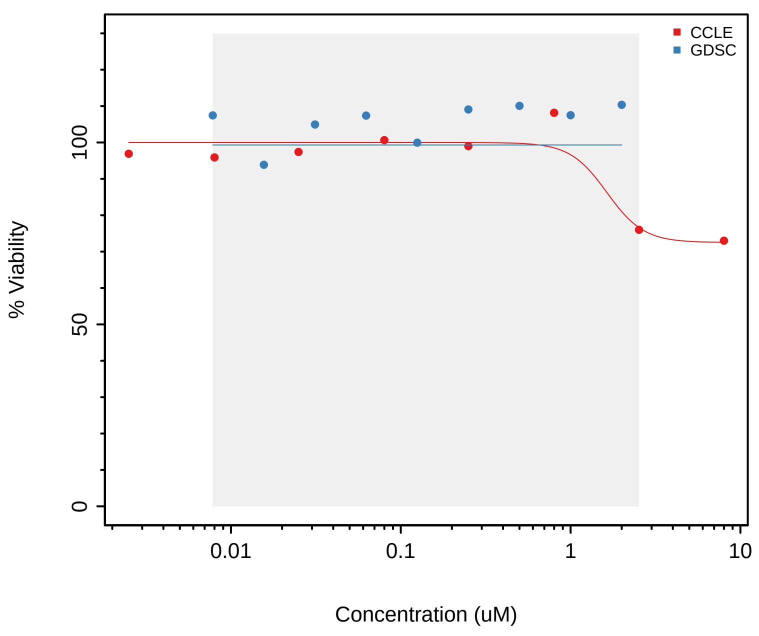

PHA−665752:JVM−3

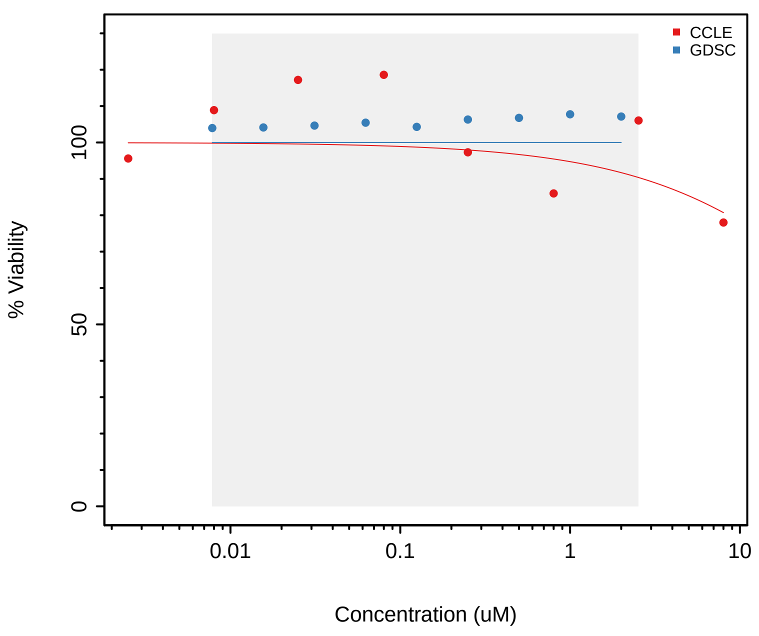

PHA−665752:KALS−1

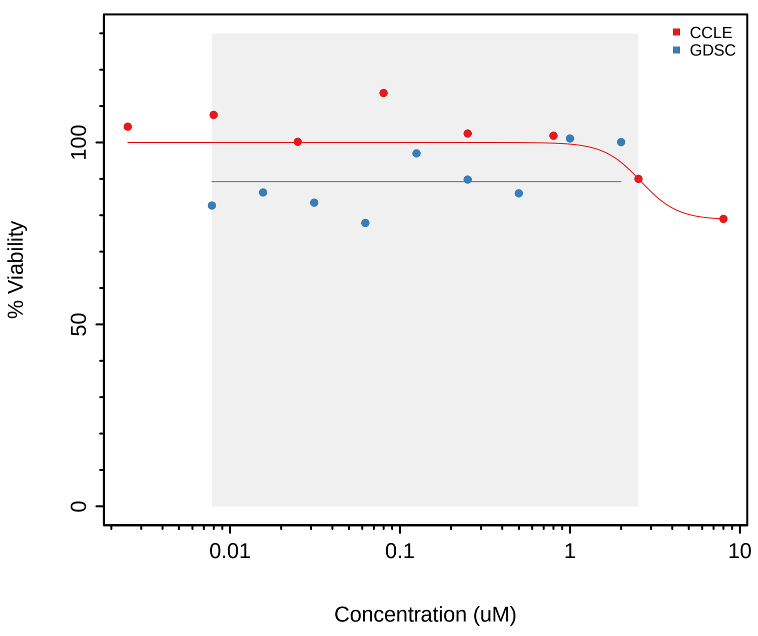

PHA−665752:KARPAS−299

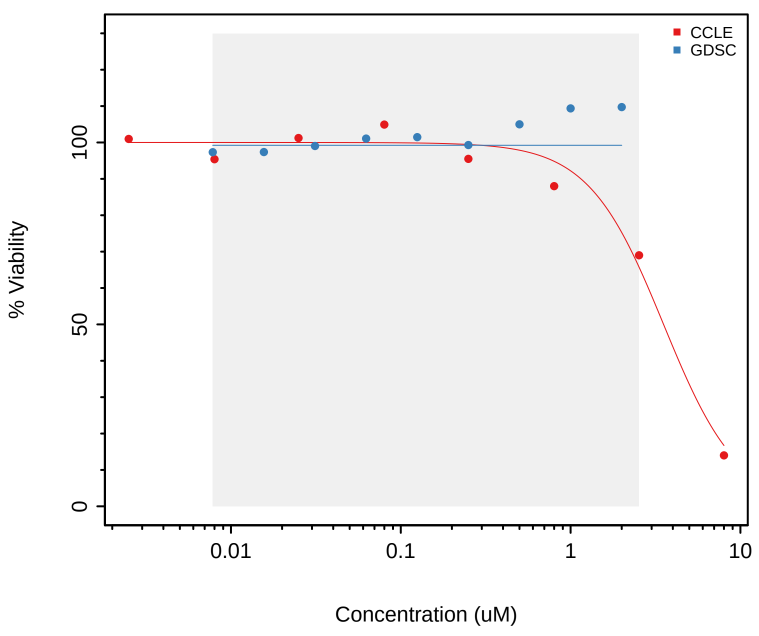

PHA−665752:KARPAS−422

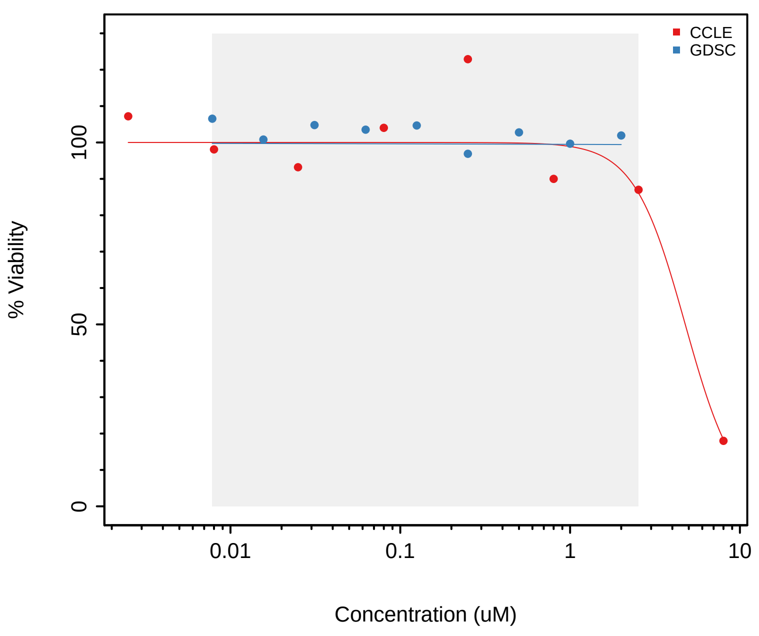

PHA−665752:KG−1

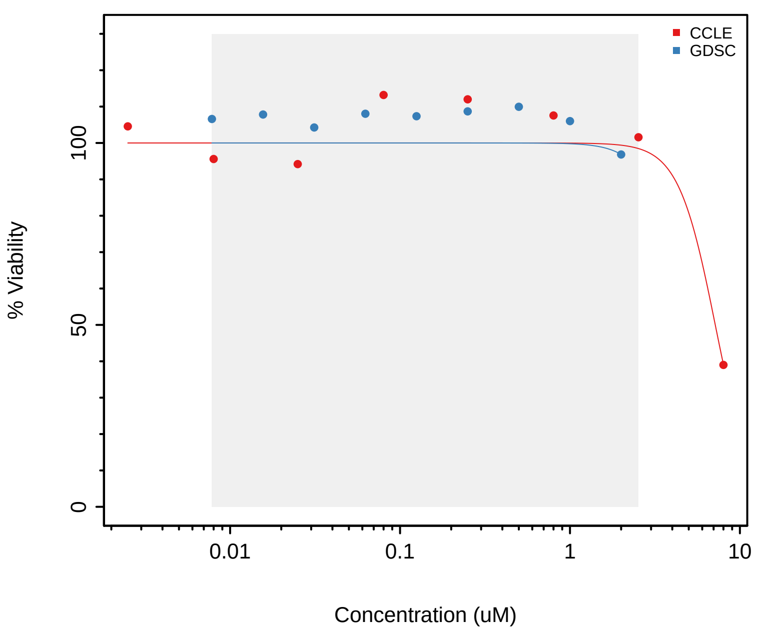

PHA−665752:KLE

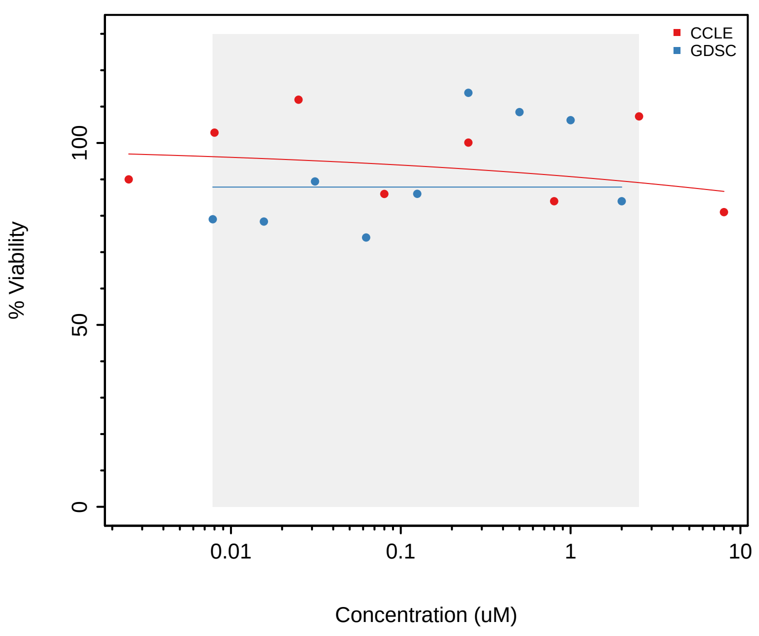

PHA−665752:KNS−42

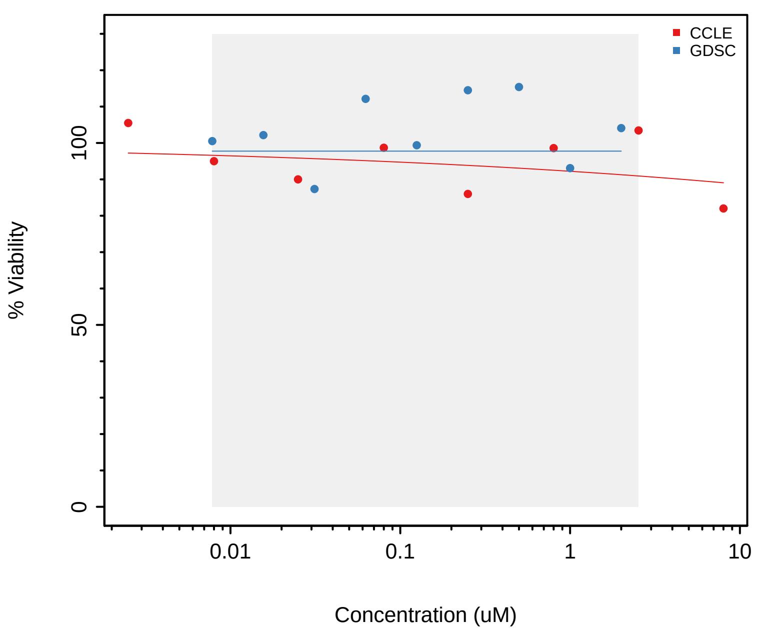

PHA−665752:KNS−81−FD

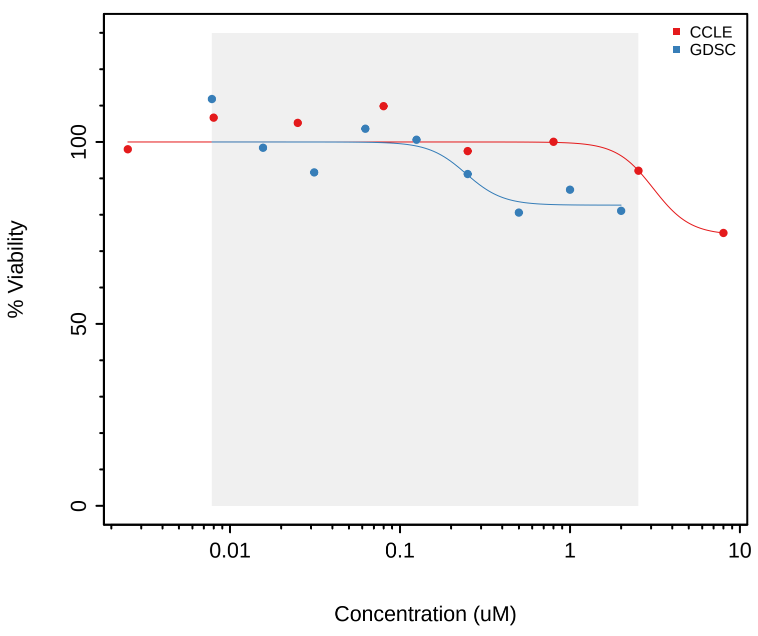

PHA−665752:K052

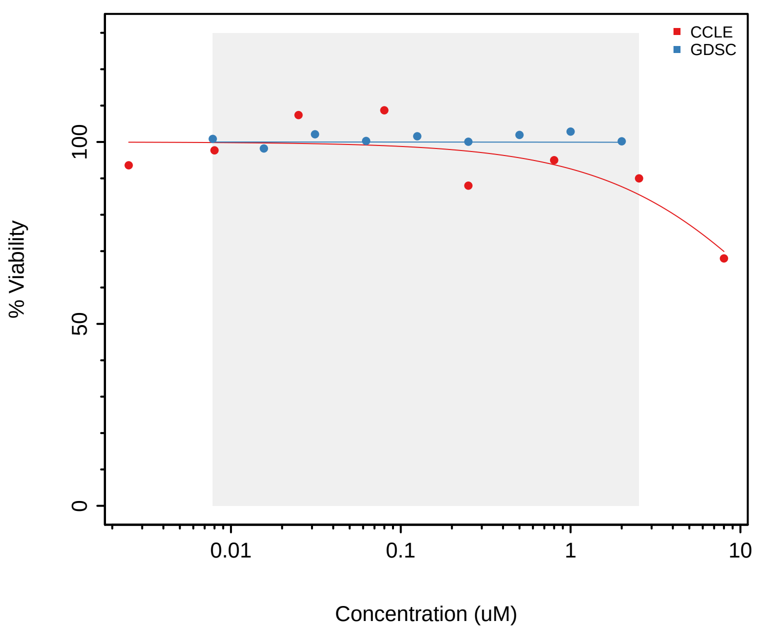

PHA−665752:KU812

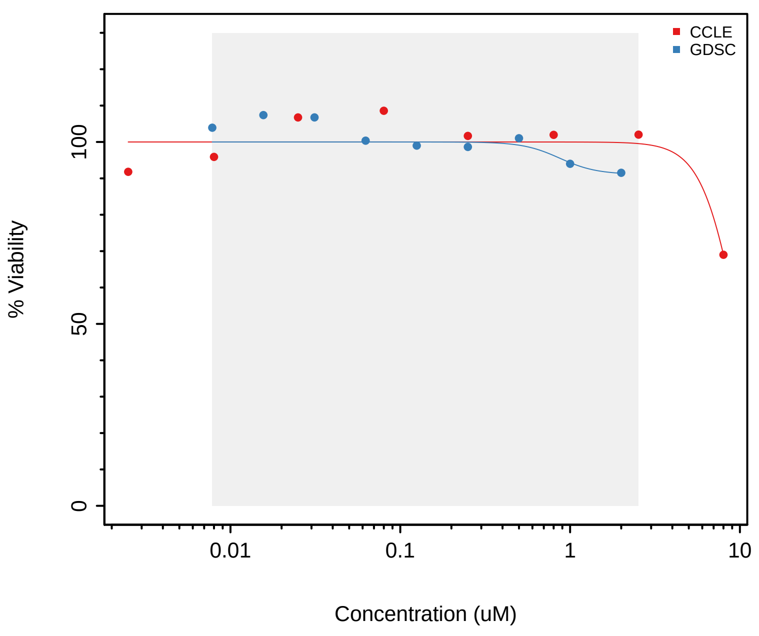

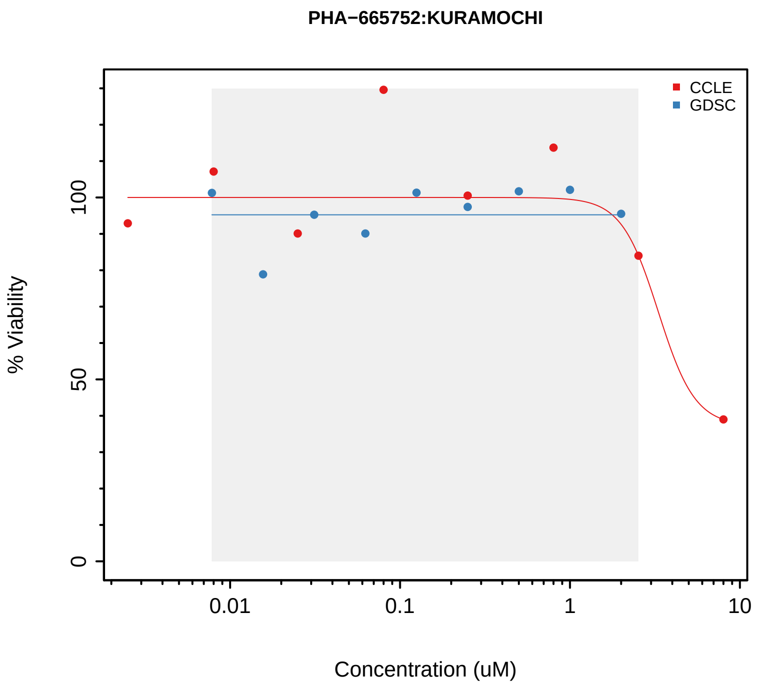
PHA−665752:KURAMOCHI
CCLE
GDSC
% Viability
0
50
100
0.01
0.1
1
10
Concentration (uM)

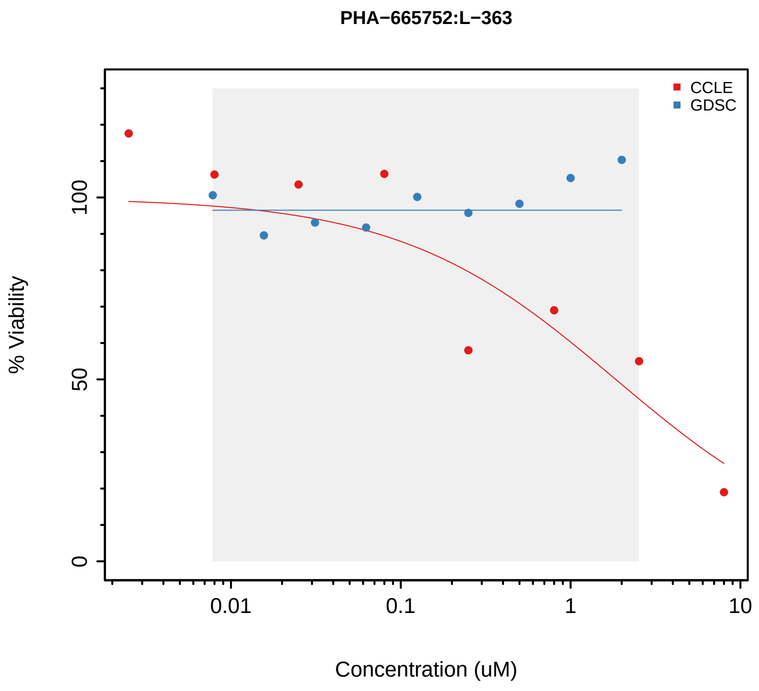
PHA−665752:L−363
CCLE
GDSC
% Viability
0
50
100
0.01
0.1
1
10
Concentration (uM)

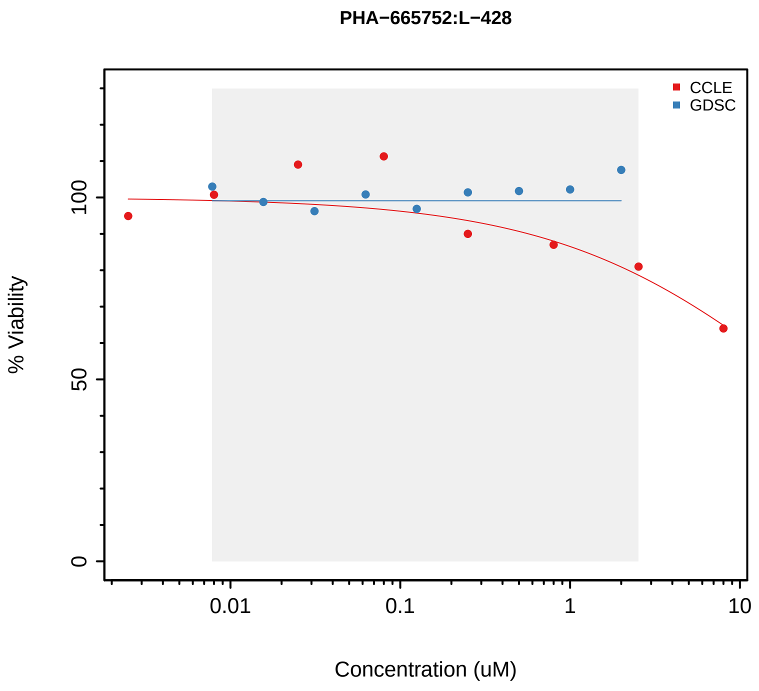
PHA−665752:L−428
CCLE
GDSC
% Viability
0
50
100
0.01
0.1
1
10
Concentration (uM)

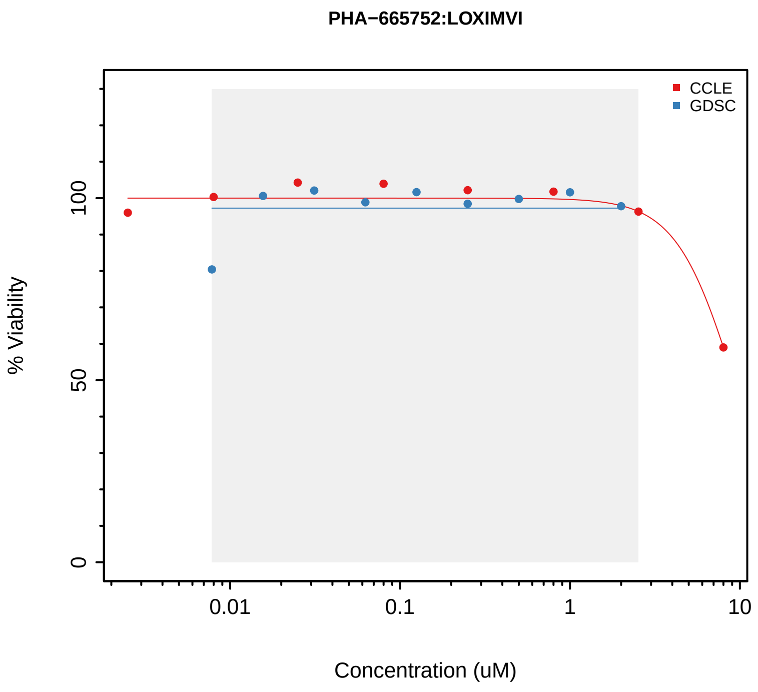
PHA−665752:LOXIMVI
CCLE
GDSC
% Viability
0
50
100
0.01
0.1
1
10
Concentration (uM)

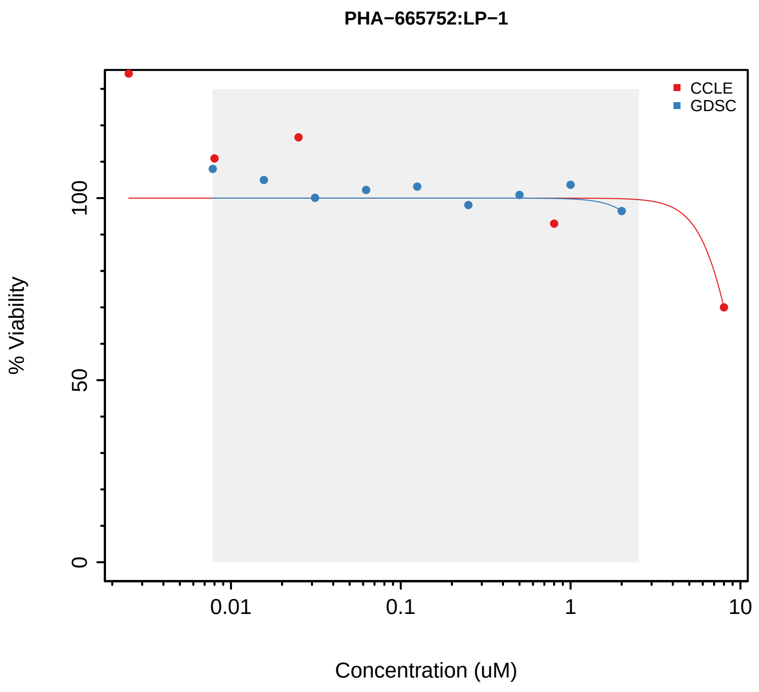
PHA−665752:LP−1
CCLE
GDSC
% Viability
0
50
100
0.01
0.1
1
10
Concentration (uM)

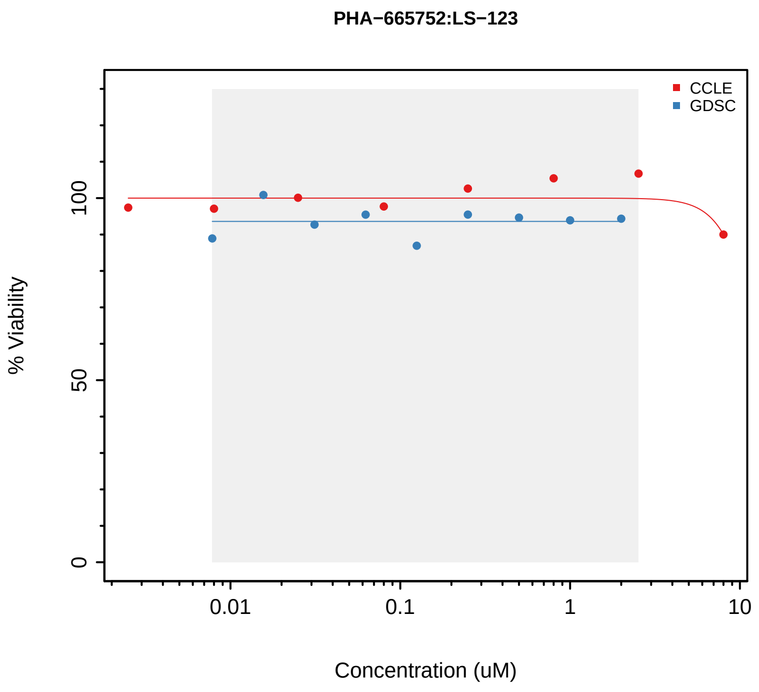
PHA−665752:LS−123
CCLE
GDSC
% Viability
0
50
100
0.01
0.1
1
10
Concentration (uM)

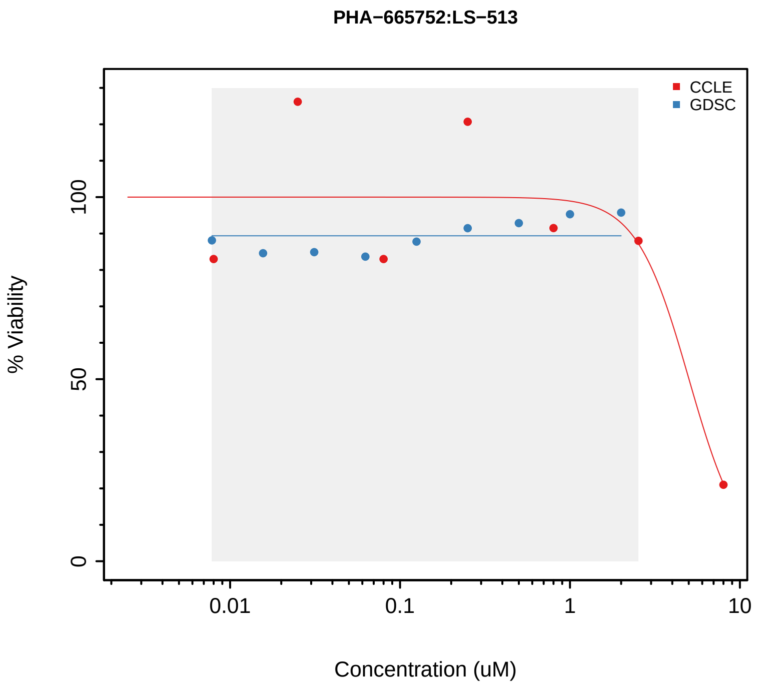
PHA−665752:LS−513
CCLE
GDSC
% Viability
0
50
100
0.01
0.1
1
10
Concentration (uM)

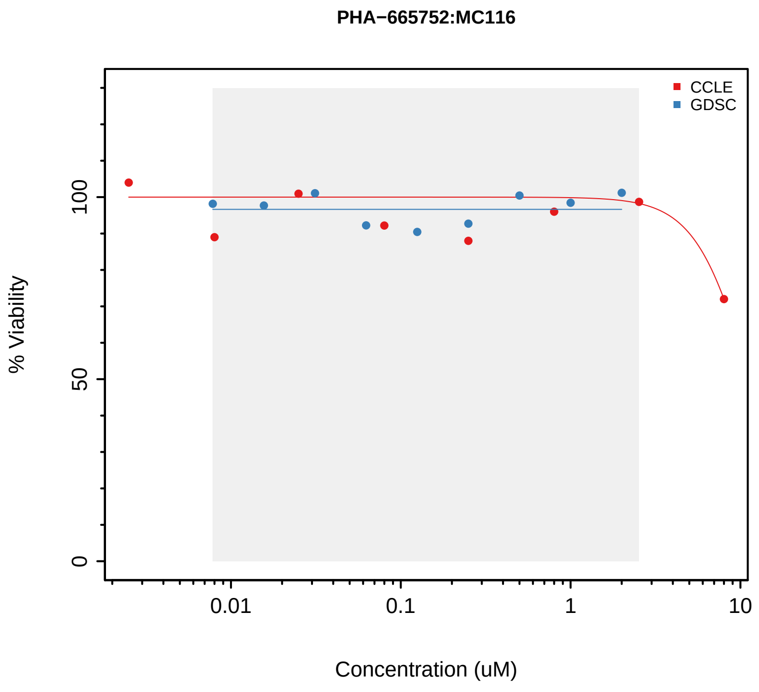
PHA−665752:MC116
CCLE
GDSC
% Viability
0
50
100
0.01
0.1
1
10
Concentration (uM)

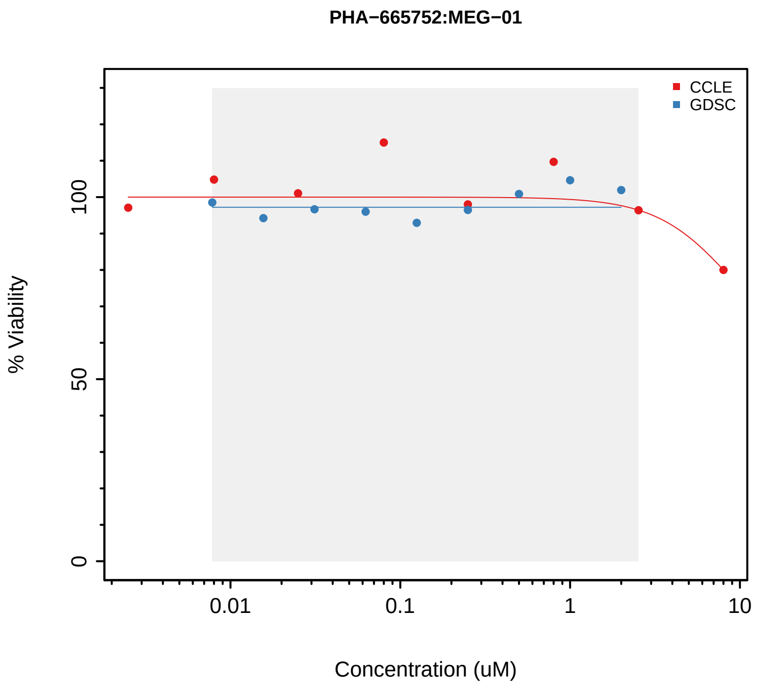
PHA−665752:MEG−01
CCLE
GDSC
% Viability
0
50
100
0.01
0.1
1
10
Concentration (uM)

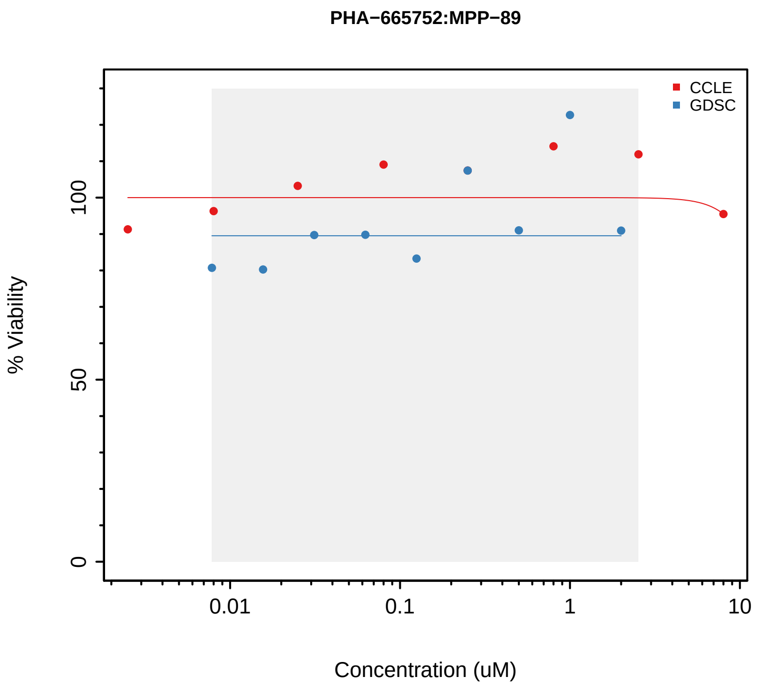
PHA−665752:MPP−89
CCLE
GDSC
% Viability
0
50
100
0.01
0.1
1
10
Concentration (uM)

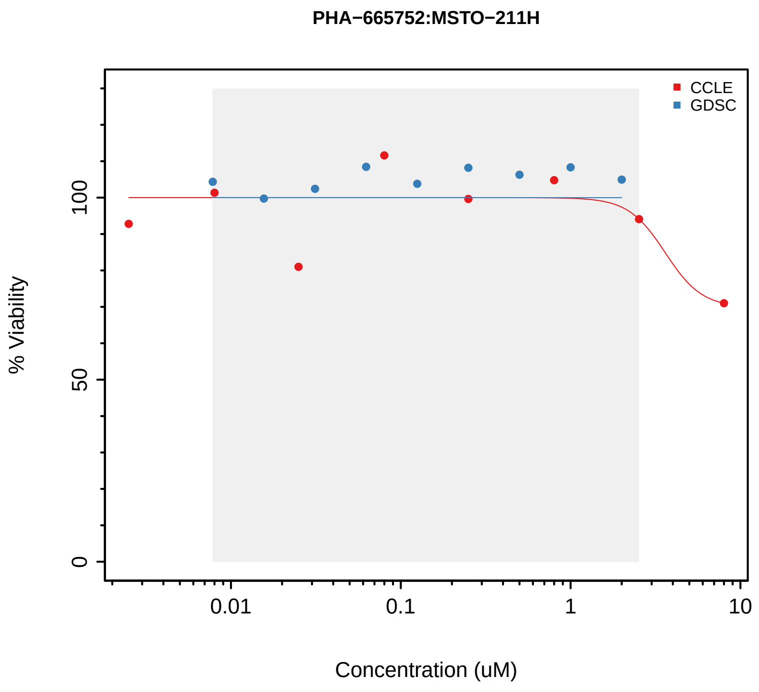
PHA−665752:MSTO−211H
CCLE
GDSC
% Viability
0
50
100
0.01
0.1
1
10
Concentration (uM)

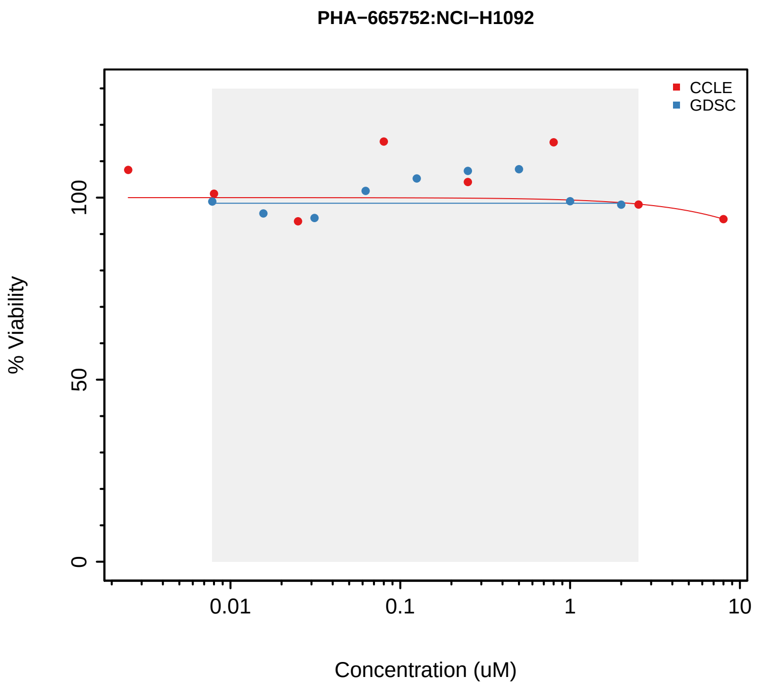
PHA−665752:NCI−H1092
CCLE
GDSC
% Viability
0
50
100
0.01
0.1
1
10
Concentration (uM)

PHA−665752:NCI−H1155

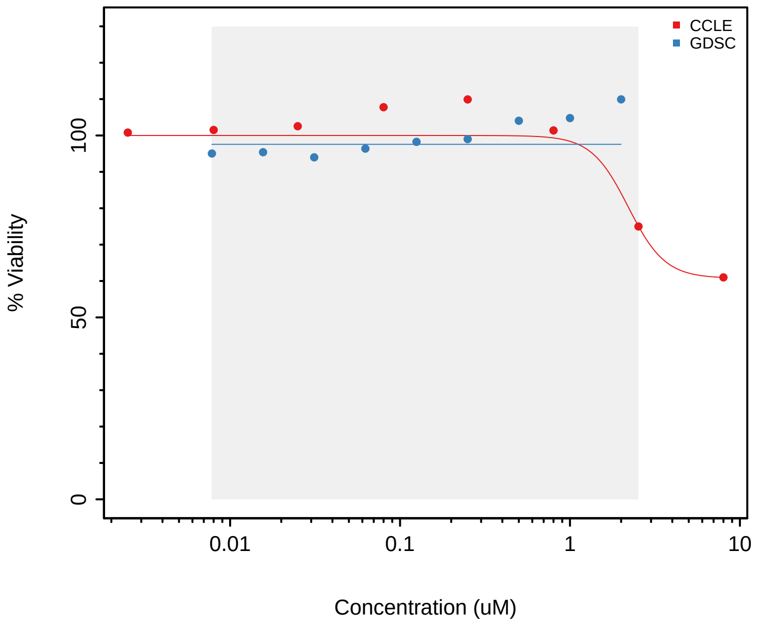

PHA−665752:NCI−H1299

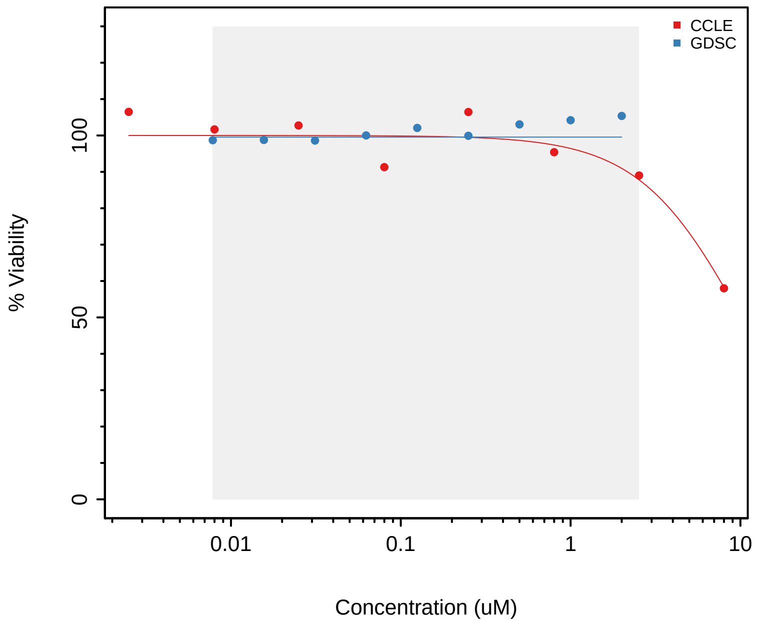

PHA−665752:NCI−H1355

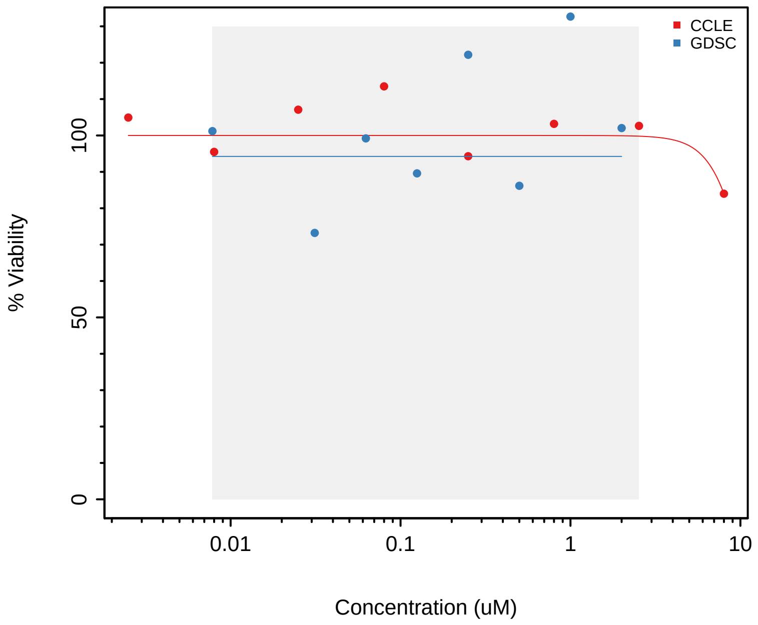

PHA−665752:NCI−H1581

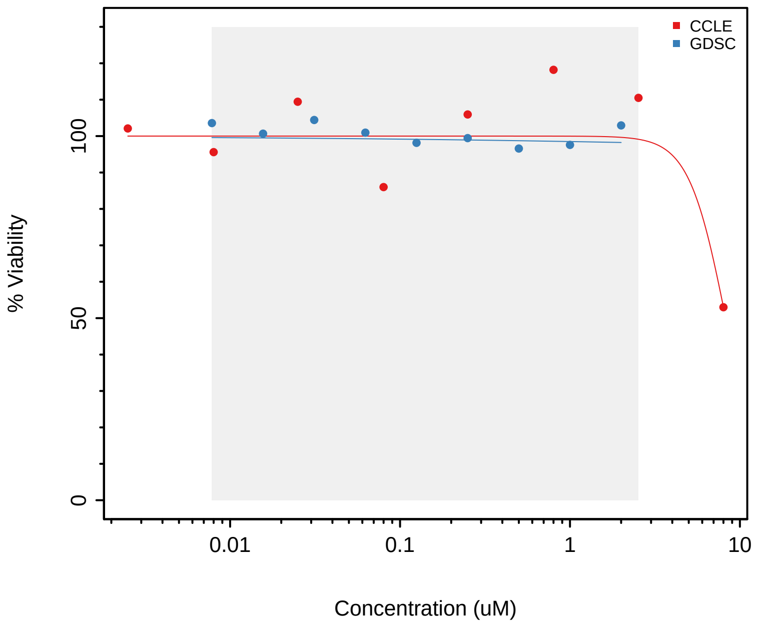

PHA−665752:NCI−H1648

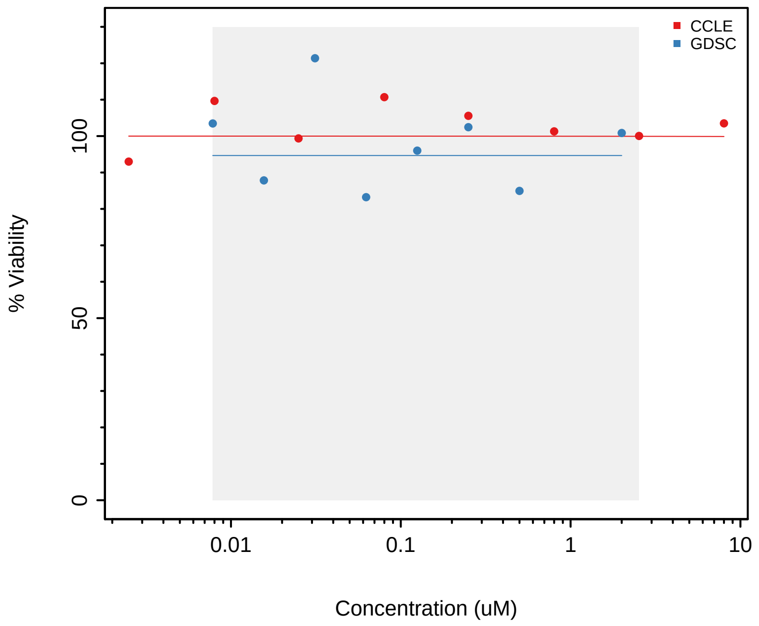

PHA−665752:NCI−H1666

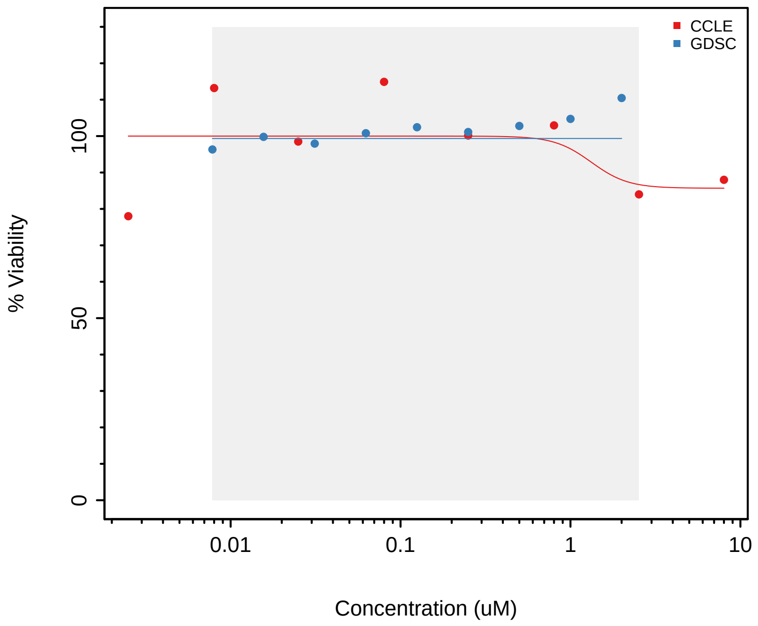

PHA−665752:NCI−H1694

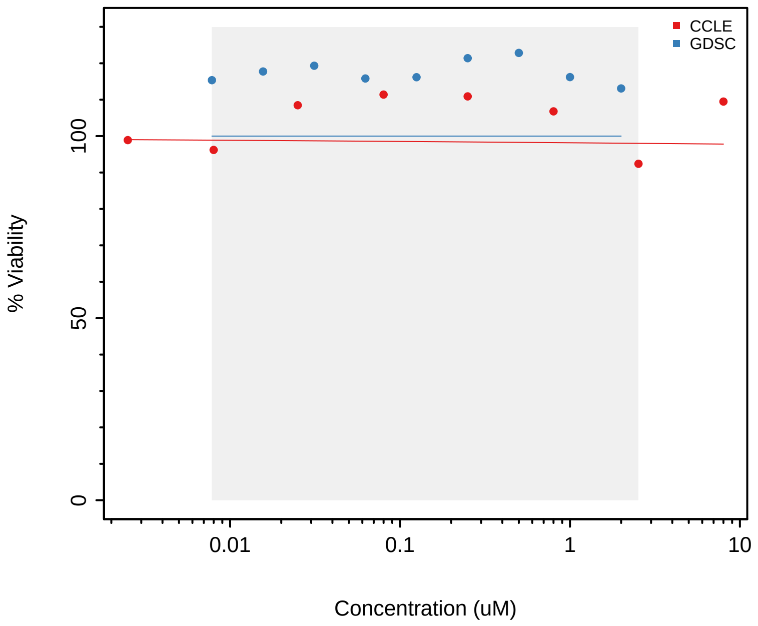

PHA−665752:NCI−H1975

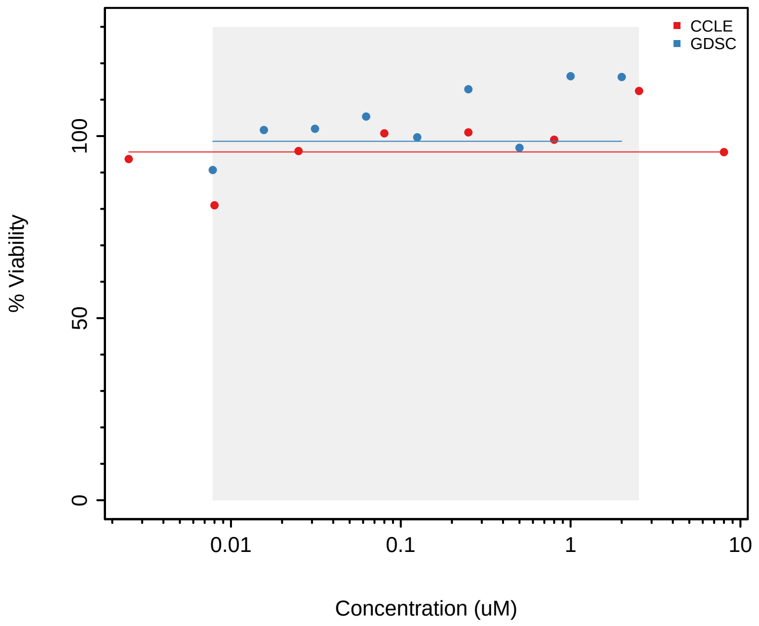

PHA−665752:NCI−H226

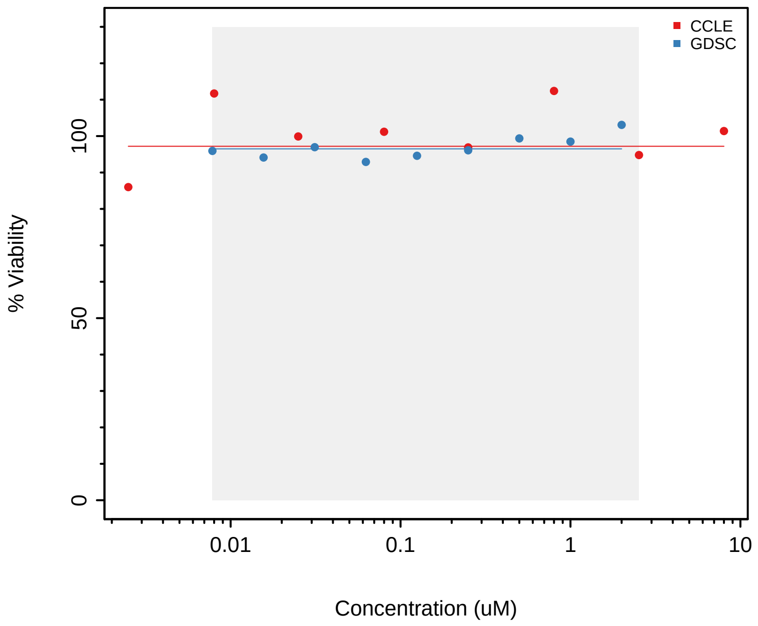

PHA−665752:NCI−H23

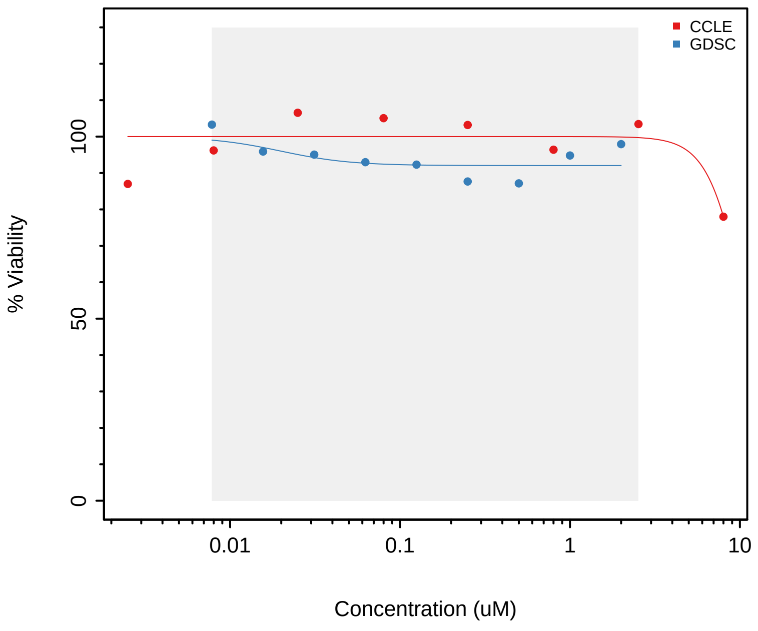

PHA−665752:NCI−H747

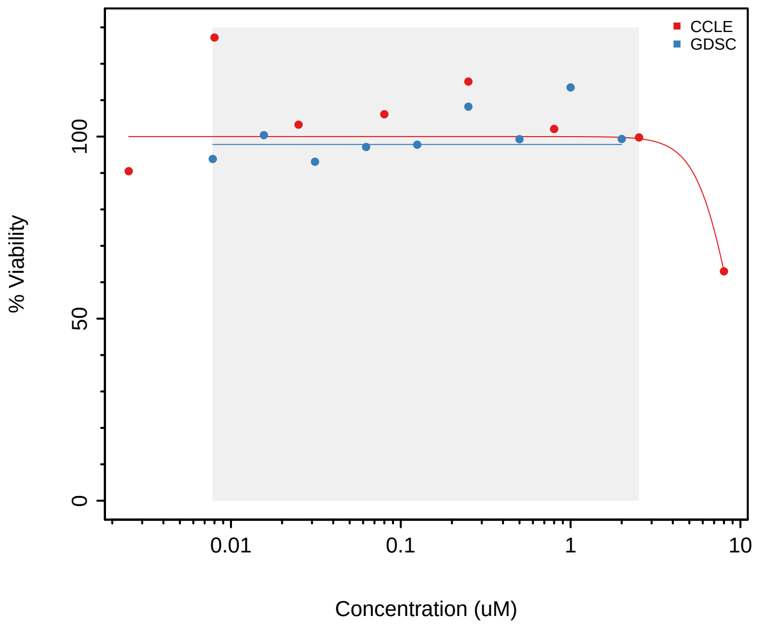

PHA−665752:OCI−AML2

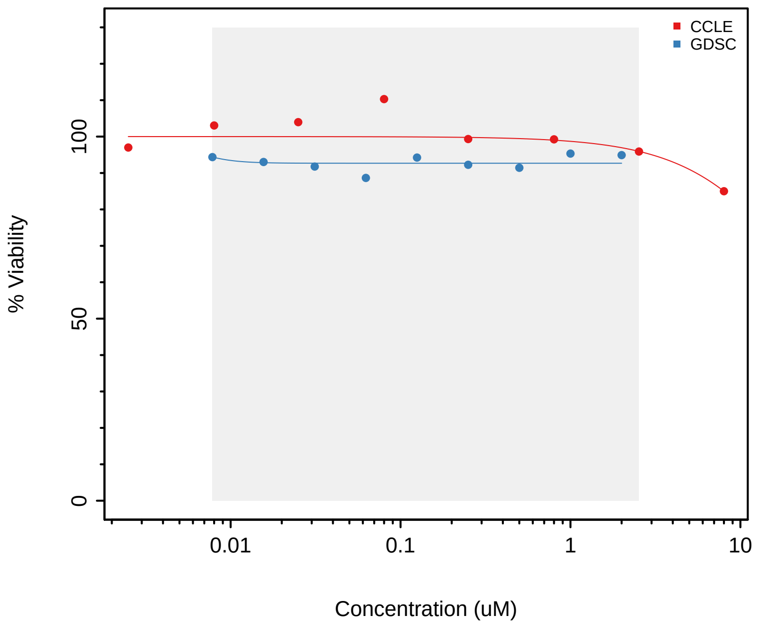

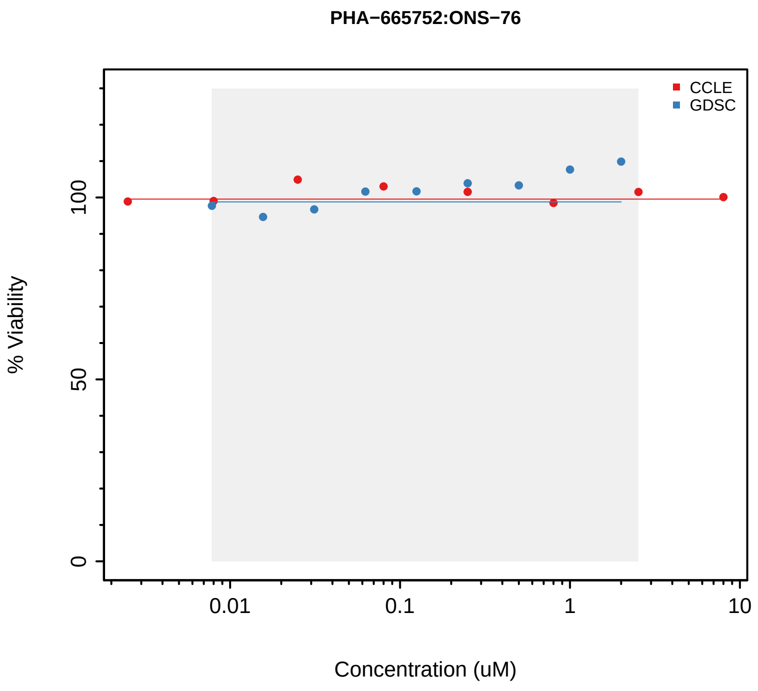
PHA−665752:ONS−76
CCLE
GDSC
% Viability
0
50
100
0.01
0.1
1
10
Concentration (uM)

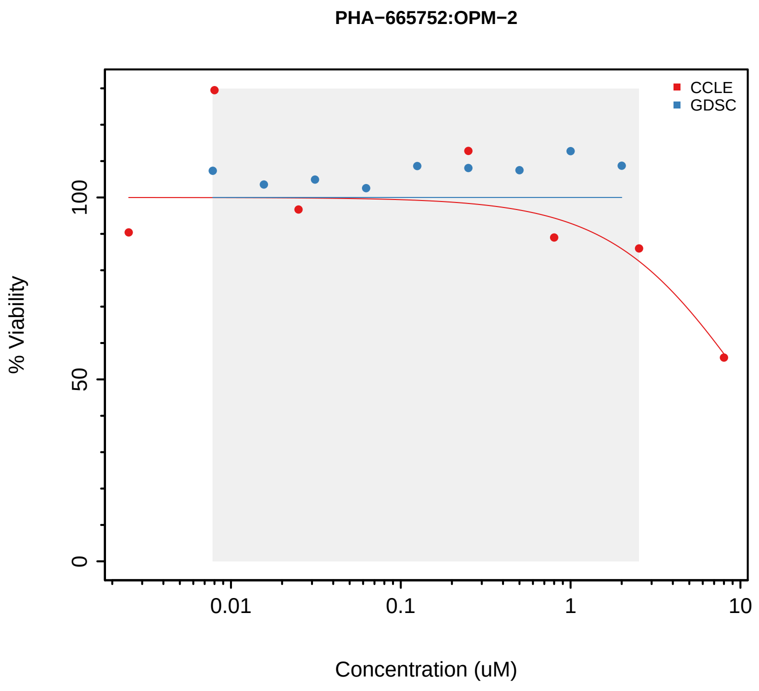
PHA−665752:OPM−2
CCLE
GDSC
% Viability
0
50
100
0.01
0.1
1
10
Concentration (uM)

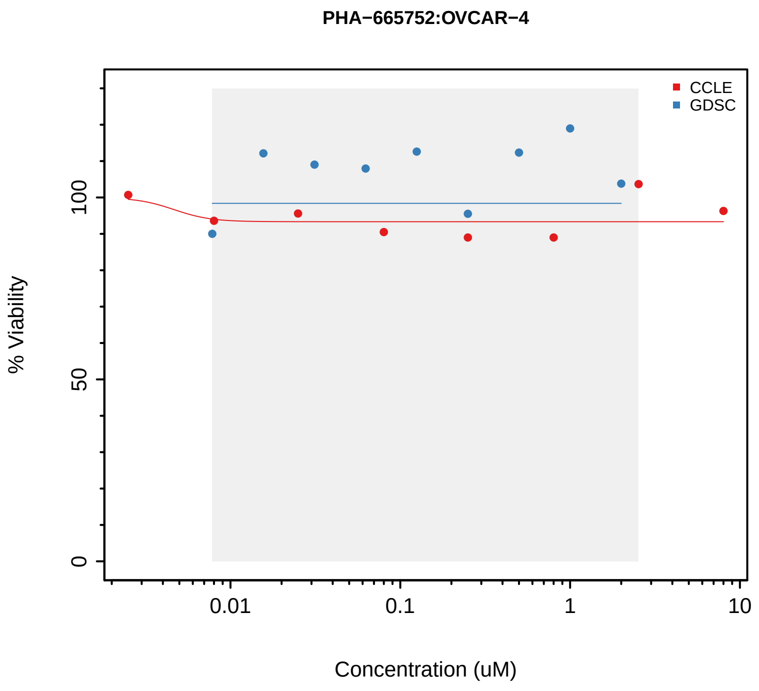
PHA−665752:OVCAR−4
CCLE
GDSC
% Viability
0
50
100
0.01
0.1
1
10
Concentration (uM)

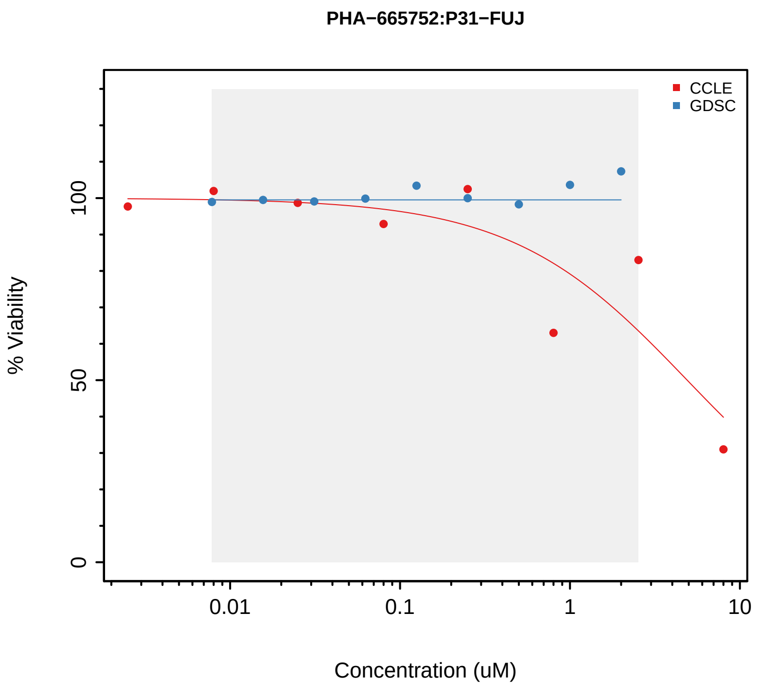
PHA−665752:P31−FUJ
CCLE
GDSC
% Viability
0
50
100
0.01
0.1
1
10
Concentration (uM)

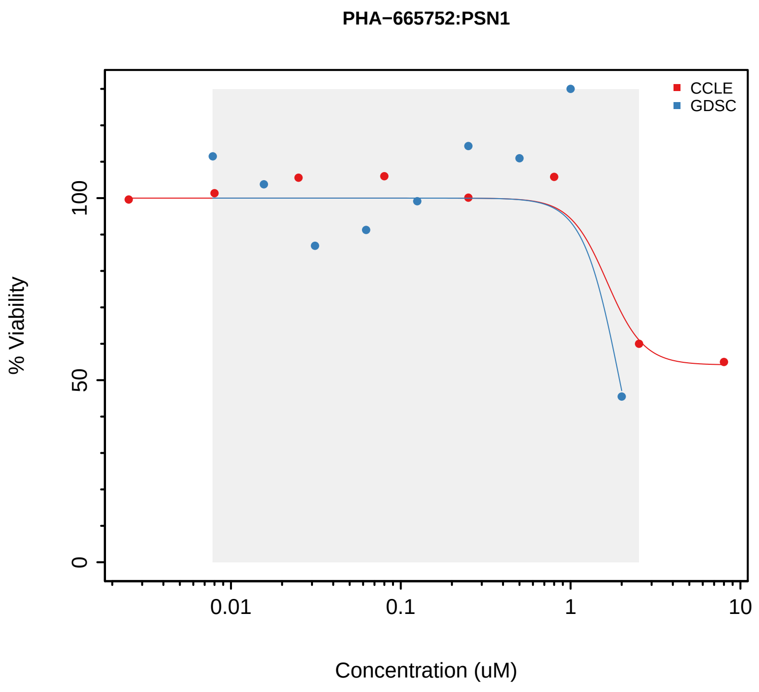
PHA−665752:PSN1
CCLE
GDSC
% Viability
0
50
100
0.01
0.1
1
10
Concentration (uM)

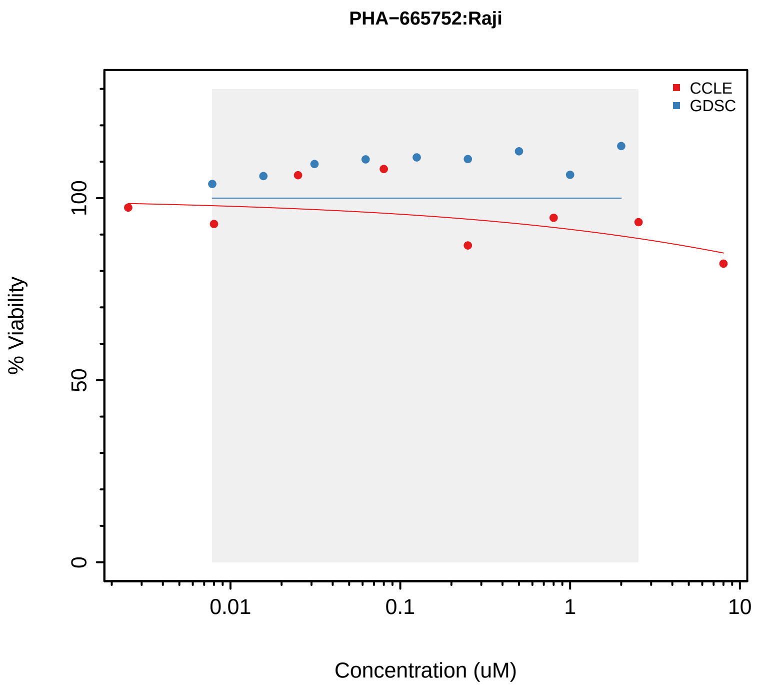
PHA−665752:Raji
CCLE
GDSC
% Viability
0
50
100
0.01
0.1
1
10
Concentration (uM)

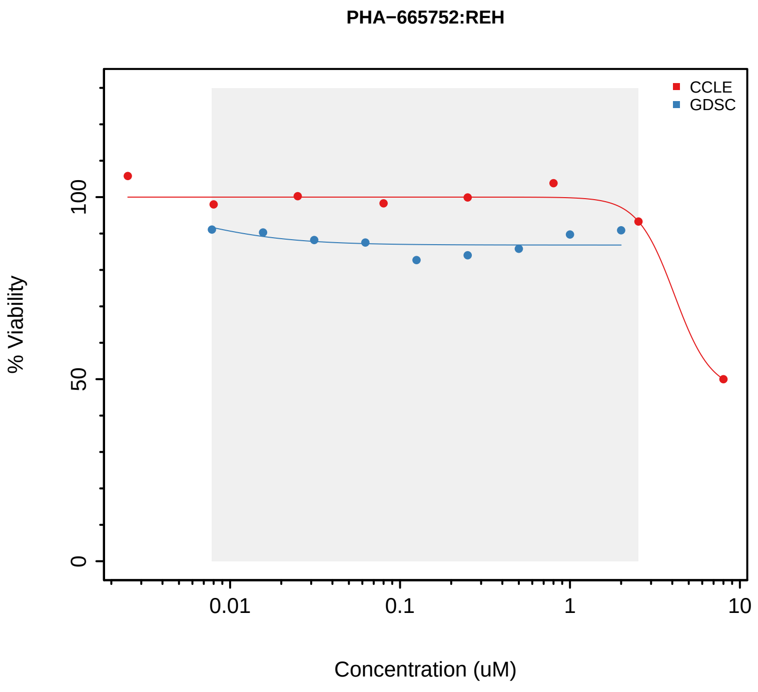
PHA−665752:REH
CCLE
GDSC
% Viability
0
50
100
0.01
0.1
1
10
Concentration (uM)

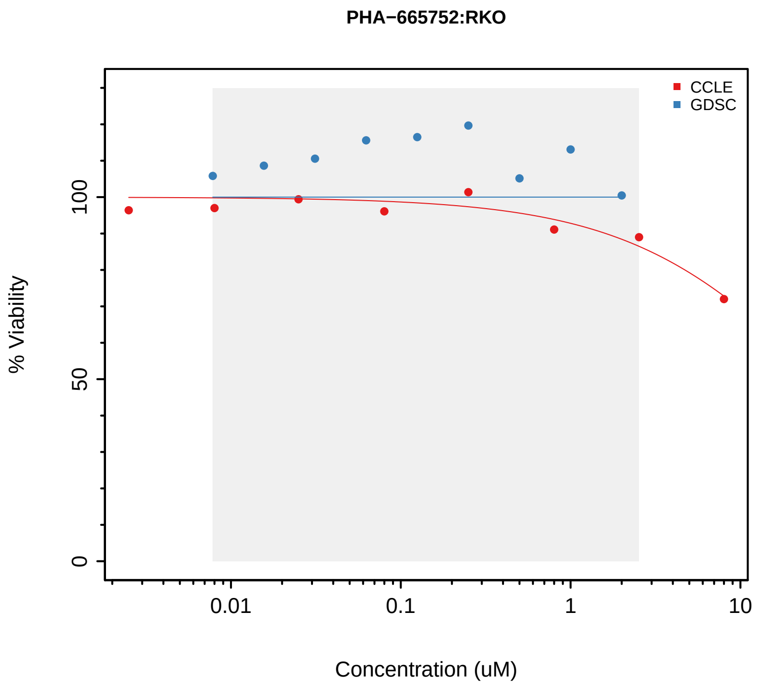
PHA−665752:RKO
CCLE
GDSC
% Viability
0
50
100
0.01
0.1
1
10
Concentration (uM)

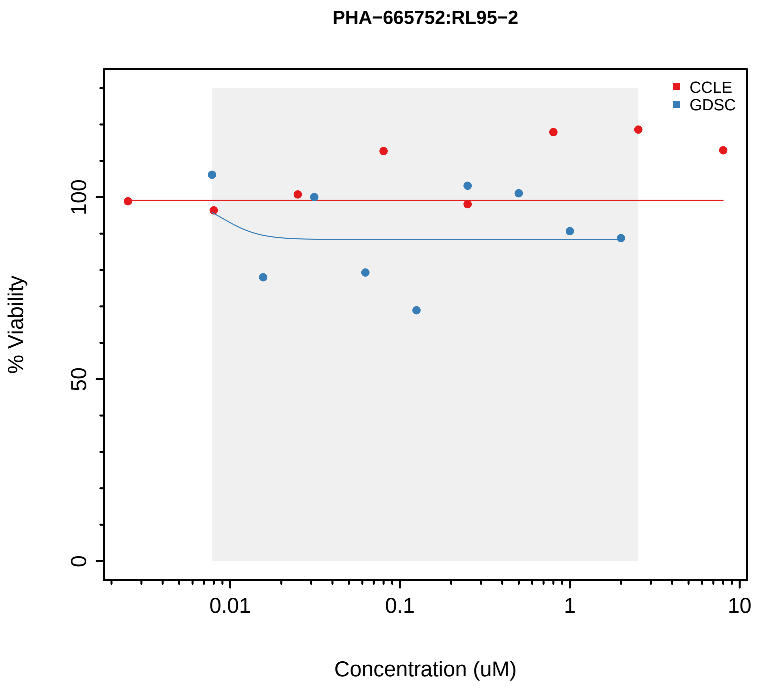
PHA−665752:RL95−2
CCLE
GDSC
% Viability
0
50
100
0.01
0.1
1
10
Concentration (uM)

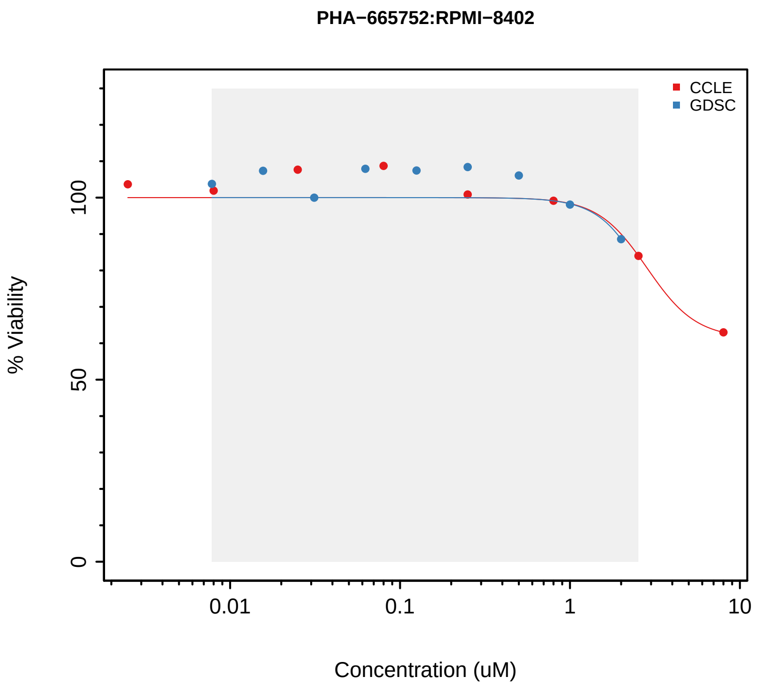
PHA−665752:RPMI−8402
CCLE
GDSC
% Viability
0
50
100
0.01
0.1
1
10
Concentration (uM)

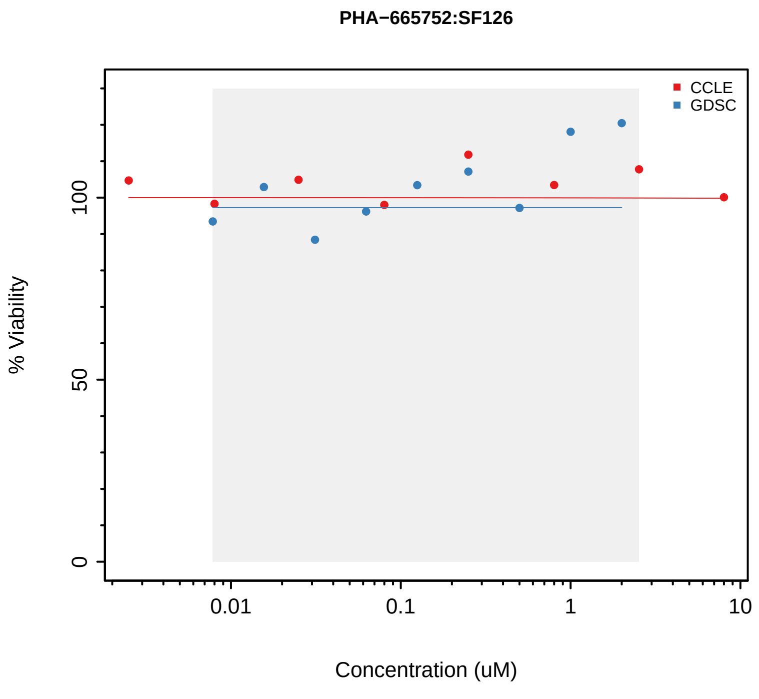
PHA−665752:SF126
CCLE
GDSC
% Viability
0
50
100
0.01
0.1
1
10
Concentration (uM)

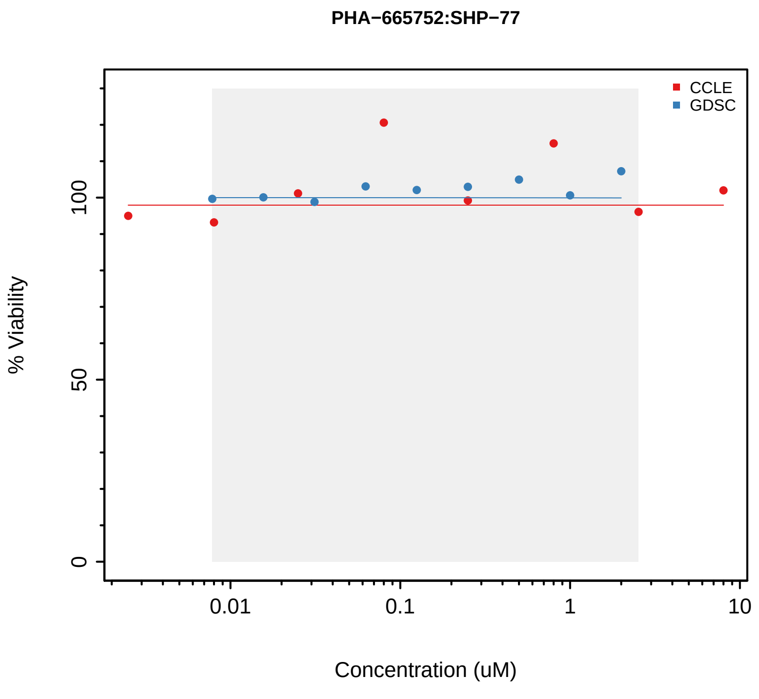
PHA−665752:SHP−77
CCLE
GDSC
% Viability
0
50
100
0.01
0.1
1
10
Concentration (uM)

PHA−665752:SIG−M5

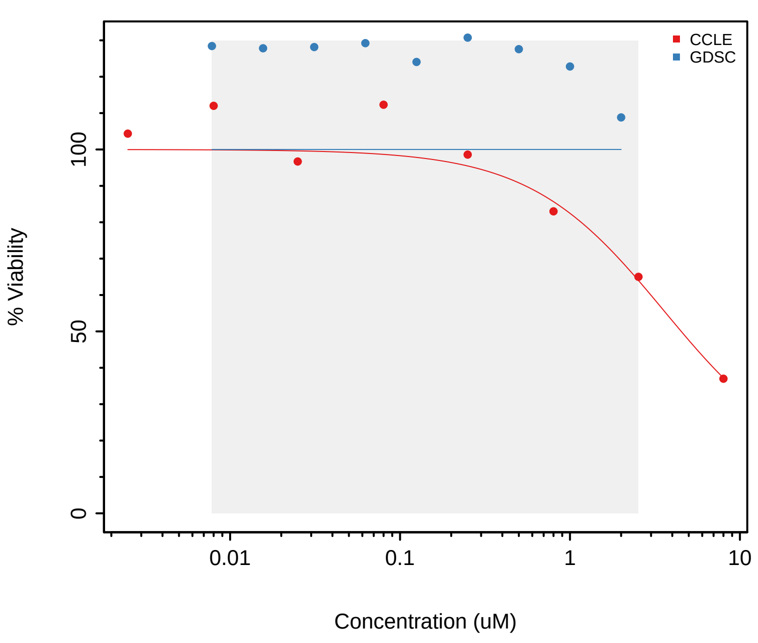

PHA−665752:SIMA

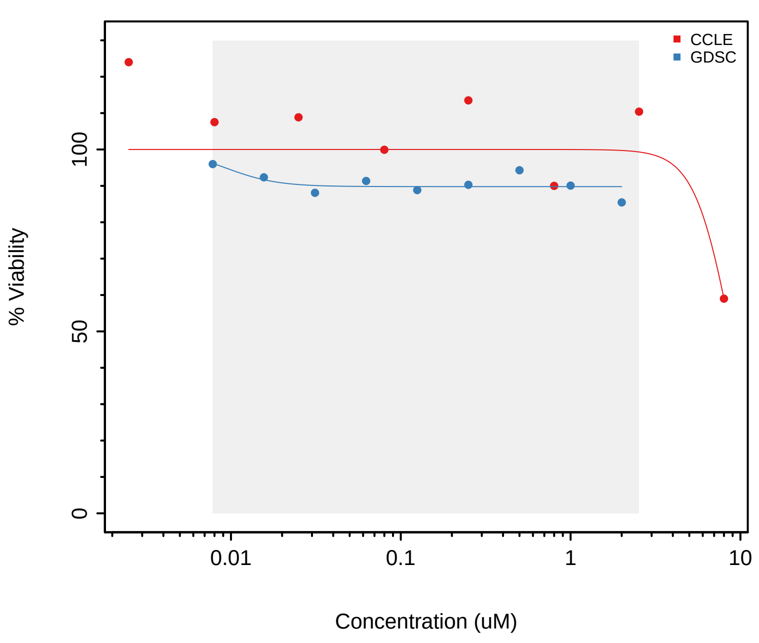

PHA−665752:SJSA−1

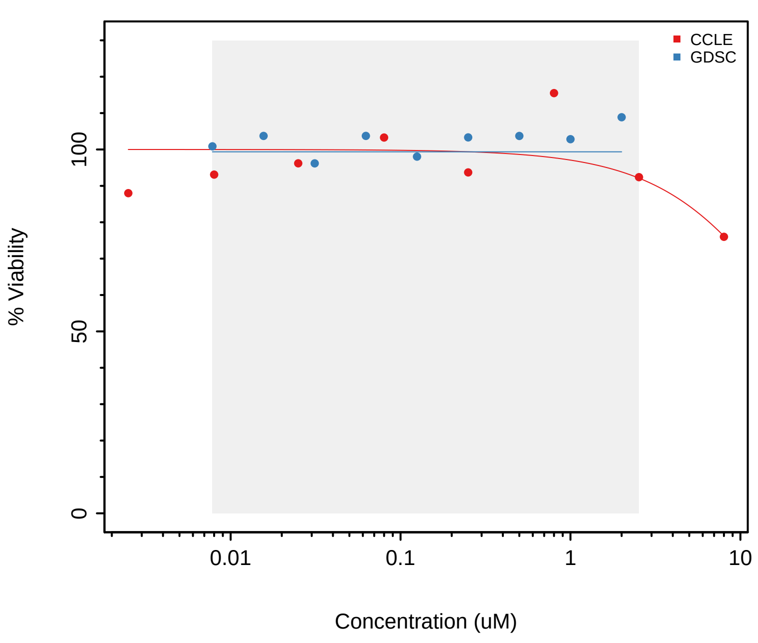

PHA−665752:SK−LMS−1

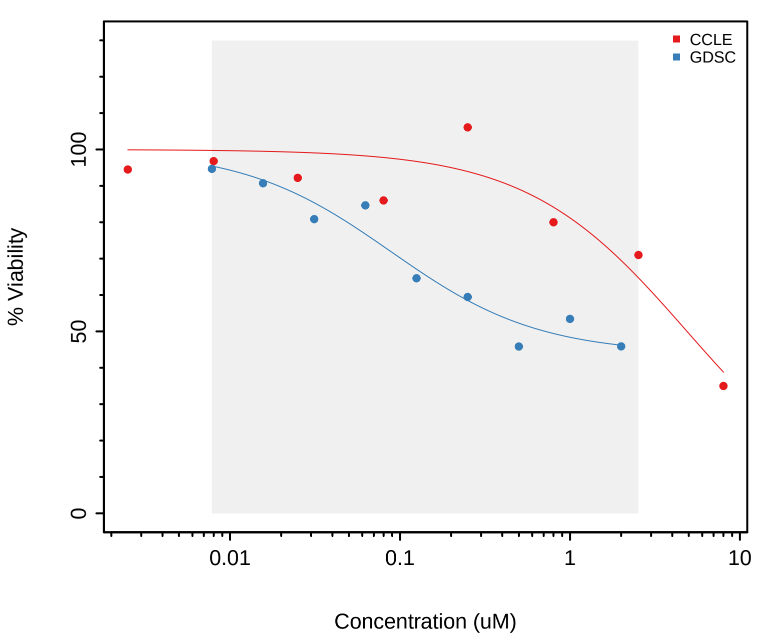

PHA−665752:SK−MEL−2

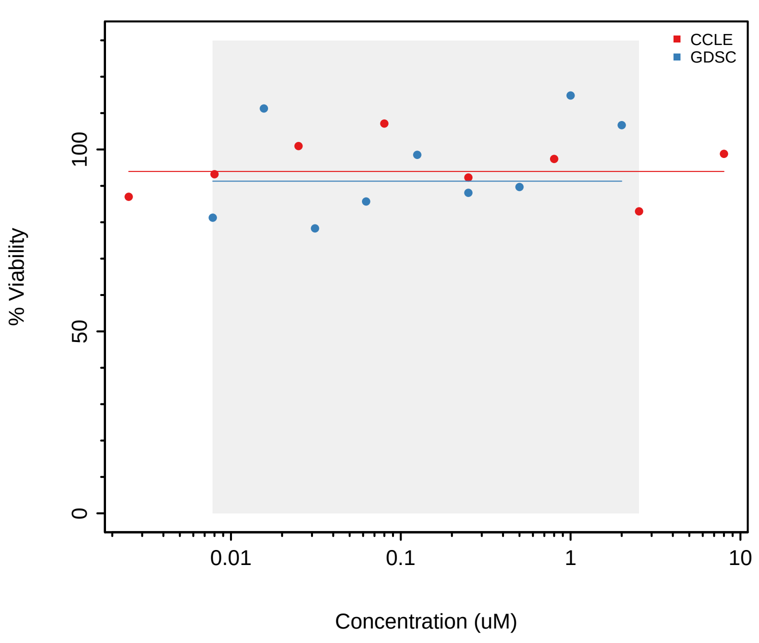

PHA−665752:SK−MM−2

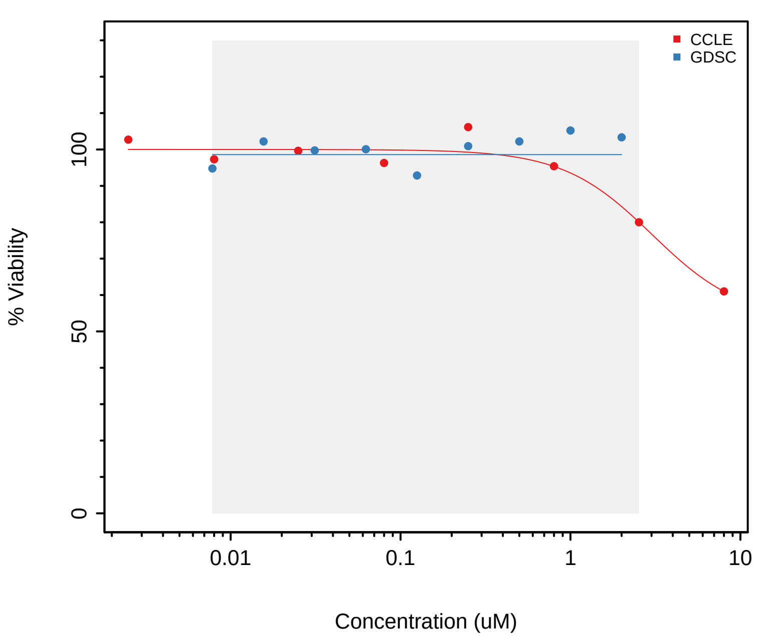

PHA−665752:SK−N−DZ

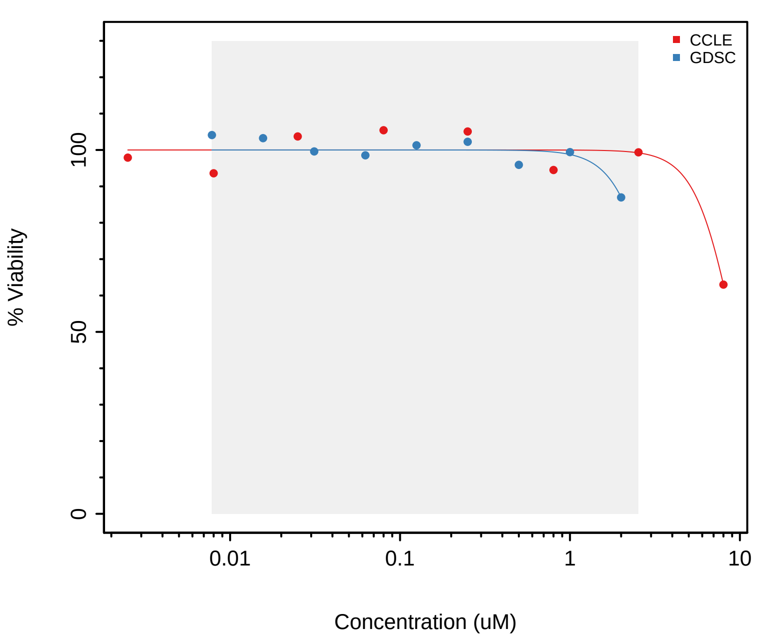

PHA−665752:SK−N−FI

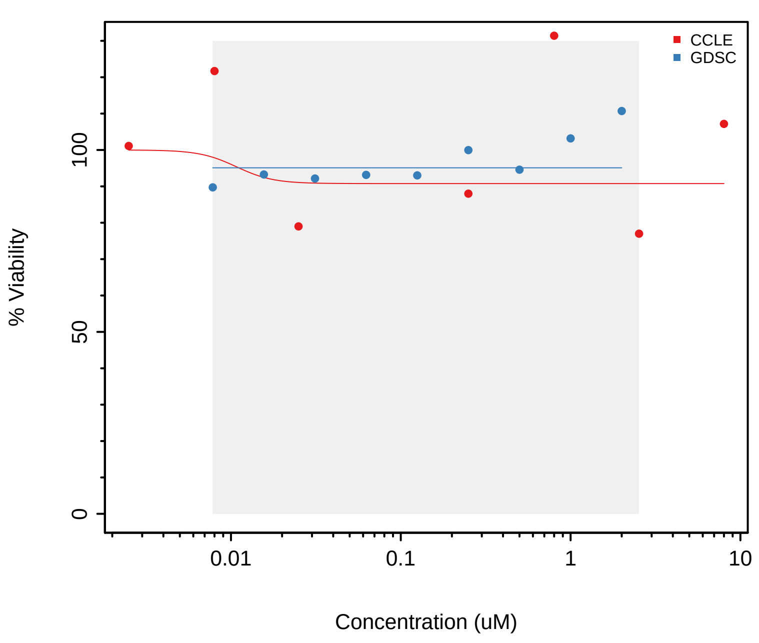

PHA−665752:NCI−SNU−1

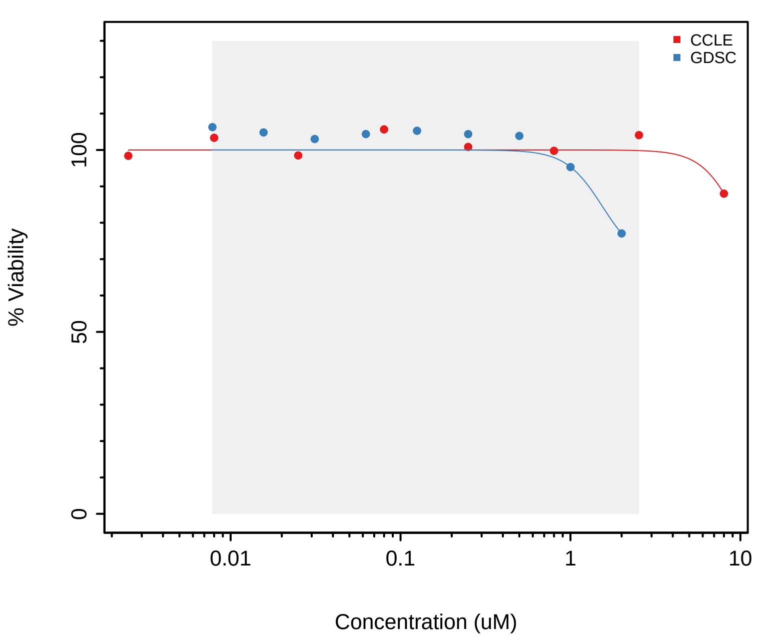

PHA−665752:NCI−SNU−16

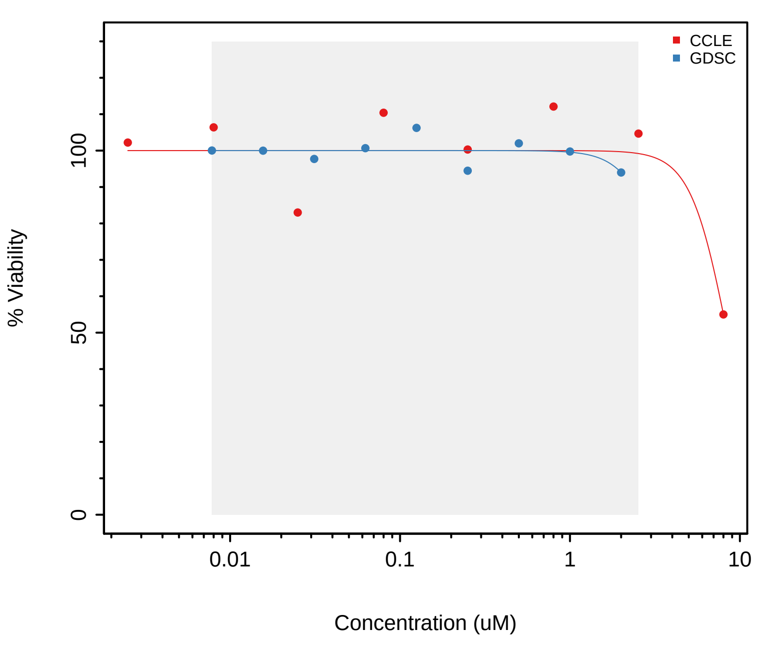

PHA−665752:SNU−C2B

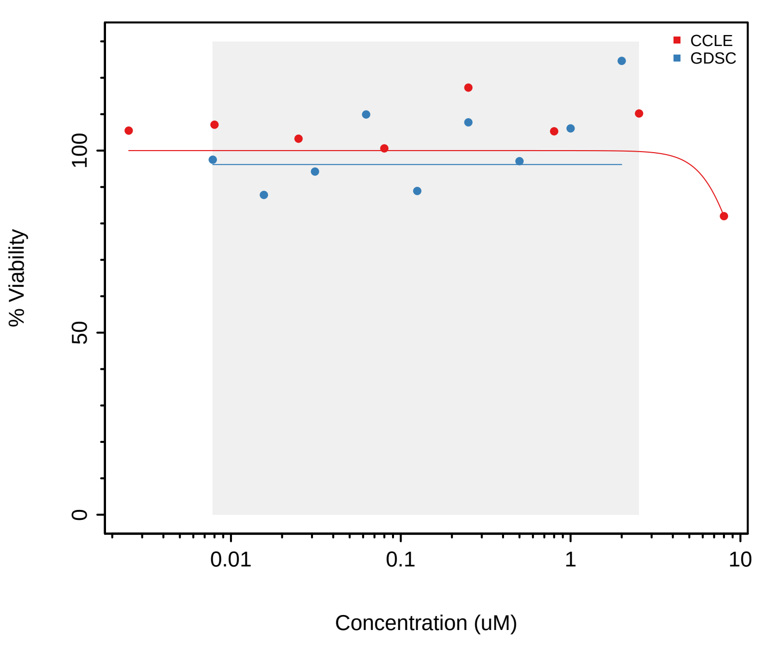

PHA−665752:SUP−T1

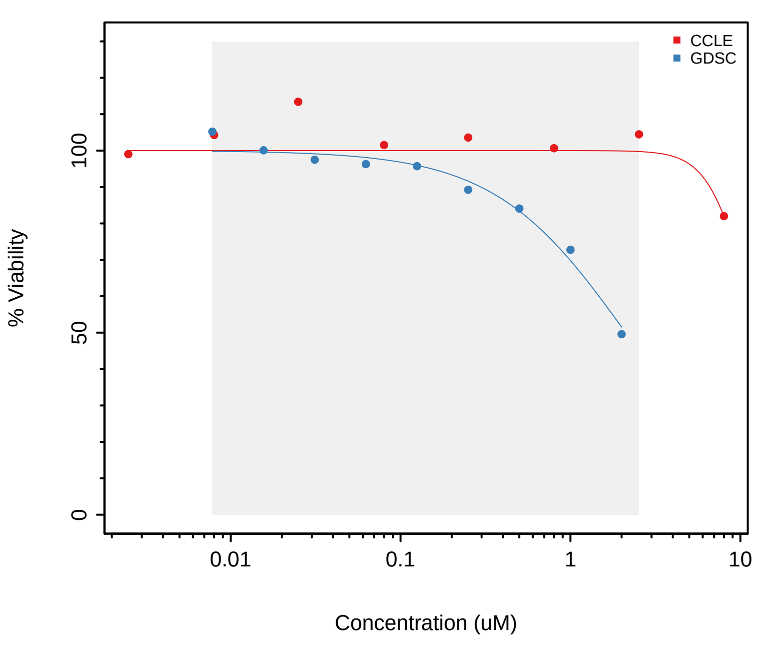

PHA−665752:TE−1

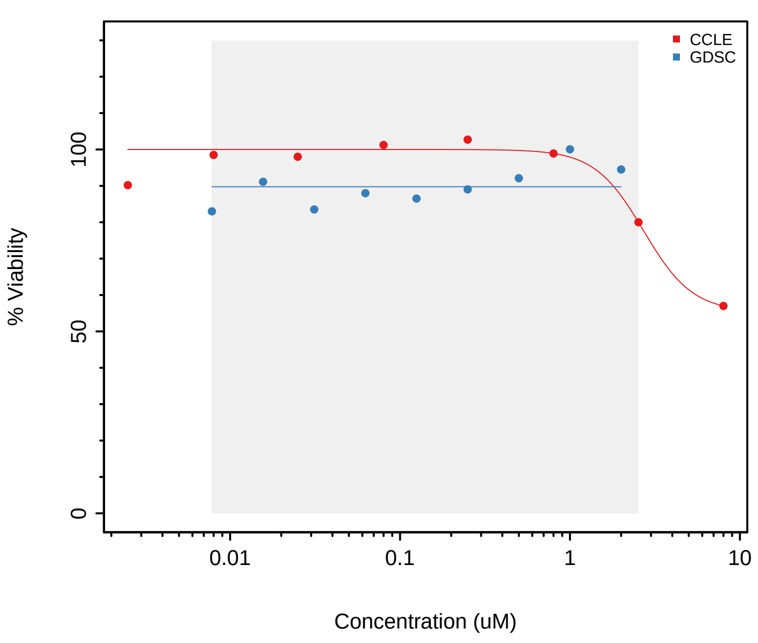

PHA−665752:TE−15

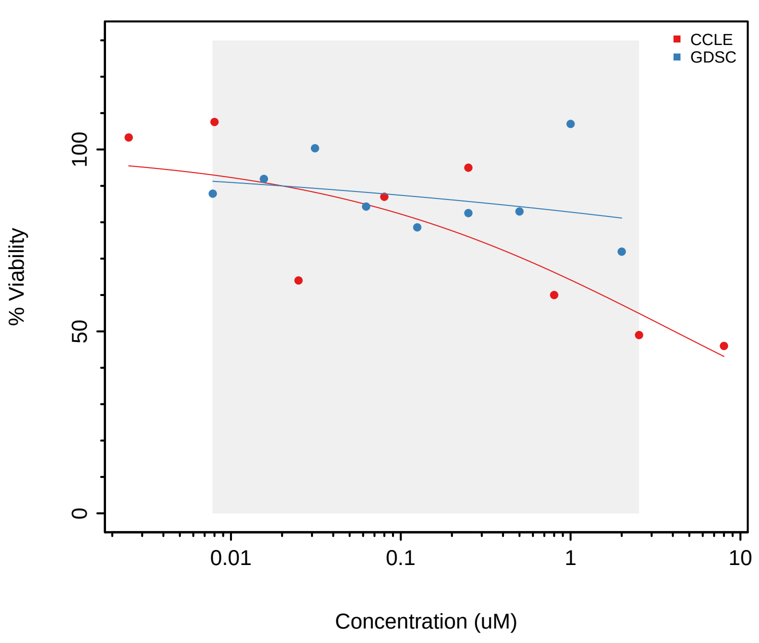

PHA−665752:TE−5

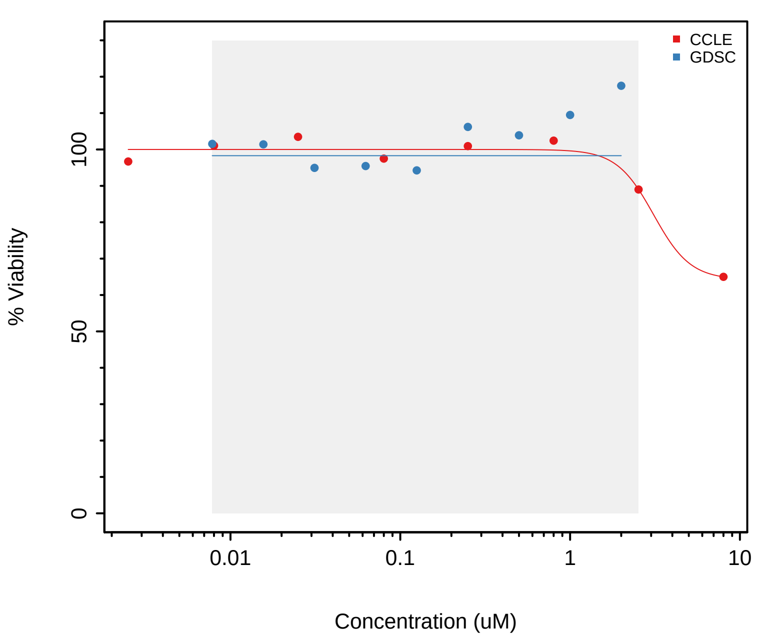

PHA−665752:TE−9

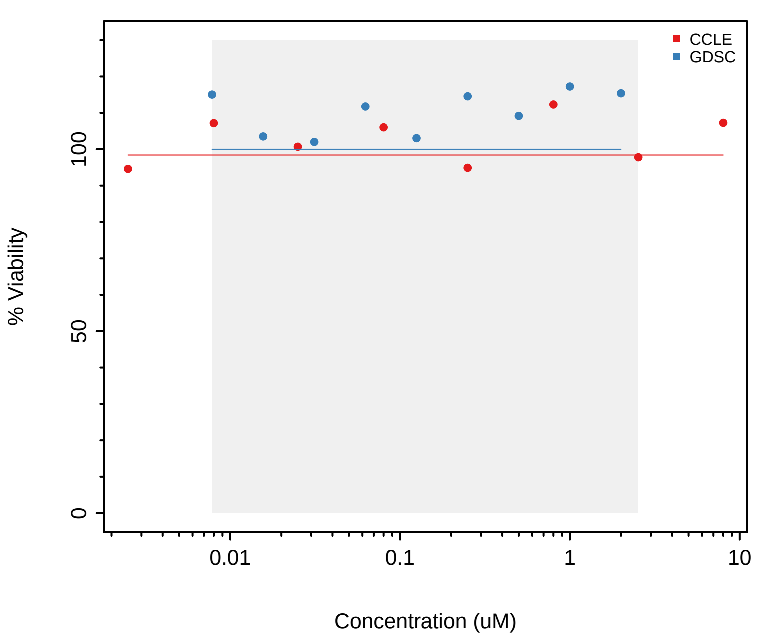

PHA−665752:U−87−MG

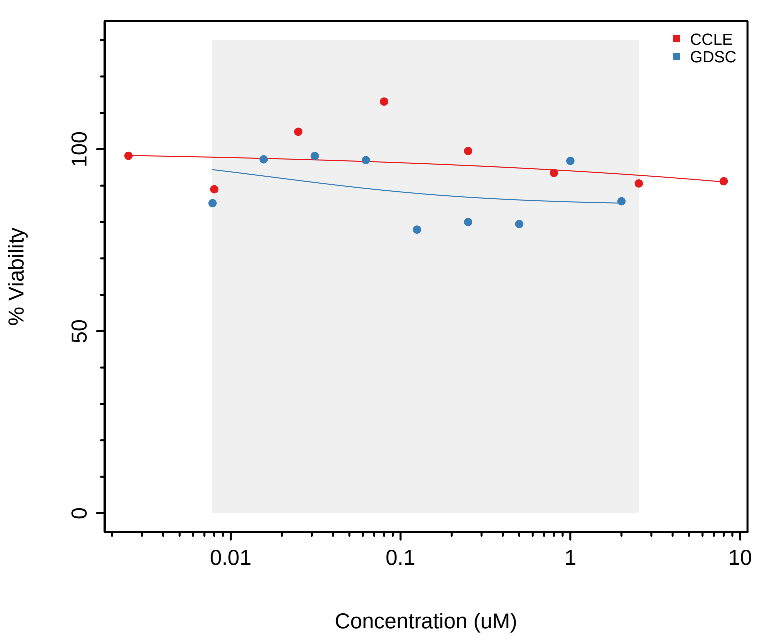

PHA−665752:UACC−257

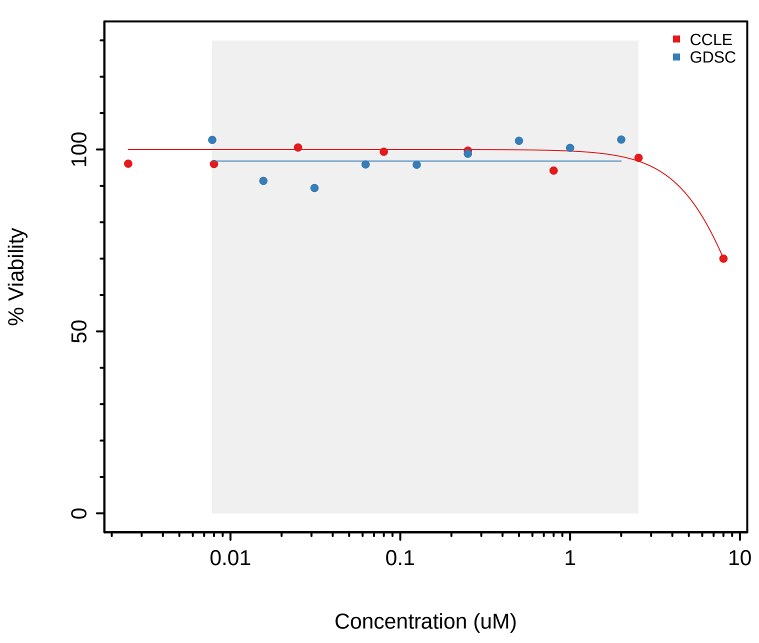

PHA−665752:UACC−812

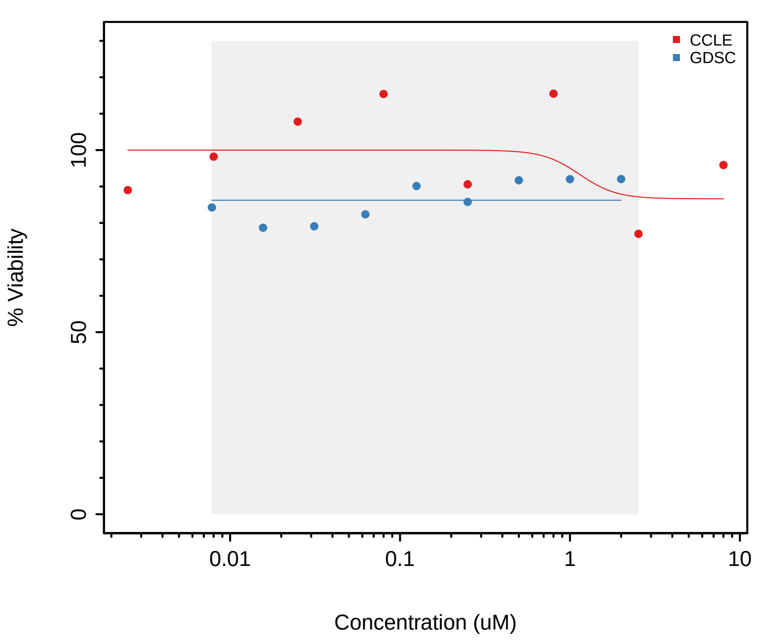

lapatinib:697

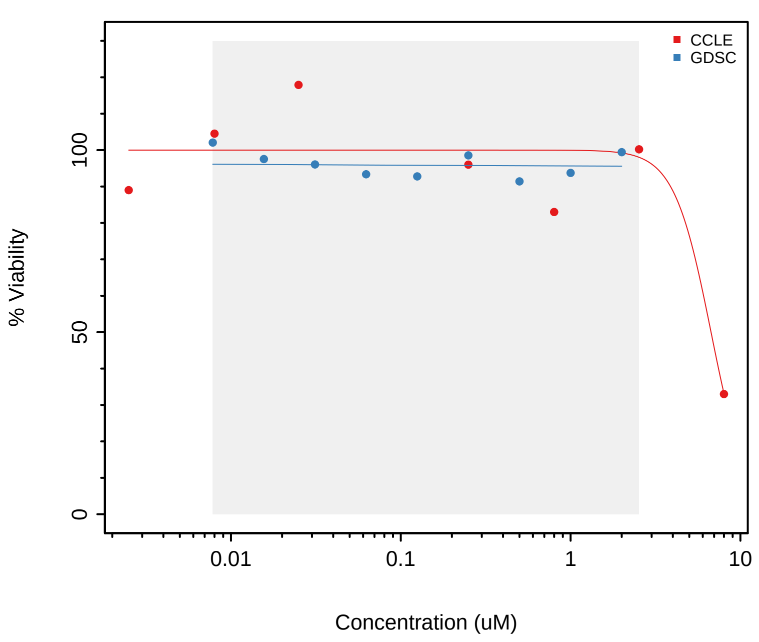

lapatinib:8−MG−BA

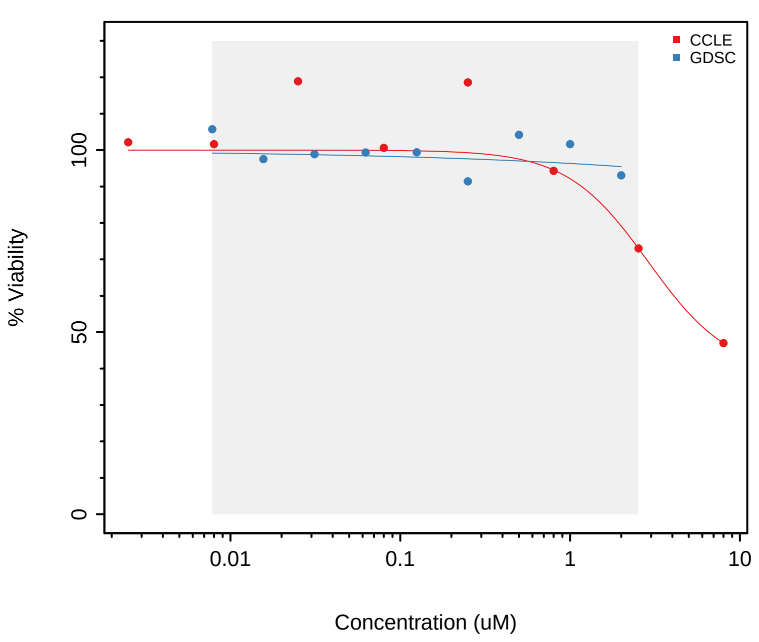

lapatinib:A253

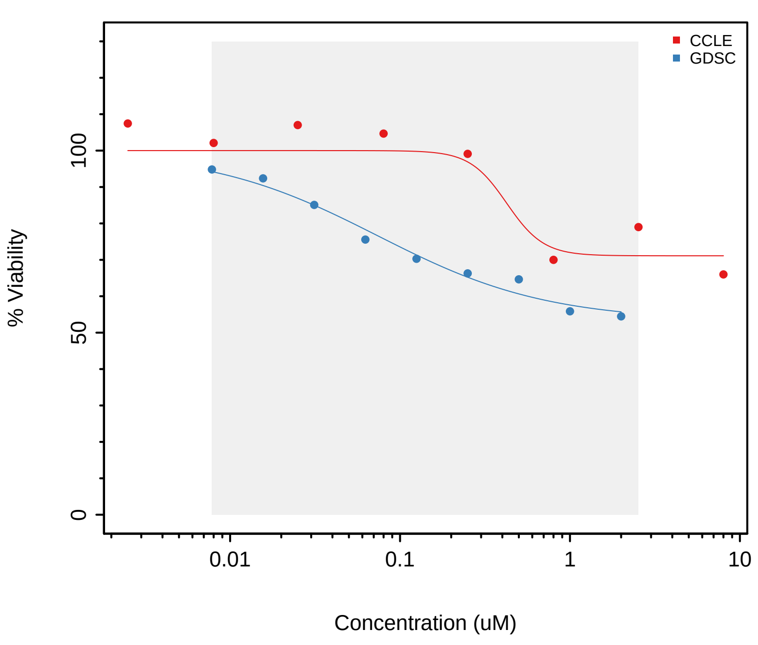

lapatinib:BL−41

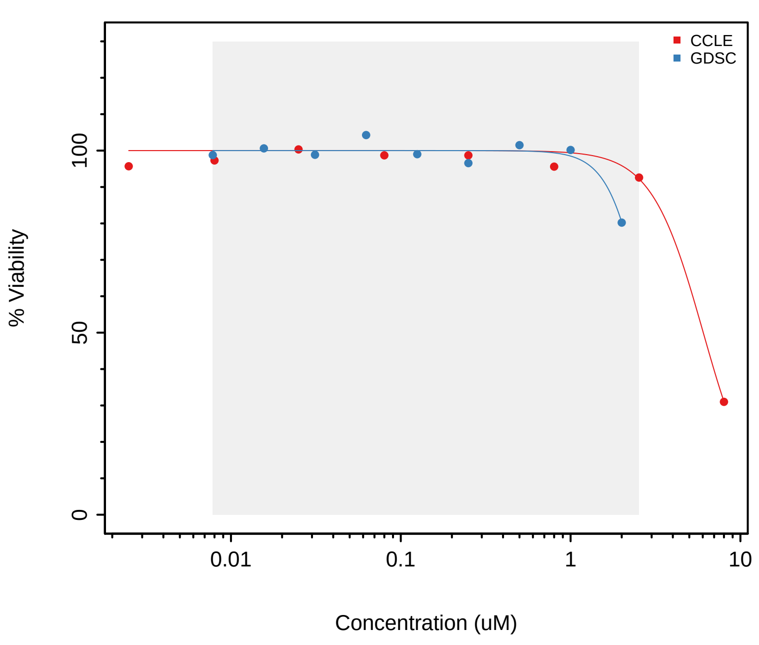

lapatinib:BL−70

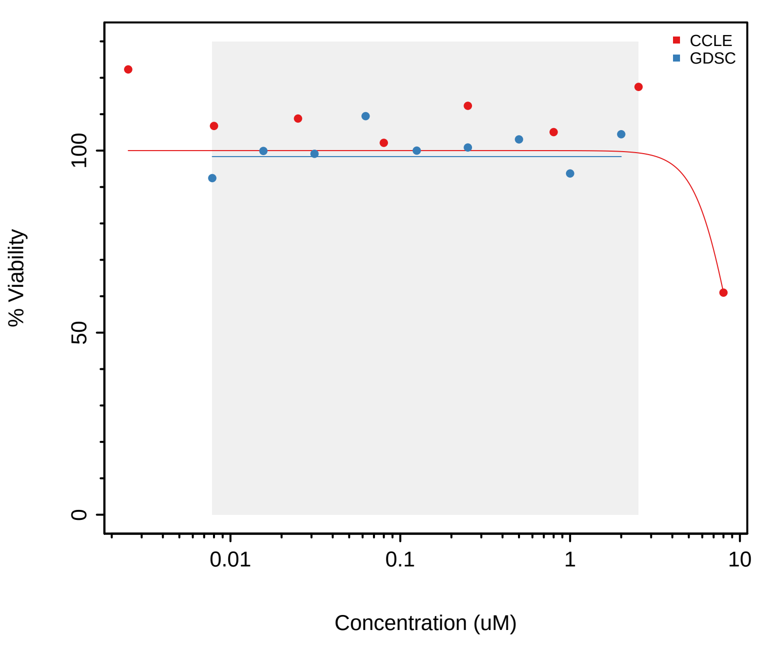

lapatinib:BT−474

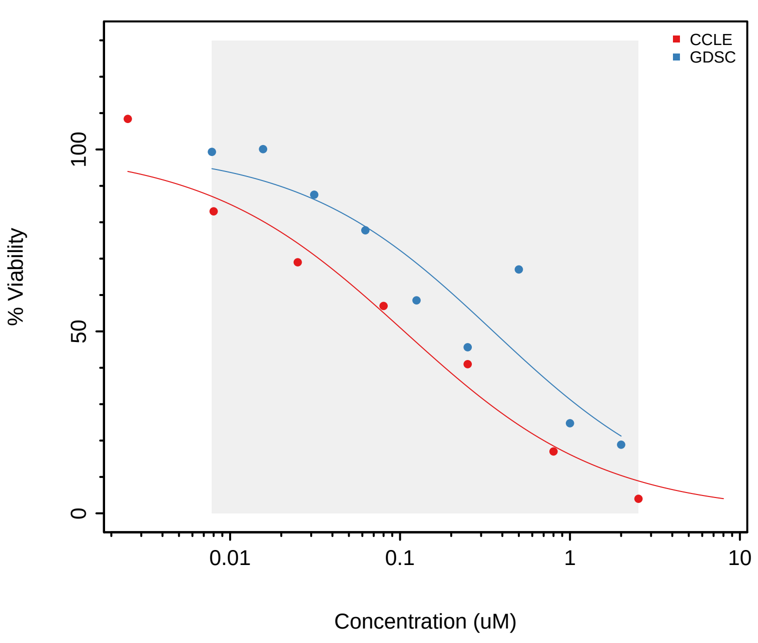

lapatinib:C2BBe1

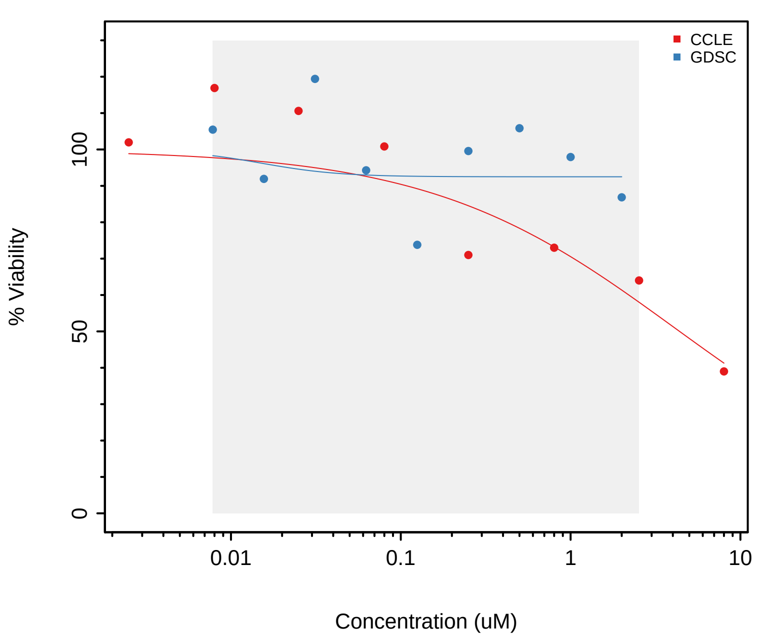

lapatinib:CAS−1

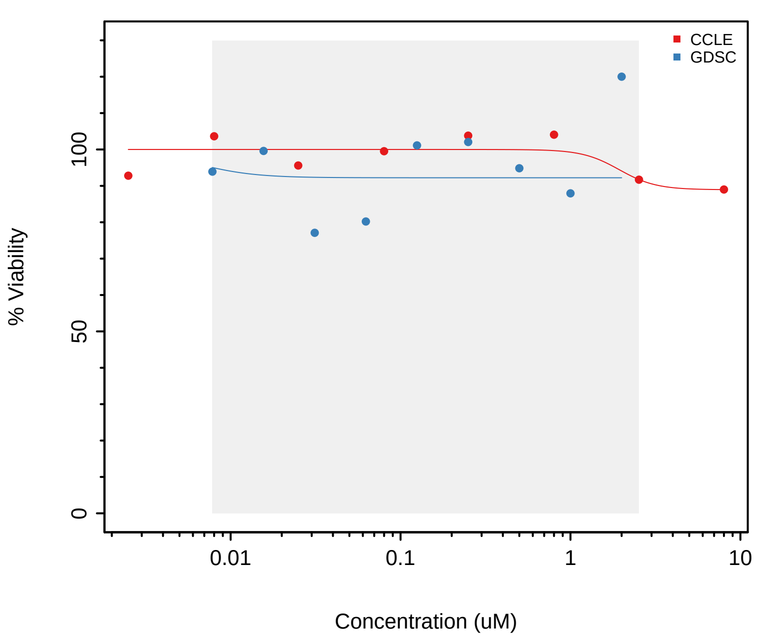

lapatinib:COLO−320−HSR

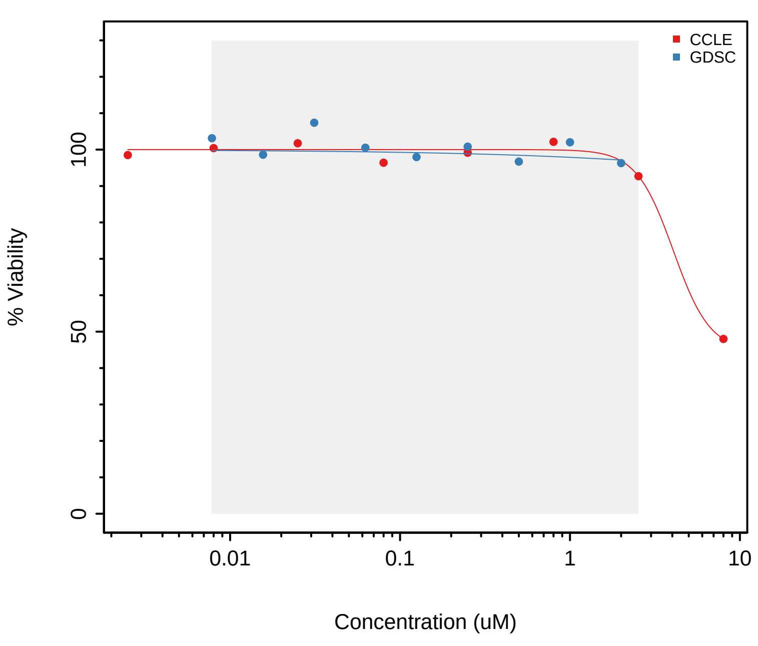

lapatinib:DEL

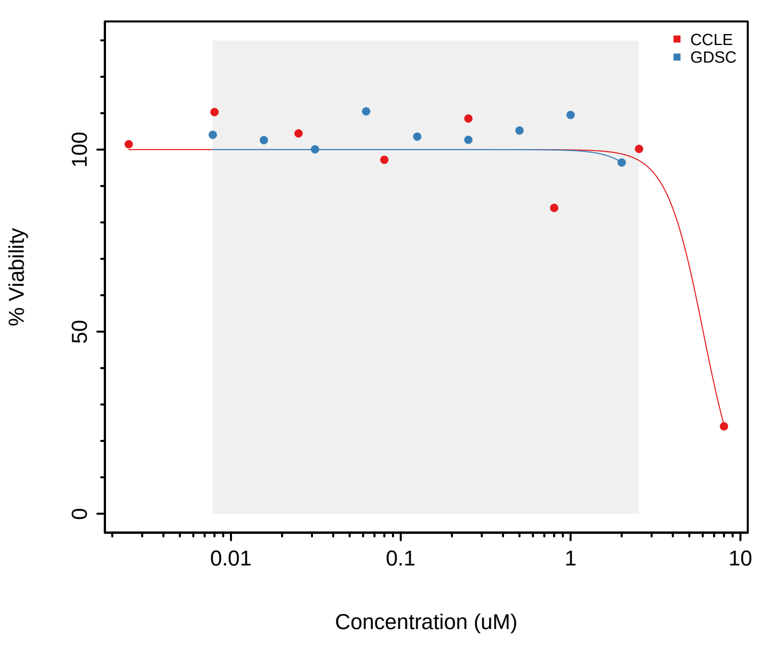

lapatinib:DMS−114

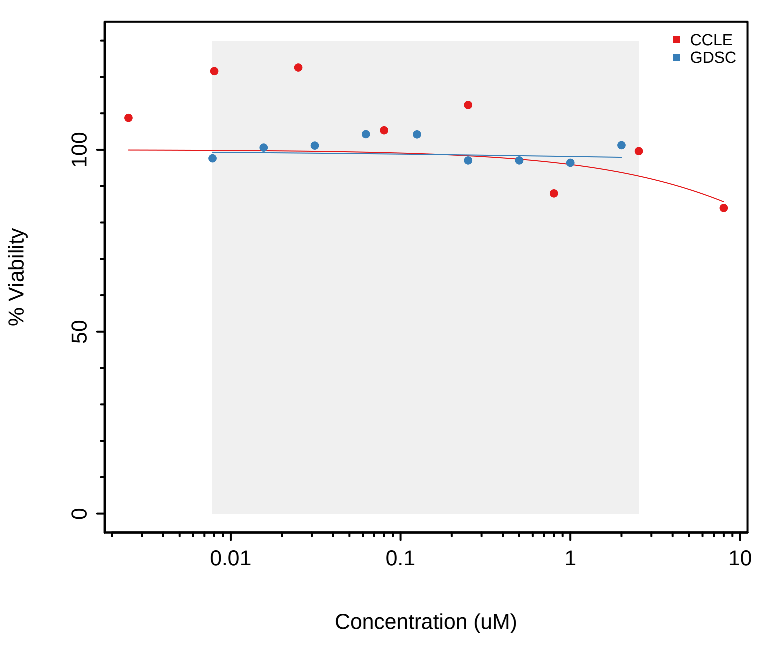

lapatinib:DOHH−2

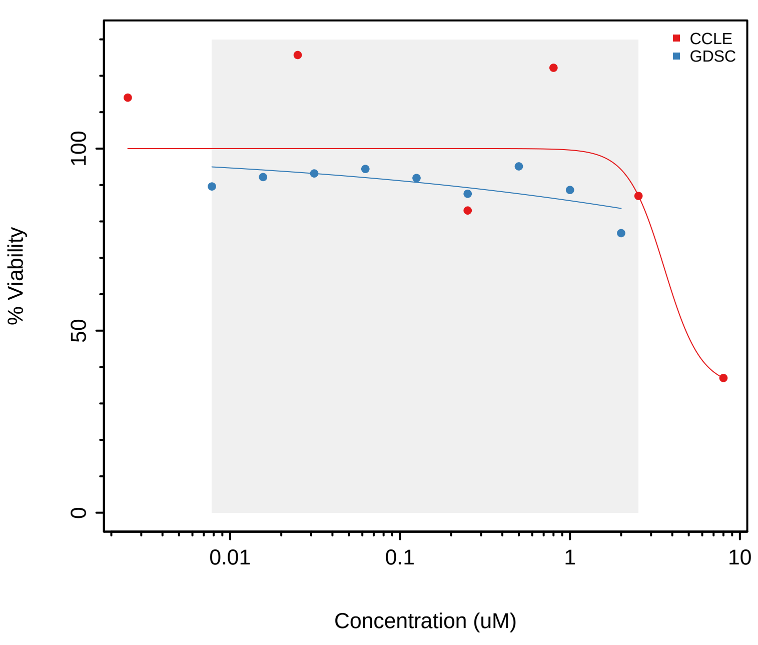

lapatinib:EB2

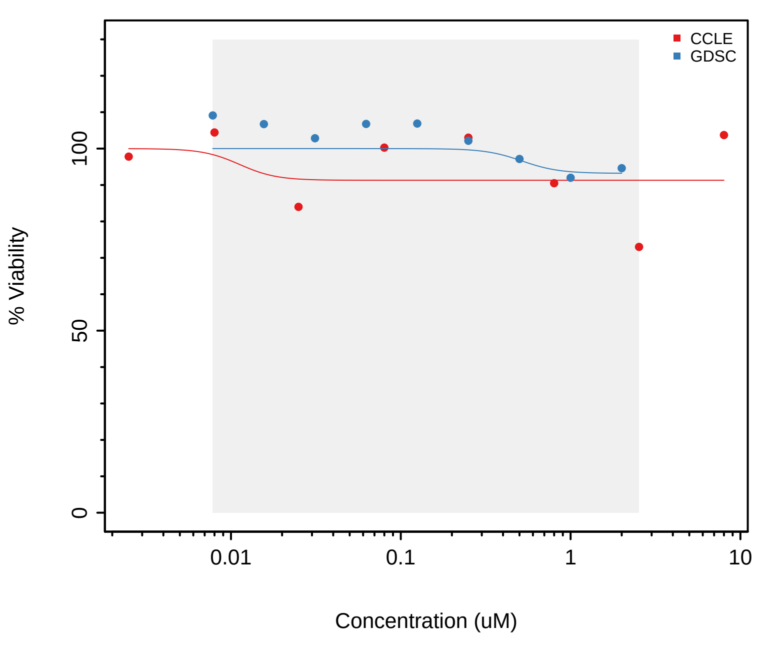

lapatinib:EM−2

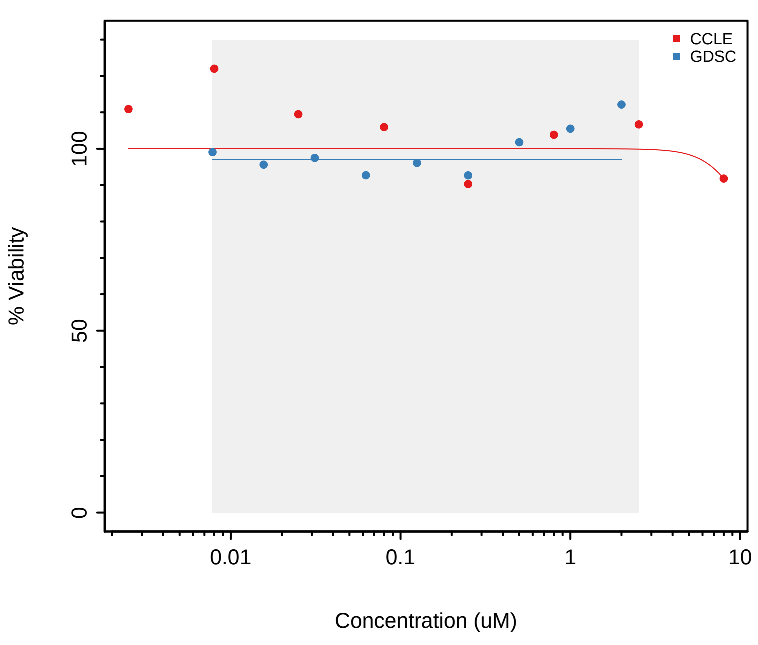

lapatinib:GB−1

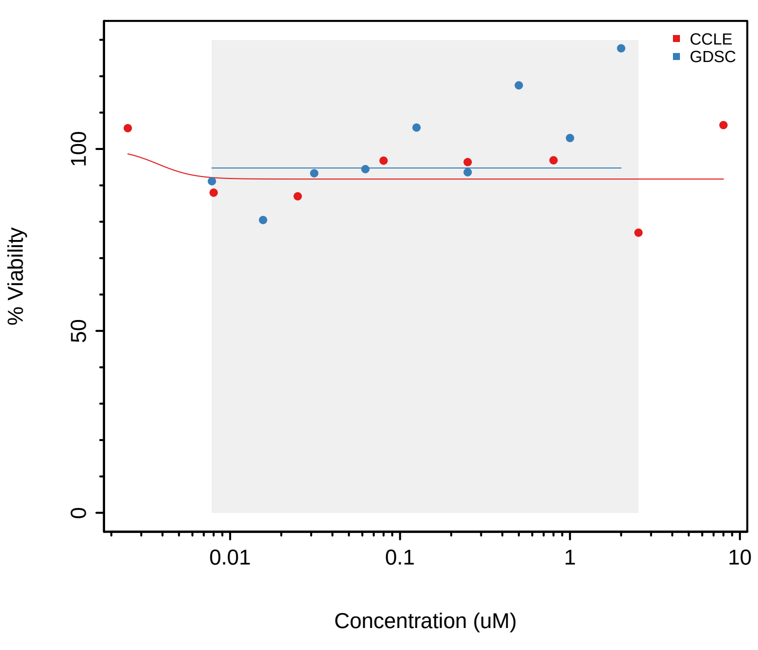

lapatinib:GCIY

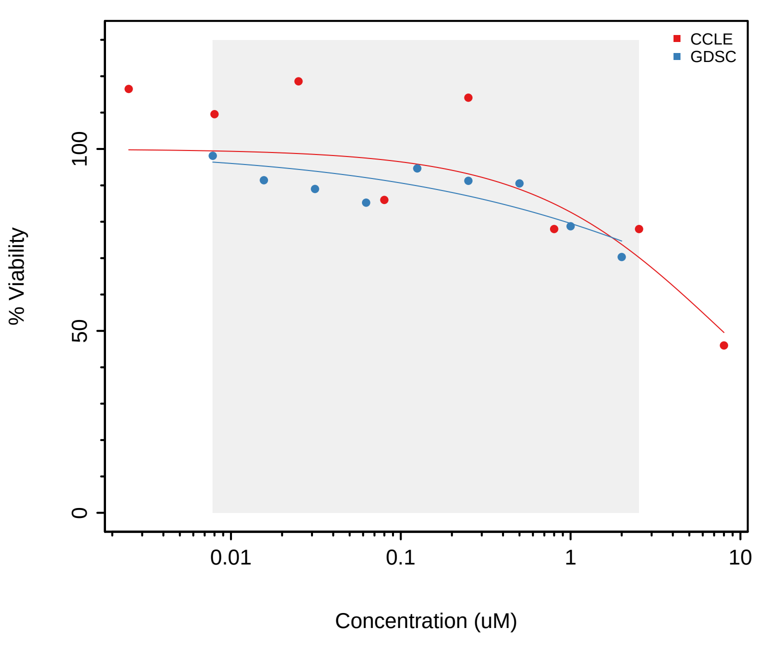

lapatinib:GI−1

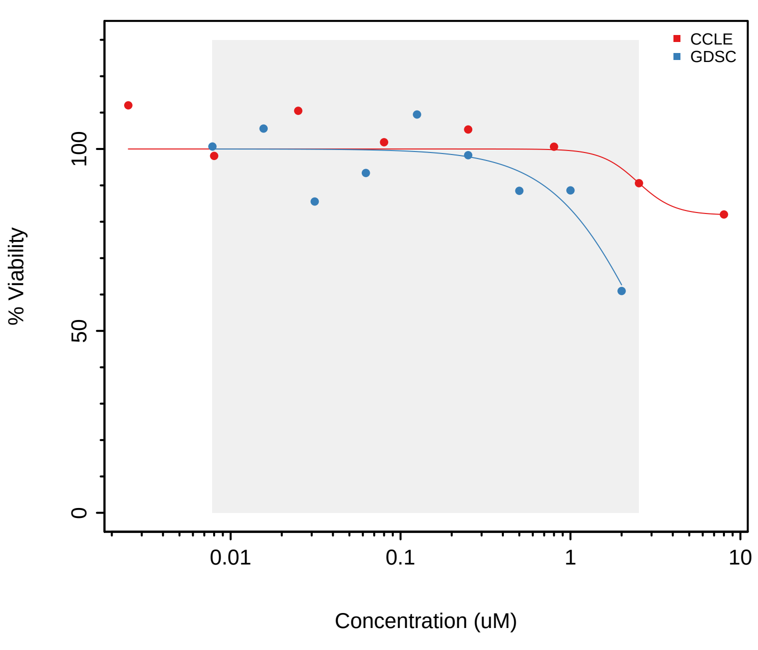

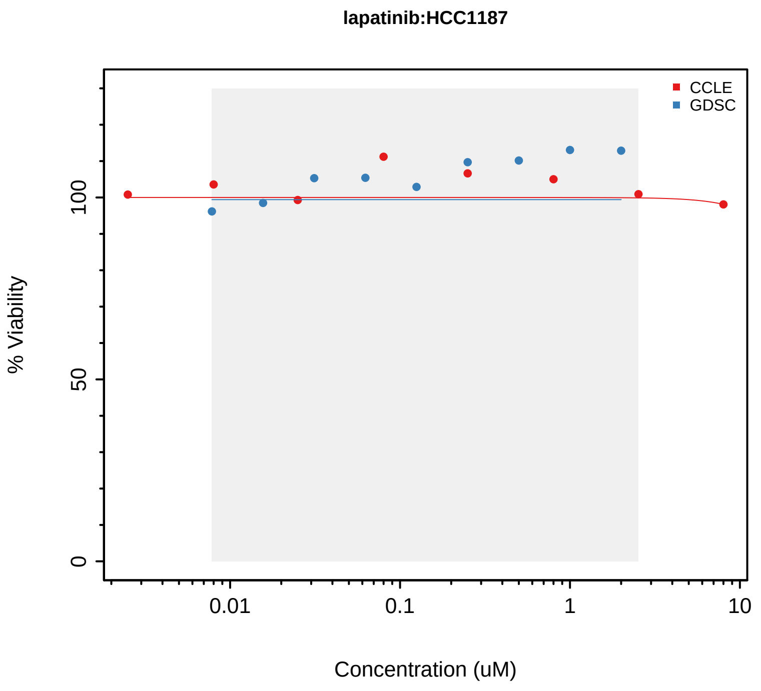
lapatinib:HCC1187
CCLE
GDSC
% Viability
0
50
100
0.01
0.1
1
10
Concentration (uM)

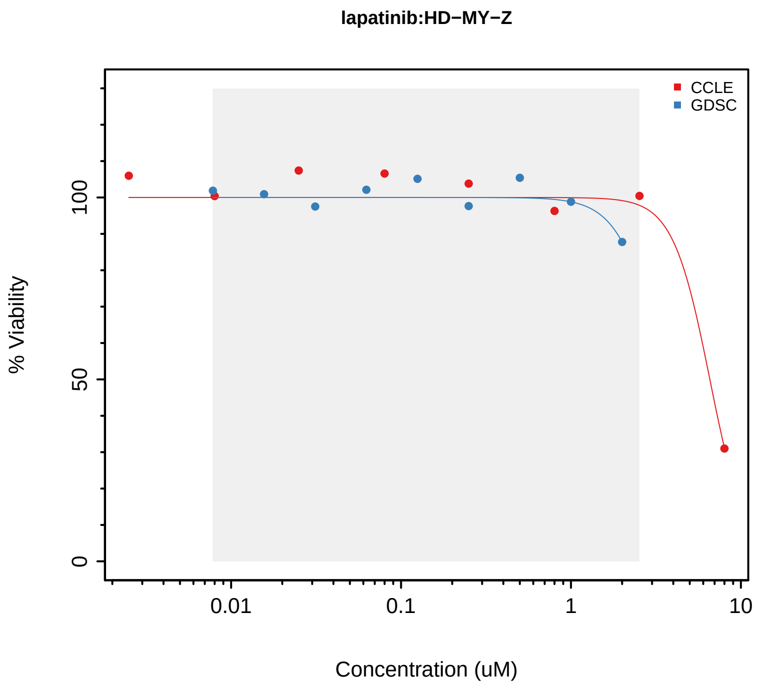
lapatinib:HD−MY−Z
CCLE
GDSC
% Viability
0
50
100
0.01
0.1
1
10
Concentration (uM)

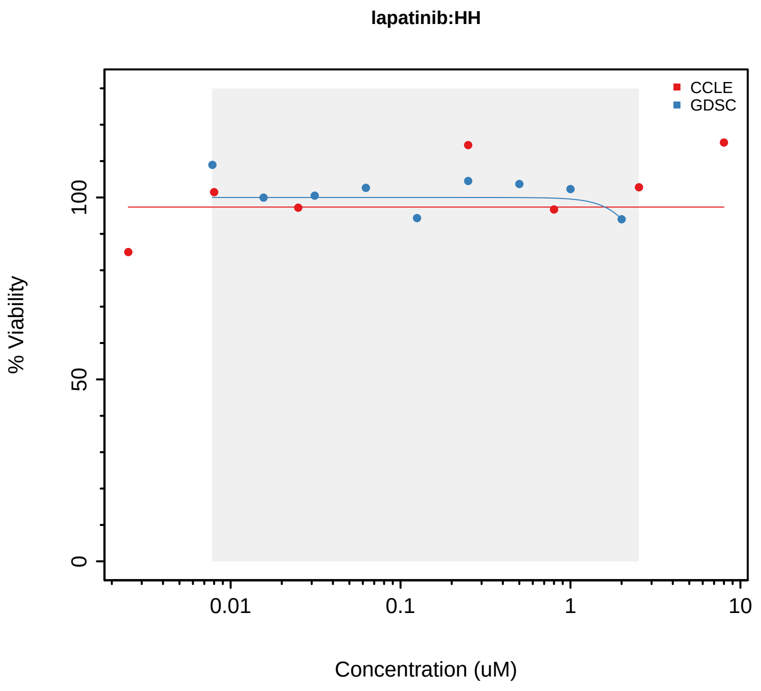
lapatinib:HH
CCLE
GDSC
% Viability
0
50
100
0.01
0.1
1
10
Concentration (uM)

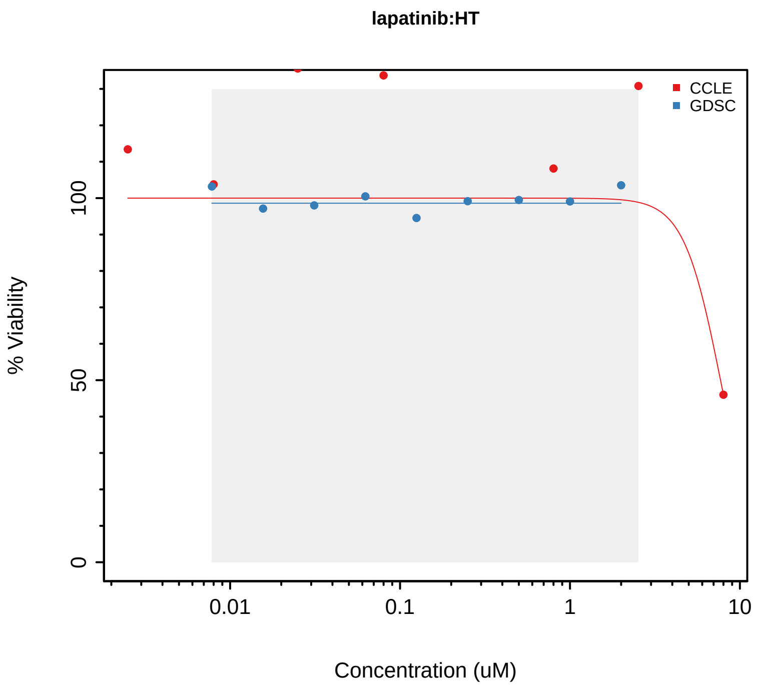
lapatinib:HT
CCLE
GDSC
% Viability
0
50
100
0.01
0.1
1
10
Concentration (uM)

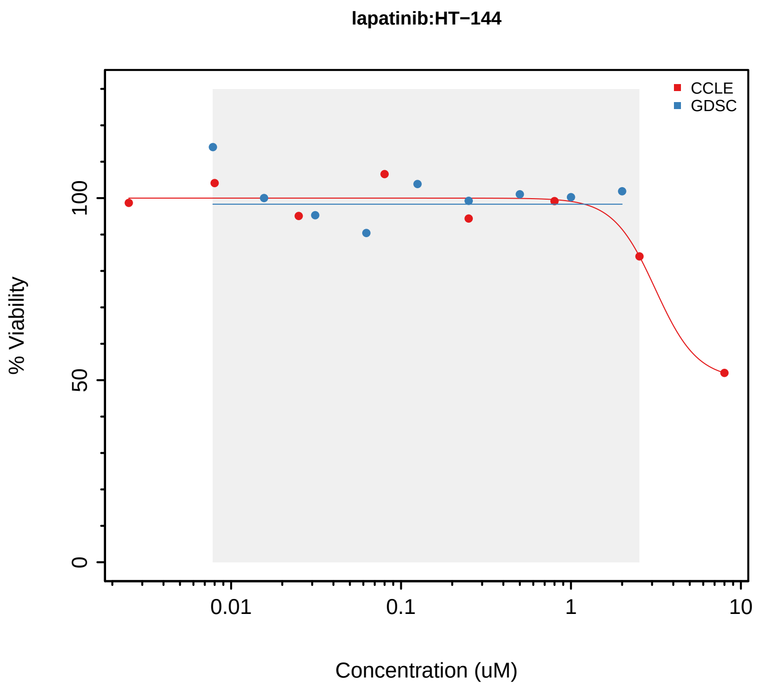
lapatinib:HT−144
CCLE
GDSC
% Viability
0
50
100
0.01
0.1
1
10
Concentration (uM)

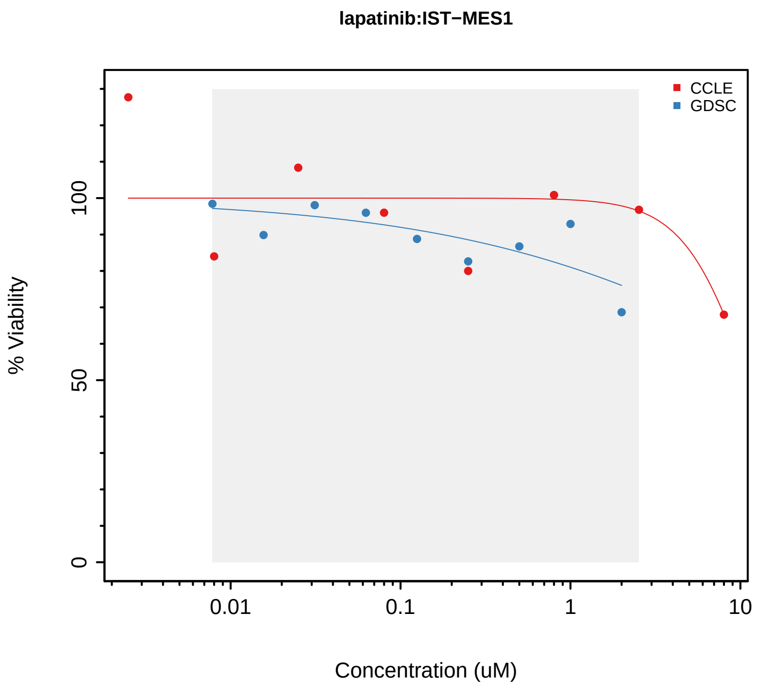
lapatinib:IST−MES1
CCLE
GDSC
% Viability
0
50
100
0.01
0.1
1
10
Concentration (uM)

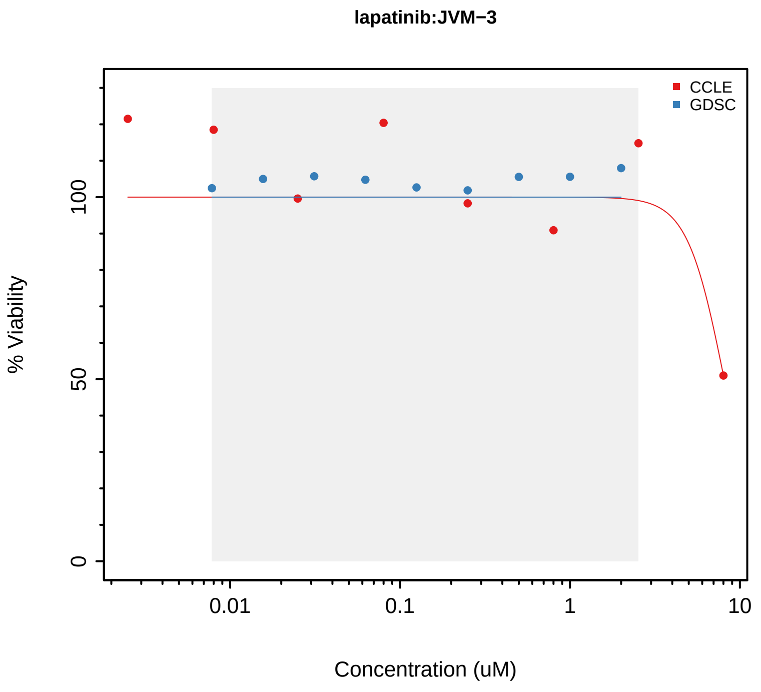
lapatinib:JVM−3
CCLE
GDSC
% Viability
0
50
100
0.01
0.1
1
10
Concentration (uM)

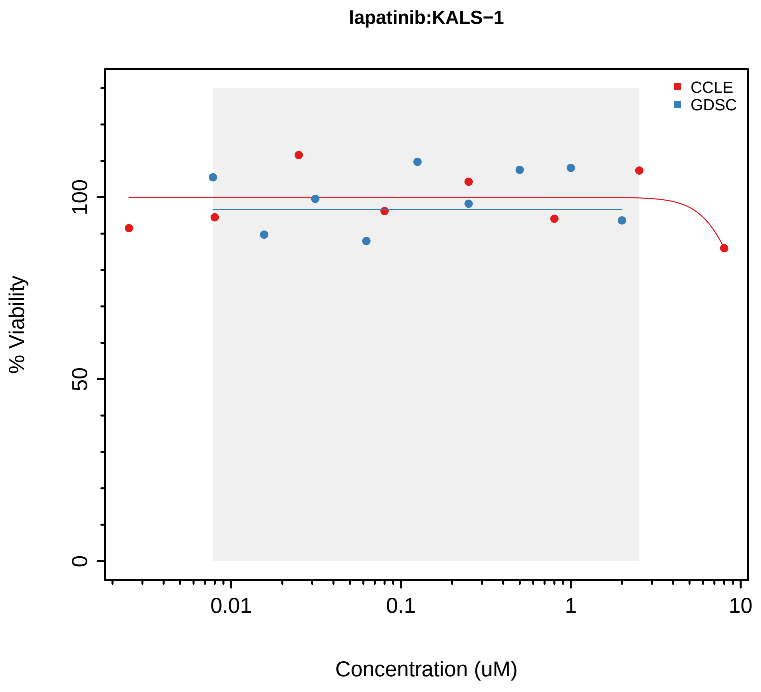
lapatinib:KALS−1
CCLE
GDSC
% Viability
0
50
100
0.01
0.1
1
10
Concentration (uM)

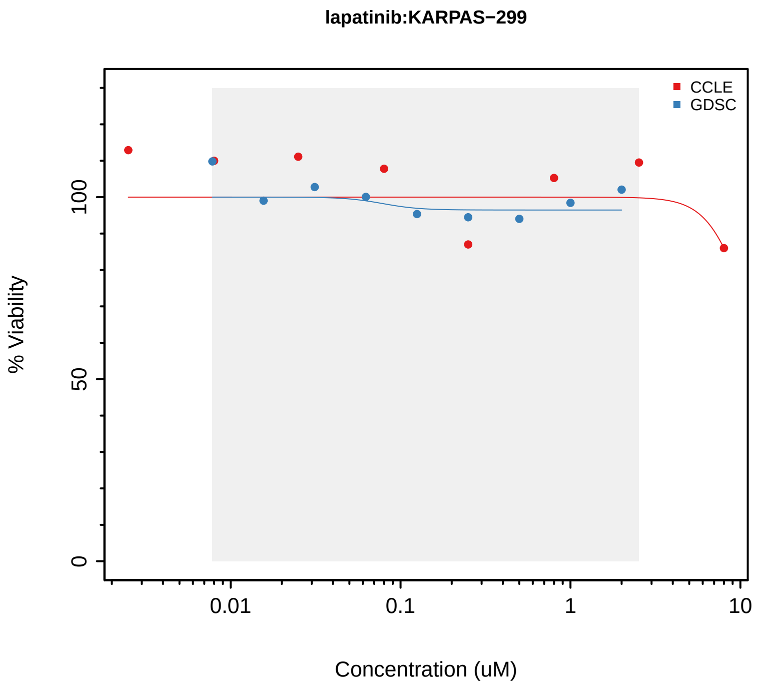
lapatinib:KARPAS−299
CCLE
GDSC
% Viability
0
50
100
0.01
0.1
1
10
Concentration (uM)

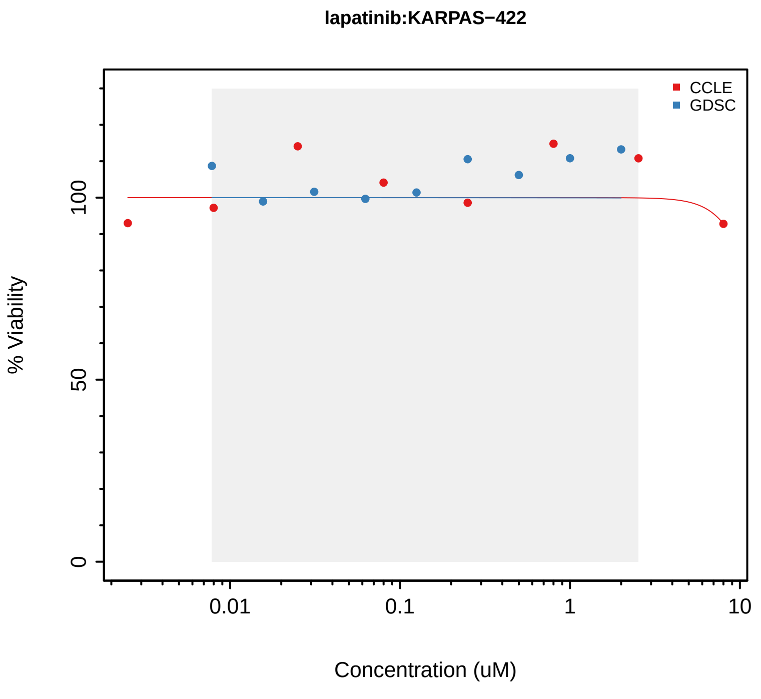
lapatinib:KARPAS−422
CCLE
GDSC
% Viability
0
50
100
0.01
0.1
1
10
Concentration (uM)

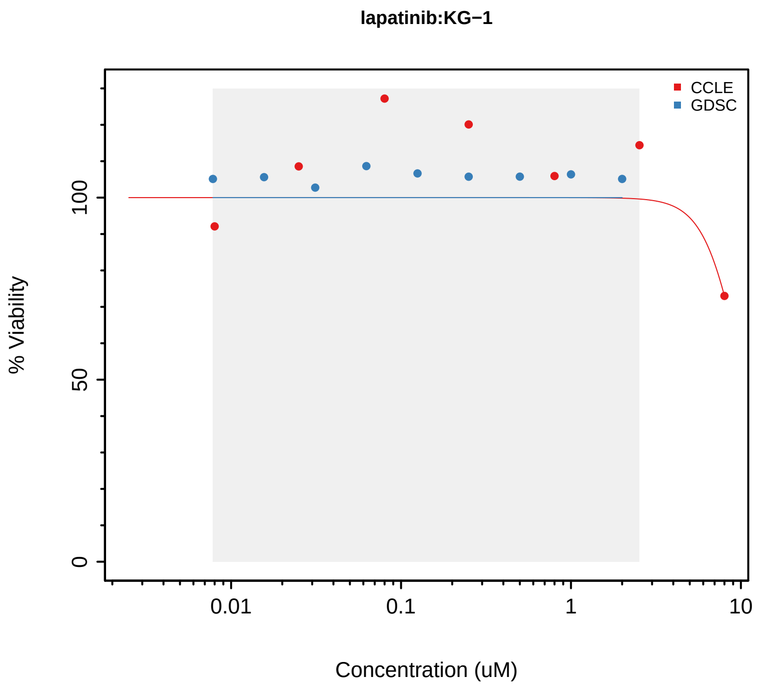
lapatinib:KG−1
CCLE
GDSC
% Viability
0
50
100
0.01
0.1
1
10
Concentration (uM)

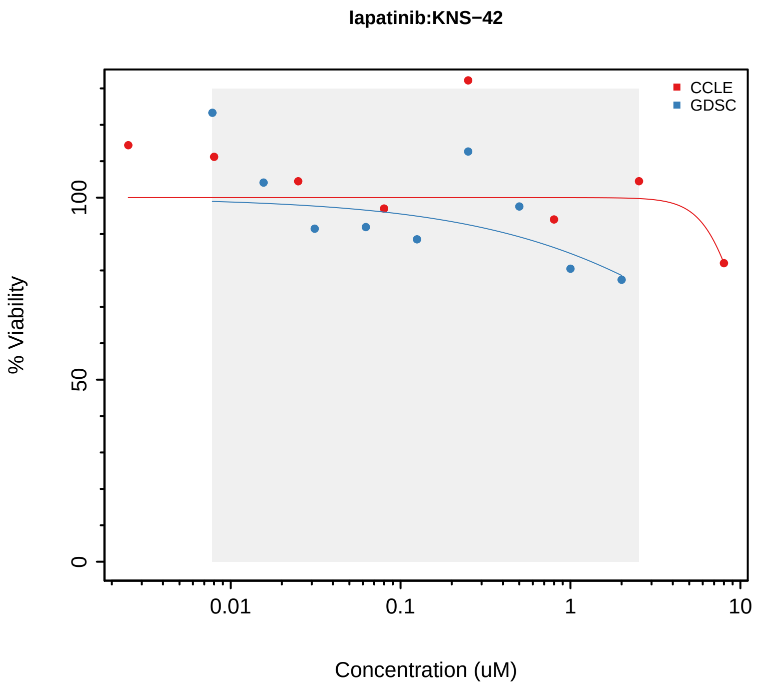
lapatinib:KNS−42
CCLE
GDSC
% Viability
0
50
100
0.01
0.1
1
10
Concentration (uM)

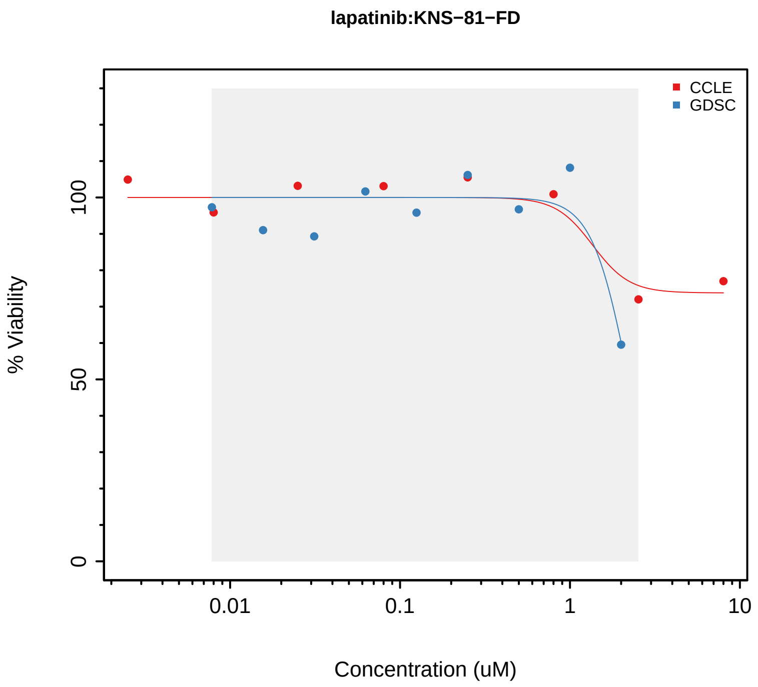
lapatinib:KNS−81−FD
CCLE
GDSC
% Viability
0
50
100
0.01
0.1
1
10
Concentration (uM)

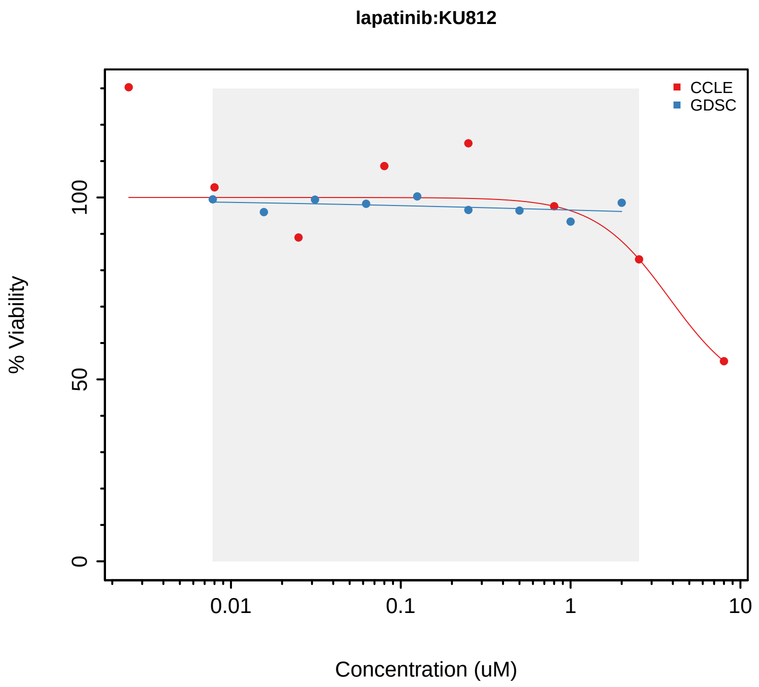
lapatinib:KU812
CCLE
GDSC
% Viability
0
50
100
0.01
0.1
1
10
Concentration (uM)

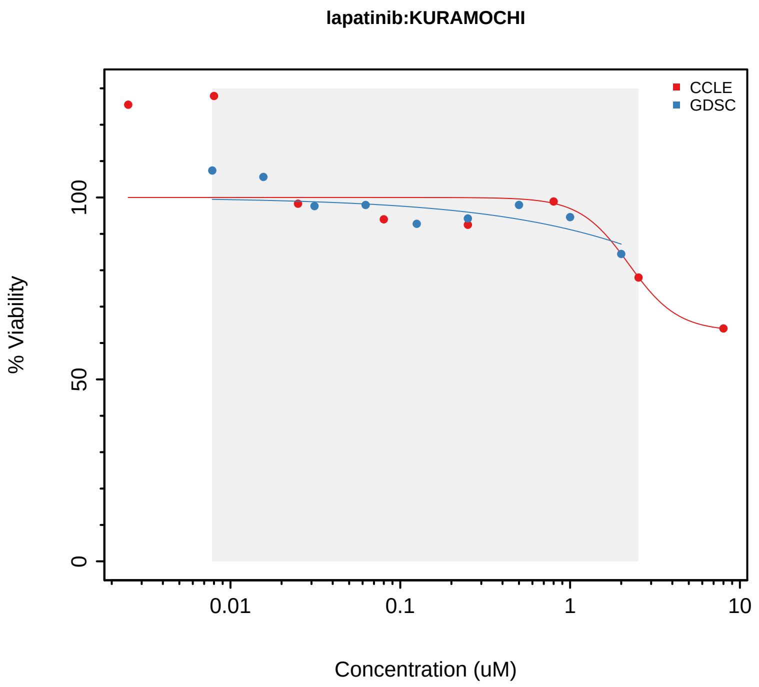
lapatinib:KURAMOCHI
CCLE
GDSC
% Viability
0
50
100
0.01
0.1
1
10
Concentration (uM)

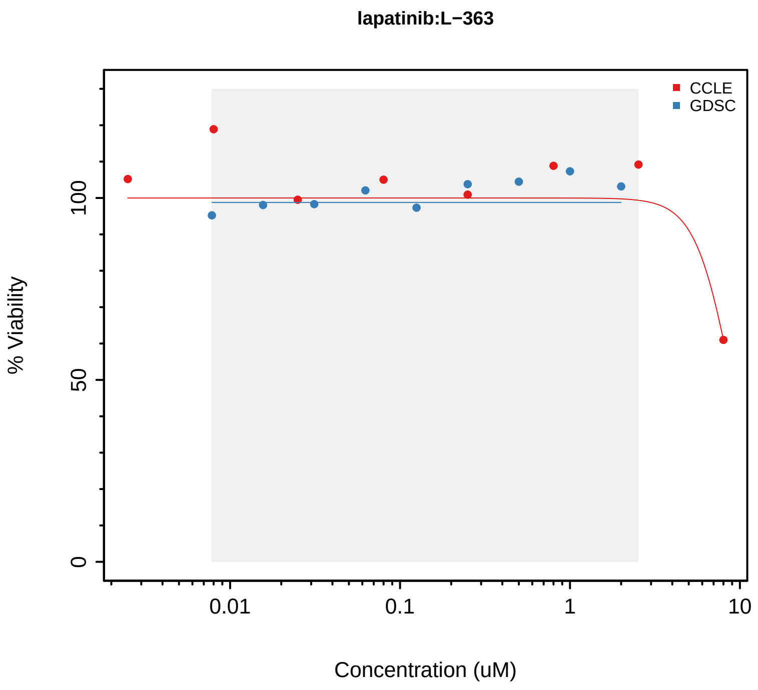
lapatinib:L−363
CCLE
GDSC
% Viability
0
50
100
0.01
0.1
1
10
Concentration (uM)

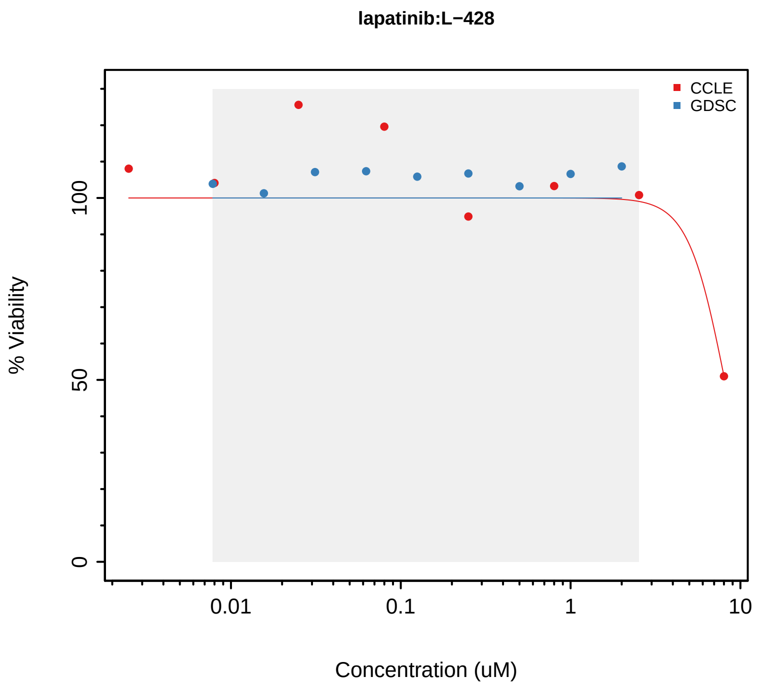
lapatinib:L−428
CCLE
GDSC
% Viability
0
50
100
0.01
0.1
1
10
Concentration (uM)

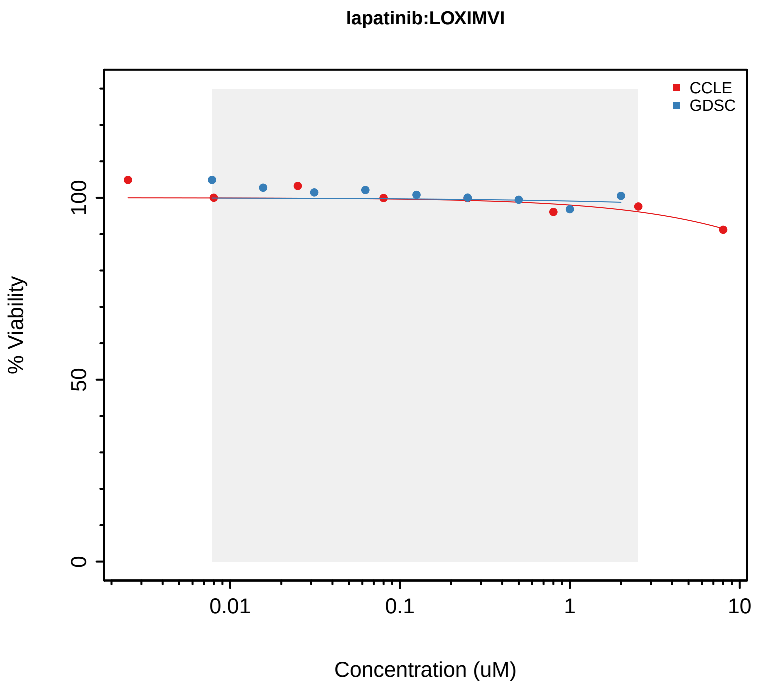
lapatinib:LOXIMVI
CCLE
GDSC
% Viability
0
50
100
0.01
0.1
1
10
Concentration (uM)

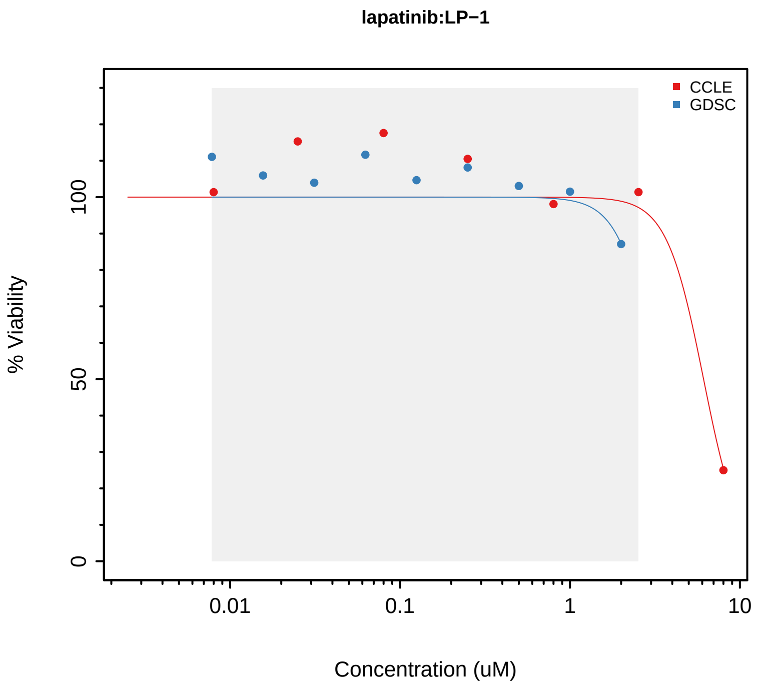
lapatinib:LP−1
CCLE
GDSC
% Viability
0
50
100
0.01
0.1
1
10
Concentration (uM)

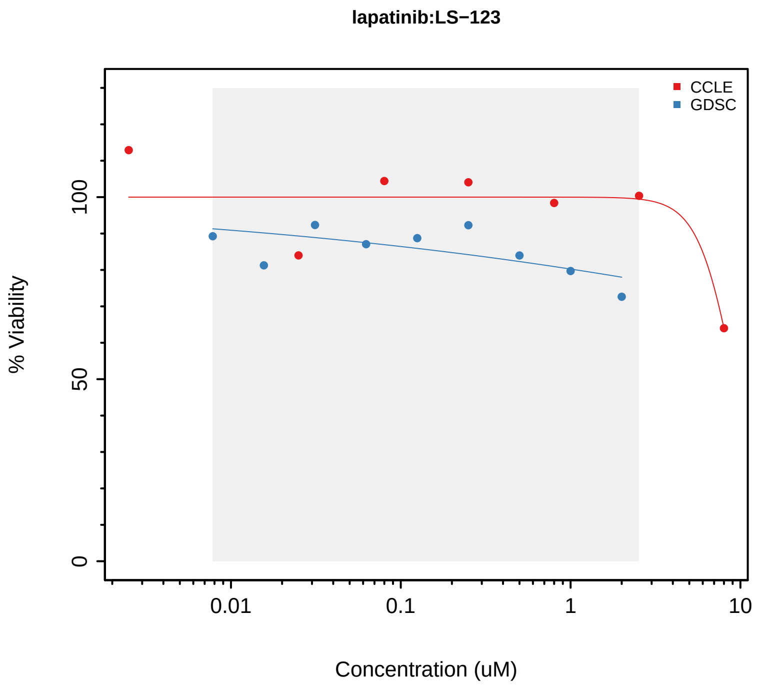
lapatinib:LS−123
CCLE
GDSC
% Viability
0
50
100
0.01
0.1
1
10
Concentration (uM)

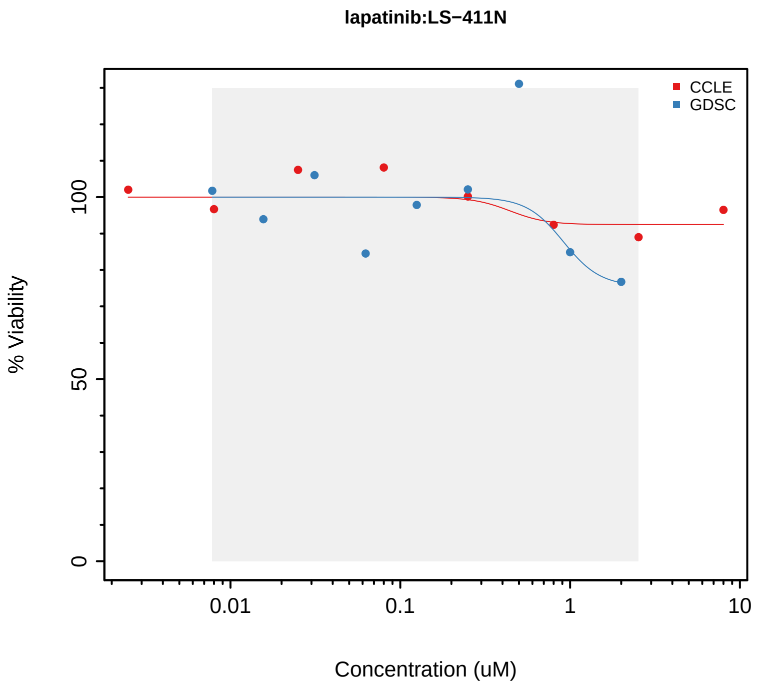
lapatinib:LS−411N
CCLE
GDSC
% Viability
0
50
100
0.01
0.1
1
10
Concentration (uM)

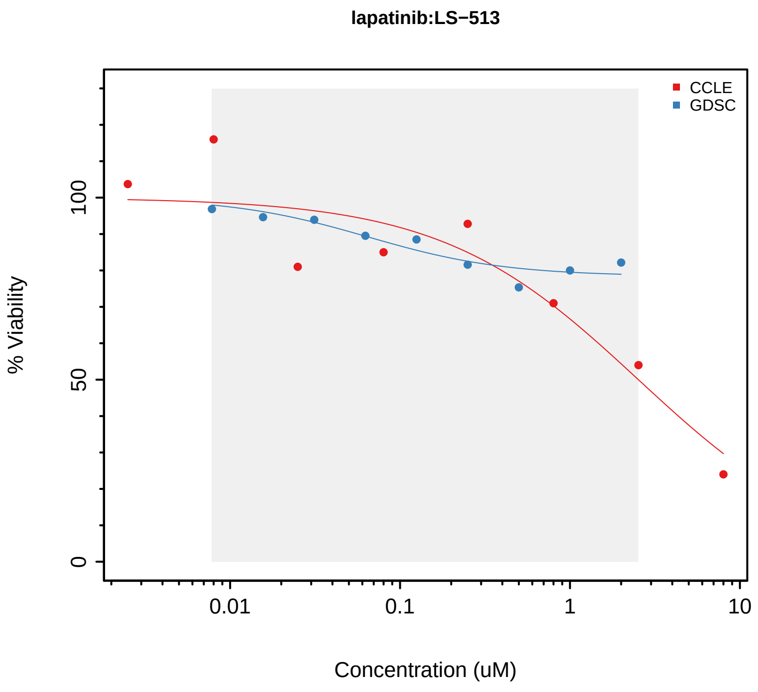
lapatinib:LS−513
CCLE
GDSC
% Viability
0
50
100
0.01
0.1
1
10
Concentration (uM)

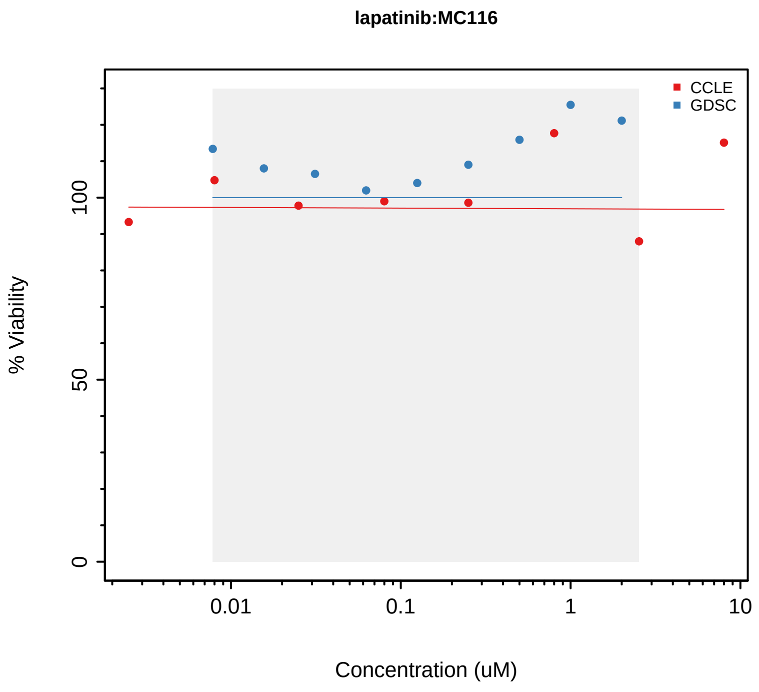
lapatinib:MC116
CCLE
GDSC
% Viability
0
50
100
0.01
0.1
1
10
Concentration (uM)

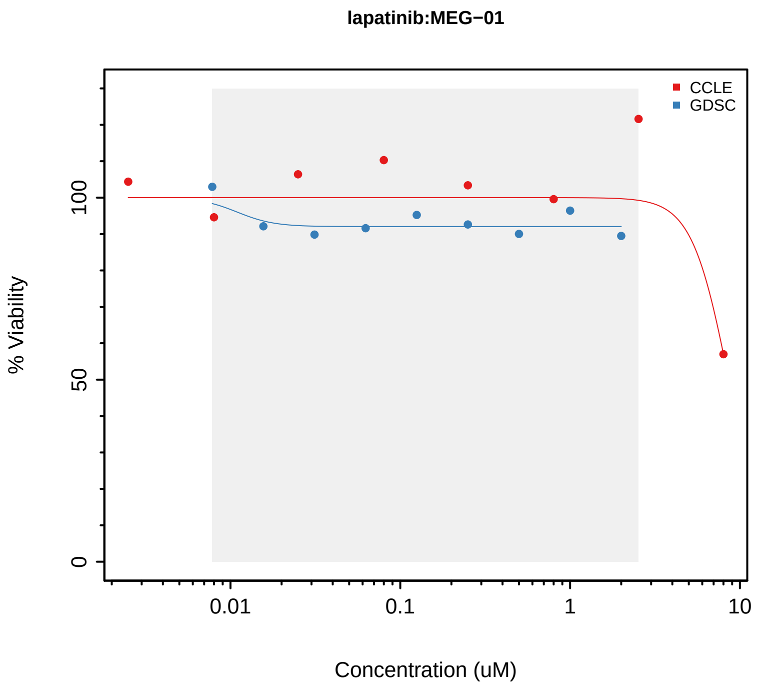
lapatinib:MEG−01
CCLE
GDSC
% Viability
0
50
100
0.01
0.1
1
10
Concentration (uM)

lapatinib:MPP−89

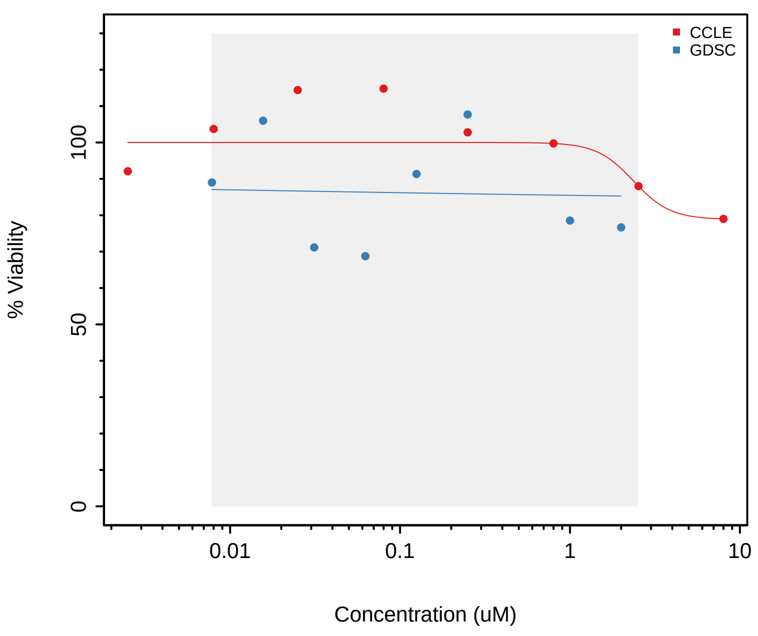

lapatinib:MSTO−211H

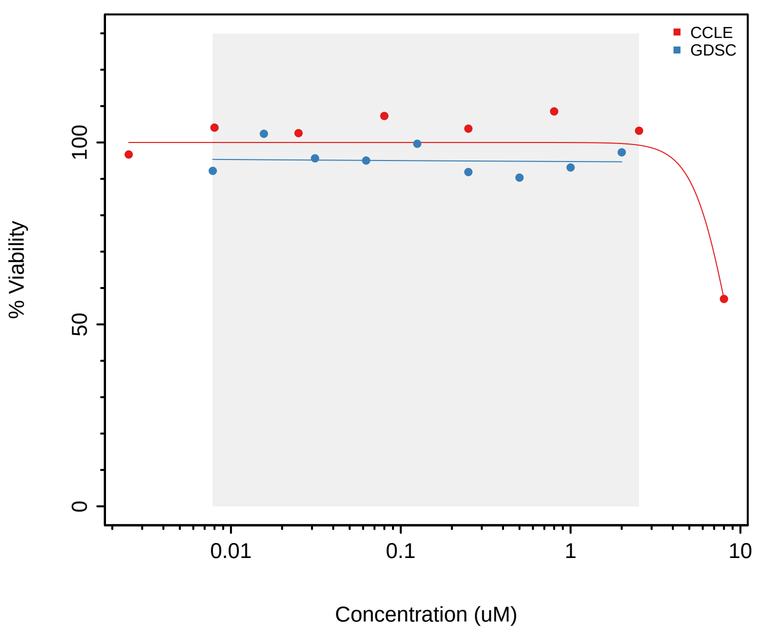

lapatinib:NCI−H1092

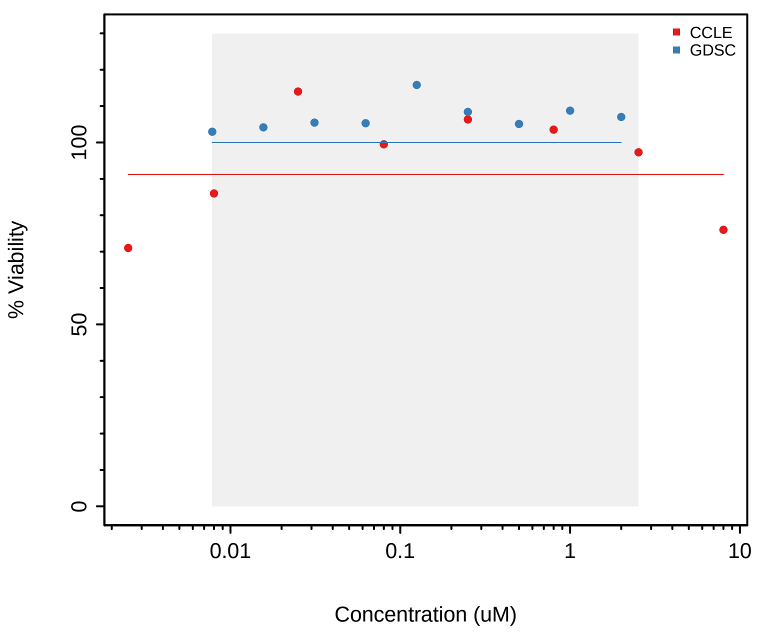

lapatinib:NCI−H1155

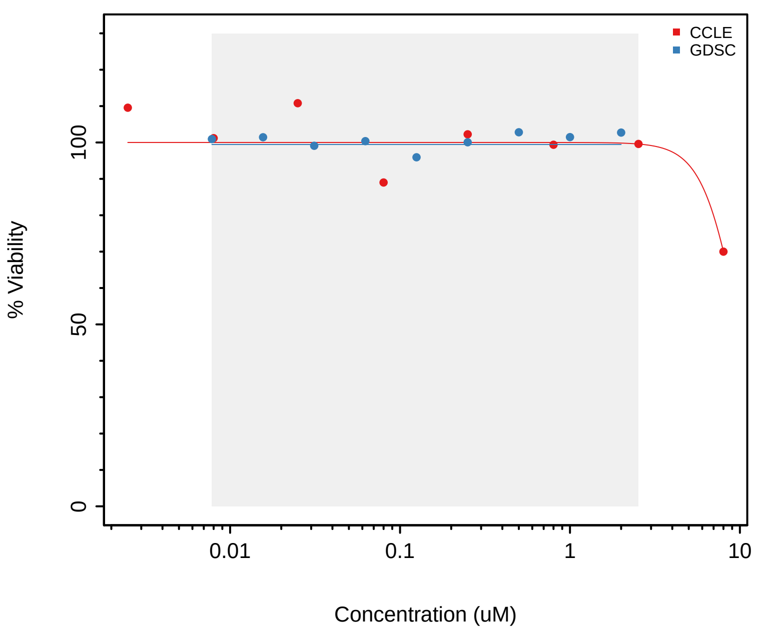

lapatinib:NCI−H1355

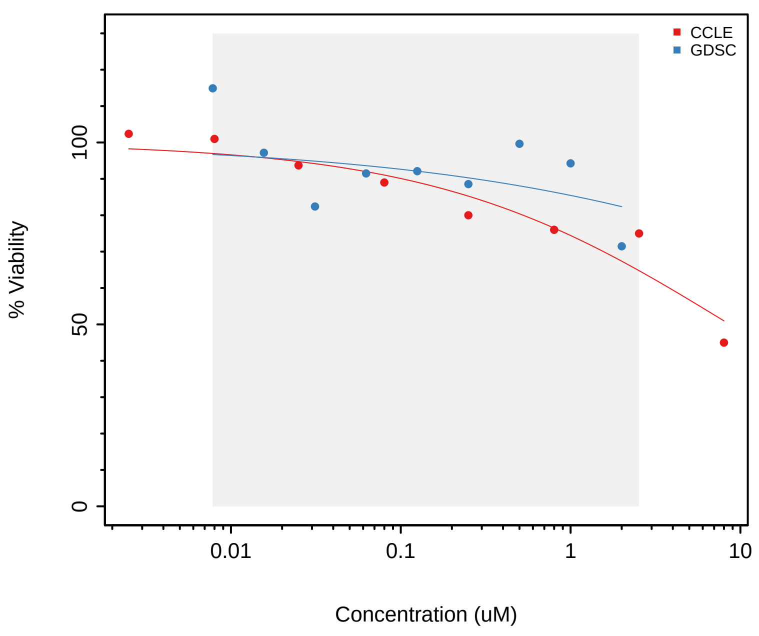

lapatinib:NCI−H1581

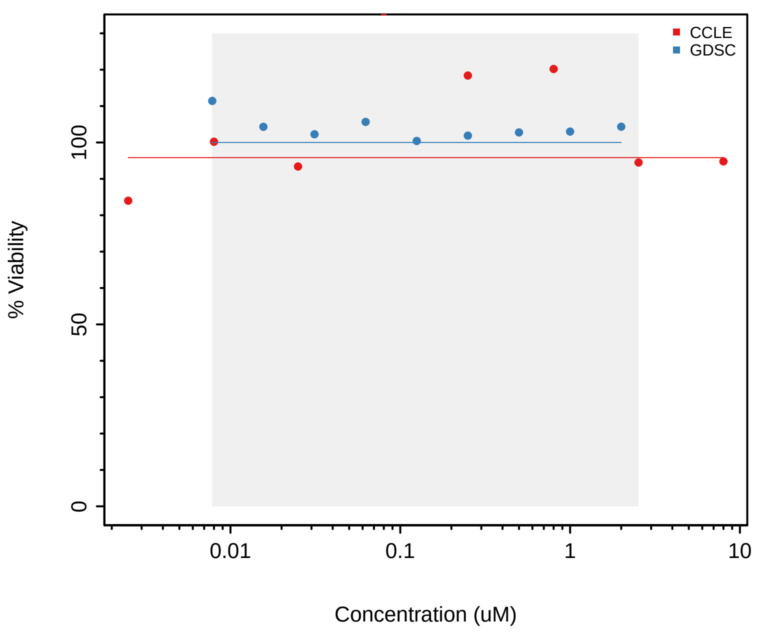

lapatinib:NCI−H1648

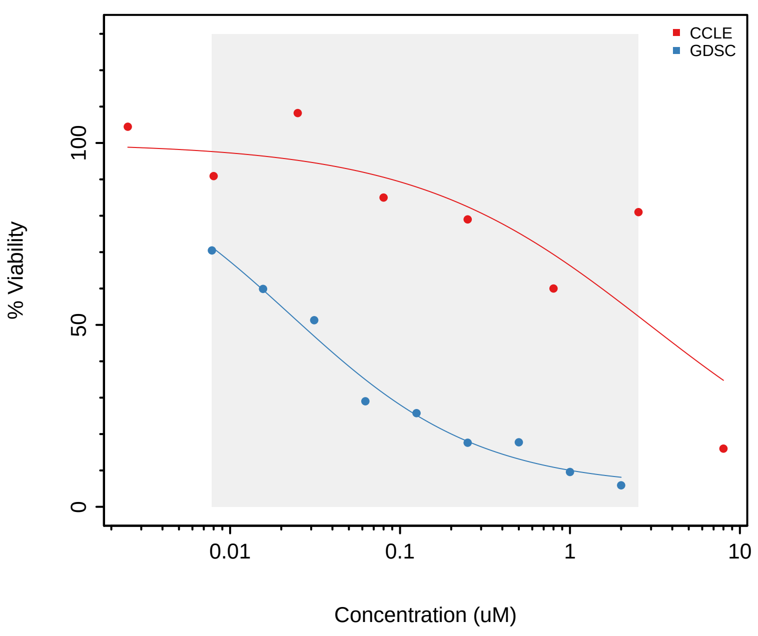

lapatinib:NCI−H1666

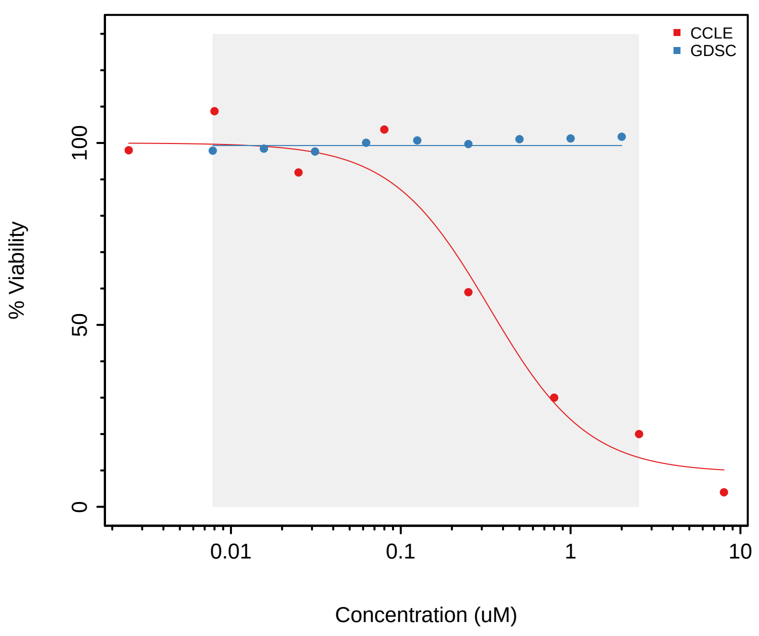

lapatinib:NCI−H1694

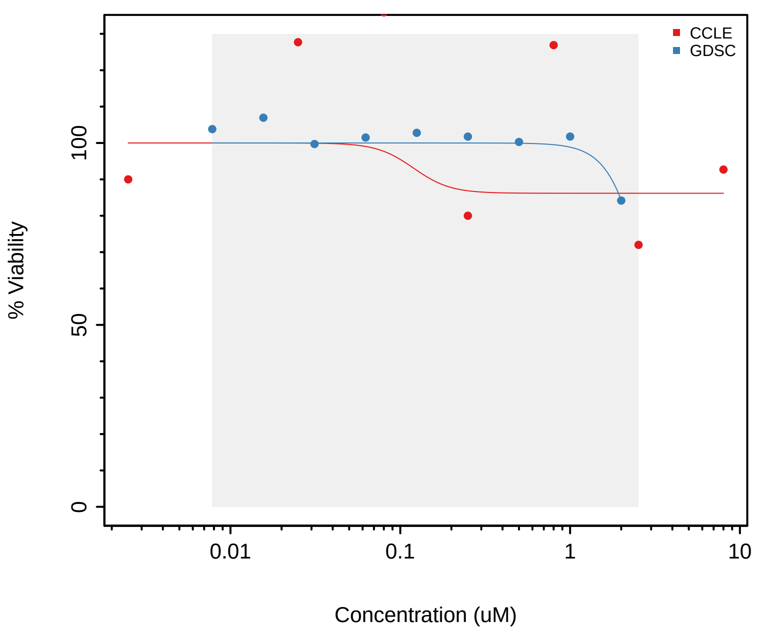

lapatinib:NCI−H1975

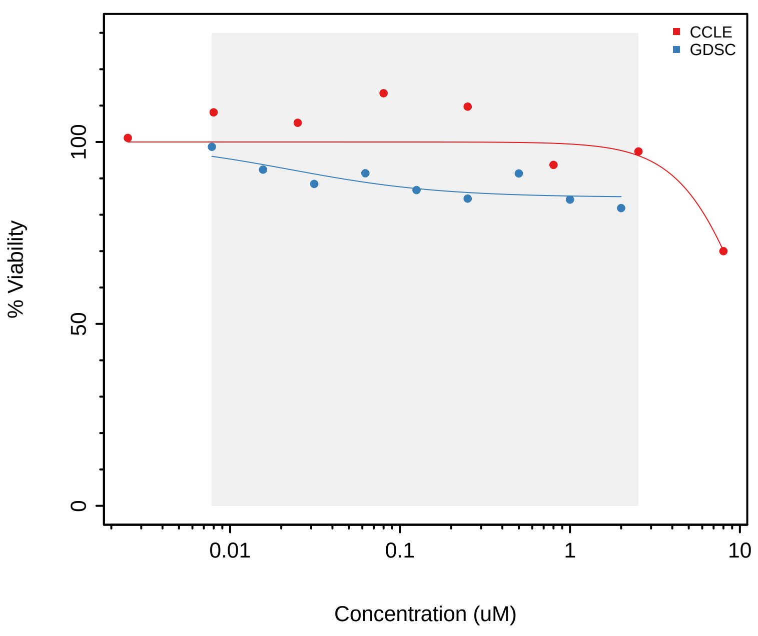

lapatinib:NCI−H226

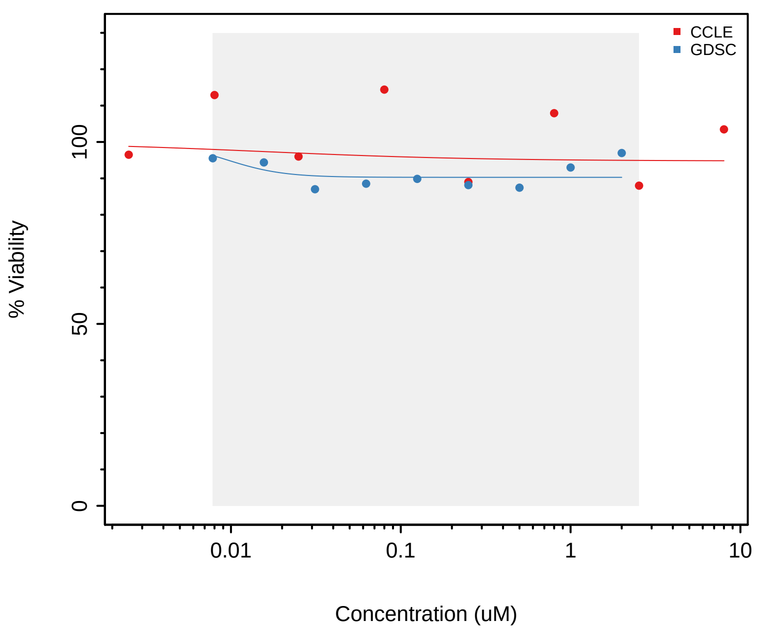

lapatinib:NCI−H23

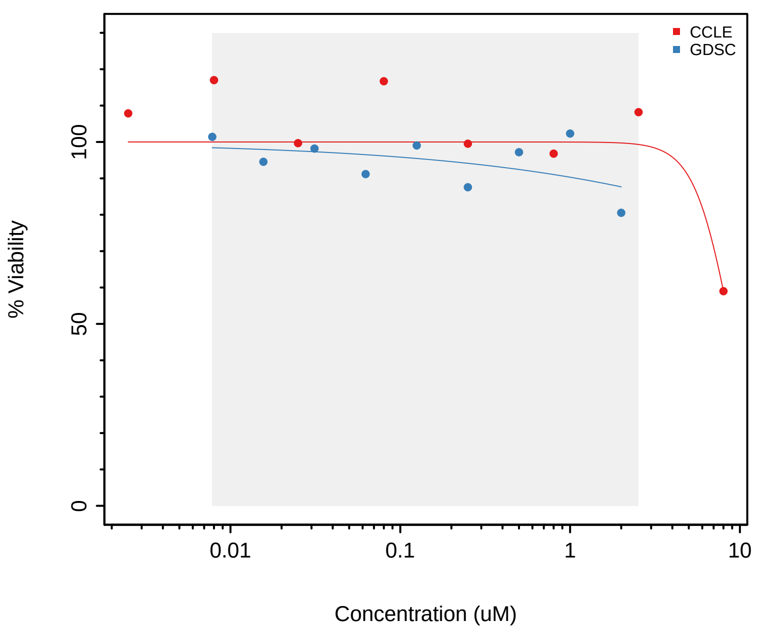

lapatinib:NCI−H747

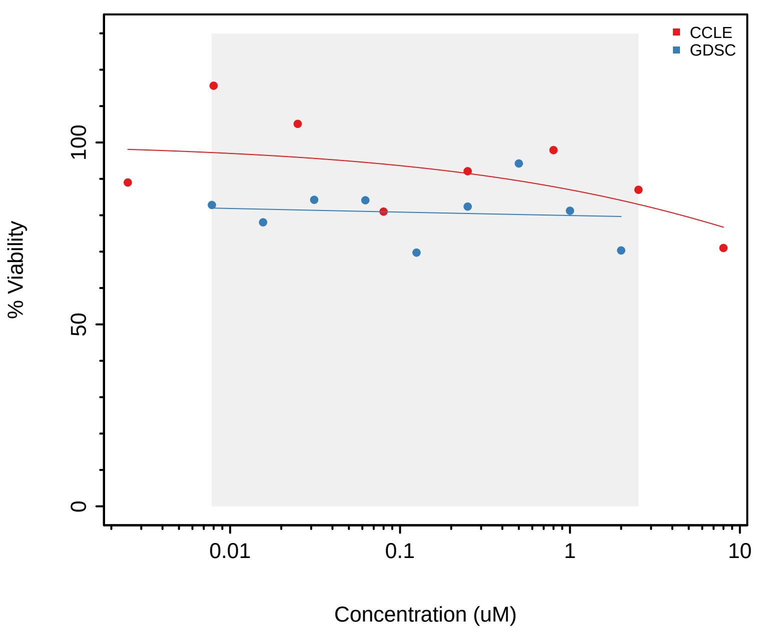

lapatinib:OCI−AML2

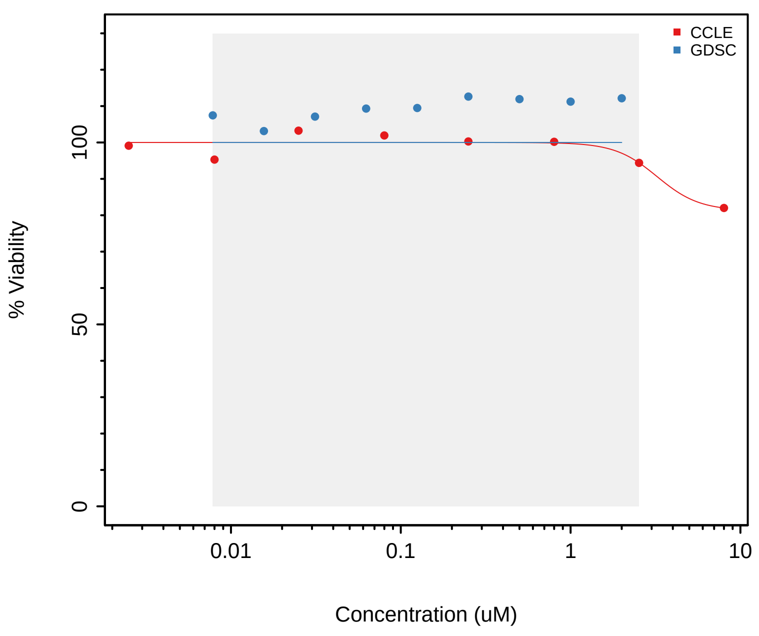

lapatinib:ONS−76

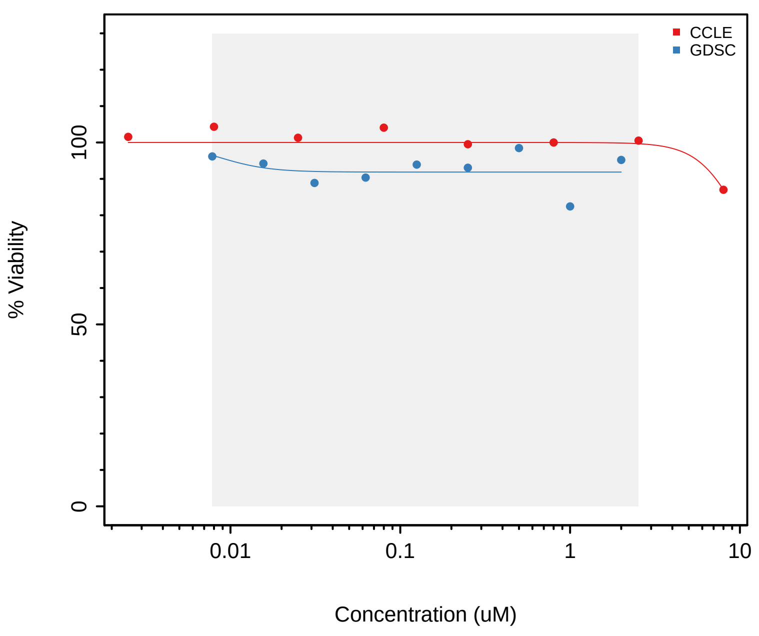

lapatinib:OPM−2

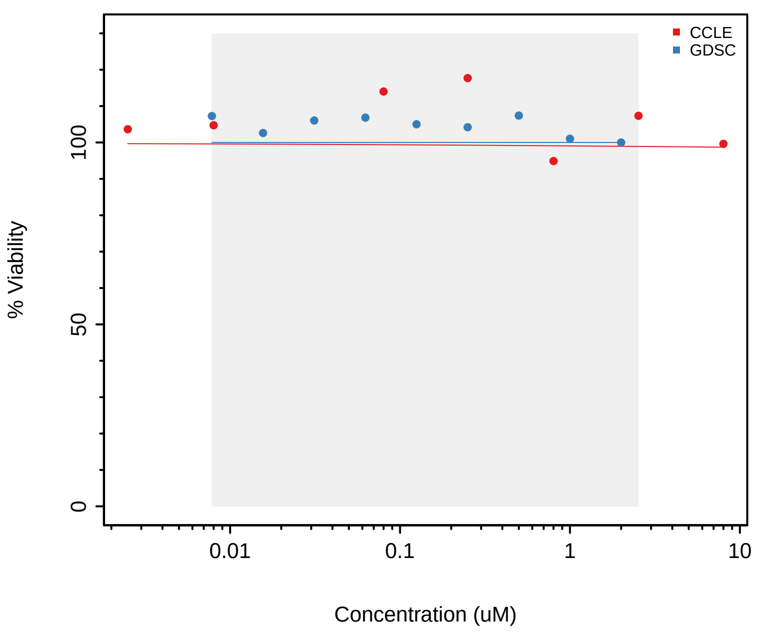

lapatinib:OVCAR−4

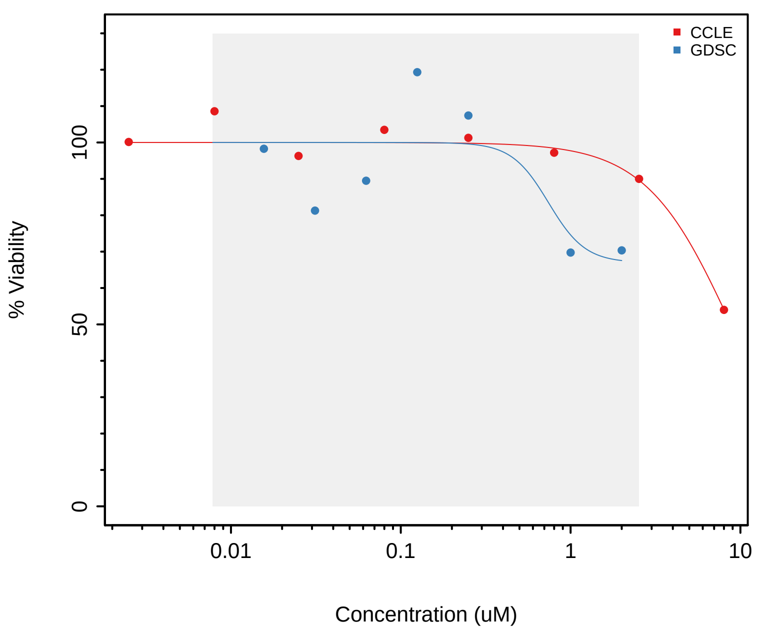

lapatinib:P31−FUJ

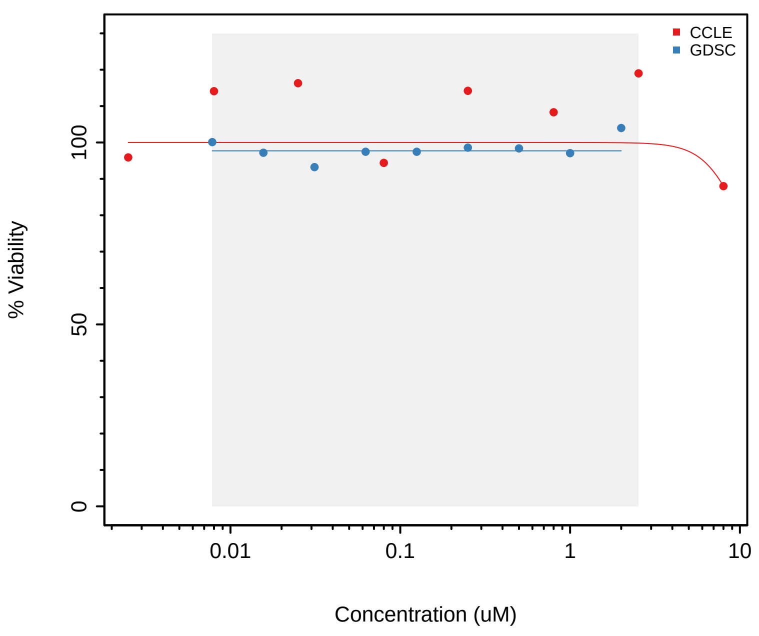

lapatinib:PSN1

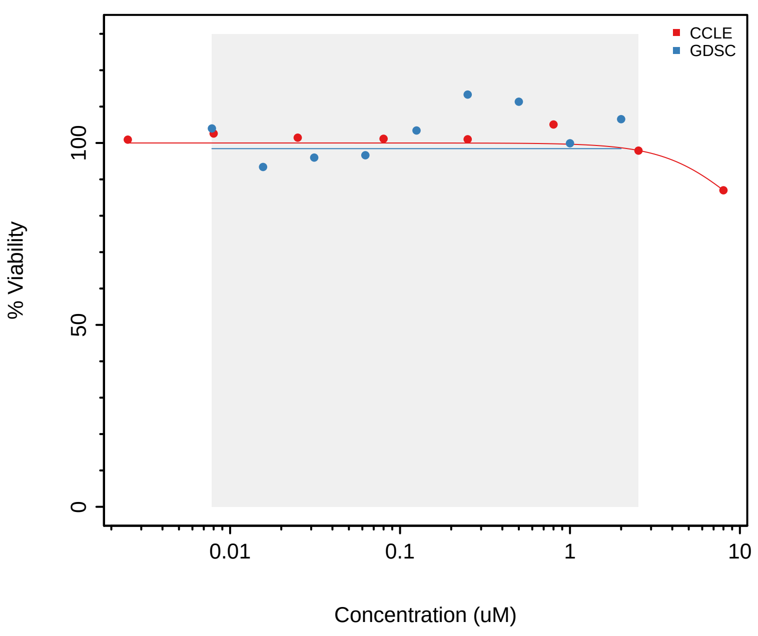

lapatinib:Raji

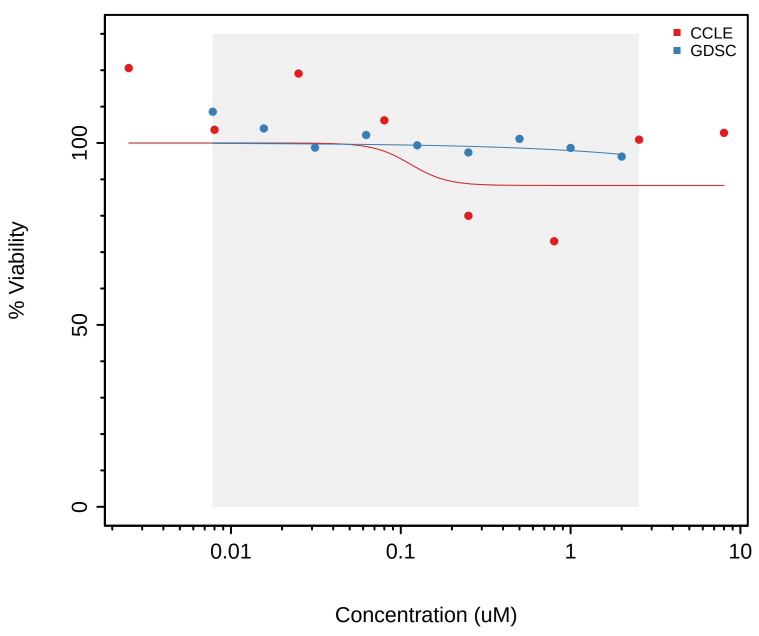

lapatinib:REH

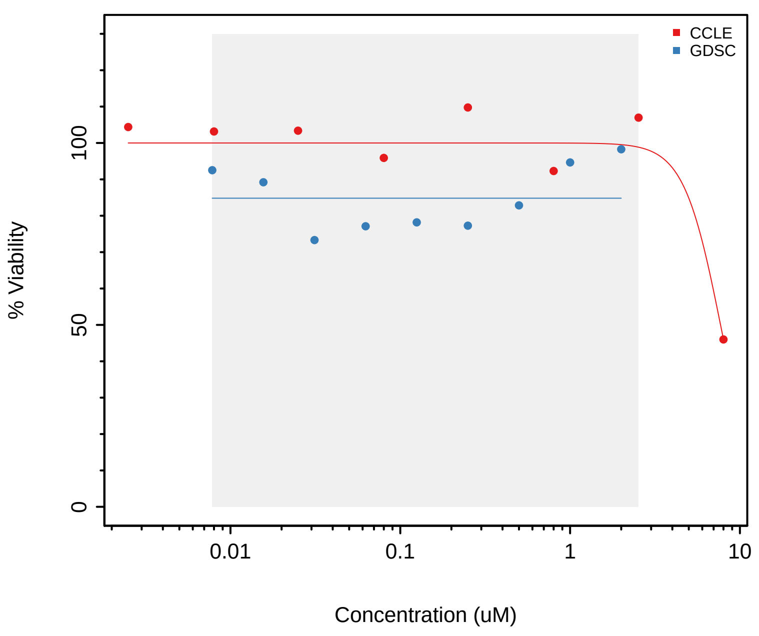

lapatinib:RKO

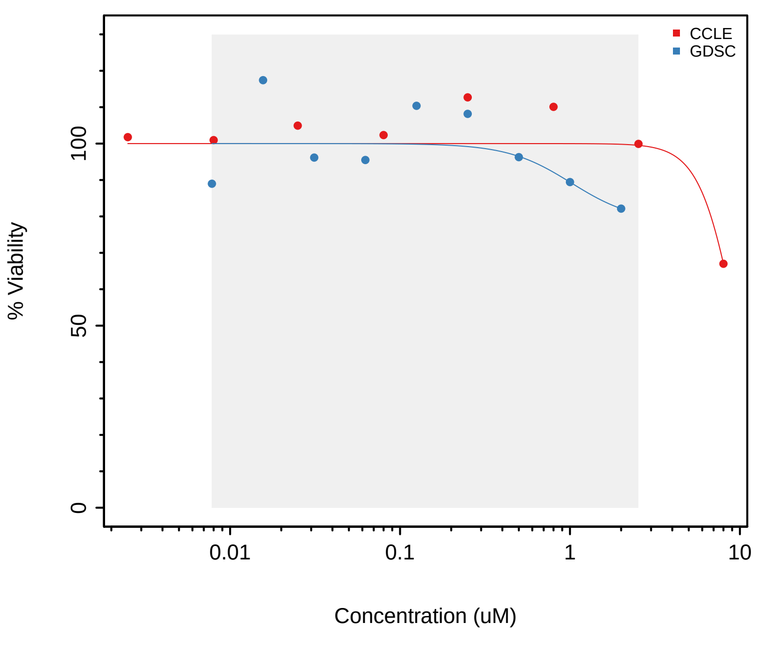

lapatinib:RL95−2

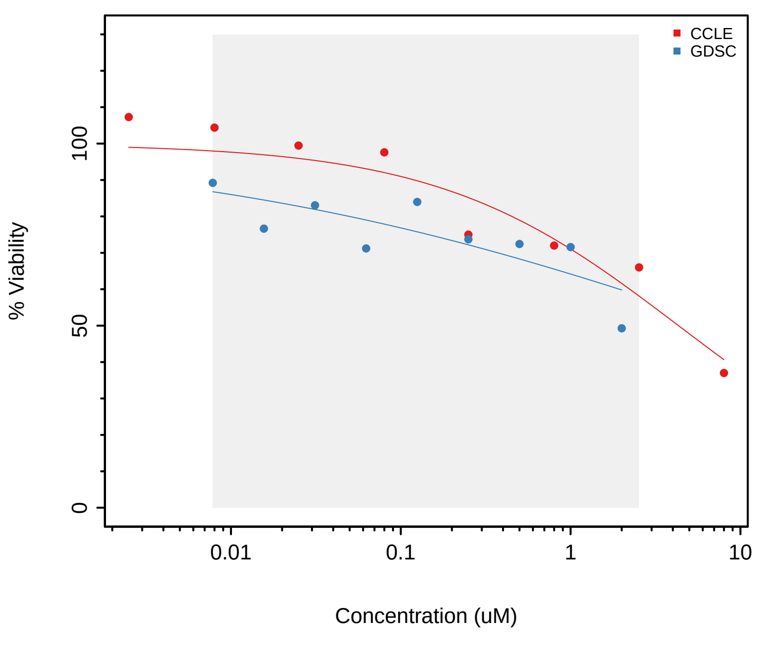

lapatinib:RPMI−8402

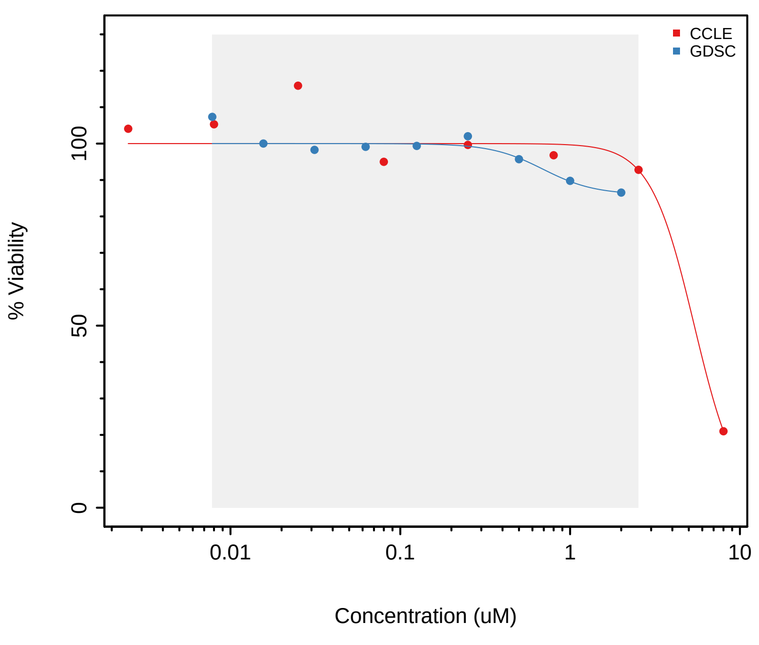

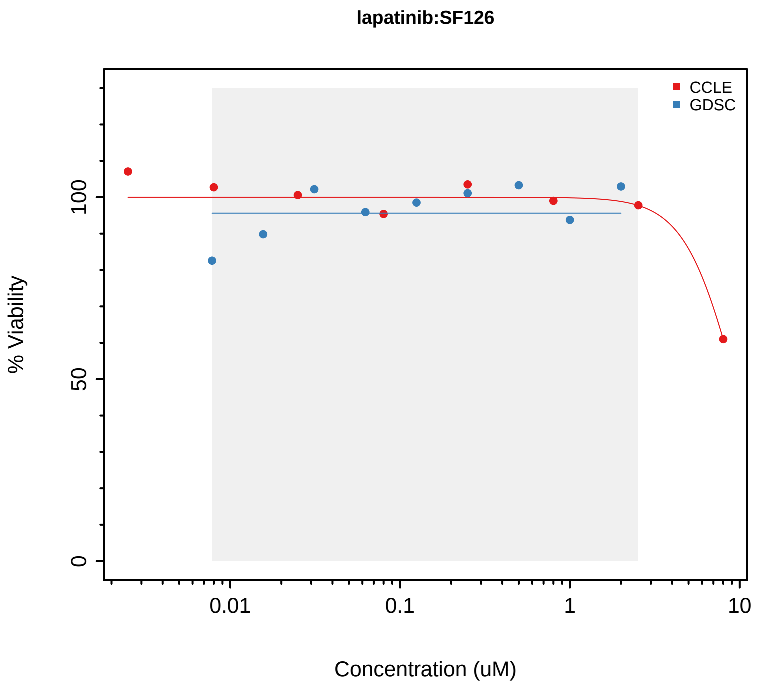
lapatinib:SF126
CCLE
GDSC
% Viability
0
50
100
0.01
0.1
1
10
Concentration (uM)

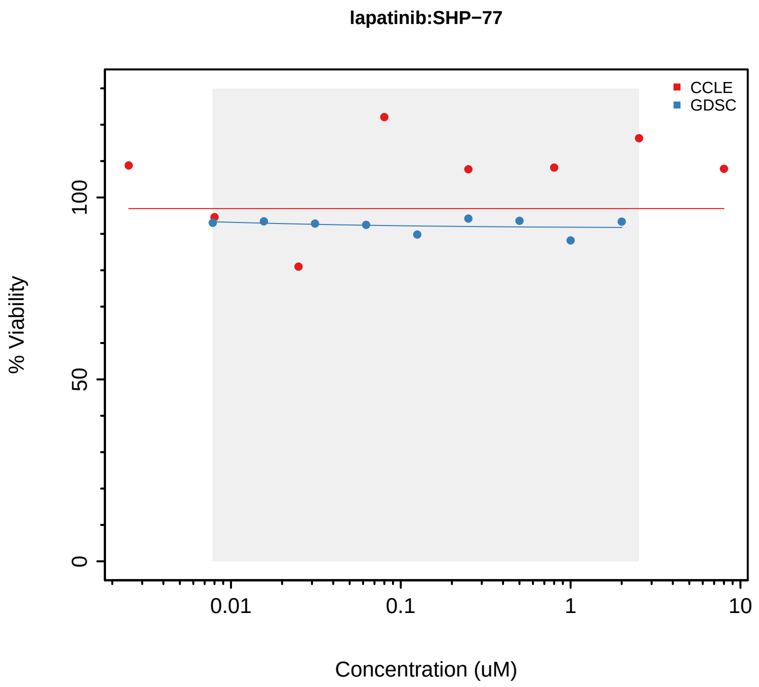
lapatinib:SHP−77
CCLE
GDSC
% Viability
0
50
100
0.01
0.1
1
10
Concentration (uM)

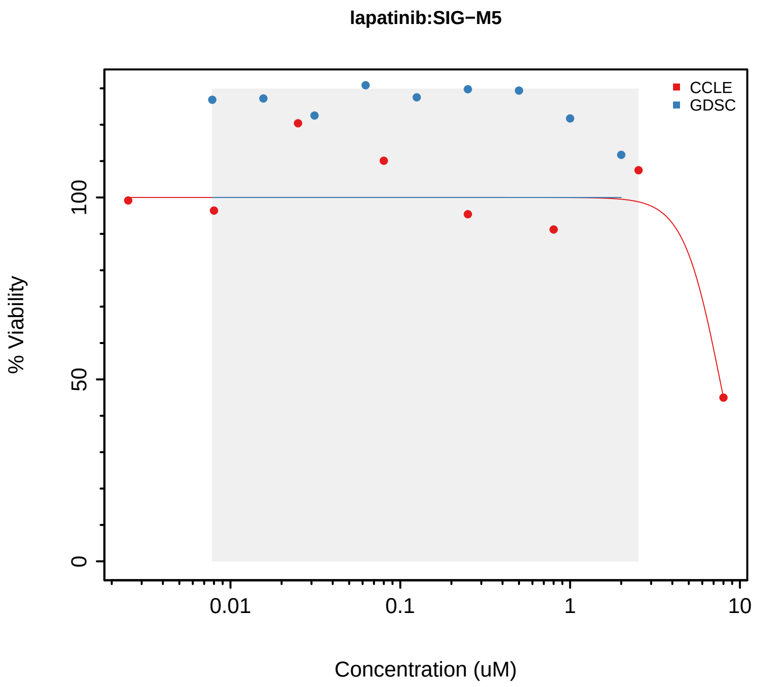
lapatinib:SIG−M5
CCLE
GDSC
% Viability
0
50
100
0.01
0.1
1
10
Concentration (uM)

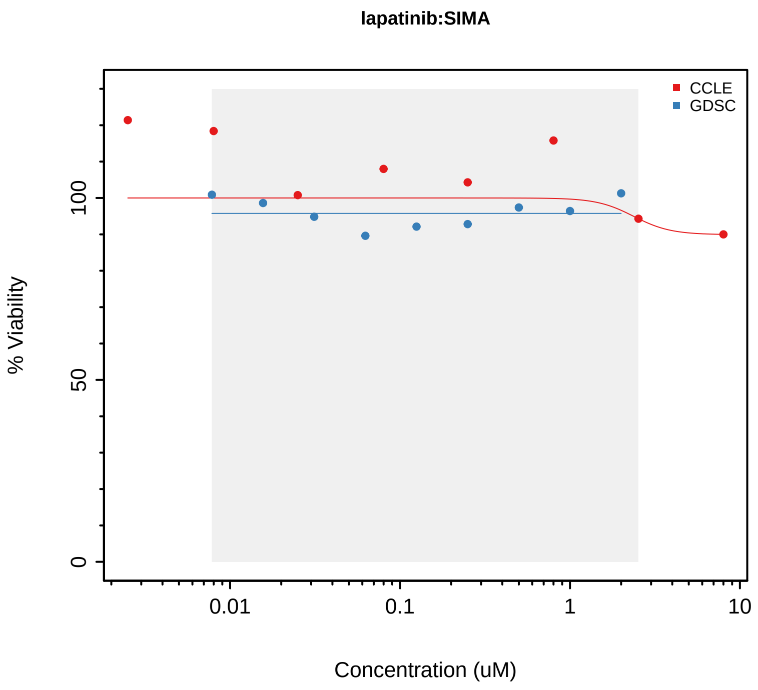
lapatinib:SIMA
CCLE
GDSC
% Viability
0
50
100
0.01
0.1
1
10
Concentration (uM)

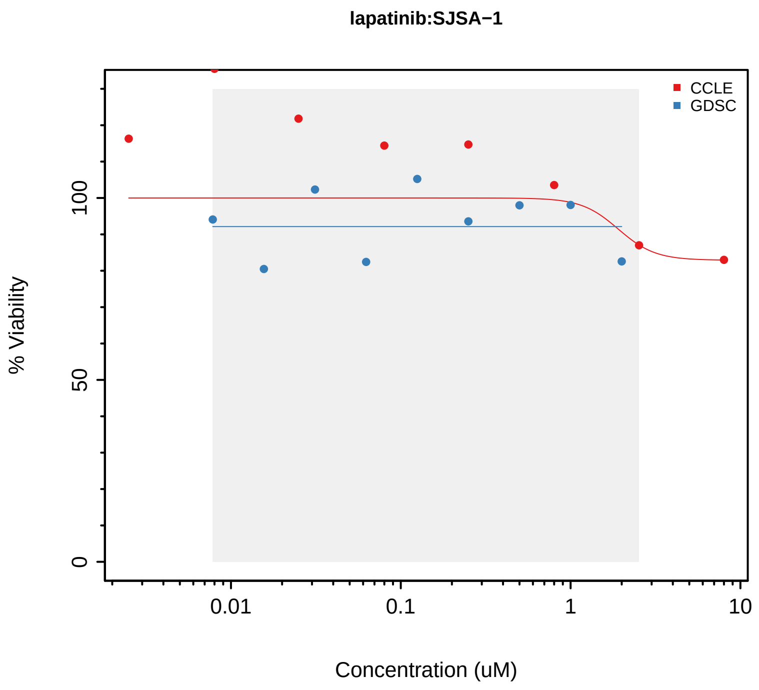
lapatinib:SJSA−1
CCLE
GDSC
% Viability
0
50
100
0.01
0.1
1
10
Concentration (uM)

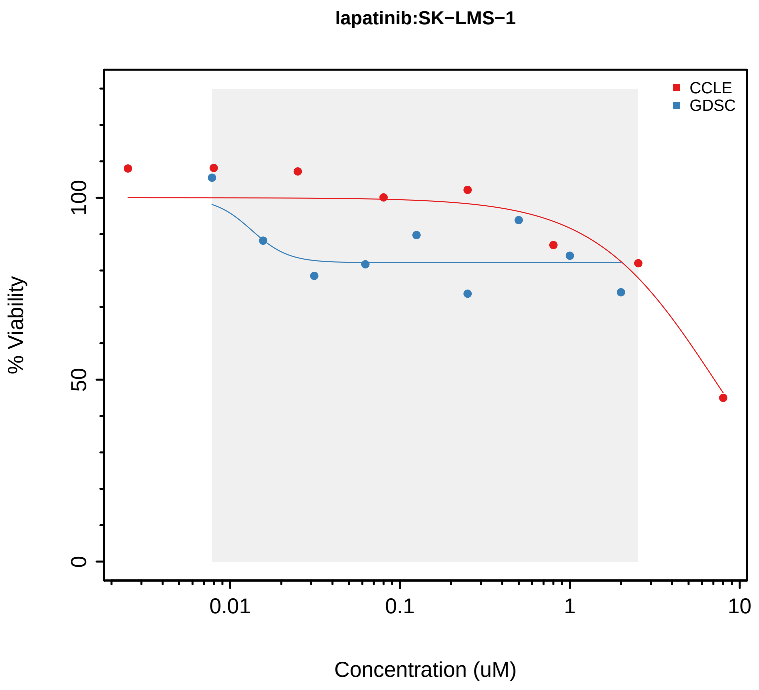
lapatinib:SK−LMS−1
CCLE
GDSC
% Viability
0
50
100
0.01
0.1
1
10
Concentration (uM)

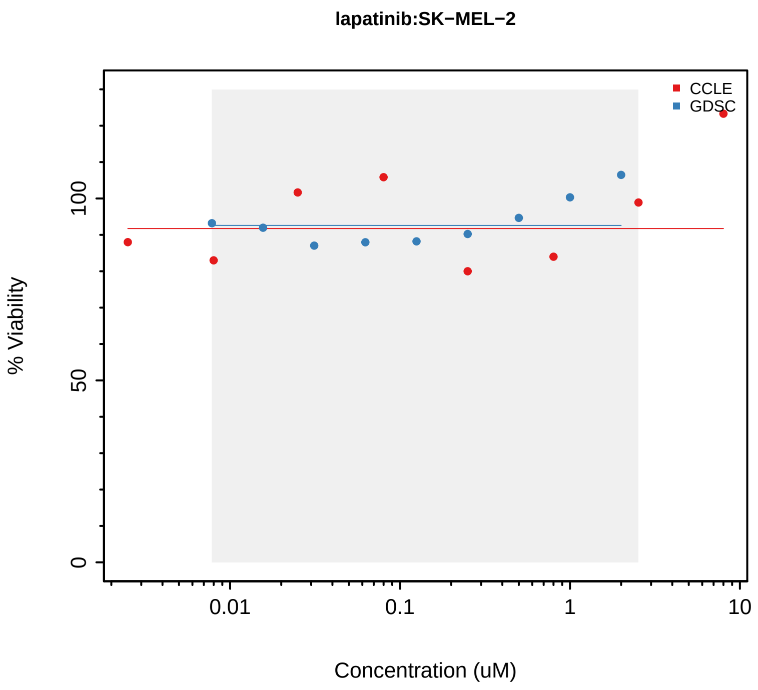
lapatinib:SK−MEL−2
CCLE
GDSC
% Viability
0
50
100
0.01
0.1
1
10
Concentration (uM)

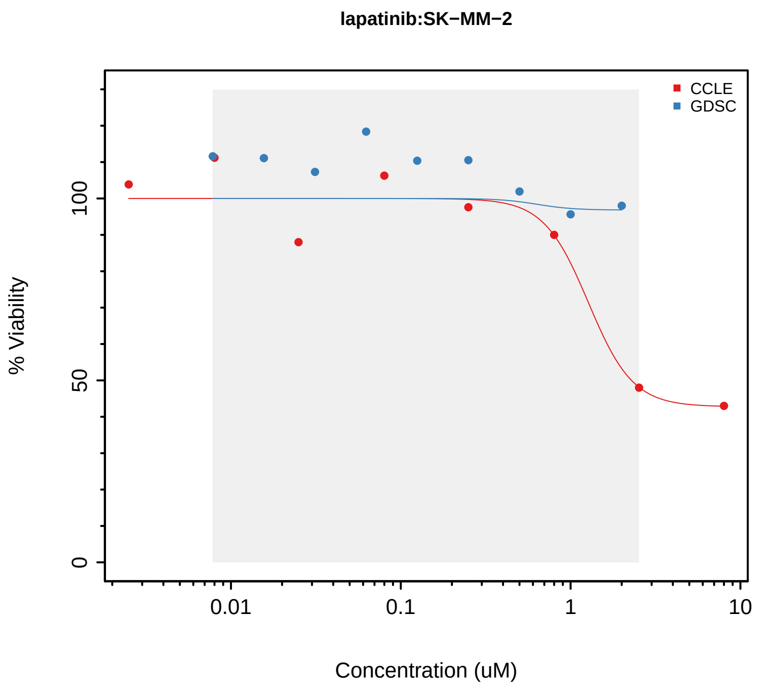
lapatinib:SK−MM−2
CCLE
GDSC
% Viability
0
50
100
0.01
0.1
1
10
Concentration (uM)

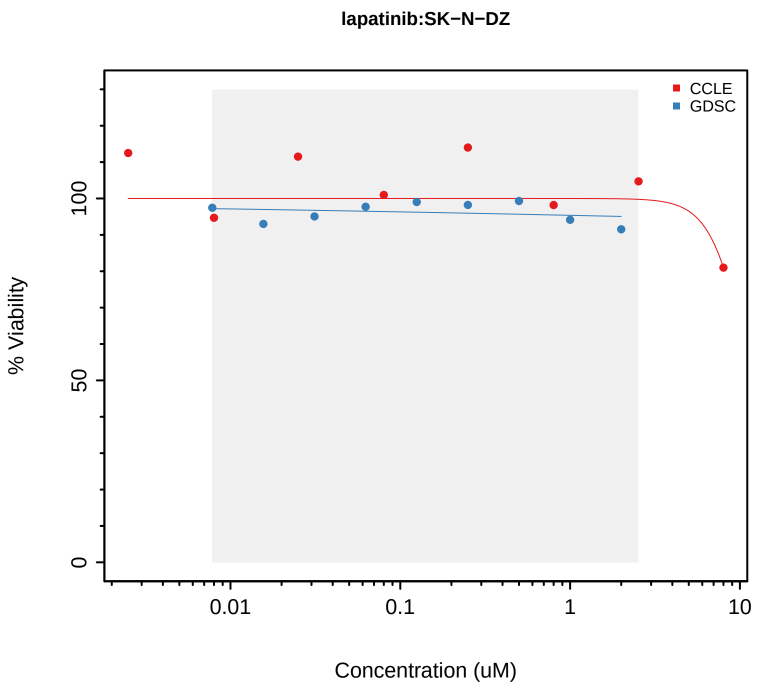
lapatinib:SK−N−DZ
CCLE
GDSC
% Viability
0
50
100
0.01
0.1
1
10
Concentration (uM)

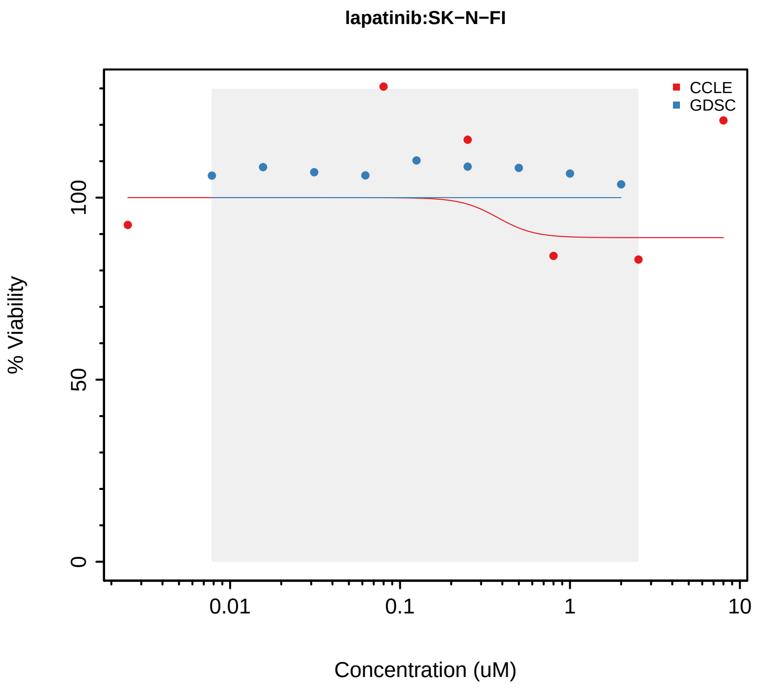
lapatinib:SK−N−FI
CCLE
GDSC
% Viability
0
50
100
0.01
0.1
1
10
Concentration (uM)

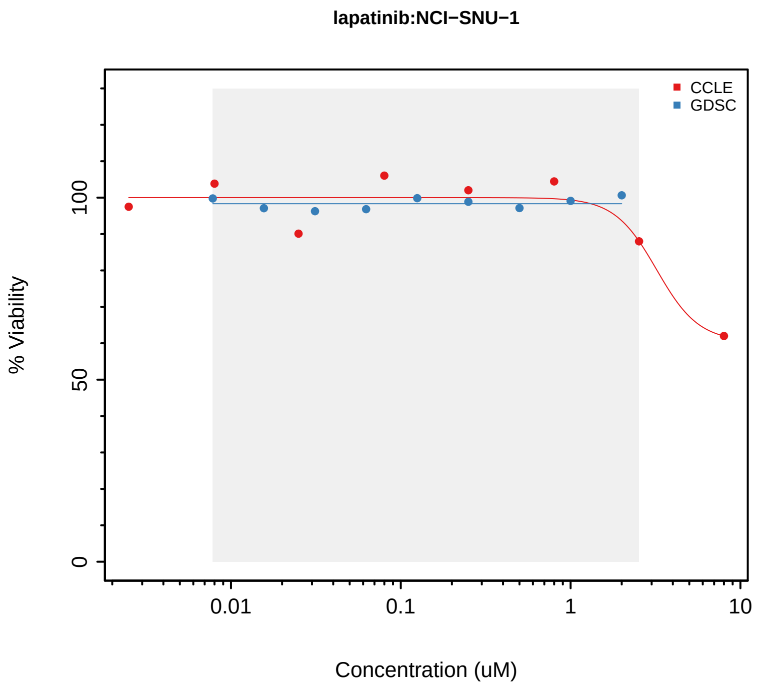
lapatinib:NCI−SNU−1
CCLE
GDSC
% Viability
0
50
100
0.01
0.1
1
10
Concentration (uM)

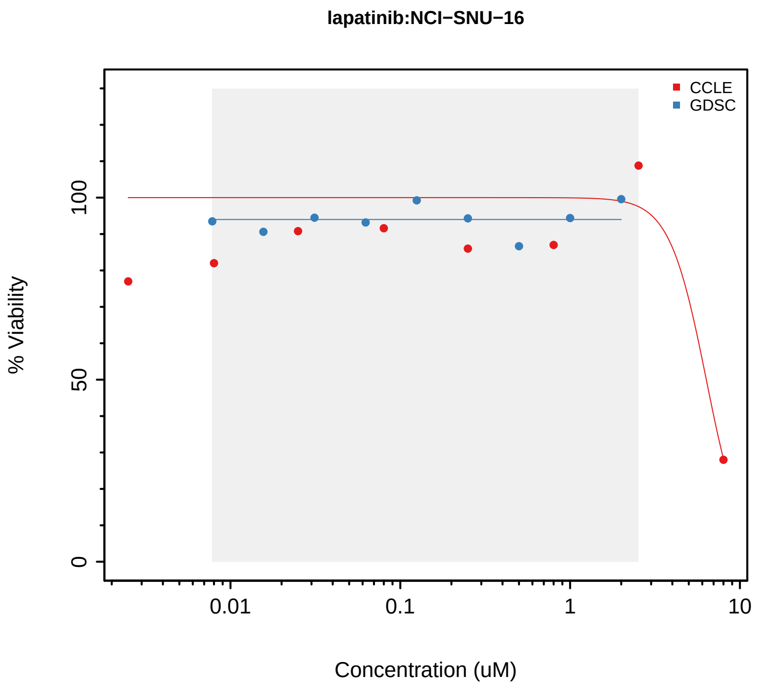
lapatinib:NCI−SNU−16
CCLE
GDSC
% Viability
0
50
100
0.01
0.1
1
10
Concentration (uM)

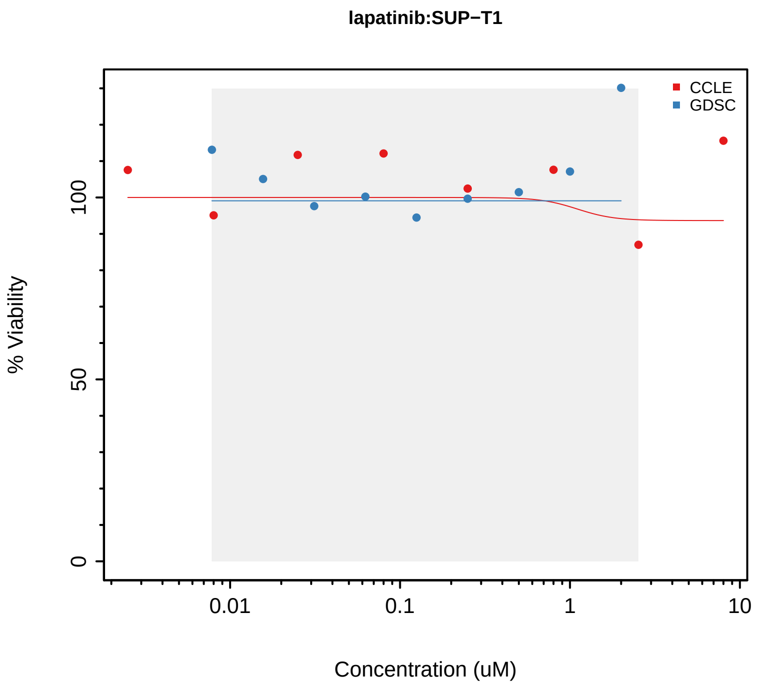
lapatinib:SUP−T1
CCLE
GDSC
% Viability
0
50
100
0.01
0.1
1
10
Concentration (uM)

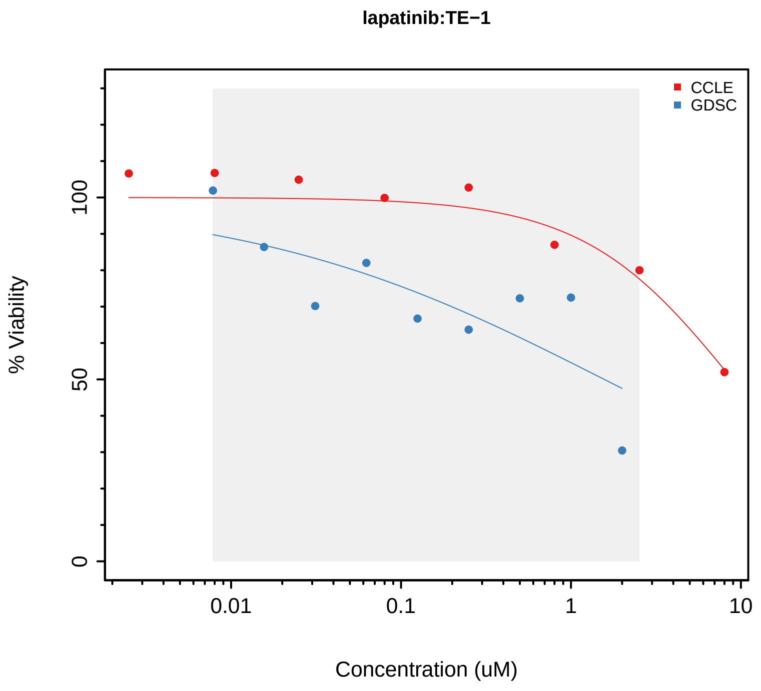
lapatinib:TE−1
CCLE
GDSC
% Viability
0
50
100
0.01
0.1
1
10
Concentration (uM)

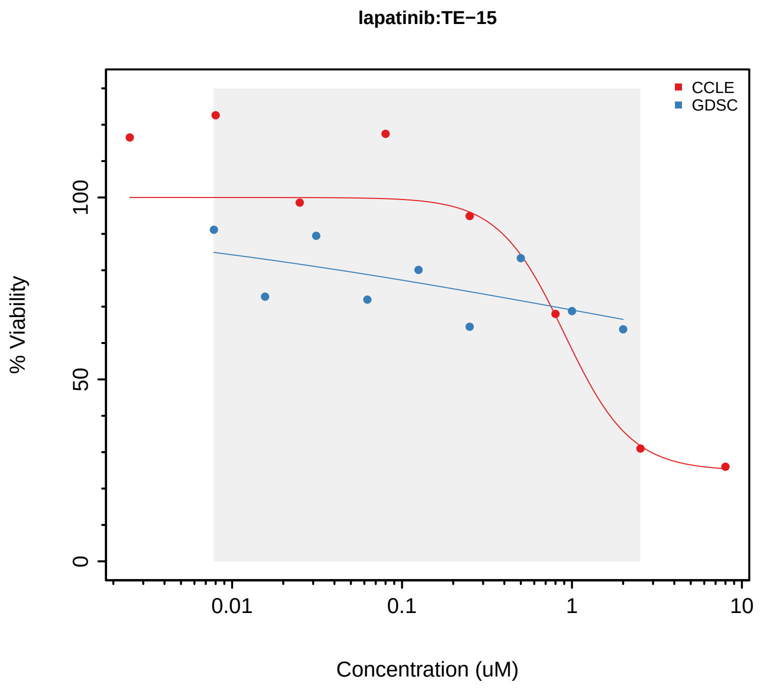
lapatinib:TE−15
CCLE
GDSC
% Viability
0
50
100
0.01
0.1
1
10
Concentration (uM)

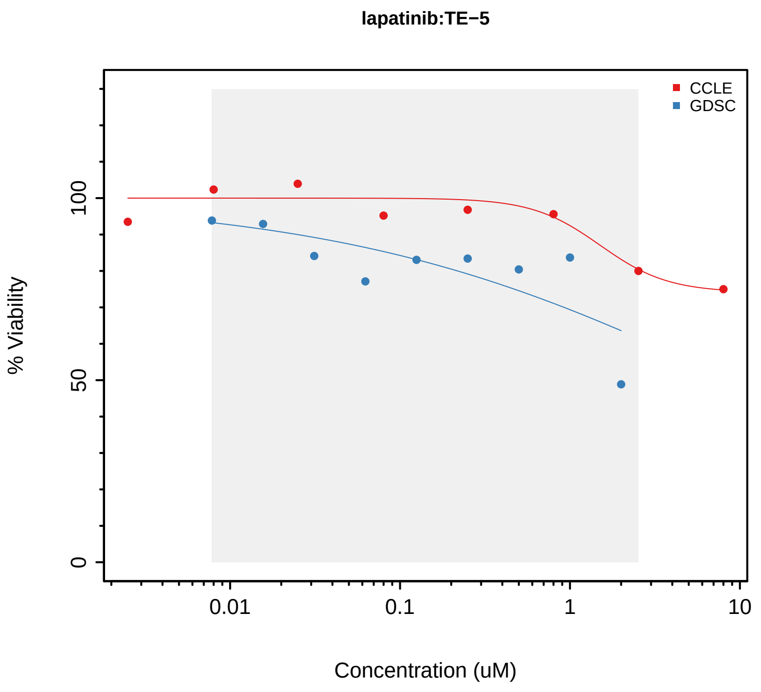
lapatinib:TE−5
CCLE
GDSC
% Viability
0
50
100
0.01
0.1
1
10
Concentration (uM)

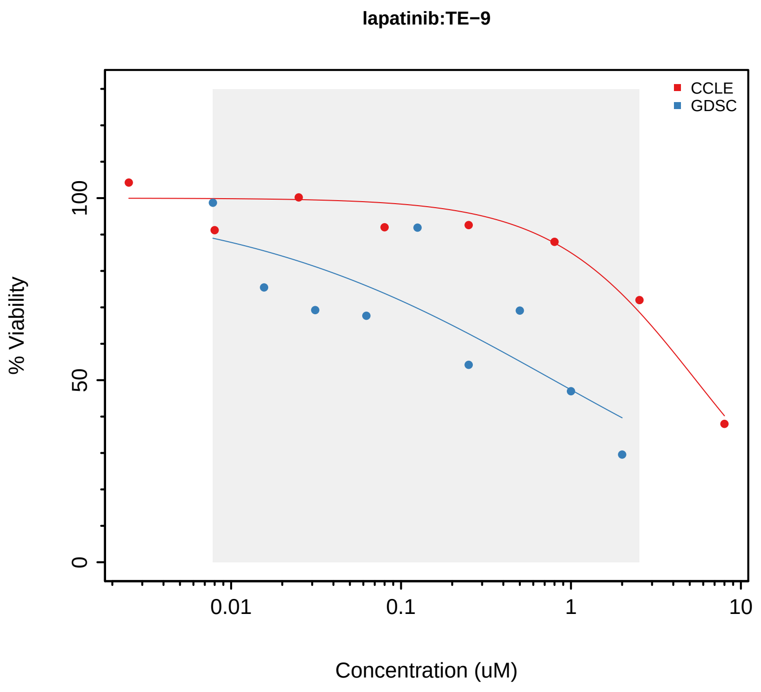
lapatinib:TE−9
CCLE
GDSC
% Viability
0
50
100
0.01
0.1
1
10
Concentration (uM)

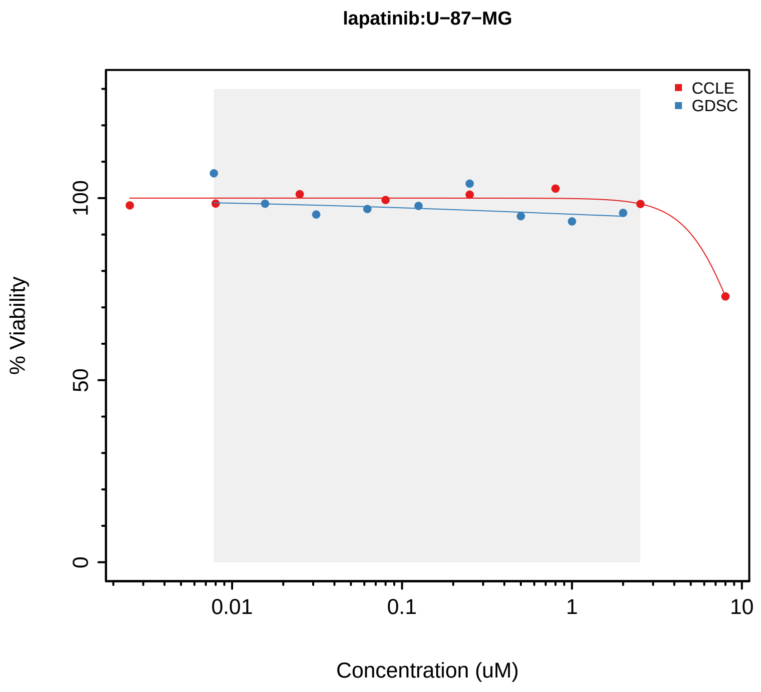
lapatinib:U−87−MG
CCLE
GDSC
% Viability
0
50
100
0.01
0.1
1
10
Concentration (uM)

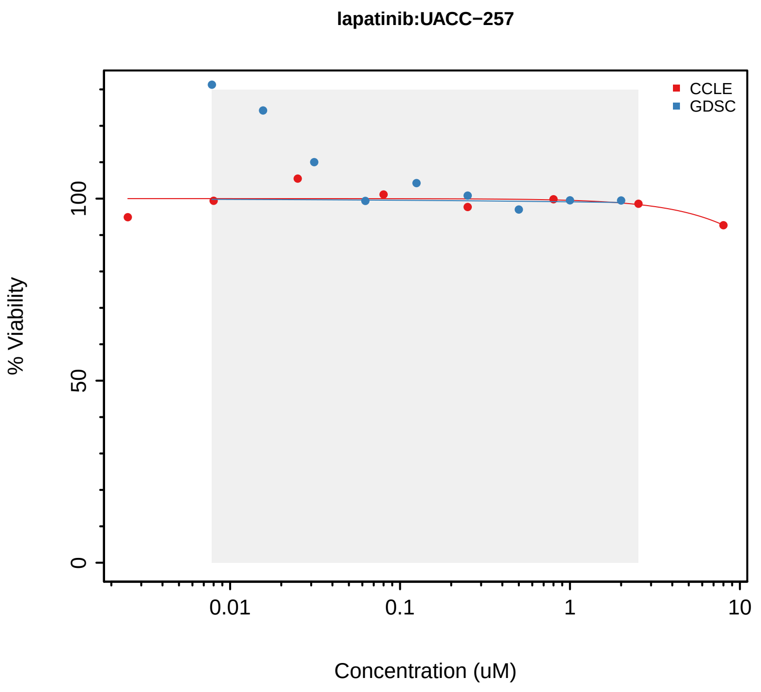
lapatinib:UACC−257
CCLE
GDSC
% Viability
0
50
100
0.01
0.1
1
10
Concentration (uM)

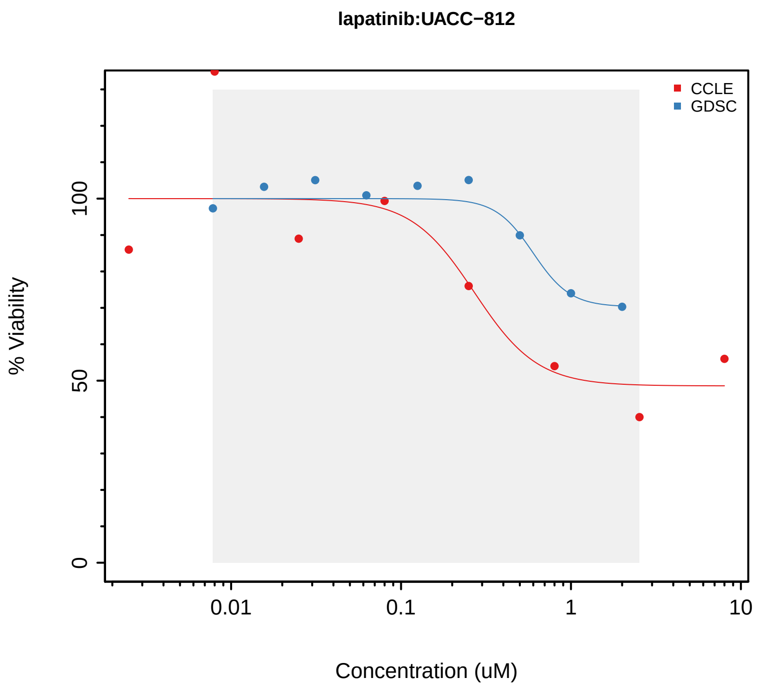
lapatinib:UACC−812
CCLE
GDSC
% Viability
0
50
100
0.01
0.1
1
10
Concentration (uM)

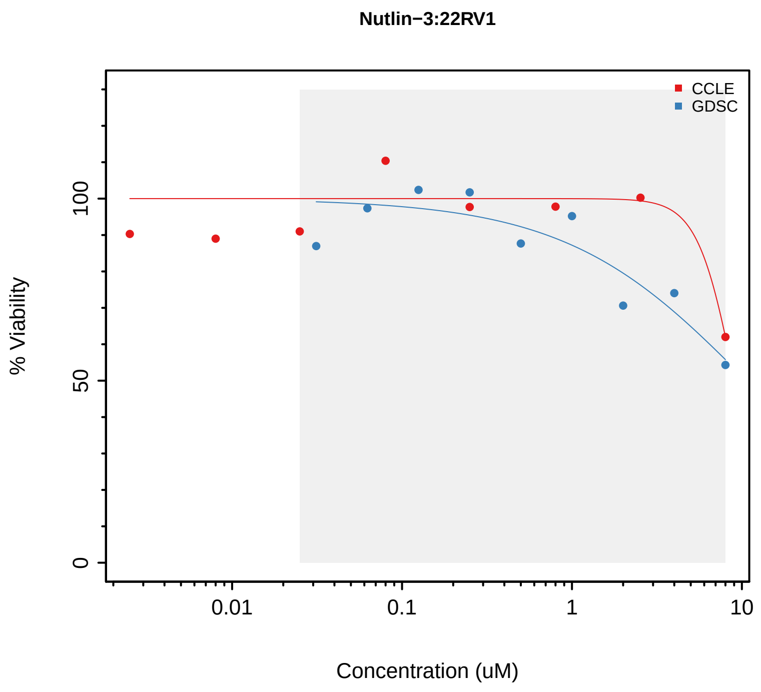
Nutlin−3:22RV1
CCLE
GDSC
% Viability
0
50
100
0.01
0.1
1
10
Concentration (uM)

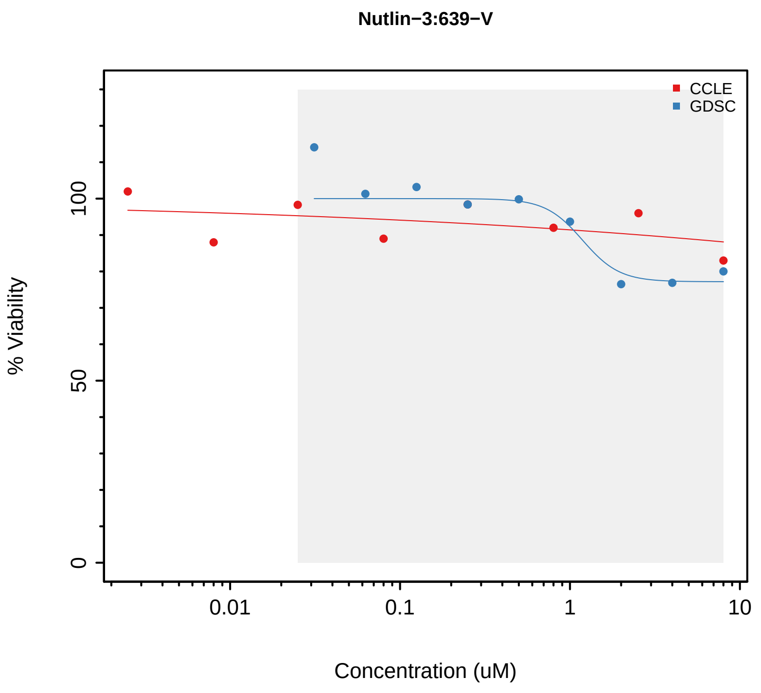
Nutlin−3:639−V
CCLE
GDSC
% Viability
0
50
100
0.01
0.1
1
10
Concentration (uM)

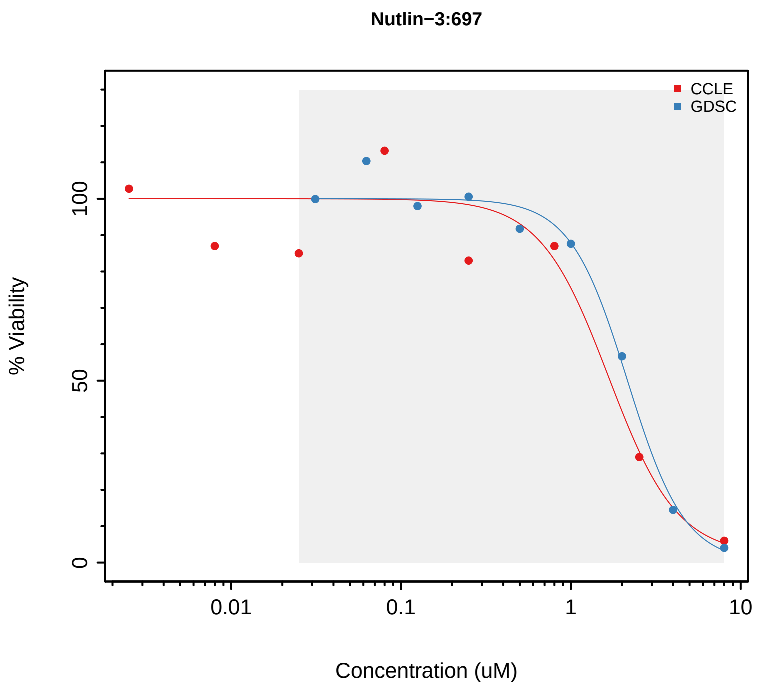
Nutlin−3:697
CCLE
GDSC
% Viability
0
50
100
0.01
0.1
1
10
Concentration (uM)

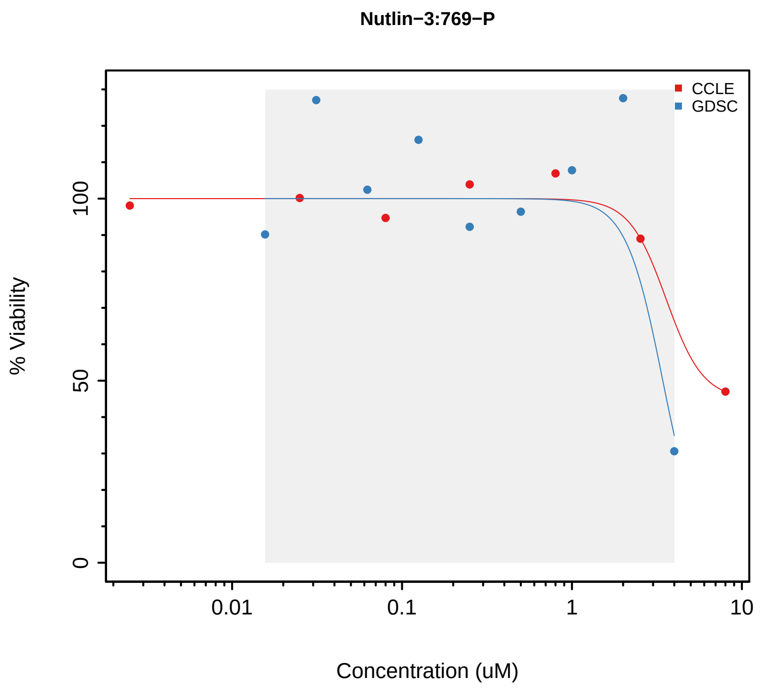
Nutlin−3:769−P
CCLE
GDSC
% Viability
0
50
100
0.01
0.1
1
10
Concentration (uM)

Nutlin−3:786−0

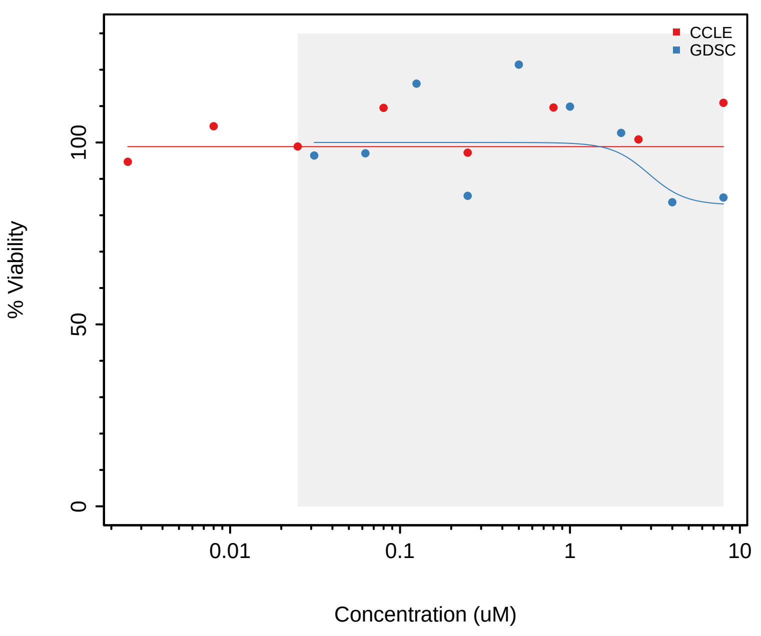

Nutlin−3:8305C

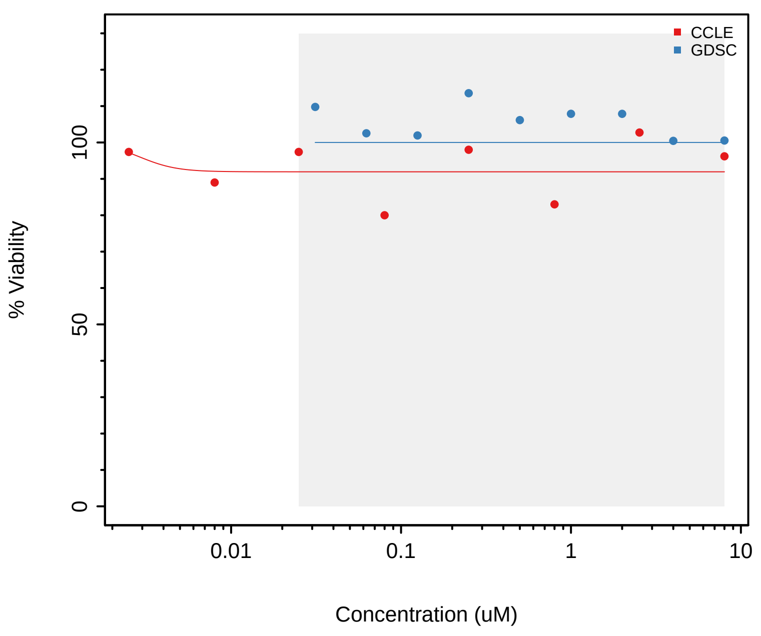

Nutlin−3:8505C

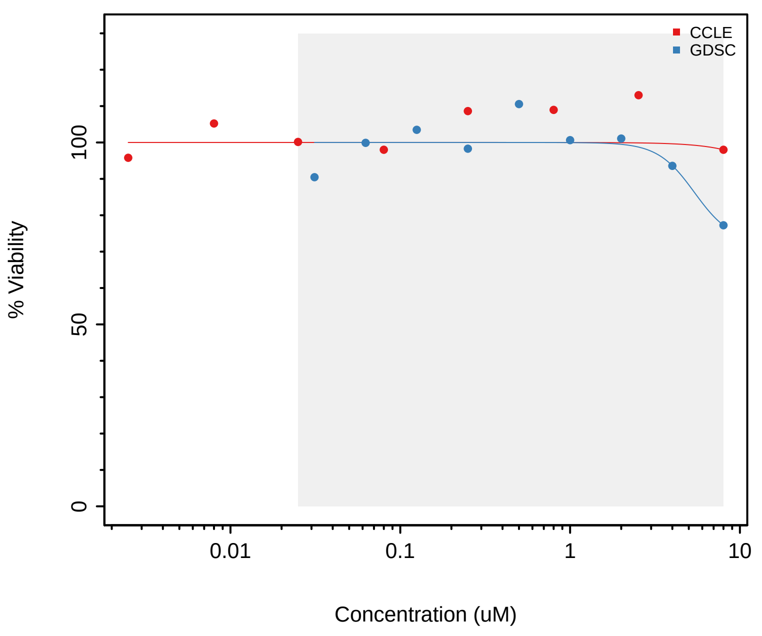

Nutlin−3:8−MG−BA

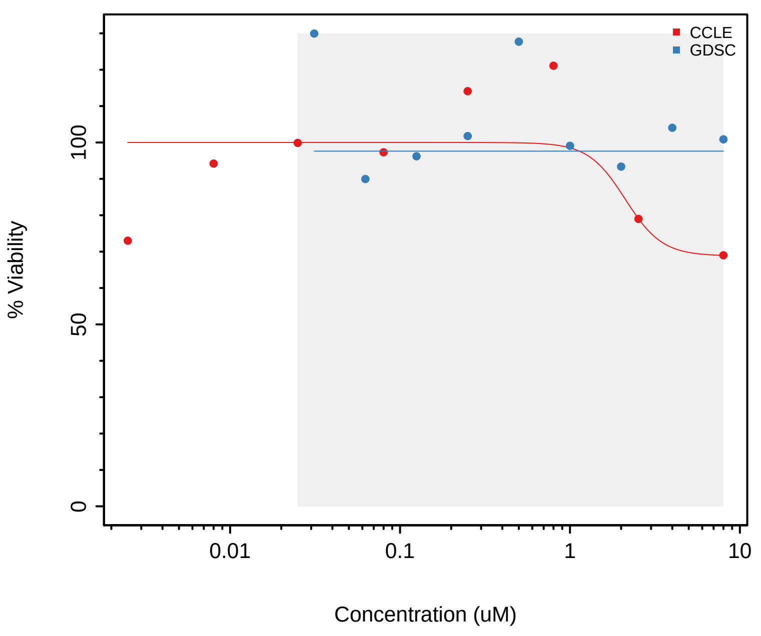

Nutlin−3:A172

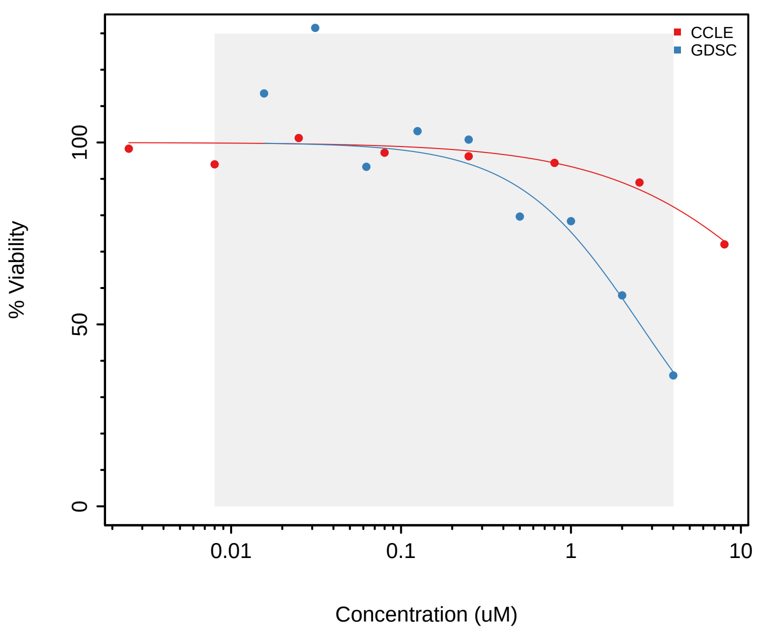

Nutlin−3:A204

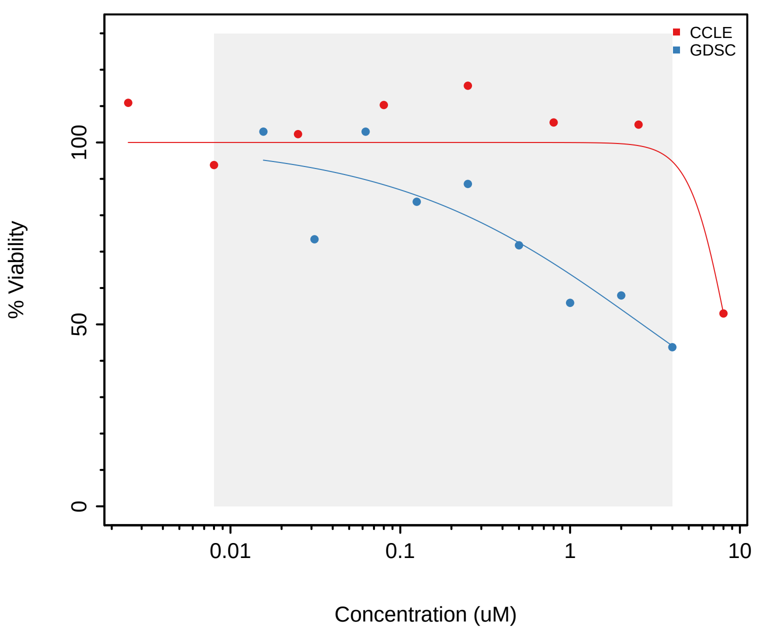

Nutlin−3:A2058

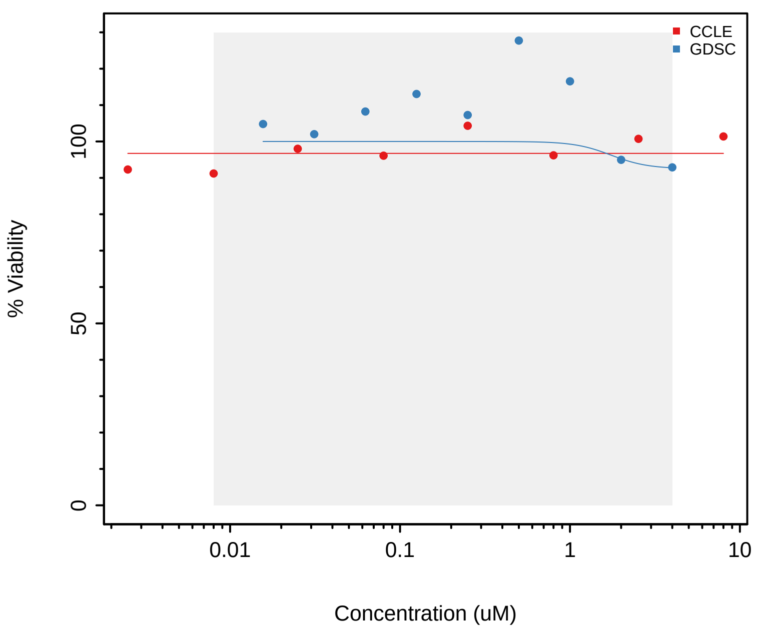

Nutlin−3:A253

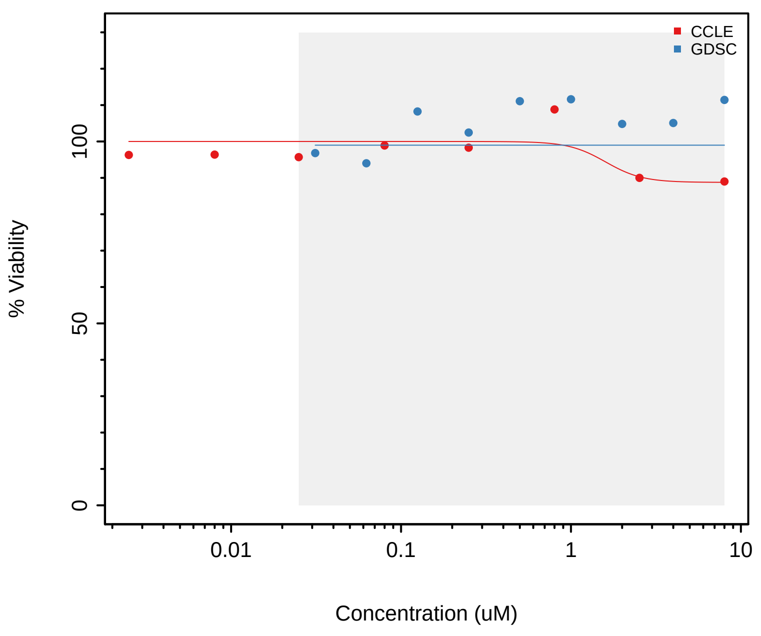

Nutlin−3:A2780

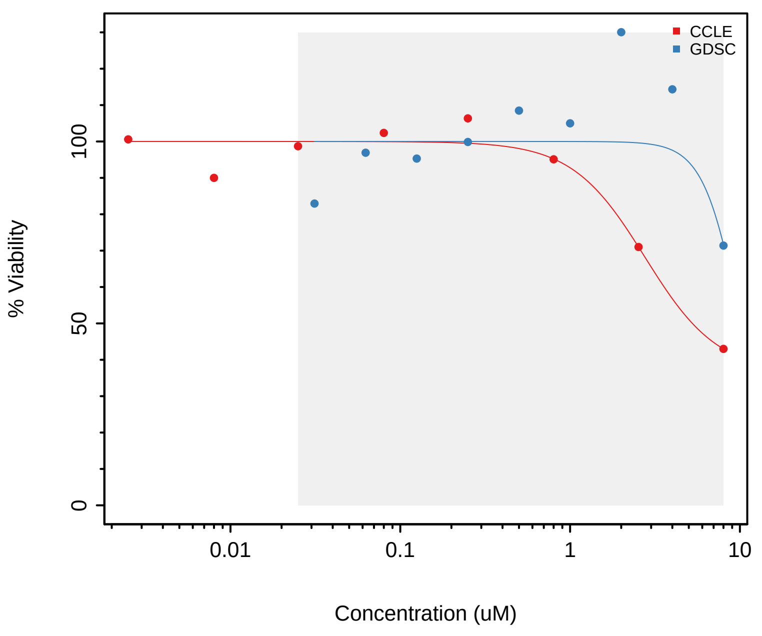

Nutlin−3:A375

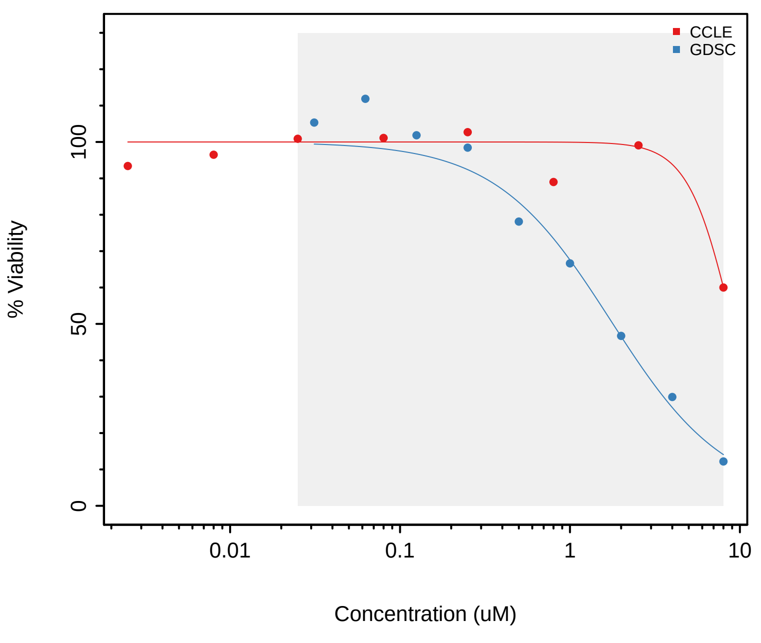

Nutlin−3:A549

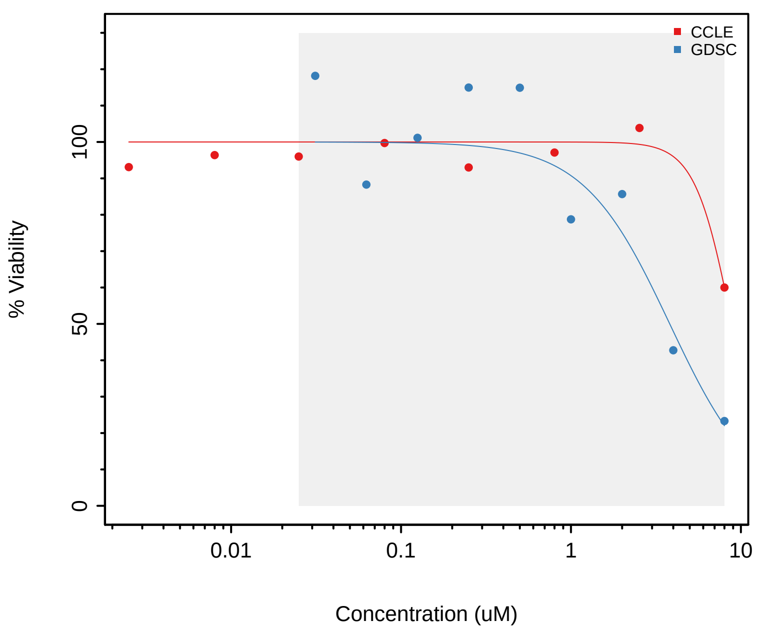

Nutlin−3:A673

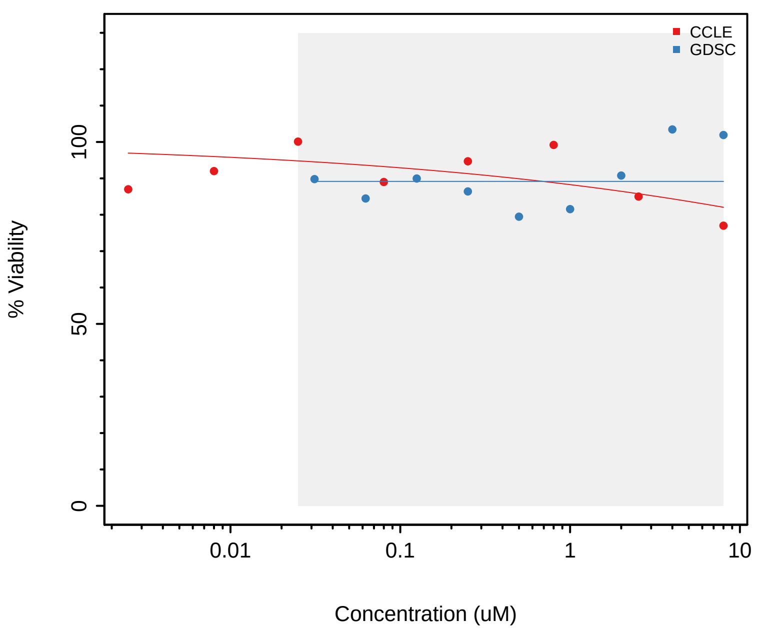

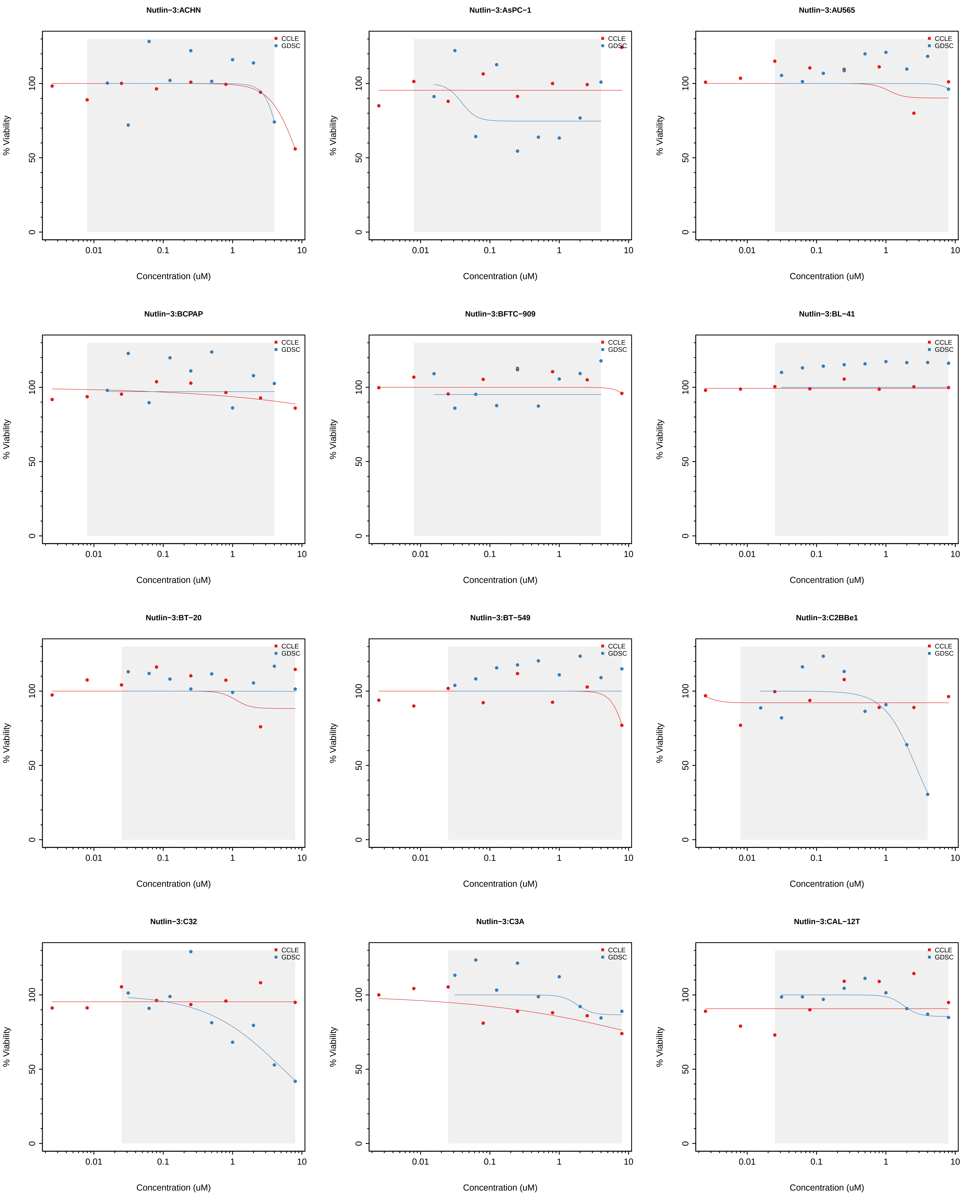
Nutlin−3:ACHN
Nutlin−3:AsPC−1
Nutlin−3:AU565
Nutlin−3:BCPAP
Nutlin−3:BFTC−909
Nutlin−3:BL−41
Nutlin−3:BT−20
Nutlin−3:BT−549
Nutlin−3:C2BBe1
Nutlin−3:C32
Nutlin−3:C3A
Nutlin−3:CAL−12T
CCLE
GDSC
% Viability
Concentration (uM)
0
50
100
0.01
0.1
1
10

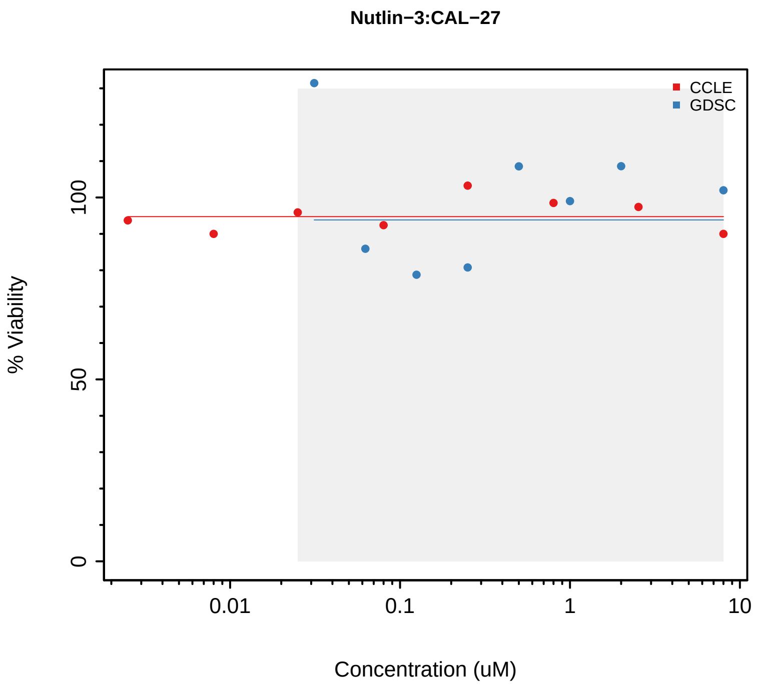
Nutlin−3:CAL−27
CCLE
GDSC
% Viability
0
50
100
0.01
0.1
1
10
Concentration (uM)

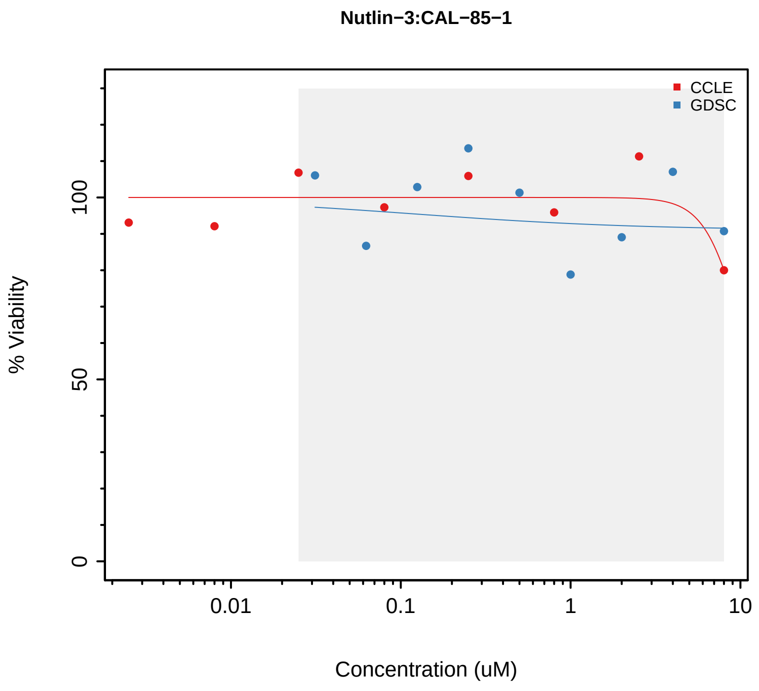
Nutlin−3:CAL−85−1
CCLE
GDSC
% Viability
0
50
100
0.01
0.1
1
10
Concentration (uM)

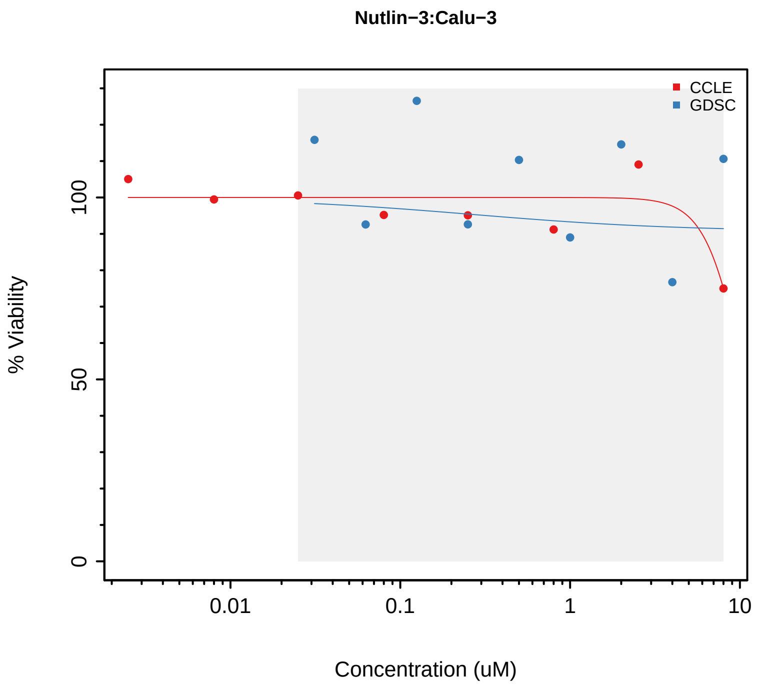
Nutlin−3:Calu−3
CCLE
GDSC
% Viability
0
50
100
0.01
0.1
1
10
Concentration (uM)

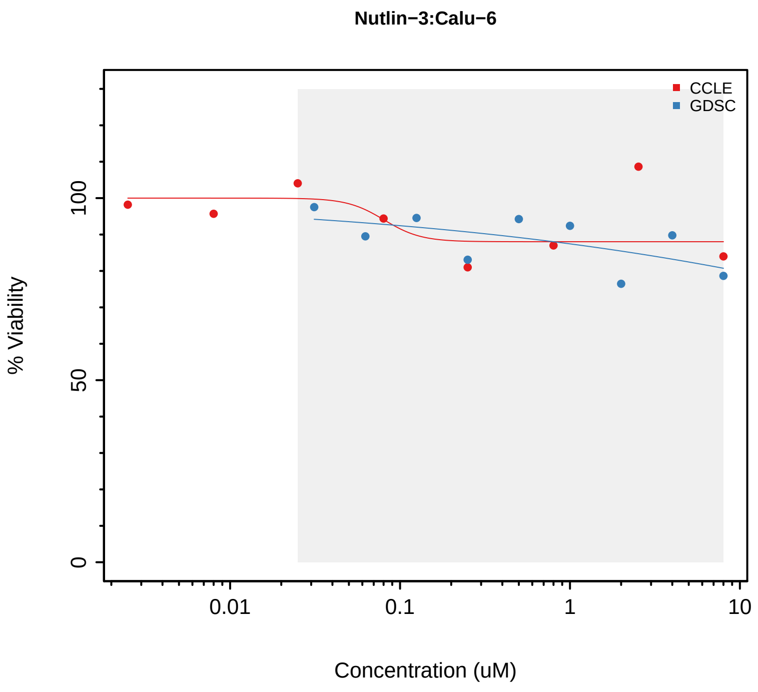
Nutlin−3:Calu−6
CCLE
GDSC
% Viability
0
50
100
0.01
0.1
1
10
Concentration (uM)

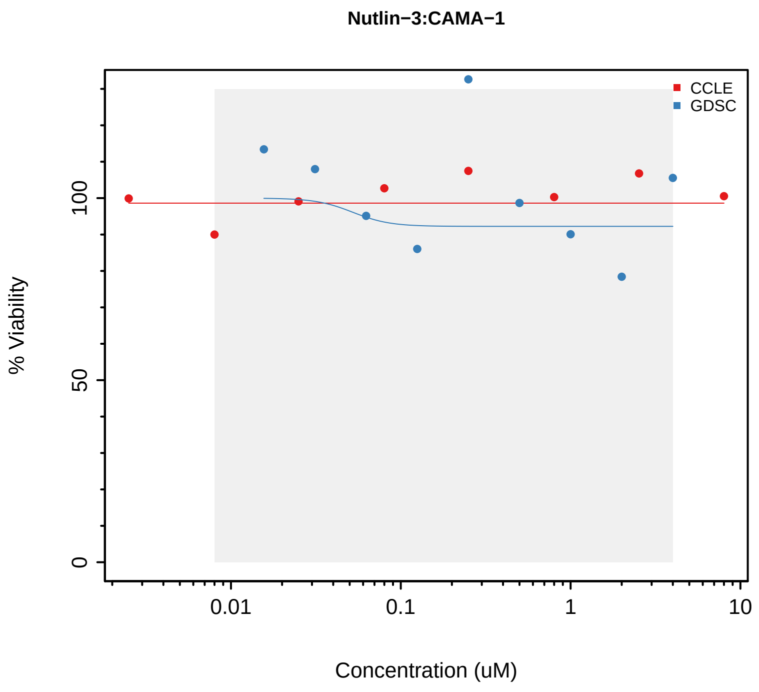
Nutlin−3:CAMA−1
CCLE
GDSC
% Viability
0
50
100
0.01
0.1
1
10
Concentration (uM)

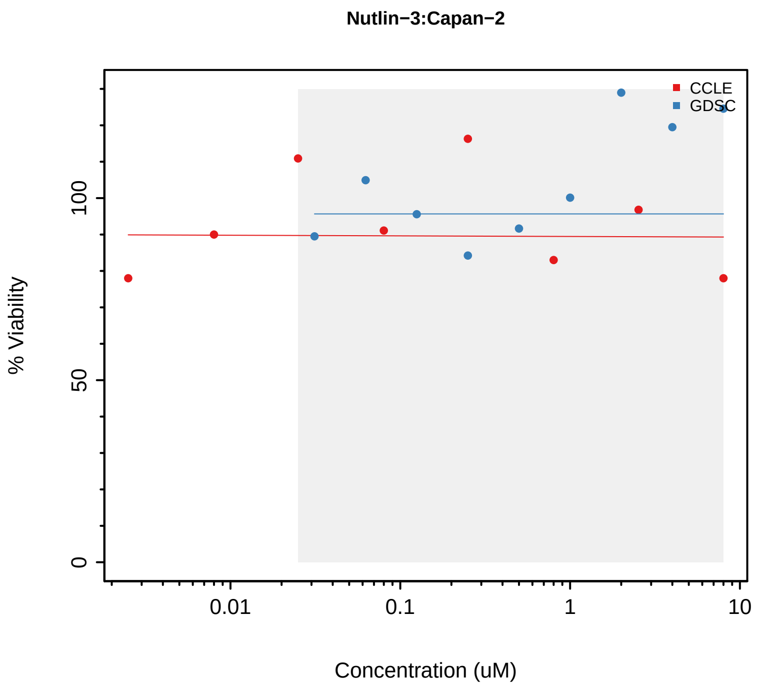
Nutlin−3:Capan−2
CCLE
GDSC
% Viability
0
50
100
0.01
0.1
1
10
Concentration (uM)

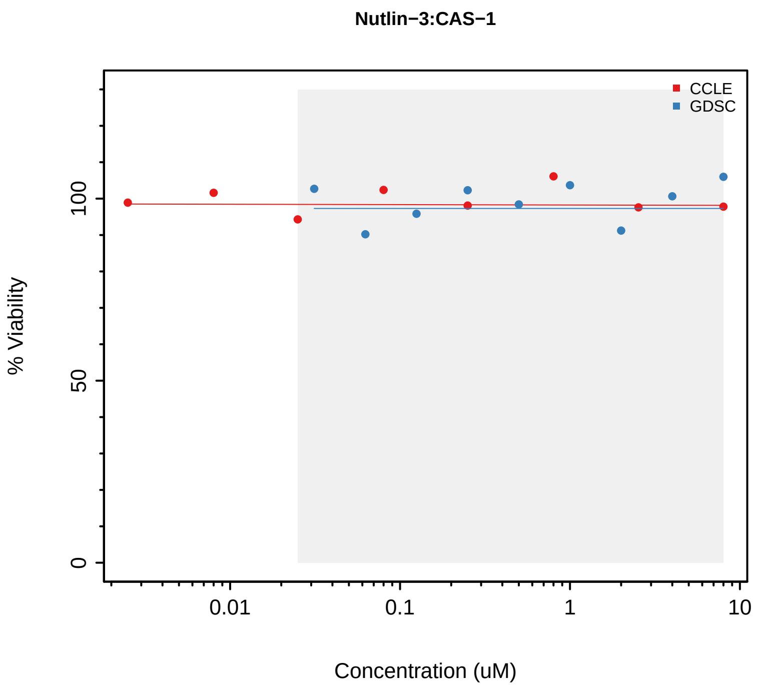
Nutlin−3:CAS−1
CCLE
GDSC
% Viability
0
50
100
0.01
0.1
1
10
Concentration (uM)

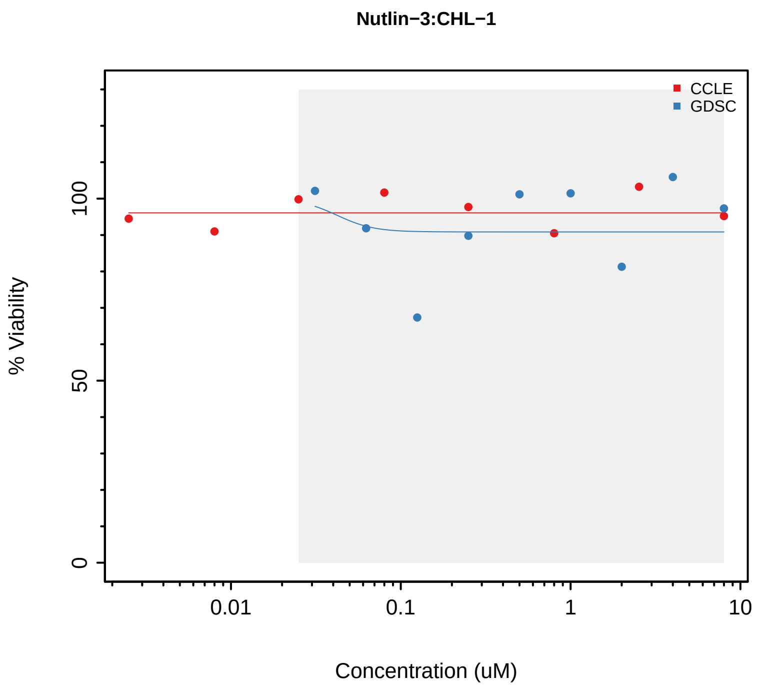
Nutlin−3:CHL−1
CCLE
GDSC
% Viability
0
50
100
0.01
0.1
1
10
Concentration (uM)

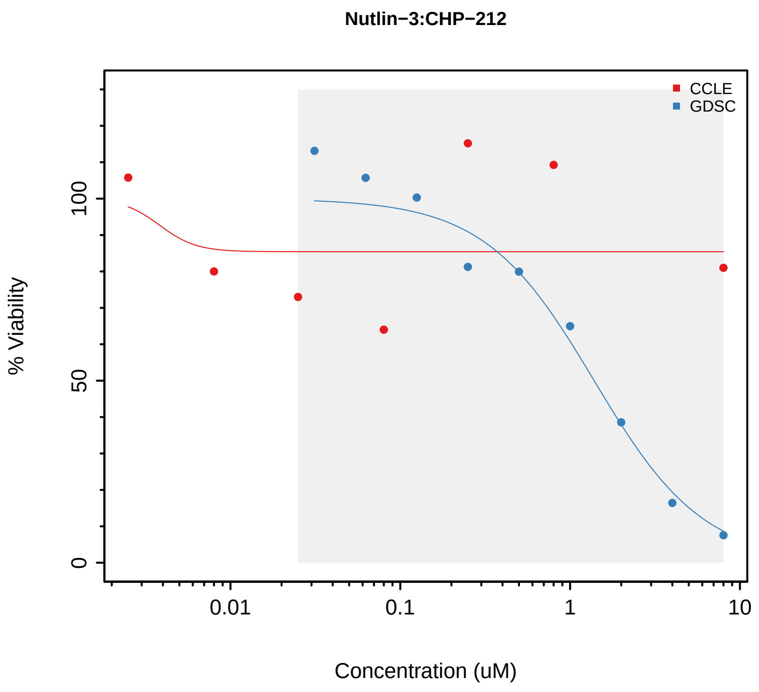
Nutlin−3:CHP−212
CCLE
GDSC
% Viability
0
50
100
0.01
0.1
1
10
Concentration (uM)

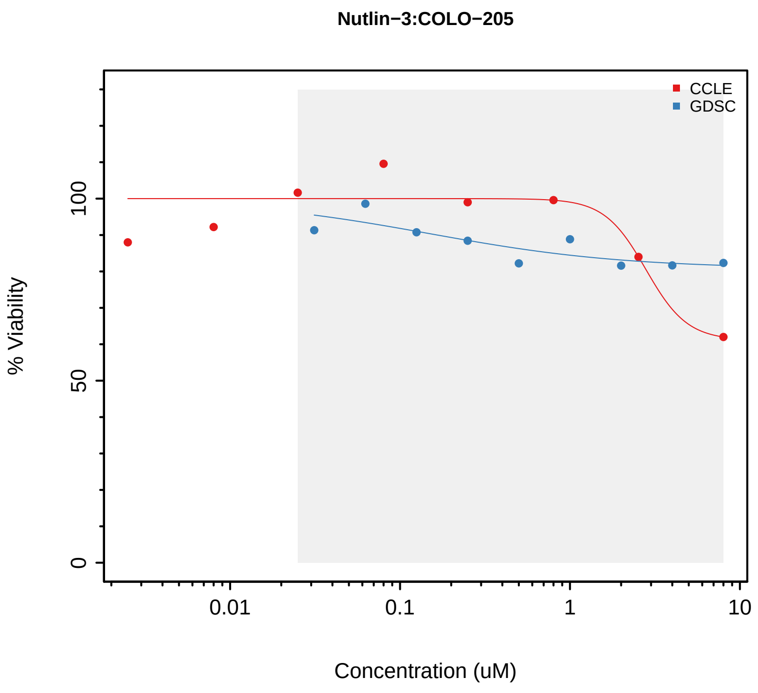
Nutlin−3:COLO−205
CCLE
GDSC
% Viability
0
50
100
0.01
0.1
1
10
Concentration (uM)

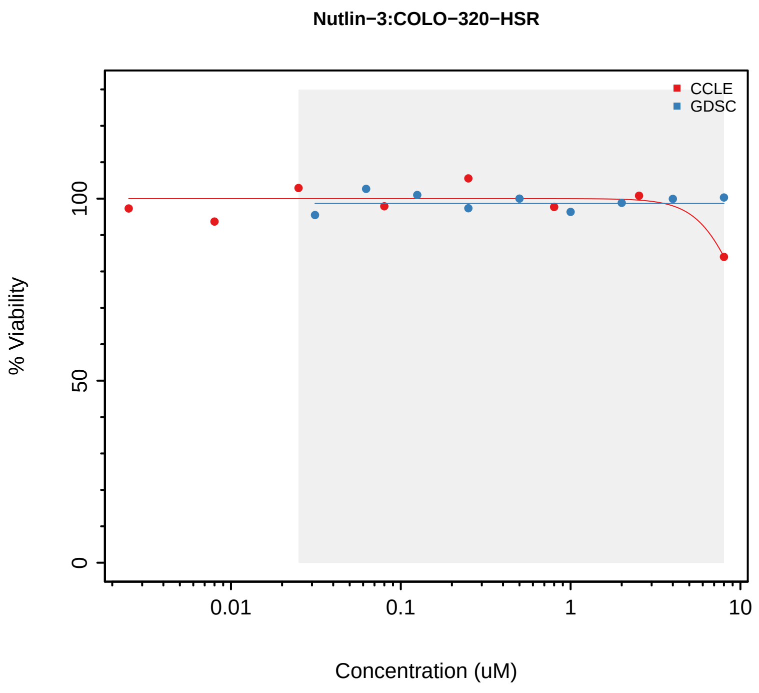
Nutlin−3:COLO−320−HSR
CCLE
GDSC
% Viability
0
50
100
0.01
0.1
1
10
Concentration (uM)

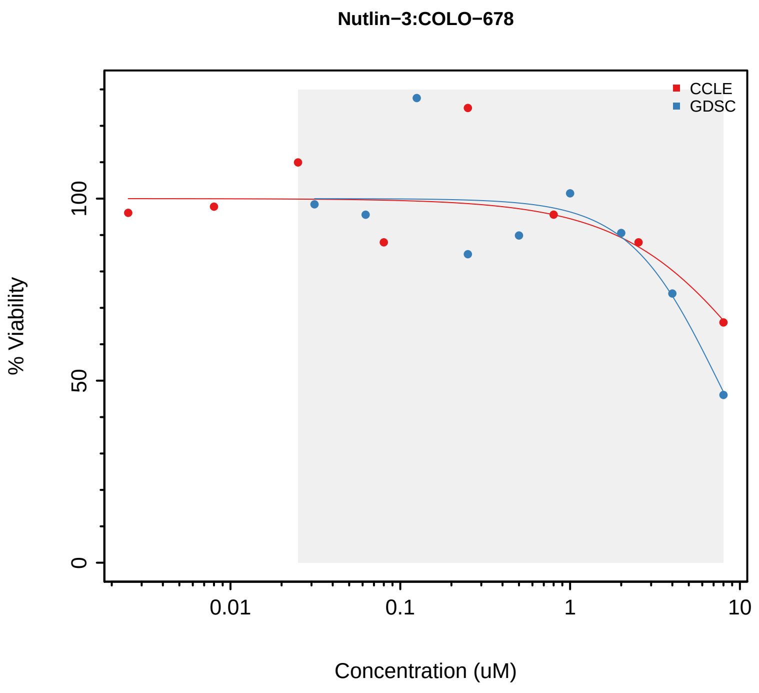
Nutlin−3:COLO−678
CCLE
GDSC
% Viability
0
50
100
0.01
0.1
1
10
Concentration (uM)

Nutlin−3:COLO−679

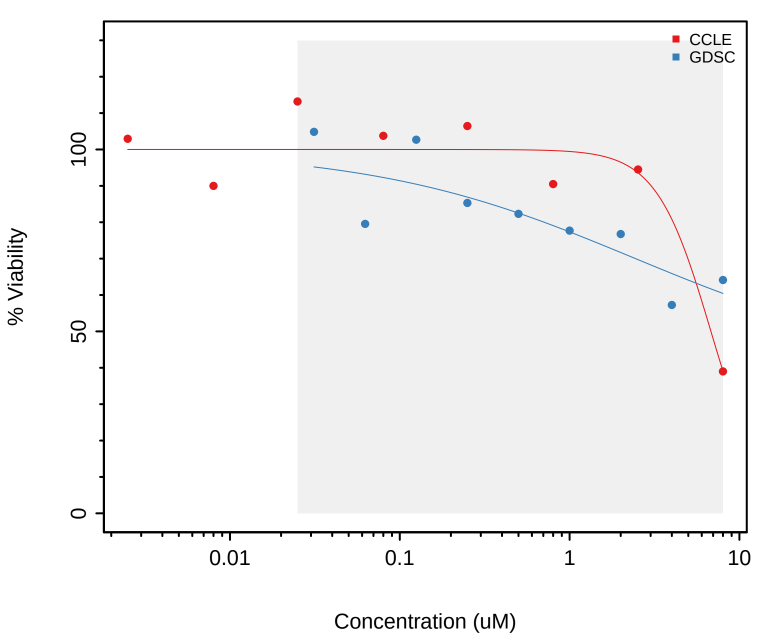

Nutlin−3:COLO−741

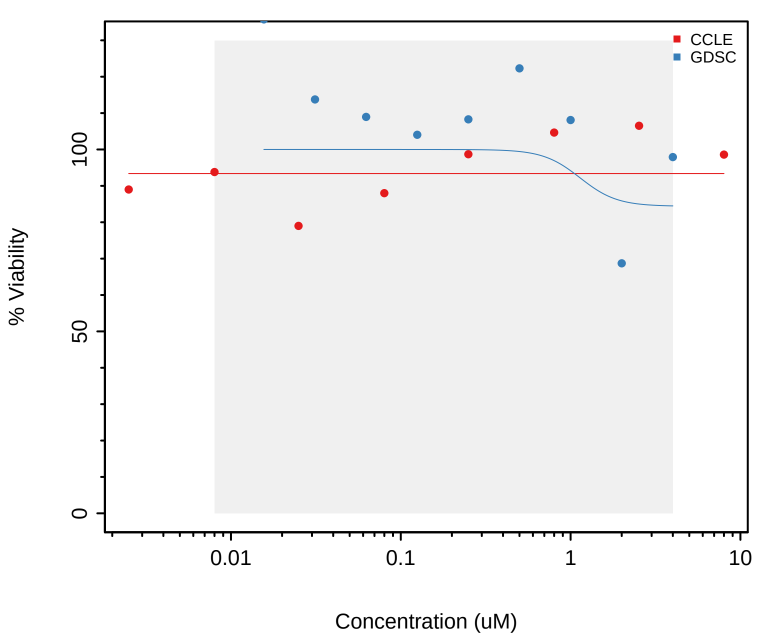

Nutlin−3:COR−L105

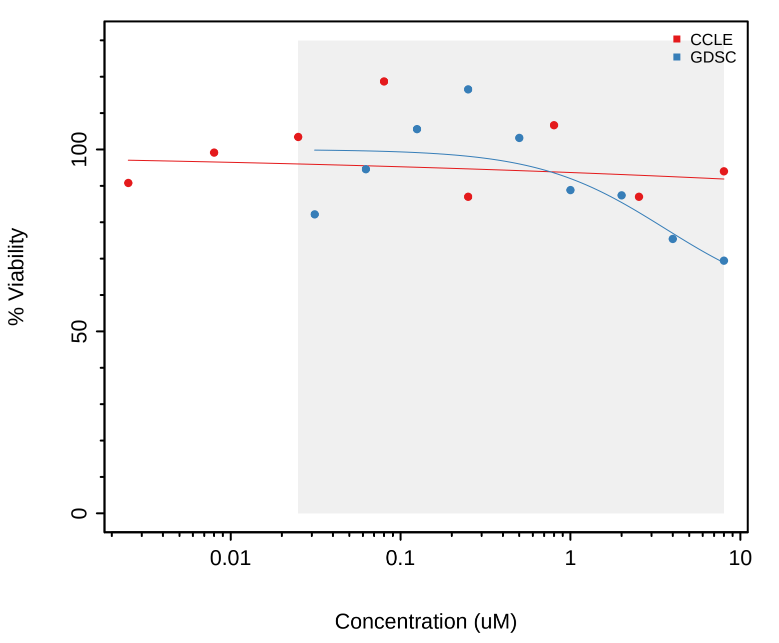

Nutlin−3:COR−L23

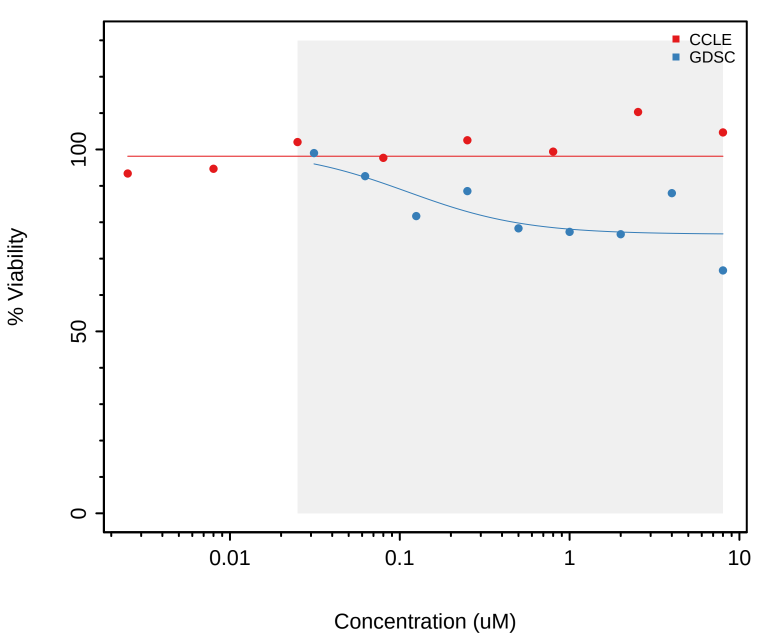

Nutlin−3:Daoy

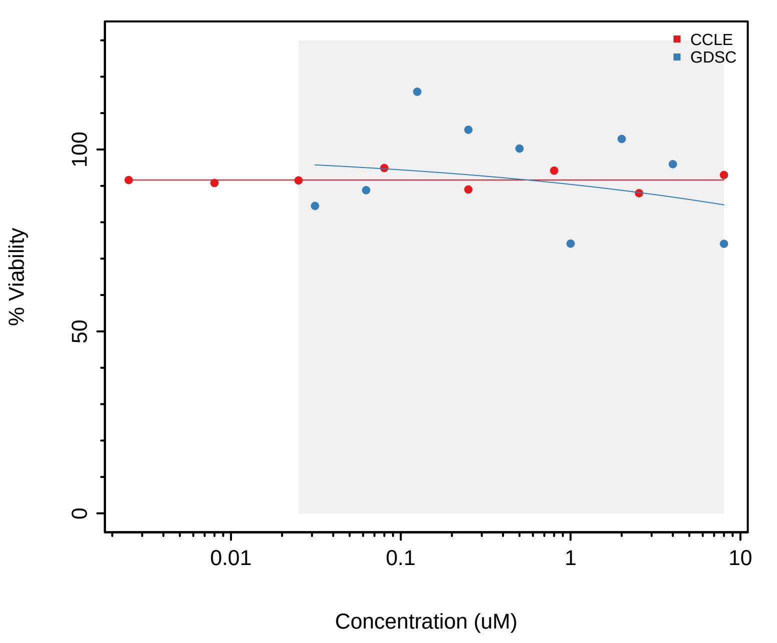

Nutlin−3:DBTRG−05MG

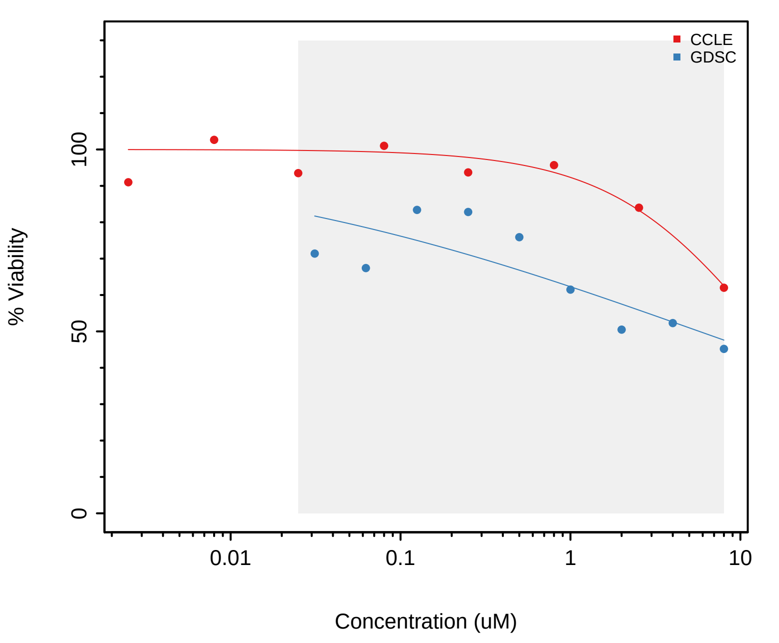

Nutlin−3:DEL

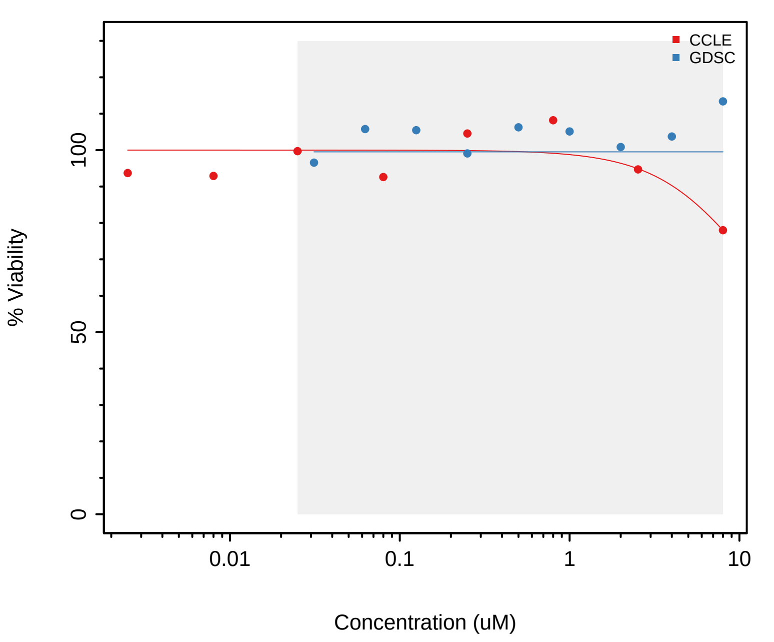

Nutlin−3:Detroit562

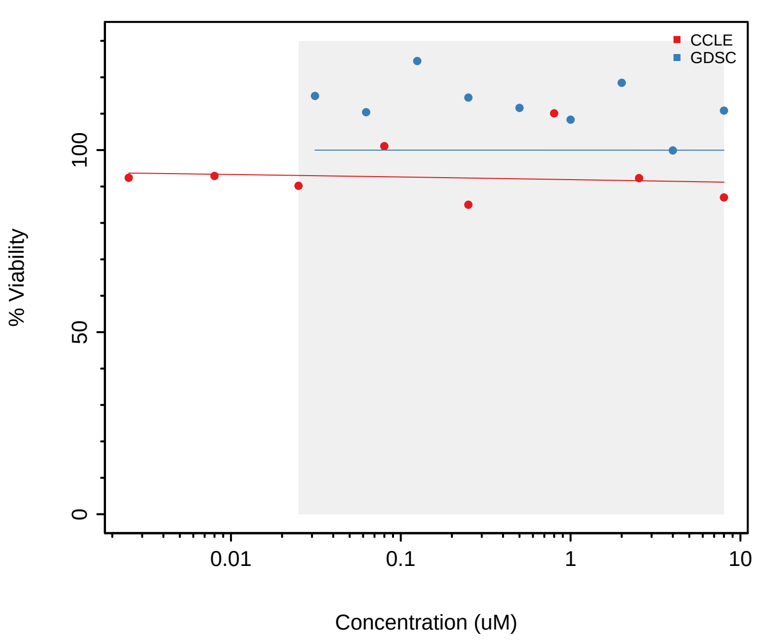

Nutlin−3:DK−MG

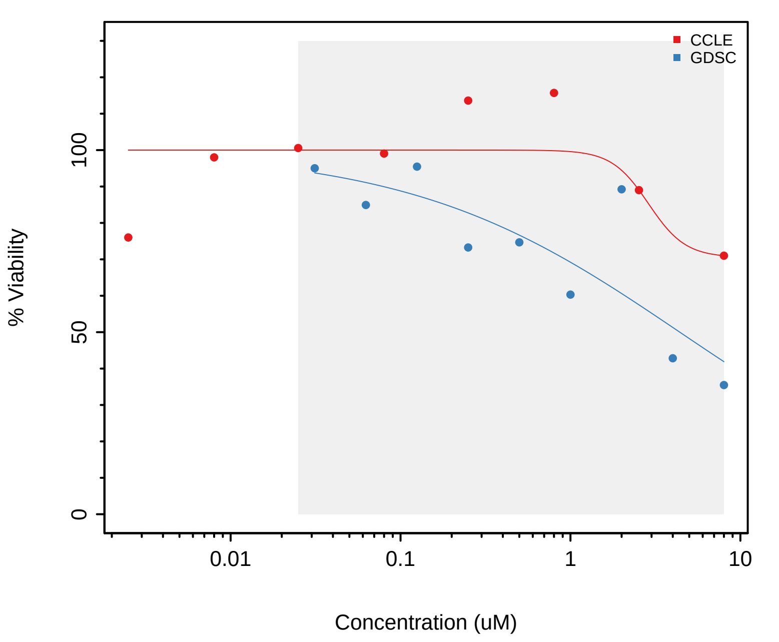

Nutlin−3:DMS−114

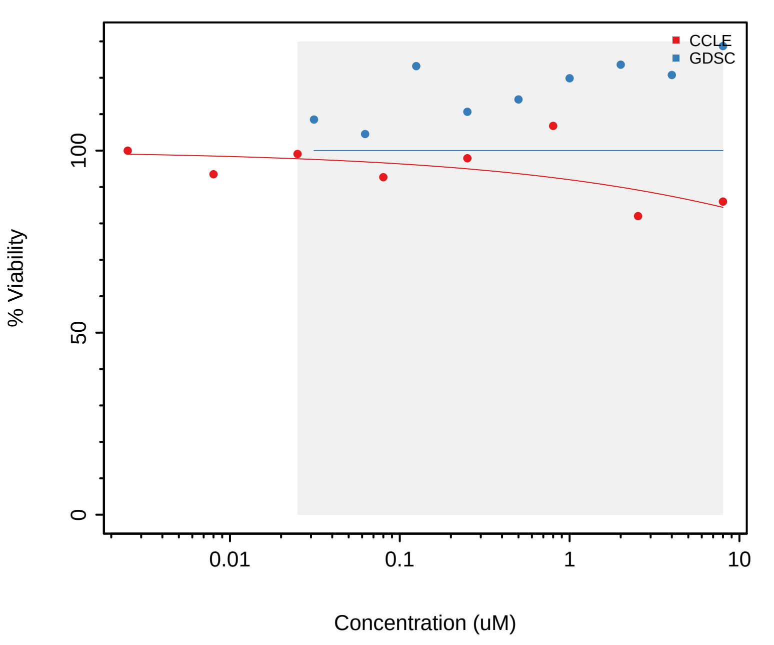

Nutlin−3:DOHH−2

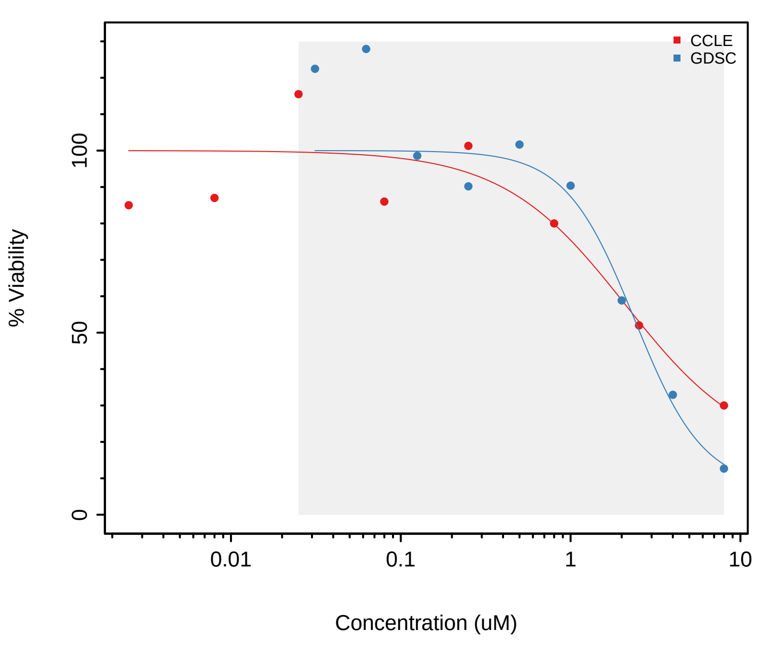

Nutlin−3:DU−145

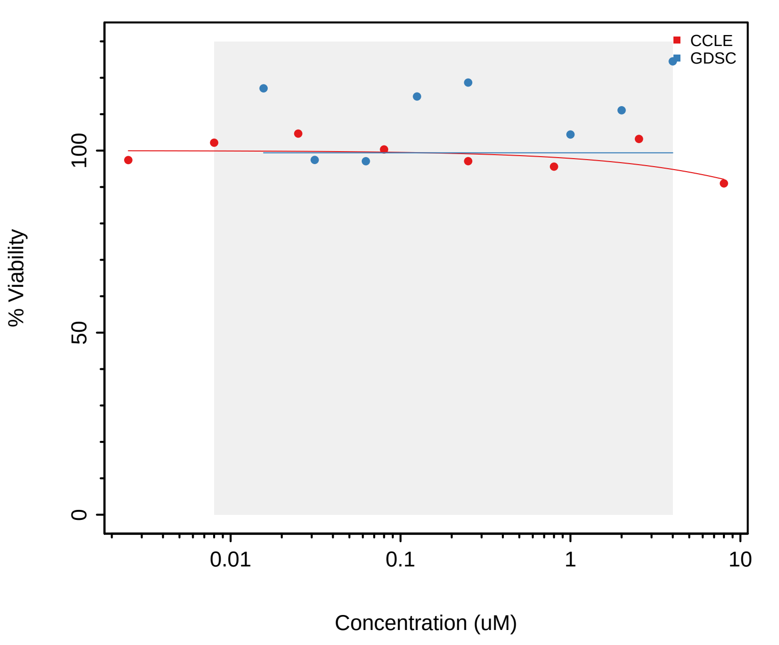

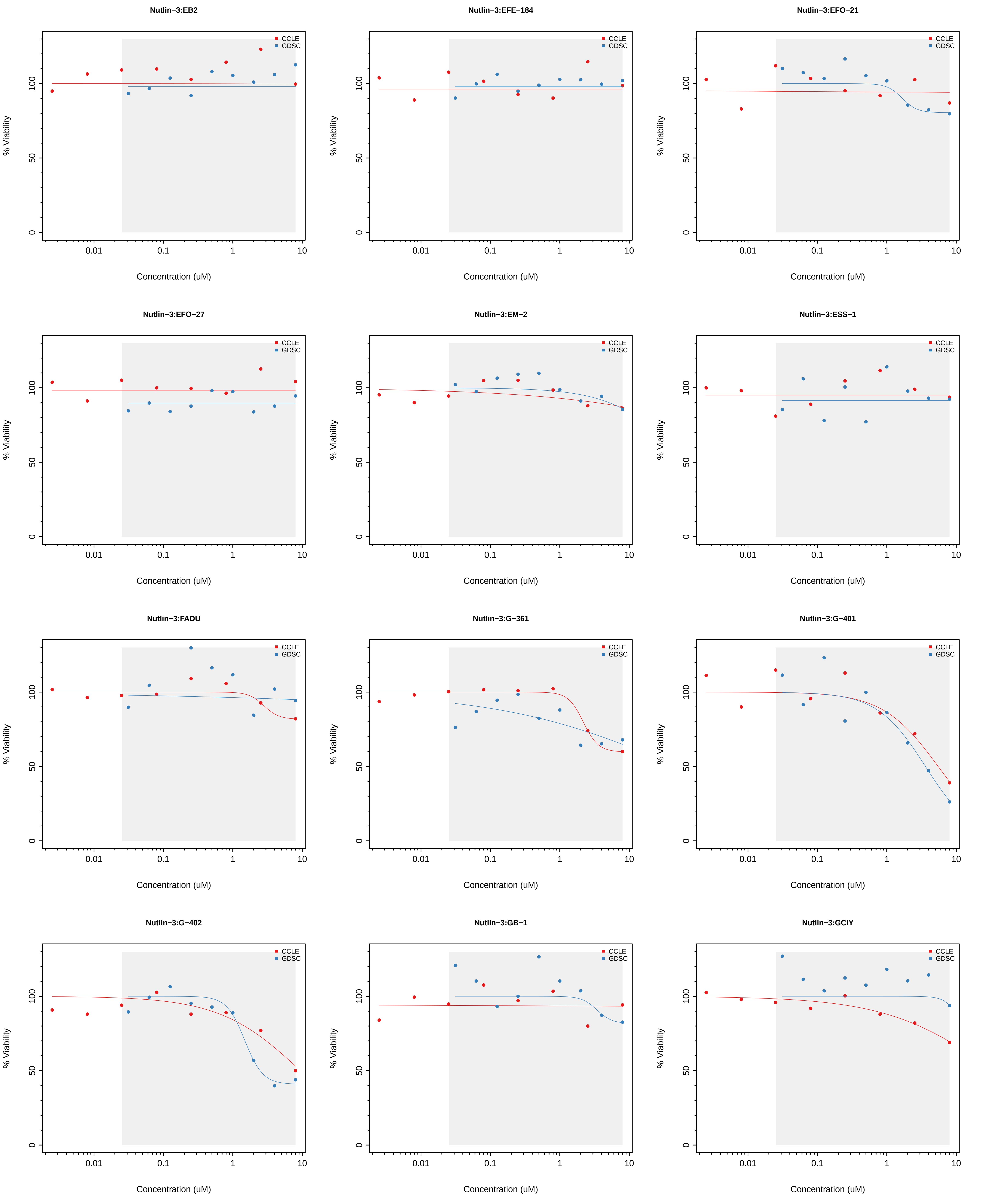
Nutlin−3:EB2
Nutlin−3:EFE−184
Nutlin−3:EFO−21
Nutlin−3:EFO−27
Nutlin−3:EM−2
Nutlin−3:ESS−1
Nutlin−3:FADU
Nutlin−3:G−361
Nutlin−3:G−401
Nutlin−3:G−402
Nutlin−3:GB−1
Nutlin−3:GCIY
CCLE
GDSC
% Viability
0
50
100
0.01
0.1
1
10
Concentration (uM)

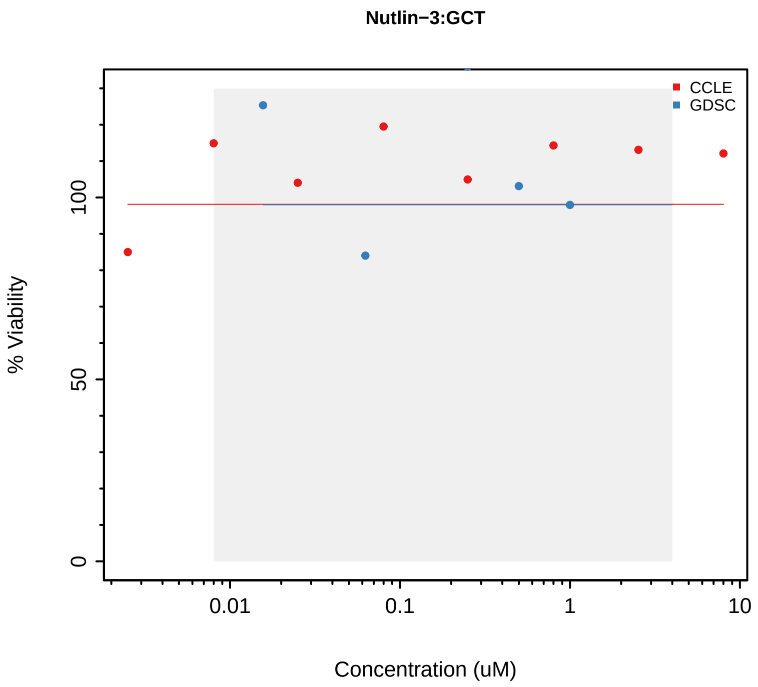
Nutlin−3:GCT
CCLE
GDSC
% Viability
0
50
100
0.01
0.1
1
10
Concentration (uM)

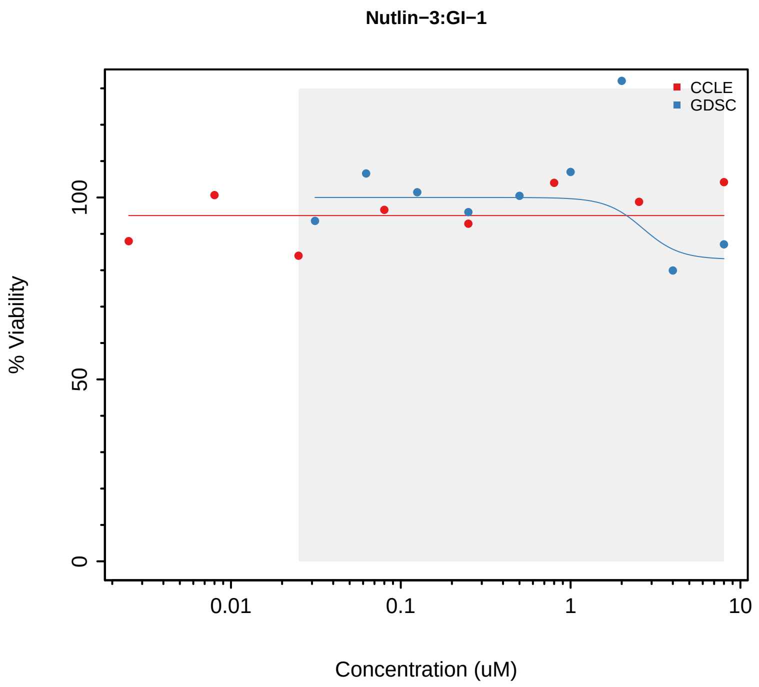
Nutlin−3:GI−1
CCLE
GDSC
% Viability
0
50
100
0.01
0.1
1
10
Concentration (uM)

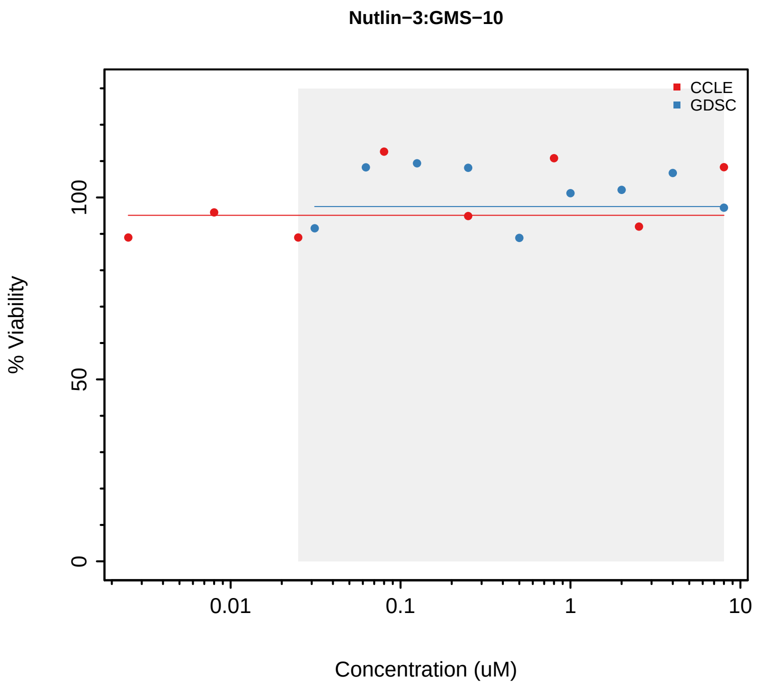
Nutlin−3:GMS−10
CCLE
GDSC
% Viability
0
50
100
0.01
0.1
1
10
Concentration (uM)

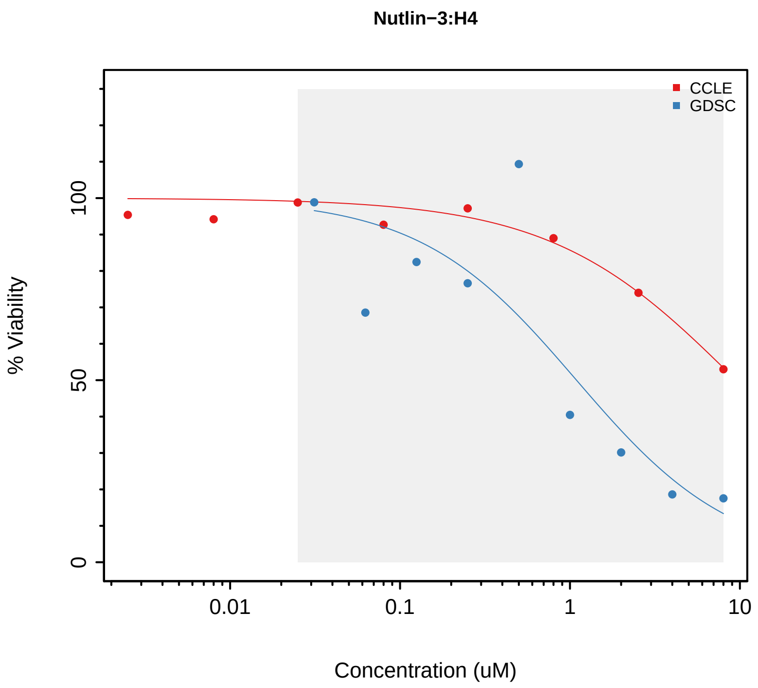
Nutlin−3:H4
CCLE
GDSC
% Viability
0
50
100
0.01
0.1
1
10
Concentration (uM)

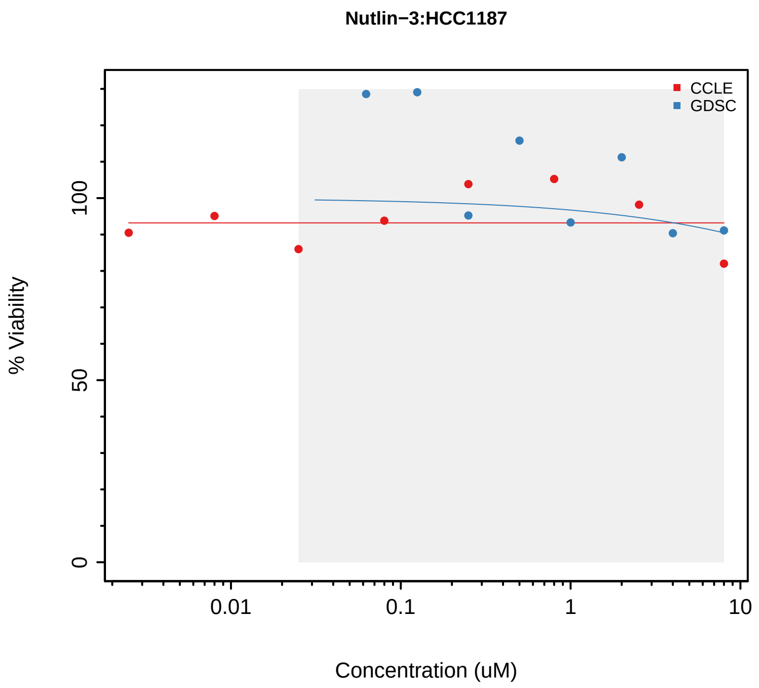
Nutlin−3:HCC1187
CCLE
GDSC
% Viability
0
50
100
0.01
0.1
1
10
Concentration (uM)

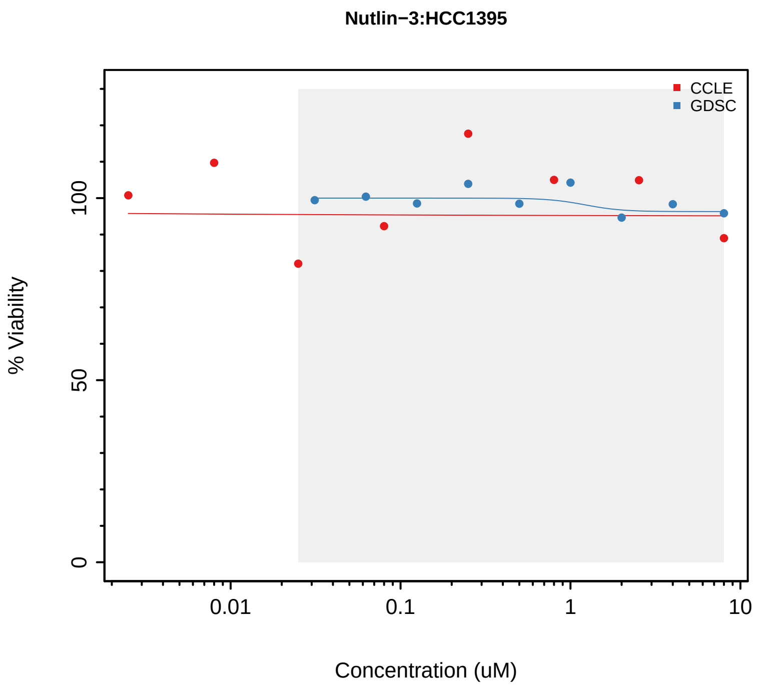
Nutlin−3:HCC1395
CCLE
GDSC
% Viability
0
50
100
0.01
0.1
1
10
Concentration (uM)

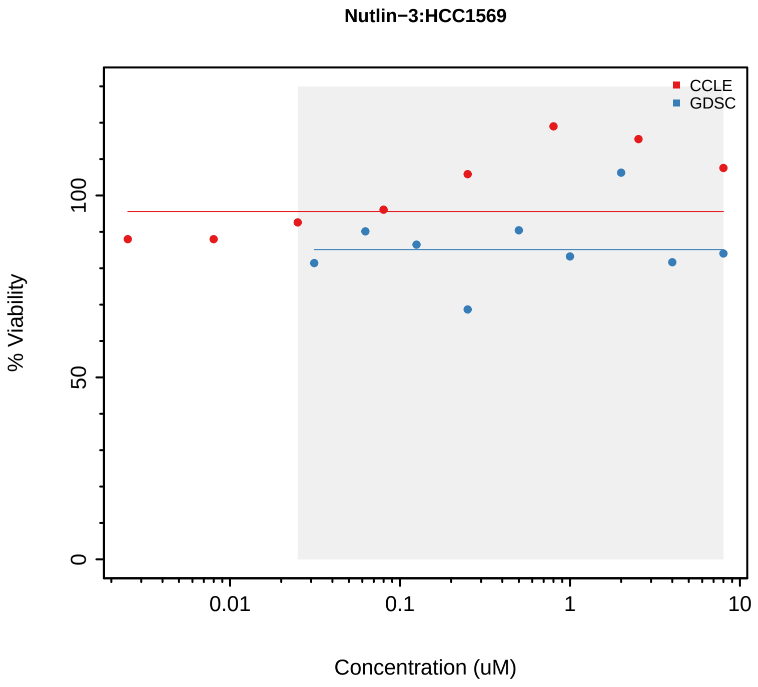
Nutlin−3:HCC1569
CCLE
GDSC
% Viability
0
50
100
0.01
0.1
1
10
Concentration (uM)

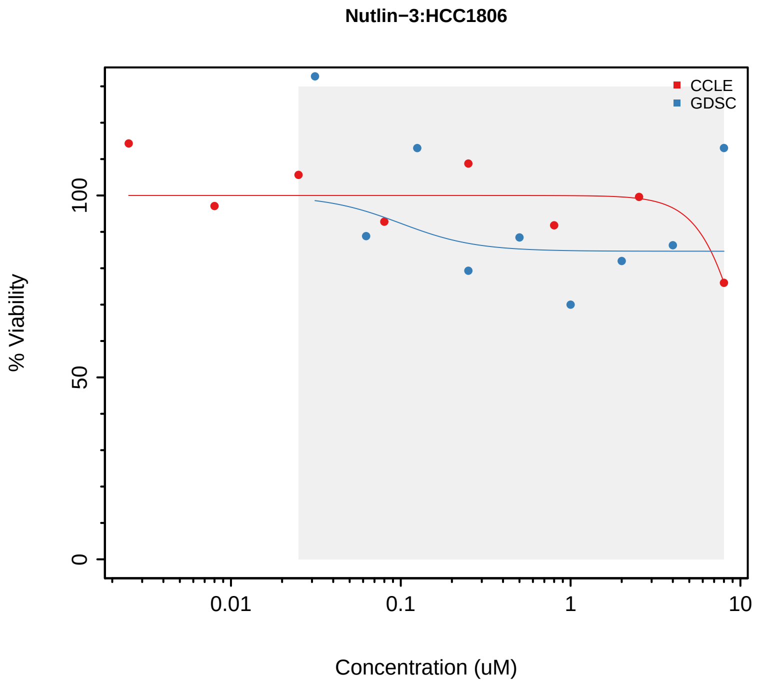
Nutlin−3:HCC1806
CCLE
GDSC
% Viability
0
50
100
0.01
0.1
1
10
Concentration (uM)

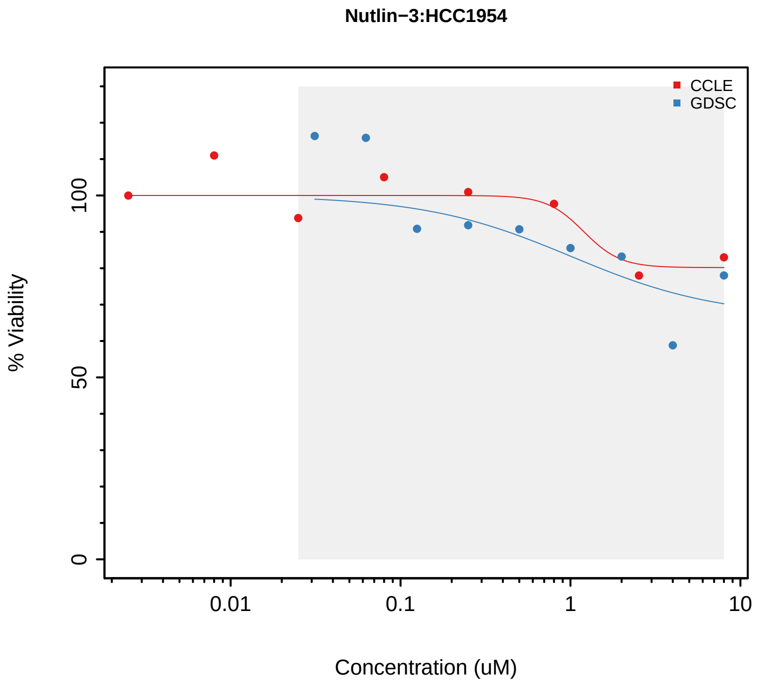
Nutlin−3:HCC1954
CCLE
GDSC
% Viability
0
50
100
0.01
0.1
1
10
Concentration (uM)

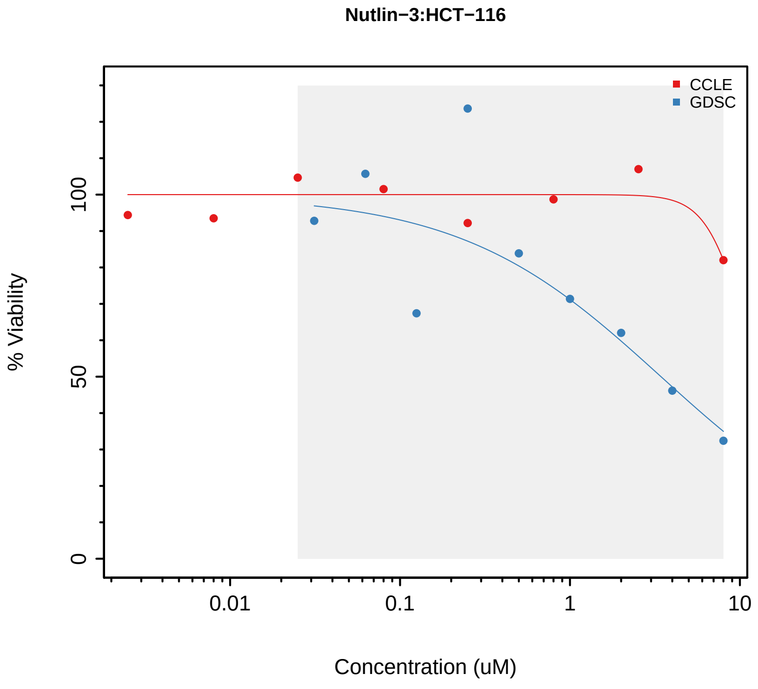
Nutlin−3:HCT−116
CCLE
GDSC
% Viability
0
50
100
0.01
0.1
1
10
Concentration (uM)

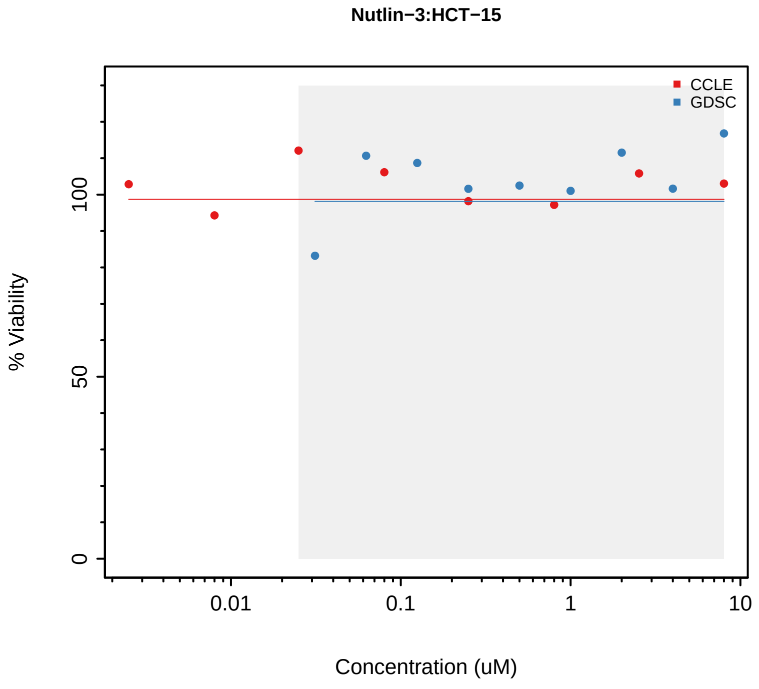
Nutlin−3:HCT−15
CCLE
GDSC
% Viability
0
50
100
0.01
0.1
1
10
Concentration (uM)

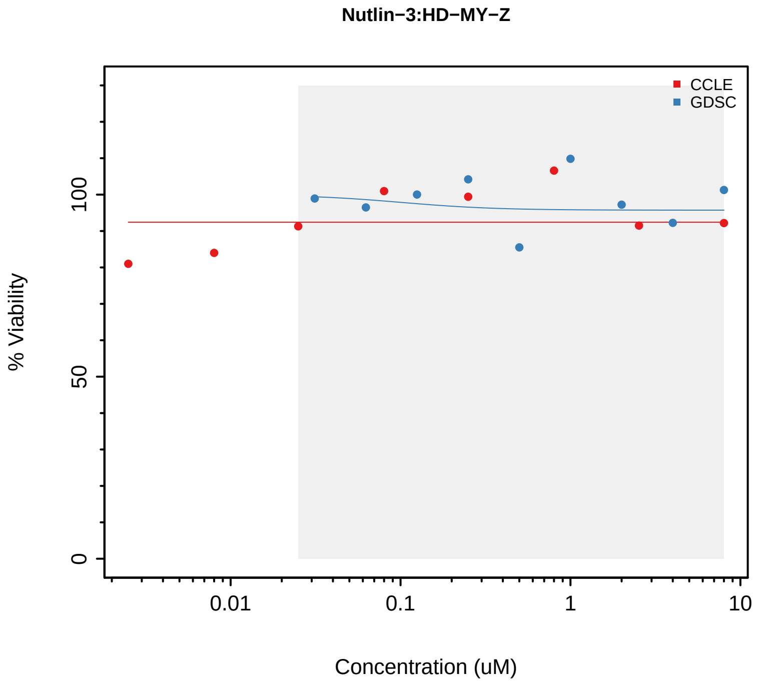
Nutlin−3:HD−MY−Z
CCLE
GDSC
% Viability
0
50
100
0.01
0.1
1
10
Concentration (uM)

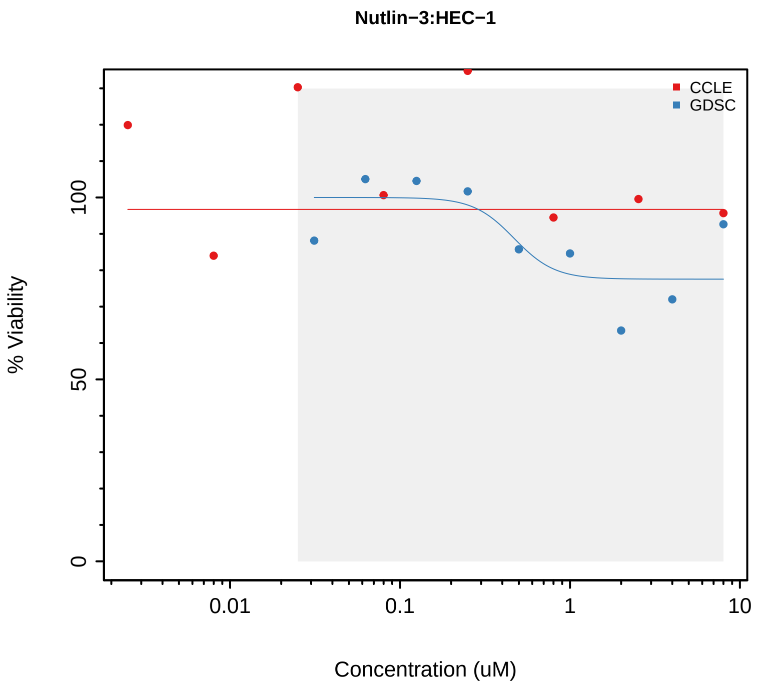
Nutlin−3:HEC−1
CCLE
GDSC
% Viability
0
50
100
0.01
0.1
1
10
Concentration (uM)
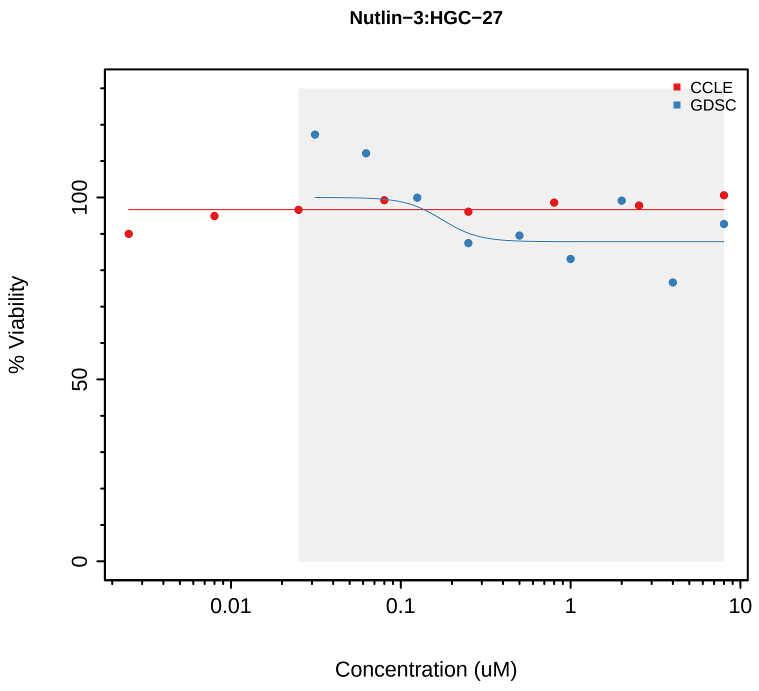
Nutlin−3:HGC−27
CCLE
GDSC
% Viability
0
50
100
0.01
0.1
1
10
Concentration (uM)
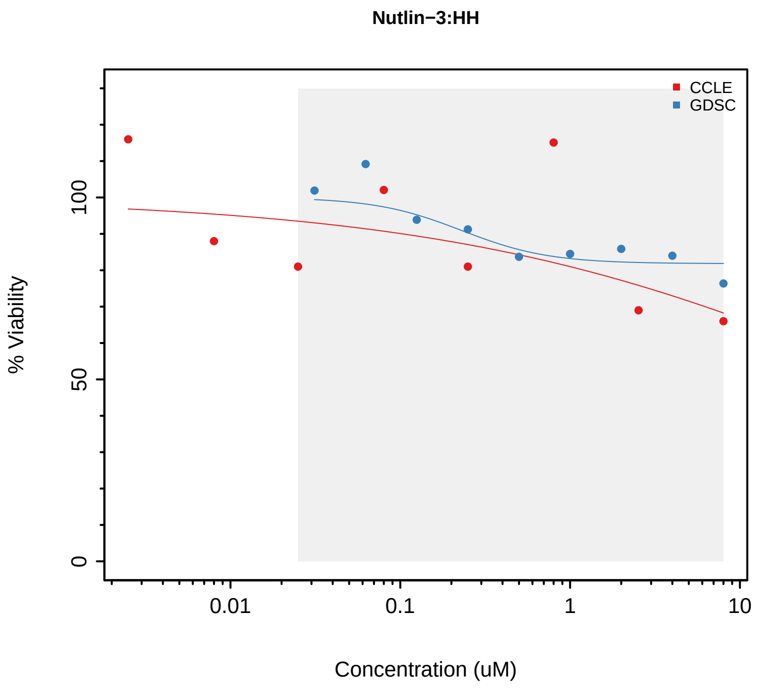
Nutlin−3:HH
CCLE
GDSC
% Viability
0
50
100
0.01
0.1
1
10
Concentration (uM)
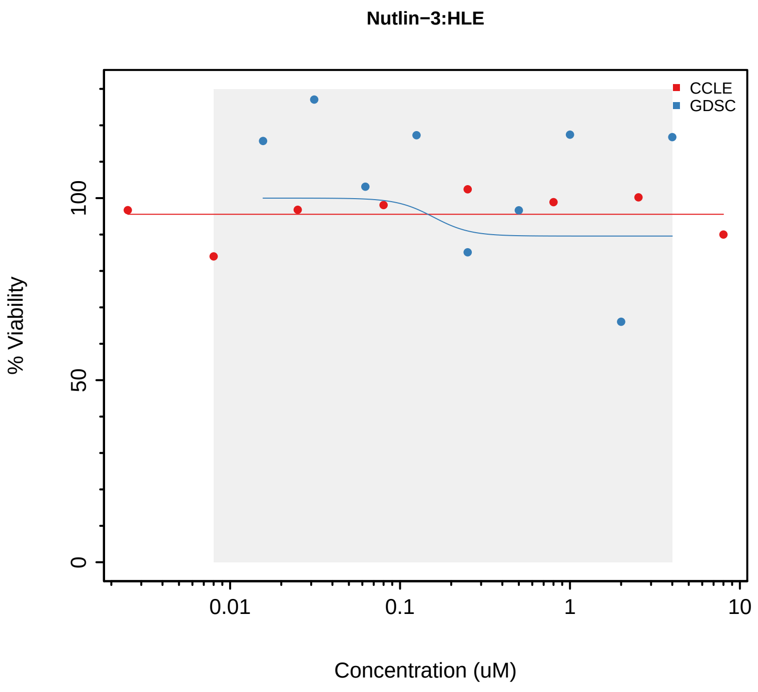
Nutlin−3:HLE
CCLE
GDSC
% Viability
0
50
100
0.01
0.1
1
10
Concentration (uM)
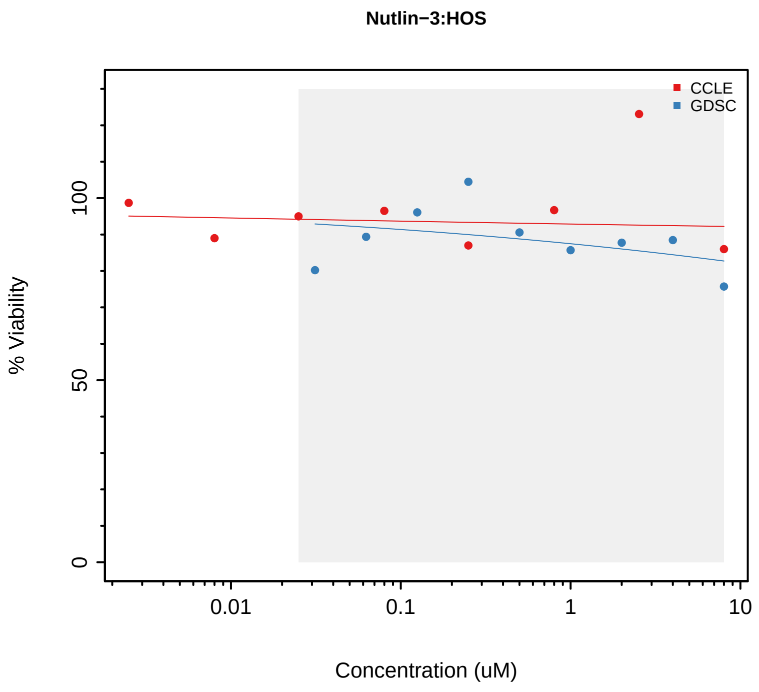
Nutlin−3:HOS
CCLE
GDSC
% Viability
0
50
100
0.01
0.1
1
10
Concentration (uM)
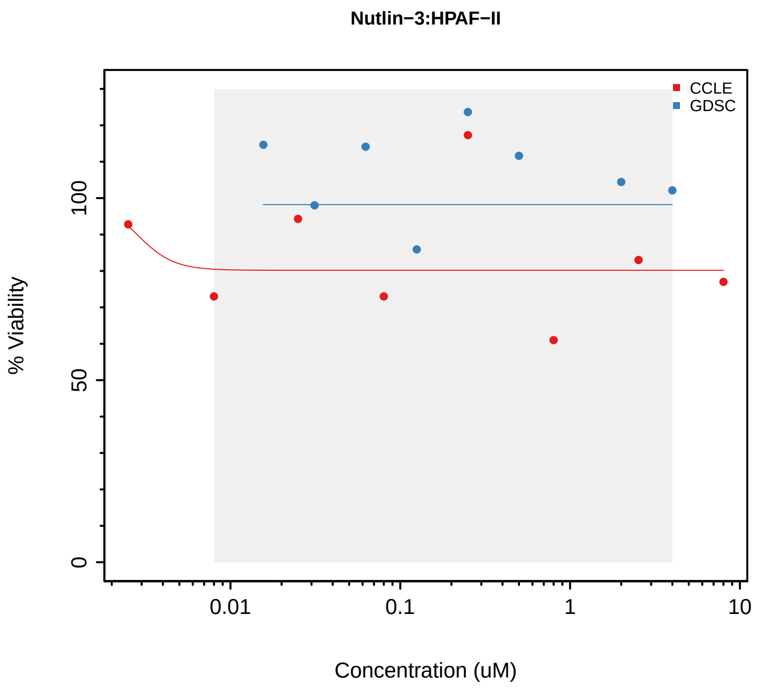
Nutlin−3:HPAF−II
CCLE
GDSC
% Viability
0
50
100
0.01
0.1
1
10
Concentration (uM)
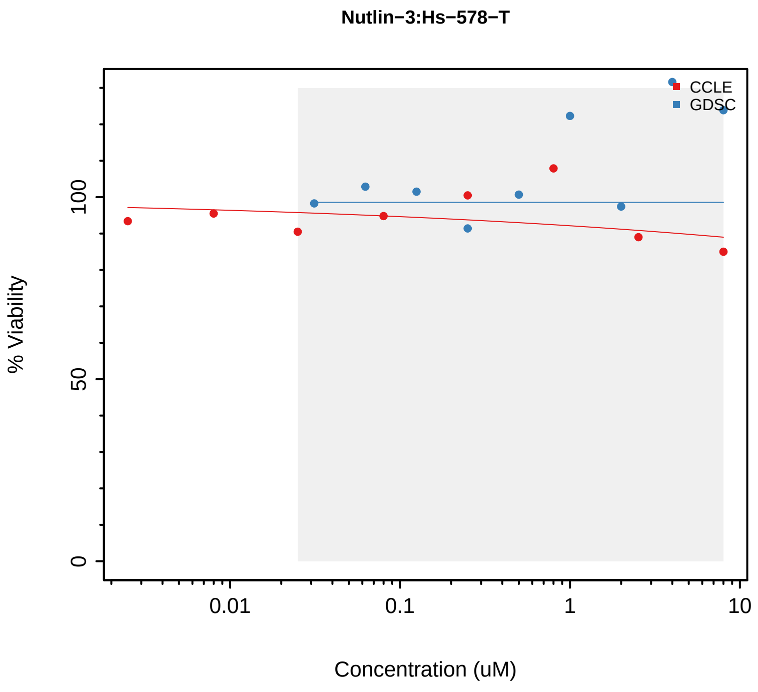
Nutlin−3:Hs−578−T
CCLE
GDSC
% Viability
0
50
100
0.01
0.1
1
10
Concentration (uM)
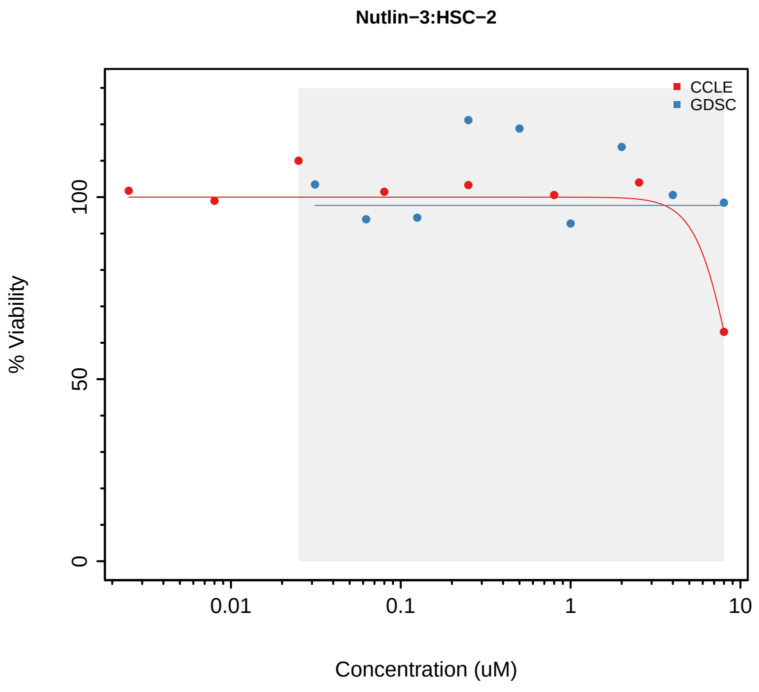
Nutlin−3:HSC−2
CCLE
GDSC
% Viability
0
50
100
0.01
0.1
1
10
Concentration (uM)
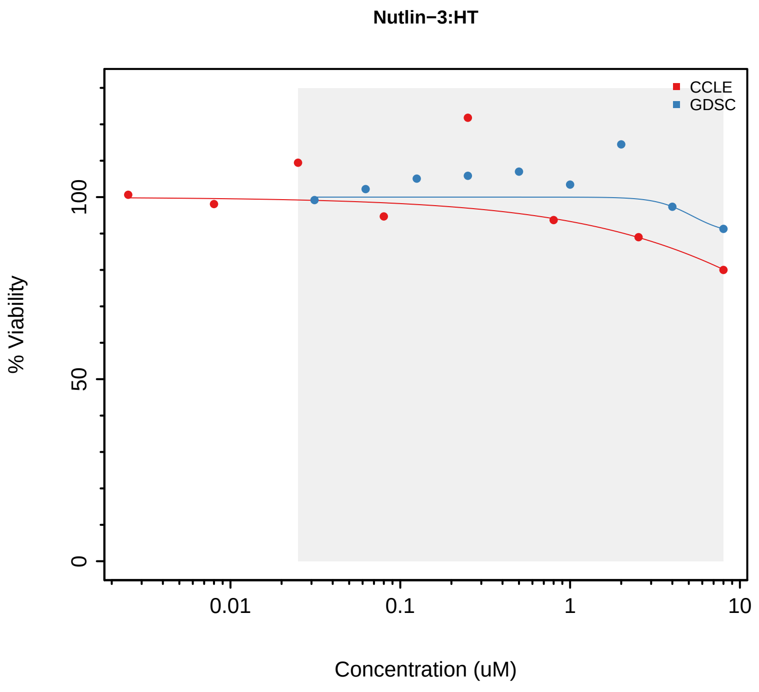
Nutlin−3:HT
CCLE
GDSC
% Viability
0
50
100
0.01
0.1
1
10
Concentration (uM)
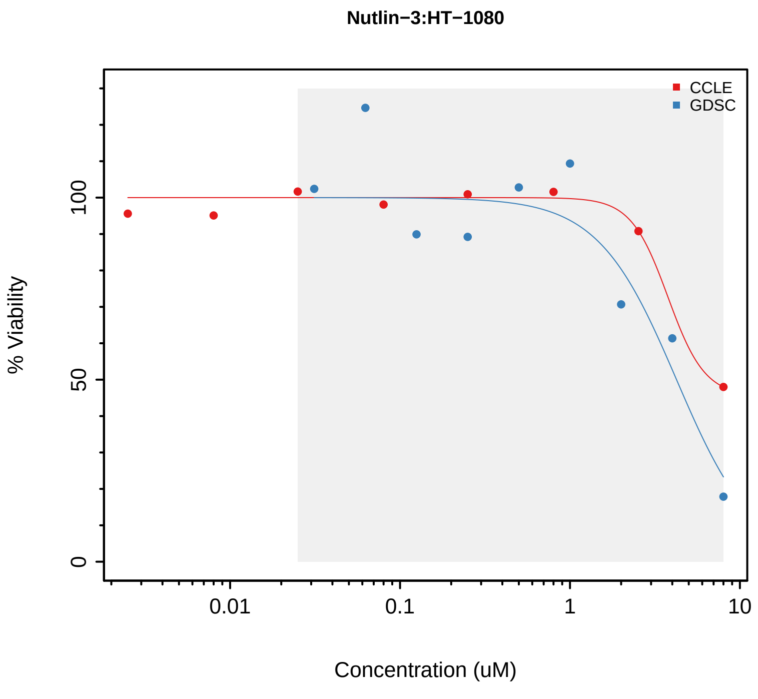
Nutlin−3:HT−1080
CCLE
GDSC
% Viability
0
50
100
0.01
0.1
1
10
Concentration (uM)
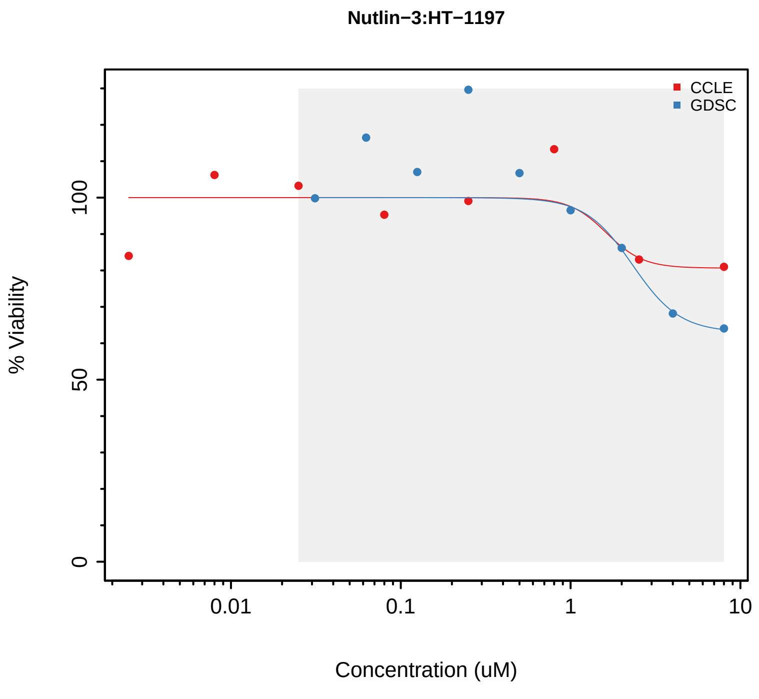
Nutlin−3:HT−1197
CCLE
GDSC
% Viability
0
50
100
0.01
0.1
1
10
Concentration (uM)
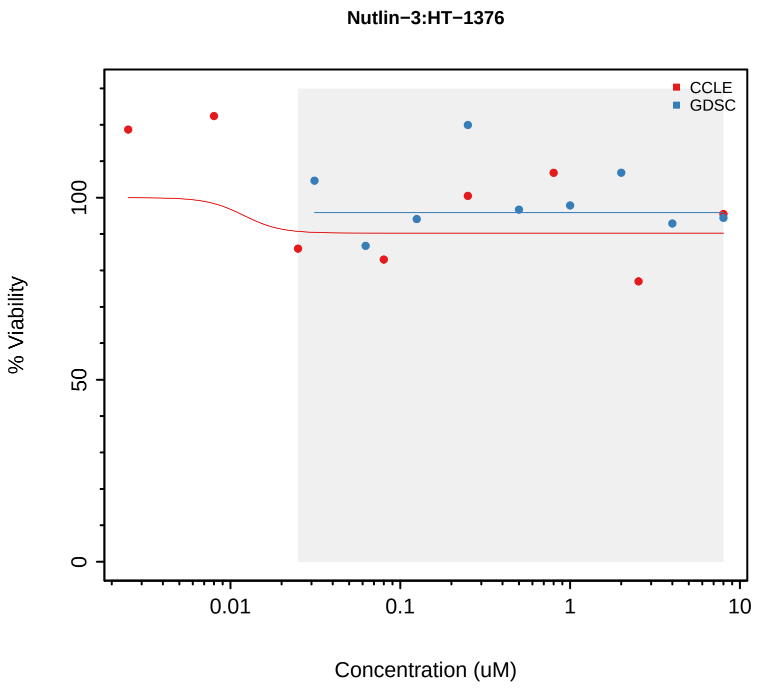
Nutlin−3:HT−1376
CCLE
GDSC
% Viability
0
50
100
0.01
0.1
1
10
Concentration (uM)

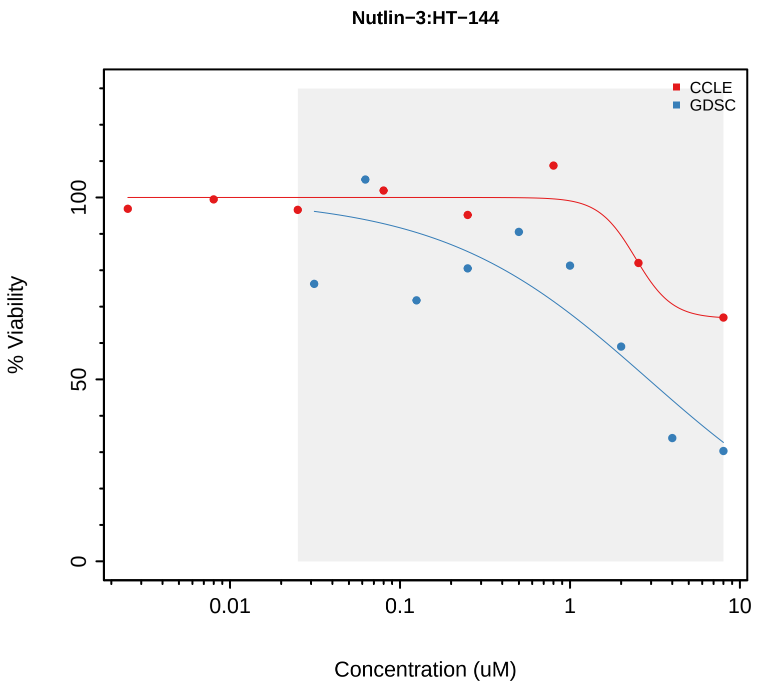
Nutlin−3:HT−144
CCLE
GDSC
% Viability
0 50 100
0.01 0.1 1 10
Concentration (uM)

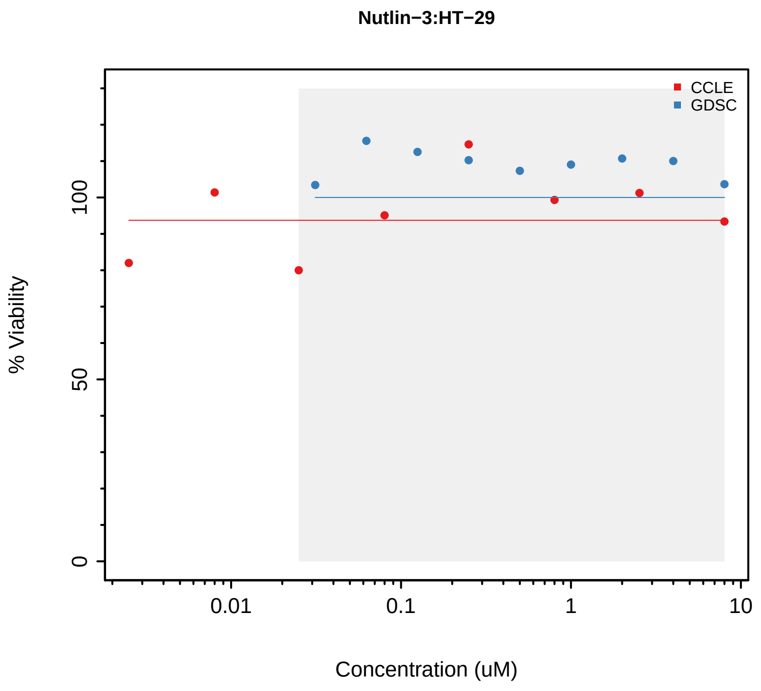
Nutlin−3:HT−29
CCLE
GDSC
% Viability
0 50 100
0.01 0.1 1 10
Concentration (uM)

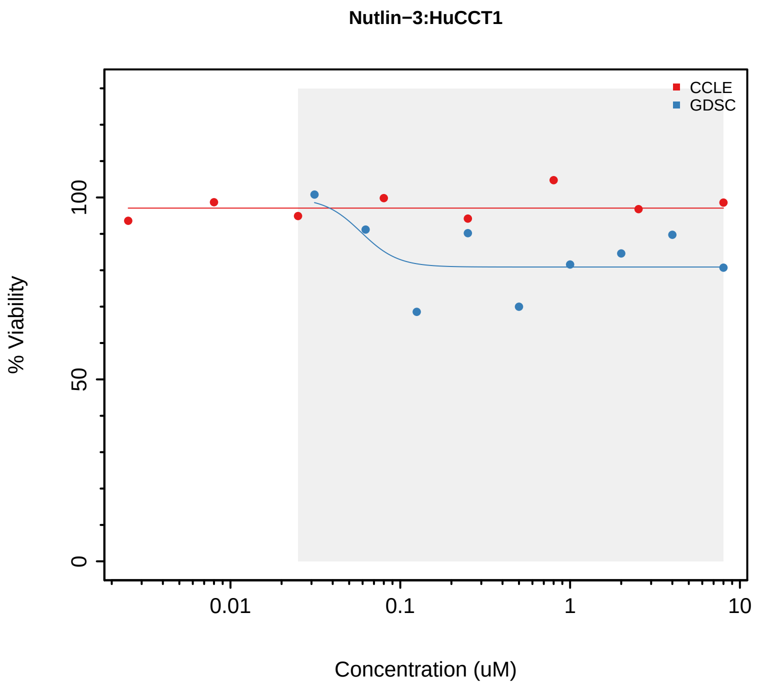
Nutlin−3:HuCCT1
CCLE
GDSC
% Viability
0 50 100
0.01 0.1 1 10
Concentration (uM)

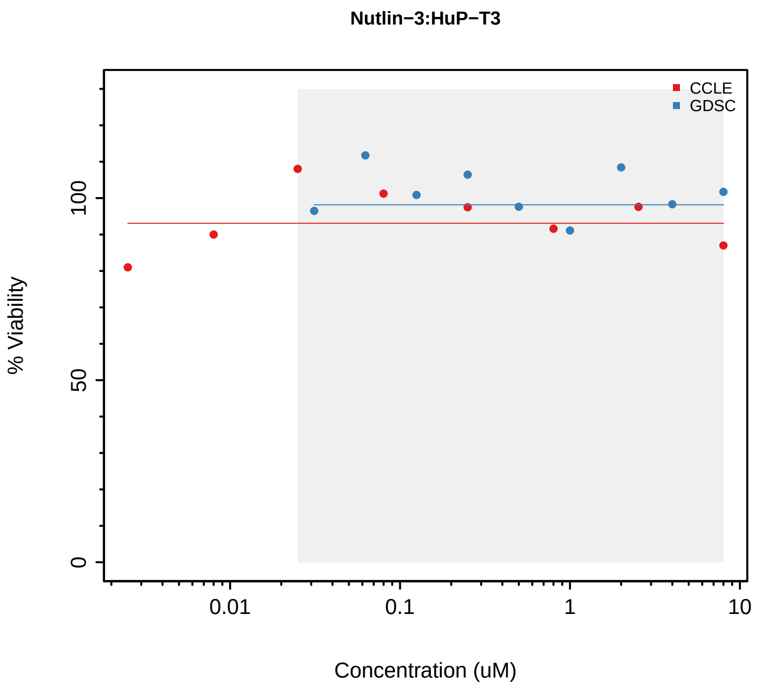
Nutlin−3:HuP−T3
CCLE
GDSC
% Viability
0 50 100
0.01 0.1 1 10
Concentration (uM)

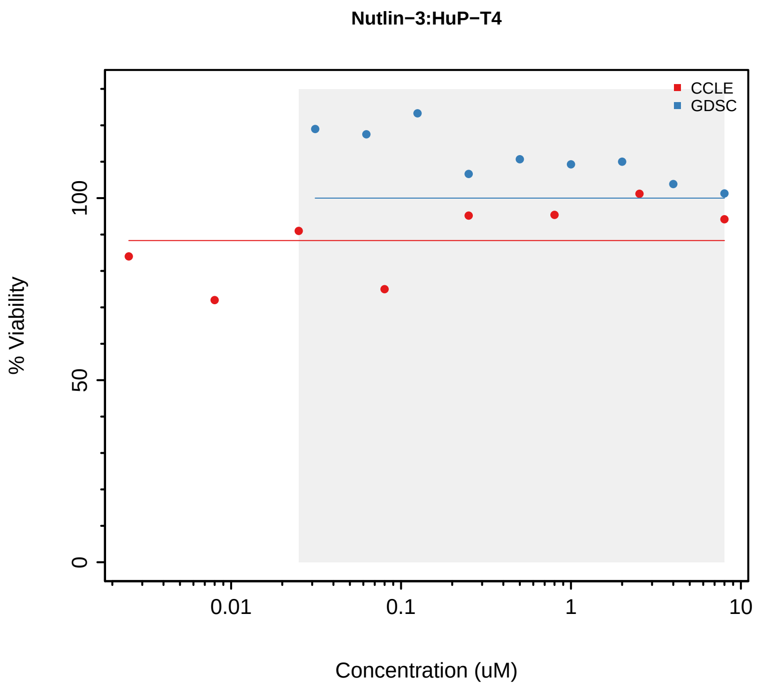
Nutlin−3:HuP−T4
CCLE
GDSC
% Viability
0 50 100
0.01 0.1 1 10
Concentration (uM)

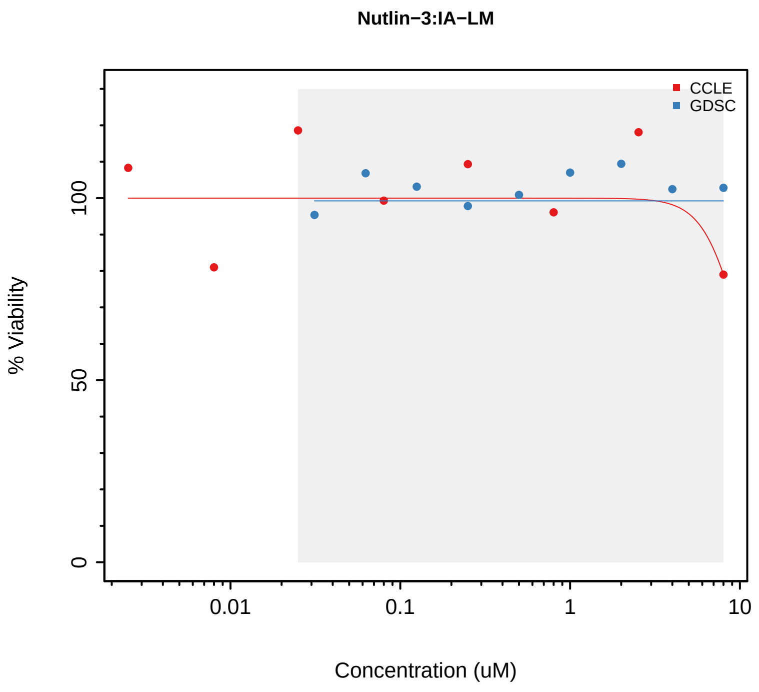
Nutlin−3:IA−LM
CCLE
GDSC
% Viability
0 50 100
0.01 0.1 1 10
Concentration (uM)

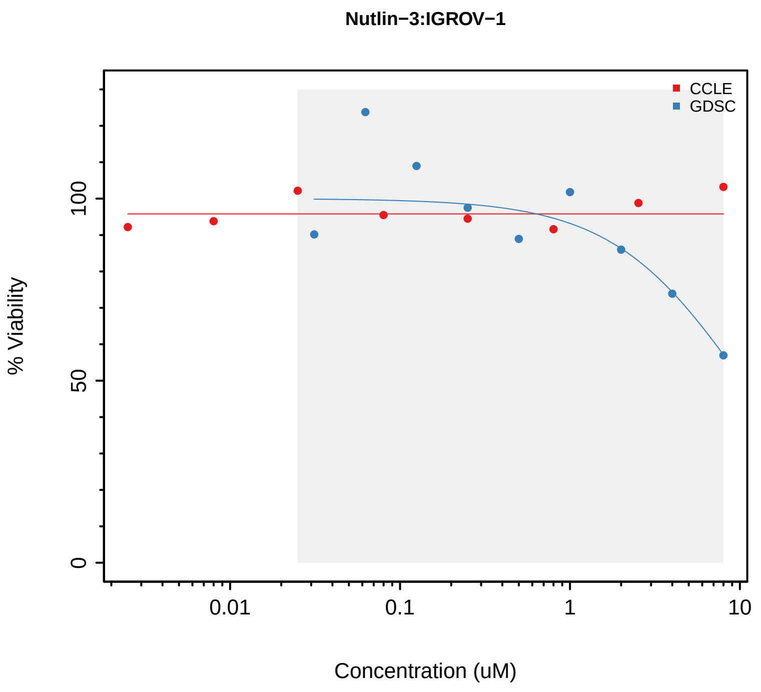
Nutlin−3:IGROV−1
CCLE
GDSC
% Viability
0 50 100
0.01 0.1 1 10
Concentration (uM)

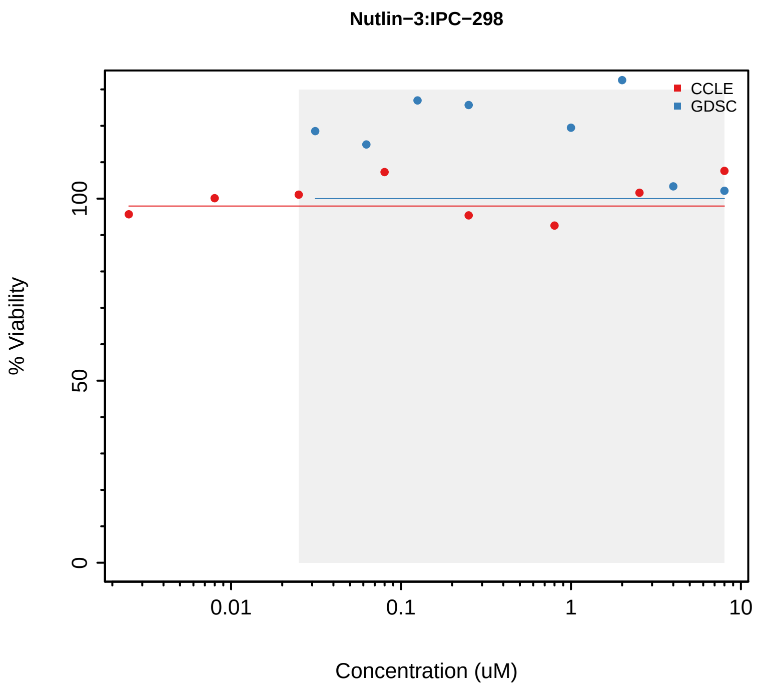
Nutlin−3:IPC−298
CCLE
GDSC
% Viability
0 50 100
0.01 0.1 1 10
Concentration (uM)

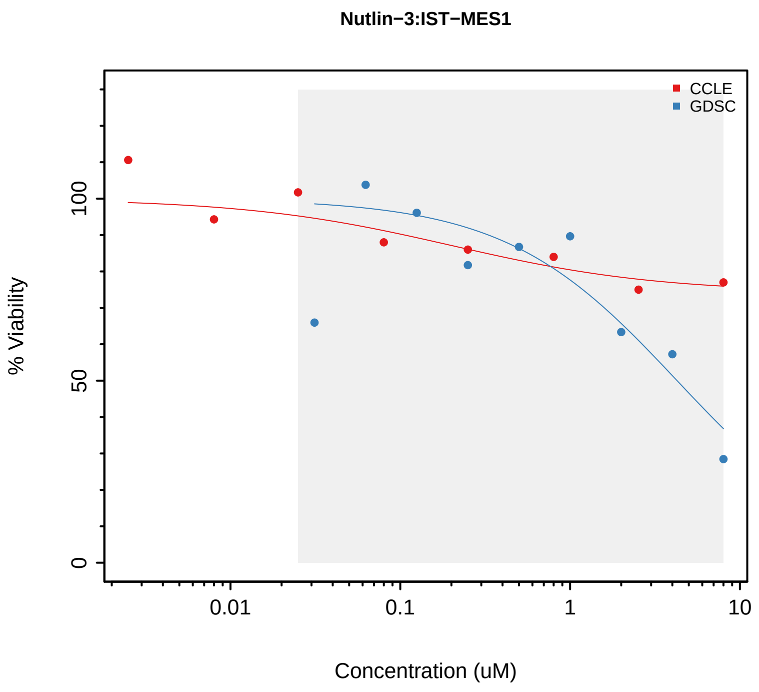
Nutlin−3:IST−MES1
CCLE
GDSC
% Viability
0 50 100
0.01 0.1 1 10
Concentration (uM)

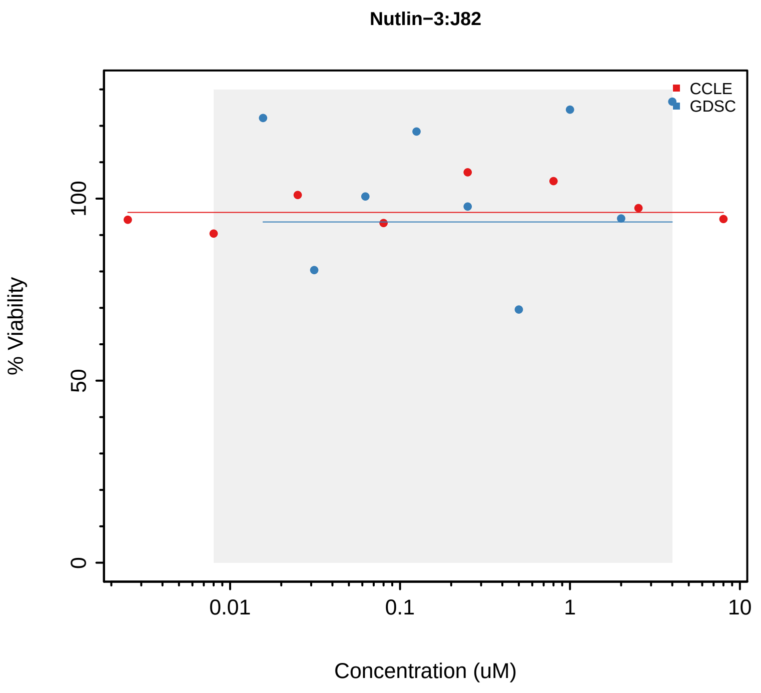
Nutlin−3:J82
CCLE
GDSC
% Viability
0 50 100
0.01 0.1 1 10
Concentration (uM)

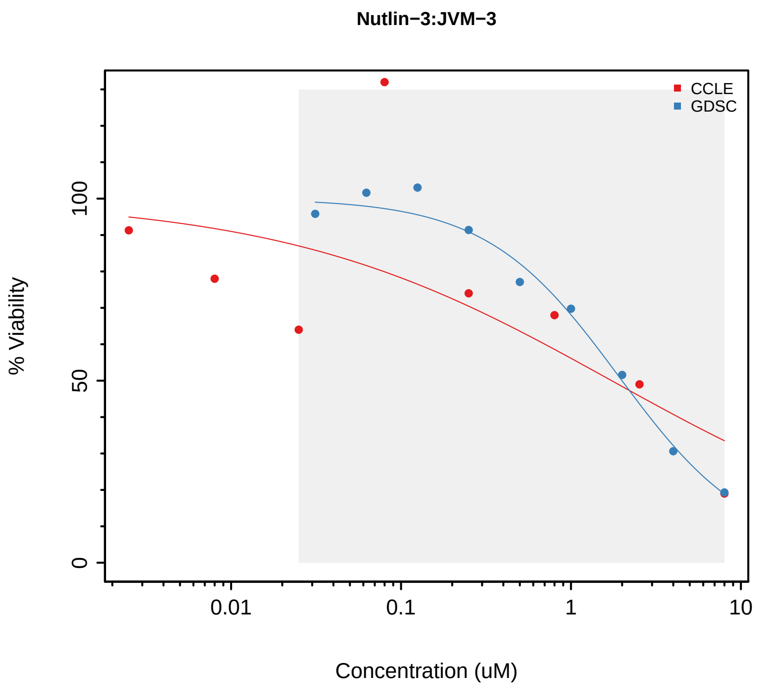
Nutlin−3:JVM−3
CCLE
GDSC
% Viability
0 50 100
0.01 0.1 1 10
Concentration (uM)

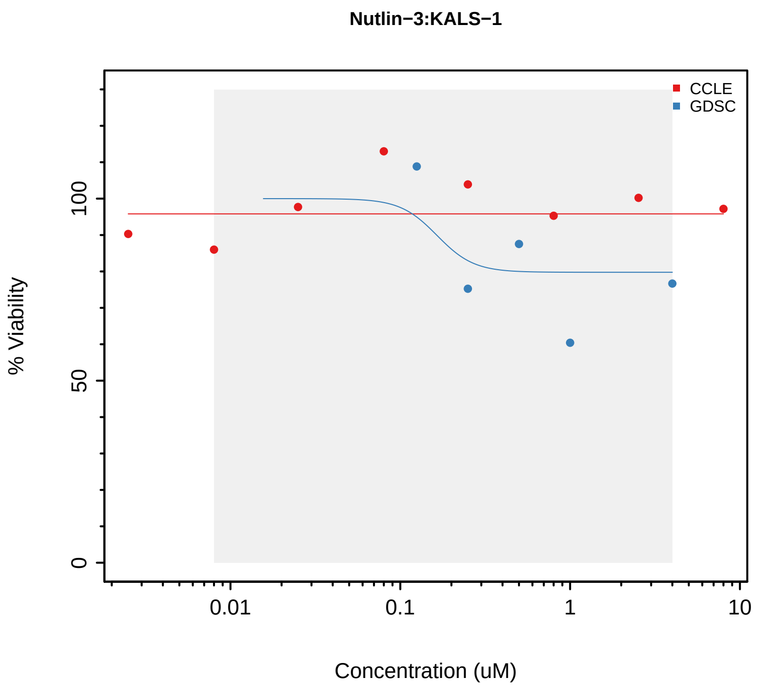
Nutlin−3:KALS−1
CCLE
GDSC
% Viability
0 50 100
0.01 0.1 1 10
Concentration (uM)

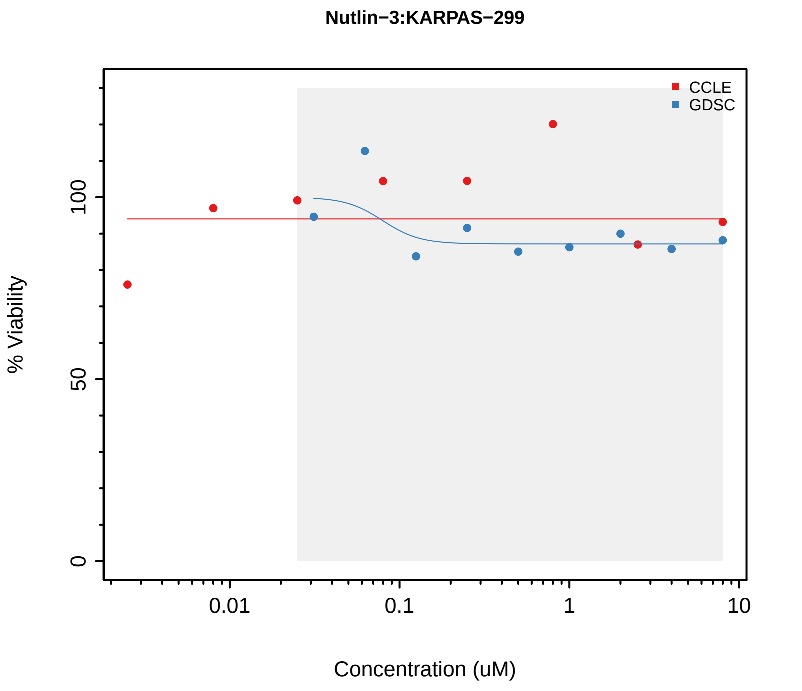
Nutlin−3:KARPAS−299
CCLE
GDSC
% Viability
Concentration (uM)
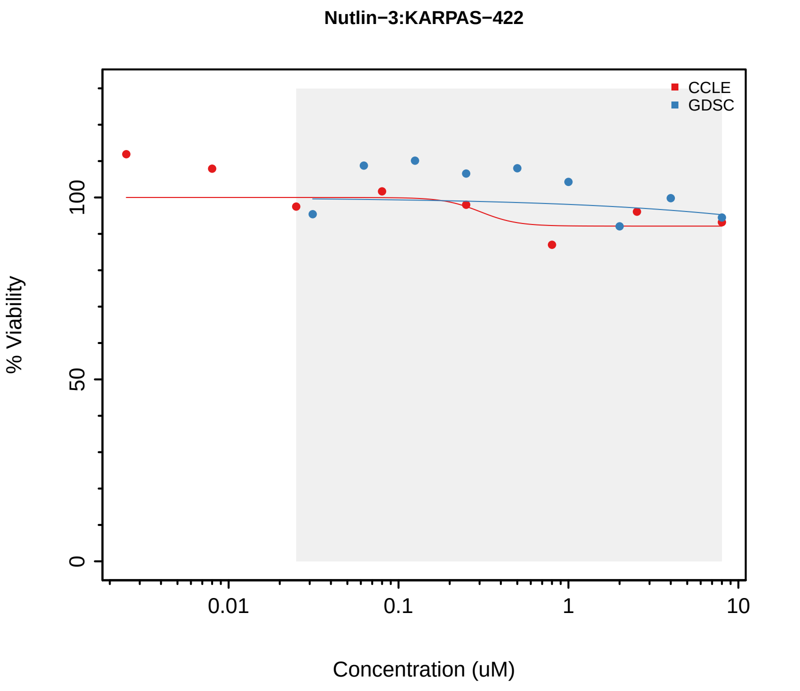
Nutlin−3:KARPAS−422
CCLE
GDSC
% Viability
Concentration (uM)
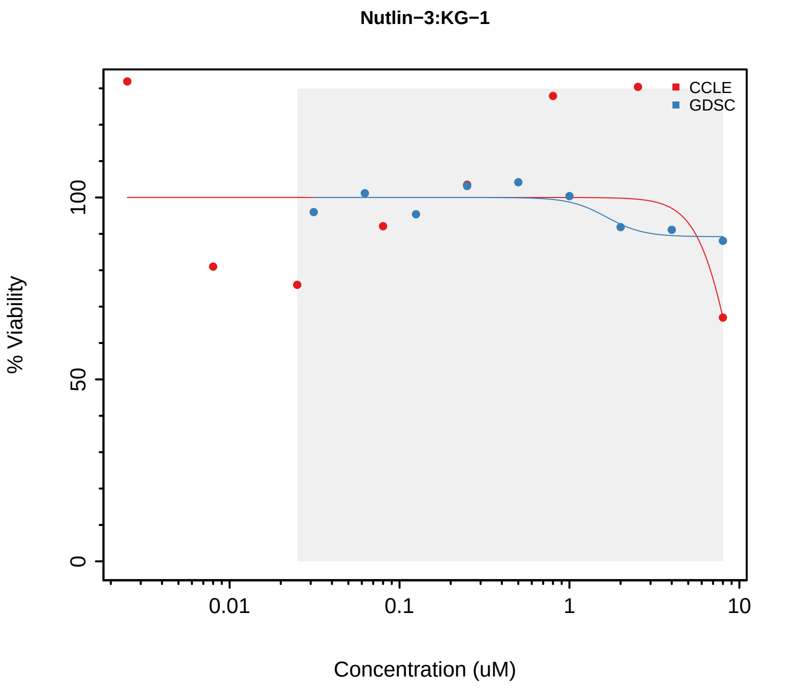
Nutlin−3:KG−1
CCLE
GDSC
% Viability
Concentration (uM)
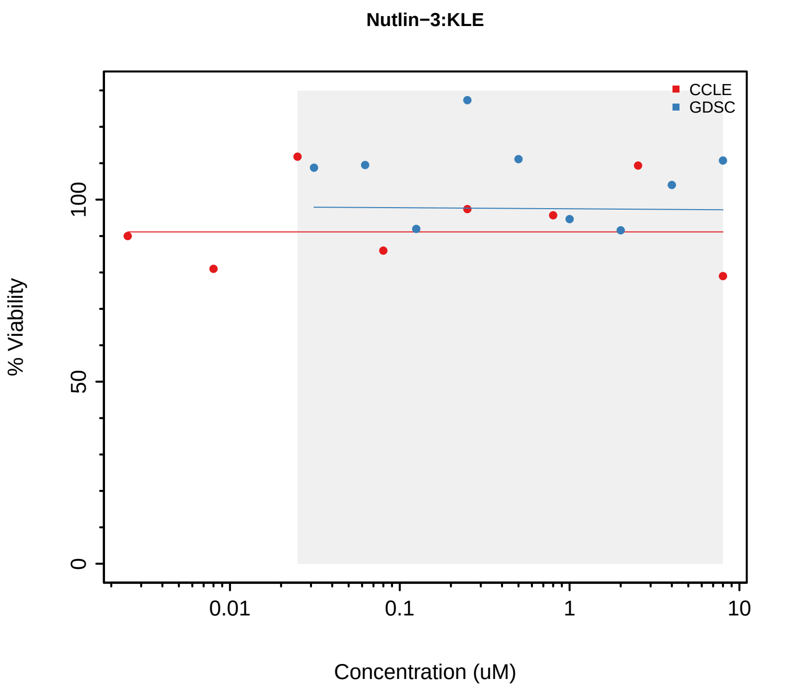
Nutlin−3:KLE
CCLE
GDSC
% Viability
Concentration (uM)
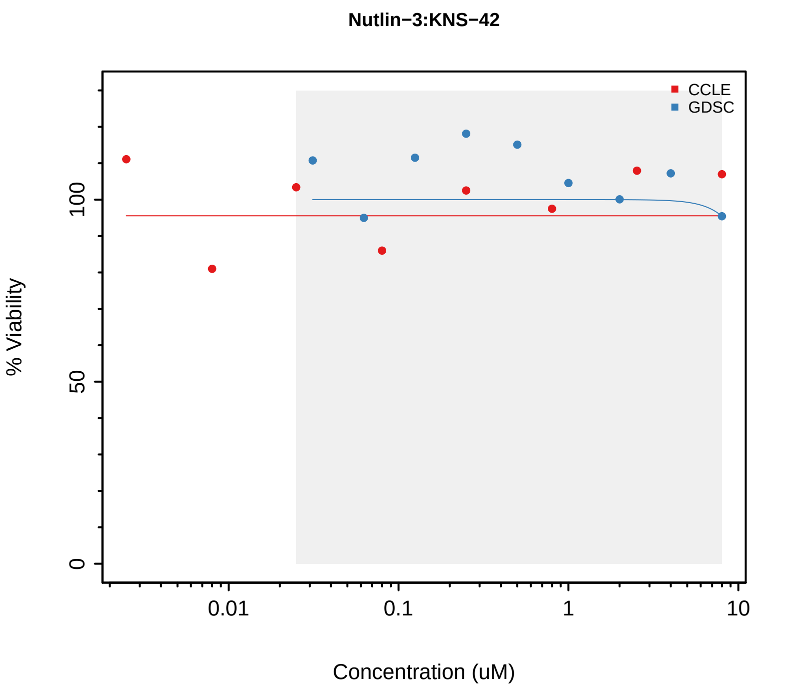
Nutlin−3:KNS−42
CCLE
GDSC
% Viability
Concentration (uM)
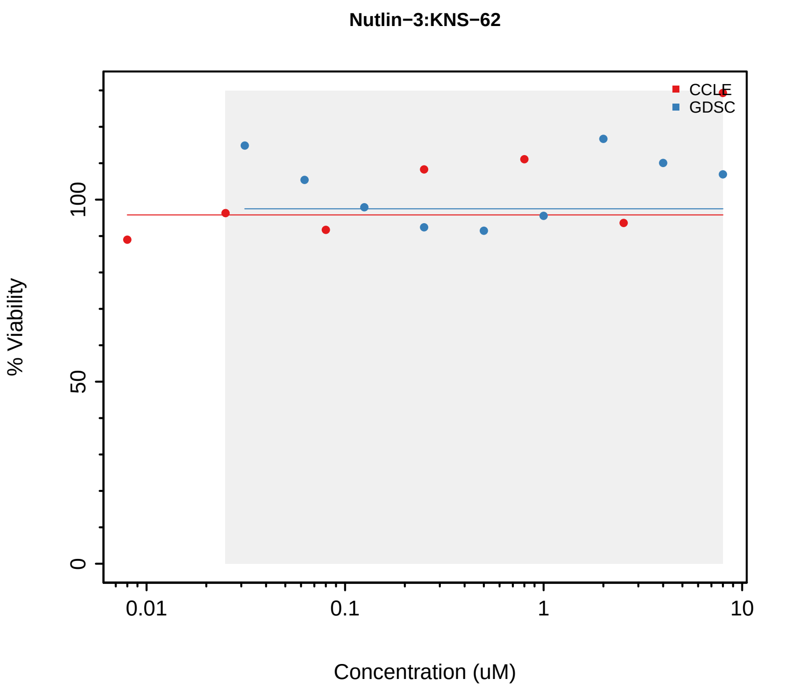
Nutlin−3:KNS−62
CCLE
GDSC
% Viability
Concentration (uM)
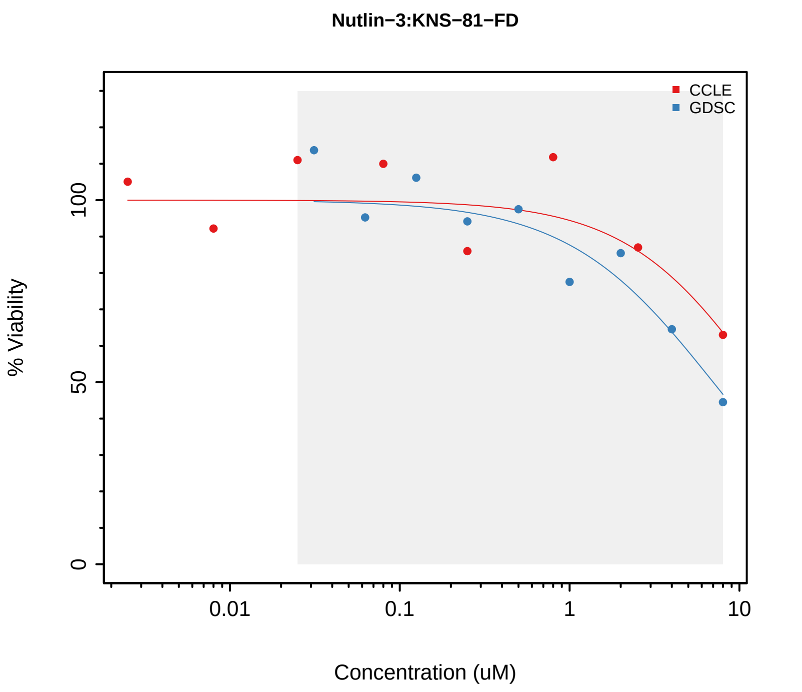
Nutlin−3:KNS−81−FD
CCLE
GDSC
% Viability
Concentration (uM)
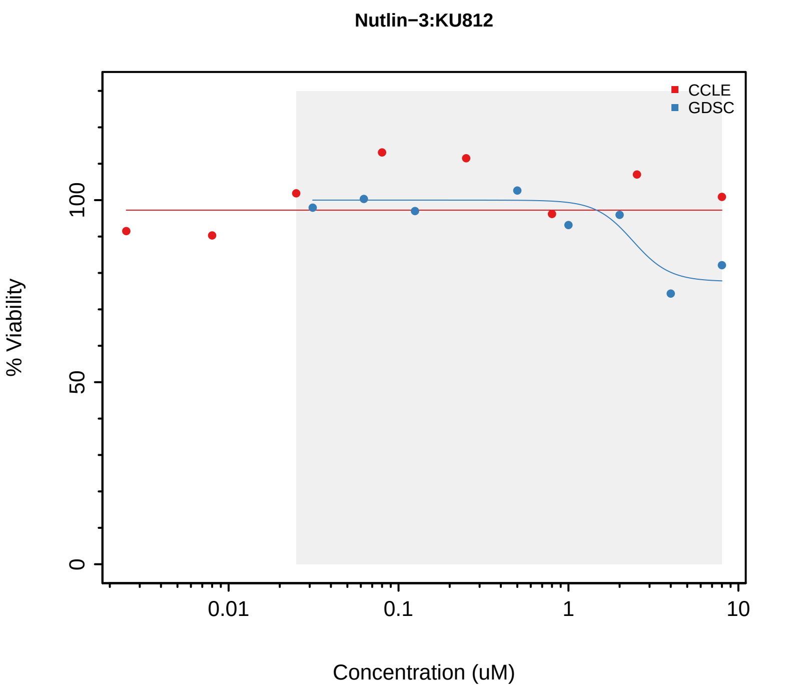
Nutlin−3:KU812
CCLE
GDSC
% Viability
Concentration (uM)
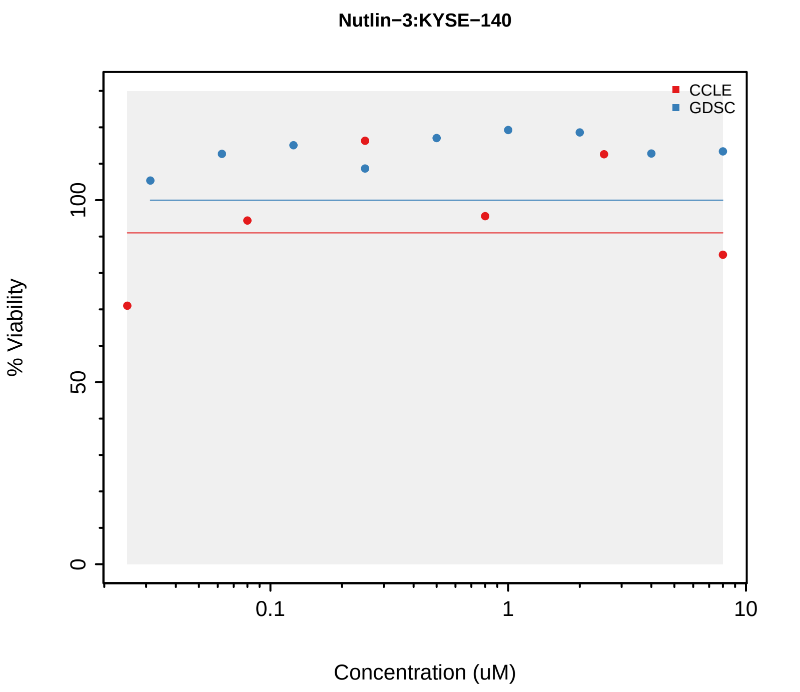
Nutlin−3:KYSE−140
CCLE
GDSC
% Viability
Concentration (uM)
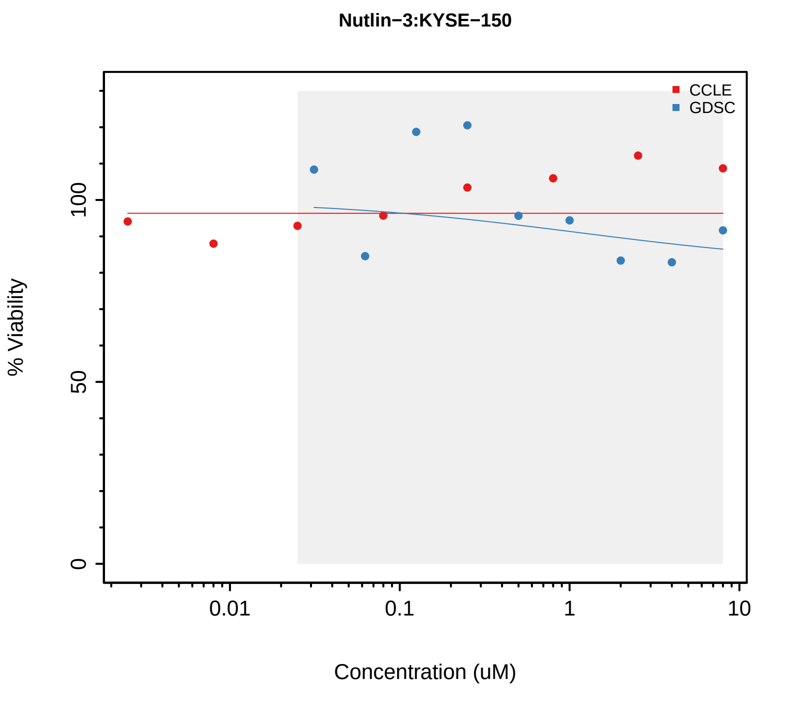
Nutlin−3:KYSE−150
CCLE
GDSC
% Viability
Concentration (uM)
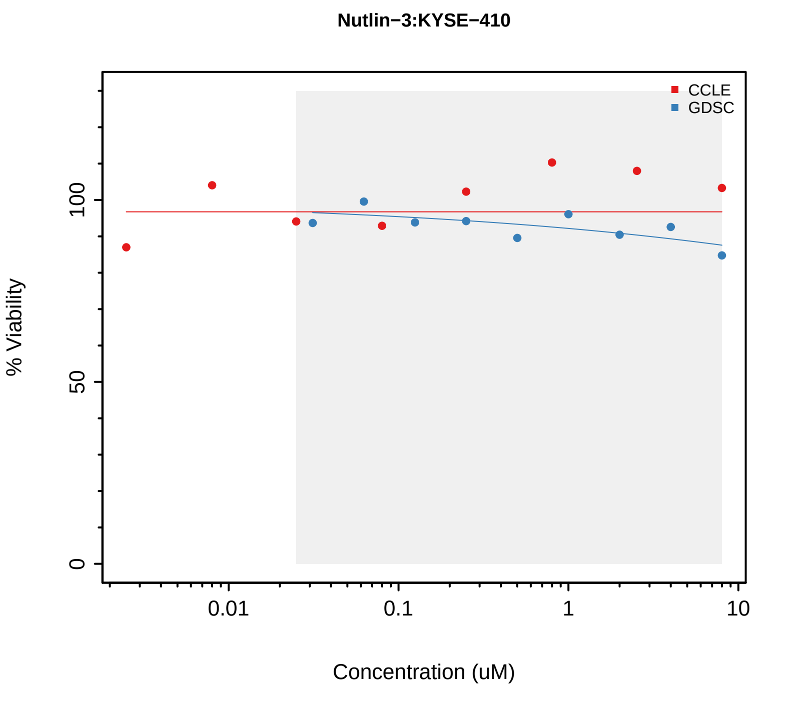
Nutlin−3:KYSE−410
CCLE
GDSC
% Viability
Concentration (uM)
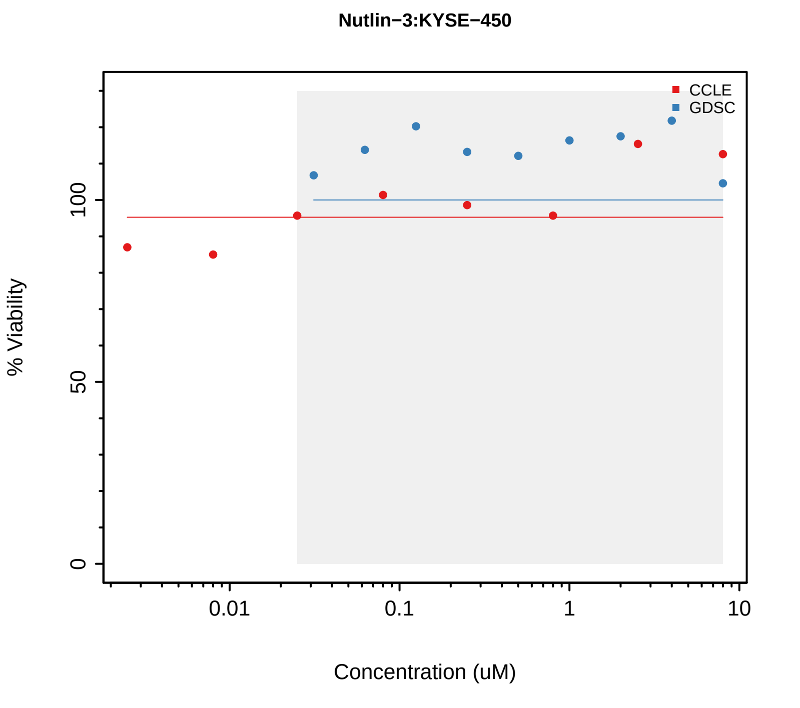
Nutlin−3:KYSE−450
CCLE
GDSC
% Viability
Concentration (uM)

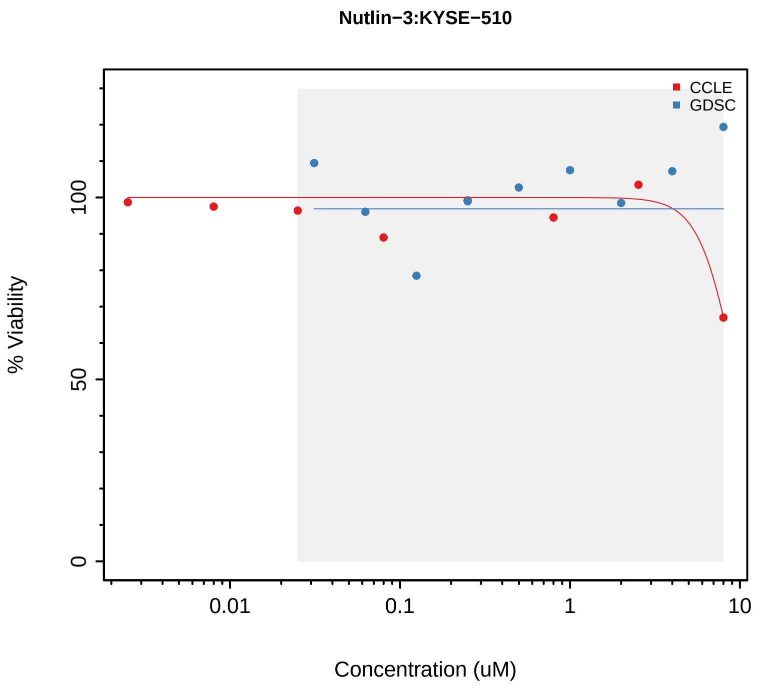
Nutlin−3:KYSE−510
CCLE
GDSC
% Viability
0
50
100
0.01
0.1
1
10
Concentration (uM)

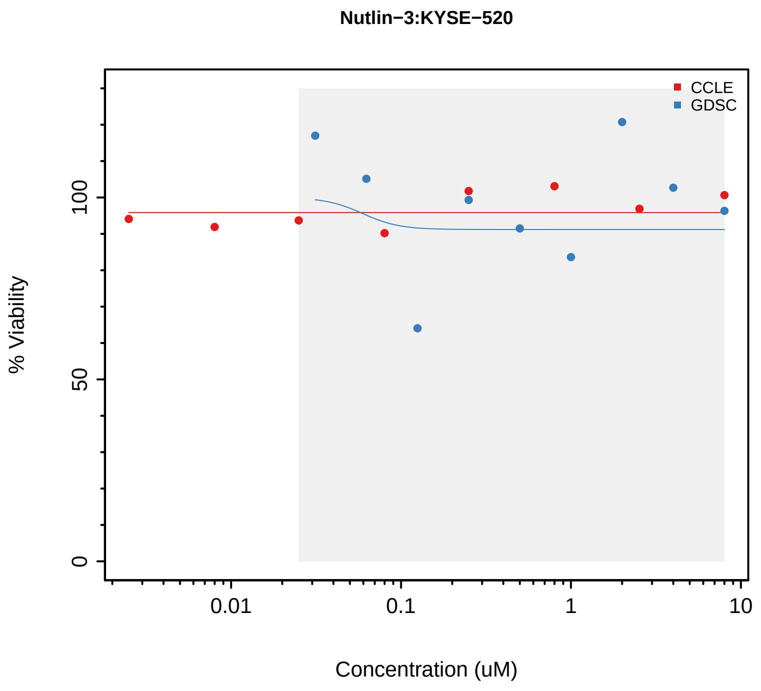
Nutlin−3:KYSE−520
CCLE
GDSC
% Viability
0
50
100
0.01
0.1
1
10
Concentration (uM)

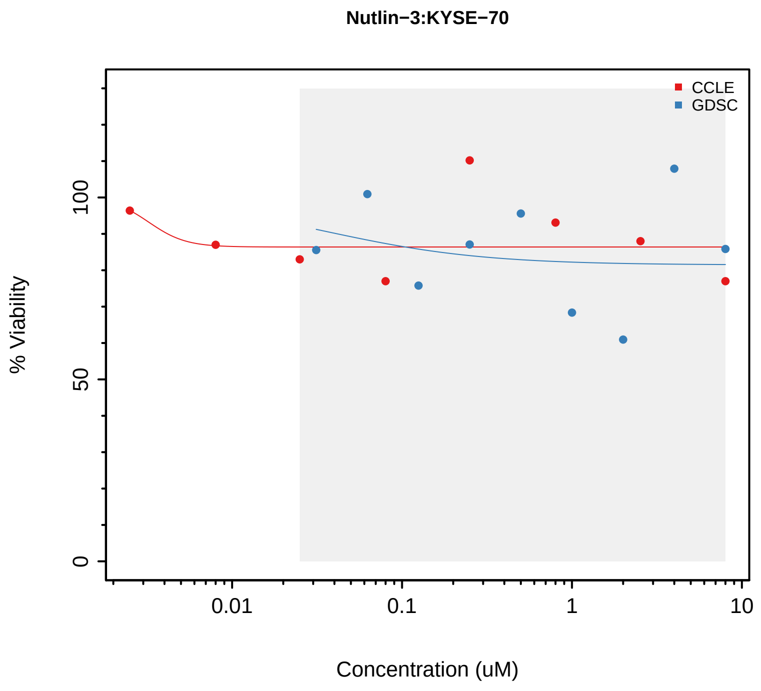
Nutlin−3:KYSE−70
CCLE
GDSC
% Viability
0
50
100
0.01
0.1
1
10
Concentration (uM)

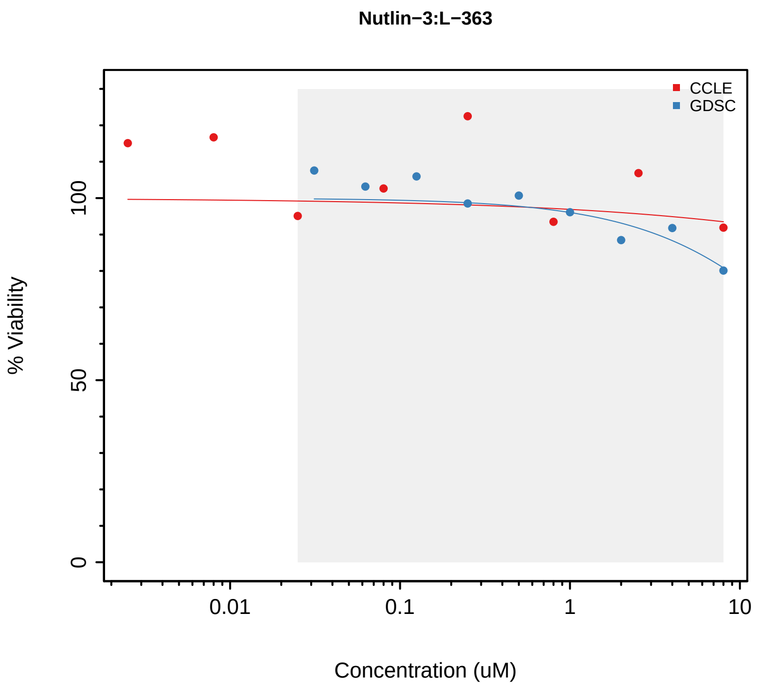
Nutlin−3:L−363
CCLE
GDSC
% Viability
0
50
100
0.01
0.1
1
10
Concentration (uM)

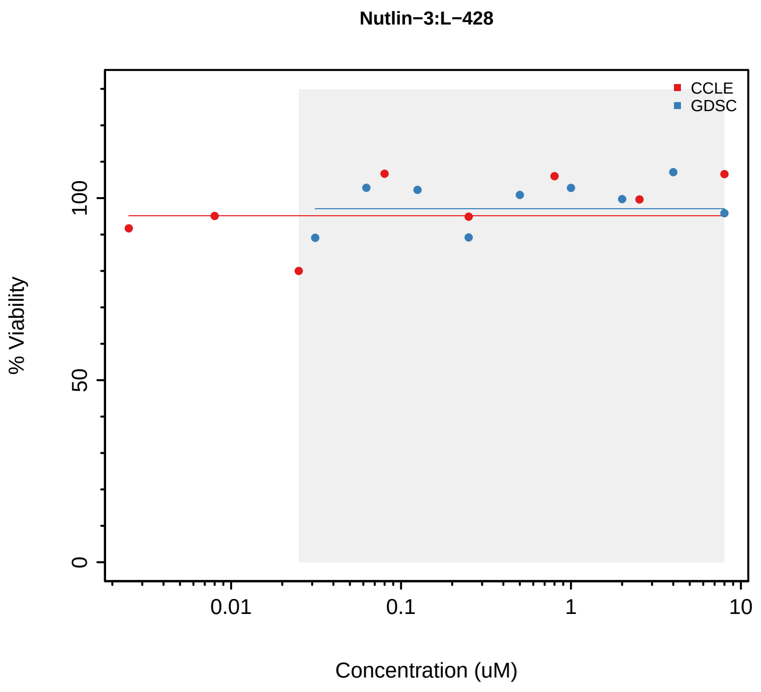
Nutlin−3:L−428
CCLE
GDSC
% Viability
0
50
100
0.01
0.1
1
10
Concentration (uM)

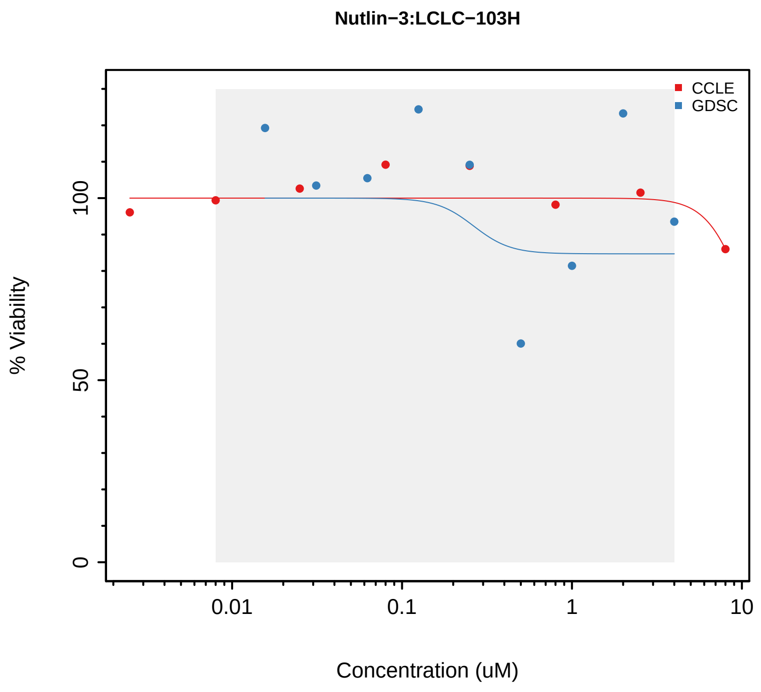
Nutlin−3:LCLC−103H
CCLE
GDSC
% Viability
0
50
100
0.01
0.1
1
10
Concentration (uM)

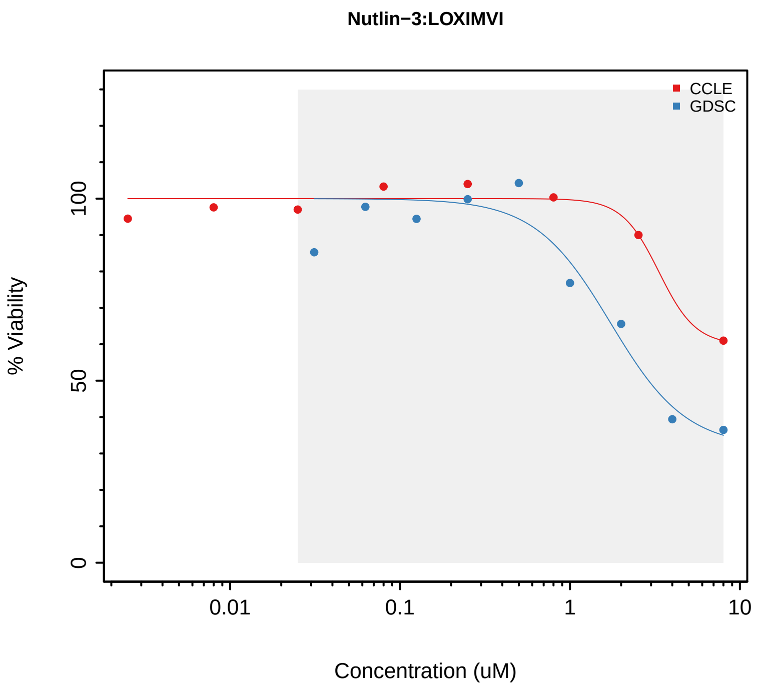
Nutlin−3:LOXIMVI
CCLE
GDSC
% Viability
0
50
100
0.01
0.1
1
10
Concentration (uM)

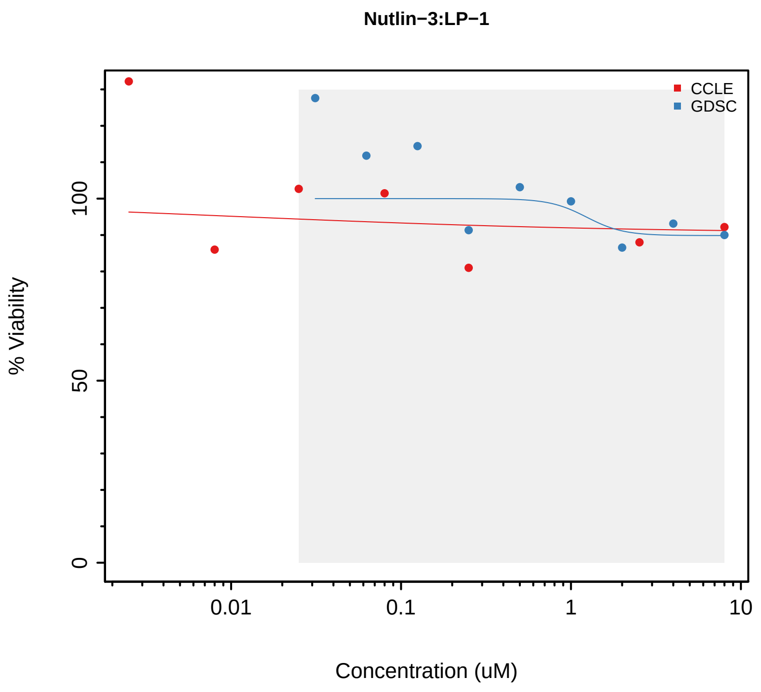
Nutlin−3:LP−1
CCLE
GDSC
% Viability
0
50
100
0.01
0.1
1
10
Concentration (uM)

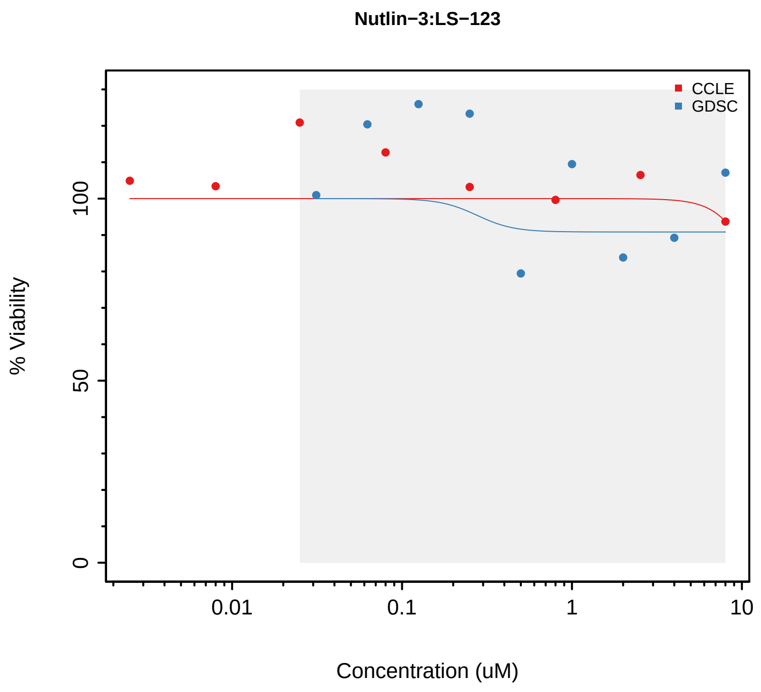
Nutlin−3:LS−123
CCLE
GDSC
% Viability
0
50
100
0.01
0.1
1
10
Concentration (uM)

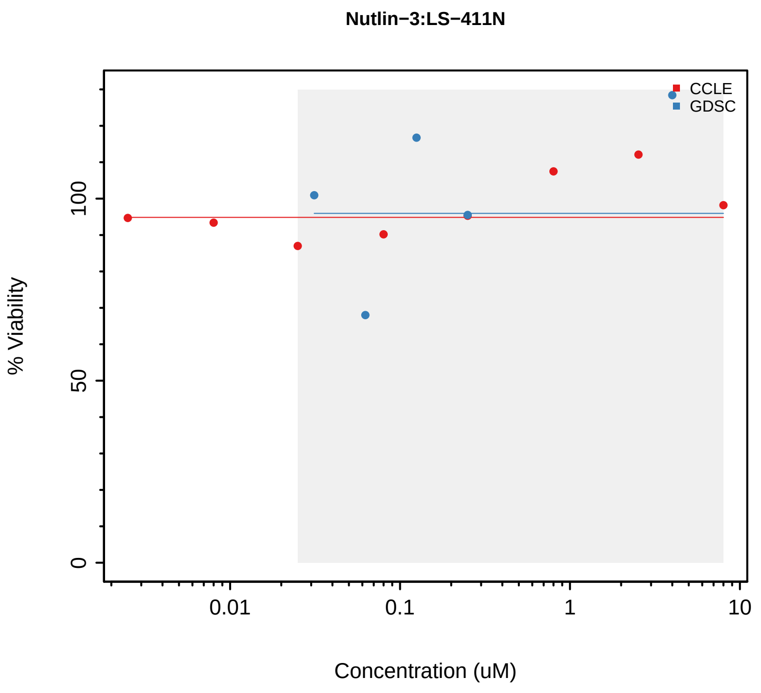
Nutlin−3:LS−411N
CCLE
GDSC
% Viability
0
50
100
0.01
0.1
1
10
Concentration (uM)

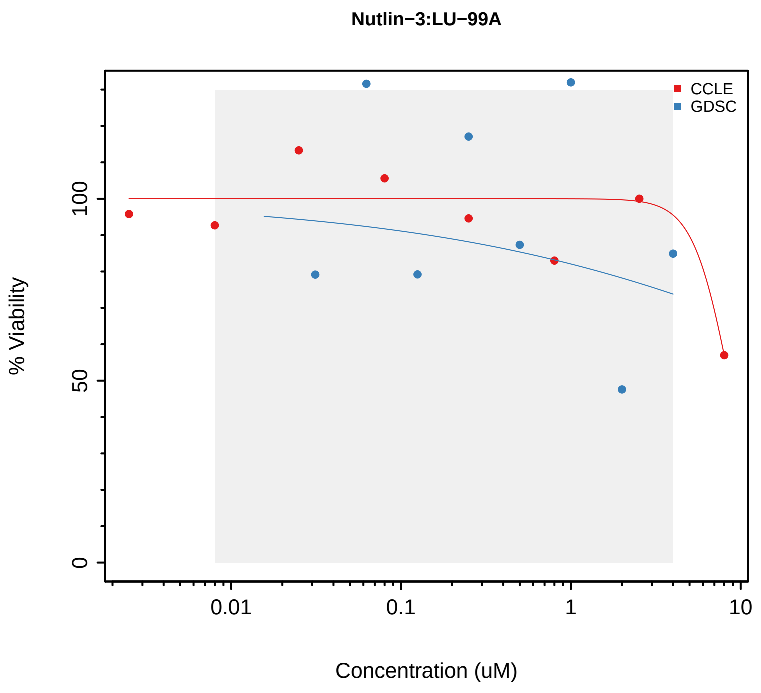
Nutlin−3:LU−99A
CCLE
GDSC
% Viability
0
50
100
0.01
0.1
1
10
Concentration (uM)

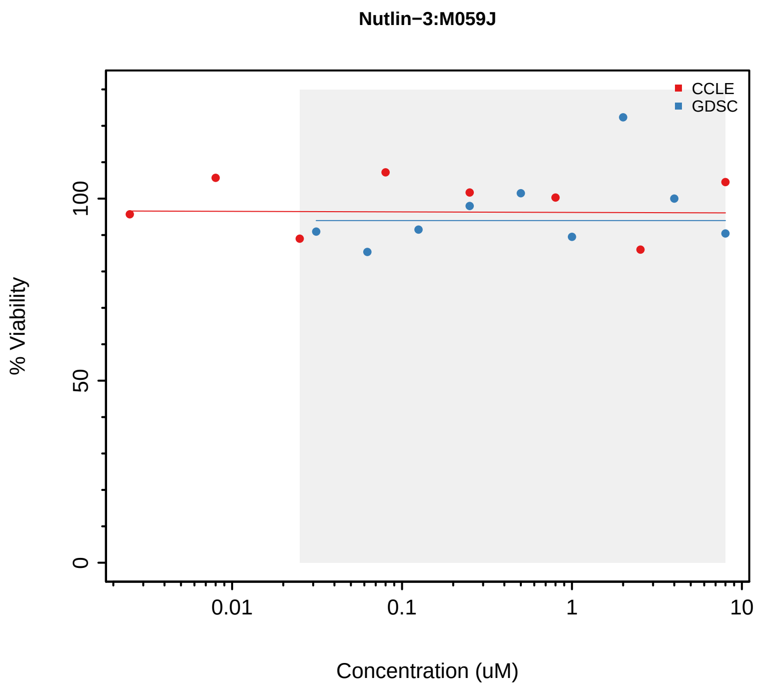
Nutlin−3:M059J
CCLE
GDSC
% Viability
0
50
100
0.01
0.1
1
10
Concentration (uM)

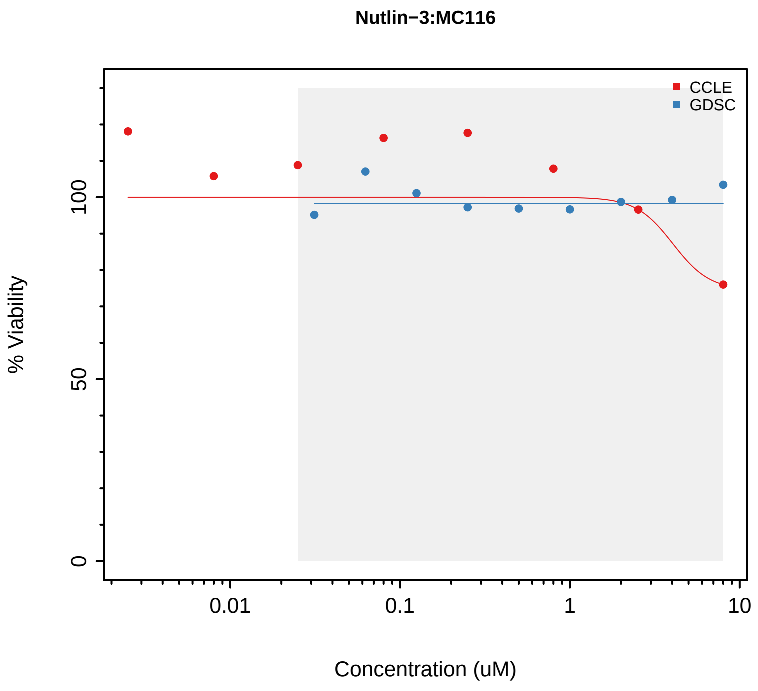
Nutlin−3:MC116
CCLE
GDSC
% Viability
0
50
100
0.01
0.1
1
10
Concentration (uM)

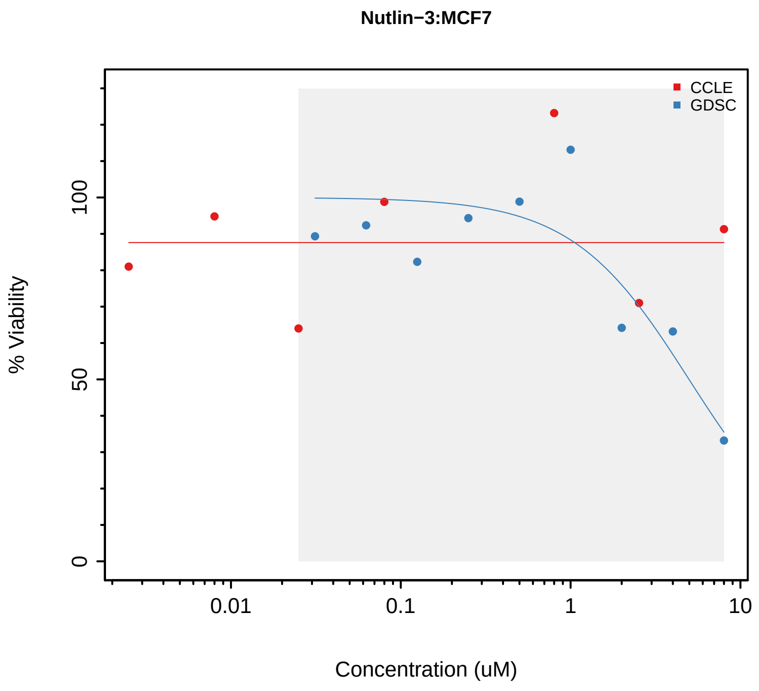
Nutlin−3:MCF7
CCLE
GDSC
% Viability
0
50
100
0.01
0.1
1
10
Concentration (uM)

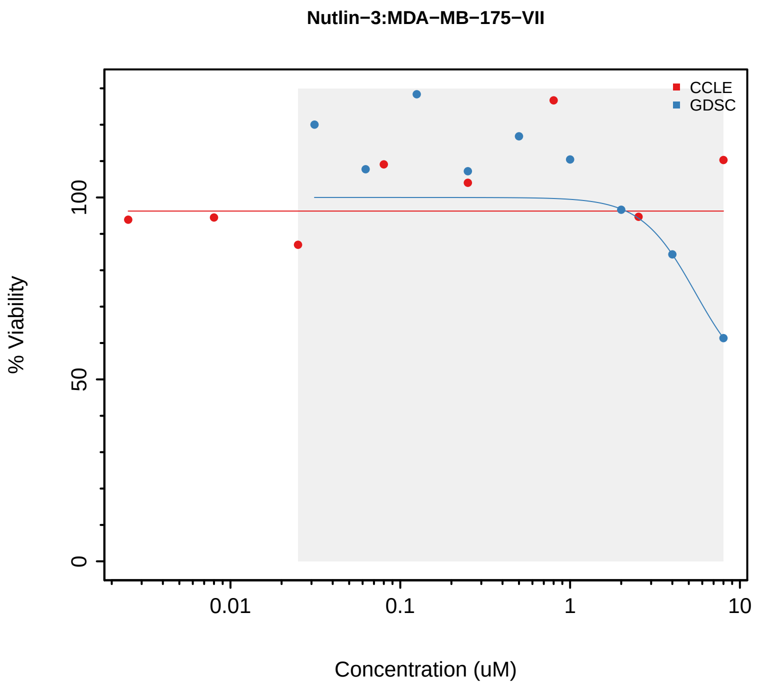
Nutlin−3:MDA−MB−175−VII
CCLE
GDSC
% Viability
0
50
100
0.01
0.1
1
10
Concentration (uM)

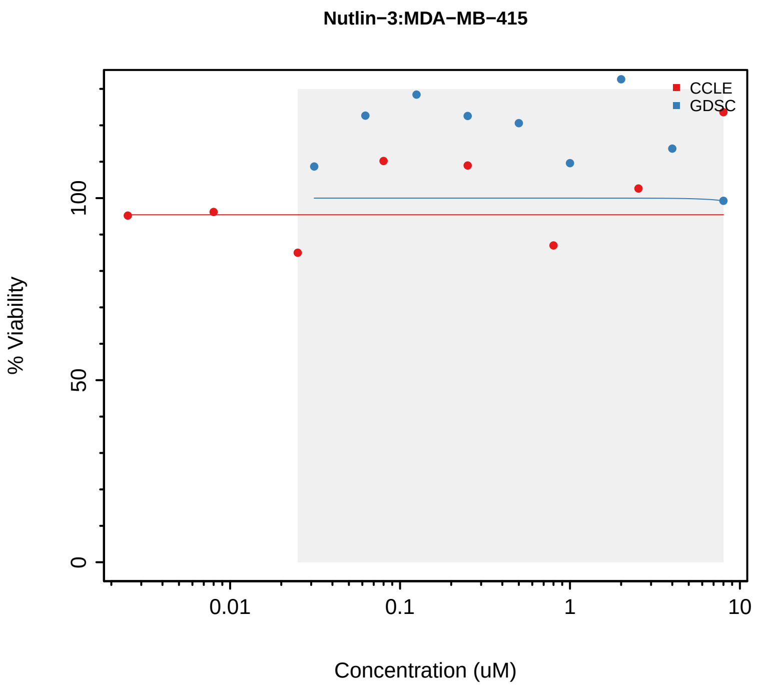
Nutlin−3:MDA−MB−415
CCLE
GDSC
% Viability
0
50
100
0.01
0.1
1
10
Concentration (uM)

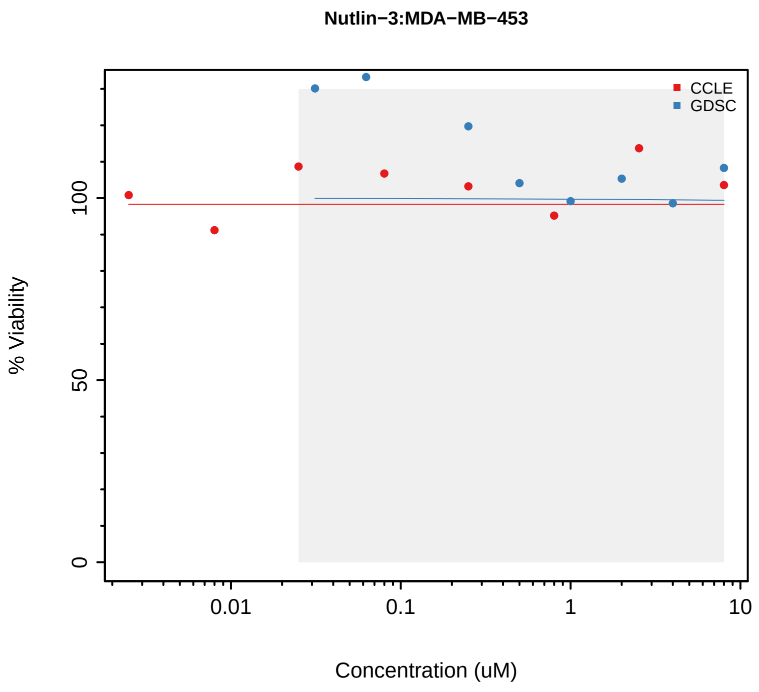
Nutlin−3:MDA−MB−453
CCLE
GDSC
% Viability
0
50
100
0.01
0.1
1
10
Concentration (uM)

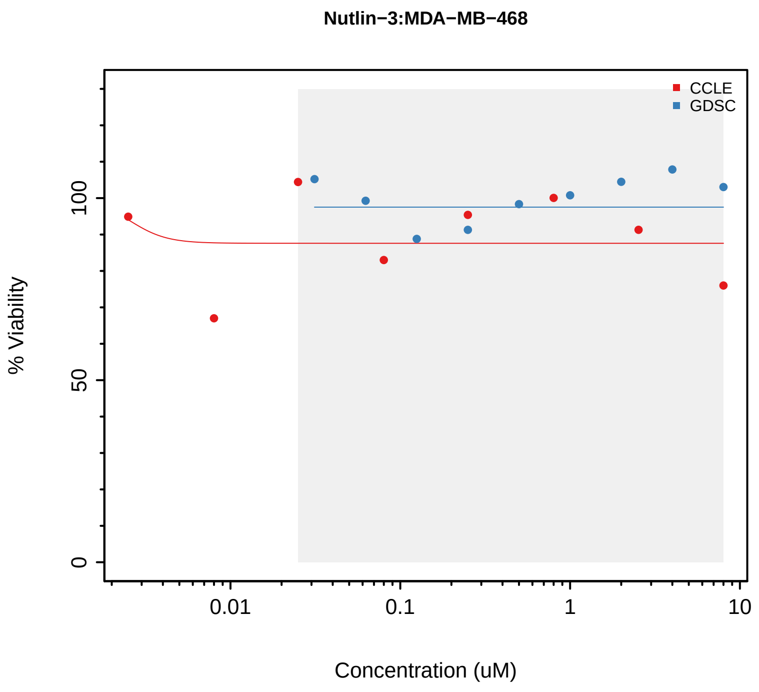
Nutlin−3:MDA−MB−468
CCLE
GDSC
% Viability
0
50
100
0.01
0.1
1
10
Concentration (uM)

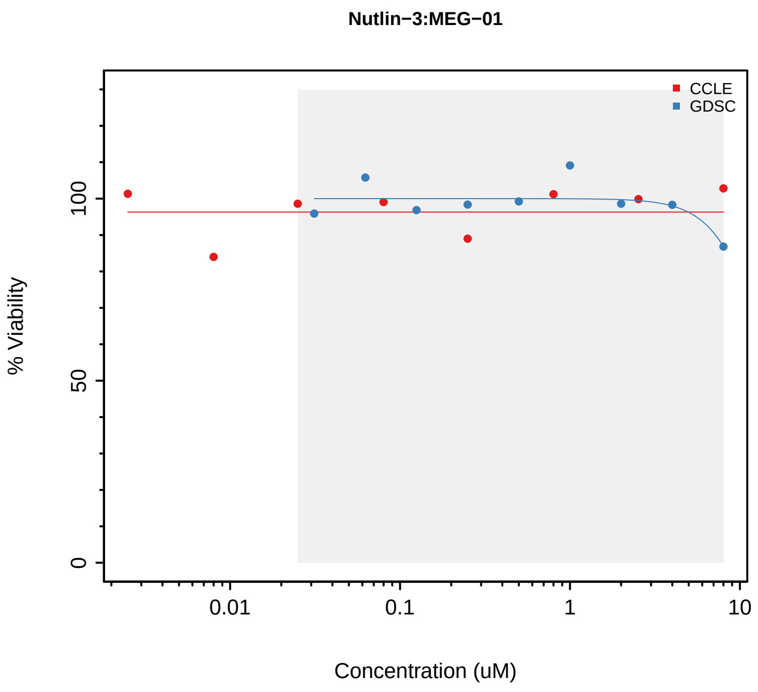
Nutlin−3:MEG−01
CCLE
GDSC
% Viability
0
50
100
0.01
0.1
1
10
Concentration (uM)

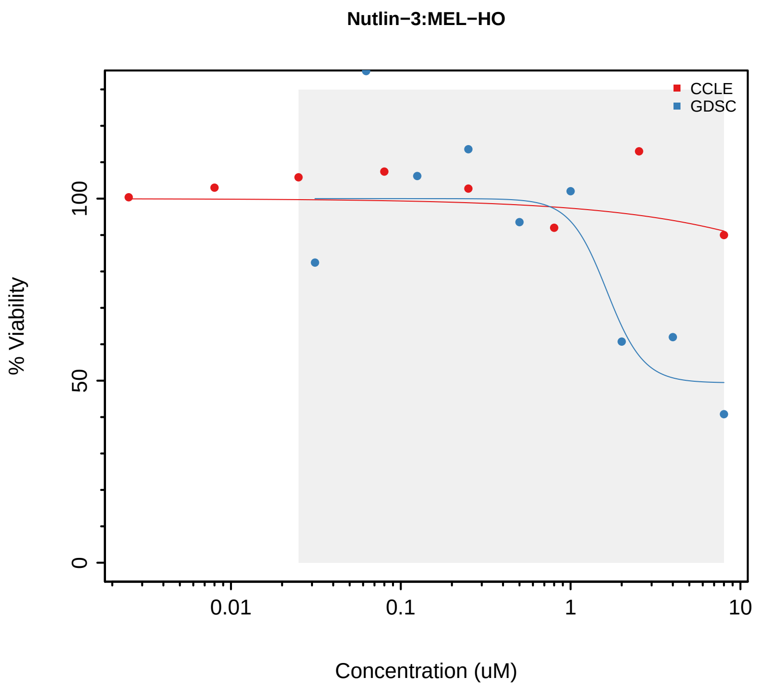
Nutlin−3:MEL−HO
CCLE
GDSC
% Viability
0
50
100
0.01
0.1
1
10
Concentration (uM)

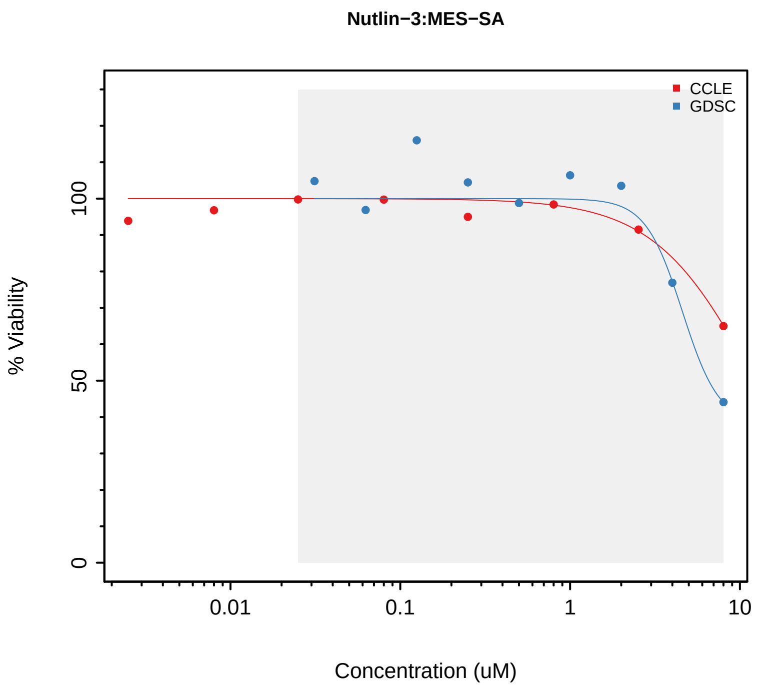
Nutlin−3:MES−SA
CCLE
GDSC
% Viability
0
50
100
0.01
0.1
1
10
Concentration (uM)

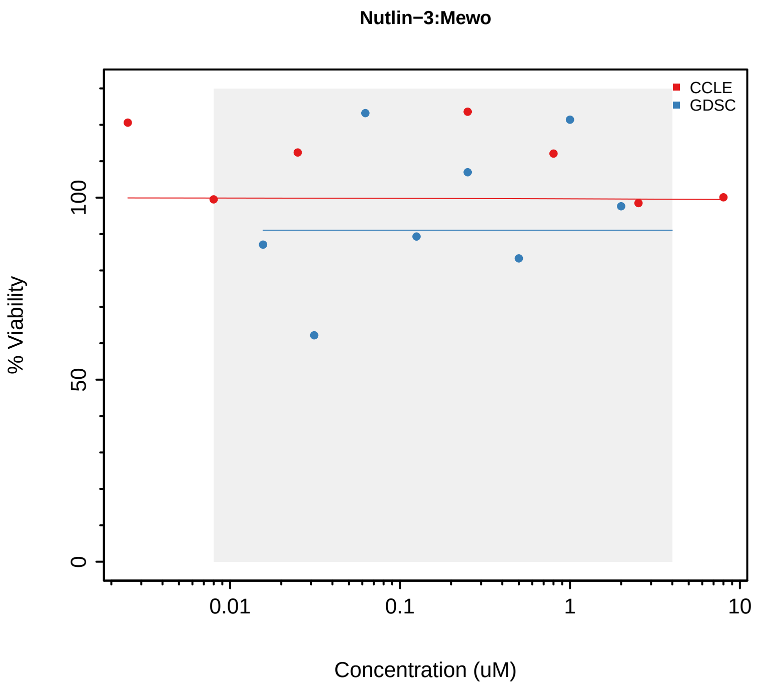
Nutlin−3:Mewo
CCLE
GDSC
% Viability
0
50
100
0.01
0.1
1
10
Concentration (uM)

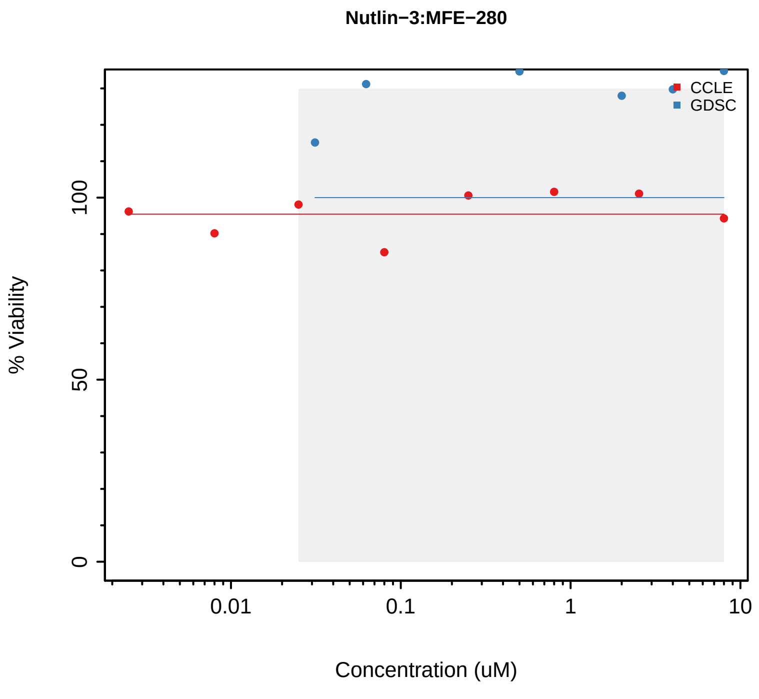
Nutlin−3:MFE−280
CCLE
GDSC
% Viability
0
50
100
0.01
0.1
1
10
Concentration (uM)

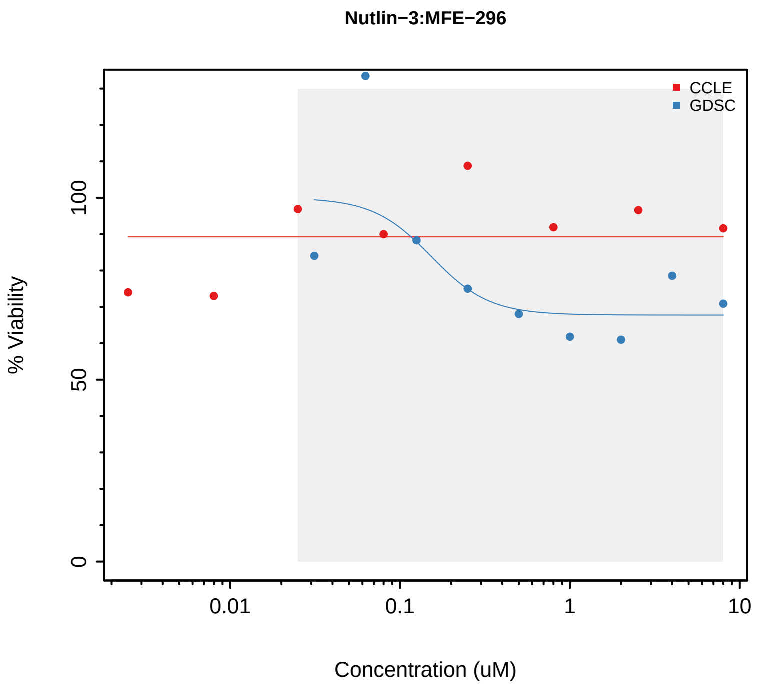
Nutlin−3:MFE−296
CCLE
GDSC
% Viability
0
50
100
0.01
0.1
1
10
Concentration (uM)

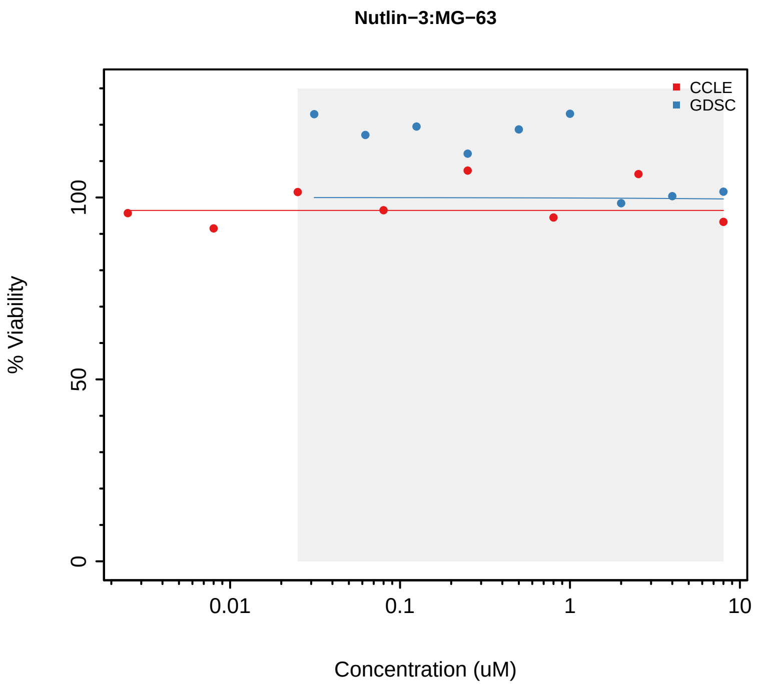
Nutlin−3:MG−63
CCLE
GDSC
% Viability
0
50
100
0.01
0.1
1
10
Concentration (uM)

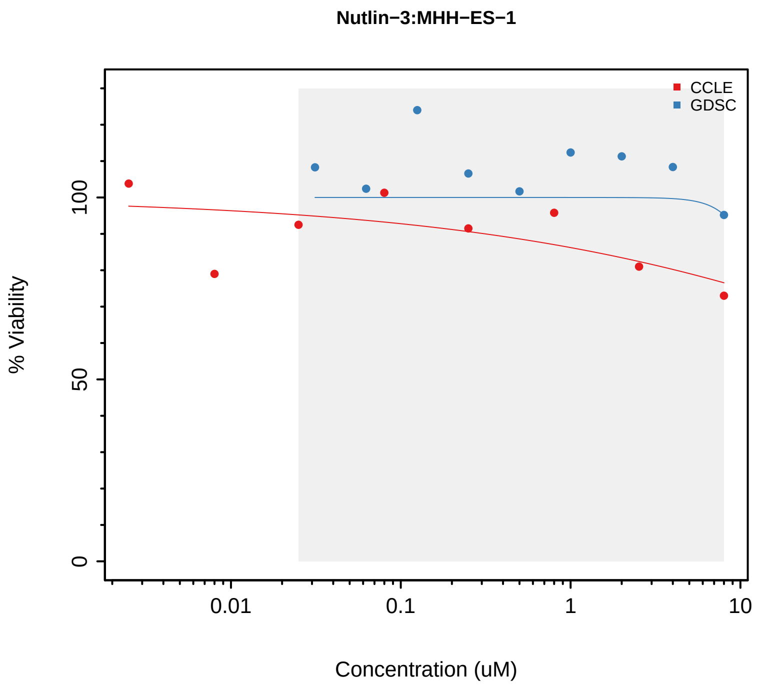
Nutlin−3:MHH−ES−1
CCLE
GDSC
% Viability
0
50
100
0.01
0.1
1
10
Concentration (uM)

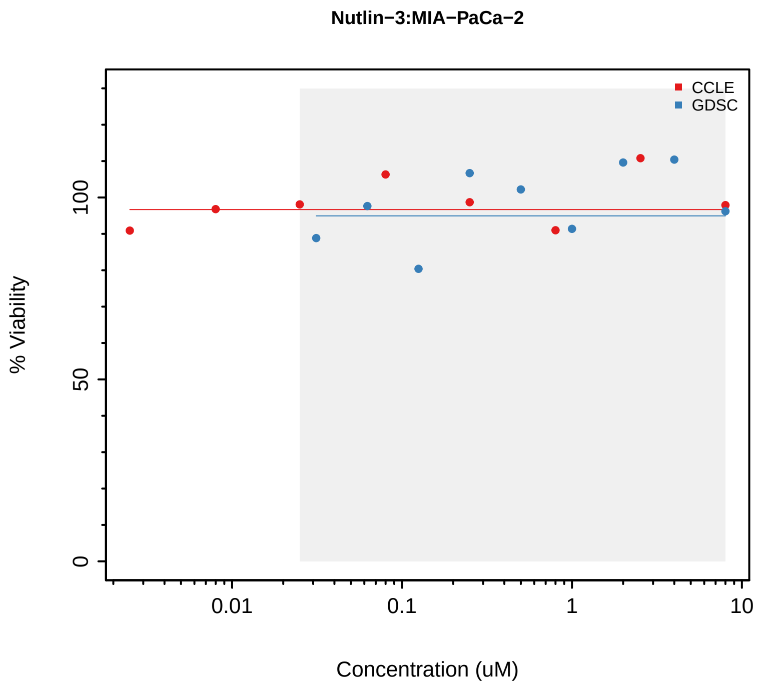
Nutlin−3:MIA−PaCa−2
CCLE
GDSC
% Viability
0
50
100
0.01
0.1
1
10
Concentration (uM)

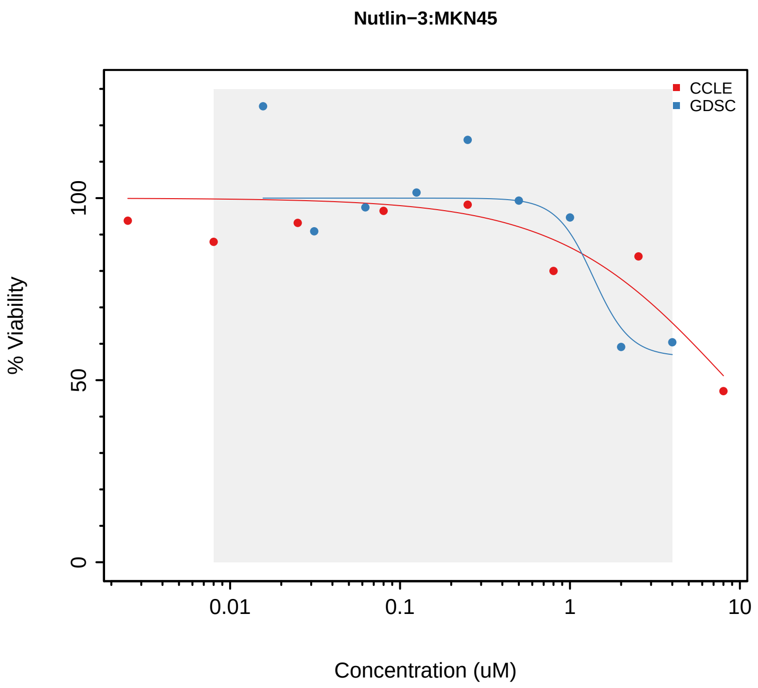
Nutlin−3:MKN45
CCLE
GDSC
% Viability
0
50
100
0.01
0.1
1
10
Concentration (uM)

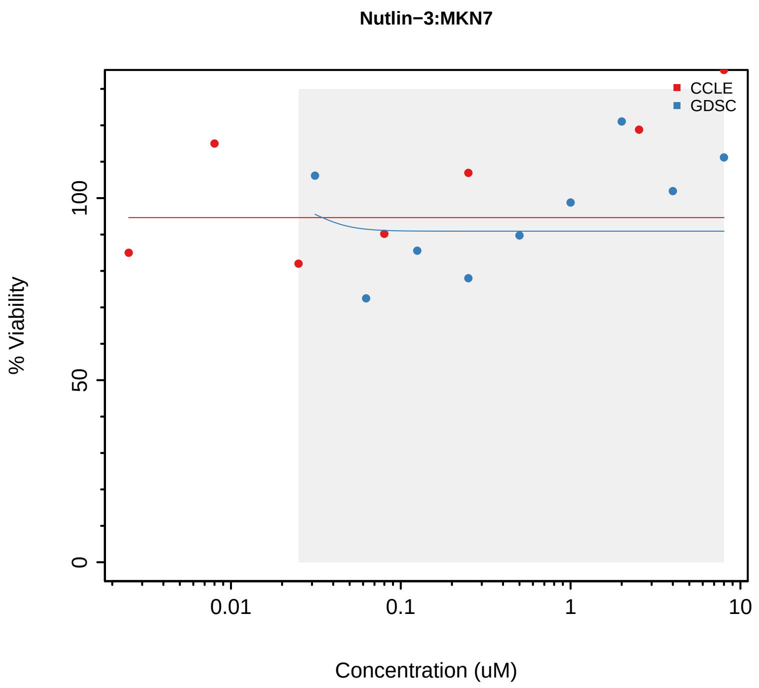
Nutlin−3:MKN7
CCLE
GDSC
% Viability
0
50
100
0.01
0.1
1
10
Concentration (uM)

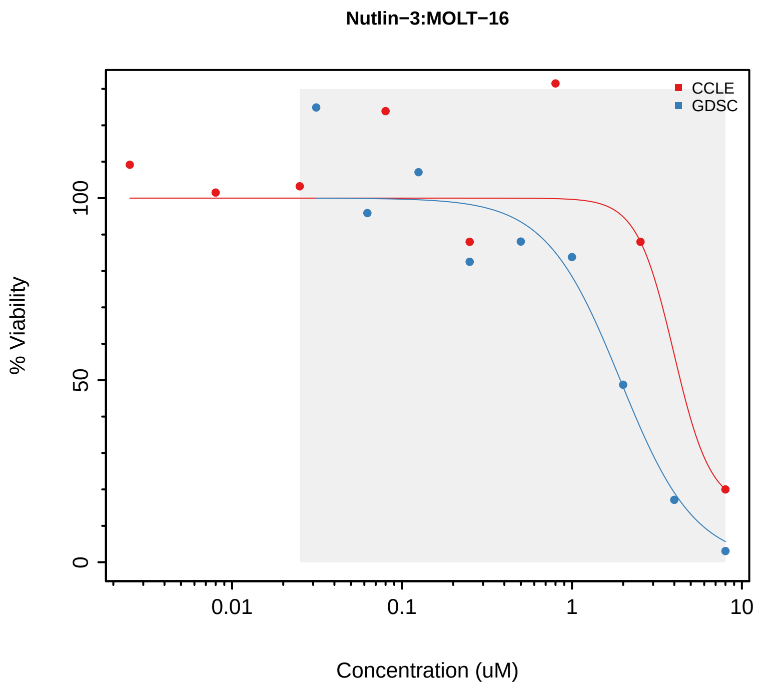
Nutlin−3:MOLT−16
CCLE
GDSC
% Viability
0
50
100
0.01
0.1
1
10
Concentration (uM)

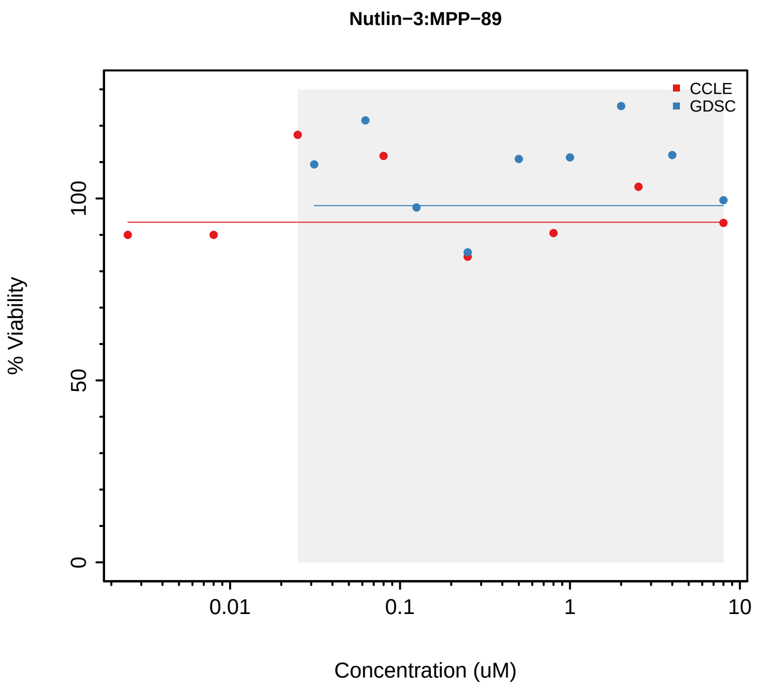
Nutlin−3:MPP−89
CCLE
GDSC
% Viability
0
50
100
0.01
0.1
1
10
Concentration (uM)

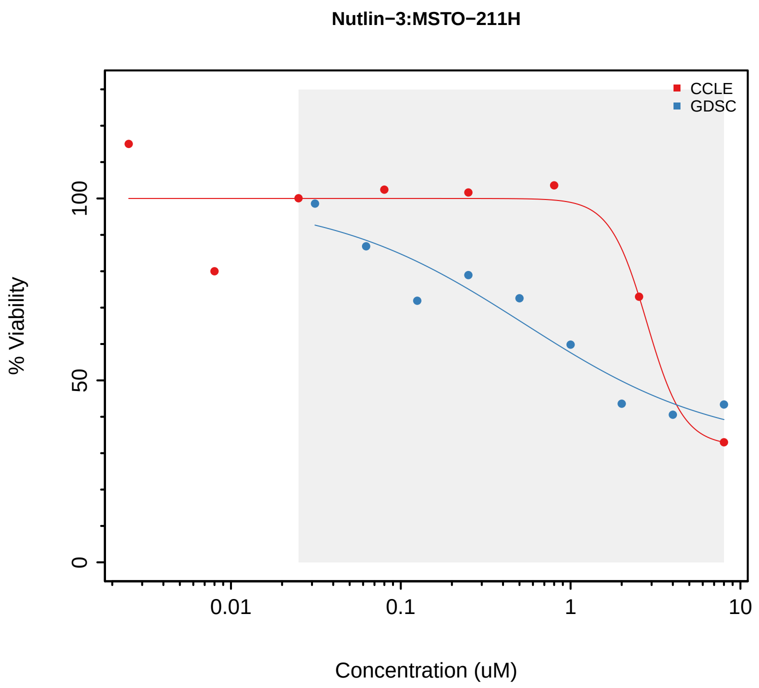
Nutlin−3:MSTO−211H
CCLE
GDSC
% Viability
0
50
100
0.01
0.1
1
10
Concentration (uM)

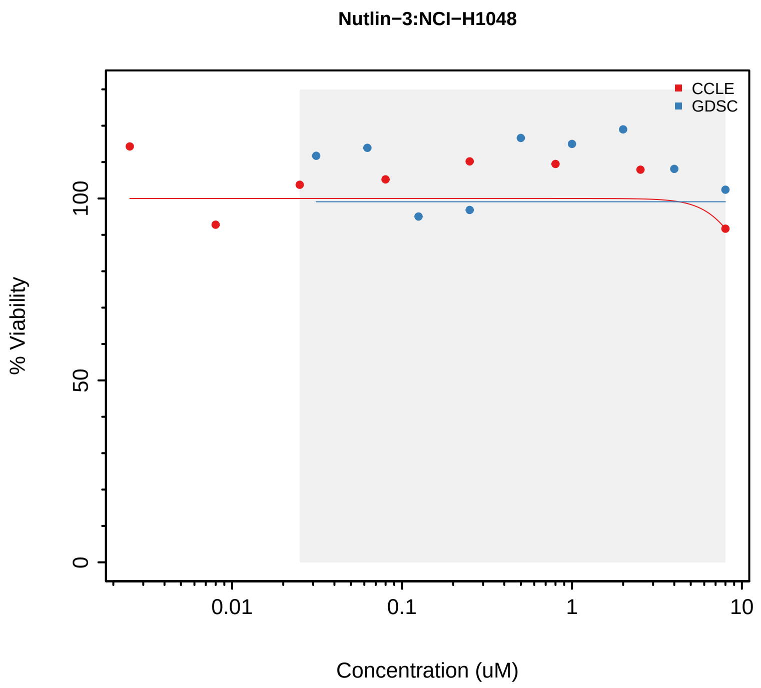
Nutlin−3:NCI−H1048
CCLE
GDSC
% Viability
0
50
100
0.01
0.1
1
10
Concentration (uM)

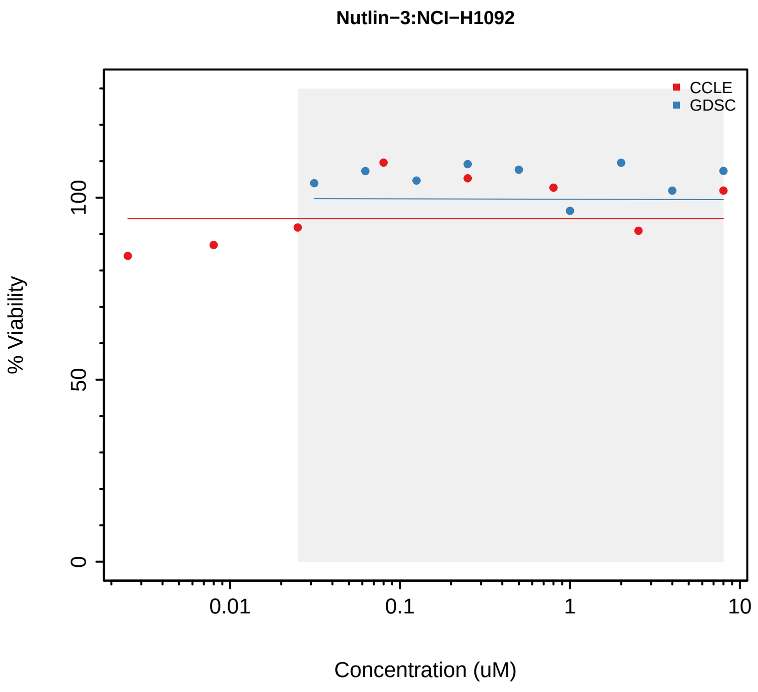
Nutlin−3:NCI−H1092
CCLE
GDSC
% Viability
0
50
100
0.01
0.1
1
10
Concentration (uM)

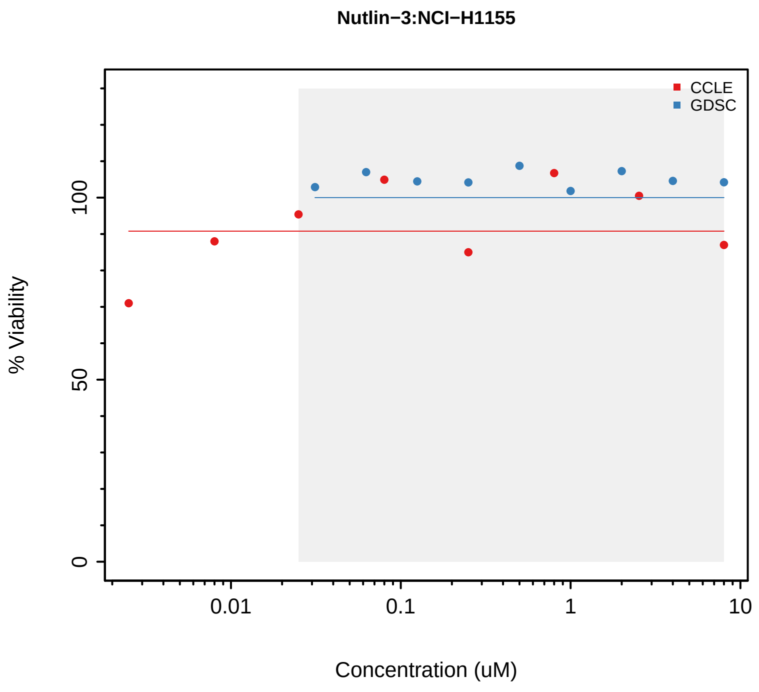
Nutlin−3:NCI−H1155
CCLE
GDSC
% Viability
0
50
100
0.01
0.1
1
10
Concentration (uM)

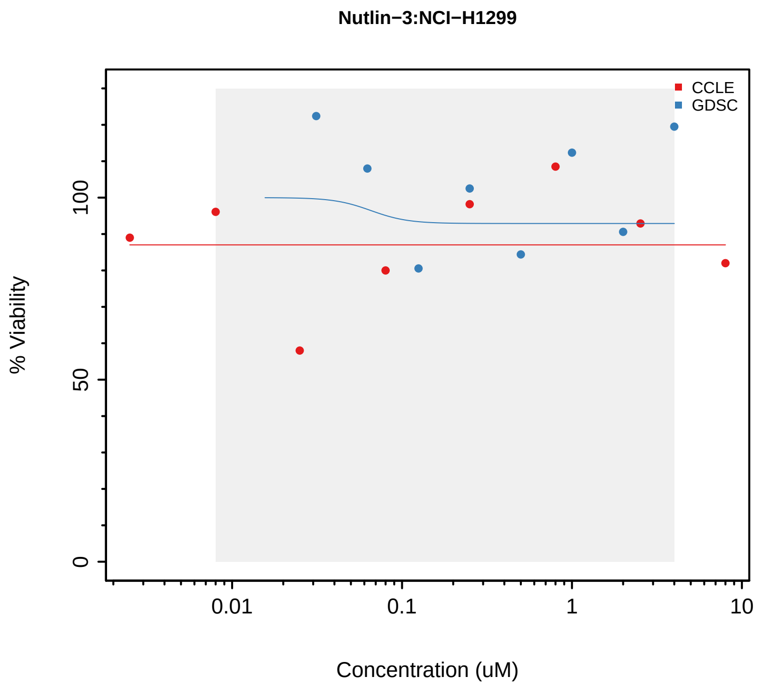
Nutlin−3:NCI−H1299
CCLE
GDSC
% Viability
0
50
100
0.01
0.1
1
10
Concentration (uM)

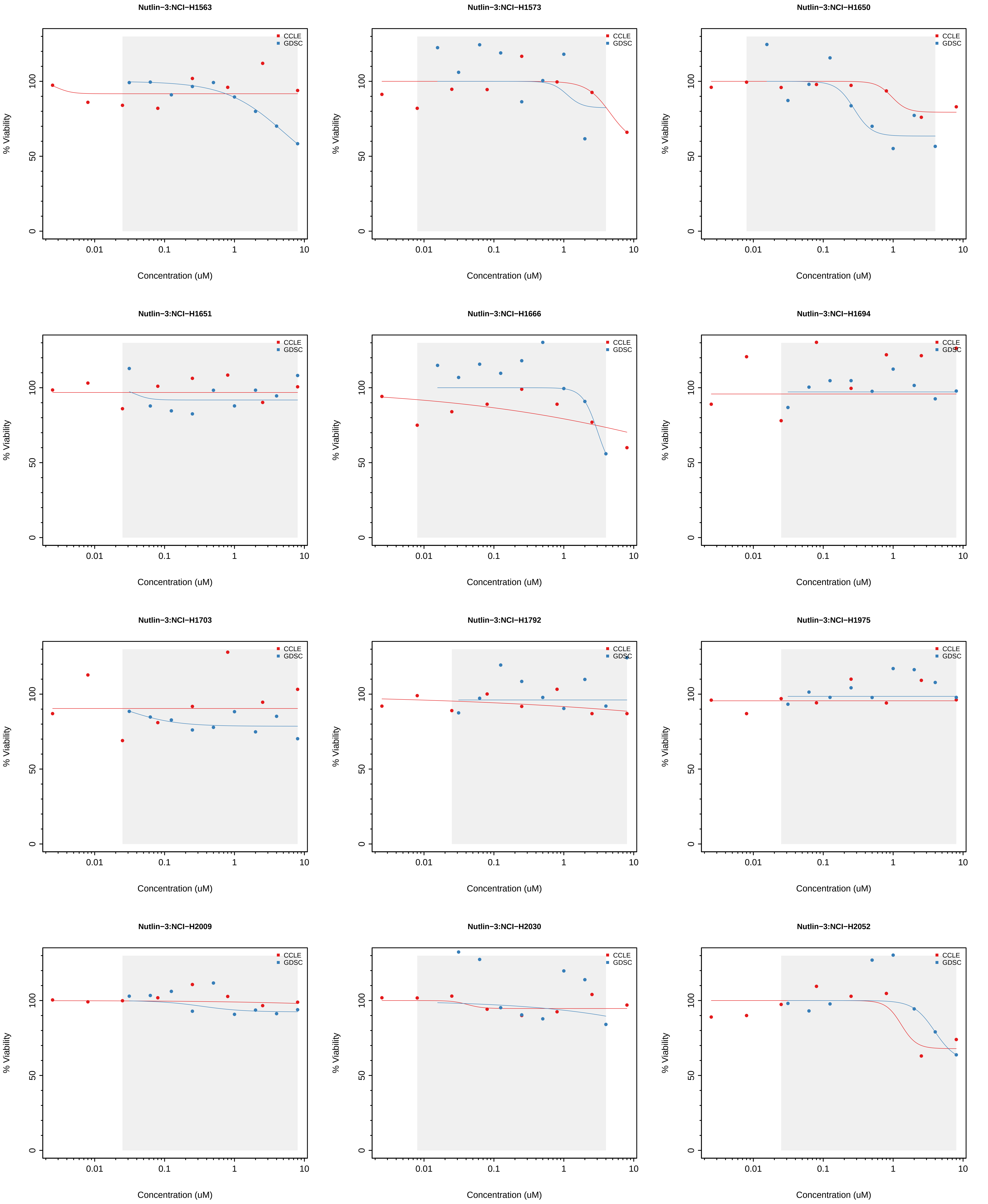
Nutlin−3:NCI−H1563
Nutlin−3:NCI−H1573
Nutlin−3:NCI−H1650
Nutlin−3:NCI−H1651
Nutlin−3:NCI−H1666
Nutlin−3:NCI−H1694
Nutlin−3:NCI−H1703
Nutlin−3:NCI−H1792
Nutlin−3:NCI−H1975
Nutlin−3:NCI−H2009
Nutlin−3:NCI−H2030
Nutlin−3:NCI−H2052
CCLE
GDSC
% Viability
0
50
100
0.01
0.1
1
10
Concentration (uM)

Nutlin−3:NCI−H2087

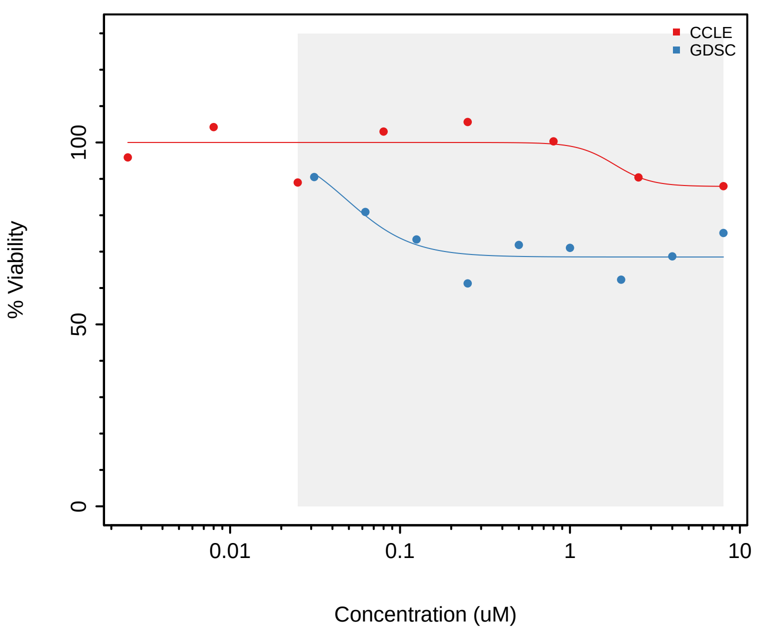

Nutlin−3:NCI−H2122

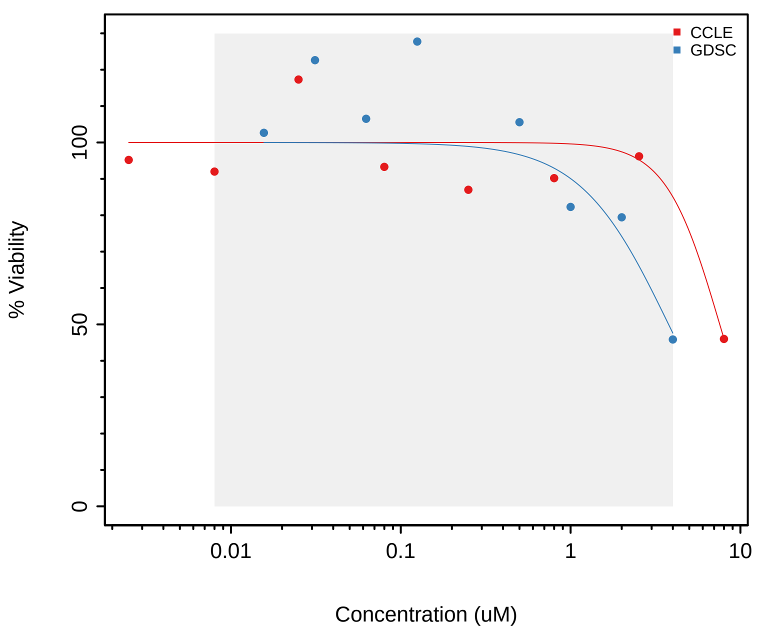

Nutlin−3:NCI−H2170

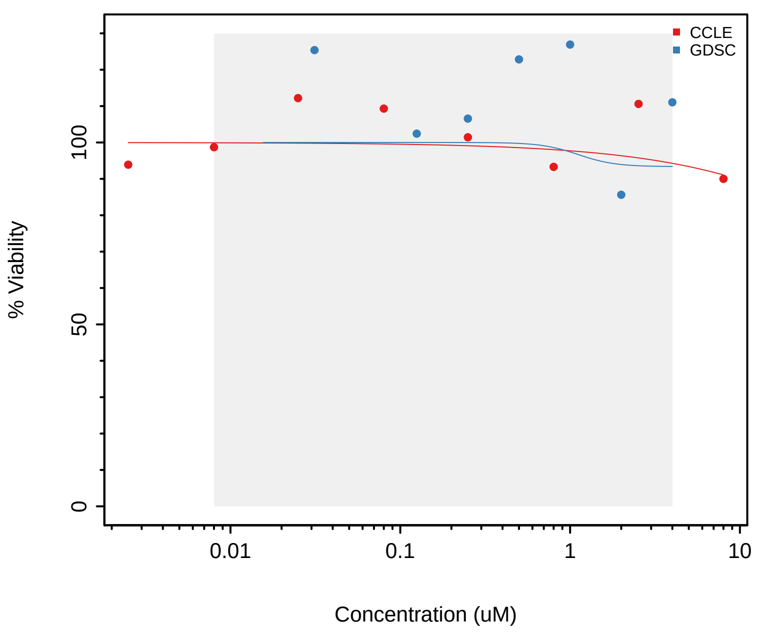

Nutlin−3:NCI−H2228

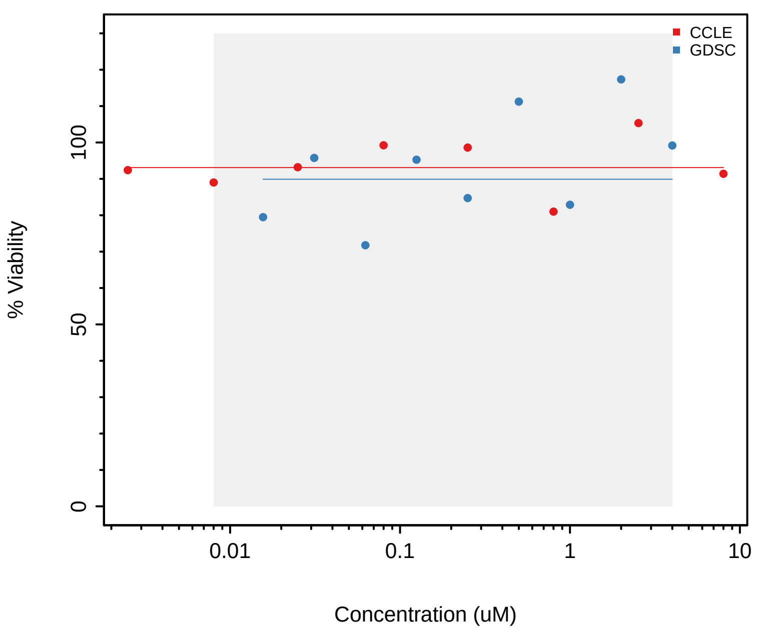

Nutlin−3:NCI−H226

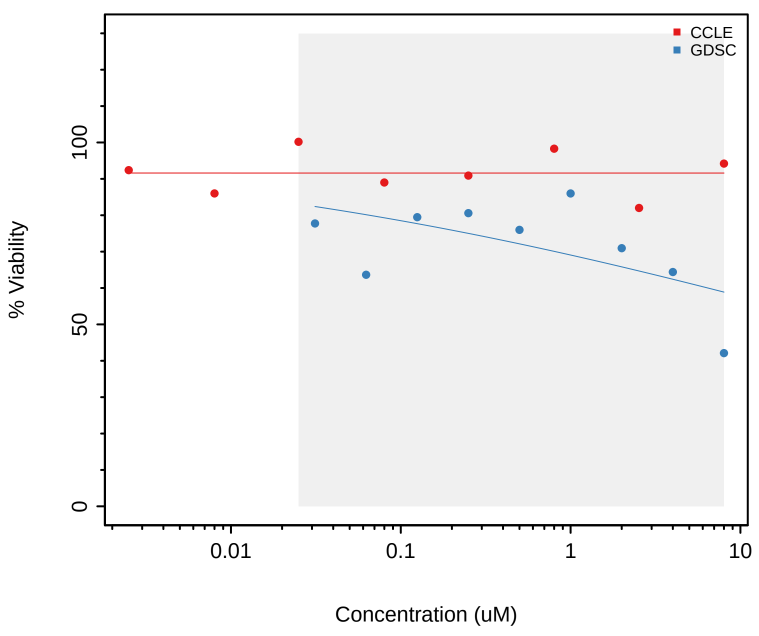

Nutlin−3:NCI−H2452

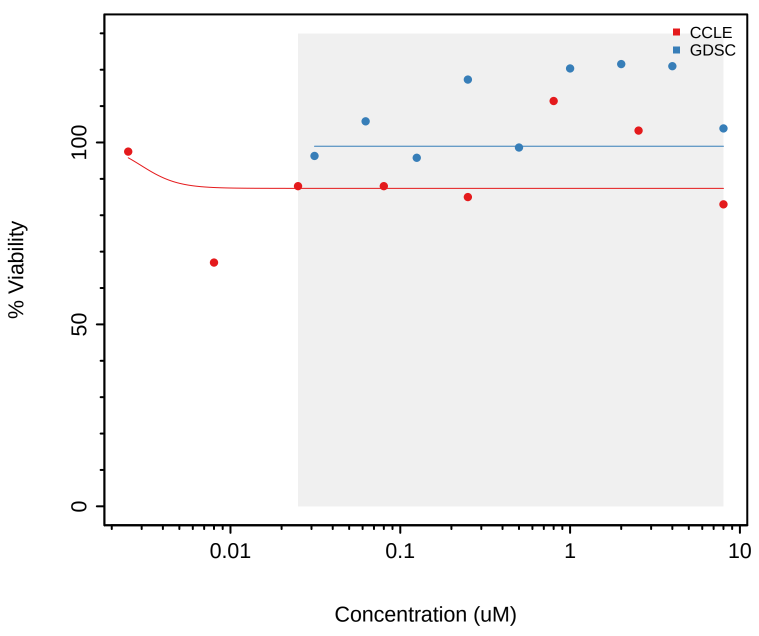

Nutlin−3:NCI−H28

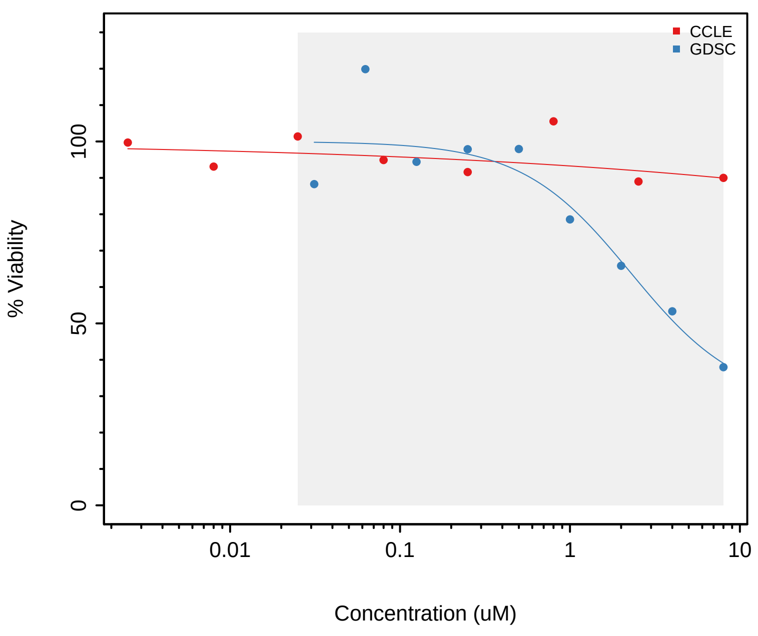

Nutlin−3:NCI−H358

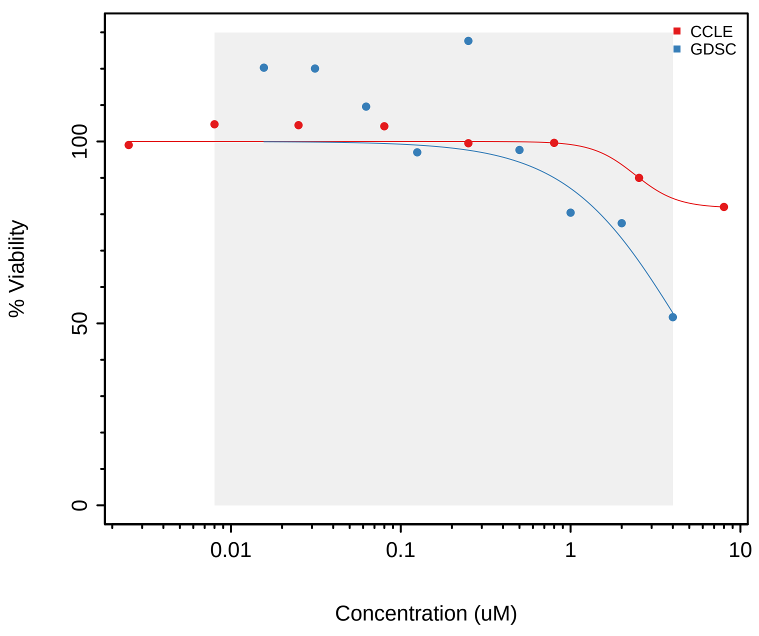

Nutlin−3:NCI−H441

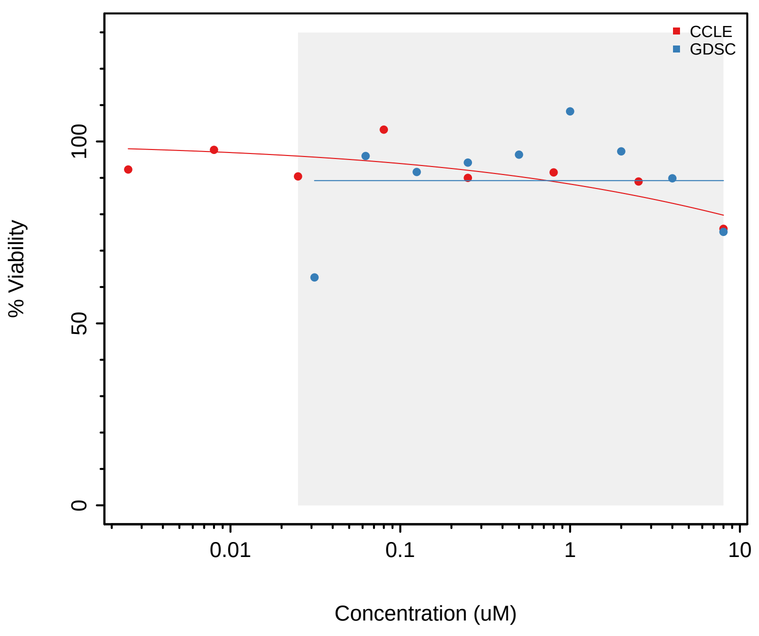

Nutlin−3:NCI−H460

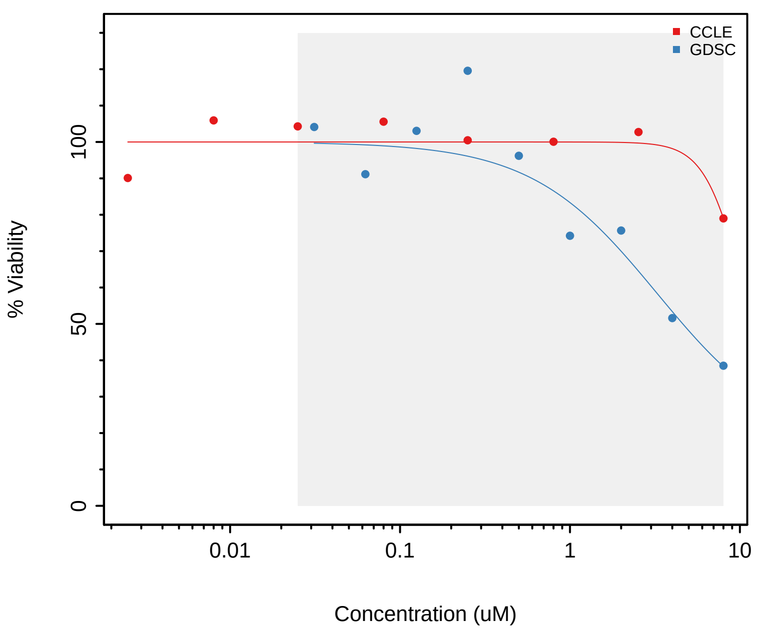

Nutlin−3:NCI−H520

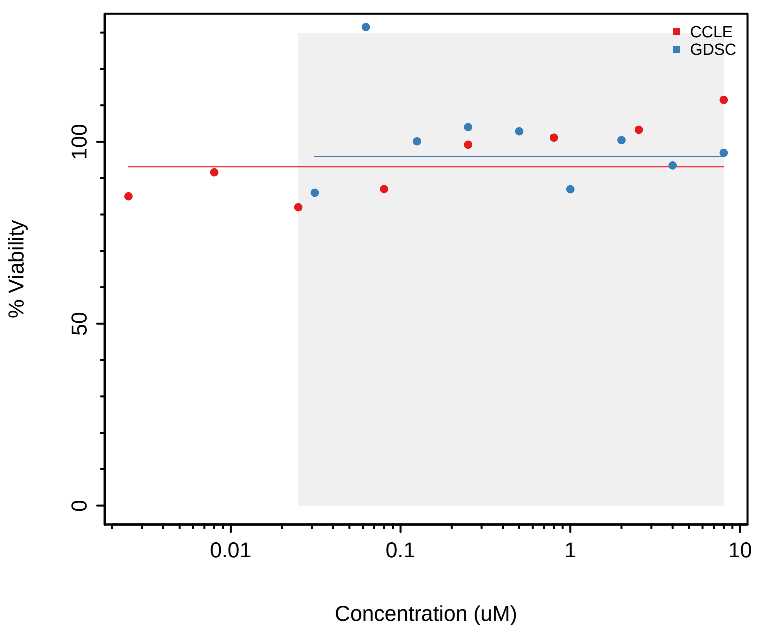

Nutlin−3:NCI−H522

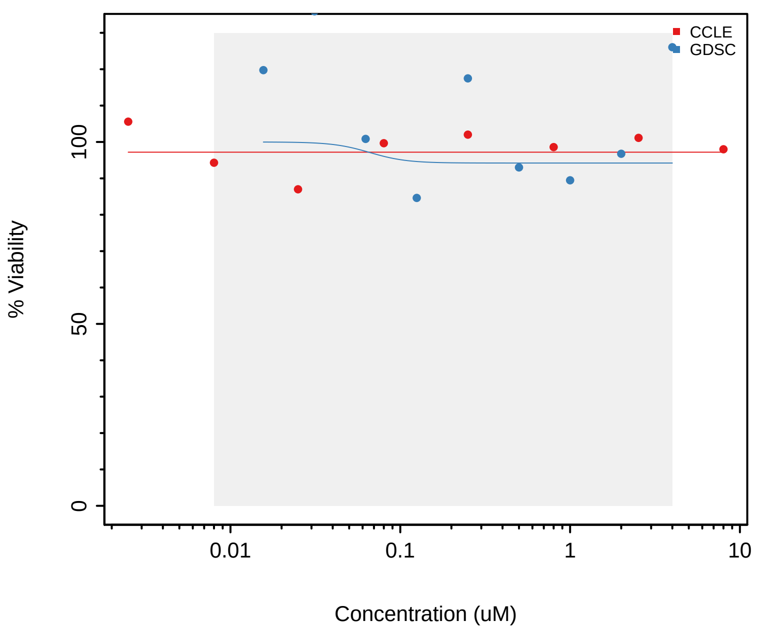

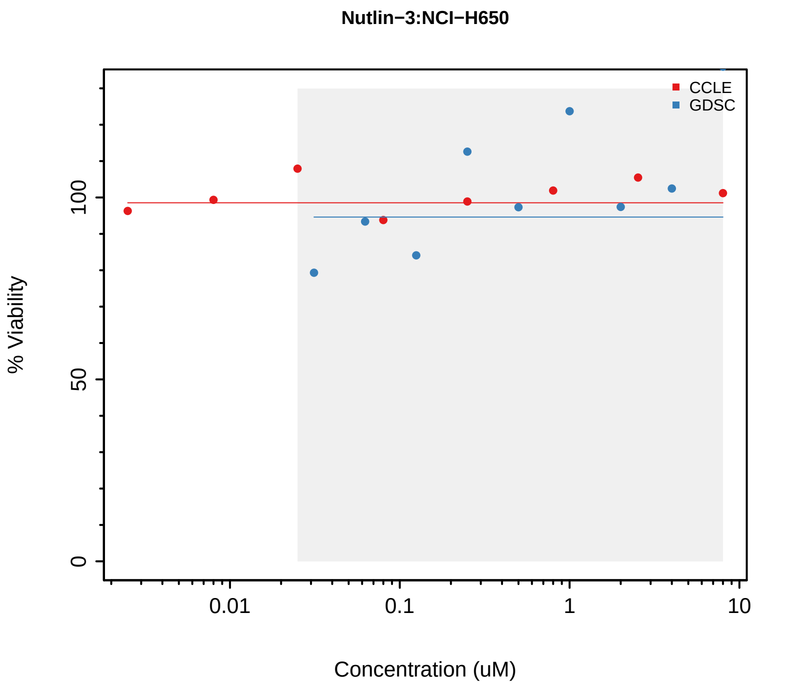
Nutlin−3:NCI−H650
CCLE
GDSC
% Viability
0
50
100
0.01
0.1
1
10
Concentration (uM)

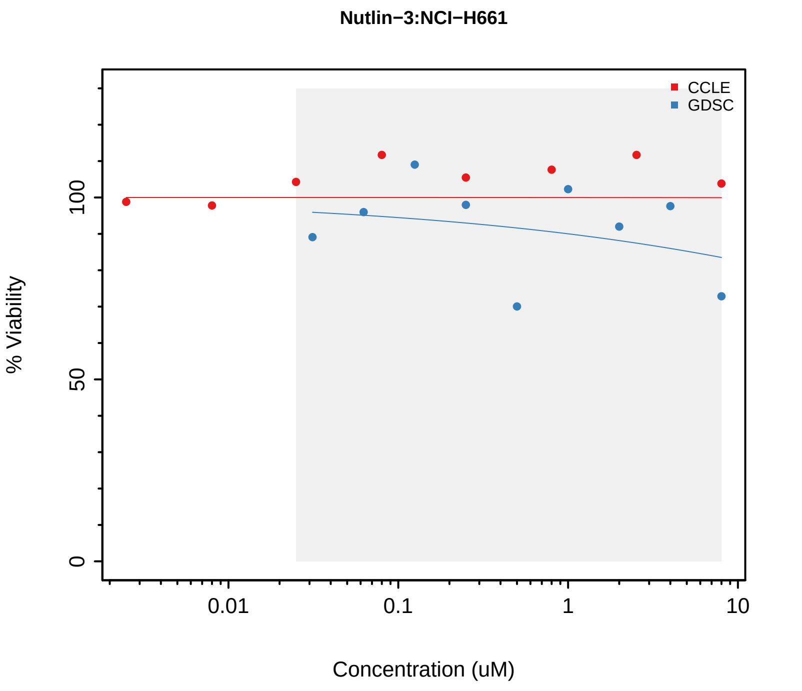
Nutlin−3:NCI−H661
CCLE
GDSC
% Viability
0
50
100
0.01
0.1
1
10
Concentration (uM)

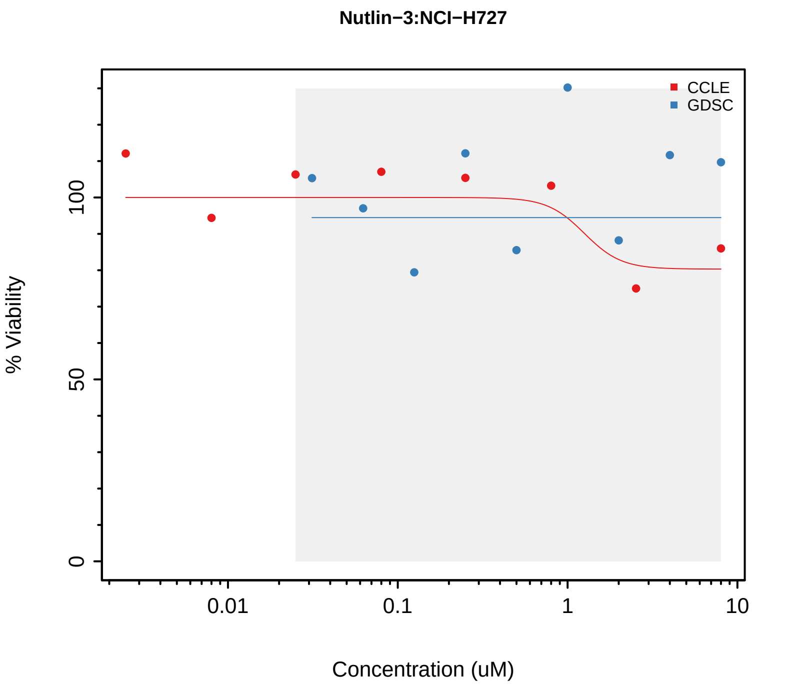
Nutlin−3:NCI−H727
CCLE
GDSC
% Viability
0
50
100
0.01
0.1
1
10
Concentration (uM)

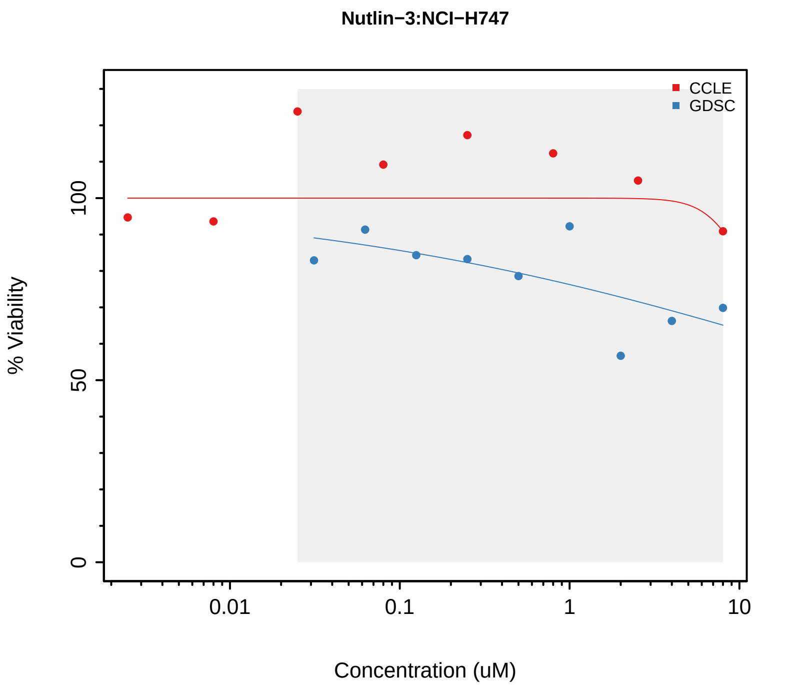
Nutlin−3:NCI−H747
CCLE
GDSC
% Viability
0
50
100
0.01
0.1
1
10
Concentration (uM)

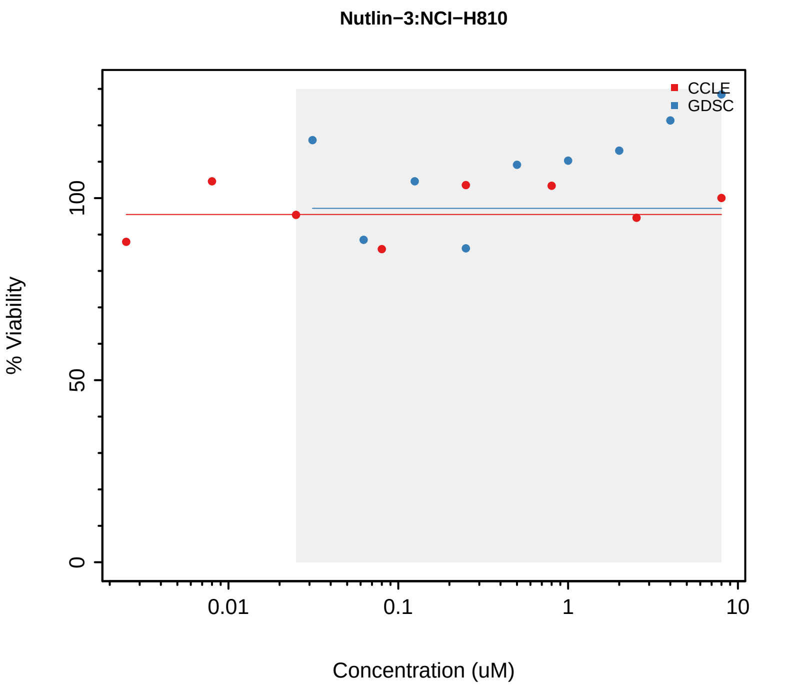
Nutlin−3:NCI−H810
CCLE
GDSC
% Viability
0
50
100
0.01
0.1
1
10
Concentration (uM)

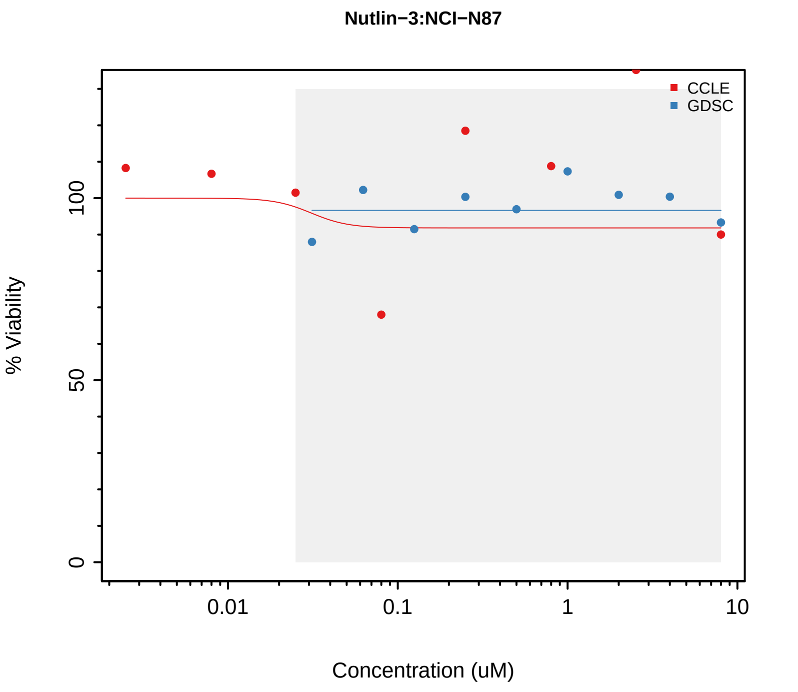
Nutlin−3:NCI−N87
CCLE
GDSC
% Viability
0
50
100
0.01
0.1
1
10
Concentration (uM)

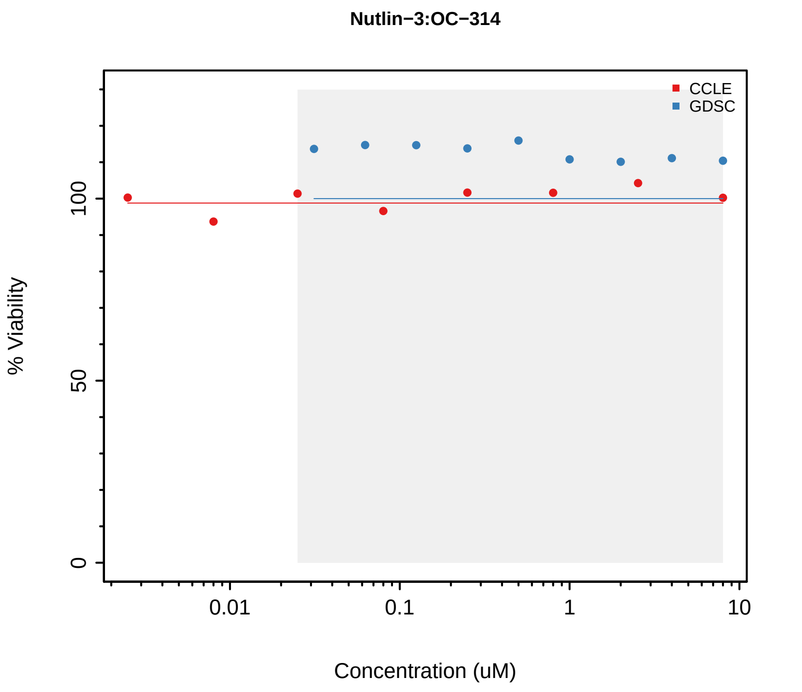
Nutlin−3:OC−314
CCLE
GDSC
% Viability
0
50
100
0.01
0.1
1
10
Concentration (uM)

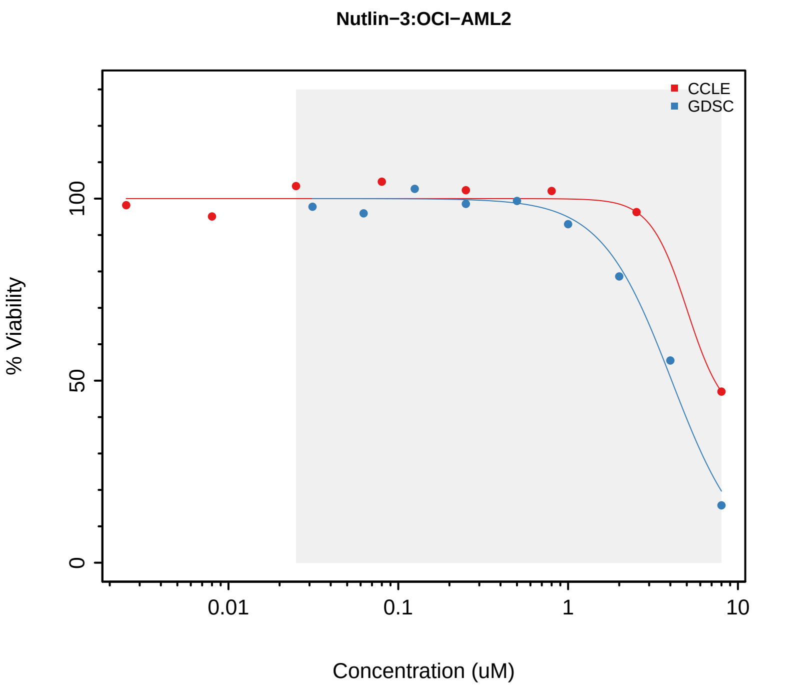
Nutlin−3:OCI−AML2
CCLE
GDSC
% Viability
0
50
100
0.01
0.1
1
10
Concentration (uM)

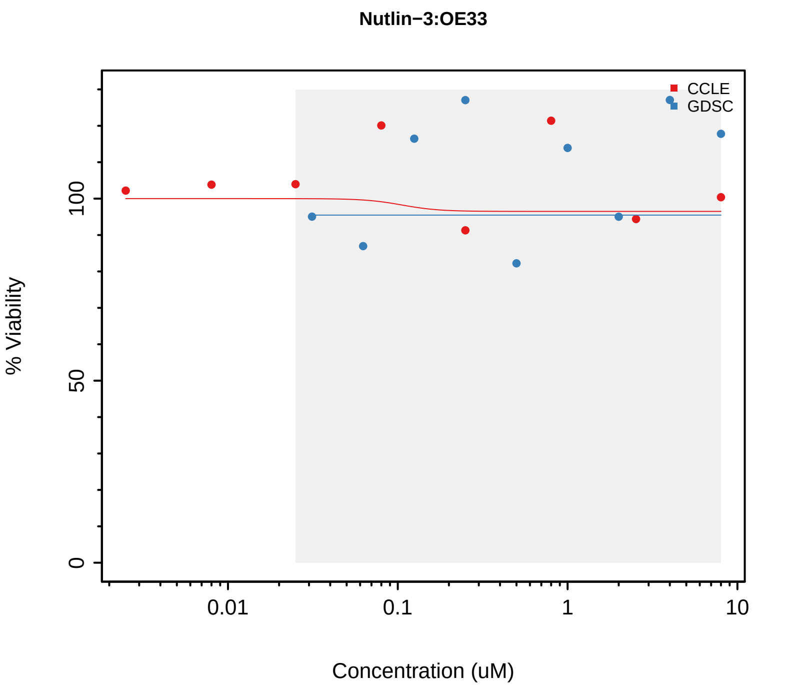
Nutlin−3:OE33
CCLE
GDSC
% Viability
0
50
100
0.01
0.1
1
10
Concentration (uM)

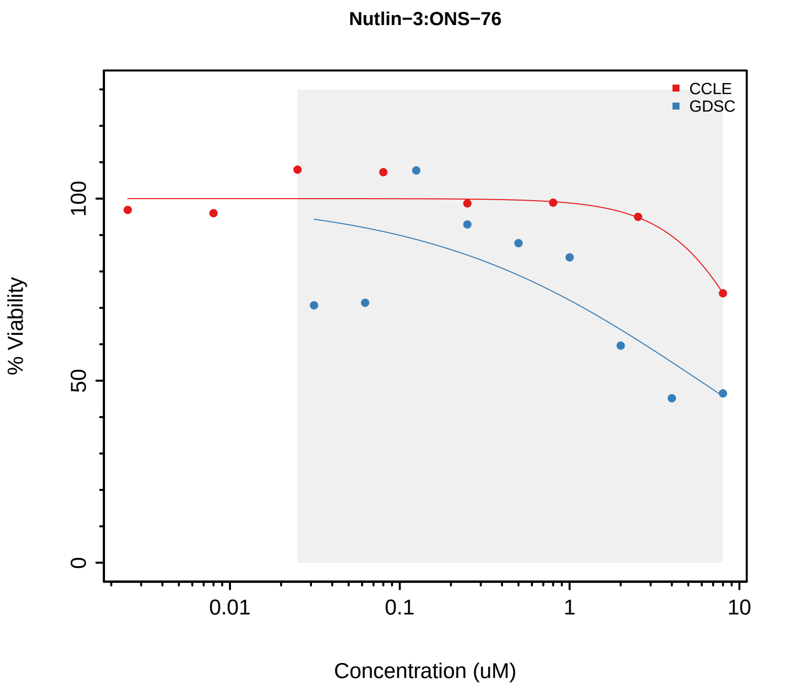
Nutlin−3:ONS−76
CCLE
GDSC
% Viability
0
50
100
0.01
0.1
1
10
Concentration (uM)

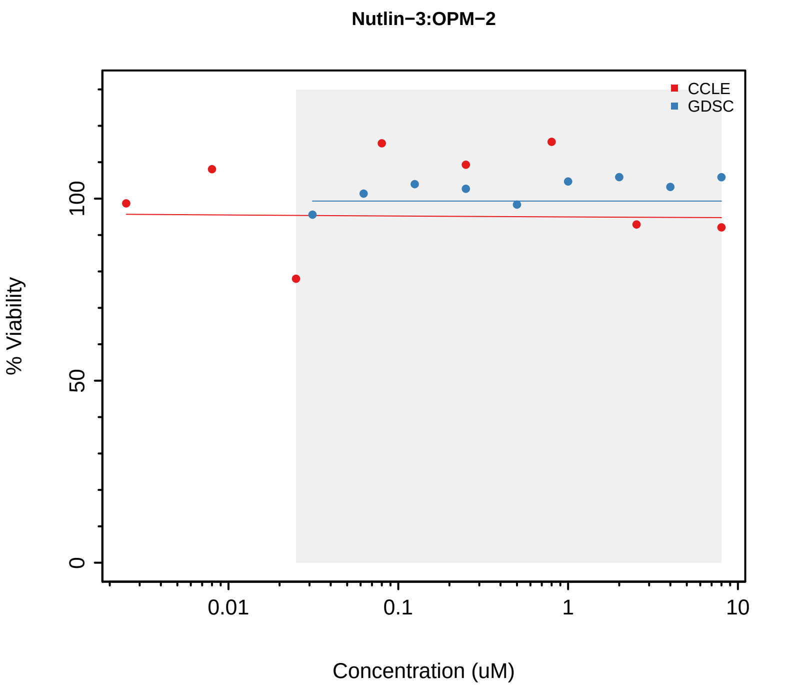
Nutlin−3:OPM−2
CCLE
GDSC
% Viability
0
50
100
0.01
0.1
1
10
Concentration (uM)

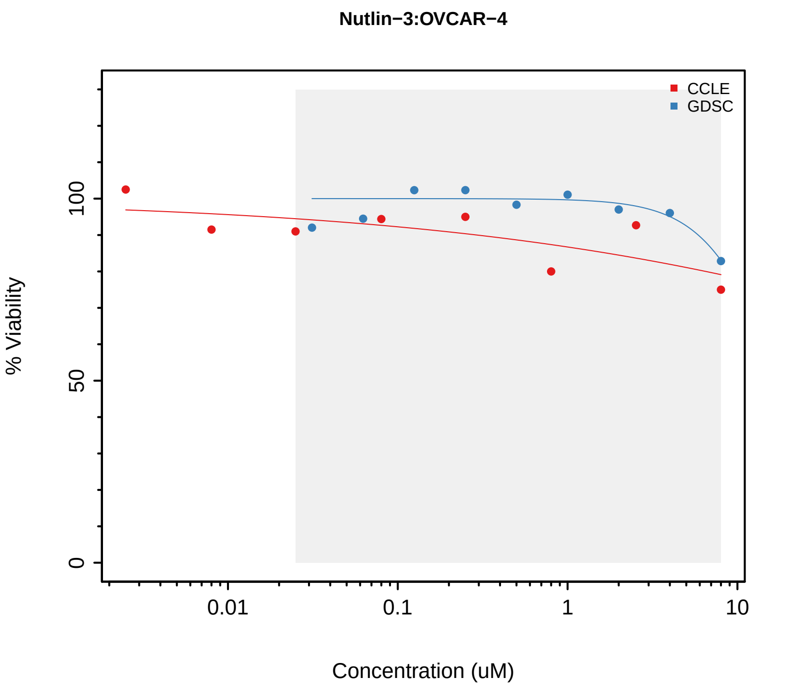
Nutlin−3:OVCAR−4
CCLE
GDSC
% Viability
0
50
100
0.01
0.1
1
10
Concentration (uM)

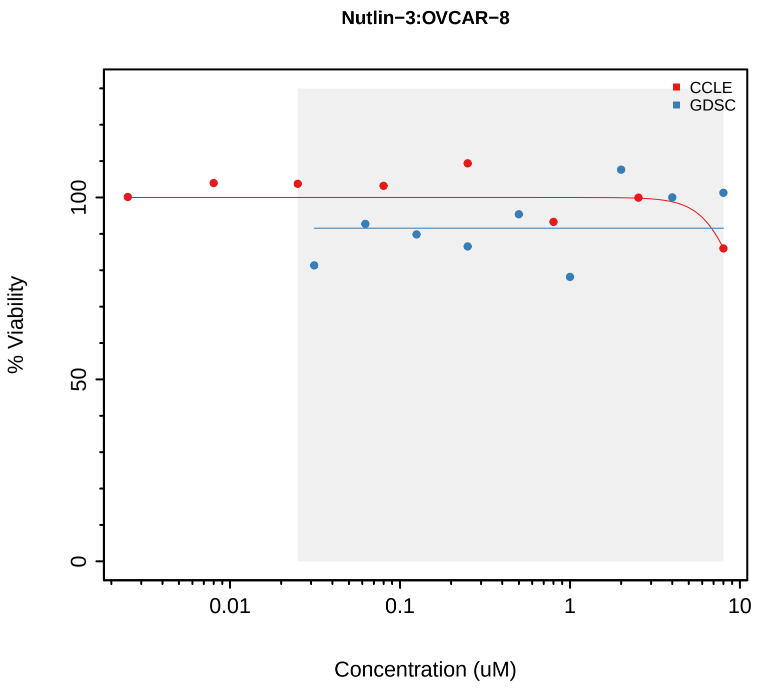
Nutlin−3:OVCAR−8
CCLE
GDSC
% Viability
0
50
100
0.01
0.1
1
10
Concentration (uM)

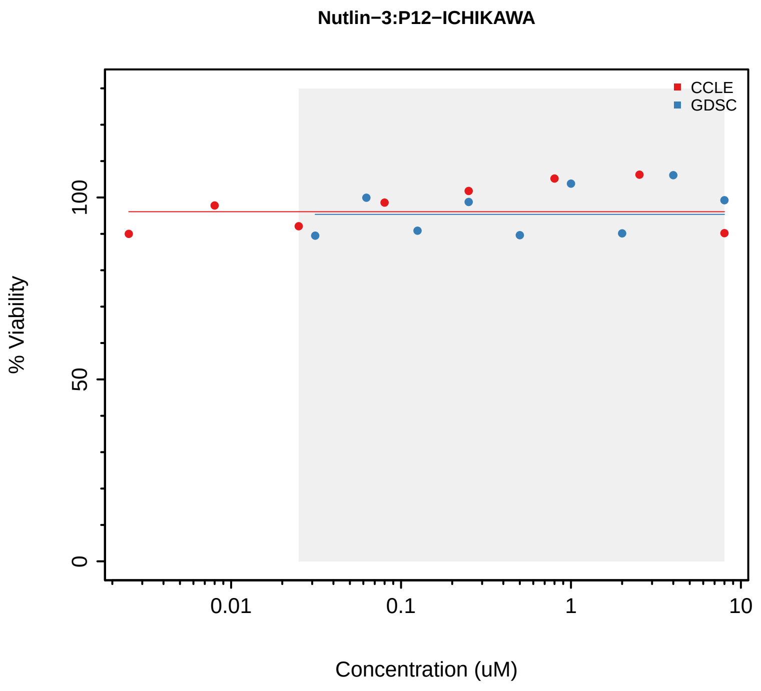
Nutlin−3:P12−ICHIKAWA
CCLE
GDSC
% Viability
0
50
100
0.01
0.1
1
10
Concentration (uM)

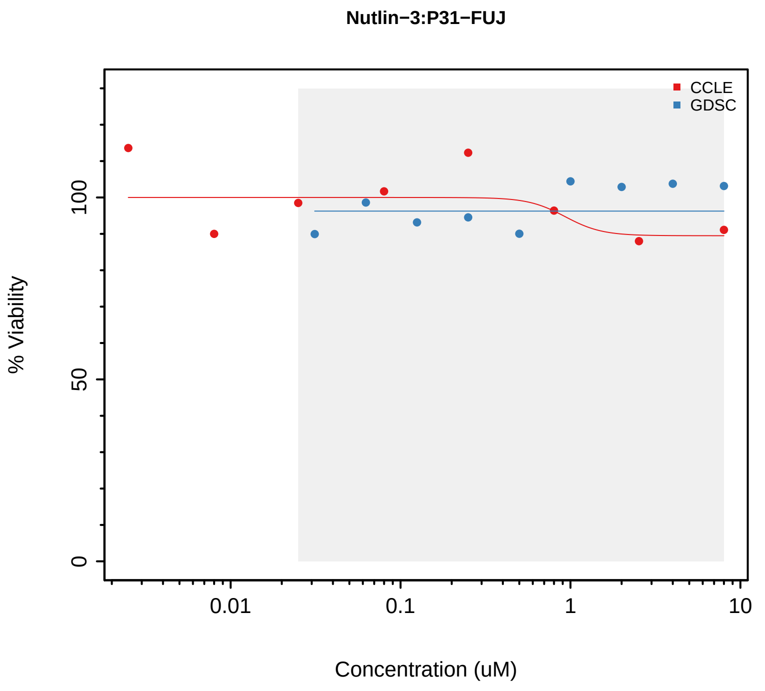
Nutlin−3:P31−FUJ
CCLE
GDSC
% Viability
0
50
100
0.01
0.1
1
10
Concentration (uM)

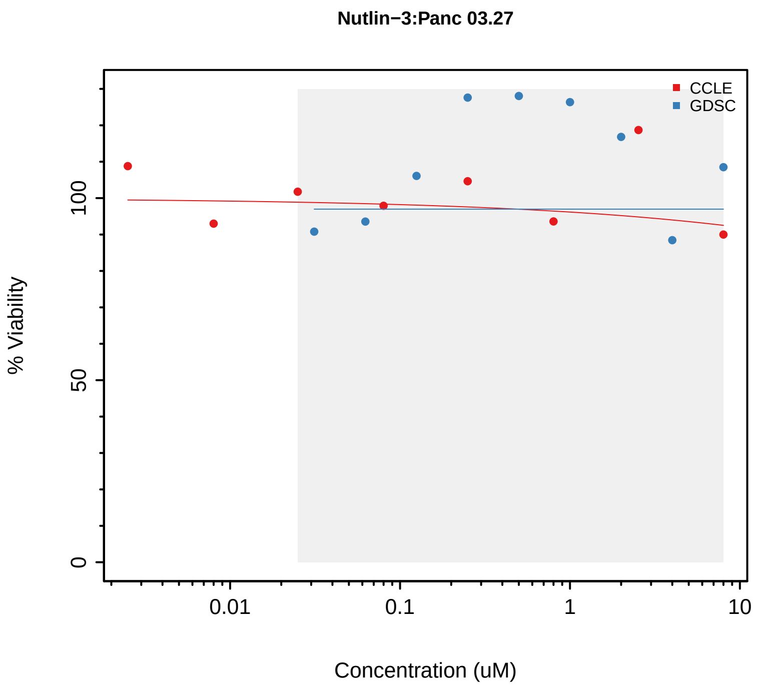
Nutlin−3:Panc 03.27
CCLE
GDSC
% Viability
0
50
100
0.01
0.1
1
10
Concentration (uM)

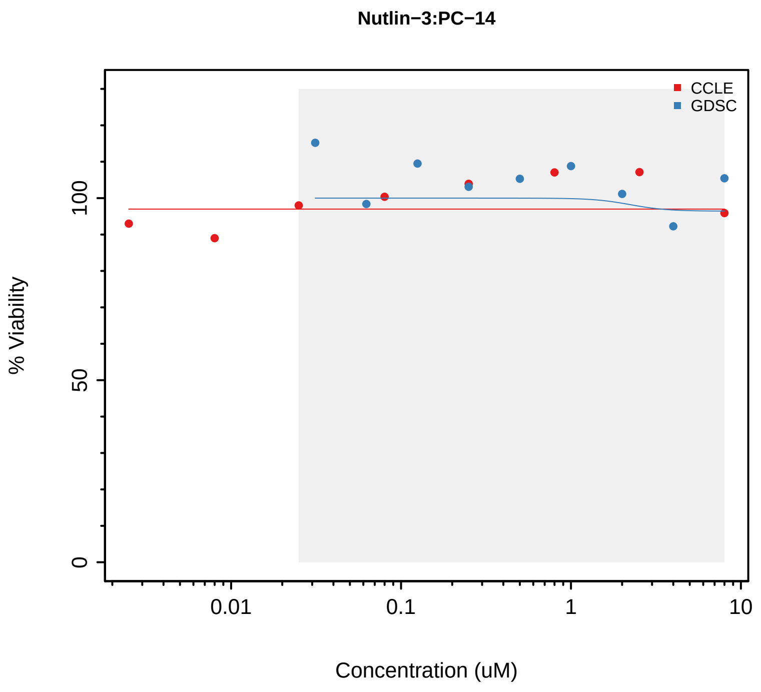
Nutlin−3:PC−14
CCLE
GDSC
% Viability
0
50
100
0.01
0.1
1
10
Concentration (uM)

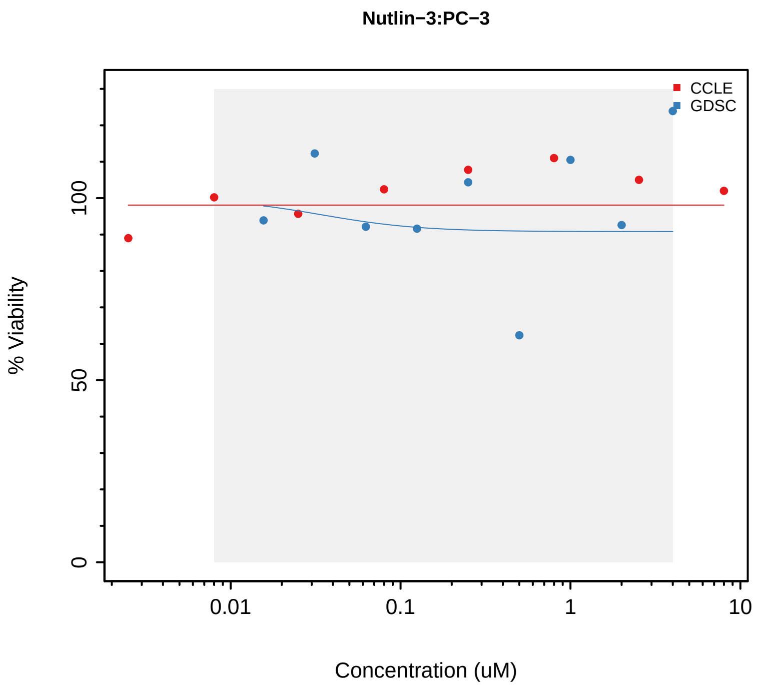
Nutlin−3:PC−3
CCLE
GDSC
% Viability
0
50
100
0.01
0.1
1
10
Concentration (uM)

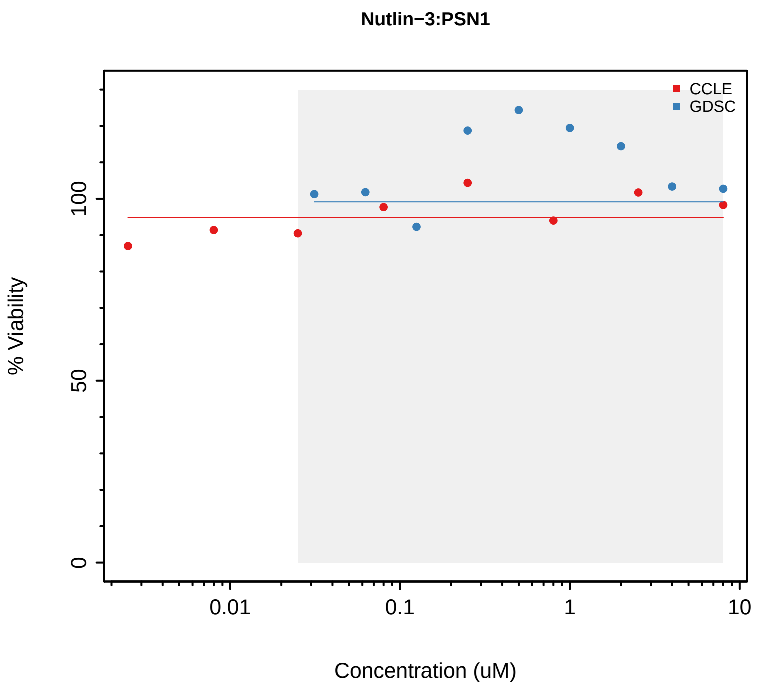
Nutlin−3:PSN1
CCLE
GDSC
% Viability
0
50
100
0.01
0.1
1
10
Concentration (uM)

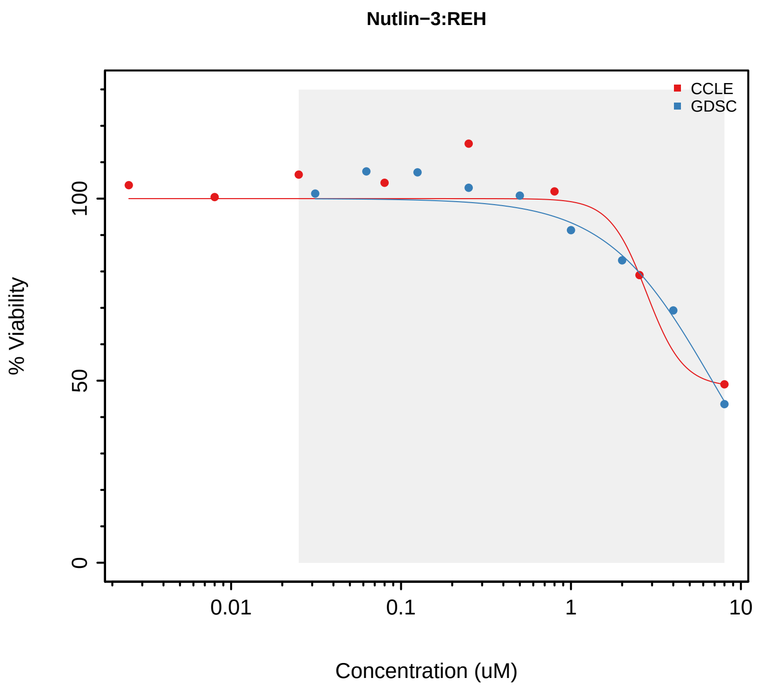
Nutlin−3:REH
CCLE
GDSC
% Viability
0
50
100
0.01
0.1
1
10
Concentration (uM)

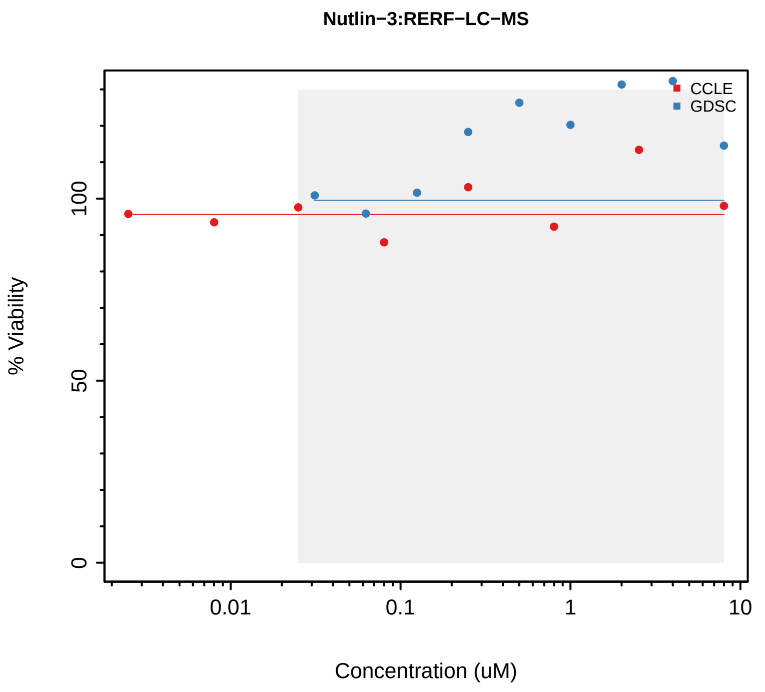
Nutlin−3:RERF−LC−MS
CCLE
GDSC
% Viability
0
50
100
0.01
0.1
1
10
Concentration (uM)

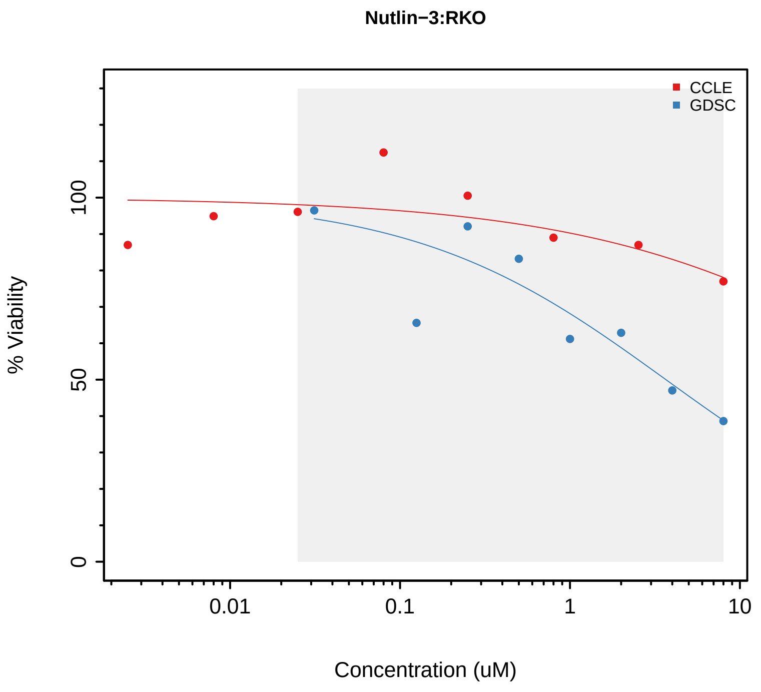
Nutlin−3:RKO
CCLE
GDSC
% Viability
0
50
100
0.01
0.1
1
10
Concentration (uM)

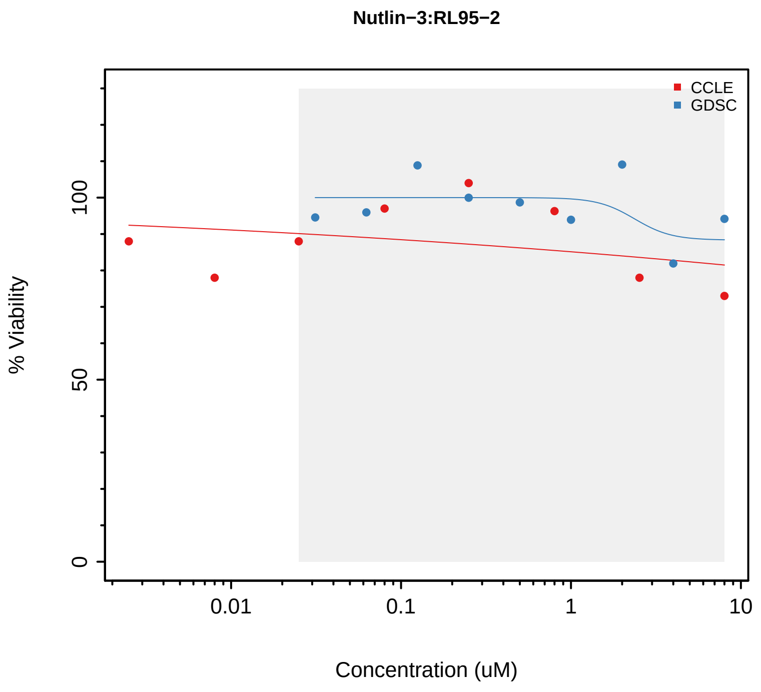
Nutlin−3:RL95−2
CCLE
GDSC
% Viability
0
50
100
0.01
0.1
1
10
Concentration (uM)

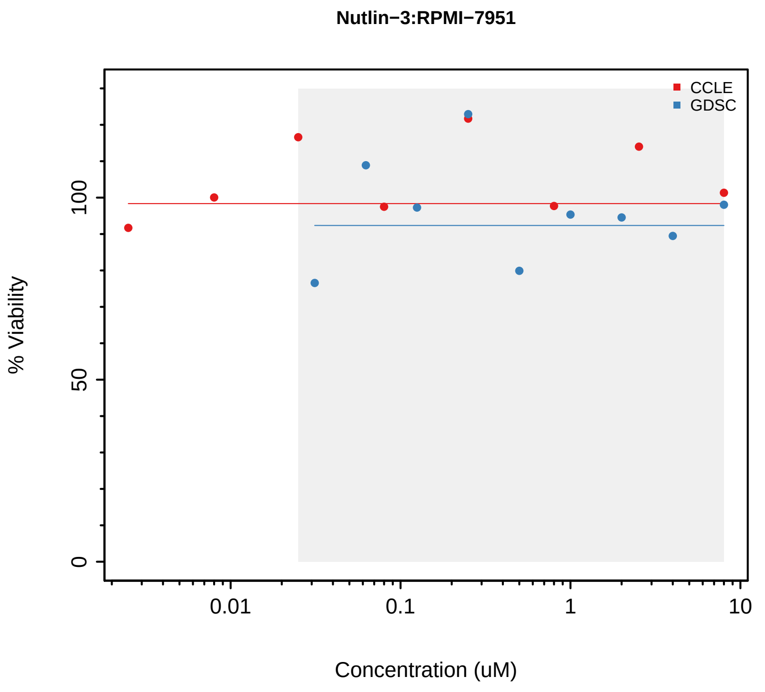
Nutlin−3:RPMI−7951
CCLE
GDSC
% Viability
0
50
100
0.01
0.1
1
10
Concentration (uM)

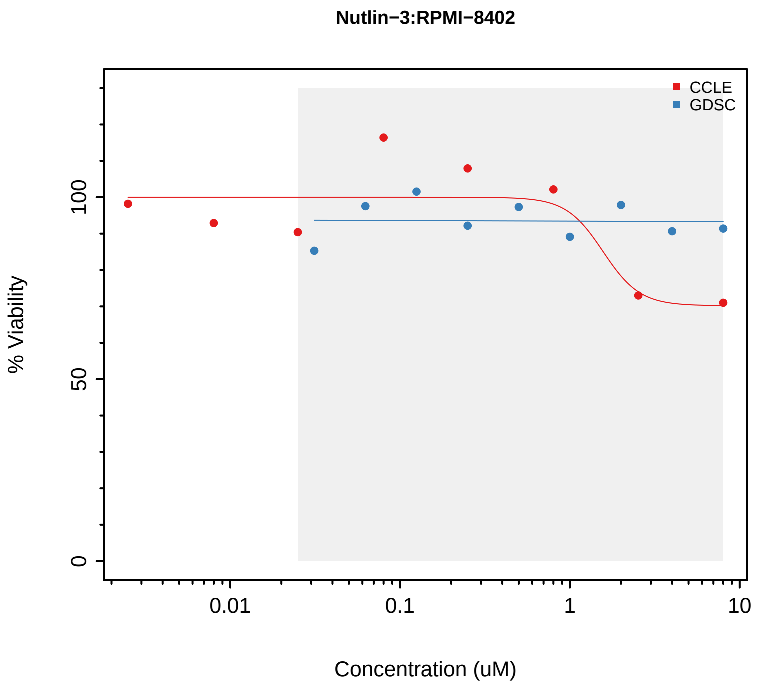
Nutlin−3:RPMI−8402
CCLE
GDSC
% Viability
0
50
100
0.01
0.1
1
10
Concentration (uM)

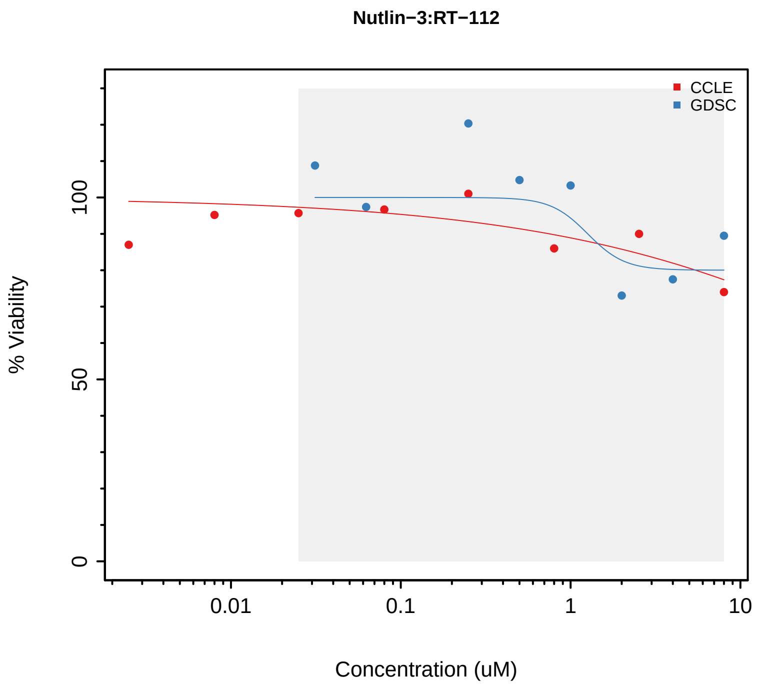
Nutlin−3:RT−112
CCLE
GDSC
% Viability
0
50
100
0.01
0.1
1
10
Concentration (uM)

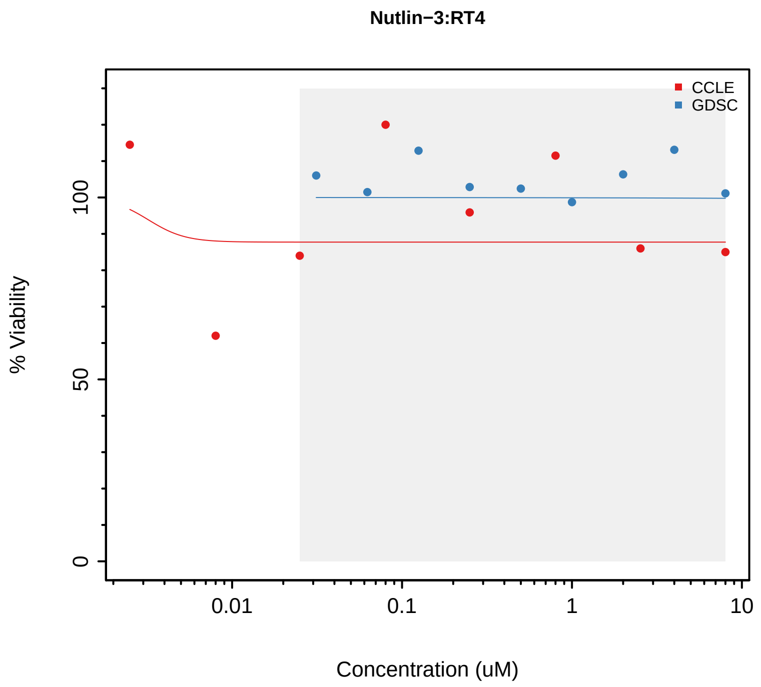
Nutlin−3:RT4
CCLE
GDSC
% Viability
0
50
100
0.01
0.1
1
10
Concentration (uM)

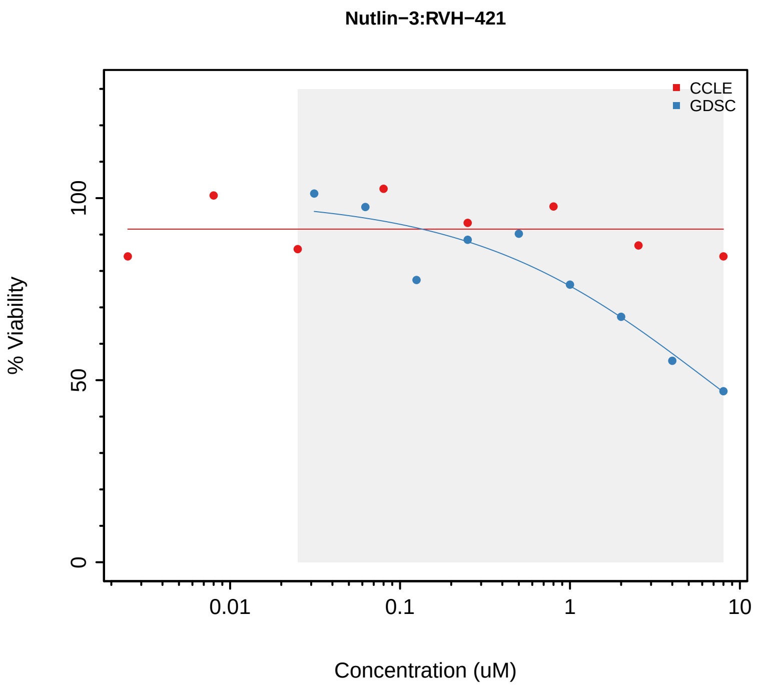
Nutlin−3:RVH−421
CCLE
GDSC
% Viability
0
50
100
0.01
0.1
1
10
Concentration (uM)

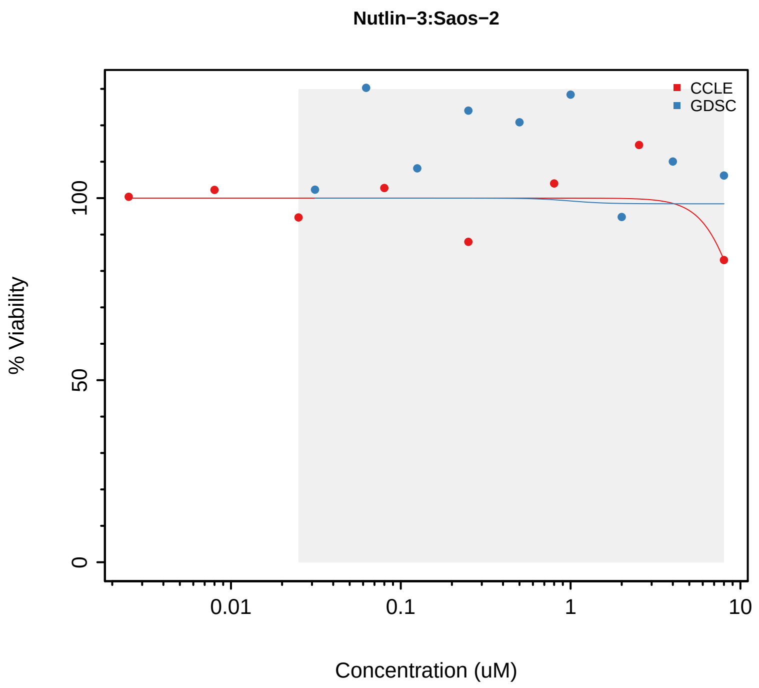
Nutlin−3:Saos−2
CCLE
GDSC
% Viability
0
50
100
0.01
0.1
1
10
Concentration (uM)

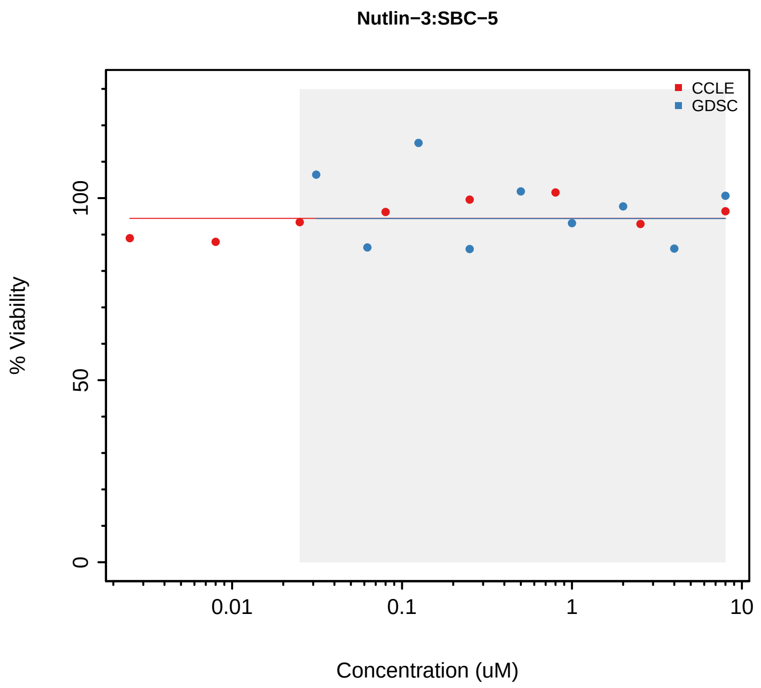
Nutlin−3:SBC−5
CCLE
GDSC
% Viability
0
50
100
0.01
0.1
1
10
Concentration (uM)

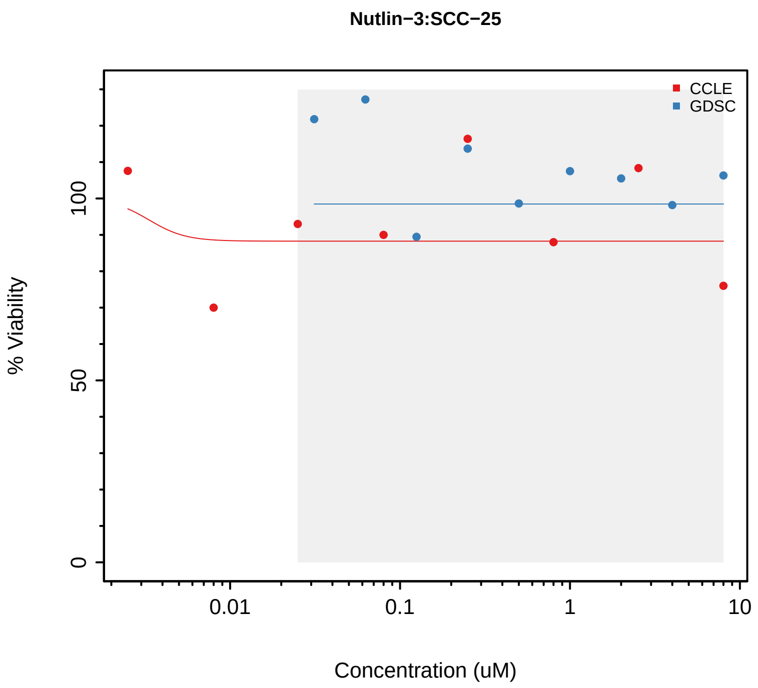
Nutlin−3:SCC−25
CCLE
GDSC
% Viability
0
50
100
0.01
0.1
1
10
Concentration (uM)

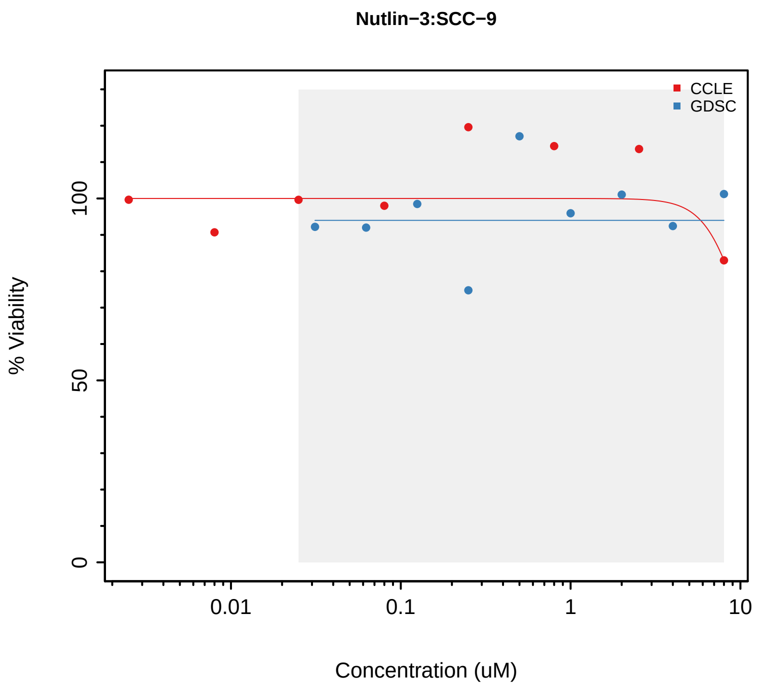
Nutlin−3:SCC−9
CCLE
GDSC
% Viability
0
50
100
0.01
0.1
1
10
Concentration (uM)

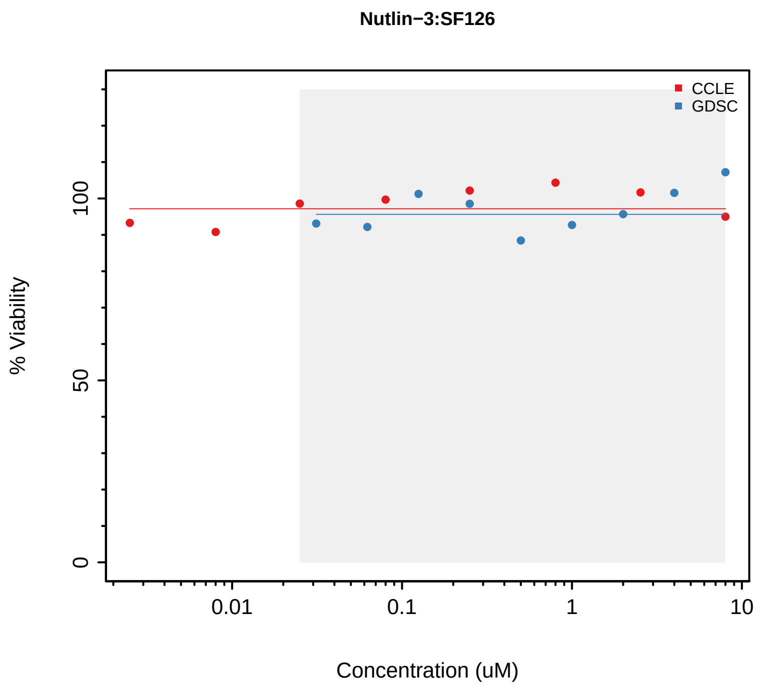
Nutlin−3:SF126
CCLE
GDSC
% Viability
0
50
100
0.01
0.1
1
10
Concentration (uM)

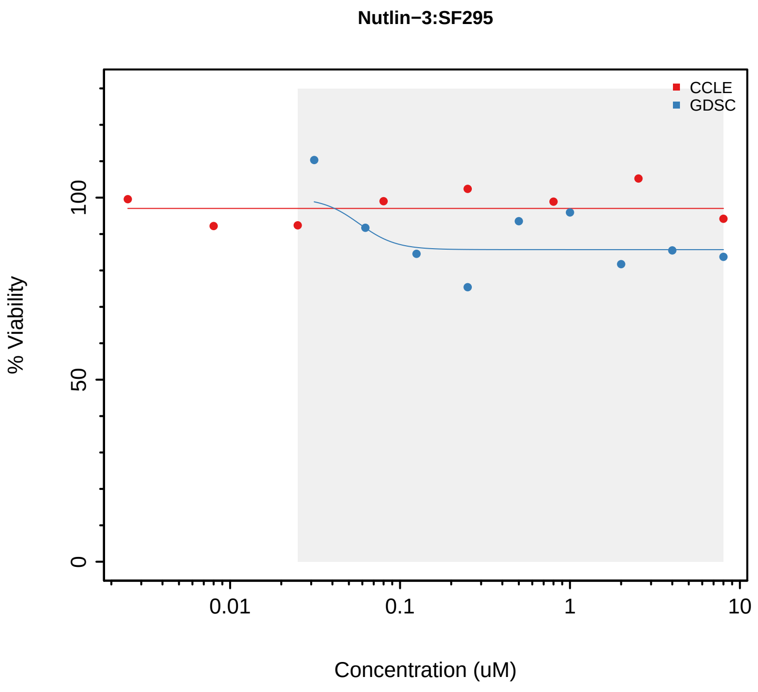
Nutlin−3:SF295
CCLE
GDSC
% Viability
0
50
100
0.01
0.1
1
10
Concentration (uM)

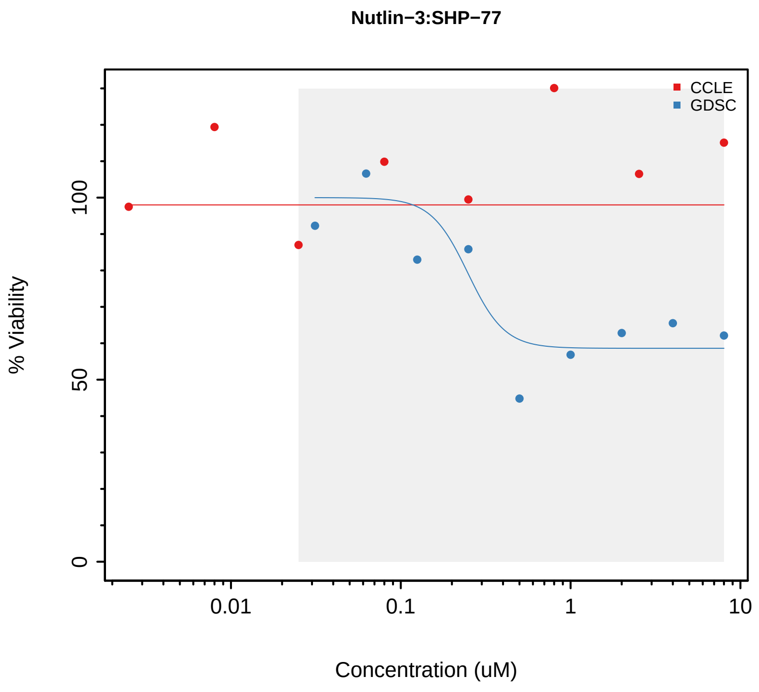
Nutlin−3:SHP−77
CCLE
GDSC
% Viability
0
50
100
0.01
0.1
1
10
Concentration (uM)

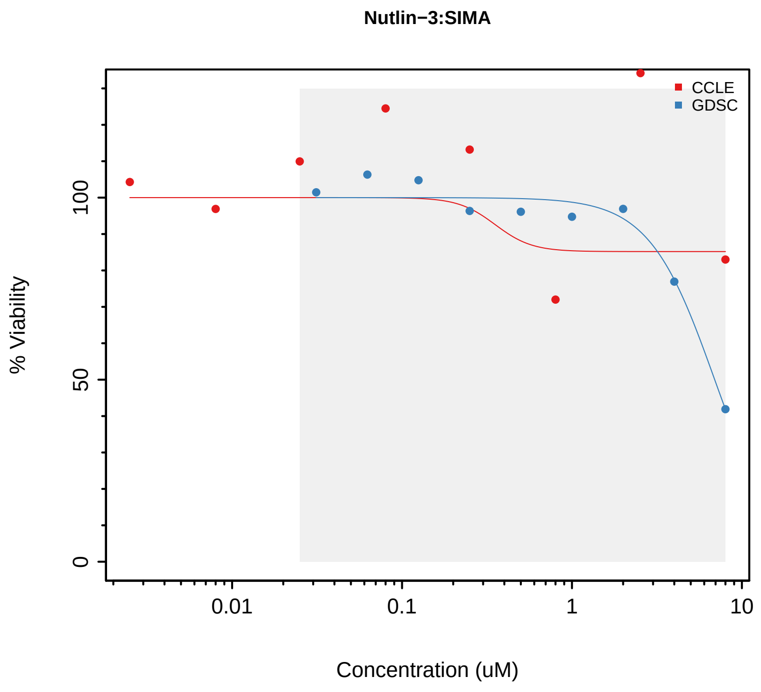
Nutlin−3:SIMA
CCLE
GDSC
% Viability
0
50
100
0.01
0.1
1
10
Concentration (uM)

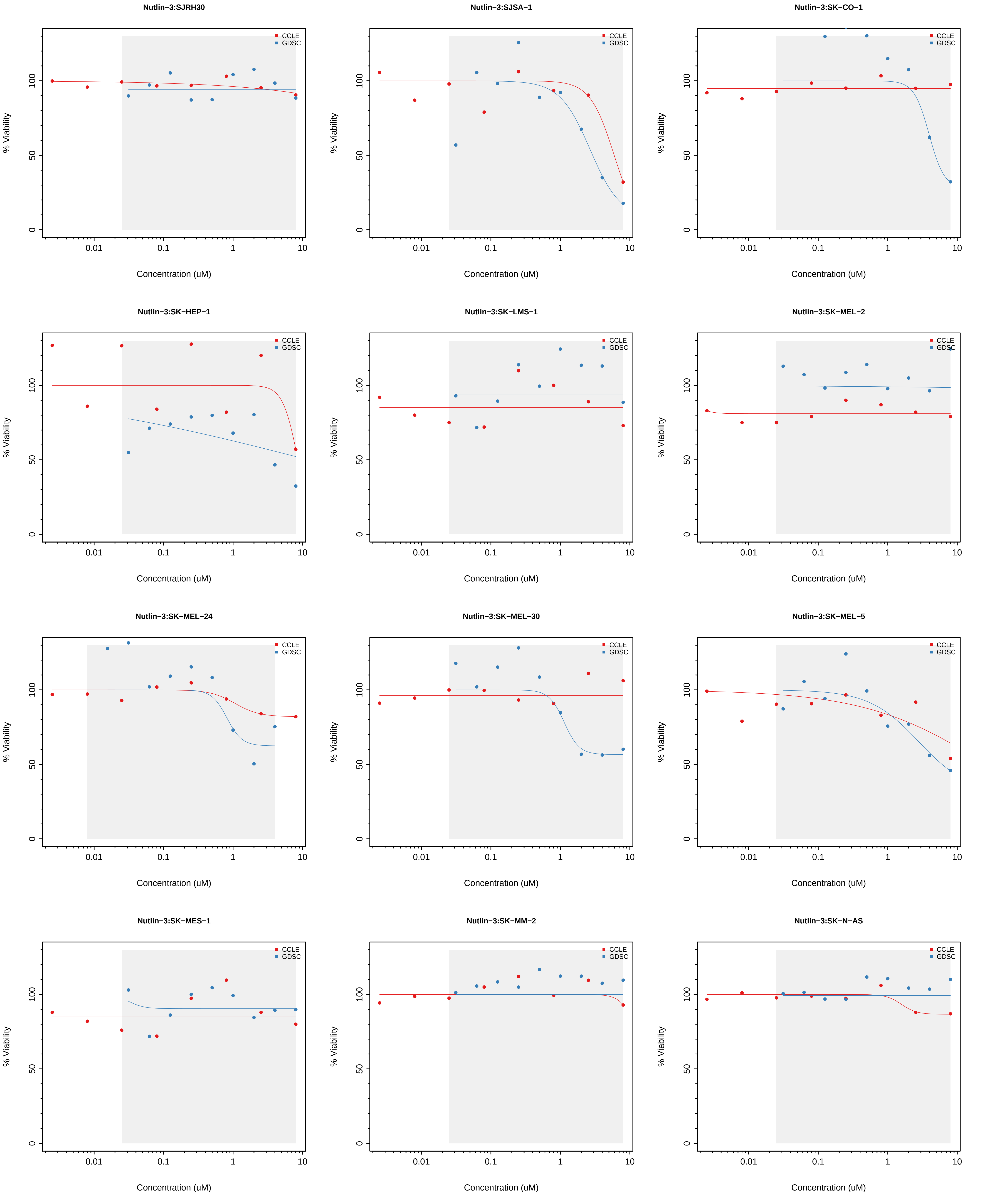
Nutlin−3:SJRH30
Nutlin−3:SJSA−1
Nutlin−3:SK−CO−1
Nutlin−3:SK−HEP−1
Nutlin−3:SK−LMS−1
Nutlin−3:SK−MEL−2
Nutlin−3:SK−MEL−24
Nutlin−3:SK−MEL−30
Nutlin−3:SK−MEL−5
Nutlin−3:SK−MES−1
Nutlin−3:SK−MM−2
Nutlin−3:SK−N−AS
CCLE
GDSC
% Viability
0
50
100
0.01
0.1
1
10
Concentration (uM)

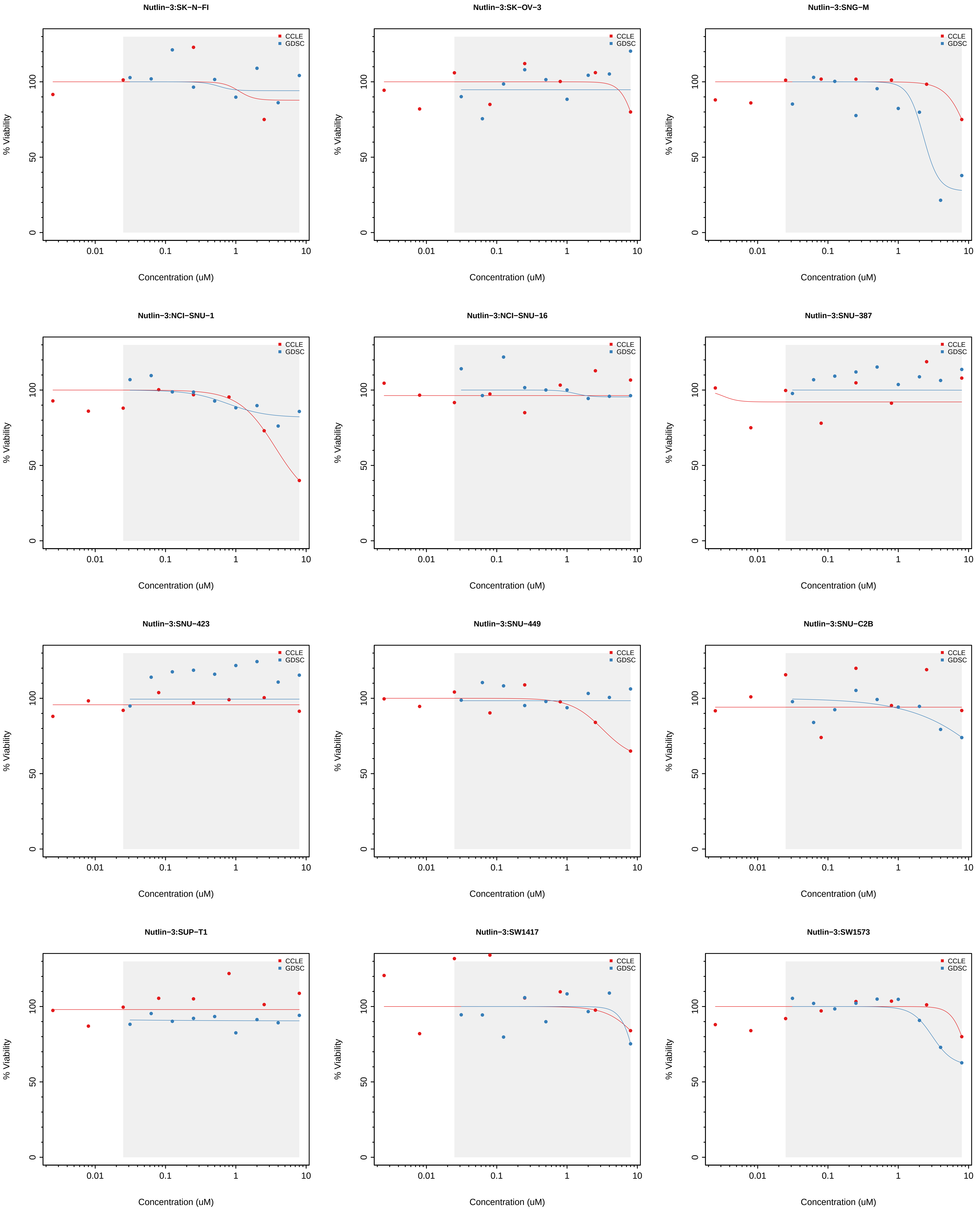
Nutlin−3:SK−N−FI
Nutlin−3:SK−OV−3
Nutlin−3:SNG−M
Nutlin−3:NCI−SNU−1
Nutlin−3:NCI−SNU−16
Nutlin−3:SNU−387
Nutlin−3:SNU−423
Nutlin−3:SNU−449
Nutlin−3:SNU−C2B
Nutlin−3:SUP−T1
Nutlin−3:SW1417
Nutlin−3:SW1573
CCLE
GDSC
% Viability
Concentration (uM)

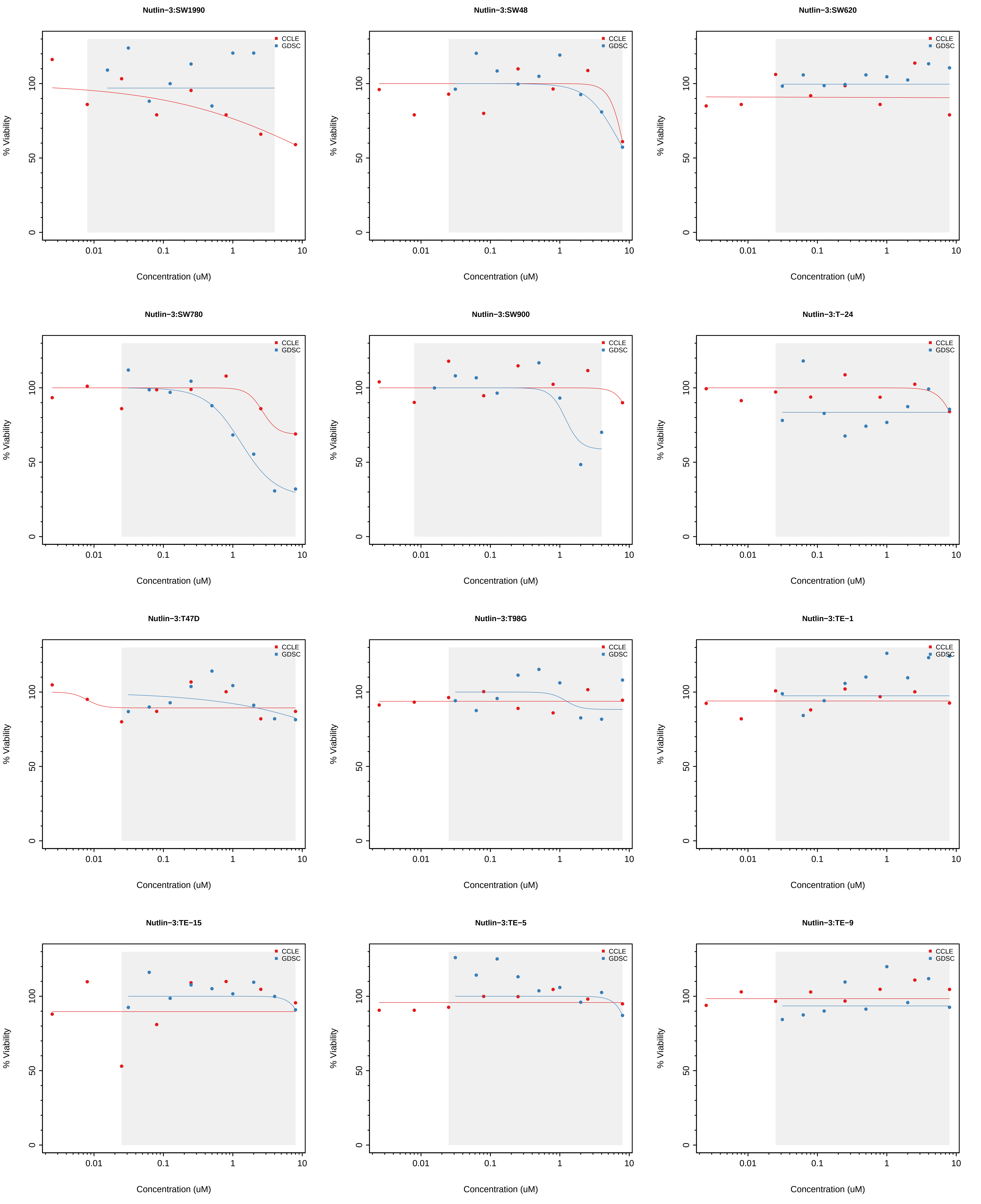
Nutlin−3:SW1990
Nutlin−3:SW48
Nutlin−3:SW620
Nutlin−3:SW780
Nutlin−3:SW900
Nutlin−3:T−24
Nutlin−3:T47D
Nutlin−3:T98G
Nutlin−3:TE−1
Nutlin−3:TE−15
Nutlin−3:TE−5
Nutlin−3:TE−9
CCLE
GDSC
% Viability
0
50
100
0.01
0.1
1
10
Concentration (uM)

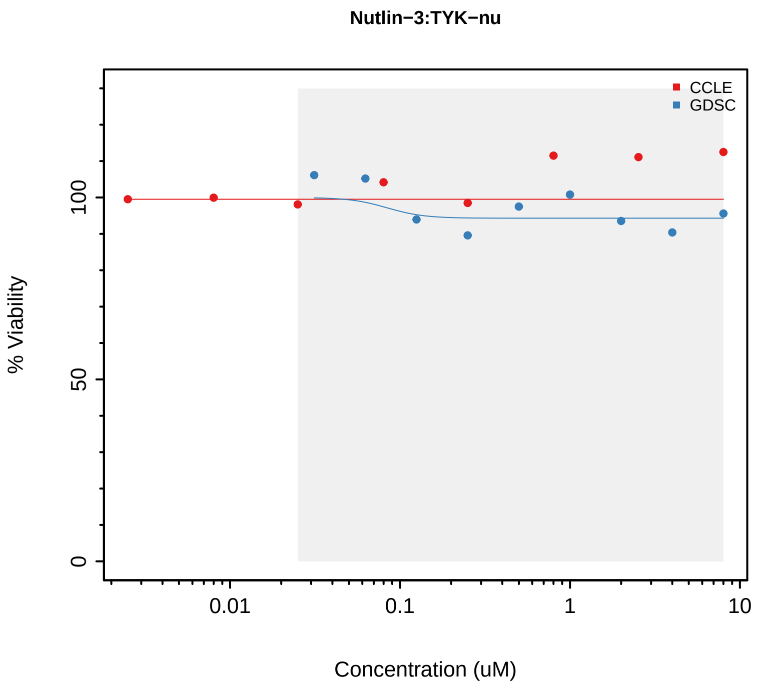
Nutlin−3:TYK−nu
CCLE
GDSC
% Viability
Concentration (uM)

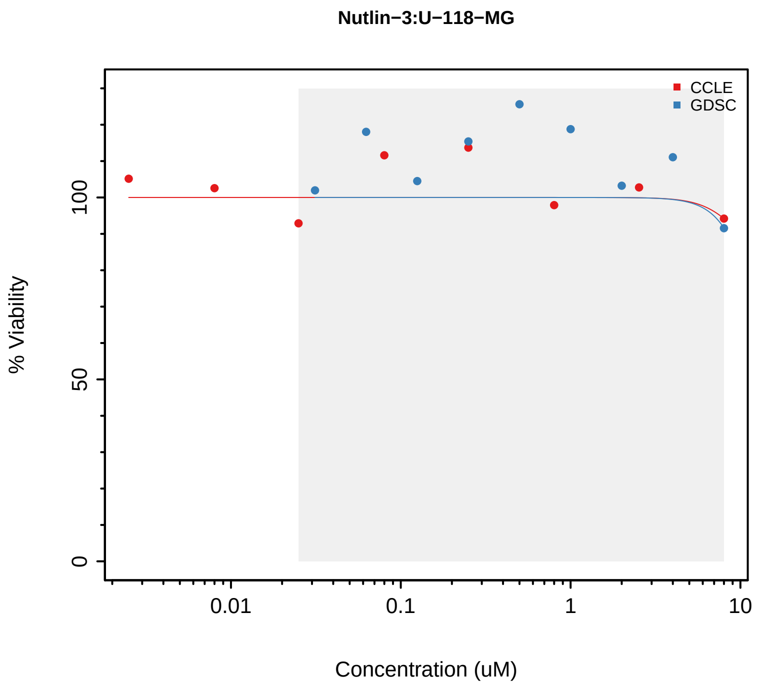
Nutlin−3:U−118−MG
CCLE
GDSC
% Viability
Concentration (uM)

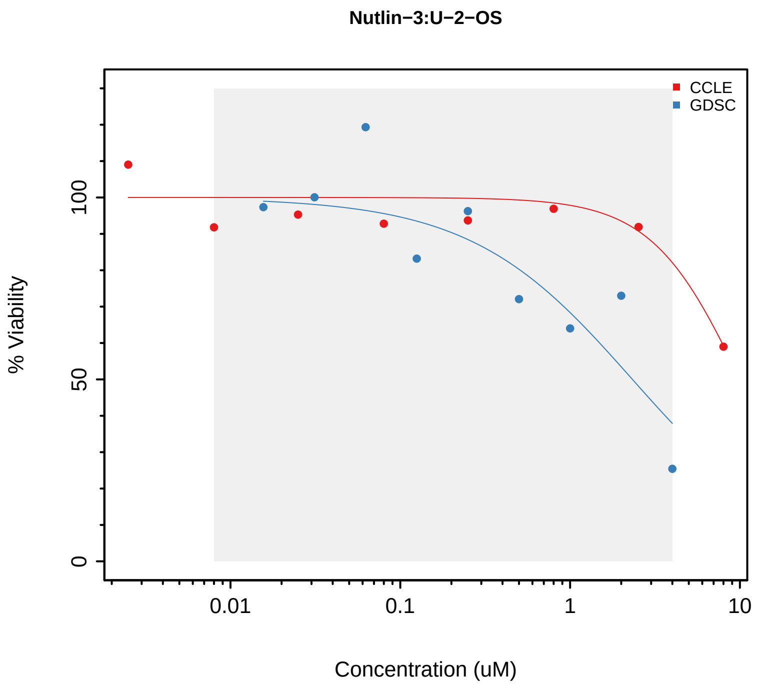
Nutlin−3:U−2−OS
CCLE
GDSC
% Viability
Concentration (uM)

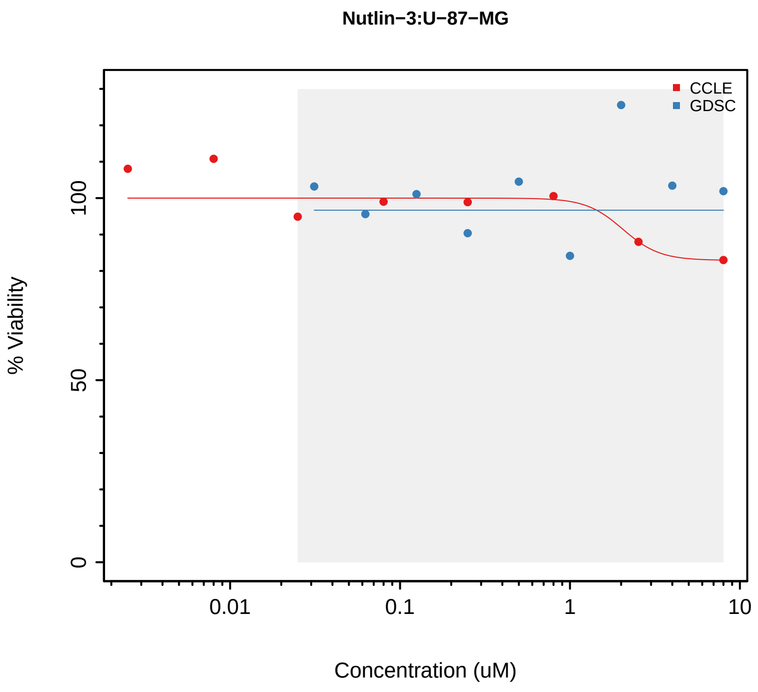
Nutlin−3:U−87−MG
CCLE
GDSC
% Viability
Concentration (uM)

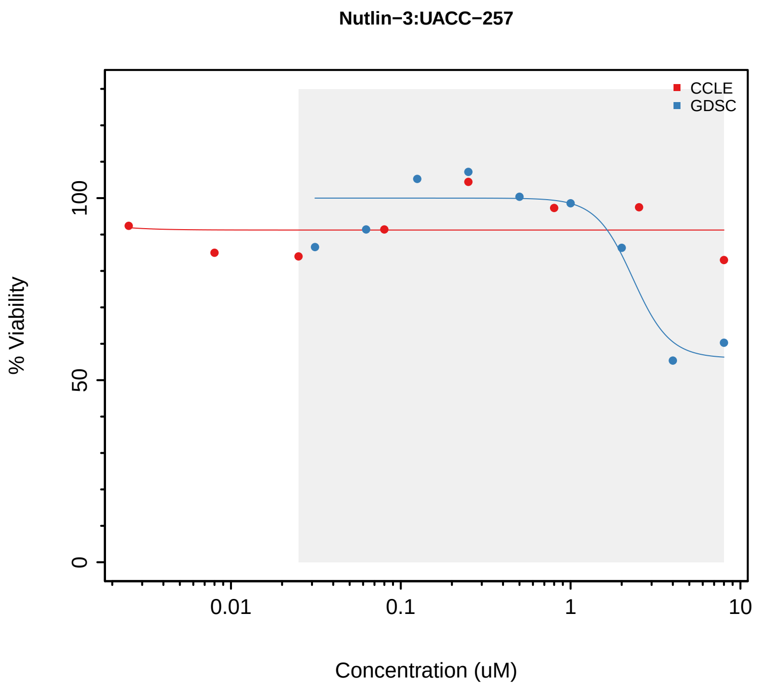
Nutlin−3:UACC−257
CCLE
GDSC
% Viability
Concentration (uM)

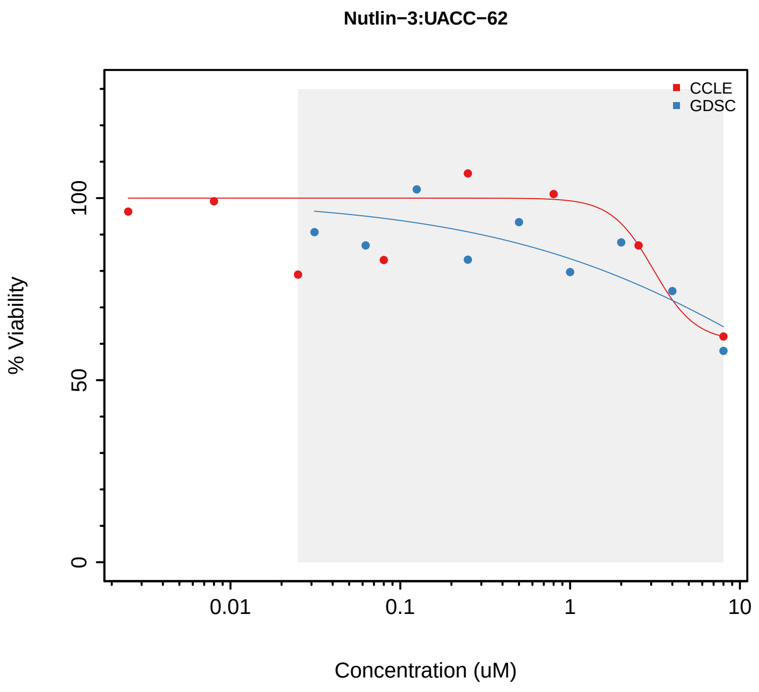
Nutlin−3:UACC−62
CCLE
GDSC
% Viability
Concentration (uM)

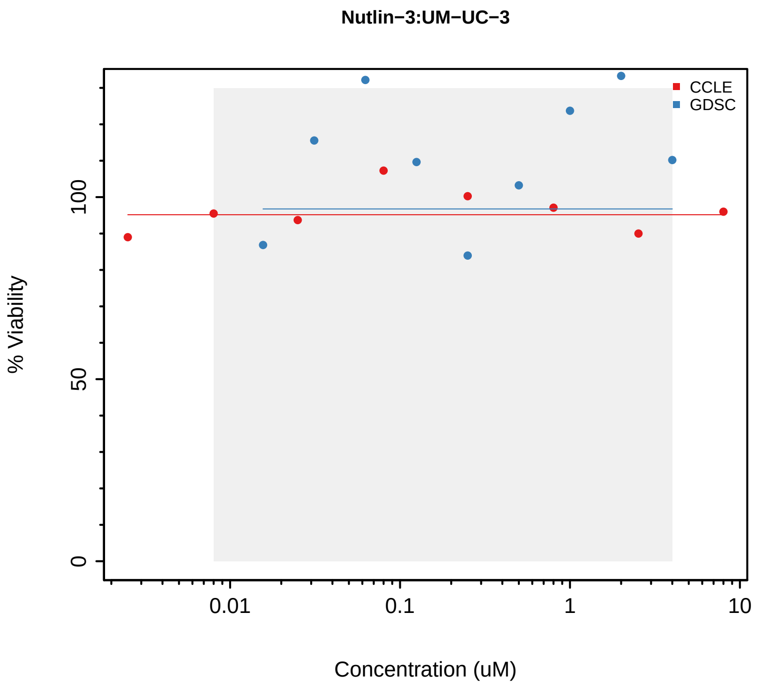
Nutlin−3:UM−UC−3
CCLE
GDSC
% Viability
Concentration (uM)

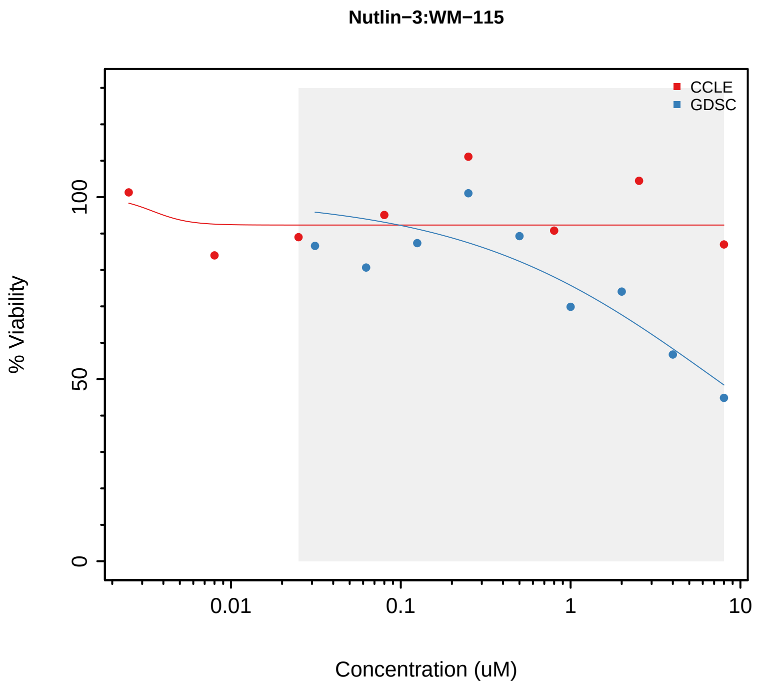
Nutlin−3:WM−115
CCLE
GDSC
% Viability
Concentration (uM)

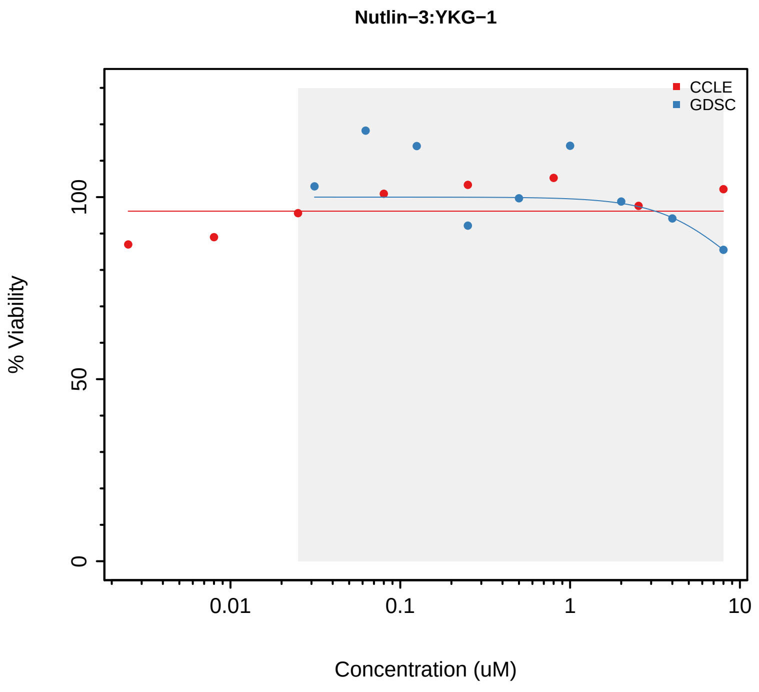
Nutlin−3:YKG−1
CCLE
GDSC
% Viability
Concentration (uM)

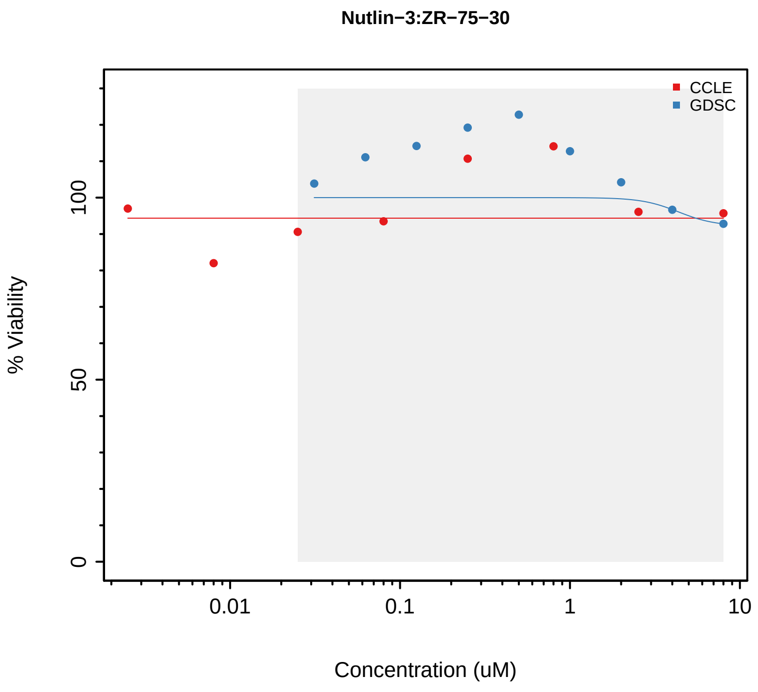
Nutlin−3:ZR−75−30
CCLE
GDSC
% Viability
Concentration (uM)

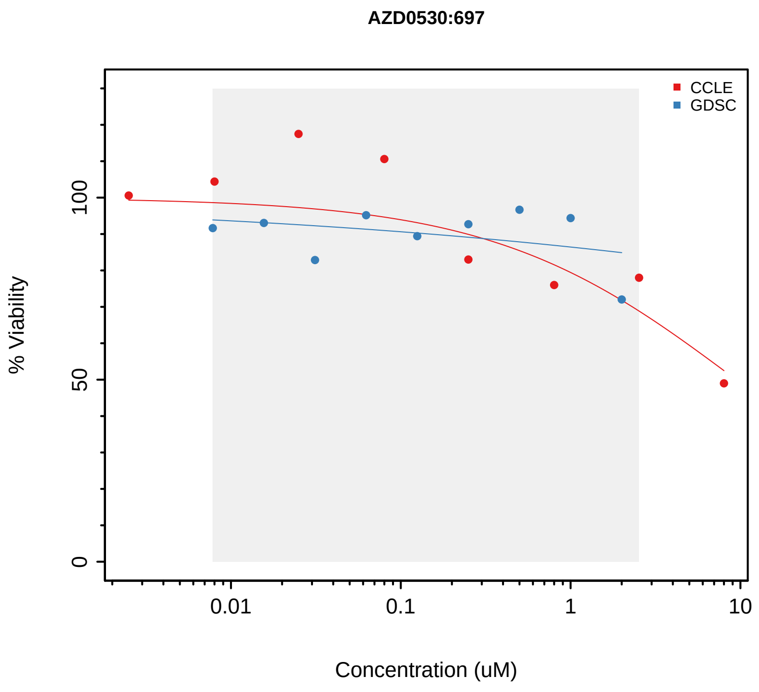
AZD0530:697
CCLE
GDSC
% Viability
Concentration (uM)

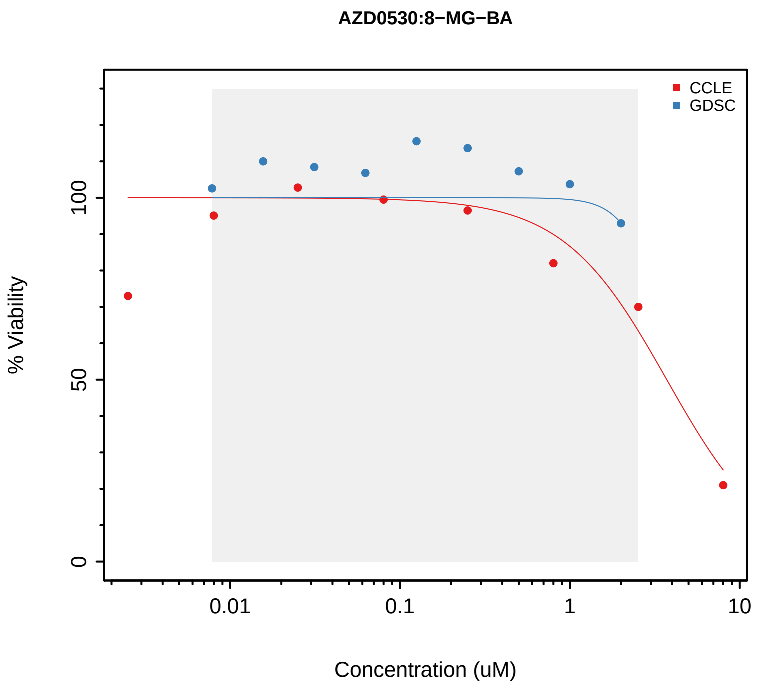
AZD0530:8−MG−BA
CCLE
GDSC
% Viability
Concentration (uM)

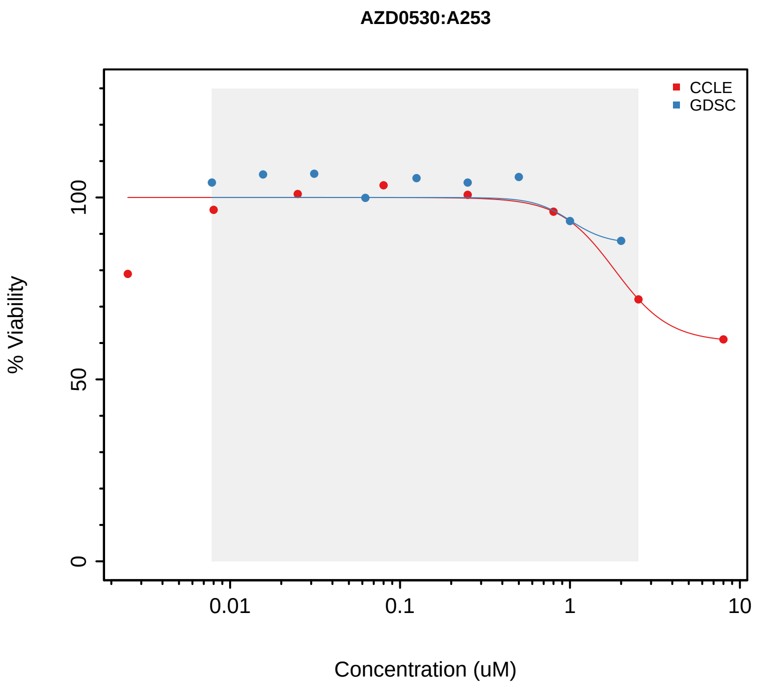
AZD0530:A253
CCLE
GDSC
% Viability
0
50
100
0.01
0.1
1
10
Concentration (uM)

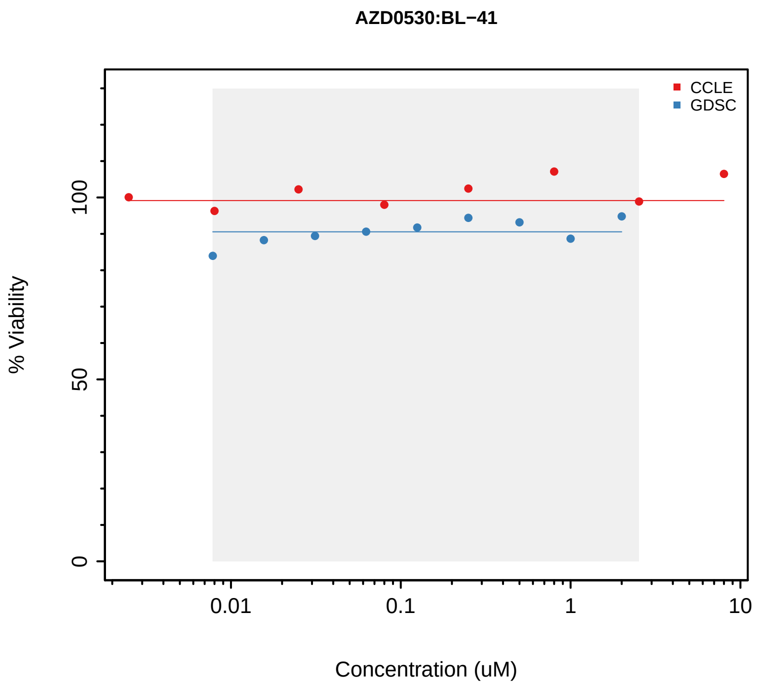
AZD0530:BL−41
CCLE
GDSC
% Viability
0
50
100
0.01
0.1
1
10
Concentration (uM)

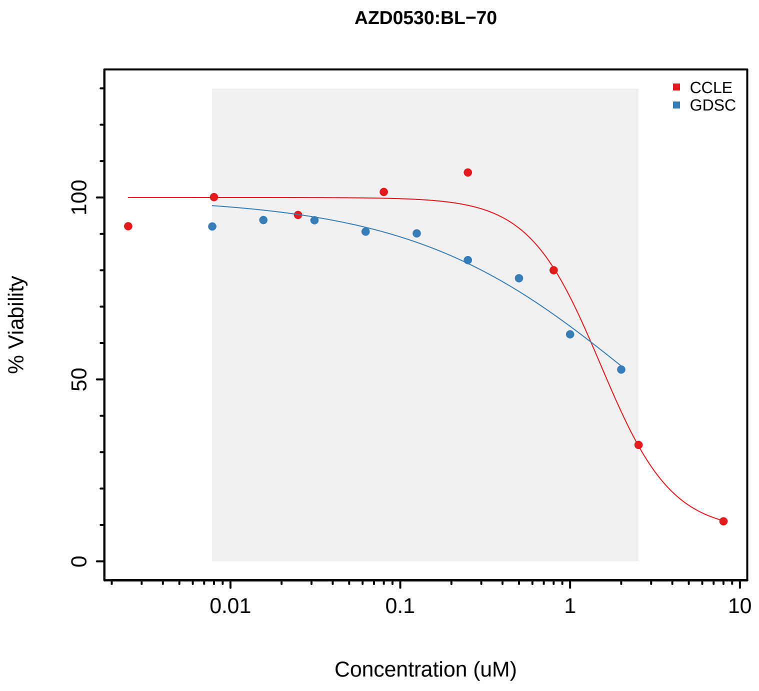
AZD0530:BL−70
CCLE
GDSC
% Viability
0
50
100
0.01
0.1
1
10
Concentration (uM)

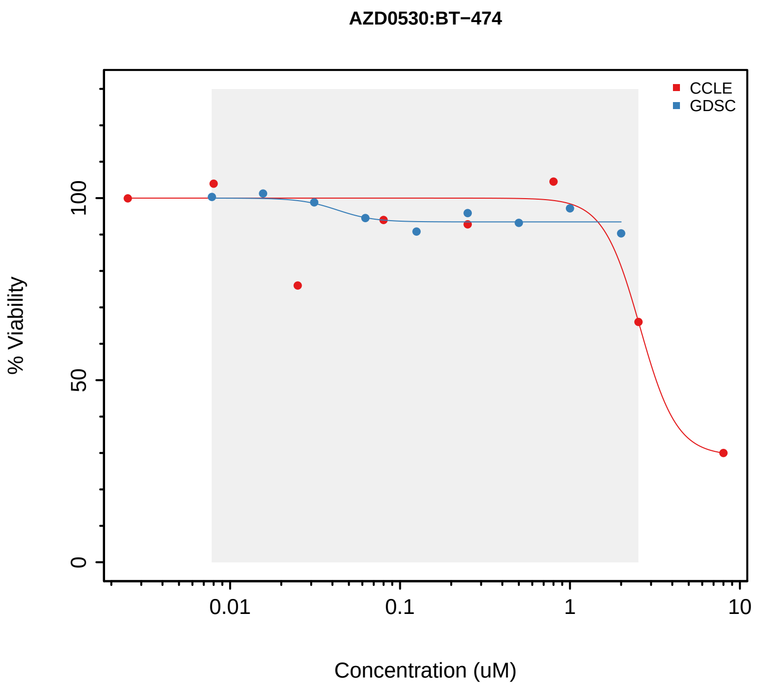
AZD0530:BT−474
CCLE
GDSC
% Viability
0
50
100
0.01
0.1
1
10
Concentration (uM)

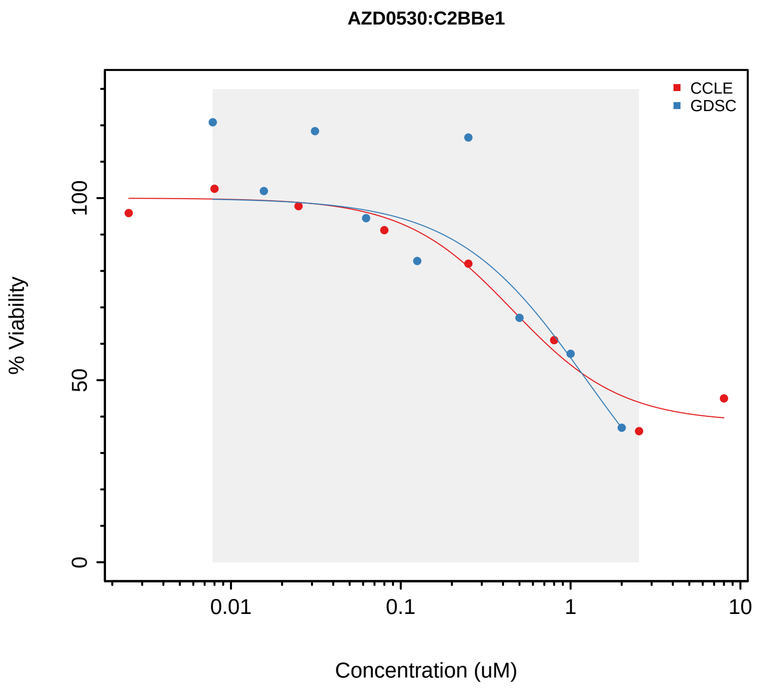
AZD0530:C2BBe1
CCLE
GDSC
% Viability
0
50
100
0.01
0.1
1
10
Concentration (uM)

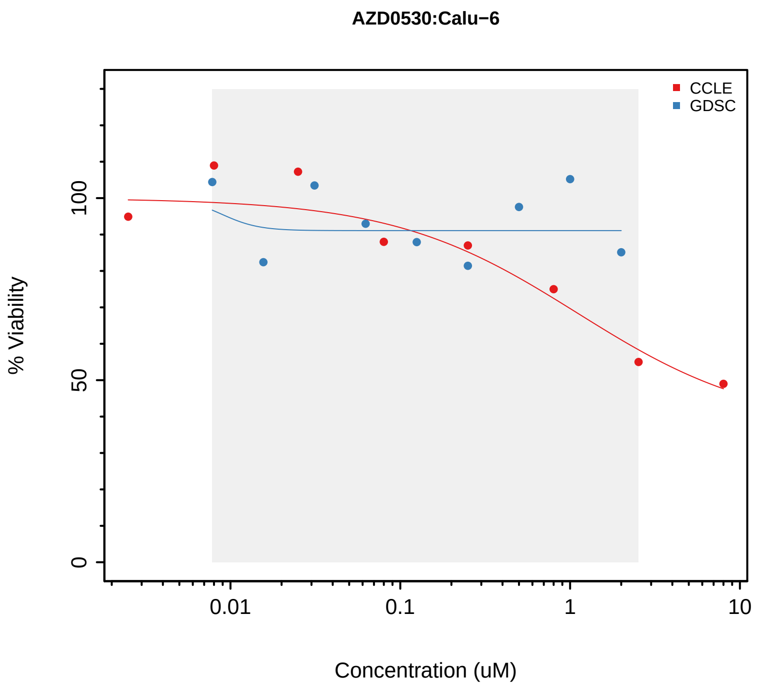
AZD0530:Calu−6
CCLE
GDSC
% Viability
0
50
100
0.01
0.1
1
10
Concentration (uM)

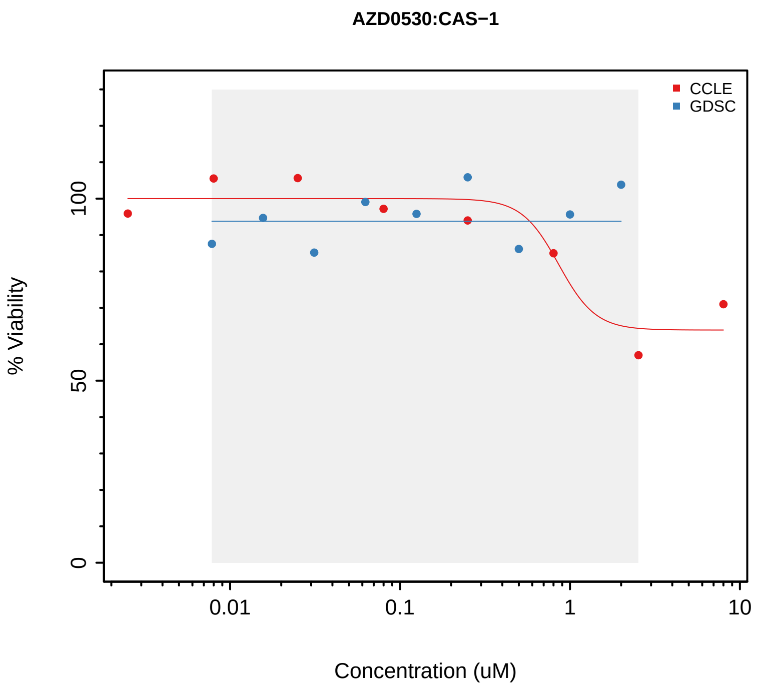
AZD0530:CAS−1
CCLE
GDSC
% Viability
0
50
100
0.01
0.1
1
10
Concentration (uM)

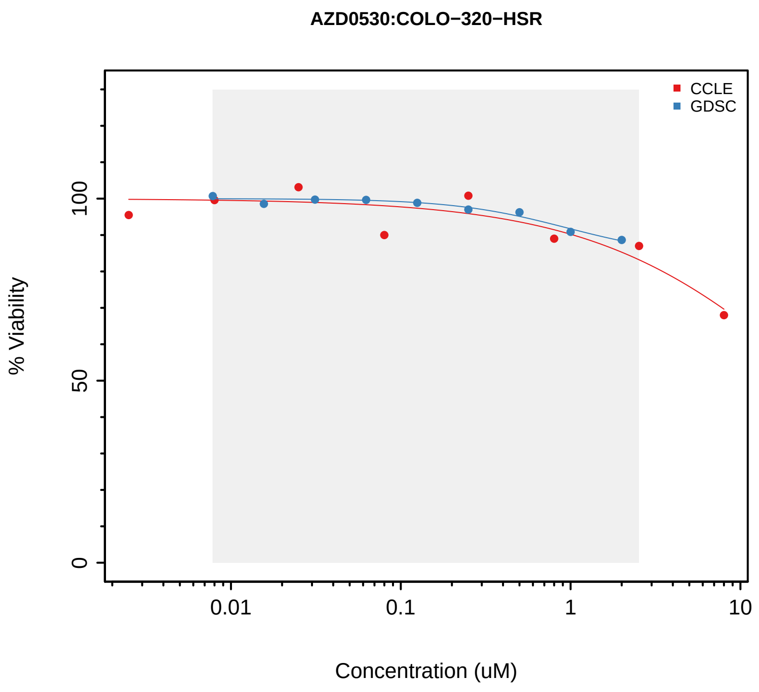
AZD0530:COLO−320−HSR
CCLE
GDSC
% Viability
0
50
100
0.01
0.1
1
10
Concentration (uM)

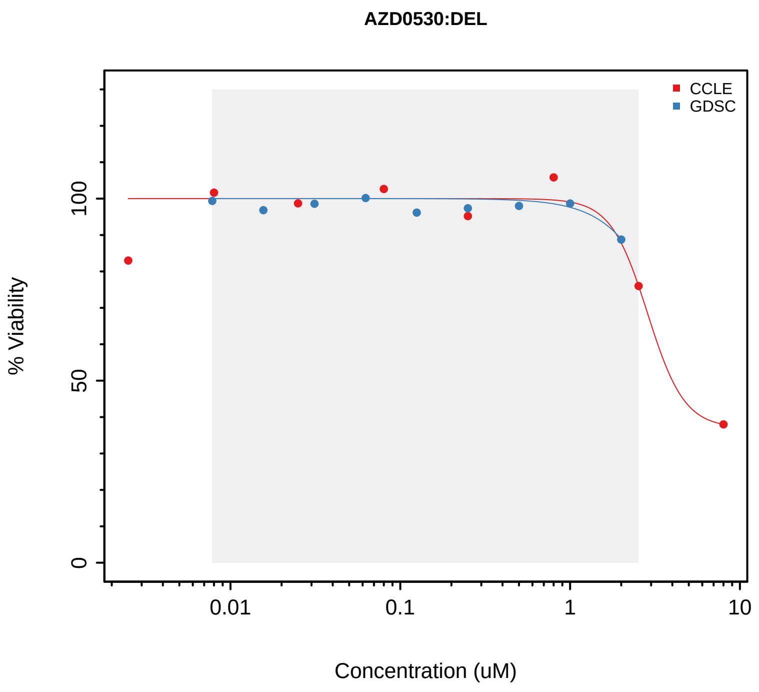
AZD0530:DEL
CCLE
GDSC
% Viability
0
50
100
0.01
0.1
1
10
Concentration (uM)

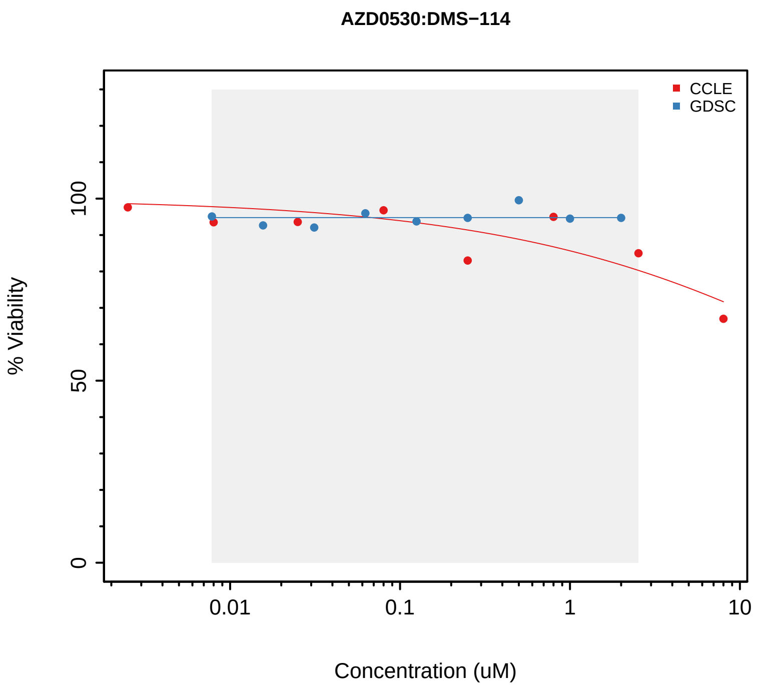
AZD0530:DMS−114
CCLE
GDSC
% Viability
0
50
100
0.01
0.1
1
10
Concentration (uM)

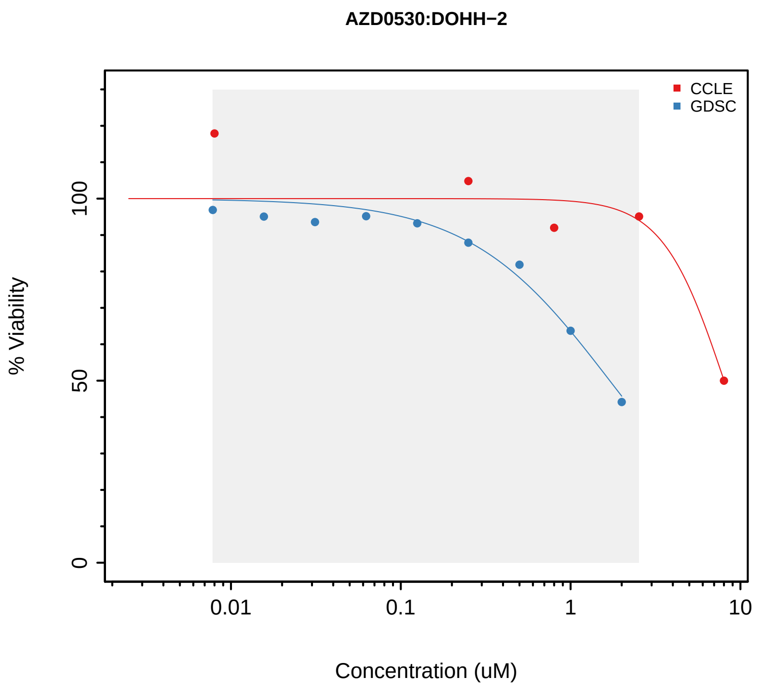
AZD0530:DOHH−2
CCLE
GDSC
% Viability
0
50
100
0.01
0.1
1
10
Concentration (uM)

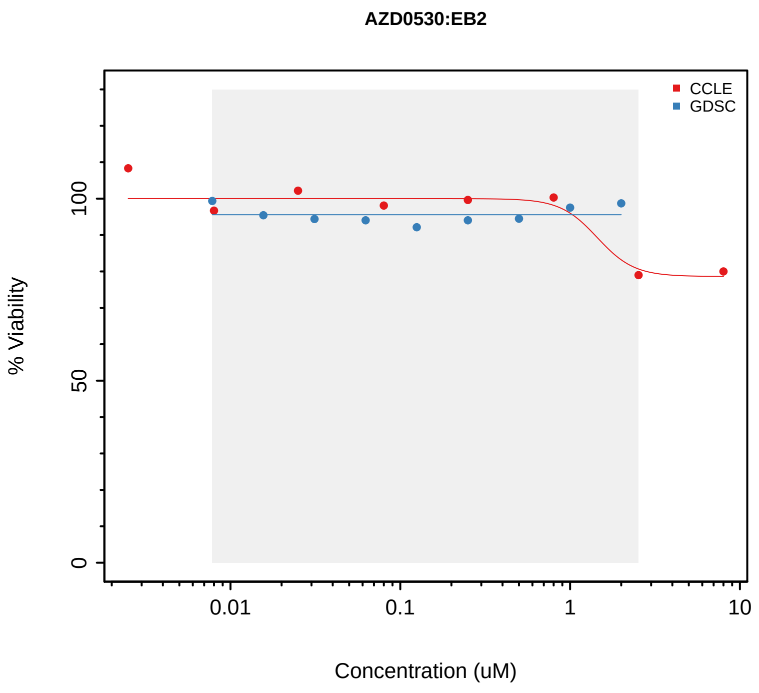
AZD0530:EB2
CCLE
GDSC
% Viability
0
50
100
0.01
0.1
1
10
Concentration (uM)

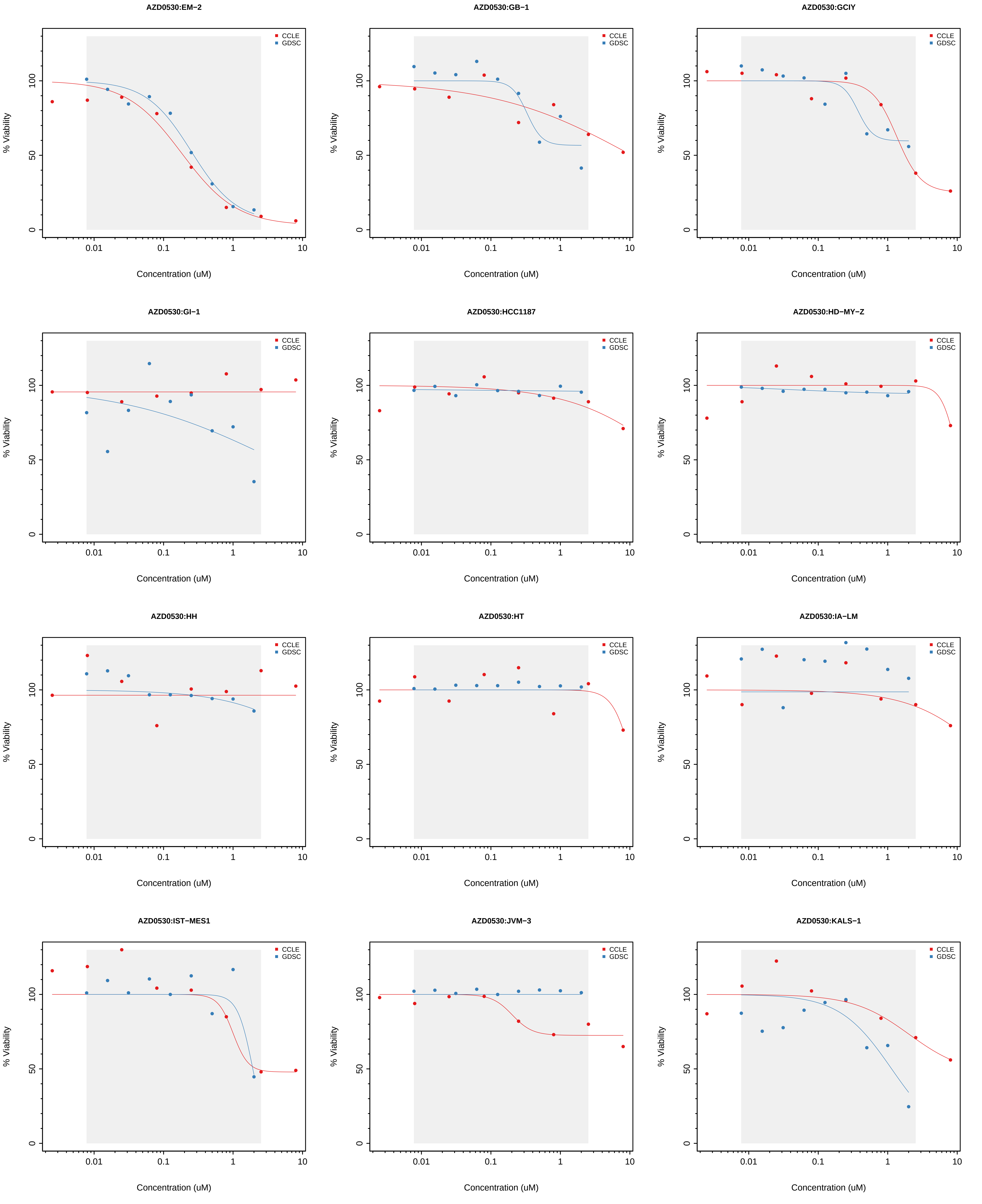

AZD0530:EM−2
AZD0530:GB−1
AZD0530:GCIY
AZD0530:GI−1
AZD0530:HCC1187
AZD0530:HD−MY−Z
AZD0530:HH
AZD0530:HT
AZD0530:IA−LM
AZD0530:IST−MES1
AZD0530:JVM−3
AZD0530:KALS−1
CCLE
GDSC
% Viability
0
50
100
0.01
0.1
1
10
Concentration (uM)

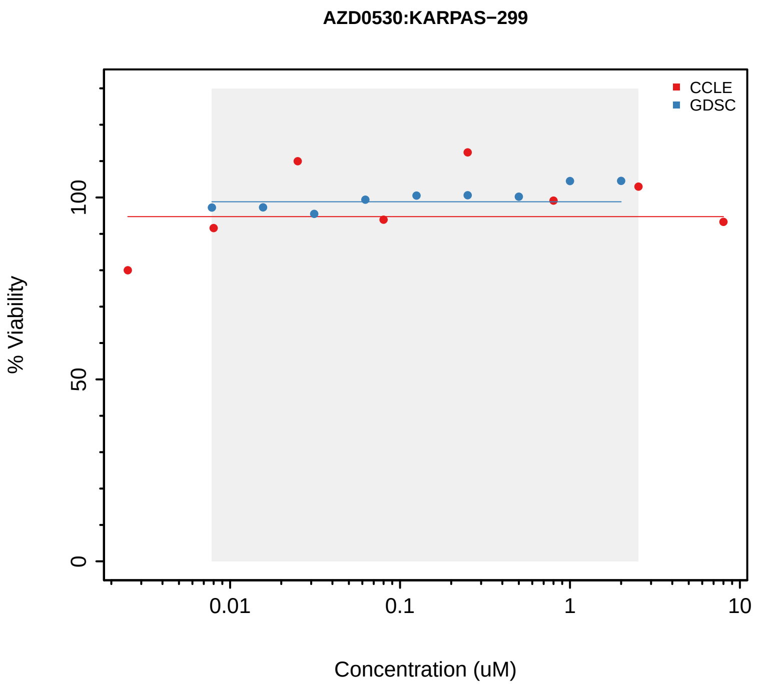
AZD0530:KARPAS−299
CCLE
GDSC
% Viability
0
50
100
0.01
0.1
1
10
Concentration (uM)

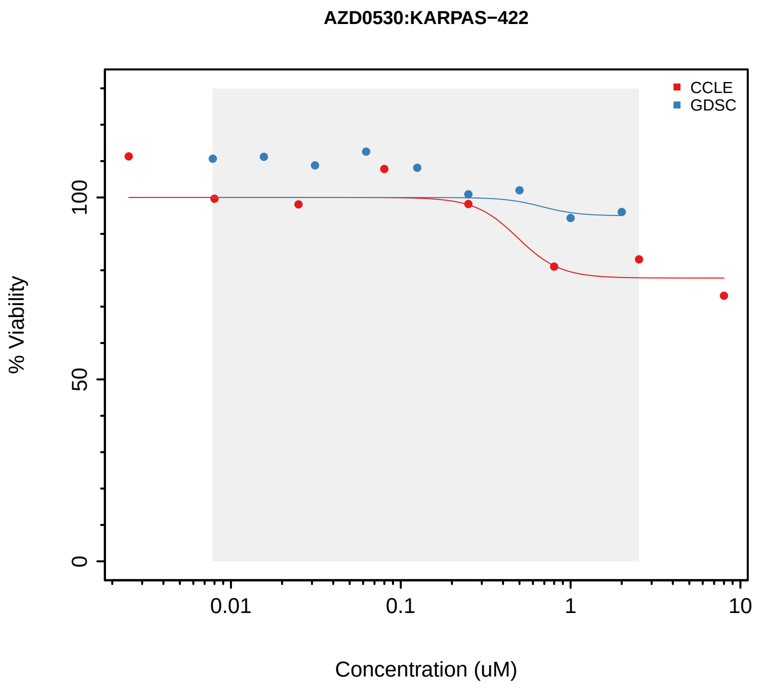
AZD0530:KARPAS−422
CCLE
GDSC
% Viability
0
50
100
0.01
0.1
1
10
Concentration (uM)

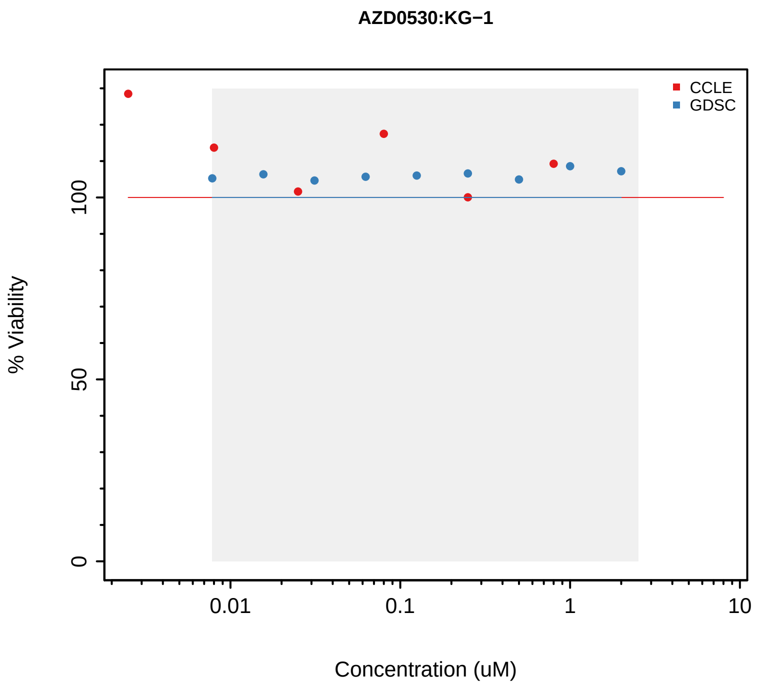
AZD0530:KG−1
CCLE
GDSC
% Viability
0
50
100
0.01
0.1
1
10
Concentration (uM)

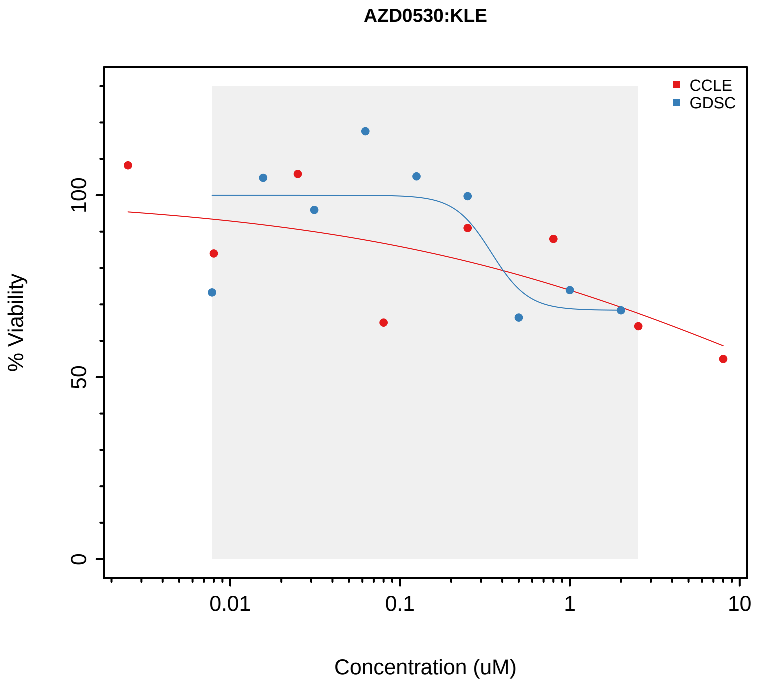
AZD0530:KLE
CCLE
GDSC
% Viability
0
50
100
0.01
0.1
1
10
Concentration (uM)

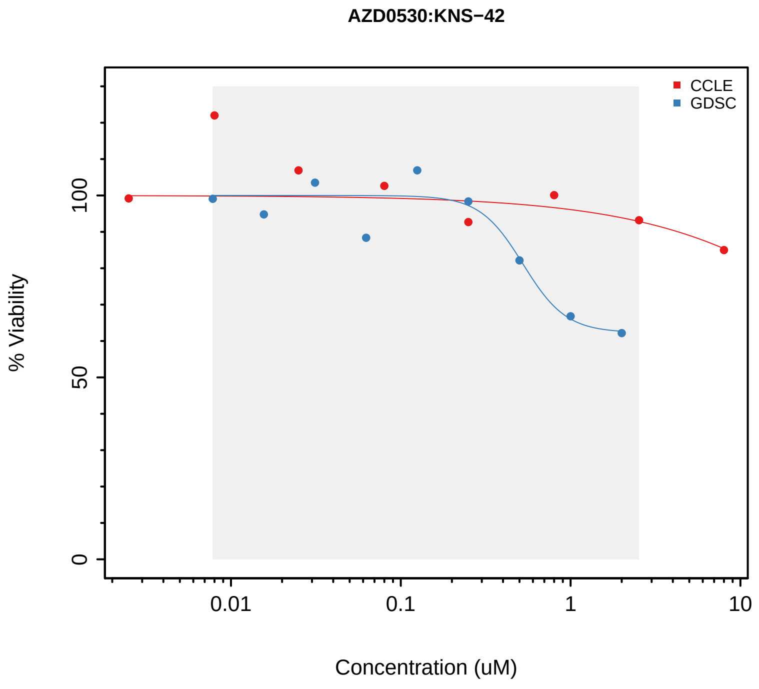
AZD0530:KNS−42
CCLE
GDSC
% Viability
0
50
100
0.01
0.1
1
10
Concentration (uM)

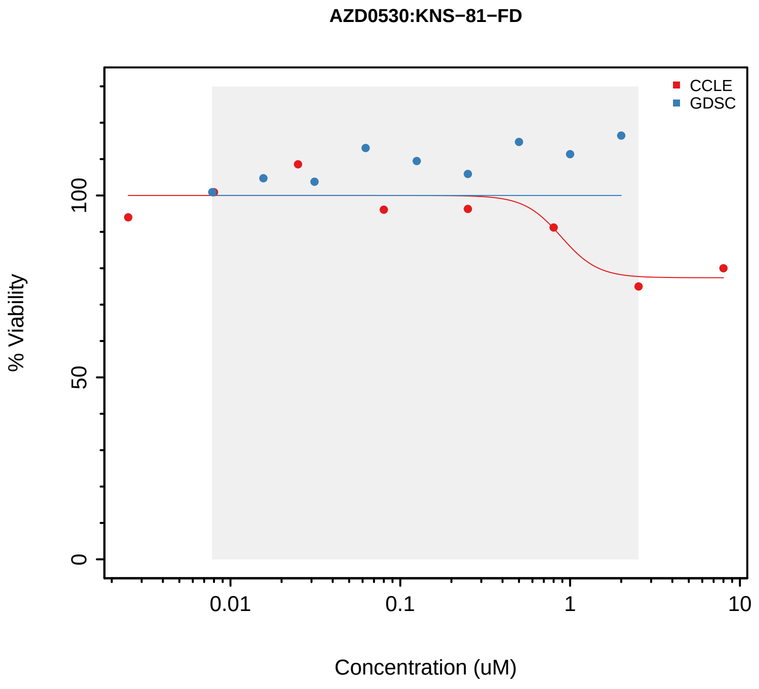
AZD0530:KNS−81−FD
CCLE
GDSC
% Viability
0
50
100
0.01
0.1
1
10
Concentration (uM)

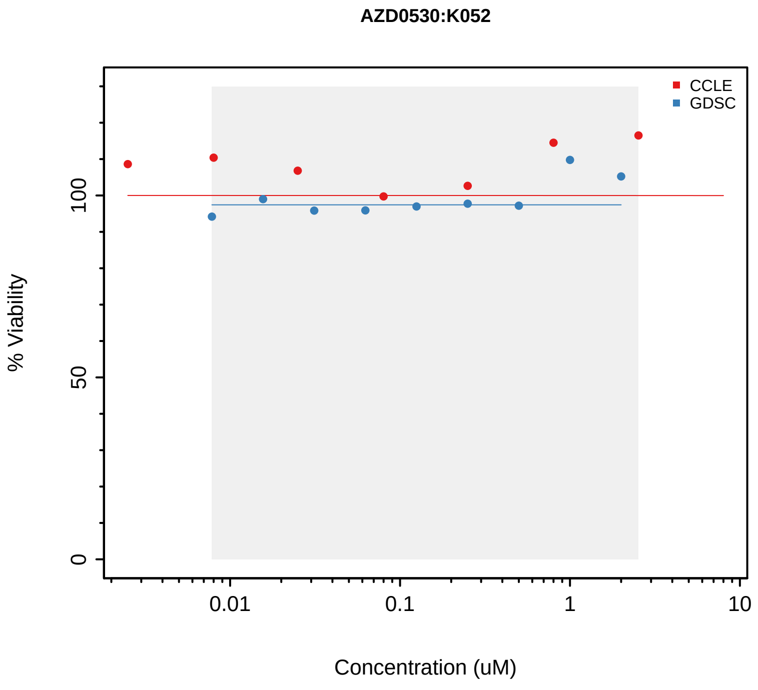
AZD0530:K052
CCLE
GDSC
% Viability
0
50
100
0.01
0.1
1
10
Concentration (uM)

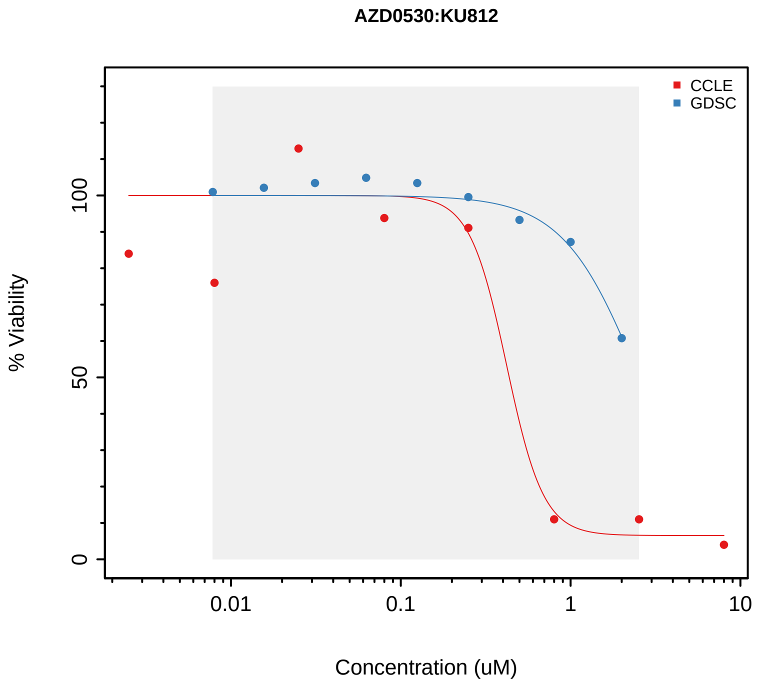
AZD0530:KU812
CCLE
GDSC
% Viability
0
50
100
0.01
0.1
1
10
Concentration (uM)

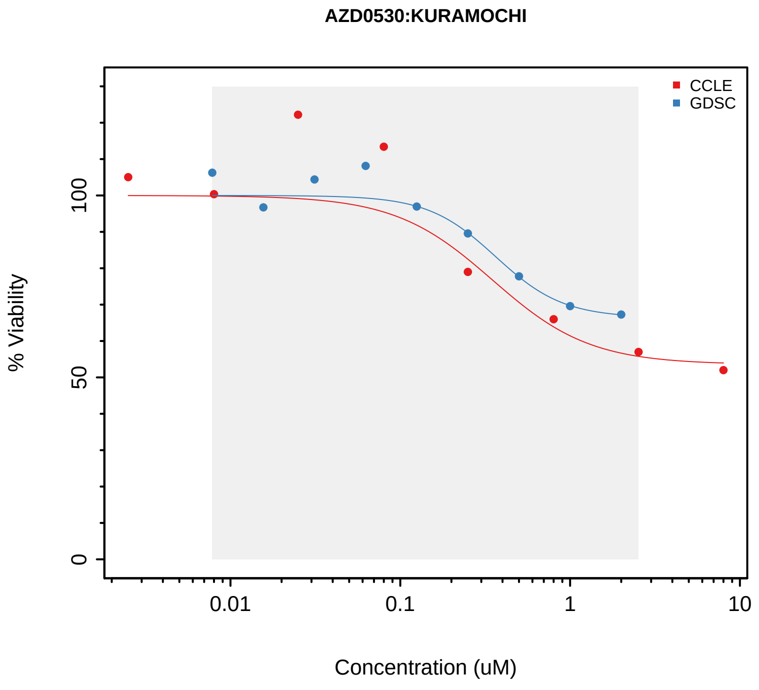
AZD0530:KURAMOCHI
CCLE
GDSC
% Viability
0
50
100
0.01
0.1
1
10
Concentration (uM)

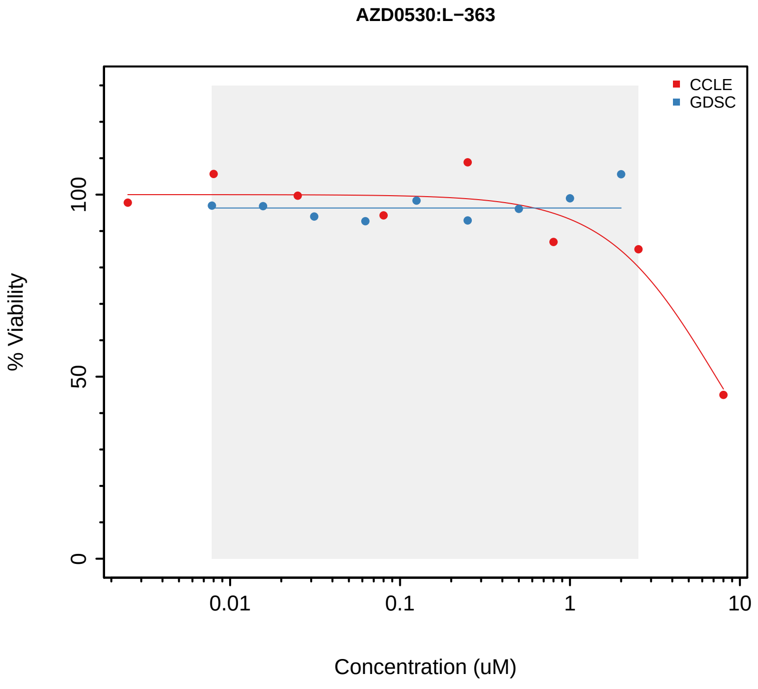
AZD0530:L−363
CCLE
GDSC
% Viability
0
50
100
0.01
0.1
1
10
Concentration (uM)

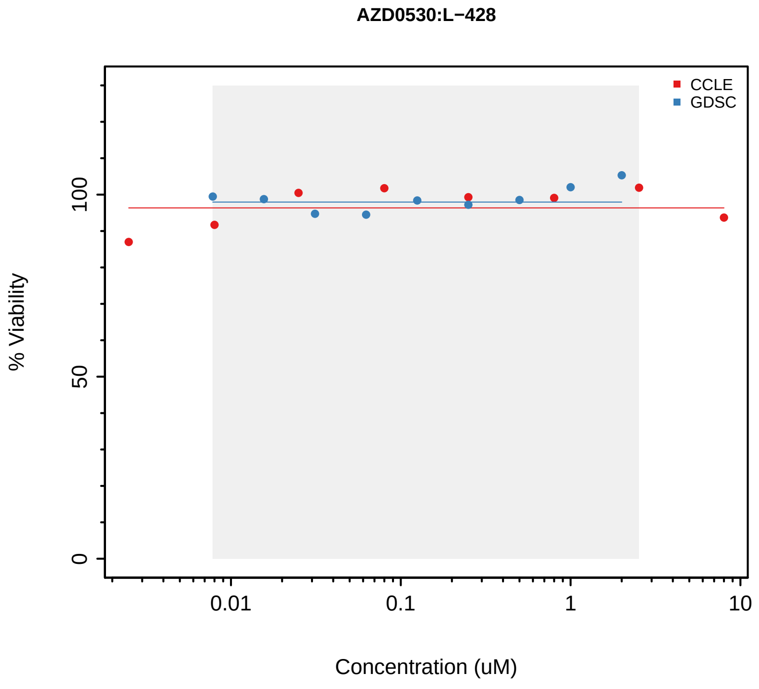
AZD0530:L−428
CCLE
GDSC
% Viability
0
50
100
0.01
0.1
1
10
Concentration (uM)

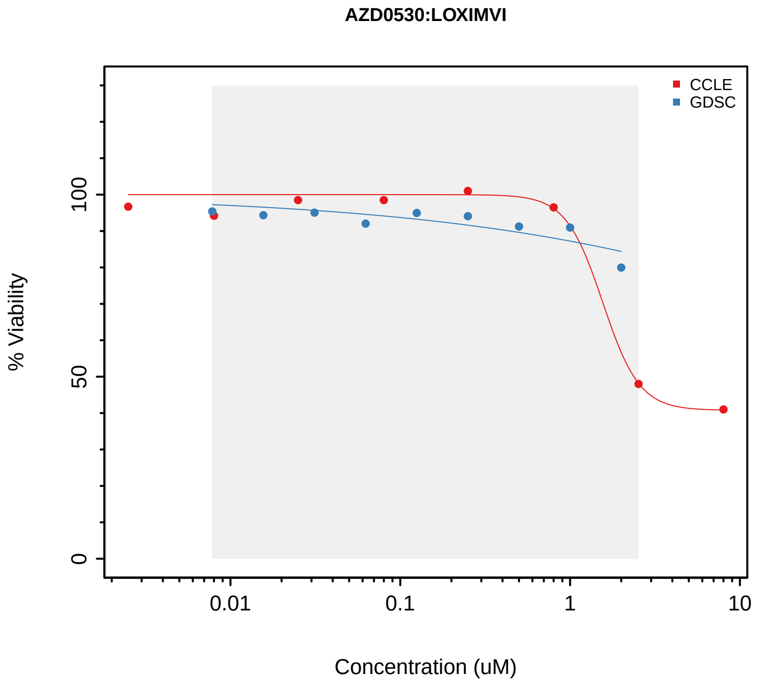
AZD0530:LOXIMVI
CCLE
GDSC
% Viability
0
50
100
0.01
0.1
1
10
Concentration (uM)

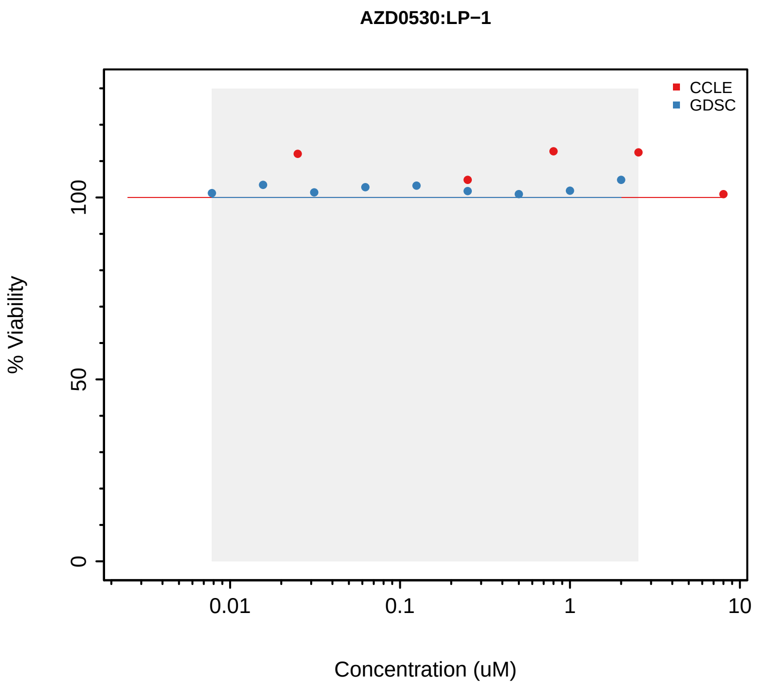
AZD0530:LP−1
CCLE
GDSC
% Viability
0
50
100
0.01
0.1
1
10
Concentration (uM)

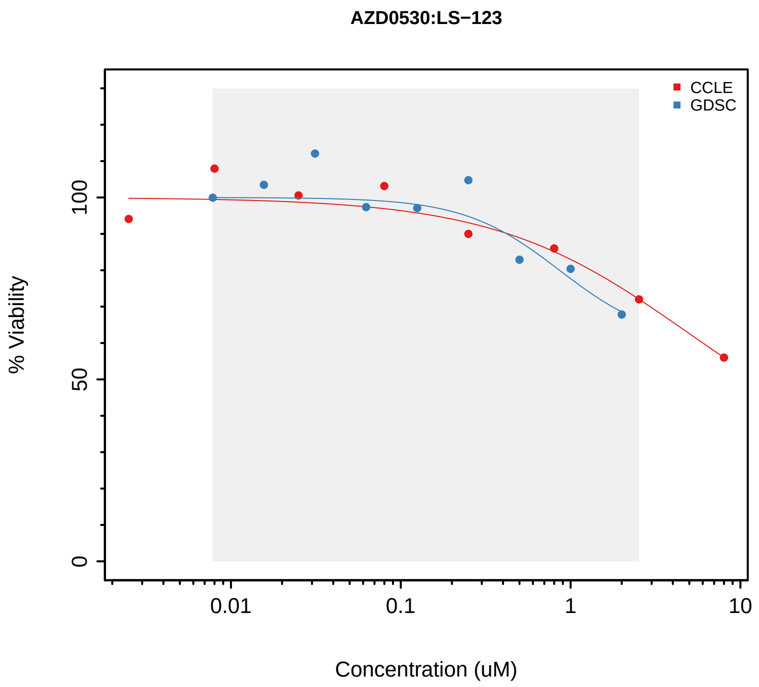
AZD0530:LS−123
CCLE
GDSC
% Viability
0
50
100
0.01
0.1
1
10
Concentration (uM)

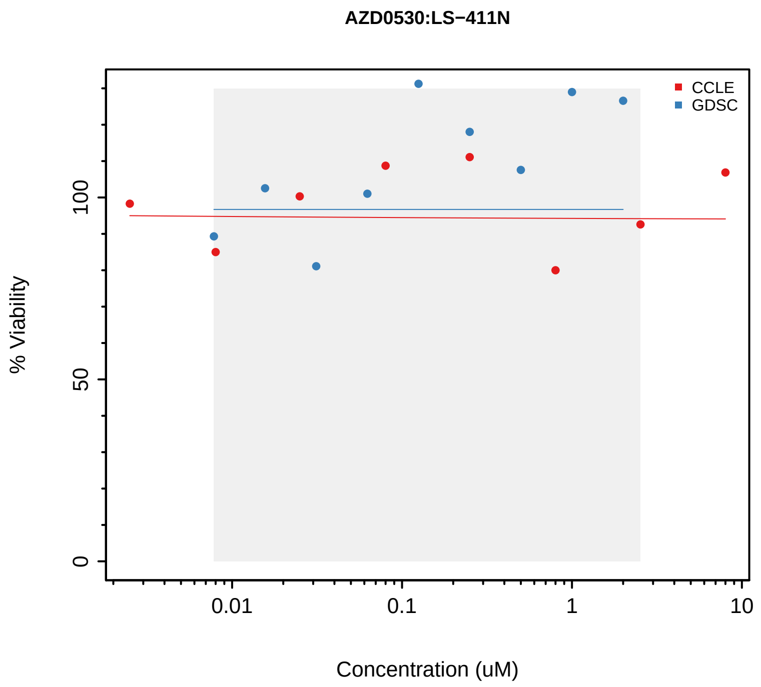
AZD0530:LS−411N
CCLE
GDSC
% Viability
0
50
100
0.01
0.1
1
10
Concentration (uM)

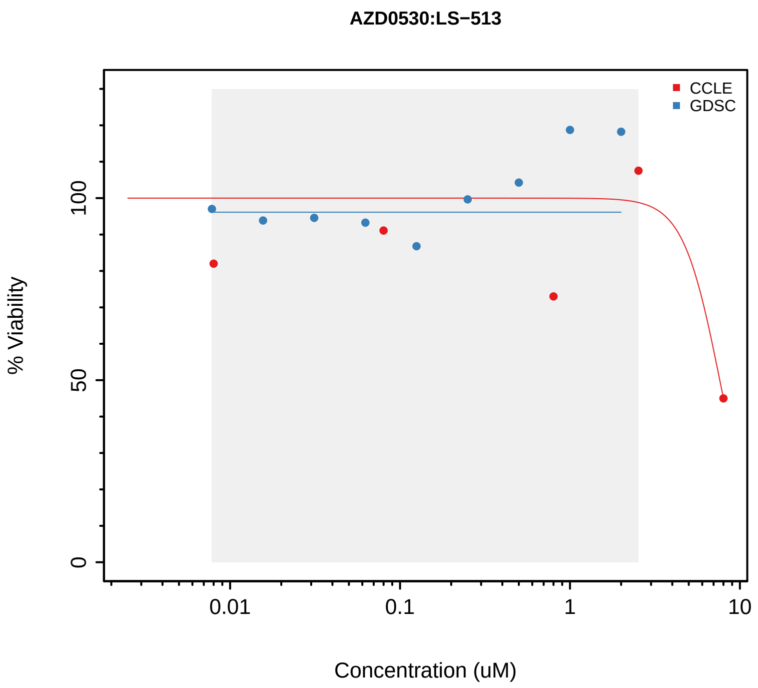
AZD0530:LS−513
CCLE
GDSC
% Viability
0
50
100
0.01
0.1
1
10
Concentration (uM)

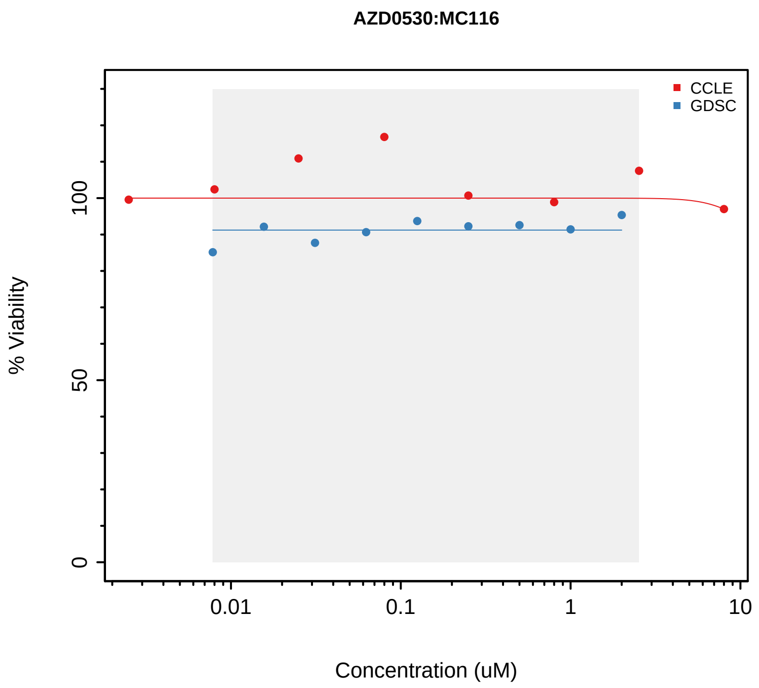
AZD0530:MC116
CCLE
GDSC
% Viability
0
50
100
0.01
0.1
1
10
Concentration (uM)

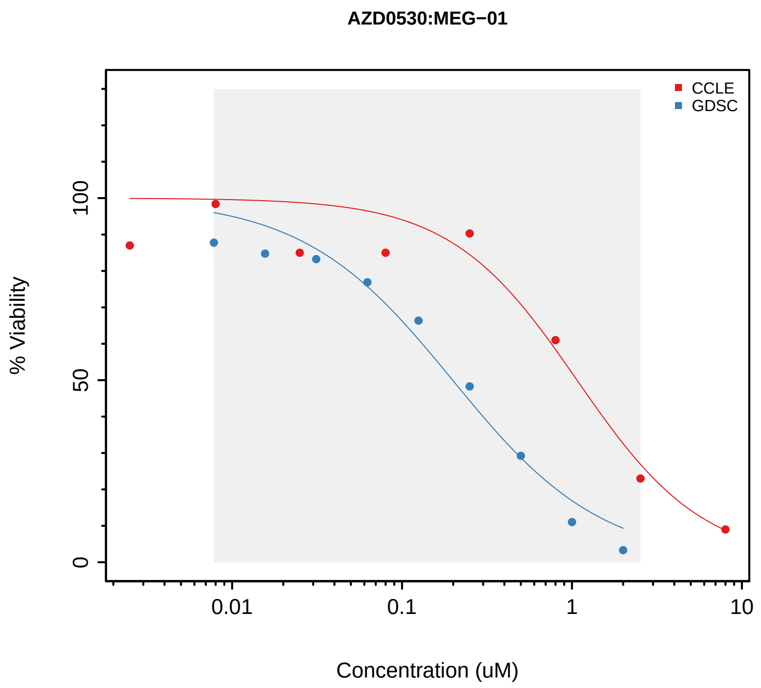
AZD0530:MEG−01
CCLE
GDSC
% Viability
0
50
100
0.01
0.1
1
10
Concentration (uM)

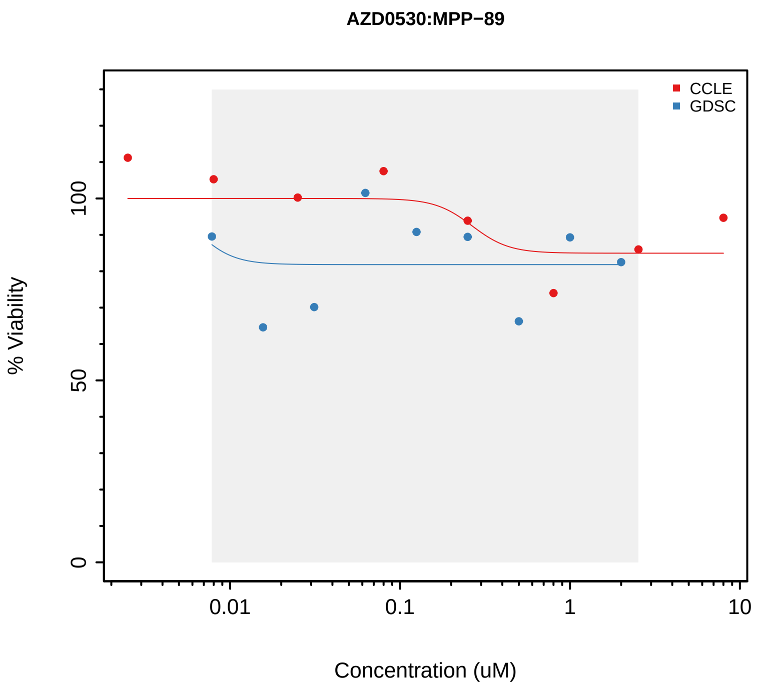
AZD0530:MPP−89
CCLE
GDSC
% Viability
0
50
100
0.01
0.1
1
10
Concentration (uM)

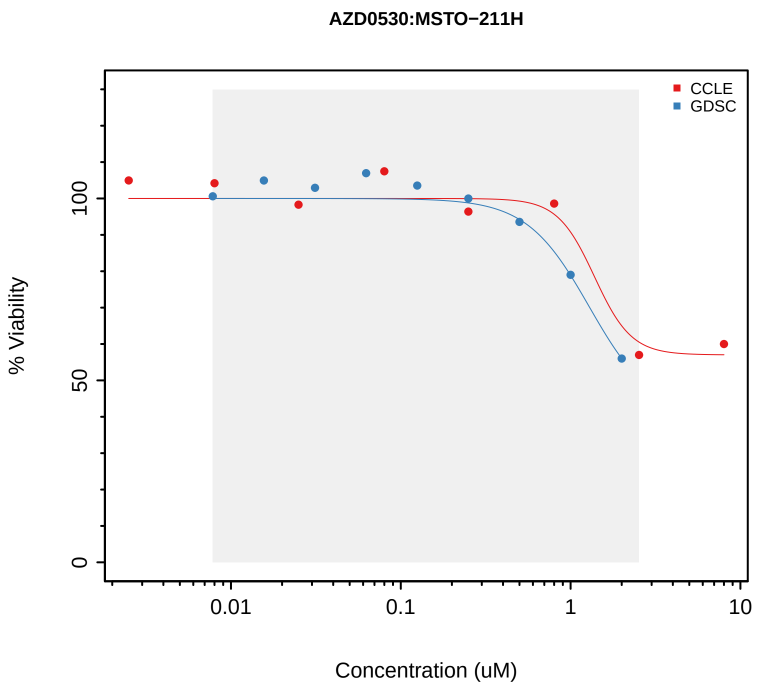
AZD0530:MSTO−211H
CCLE
GDSC
% Viability
0
50
100
0.01
0.1
1
10
Concentration (uM)

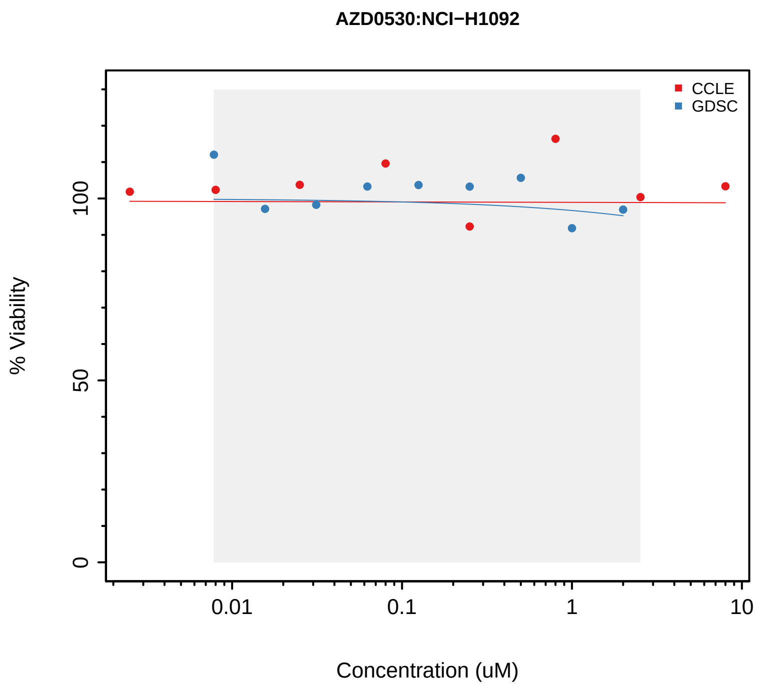
AZD0530:NCI−H1092
CCLE
GDSC
% Viability
0
50
100
0.01
0.1
1
10
Concentration (uM)

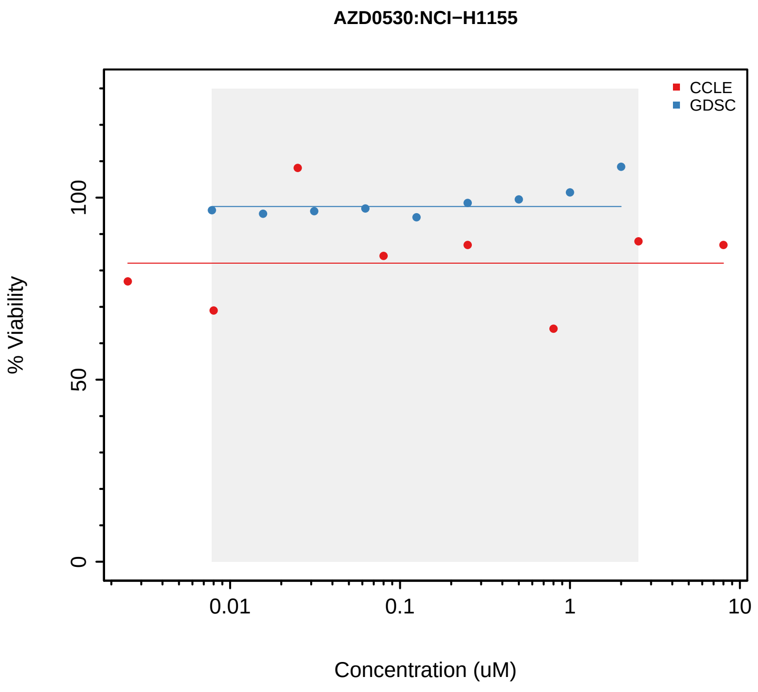
AZD0530:NCI−H1155
CCLE
GDSC
% Viability
0
50
100
0.01
0.1
1
10
Concentration (uM)

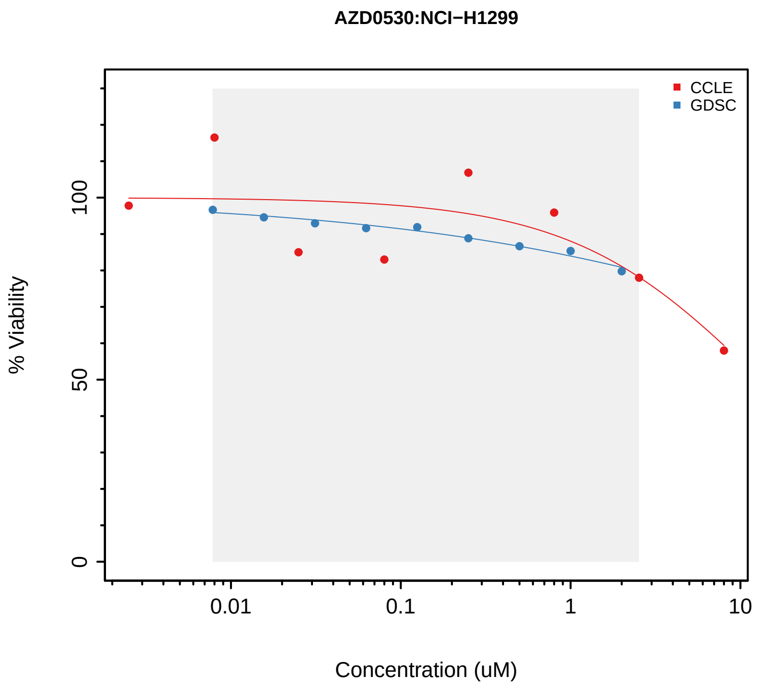
AZD0530:NCI−H1299
CCLE
GDSC
% Viability
0
50
100
0.01
0.1
1
10
Concentration (uM)

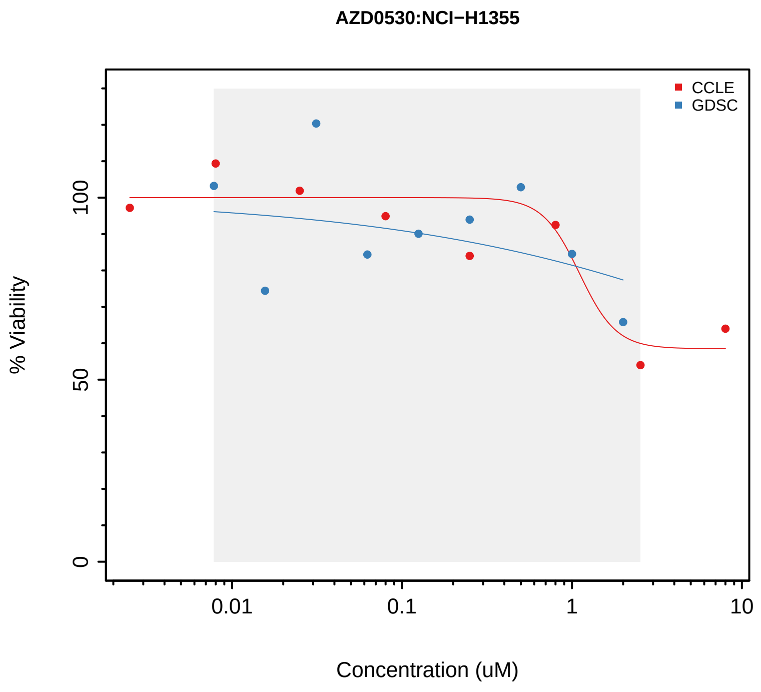
AZD0530:NCI−H1355
CCLE
GDSC
% Viability
0
50
100
0.01
0.1
1
10
Concentration (uM)

AZD0530:NCI−H1581

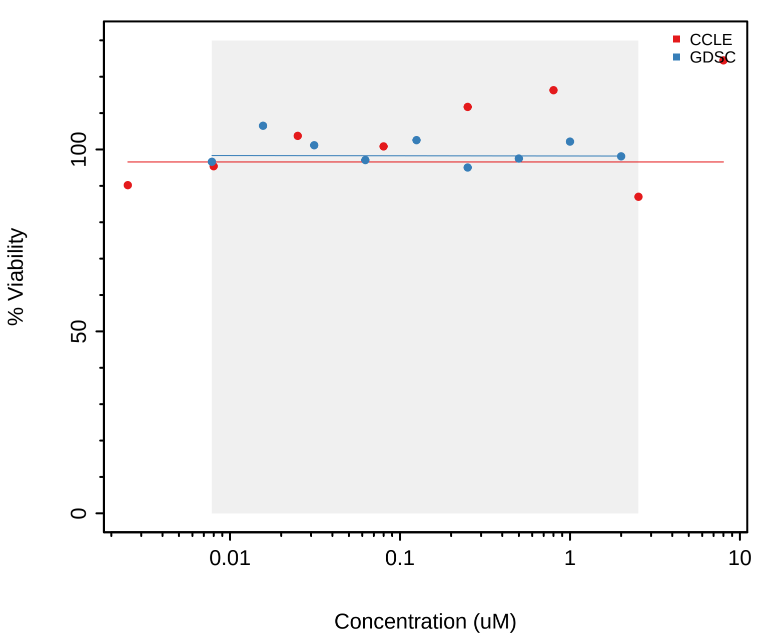

AZD0530:NCI−H1648

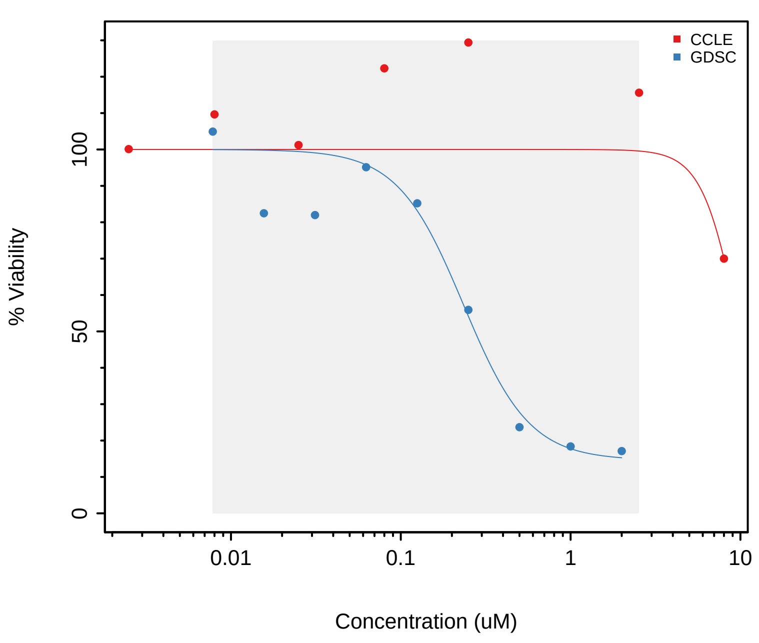

AZD0530:NCI−H1666

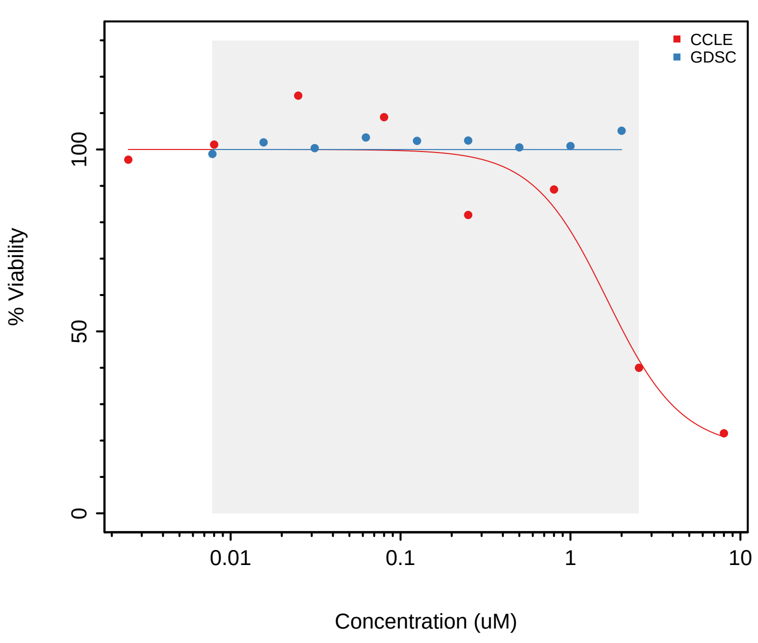

AZD0530:NCI−H1694

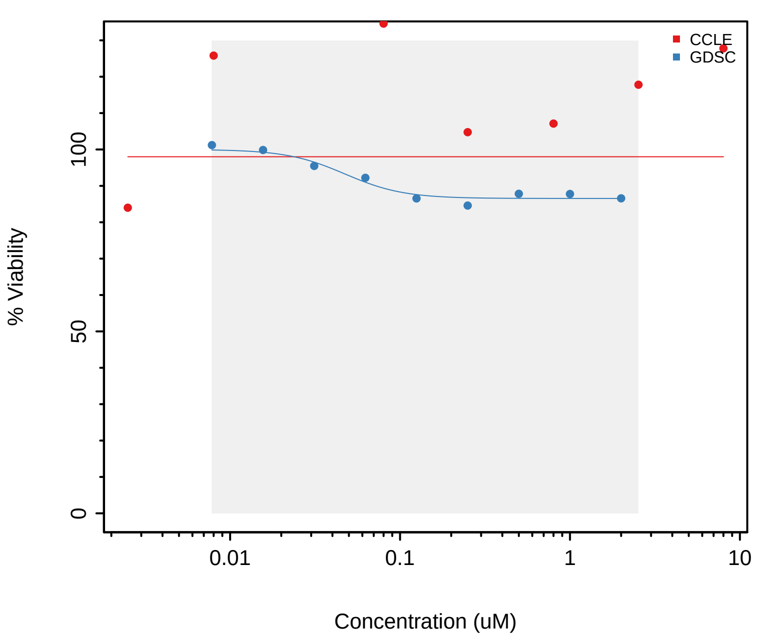

AZD0530:NCI−H1975

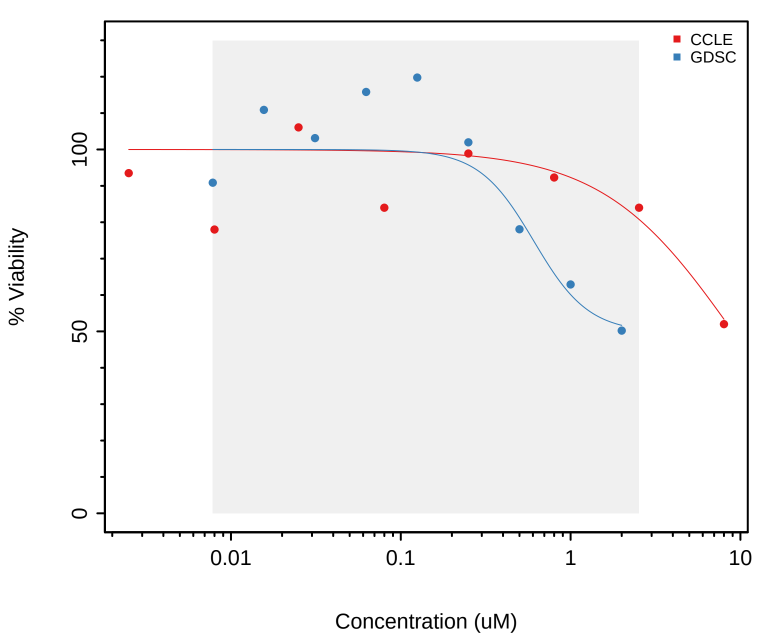

AZD0530:NCI−H226

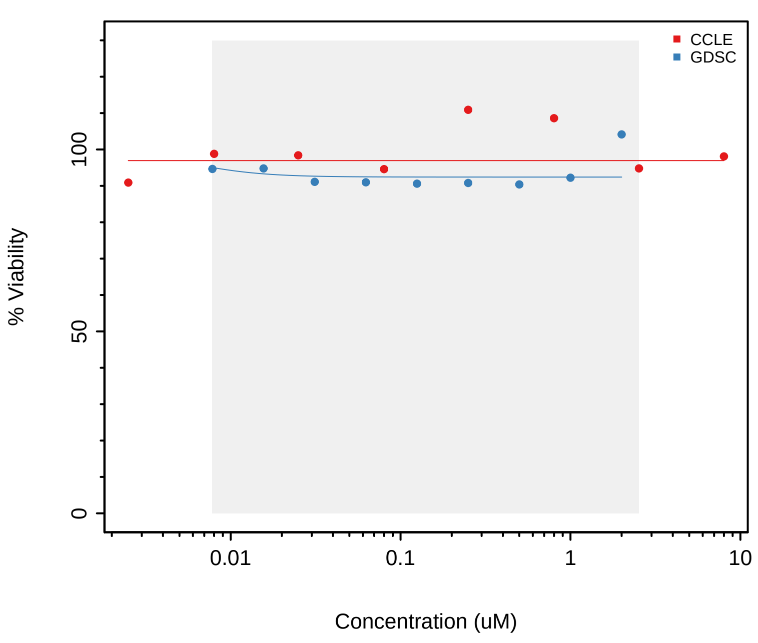

AZD0530:NCI−H23

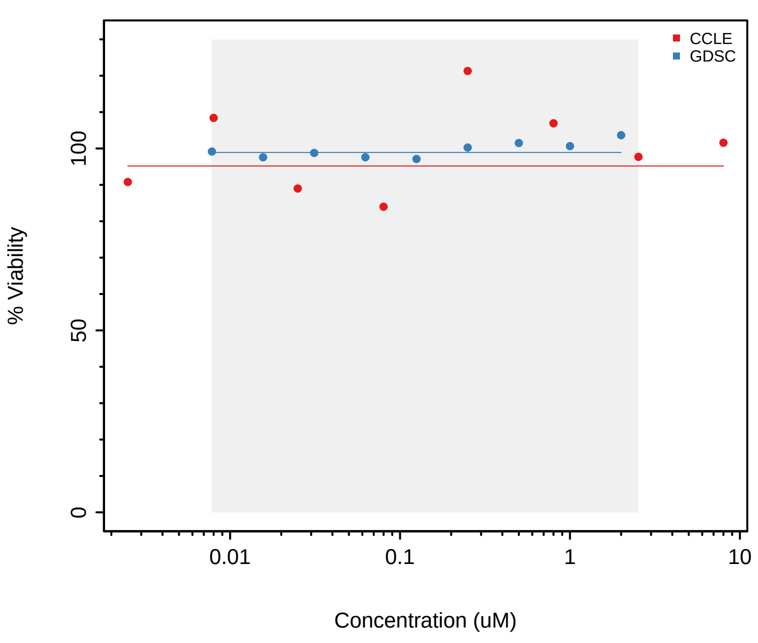

AZD0530:NCI−H747

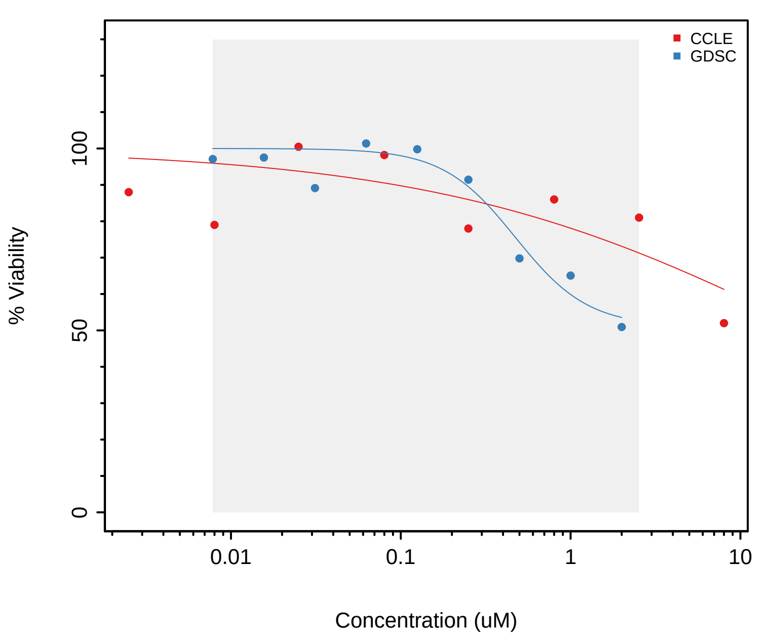

AZD0530:OCI−AML2

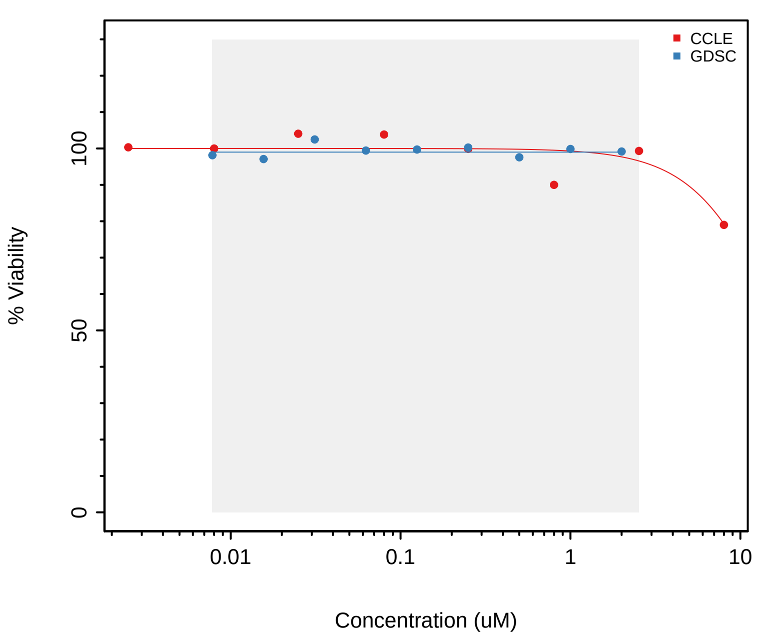

AZD0530:ONS−76

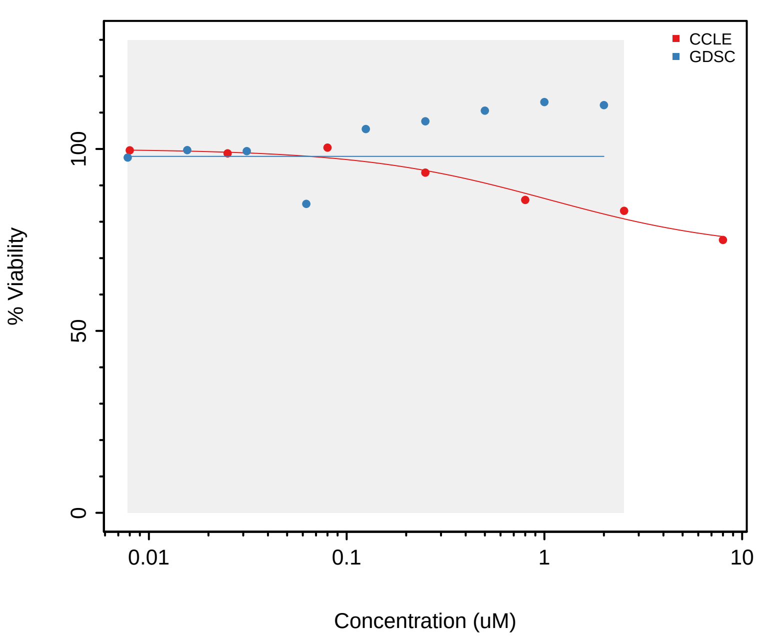

AZD0530:OPM−2

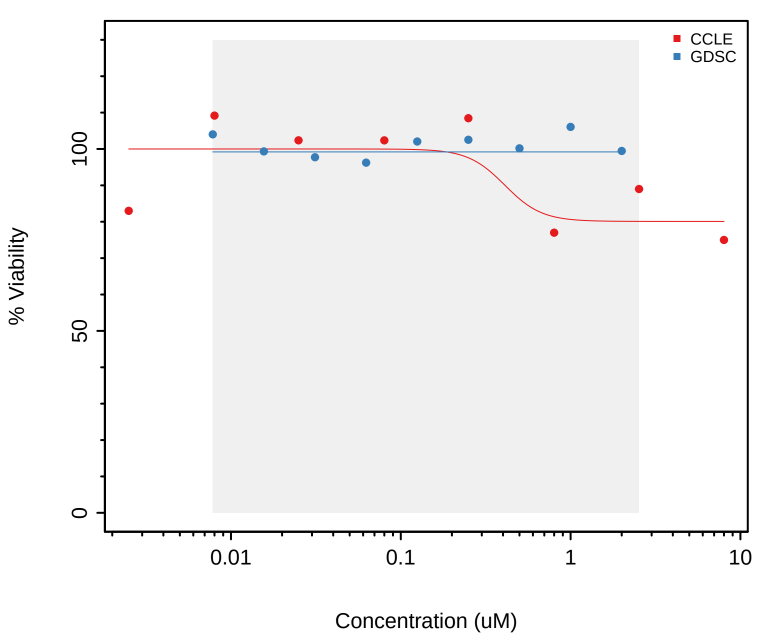

AZD0530:OVCAR−4

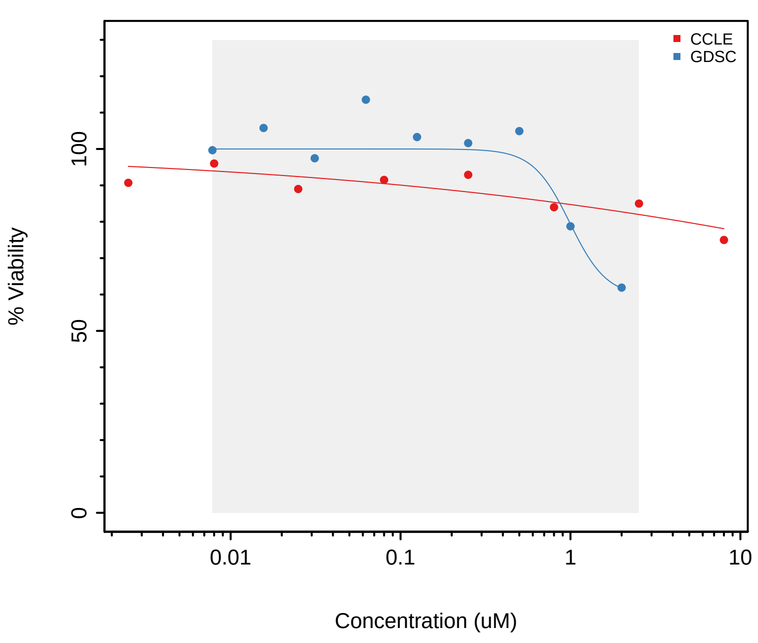

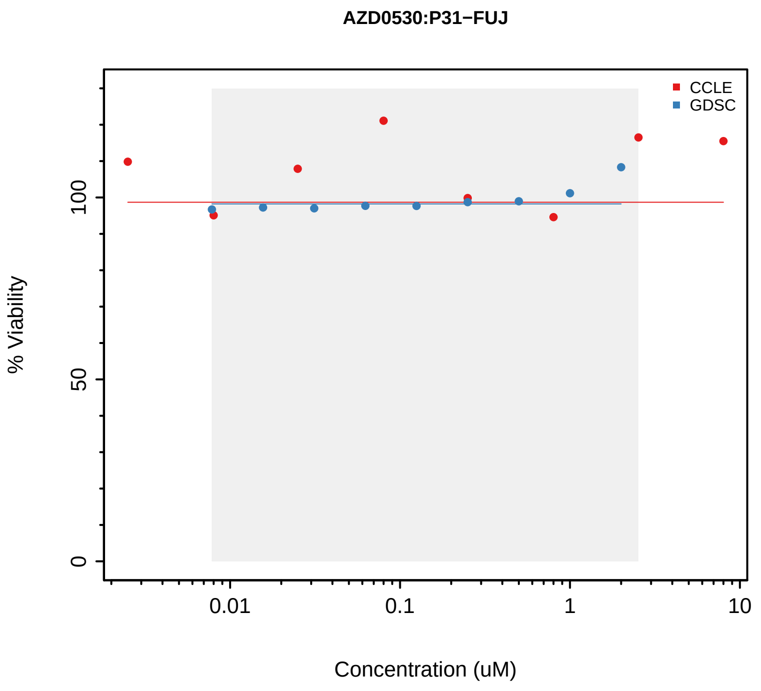
AZD0530:P31−FUJ
CCLE
GDSC
% Viability
0
50
100
0.01
0.1
1
10
Concentration (uM)

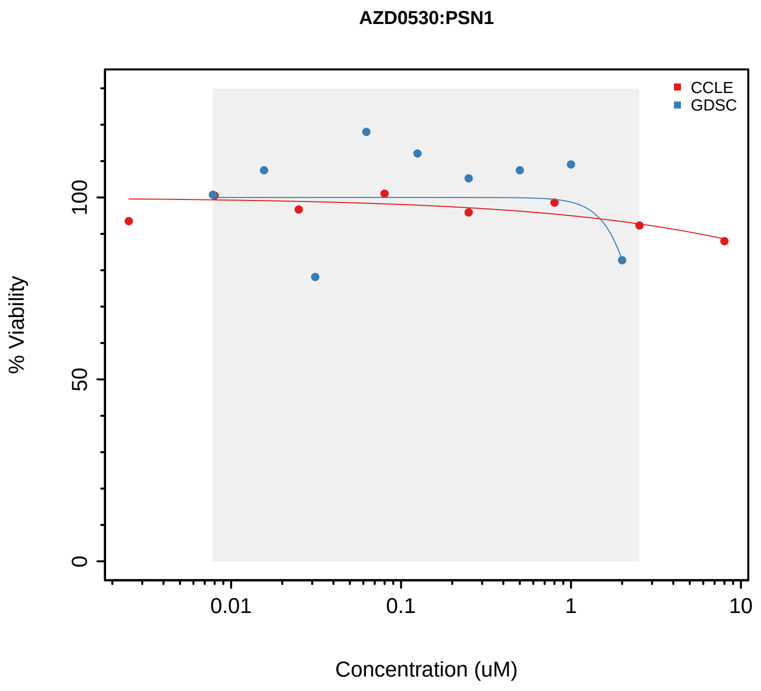
AZD0530:PSN1
CCLE
GDSC
% Viability
0
50
100
0.01
0.1
1
10
Concentration (uM)

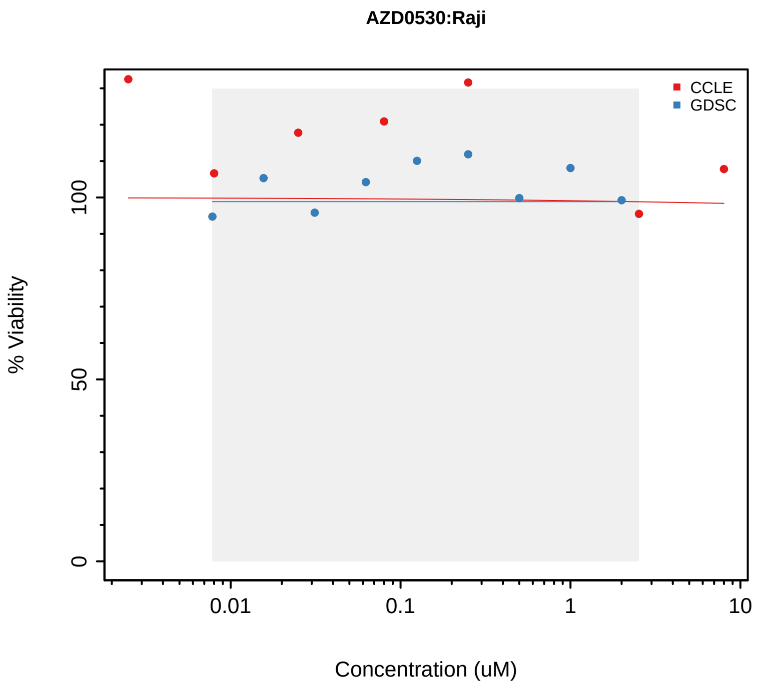
AZD0530:Raji
CCLE
GDSC
% Viability
0
50
100
0.01
0.1
1
10
Concentration (uM)

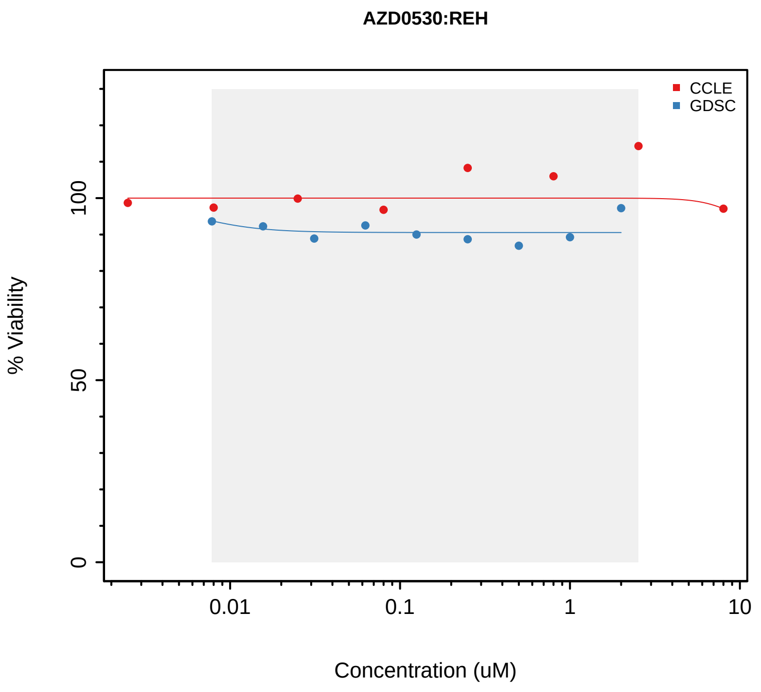
AZD0530:REH
CCLE
GDSC
% Viability
0
50
100
0.01
0.1
1
10
Concentration (uM)

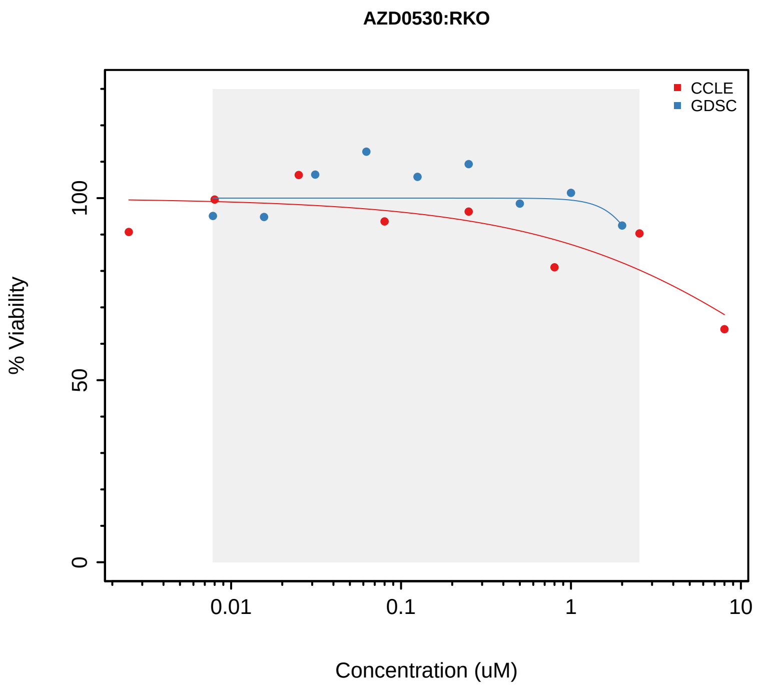
AZD0530:RKO
CCLE
GDSC
% Viability
0
50
100
0.01
0.1
1
10
Concentration (uM)

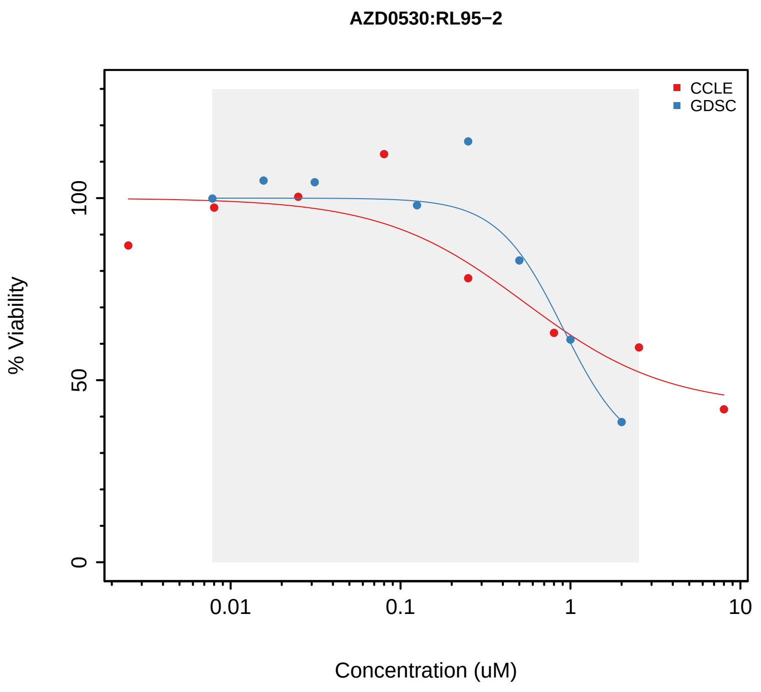
AZD0530:RL95−2
CCLE
GDSC
% Viability
0
50
100
0.01
0.1
1
10
Concentration (uM)

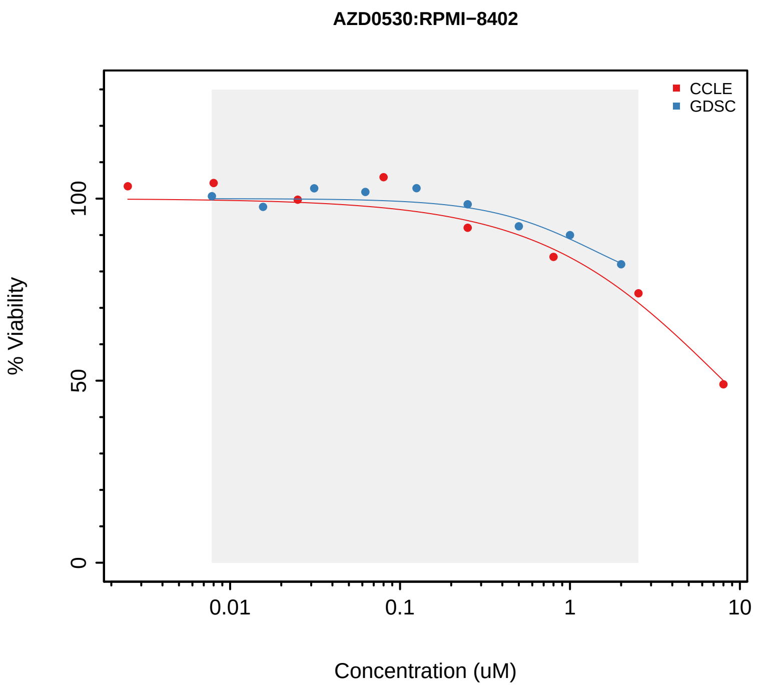
AZD0530:RPMI−8402
CCLE
GDSC
% Viability
0
50
100
0.01
0.1
1
10
Concentration (uM)

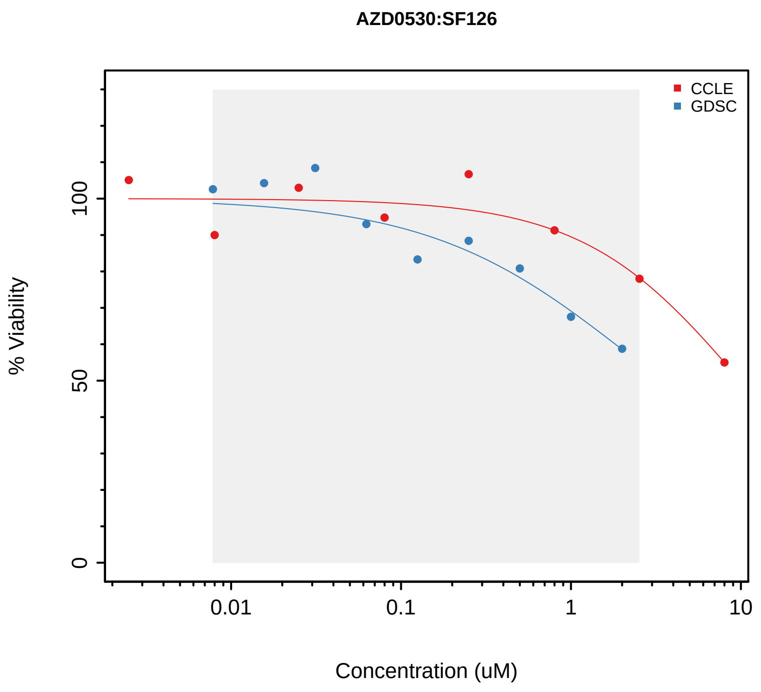
AZD0530:SF126
CCLE
GDSC
% Viability
0
50
100
0.01
0.1
1
10
Concentration (uM)

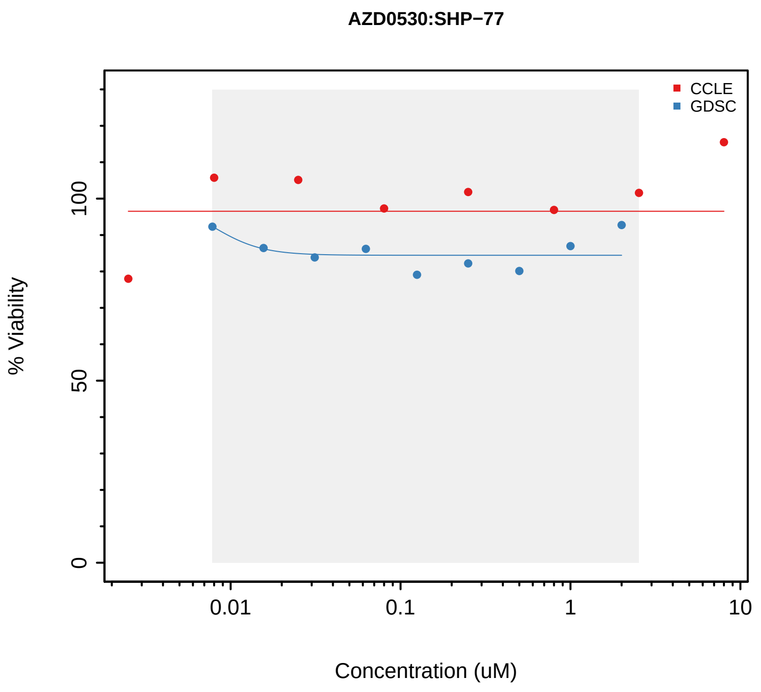
AZD0530:SHP−77
CCLE
GDSC
% Viability
0
50
100
0.01
0.1
1
10
Concentration (uM)

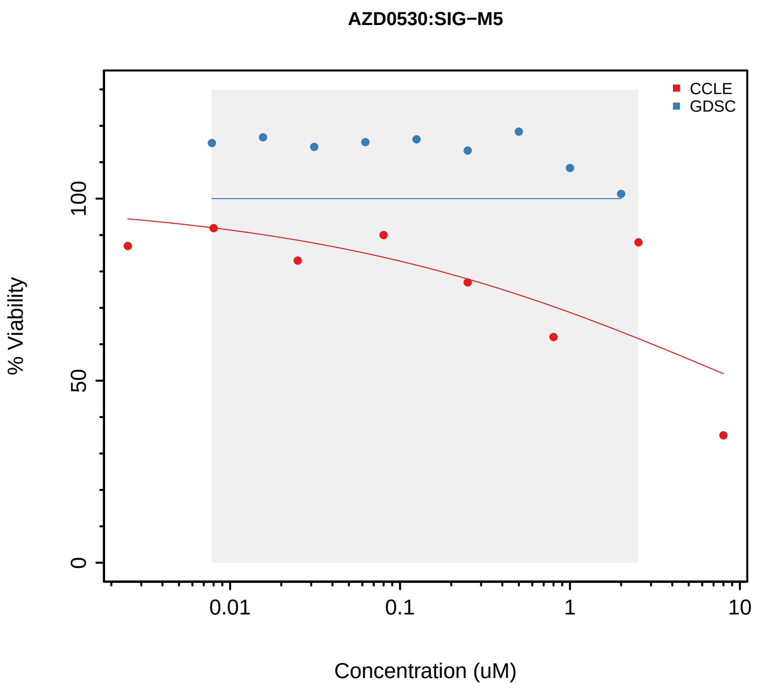
AZD0530:SIG−M5
CCLE
GDSC
% Viability
0
50
100
0.01
0.1
1
10
Concentration (uM)

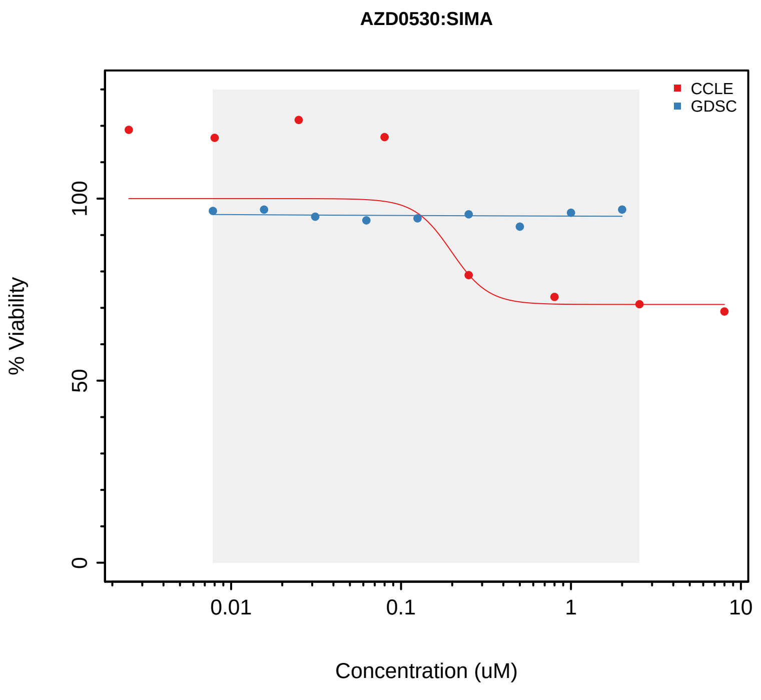
AZD0530:SIMA
CCLE
GDSC
% Viability
0
50
100
0.01
0.1
1
10
Concentration (uM)

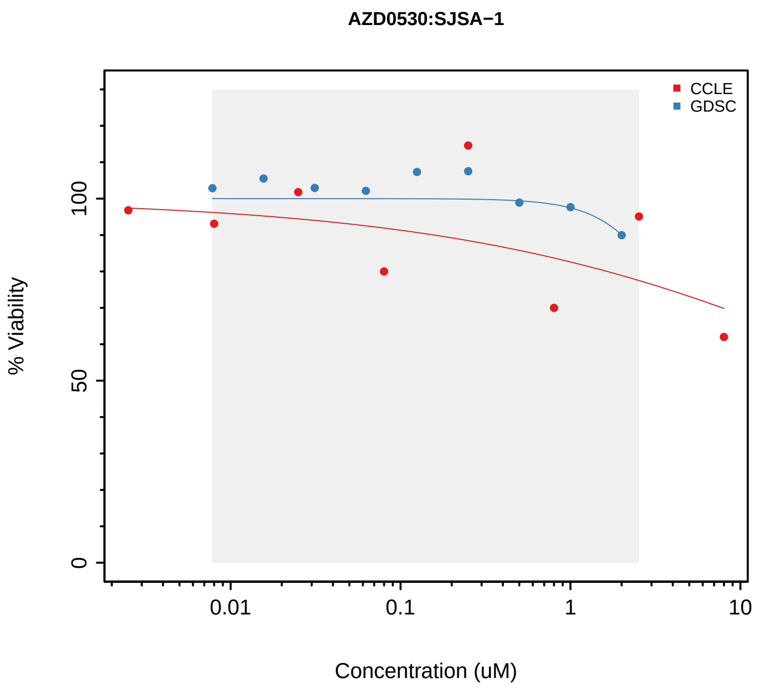
AZD0530:SJSA−1
CCLE
GDSC
% Viability
0
50
100
0.01
0.1
1
10
Concentration (uM)

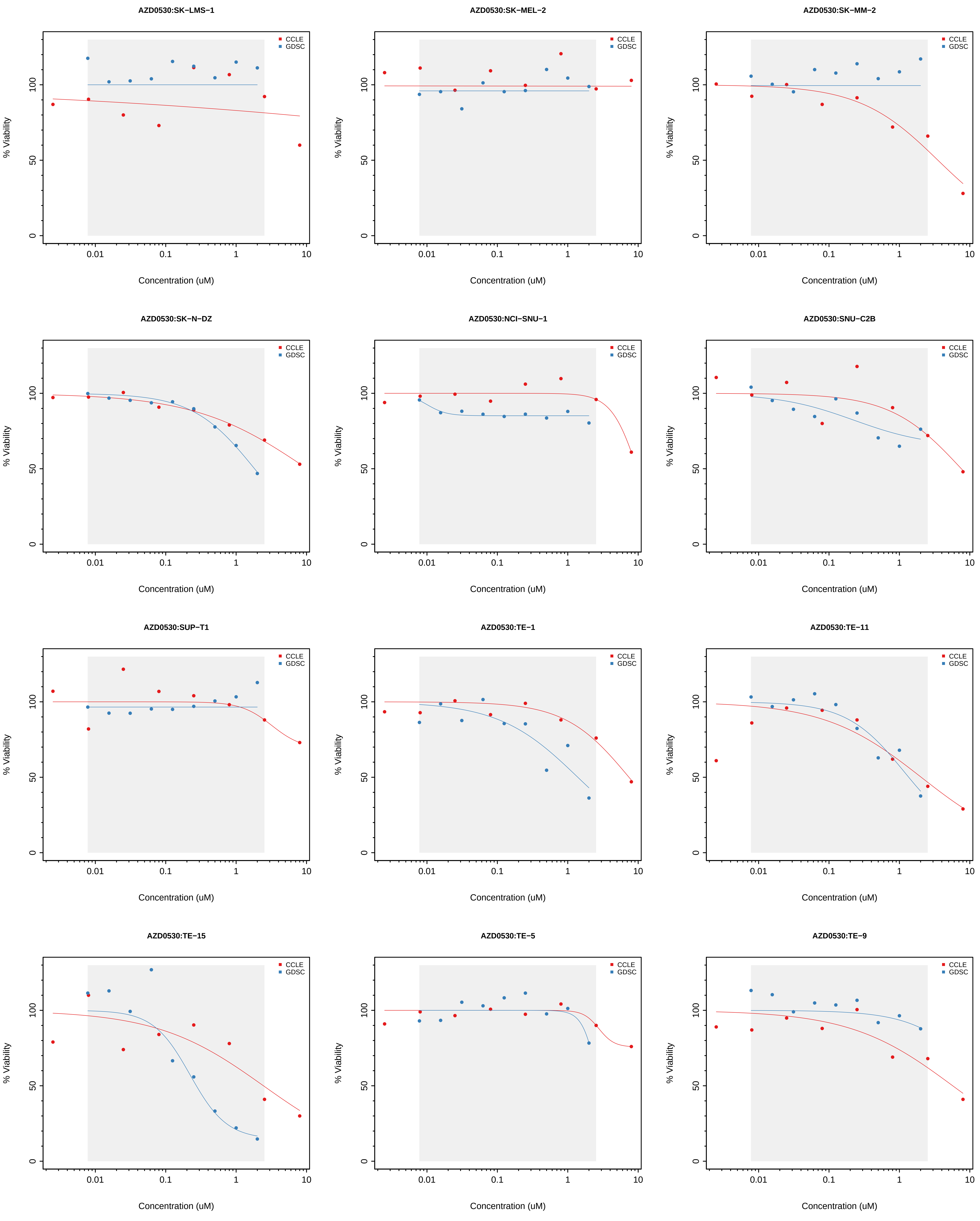
AZD0530:SK−LMS−1
AZD0530:SK−MEL−2
AZD0530:SK−MM−2
AZD0530:SK−N−DZ
AZD0530:NCI−SNU−1
AZD0530:SNU−C2B
AZD0530:SUP−T1
AZD0530:TE−1
AZD0530:TE−11
AZD0530:TE−15
AZD0530:TE−5
AZD0530:TE−9
CCLE
GDSC
% Viability
0
50
100
0.01
0.1
1
10
Concentration (uM)

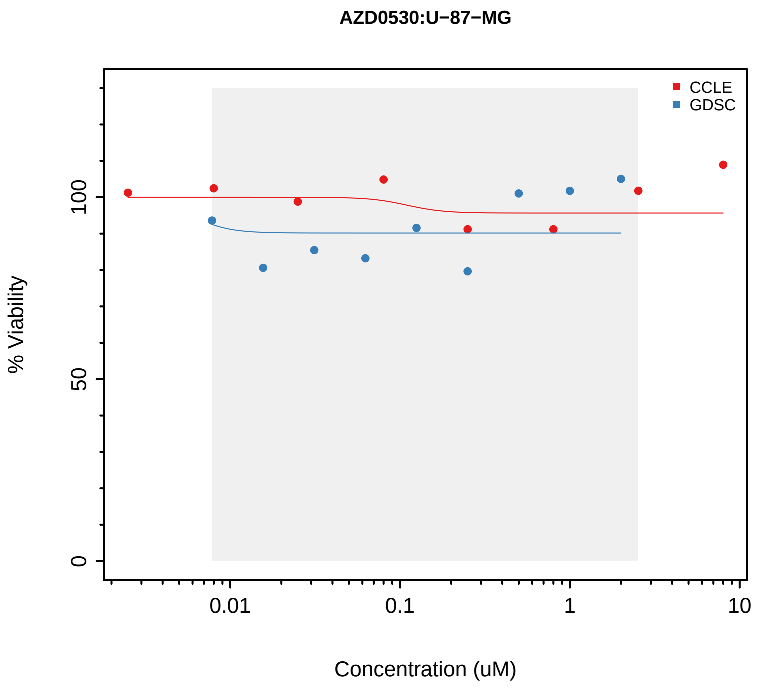
AZD0530:U−87−MG
CCLE
GDSC
% Viability
0
50
100
0.01
0.1
1
10
Concentration (uM)

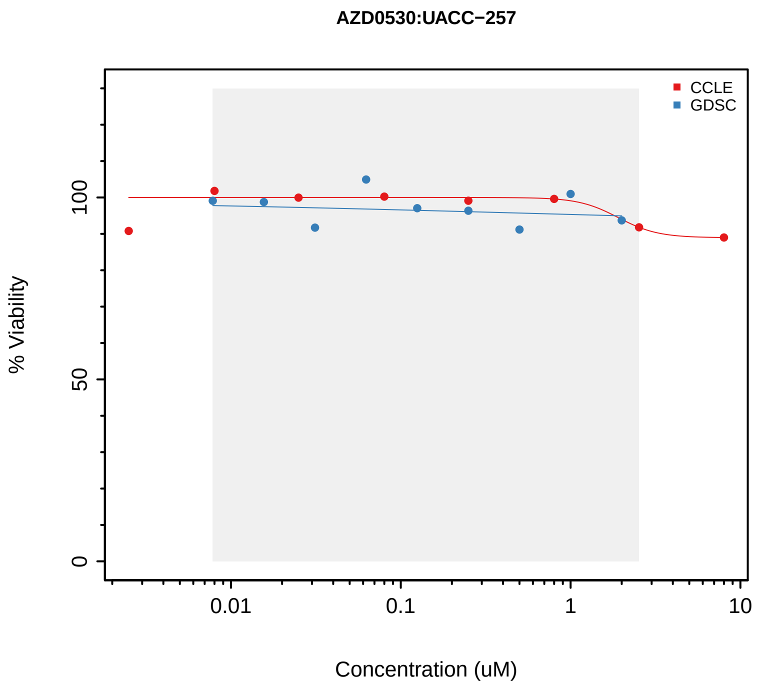
AZD0530:UACC−257
CCLE
GDSC
% Viability
0
50
100
0.01
0.1
1
10
Concentration (uM)

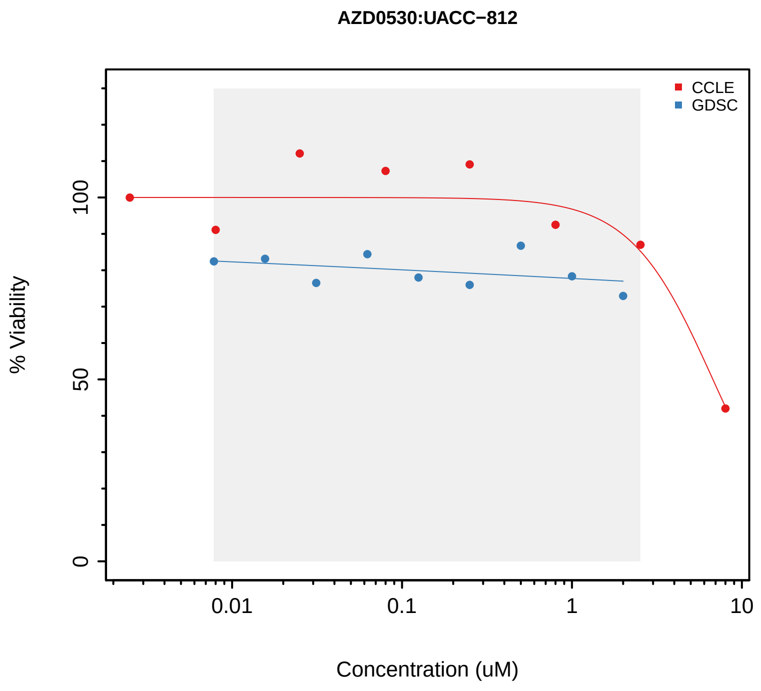
AZD0530:UACC−812
CCLE
GDSC
% Viability
0
50
100
0.01
0.1
1
10
Concentration (uM)

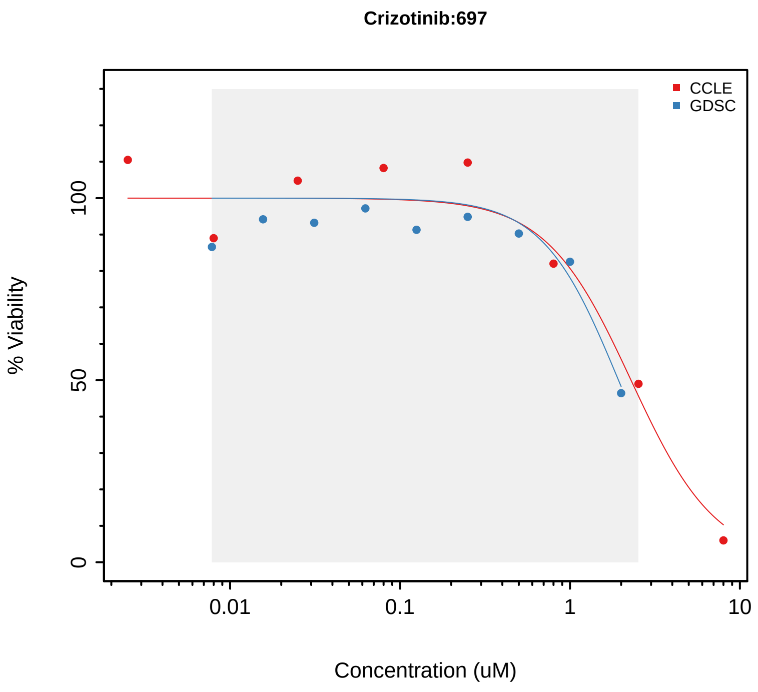
Crizotinib:697
CCLE
GDSC
% Viability
0
50
100
0.01
0.1
1
10
Concentration (uM)

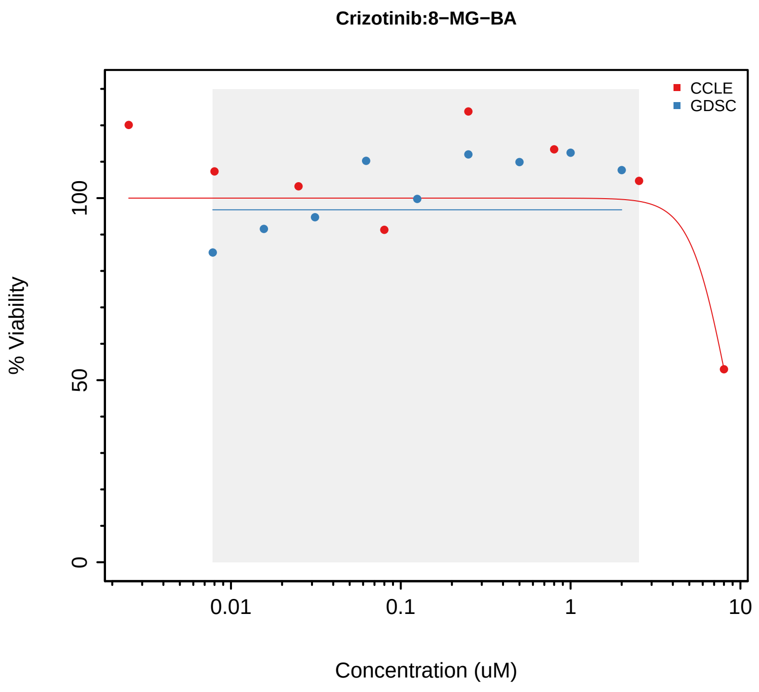
Crizotinib:8−MG−BA
CCLE
GDSC
% Viability
0
50
100
0.01
0.1
1
10
Concentration (uM)

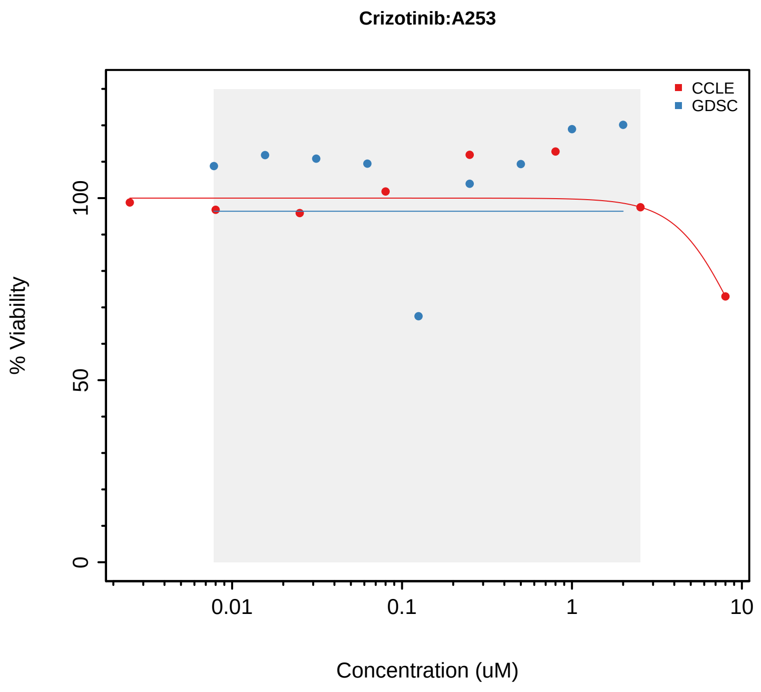
Crizotinib:A253
CCLE
GDSC
% Viability
0
50
100
0.01
0.1
1
10
Concentration (uM)

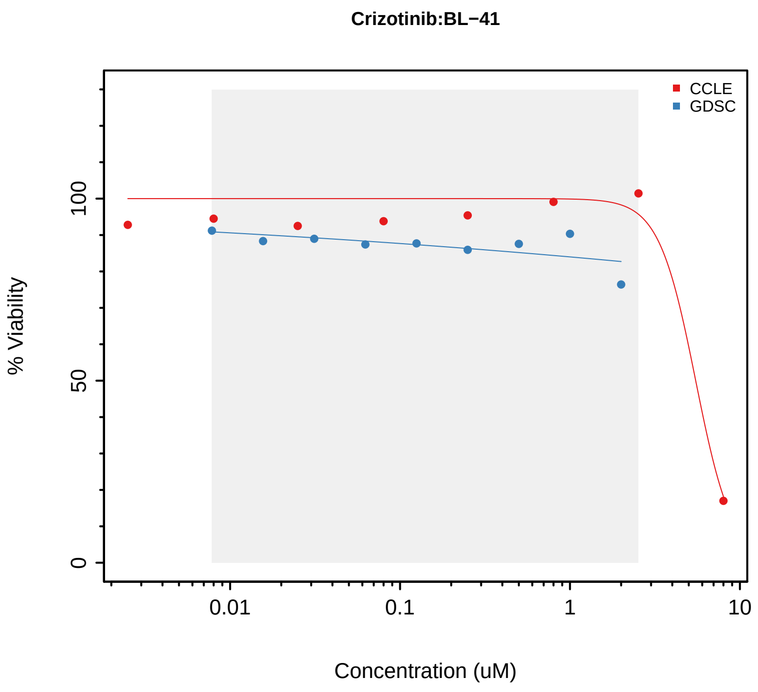
Crizotinib:BL−41
CCLE
GDSC
% Viability
0
50
100
0.01
0.1
1
10
Concentration (uM)

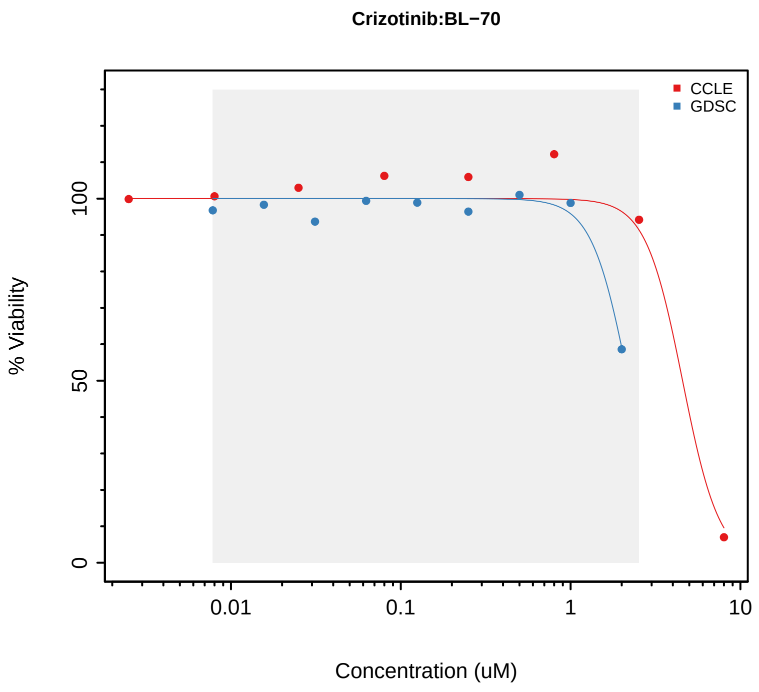
Crizotinib:BL−70
CCLE
GDSC
% Viability
0
50
100
0.01
0.1
1
10
Concentration (uM)

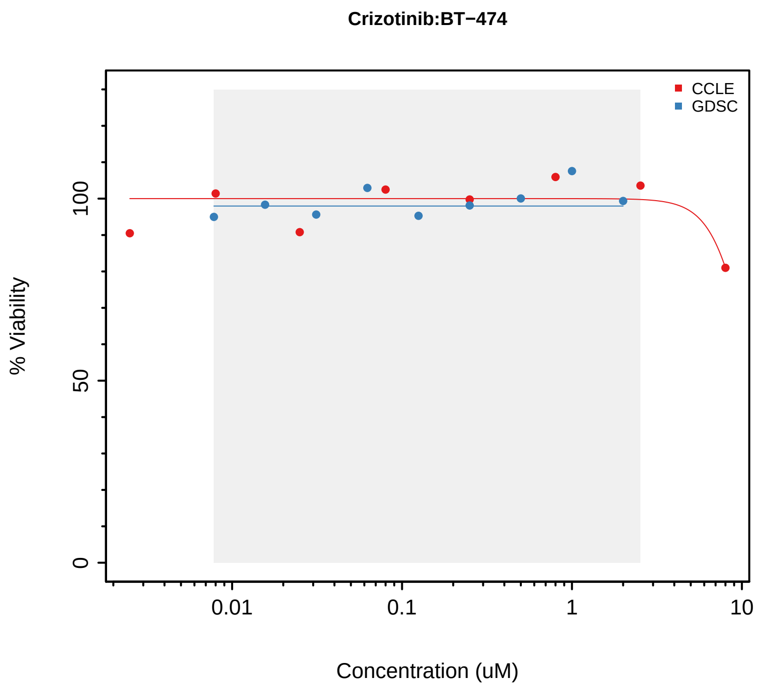
Crizotinib:BT−474
CCLE
GDSC
% Viability
0
50
100
0.01
0.1
1
10
Concentration (uM)

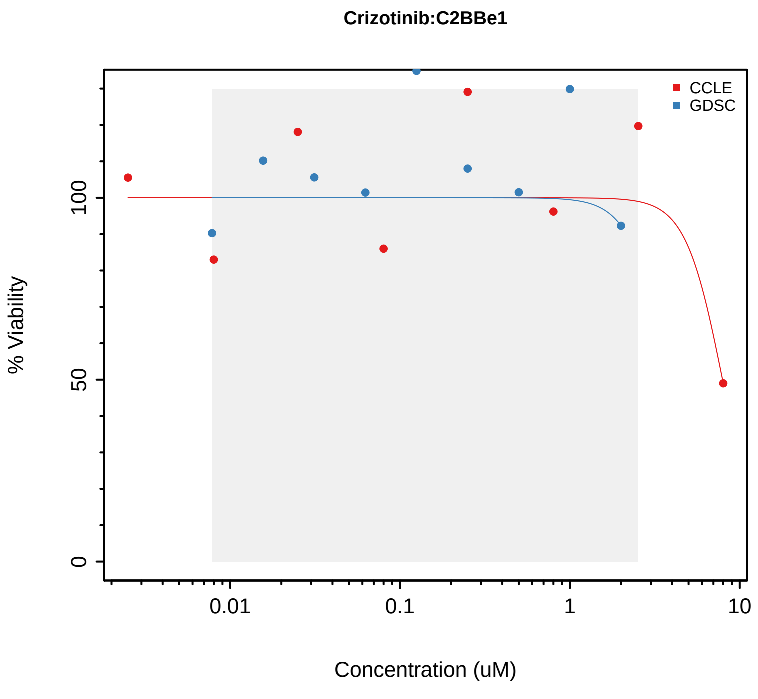
Crizotinib:C2BBe1
CCLE
GDSC
% Viability
0
50
100
0.01
0.1
1
10
Concentration (uM)

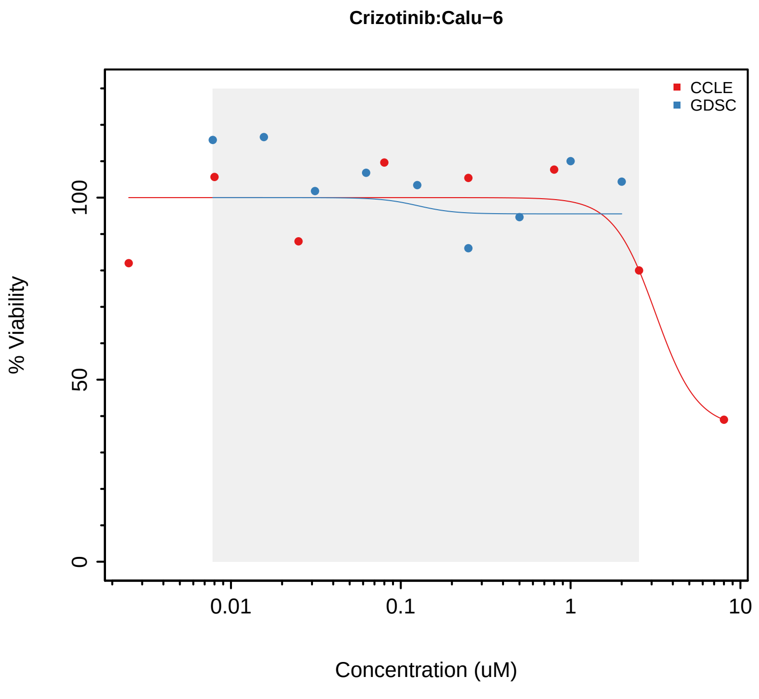
Crizotinib:Calu−6
CCLE
GDSC
% Viability
0
50
100
0.01
0.1
1
10
Concentration (uM)

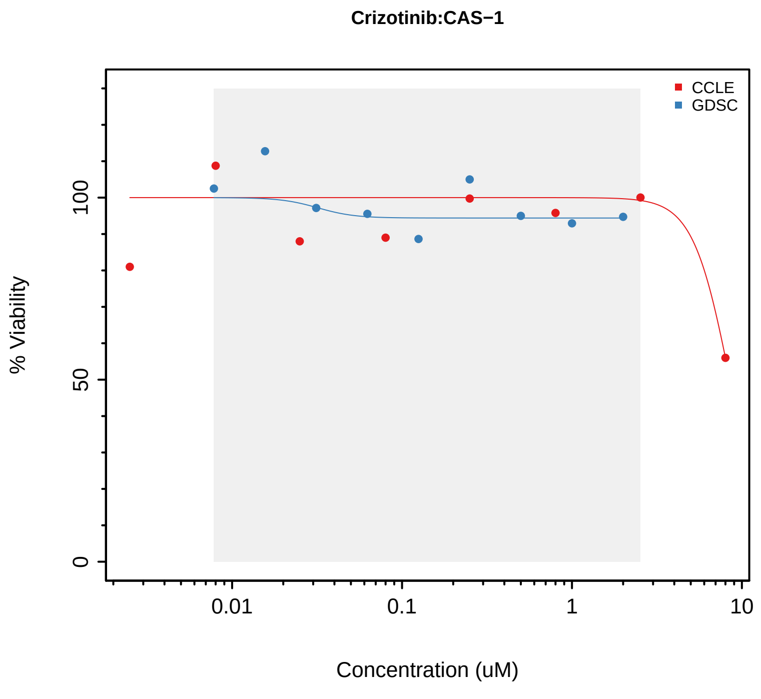
Crizotinib:CAS−1
CCLE
GDSC
% Viability
0
50
100
0.01
0.1
1
10
Concentration (uM)

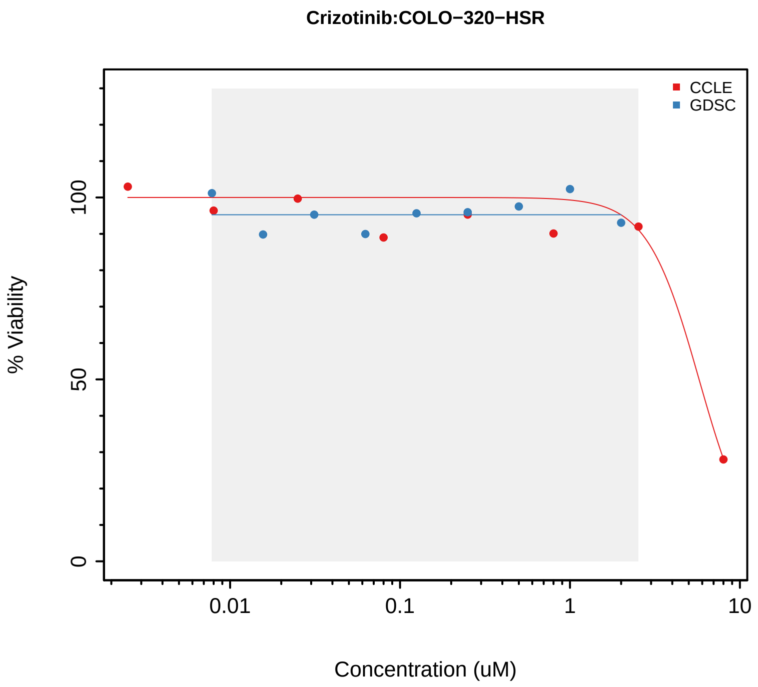
Crizotinib:COLO−320−HSR
CCLE
GDSC
% Viability
0 50 100
0.01 0.1 1 10
Concentration (uM)

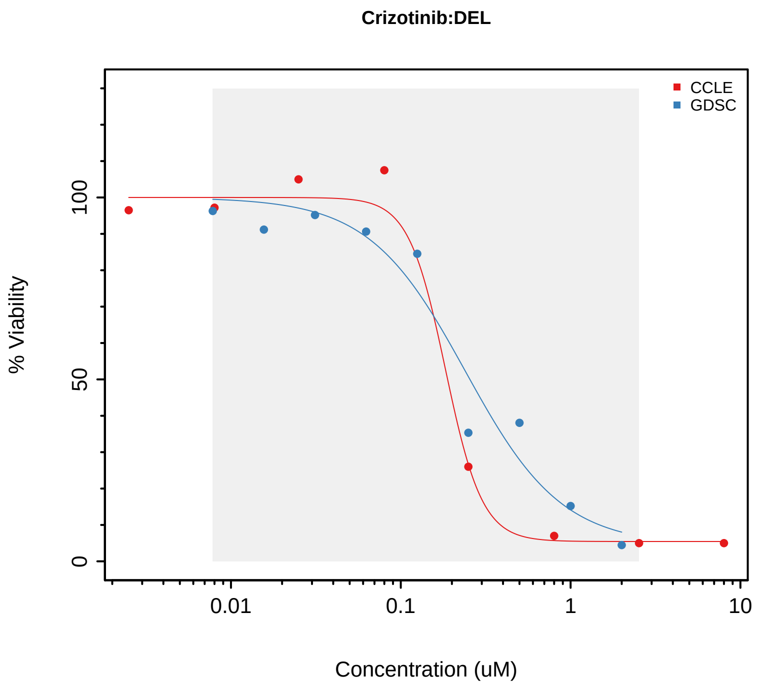
Crizotinib:DEL
CCLE
GDSC
% Viability
0 50 100
0.01 0.1 1 10
Concentration (uM)

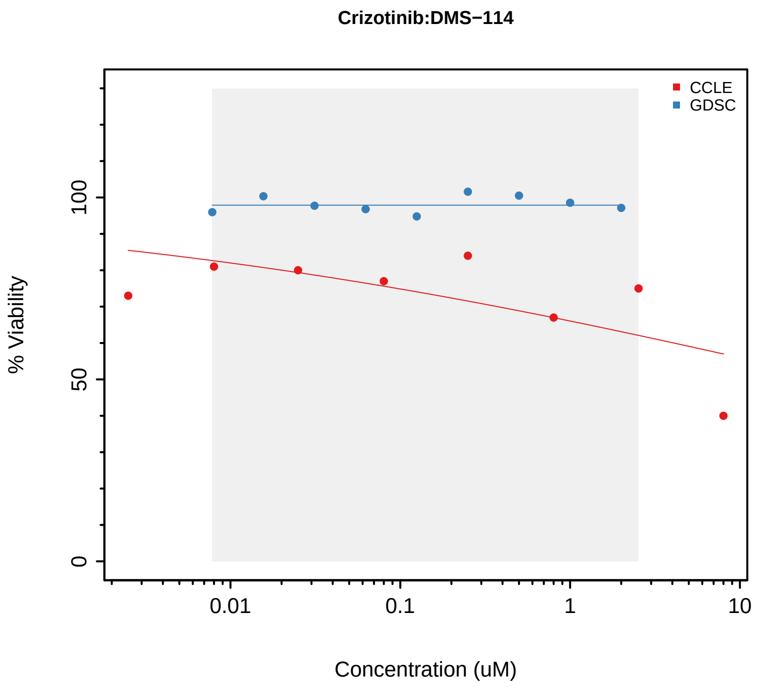
Crizotinib:DMS−114
CCLE
GDSC
% Viability
0 50 100
0.01 0.1 1 10
Concentration (uM)

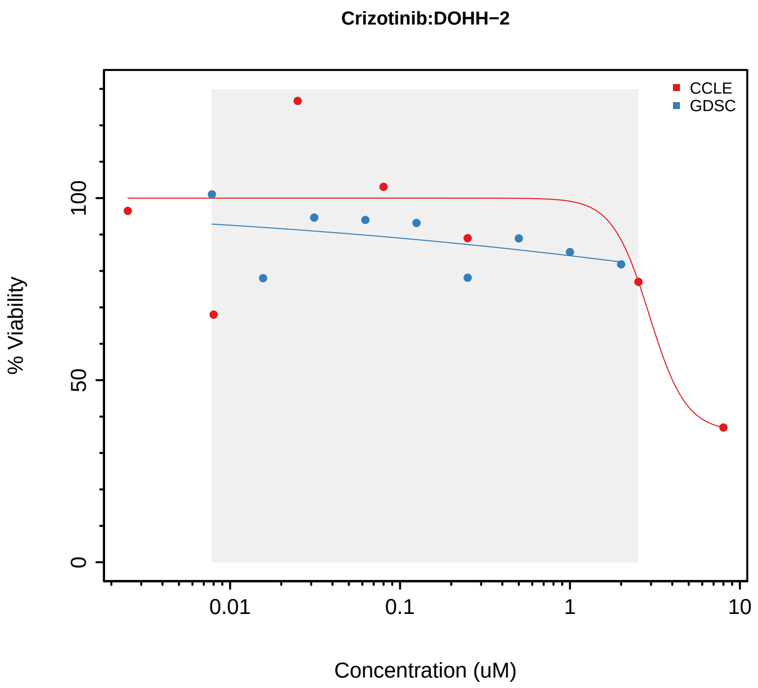
Crizotinib:DOHH−2
CCLE
GDSC
% Viability
0 50 100
0.01 0.1 1 10
Concentration (uM)

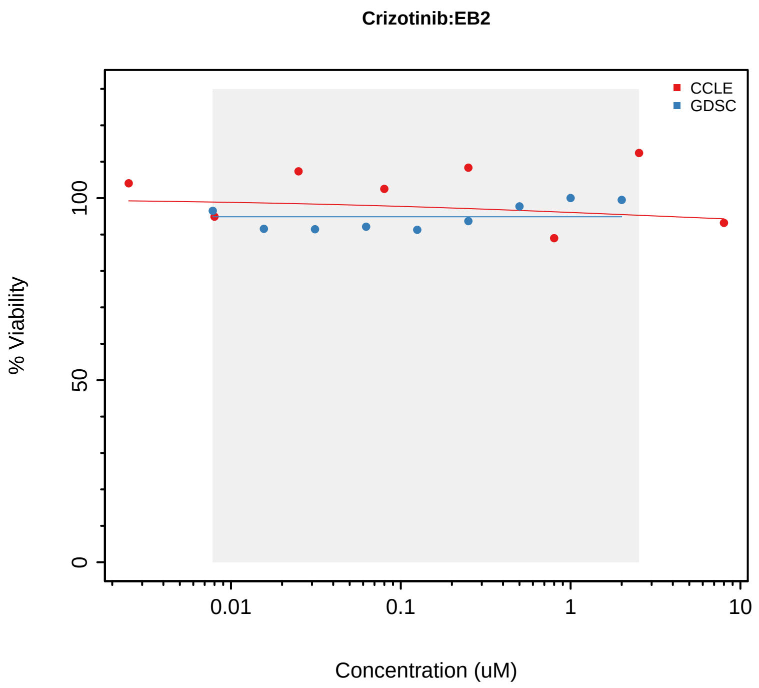
Crizotinib:EB2
CCLE
GDSC
% Viability
0 50 100
0.01 0.1 1 10
Concentration (uM)

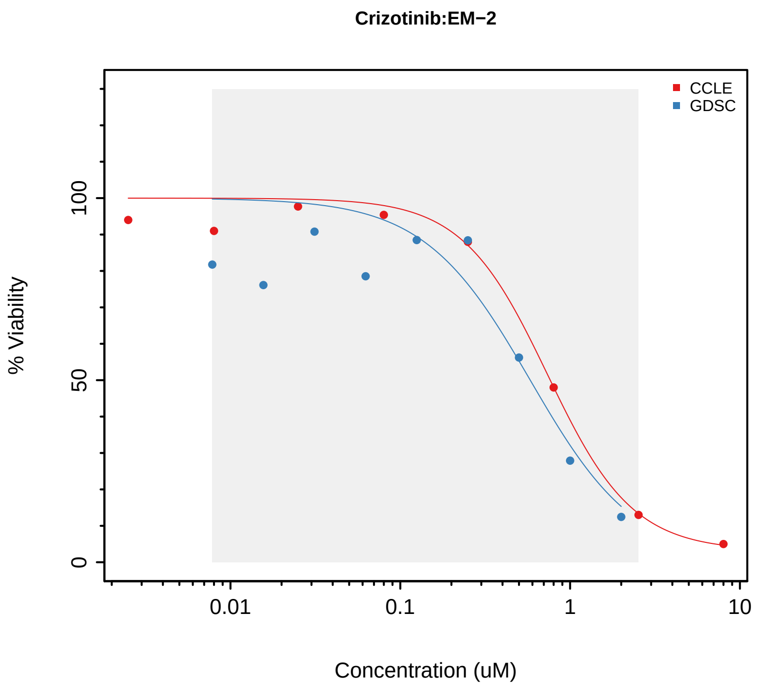
Crizotinib:EM−2
CCLE
GDSC
% Viability
0 50 100
0.01 0.1 1 10
Concentration (uM)

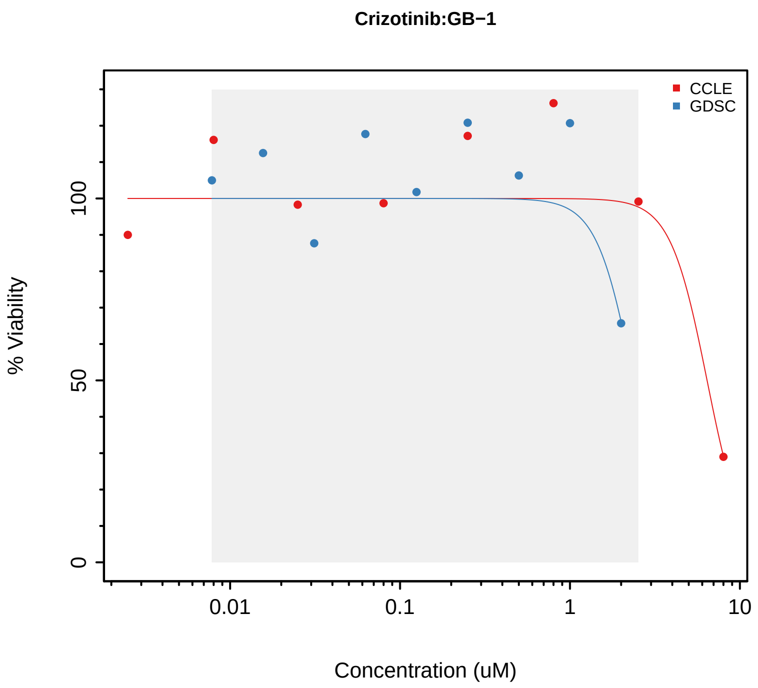
Crizotinib:GB−1
CCLE
GDSC
% Viability
0 50 100
0.01 0.1 1 10
Concentration (uM)

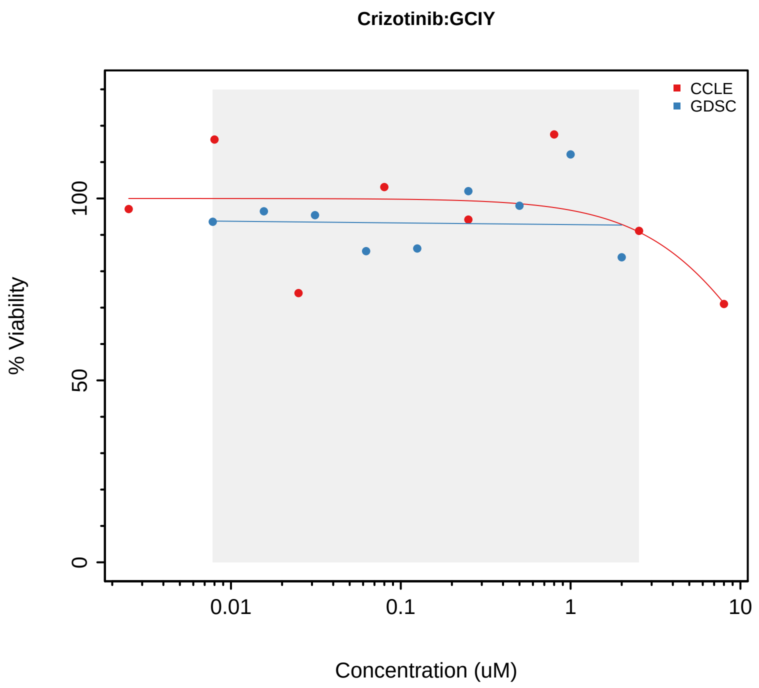
Crizotinib:GCIY
CCLE
GDSC
% Viability
0 50 100
0.01 0.1 1 10
Concentration (uM)

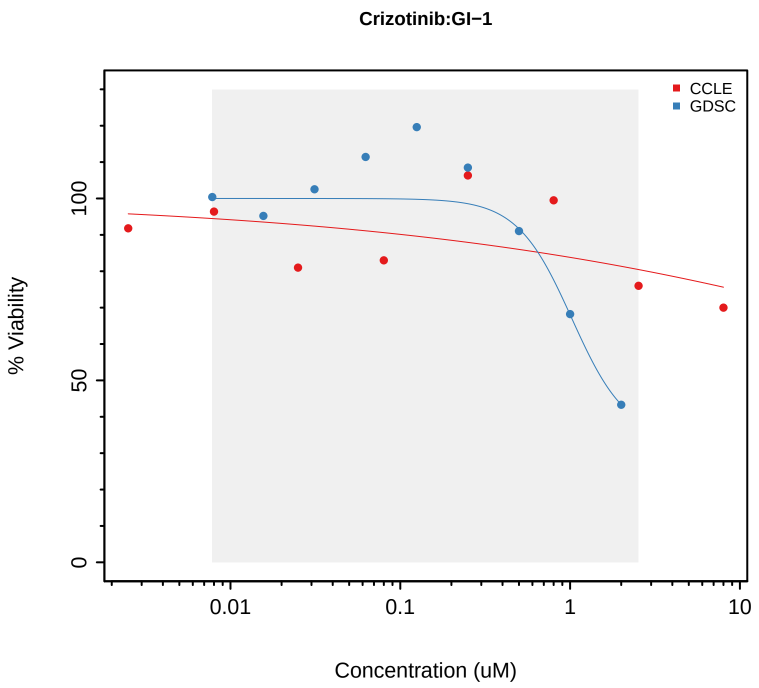
Crizotinib:GI−1
CCLE
GDSC
% Viability
0 50 100
0.01 0.1 1 10
Concentration (uM)

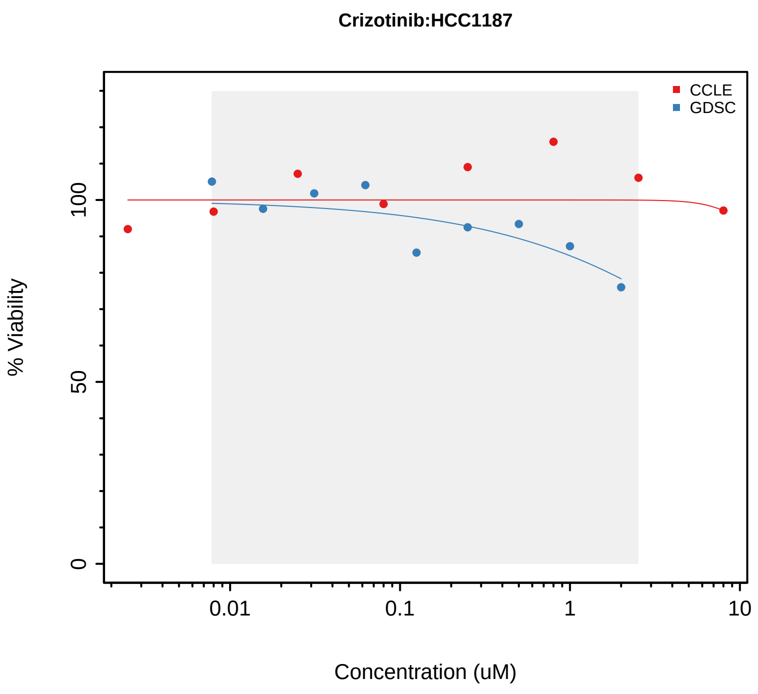
Crizotinib:HCC1187
CCLE
GDSC
% Viability
0 50 100
0.01 0.1 1 10
Concentration (uM)

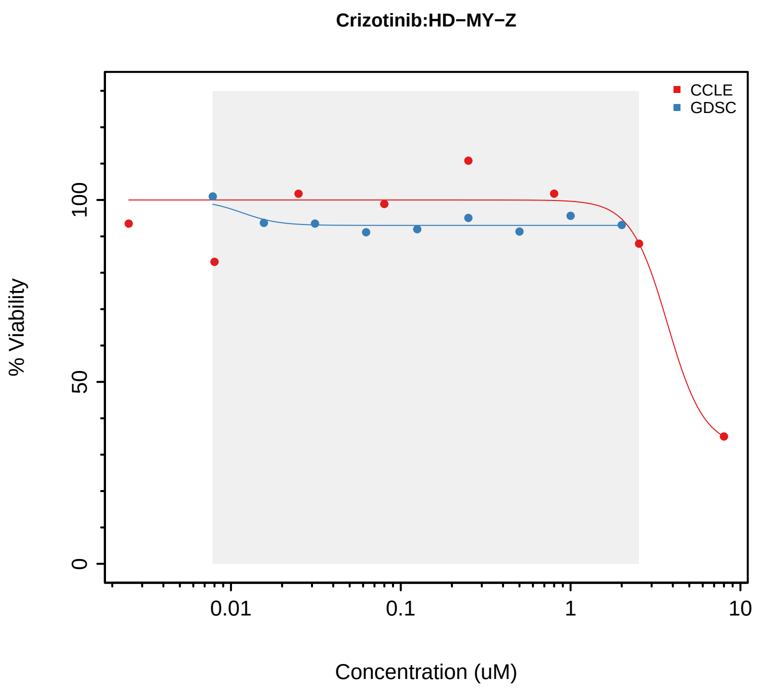
Crizotinib:HD−MY−Z
CCLE
GDSC
% Viability
0 50 100
0.01 0.1 1 10
Concentration (uM)

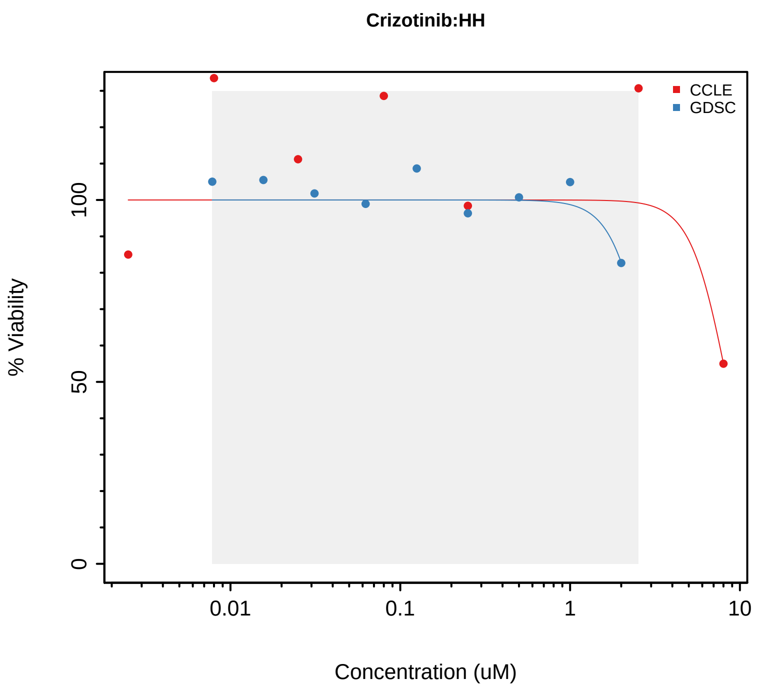
Crizotinib:HH
CCLE
GDSC
% Viability
0 50 100
0.01 0.1 1 10
Concentration (uM)

Crizotinib:HT

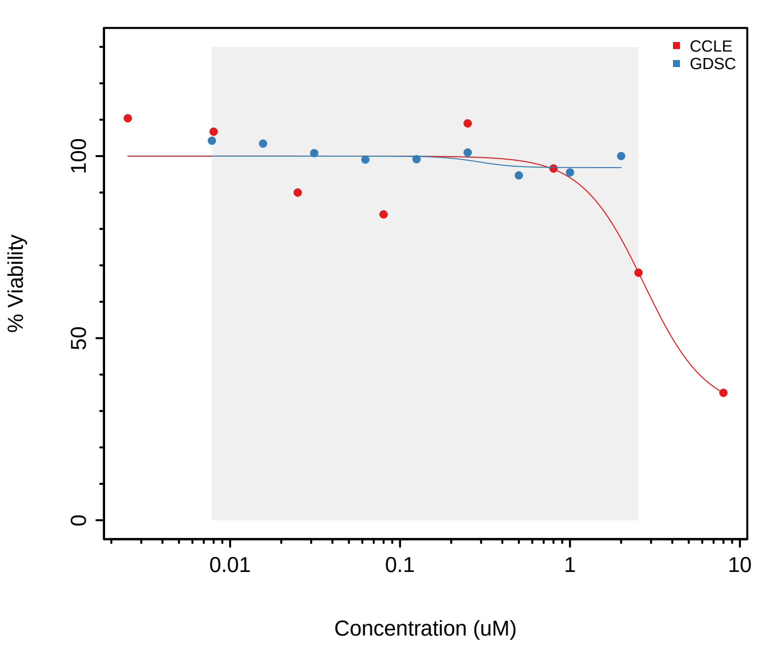

Crizotinib:HT−144

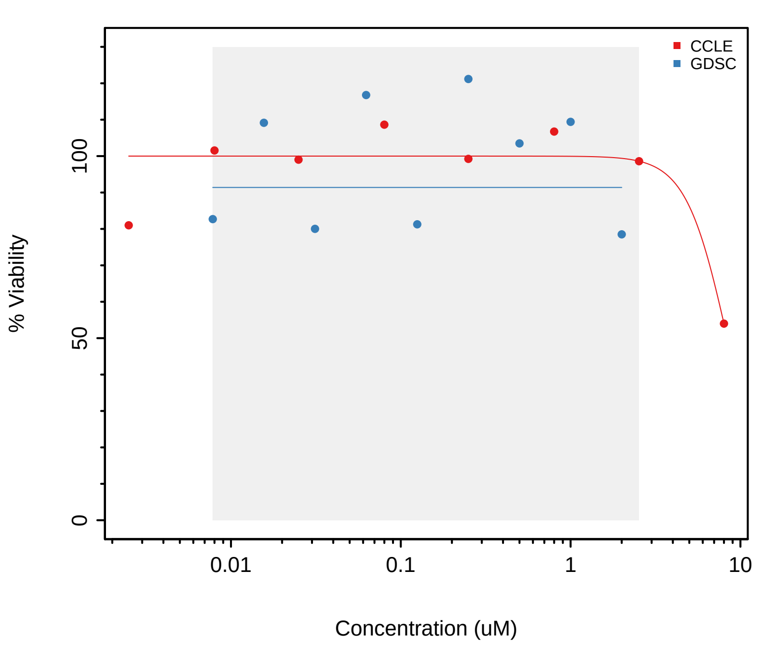

Crizotinib:IA−LM

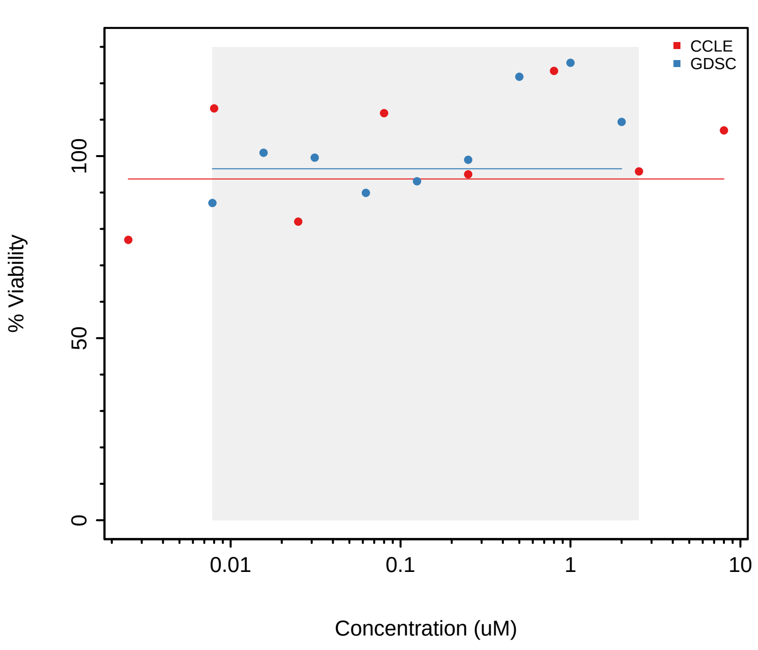

Crizotinib:IST−MES1

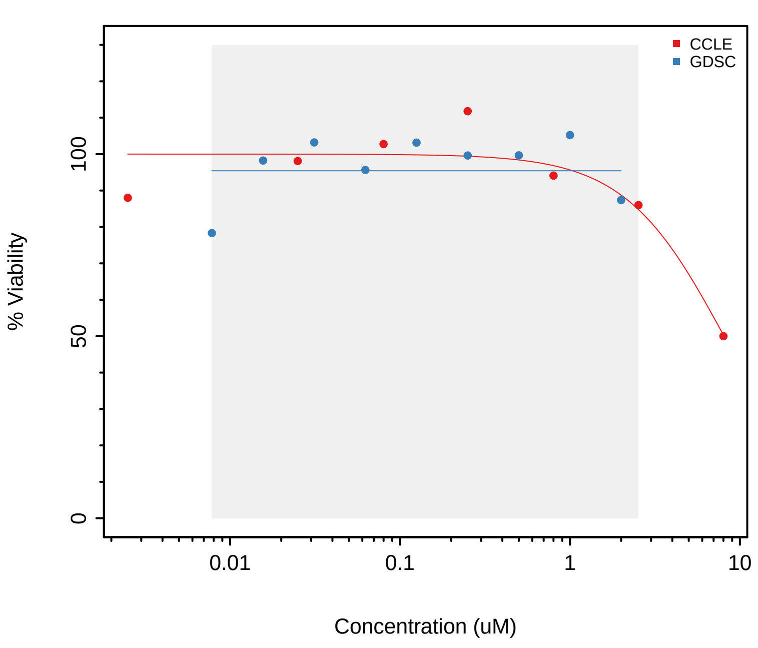

Crizotinib:JVM−3

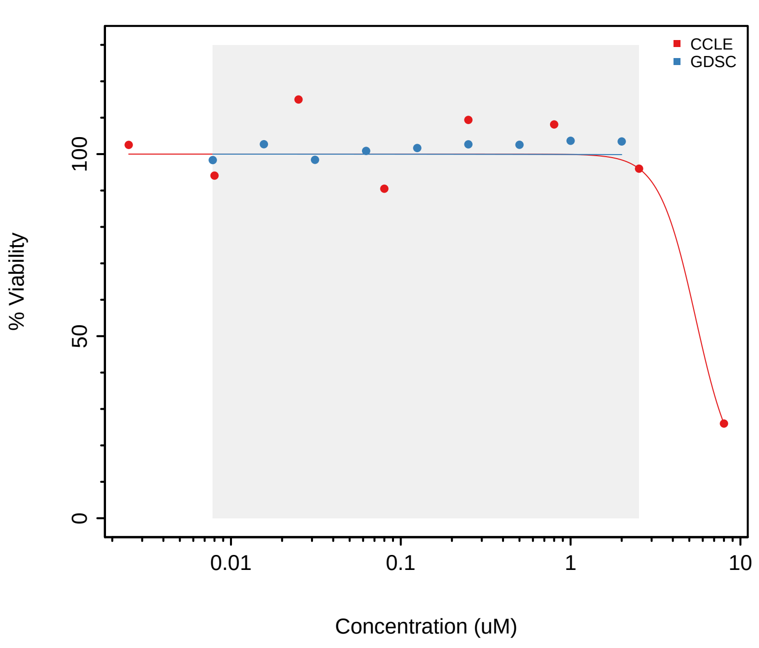

Crizotinib:KALS−1

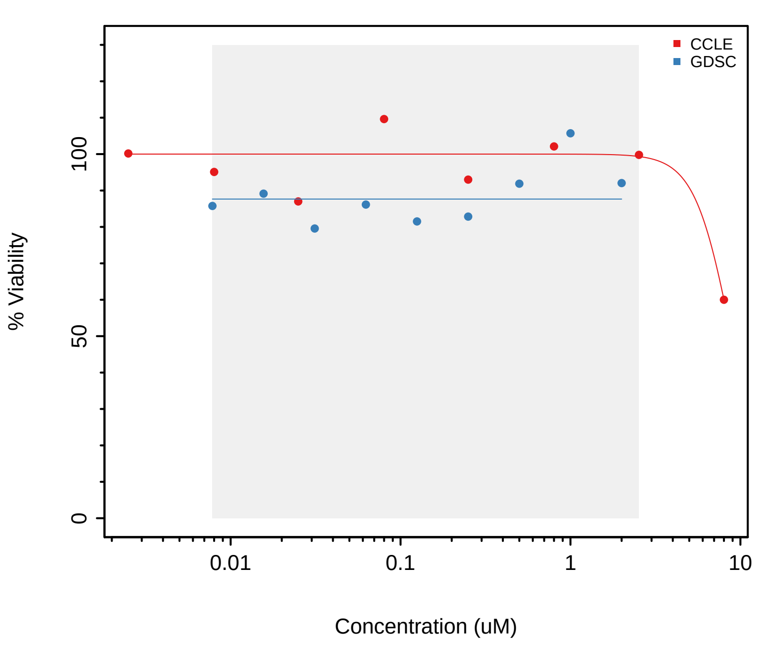

Crizotinib:KARPAS−299

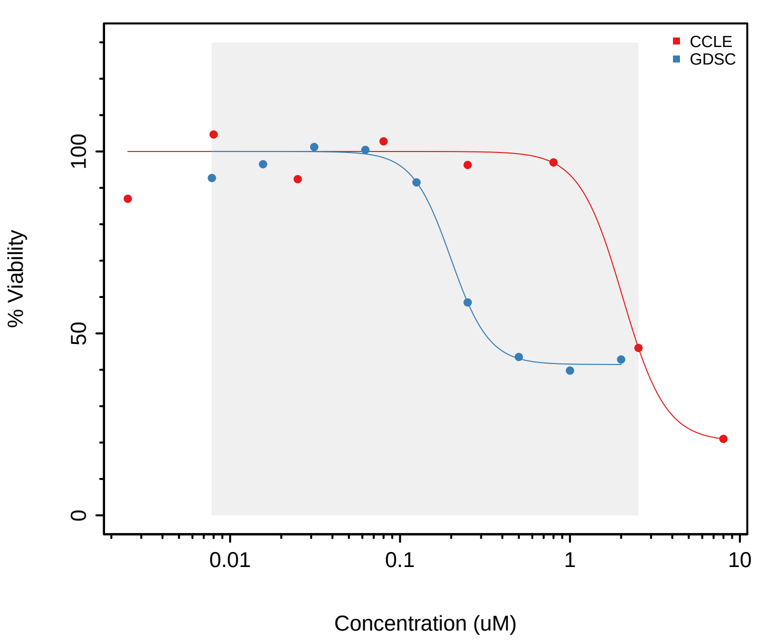

Crizotinib:KARPAS−422

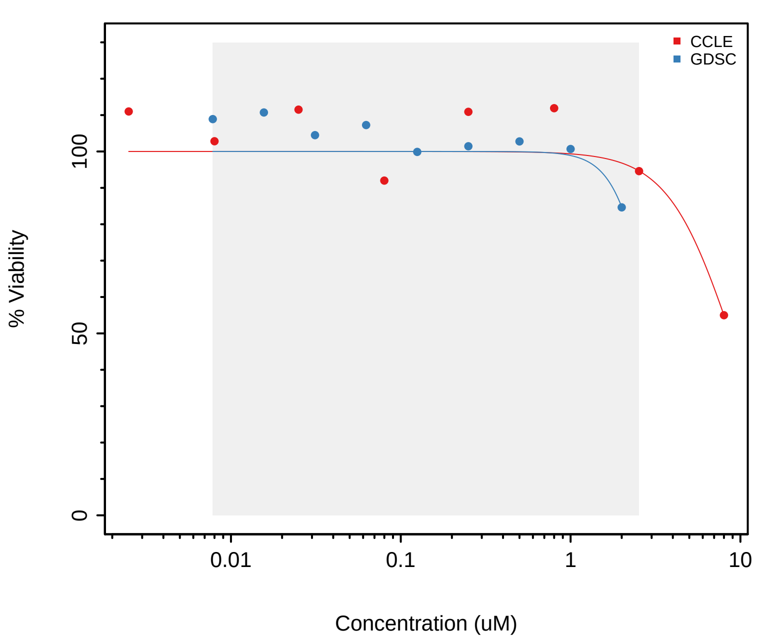

Crizotinib:KG−1

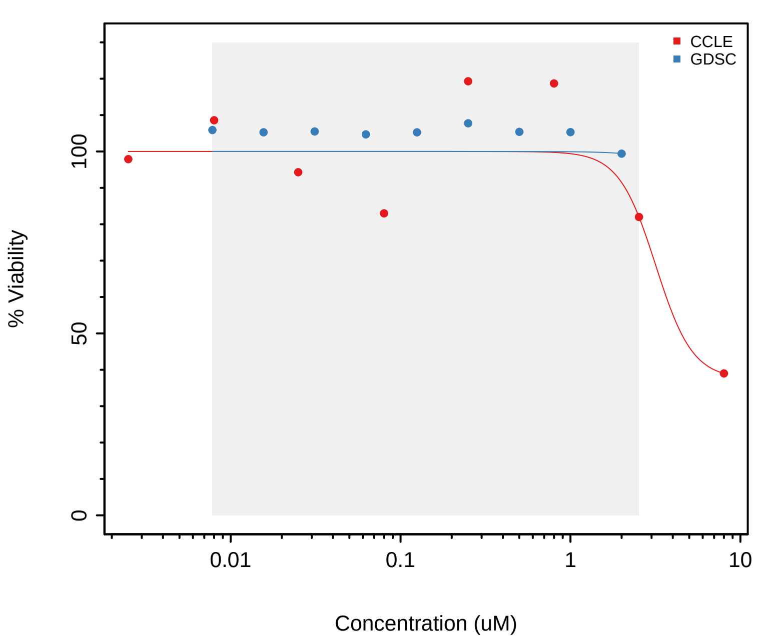

Crizotinib:KLE

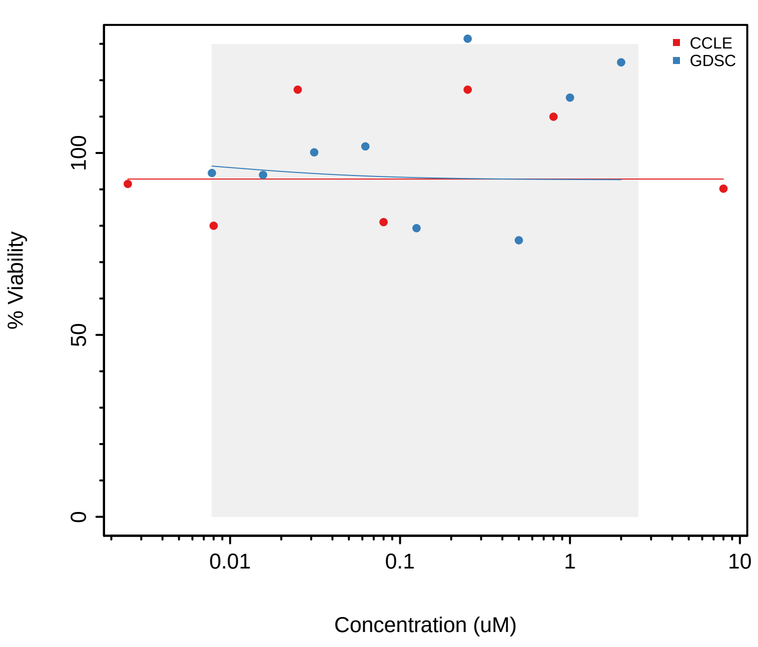

Crizotinib:KNS−42

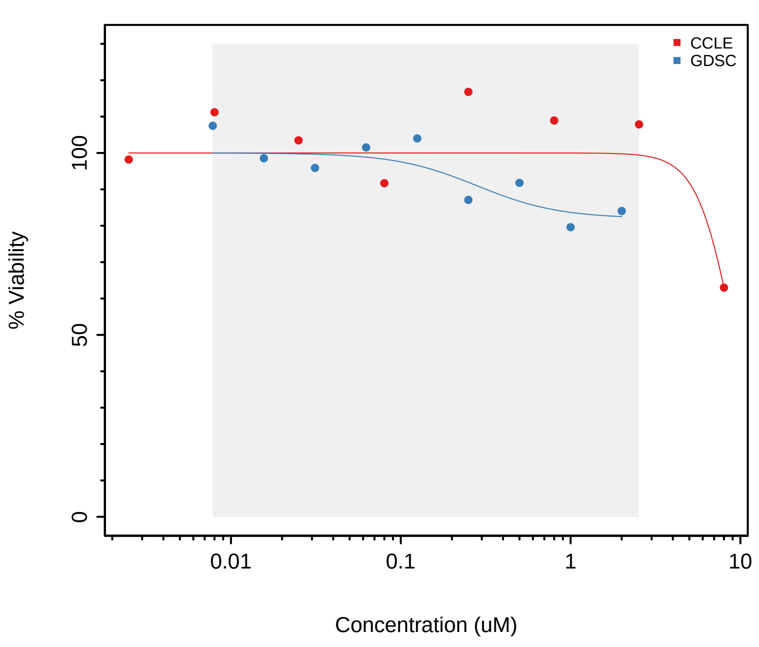

Crizotinib:KNS−81−FD

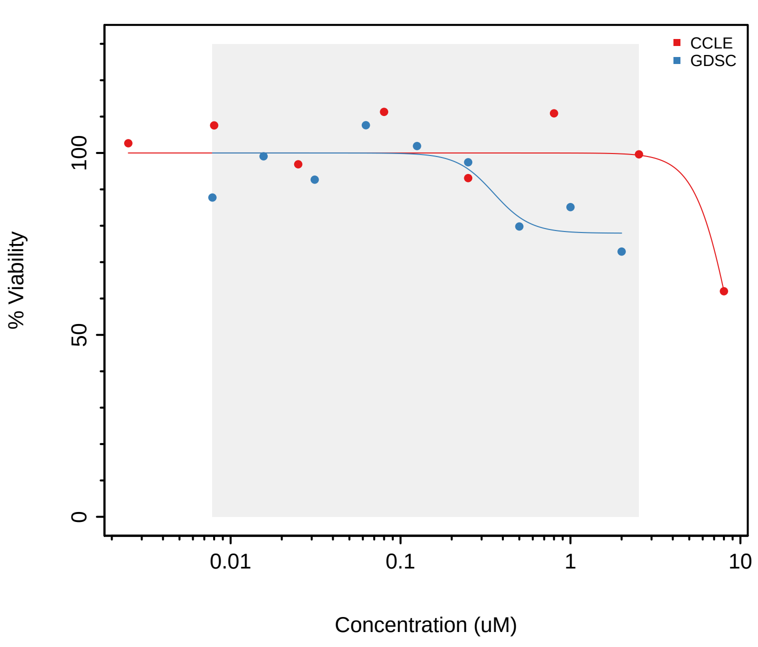

Crizotinib:K052

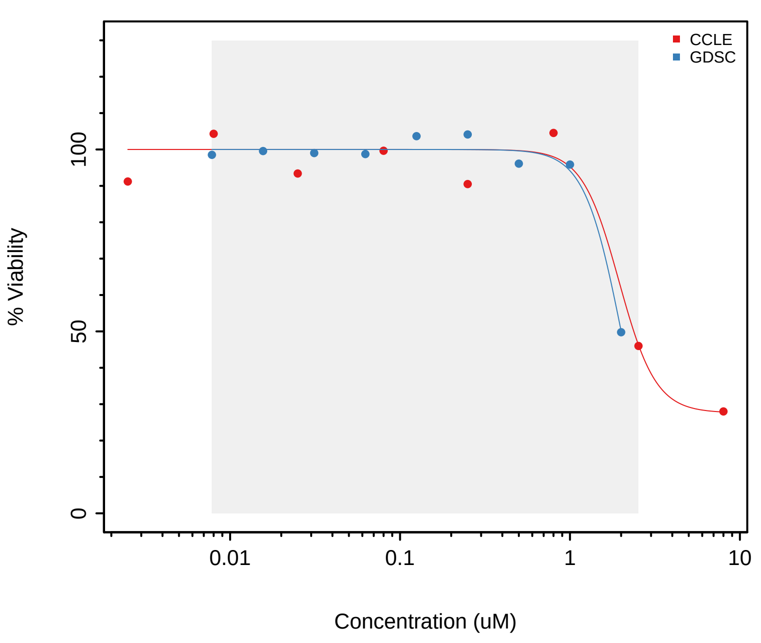

Crizotinib:KU812

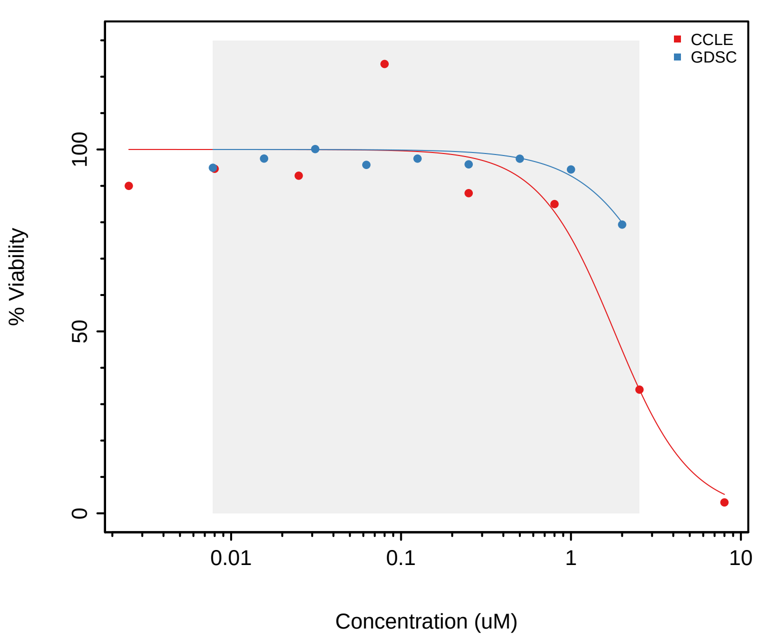

Crizotinib:KURAMOCHI

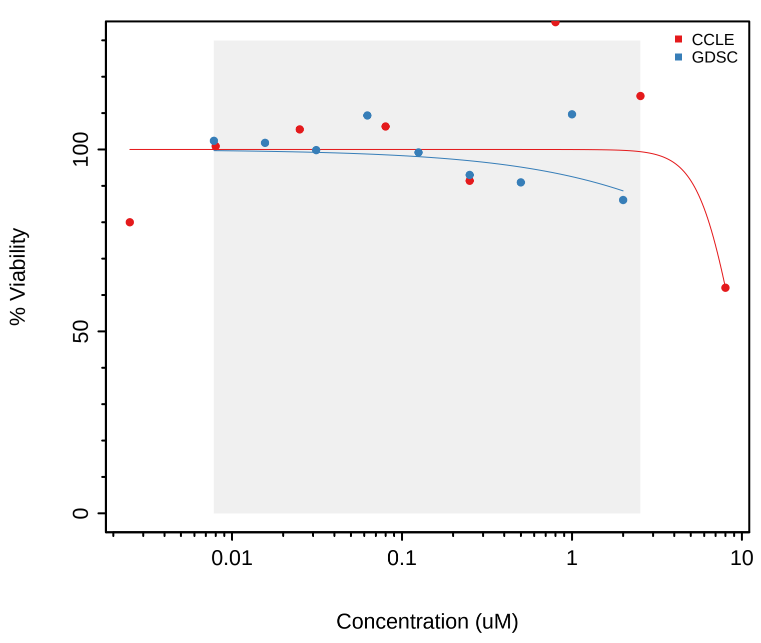

Crizotinib:L−363

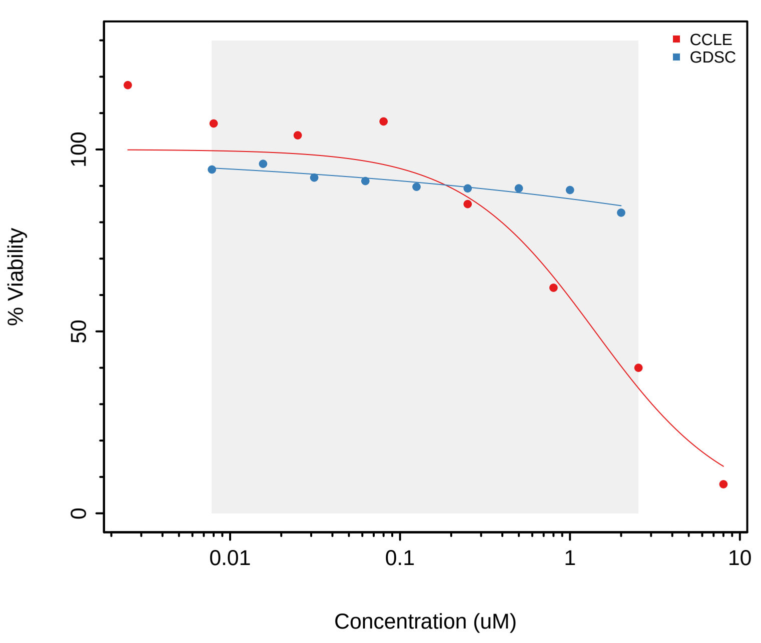

Crizotinib:L−428

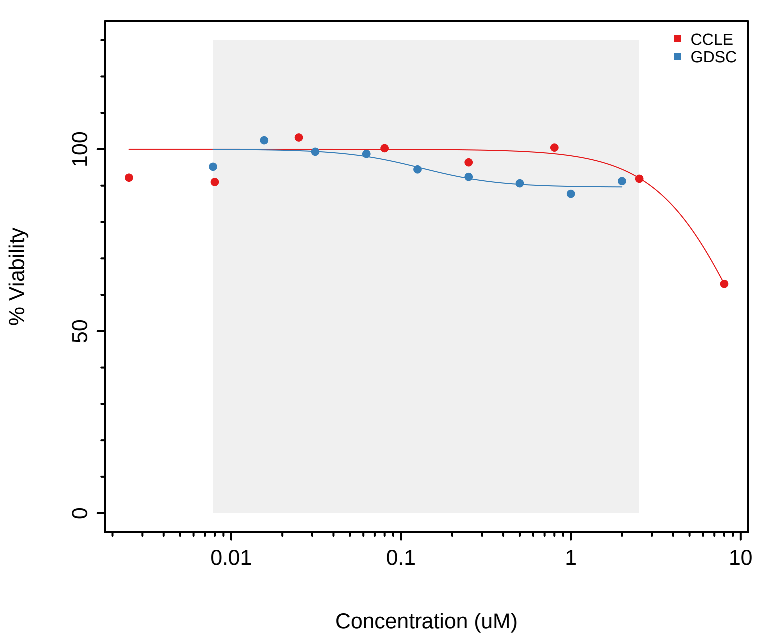

Crizotinib:LOXIMVI

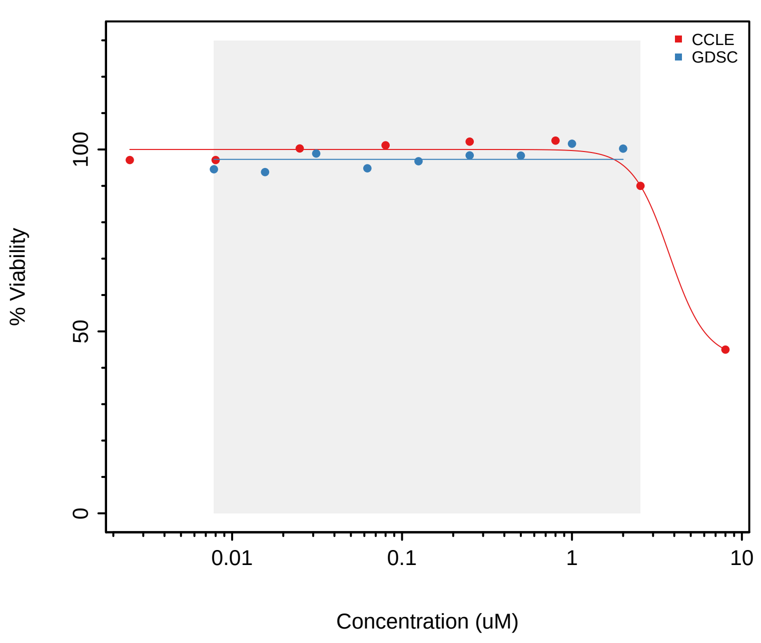

Crizotinib:LP−1

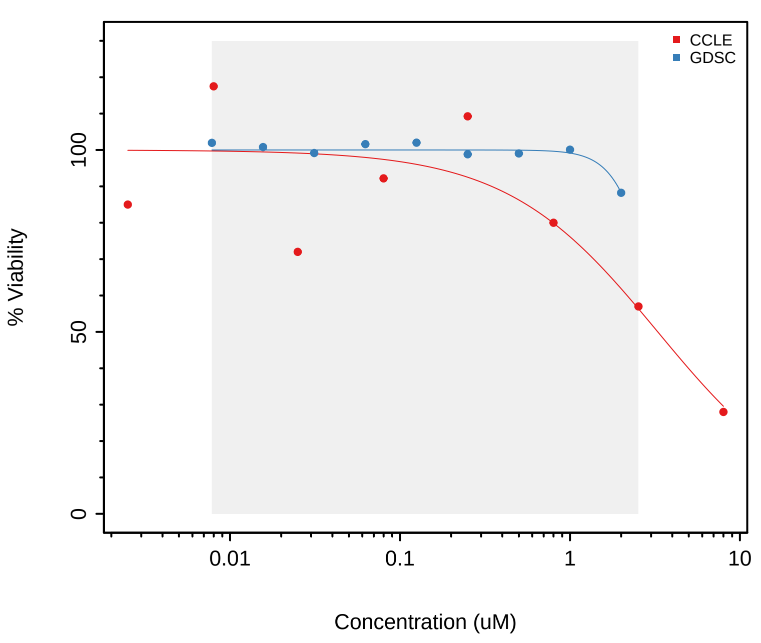

Crizotinib:LS−123

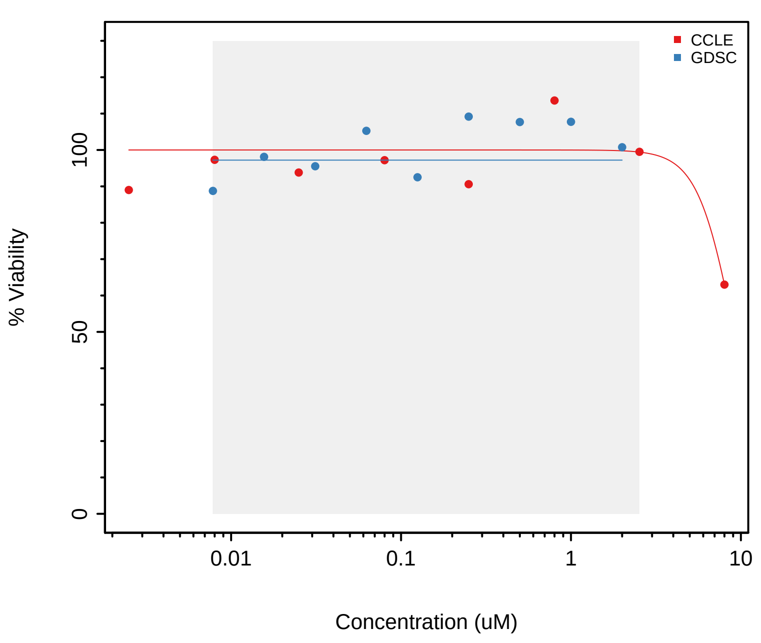

Crizotinib:LS−411N

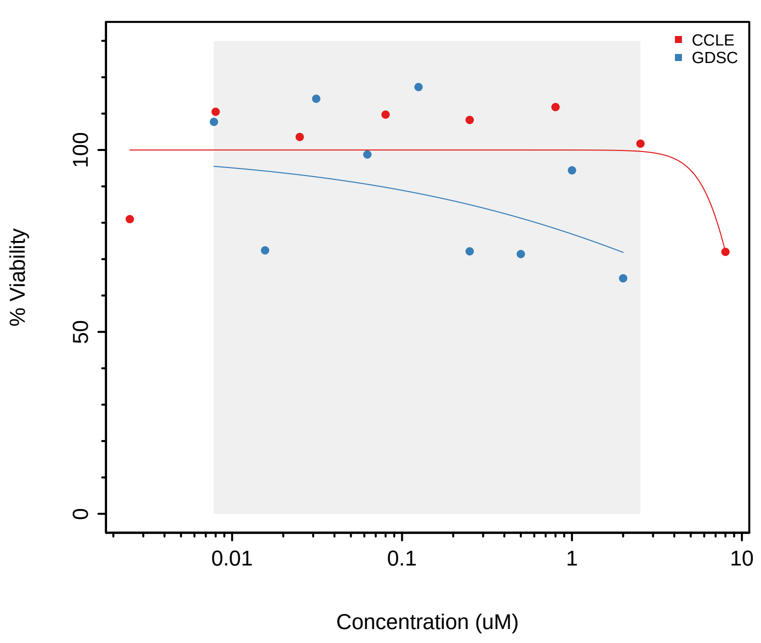

Crizotinib:LS−513

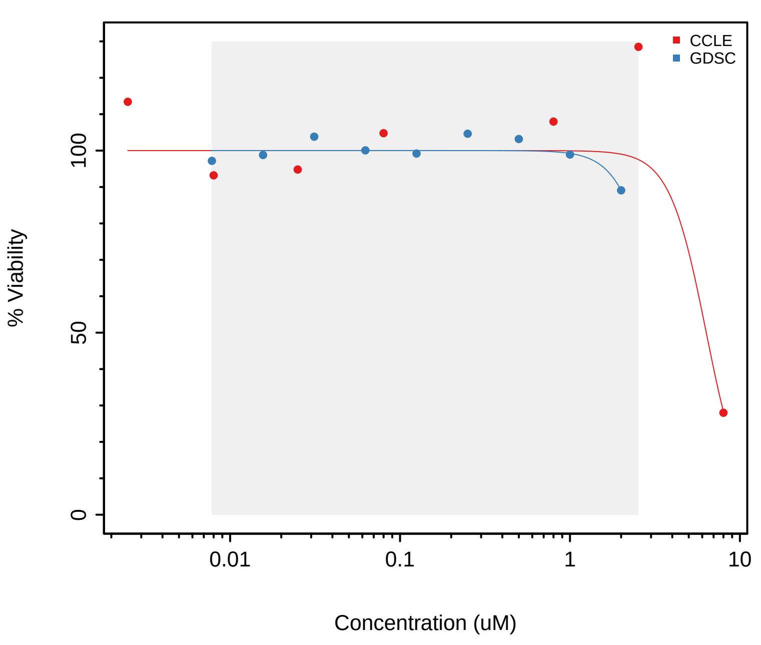

Crizotinib:MC116

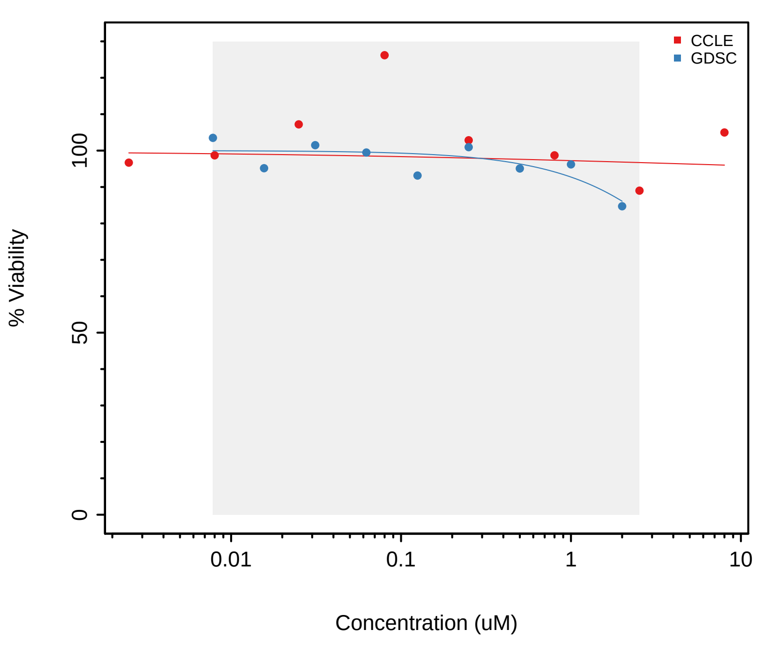

Crizotinib:MEG−01

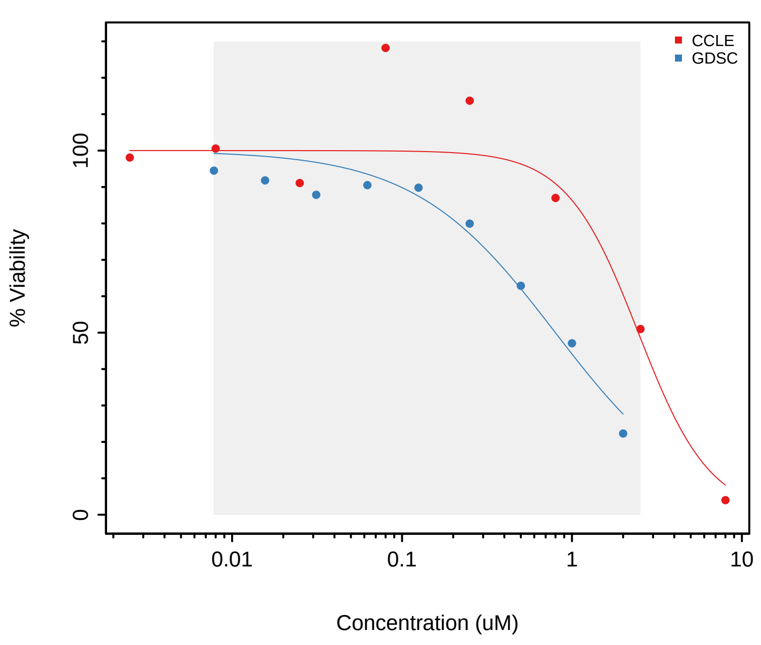

Crizotinib:MSTO−211H

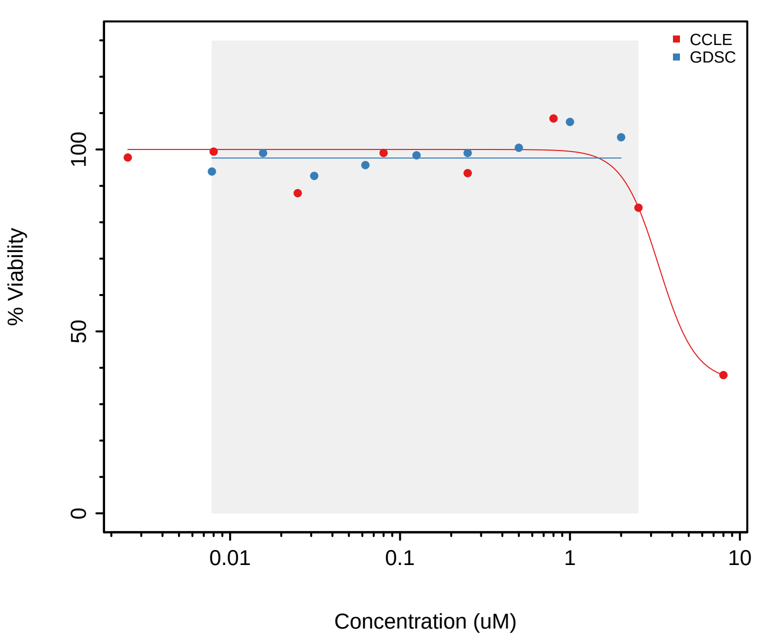

Crizotinib:NCI−H1092

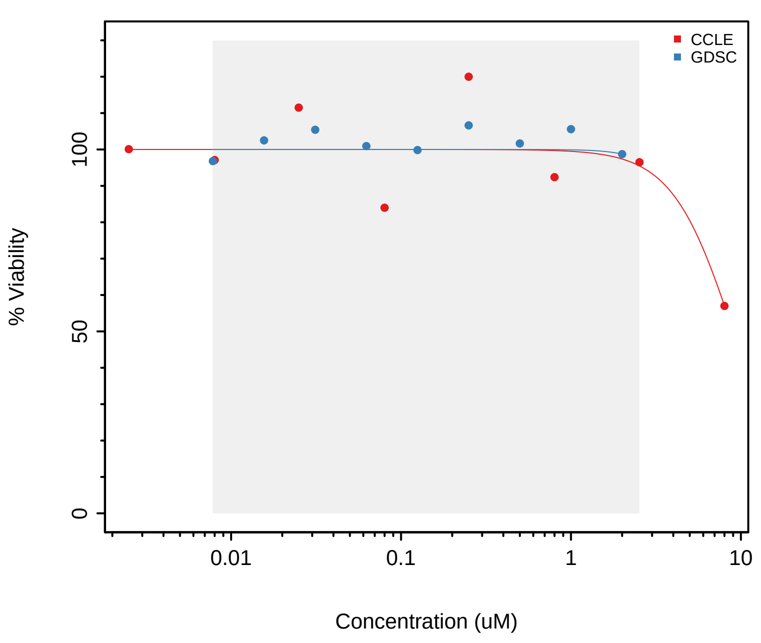

Crizotinib:NCI−H1155

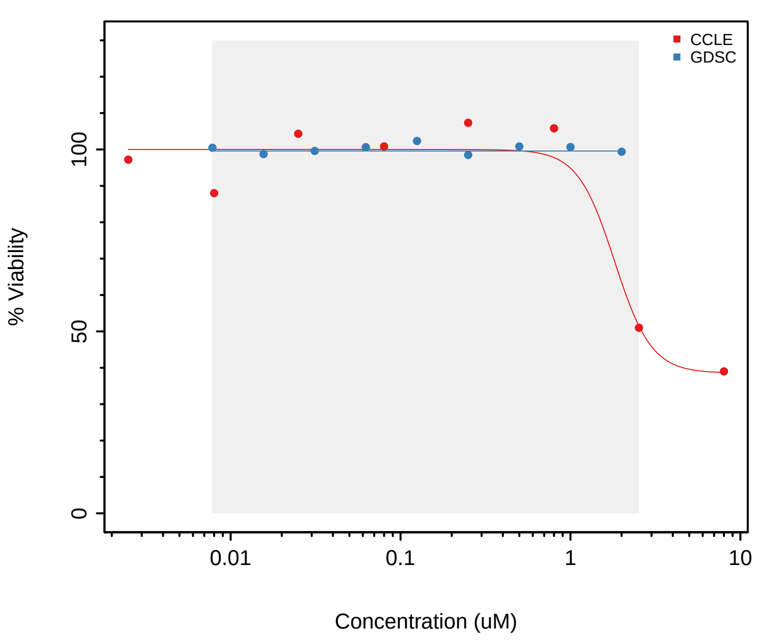

Crizotinib:NCI−H1299

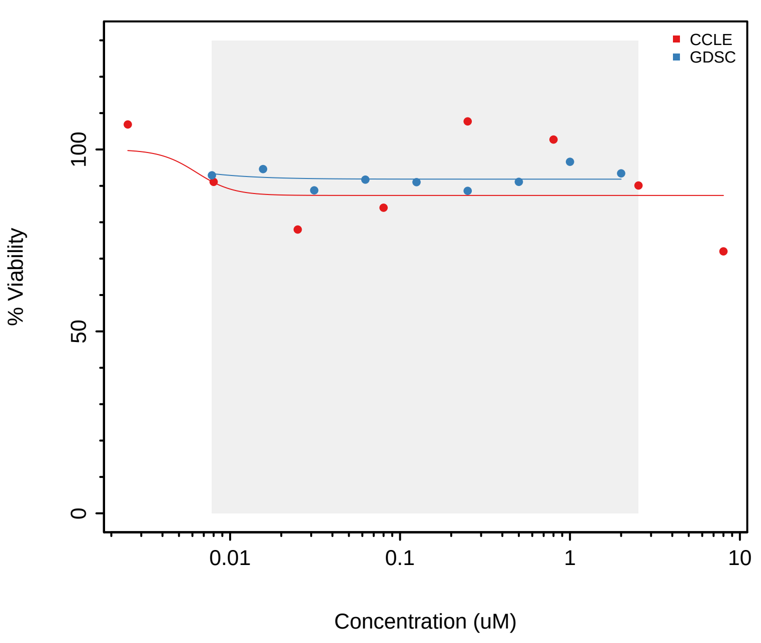

Crizotinib:NCI−H1355

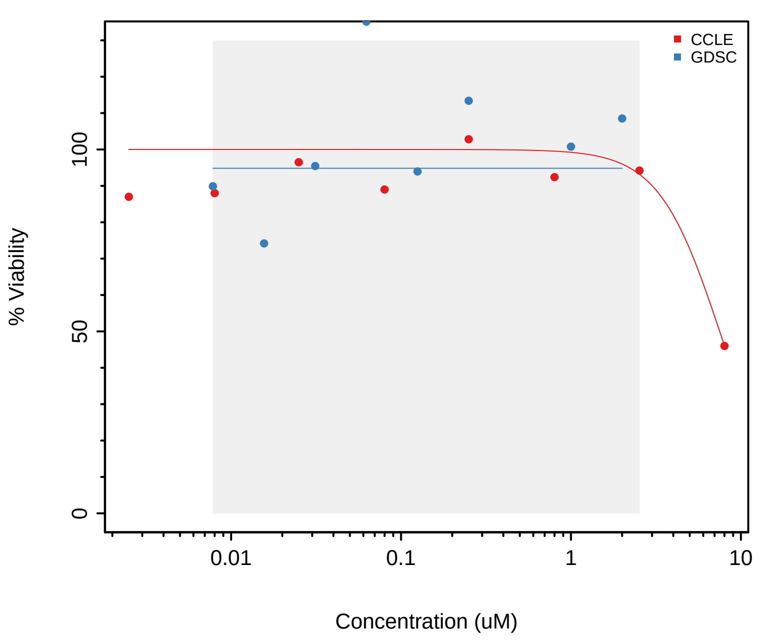

Crizotinib:NCI−H1581

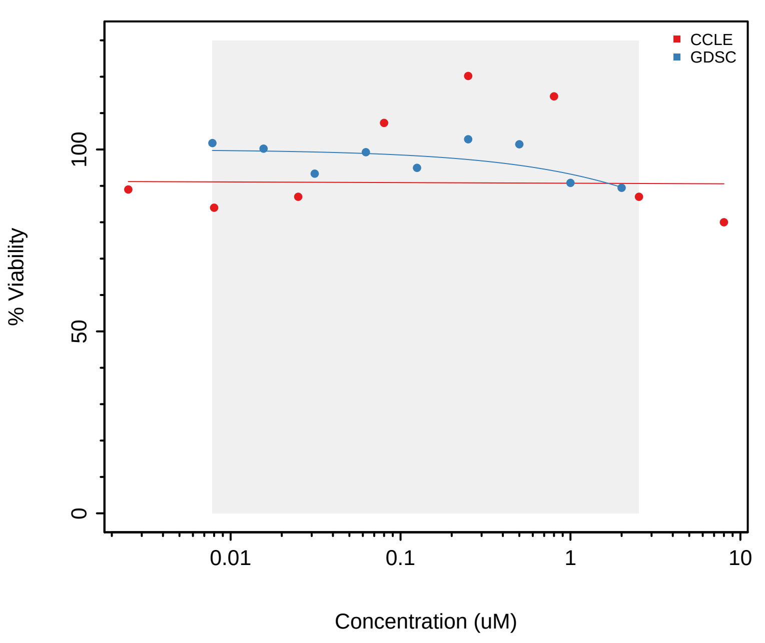

Crizotinib:NCI−H1648

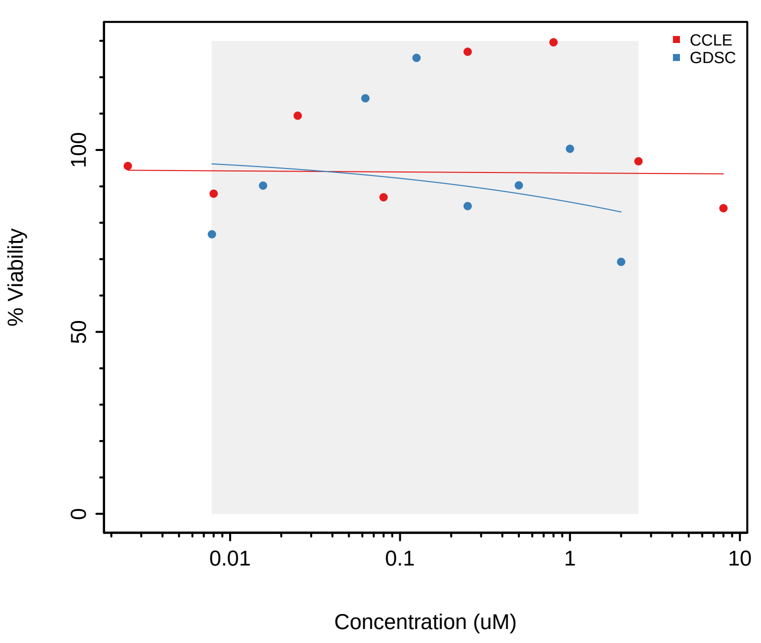

Crizotinib:NCI−H1666

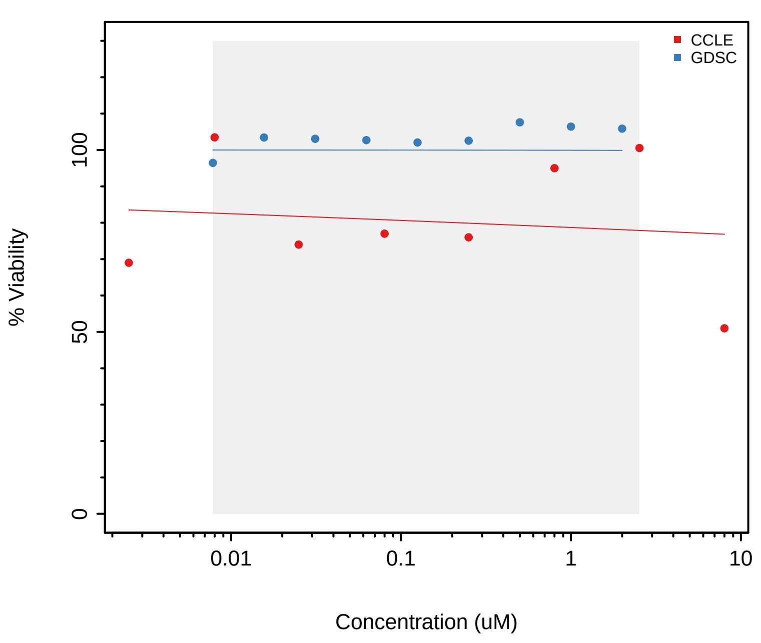

Crizotinib:NCI−H1694

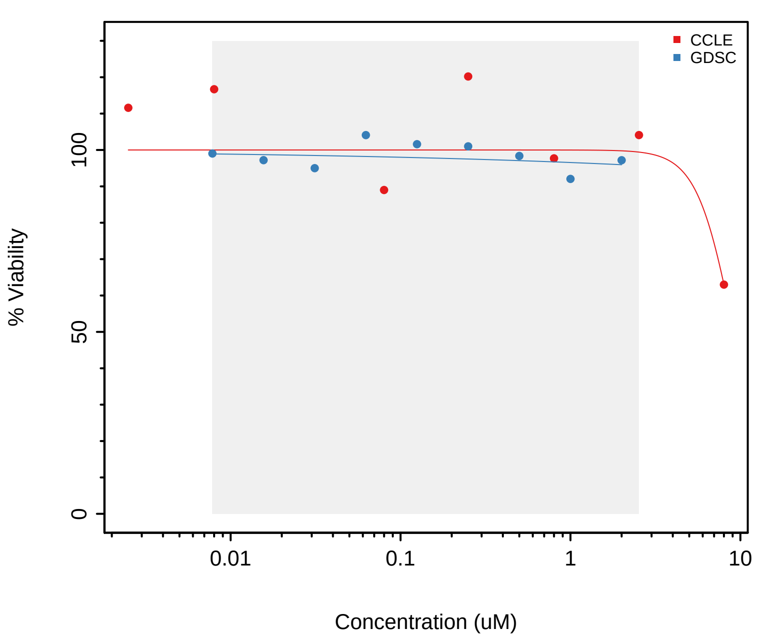

Crizotinib:NCI−H1975

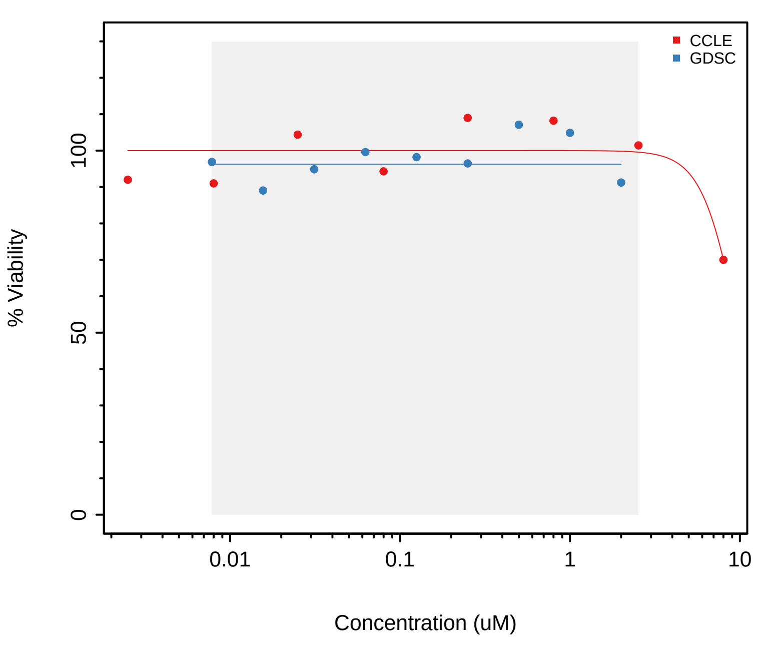

Crizotinib:NCI−H226

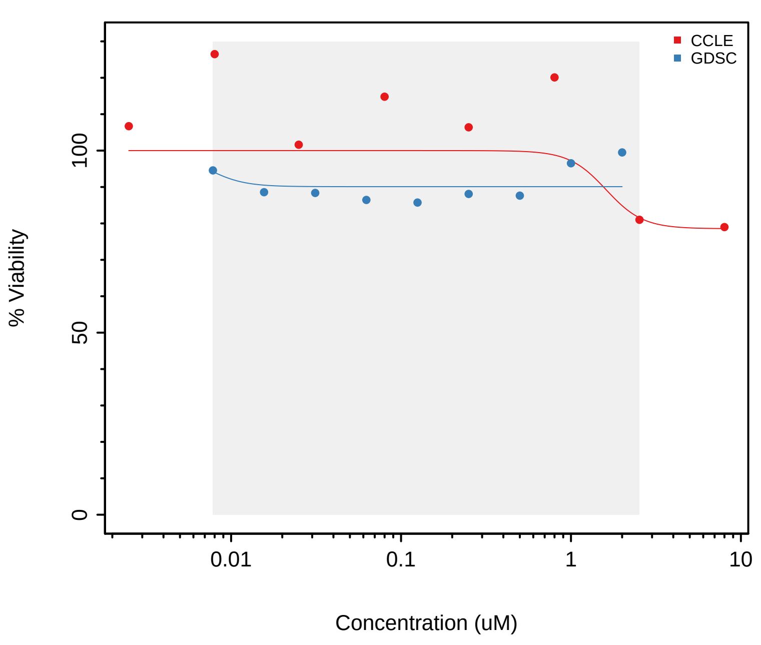

Crizotinib:NCI−H23

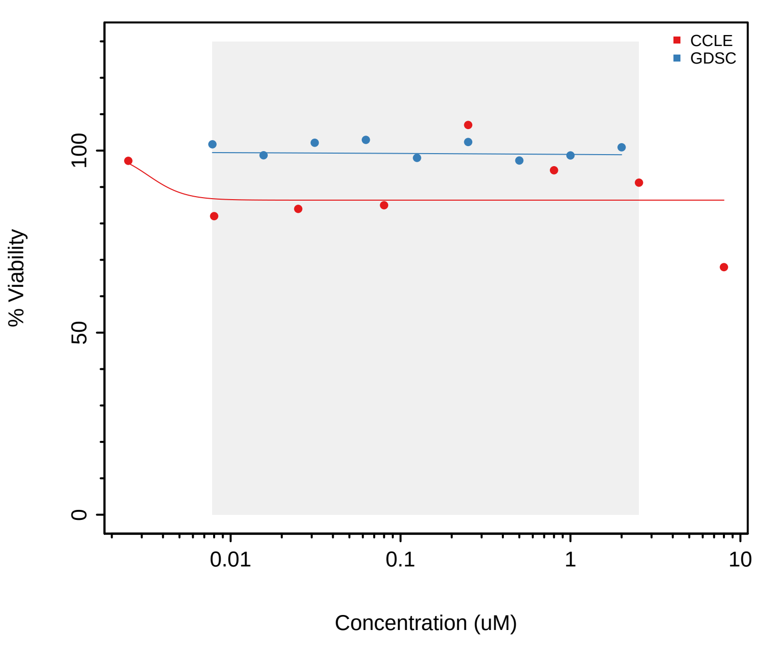

Crizotinib:NCI−H747

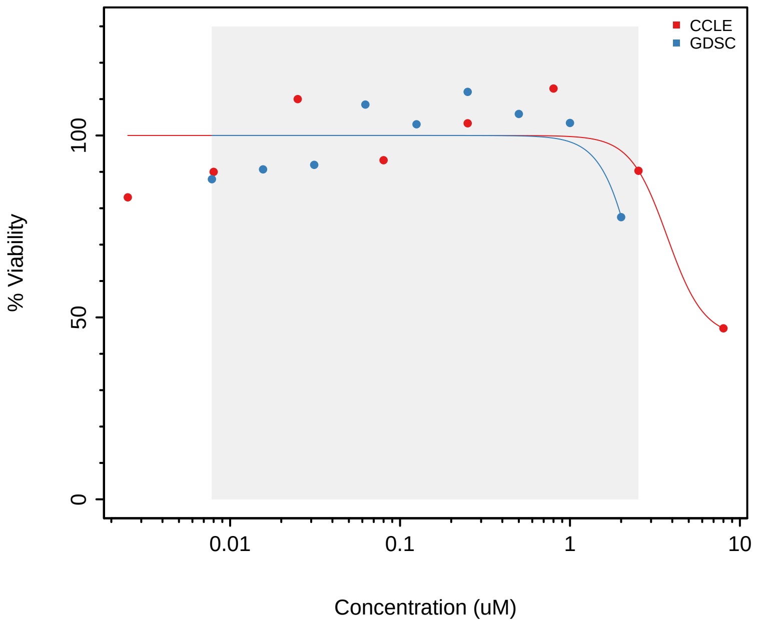

Crizotinib:OCI−AML2

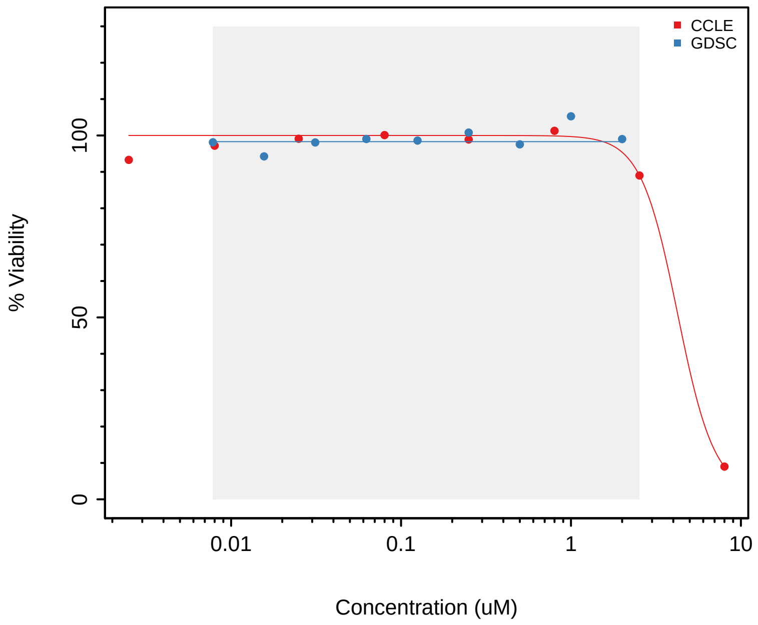

Crizotinib:ONS−76

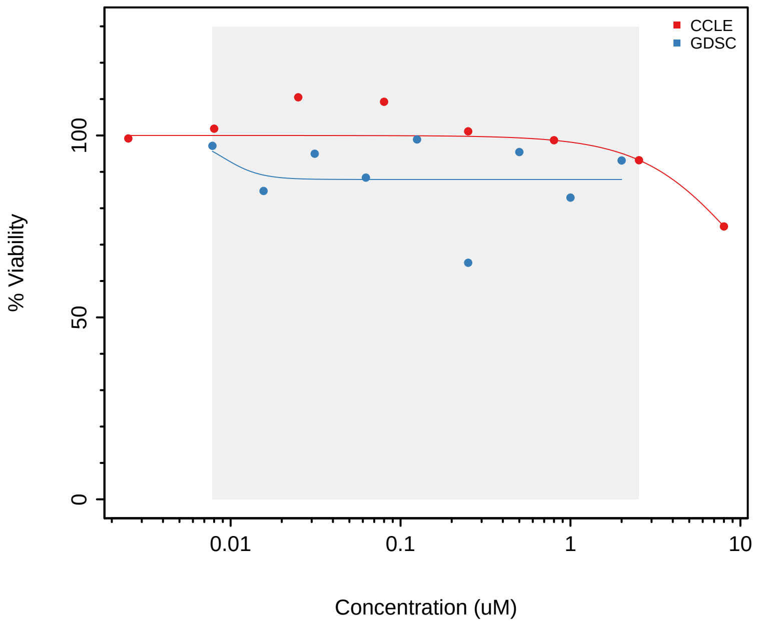

Crizotinib:OPM−2

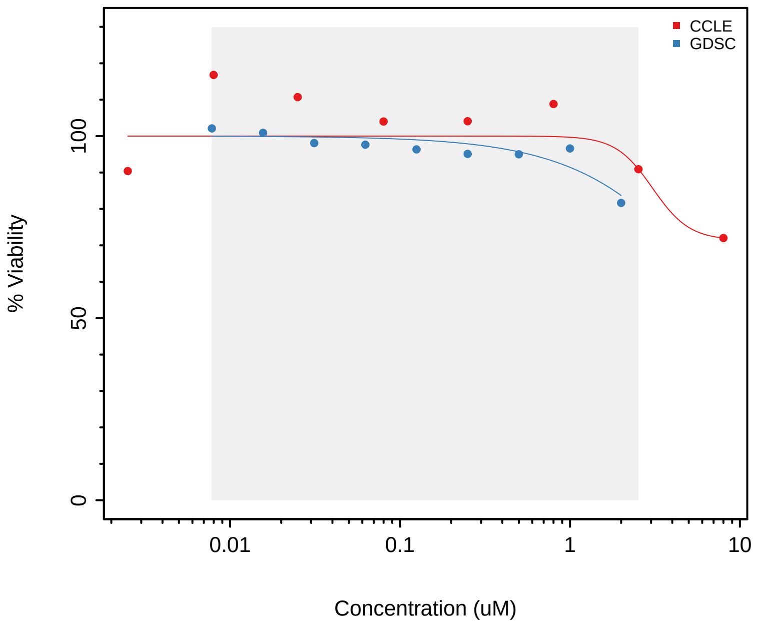

Crizotinib:OVCAR−4

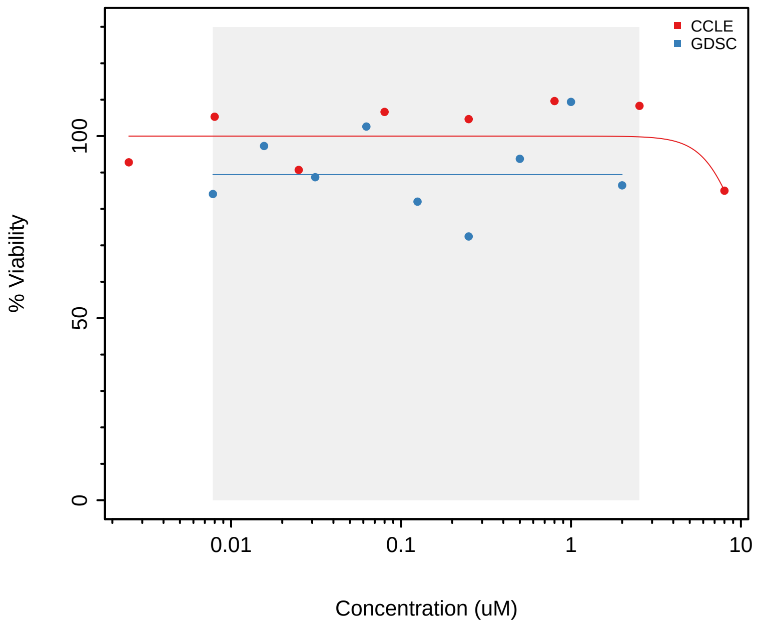

Crizotinib:P31−FUJ

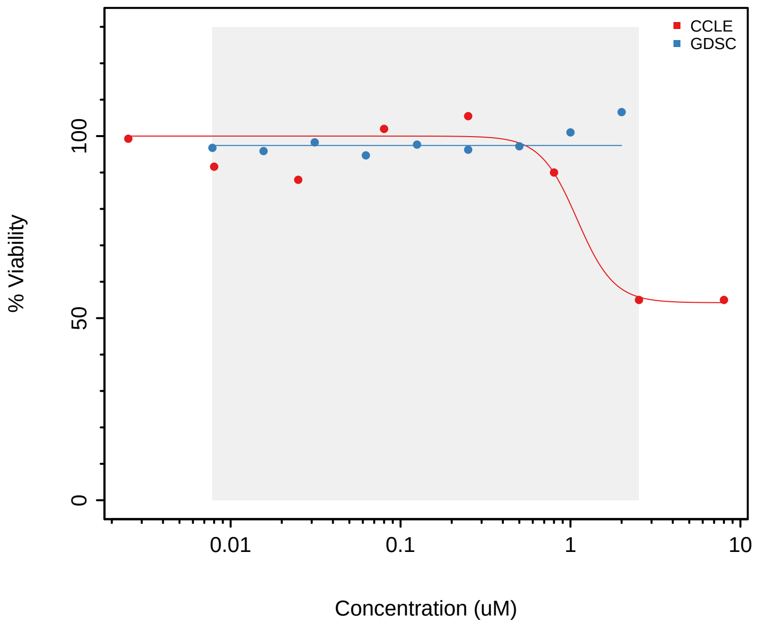

Crizotinib:PSN1

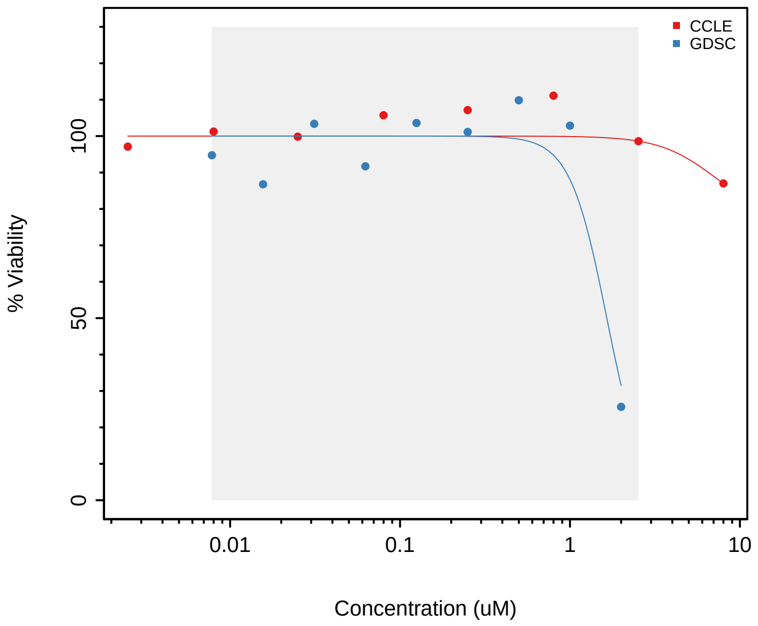

Crizotinib:Raji

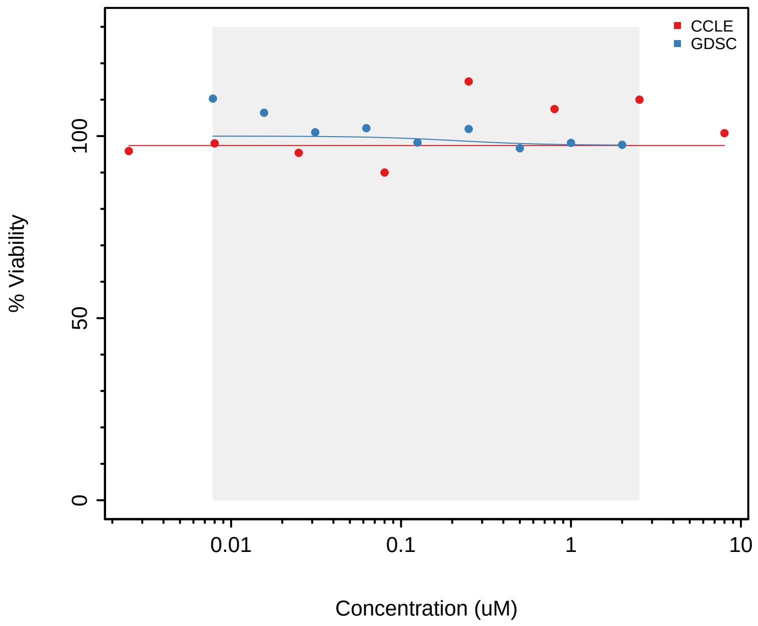

Crizotinib:REH

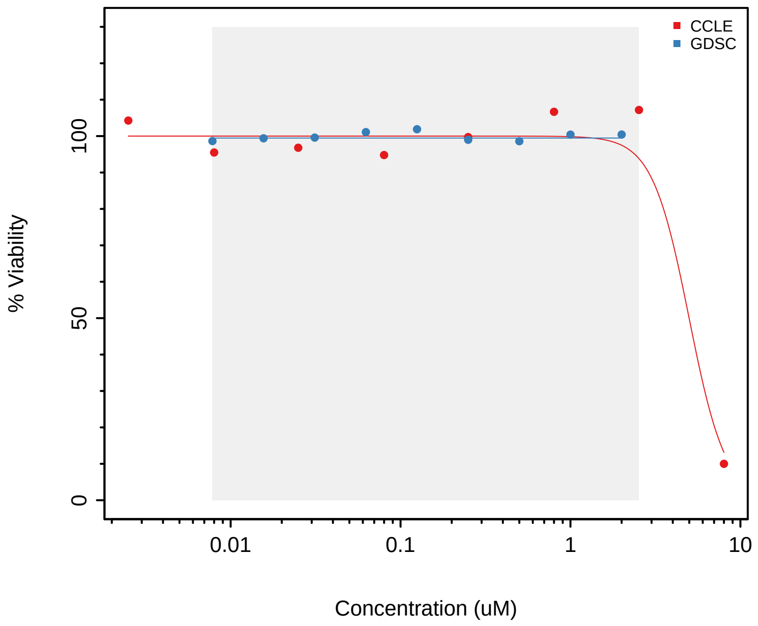

Crizotinib:RKO

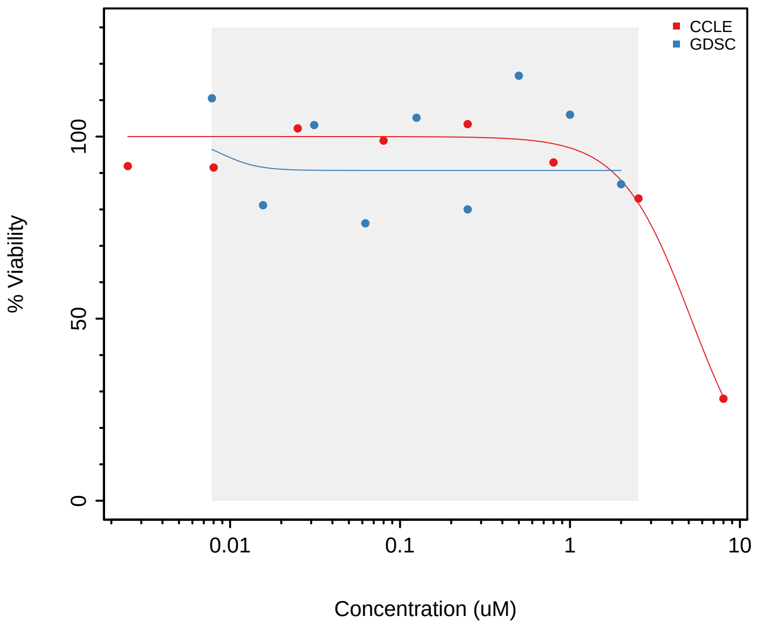

Crizotinib:RL95−2

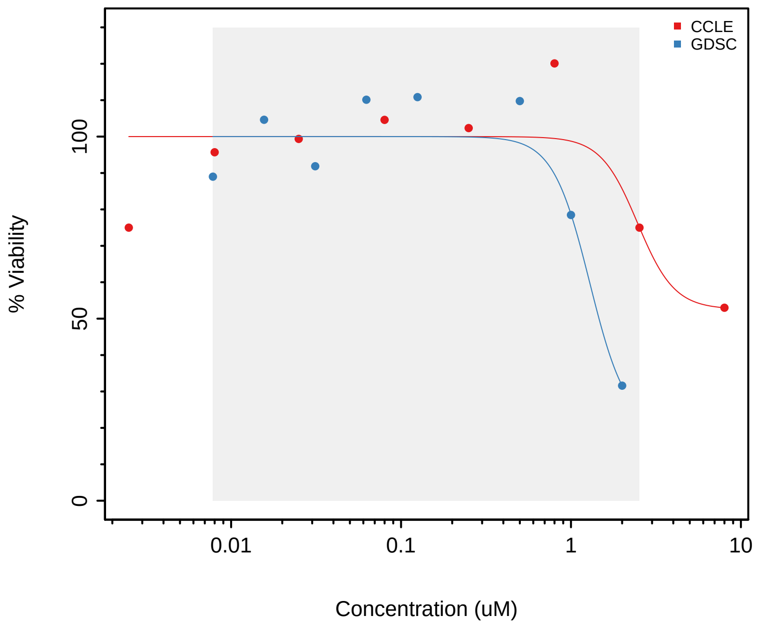

Crizotinib:RPMI−8402

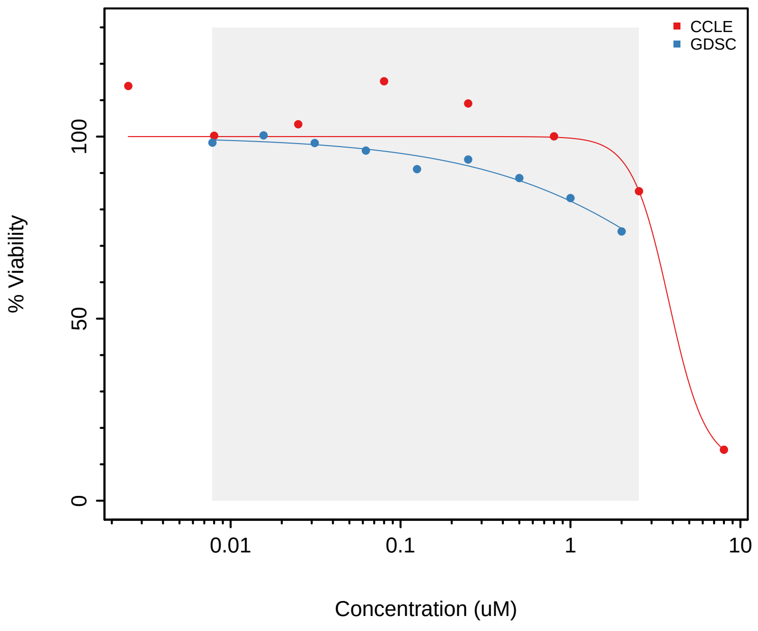

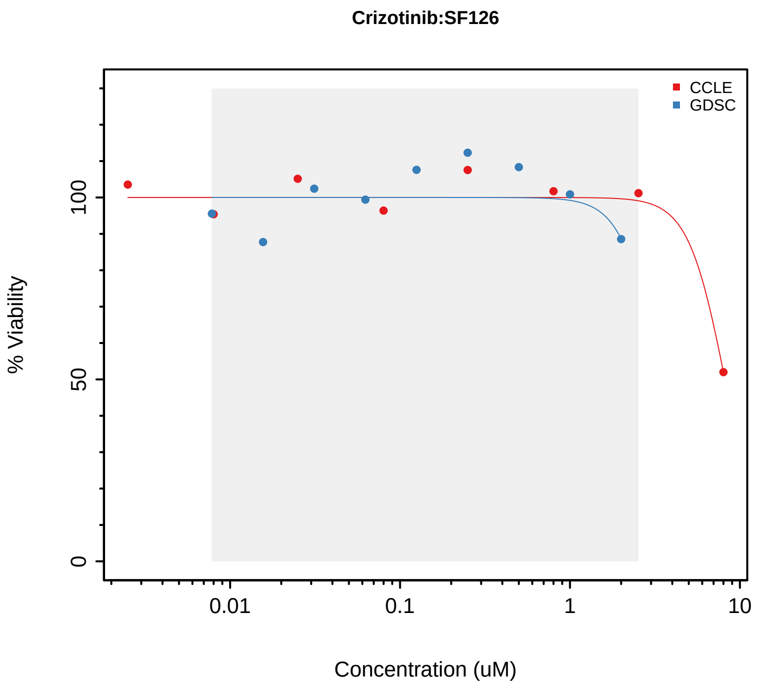
Crizotinib:SF126
CCLE
GDSC
% Viability
Concentration (uM)

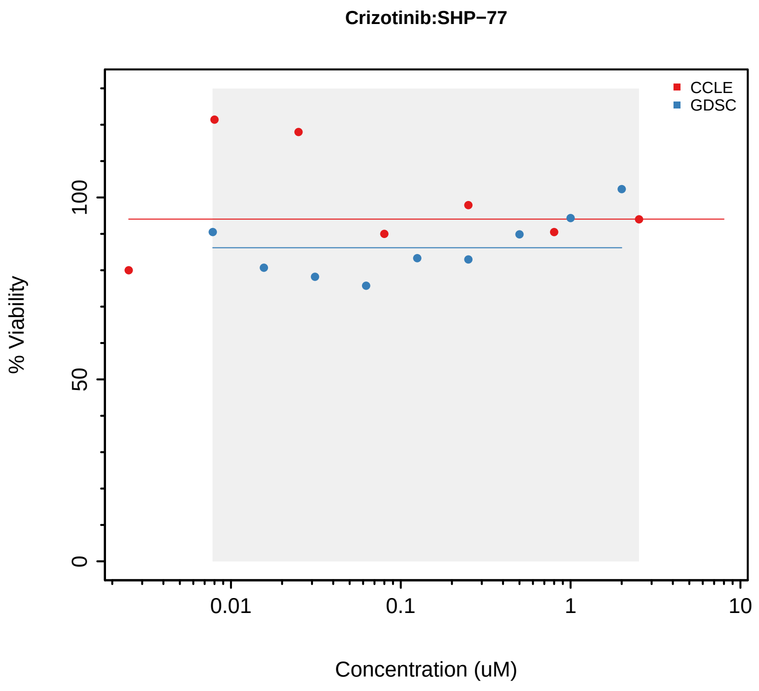
Crizotinib:SHP−77
CCLE
GDSC
% Viability
Concentration (uM)

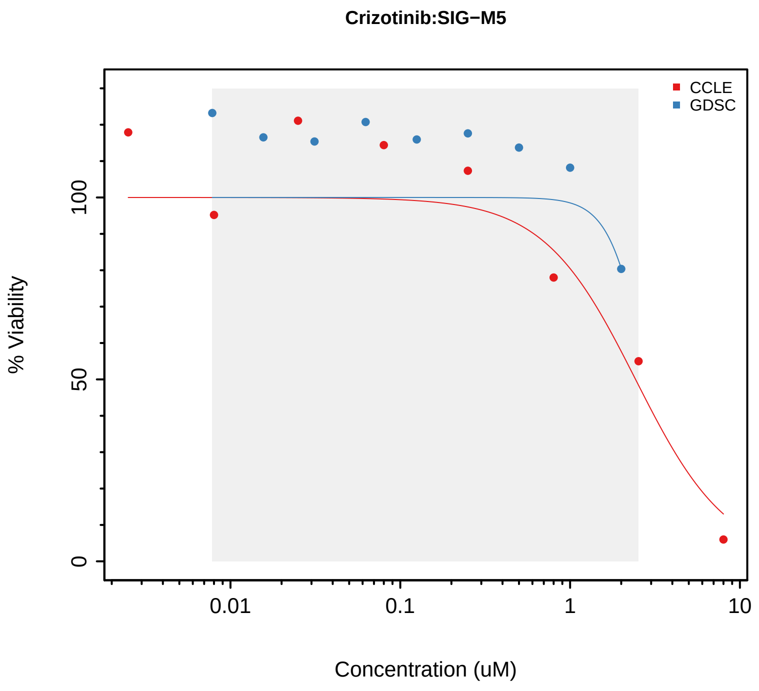
Crizotinib:SIG−M5
CCLE
GDSC
% Viability
Concentration (uM)

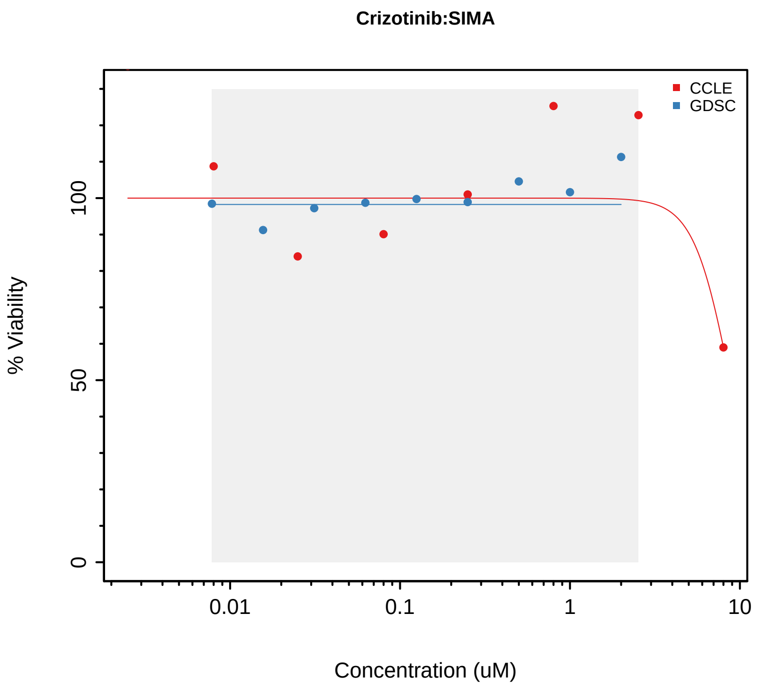
Crizotinib:SIMA
CCLE
GDSC
% Viability
Concentration (uM)

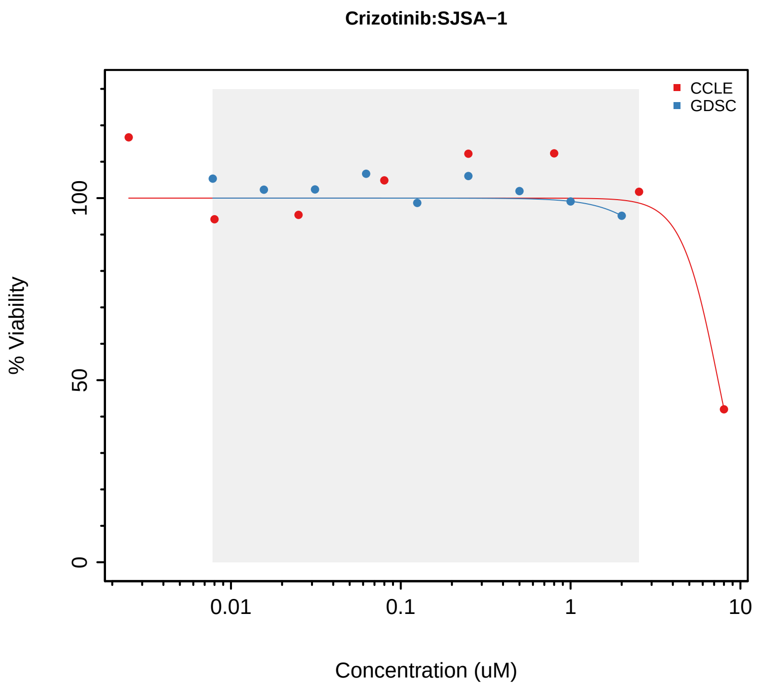
Crizotinib:SJSA−1
CCLE
GDSC
% Viability
Concentration (uM)

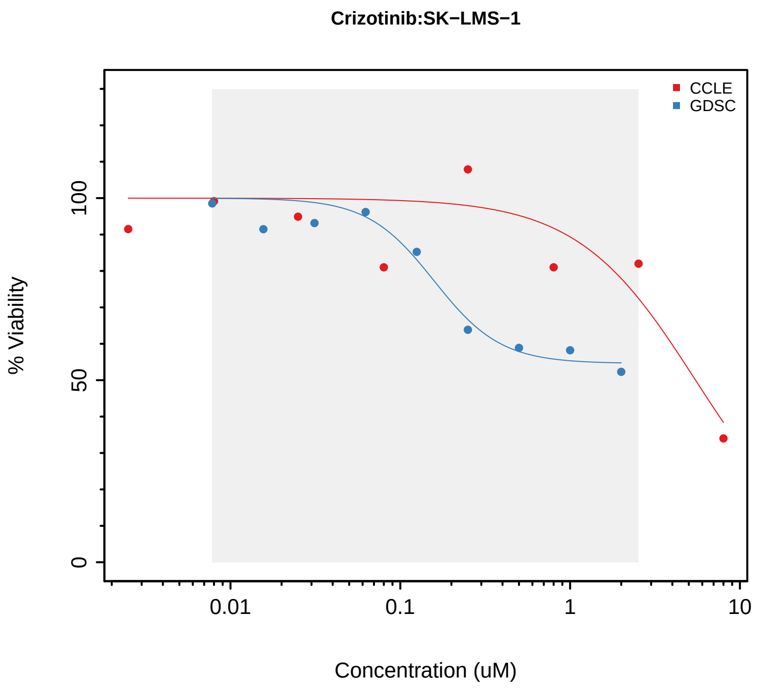
Crizotinib:SK−LMS−1
CCLE
GDSC
% Viability
Concentration (uM)

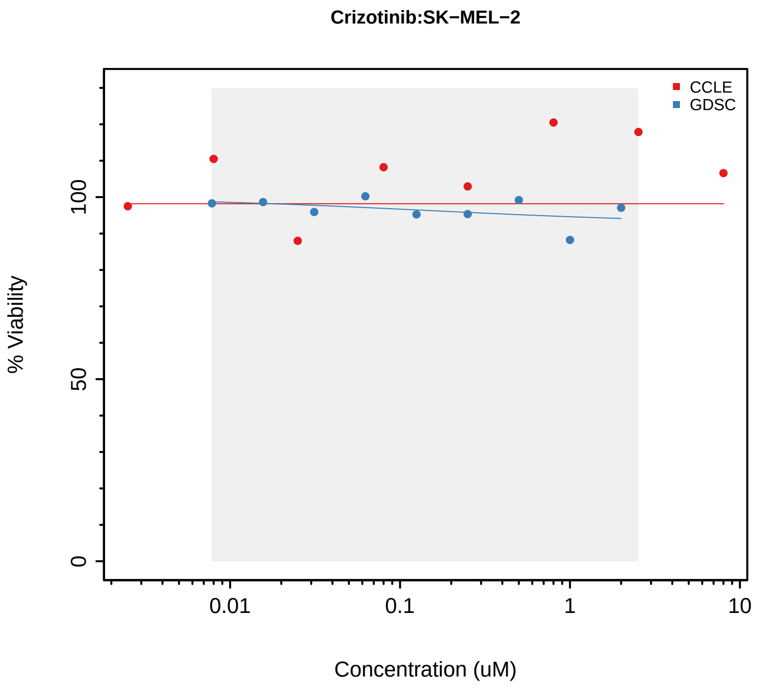
Crizotinib:SK−MEL−2
CCLE
GDSC
% Viability
Concentration (uM)

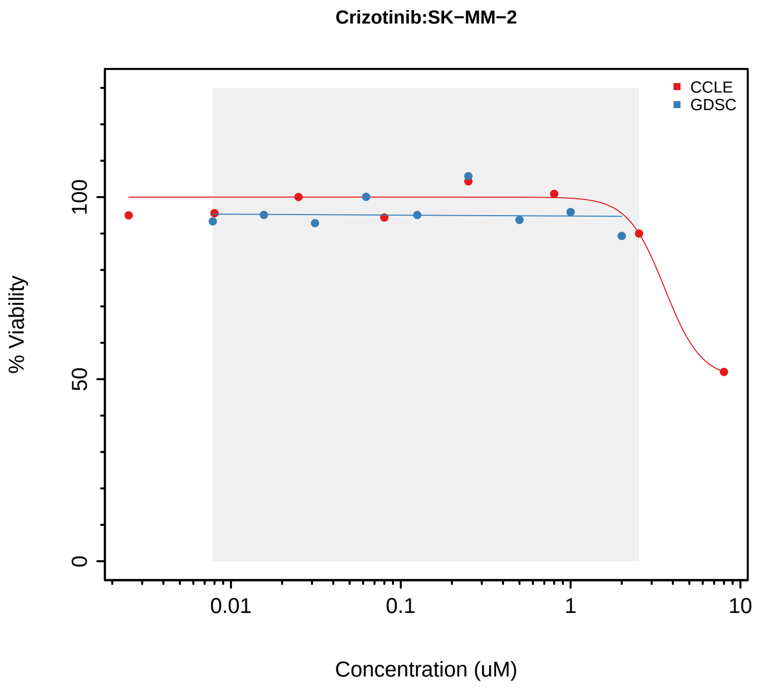
Crizotinib:SK−MM−2
CCLE
GDSC
% Viability
Concentration (uM)

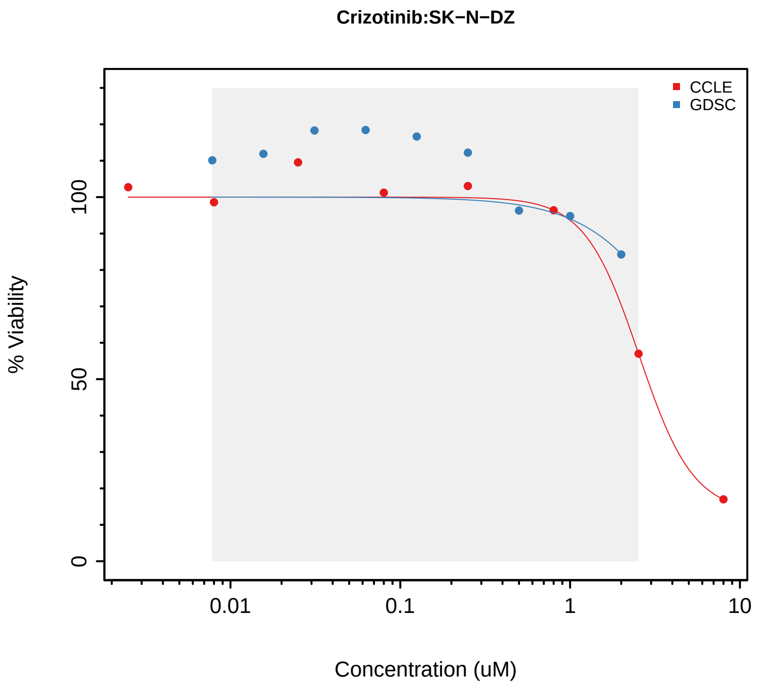
Crizotinib:SK−N−DZ
CCLE
GDSC
% Viability
Concentration (uM)

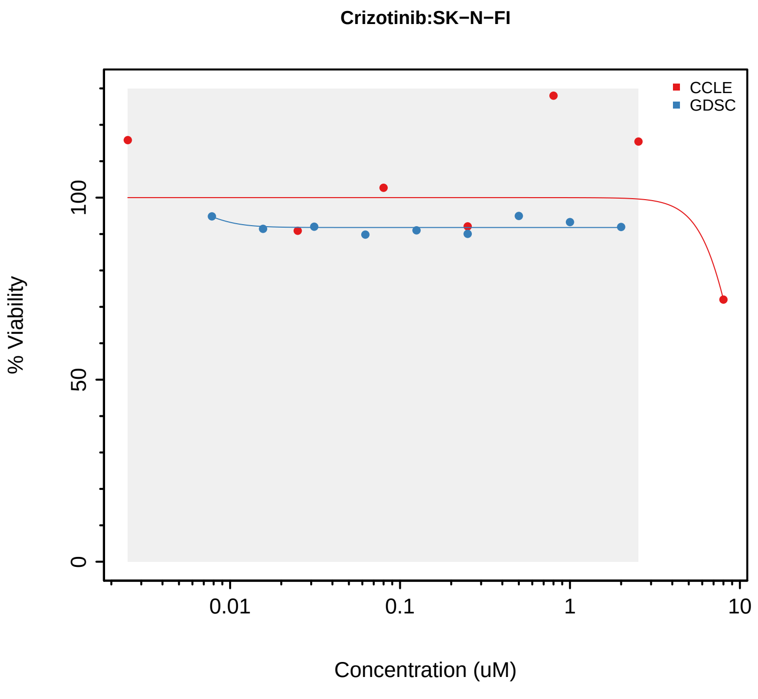
Crizotinib:SK−N−FI
CCLE
GDSC
% Viability
Concentration (uM)

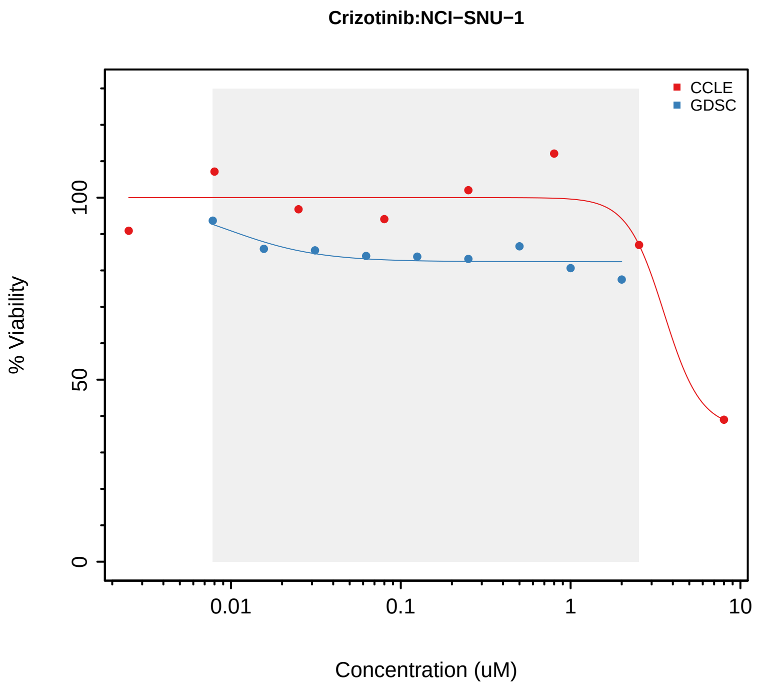
Crizotinib:NCI−SNU−1
CCLE
GDSC
% Viability
Concentration (uM)

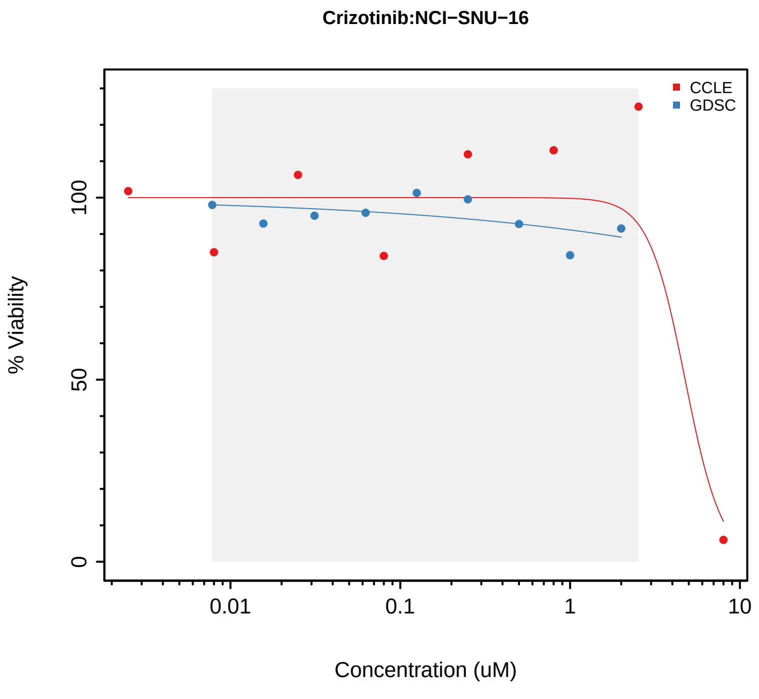
Crizotinib:NCI−SNU−16
CCLE
GDSC
% Viability
Concentration (uM)

Crizotinib:SNU−C2B

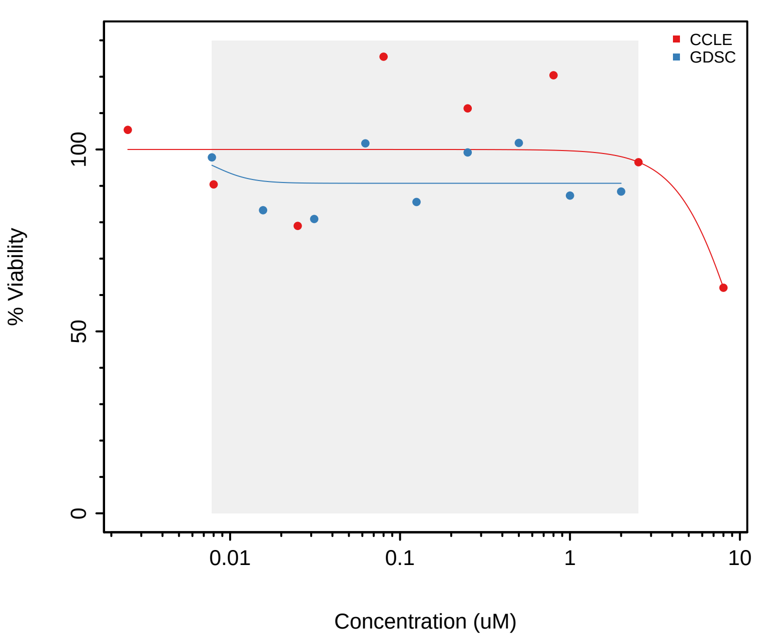

Crizotinib:SUP−T1

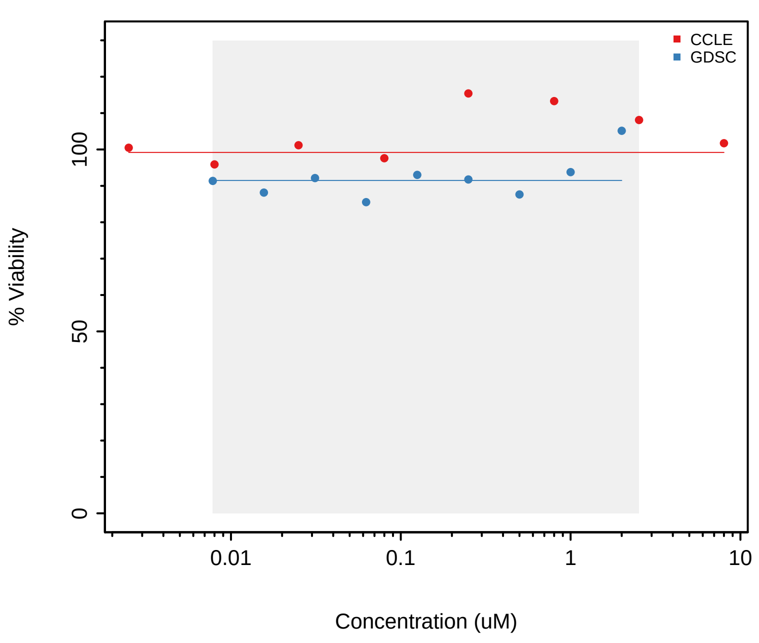

Crizotinib:TE−1

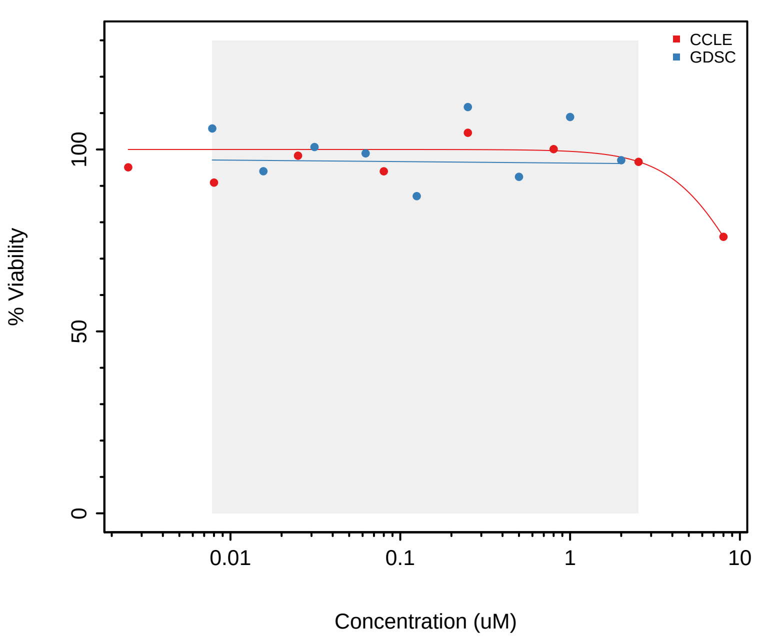

Crizotinib:TE−11

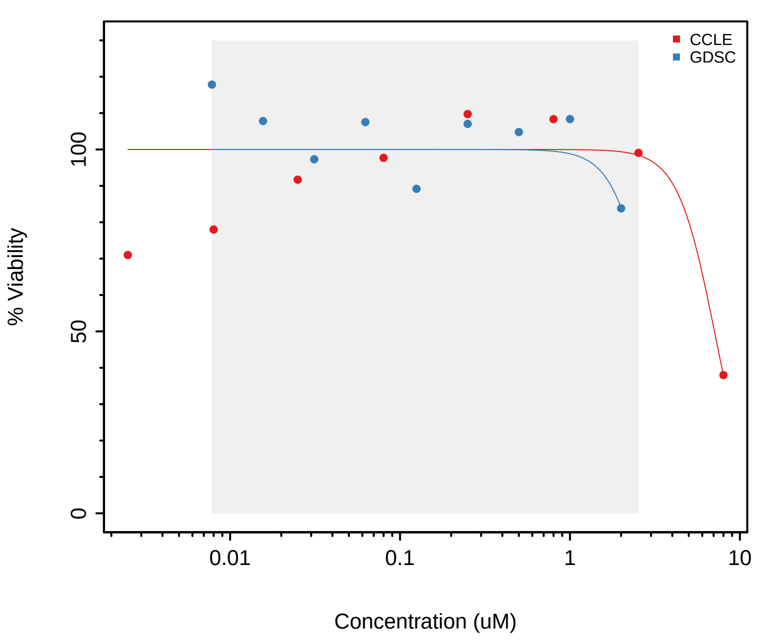

Crizotinib:TE−15

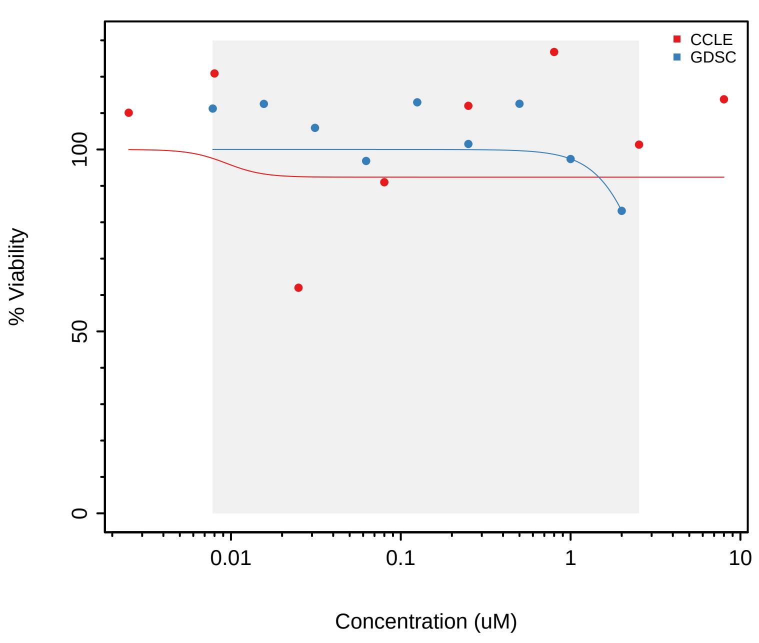

Crizotinib:TE−5

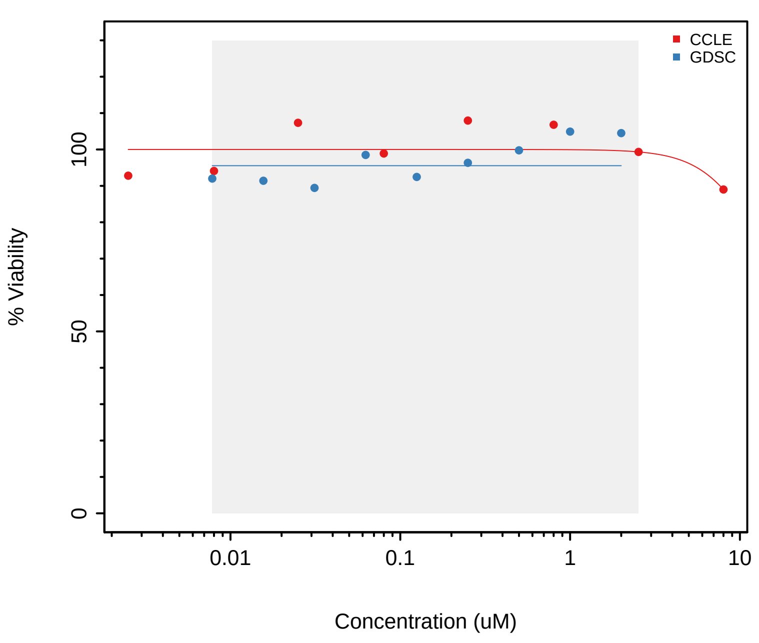

Crizotinib:TE−9

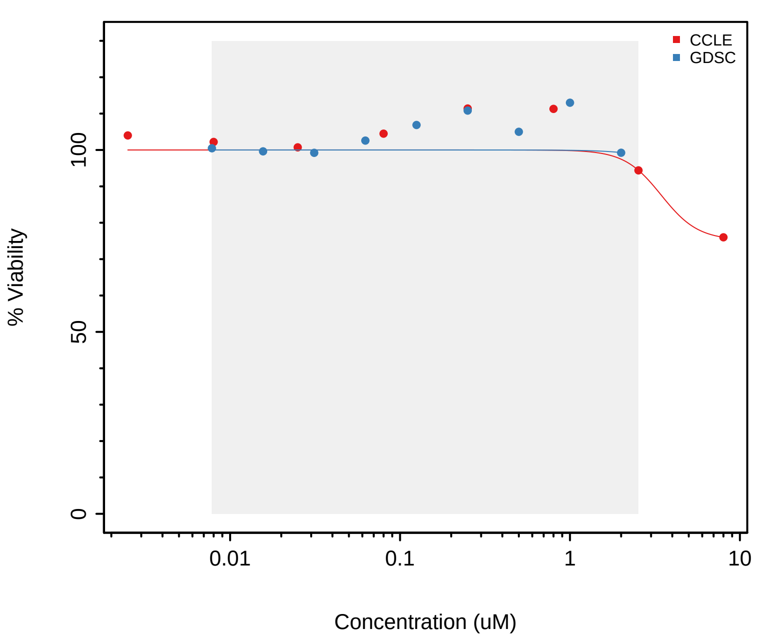

Crizotinib:U−87−MG

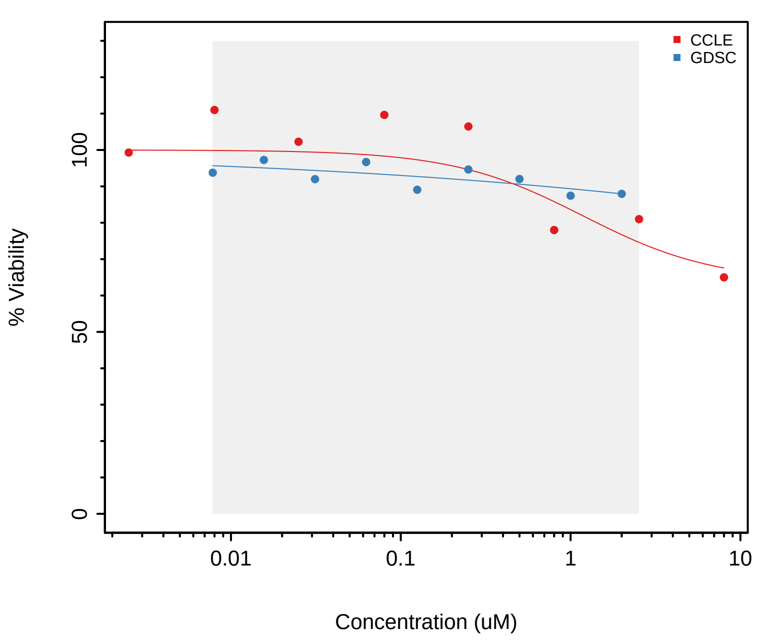

Crizotinib:UACC−257

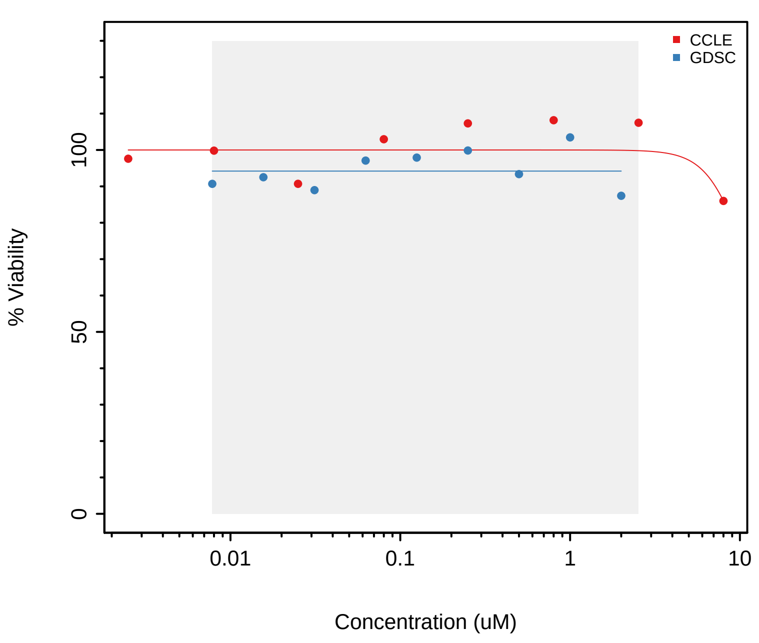

Crizotinib:UACC−812

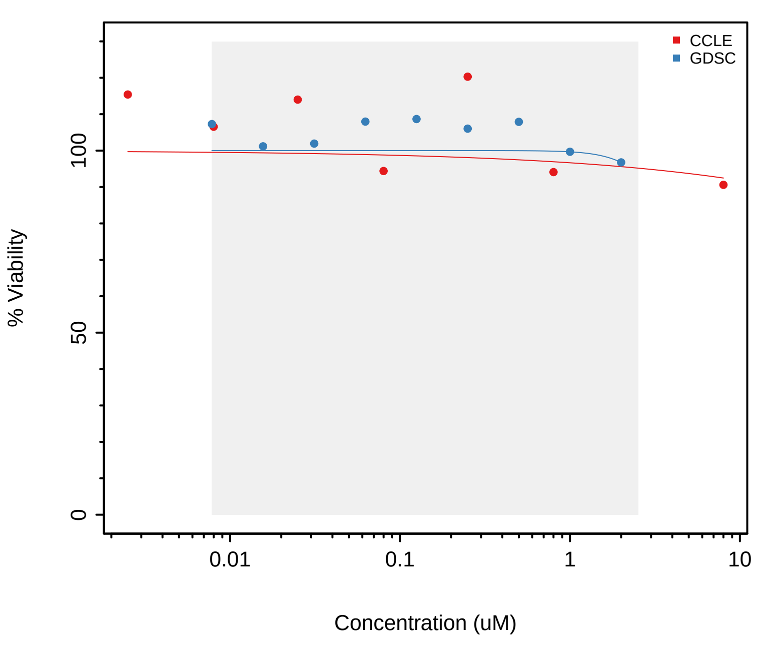

Sorafenib:697

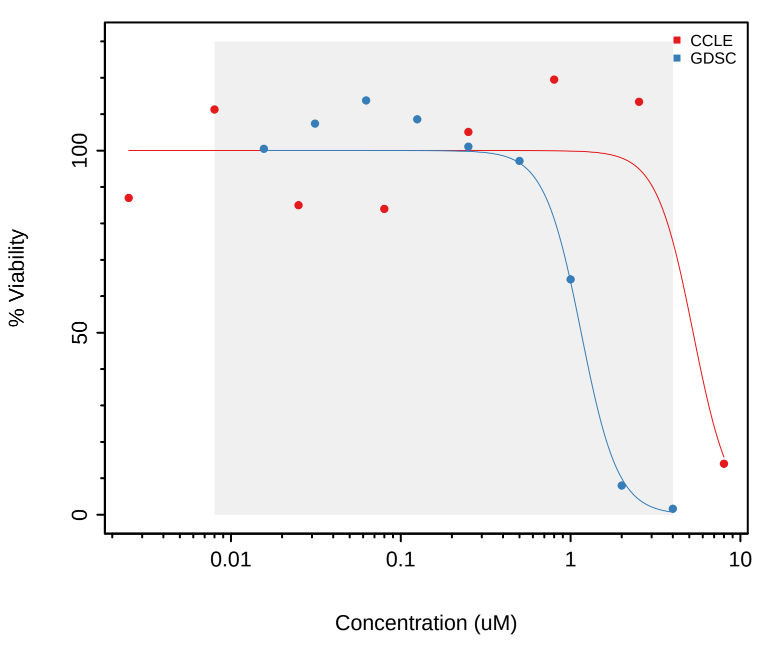

Sorafenib:8−MG−BA

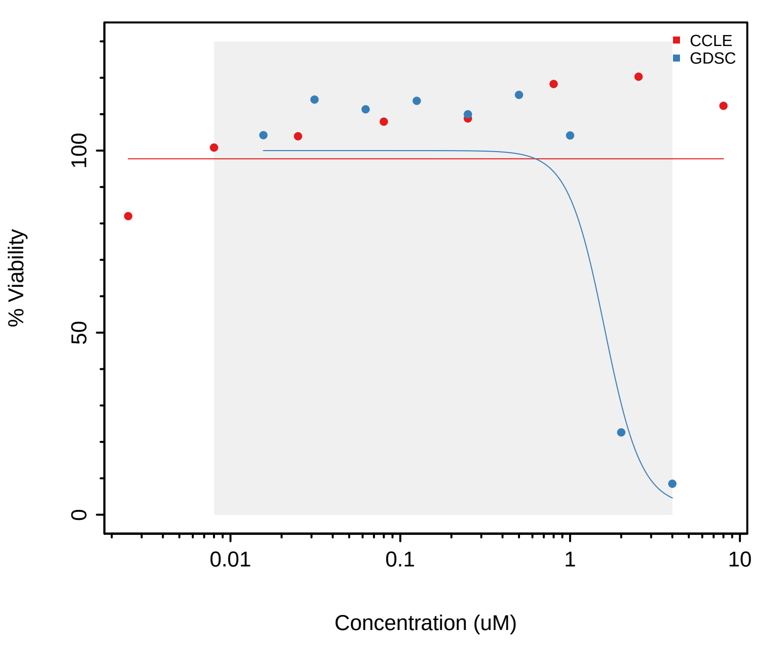

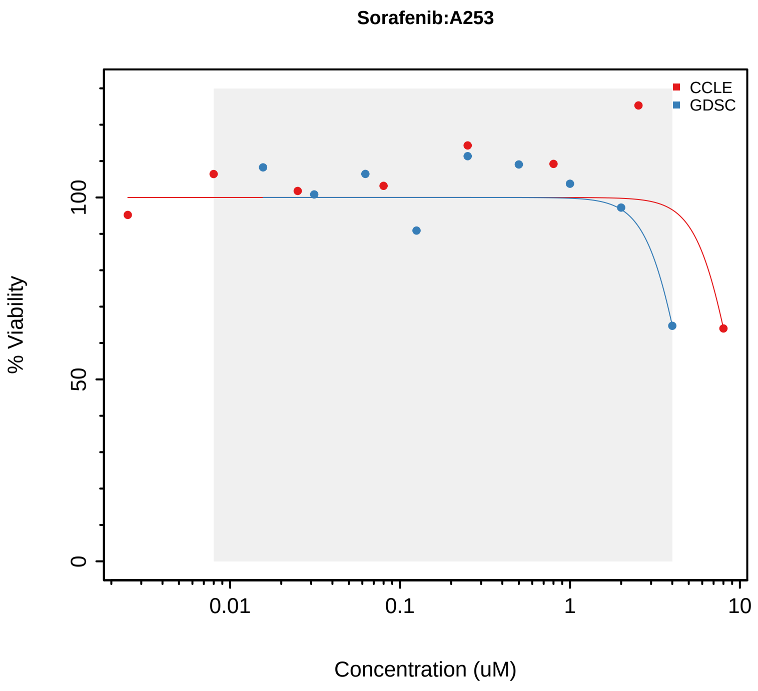
Sorafenib:A253
CCLE
GDSC
% Viability
Concentration (uM)

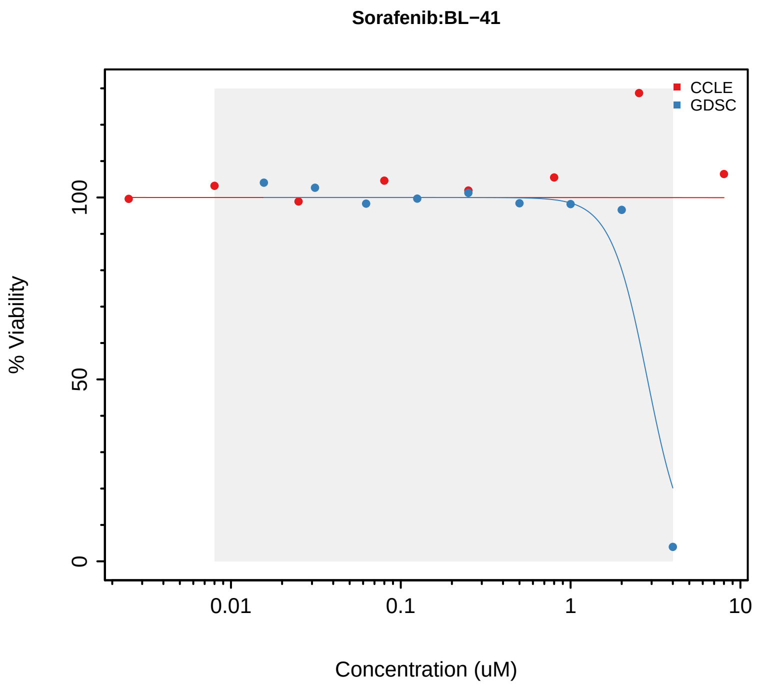
Sorafenib:BL−41
CCLE
GDSC
% Viability
Concentration (uM)

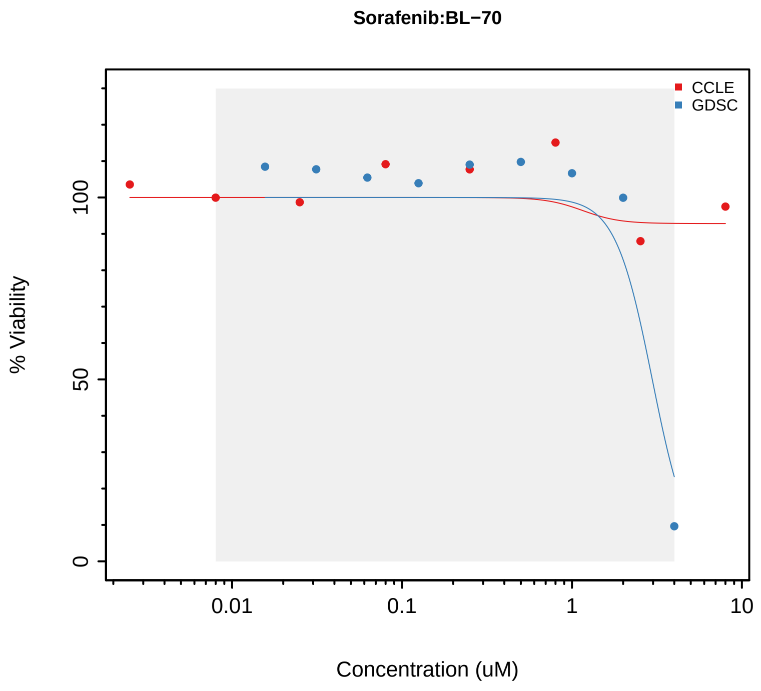
Sorafenib:BL−70
CCLE
GDSC
% Viability
Concentration (uM)

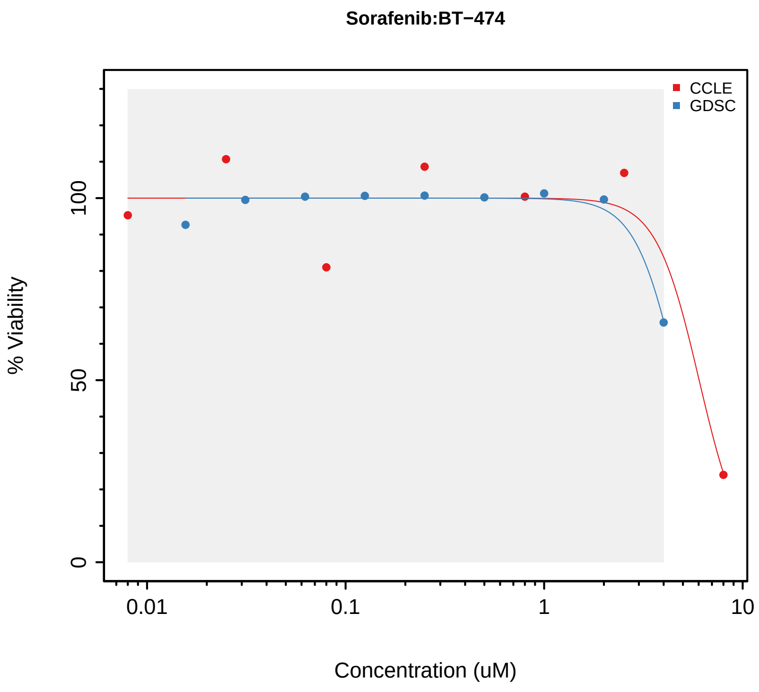
Sorafenib:BT−474
CCLE
GDSC
% Viability
Concentration (uM)

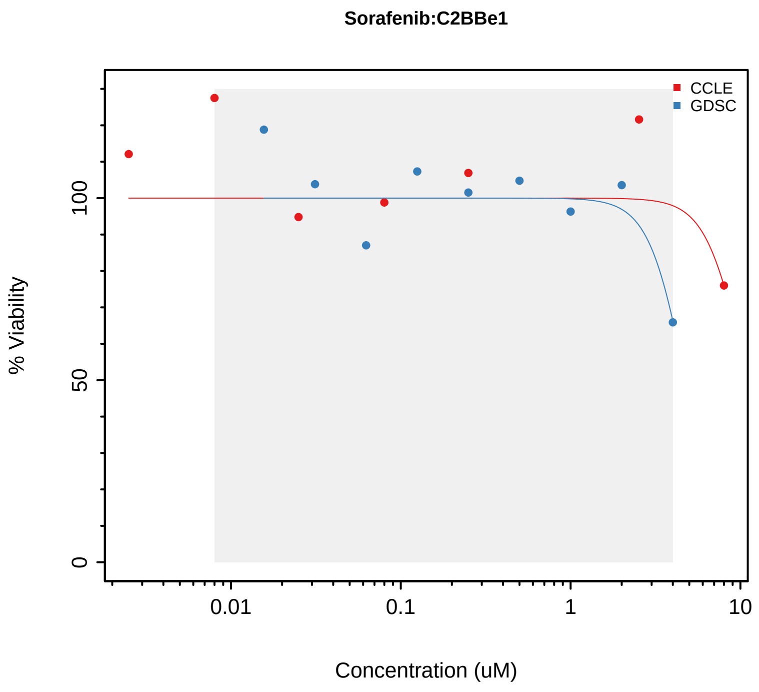
Sorafenib:C2BBe1
CCLE
GDSC
% Viability
Concentration (uM)

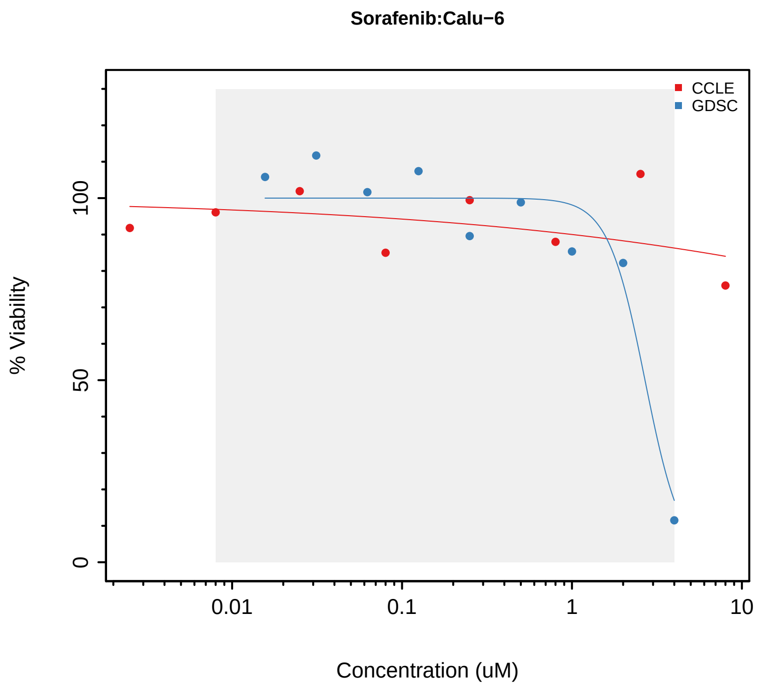
Sorafenib:Calu−6
CCLE
GDSC
% Viability
Concentration (uM)

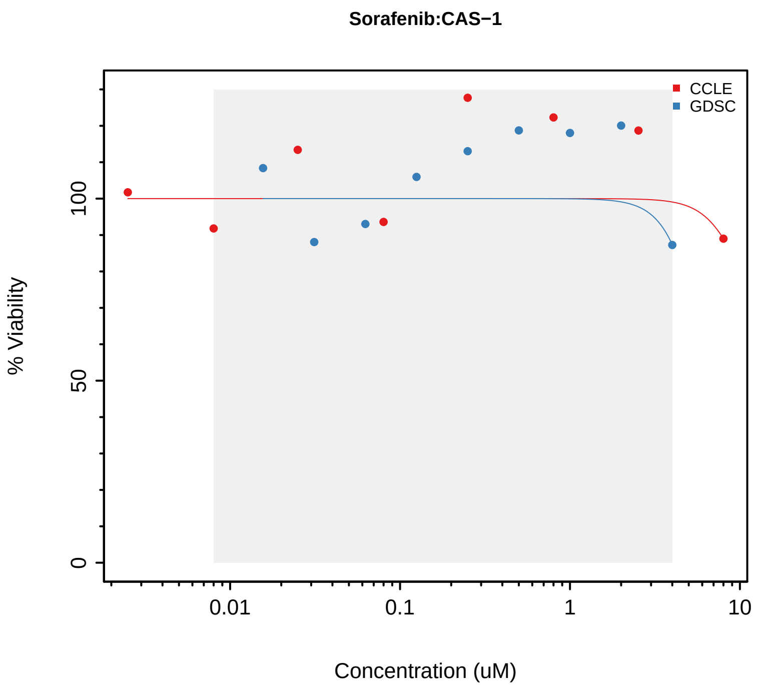
Sorafenib:CAS−1
CCLE
GDSC
% Viability
Concentration (uM)

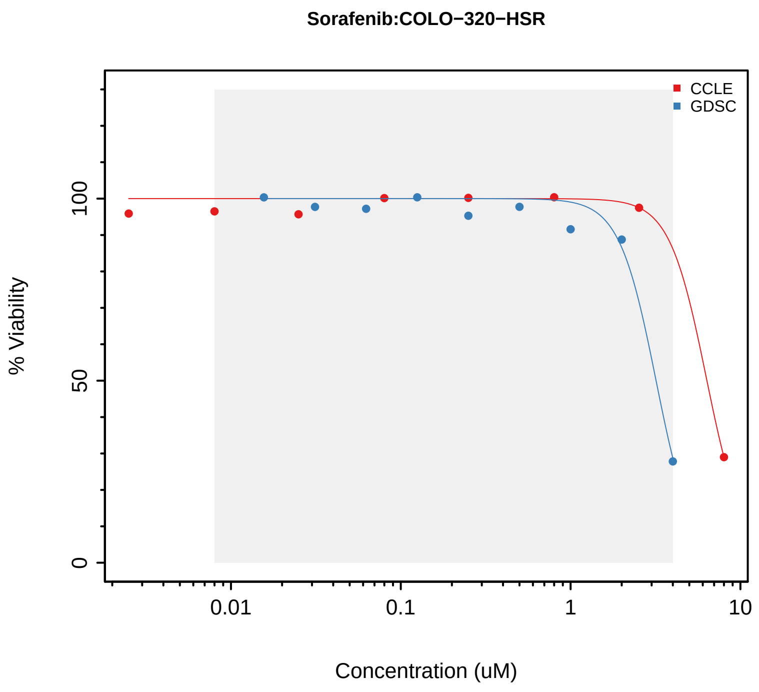
Sorafenib:COLO−320−HSR
CCLE
GDSC
% Viability
Concentration (uM)

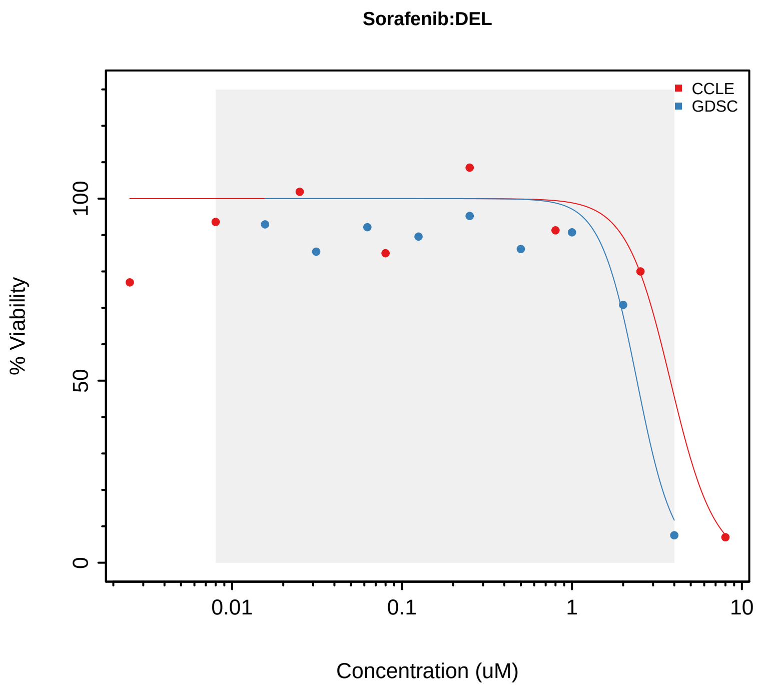
Sorafenib:DEL
CCLE
GDSC
% Viability
Concentration (uM)

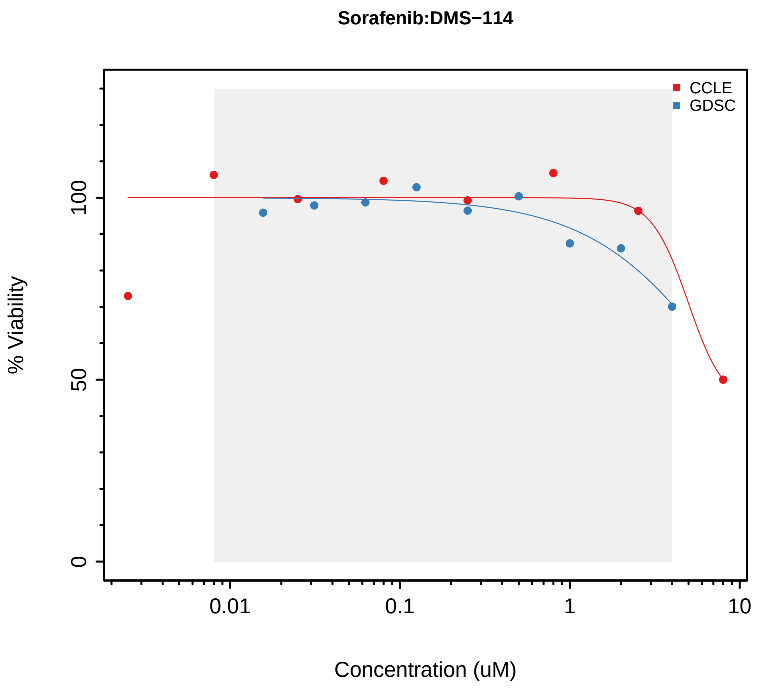
Sorafenib:DMS−114
CCLE
GDSC
% Viability
Concentration (uM)

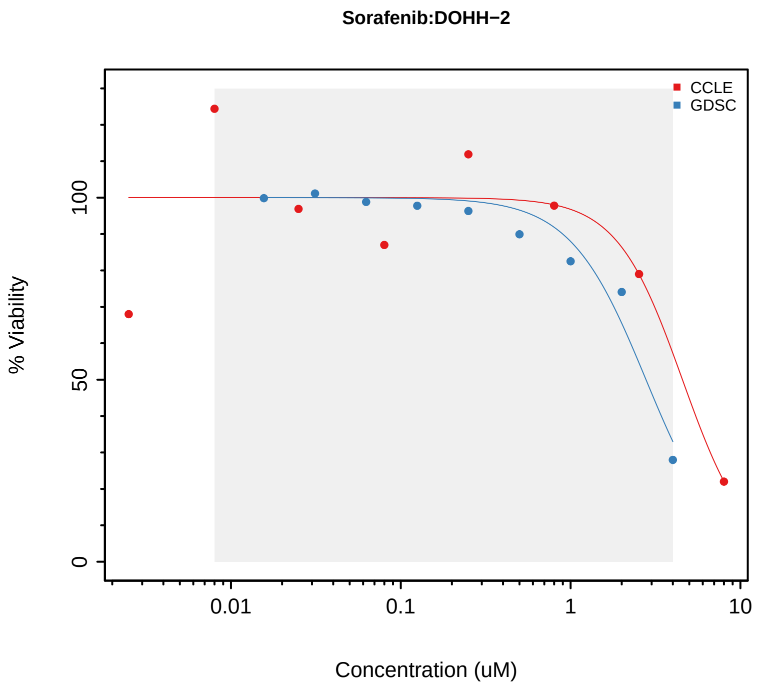
Sorafenib:DOHH−2
CCLE
GDSC
% Viability
Concentration (uM)

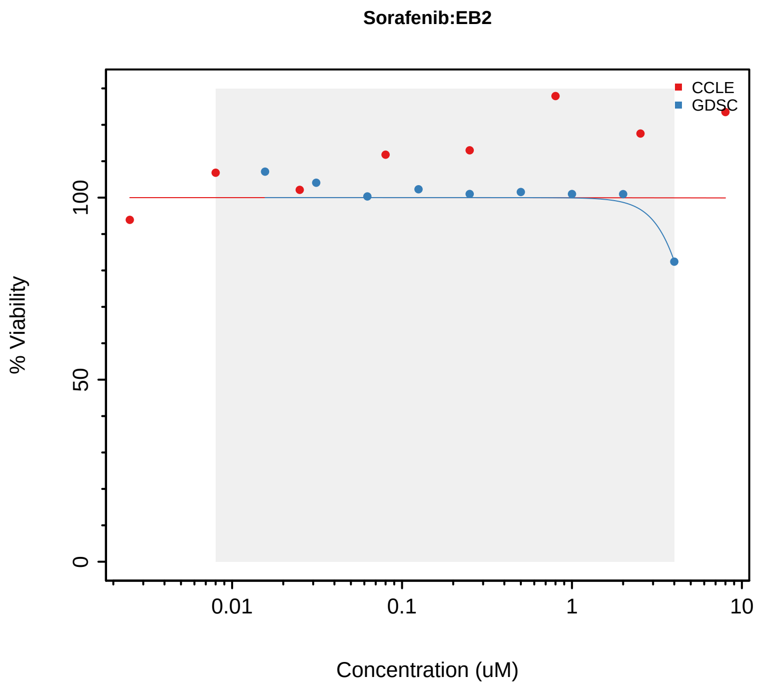
Sorafenib:EB2
CCLE
GDSC
% Viability
Concentration (uM)

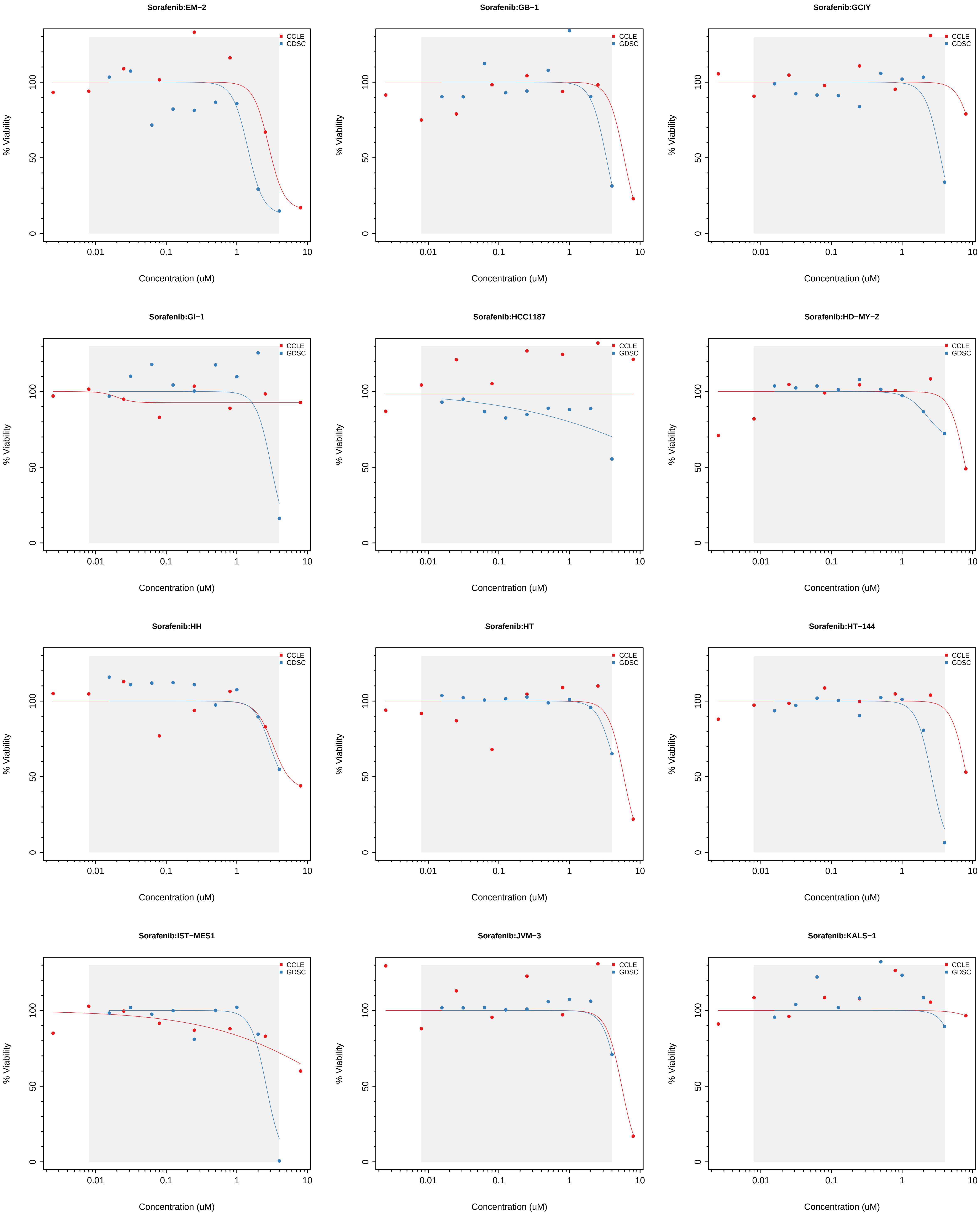

Sorafenib:EM−2
Sorafenib:GB−1
Sorafenib:GCIY
Sorafenib:GI−1
Sorafenib:HCC1187
Sorafenib:HD−MY−Z
Sorafenib:HH
Sorafenib:HT
Sorafenib:HT−144
Sorafenib:IST−MES1
Sorafenib:JVM−3
Sorafenib:KALS−1
CCLE
GDSC
% Viability
0
50
100
0.01
0.1
1
10
Concentration (uM)

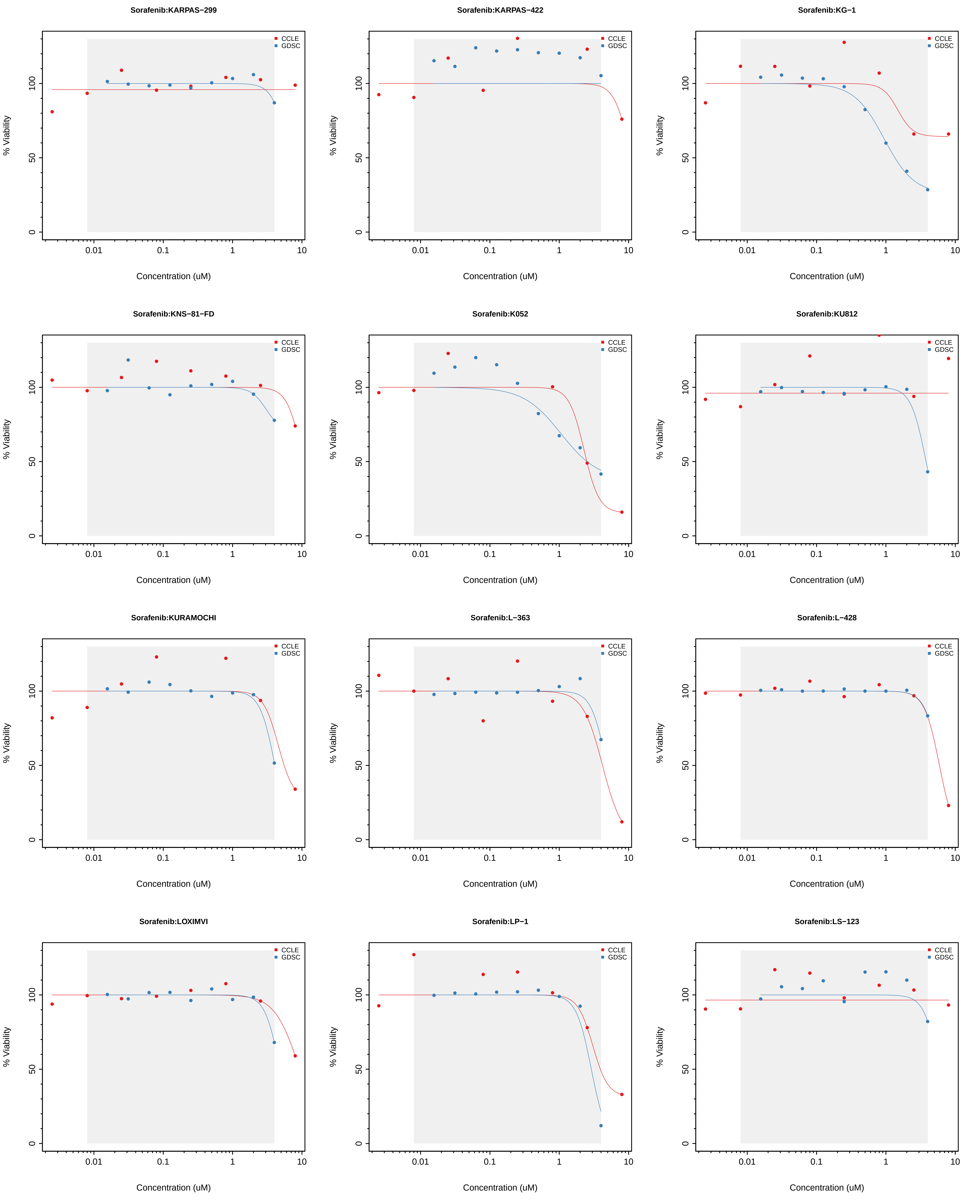
Sorafenib:KARPAS−299
Sorafenib:KARPAS−422
Sorafenib:KG−1
Sorafenib:KNS−81−FD
Sorafenib:K052
Sorafenib:KU812
Sorafenib:KURAMOCHI
Sorafenib:L−363
Sorafenib:L−428
Sorafenib:LOXIMVI
Sorafenib:LP−1
Sorafenib:LS−123
CCLE
GDSC
% Viability
0
50
100
0.01
0.1
1
10
Concentration (uM)

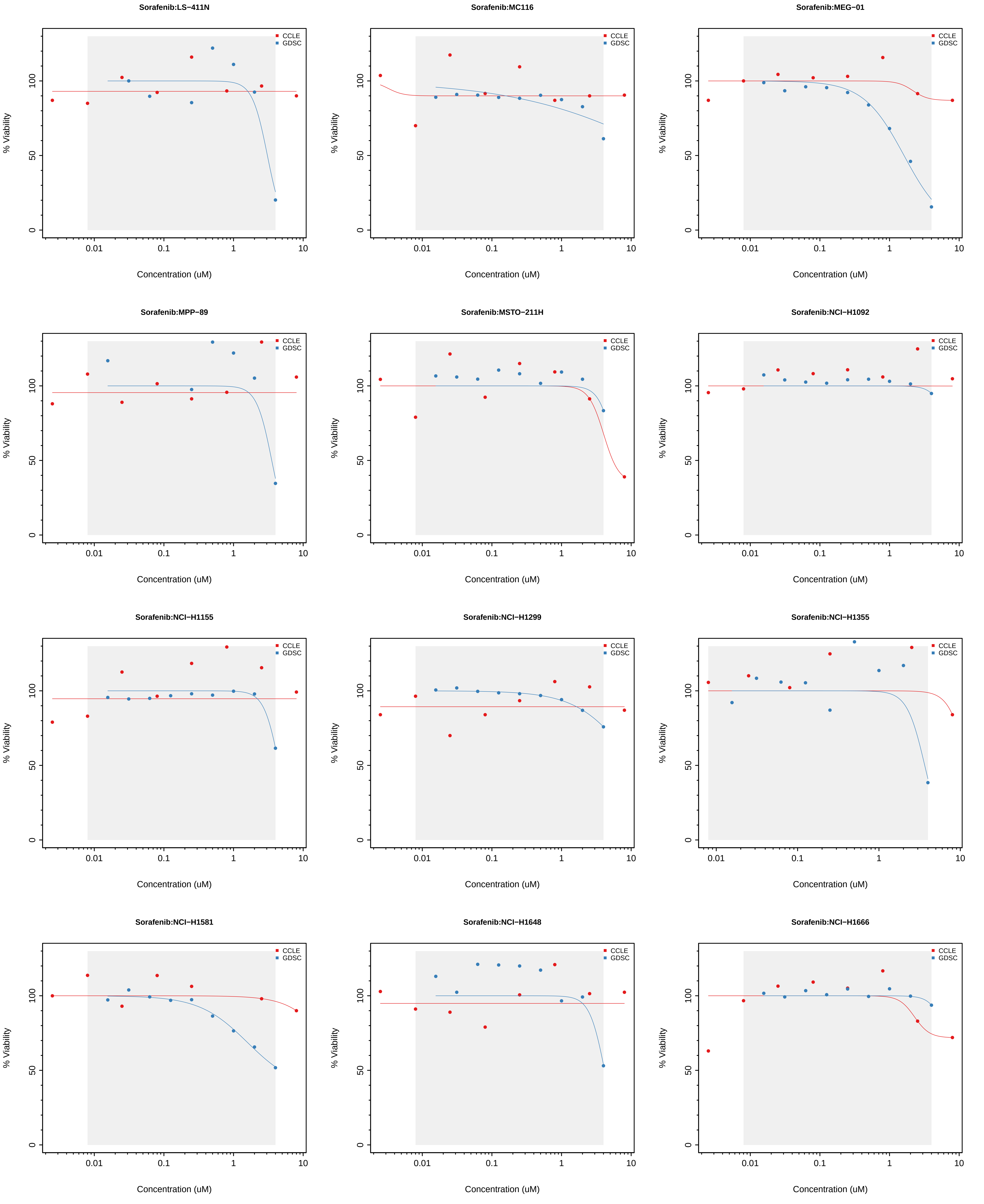

Sorafenib:LS−411N
Sorafenib:MC116
Sorafenib:MEG−01
Sorafenib:MPP−89
Sorafenib:MSTO−211H
Sorafenib:NCI−H1092
Sorafenib:NCI−H1155
Sorafenib:NCI−H1299
Sorafenib:NCI−H1355
Sorafenib:NCI−H1581
Sorafenib:NCI−H1648
Sorafenib:NCI−H1666
CCLE
GDSC
% Viability
Concentration (uM)
0
50
100
0.01
0.1
1
10

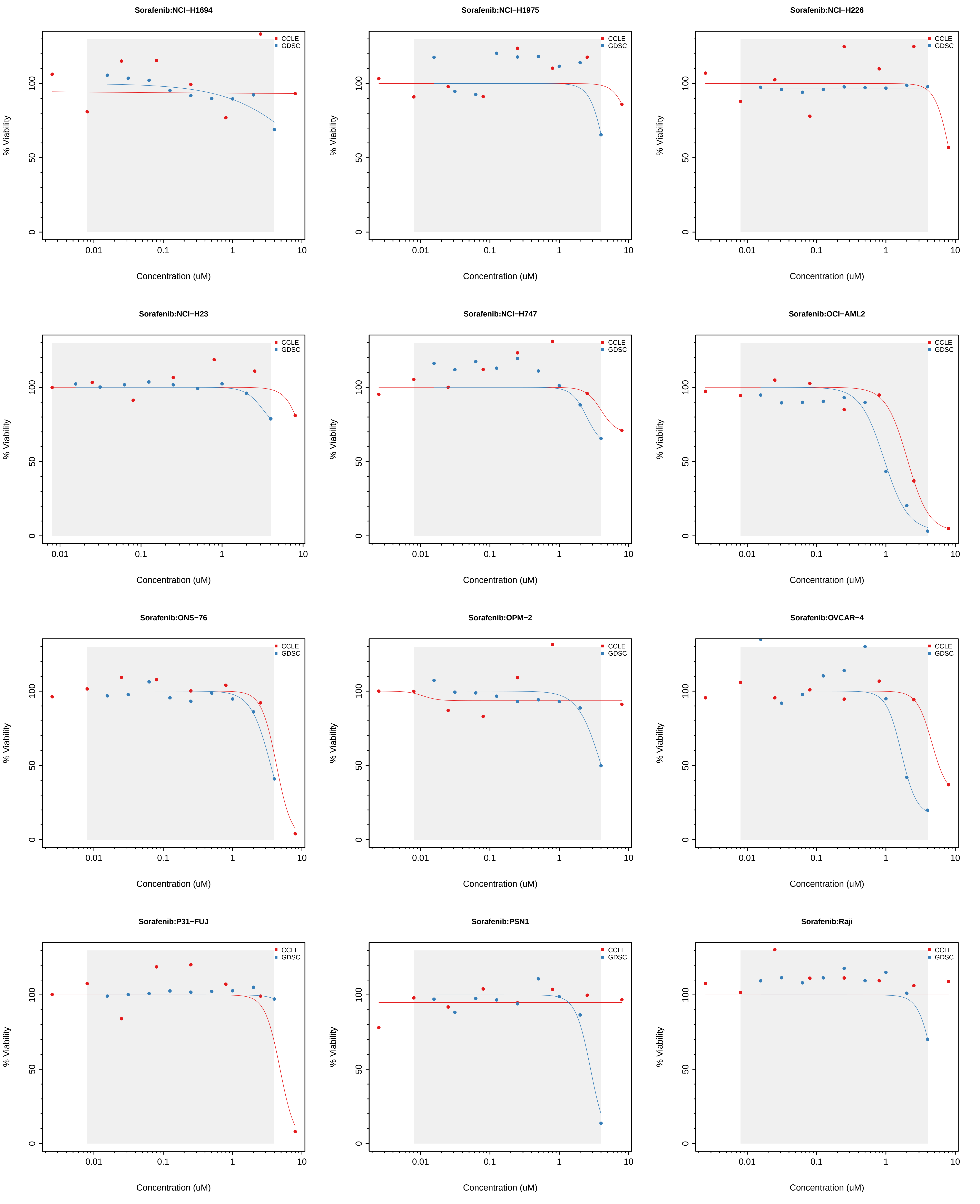
Sorafenib:NCI−H1694
Sorafenib:NCI−H1975
Sorafenib:NCI−H226
Sorafenib:NCI−H23
Sorafenib:NCI−H747
Sorafenib:OCI−AML2
Sorafenib:ONS−76
Sorafenib:OPM−2
Sorafenib:OVCAR−4
Sorafenib:P31−FUJ
Sorafenib:PSN1
Sorafenib:Raji
CCLE
GDSC
% Viability
Concentration (uM)
0
50
100
0.01
0.1
1
10

Sorafenib:REH

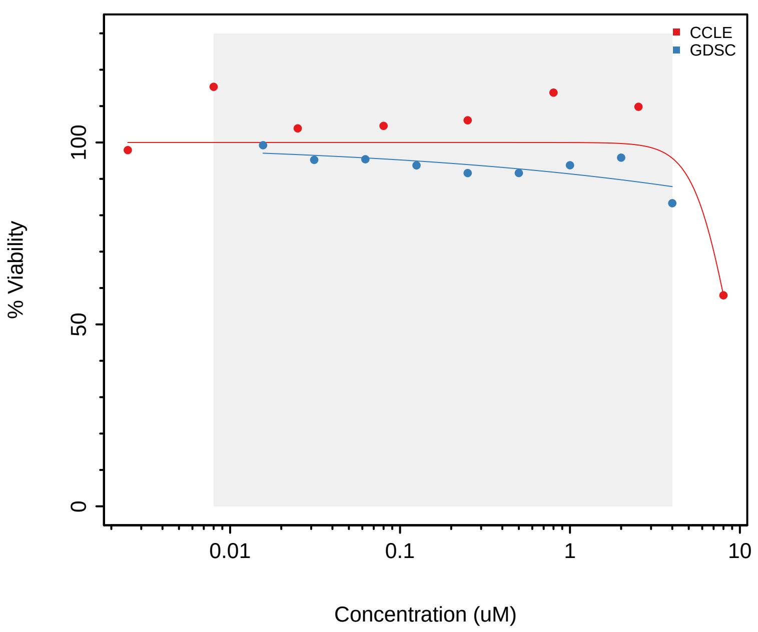

Sorafenib:RKO

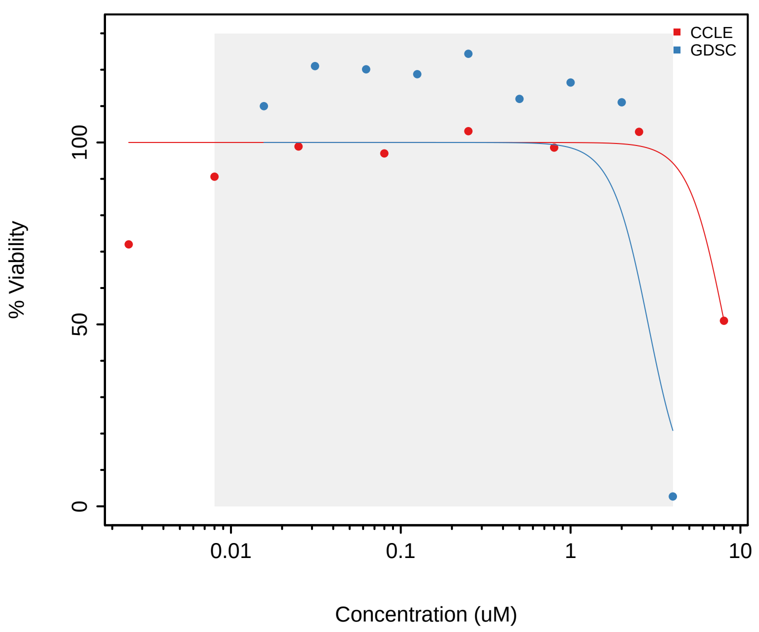

Sorafenib:RL95−2

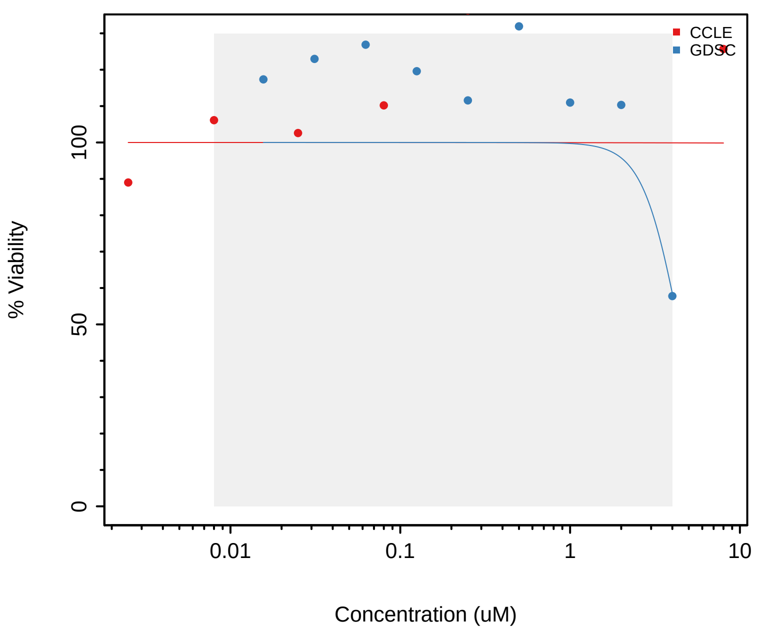

Sorafenib:RPMI−8402

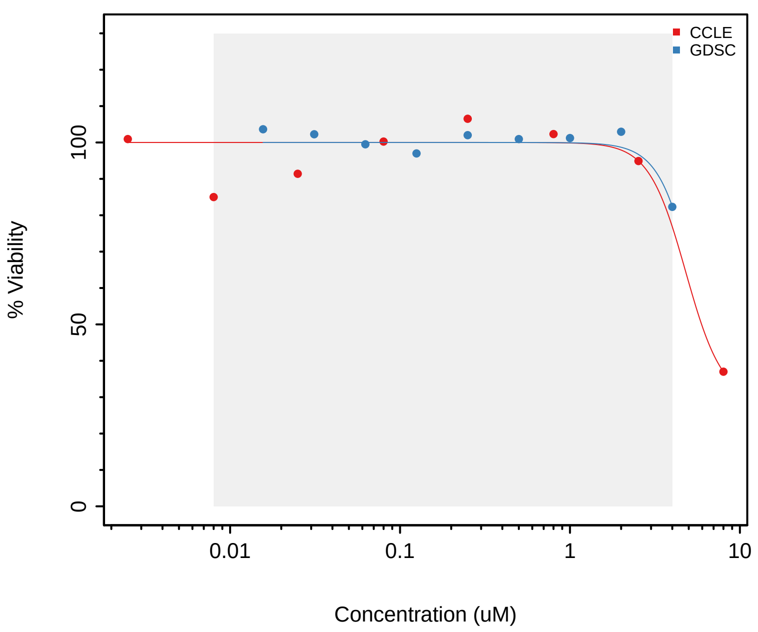

Sorafenib:SHP−77

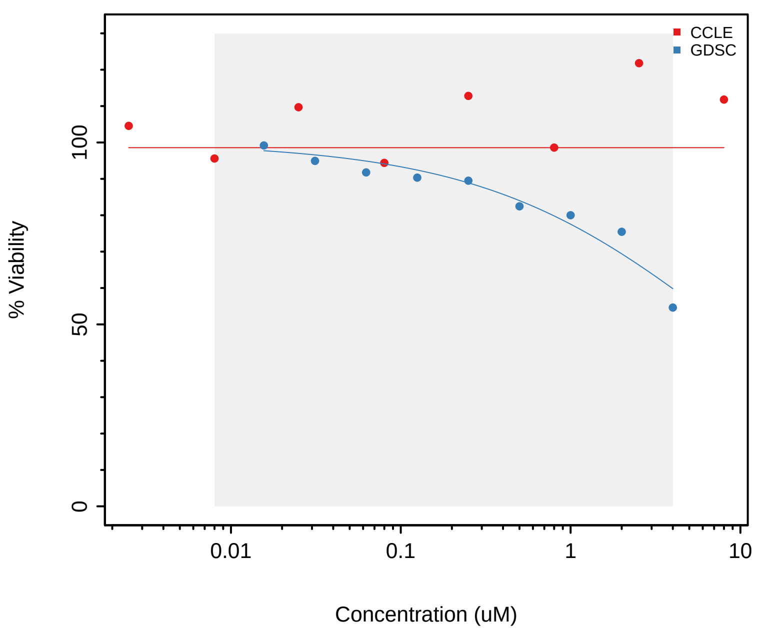

Sorafenib:SIG−M5

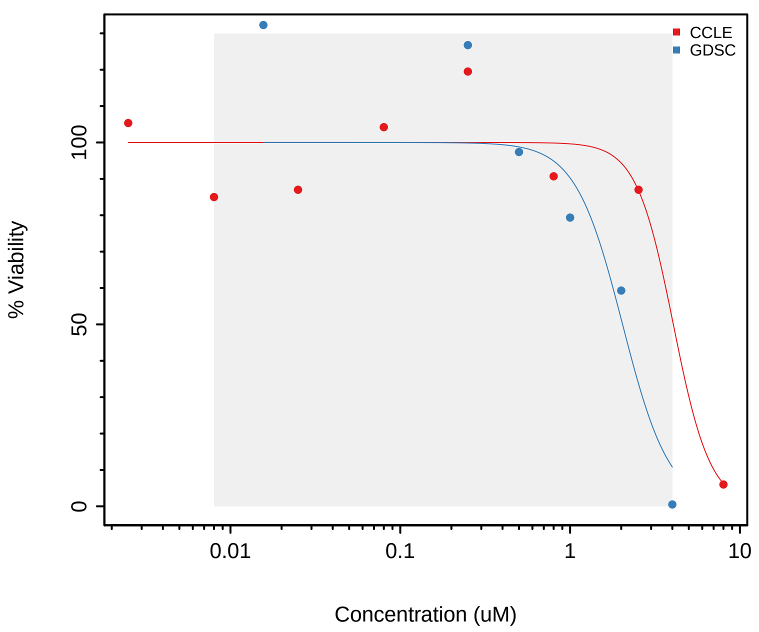

Sorafenib:SIMA

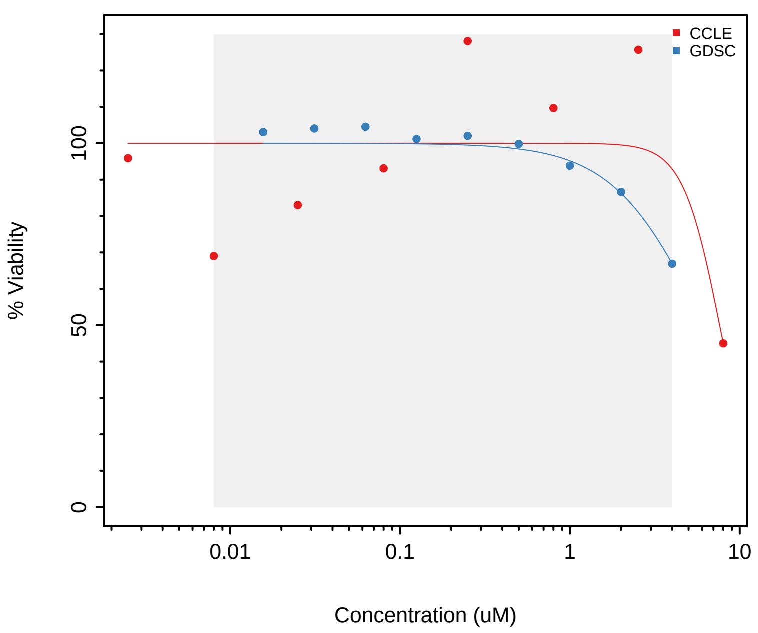

Sorafenib:SJSA−1

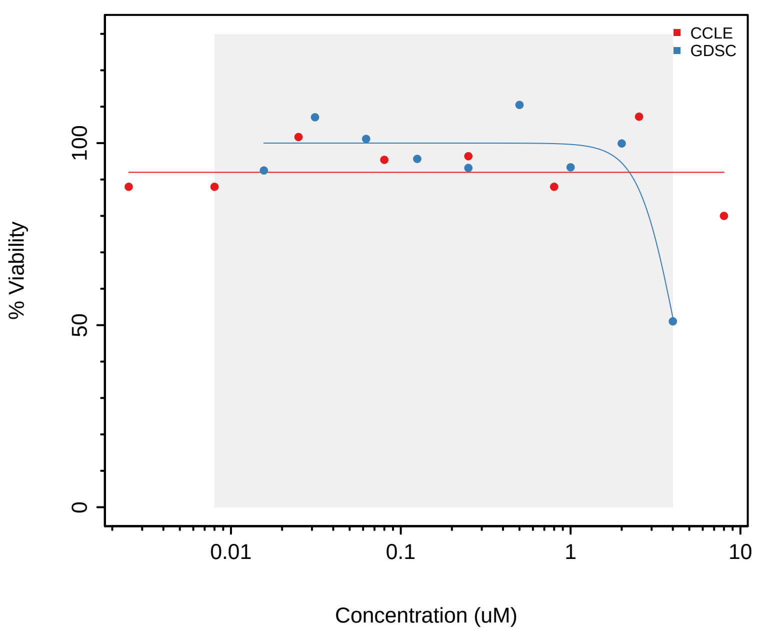

Sorafenib:SK−LMS−1

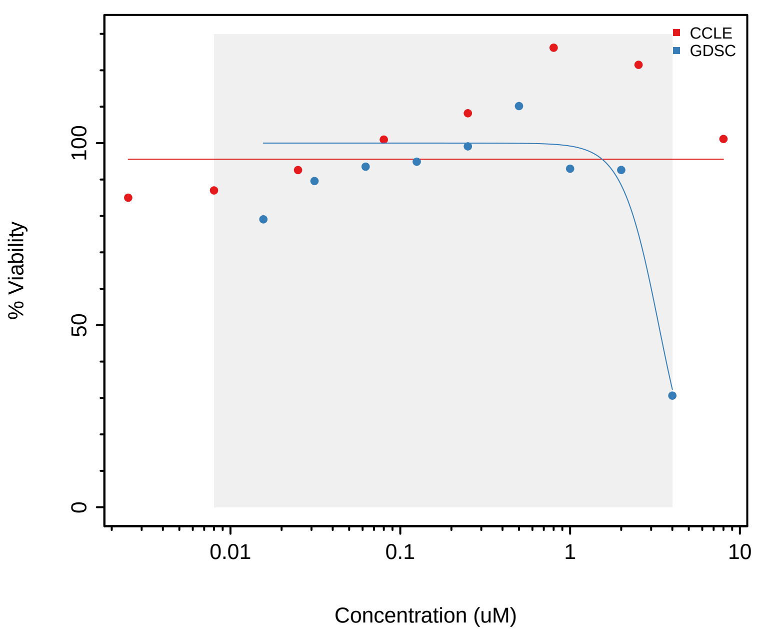

Sorafenib:SK−MEL−2

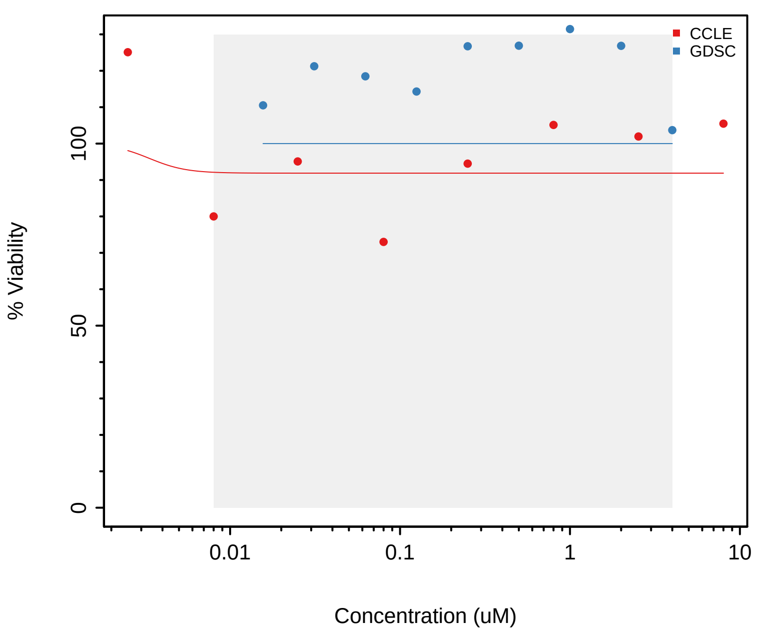

Sorafenib:SK−MM−2

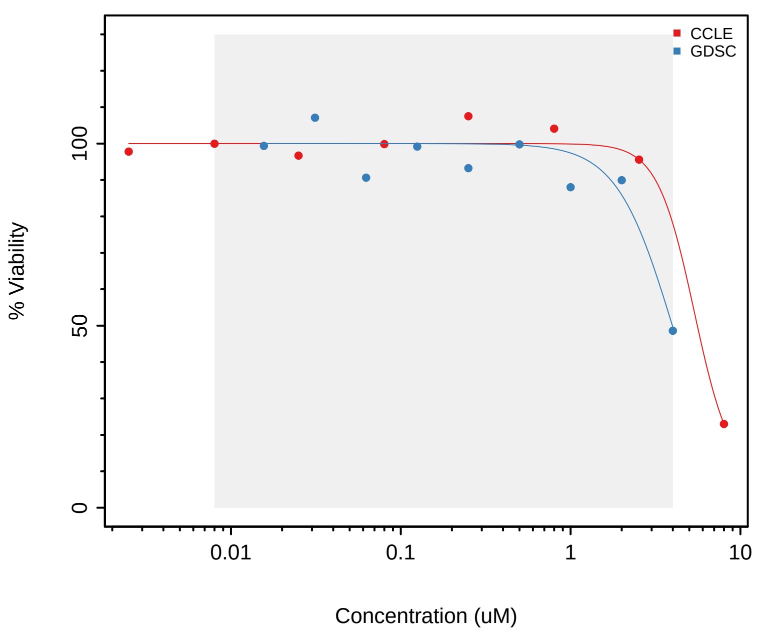

Sorafenib:SK−N−DZ

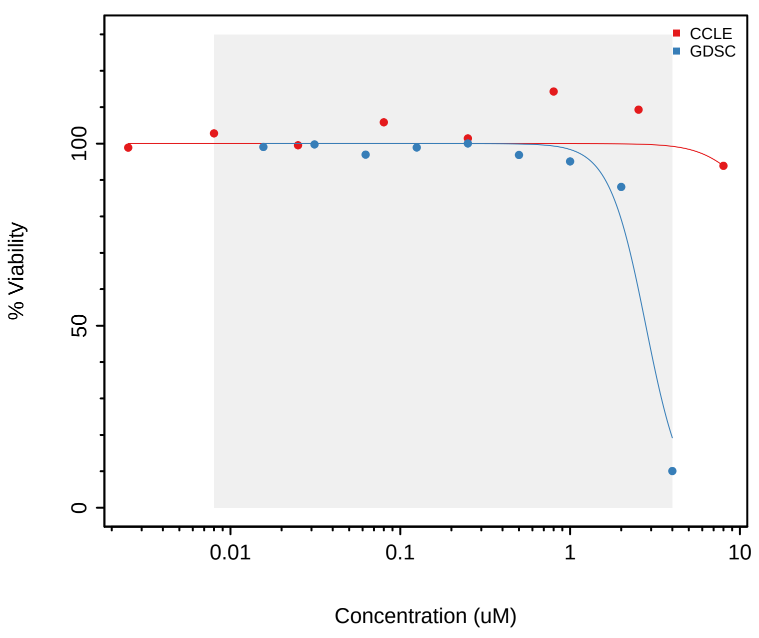

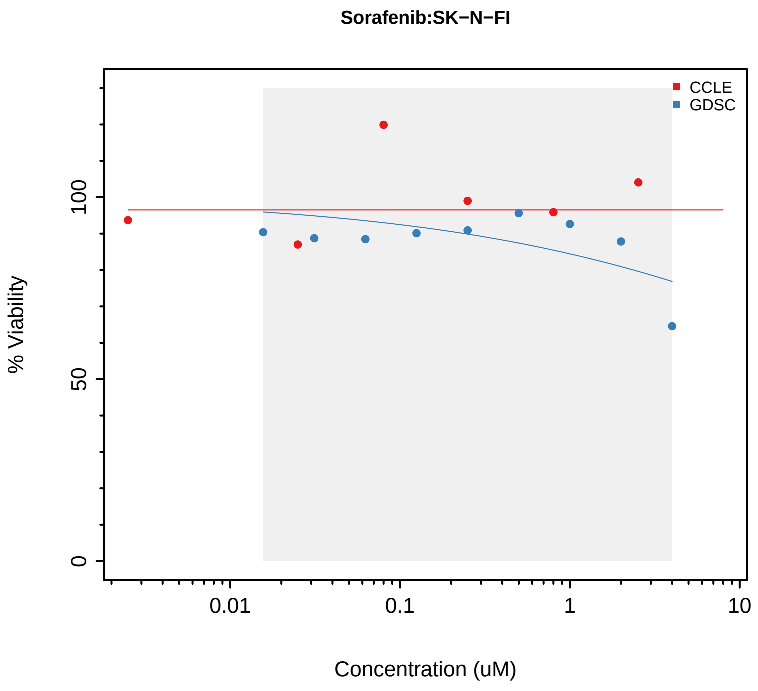
Sorafenib:SK−N−FI
CCLE
GDSC
% Viability
0
50
100
0.01
0.1
1
10
Concentration (uM)

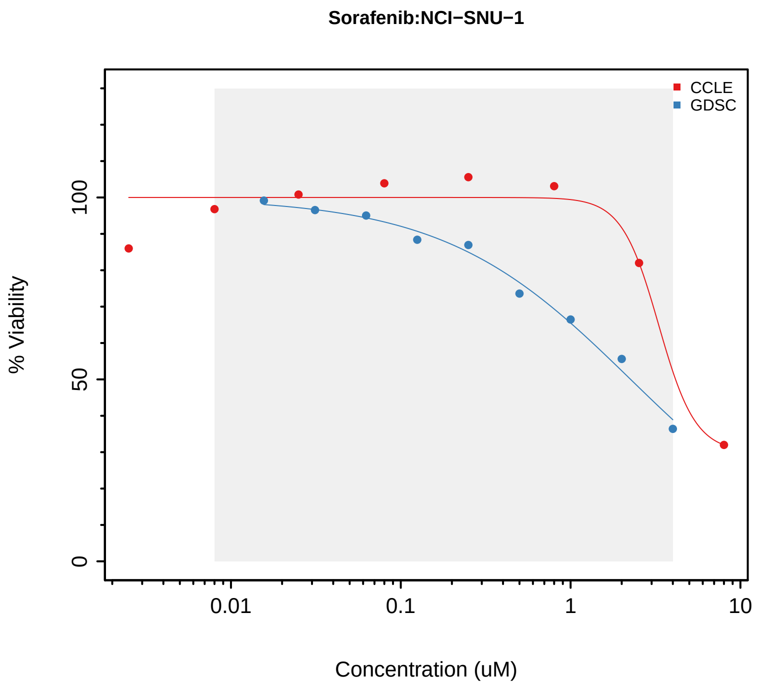
Sorafenib:NCI−SNU−1
CCLE
GDSC
% Viability
0
50
100
0.01
0.1
1
10
Concentration (uM)

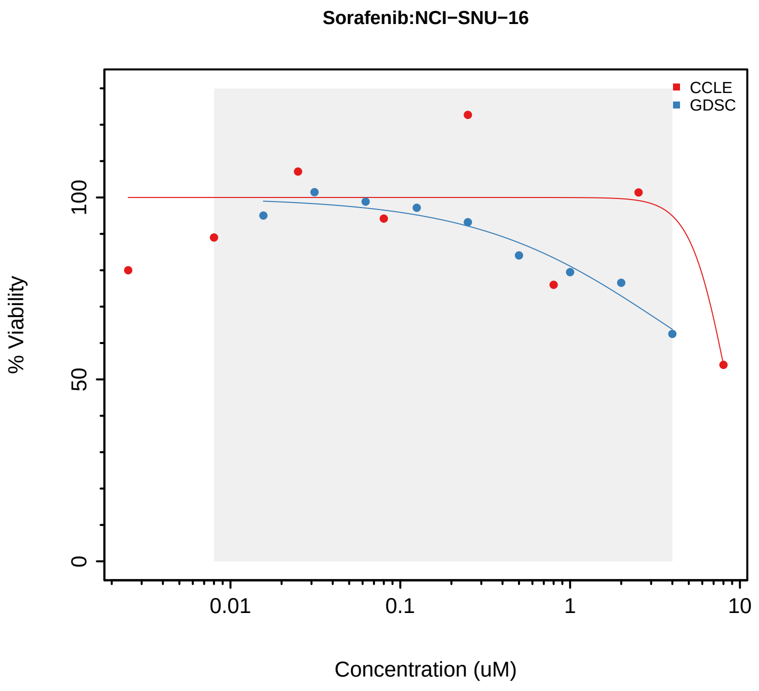
Sorafenib:NCI−SNU−16
CCLE
GDSC
% Viability
0
50
100
0.01
0.1
1
10
Concentration (uM)

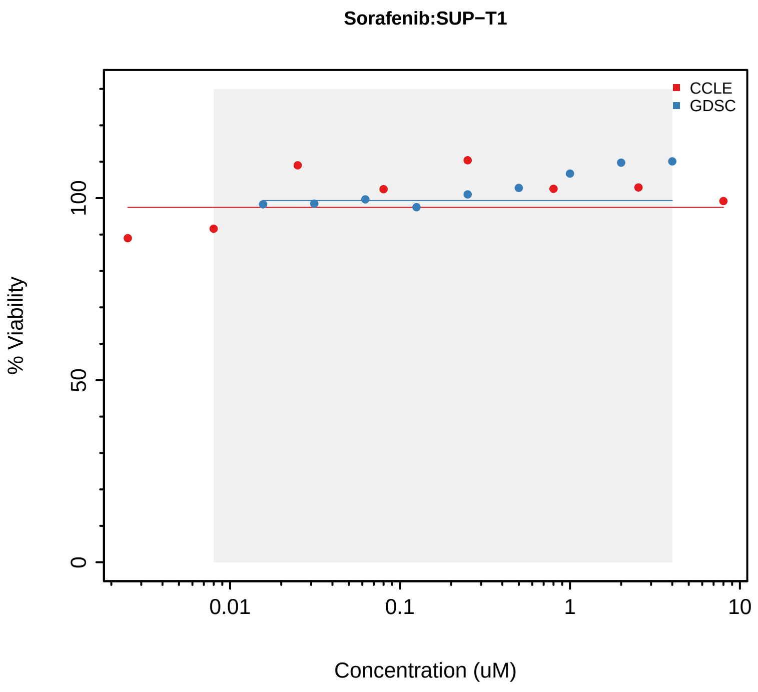
Sorafenib:SUP−T1
CCLE
GDSC
% Viability
0
50
100
0.01
0.1
1
10
Concentration (uM)

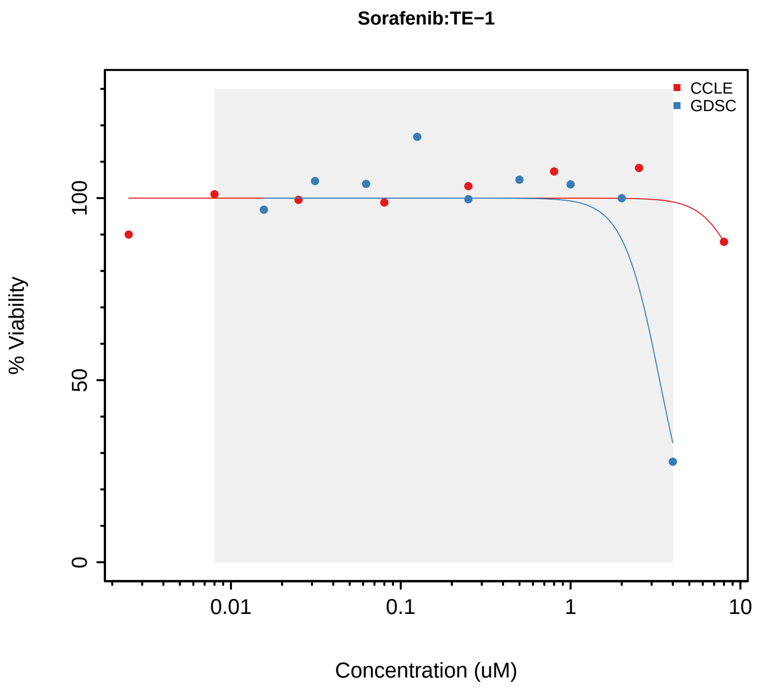
Sorafenib:TE−1
CCLE
GDSC
% Viability
0
50
100
0.01
0.1
1
10
Concentration (uM)

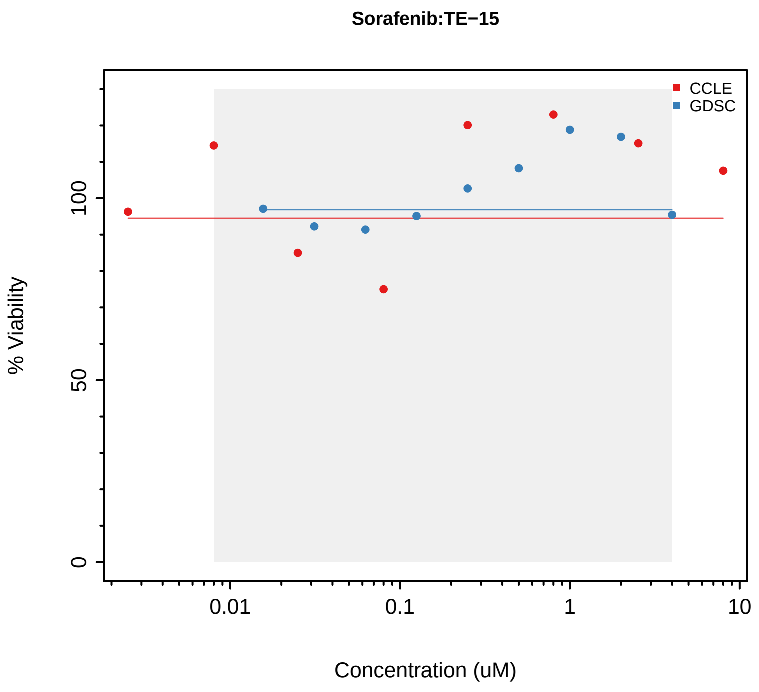
Sorafenib:TE−15
CCLE
GDSC
% Viability
0
50
100
0.01
0.1
1
10
Concentration (uM)

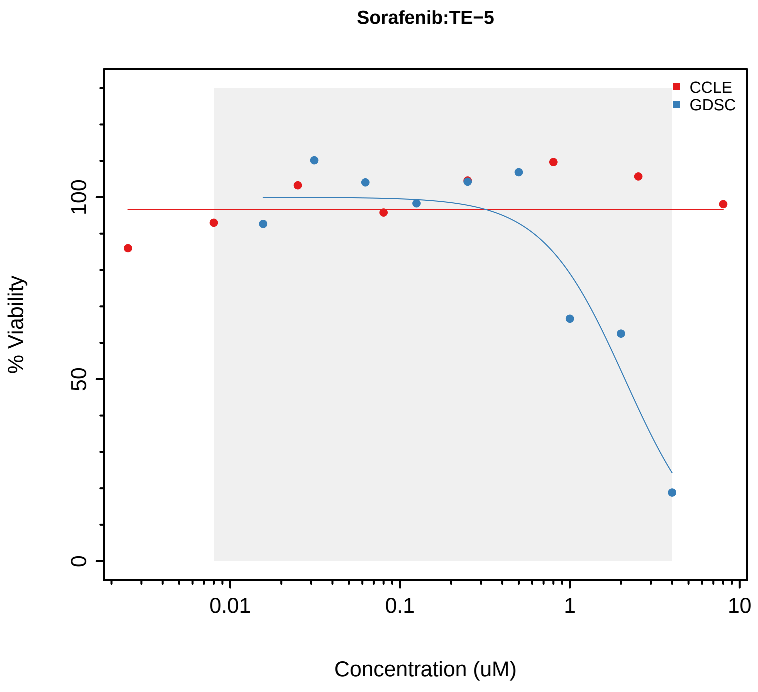
Sorafenib:TE−5
CCLE
GDSC
% Viability
0
50
100
0.01
0.1
1
10
Concentration (uM)

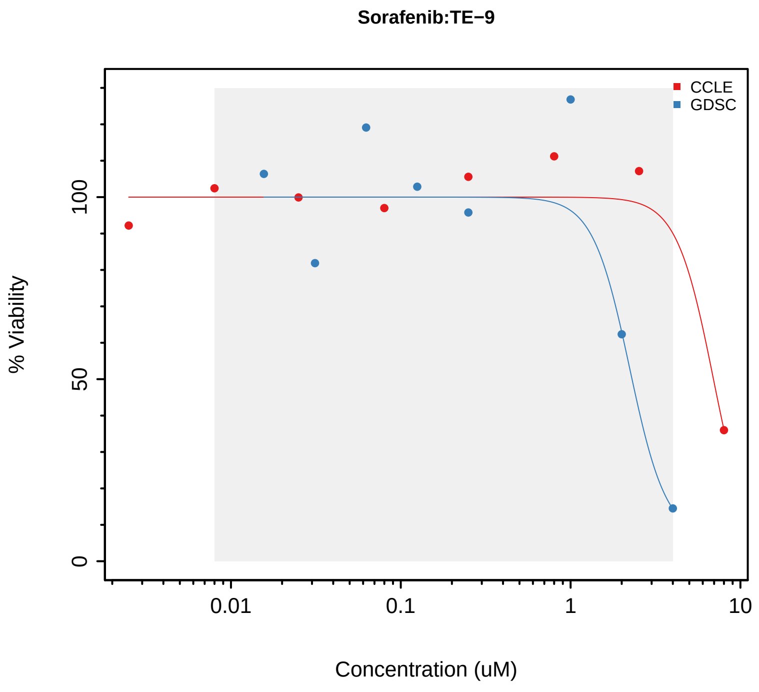
Sorafenib:TE−9
CCLE
GDSC
% Viability
0
50
100
0.01
0.1
1
10
Concentration (uM)

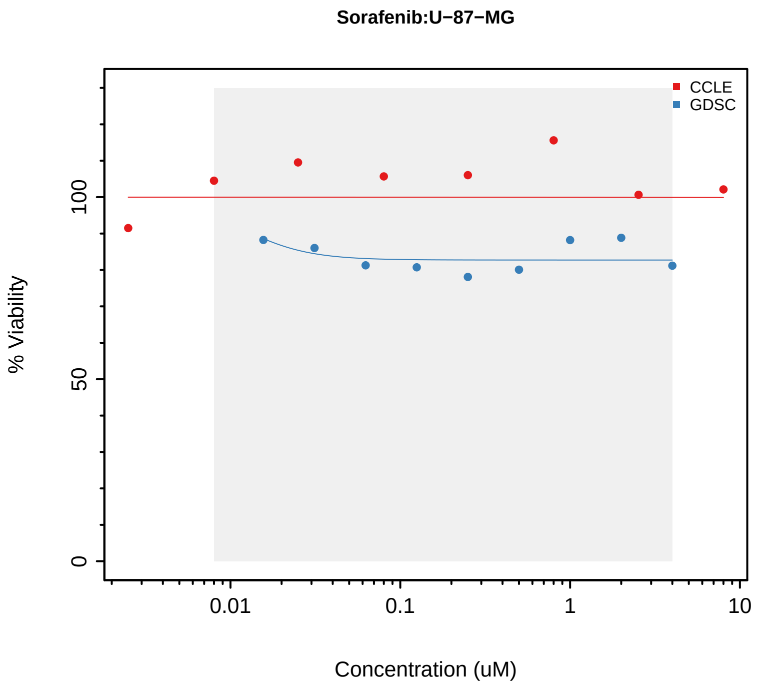
Sorafenib:U−87−MG
CCLE
GDSC
% Viability
0
50
100
0.01
0.1
1
10
Concentration (uM)

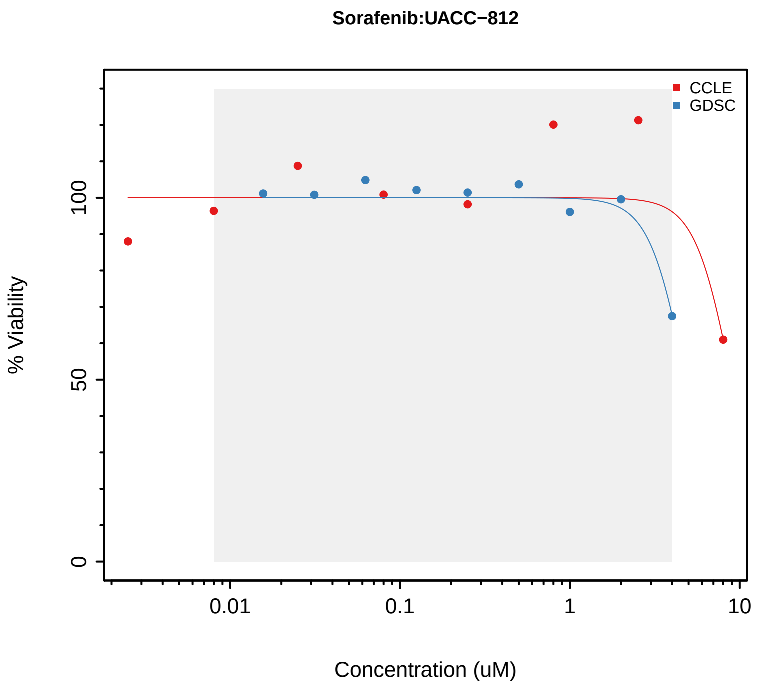
Sorafenib:UACC−812
CCLE
GDSC
% Viability
0
50
100
0.01
0.1
1
10
Concentration (uM)

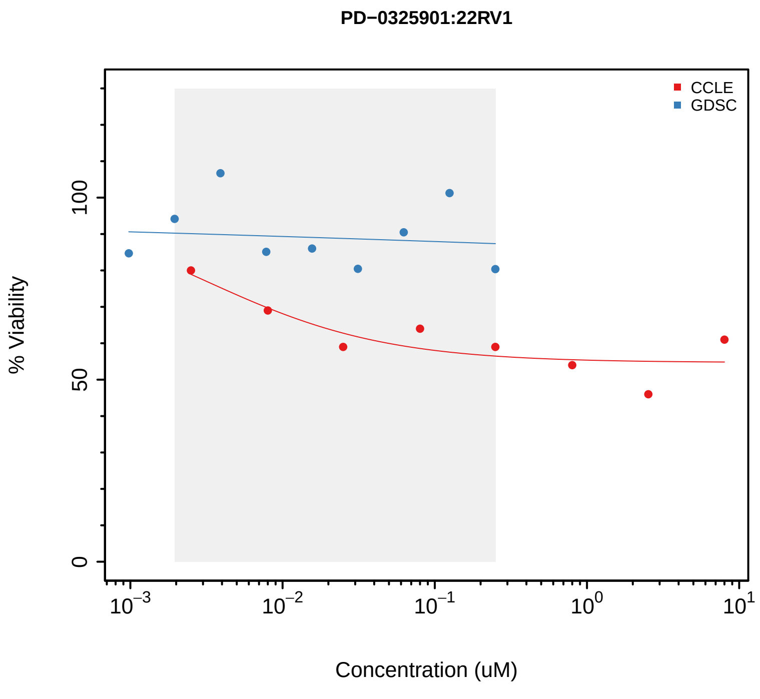
PD−0325901:22RV1
CCLE
GDSC
% Viability
0
50
100
10^{-3}
10^{-2}
10^{-1}
10^{0}
10^{1}
Concentration (uM)

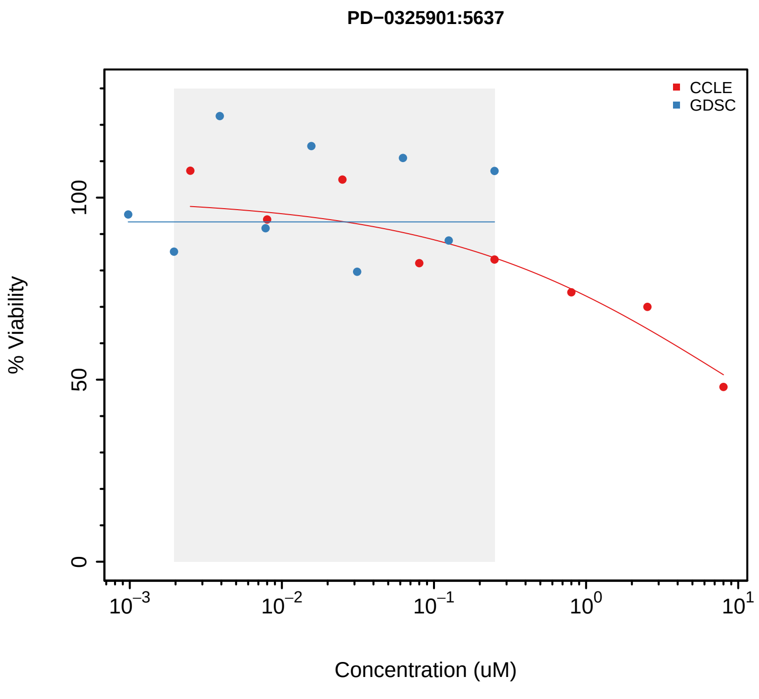
PD−0325901:5637
CCLE
GDSC
% Viability
0
50
100
10^{-3}
10^{-2}
10^{-1}
10^{0}
10^{1}
Concentration (uM)

PD−0325901:639−V

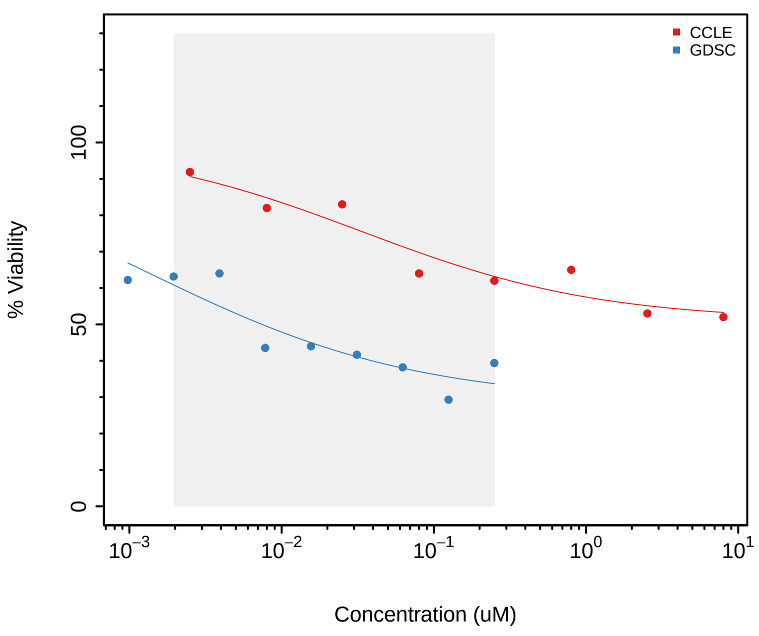

PD−0325901:697

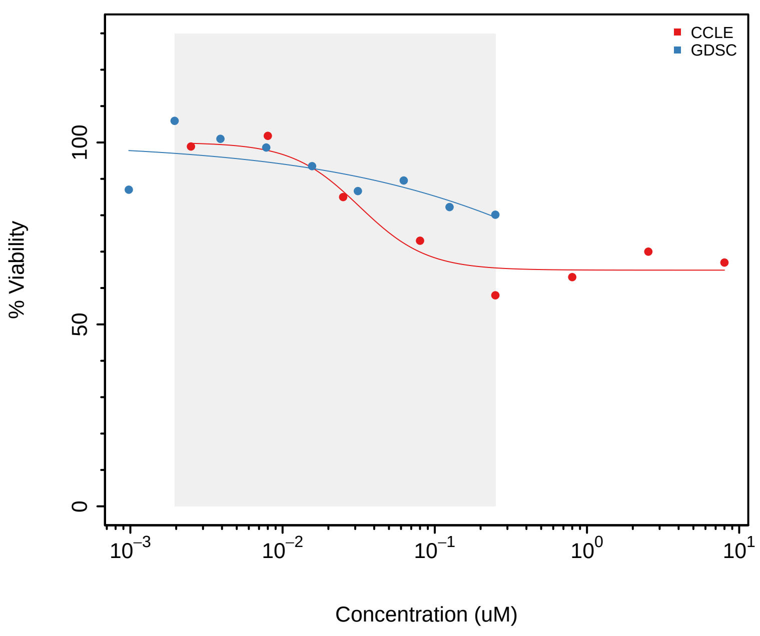

PD−0325901:769−P

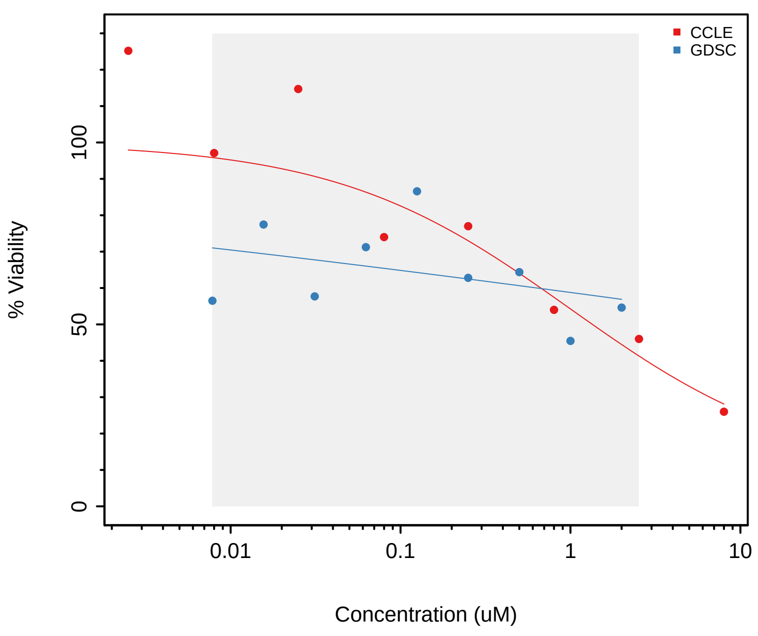

PD−0325901:786−0

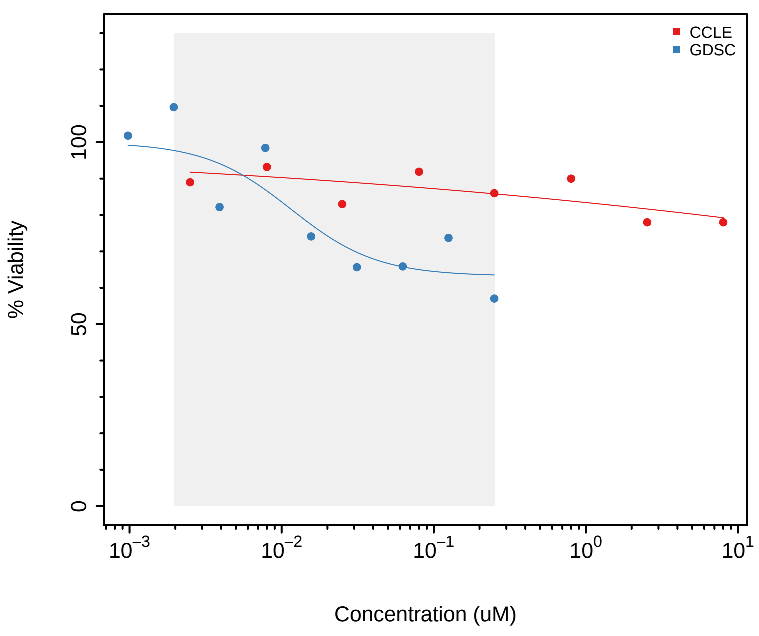

PD−0325901:8305C

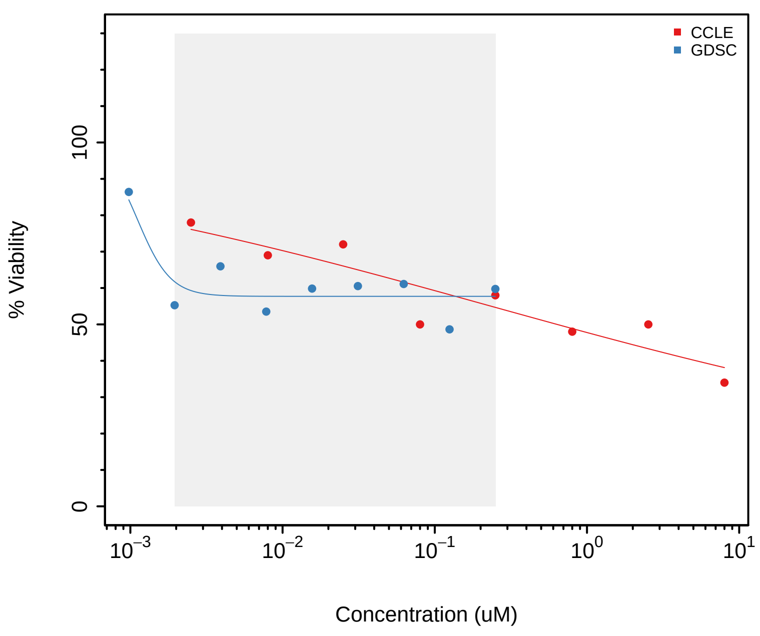

PD−0325901:8505C

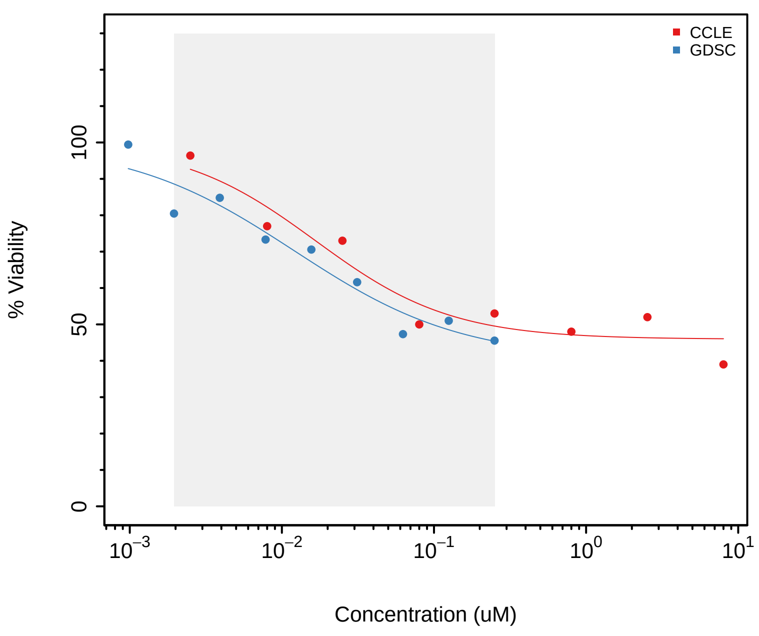

PD−0325901:8−MG−BA

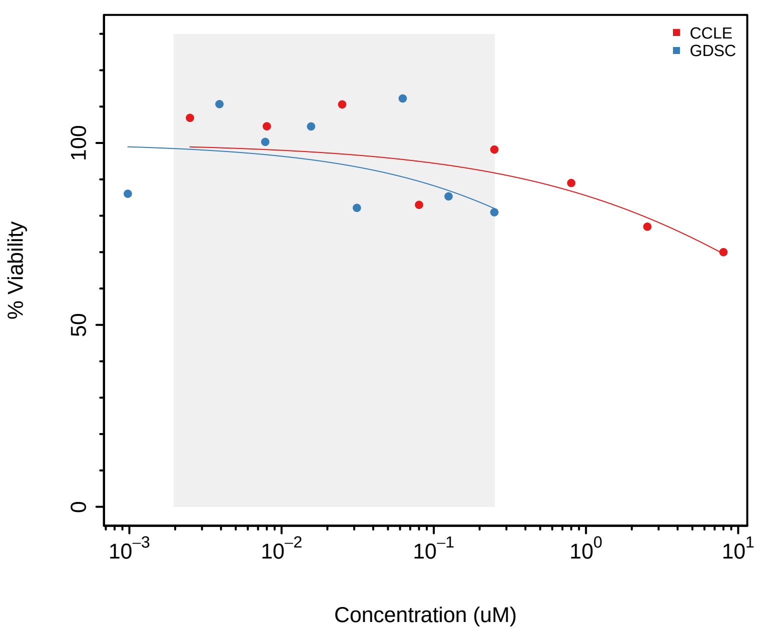

PD−0325901:A204

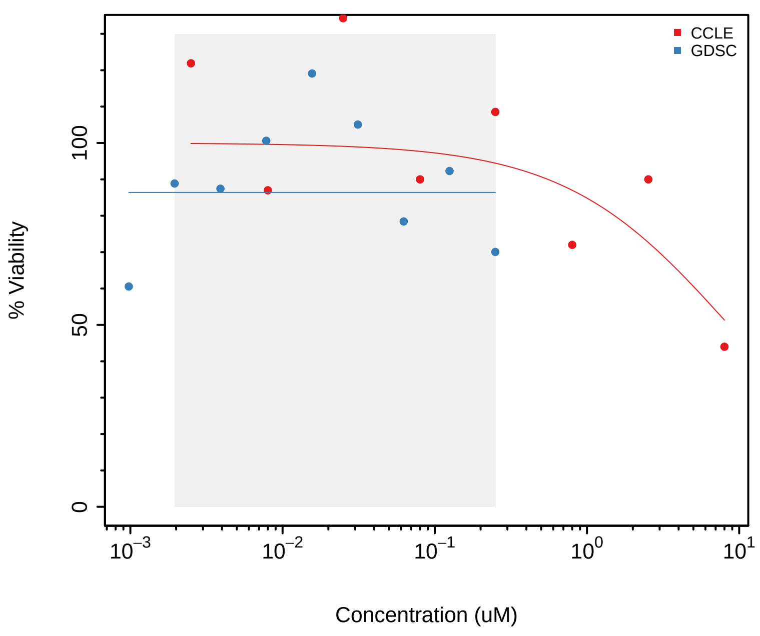

PD−0325901:A2058

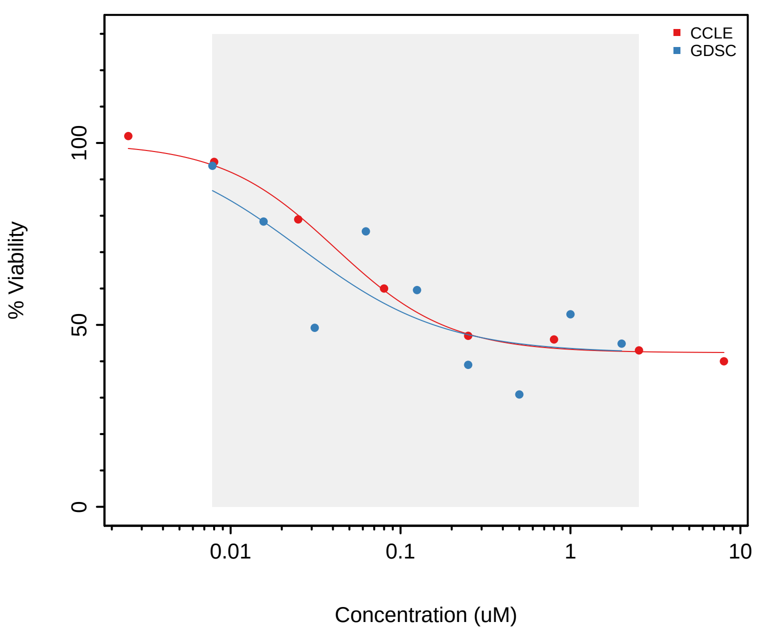

PD−0325901:A253

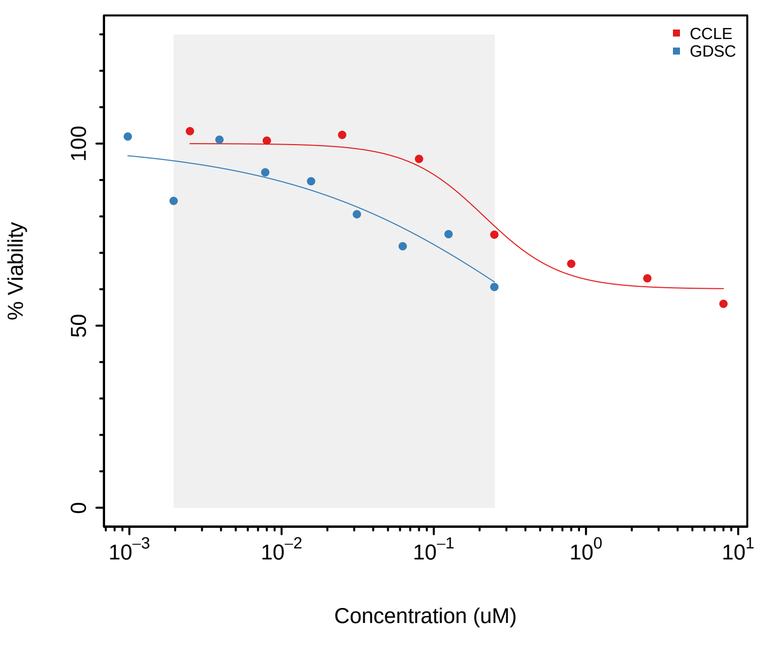

PD−0325901:A2780

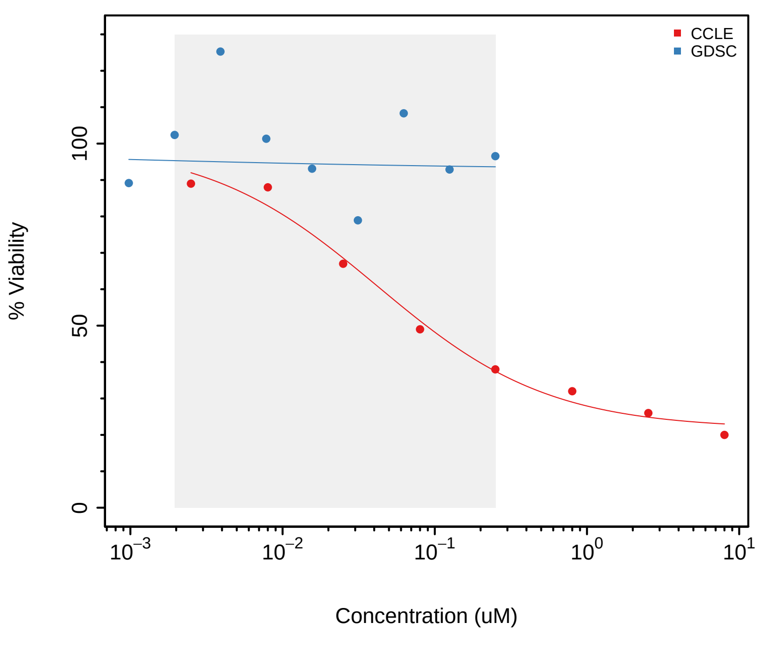

PD−0325901:A375

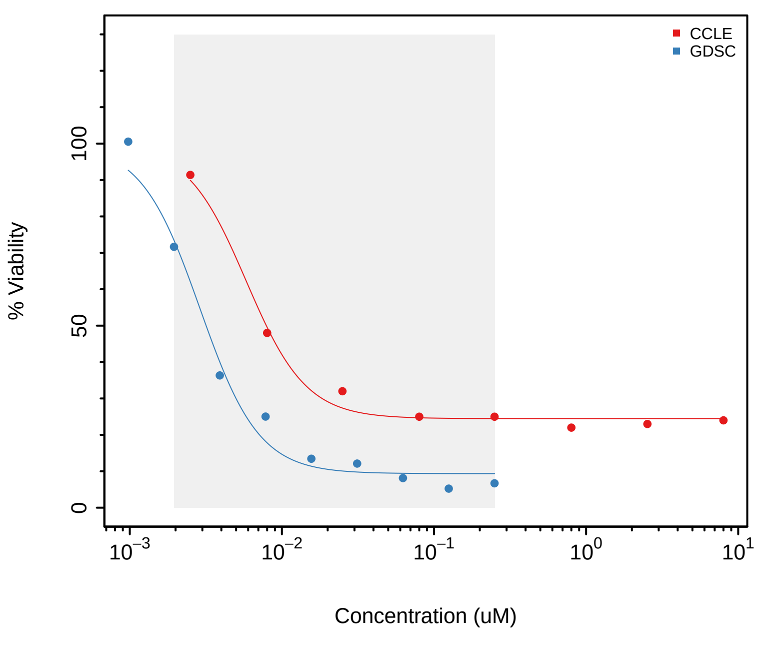

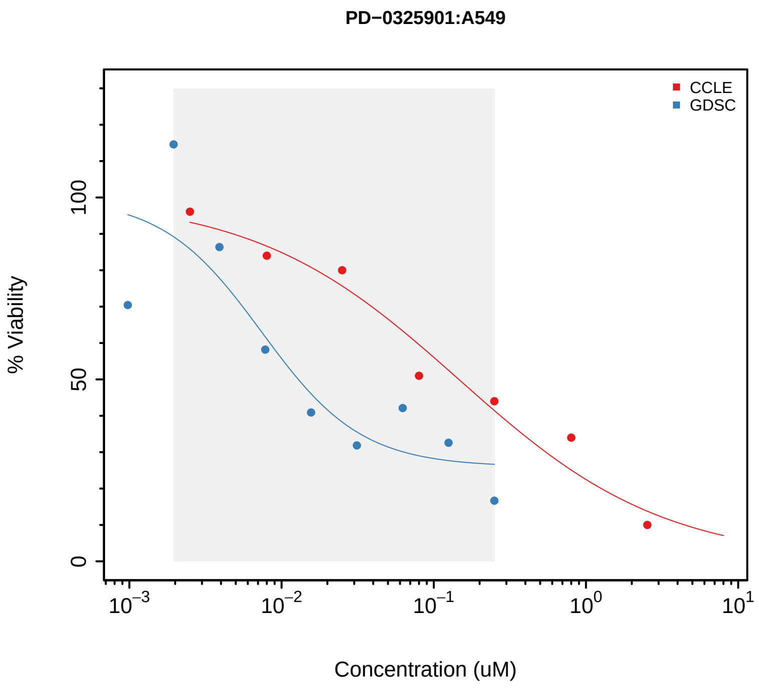
PD−0325901:A549
CCLE
GDSC
% Viability
Concentration (uM)

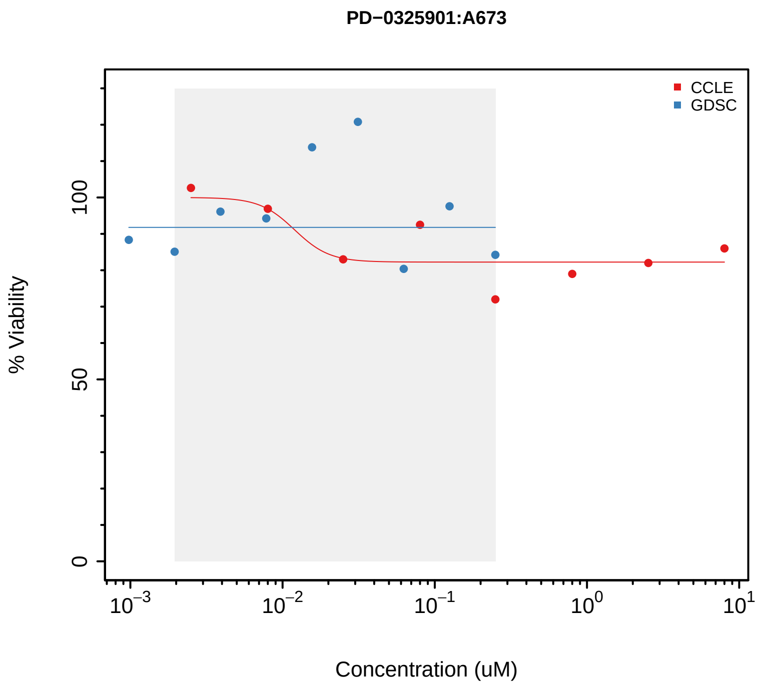
PD−0325901:A673
CCLE
GDSC
% Viability
Concentration (uM)

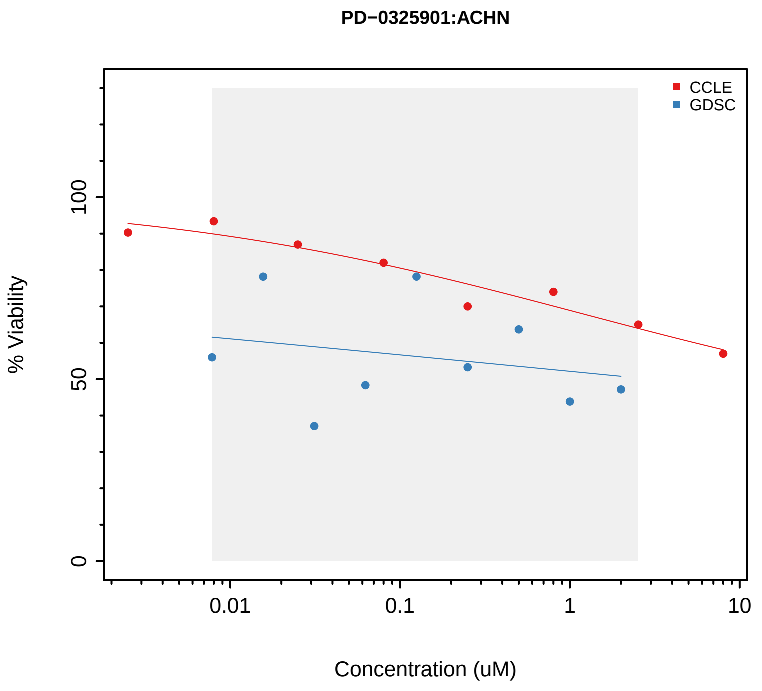
PD−0325901:ACHN
CCLE
GDSC
% Viability
Concentration (uM)

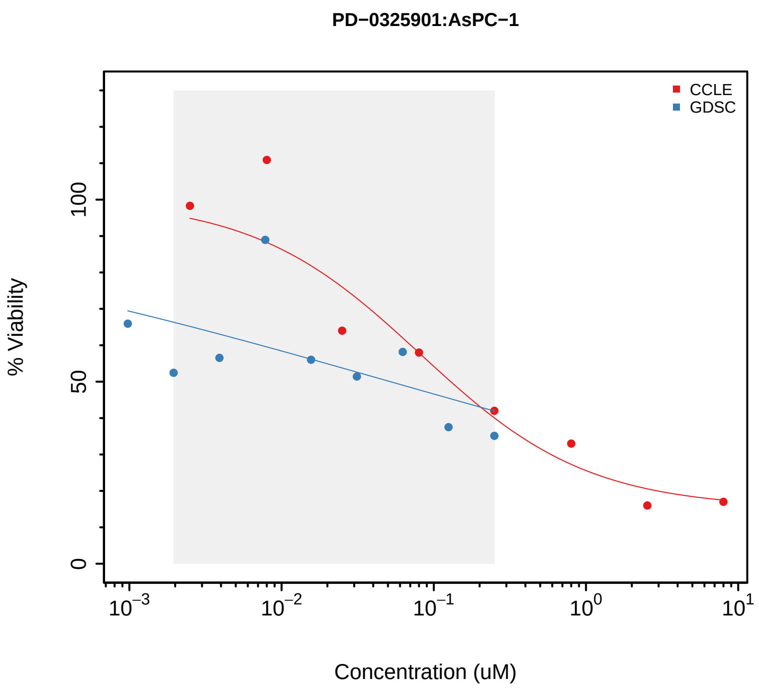
PD−0325901:AsPC−1
CCLE
GDSC
% Viability
Concentration (uM)

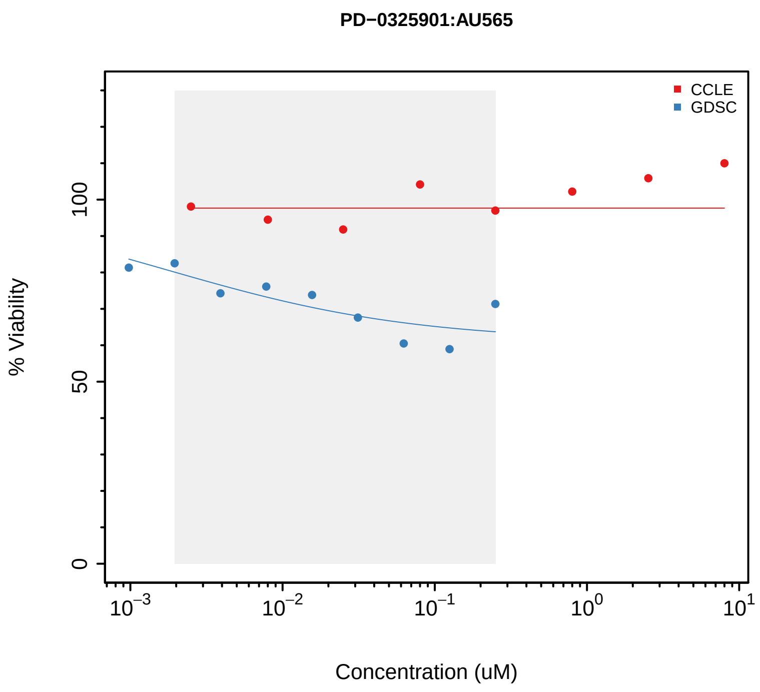
PD−0325901:AU565
CCLE
GDSC
% Viability
Concentration (uM)

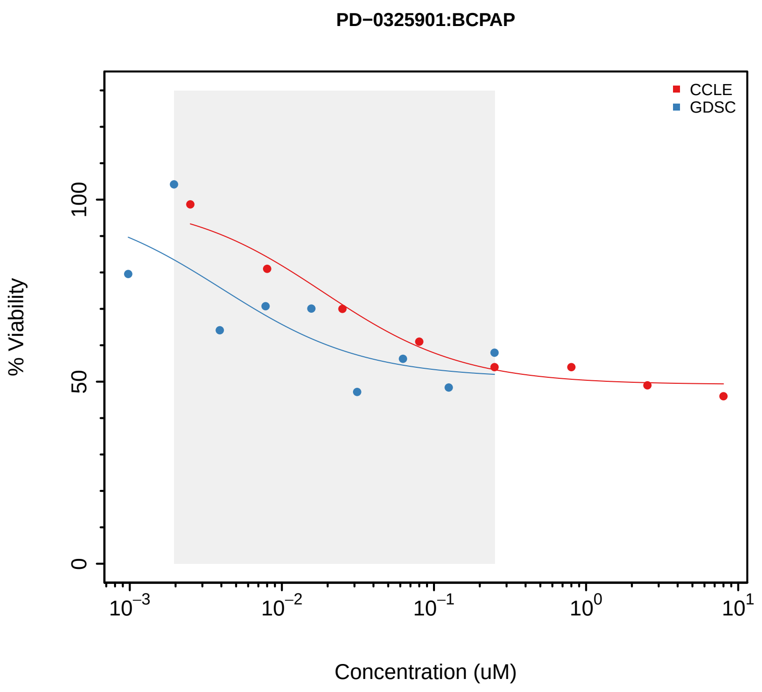
PD−0325901:BCPAP
CCLE
GDSC
% Viability
Concentration (uM)

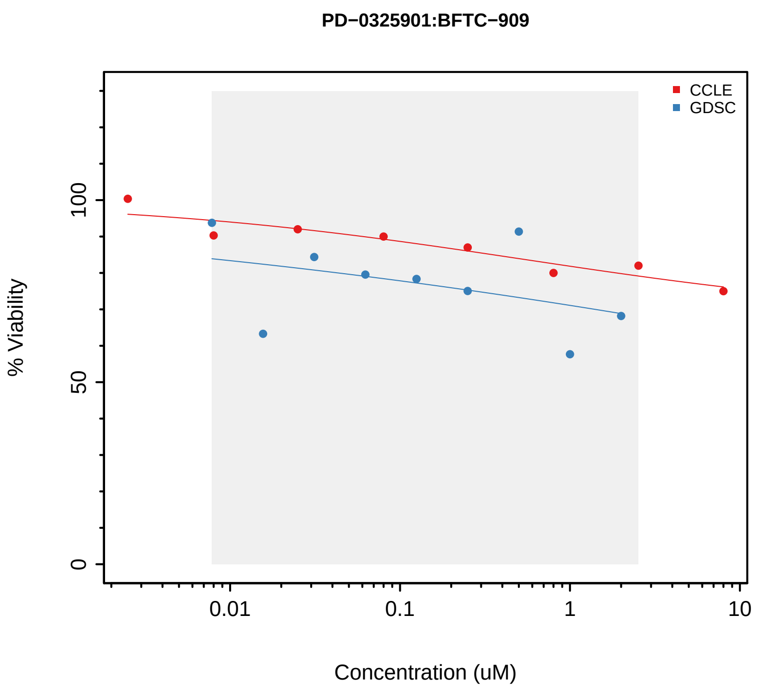
PD−0325901:BFTC−909
CCLE
GDSC
% Viability
Concentration (uM)

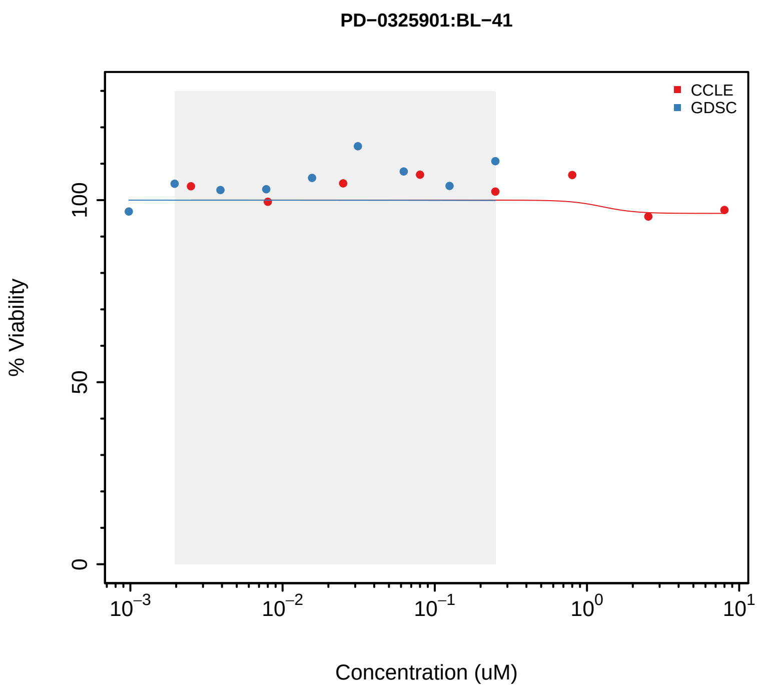
PD−0325901:BL−41
CCLE
GDSC
% Viability
Concentration (uM)

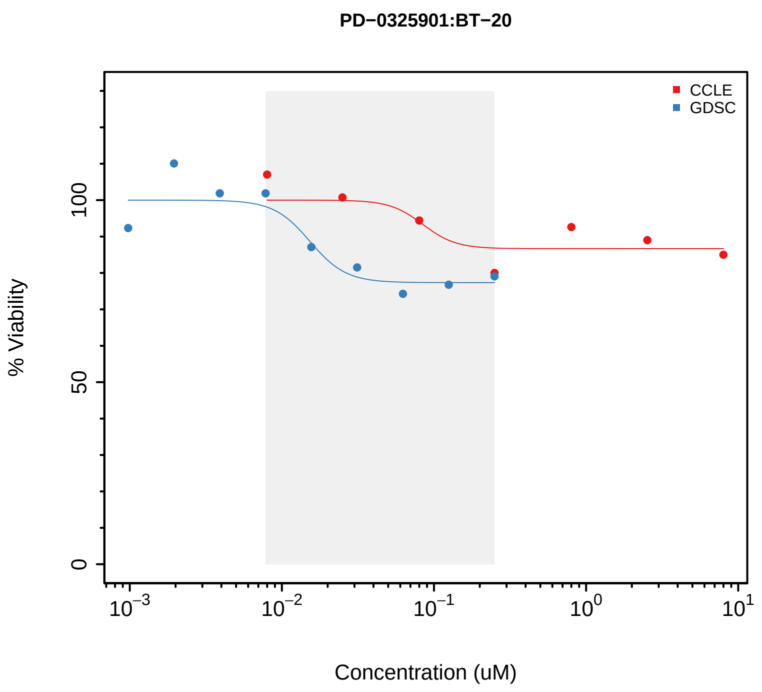
PD−0325901:BT−20
CCLE
GDSC
% Viability
Concentration (uM)

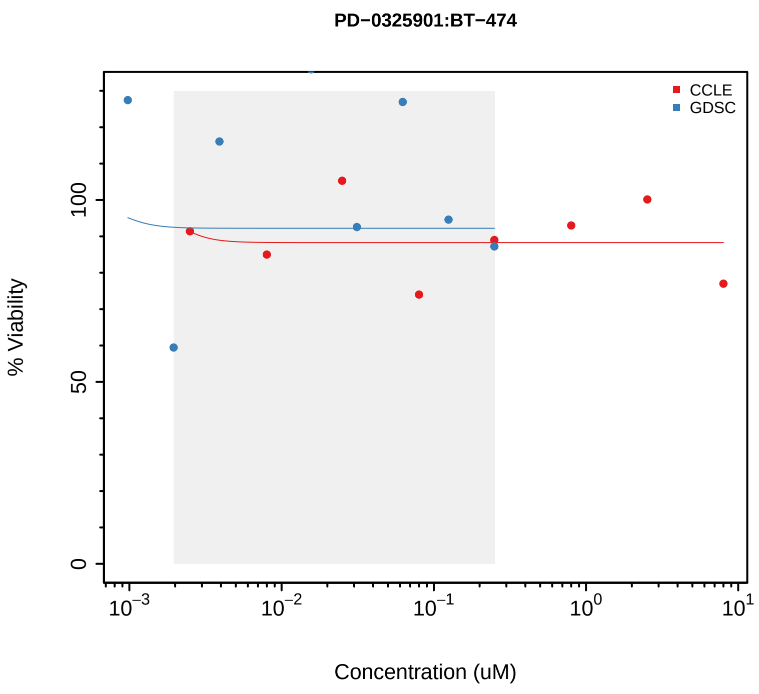
PD−0325901:BT−474
CCLE
GDSC
% Viability
Concentration (uM)

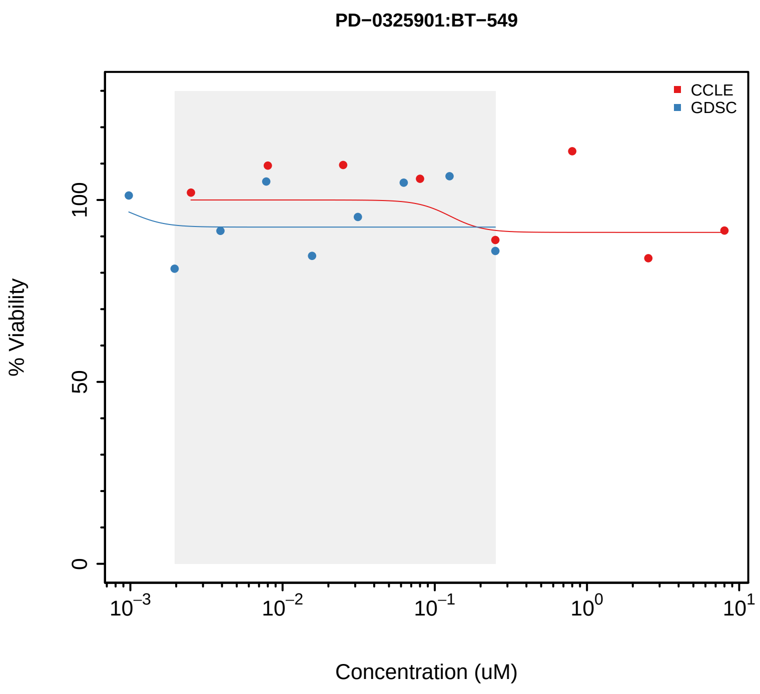
PD−0325901:BT−549
CCLE
GDSC
% Viability
Concentration (uM)

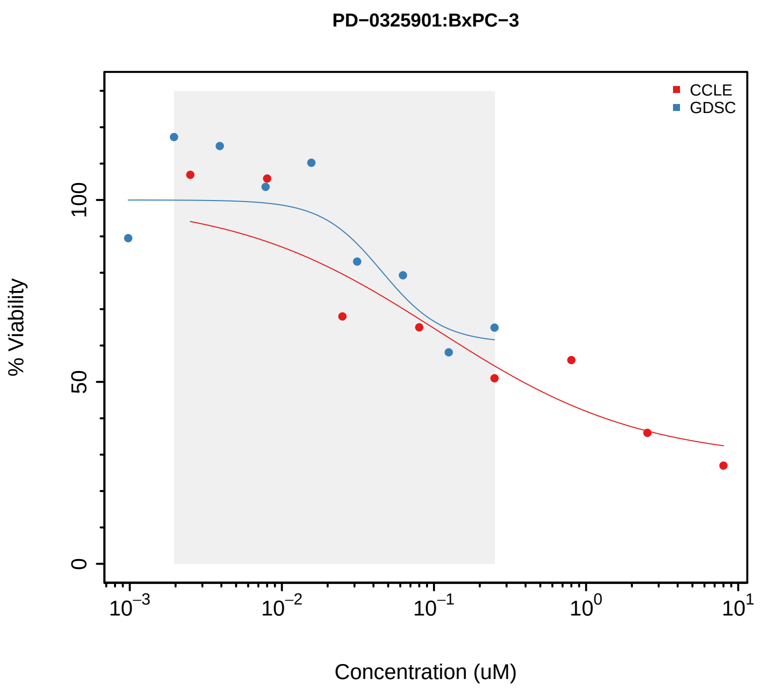
PD−0325901:BxPC−3
CCLE
GDSC
% Viability
Concentration (uM)

PD−0325901:C2BBe1

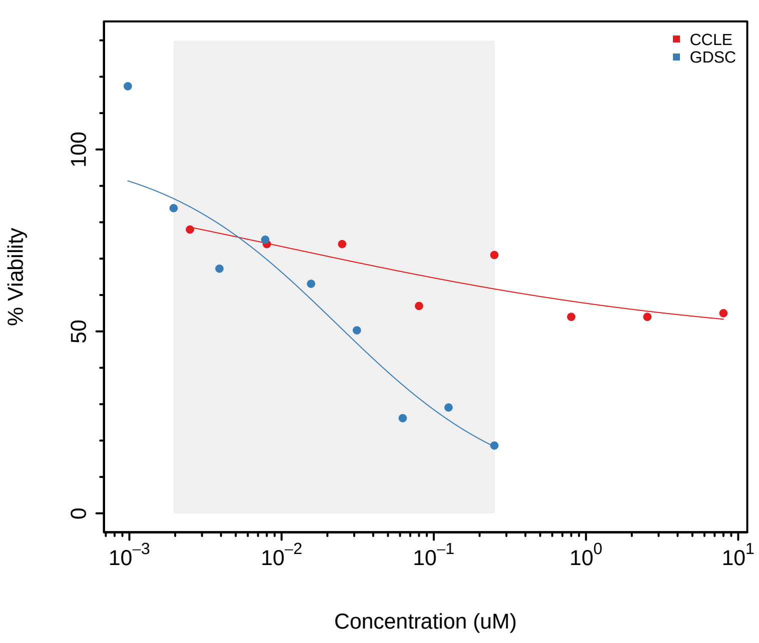

PD−0325901:C32

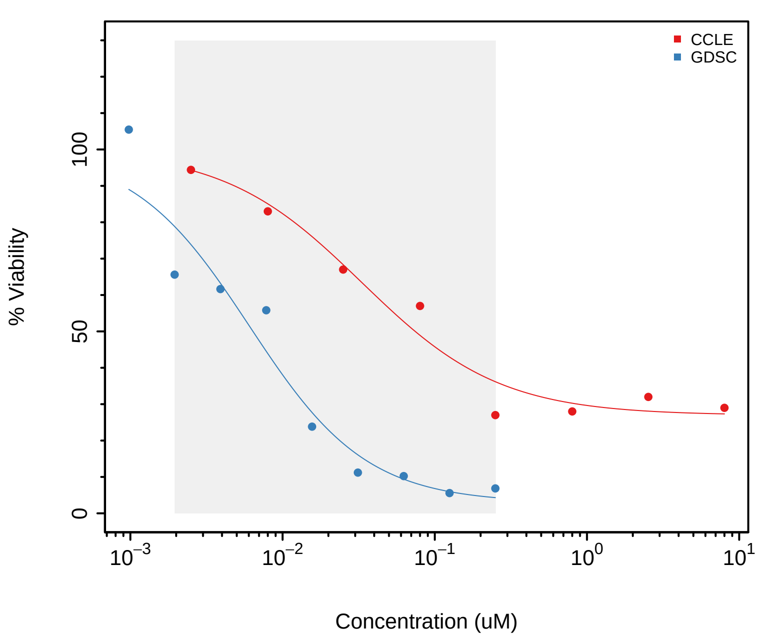

PD−0325901:CAL−12T

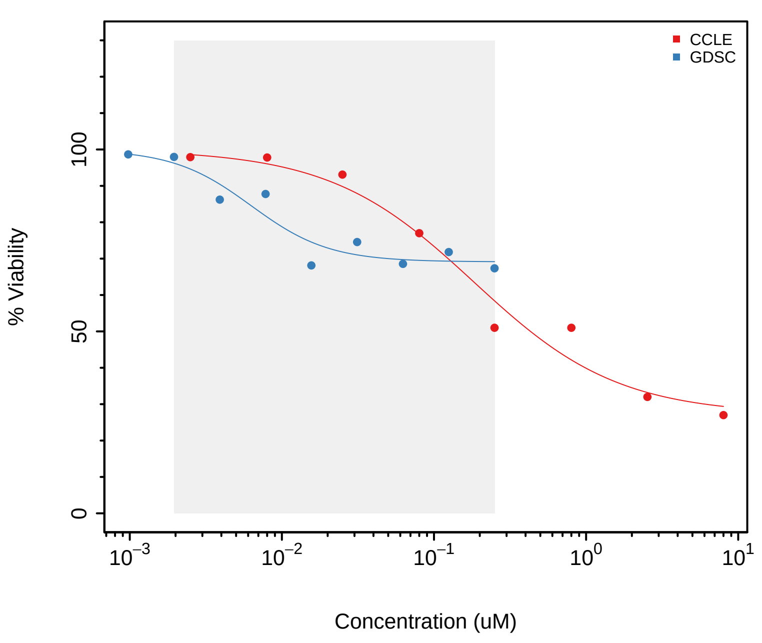

PD−0325901:CAL−27

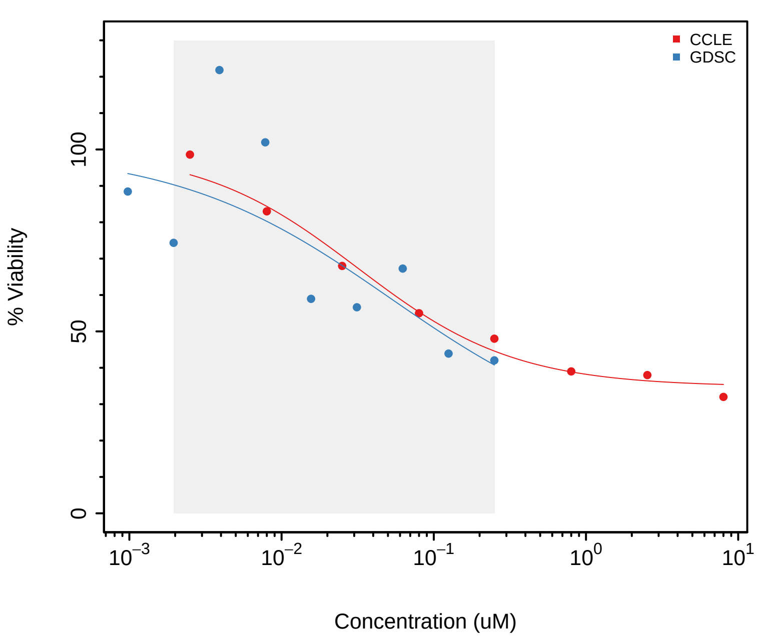

PD−0325901:CAL−85−1

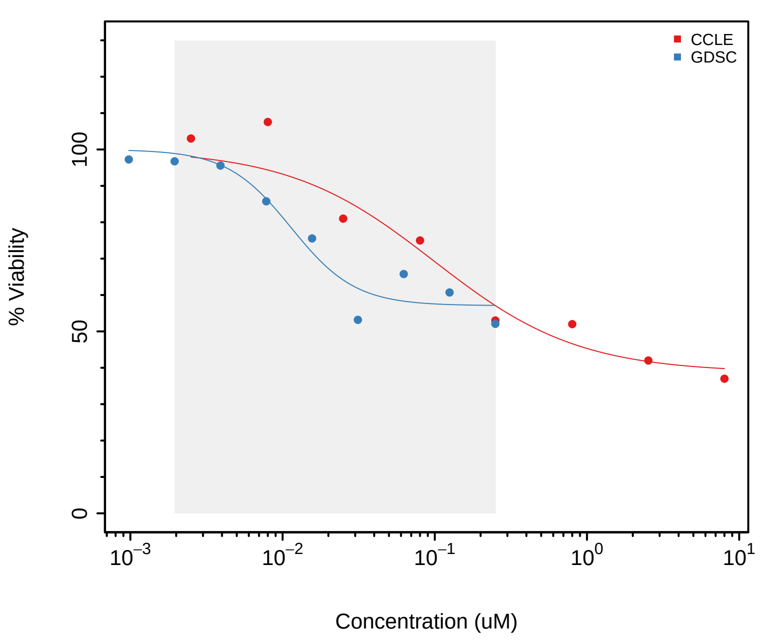

PD−0325901:Calu−3

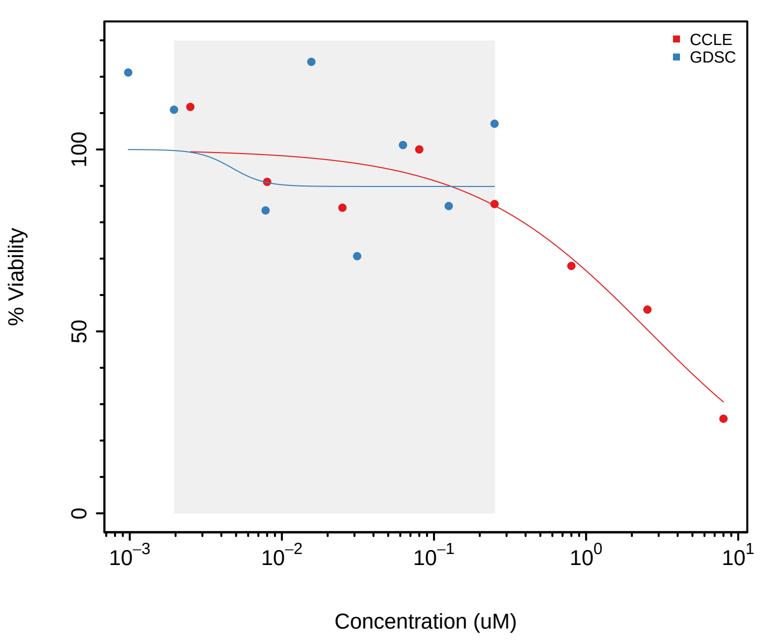

PD−0325901:Calu−6

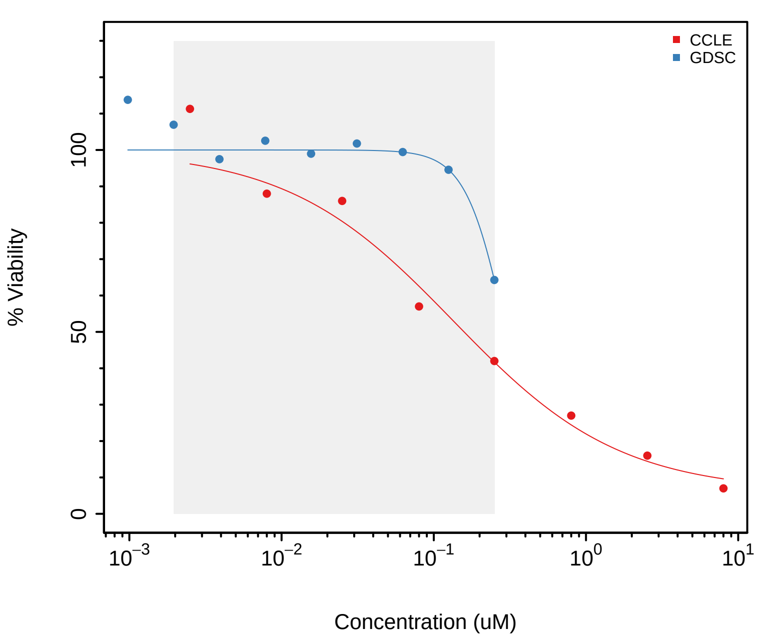

PD−0325901:CAMA−1

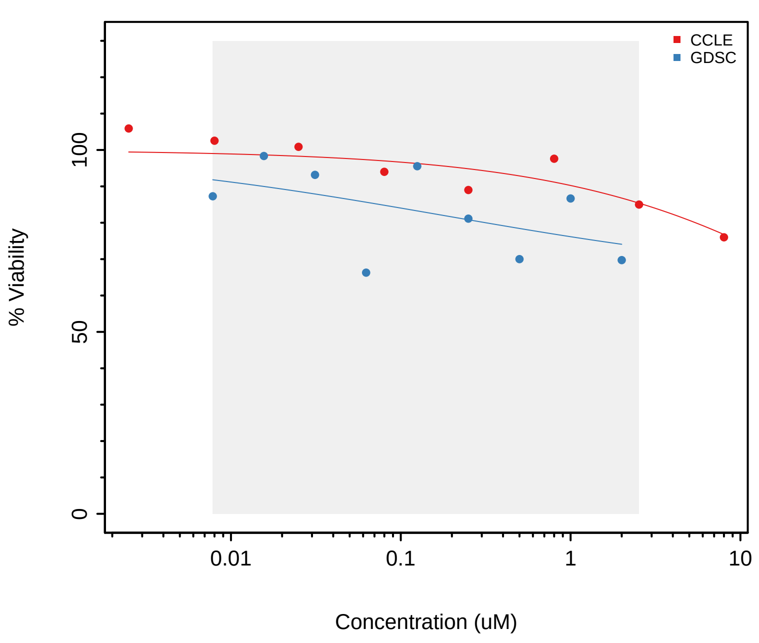

PD−0325901:Capan−2

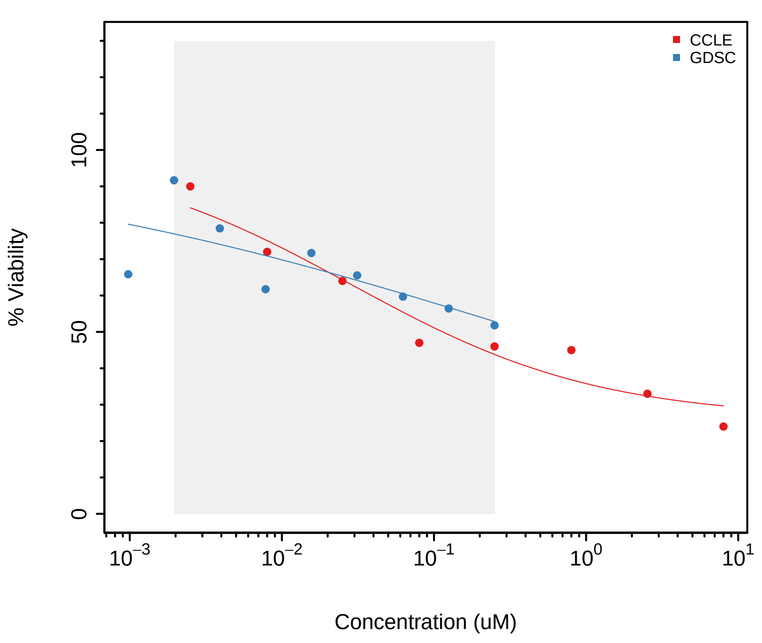

PD−0325901:CAS−1

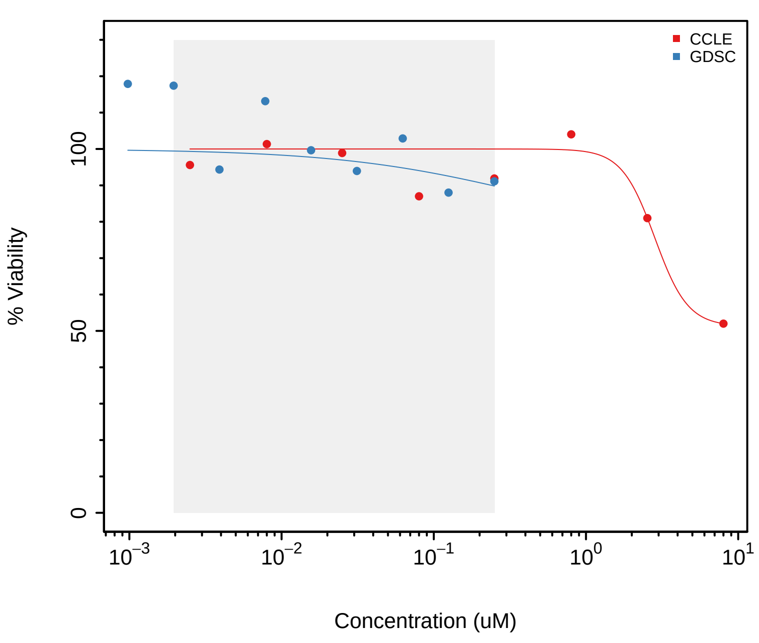

PD−0325901:CHL−1

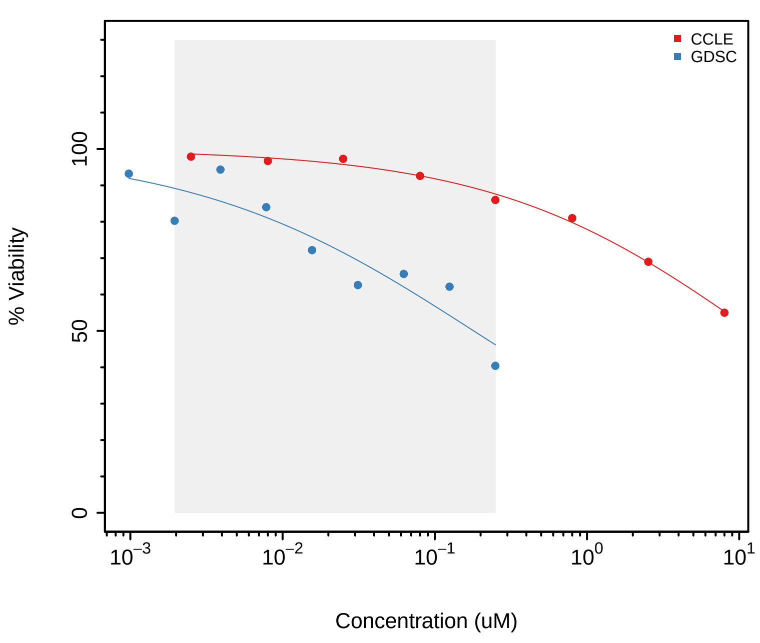

PD−0325901:CHP−212

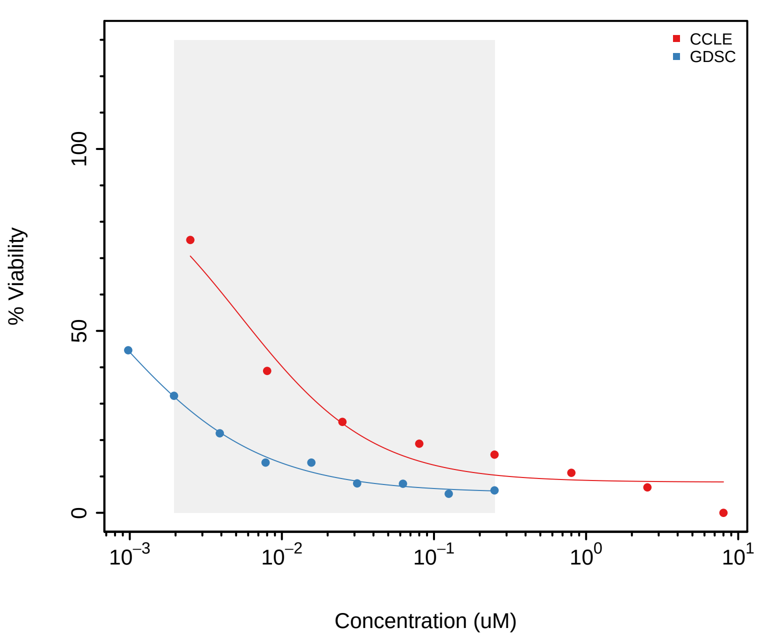

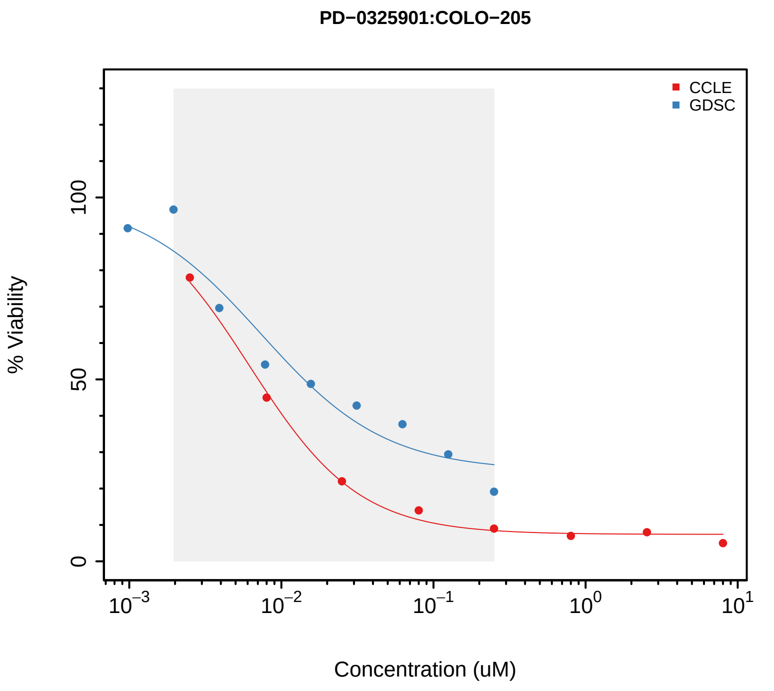
PD−0325901:COLO−205
CCLE
GDSC
% Viability
0
50
100
Concentration (uM)
10^{-3}
10^{-2}
10^{-1}
10^{0}
10^{1}

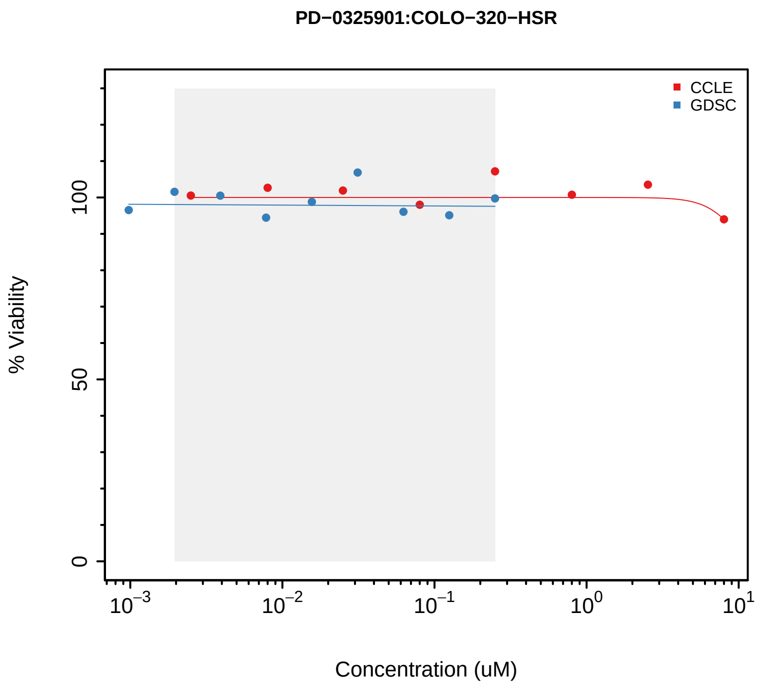
PD−0325901:COLO−320−HSR
CCLE
GDSC
% Viability
0
50
100
Concentration (uM)
10^{-3}
10^{-2}
10^{-1}
10^{0}
10^{1}

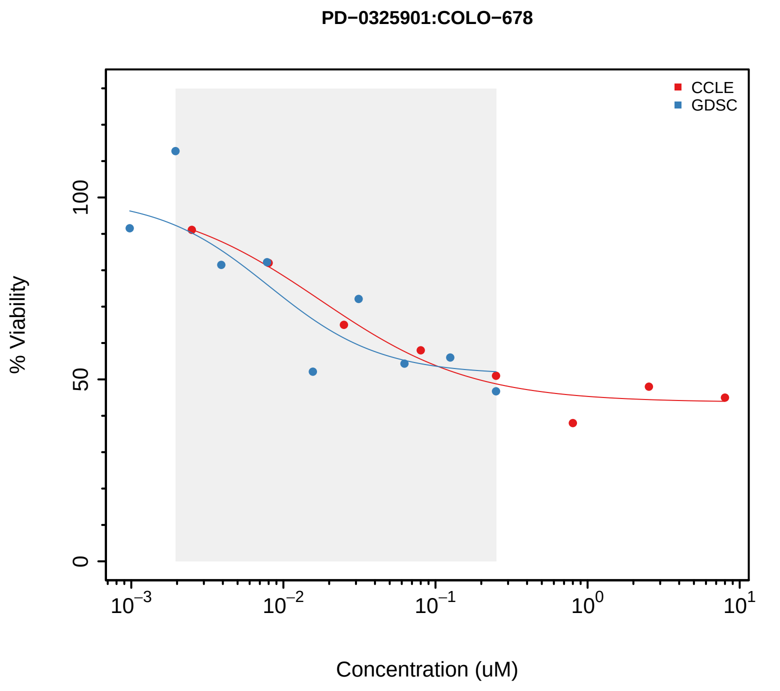
PD−0325901:COLO−678
CCLE
GDSC
% Viability
0
50
100
Concentration (uM)
10^{-3}
10^{-2}
10^{-1}
10^{0}
10^{1}

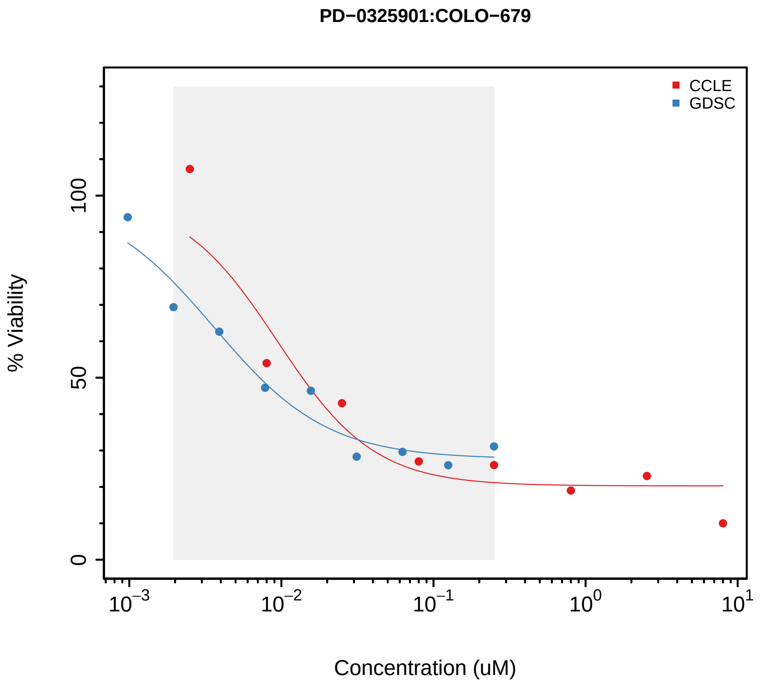
PD−0325901:COLO−679
CCLE
GDSC
% Viability
0
50
100
Concentration (uM)
10^{-3}
10^{-2}
10^{-1}
10^{0}
10^{1}

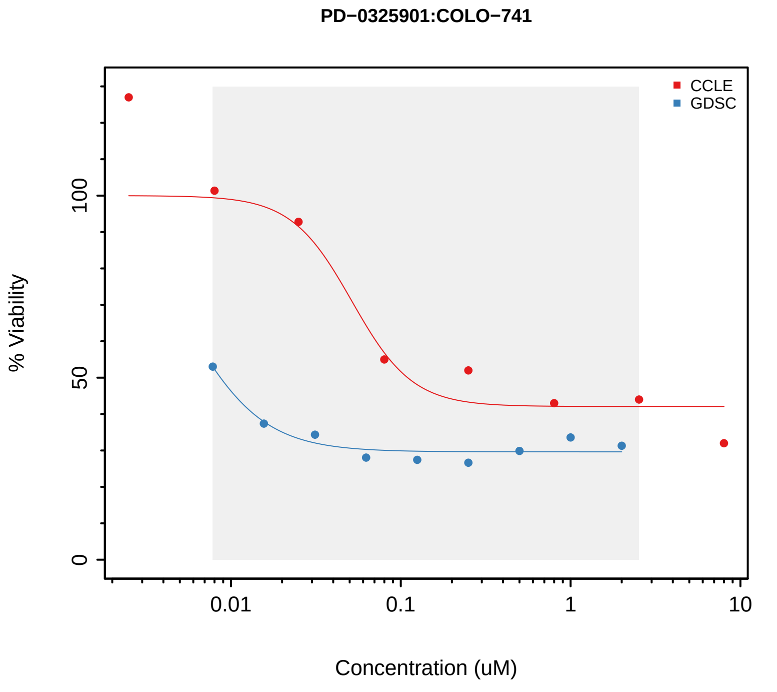
PD−0325901:COLO−741
CCLE
GDSC
% Viability
0
50
100
Concentration (uM)
0.01
0.1
1
10

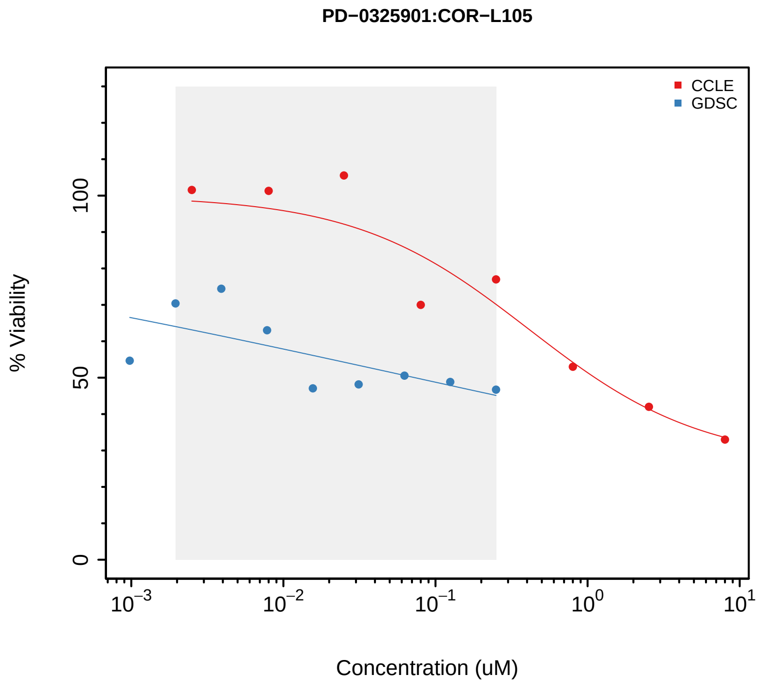
PD−0325901:COR−L105
CCLE
GDSC
% Viability
0
50
100
Concentration (uM)
10^{-3}
10^{-2}
10^{-1}
10^{0}
10^{1}

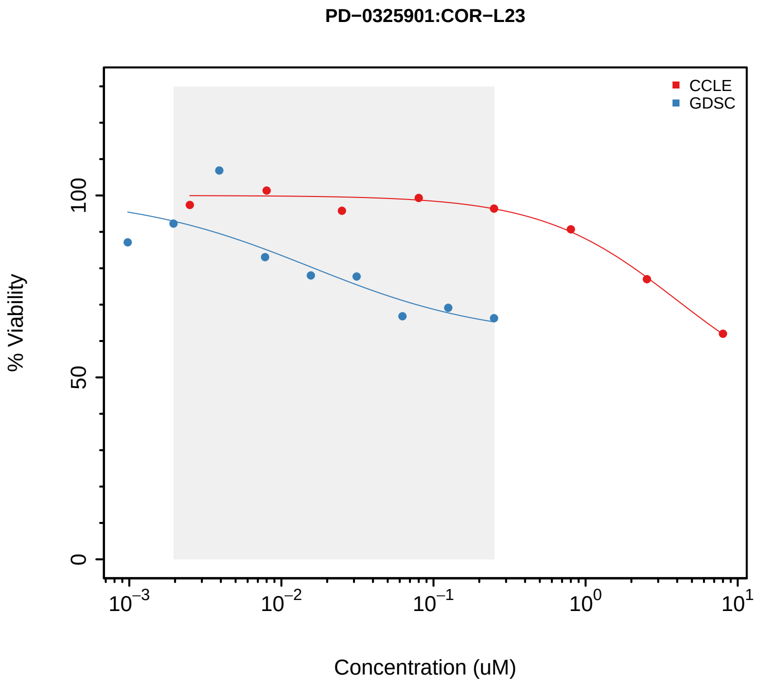
PD−0325901:COR−L23
CCLE
GDSC
% Viability
0
50
100
Concentration (uM)
10^{-3}
10^{-2}
10^{-1}
10^{0}
10^{1}

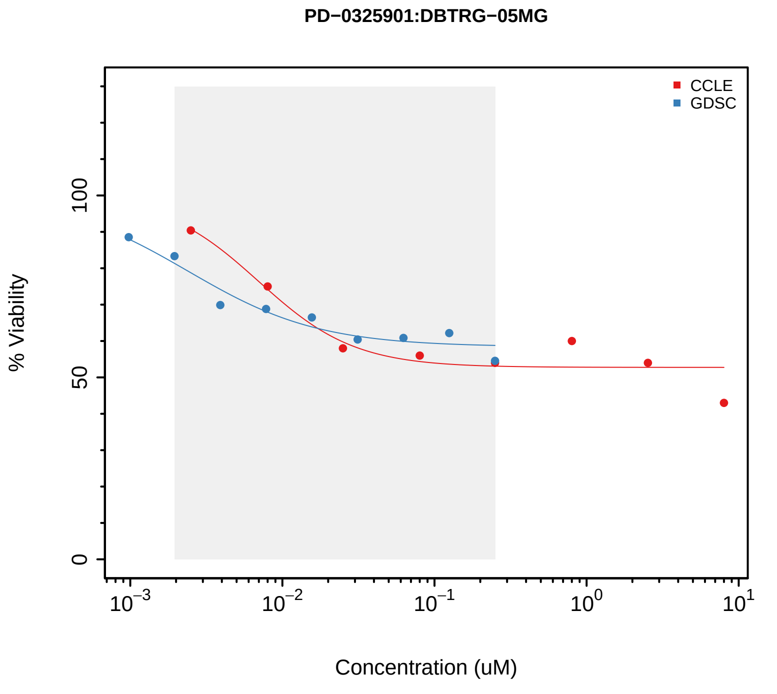
PD−0325901:DBTRG−05MG
CCLE
GDSC
% Viability
0
50
100
Concentration (uM)
10^{-3}
10^{-2}
10^{-1}
10^{0}
10^{1}

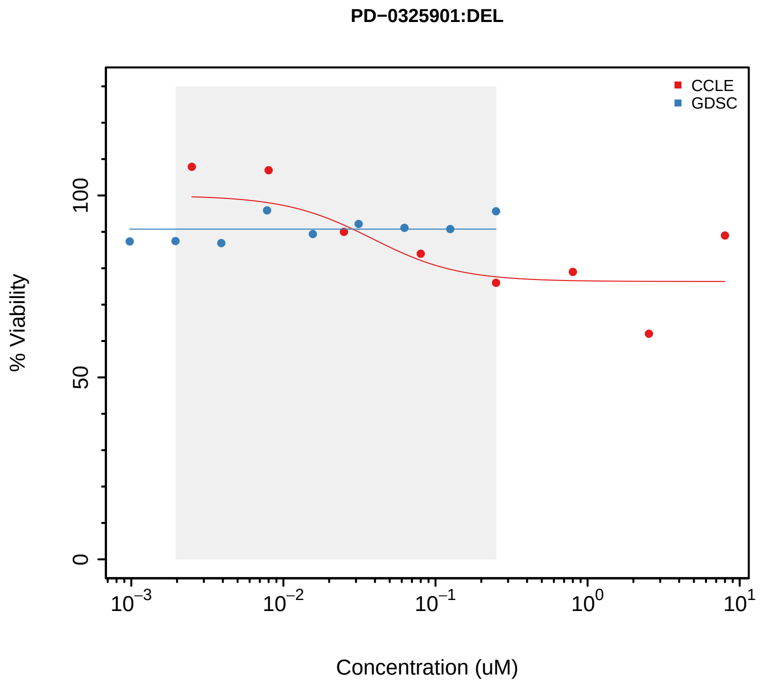
PD−0325901:DEL
CCLE
GDSC
% Viability
0
50
100
Concentration (uM)
10^{-3}
10^{-2}
10^{-1}
10^{0}
10^{1}

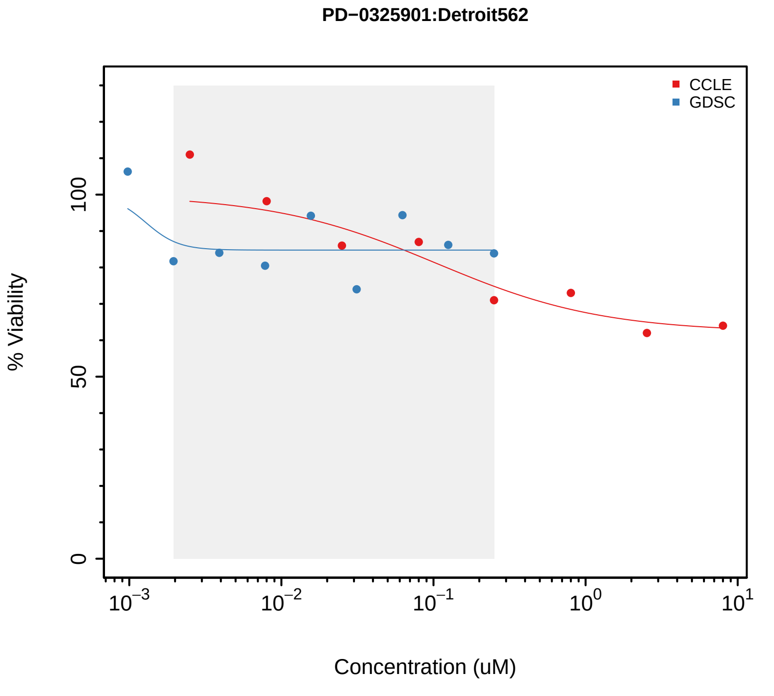
PD−0325901:Detroit562
CCLE
GDSC
% Viability
0
50
100
Concentration (uM)
10^{-3}
10^{-2}
10^{-1}
10^{0}
10^{1}

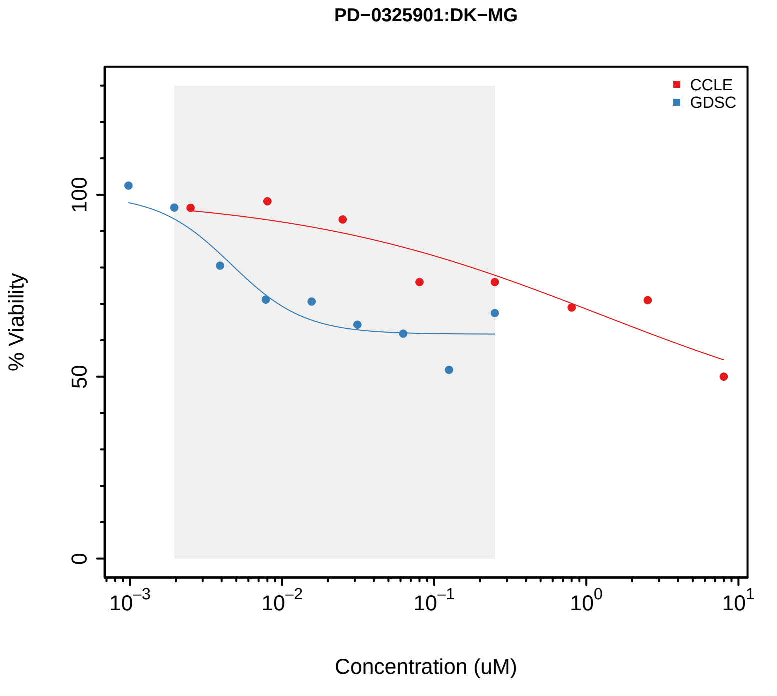
PD−0325901:DK−MG
CCLE
GDSC
% Viability
0
50
100
Concentration (uM)
10^{-3}
10^{-2}
10^{-1}
10^{0}
10^{1}

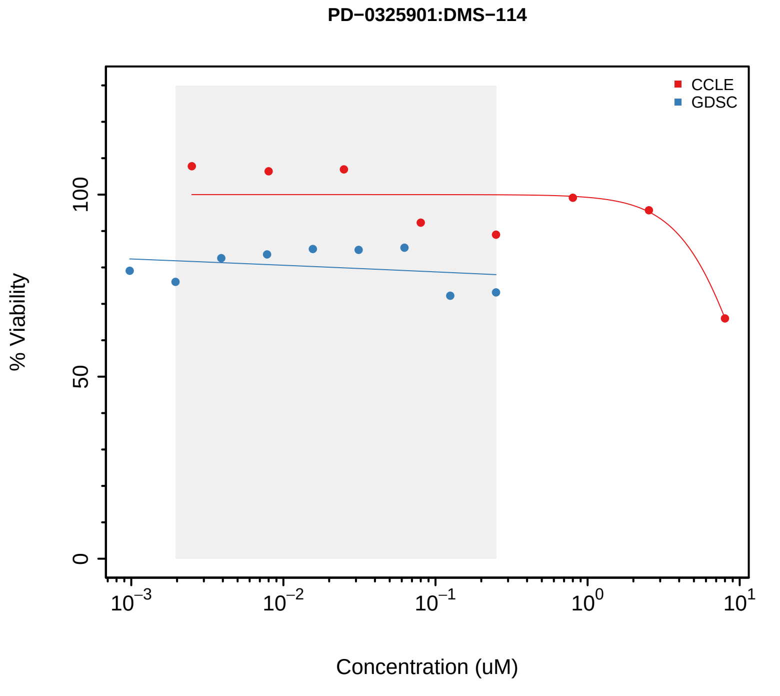
PD−0325901:DMS−114
CCLE
GDSC
% Viability
0
50
100
Concentration (uM)
10^{-3}
10^{-2}
10^{-1}
10^{0}
10^{1}

PD−0325901:DOHH−2

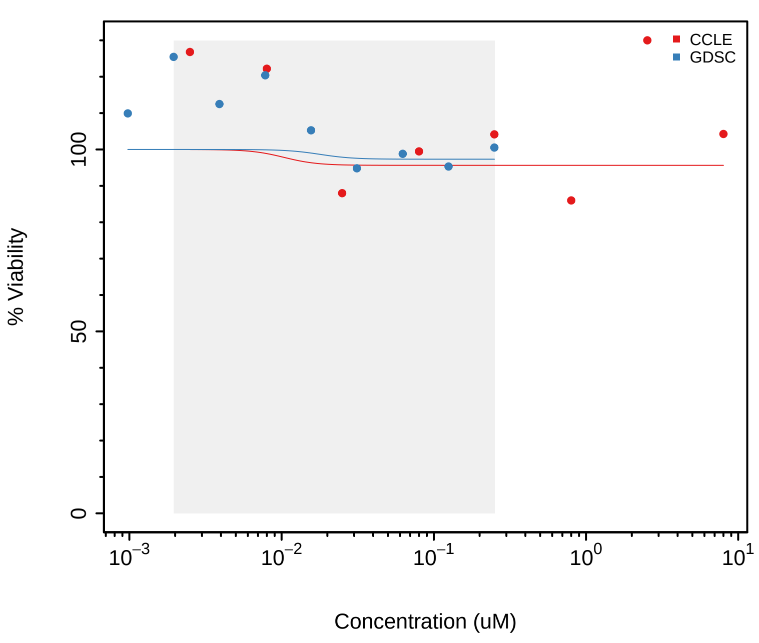

PD−0325901:DU−145

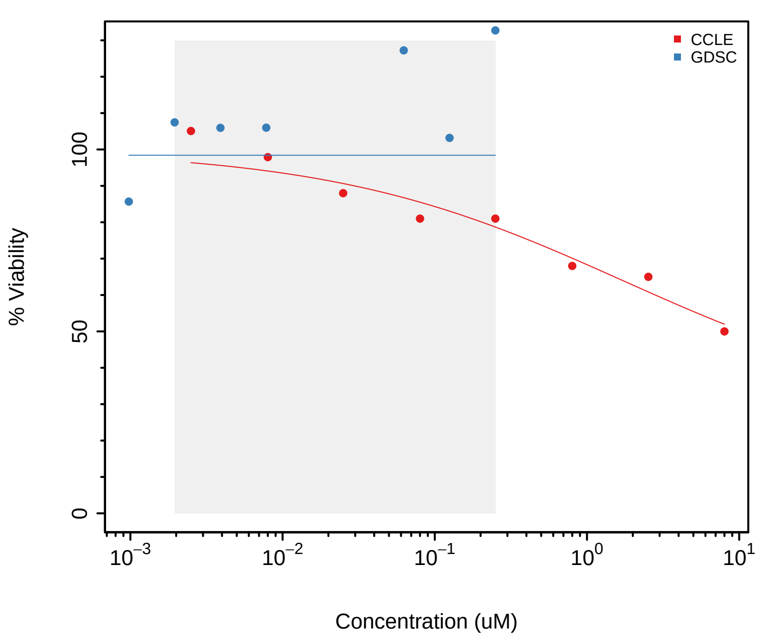

PD−0325901:EB2

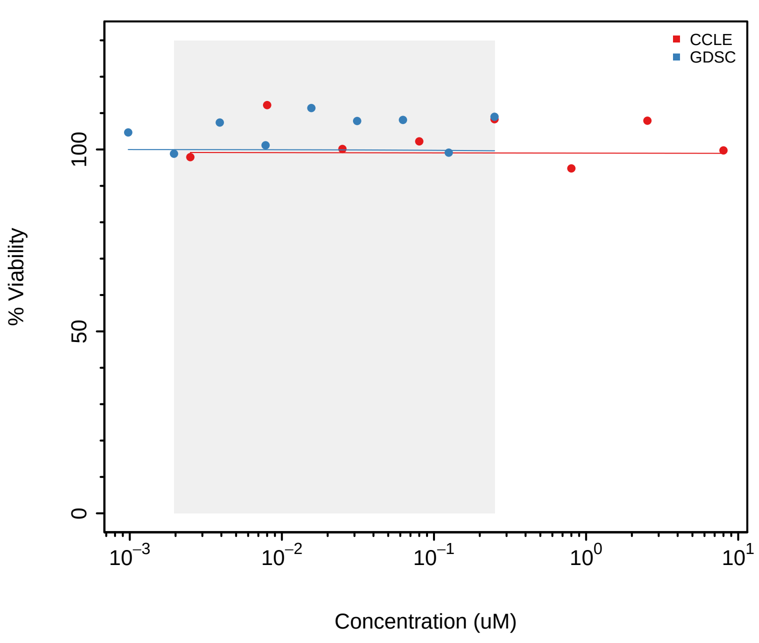

PD−0325901:EFE−184

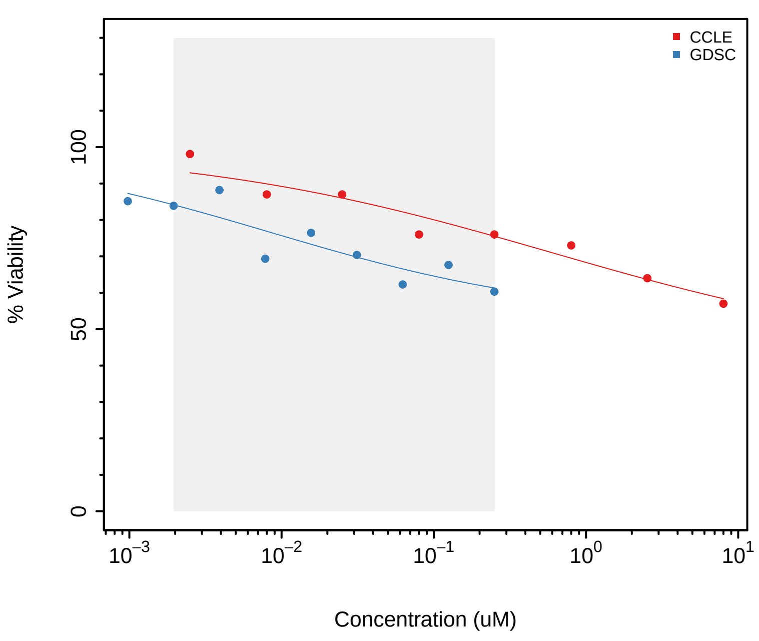

PD−0325901:EFO−21

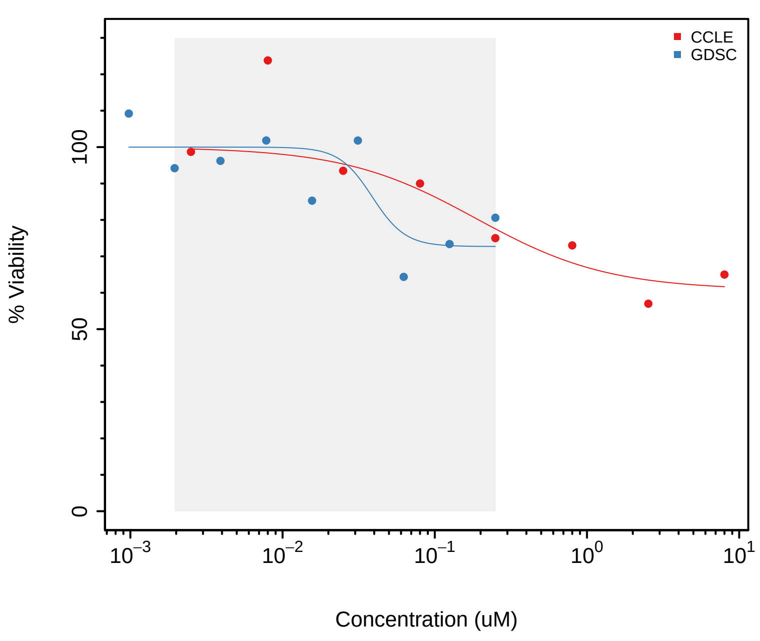

PD−0325901:EFO−27

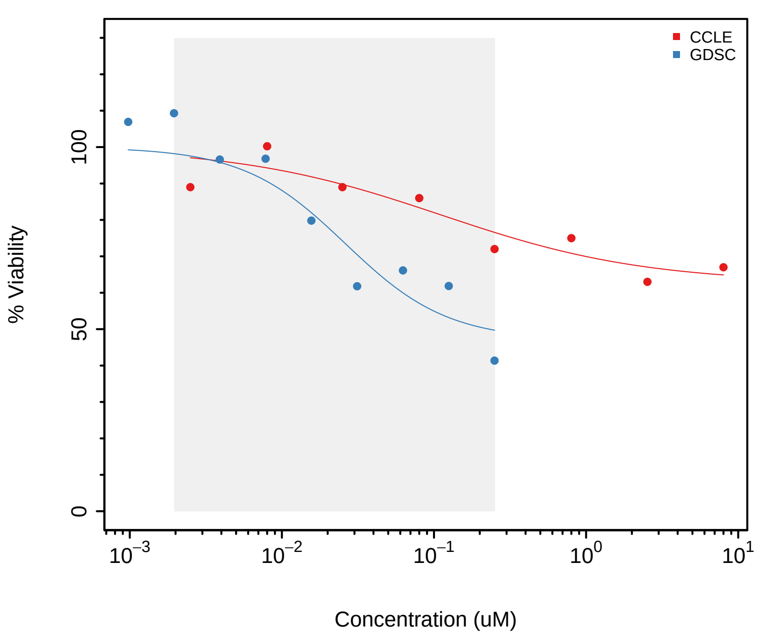

PD−0325901:EM−2

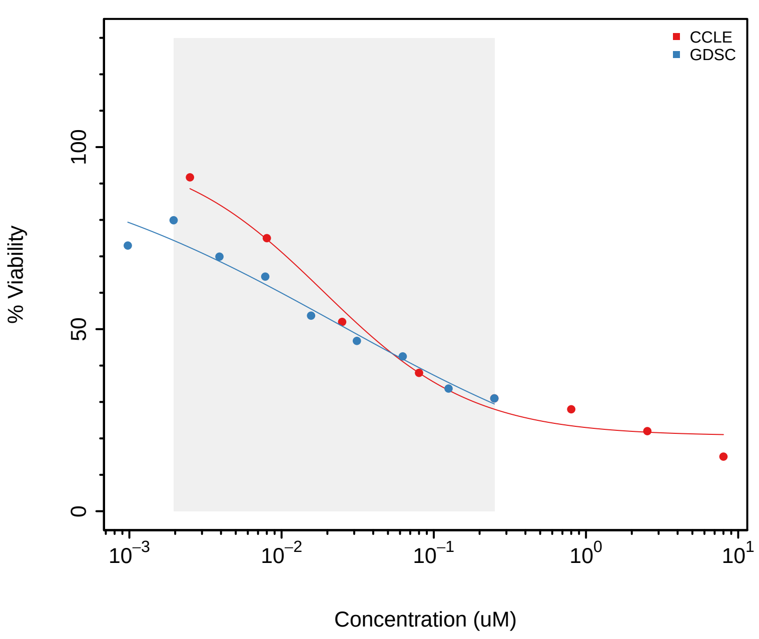

PD−0325901:ESS−1

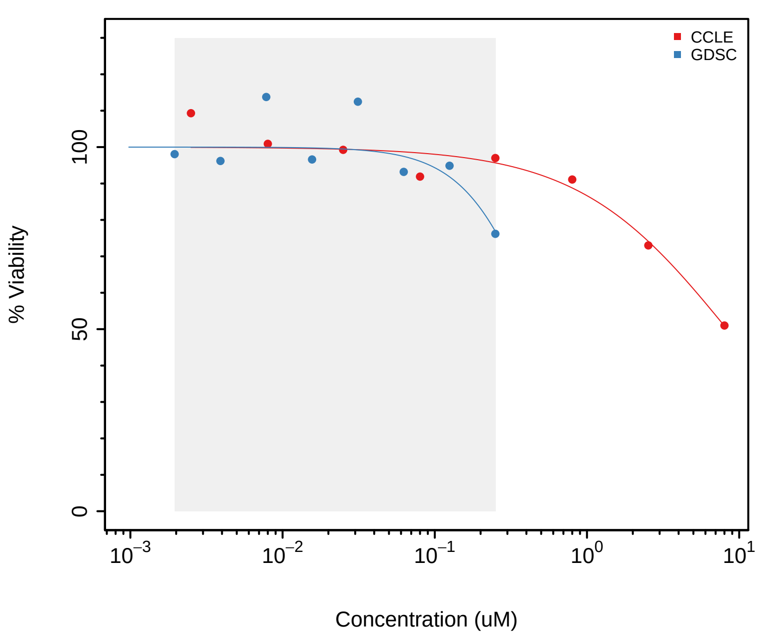

PD−0325901:FADU

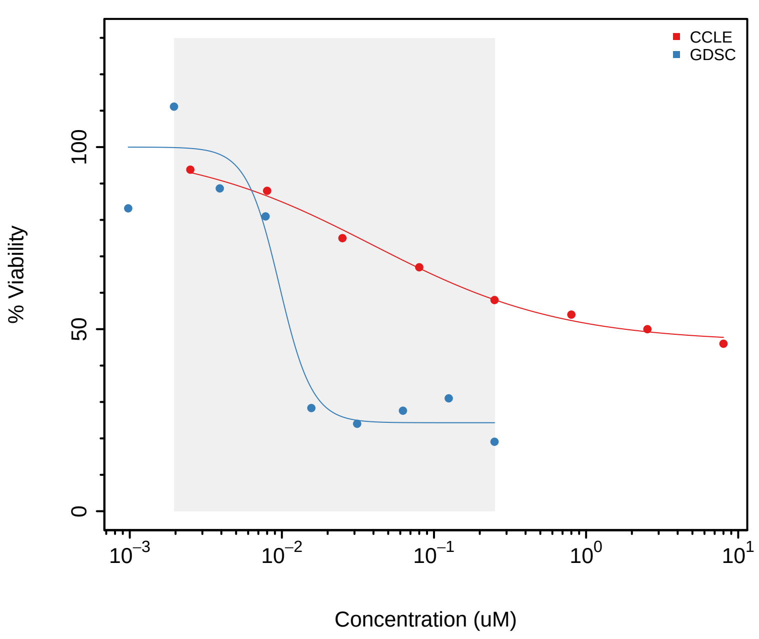

PD−0325901:G−361

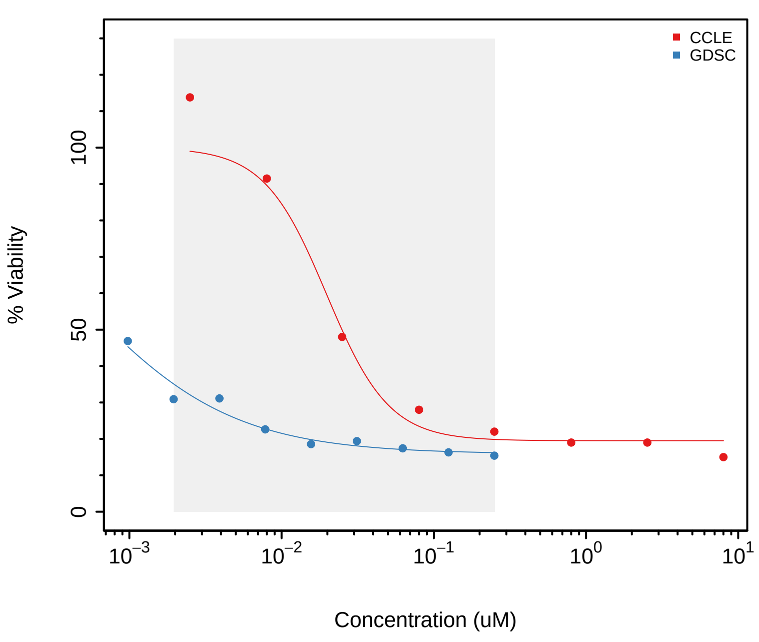

PD−0325901:G−401

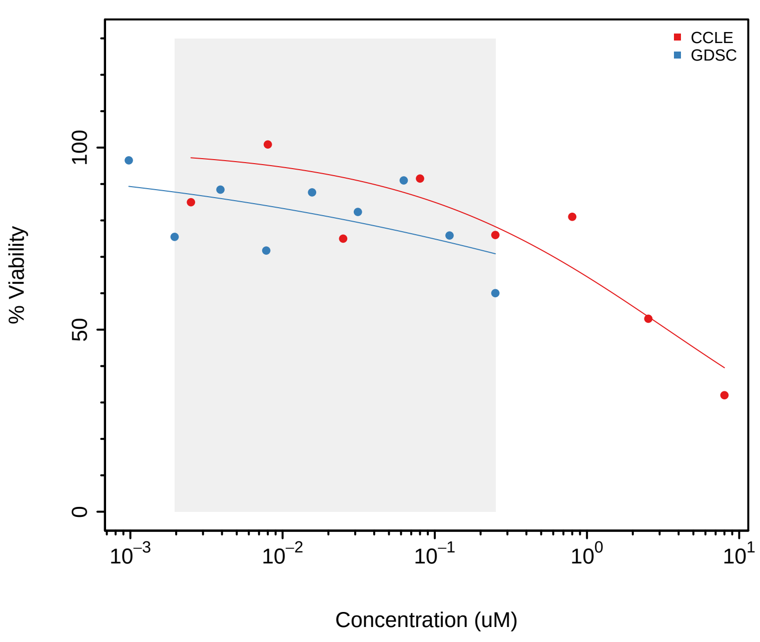

PD−0325901:G−402

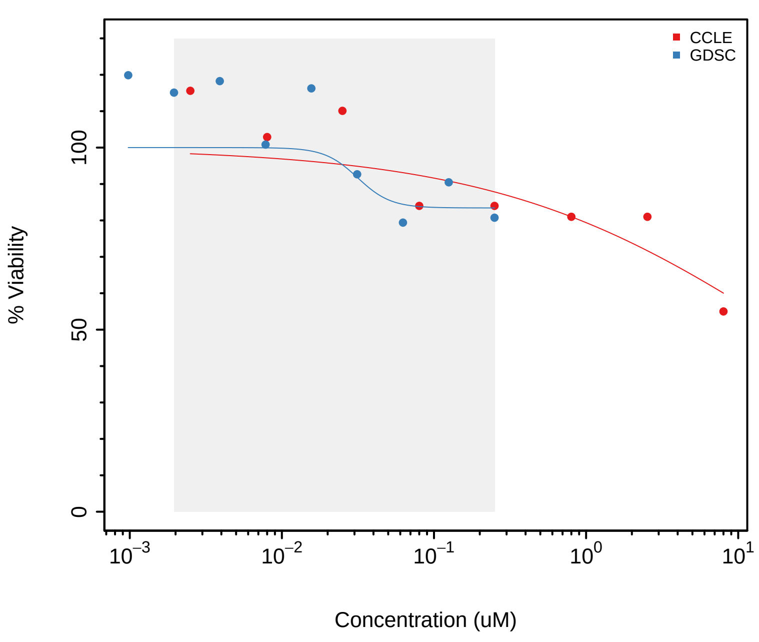

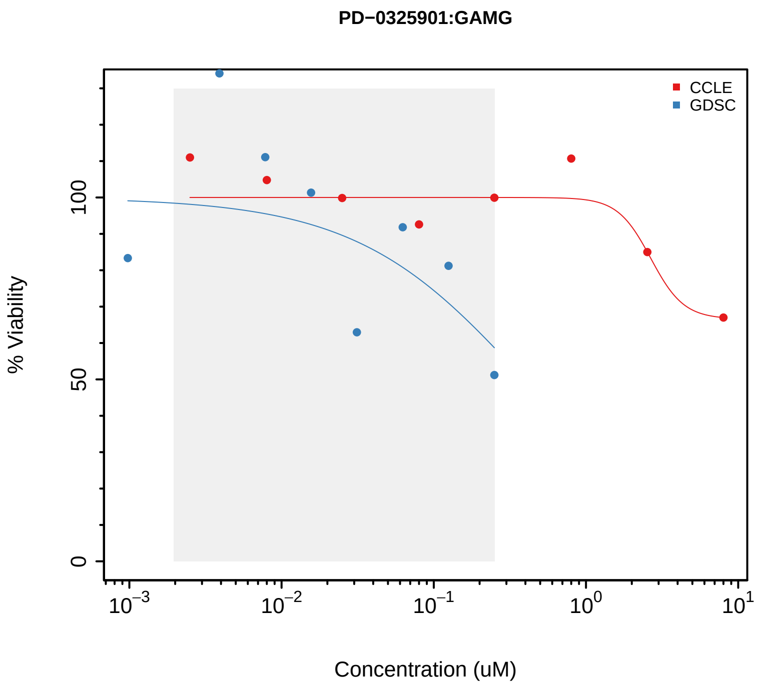
PD−0325901:GAMG
CCLE
GDSC
% Viability
Concentration (uM)

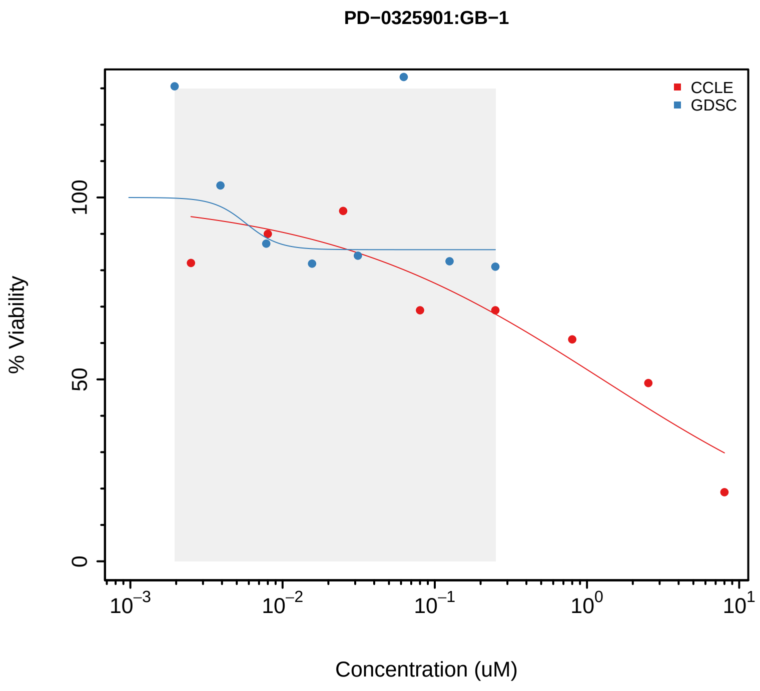
PD−0325901:GB−1
CCLE
GDSC
% Viability
Concentration (uM)

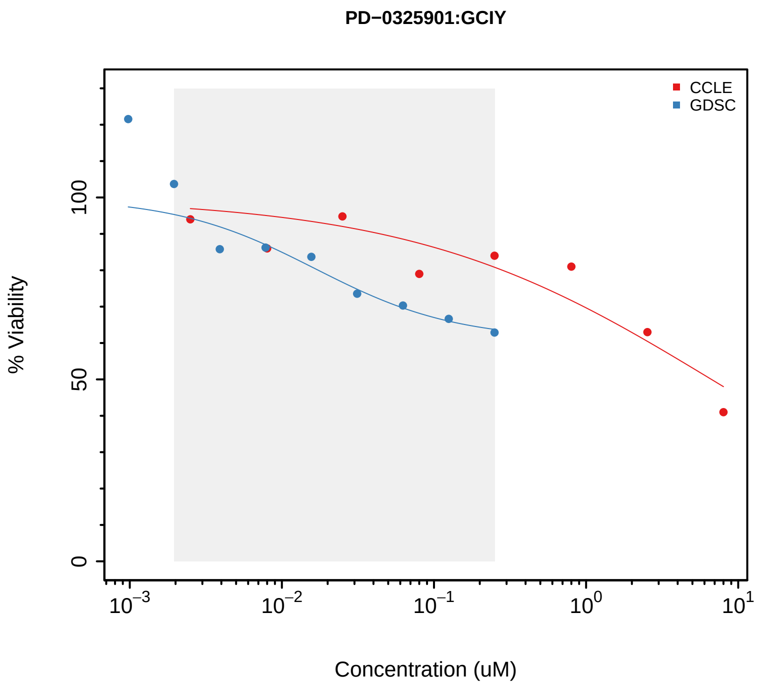
PD−0325901:GCIY
CCLE
GDSC
% Viability
Concentration (uM)

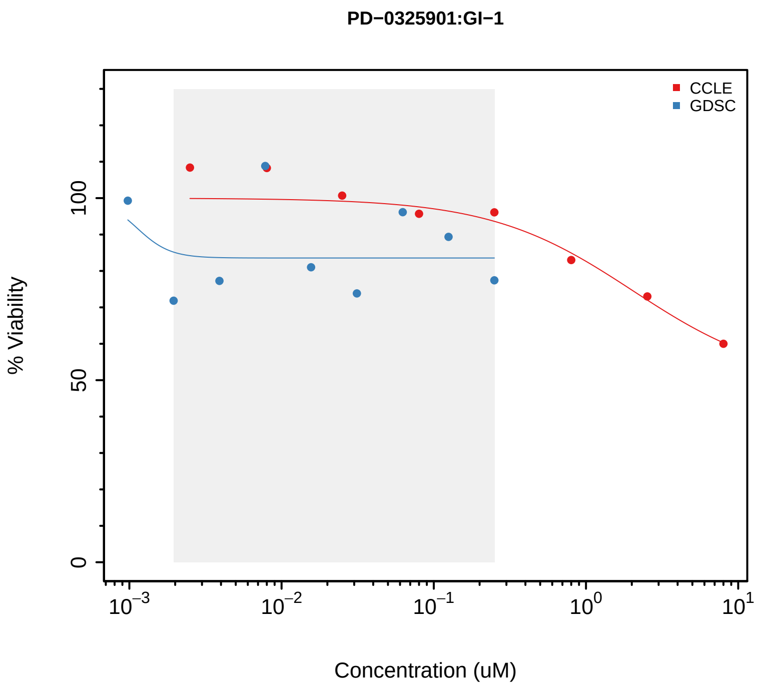
PD−0325901:GI−1
CCLE
GDSC
% Viability
Concentration (uM)

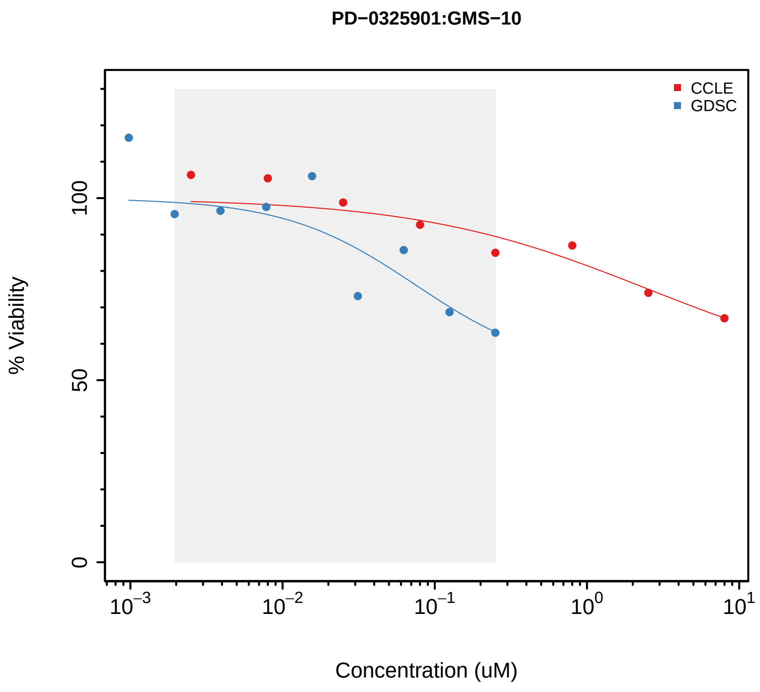
PD−0325901:GMS−10
CCLE
GDSC
% Viability
Concentration (uM)

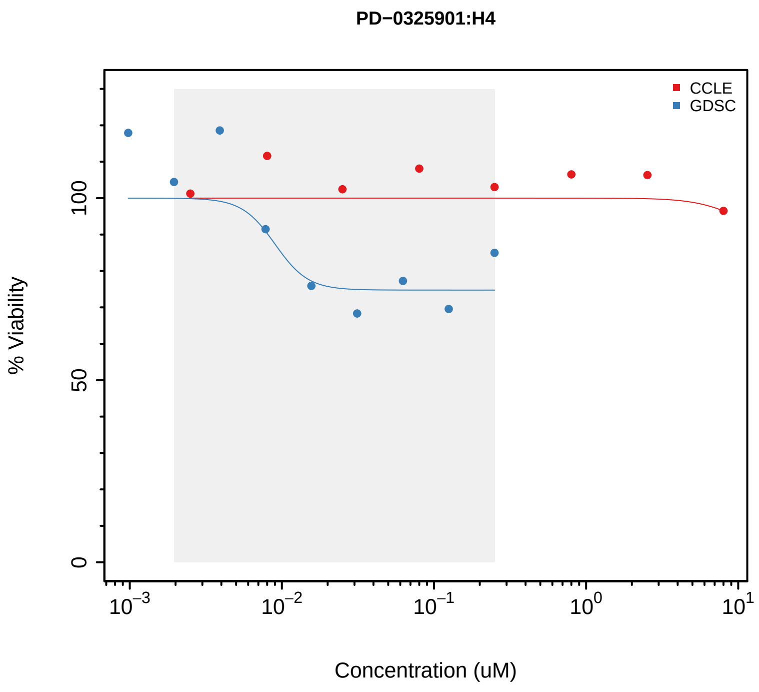
PD−0325901:H4
CCLE
GDSC
% Viability
Concentration (uM)

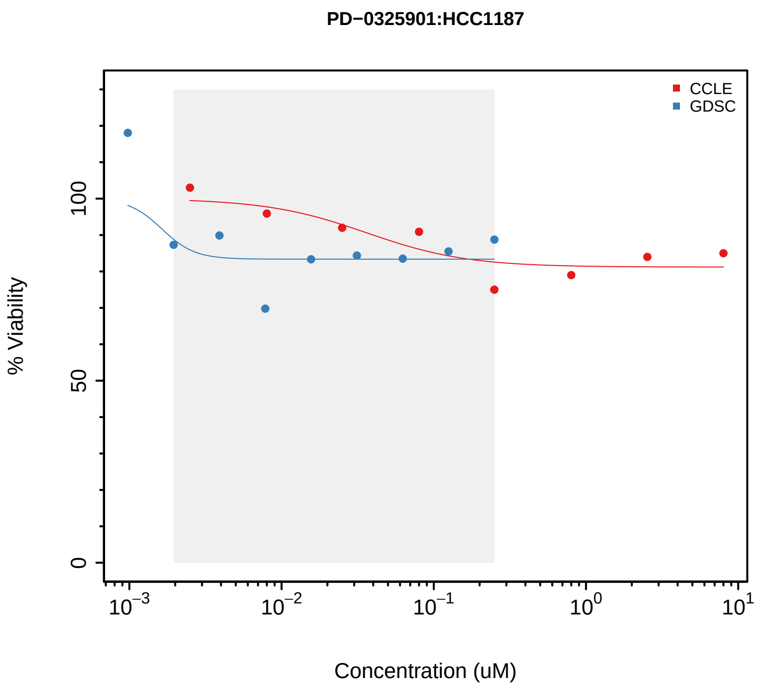
PD−0325901:HCC1187
CCLE
GDSC
% Viability
Concentration (uM)

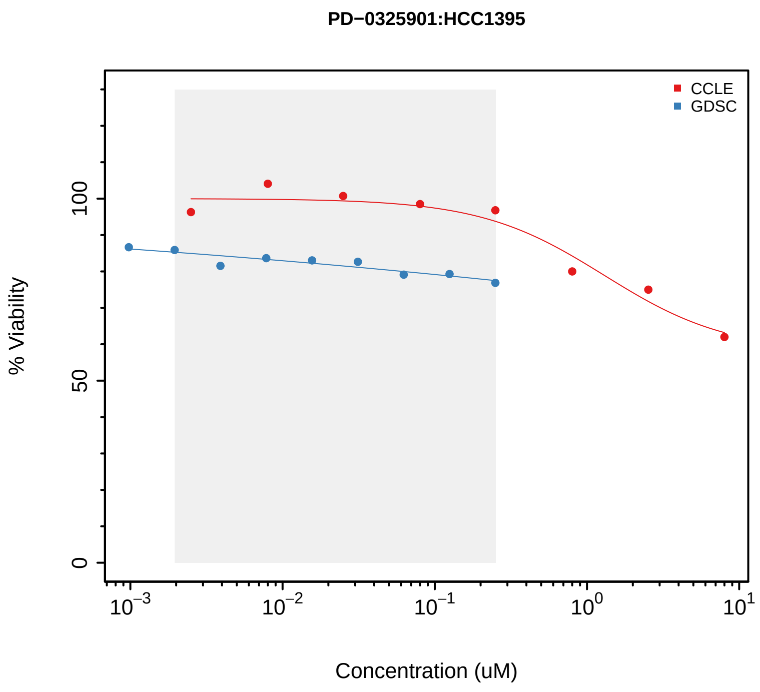
PD−0325901:HCC1395
CCLE
GDSC
% Viability
Concentration (uM)

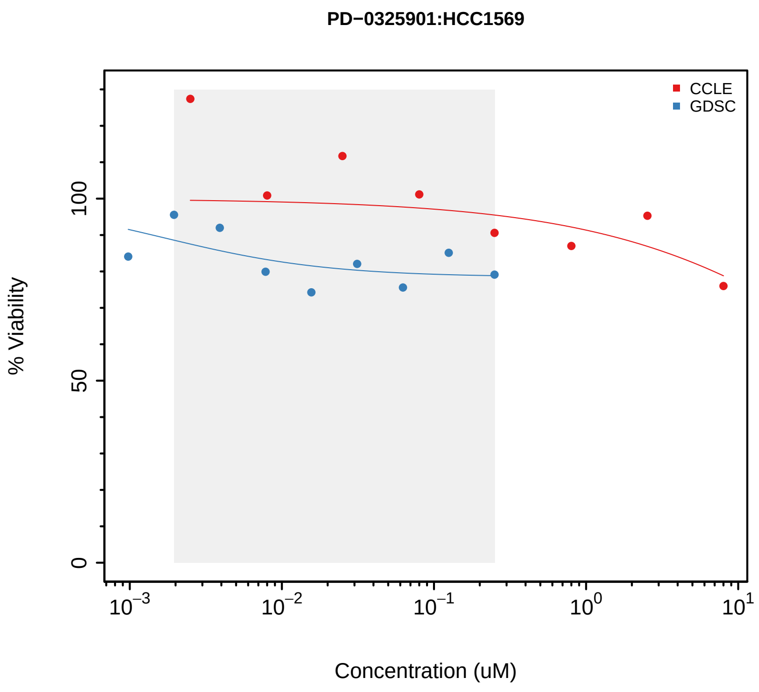
PD−0325901:HCC1569
CCLE
GDSC
% Viability
Concentration (uM)

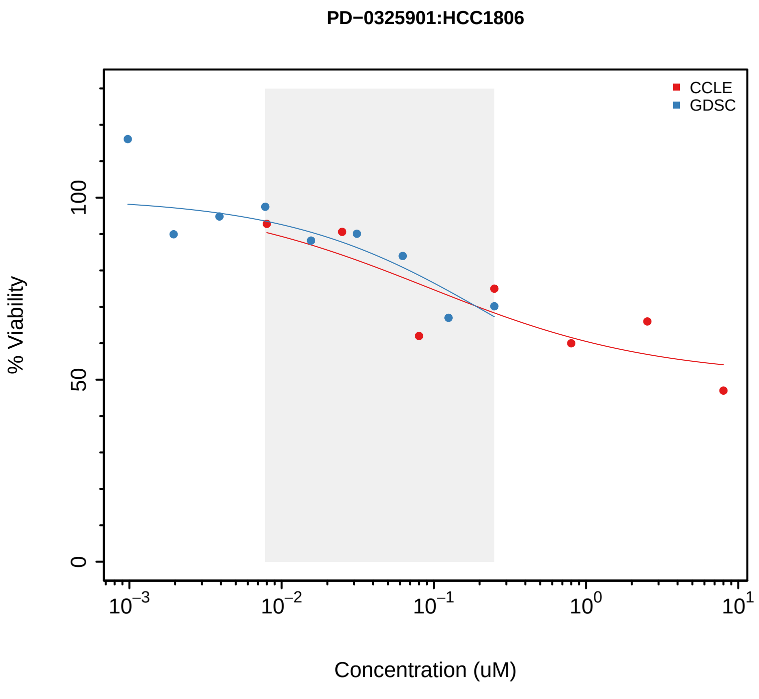
PD−0325901:HCC1806
CCLE
GDSC
% Viability
Concentration (uM)

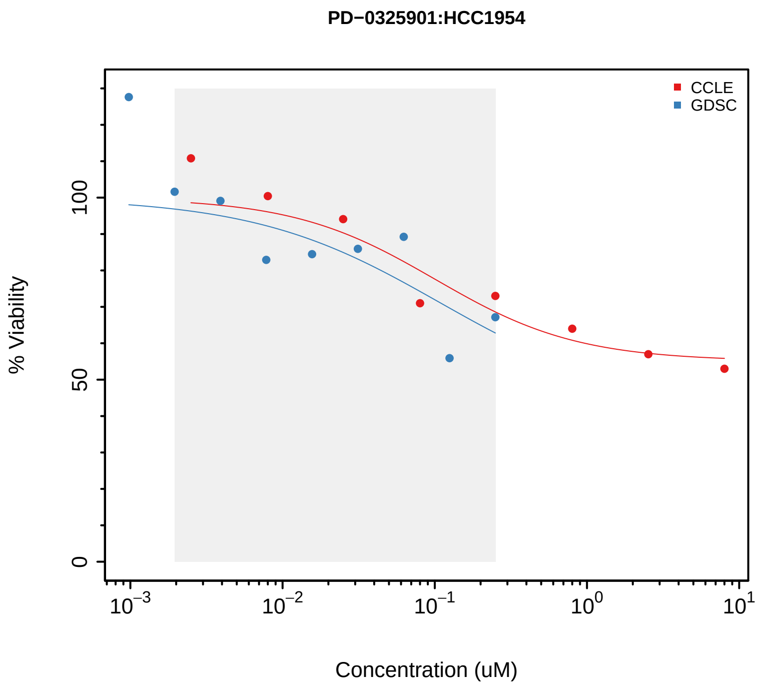
PD−0325901:HCC1954
CCLE
GDSC
% Viability
Concentration (uM)

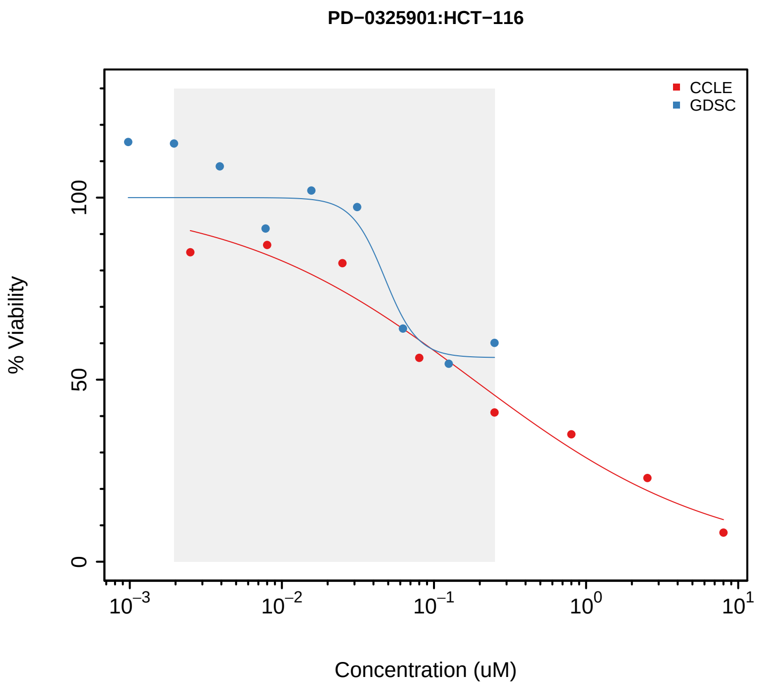
PD−0325901:HCT−116
CCLE
GDSC
% Viability
Concentration (uM)

PD−0325901:HCT−15

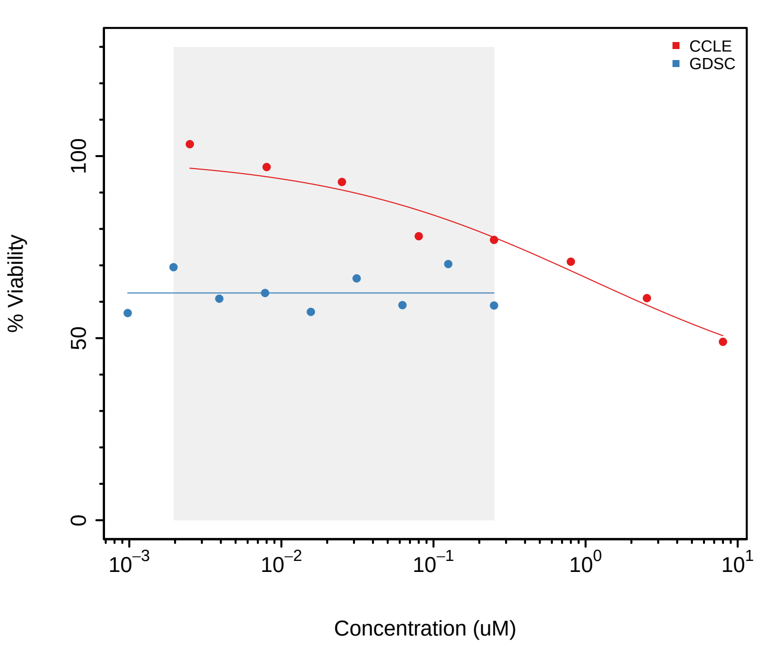

PD−0325901:HD−MY−Z

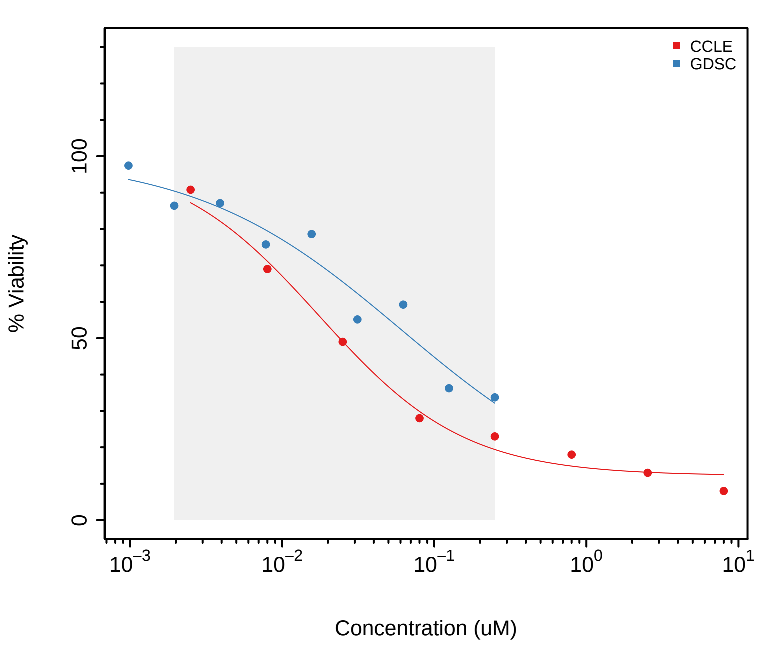

PD−0325901:HEC−1

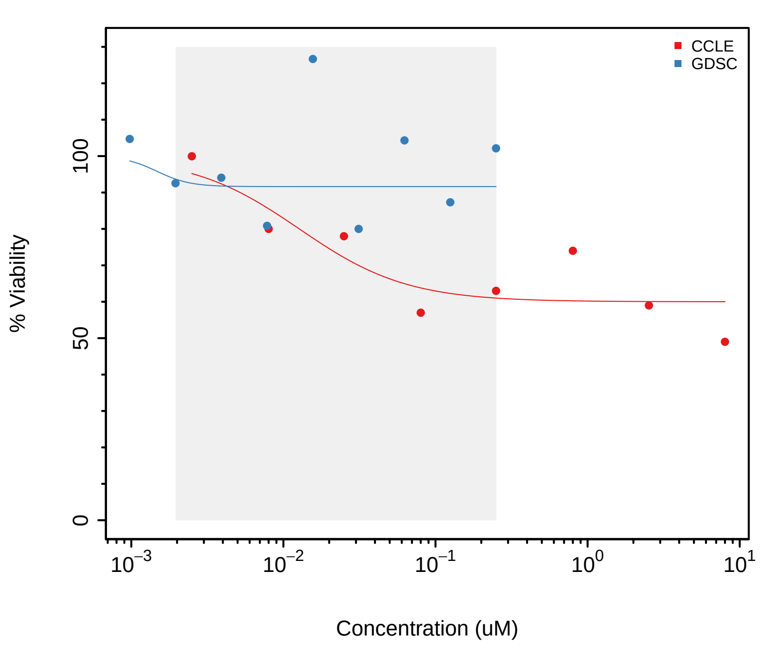

PD−0325901:HGC−27

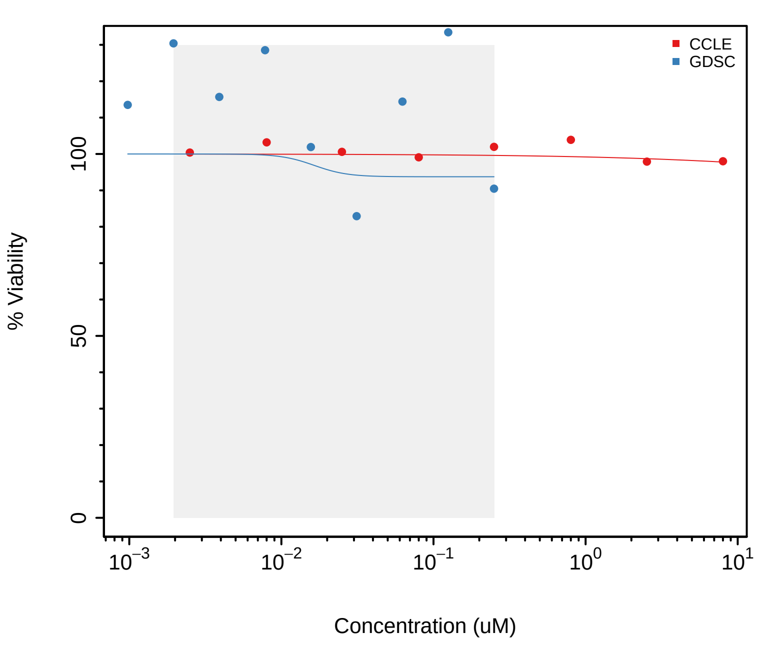

PD−0325901:HH

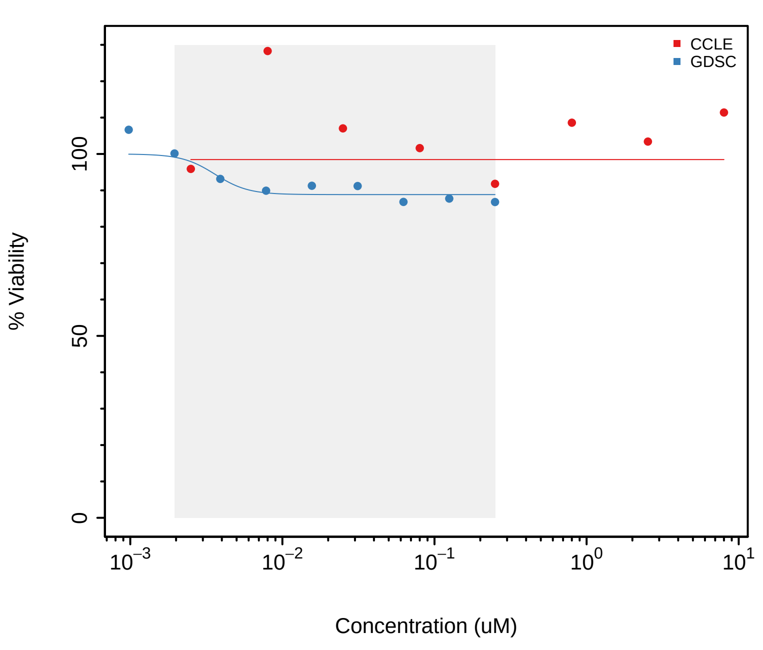

PD−0325901:HLE

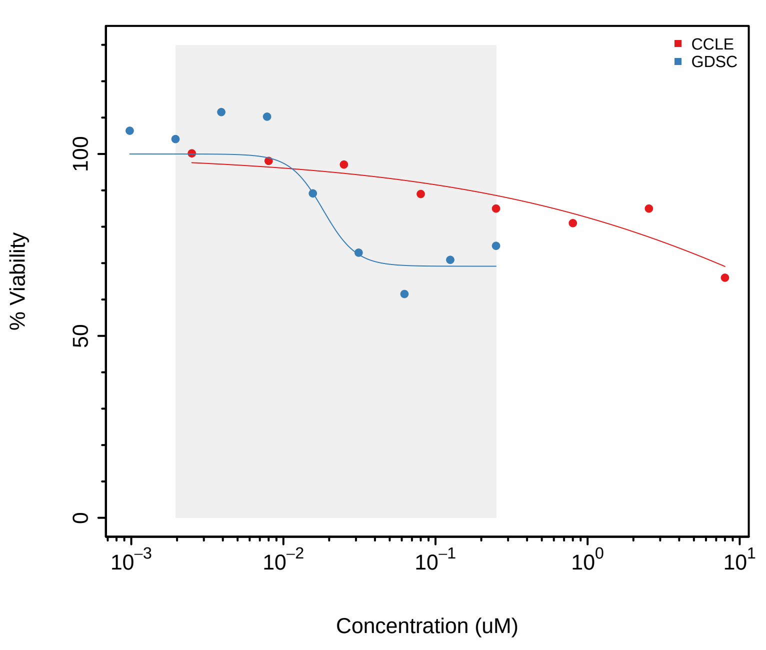

PD−0325901:HOS

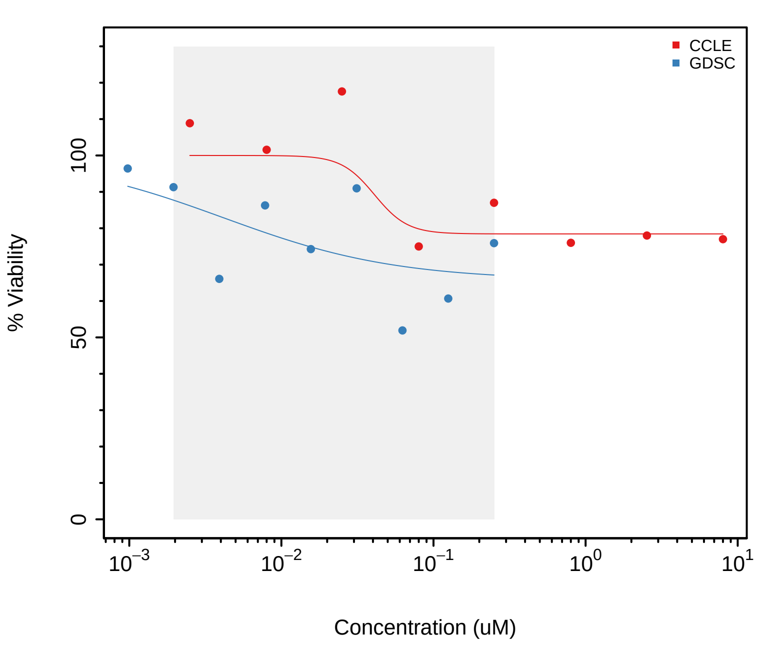

PD−0325901:HPAF−II

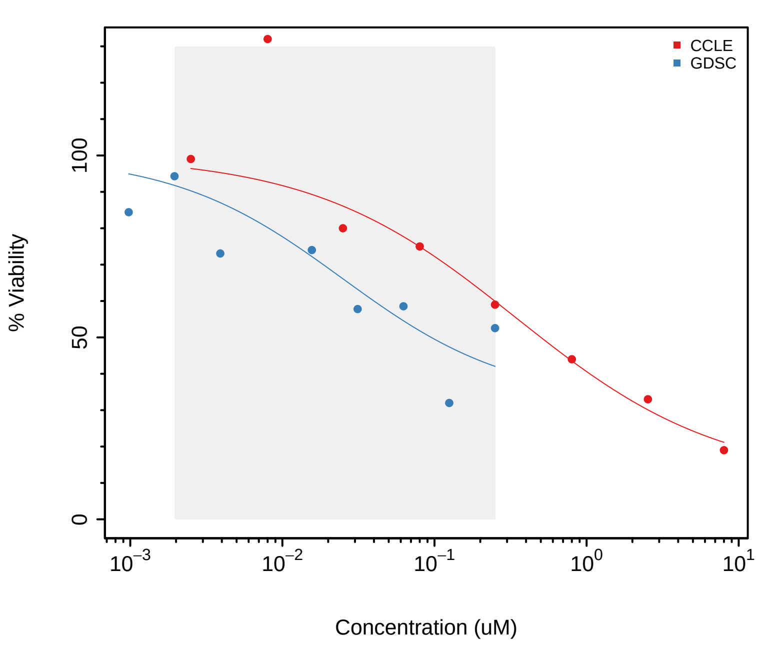

PD−0325901:Hs−578−T

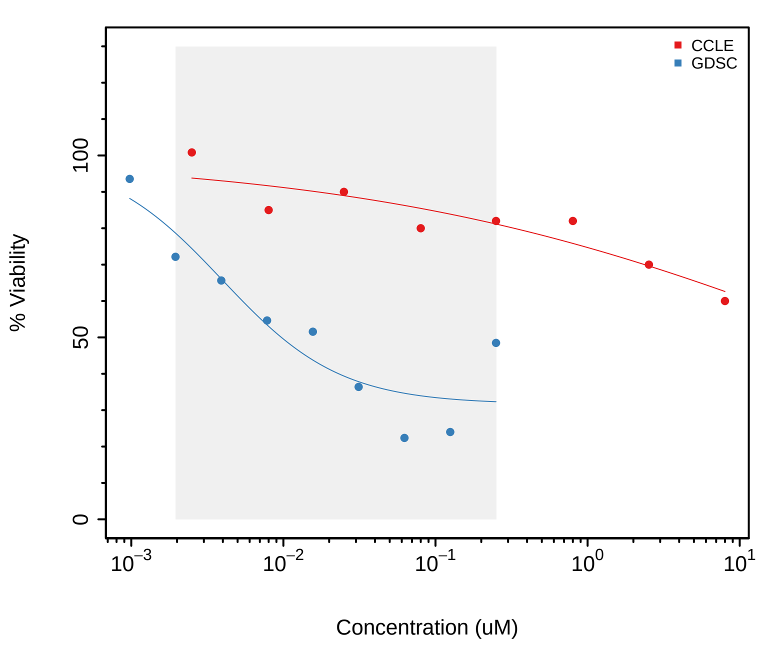

PD−0325901:HSC−2

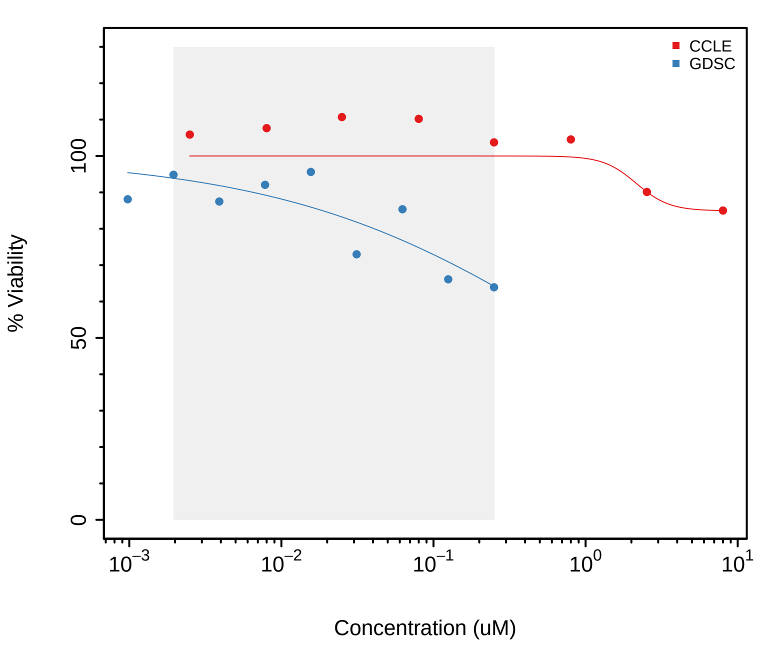

PD−0325901:HT

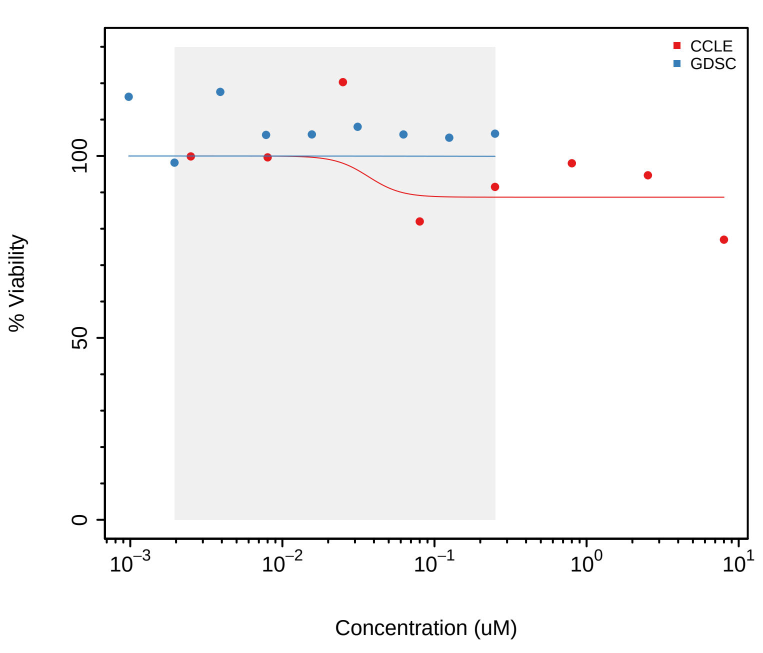

PD−0325901:HT−1080

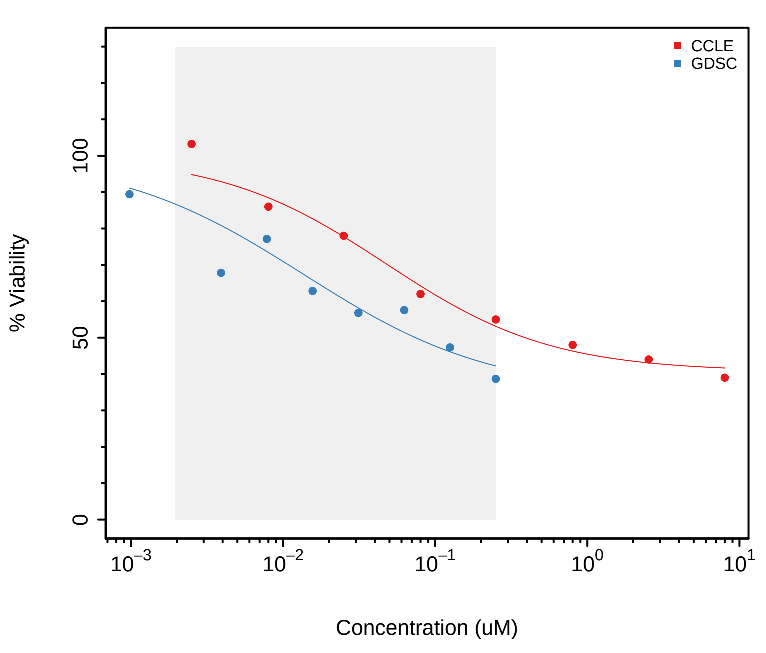

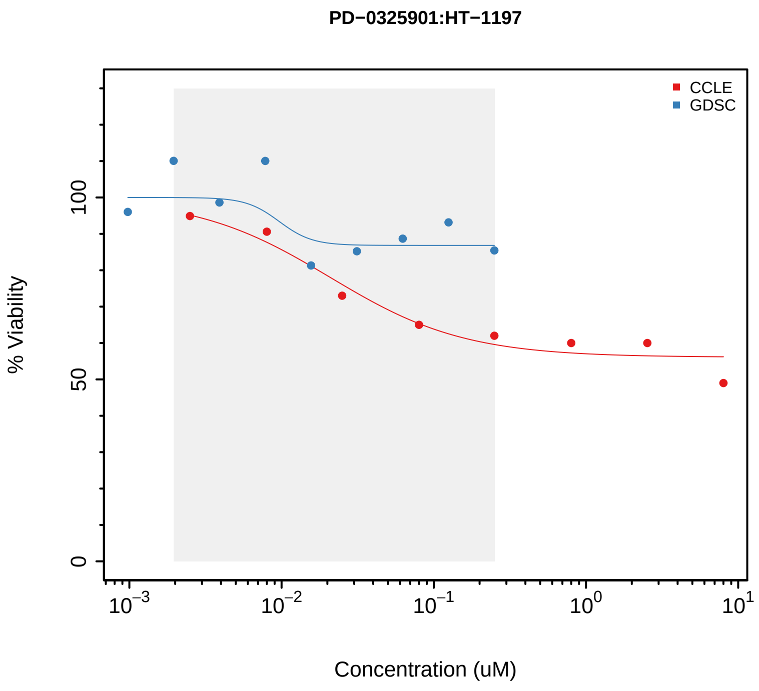
PD−0325901:HT−1197
CCLE
GDSC
% Viability
Concentration (uM)

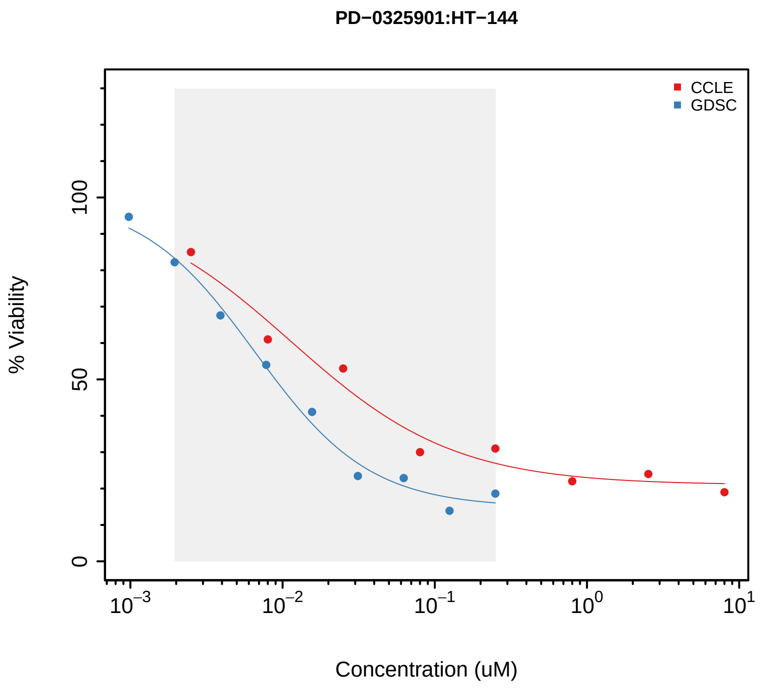
PD−0325901:HT−144
CCLE
GDSC
% Viability
Concentration (uM)

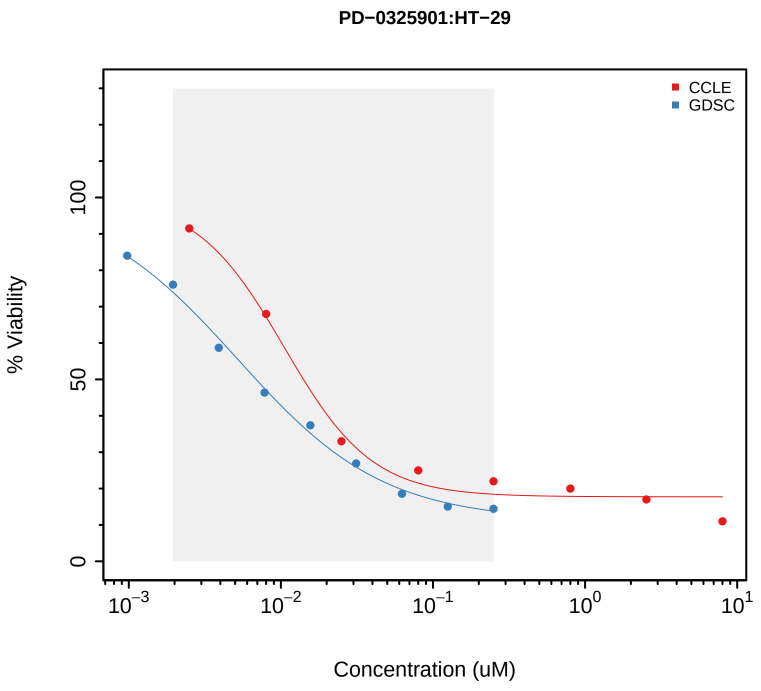
PD−0325901:HT−29
CCLE
GDSC
% Viability
Concentration (uM)

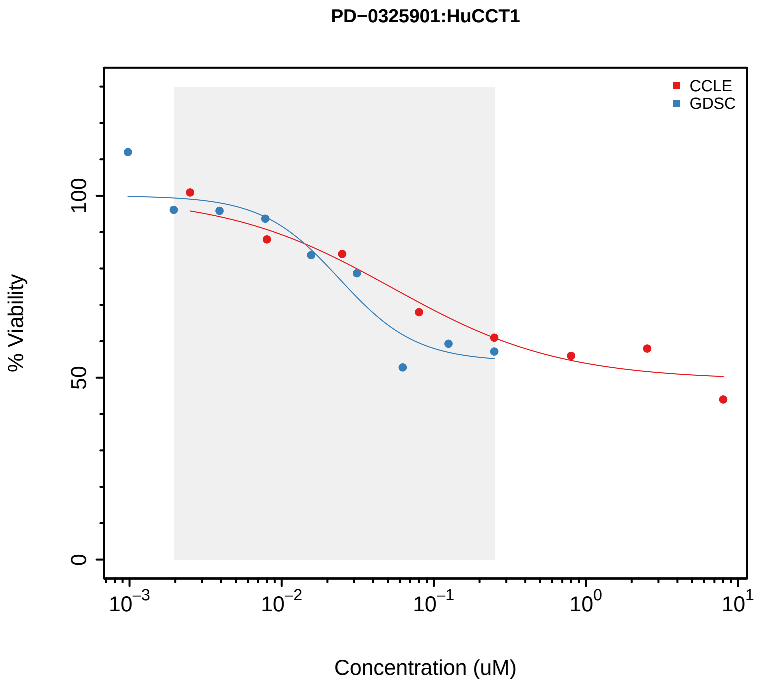
PD−0325901:HuCCT1
CCLE
GDSC
% Viability
Concentration (uM)

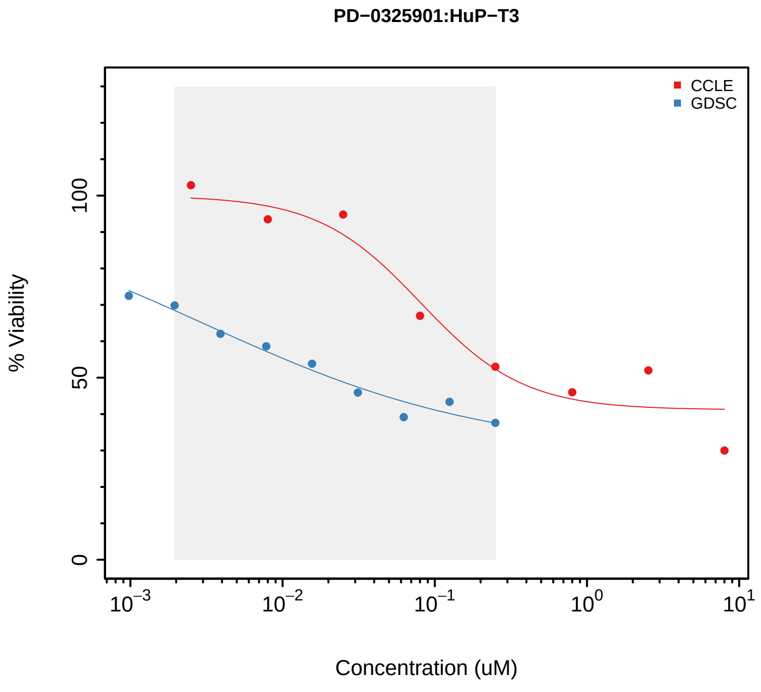
PD−0325901:HuP−T3
CCLE
GDSC
% Viability
Concentration (uM)

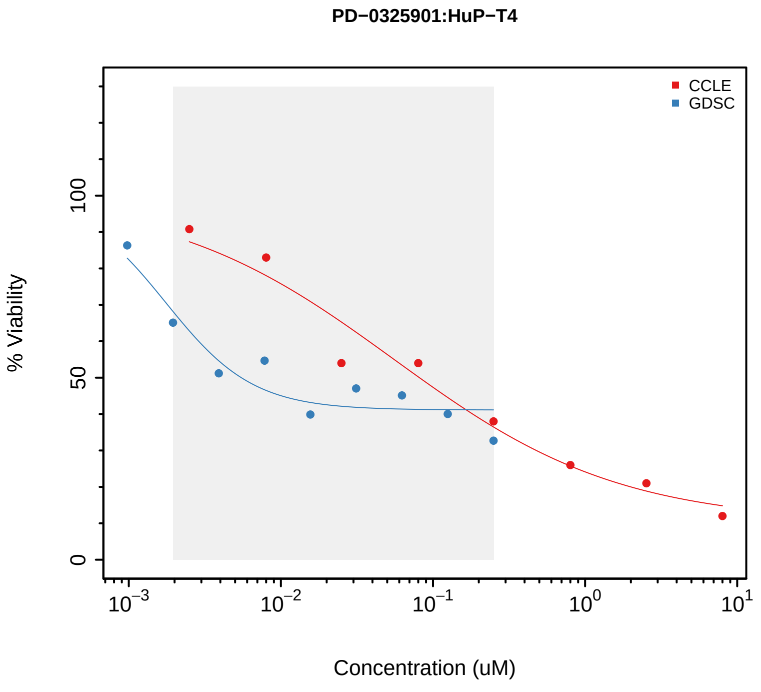
PD−0325901:HuP−T4
CCLE
GDSC
% Viability
Concentration (uM)

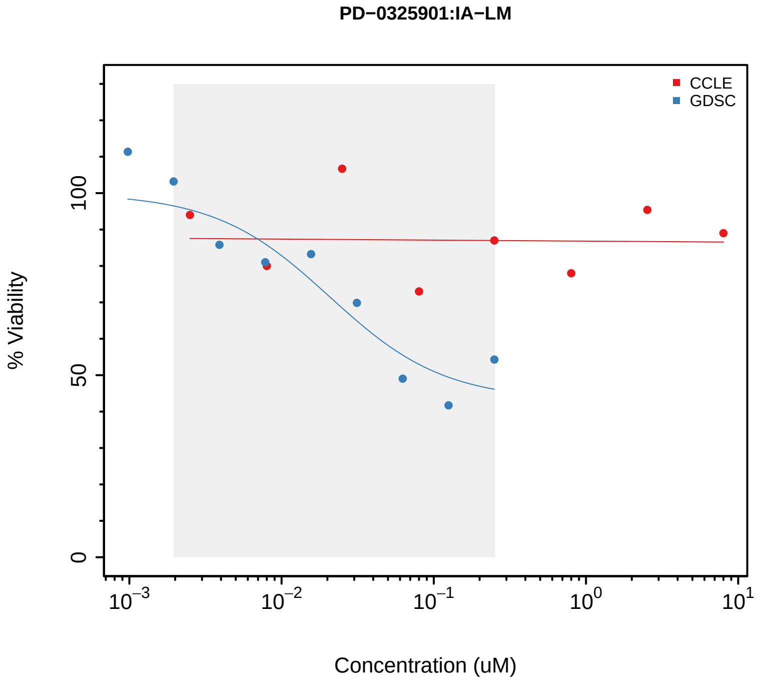
PD−0325901:IA−LM
CCLE
GDSC
% Viability
Concentration (uM)

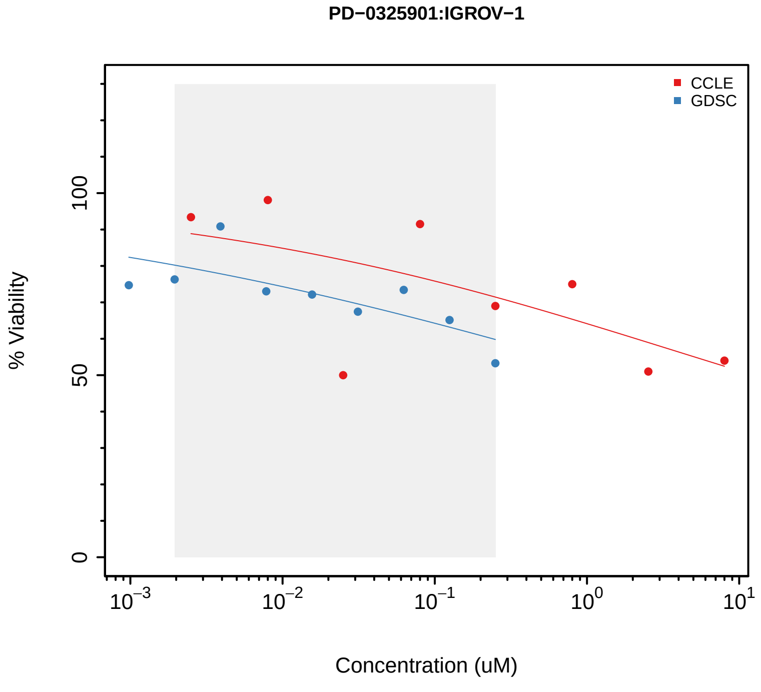
PD−0325901:IGROV−1
CCLE
GDSC
% Viability
Concentration (uM)

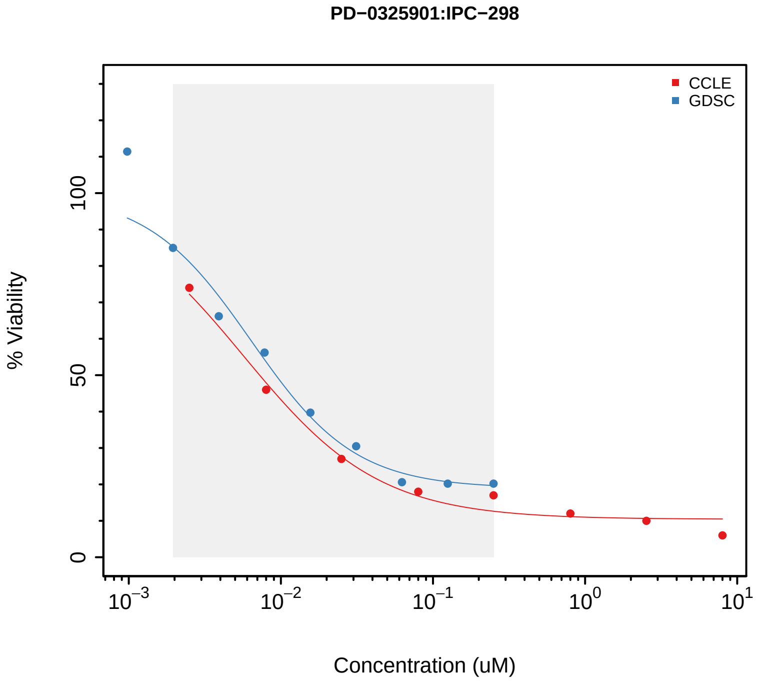
PD−0325901:IPC−298
CCLE
GDSC
% Viability
Concentration (uM)

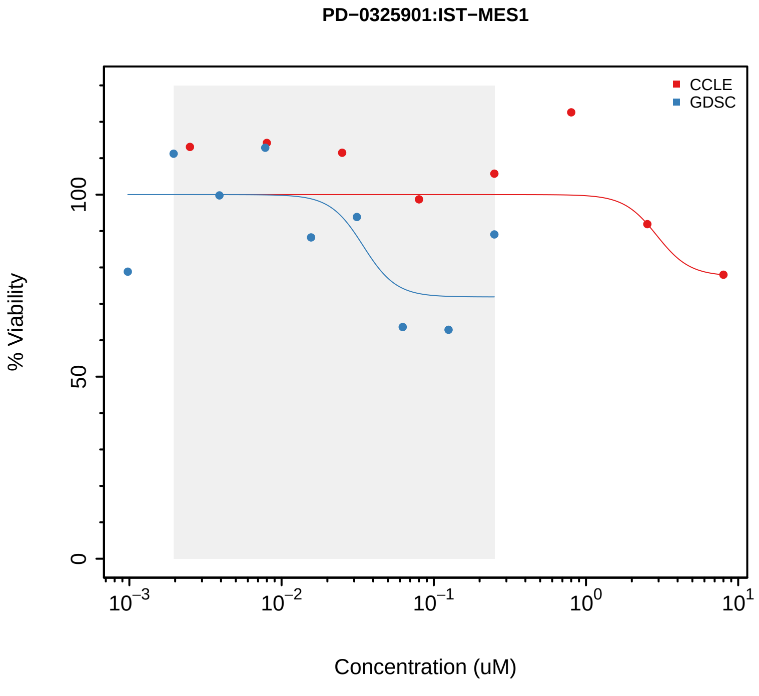
PD−0325901:IST−MES1
CCLE
GDSC
% Viability
Concentration (uM)

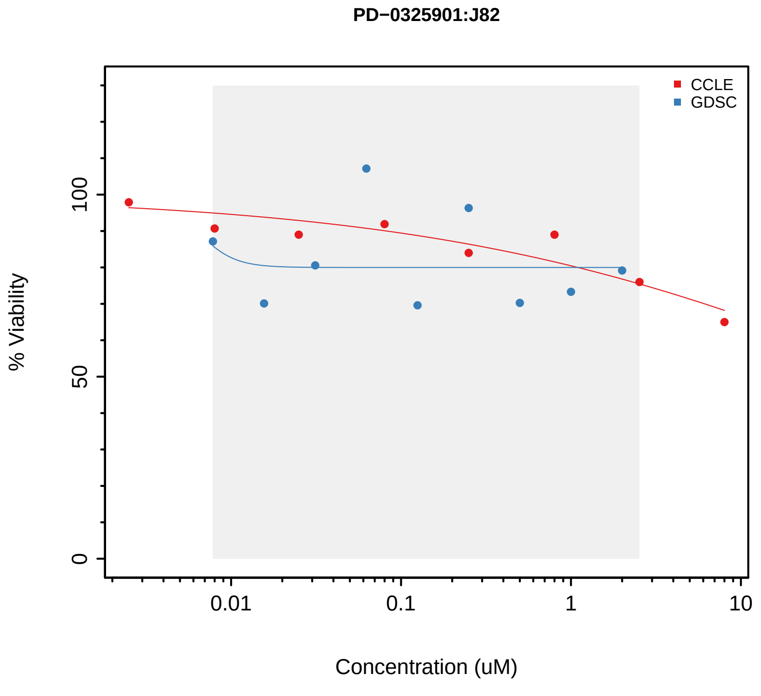
PD−0325901:J82
CCLE
GDSC
% Viability
Concentration (uM)

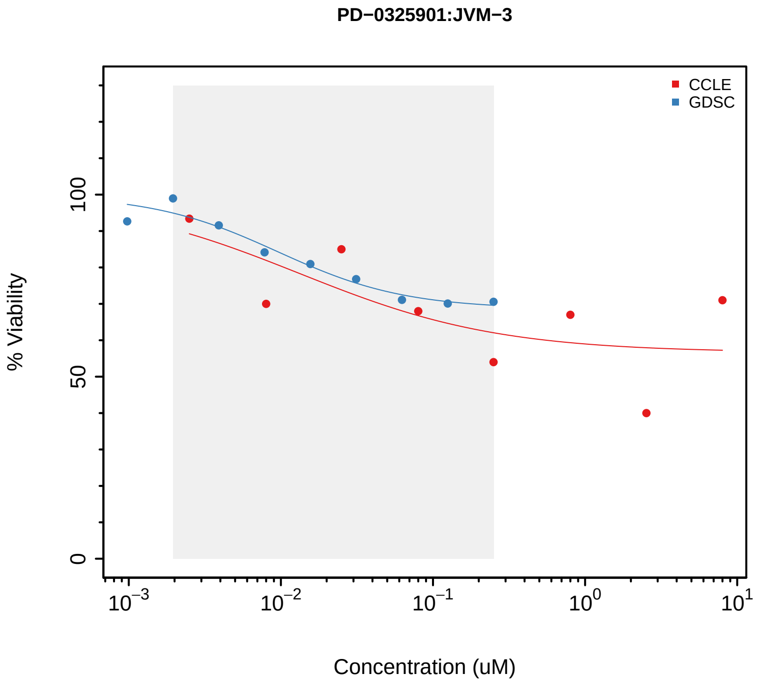
PD−0325901:JVM−3
CCLE
GDSC
% Viability
Concentration (uM)

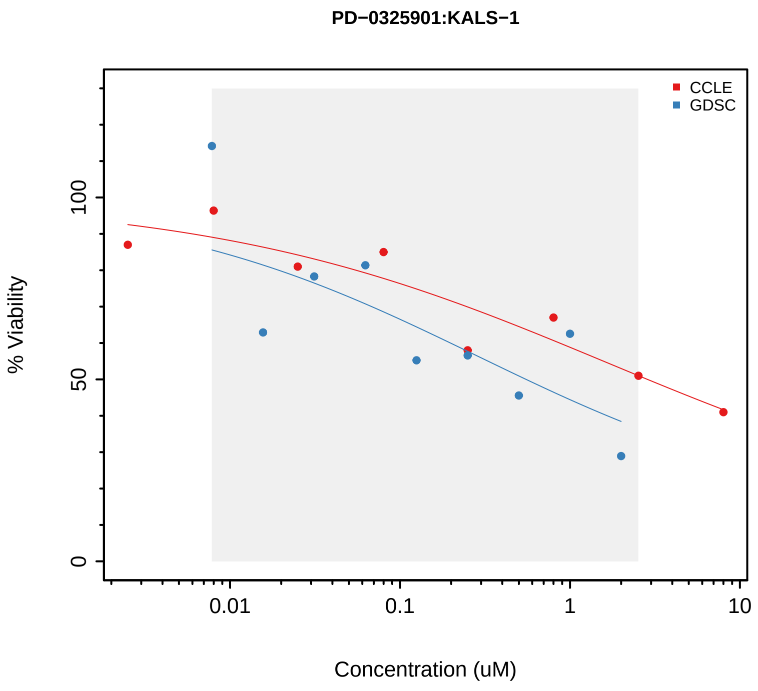
PD−0325901:KALS−1
CCLE
GDSC
% Viability
Concentration (uM)

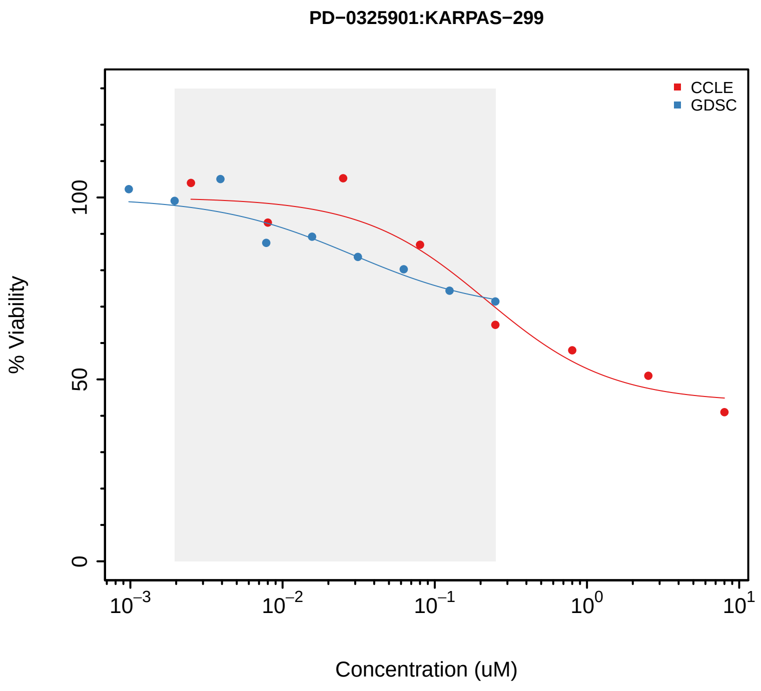
PD−0325901:KARPAS−299
CCLE
GDSC
% Viability
Concentration (uM)

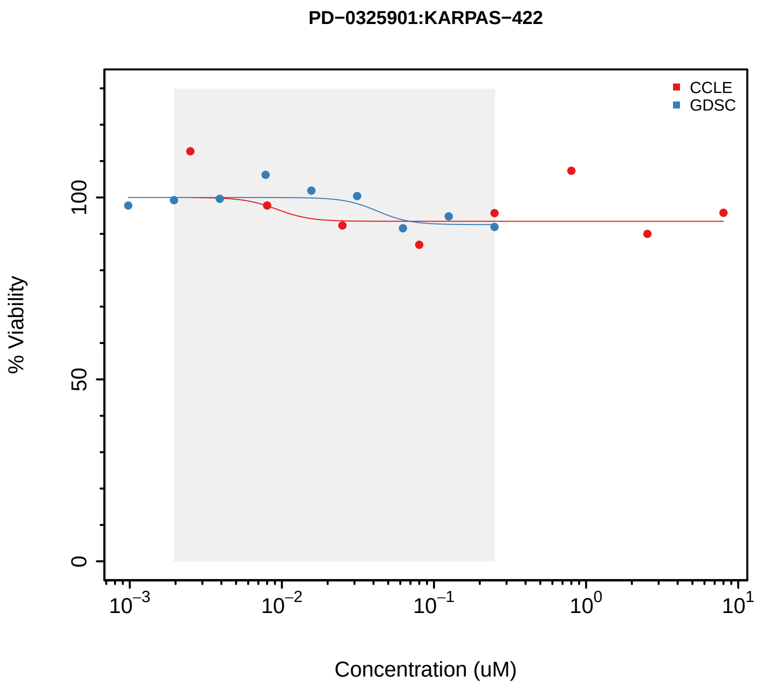
PD−0325901:KARPAS−422
CCLE
GDSC
% Viability
Concentration (uM)

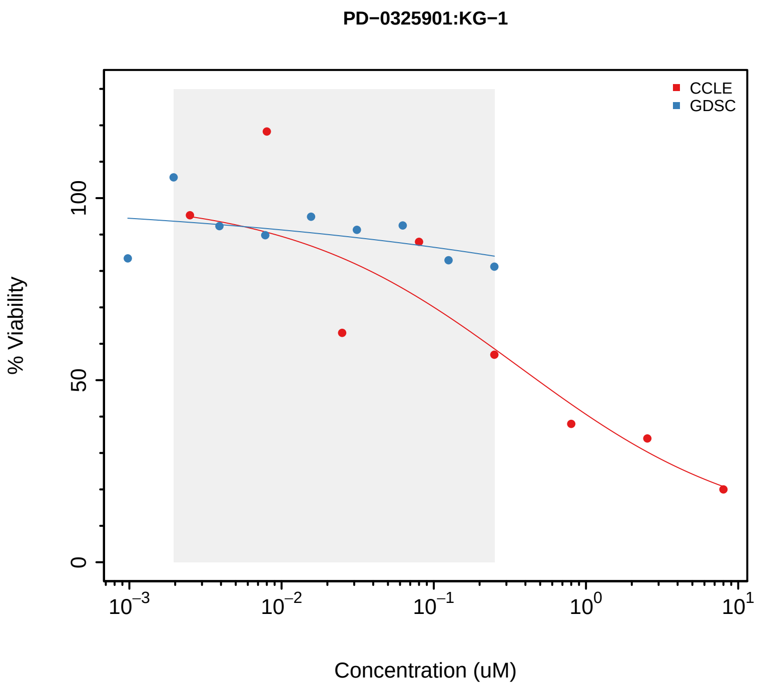
PD−0325901:KG−1
CCLE
GDSC
% Viability
Concentration (uM)

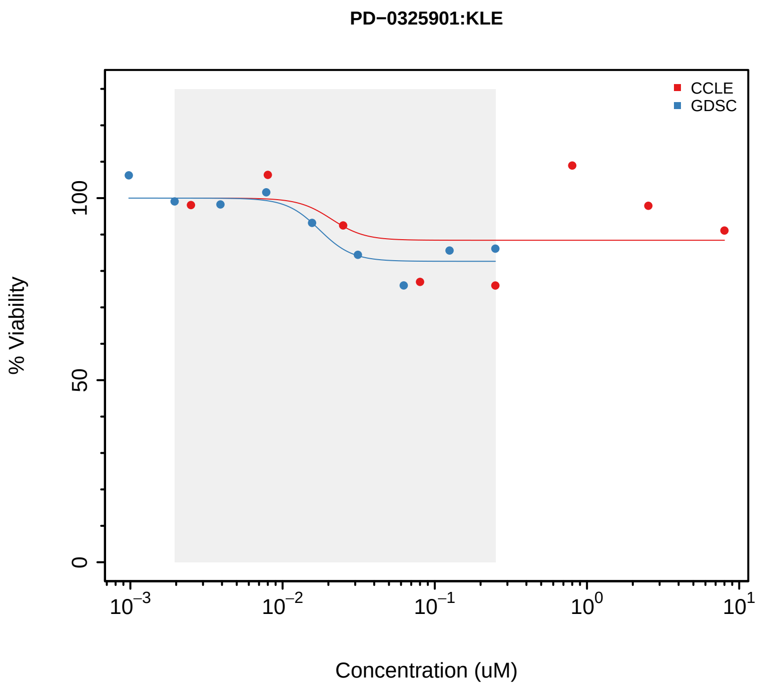
PD−0325901:KLE
CCLE
GDSC
% Viability
Concentration (uM)

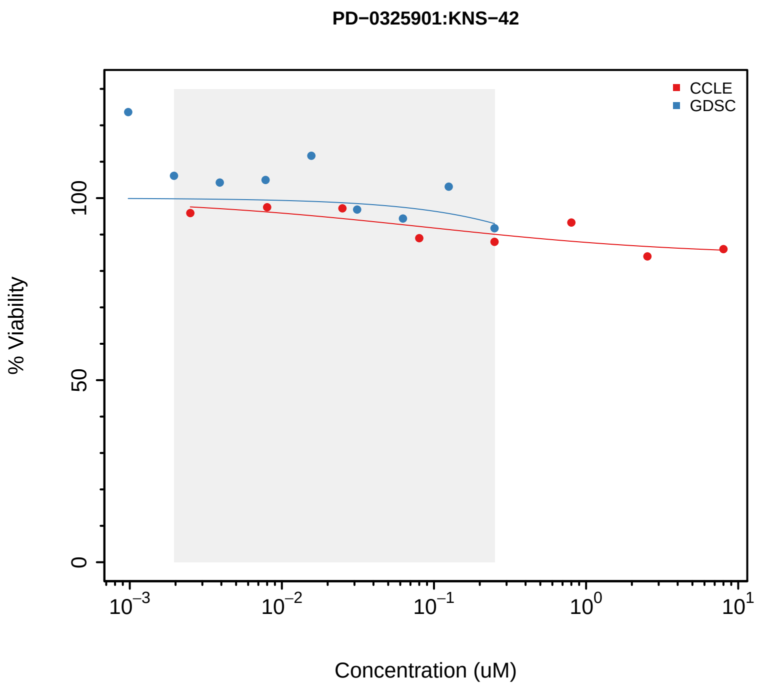
PD−0325901:KNS−42
CCLE
GDSC
% Viability
Concentration (uM)

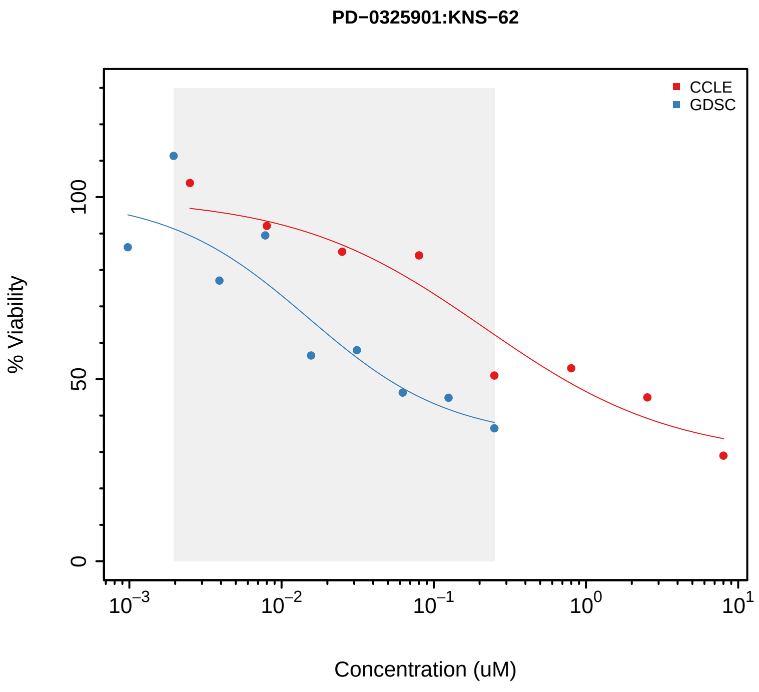
PD−0325901:KNS−62
CCLE
GDSC
% Viability
Concentration (uM)

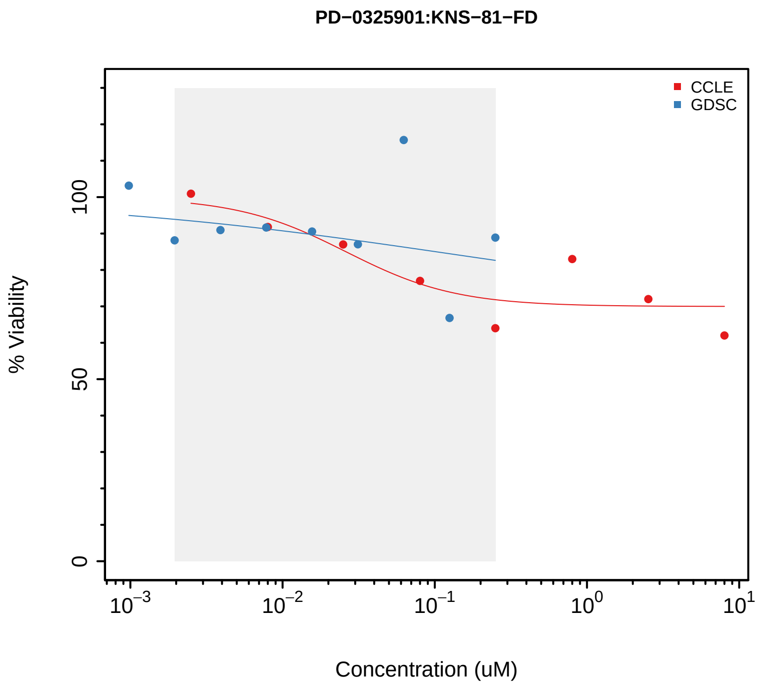
PD−0325901:KNS−81−FD
CCLE
GDSC
% Viability
Concentration (uM)

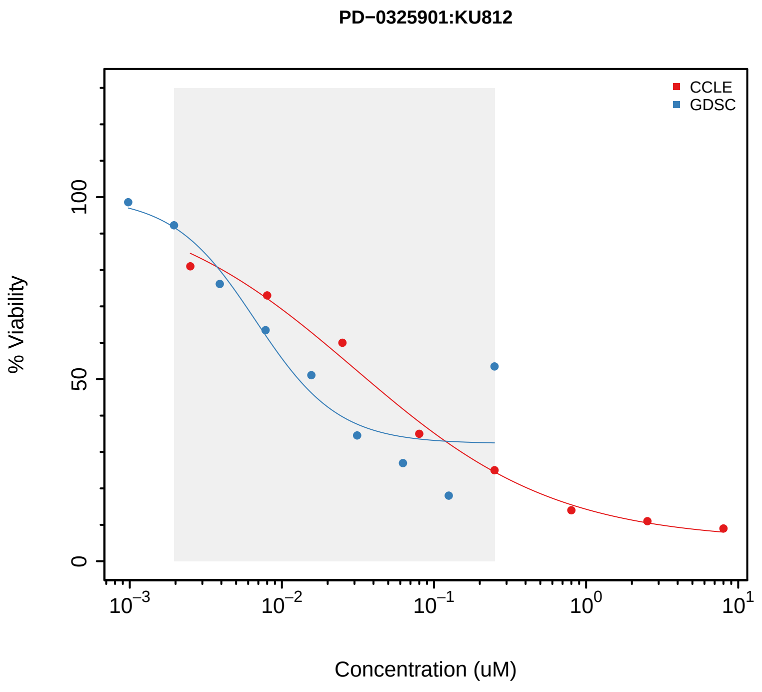
PD−0325901:KU812
CCLE
GDSC
% Viability
Concentration (uM)

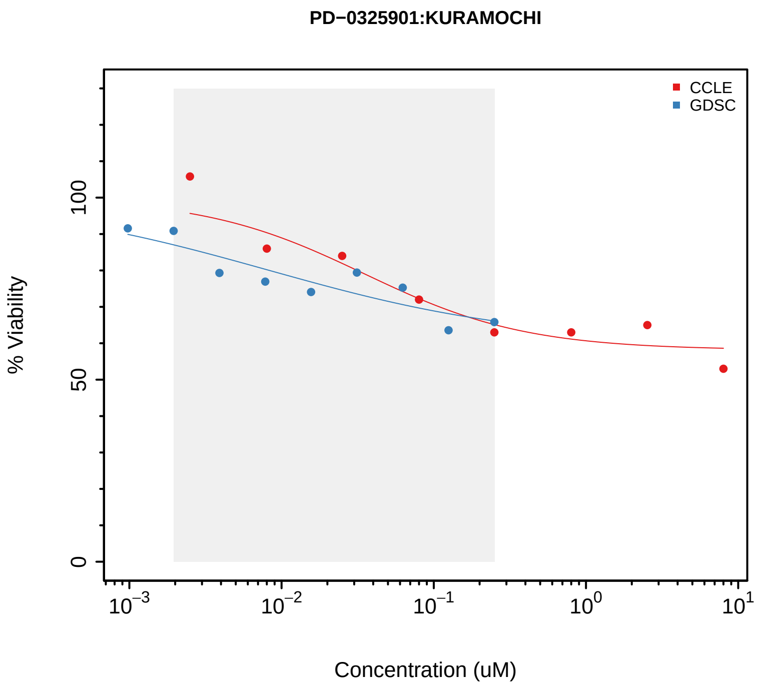
PD−0325901:KURAMOCHI
CCLE
GDSC
% Viability
Concentration (uM)

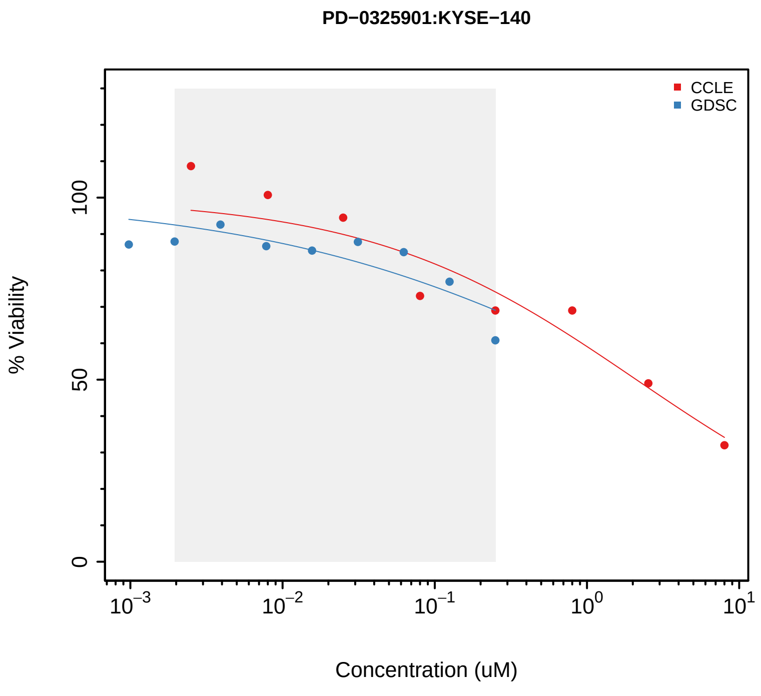
PD−0325901:KYSE−140
CCLE
GDSC
% Viability
Concentration (uM)

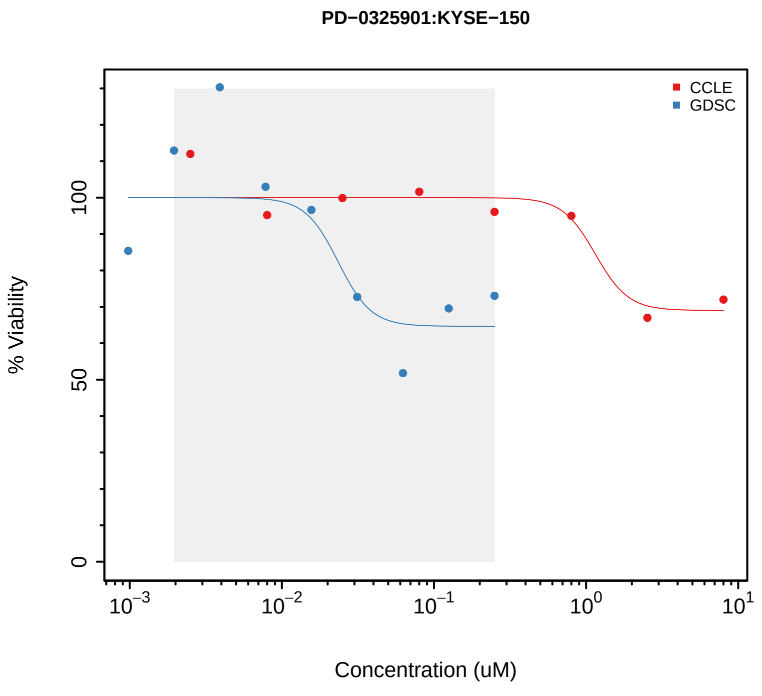
PD−0325901:KYSE−150
CCLE
GDSC
% Viability
Concentration (uM)

PD−0325901:KYSE−410

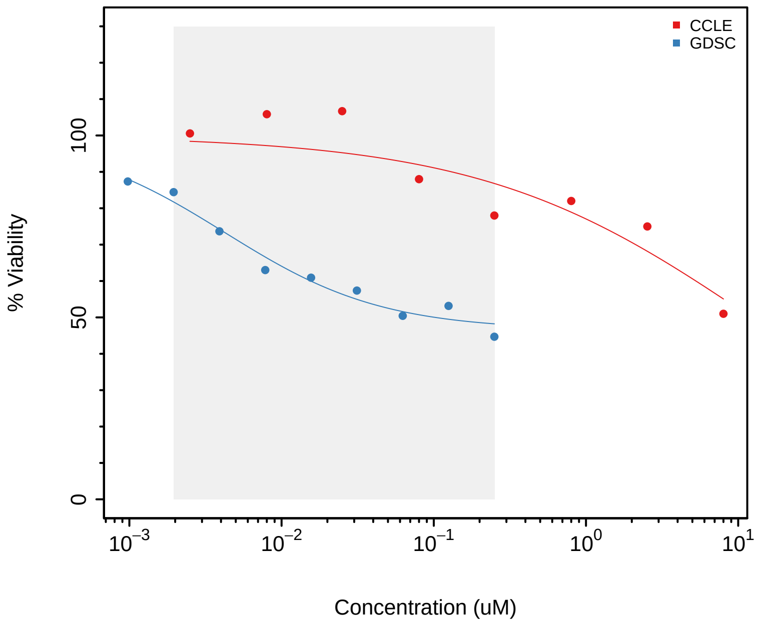

PD−0325901:KYSE−450

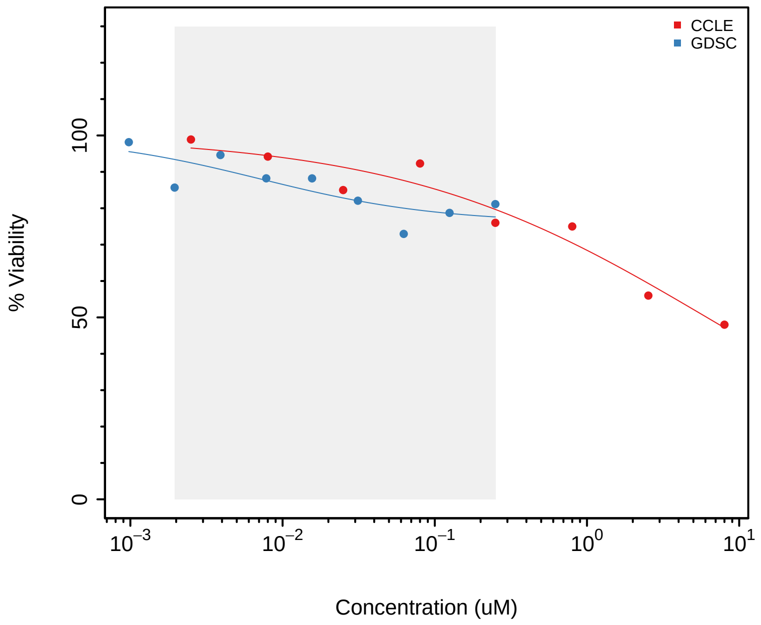

PD−0325901:KYSE−520

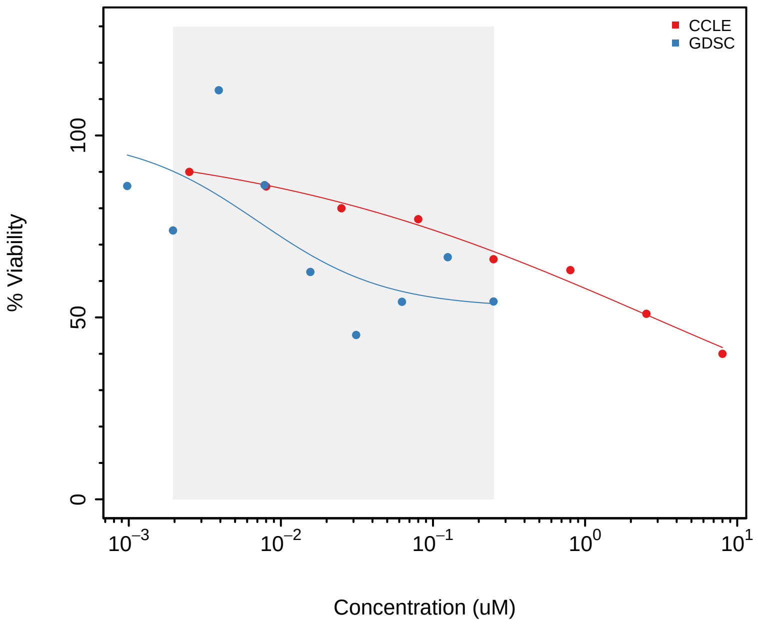

PD−0325901:L−363

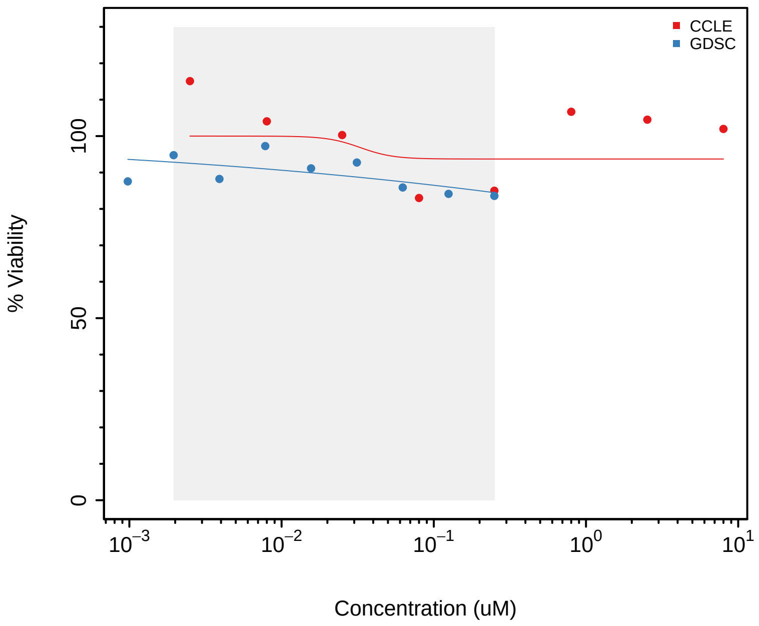

PD−0325901:L−428

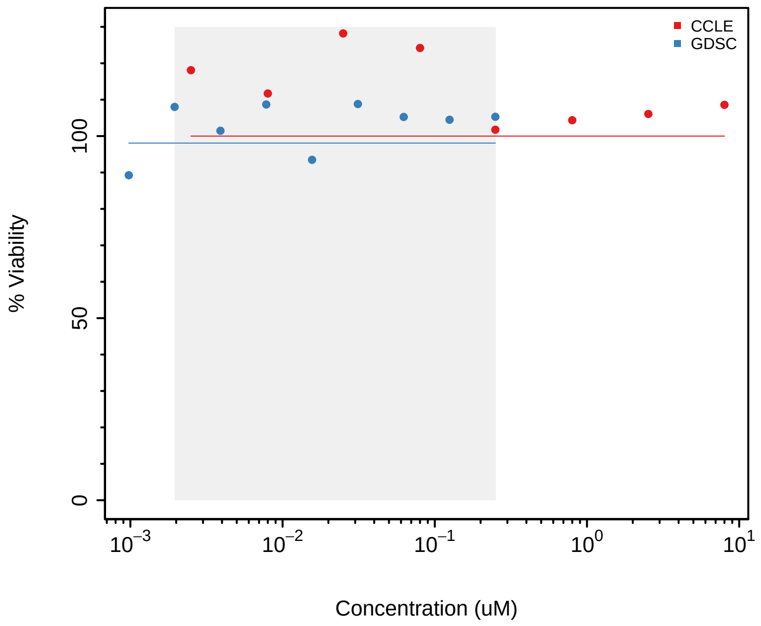

PD−0325901:LOXIMVI

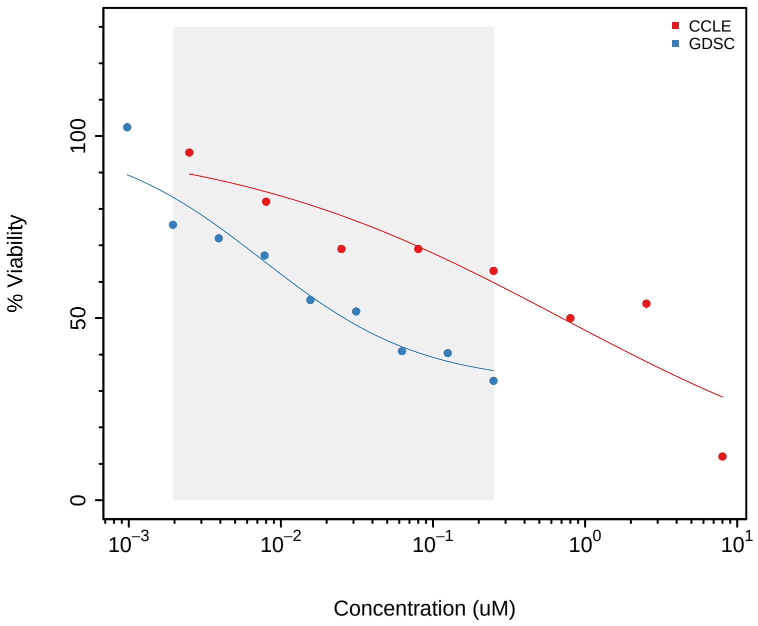

PD−0325901:LP−1

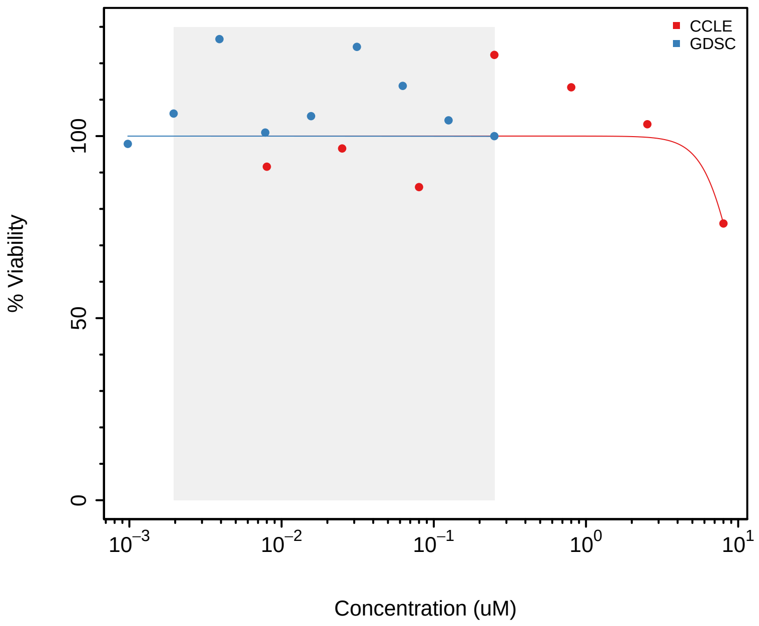

PD−0325901:LS−123

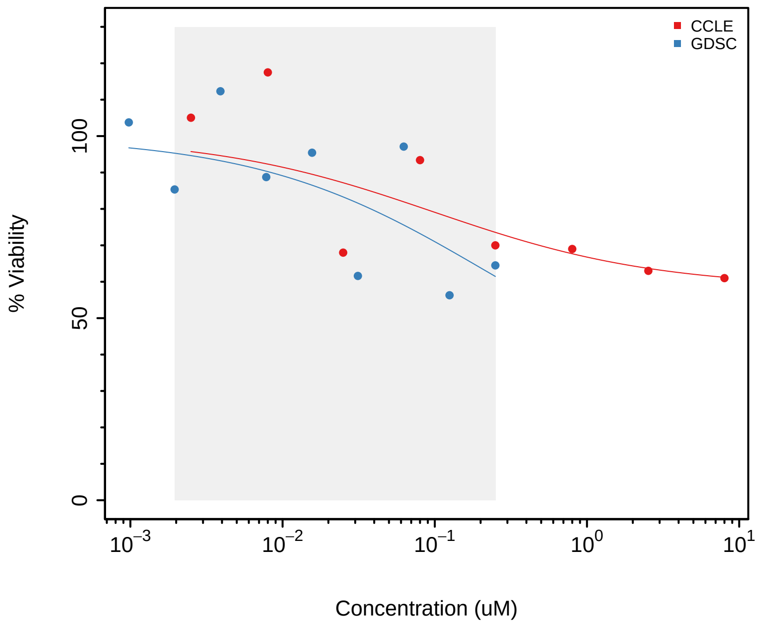

PD−0325901:LS−411N

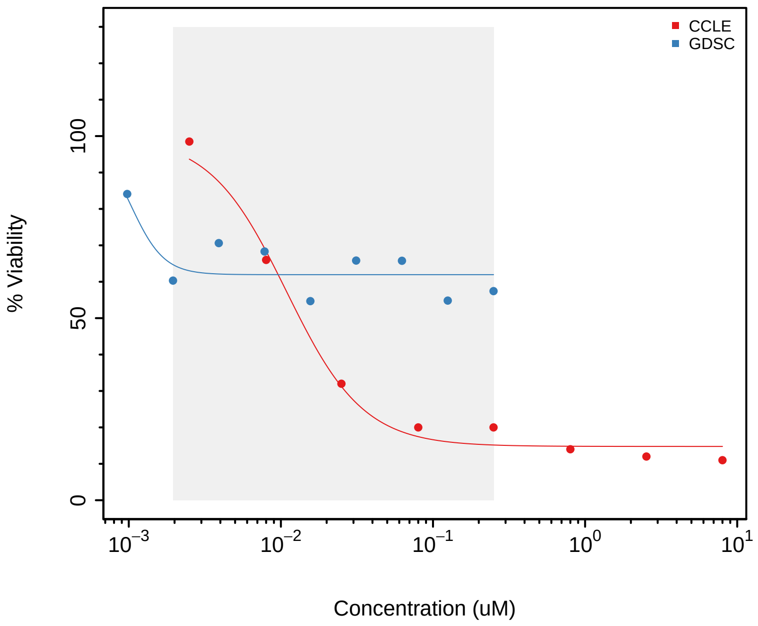

PD−0325901:LS−513

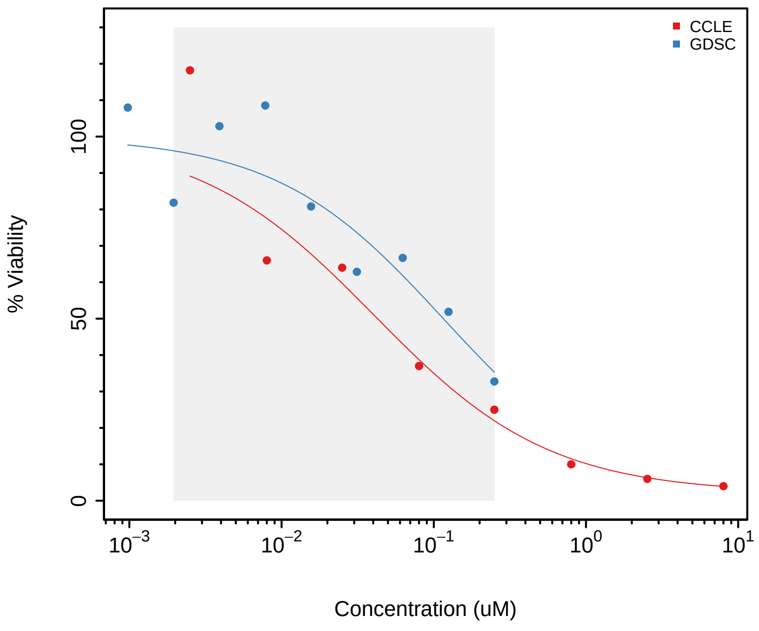

PD−0325901:M059J

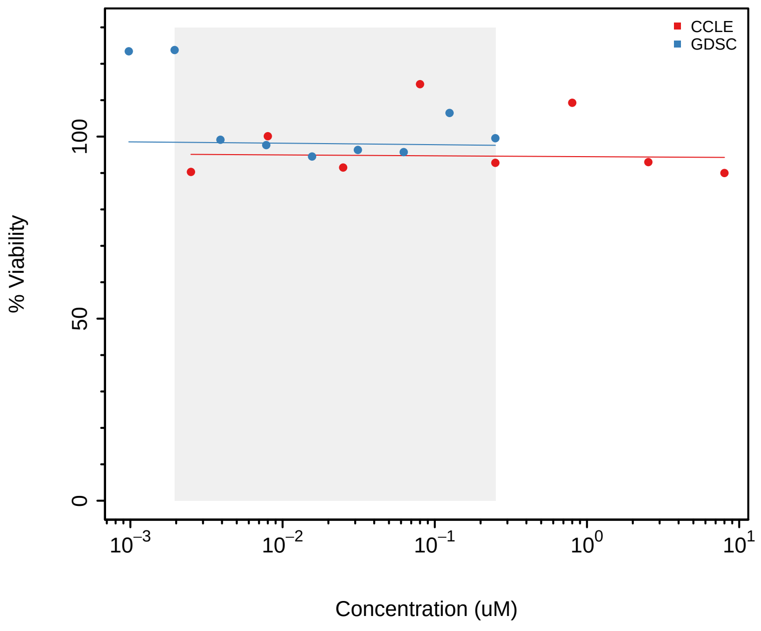

PD−0325901:MC116

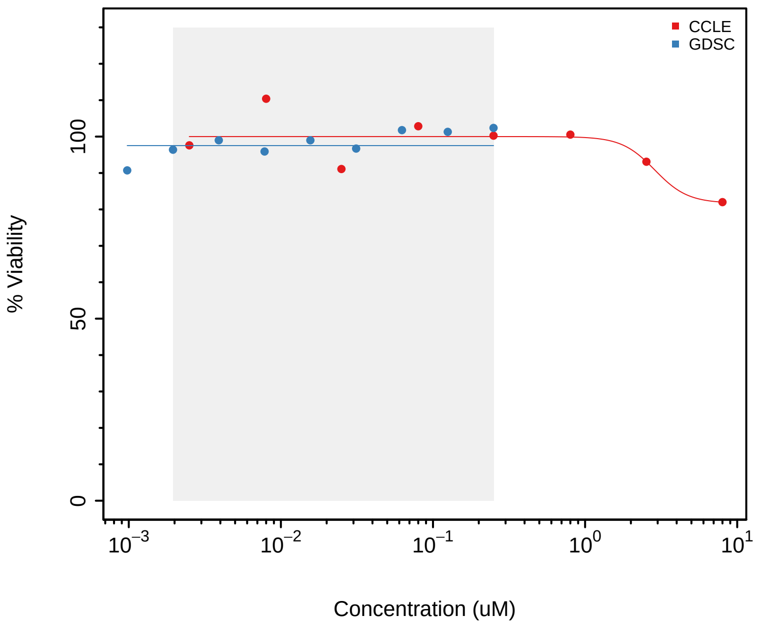

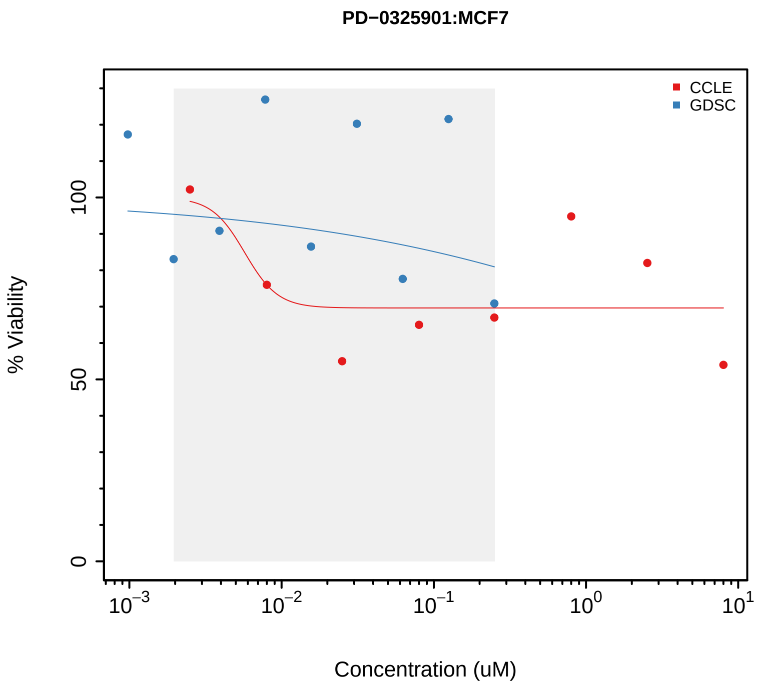
PD−0325901:MCF7
CCLE
GDSC
% Viability
Concentration (uM)

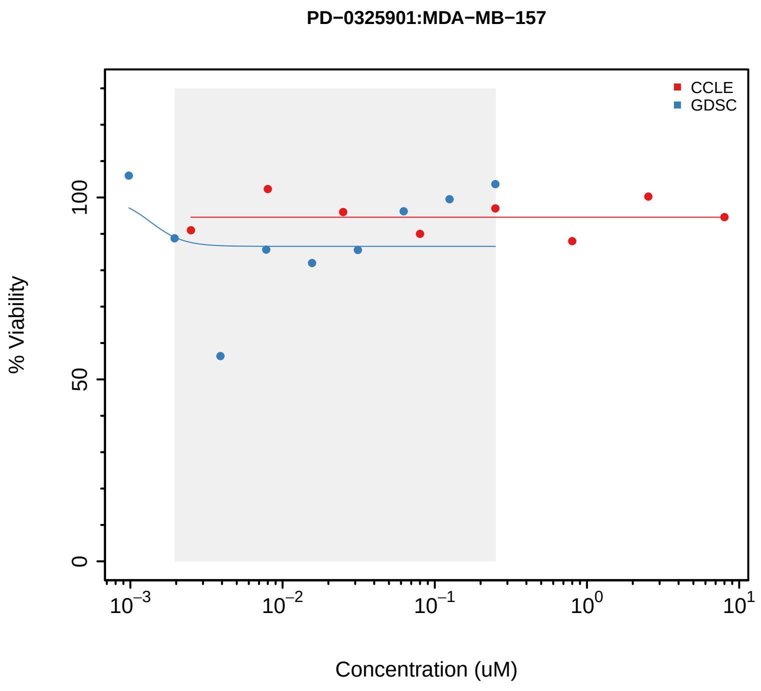
PD−0325901:MDA−MB−157
CCLE
GDSC
% Viability
Concentration (uM)

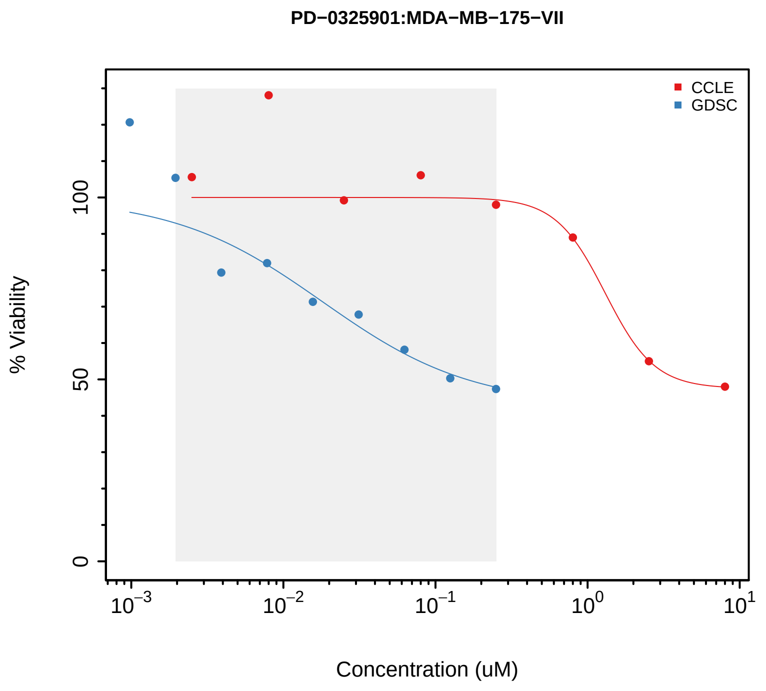
PD−0325901:MDA−MB−175−VII
CCLE
GDSC
% Viability
Concentration (uM)

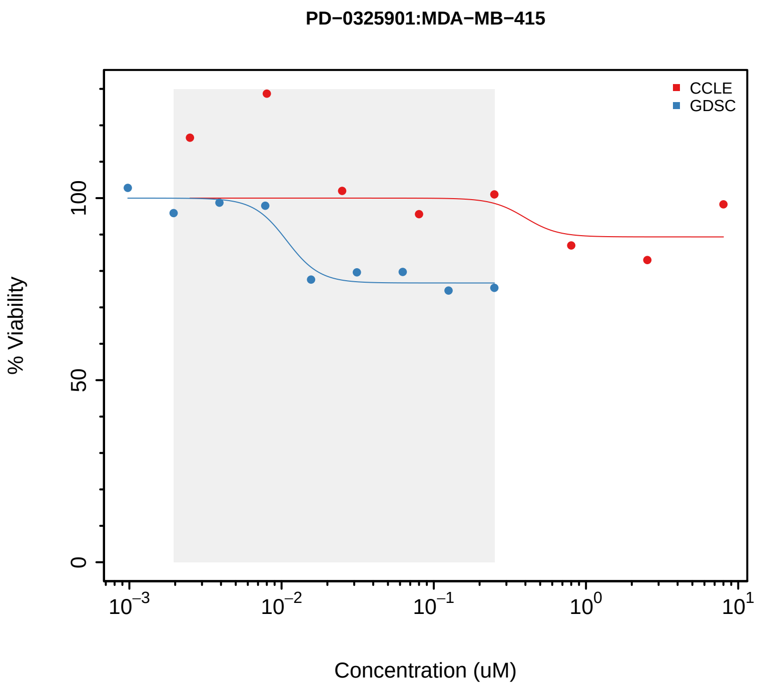
PD−0325901:MDA−MB−415
CCLE
GDSC
% Viability
Concentration (uM)

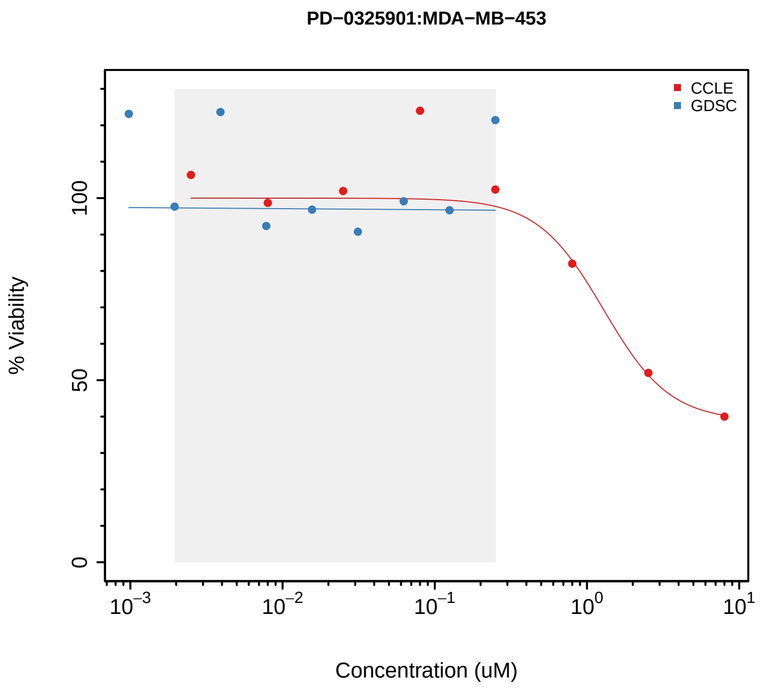
PD−0325901:MDA−MB−453
CCLE
GDSC
% Viability
Concentration (uM)

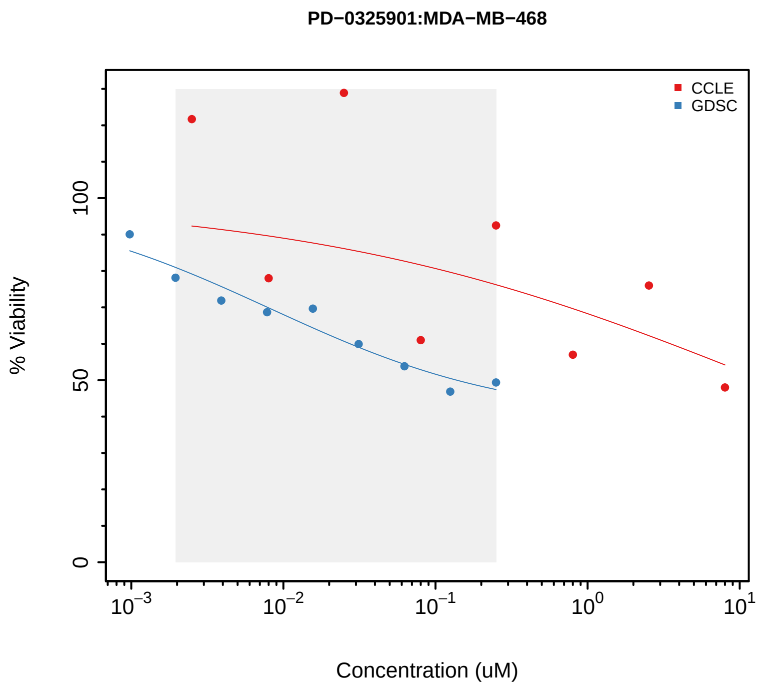
PD−0325901:MDA−MB−468
CCLE
GDSC
% Viability
Concentration (uM)

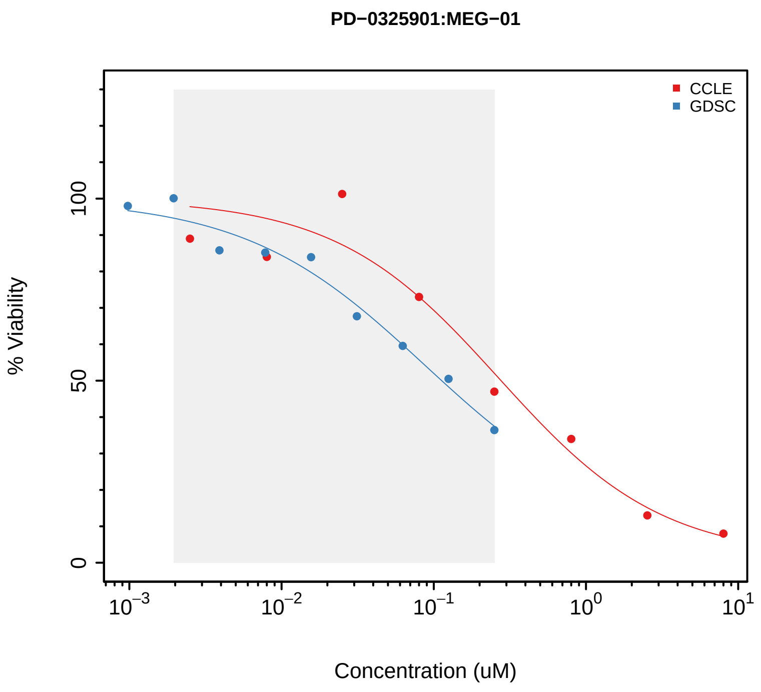
PD−0325901:MEG−01
CCLE
GDSC
% Viability
Concentration (uM)

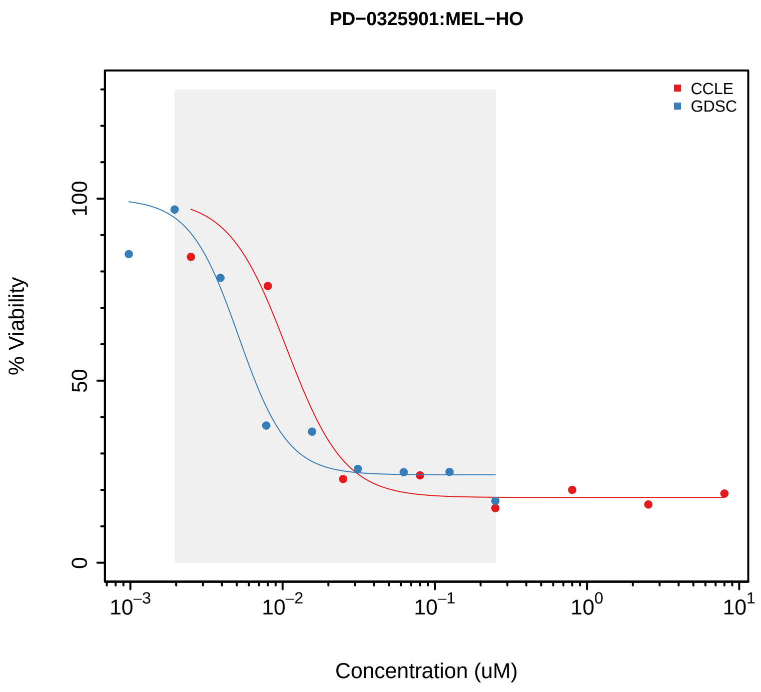
PD−0325901:MEL−HO
CCLE
GDSC
% Viability
Concentration (uM)

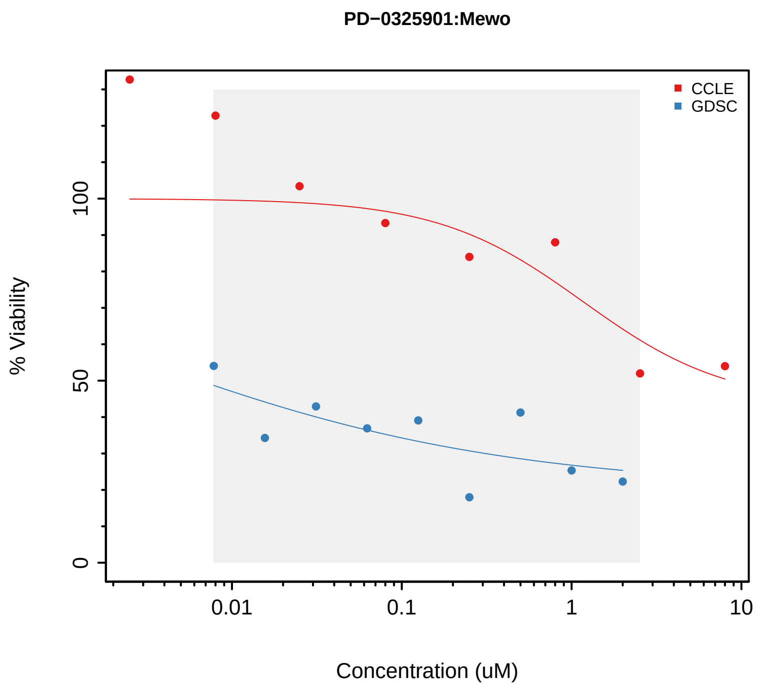
PD−0325901:Mewo
CCLE
GDSC
% Viability
Concentration (uM)

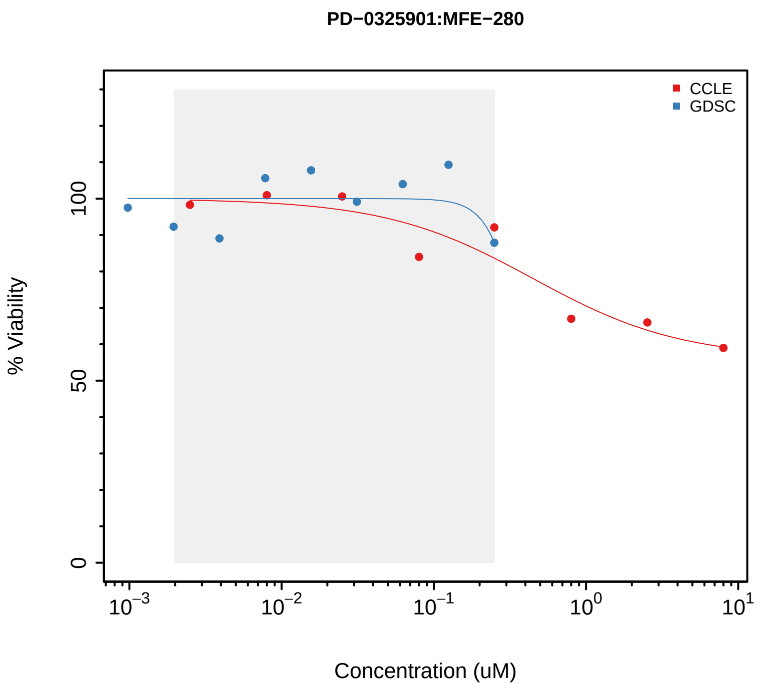
PD−0325901:MFE−280
CCLE
GDSC
% Viability
Concentration (uM)

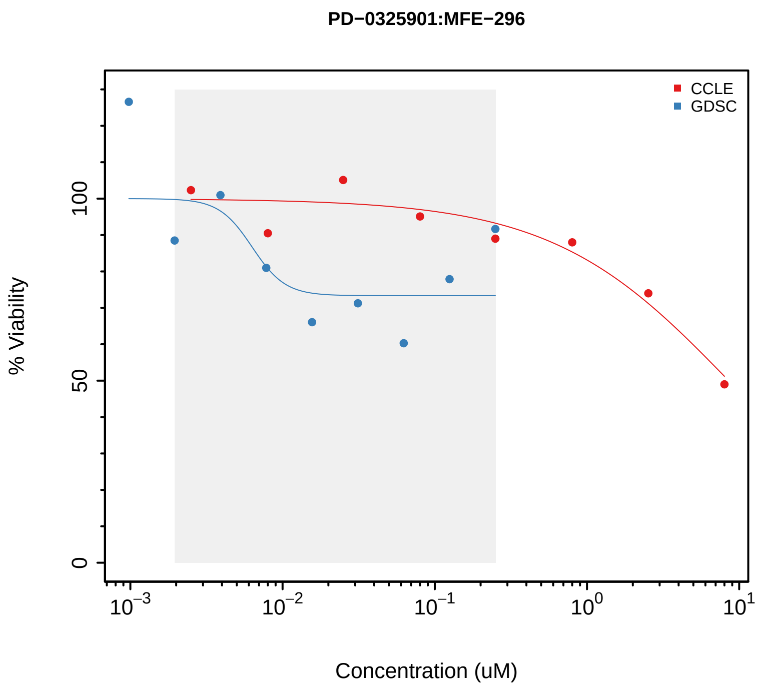
PD−0325901:MFE−296
CCLE
GDSC
% Viability
Concentration (uM)

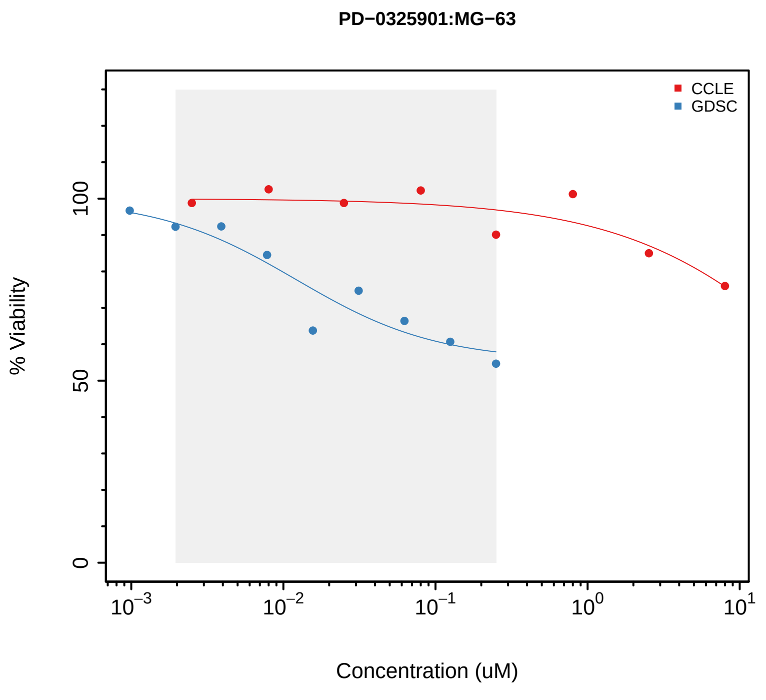
PD−0325901:MG−63
CCLE
GDSC
% Viability
Concentration (uM)

PD−0325901:MHH−ES−1

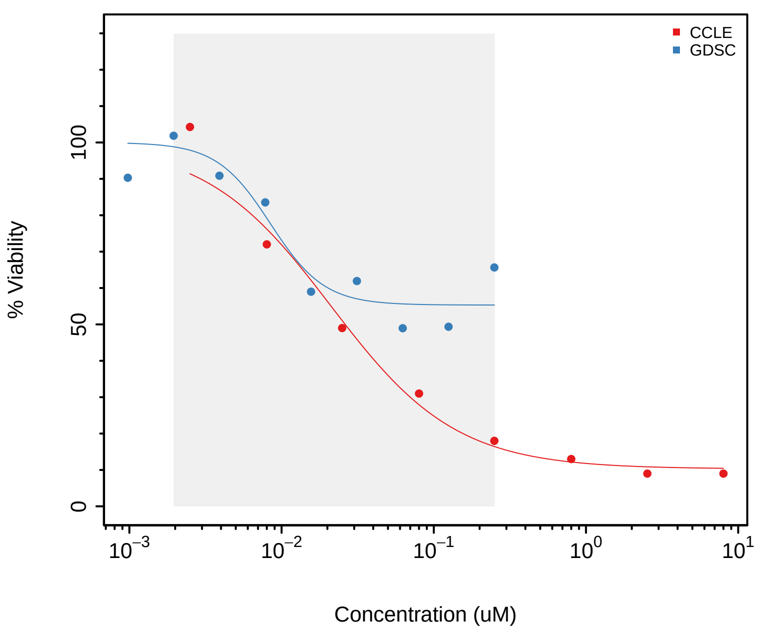

PD−0325901:MIA−PaCa−2

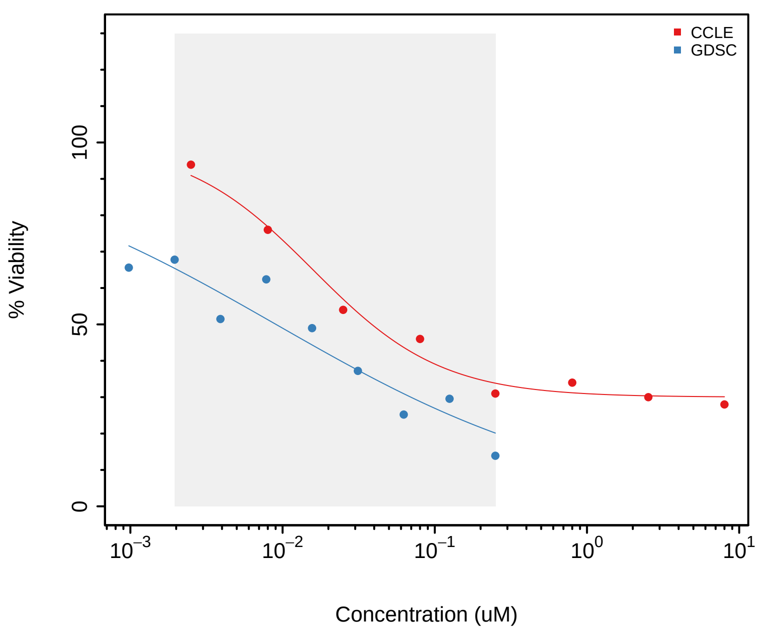

PD−0325901:MKN7

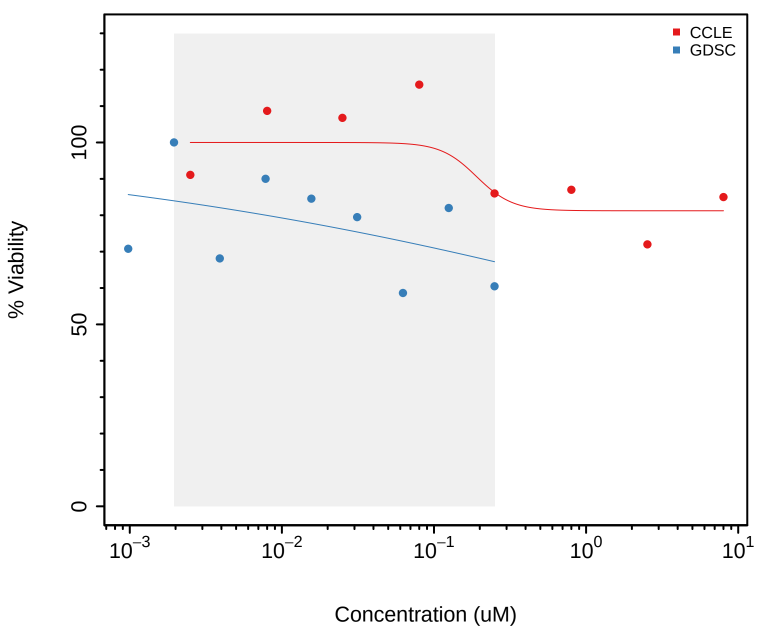

PD−0325901:MOLT−16

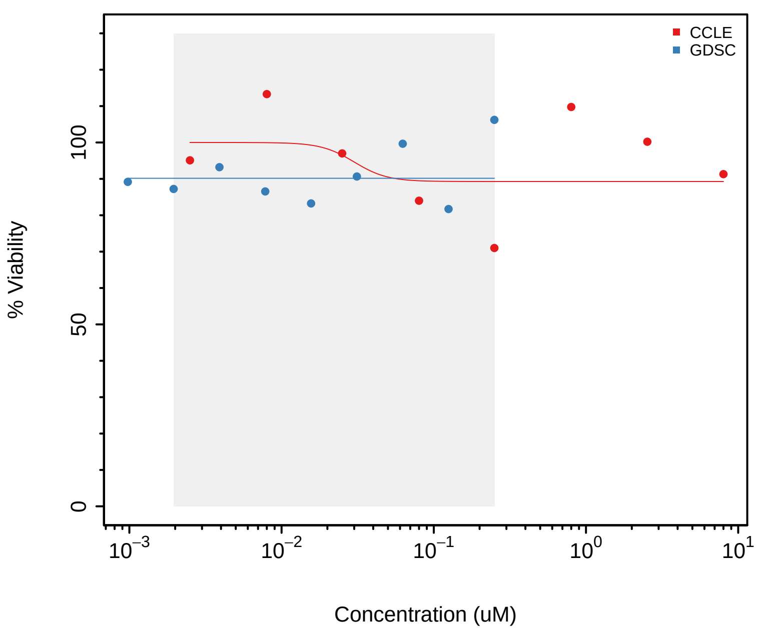

PD−0325901:MPP−89

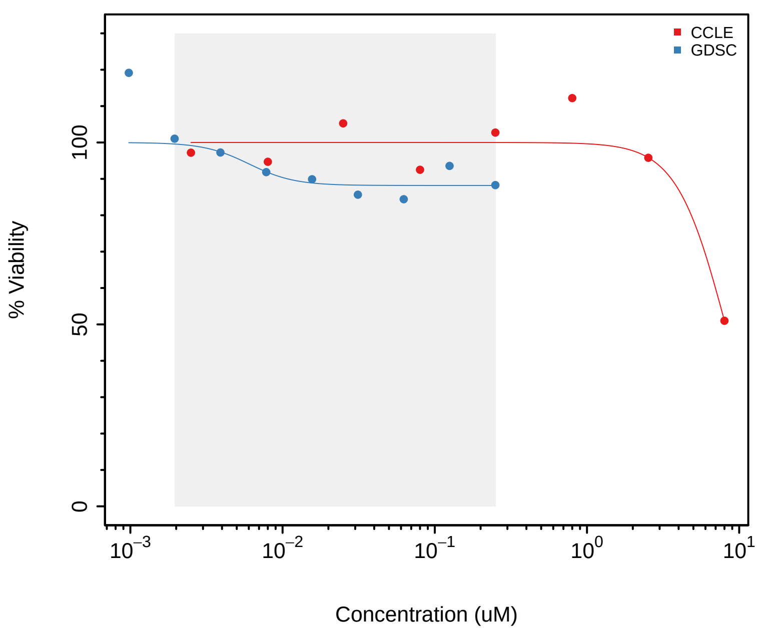

PD−0325901:MSTO−211H

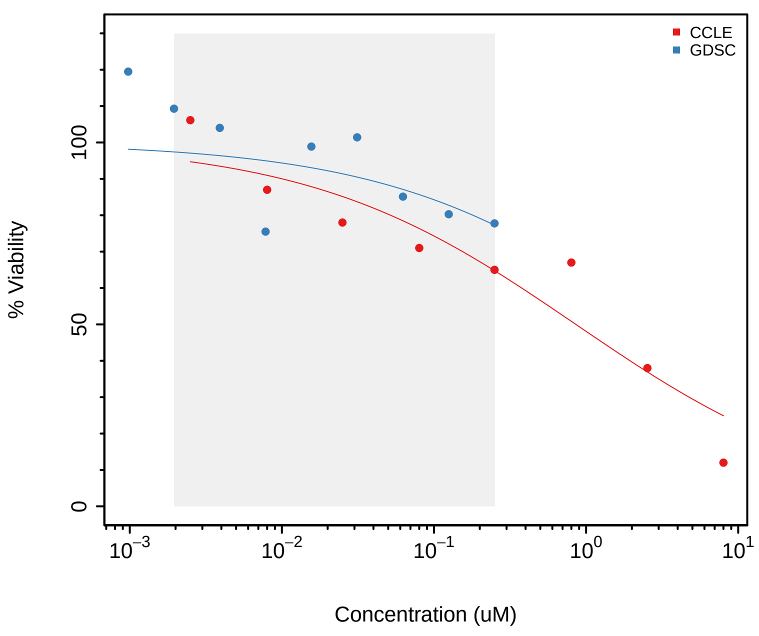

PD−0325901:NCI−H1048

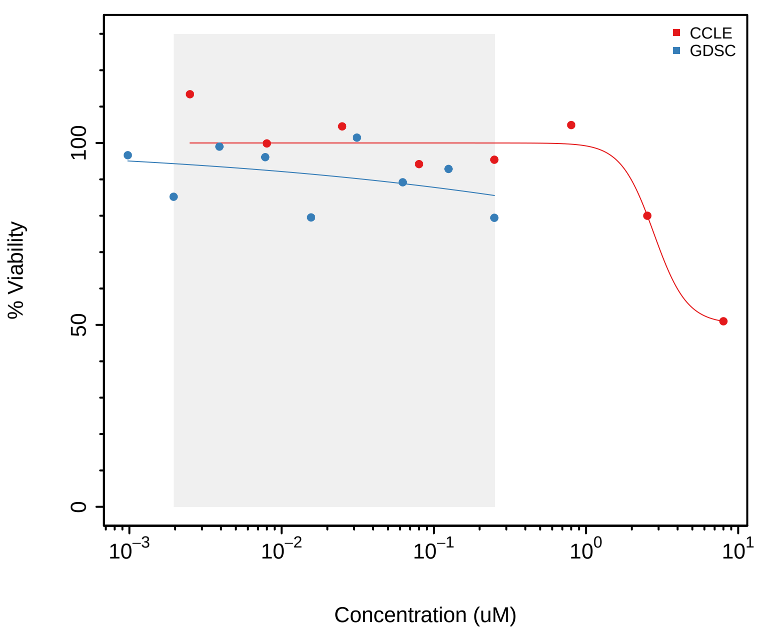

PD−0325901:NCI−H1092

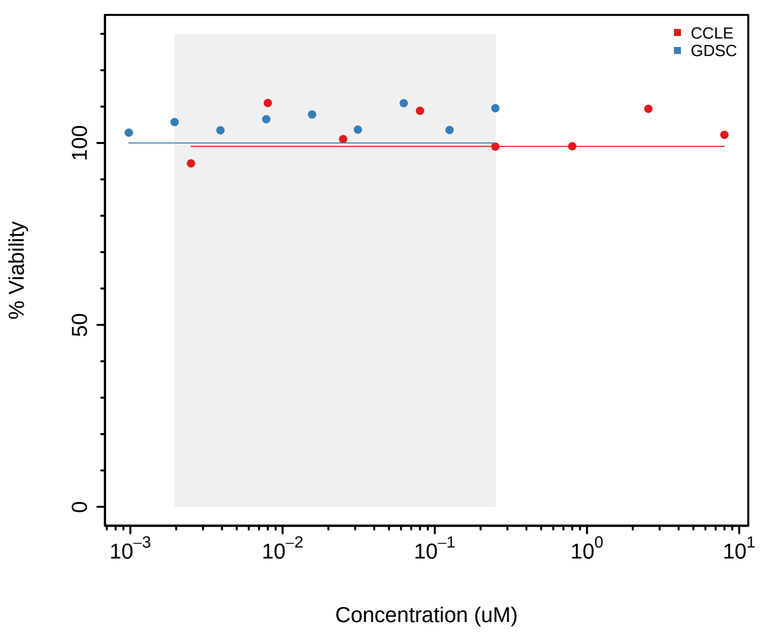

PD−0325901:NCI−H1155

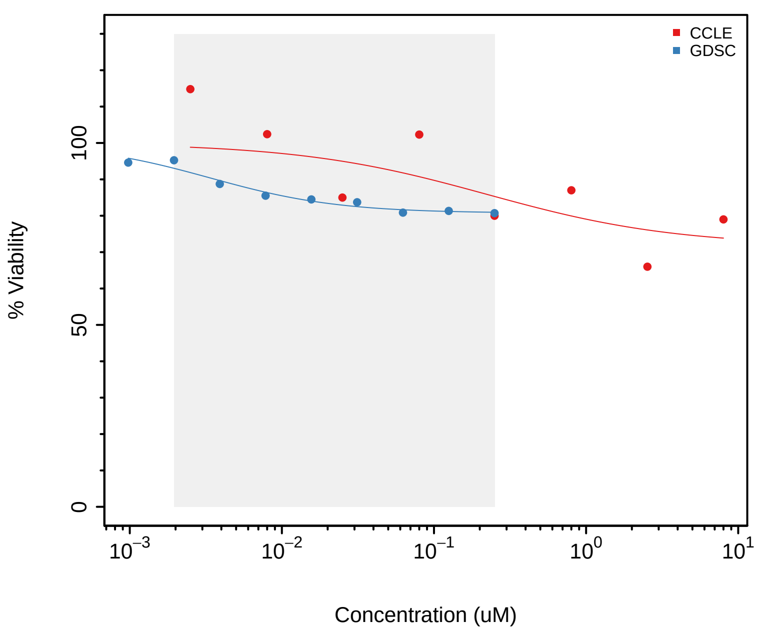

PD−0325901:NCI−H1299

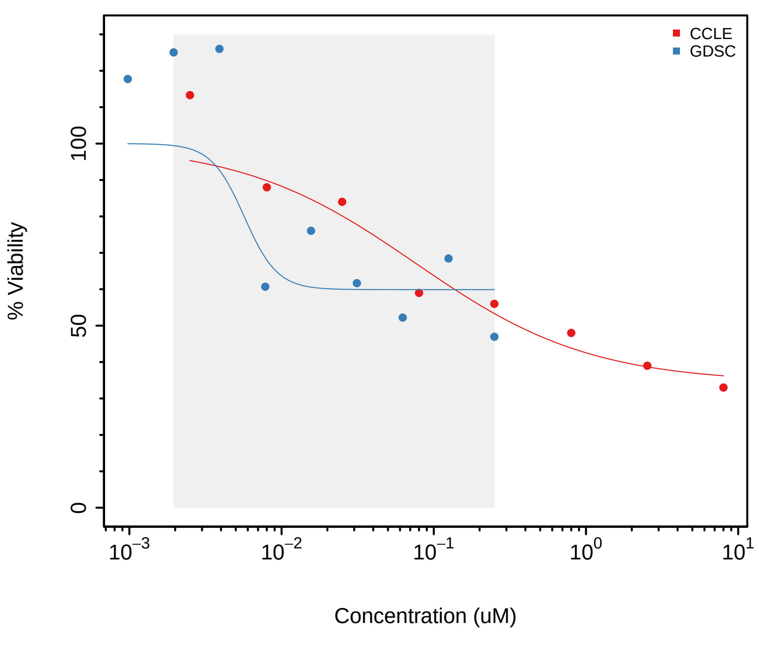

PD−0325901:NCI−H1563

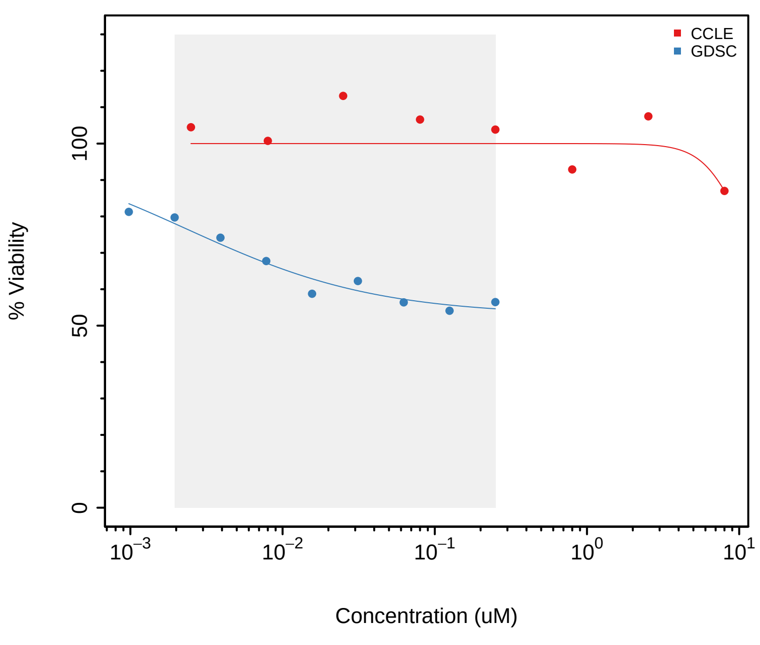

PD−0325901:NCI−H1648

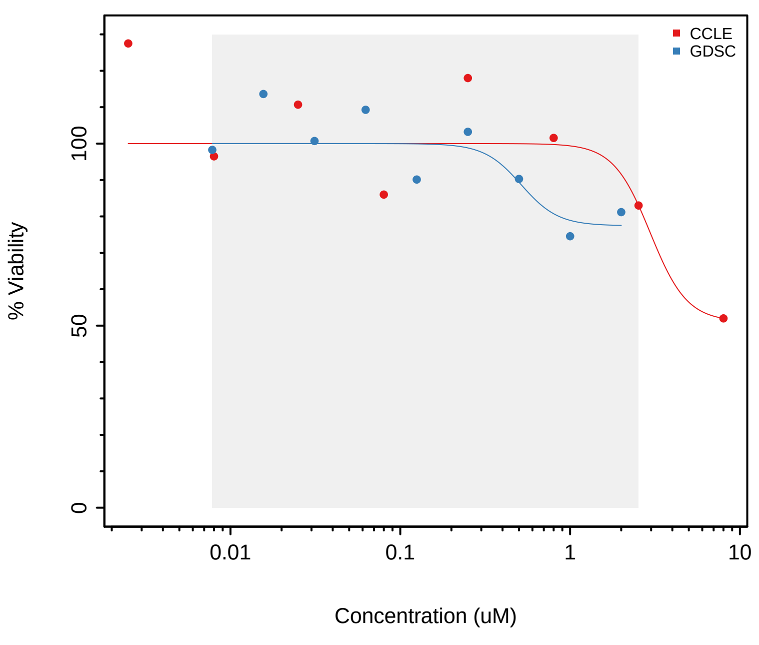

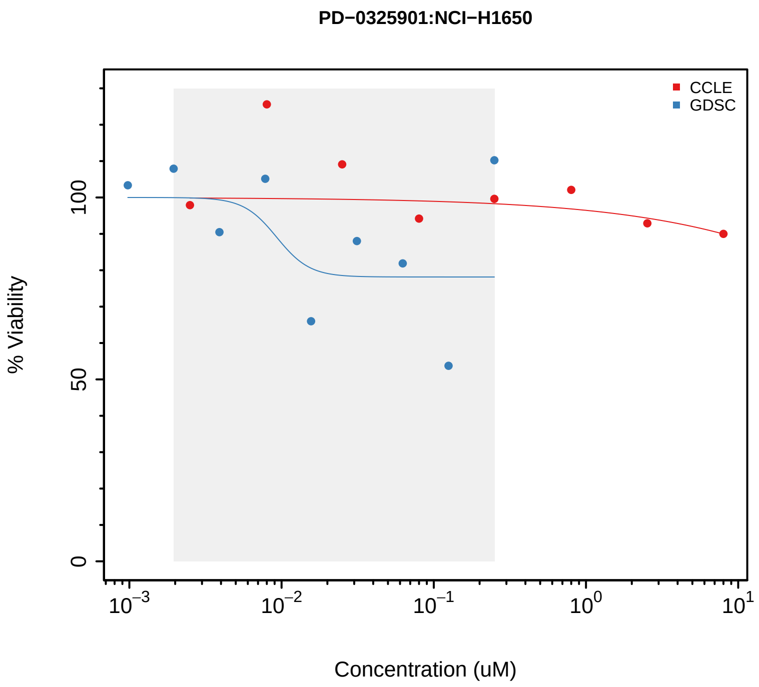
PD−0325901:NCI−H1650
CCLE
GDSC
% Viability
Concentration (uM)

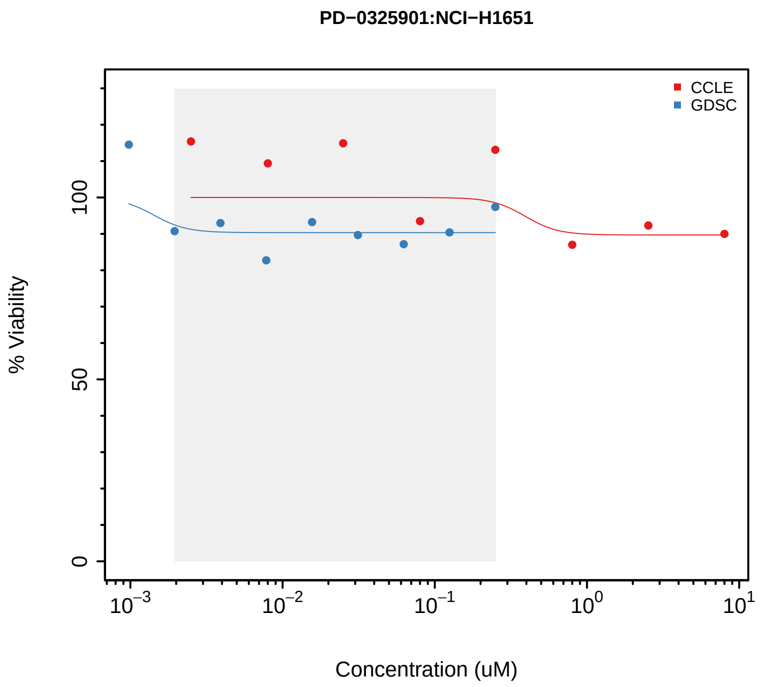
PD−0325901:NCI−H1651
CCLE
GDSC
% Viability
Concentration (uM)

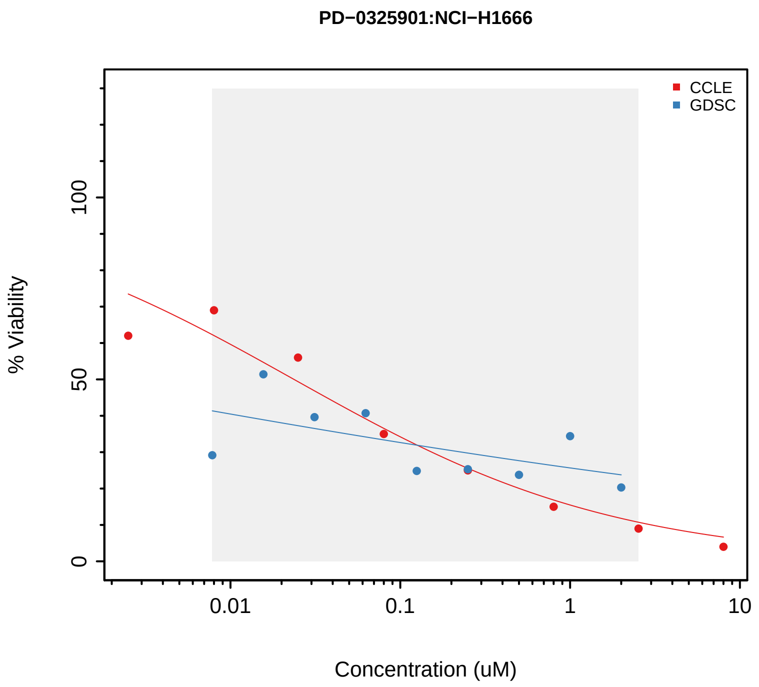
PD−0325901:NCI−H1666
CCLE
GDSC
% Viability
Concentration (uM)

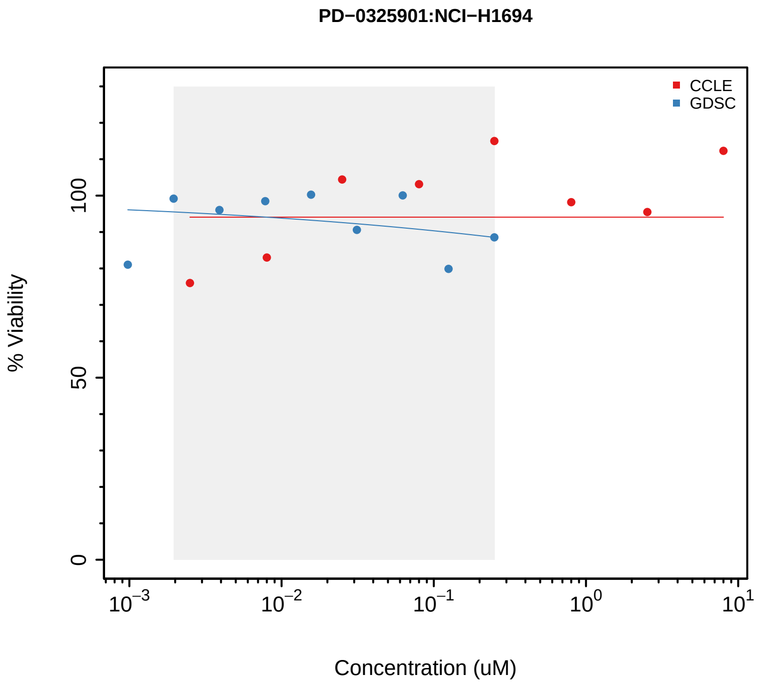
PD−0325901:NCI−H1694
CCLE
GDSC
% Viability
Concentration (uM)

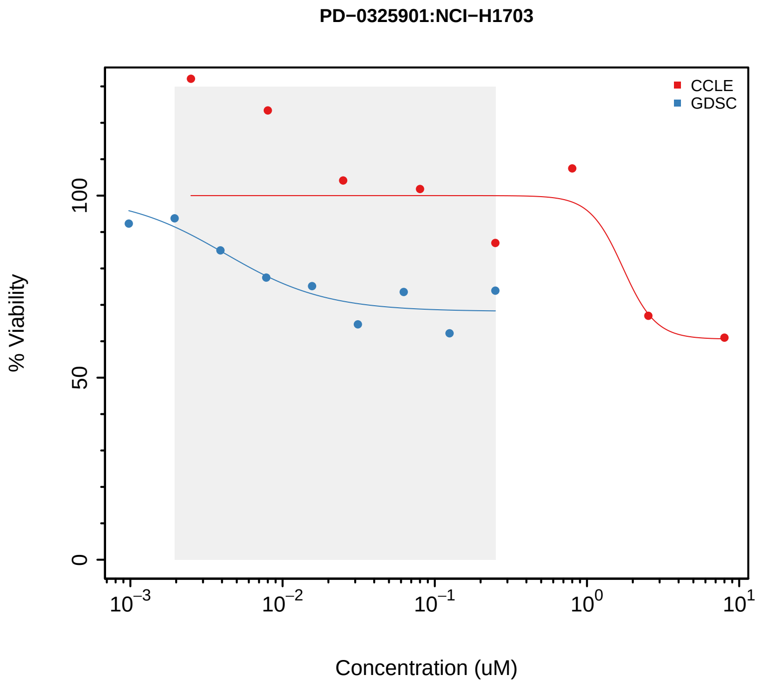
PD−0325901:NCI−H1703
CCLE
GDSC
% Viability
Concentration (uM)

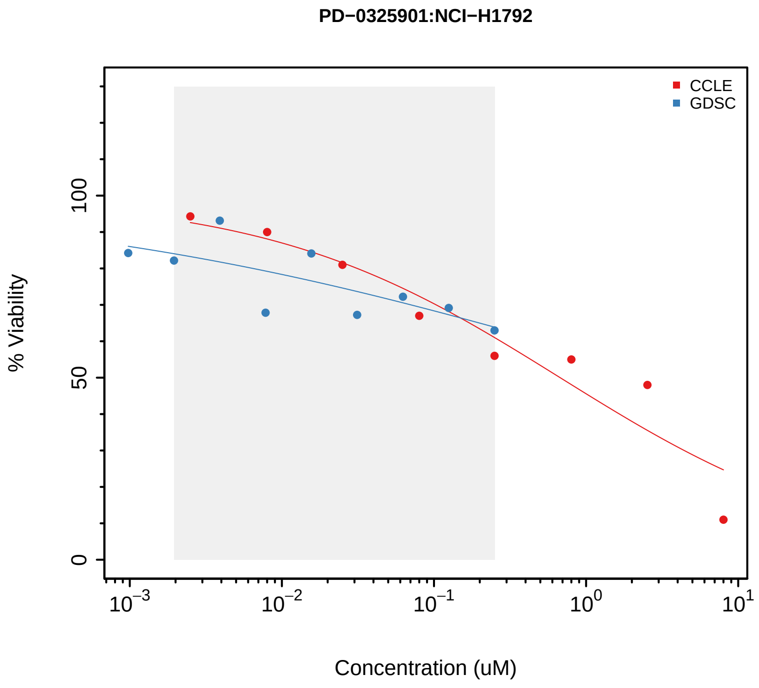
PD−0325901:NCI−H1792
CCLE
GDSC
% Viability
Concentration (uM)

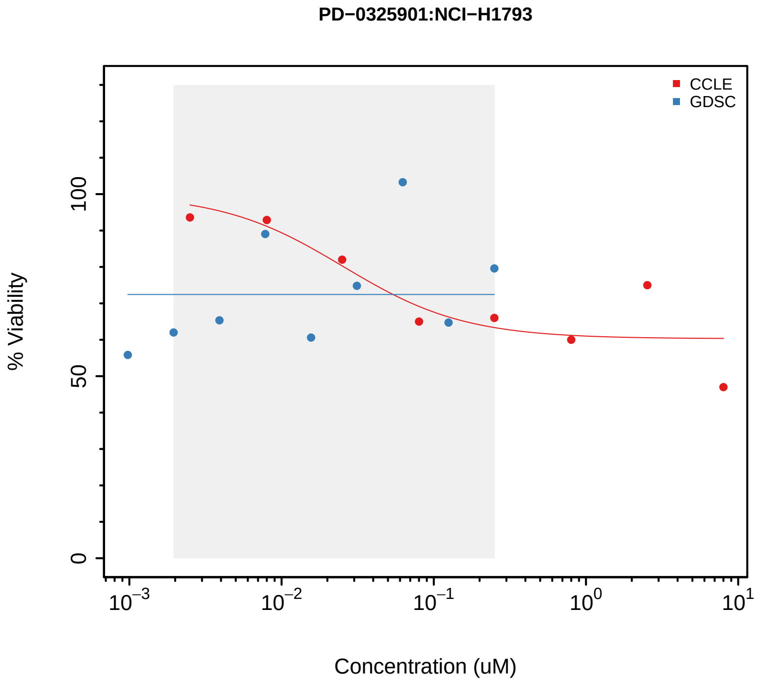
PD−0325901:NCI−H1793
CCLE
GDSC
% Viability
Concentration (uM)

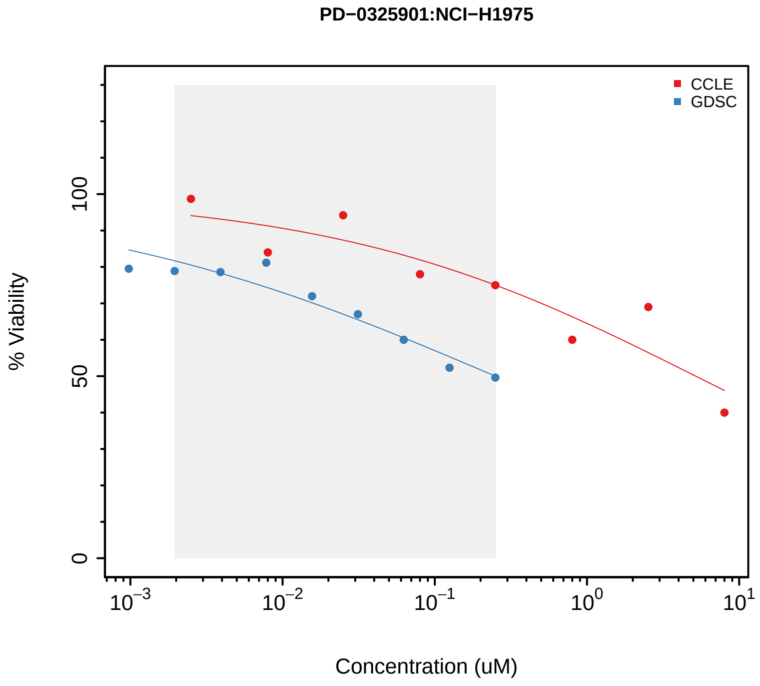
PD−0325901:NCI−H1975
CCLE
GDSC
% Viability
Concentration (uM)

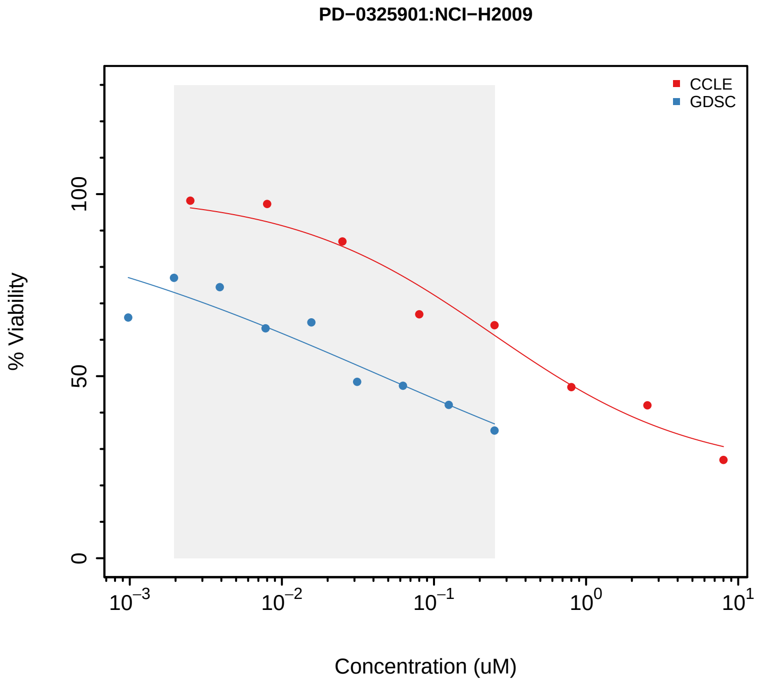
PD−0325901:NCI−H2009
CCLE
GDSC
% Viability
Concentration (uM)

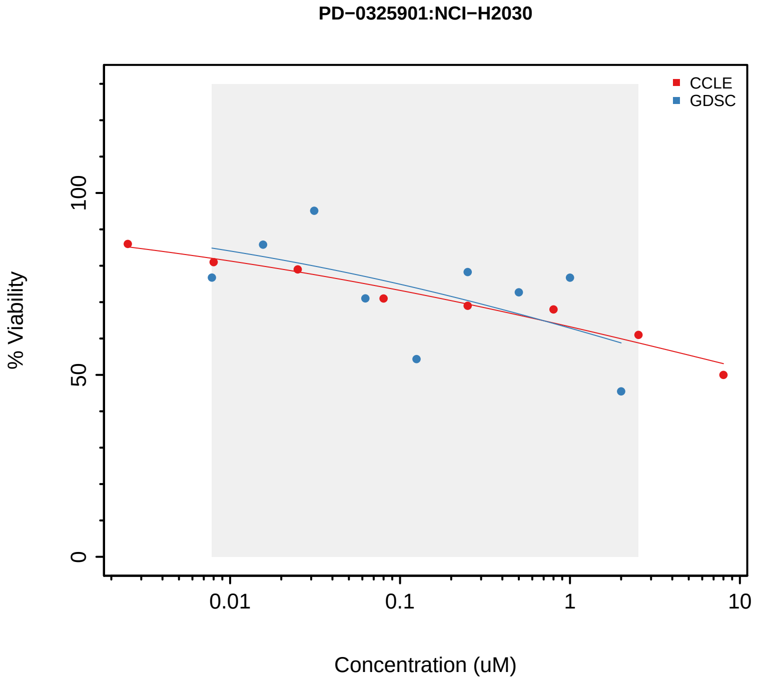
PD−0325901:NCI−H2030
CCLE
GDSC
% Viability
Concentration (uM)

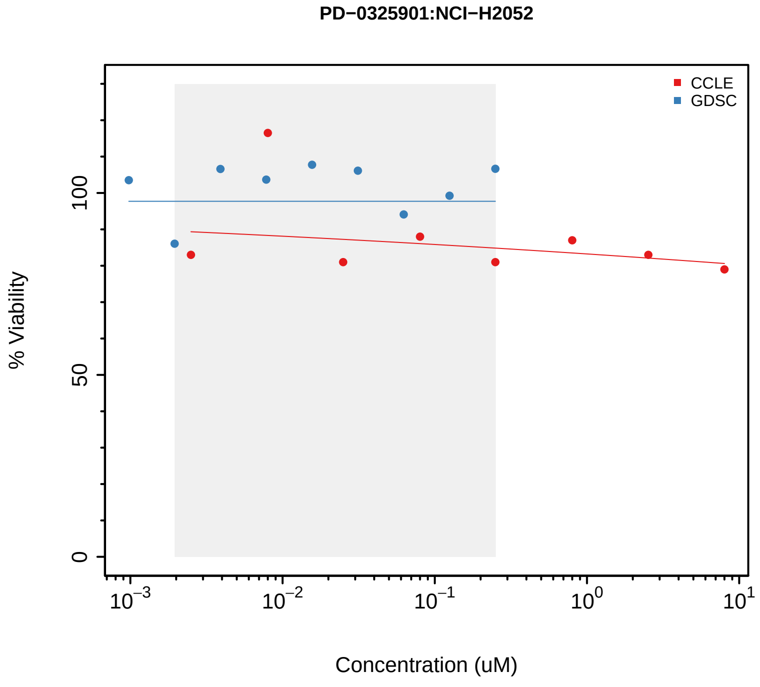
PD−0325901:NCI−H2052
CCLE
GDSC
% Viability
Concentration (uM)

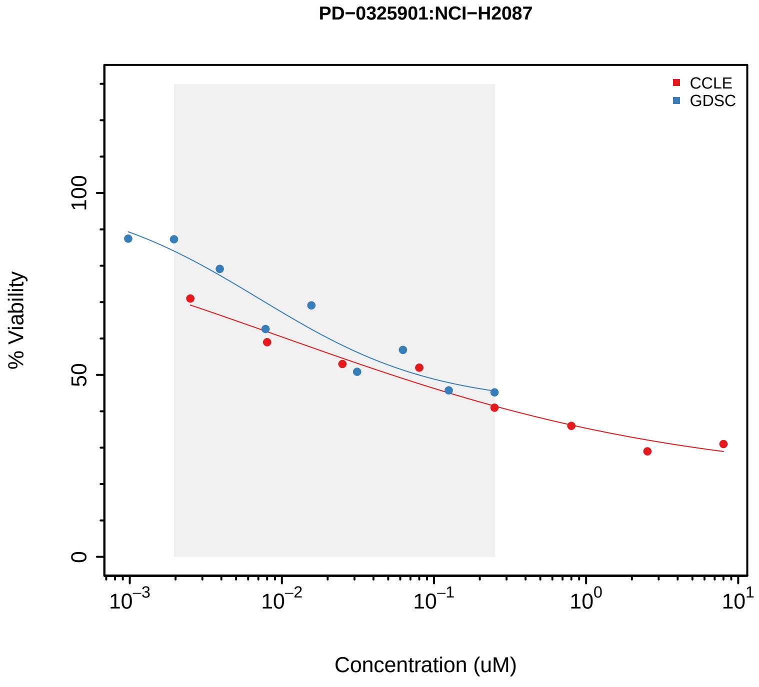
PD−0325901:NCI−H2087
CCLE
GDSC
% Viability
Concentration (uM)

PD−0325901:NCI−H2122

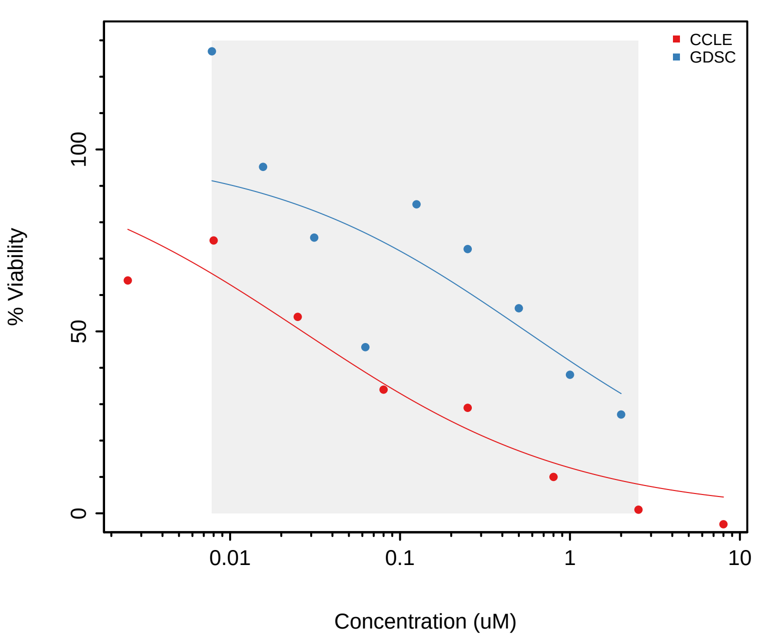

PD−0325901:NCI−H2170

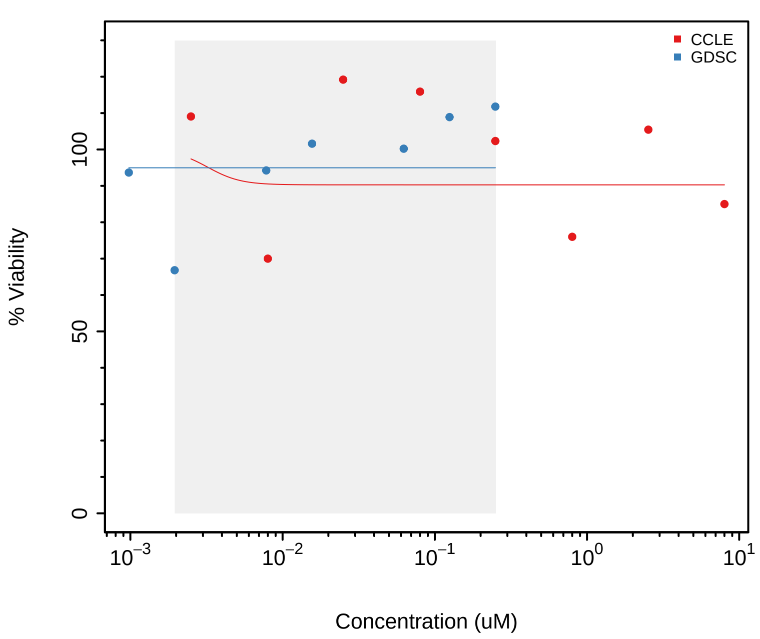

PD−0325901:NCI−H226

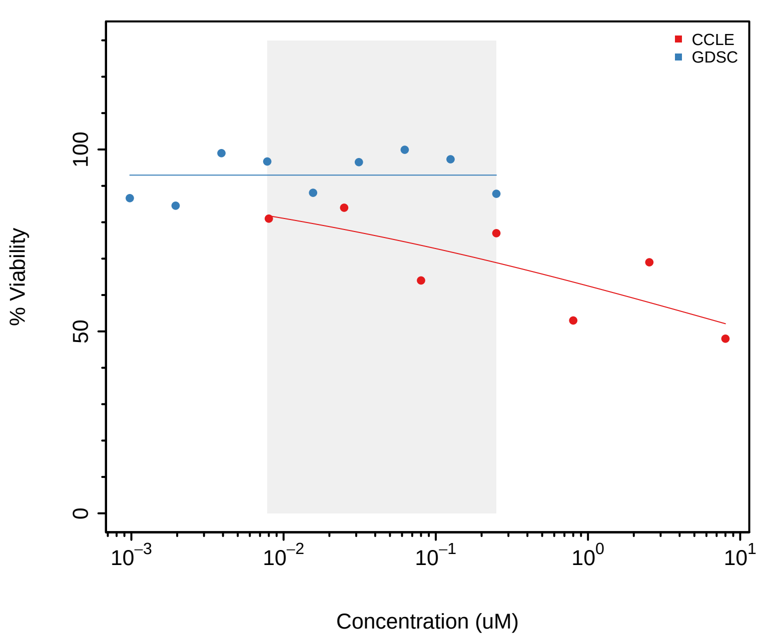

PD−0325901:NCI−H23

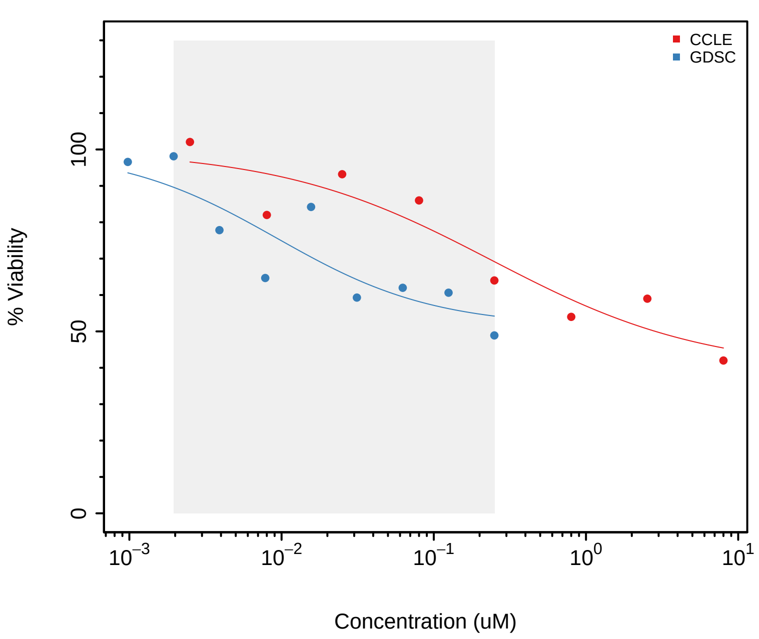

PD−0325901:NCI−H28

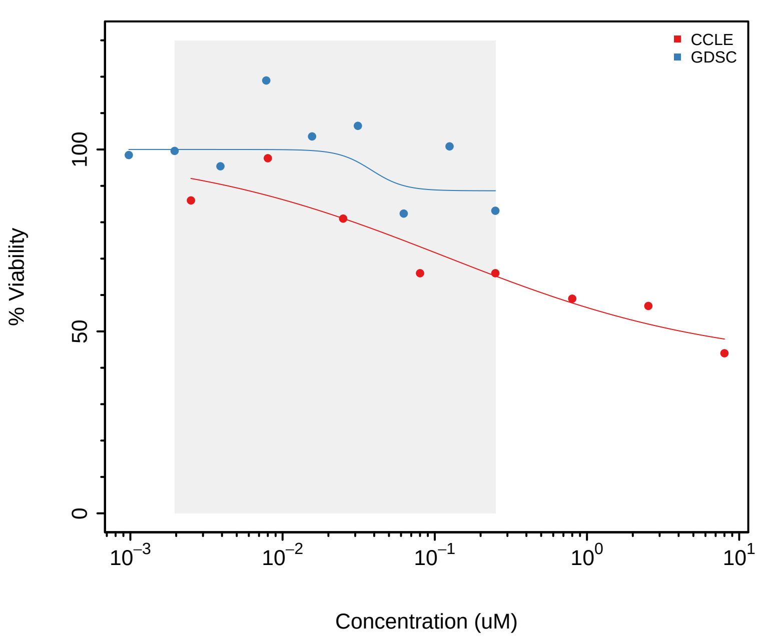

PD−0325901:NCI−H358

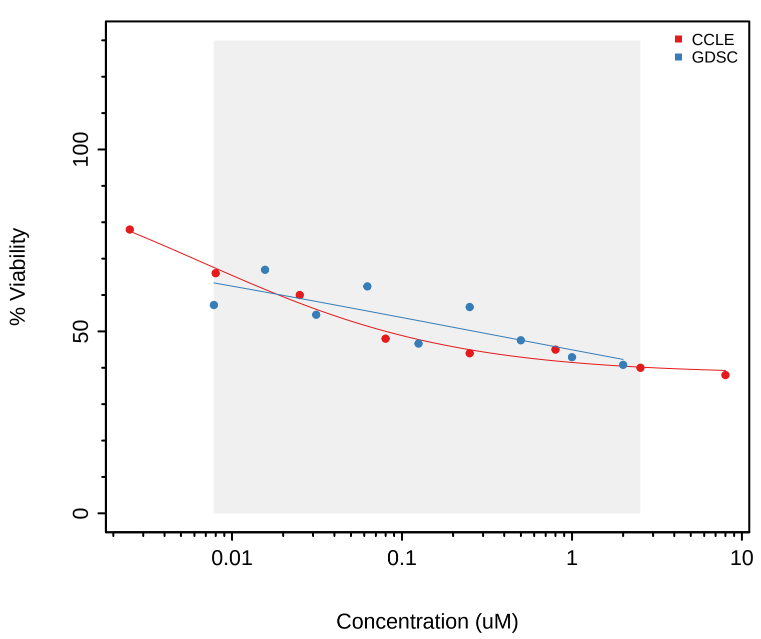

PD−0325901:NCI−H441

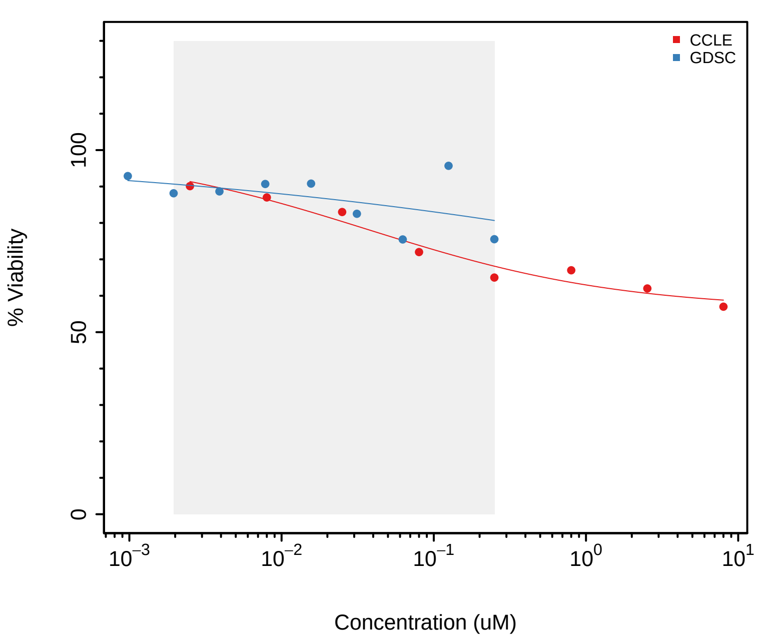

PD−0325901:NCI−H460

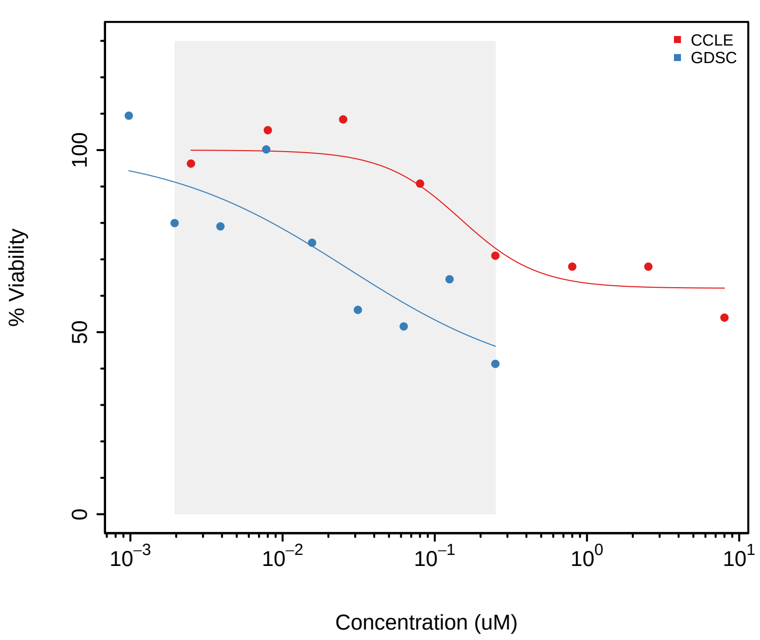

PD−0325901:NCI−H520

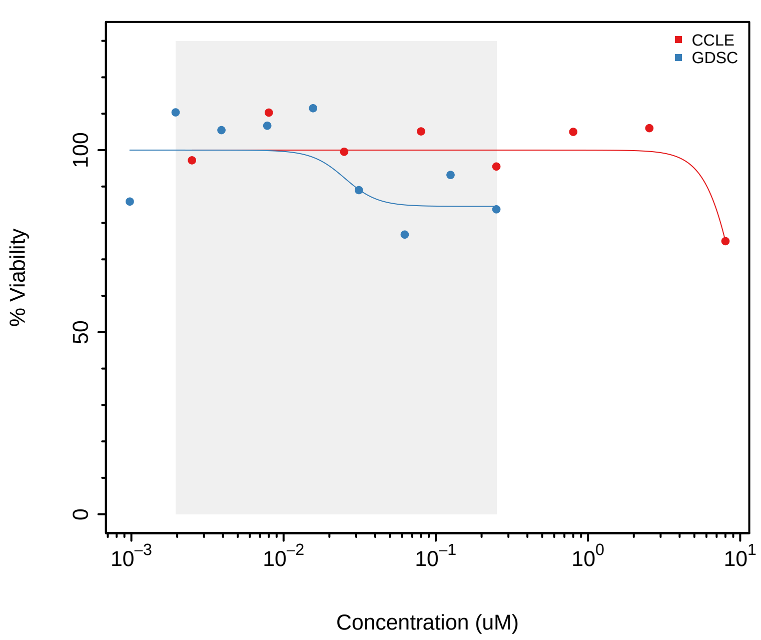

PD−0325901:NCI−H522

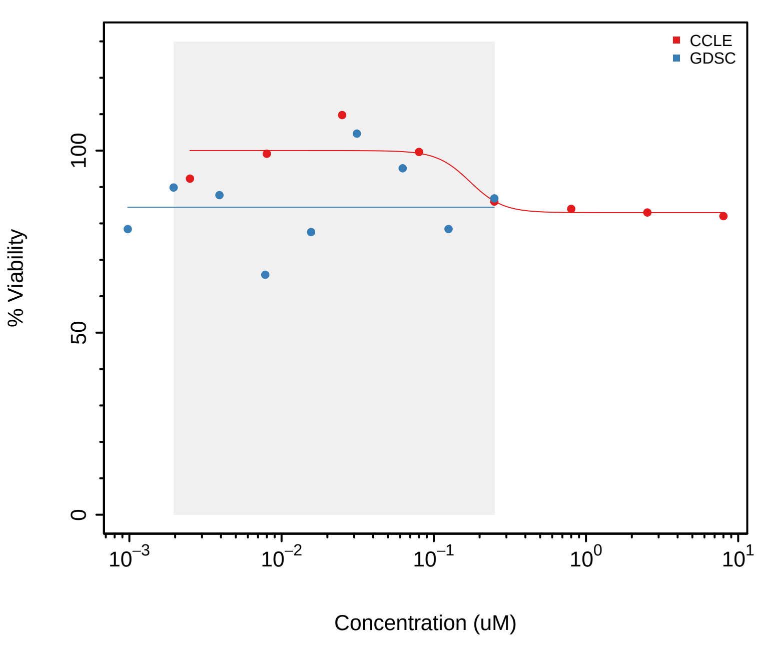

PD−0325901:NCI−H650

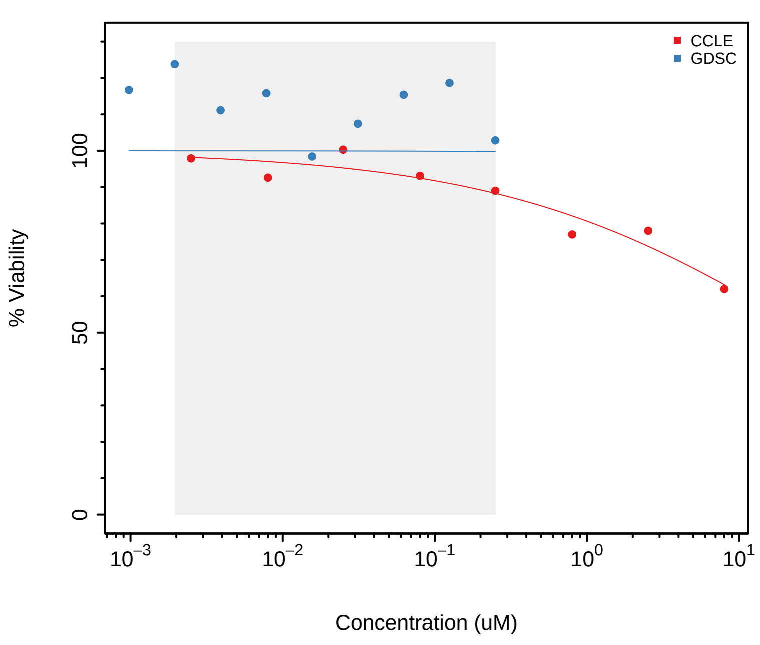

PD−0325901:NCI−H661

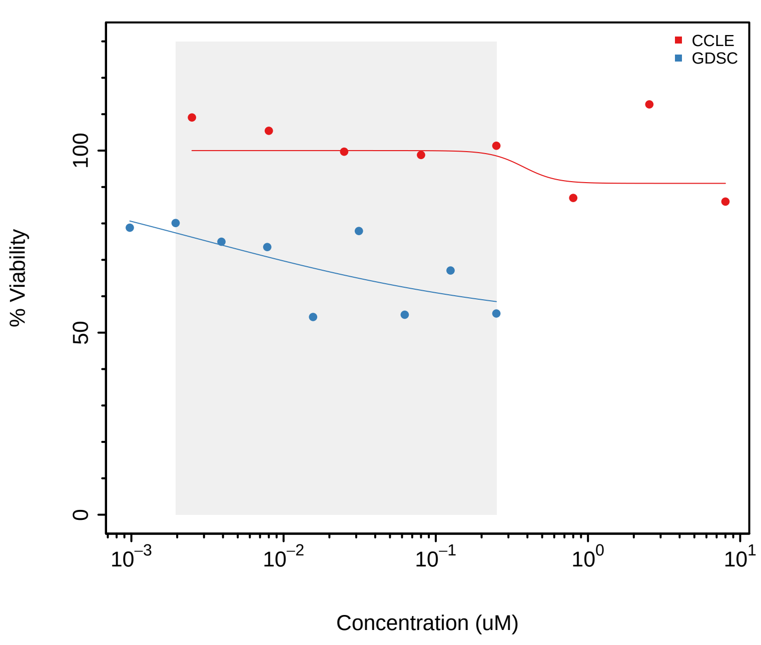

PD−0325901:NCI−H747

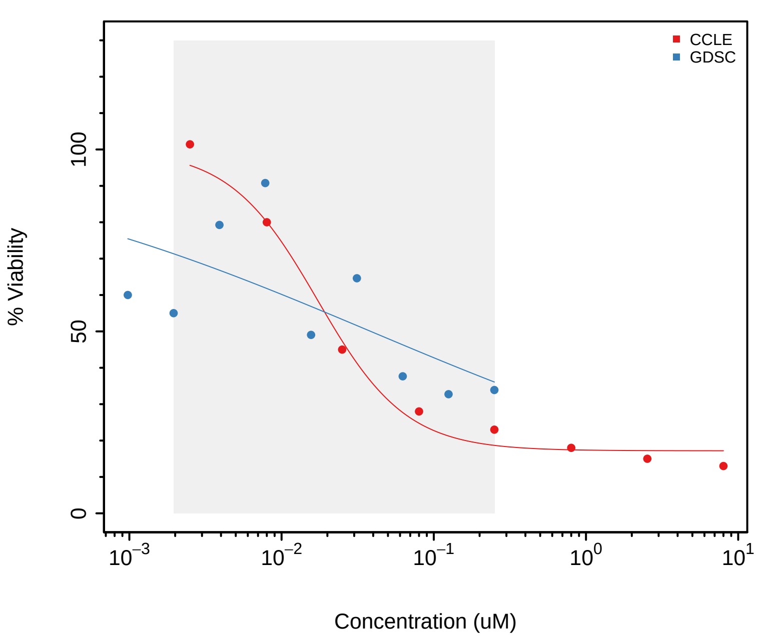

PD−0325901:NCI−H810

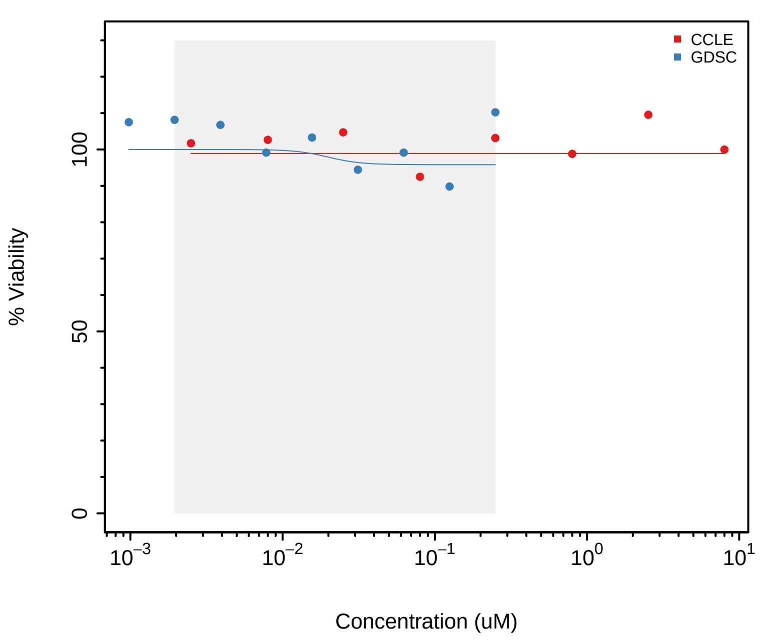

PD−0325901:NCI−N87

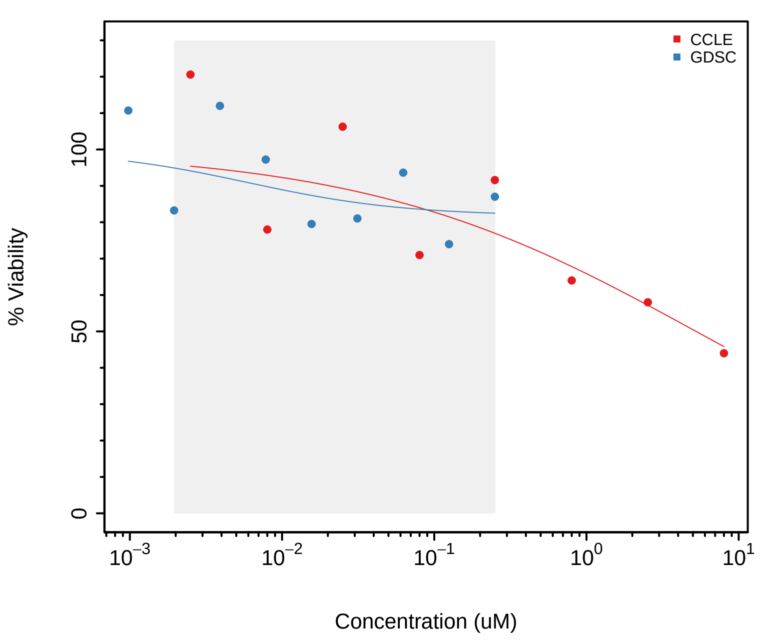

PD−0325901:OVCAR−3

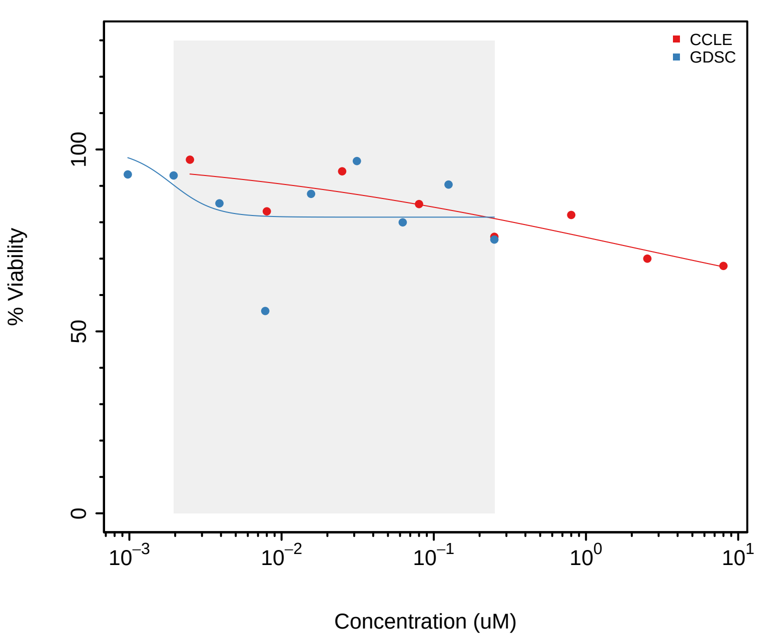

PD−0325901:NUGC−3

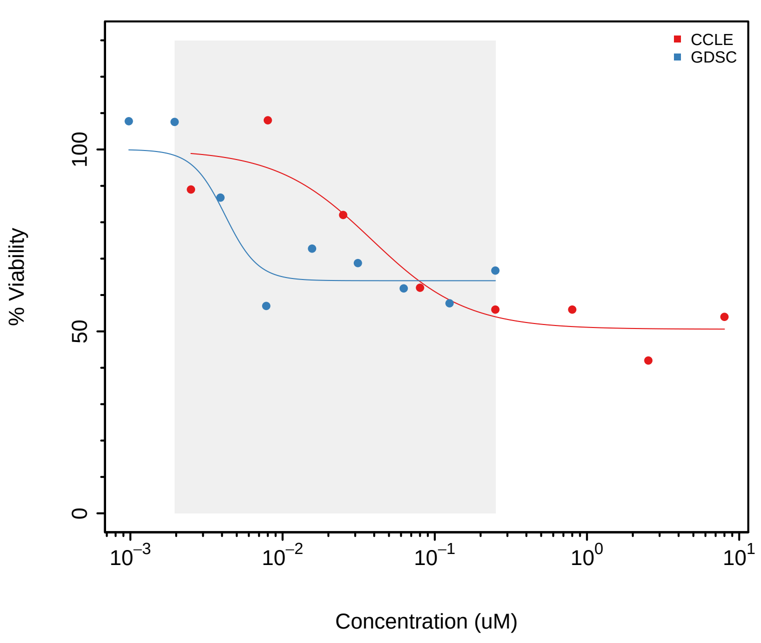

PD−0325901:OC−314

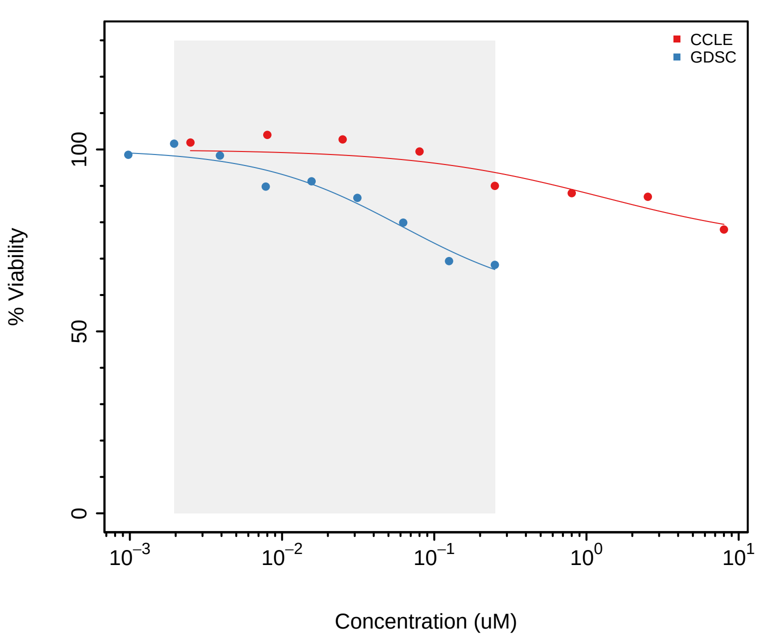

PD−0325901:OCI−AML2

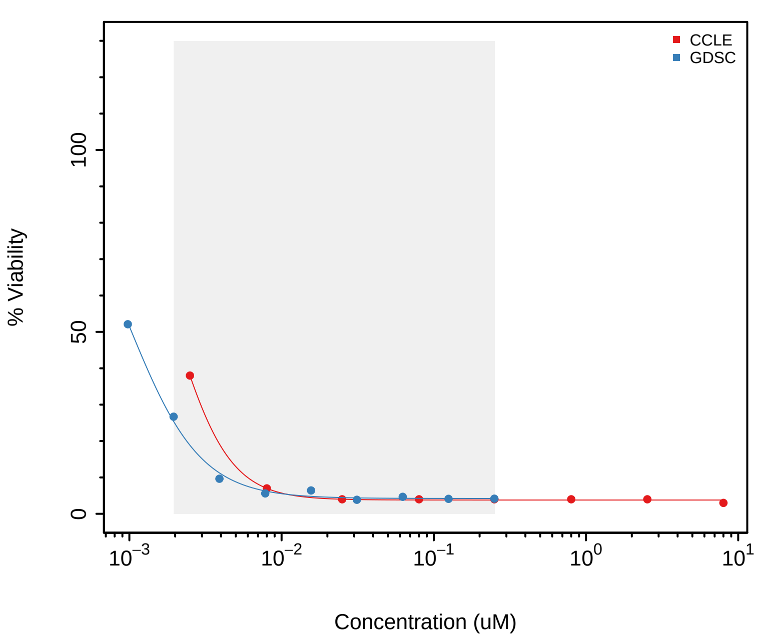

PD−0325901:OE33

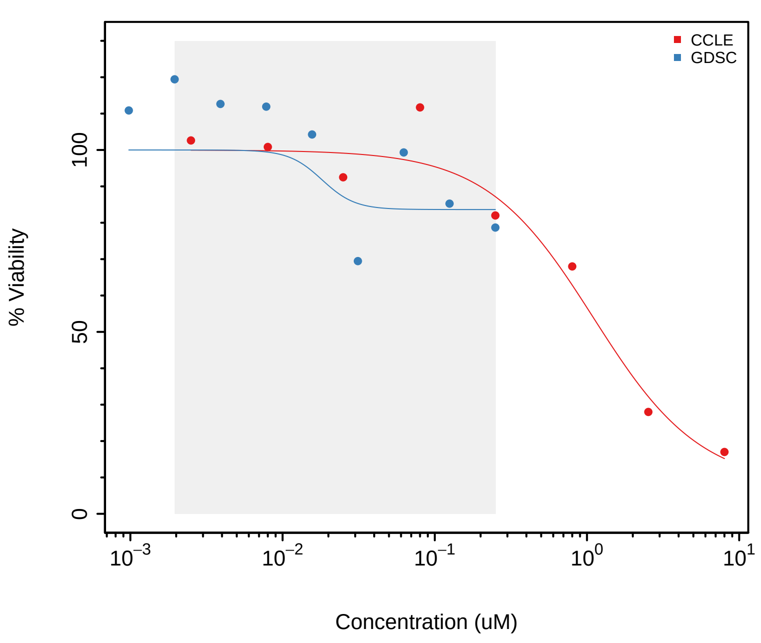

PD−0325901:ONS−76

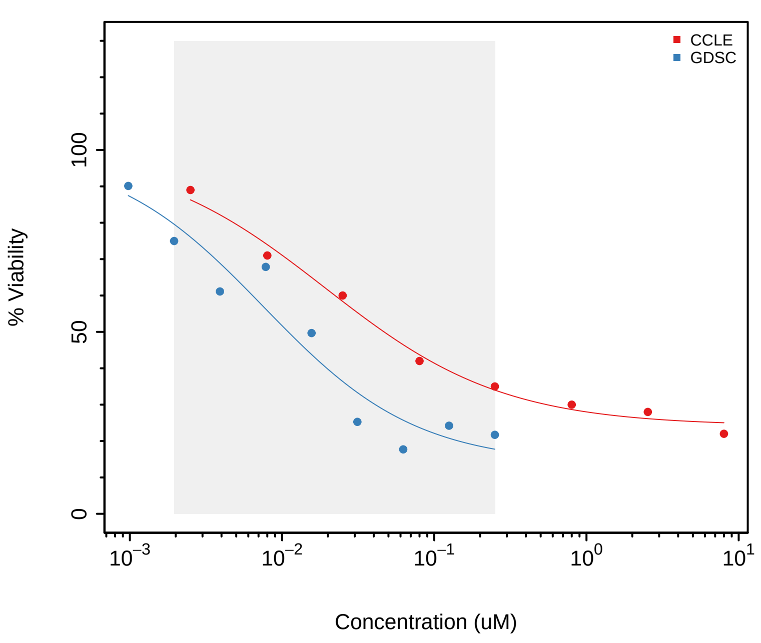

PD−0325901:OPM−2

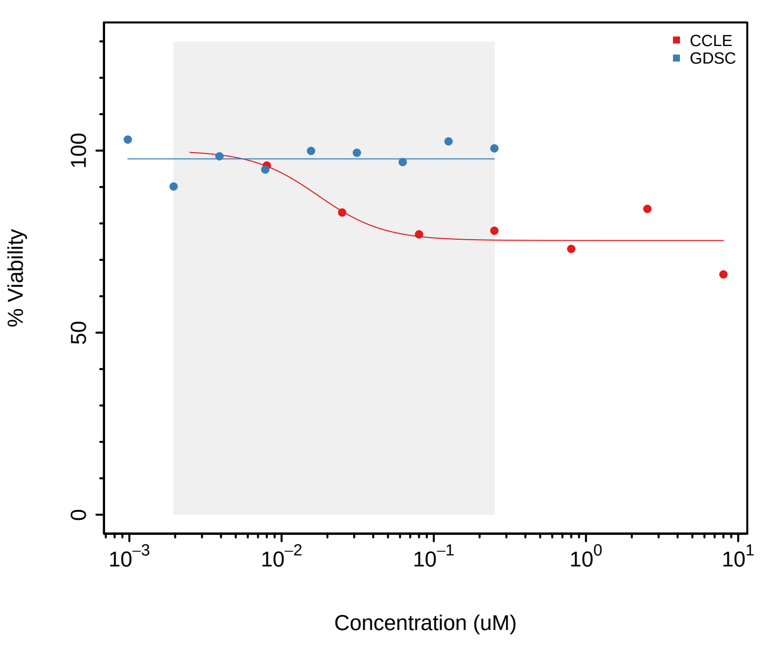

PD−0325901:OVCAR−4

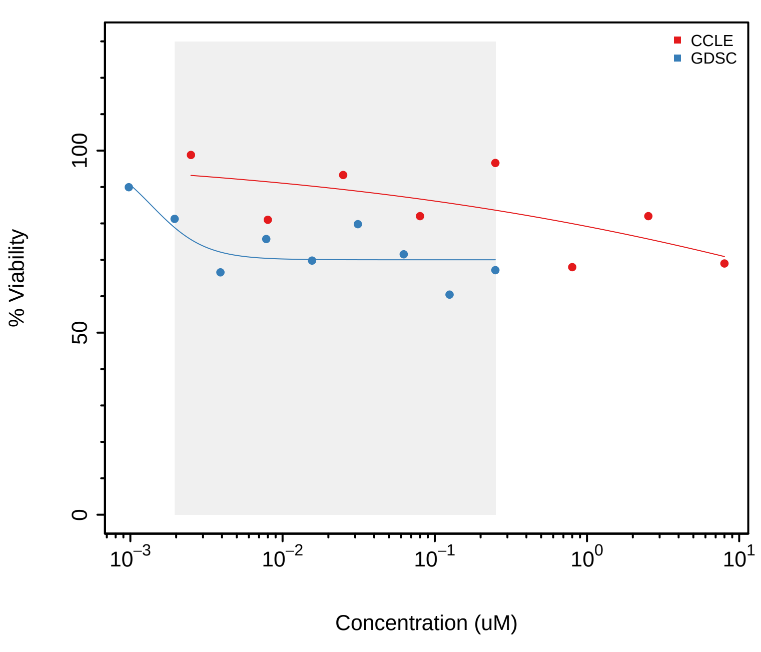

PD−0325901:OVCAR−8

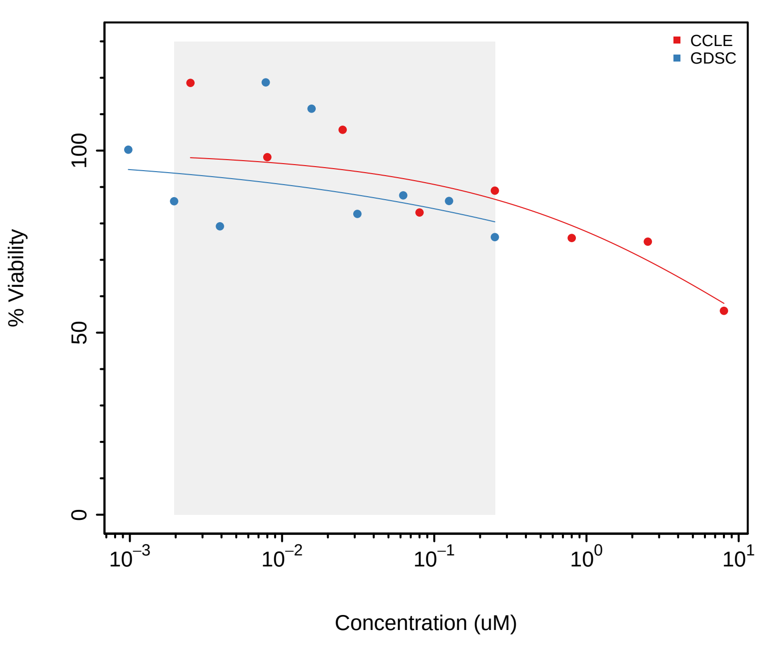

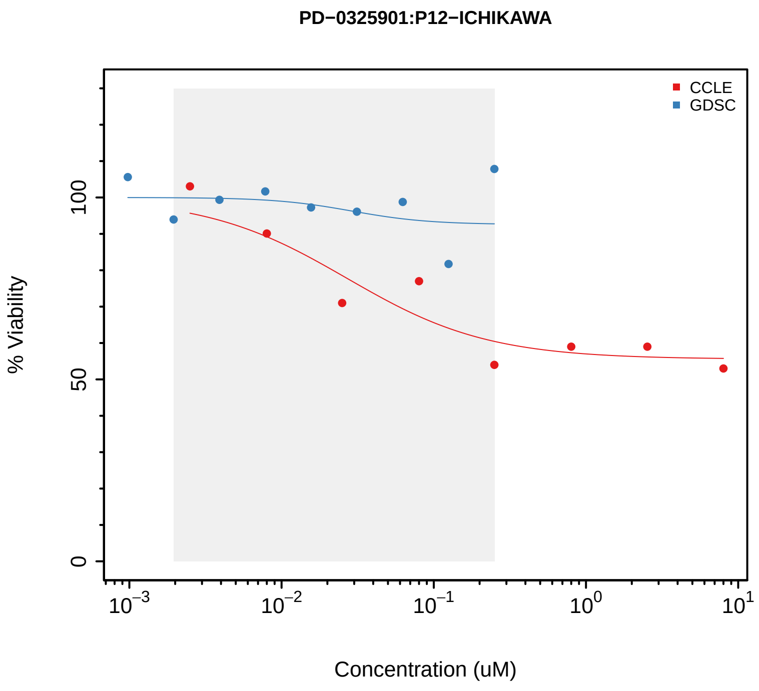
PD−0325901:P12−ICHIKAWA
CCLE
GDSC
% Viability
0
50
100
10^{-3} 10^{-2} 10^{-1} 10^{0} 10^{1}
Concentration (uM)

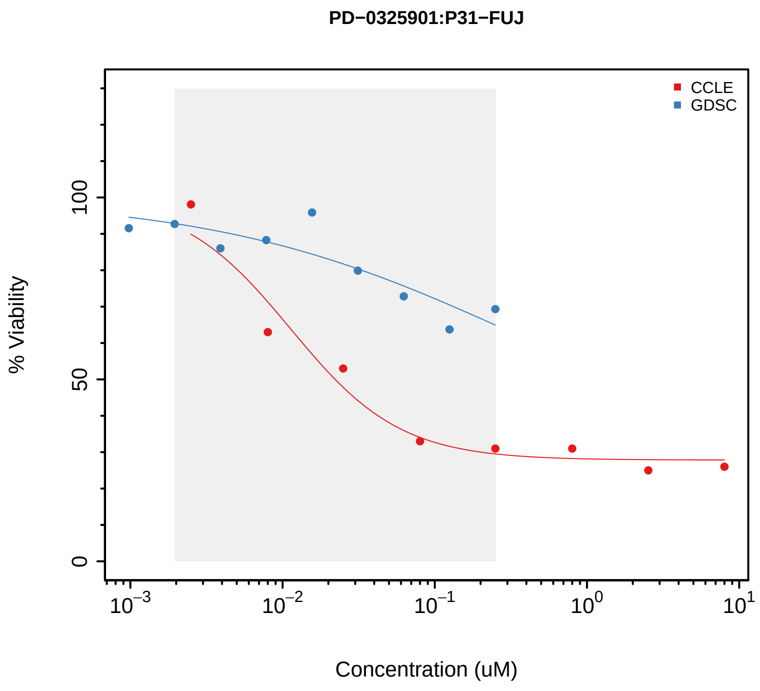
PD−0325901:P31−FUJ
CCLE
GDSC
% Viability
0
50
100
10^{-3} 10^{-2} 10^{-1} 10^{0} 10^{1}
Concentration (uM)

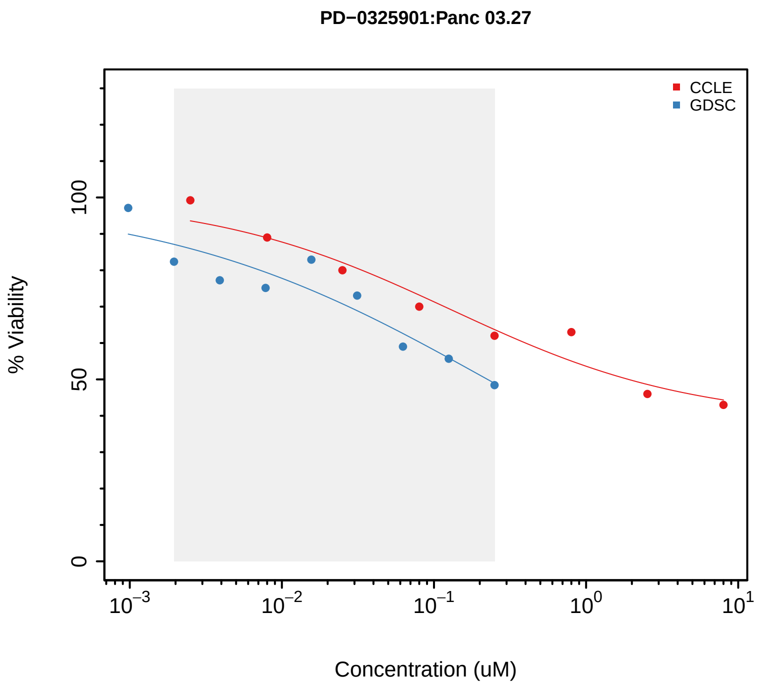
PD−0325901:Panc 03.27
CCLE
GDSC
% Viability
0
50
100
10^{-3} 10^{-2} 10^{-1} 10^{0} 10^{1}
Concentration (uM)

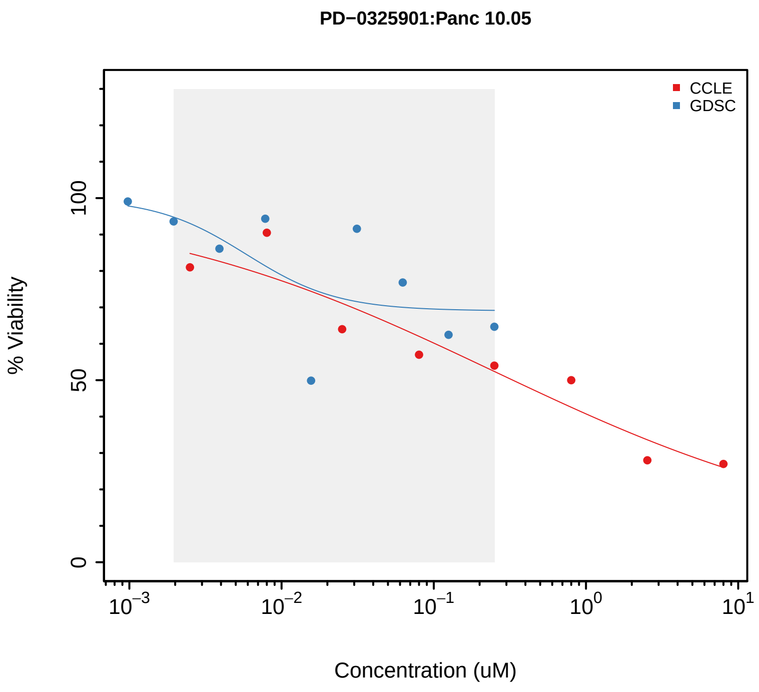
PD−0325901:Panc 10.05
CCLE
GDSC
% Viability
0
50
100
10^{-3} 10^{-2} 10^{-1} 10^{0} 10^{1}
Concentration (uM)

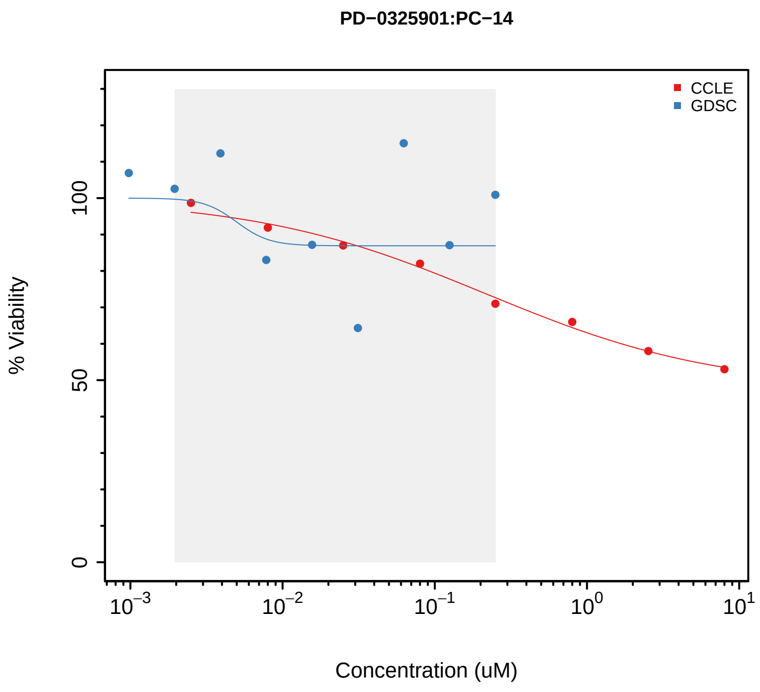
PD−0325901:PC−14
CCLE
GDSC
% Viability
0
50
100
10^{-3} 10^{-2} 10^{-1} 10^{0} 10^{1}
Concentration (uM)

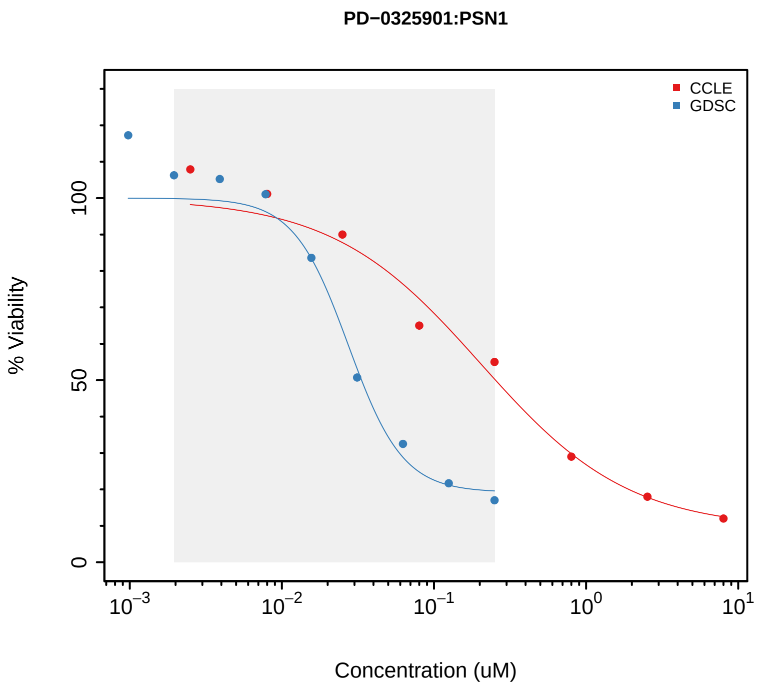
PD−0325901:PSN1
CCLE
GDSC
% Viability
0
50
100
10^{-3} 10^{-2} 10^{-1} 10^{0} 10^{1}
Concentration (uM)

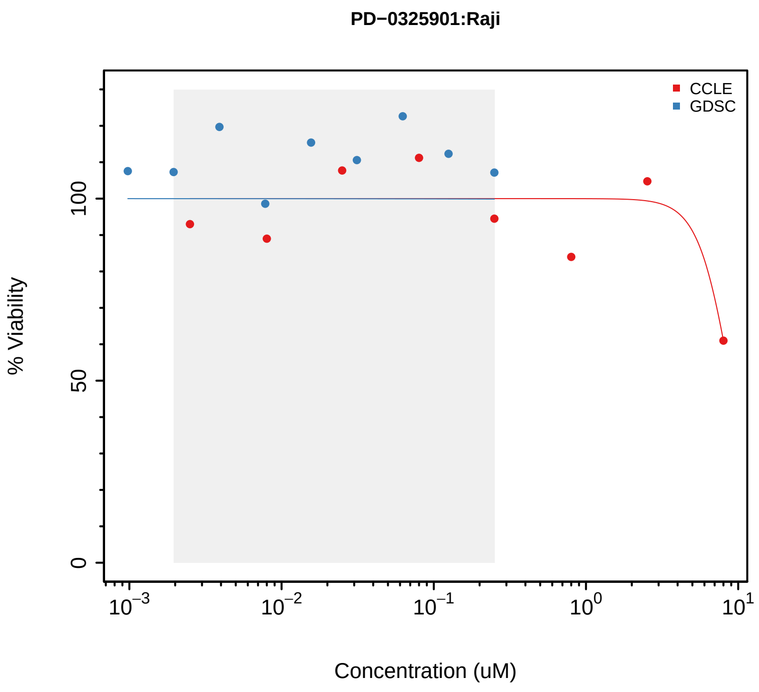
PD−0325901:Raji
CCLE
GDSC
% Viability
0
50
100
10^{-3} 10^{-2} 10^{-1} 10^{0} 10^{1}
Concentration (uM)

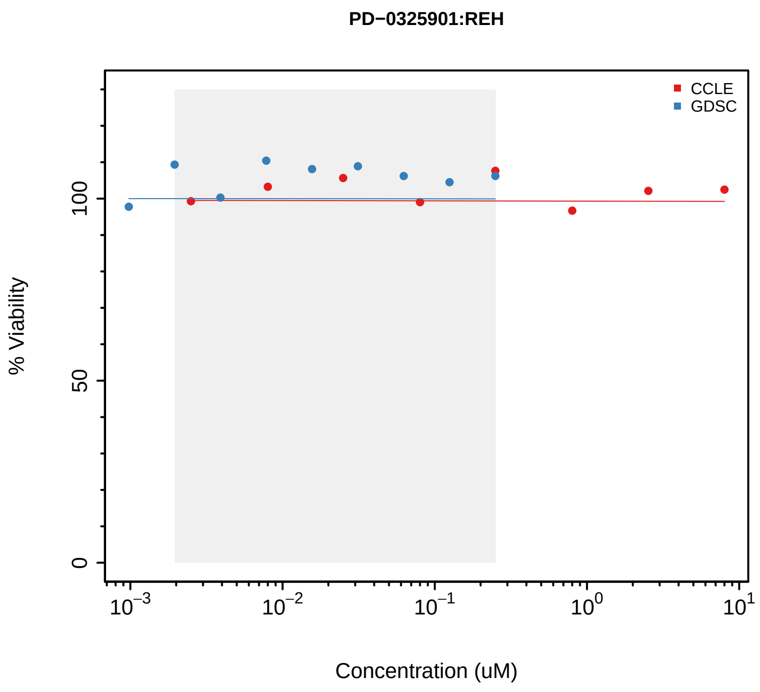
PD−0325901:REH
CCLE
GDSC
% Viability
0
50
100
10^{-3} 10^{-2} 10^{-1} 10^{0} 10^{1}
Concentration (uM)

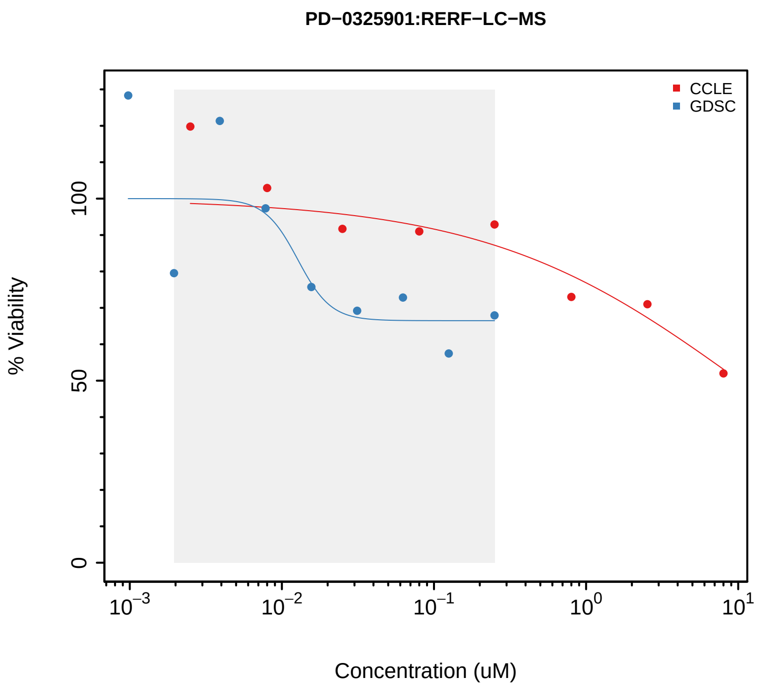
PD−0325901:RERF−LC−MS
CCLE
GDSC
% Viability
0
50
100
10^{-3} 10^{-2} 10^{-1} 10^{0} 10^{1}
Concentration (uM)

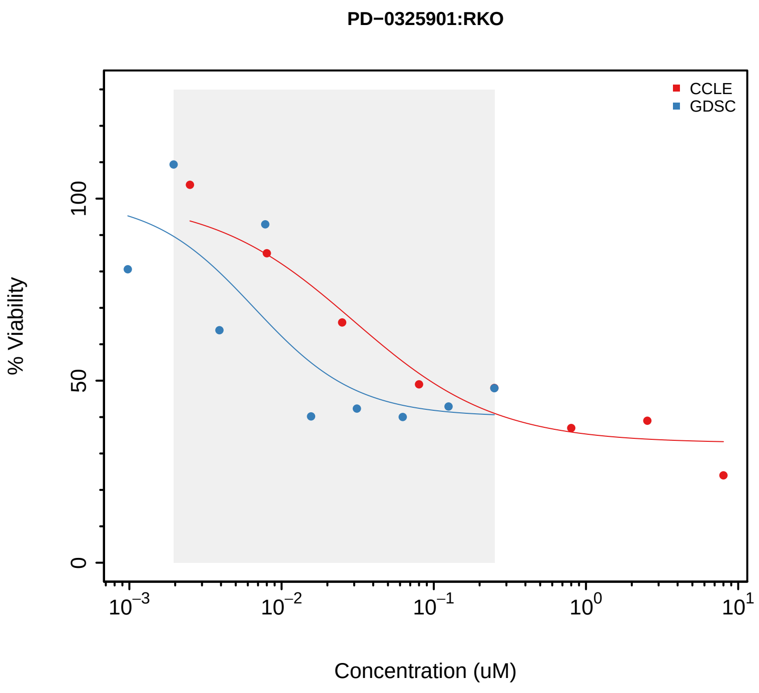
PD−0325901:RKO
CCLE
GDSC
% Viability
0
50
100
10^{-3} 10^{-2} 10^{-1} 10^{0} 10^{1}
Concentration (uM)

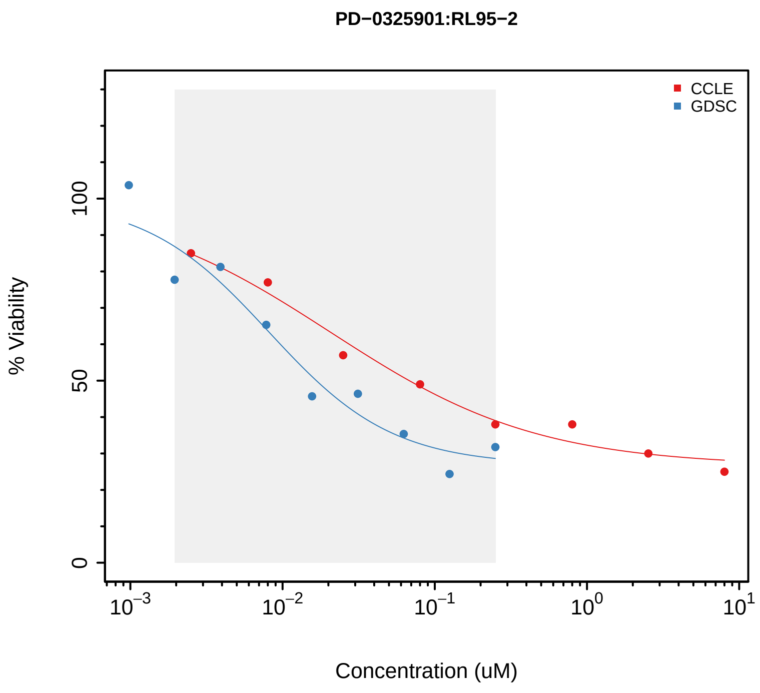
PD−0325901:RL95−2
CCLE
GDSC
% Viability
0
50
100
10^{-3} 10^{-2} 10^{-1} 10^{0} 10^{1}
Concentration (uM)

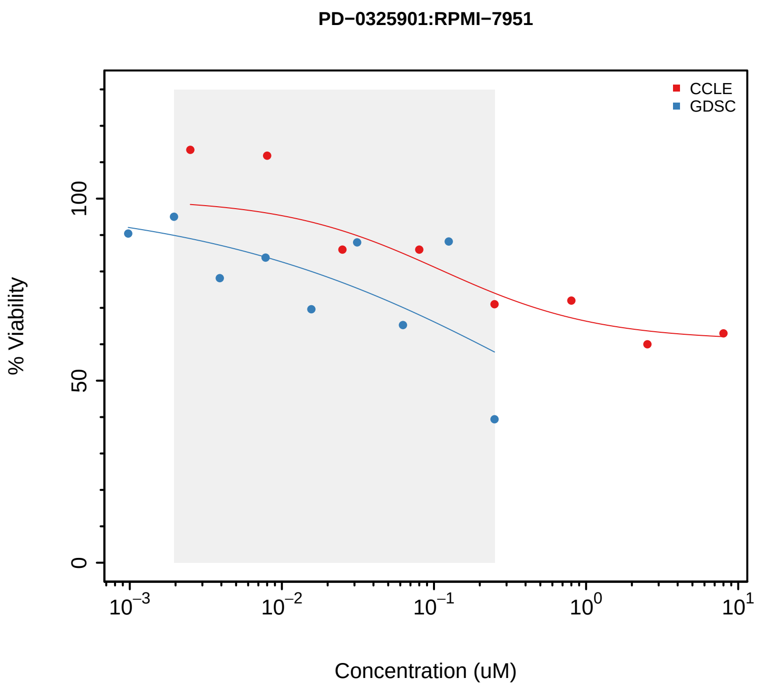
PD−0325901:RPMI−7951
CCLE
GDSC
% Viability
0
50
100
10^{-3} 10^{-2} 10^{-1} 10^{0} 10^{1}
Concentration (uM)

PD−0325901:RPMI−8402

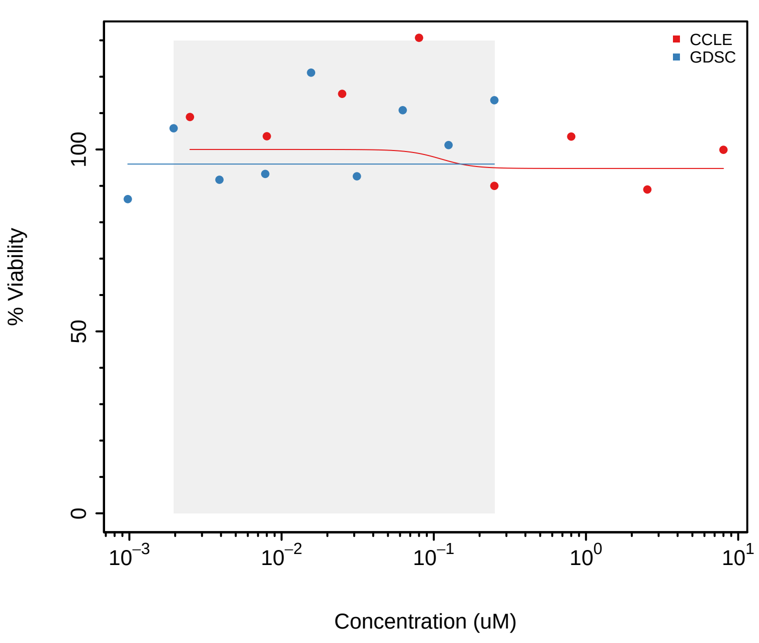

PD−0325901:RT−112

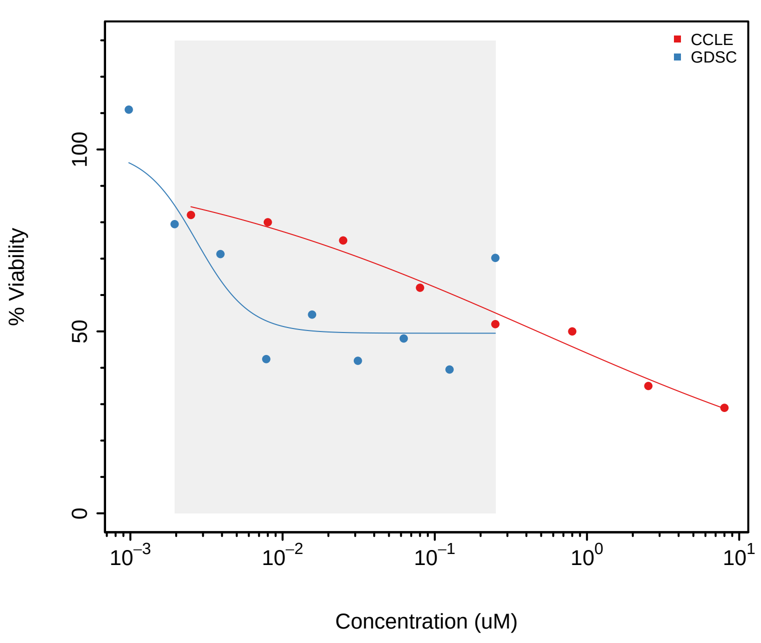

PD−0325901:RVH−421

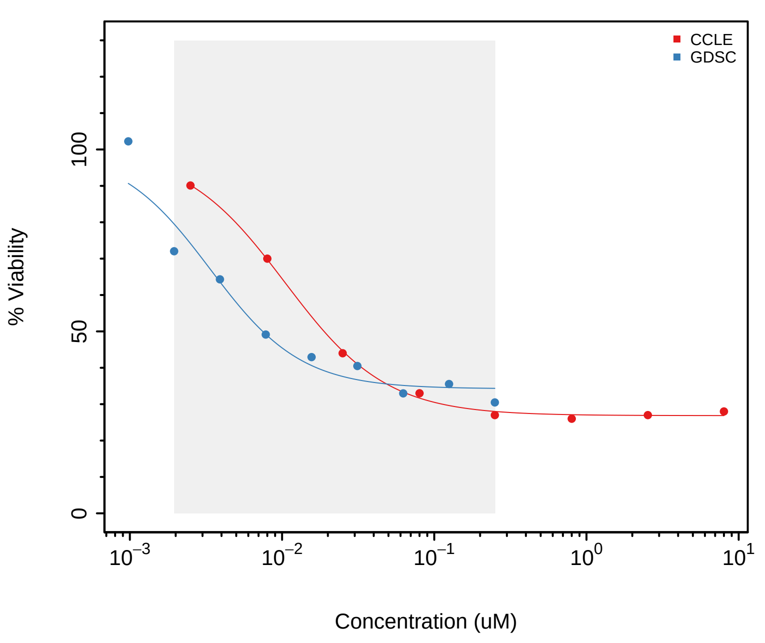

PD−0325901:Saos−2

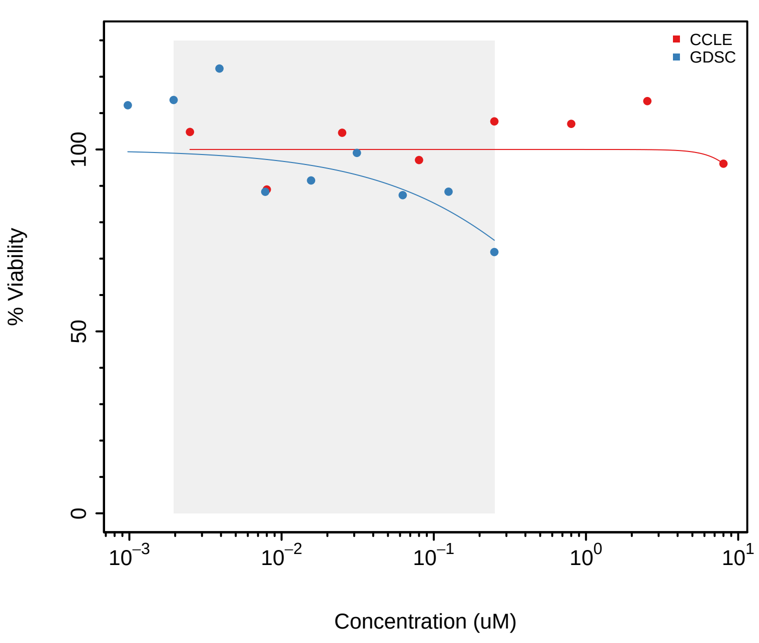

PD−0325901:SBC−5

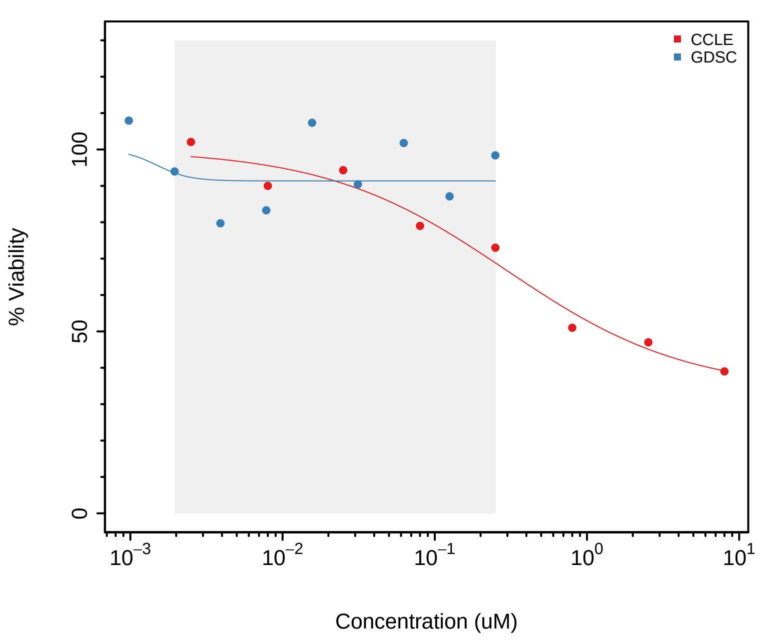

PD−0325901:SCC−25

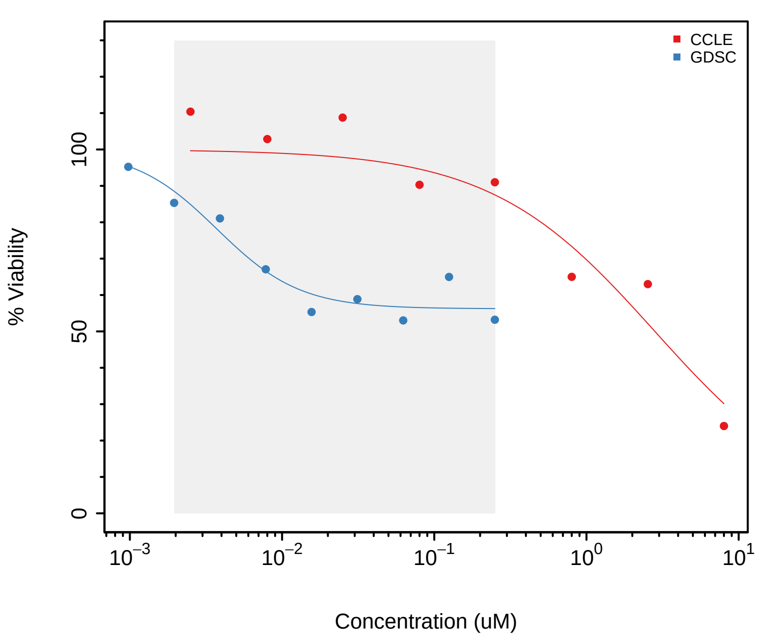

PD−0325901:SCC−9

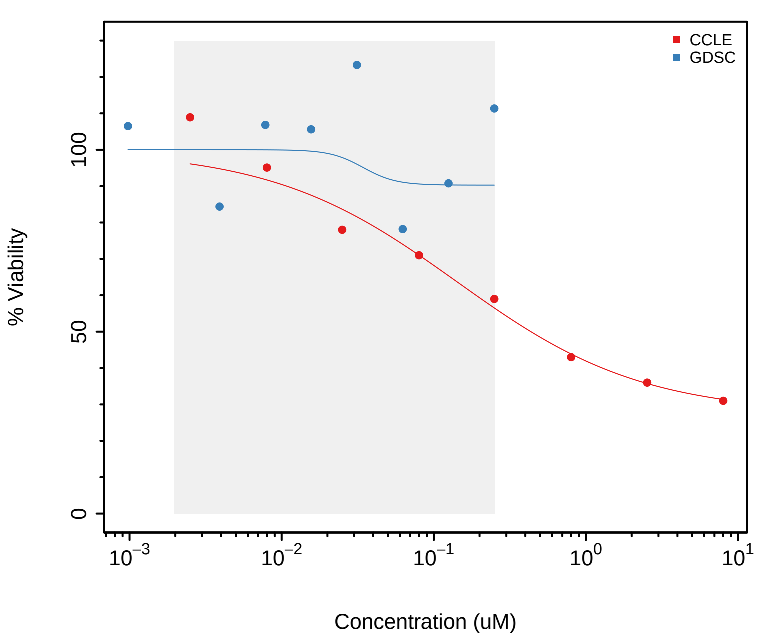

PD−0325901:SF126

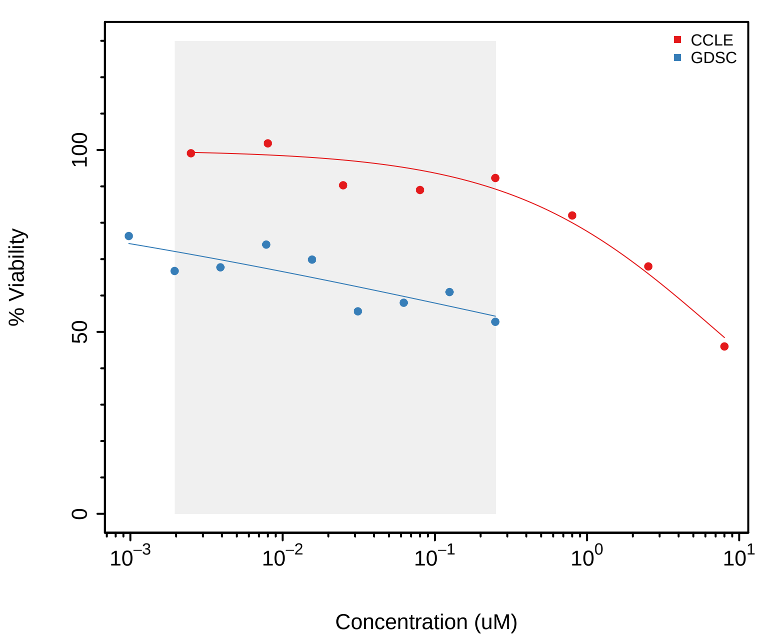

PD−0325901:SF295

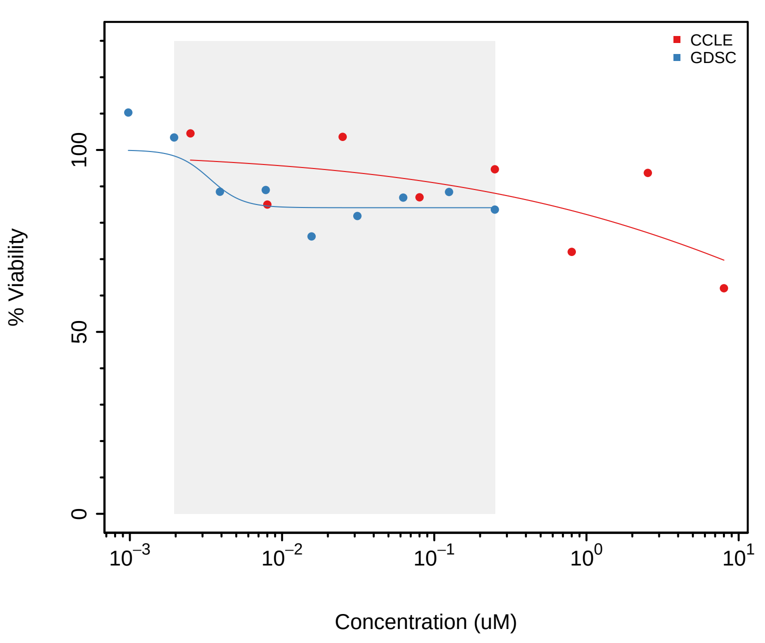

PD−0325901:SHP−77

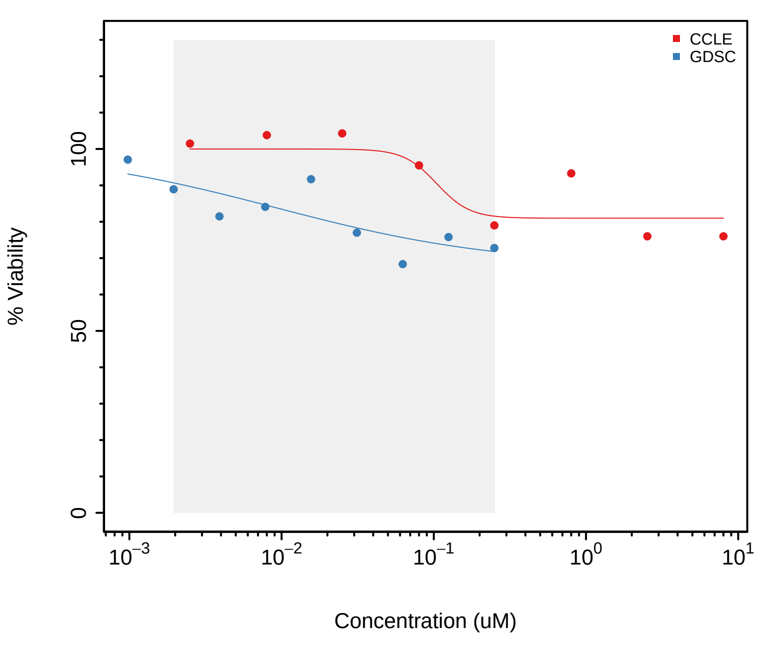

PD−0325901:SIMA

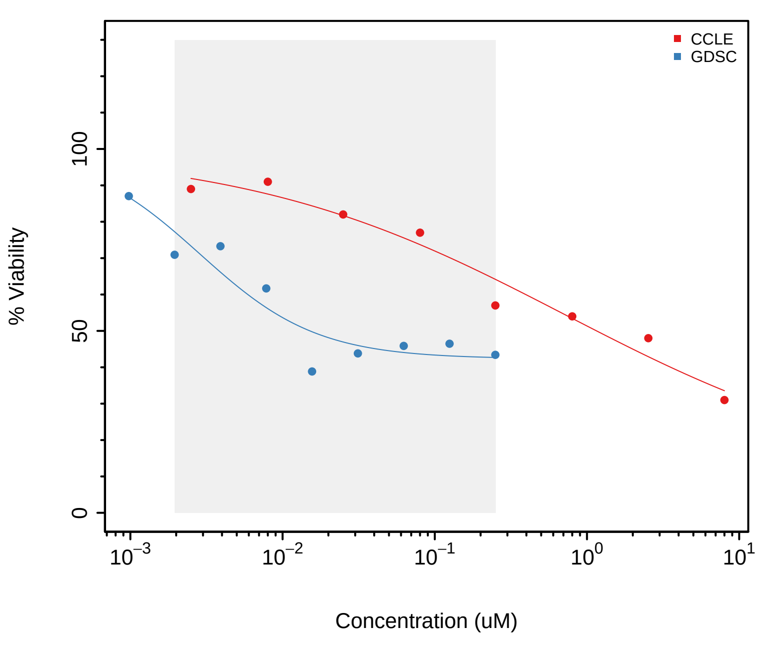

PD−0325901:SJRH30

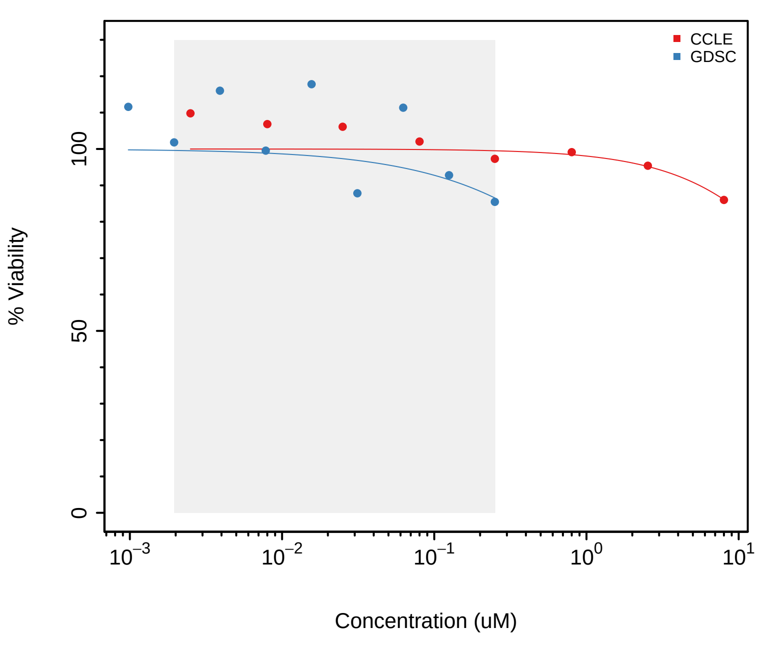

PD−0325901:SJSA−1

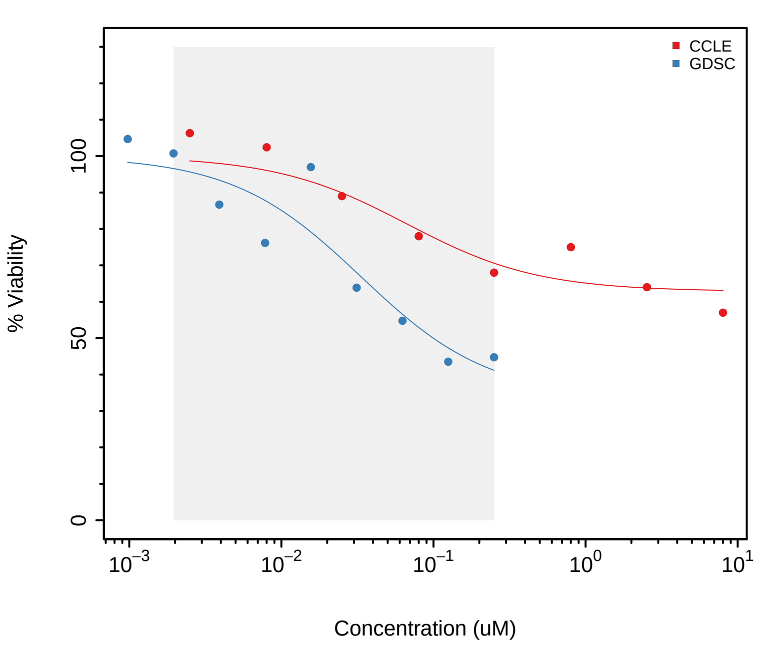

PD−0325901:SK−CO−1

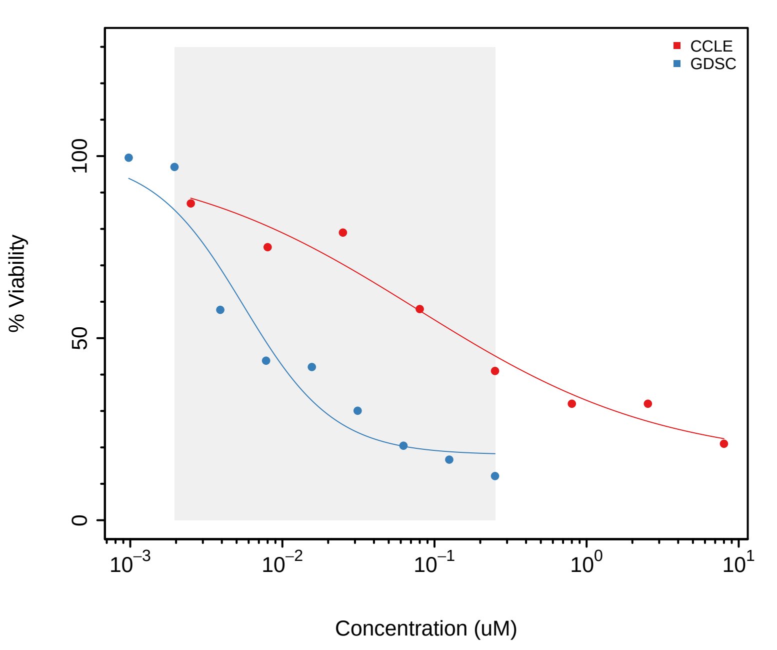

PD−0325901:SK−HEP−1

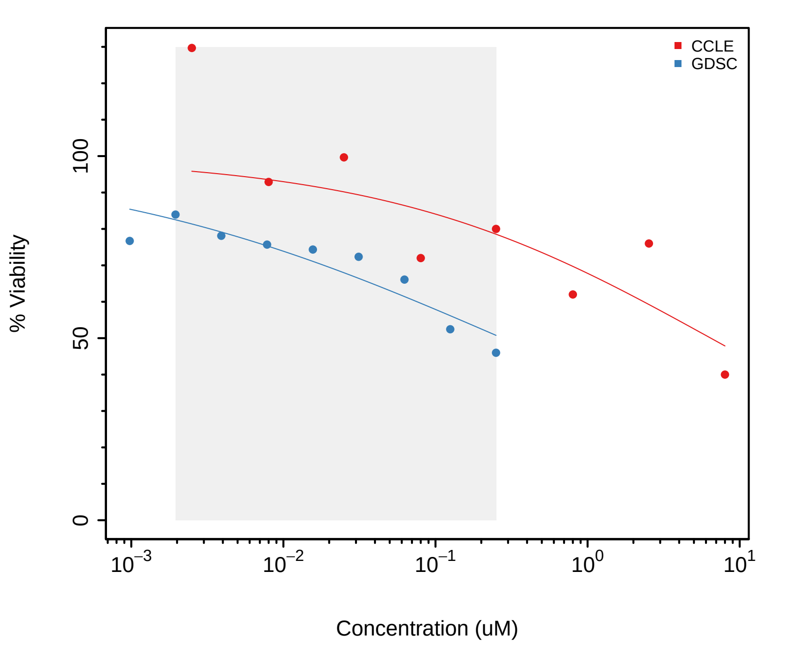

PD−0325901:SK−LMS−1

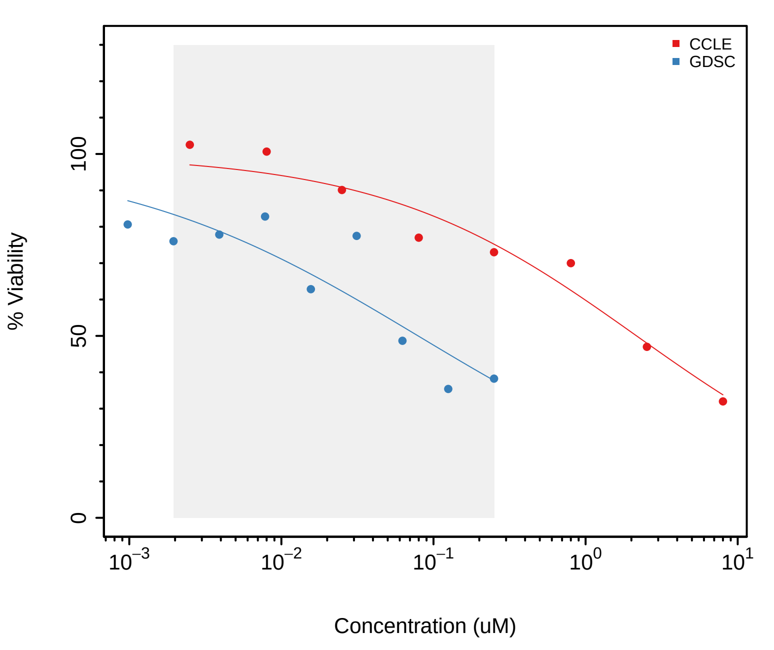

PD−0325901:SK−LU−1

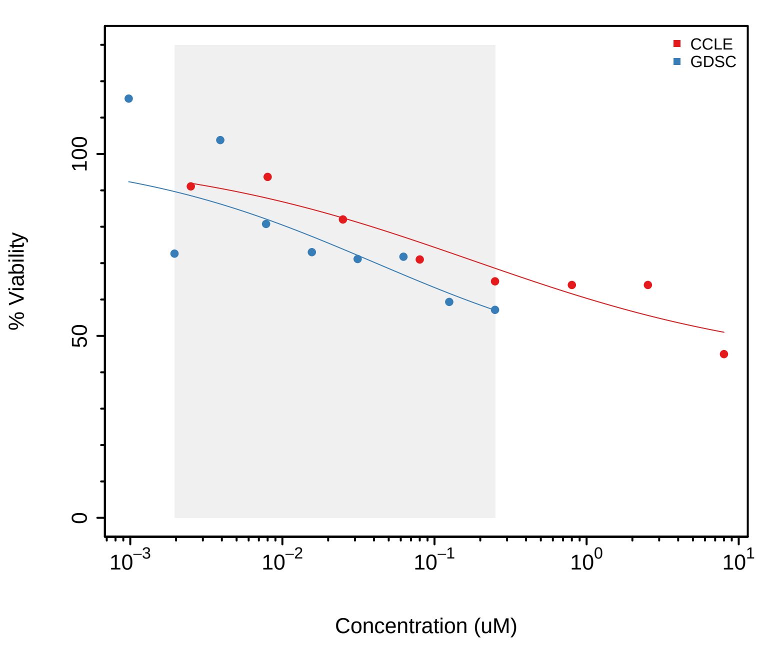

PD−0325901:SK−MEL−2

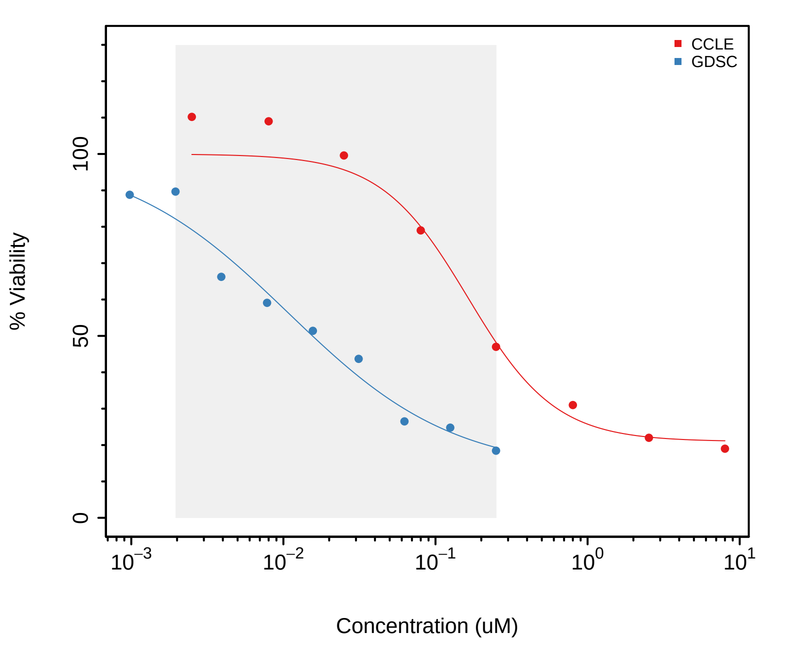

PD−0325901:SK−MEL−24

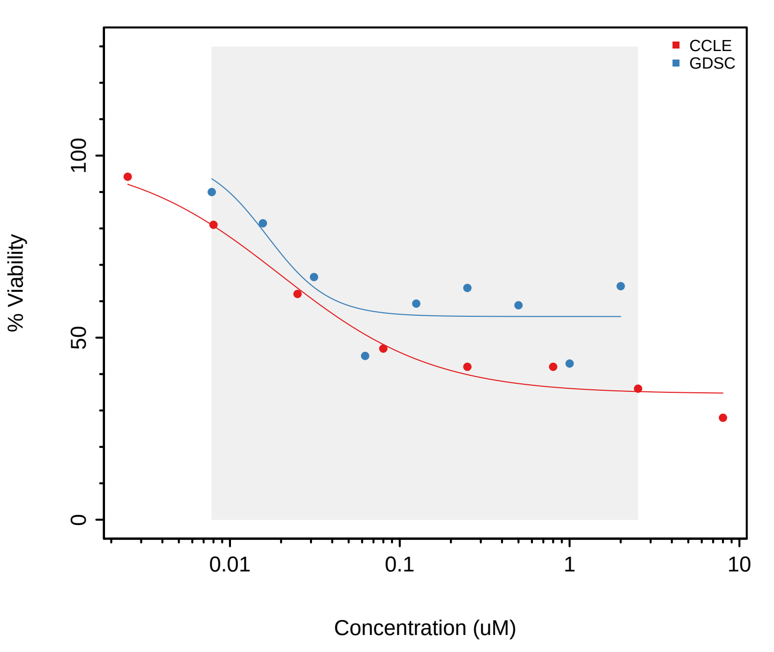

PD−0325901:SK−MEL−30

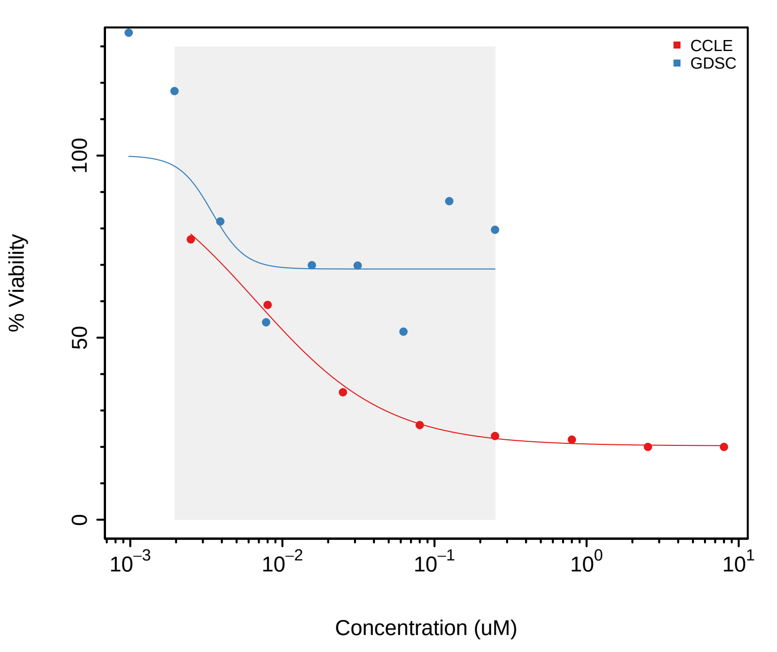

PD−0325901:SK−MEL−5

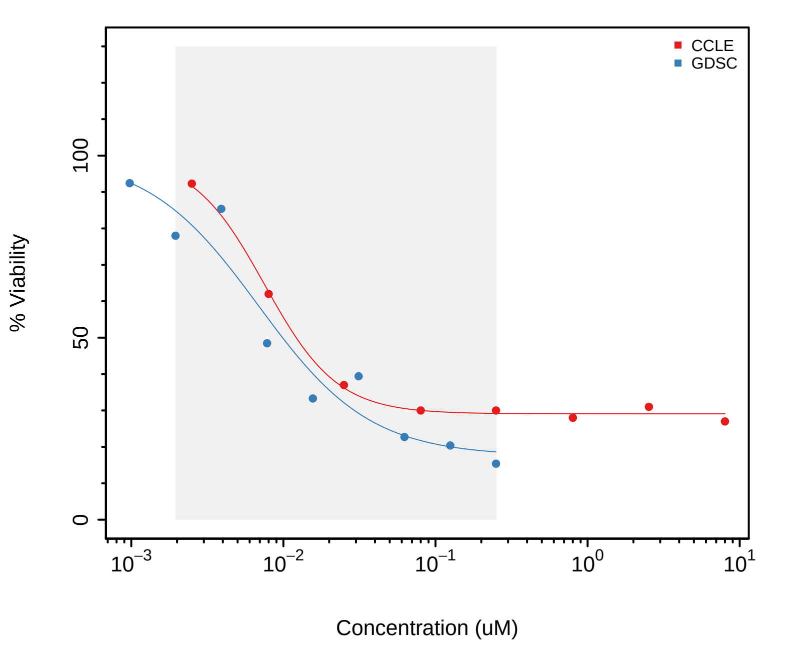

PD−0325901:SK−MES−1

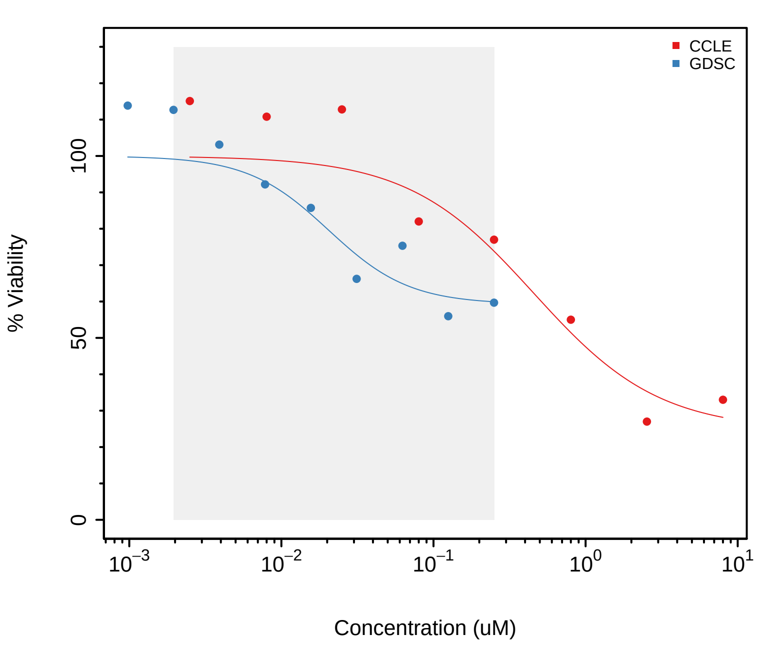

PD−0325901:SK−MM−2

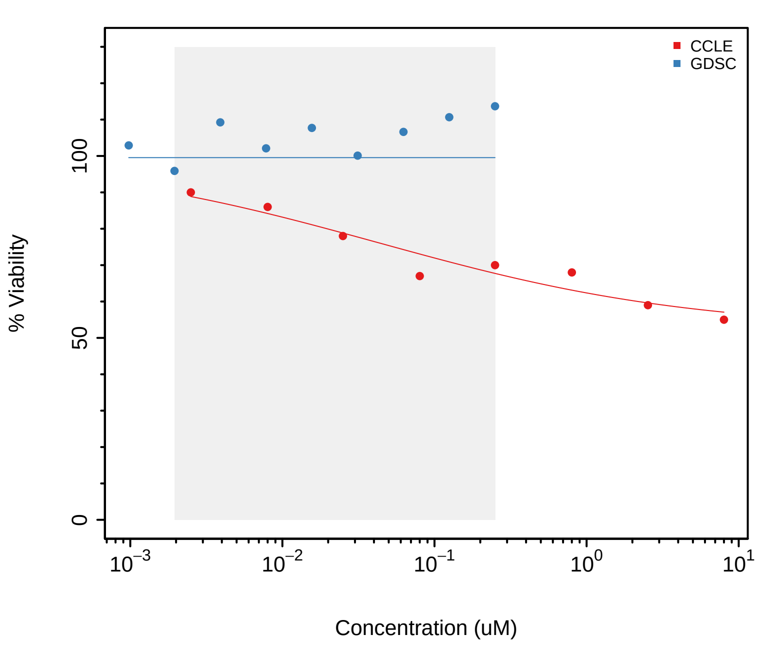

PD−0325901:SK−N−AS

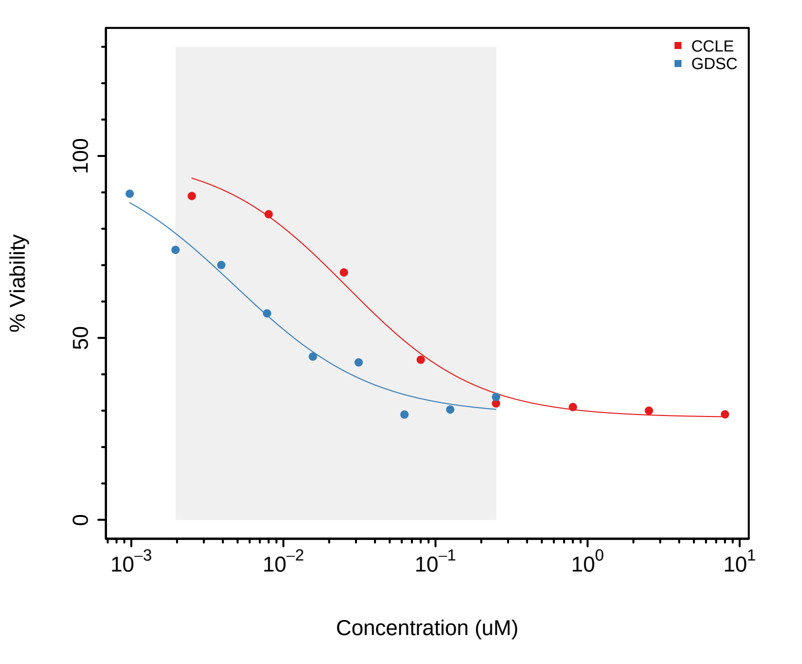

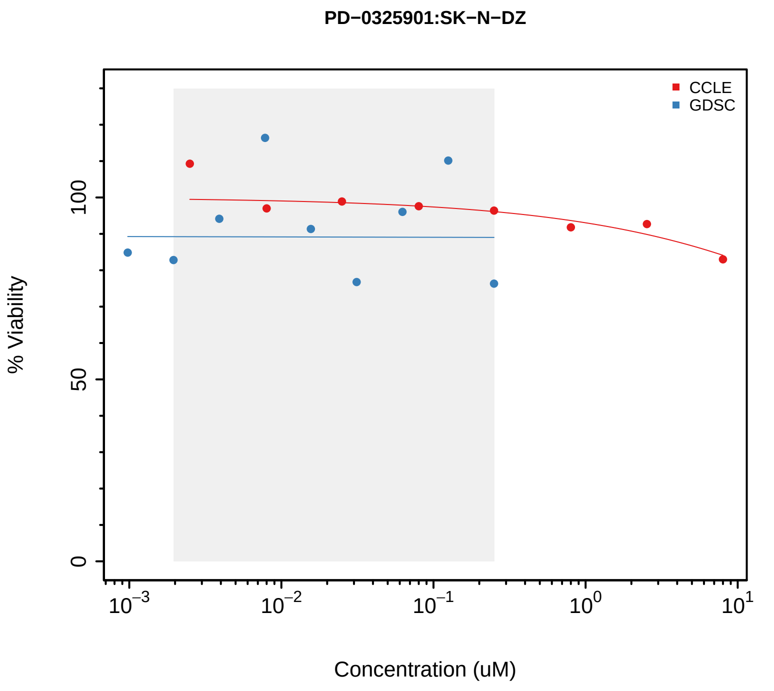
PD−0325901:SK−N−DZ
CCLE
GDSC
% Viability
Concentration (uM)

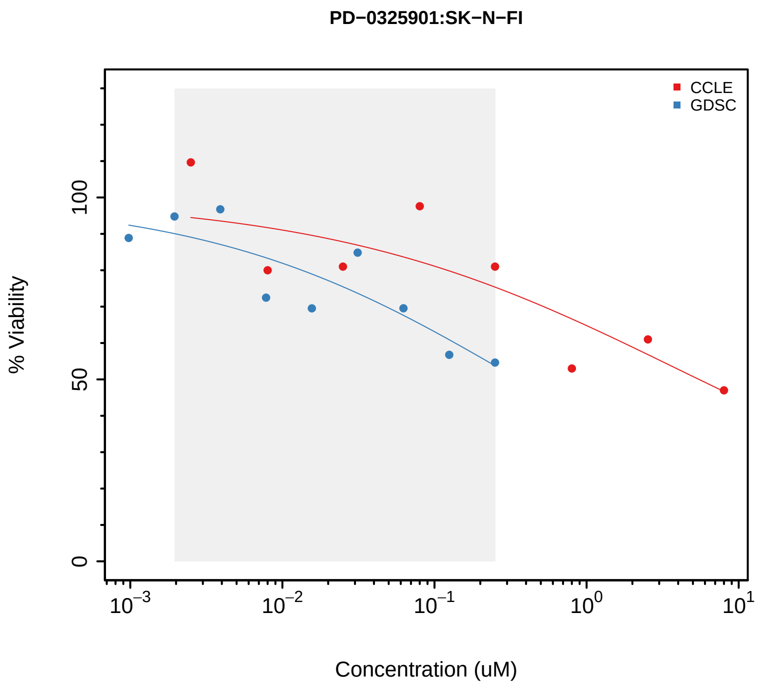
PD−0325901:SK−N−FI
CCLE
GDSC
% Viability
Concentration (uM)

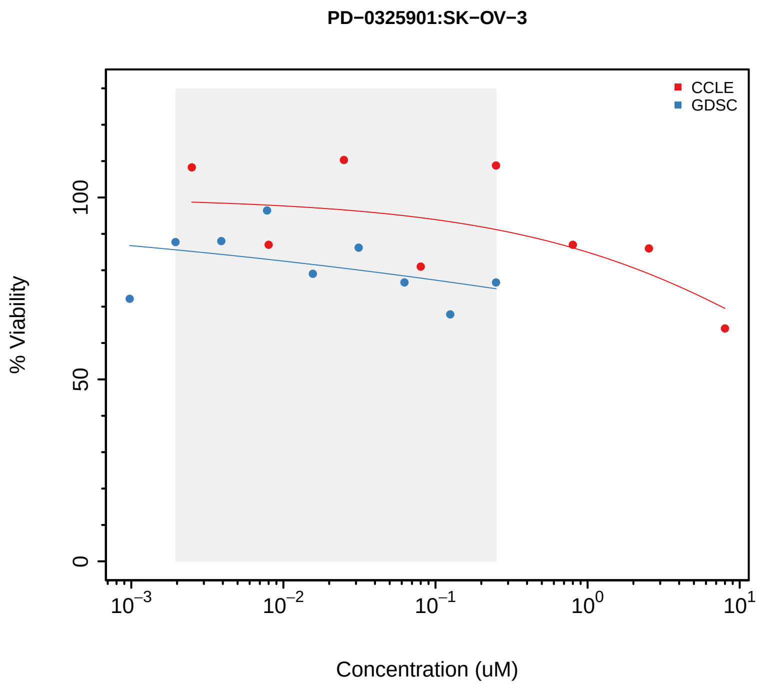
PD−0325901:SK−OV−3
CCLE
GDSC
% Viability
Concentration (uM)

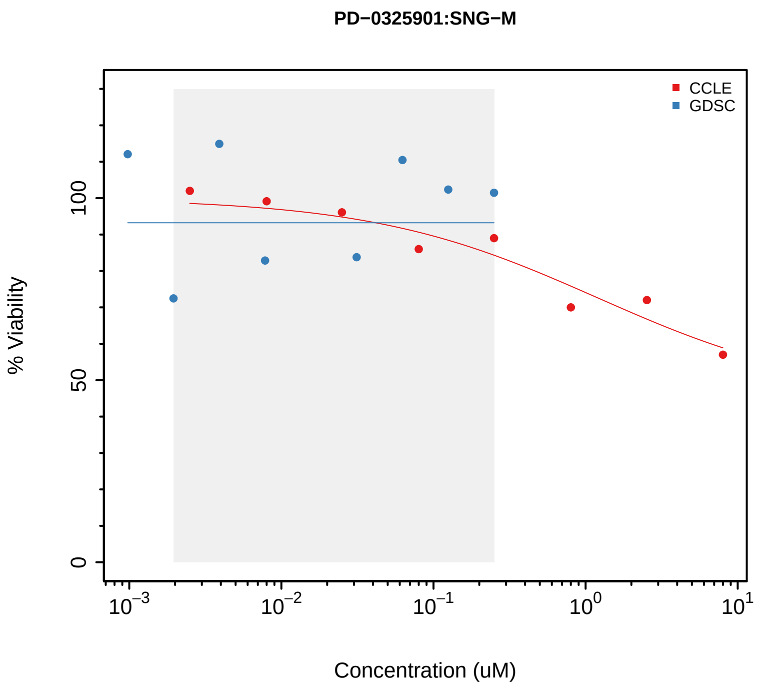
PD−0325901:SNG−M
CCLE
GDSC
% Viability
Concentration (uM)

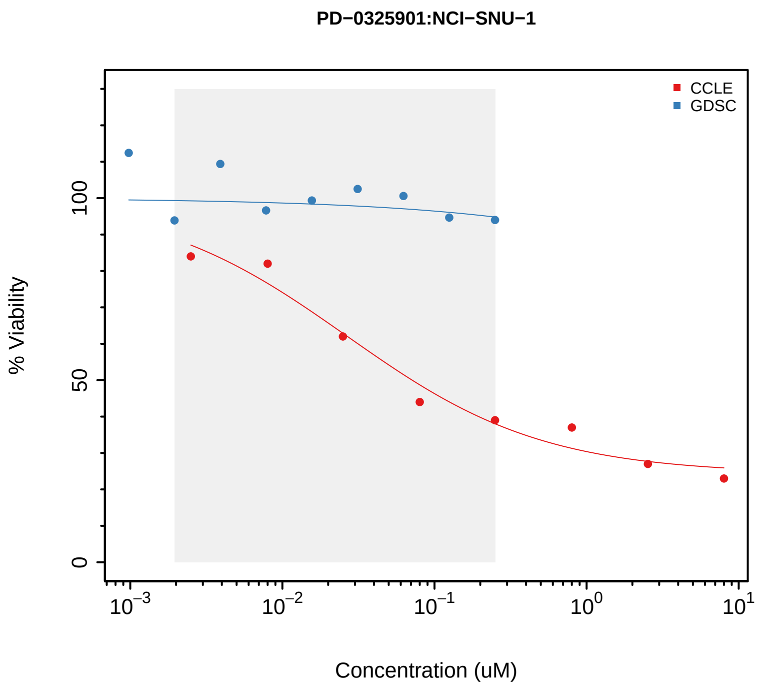
PD−0325901:NCI−SNU−1
CCLE
GDSC
% Viability
Concentration (uM)

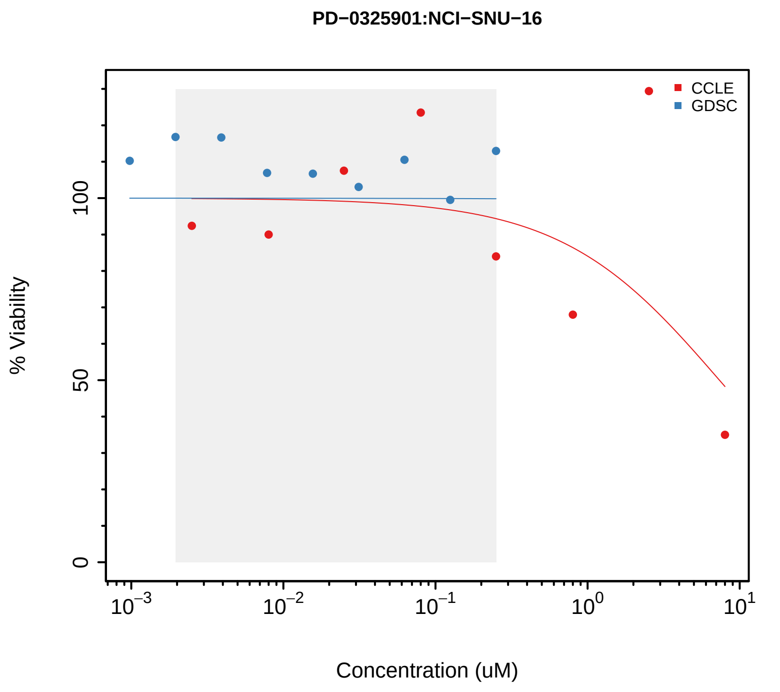
PD−0325901:NCI−SNU−16
CCLE
GDSC
% Viability
Concentration (uM)

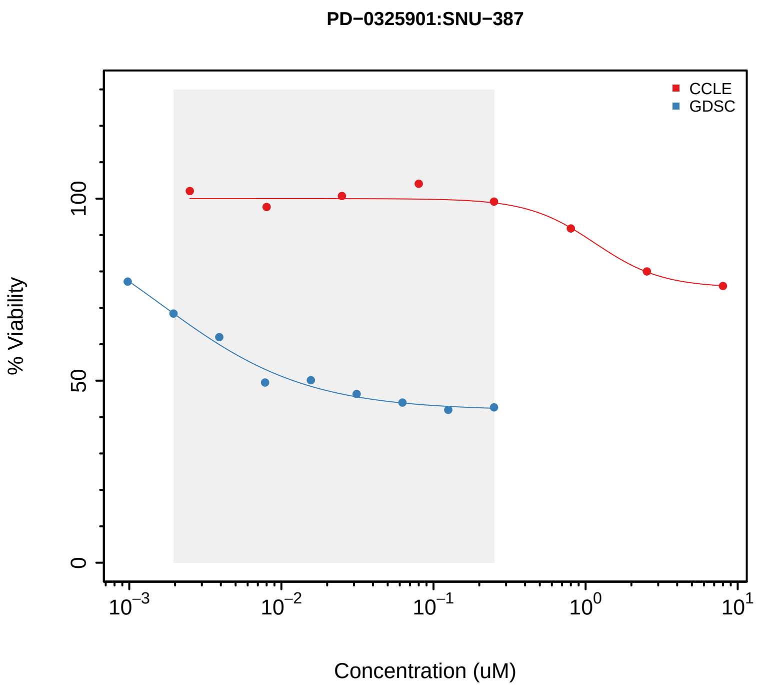
PD−0325901:SNU−387
CCLE
GDSC
% Viability
Concentration (uM)

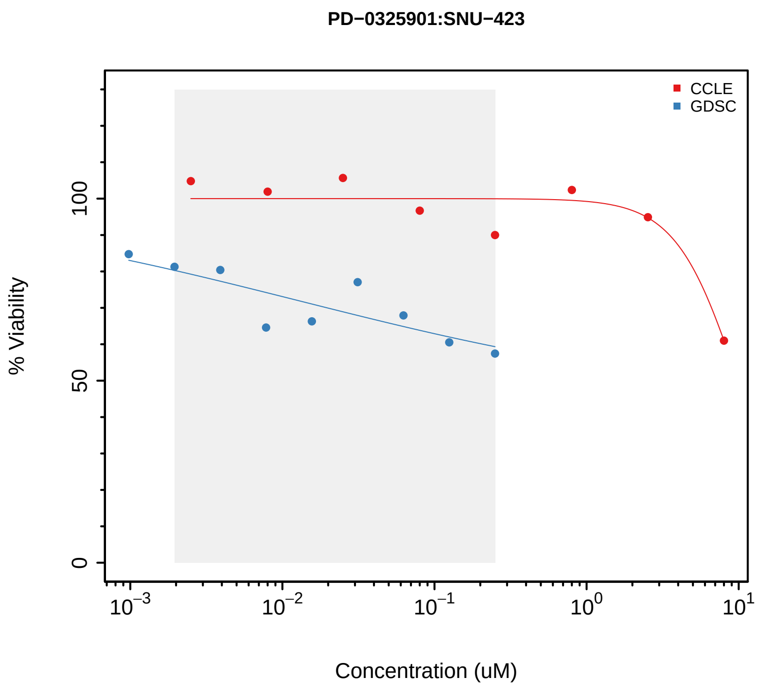
PD−0325901:SNU−423
CCLE
GDSC
% Viability
Concentration (uM)

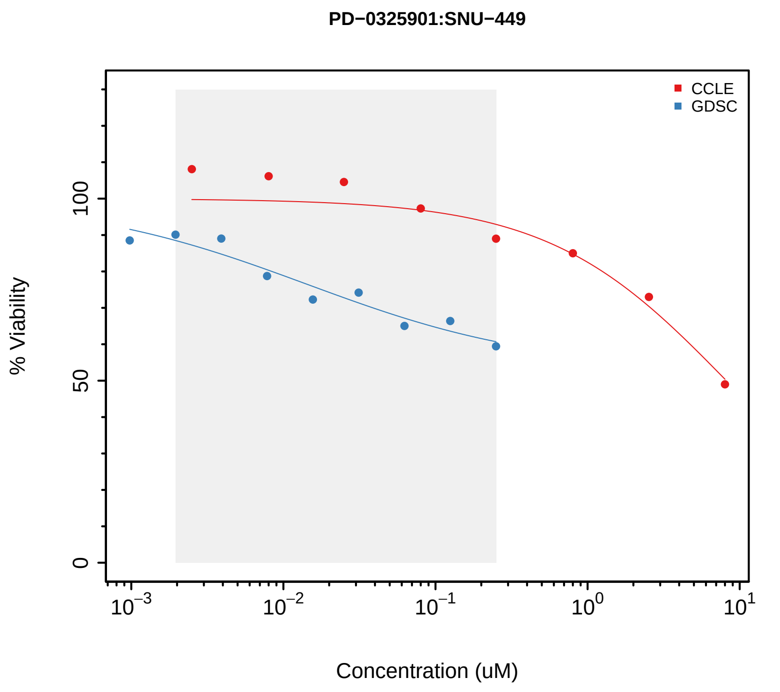
PD−0325901:SNU−449
CCLE
GDSC
% Viability
Concentration (uM)

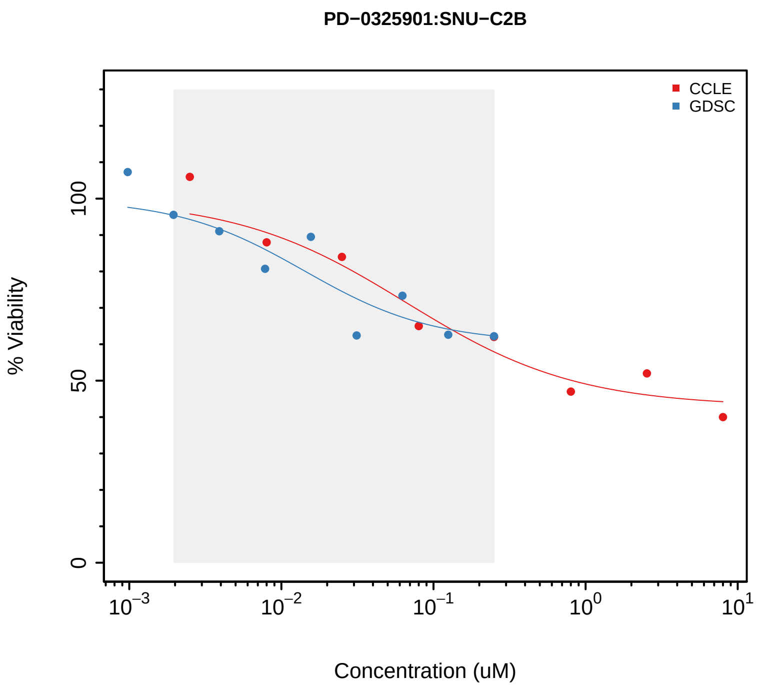
PD−0325901:SNU−C2B
CCLE
GDSC
% Viability
Concentration (uM)

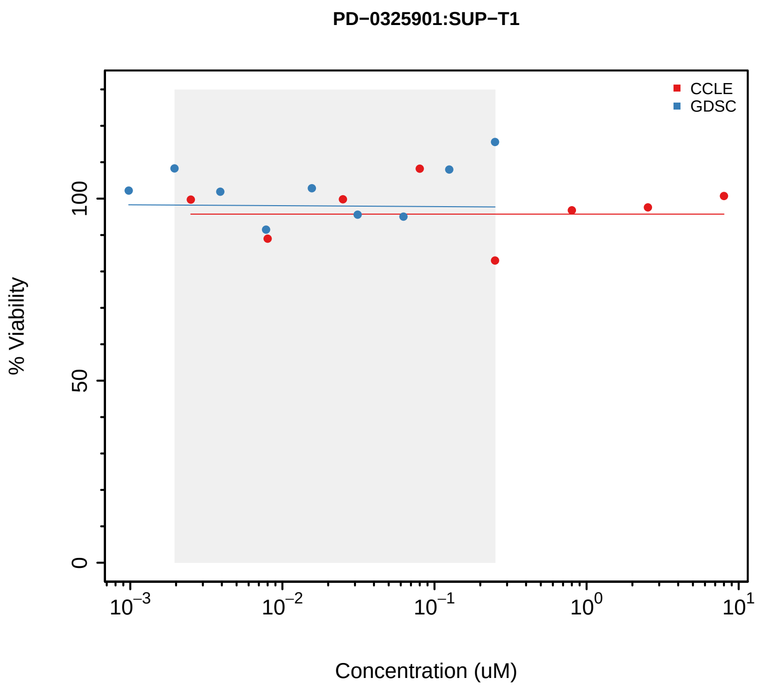
PD−0325901:SUP−T1
CCLE
GDSC
% Viability
Concentration (uM)

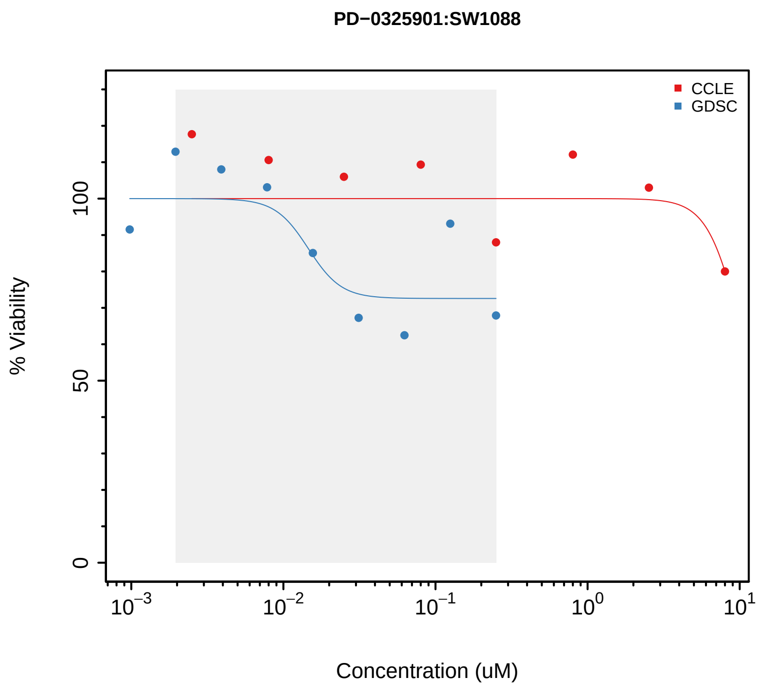
PD−0325901:SW1088
CCLE
GDSC
% Viability
Concentration (uM)

PD−0325901:SW1417

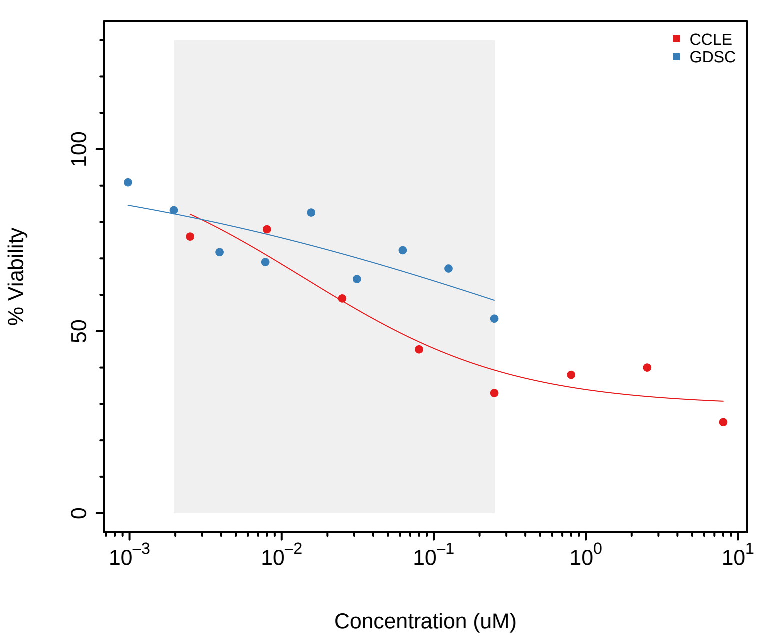

PD−0325901:SW1573

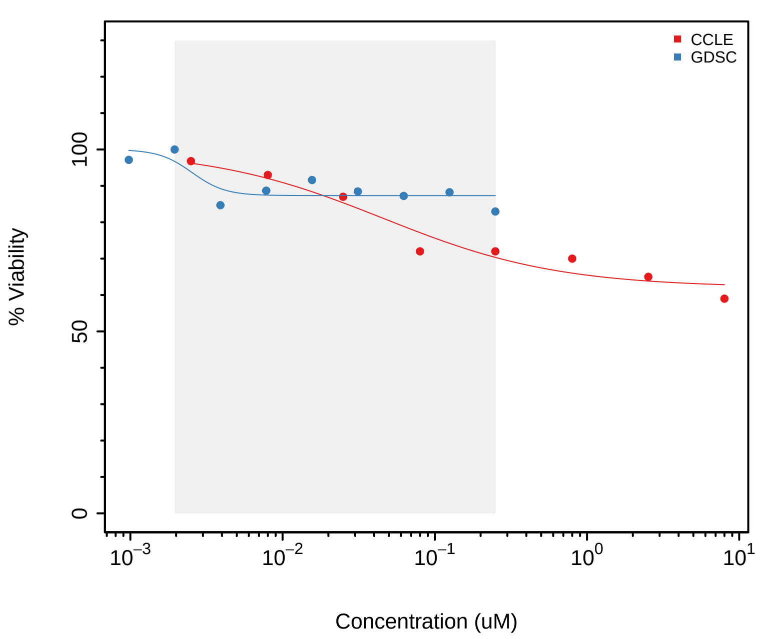

PD−0325901:SW1990

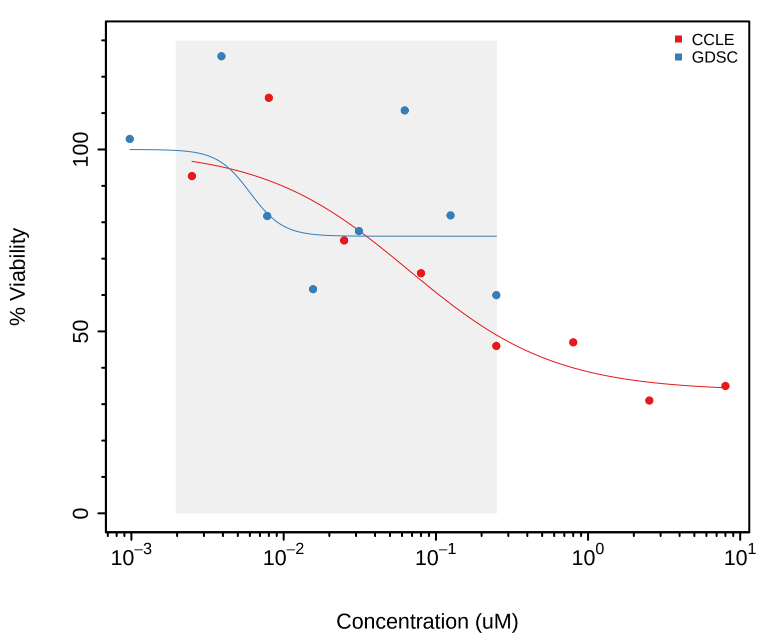

PD−0325901:SW48

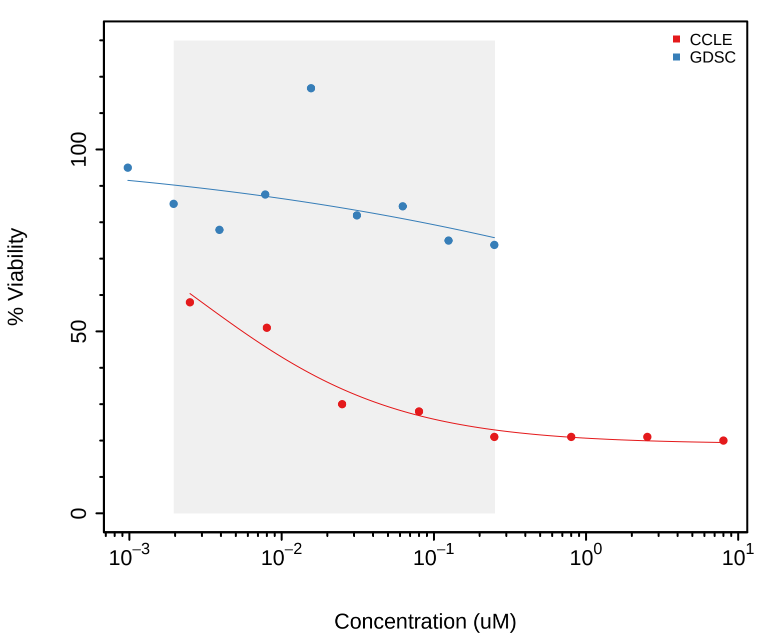

PD−0325901:SW620

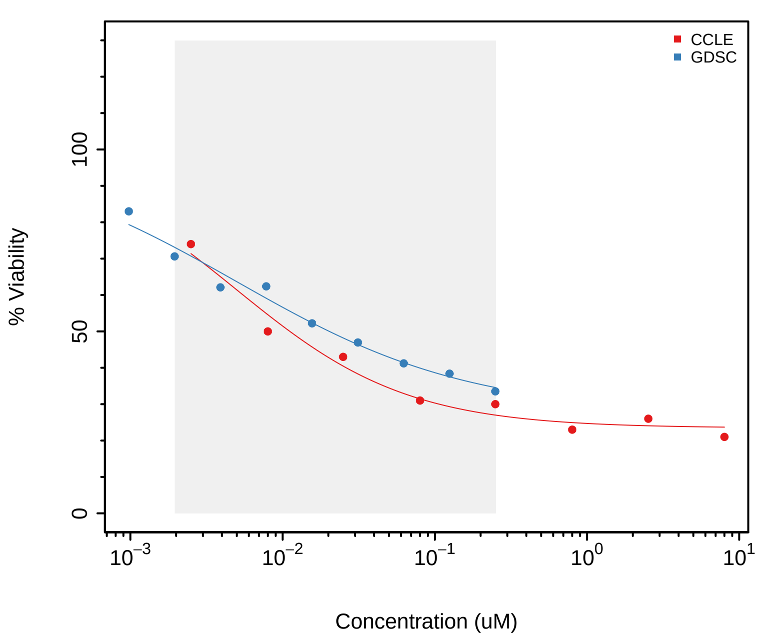

PD−0325901:SW780

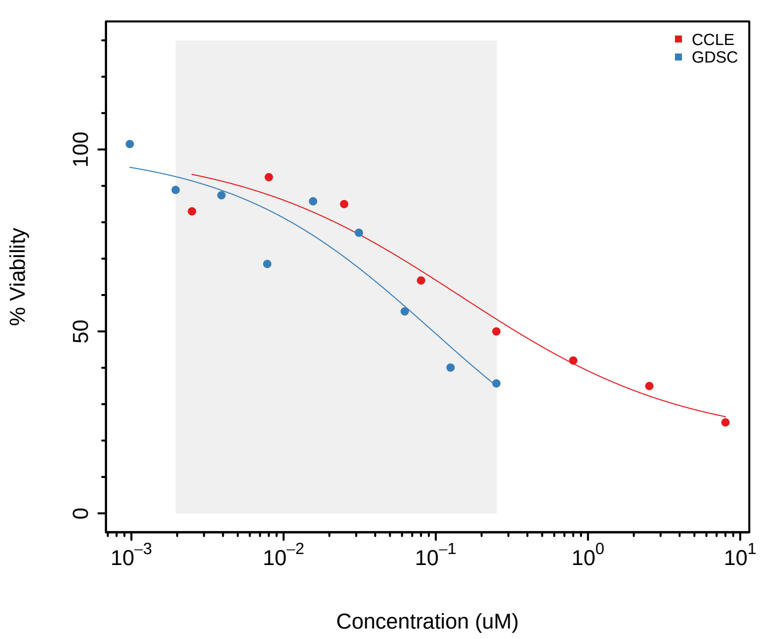

PD−0325901:SW900

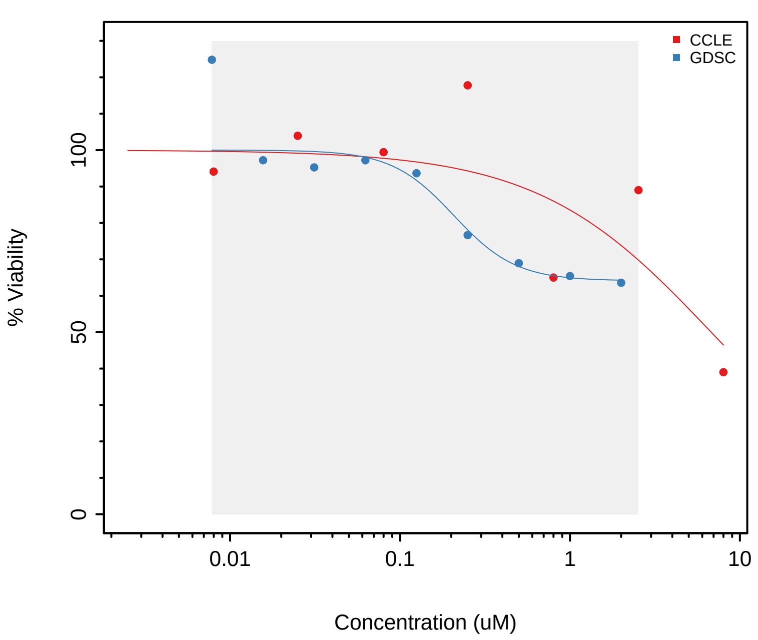

PD−0325901:T−24

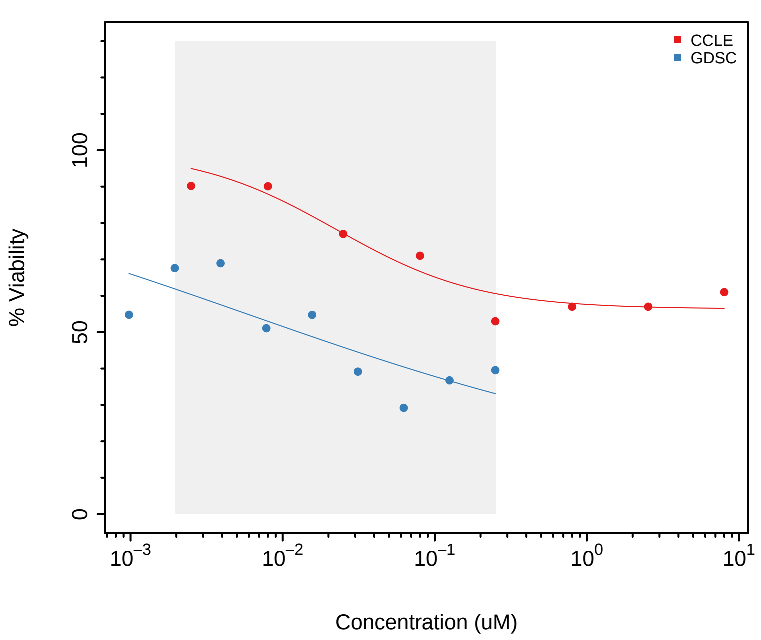

PD−0325901:T47D

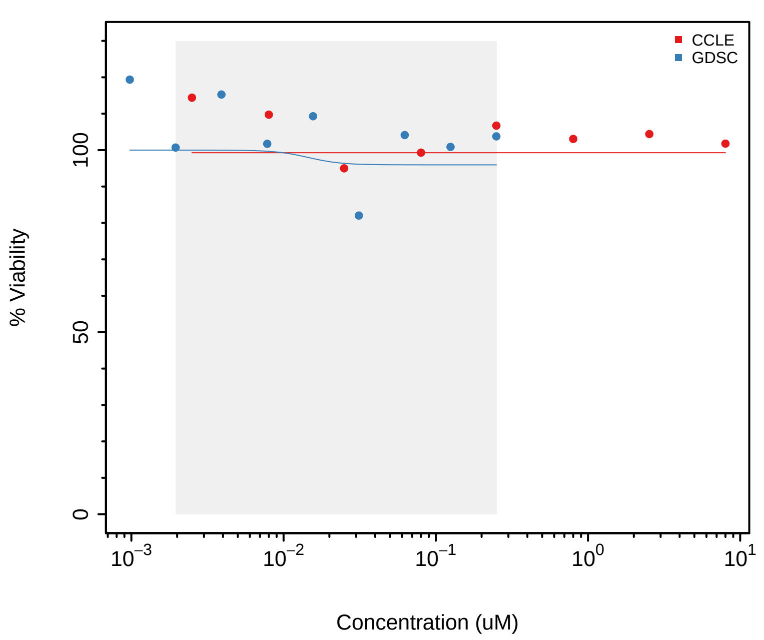

PD−0325901:T84

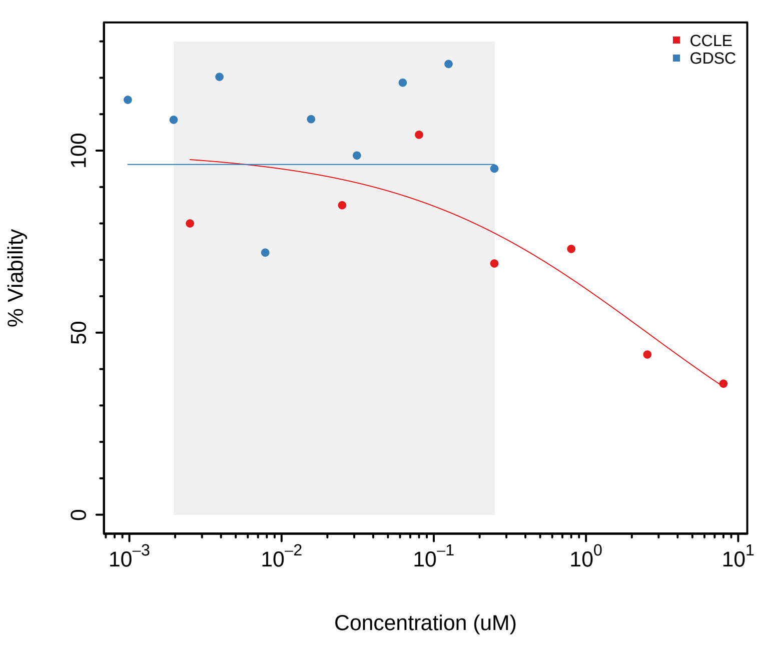

PD−0325901:T98G

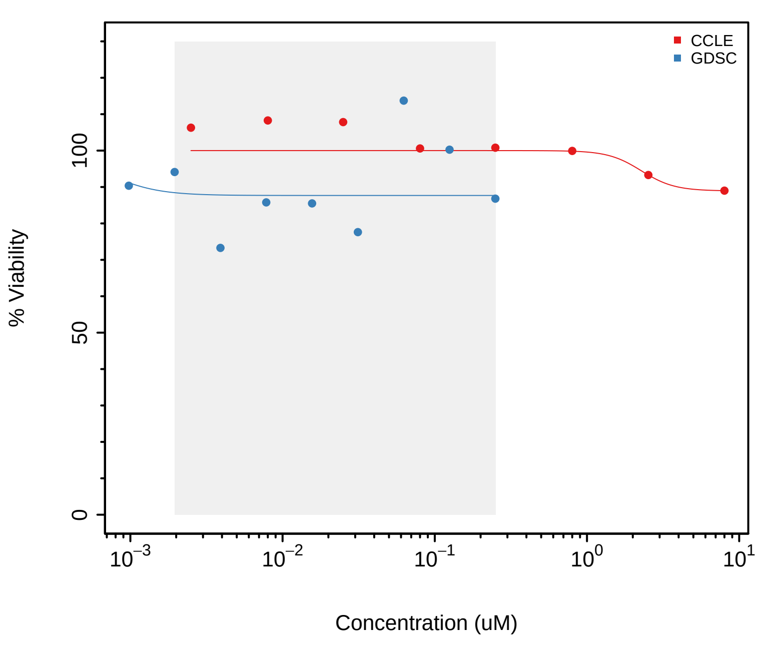

PD−0325901:TE−1

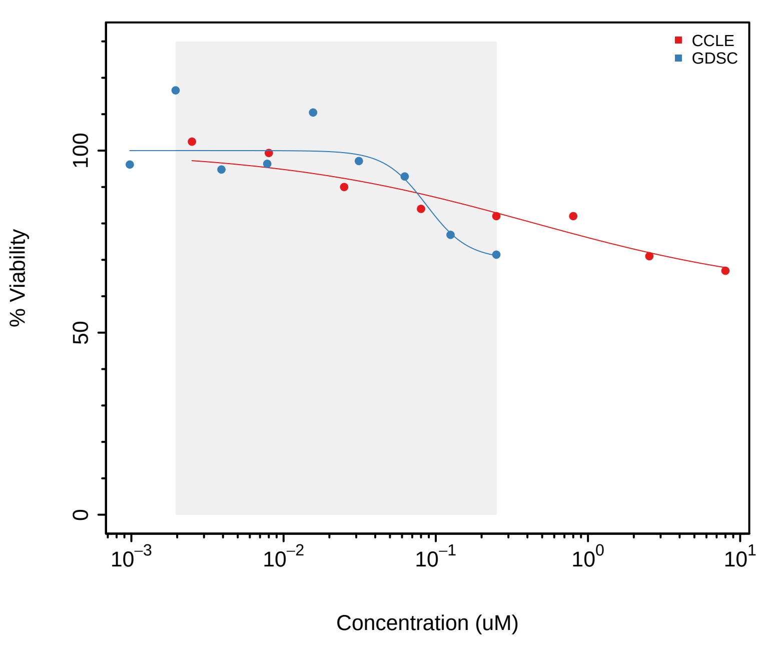

PD−0325901:TE−11

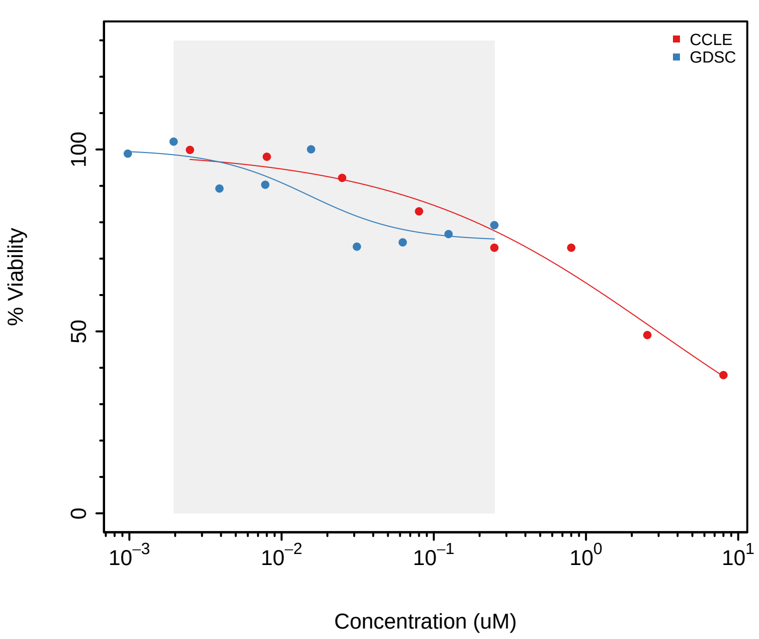

PD−0325901:TE−15

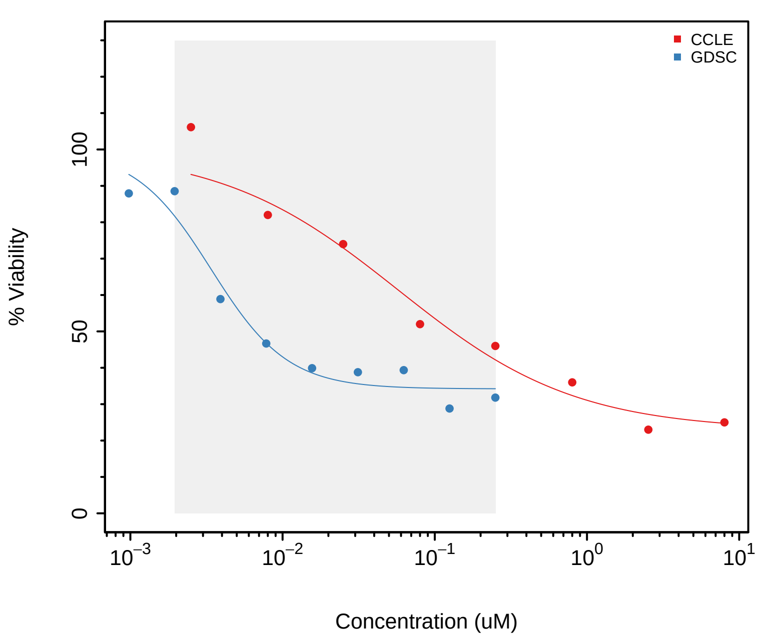

PD−0325901:TE−5

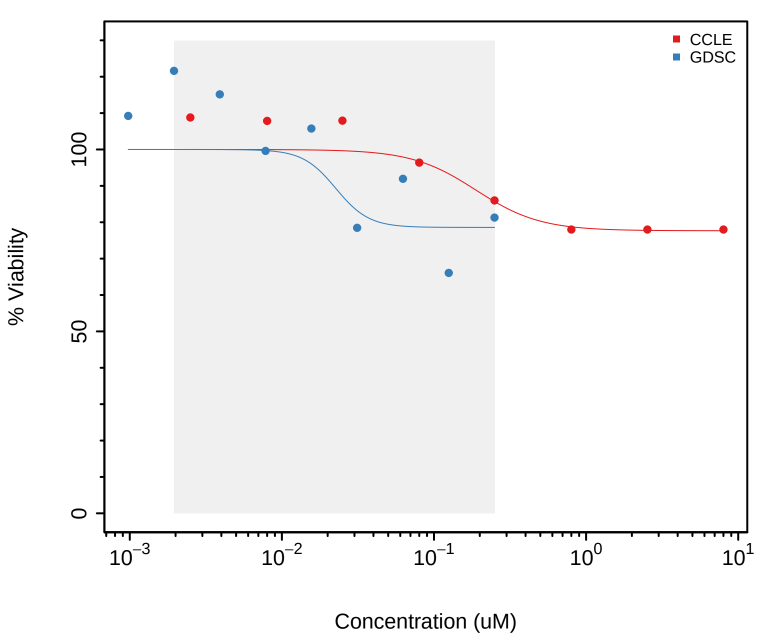

PD−0325901:TE−9

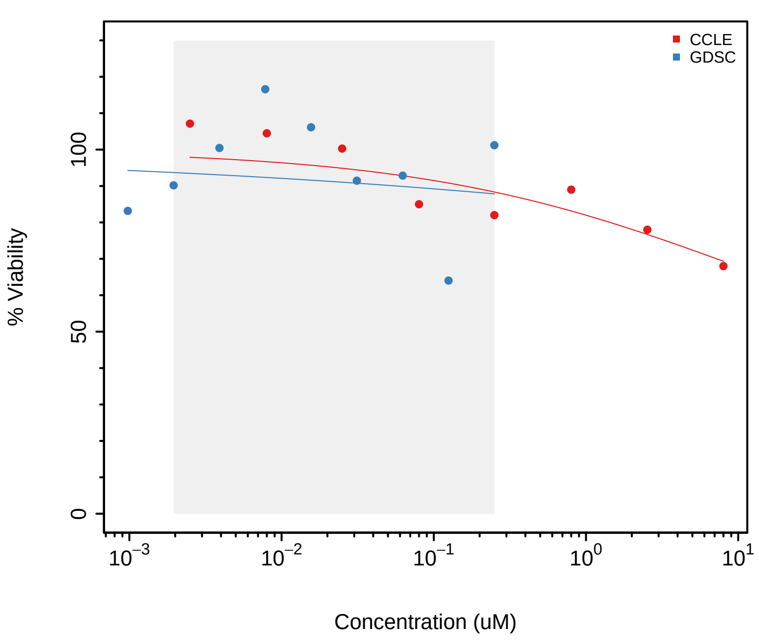

PD−0325901:TYK−nu

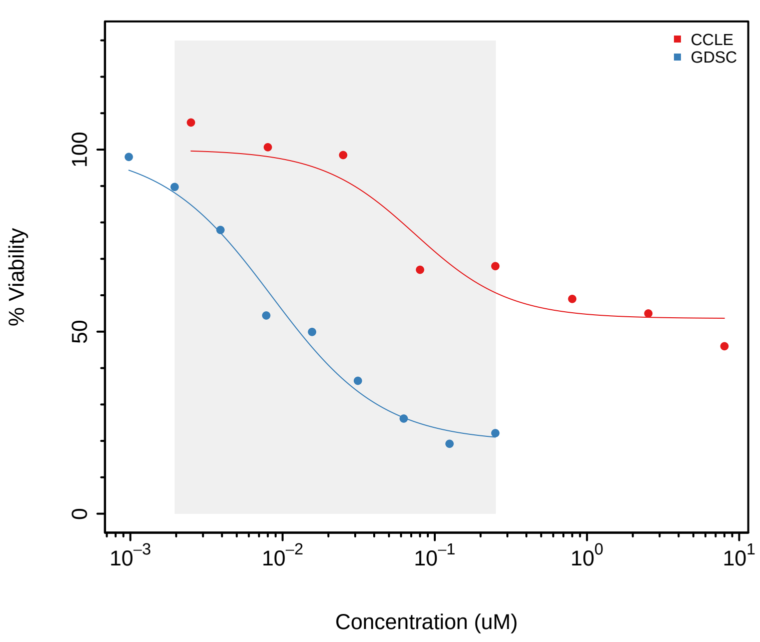

PD−0325901:U−118−MG

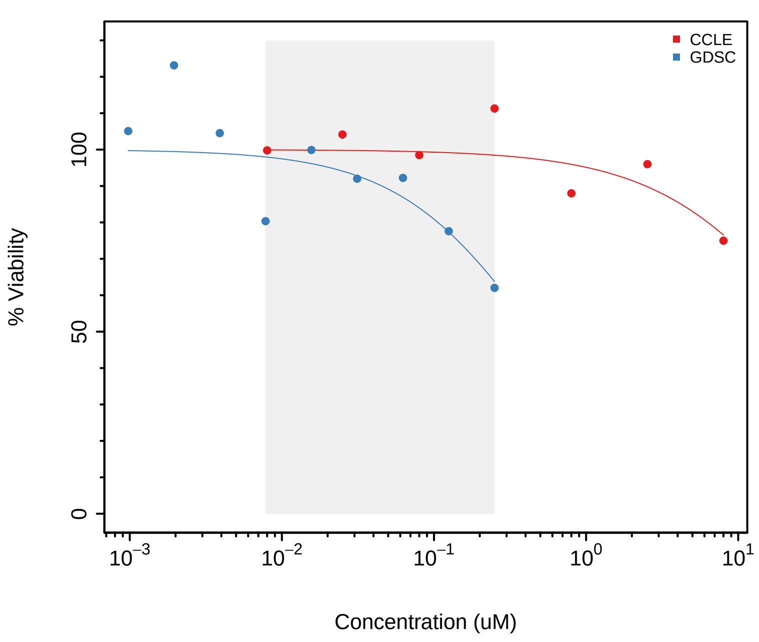

PD−0325901:U−2−OS

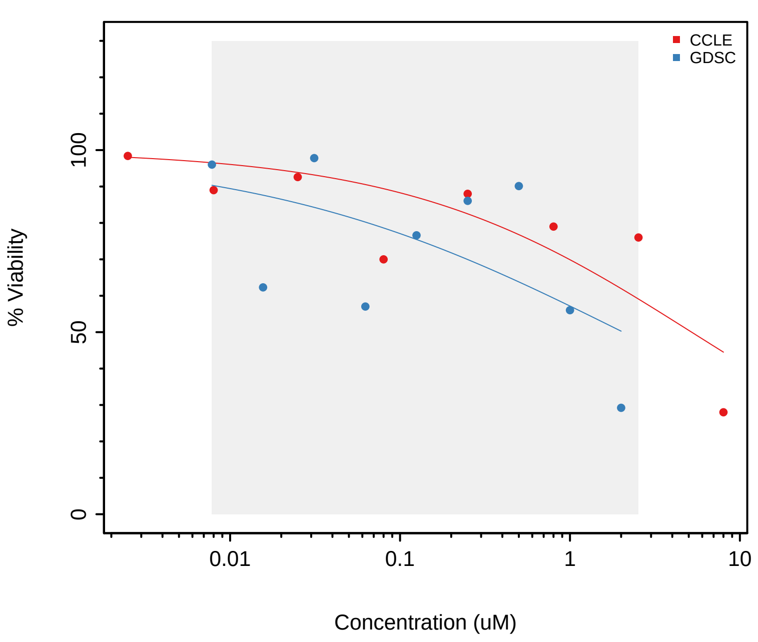

PD−0325901:U−87−MG

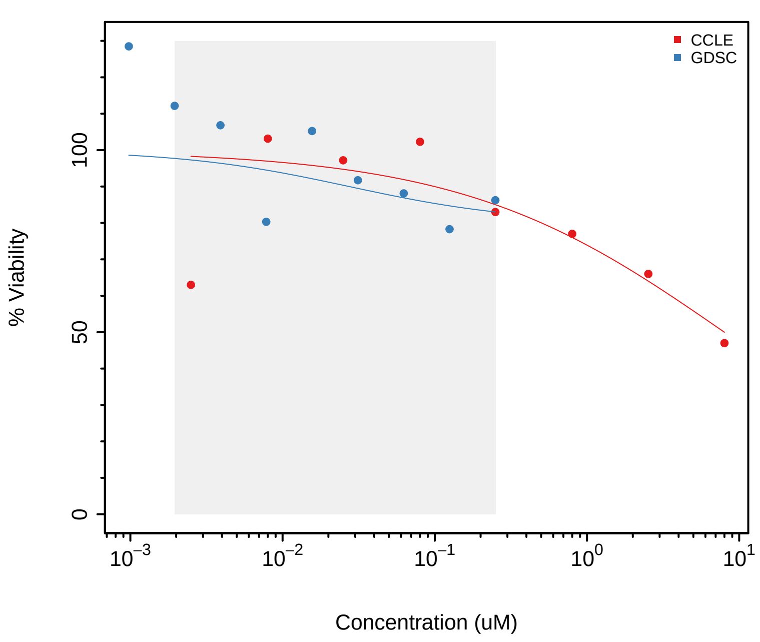

PD−0325901:UACC−257

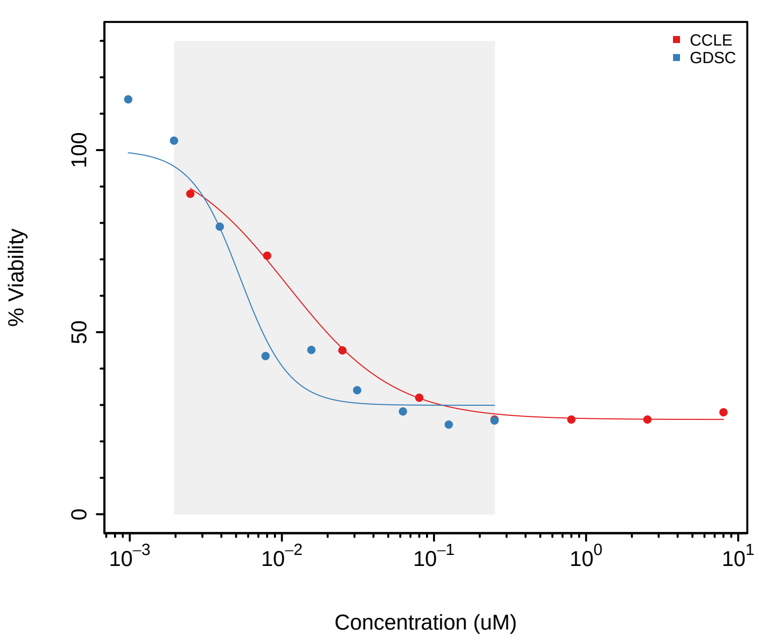

PD−0325901:UACC−62

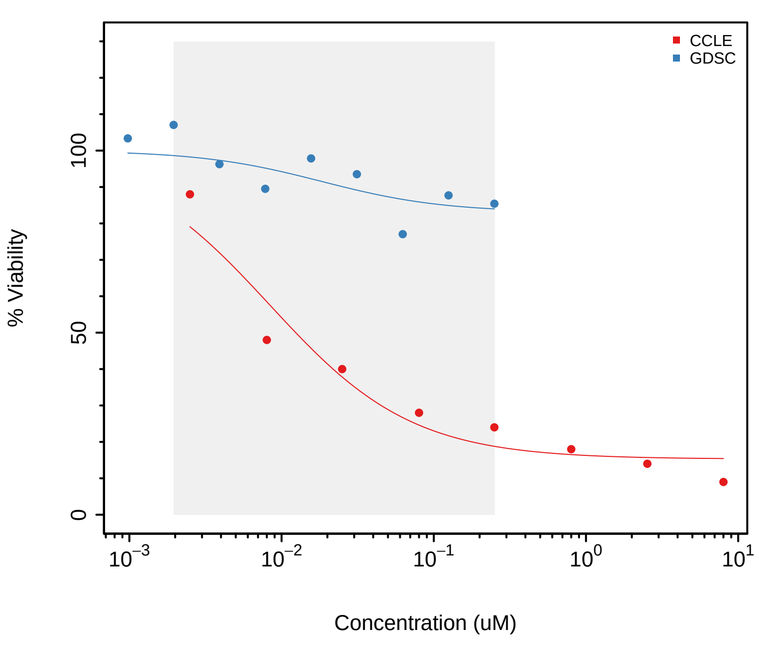

PD−0325901:UM−UC−3

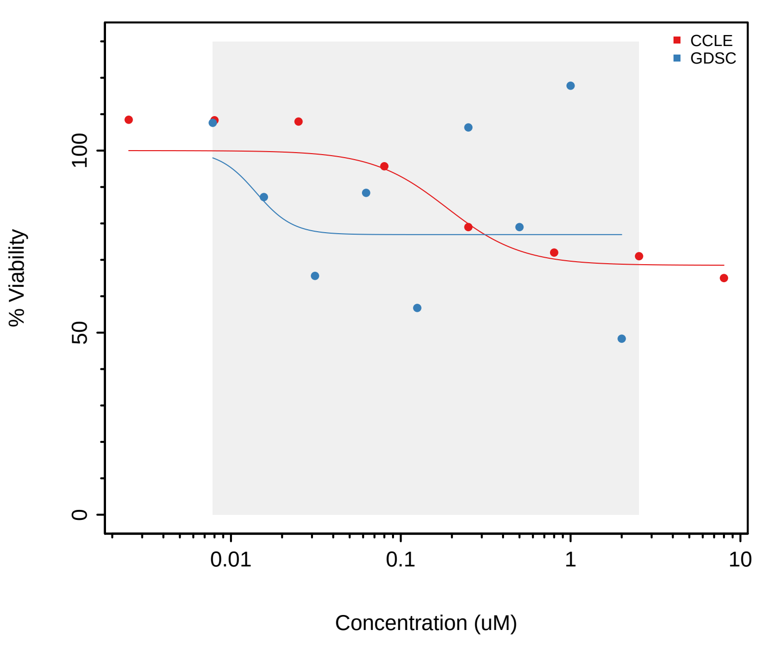

PD−0325901:VMRC−RCZ

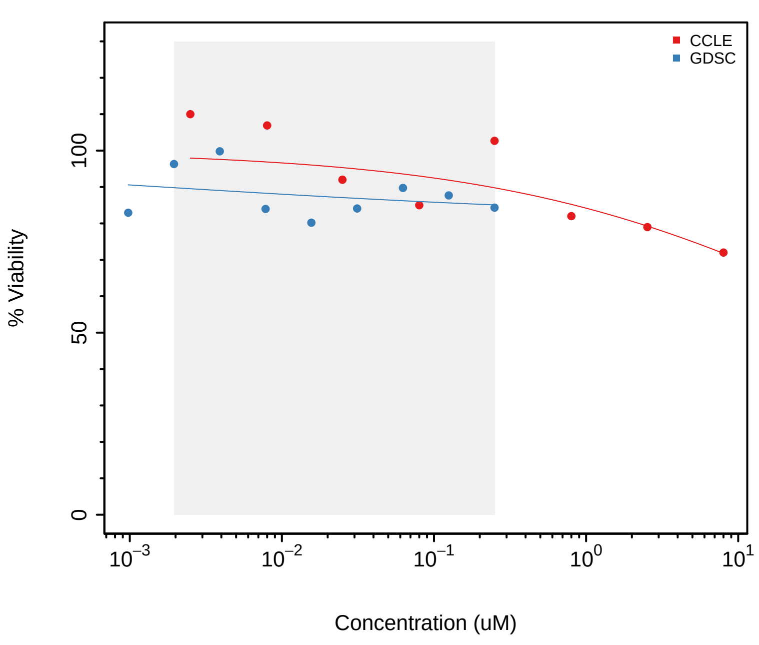

PD−0325901:WM−115

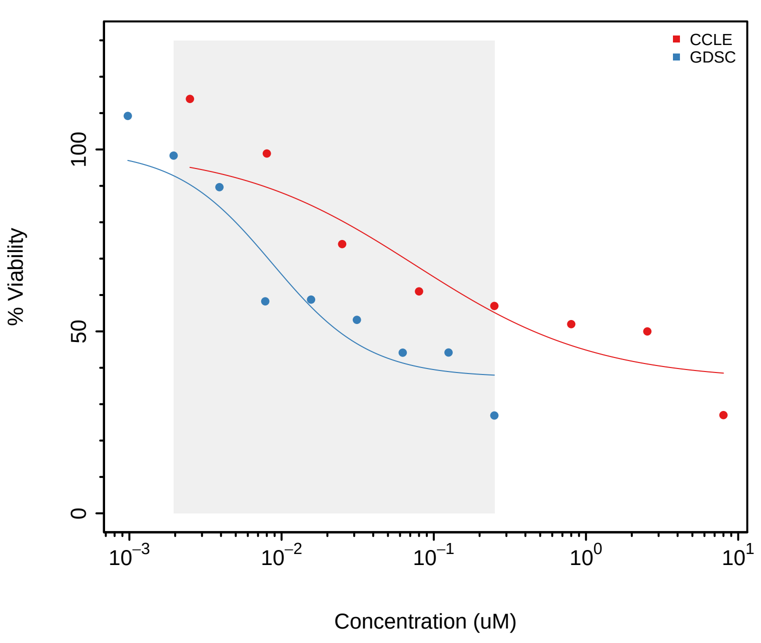

PD−0325901:YKG−1

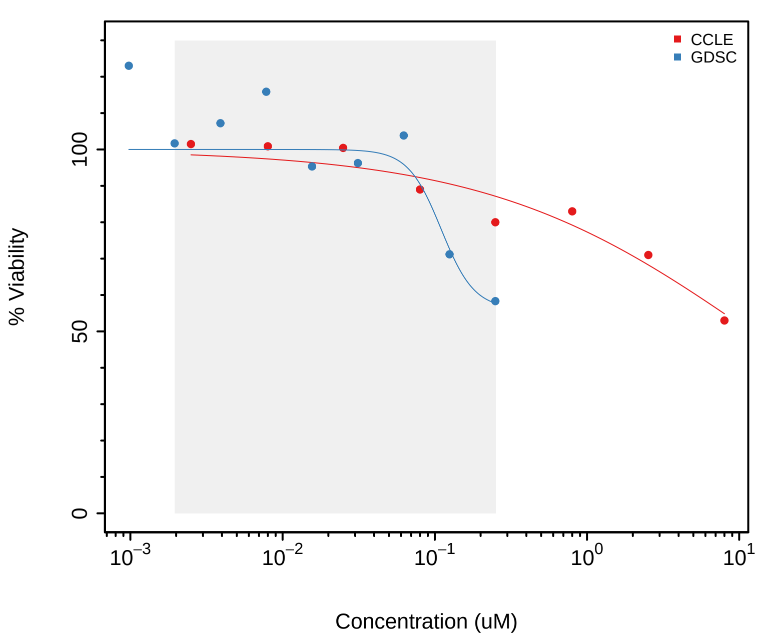

PD−0325901:ZR−75−30

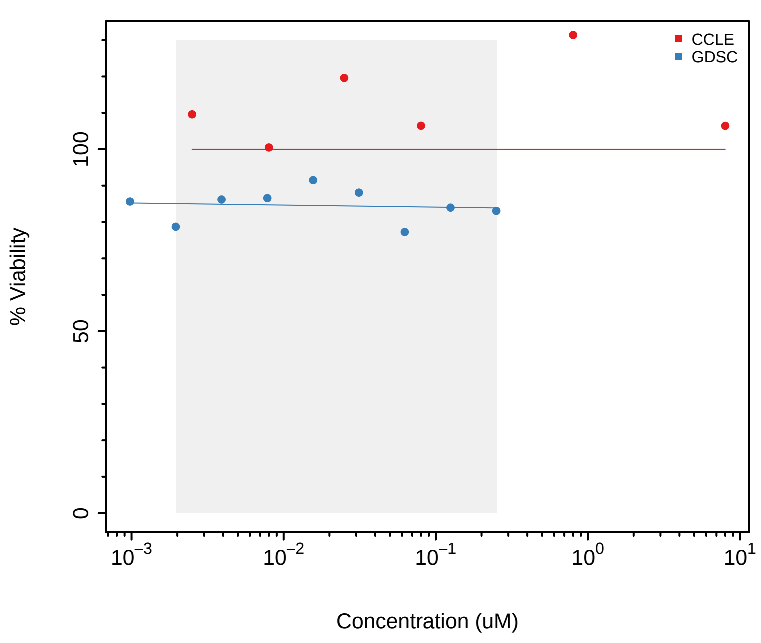

PD−0332991:22RV1

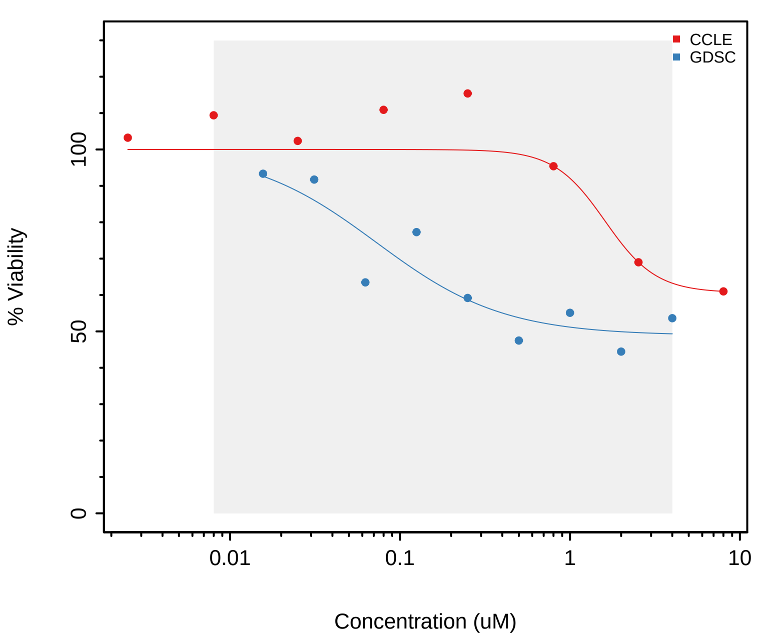

PD−0332991:5637

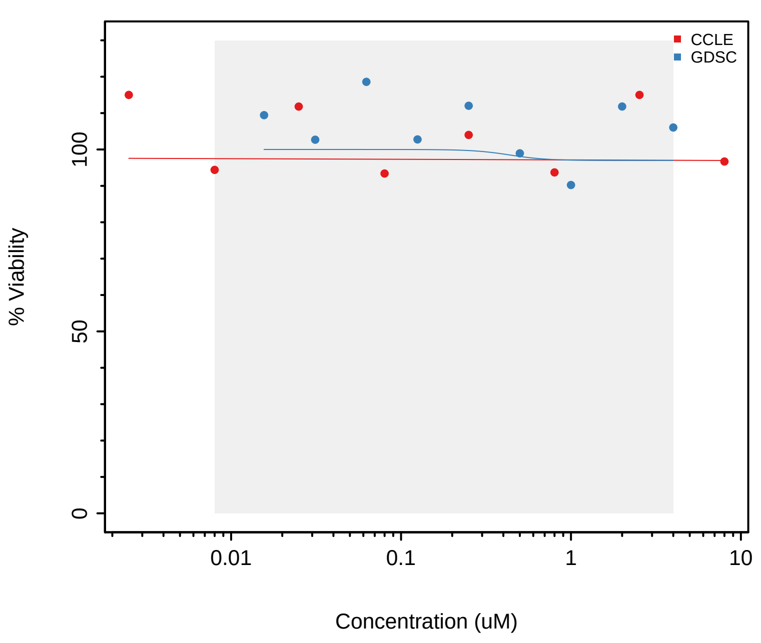

PD−0332991:639−V

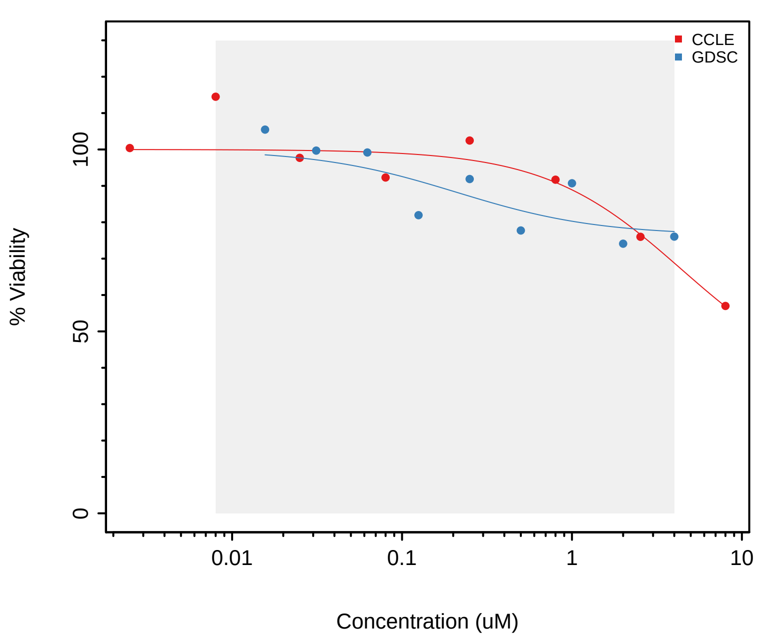

PD−0332991:697

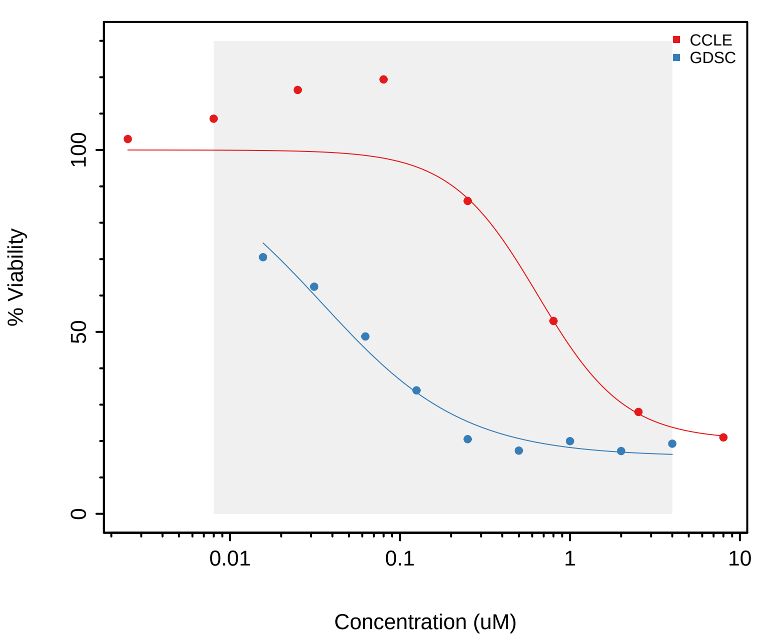

PD−0332991:786−0

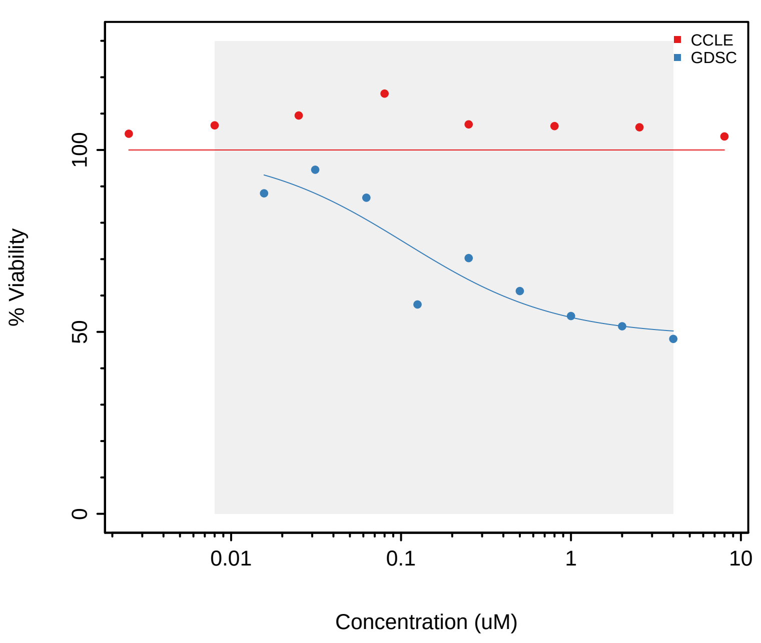

PD−0332991:8305C

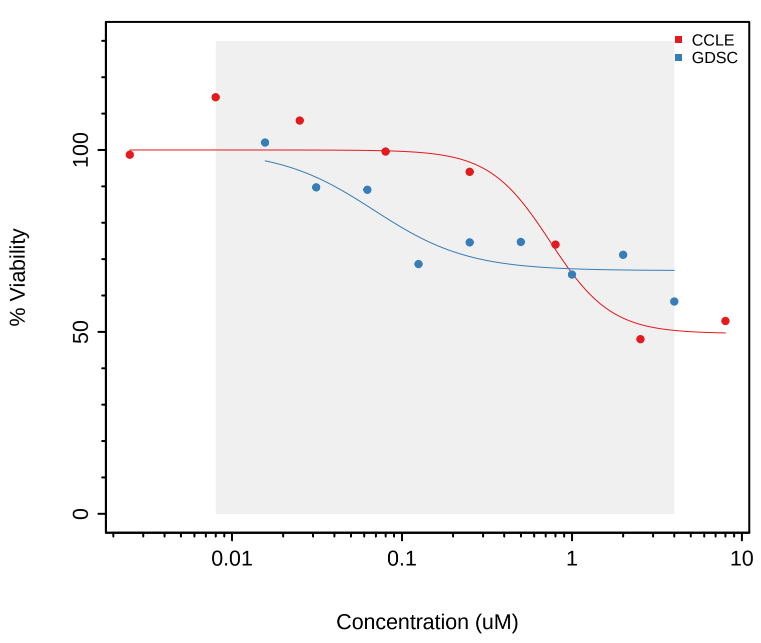

PD−0332991:8505C

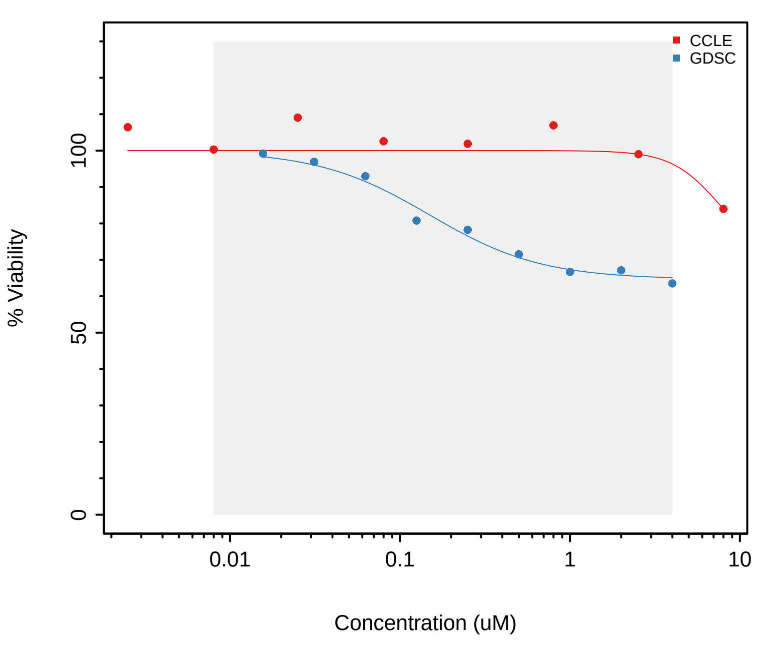

PD−0332991:A204

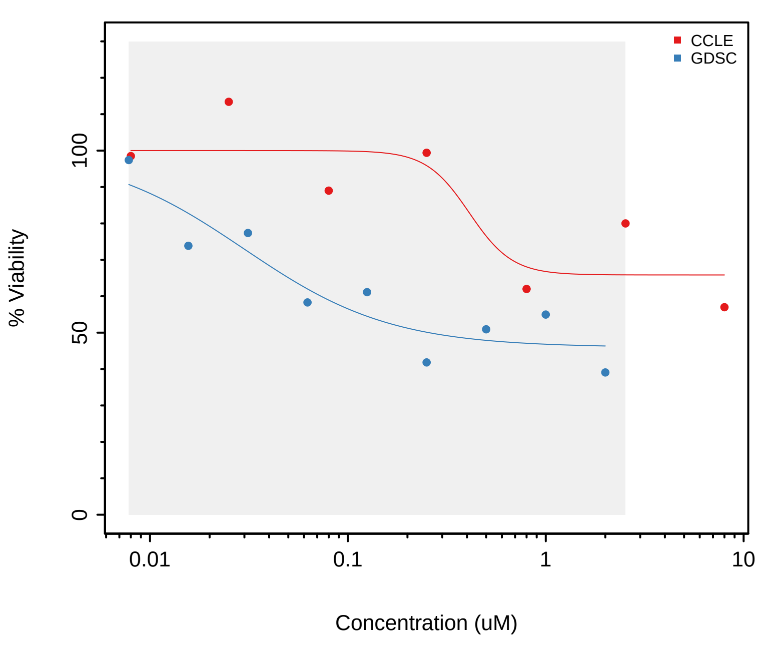

PD−0332991:A253

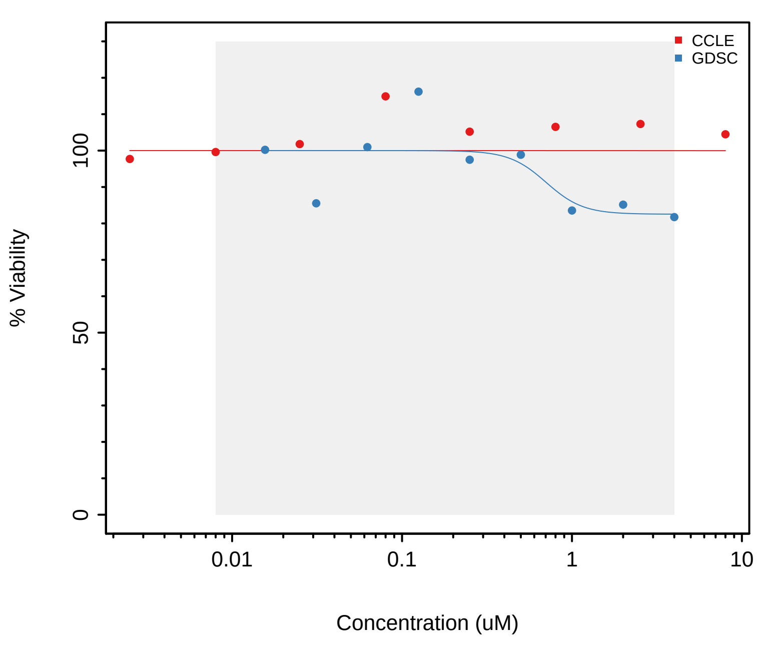

PD−0332991:A2780

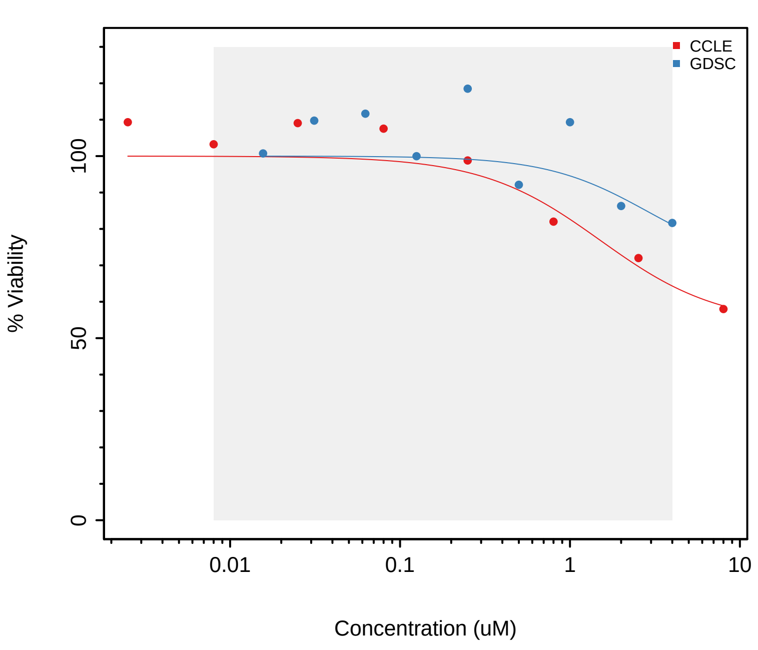

PD−0332991:A375

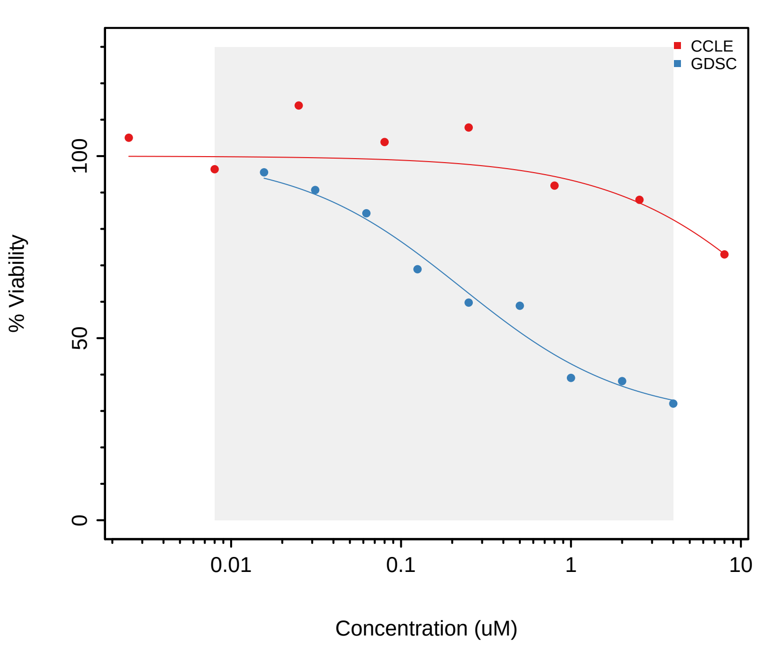

PD−0332991:A549

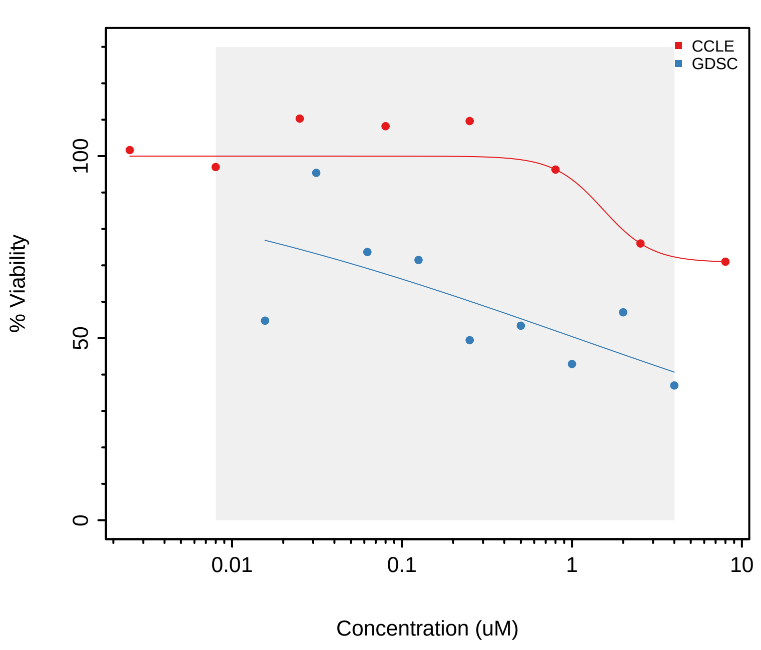

PD−0332991:A673

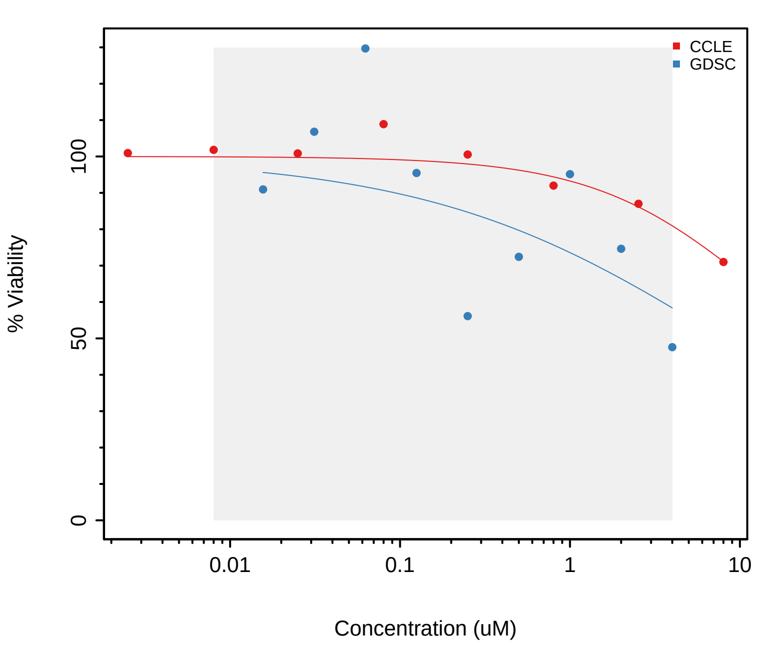

PD−0332991:AsPC−1

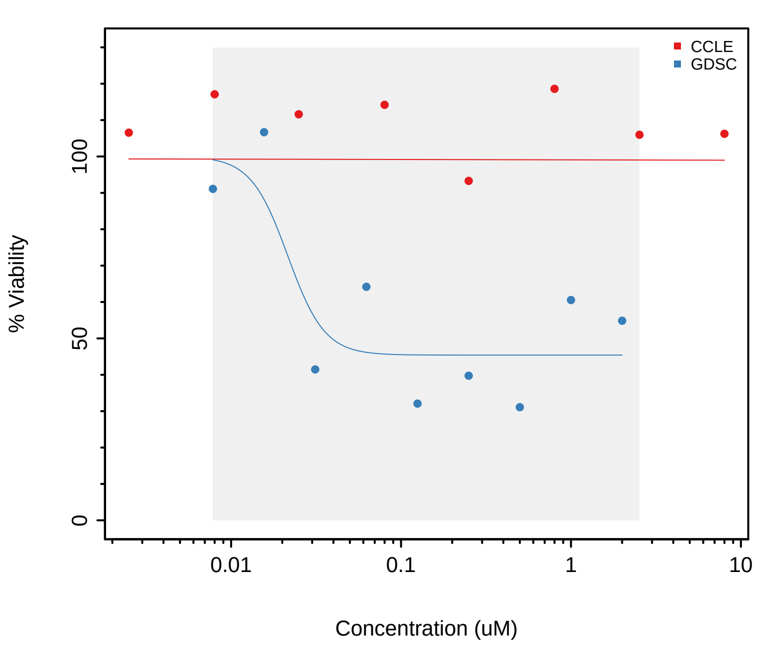

PD−0332991:AU565

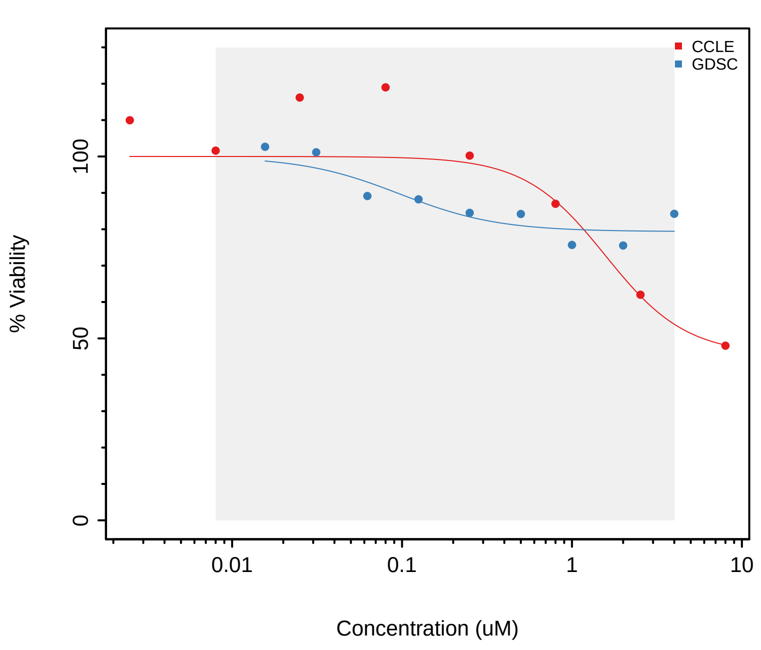

PD−0332991:BCPAP

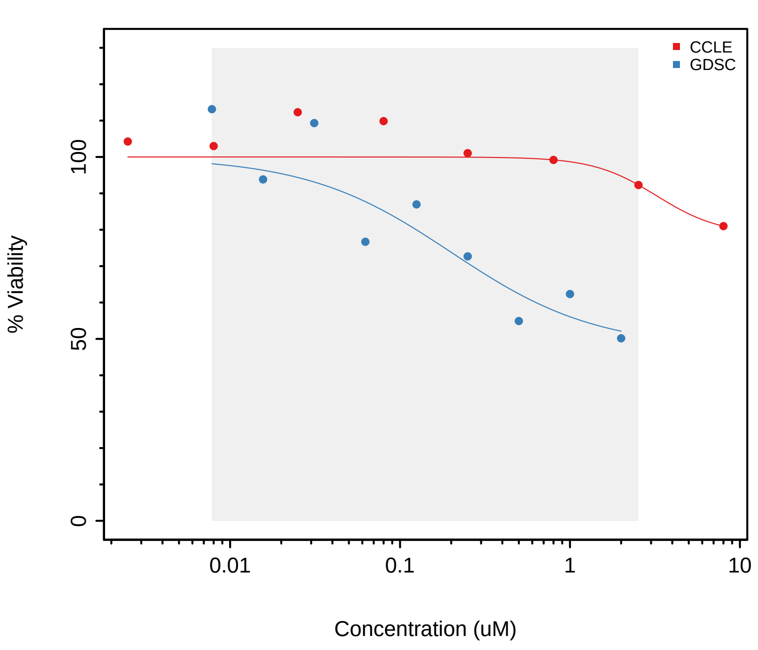

PD−0332991:BFTC−909

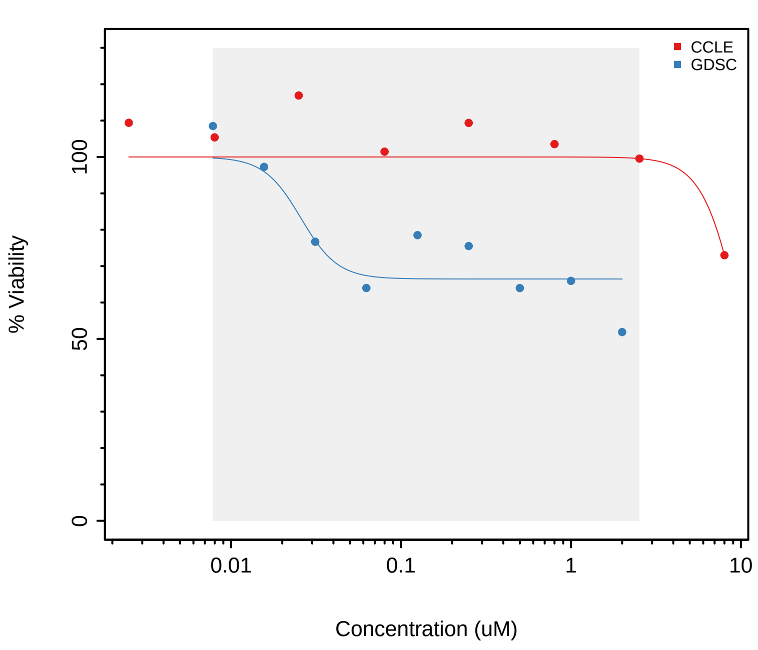

PD−0332991:BL−41

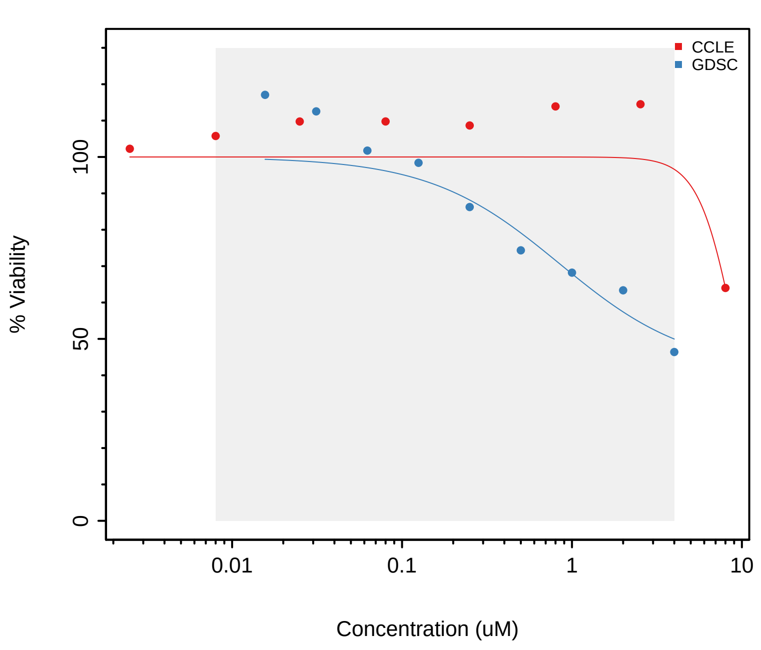

PD−0332991:BT−20

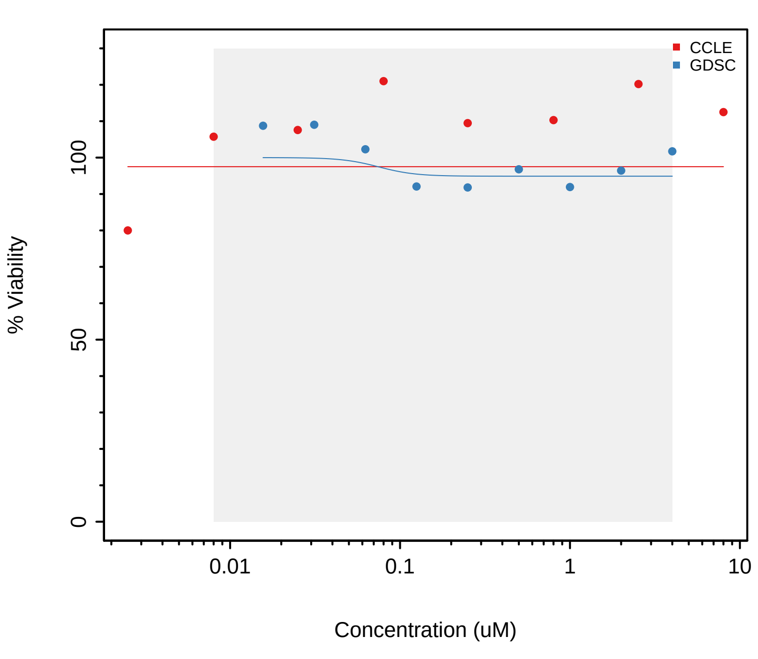

PD−0332991:BT−474

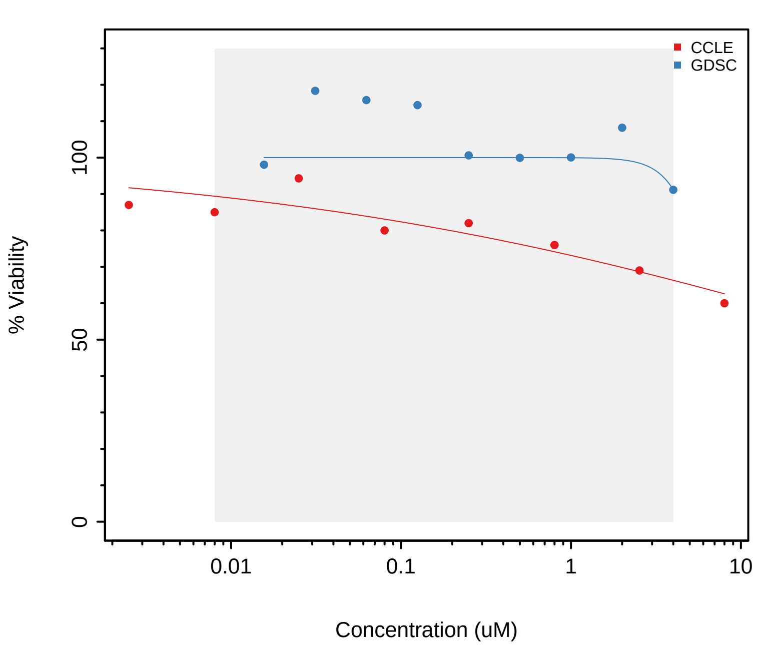

PD−0332991:BT−549

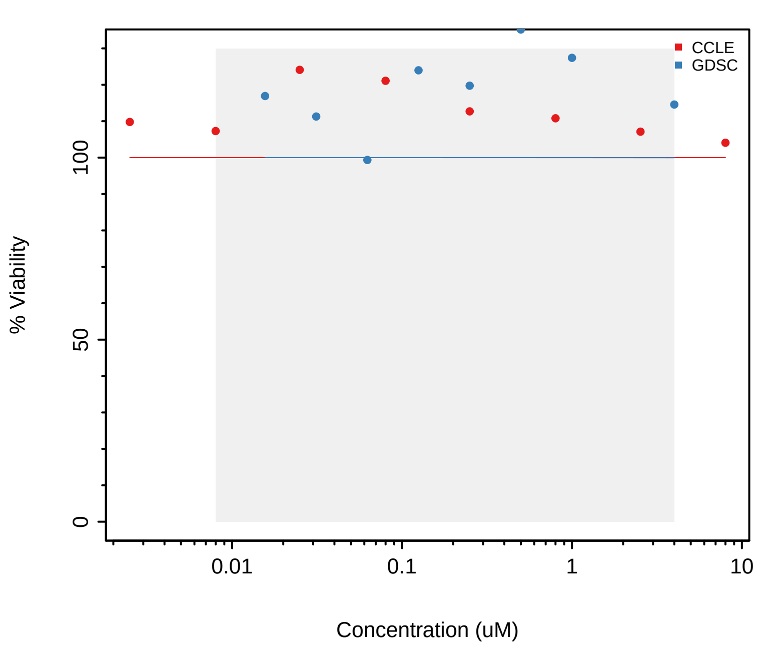

PD−0332991:C2BBe1

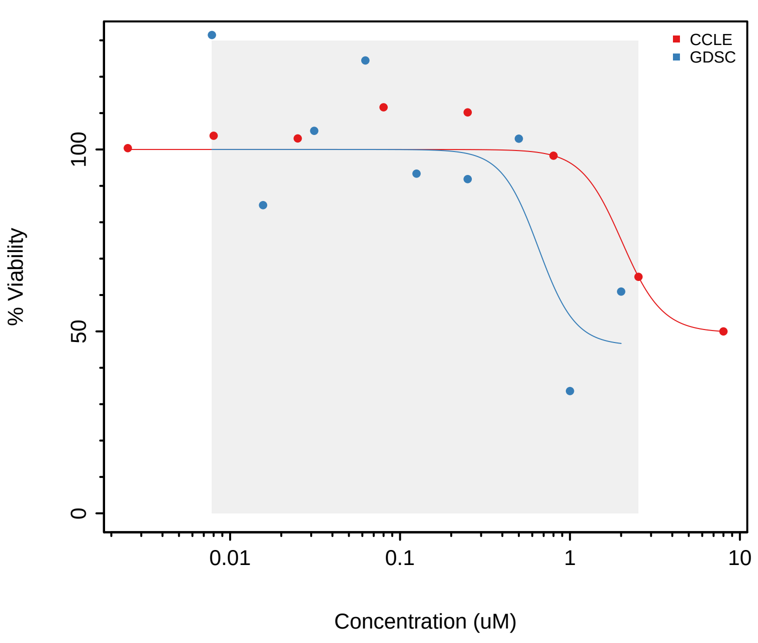

PD−0332991:C32

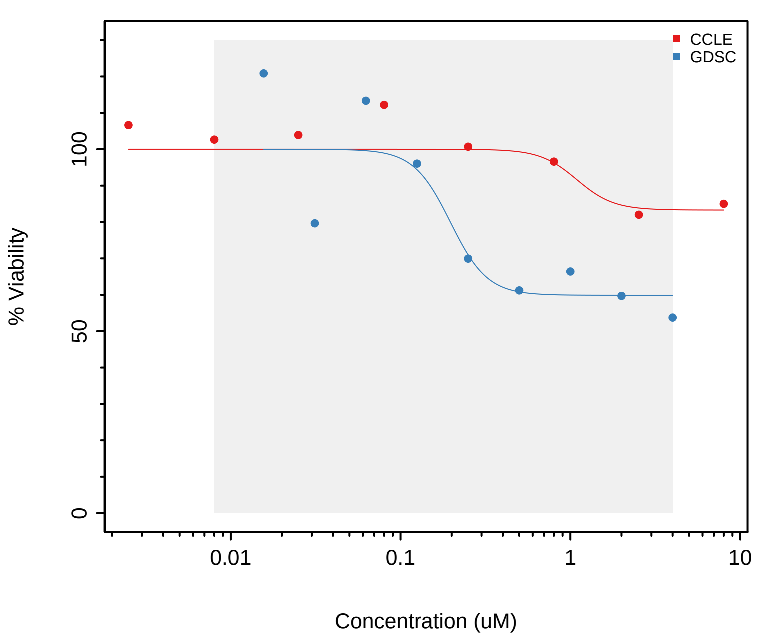

PD−0332991:C3A

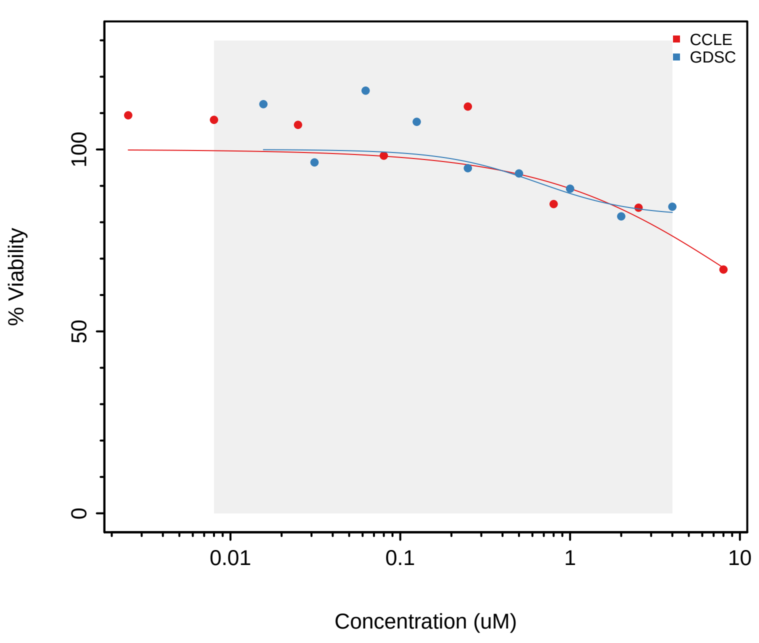

PD−0332991:CAL−27

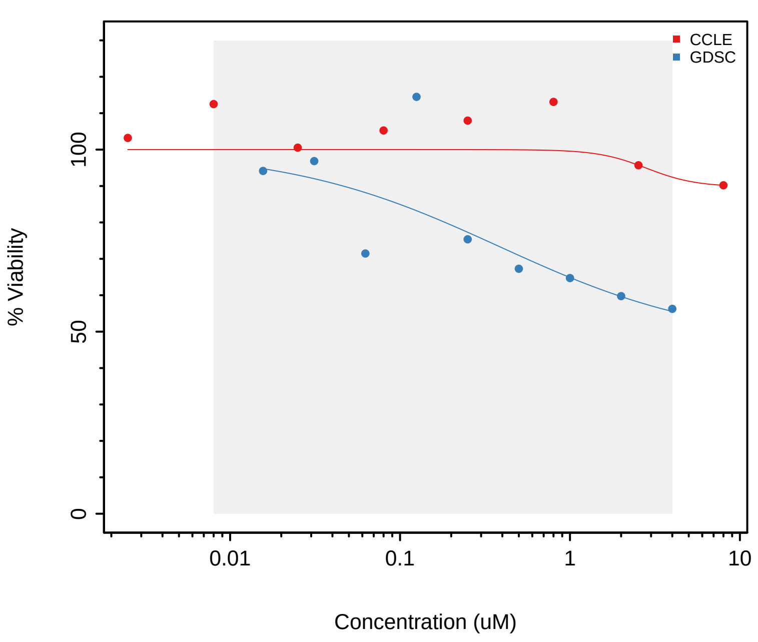

PD−0332991:CAL−85−1

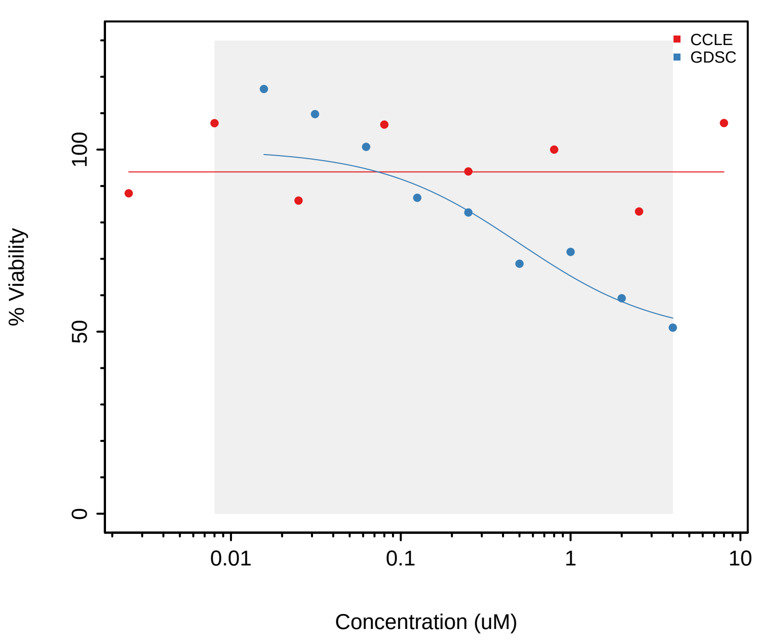

PD−0332991:Calu−3

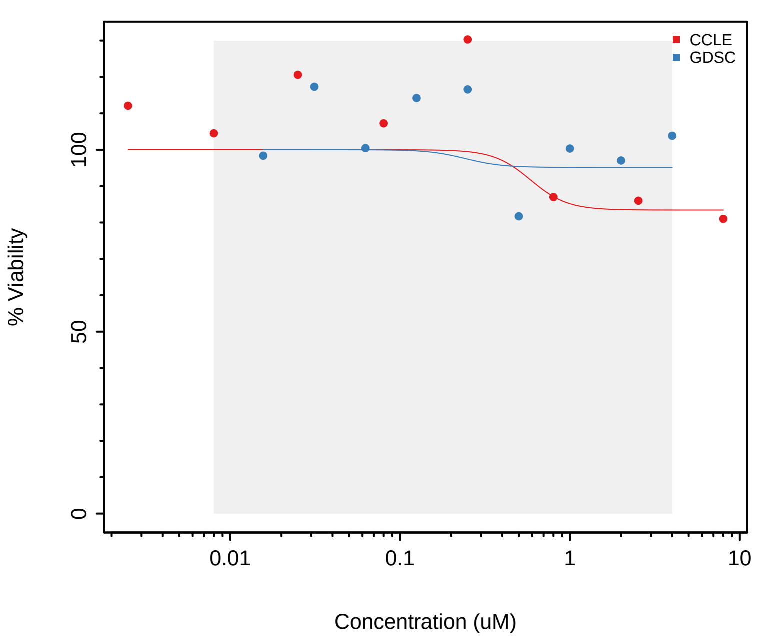

PD−0332991:CAMA−1

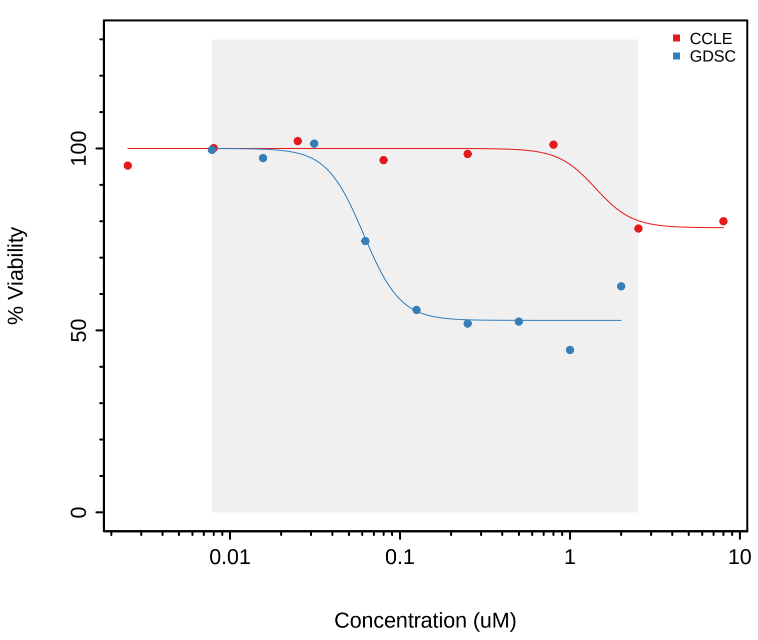

PD−0332991:Capan−2

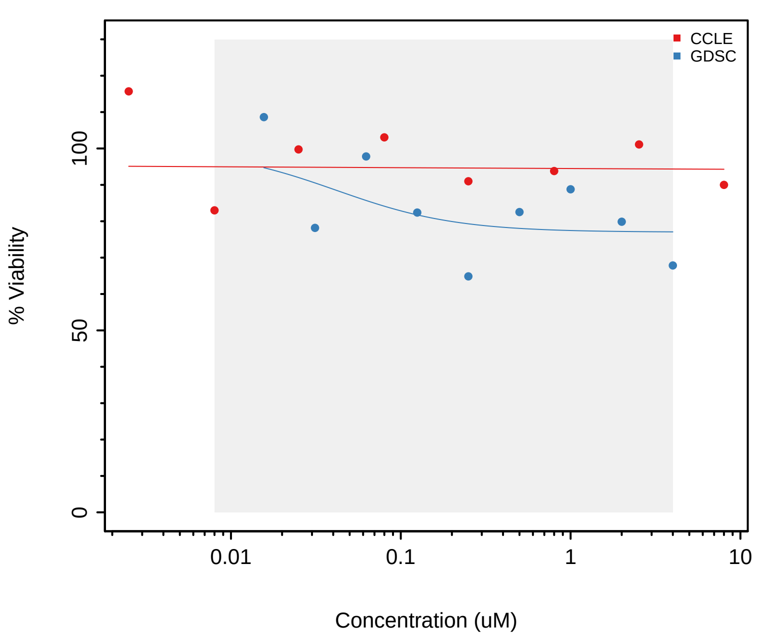

PD−0332991:CAS−1

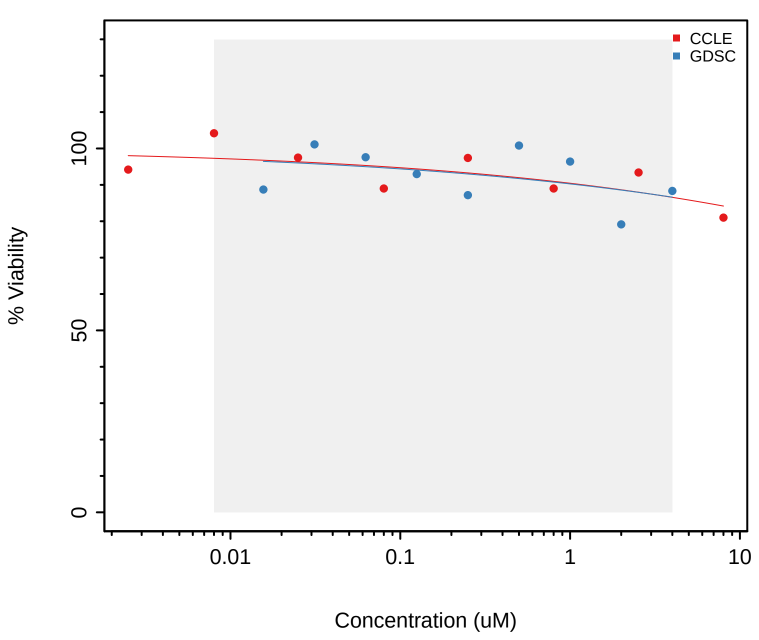

PD−0332991:CHL−1

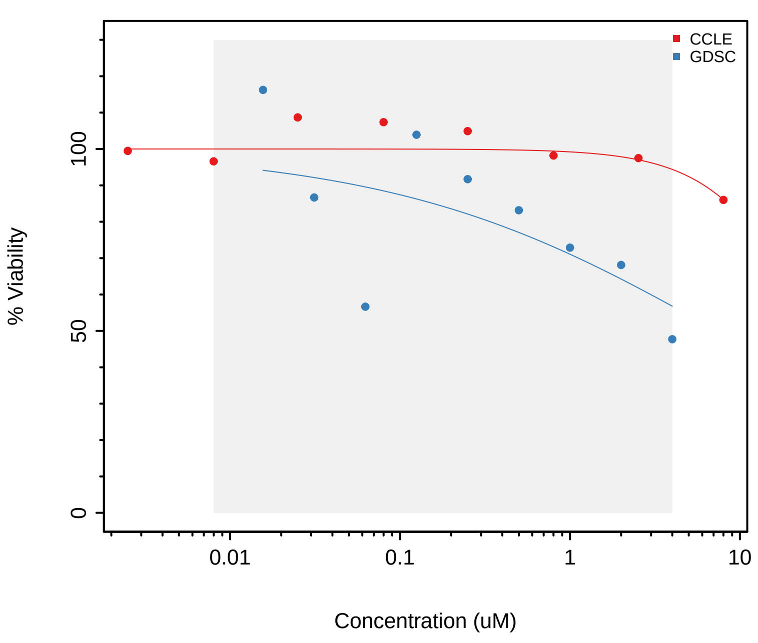

PD−0332991:COLO−205

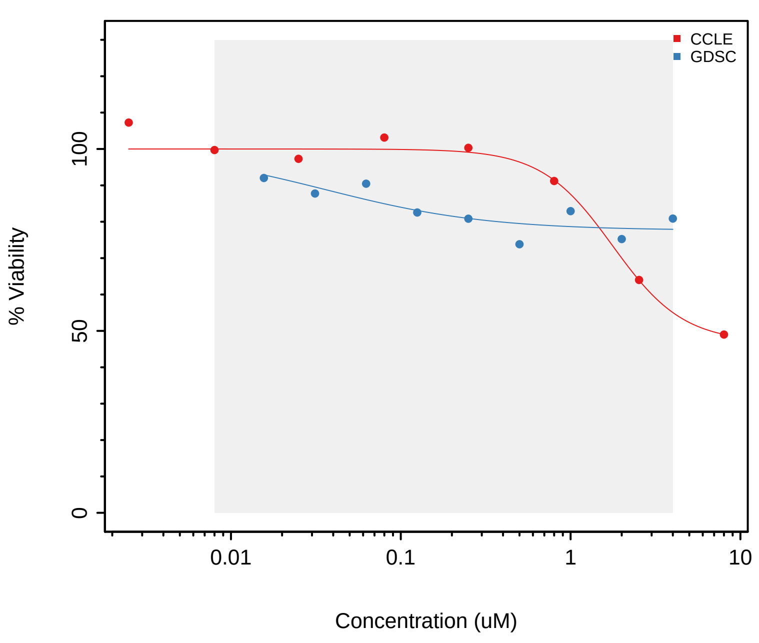

PD−0332991:COLO−320−HSR

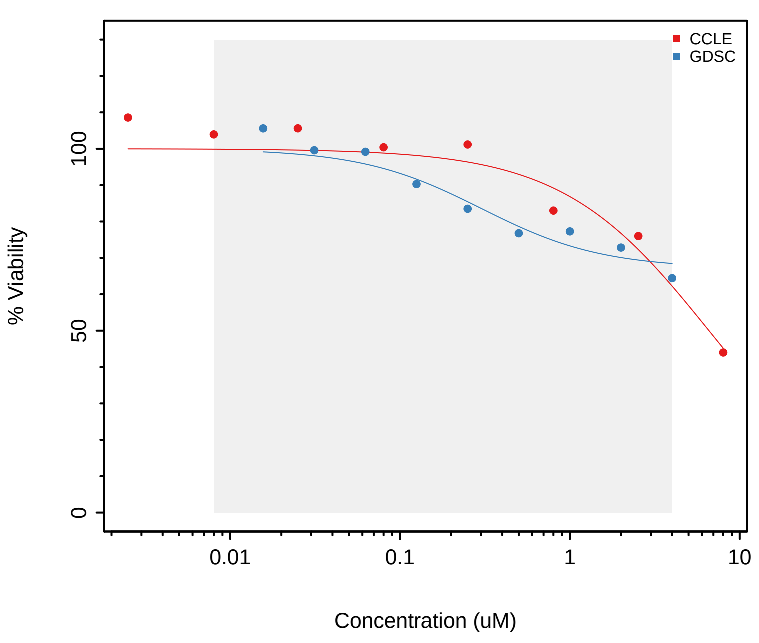

PD−0332991:COLO−678

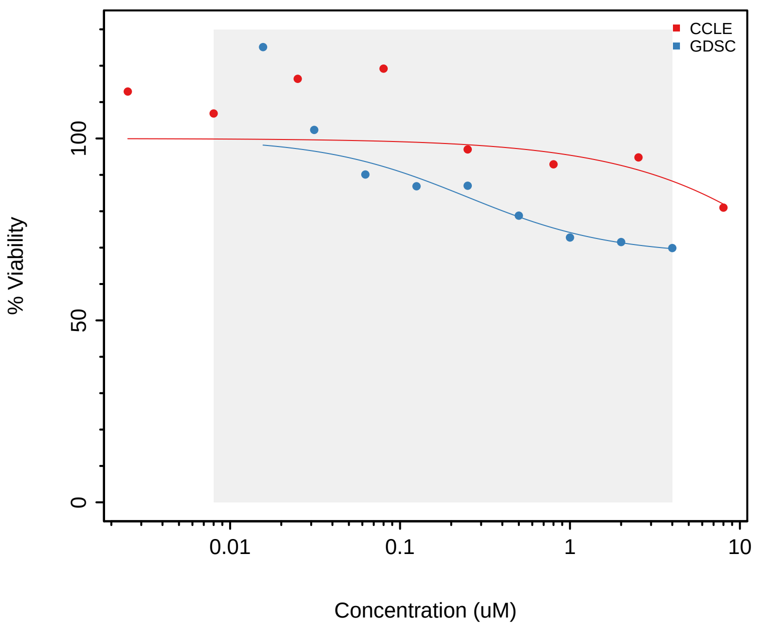

PD−0332991:COLO−679

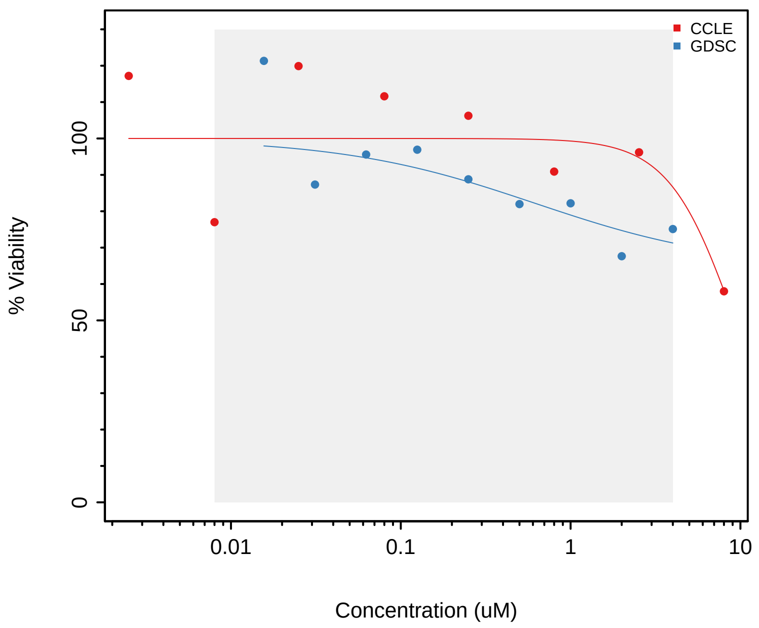

PD−0332991:COR−L23

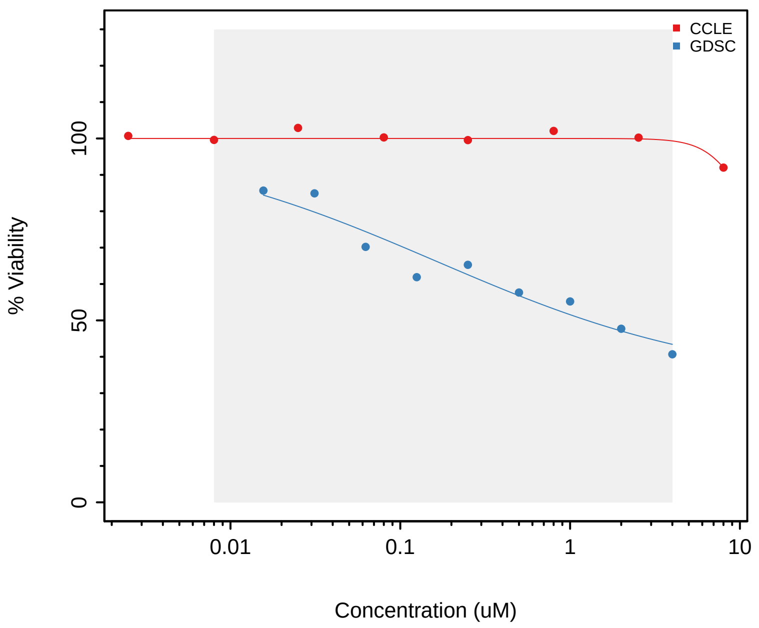

PD−0332991:DBTRG−05MG

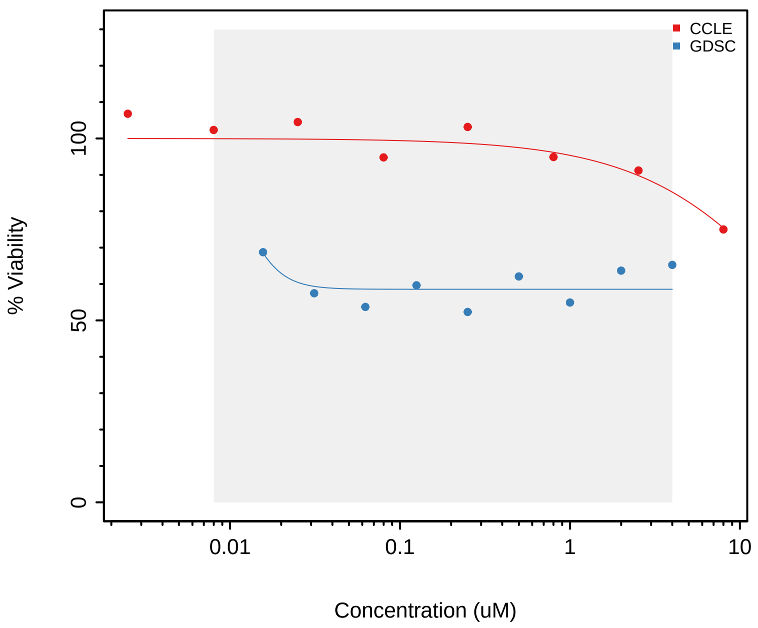

PD−0332991:DEL

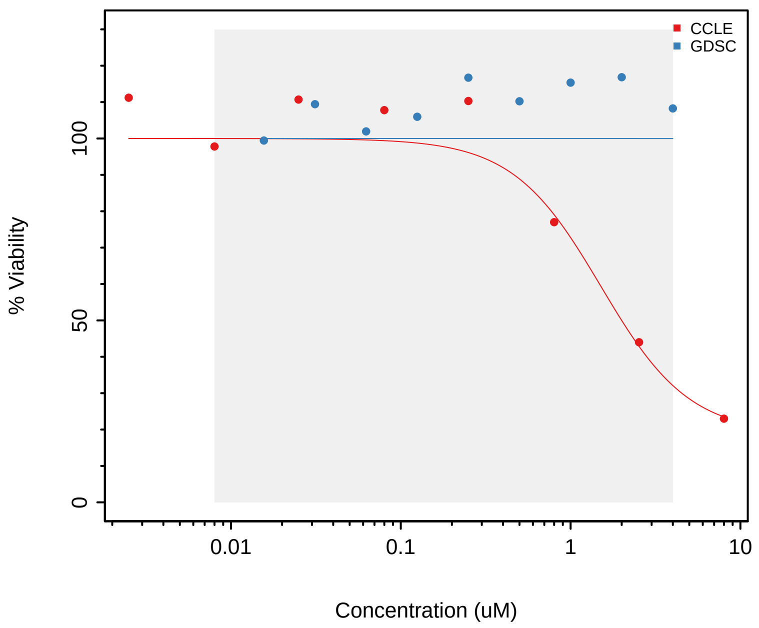

PD−0332991:Detroit562

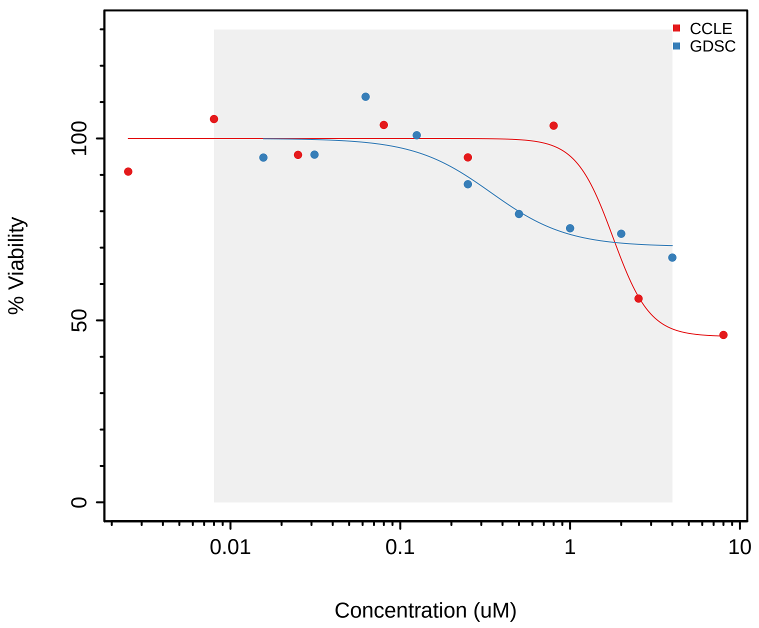

PD−0332991:DK−MG

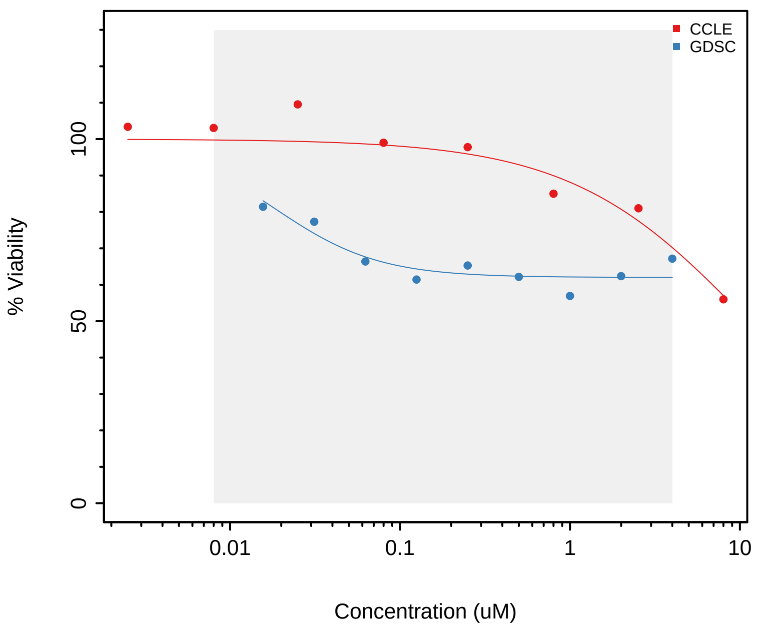

PD−0332991:DMS−114

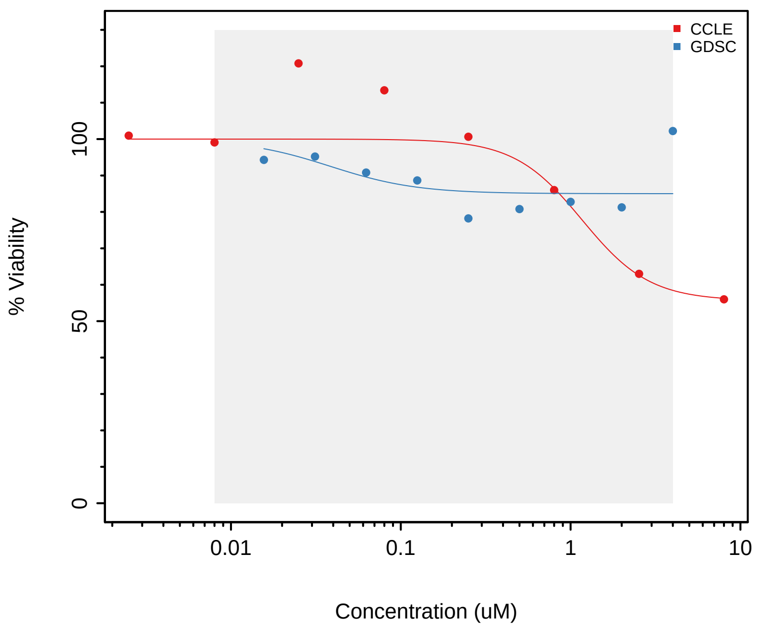

PD−0332991:DOHH−2

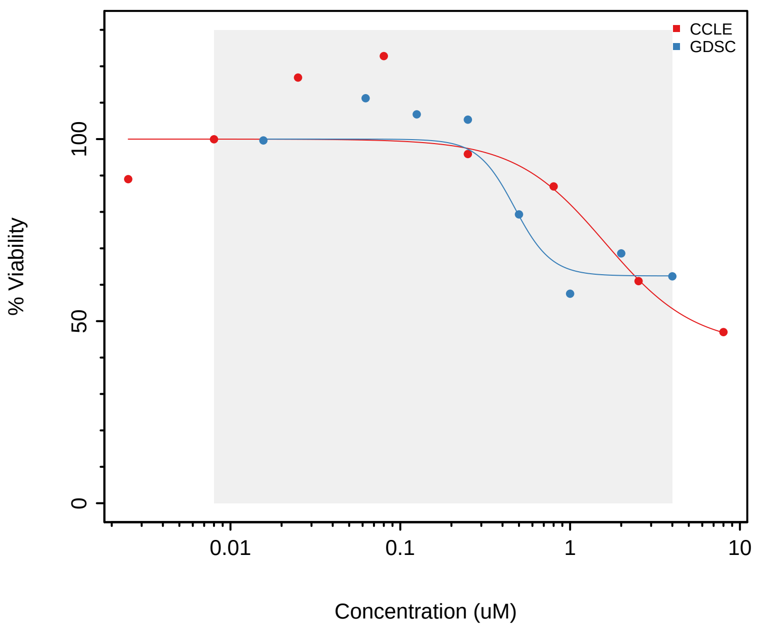

PD−0332991:DU−145

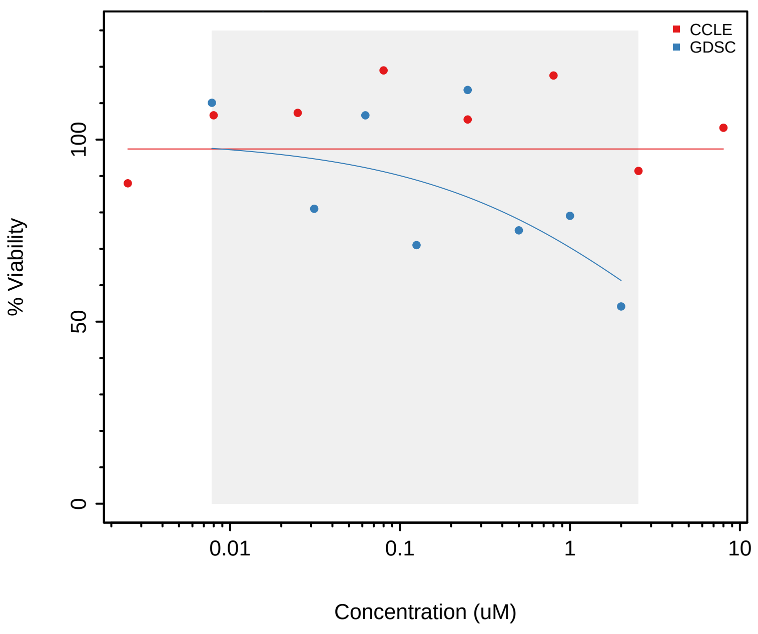

PD−0332991:EB2

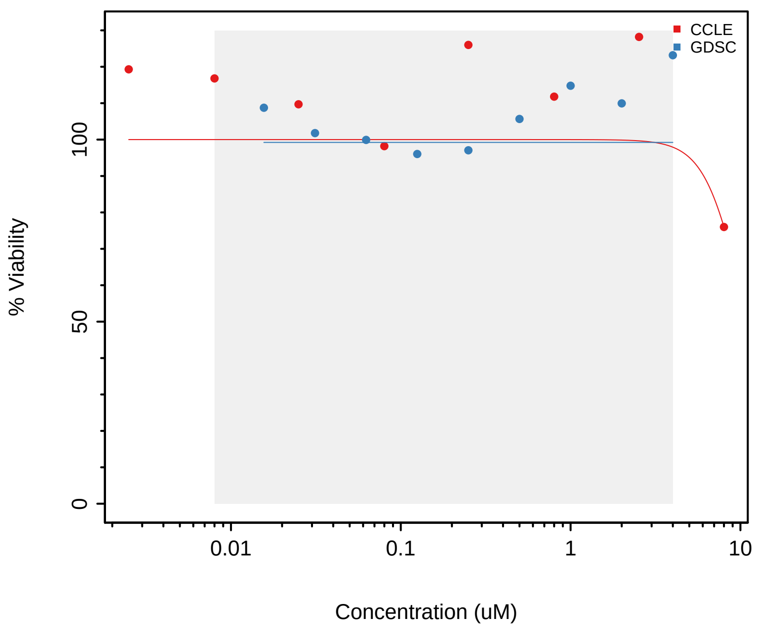

PD−0332991:EFE−184

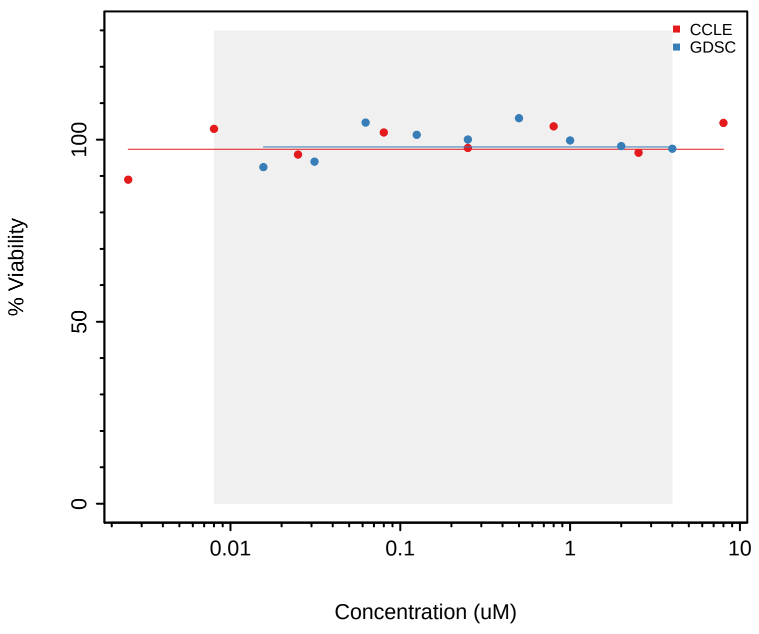

PD−0332991:EFO−21

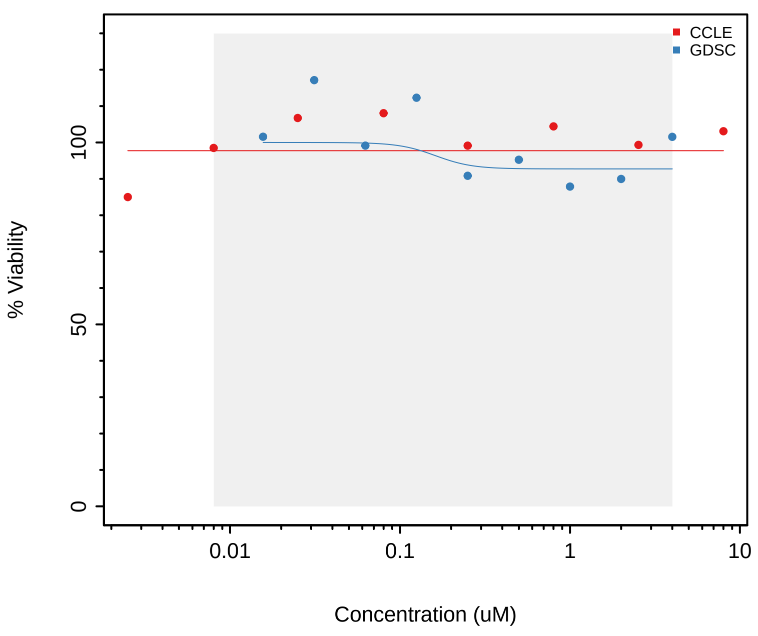

PD−0332991:EFO−27

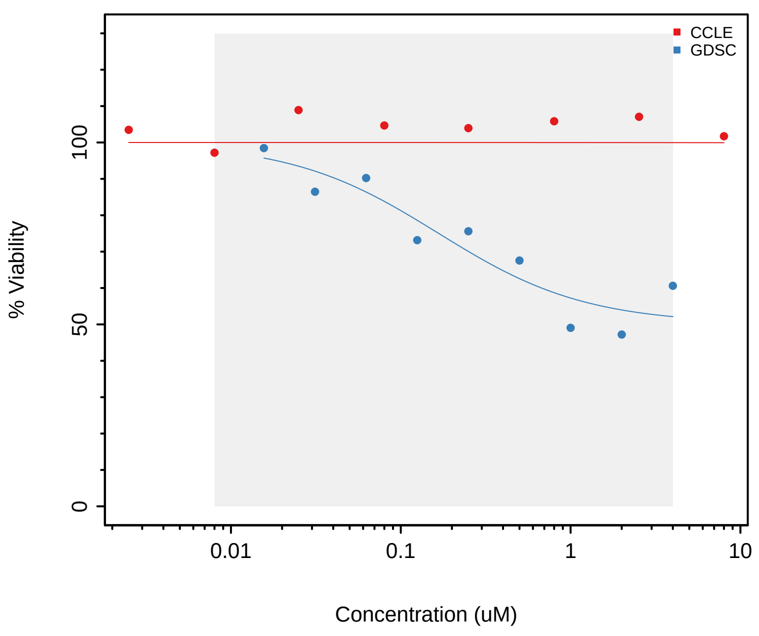

PD−0332991:EM−2

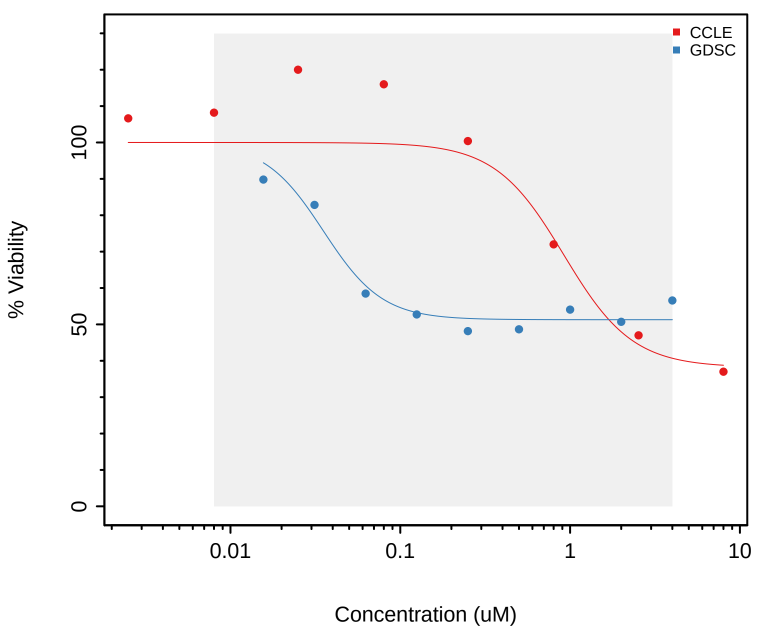

PD−0332991:FADU

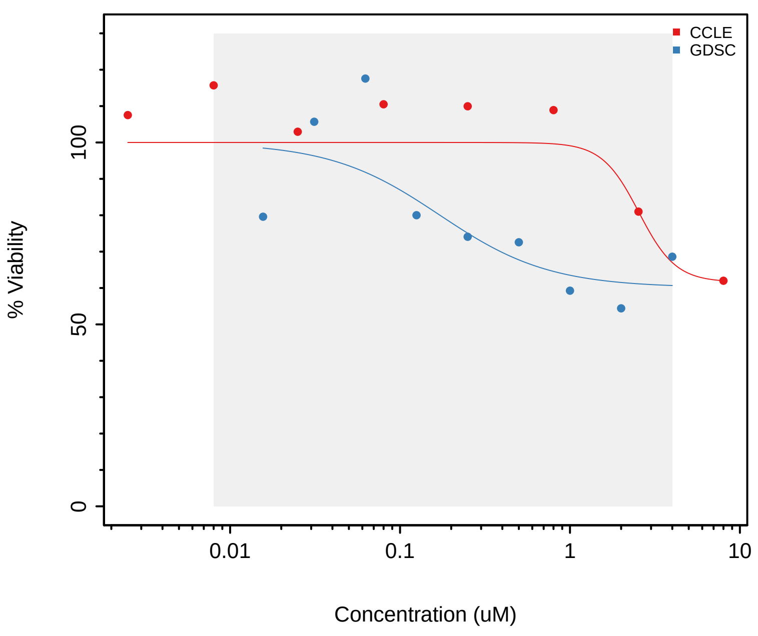

PD−0332991:G−361

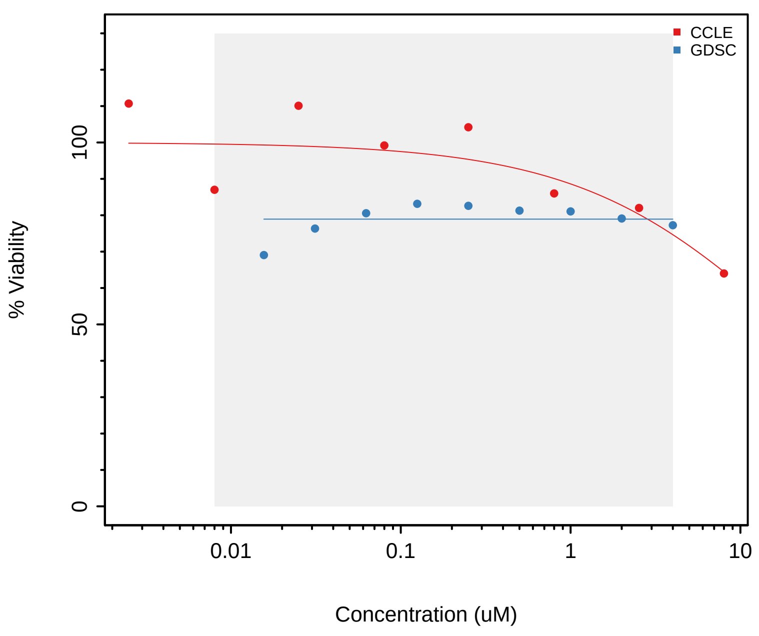

PD−0332991:G−401

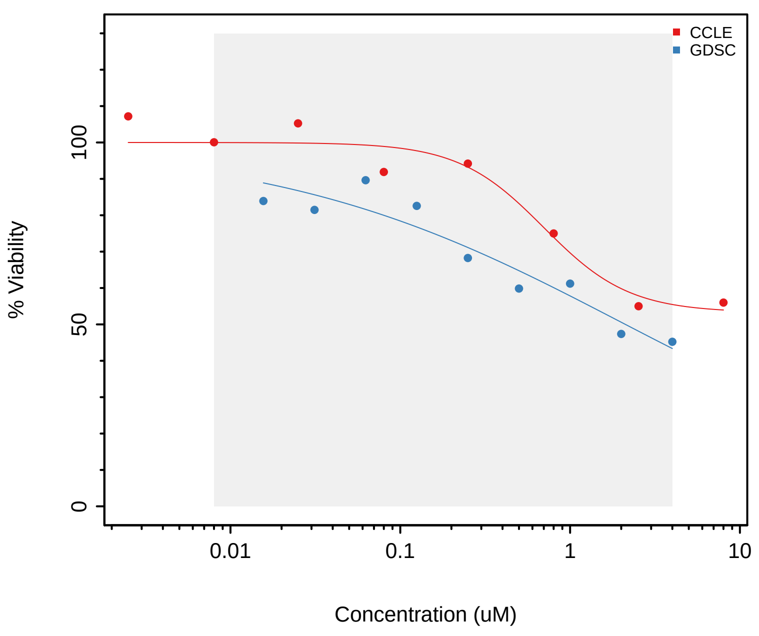

PD−0332991:GB−1

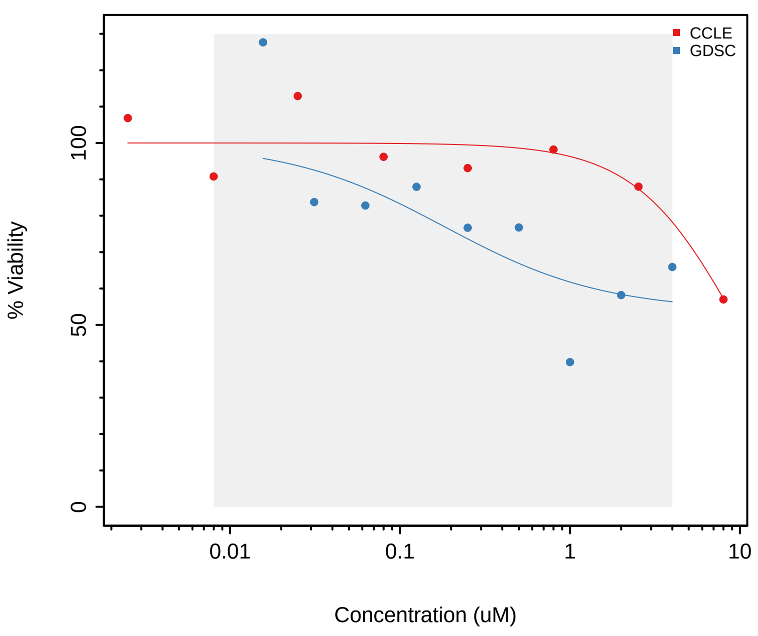

PD−0332991:GCIY

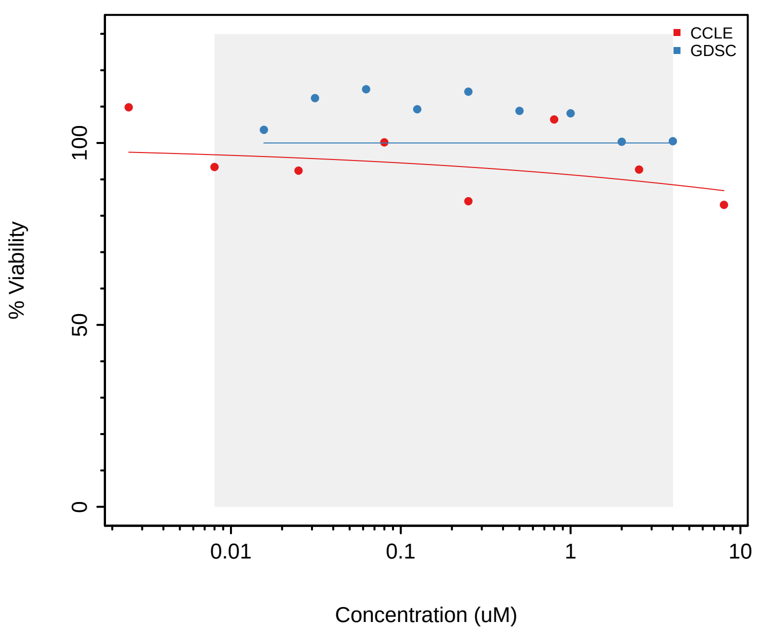

PD−0332991:GCT

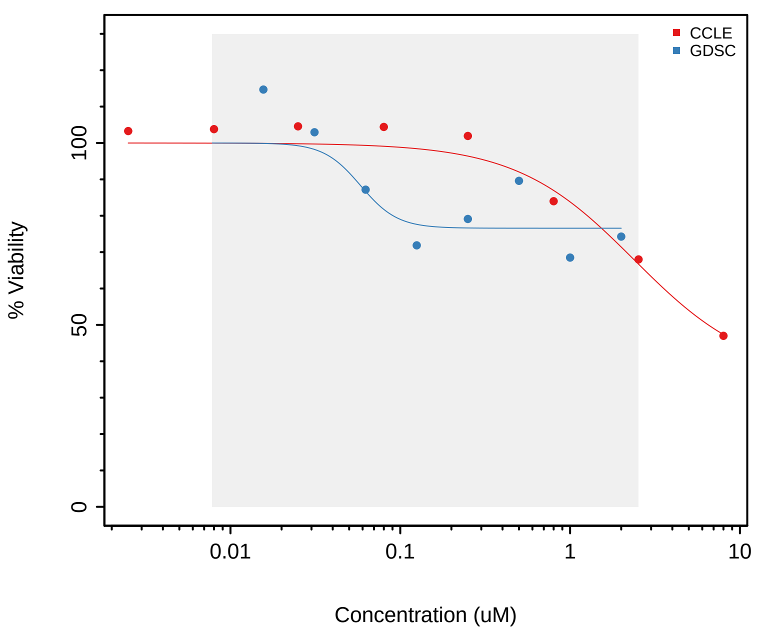

PD−0332991:GMS−10

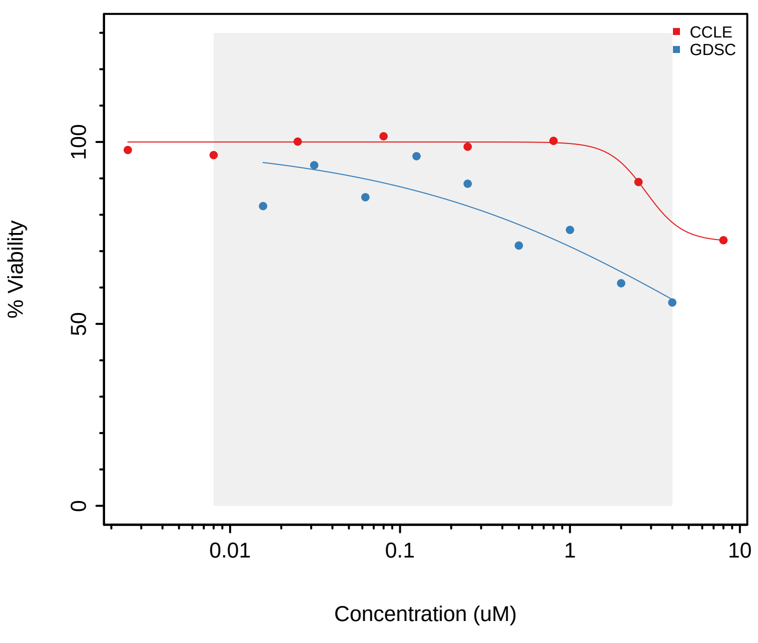

PD−0332991:H4

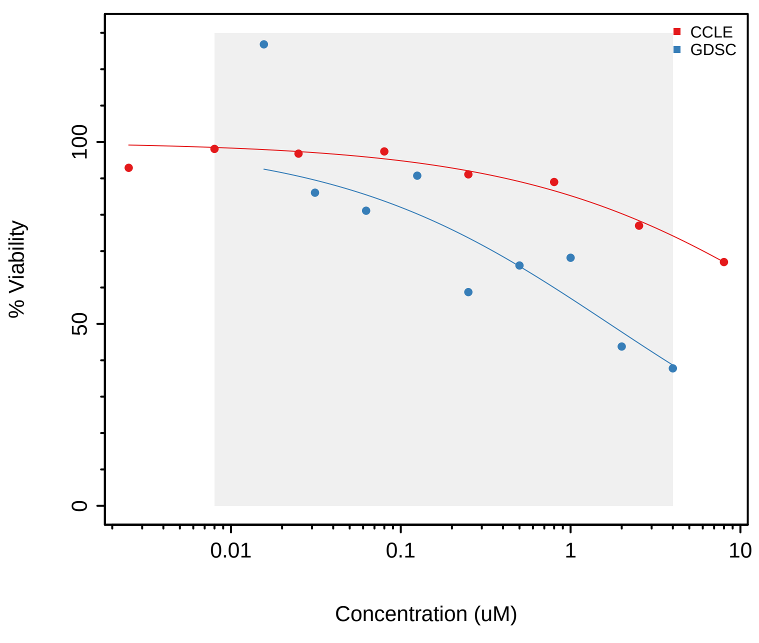

PD−0332991:HCC1187

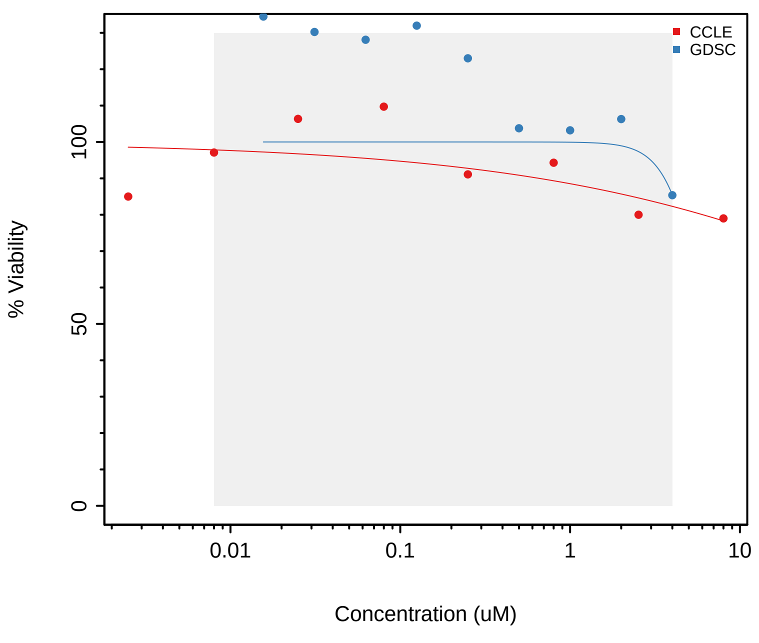

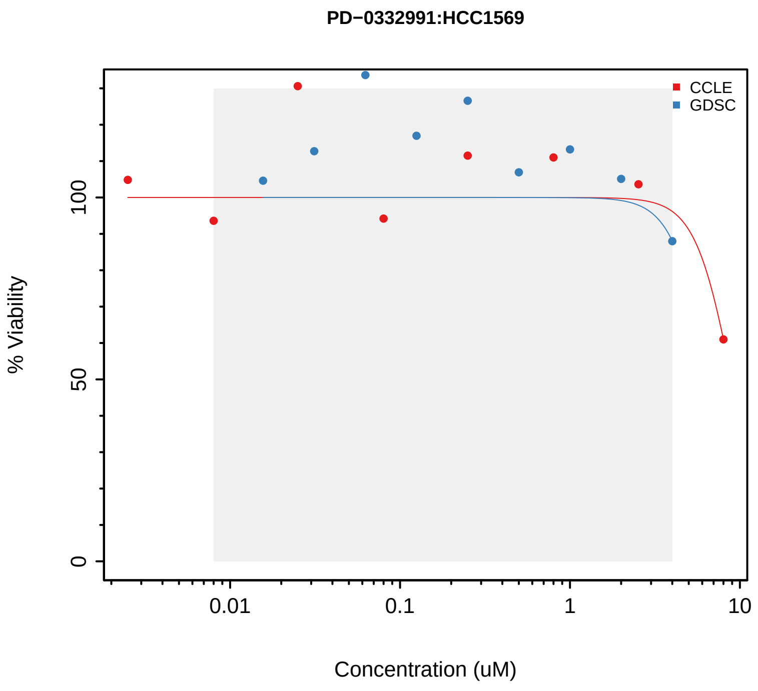
PD−0332991:HCC1569
CCLE
GDSC
% Viability
0
50
100
0.01
0.1
1
10
Concentration (uM)

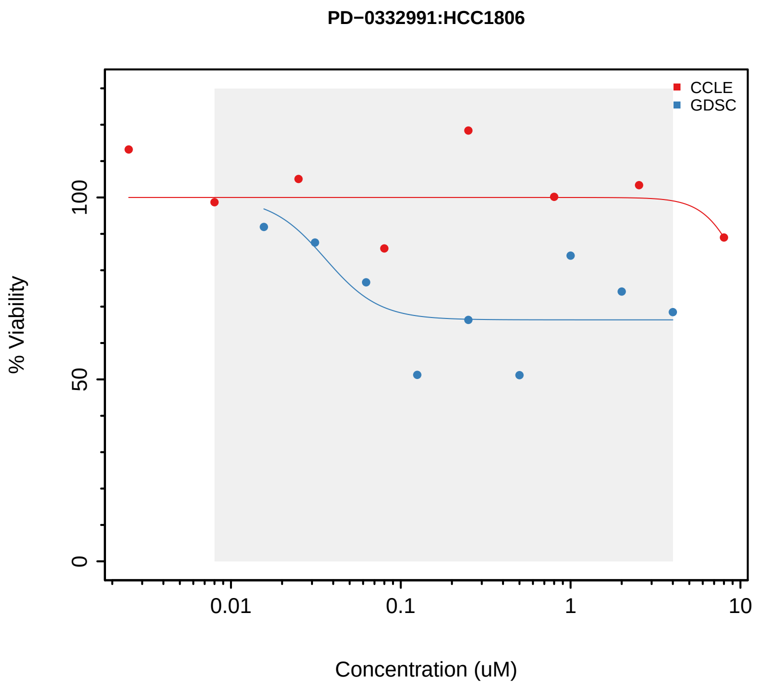
PD−0332991:HCC1806
CCLE
GDSC
% Viability
0
50
100
0.01
0.1
1
10
Concentration (uM)

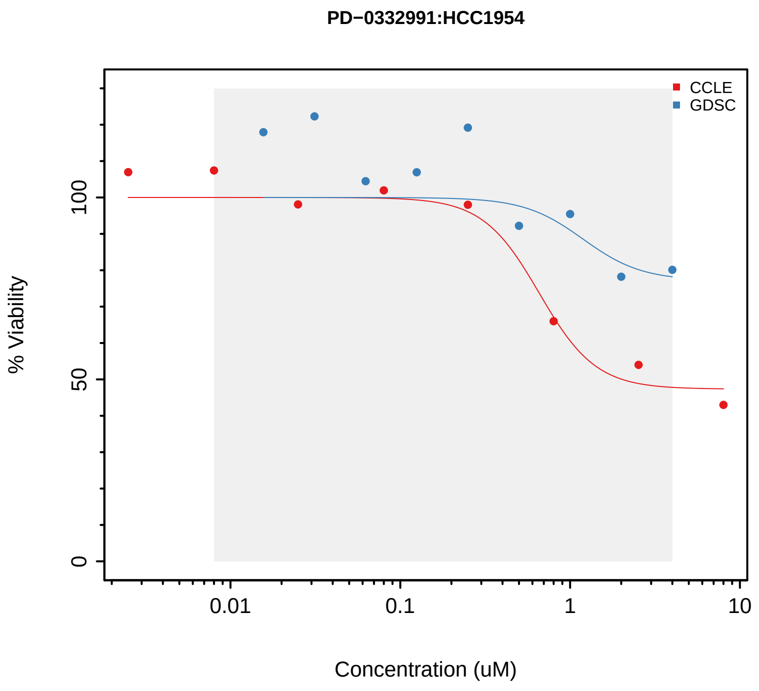
PD−0332991:HCC1954
CCLE
GDSC
% Viability
0
50
100
0.01
0.1
1
10
Concentration (uM)

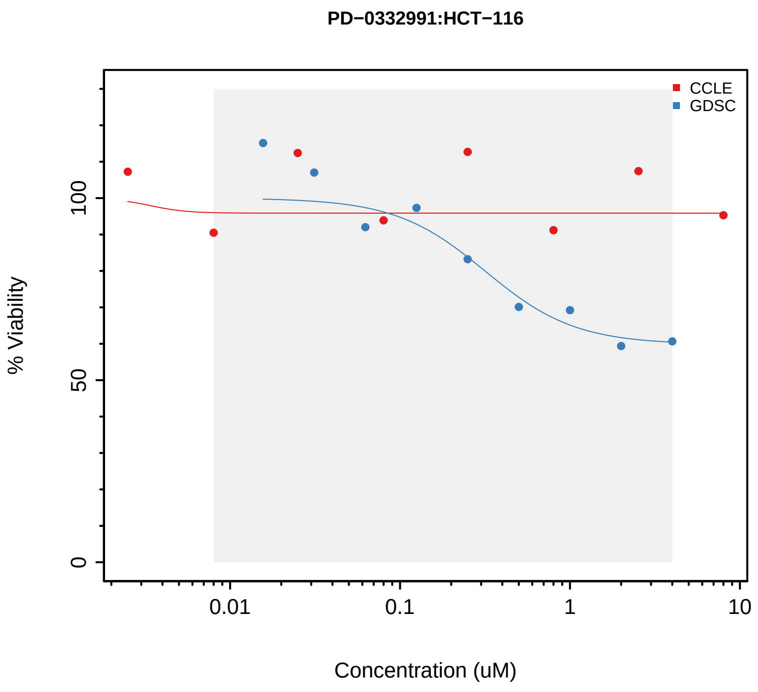
PD−0332991:HCT−116
CCLE
GDSC
% Viability
0
50
100
0.01
0.1
1
10
Concentration (uM)

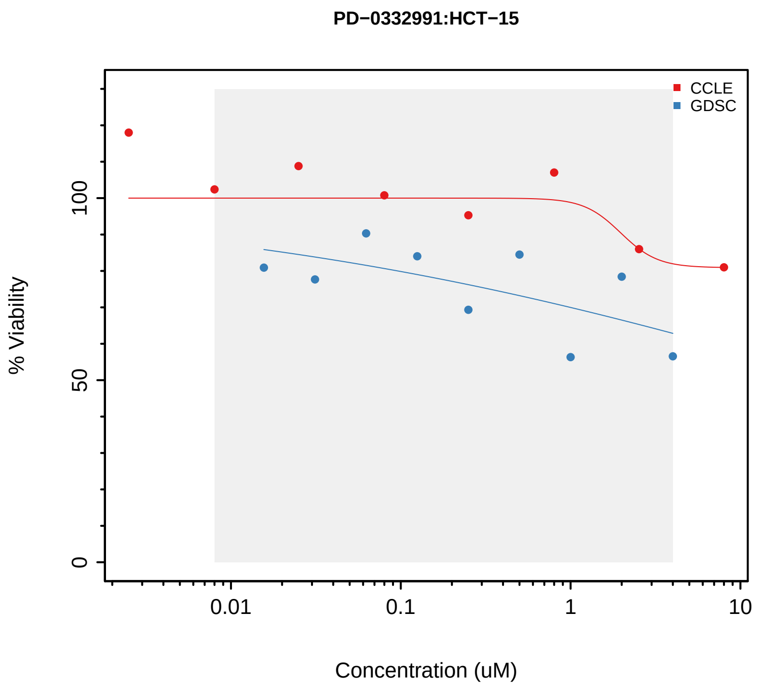
PD−0332991:HCT−15
CCLE
GDSC
% Viability
0
50
100
0.01
0.1
1
10
Concentration (uM)

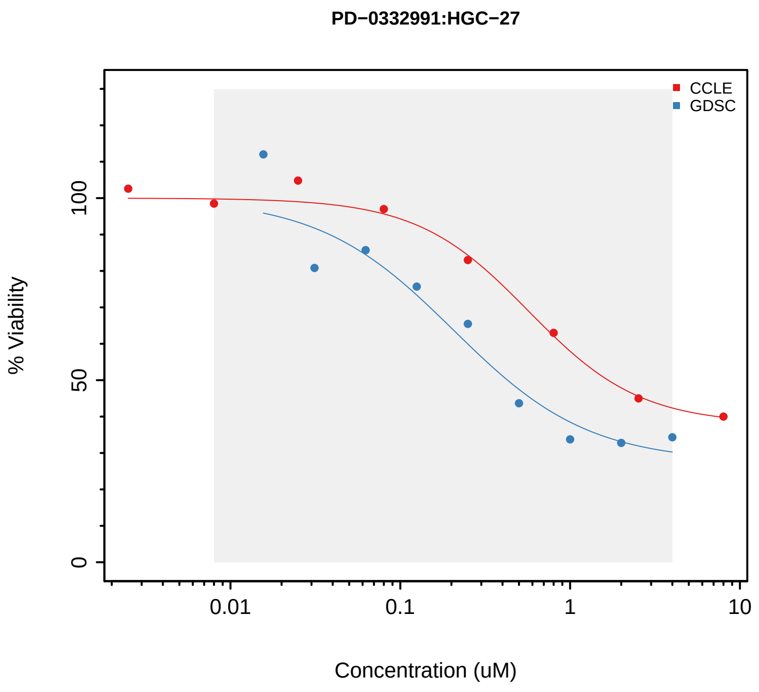
PD−0332991:HGC−27
CCLE
GDSC
% Viability
0
50
100
0.01
0.1
1
10
Concentration (uM)

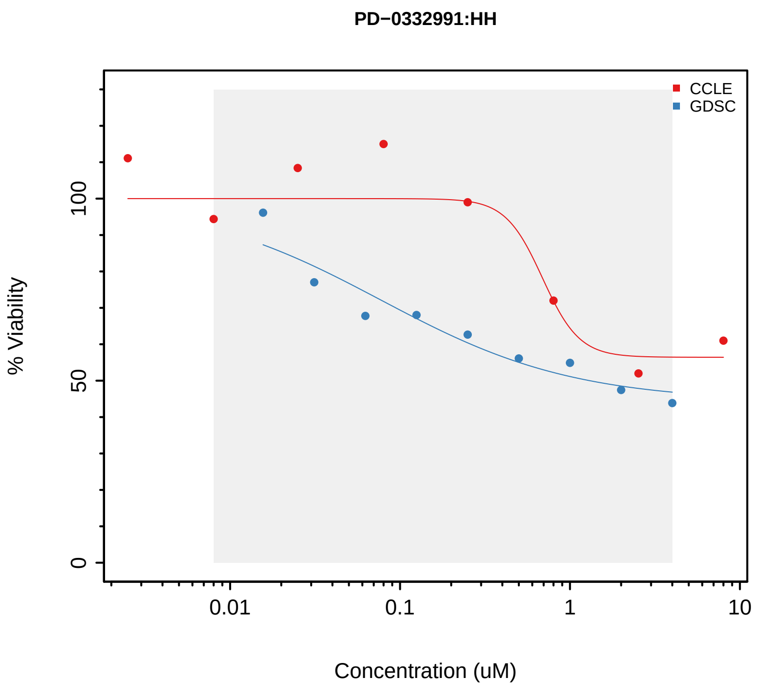
PD−0332991:HH
CCLE
GDSC
% Viability
0
50
100
0.01
0.1
1
10
Concentration (uM)

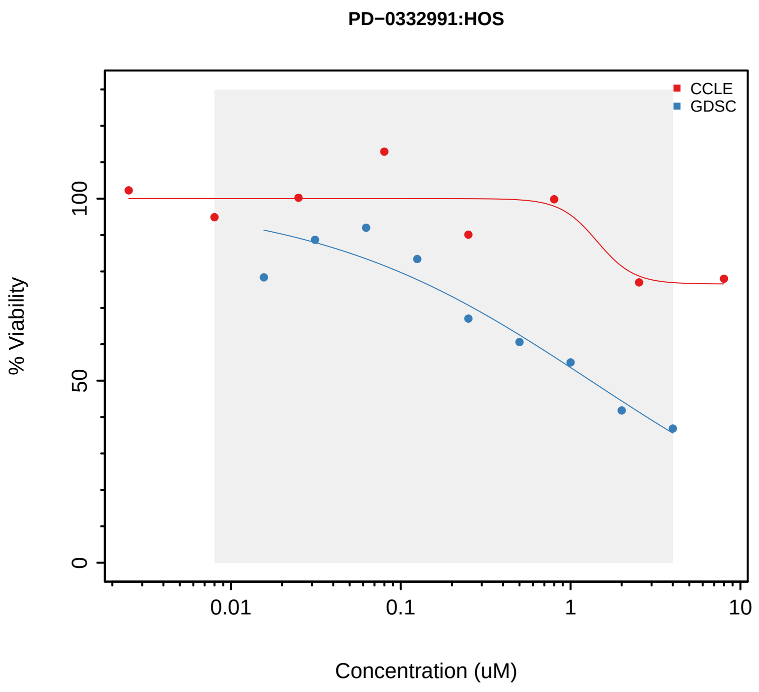
PD−0332991:HOS
CCLE
GDSC
% Viability
0
50
100
0.01
0.1
1
10
Concentration (uM)

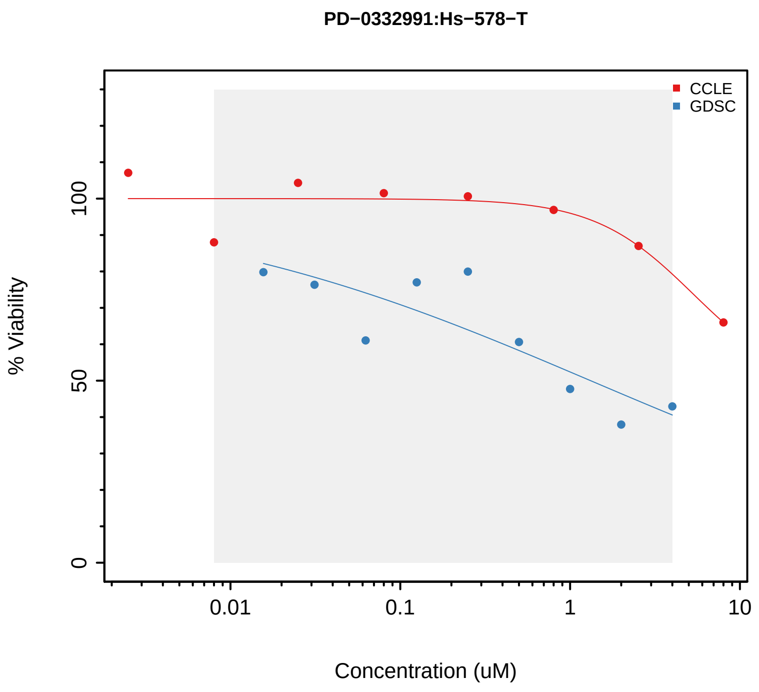
PD−0332991:Hs−578−T
CCLE
GDSC
% Viability
0
50
100
0.01
0.1
1
10
Concentration (uM)

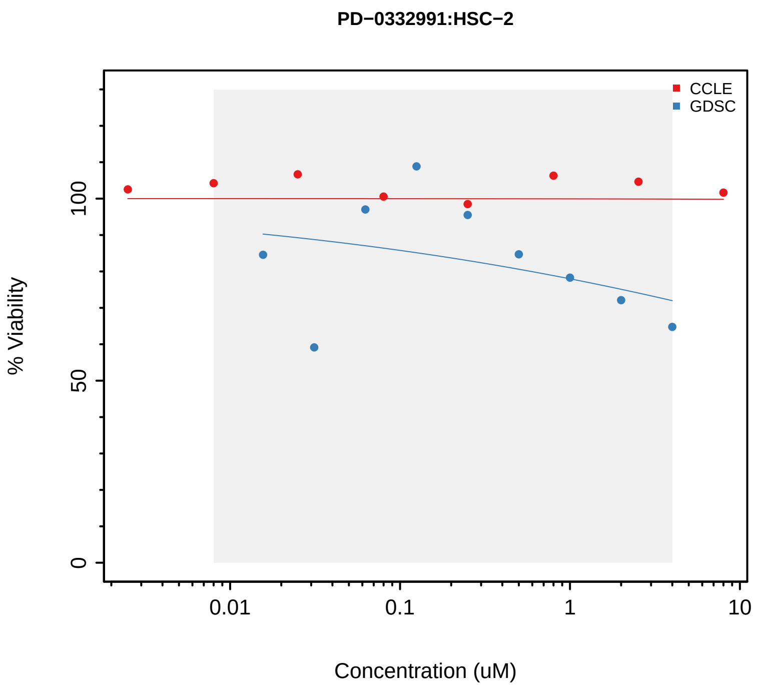
PD−0332991:HSC−2
CCLE
GDSC
% Viability
0
50
100
0.01
0.1
1
10
Concentration (uM)

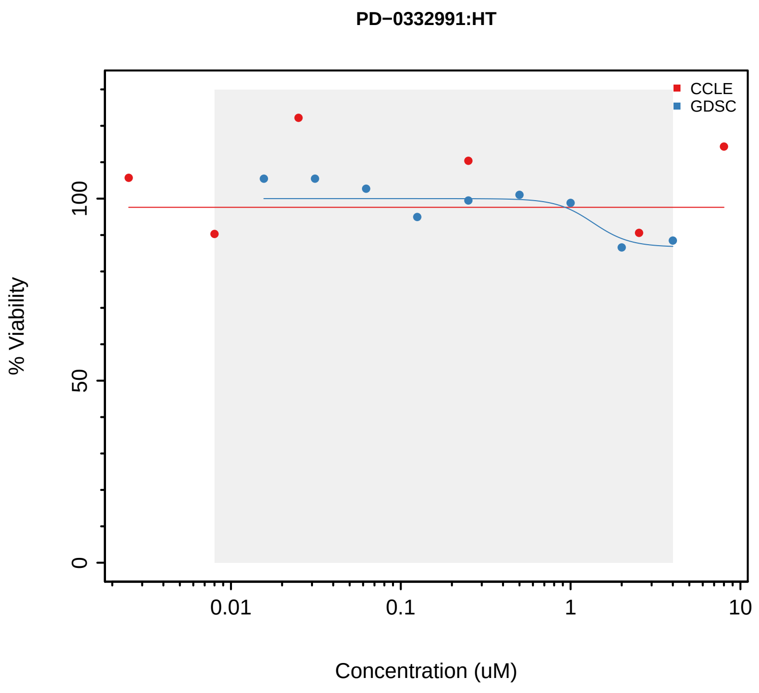
PD−0332991:HT
CCLE
GDSC
% Viability
0
50
100
0.01
0.1
1
10
Concentration (uM)

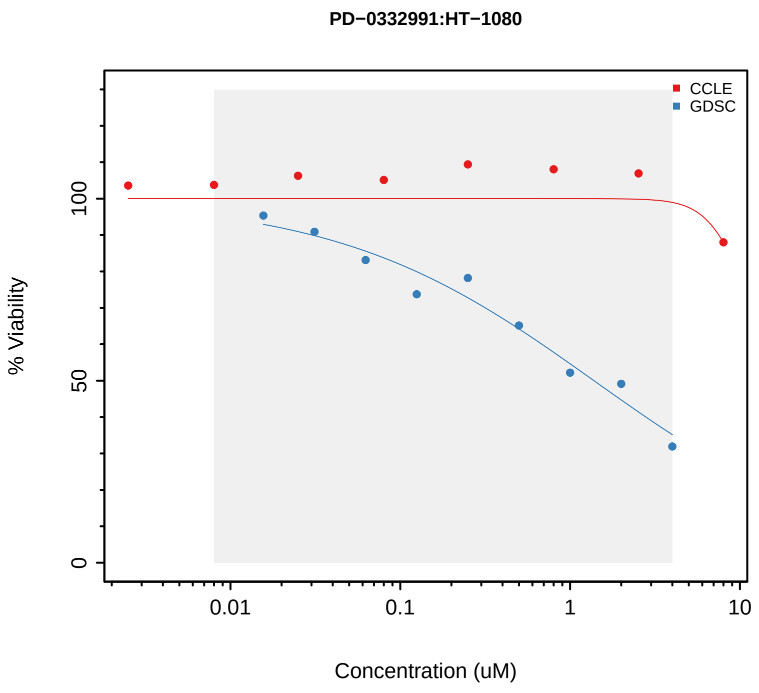
PD−0332991:HT−1080
CCLE
GDSC
% Viability
0
50
100
0.01
0.1
1
10
Concentration (uM)

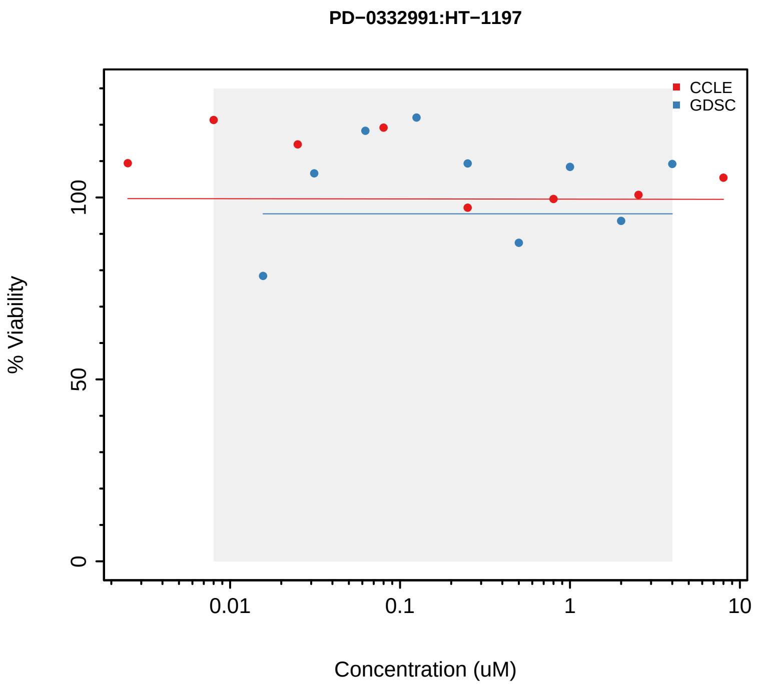
PD−0332991:HT−1197
CCLE
GDSC
% Viability
0
50
100
0.01
0.1
1
10
Concentration (uM)

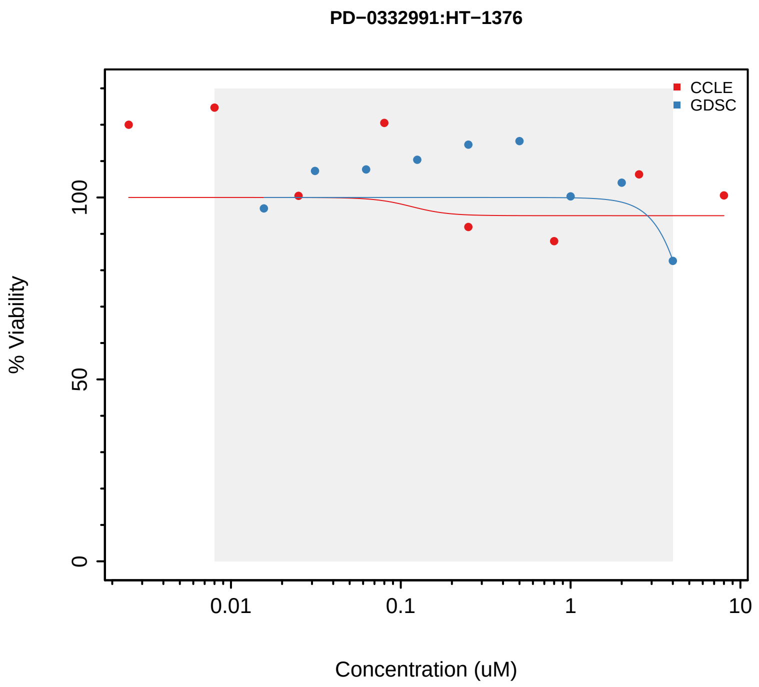
PD−0332991:HT−1376
CCLE
GDSC
% Viability
0
50
100
0.01
0.1
1
10
Concentration (uM)

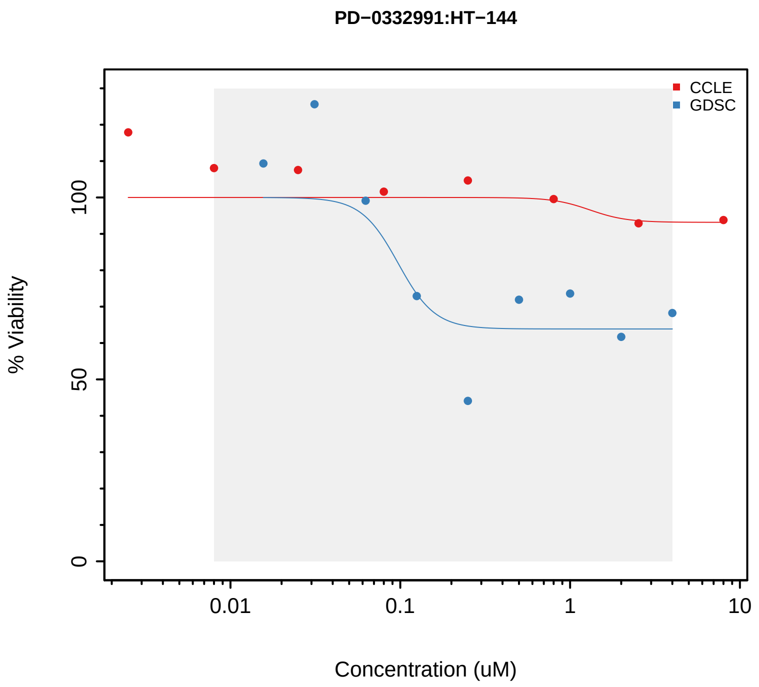
PD−0332991:HT−144
CCLE
GDSC
% Viability
0
50
100
0.01
0.1
1
10
Concentration (uM)

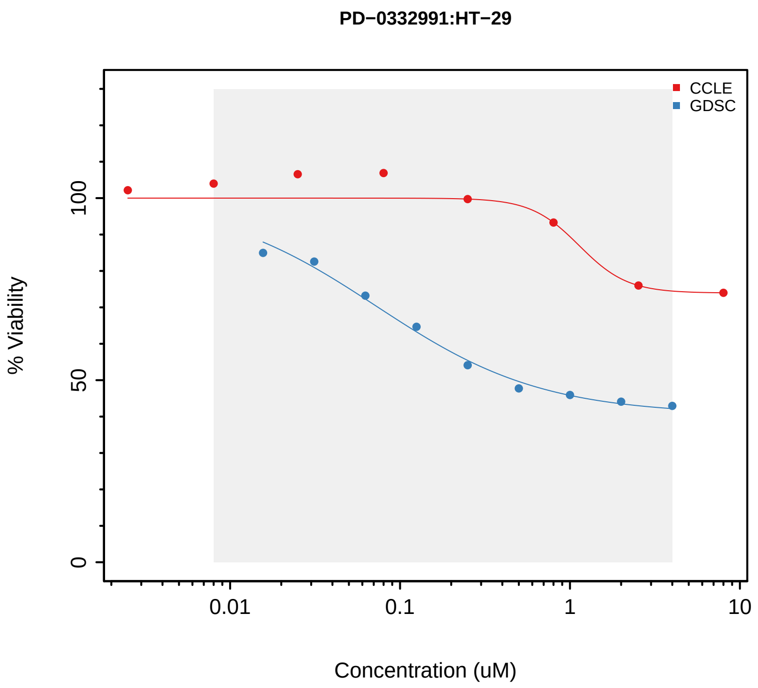
PD−0332991:HT−29
CCLE
GDSC
% Viability
0
50
100
0.01
0.1
1
10
Concentration (uM)

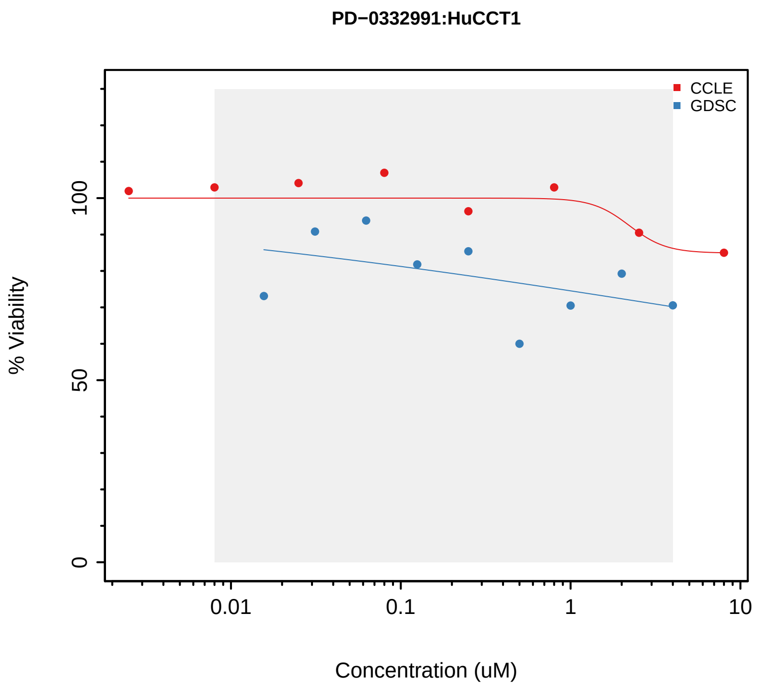
PD−0332991:HuCCT1
CCLE
GDSC
% Viability
0
50
100
0.01
0.1
1
10
Concentration (uM)

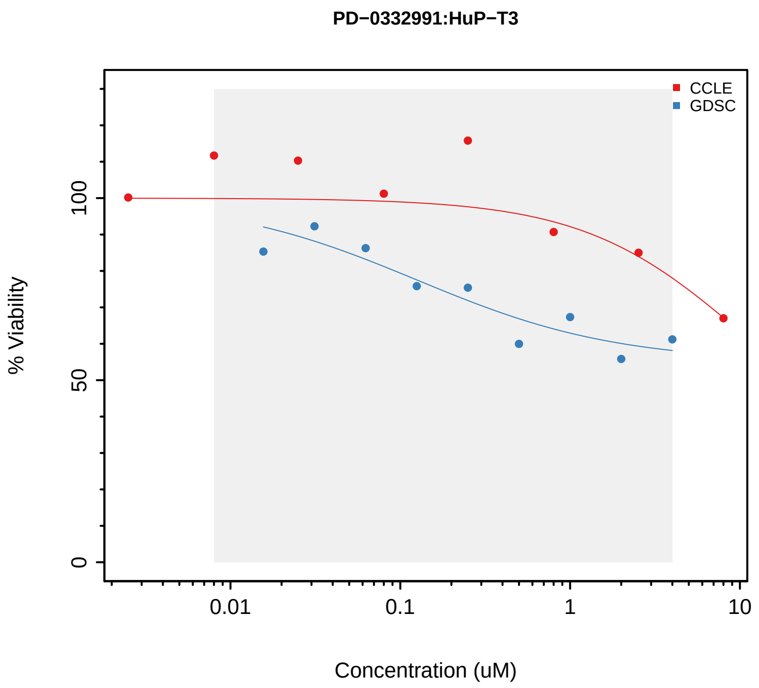
PD−0332991:HuP−T3
CCLE
GDSC
% Viability
0
50
100
0.01
0.1
1
10
Concentration (uM)

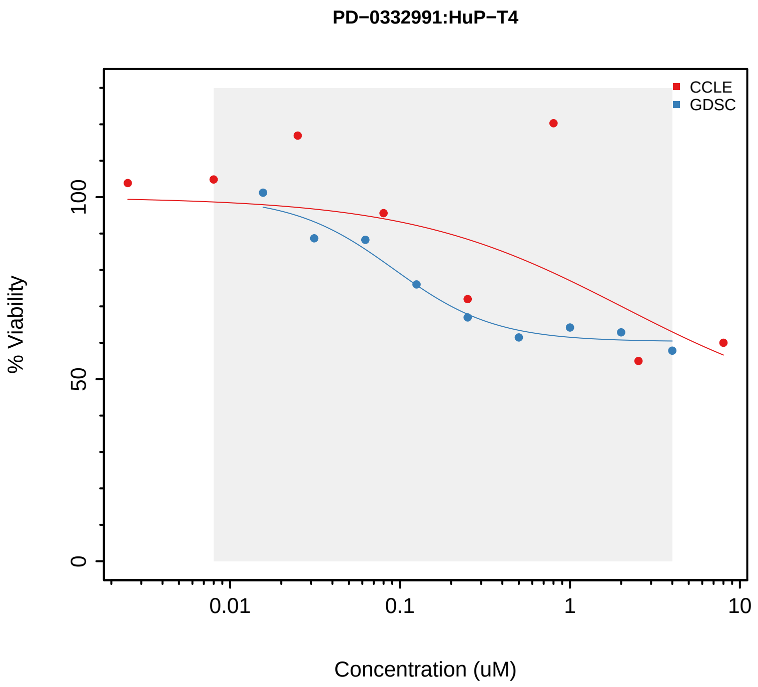
PD−0332991:HuP−T4
CCLE
GDSC
% Viability
0
50
100
0.01
0.1
1
10
Concentration (uM)

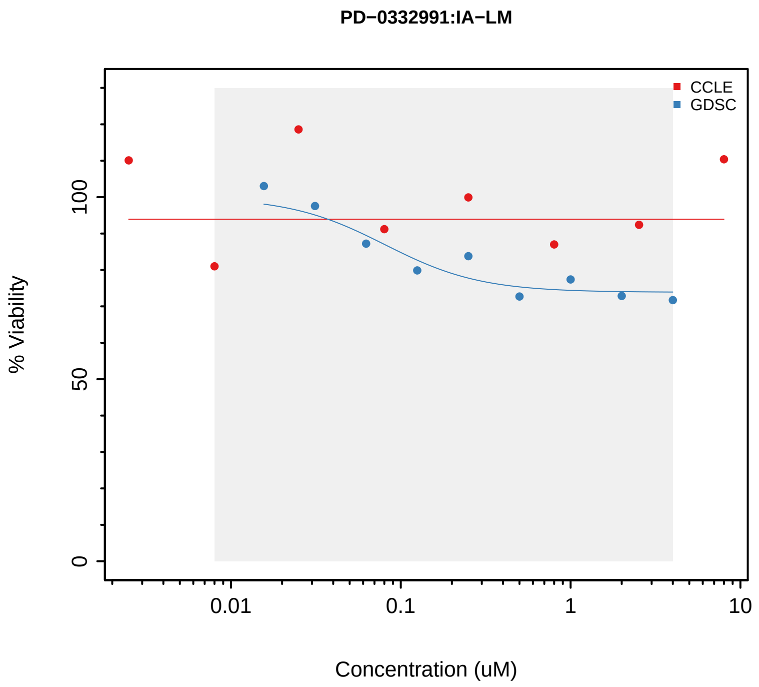
PD−0332991:IA−LM
CCLE
GDSC
% Viability
0
50
100
0.01
0.1
1
10
Concentration (uM)

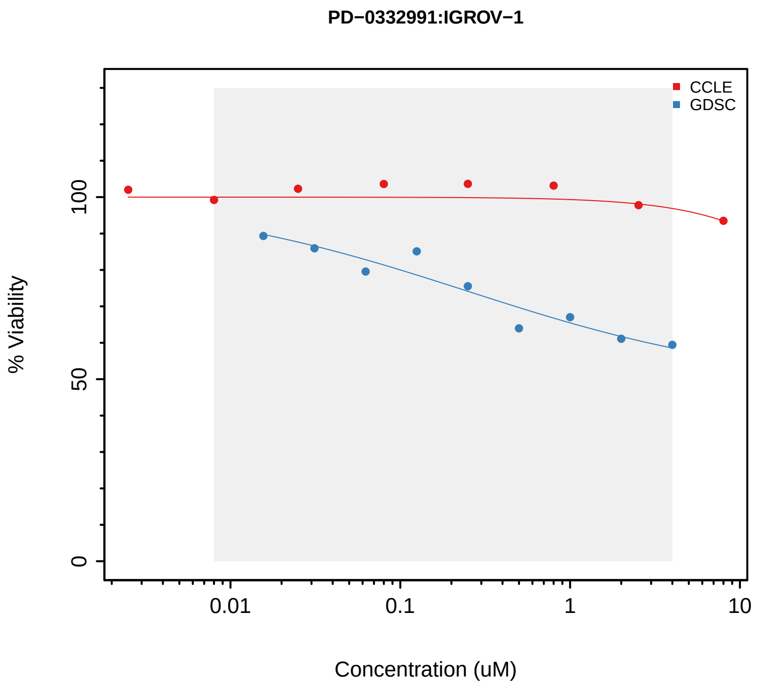
PD−0332991:IGROV−1
CCLE
GDSC
% Viability
0
50
100
0.01
0.1
1
10
Concentration (uM)

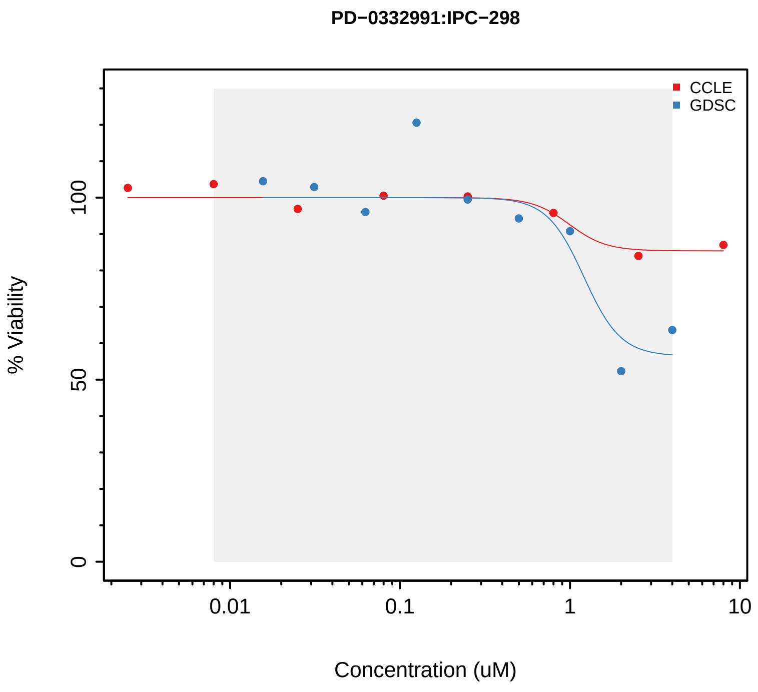
PD−0332991:IPC−298
CCLE
GDSC
% Viability
0
50
100
0.01
0.1
1
10
Concentration (uM)

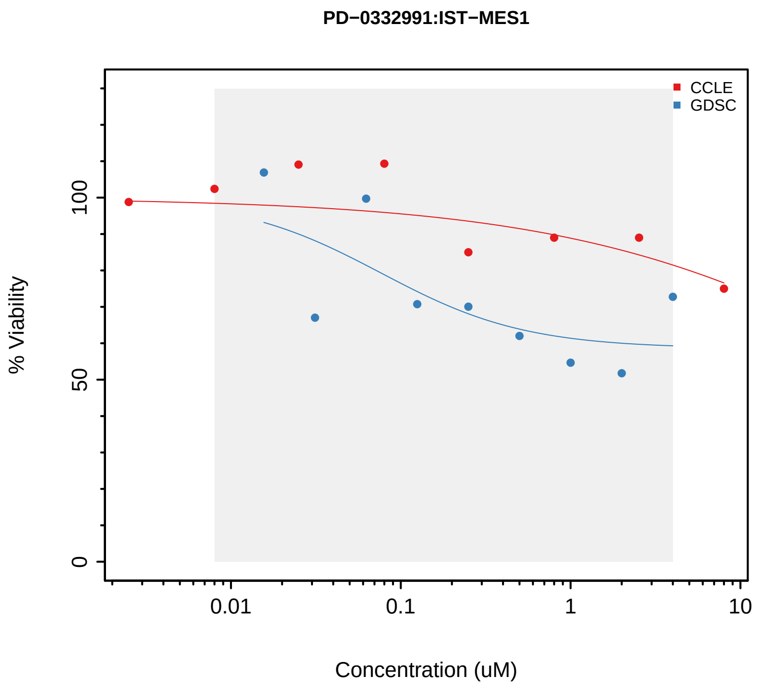
PD−0332991:IST−MES1
CCLE
GDSC
% Viability
0
50
100
0.01
0.1
1
10
Concentration (uM)

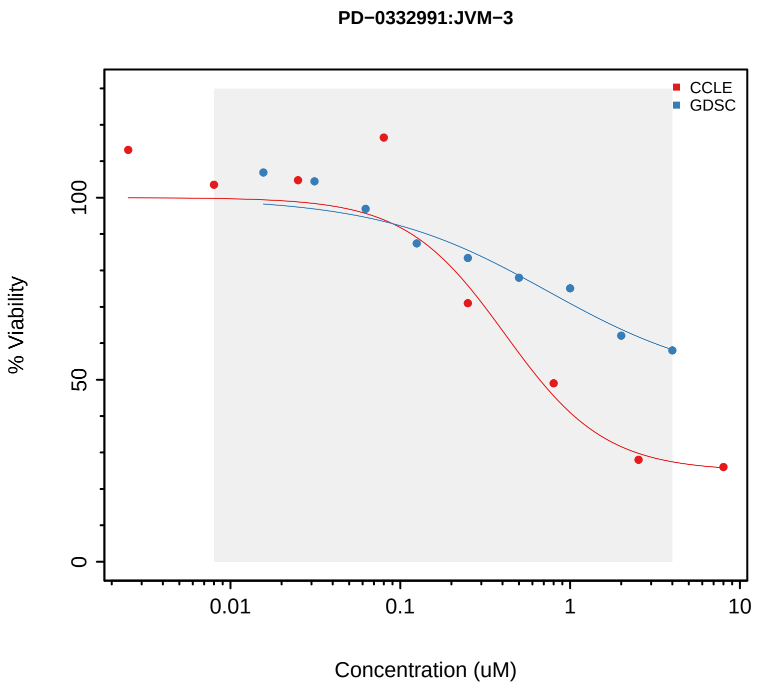
PD−0332991:JVM−3
CCLE
GDSC
% Viability
0
50
100
0.01
0.1
1
10
Concentration (uM)

PD−0332991:KARPAS−299

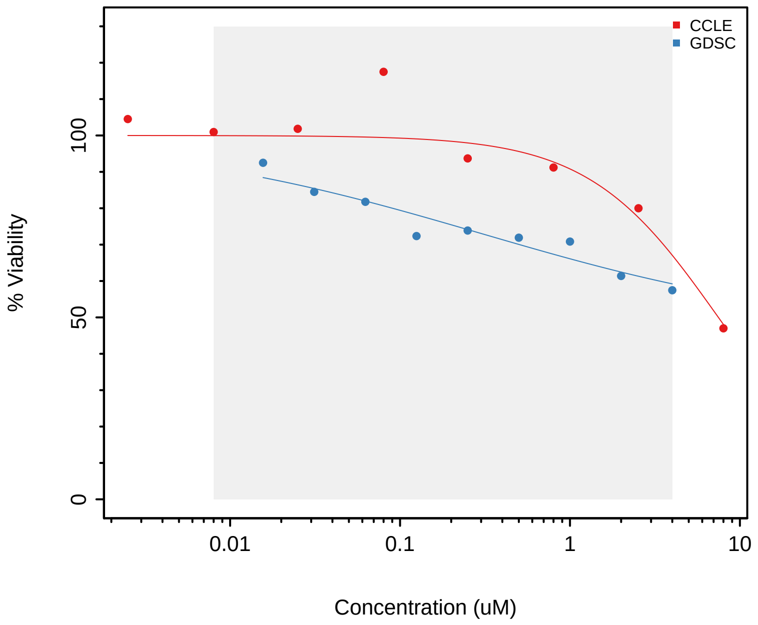

PD−0332991:KARPAS−422

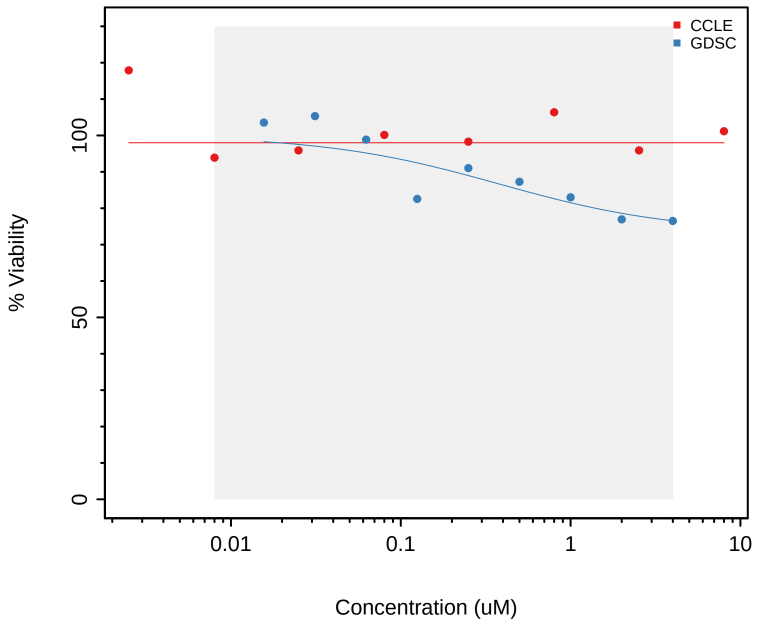

PD−0332991:KG−1

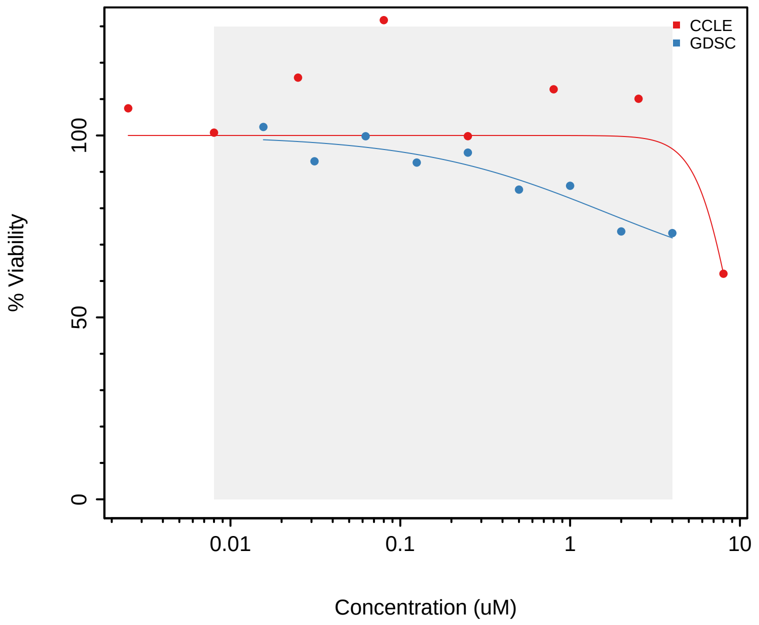

PD−0332991:KLE

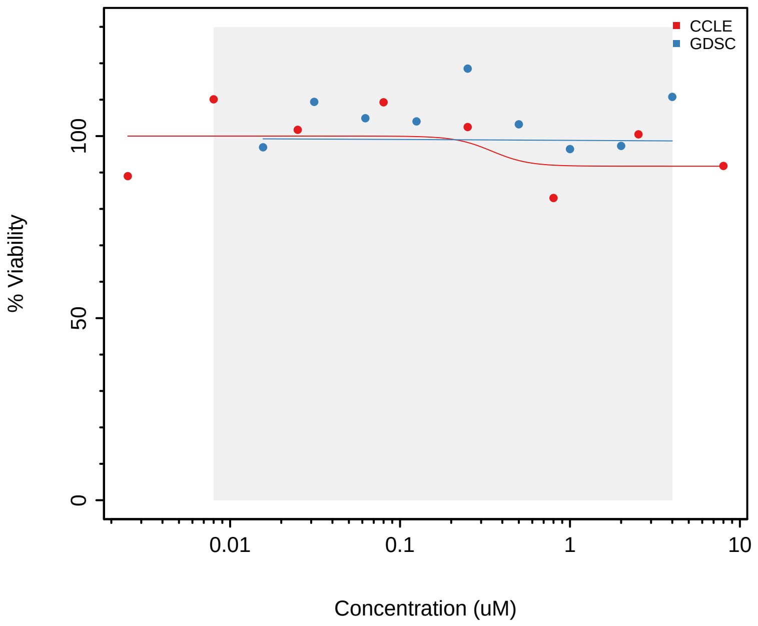

PD−0332991:KNS−42

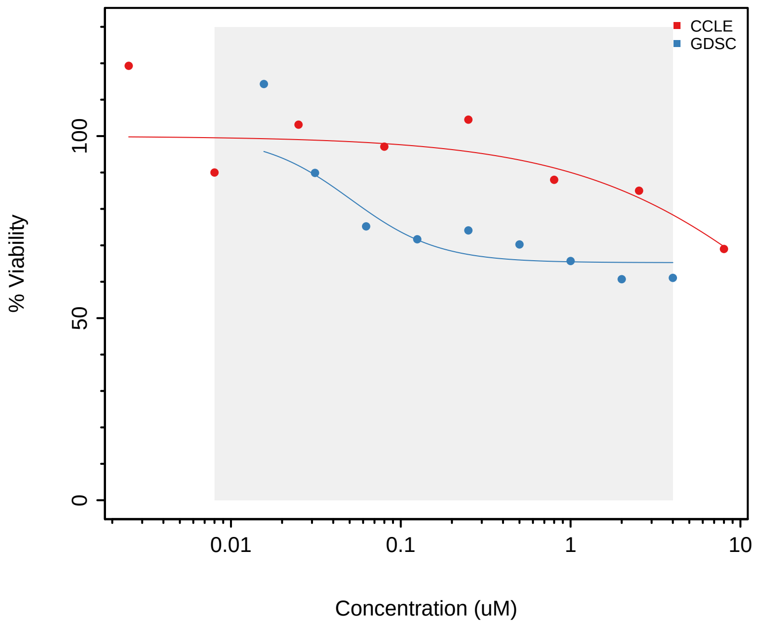

PD−0332991:KNS−81−FD

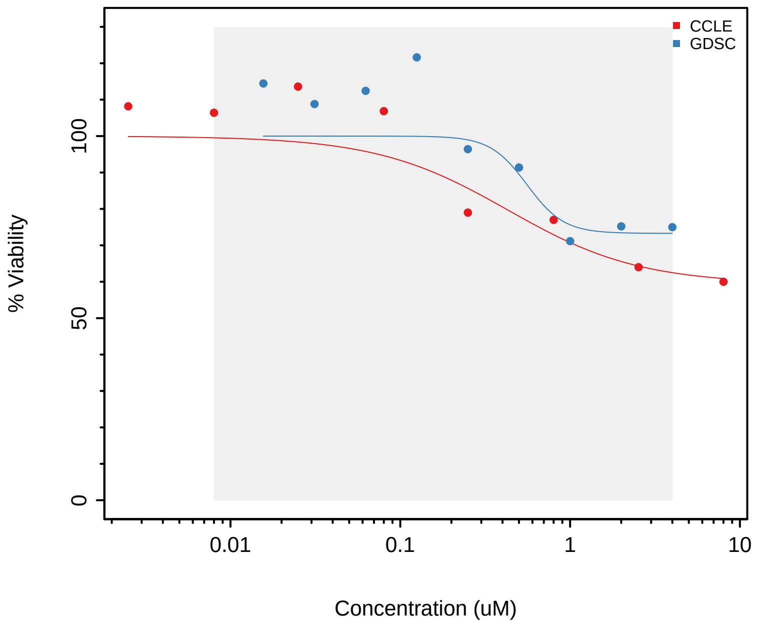

PD−0332991:KU812

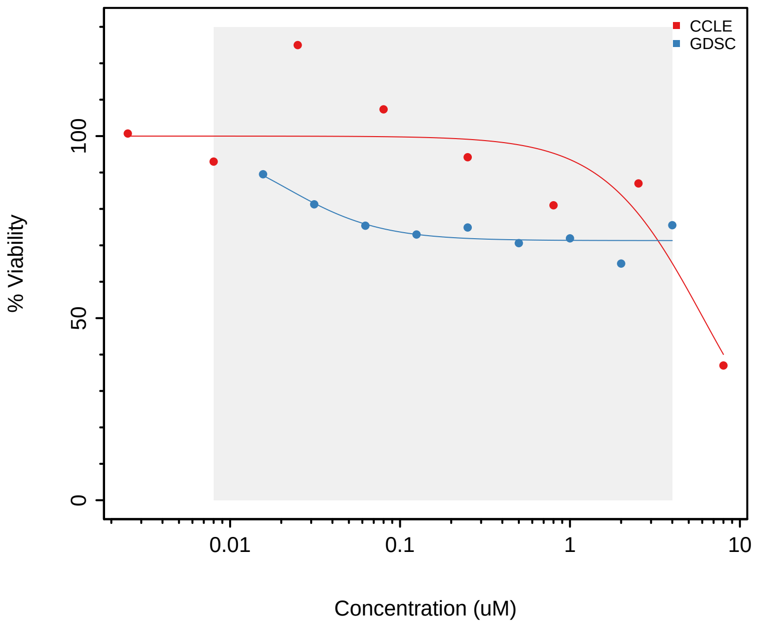

PD−0332991:KURAMOCHI

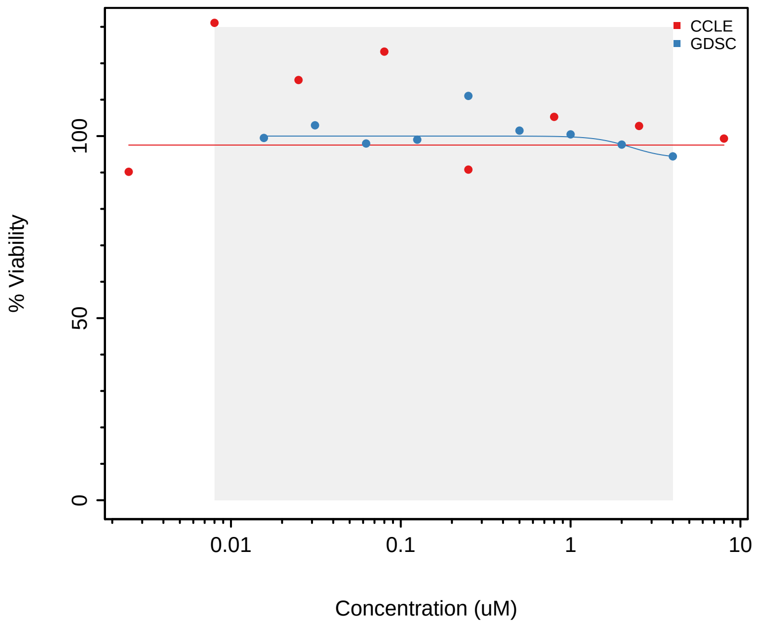

PD−0332991:KYSE−410

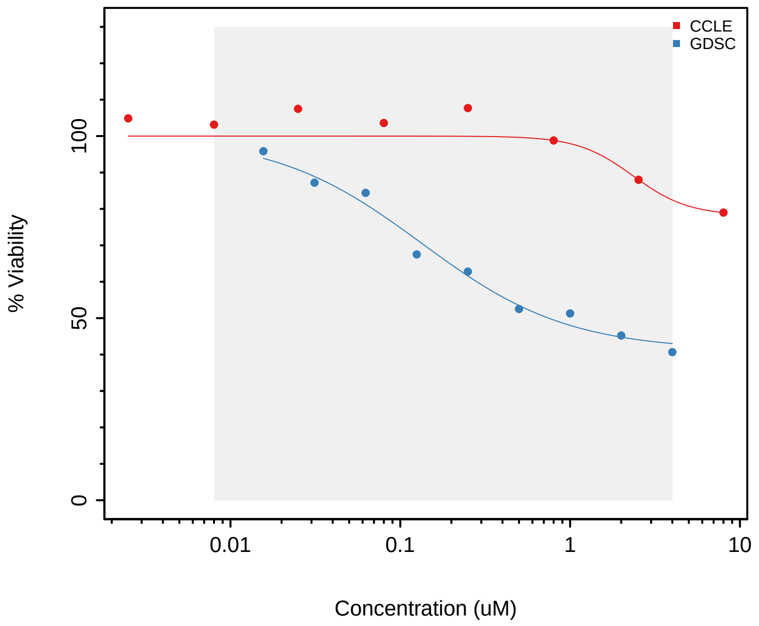

PD−0332991:L−363

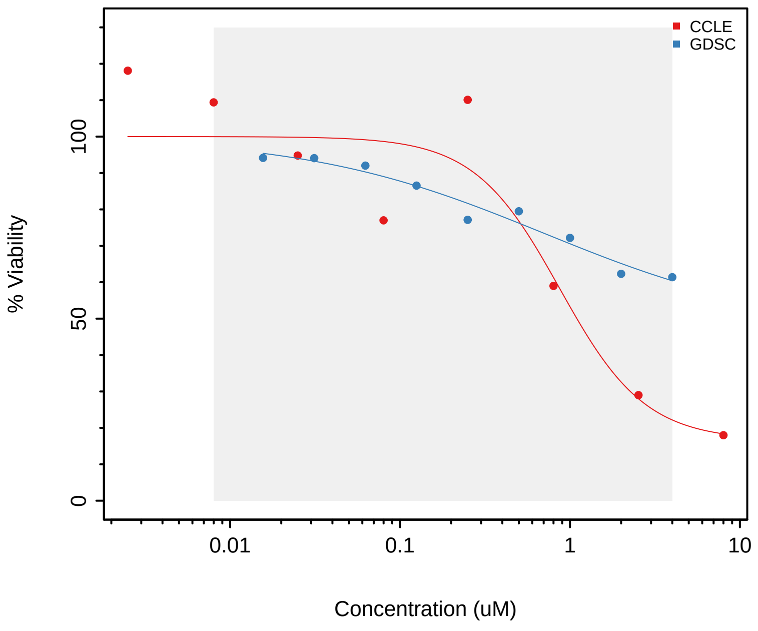

PD−0332991:L−428

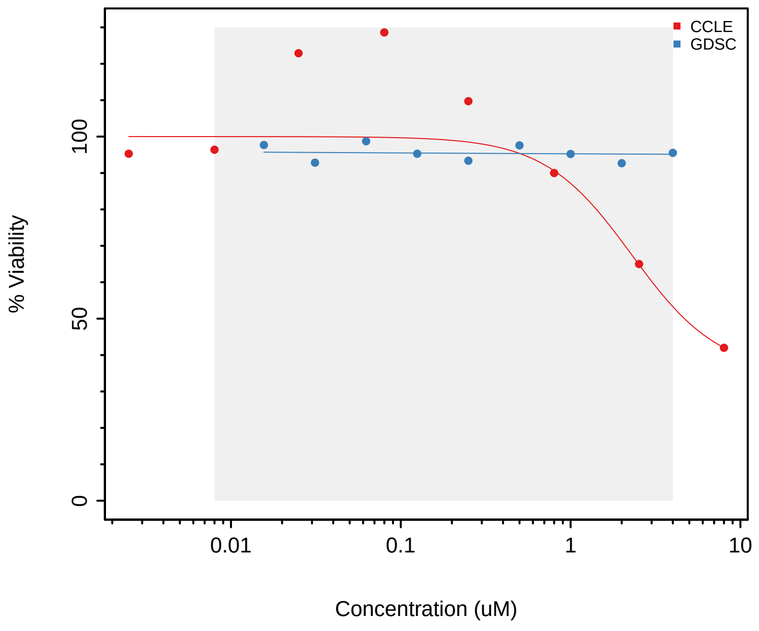

PD−0332991:LCLC−103H

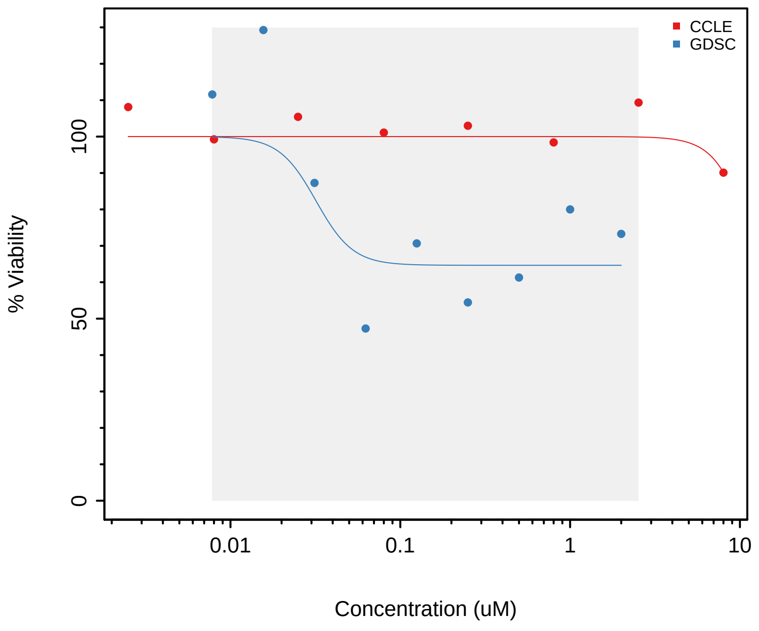

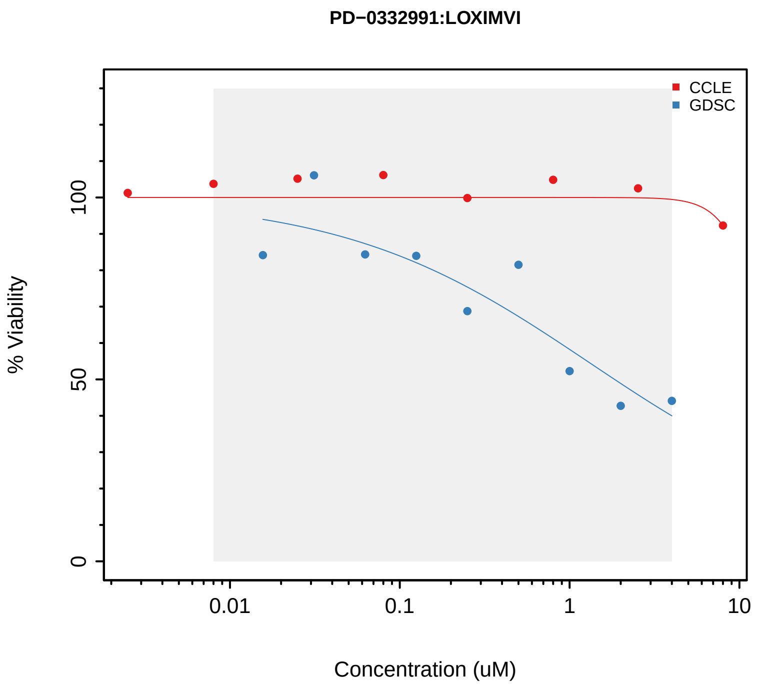
PD−0332991:LOXIMVI
CCLE
GDSC
% Viability
0
50
100
0.01
0.1
1
10
Concentration (uM)

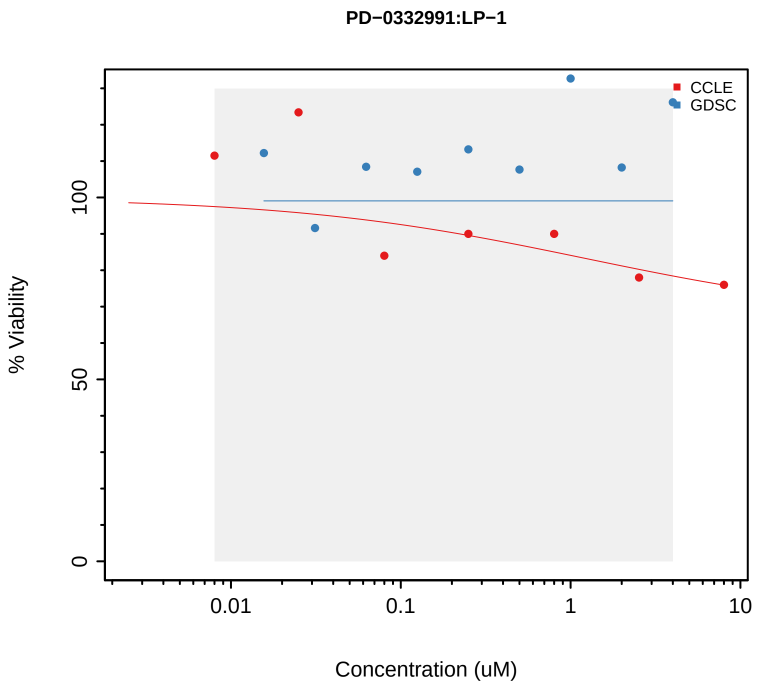
PD−0332991:LP−1
CCLE
GDSC
% Viability
0
50
100
0.01
0.1
1
10
Concentration (uM)

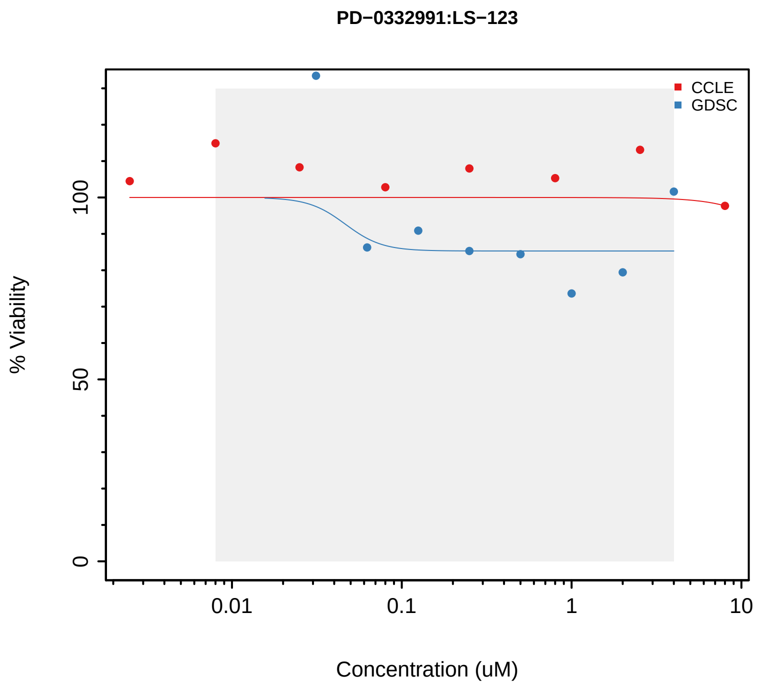
PD−0332991:LS−123
CCLE
GDSC
% Viability
0
50
100
0.01
0.1
1
10
Concentration (uM)

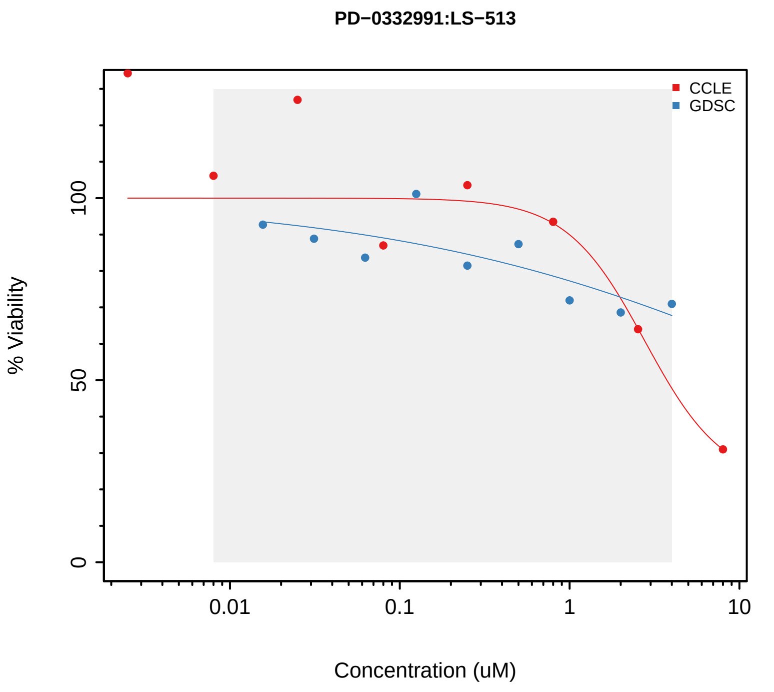
PD−0332991:LS−513
CCLE
GDSC
% Viability
0
50
100
0.01
0.1
1
10
Concentration (uM)

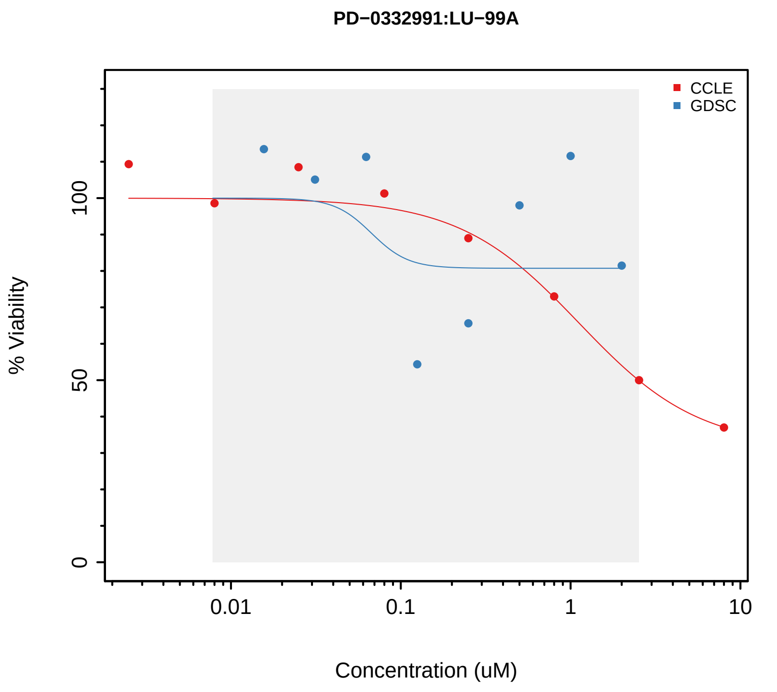
PD−0332991:LU−99A
CCLE
GDSC
% Viability
0
50
100
0.01
0.1
1
10
Concentration (uM)

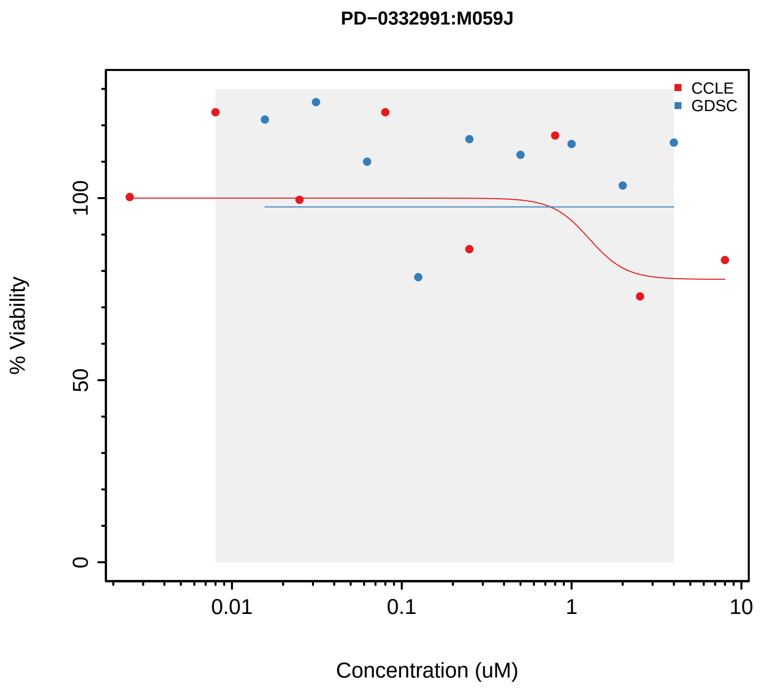
PD−0332991:M059J
CCLE
GDSC
% Viability
0
50
100
0.01
0.1
1
10
Concentration (uM)

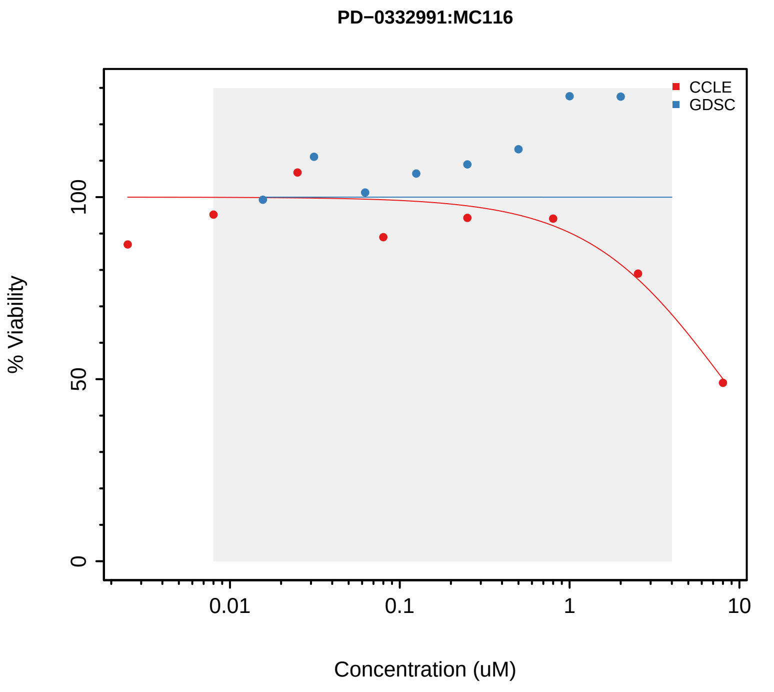
PD−0332991:MC116
CCLE
GDSC
% Viability
0
50
100
0.01
0.1
1
10
Concentration (uM)

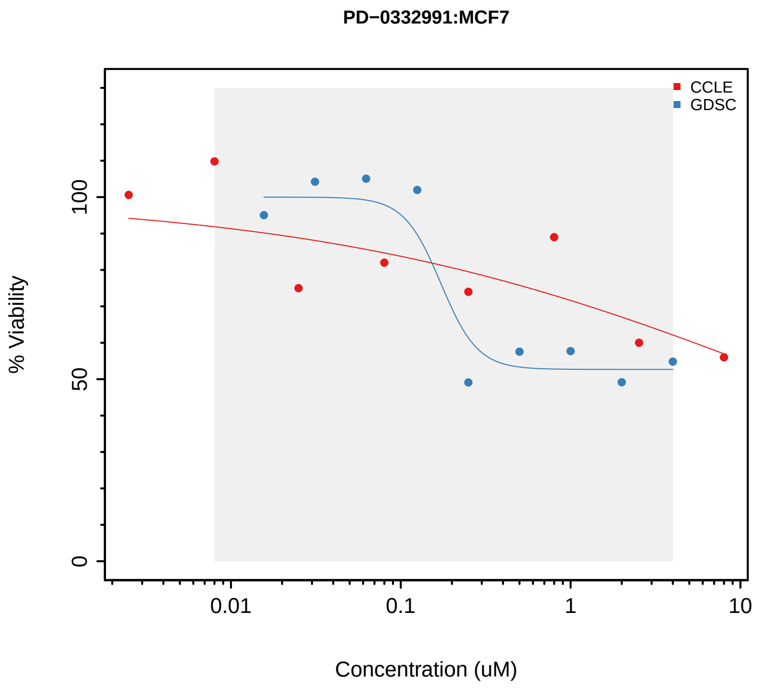
PD−0332991:MCF7
CCLE
GDSC
% Viability
0
50
100
0.01
0.1
1
10
Concentration (uM)

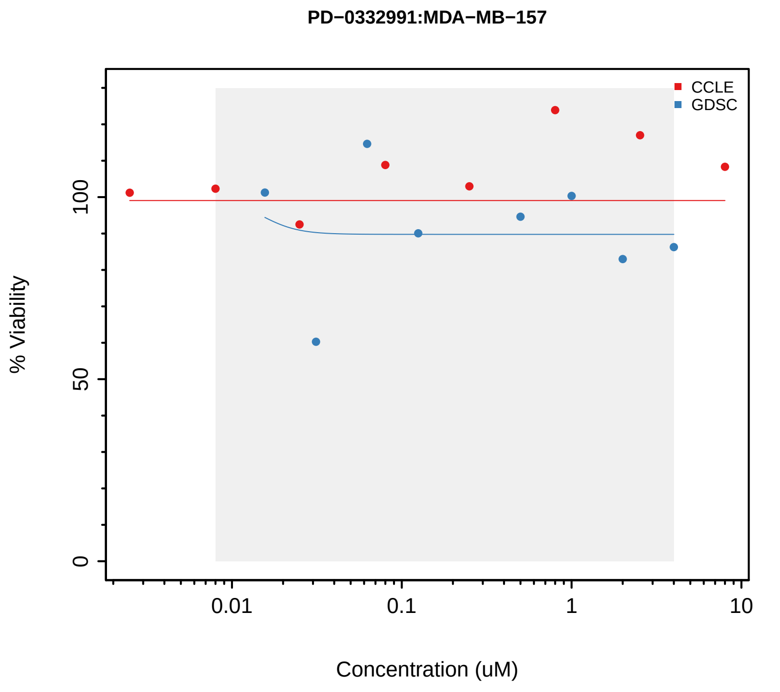
PD−0332991:MDA−MB−157
CCLE
GDSC
% Viability
0
50
100
0.01
0.1
1
10
Concentration (uM)

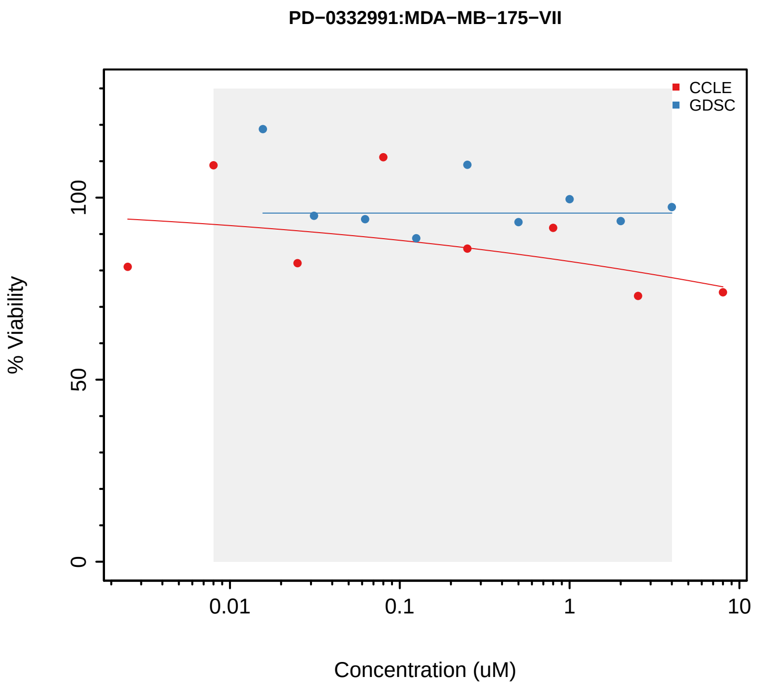
PD−0332991:MDA−MB−175−VII
CCLE
GDSC
% Viability
0
50
100
0.01
0.1
1
10
Concentration (uM)

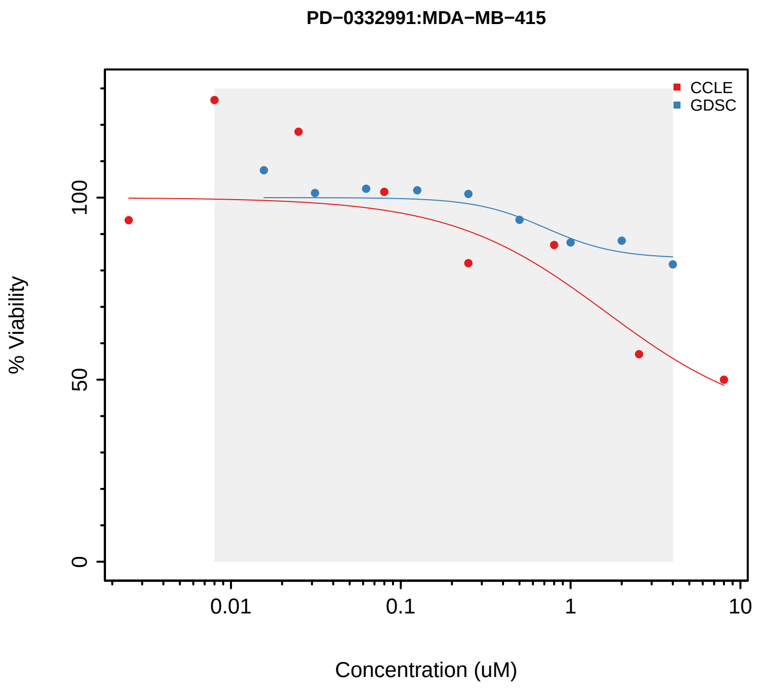
PD−0332991:MDA−MB−415
CCLE
GDSC
% Viability
0
50
100
0.01
0.1
1
10
Concentration (uM)

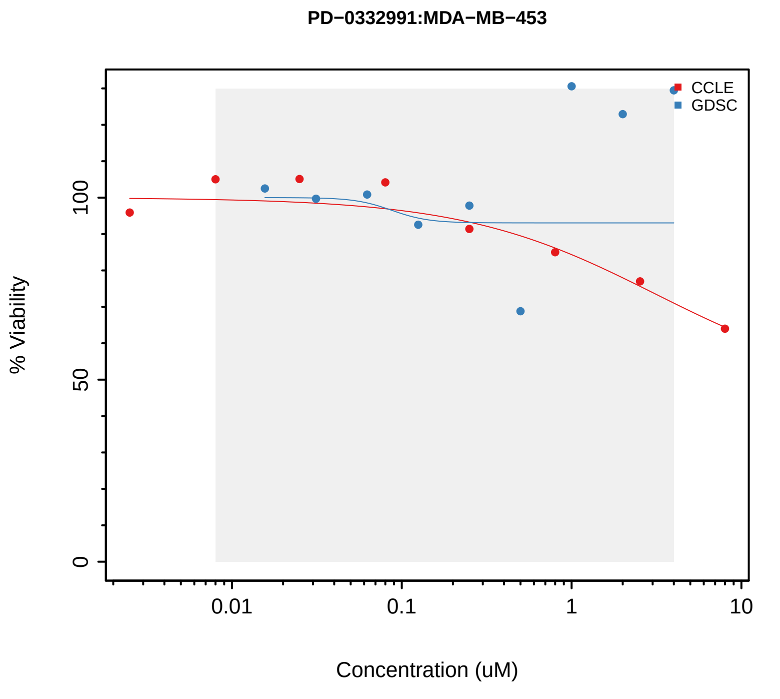
PD−0332991:MDA−MB−453
CCLE
GDSC
% Viability
0
50
100
0.01
0.1
1
10
Concentration (uM)

PD−0332991:MEG−01

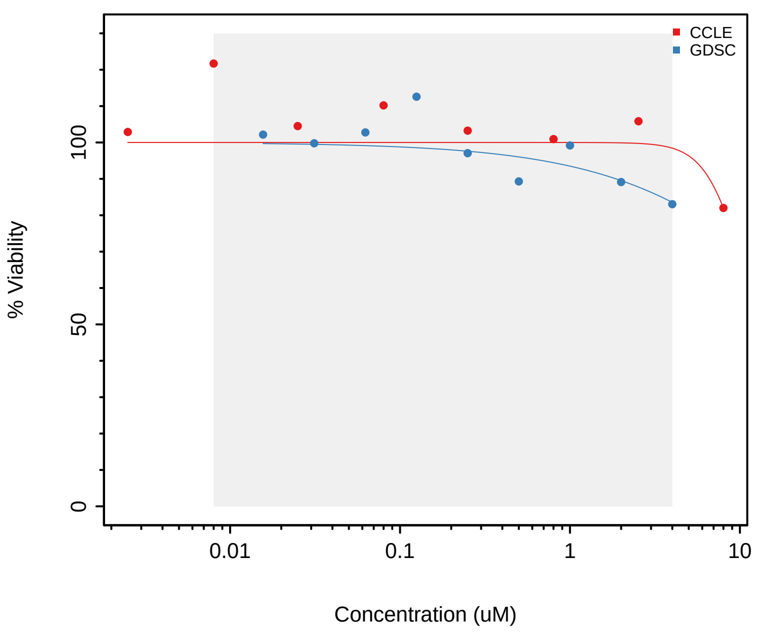

PD−0332991:MEL−HO

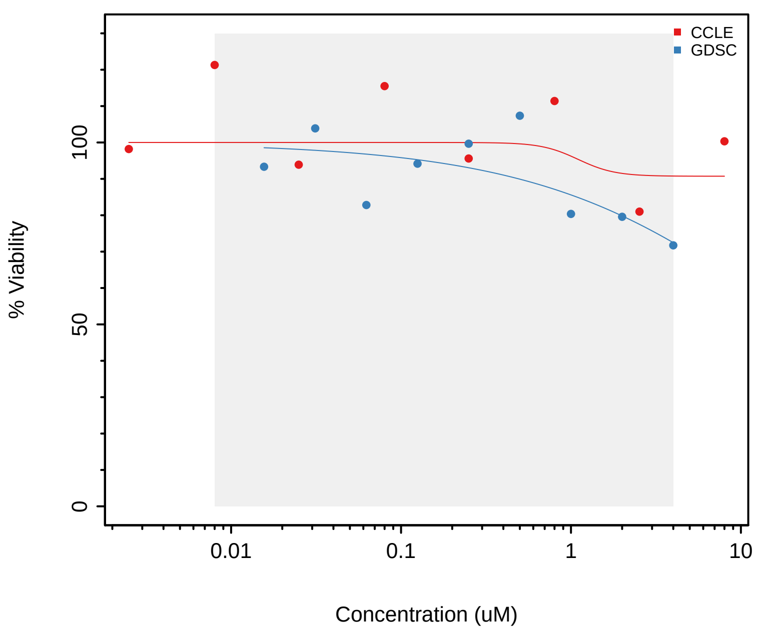

PD−0332991:MES−SA

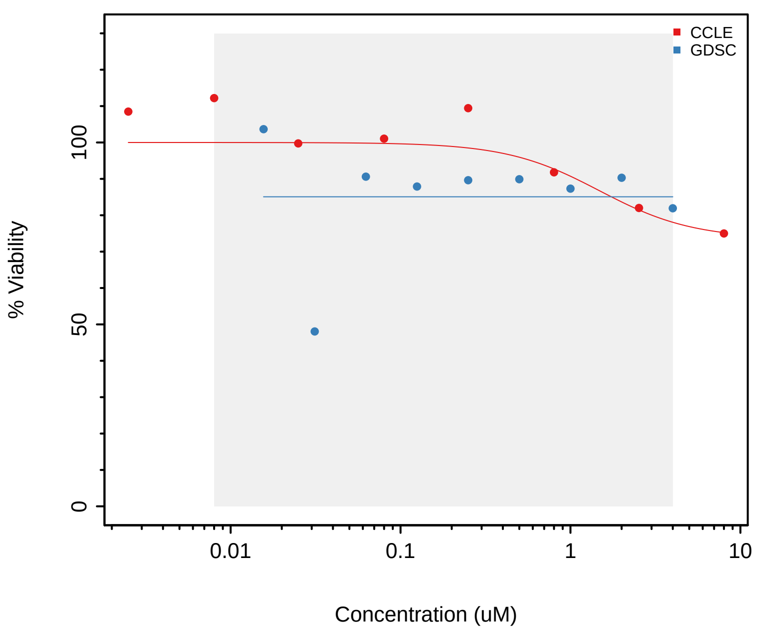

PD−0332991:Mewo

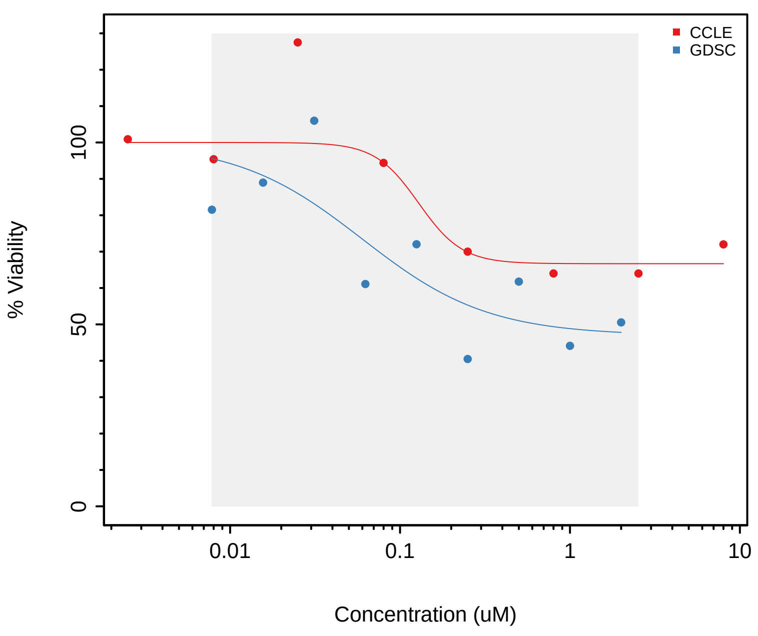

PD−0332991:MFE−280

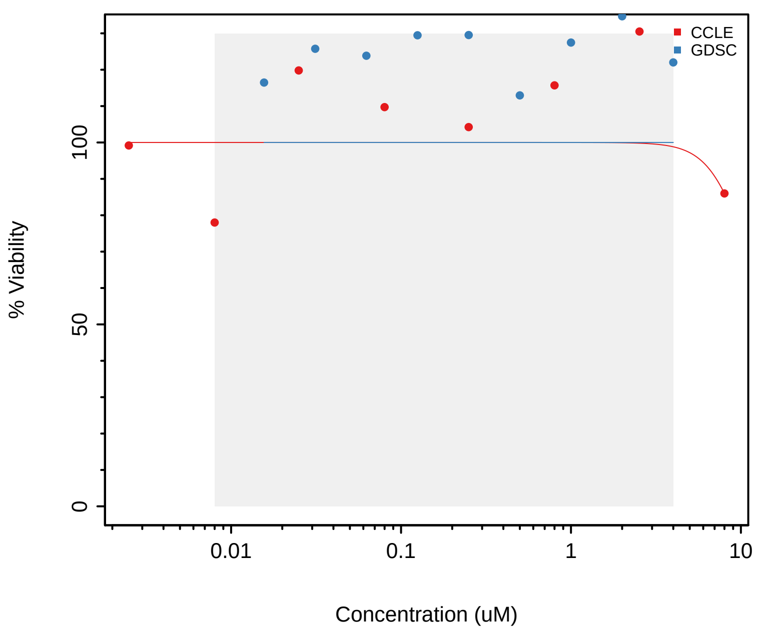

PD−0332991:MG−63

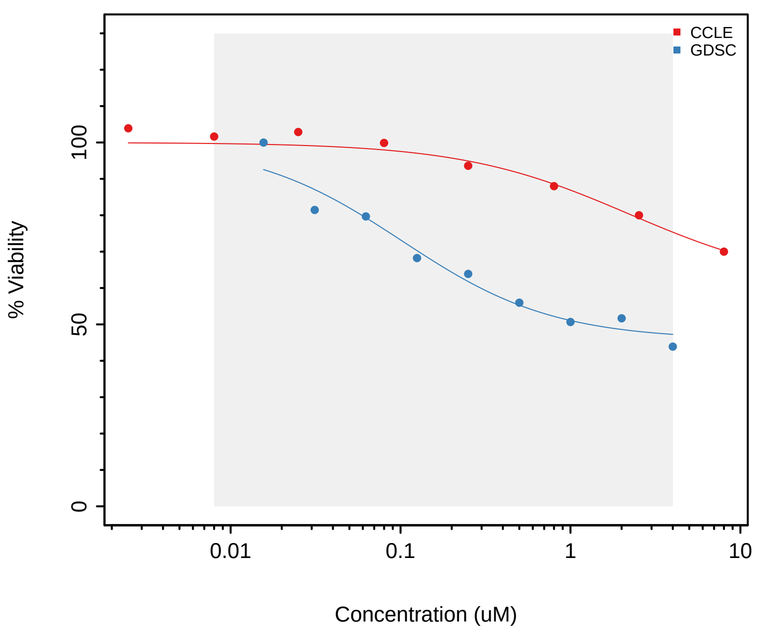

PD−0332991:MHH−ES−1

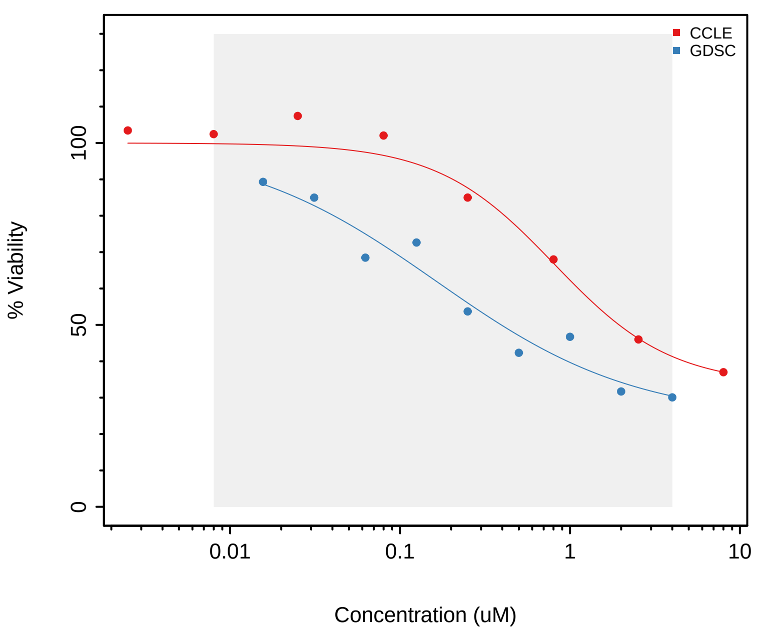

PD−0332991:MIA−PaCa−2

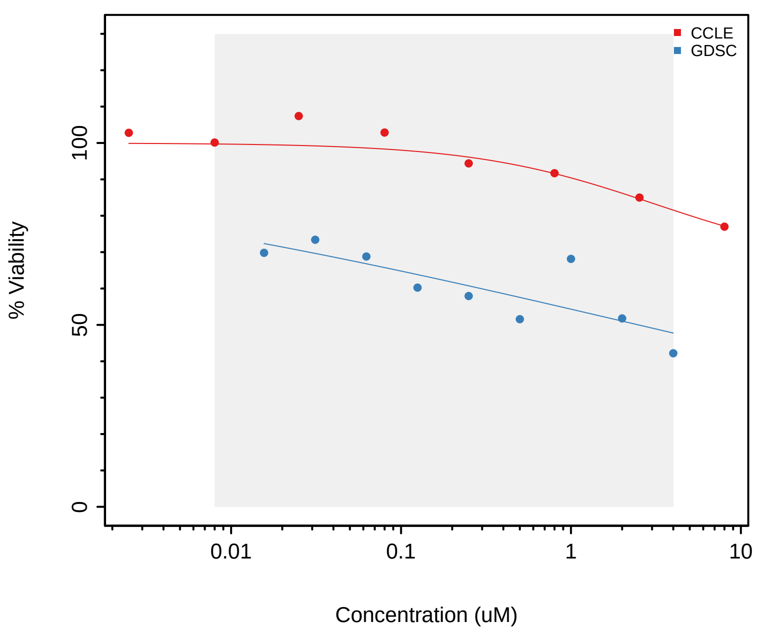

PD−0332991:MOLT−16

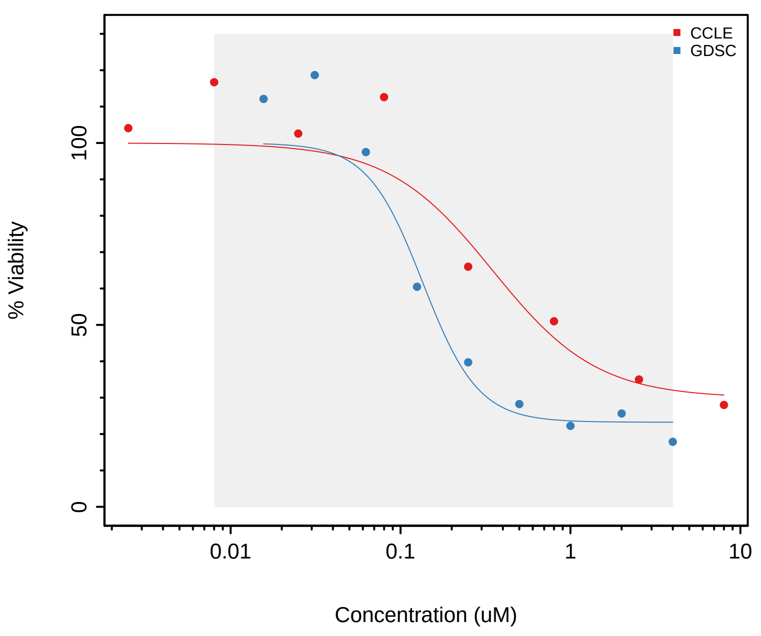

PD−0332991:MPP−89

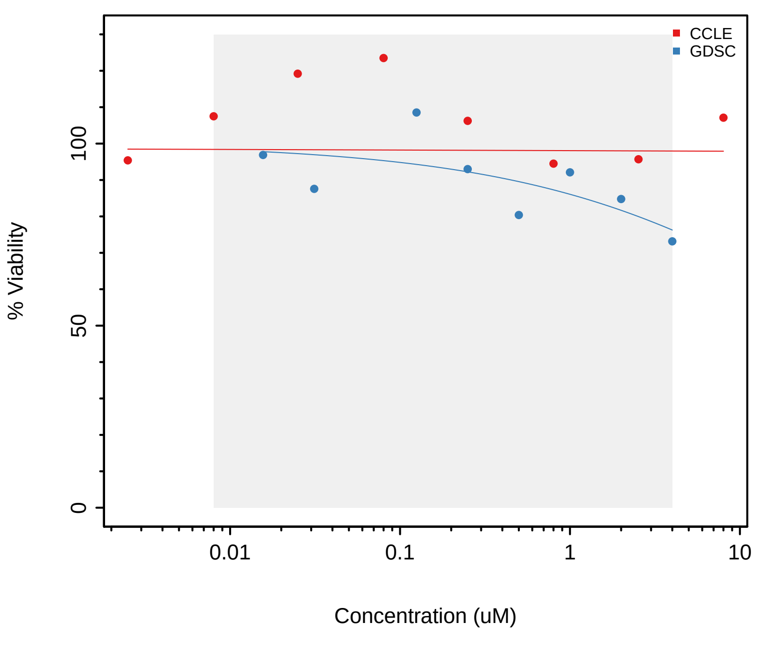

PD−0332991:MSTO−211H

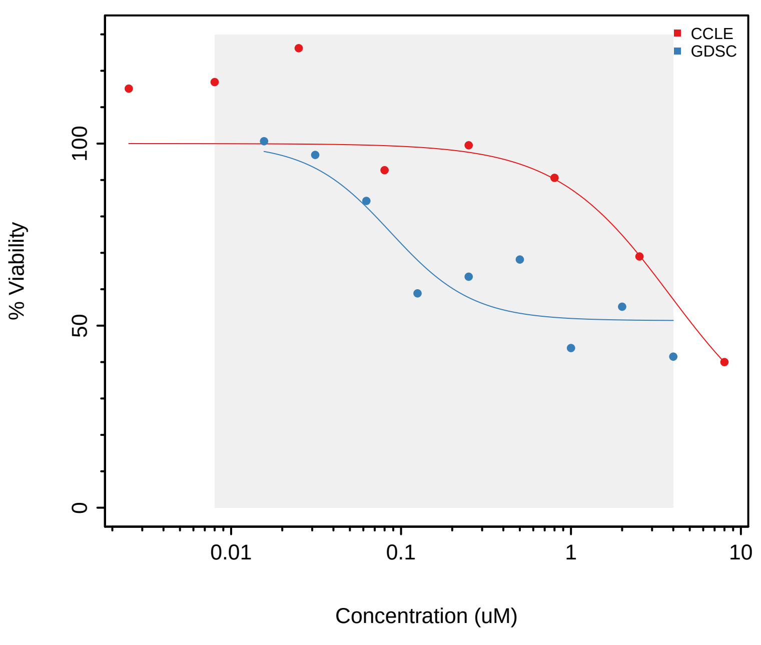

PD−0332991:NCI−H1048

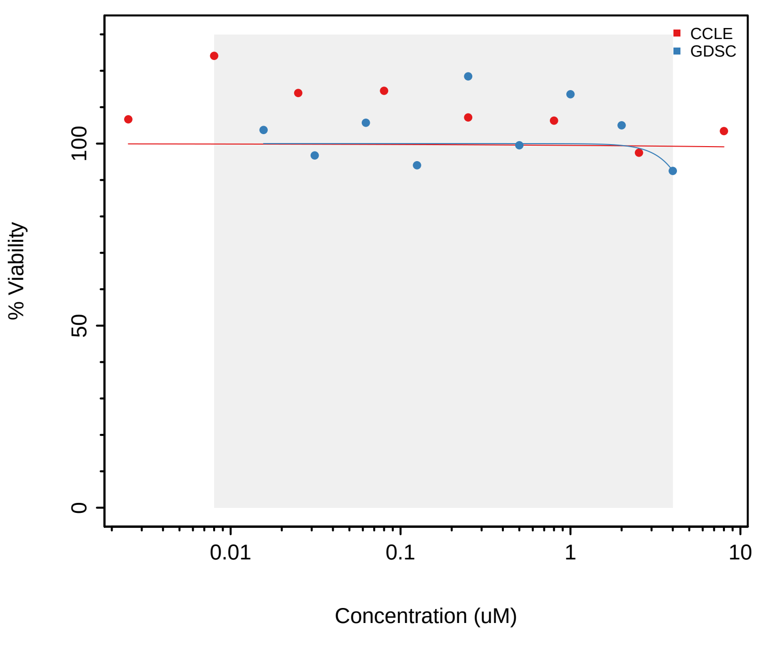

PD−0332991:NCI−H1092

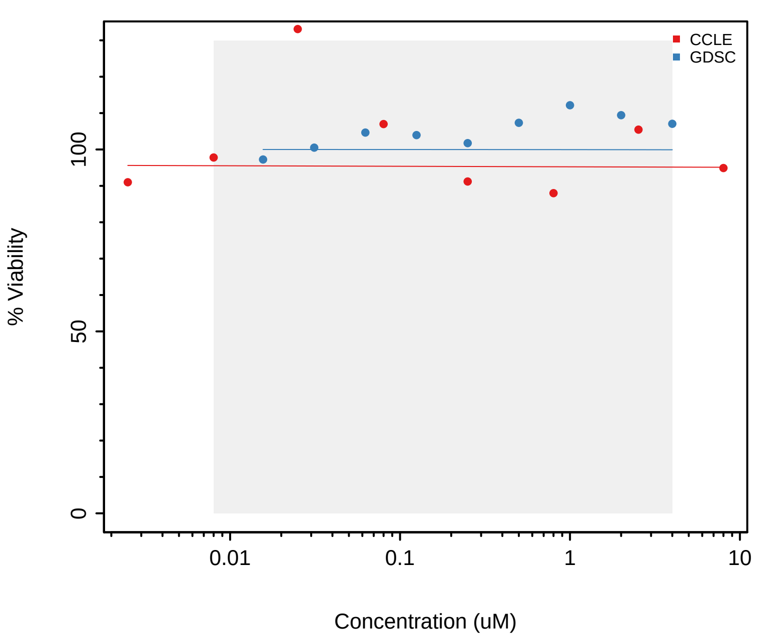

PD−0332991:NCI−H1355

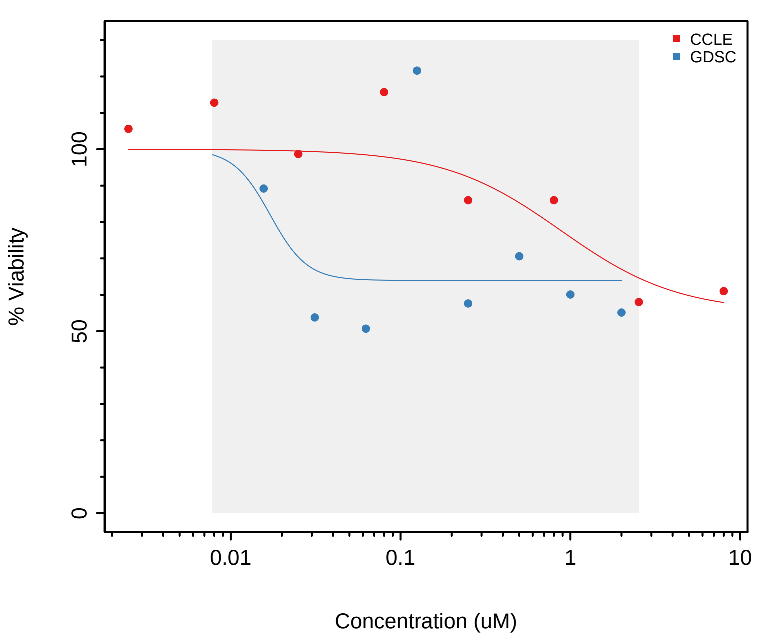

PD−0332991:NCI−H1563

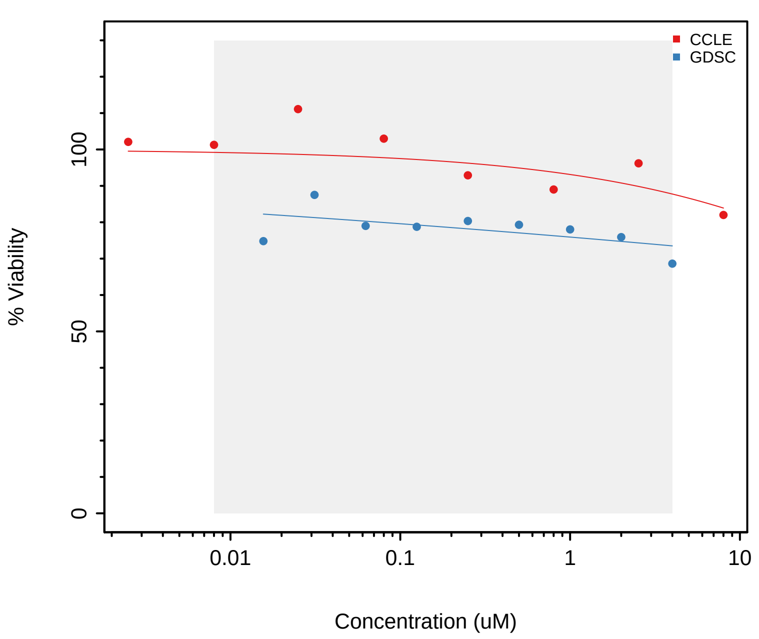

PD−0332991:NCI−H1573

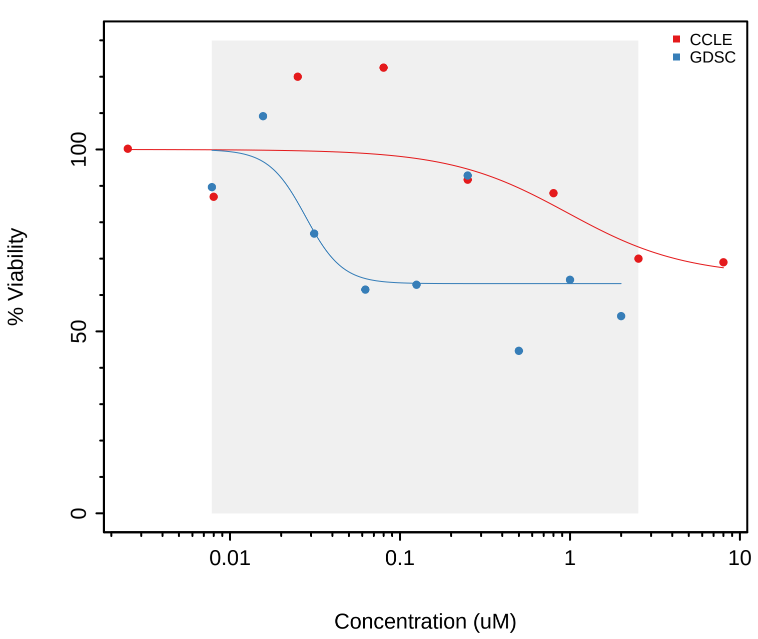

PD−0332991:NCI−H1581

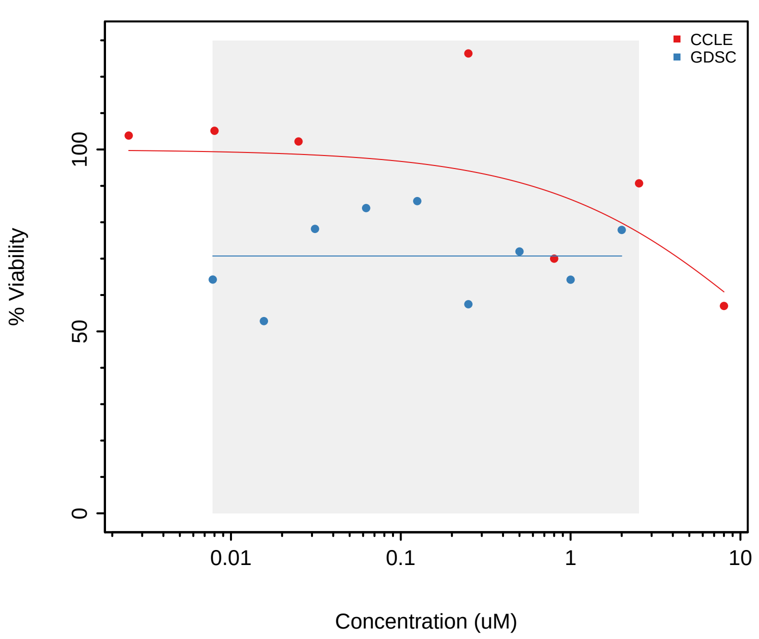

PD−0332991:NCI−H1650

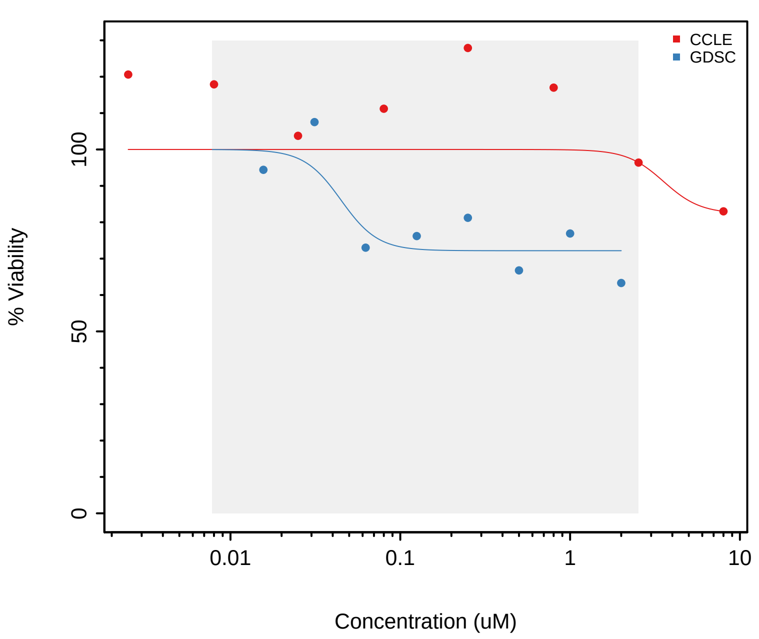

PD−0332991:NCI−H1651

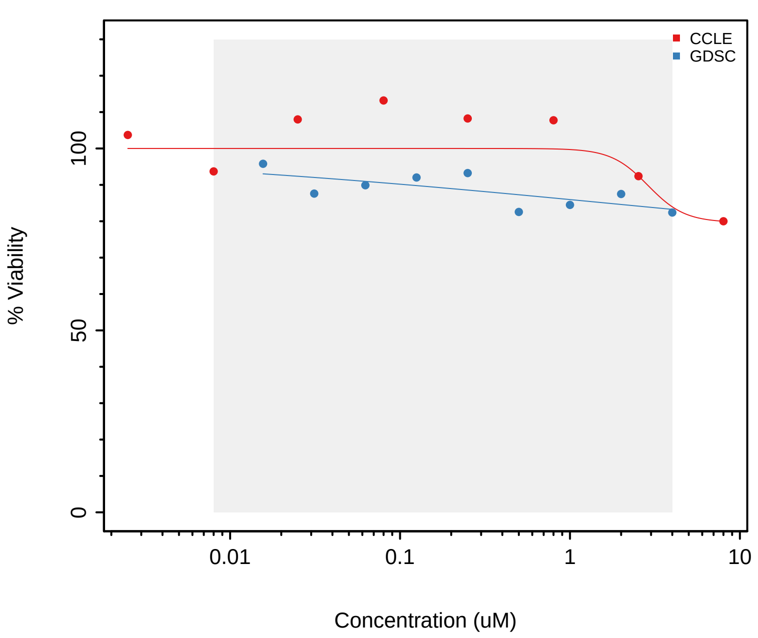

PD−0332991:NCI−H1694

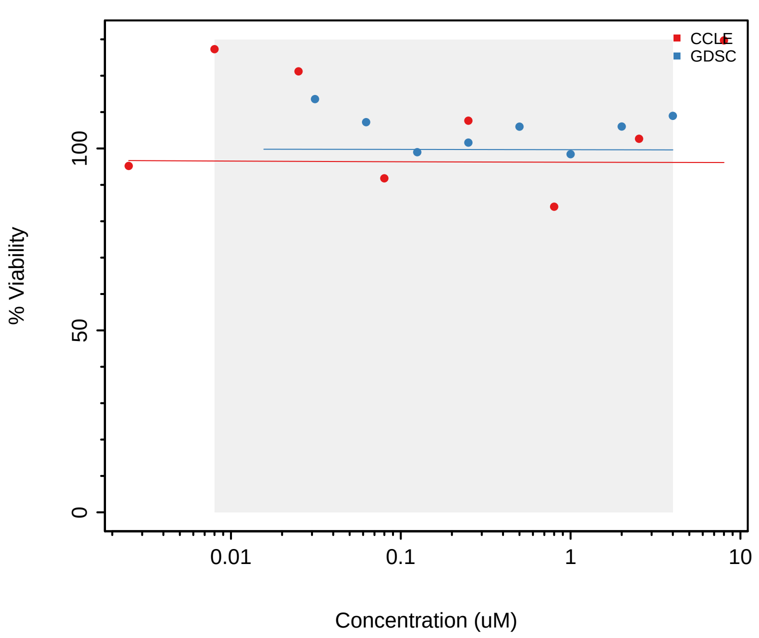

PD−0332991:NCI−H1792

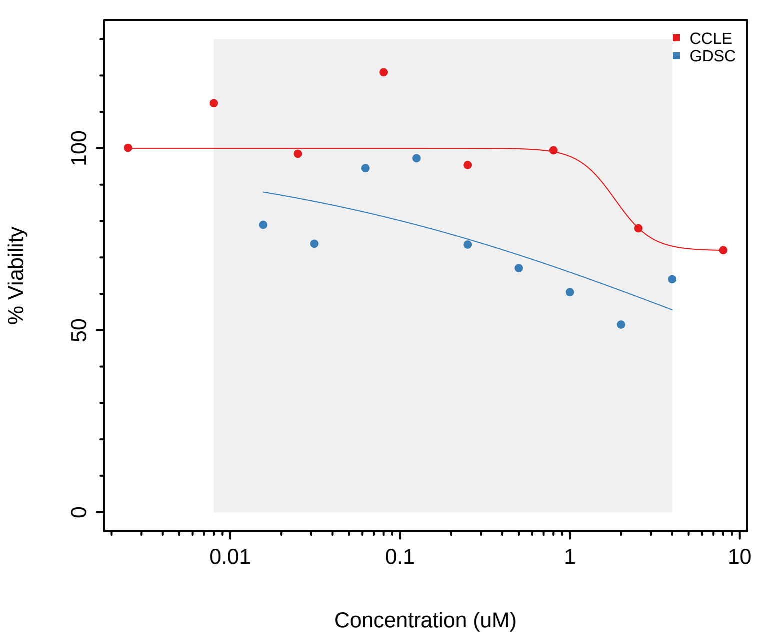

PD−0332991:NCI−H1975

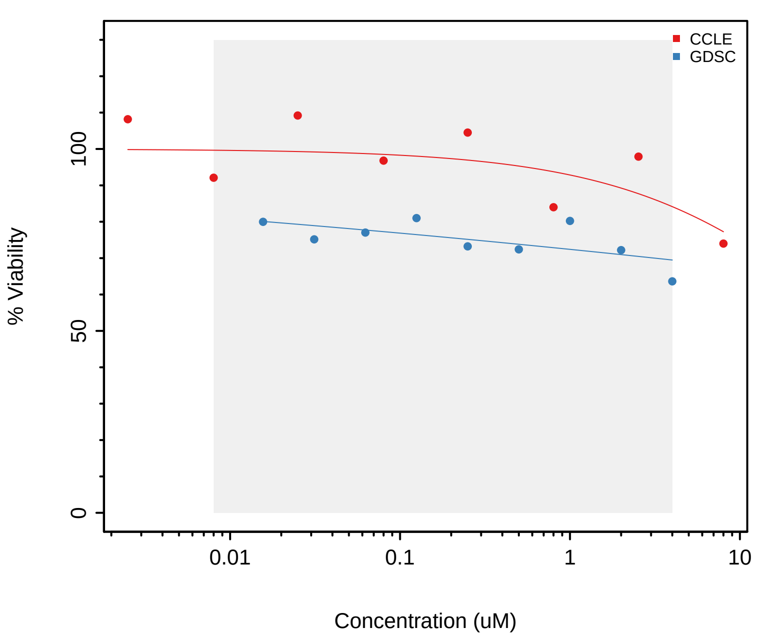

PD−0332991:NCI−H2009

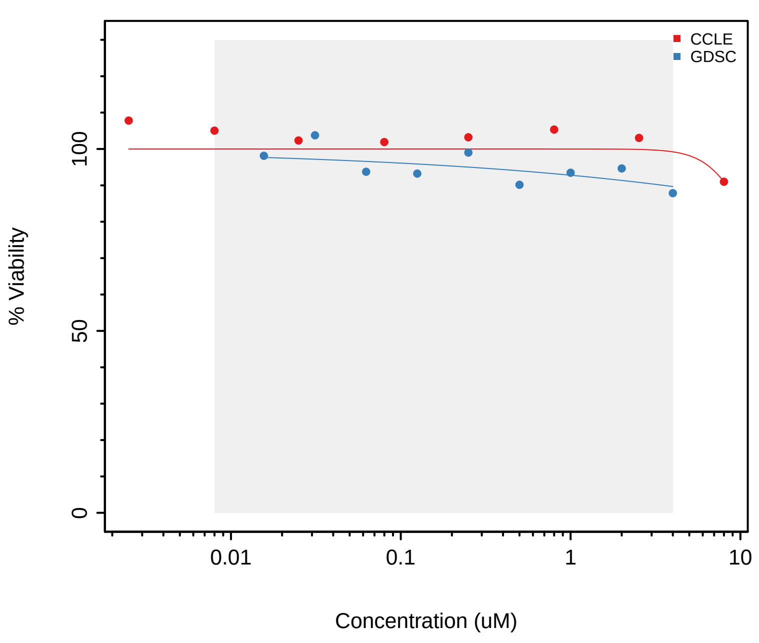

PD−0332991:NCI−H2052

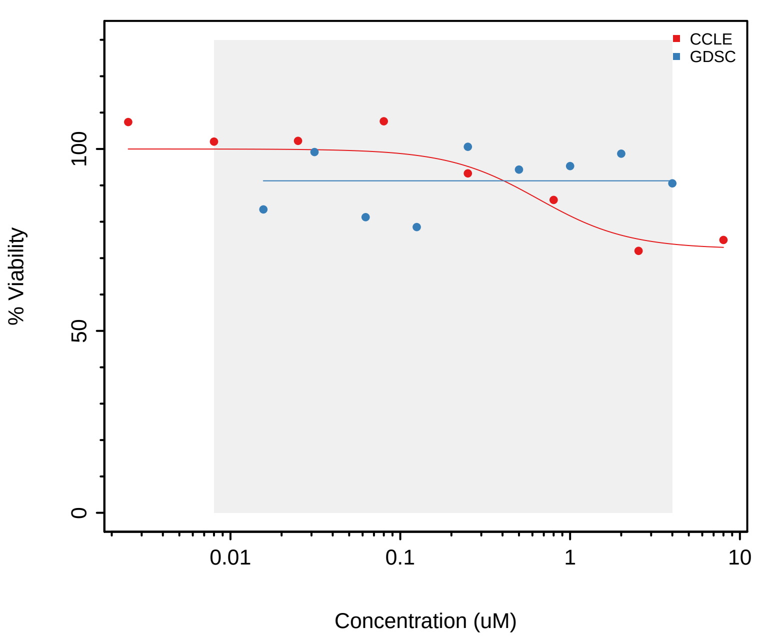

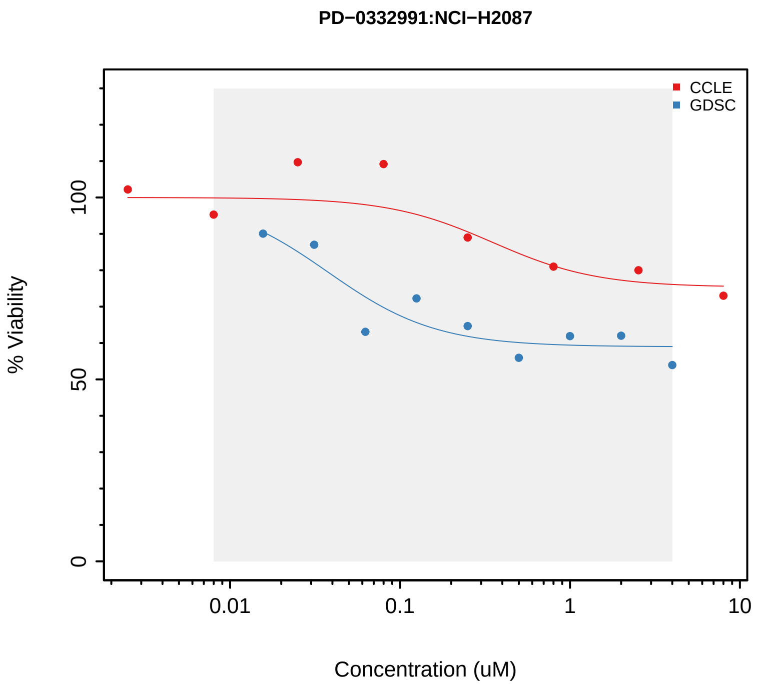
PD−0332991:NCI−H2087
CCLE
GDSC
% Viability
0
50
100
0.01
0.1
1
10
Concentration (uM)

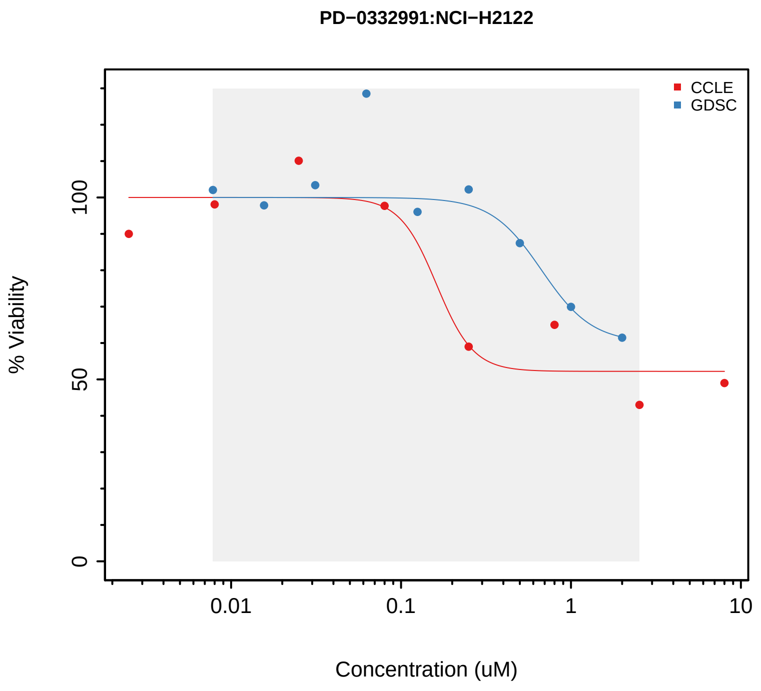
PD−0332991:NCI−H2122
CCLE
GDSC
% Viability
0
50
100
0.01
0.1
1
10
Concentration (uM)

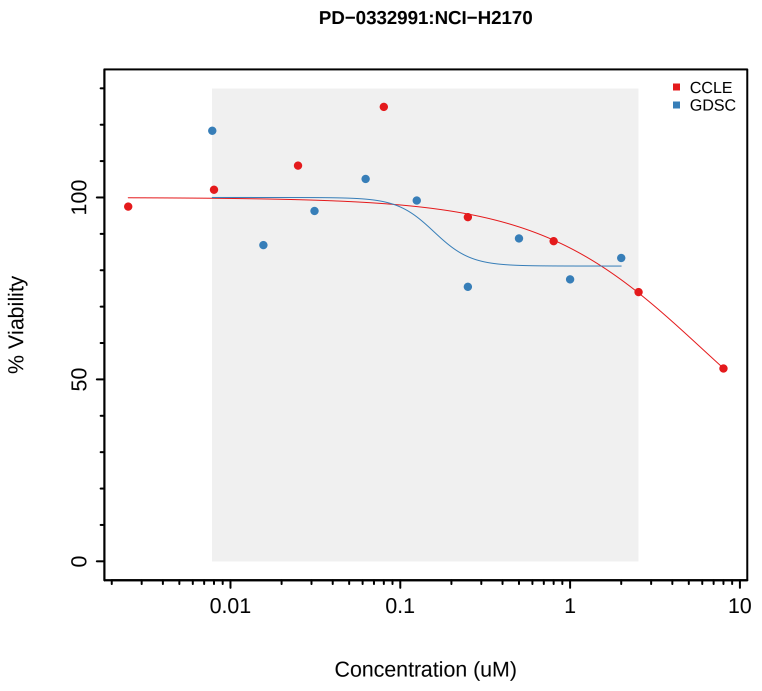
PD−0332991:NCI−H2170
CCLE
GDSC
% Viability
0
50
100
0.01
0.1
1
10
Concentration (uM)

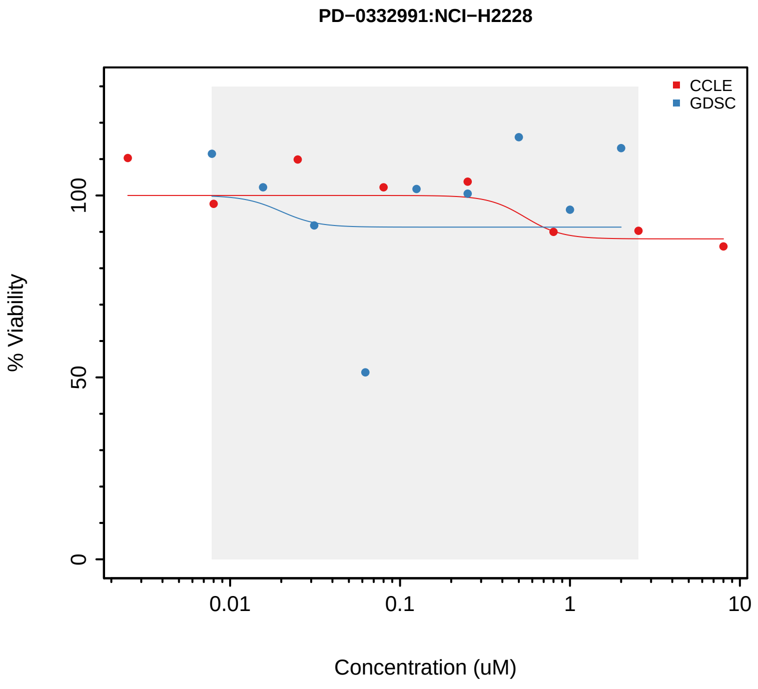
PD−0332991:NCI−H2228
CCLE
GDSC
% Viability
0
50
100
0.01
0.1
1
10
Concentration (uM)

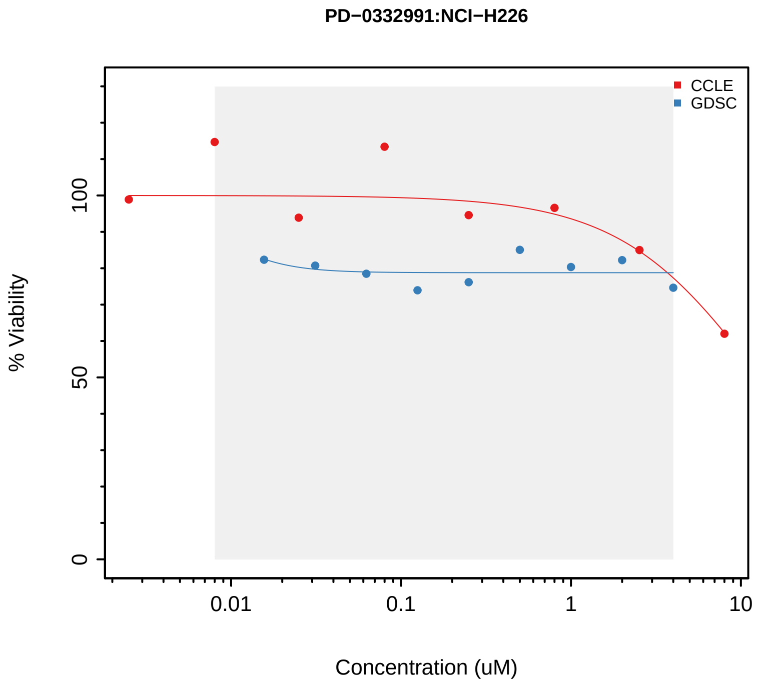
PD−0332991:NCI−H226
CCLE
GDSC
% Viability
0
50
100
0.01
0.1
1
10
Concentration (uM)

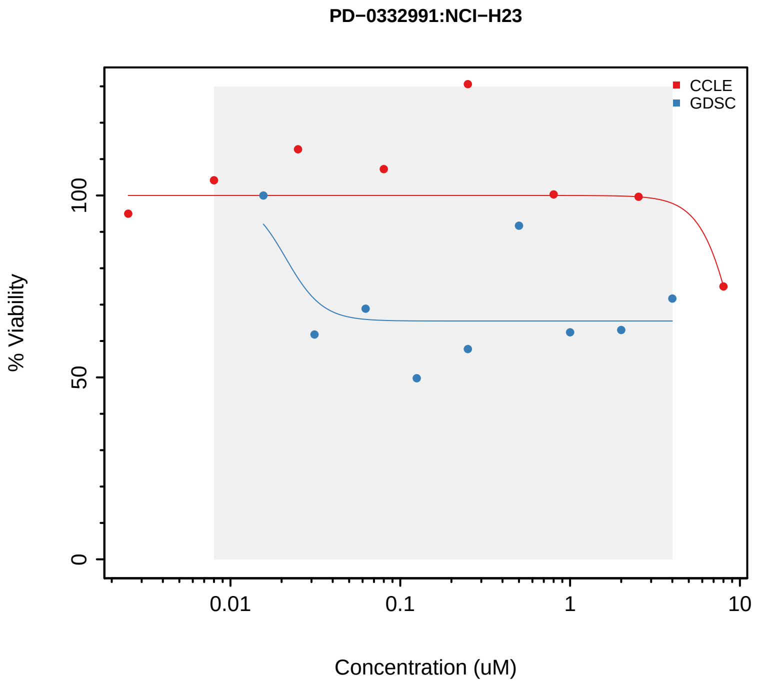
PD−0332991:NCI−H23
CCLE
GDSC
% Viability
0
50
100
0.01
0.1
1
10
Concentration (uM)

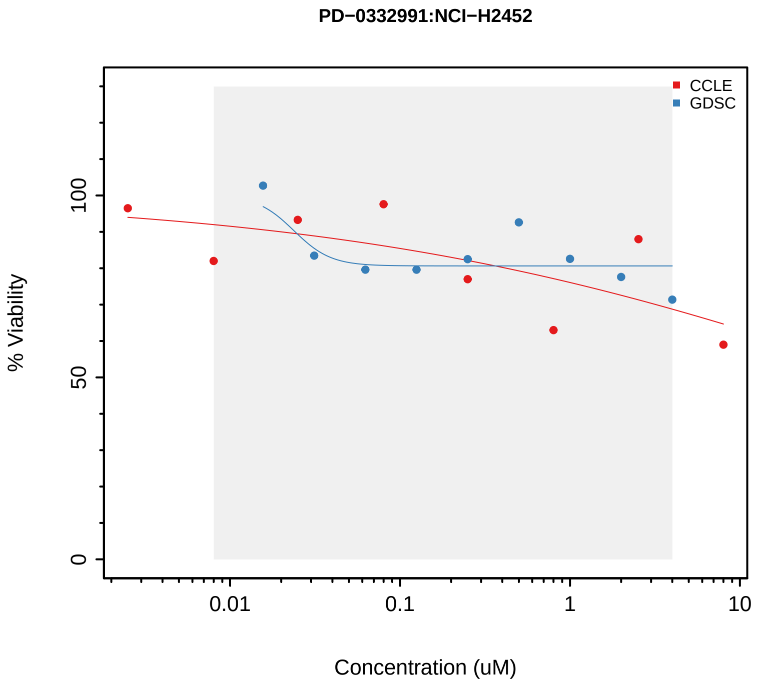
PD−0332991:NCI−H2452
CCLE
GDSC
% Viability
0
50
100
0.01
0.1
1
10
Concentration (uM)

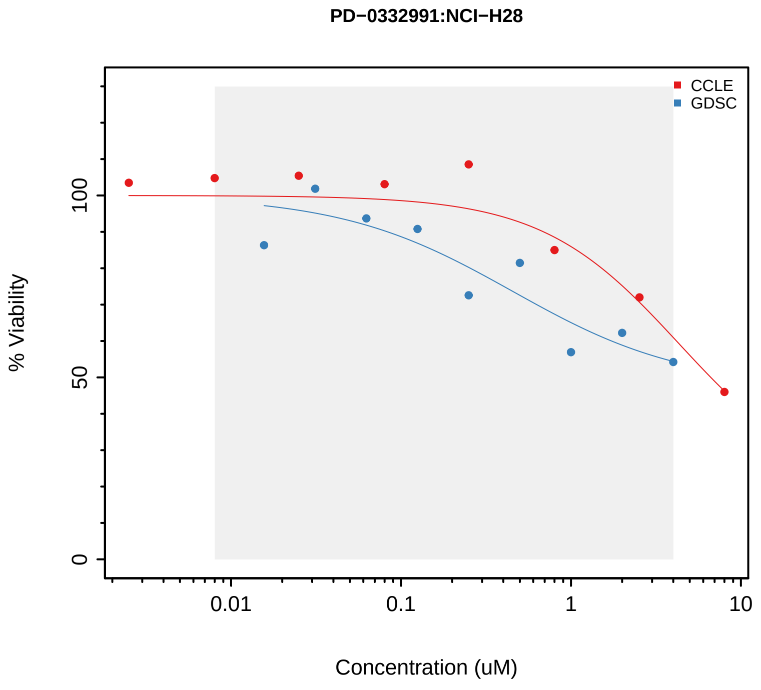
PD−0332991:NCI−H28
CCLE
GDSC
% Viability
0
50
100
0.01
0.1
1
10
Concentration (uM)

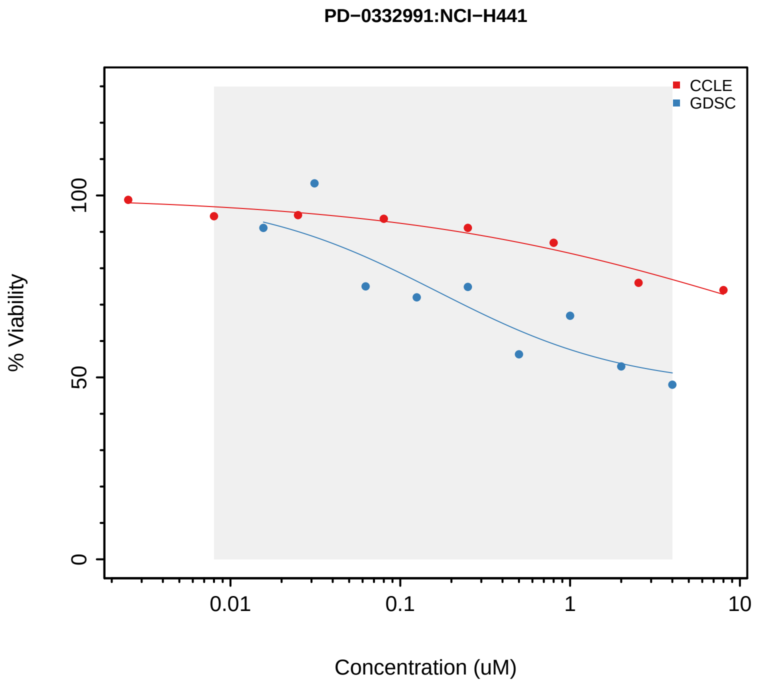
PD−0332991:NCI−H441
CCLE
GDSC
% Viability
0
50
100
0.01
0.1
1
10
Concentration (uM)

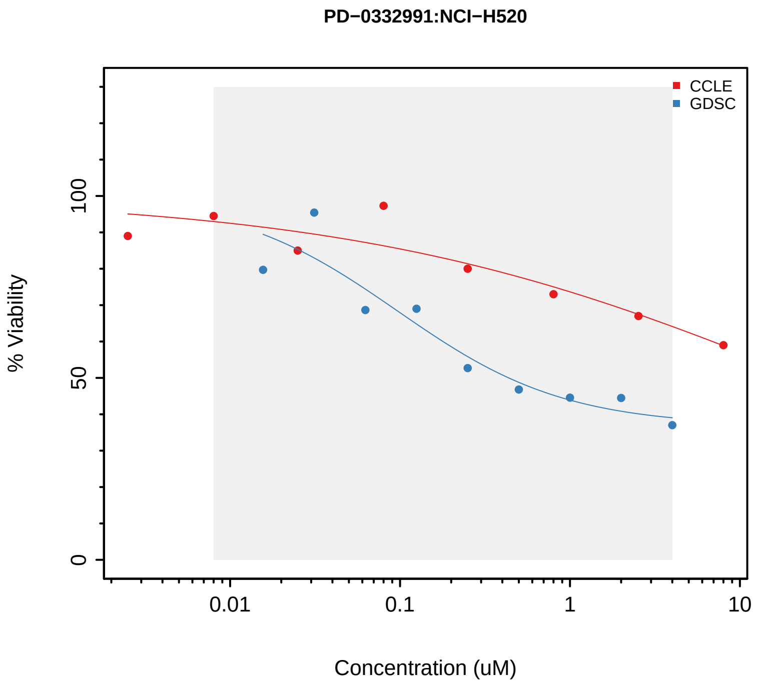
PD−0332991:NCI−H520
CCLE
GDSC
% Viability
0
50
100
0.01
0.1
1
10
Concentration (uM)

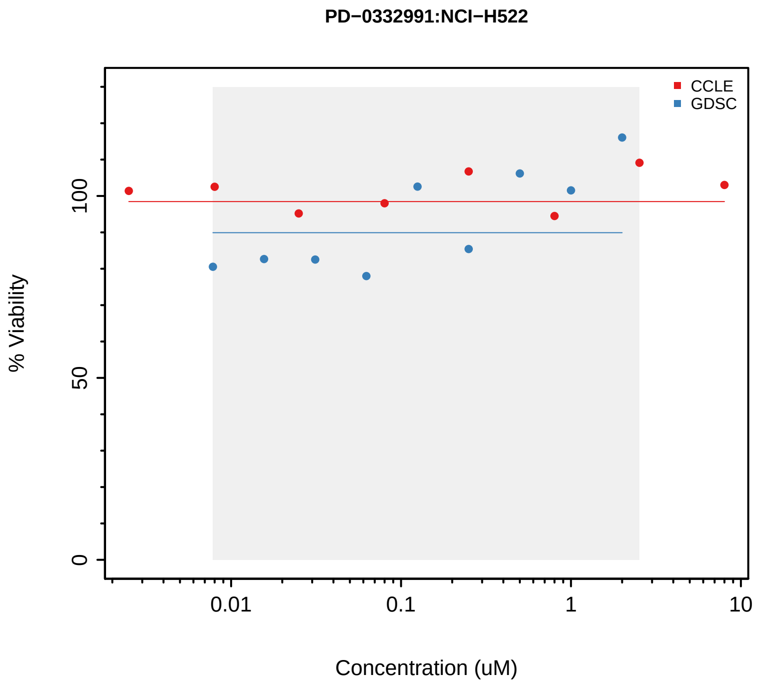
PD−0332991:NCI−H522
CCLE
GDSC
% Viability
0
50
100
0.01
0.1
1
10
Concentration (uM)

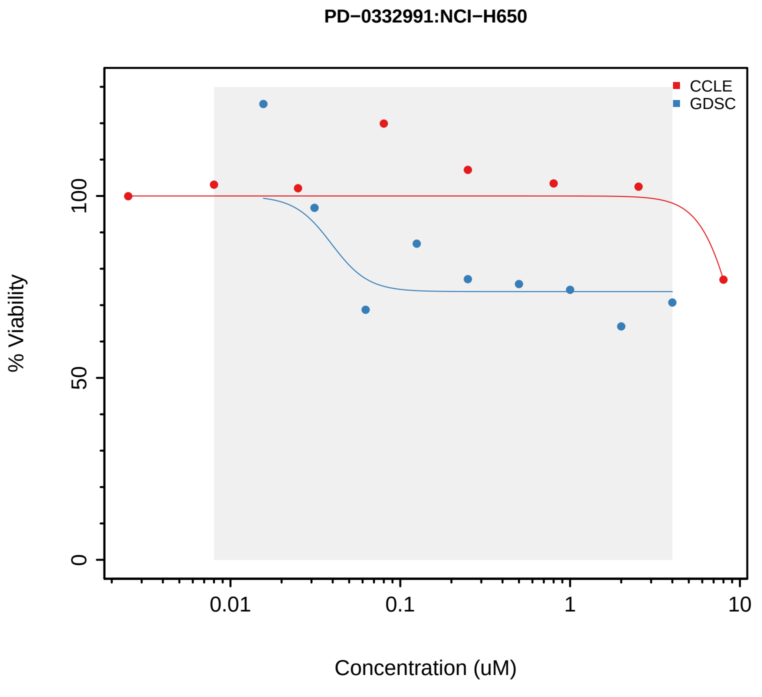
PD−0332991:NCI−H650
CCLE
GDSC
% Viability
0
50
100
0.01
0.1
1
10
Concentration (uM)

PD−0332991:NCI−H661

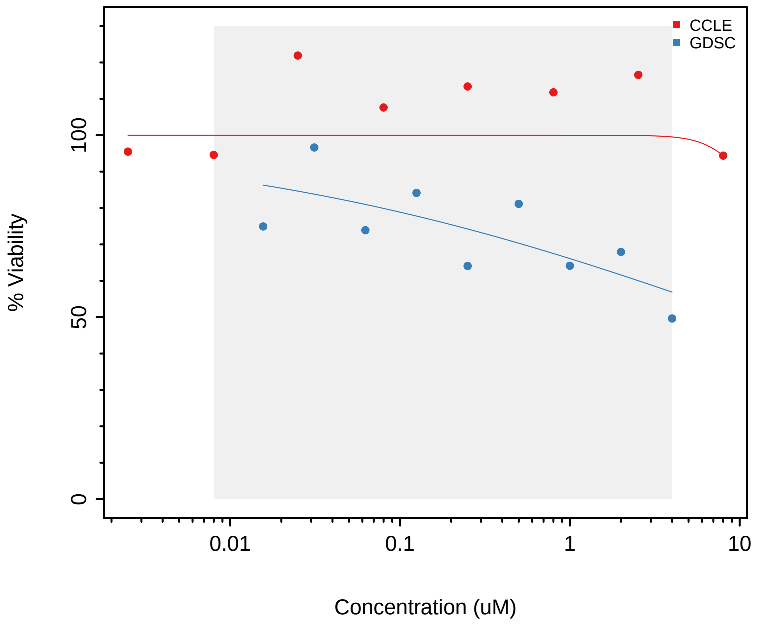

PD−0332991:NCI−H747

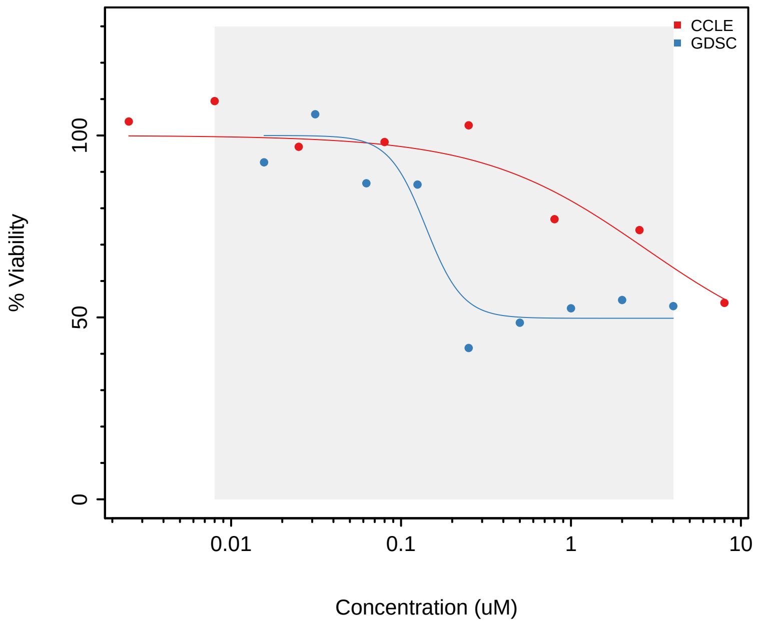

PD−0332991:NCI−H810

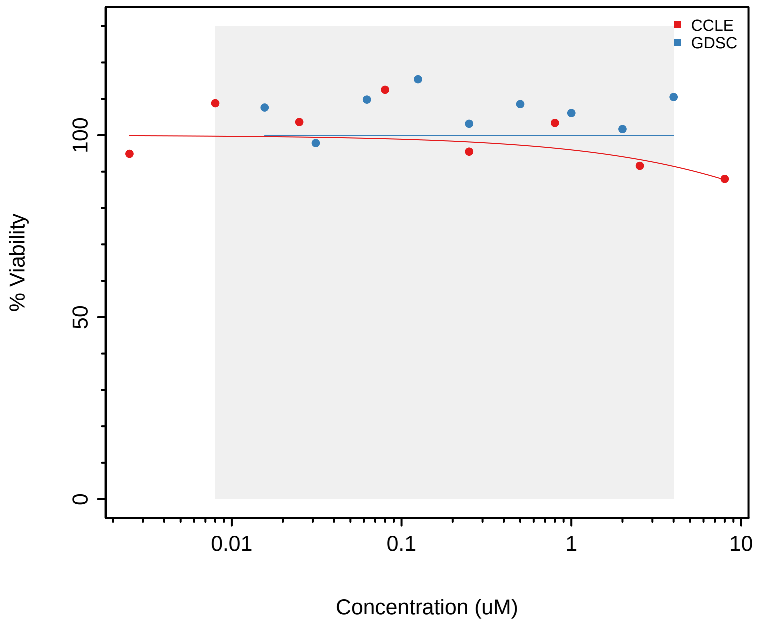

PD−0332991:NCI−N87

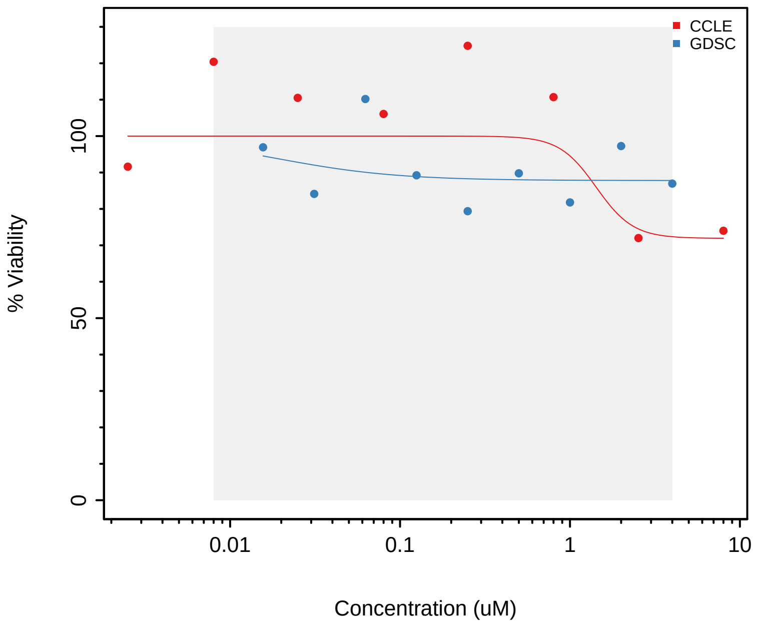

PD−0332991:OVCAR−3

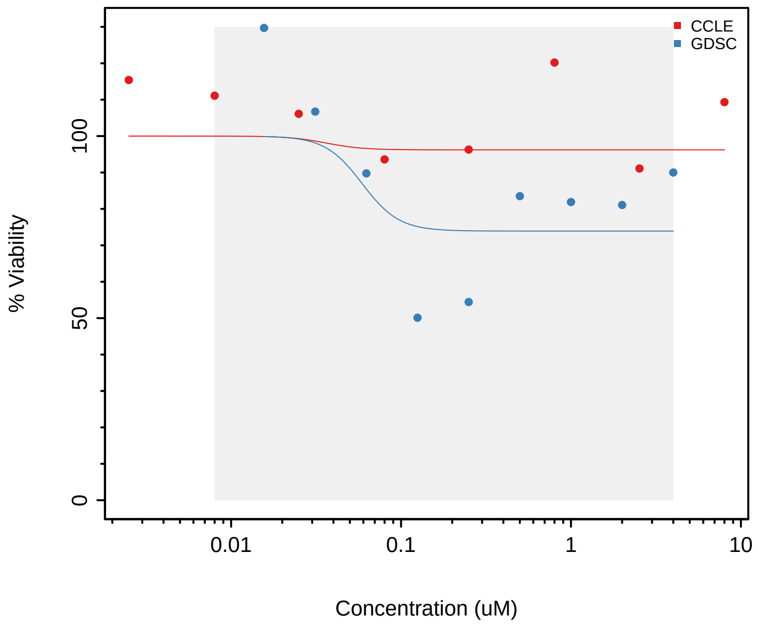

PD−0332991:NUGC−3

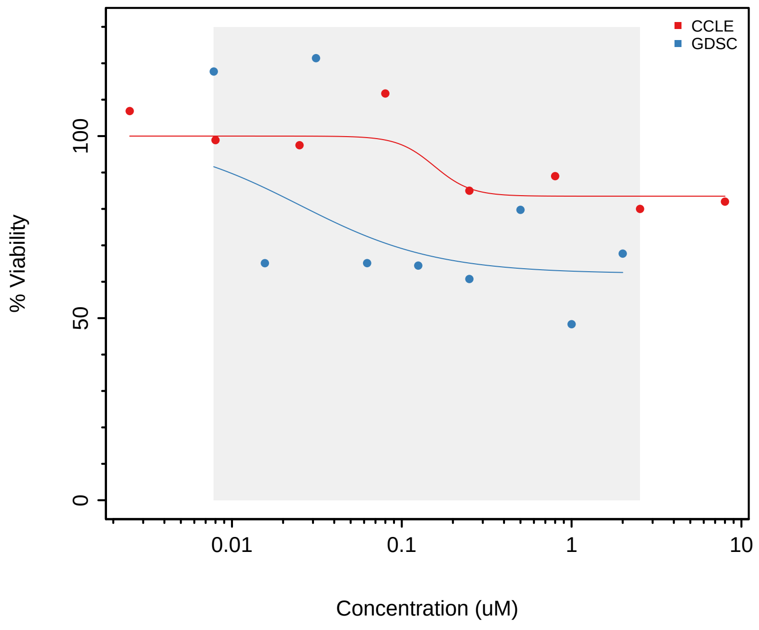

PD−0332991:OC−314

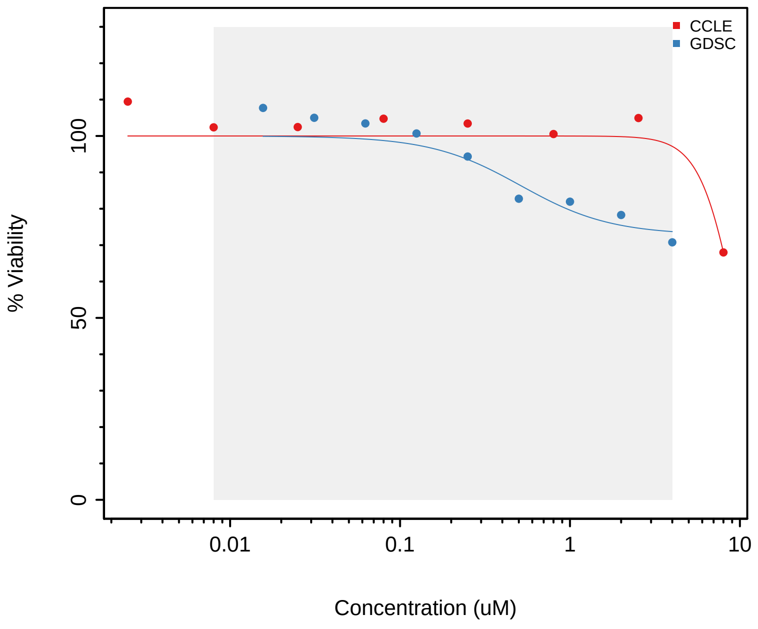

PD−0332991:OCI−AML2

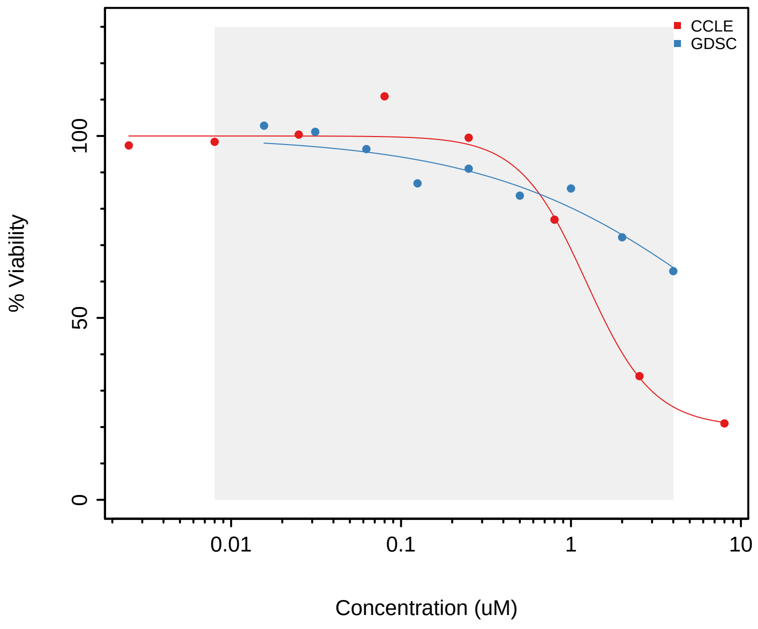

PD−0332991:OE33

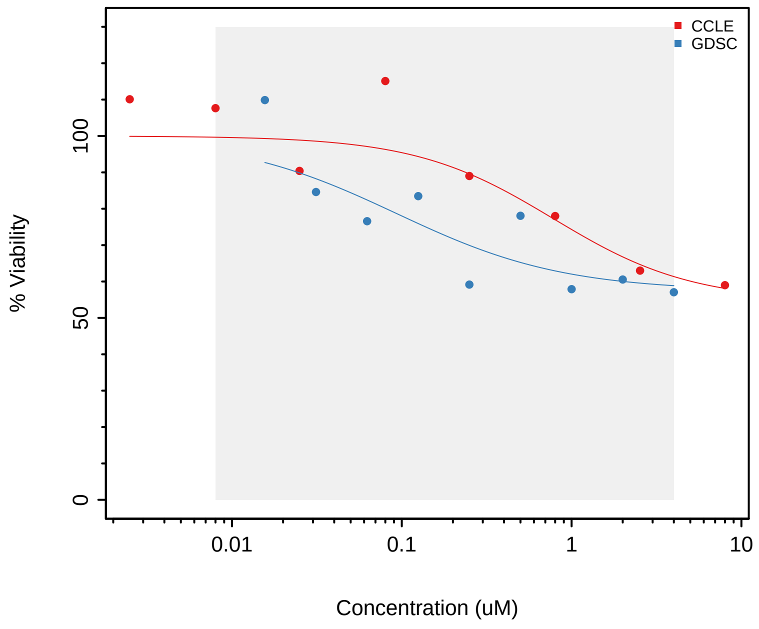

PD−0332991:ONS−76

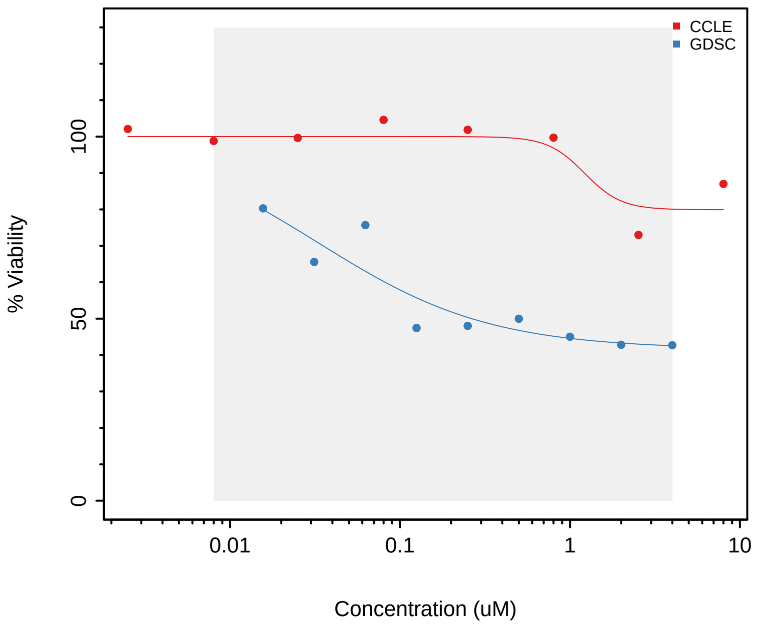

PD−0332991:OPM−2

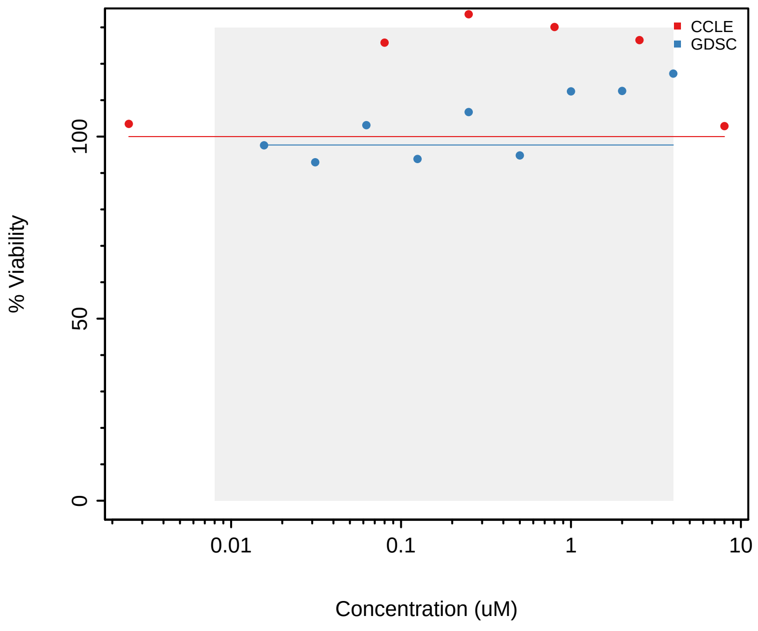

PD−0332991:OVCAR−4

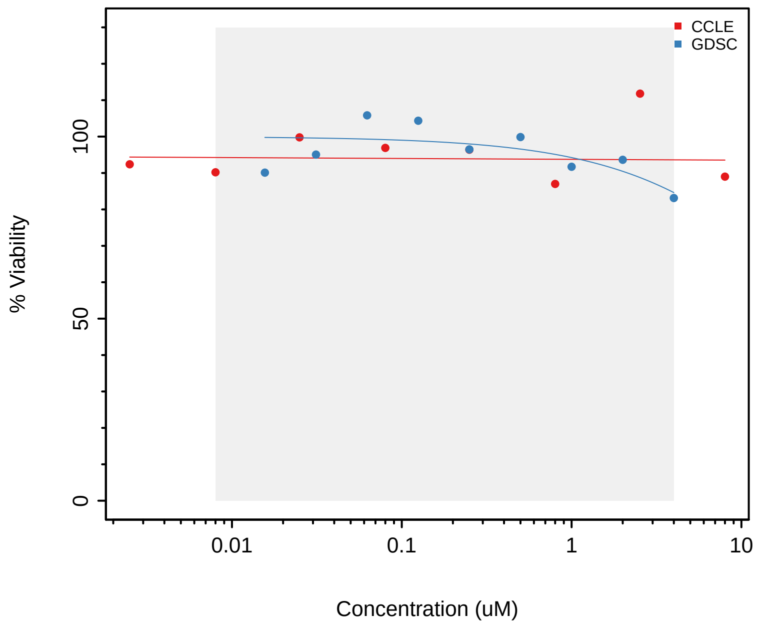

PD−0332991:OVCAR−8

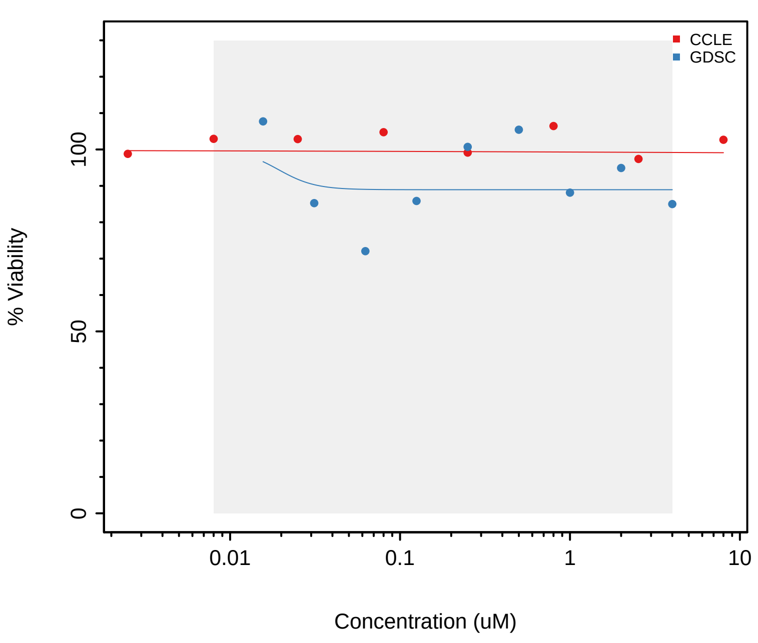

PD−0332991:P12−ICHIKAWA

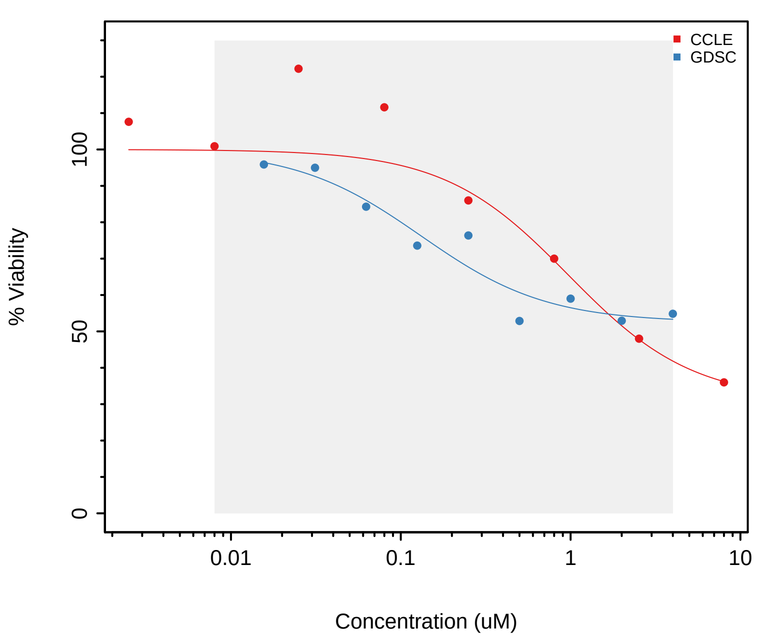

PD−0332991:P31−FUJ

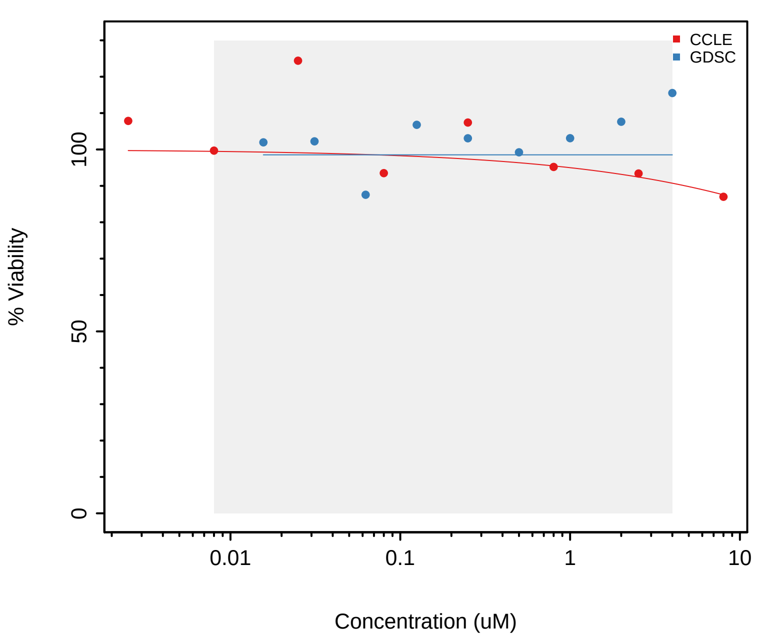

PD−0332991:Panc 03.27

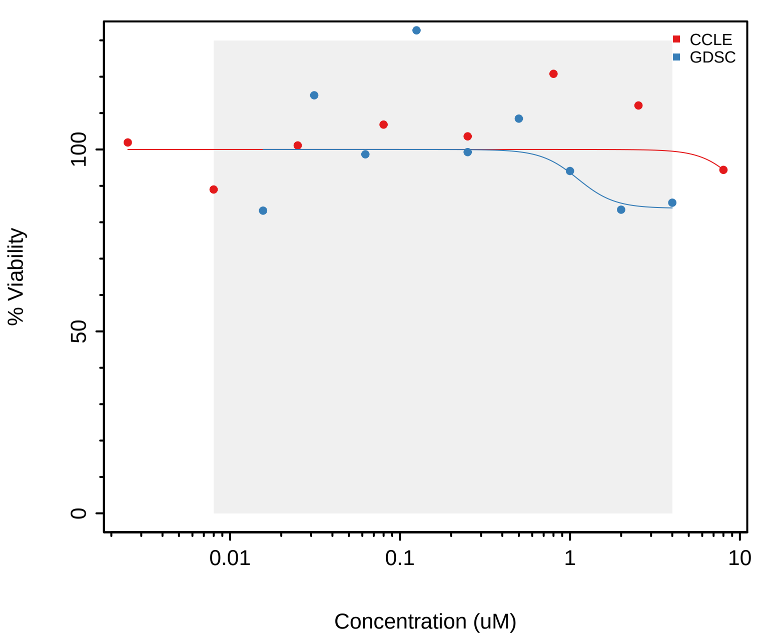

PD−0332991:Raji

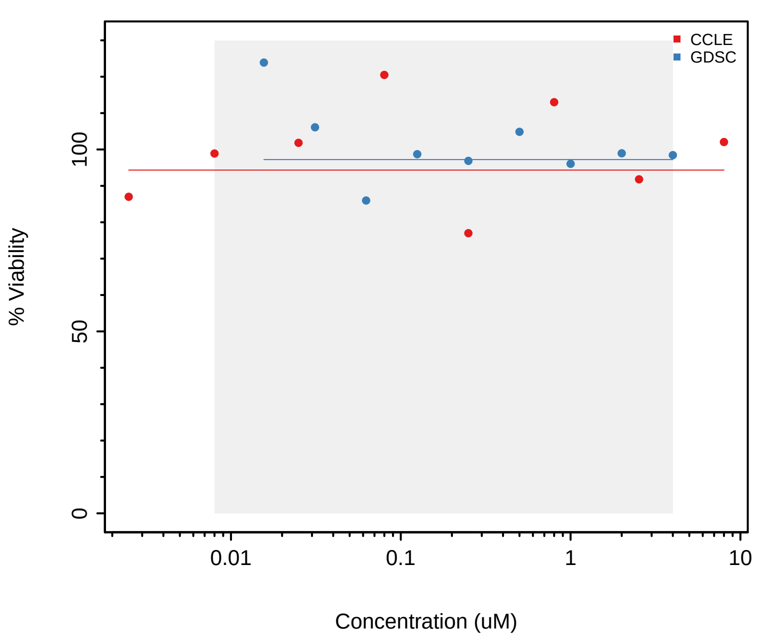

PD−0332991:RD

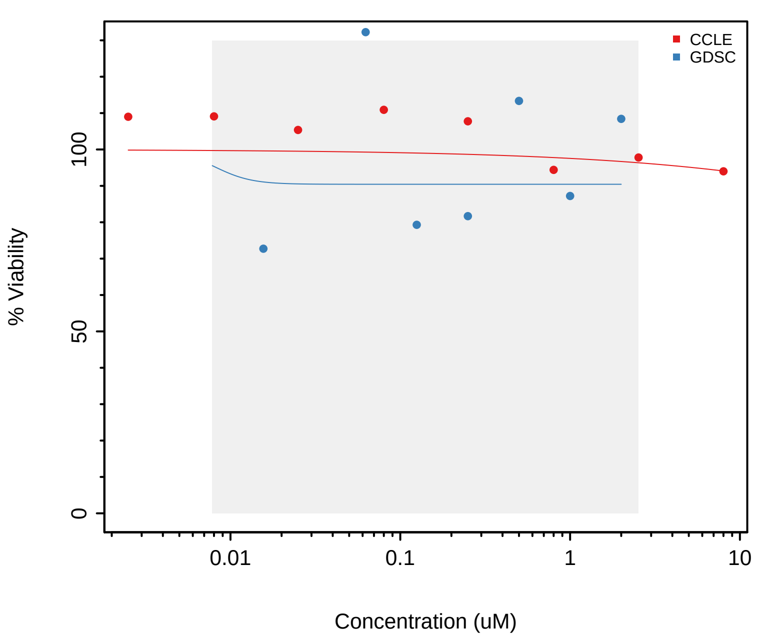

PD−0332991:REH

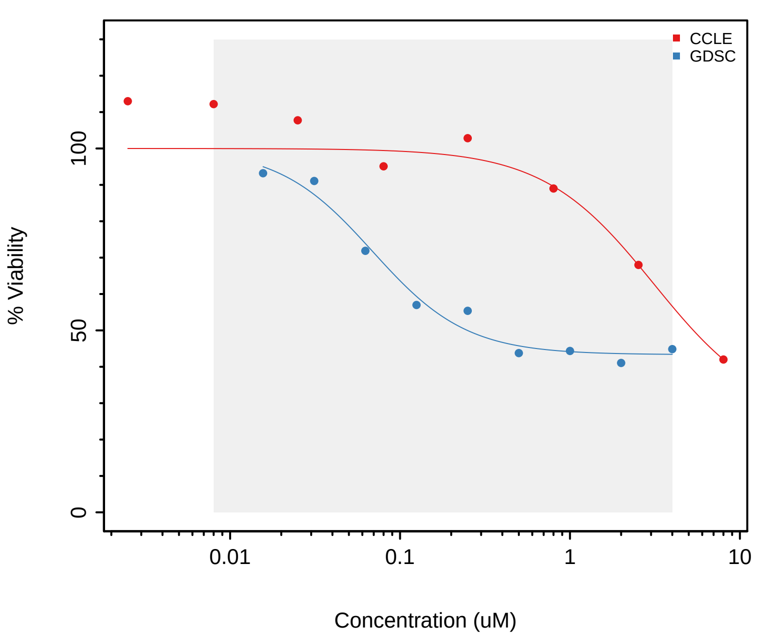

PD−0332991:RERF−LC−MS

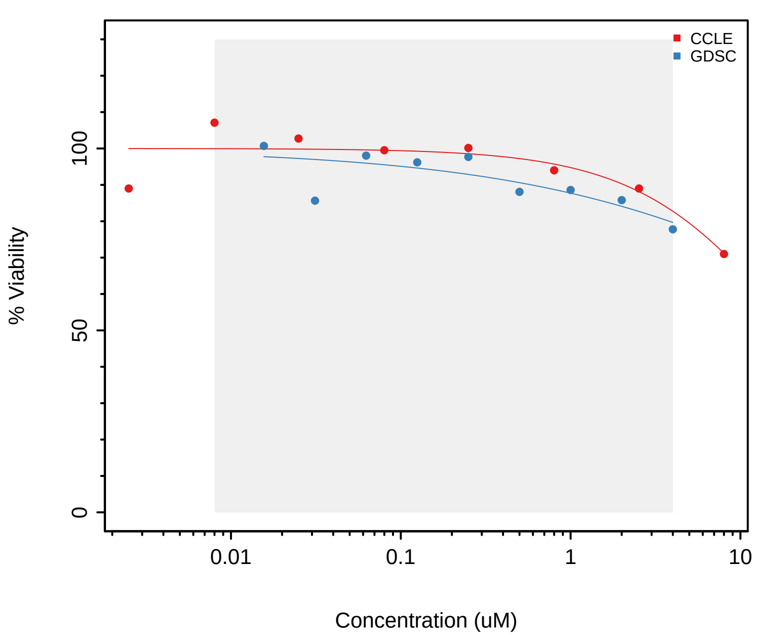

PD−0332991:RKO

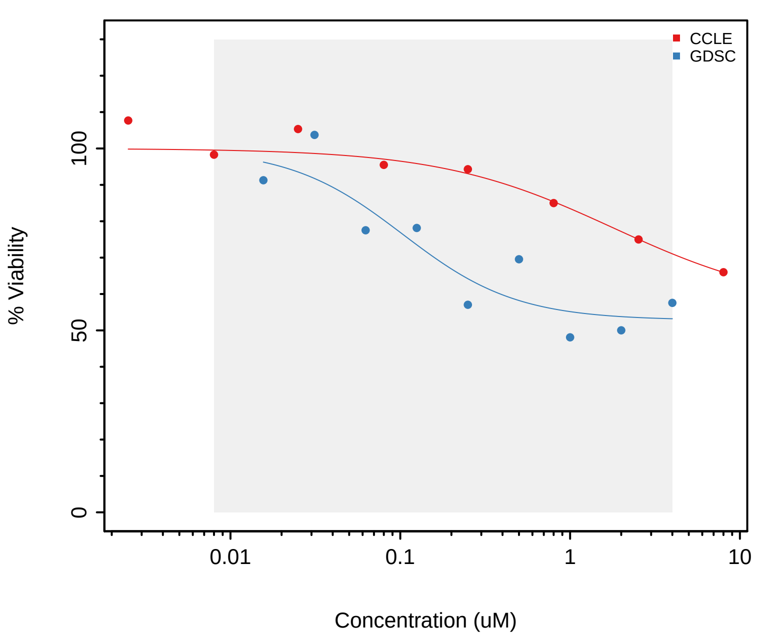

PD−0332991:RL95−2

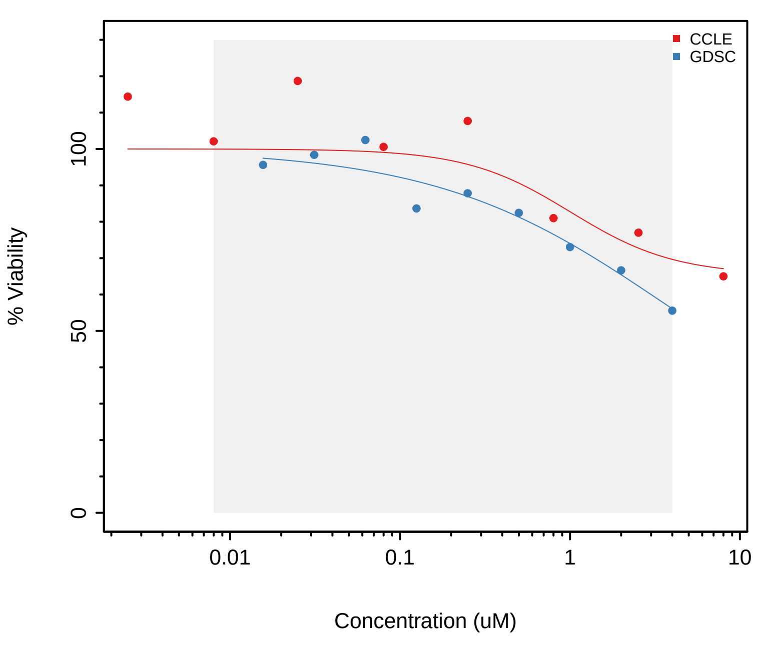

PD−0332991:RPMI−8402

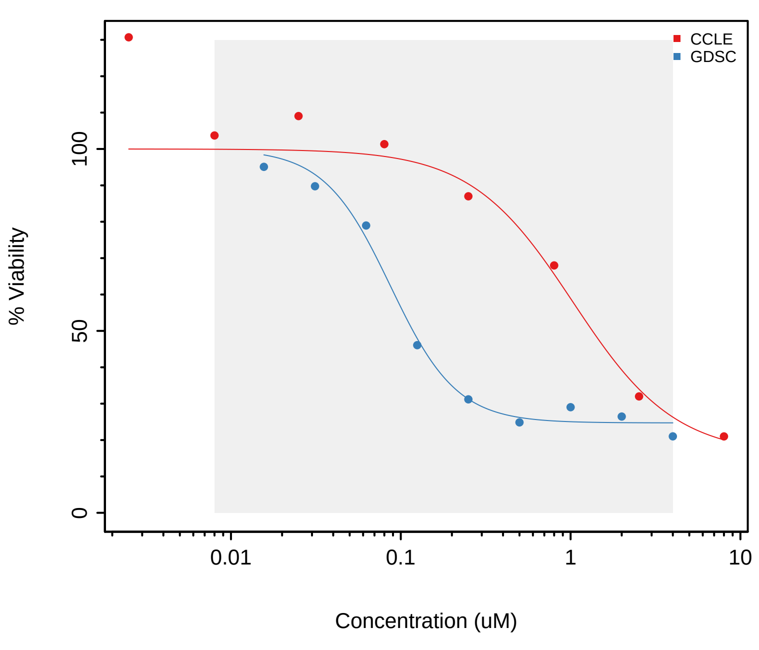

PD−0332991:RT−112

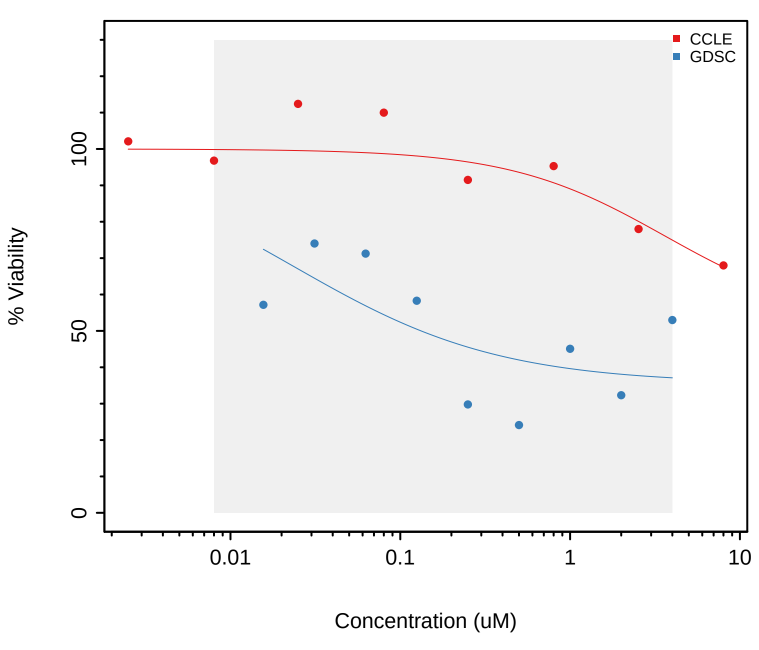

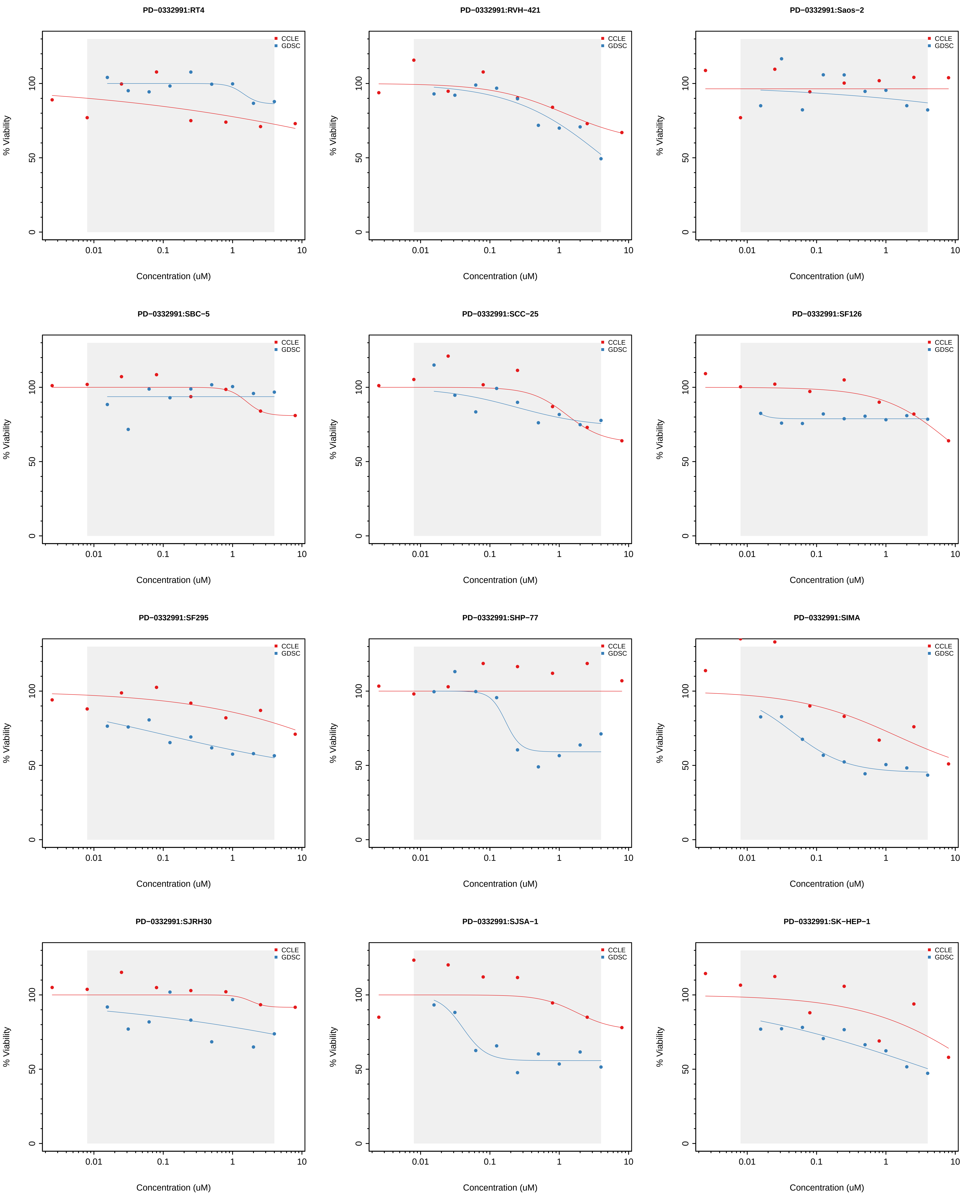
PD−0332991:RT4
PD−0332991:RVH−421
PD−0332991:Saos−2
PD−0332991:SBC−5
PD−0332991:SCC−25
PD−0332991:SF126
PD−0332991:SF295
PD−0332991:SHP−77
PD−0332991:SIMA
PD−0332991:SJRH30
PD−0332991:SJSA−1
PD−0332991:SK−HEP−1
CCLE
GDSC
% Viability
0
50
100
Concentration (uM)
0.01
0.1
1
10

PD−0332991:SK−LU−1

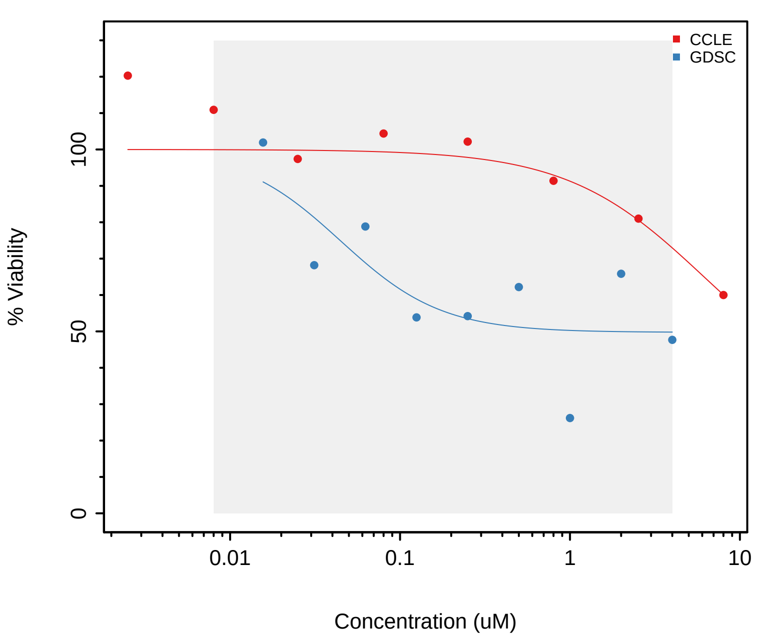

PD−0332991:SK−MEL−2

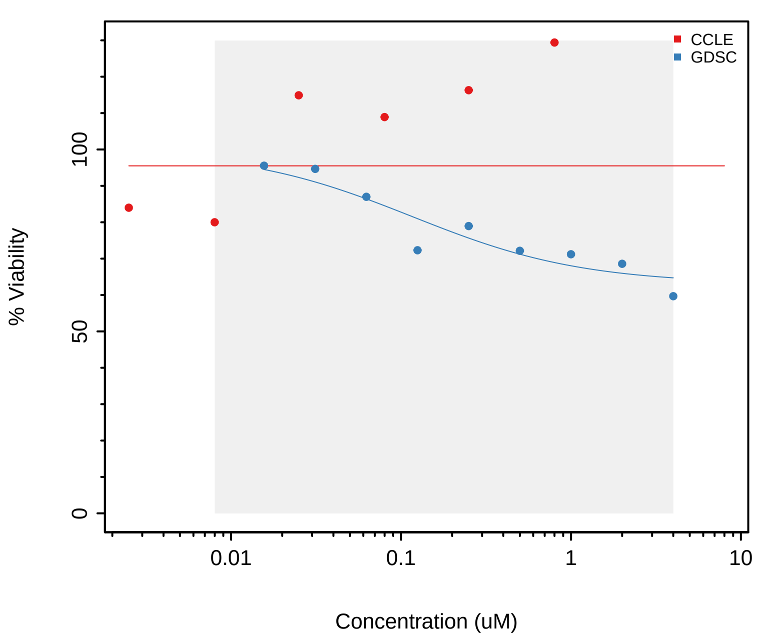

PD−0332991:SK−MEL−30

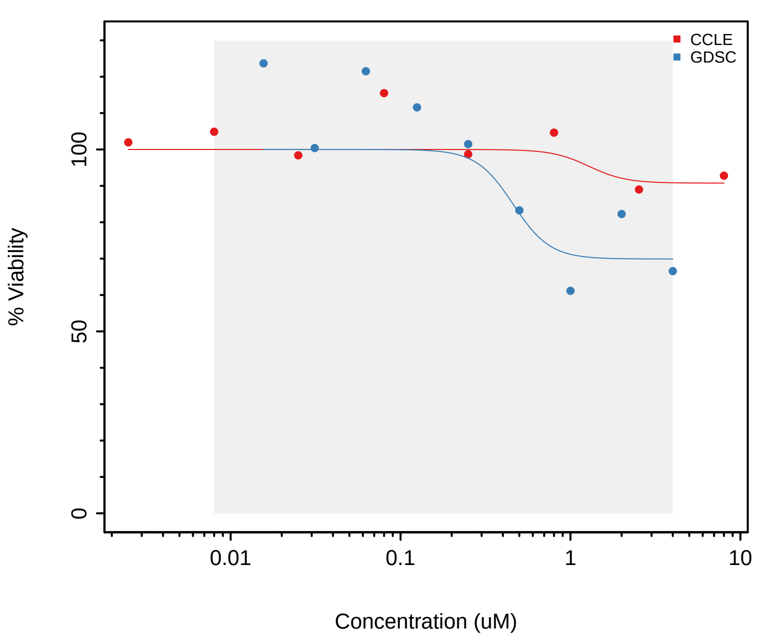

PD−0332991:SK−MEL−5

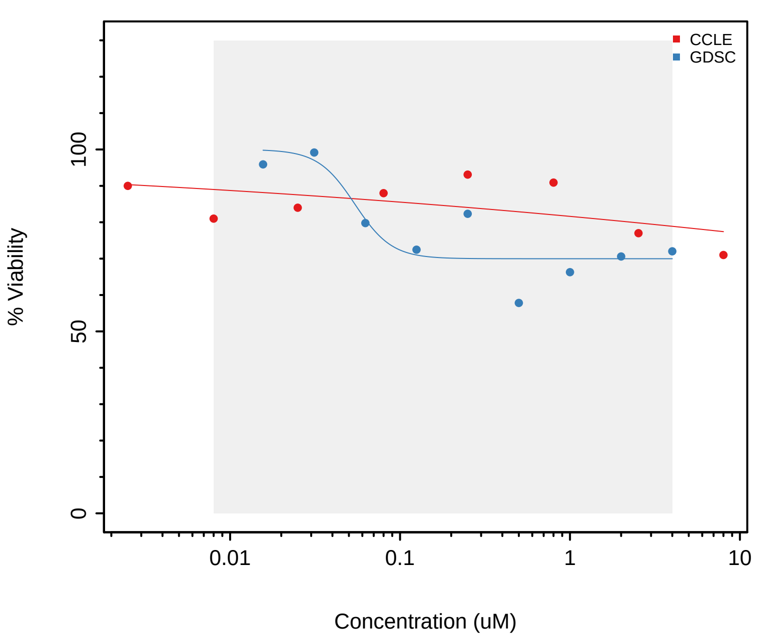

PD−0332991:SK−MM−2

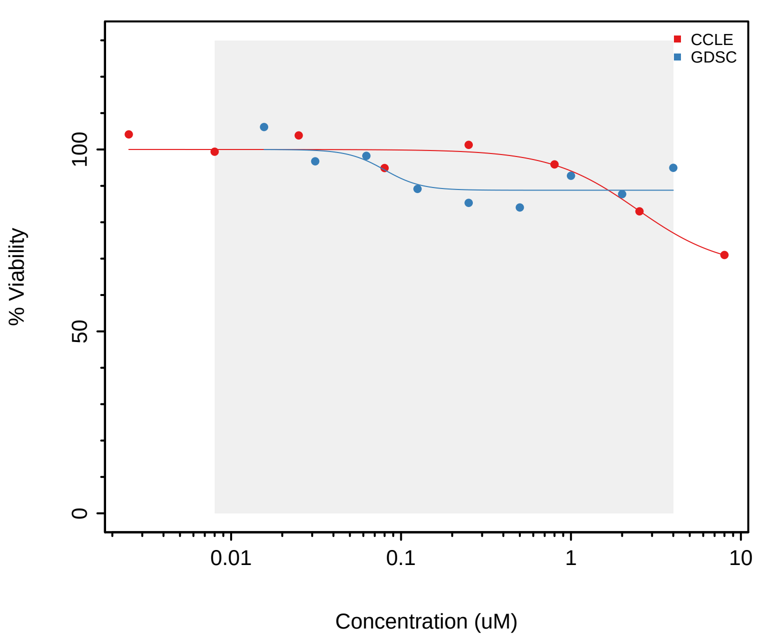

PD−0332991:SK−N−AS

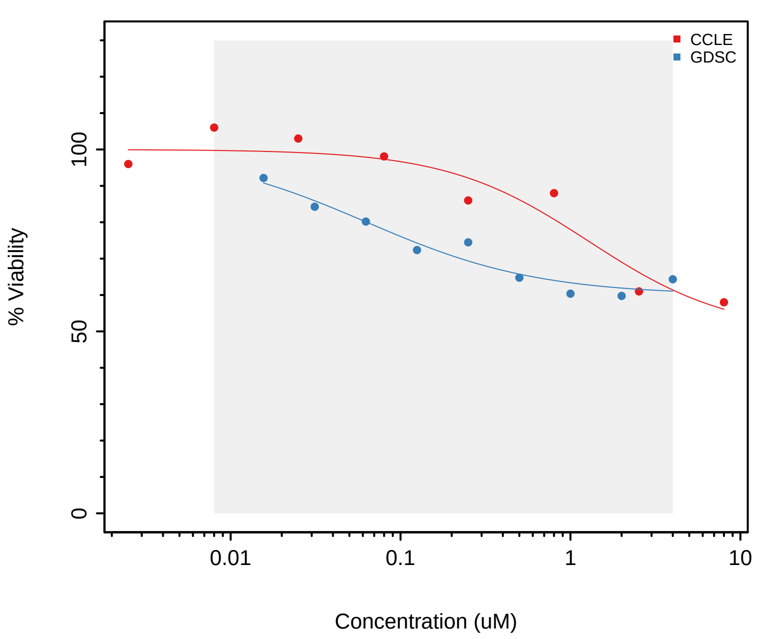

PD−0332991:SK−OV−3

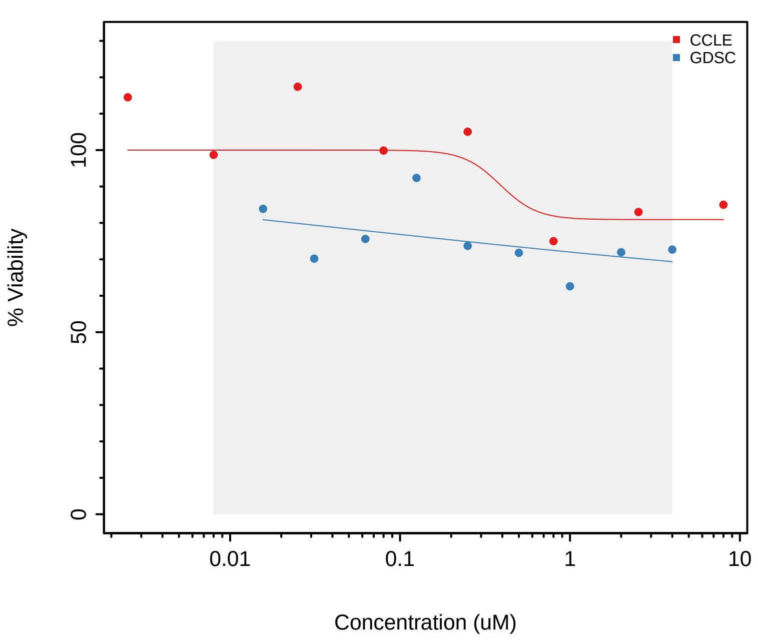

PD−0332991:SNG−M

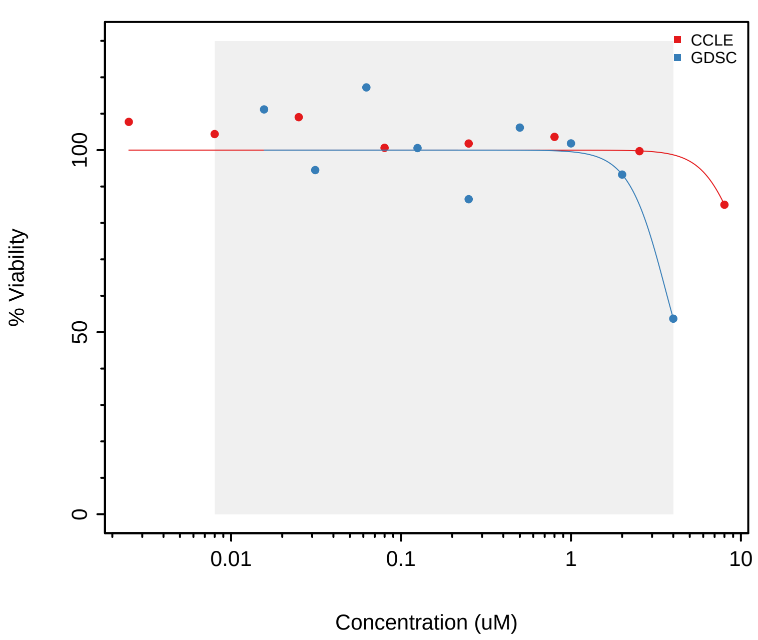

PD−0332991:NCI−SNU−1

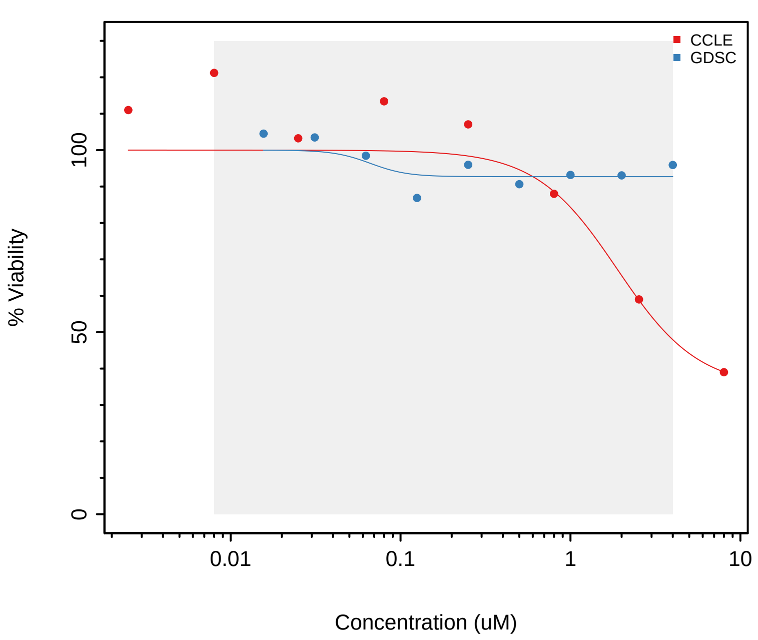

PD−0332991:NCI−SNU−16

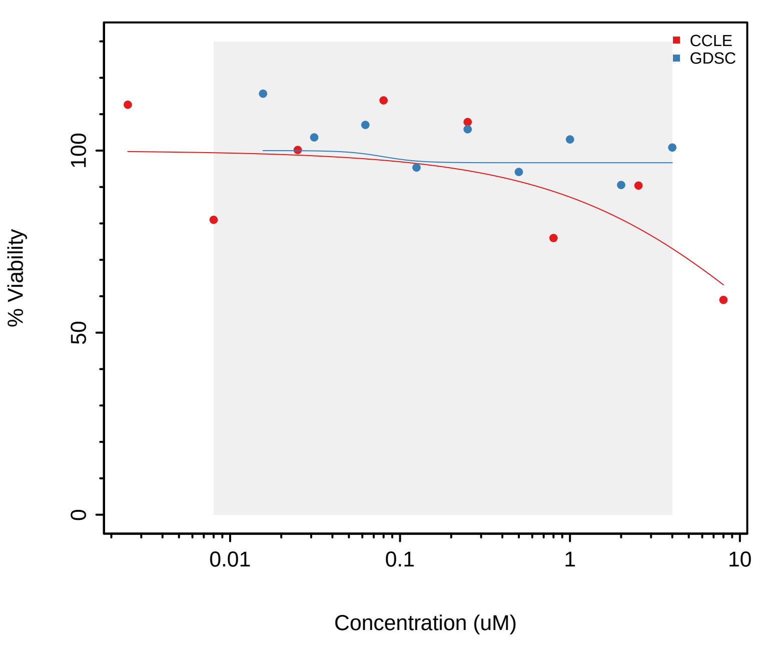

PD−0332991:SNU−423

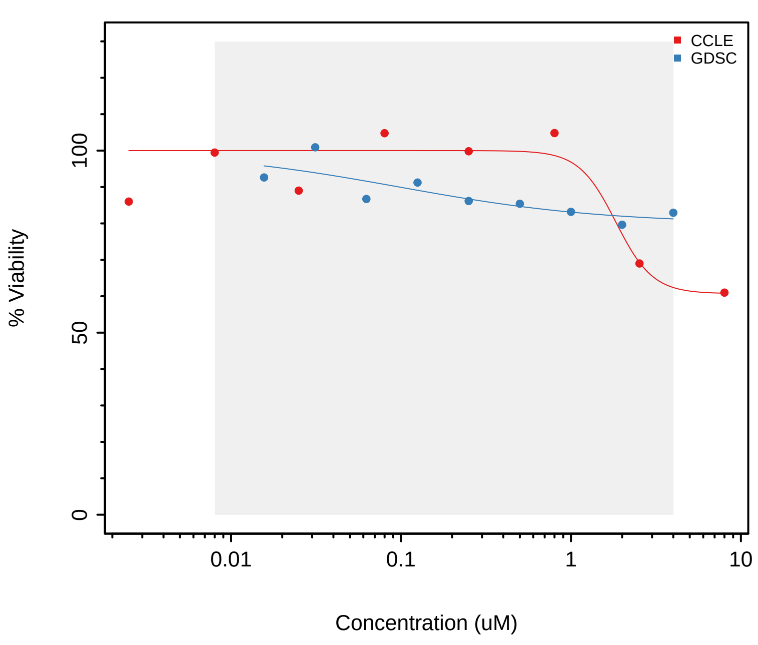

PD−0332991:SNU−449

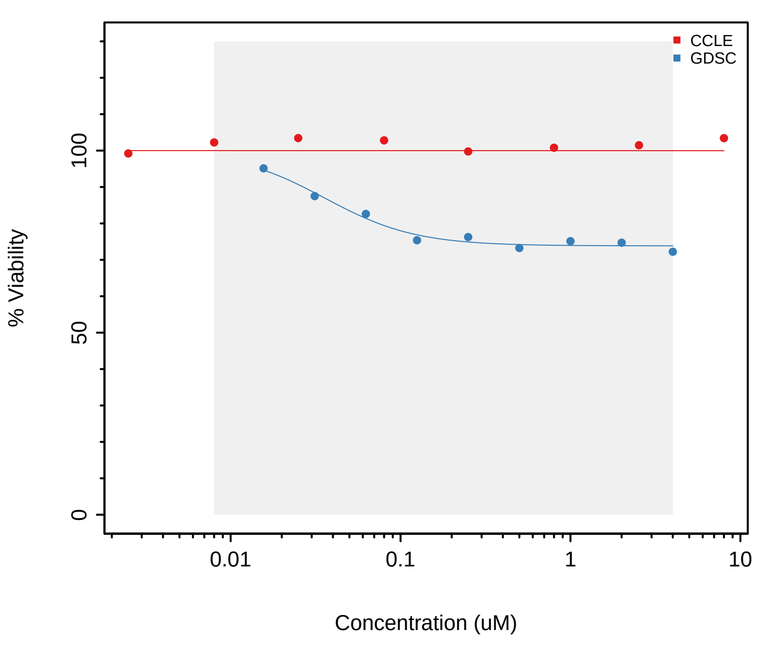

PD−0332991:SNU−C2B

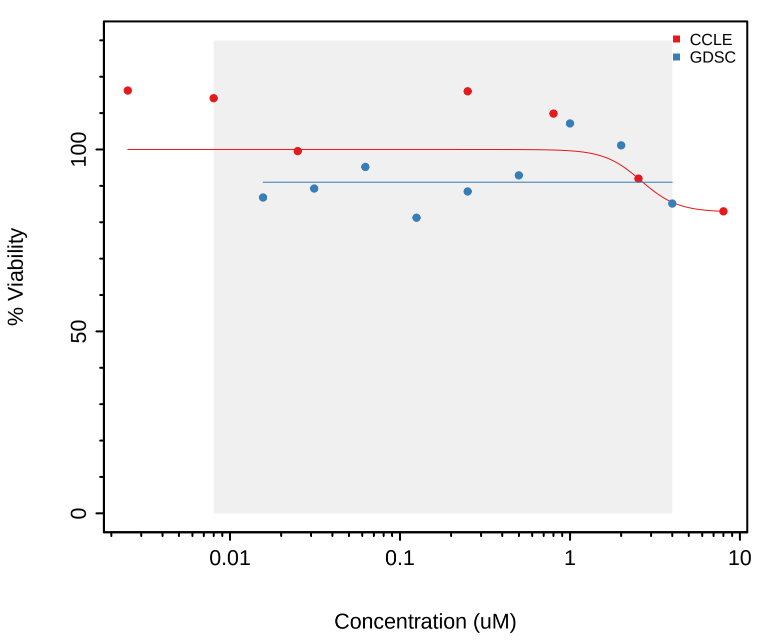

PD−0332991:SUP−T1

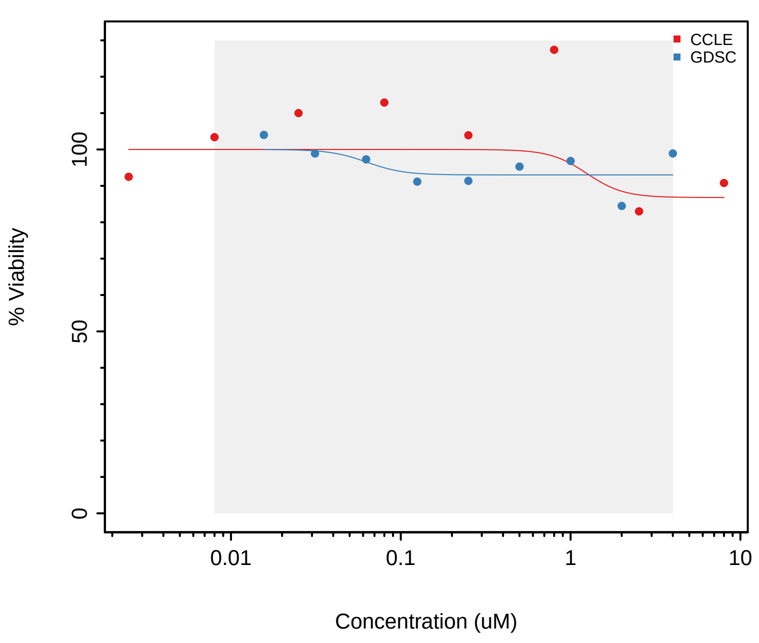

PD−0332991:SW1088

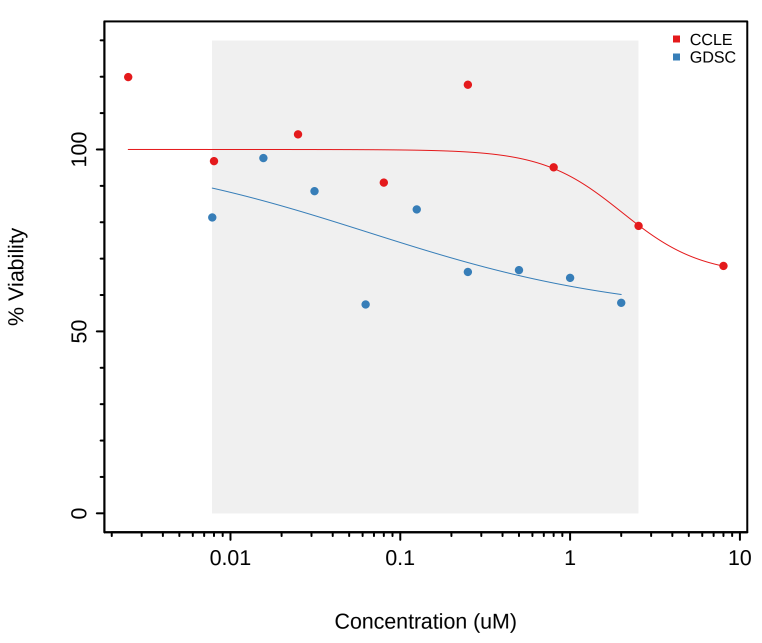

PD−0332991:SW1417

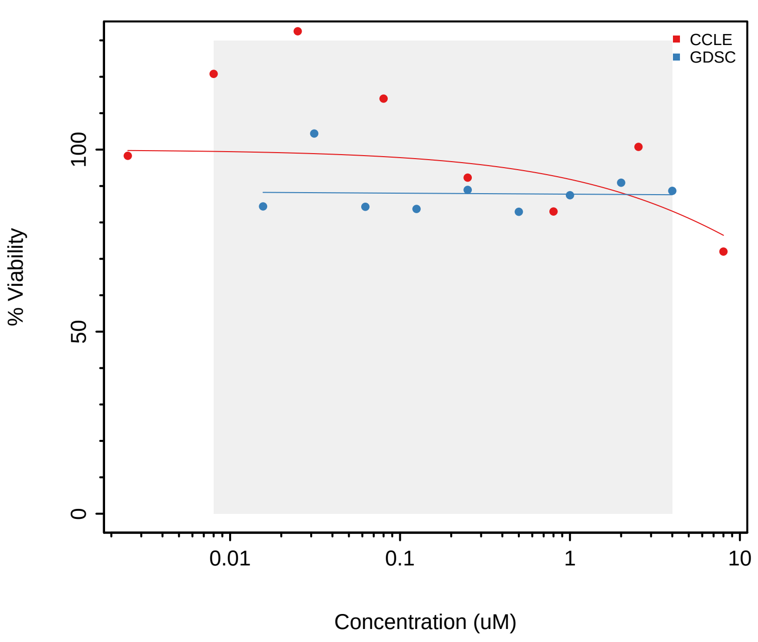

PD−0332991:SW1990

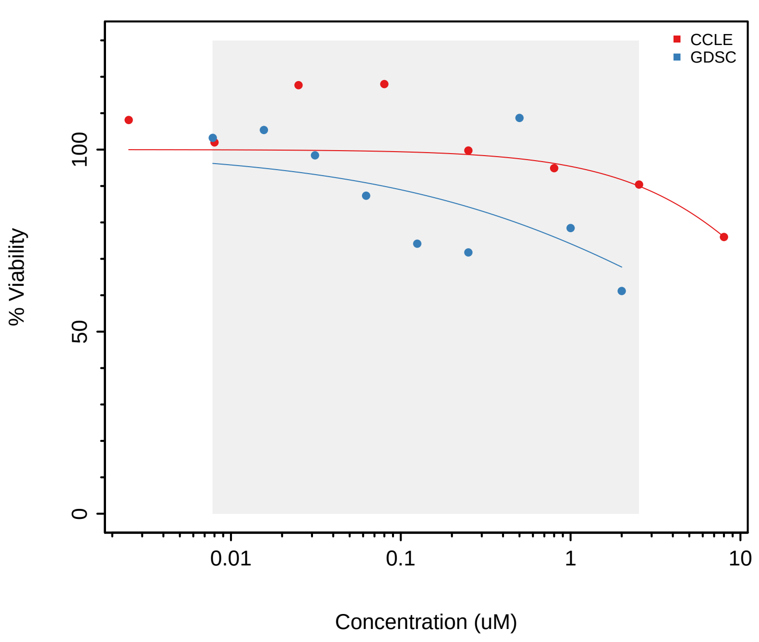

PD−0332991:SW48

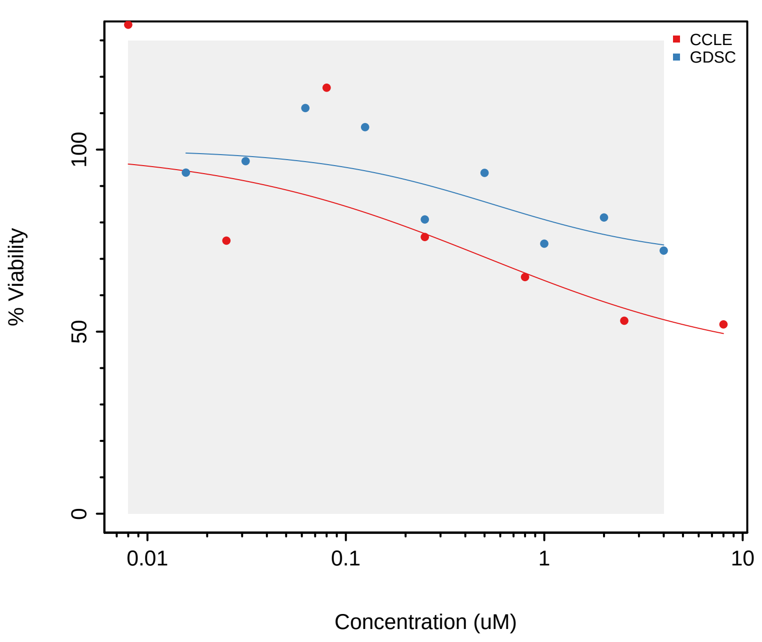

PD−0332991:SW620

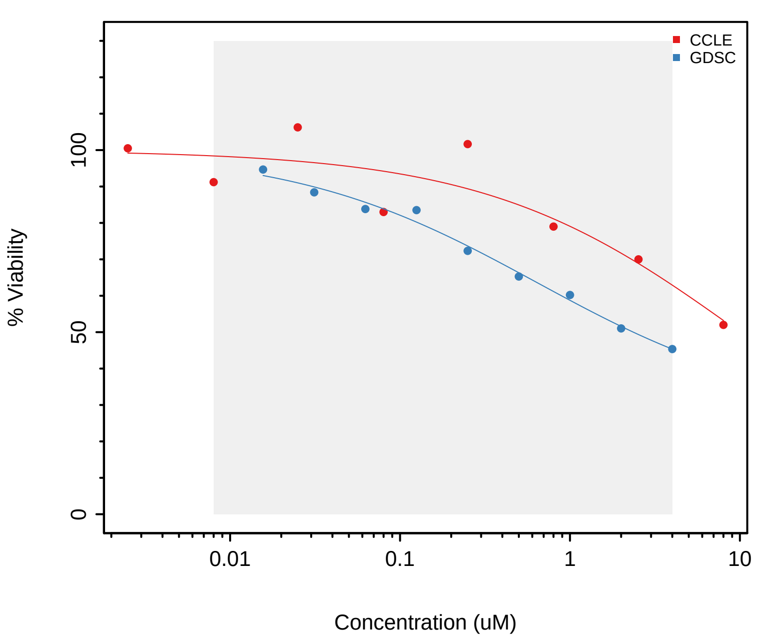

PD−0332991:SW780

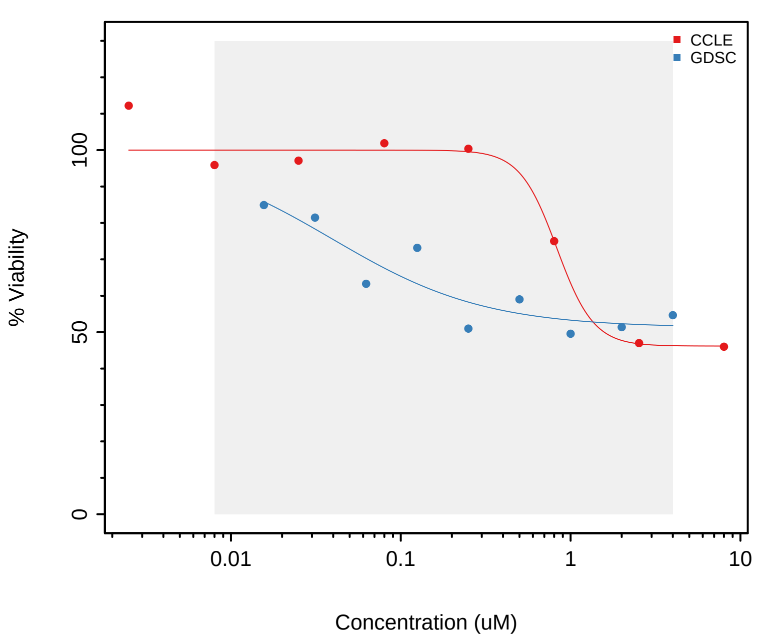

PD−0332991:T−24

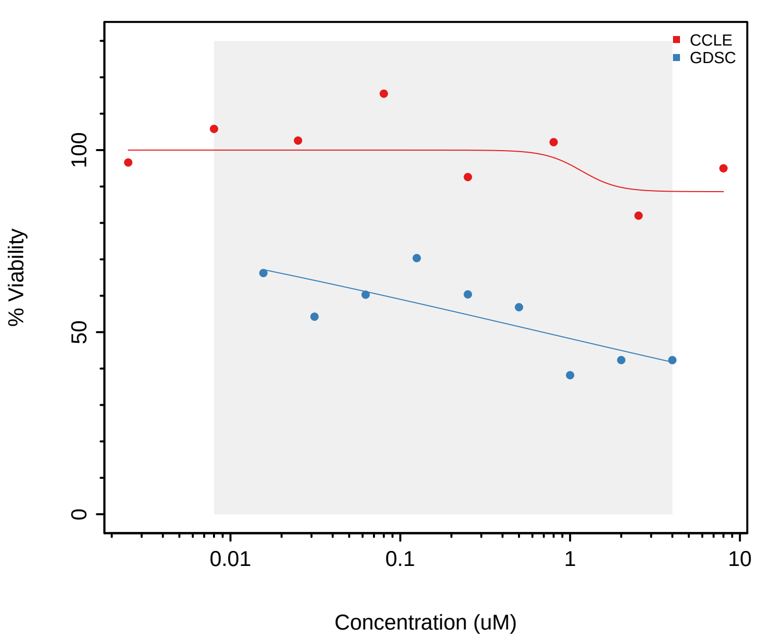

PD−0332991:T47D

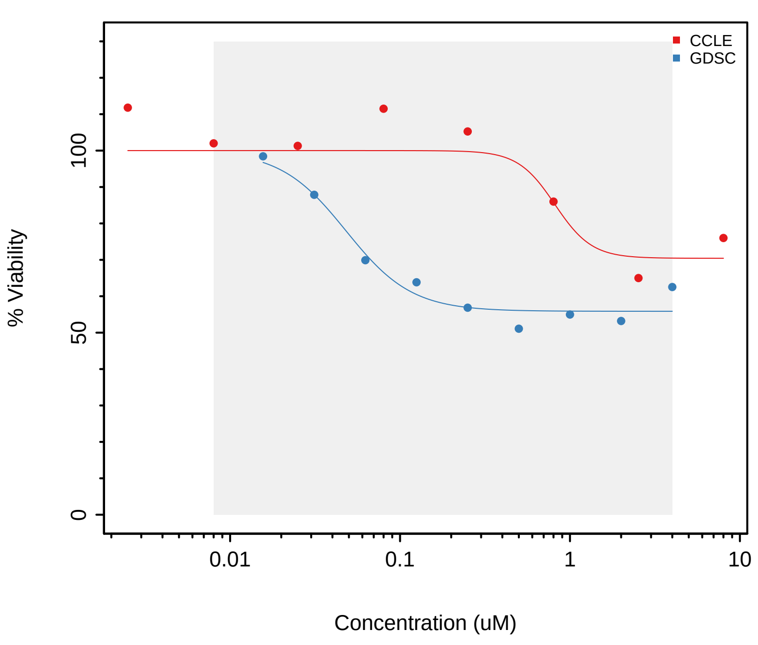

PD−0332991:T84

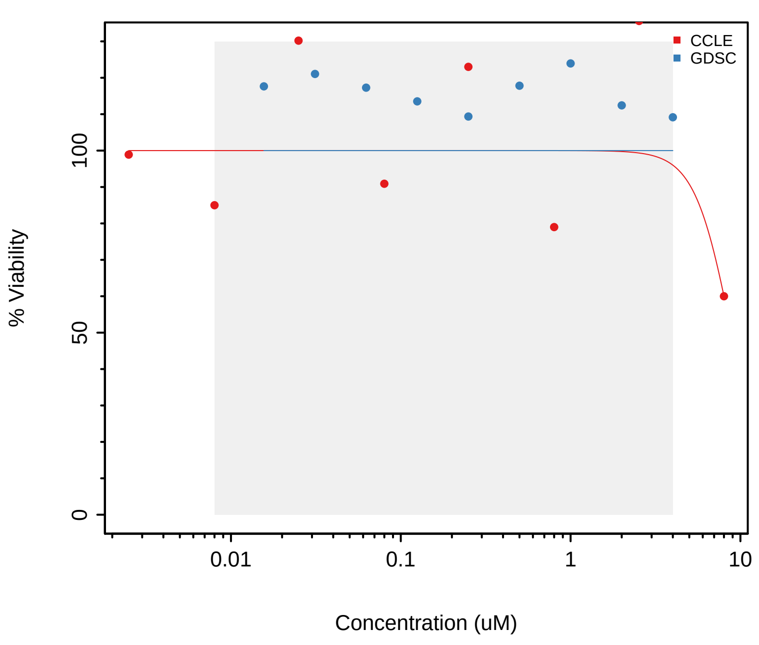

PD−0332991:T98G

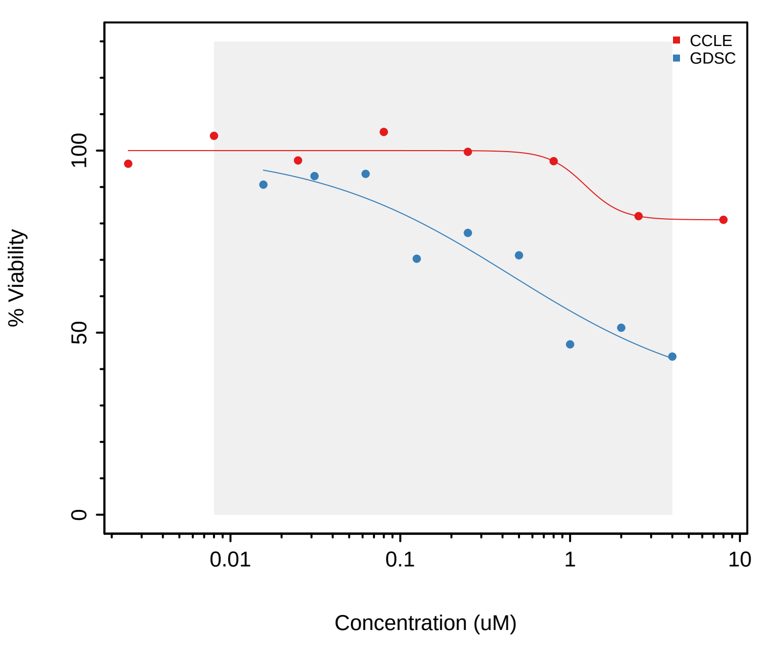

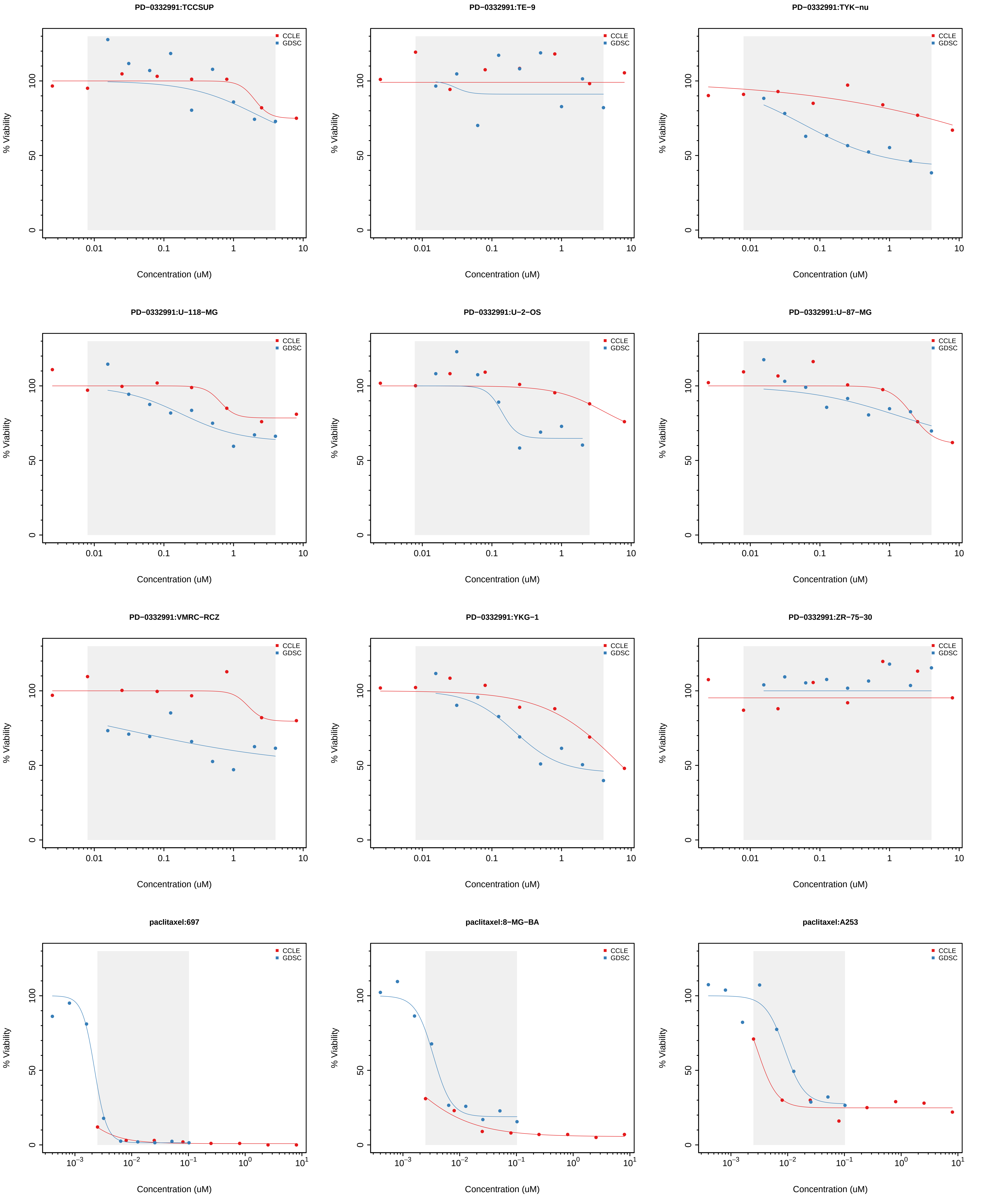
PD−0332991:TCCSUP
PD−0332991:TE−9
PD−0332991:TYK−nu
PD−0332991:U−118−MG
PD−0332991:U−2−OS
PD−0332991:U−87−MG
PD−0332991:VMRC−RCZ
PD−0332991:YKG−1
PD−0332991:ZR−75−30
paclitaxel:697
paclitaxel:8−MG−BA
paclitaxel:A253
CCLE
GDSC
% Viability
Concentration (uM)

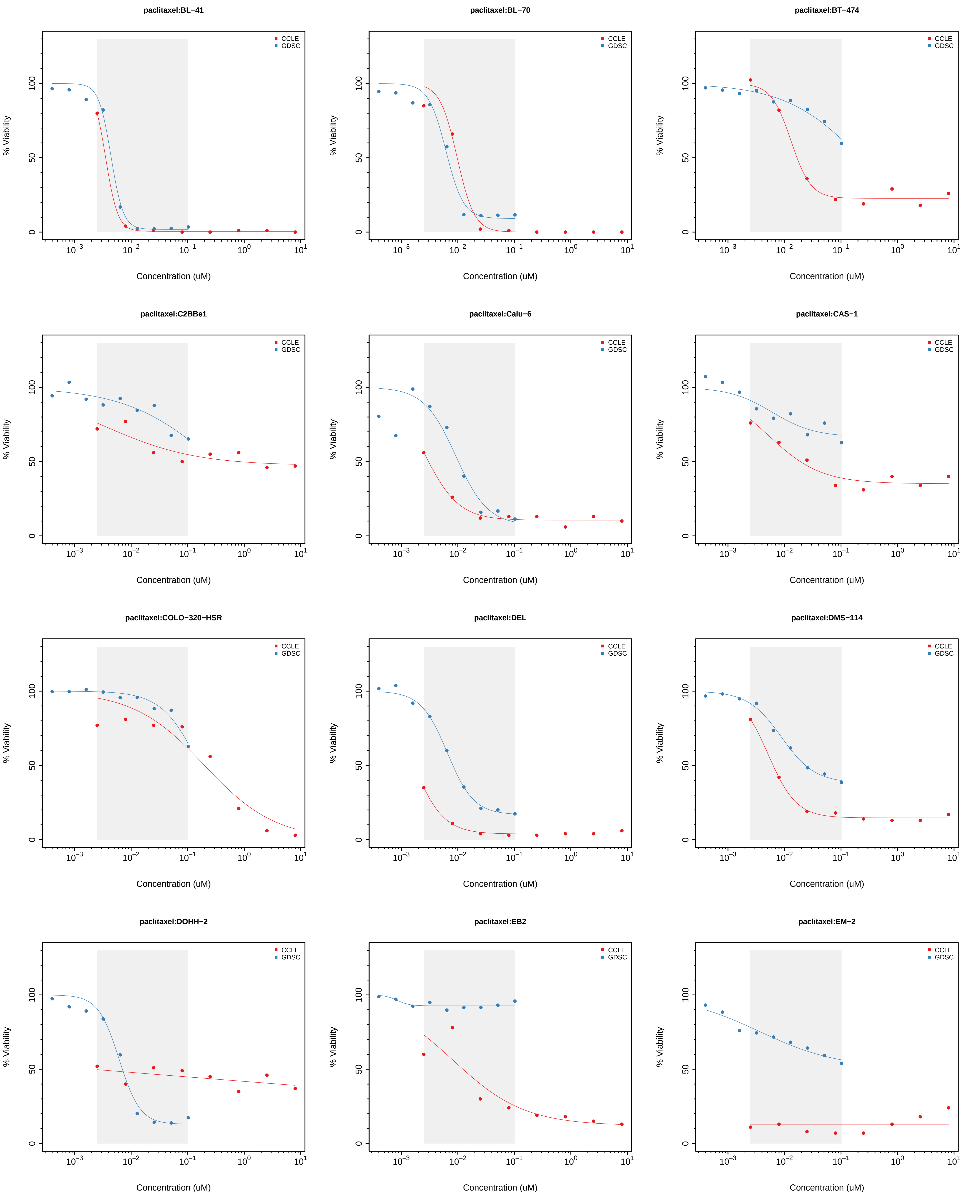
paclitaxel:BL−41
paclitaxel:BL−70
paclitaxel:BT−474
paclitaxel:C2BBe1
paclitaxel:Calu−6
paclitaxel:CAS−1
paclitaxel:COLO−320−HSR
paclitaxel:DEL
paclitaxel:DMS−114
paclitaxel:DOHH−2
paclitaxel:EB2
paclitaxel:EM−2
CCLE
GDSC
% Viability
Concentration (uM)
0
50
100
10−3
10−2
10−1
100
101

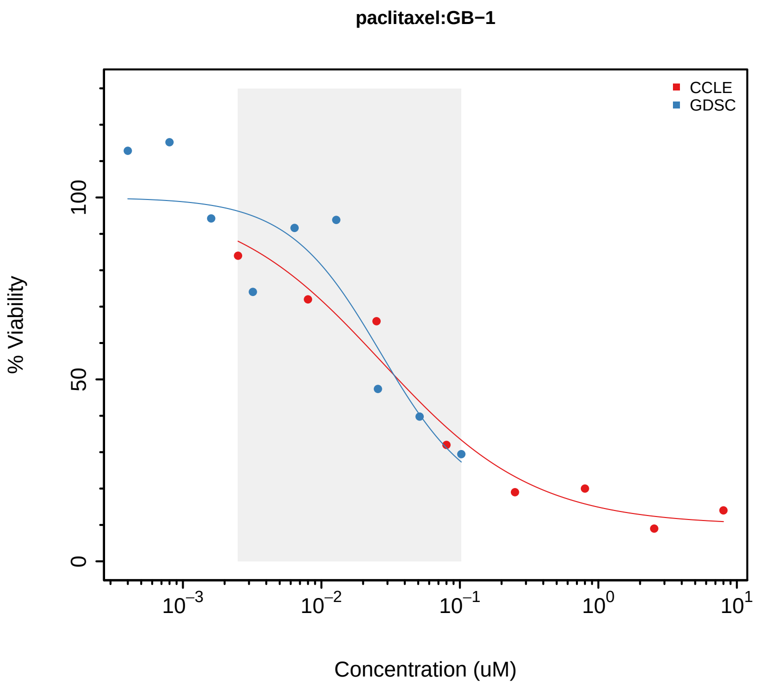
paclitaxel:GB−1
CCLE
GDSC
% Viability
Concentration (uM)

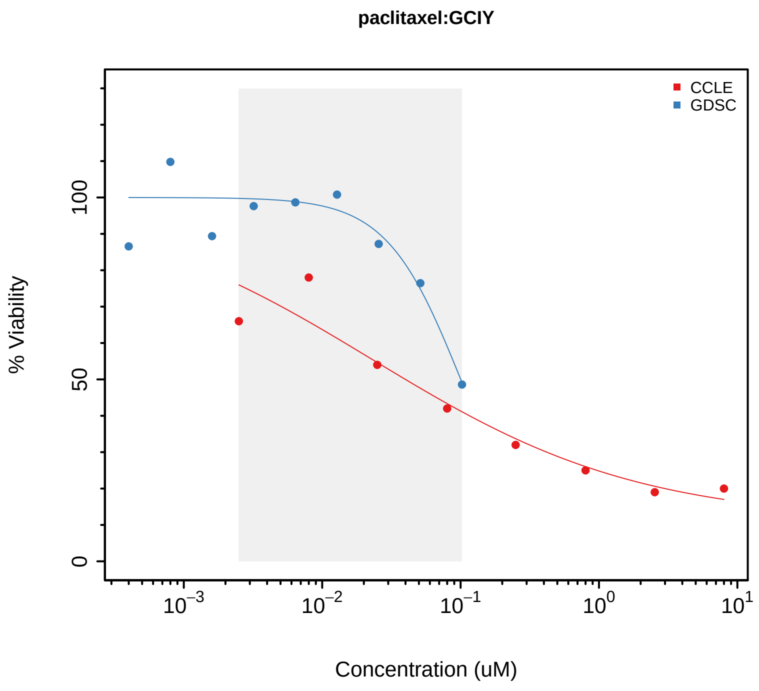
paclitaxel:GCIY
CCLE
GDSC
% Viability
Concentration (uM)

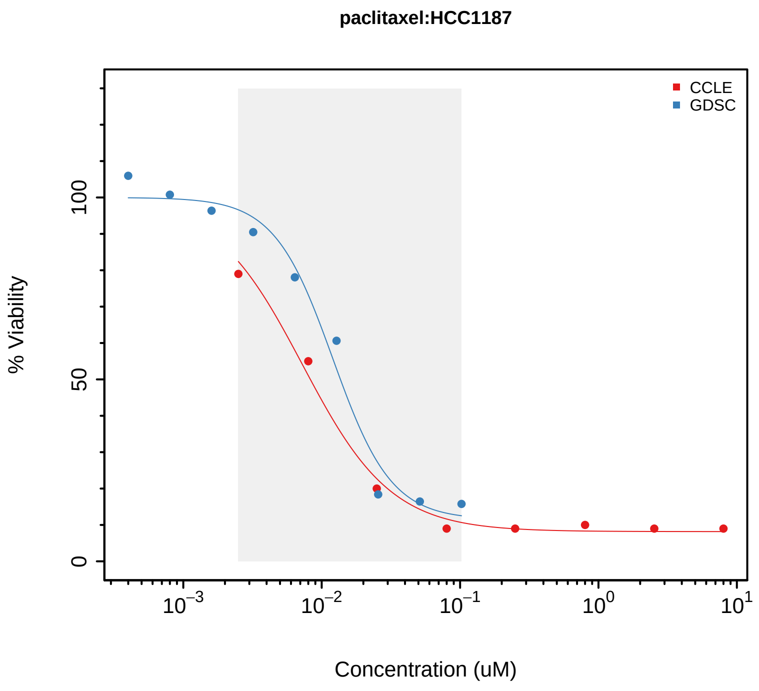
paclitaxel:HCC1187
CCLE
GDSC
% Viability
Concentration (uM)

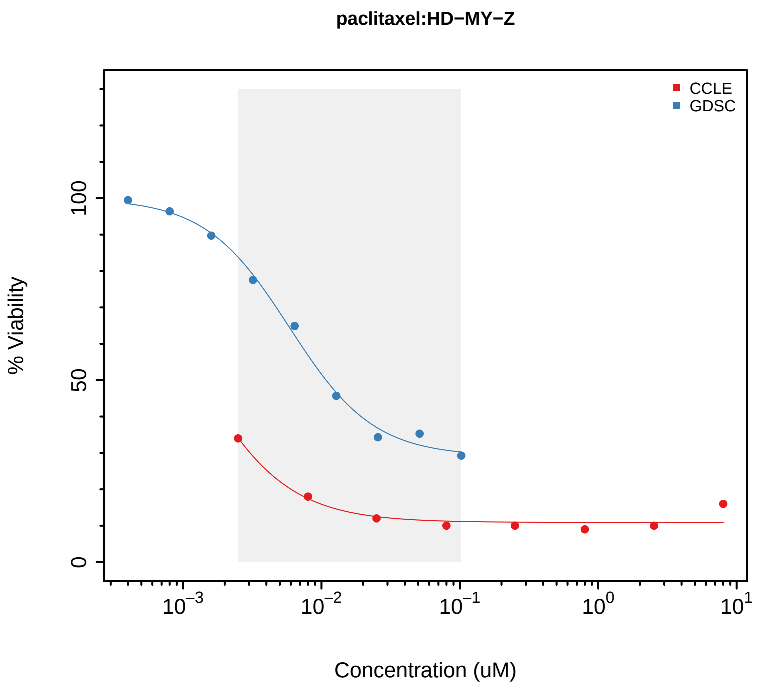
paclitaxel:HD−MY−Z
CCLE
GDSC
% Viability
Concentration (uM)

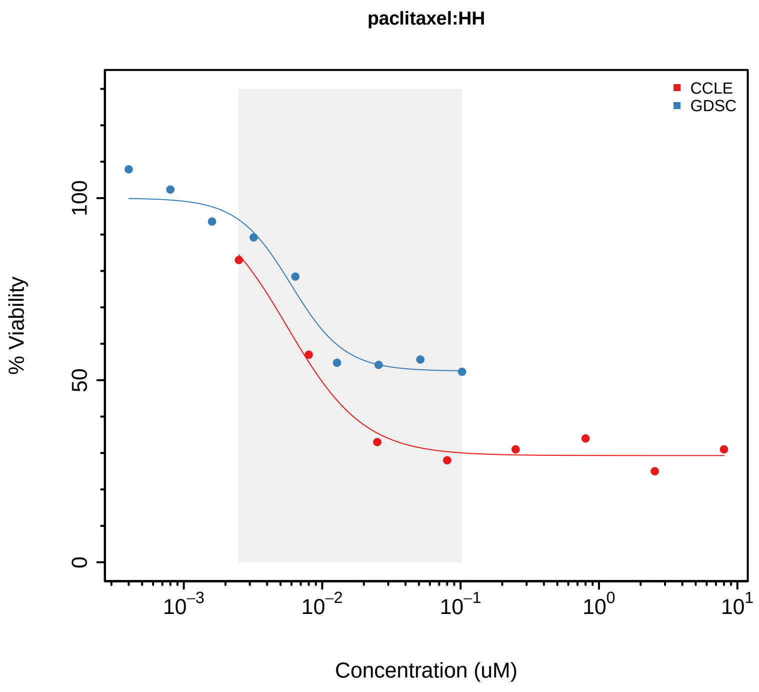
paclitaxel:HH
CCLE
GDSC
% Viability
Concentration (uM)

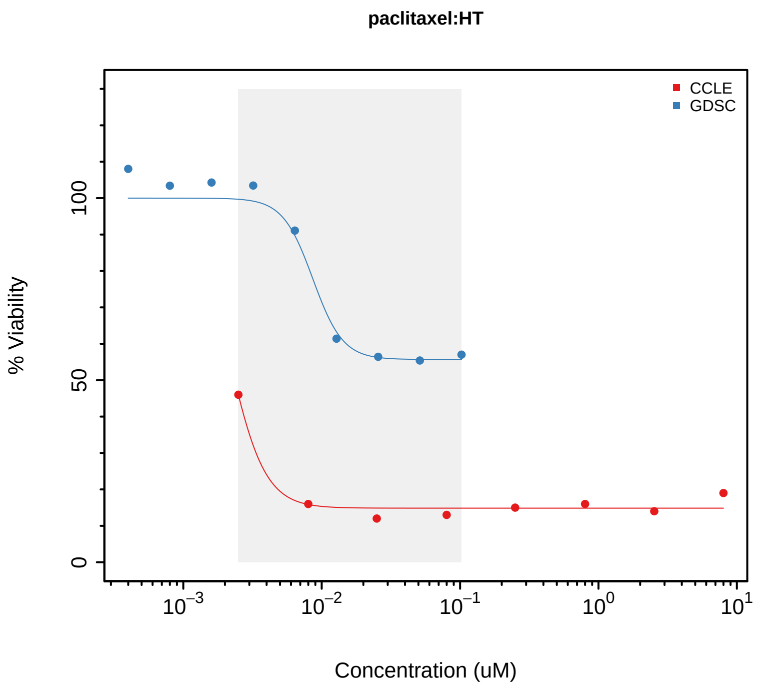
paclitaxel:HT
CCLE
GDSC
% Viability
Concentration (uM)

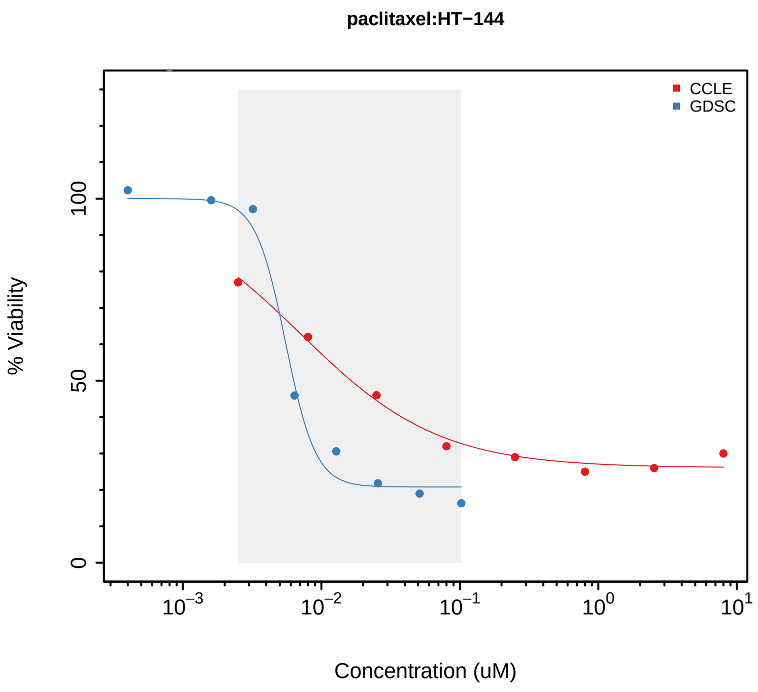
paclitaxel:HT−144
CCLE
GDSC
% Viability
Concentration (uM)

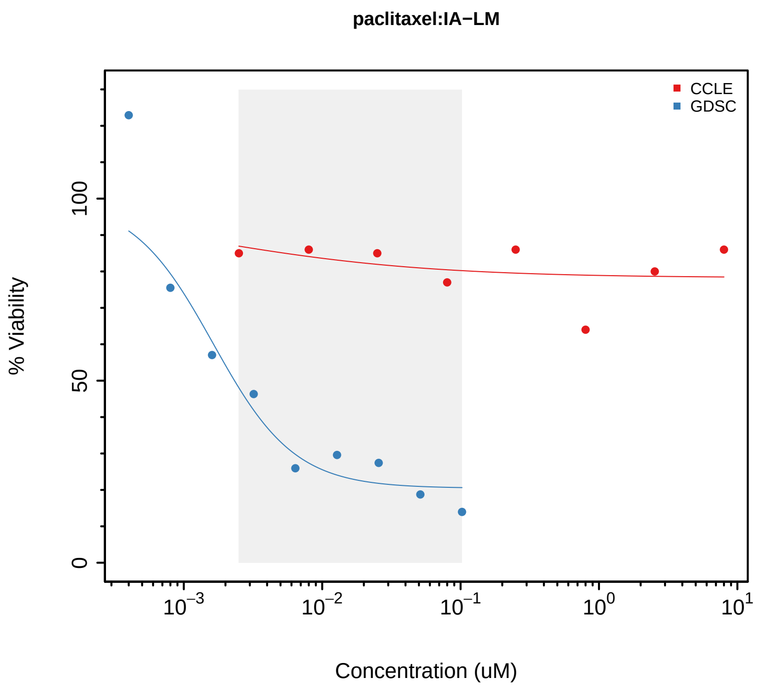
paclitaxel:IA−LM
CCLE
GDSC
% Viability
Concentration (uM)

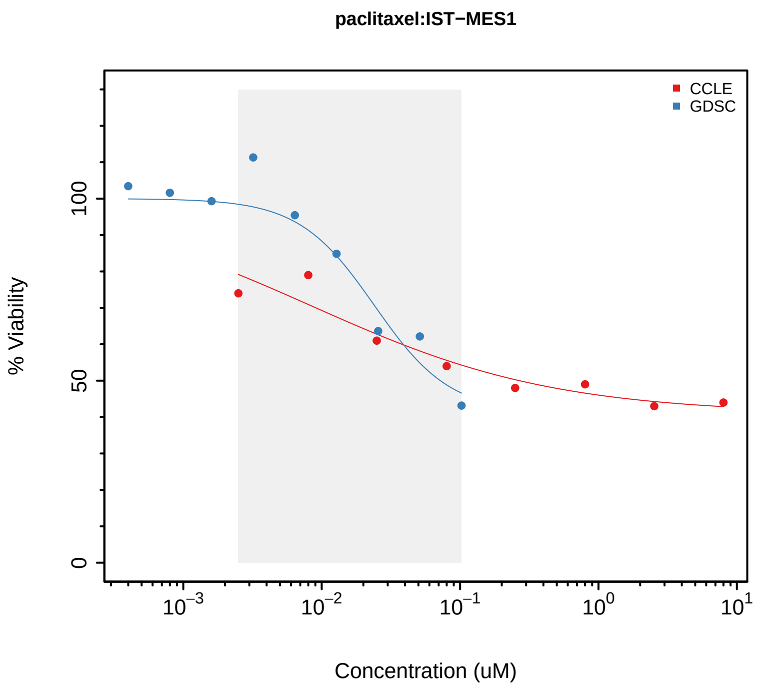
paclitaxel:IST−MES1
CCLE
GDSC
% Viability
Concentration (uM)

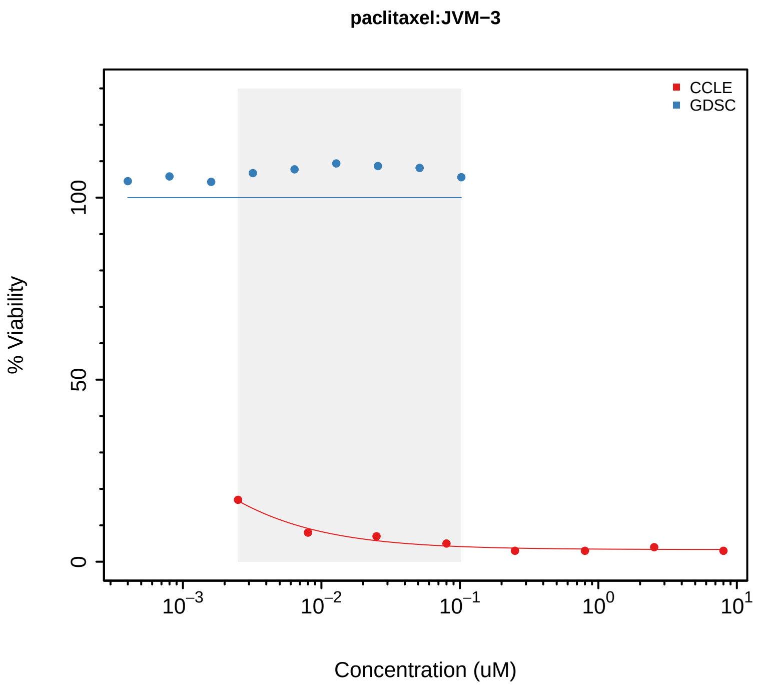
paclitaxel:JVM−3
CCLE
GDSC
% Viability
Concentration (uM)

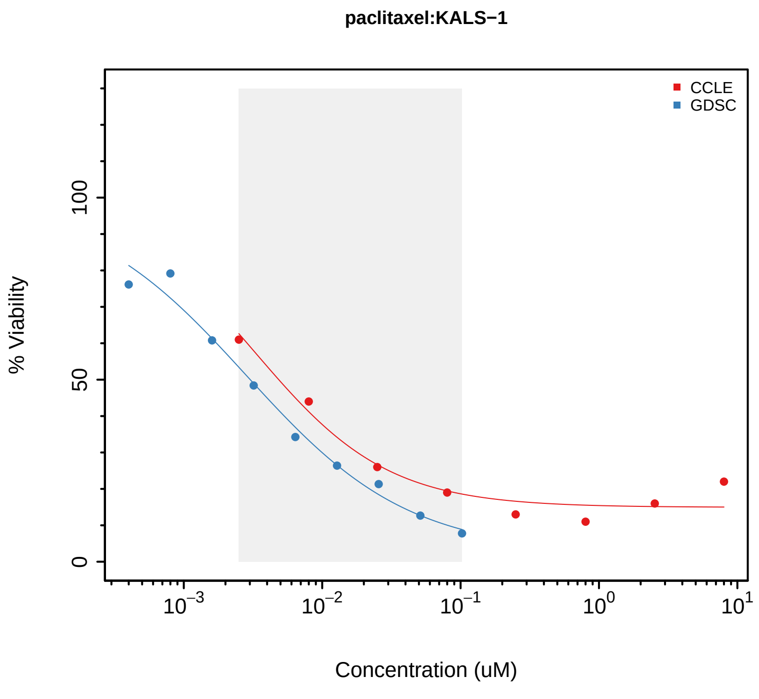
paclitaxel:KALS−1
CCLE
GDSC
% Viability
Concentration (uM)

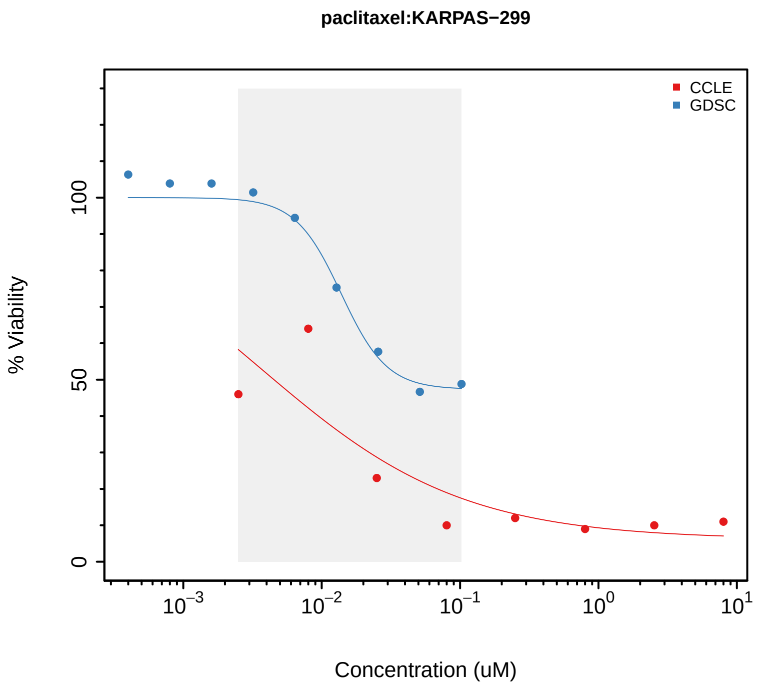
paclitaxel:KARPAS−299
CCLE
GDSC
% Viability
Concentration (uM)

paclitaxel:KARPAS−422

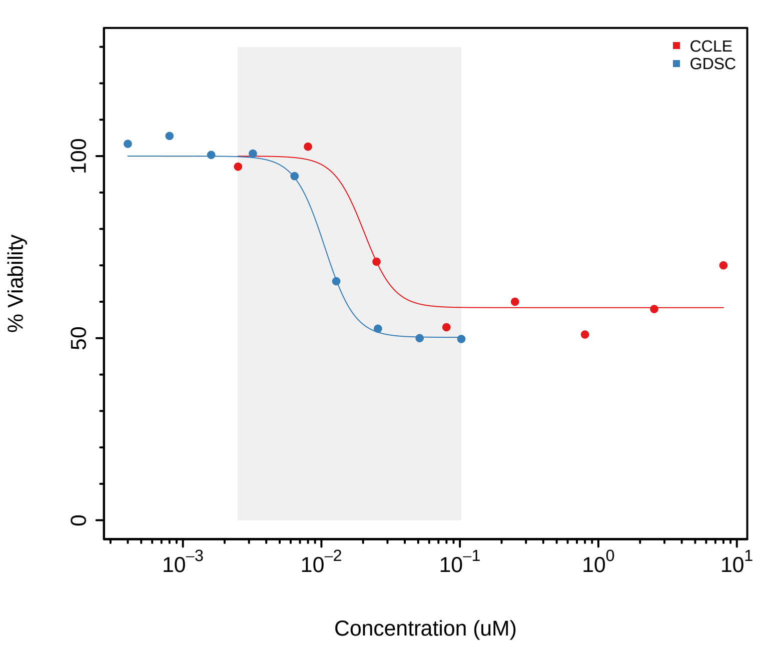

paclitaxel:KG−1

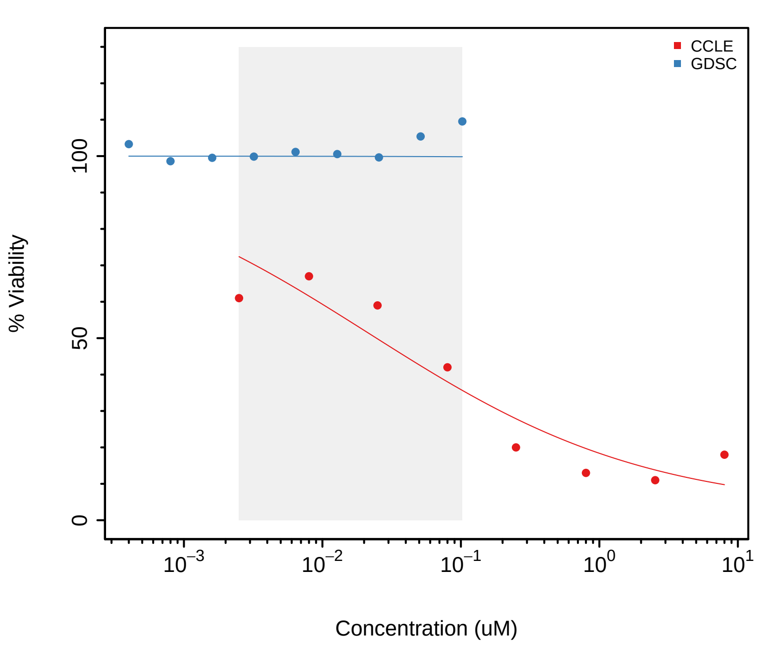

paclitaxel:KLE

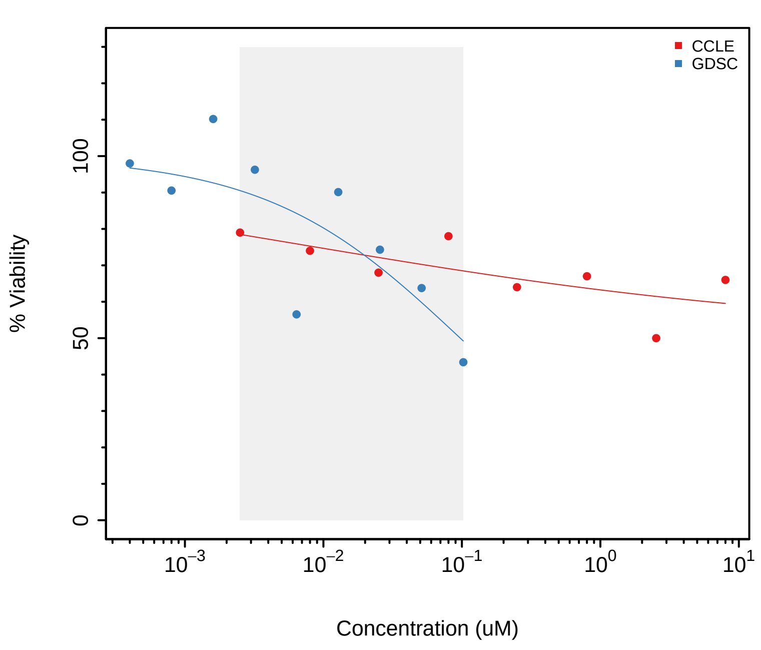

paclitaxel:KNS−42

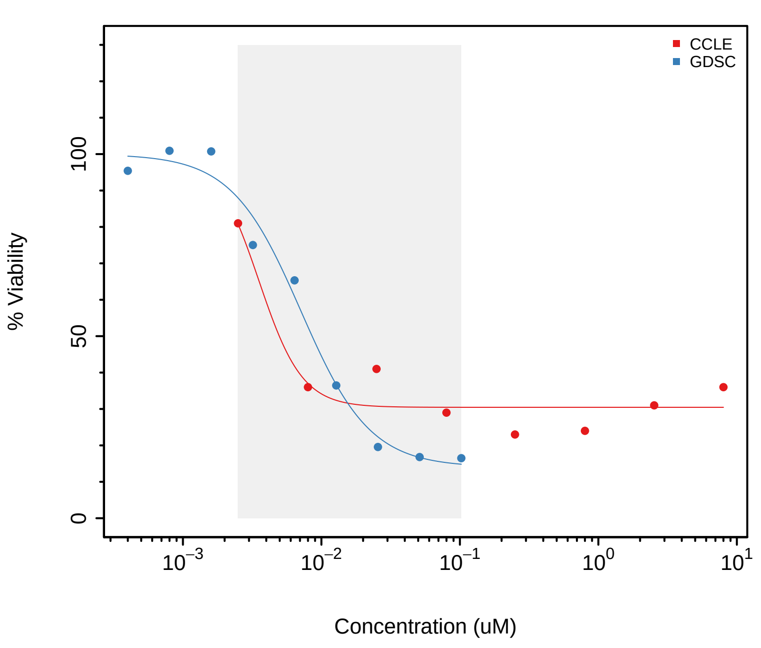

paclitaxel:KNS−81−FD

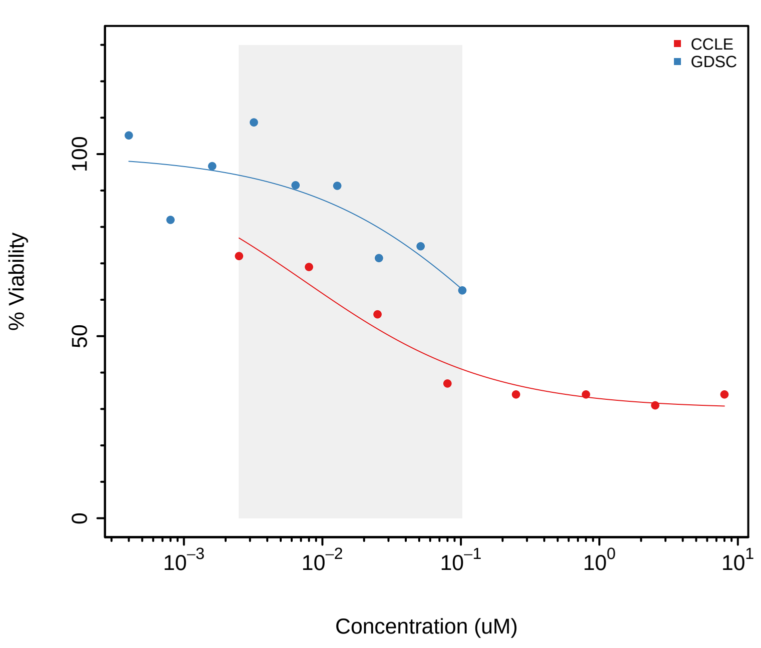

paclitaxel:K052

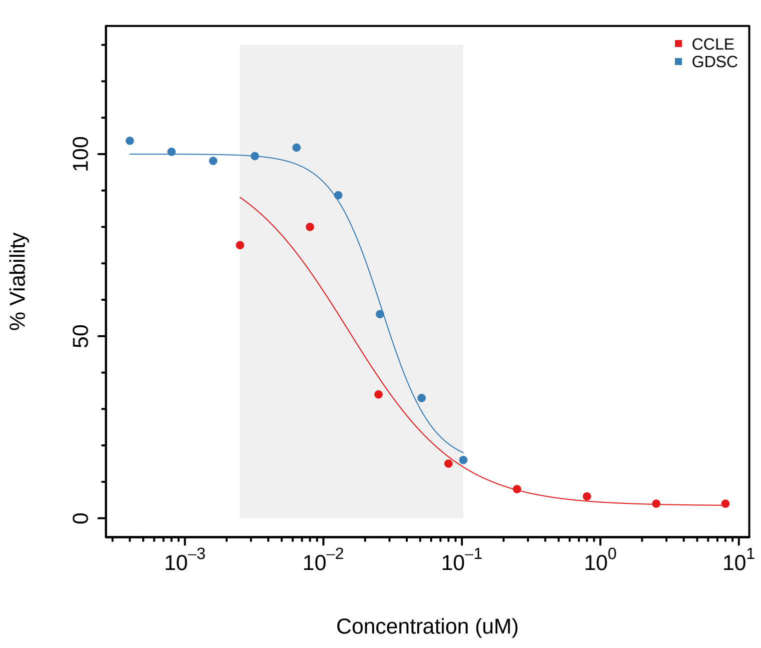

paclitaxel:KU812

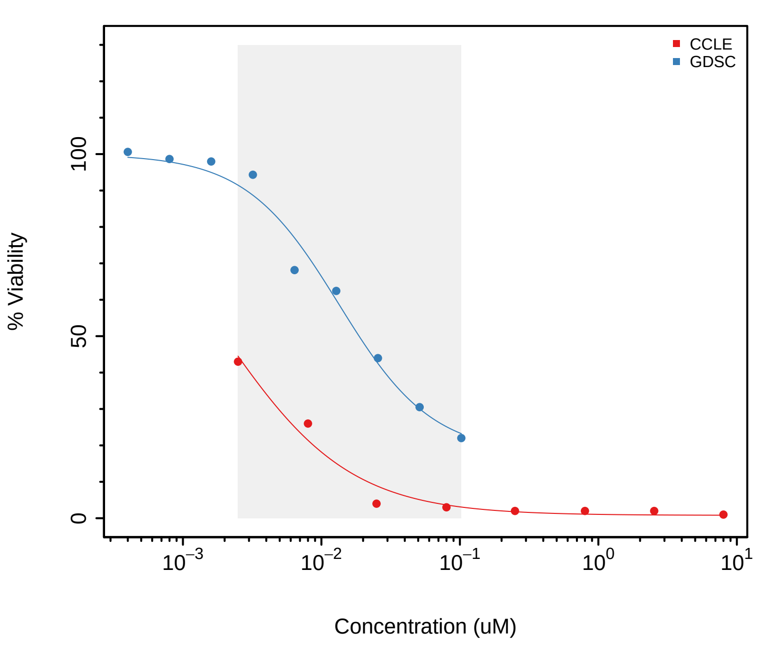

paclitaxel:KURAMOCHI

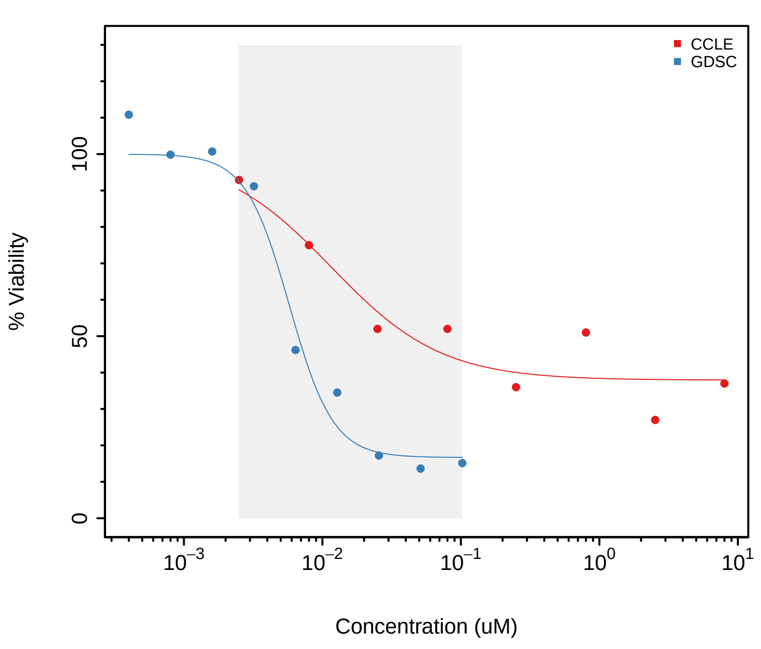

paclitaxel:L−363

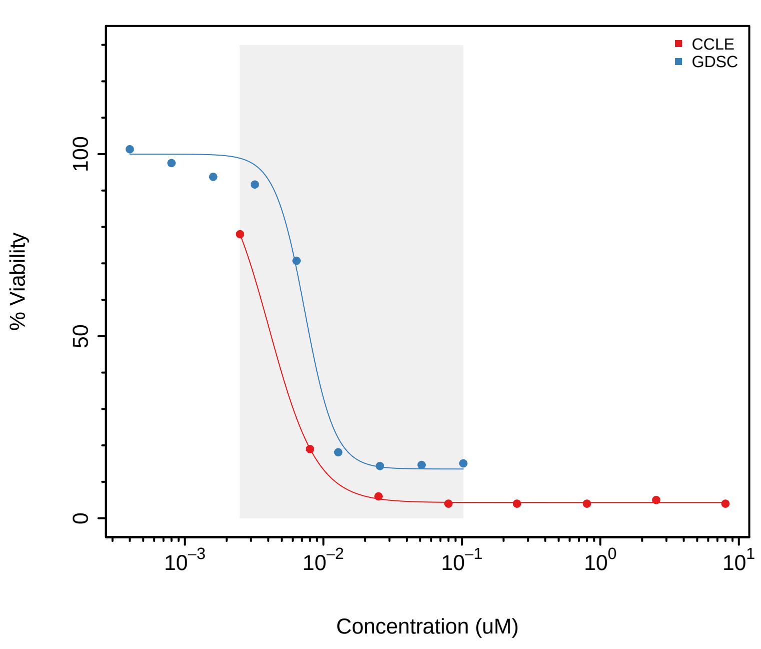

paclitaxel:L−428

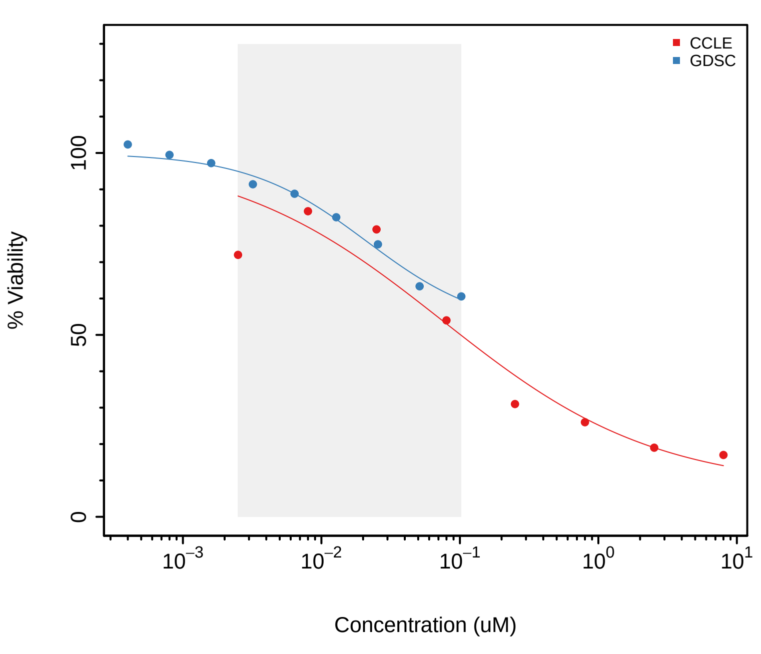

paclitaxel:LOXIMVI

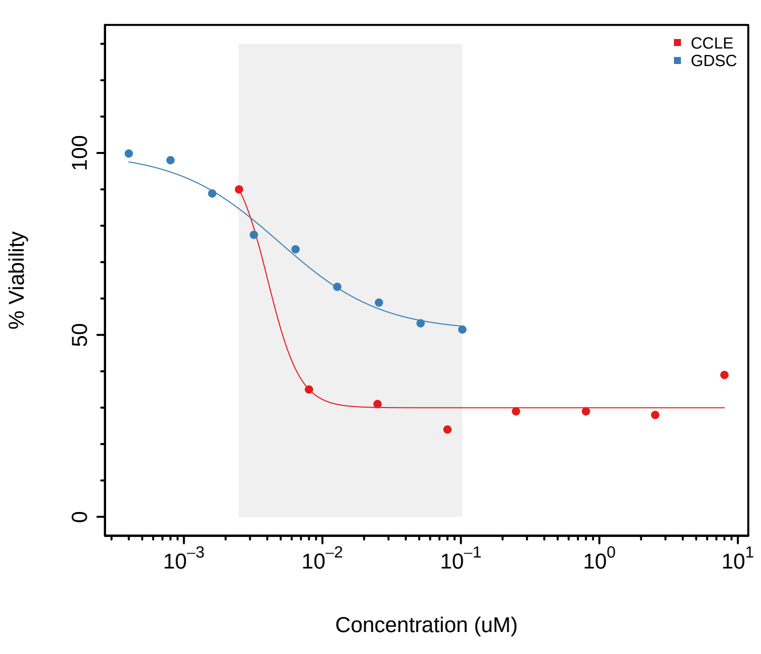

paclitaxel:LP−1

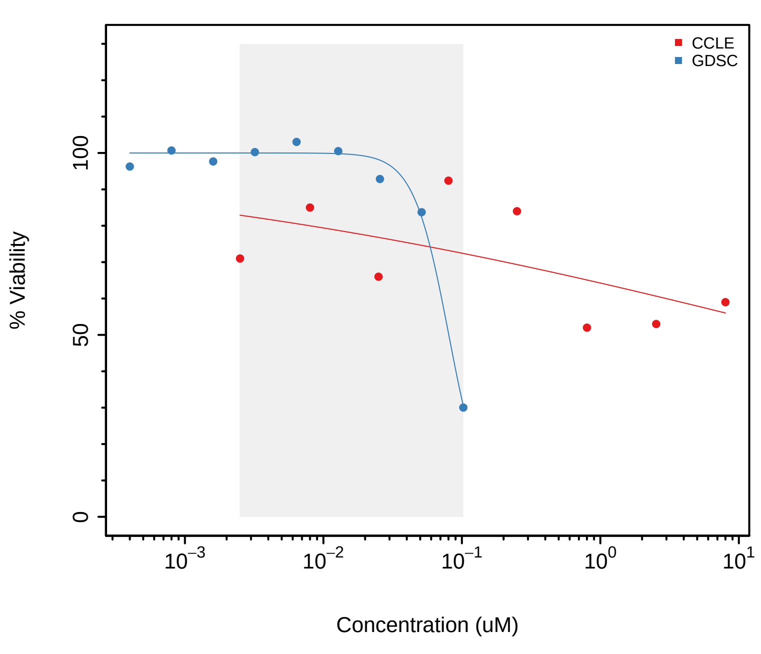

paclitaxel:LS−123

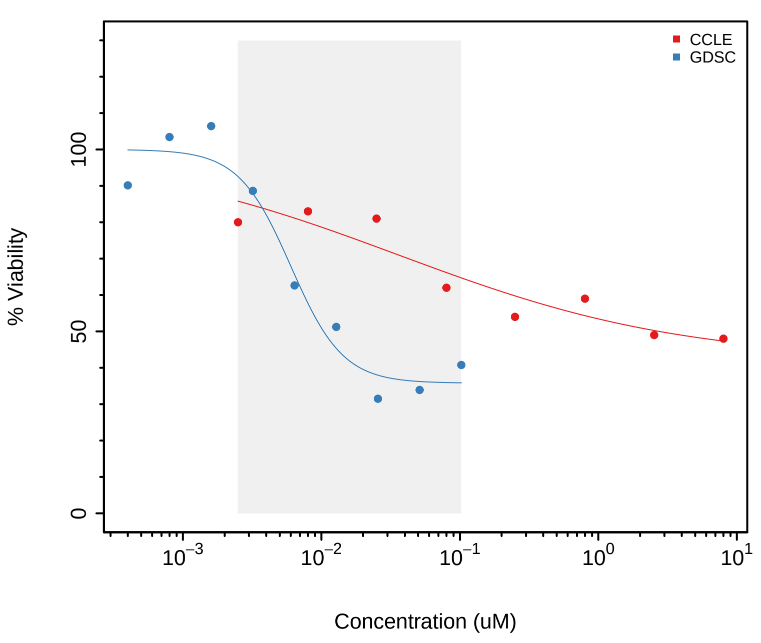

paclitaxel:LS−411N

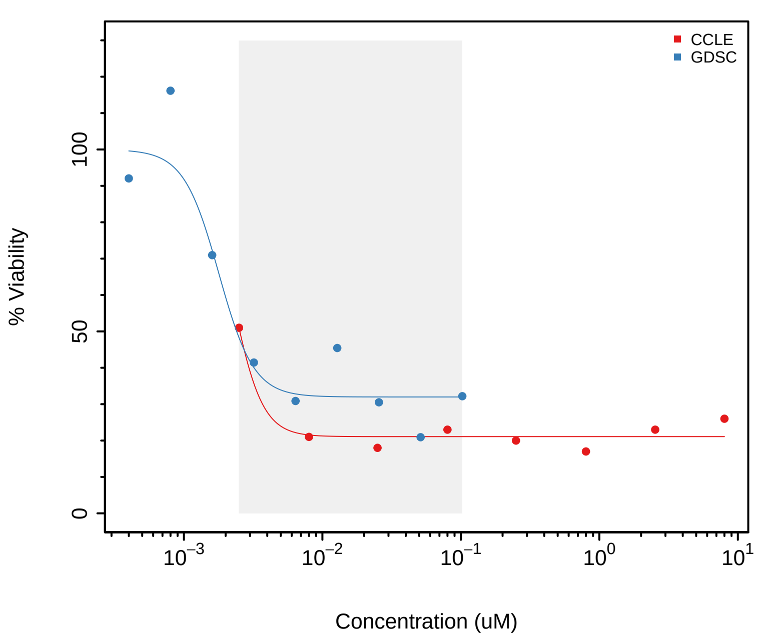

paclitaxel:LS−513

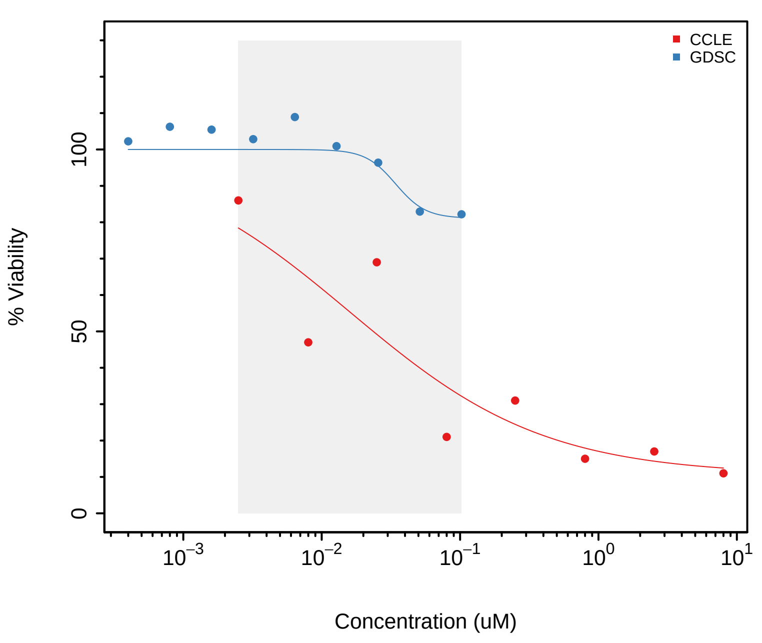

paclitaxel:MC116

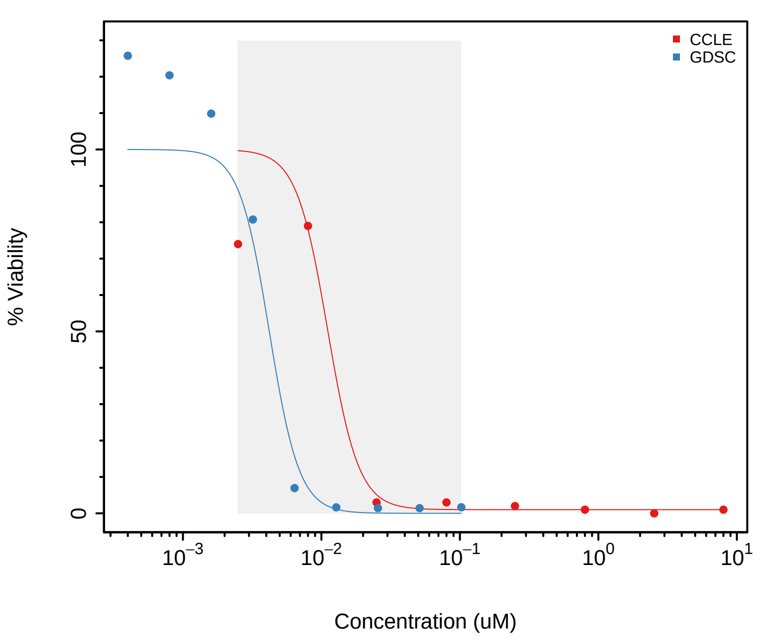

paclitaxel:MEG−01

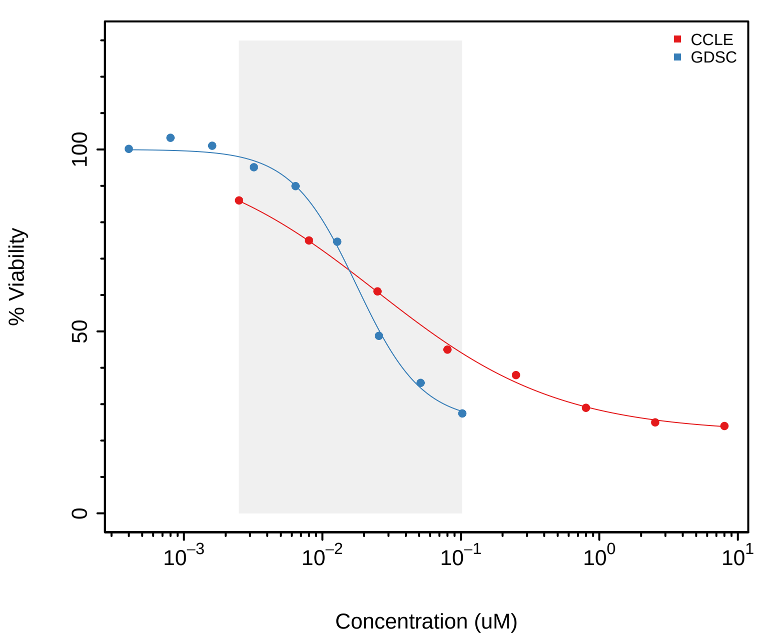

paclitaxel:MPP−89

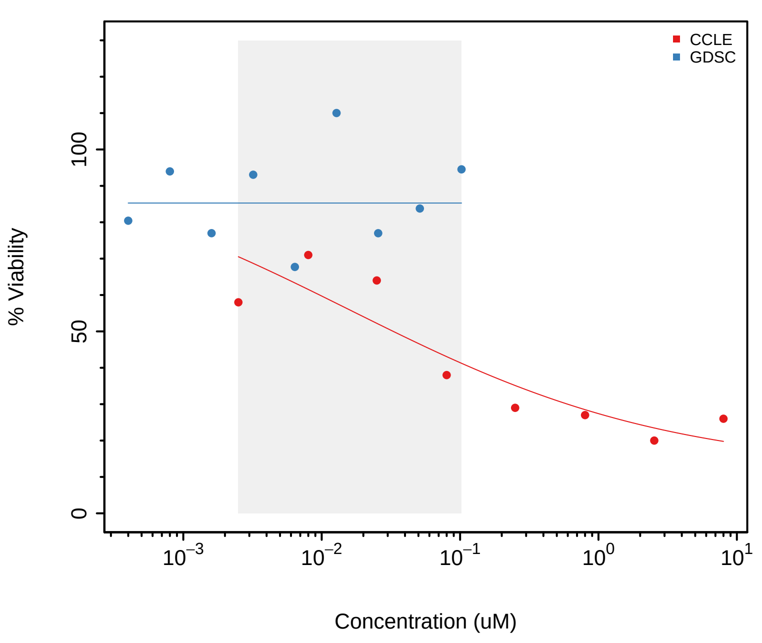

paclitaxel:MSTO−211H

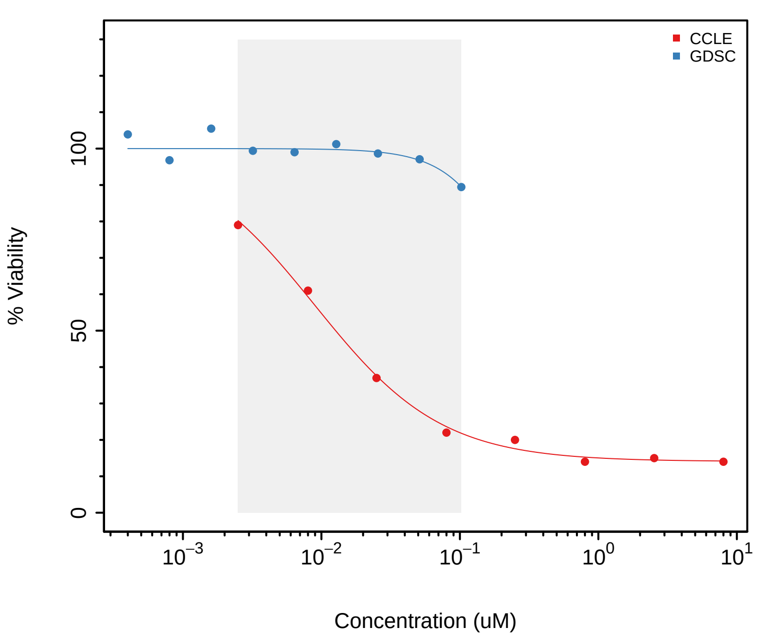

paclitaxel:NCI−H1092

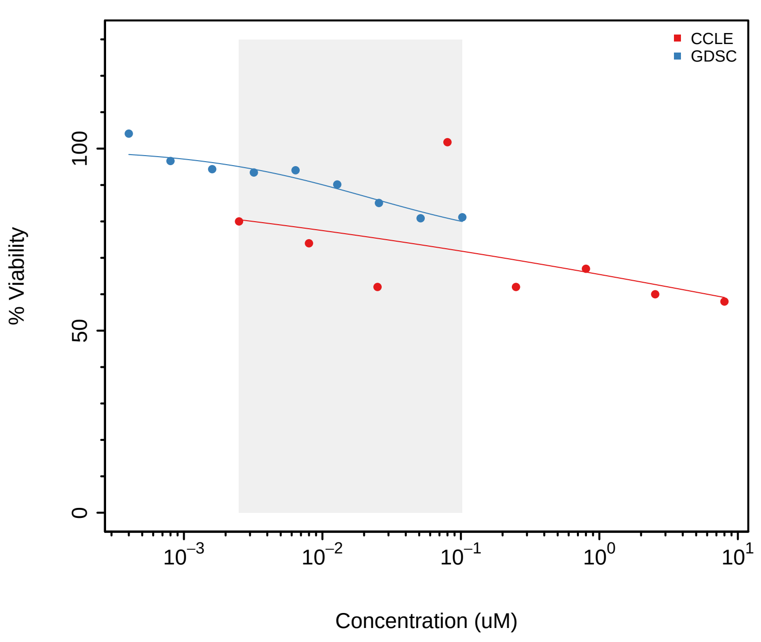

paclitaxel:NCI−H1155

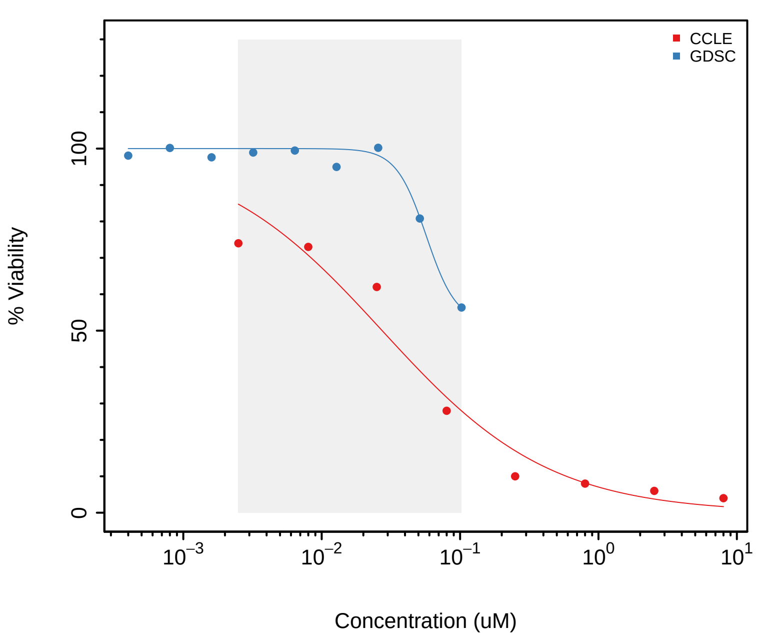

paclitaxel:NCI−H1355

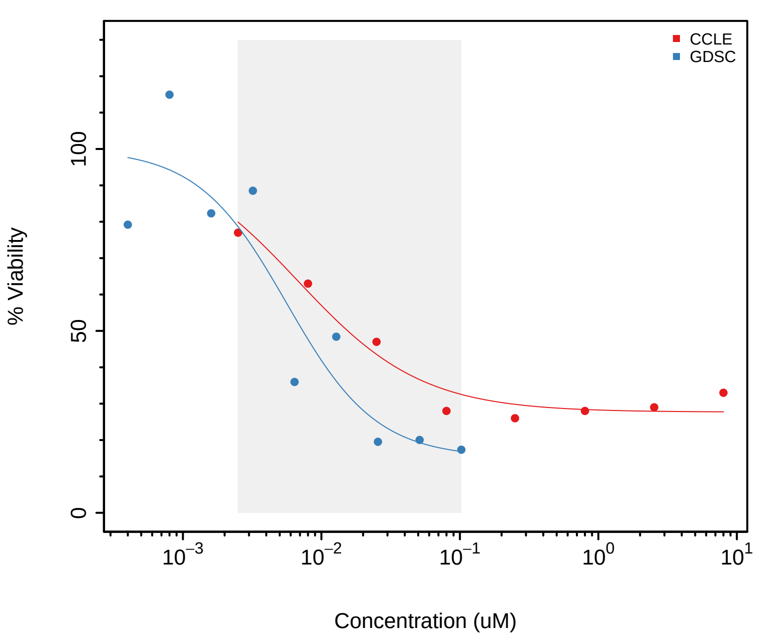

paclitaxel:NCI−H1581

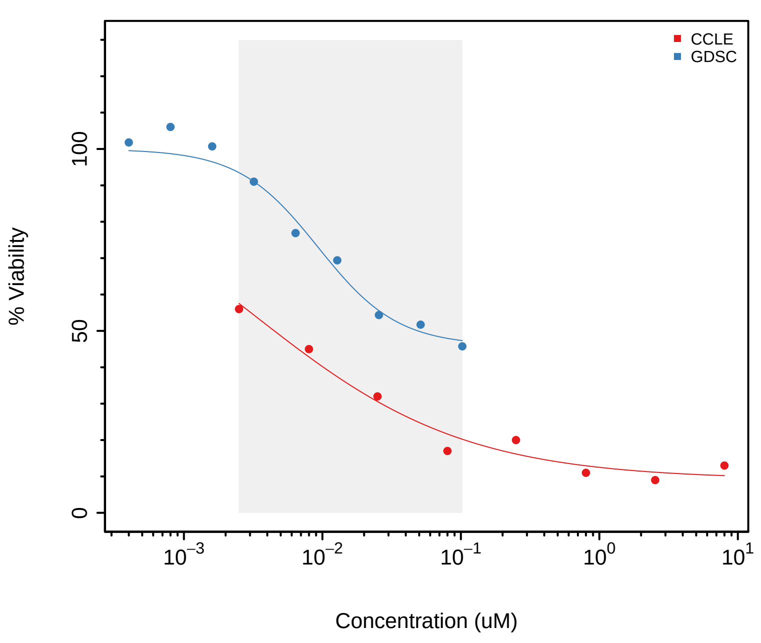

paclitaxel:NCI−H1648

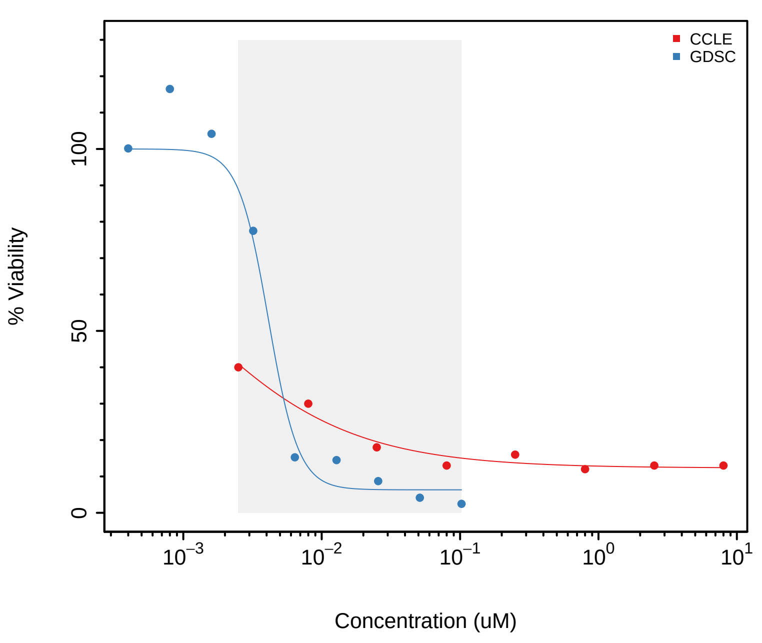

paclitaxel:NCI−H1666

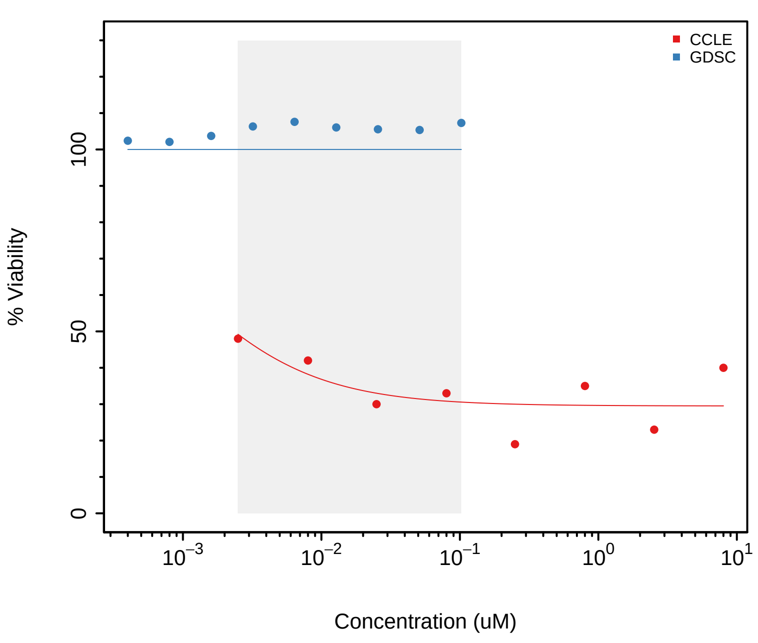

paclitaxel:NCI−H1694

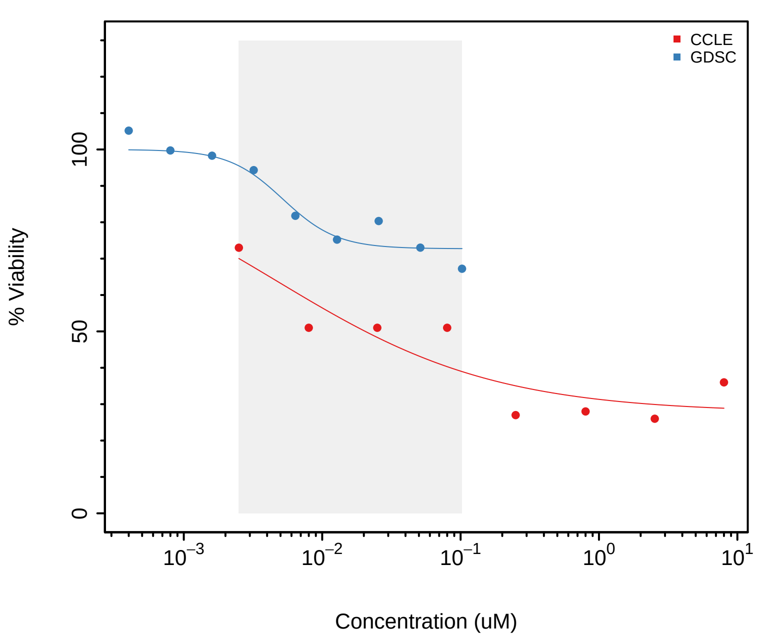

paclitaxel:NCI−H1975

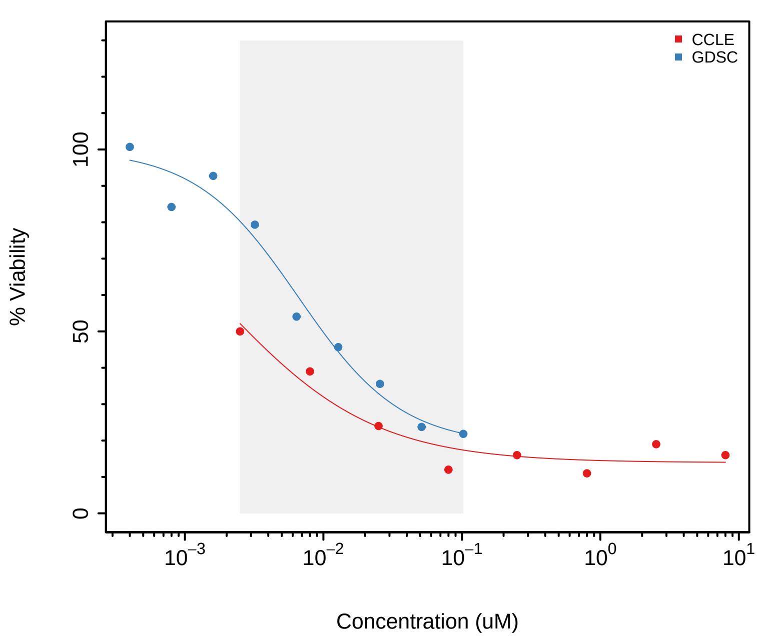

paclitaxel:NCI−H226

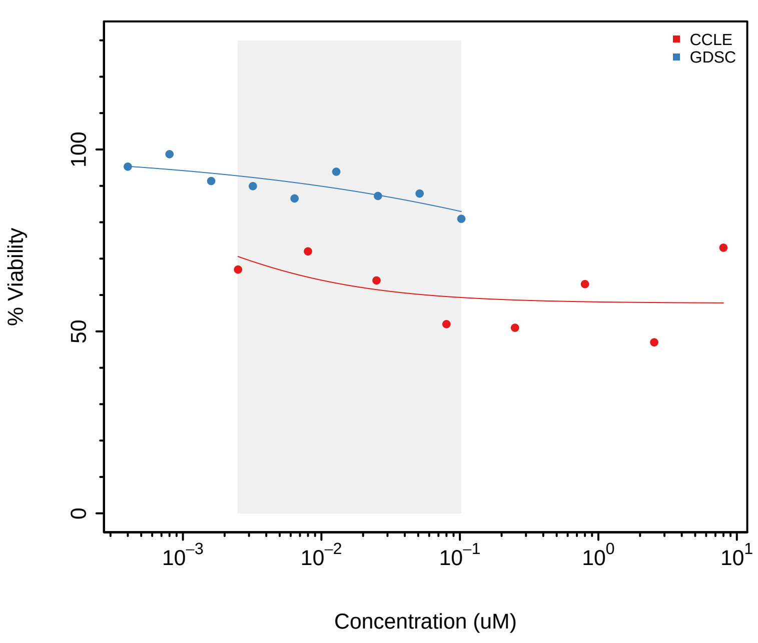

paclitaxel:NCI−H23

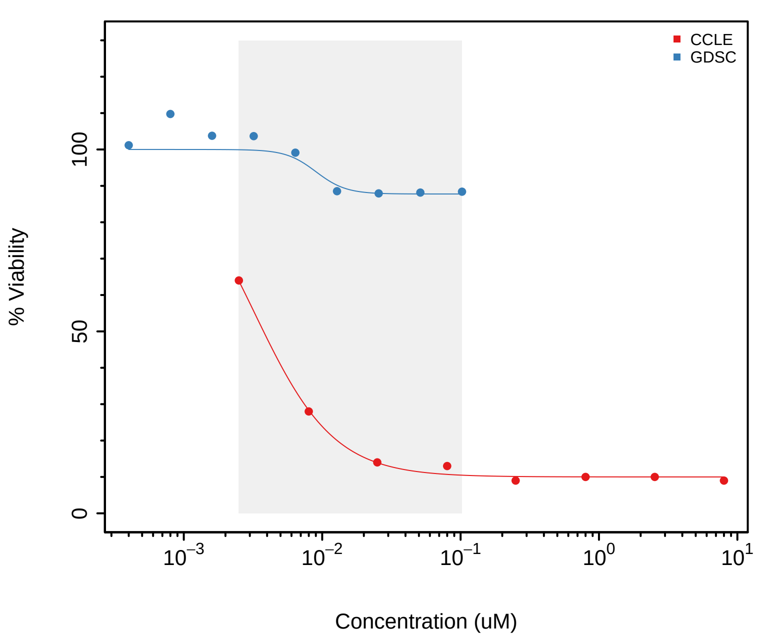

paclitaxel:NCI−H747

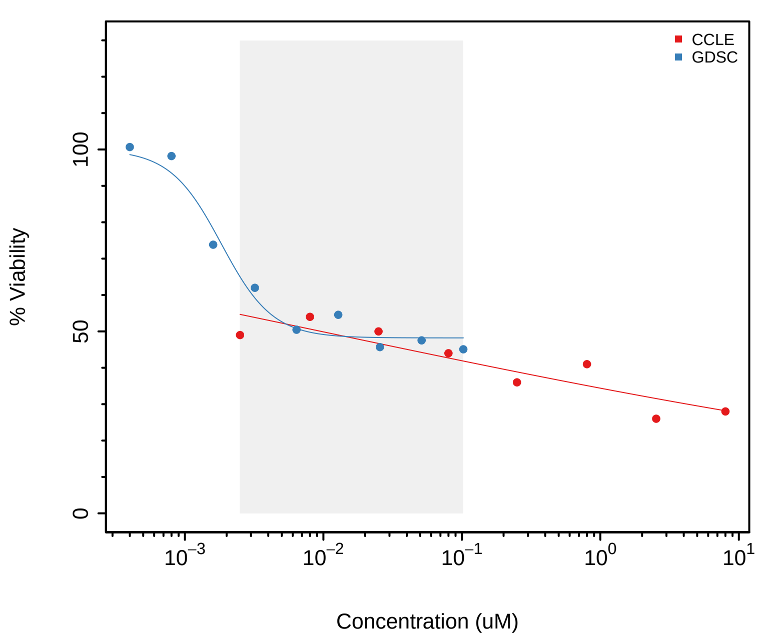

paclitaxel:OCI−AML2

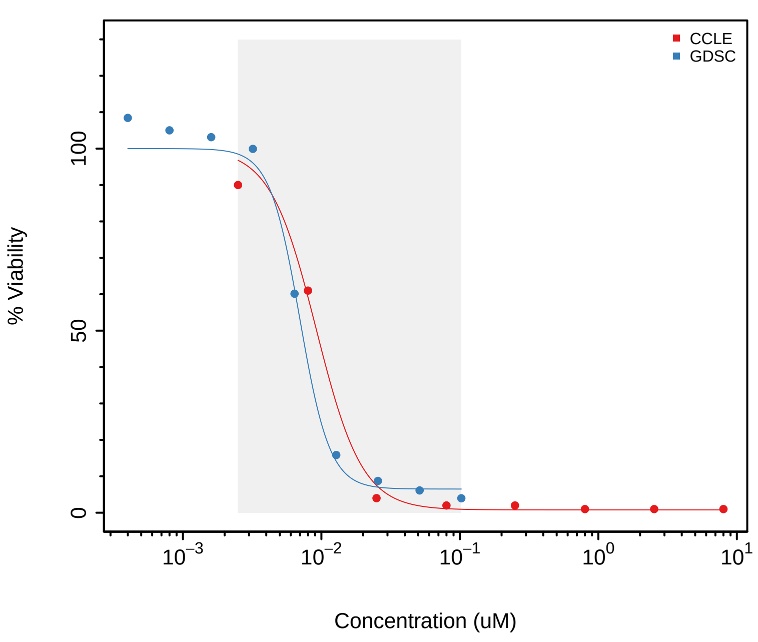

paclitaxel:OPM−2

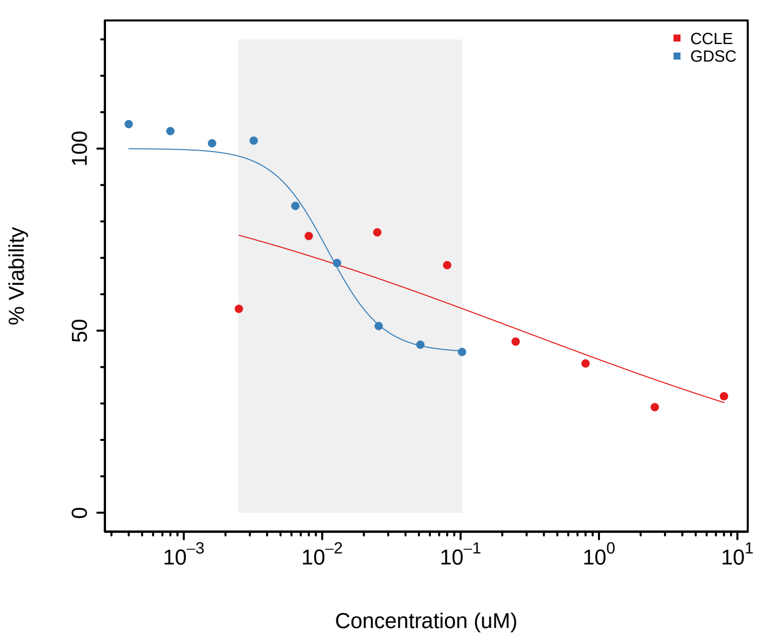

paclitaxel:OVCAR−4

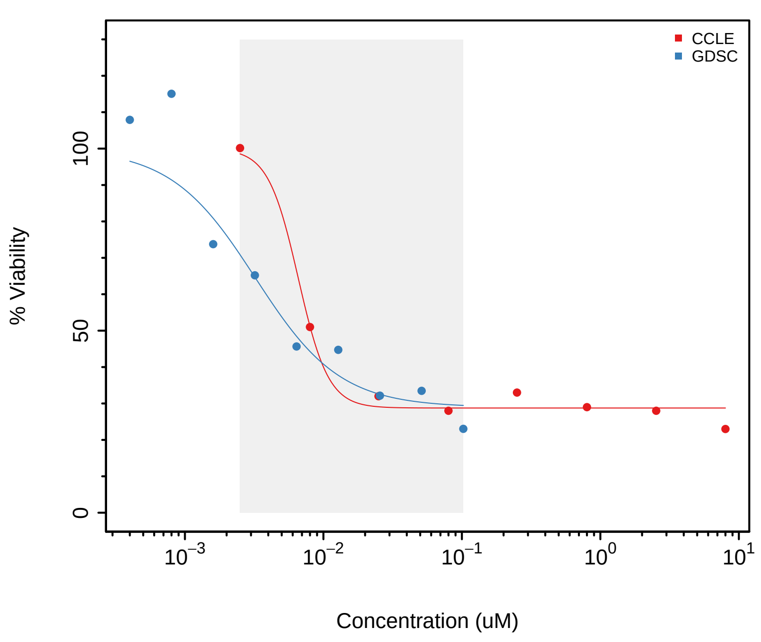

paclitaxel:P31−FUJ

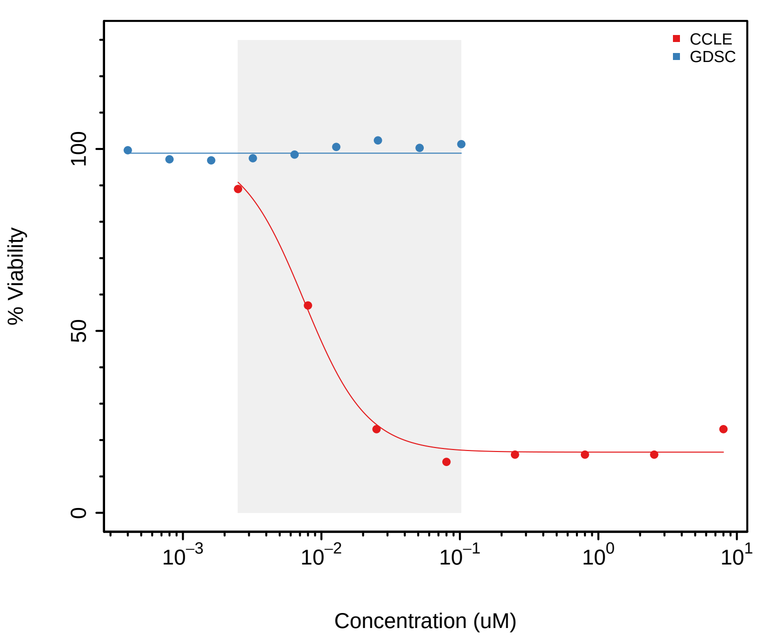

paclitaxel:PSN1

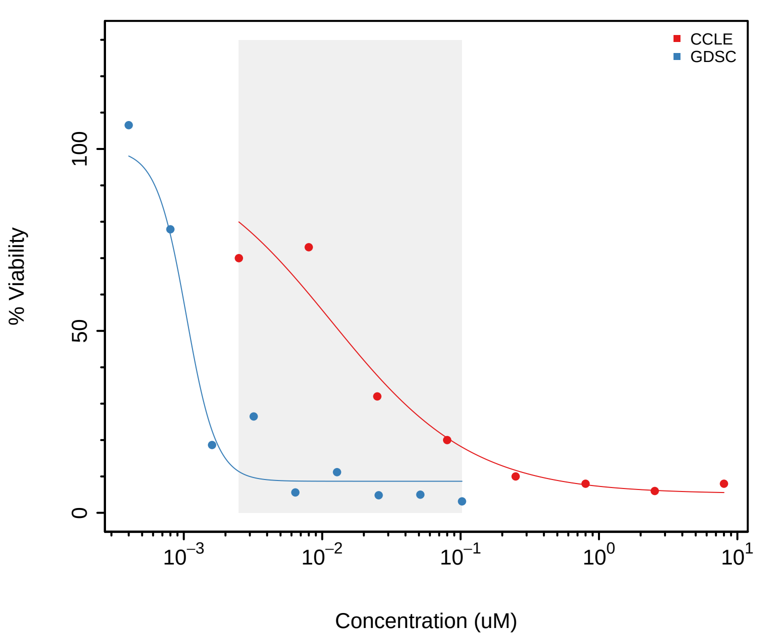

paclitaxel:Raji

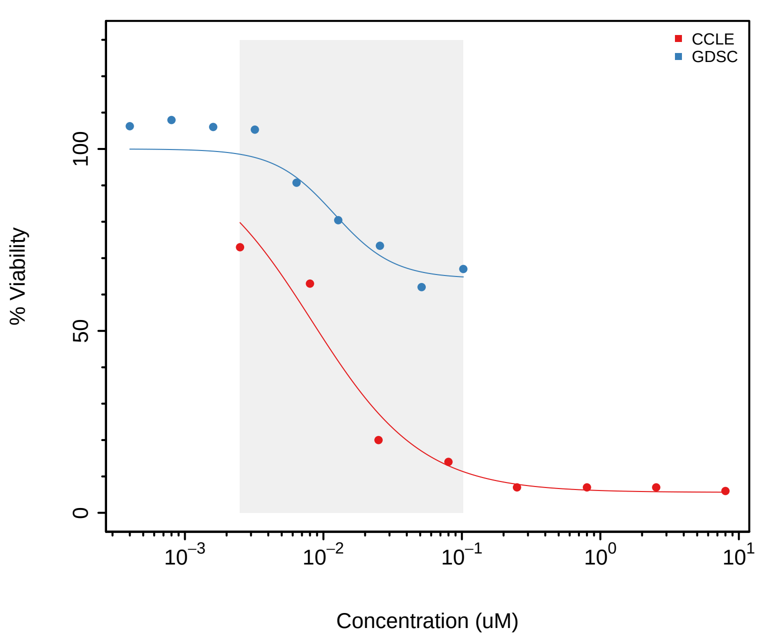

paclitaxel:REH

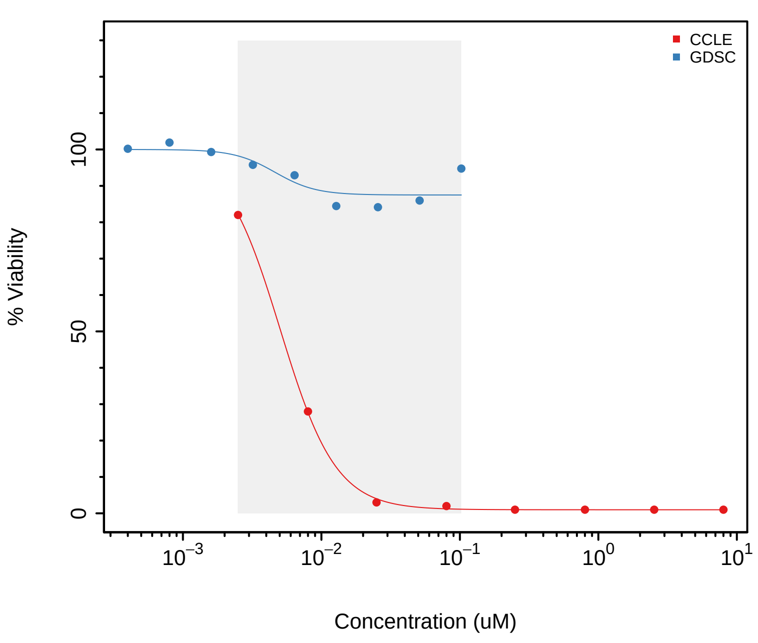

paclitaxel:RKO

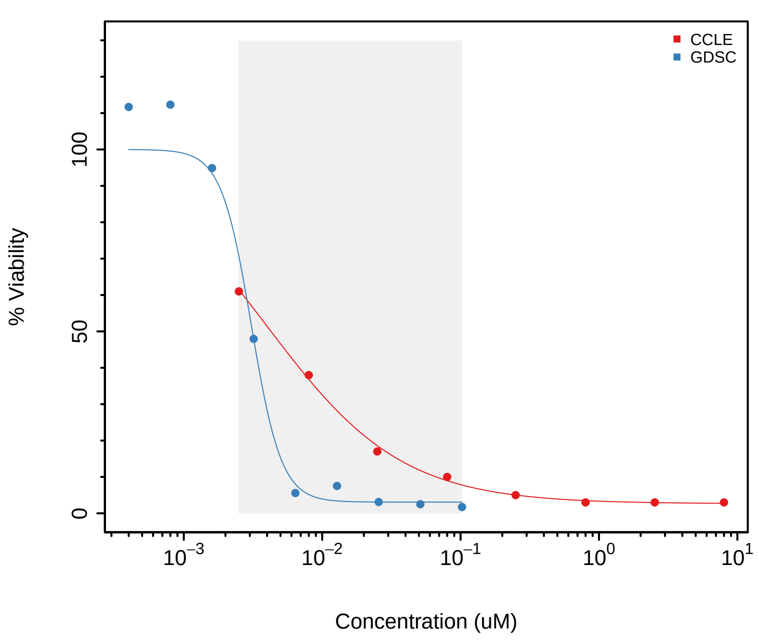

paclitaxel:RL95−2

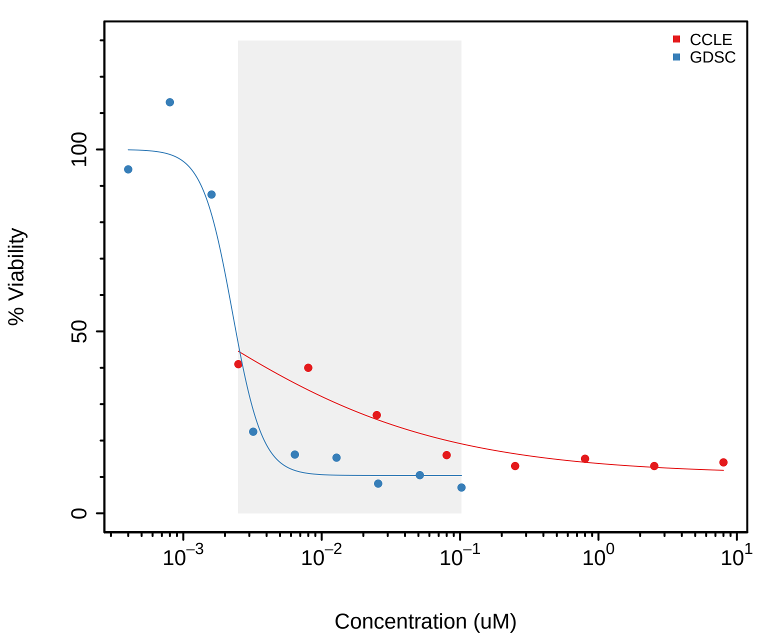

paclitaxel:RPMI−8402

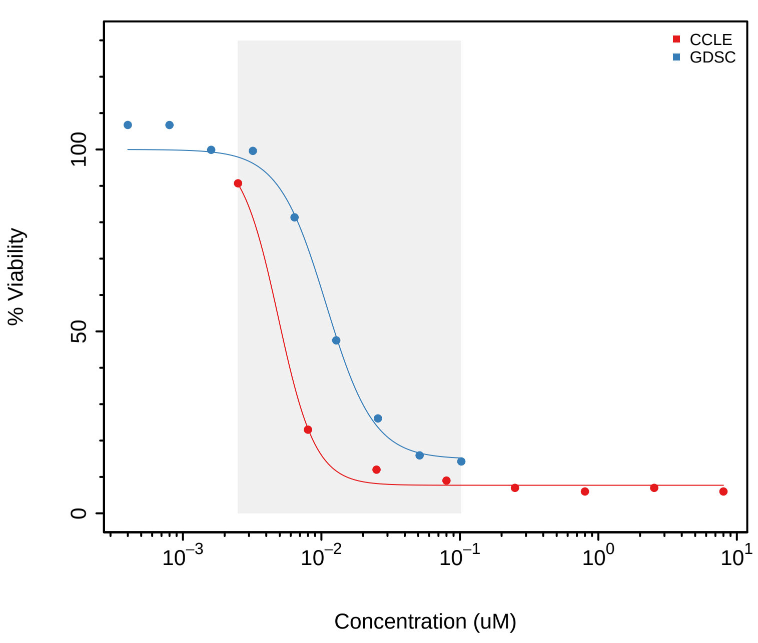

paclitaxel:SF126

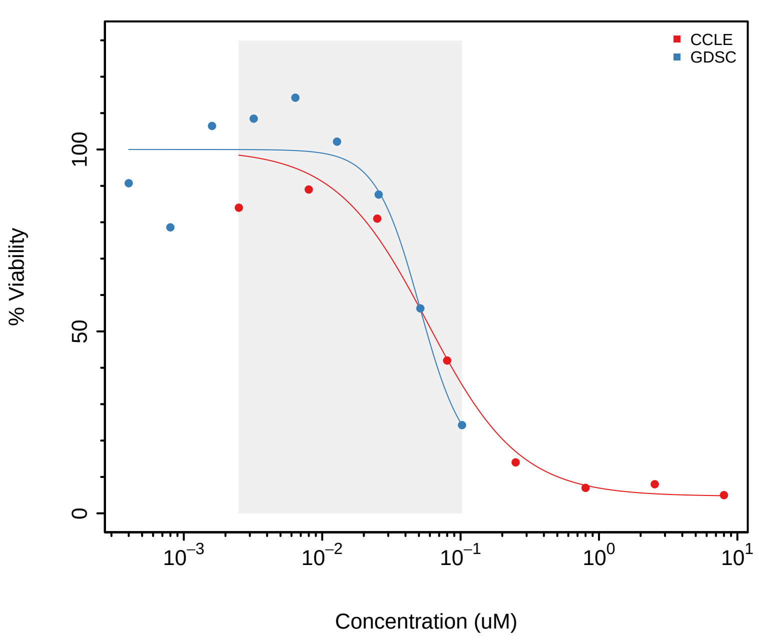

paclitaxel:SHP−77

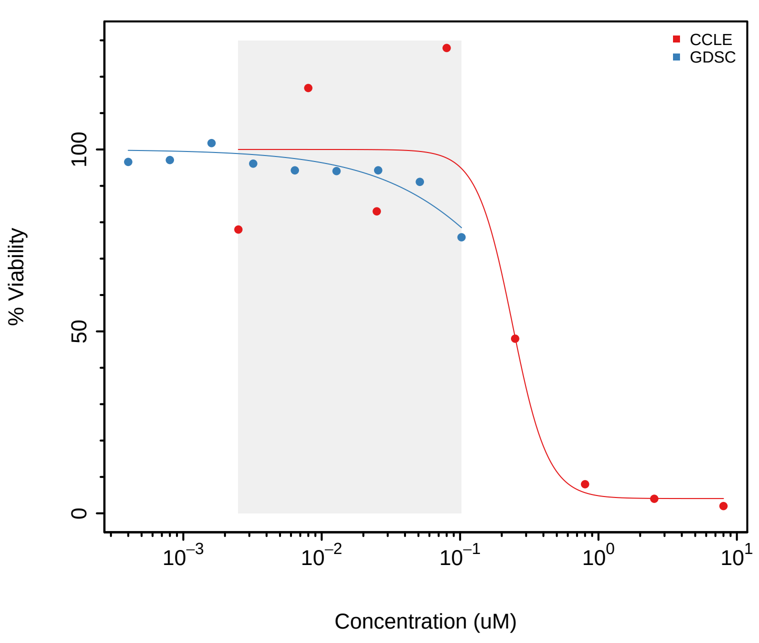

paclitaxel:SIG−M5

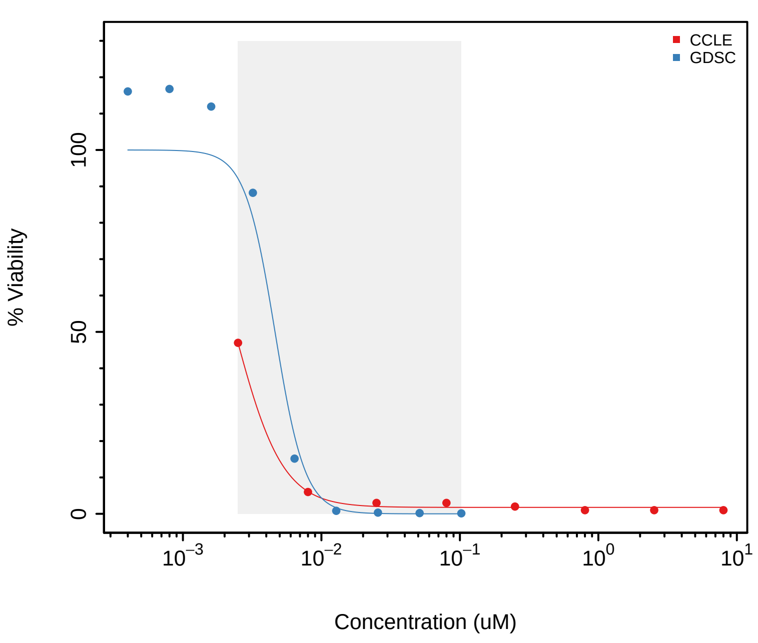

paclitaxel:SIMA

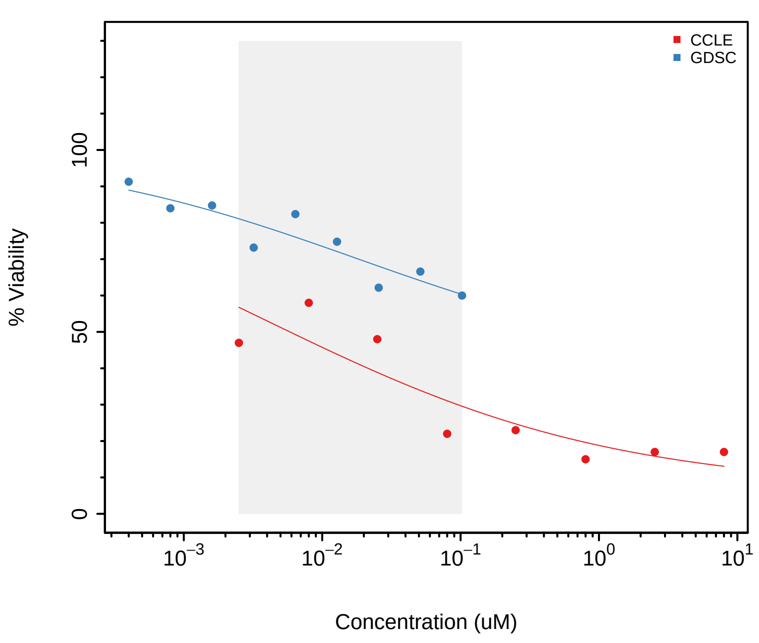

paclitaxel:SJSA−1

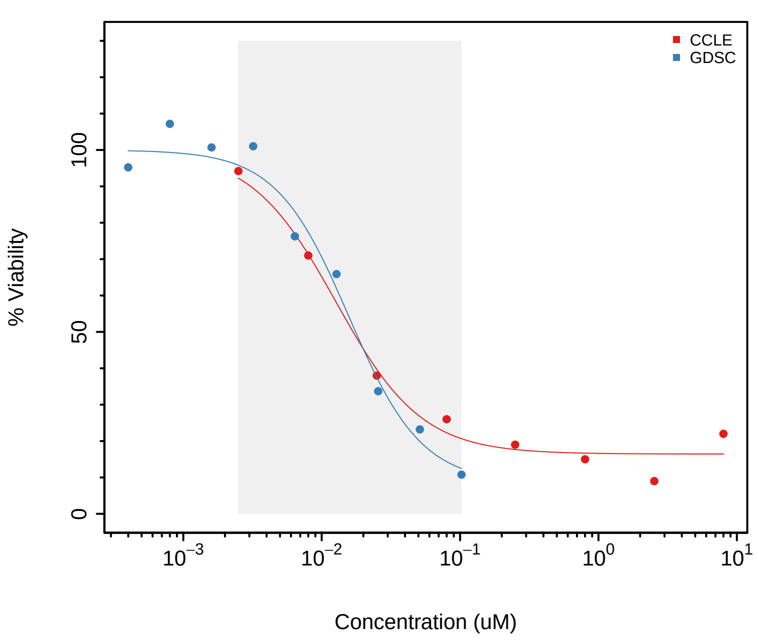

paclitaxel:SK−LMS−1

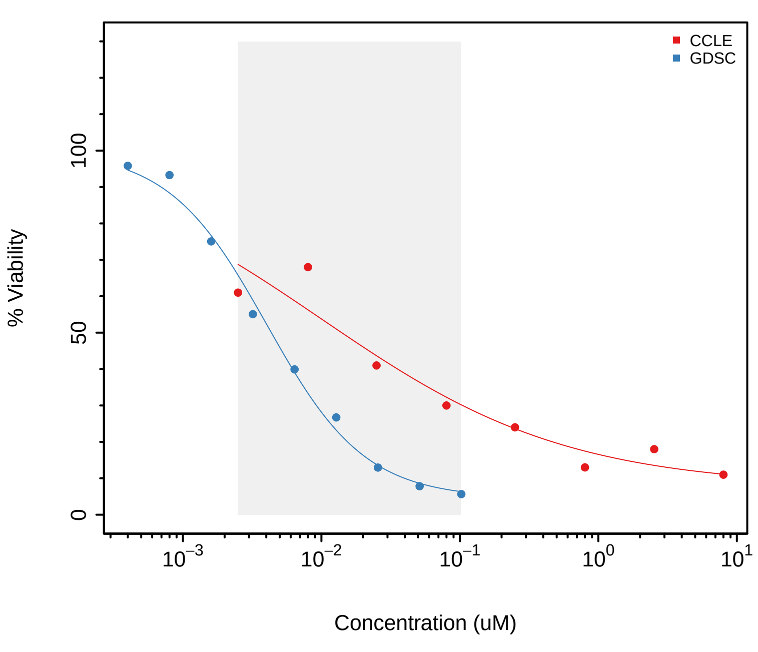

paclitaxel:SK−MEL−2

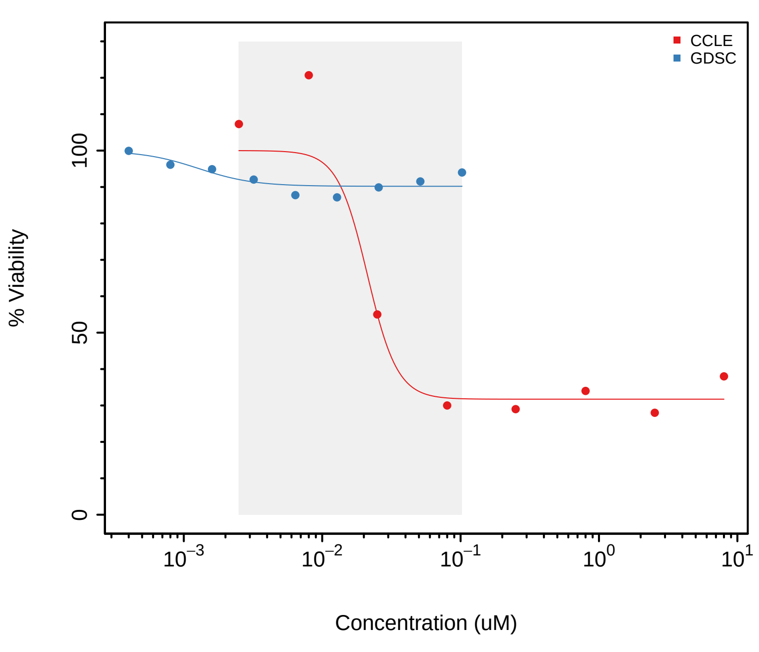

paclitaxel:SK−MM−2

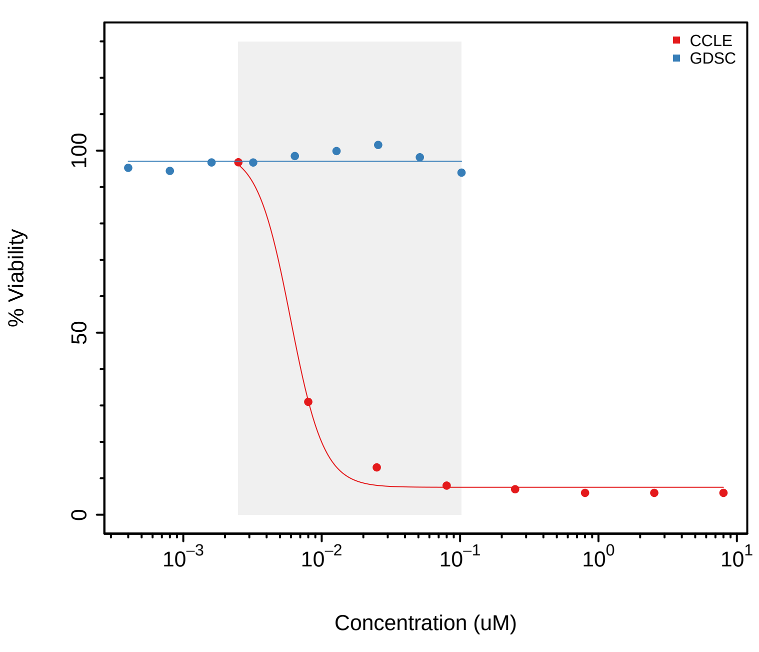

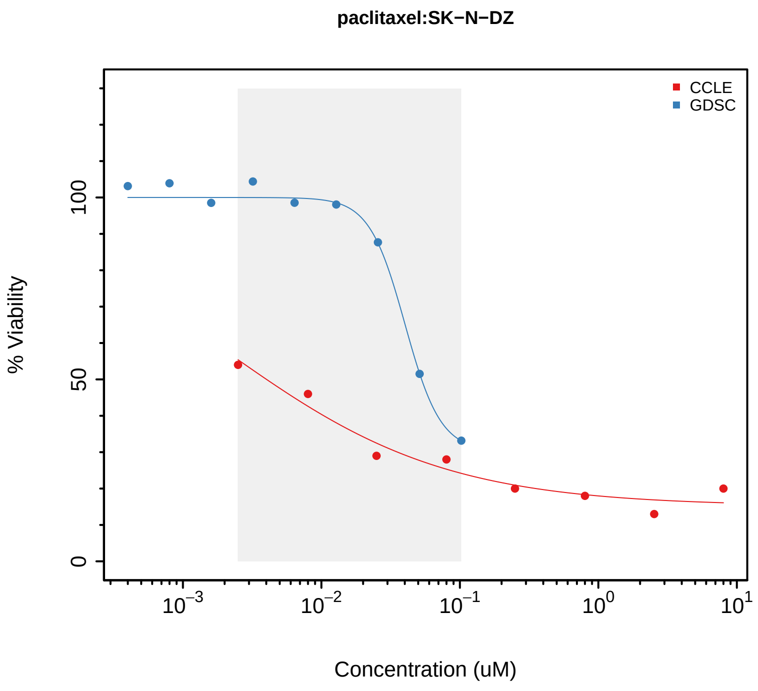
paclitaxel:SK−N−DZ
CCLE
GDSC
% Viability
0
50
100
10^{-3} 10^{-2} 10^{-1} 10^{0} 10^{1}
Concentration (uM)

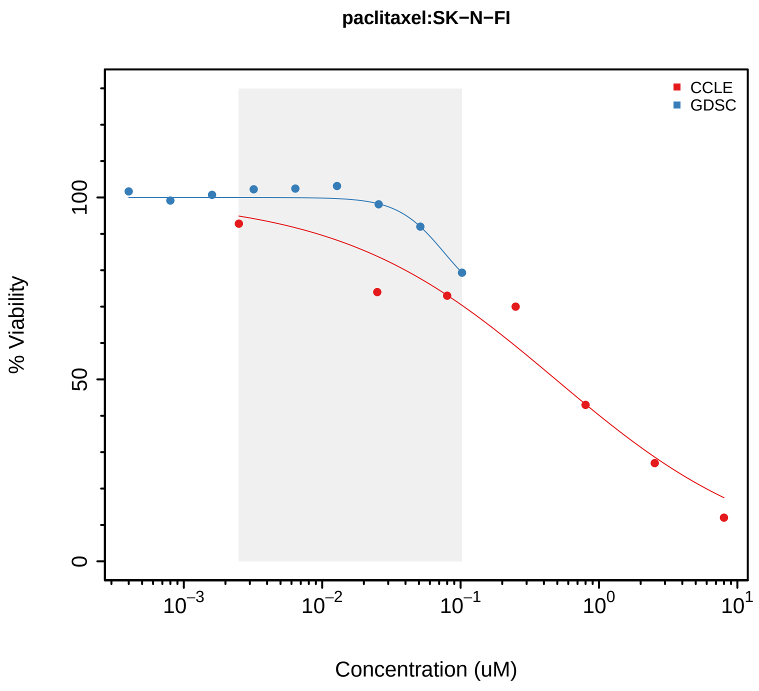
paclitaxel:SK−N−FI
CCLE
GDSC
% Viability
0
50
100
10^{-3} 10^{-2} 10^{-1} 10^{0} 10^{1}
Concentration (uM)

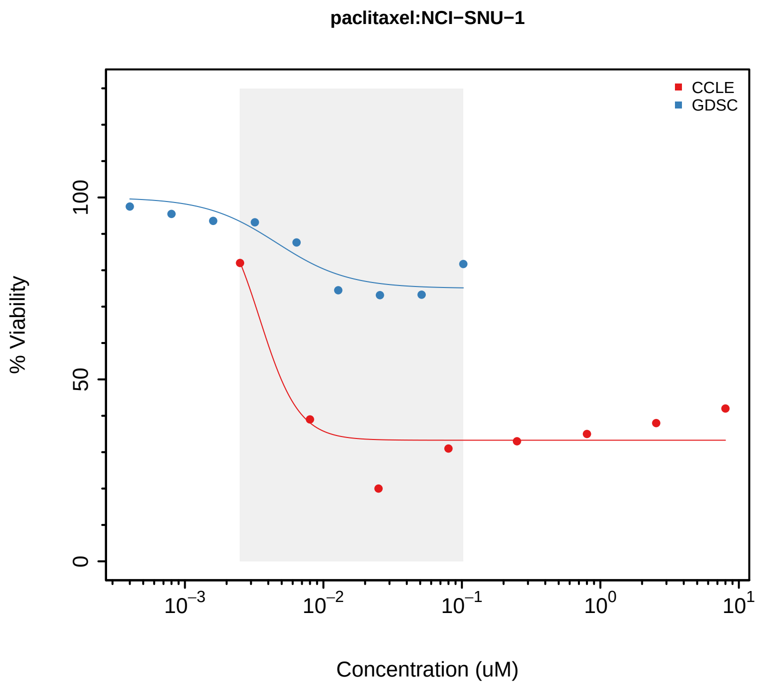
paclitaxel:NCI−SNU−1
CCLE
GDSC
% Viability
0
50
100
10^{-3} 10^{-2} 10^{-1} 10^{0} 10^{1}
Concentration (uM)

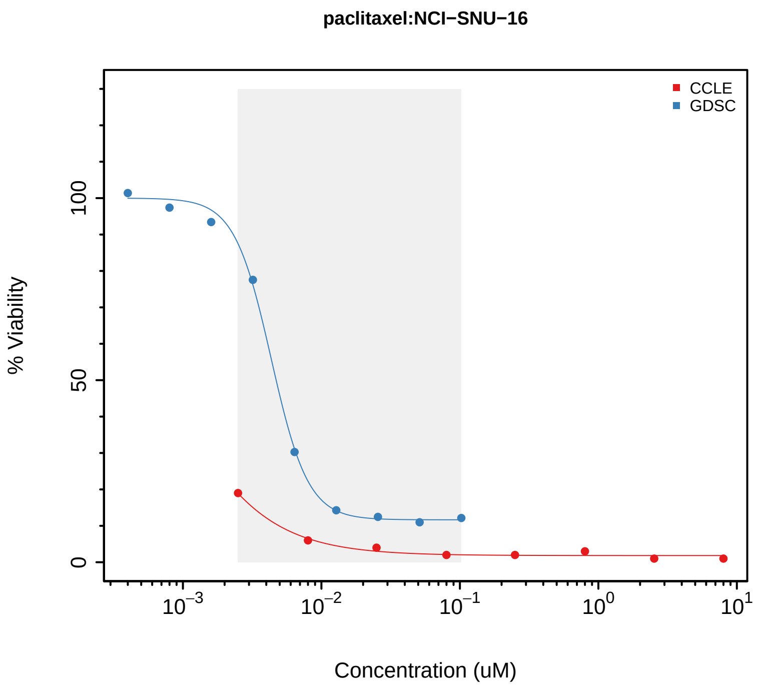
paclitaxel:NCI−SNU−16
CCLE
GDSC
% Viability
0
50
100
10^{-3} 10^{-2} 10^{-1} 10^{0} 10^{1}
Concentration (uM)

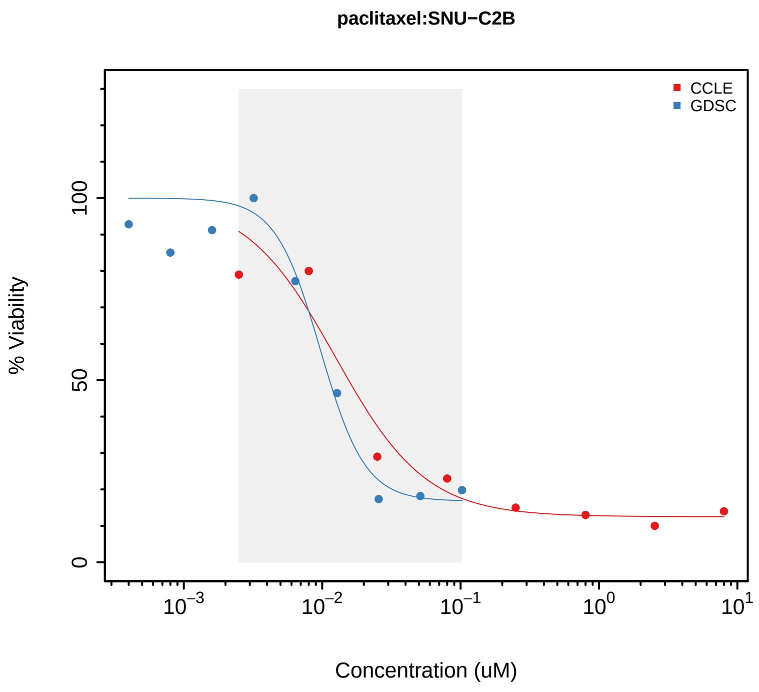
paclitaxel:SNU−C2B
CCLE
GDSC
% Viability
0
50
100
10^{-3} 10^{-2} 10^{-1} 10^{0} 10^{1}
Concentration (uM)

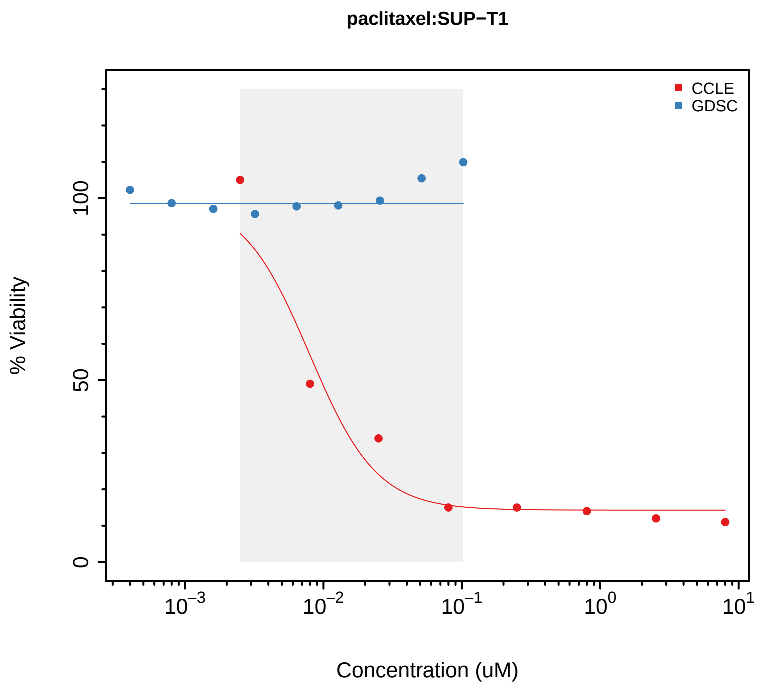
paclitaxel:SUP−T1
CCLE
GDSC
% Viability
0
50
100
10^{-3} 10^{-2} 10^{-1} 10^{0} 10^{1}
Concentration (uM)

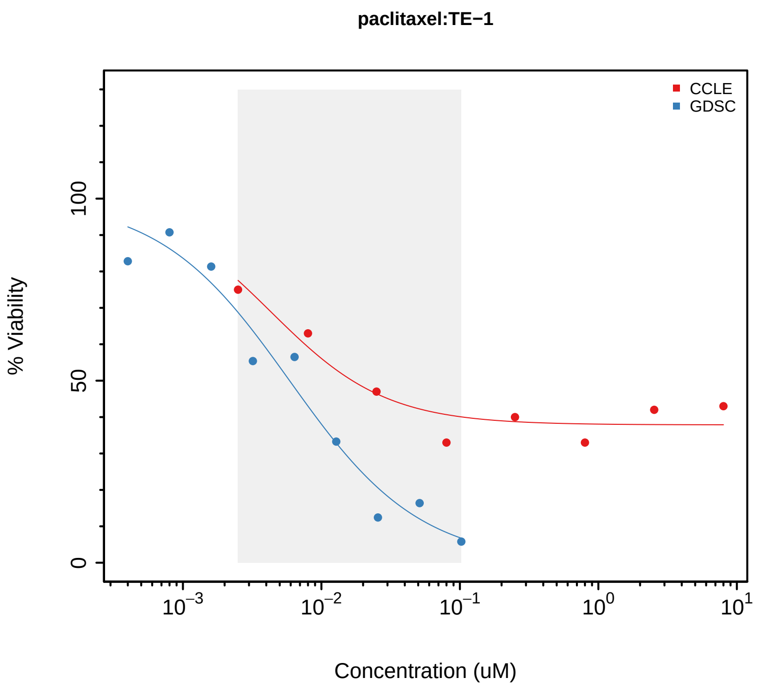
paclitaxel:TE−1
CCLE
GDSC
% Viability
0
50
100
10^{-3} 10^{-2} 10^{-1} 10^{0} 10^{1}
Concentration (uM)

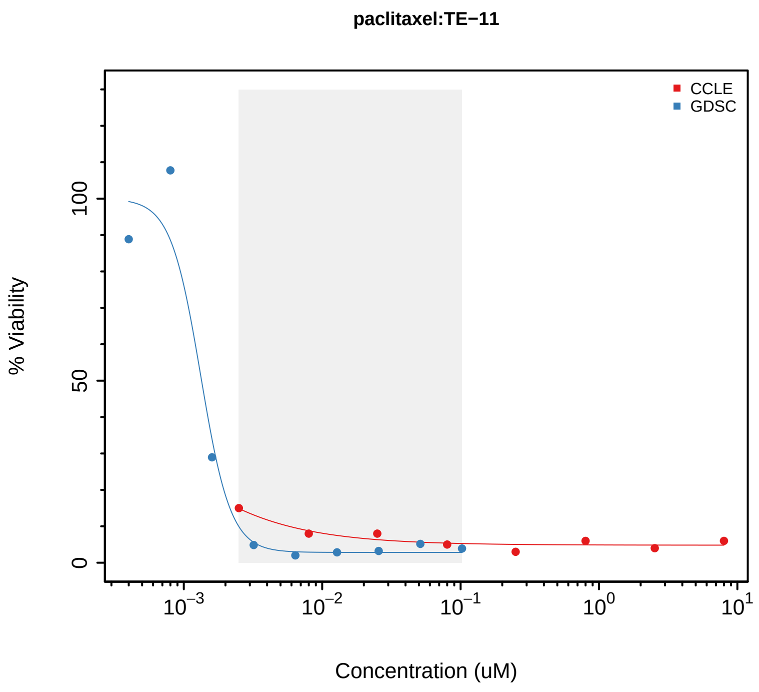
paclitaxel:TE−11
CCLE
GDSC
% Viability
0
50
100
10^{-3} 10^{-2} 10^{-1} 10^{0} 10^{1}
Concentration (uM)

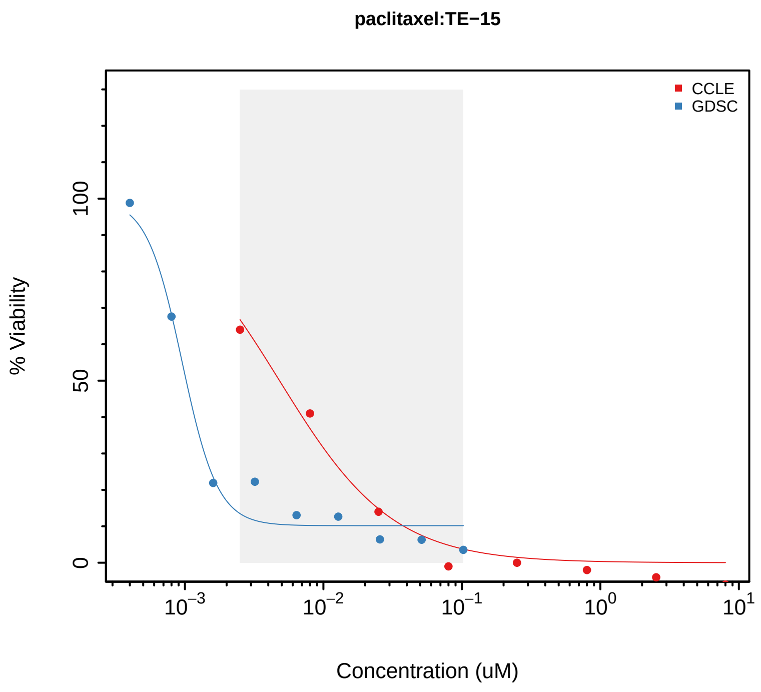
paclitaxel:TE−15
CCLE
GDSC
% Viability
0
50
100
10^{-3} 10^{-2} 10^{-1} 10^{0} 10^{1}
Concentration (uM)

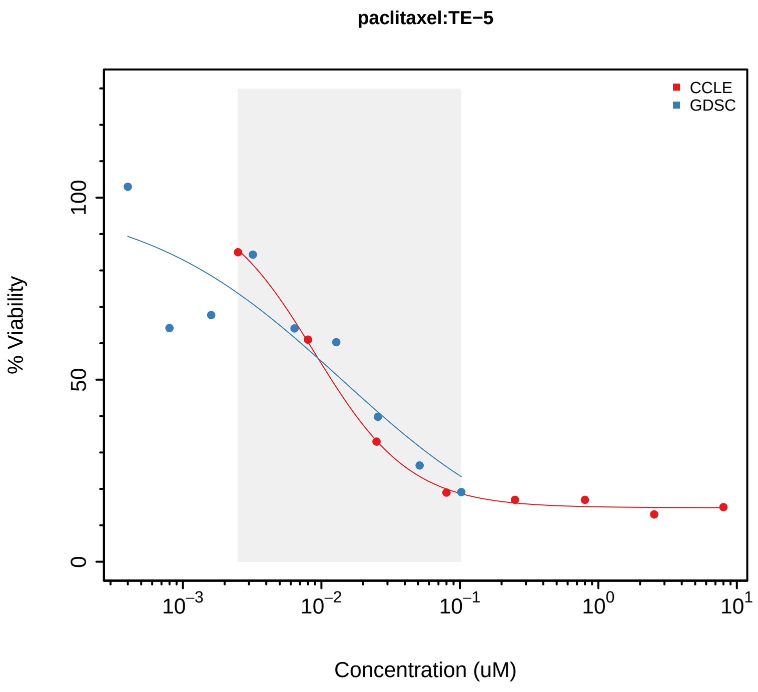
paclitaxel:TE−5
CCLE
GDSC
% Viability
0
50
100
10^{-3} 10^{-2} 10^{-1} 10^{0} 10^{1}
Concentration (uM)

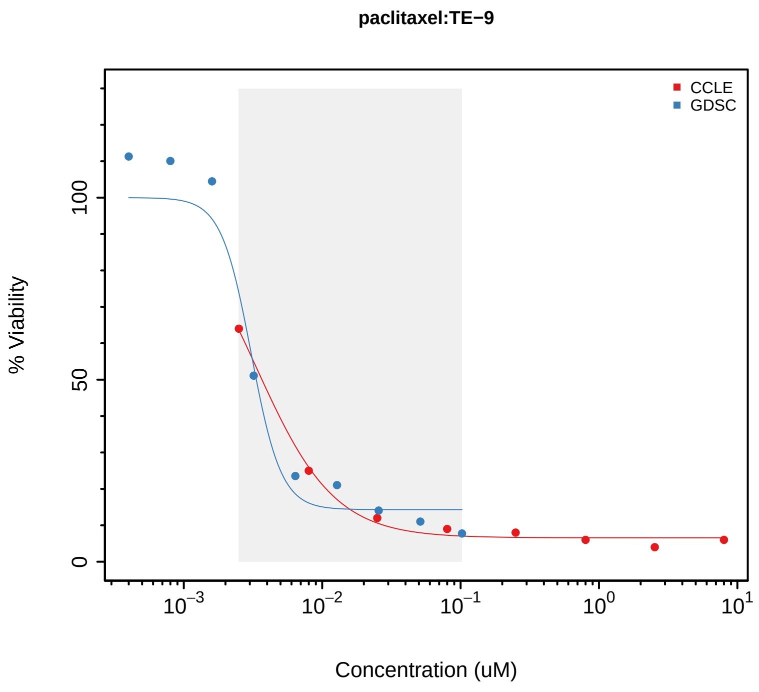
paclitaxel:TE−9
CCLE
GDSC
% Viability
0
50
100
10^{-3} 10^{-2} 10^{-1} 10^{0} 10^{1}
Concentration (uM)

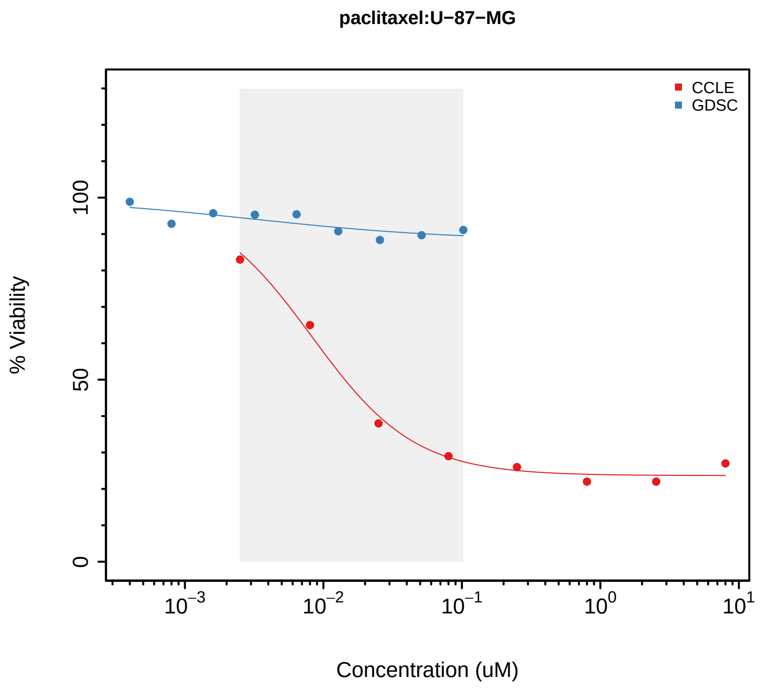
paclitaxel:U−87−MG
CCLE
GDSC
% Viability
0
50
100
10^{-3} 10^{-2} 10^{-1} 10^{0} 10^{1}
Concentration (uM)

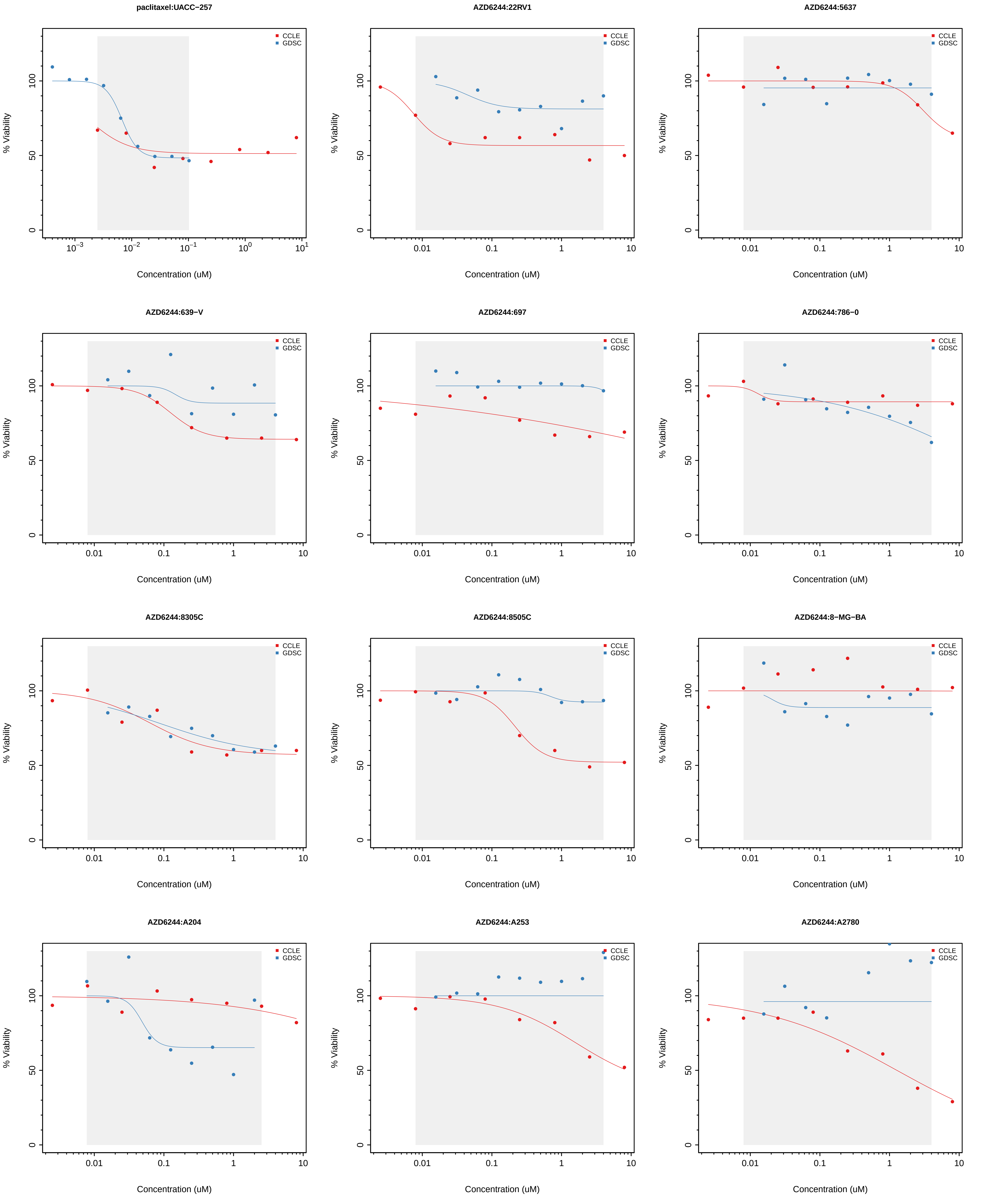
paclitaxel:UACC−257
AZD6244:22RV1
AZD6244:5637
AZD6244:639−V
AZD6244:697
AZD6244:786−0
AZD6244:8305C
AZD6244:8505C
AZD6244:8−MG−BA
AZD6244:A204
AZD6244:A253
AZD6244:A2780
CCLE
GDSC
% Viability
Concentration (uM)
0
50
100
10^{-3}
10^{-2}
10^{-1}
10^{0}
10^{1}
0.01
0.1
1
10

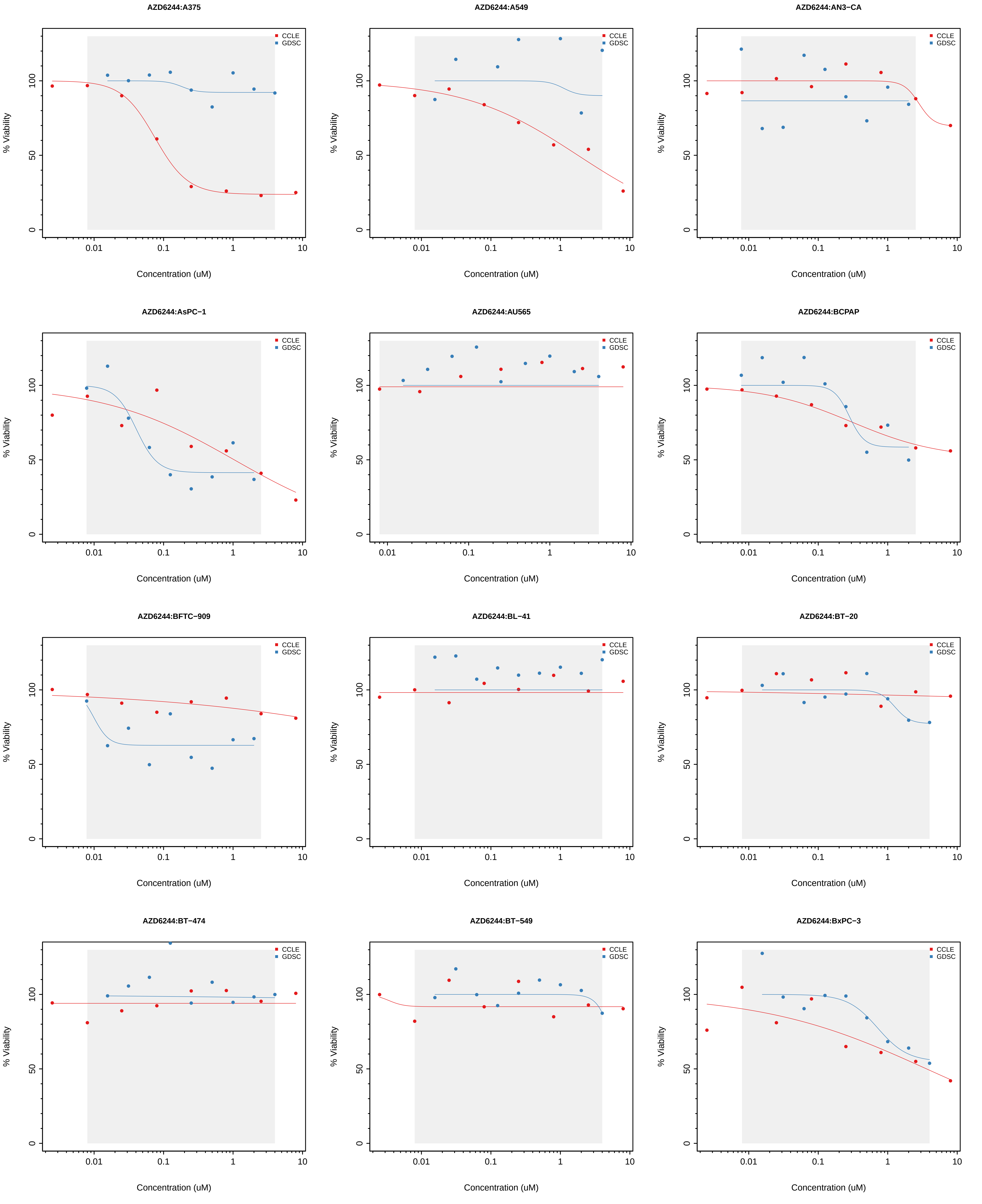
AZD6244:A375
AZD6244:A549
AZD6244:AN3−CA
AZD6244:AsPC−1
AZD6244:AU565
AZD6244:BCPAP
AZD6244:BFTC−909
AZD6244:BL−41
AZD6244:BT−20
AZD6244:BT−474
AZD6244:BT−549
AZD6244:BxPC−3
CCLE
GDSC
% Viability
0
50
100
Concentration (uM)
0.01
0.1
1
10

AZD6244:C2BBe1

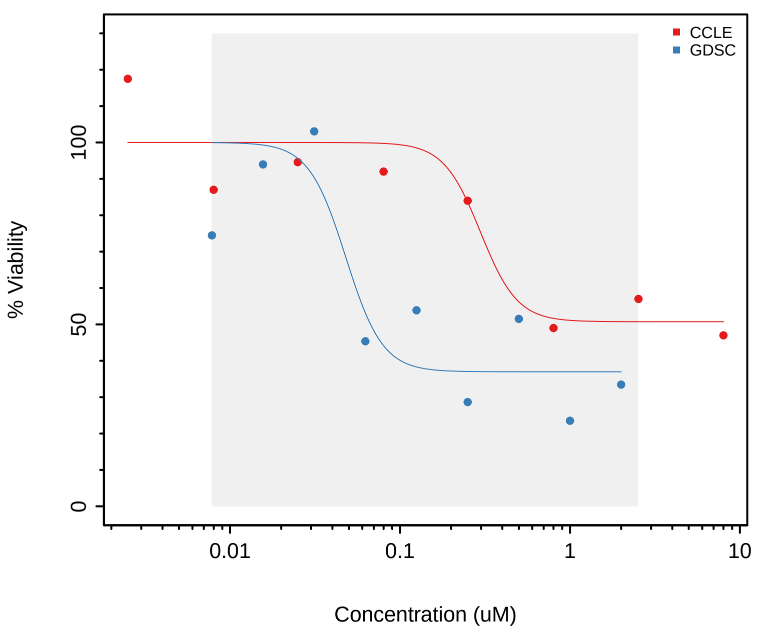

AZD6244:C32

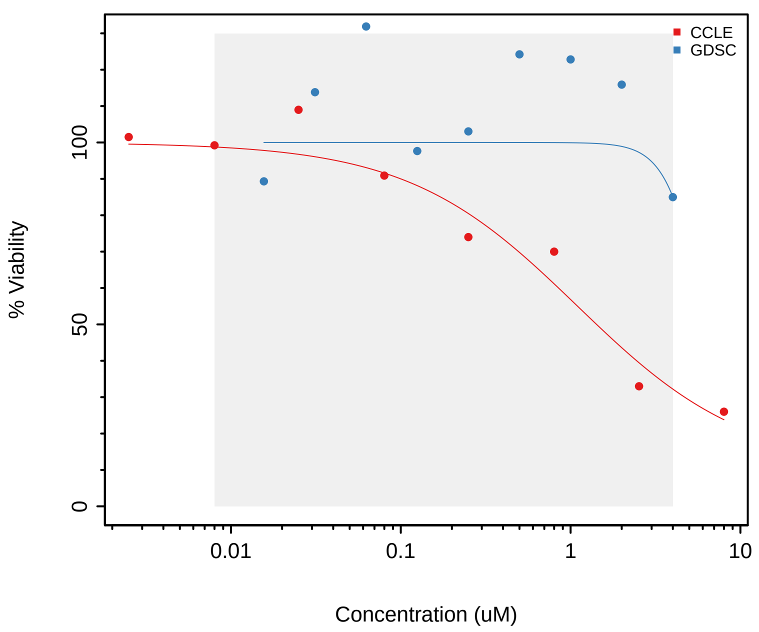

AZD6244:C3A

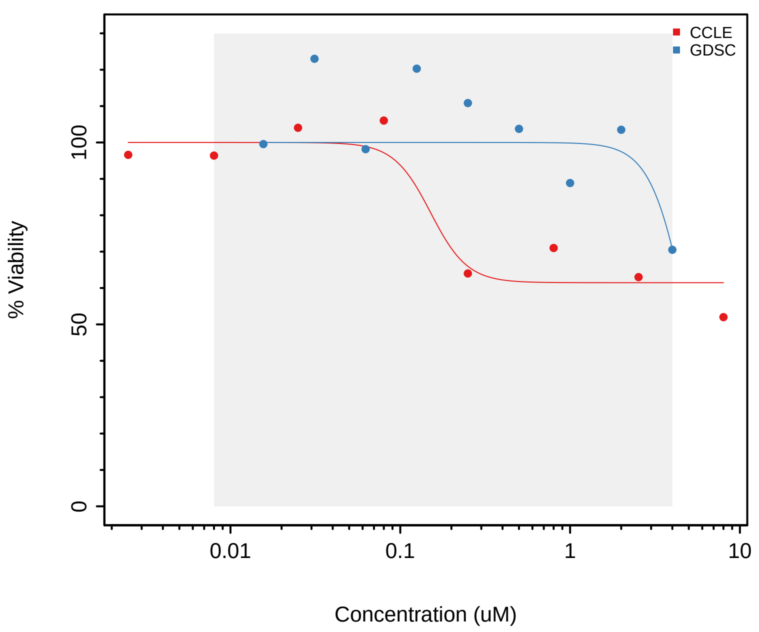

AZD6244:CAL−12T

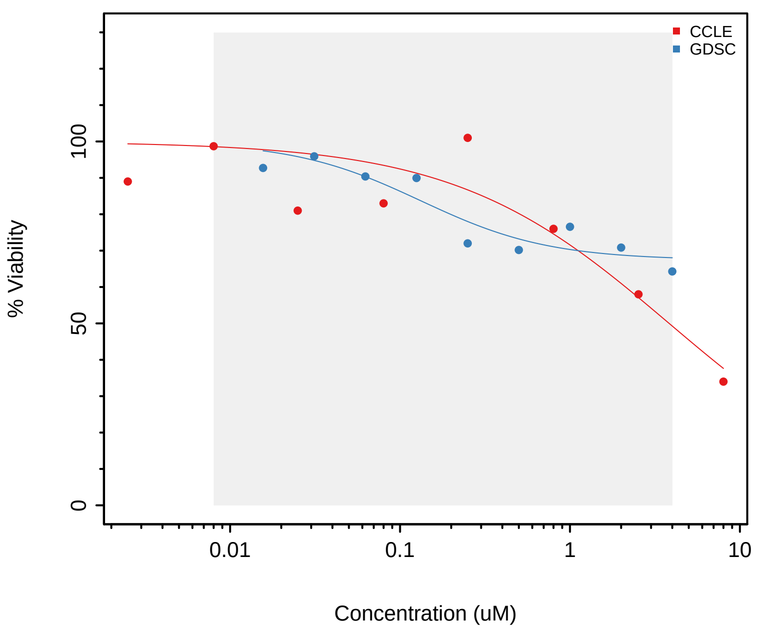

AZD6244:CAL−27

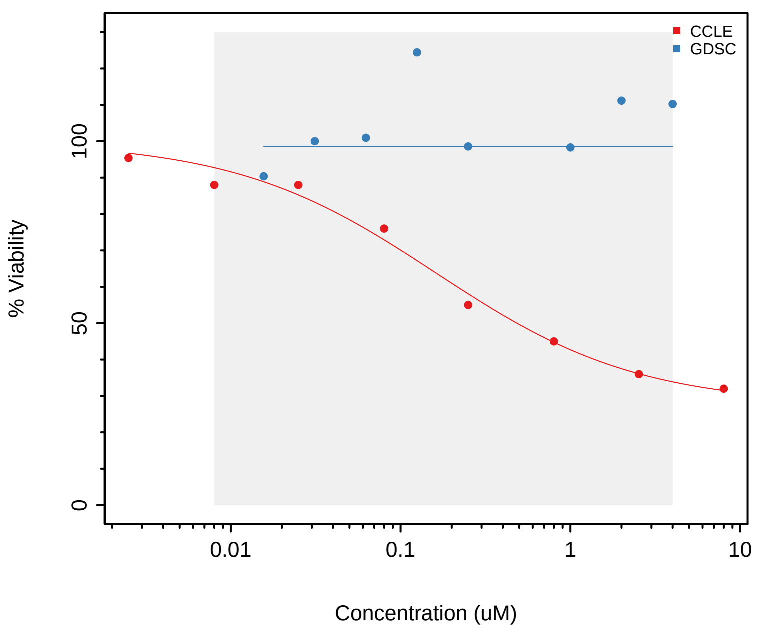

AZD6244:CAL−85−1

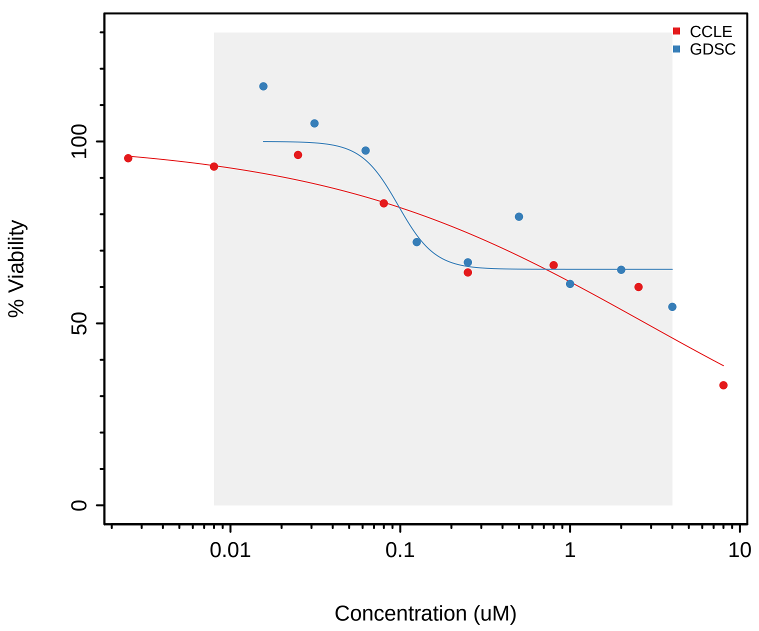

AZD6244:Calu−3

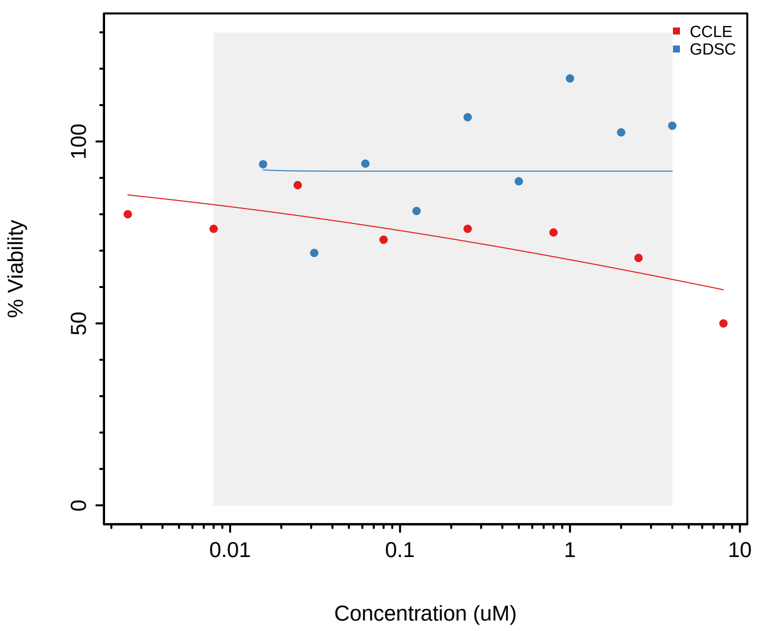

AZD6244:Calu−6

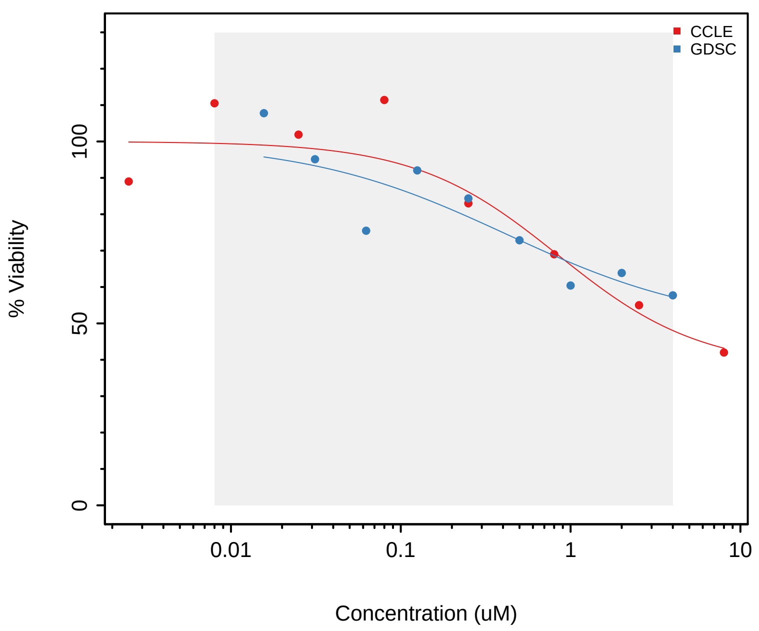

AZD6244:CAMA−1

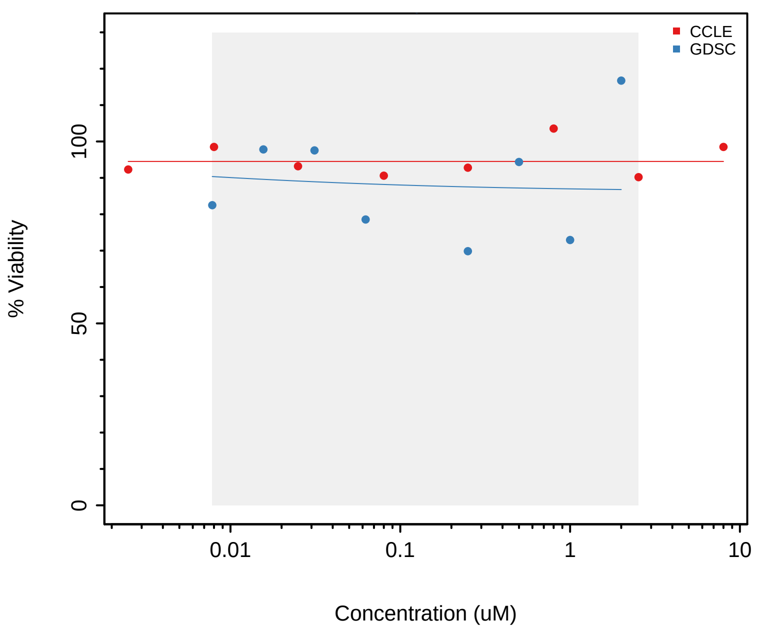

AZD6244:Capan−2

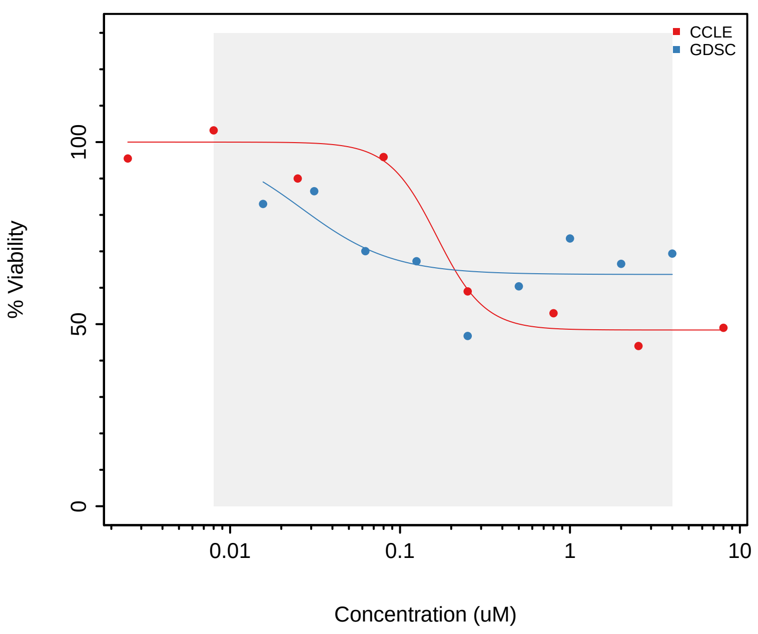

AZD6244:CAS−1

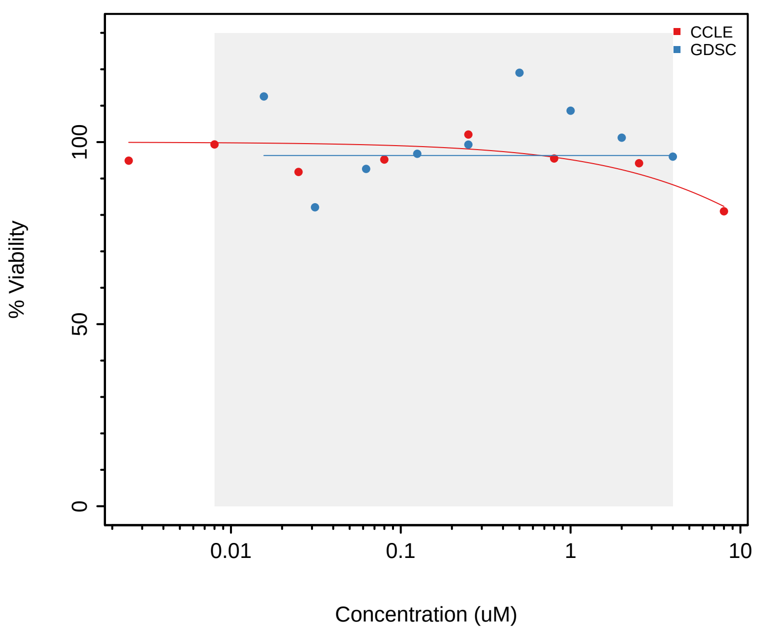

AZD6244:CHL−1

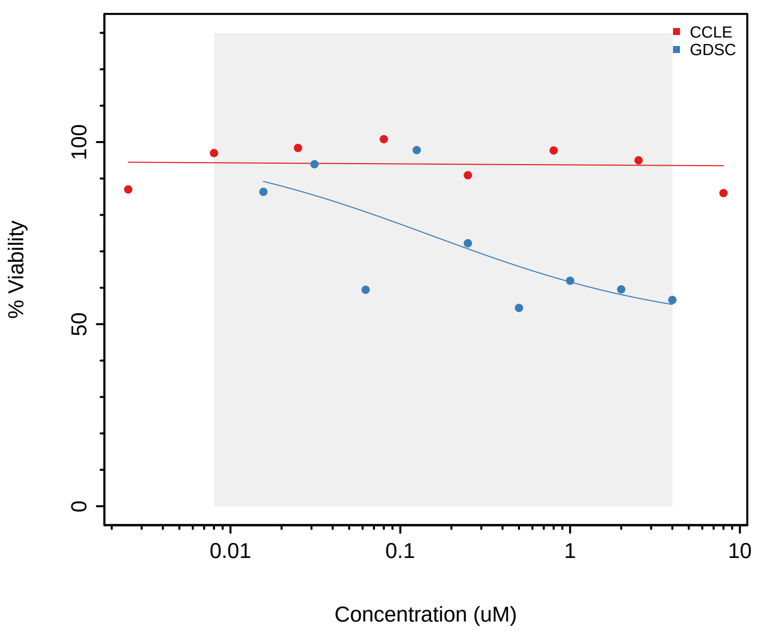

AZD6244:CHP−212

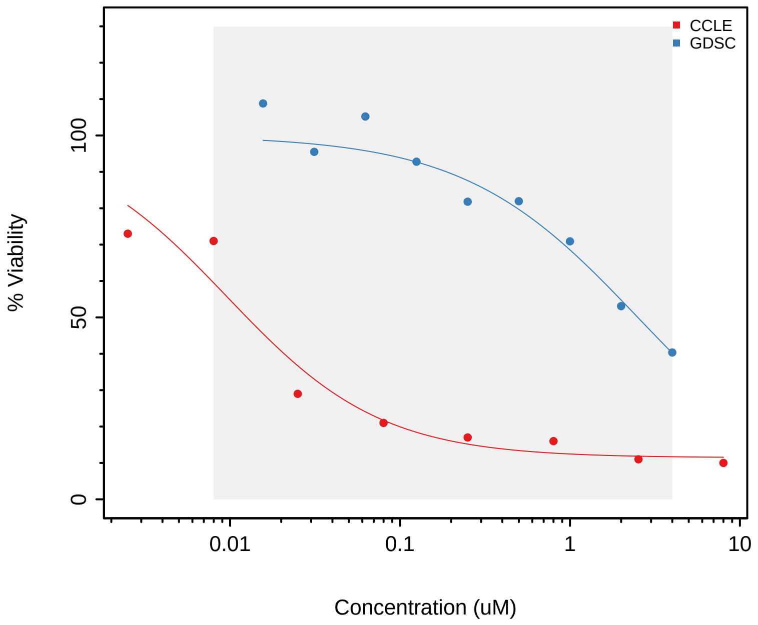

AZD6244:COLO−205

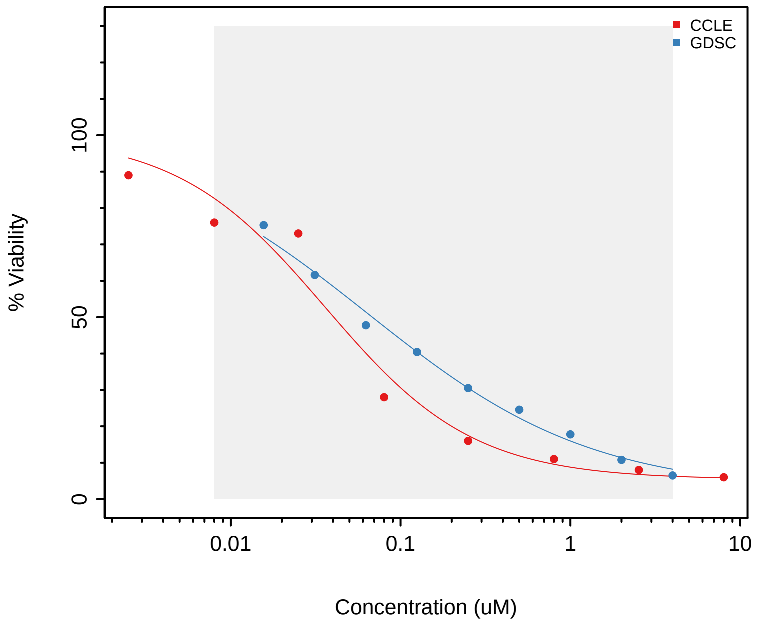

AZD6244:COLO−320−HSR

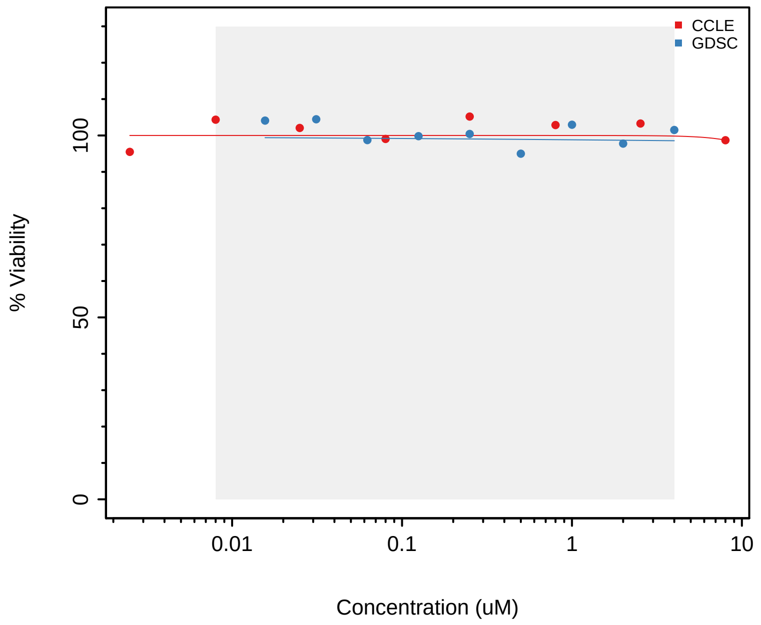

AZD6244:COLO−678

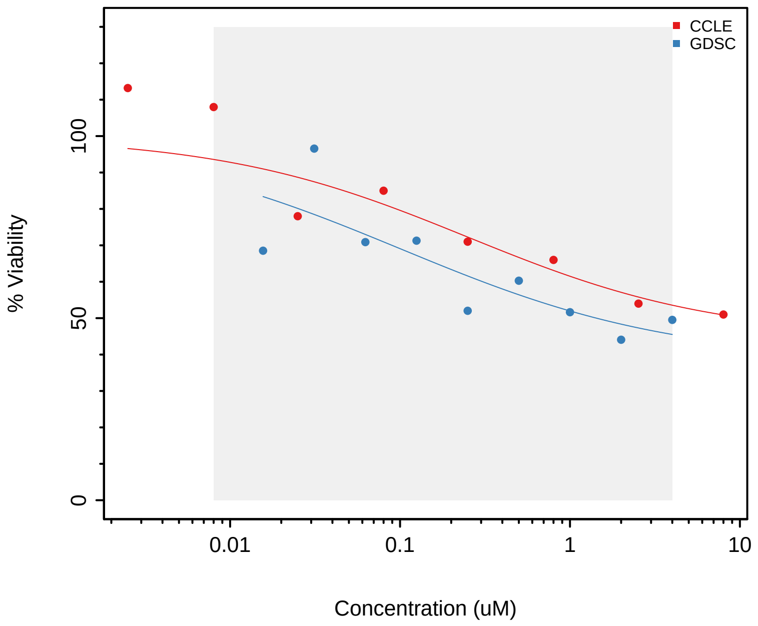

AZD6244:COLO−679

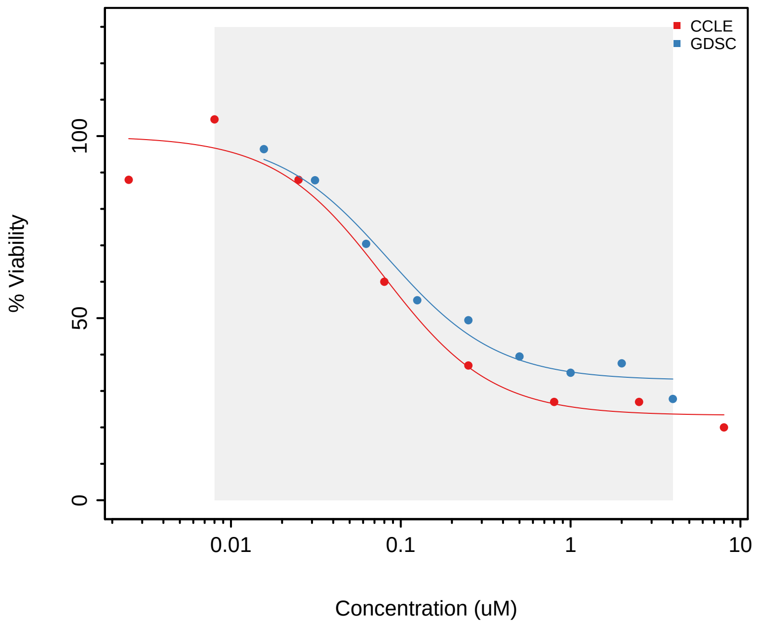

AZD6244:COLO−741

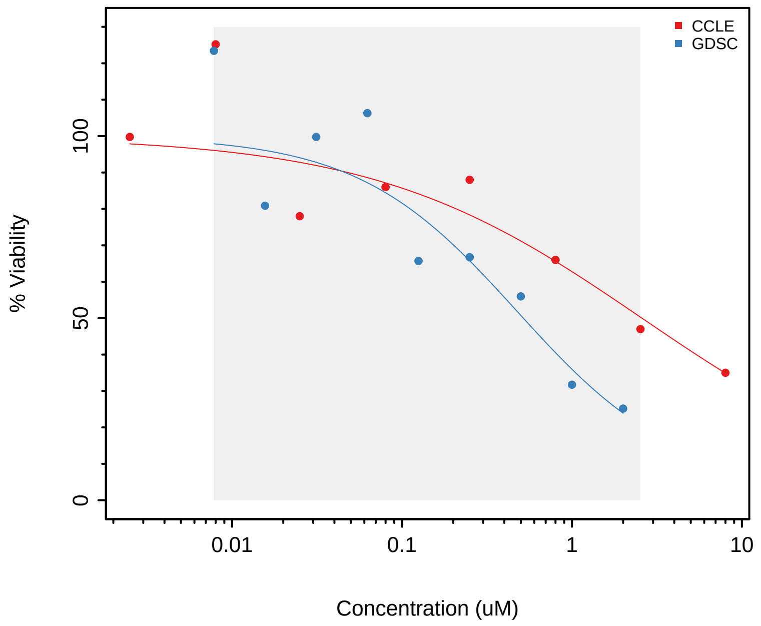

AZD6244:COR−L105

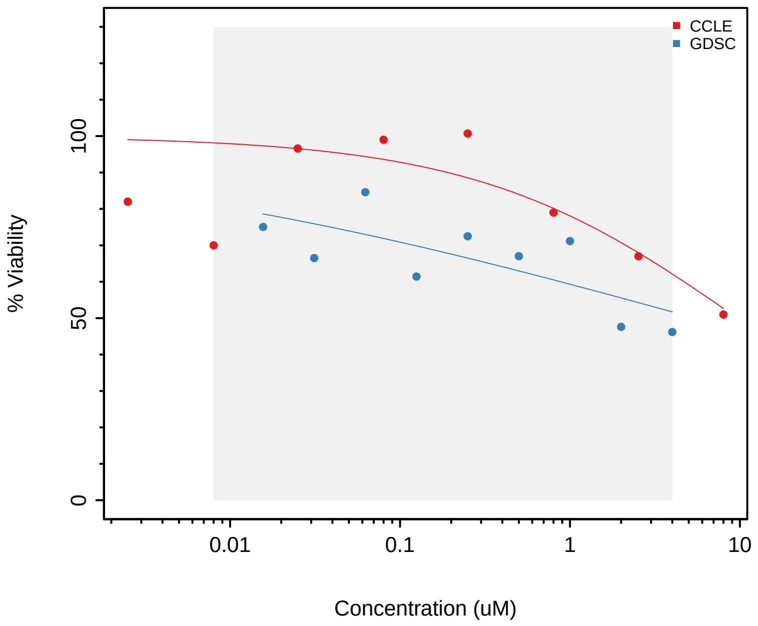

AZD6244:COR−L23

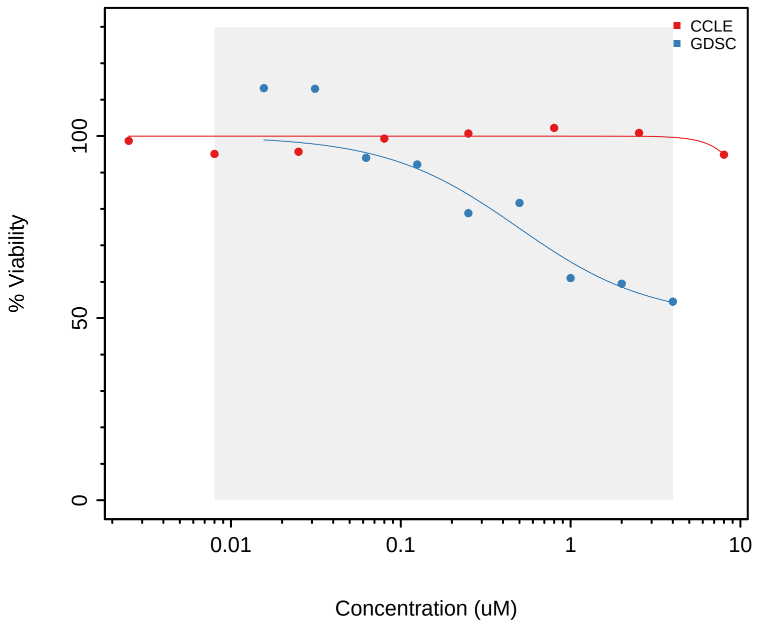

AZD6244:DBTRG−05MG

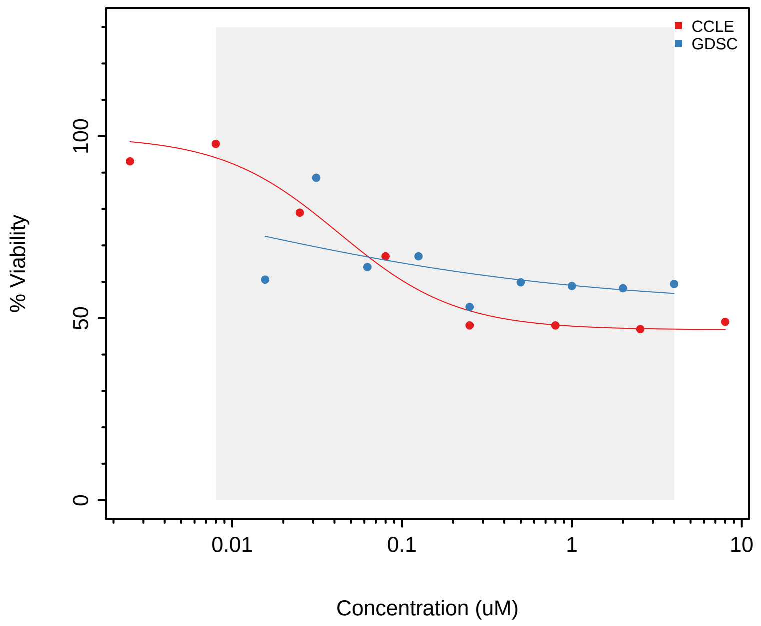

AZD6244:DEL

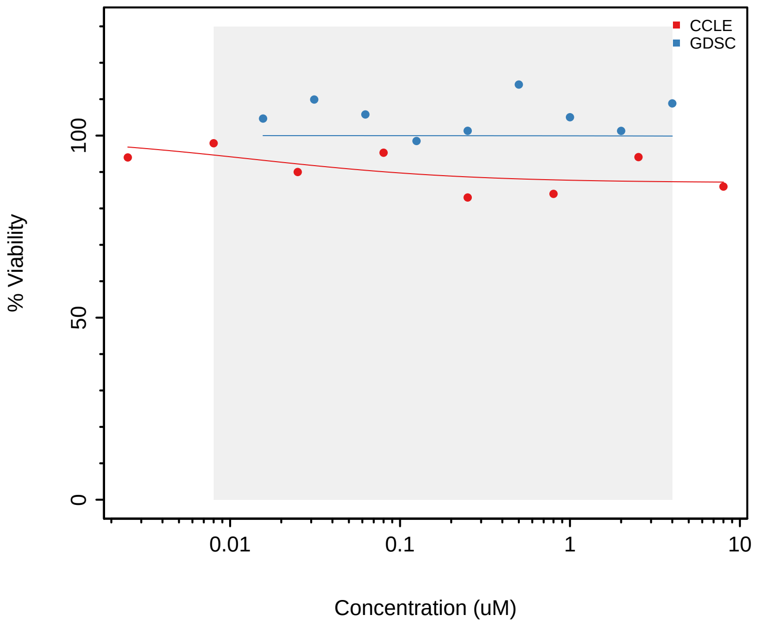

AZD6244:Detroit562

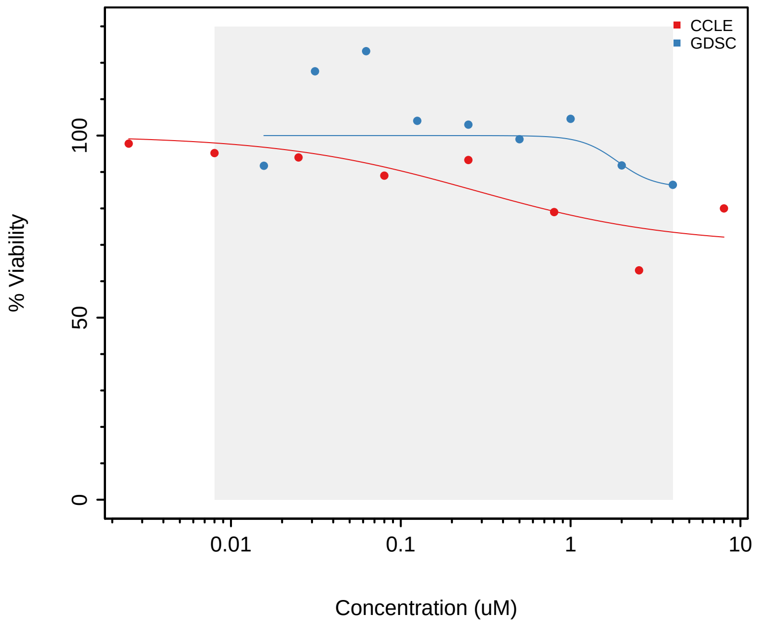

AZD6244:DK−MG

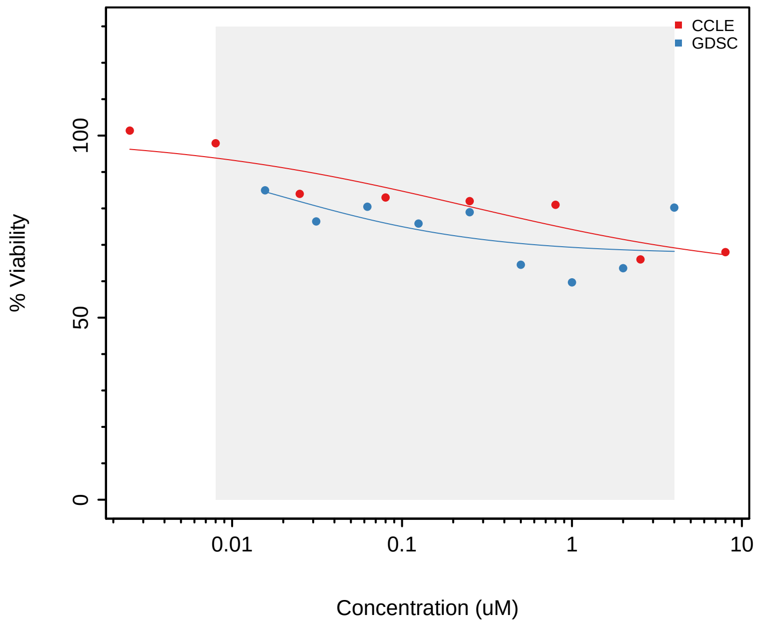

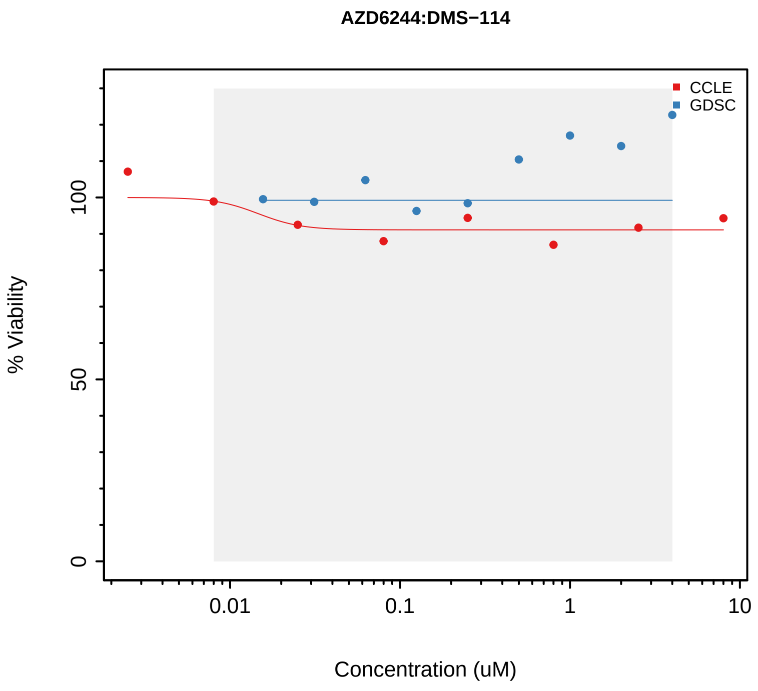
AZD6244:DMS−114
CCLE
GDSC
% Viability
Concentration (uM)

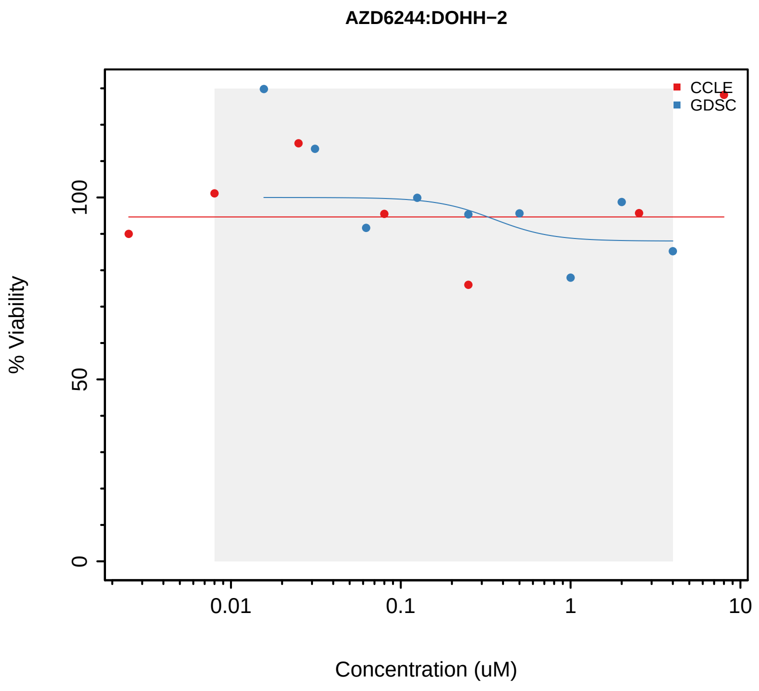
AZD6244:DOHH−2
CCLE
GDSC
% Viability
Concentration (uM)

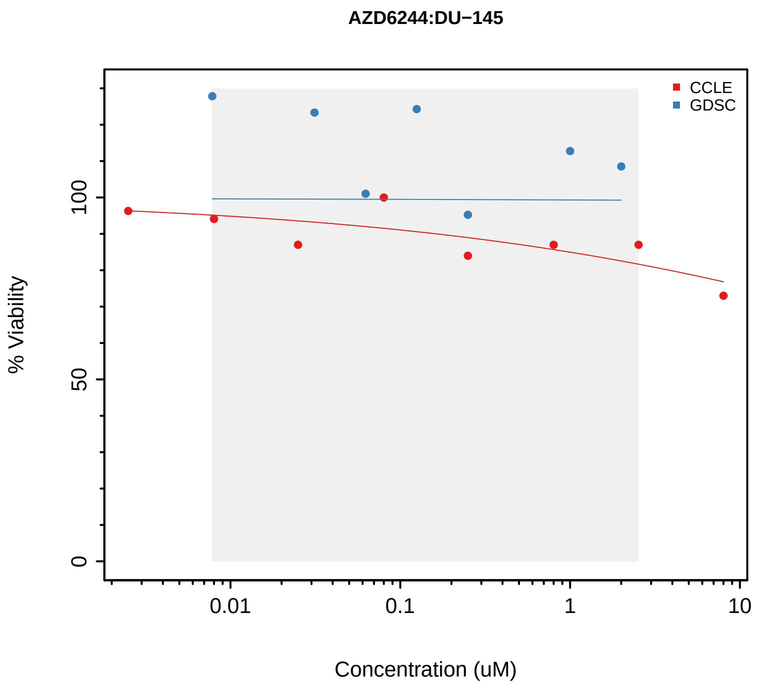
AZD6244:DU−145
CCLE
GDSC
% Viability
Concentration (uM)

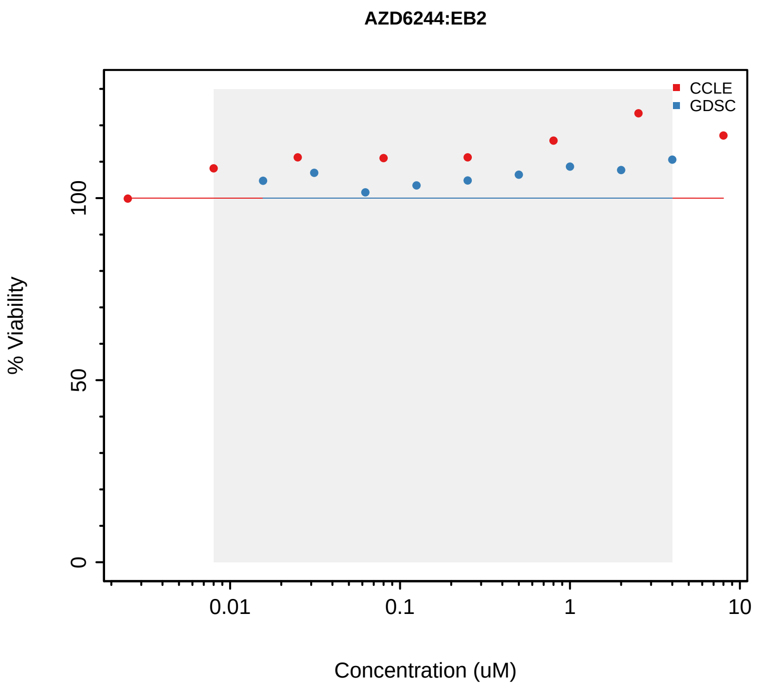
AZD6244:EB2
CCLE
GDSC
% Viability
Concentration (uM)

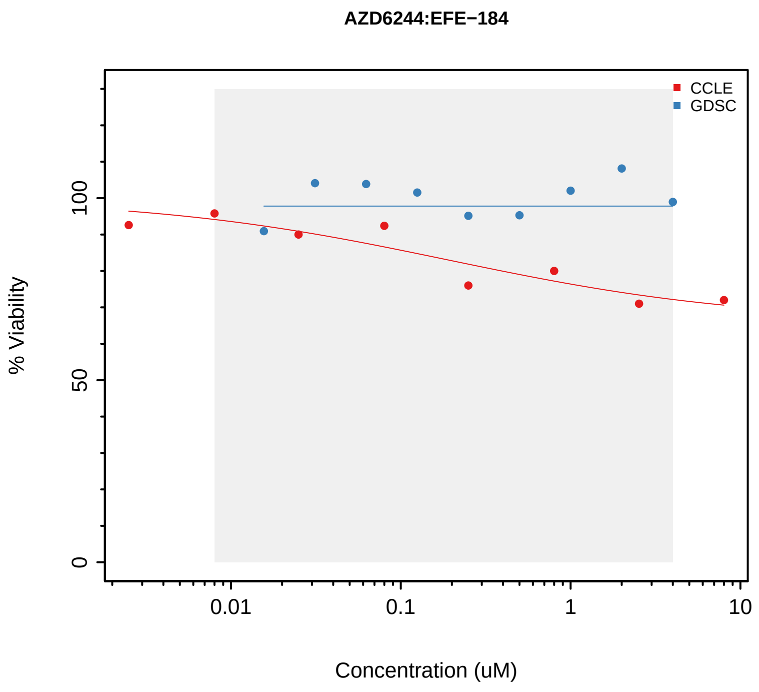
AZD6244:EFE−184
CCLE
GDSC
% Viability
Concentration (uM)

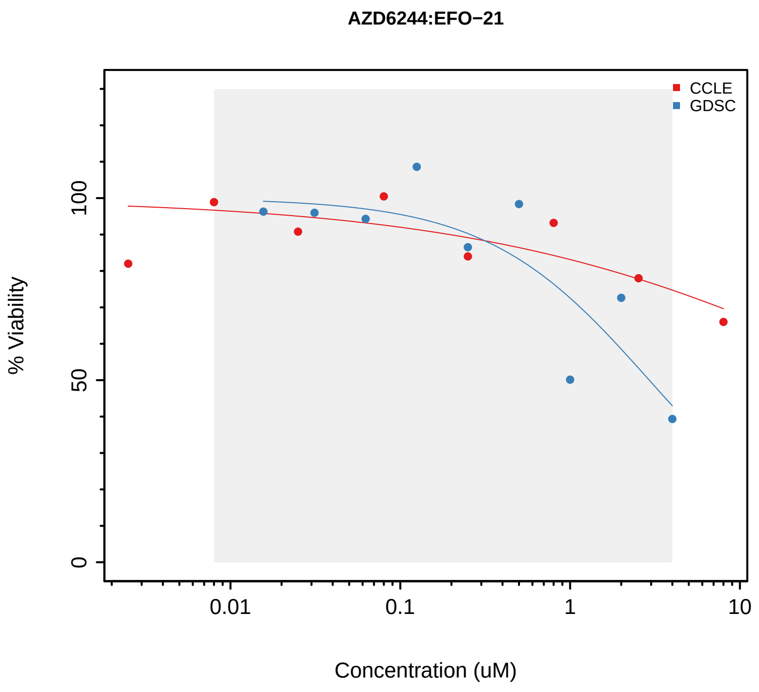
AZD6244:EFO−21
CCLE
GDSC
% Viability
Concentration (uM)

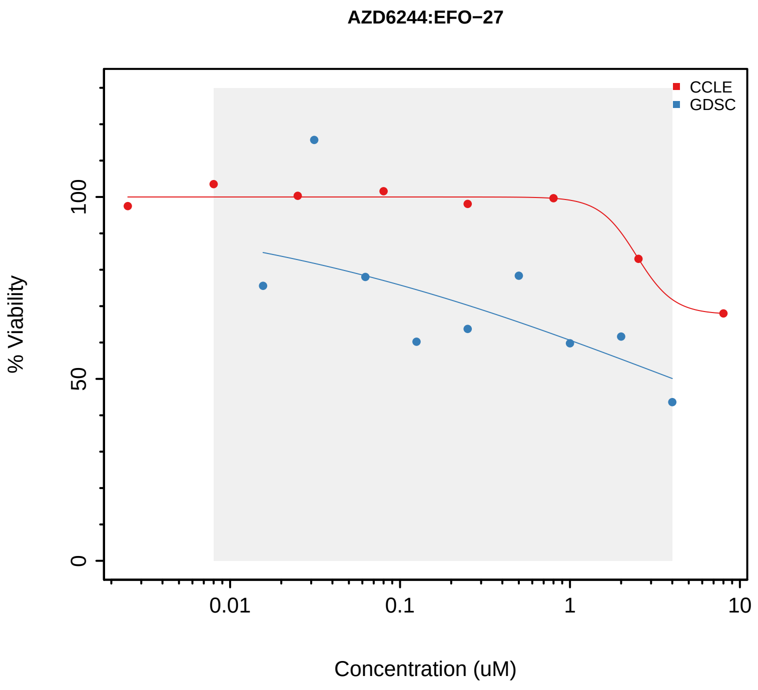
AZD6244:EFO−27
CCLE
GDSC
% Viability
Concentration (uM)

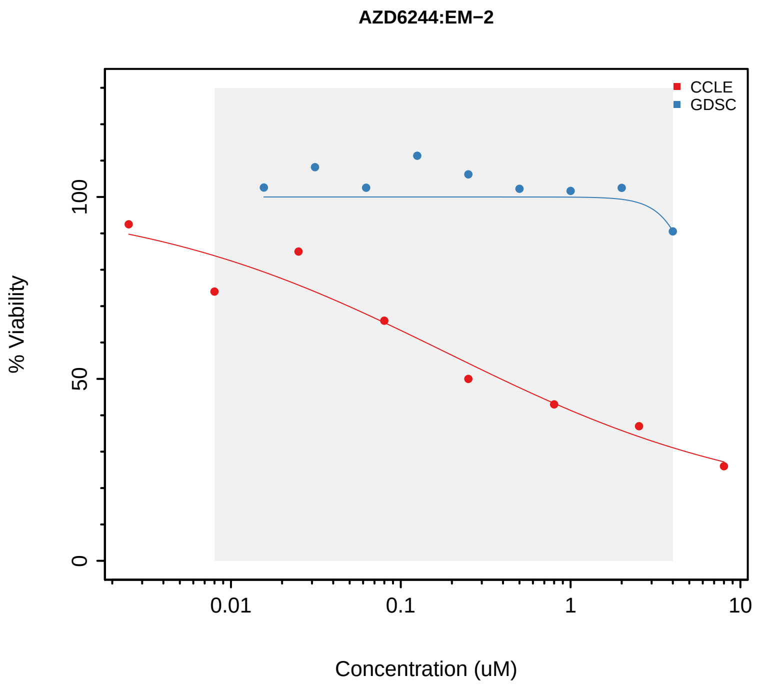
AZD6244:EM−2
CCLE
GDSC
% Viability
Concentration (uM)

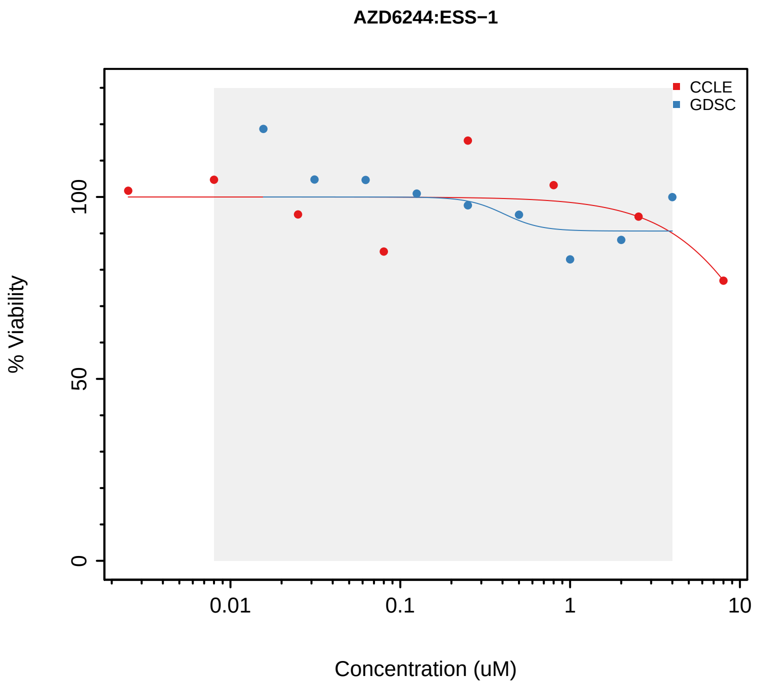
AZD6244:ESS−1
CCLE
GDSC
% Viability
Concentration (uM)

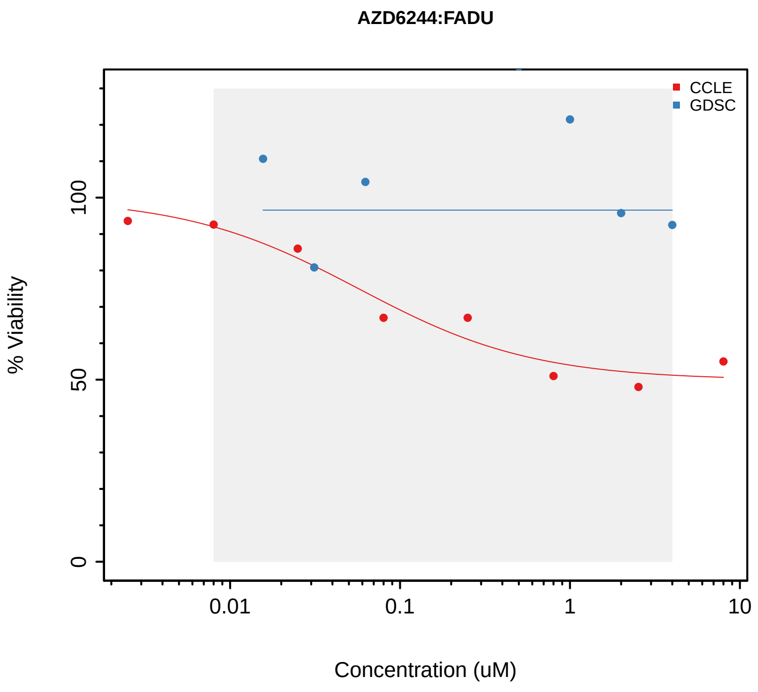
AZD6244:FADU
CCLE
GDSC
% Viability
Concentration (uM)

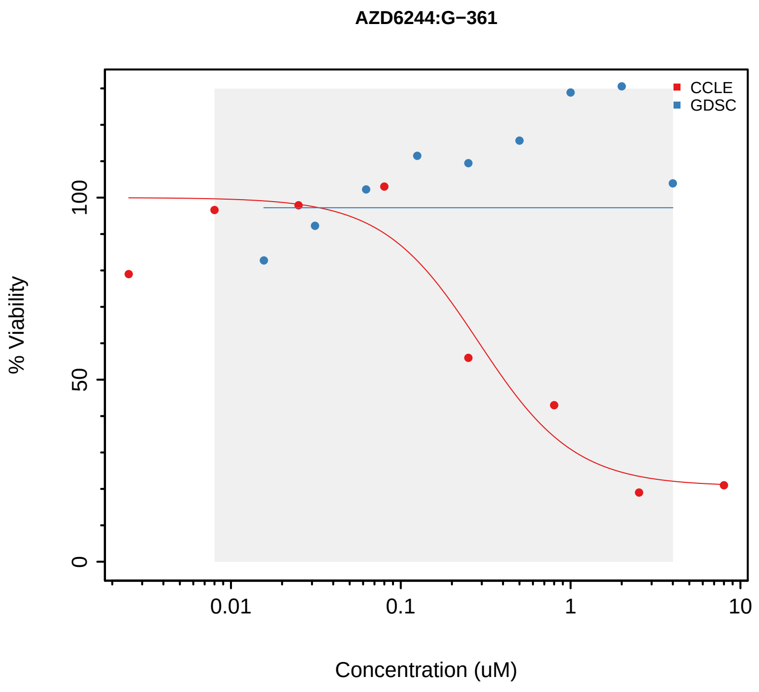
AZD6244:G−361
CCLE
GDSC
% Viability
Concentration (uM)

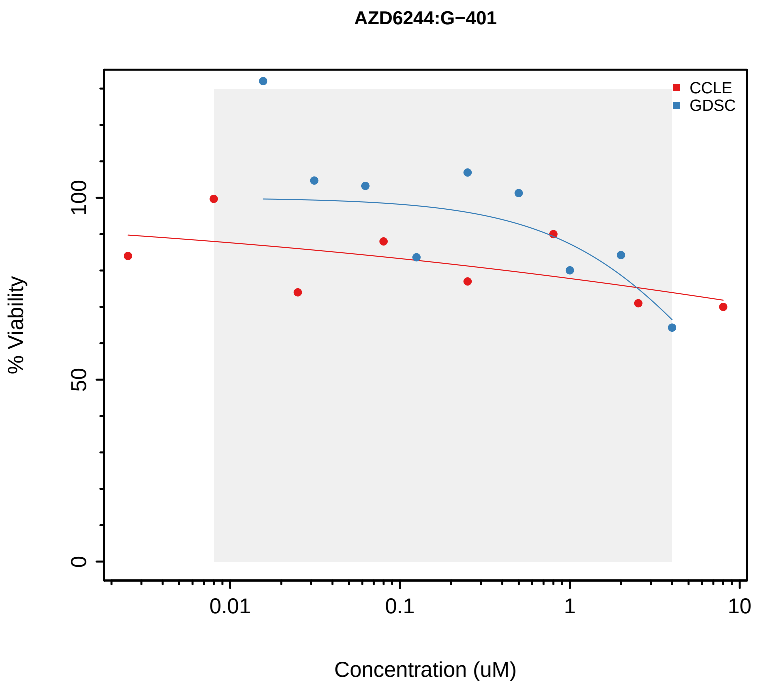
AZD6244:G−401
CCLE
GDSC
% Viability
Concentration (uM)

AZD6244:GAMG

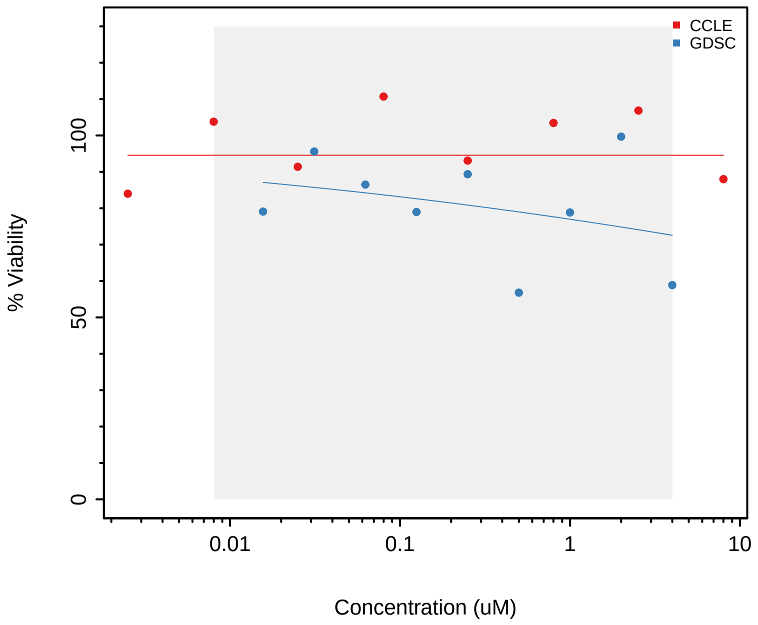

AZD6244:GB−1

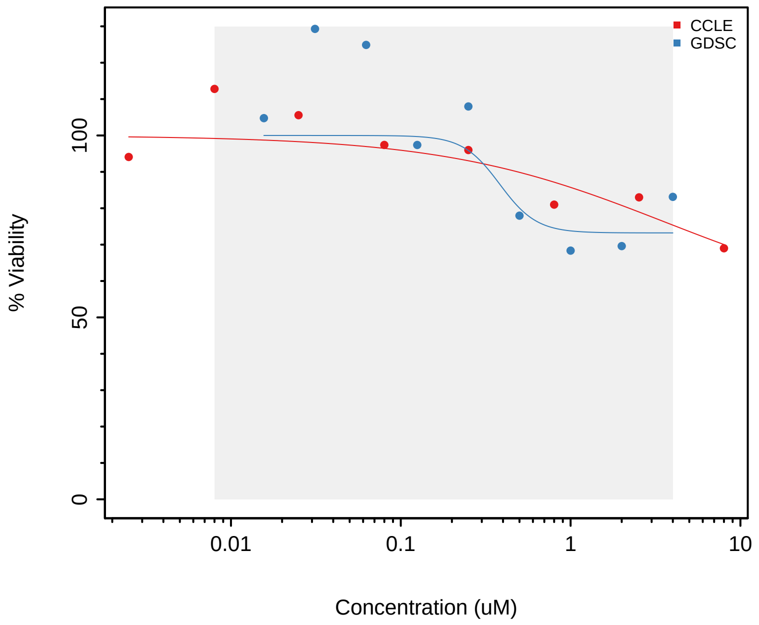

AZD6244:GCIY

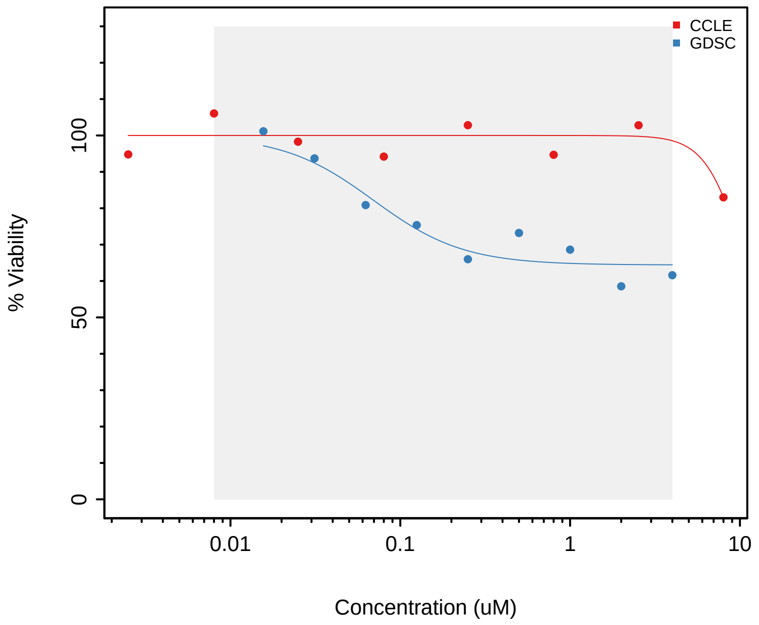

AZD6244:GCT

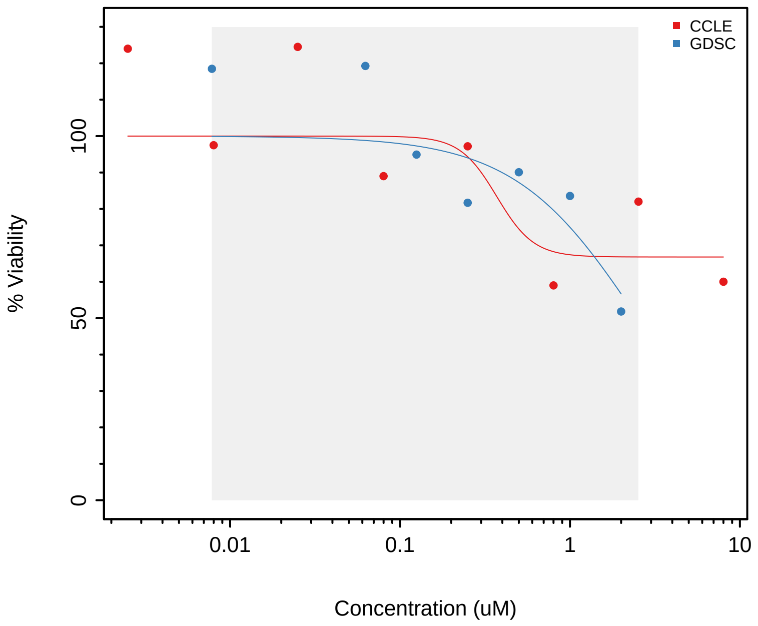

AZD6244:GI−1

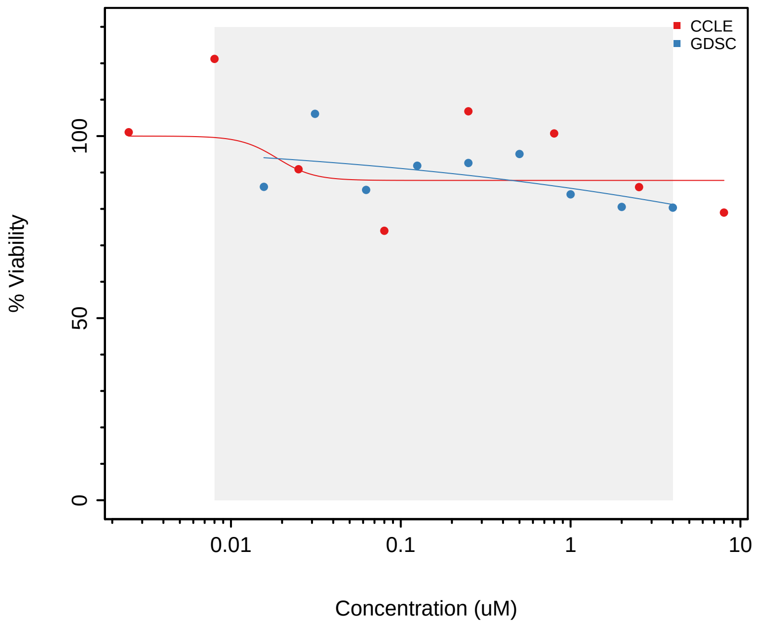

AZD6244:GMS−10

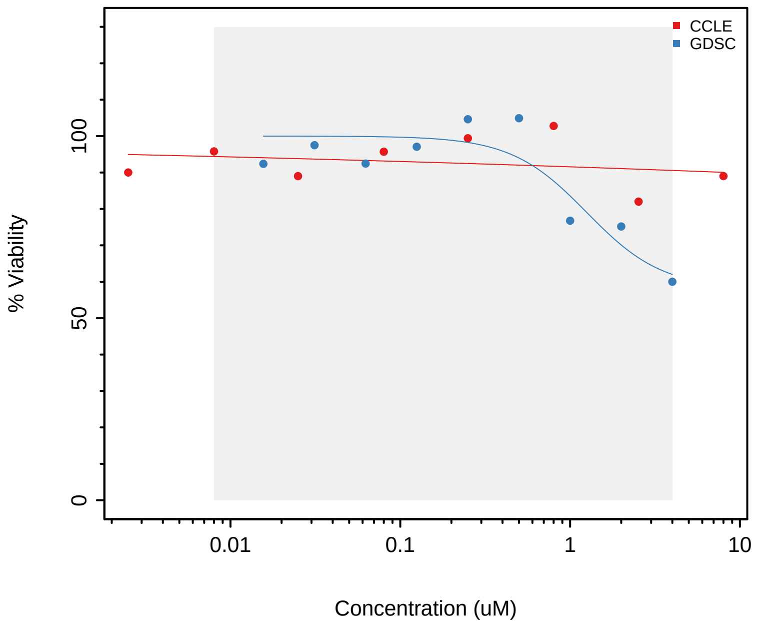

AZD6244:H4

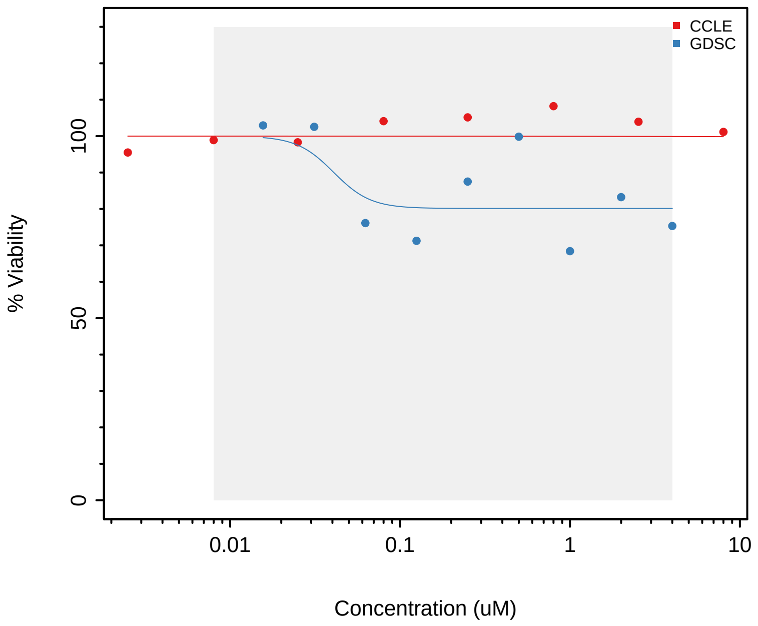

AZD6244:HCC1187

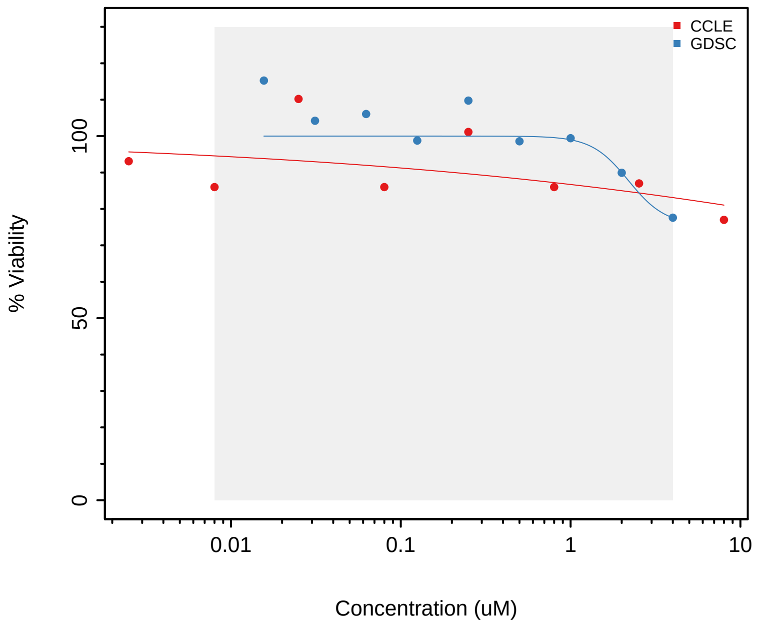

AZD6244:HCC1395

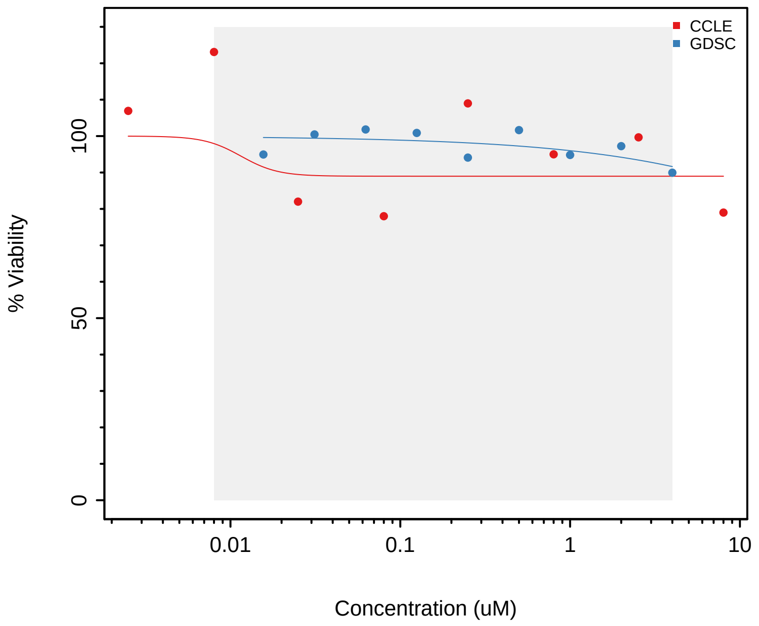

AZD6244:HCC1569

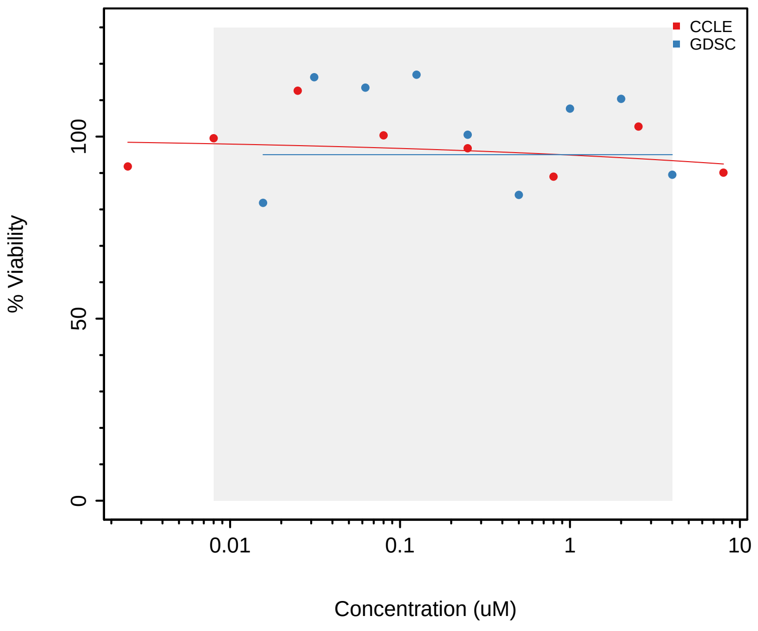

AZD6244:HCC1954

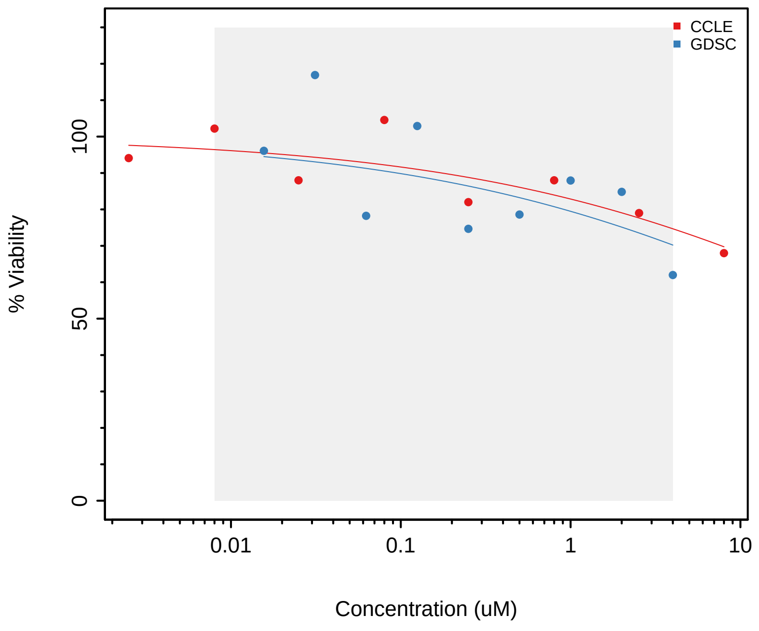

AZD6244:HCT−116

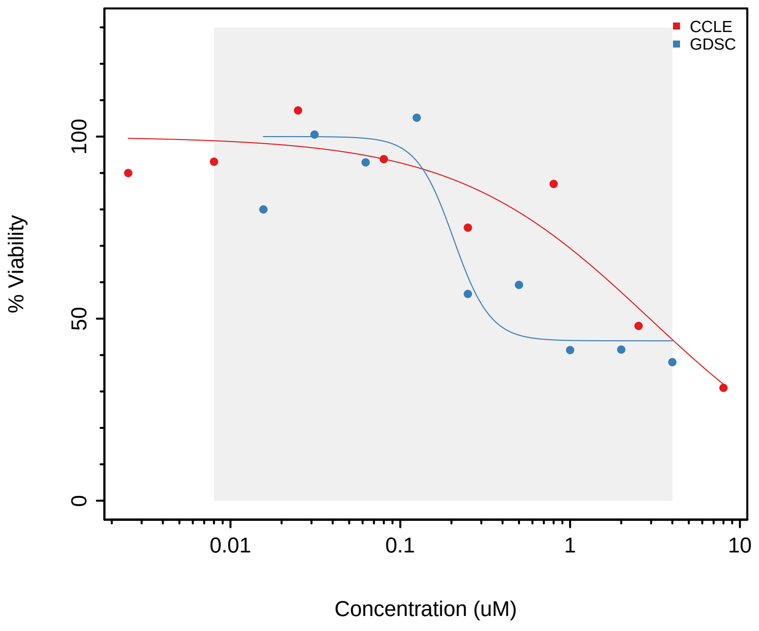

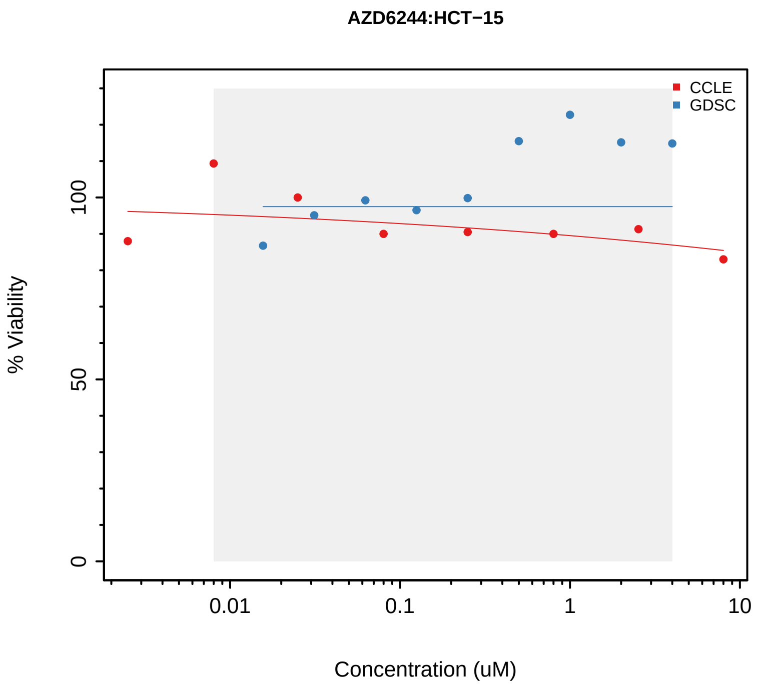
AZD6244:HCT−15
CCLE
GDSC
% Viability
Concentration (uM)

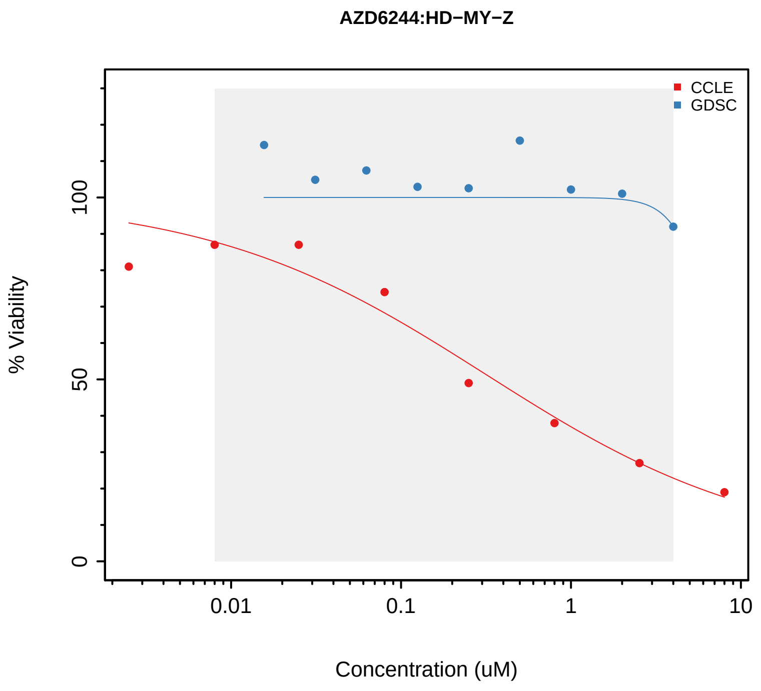
AZD6244:HD−MY−Z
CCLE
GDSC
% Viability
Concentration (uM)

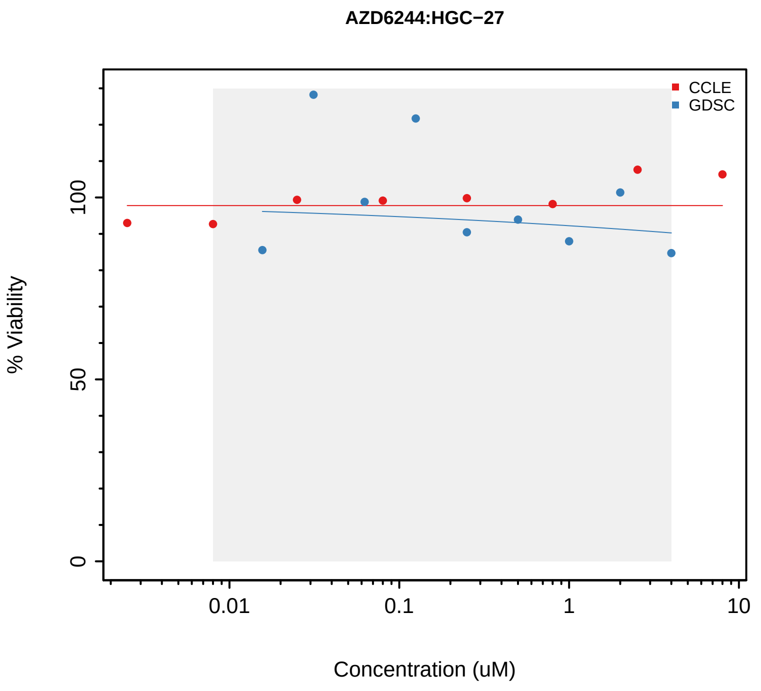
AZD6244:HGC−27
CCLE
GDSC
% Viability
Concentration (uM)

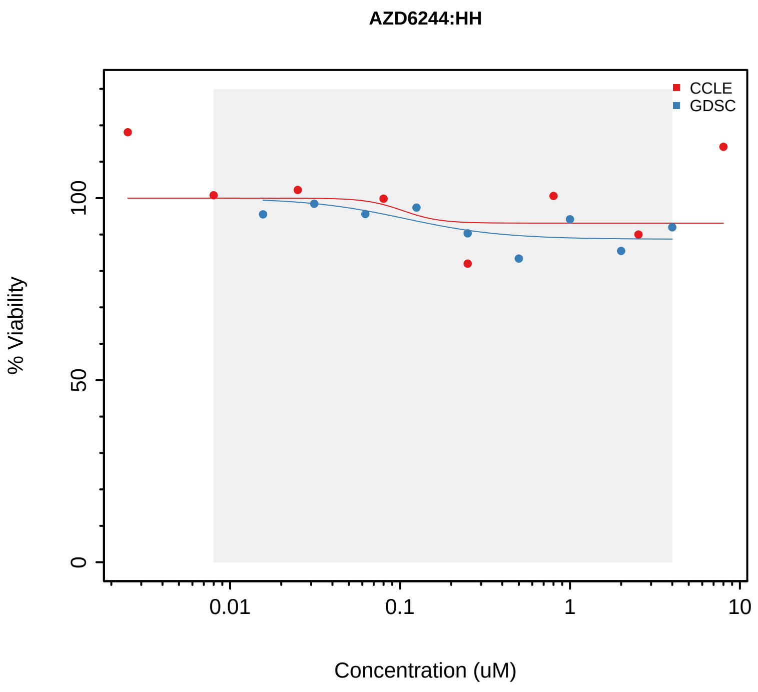
AZD6244:HH
CCLE
GDSC
% Viability
Concentration (uM)

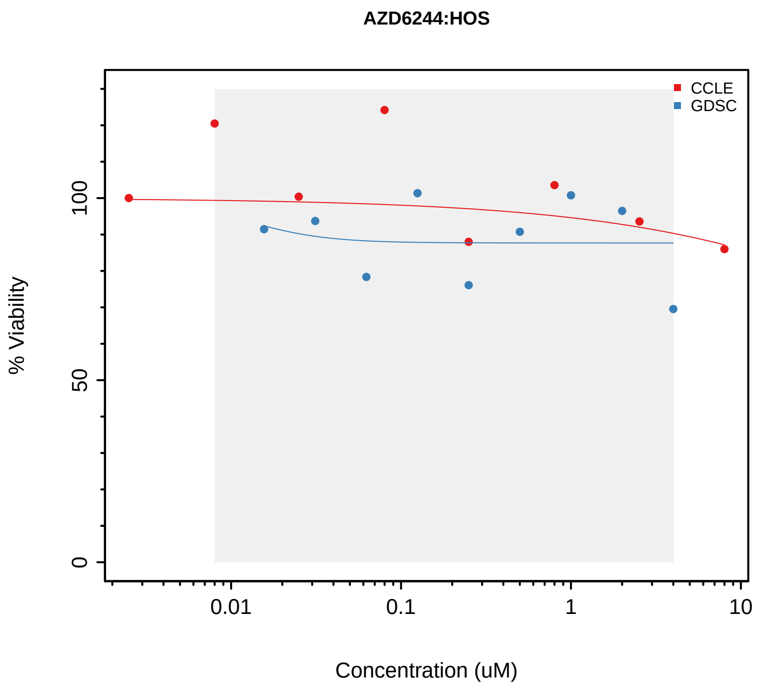
AZD6244:HOS
CCLE
GDSC
% Viability
Concentration (uM)

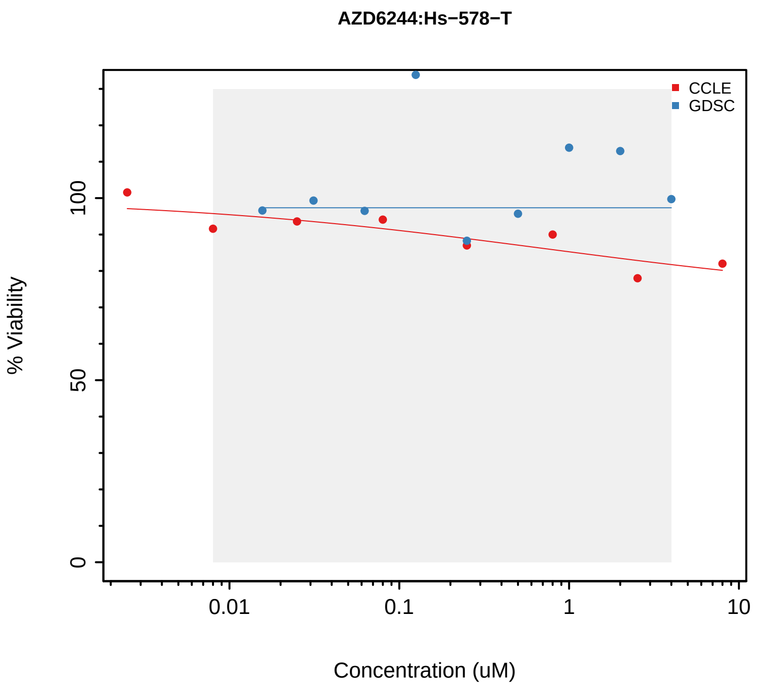
AZD6244:Hs−578−T
CCLE
GDSC
% Viability
Concentration (uM)

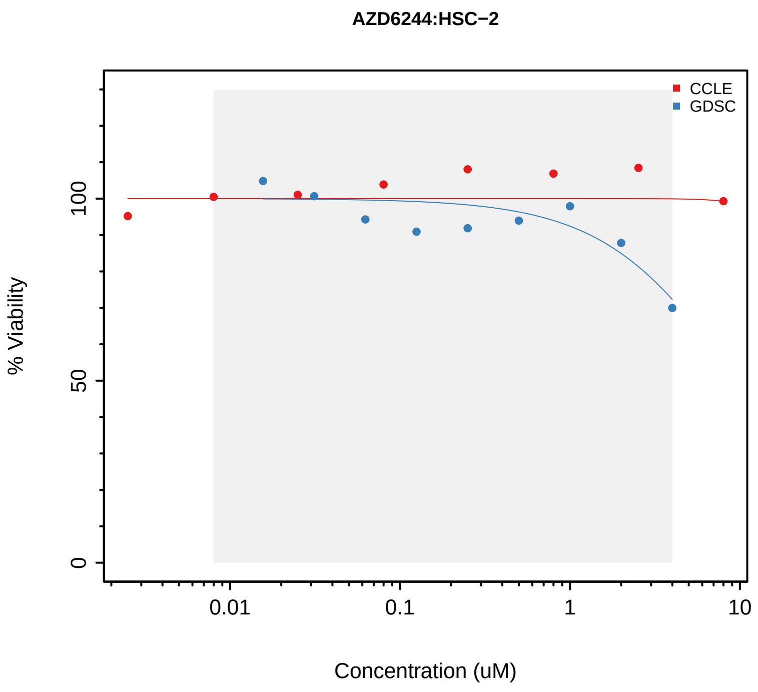
AZD6244:HSC−2
CCLE
GDSC
% Viability
Concentration (uM)

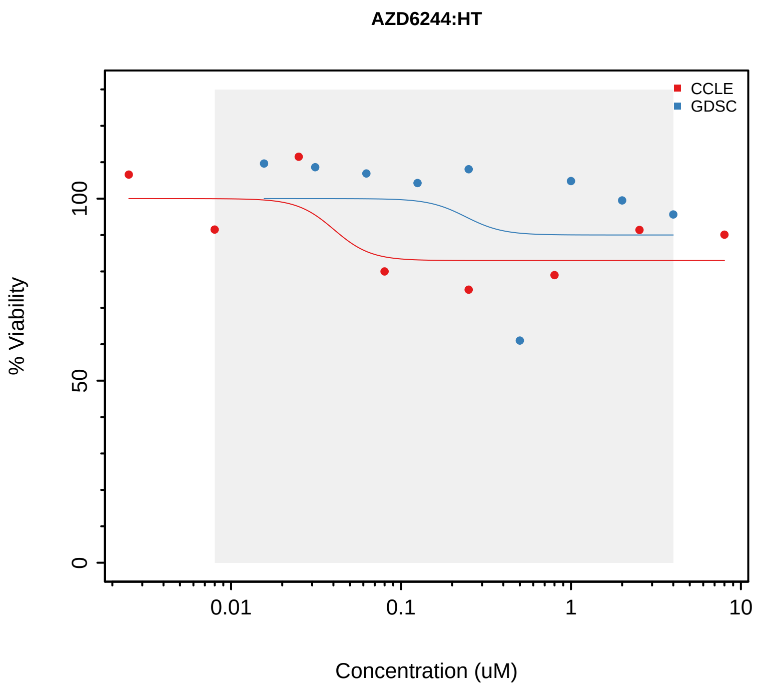
AZD6244:HT
CCLE
GDSC
% Viability
Concentration (uM)

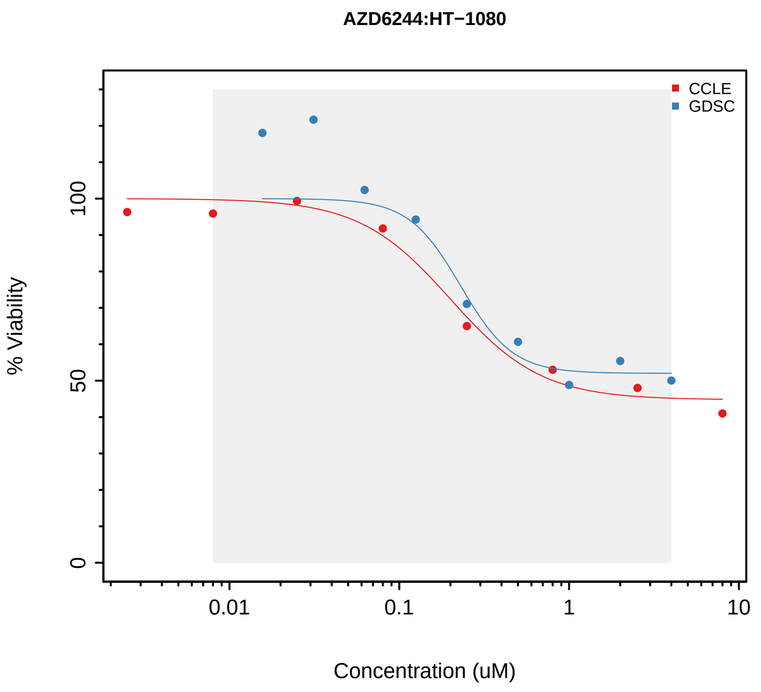
AZD6244:HT−1080
CCLE
GDSC
% Viability
Concentration (uM)

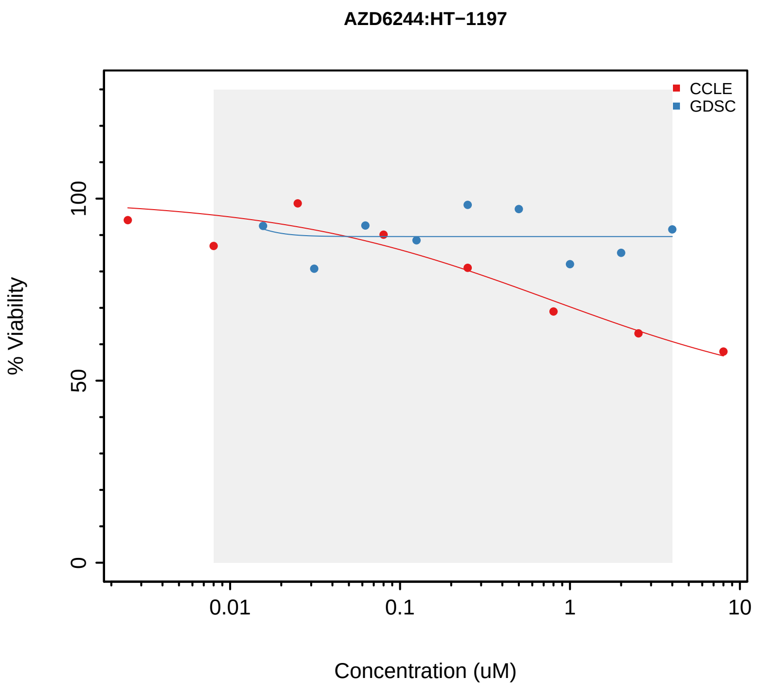
AZD6244:HT−1197
CCLE
GDSC
% Viability
Concentration (uM)

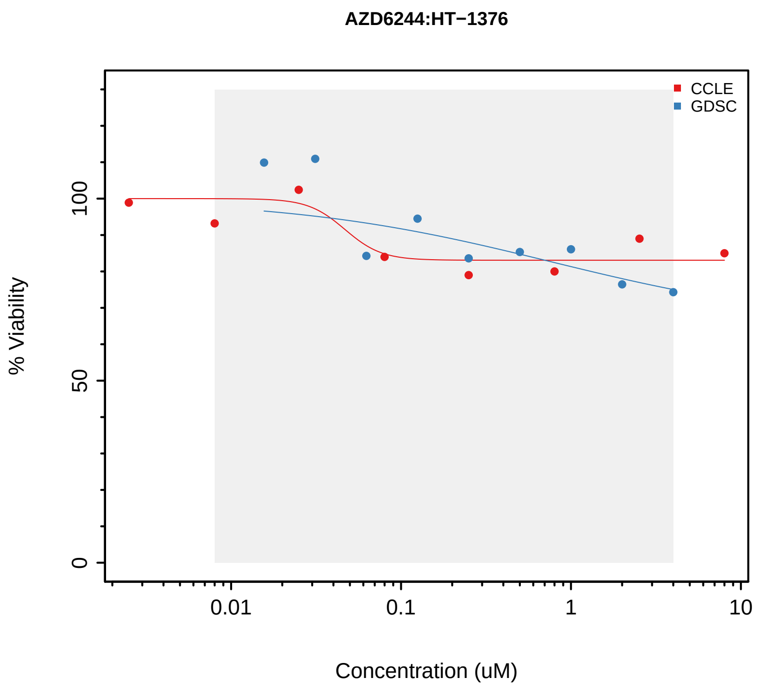
AZD6244:HT−1376
CCLE
GDSC
% Viability
Concentration (uM)

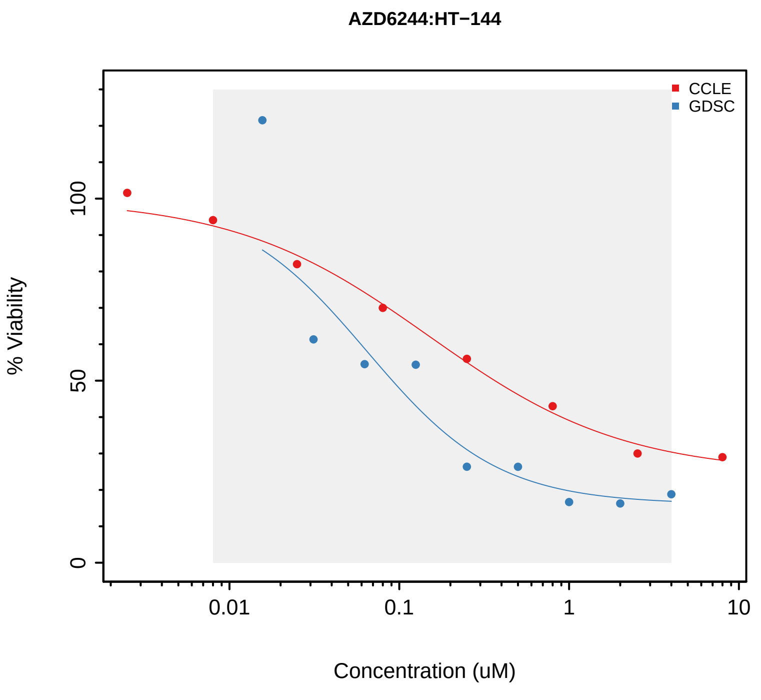
AZD6244:HT−144
CCLE
GDSC
% Viability
Concentration (uM)

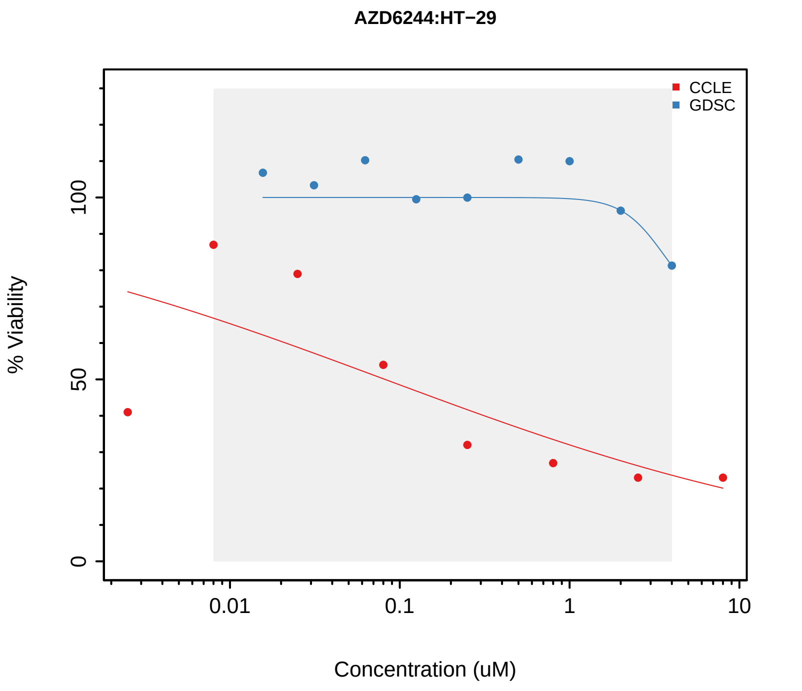
AZD6244:HT−29
CCLE
GDSC
% Viability
0
50
100
0.01
0.1
1
10
Concentration (uM)

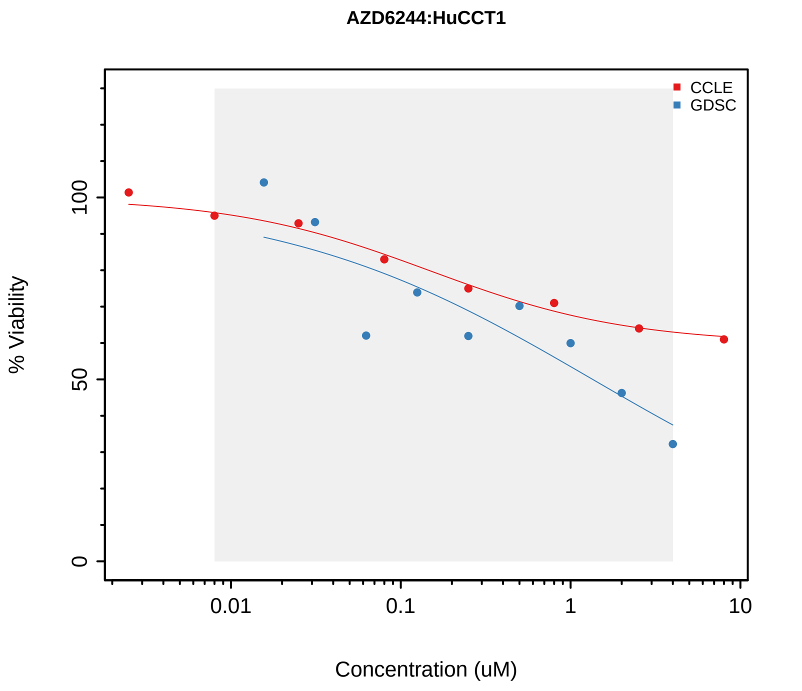
AZD6244:HuCCT1
CCLE
GDSC
% Viability
0
50
100
0.01
0.1
1
10
Concentration (uM)

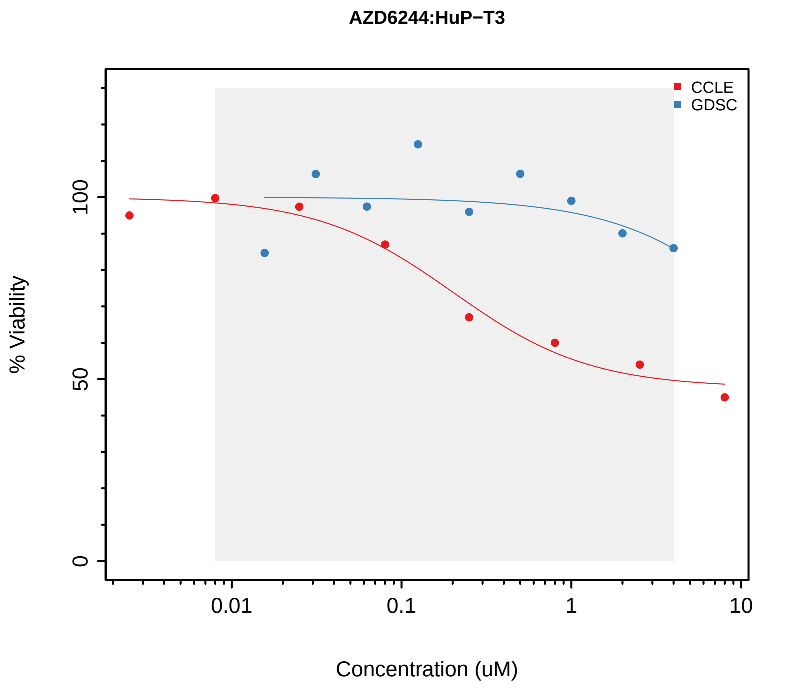
AZD6244:HuP−T3
CCLE
GDSC
% Viability
0
50
100
0.01
0.1
1
10
Concentration (uM)

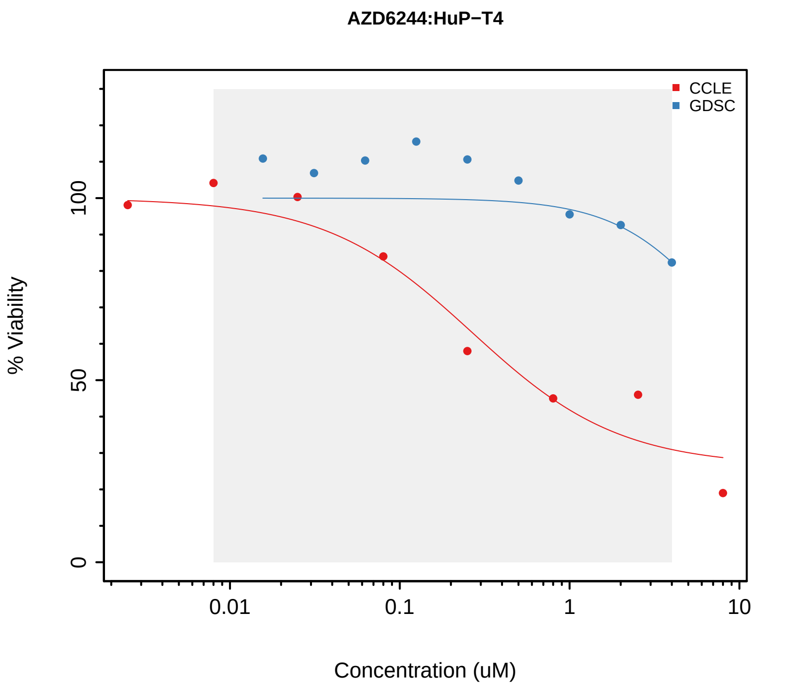
AZD6244:HuP−T4
CCLE
GDSC
% Viability
0
50
100
0.01
0.1
1
10
Concentration (uM)

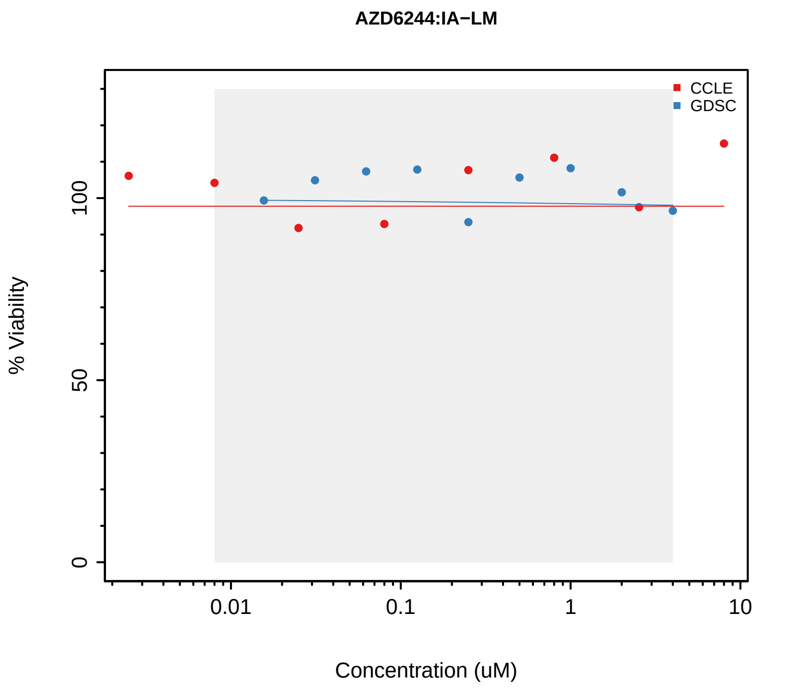
AZD6244:IA−LM
CCLE
GDSC
% Viability
0
50
100
0.01
0.1
1
10
Concentration (uM)

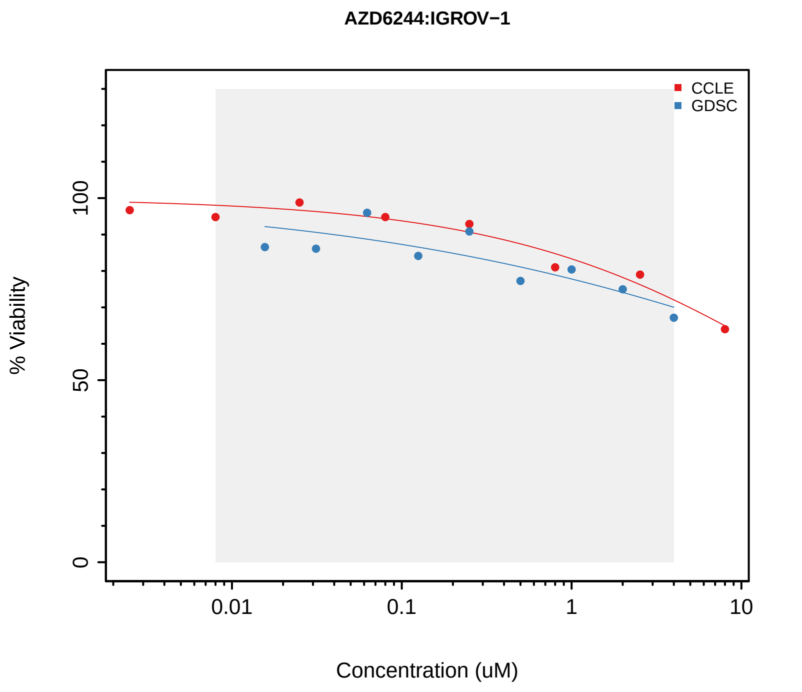
AZD6244:IGROV−1
CCLE
GDSC
% Viability
0
50
100
0.01
0.1
1
10
Concentration (uM)

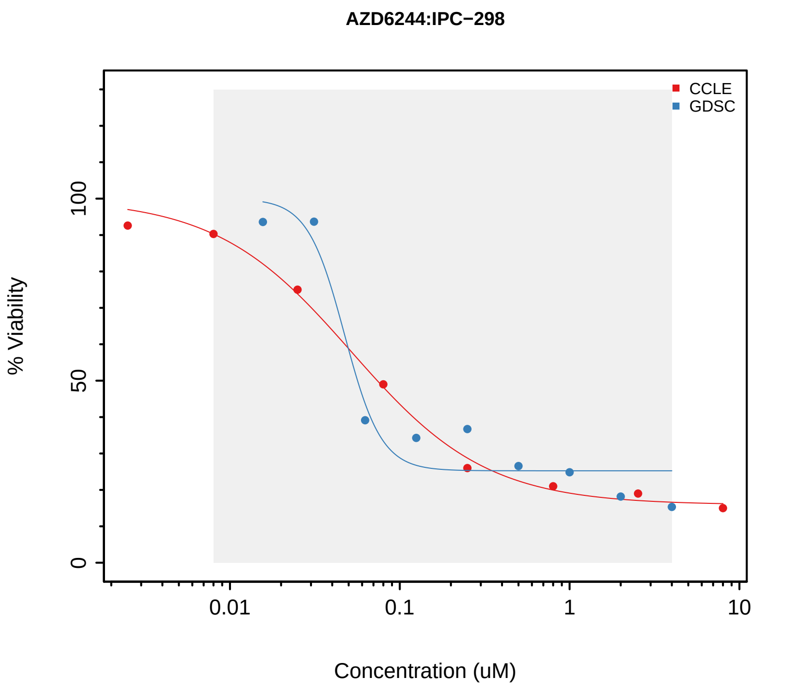
AZD6244:IPC−298
CCLE
GDSC
% Viability
0
50
100
0.01
0.1
1
10
Concentration (uM)

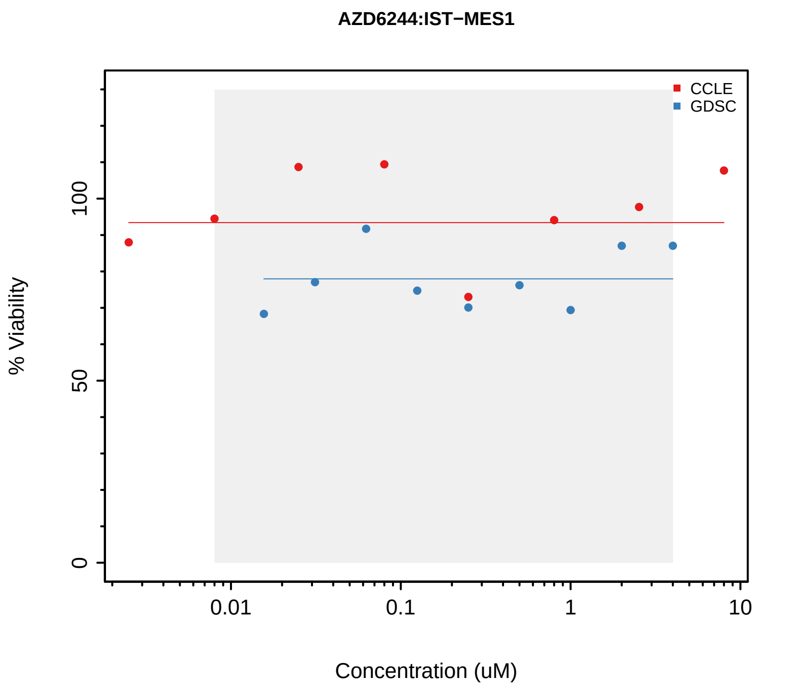
AZD6244:IST−MES1
CCLE
GDSC
% Viability
0
50
100
0.01
0.1
1
10
Concentration (uM)

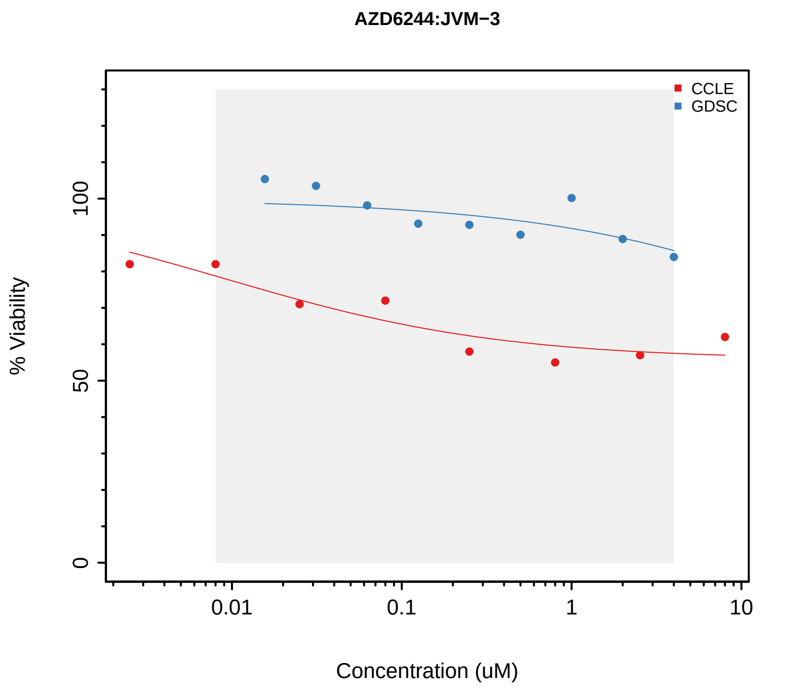
AZD6244:JVM−3
CCLE
GDSC
% Viability
0
50
100
0.01
0.1
1
10
Concentration (uM)

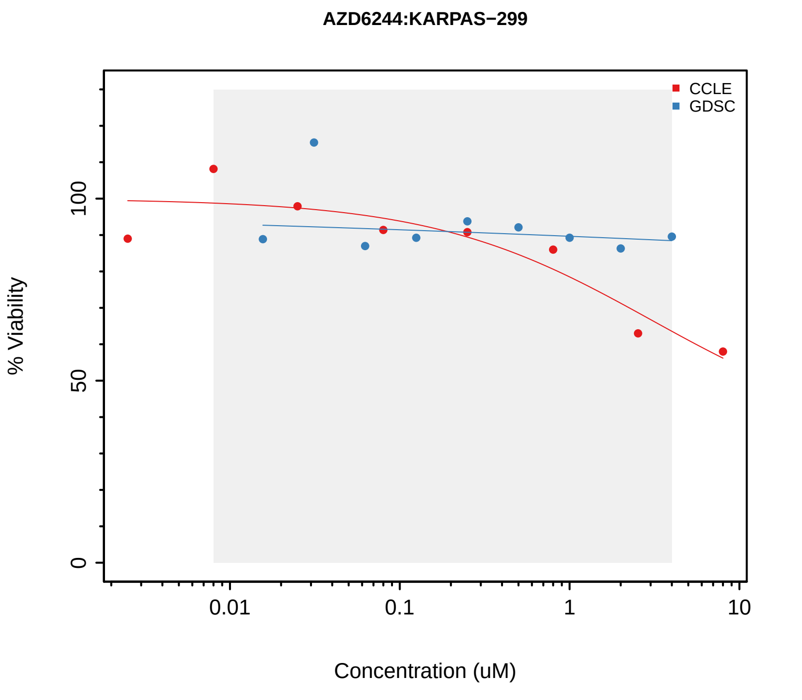
AZD6244:KARPAS−299
CCLE
GDSC
% Viability
0
50
100
0.01
0.1
1
10
Concentration (uM)

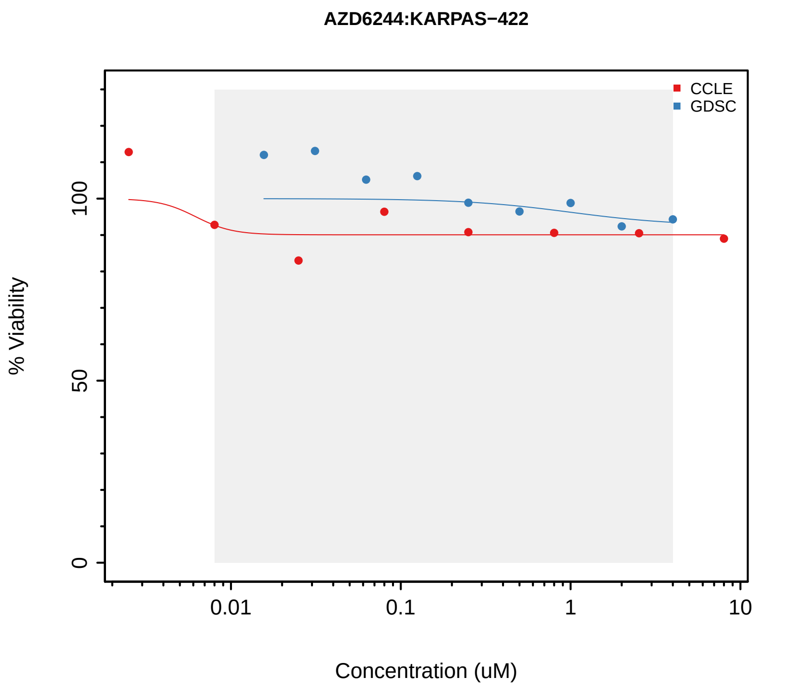
AZD6244:KARPAS−422
CCLE
GDSC
% Viability
0
50
100
0.01
0.1
1
10
Concentration (uM)

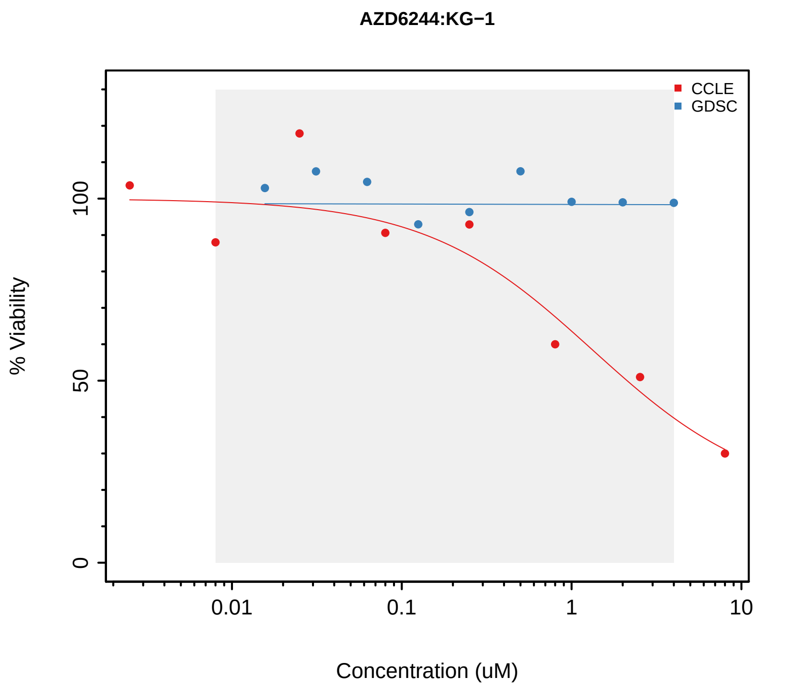
AZD6244:KG−1
CCLE
GDSC
% Viability
0
50
100
0.01
0.1
1
10
Concentration (uM)

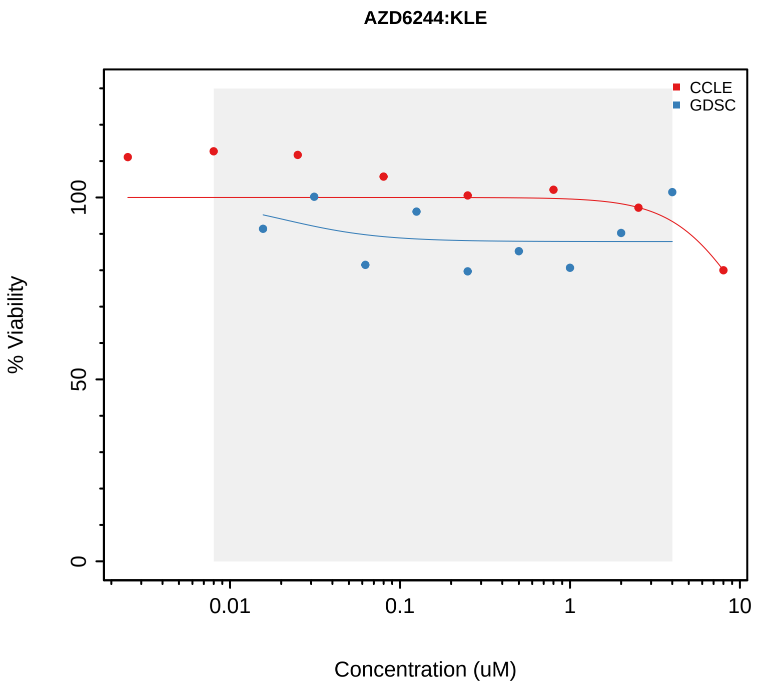
AZD6244:KLE
CCLE
GDSC
% Viability
0
50
100
0.01
0.1
1
10
Concentration (uM)

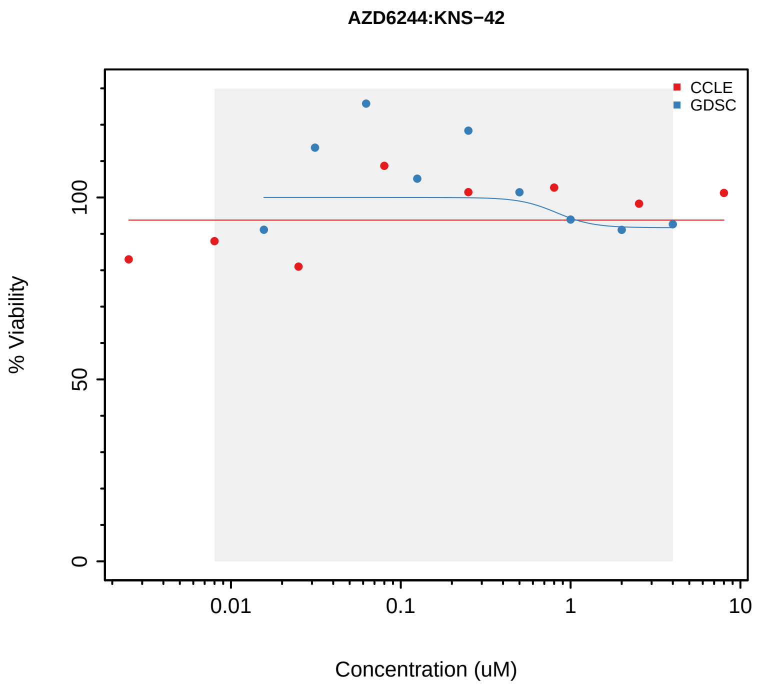
AZD6244:KNS−42
CCLE
GDSC
% Viability
0
50
100
0.01
0.1
1
10
Concentration (uM)

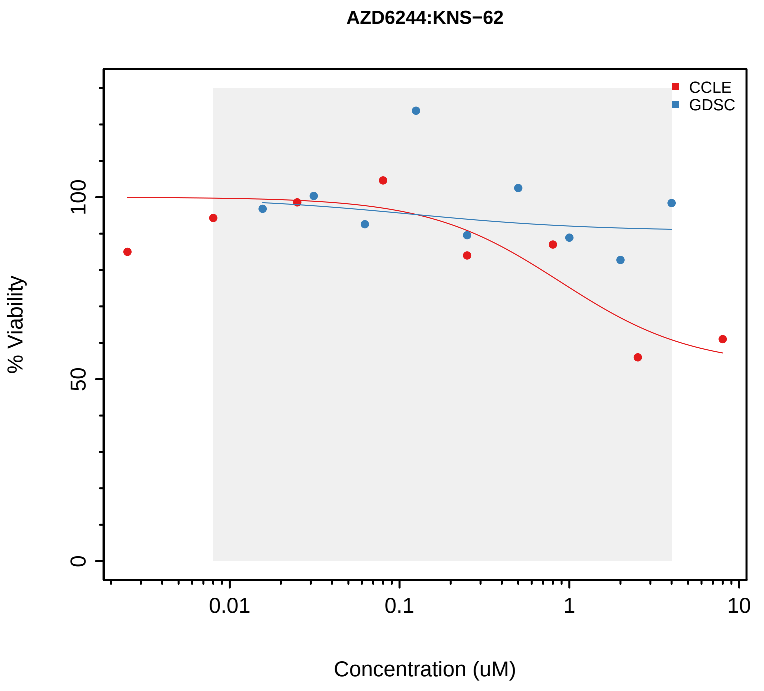
AZD6244:KNS−62
CCLE
GDSC
% Viability
0
50
100
0.01
0.1
1
10
Concentration (uM)

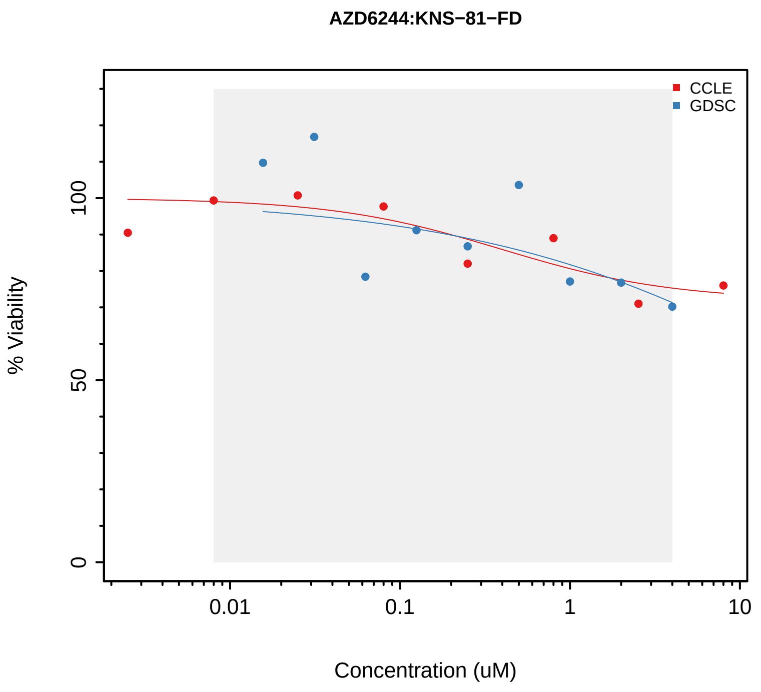
AZD6244:KNS−81−FD
CCLE
GDSC
% Viability
0
50
100
0.01
0.1
1
10
Concentration (uM)

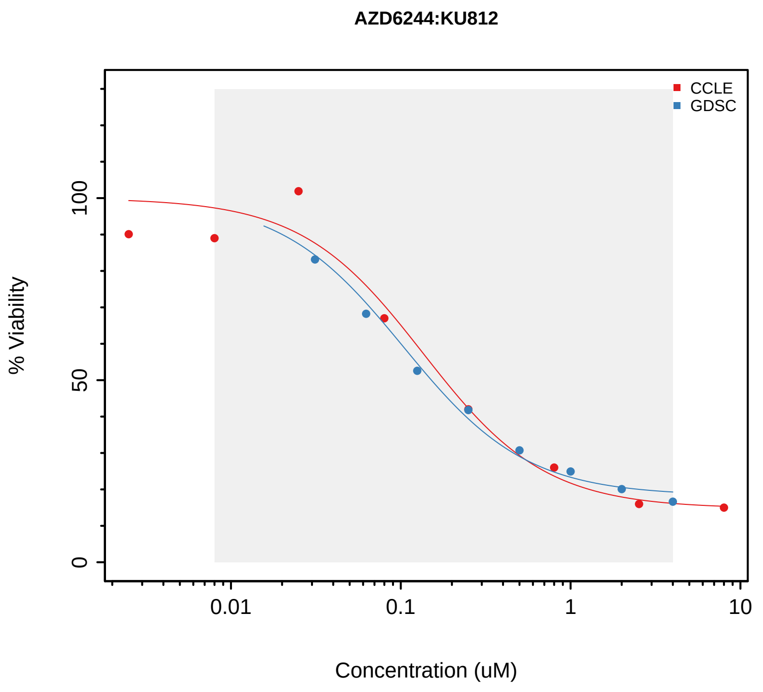
AZD6244:KU812
CCLE
GDSC
% Viability
0
50
100
0.01
0.1
1
10
Concentration (uM)

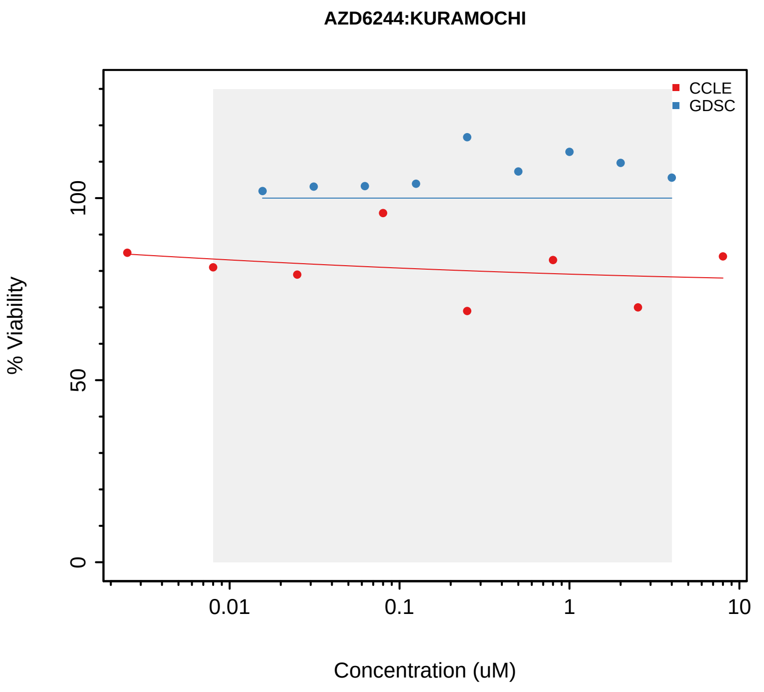
AZD6244:KURAMOCHI
CCLE
GDSC
% Viability
0
50
100
0.01
0.1
1
10
Concentration (uM)

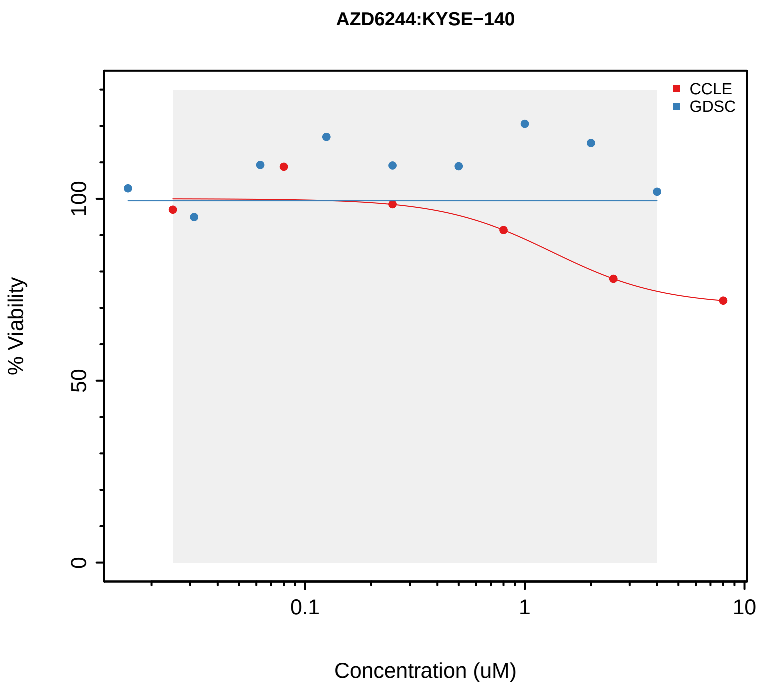
AZD6244:KYSE−140
CCLE
GDSC
% Viability
0
50
100
0.1
1
10
Concentration (uM)

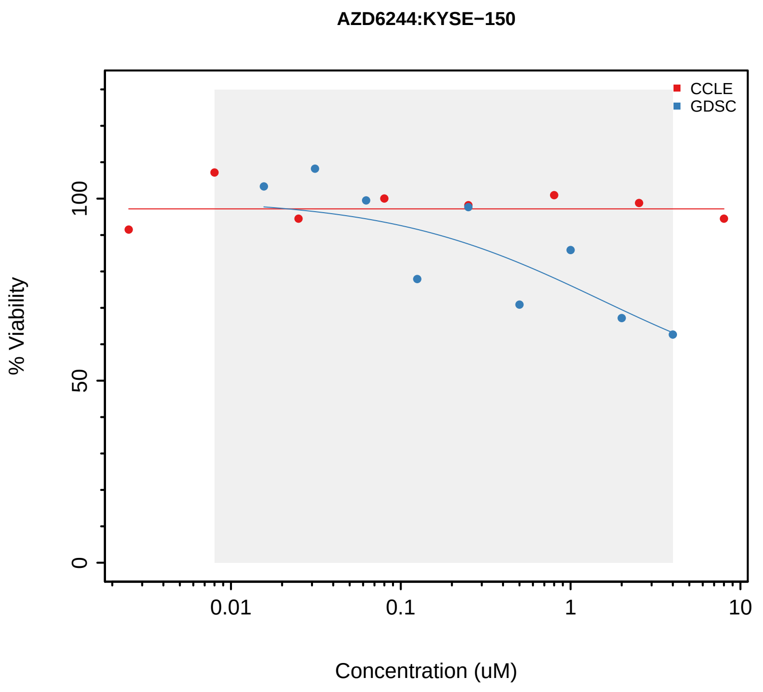
AZD6244:KYSE−150
CCLE
GDSC
% Viability
0
50
100
0.01
0.1
1
10
Concentration (uM)

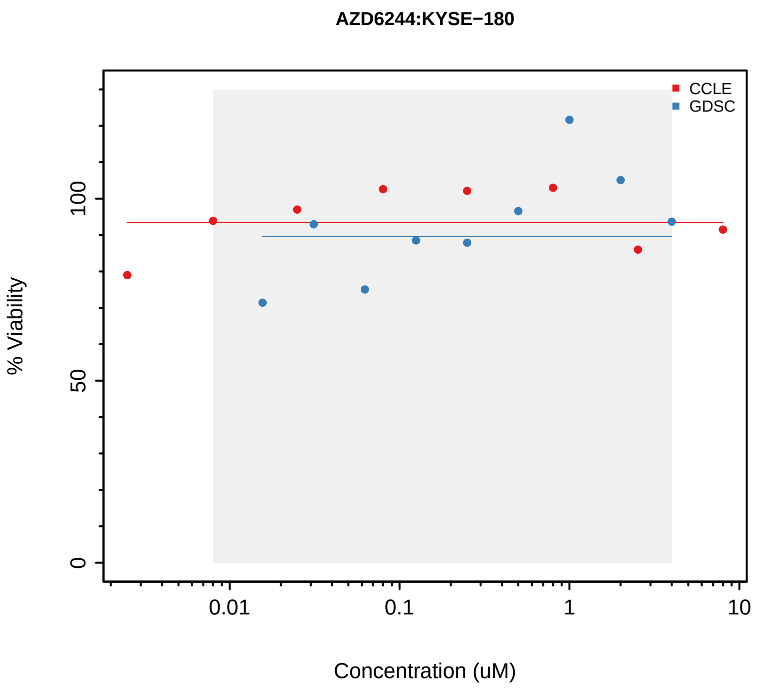
AZD6244:KYSE−180
CCLE
GDSC
% Viability
0
50
100
0.01
0.1
1
10
Concentration (uM)

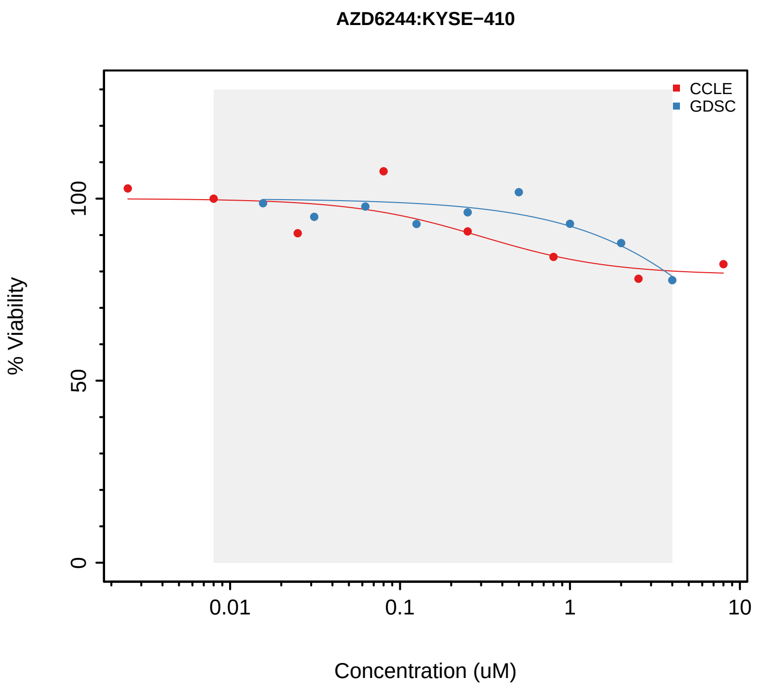
AZD6244:KYSE−410
CCLE
GDSC
% Viability
0
50
100
0.01
0.1
1
10
Concentration (uM)

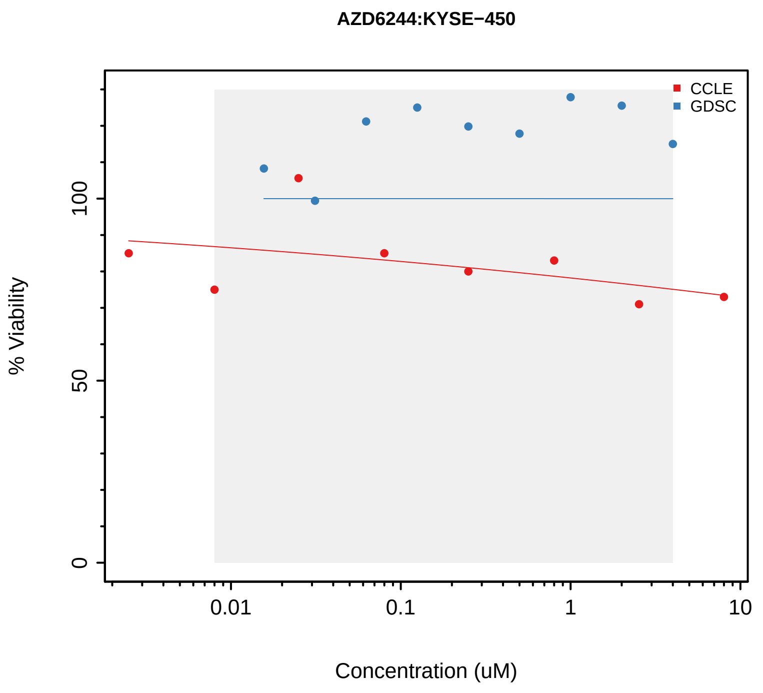
AZD6244:KYSE−450
CCLE
GDSC
% Viability
0
50
100
0.01
0.1
1
10
Concentration (uM)

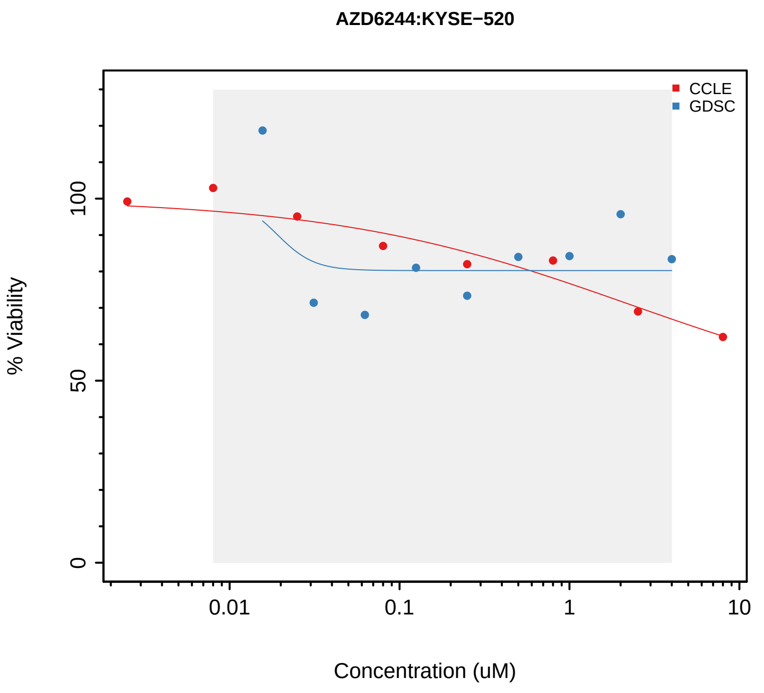
AZD6244:KYSE−520
CCLE
GDSC
% Viability
0
50
100
0.01
0.1
1
10
Concentration (uM)

AZD6244:KYSE−70

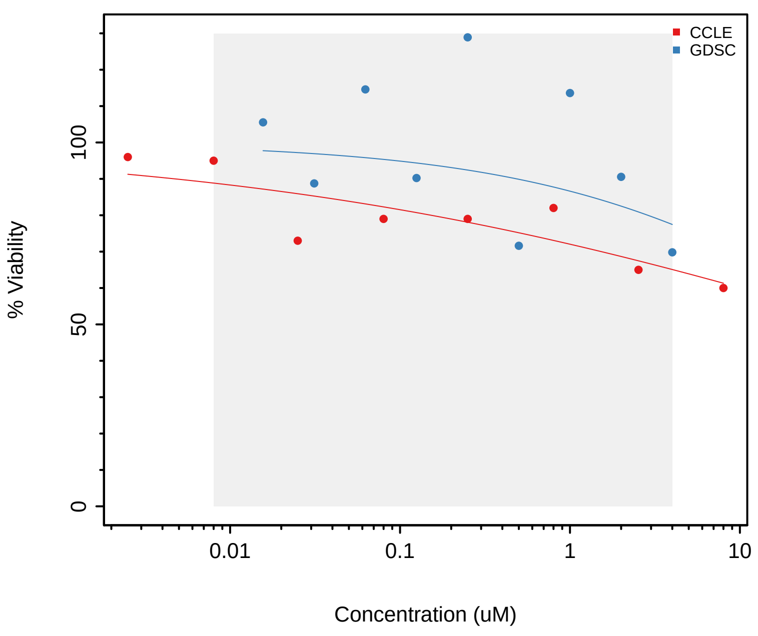

AZD6244:L−363

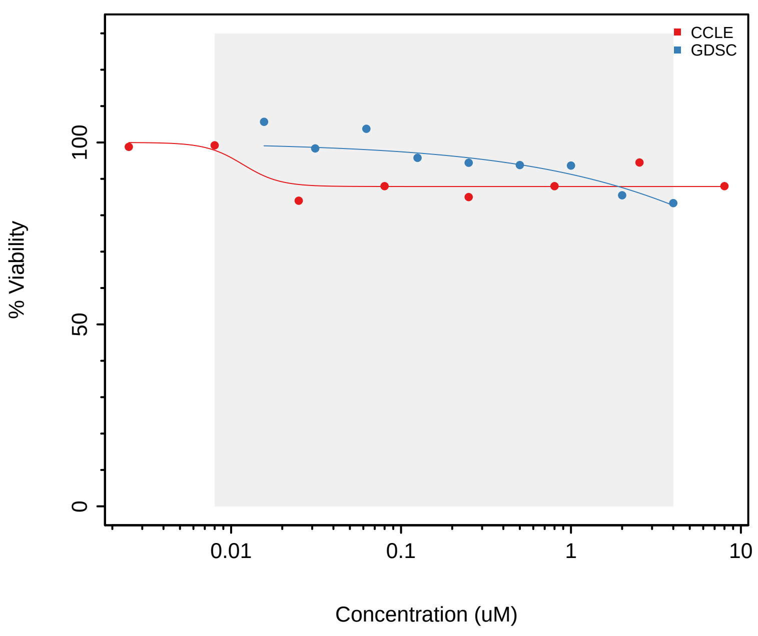

AZD6244:L−428

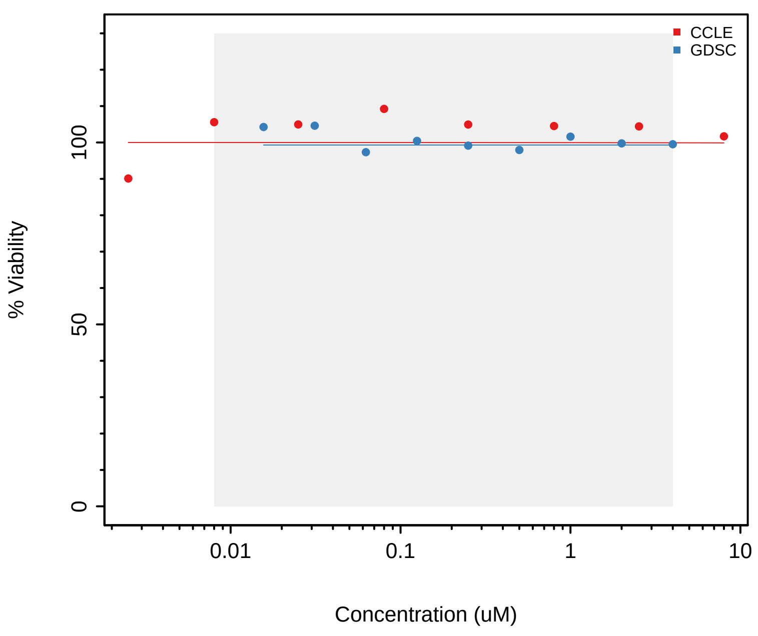

AZD6244:LCLC−103H

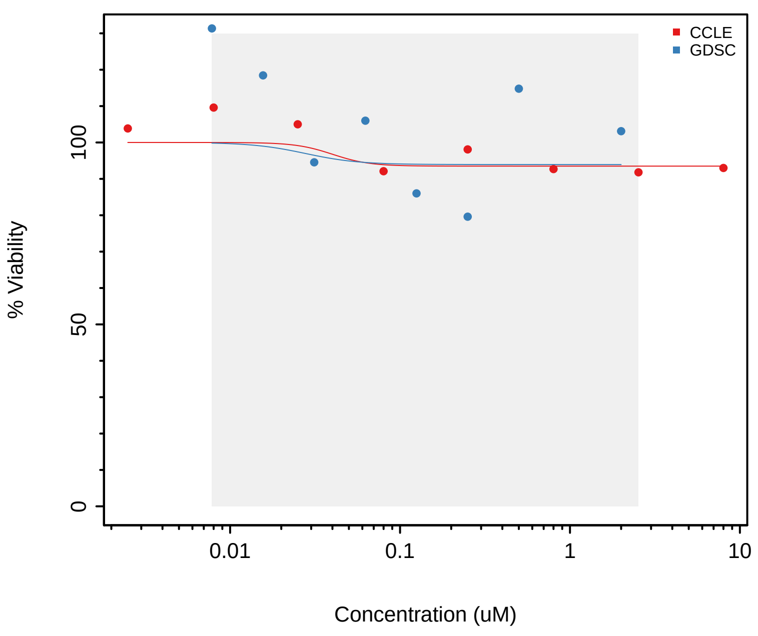

AZD6244:LOXIMVI

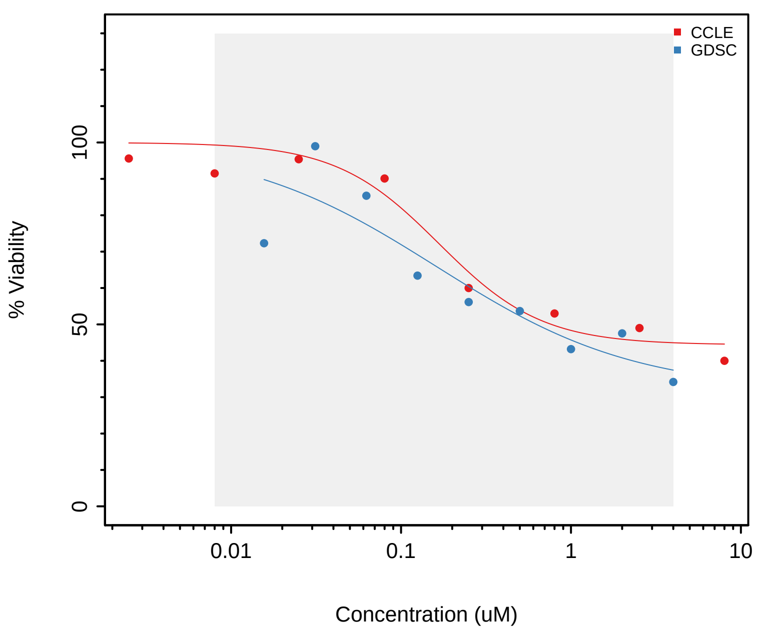

AZD6244:LP−1

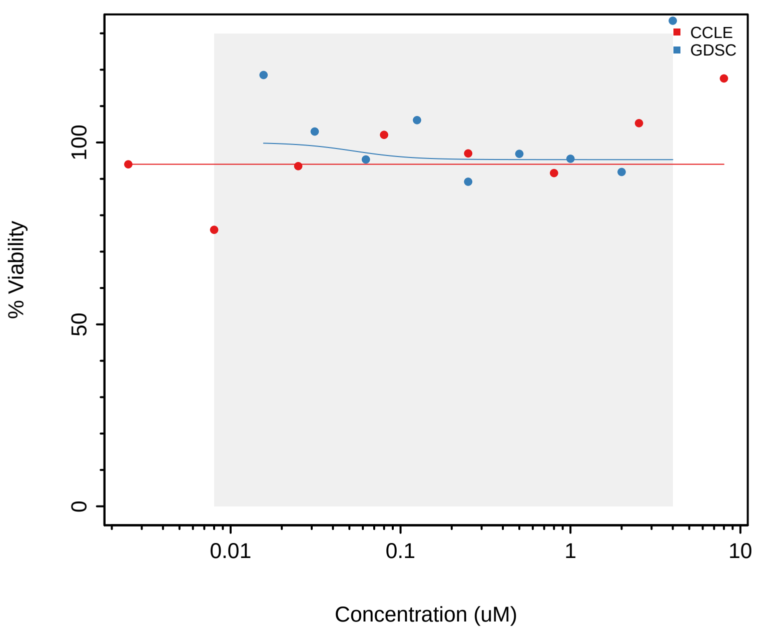

AZD6244:LS−123

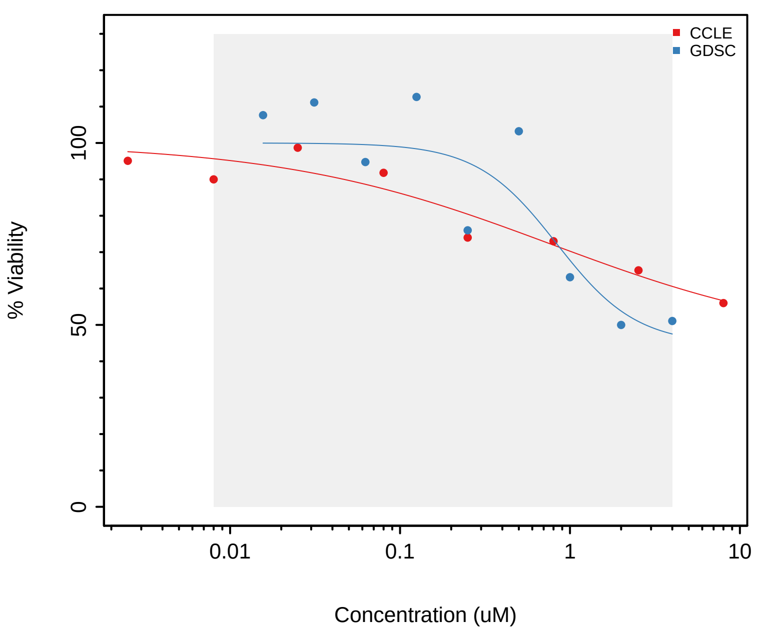

AZD6244:LS−411N

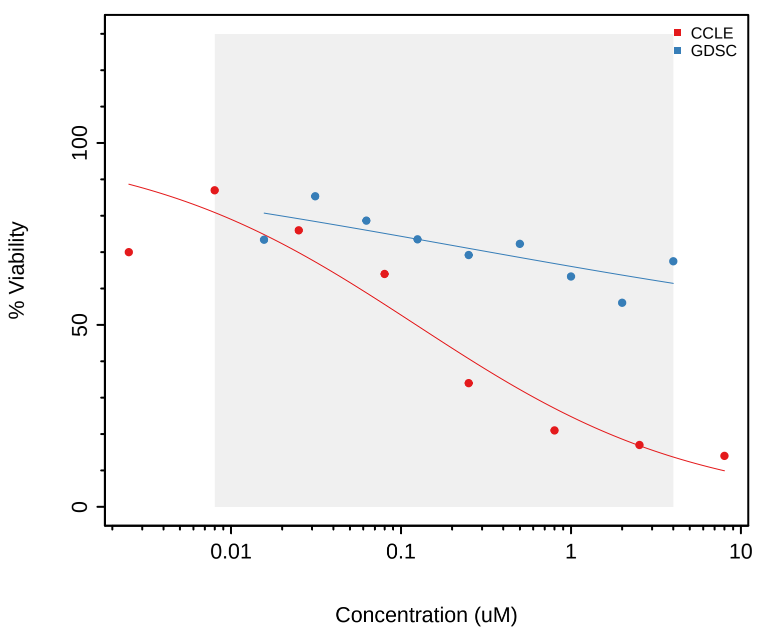

AZD6244:LS−513

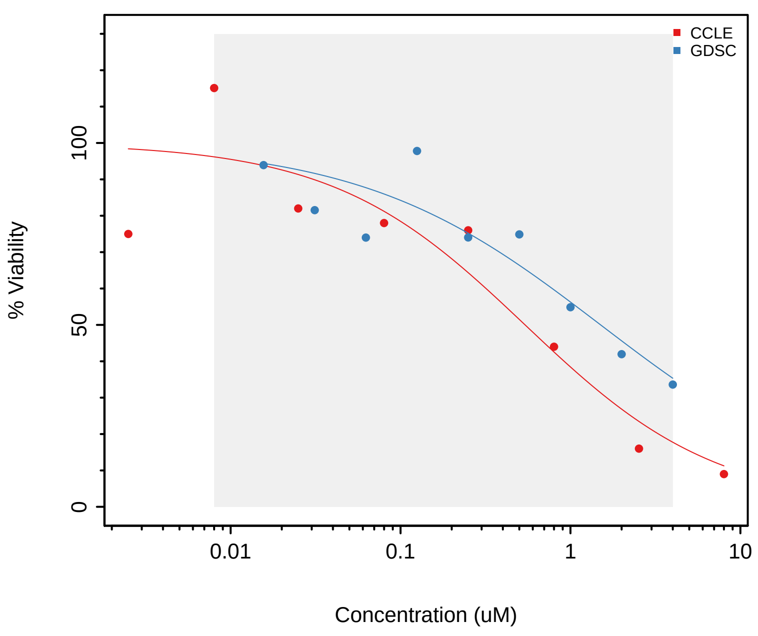

AZD6244:LU−99A

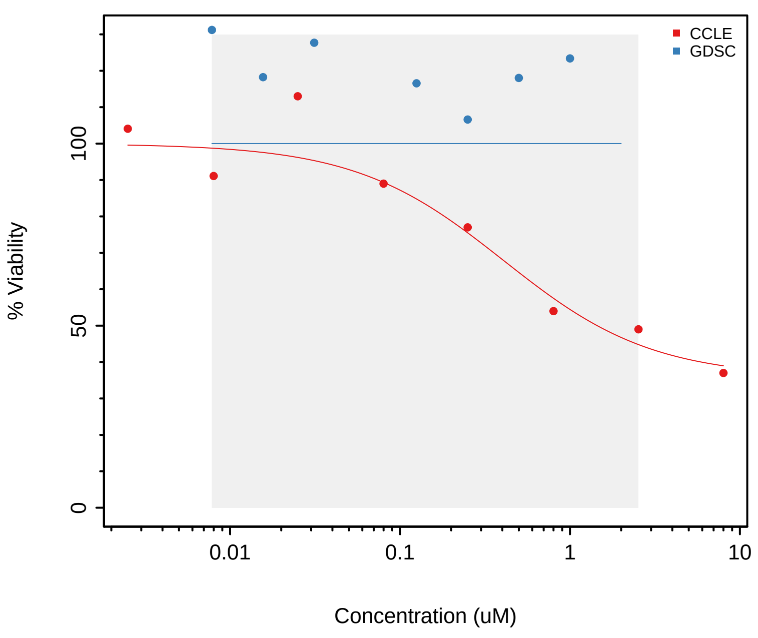

AZD6244:M059J

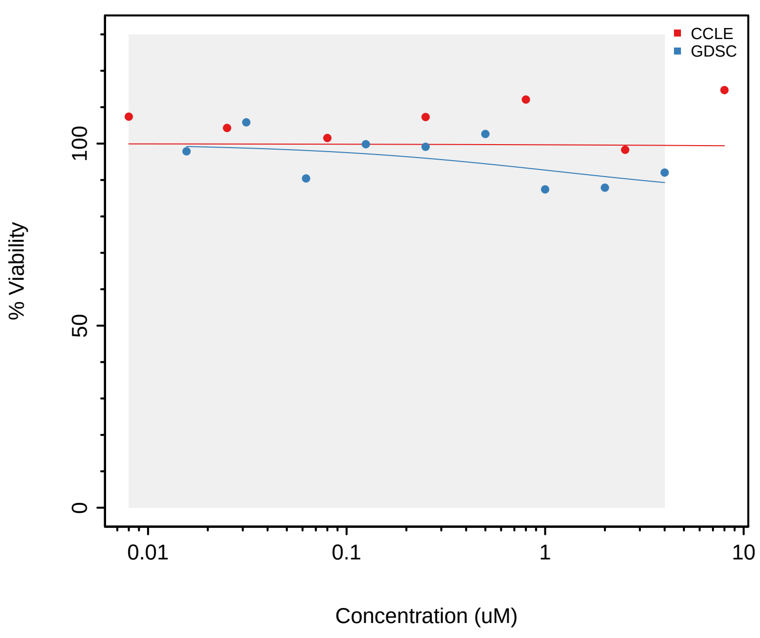

AZD6244:MC116

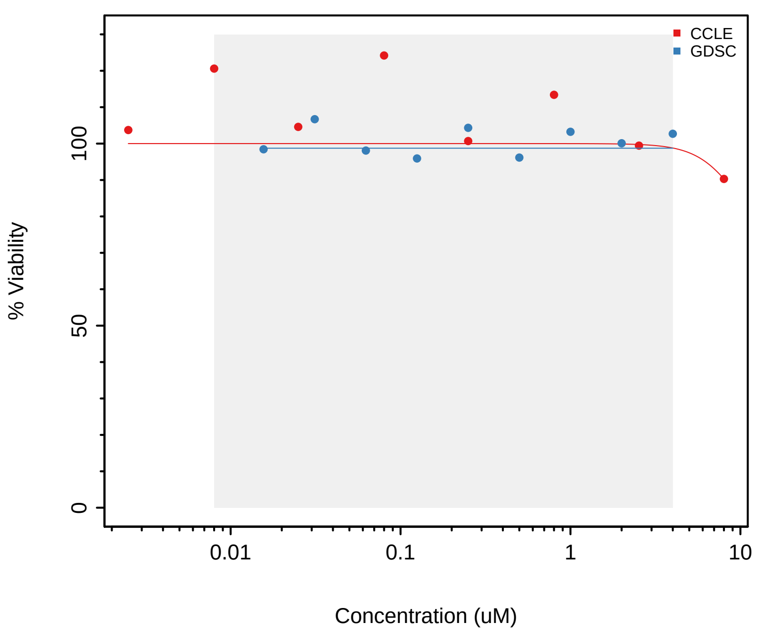

AZD6244:MDA−MB−175−VII

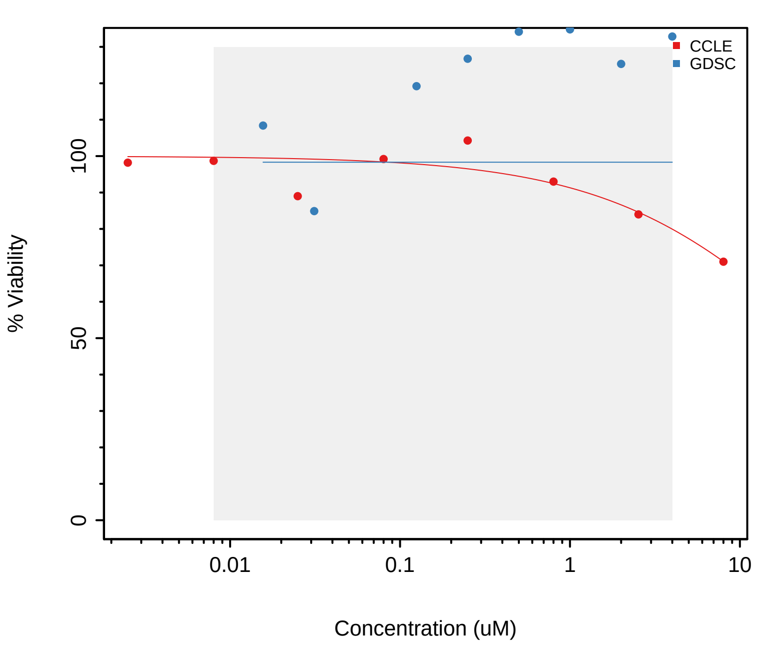

AZD6244:MDA−MB−415

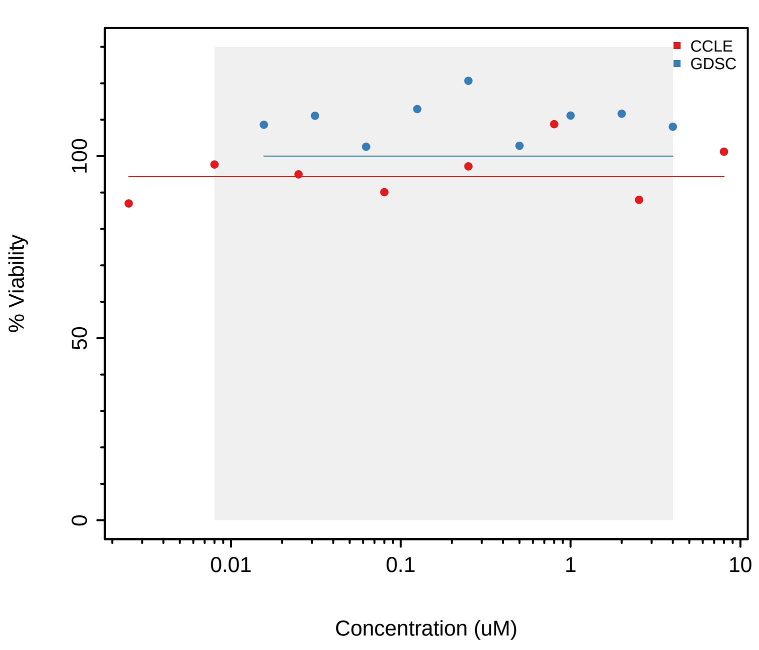

AZD6244:MDA−MB−453

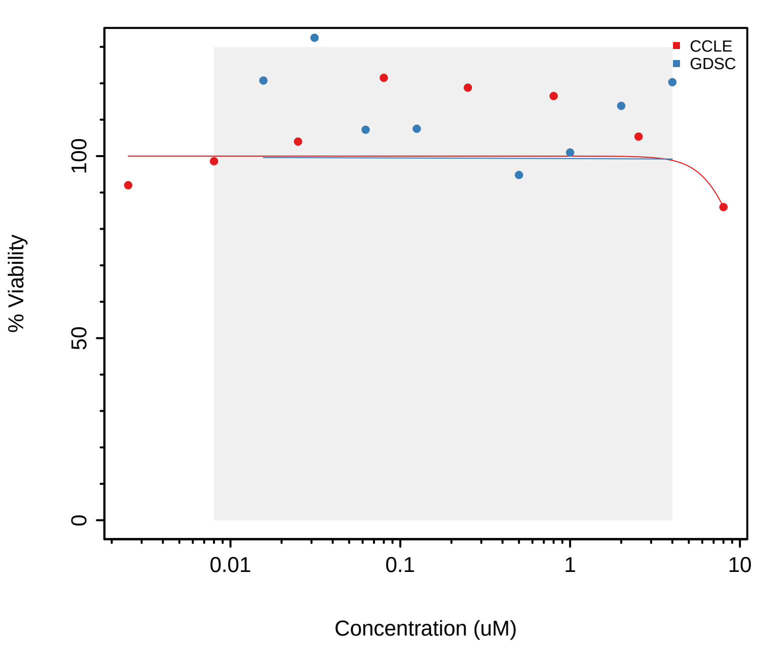

AZD6244:MDA−MB−468

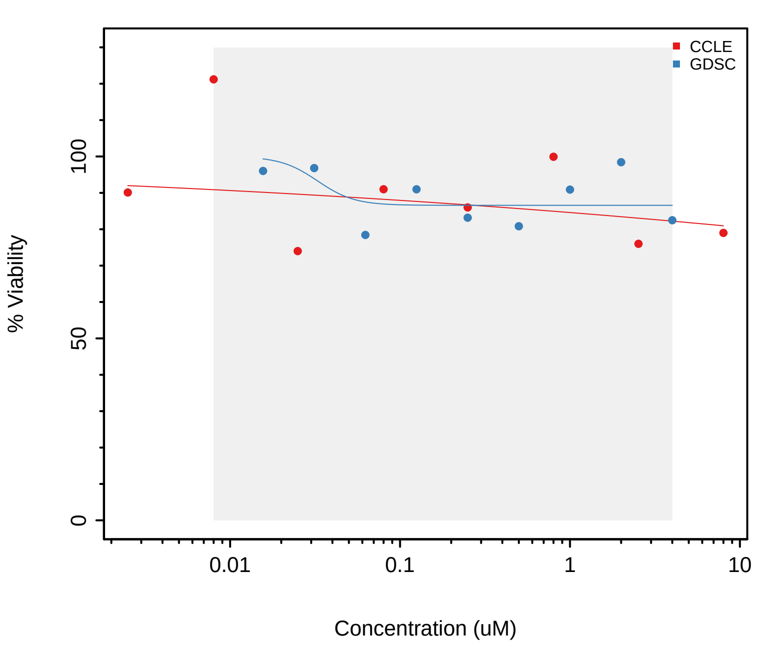

AZD6244:MEG−01

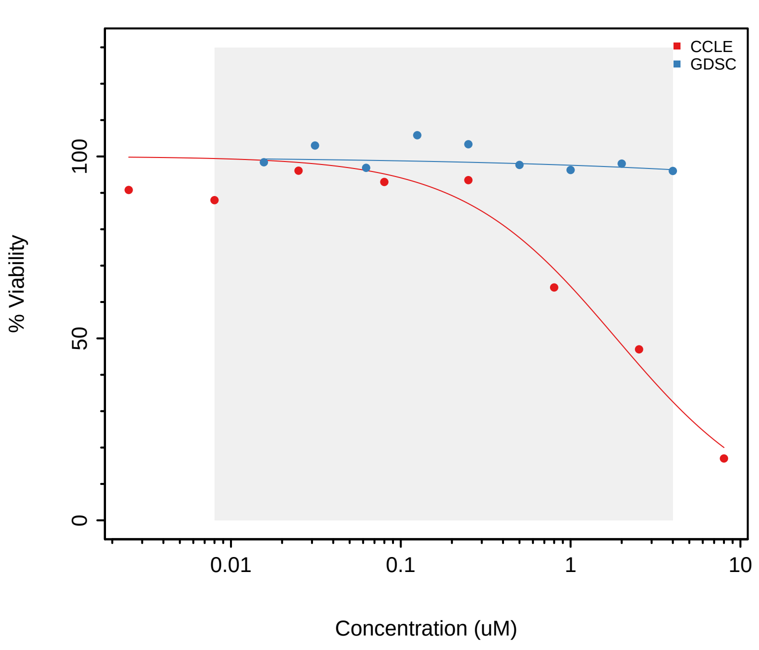

AZD6244:MEL−HO

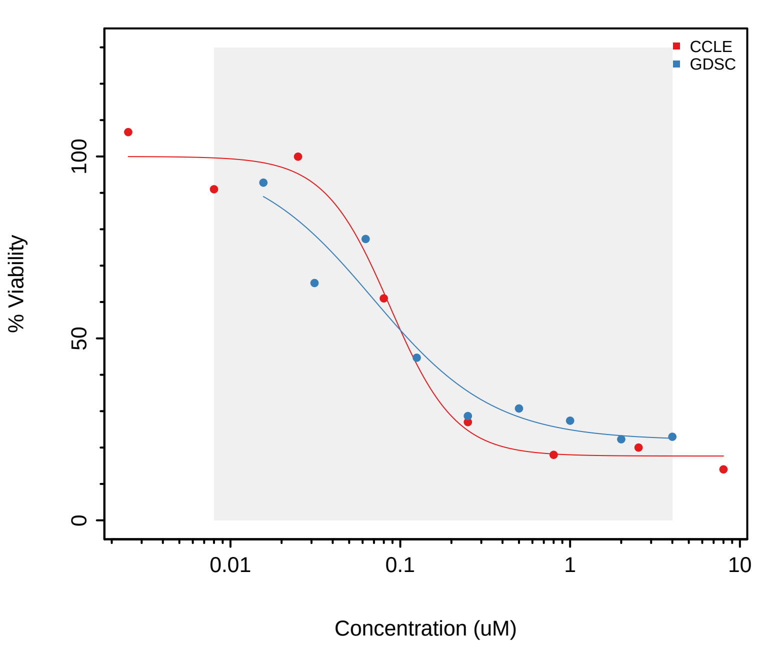

AZD6244:Mewo

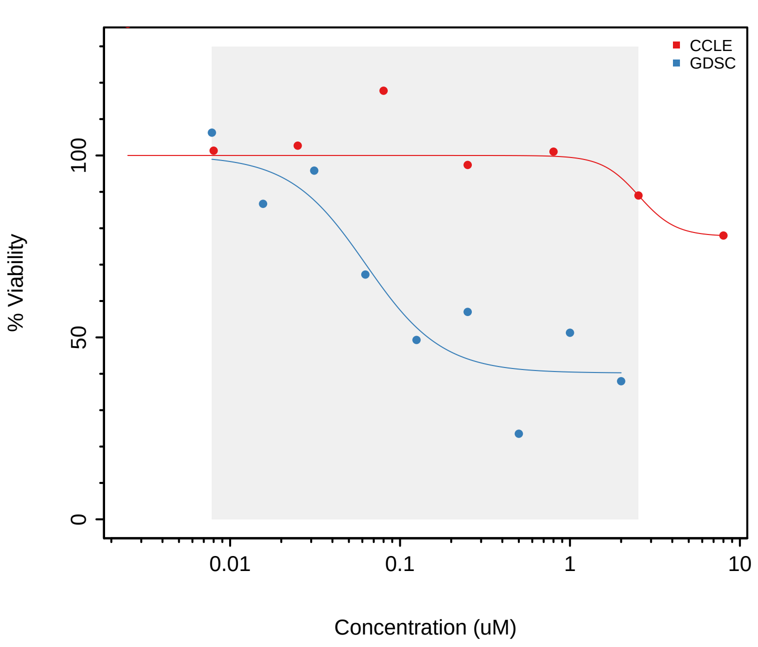

AZD6244:MFE−280

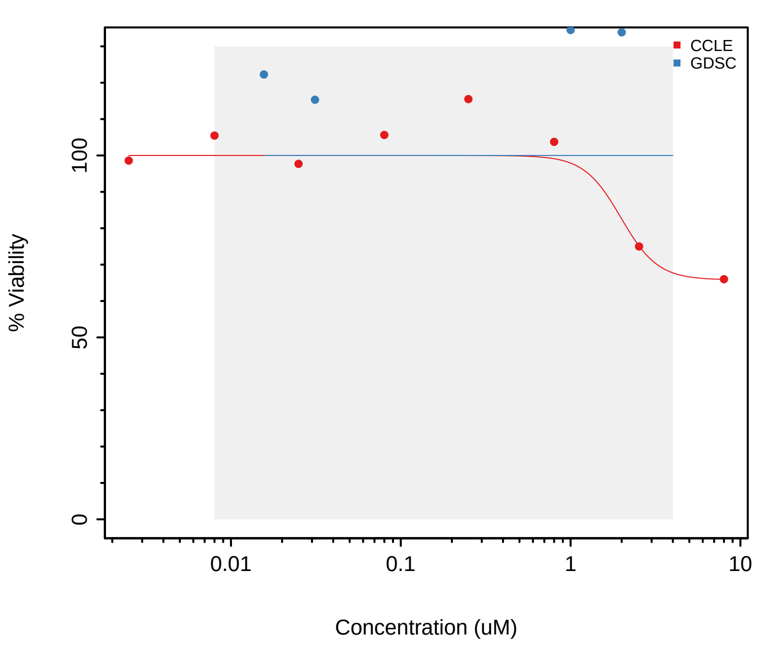

AZD6244:MFE−296

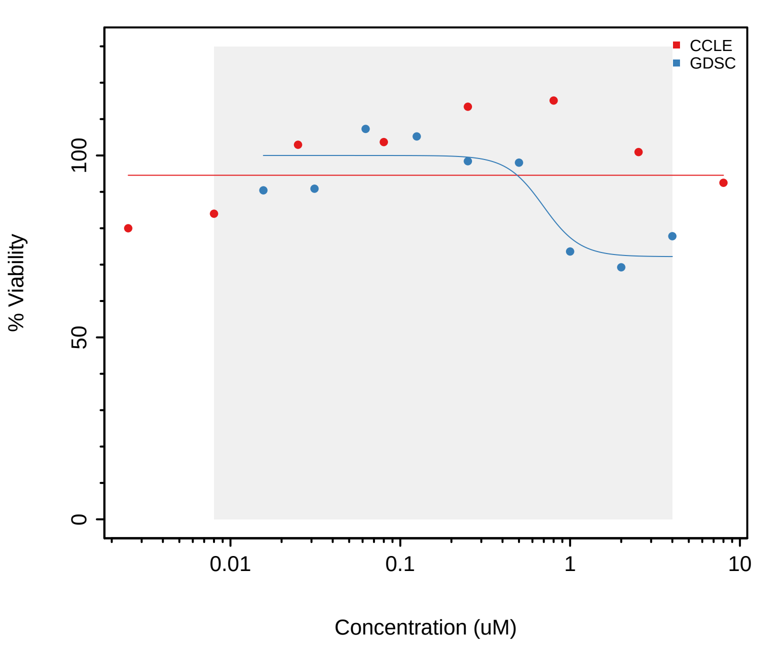

AZD6244:MG−63

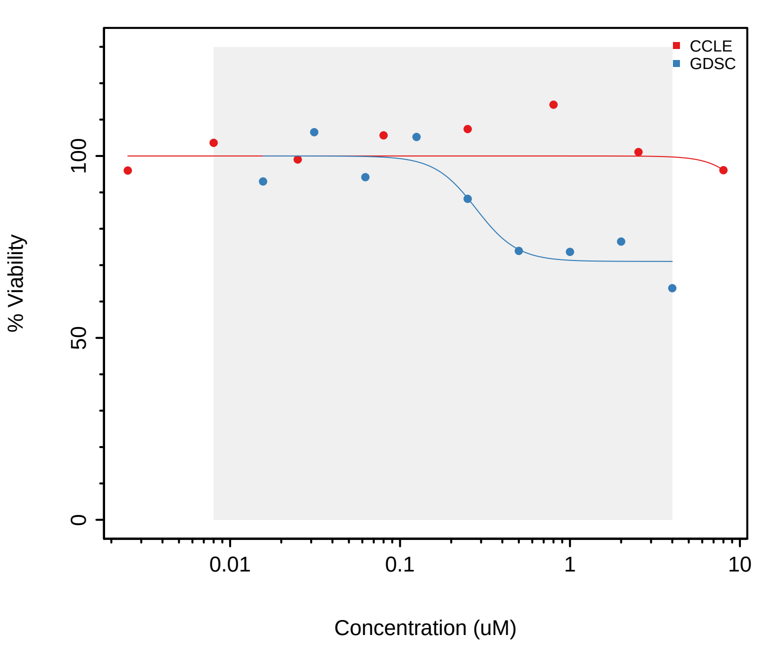

AZD6244:MHH−ES−1

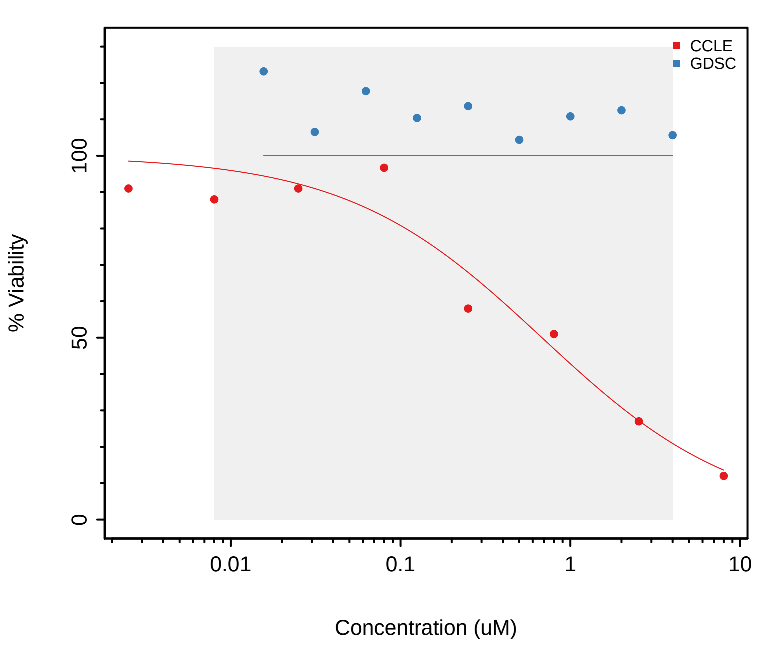

AZD6244:MIA−PaCa−2

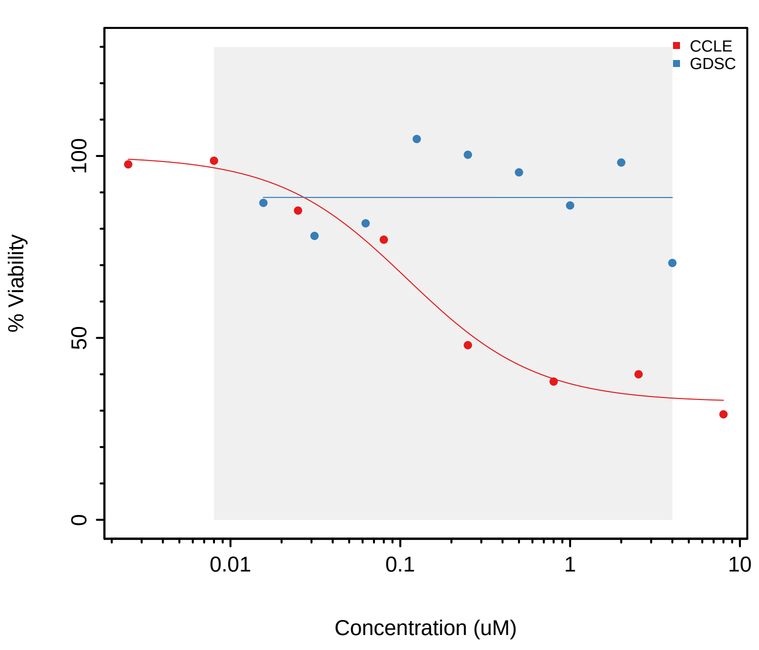

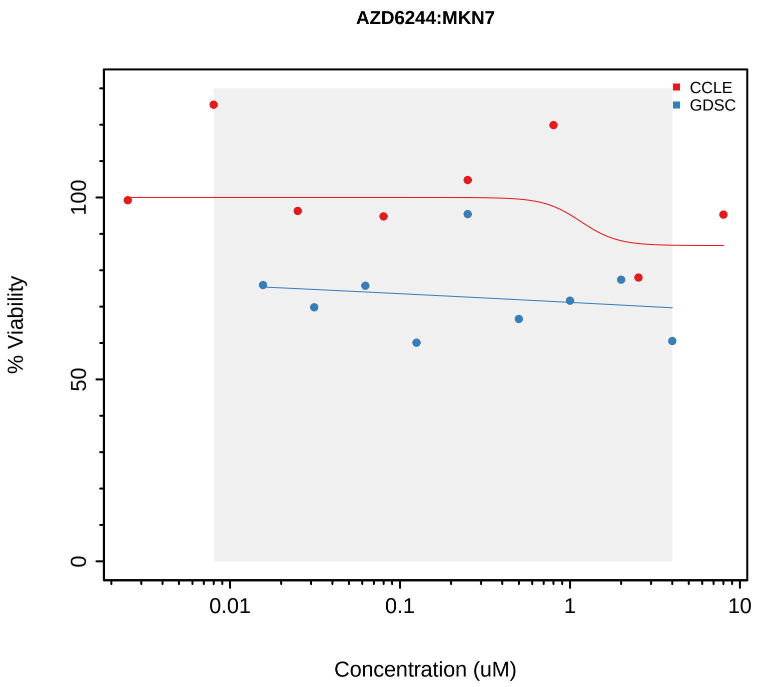
AZD6244:MKN7
CCLE
GDSC
% Viability
Concentration (uM)

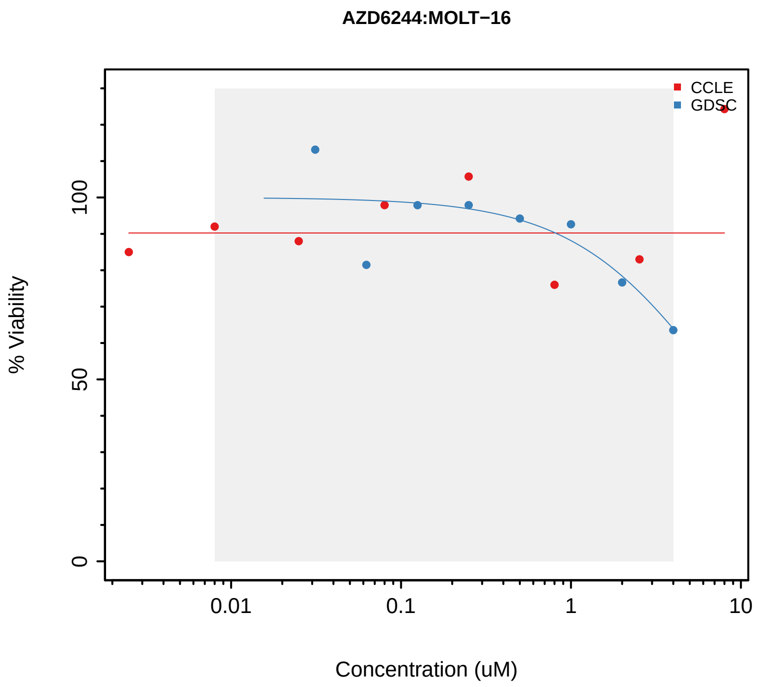
AZD6244:MOLT−16
CCLE
GDSC
% Viability
Concentration (uM)

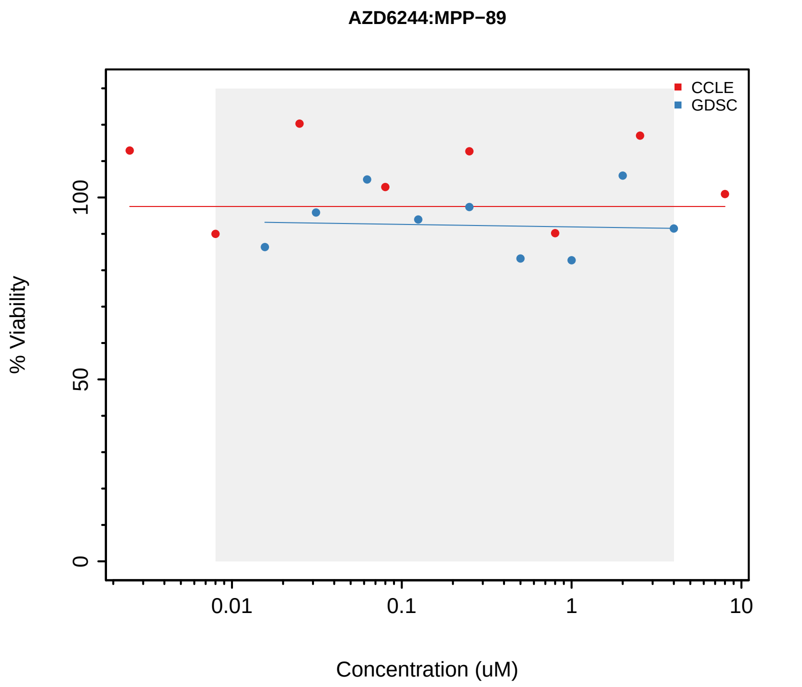
AZD6244:MPP−89
CCLE
GDSC
% Viability
Concentration (uM)

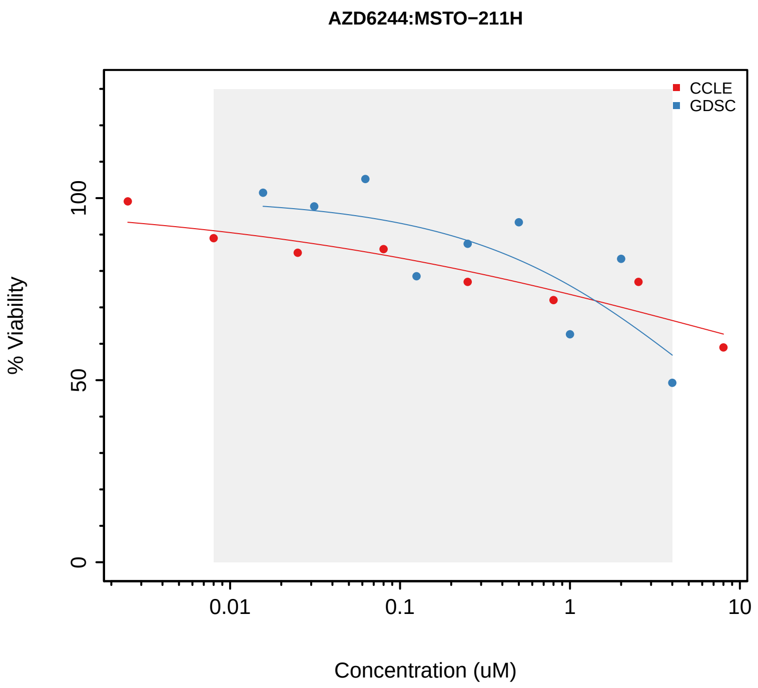
AZD6244:MSTO−211H
CCLE
GDSC
% Viability
Concentration (uM)

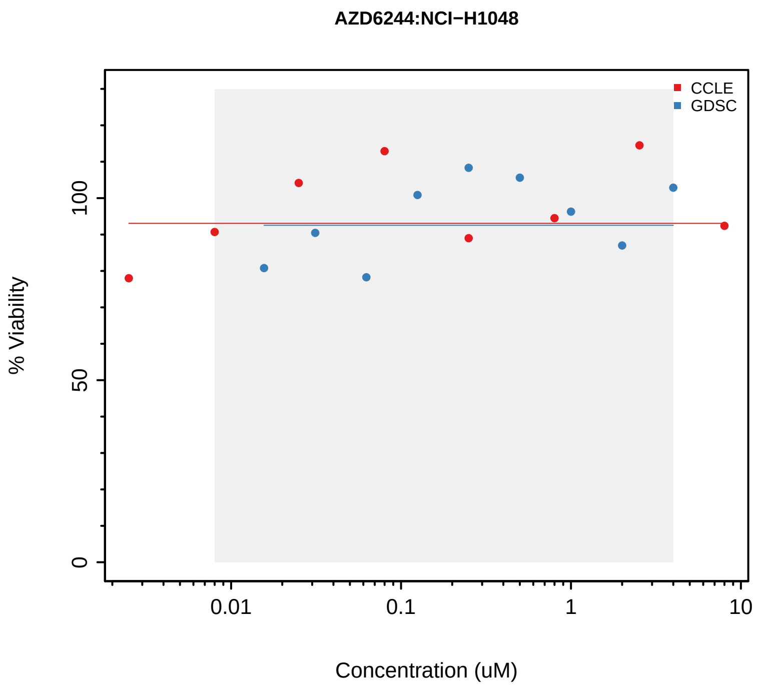
AZD6244:NCI−H1048
CCLE
GDSC
% Viability
Concentration (uM)

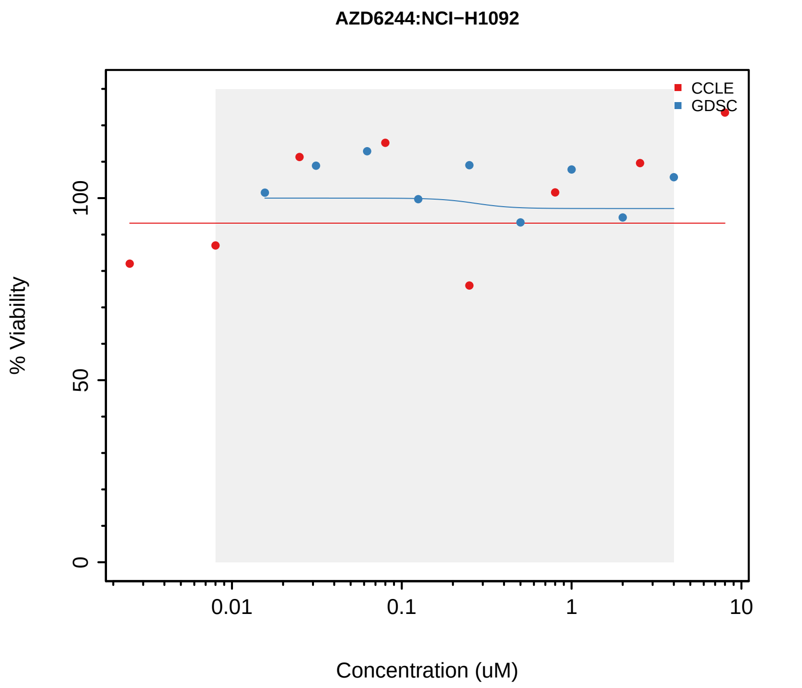
AZD6244:NCI−H1092
CCLE
GDSC
% Viability
Concentration (uM)

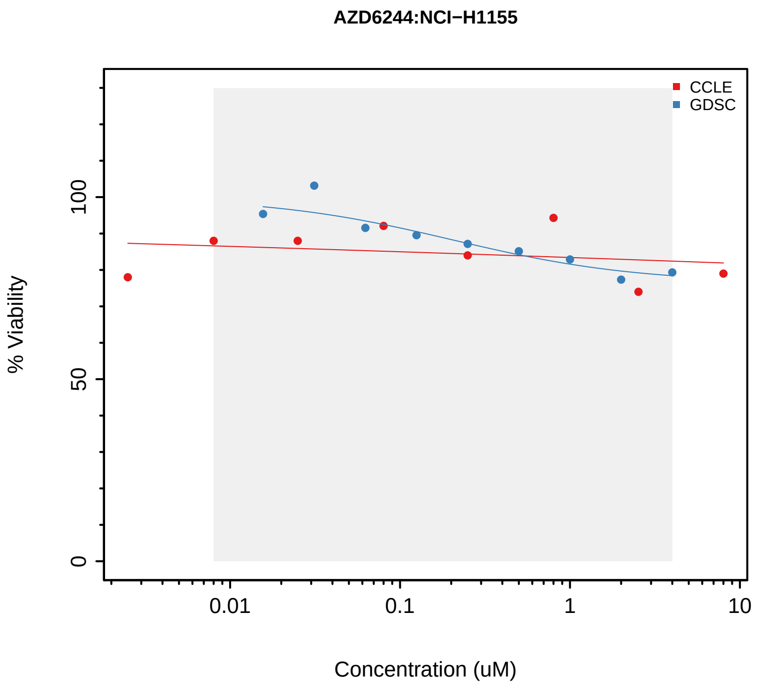
AZD6244:NCI−H1155
CCLE
GDSC
% Viability
Concentration (uM)

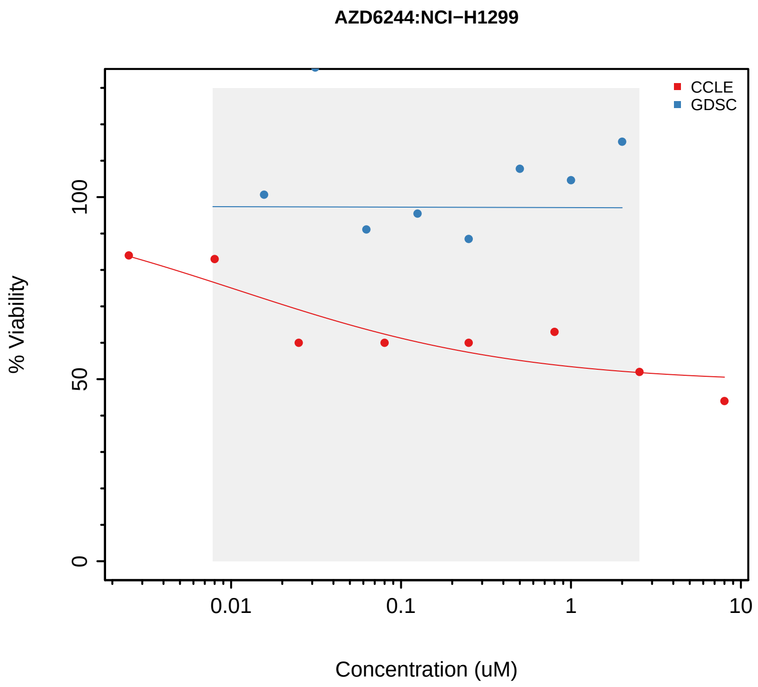
AZD6244:NCI−H1299
CCLE
GDSC
% Viability
Concentration (uM)

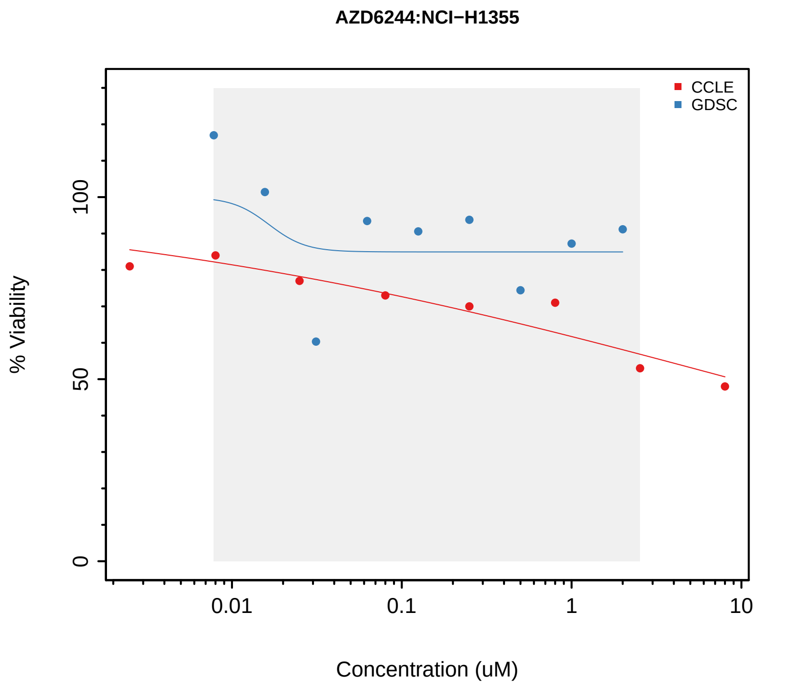
AZD6244:NCI−H1355
CCLE
GDSC
% Viability
Concentration (uM)

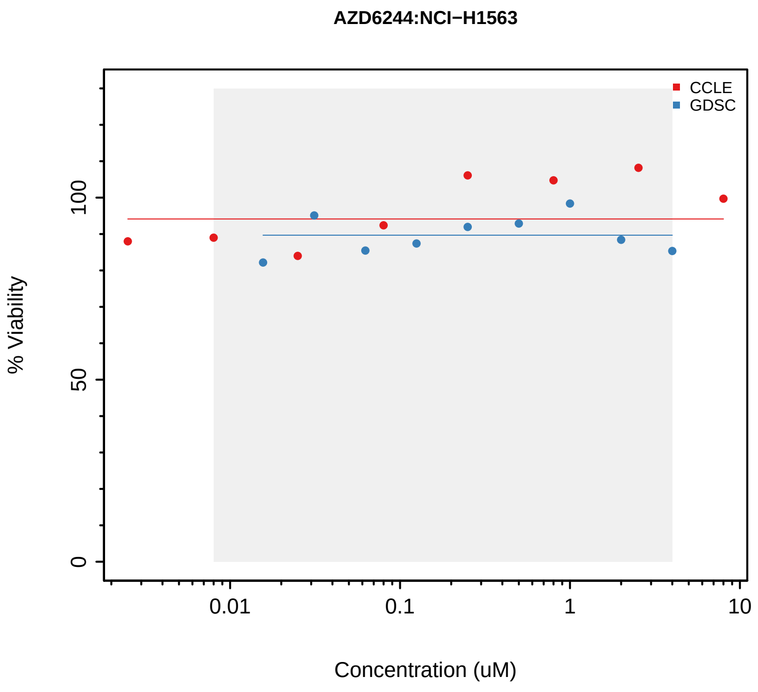
AZD6244:NCI−H1563
CCLE
GDSC
% Viability
Concentration (uM)

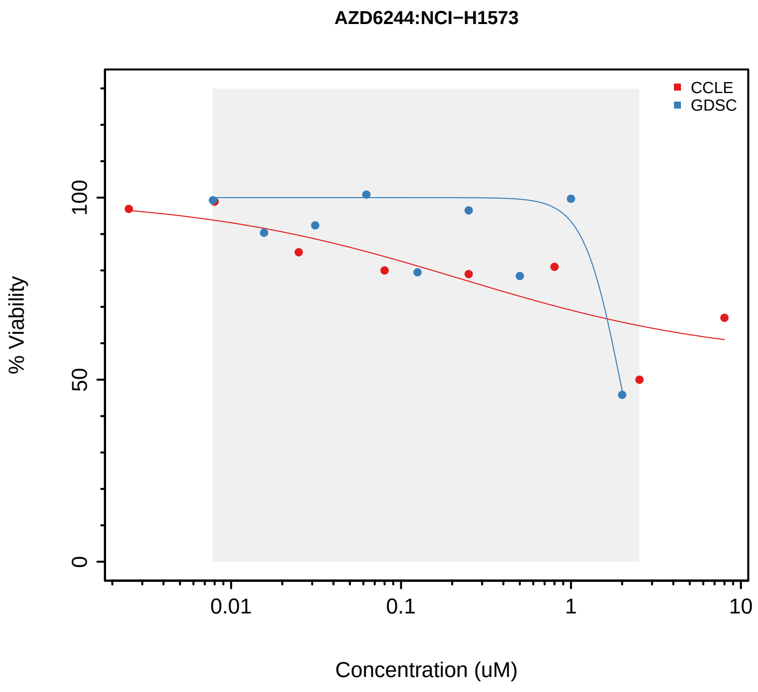
AZD6244:NCI−H1573
CCLE
GDSC
% Viability
Concentration (uM)

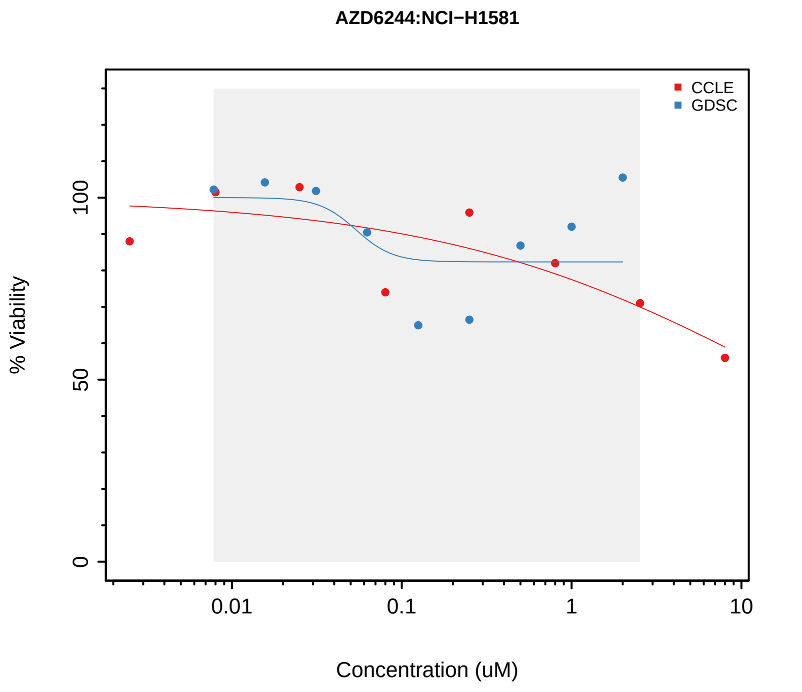
AZD6244:NCI−H1581
CCLE
GDSC
% Viability
Concentration (uM)

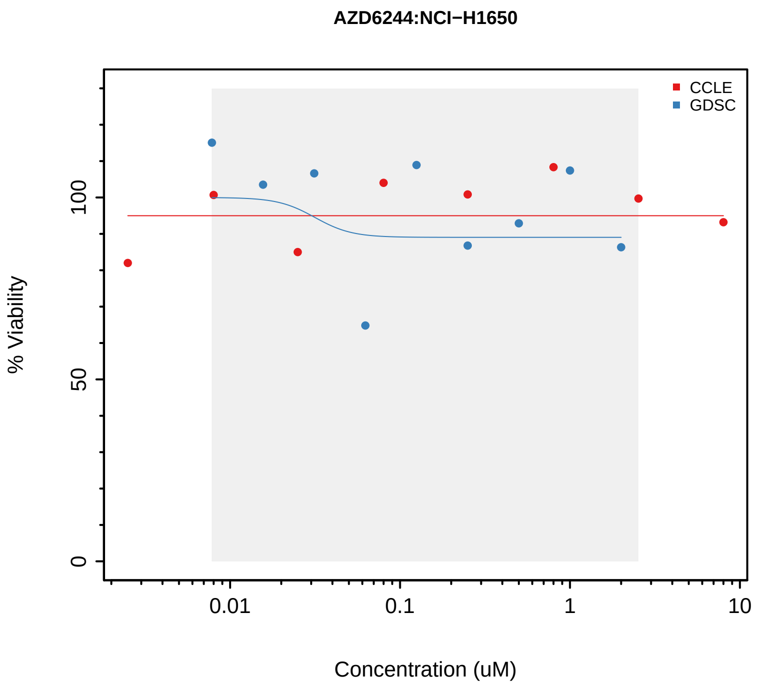
AZD6244:NCI−H1650
CCLE
GDSC
% Viability
0
50
100
0.01
0.1
1
10
Concentration (uM)

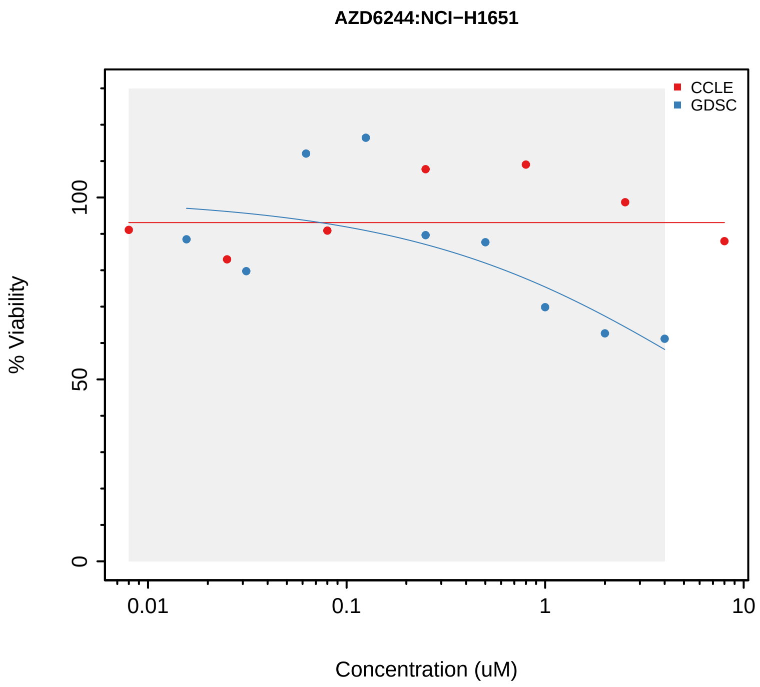
AZD6244:NCI−H1651
CCLE
GDSC
% Viability
0
50
100
0.01
0.1
1
10
Concentration (uM)

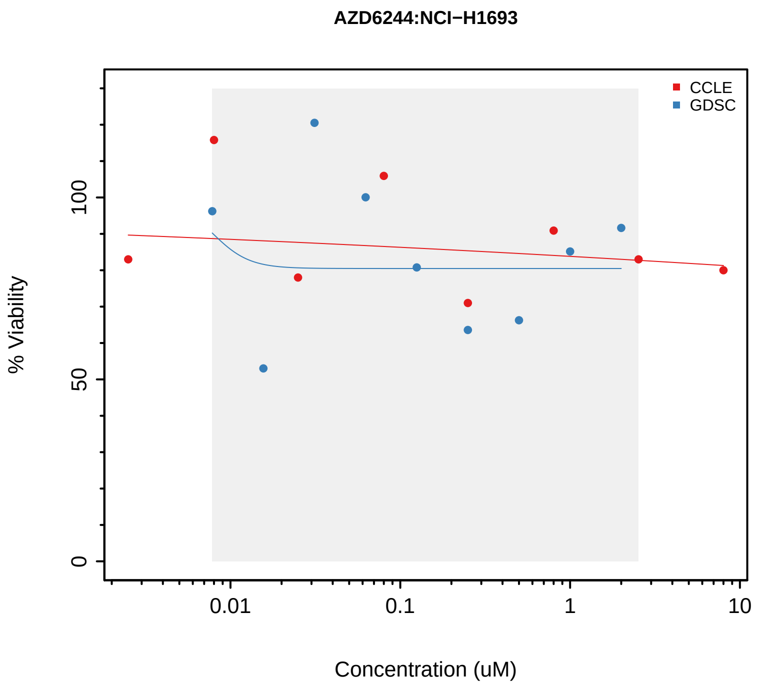
AZD6244:NCI−H1693
CCLE
GDSC
% Viability
0
50
100
0.01
0.1
1
10
Concentration (uM)

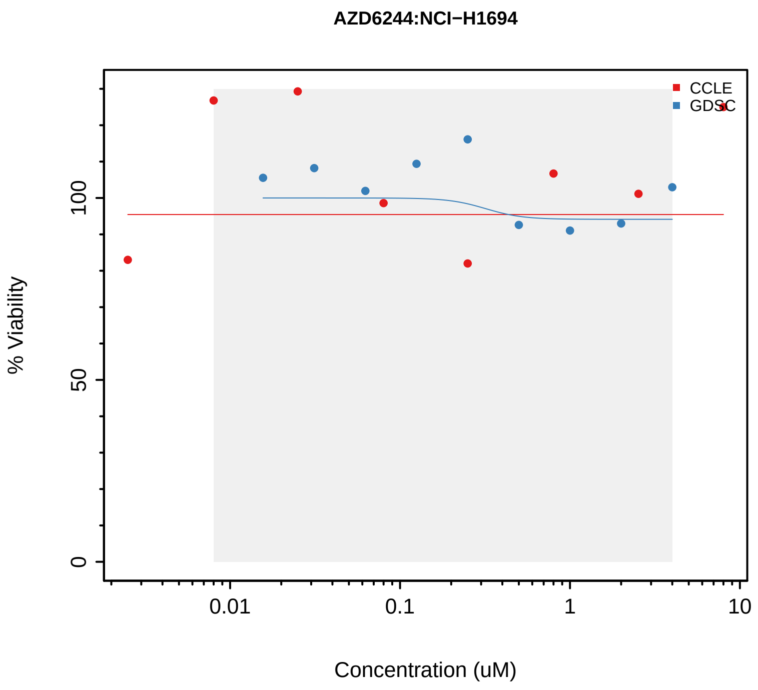
AZD6244:NCI−H1694
CCLE
GDSC
% Viability
0
50
100
0.01
0.1
1
10
Concentration (uM)

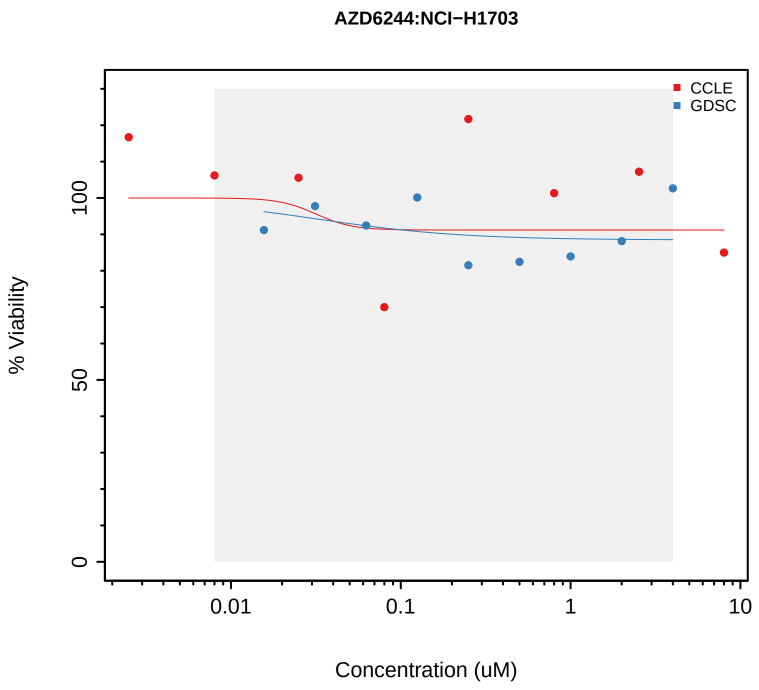
AZD6244:NCI−H1703
CCLE
GDSC
% Viability
0
50
100
0.01
0.1
1
10
Concentration (uM)

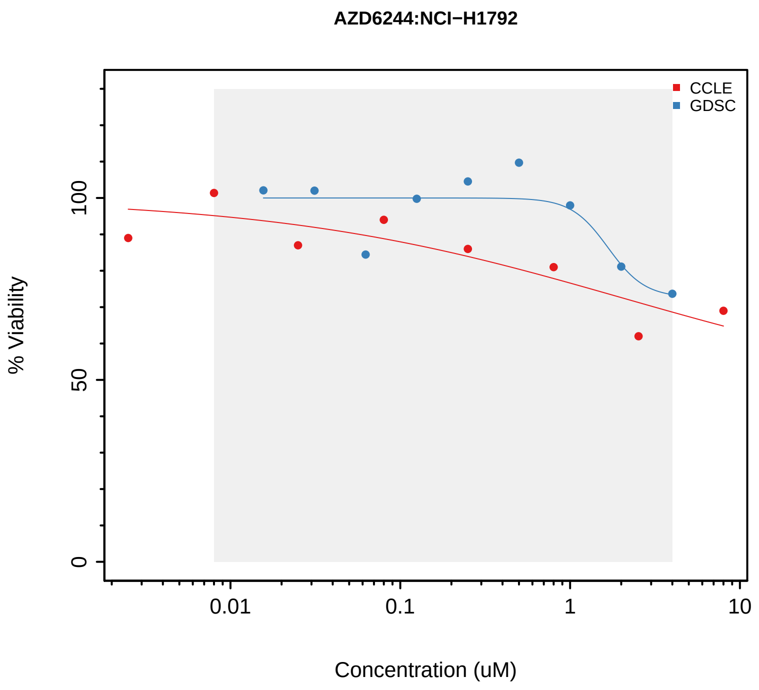
AZD6244:NCI−H1792
CCLE
GDSC
% Viability
0
50
100
0.01
0.1
1
10
Concentration (uM)

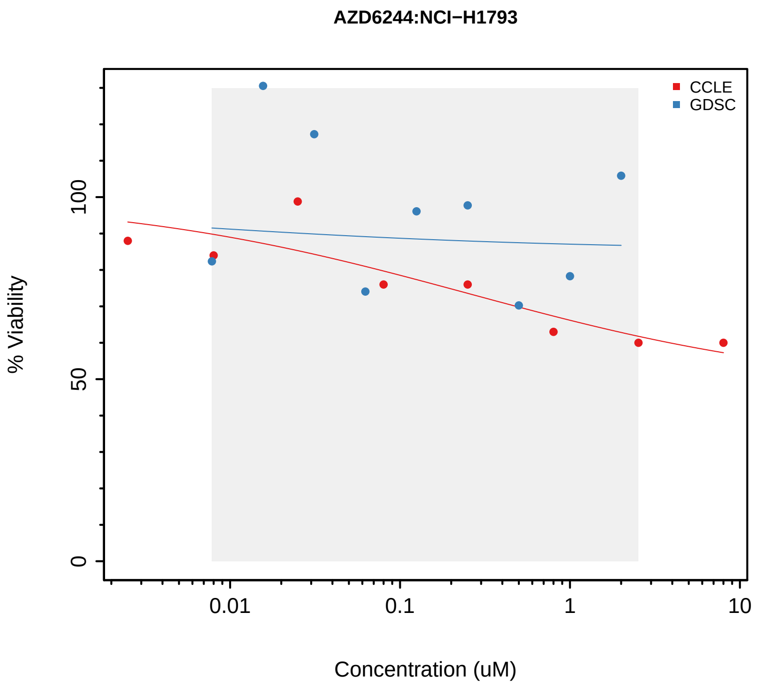
AZD6244:NCI−H1793
CCLE
GDSC
% Viability
0
50
100
0.01
0.1
1
10
Concentration (uM)

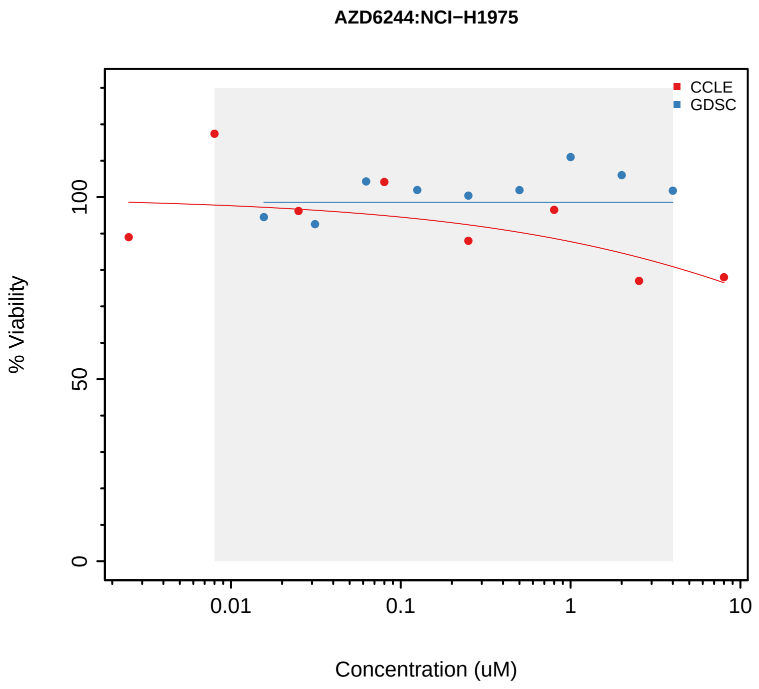
AZD6244:NCI−H1975
CCLE
GDSC
% Viability
0
50
100
0.01
0.1
1
10
Concentration (uM)

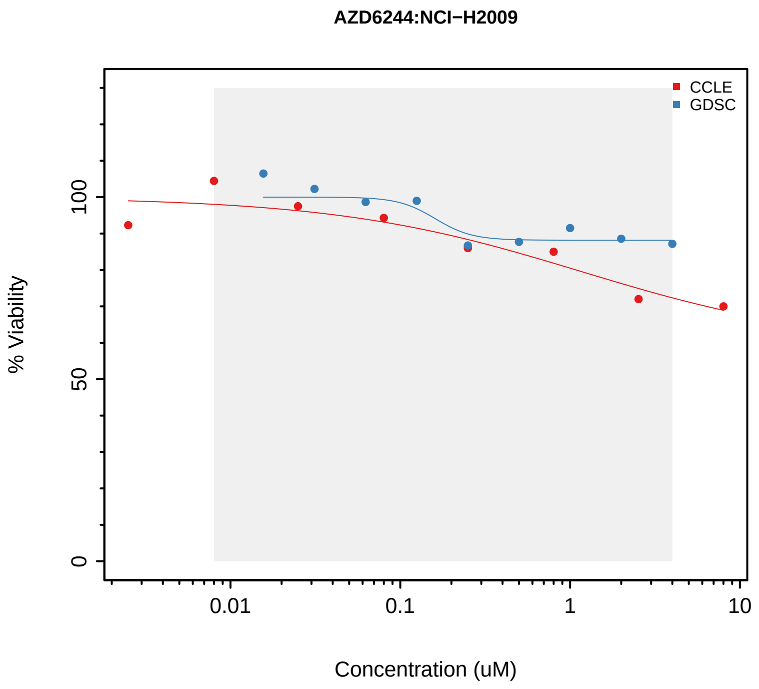
AZD6244:NCI−H2009
CCLE
GDSC
% Viability
0
50
100
0.01
0.1
1
10
Concentration (uM)

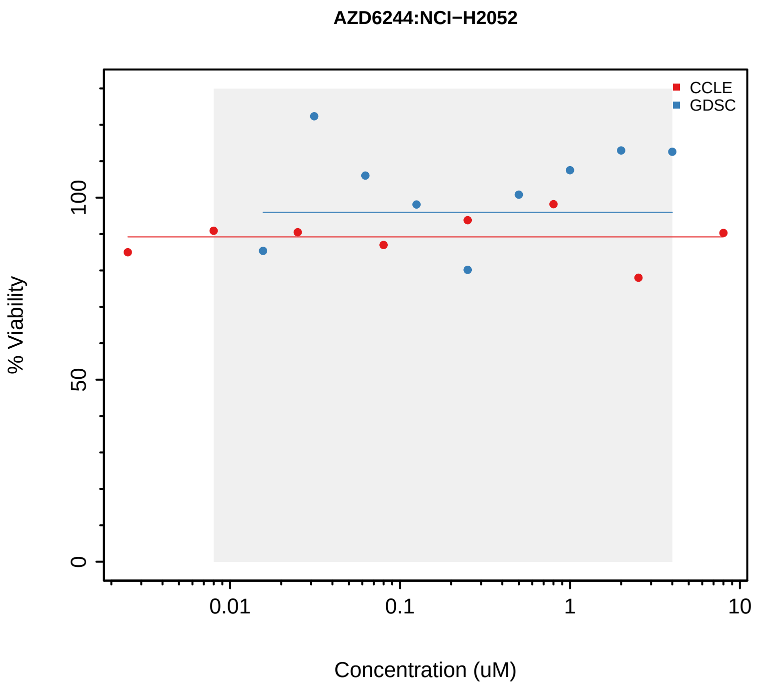
AZD6244:NCI−H2052
CCLE
GDSC
% Viability
0
50
100
0.01
0.1
1
10
Concentration (uM)

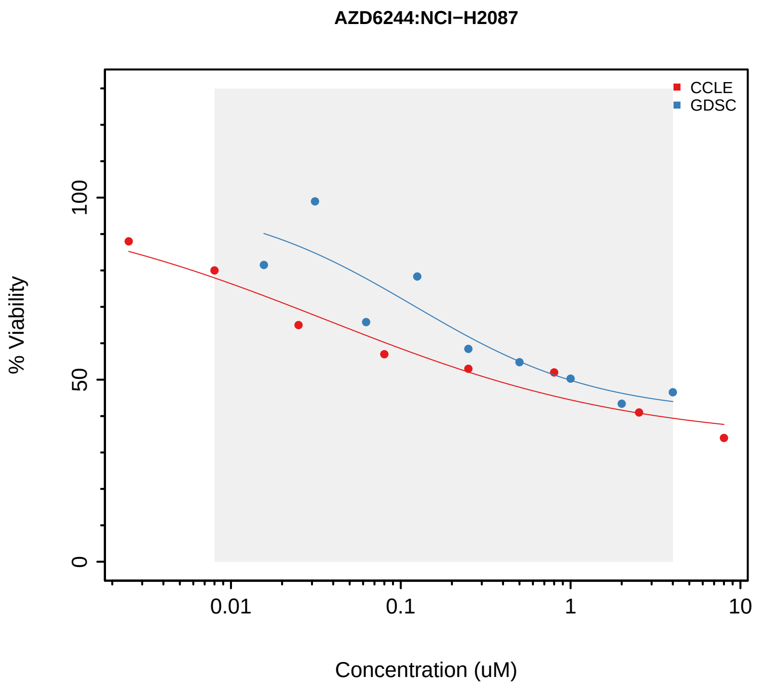
AZD6244:NCI−H2087
CCLE
GDSC
% Viability
0
50
100
0.01
0.1
1
10
Concentration (uM)

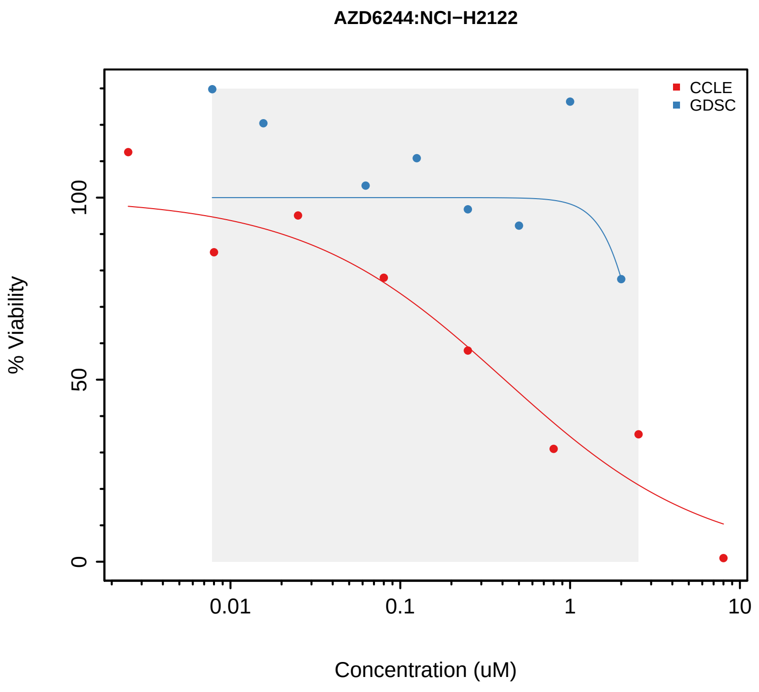
AZD6244:NCI−H2122
CCLE
GDSC
% Viability
0
50
100
0.01
0.1
1
10
Concentration (uM)

AZD6244:NCI−H2170

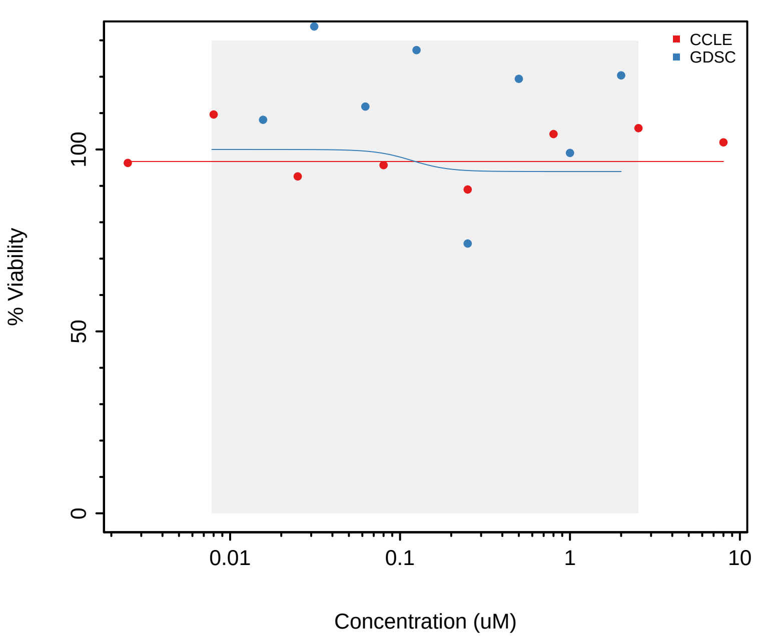

AZD6244:NCI−H226

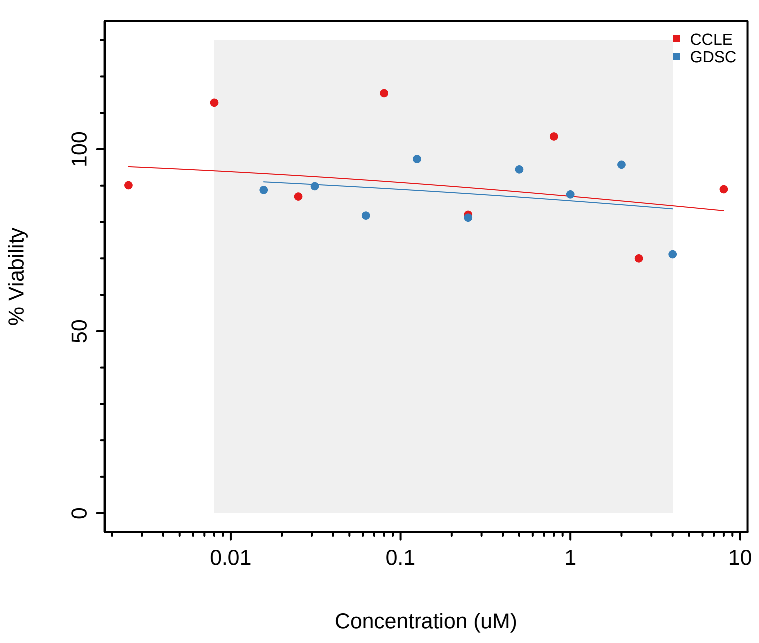

AZD6244:NCI−H23

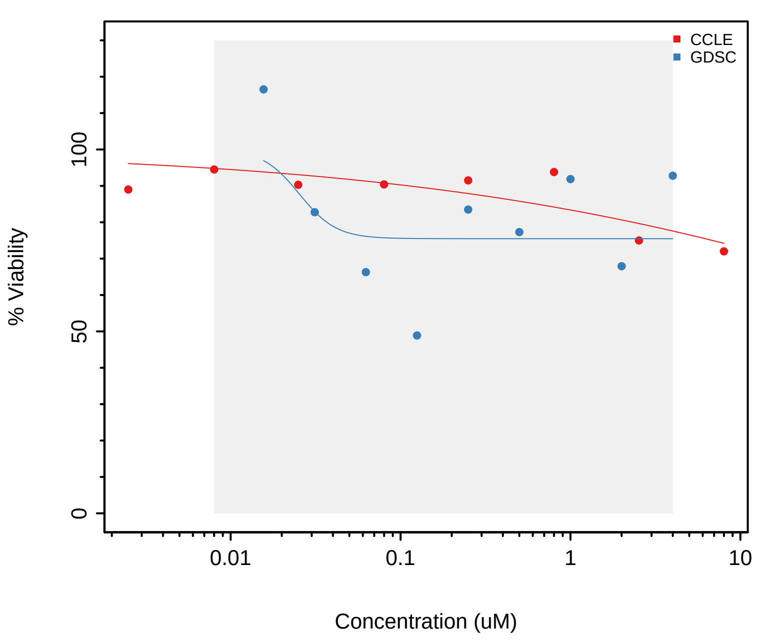

AZD6244:NCI−H2452

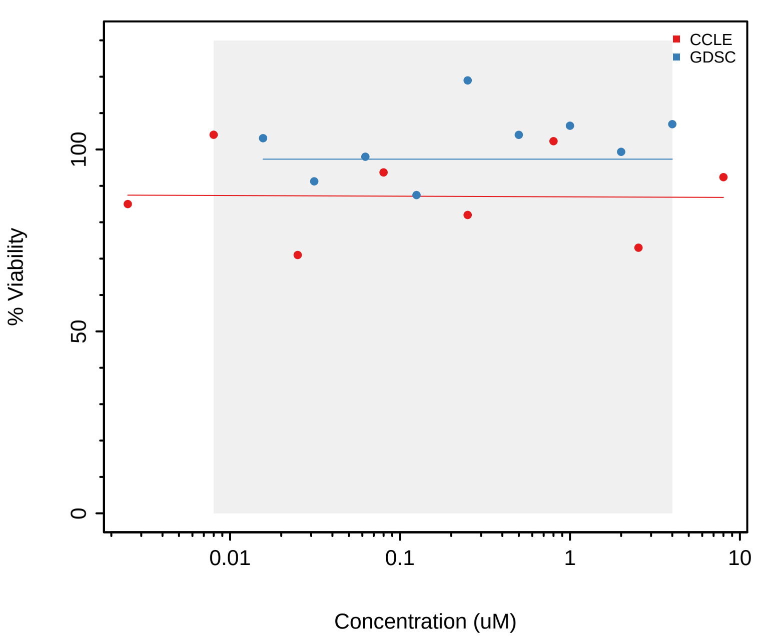

AZD6244:NCI−H28

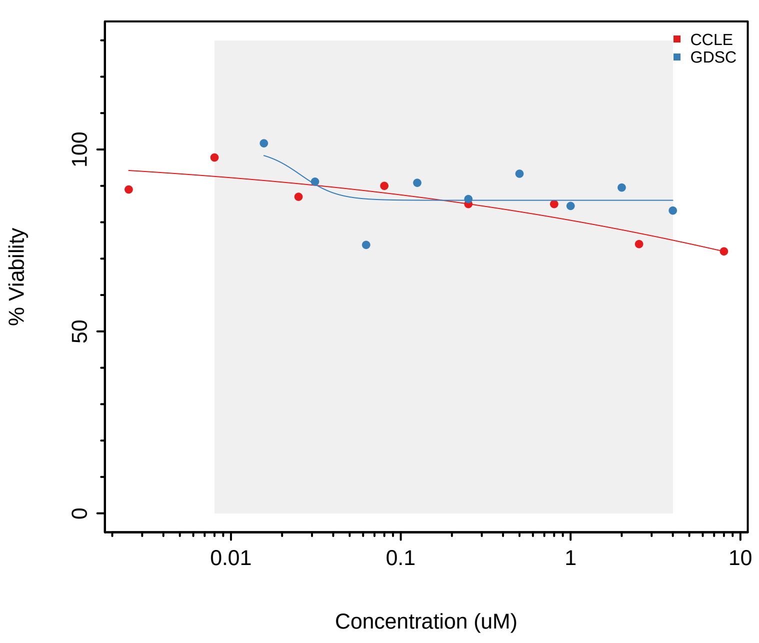

AZD6244:NCI−H441

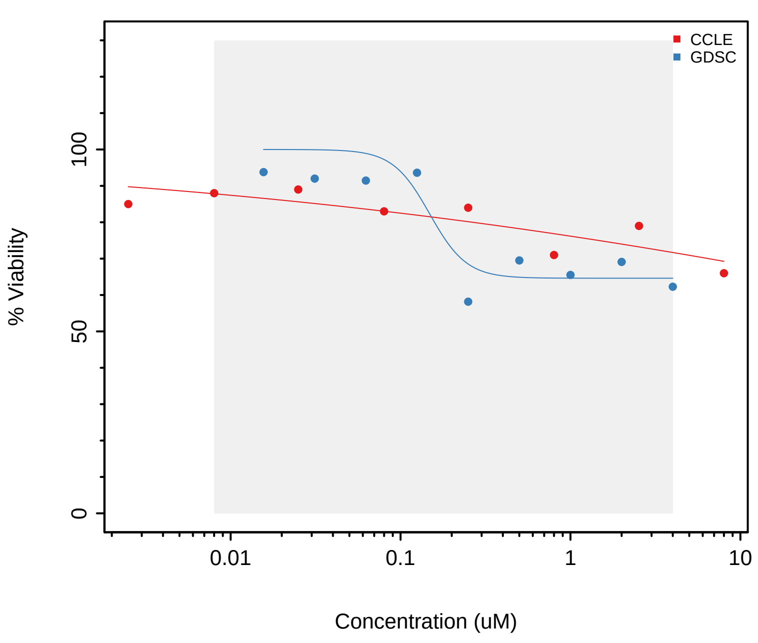

AZD6244:NCI−H460

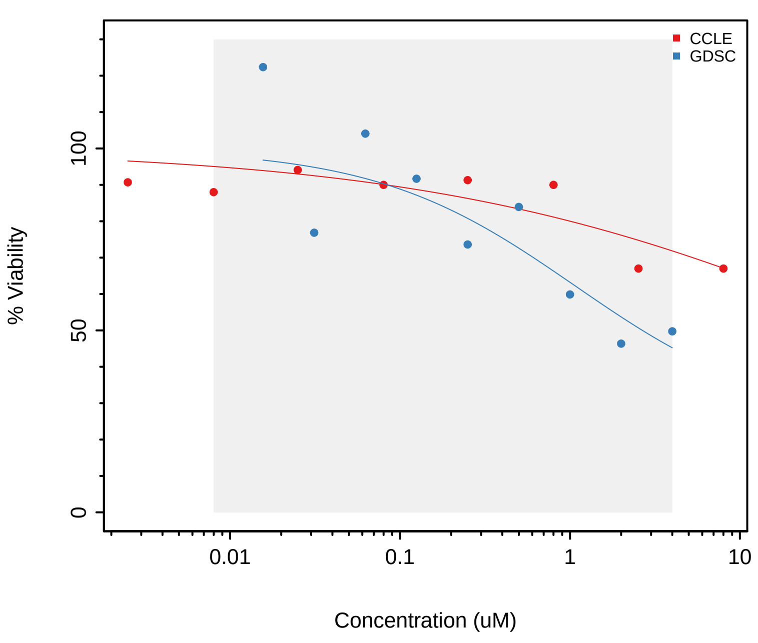

AZD6244:NCI−H520

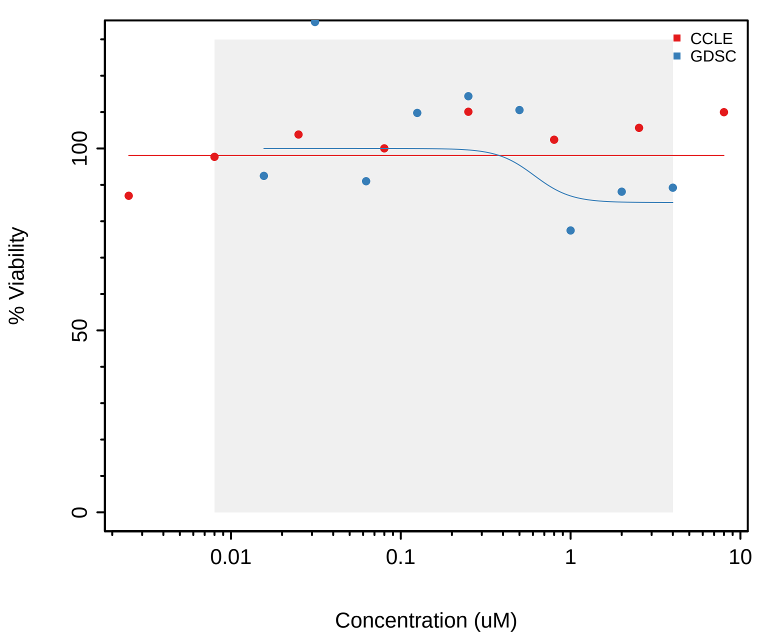

AZD6244:NCI−H522

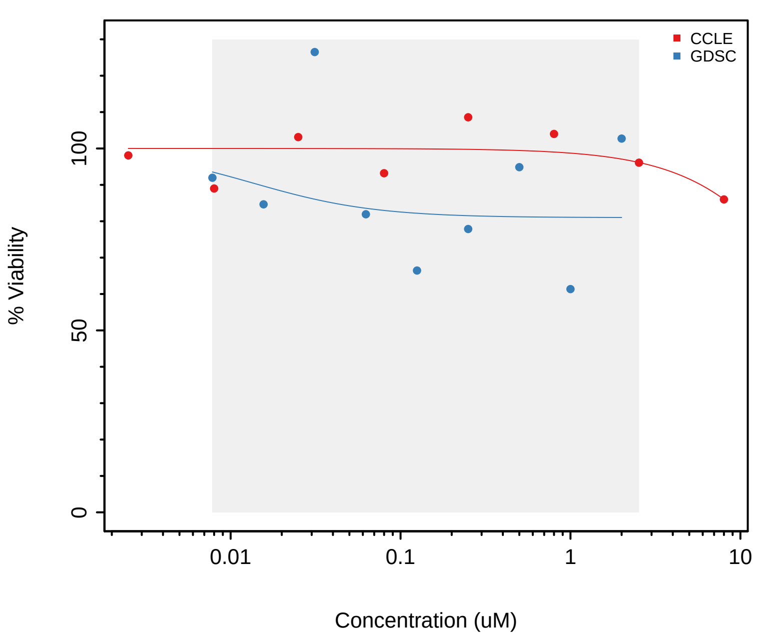

AZD6244:NCI−H650

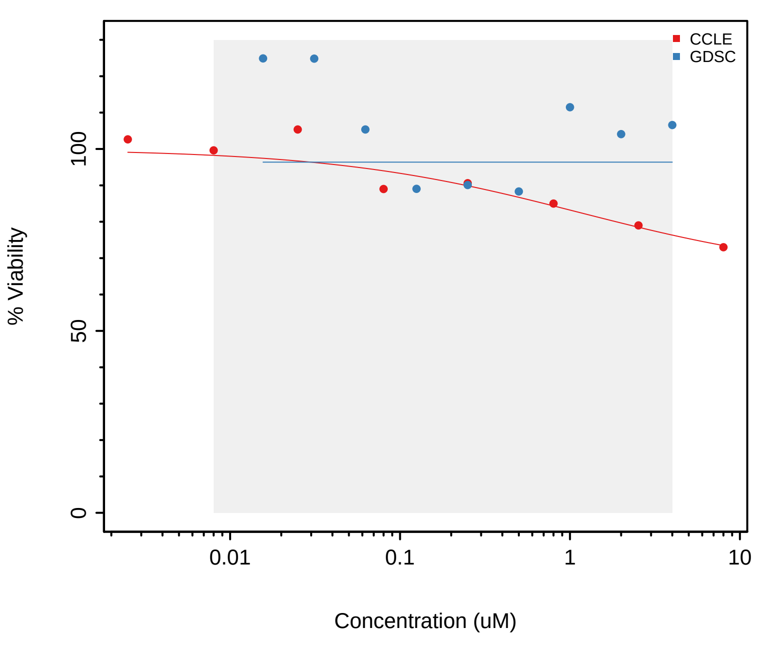

AZD6244:NCI−H661

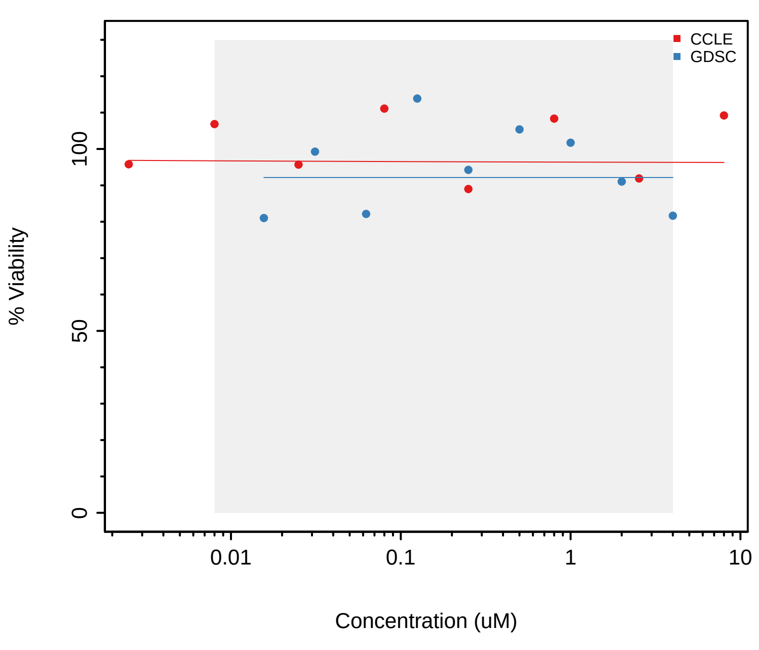

AZD6244:NCI−H727

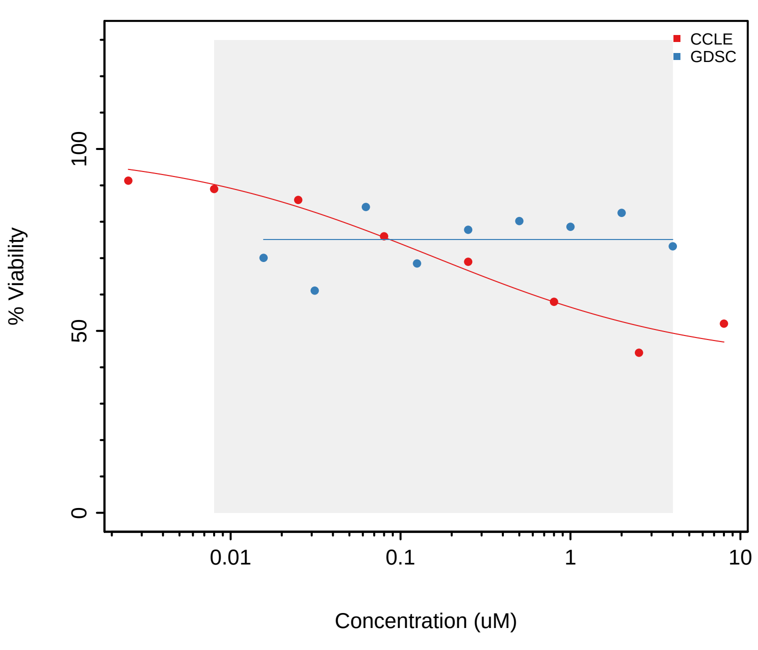

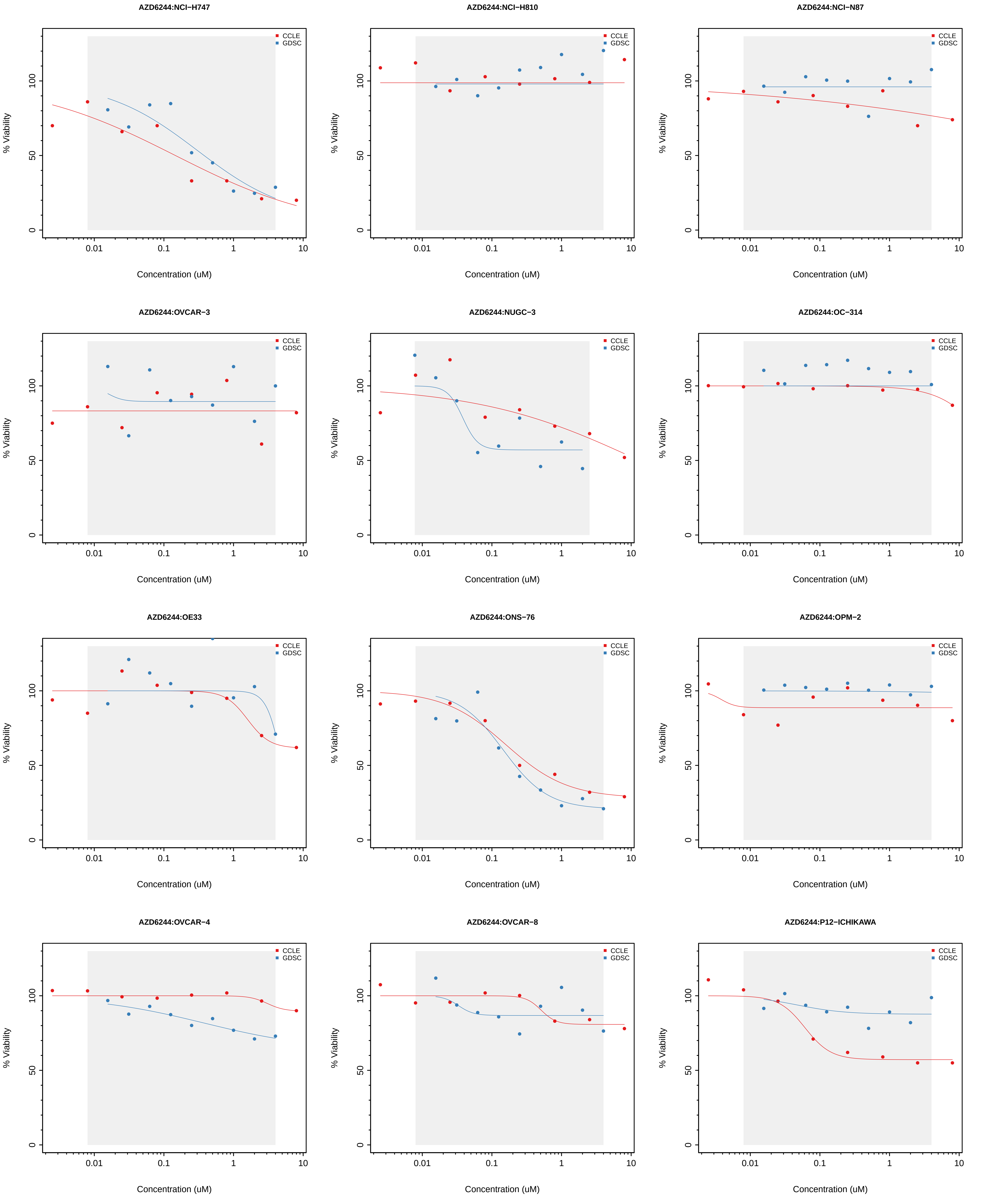
AZD6244:NCI−H747
AZD6244:NCI−H810
AZD6244:NCI−N87
AZD6244:OVCAR−3
AZD6244:NUGC−3
AZD6244:OC−314
AZD6244:OE33
AZD6244:ONS−76
AZD6244:OPM−2
AZD6244:OVCAR−4
AZD6244:OVCAR−8
AZD6244:P12−ICHIKAWA
CCLE
GDSC
% Viability
0
50
100
Concentration (uM)
0.01
0.1
1
10

AZD6244:P31−FUJ

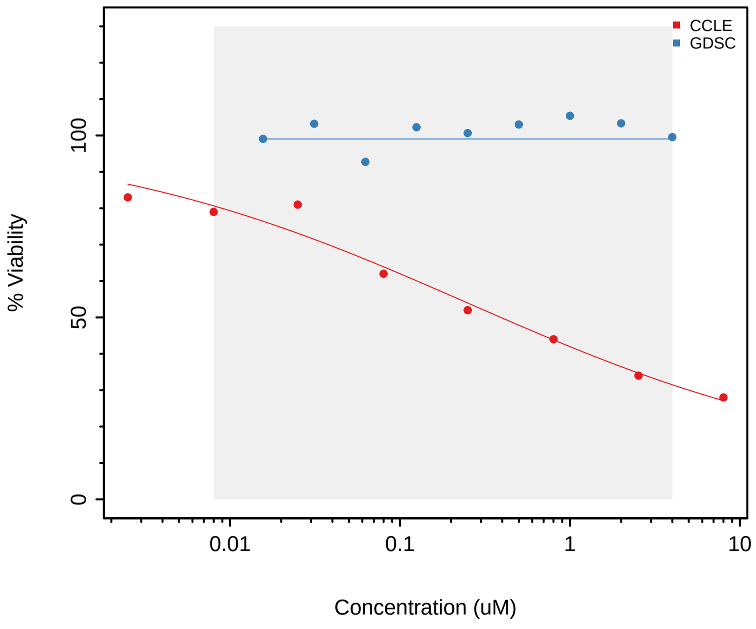

AZD6244:Panc 03.27

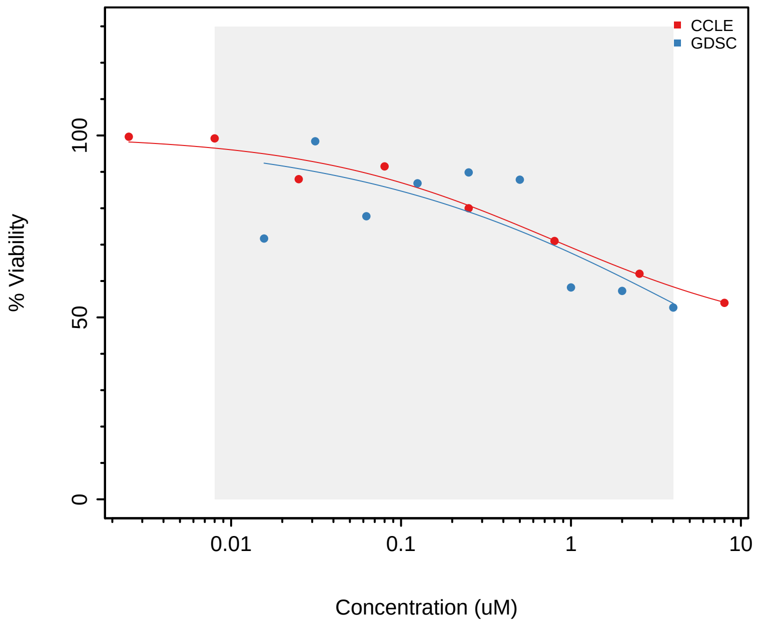

AZD6244:Panc 10.05

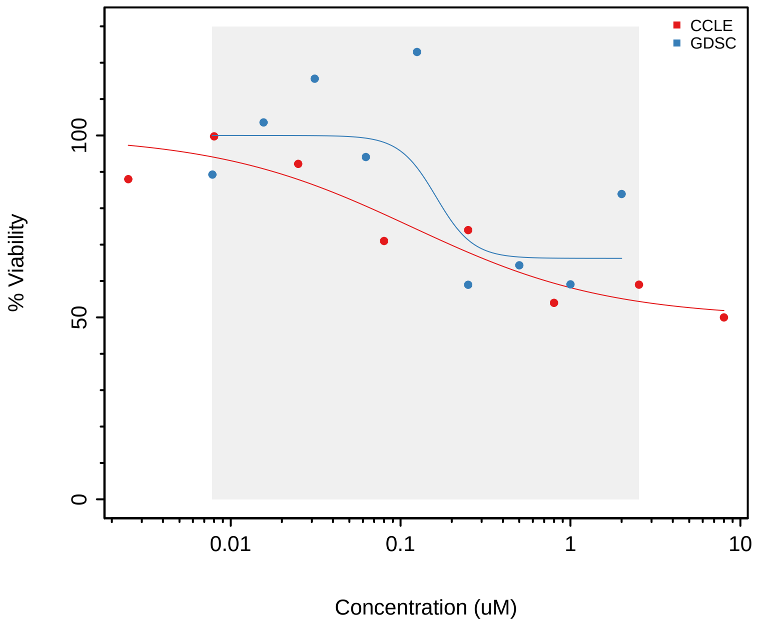

AZD6244:PC−14

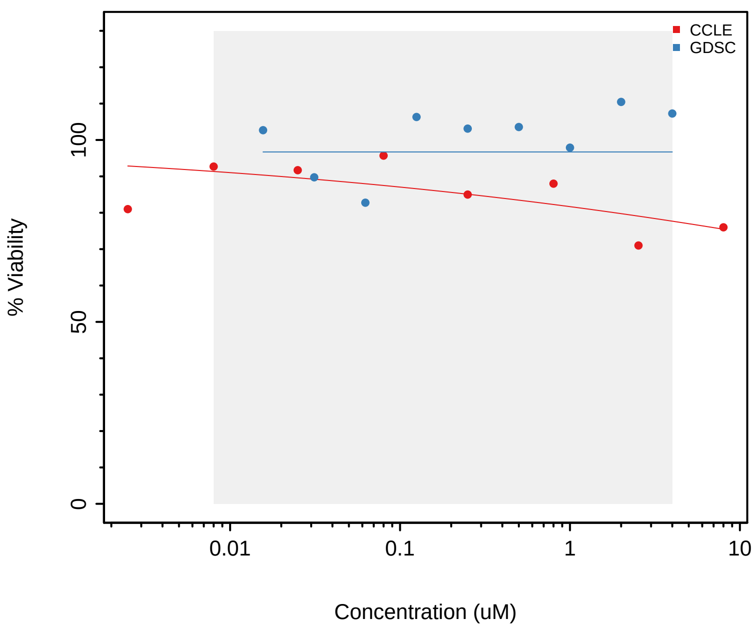

AZD6244:PSN1

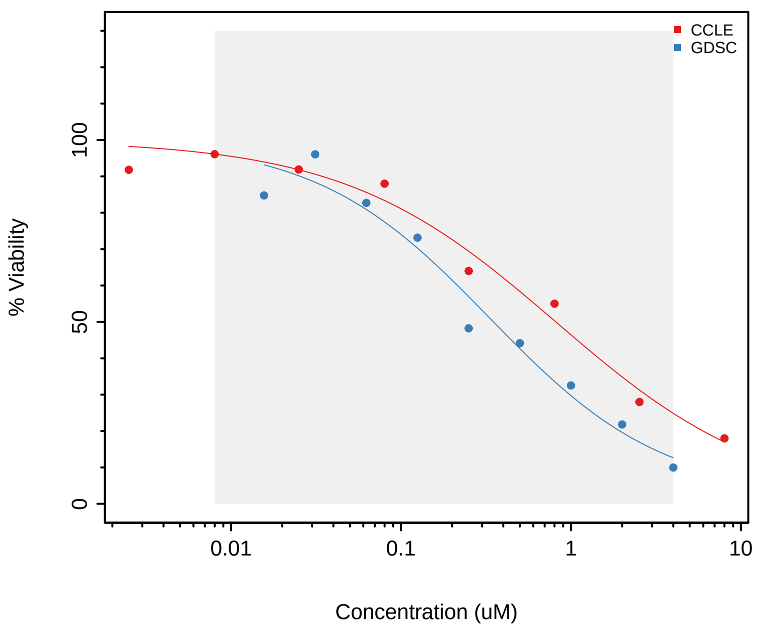

AZD6244:Raji

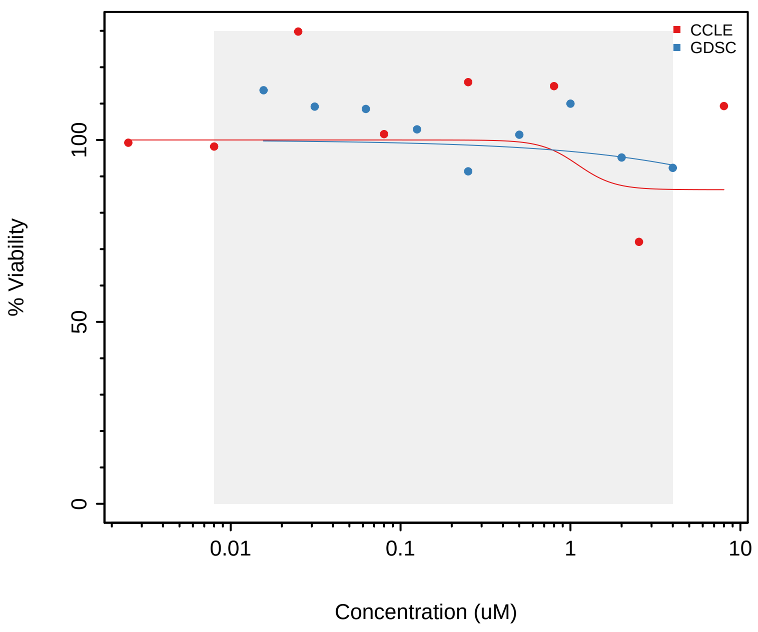

AZD6244:REH

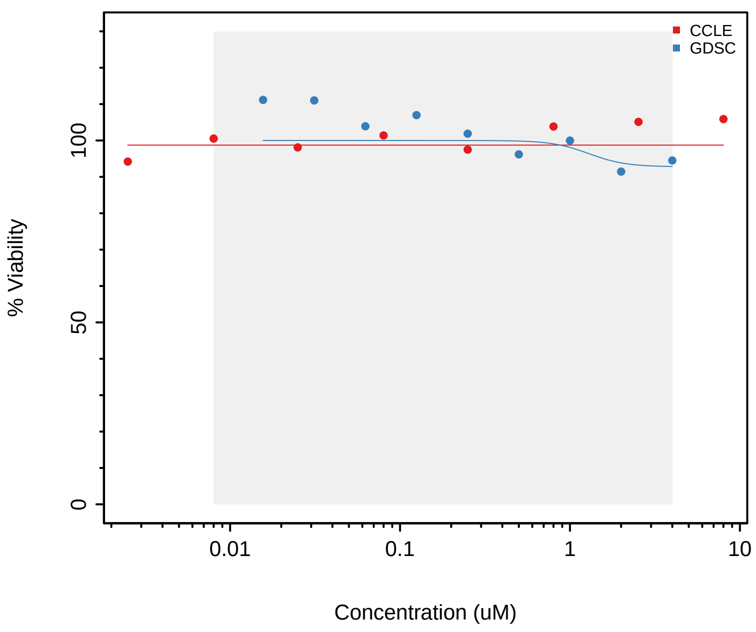

AZD6244:RKO

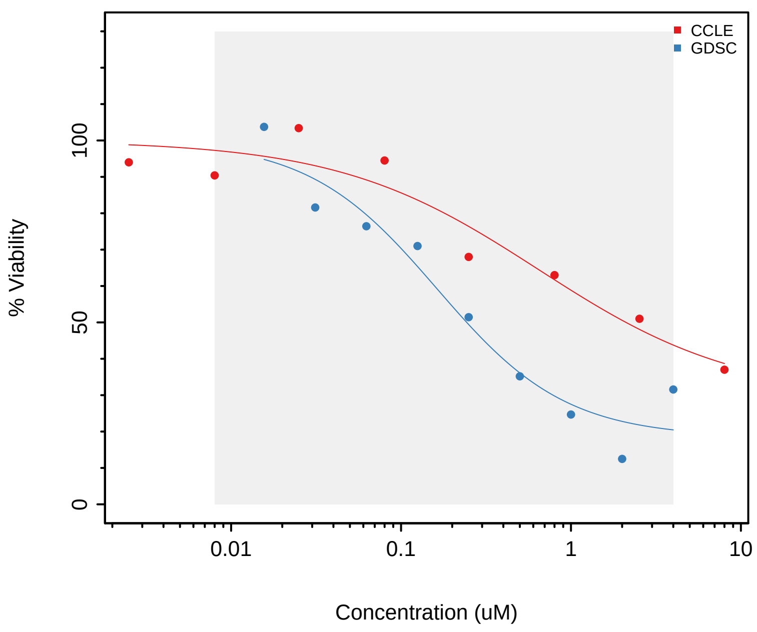

AZD6244:RL95−2

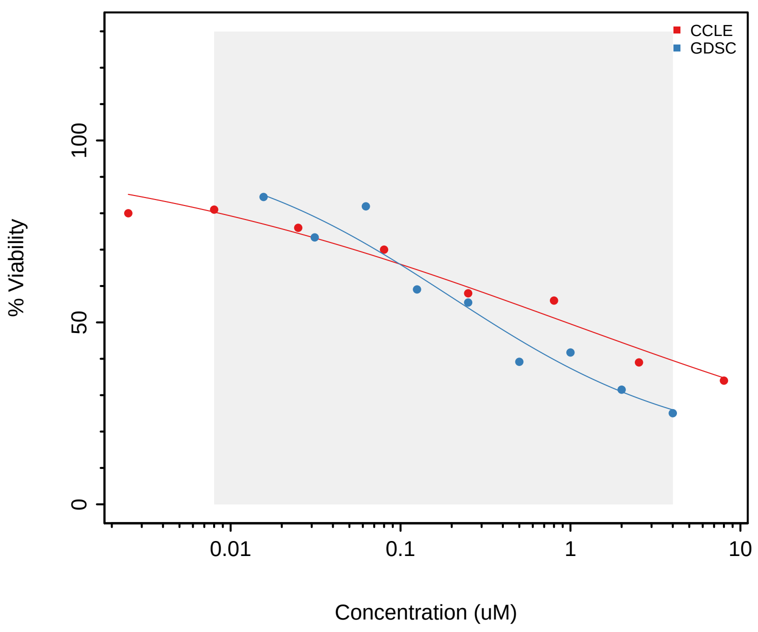

AZD6244:RPMI−7951

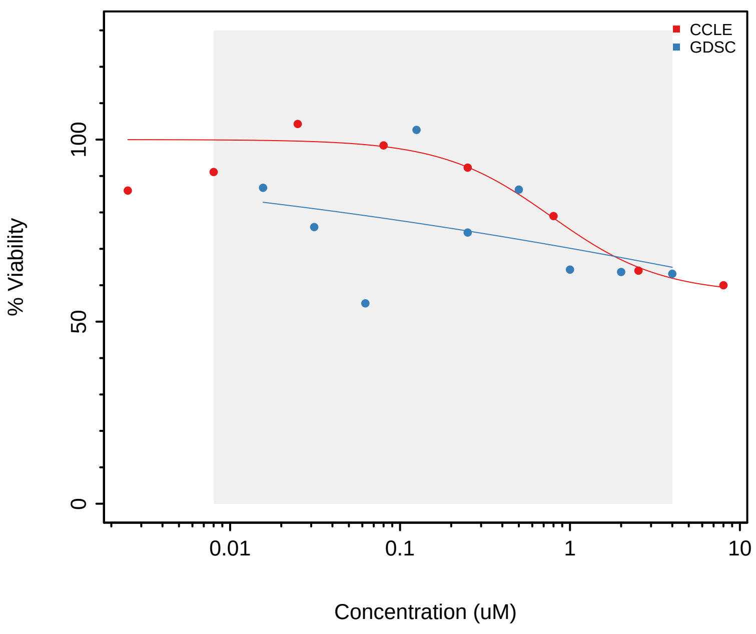

AZD6244:RPMI−8402

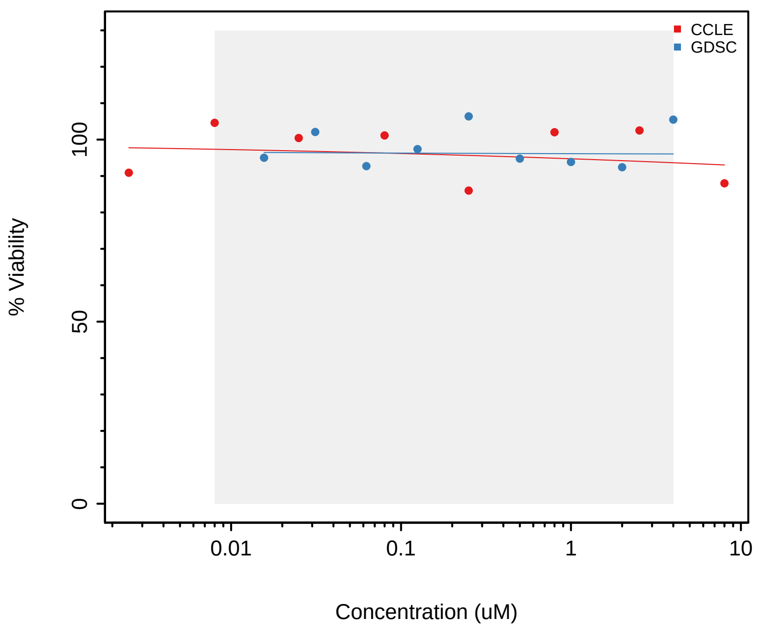

AZD6244:RT−112

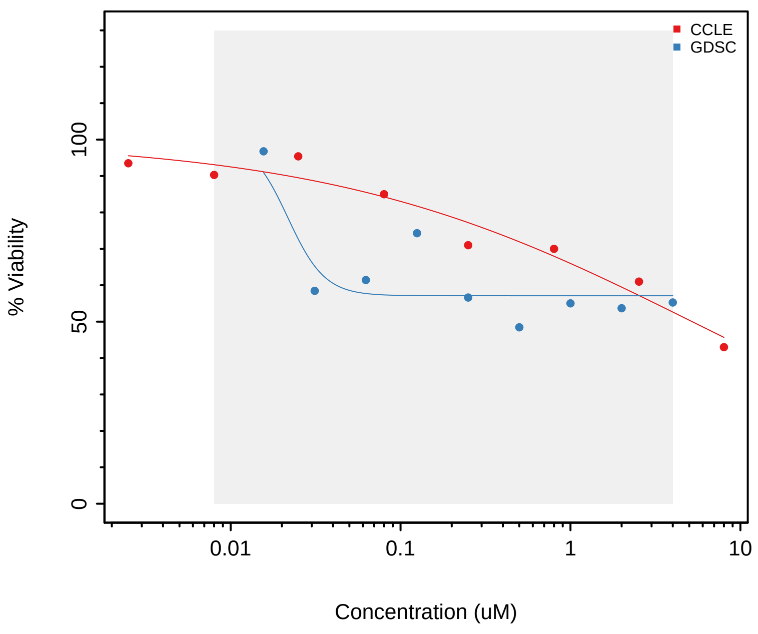

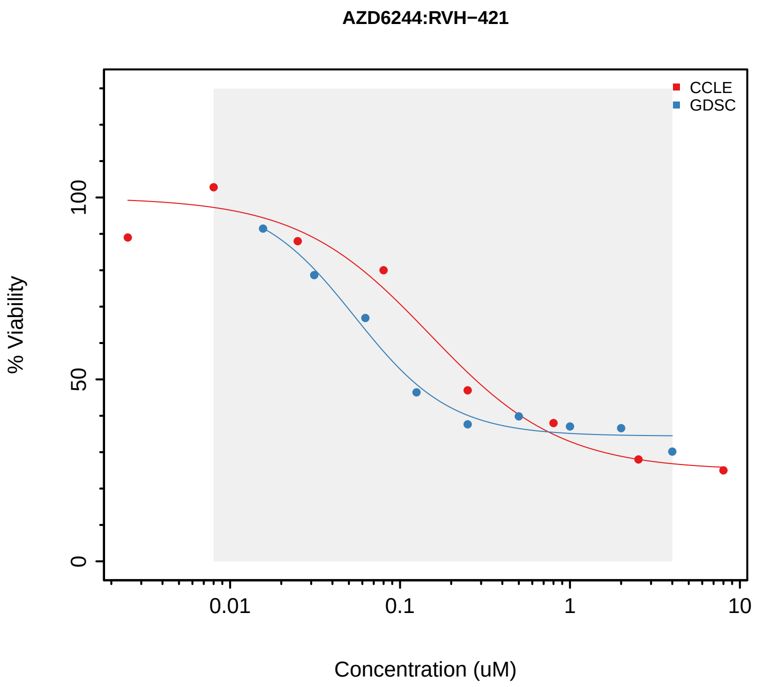
AZD6244:RVH−421
CCLE
GDSC
% Viability
Concentration (uM)

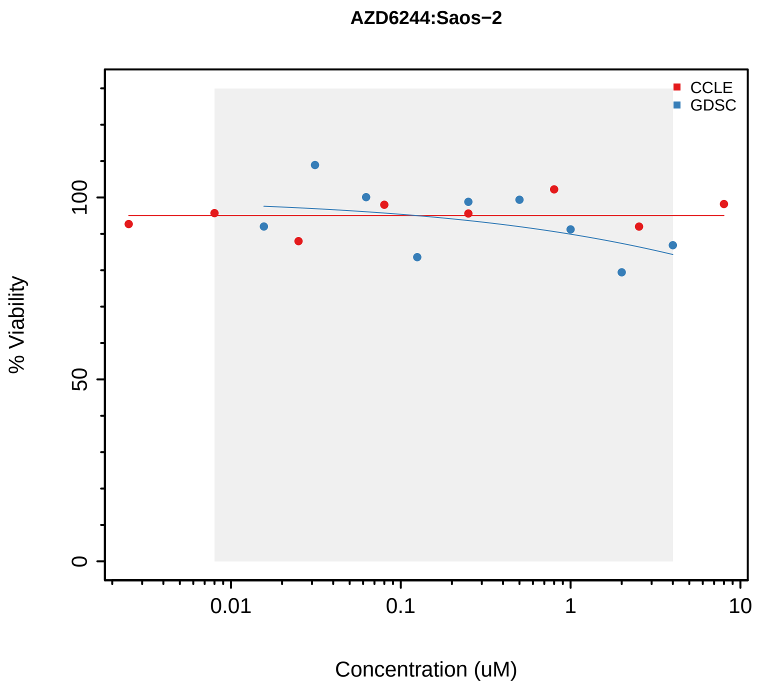
AZD6244:Saos−2
CCLE
GDSC
% Viability
Concentration (uM)

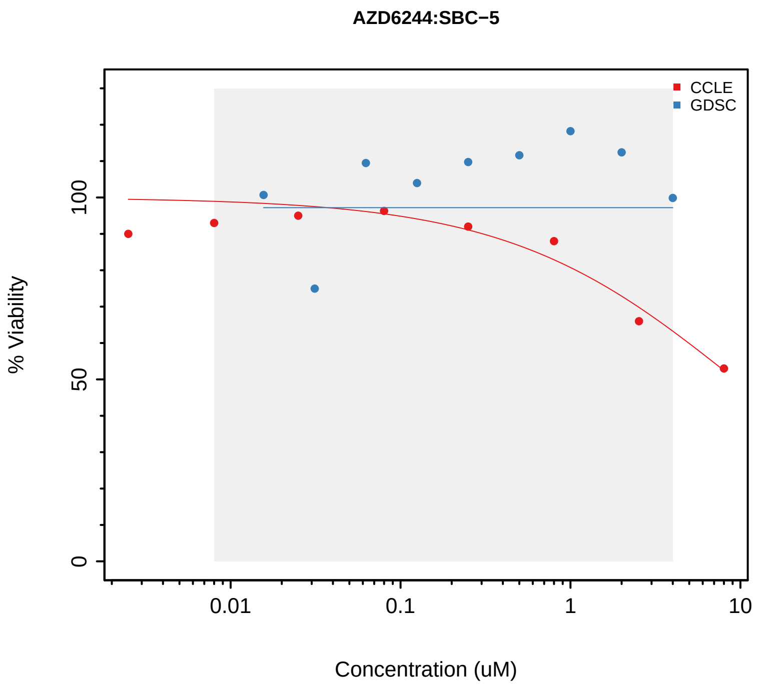
AZD6244:SBC−5
CCLE
GDSC
% Viability
Concentration (uM)

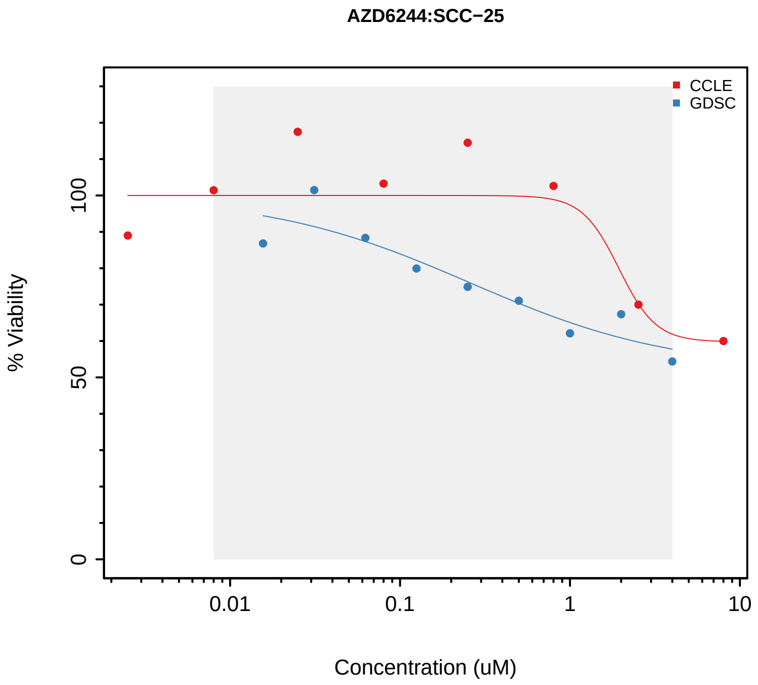
AZD6244:SCC−25
CCLE
GDSC
% Viability
Concentration (uM)

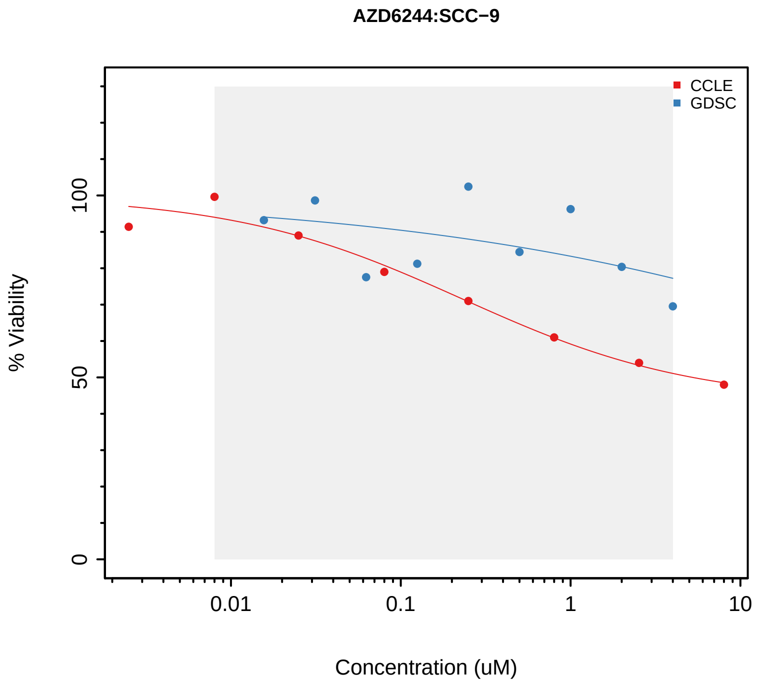
AZD6244:SCC−9
CCLE
GDSC
% Viability
Concentration (uM)

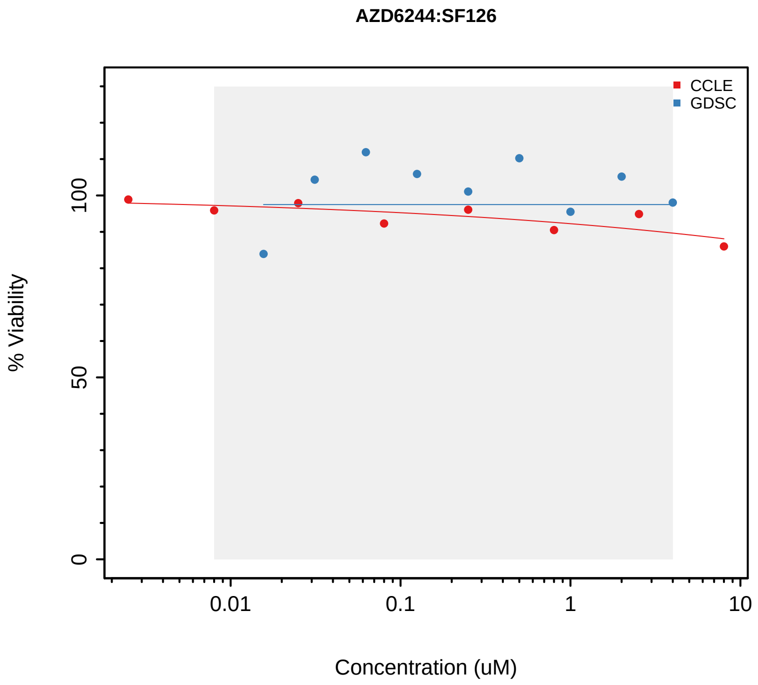
AZD6244:SF126
CCLE
GDSC
% Viability
Concentration (uM)

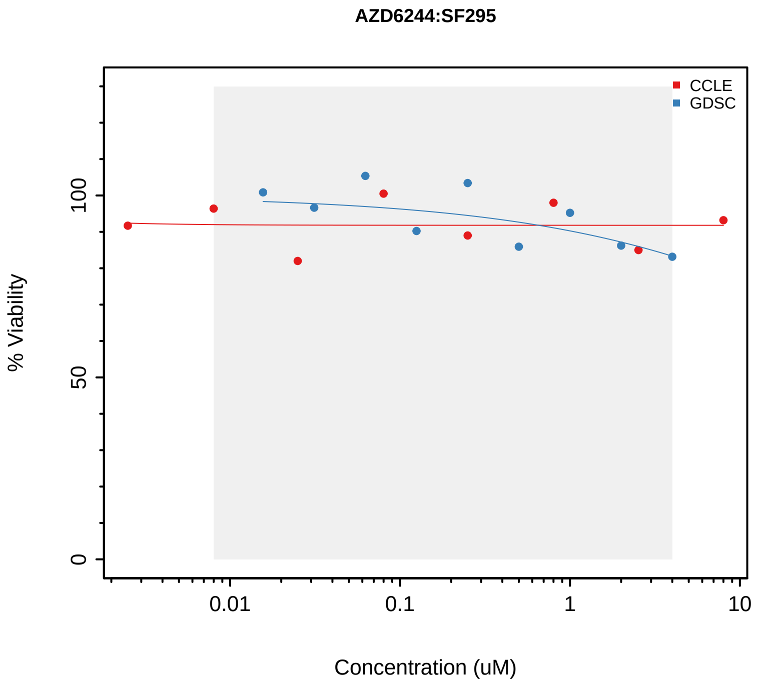
AZD6244:SF295
CCLE
GDSC
% Viability
Concentration (uM)

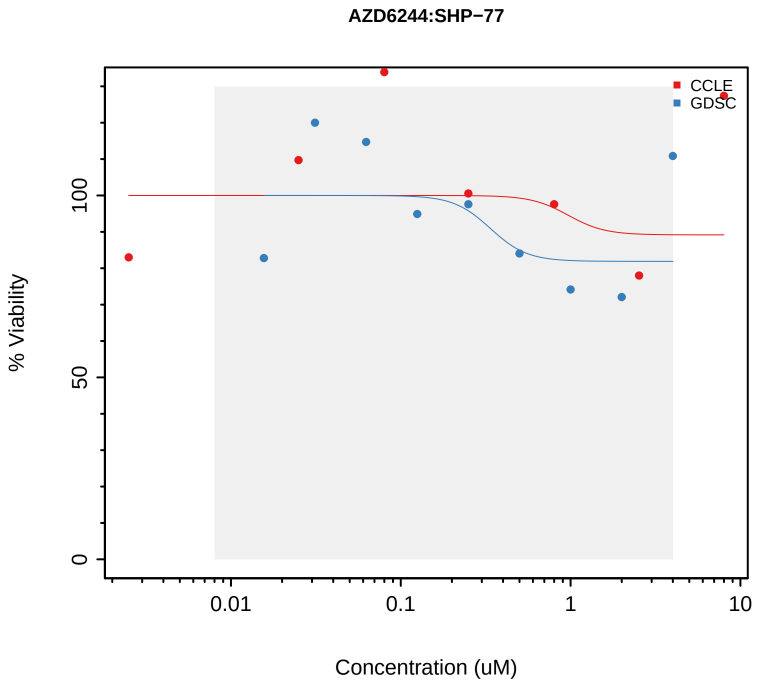
AZD6244:SHP−77
CCLE
GDSC
% Viability
Concentration (uM)

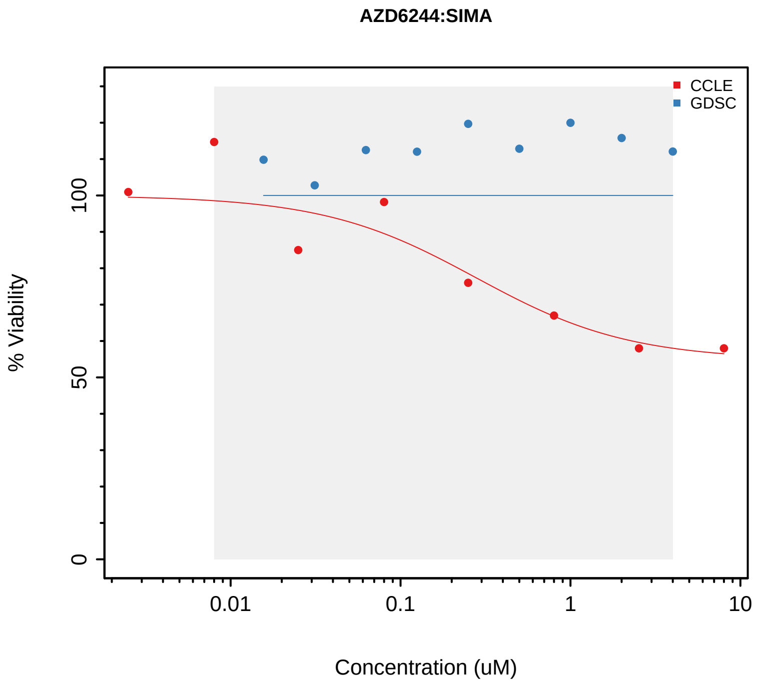
AZD6244:SIMA
CCLE
GDSC
% Viability
Concentration (uM)

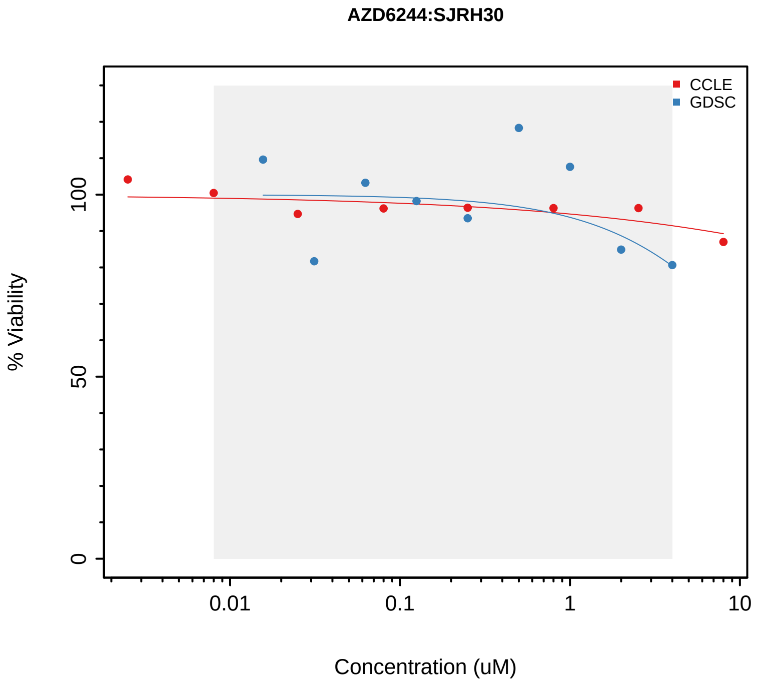
AZD6244:SJRH30
CCLE
GDSC
% Viability
Concentration (uM)

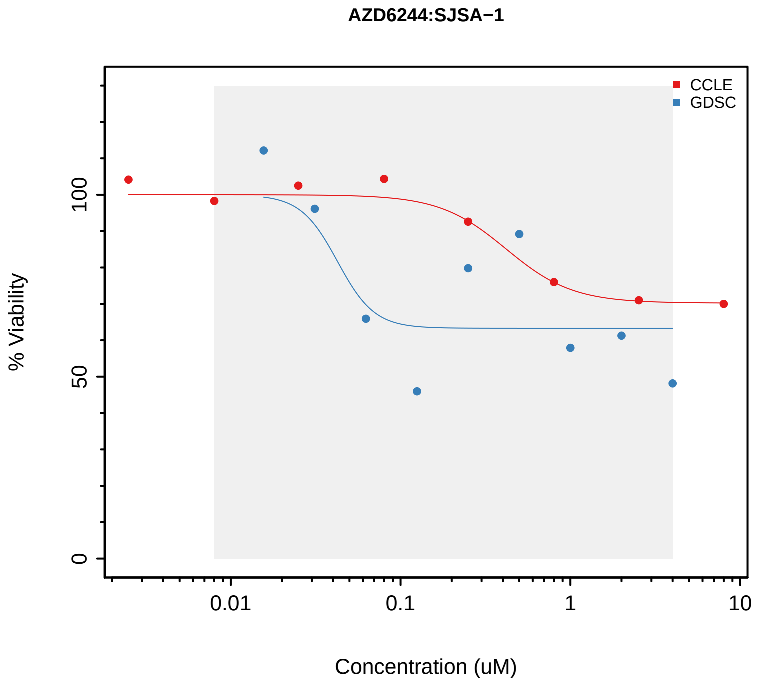
AZD6244:SJSA−1
CCLE
GDSC
% Viability
Concentration (uM)

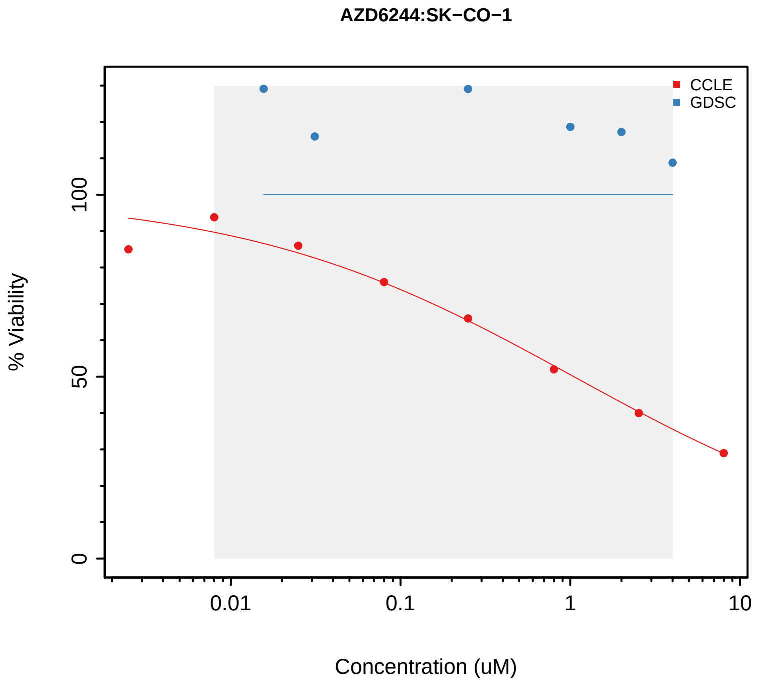
AZD6244:SK−CO−1
CCLE
GDSC
% Viability
Concentration (uM)

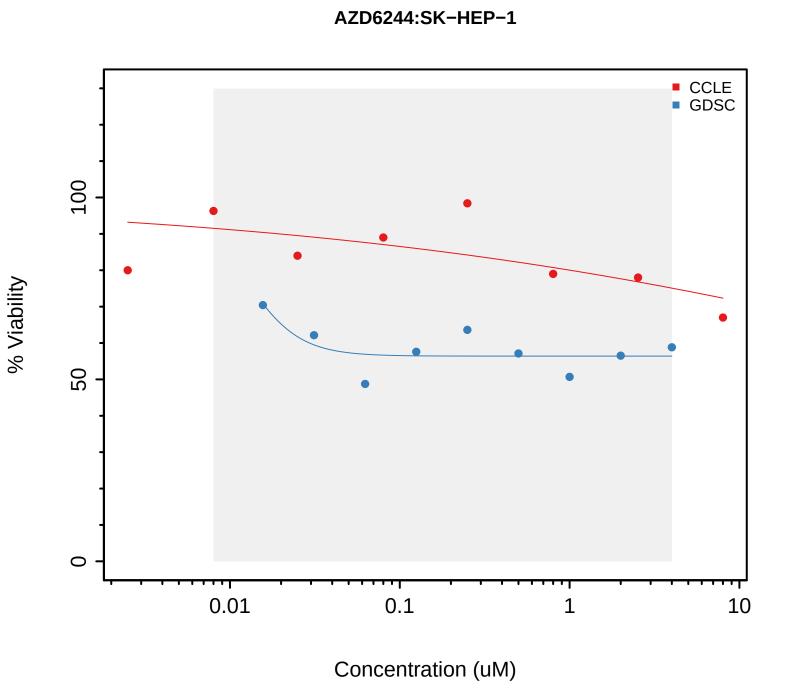
AZD6244:SK−HEP−1
CCLE
GDSC
% Viability
0 50 100
0.01 0.1 1 10
Concentration (uM)

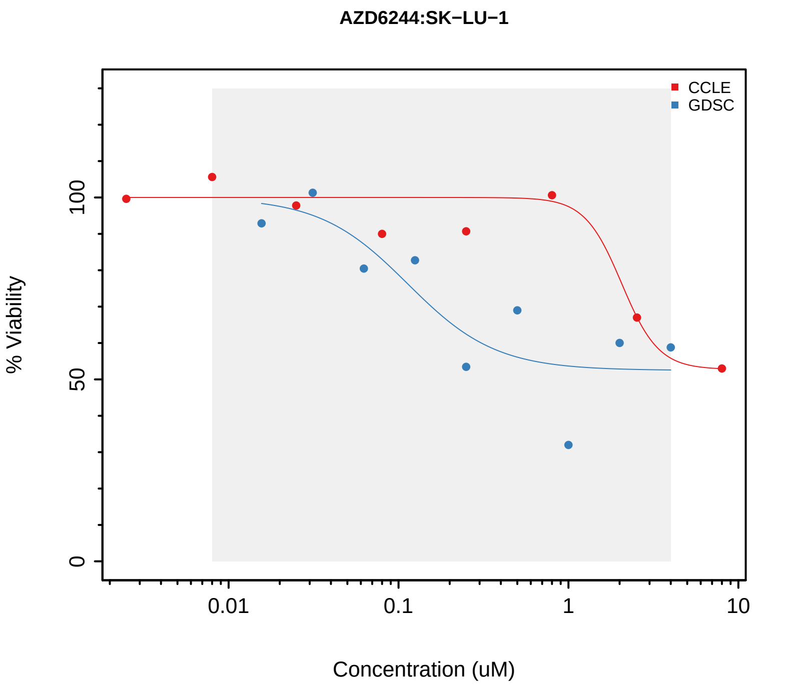
AZD6244:SK−LU−1
CCLE
GDSC
% Viability
0 50 100
0.01 0.1 1 10
Concentration (uM)

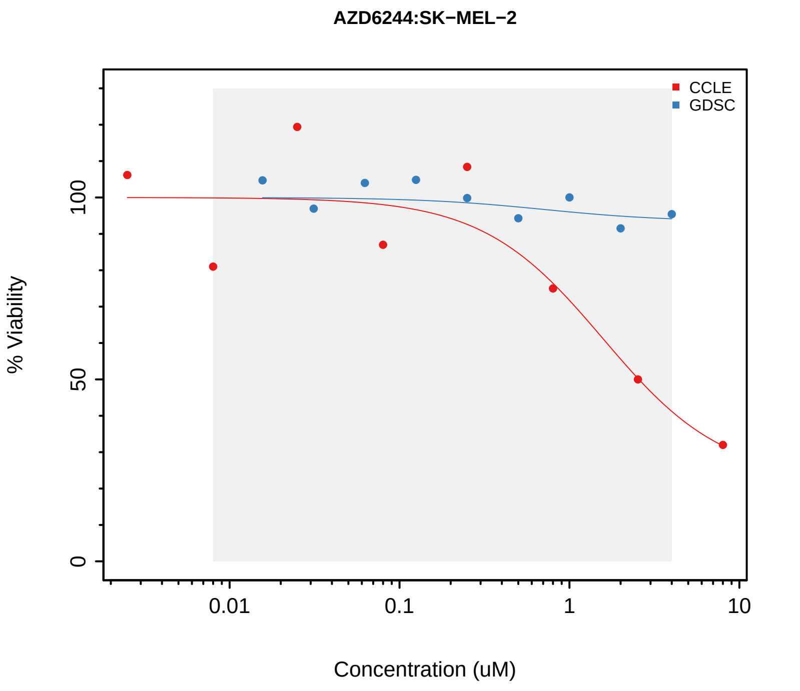
AZD6244:SK−MEL−2
CCLE
GDSC
% Viability
0 50 100
0.01 0.1 1 10
Concentration (uM)

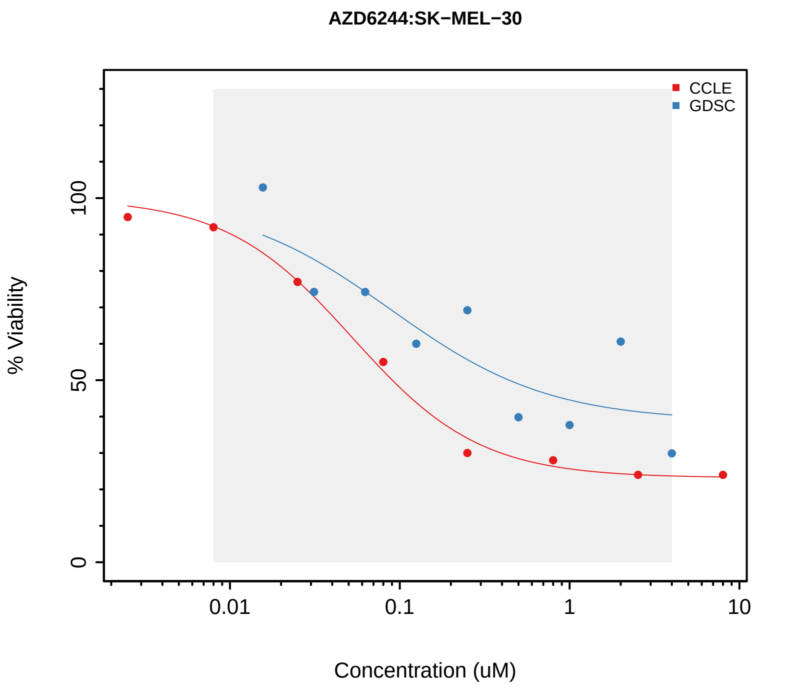
AZD6244:SK−MEL−30
CCLE
GDSC
% Viability
0 50 100
0.01 0.1 1 10
Concentration (uM)

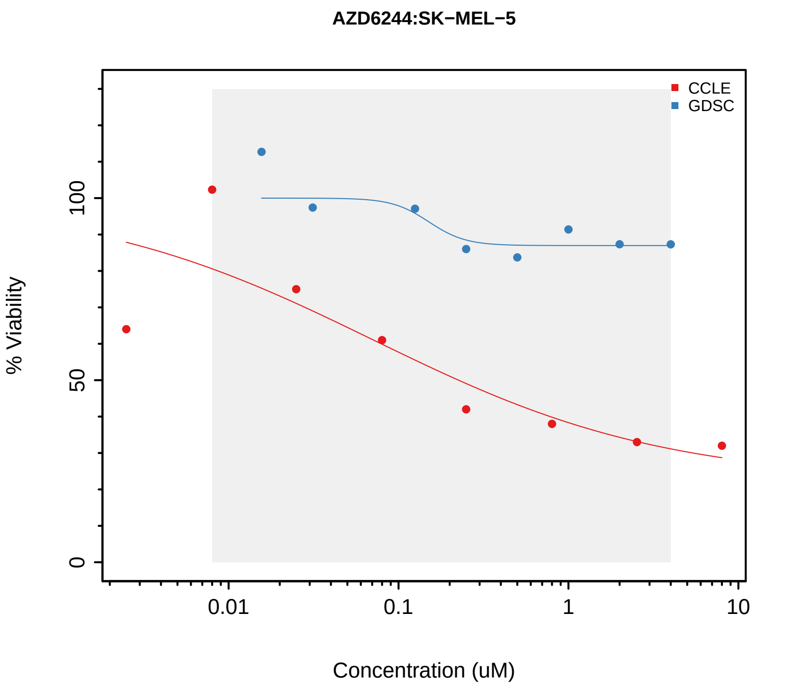
AZD6244:SK−MEL−5
CCLE
GDSC
% Viability
0 50 100
0.01 0.1 1 10
Concentration (uM)

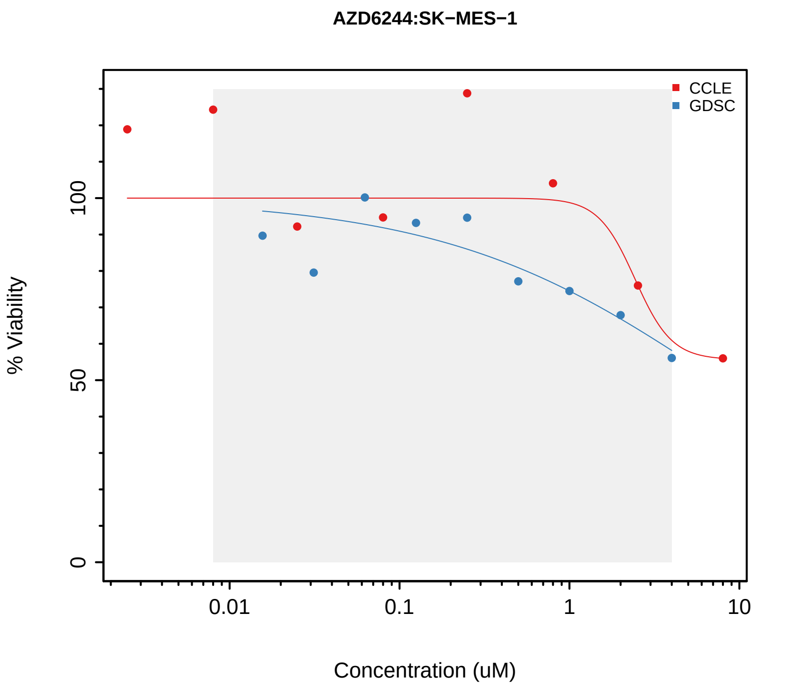
AZD6244:SK−MES−1
CCLE
GDSC
% Viability
0 50 100
0.01 0.1 1 10
Concentration (uM)

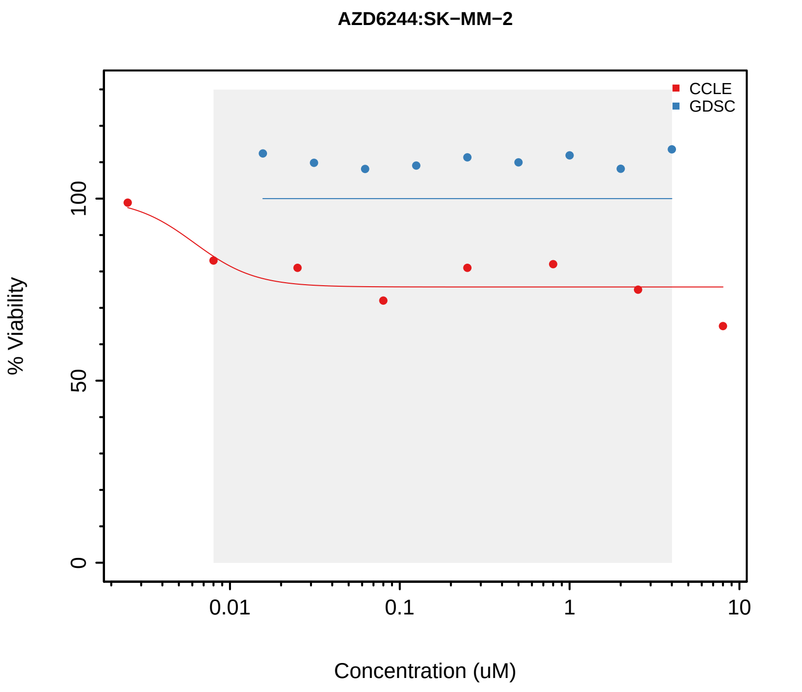
AZD6244:SK−MM−2
CCLE
GDSC
% Viability
0 50 100
0.01 0.1 1 10
Concentration (uM)

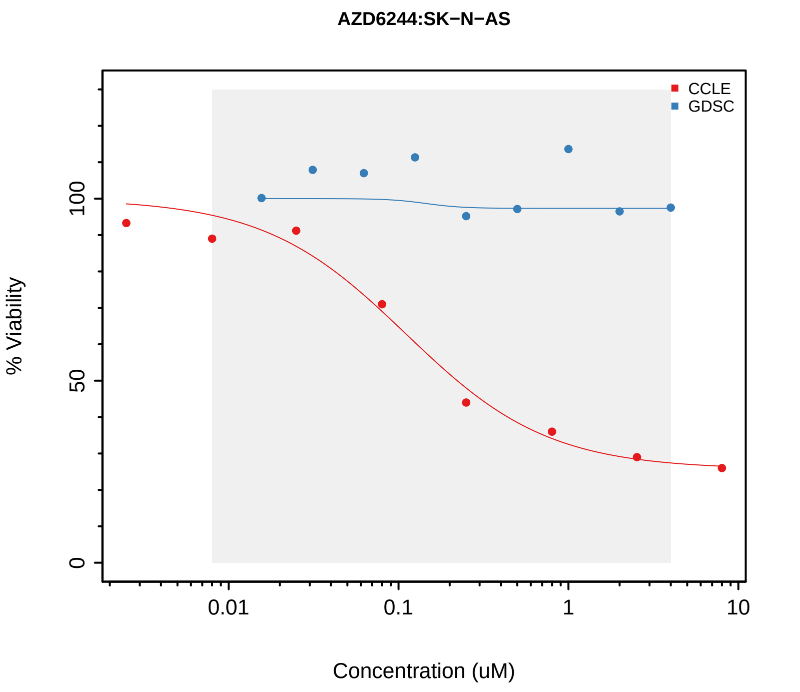
AZD6244:SK−N−AS
CCLE
GDSC
% Viability
0 50 100
0.01 0.1 1 10
Concentration (uM)

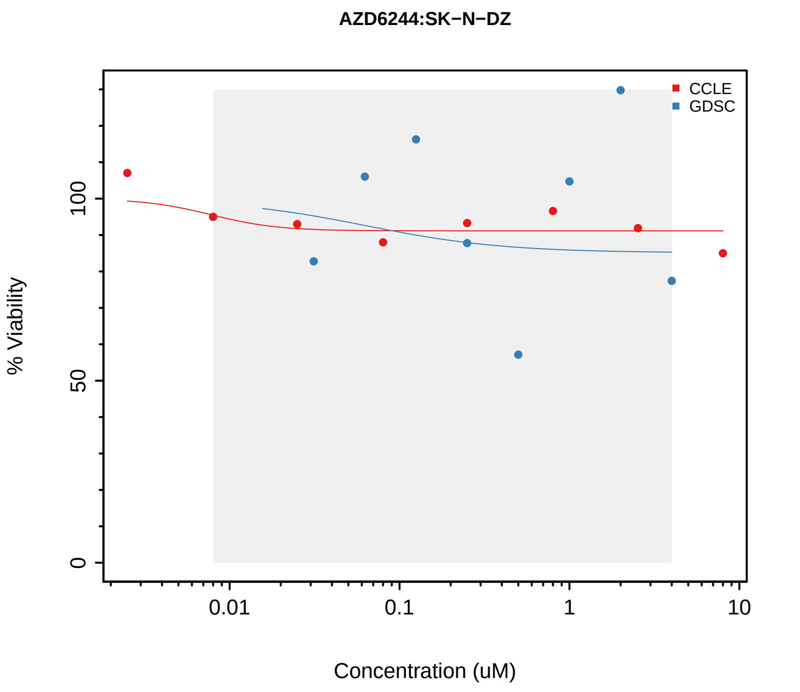
AZD6244:SK−N−DZ
CCLE
GDSC
% Viability
0 50 100
0.01 0.1 1 10
Concentration (uM)

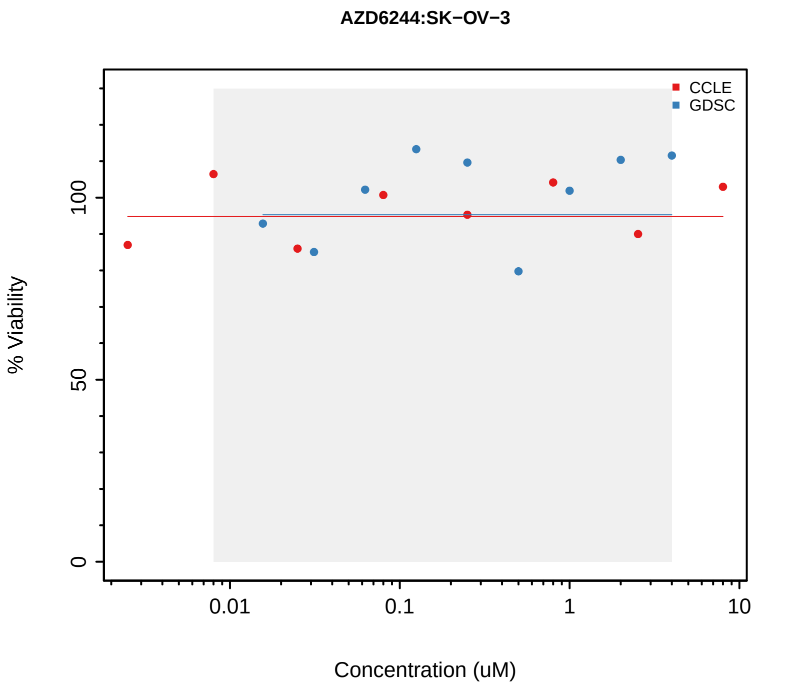
AZD6244:SK−OV−3
CCLE
GDSC
% Viability
0 50 100
0.01 0.1 1 10
Concentration (uM)

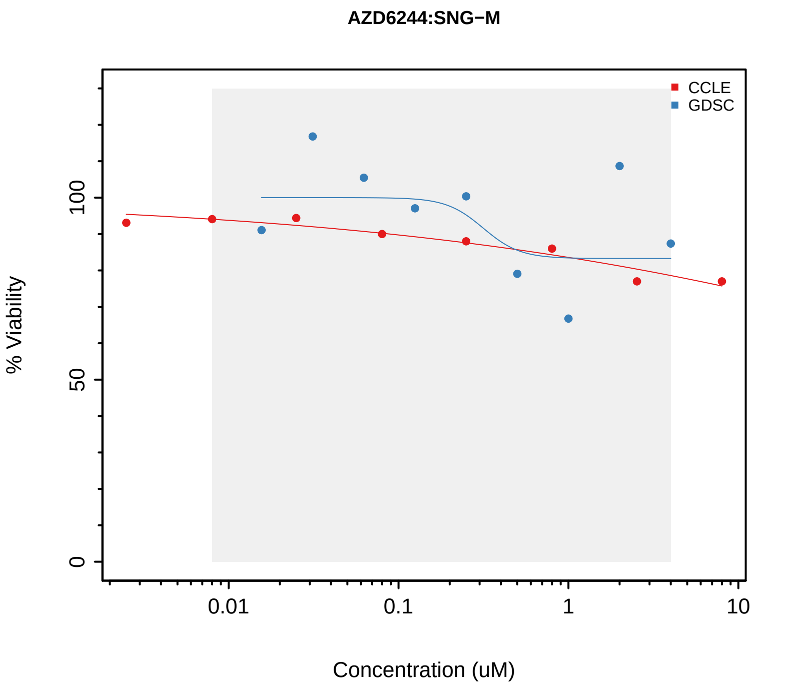
AZD6244:SNG−M
CCLE
GDSC
% Viability
0 50 100
0.01 0.1 1 10
Concentration (uM)

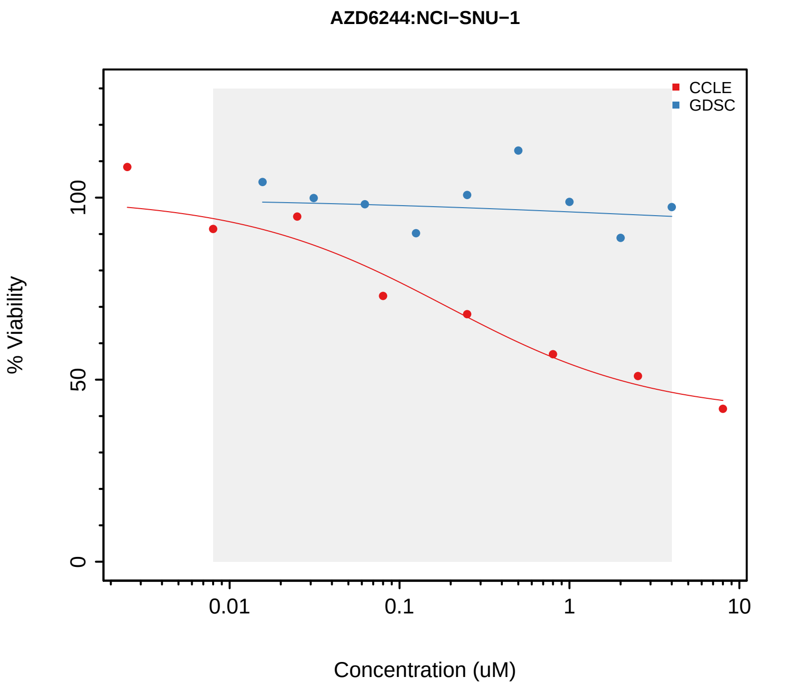
AZD6244:NCI−SNU−1
CCLE
GDSC
% Viability
0 50 100
0.01 0.1 1 10
Concentration (uM)

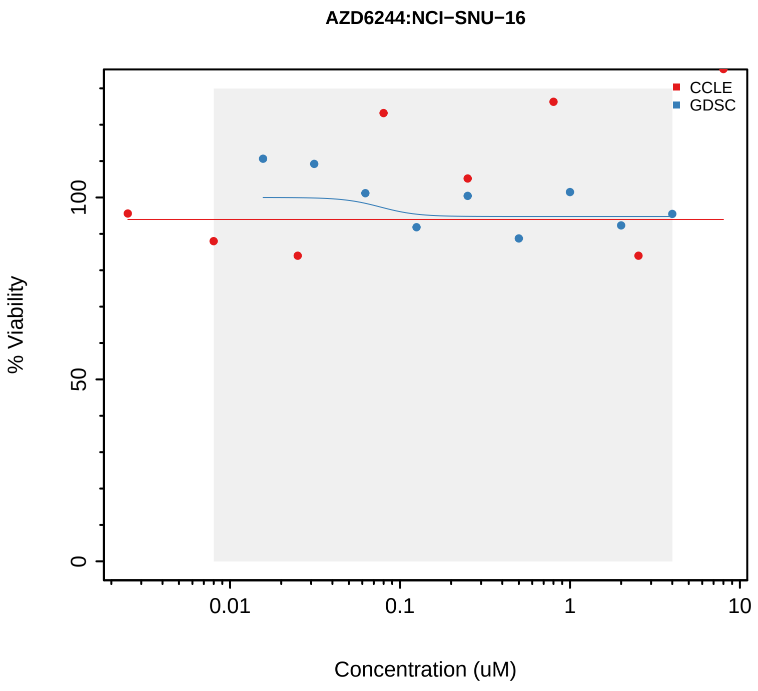
AZD6244:NCI−SNU−16
CCLE
GDSC
% Viability
0
50
100
0.01
0.1
1
10
Concentration (uM)

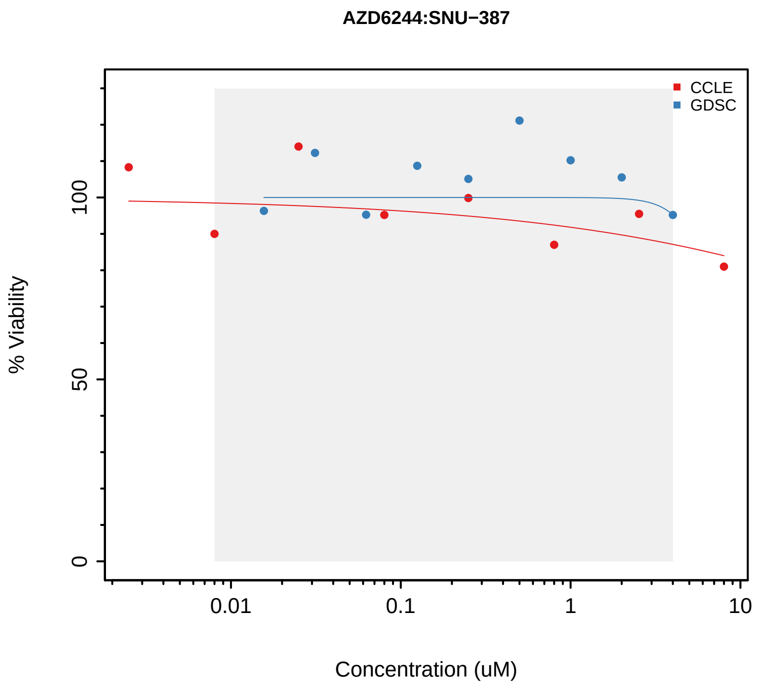
AZD6244:SNU−387
CCLE
GDSC
% Viability
0
50
100
0.01
0.1
1
10
Concentration (uM)

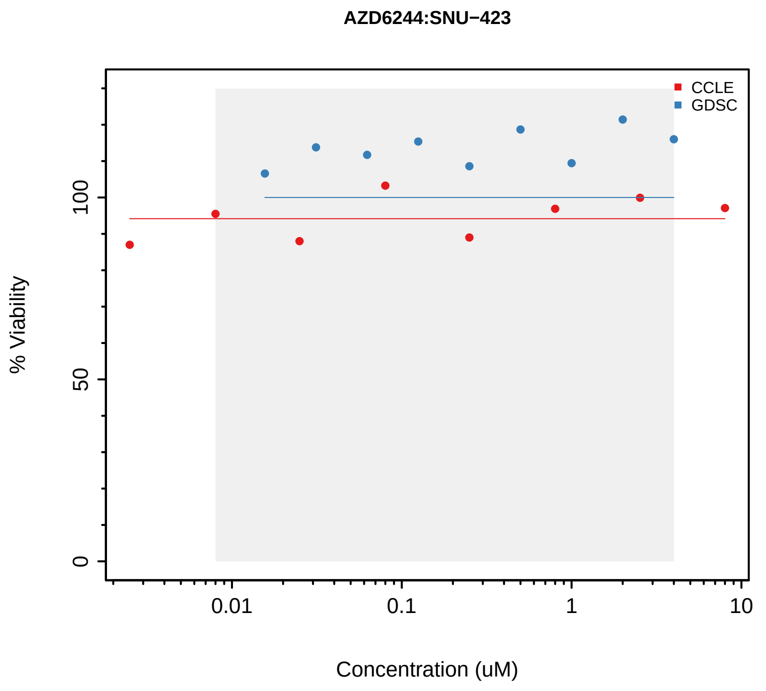
AZD6244:SNU−423
CCLE
GDSC
% Viability
0
50
100
0.01
0.1
1
10
Concentration (uM)

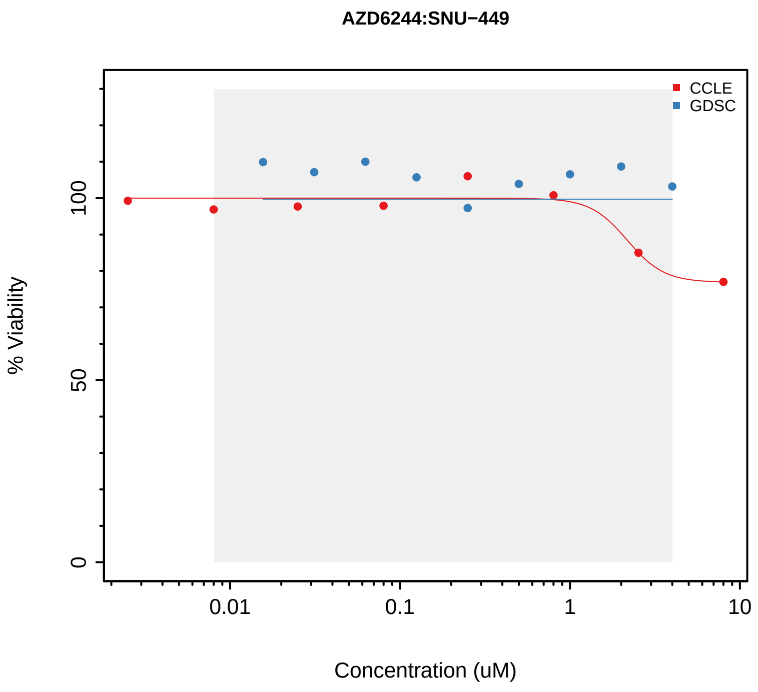
AZD6244:SNU−449
CCLE
GDSC
% Viability
0
50
100
0.01
0.1
1
10
Concentration (uM)

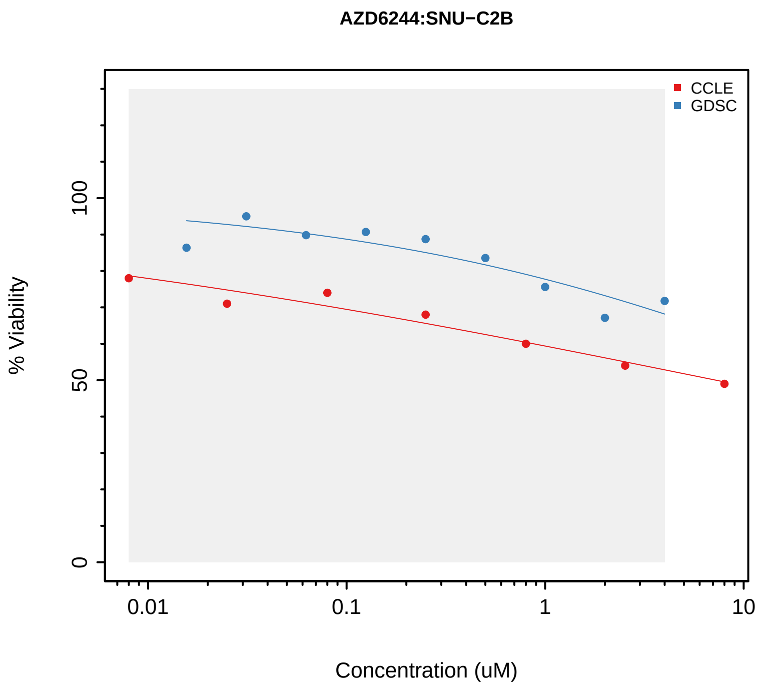
AZD6244:SNU−C2B
CCLE
GDSC
% Viability
0
50
100
0.01
0.1
1
10
Concentration (uM)

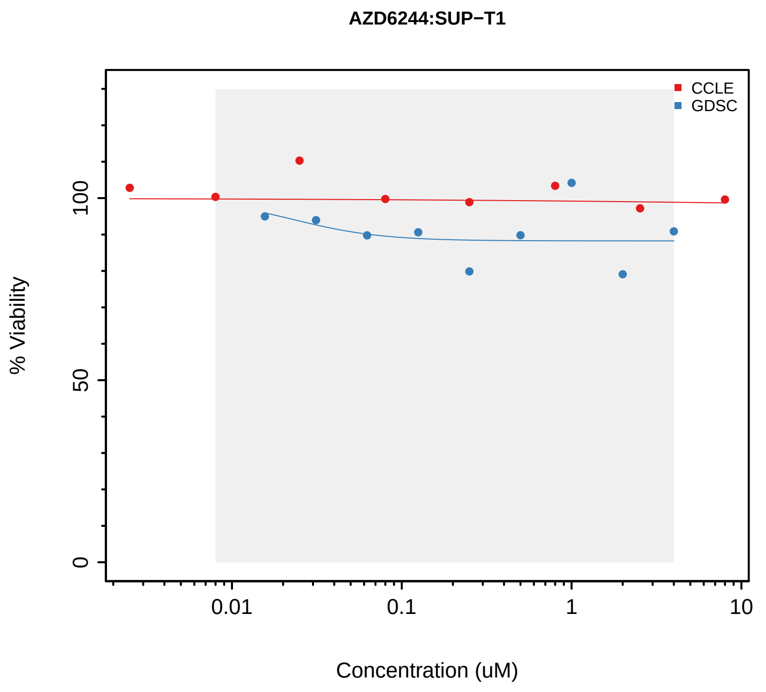
AZD6244:SUP−T1
CCLE
GDSC
% Viability
0
50
100
0.01
0.1
1
10
Concentration (uM)

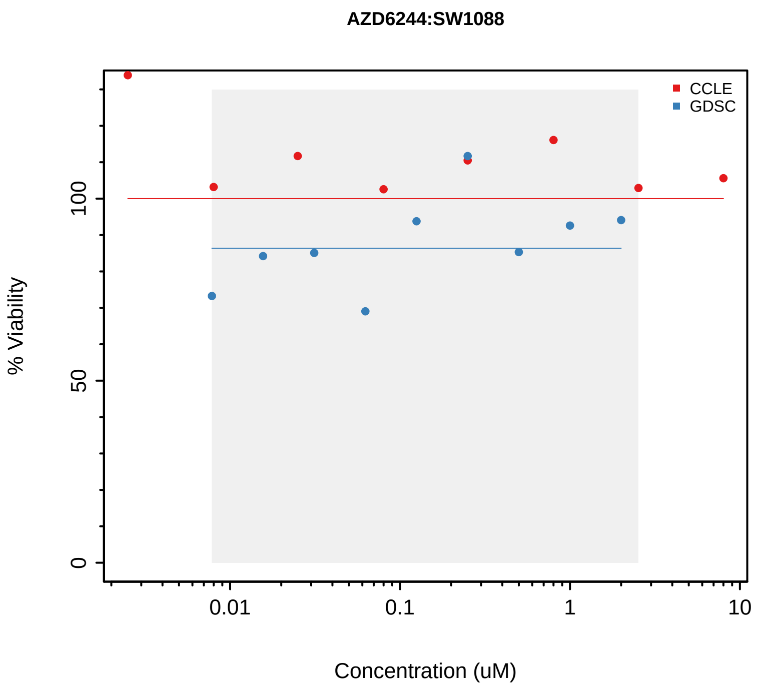
AZD6244:SW1088
CCLE
GDSC
% Viability
0
50
100
0.01
0.1
1
10
Concentration (uM)

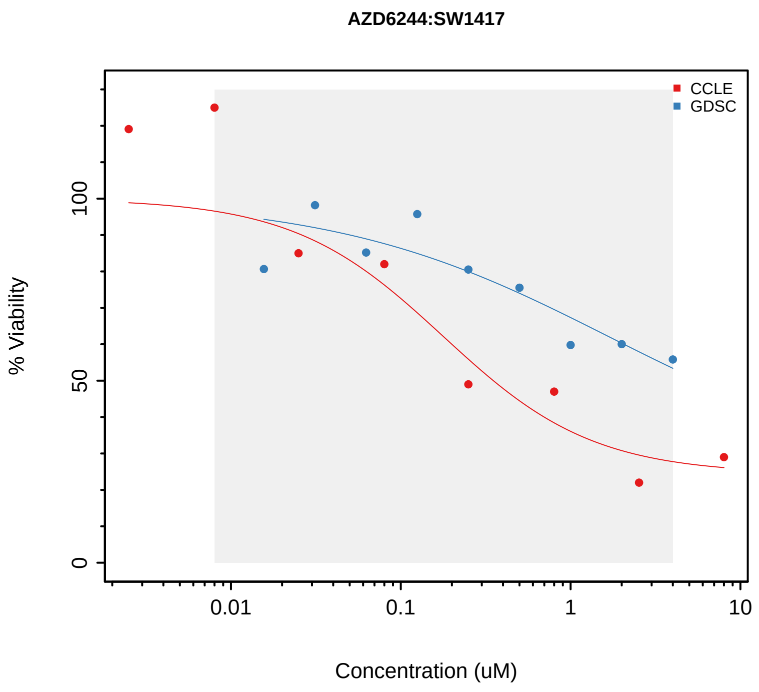
AZD6244:SW1417
CCLE
GDSC
% Viability
0
50
100
0.01
0.1
1
10
Concentration (uM)

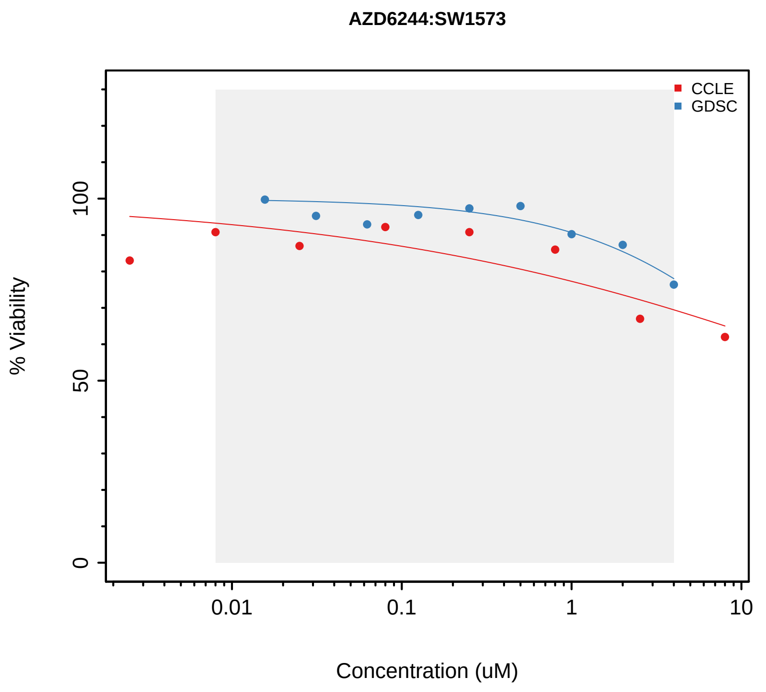
AZD6244:SW1573
CCLE
GDSC
% Viability
0
50
100
0.01
0.1
1
10
Concentration (uM)

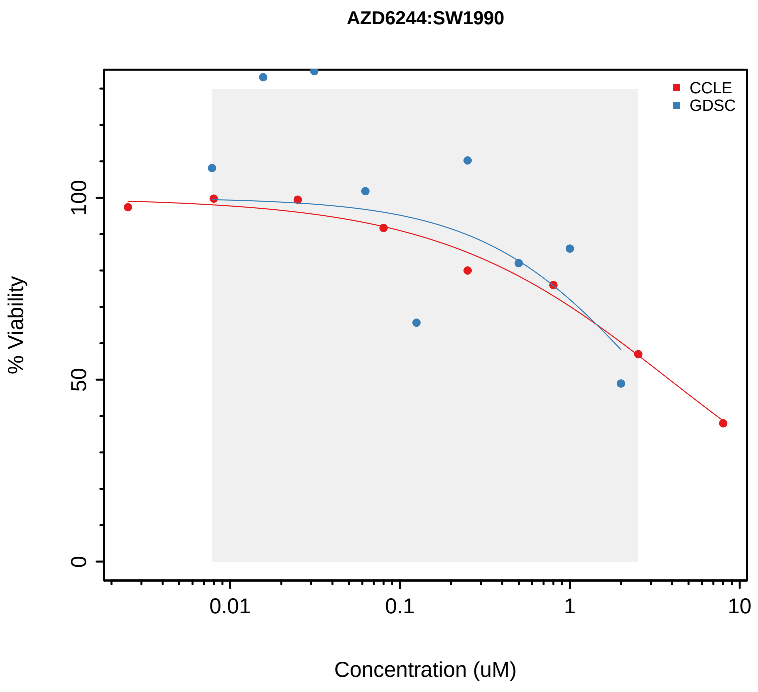
AZD6244:SW1990
CCLE
GDSC
% Viability
0
50
100
0.01
0.1
1
10
Concentration (uM)

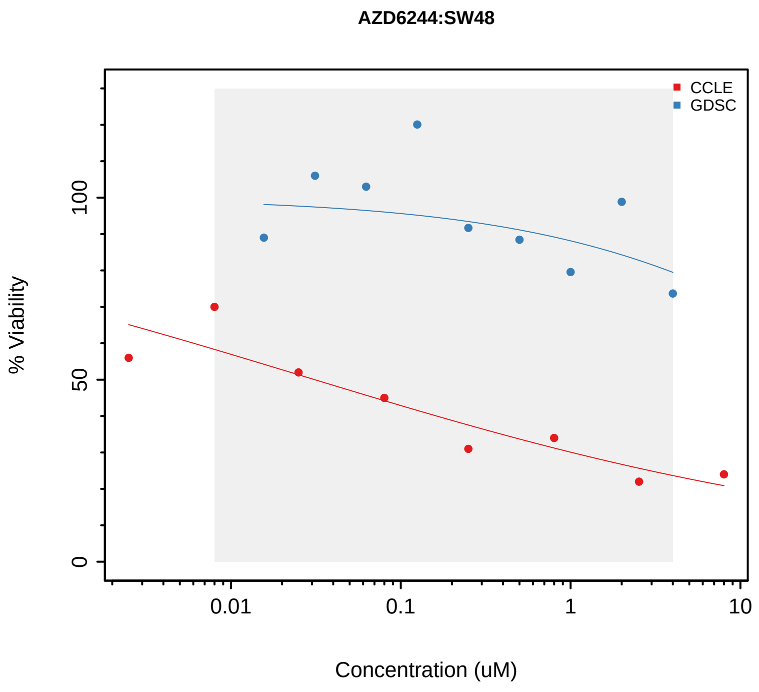
AZD6244:SW48
CCLE
GDSC
% Viability
0
50
100
0.01
0.1
1
10
Concentration (uM)

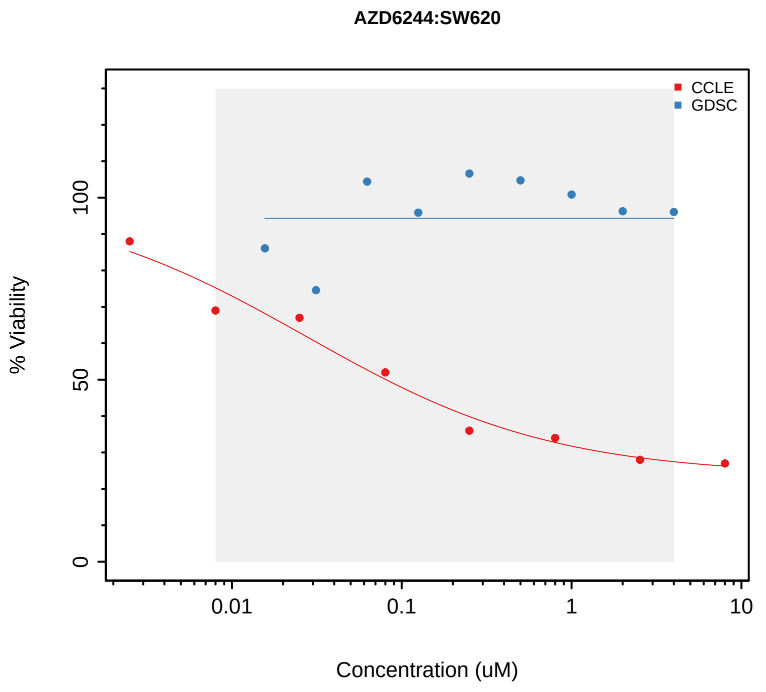
AZD6244:SW620
CCLE
GDSC
% Viability
0
50
100
0.01
0.1
1
10
Concentration (uM)

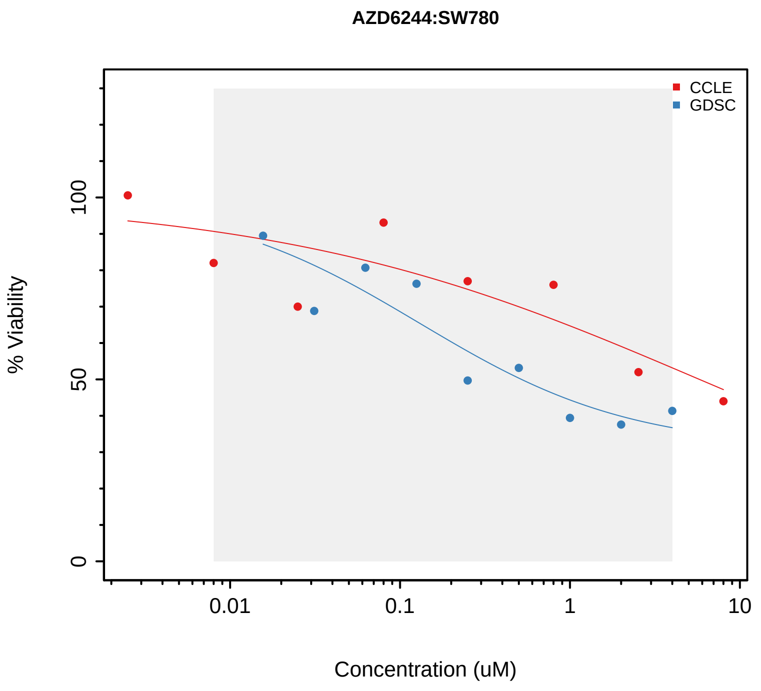
AZD6244:SW780
CCLE
GDSC
% Viability
0
50
100
0.01
0.1
1
10
Concentration (uM)

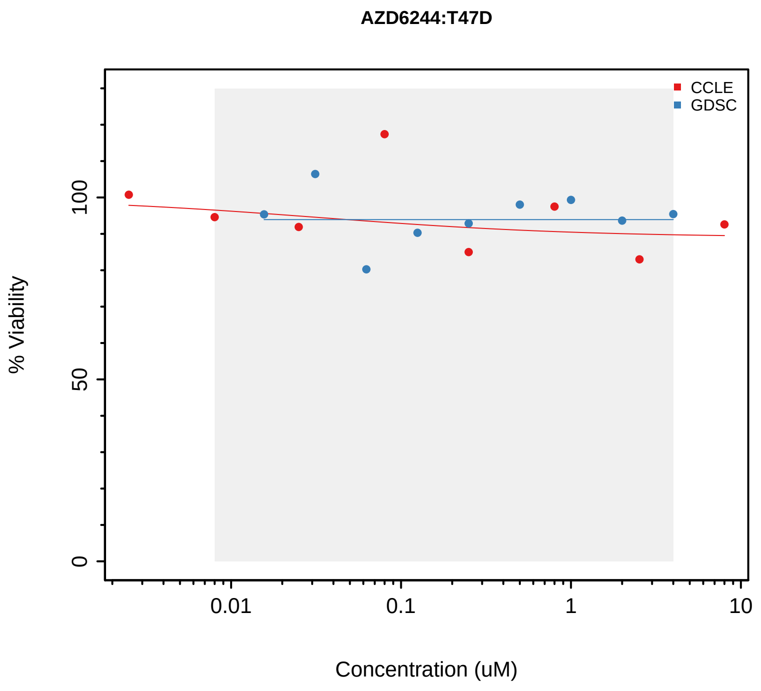
AZD6244:T47D
CCLE
GDSC
% Viability
0
50
100
0.01
0.1
1
10
Concentration (uM)

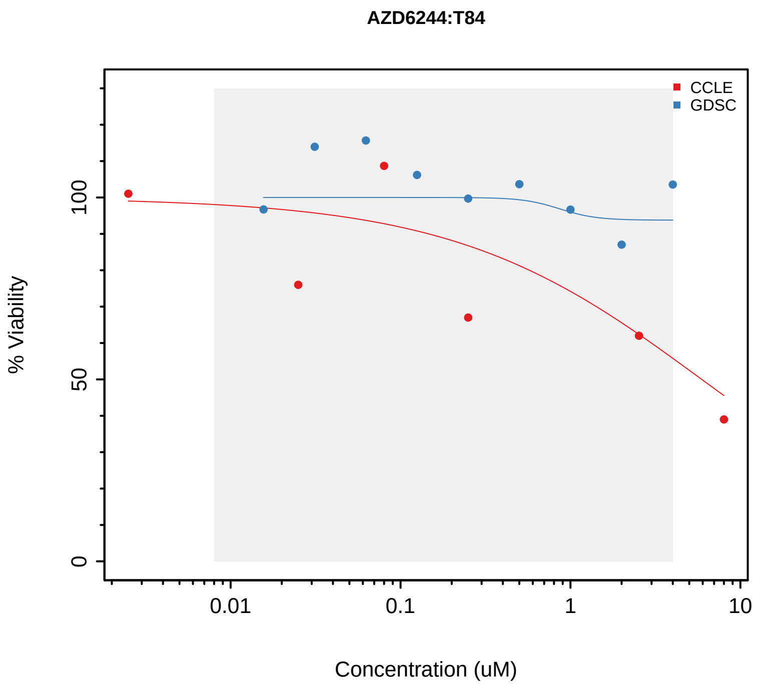
AZD6244:T84
CCLE
GDSC
% Viability
0
50
100
0.01
0.1
1
10
Concentration (uM)

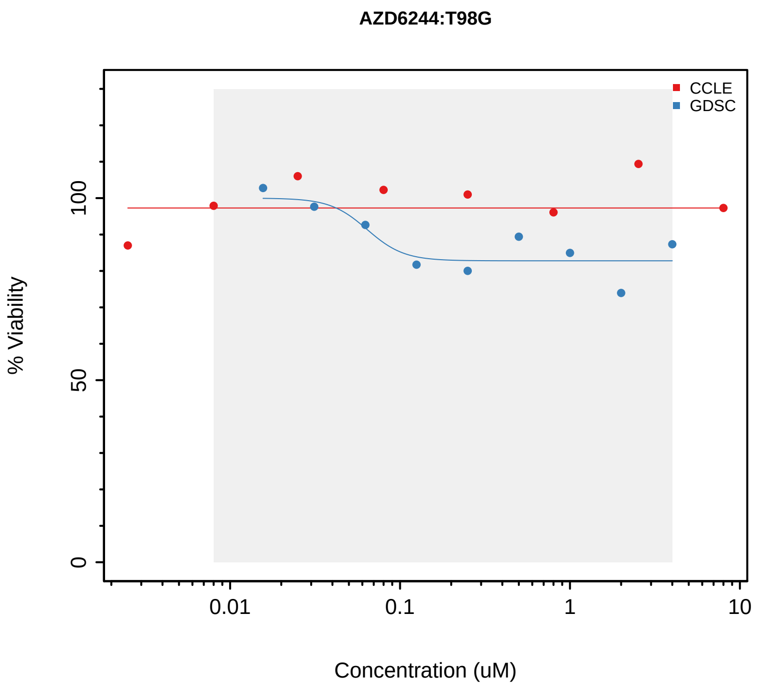
AZD6244:T98G
CCLE
GDSC
% Viability
0
50
100
0.01
0.1
1
10
Concentration (uM)

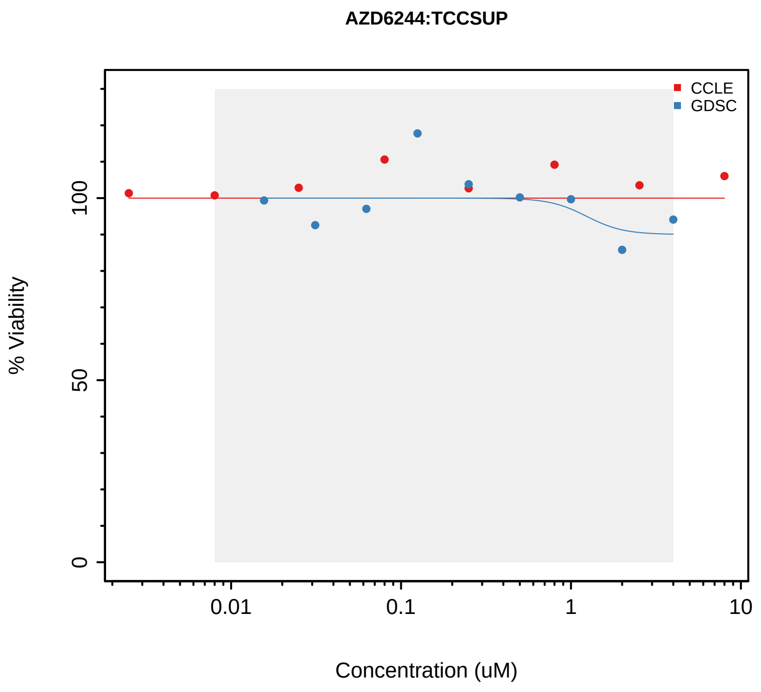
AZD6244:TCCSUP
CCLE
GDSC
% Viability
0
50
100
0.01
0.1
1
10
Concentration (uM)

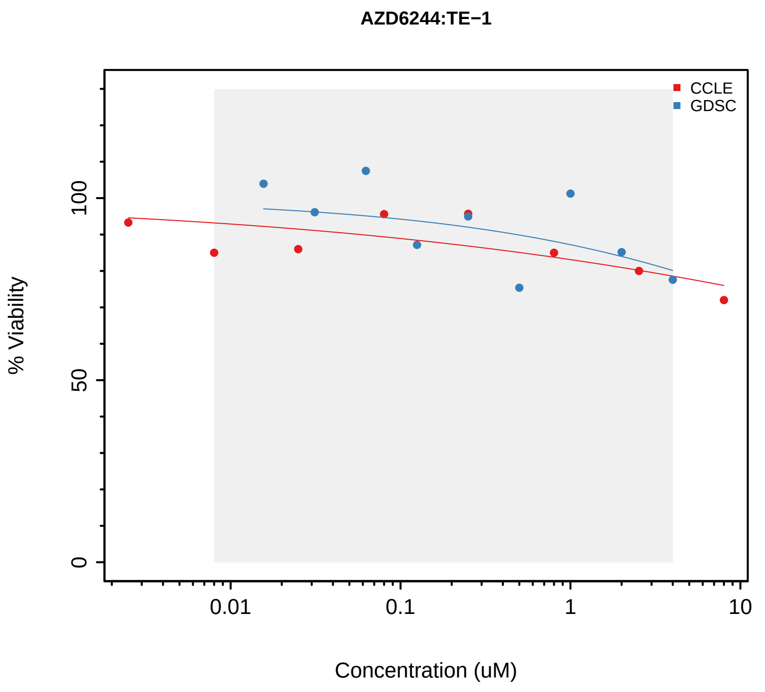
AZD6244:TE−1
CCLE
GDSC
% Viability
0
50
100
0.01
0.1
1
10
Concentration (uM)

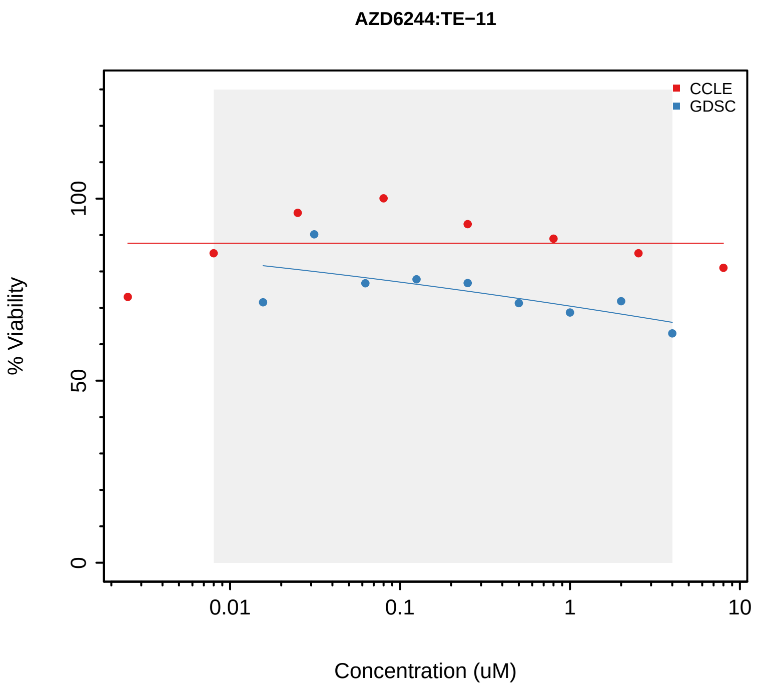
AZD6244:TE−11
CCLE
GDSC
% Viability
0
50
100
0.01
0.1
1
10
Concentration (uM)

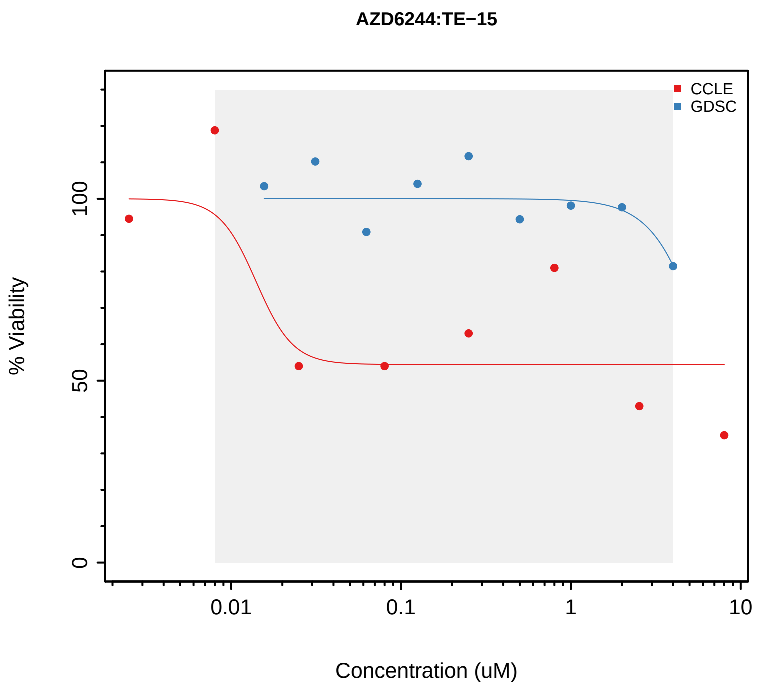
AZD6244:TE−15
CCLE
GDSC
% Viability
0
50
100
0.01
0.1
1
10
Concentration (uM)

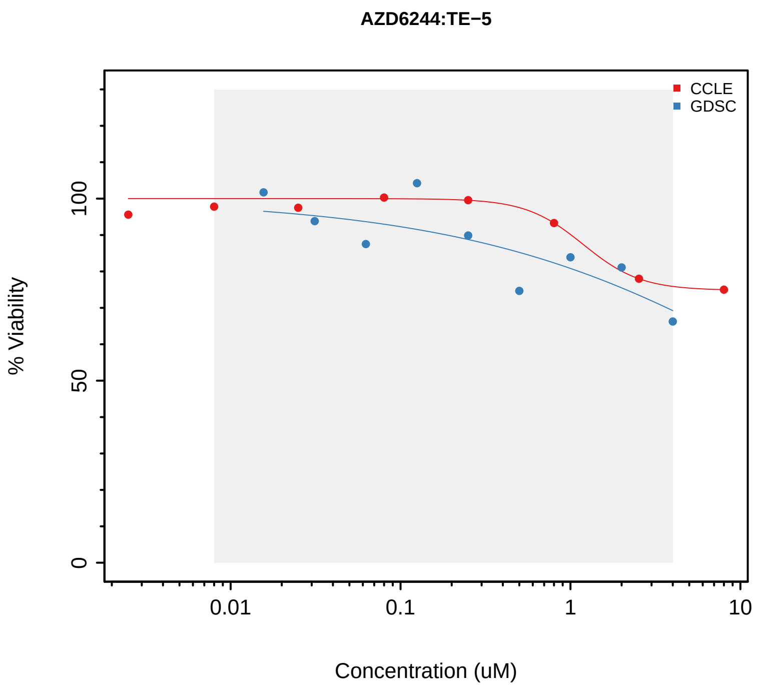
AZD6244:TE−5
CCLE
GDSC
% Viability
0
50
100
0.01
0.1
1
10
Concentration (uM)

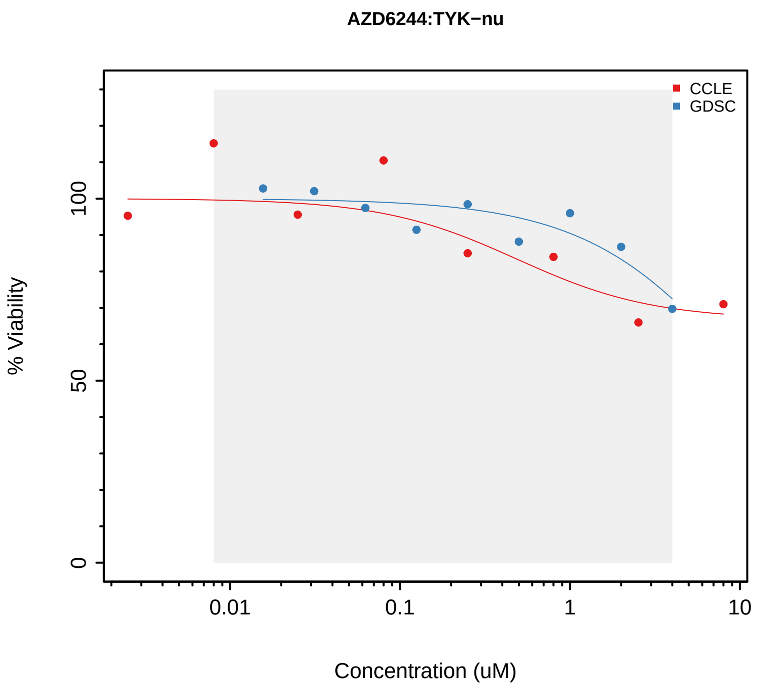
AZD6244:TYK−nu
CCLE
GDSC
% Viability
0
50
100
0.01
0.1
1
10
Concentration (uM)

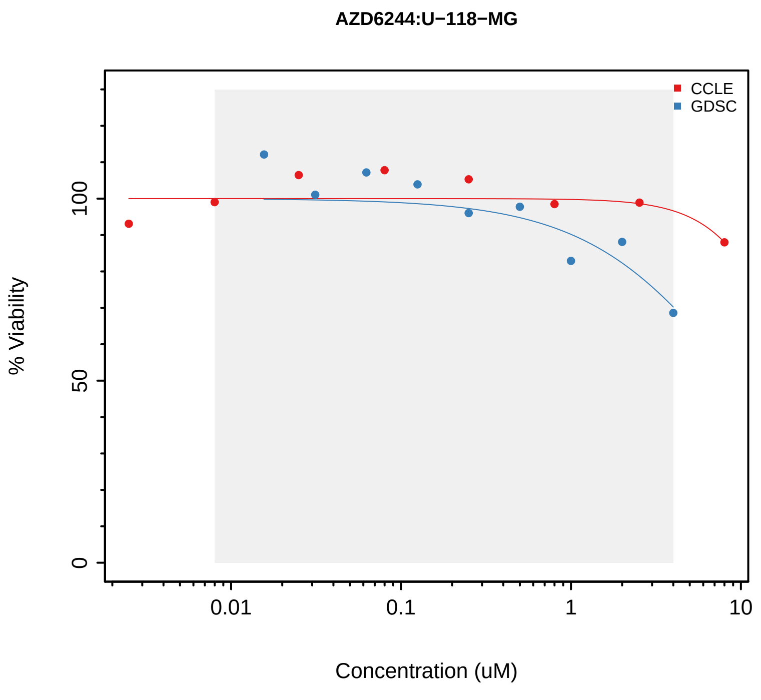
AZD6244:U−118−MG
CCLE
GDSC
% Viability
0
50
100
0.01
0.1
1
10
Concentration (uM)

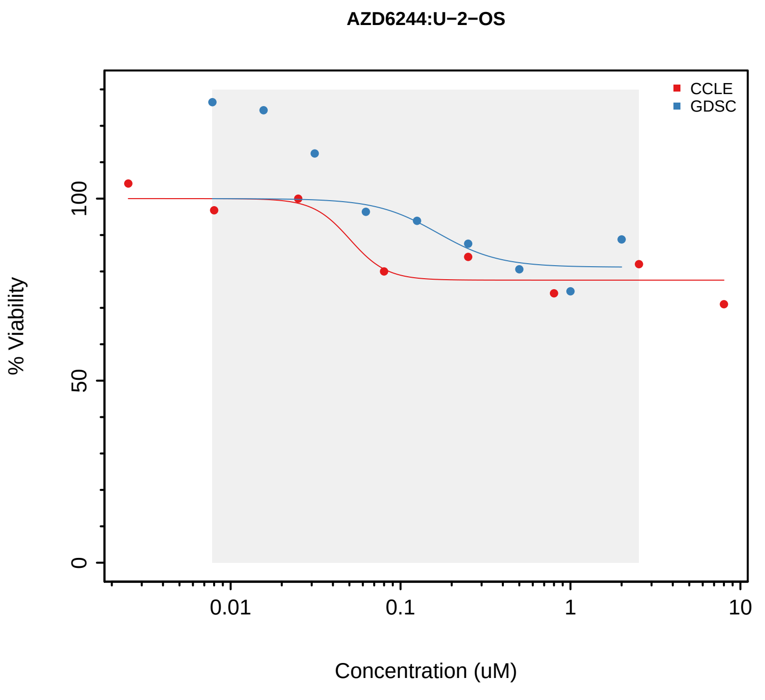
AZD6244:U−2−OS
CCLE
GDSC
% Viability
0
50
100
0.01
0.1
1
10
Concentration (uM)

AZD6244:U−87−MG

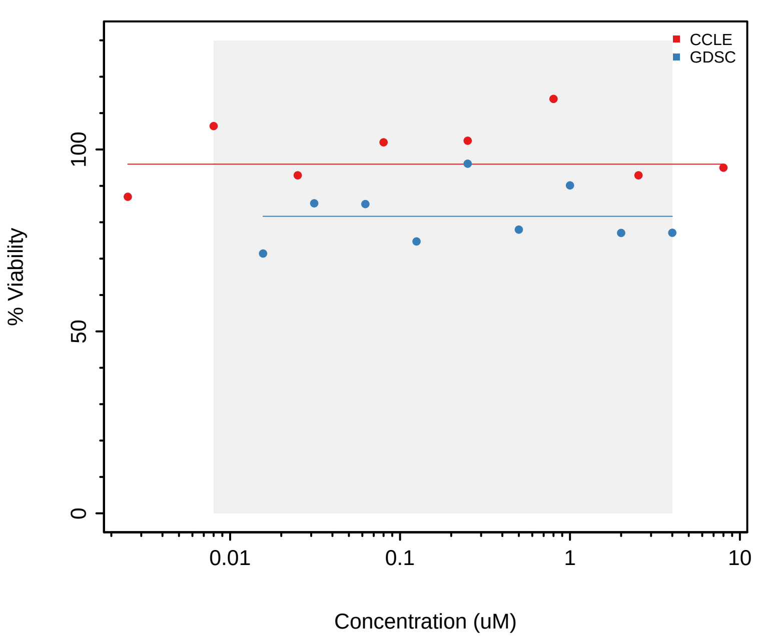

AZD6244:UACC−257

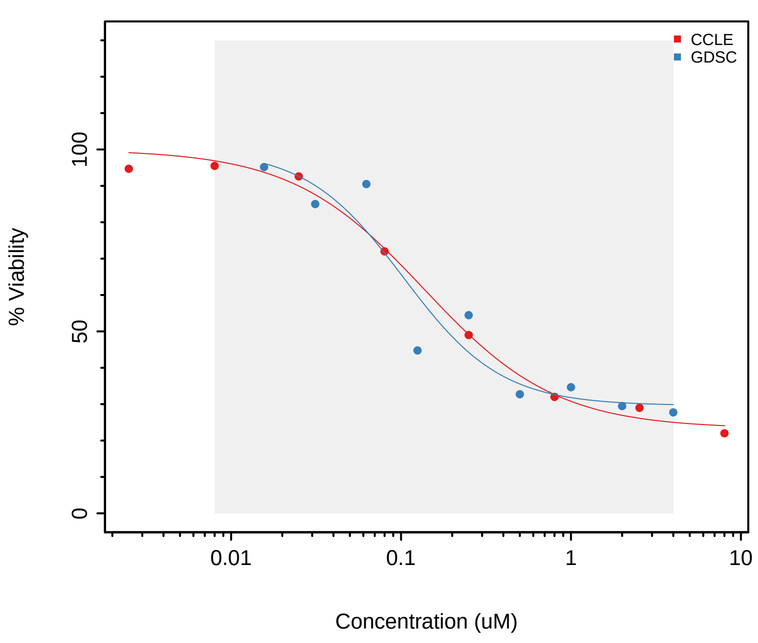

AZD6244:UACC−62

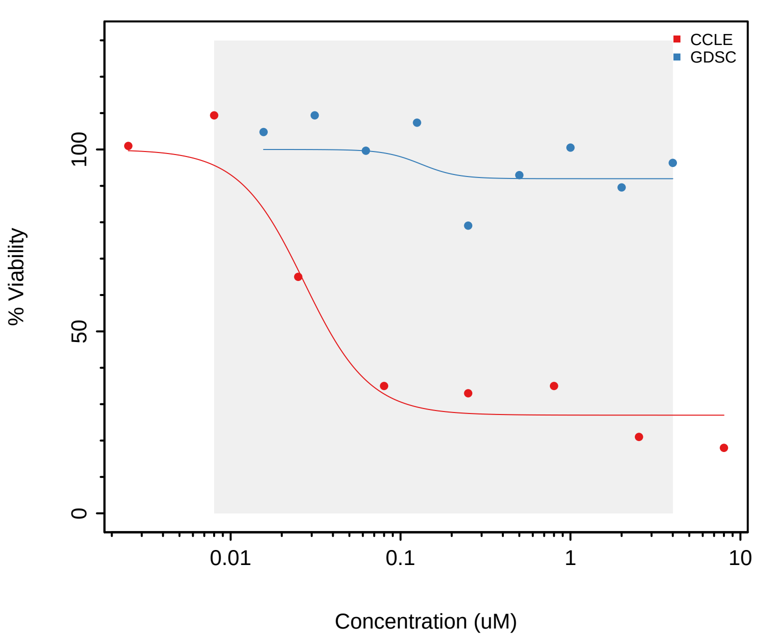

AZD6244:VMRC−RCZ

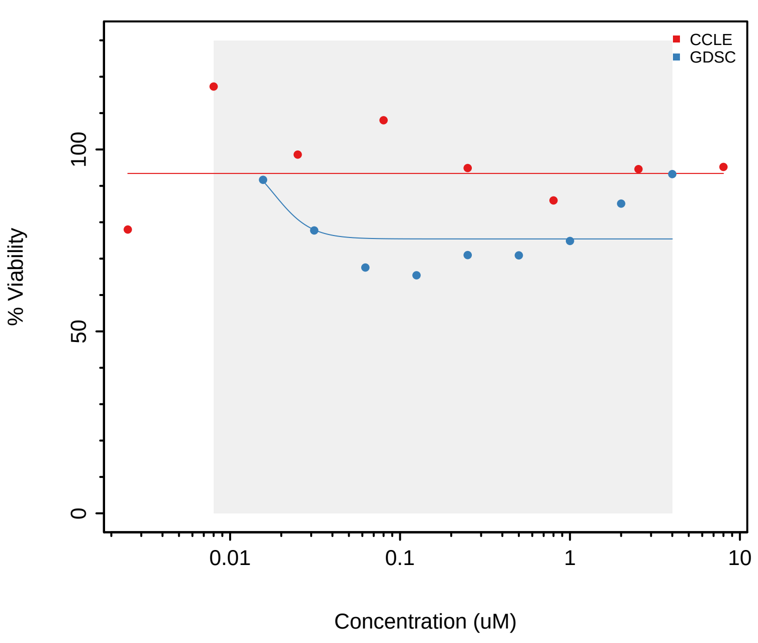

AZD6244:WM−115

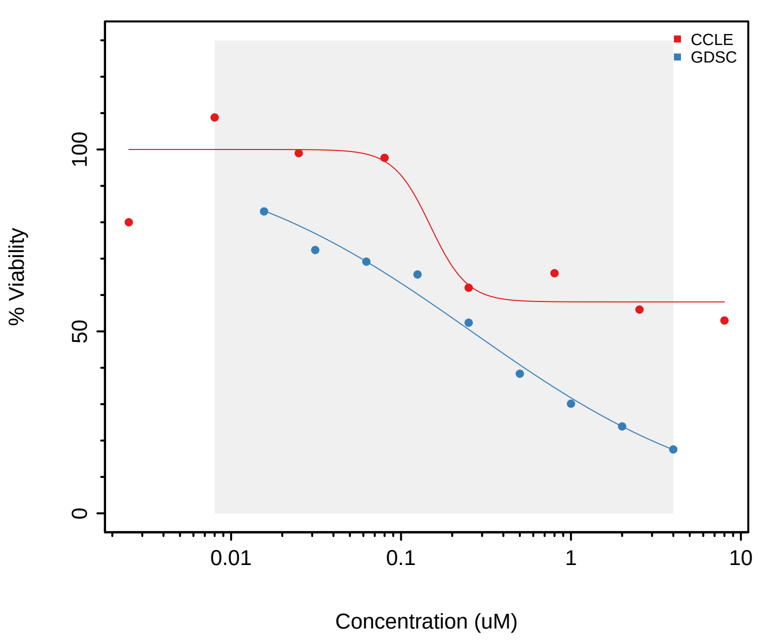

AZD6244:YKG−1

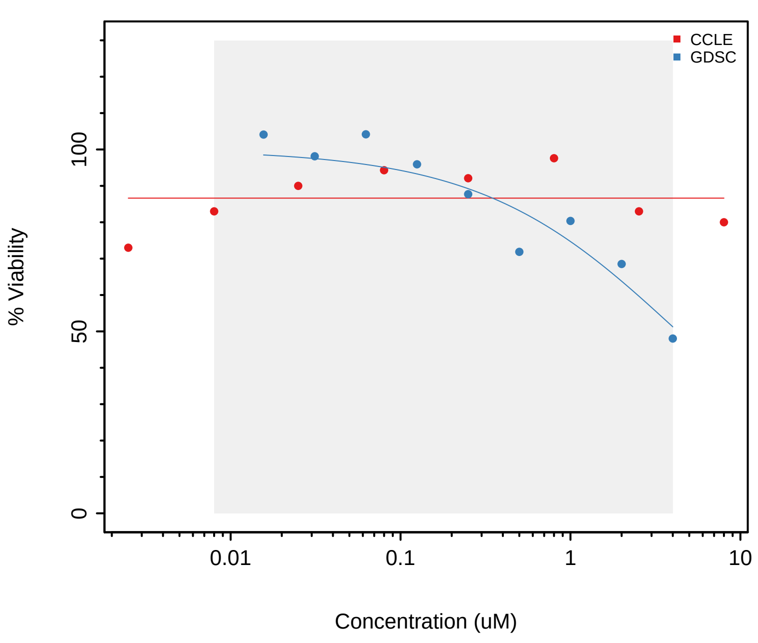

AZD6244:ZR−75−30

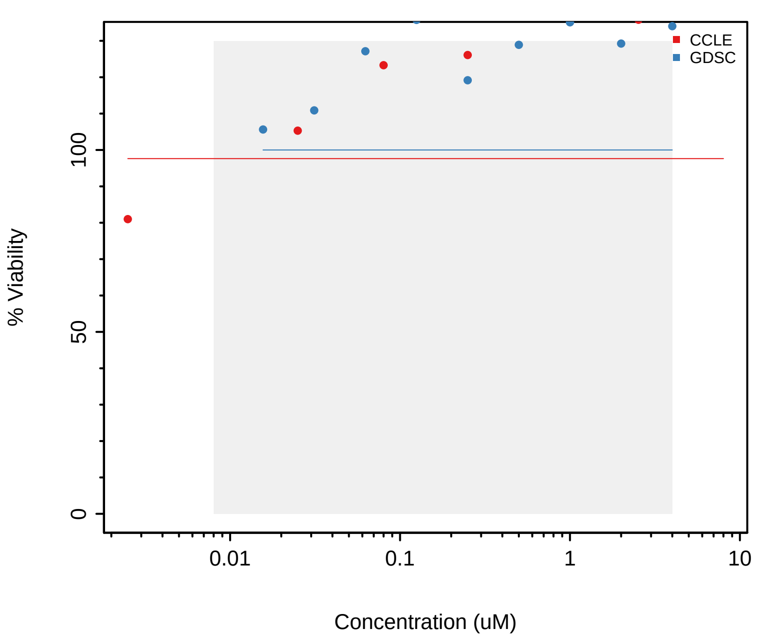

PLX4720:22RV1

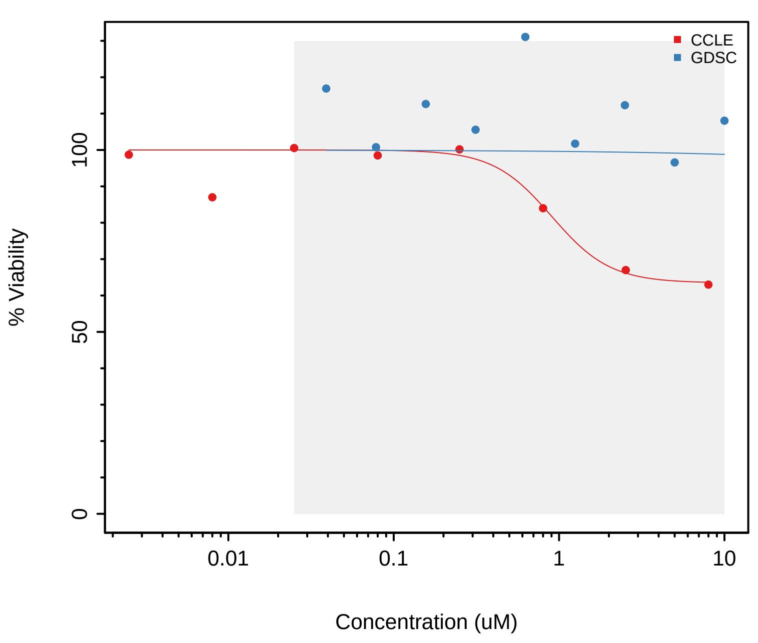

PLX4720:5637

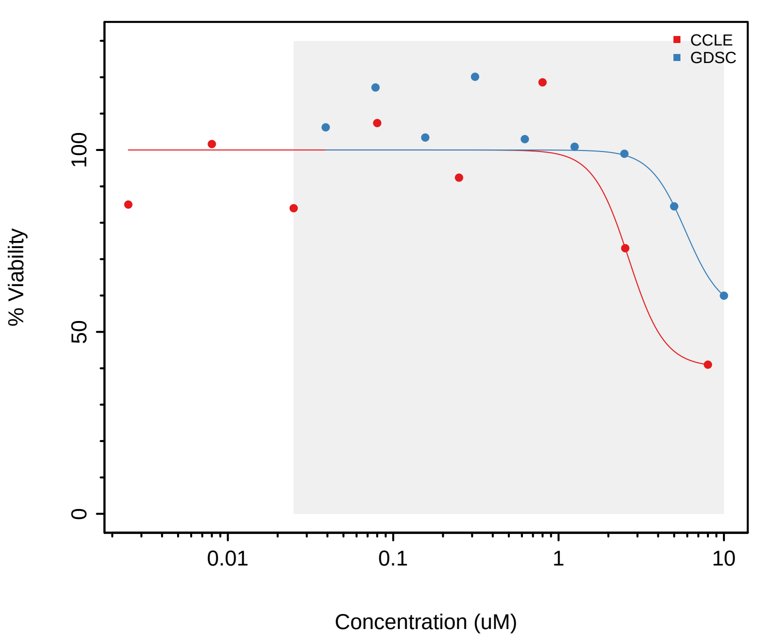

PLX4720:639−V

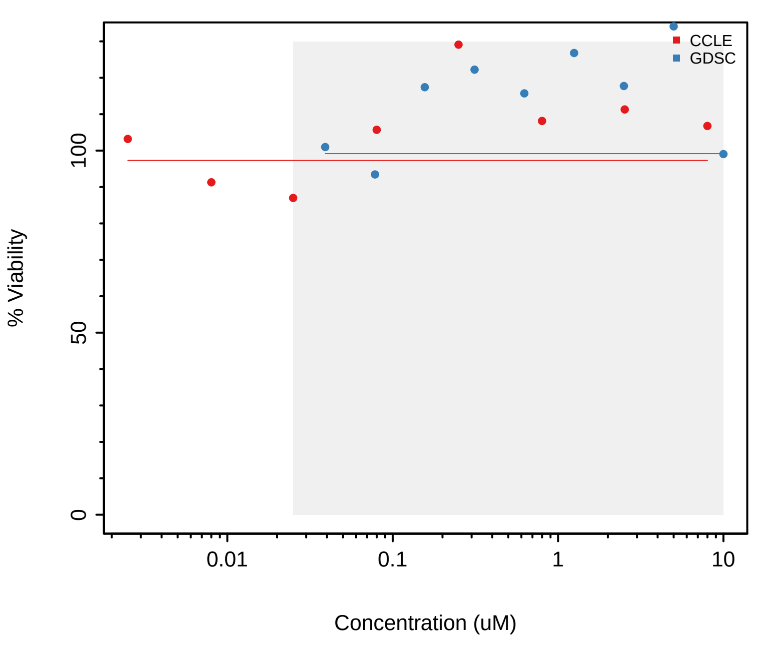

PLX4720:697

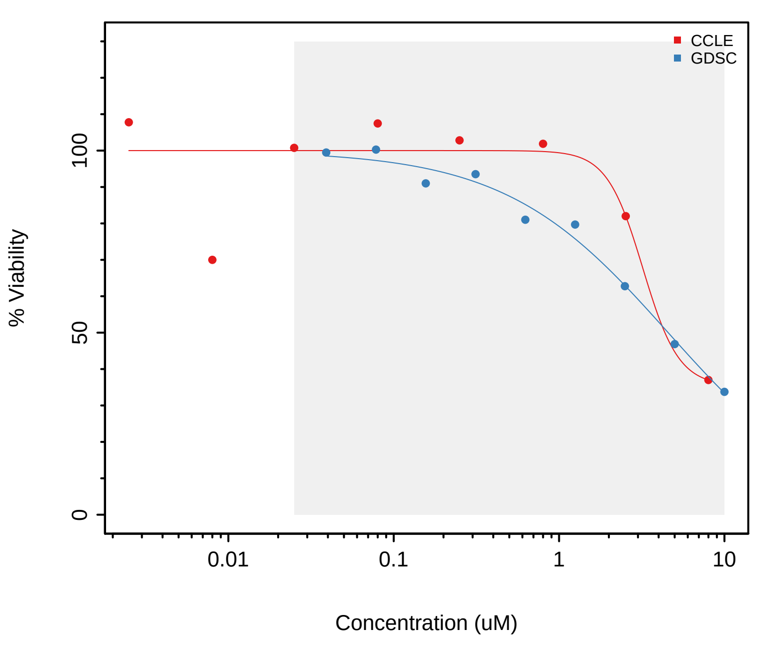

PLX4720:769−P

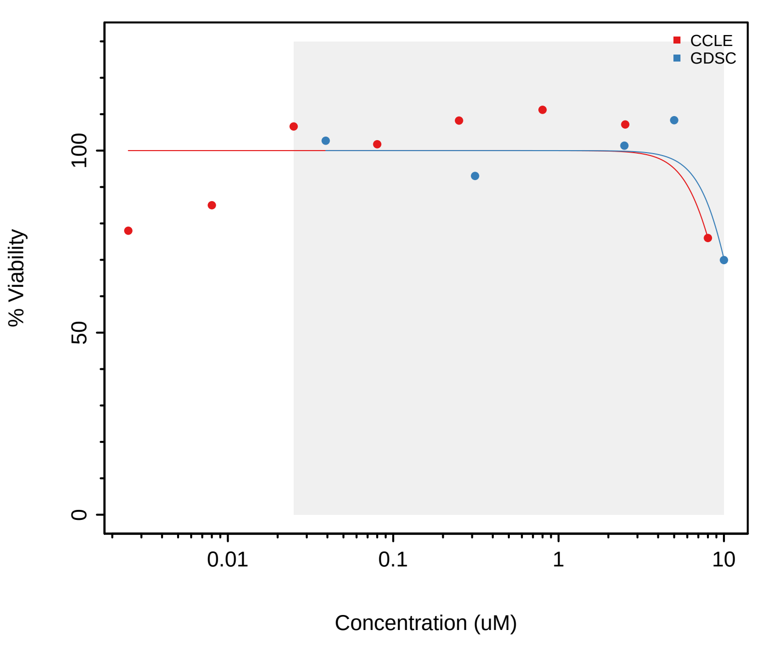

PLX4720:8305C

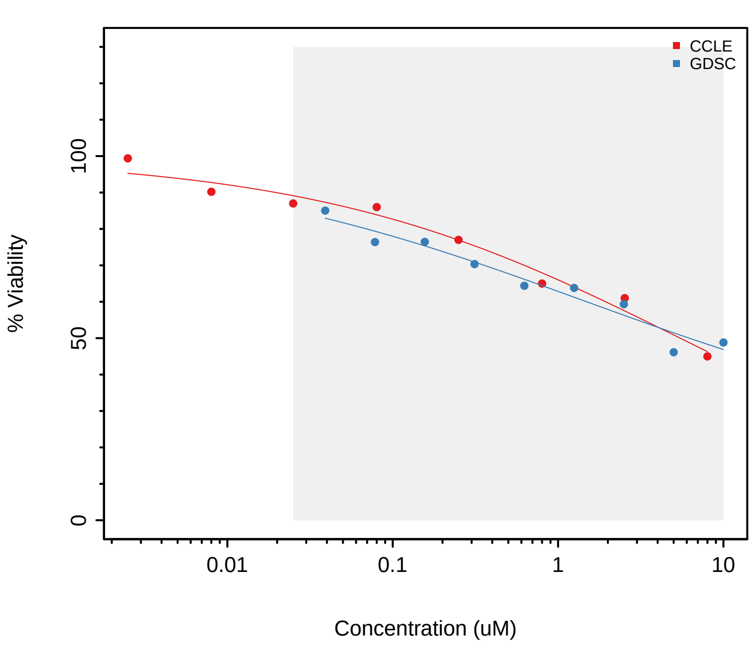

PLX4720:8505C

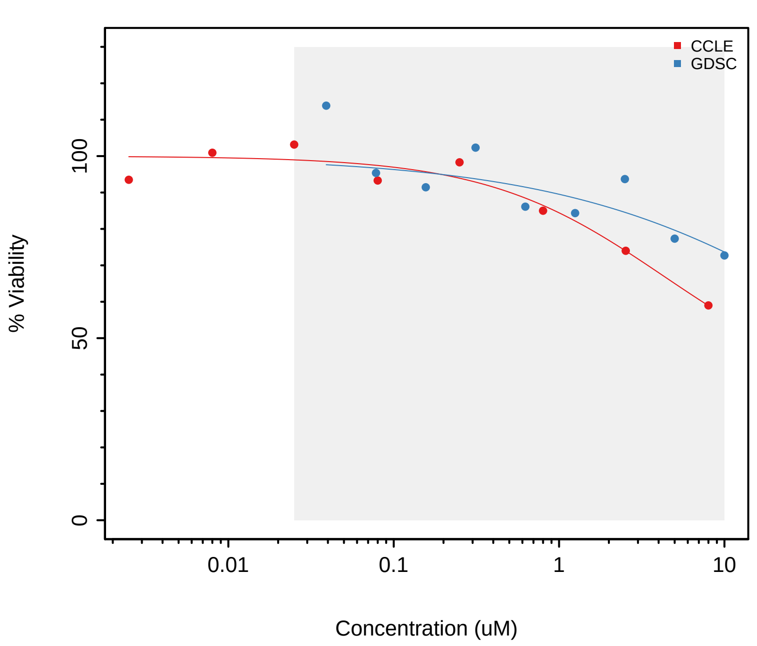

PLX4720:8−MG−BA

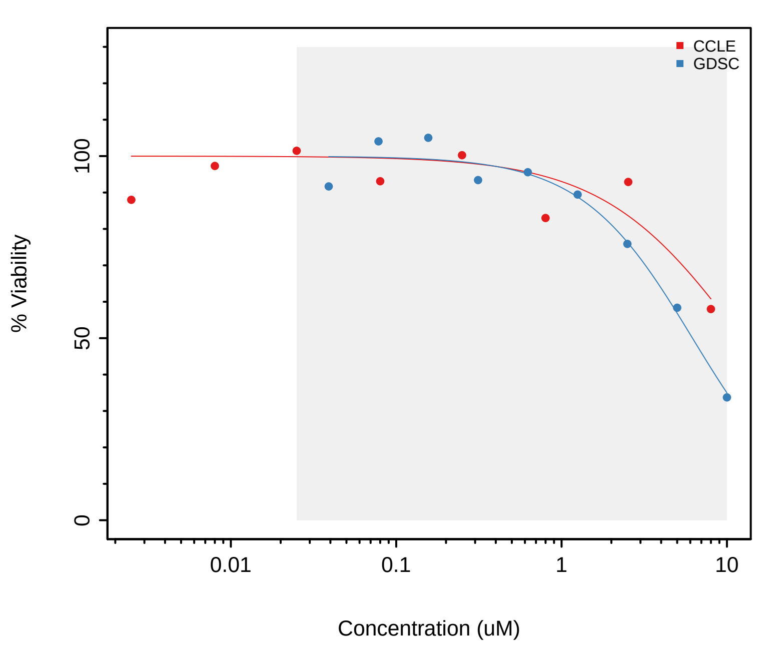

PLX4720:A172

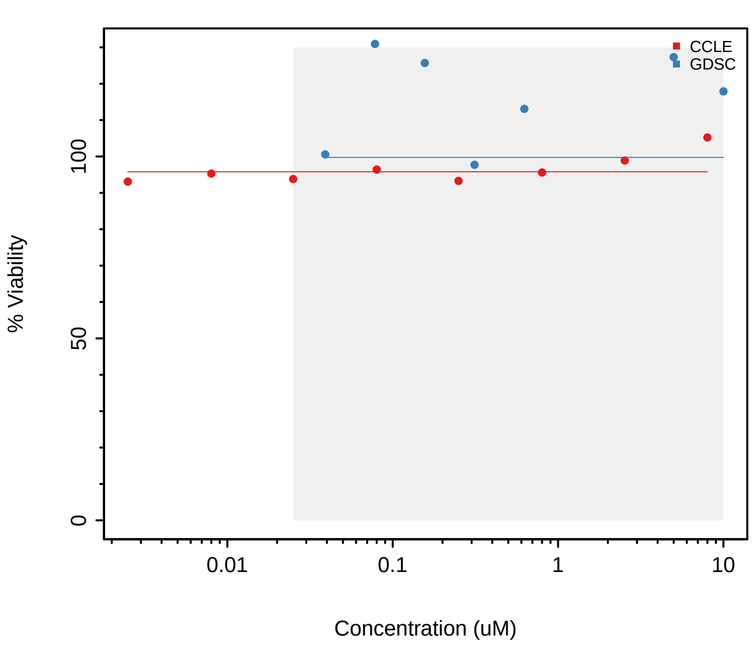

PLX4720:A204

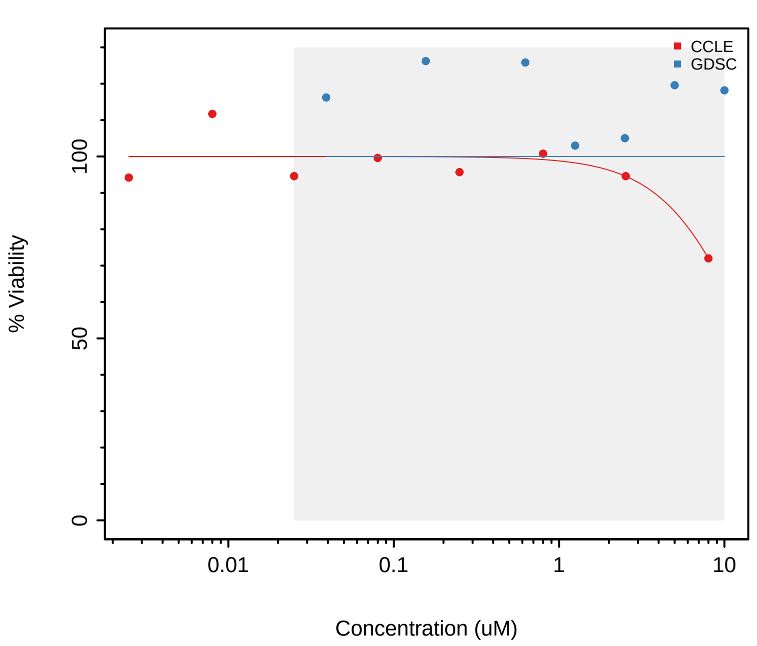

PLX4720:A2058

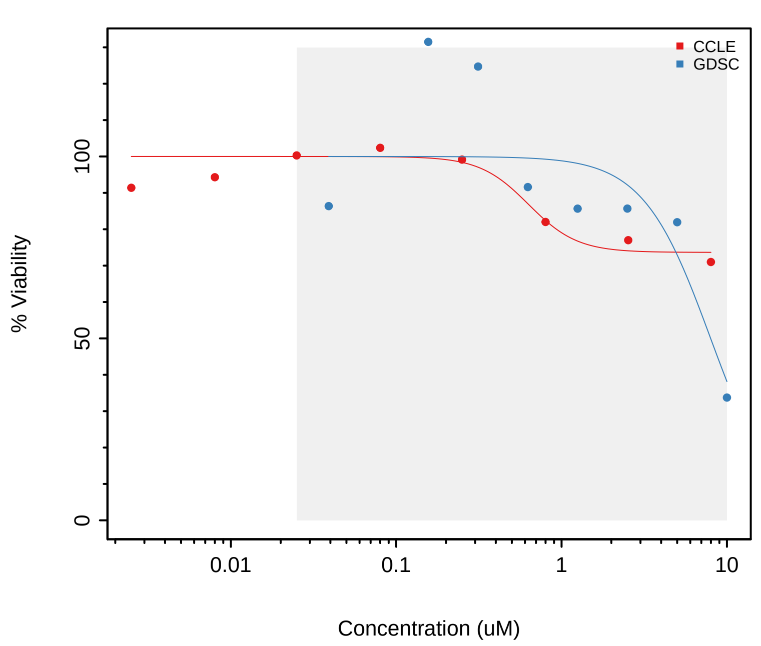

PLX4720:A253

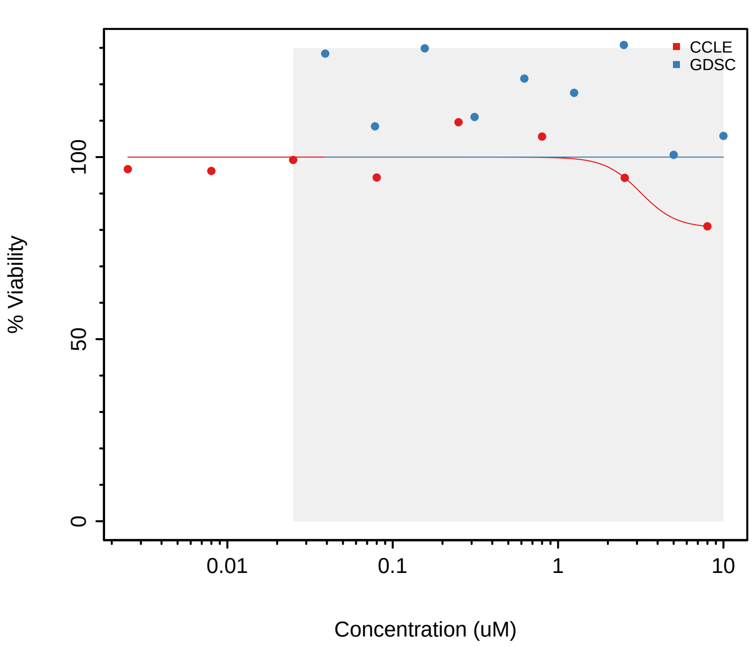

PLX4720:A2780

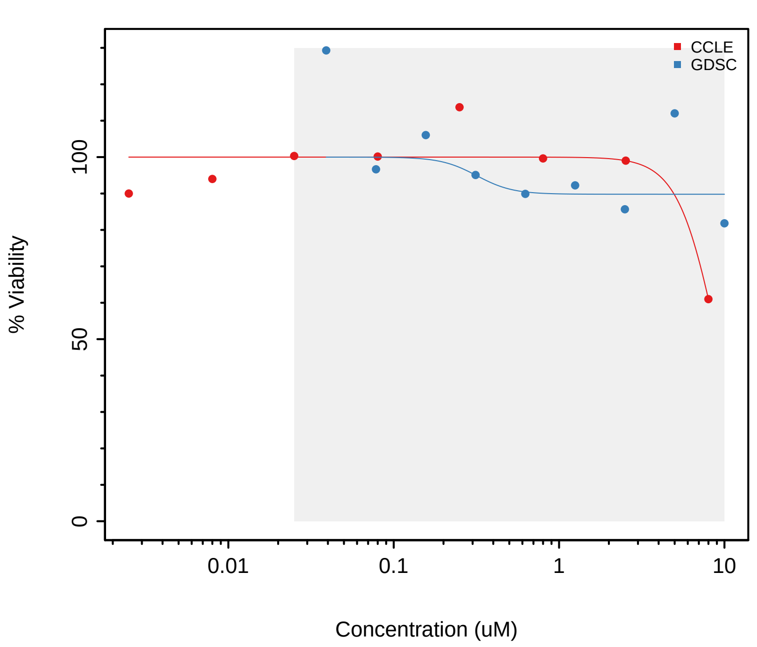

PLX4720:A375

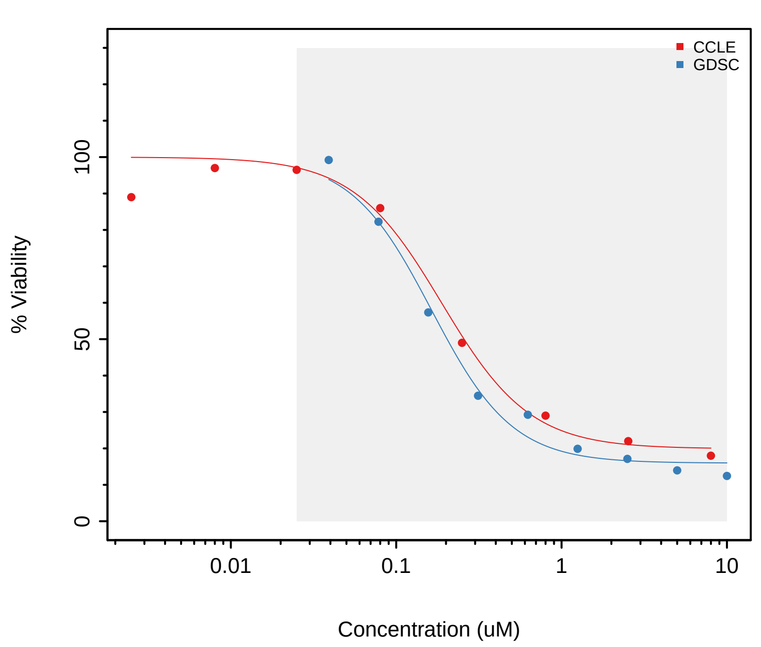

PLX4720:A549

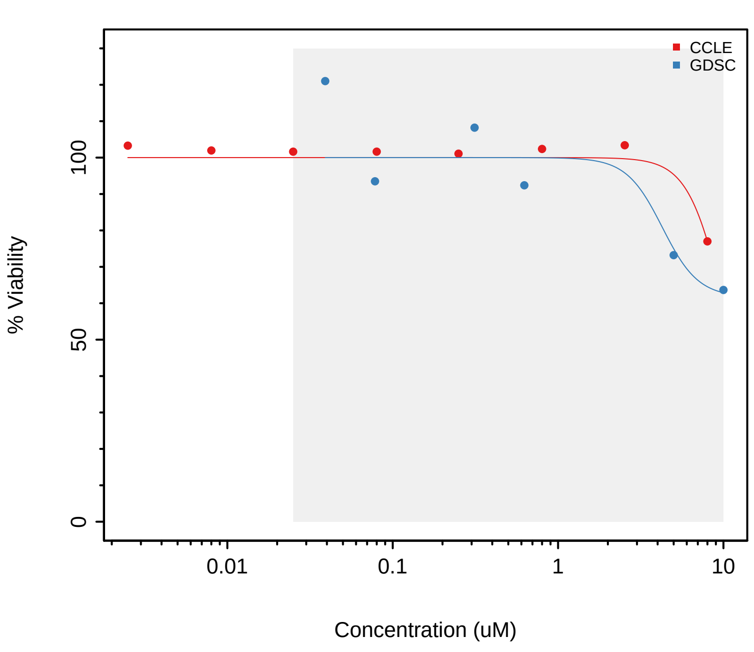

PLX4720:AsPC−1

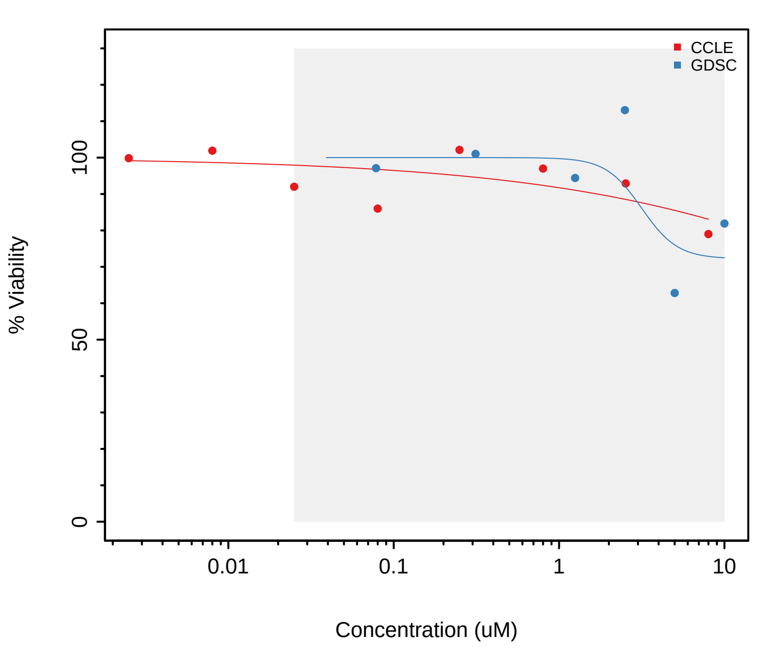

PLX4720:AU565

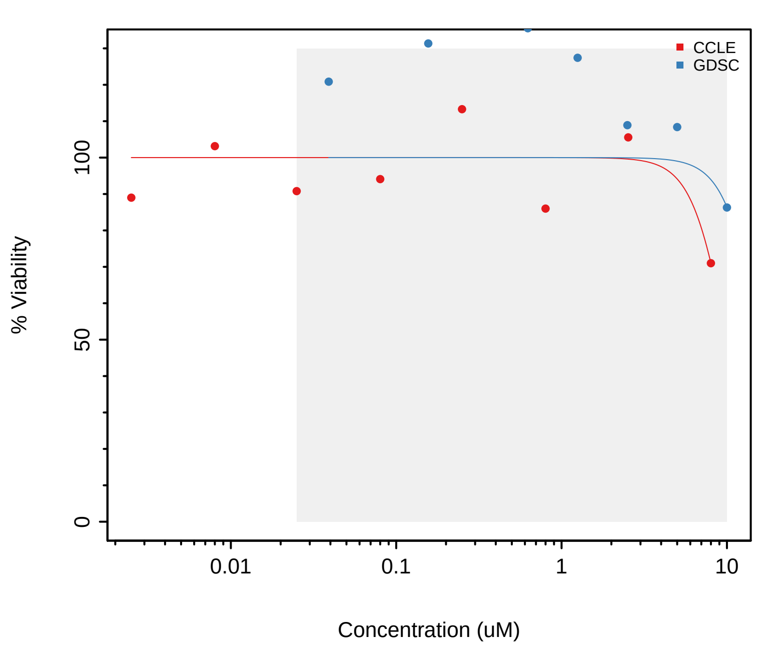

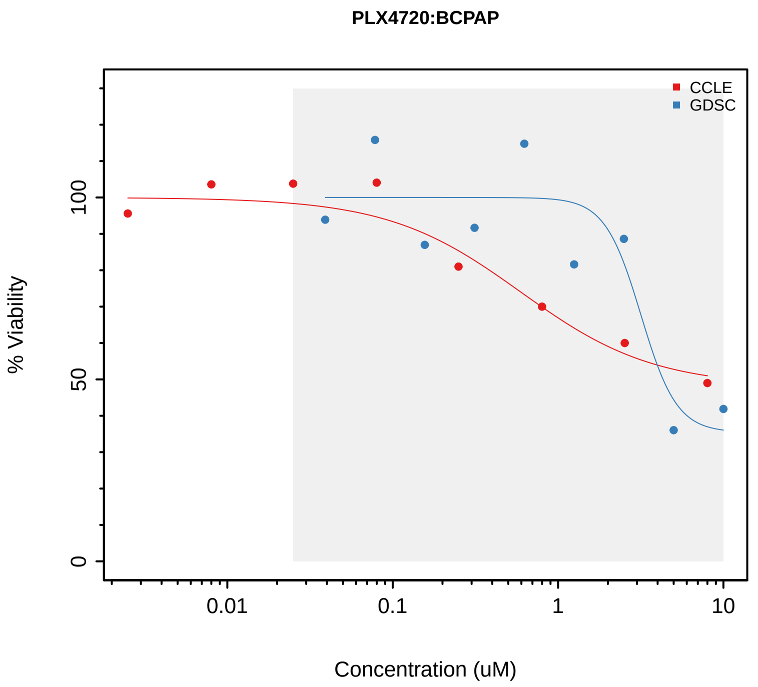
PLX4720:BCPAP
CCLE
GDSC
% Viability
0 50 100
0.01 0.1 1 10
Concentration (uM)

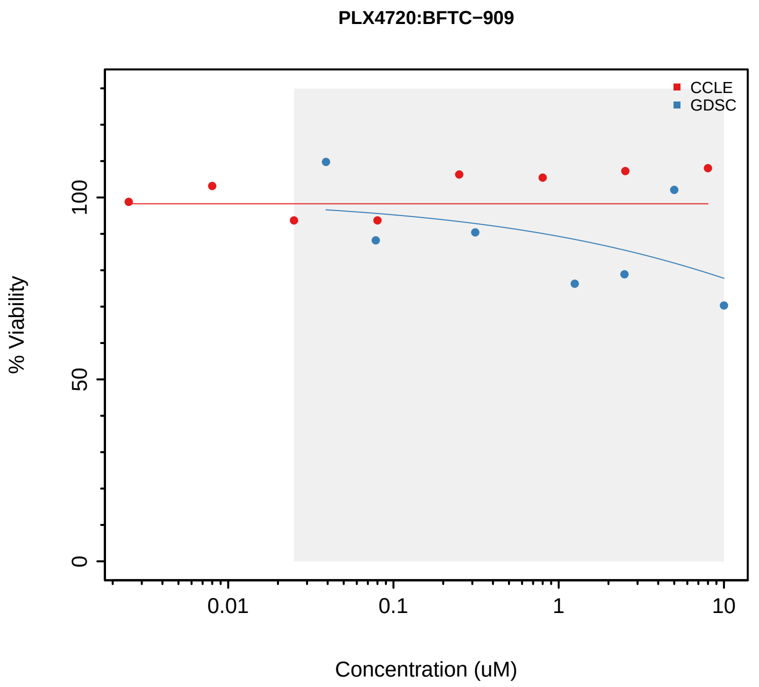
PLX4720:BFTC−909
CCLE
GDSC
% Viability
0 50 100
0.01 0.1 1 10
Concentration (uM)

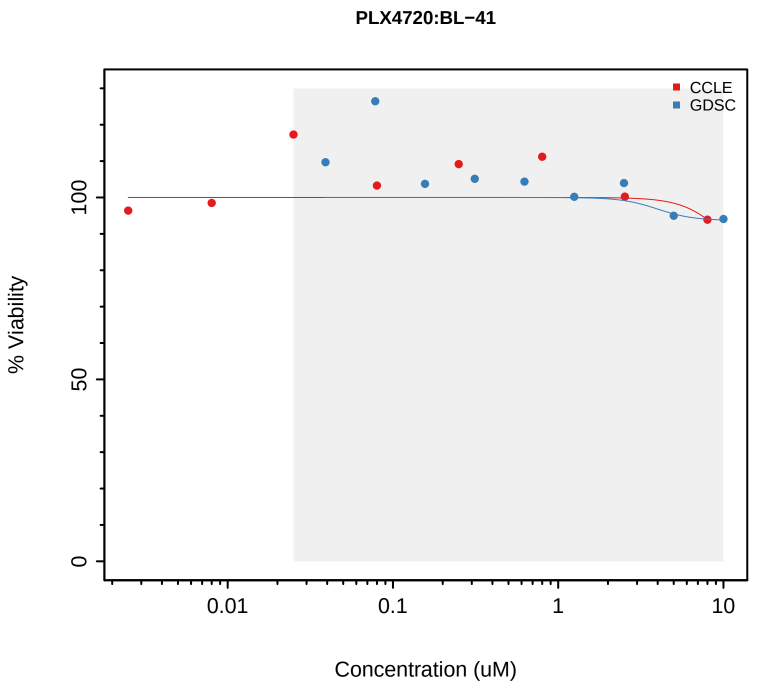
PLX4720:BL−41
CCLE
GDSC
% Viability
0 50 100
0.01 0.1 1 10
Concentration (uM)

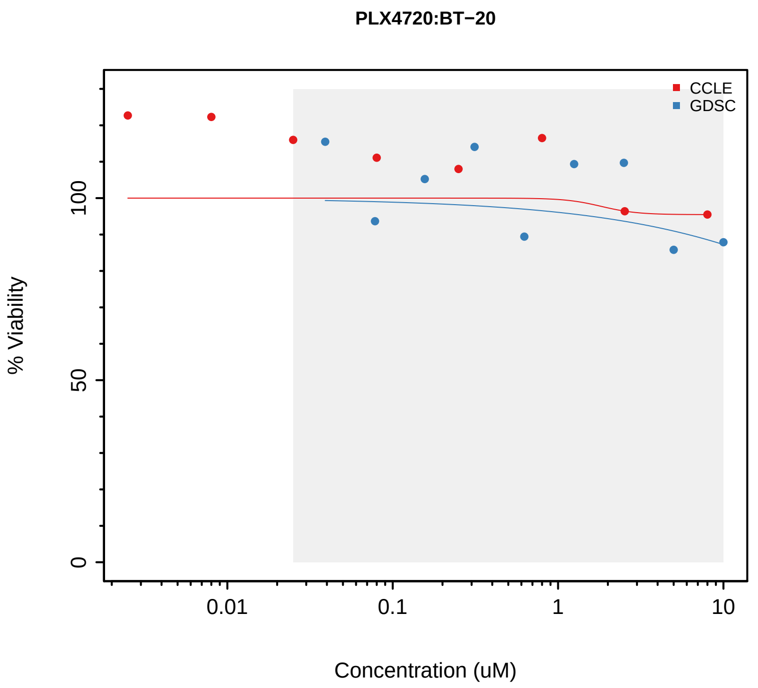
PLX4720:BT−20
CCLE
GDSC
% Viability
0 50 100
0.01 0.1 1 10
Concentration (uM)

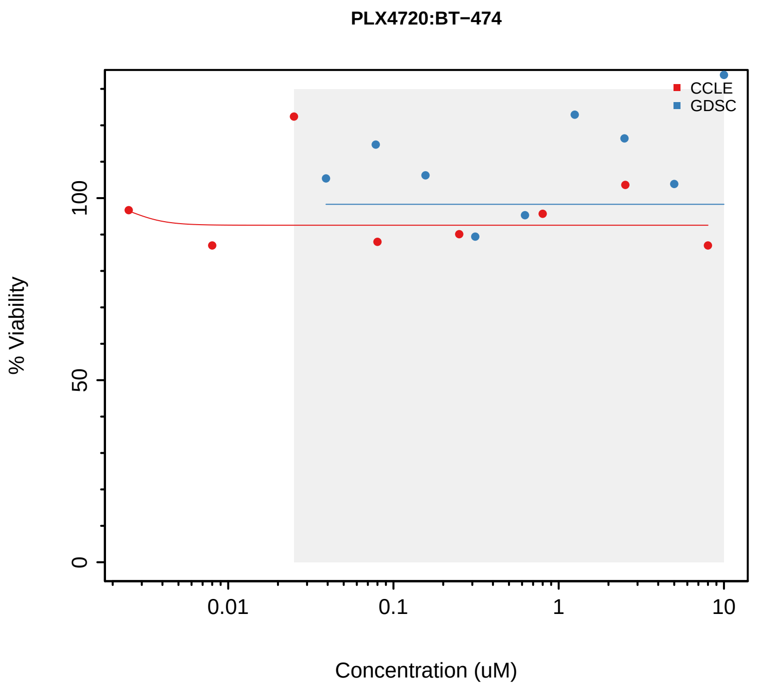
PLX4720:BT−474
CCLE
GDSC
% Viability
0 50 100
0.01 0.1 1 10
Concentration (uM)

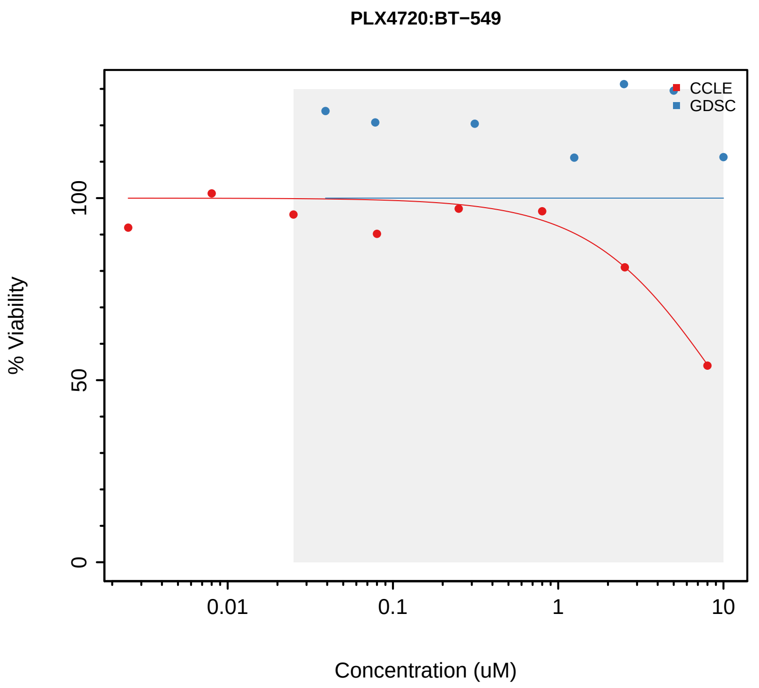
PLX4720:BT−549
CCLE
GDSC
% Viability
0 50 100
0.01 0.1 1 10
Concentration (uM)

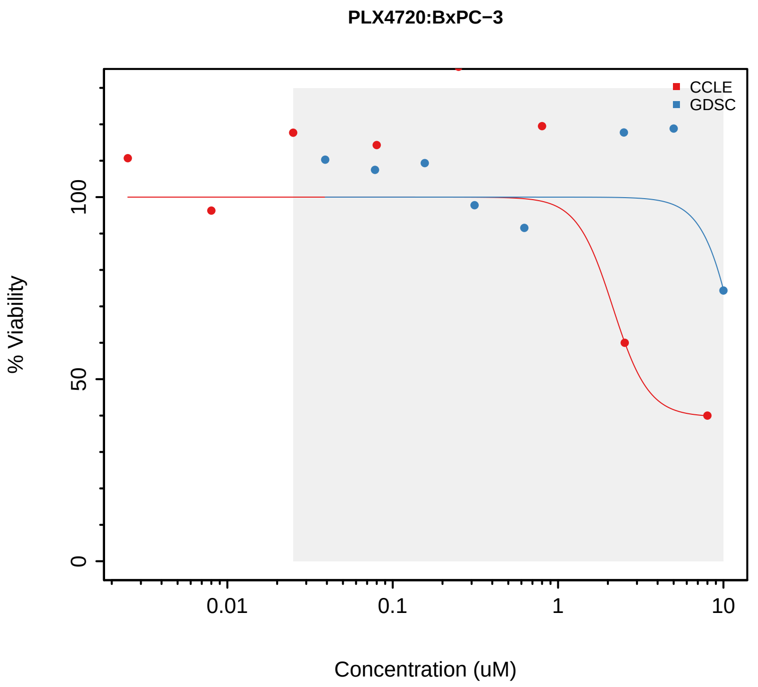
PLX4720:BxPC−3
CCLE
GDSC
% Viability
0 50 100
0.01 0.1 1 10
Concentration (uM)

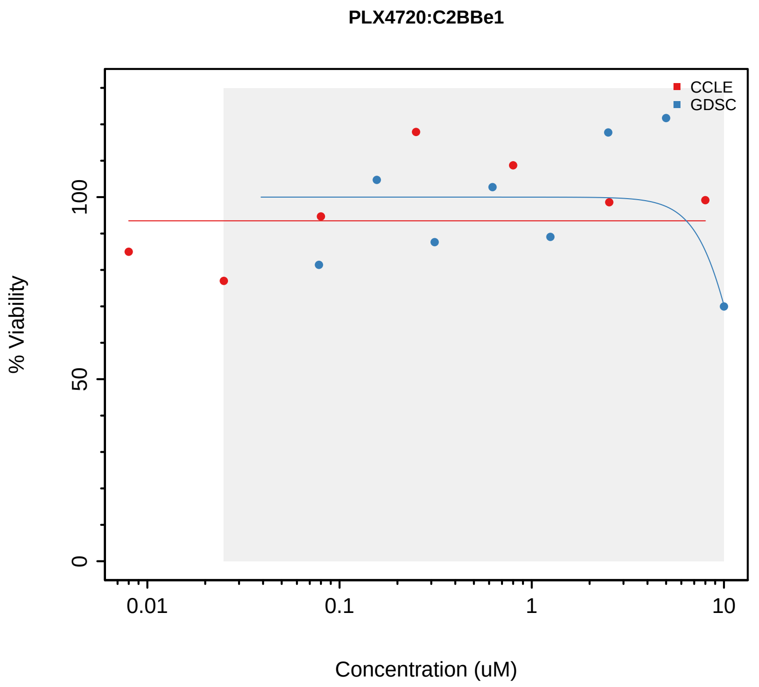
PLX4720:C2BBe1
CCLE
GDSC
% Viability
0 50 100
0.01 0.1 1 10
Concentration (uM)

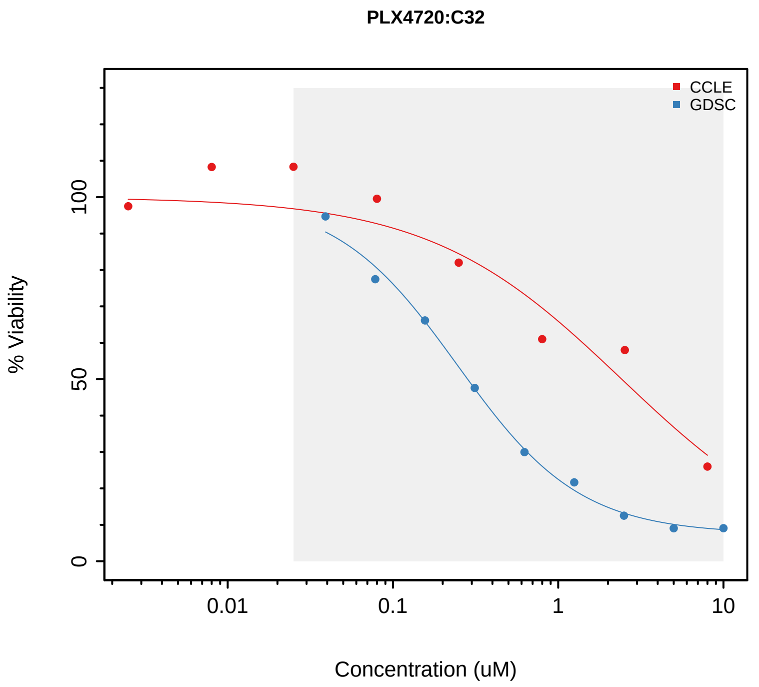
PLX4720:C32
CCLE
GDSC
% Viability
0 50 100
0.01 0.1 1 10
Concentration (uM)

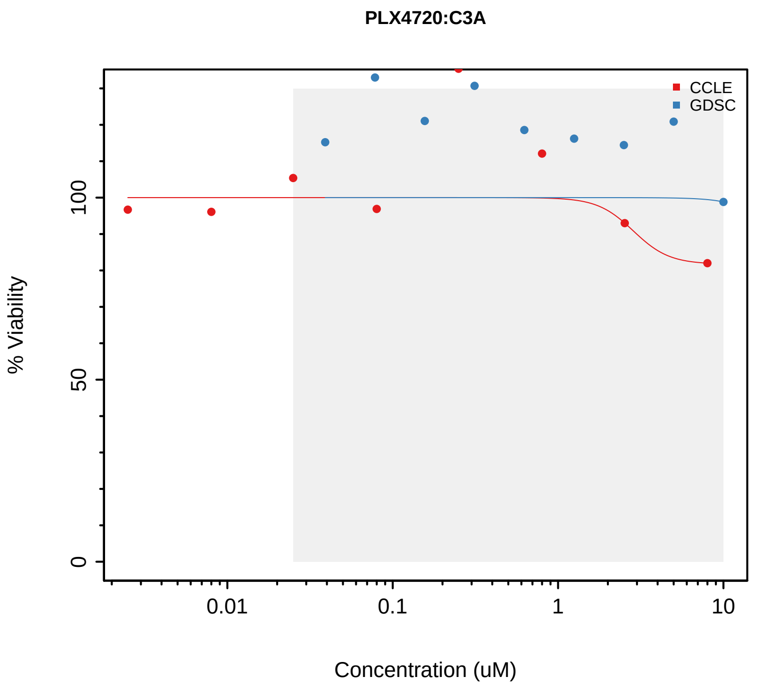
PLX4720:C3A
CCLE
GDSC
% Viability
0 50 100
0.01 0.1 1 10
Concentration (uM)

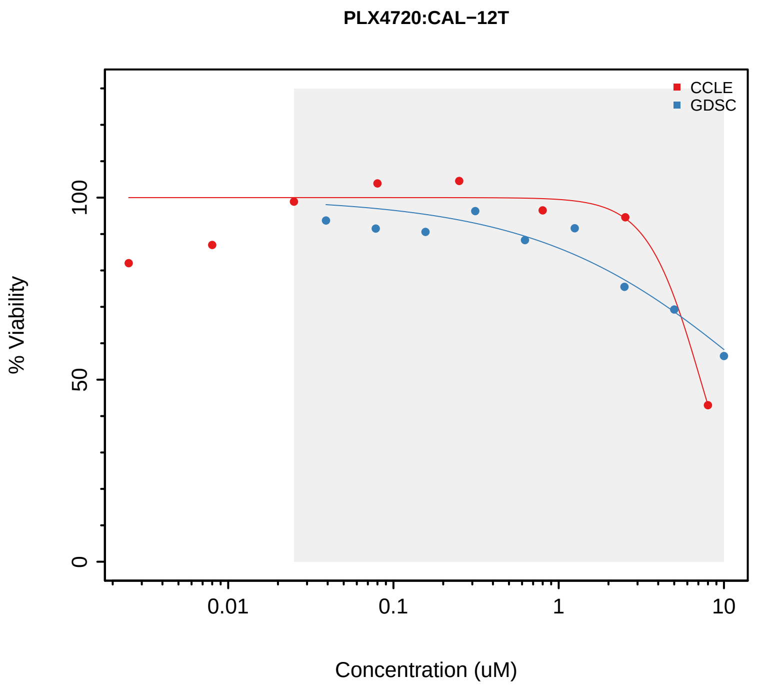
PLX4720:CAL−12T
CCLE
GDSC
% Viability
0 50 100
0.01 0.1 1 10
Concentration (uM)

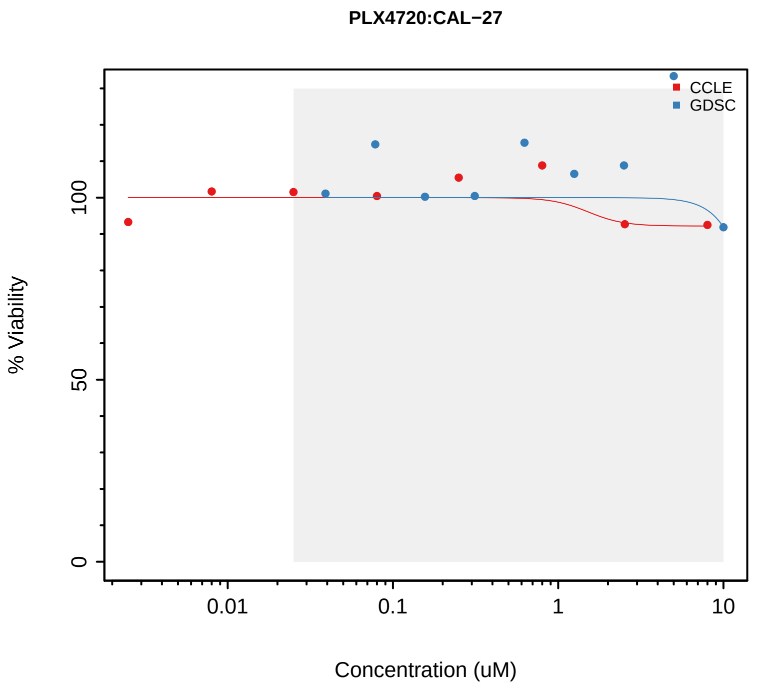
PLX4720:CAL−27
CCLE
GDSC
% Viability
0 50 100
0.01 0.1 1 10
Concentration (uM)

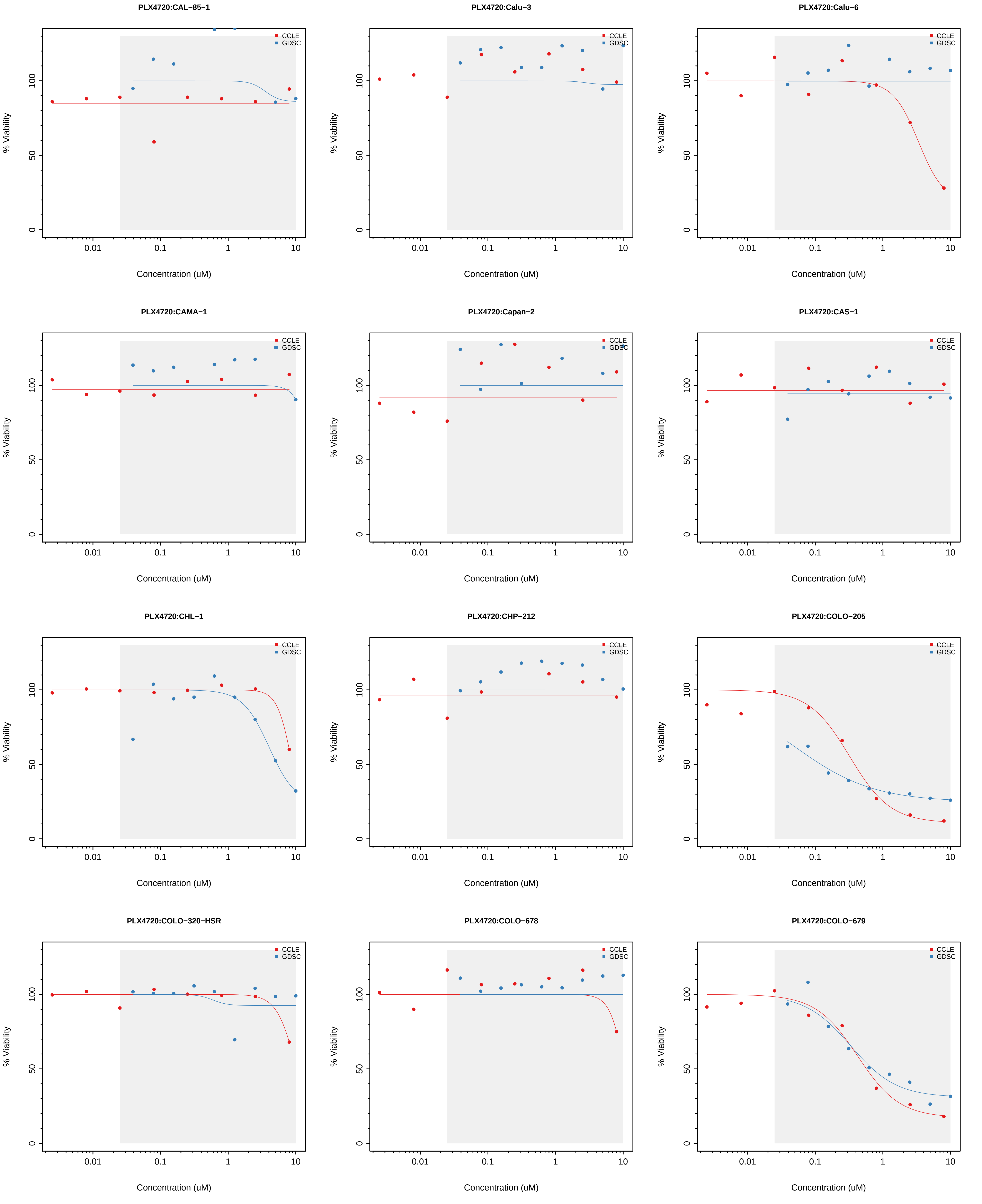
PLX4720:CAL−85−1
PLX4720:Calu−3
PLX4720:Calu−6
PLX4720:CAMA−1
PLX4720:Capan−2
PLX4720:CAS−1
PLX4720:CHL−1
PLX4720:CHP−212
PLX4720:COLO−205
PLX4720:COLO−320−HSR
PLX4720:COLO−678
PLX4720:COLO−679
CCLE
GDSC
% Viability
Concentration (uM)
0
50
100
0.01
0.1
1
10

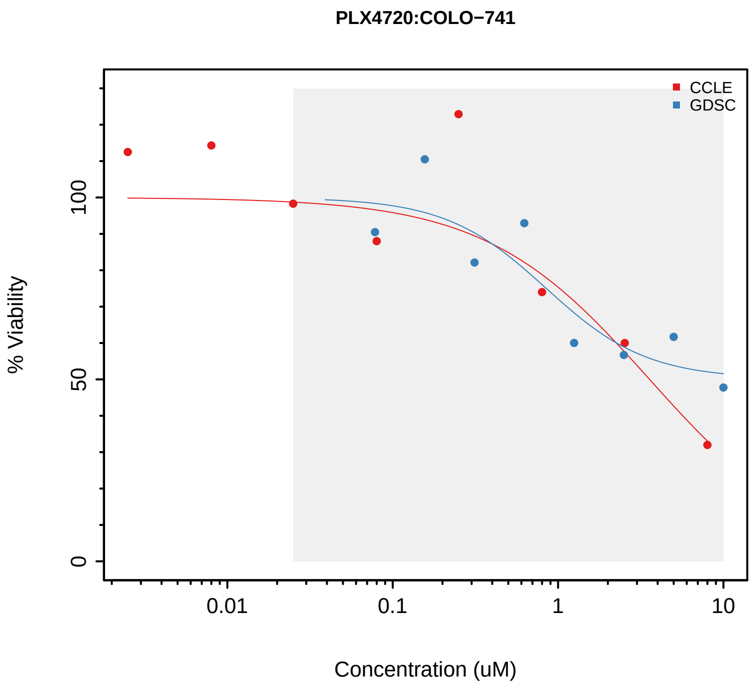
PLX4720:COLO−741
CCLE
GDSC
% Viability
0
50
100
0.01
0.1
1
10
Concentration (uM)

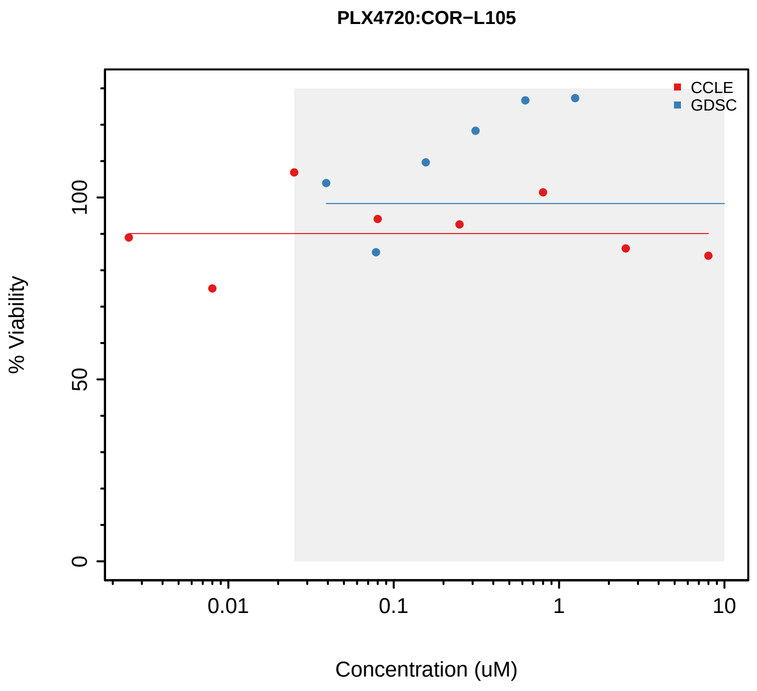
PLX4720:COR−L105
CCLE
GDSC
% Viability
0
50
100
0.01
0.1
1
10
Concentration (uM)

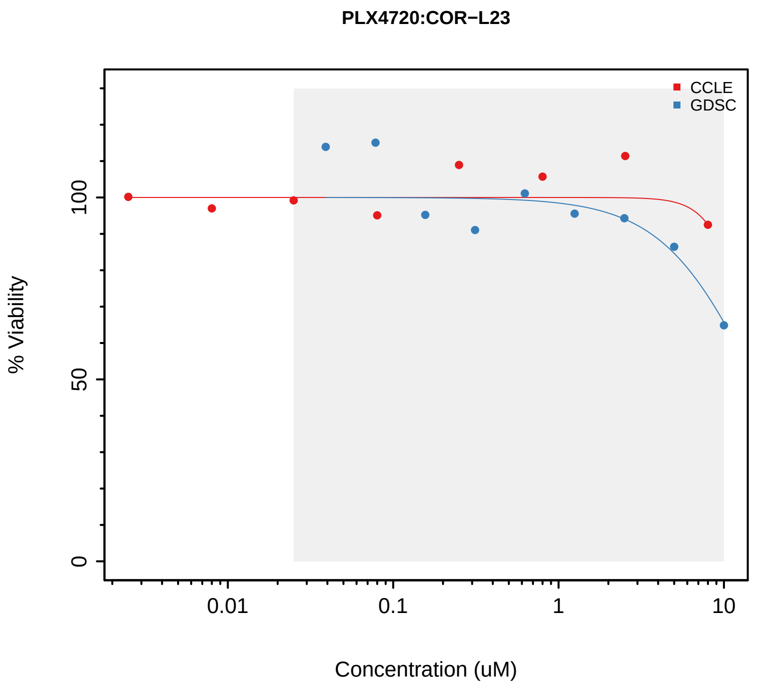
PLX4720:COR−L23
CCLE
GDSC
% Viability
0
50
100
0.01
0.1
1
10
Concentration (uM)

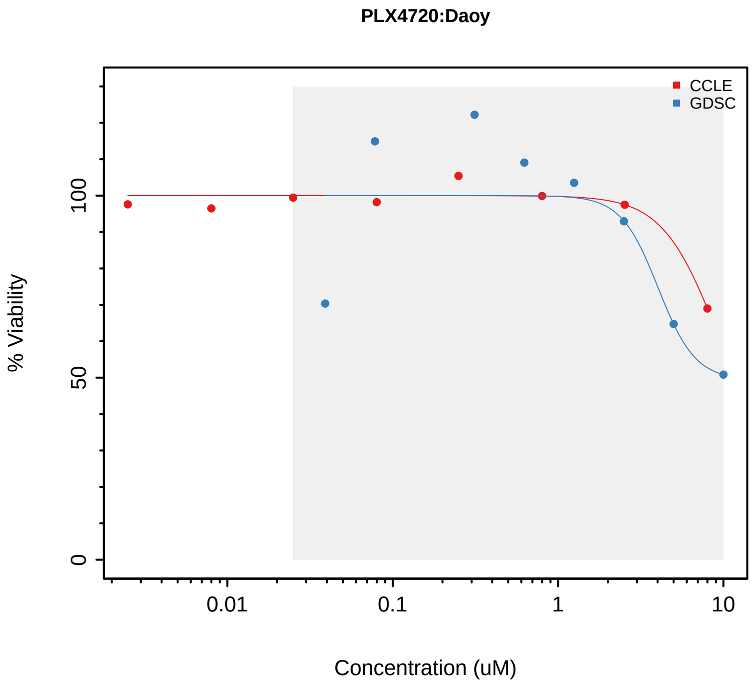
PLX4720:Daoy
CCLE
GDSC
% Viability
0
50
100
0.01
0.1
1
10
Concentration (uM)

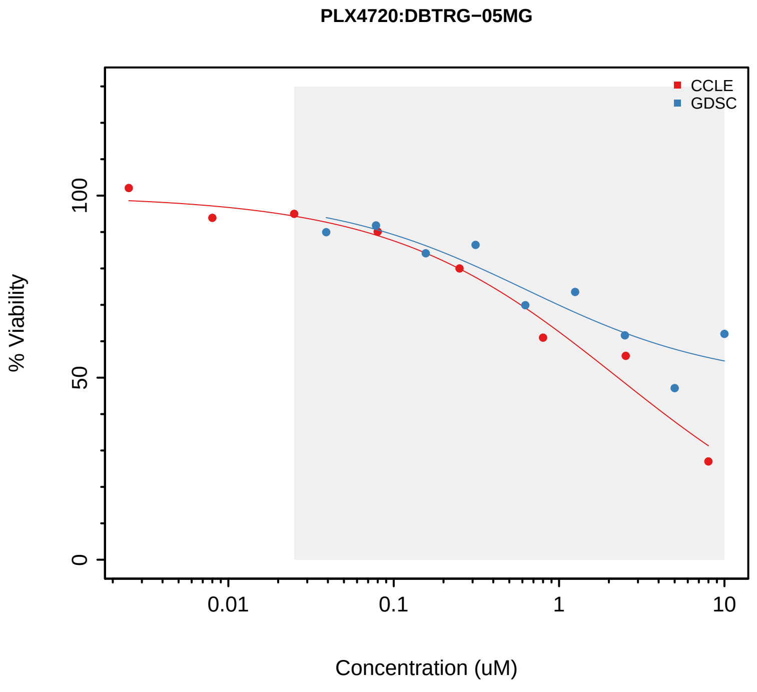
PLX4720:DBTRG−05MG
CCLE
GDSC
% Viability
0
50
100
0.01
0.1
1
10
Concentration (uM)

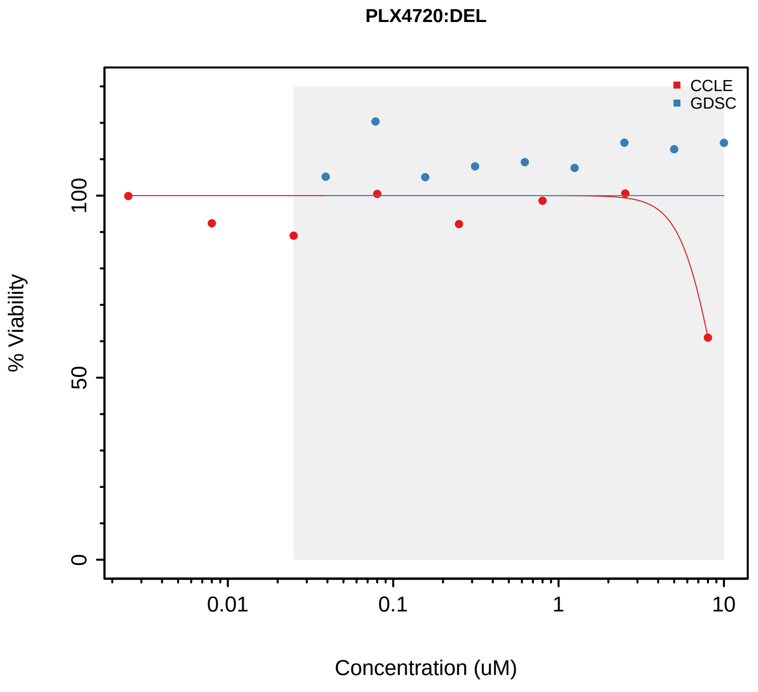
PLX4720:DEL
CCLE
GDSC
% Viability
0
50
100
0.01
0.1
1
10
Concentration (uM)

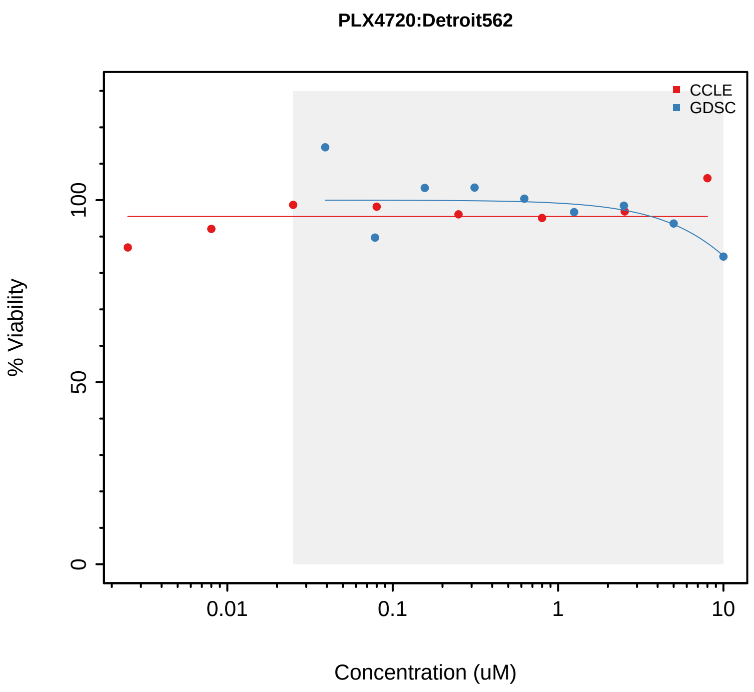
PLX4720:Detroit562
CCLE
GDSC
% Viability
0
50
100
0.01
0.1
1
10
Concentration (uM)

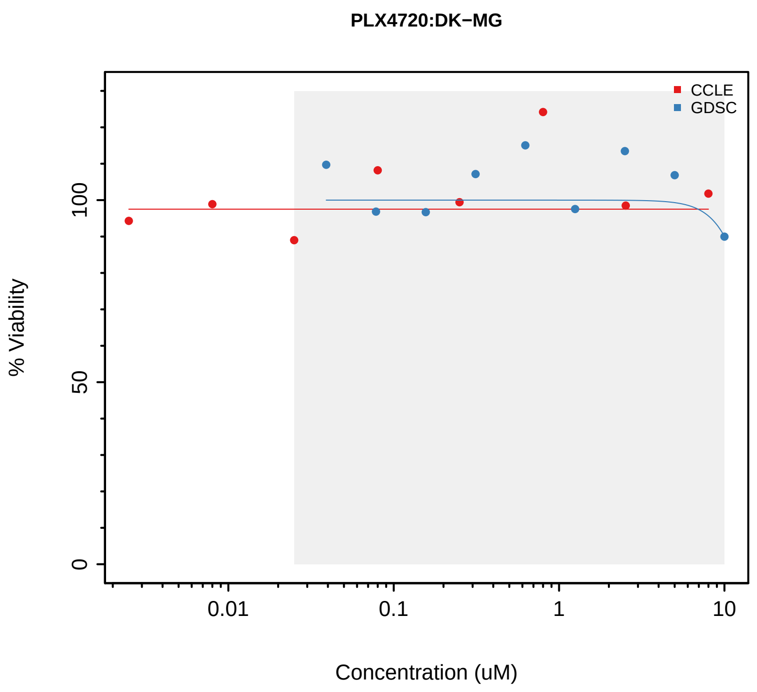
PLX4720:DK−MG
CCLE
GDSC
% Viability
0
50
100
0.01
0.1
1
10
Concentration (uM)

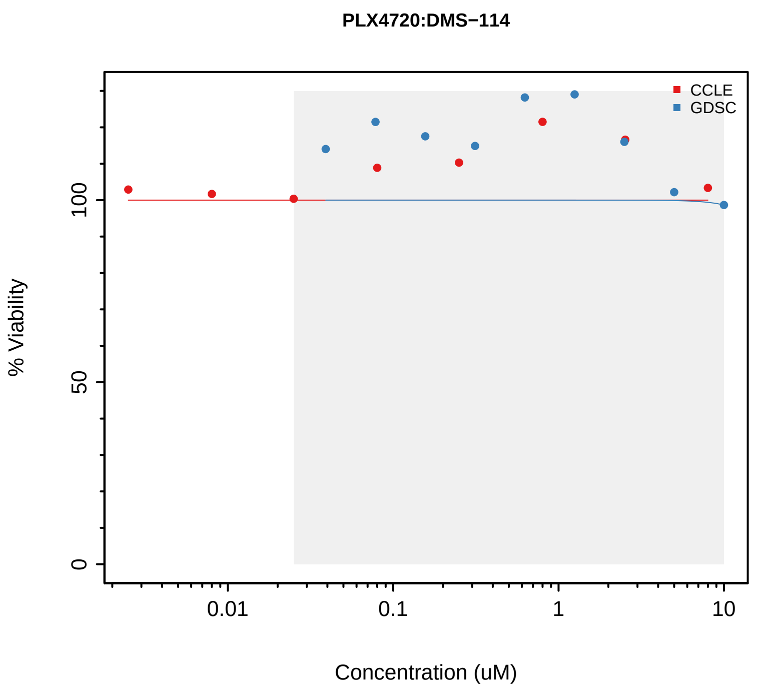
PLX4720:DMS−114
CCLE
GDSC
% Viability
0
50
100
0.01
0.1
1
10
Concentration (uM)

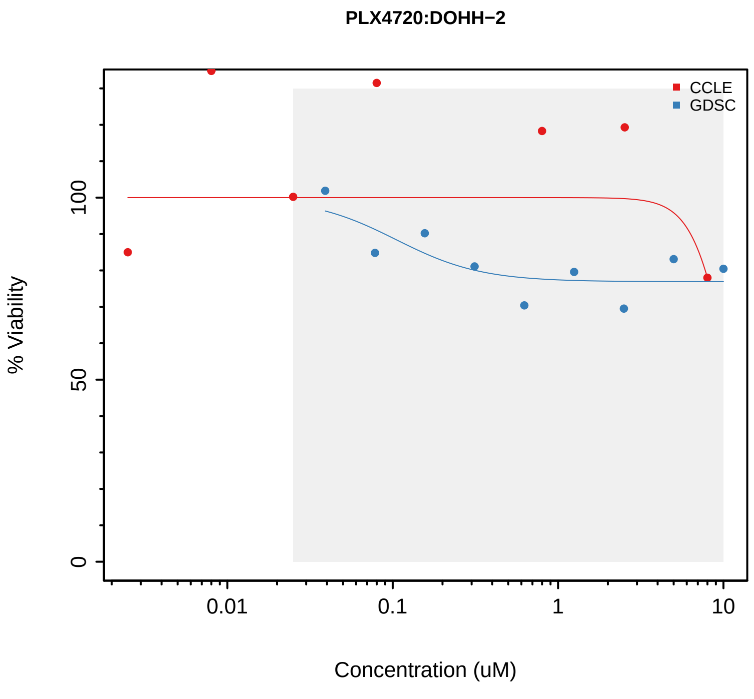
PLX4720:DOHH−2
CCLE
GDSC
% Viability
0
50
100
0.01
0.1
1
10
Concentration (uM)

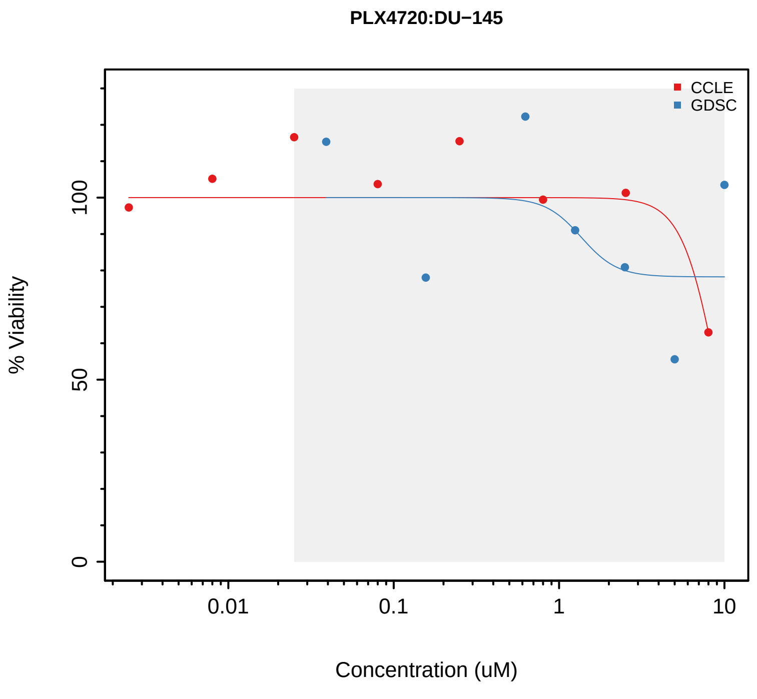
PLX4720:DU−145
CCLE
GDSC
% Viability
0
50
100
0.01
0.1
1
10
Concentration (uM)

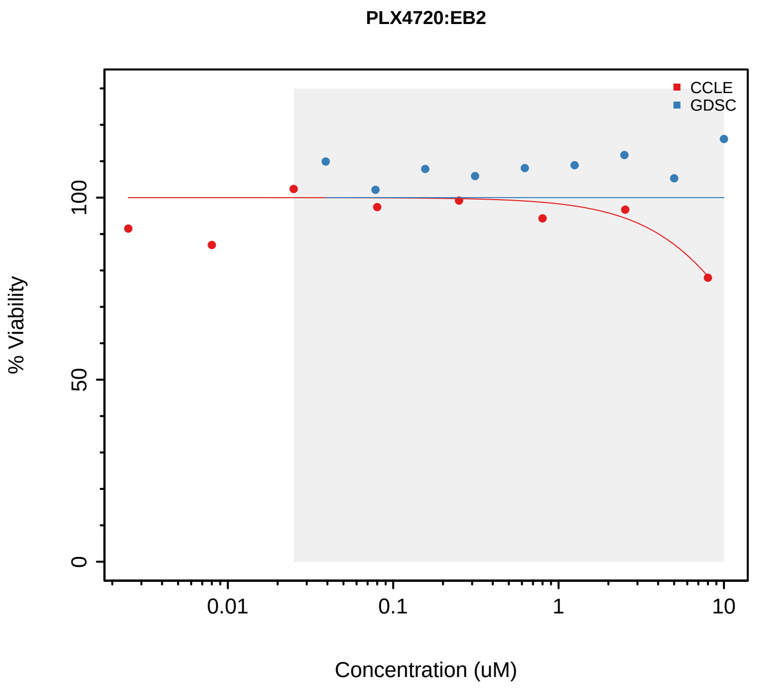
PLX4720:EB2
CCLE
GDSC
% Viability
0
50
100
0.01
0.1
1
10
Concentration (uM)

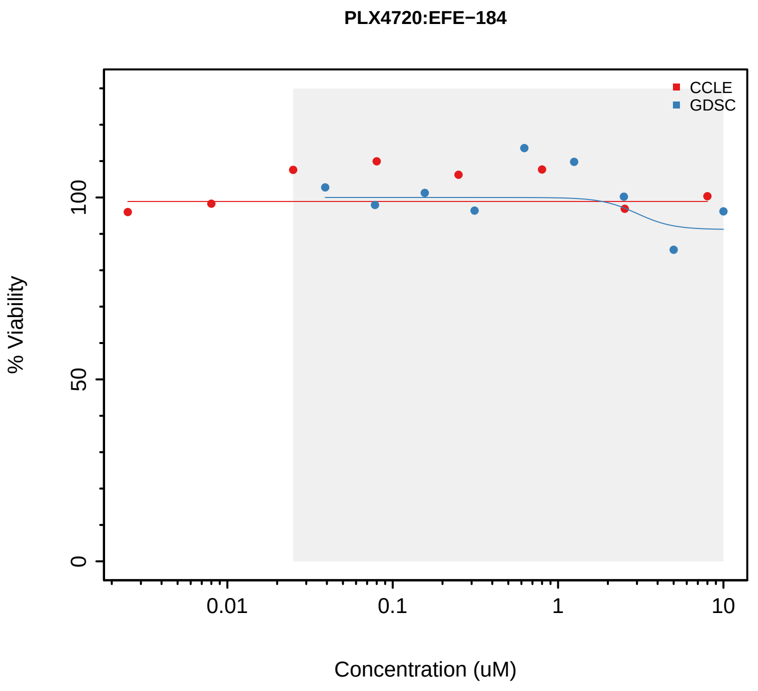
PLX4720:EFE−184
CCLE
GDSC
% Viability
0
50
100
0.01
0.1
1
10
Concentration (uM)

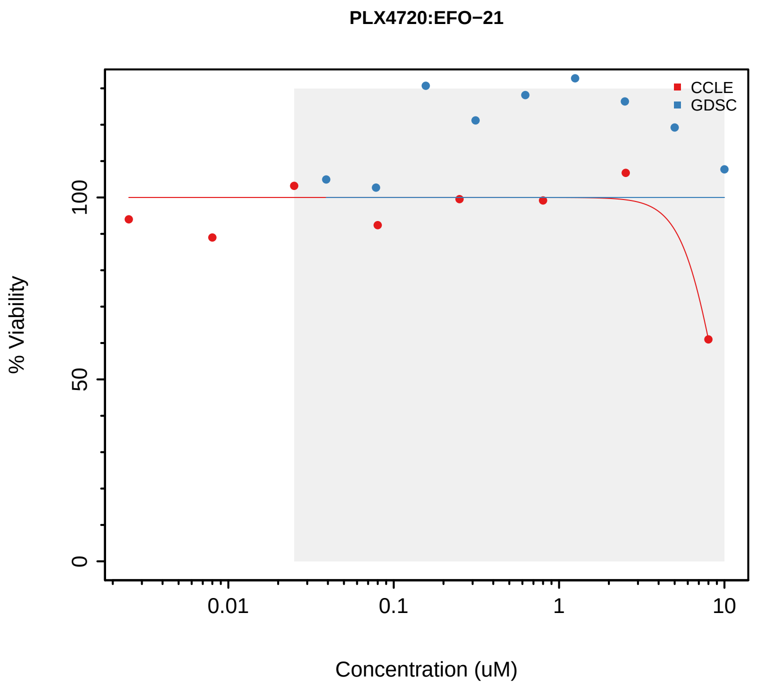
PLX4720:EFO−21
CCLE
GDSC
% Viability
0
50
100
0.01
0.1
1
10
Concentration (uM)

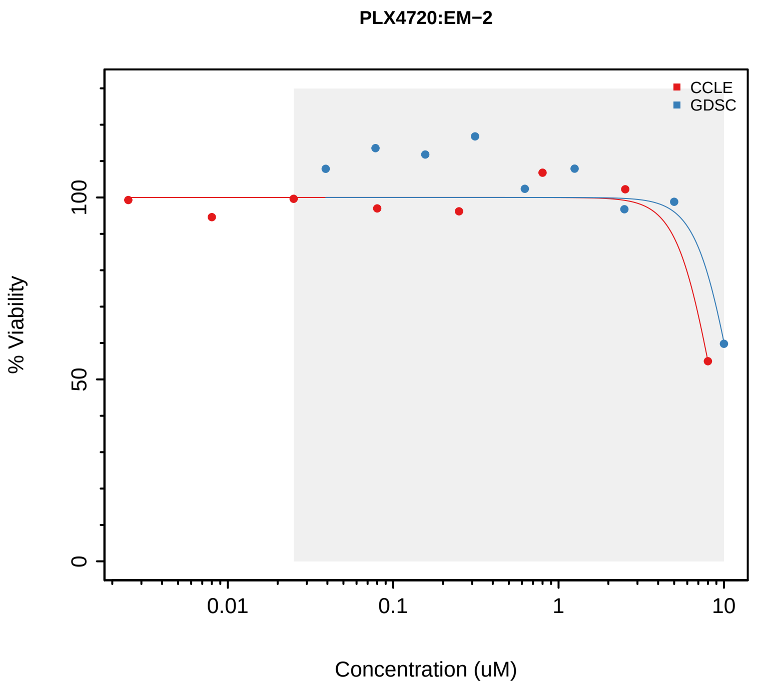
PLX4720:EM−2
CCLE
GDSC
% Viability
0
50
100
0.01
0.1
1
10
Concentration (uM)

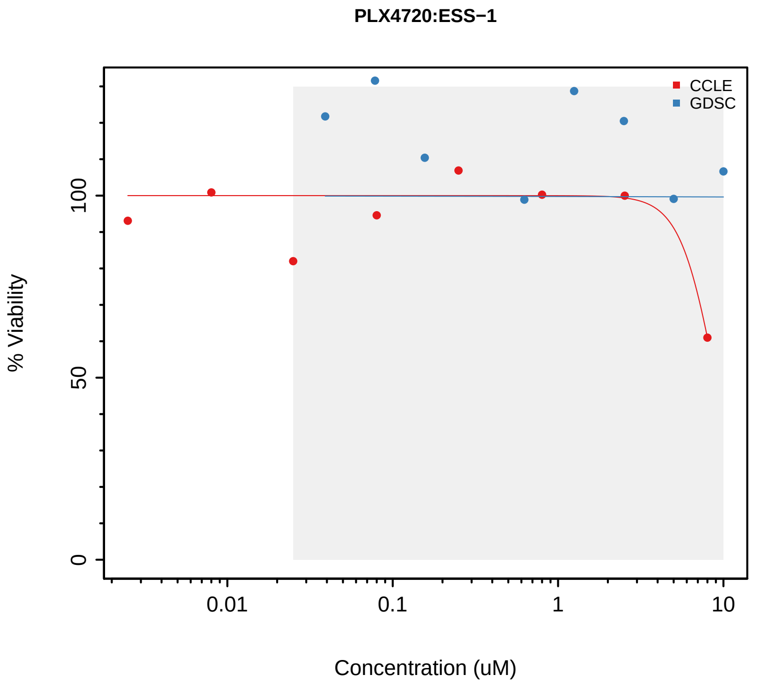
PLX4720:ESS−1
CCLE
GDSC
% Viability
0
50
100
0.01
0.1
1
10
Concentration (uM)

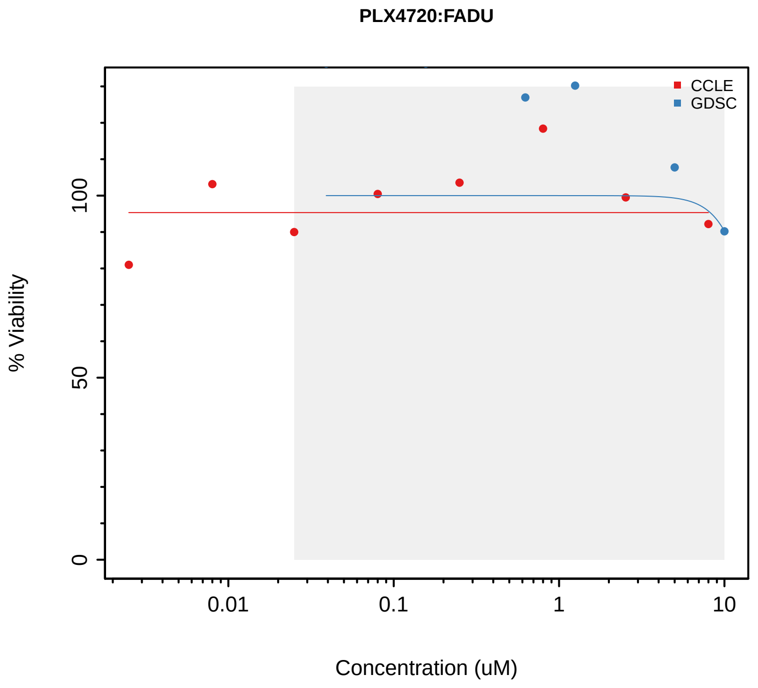
PLX4720:FADU
CCLE
GDSC
% Viability
0
50
100
0.01
0.1
1
10
Concentration (uM)

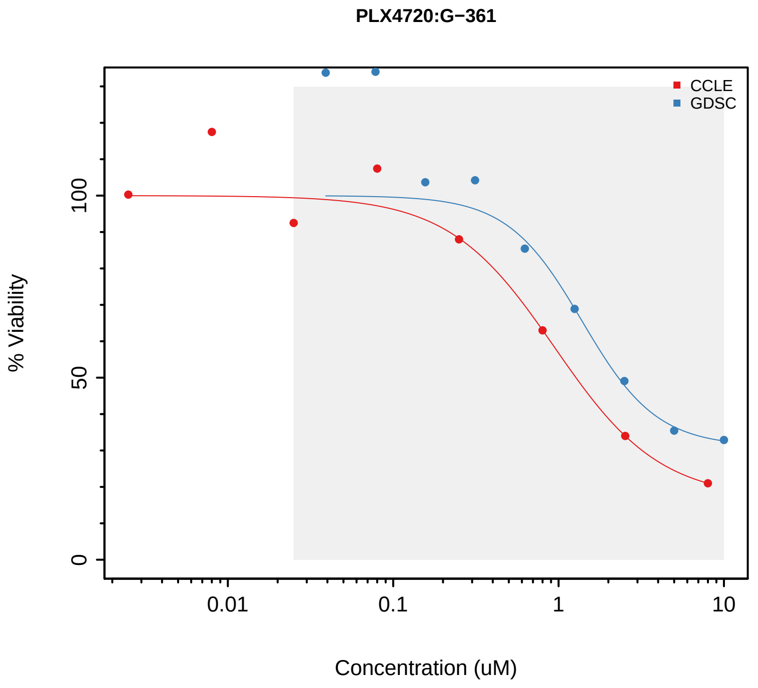
PLX4720:G−361
CCLE
GDSC
% Viability
0
50
100
0.01
0.1
1
10
Concentration (uM)

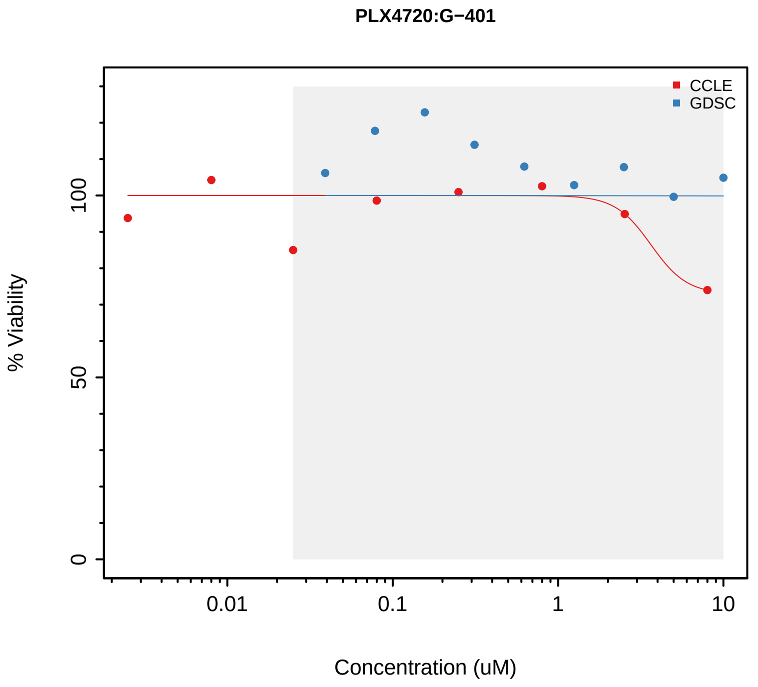
PLX4720:G−401
CCLE
GDSC
% Viability
0
50
100
0.01
0.1
1
10
Concentration (uM)

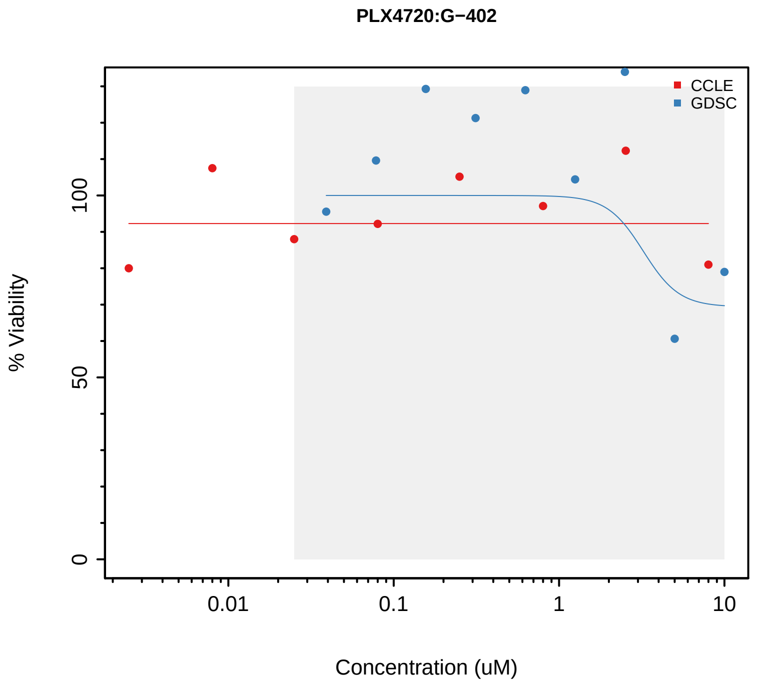
PLX4720:G−402
CCLE
GDSC
% Viability
0
50
100
0.01
0.1
1
10
Concentration (uM)

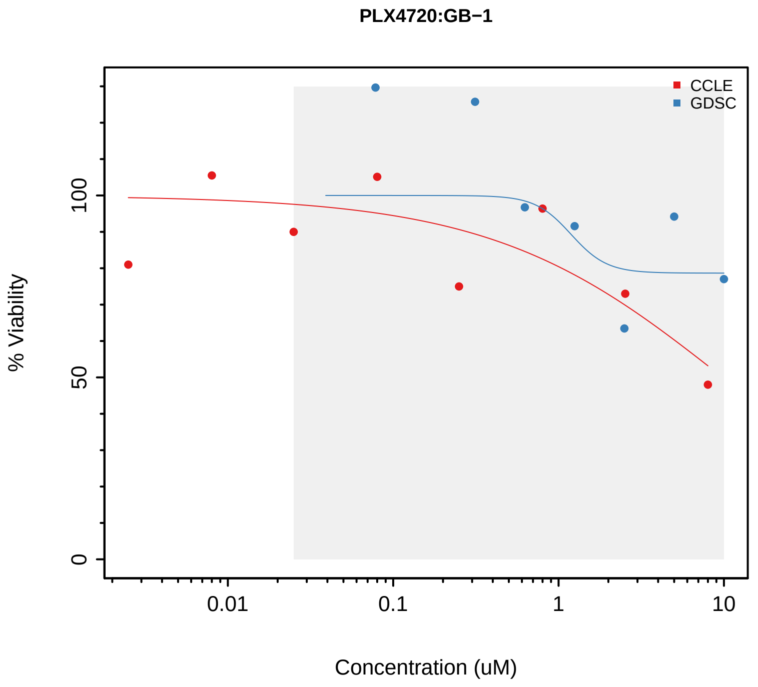
PLX4720:GB−1
CCLE
GDSC
% Viability
0
50
100
0.01
0.1
1
10
Concentration (uM)

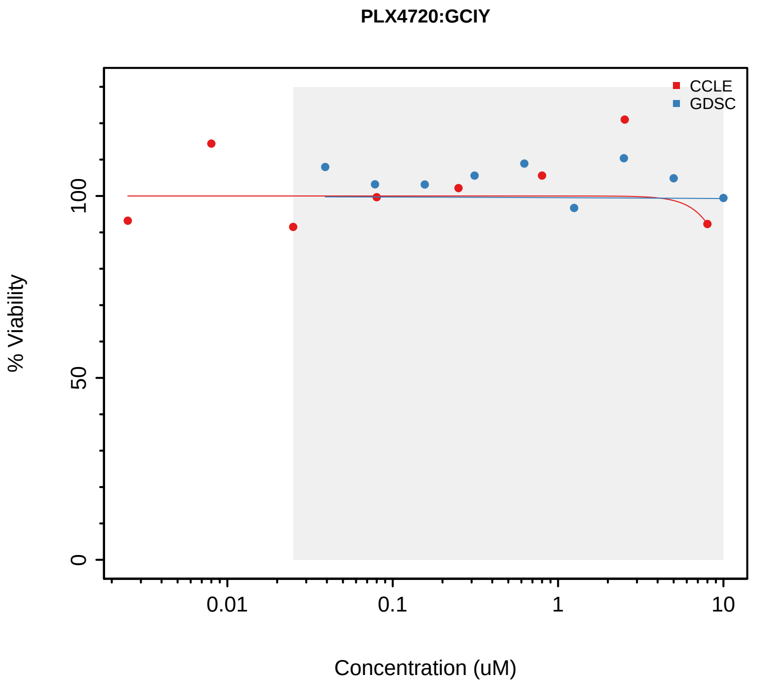
PLX4720:GCIY
CCLE
GDSC
% Viability
0
50
100
0.01
0.1
1
10
Concentration (uM)

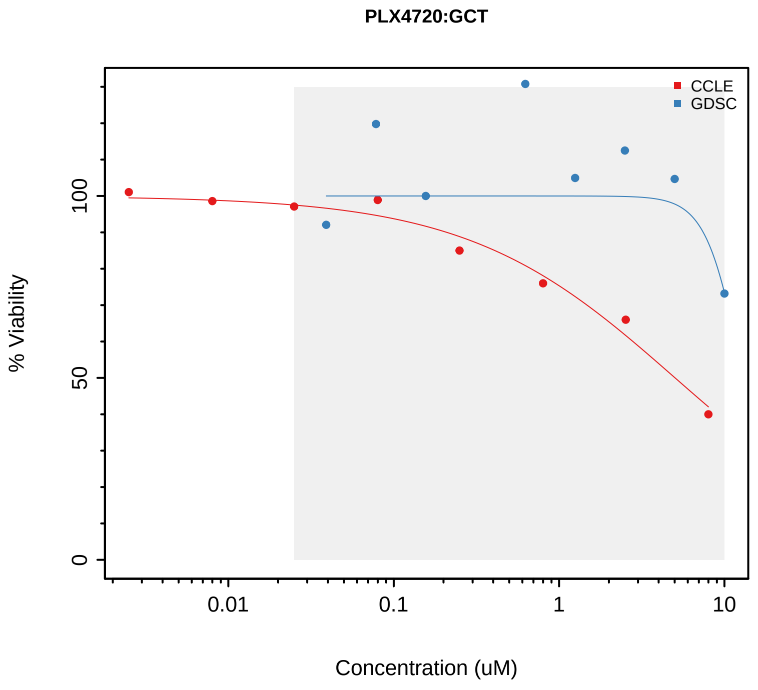
PLX4720:GCT
CCLE
GDSC
% Viability
0
50
100
0.01
0.1
1
10
Concentration (uM)

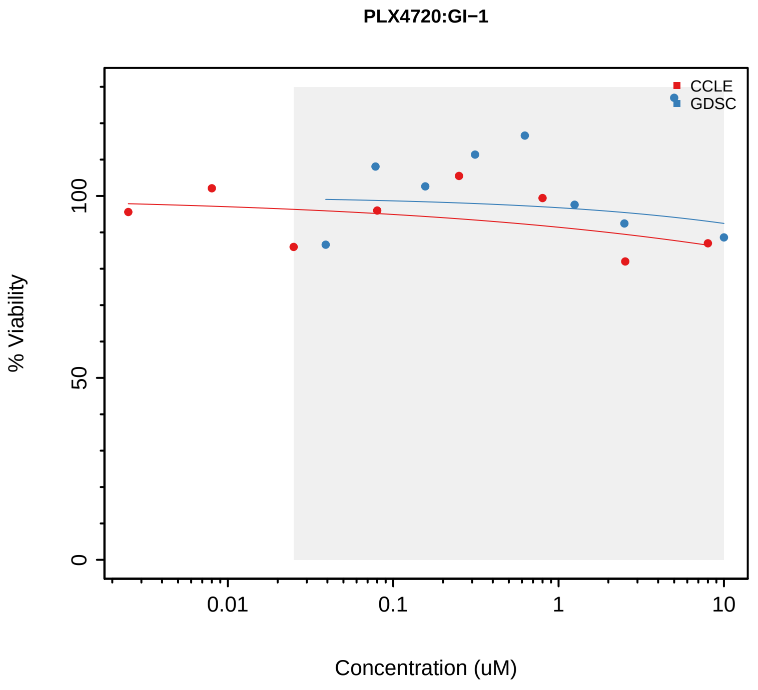
PLX4720:GI−1
CCLE
GDSC
% Viability
0
50
100
0.01
0.1
1
10
Concentration (uM)

PLX4720:GMS−10

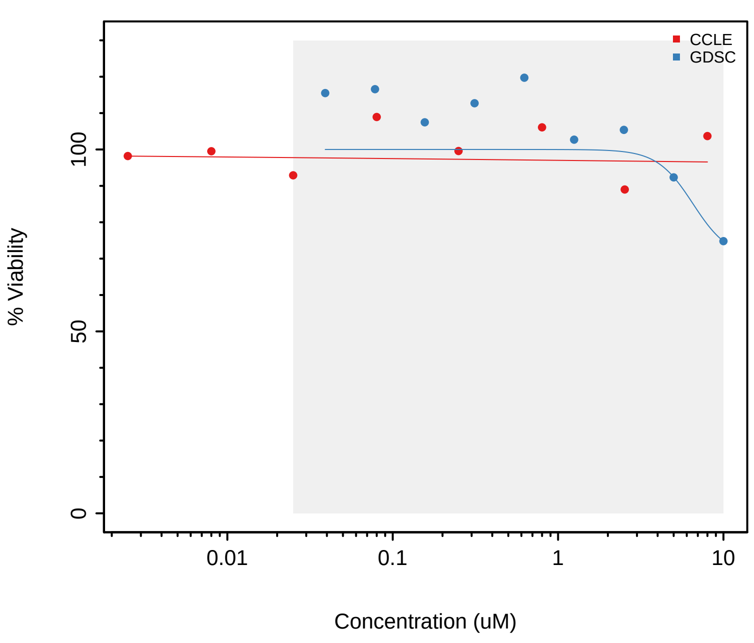

PLX4720:H4

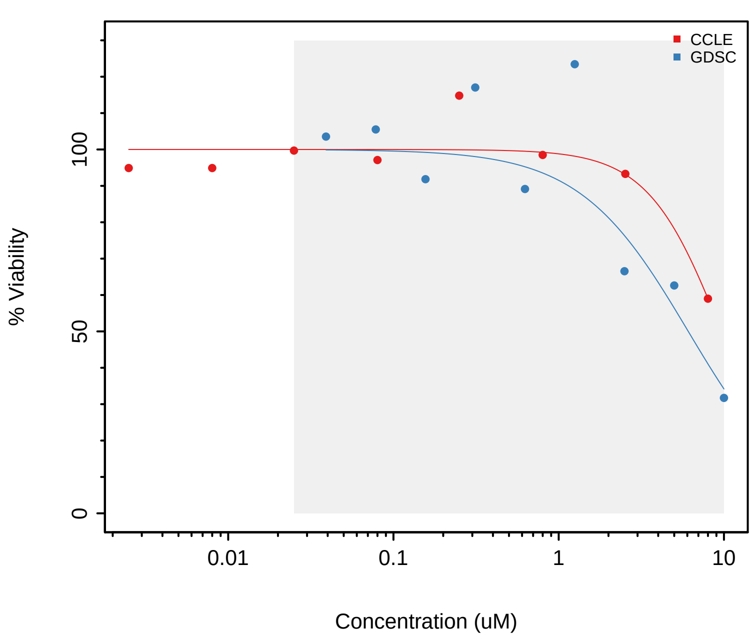

PLX4720:HCC1187

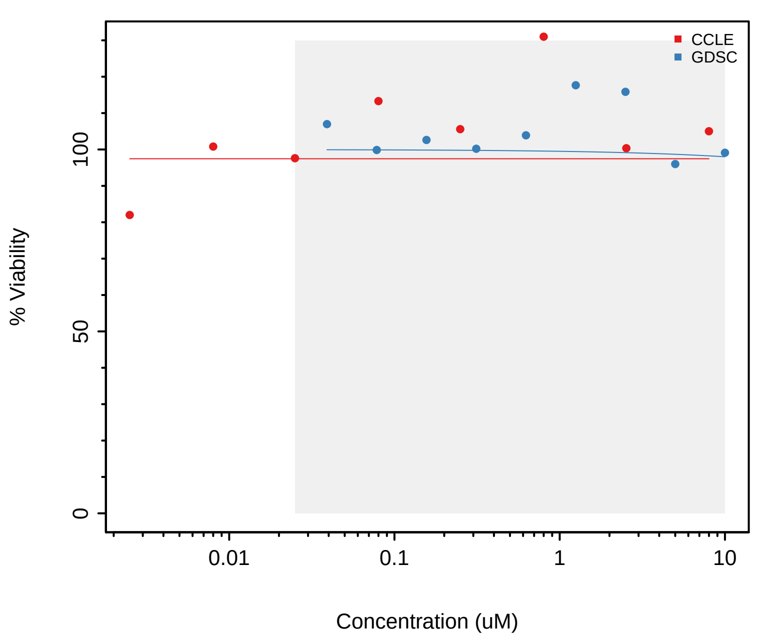

PLX4720:HCC1395

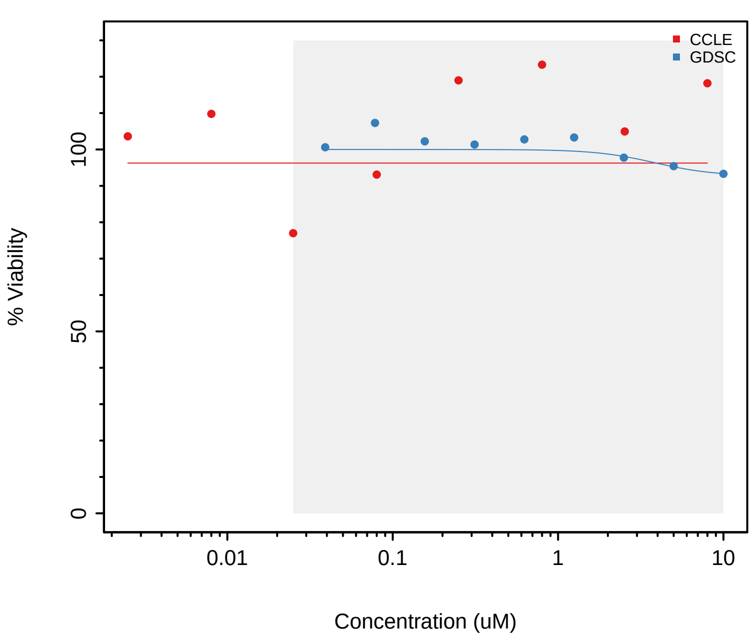

PLX4720:HCC1569

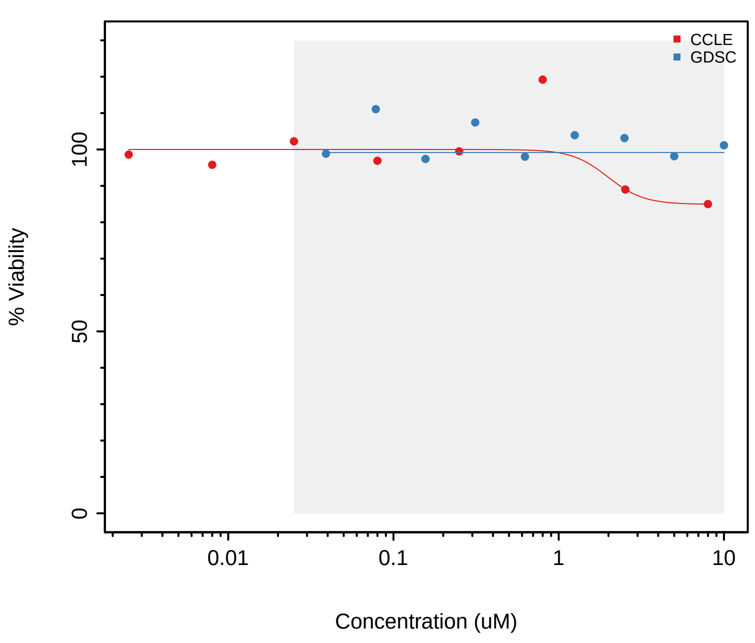

PLX4720:HCC1806

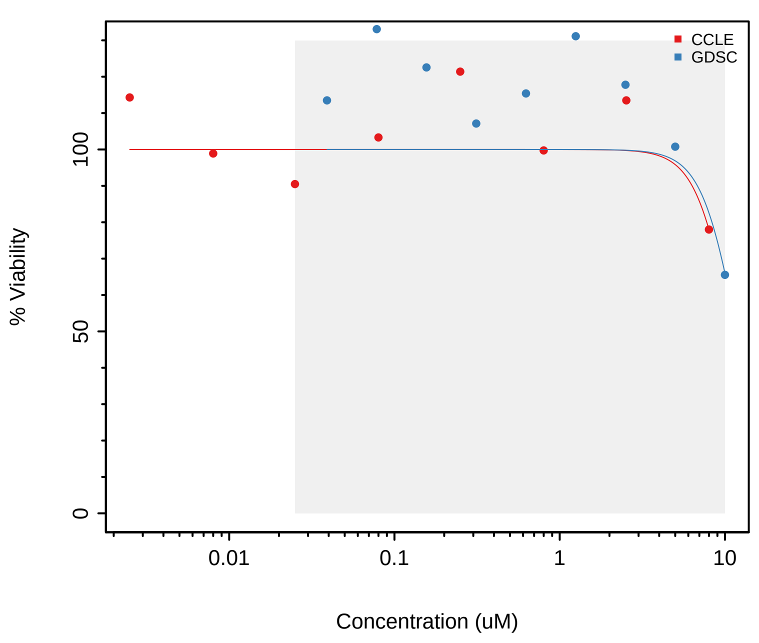

PLX4720:HCC1954

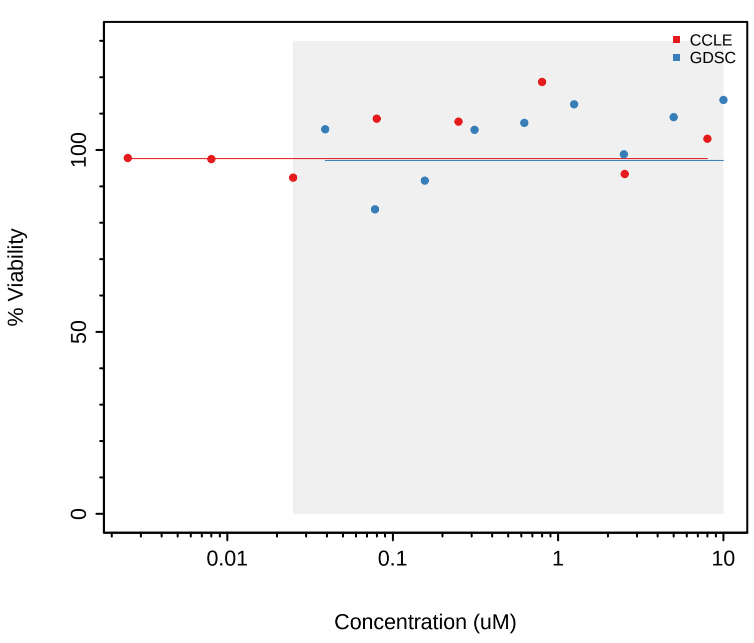

PLX4720:HCT−116

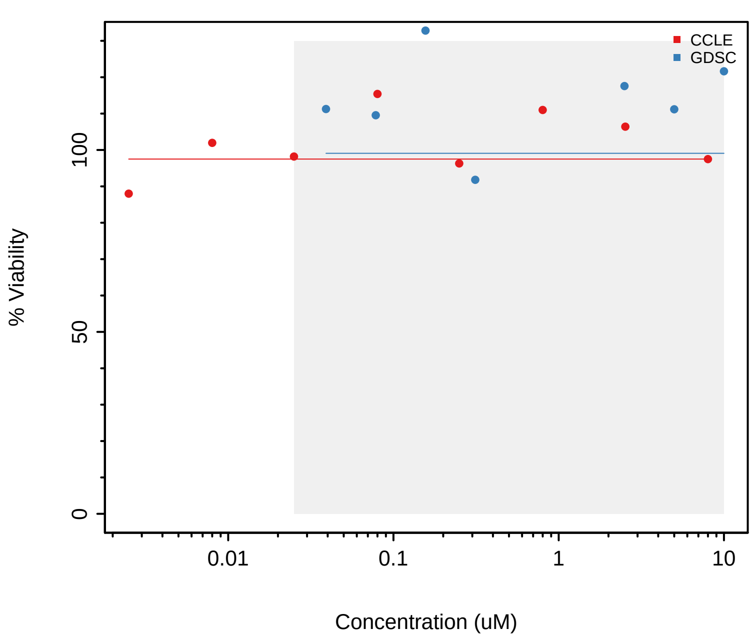

PLX4720:HCT−15

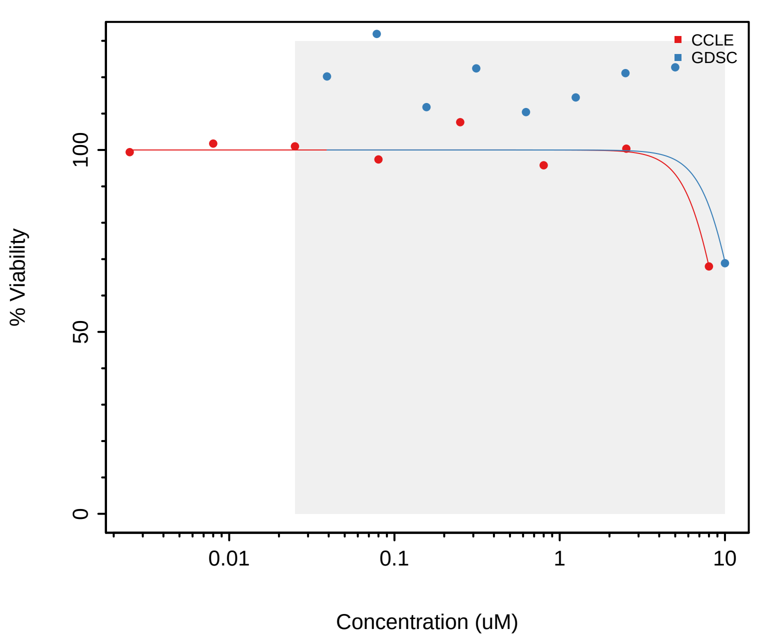

PLX4720:HD−MY−Z

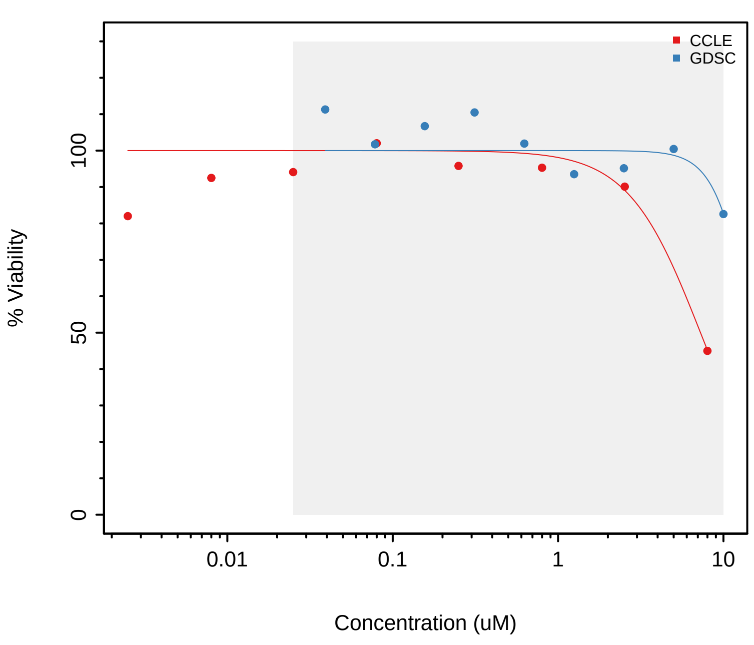

PLX4720:HEC−1

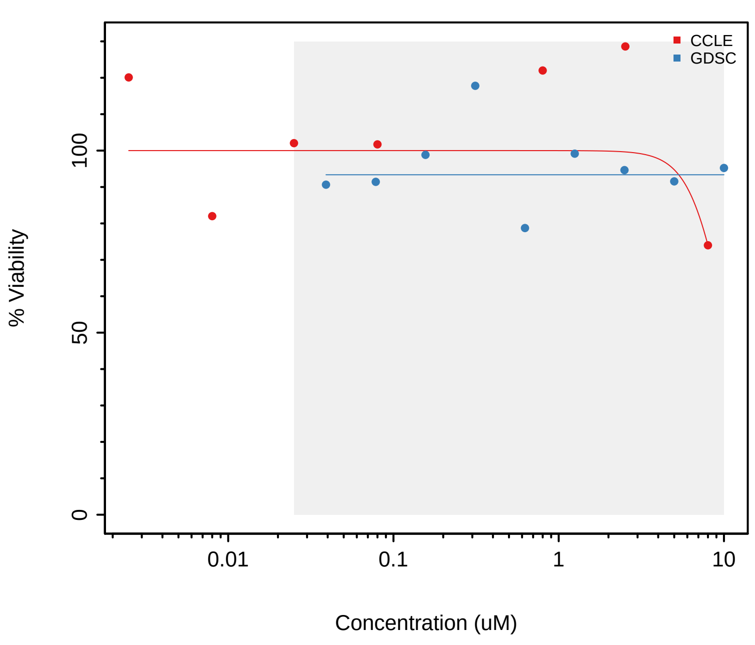

PLX4720:HGC−27

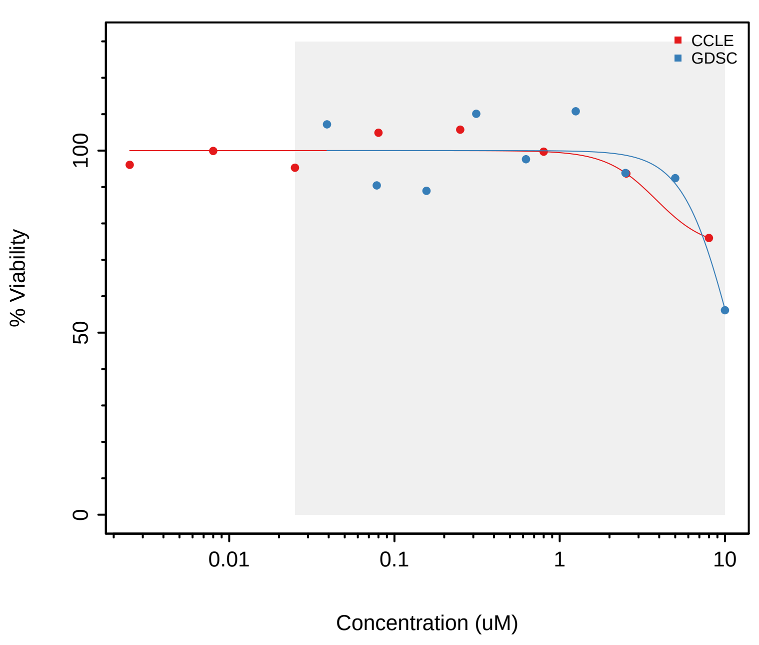

PLX4720:HH

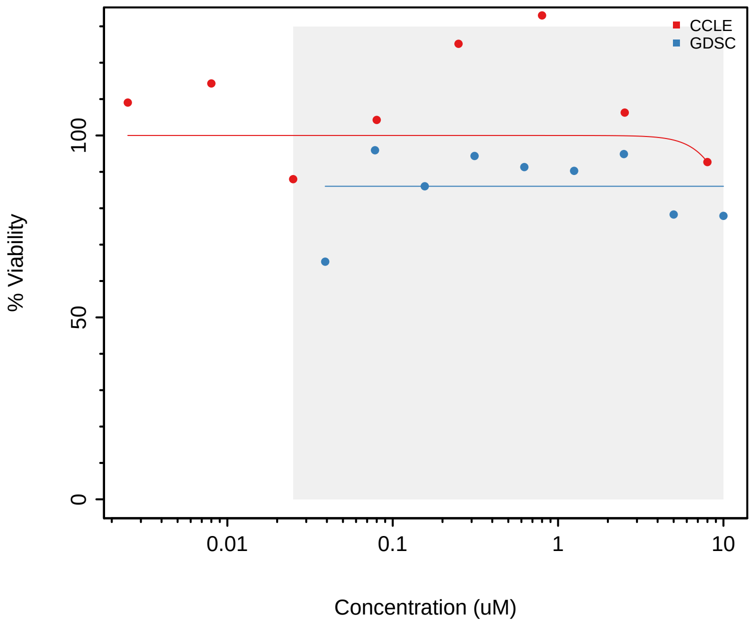

PLX4720:HLE

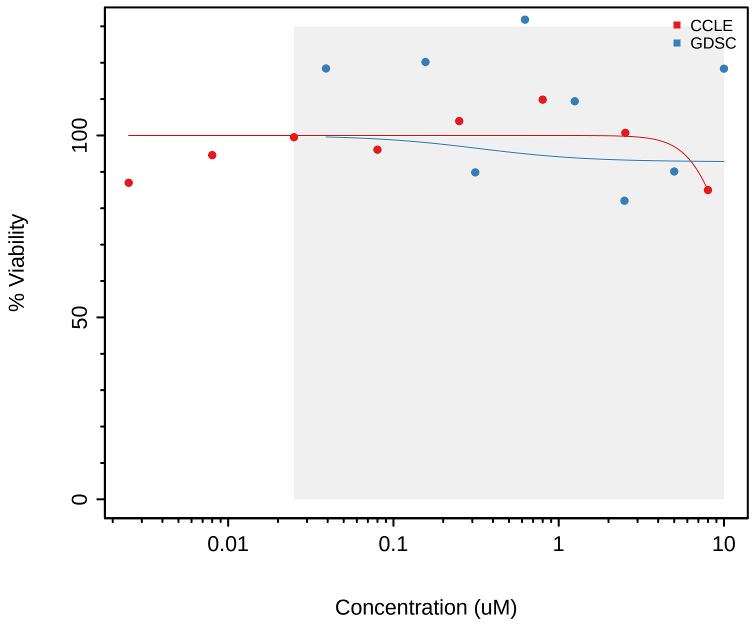

PLX4720:HOS

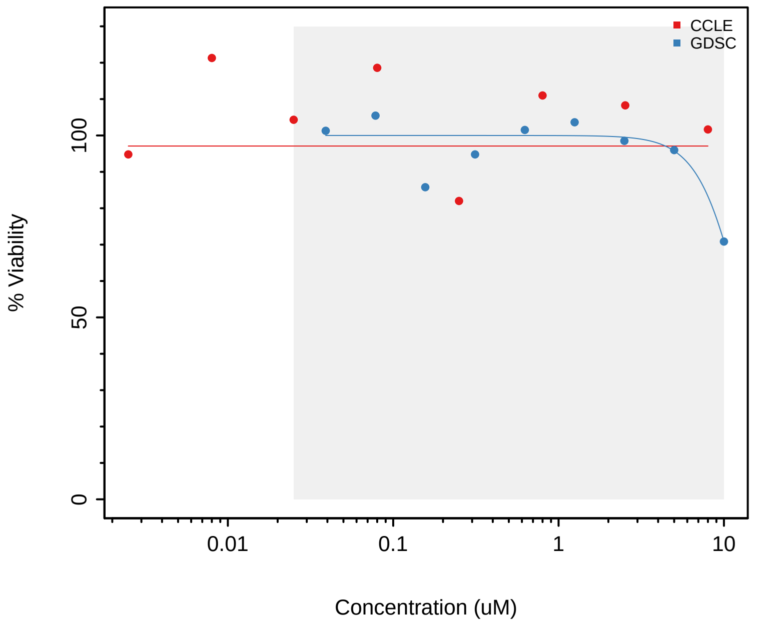

PLX4720:HPAF−II

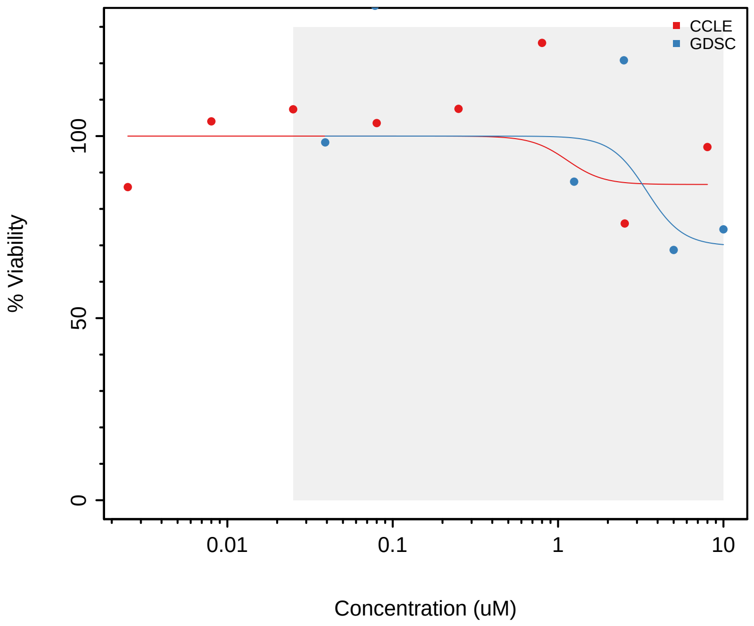

PLX4720:Hs−578−T

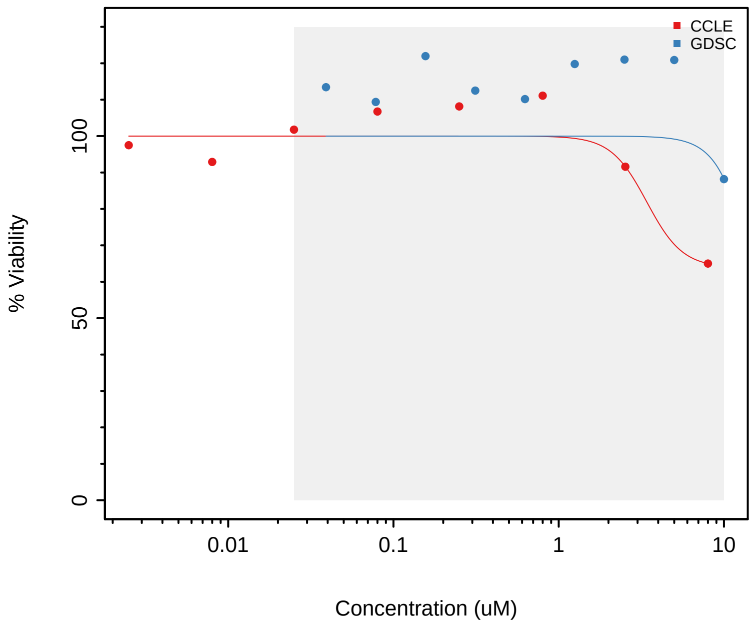

PLX4720:HSC−2

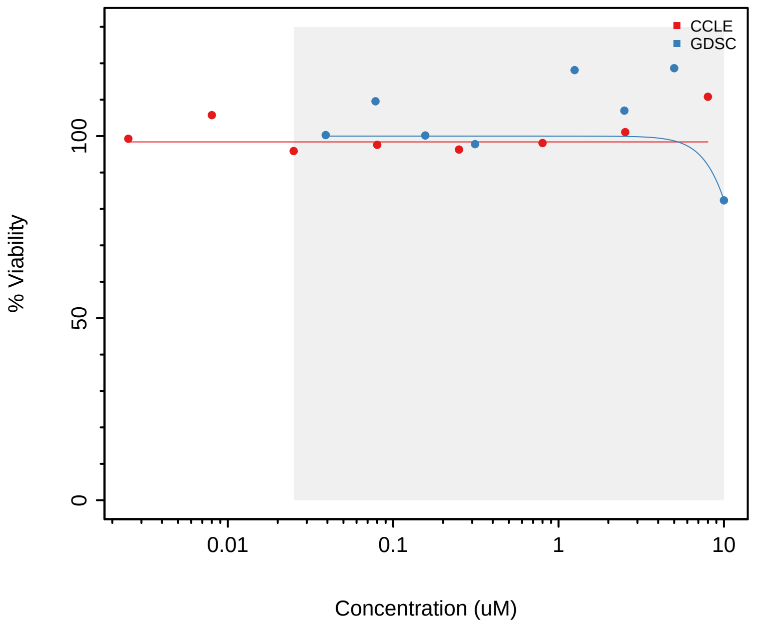

PLX4720:HT

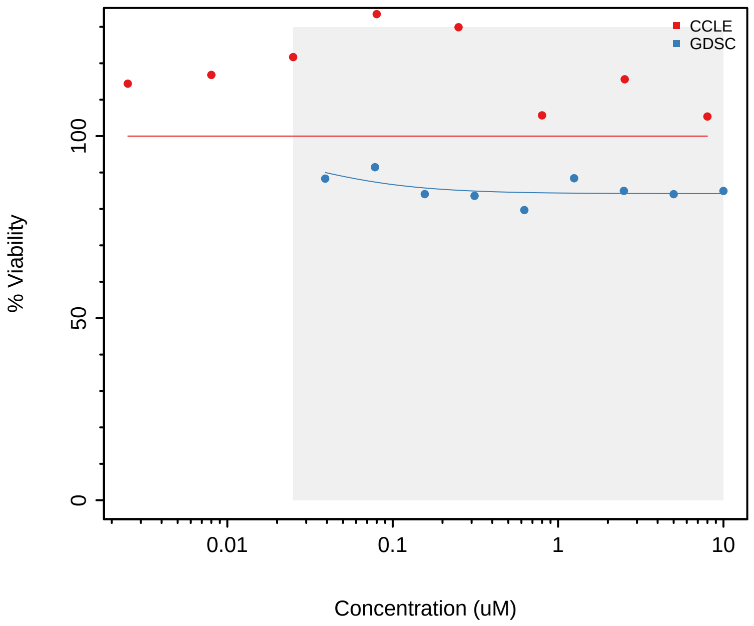

PLX4720:HT−1080

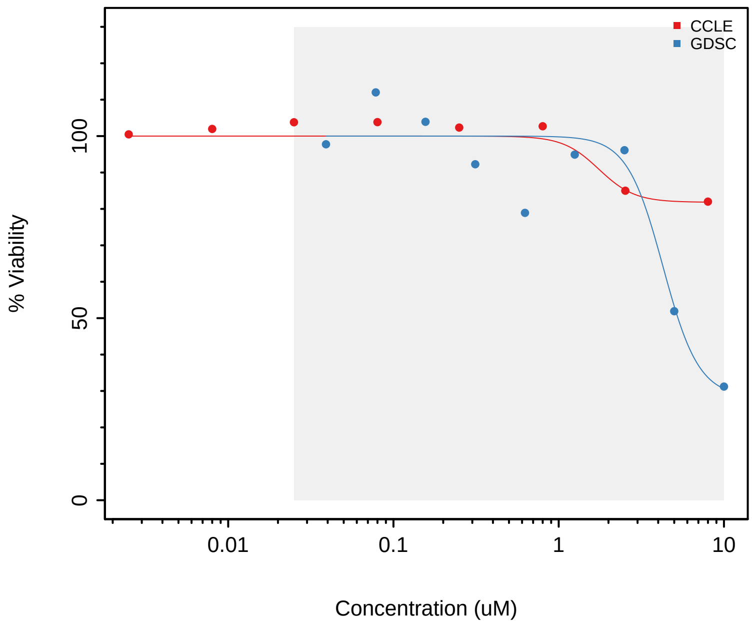

PLX4720:HT−1197

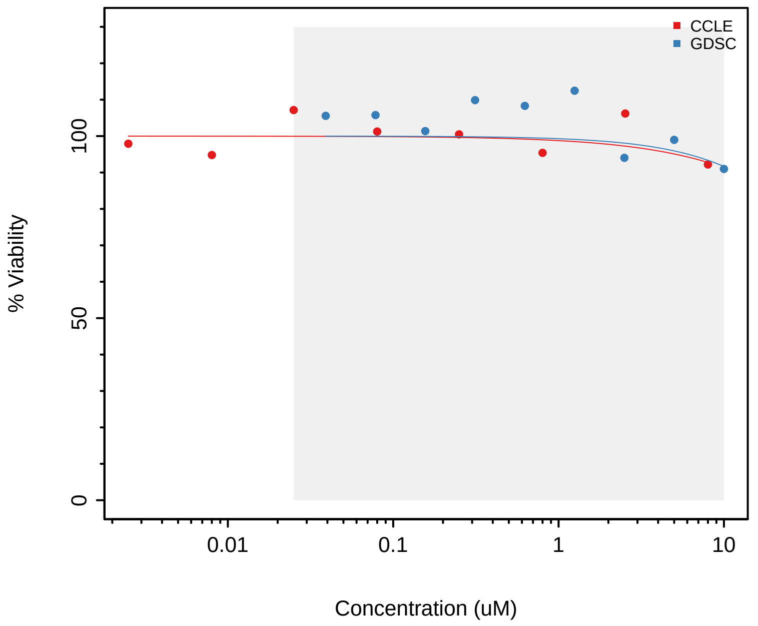

PLX4720:HT−1376

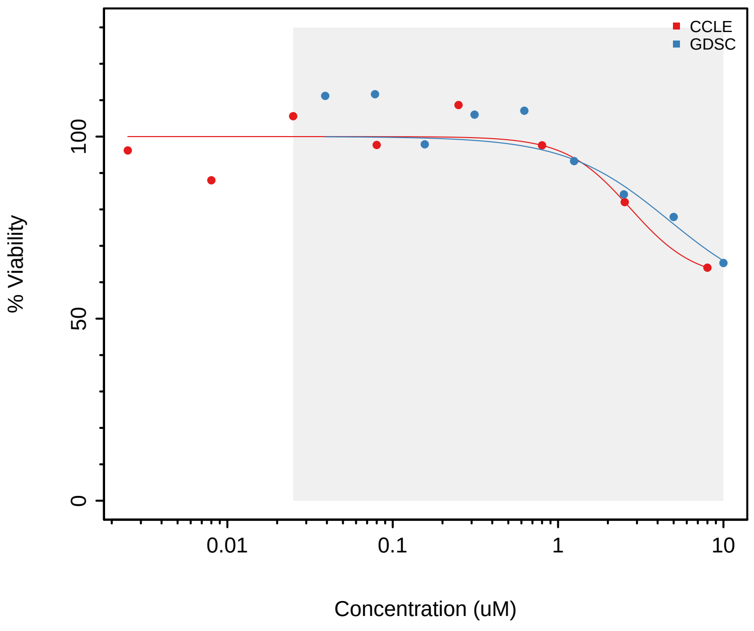

PLX4720:HT−144

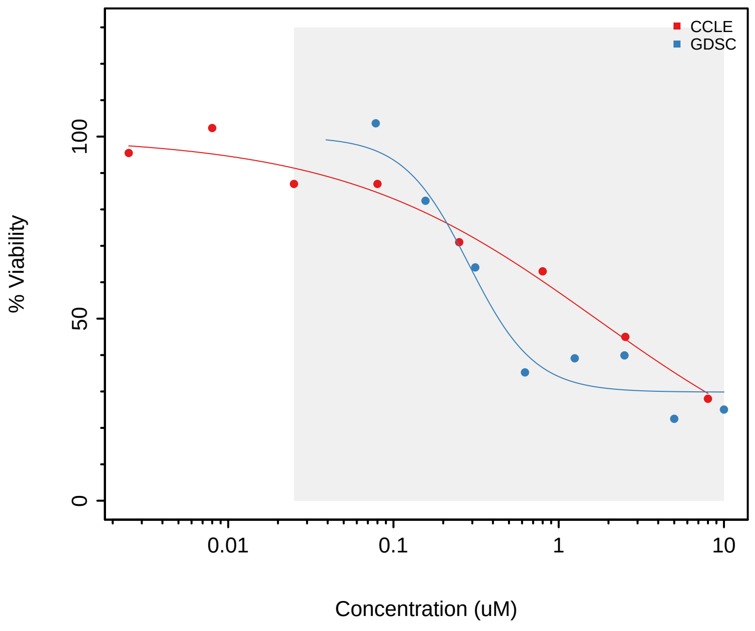

PLX4720:HT−29

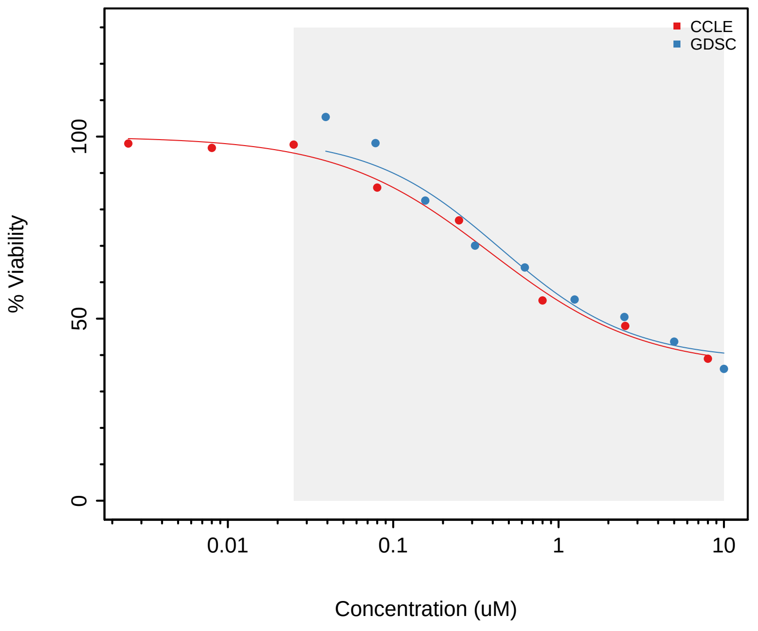

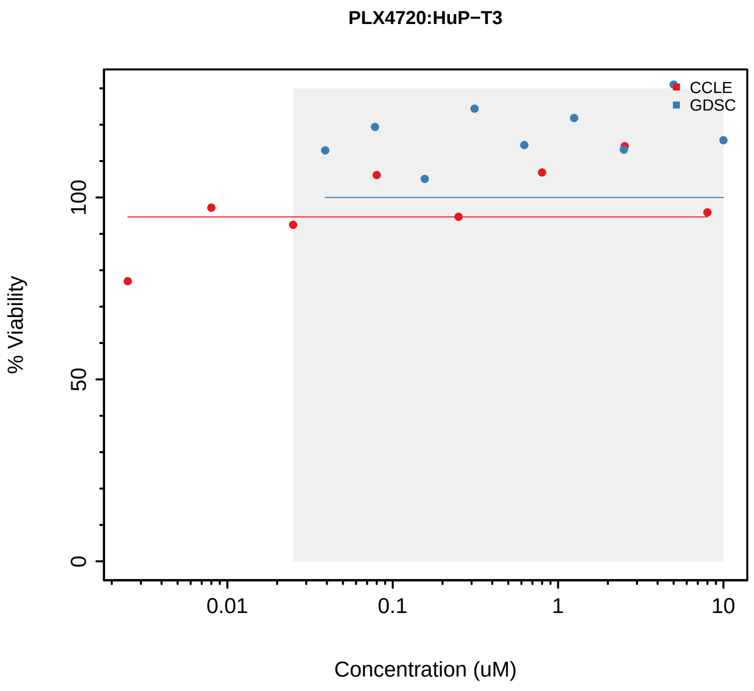
PLX4720:HuP−T3
CCLE
GDSC
% Viability
0
50
100
0.01
0.1
1
10
Concentration (uM)

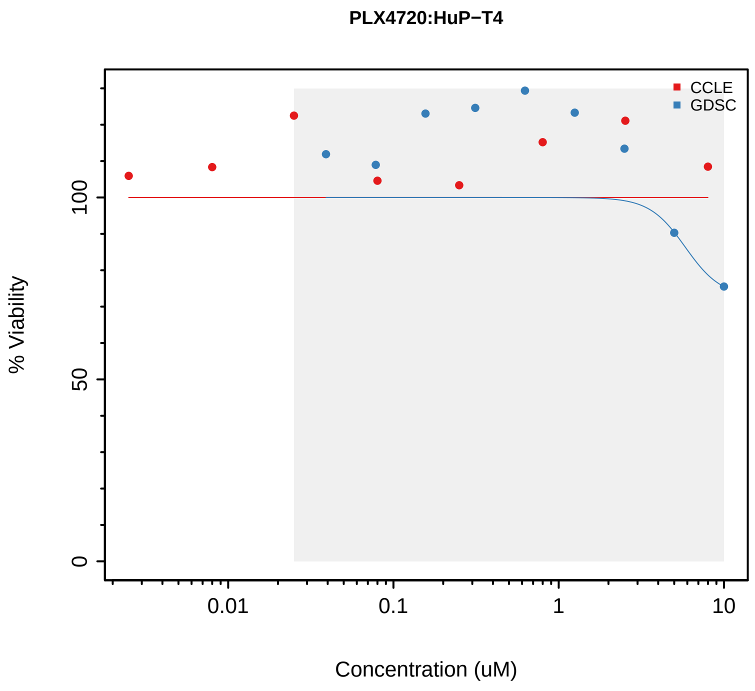
PLX4720:HuP−T4
CCLE
GDSC
% Viability
0
50
100
0.01
0.1
1
10
Concentration (uM)

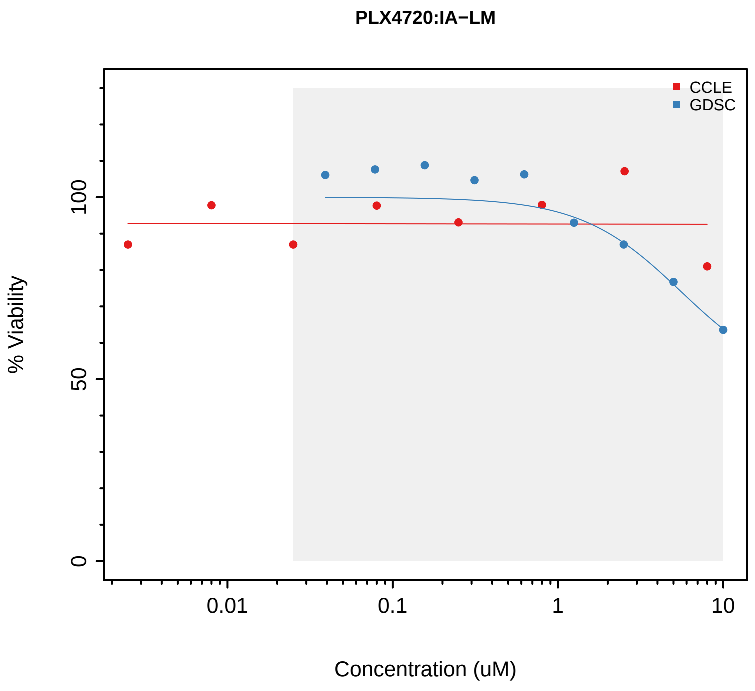
PLX4720:IA−LM
CCLE
GDSC
% Viability
0
50
100
0.01
0.1
1
10
Concentration (uM)

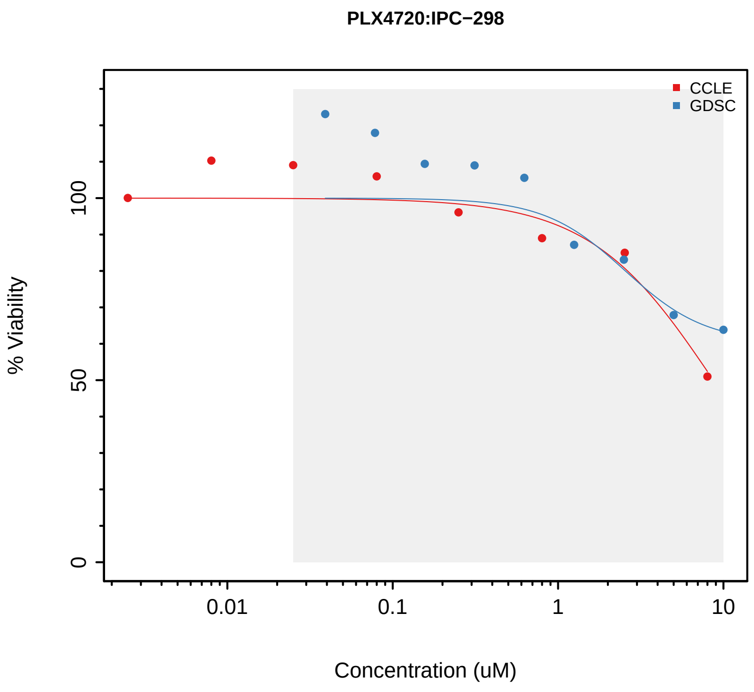
PLX4720:IPC−298
CCLE
GDSC
% Viability
0
50
100
0.01
0.1
1
10
Concentration (uM)

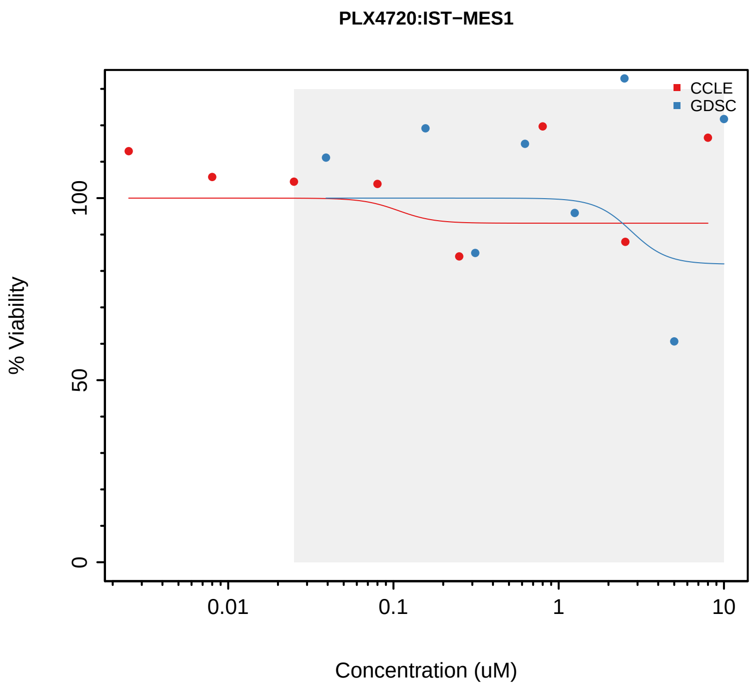
PLX4720:IST−MES1
CCLE
GDSC
% Viability
0
50
100
0.01
0.1
1
10
Concentration (uM)

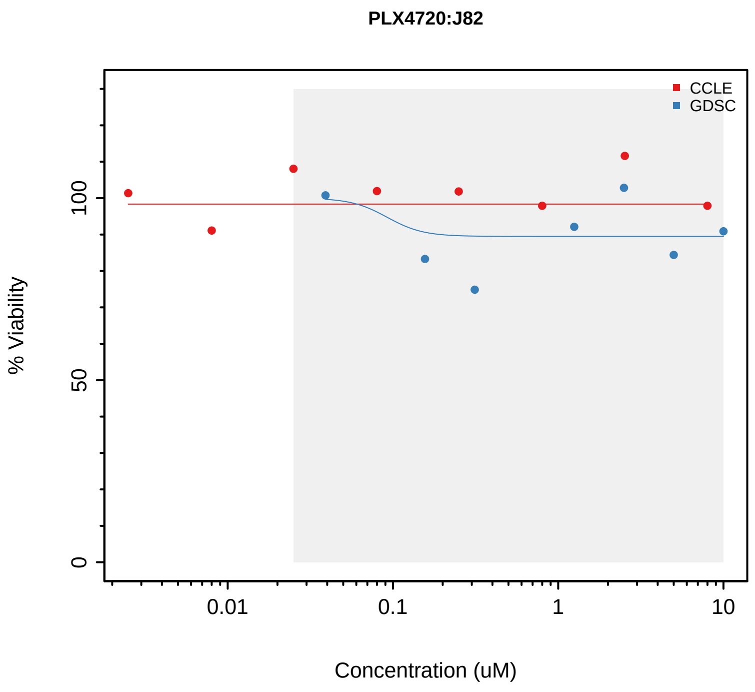
PLX4720:J82
CCLE
GDSC
% Viability
0
50
100
0.01
0.1
1
10
Concentration (uM)

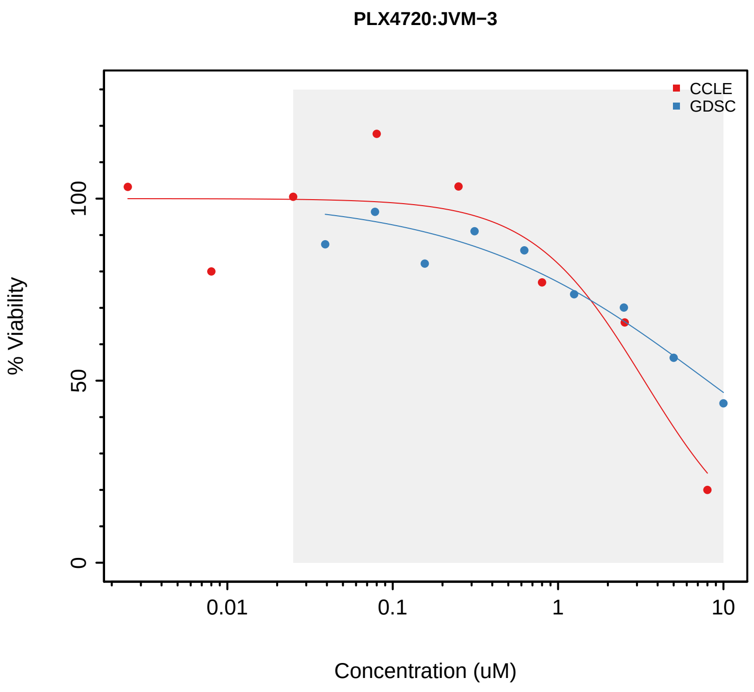
PLX4720:JVM−3
CCLE
GDSC
% Viability
0
50
100
0.01
0.1
1
10
Concentration (uM)

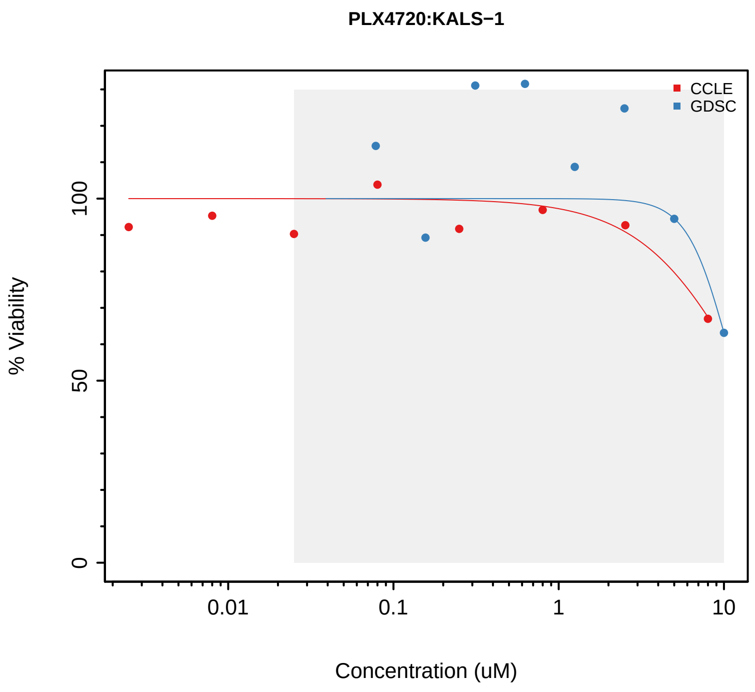
PLX4720:KALS−1
CCLE
GDSC
% Viability
0
50
100
0.01
0.1
1
10
Concentration (uM)

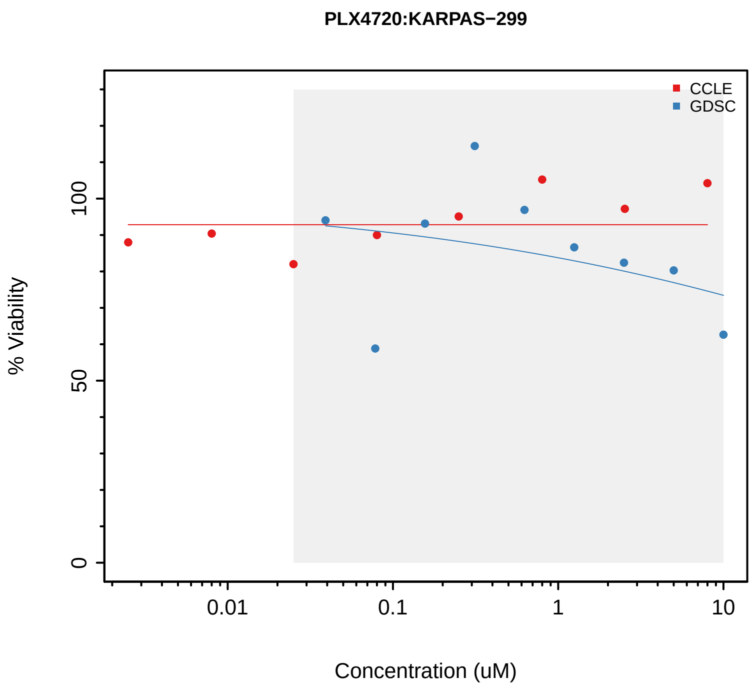
PLX4720:KARPAS−299
CCLE
GDSC
% Viability
0
50
100
0.01
0.1
1
10
Concentration (uM)

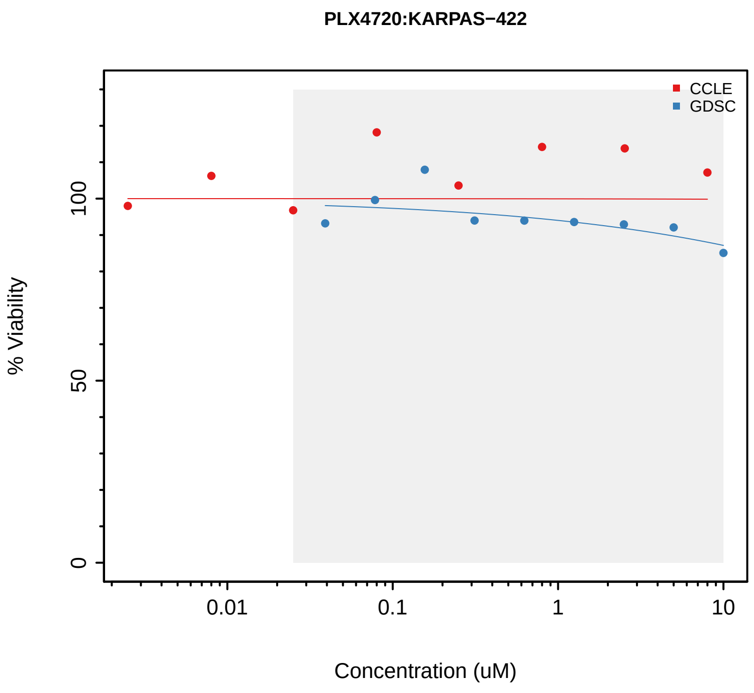
PLX4720:KARPAS−422
CCLE
GDSC
% Viability
0
50
100
0.01
0.1
1
10
Concentration (uM)

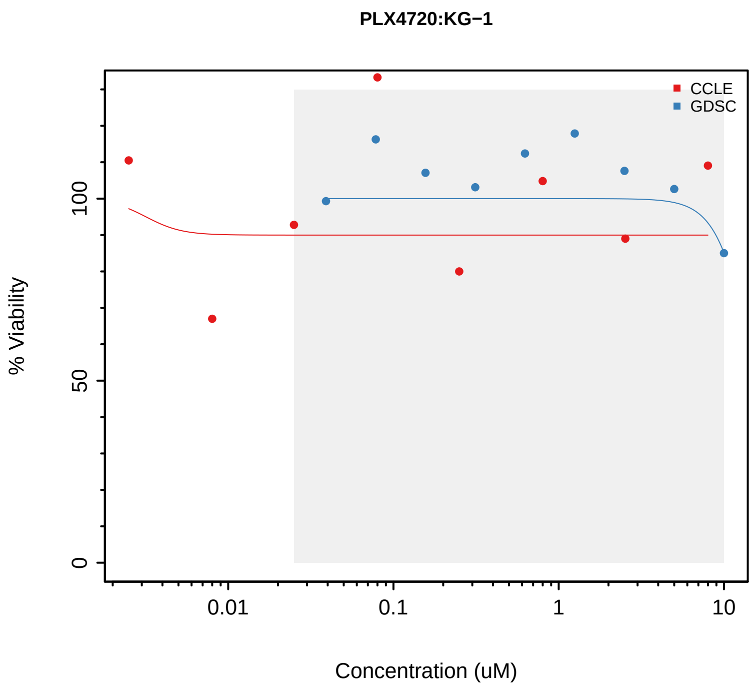
PLX4720:KG−1
CCLE
GDSC
% Viability
0
50
100
0.01
0.1
1
10
Concentration (uM)

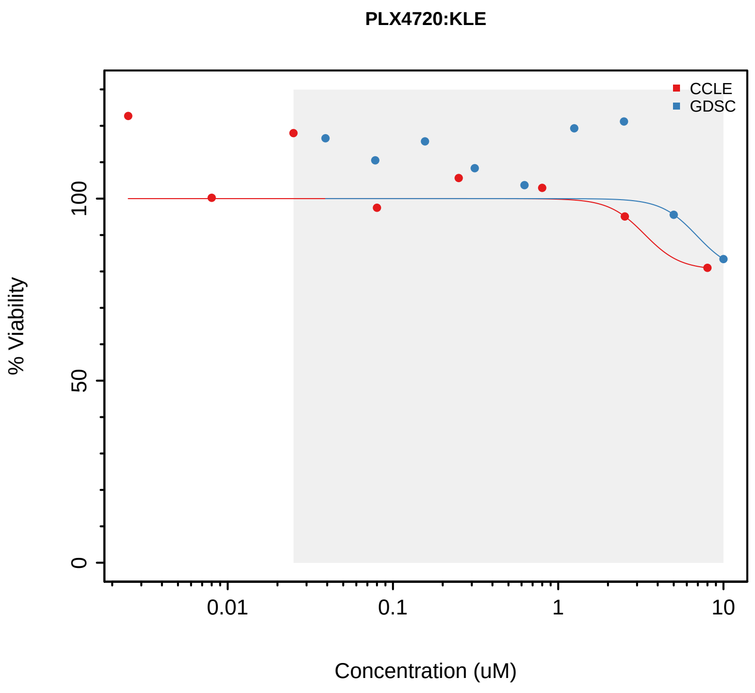
PLX4720:KLE
CCLE
GDSC
% Viability
0
50
100
0.01
0.1
1
10
Concentration (uM)

PLX4720:KNS−42

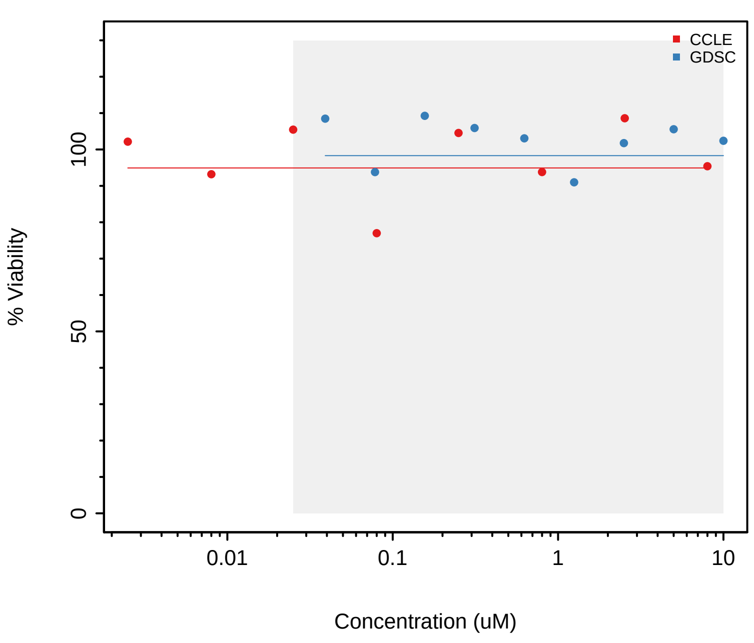

PLX4720:KNS−62

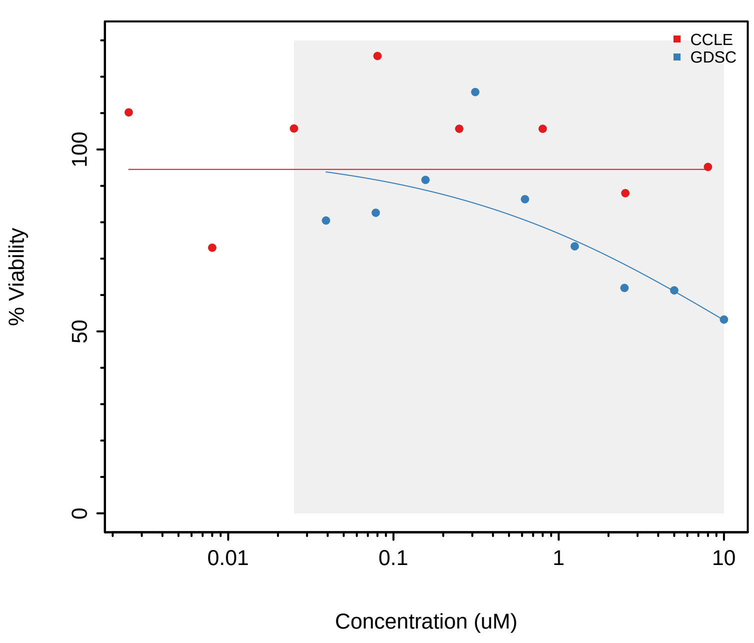

PLX4720:KNS−81−FD

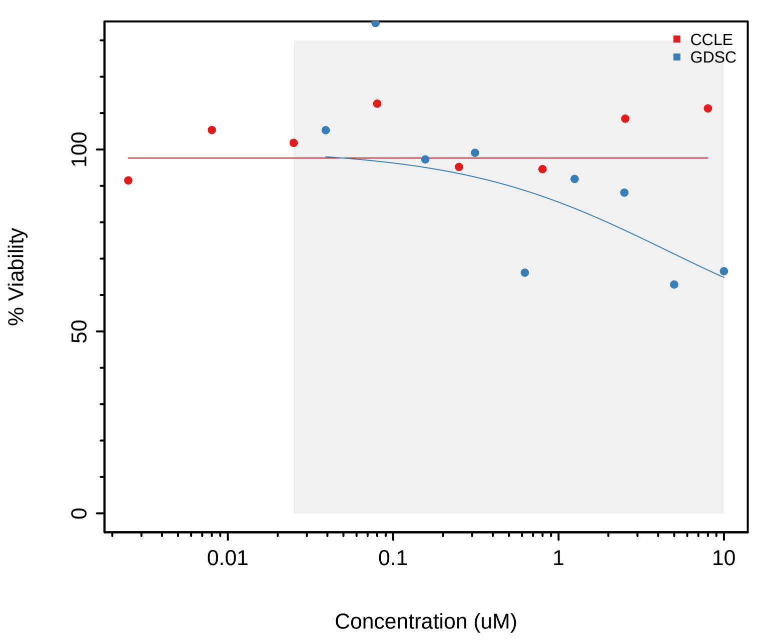

PLX4720:KP−4

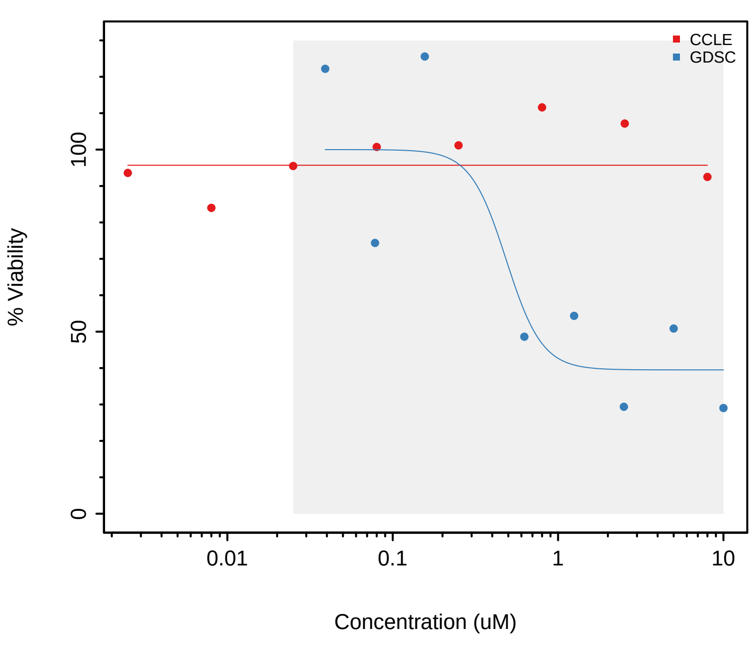

PLX4720:KU812

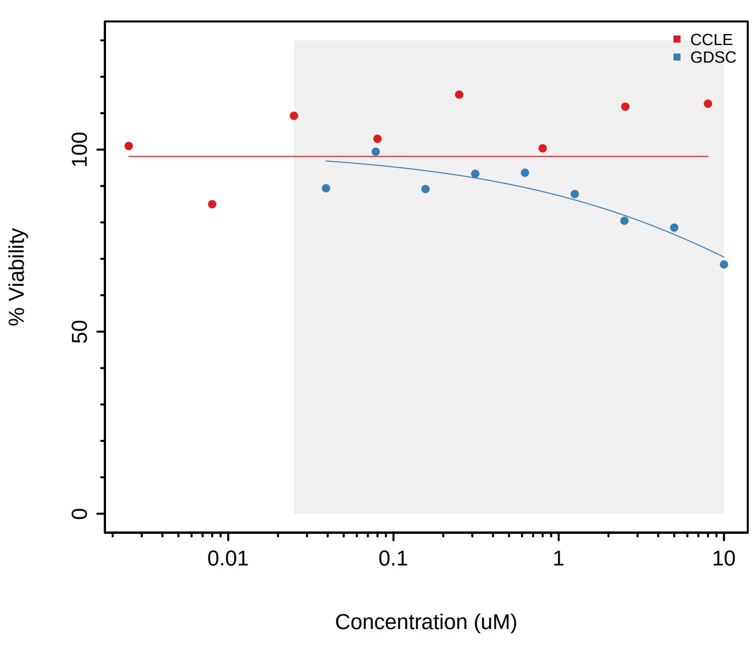

PLX4720:KURAMOCHI

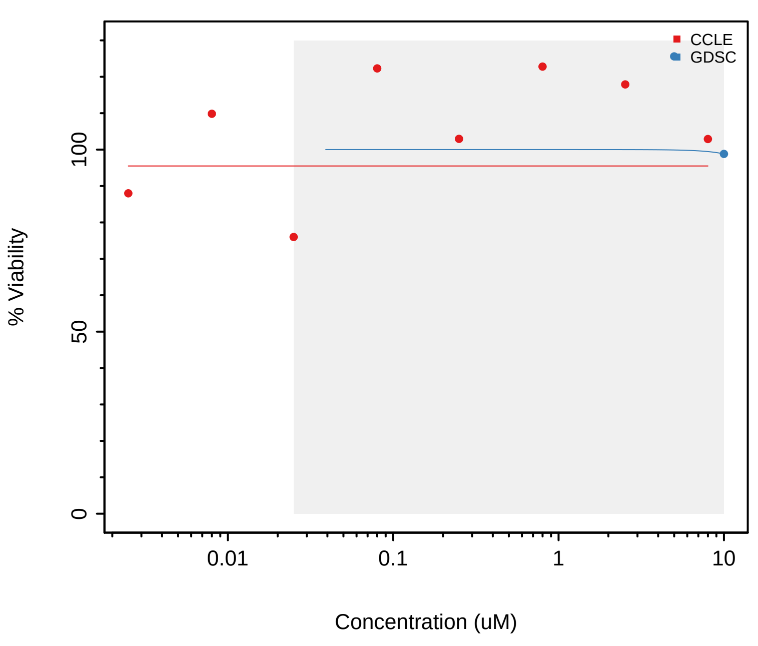

PLX4720:KYSE−140

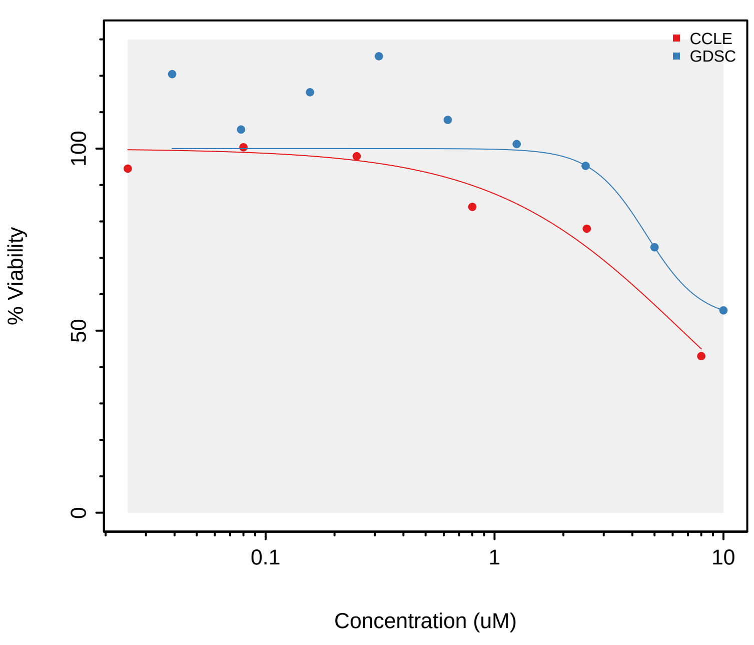

PLX4720:KYSE−150

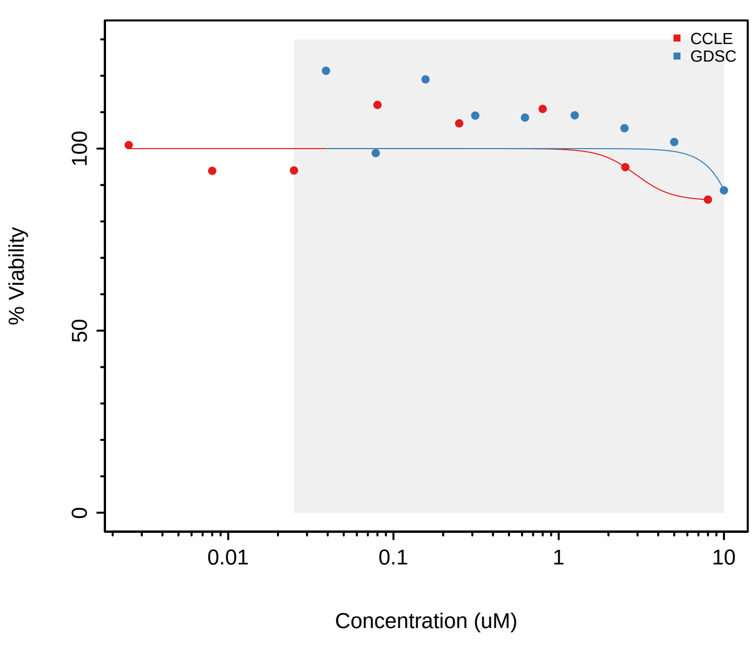

PLX4720:KYSE−410

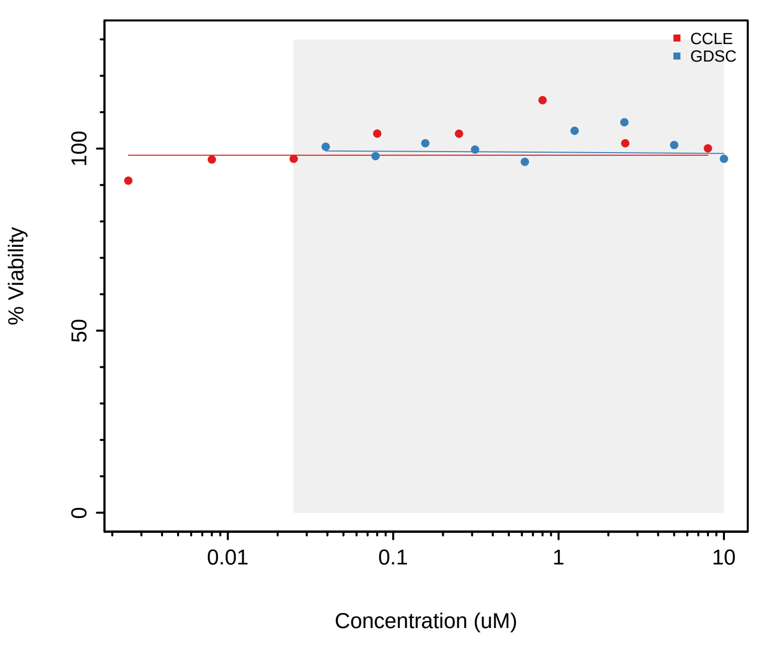

PLX4720:KYSE−450

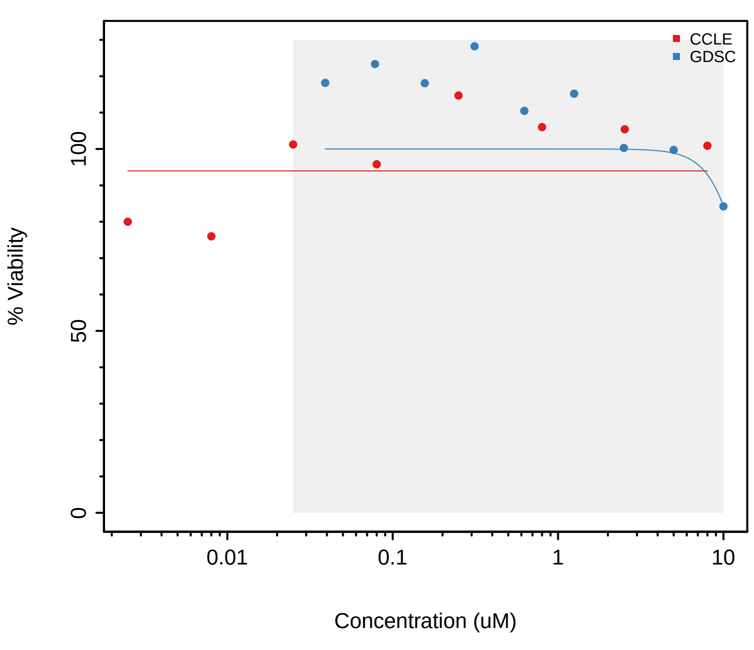

PLX4720:KYSE−520

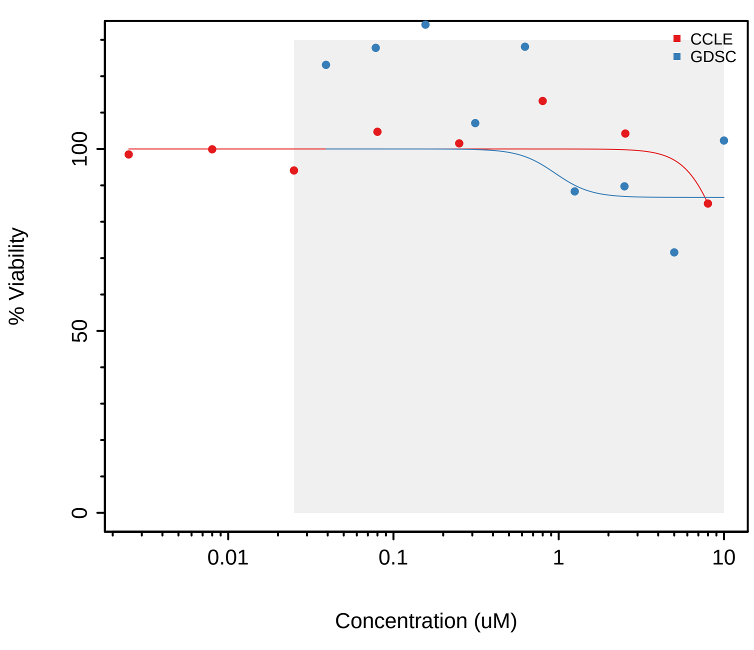

PLX4720:KYSE−70

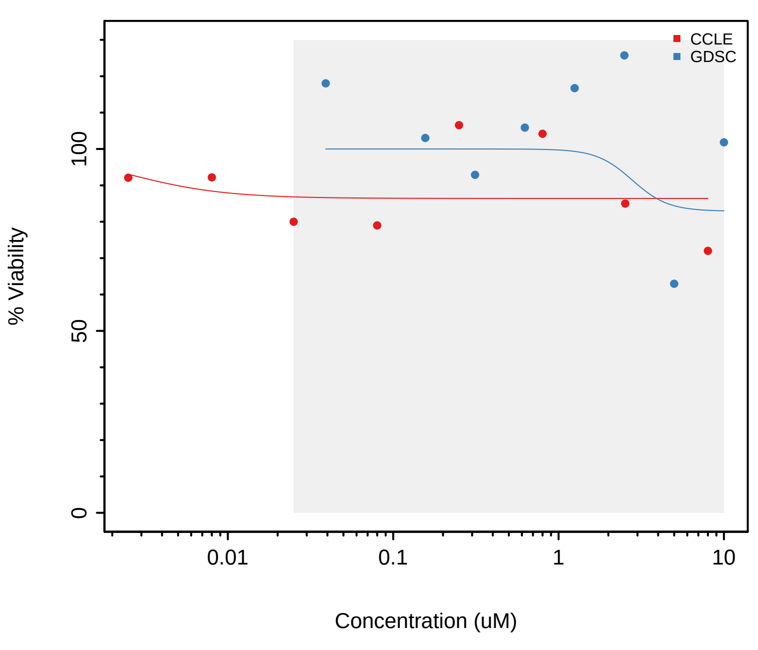

PLX4720:L−363

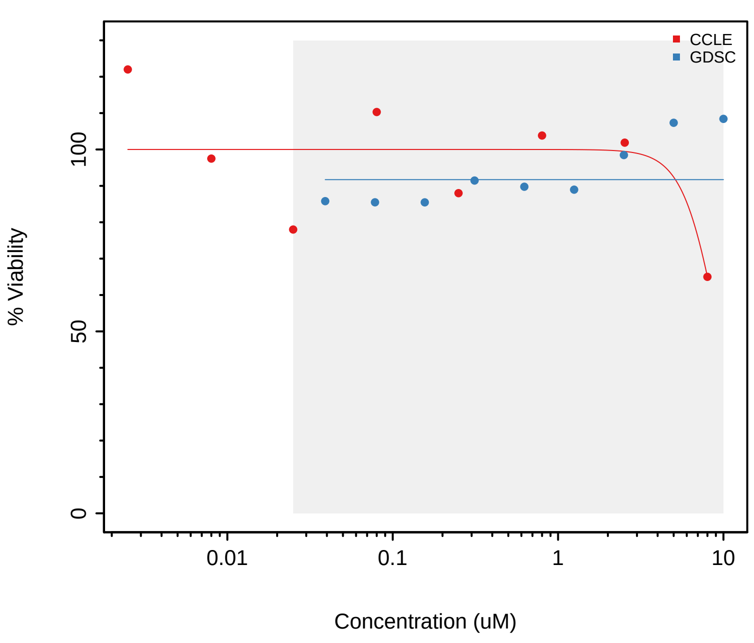

PLX4720:L−428

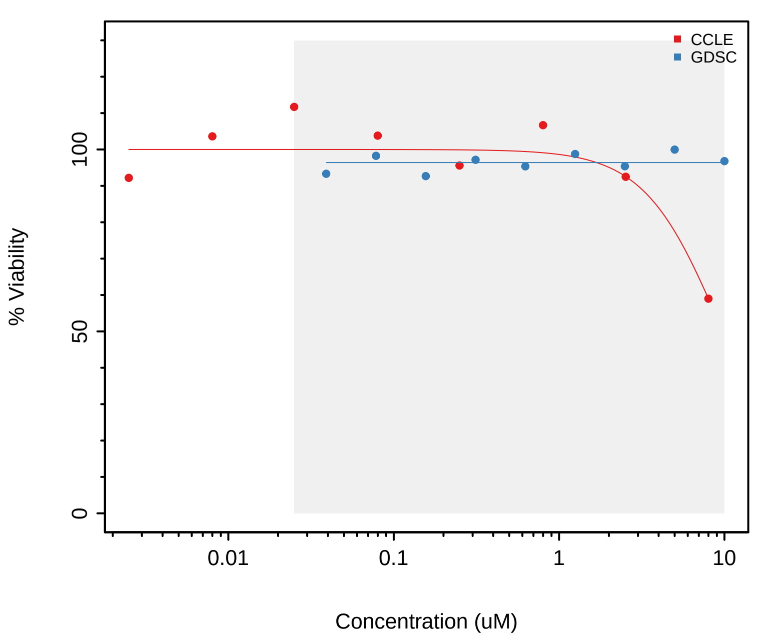

PLX4720:LCLC−103H

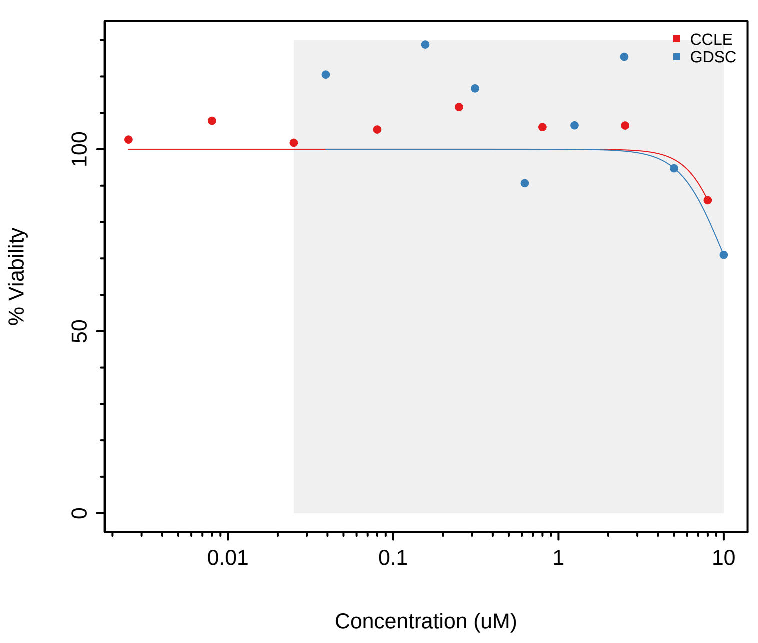

PLX4720:LOXIMVI

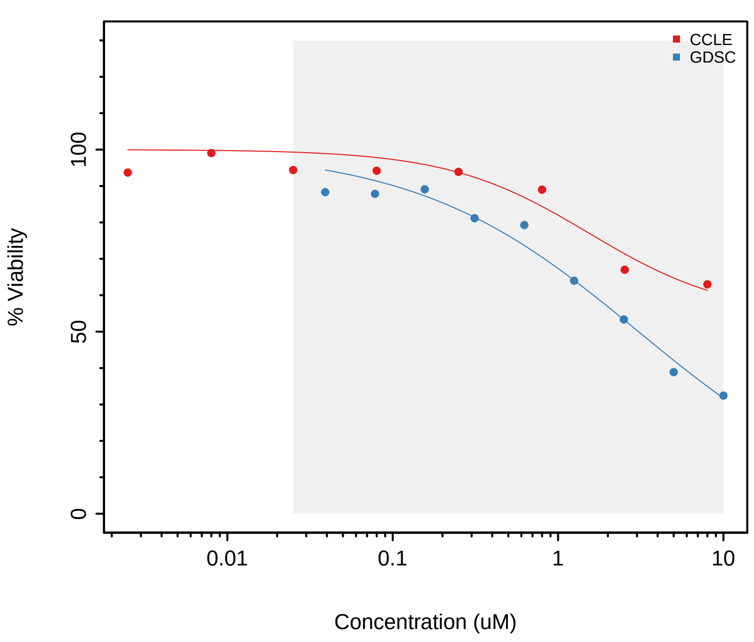

PLX4720:LP−1

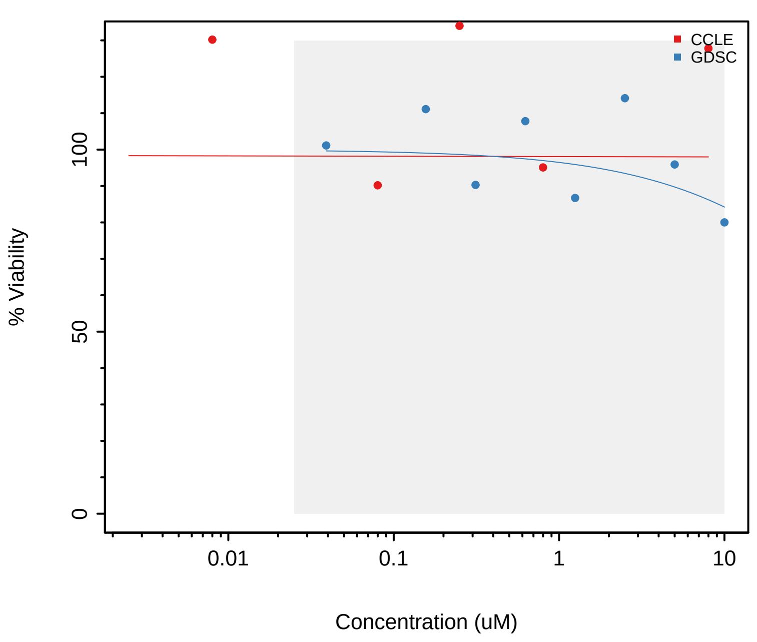

PLX4720:LS−123

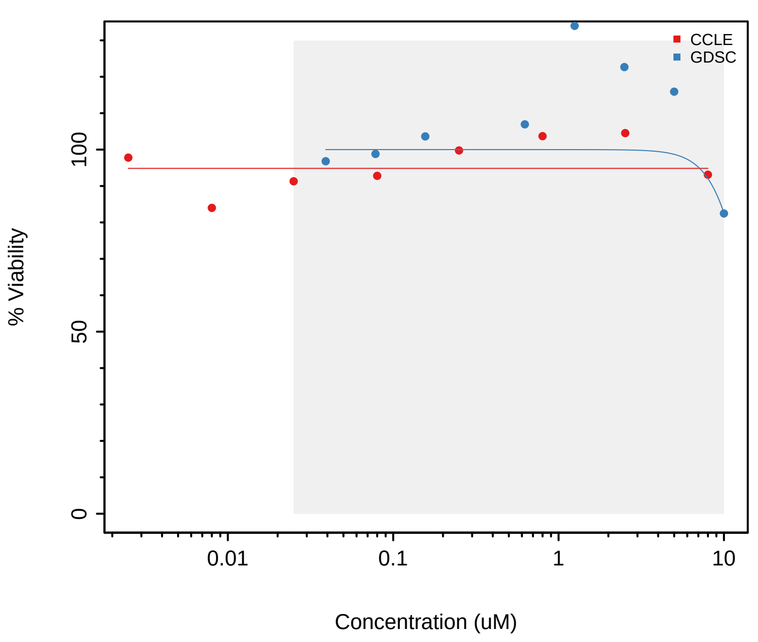

PLX4720:LS−411N

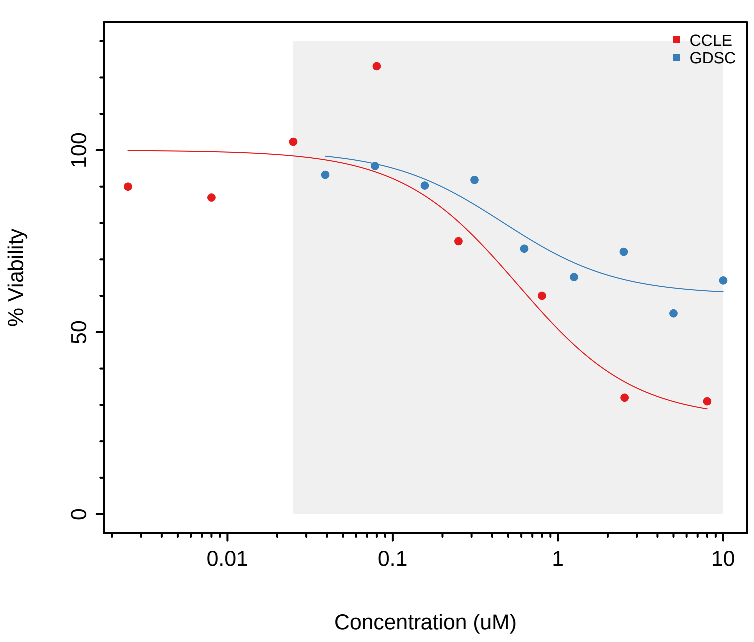

PLX4720:LS−513

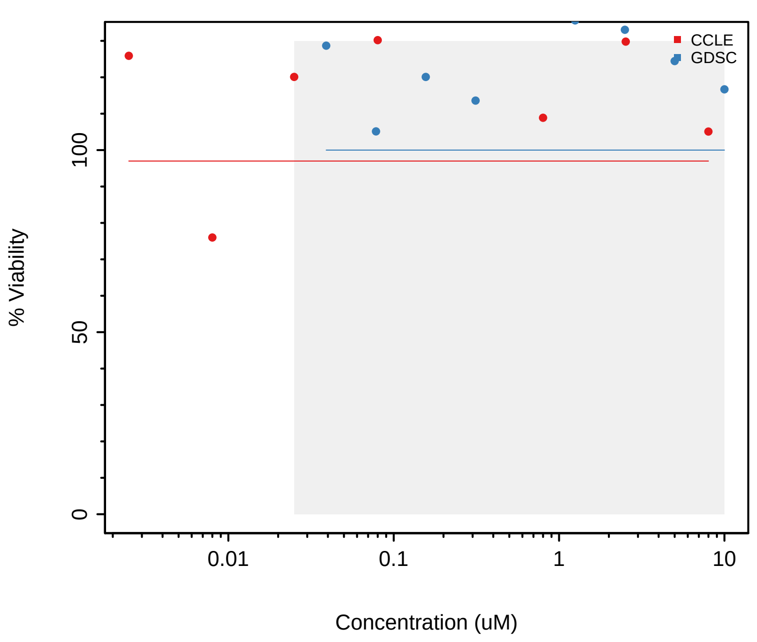

PLX4720:LU−99A

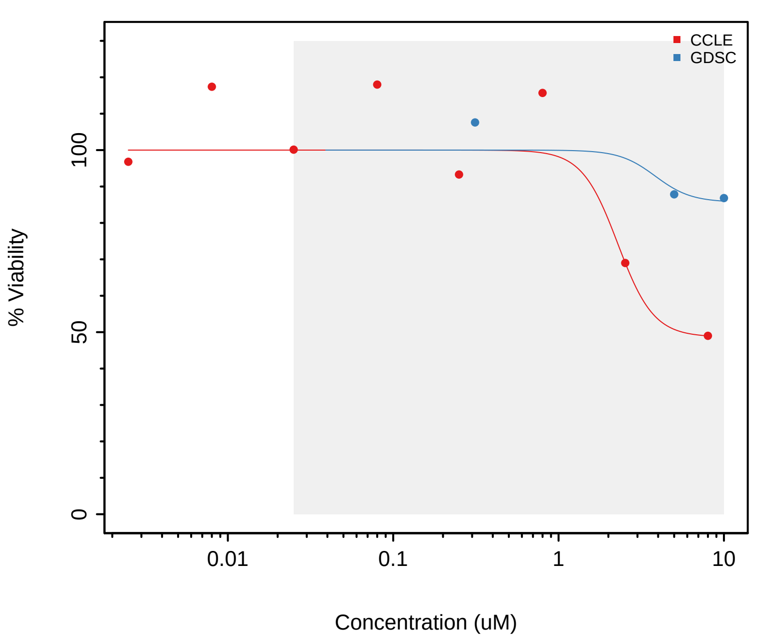

PLX4720:M059J

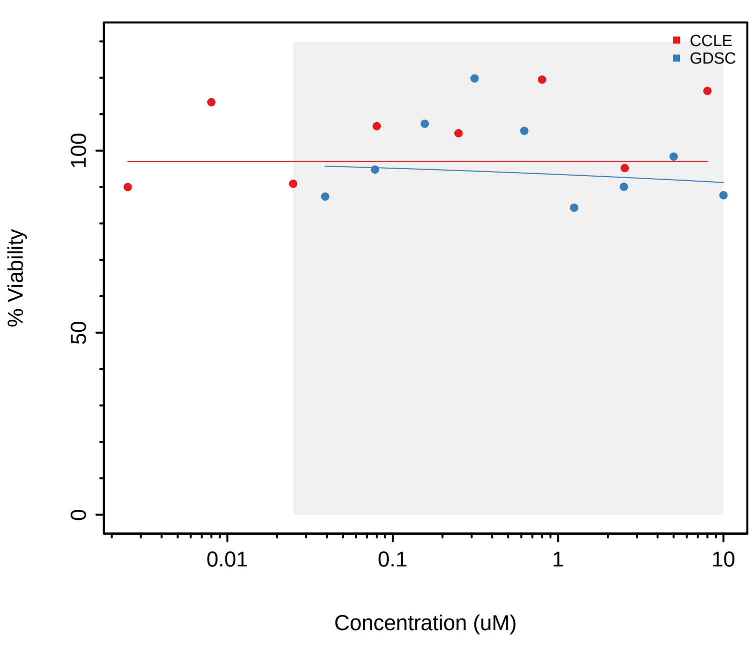

PLX4720:MC116

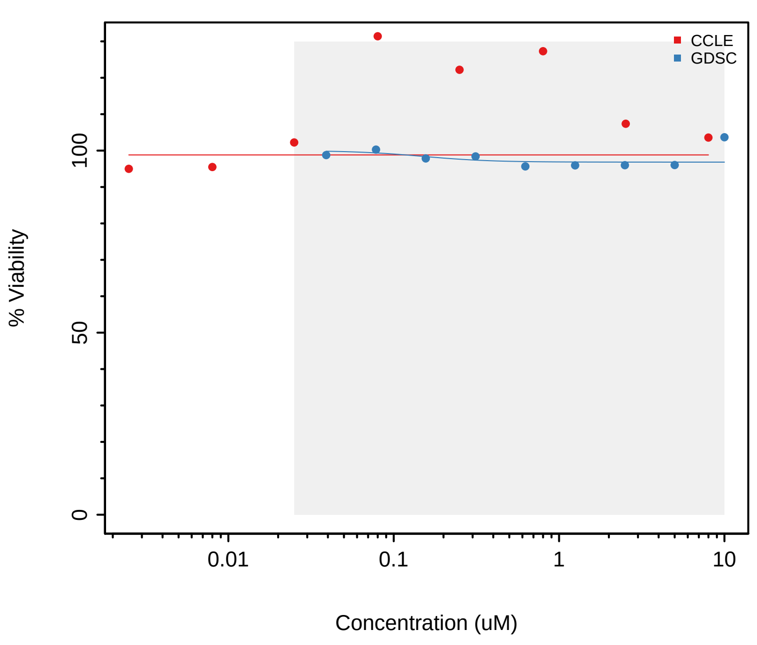

PLX4720:MDA−MB−157

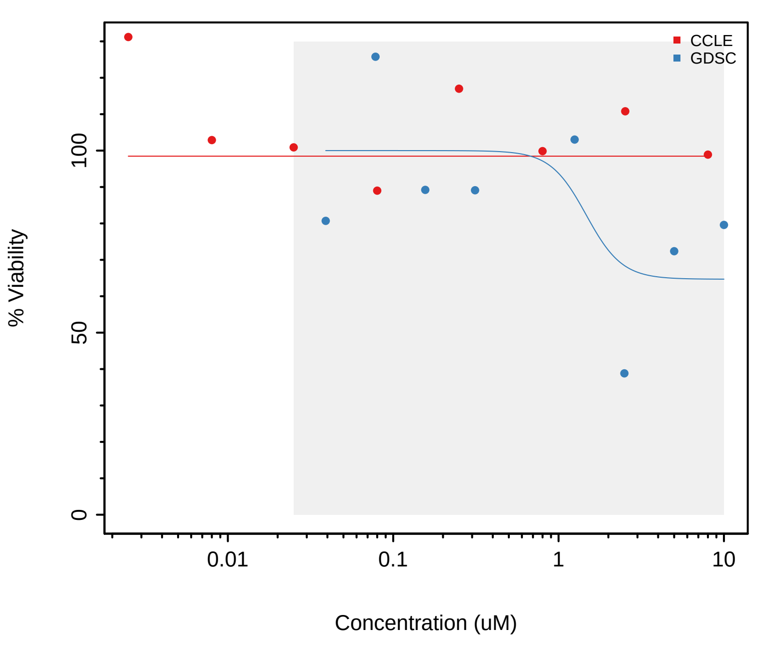

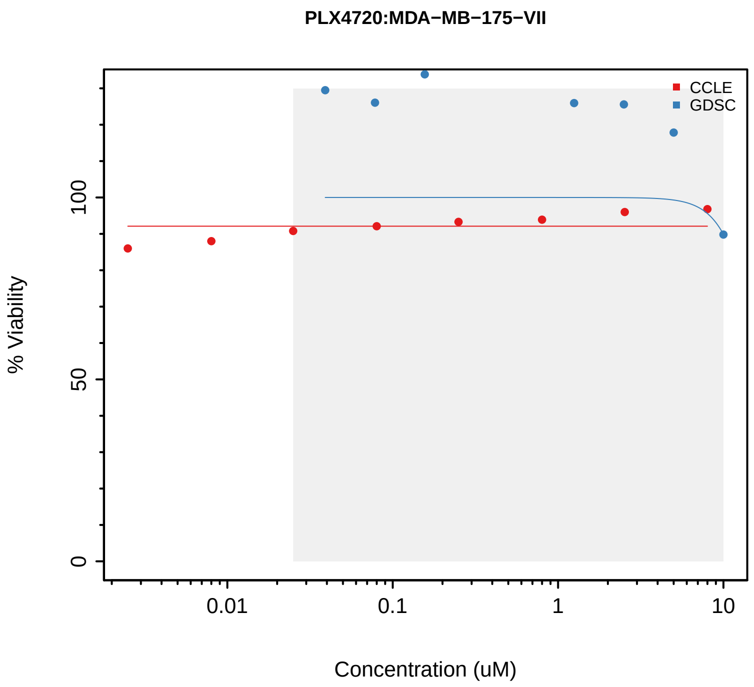
PLX4720:MDA−MB−175−VII
CCLE
GDSC
% Viability
0
50
100
0.01
0.1
1
10
Concentration (uM)
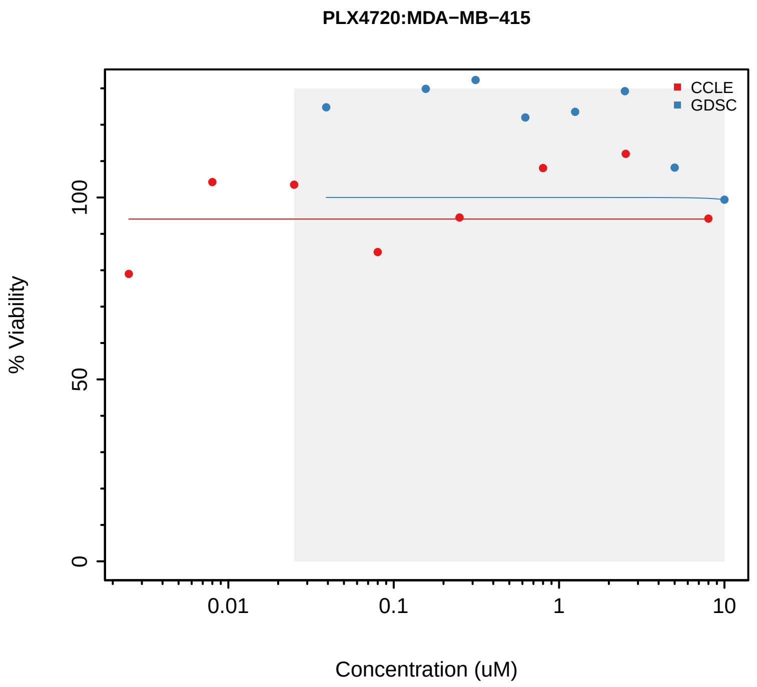
PLX4720:MDA−MB−415
CCLE
GDSC
% Viability
0
50
100
0.01
0.1
1
10
Concentration (uM)
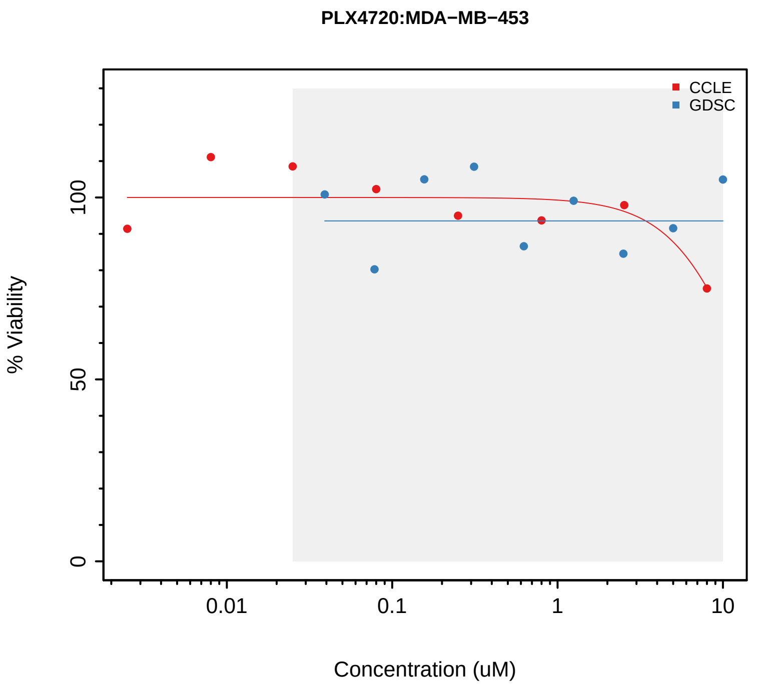
PLX4720:MDA−MB−453
CCLE
GDSC
% Viability
0
50
100
0.01
0.1
1
10
Concentration (uM)
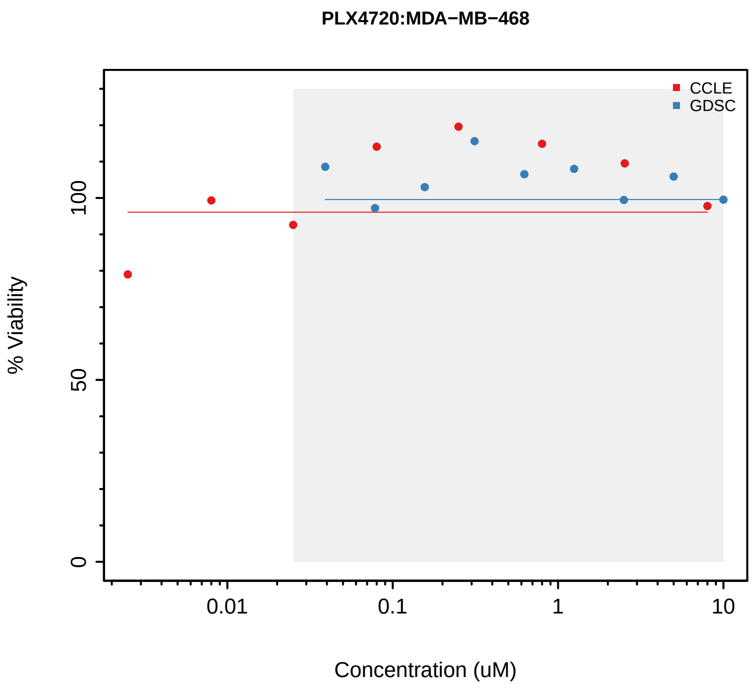
PLX4720:MDA−MB−468
CCLE
GDSC
% Viability
0
50
100
0.01
0.1
1
10
Concentration (uM)
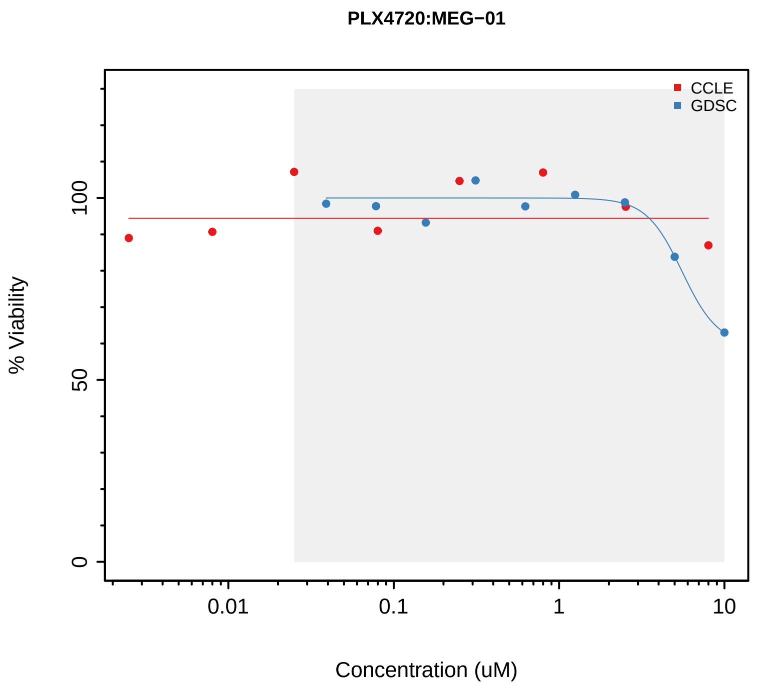
PLX4720:MEG−01
CCLE
GDSC
% Viability
0
50
100
0.01
0.1
1
10
Concentration (uM)
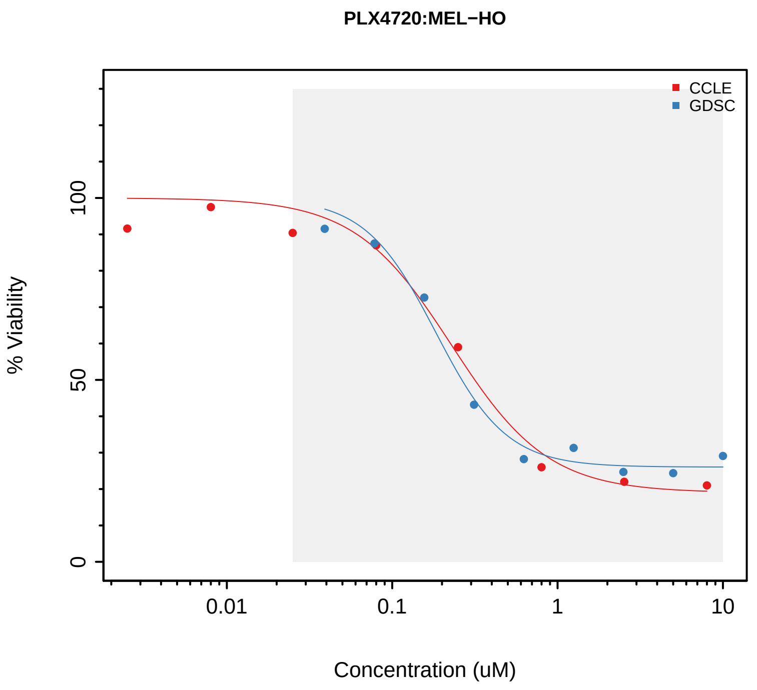
PLX4720:MEL−HO
CCLE
GDSC
% Viability
0
50
100
0.01
0.1
1
10
Concentration (uM)
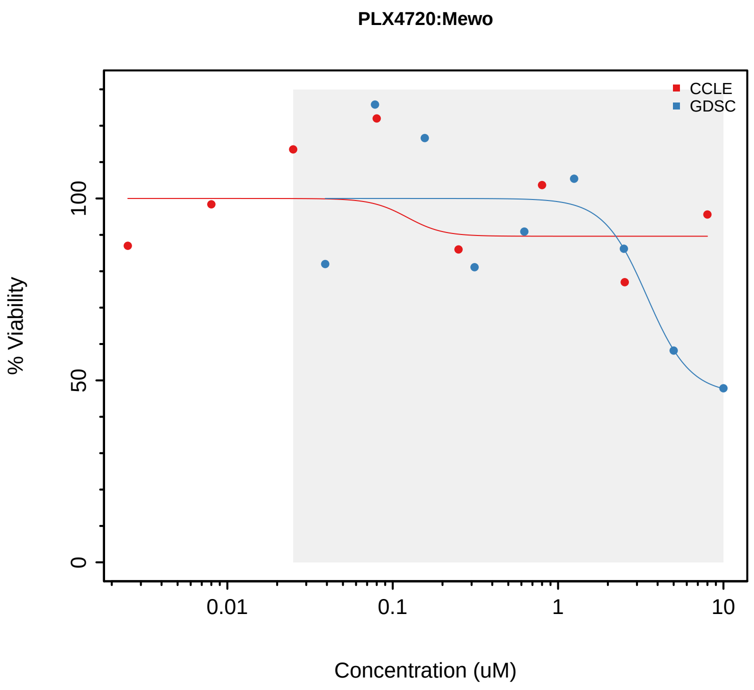
PLX4720:Mewo
CCLE
GDSC
% Viability
0
50
100
0.01
0.1
1
10
Concentration (uM)
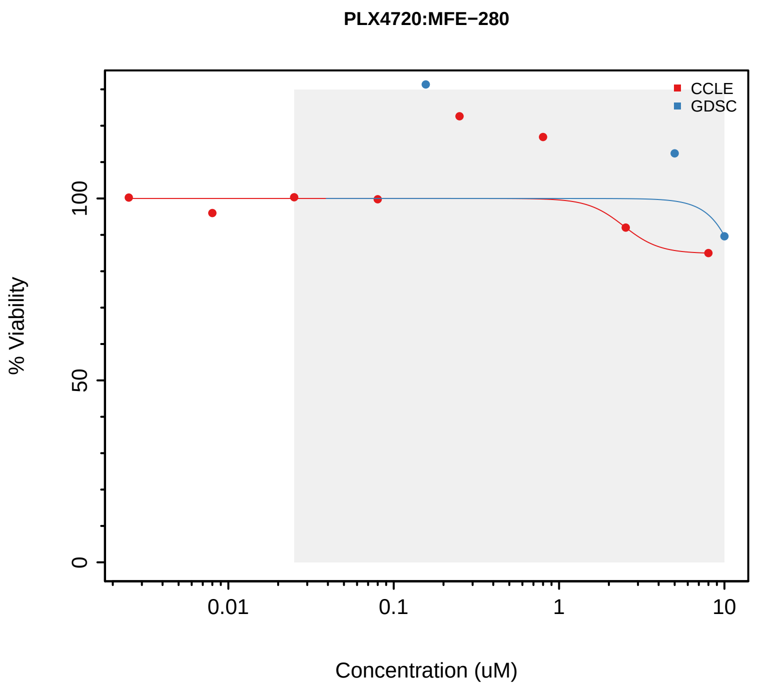
PLX4720:MFE−280
CCLE
GDSC
% Viability
0
50
100
0.01
0.1
1
10
Concentration (uM)
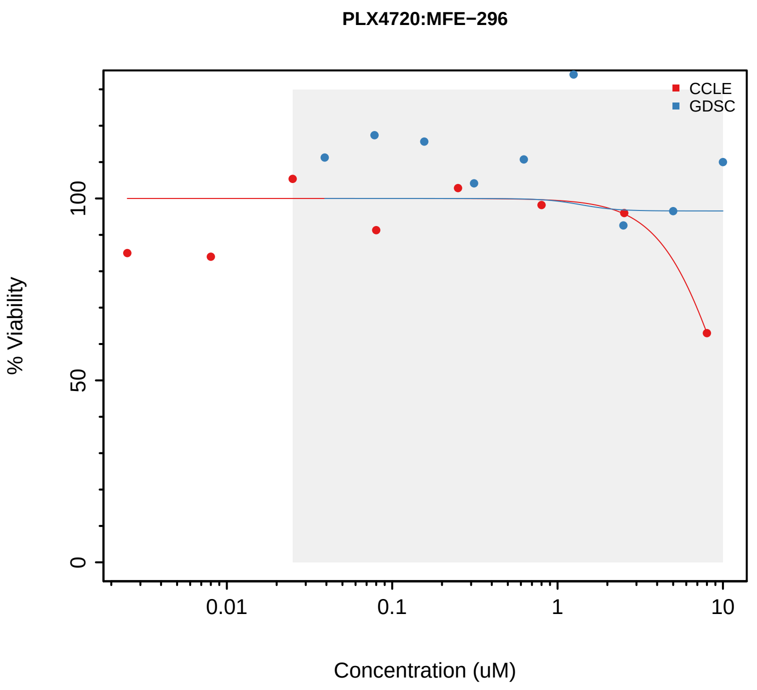
PLX4720:MFE−296
CCLE
GDSC
% Viability
0
50
100
0.01
0.1
1
10
Concentration (uM)
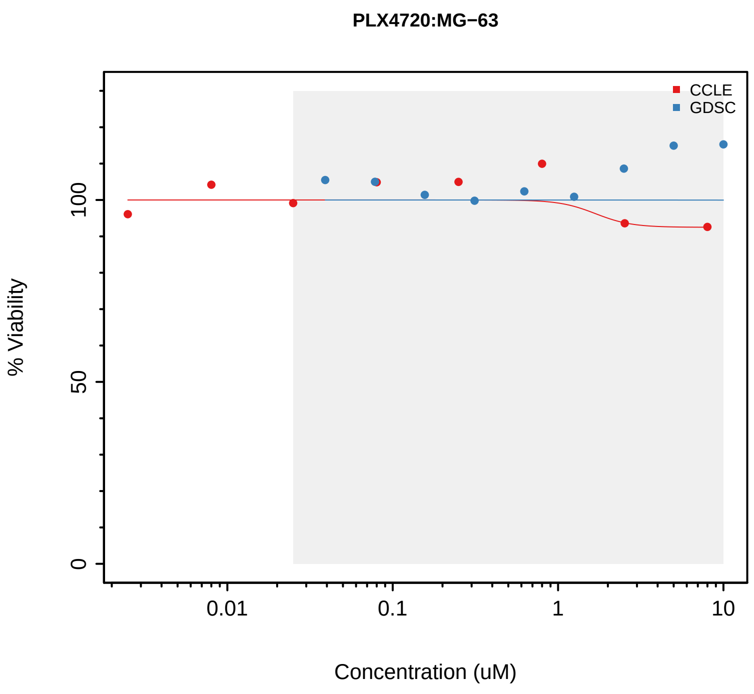
PLX4720:MG−63
CCLE
GDSC
% Viability
0
50
100
0.01
0.1
1
10
Concentration (uM)
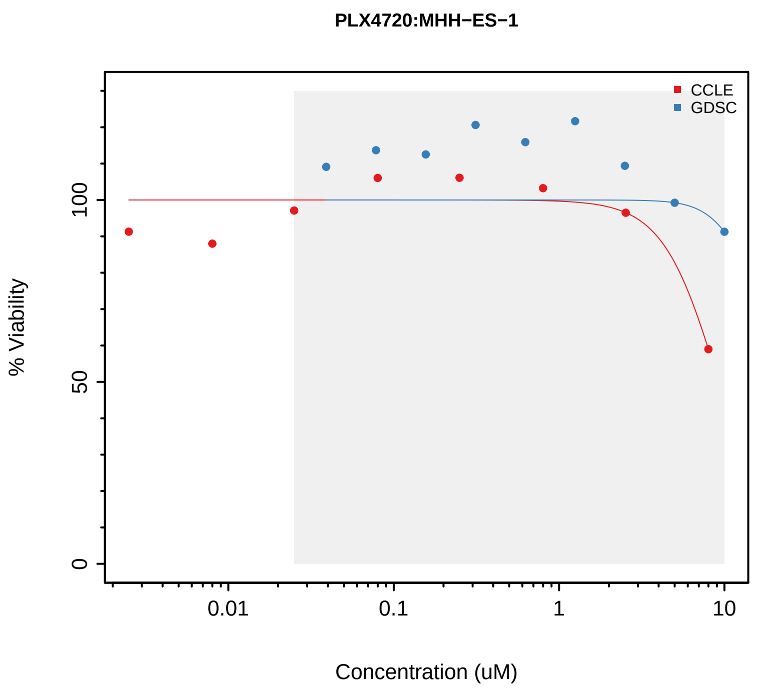
PLX4720:MHH−ES−1
CCLE
GDSC
% Viability
0
50
100
0.01
0.1
1
10
Concentration (uM)
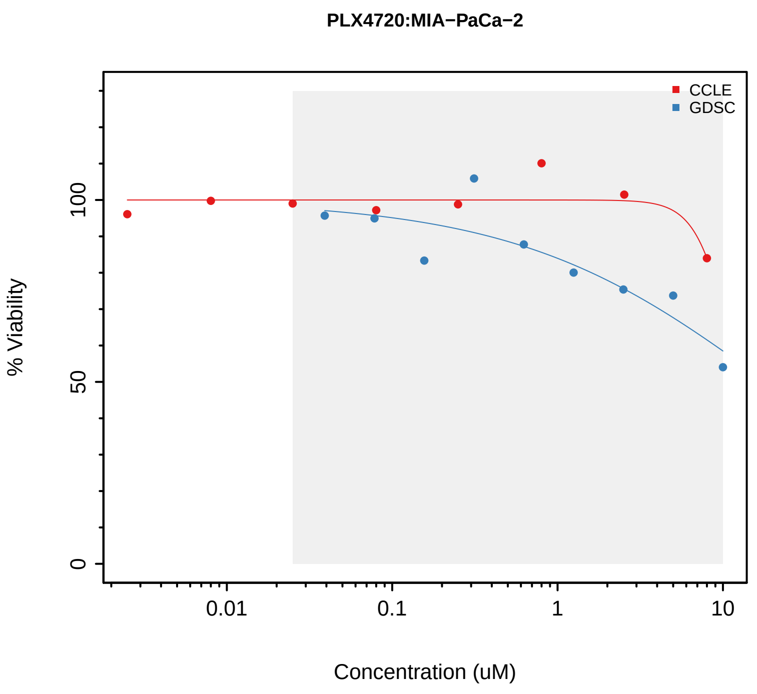
PLX4720:MIA−PaCa−2
CCLE
GDSC
% Viability
0
50
100
0.01
0.1
1
10
Concentration (uM)

PLX4720:MKN45

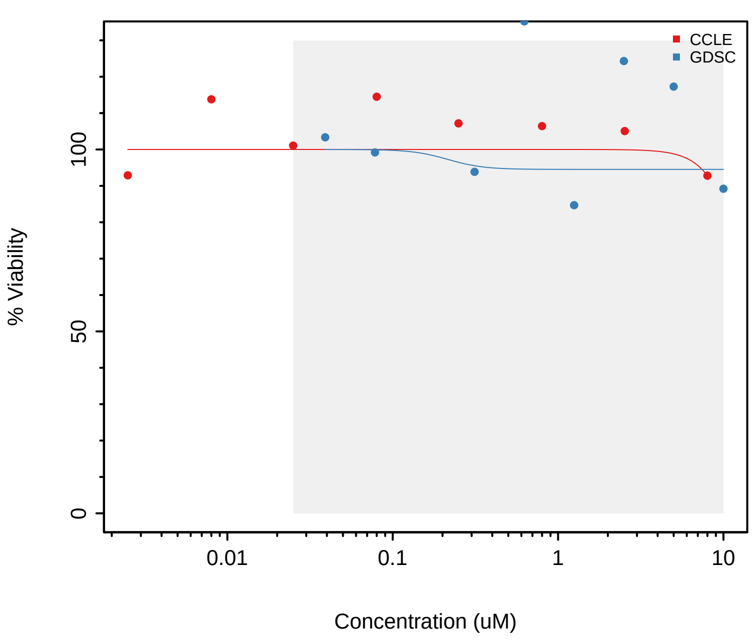

PLX4720:MKN7

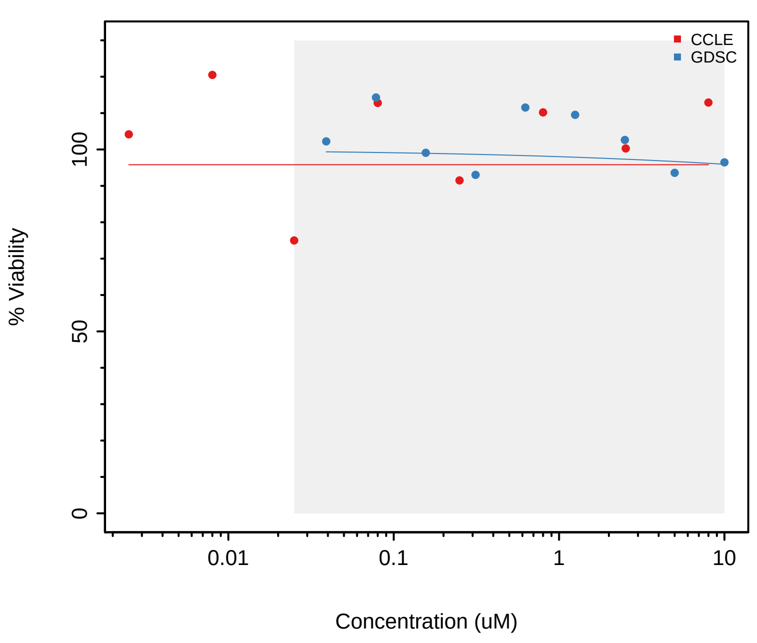

PLX4720:MOLT−16

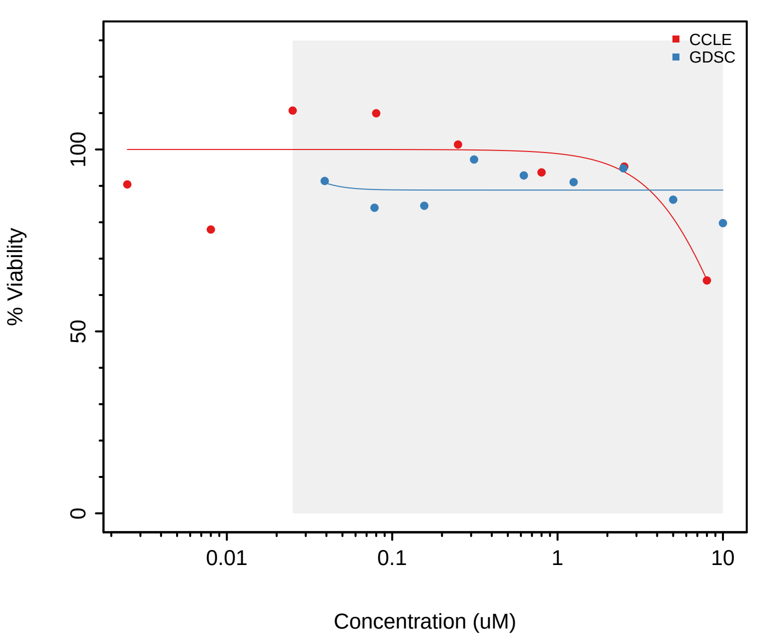

PLX4720:MPP−89

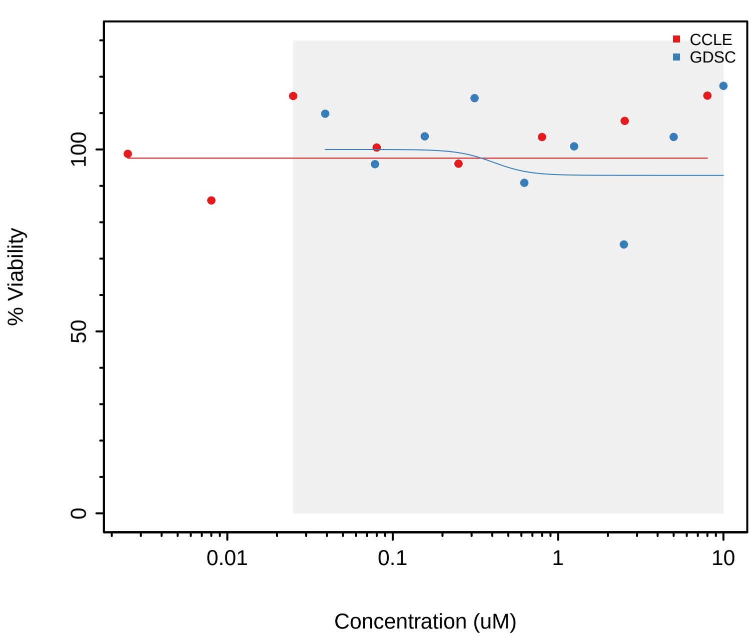

PLX4720:MSTO−211H

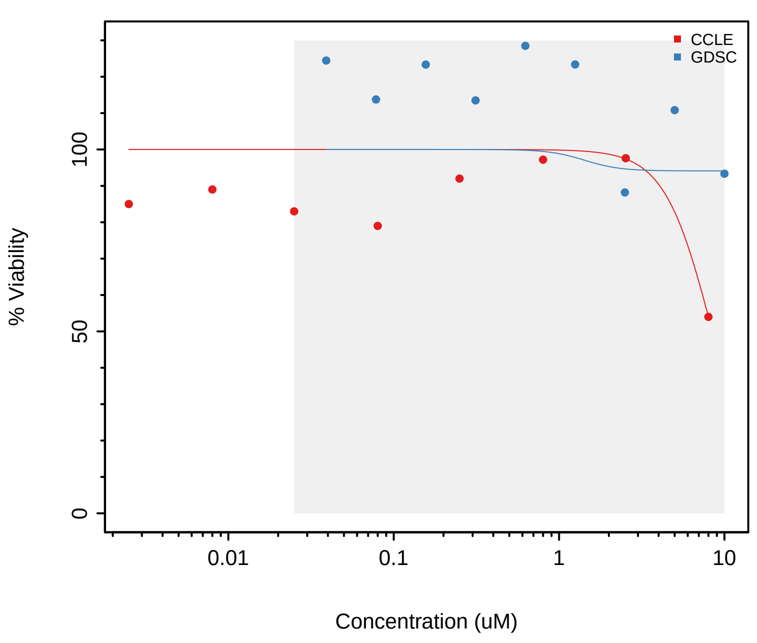

PLX4720:NCI−H1048

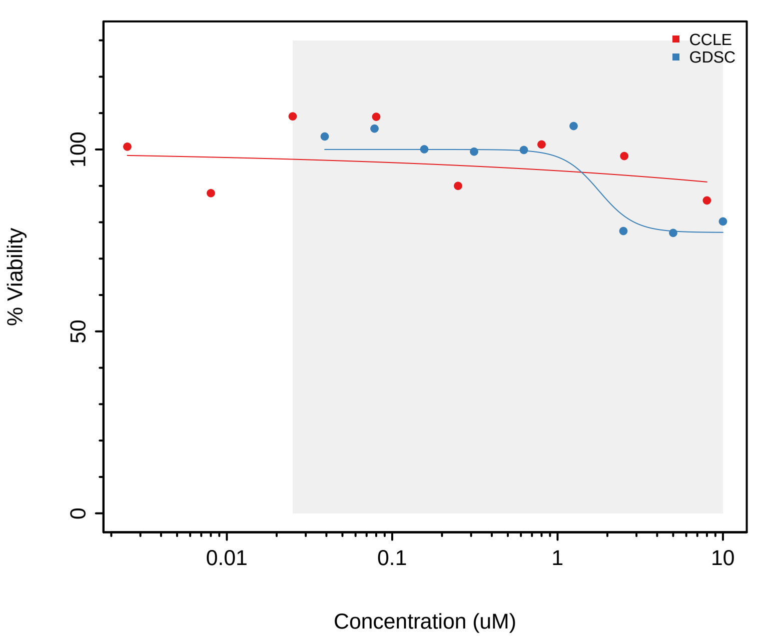

PLX4720:NCI−H1092

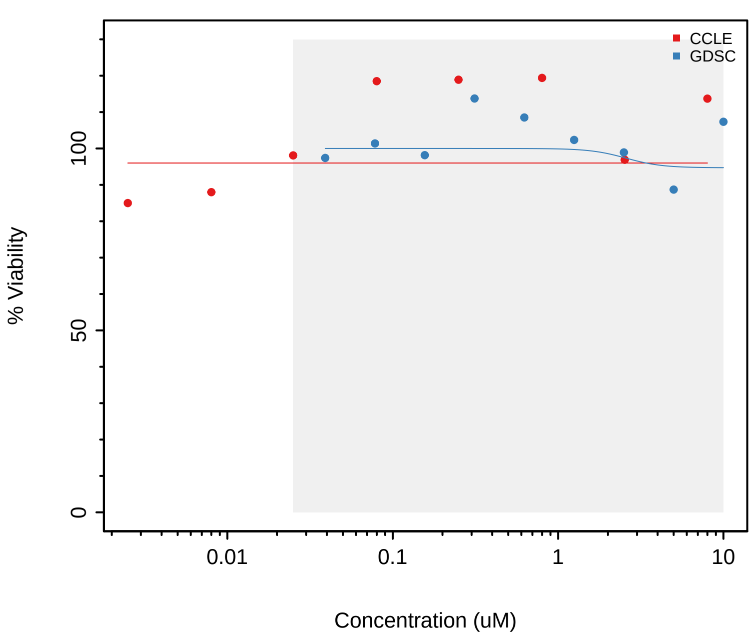

PLX4720:NCI−H1155

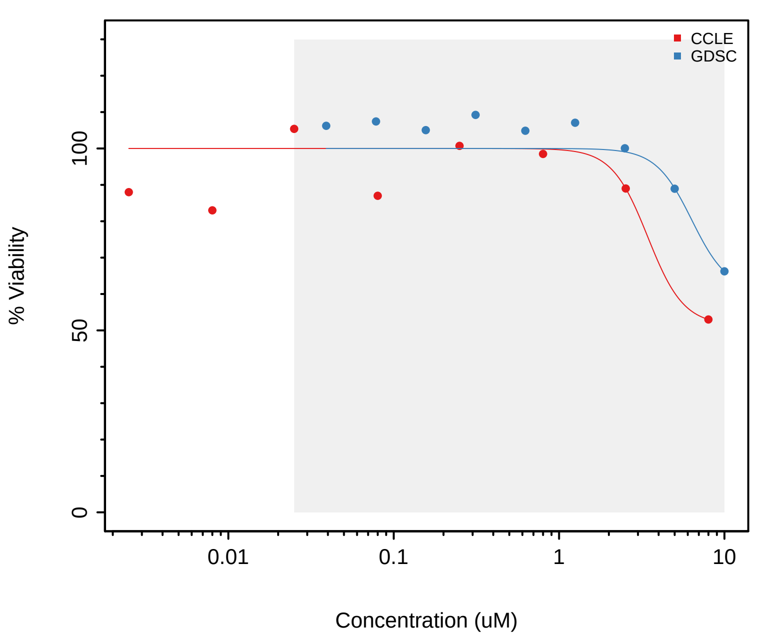

PLX4720:NCI−H1299

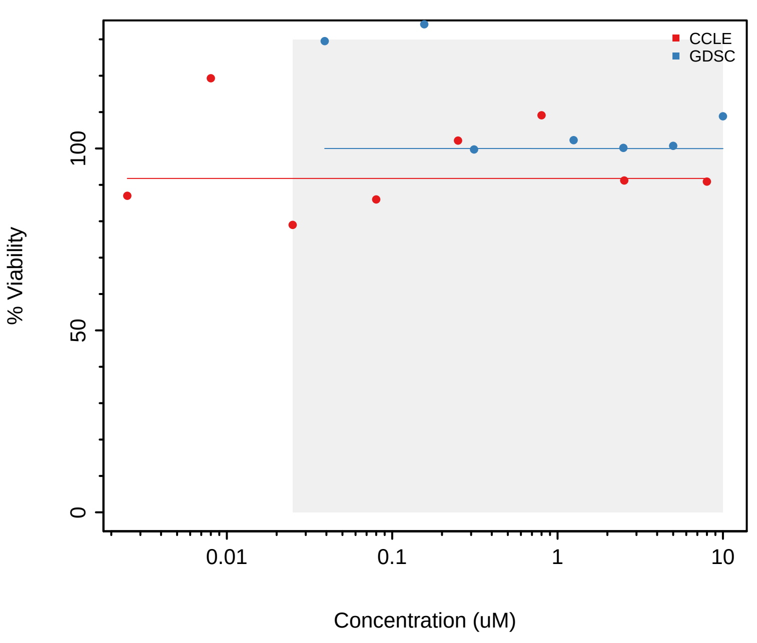

PLX4720:NCI−H1355

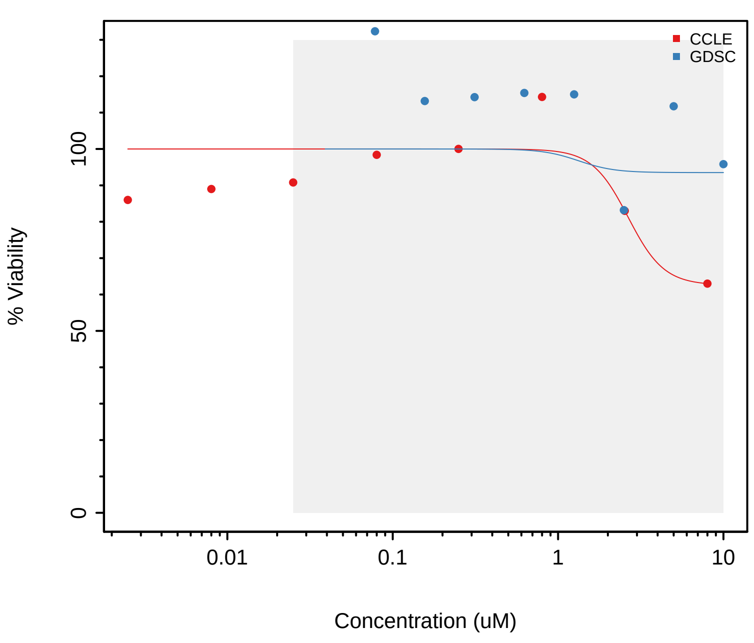

PLX4720:NCI−H1563

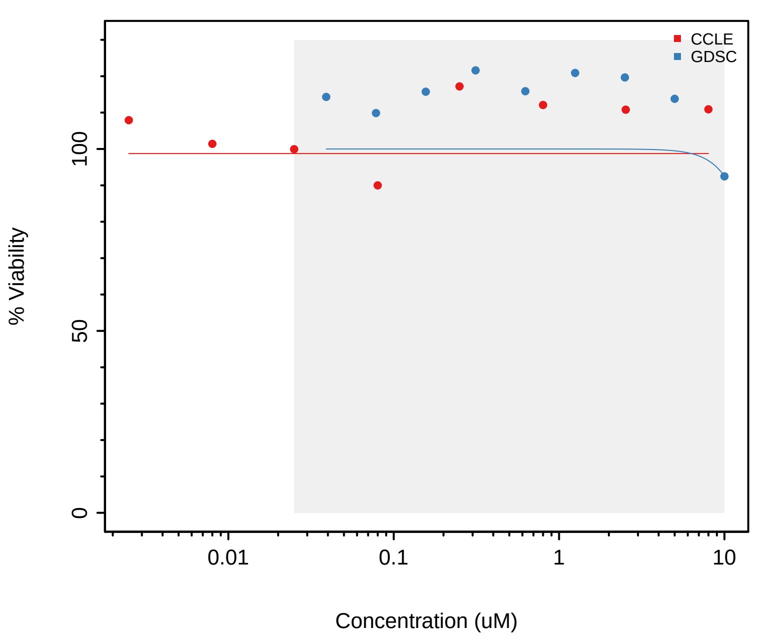

PLX4720:NCI−H1573

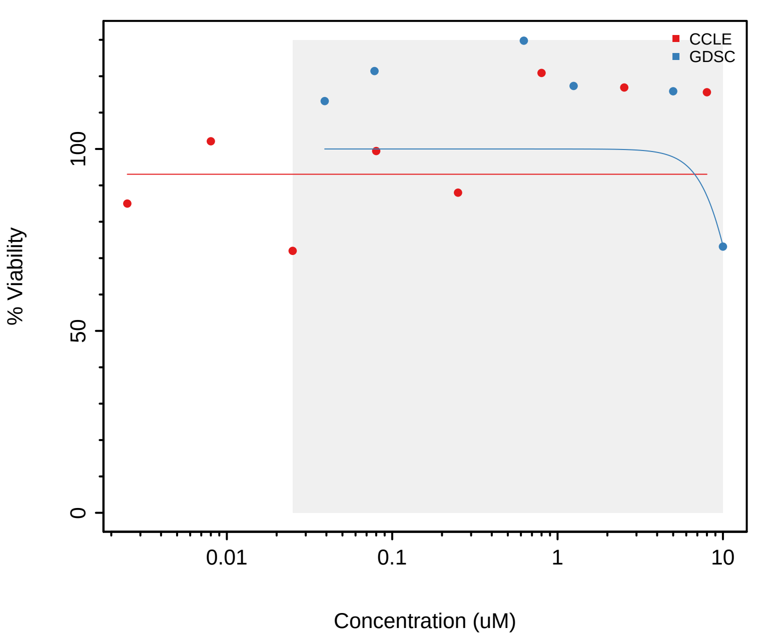

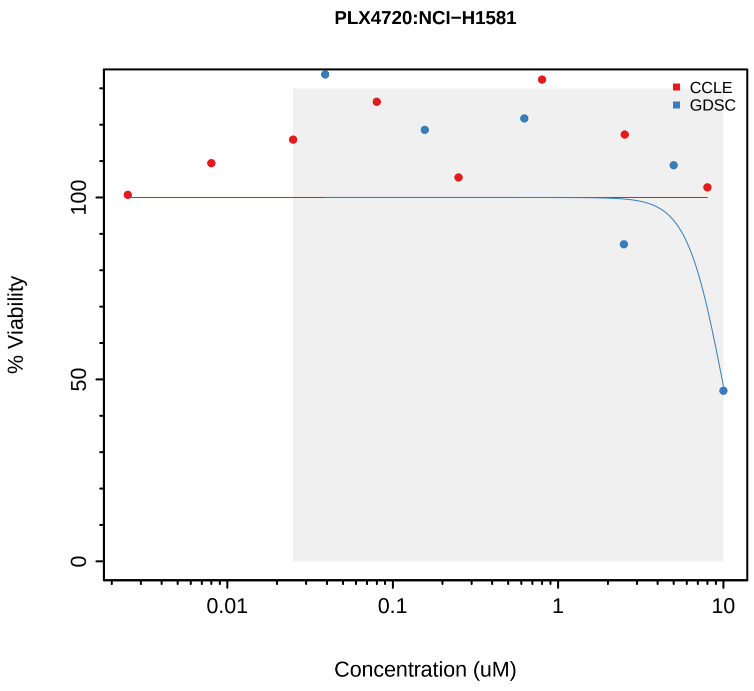
PLX4720:NCI−H1581
CCLE
GDSC
% Viability
0
50
100
0.01
0.1
1
10
Concentration (uM)

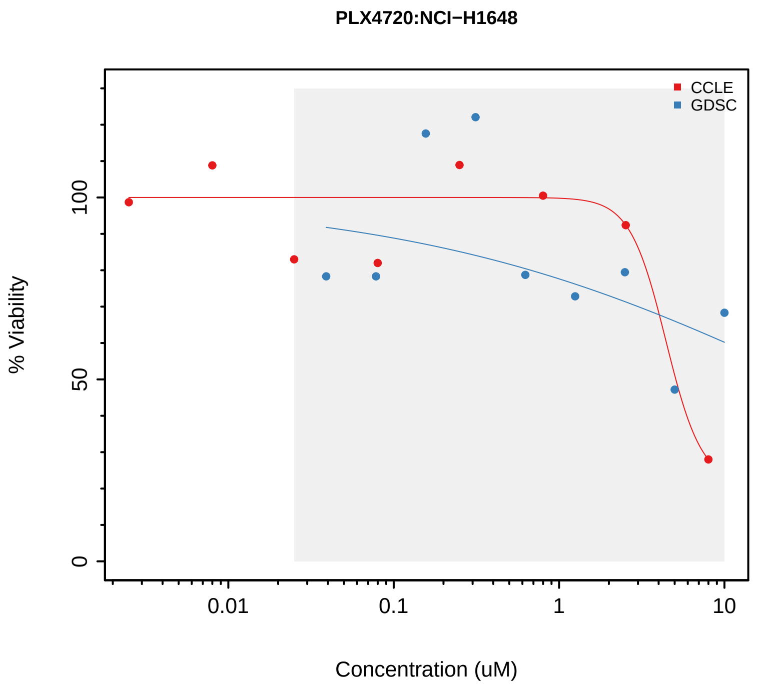
PLX4720:NCI−H1648
CCLE
GDSC
% Viability
0
50
100
0.01
0.1
1
10
Concentration (uM)

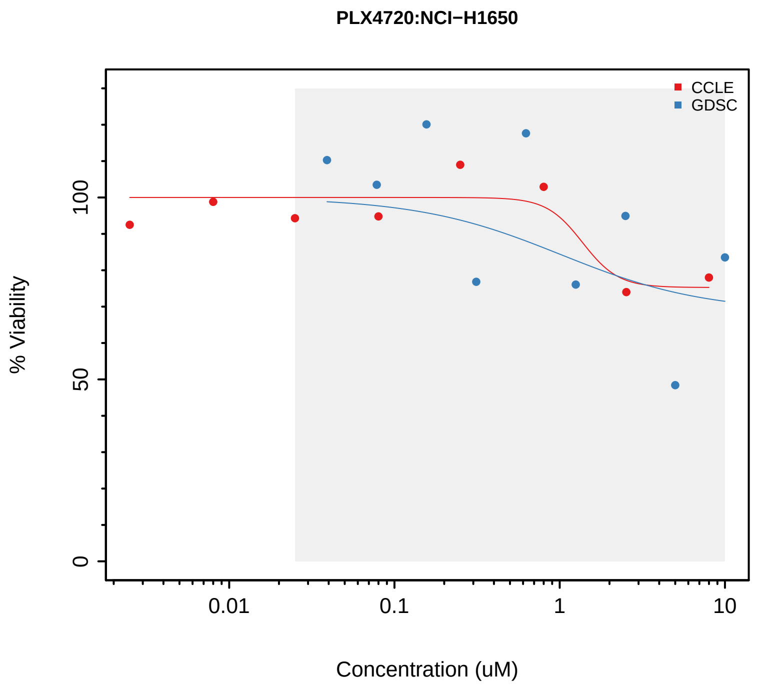
PLX4720:NCI−H1650
CCLE
GDSC
% Viability
0
50
100
0.01
0.1
1
10
Concentration (uM)

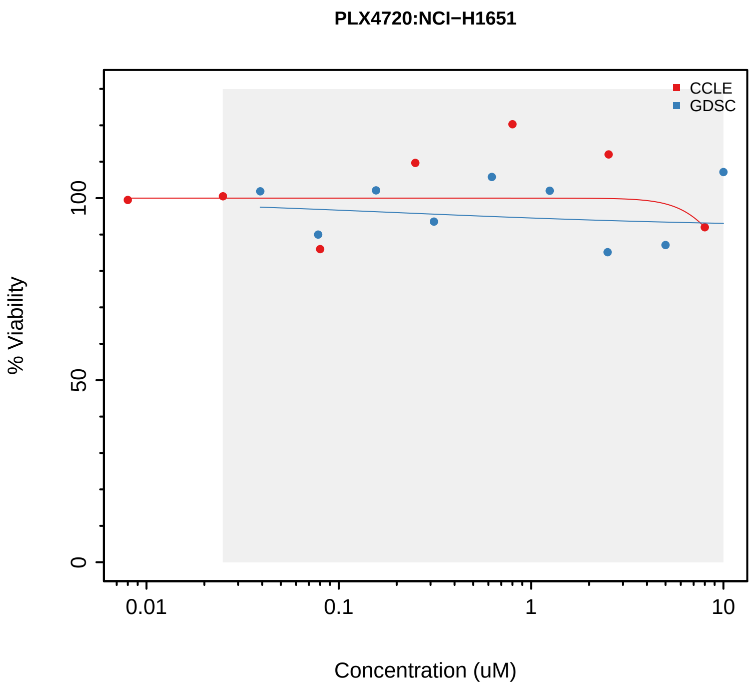
PLX4720:NCI−H1651
CCLE
GDSC
% Viability
0
50
100
0.01
0.1
1
10
Concentration (uM)

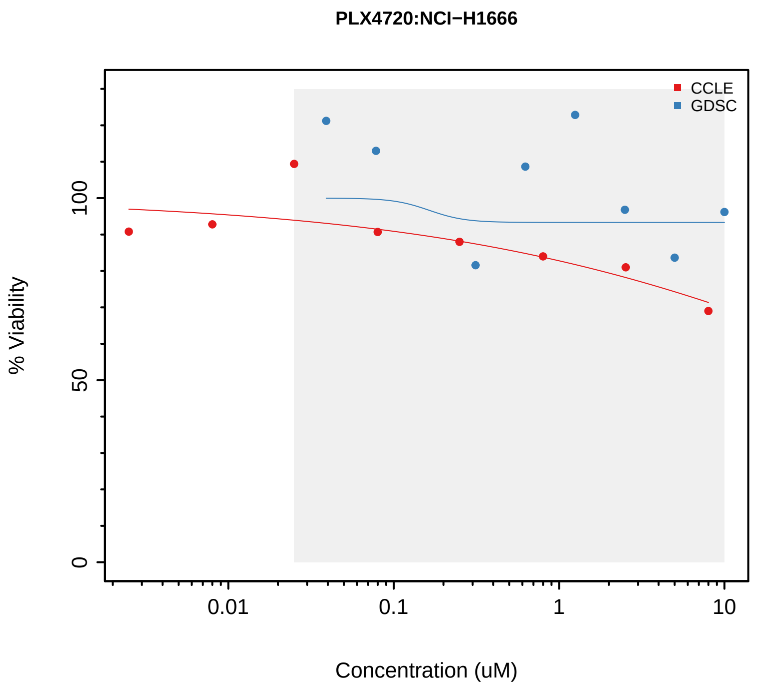
PLX4720:NCI−H1666
CCLE
GDSC
% Viability
0
50
100
0.01
0.1
1
10
Concentration (uM)

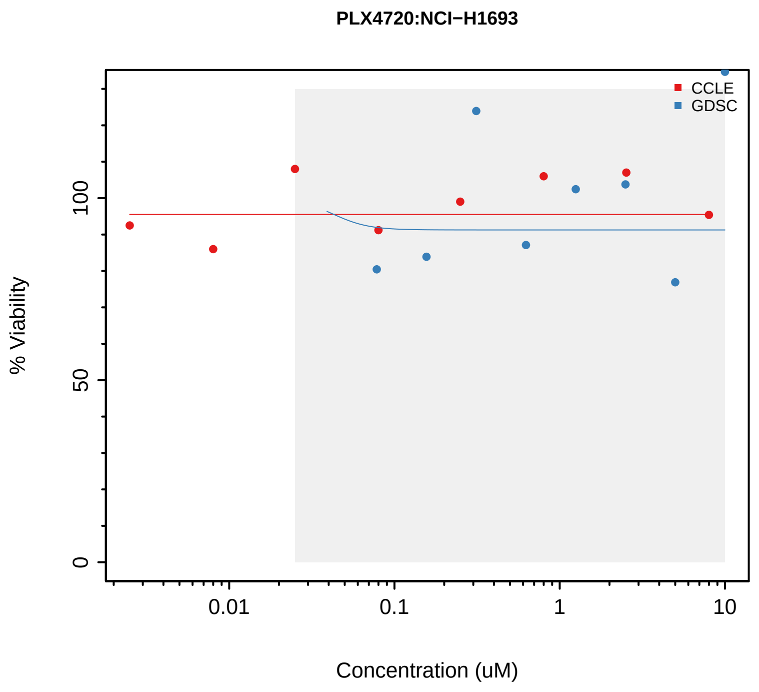
PLX4720:NCI−H1693
CCLE
GDSC
% Viability
0
50
100
0.01
0.1
1
10
Concentration (uM)

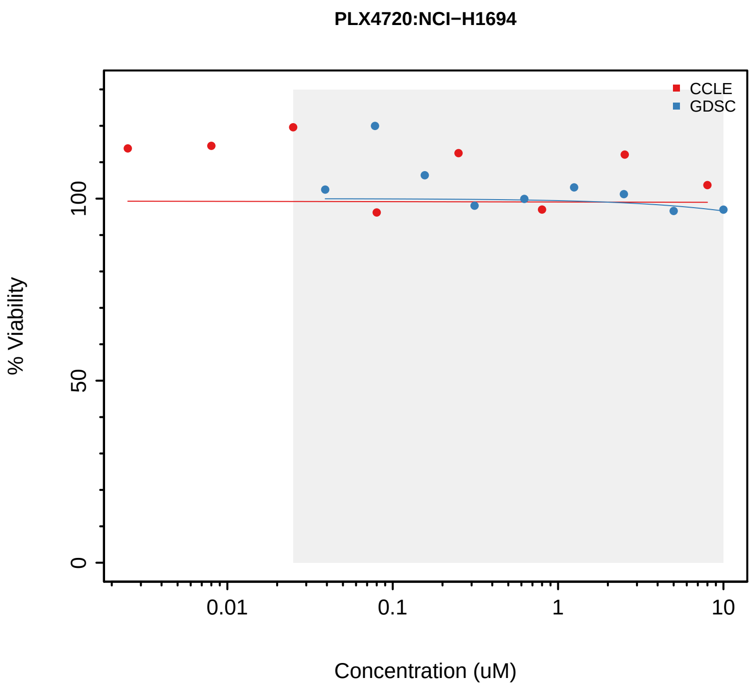
PLX4720:NCI−H1694
CCLE
GDSC
% Viability
0
50
100
0.01
0.1
1
10
Concentration (uM)

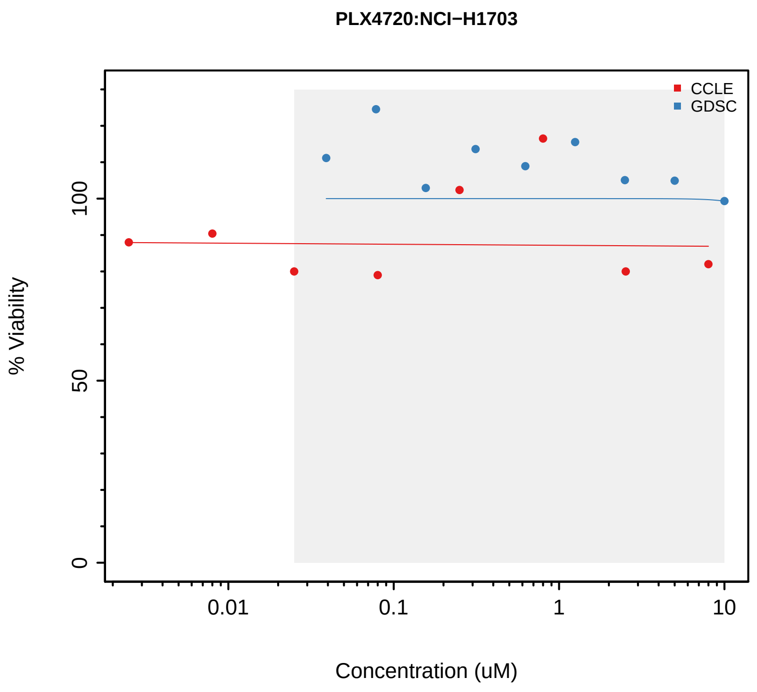
PLX4720:NCI−H1703
CCLE
GDSC
% Viability
0
50
100
0.01
0.1
1
10
Concentration (uM)

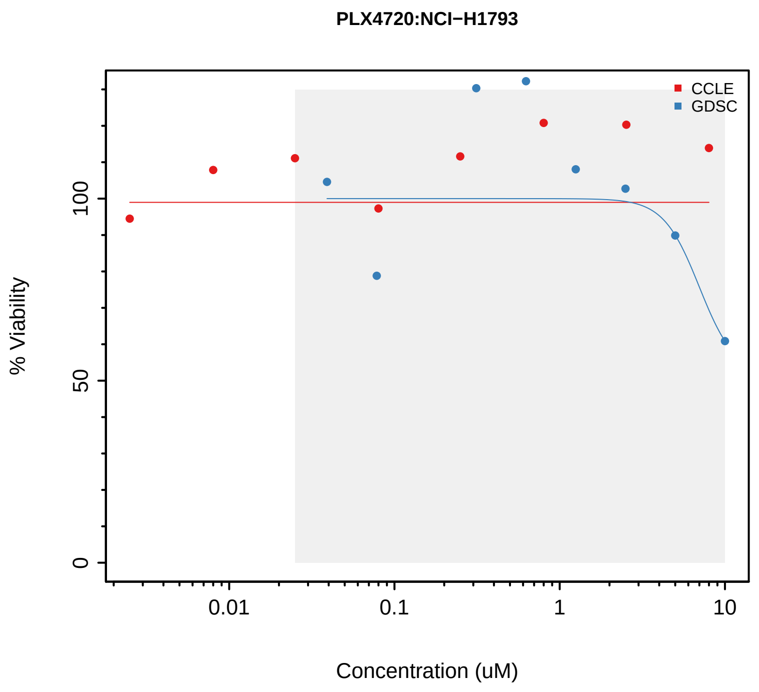
PLX4720:NCI−H1793
CCLE
GDSC
% Viability
0
50
100
0.01
0.1
1
10
Concentration (uM)

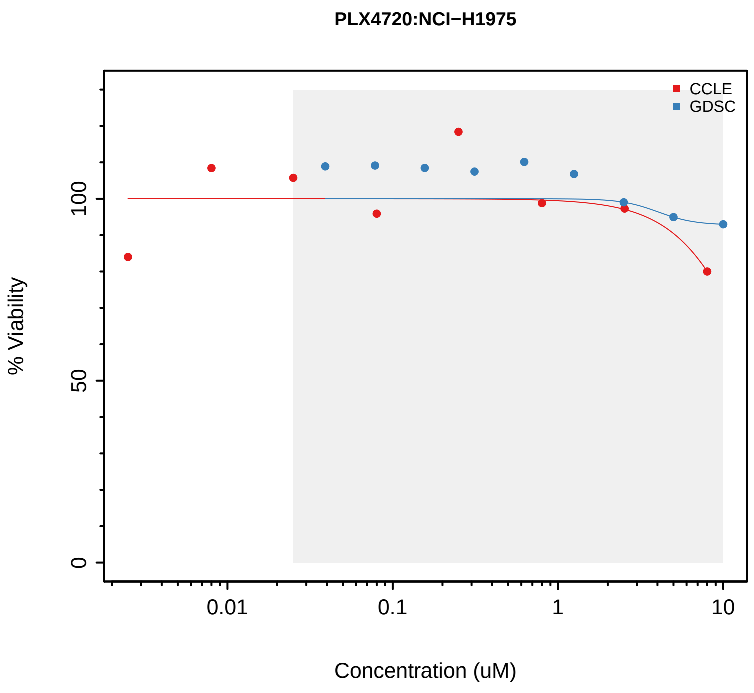
PLX4720:NCI−H1975
CCLE
GDSC
% Viability
0
50
100
0.01
0.1
1
10
Concentration (uM)

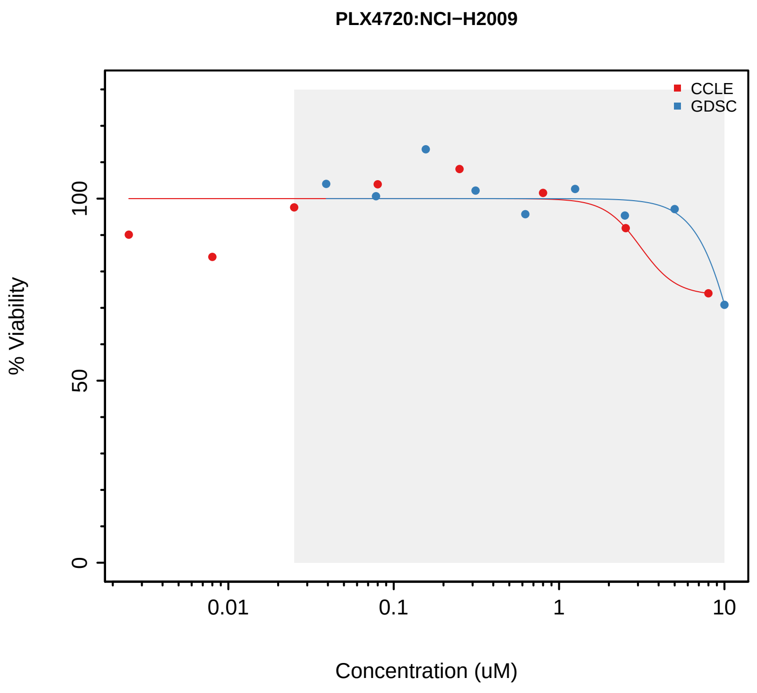
PLX4720:NCI−H2009
CCLE
GDSC
% Viability
0
50
100
0.01
0.1
1
10
Concentration (uM)

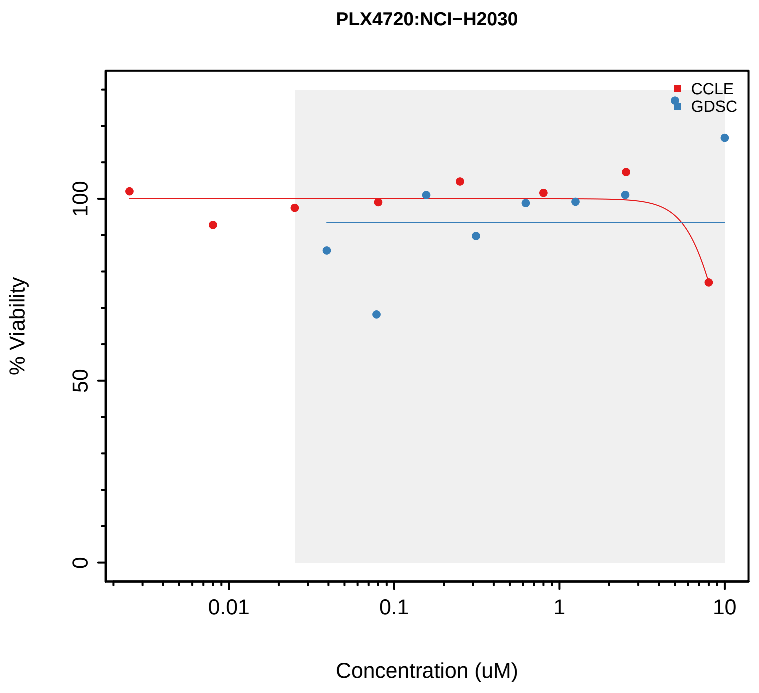
PLX4720:NCI−H2030
CCLE
GDSC
% Viability
0
50
100
0.01
0.1
1
10
Concentration (uM)

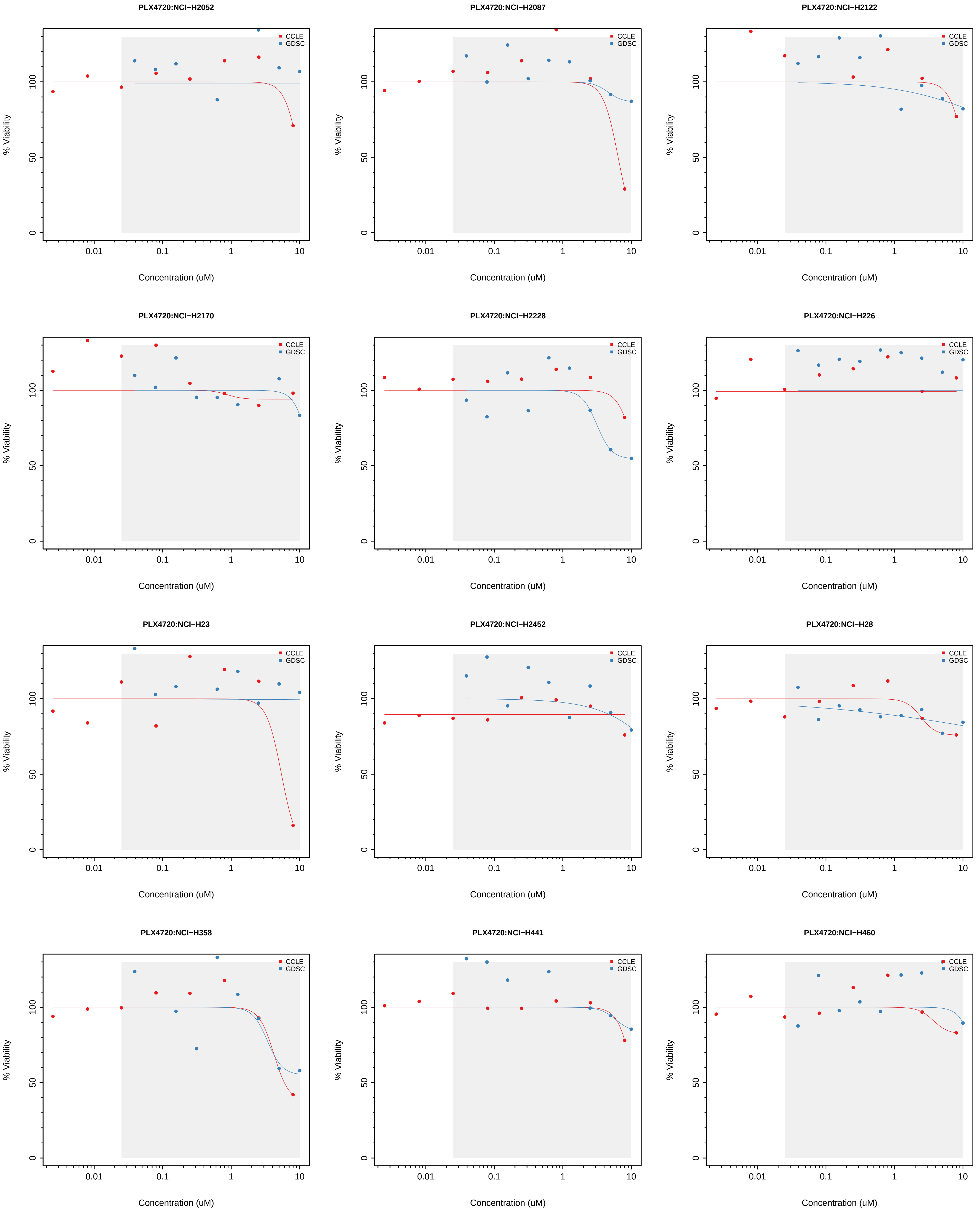
PLX4720:NCI−H2052
PLX4720:NCI−H2087
PLX4720:NCI−H2122
PLX4720:NCI−H2170
PLX4720:NCI−H2228
PLX4720:NCI−H226
PLX4720:NCI−H23
PLX4720:NCI−H2452
PLX4720:NCI−H28
PLX4720:NCI−H358
PLX4720:NCI−H441
PLX4720:NCI−H460
CCLE
GDSC
% Viability
Concentration (uM)
0
50
100
0.01
0.1
1
10

PLX4720:NCI−H520

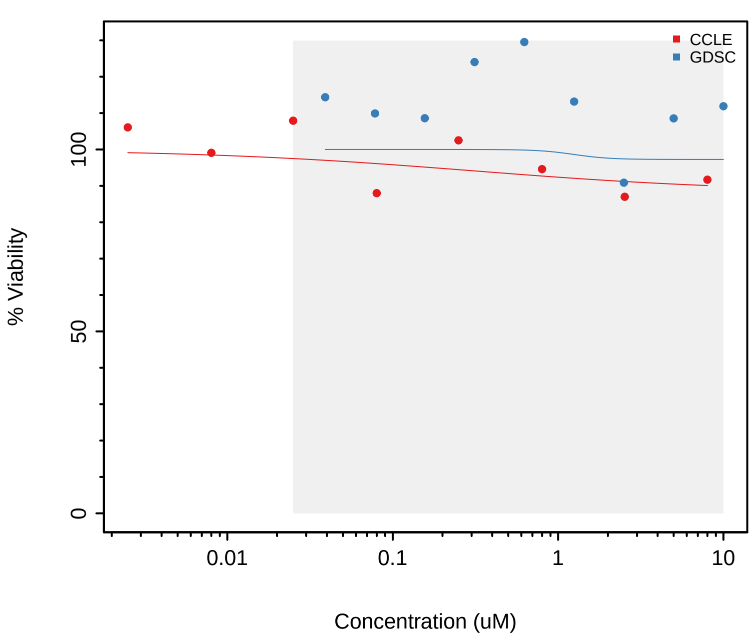

PLX4720:NCI−H522

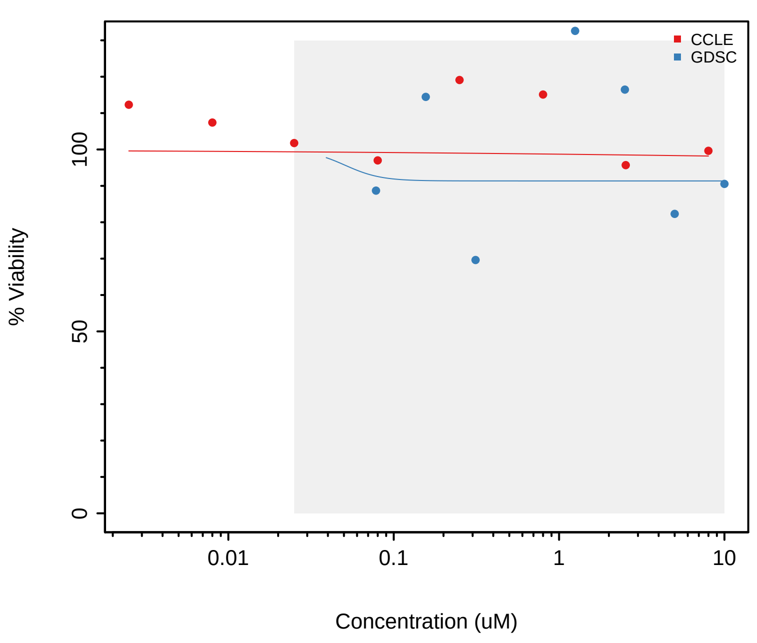

PLX4720:NCI−H650

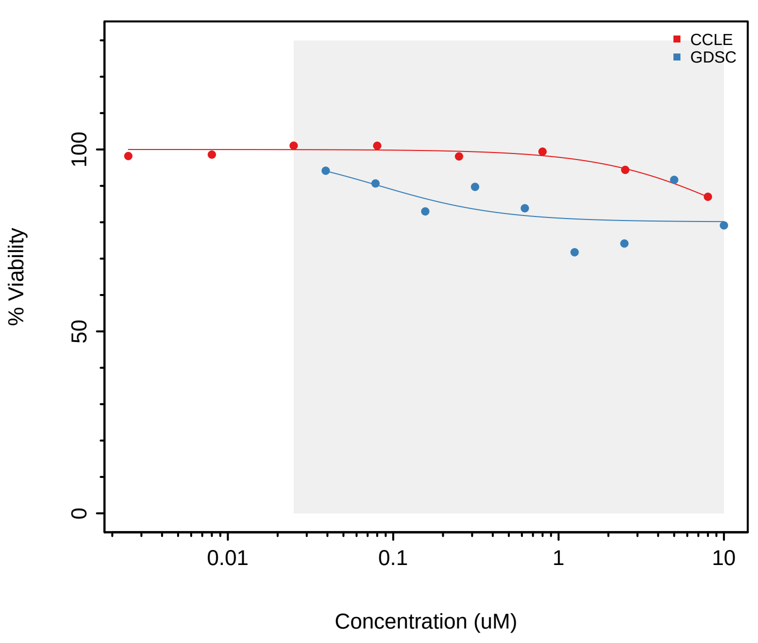

PLX4720:NCI−H661

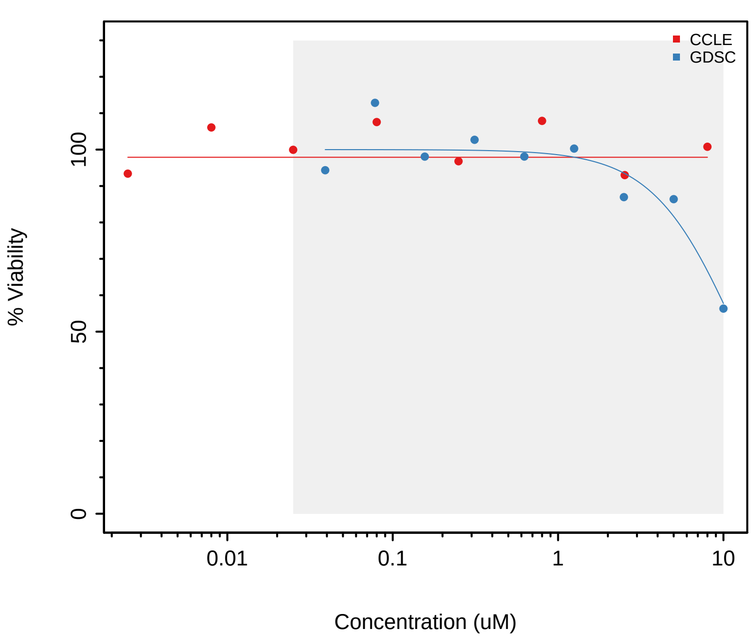

PLX4720:NCI−H747

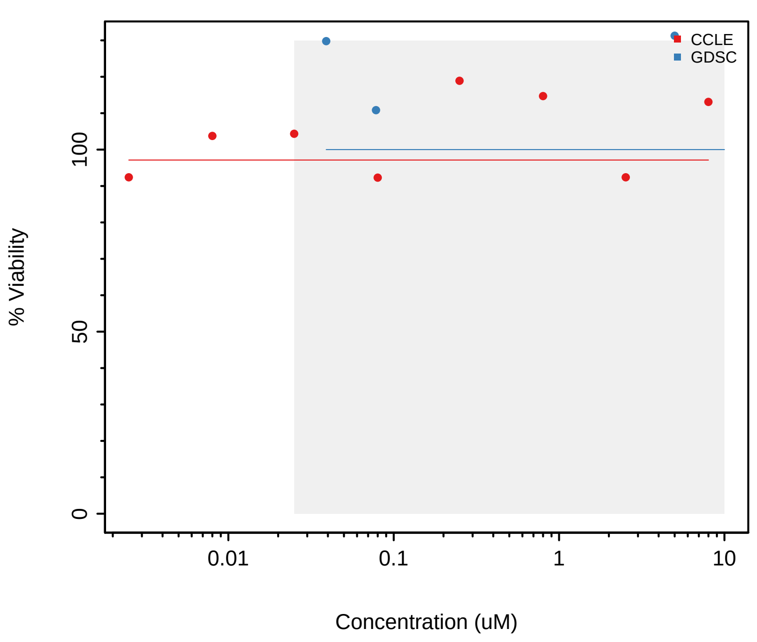

PLX4720:NCI−H810

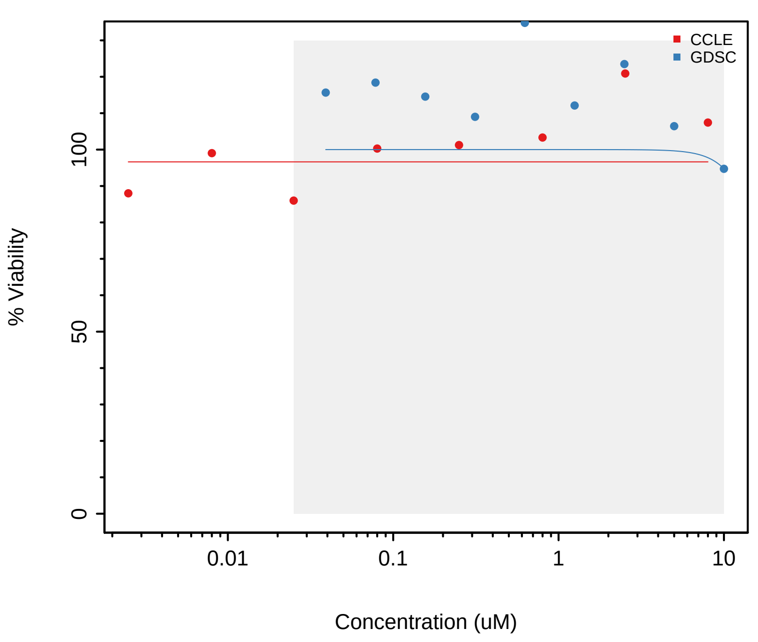

PLX4720:NCI−N87

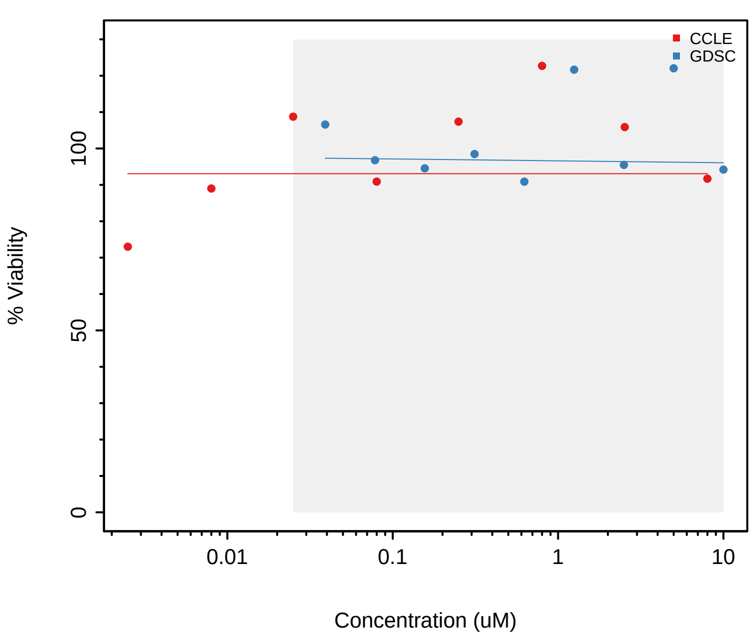

PLX4720:OVCAR−3

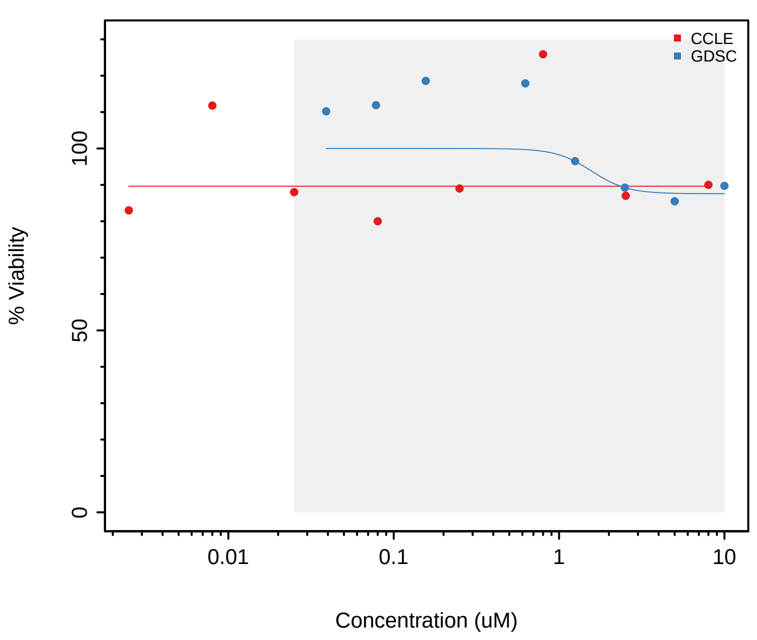

PLX4720:NUGC−3

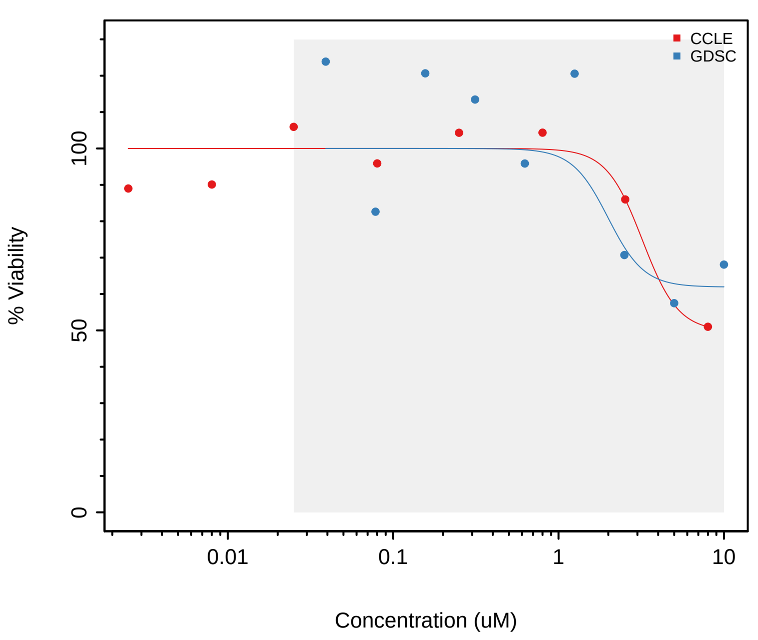

PLX4720:OC−314

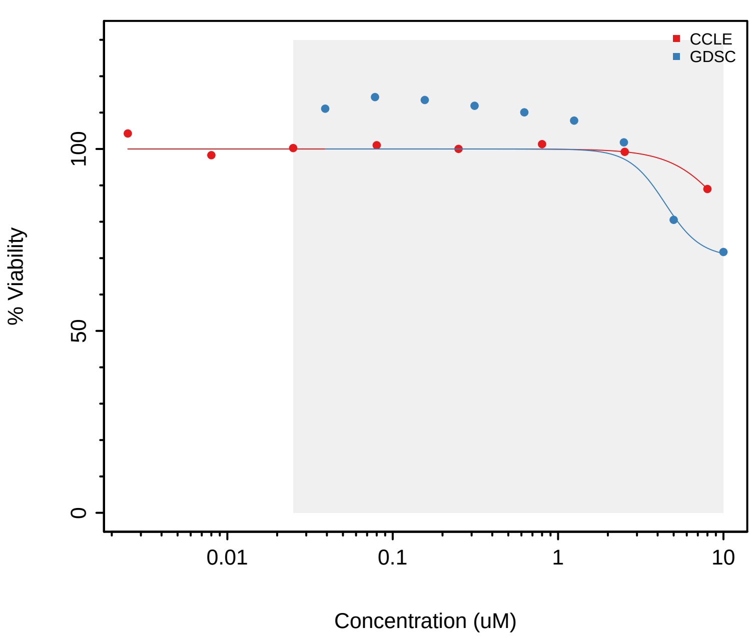

PLX4720:OCI−AML2

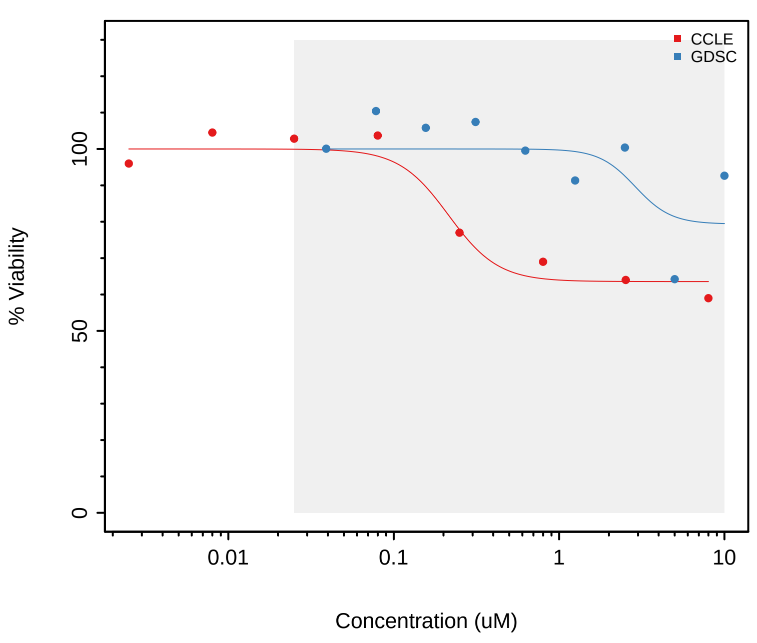

PLX4720:OE33

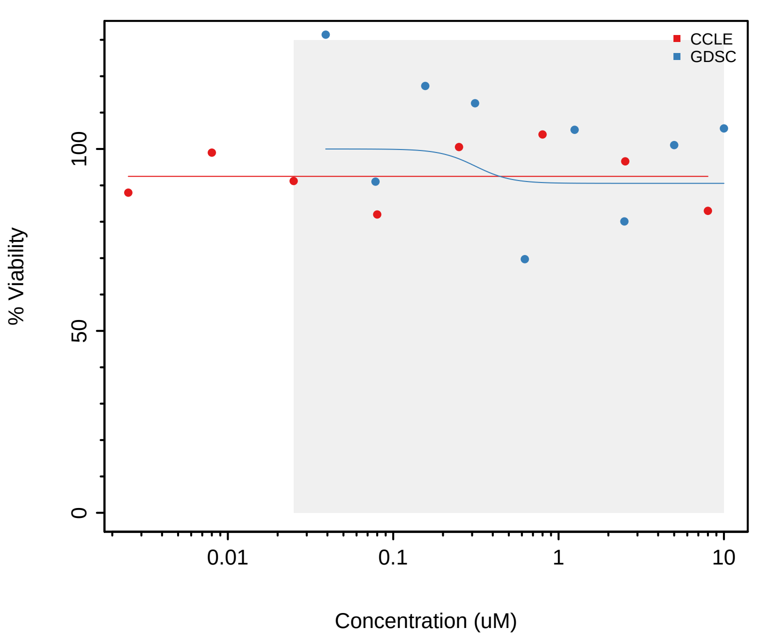

PLX4720:ONS−76

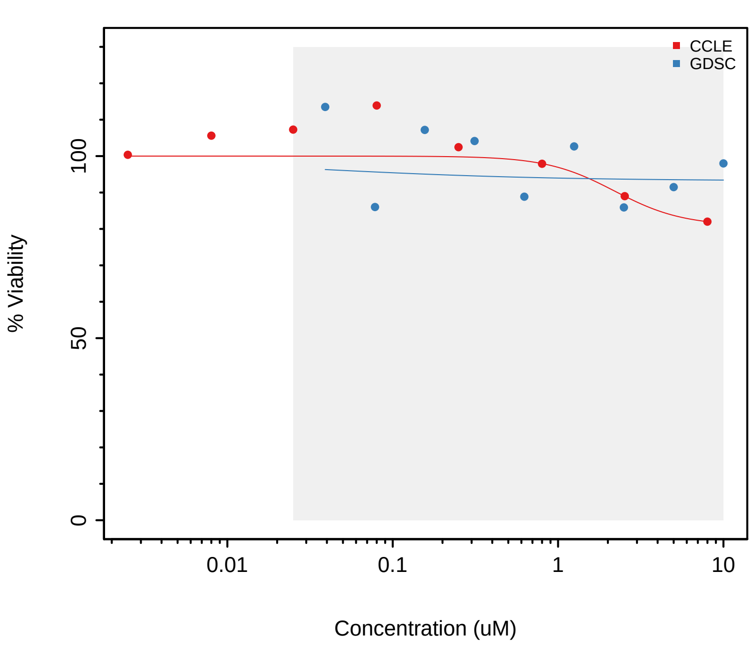

PLX4720:OPM−2

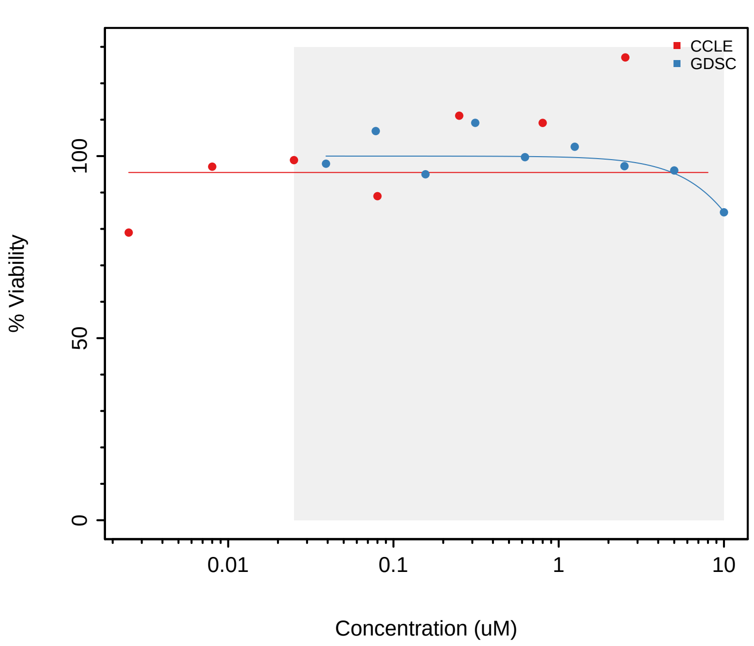

PLX4720:OVCAR−4

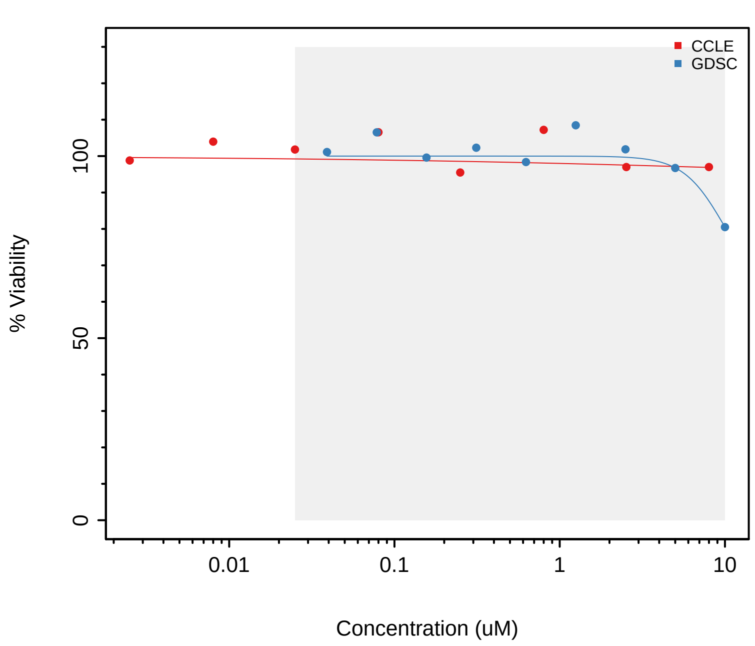

PLX4720:OVCAR−8

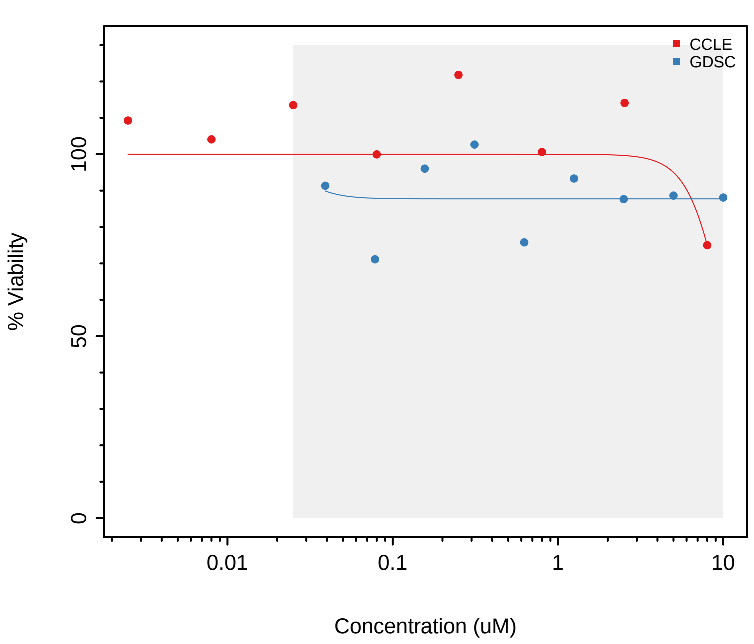

PLX4720:P12−ICHIKAWA

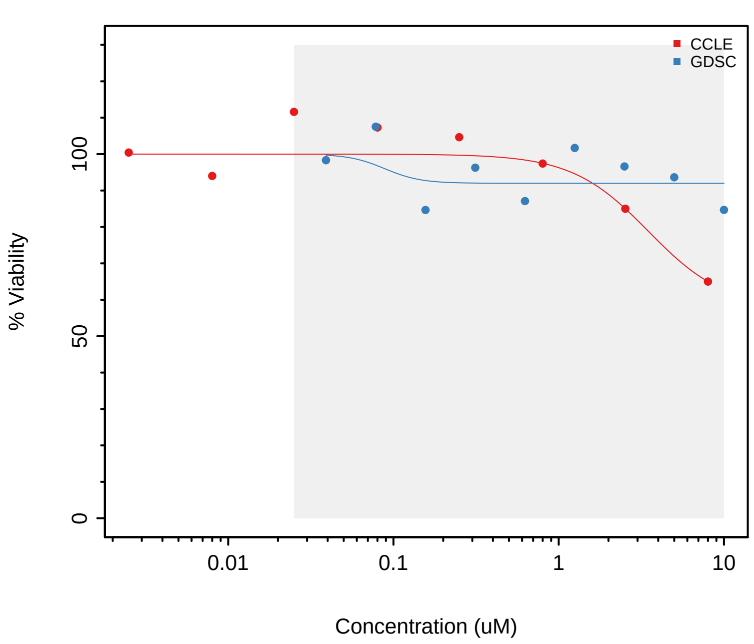

PLX4720:P31−FUJ

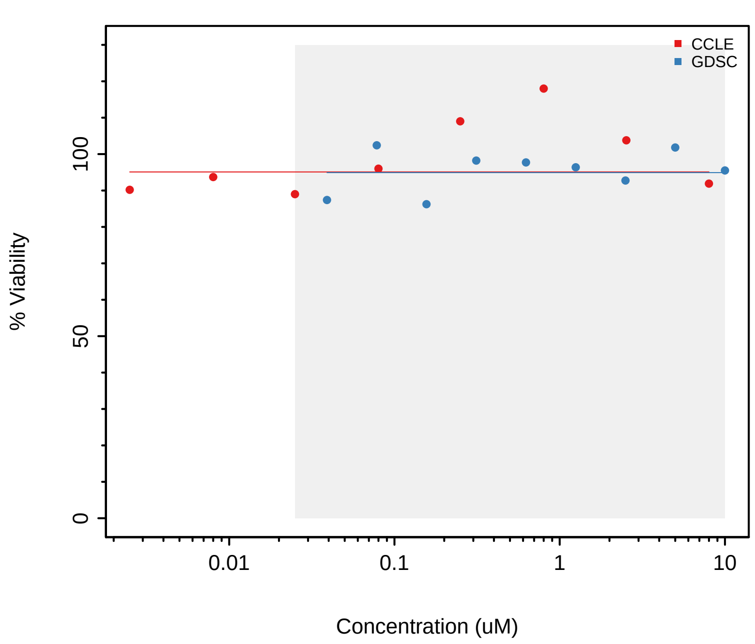

PLX4720:Panc 03.27

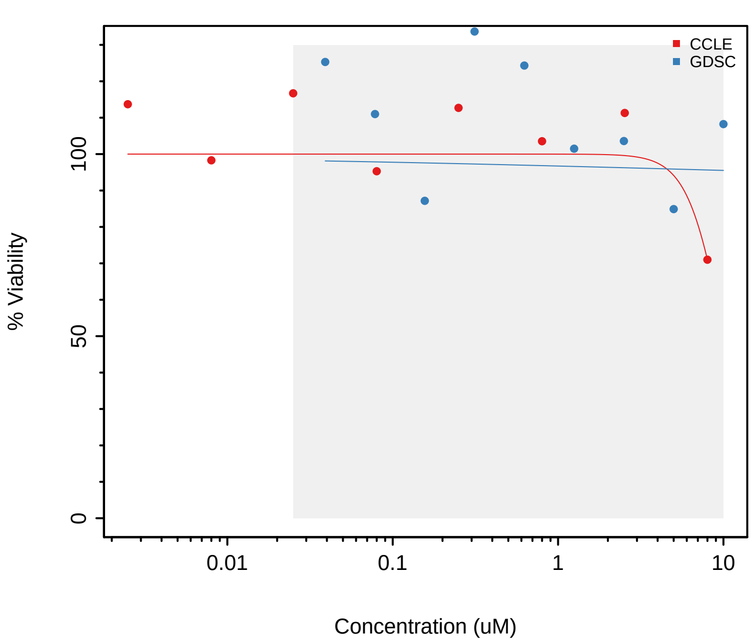

PLX4720:Panc 10.05

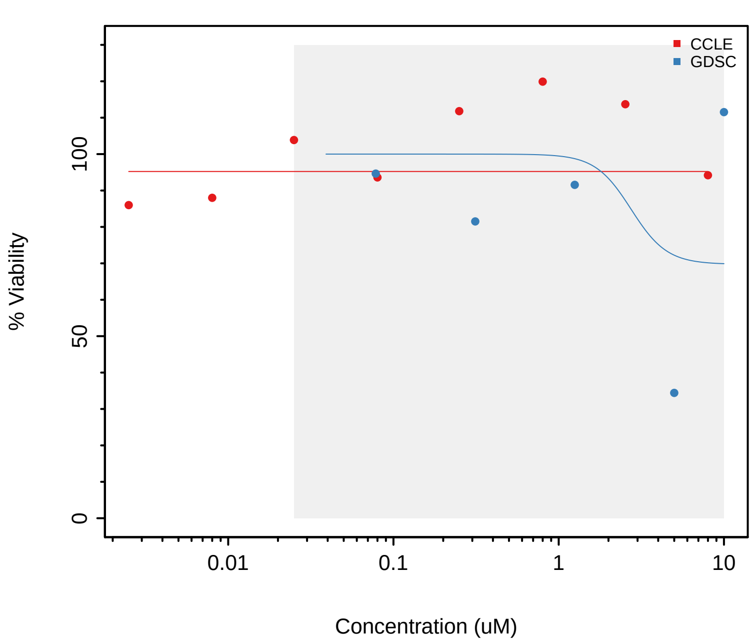

PLX4720:PC−14

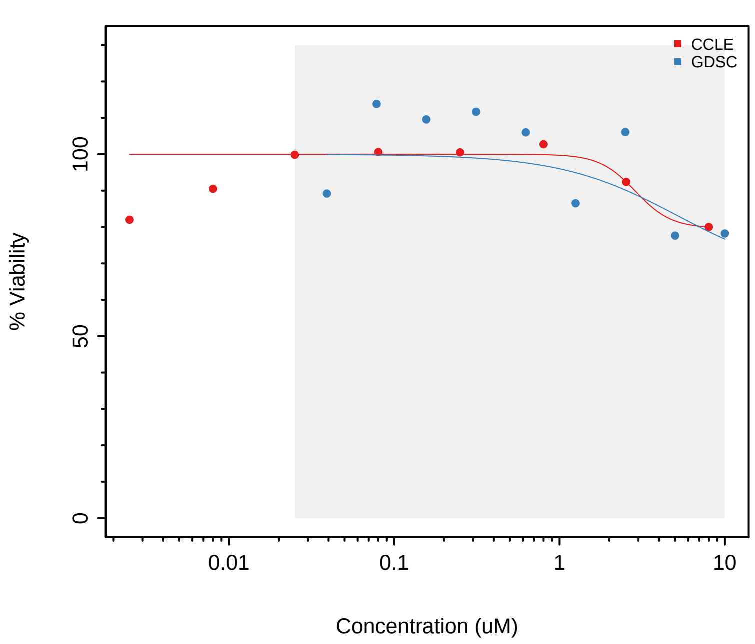

PLX4720:PSN1

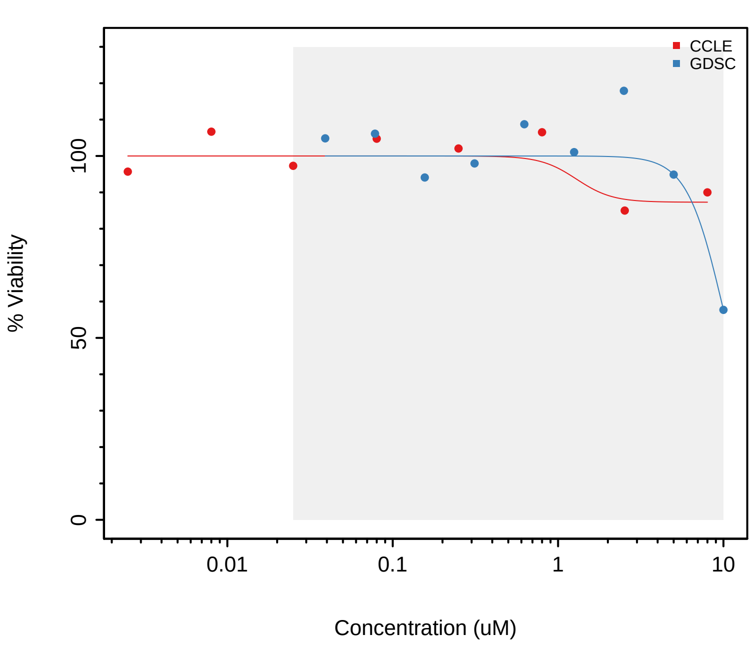

PLX4720:Raji

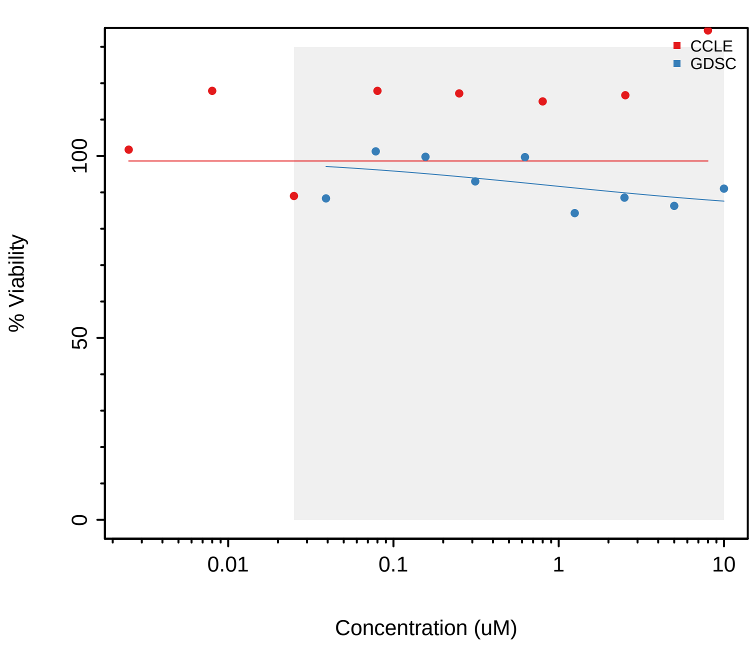

PLX4720:RD

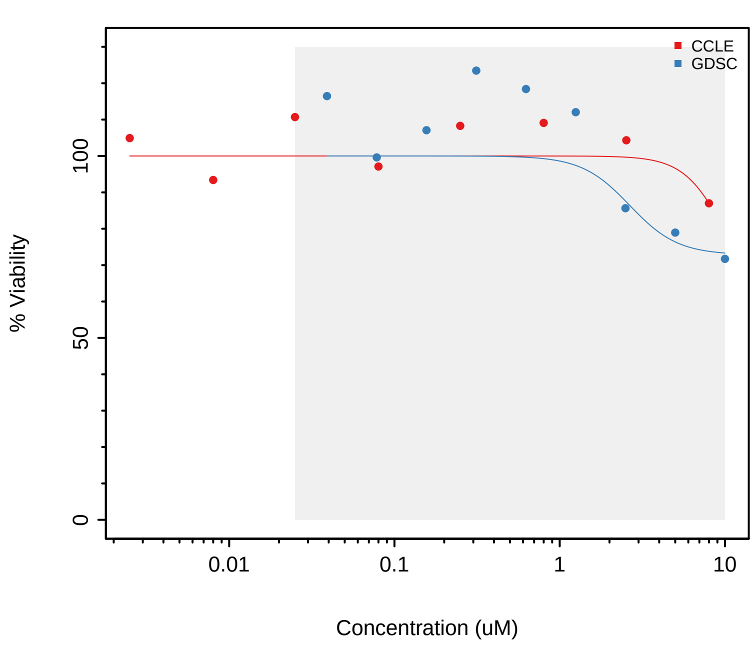

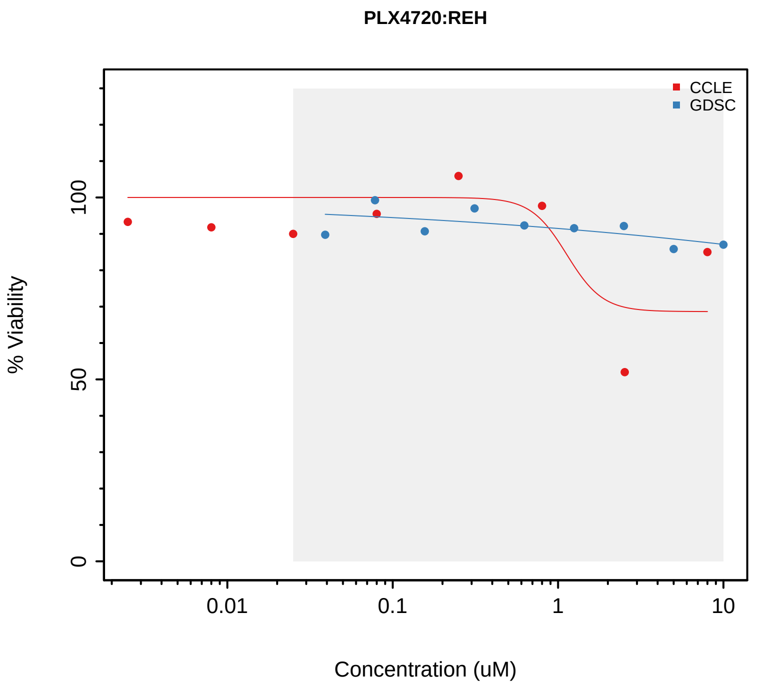
PLX4720:REH
CCLE
GDSC
% Viability
0
50
100
0.01
0.1
1
10
Concentration (uM)
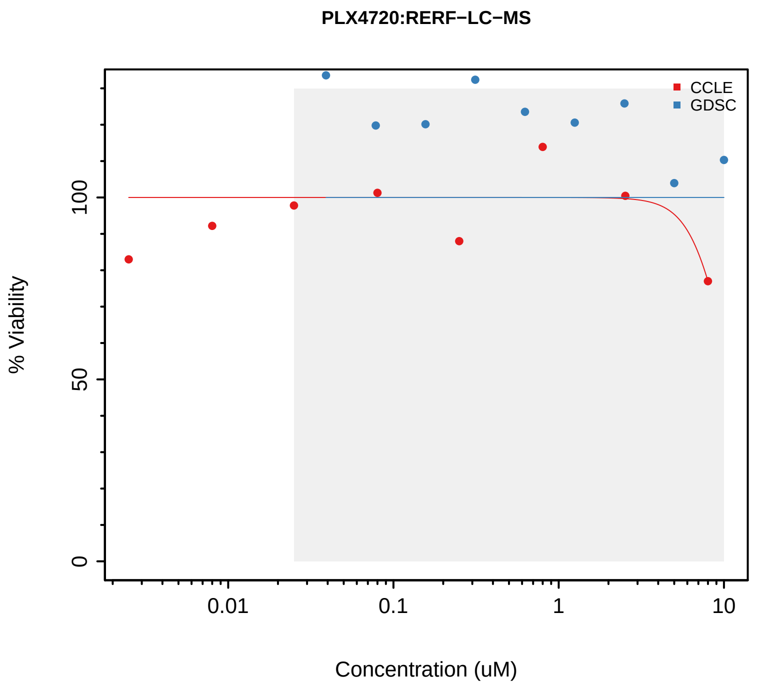
PLX4720:RERF−LC−MS
CCLE
GDSC
% Viability
0
50
100
0.01
0.1
1
10
Concentration (uM)
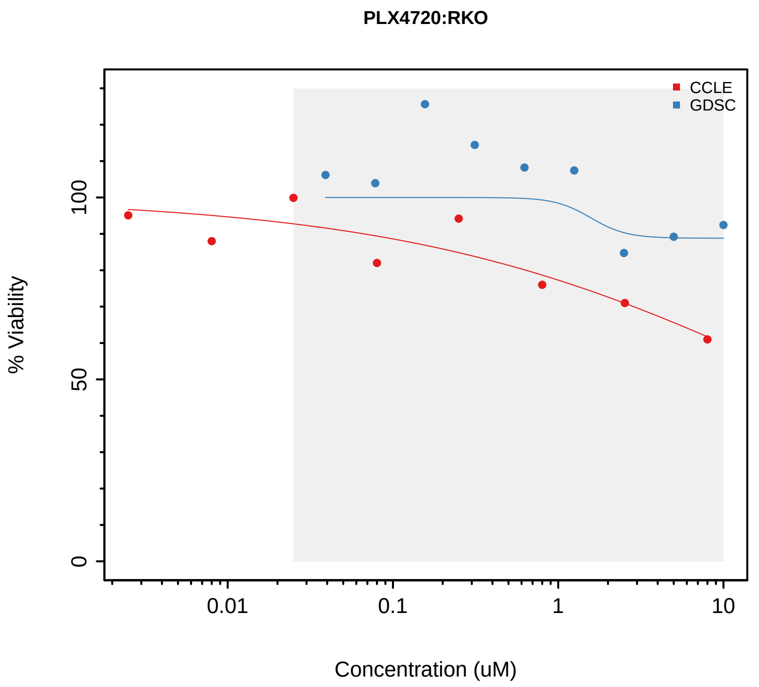
PLX4720:RKO
CCLE
GDSC
% Viability
0
50
100
0.01
0.1
1
10
Concentration (uM)
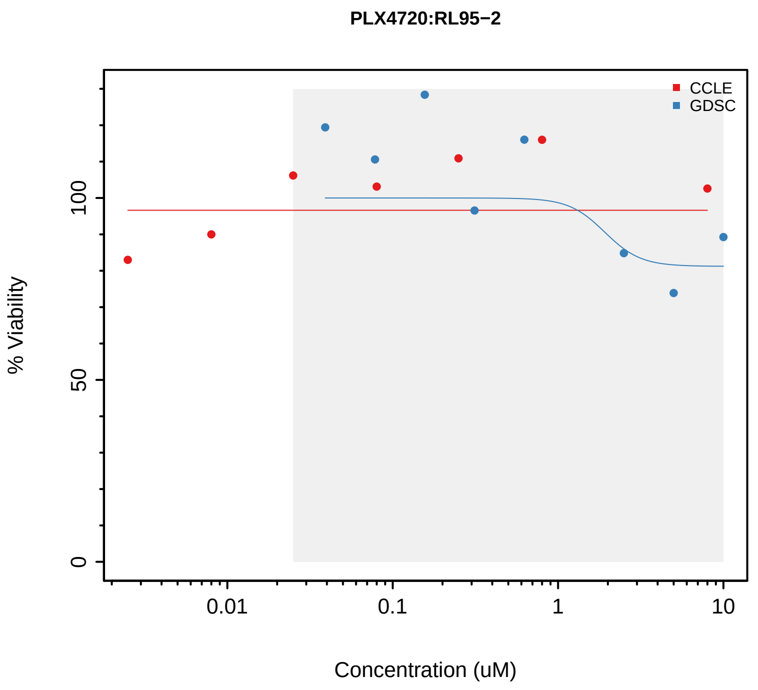
PLX4720:RL95−2
CCLE
GDSC
% Viability
0
50
100
0.01
0.1
1
10
Concentration (uM)
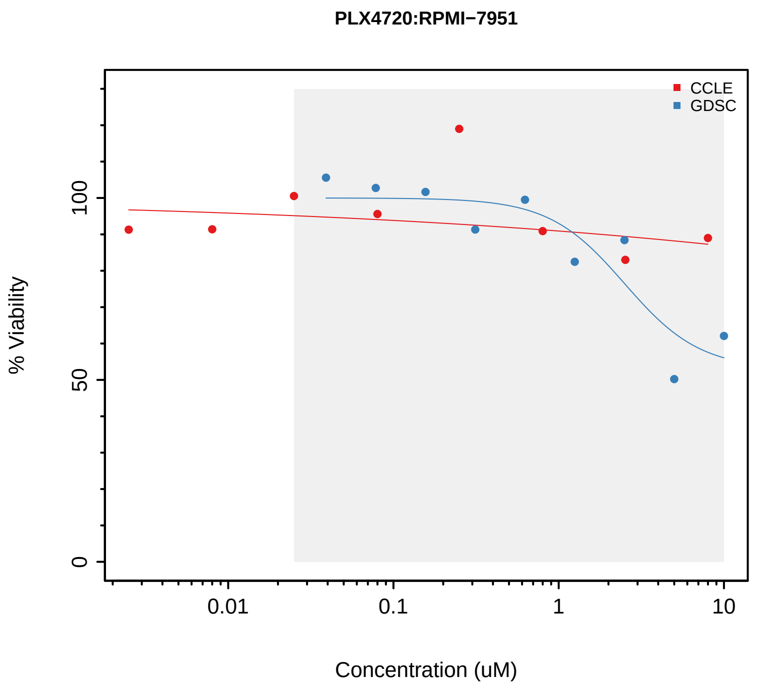
PLX4720:RPMI−7951
CCLE
GDSC
% Viability
0
50
100
0.01
0.1
1
10
Concentration (uM)
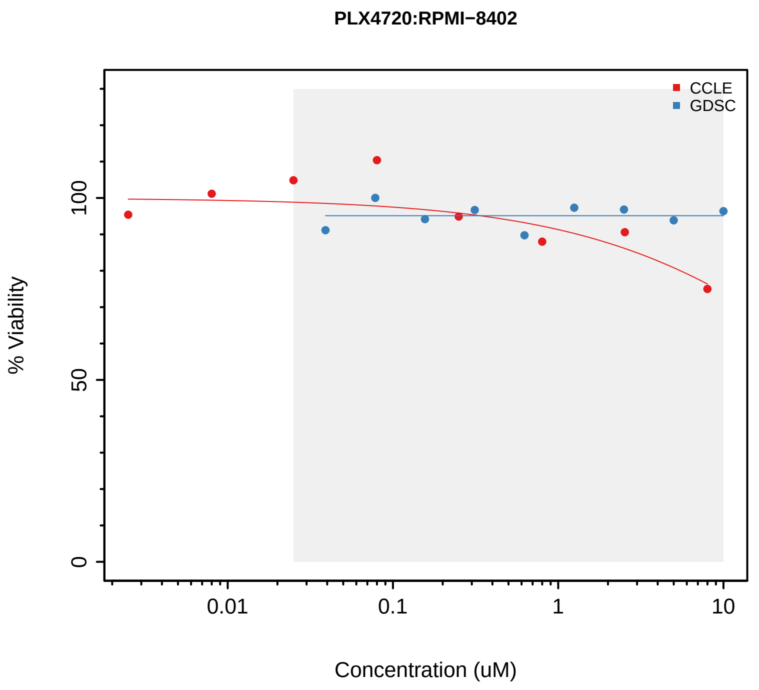
PLX4720:RPMI−8402
CCLE
GDSC
% Viability
0
50
100
0.01
0.1
1
10
Concentration (uM)
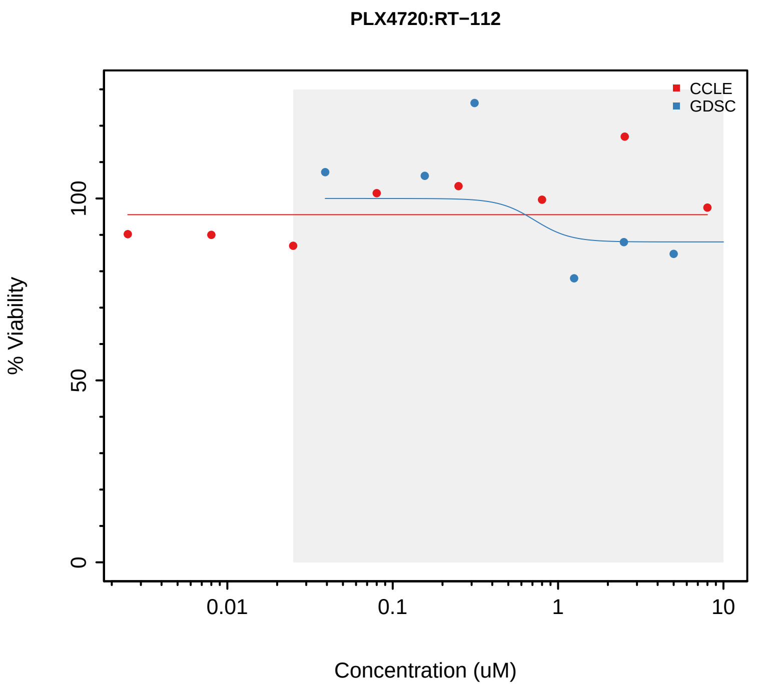
PLX4720:RT−112
CCLE
GDSC
% Viability
0
50
100
0.01
0.1
1
10
Concentration (uM)
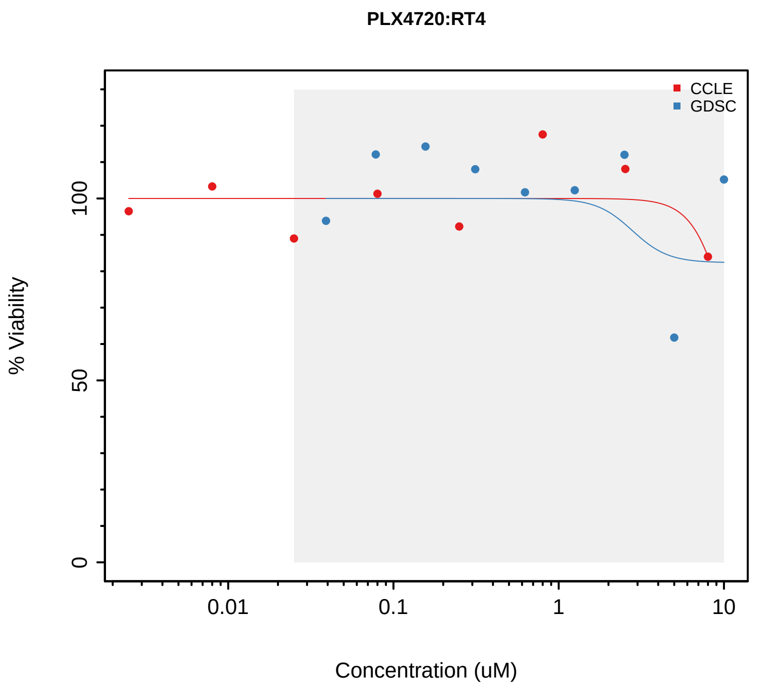
PLX4720:RT4
CCLE
GDSC
% Viability
0
50
100
0.01
0.1
1
10
Concentration (uM)
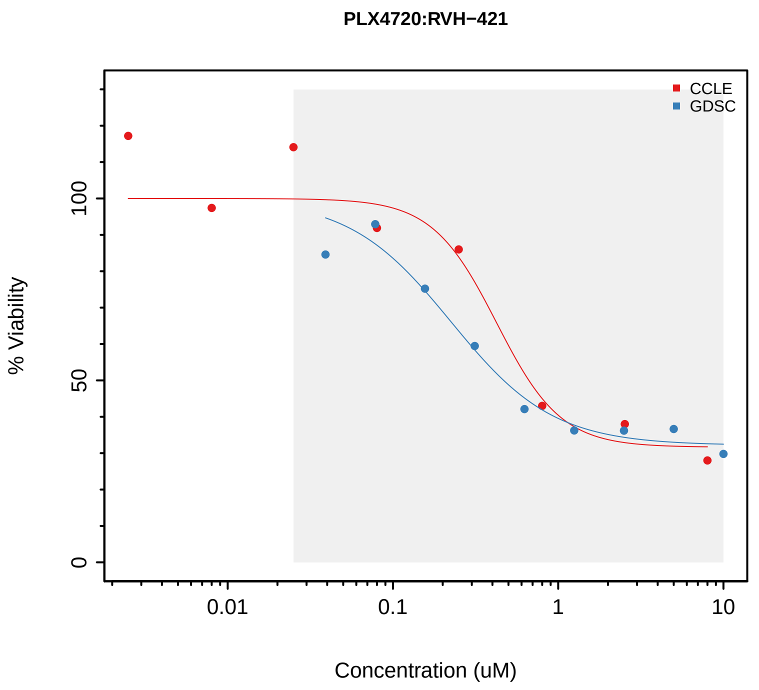
PLX4720:RVH−421
CCLE
GDSC
% Viability
0
50
100
0.01
0.1
1
10
Concentration (uM)
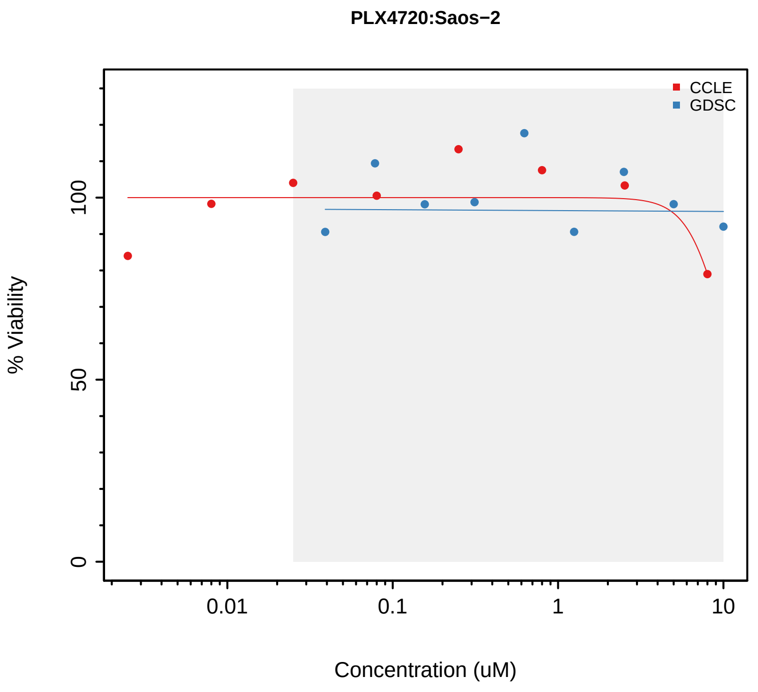
PLX4720:Saos−2
CCLE
GDSC
% Viability
0
50
100
0.01
0.1
1
10
Concentration (uM)
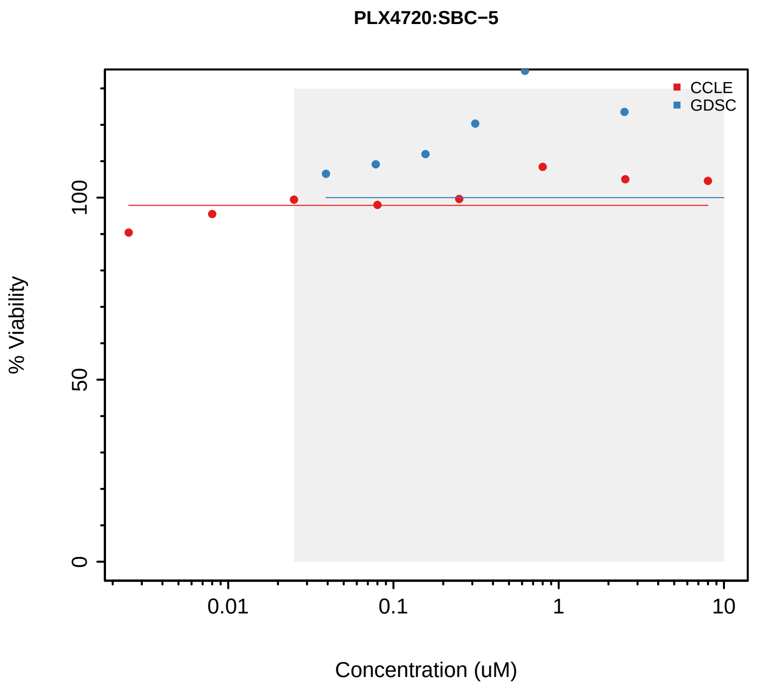
PLX4720:SBC−5
CCLE
GDSC
% Viability
0
50
100
0.01
0.1
1
10
Concentration (uM)
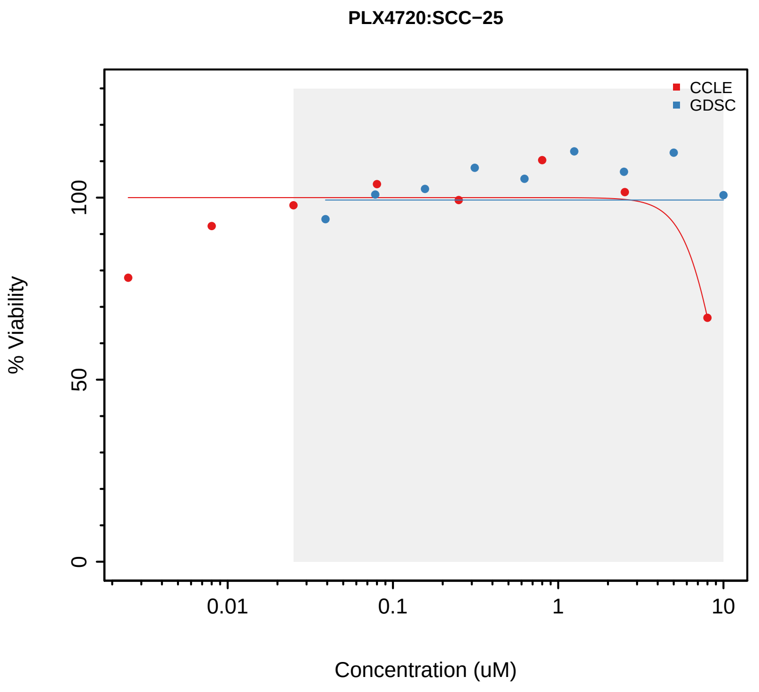
PLX4720:SCC−25
CCLE
GDSC
% Viability
0
50
100
0.01
0.1
1
10
Concentration (uM)

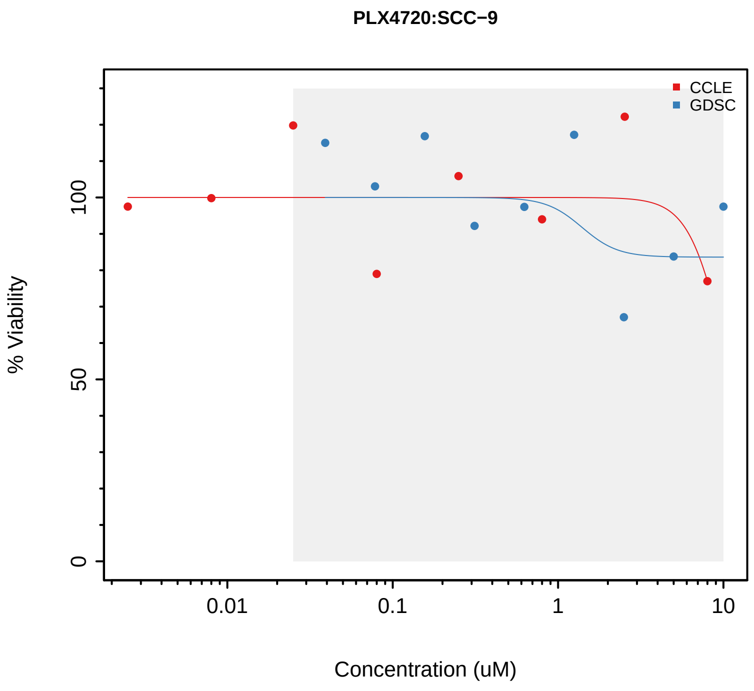
PLX4720:SCC−9
CCLE
GDSC
% Viability
0
50
100
0.01
0.1
1
10
Concentration (uM)
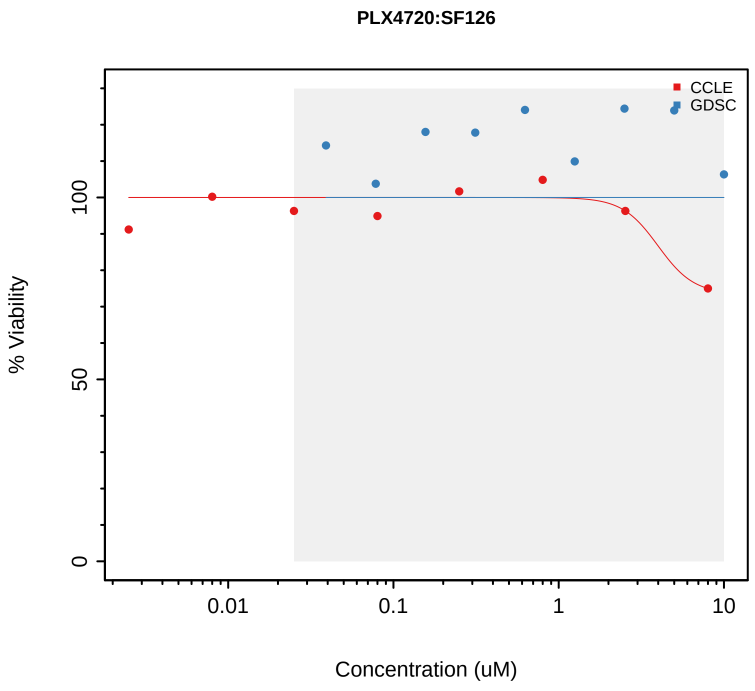
PLX4720:SF126
CCLE
GDSC
% Viability
0
50
100
0.01
0.1
1
10
Concentration (uM)
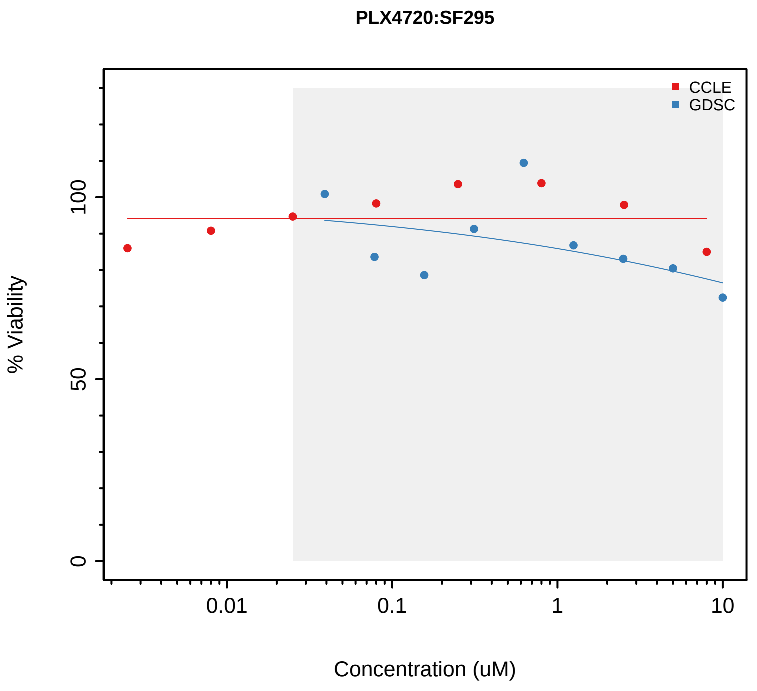
PLX4720:SF295
CCLE
GDSC
% Viability
0
50
100
0.01
0.1
1
10
Concentration (uM)
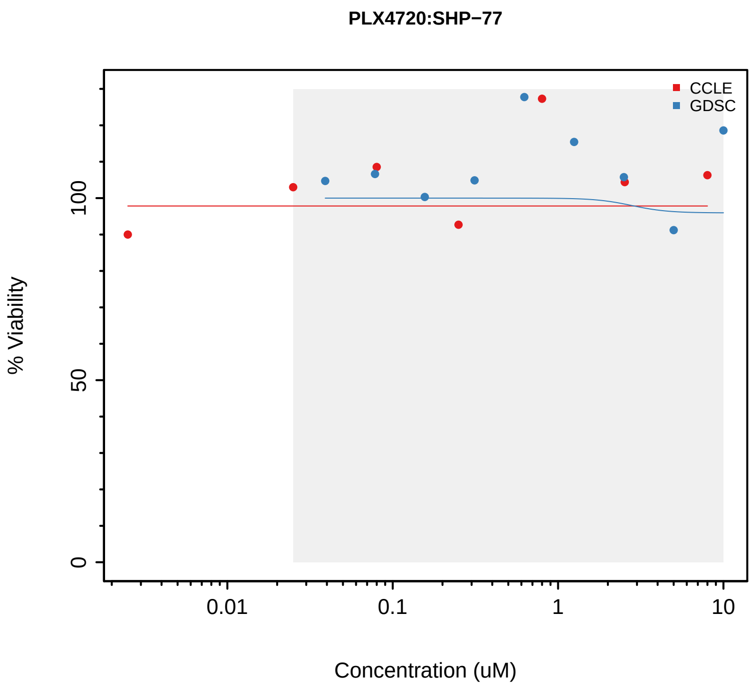
PLX4720:SHP−77
CCLE
GDSC
% Viability
0
50
100
0.01
0.1
1
10
Concentration (uM)
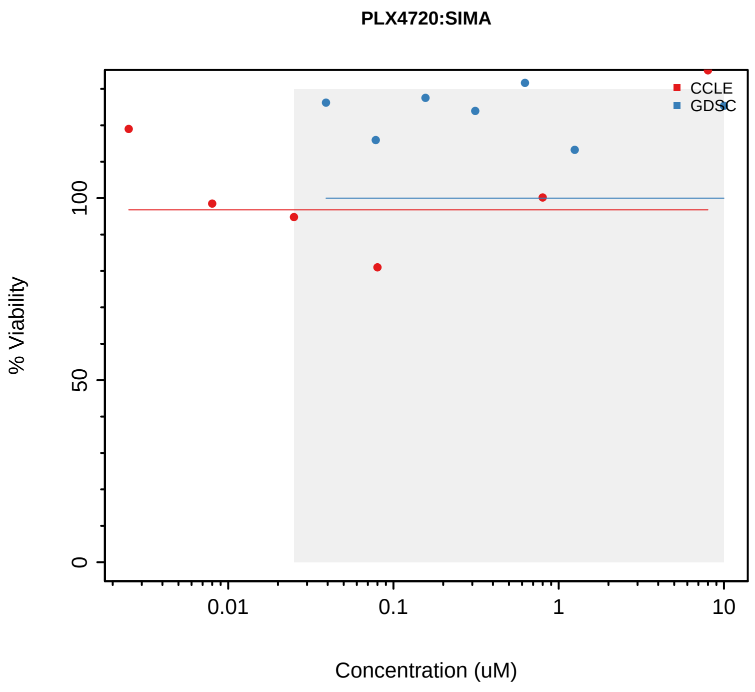
PLX4720:SIMA
CCLE
GDSC
% Viability
0
50
100
0.01
0.1
1
10
Concentration (uM)
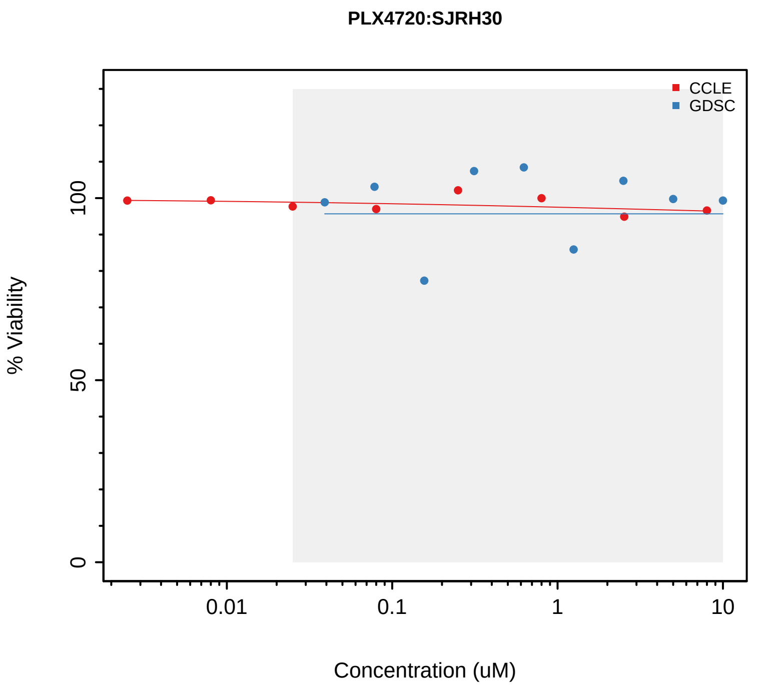
PLX4720:SJRH30
CCLE
GDSC
% Viability
0
50
100
0.01
0.1
1
10
Concentration (uM)
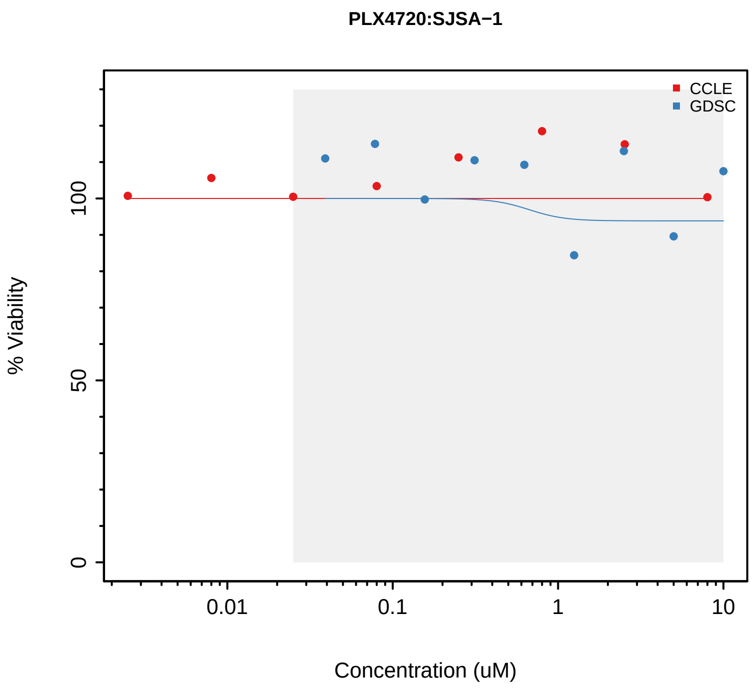
PLX4720:SJSA−1
CCLE
GDSC
% Viability
0
50
100
0.01
0.1
1
10
Concentration (uM)
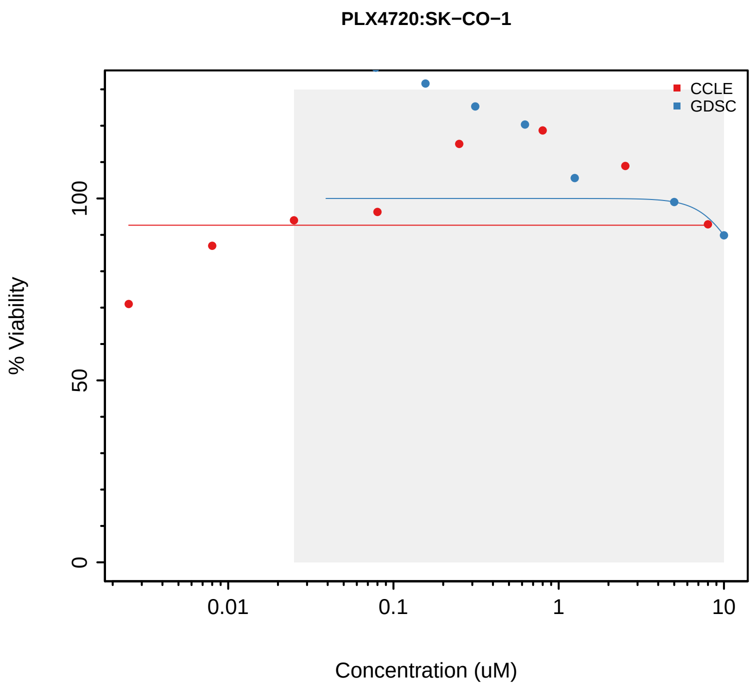
PLX4720:SK−CO−1
CCLE
GDSC
% Viability
0
50
100
0.01
0.1
1
10
Concentration (uM)
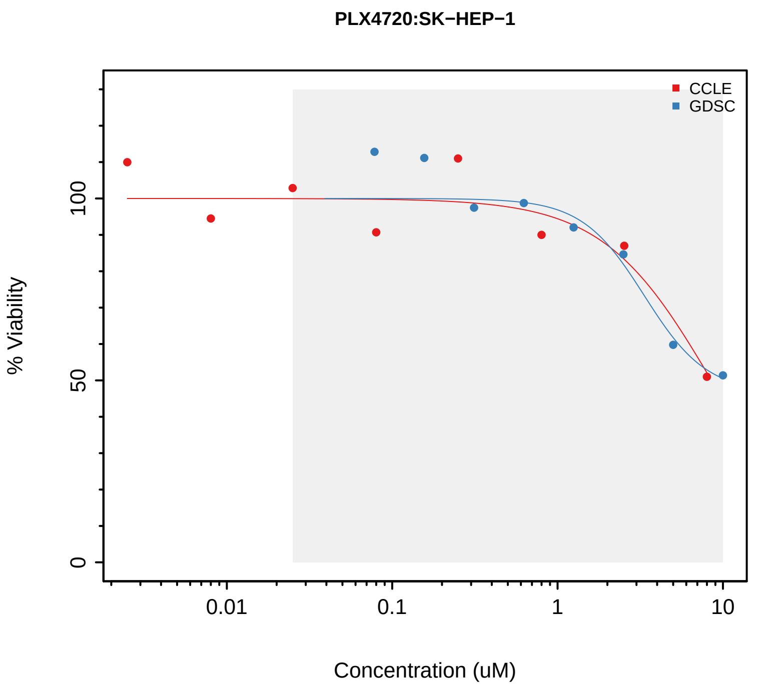
PLX4720:SK−HEP−1
CCLE
GDSC
% Viability
0
50
100
0.01
0.1
1
10
Concentration (uM)
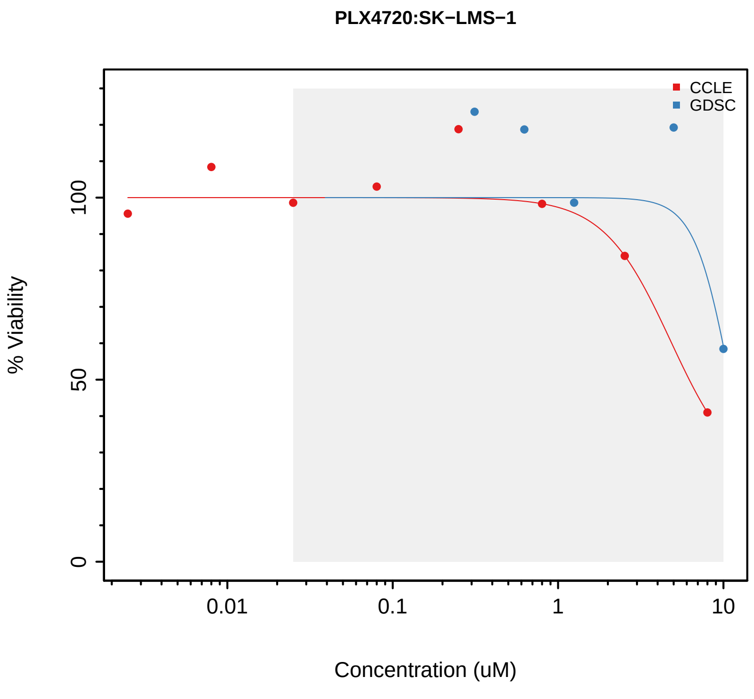
PLX4720:SK−LMS−1
CCLE
GDSC
% Viability
0
50
100
0.01
0.1
1
10
Concentration (uM)
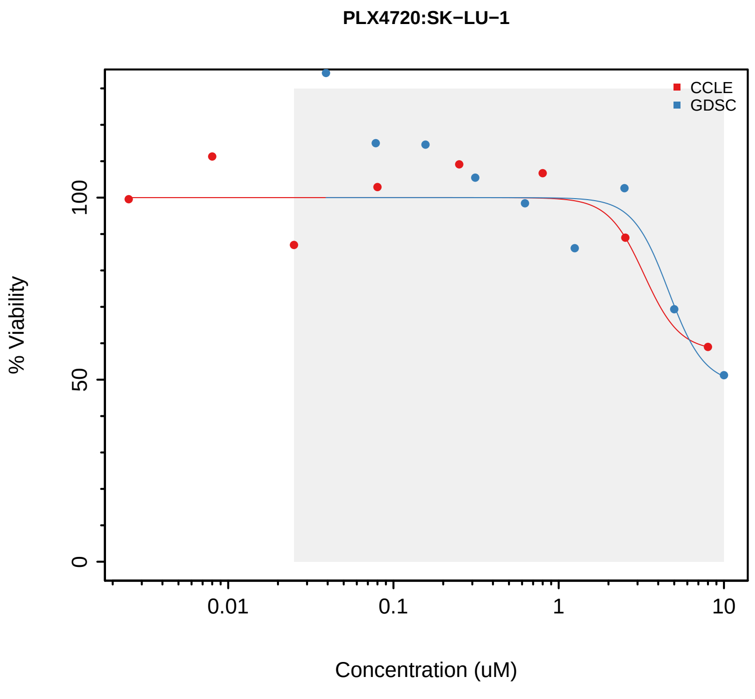
PLX4720:SK−LU−1
CCLE
GDSC
% Viability
0
50
100
0.01
0.1
1
10
Concentration (uM)
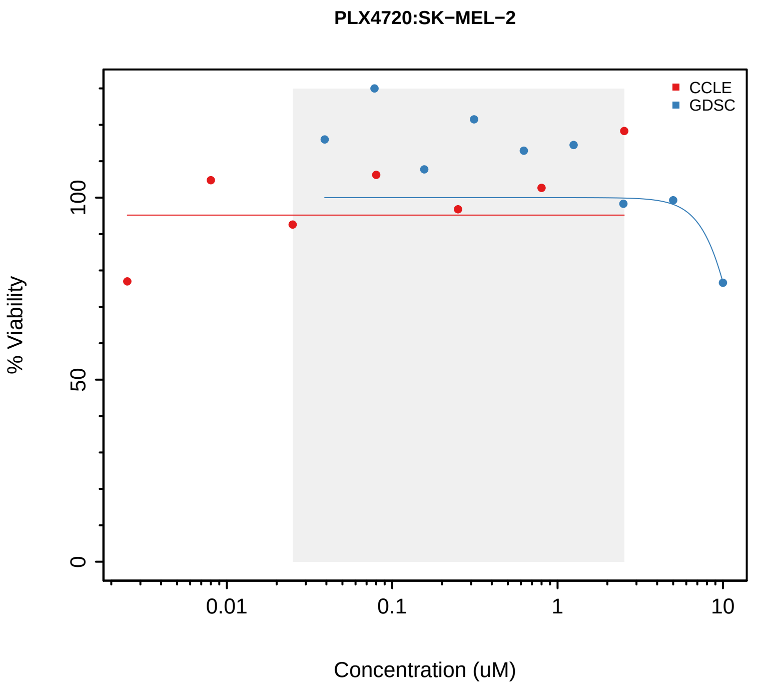
PLX4720:SK−MEL−2
CCLE
GDSC
% Viability
0
50
100
0.01
0.1
1
10
Concentration (uM)

PLX4720:SK−MEL−24

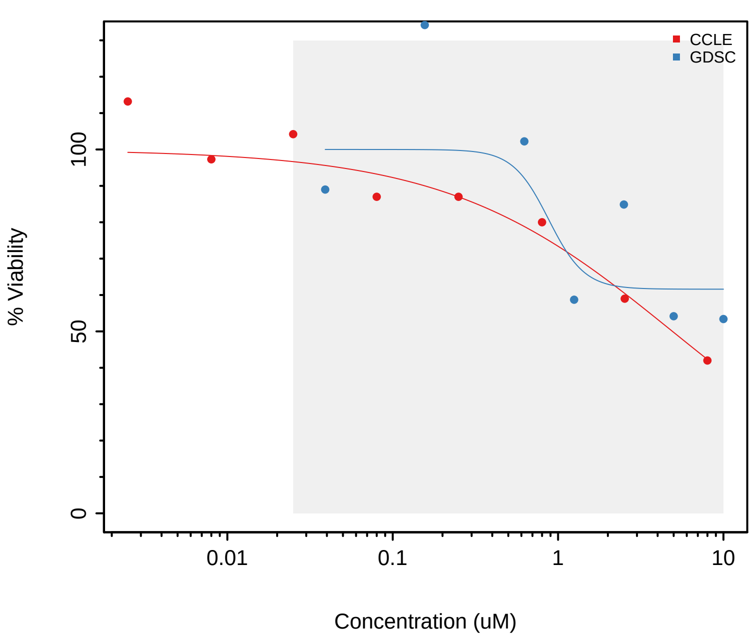

PLX4720:SK−MEL−30

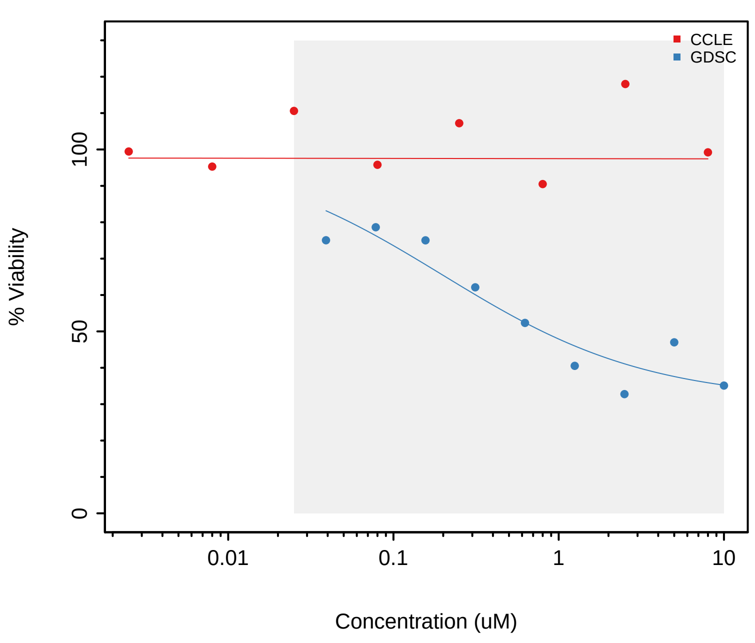

PLX4720:SK−MEL−5

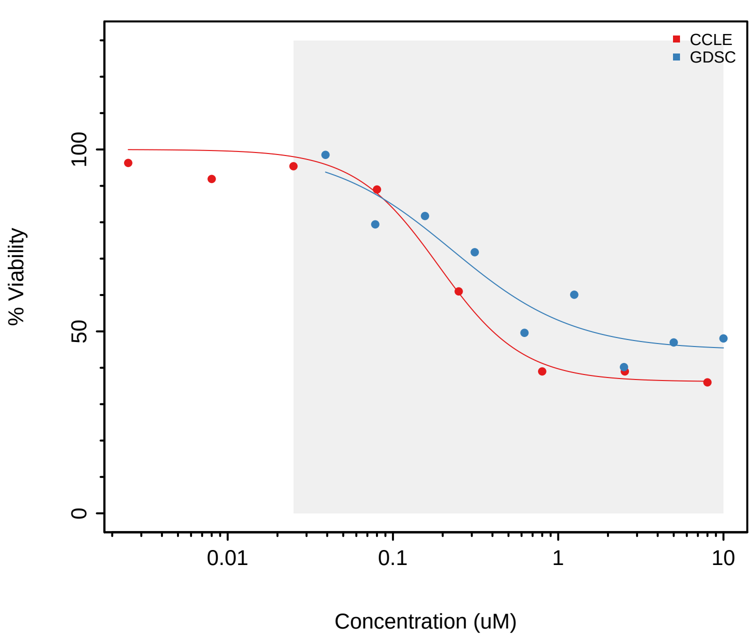

PLX4720:SK−MES−1

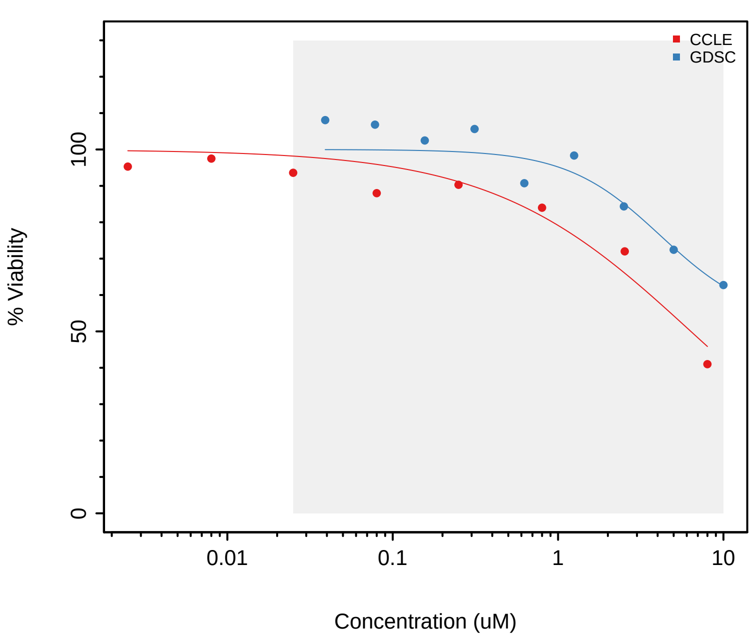

PLX4720:SK−MM−2

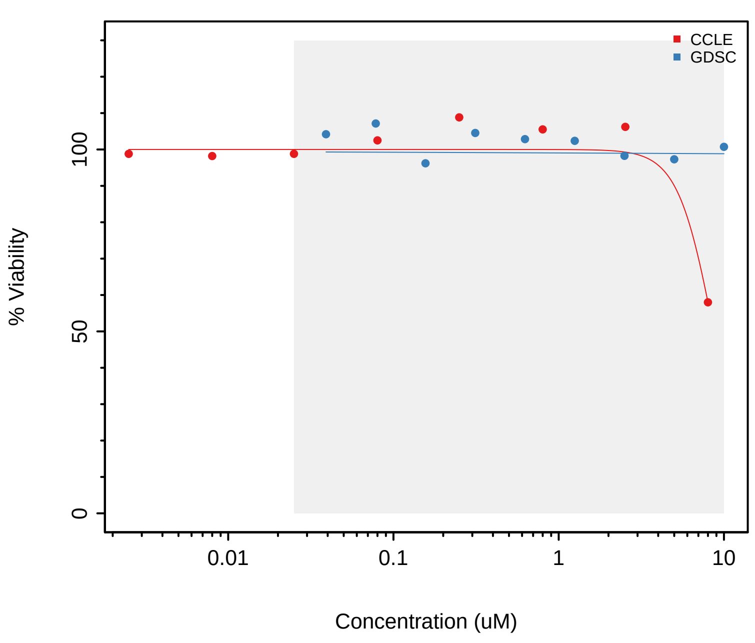

PLX4720:SK−N−AS

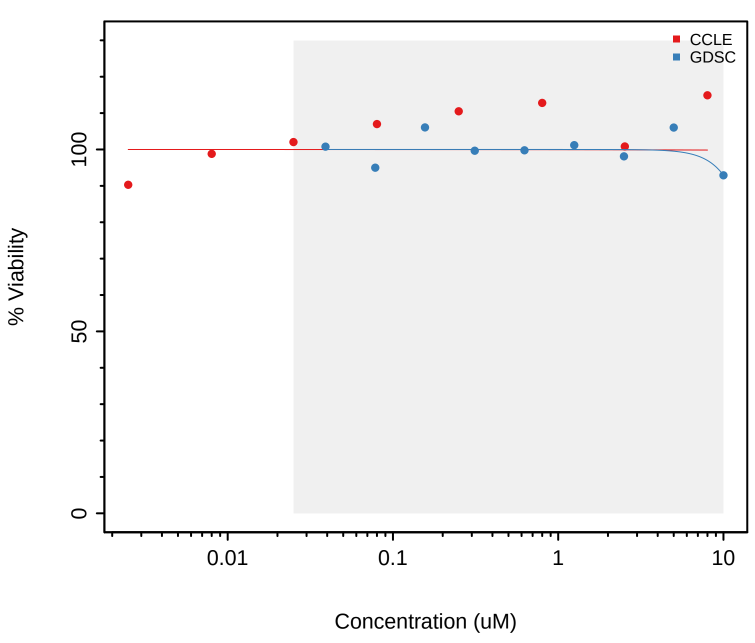

PLX4720:SK−N−DZ

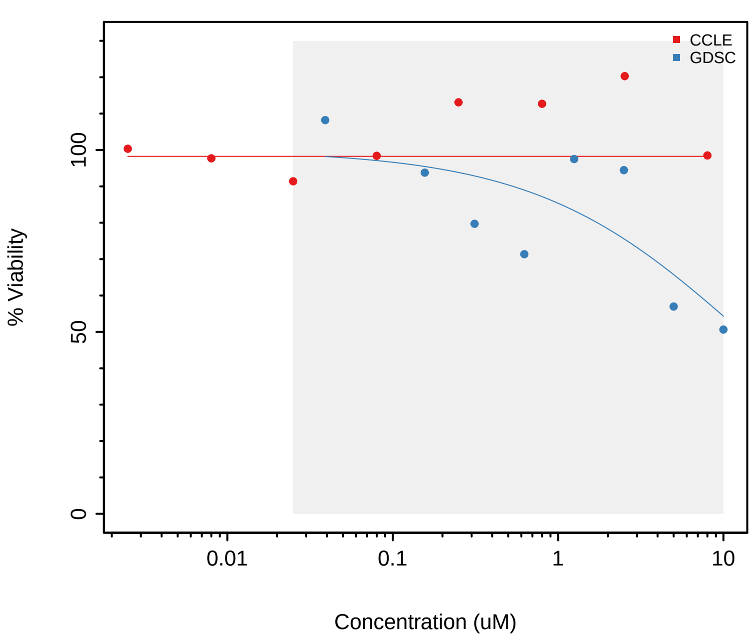

PLX4720:SK−N−FI

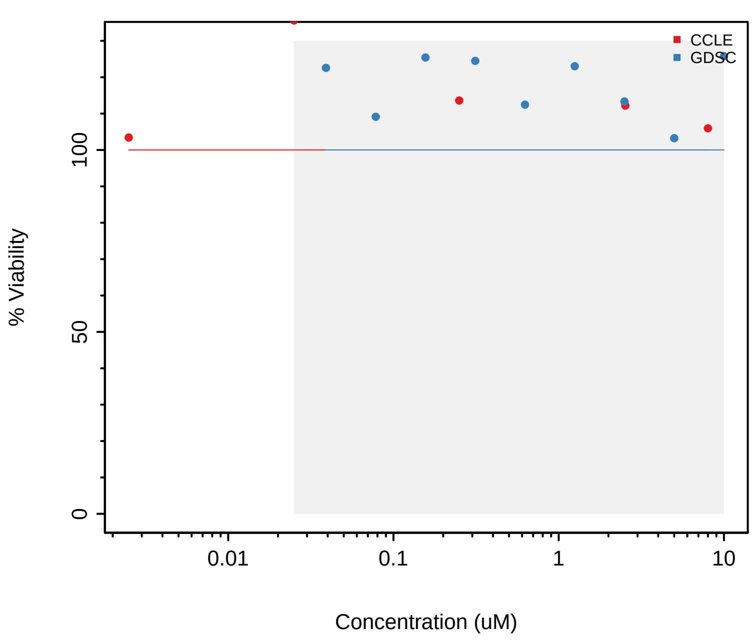

PLX4720:SK−OV−3

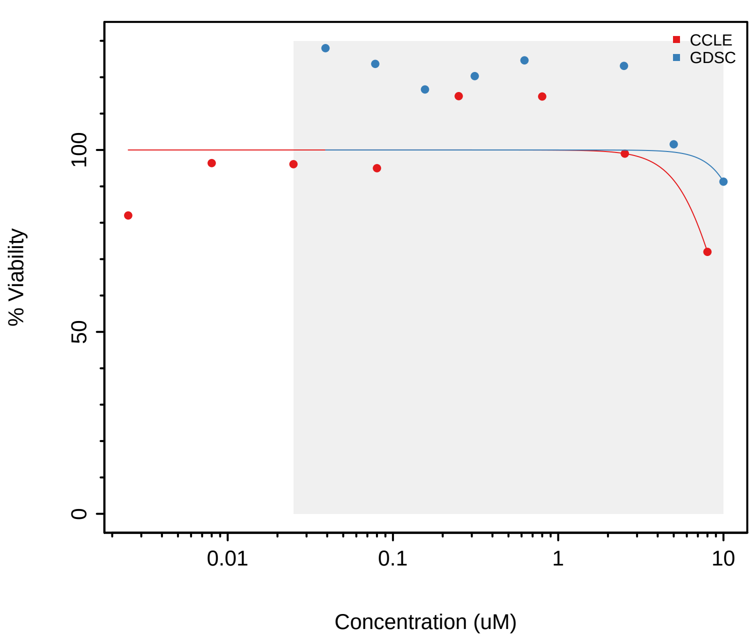

PLX4720:SNG−M

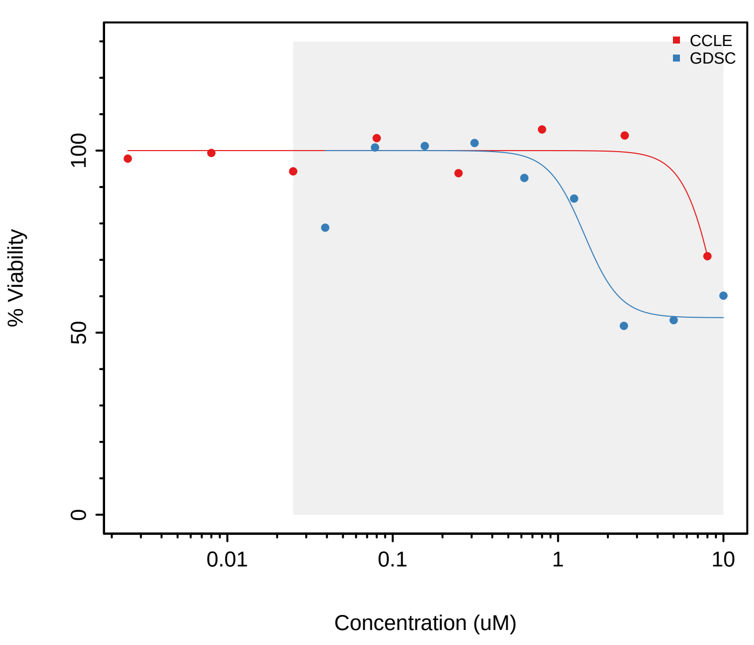

PLX4720:NCI−SNU−1

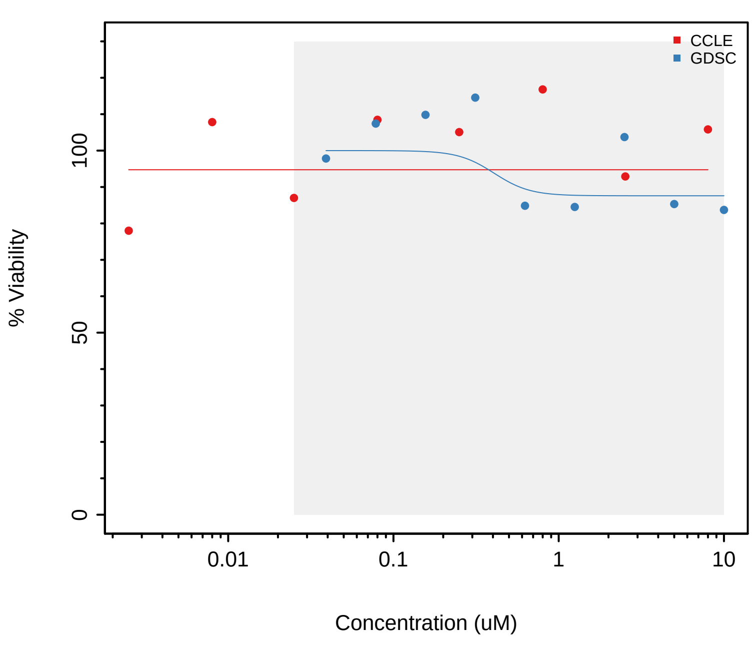

PLX4720:NCI−SNU−16

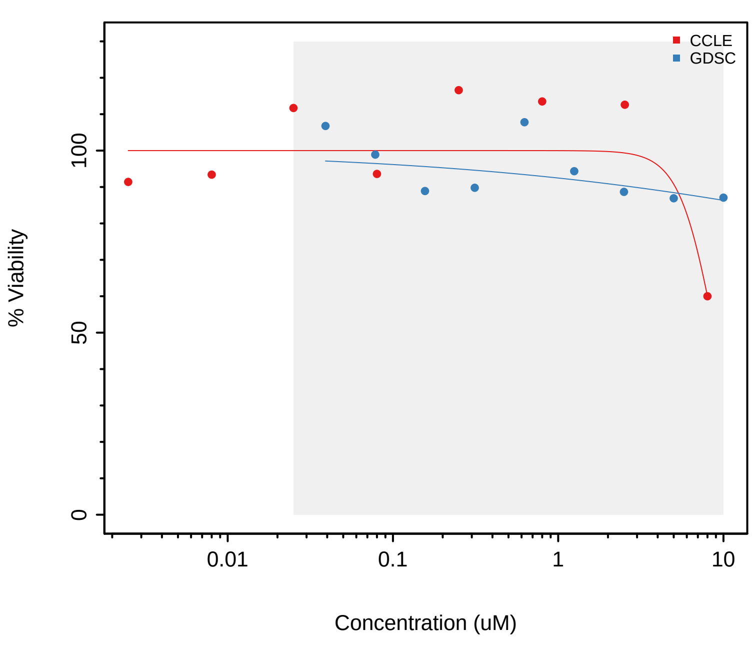

PLX4720:SNU−387

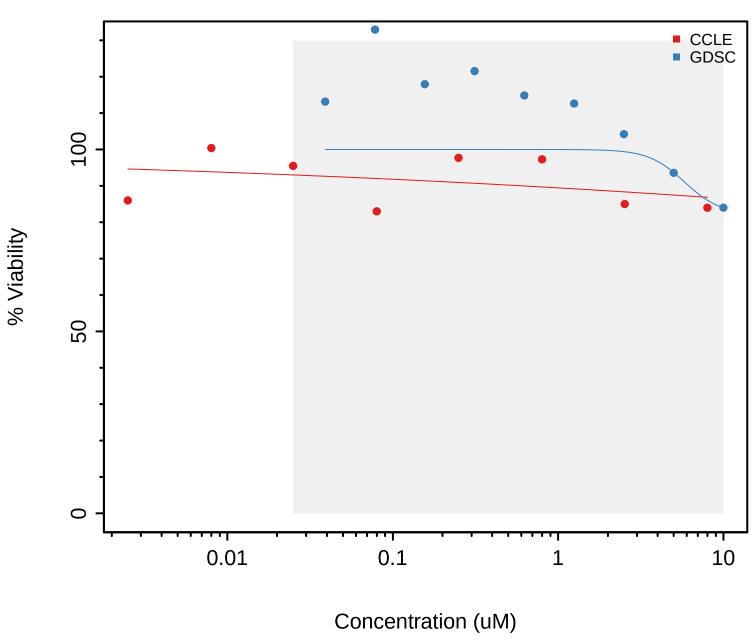

PLX4720:SNU−423

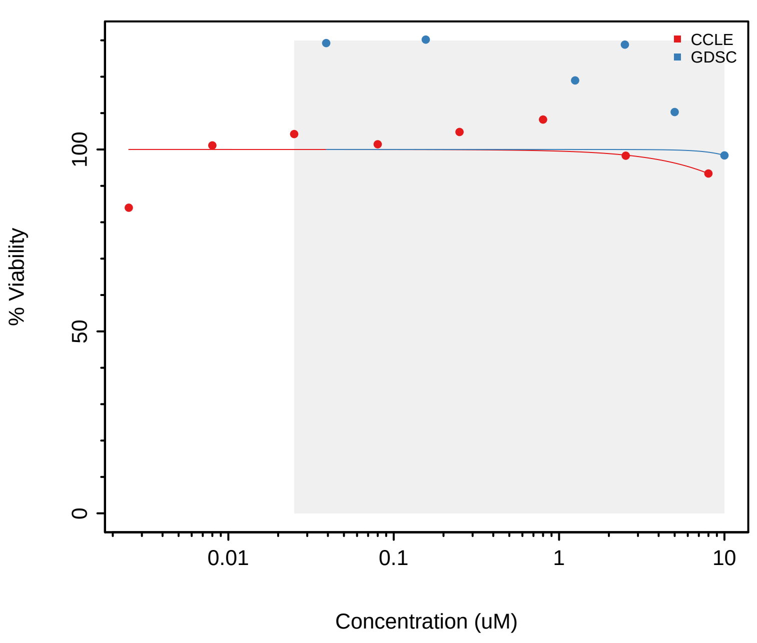

PLX4720:SNU−449

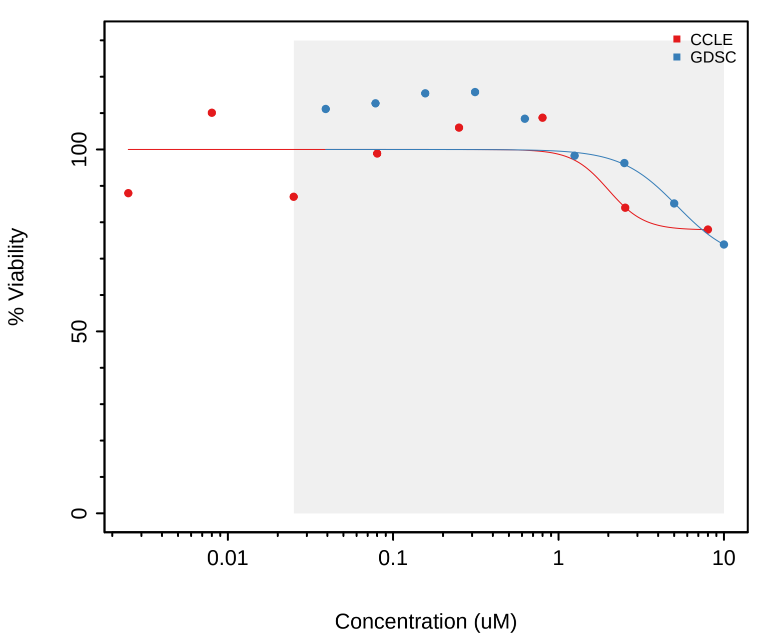

PLX4720:SNU−C2B

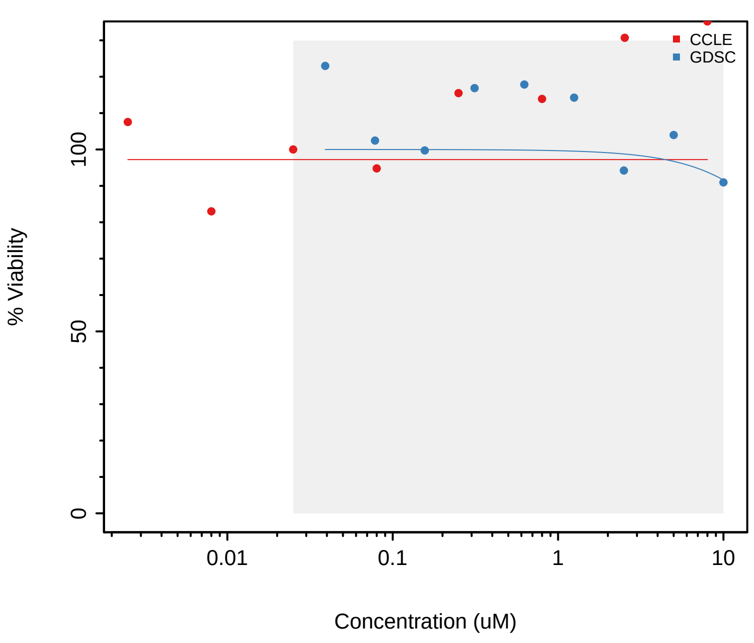

PLX4720:SUP−T1

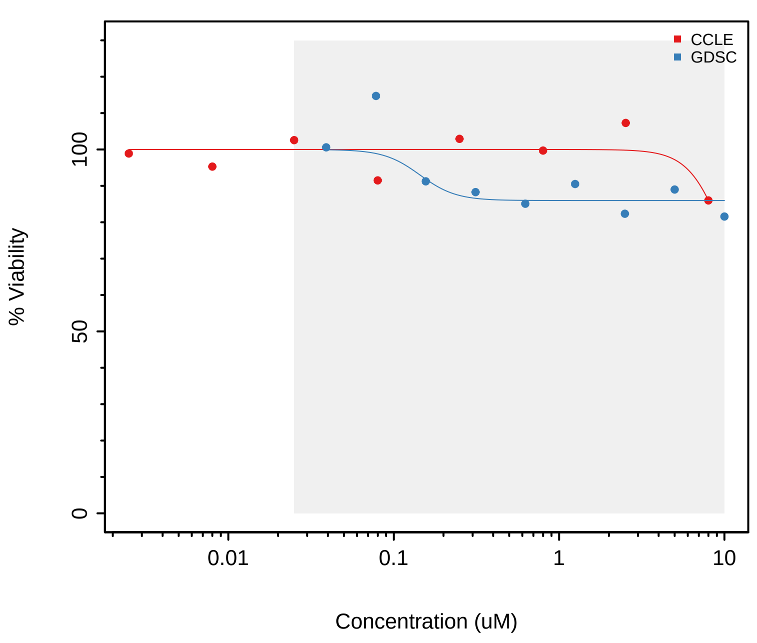

PLX4720:SW1088

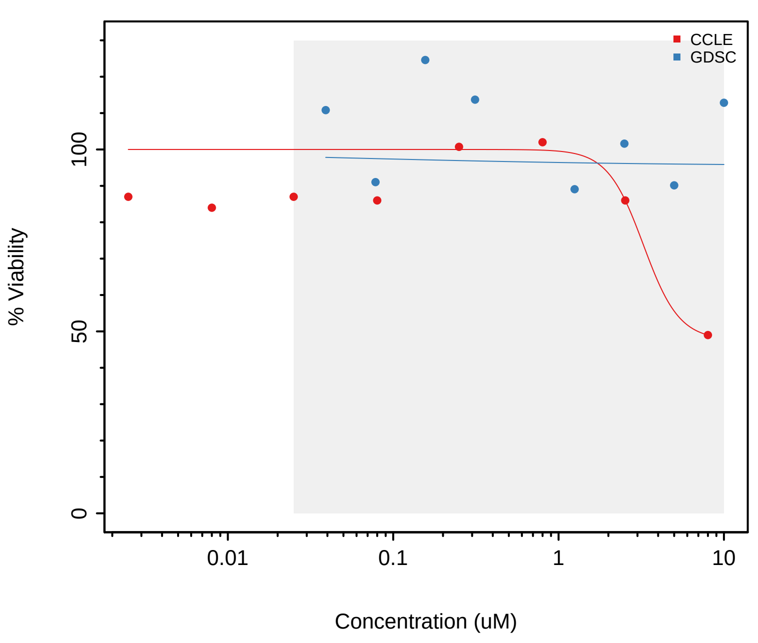

PLX4720:SW1417

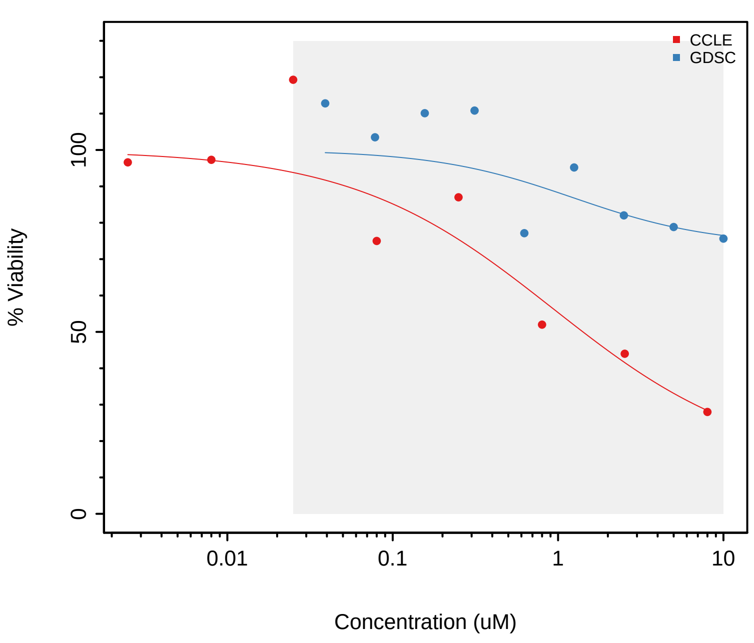

PLX4720:SW1573

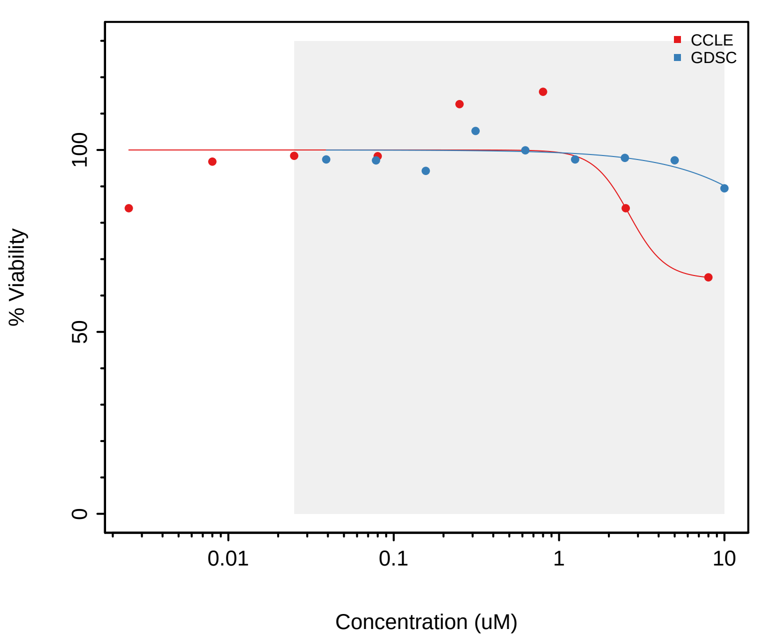

PLX4720:SW1990

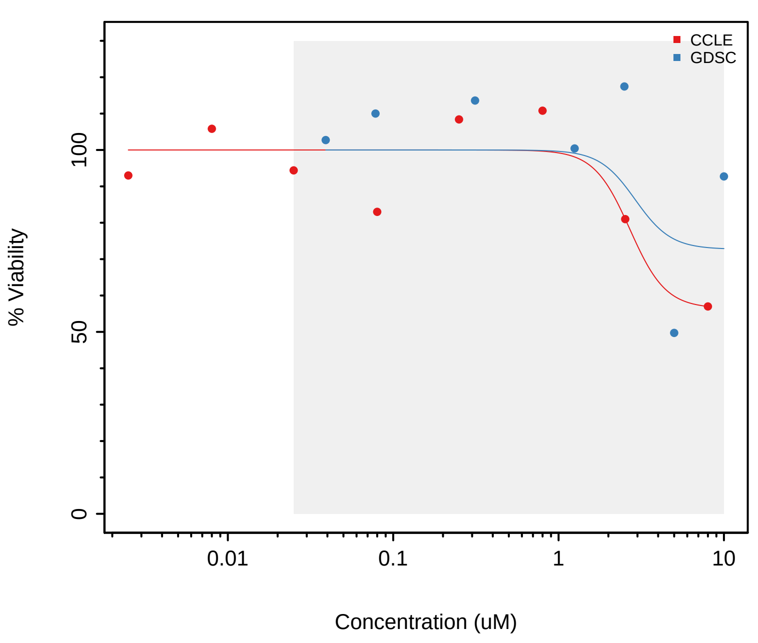

PLX4720:SW48

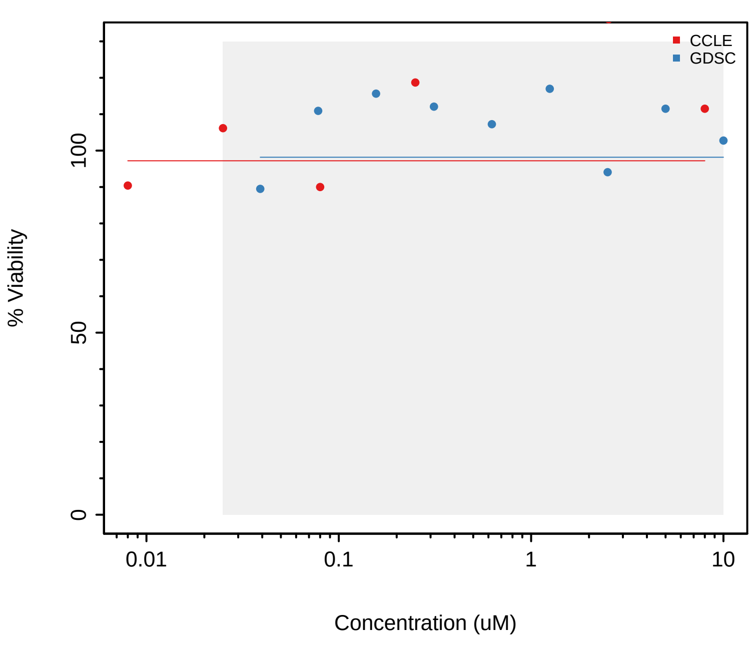

PLX4720:SW620

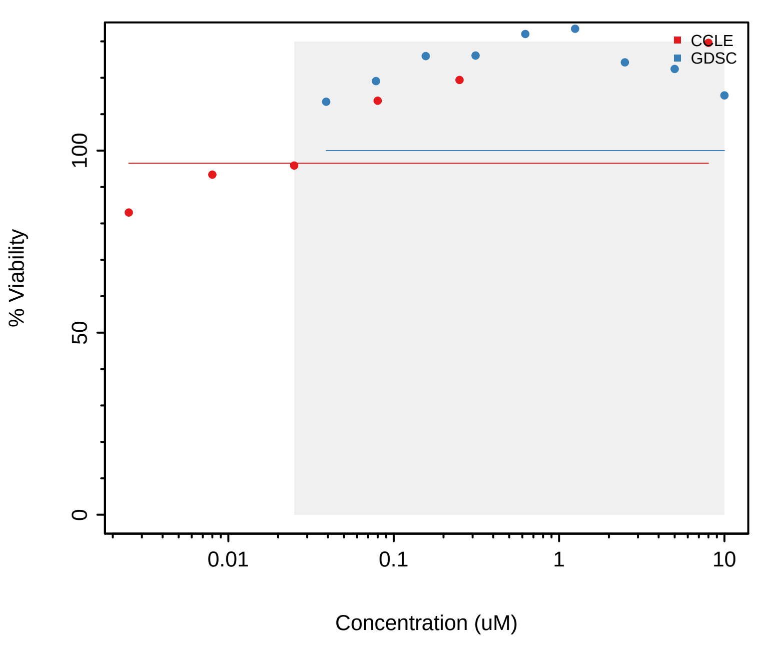

PLX4720:SW780

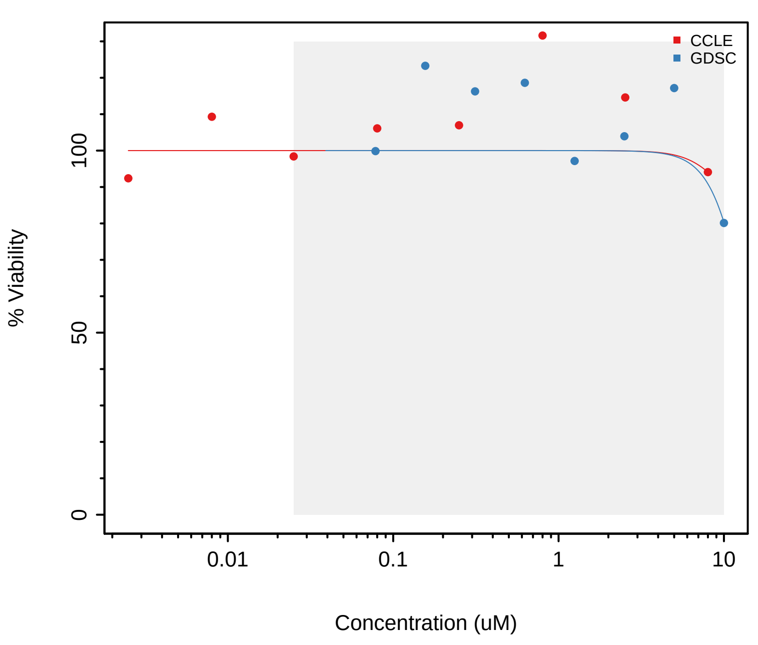

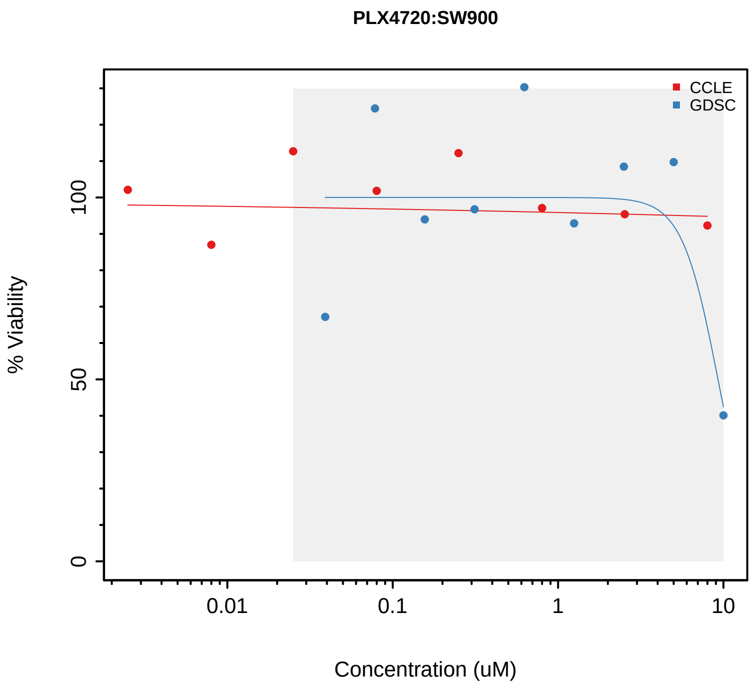
PLX4720:SW900
CCLE
GDSC
% Viability
Concentration (uM)

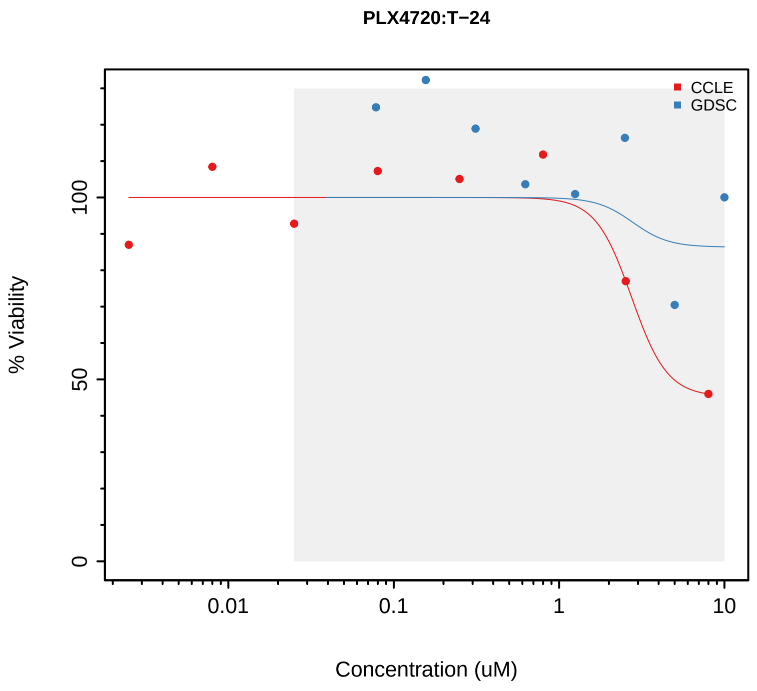
PLX4720:T−24
CCLE
GDSC
% Viability
Concentration (uM)

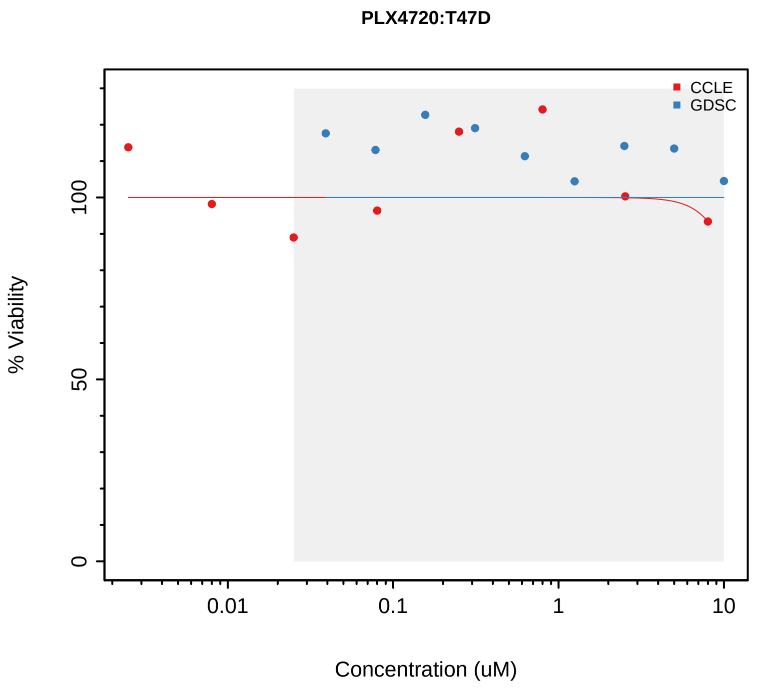
PLX4720:T47D
CCLE
GDSC
% Viability
Concentration (uM)

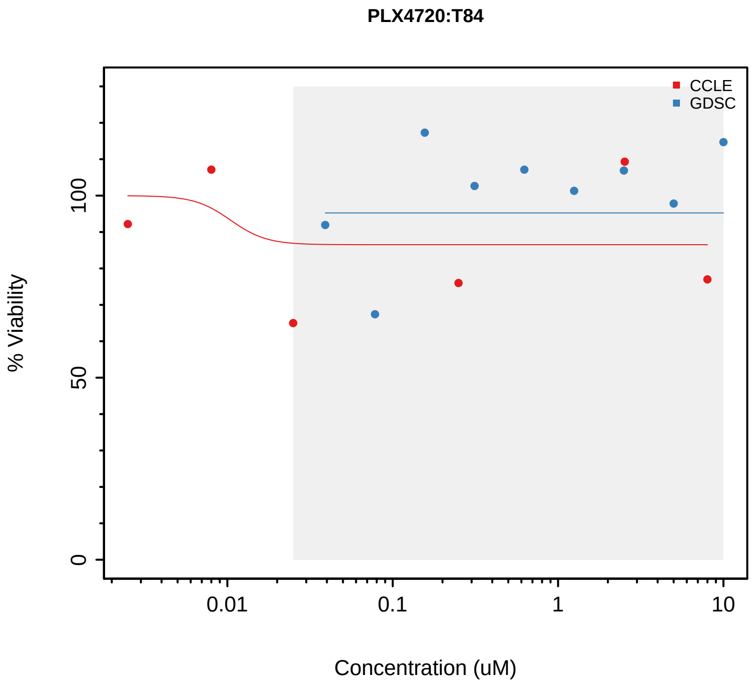
PLX4720:T84
CCLE
GDSC
% Viability
Concentration (uM)

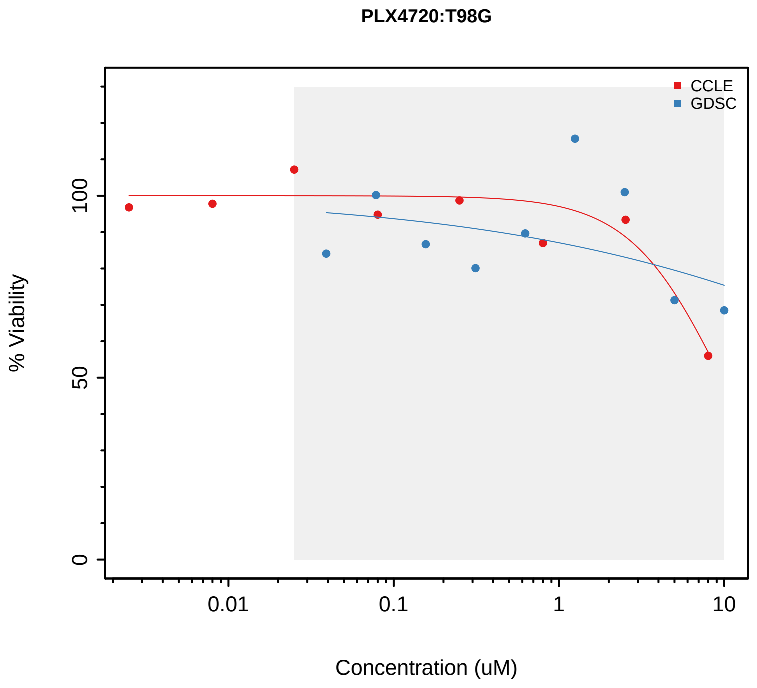
PLX4720:T98G
CCLE
GDSC
% Viability
Concentration (uM)

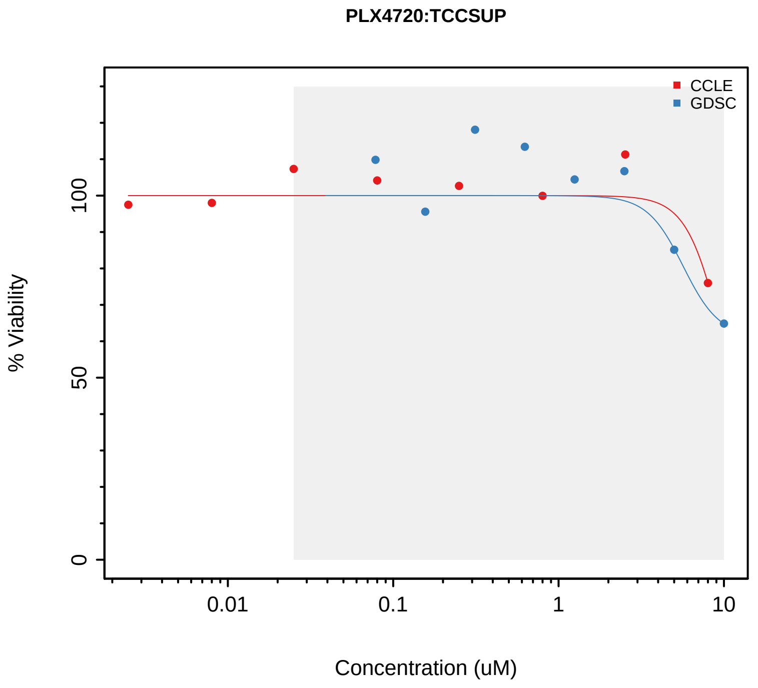
PLX4720:TCCSUP
CCLE
GDSC
% Viability
Concentration (uM)

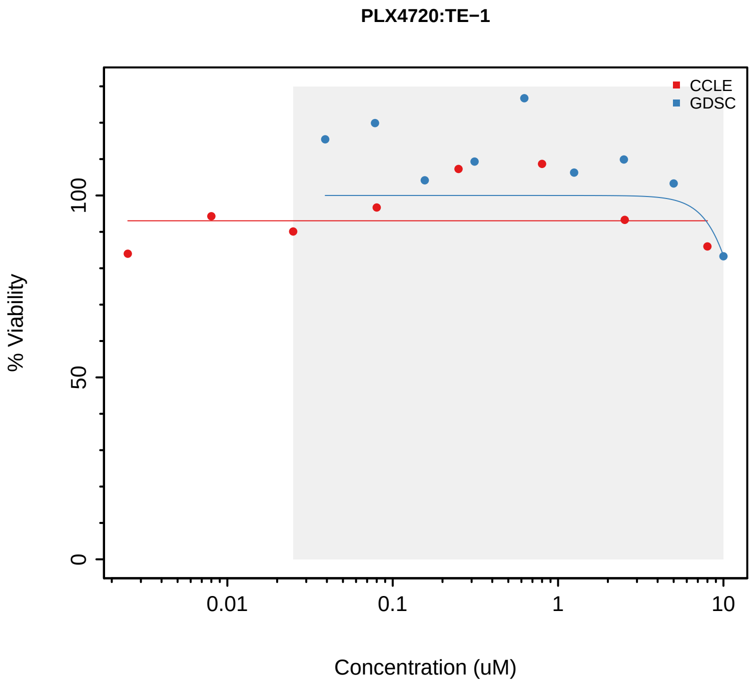
PLX4720:TE−1
CCLE
GDSC
% Viability
Concentration (uM)

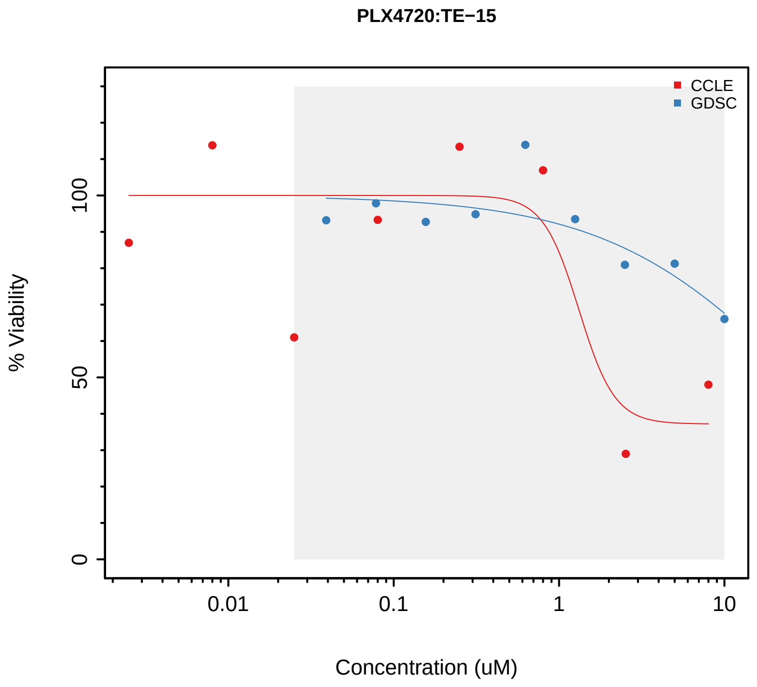
PLX4720:TE−15
CCLE
GDSC
% Viability
Concentration (uM)

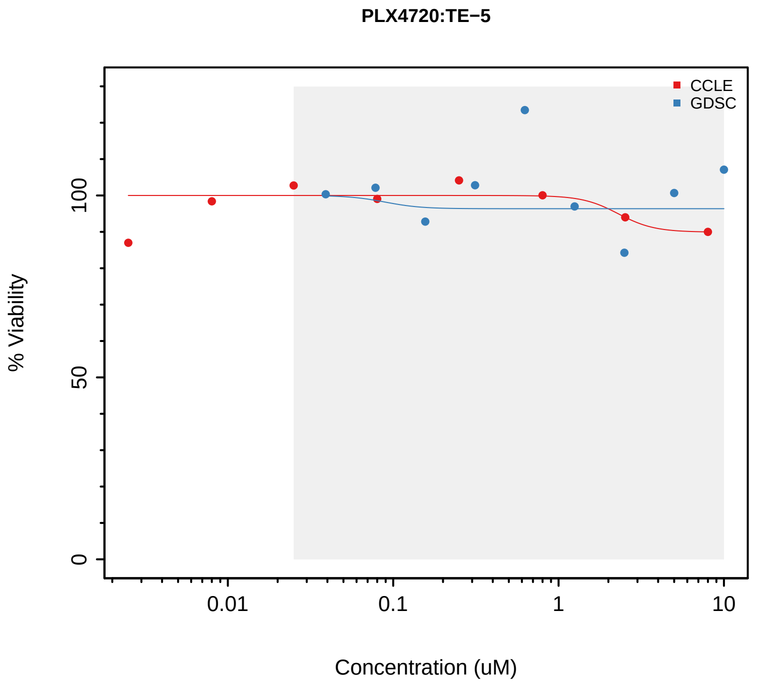
PLX4720:TE−5
CCLE
GDSC
% Viability
Concentration (uM)

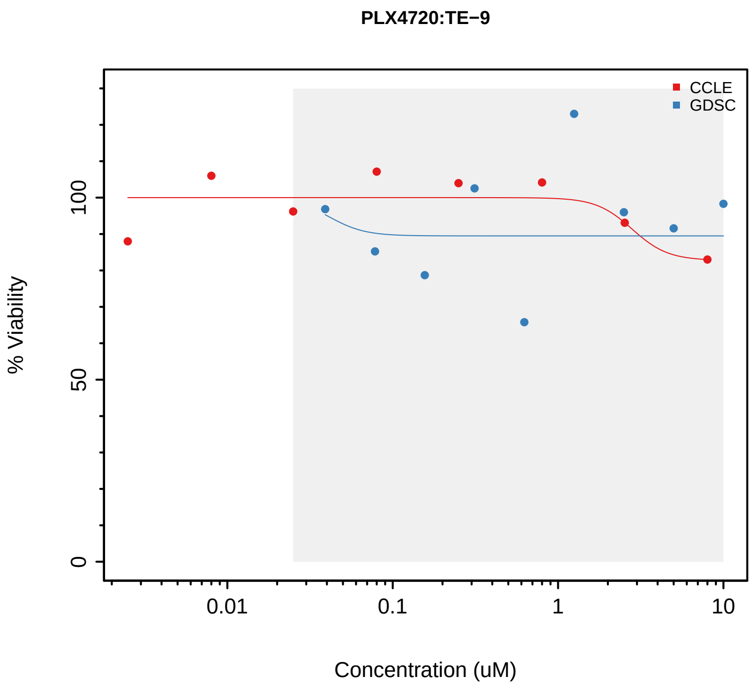
PLX4720:TE−9
CCLE
GDSC
% Viability
Concentration (uM)

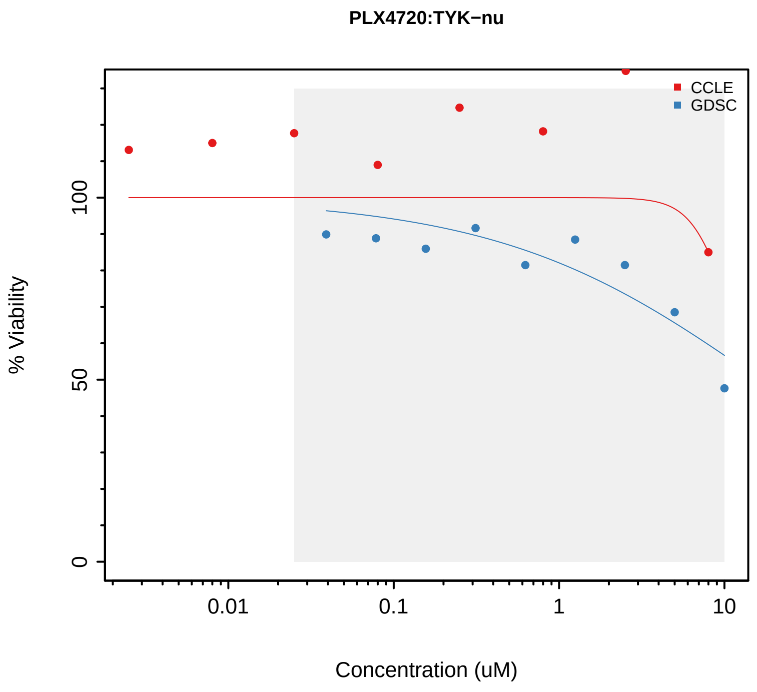
PLX4720:TYK−nu
CCLE
GDSC
% Viability
Concentration (uM)

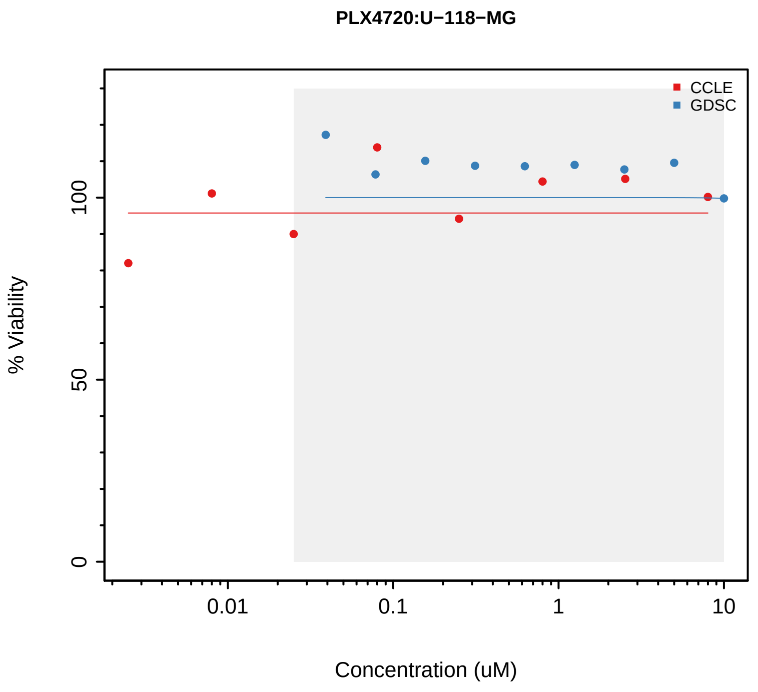
PLX4720:U−118−MG
CCLE
GDSC
% Viability
Concentration (uM)

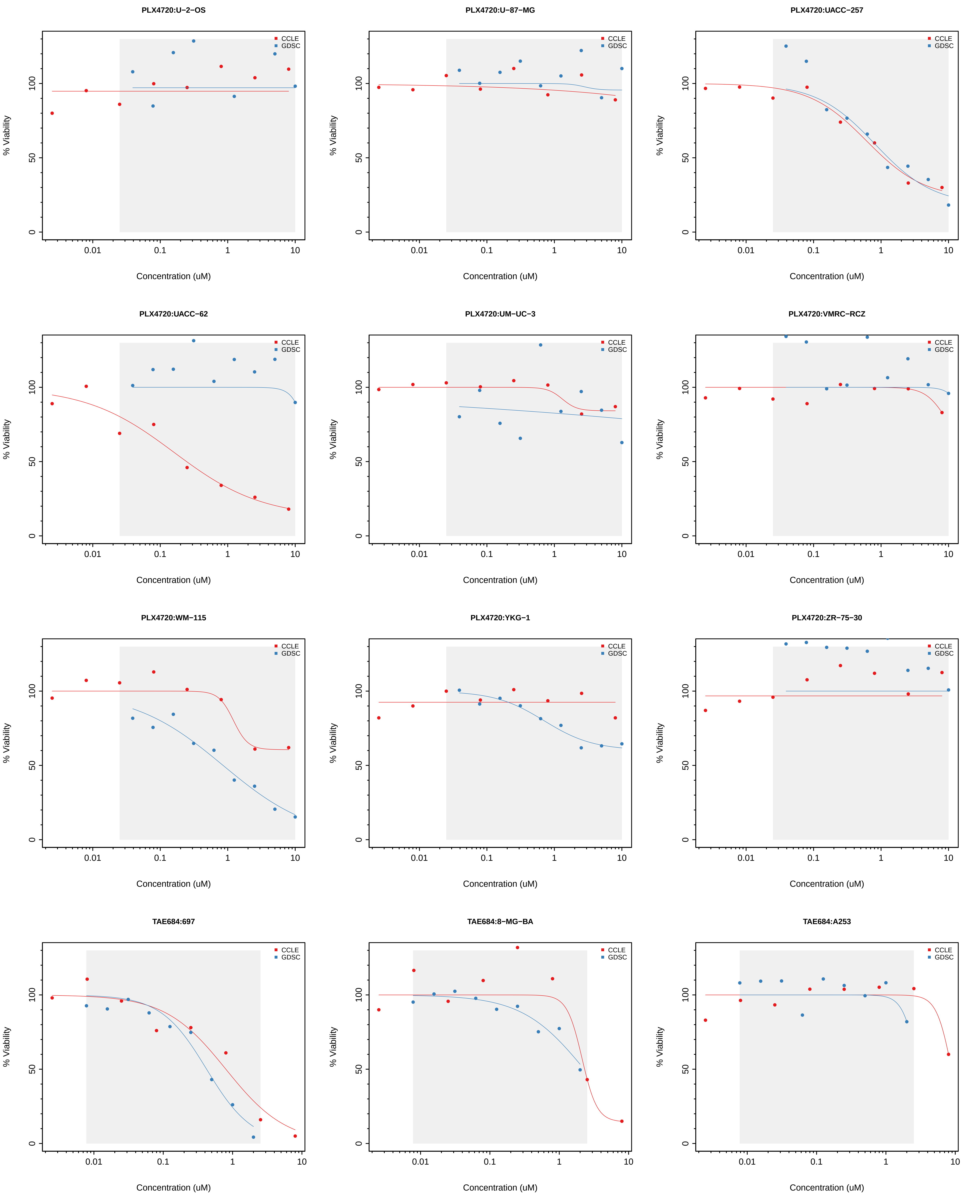
PLX4720:U−2−OS
PLX4720:U−87−MG
PLX4720:UACC−257
PLX4720:UACC−62
PLX4720:UM−UC−3
PLX4720:VMRC−RCZ
PLX4720:WM−115
PLX4720:YKG−1
PLX4720:ZR−75−30
TAE684:697
TAE684:8−MG−BA
TAE684:A253
CCLE
GDSC
% Viability
Concentration (uM)
0
50
100
0.01
0.1
1
10

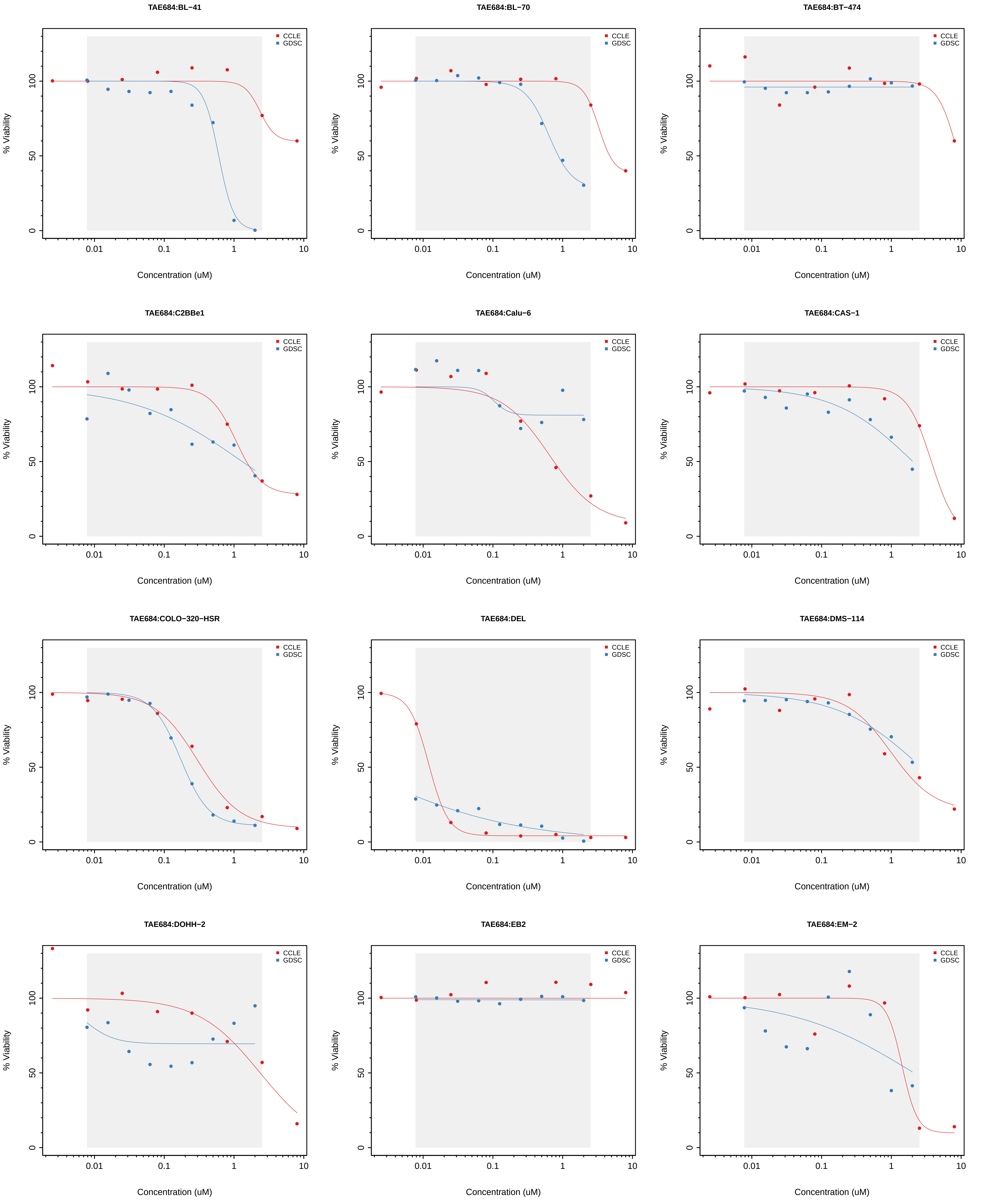
TAE684:BL−41
TAE684:BL−70
TAE684:BT−474
TAE684:C2BBe1
TAE684:Calu−6
TAE684:CAS−1
TAE684:COLO−320−HSR
TAE684:DEL
TAE684:DMS−114
TAE684:DOHH−2
TAE684:EB2
TAE684:EM−2
CCLE
GDSC
% Viability
0
50
100
Concentration (uM)
0.01
0.1
1
10

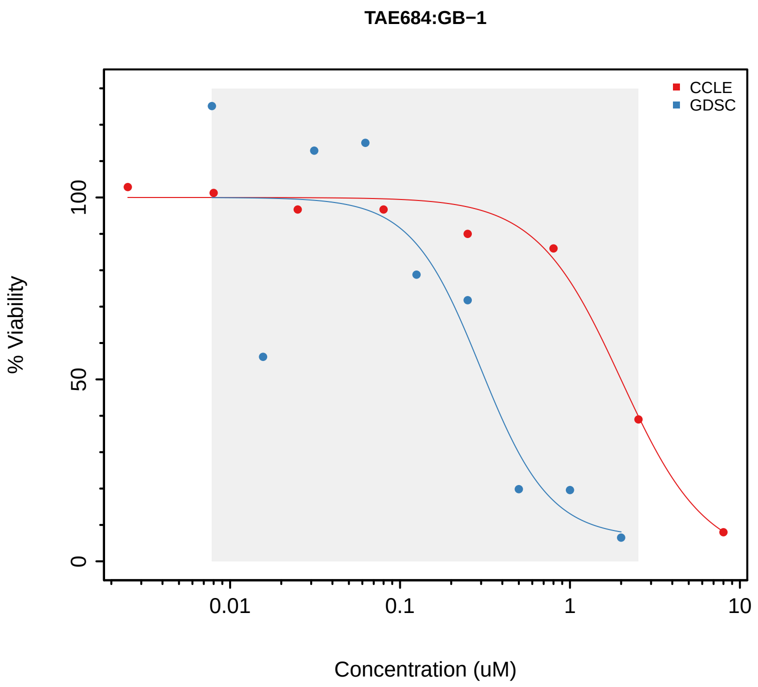
TAE684:GB−1
CCLE
GDSC
% Viability
0
50
100
0.01
0.1
1
10
Concentration (uM)

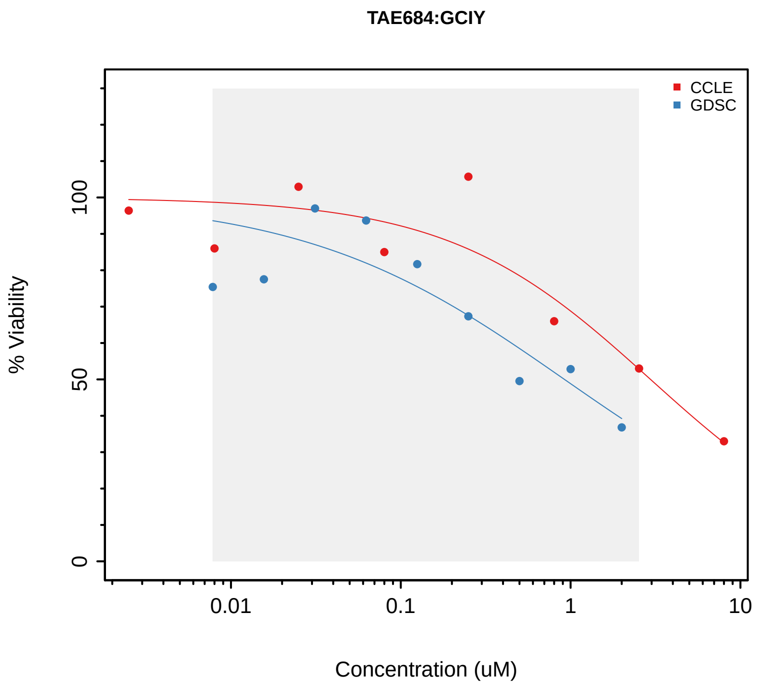
TAE684:GCIY
CCLE
GDSC
% Viability
0
50
100
0.01
0.1
1
10
Concentration (uM)

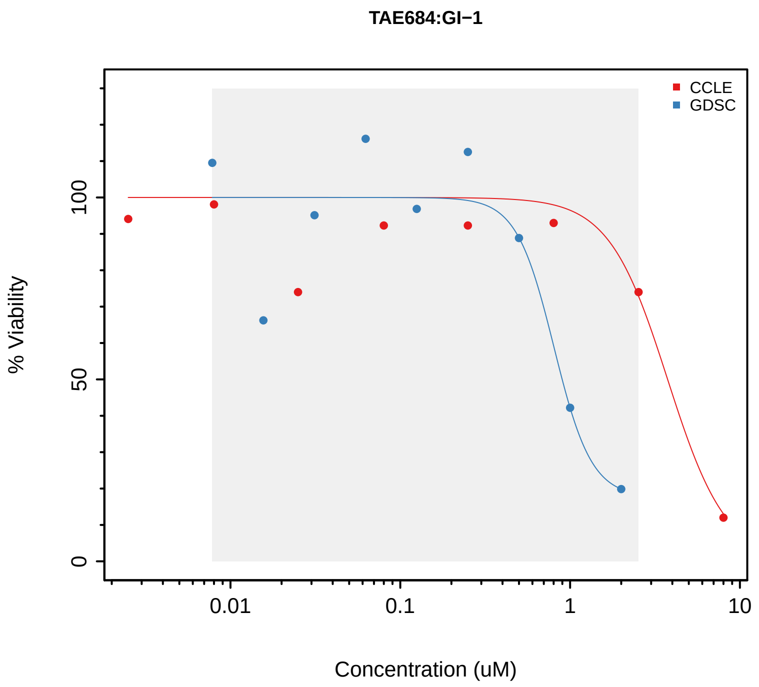
TAE684:GI−1
CCLE
GDSC
% Viability
0
50
100
0.01
0.1
1
10
Concentration (uM)

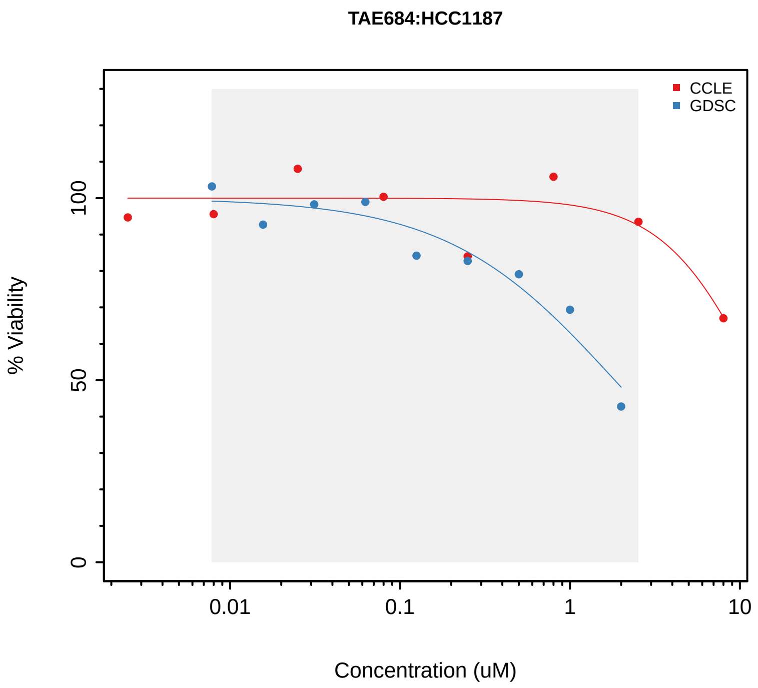
TAE684:HCC1187
CCLE
GDSC
% Viability
0
50
100
0.01
0.1
1
10
Concentration (uM)

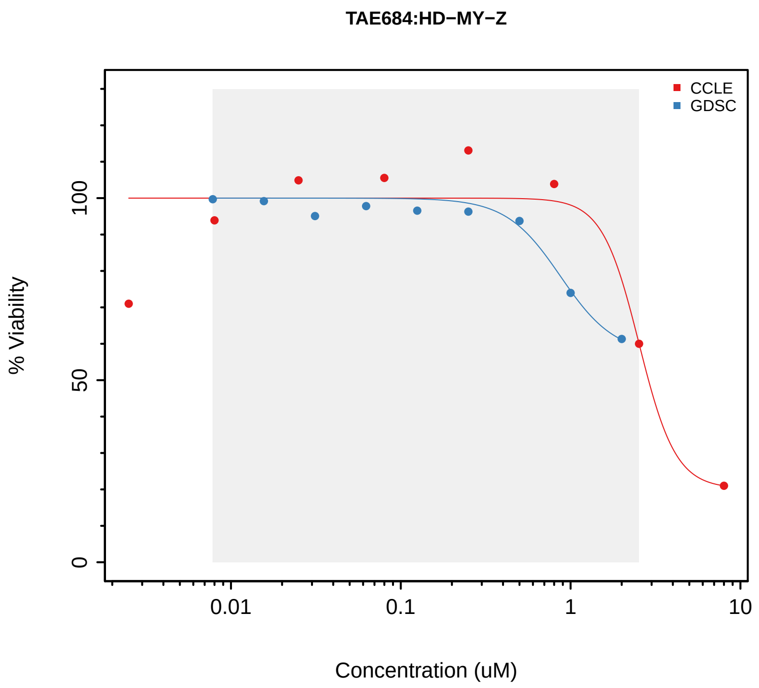
TAE684:HD−MY−Z
CCLE
GDSC
% Viability
0
50
100
0.01
0.1
1
10
Concentration (uM)

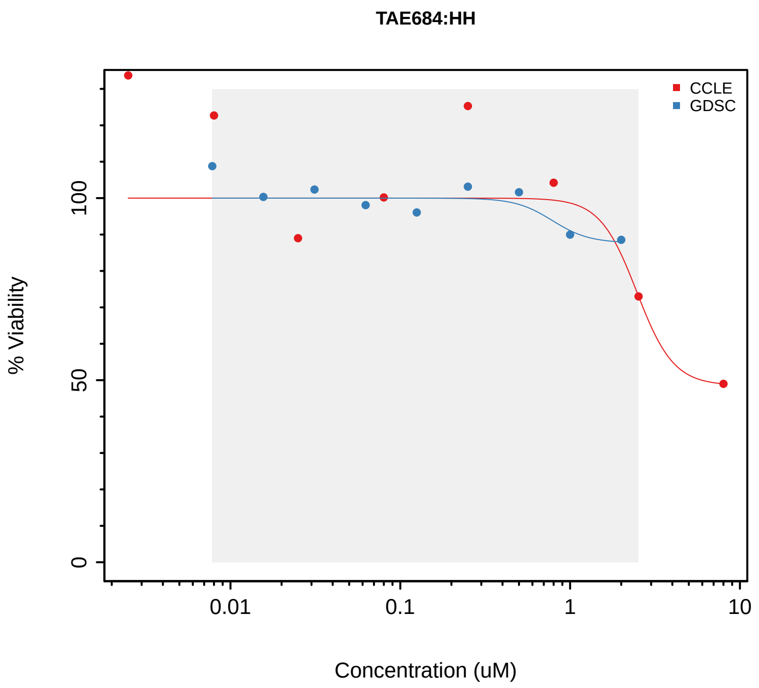
TAE684:HH
CCLE
GDSC
% Viability
0
50
100
0.01
0.1
1
10
Concentration (uM)

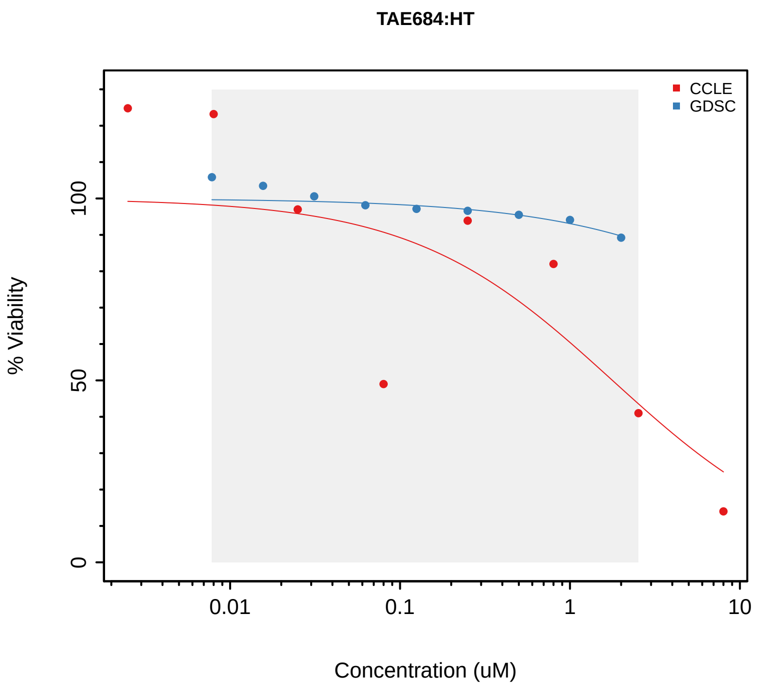
TAE684:HT
CCLE
GDSC
% Viability
0
50
100
0.01
0.1
1
10
Concentration (uM)

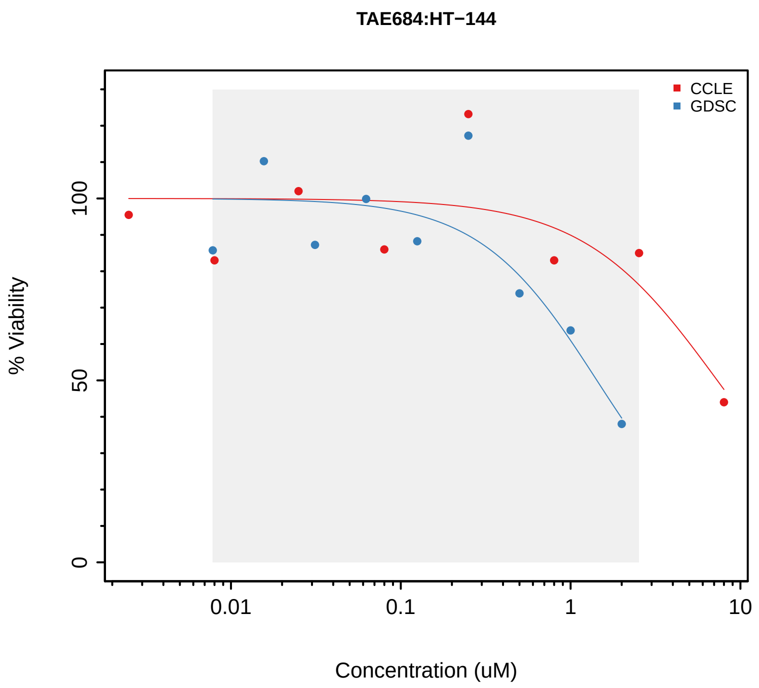
TAE684:HT−144
CCLE
GDSC
% Viability
0
50
100
0.01
0.1
1
10
Concentration (uM)

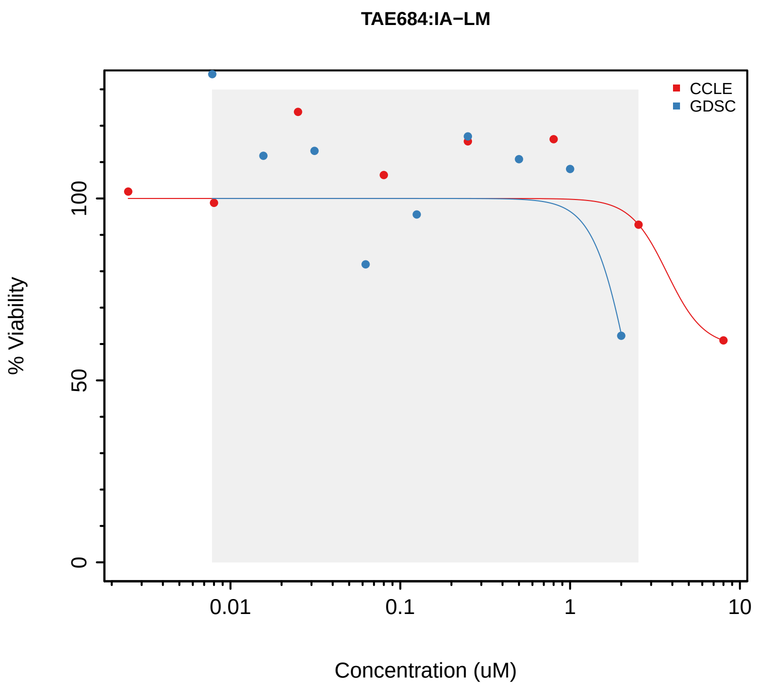
TAE684:IA−LM
CCLE
GDSC
% Viability
0
50
100
0.01
0.1
1
10
Concentration (uM)

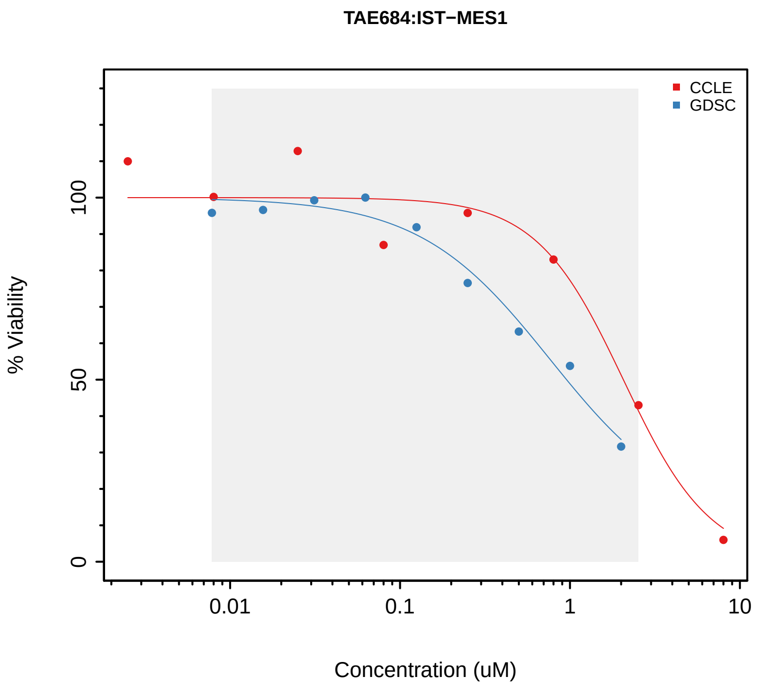
TAE684:IST−MES1
CCLE
GDSC
% Viability
0
50
100
0.01
0.1
1
10
Concentration (uM)

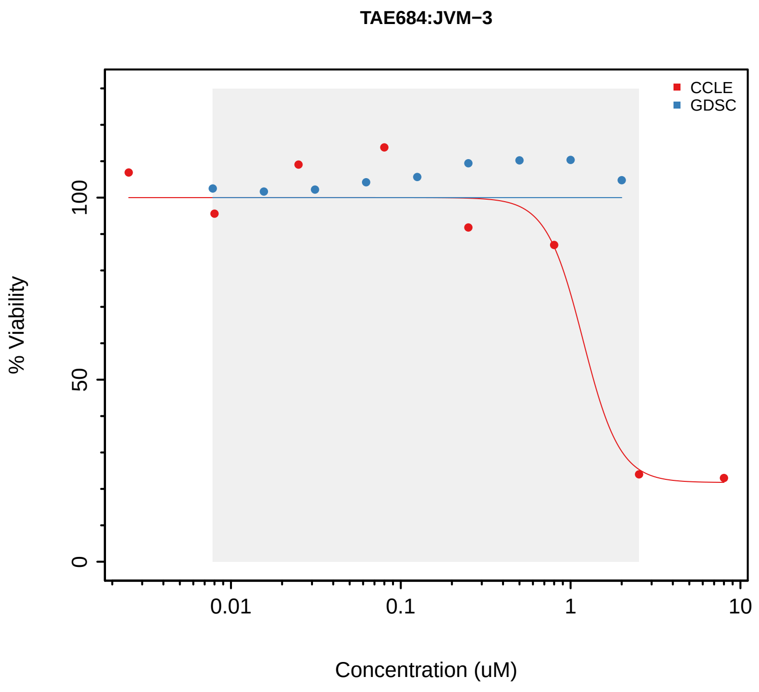
TAE684:JVM−3
CCLE
GDSC
% Viability
0
50
100
0.01
0.1
1
10
Concentration (uM)

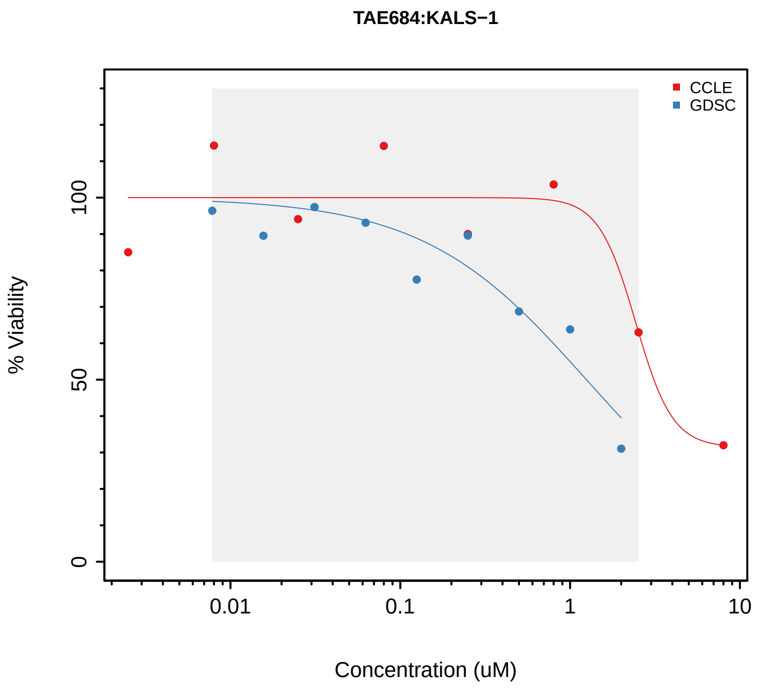
TAE684:KALS−1
CCLE
GDSC
% Viability
0
50
100
0.01
0.1
1
10
Concentration (uM)

TAE684:KARPAS−299

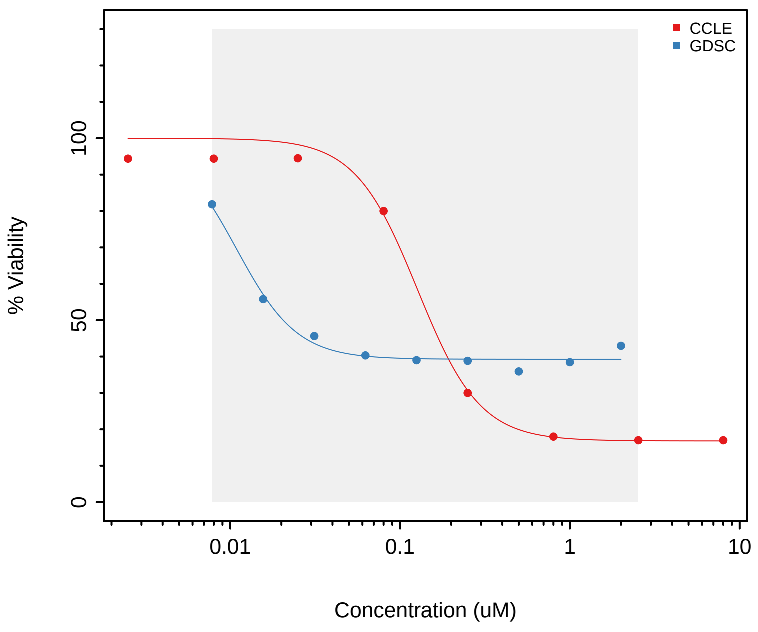

TAE684:KARPAS−422

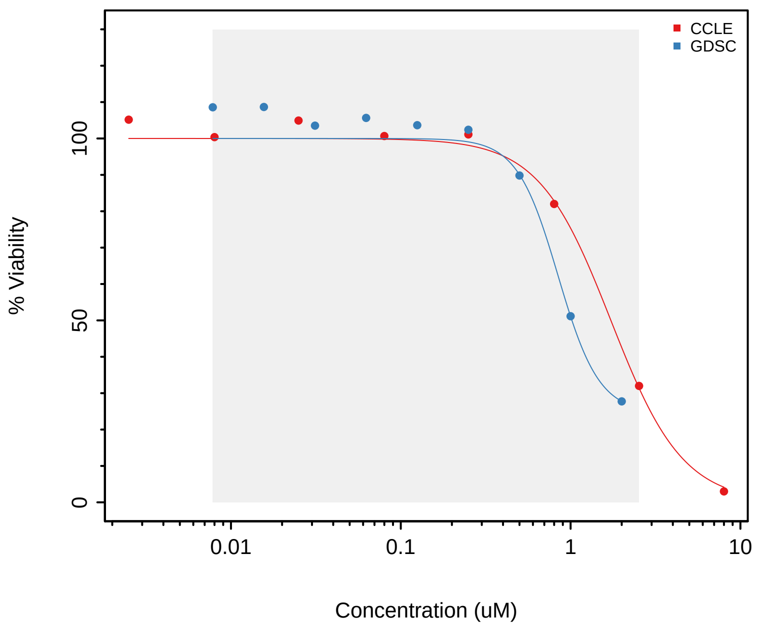

TAE684:KG−1

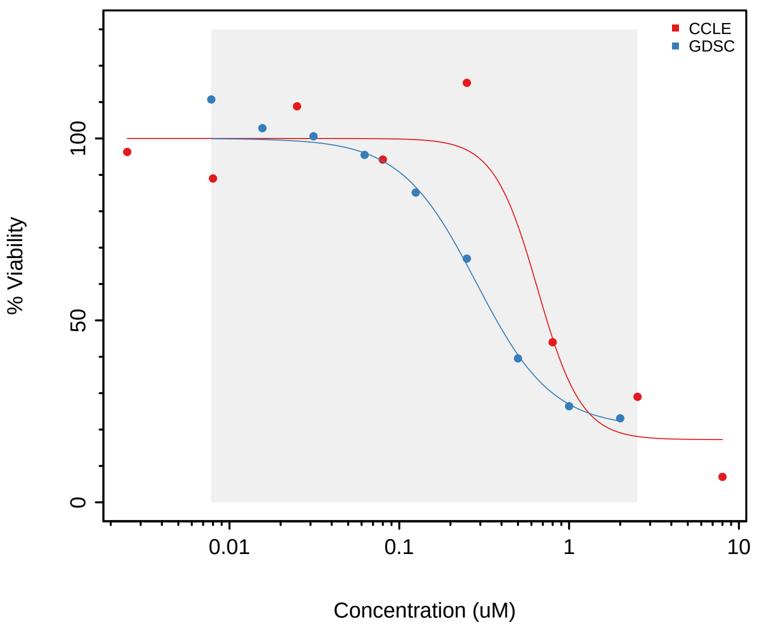

TAE684:KLE

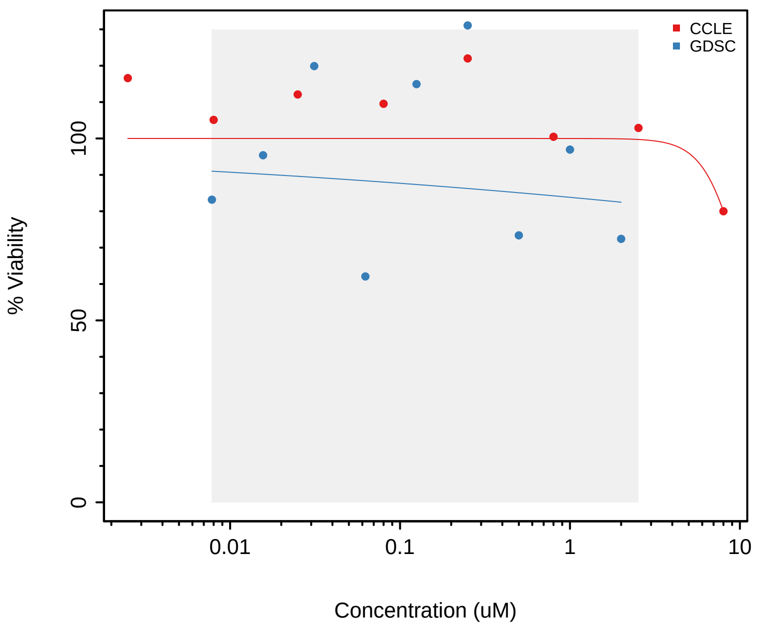

TAE684:KNS−42

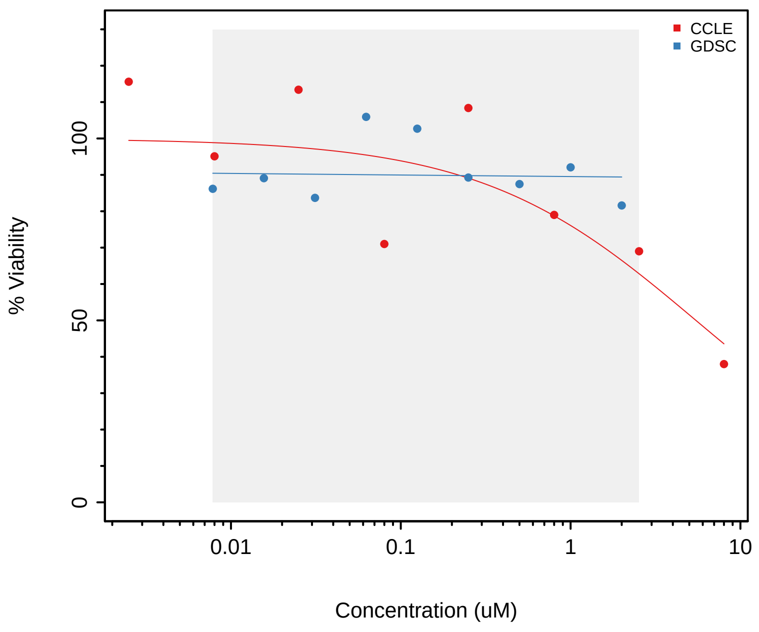

TAE684:KNS−81−FD

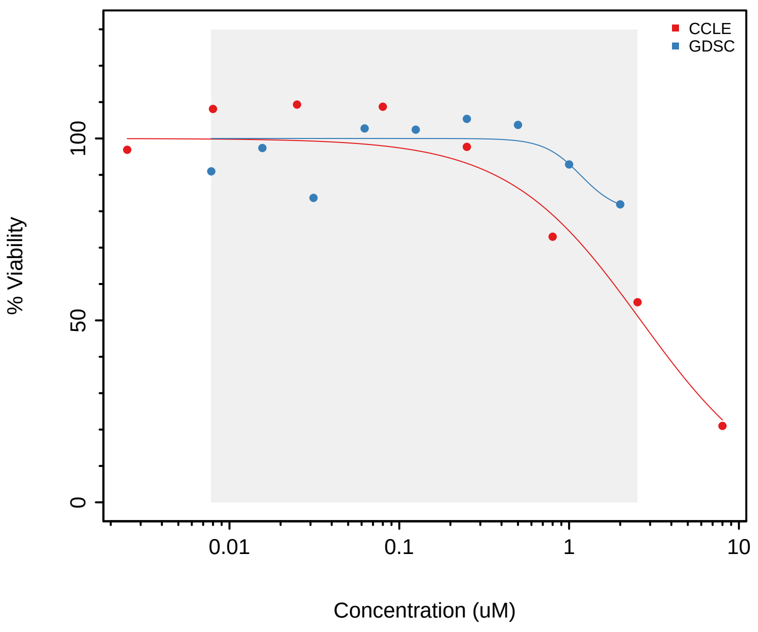

TAE684:K052

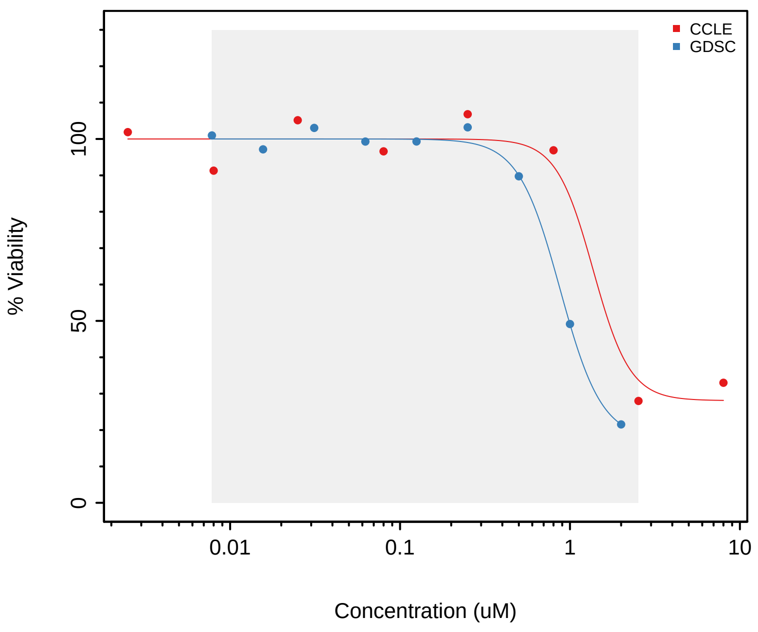

TAE684:KU812

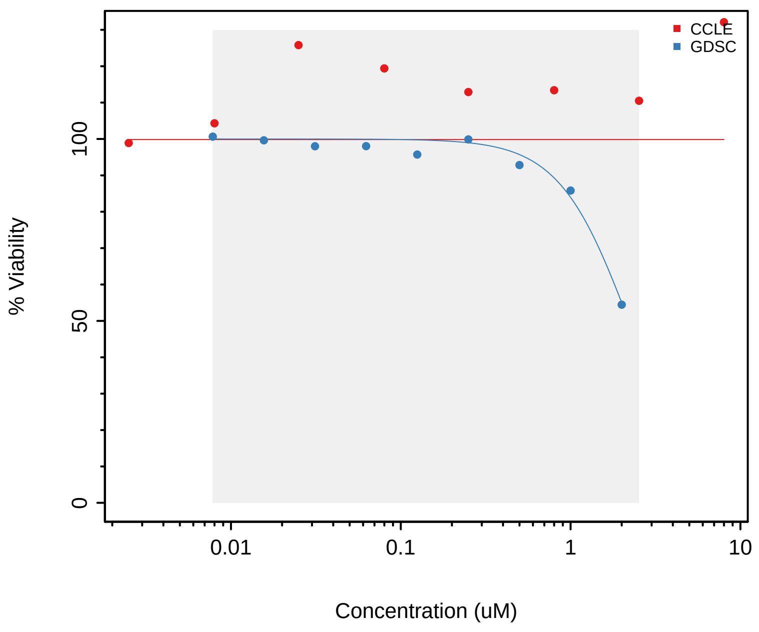

TAE684:KURAMOCHI

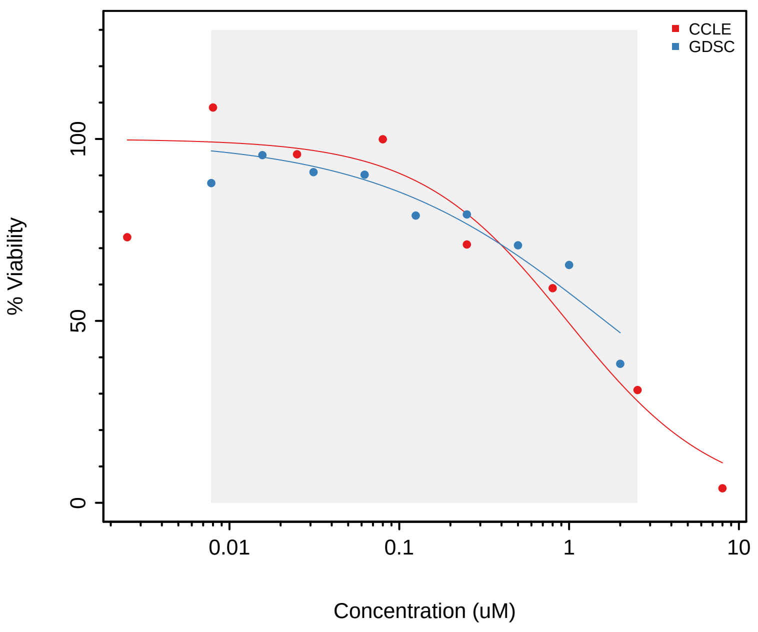

TAE684:L−363

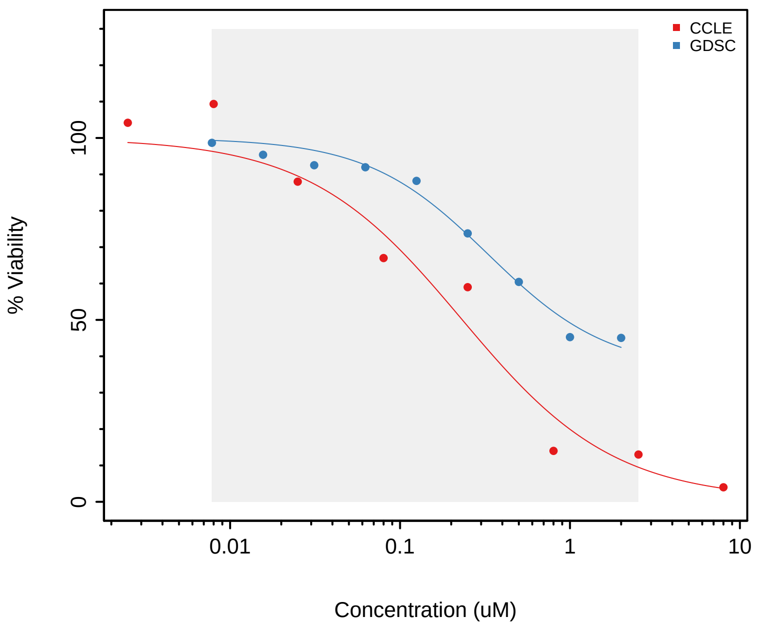

TAE684:L−428

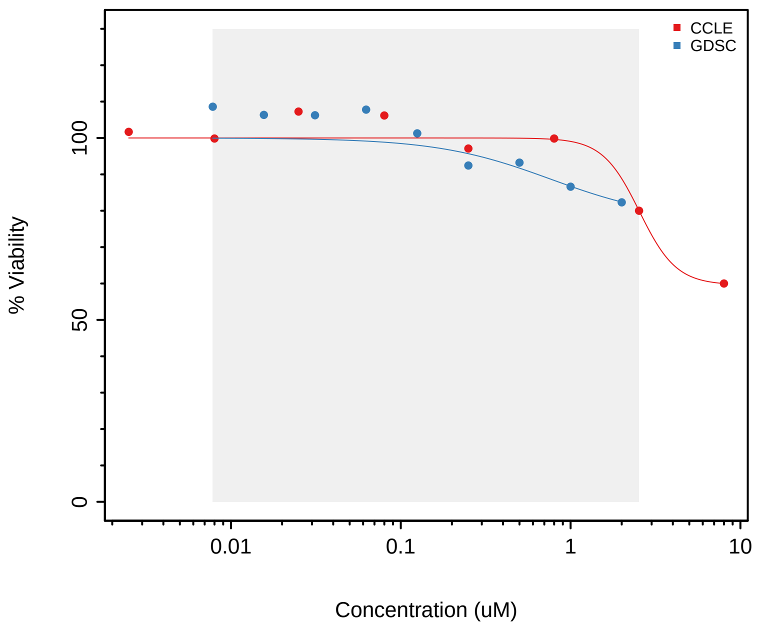

TAE684:LOXIMVI

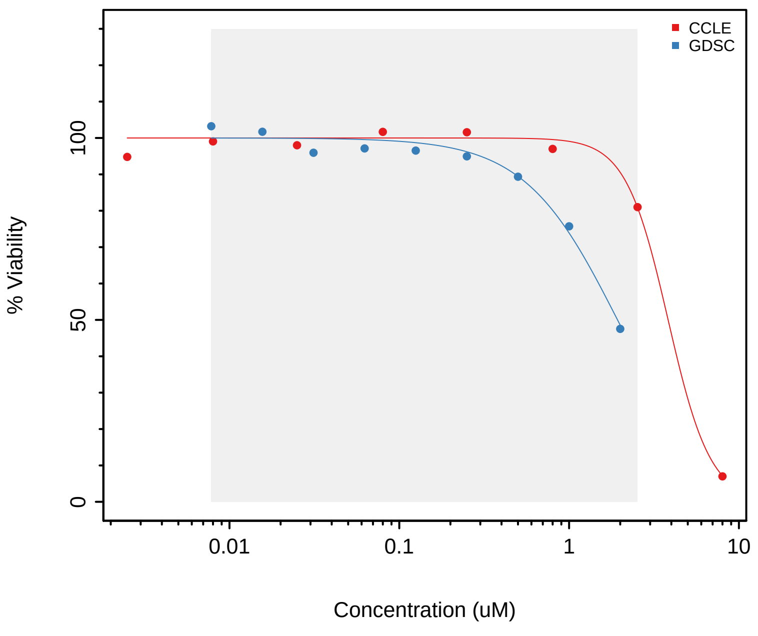

TAE684:LP−1

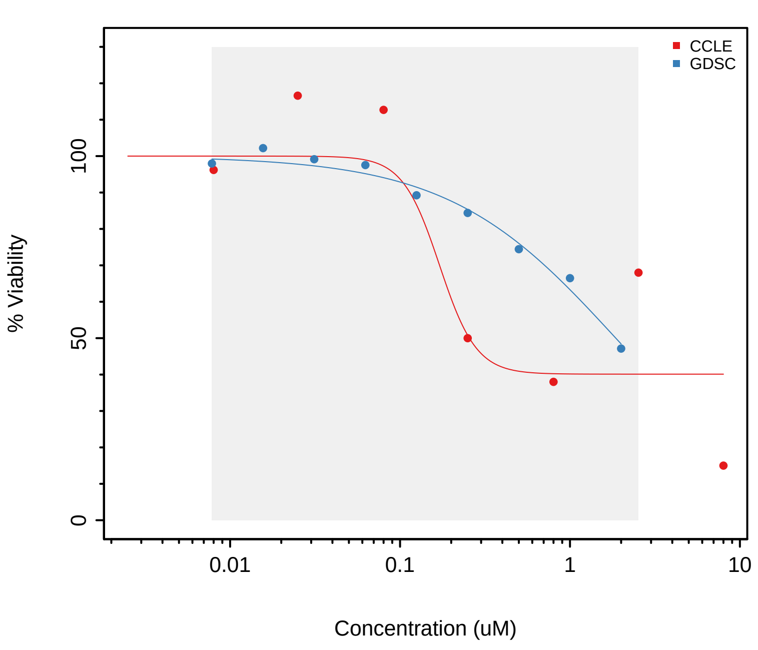

TAE684:LS−123

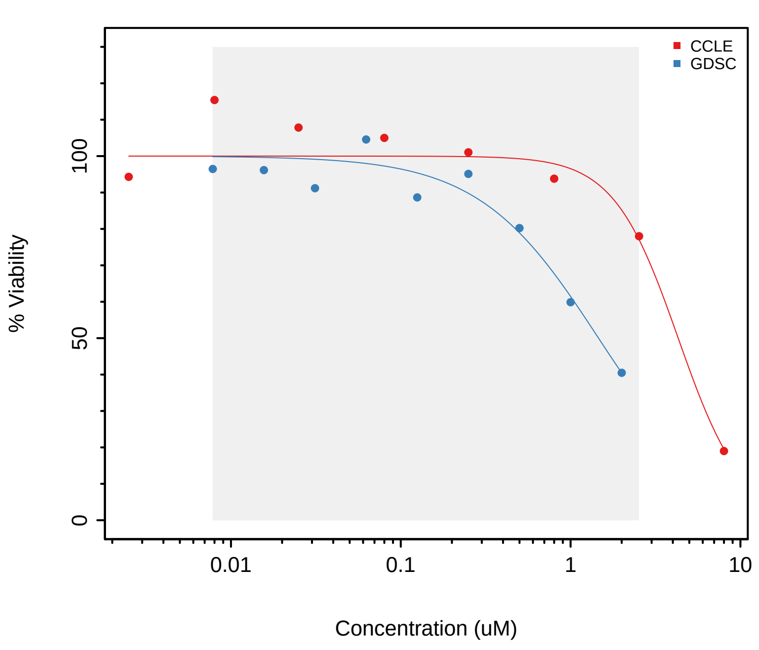

TAE684:LS−411N

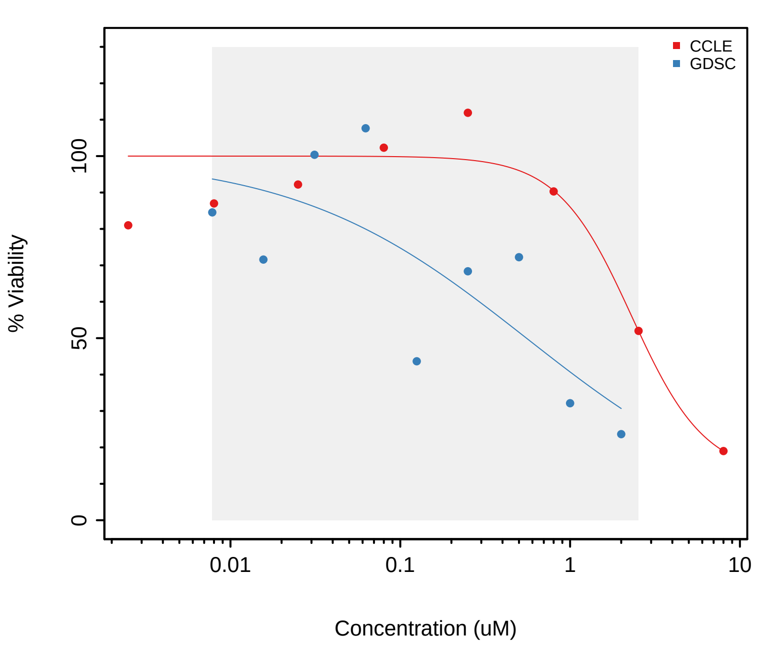

TAE684:LS−513

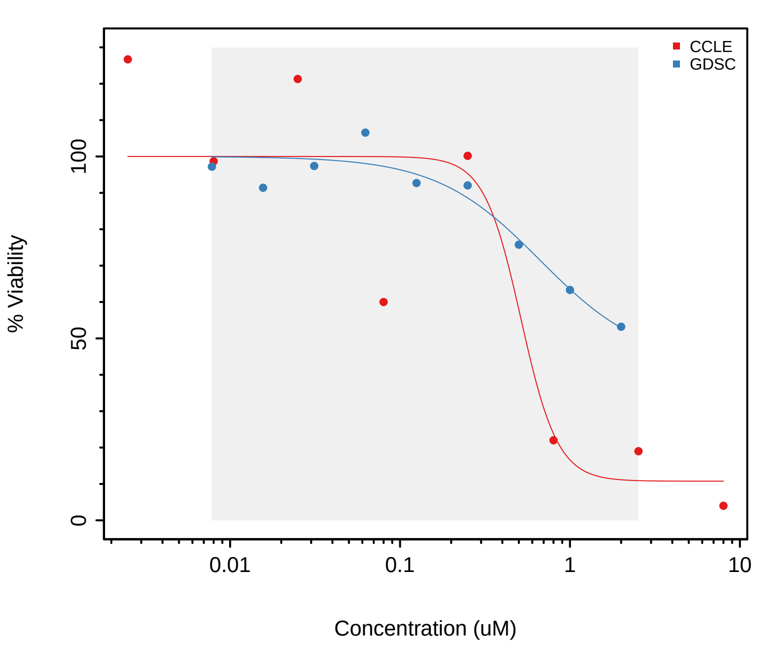

TAE684:MC116

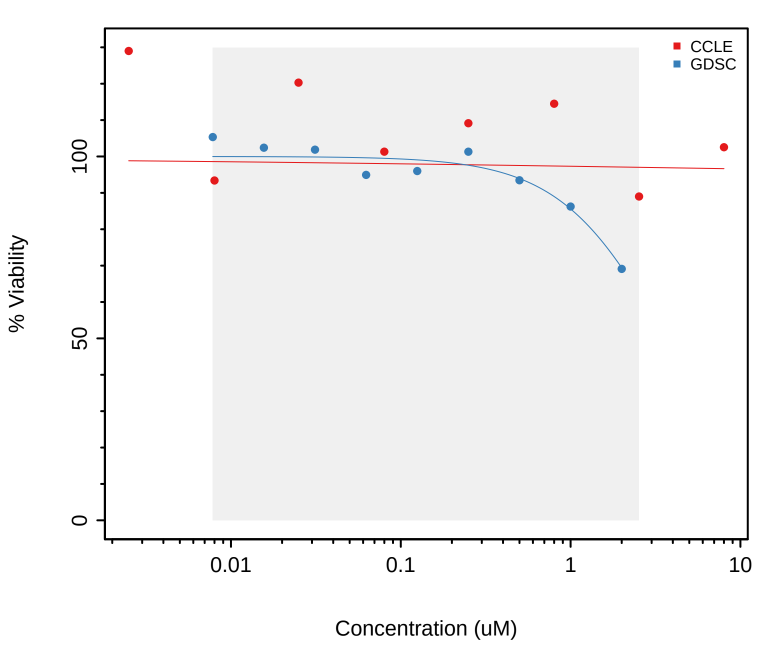

TAE684:MEG−01

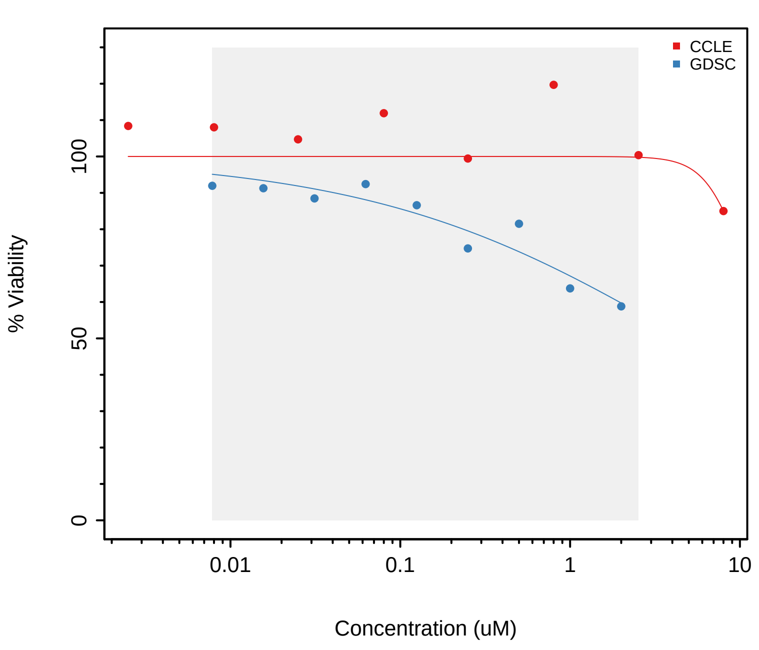

TAE684:MPP−89

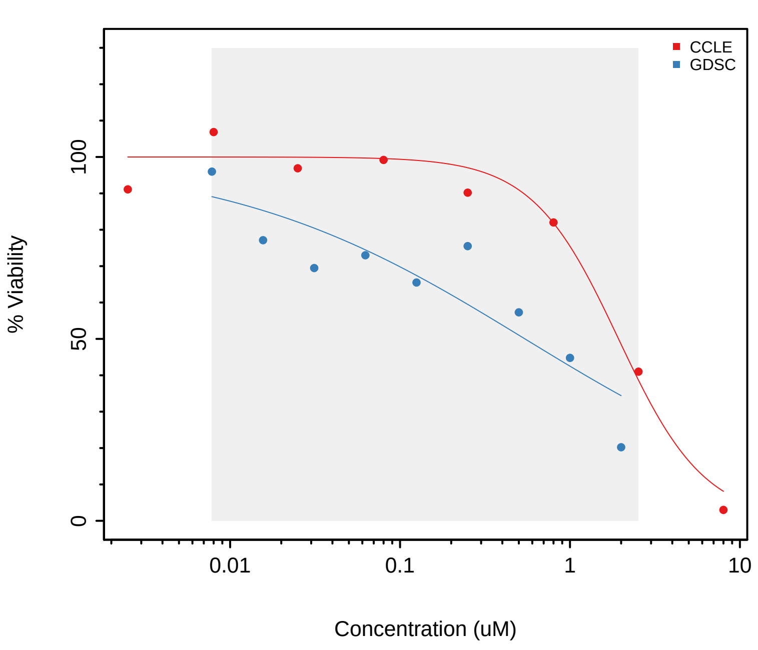

TAE684:MSTO−211H

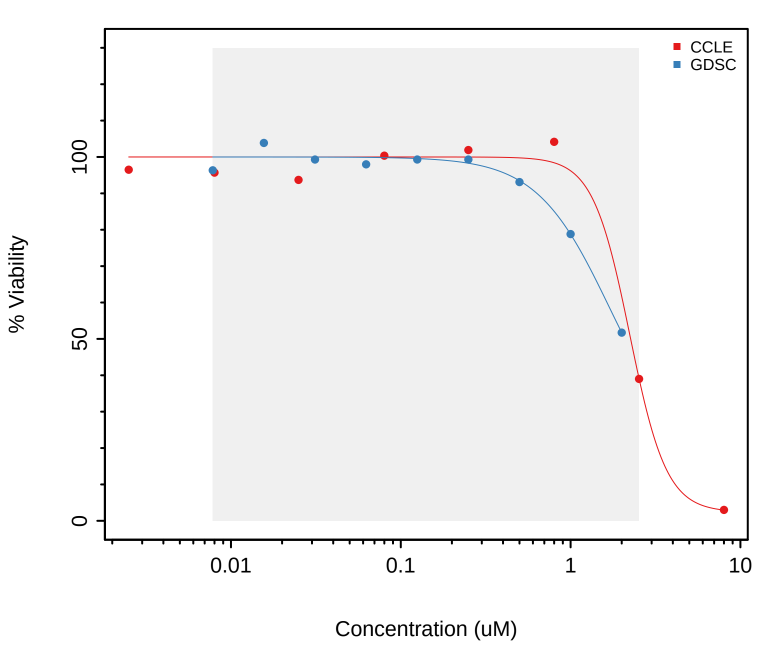

TAE684:NCI−H1092

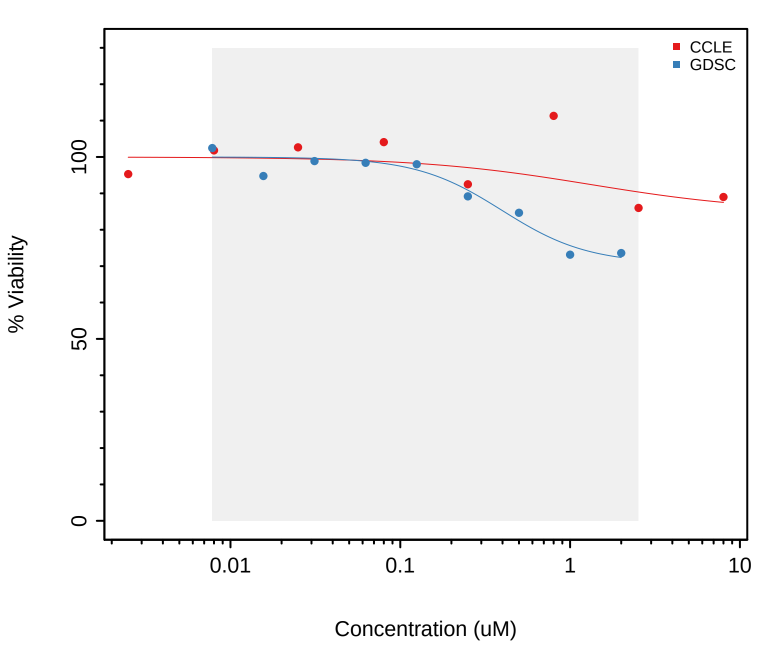

TAE684:NCI−H1155

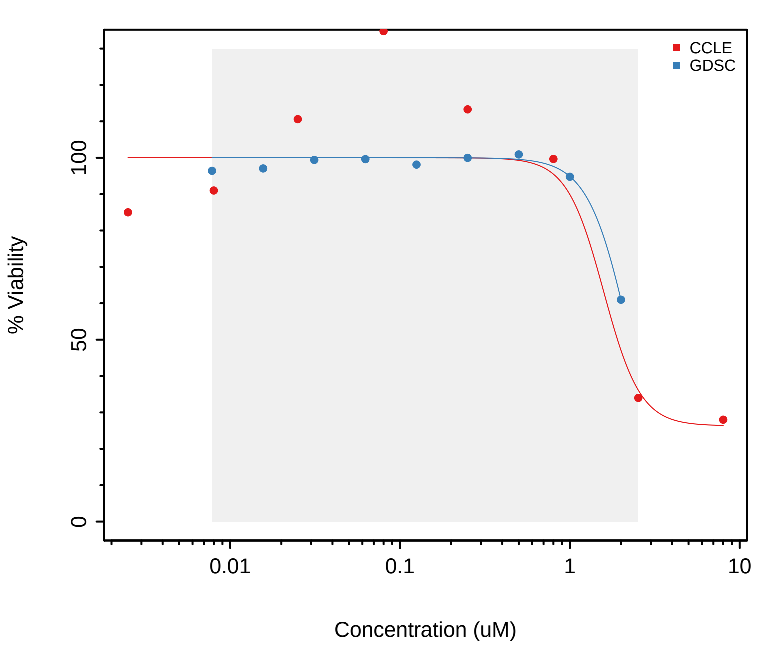

TAE684:NCI−H1299

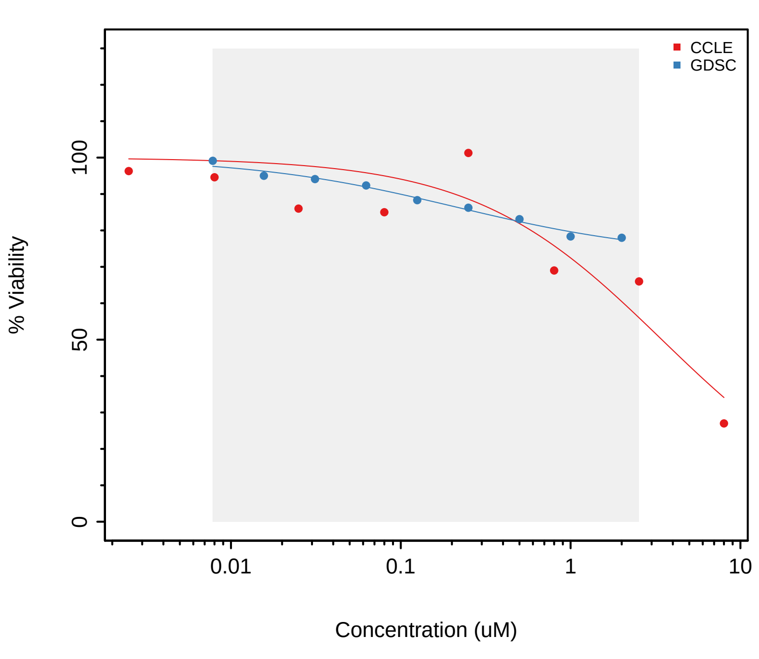

TAE684:NCI−H1355

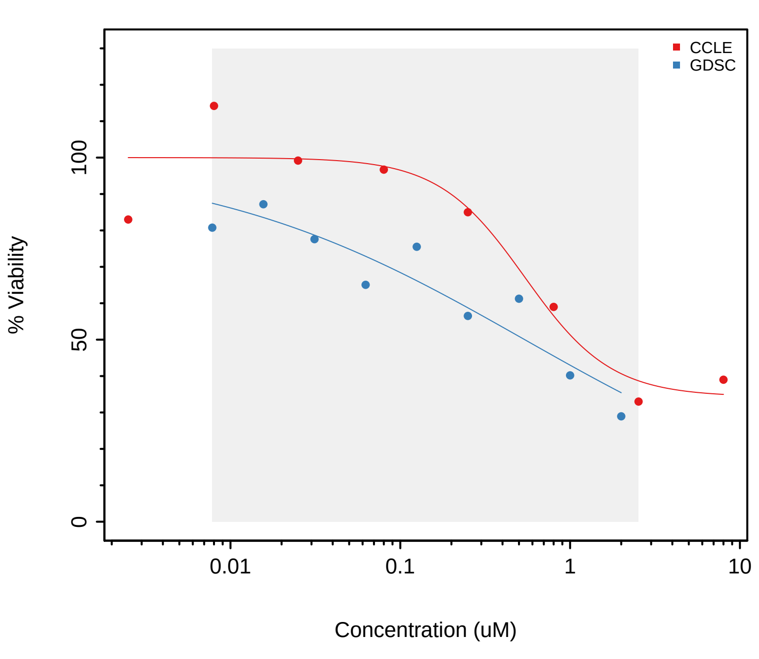

TAE684:NCI−H1581

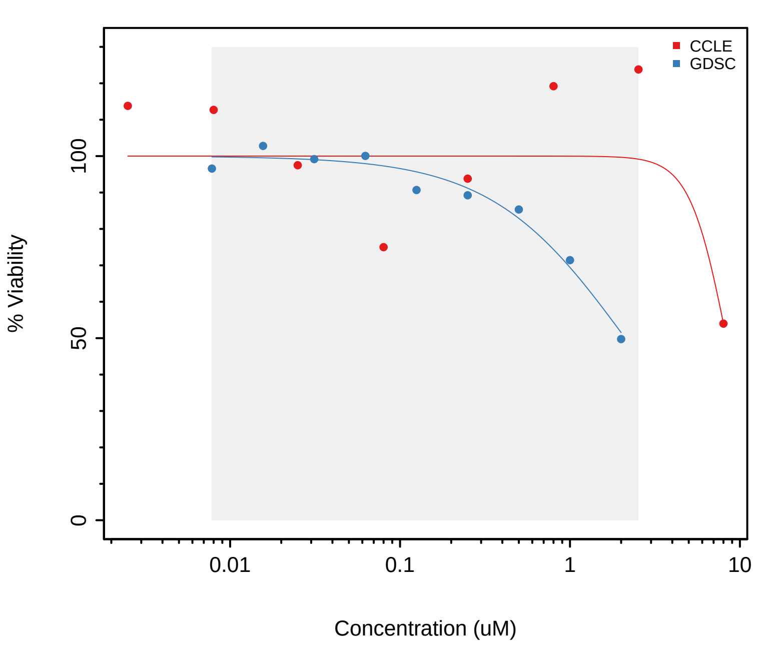

TAE684:NCI−H1648

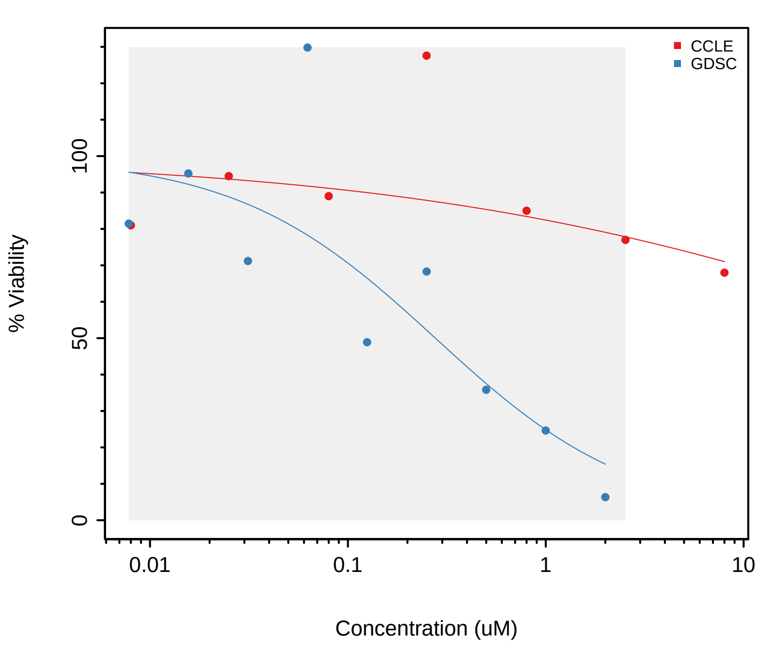

TAE684:NCI−H1666

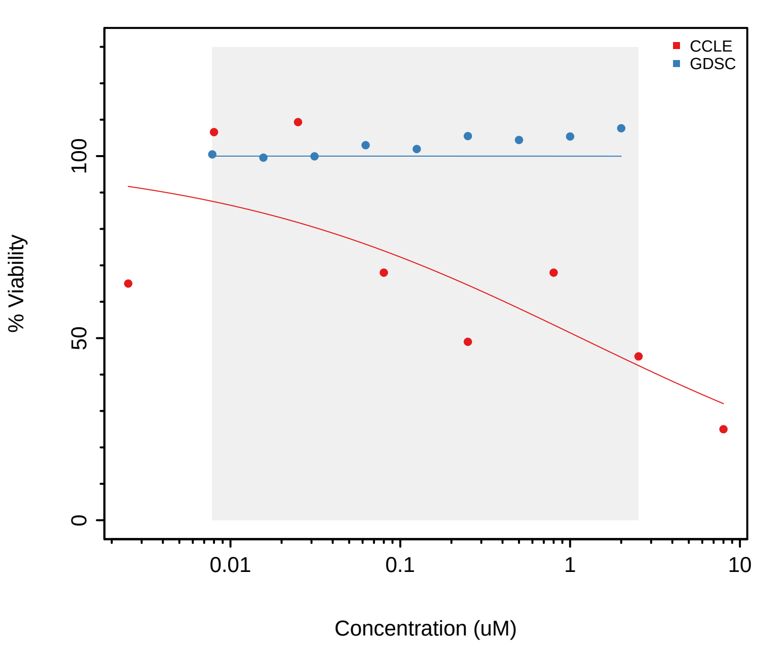

TAE684:NCI−H1694

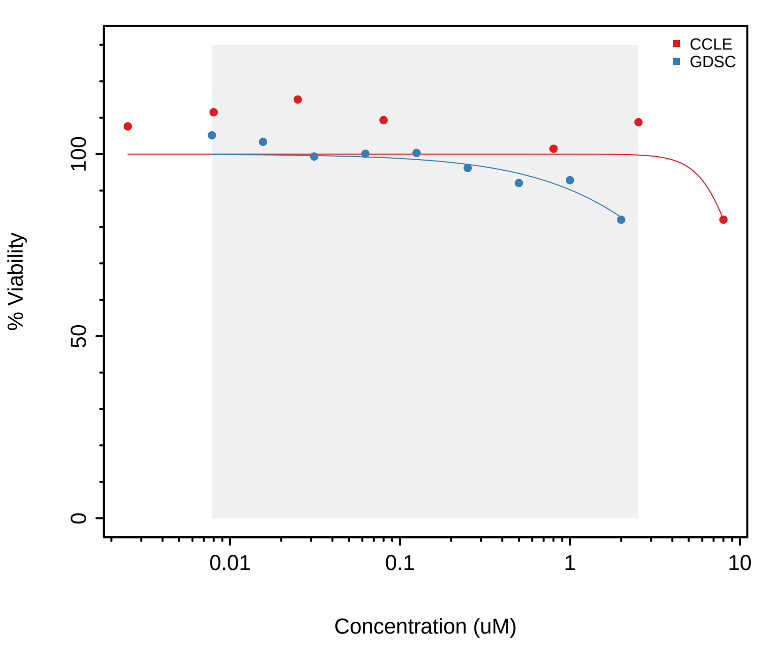

TAE684:NCI−H1975

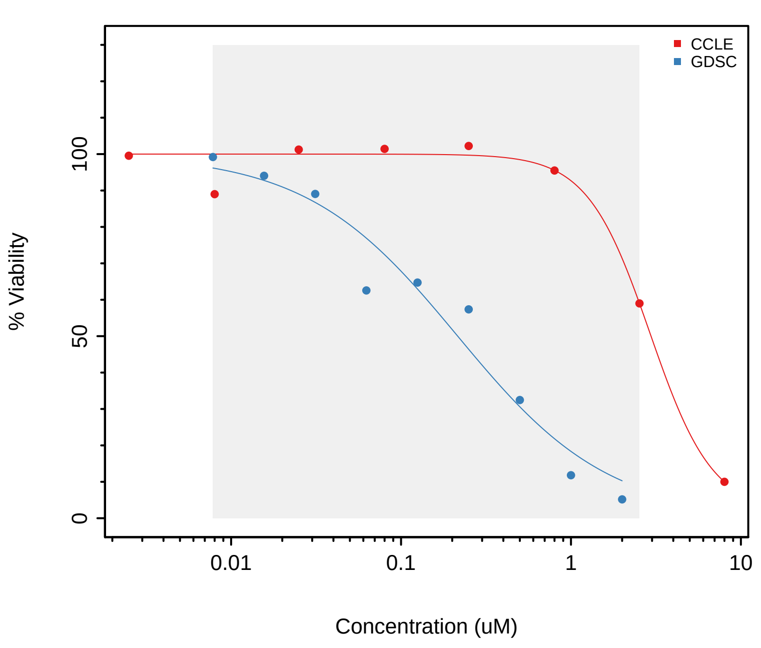

TAE684:NCI−H226

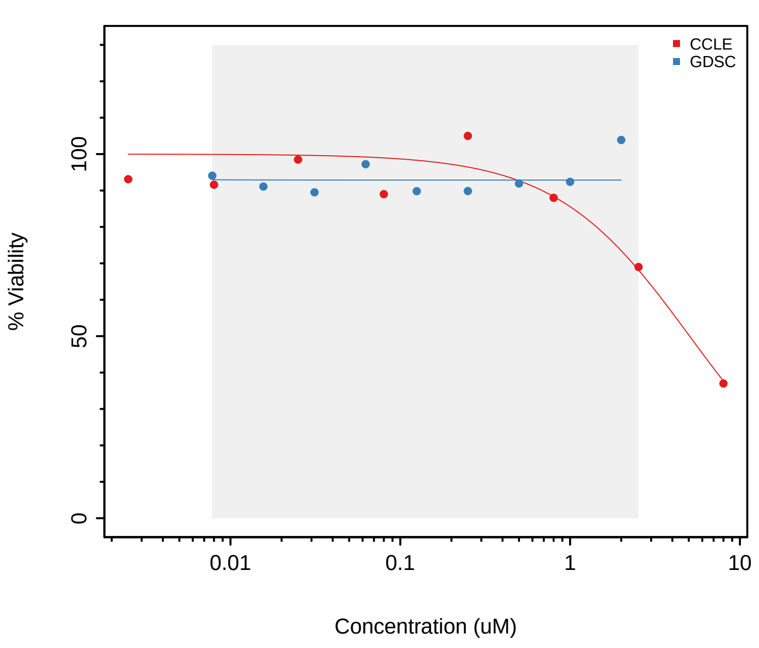

TAE684:NCI−H23

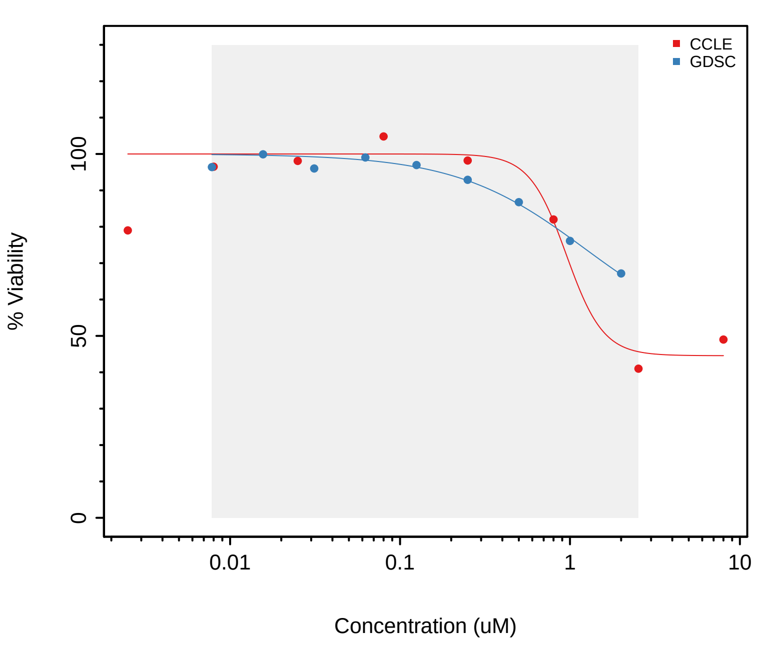

TAE684:NCI−H747

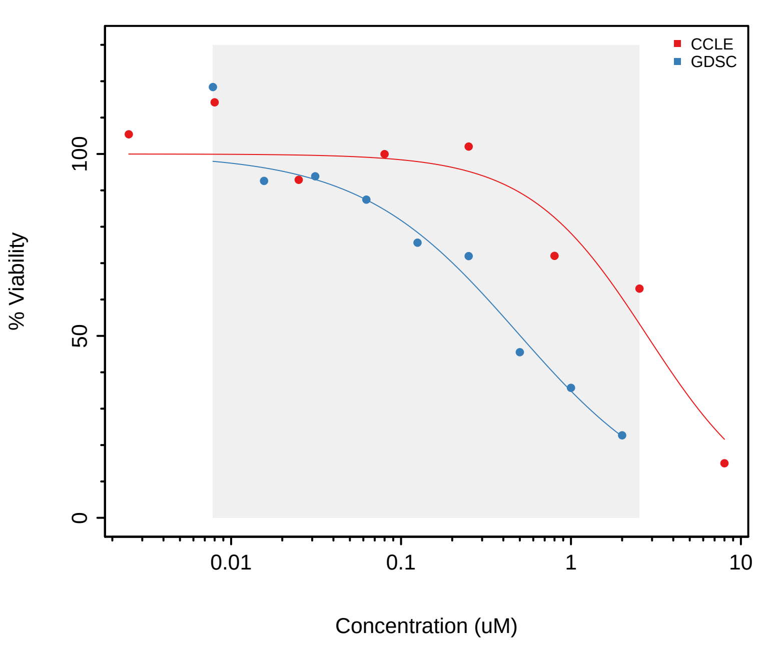

TAE684:OCI−AML2

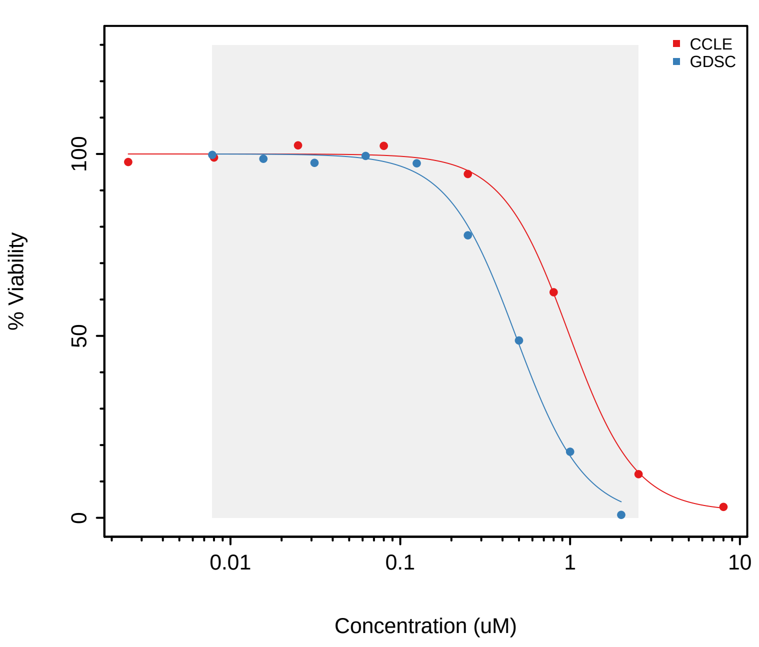

TAE684:ONS−76

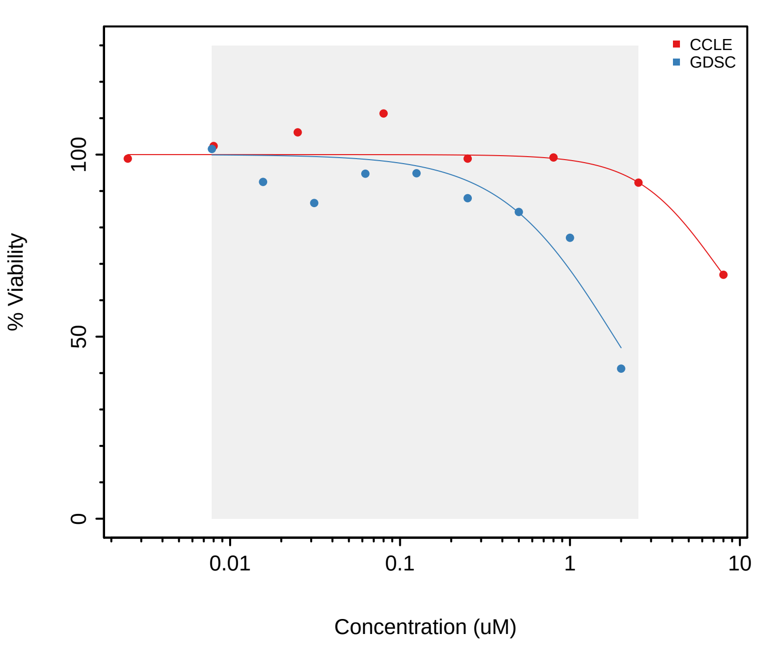

TAE684:OPM−2

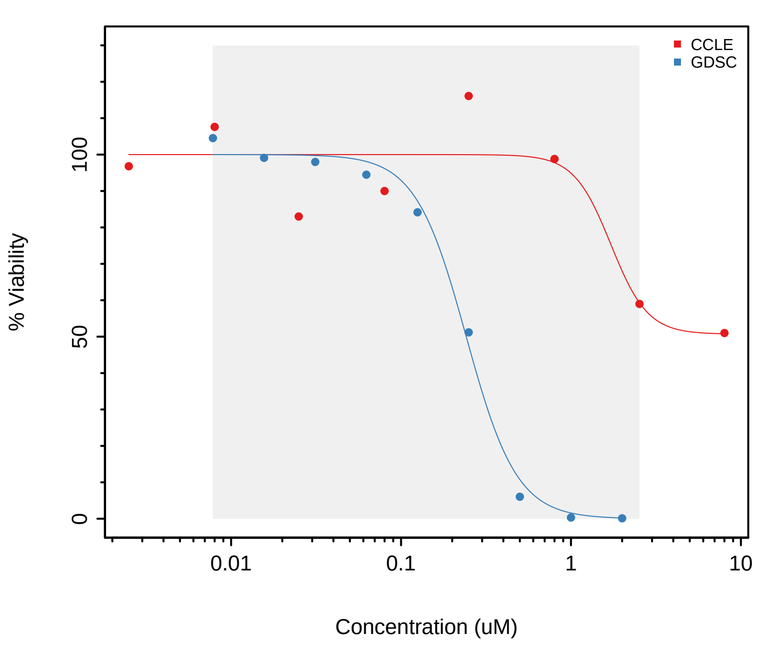

TAE684:OVCAR−4

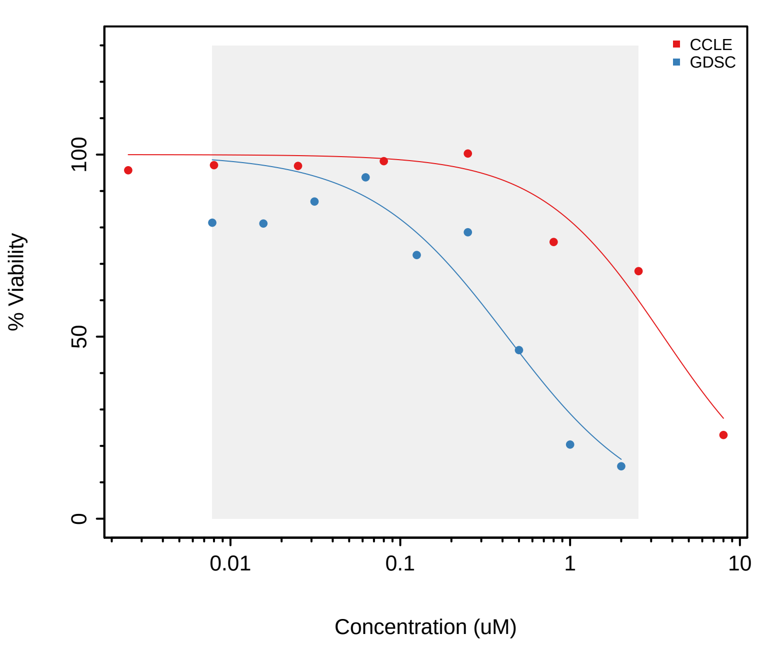

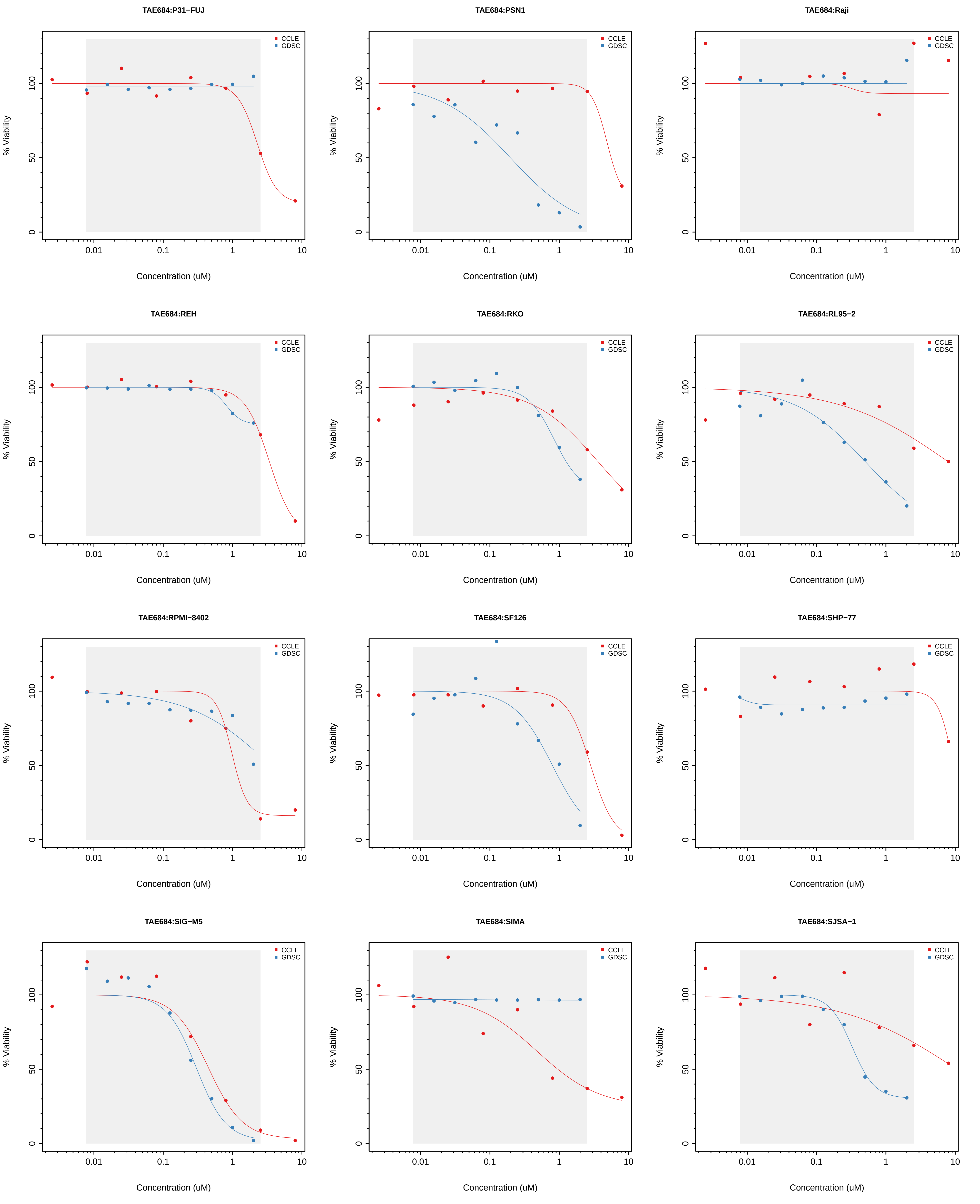
TAE684:P31−FUJ
TAE684:PSN1
TAE684:Raji
TAE684:REH
TAE684:RKO
TAE684:RL95−2
TAE684:RPMI−8402
TAE684:SF126
TAE684:SHP−77
TAE684:SIG−M5
TAE684:SIMA
TAE684:SJSA−1
CCLE
GDSC
% Viability
0
50
100
0.01
0.1
1
10
Concentration (uM)

TAE684:SK−LMS−1

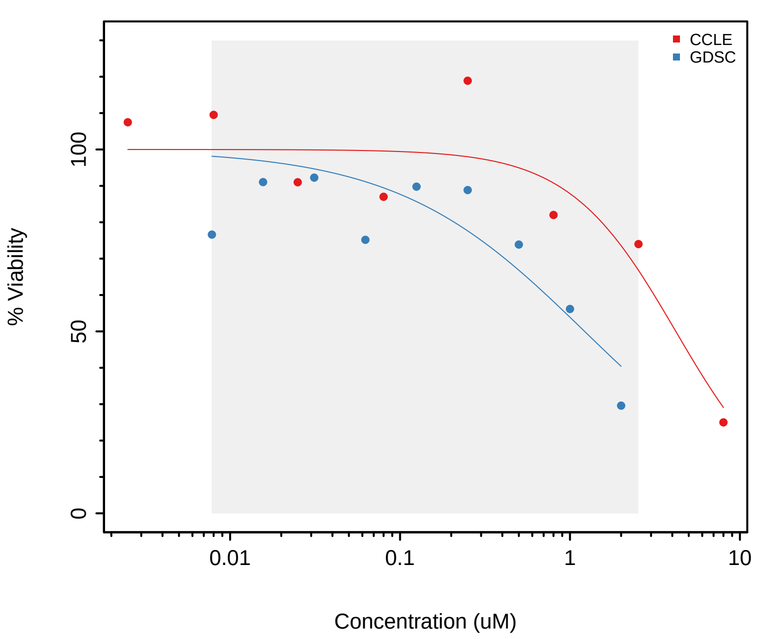

TAE684:SK−MEL−2

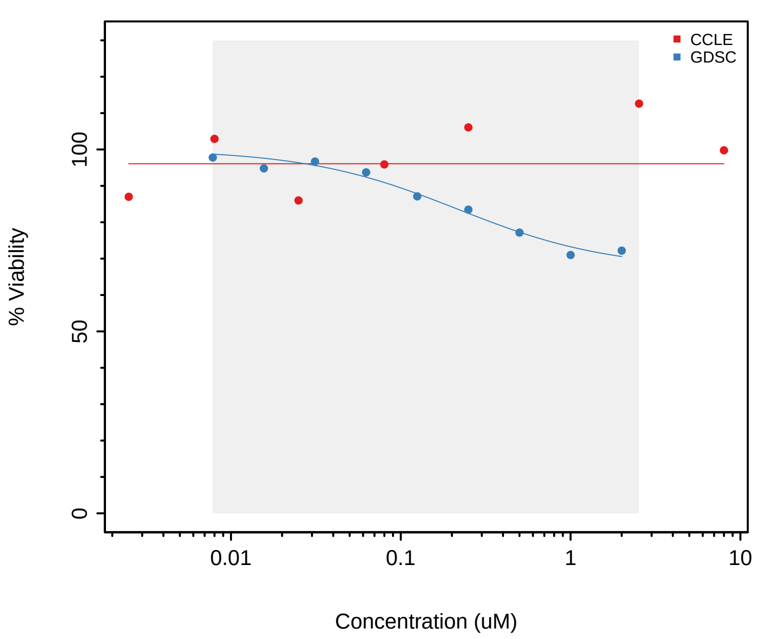

TAE684:SK−MM−2

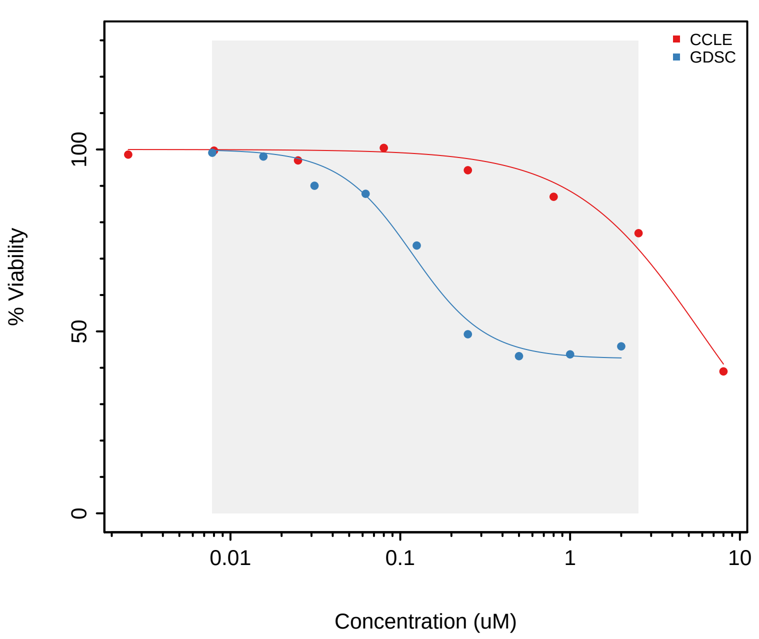

TAE684:SK−N−DZ

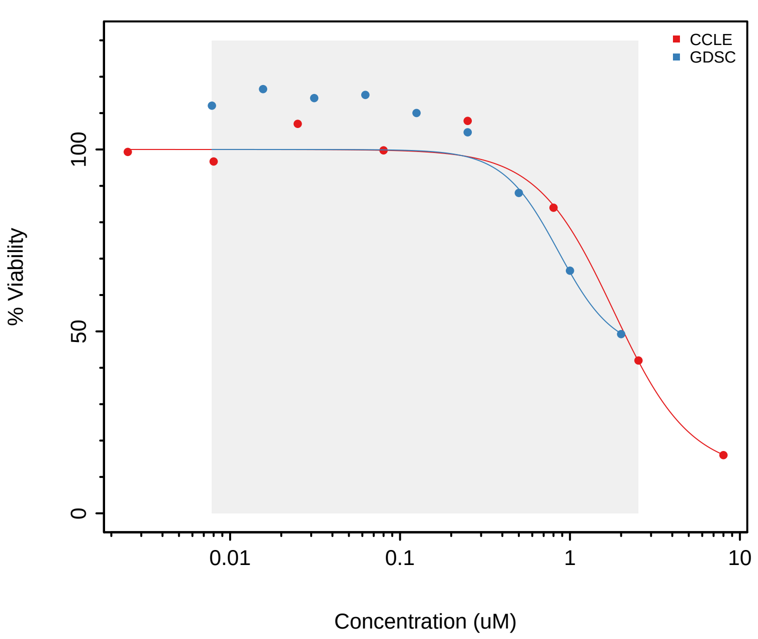

TAE684:NCI−SNU−1

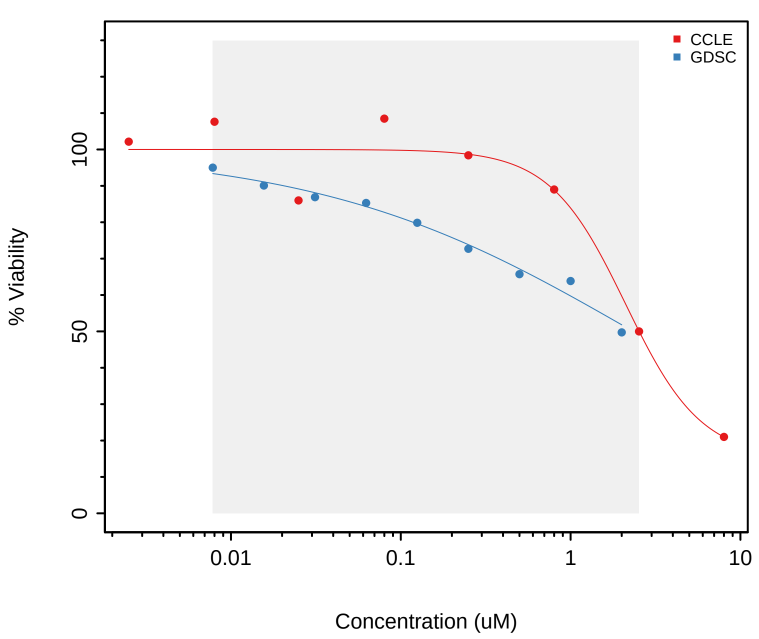

TAE684:NCI−SNU−16

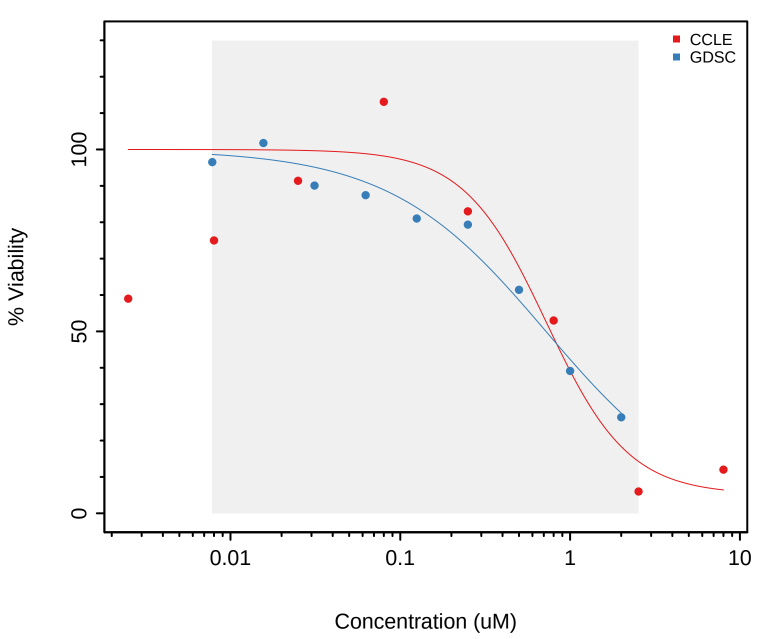

TAE684:SNU−C2B

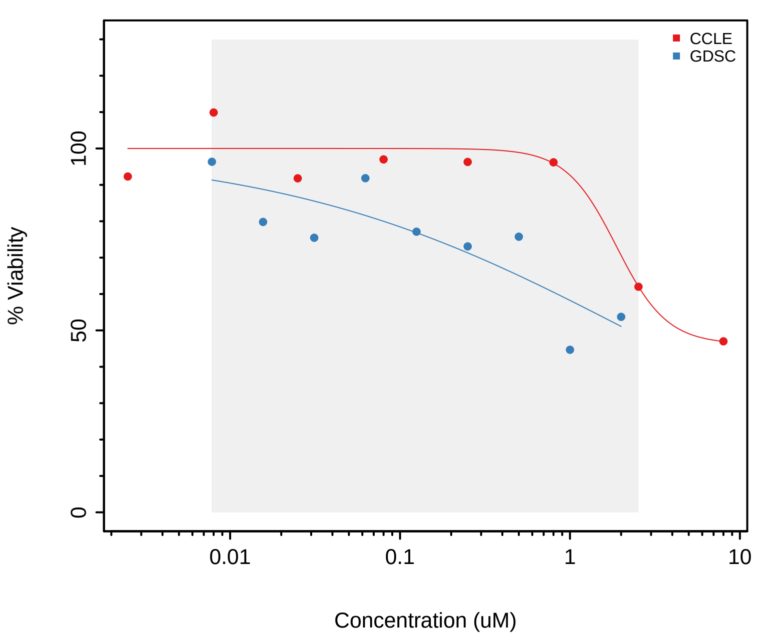

TAE684:SUP−T1

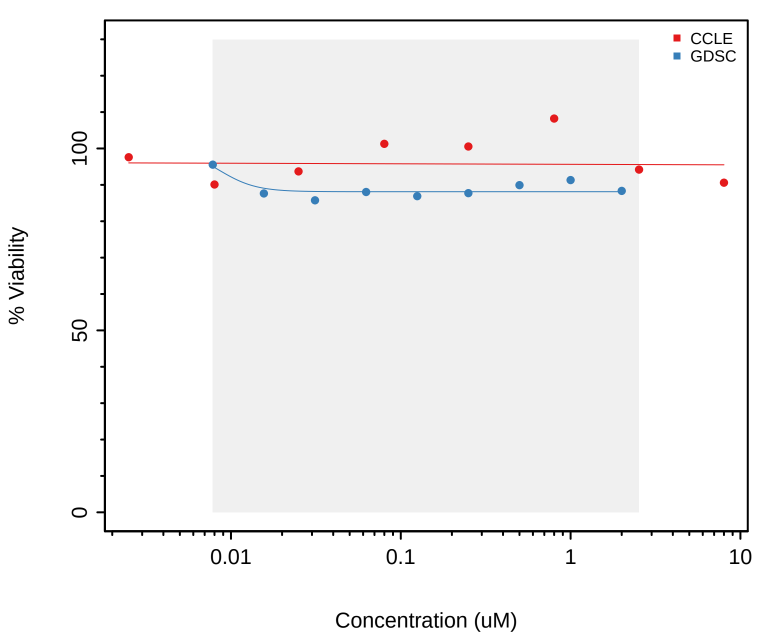

TAE684:TE−1

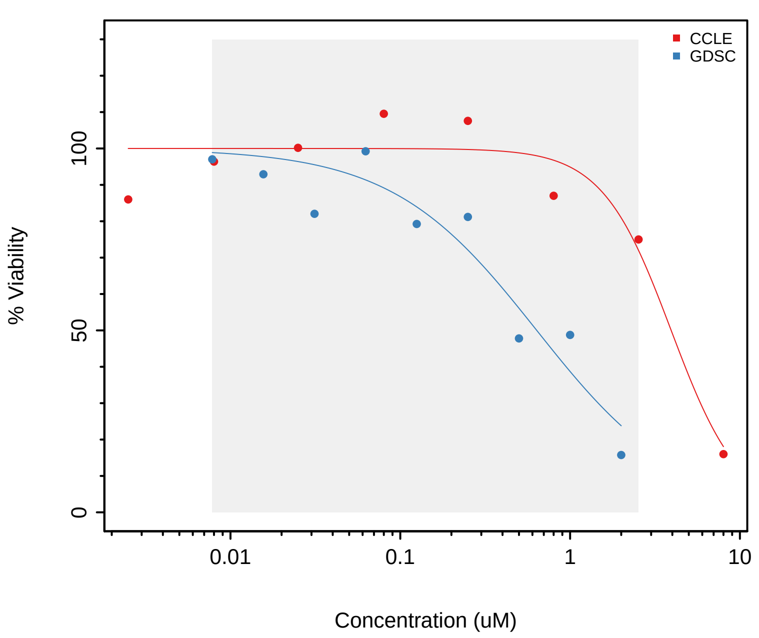

TAE684:TE−15

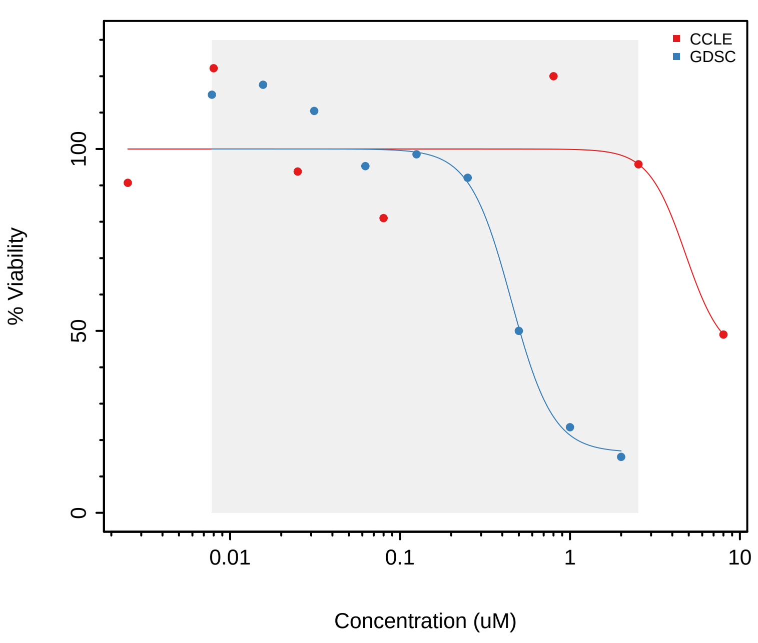

TAE684:TE−5

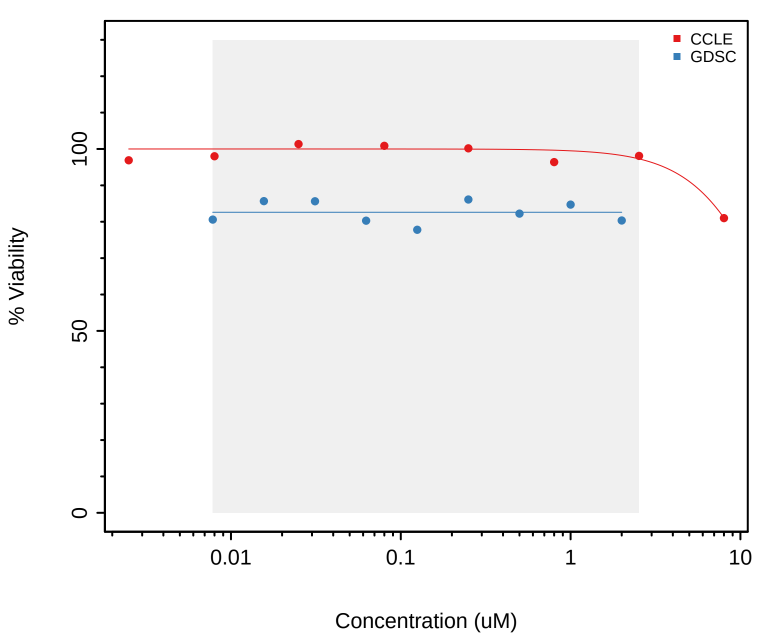

TAE684:TE−9

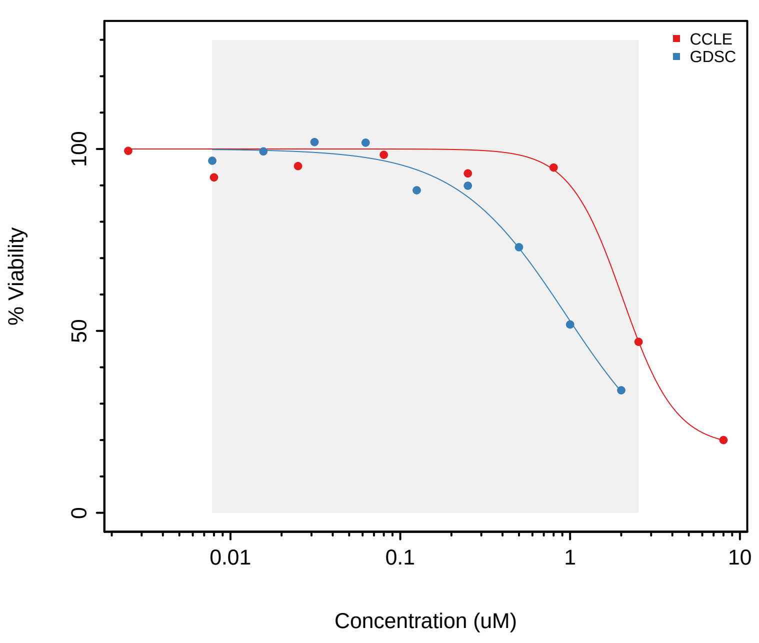

TAE684:U−87−MG

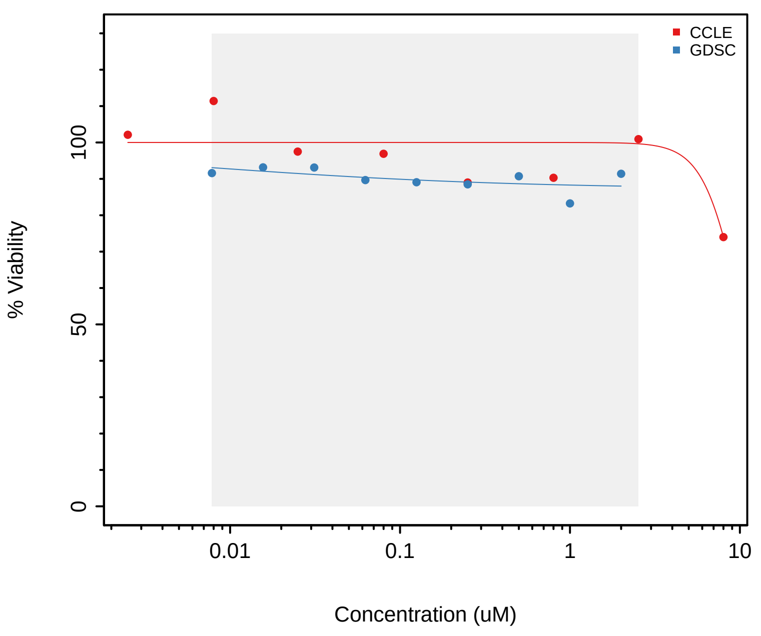

TAE684:UACC−257

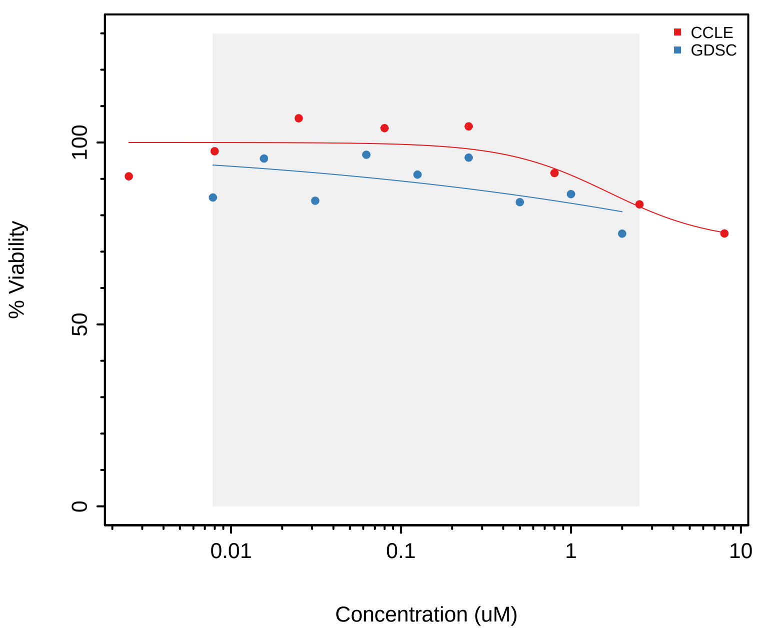

TAE684:UACC−812

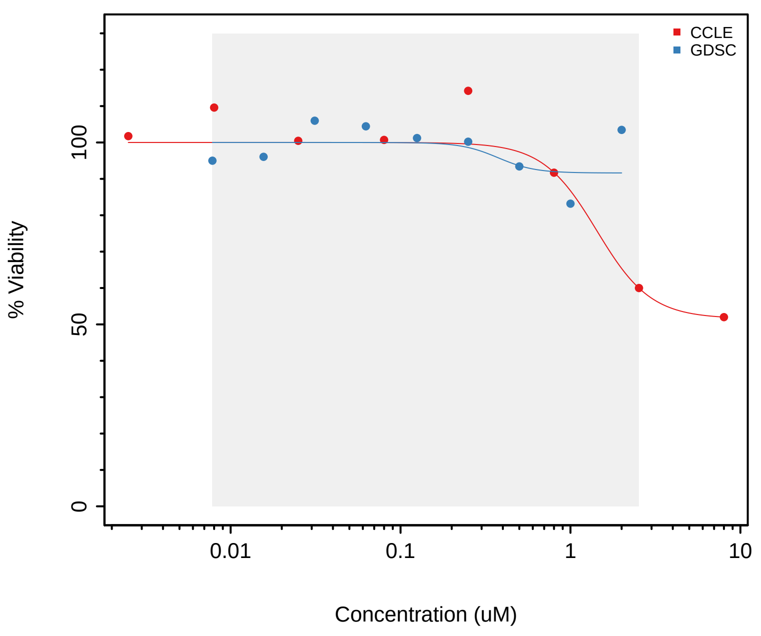

Erlotinib:697

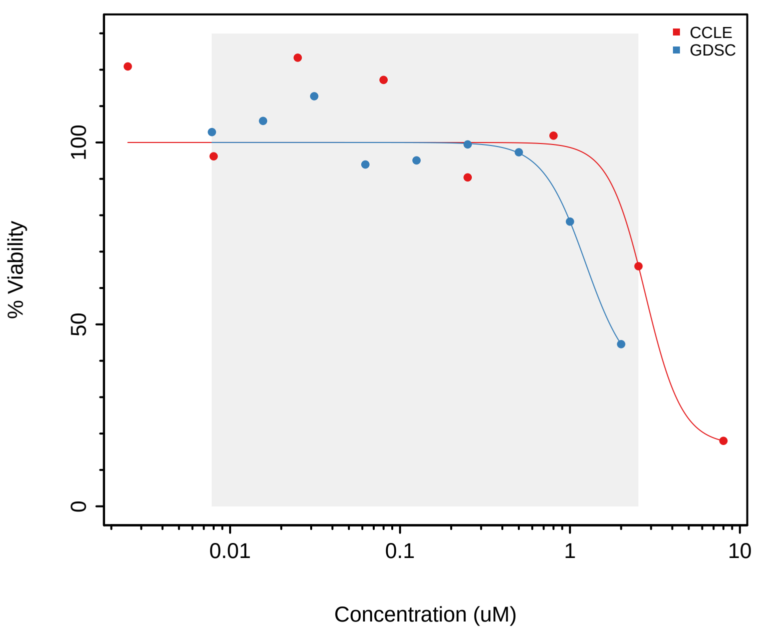

Erlotinib:8−MG−BA

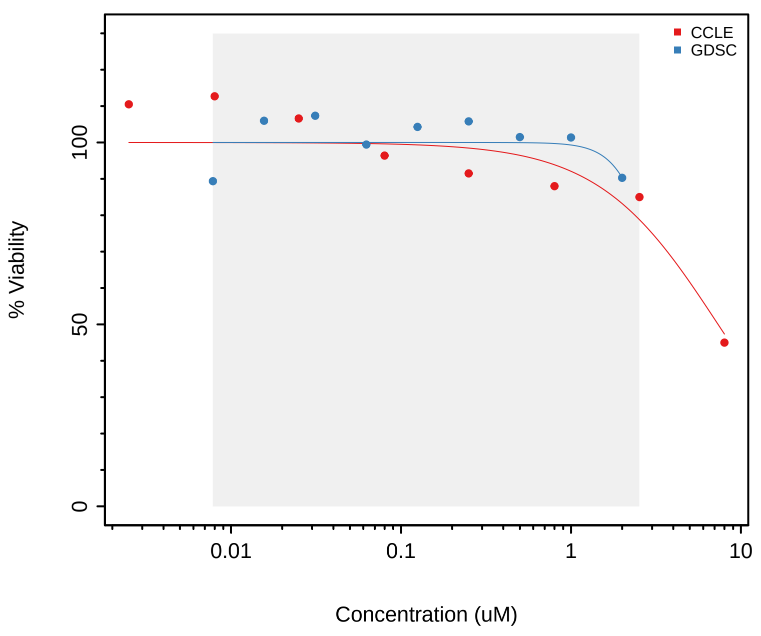

Erlotinib:A253

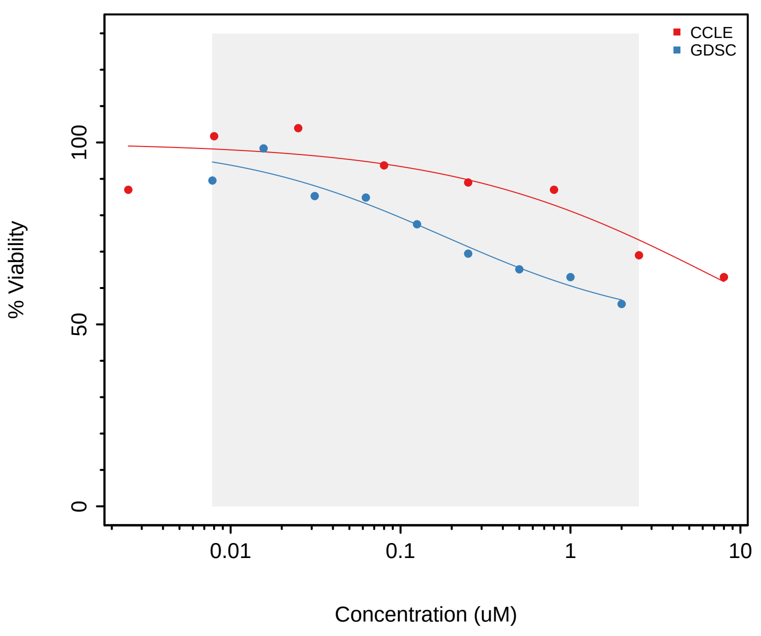

Erlotinib:BL−41

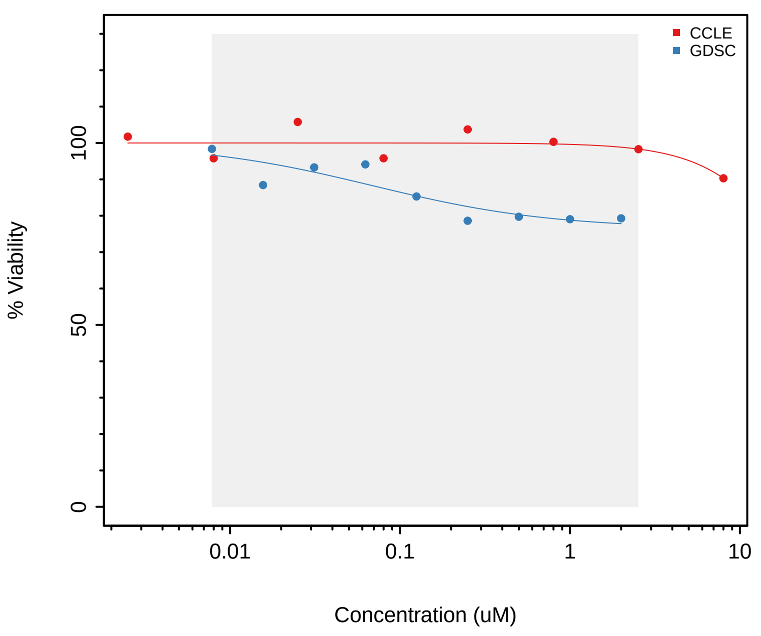

Erlotinib:BL−70

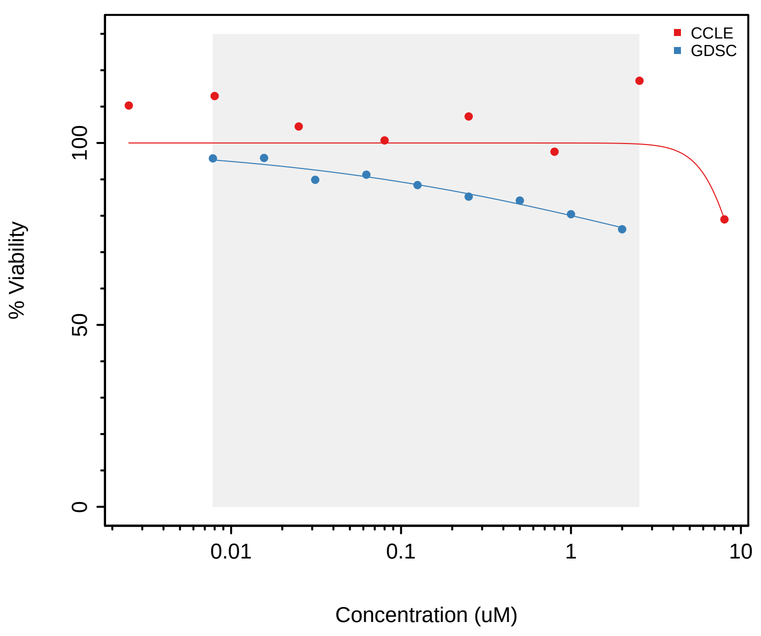

Erlotinib:BT−474

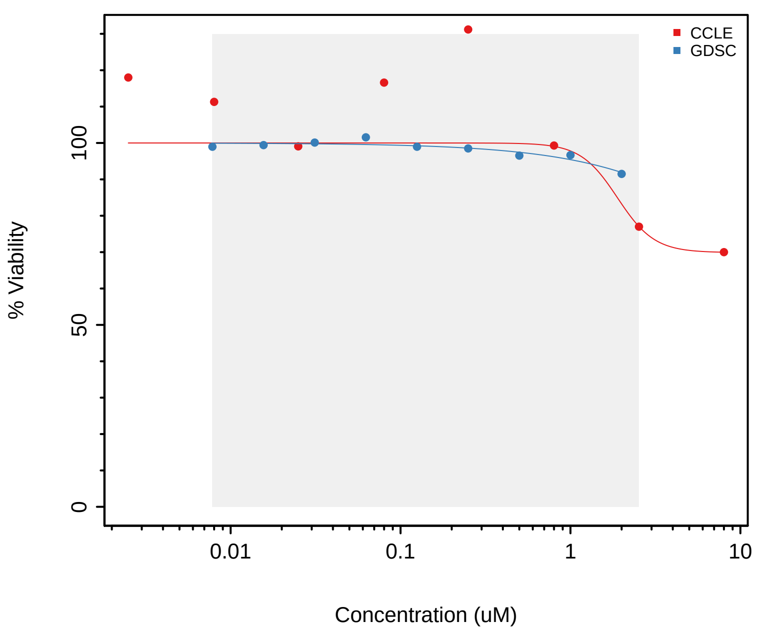

Erlotinib:C2BBe1

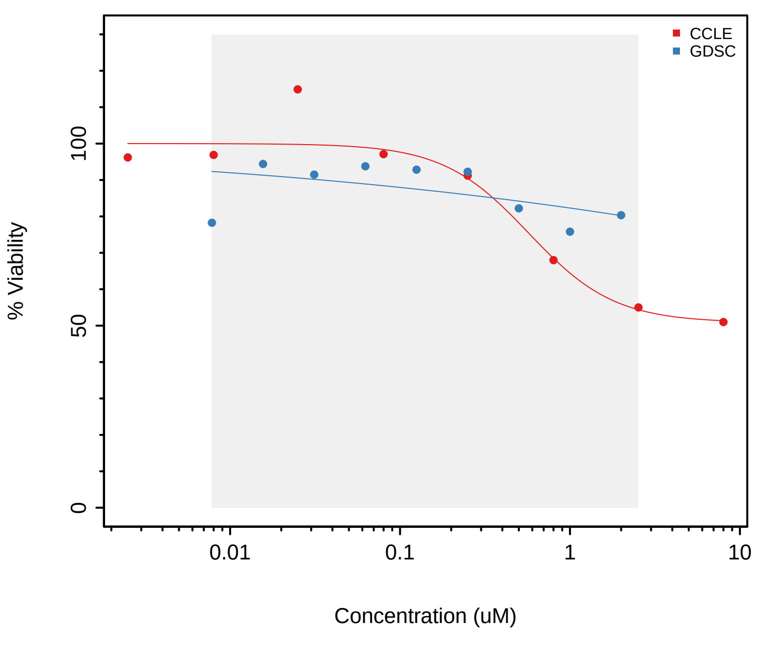

Erlotinib:Calu−6

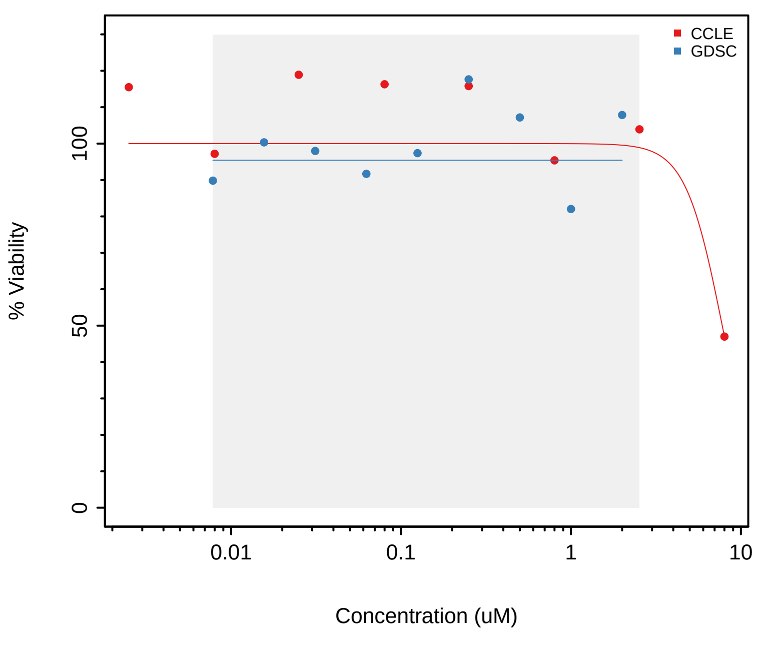

Erlotinib:COLO−320−HSR

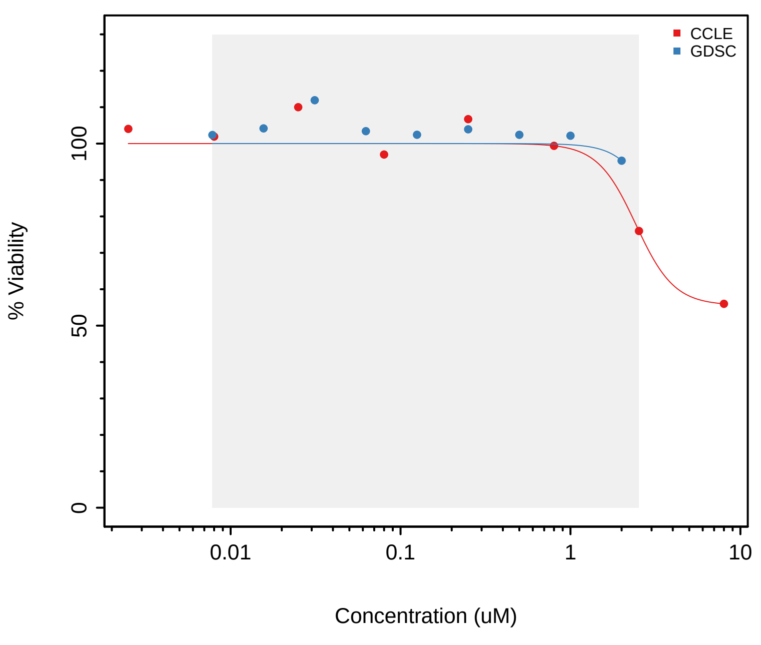

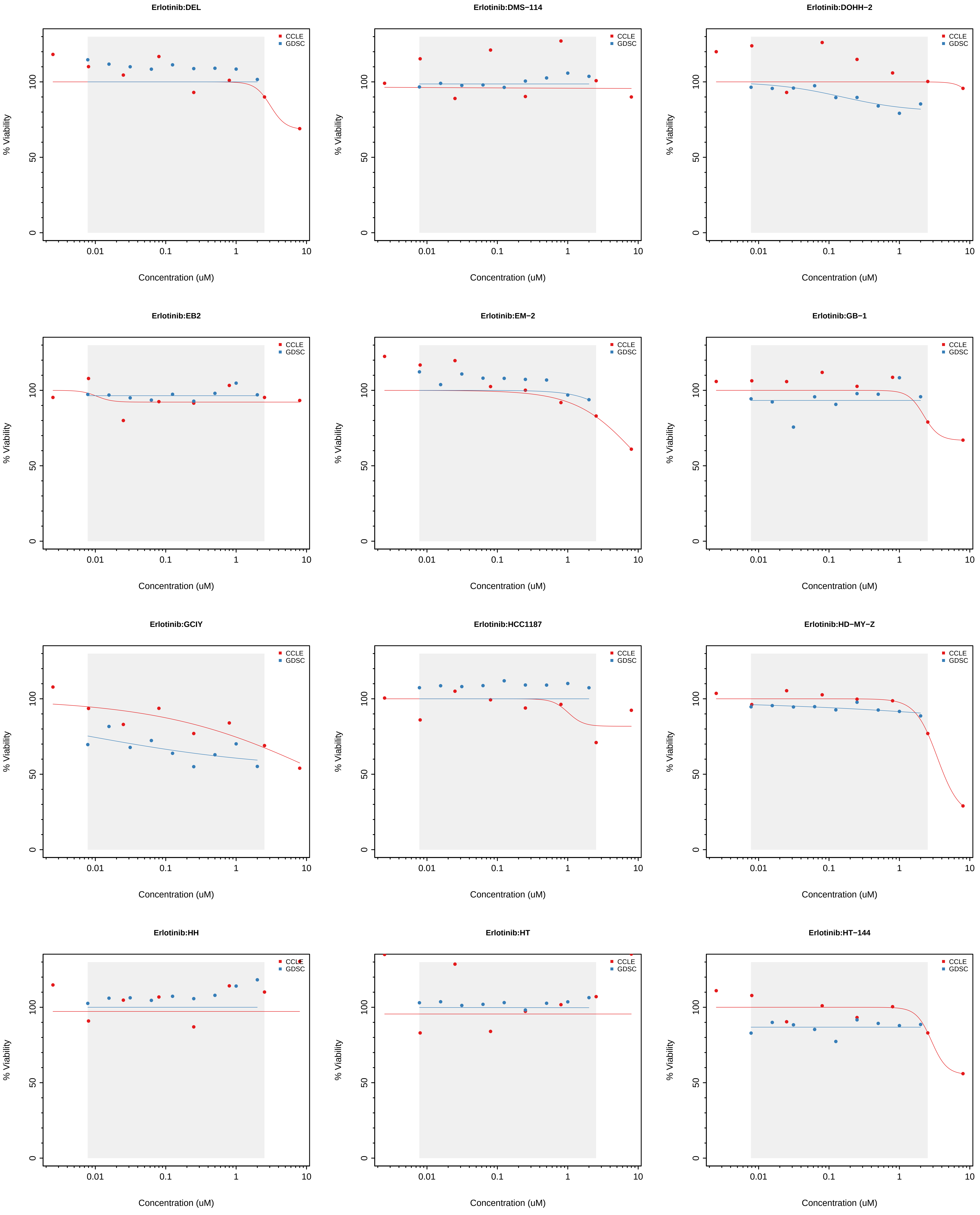
Erlotinib:DEL
Erlotinib:DMS−114
Erlotinib:DOHH−2
Erlotinib:EB2
Erlotinib:EM−2
Erlotinib:GB−1
Erlotinib:GCIY
Erlotinib:HCC1187
Erlotinib:HD−MY−Z
Erlotinib:HH
Erlotinib:HT
Erlotinib:HT−144
CCLE
GDSC
% Viability
Concentration (uM)
0
50
100
0.01
0.1
1
10

Erlotinib:IST−MES1

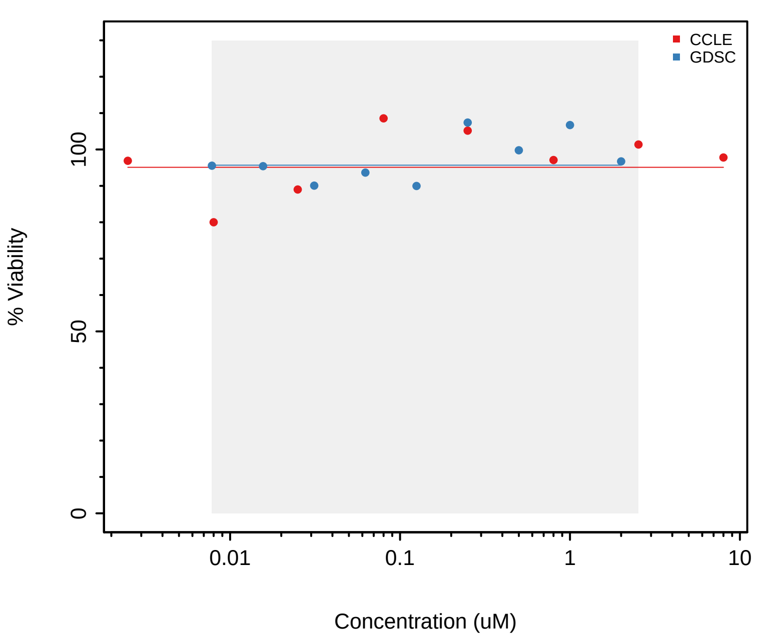

Erlotinib:JVM−3

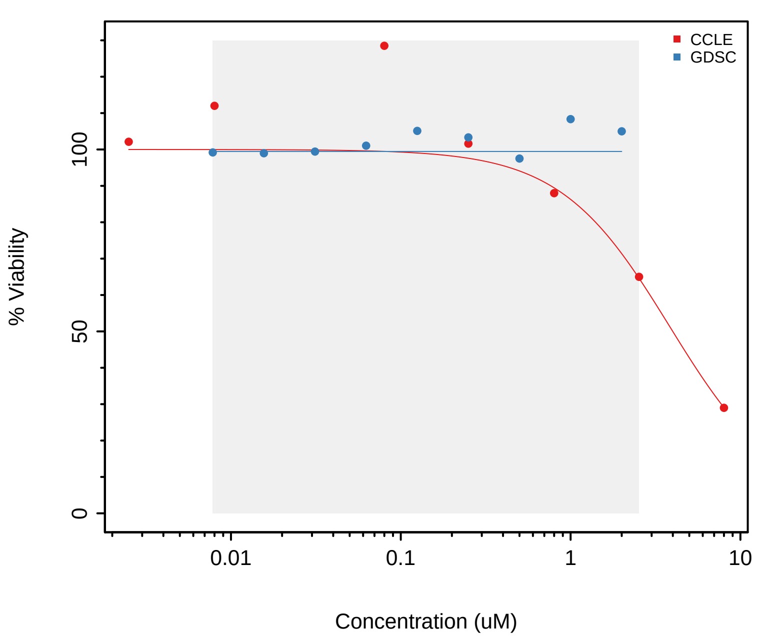

Erlotinib:KALS−1

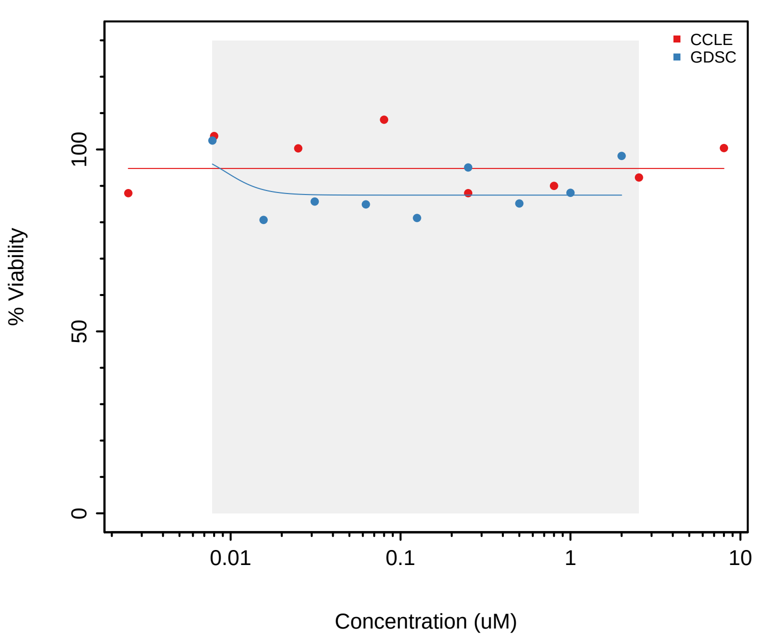

Erlotinib:KARPAS−299

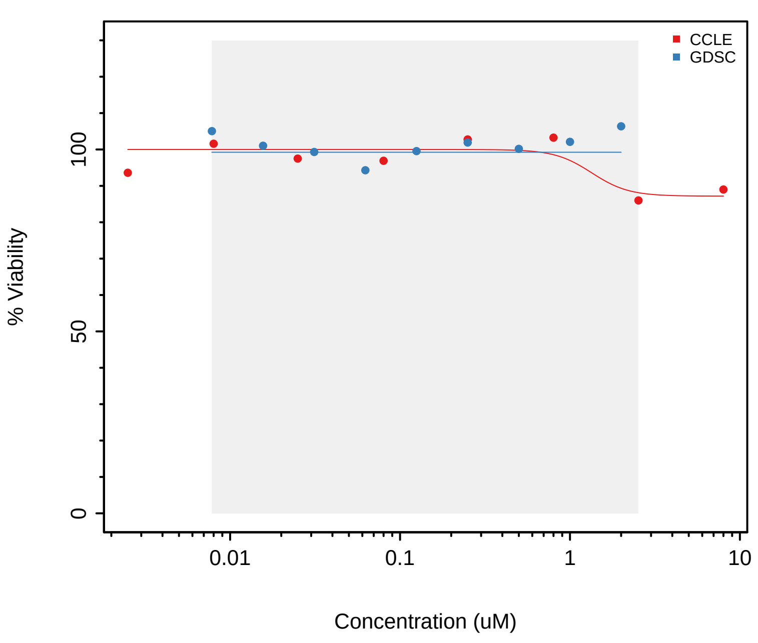

Erlotinib:KARPAS−422

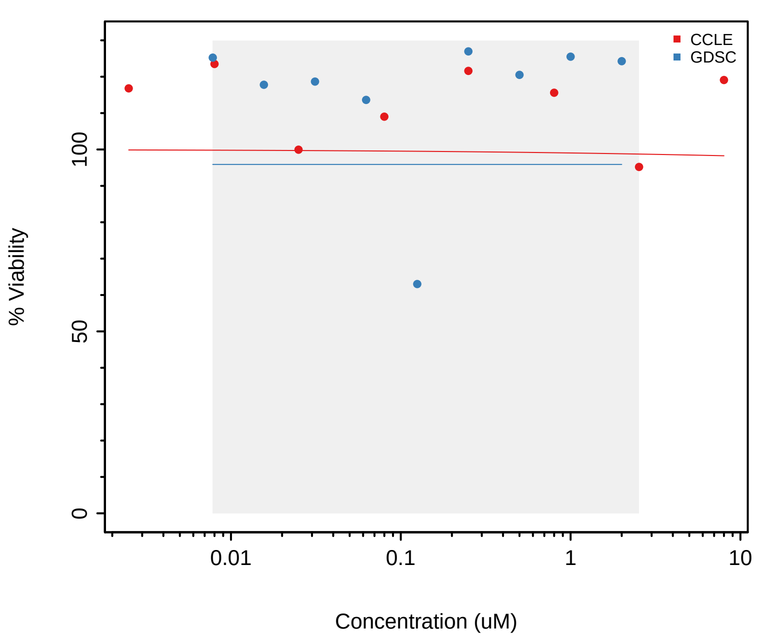

Erlotinib:KG−1

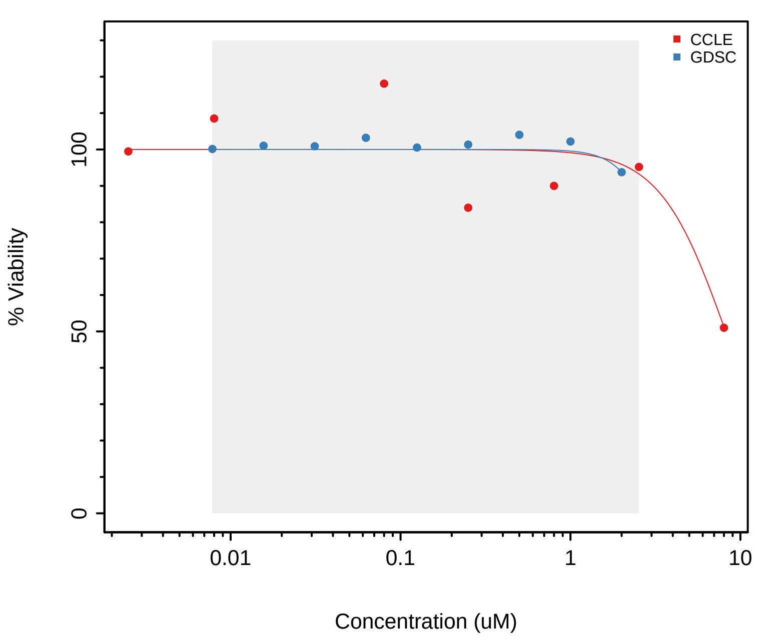

Erlotinib:KNS−42

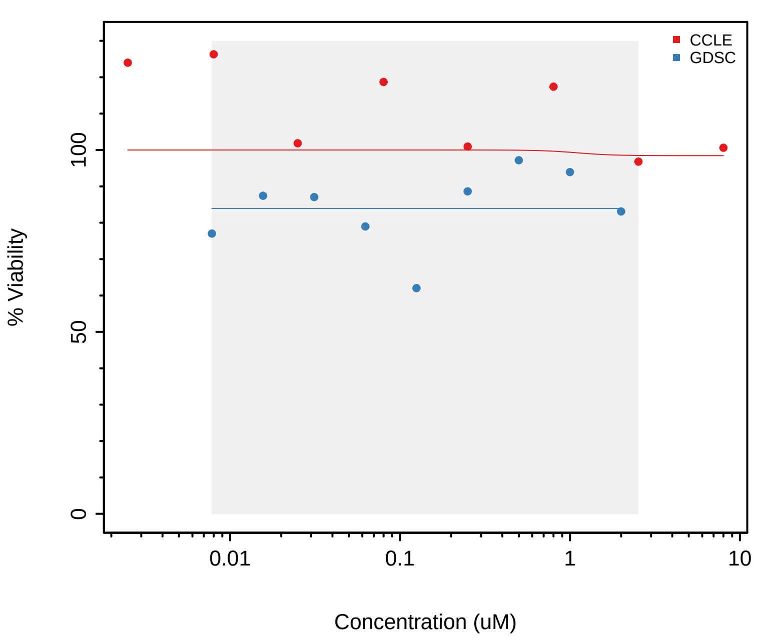

Erlotinib:KNS−81−FD

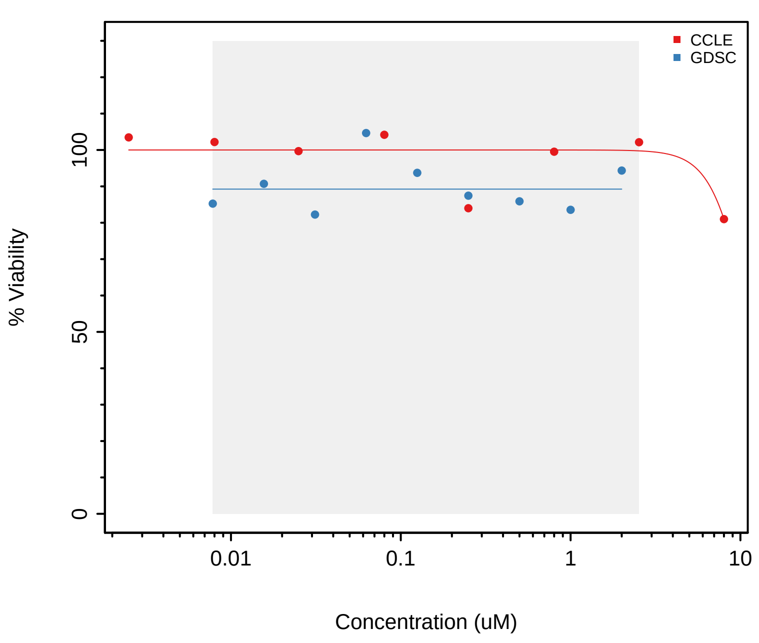

Erlotinib:KU812

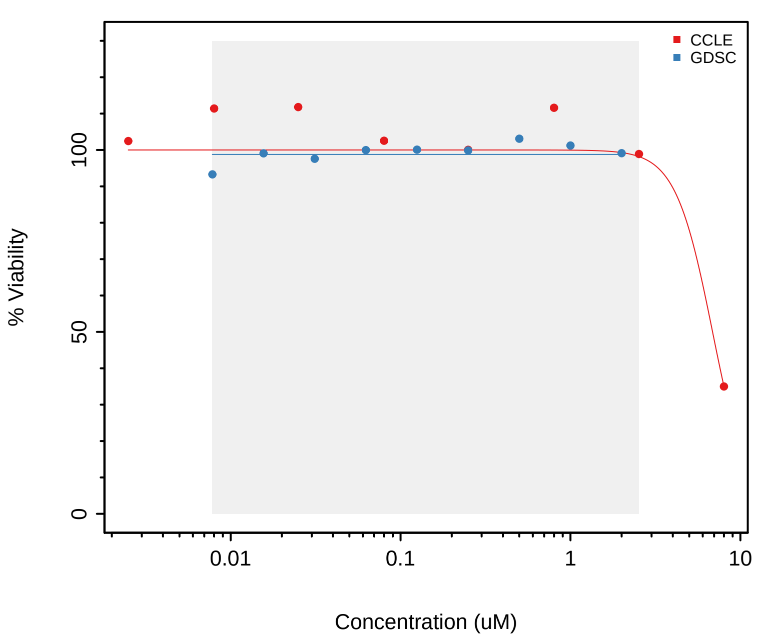

Erlotinib:KURAMOCHI

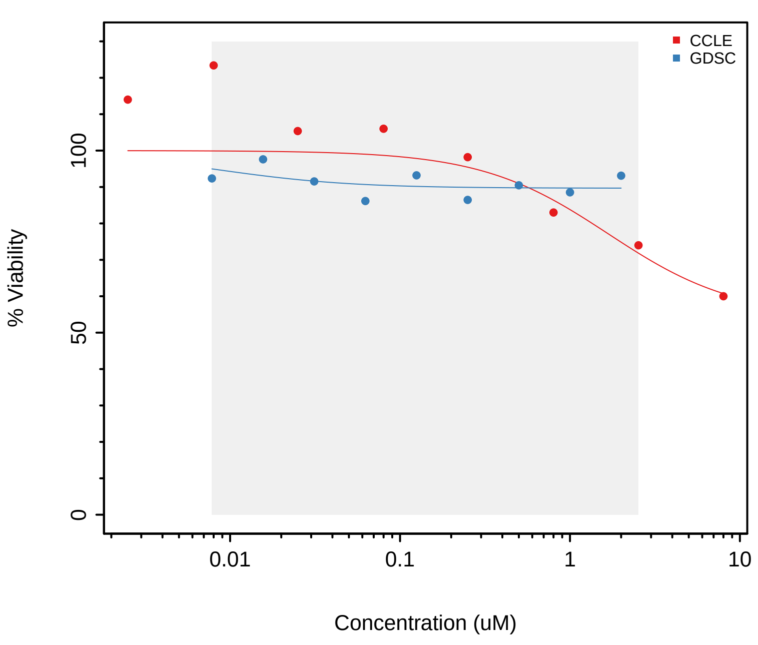

Erlotinib:L−363

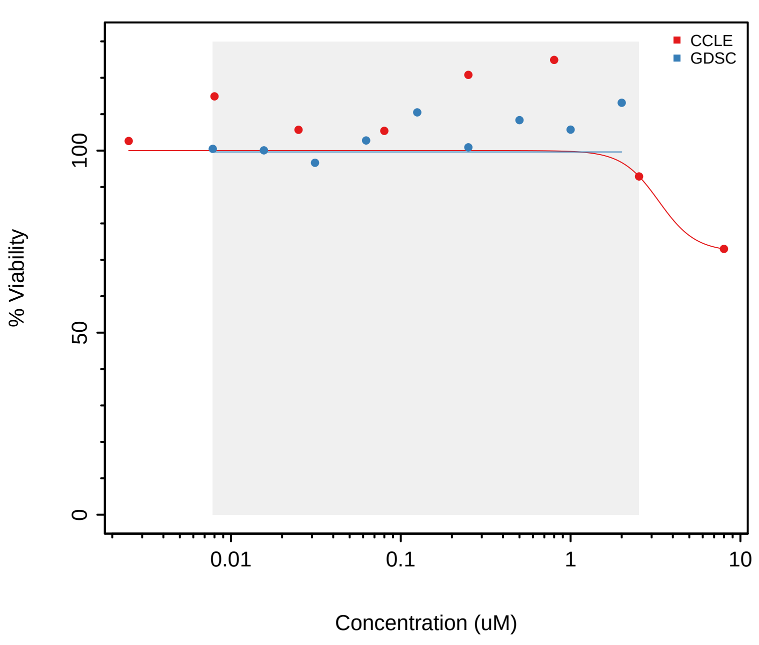

Erlotinib:L−428

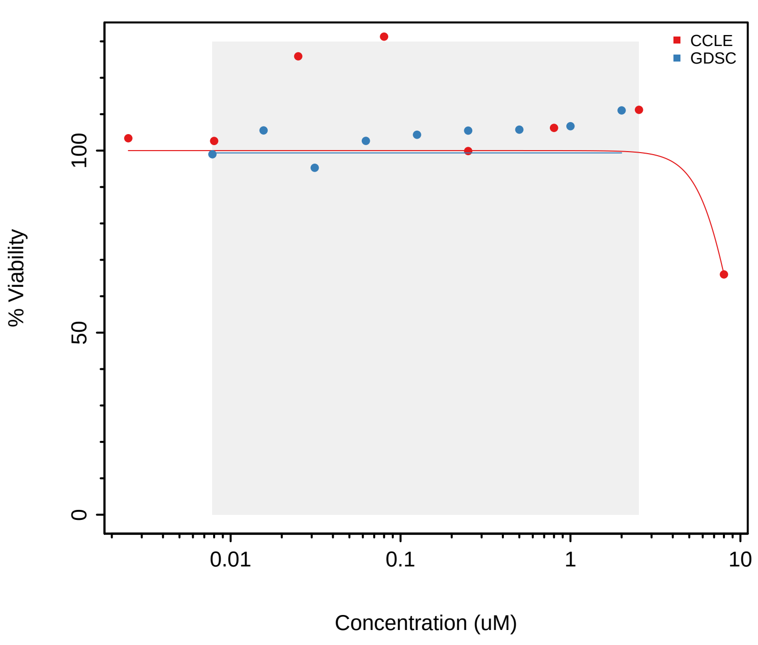

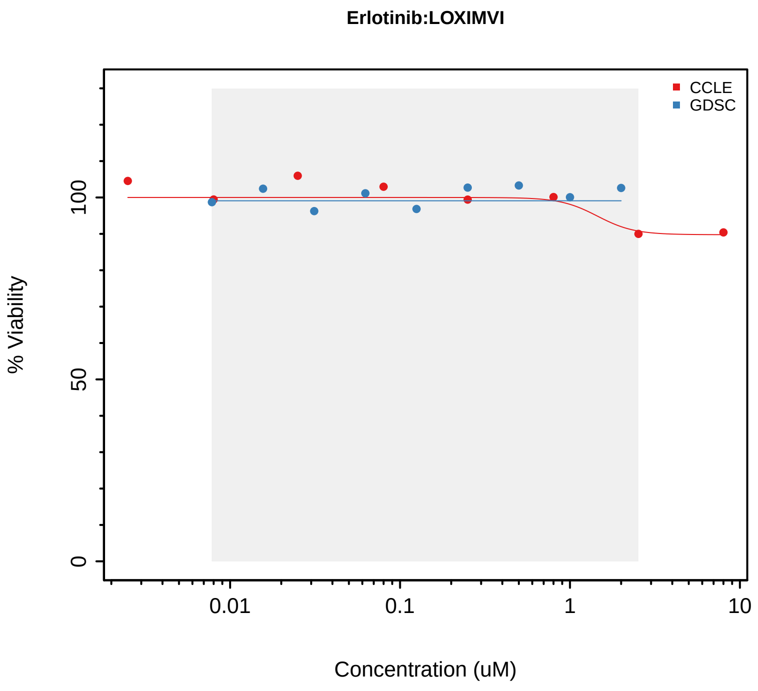
Erlotinib:LOXIMVI
CCLE
GDSC
% Viability
Concentration (uM)

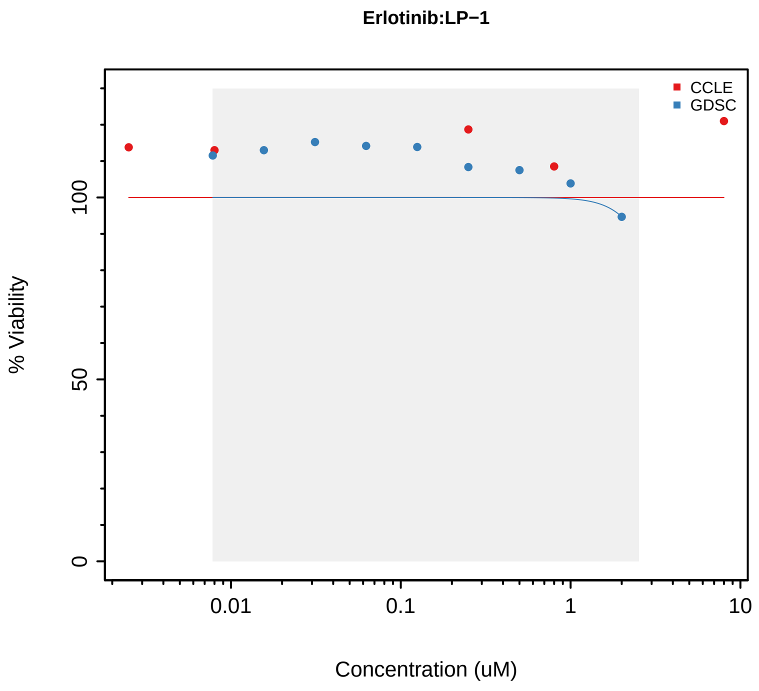
Erlotinib:LP−1
CCLE
GDSC
% Viability
Concentration (uM)

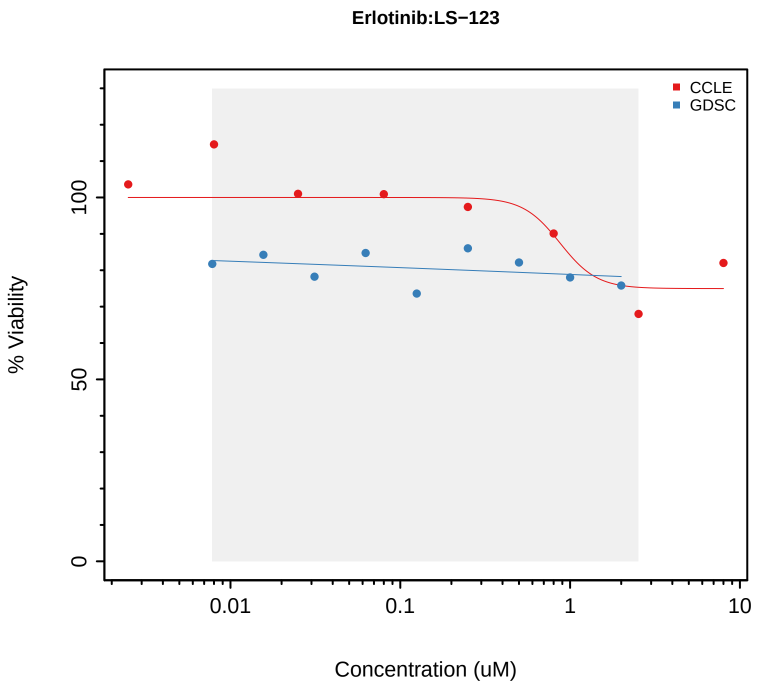
Erlotinib:LS−123
CCLE
GDSC
% Viability
Concentration (uM)

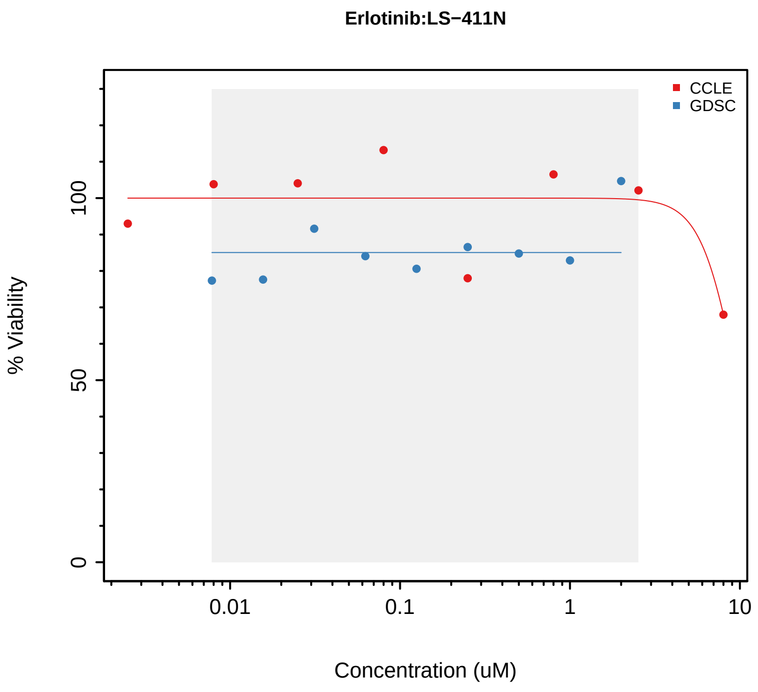
Erlotinib:LS−411N
CCLE
GDSC
% Viability
Concentration (uM)

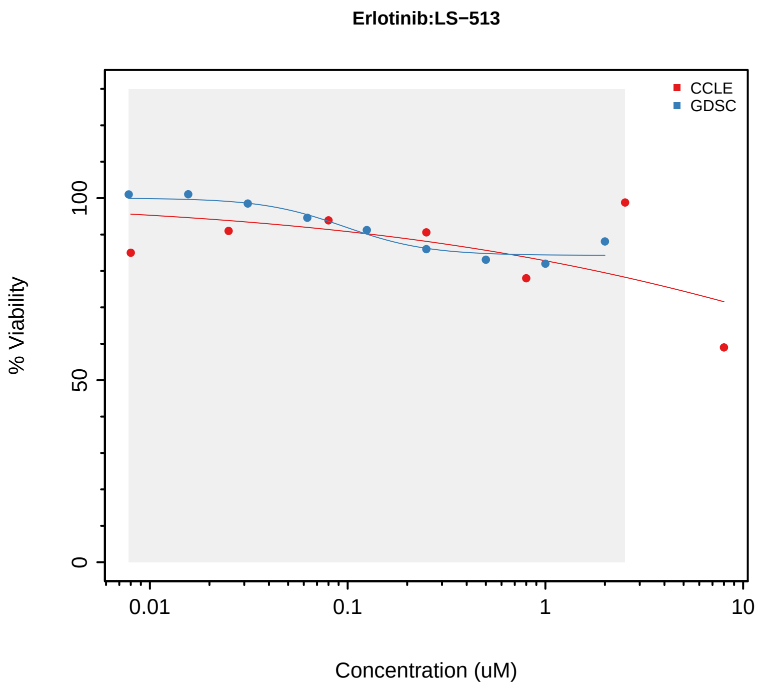
Erlotinib:LS−513
CCLE
GDSC
% Viability
Concentration (uM)

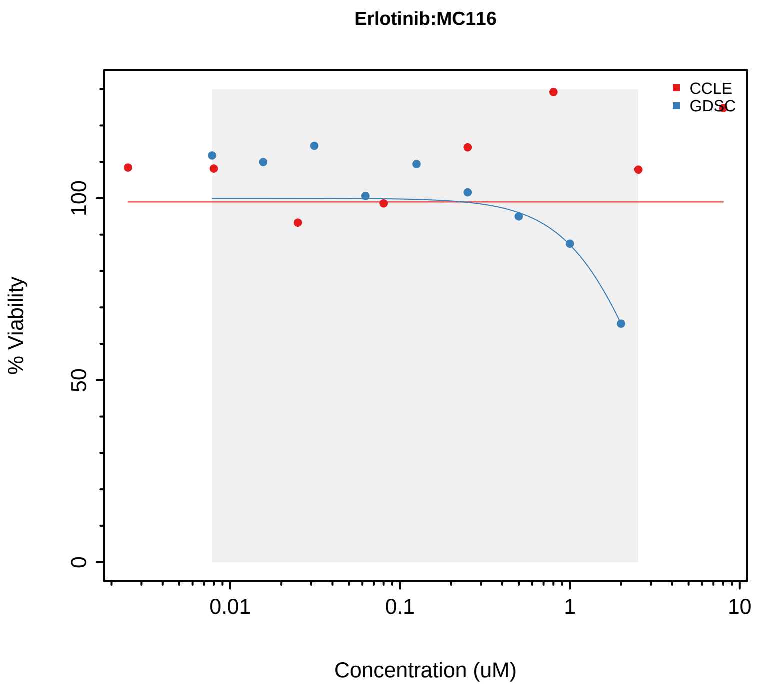
Erlotinib:MC116
CCLE
GDSC
% Viability
Concentration (uM)

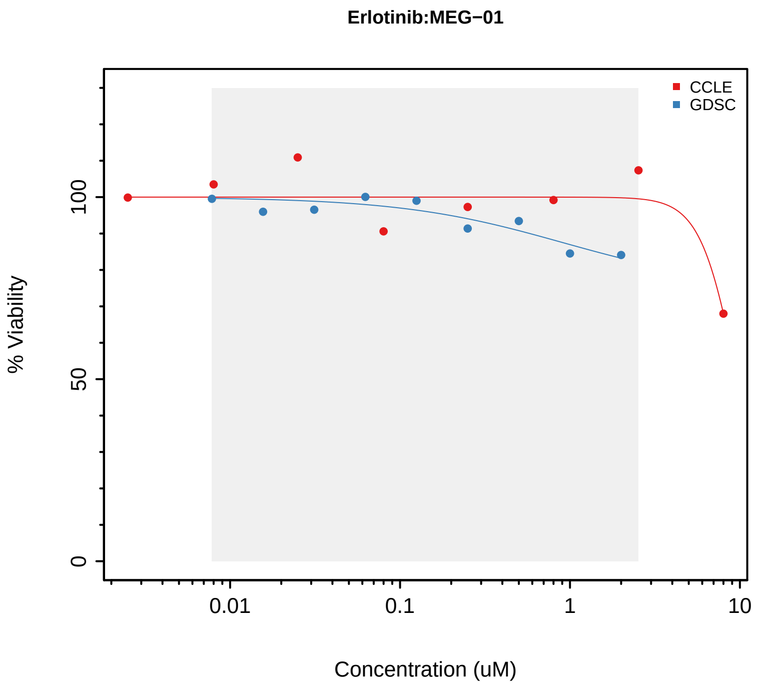
Erlotinib:MEG−01
CCLE
GDSC
% Viability
Concentration (uM)

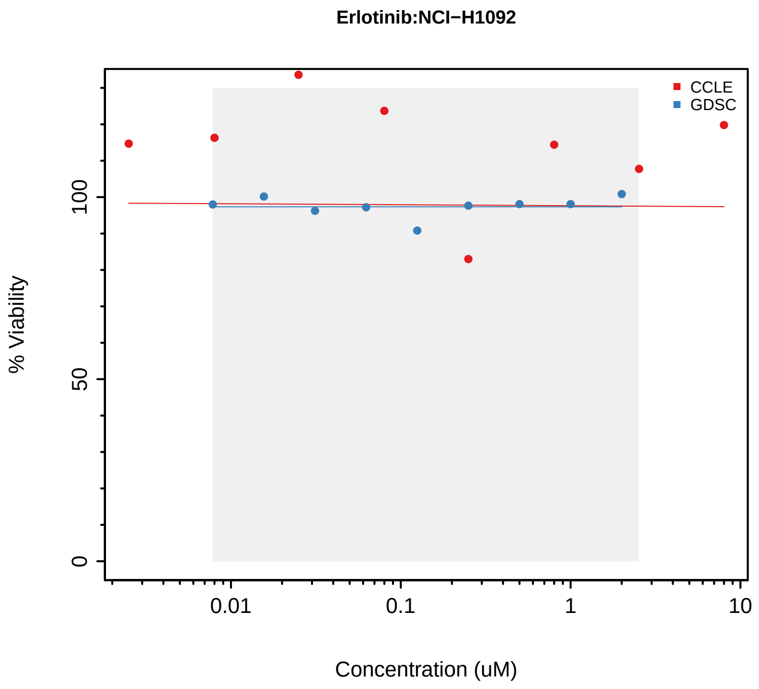
Erlotinib:NCI−H1092
CCLE
GDSC
% Viability
Concentration (uM)

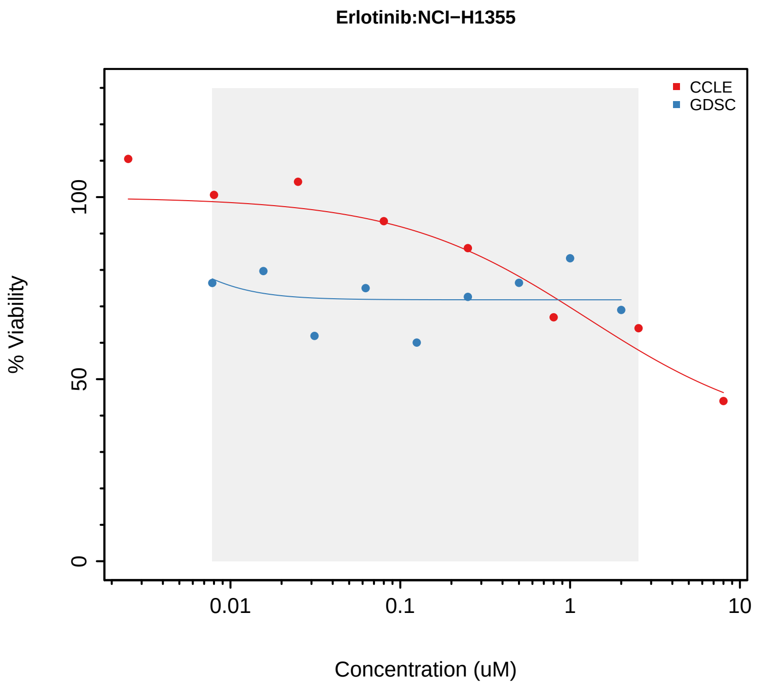
Erlotinib:NCI−H1355
CCLE
GDSC
% Viability
Concentration (uM)

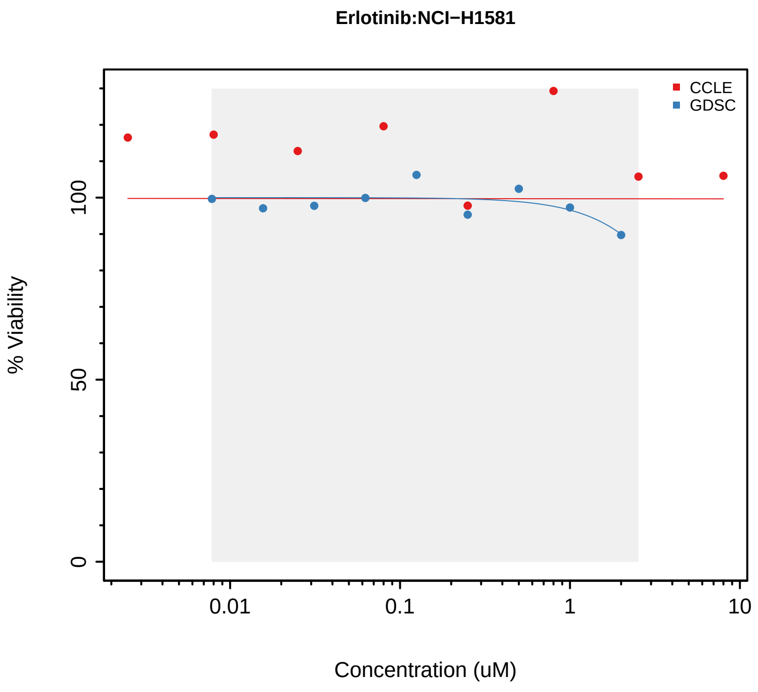
Erlotinib:NCI−H1581
CCLE
GDSC
% Viability
Concentration (uM)

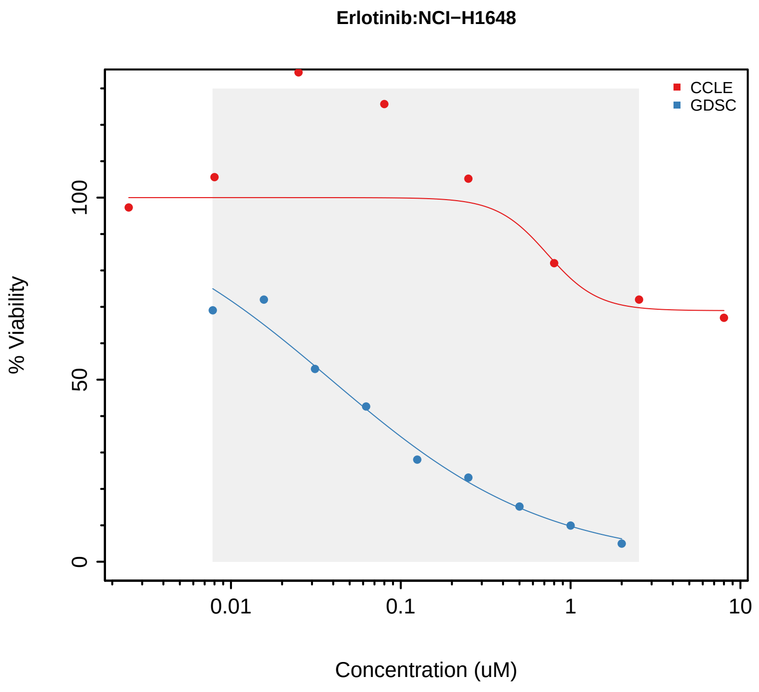
Erlotinib:NCI−H1648
CCLE
GDSC
% Viability
Concentration (uM)

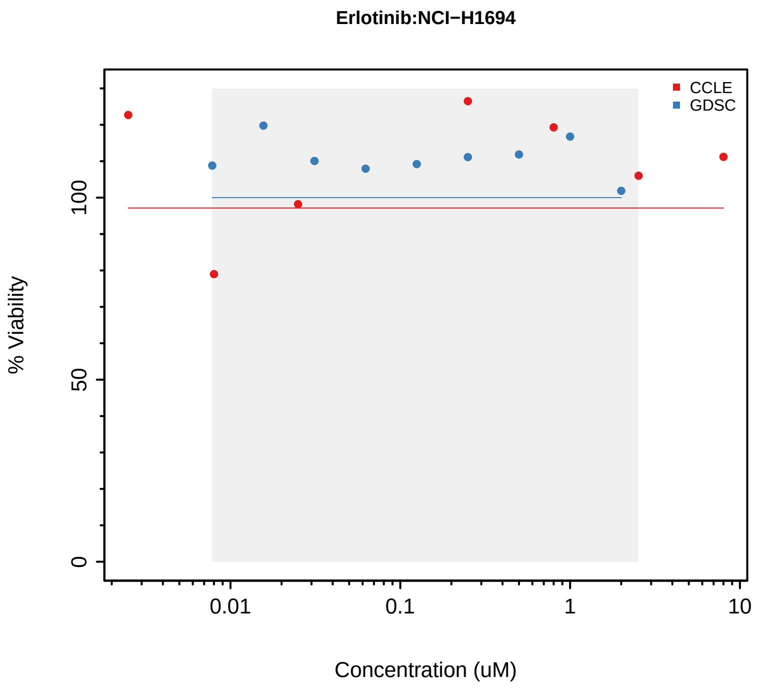
Erlotinib:NCI−H1694
CCLE
GDSC
% Viability
Concentration (uM)

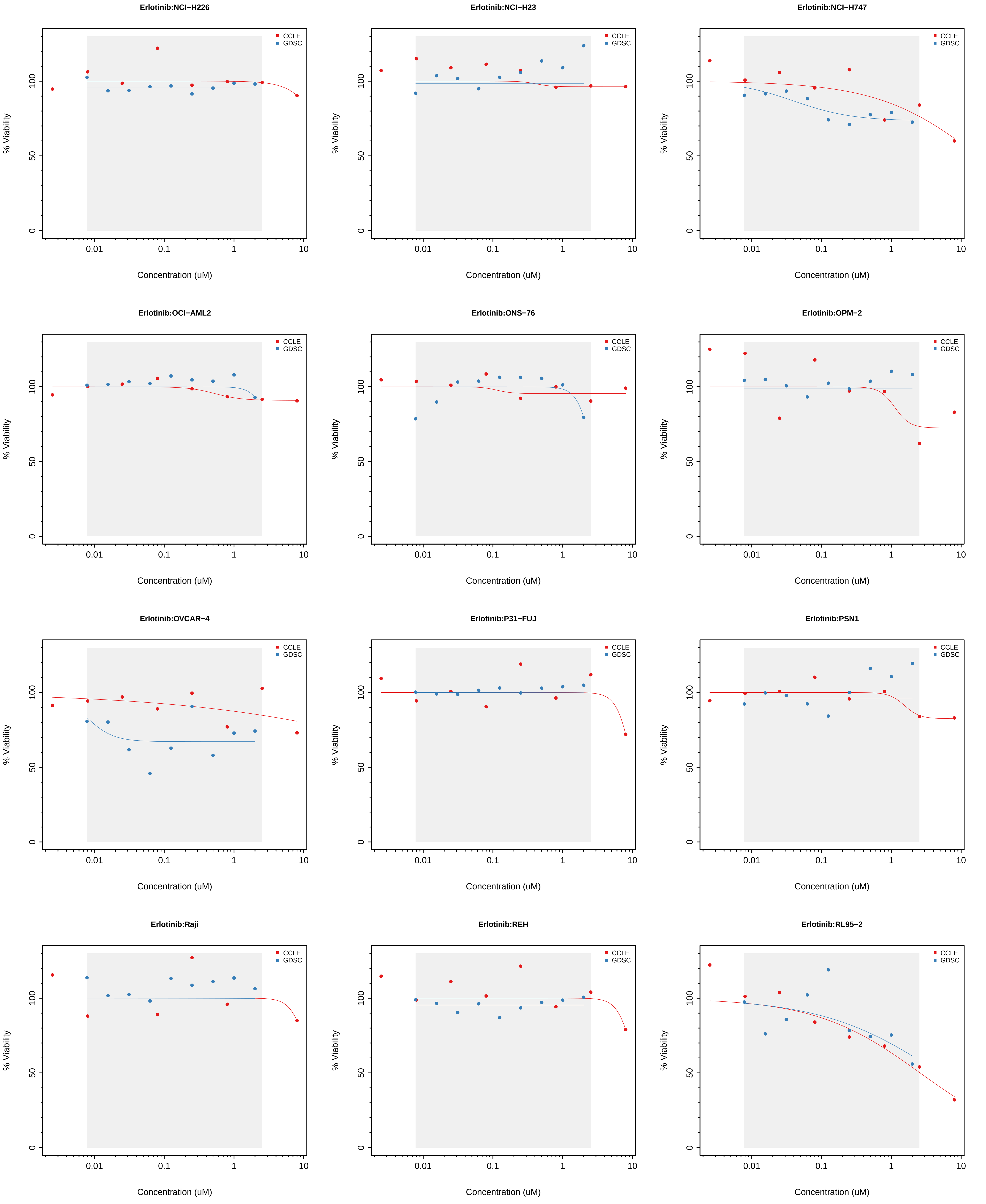
Erlotinib:NCI−H226
Erlotinib:NCI−H23
Erlotinib:NCI−H747
Erlotinib:OCI−AML2
Erlotinib:ONS−76
Erlotinib:OPM−2
Erlotinib:OVCAR−4
Erlotinib:P31−FUJ
Erlotinib:PSN1
Erlotinib:Raji
Erlotinib:REH
Erlotinib:RL95−2
CCLE
GDSC
% Viability
0
50
100
0.01
0.1
1
10
Concentration (uM)

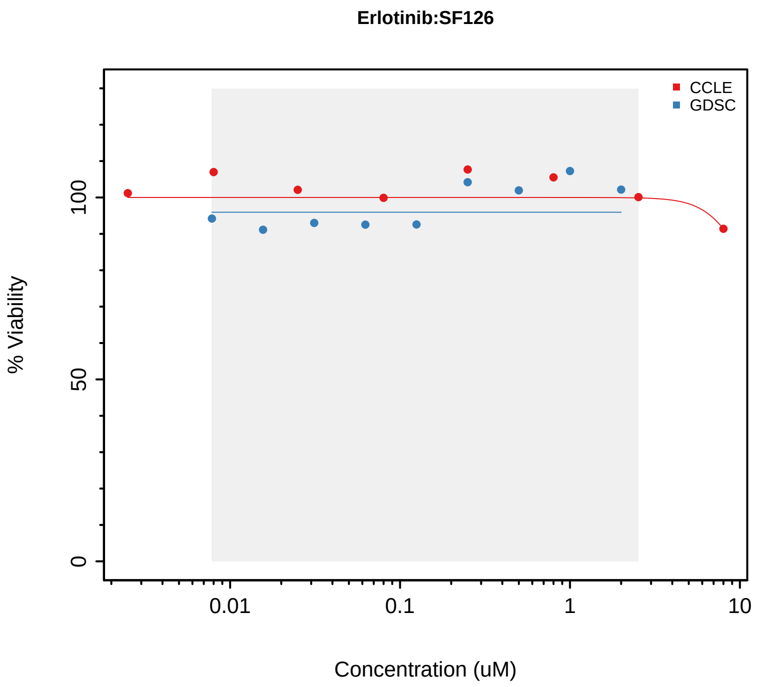
Erlotinib:SF126
CCLE
GDSC
% Viability
0
50
100
0.01
0.1
1
10
Concentration (uM)

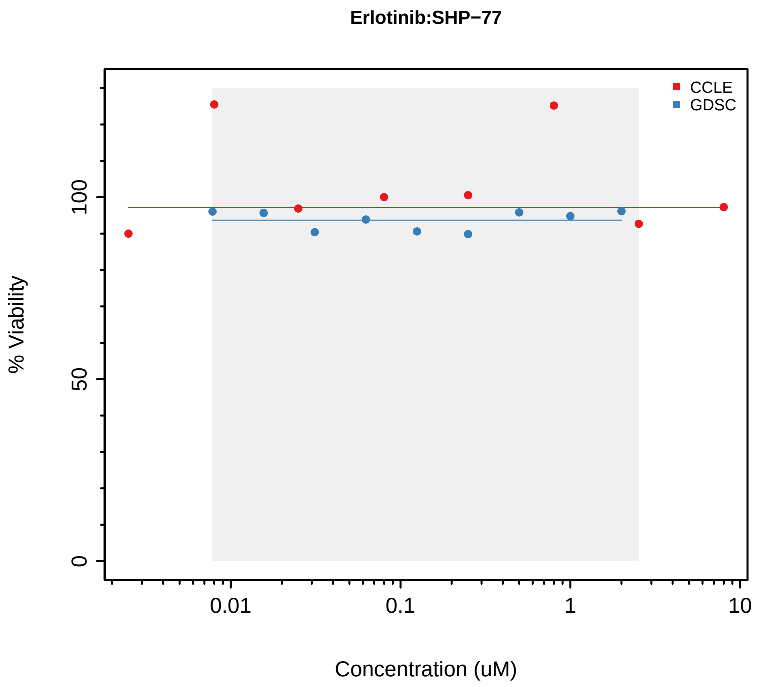
Erlotinib:SHP−77
CCLE
GDSC
% Viability
0
50
100
0.01
0.1
1
10
Concentration (uM)

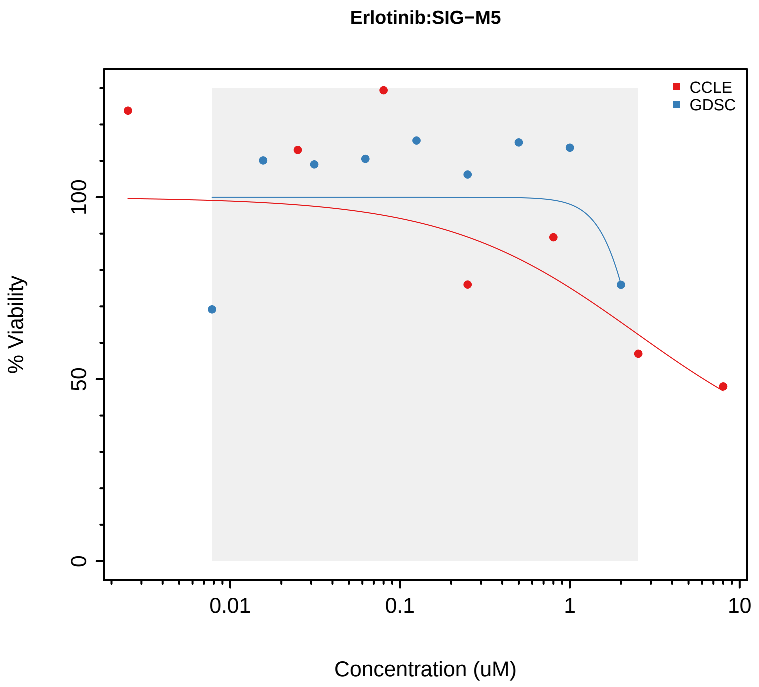
Erlotinib:SIG−M5
CCLE
GDSC
% Viability
0
50
100
0.01
0.1
1
10
Concentration (uM)

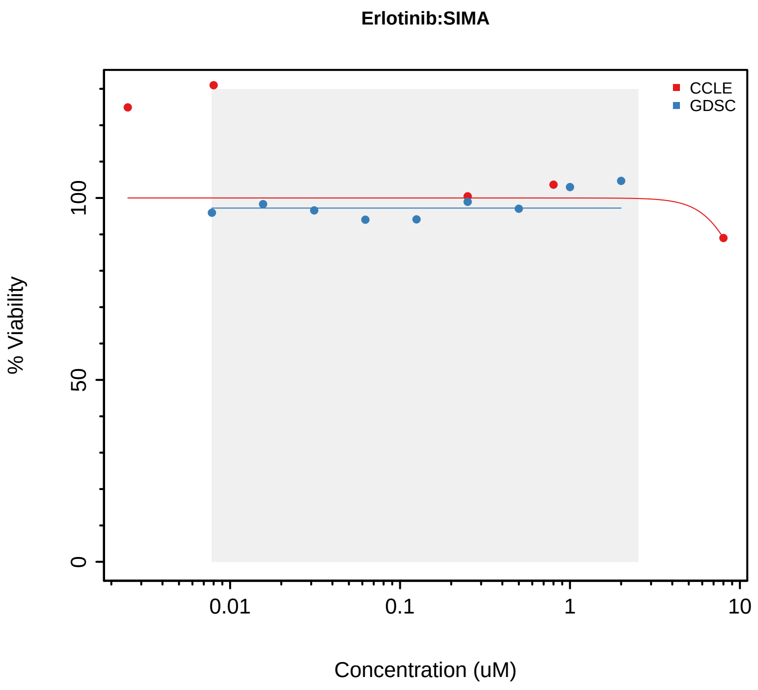
Erlotinib:SIMA
CCLE
GDSC
% Viability
0
50
100
0.01
0.1
1
10
Concentration (uM)

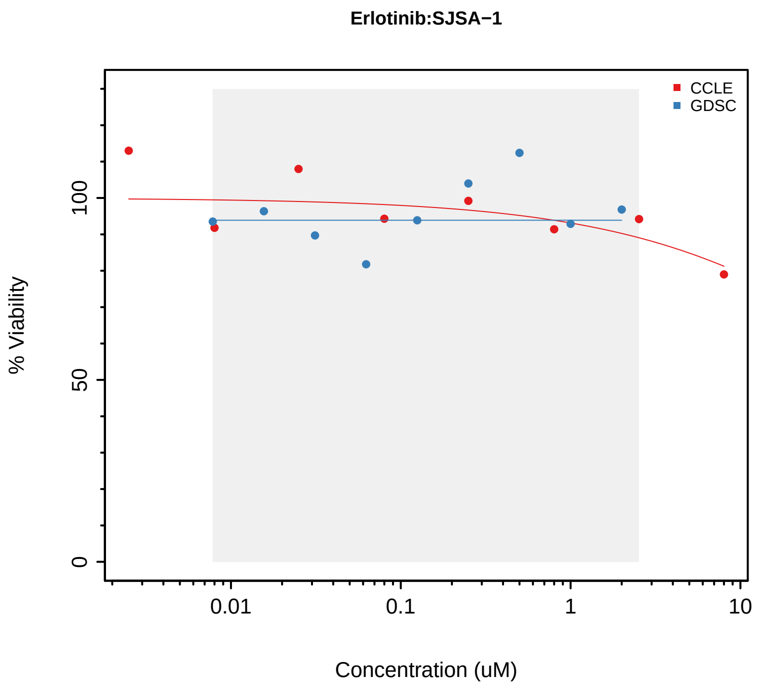
Erlotinib:SJSA−1
CCLE
GDSC
% Viability
0
50
100
0.01
0.1
1
10
Concentration (uM)

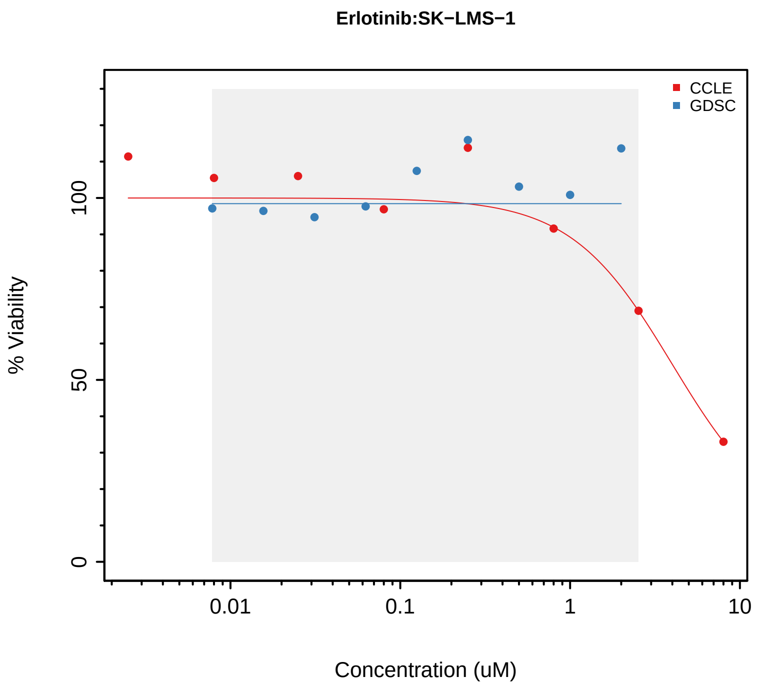
Erlotinib:SK−LMS−1
CCLE
GDSC
% Viability
0
50
100
0.01
0.1
1
10
Concentration (uM)

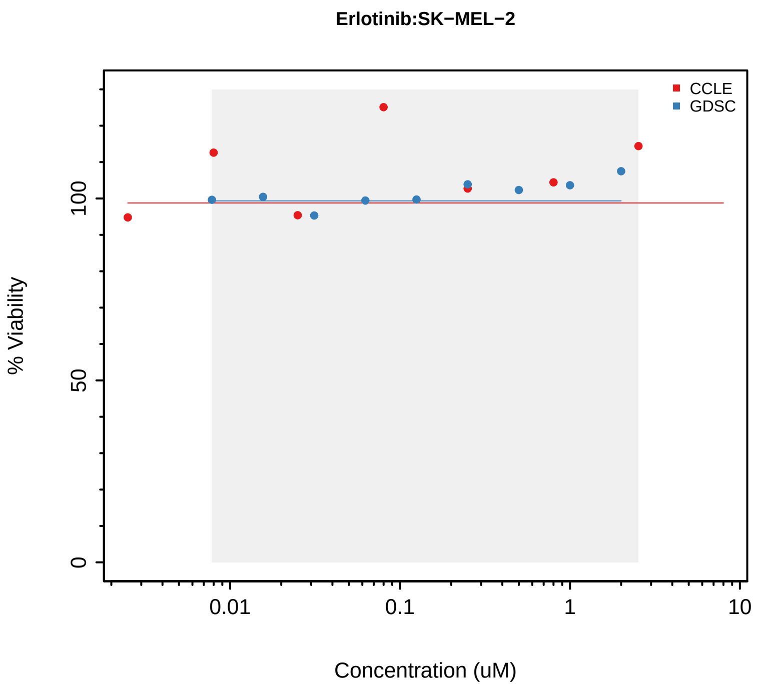
Erlotinib:SK−MEL−2
CCLE
GDSC
% Viability
0
50
100
0.01
0.1
1
10
Concentration (uM)

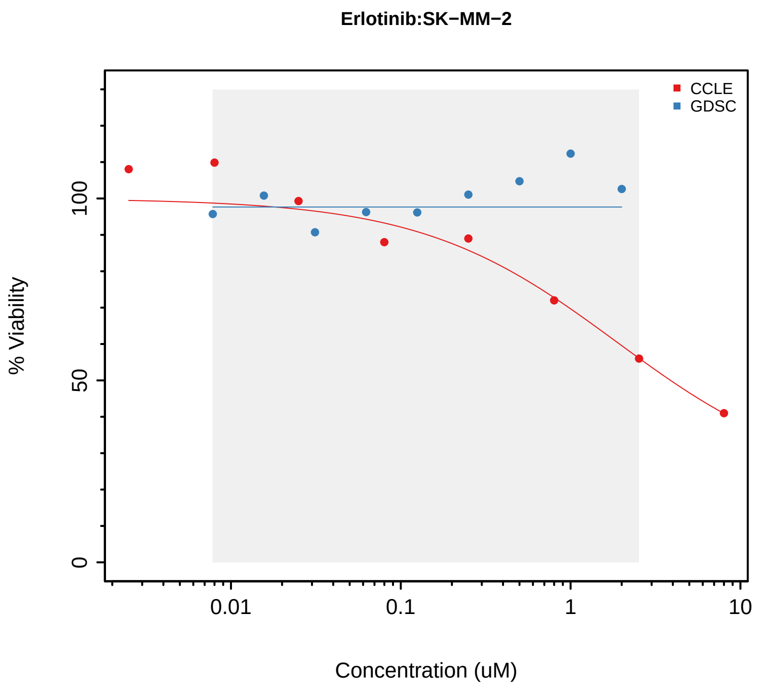
Erlotinib:SK−MM−2
CCLE
GDSC
% Viability
0
50
100
0.01
0.1
1
10
Concentration (uM)

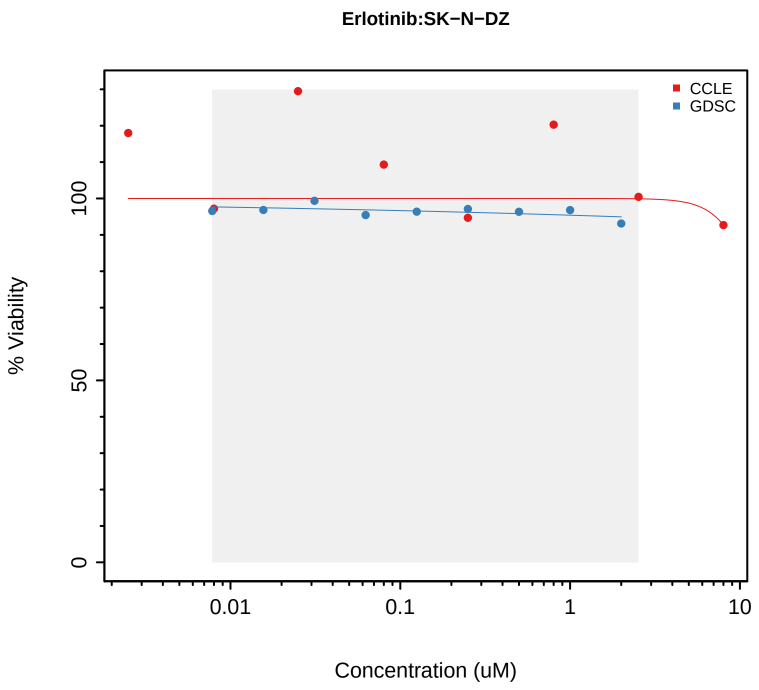
Erlotinib:SK−N−DZ
CCLE
GDSC
% Viability
0
50
100
0.01
0.1
1
10
Concentration (uM)

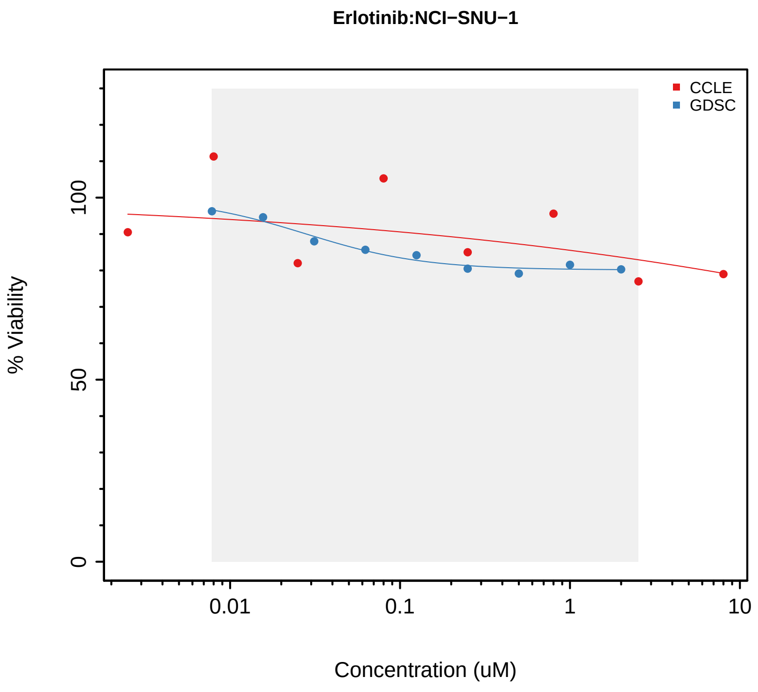
Erlotinib:NCI−SNU−1
CCLE
GDSC
% Viability
0
50
100
0.01
0.1
1
10
Concentration (uM)

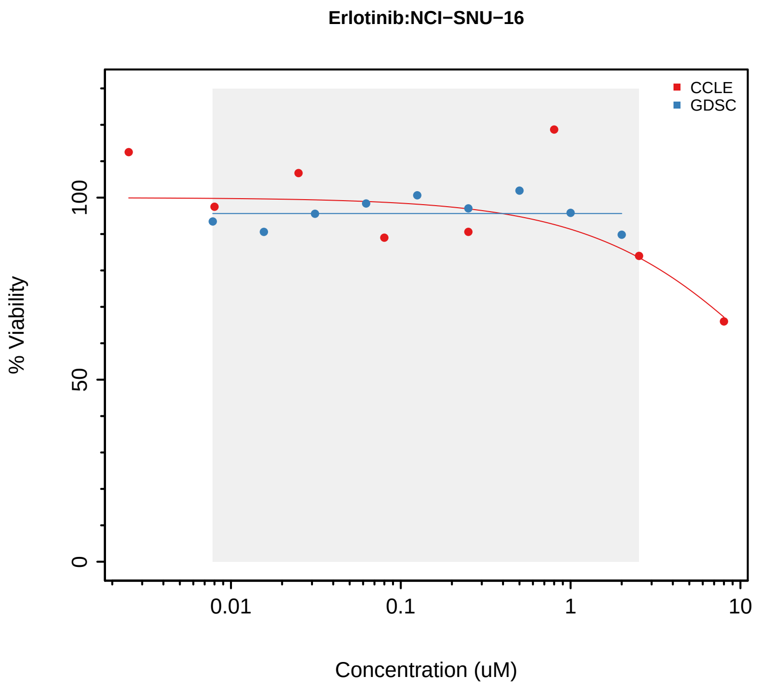
Erlotinib:NCI−SNU−16
CCLE
GDSC
% Viability
0
50
100
0.01
0.1
1
10
Concentration (uM)

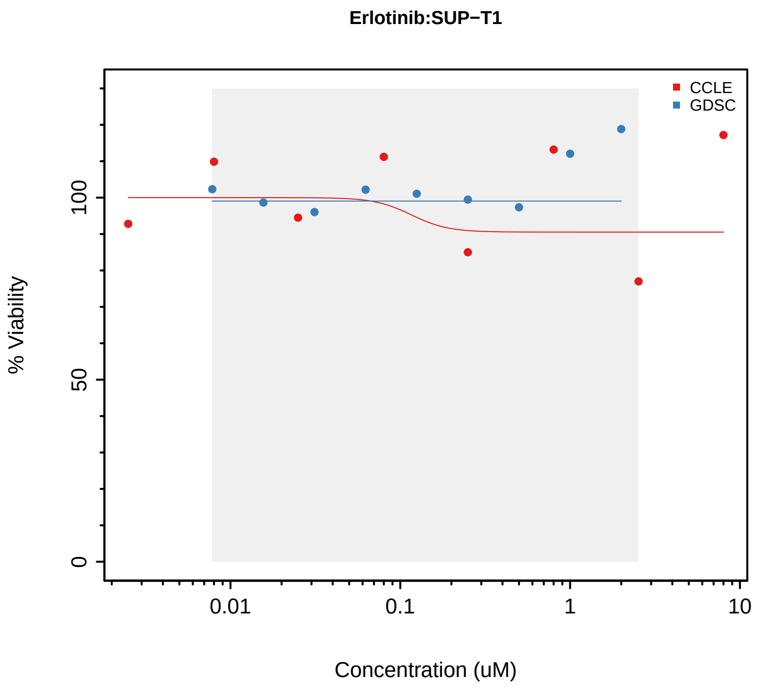
Erlotinib:SUP−T1
CCLE
GDSC
% Viability
0
50
100
0.01
0.1
1
10
Concentration (uM)

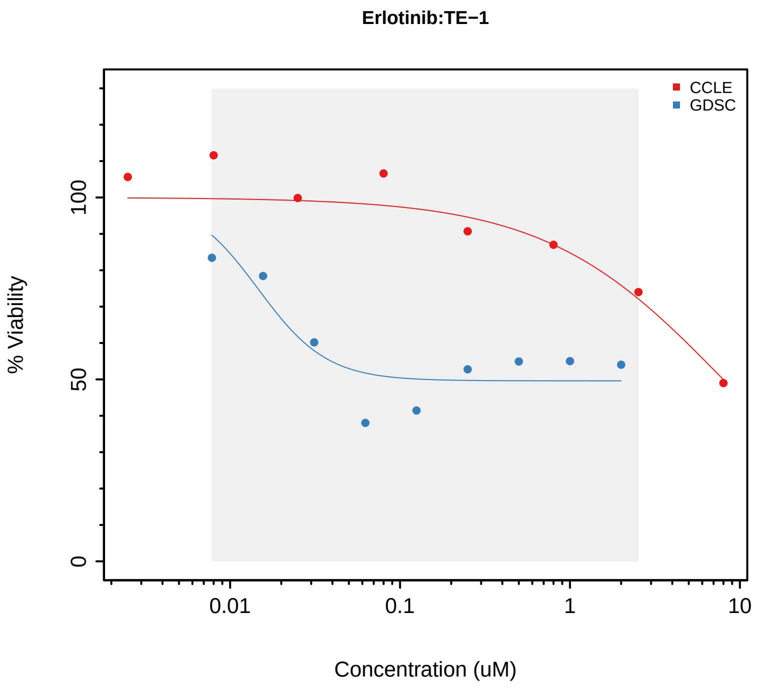
Erlotinib:TE−1
CCLE
GDSC
% Viability
0
50
100
0.01
0.1
1
10
Concentration (uM)

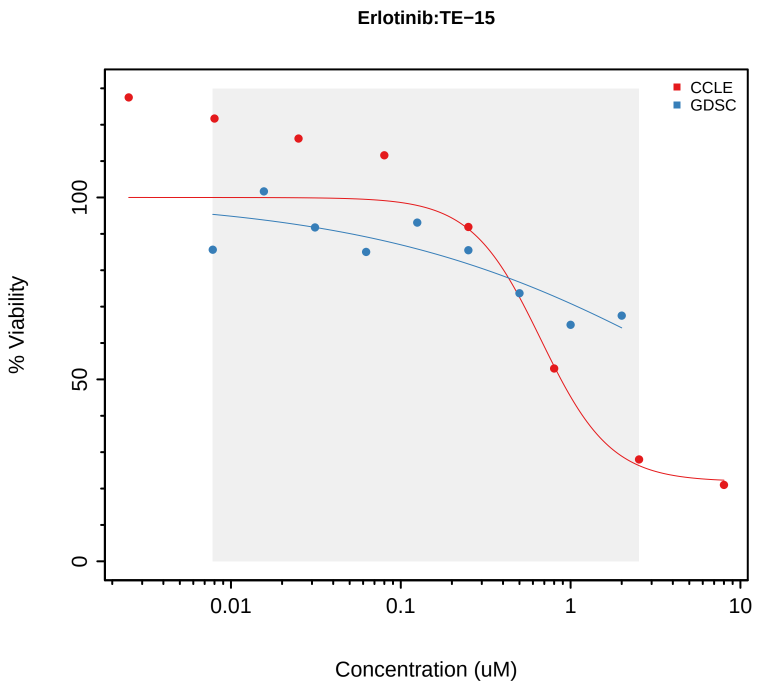
Erlotinib:TE−15
CCLE
GDSC
% Viability
0
50
100
0.01
0.1
1
10
Concentration (uM)

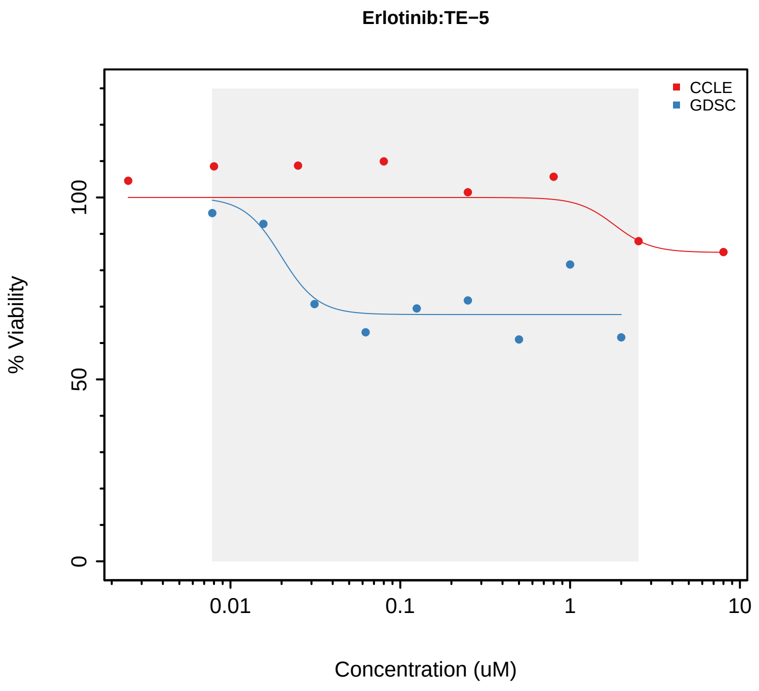
Erlotinib:TE−5
CCLE
GDSC
% Viability
0
50
100
0.01
0.1
1
10
Concentration (uM)

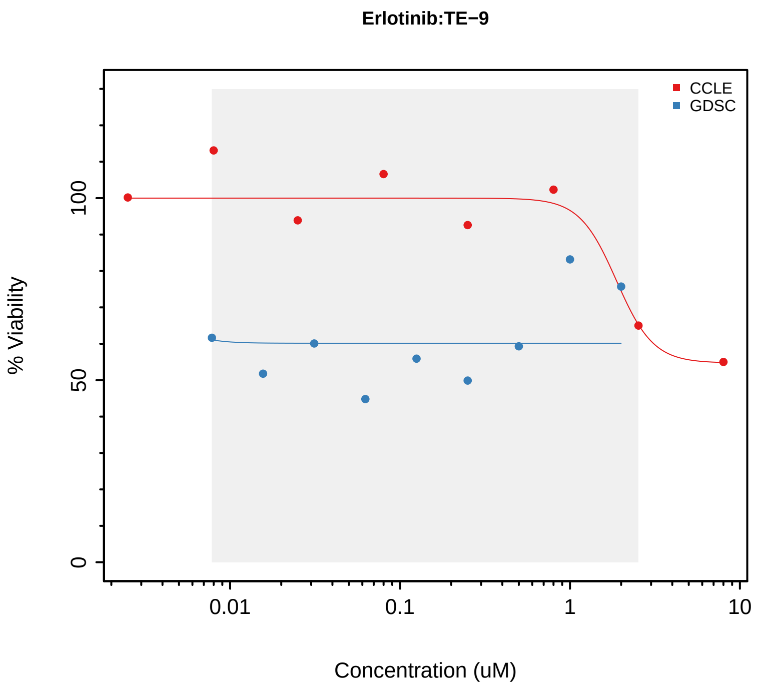
Erlotinib:TE−9
CCLE
GDSC
% Viability
0
50
100
0.01
0.1
1
10
Concentration (uM)

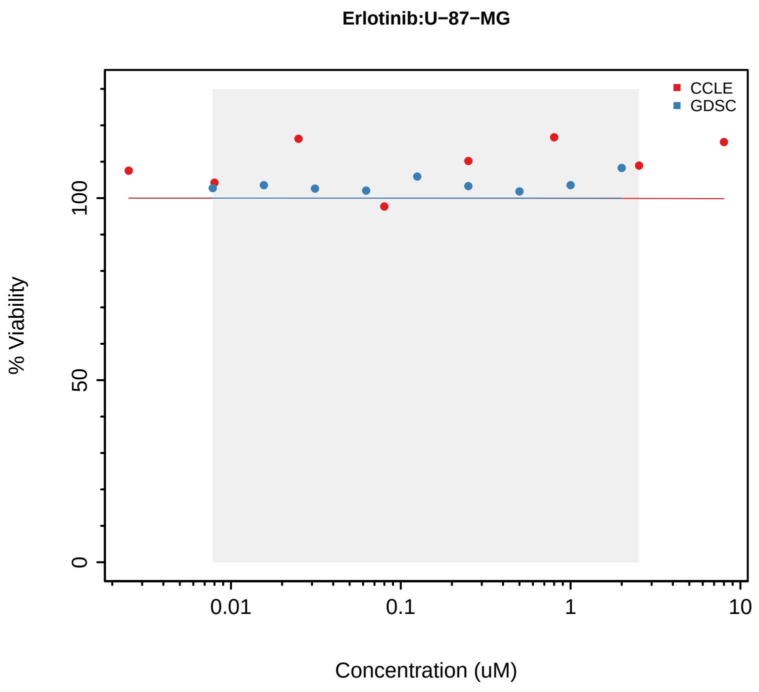
Erlotinib:U−87−MG
CCLE
GDSC
% Viability
0
50
100
0.01
0.1
1
10
Concentration (uM)

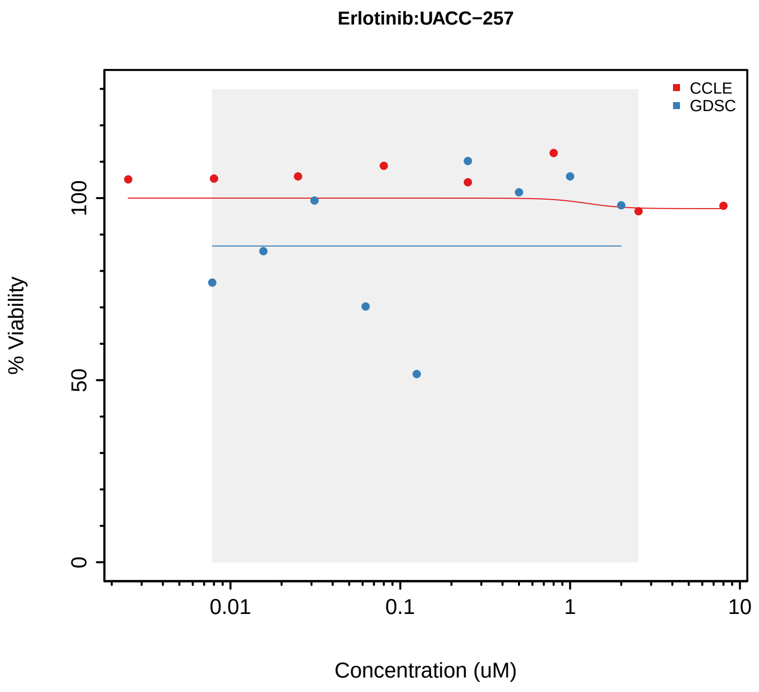
Erlotinib:UACC−257
CCLE
GDSC
% Viability
0
50
100
0.01
0.1
1
10
Concentration (uM)

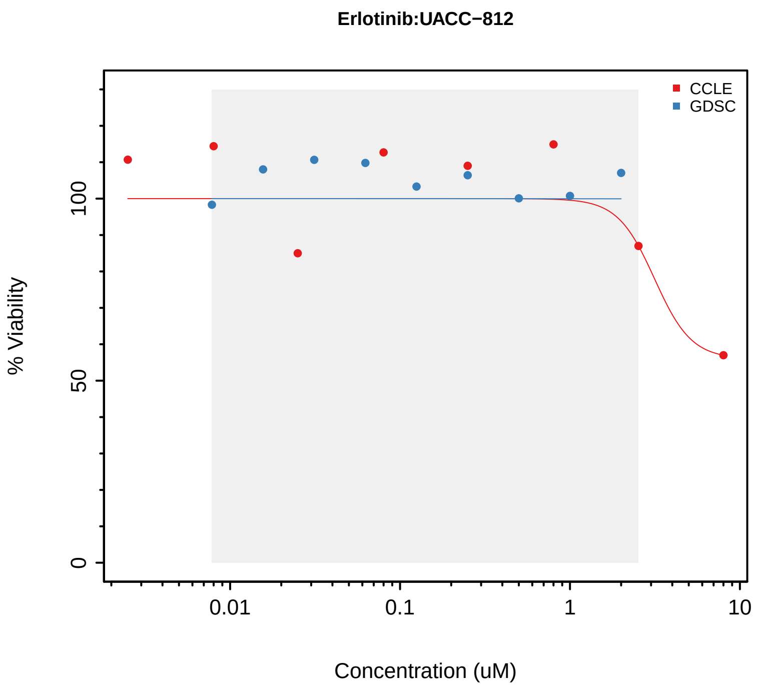
Erlotinib:UACC−812
CCLE
GDSC
% Viability
0
50
100
0.01
0.1
1
10
Concentration (uM)
