# Supplementary material for: Revisiting inconsistency in large pharmacogenomic studies
Source: F1000Res. 2017 Aug 11;5:2333. Originally published 2016 Sep 16. [Version 3] doi: 10.12688/f1000research.9611.3 (PMC5580432; doi:10.12688/f1000research.9611.3)
Supplement: Supplementary file 3 [file f1000research-5-13399-s0002.tgz › c256fb6e-04f3-48e7-bf9d-a41e91f393da.pdf]

AZD6482:UO31

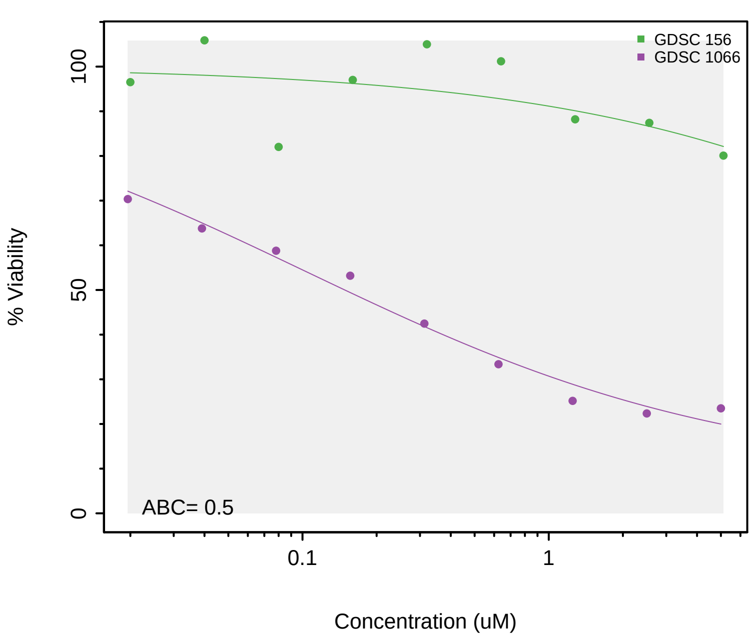

AZD6482:A498

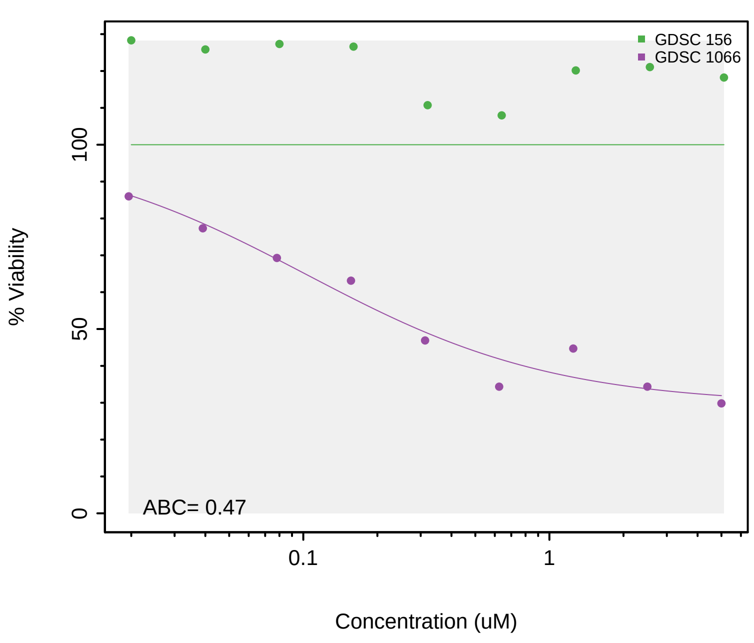

AZD6482:KURAMOCHI

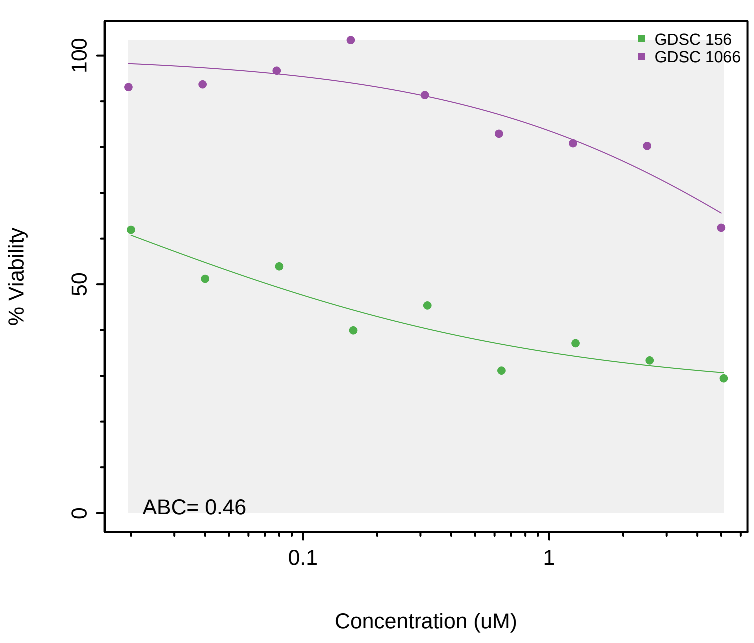

AZD6482:RPMI−8866

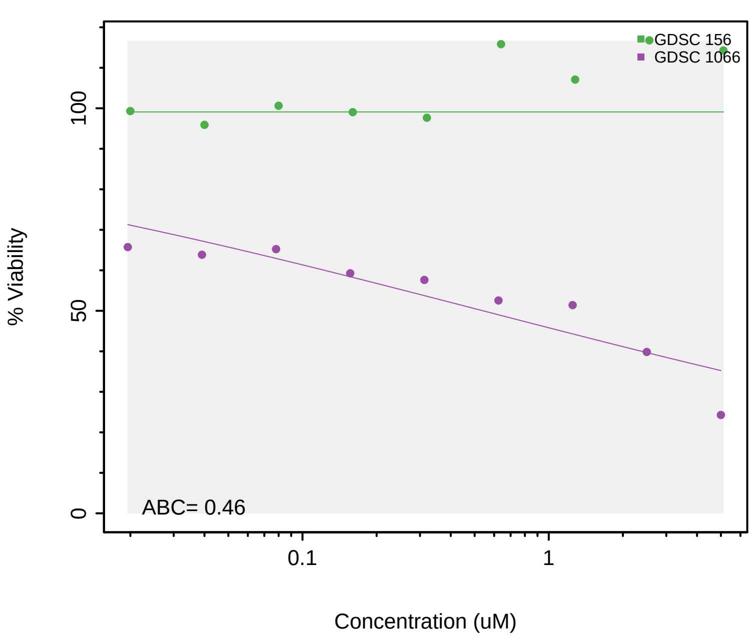

AZD6482:DG−75

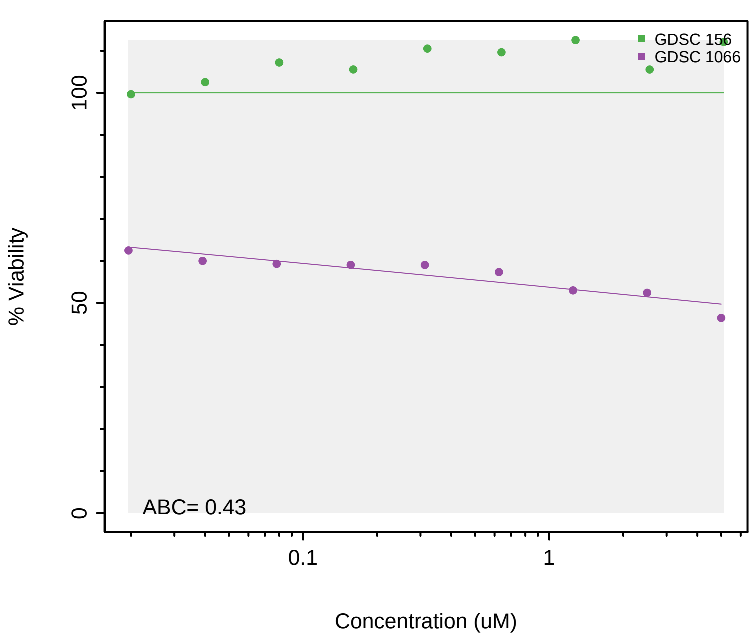

AZD6482:J82

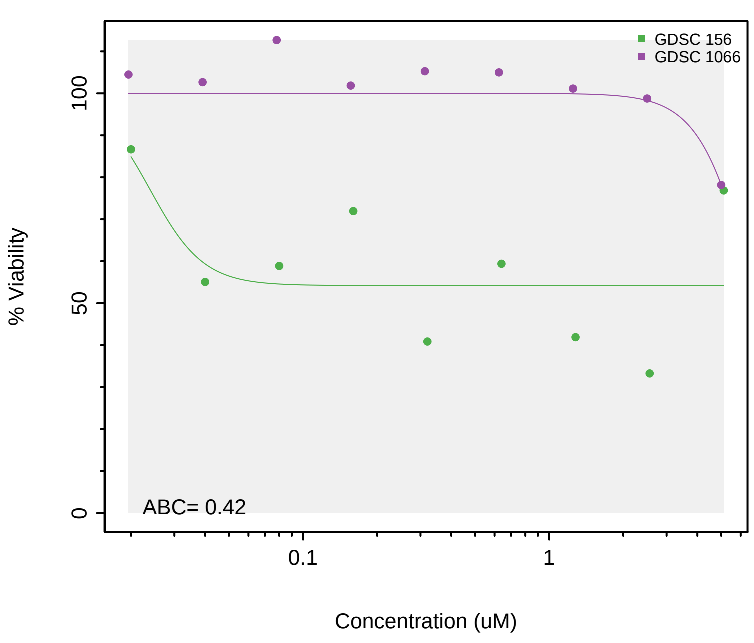

AZD6482:LB996−RCC

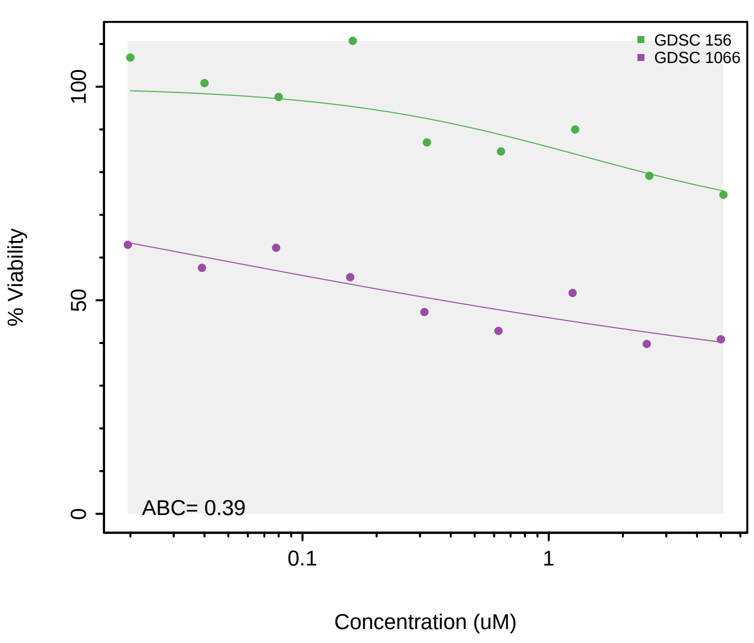

AZD6482:EoL−1−cell

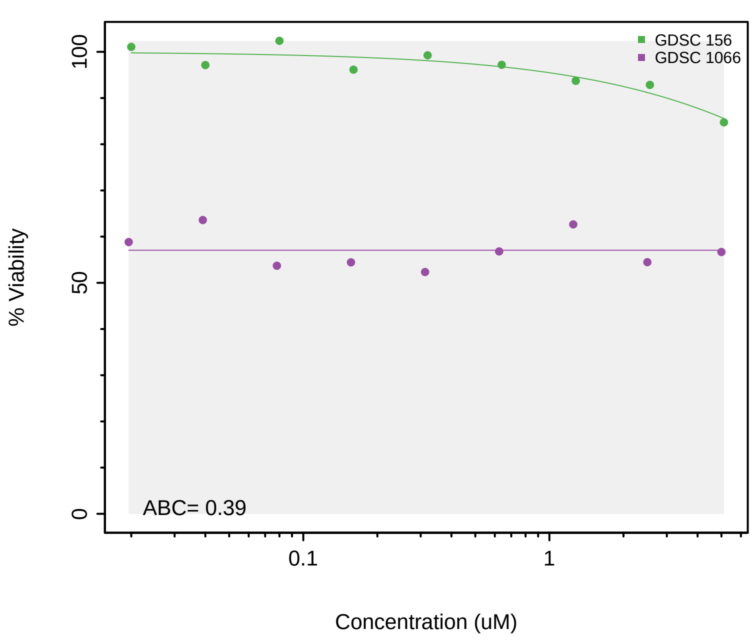

AZD6482:NCI−H2291

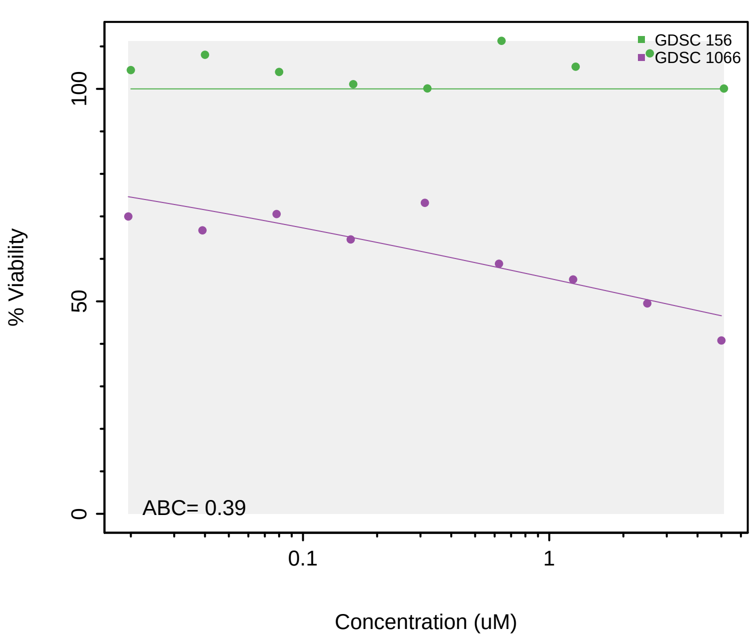

AZD6482:CCF−STTG1

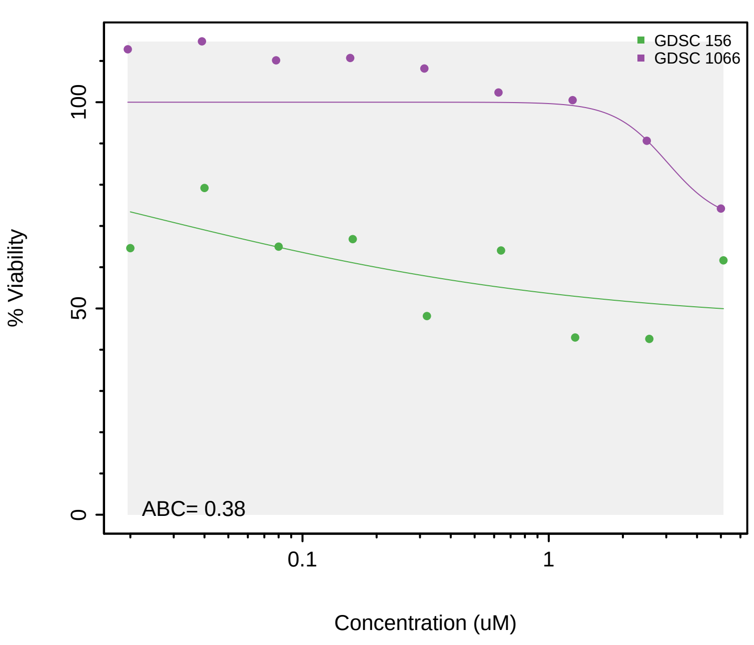

AZD6482:RXF393

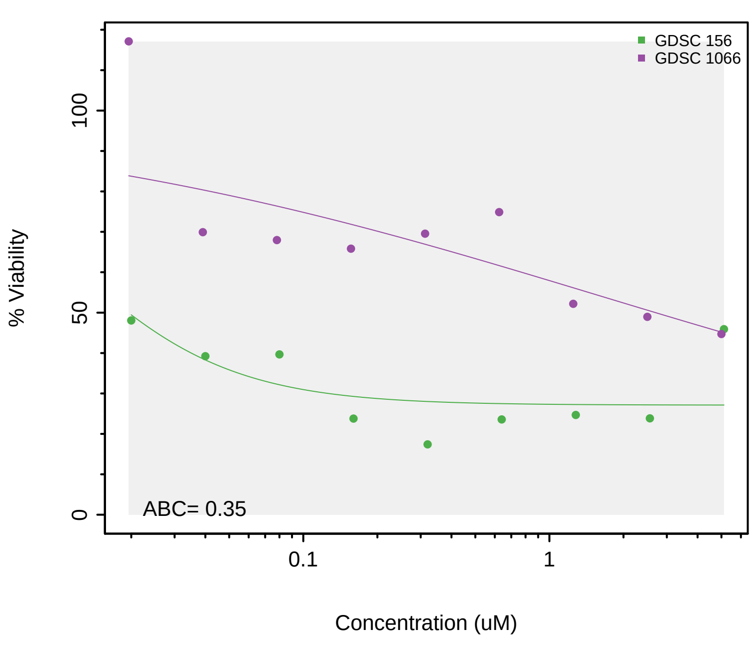

AZD6482:DU−145

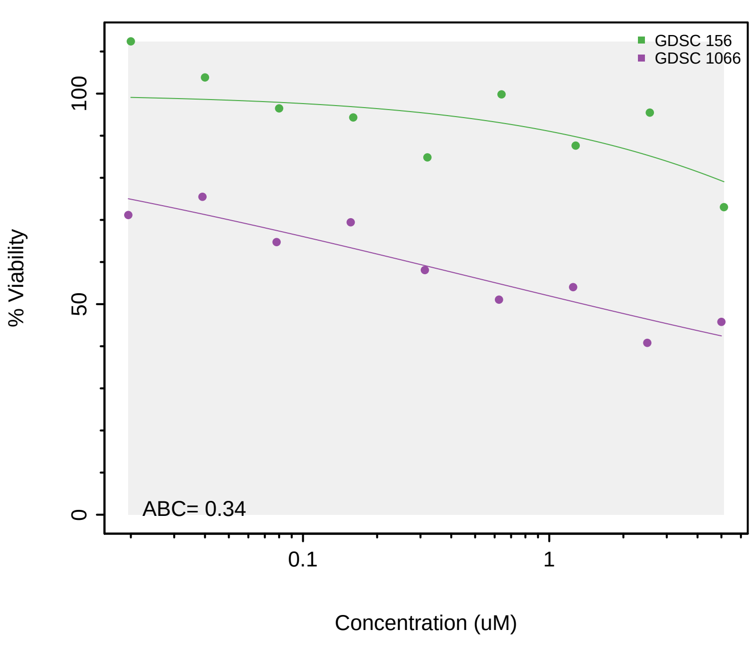

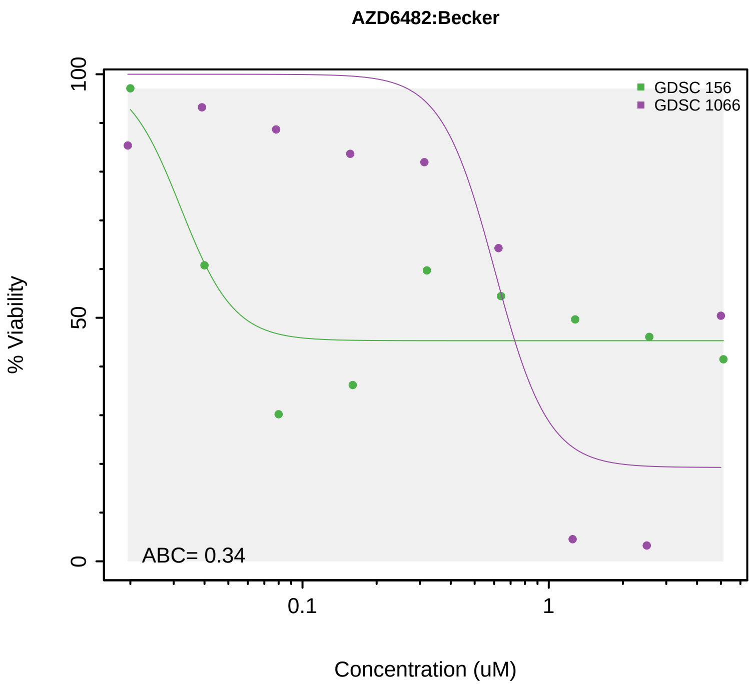
AZD6482:Becker
% Viability
Concentration (uM)
GDSC 156
GDSC 1066
ABC= 0.34

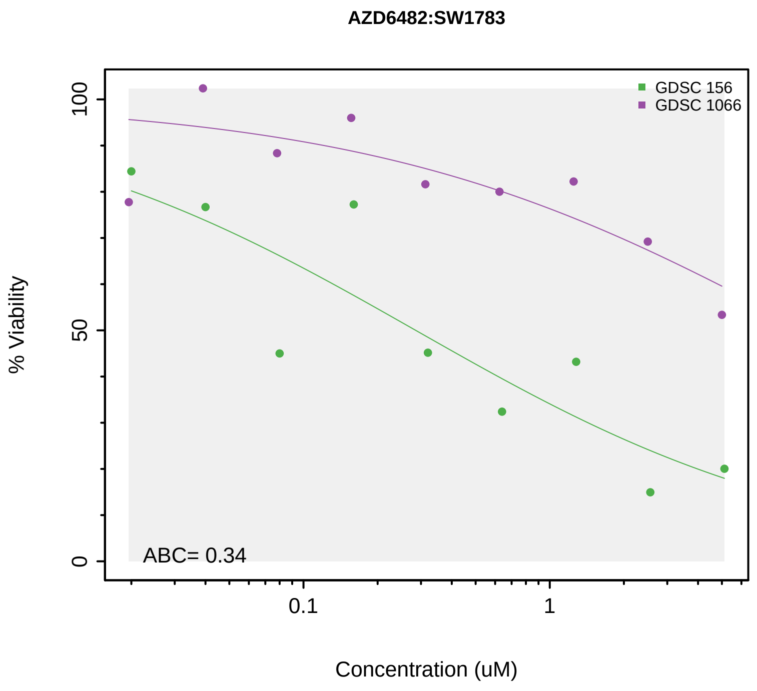
AZD6482:SW1783
% Viability
Concentration (uM)
GDSC 156
GDSC 1066
ABC= 0.34

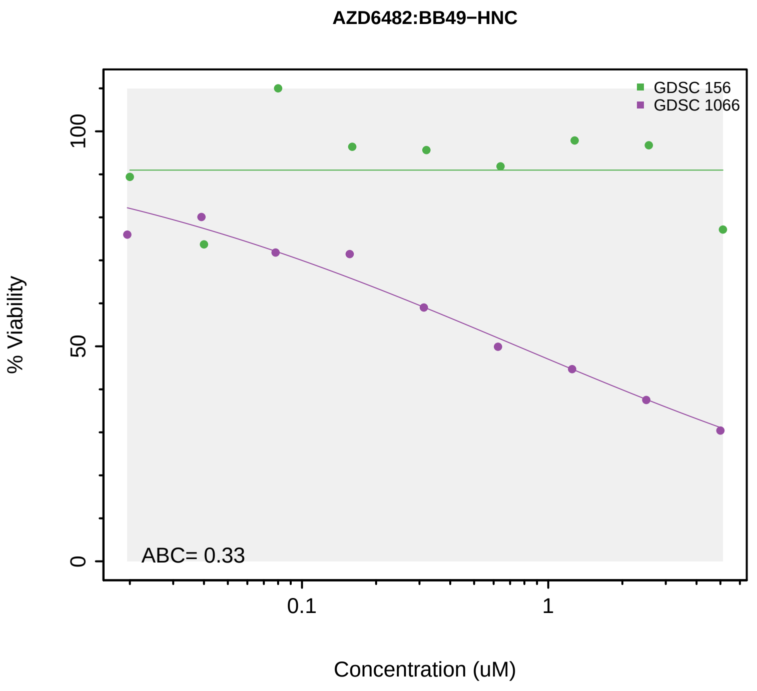
AZD6482:BB49−HNC
% Viability
Concentration (uM)
GDSC 156
GDSC 1066
ABC= 0.33

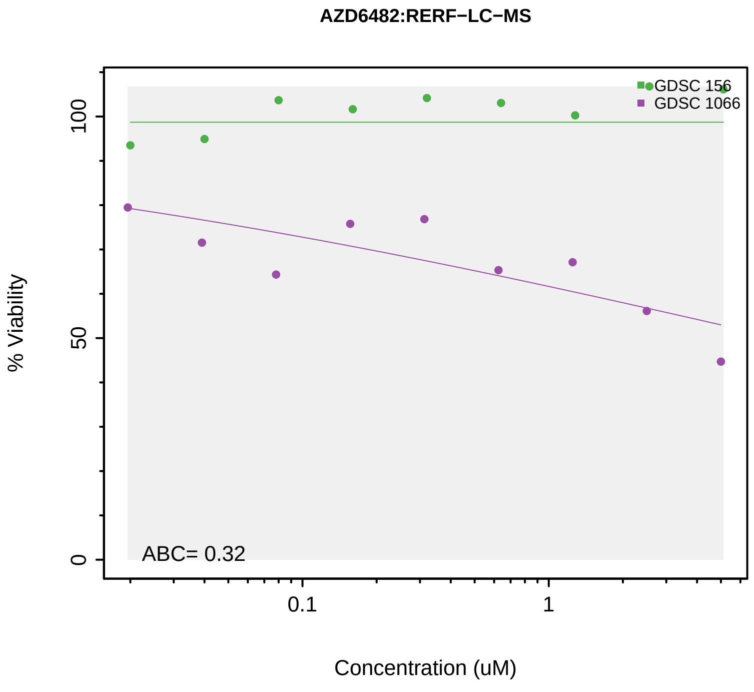
AZD6482:RERF−LC−MS
% Viability
Concentration (uM)
GDSC 156
GDSC 1066
ABC= 0.32

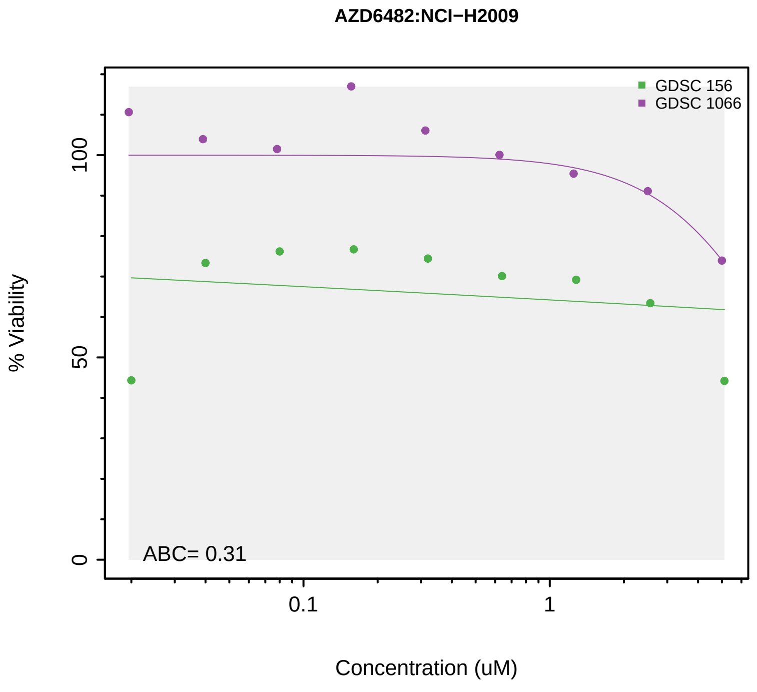
AZD6482:NCI−H2009
% Viability
Concentration (uM)
GDSC 156
GDSC 1066
ABC= 0.31

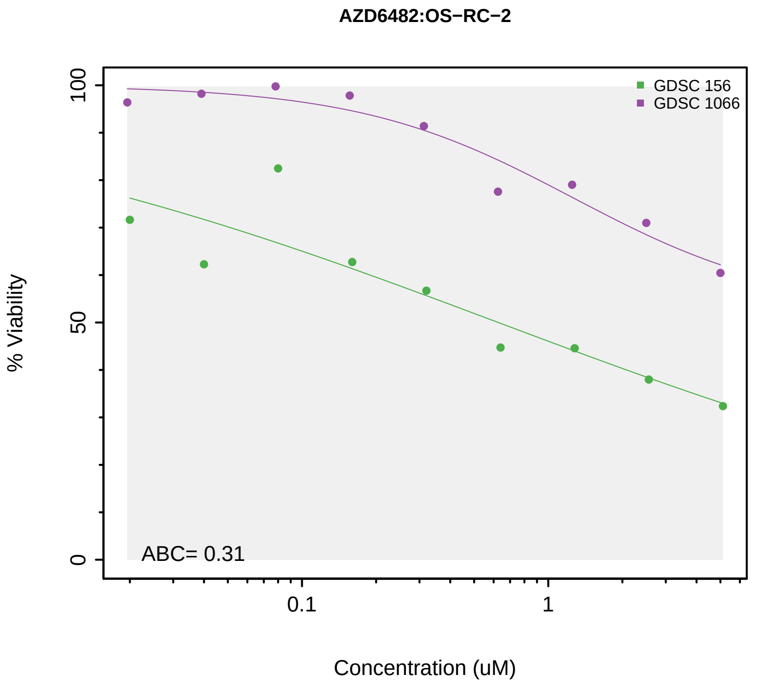
AZD6482:OS−RC−2
% Viability
Concentration (uM)
GDSC 156
GDSC 1066
ABC= 0.31

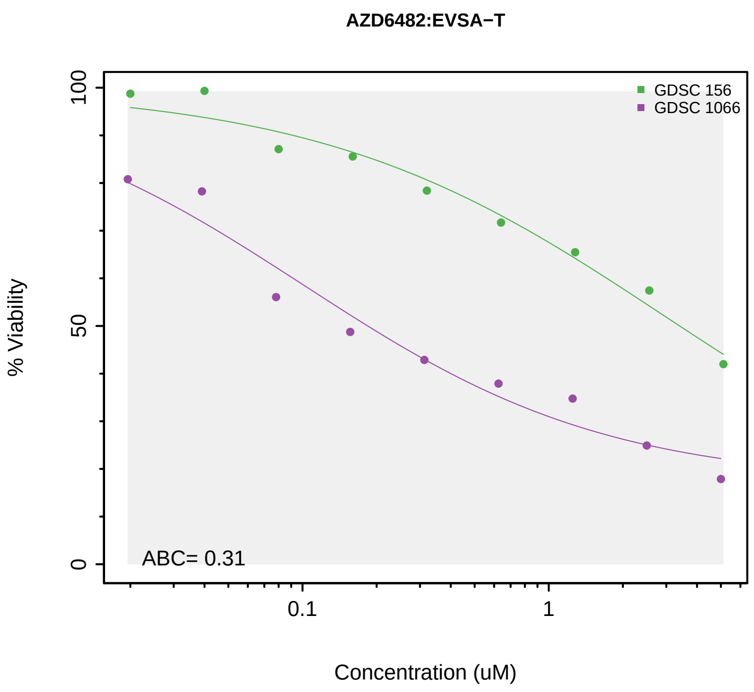
AZD6482:EVSA−T
% Viability
Concentration (uM)
GDSC 156
GDSC 1066
ABC= 0.31

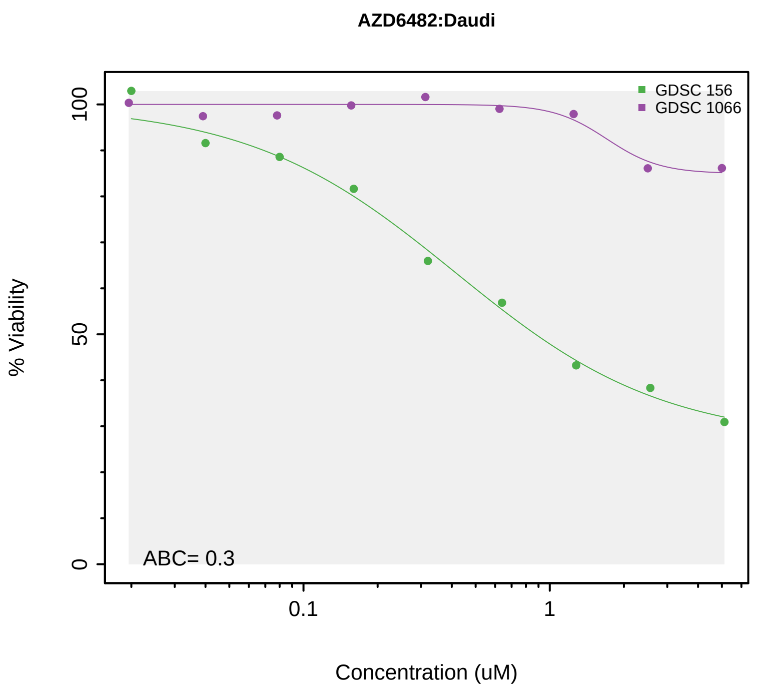
AZD6482:Daudi
% Viability
Concentration (uM)
GDSC 156
GDSC 1066
ABC= 0.3

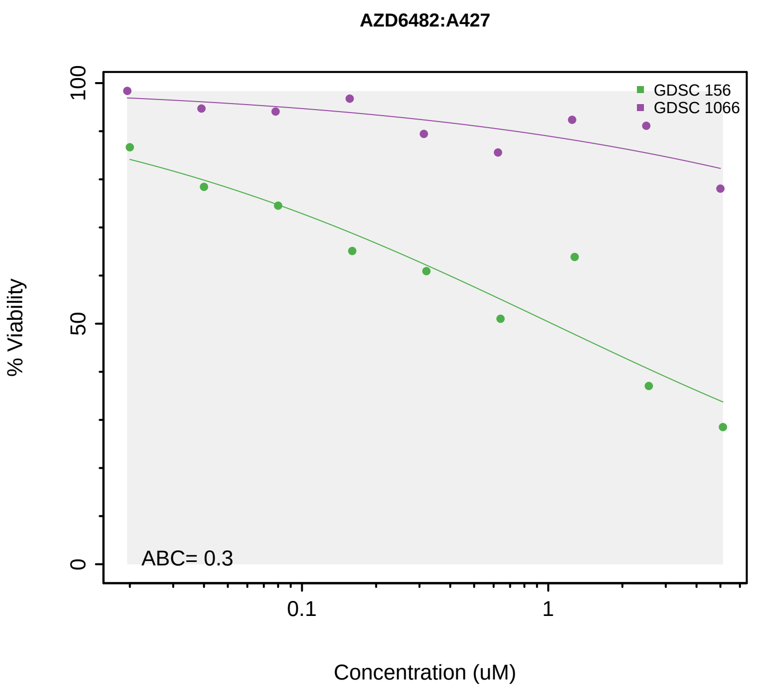
AZD6482:A427
% Viability
Concentration (uM)
GDSC 156
GDSC 1066
ABC= 0.3

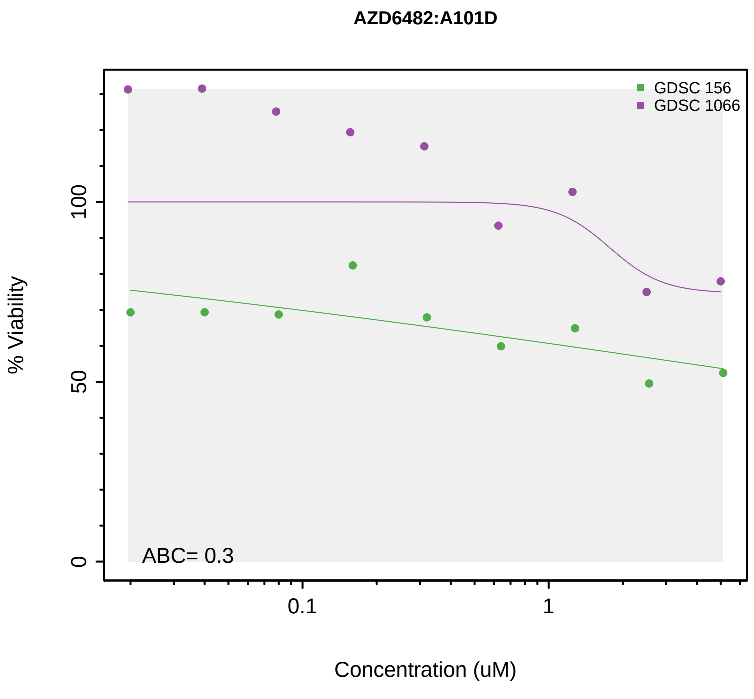
AZD6482:A101D
% Viability
Concentration (uM)
GDSC 156
GDSC 1066
ABC= 0.3

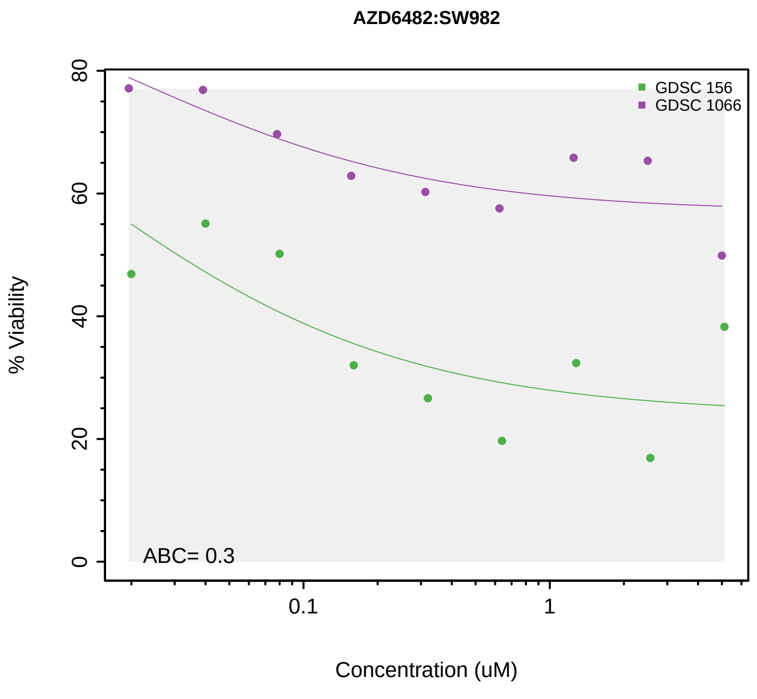
AZD6482:SW982
% Viability
Concentration (uM)
GDSC 156
GDSC 1066
ABC= 0.3

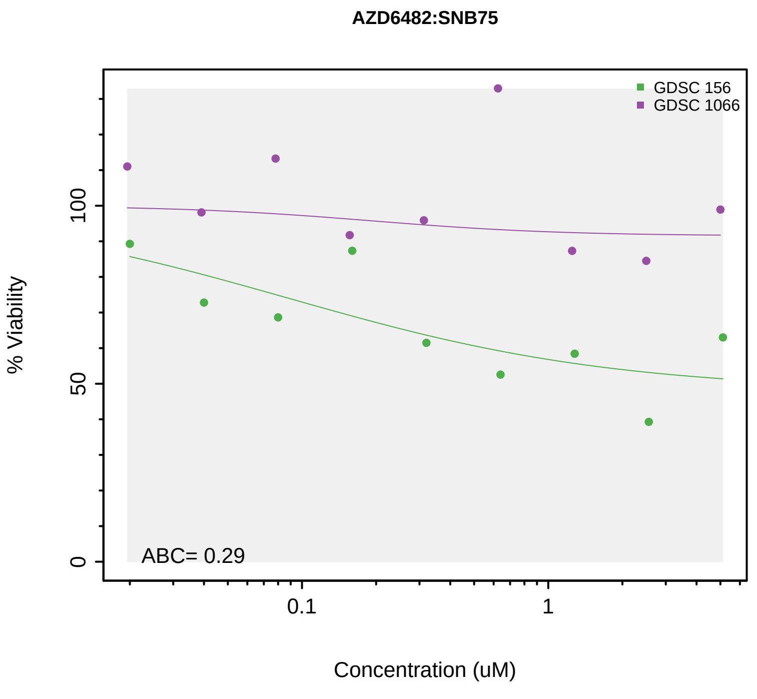
AZD6482:SNB75
% Viability
Concentration (uM)
GDSC 156
GDSC 1066
ABC= 0.29

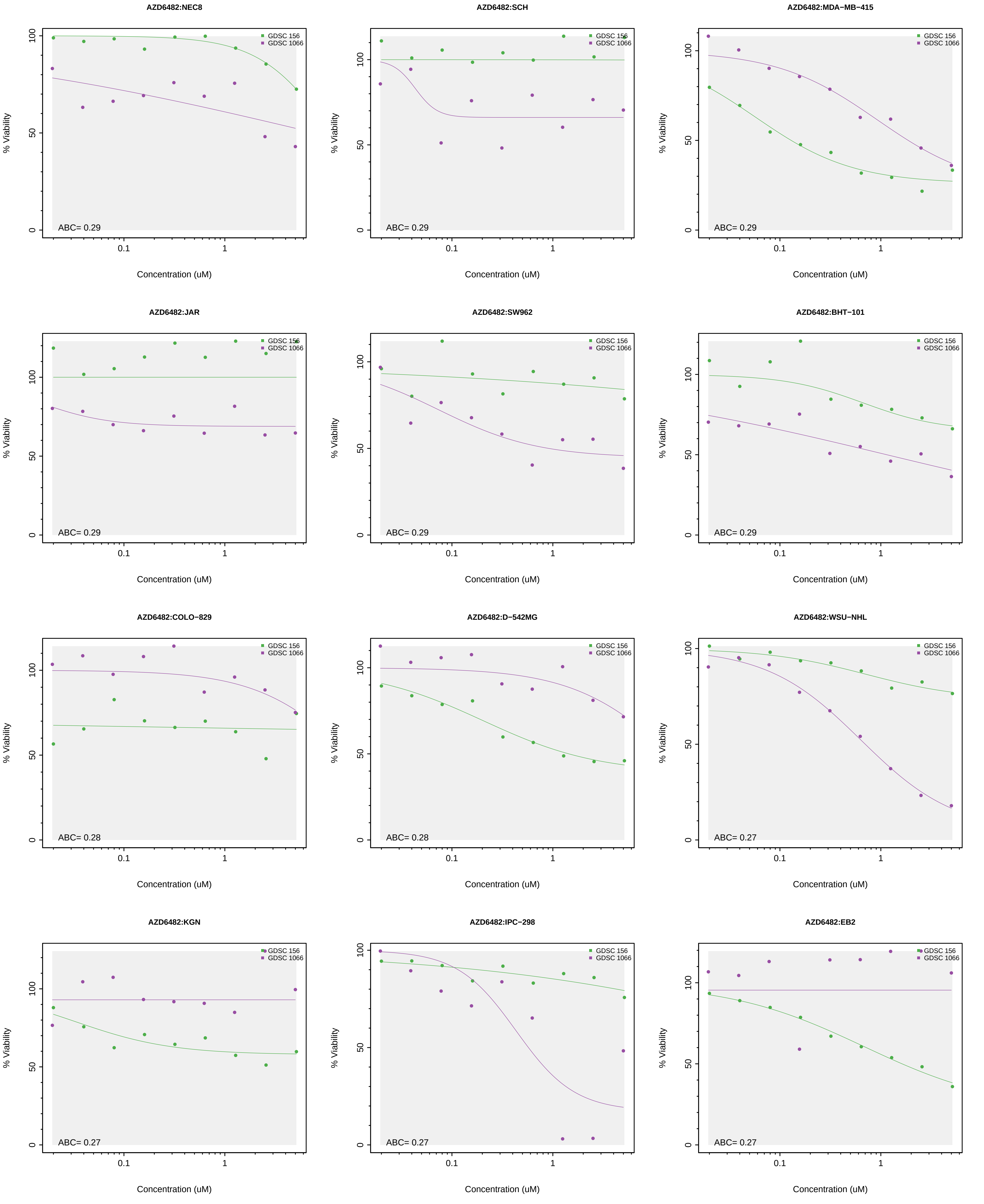

AZD6482:NEC8
ABC= 0.29
AZD6482:SCH
ABC= 0.29
AZD6482:MDA−MB−415
ABC= 0.29
AZD6482:JAR
ABC= 0.29
AZD6482:SW962
ABC= 0.29
AZD6482:BHT−101
ABC= 0.29
AZD6482:COLO−829
ABC= 0.28
AZD6482:D−542MG
ABC= 0.28
AZD6482:WSU−NHL
ABC= 0.27
AZD6482:KGN
ABC= 0.27
AZD6482:IPC−298
ABC= 0.27
AZD6482:EB2
ABC= 0.27
GDSC 156
GDSC 1066
% Viability
Concentration (uM)
0
50
100
0.1
1

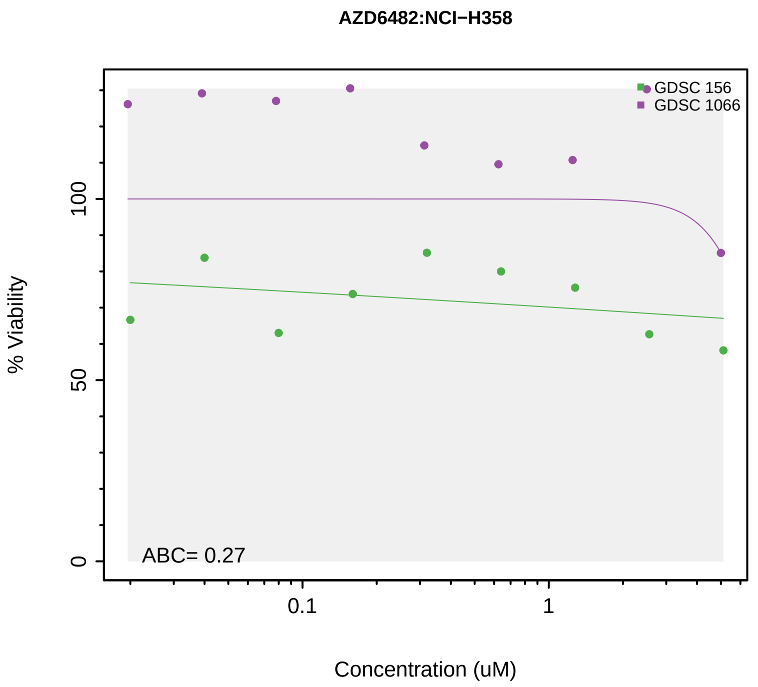
AZD6482:NCI−H358
GDSC 156
GDSC 1066
ABC= 0.27
% Viability
Concentration (uM)

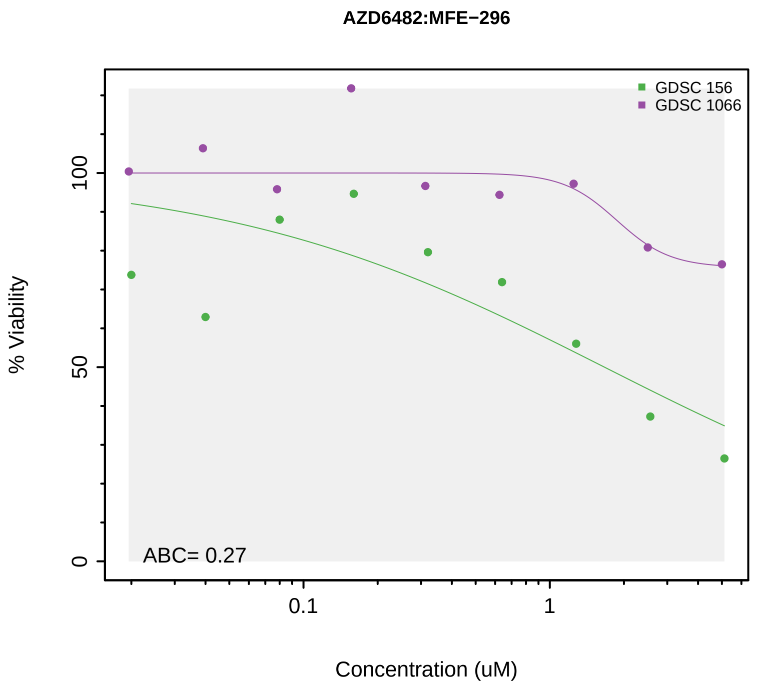
AZD6482:MFE−296
GDSC 156
GDSC 1066
ABC= 0.27
% Viability
Concentration (uM)

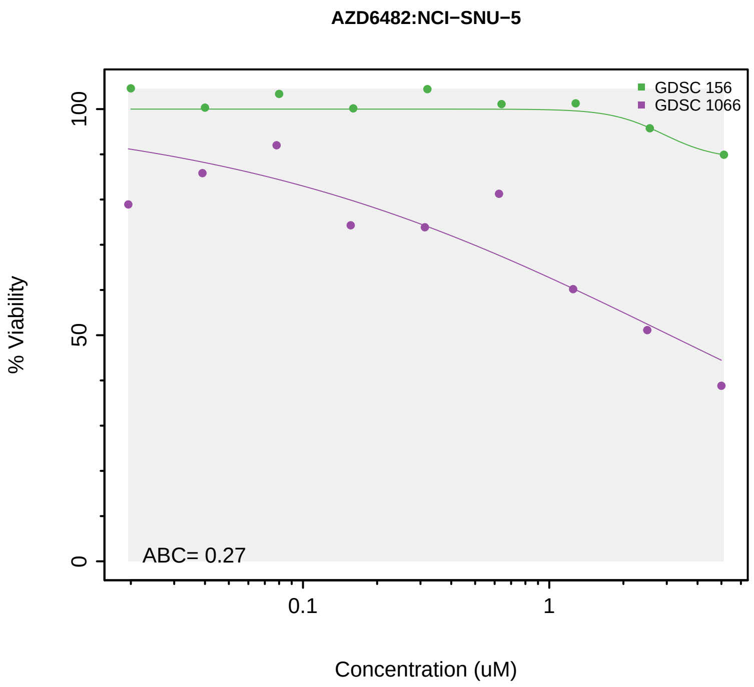
AZD6482:NCI−SNU−5
GDSC 156
GDSC 1066
ABC= 0.27
% Viability
Concentration (uM)

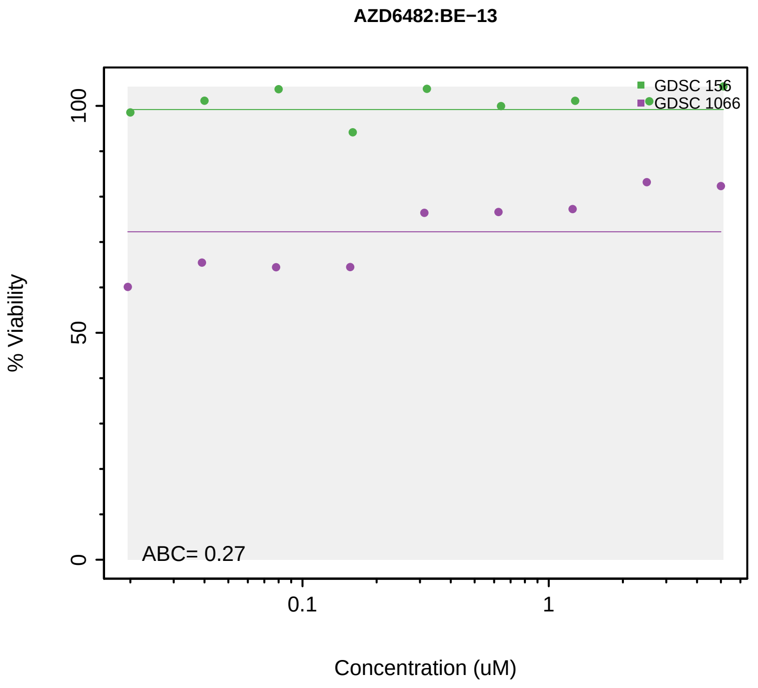
AZD6482:BE−13
GDSC 156
GDSC 1066
ABC= 0.27
% Viability
Concentration (uM)

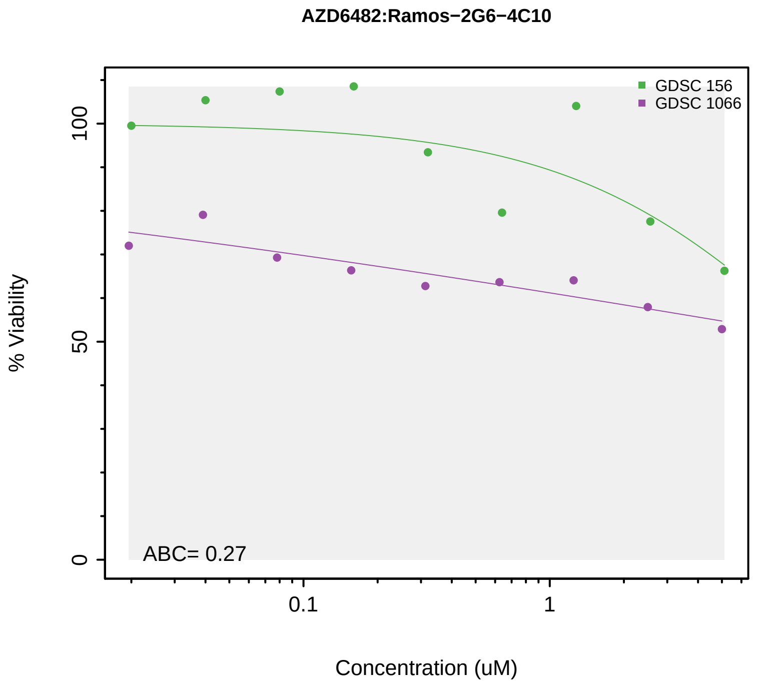
AZD6482:Ramos−2G6−4C10
GDSC 156
GDSC 1066
ABC= 0.27
% Viability
Concentration (uM)

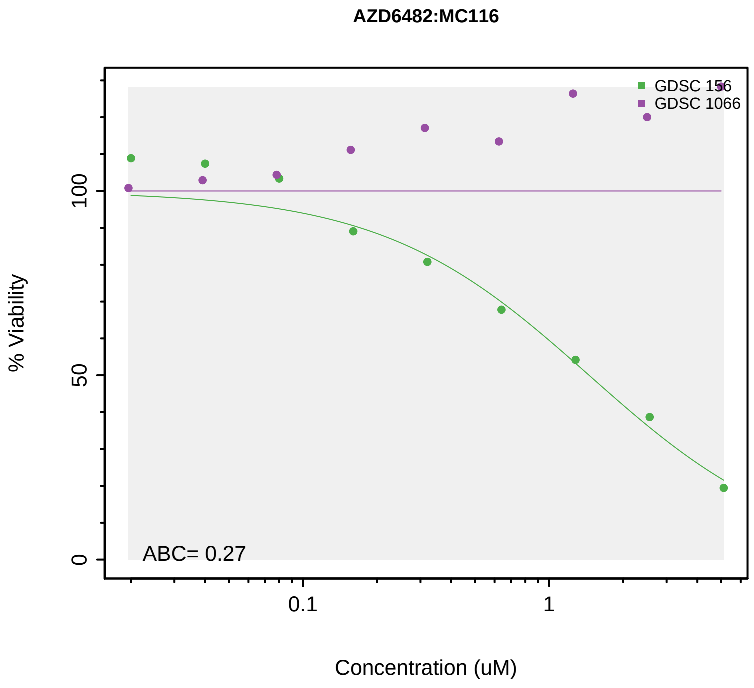
AZD6482:MC116
GDSC 156
GDSC 1066
ABC= 0.27
% Viability
Concentration (uM)

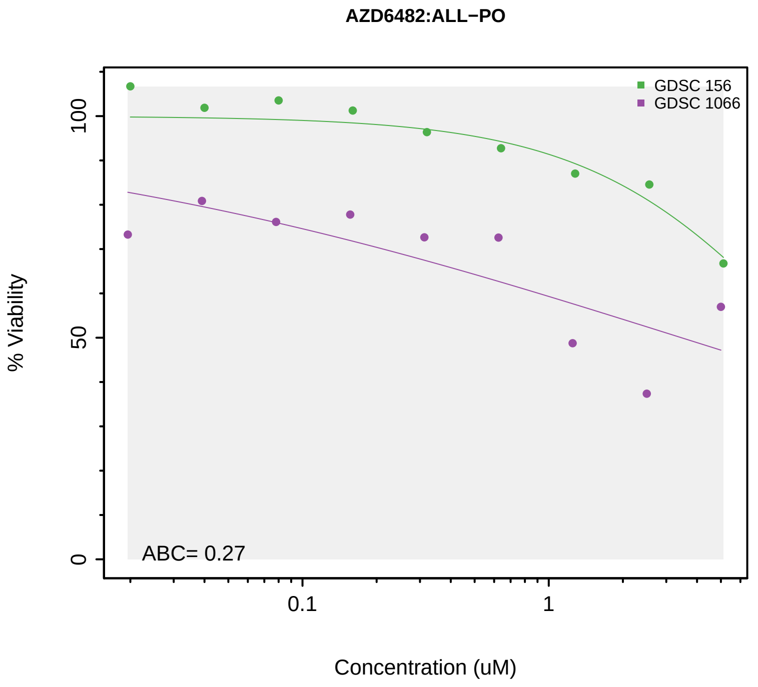
AZD6482:ALL−PO
GDSC 156
GDSC 1066
ABC= 0.27
% Viability
Concentration (uM)

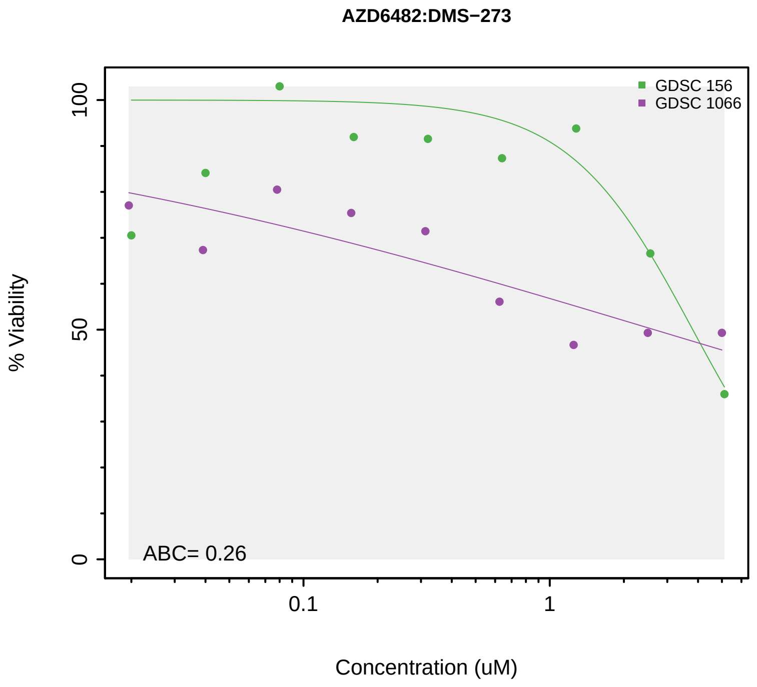
AZD6482:DMS−273
GDSC 156
GDSC 1066
ABC= 0.26
% Viability
Concentration (uM)

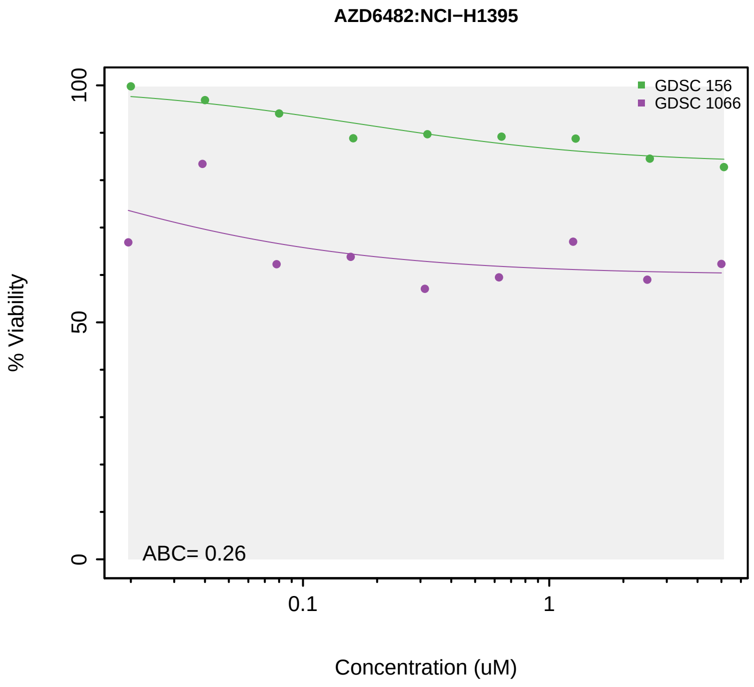
AZD6482:NCI−H1395
GDSC 156
GDSC 1066
ABC= 0.26
% Viability
Concentration (uM)

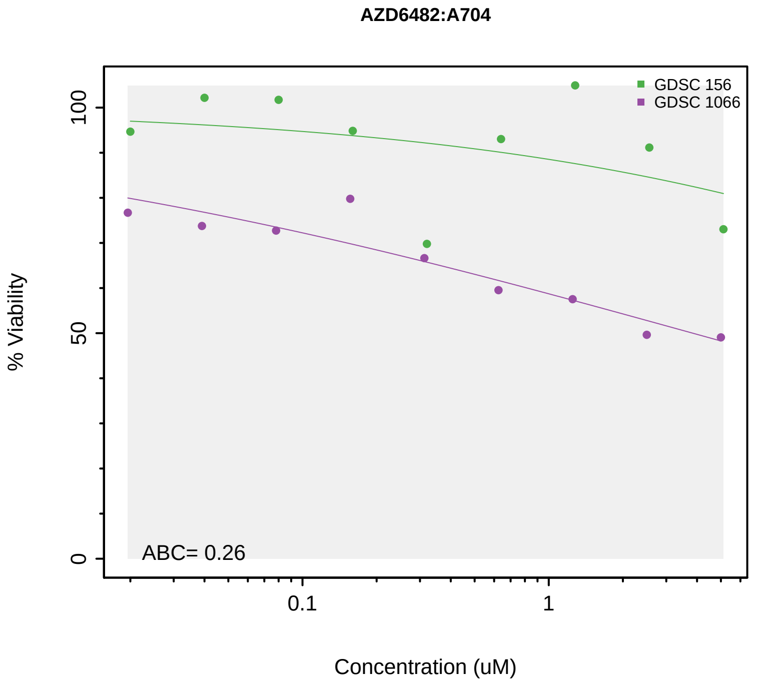
AZD6482:A704
GDSC 156
GDSC 1066
ABC= 0.26
% Viability
Concentration (uM)

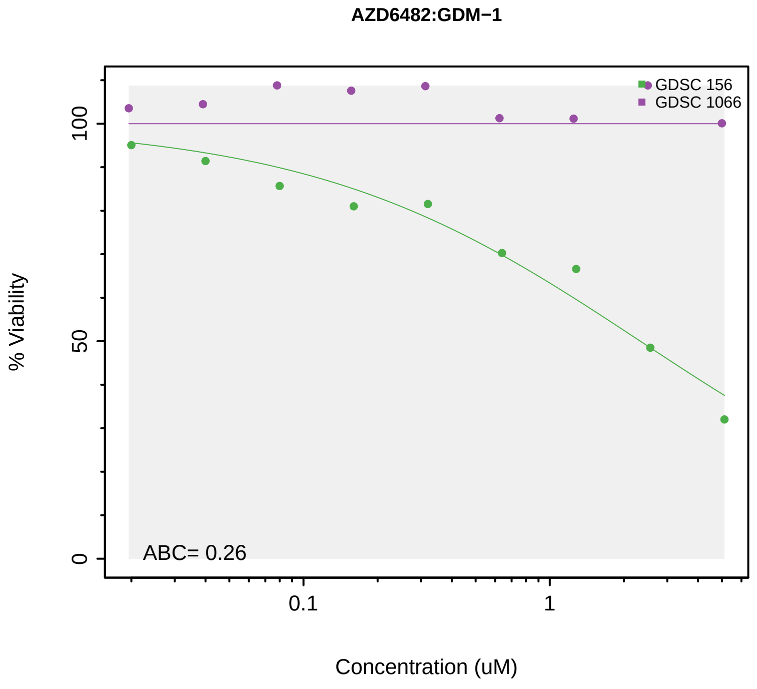
AZD6482:GDM−1
GDSC 156
GDSC 1066
ABC= 0.26
% Viability
Concentration (uM)

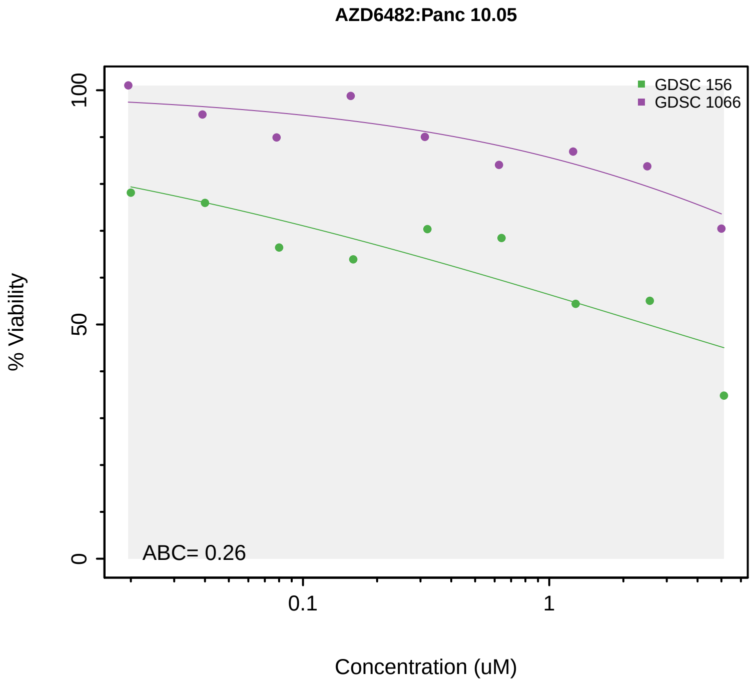
AZD6482:Panc 10.05
GDSC 156
GDSC 1066
ABC= 0.26
% Viability
Concentration (uM)

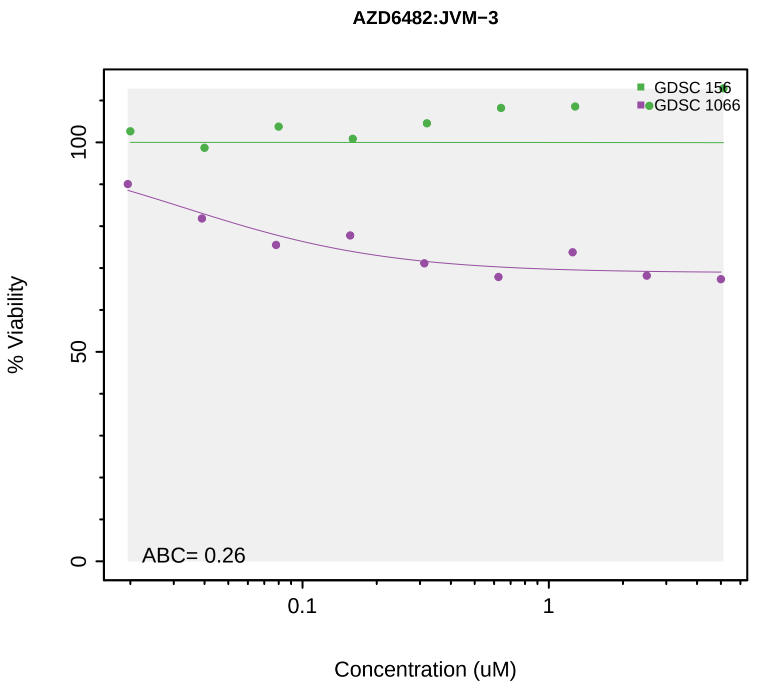
AZD6482:JVM−3
GDSC 156
GDSC 1066
ABC= 0.26
% Viability
0
50
100
0.1
1
Concentration (uM)

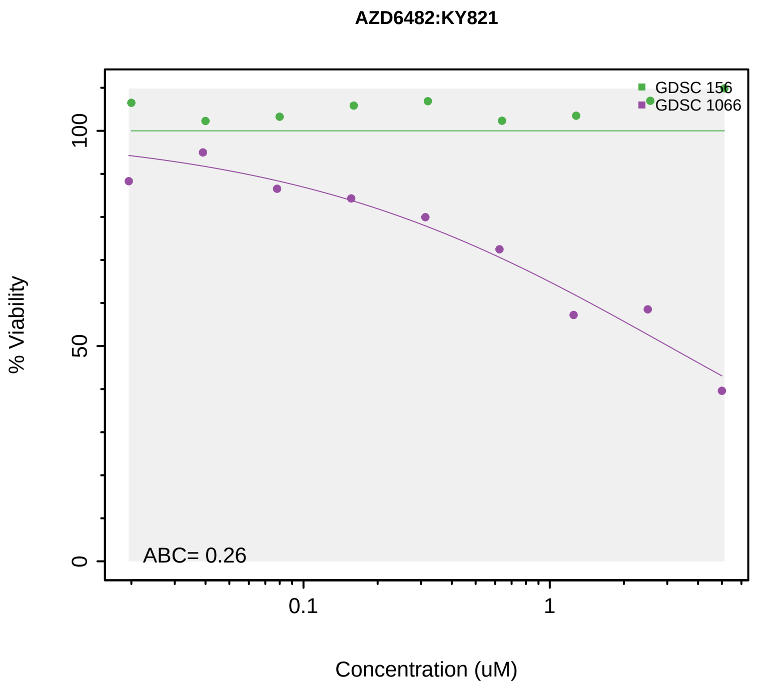
AZD6482:KY821
GDSC 156
GDSC 1066
ABC= 0.26
% Viability
0
50
100
0.1
1
Concentration (uM)

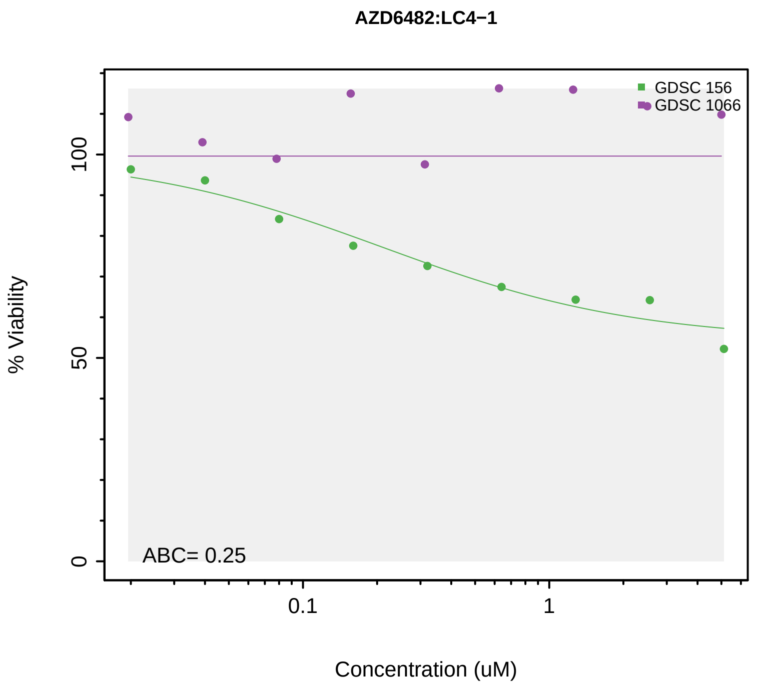
AZD6482:LC4−1
GDSC 156
GDSC 1066
ABC= 0.25
% Viability
0
50
100
0.1
1
Concentration (uM)

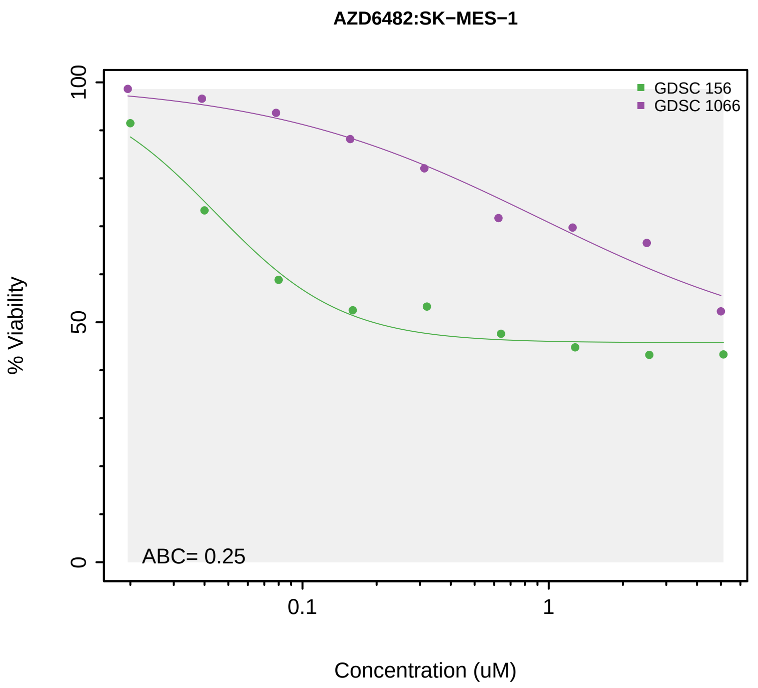
AZD6482:SK−MES−1
GDSC 156
GDSC 1066
ABC= 0.25
% Viability
0
50
100
0.1
1
Concentration (uM)

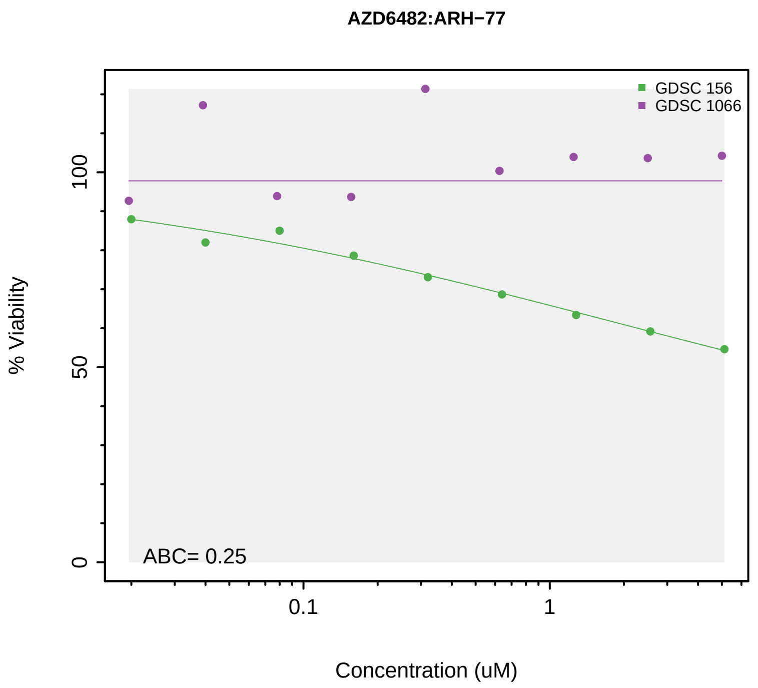
AZD6482:ARH−77
GDSC 156
GDSC 1066
ABC= 0.25
% Viability
0
50
100
0.1
1
Concentration (uM)

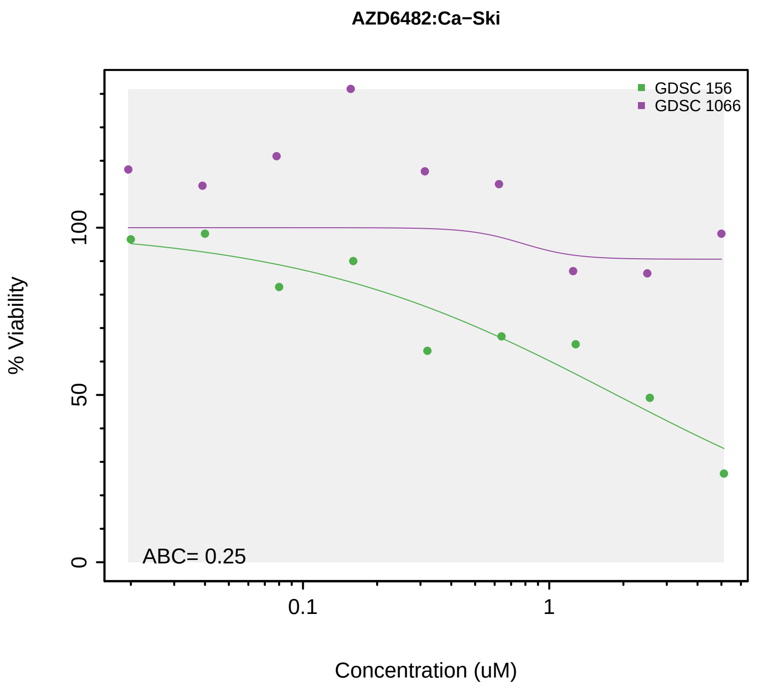
AZD6482:Ca−Ski
GDSC 156
GDSC 1066
ABC= 0.25
% Viability
0
50
100
0.1
1
Concentration (uM)

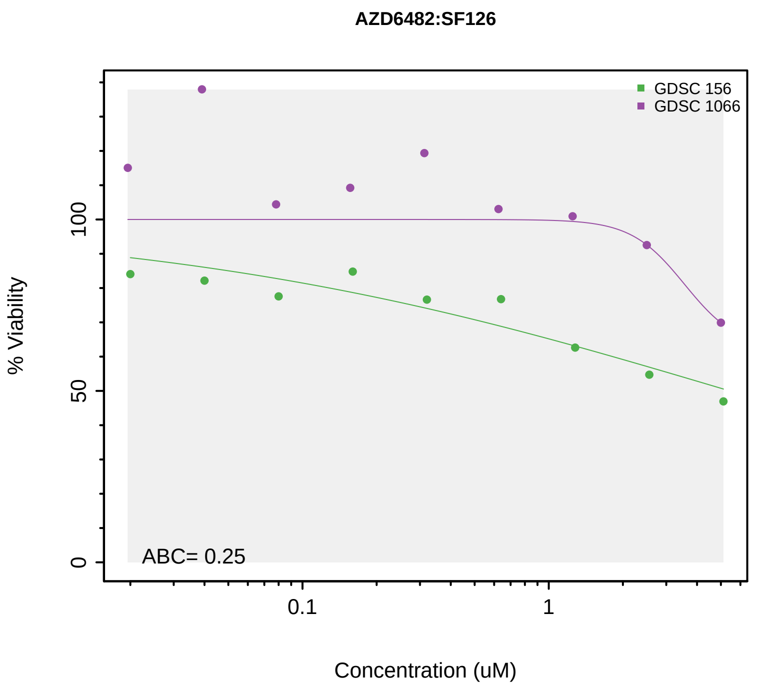
AZD6482:SF126
GDSC 156
GDSC 1066
ABC= 0.25
% Viability
0
50
100
0.1
1
Concentration (uM)

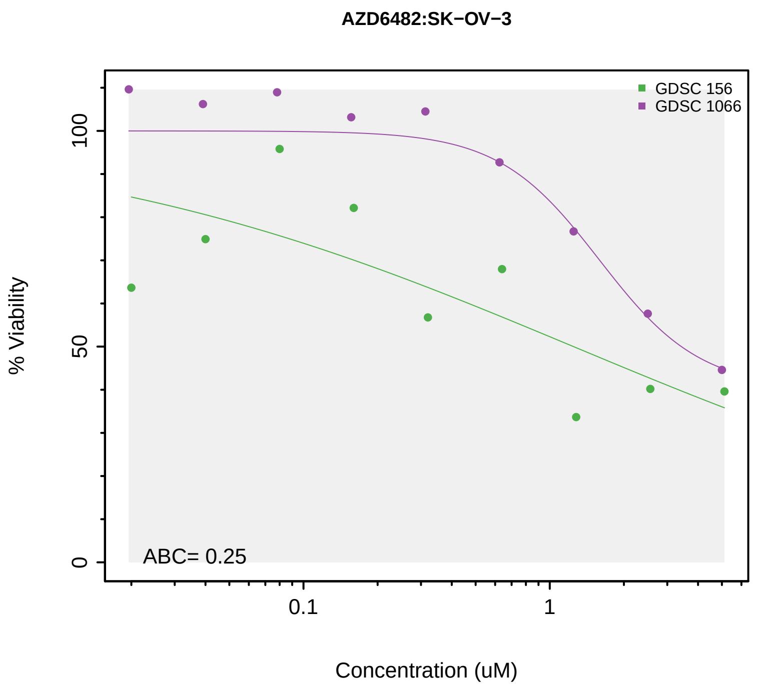
AZD6482:SK−OV−3
GDSC 156
GDSC 1066
ABC= 0.25
% Viability
0
50
100
0.1
1
Concentration (uM)

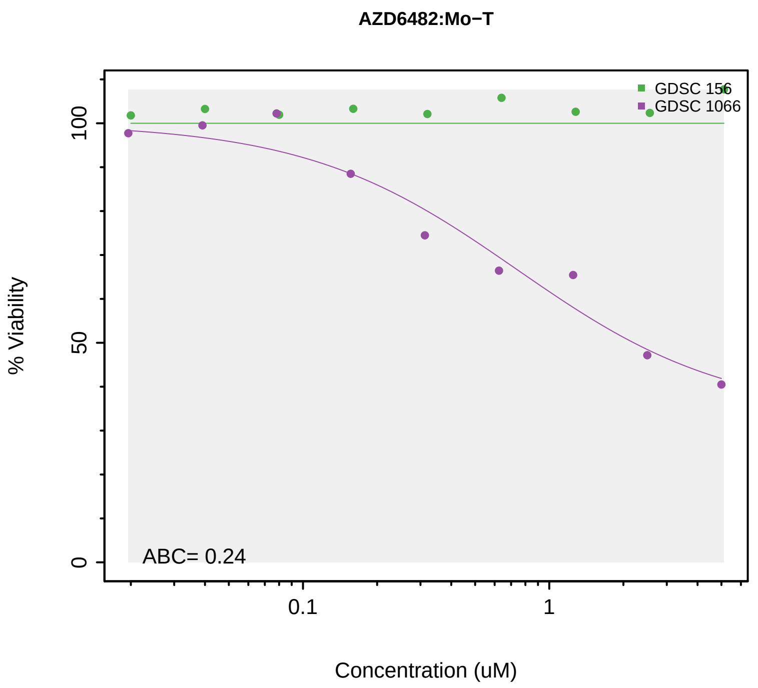
AZD6482:Mo−T
GDSC 156
GDSC 1066
ABC= 0.24
% Viability
0
50
100
0.1
1
Concentration (uM)

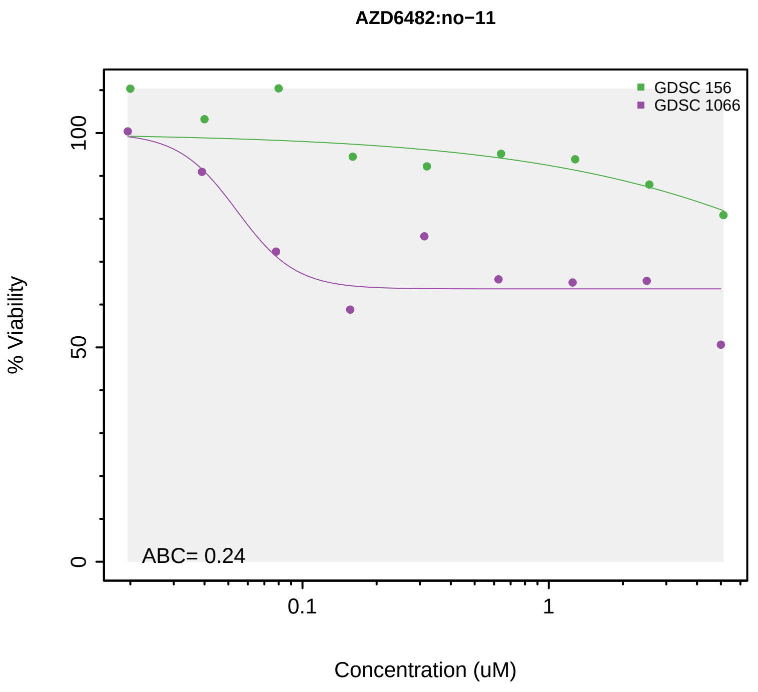
AZD6482:no−11
GDSC 156
GDSC 1066
ABC= 0.24
% Viability
0
50
100
0.1
1
Concentration (uM)

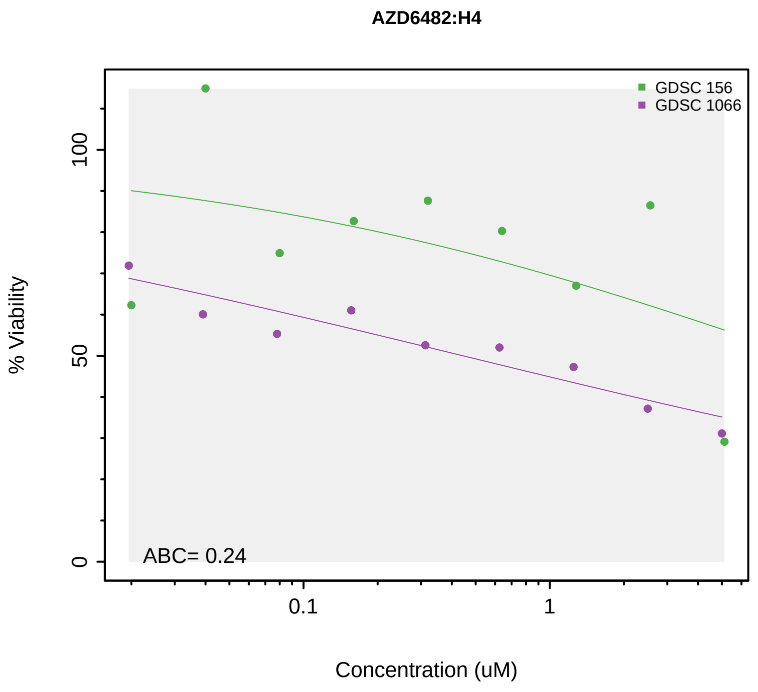
AZD6482:H4
GDSC 156
GDSC 1066
ABC= 0.24
% Viability
0
50
100
0.1
1
Concentration (uM)

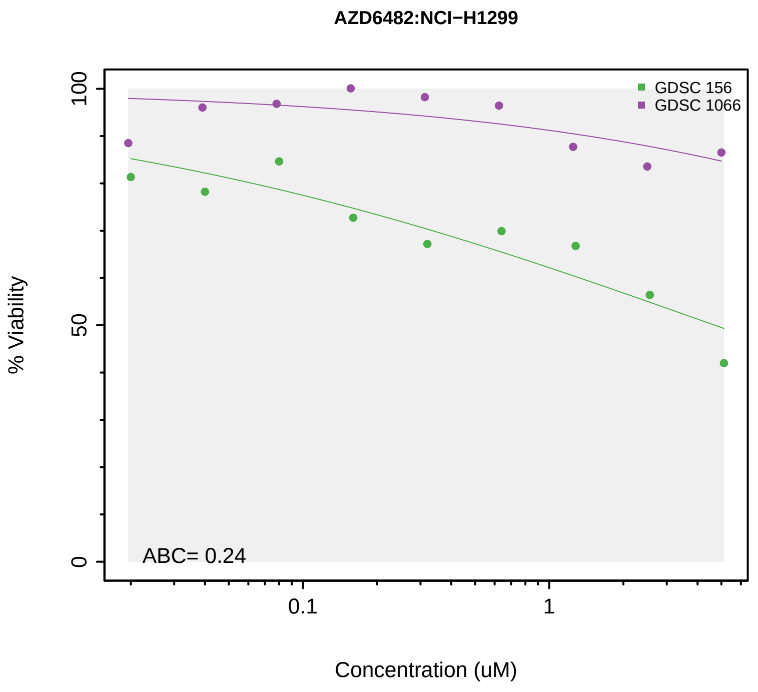
AZD6482:NCI−H1299
GDSC 156
GDSC 1066
ABC= 0.24
% Viability
0
50
100
0.1
1
Concentration (uM)

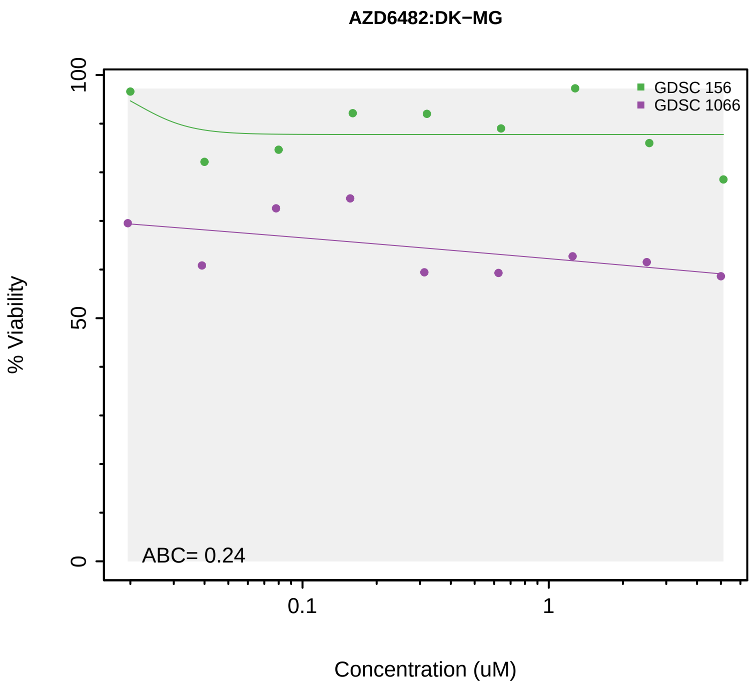
AZD6482:DK−MG
GDSC 156
GDSC 1066
ABC= 0.24
% Viability
Concentration (uM)

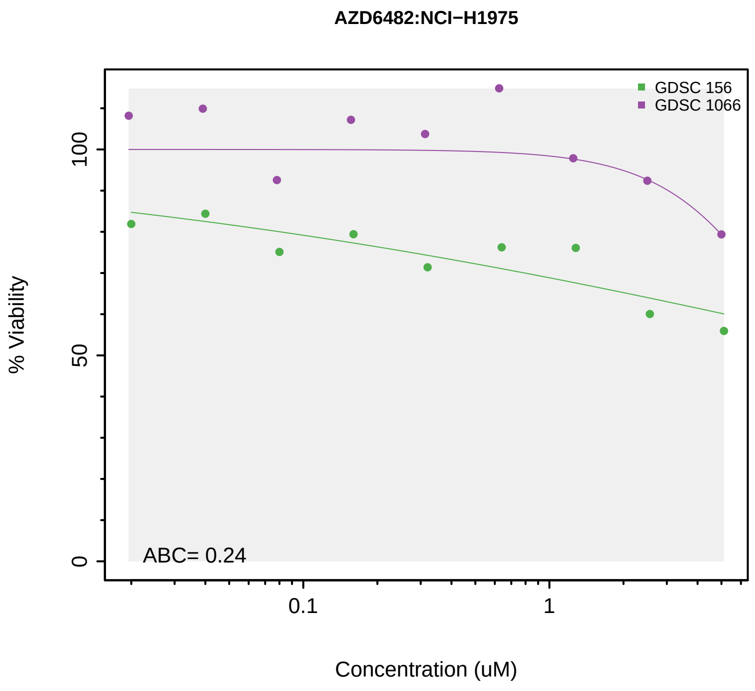
AZD6482:NCI−H1975
GDSC 156
GDSC 1066
ABC= 0.24
% Viability
Concentration (uM)

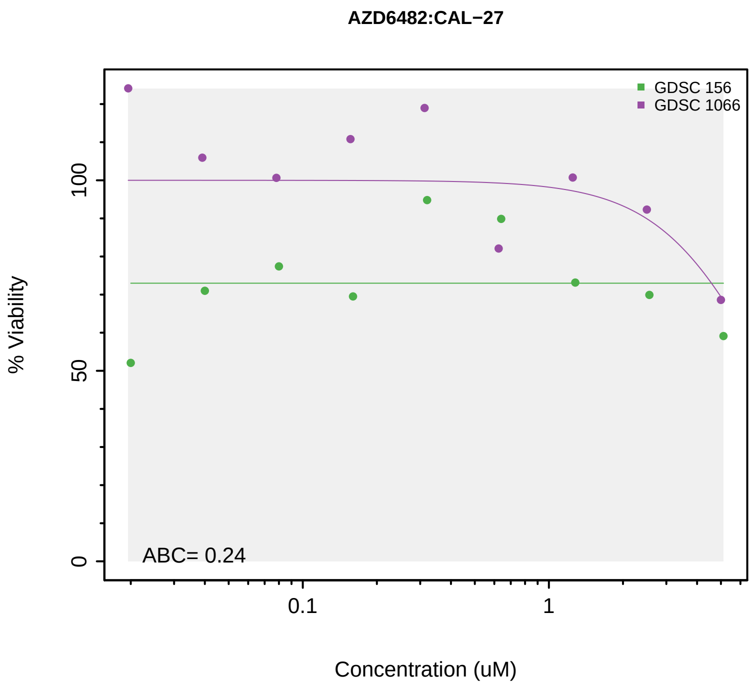
AZD6482:CAL−27
GDSC 156
GDSC 1066
ABC= 0.24
% Viability
Concentration (uM)

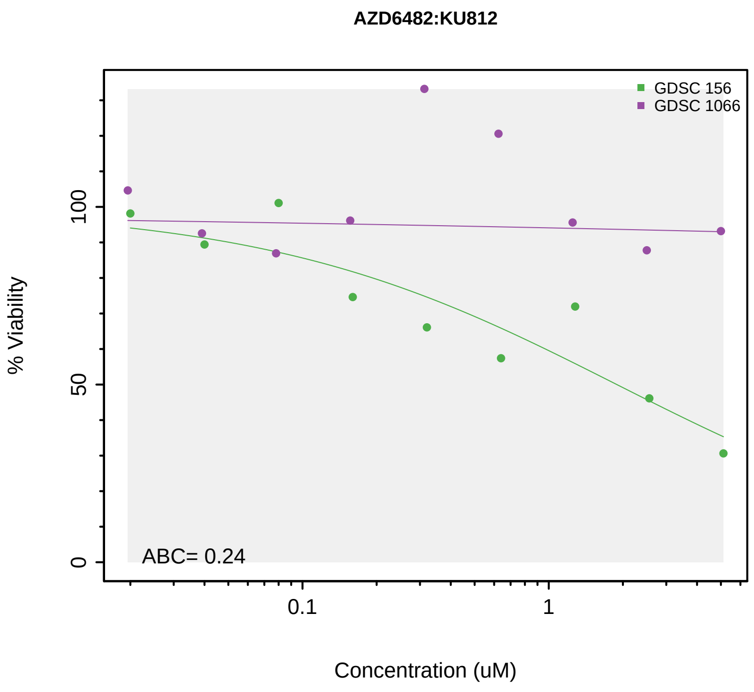
AZD6482:KU812
GDSC 156
GDSC 1066
ABC= 0.24
% Viability
Concentration (uM)

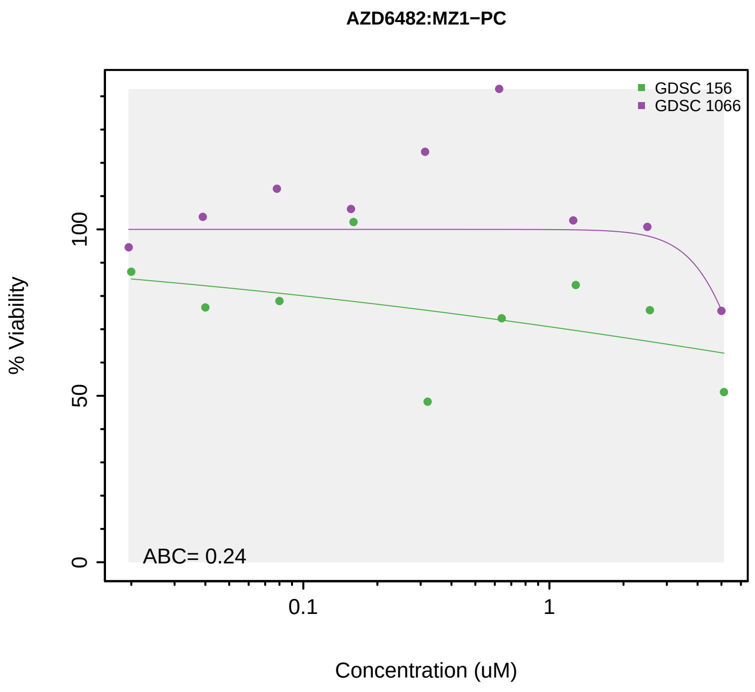
AZD6482:MZ1−PC
GDSC 156
GDSC 1066
ABC= 0.24
% Viability
Concentration (uM)

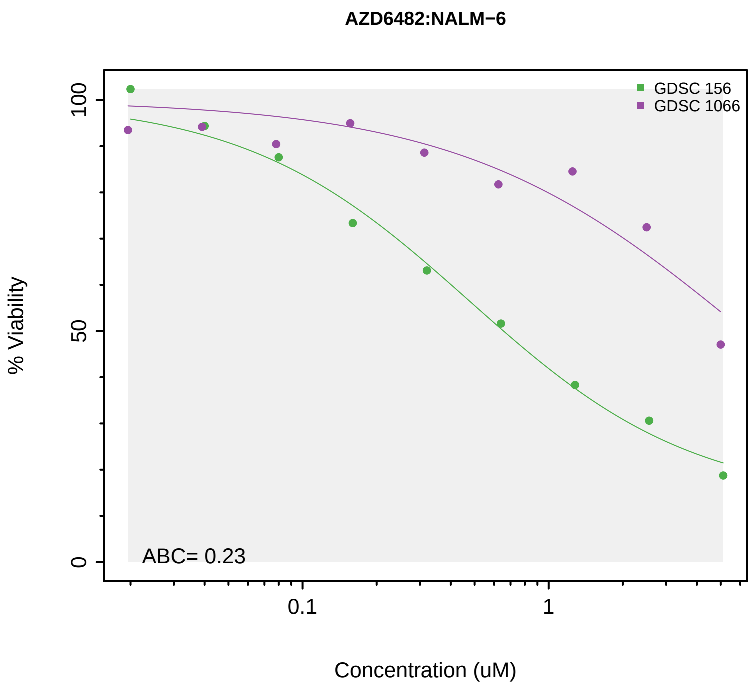
AZD6482:NALM−6
GDSC 156
GDSC 1066
ABC= 0.23
% Viability
Concentration (uM)

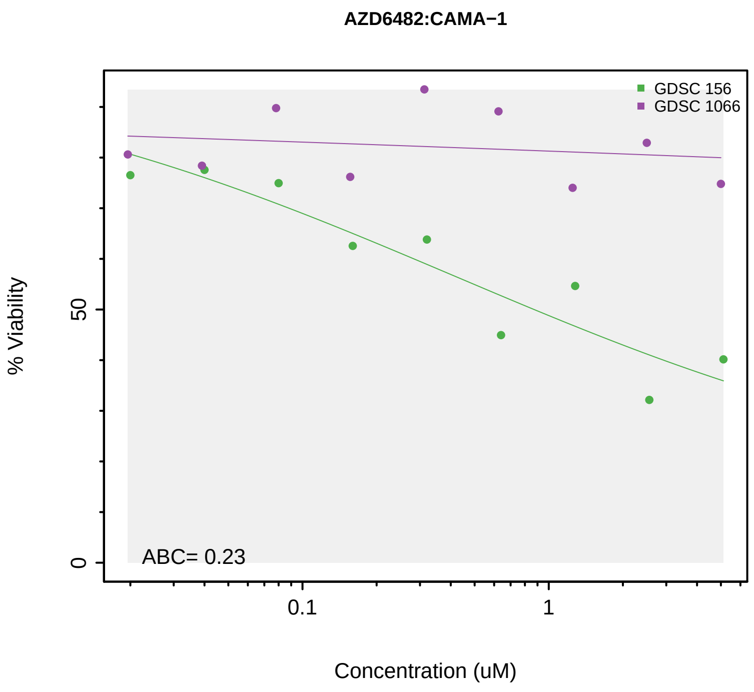
AZD6482:CAMA−1
GDSC 156
GDSC 1066
ABC= 0.23
% Viability
Concentration (uM)

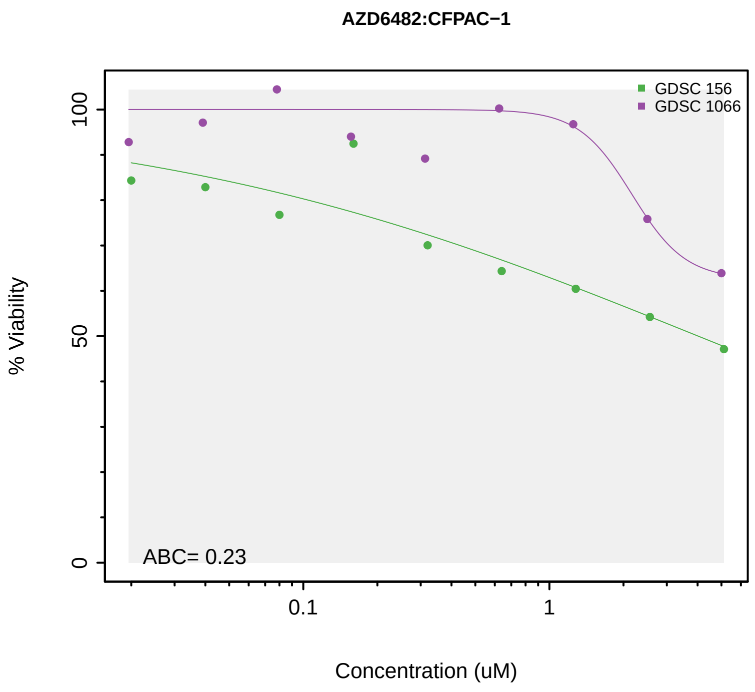
AZD6482:CFPAC−1
GDSC 156
GDSC 1066
ABC= 0.23
% Viability
Concentration (uM)

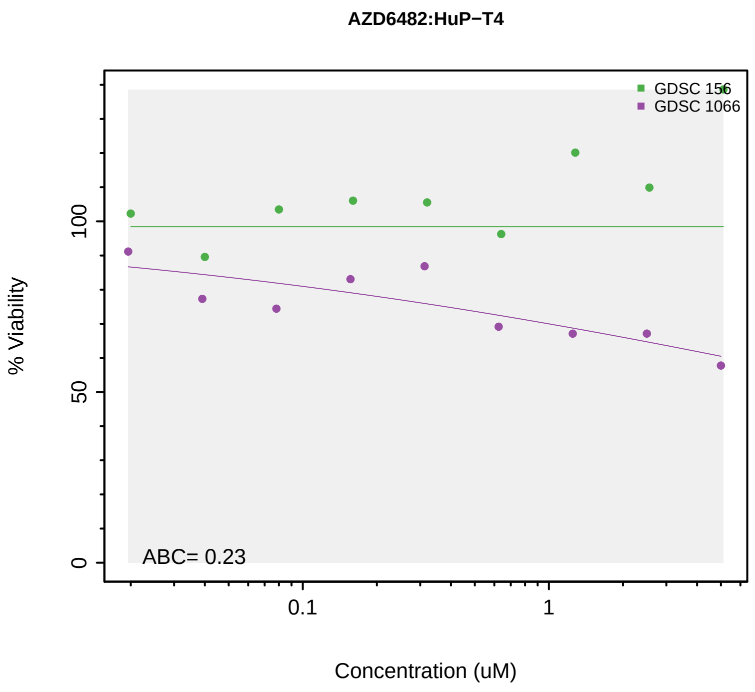
AZD6482:HuP−T4
GDSC 156
GDSC 1066
ABC= 0.23
% Viability
Concentration (uM)

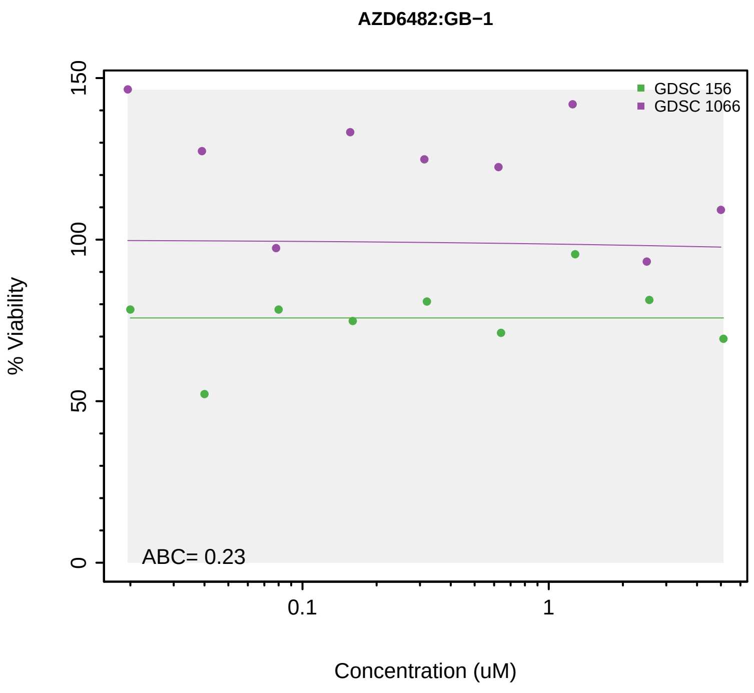
AZD6482:GB−1
GDSC 156
GDSC 1066
ABC= 0.23
% Viability
Concentration (uM)

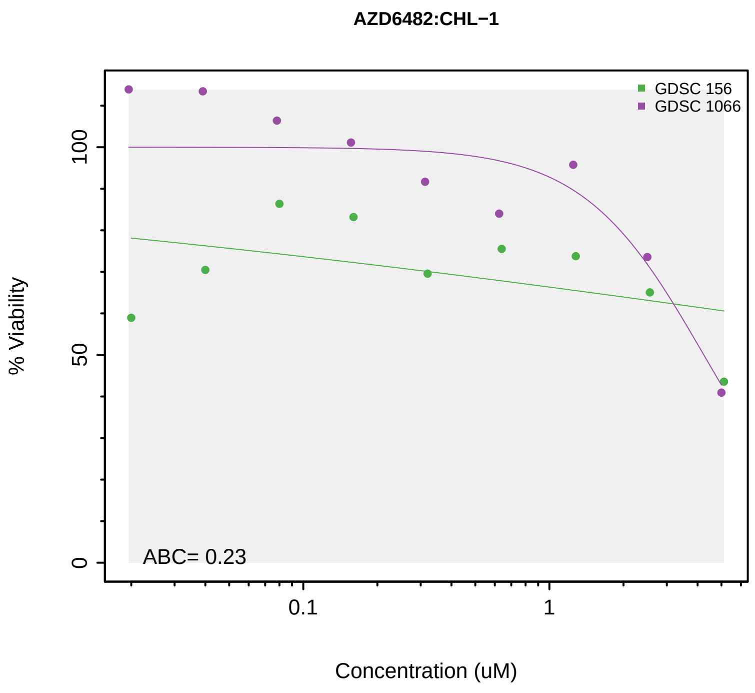
AZD6482:CHL−1
GDSC 156
GDSC 1066
ABC= 0.23
% Viability
Concentration (uM)

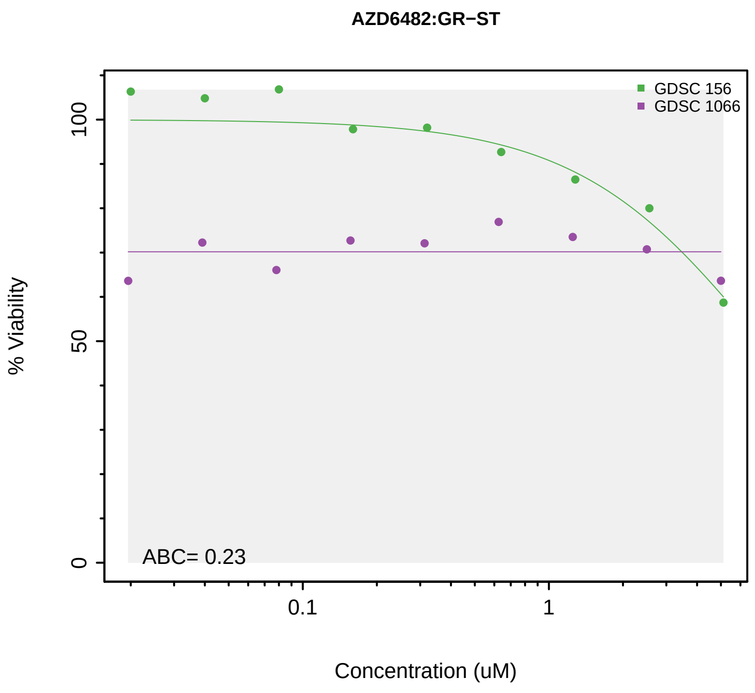
AZD6482:GR−ST
GDSC 156
GDSC 1066
ABC= 0.23
% Viability
Concentration (uM)

AZD6482:CGTH−W−1

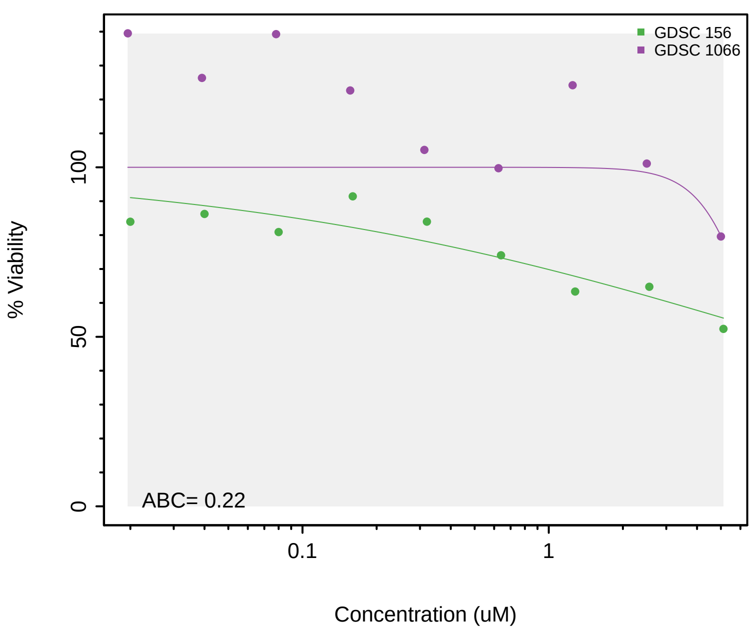

AZD6482:SF295

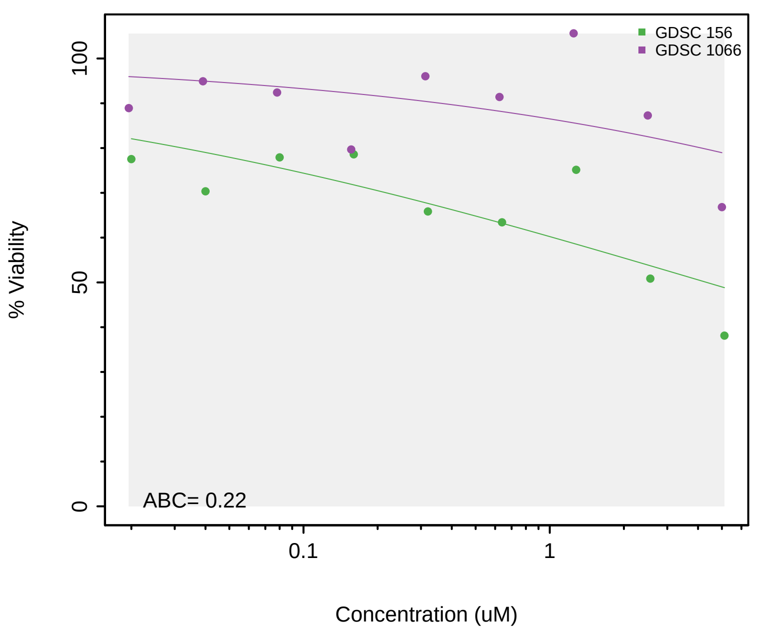

AZD6482:NCI−H1793

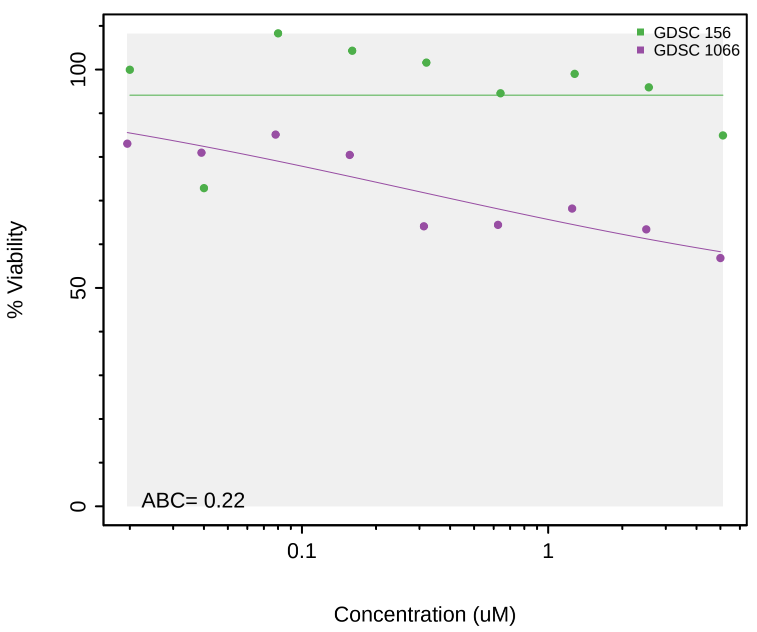

AZD6482:A431

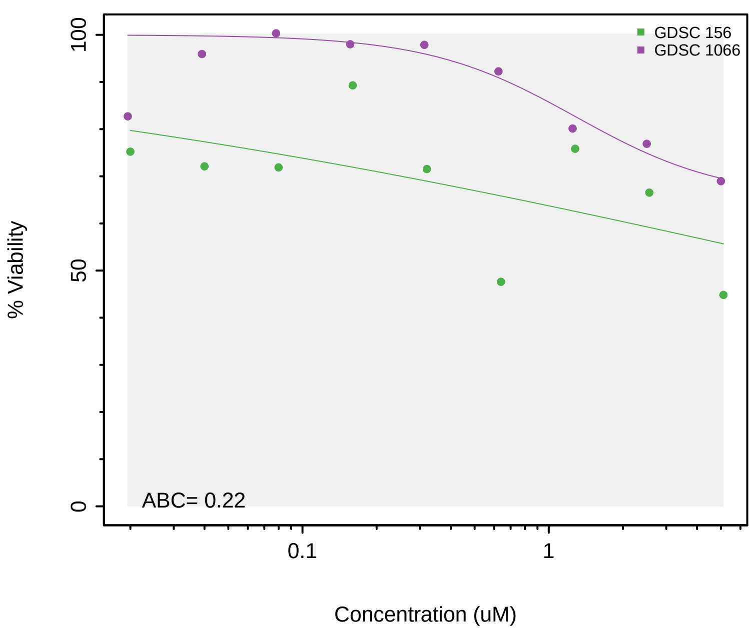

AZD6482:RCM−1

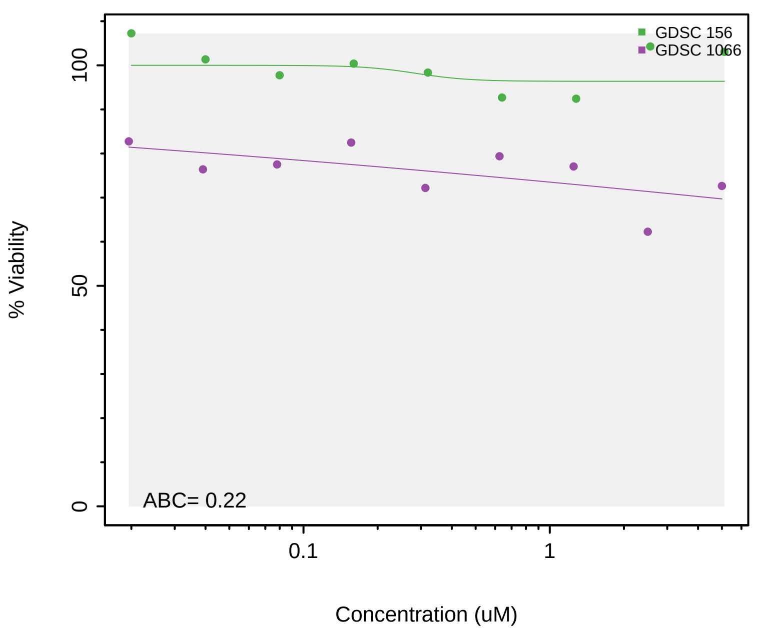

AZD6482:HCT−116

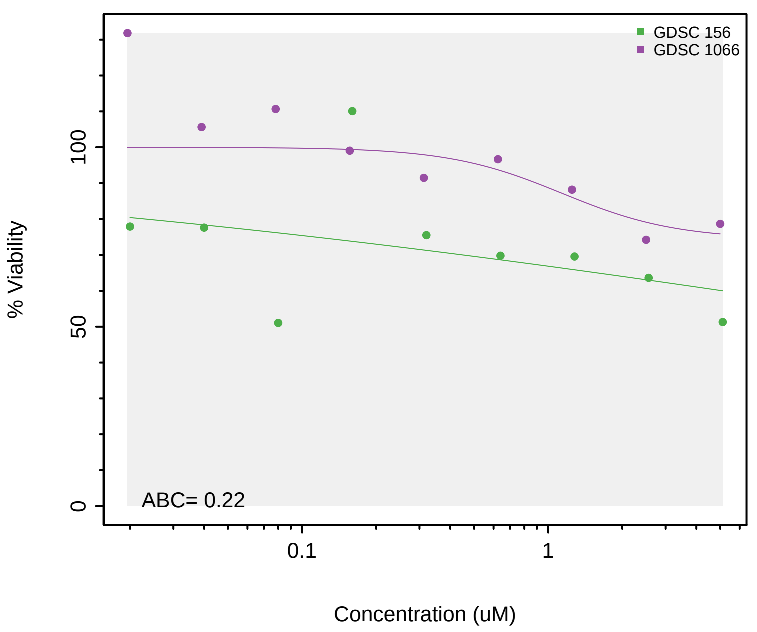

AZD6482:LoVo

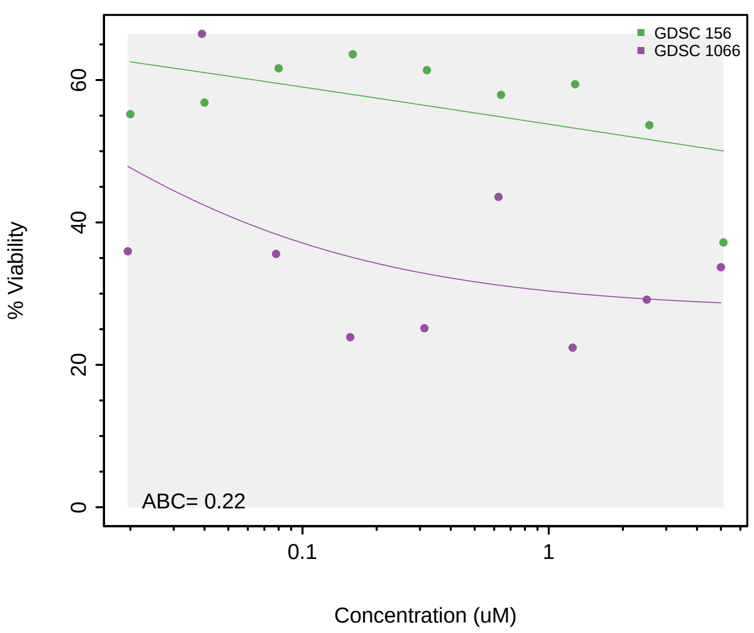

AZD6482:G−402

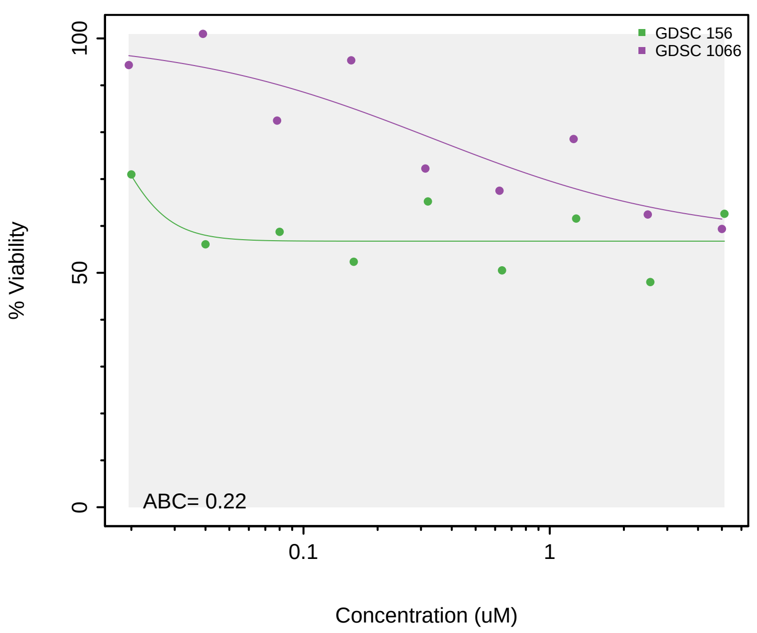

AZD6482:NCI−H2452

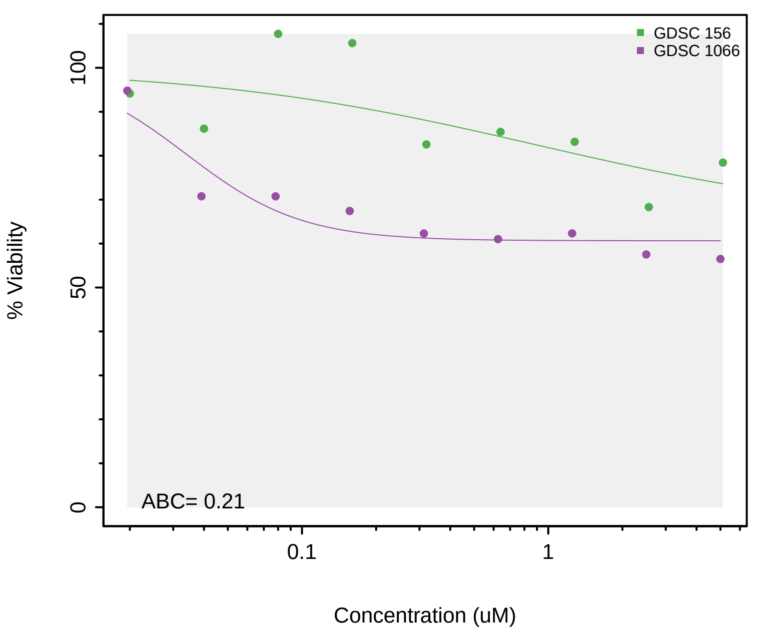

AZD6482:MDA−MB−453

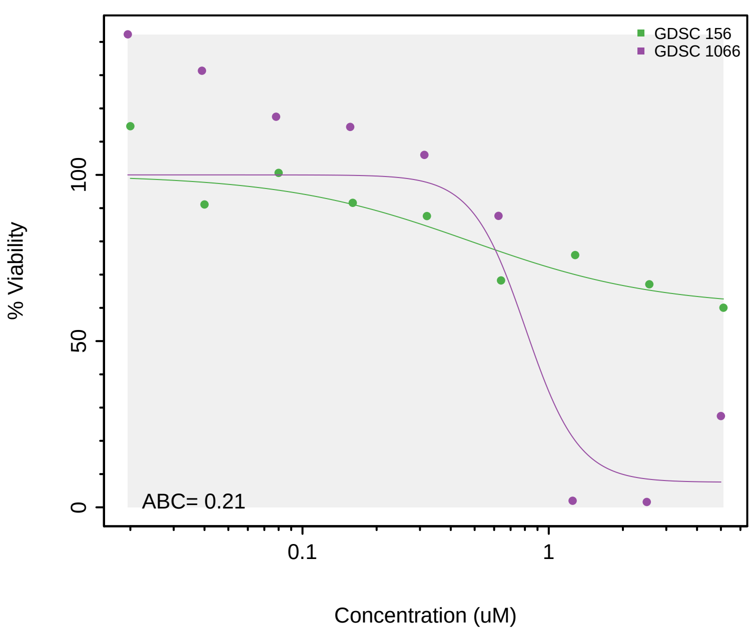

AZD6482:RPMI−2650

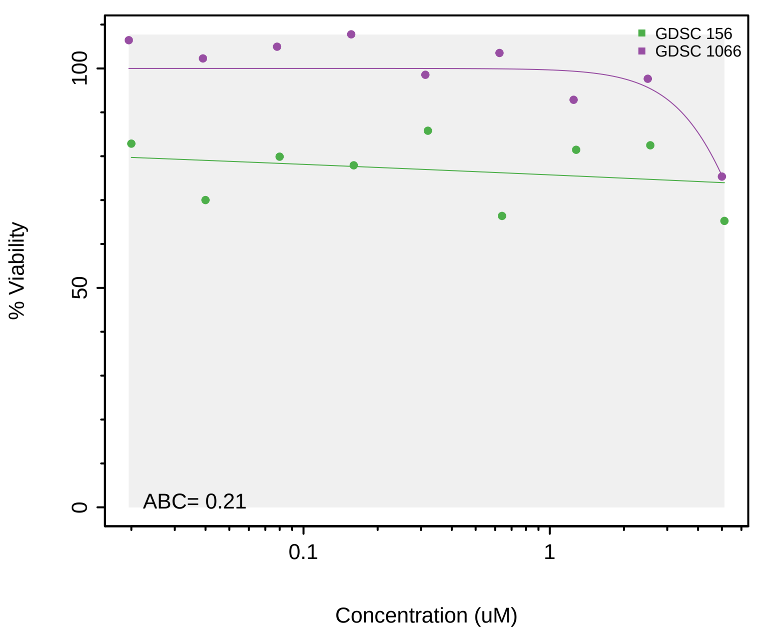

AZD6482:ES6

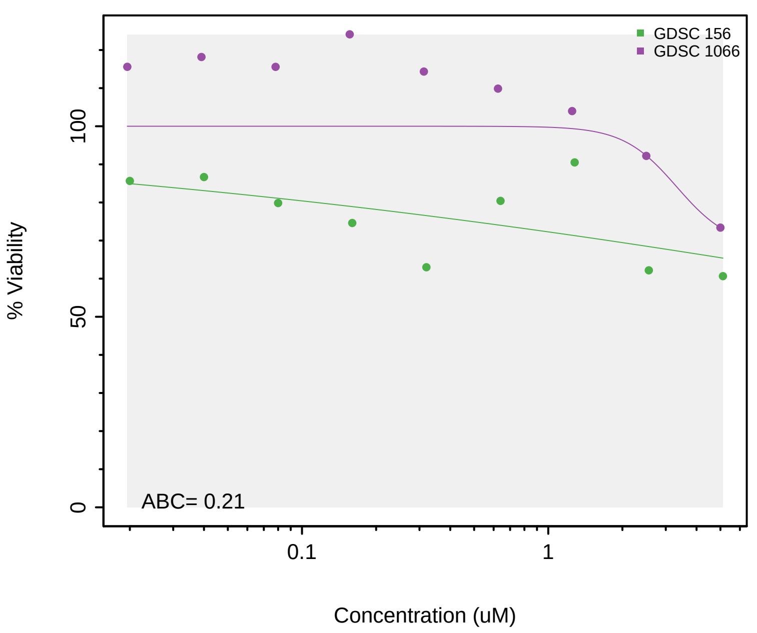

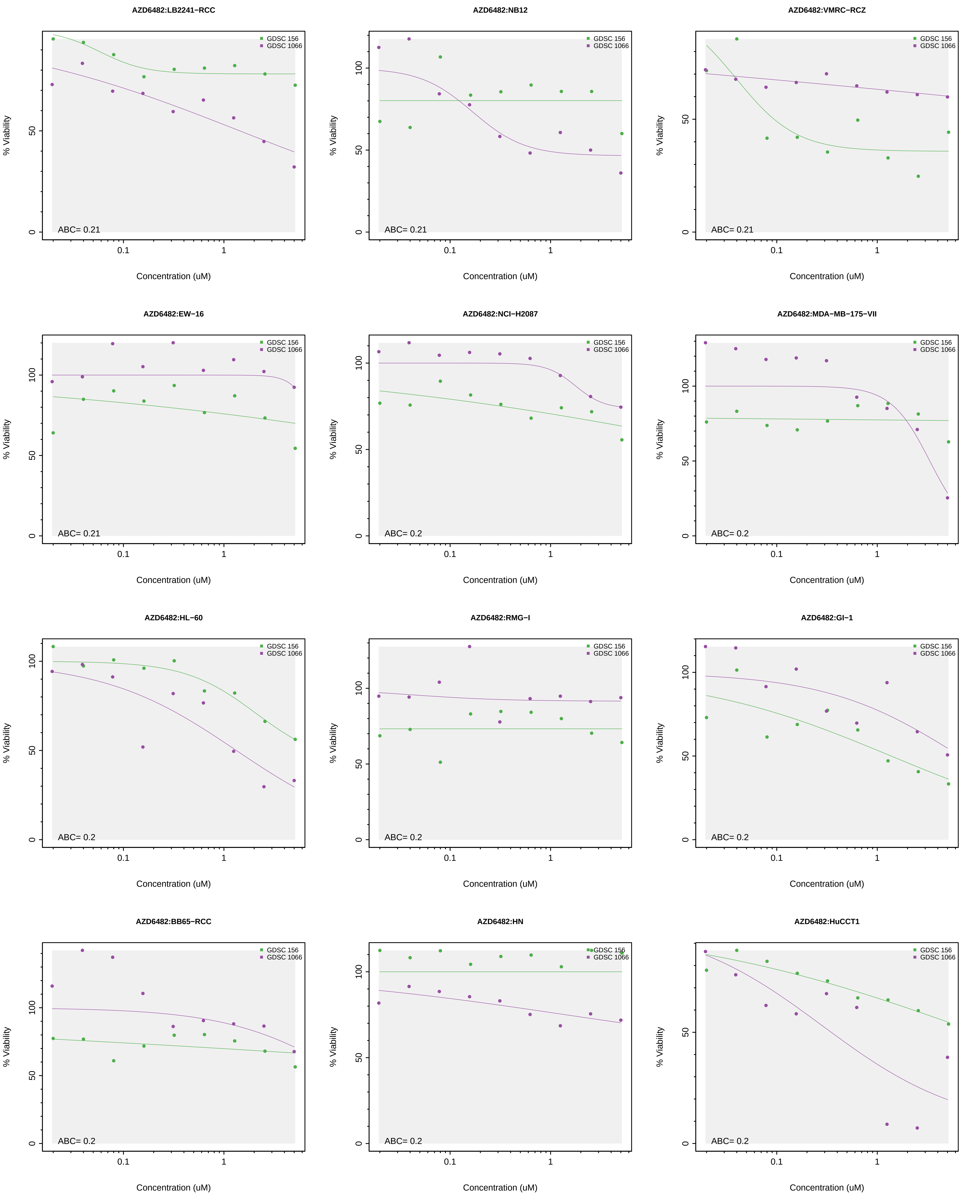
AZD6482:LB2241−RCC
AZD6482:NB12
AZD6482:VMRC−RCZ
AZD6482:EW−16
AZD6482:NCI−H2087
AZD6482:MDA−MB−175−VII
AZD6482:HL−60
AZD6482:RMG−I
AZD6482:GI−1
AZD6482:BB65−RCC
AZD6482:HN
AZD6482:HuCCT1
GDSC 156
GDSC 1066
% Viability
Concentration (uM)
ABC= 0.21
ABC= 0.21
ABC= 0.21
ABC= 0.21
ABC= 0.2
ABC= 0.2
ABC= 0.2
ABC= 0.2
ABC= 0.2
ABC= 0.2
ABC= 0.2
ABC= 0.2

AZD6482:Calu−3

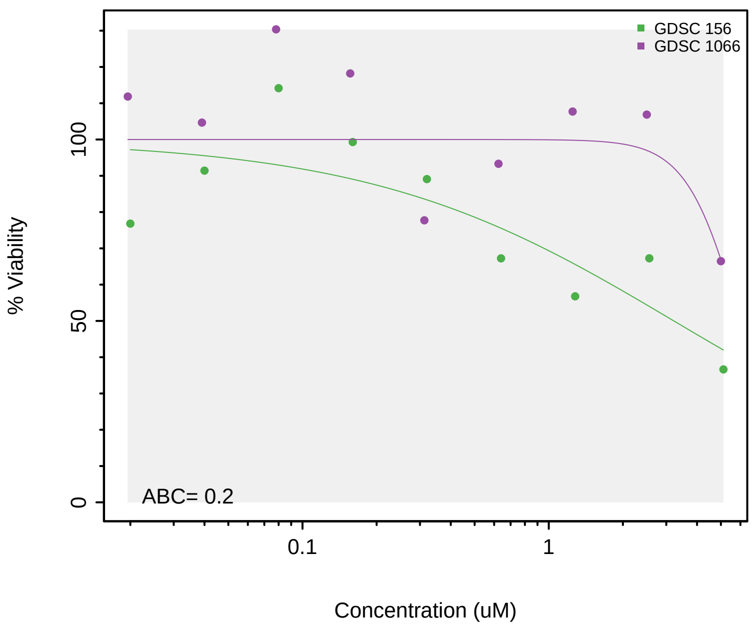

AZD6482:D−283MED

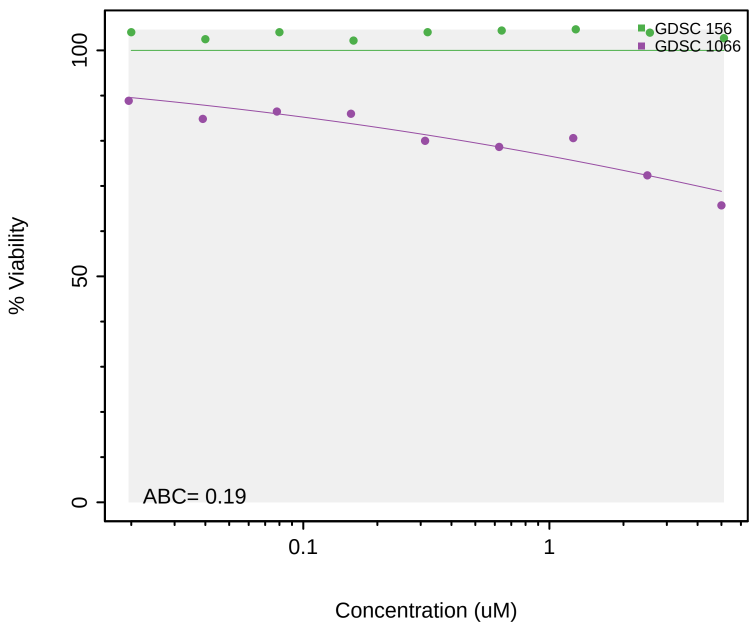

AZD6482:CaR−1

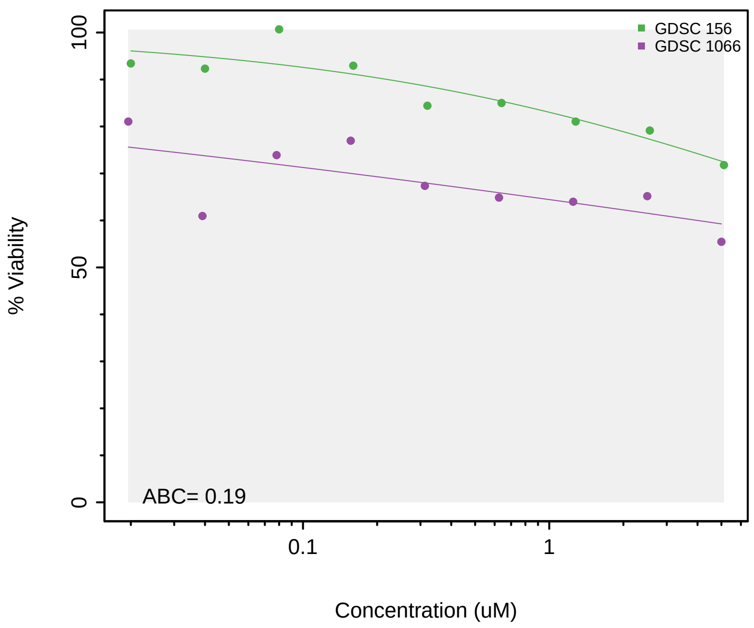

AZD6482:HC−1

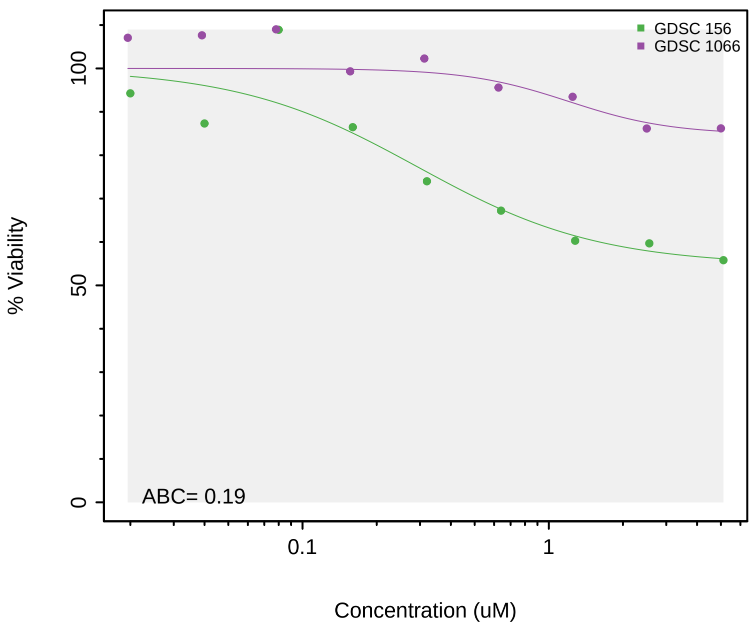

AZD6482:SK−N−DZ

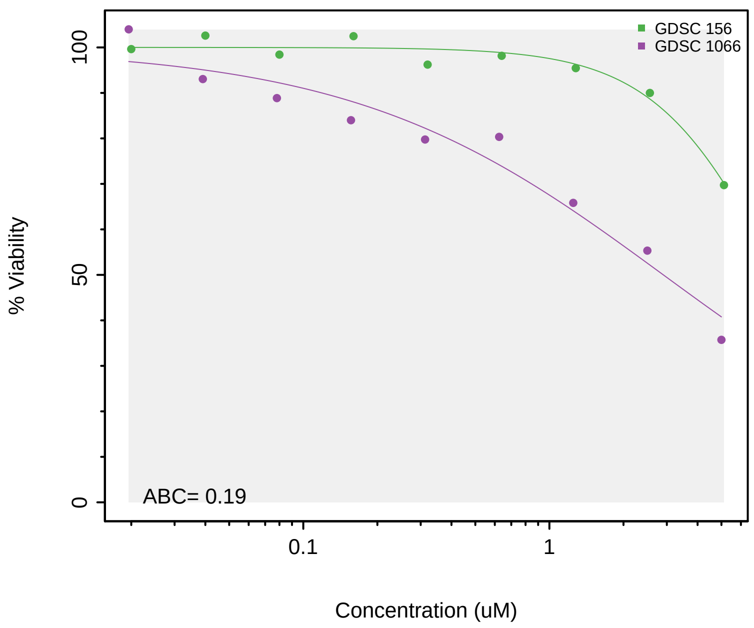

AZD6482:HH

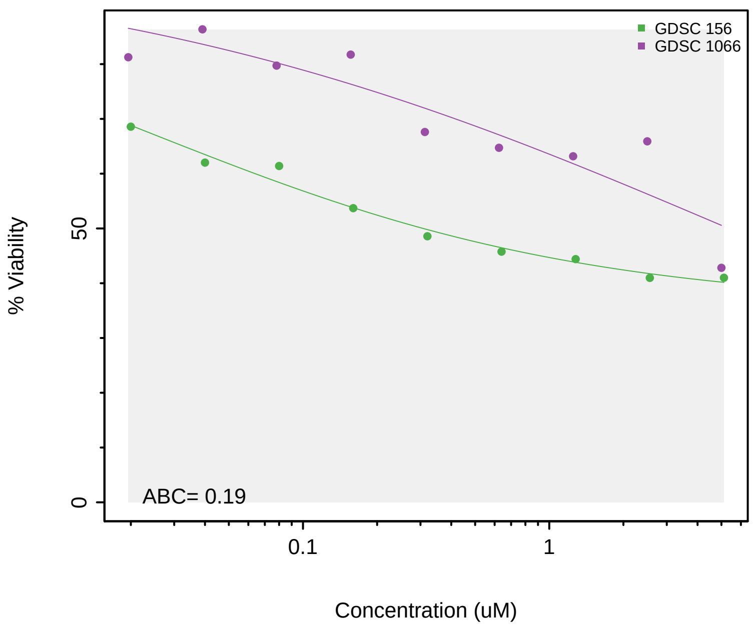

AZD6482:HGC−27

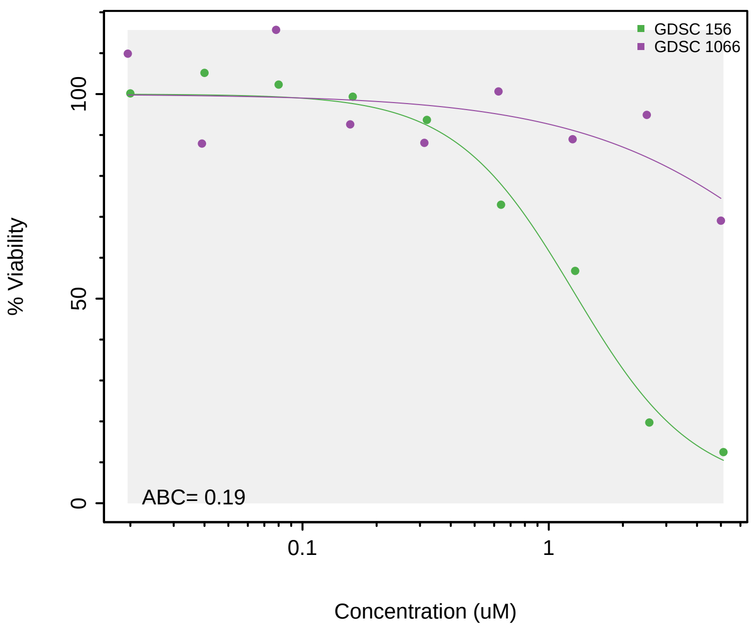

AZD6482:EKVX

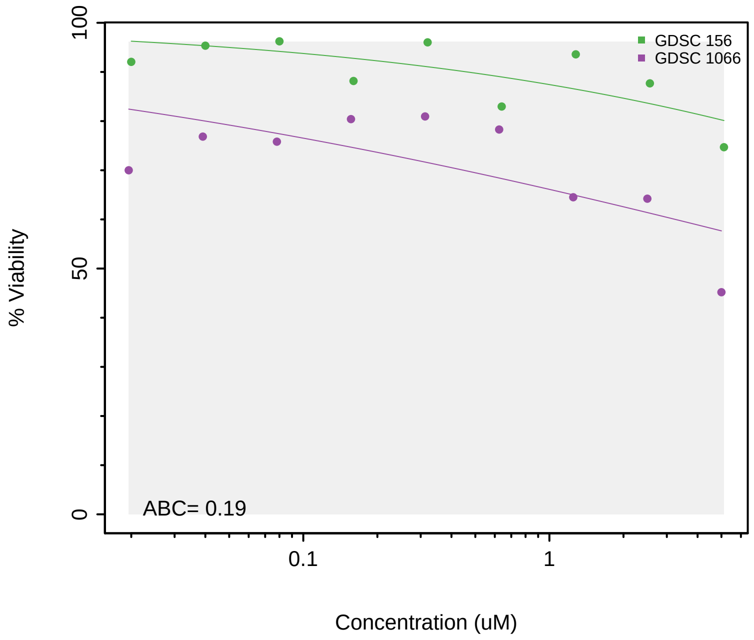

AZD6482:GCIY

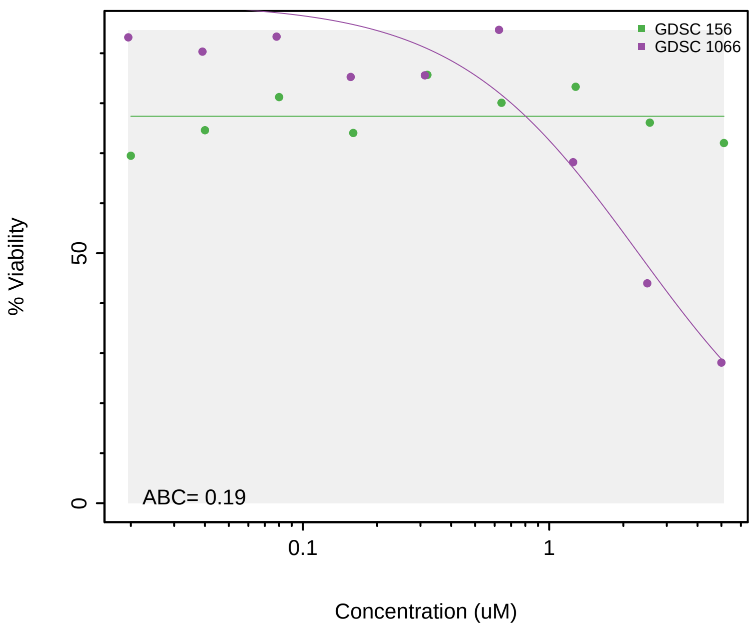

AZD6482:U251

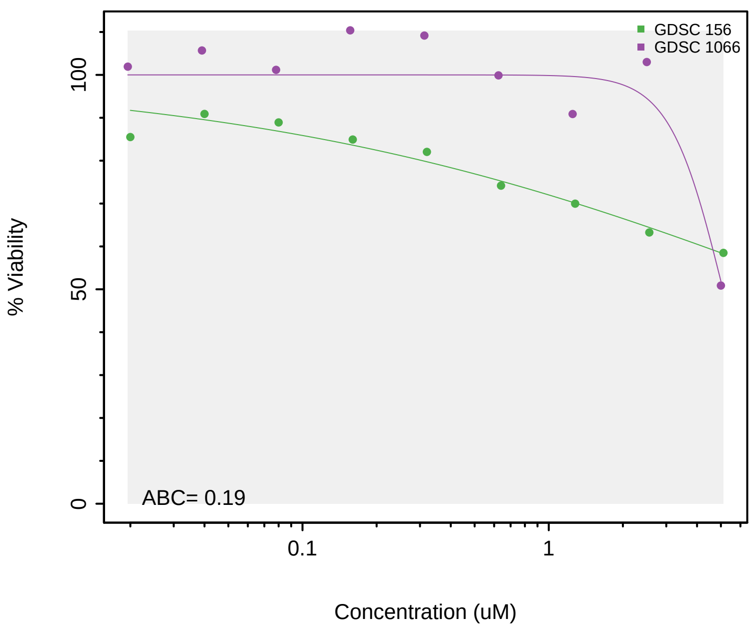

AZD6482:NCI−H661

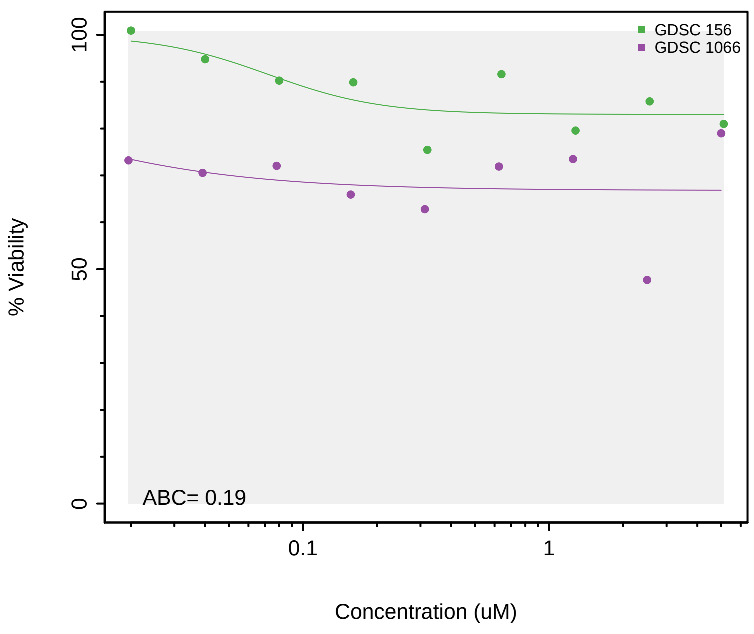

AZD6482:HT−1080

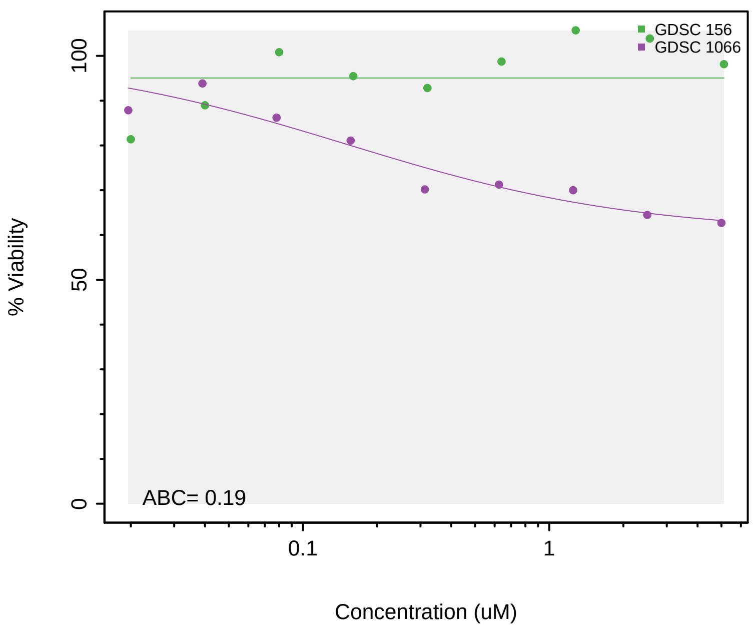

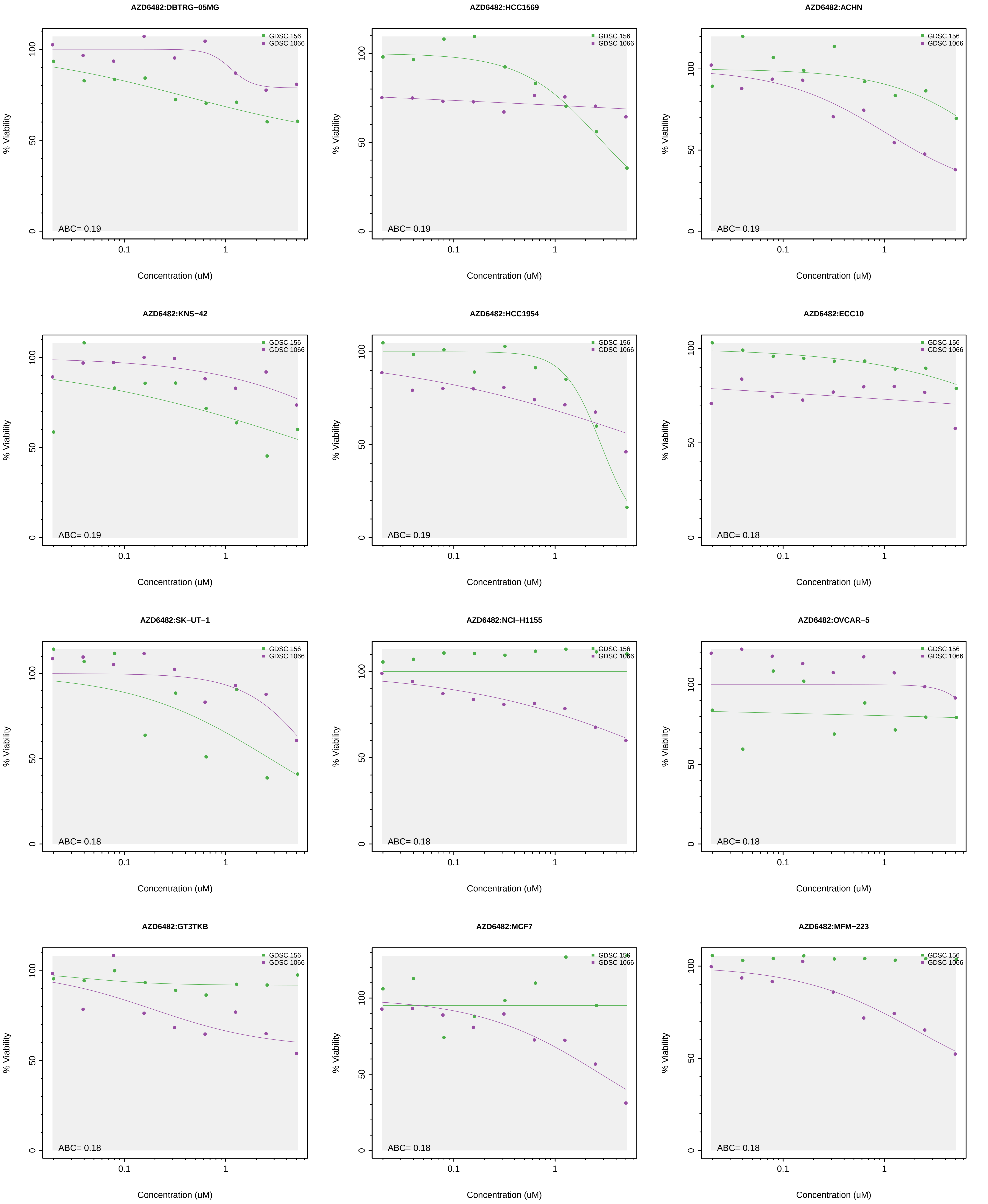
AZD6482:DBTRG−05MG
AZD6482:HCC1569
AZD6482:ACHN
AZD6482:KNS−42
AZD6482:HCC1954
AZD6482:ECC10
AZD6482:SK−UT−1
AZD6482:NCI−H1155
AZD6482:OVCAR−5
AZD6482:GT3TKB
AZD6482:MCF7
AZD6482:MFM−223
GDSC 156
GDSC 1066
% Viability
Concentration (uM)
ABC= 0.19
ABC= 0.18

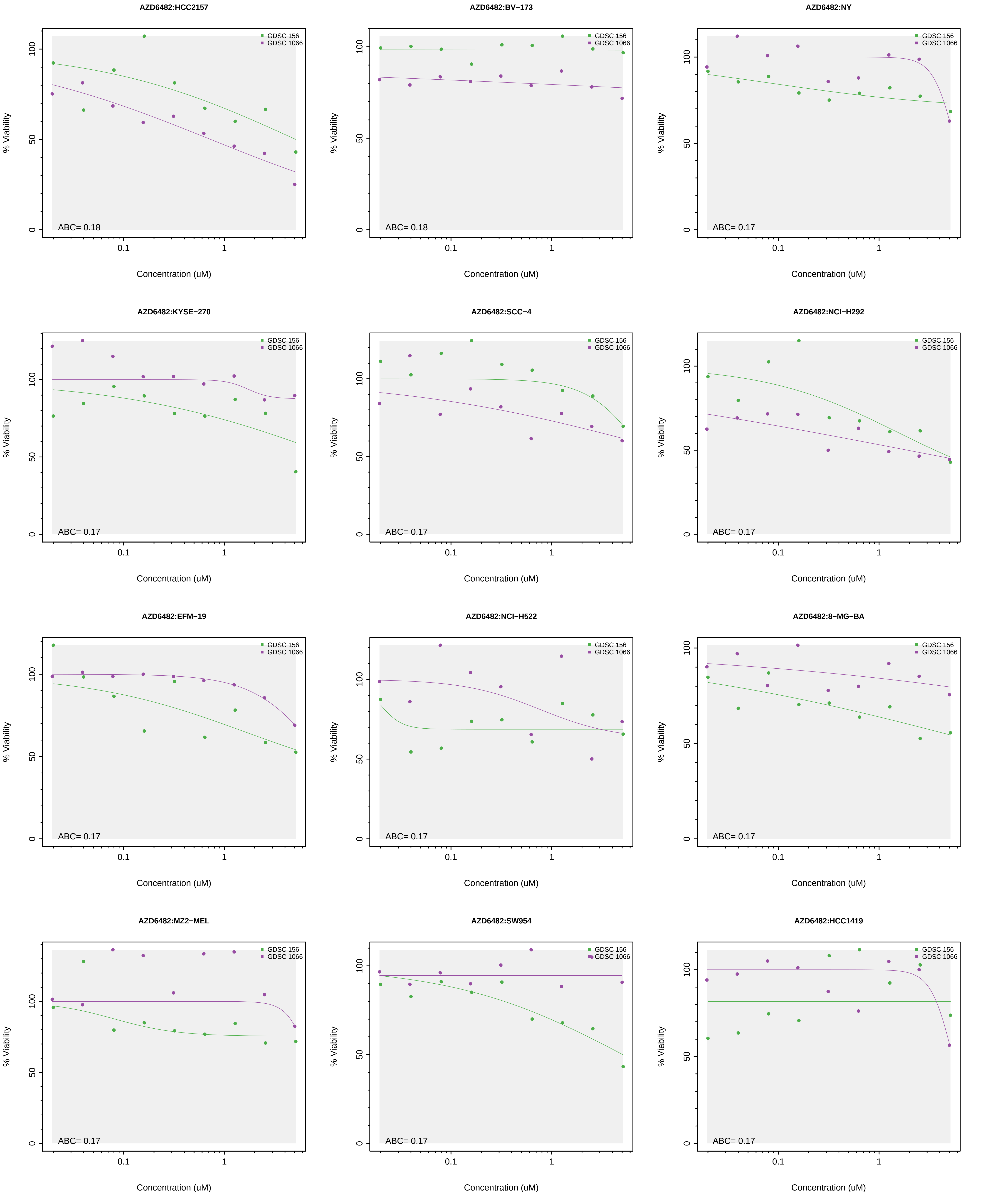
AZD6482:HCC2157
GDSC 156
GDSC 1066
ABC= 0.18
% Viability
Concentration (uM)
AZD6482:BV−173
GDSC 156
GDSC 1066
ABC= 0.18
% Viability
Concentration (uM)
AZD6482:NY
GDSC 156
GDSC 1066
ABC= 0.17
% Viability
Concentration (uM)
AZD6482:KYSE−270
GDSC 156
GDSC 1066
ABC= 0.17
% Viability
Concentration (uM)
AZD6482:SCC−4
GDSC 156
GDSC 1066
ABC= 0.17
% Viability
Concentration (uM)
AZD6482:NCI−H292
GDSC 156
GDSC 1066
ABC= 0.17
% Viability
Concentration (uM)
AZD6482:EFM−19
GDSC 156
GDSC 1066
ABC= 0.17
% Viability
Concentration (uM)
AZD6482:NCI−H522
GDSC 156
GDSC 1066
ABC= 0.17
% Viability
Concentration (uM)
AZD6482:8−MG−BA
GDSC 156
GDSC 1066
ABC= 0.17
% Viability
Concentration (uM)
AZD6482:MZ2−MEL
GDSC 156
GDSC 1066
ABC= 0.17
% Viability
Concentration (uM)
AZD6482:SW954
GDSC 156
GDSC 1066
ABC= 0.17
% Viability
Concentration (uM)
AZD6482:HCC1419
GDSC 156
GDSC 1066
ABC= 0.17
% Viability
Concentration (uM)

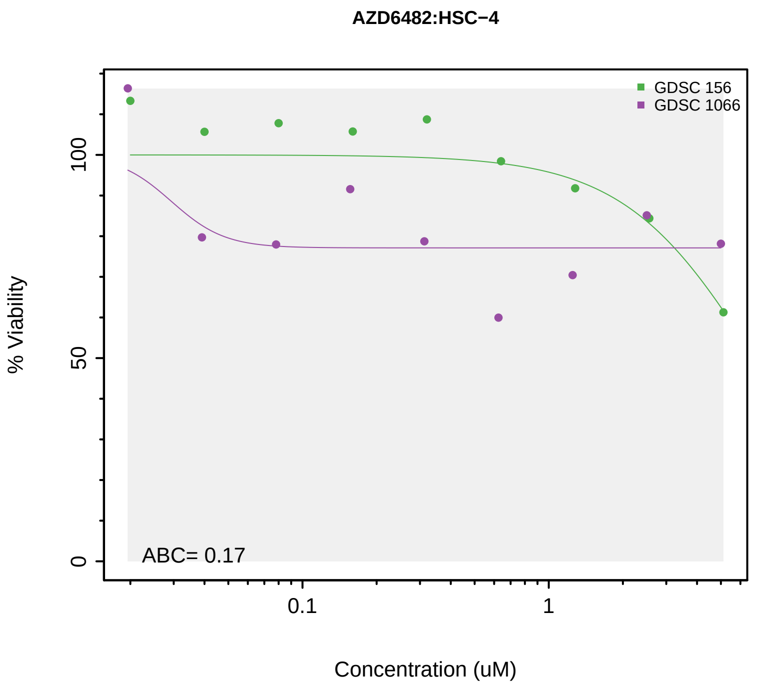
AZD6482:HSC−4
GDSC 156
GDSC 1066
ABC= 0.17
% Viability
Concentration (uM)

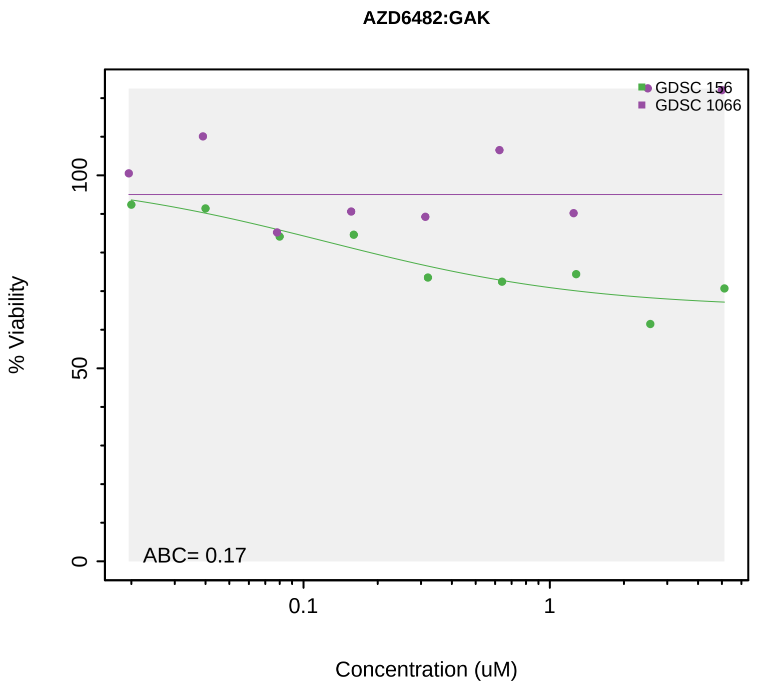
AZD6482:GAK
GDSC 156
GDSC 1066
ABC= 0.17
% Viability
Concentration (uM)

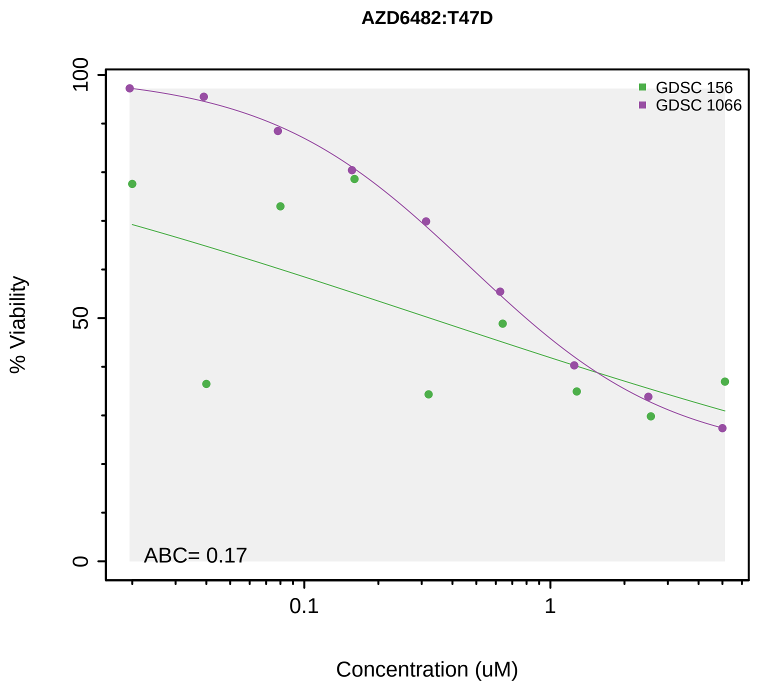
AZD6482:T47D
GDSC 156
GDSC 1066
ABC= 0.17
% Viability
Concentration (uM)

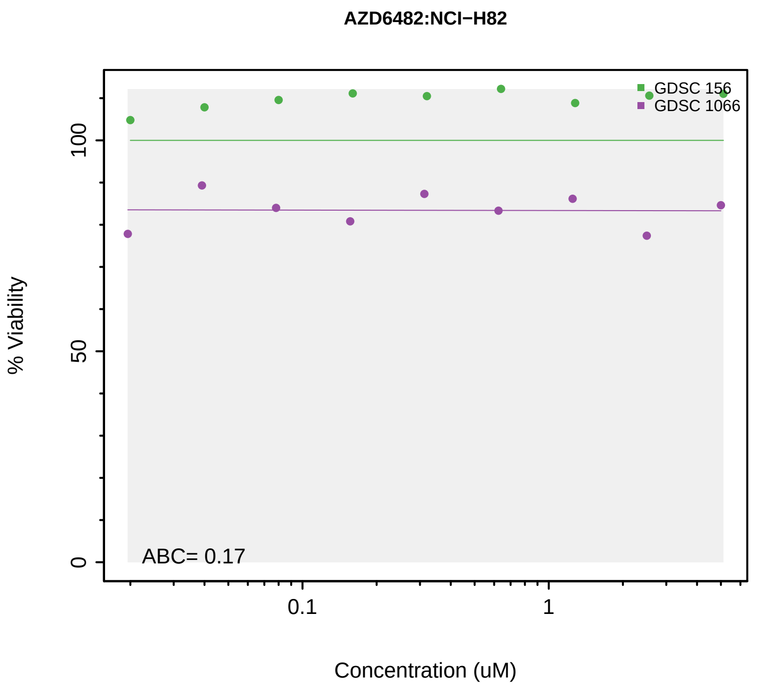
AZD6482:NCI−H82
GDSC 156
GDSC 1066
ABC= 0.17
% Viability
Concentration (uM)

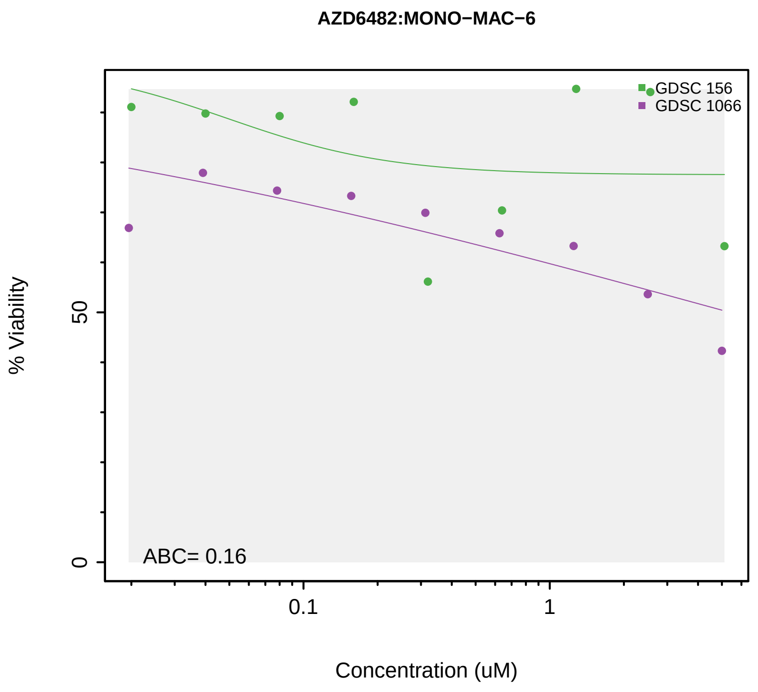
AZD6482:MONO−MAC−6
GDSC 156
GDSC 1066
ABC= 0.16
% Viability
Concentration (uM)

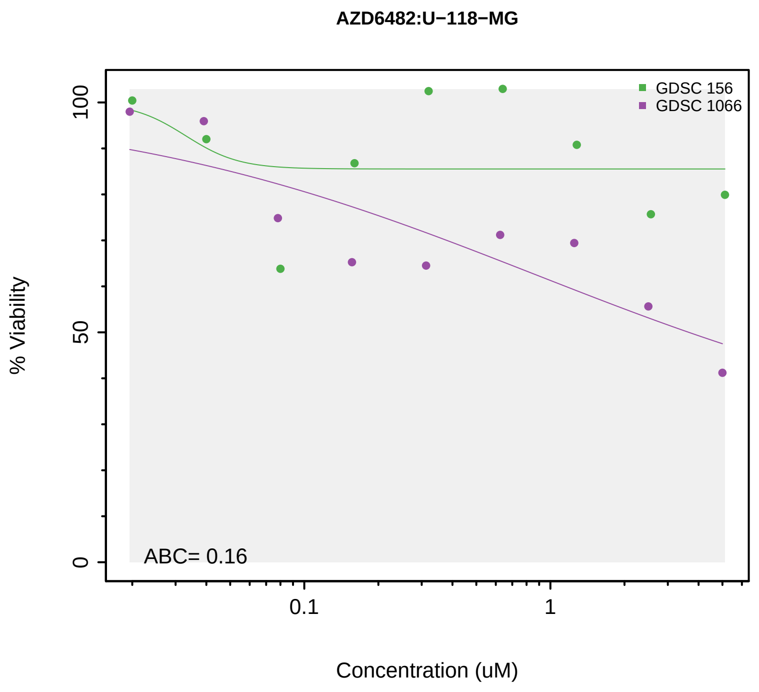
AZD6482:U−118−MG
GDSC 156
GDSC 1066
ABC= 0.16
% Viability
Concentration (uM)

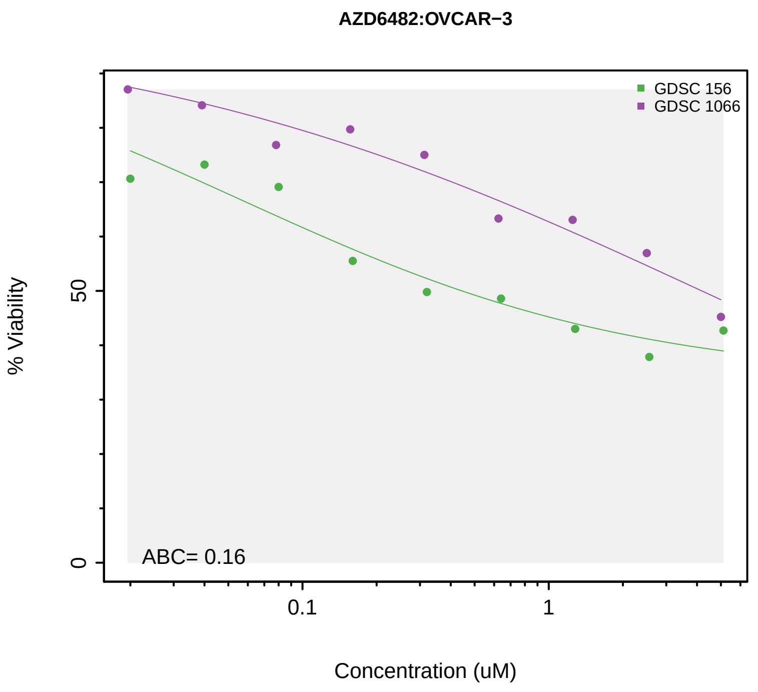
AZD6482:OVCAR−3
GDSC 156
GDSC 1066
ABC= 0.16
% Viability
Concentration (uM)

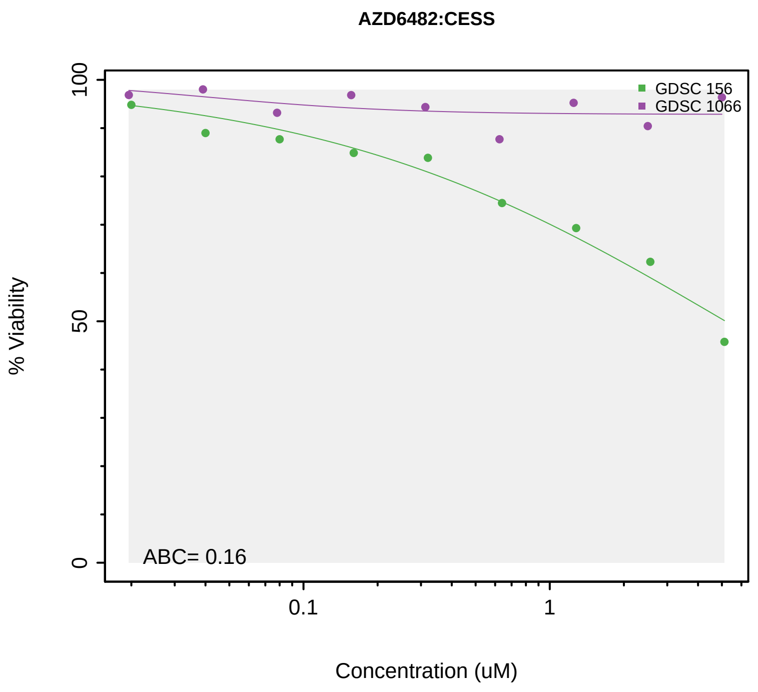
AZD6482:CESS
GDSC 156
GDSC 1066
ABC= 0.16
% Viability
Concentration (uM)

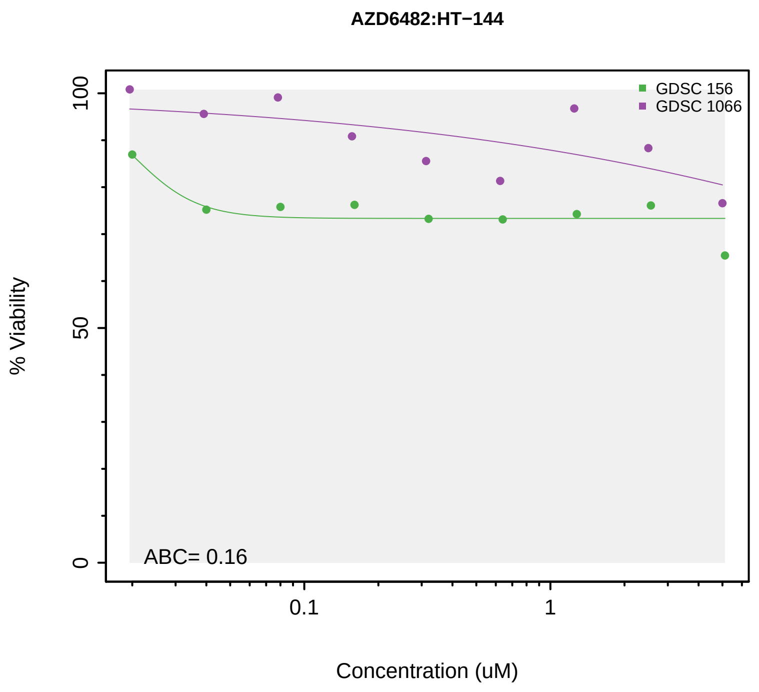
AZD6482:HT−144
GDSC 156
GDSC 1066
ABC= 0.16
% Viability
Concentration (uM)

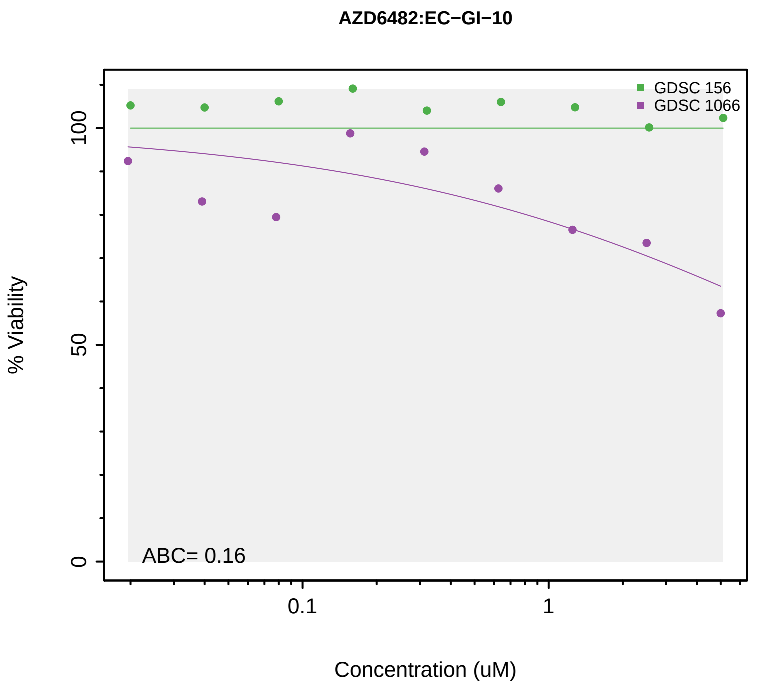
AZD6482:EC−GI−10
GDSC 156
GDSC 1066
ABC= 0.16
% Viability
Concentration (uM)

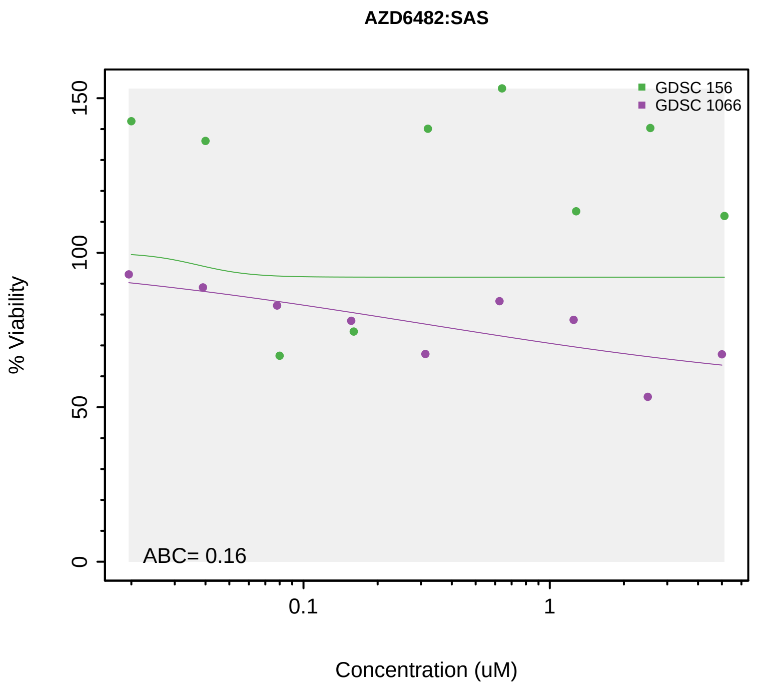
AZD6482:SAS
GDSC 156
GDSC 1066
ABC= 0.16
% Viability
Concentration (uM)

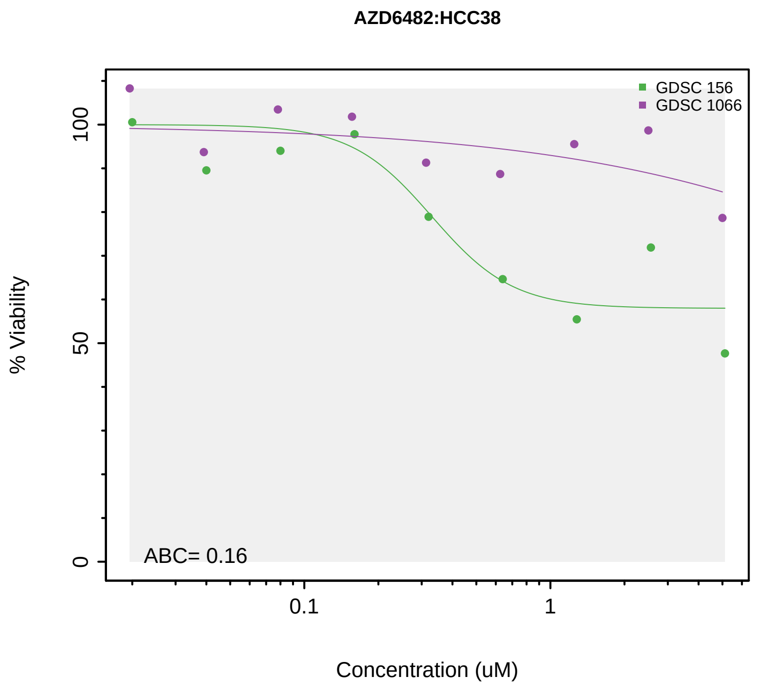
AZD6482:HCC38
GDSC 156
GDSC 1066
ABC= 0.16
% Viability
Concentration (uM)

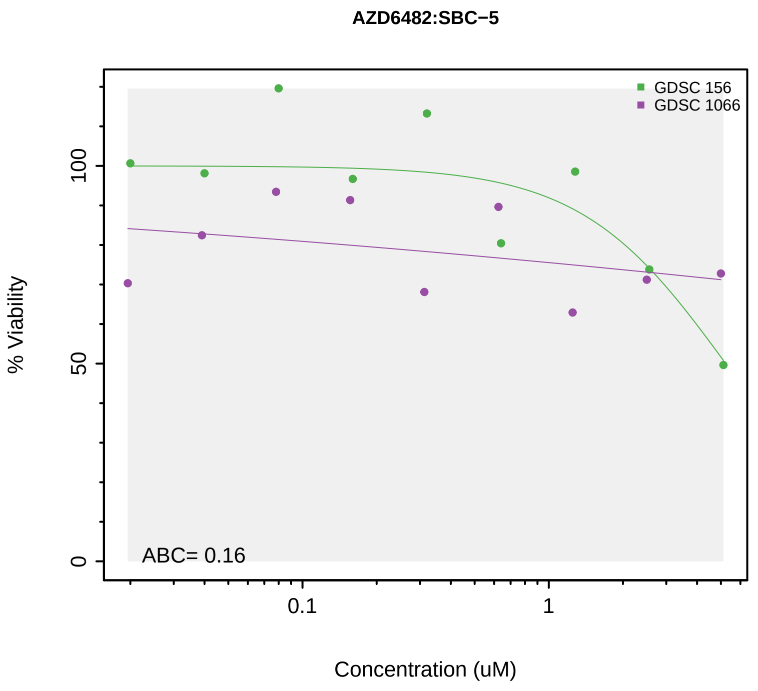
AZD6482:SBC−5
GDSC 156
GDSC 1066
ABC= 0.16
% Viability
0
50
100
0.1
1
Concentration (uM)

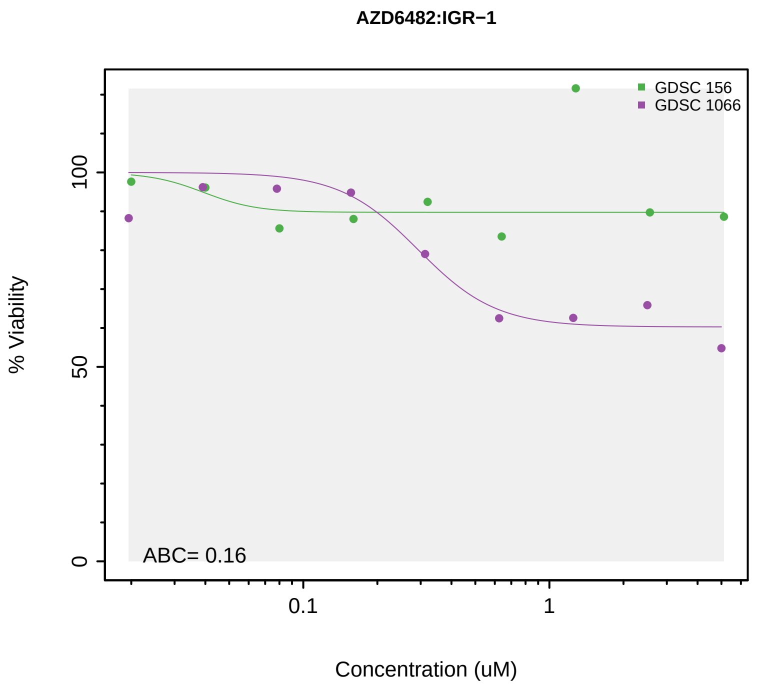
AZD6482:IGR−1
GDSC 156
GDSC 1066
ABC= 0.16
% Viability
0
50
100
0.1
1
Concentration (uM)

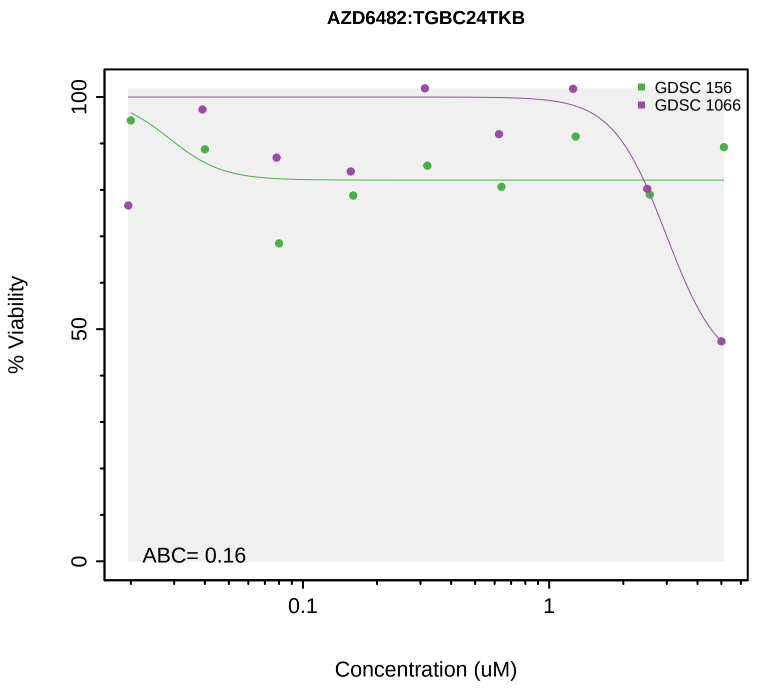
AZD6482:TGBC24TKB
GDSC 156
GDSC 1066
ABC= 0.16
% Viability
0
50
100
0.1
1
Concentration (uM)

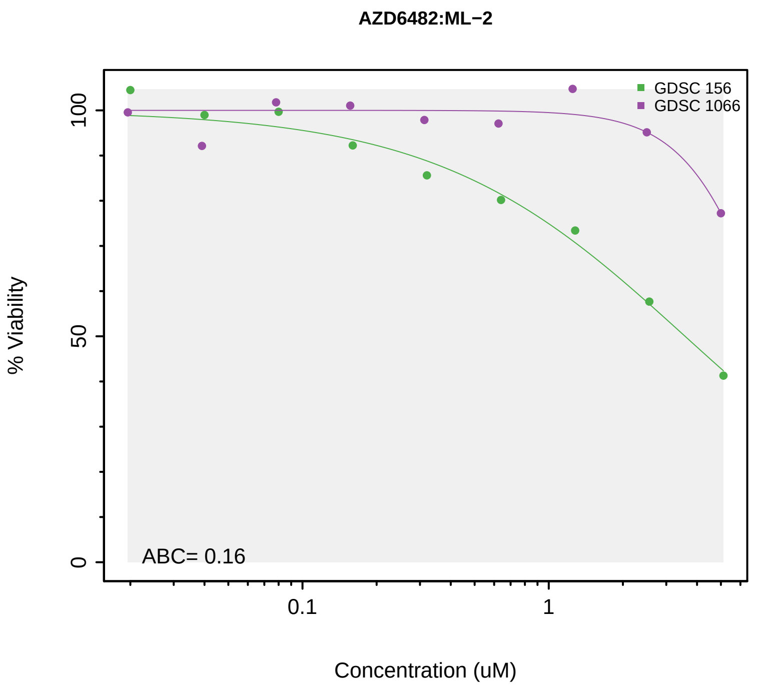
AZD6482:ML−2
GDSC 156
GDSC 1066
ABC= 0.16
% Viability
0
50
100
0.1
1
Concentration (uM)

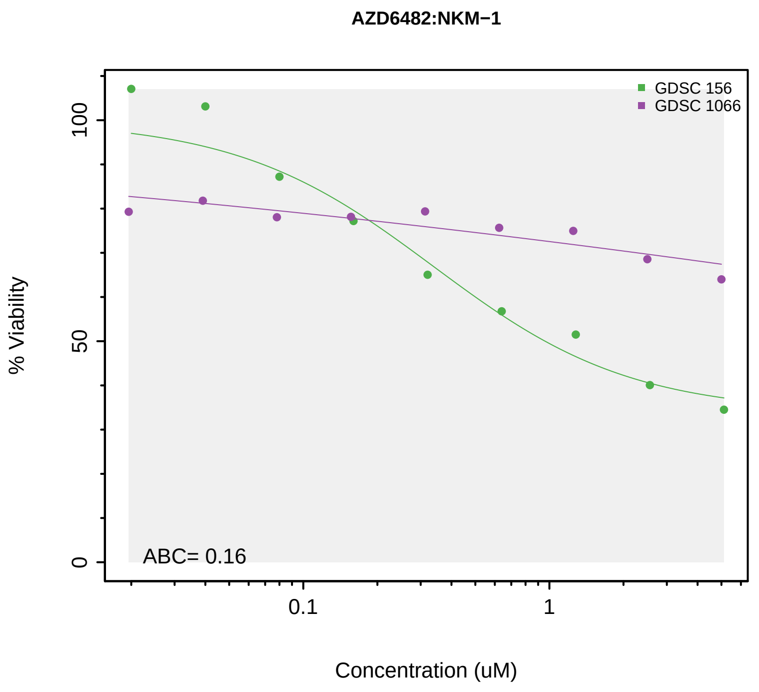
AZD6482:NKM−1
GDSC 156
GDSC 1066
ABC= 0.16
% Viability
0
50
100
0.1
1
Concentration (uM)

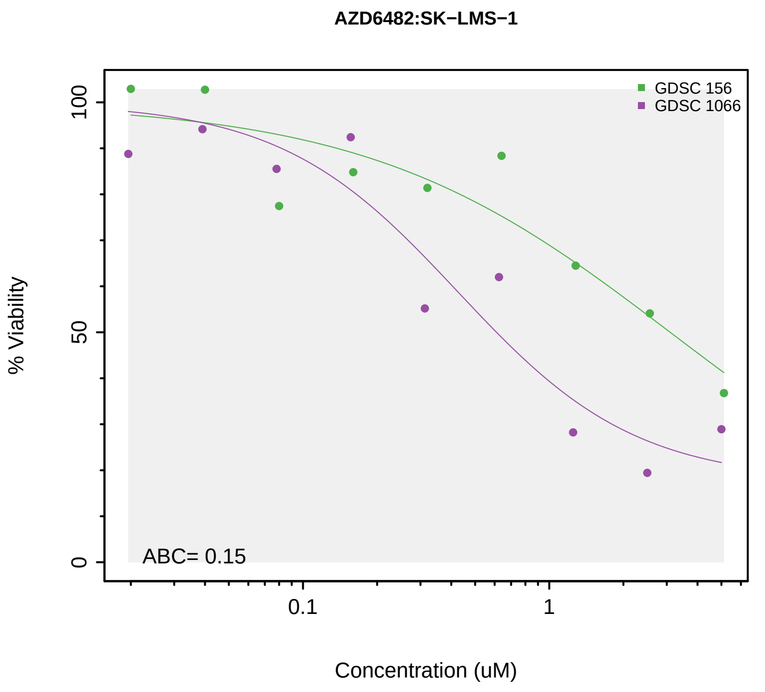
AZD6482:SK−LMS−1
GDSC 156
GDSC 1066
ABC= 0.15
% Viability
0
50
100
0.1
1
Concentration (uM)

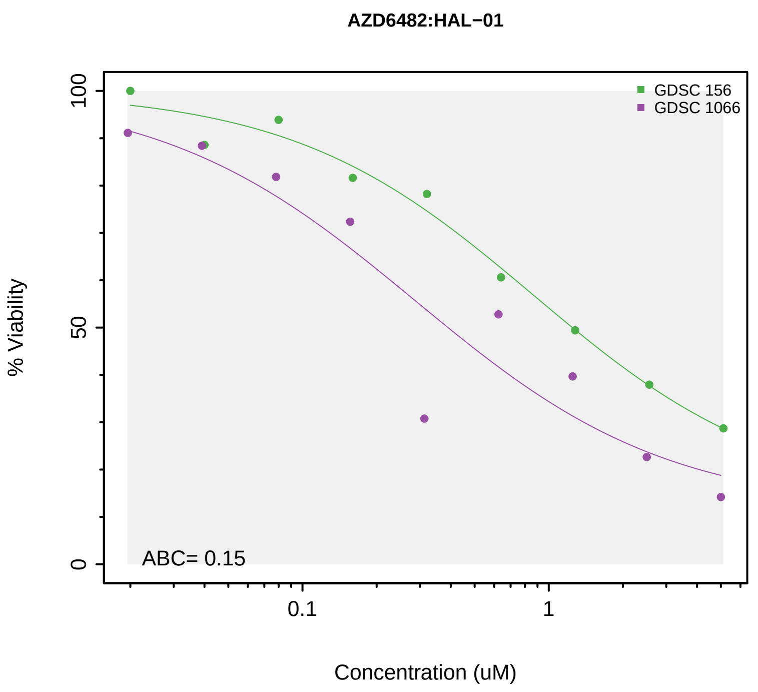
AZD6482:HAL−01
GDSC 156
GDSC 1066
ABC= 0.15
% Viability
0
50
100
0.1
1
Concentration (uM)

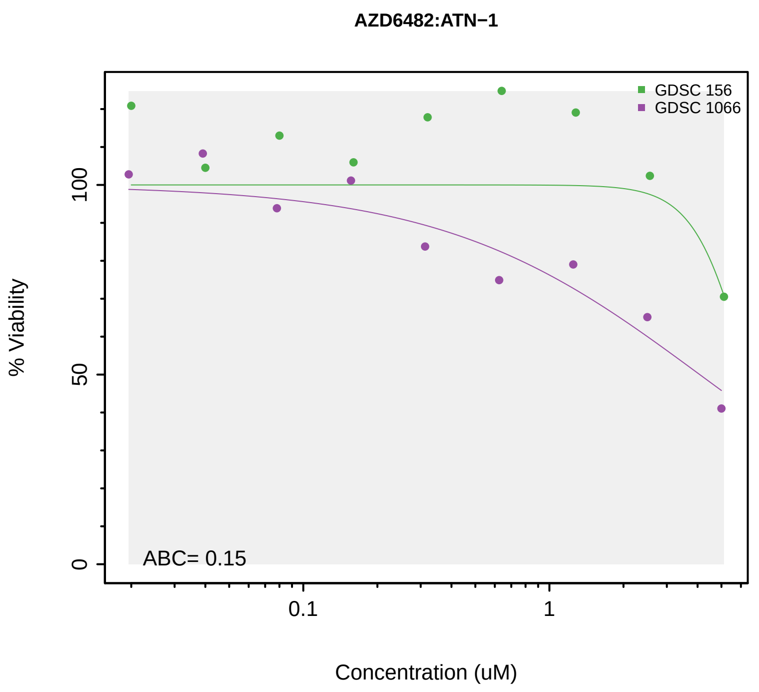
AZD6482:ATN−1
GDSC 156
GDSC 1066
ABC= 0.15
% Viability
0
50
100
0.1
1
Concentration (uM)

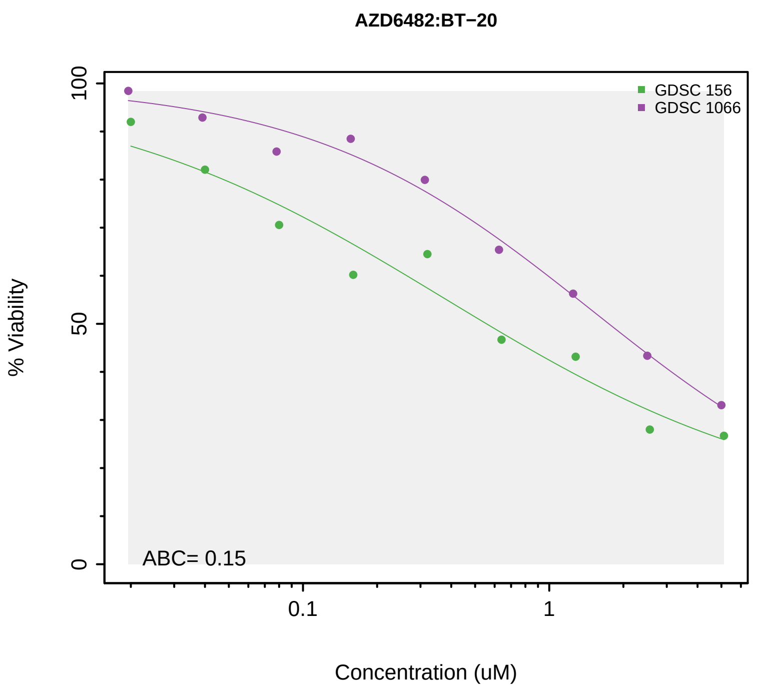
AZD6482:BT−20
GDSC 156
GDSC 1066
ABC= 0.15
% Viability
0
50
100
0.1
1
Concentration (uM)

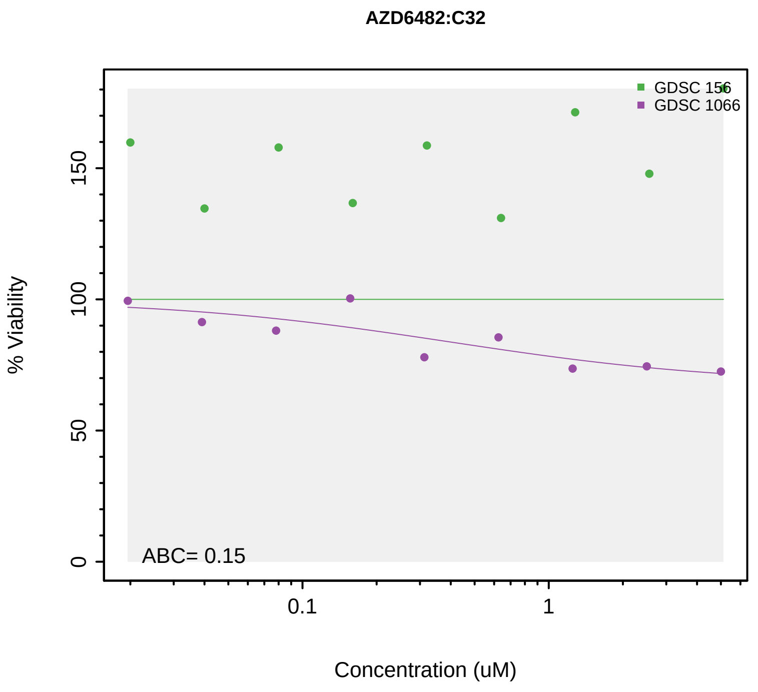
AZD6482:C32
GDSC 156
GDSC 1066
ABC= 0.15
% Viability
0
50
100
150
0.1
1
Concentration (uM)

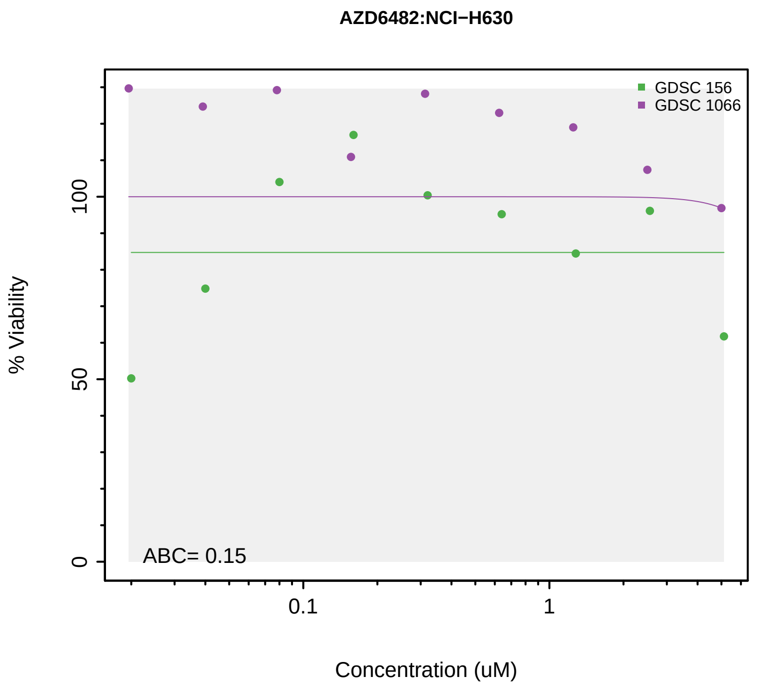
AZD6482:NCI−H630
GDSC 156
GDSC 1066
ABC= 0.15
% Viability
0
50
100
0.1
1
Concentration (uM)

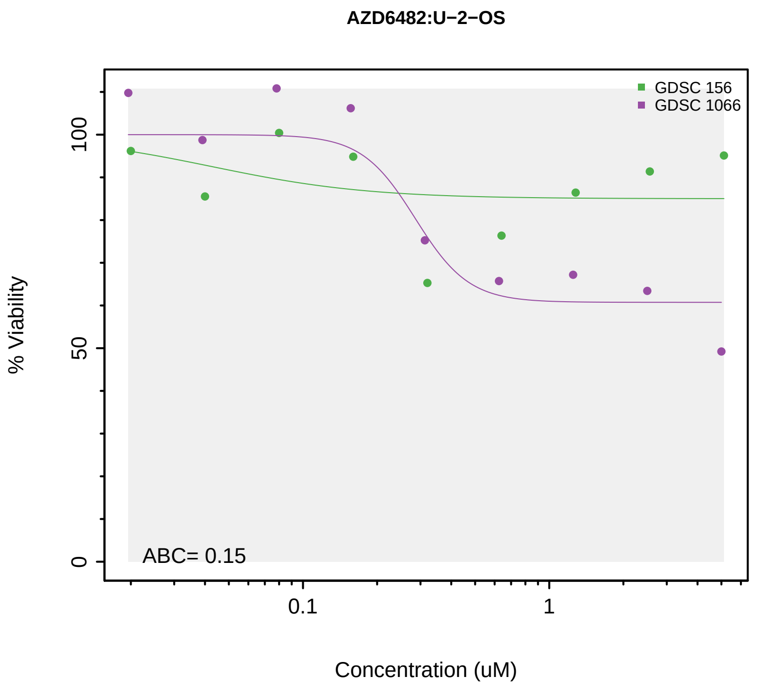
AZD6482:U−2−OS
GDSC 156
GDSC 1066
ABC= 0.15
% Viability
0
50
100
0.1
1
Concentration (uM)

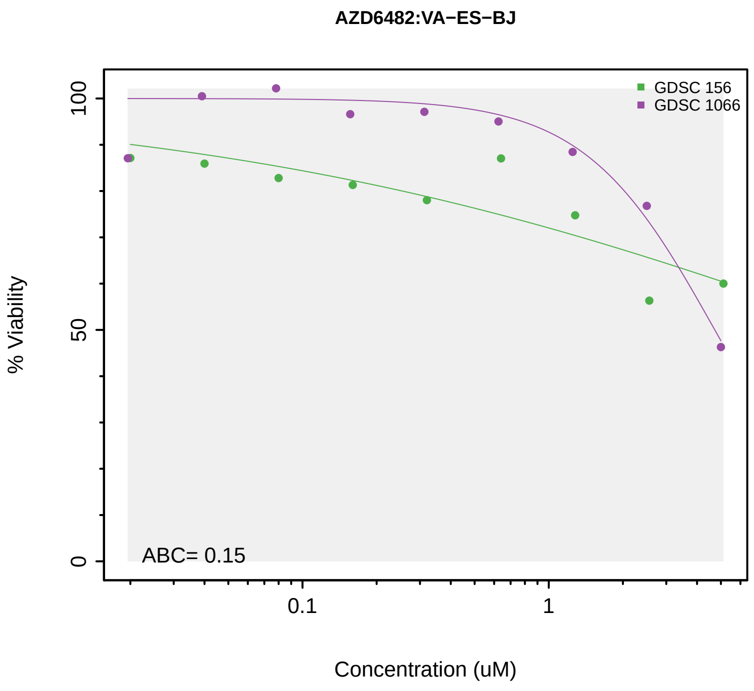
AZD6482:VA−ES−BJ
GDSC 156
GDSC 1066
ABC= 0.15
% Viability
Concentration (uM)

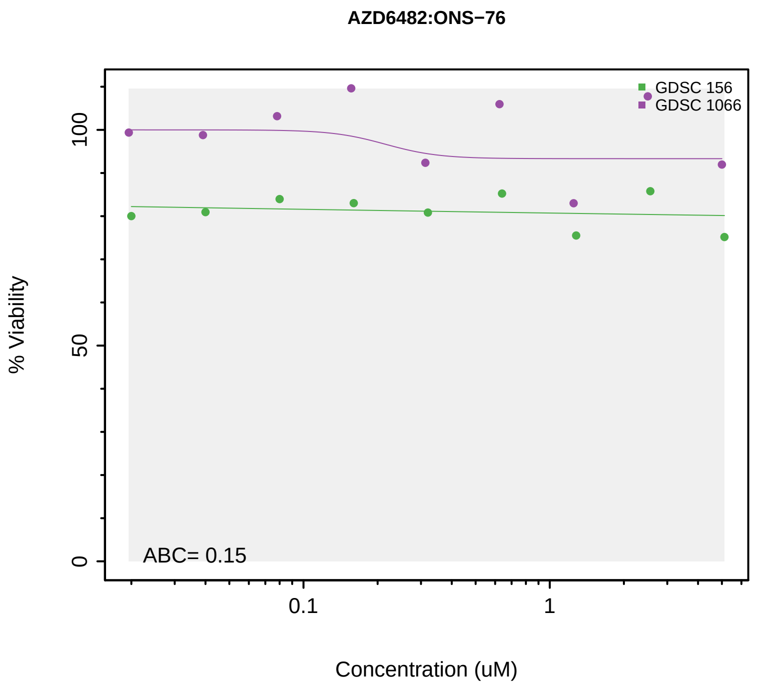
AZD6482:ONS−76
GDSC 156
GDSC 1066
ABC= 0.15
% Viability
Concentration (uM)

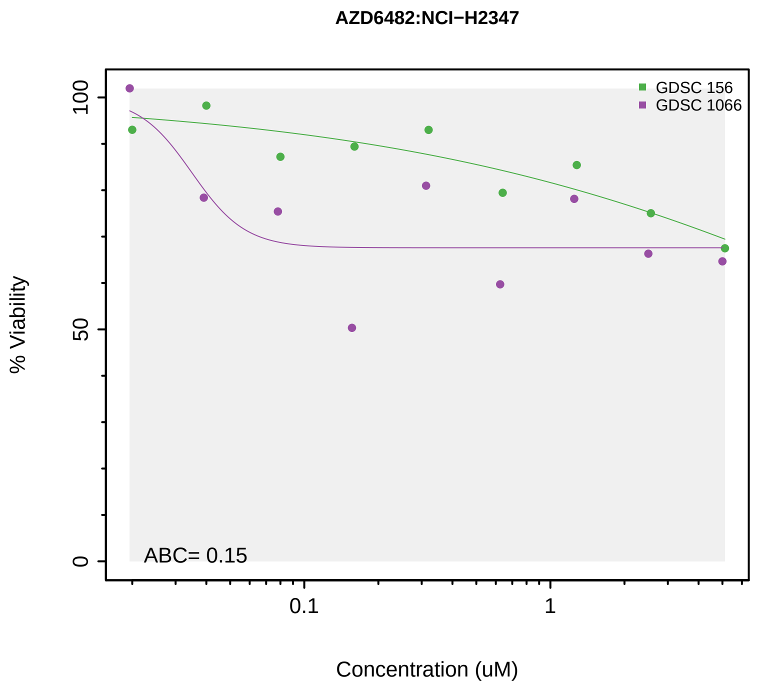
AZD6482:NCI−H2347
GDSC 156
GDSC 1066
ABC= 0.15
% Viability
Concentration (uM)

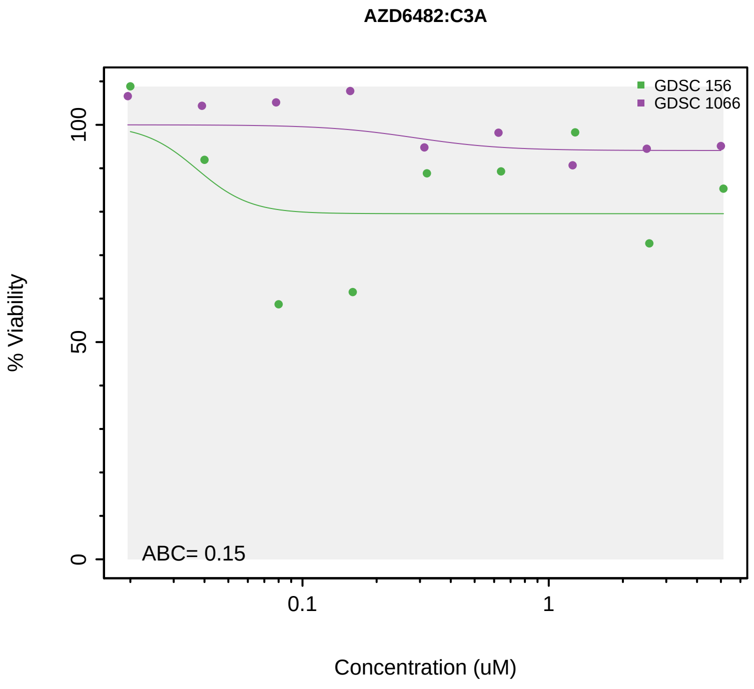
AZD6482:C3A
GDSC 156
GDSC 1066
ABC= 0.15
% Viability
Concentration (uM)

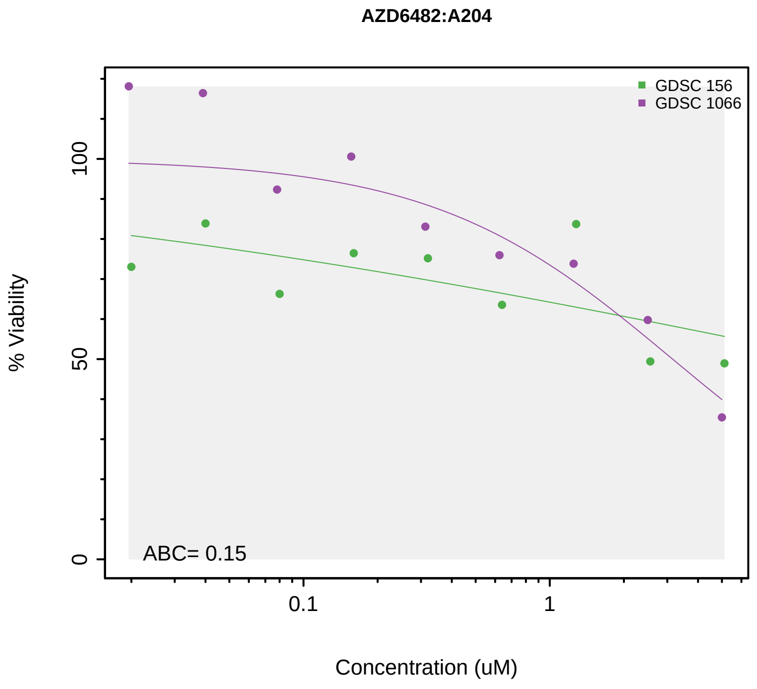
AZD6482:A204
GDSC 156
GDSC 1066
ABC= 0.15
% Viability
Concentration (uM)

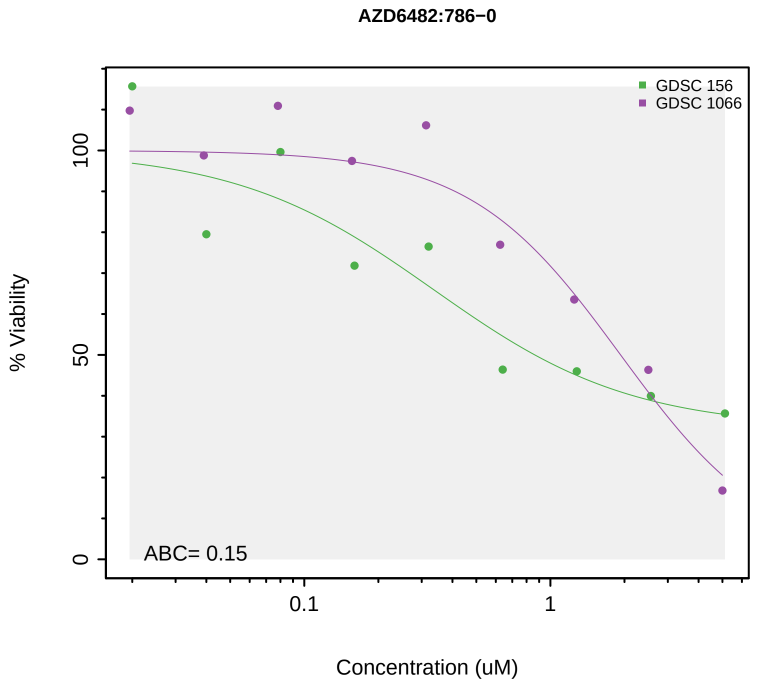
AZD6482:786−0
GDSC 156
GDSC 1066
ABC= 0.15
% Viability
Concentration (uM)

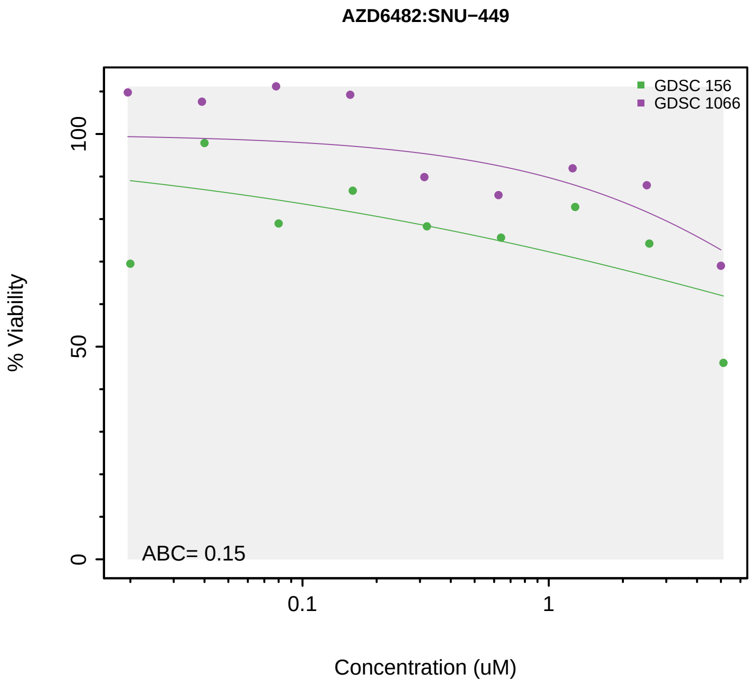
AZD6482:SNU−449
GDSC 156
GDSC 1066
ABC= 0.15
% Viability
Concentration (uM)

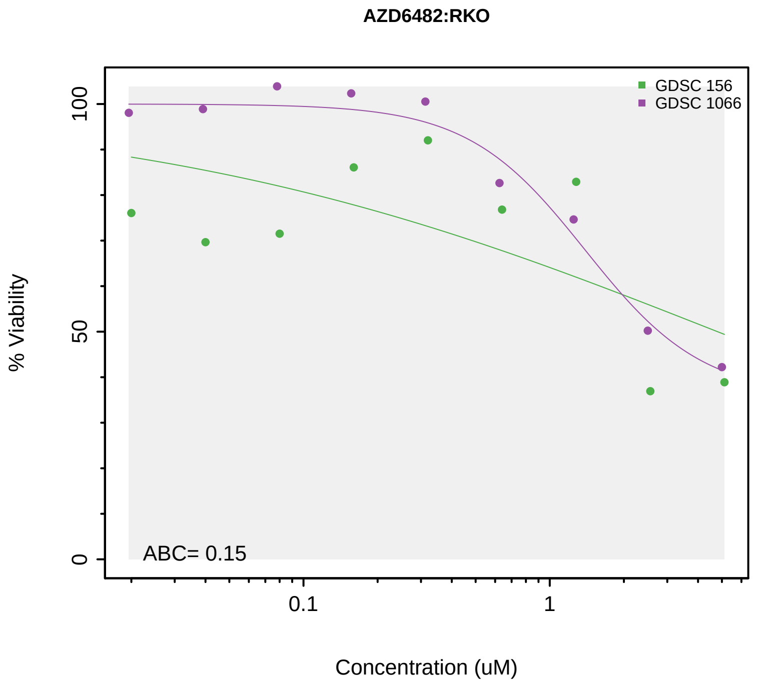
AZD6482:RKO
GDSC 156
GDSC 1066
ABC= 0.15
% Viability
Concentration (uM)

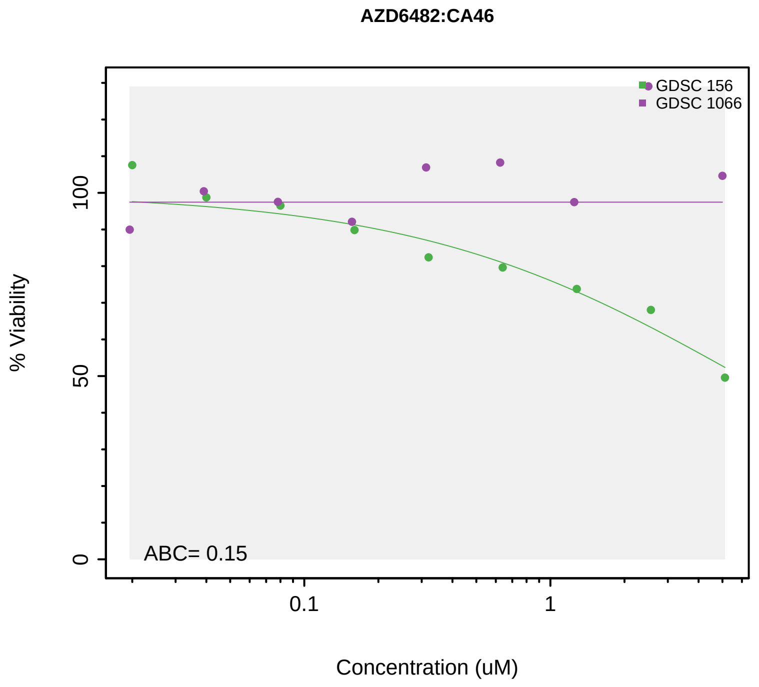
AZD6482:CA46
GDSC 156
GDSC 1066
ABC= 0.15
% Viability
Concentration (uM)

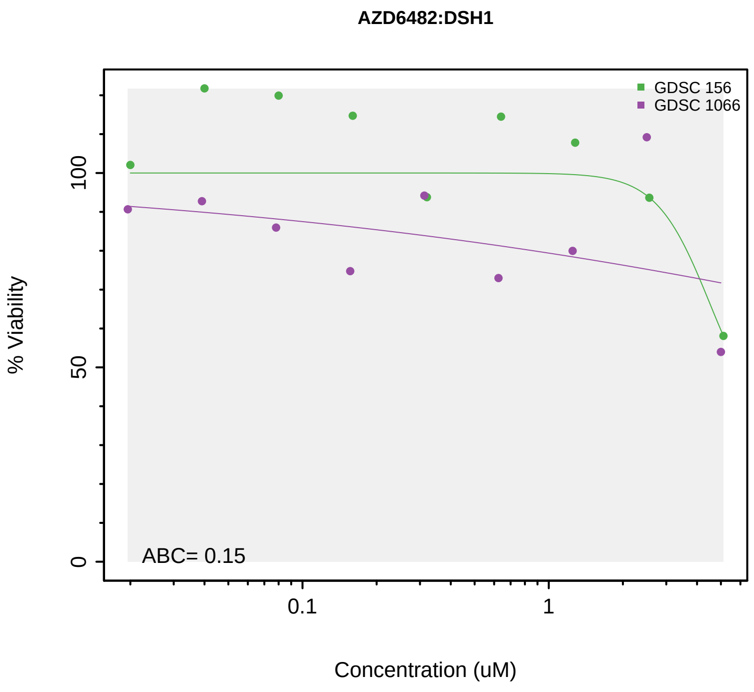
AZD6482:DSH1
GDSC 156
GDSC 1066
ABC= 0.15
% Viability
Concentration (uM)

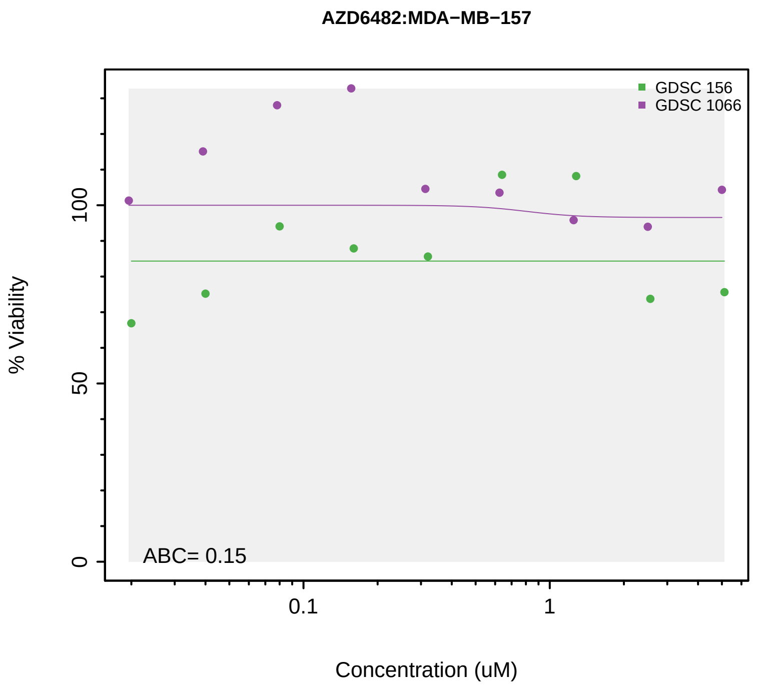
AZD6482:MDA−MB−157
GDSC 156
GDSC 1066
ABC= 0.15
% Viability
Concentration (uM)

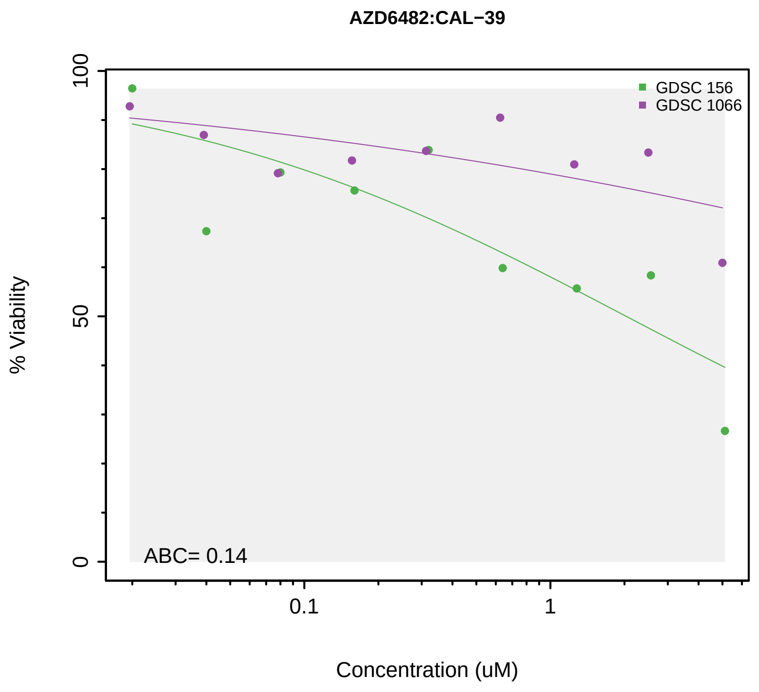
AZD6482:CAL−39
GDSC 156
GDSC 1066
ABC= 0.14
% Viability
Concentration (uM)

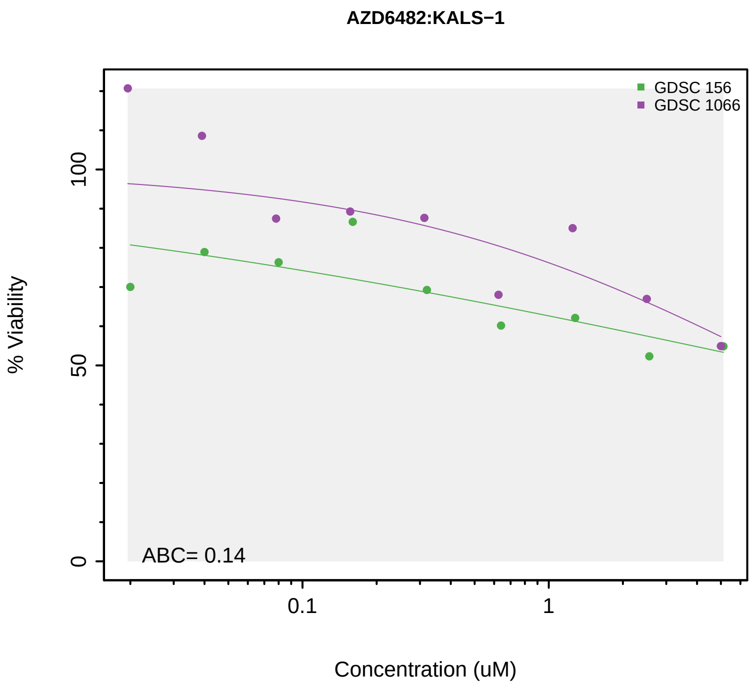
AZD6482:KALS−1
GDSC 156
GDSC 1066
ABC= 0.14
% Viability
Concentration (uM)

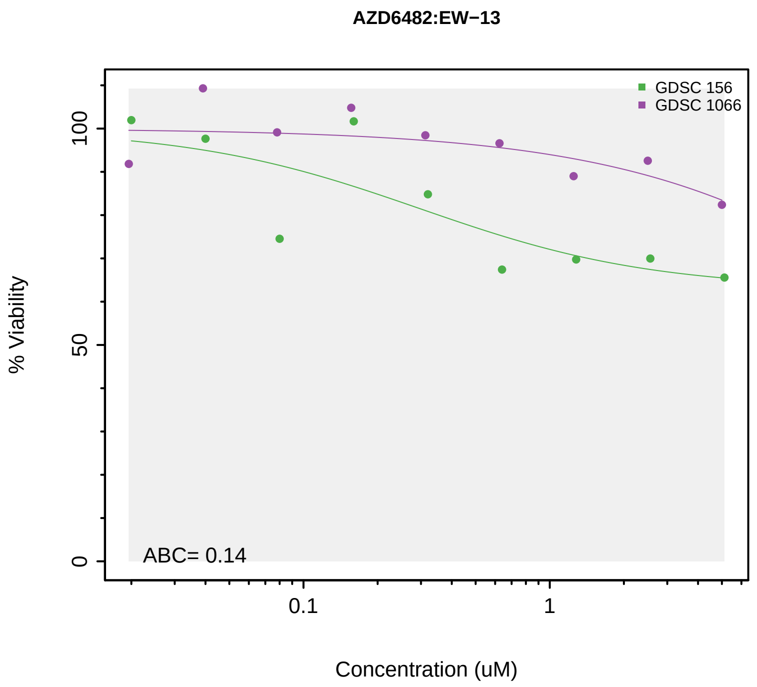
AZD6482:EW−13
GDSC 156
GDSC 1066
ABC= 0.14
% Viability
Concentration (uM)

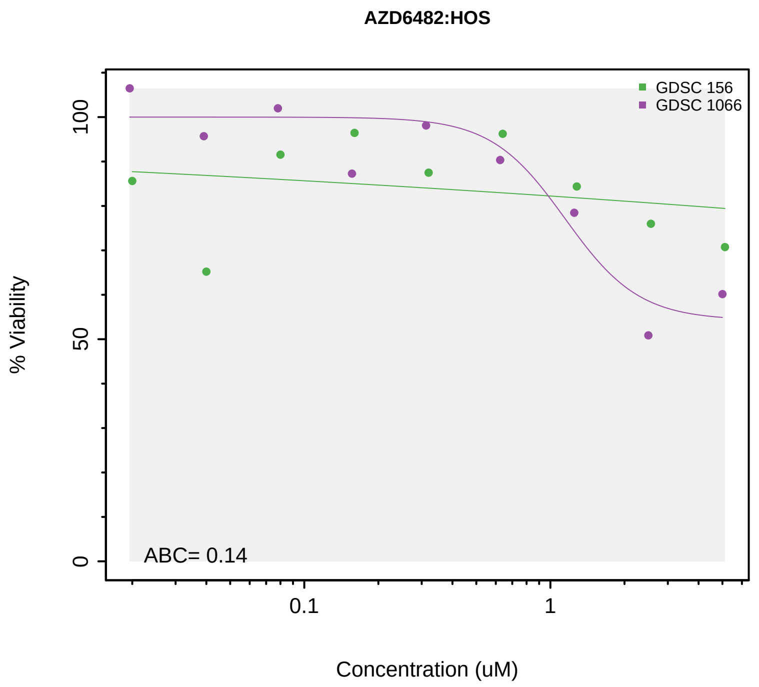
AZD6482:HOS
GDSC 156
GDSC 1066
ABC= 0.14
% Viability
Concentration (uM)

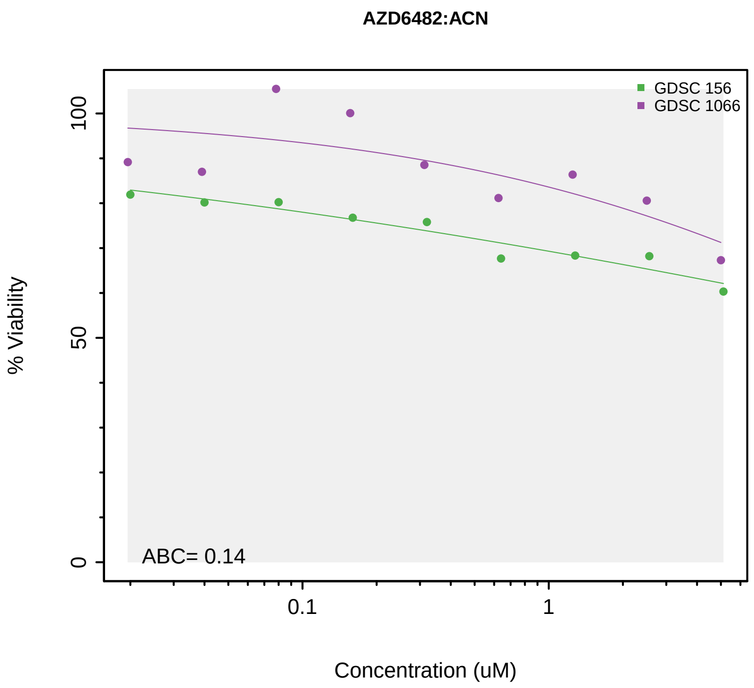
AZD6482:ACN
GDSC 156
GDSC 1066
ABC= 0.14
% Viability
Concentration (uM)

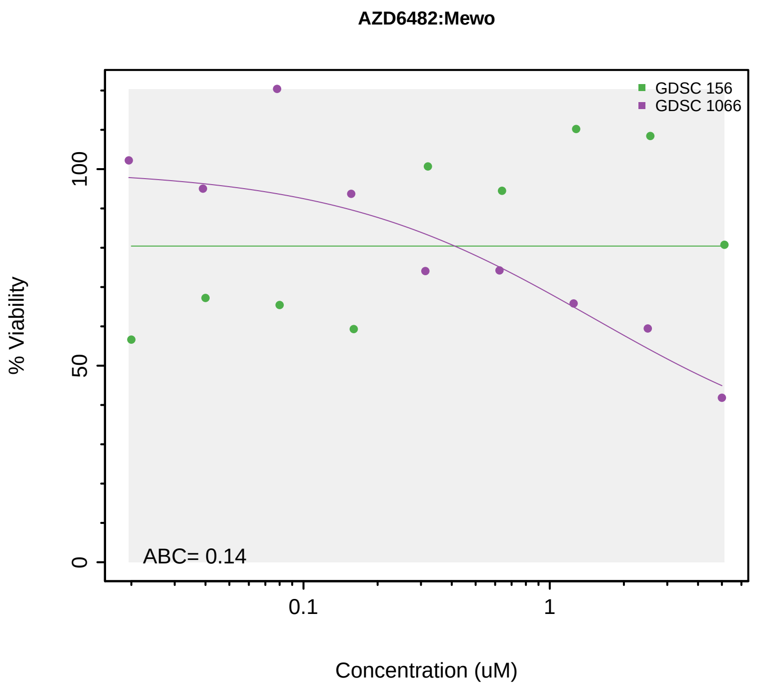
AZD6482:Mewo
GDSC 156
GDSC 1066
ABC= 0.14
% Viability
Concentration (uM)

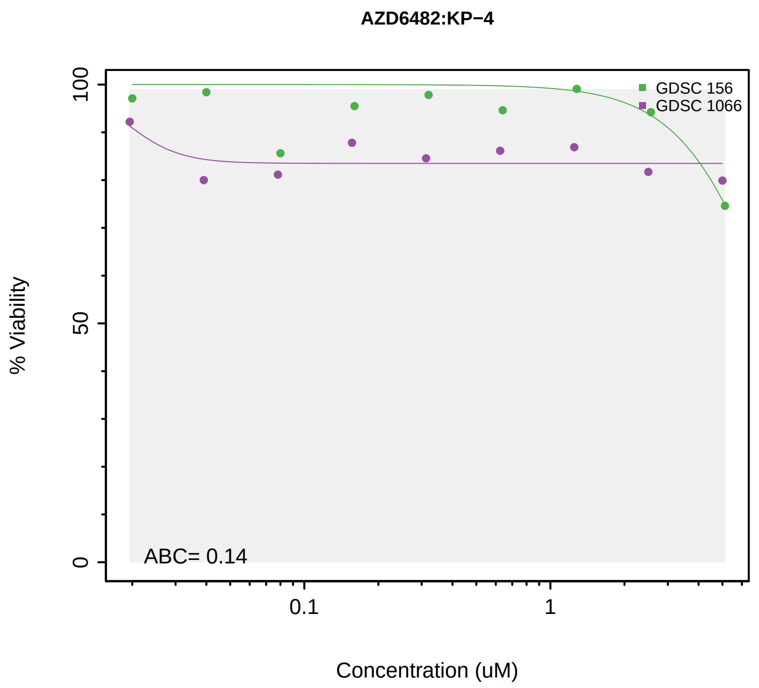
AZD6482:KP−4
GDSC 156
GDSC 1066
ABC= 0.14
% Viability
Concentration (uM)

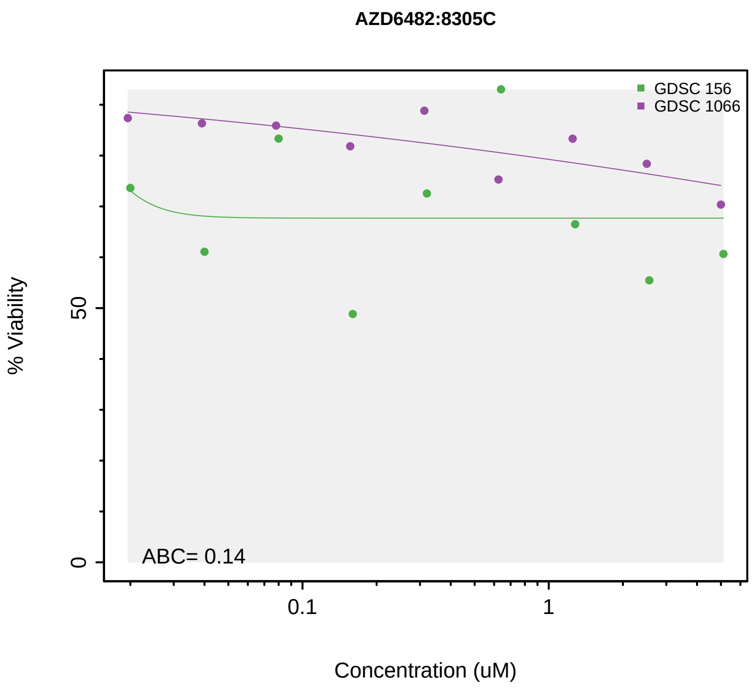
AZD6482:8305C
GDSC 156
GDSC 1066
ABC= 0.14
% Viability
Concentration (uM)

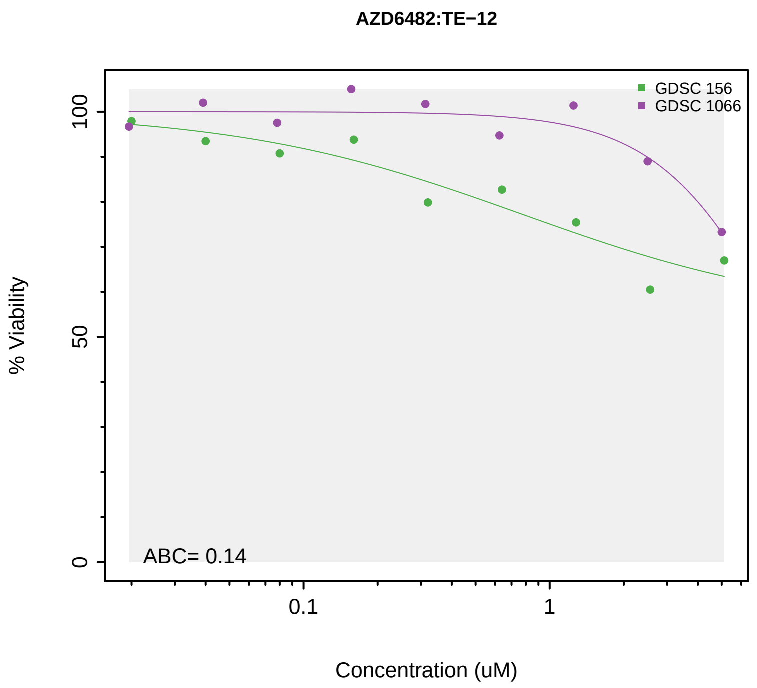
AZD6482:TE−12
GDSC 156
GDSC 1066
ABC= 0.14
% Viability
Concentration (uM)

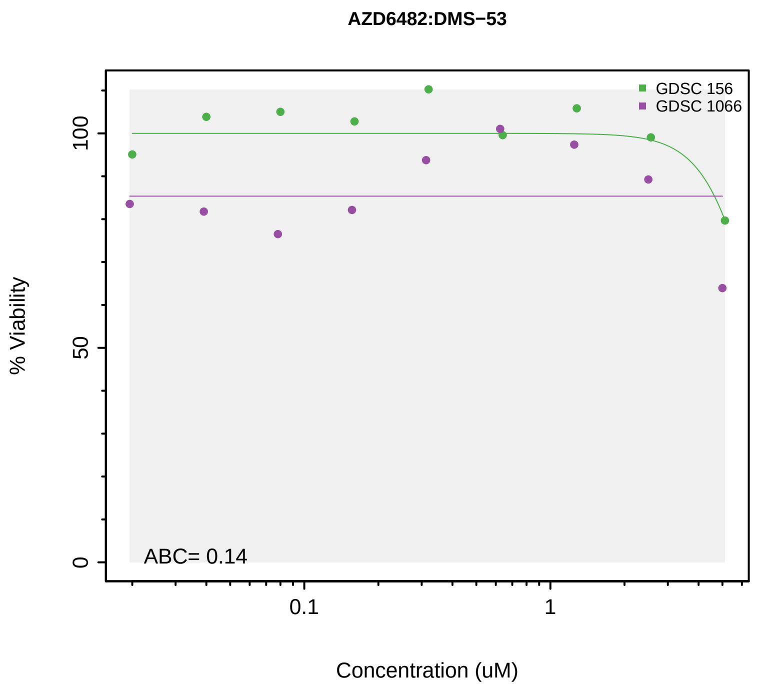
AZD6482:DMS−53
GDSC 156
GDSC 1066
ABC= 0.14
% Viability
Concentration (uM)

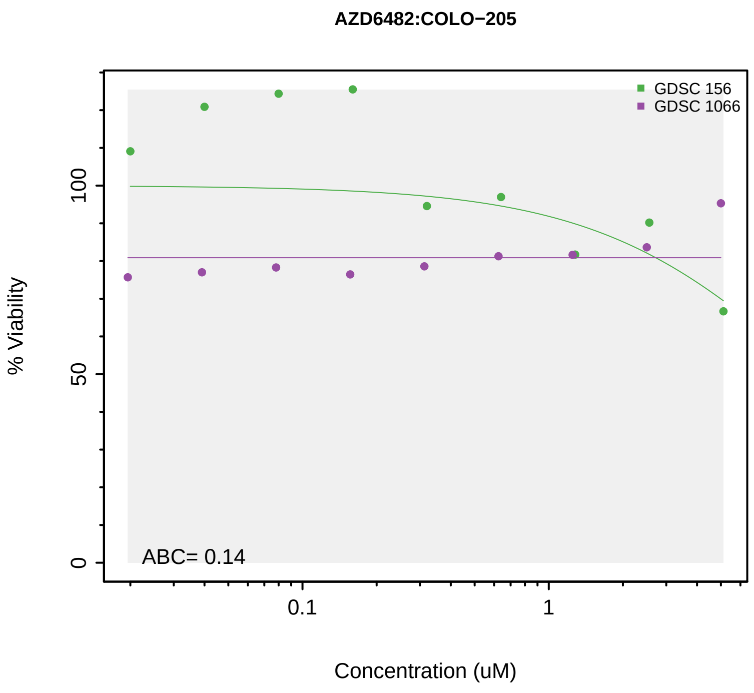
AZD6482:COLO−205
GDSC 156
GDSC 1066
ABC= 0.14
% Viability
Concentration (uM)

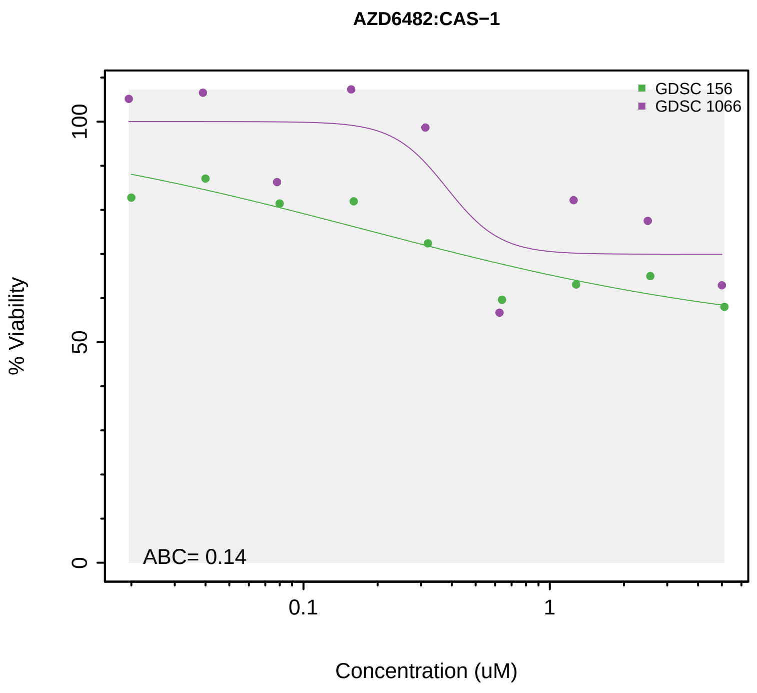
AZD6482:CAS−1
GDSC 156
GDSC 1066
ABC= 0.14
% Viability
Concentration (uM)

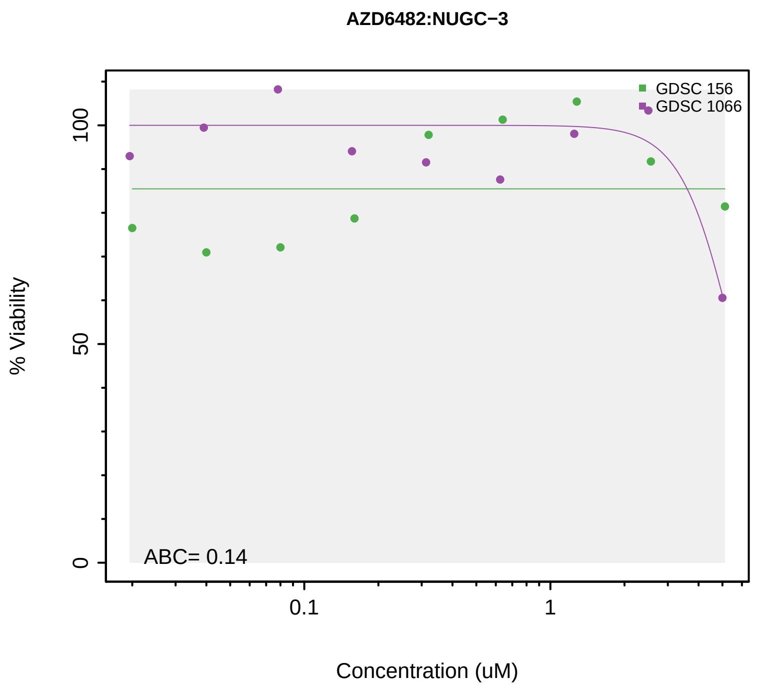
AZD6482:NUGC−3
GDSC 156
GDSC 1066
ABC= 0.14
% Viability
Concentration (uM)

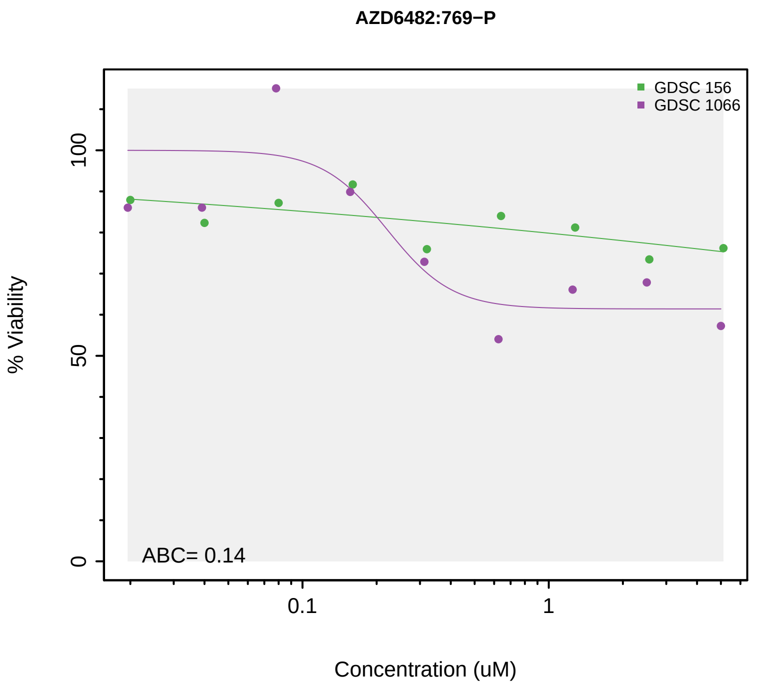
AZD6482:769−P
GDSC 156
GDSC 1066
ABC= 0.14
% Viability
Concentration (uM)

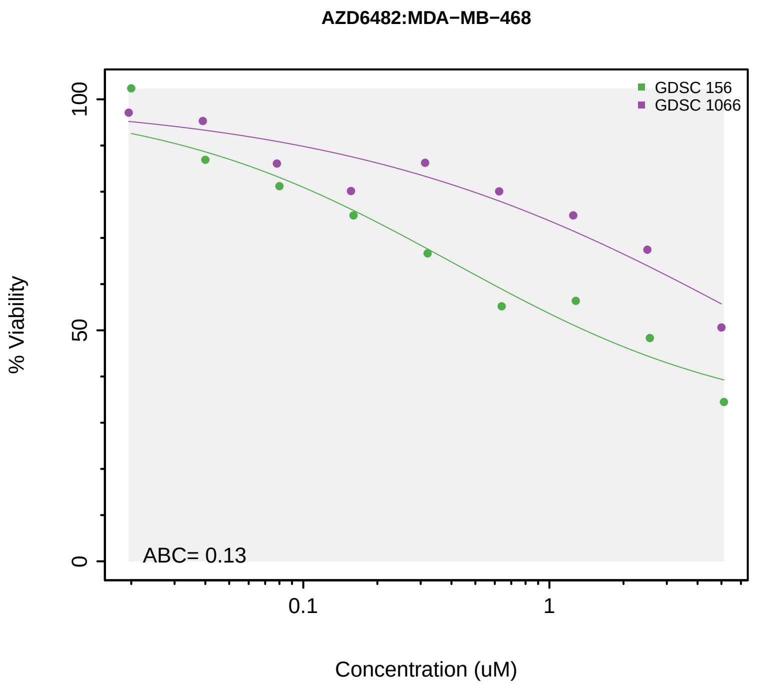
AZD6482:MDA−MB−468
GDSC 156
GDSC 1066
ABC= 0.13
% Viability
Concentration (uM)

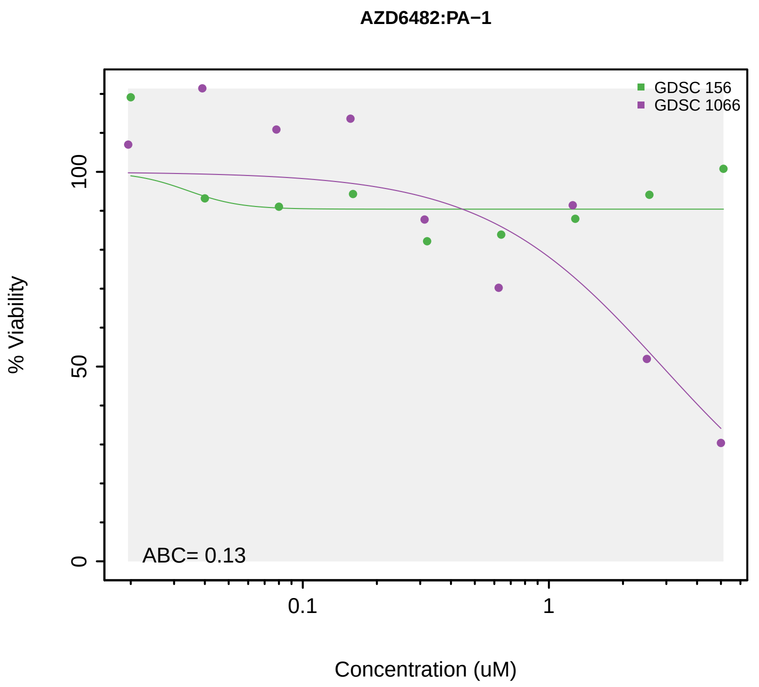
AZD6482:PA−1
GDSC 156
GDSC 1066
ABC= 0.13
% Viability
Concentration (uM)

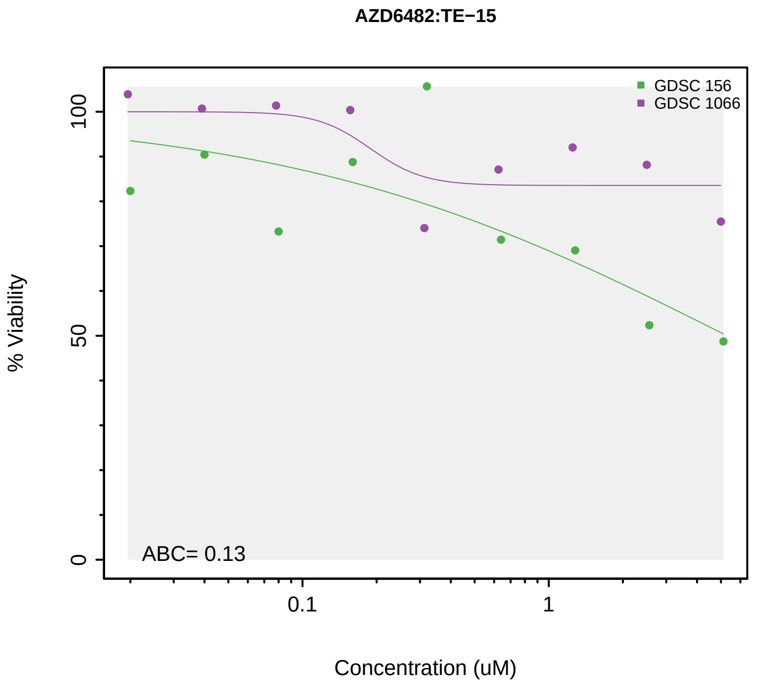
AZD6482:TE−15
GDSC 156
GDSC 1066
ABC= 0.13
% Viability
Concentration (uM)

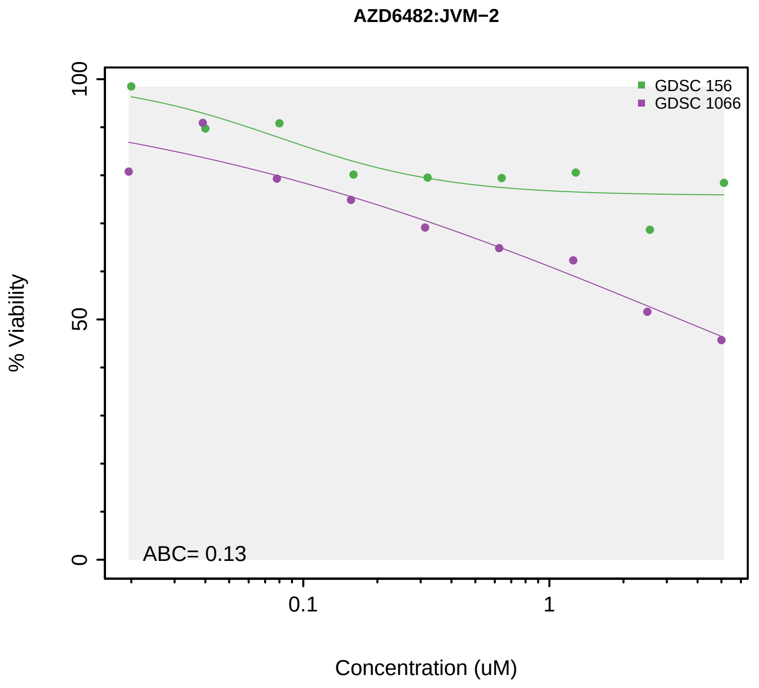
AZD6482:JVM−2
GDSC 156
GDSC 1066
ABC= 0.13
% Viability
Concentration (uM)

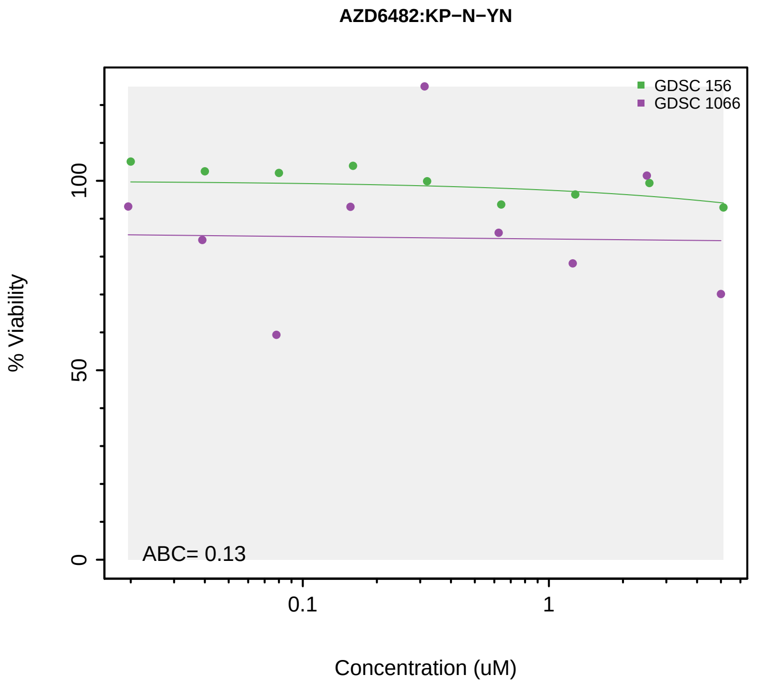
AZD6482:KP−N−YN
GDSC 156
GDSC 1066
ABC= 0.13
% Viability
Concentration (uM)

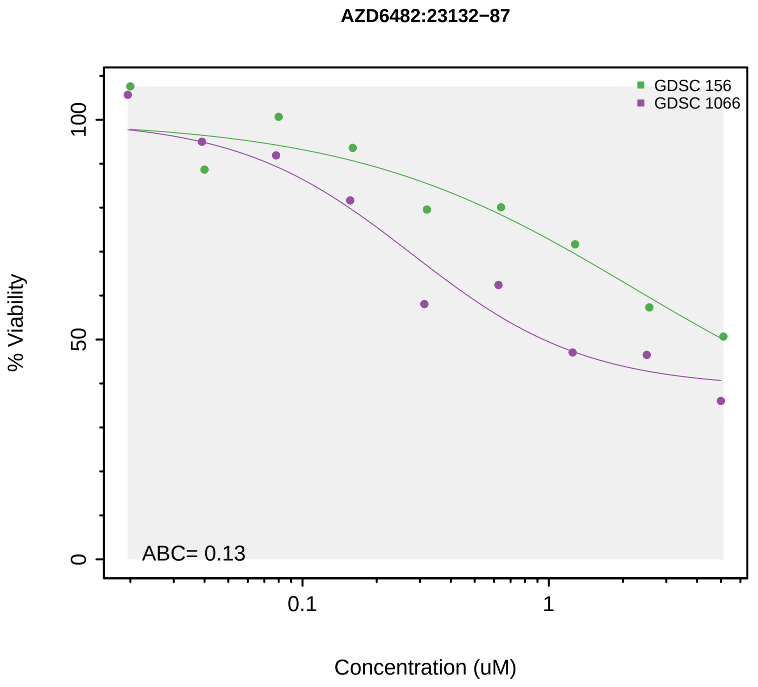
AZD6482:23132−87
GDSC 156
GDSC 1066
ABC= 0.13
% Viability
Concentration (uM)

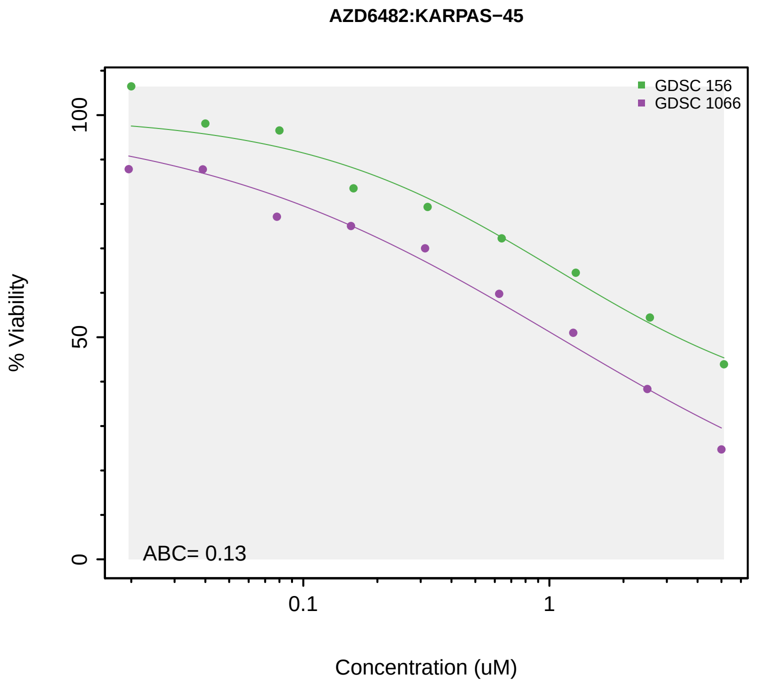
AZD6482:KARPAS−45
GDSC 156
GDSC 1066
ABC= 0.13
% Viability
Concentration (uM)

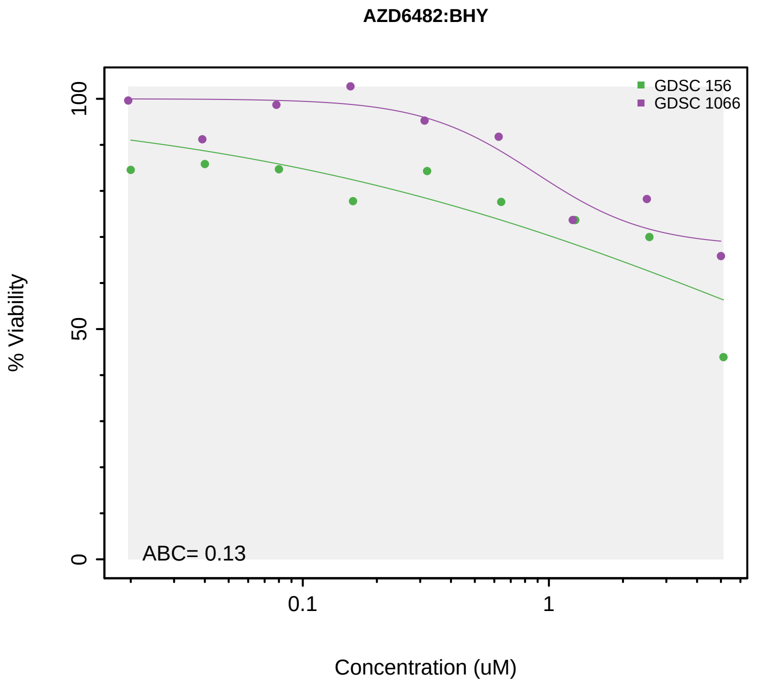
AZD6482:BHY
GDSC 156
GDSC 1066
ABC= 0.13
% Viability
Concentration (uM)

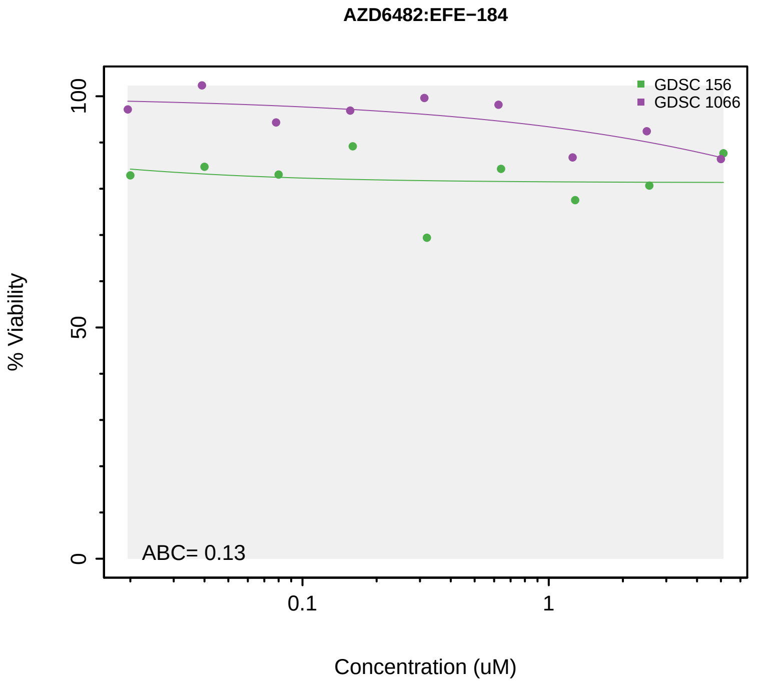
AZD6482:EFE−184
GDSC 156
GDSC 1066
ABC= 0.13
% Viability
Concentration (uM)

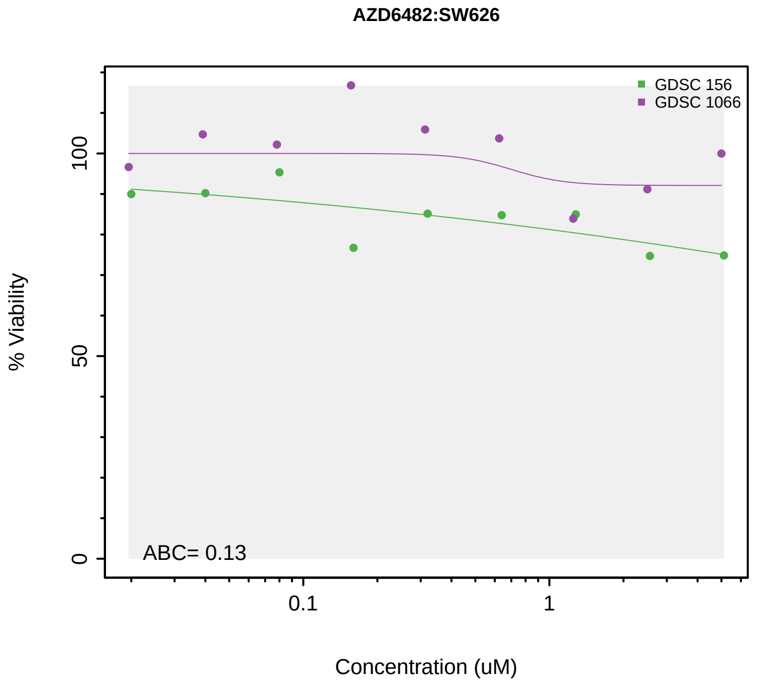
AZD6482:SW626
GDSC 156
GDSC 1066
ABC= 0.13
% Viability
Concentration (uM)

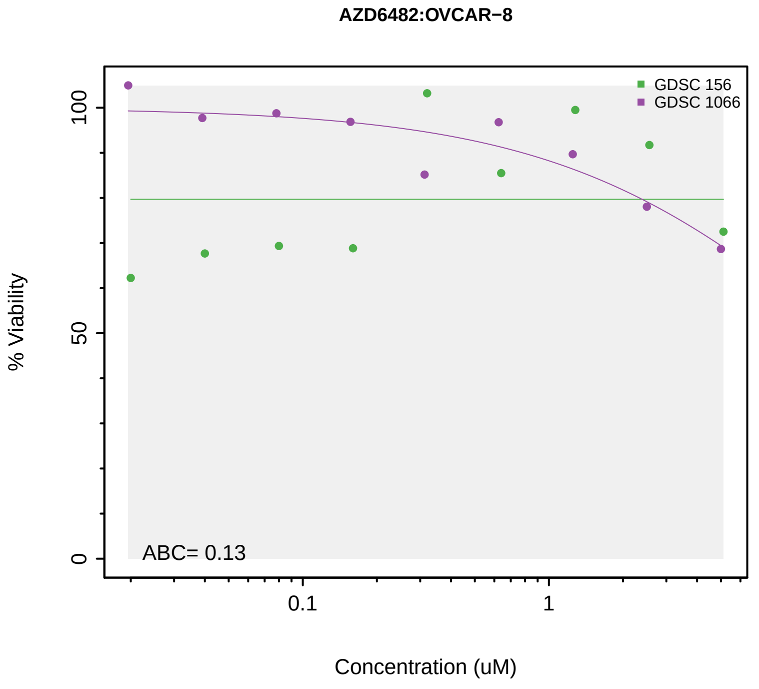
AZD6482:OVCAR−8
GDSC 156
GDSC 1066
ABC= 0.13
% Viability
Concentration (uM)

AZD6482:NCI−H1623

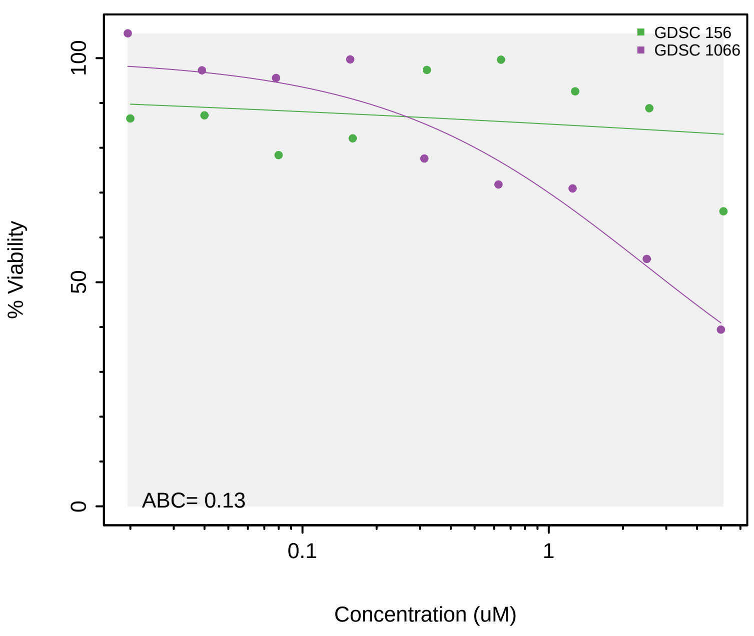

AZD6482:KNS−81−FD

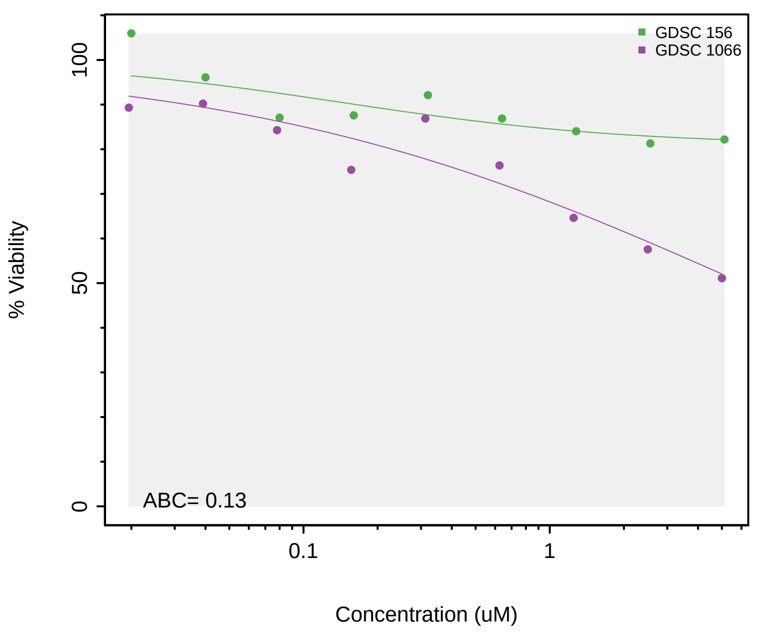

AZD6482:MHH−ES−1

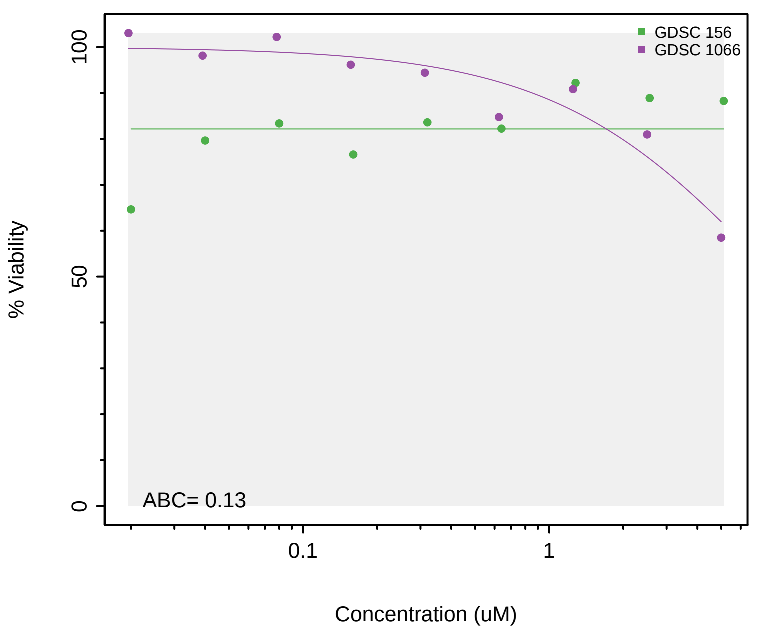

AZD6482:CMK

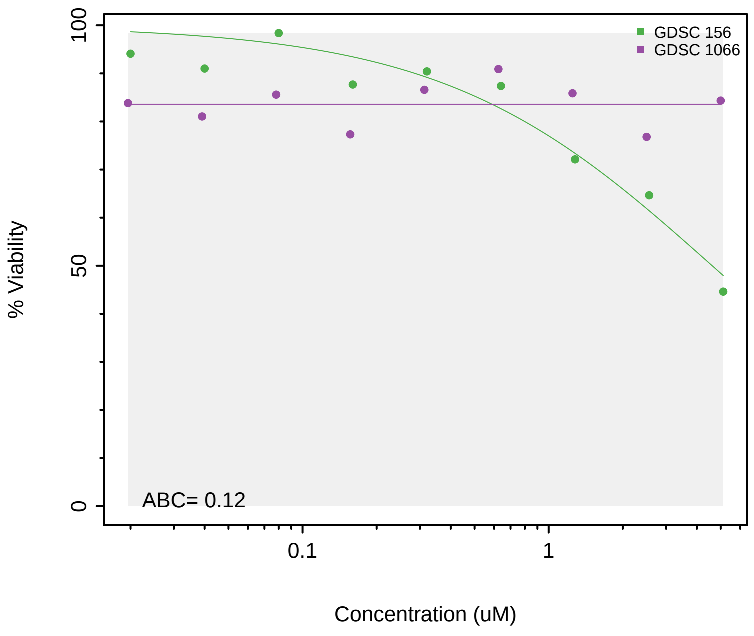

AZD6482:KMOE−2

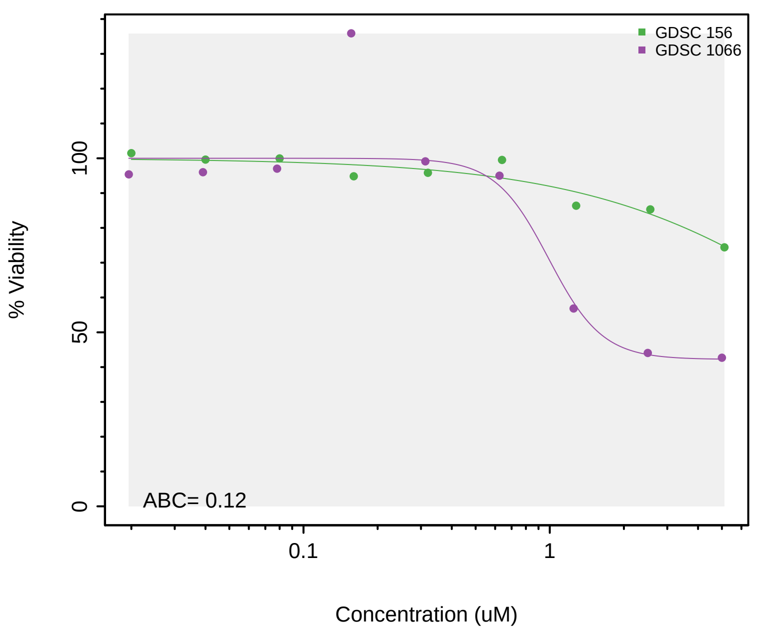

AZD6482:SK−NEP−1

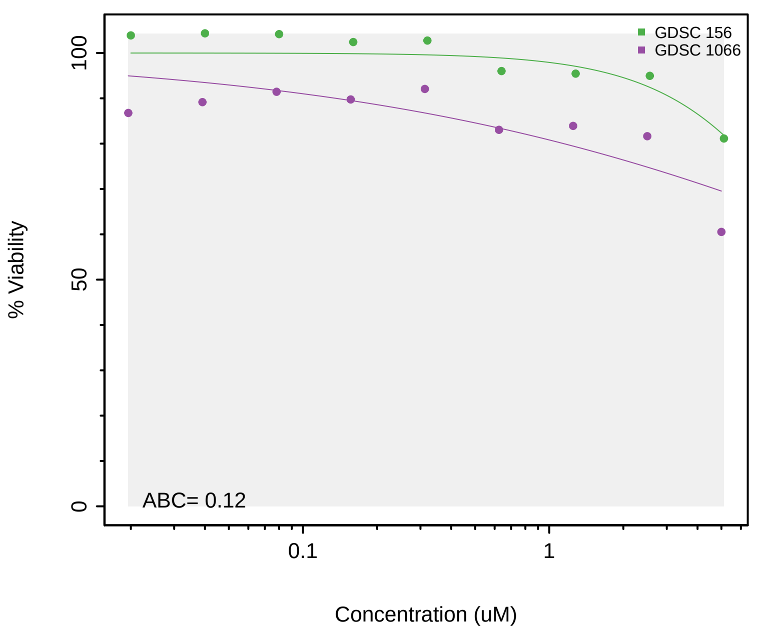

AZD6482:Hs−578−T

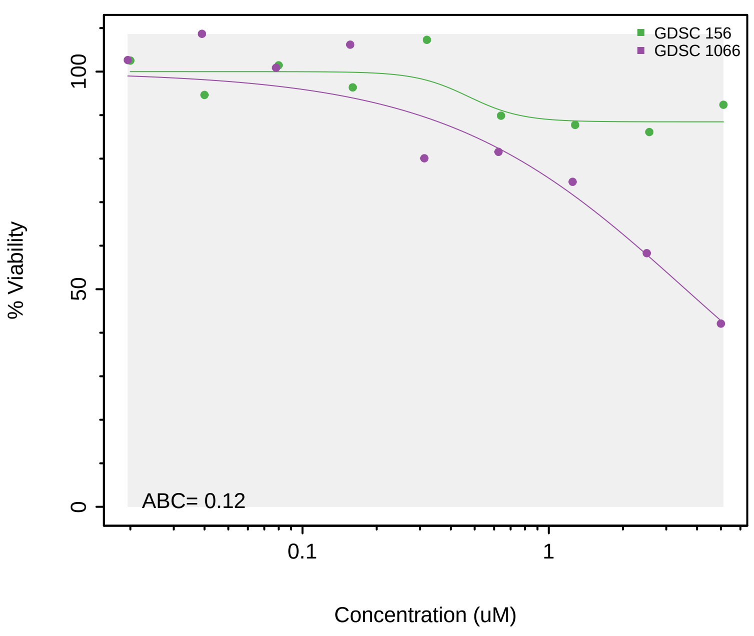

AZD6482:J−RT3−T3−5

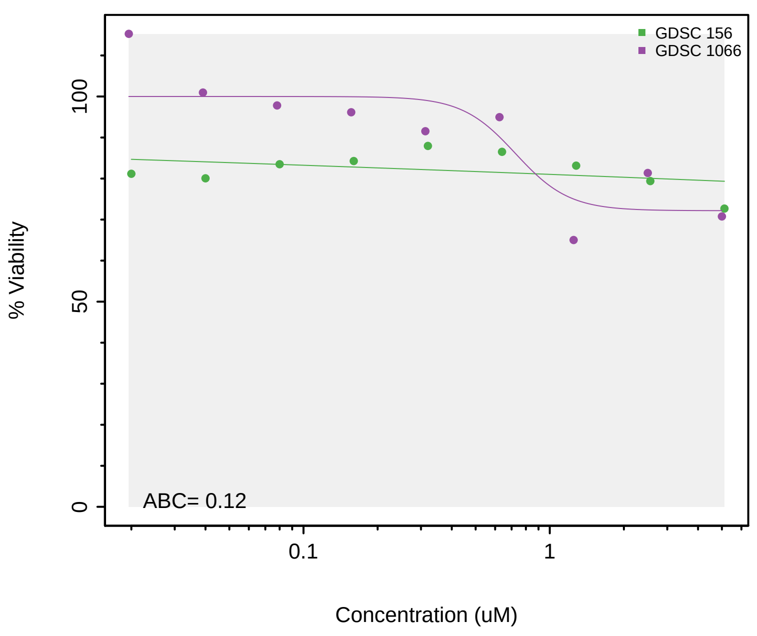

AZD6482:NCI−H520

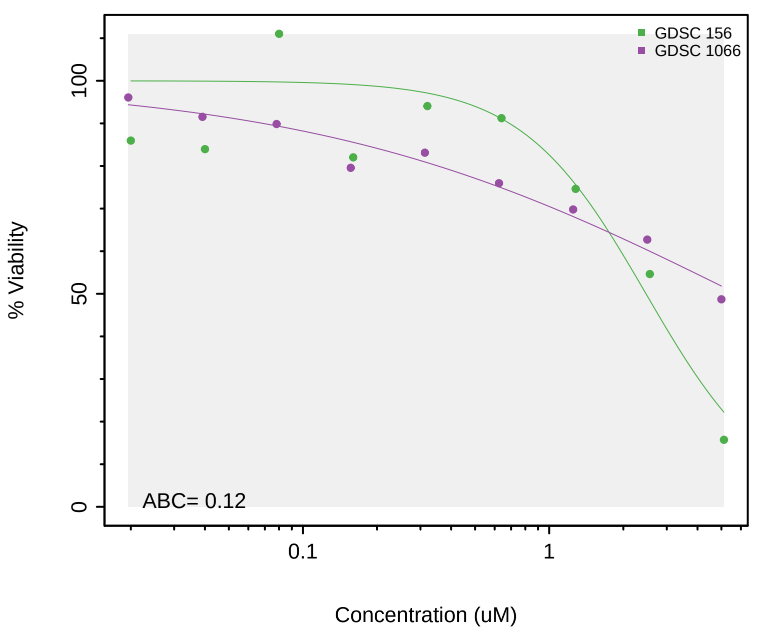

AZD6482:SNU−387

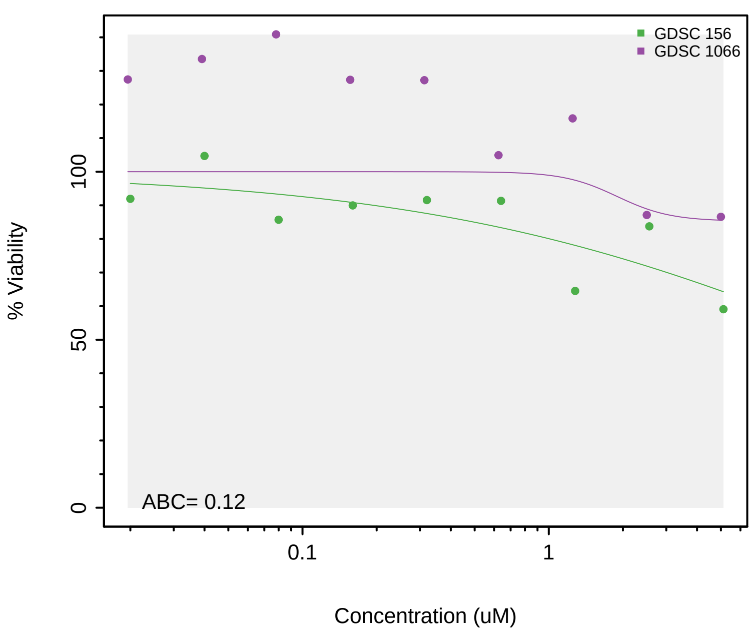

AZD6482:GAMG

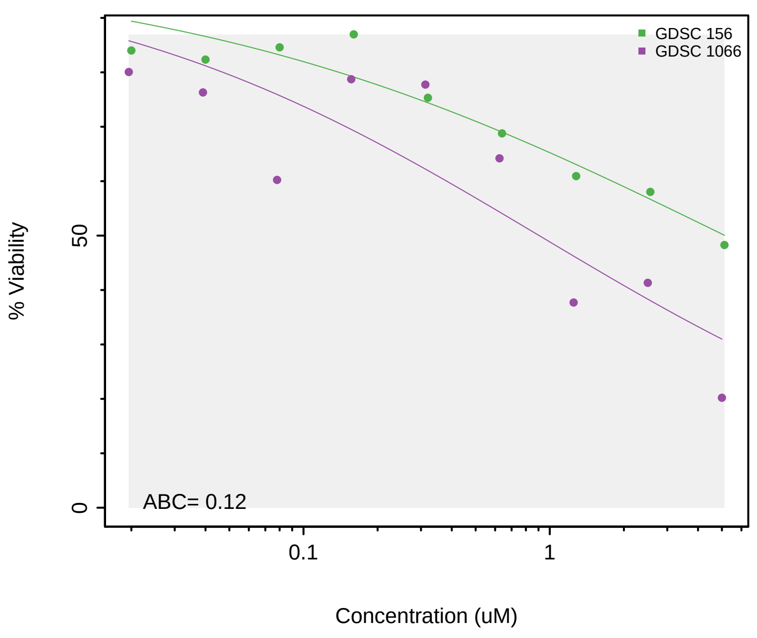

AZD6482:NCI−H446

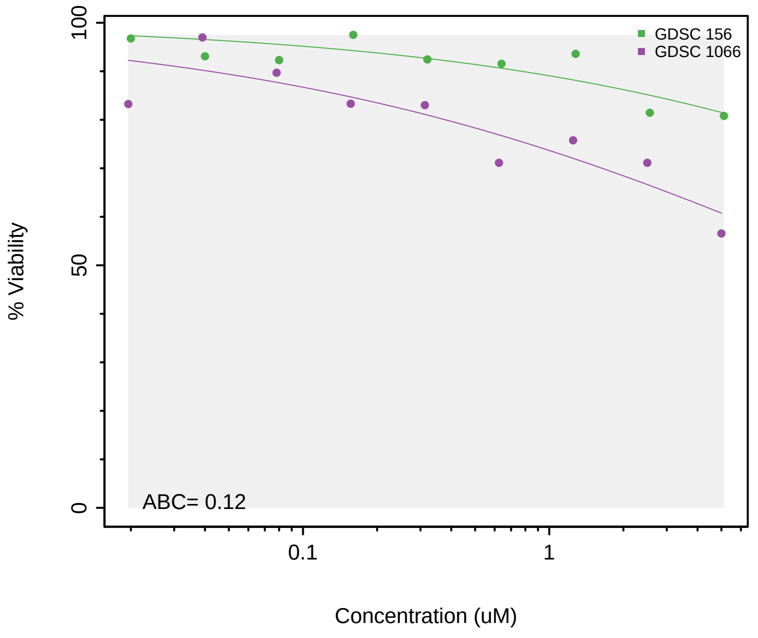

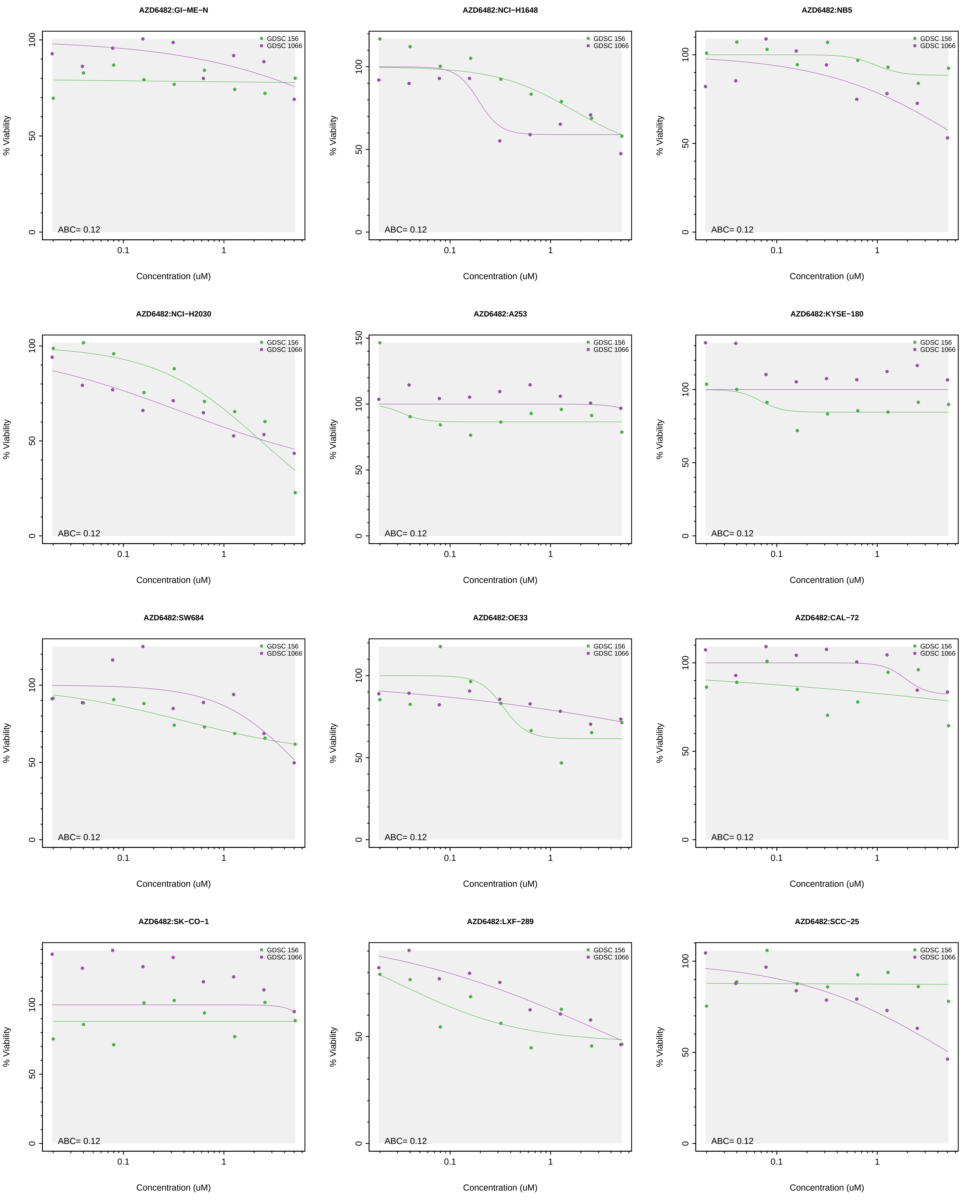
AZD6482:GI−ME−N
AZD6482:NCI−H1648
AZD6482:NB5
AZD6482:NCI−H2030
AZD6482:A253
AZD6482:KYSE−180
AZD6482:SW684
AZD6482:OE33
AZD6482:CAL−72
AZD6482:SK−CO−1
AZD6482:LXF−289
AZD6482:SCC−25
GDSC 156
GDSC 1066
ABC= 0.12
% Viability
Concentration (uM)
0.1
1
0
50
100
150

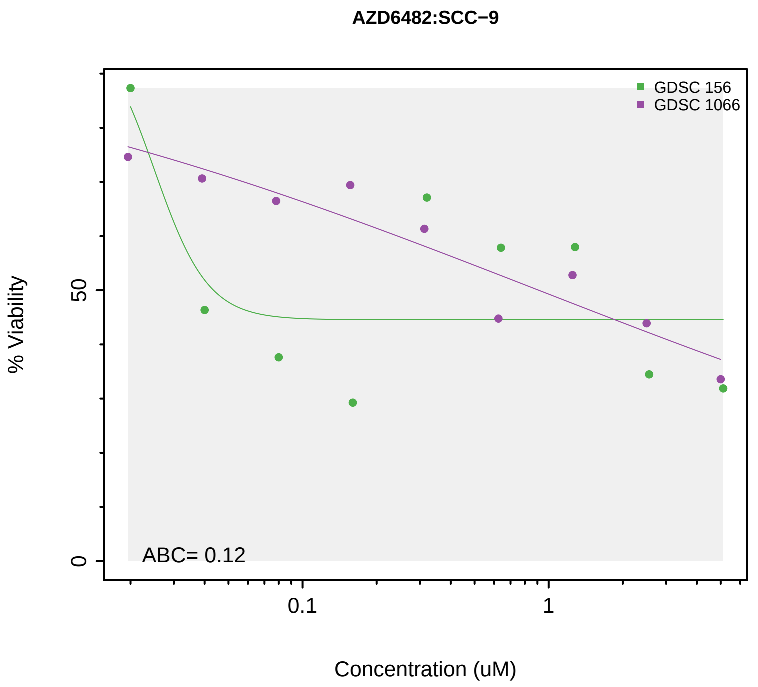
AZD6482:SCC−9
GDSC 156
GDSC 1066
% Viability
0
50
0.1
1
Concentration (uM)
ABC= 0.12

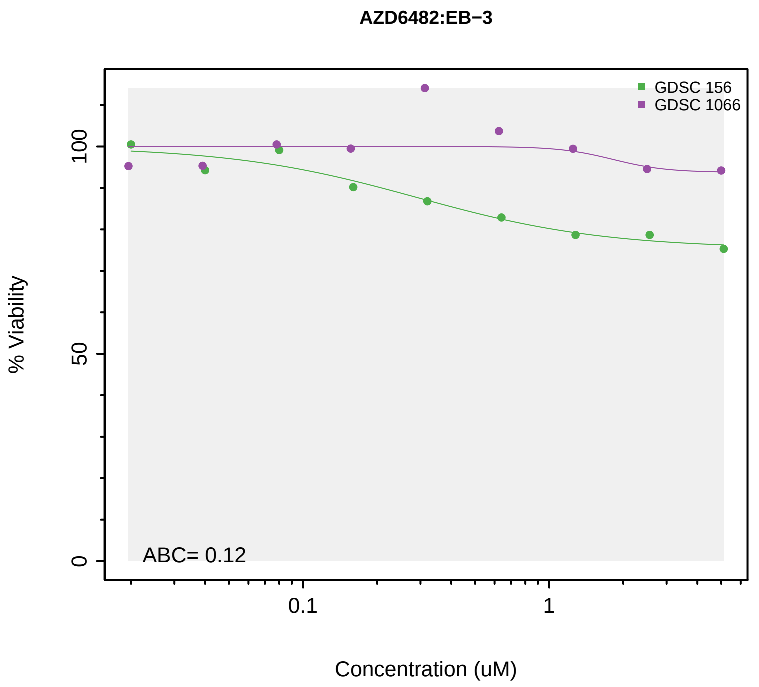
AZD6482:EB−3
GDSC 156
GDSC 1066
% Viability
0
50
100
0.1
1
Concentration (uM)
ABC= 0.12

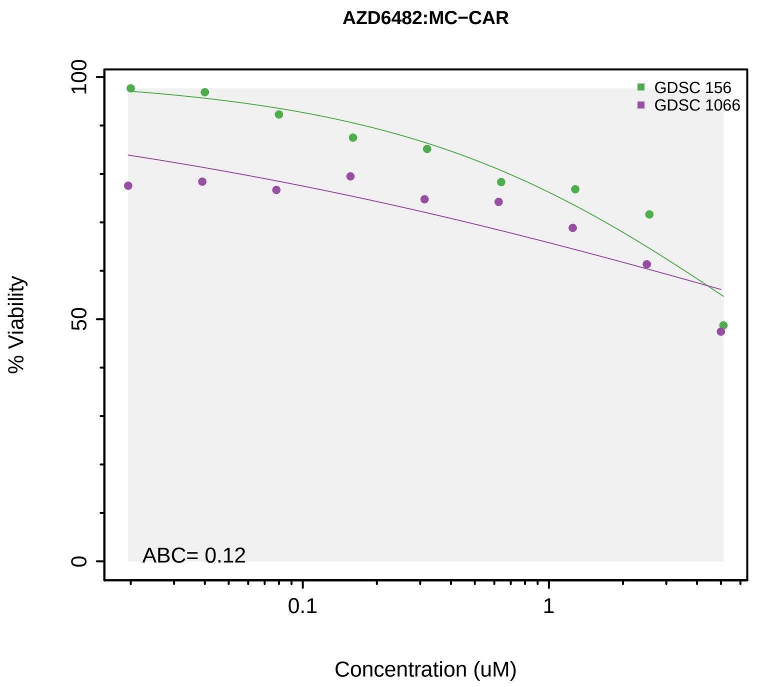
AZD6482:MC−CAR
GDSC 156
GDSC 1066
% Viability
0
50
100
0.1
1
Concentration (uM)
ABC= 0.12

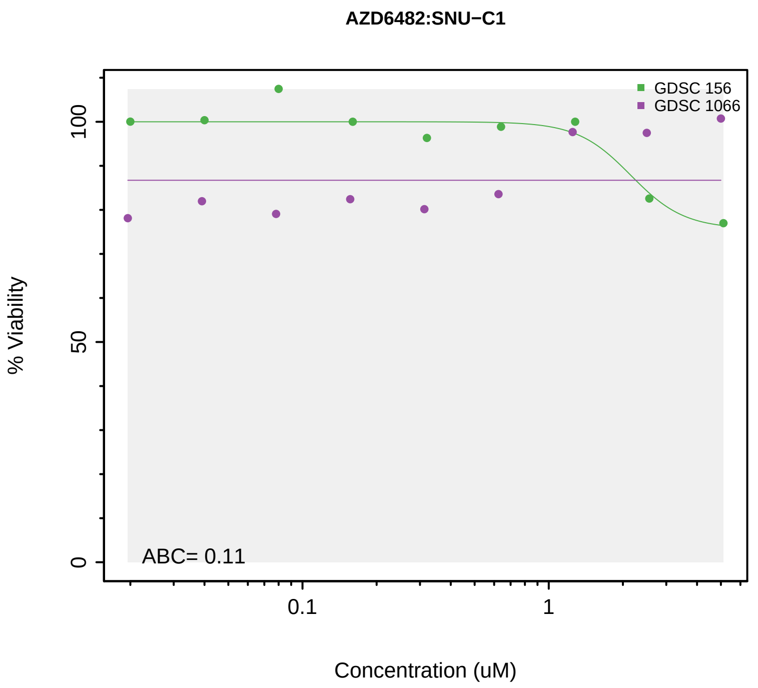
AZD6482:SNU−C1
GDSC 156
GDSC 1066
% Viability
0
50
100
0.1
1
Concentration (uM)
ABC= 0.11

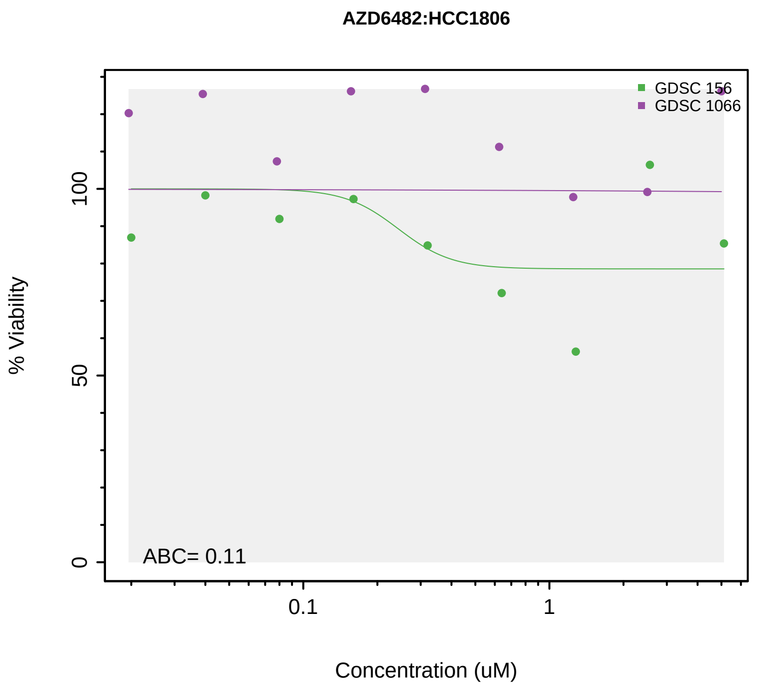
AZD6482:HCC1806
GDSC 156
GDSC 1066
% Viability
0
50
100
0.1
1
Concentration (uM)
ABC= 0.11

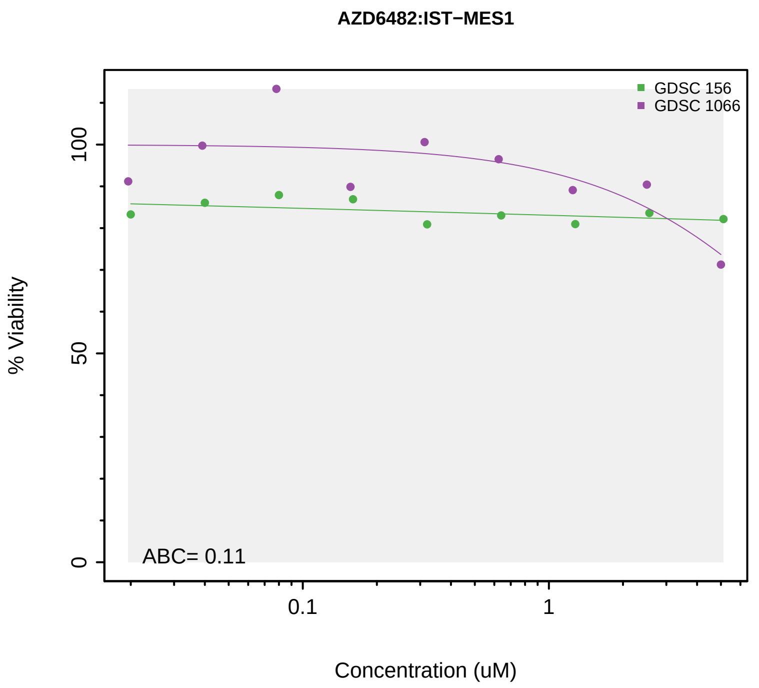
AZD6482:IST−MES1
GDSC 156
GDSC 1066
% Viability
0
50
100
0.1
1
Concentration (uM)
ABC= 0.11

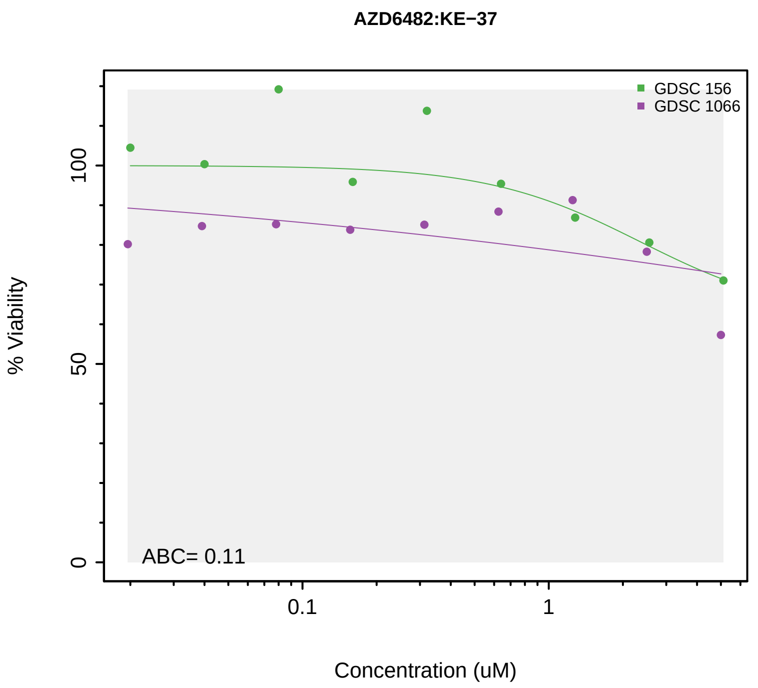
AZD6482:KE−37
GDSC 156
GDSC 1066
% Viability
0
50
100
0.1
1
Concentration (uM)
ABC= 0.11

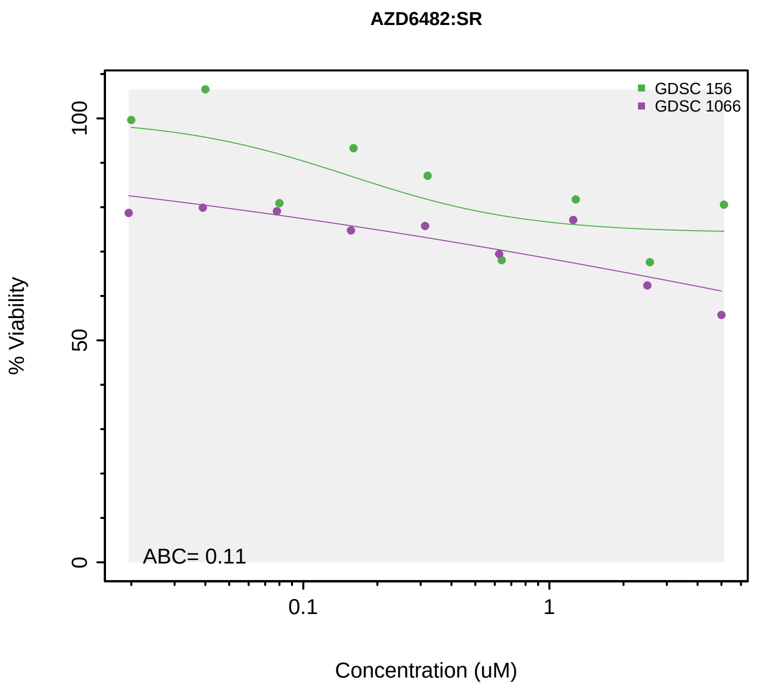
AZD6482:SR
GDSC 156
GDSC 1066
% Viability
0
50
100
0.1
1
Concentration (uM)
ABC= 0.11

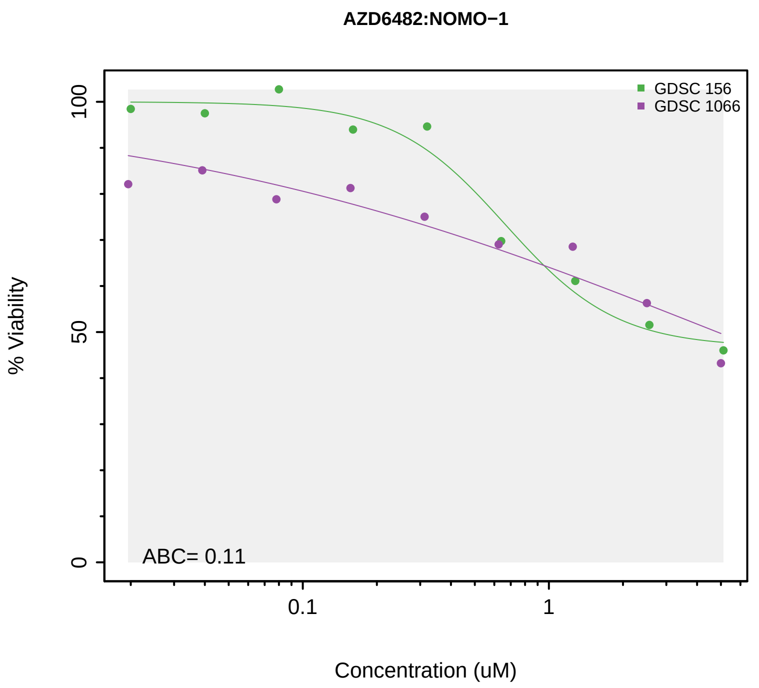
AZD6482:NOMO−1
GDSC 156
GDSC 1066
% Viability
0
50
100
0.1
1
Concentration (uM)
ABC= 0.11

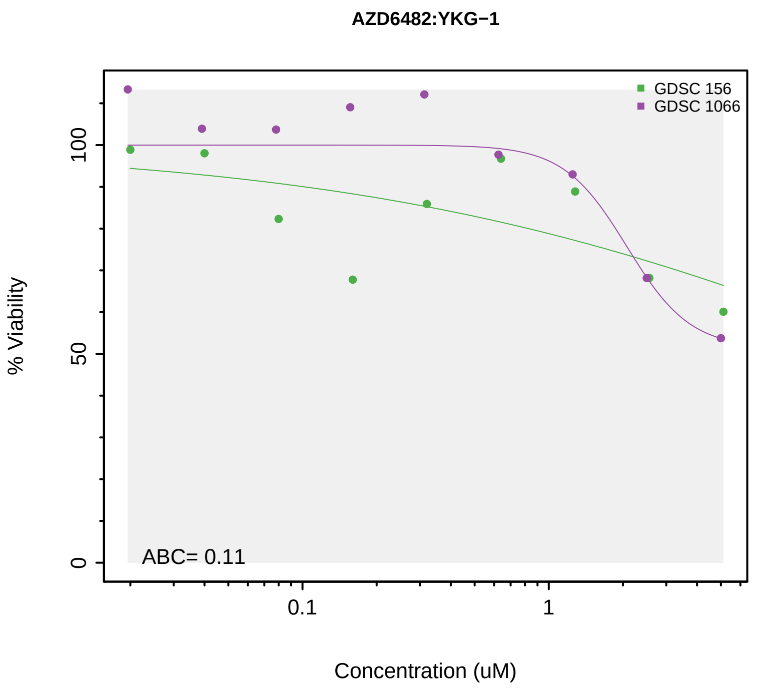
AZD6482:YKG−1
GDSC 156
GDSC 1066
% Viability
0
50
100
0.1
1
Concentration (uM)
ABC= 0.11

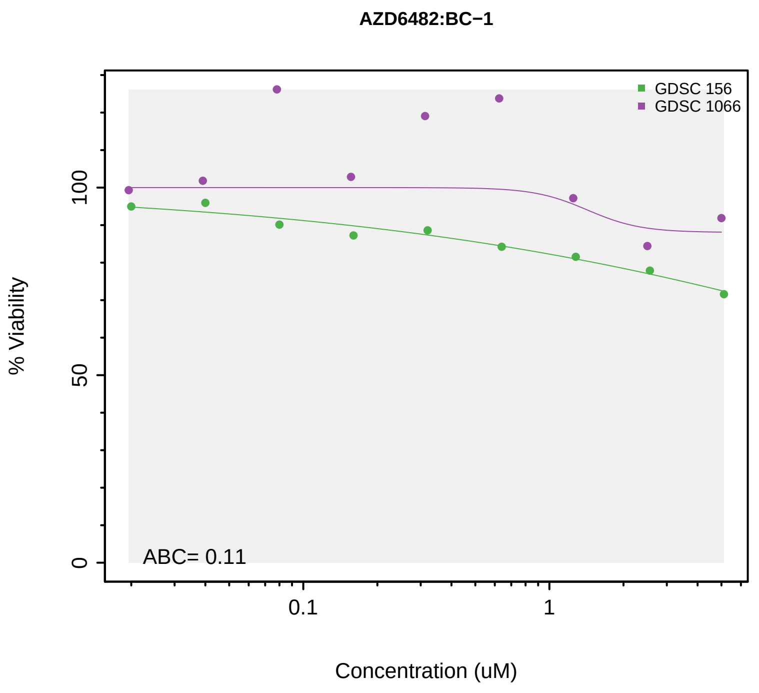
AZD6482:BC−1
GDSC 156
GDSC 1066
% Viability
0
50
100
0.1
1
Concentration (uM)
ABC= 0.11

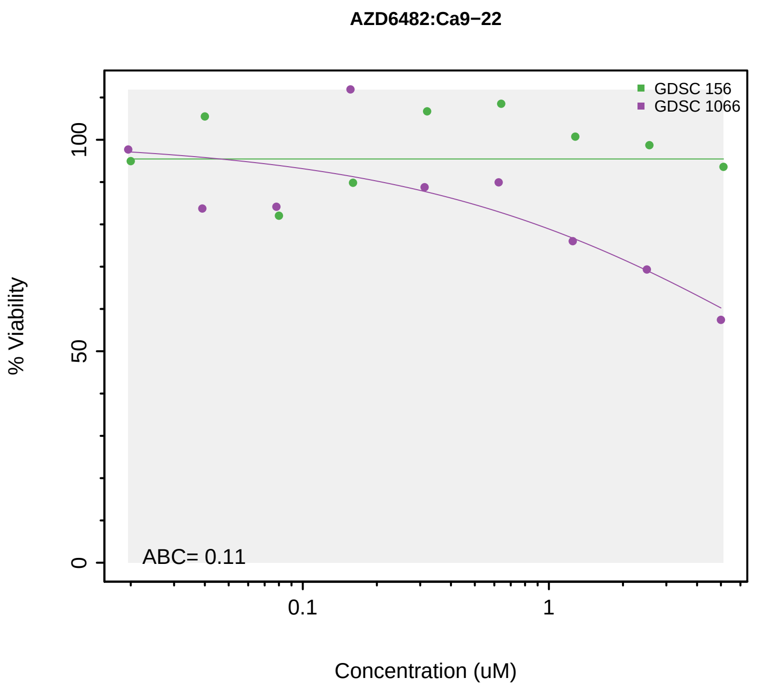
AZD6482:Ca9−22
GDSC 156
GDSC 1066
% Viability
0
50
100
0.1
1
Concentration (uM)
ABC= 0.11

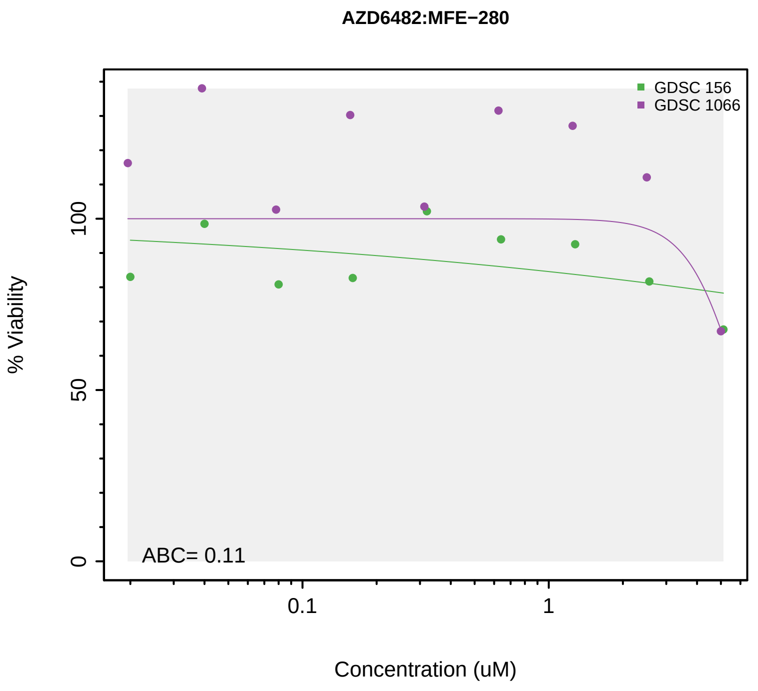
AZD6482:MFE−280
GDSC 156
GDSC 1066
ABC= 0.11
% Viability
Concentration (uM)
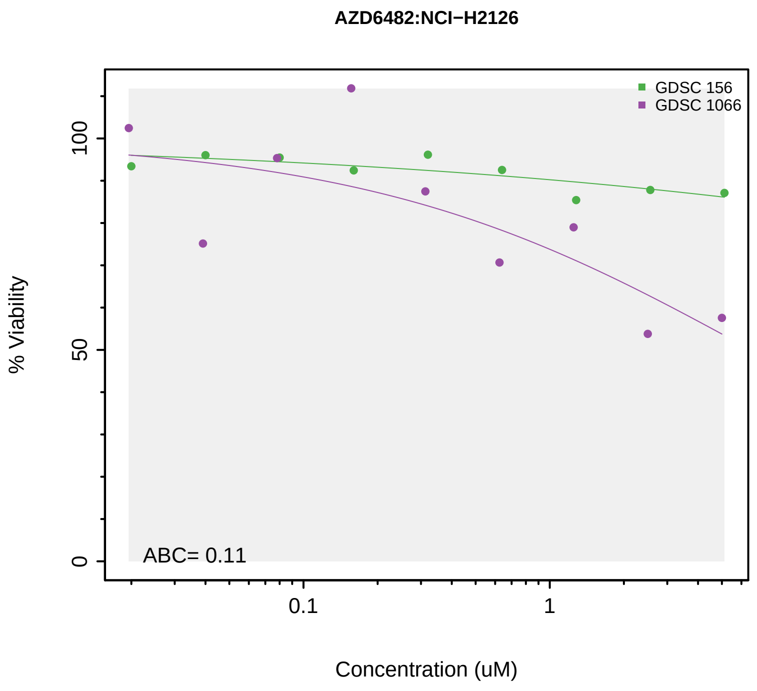
AZD6482:NCI−H2126
GDSC 156
GDSC 1066
ABC= 0.11
% Viability
Concentration (uM)
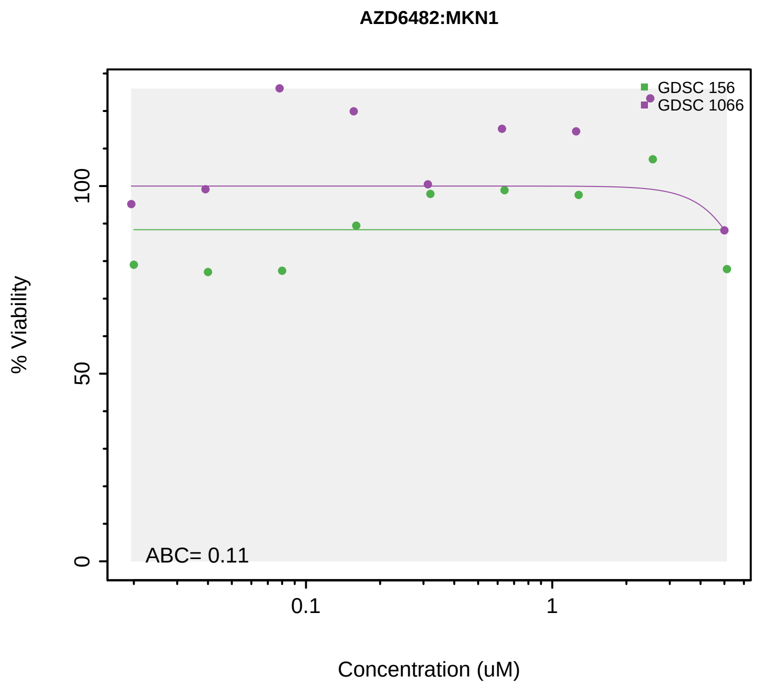
AZD6482:MKN1
GDSC 156
GDSC 1066
ABC= 0.11
% Viability
Concentration (uM)
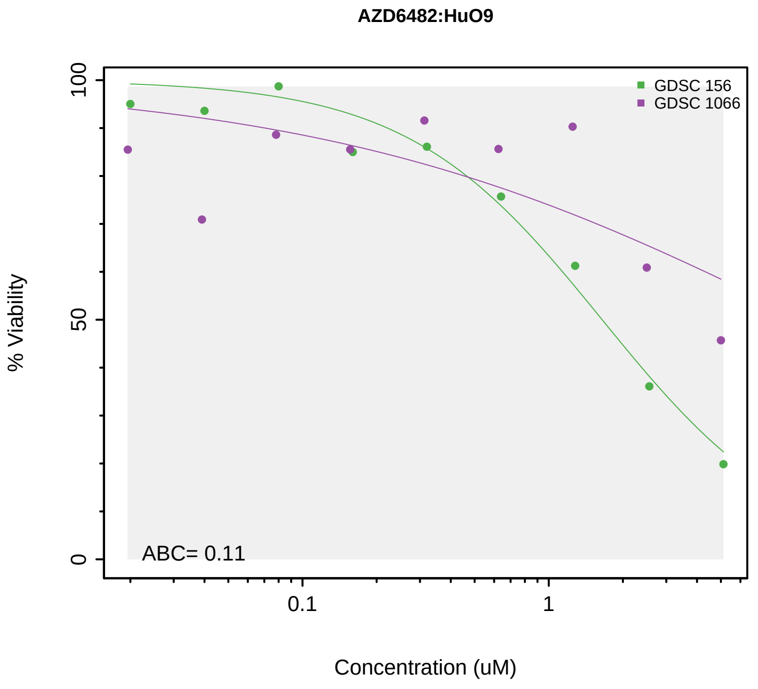
AZD6482:HuO9
GDSC 156
GDSC 1066
ABC= 0.11
% Viability
Concentration (uM)
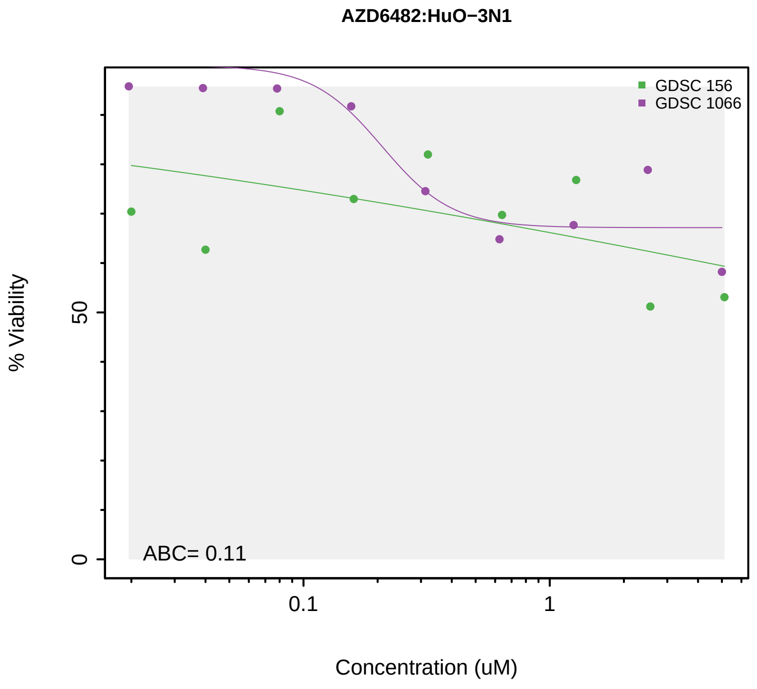
AZD6482:HuO−3N1
GDSC 156
GDSC 1066
ABC= 0.11
% Viability
Concentration (uM)
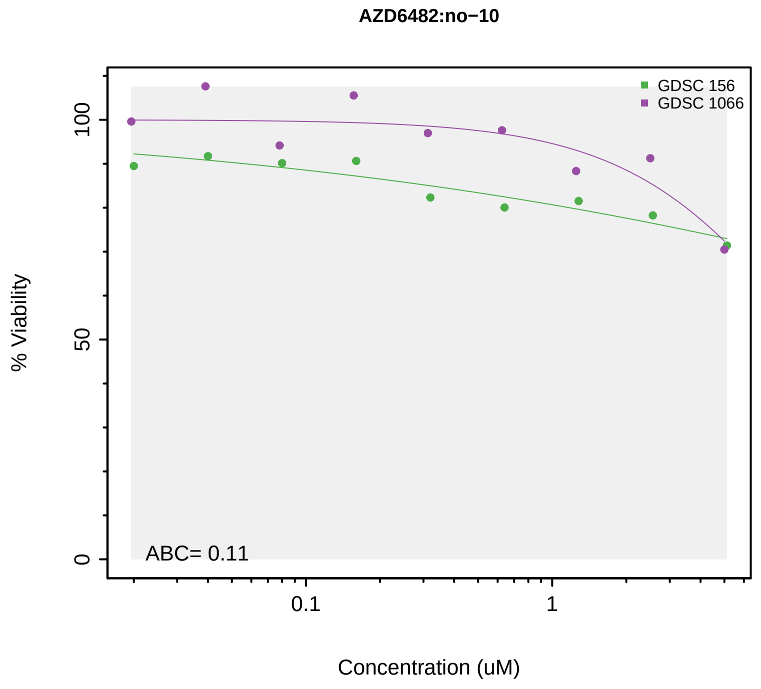
AZD6482:no−10
GDSC 156
GDSC 1066
ABC= 0.11
% Viability
Concentration (uM)
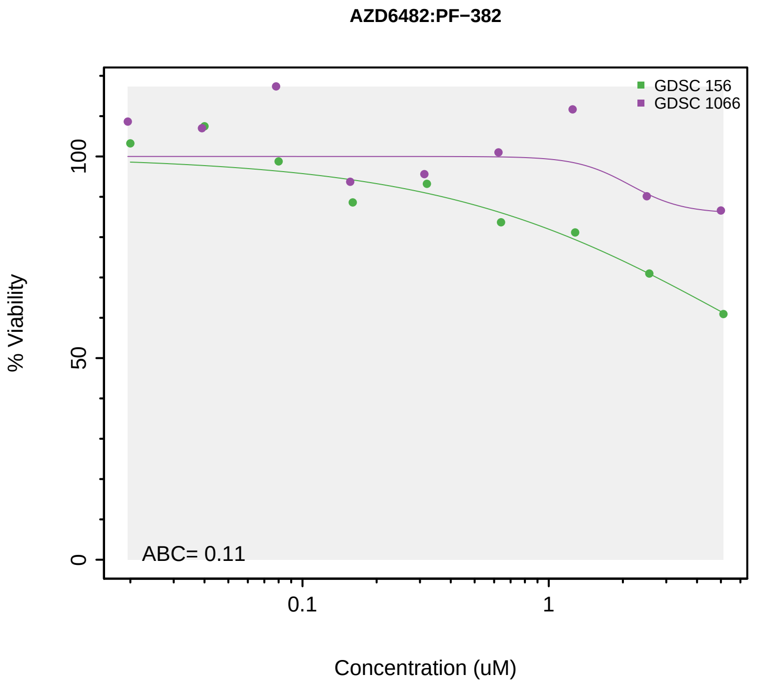
AZD6482:PF−382
GDSC 156
GDSC 1066
ABC= 0.11
% Viability
Concentration (uM)
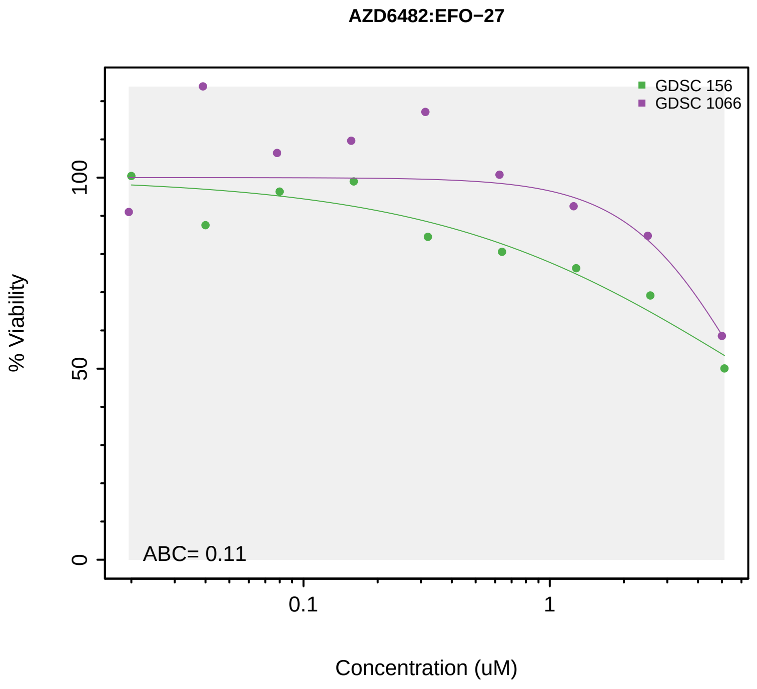
AZD6482:EFO−27
GDSC 156
GDSC 1066
ABC= 0.11
% Viability
Concentration (uM)
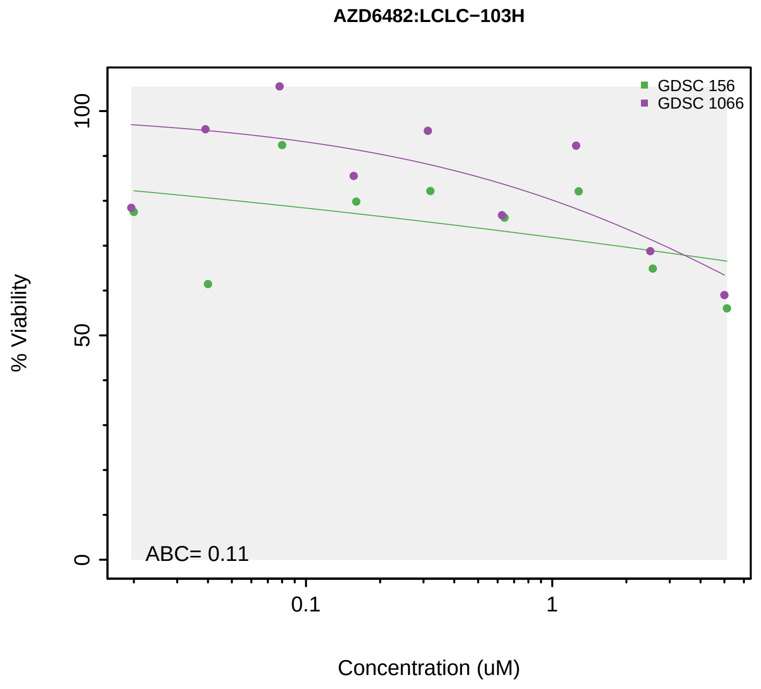
AZD6482:LCLC−103H
GDSC 156
GDSC 1066
ABC= 0.11
% Viability
Concentration (uM)
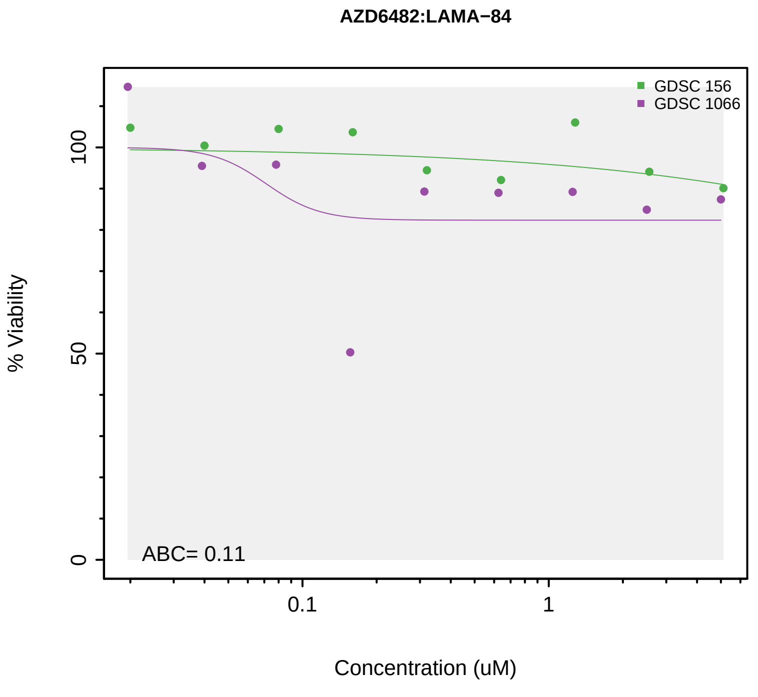
AZD6482:LAMA−84
GDSC 156
GDSC 1066
ABC= 0.11
% Viability
Concentration (uM)
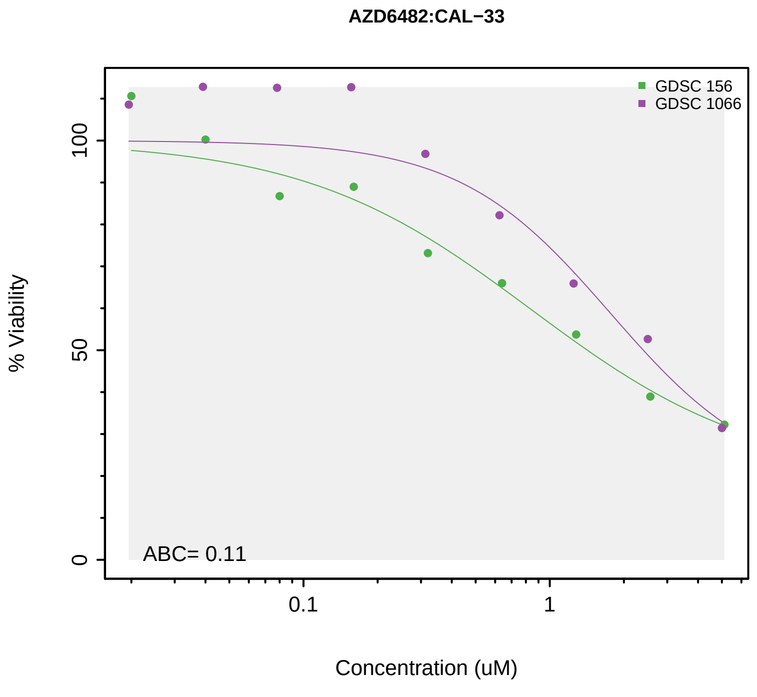
AZD6482:CAL−33
GDSC 156
GDSC 1066
ABC= 0.11
% Viability
Concentration (uM)
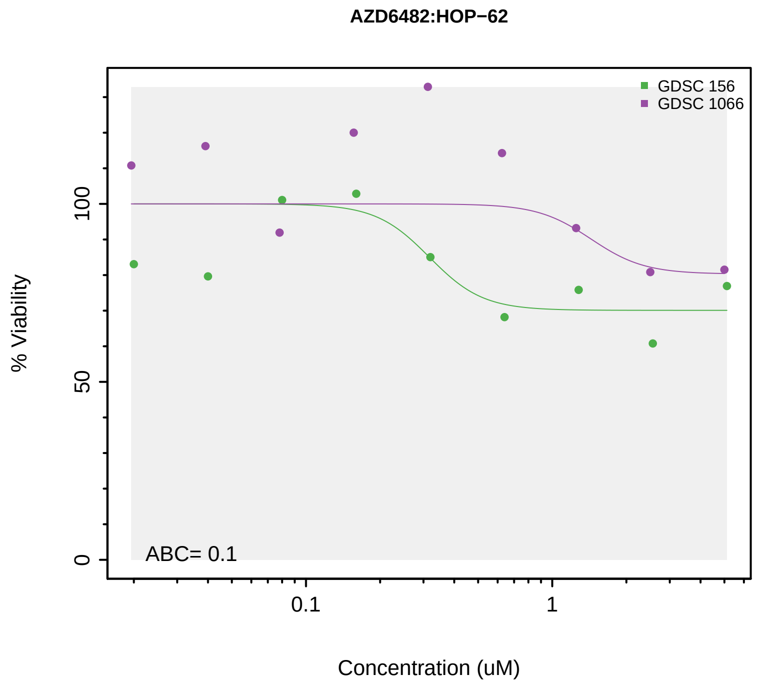
AZD6482:HOP−62
GDSC 156
GDSC 1066
ABC= 0.1
% Viability
Concentration (uM)

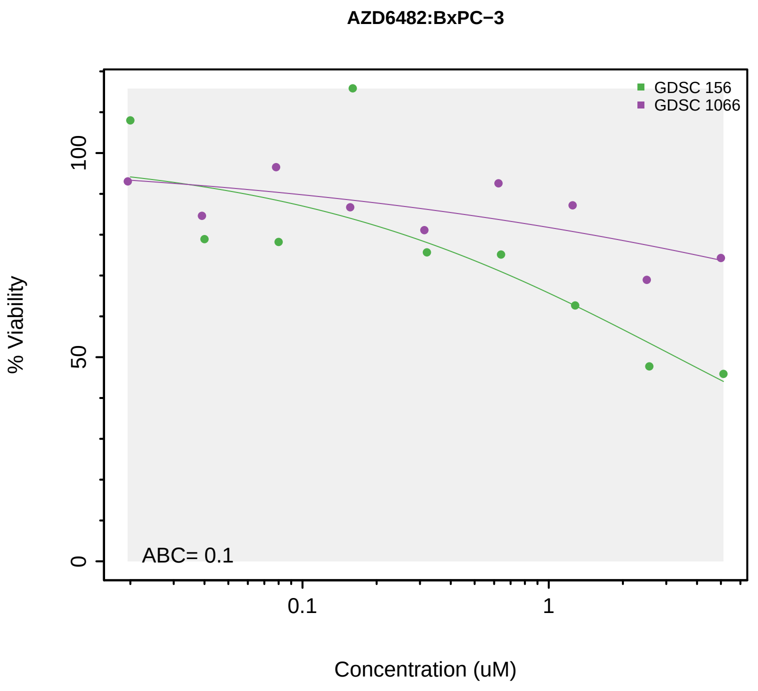
AZD6482:BxPC−3
GDSC 156
GDSC 1066
ABC= 0.1
% Viability
Concentration (uM)
0
50
100
0.1
1

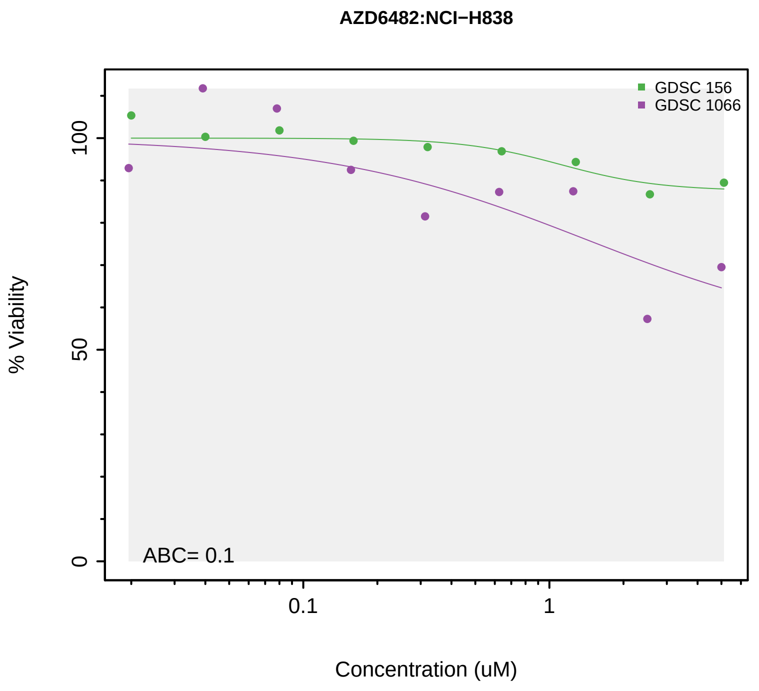
AZD6482:NCI−H838
GDSC 156
GDSC 1066
ABC= 0.1
% Viability
Concentration (uM)
0
50
100
0.1
1

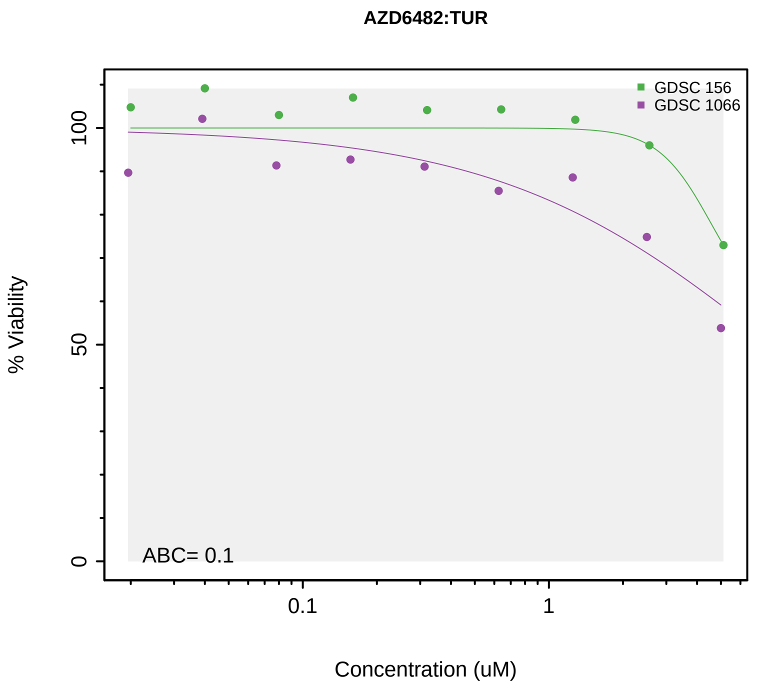
AZD6482:TUR
GDSC 156
GDSC 1066
ABC= 0.1
% Viability
Concentration (uM)
0
50
100
0.1
1

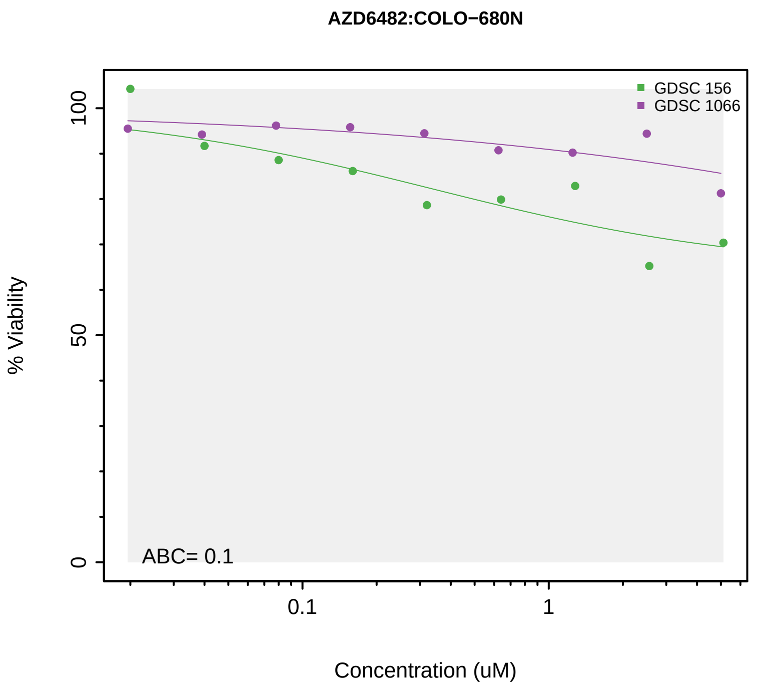
AZD6482:COLO−680N
GDSC 156
GDSC 1066
ABC= 0.1
% Viability
Concentration (uM)
0
50
100
0.1
1

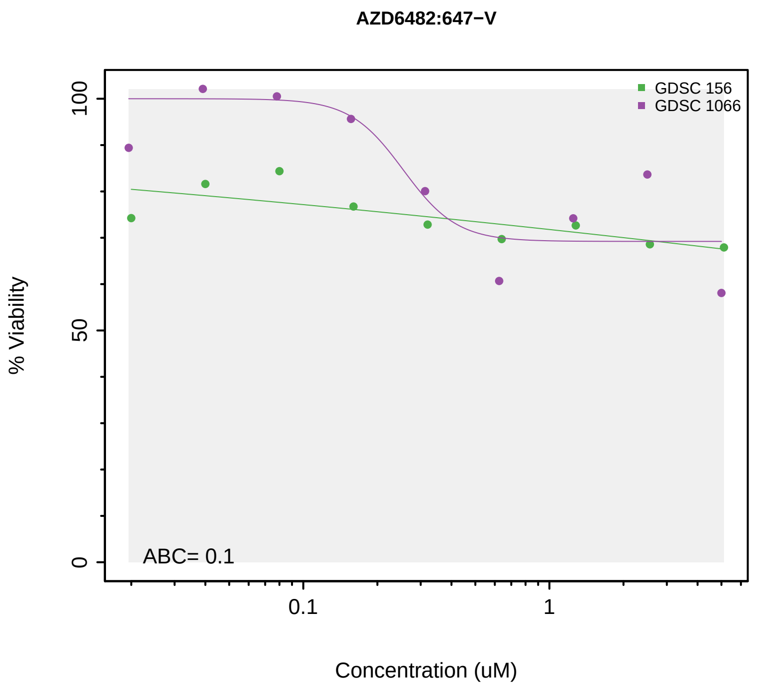
AZD6482:647−V
GDSC 156
GDSC 1066
ABC= 0.1
% Viability
Concentration (uM)
0
50
100
0.1
1

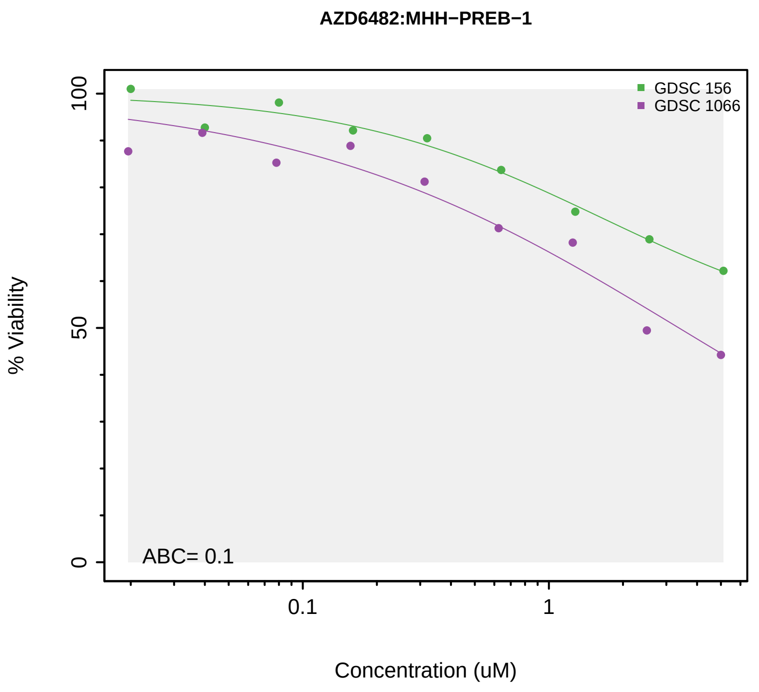
AZD6482:MHH−PREB−1
GDSC 156
GDSC 1066
ABC= 0.1
% Viability
Concentration (uM)
0
50
100
0.1
1

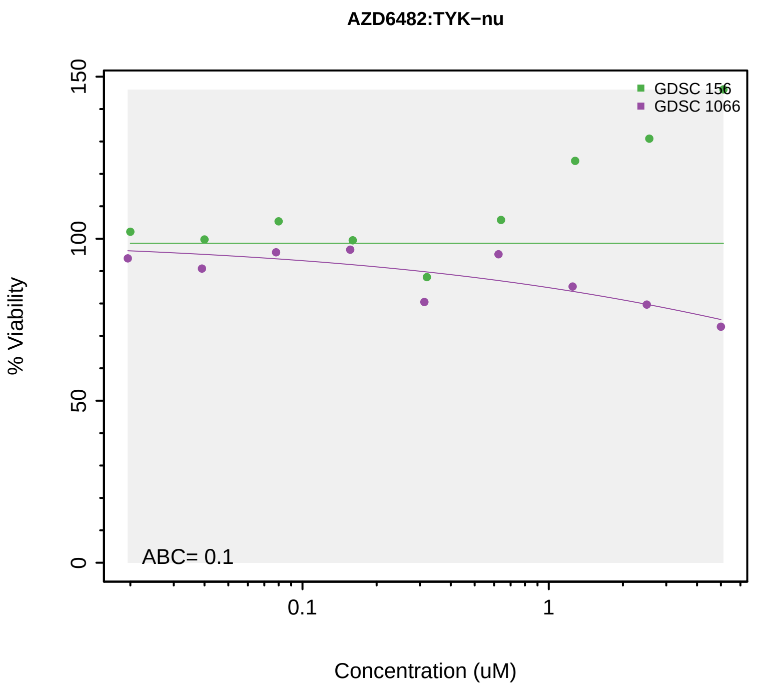
AZD6482:TYK−nu
GDSC 156
GDSC 1066
ABC= 0.1
% Viability
Concentration (uM)
0
50
100
150
0.1
1

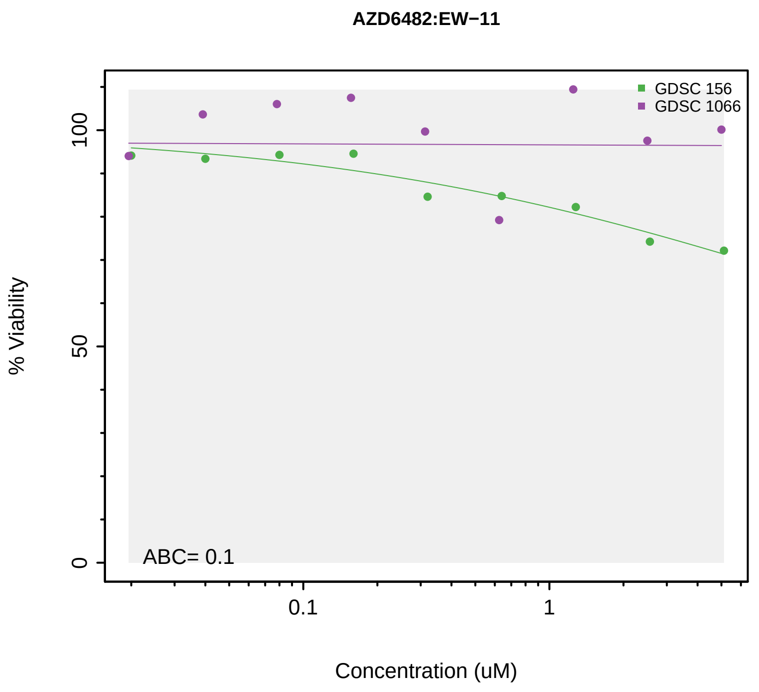
AZD6482:EW−11
GDSC 156
GDSC 1066
ABC= 0.1
% Viability
Concentration (uM)
0
50
100
0.1
1

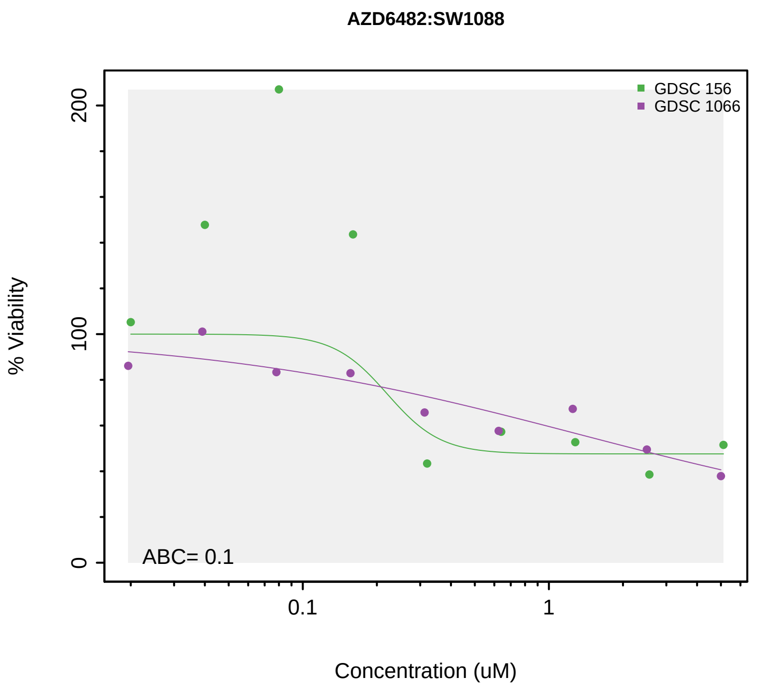
AZD6482:SW1088
GDSC 156
GDSC 1066
ABC= 0.1
% Viability
Concentration (uM)
0
100
200
0.1
1

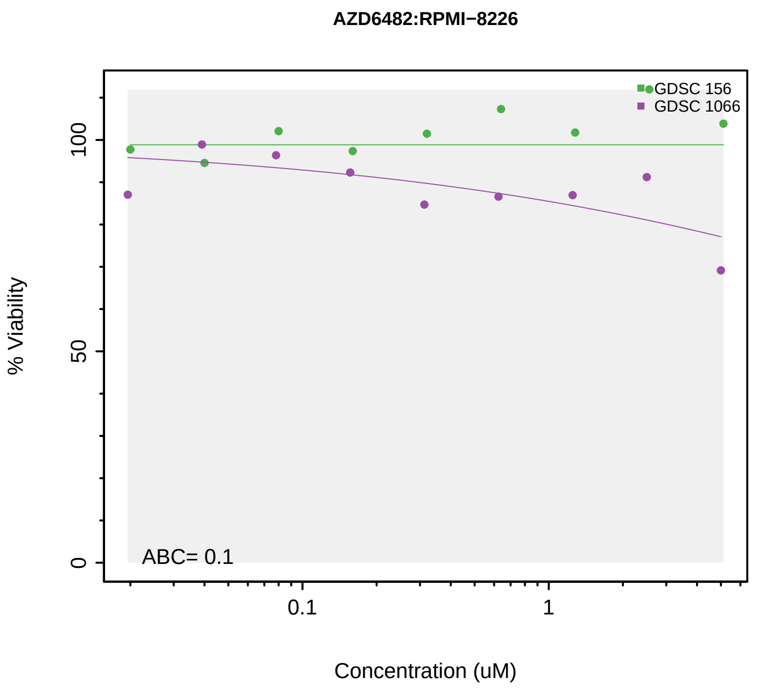
AZD6482:RPMI−8226
GDSC 156
GDSC 1066
ABC= 0.1
% Viability
Concentration (uM)
0
50
100
0.1
1

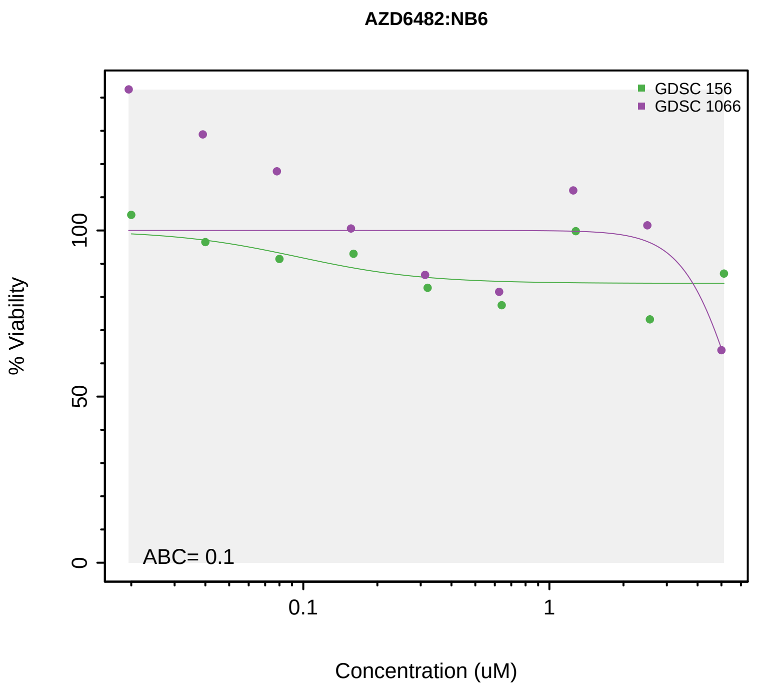
AZD6482:NB6
GDSC 156
GDSC 1066
ABC= 0.1
% Viability
Concentration (uM)
0
50
100
0.1
1

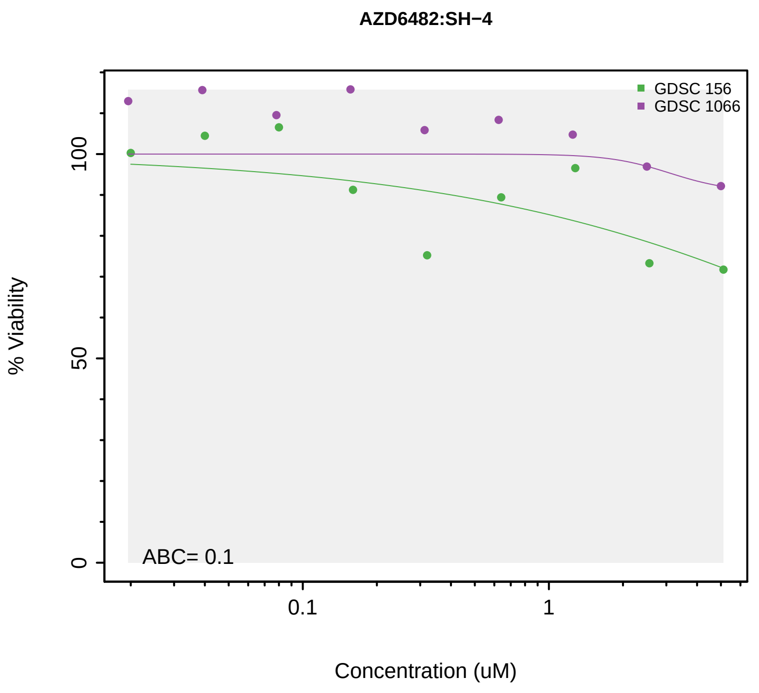
AZD6482:SH−4
GDSC 156
GDSC 1066
ABC= 0.1
% Viability
Concentration (uM)
0
50
100
0.1
1

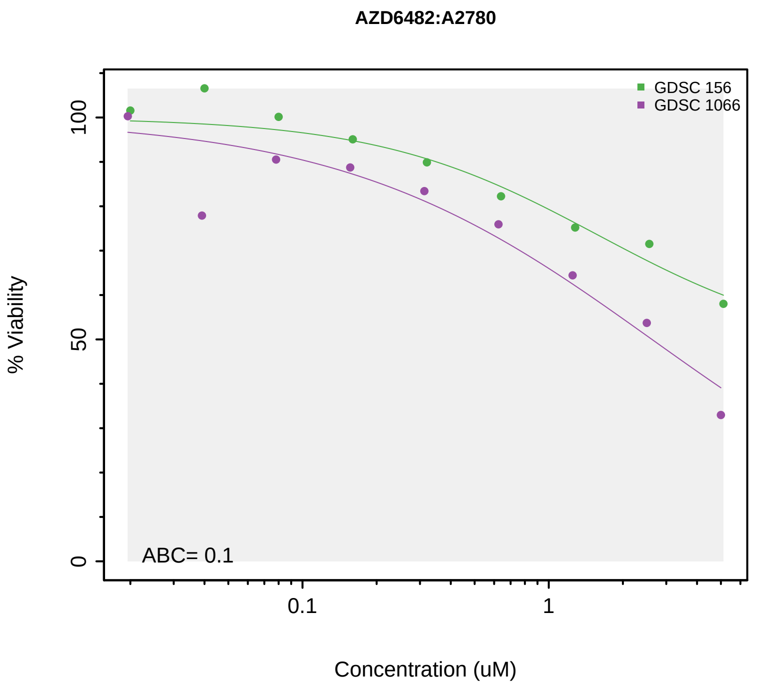
AZD6482:A2780
GDSC 156
GDSC 1066
ABC= 0.1
% Viability
0
50
100
0.1
1
Concentration (uM)

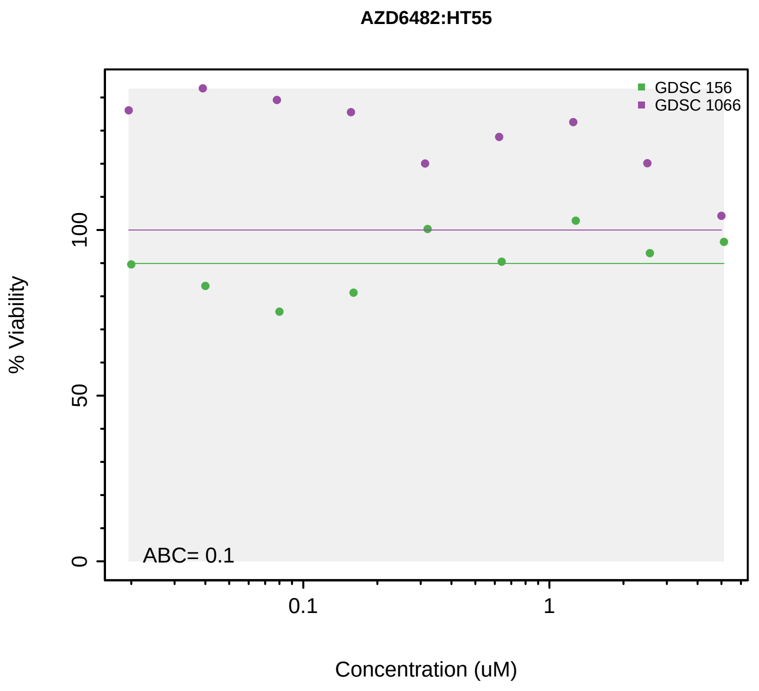
AZD6482:HT55
GDSC 156
GDSC 1066
ABC= 0.1
% Viability
0
50
100
0.1
1
Concentration (uM)

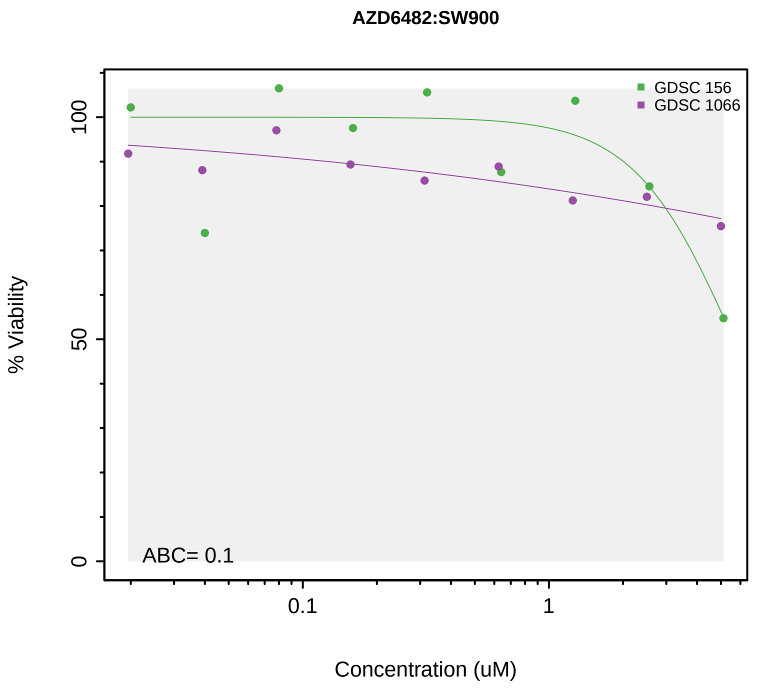
AZD6482:SW900
GDSC 156
GDSC 1066
ABC= 0.1
% Viability
0
50
100
0.1
1
Concentration (uM)

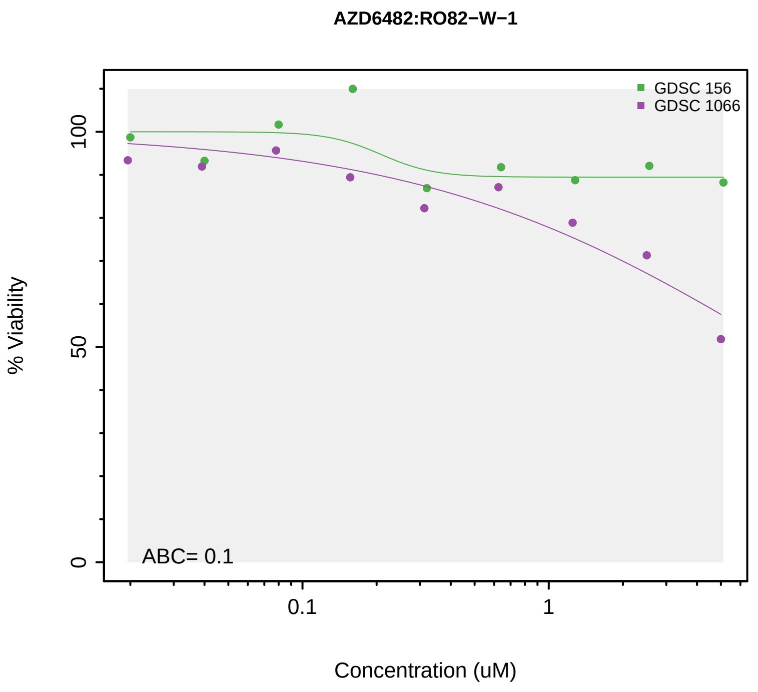
AZD6482:RO82−W−1
GDSC 156
GDSC 1066
ABC= 0.1
% Viability
0
50
100
0.1
1
Concentration (uM)

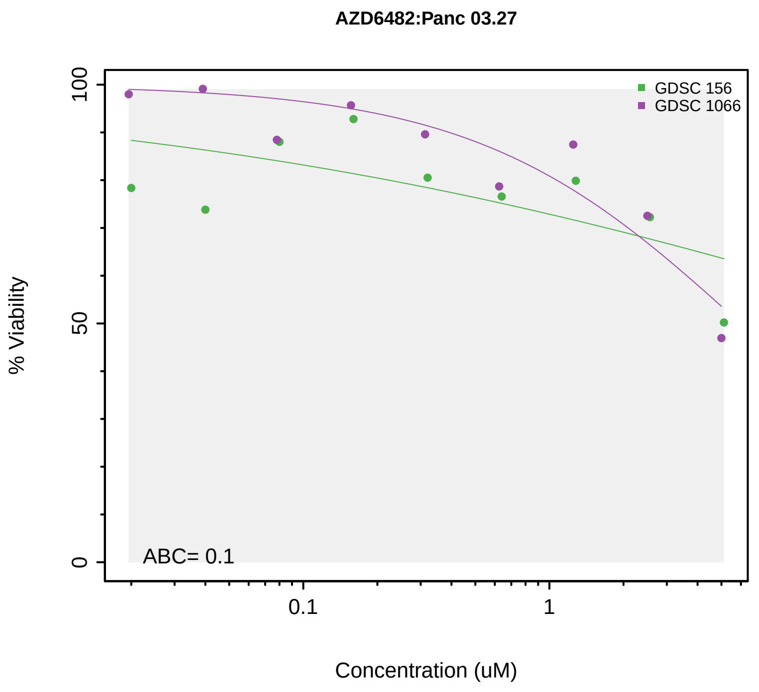
AZD6482:Panc 03.27
GDSC 156
GDSC 1066
ABC= 0.1
% Viability
0
50
100
0.1
1
Concentration (uM)

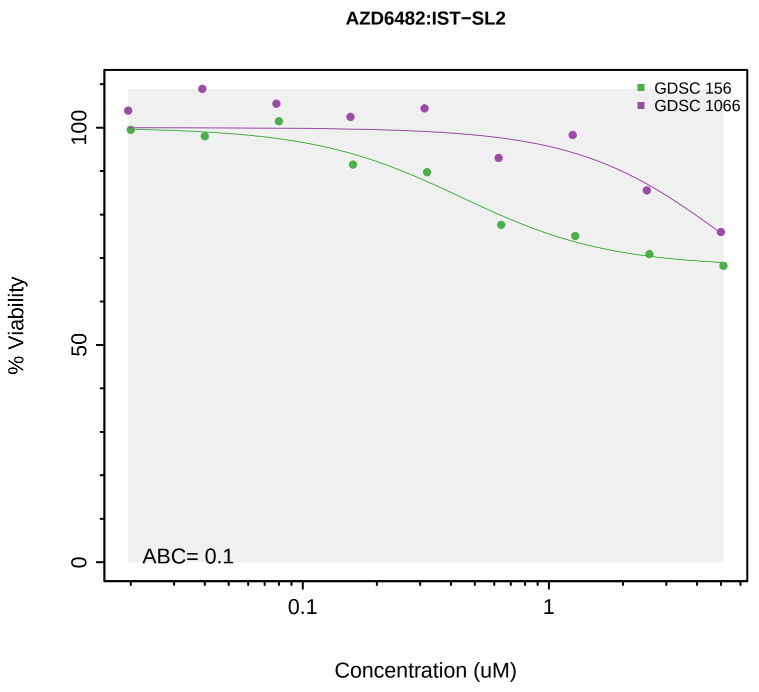
AZD6482:IST−SL2
GDSC 156
GDSC 1066
ABC= 0.1
% Viability
0
50
100
0.1
1
Concentration (uM)

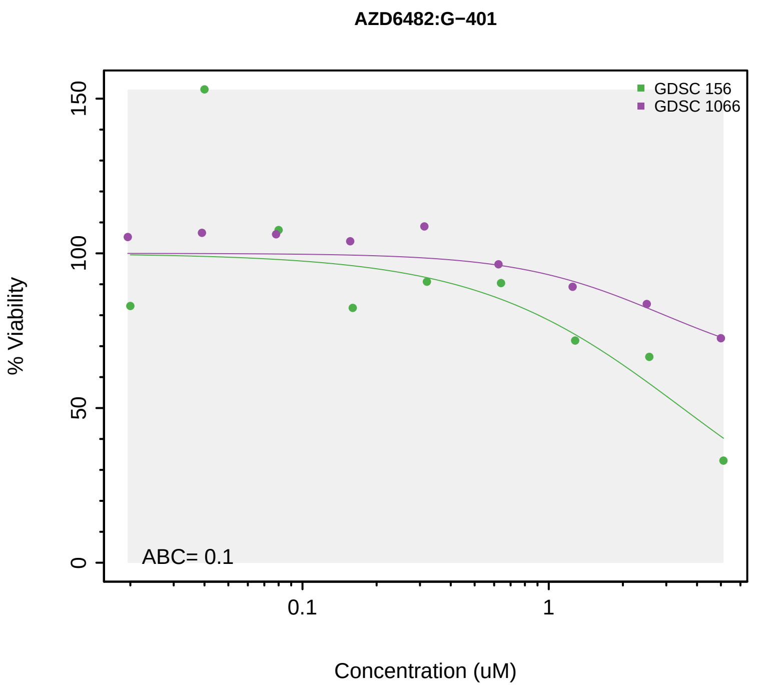
AZD6482:G−401
GDSC 156
GDSC 1066
ABC= 0.1
% Viability
0
50
100
150
0.1
1
Concentration (uM)

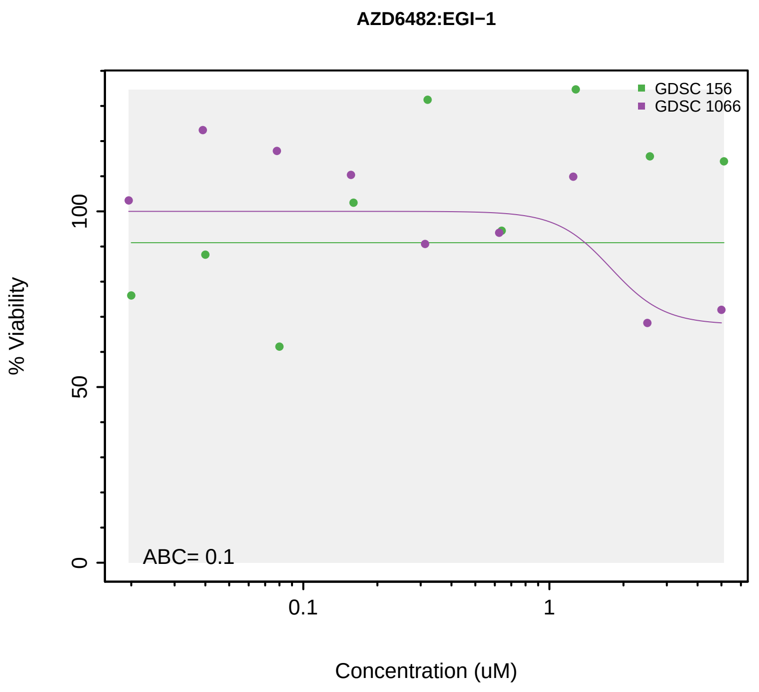
AZD6482:EGI−1
GDSC 156
GDSC 1066
ABC= 0.1
% Viability
0
50
100
0.1
1
Concentration (uM)

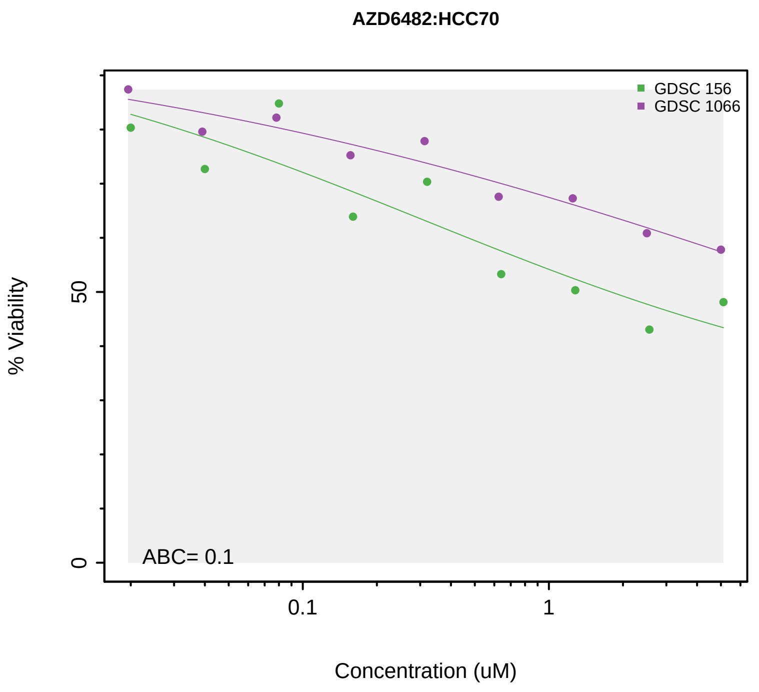
AZD6482:HCC70
GDSC 156
GDSC 1066
ABC= 0.1
% Viability
0
50
0.1
1
Concentration (uM)

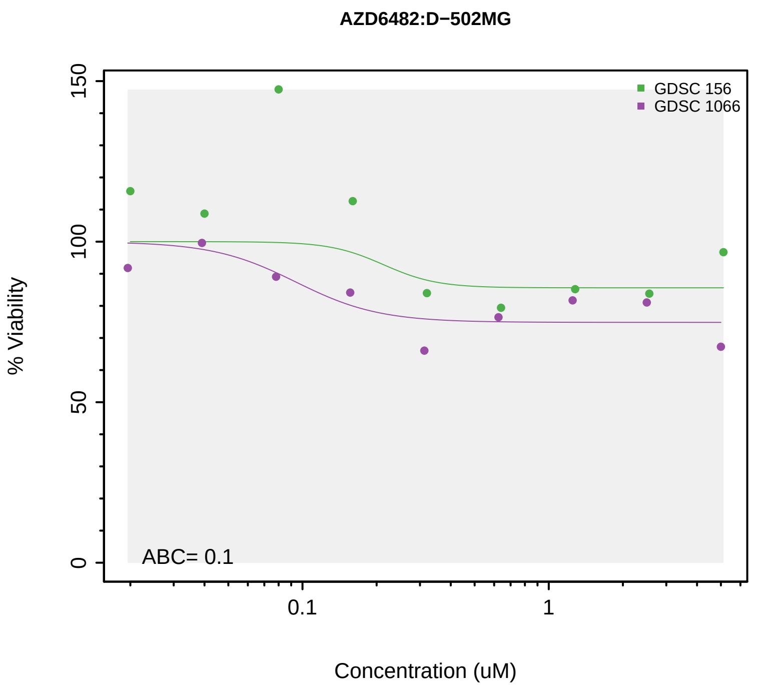
AZD6482:D−502MG
GDSC 156
GDSC 1066
ABC= 0.1
% Viability
0
50
100
150
0.1
1
Concentration (uM)

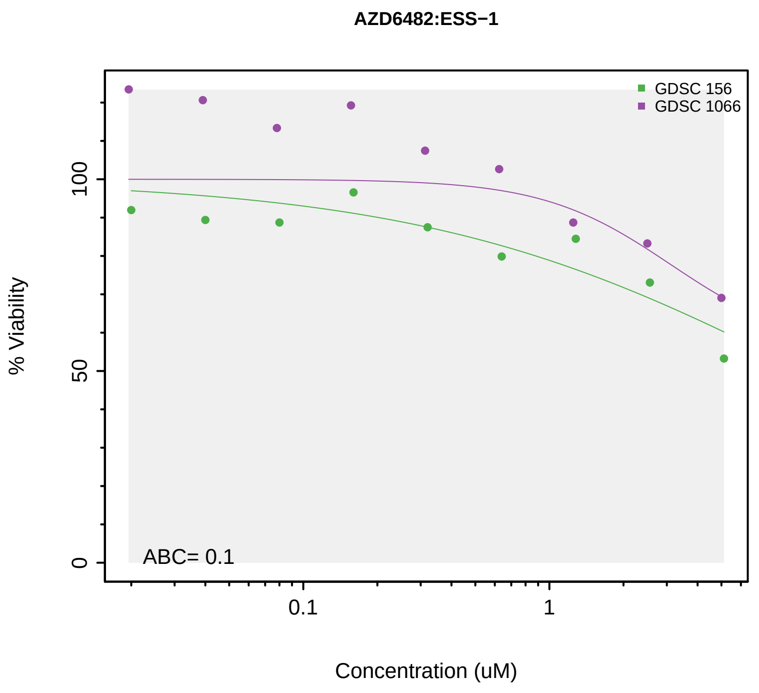
AZD6482:ESS−1
GDSC 156
GDSC 1066
ABC= 0.1
% Viability
0
50
100
0.1
1
Concentration (uM)

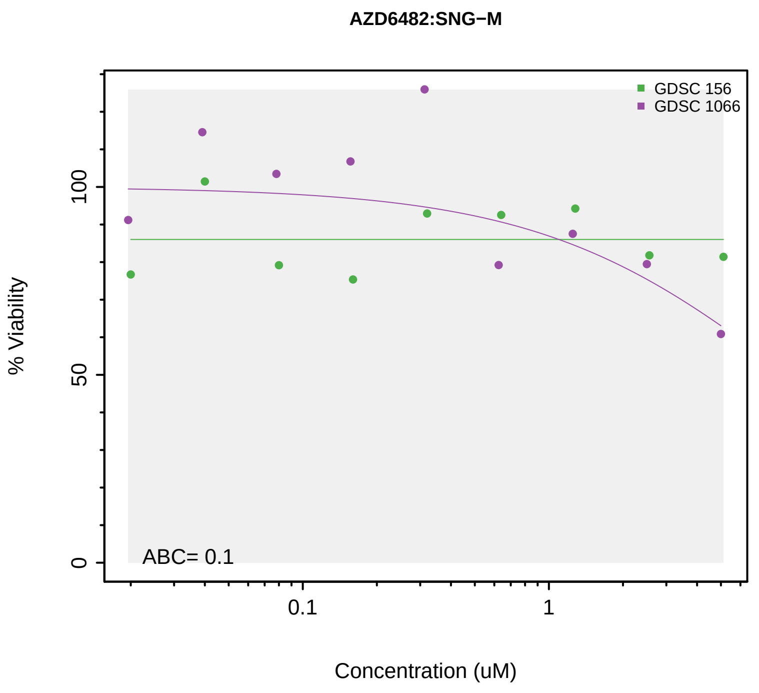
AZD6482:SNG−M
GDSC 156
GDSC 1066
ABC= 0.1
% Viability
0
50
100
0.1
1
Concentration (uM)

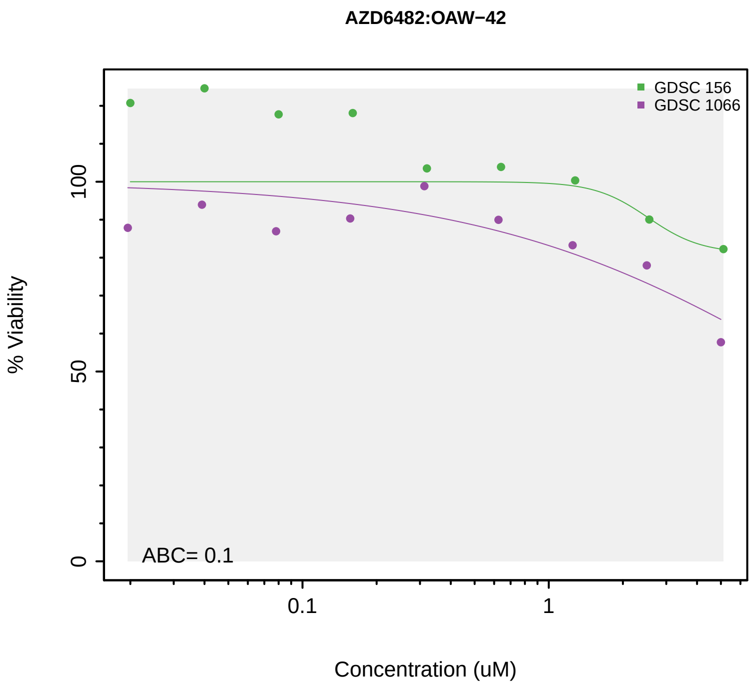
AZD6482:OAW−42
GDSC 156
GDSC 1066
% Viability
Concentration (uM)
ABC= 0.1

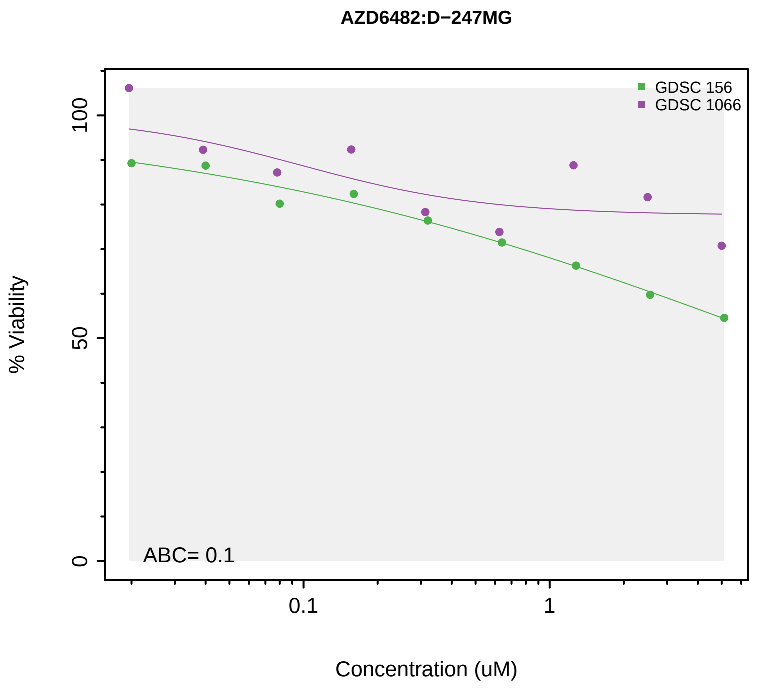
AZD6482:D−247MG
GDSC 156
GDSC 1066
% Viability
Concentration (uM)
ABC= 0.1

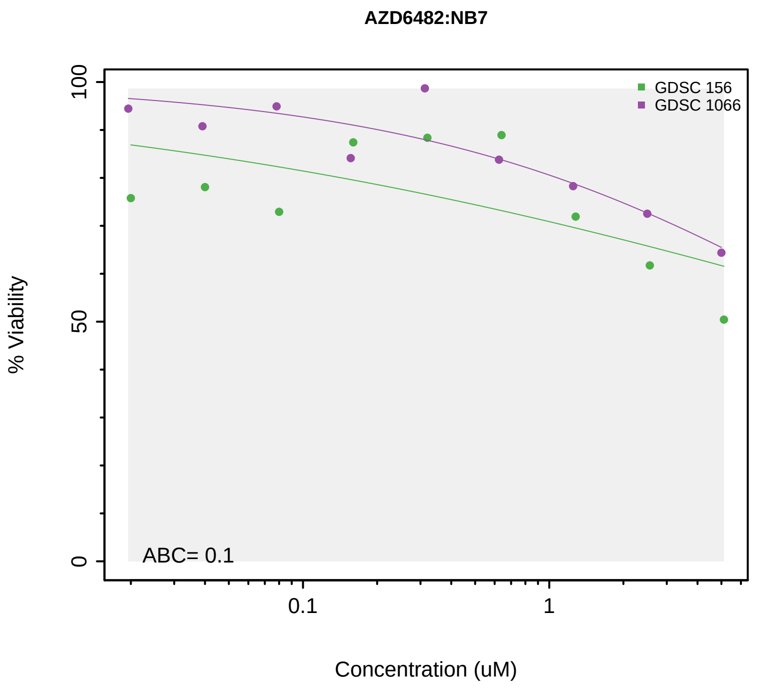
AZD6482:NB7
GDSC 156
GDSC 1066
% Viability
Concentration (uM)
ABC= 0.1

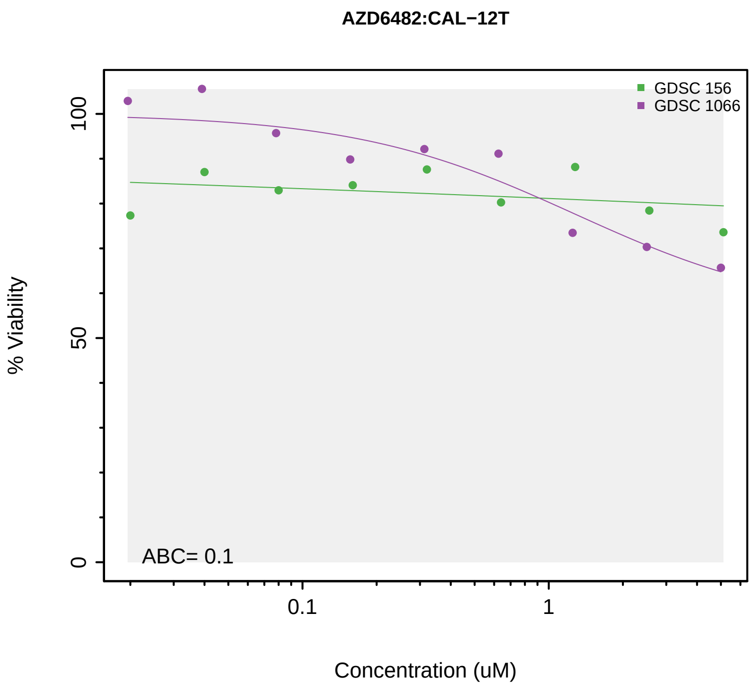
AZD6482:CAL−12T
GDSC 156
GDSC 1066
% Viability
Concentration (uM)
ABC= 0.1

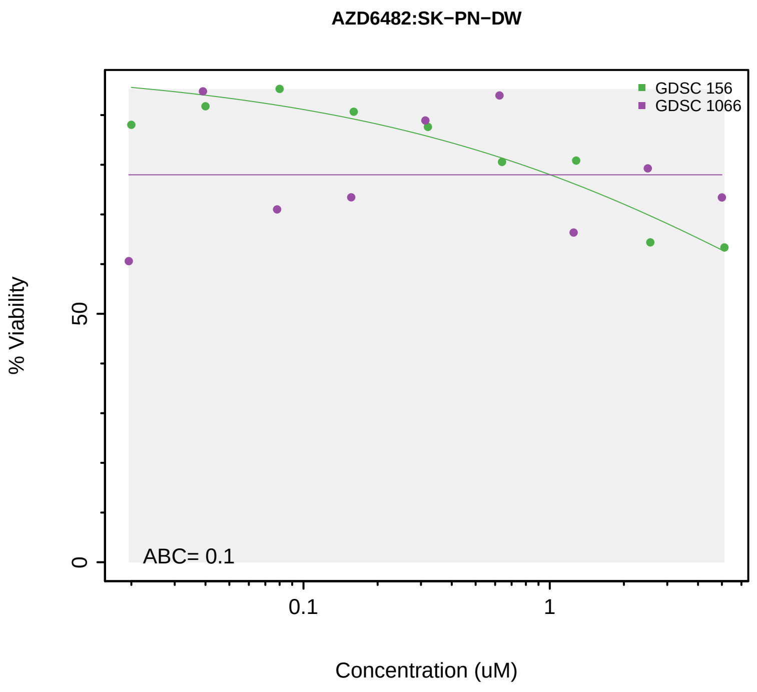
AZD6482:SK−PN−DW
GDSC 156
GDSC 1066
% Viability
Concentration (uM)
ABC= 0.1

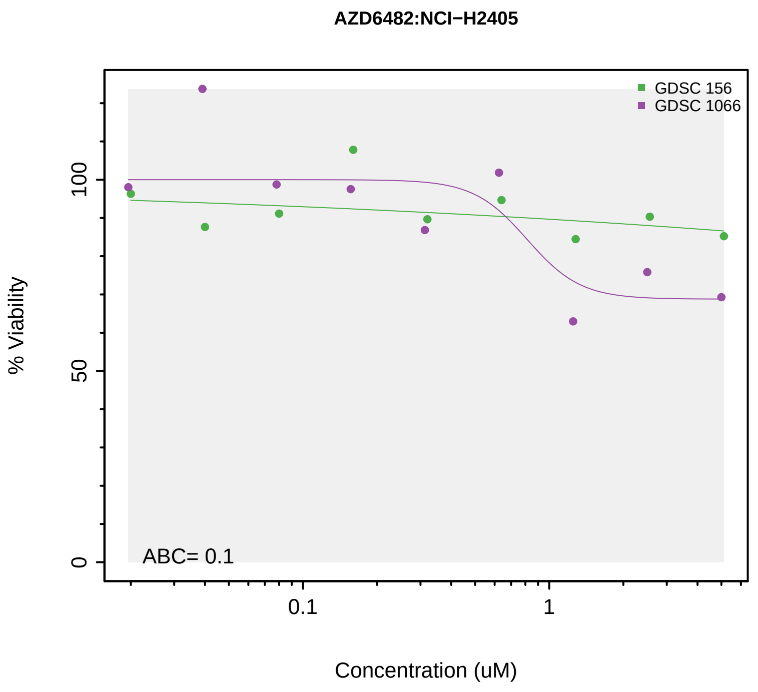
AZD6482:NCI−H2405
GDSC 156
GDSC 1066
% Viability
Concentration (uM)
ABC= 0.1

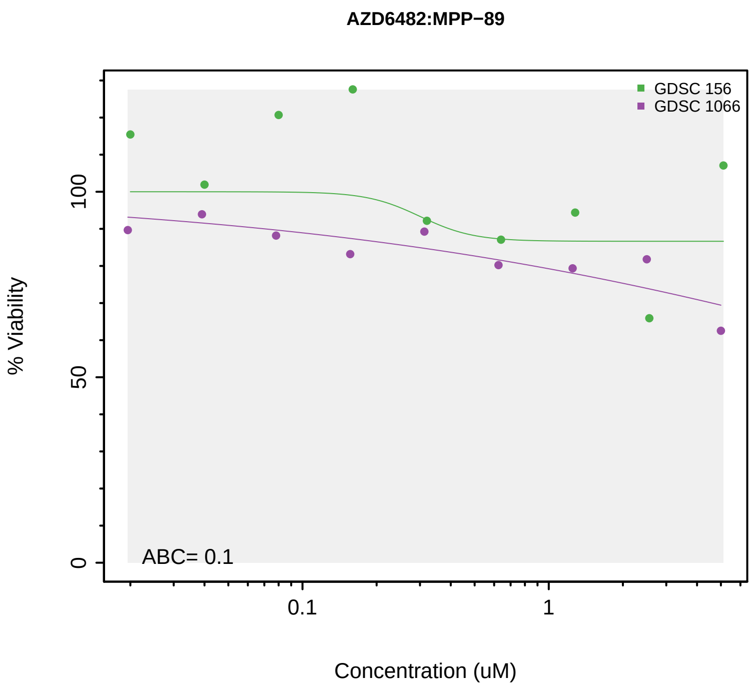
AZD6482:MPP−89
GDSC 156
GDSC 1066
% Viability
Concentration (uM)
ABC= 0.1

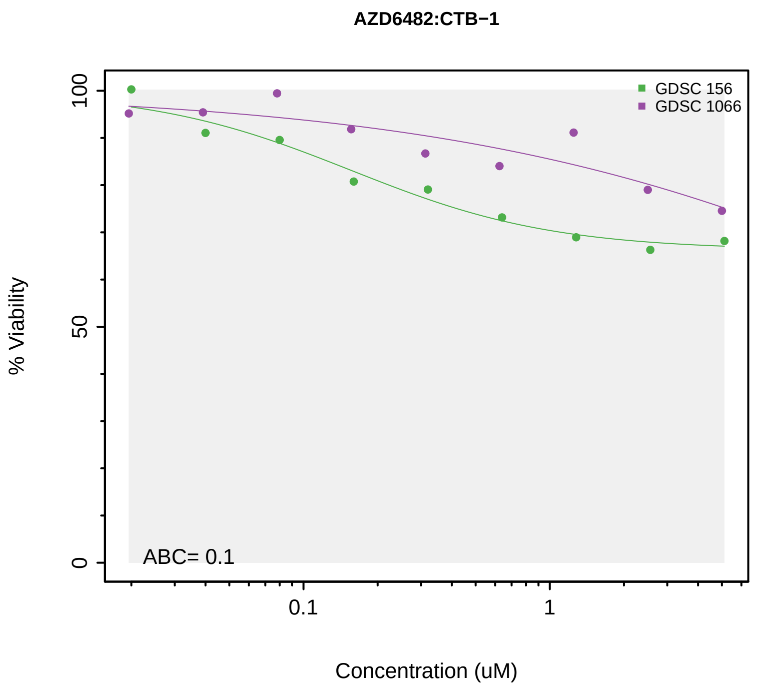
AZD6482:CTB−1
GDSC 156
GDSC 1066
% Viability
Concentration (uM)
ABC= 0.1

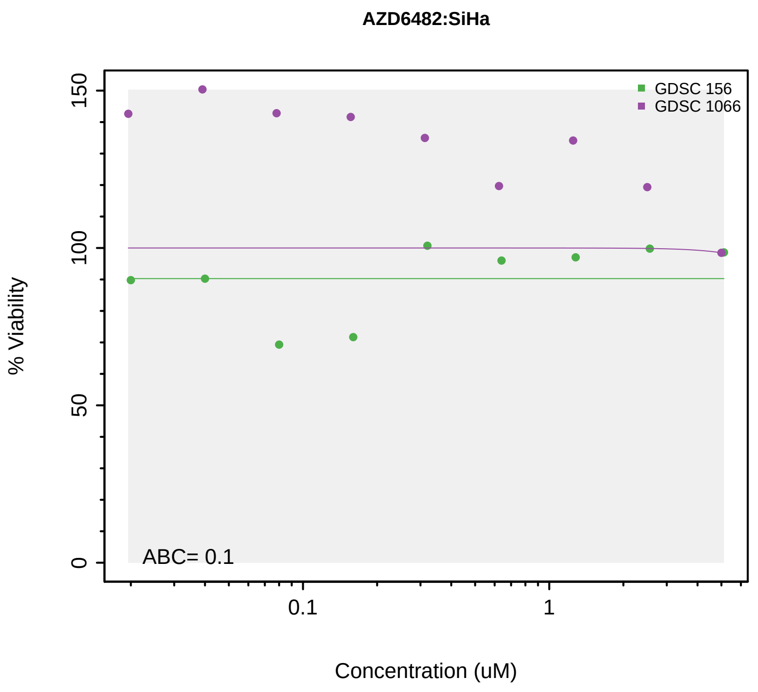
AZD6482:SiHa
GDSC 156
GDSC 1066
% Viability
Concentration (uM)
ABC= 0.1

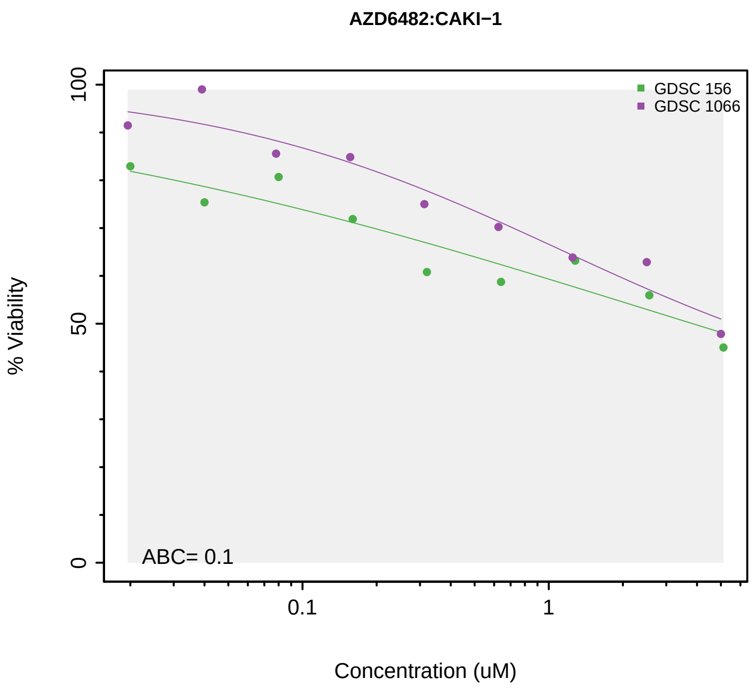
AZD6482:CAKI−1
GDSC 156
GDSC 1066
% Viability
Concentration (uM)
ABC= 0.1

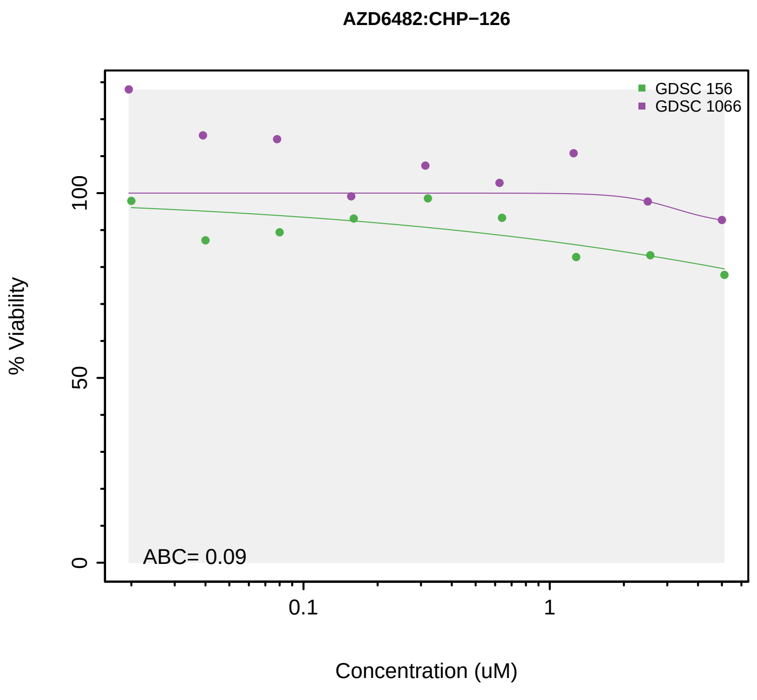
AZD6482:CHP−126
GDSC 156
GDSC 1066
% Viability
Concentration (uM)
ABC= 0.09

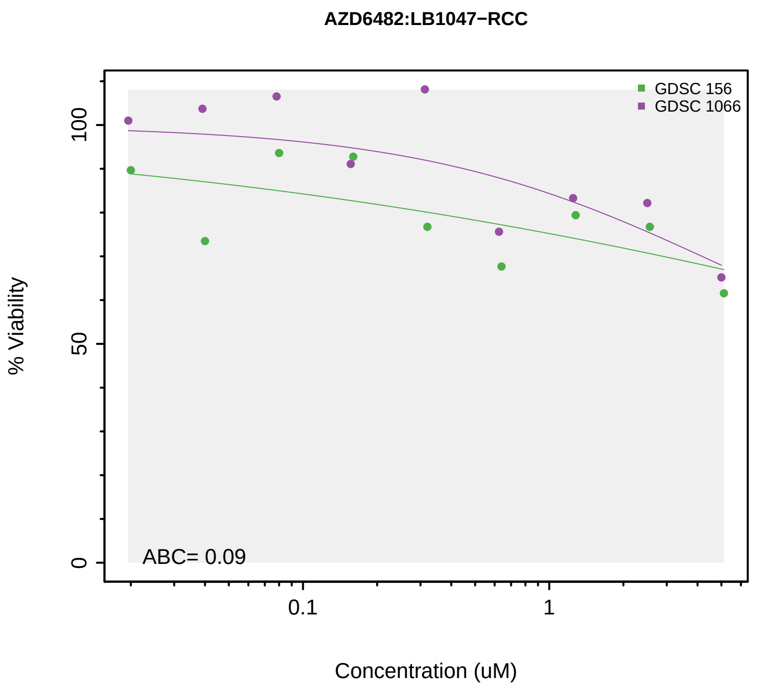
AZD6482:LB1047−RCC
GDSC 156
GDSC 1066
% Viability
Concentration (uM)
ABC= 0.09

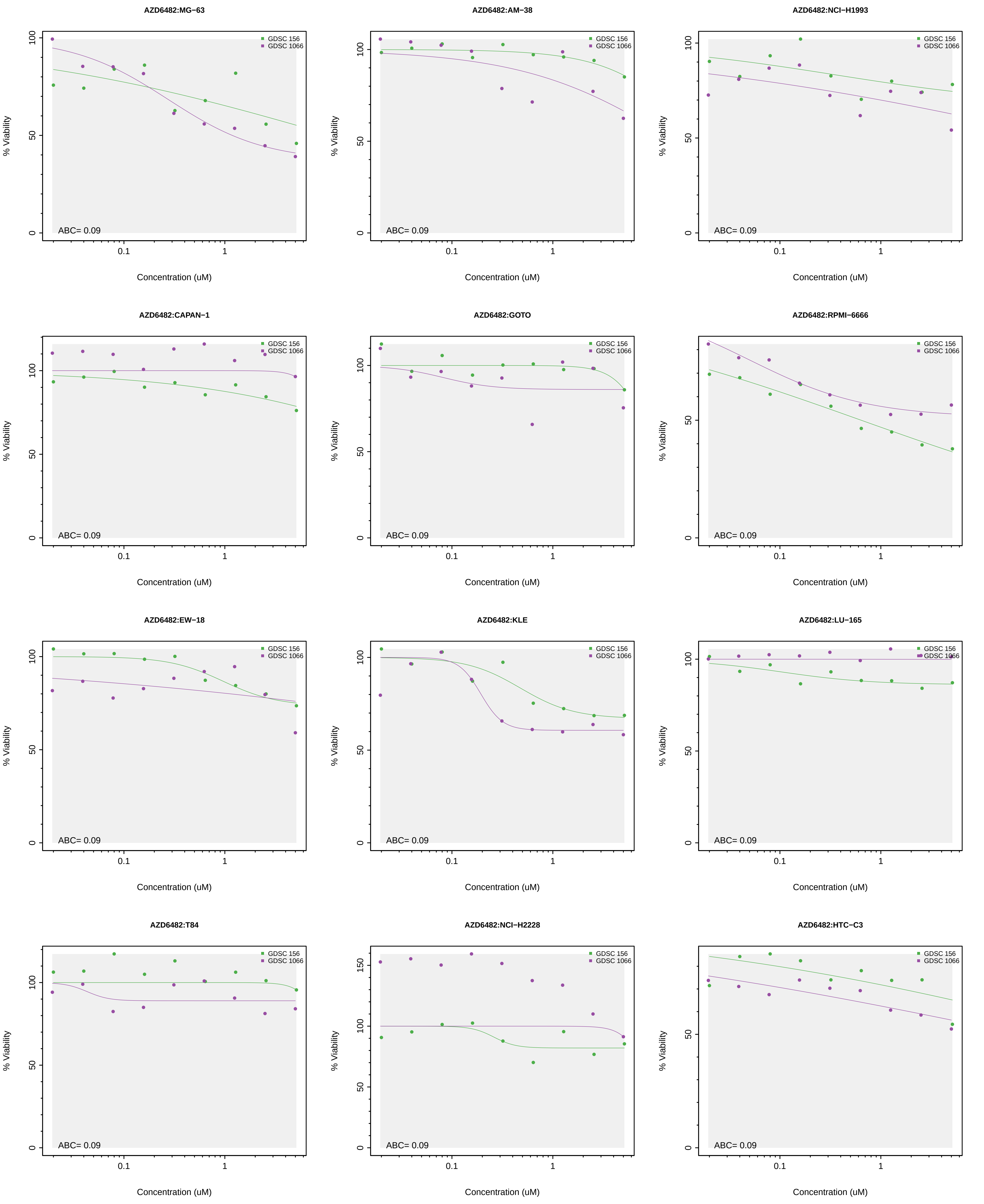
AZD6482:MG−63
AZD6482:AM−38
AZD6482:NCI−H1993
AZD6482:CAPAN−1
AZD6482:GOTO
AZD6482:RPMI−6666
AZD6482:EW−18
AZD6482:KLE
AZD6482:LU−165
AZD6482:T84
AZD6482:NCI−H2228
AZD6482:HTC−C3
GDSC 156
GDSC 1066
ABC= 0.09
% Viability
Concentration (uM)
0
50
100
150
0.1
1

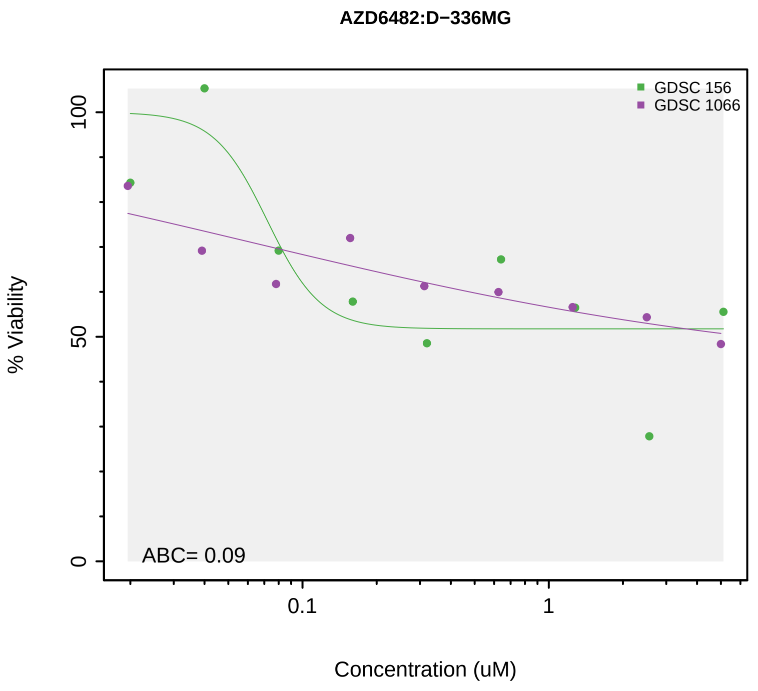
AZD6482:D−336MG
GDSC 156
GDSC 1066
ABC= 0.09
% Viability
0
50
100
0.1
1
Concentration (uM)

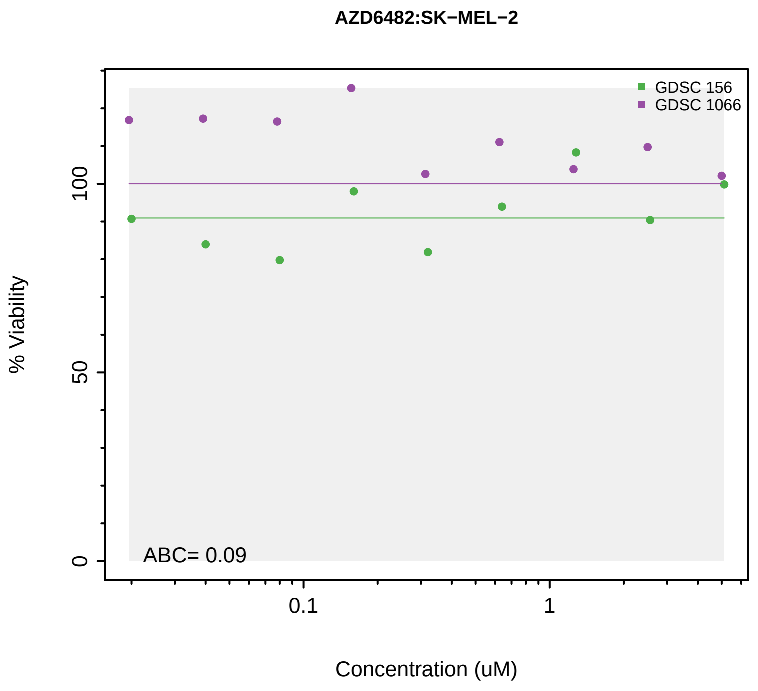
AZD6482:SK−MEL−2
GDSC 156
GDSC 1066
ABC= 0.09
% Viability
0
50
100
0.1
1
Concentration (uM)

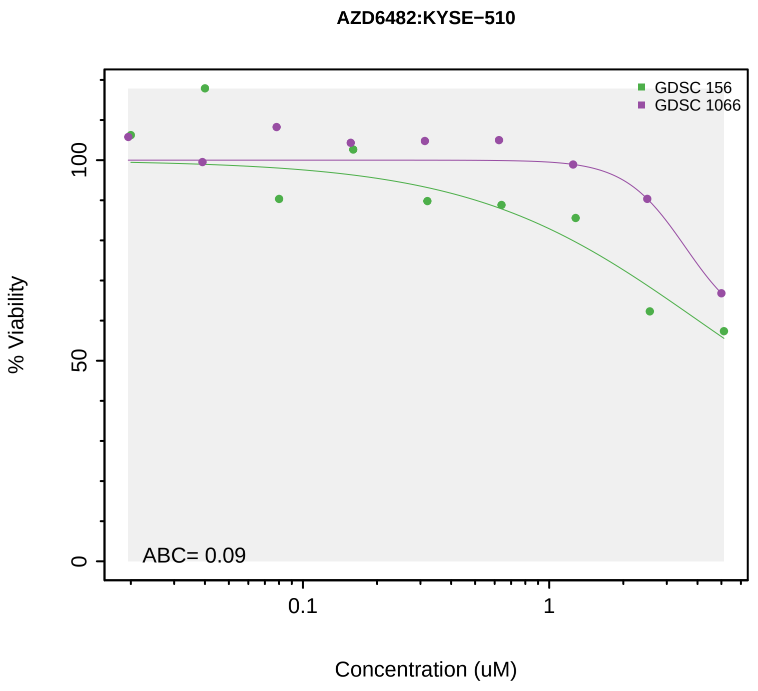
AZD6482:KYSE−510
GDSC 156
GDSC 1066
ABC= 0.09
% Viability
0
50
100
0.1
1
Concentration (uM)

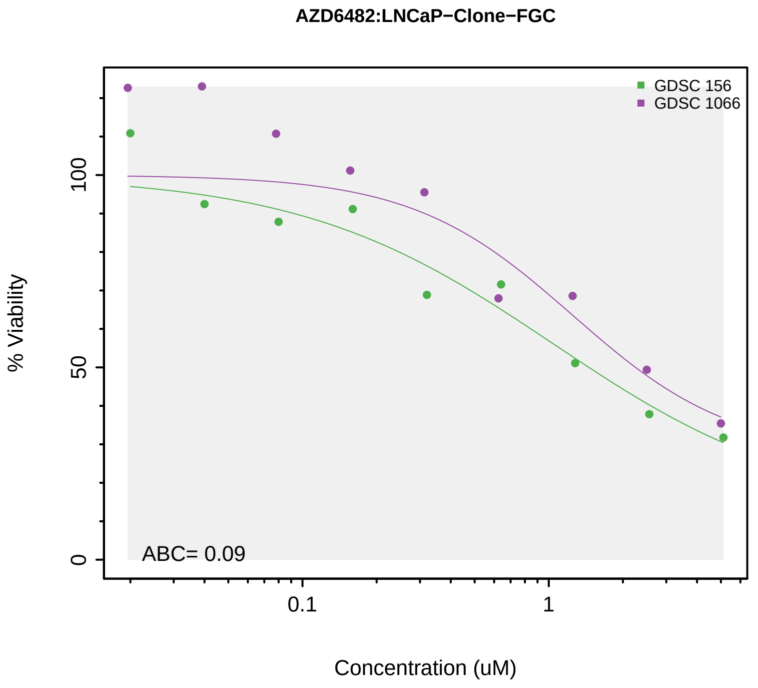
AZD6482:LNCaP−Clone−FGC
GDSC 156
GDSC 1066
ABC= 0.09
% Viability
0
50
100
0.1
1
Concentration (uM)

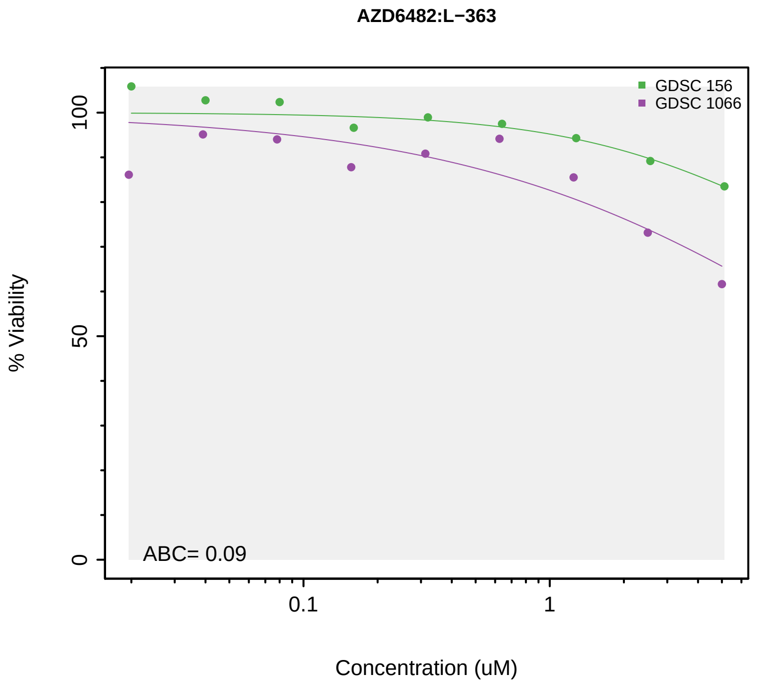
AZD6482:L−363
GDSC 156
GDSC 1066
ABC= 0.09
% Viability
0
50
100
0.1
1
Concentration (uM)

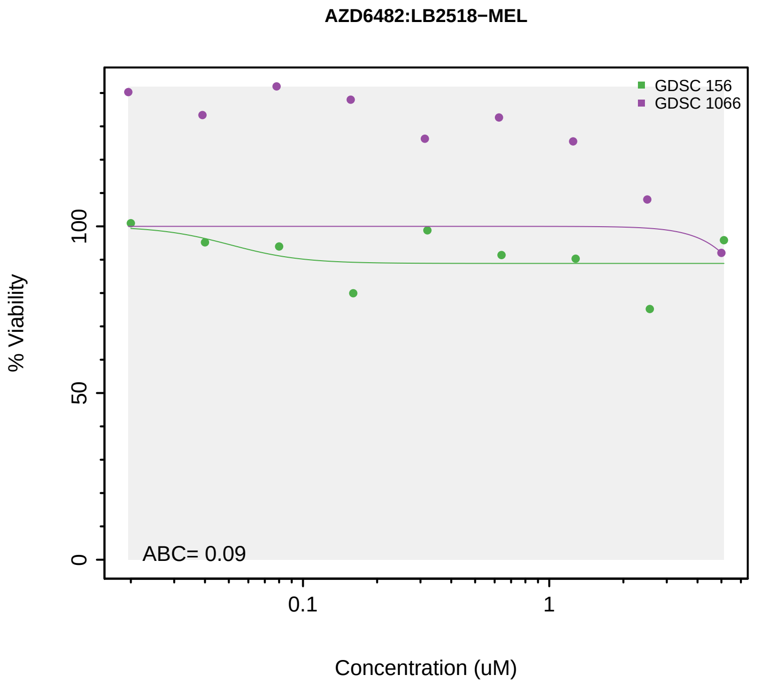
AZD6482:LB2518−MEL
GDSC 156
GDSC 1066
ABC= 0.09
% Viability
0
50
100
0.1
1
Concentration (uM)

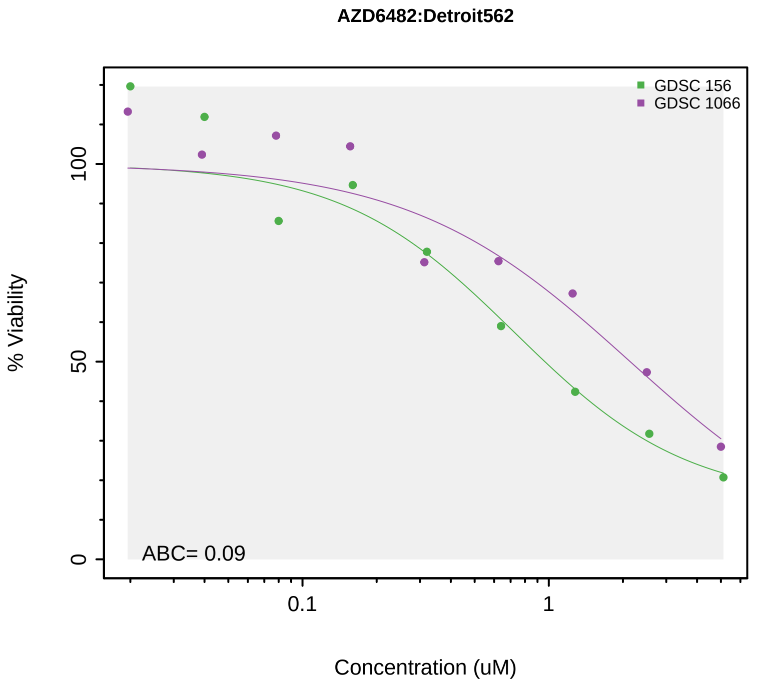
AZD6482:Detroit562
GDSC 156
GDSC 1066
ABC= 0.09
% Viability
0
50
100
0.1
1
Concentration (uM)

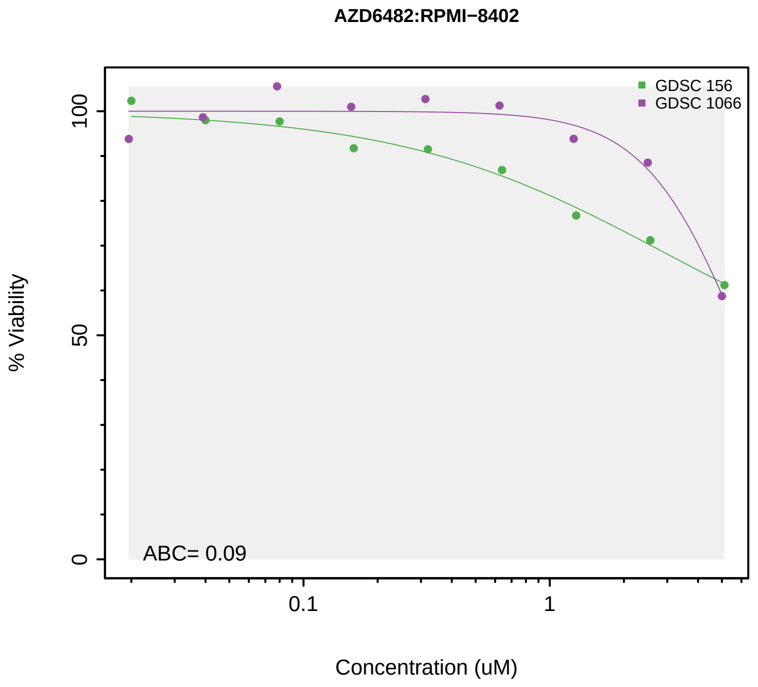
AZD6482:RPMI−8402
GDSC 156
GDSC 1066
ABC= 0.09
% Viability
0
50
100
0.1
1
Concentration (uM)

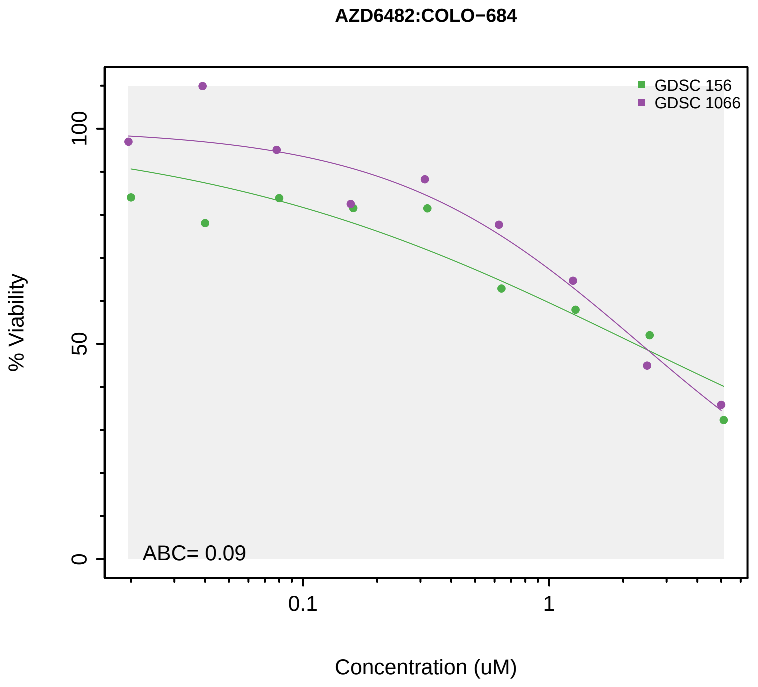
AZD6482:COLO−684
GDSC 156
GDSC 1066
ABC= 0.09
% Viability
0
50
100
0.1
1
Concentration (uM)

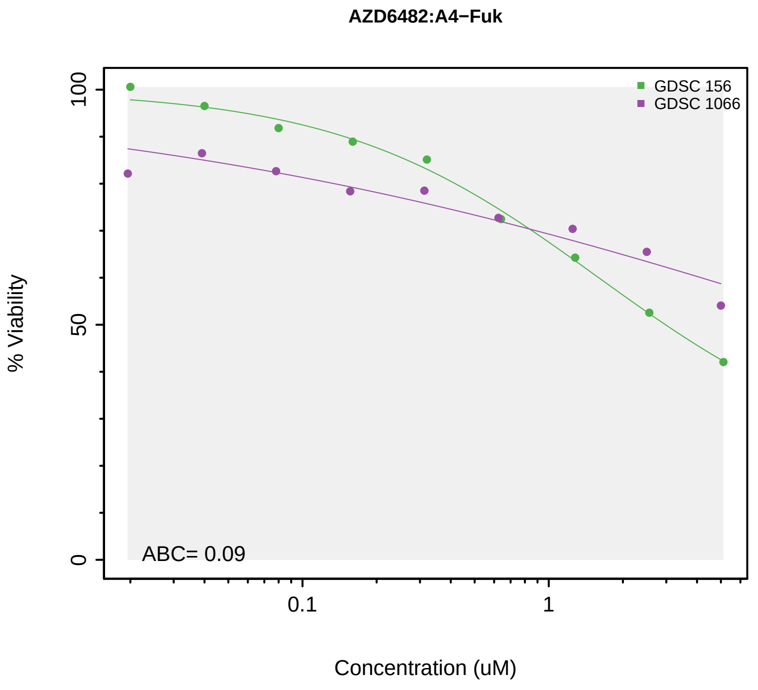
AZD6482:A4−Fuk
GDSC 156
GDSC 1066
ABC= 0.09
% Viability
0
50
100
0.1
1
Concentration (uM)

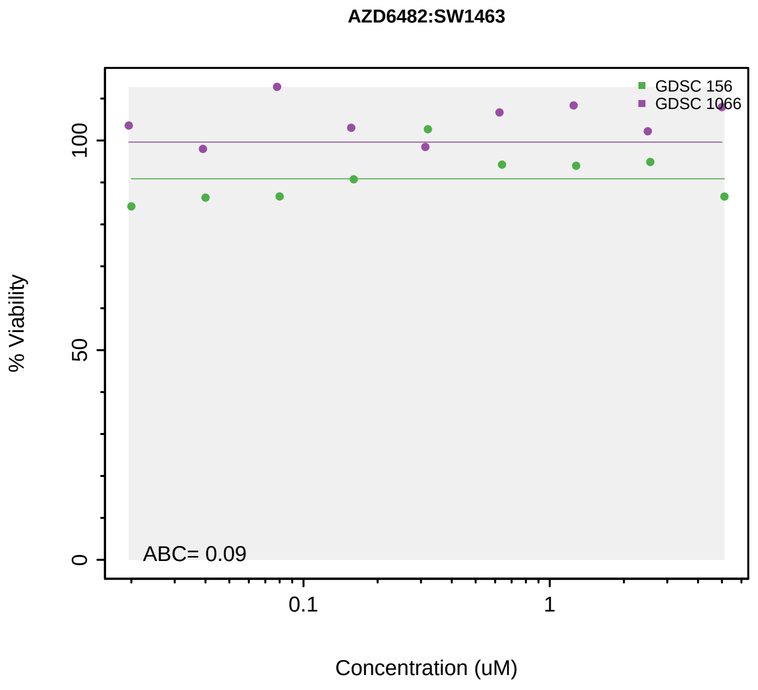
AZD6482:SW1463
GDSC 156
GDSC 1066
ABC= 0.09
% Viability
0
50
100
0.1
1
Concentration (uM)

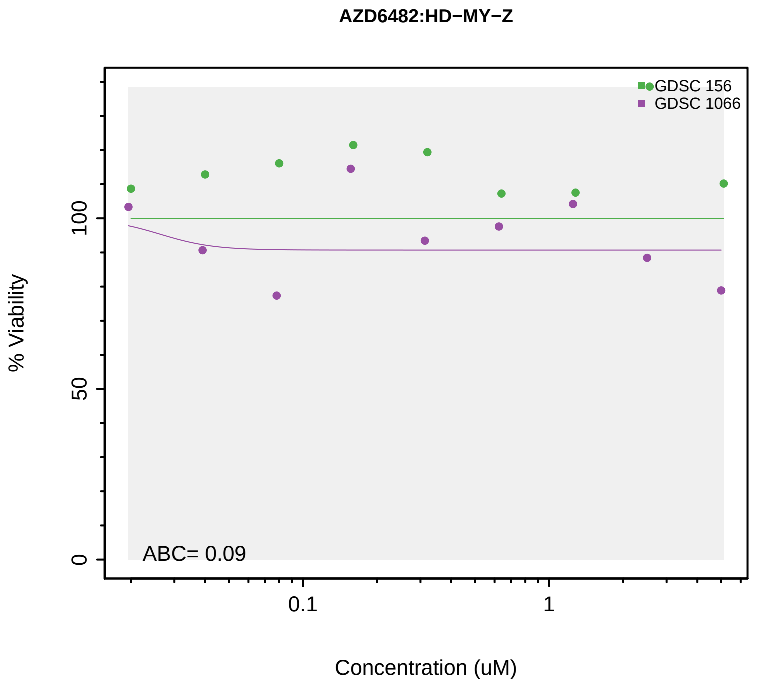
AZD6482:HD−MY−Z
GDSC 156
GDSC 1066
ABC= 0.09
% Viability
0
50
100
0.1
1
Concentration (uM)

AZD6482:SUP−T1

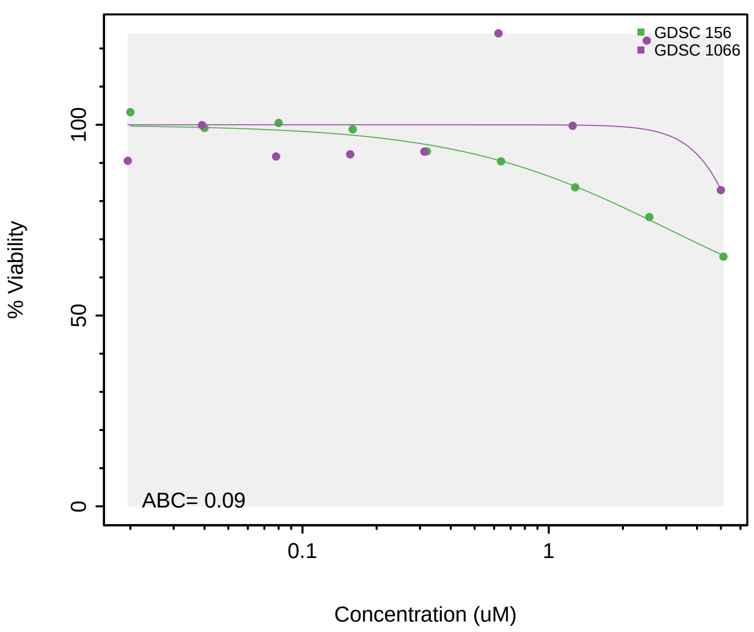

AZD6482:NCI−H1792

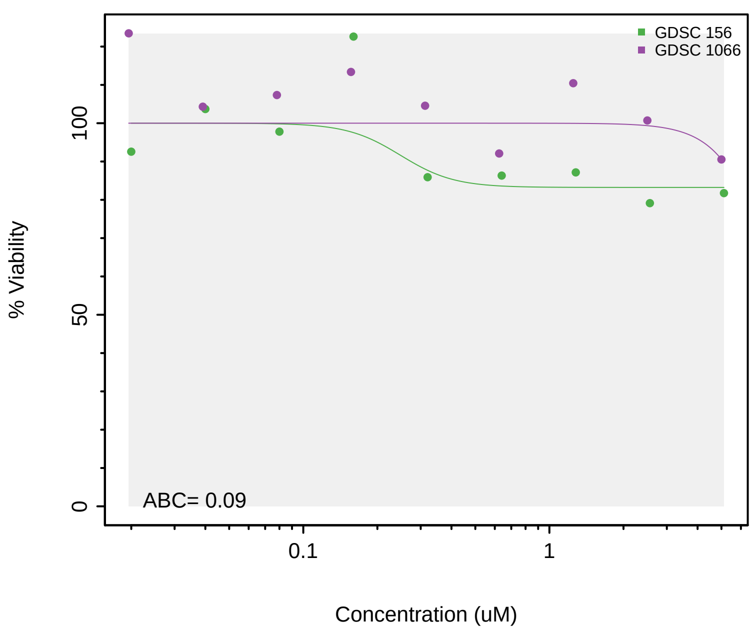

AZD6482:OCUB−M

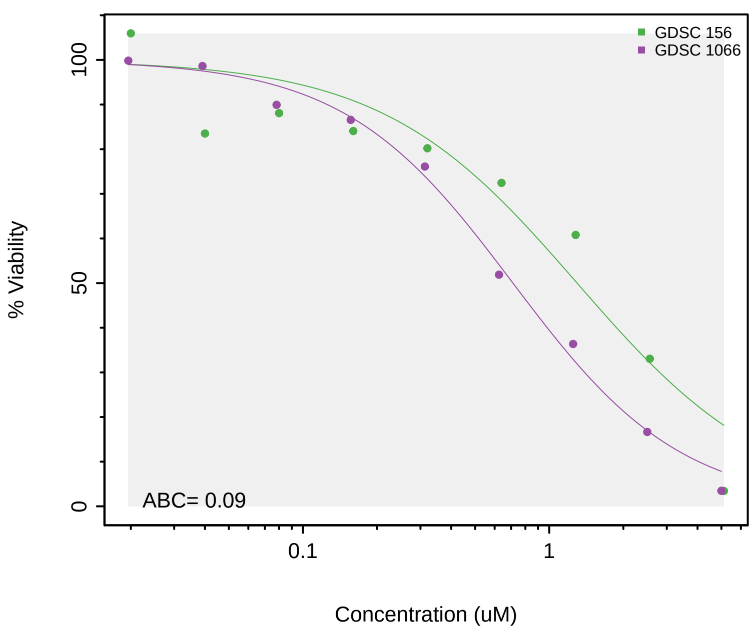

AZD6482:JiyoyeP−2003

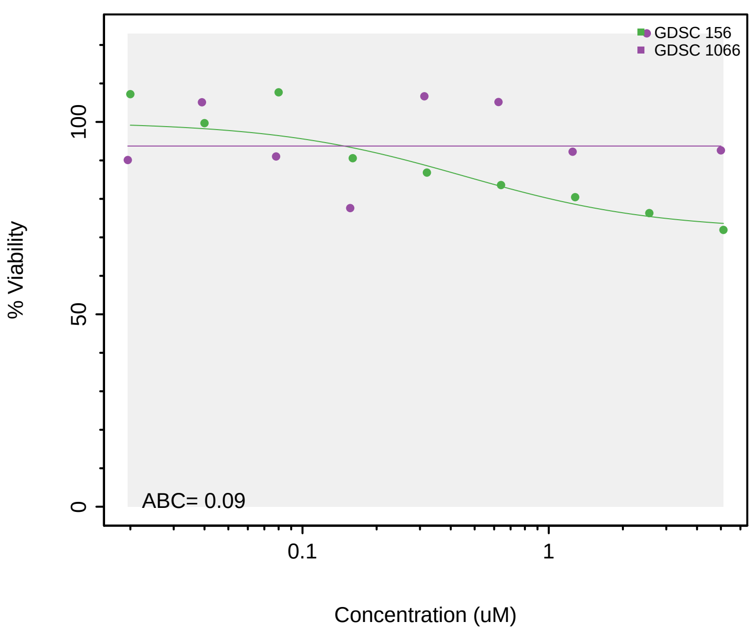

AZD6482:BFTC−909

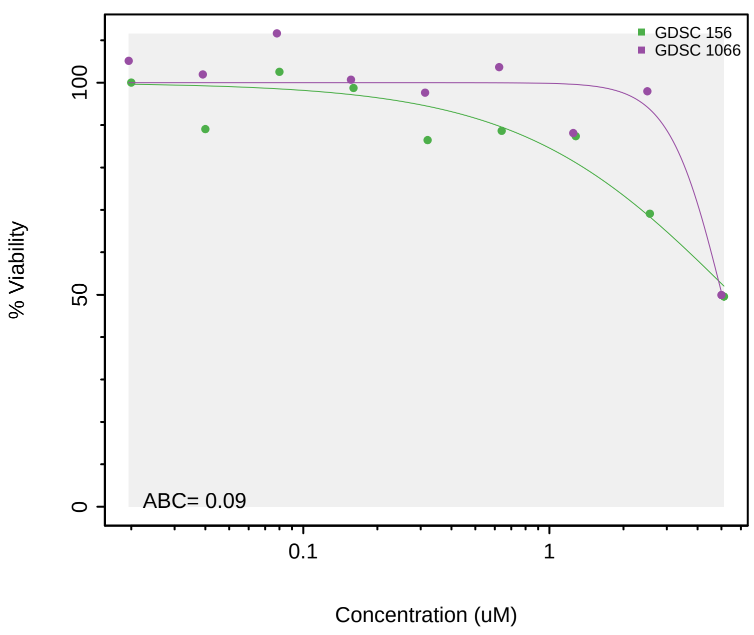

AZD6482:FTC−133

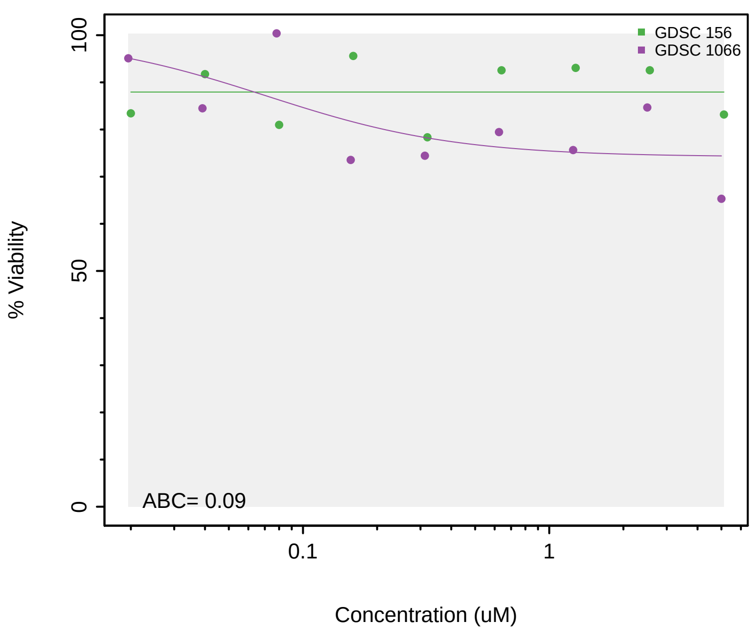

AZD6482:EW−3

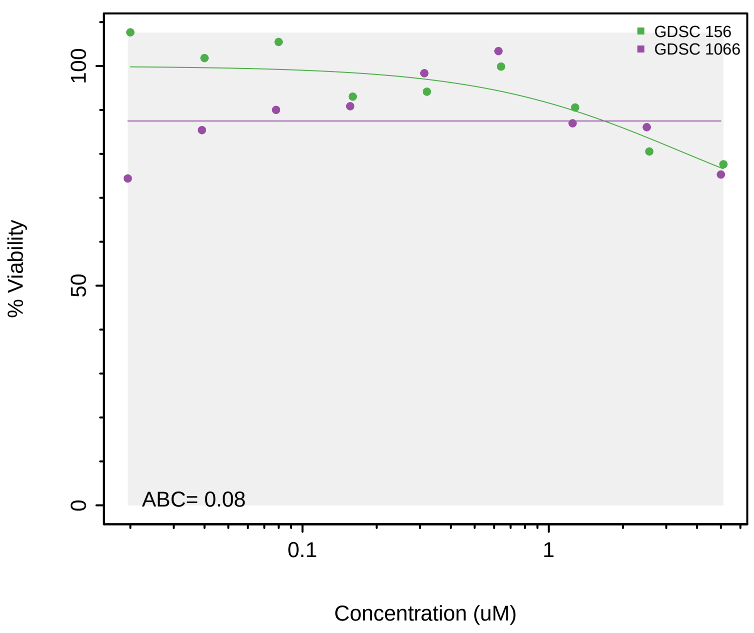

AZD6482:IMR−5

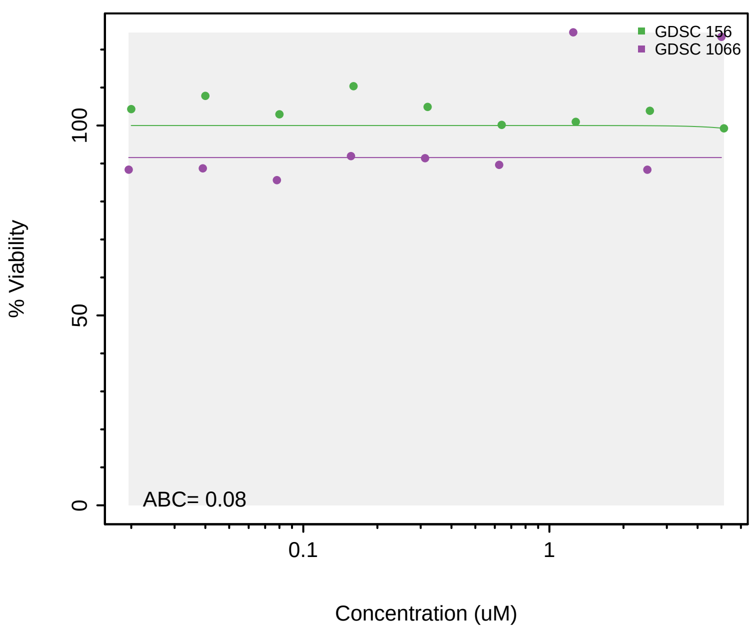

AZD6482:TE−10

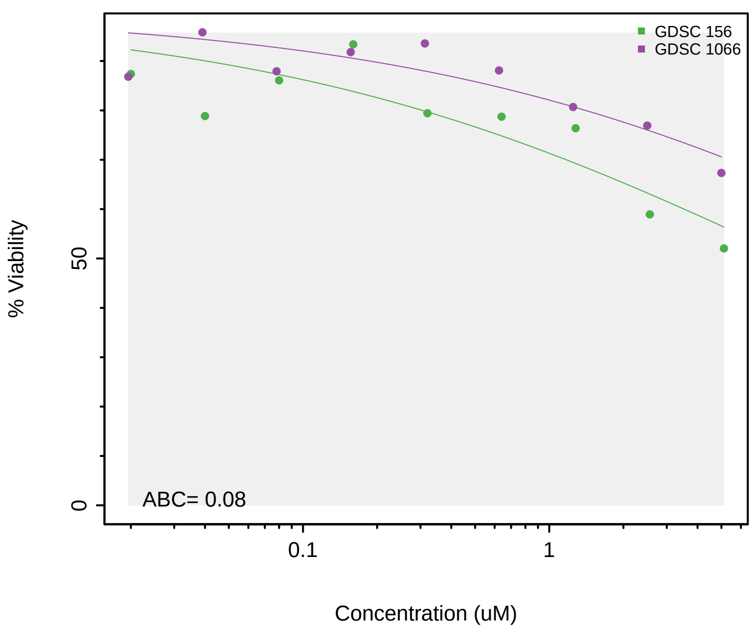

AZD6482:LS−1034

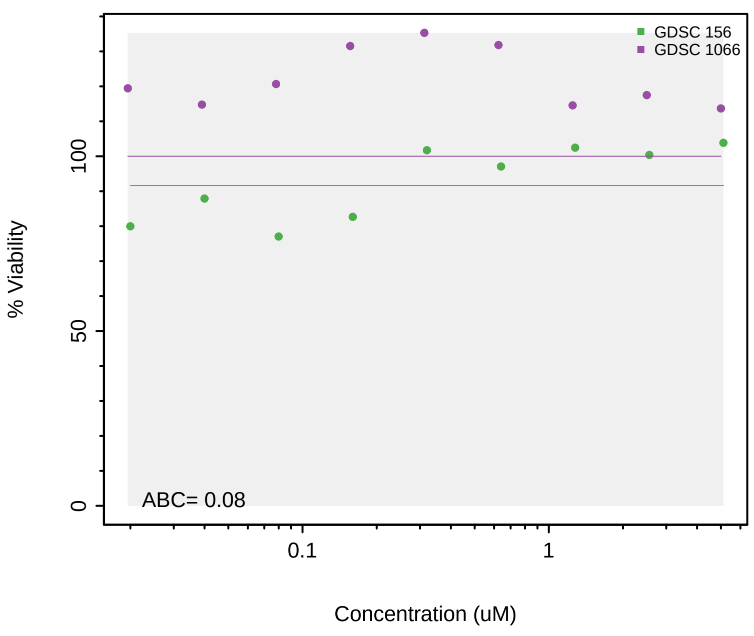

AZD6482:LC−2−ad

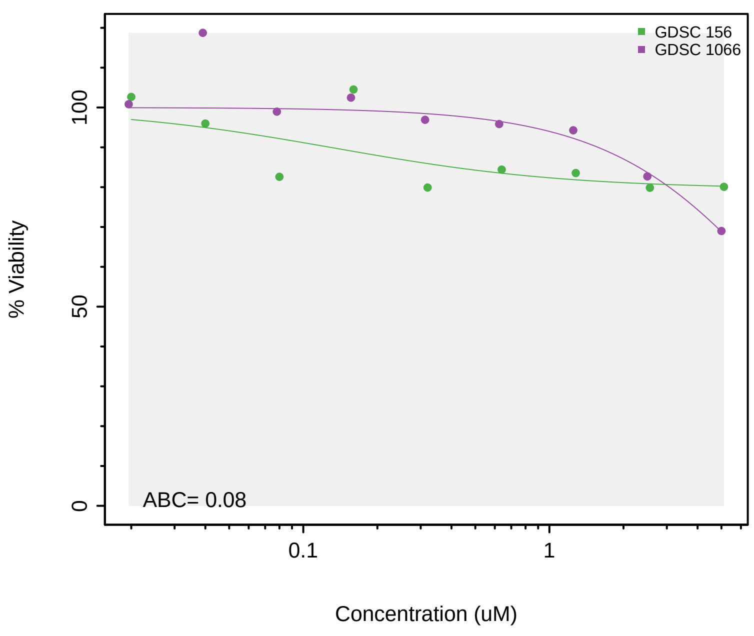

AZD6482:T98G

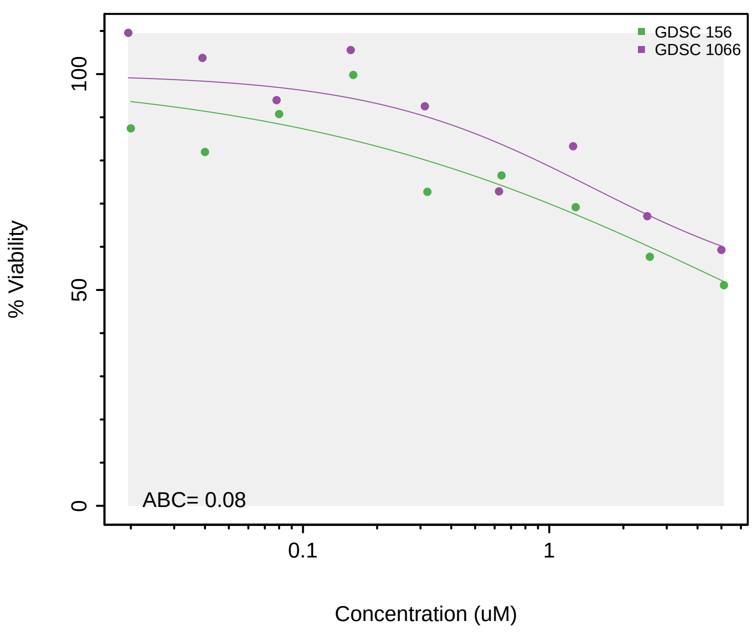

AZD6482:TE−8

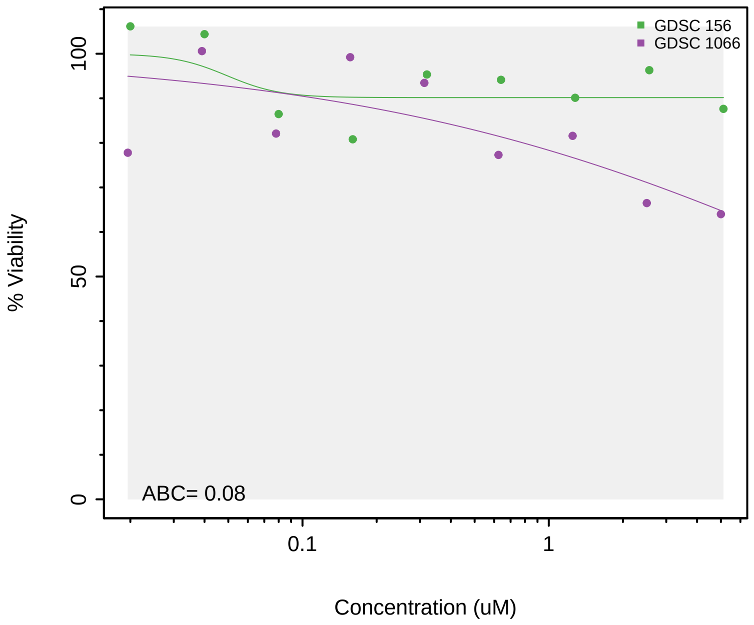

AZD6482:COLO−824

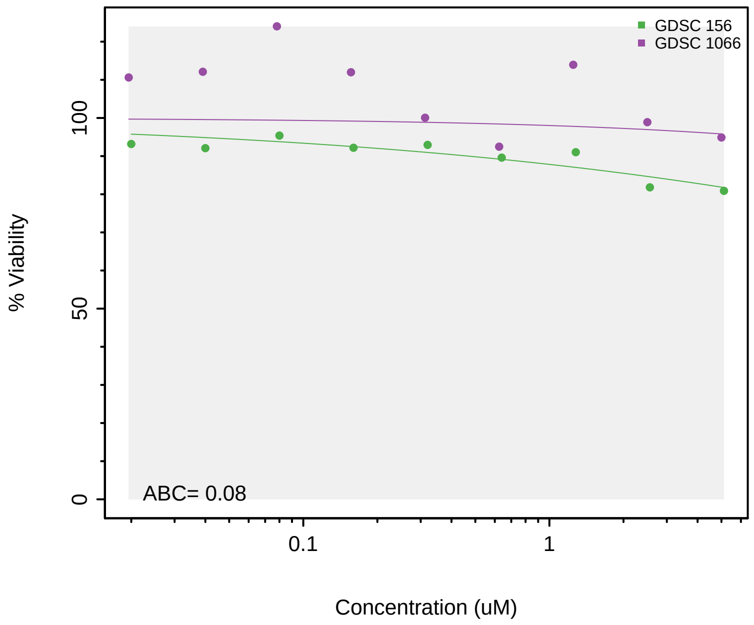

AZD6482:SW1573

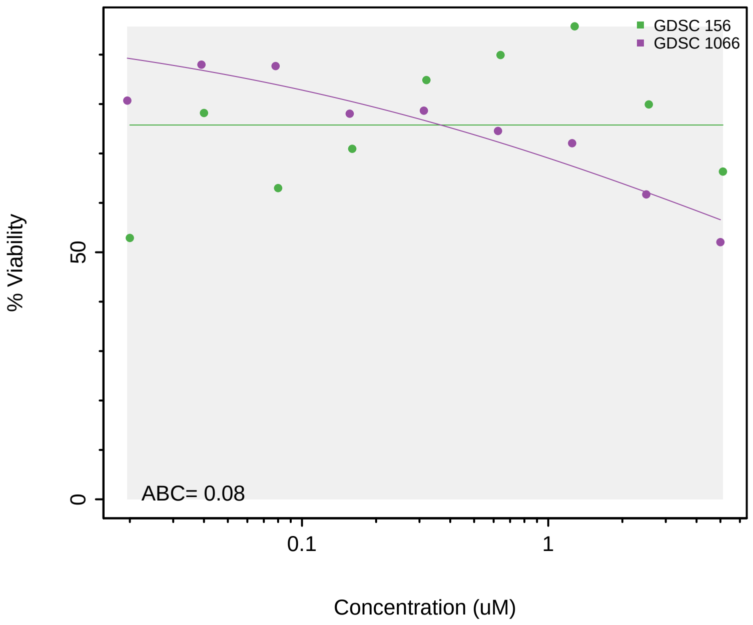

AZD6482:NB1

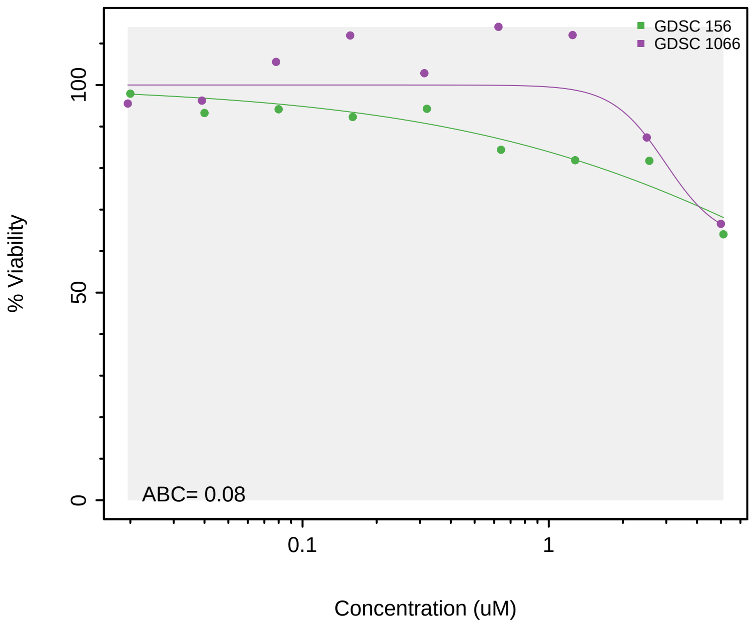

AZD6482:IM−9

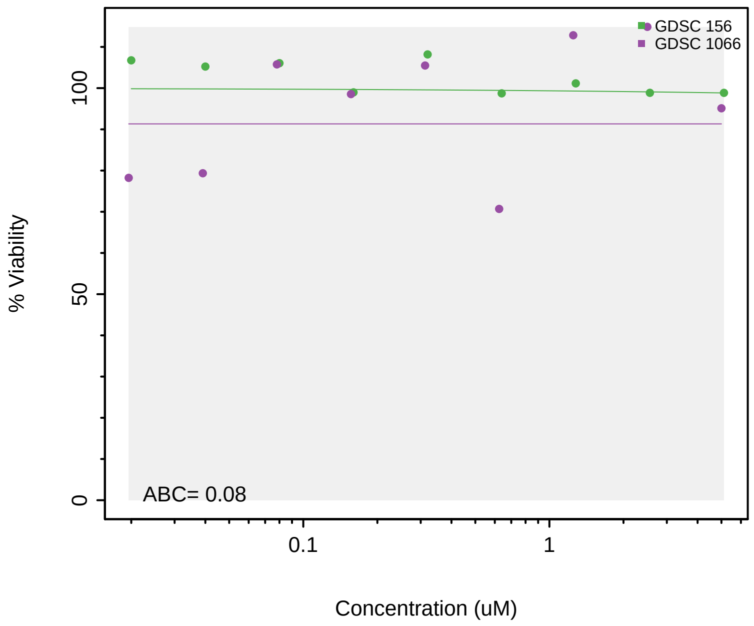

AZD6482:THP−1

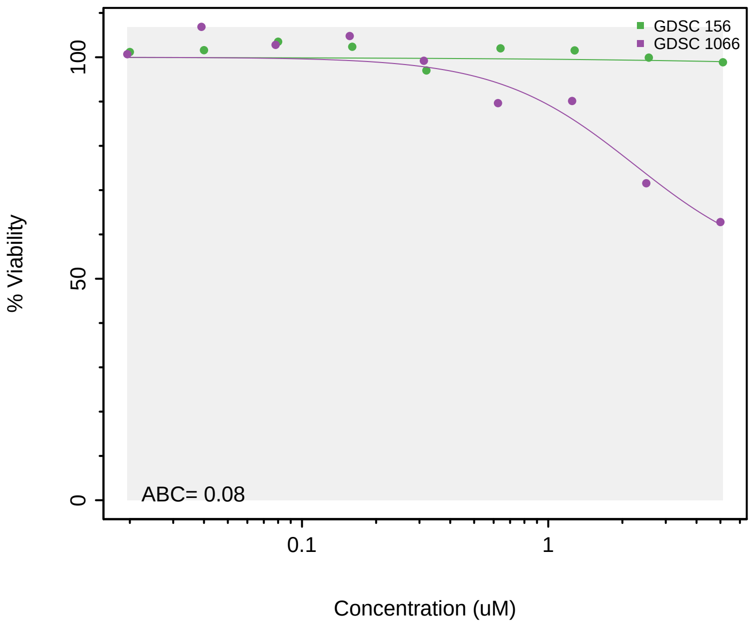

AZD6482:MFH−ino

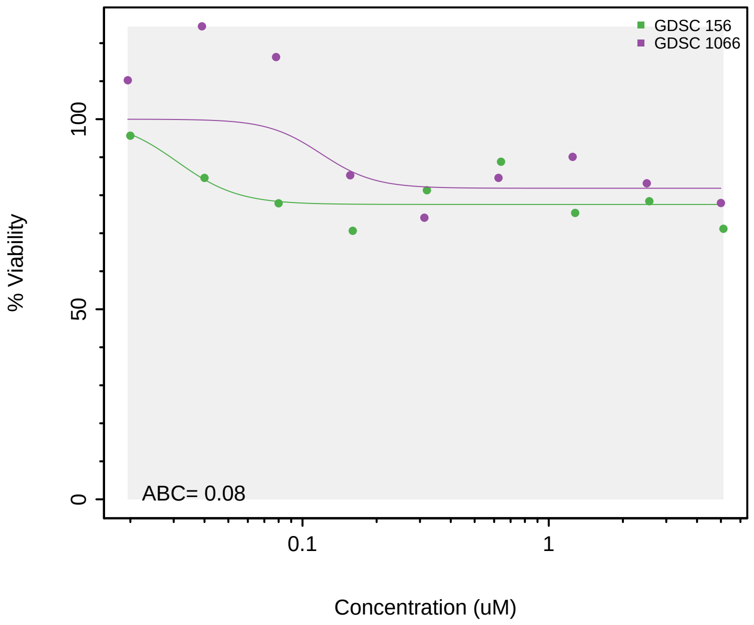

AZD6482:K5

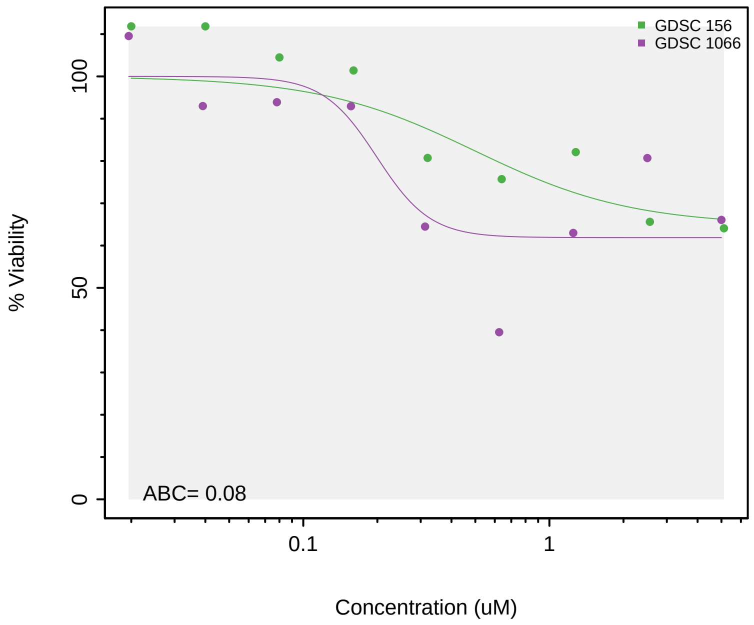

AZD6482:ES7

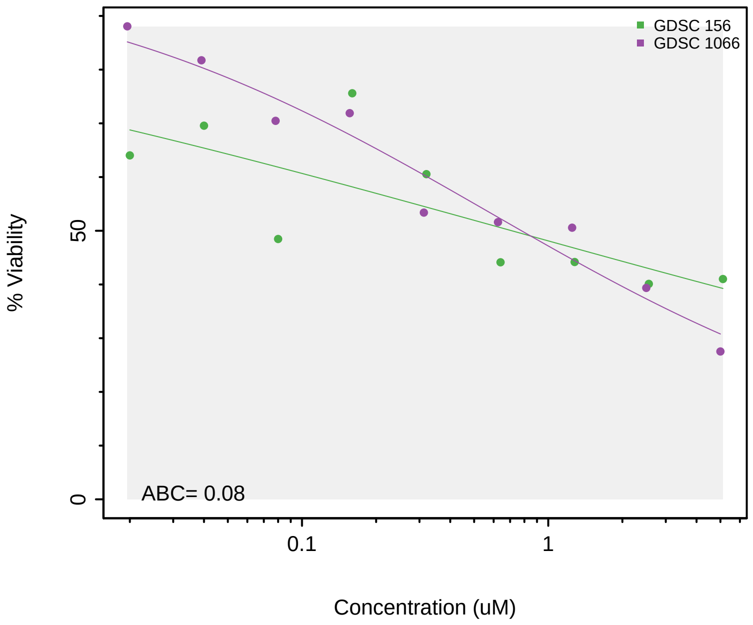

AZD6482:SW872

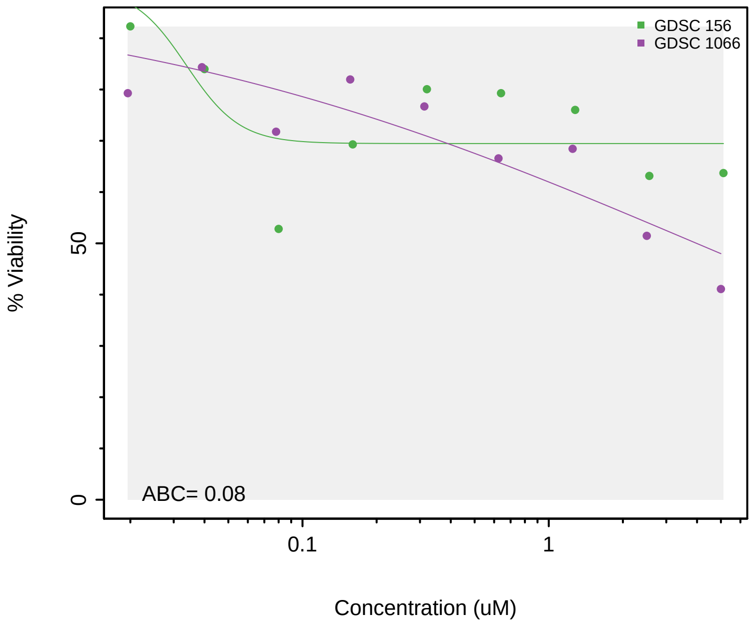

AZD6482:CP50−MEL−B

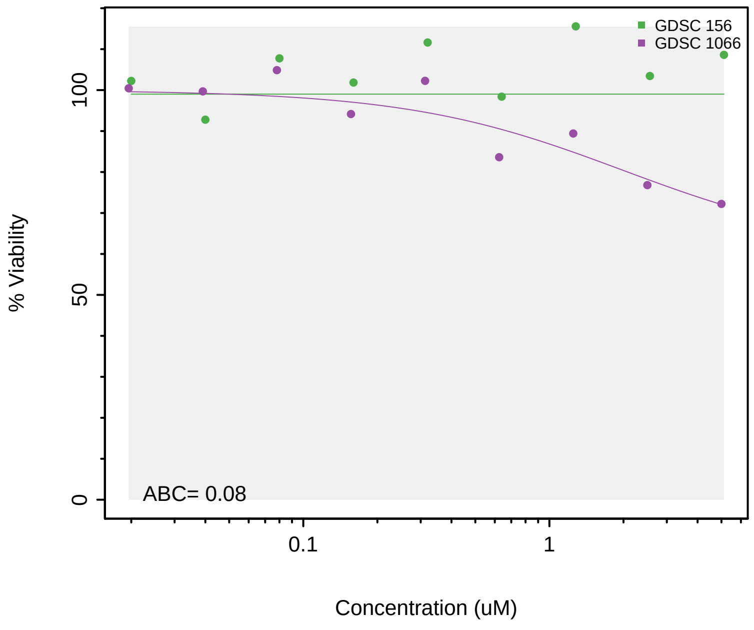

AZD6482:ES8

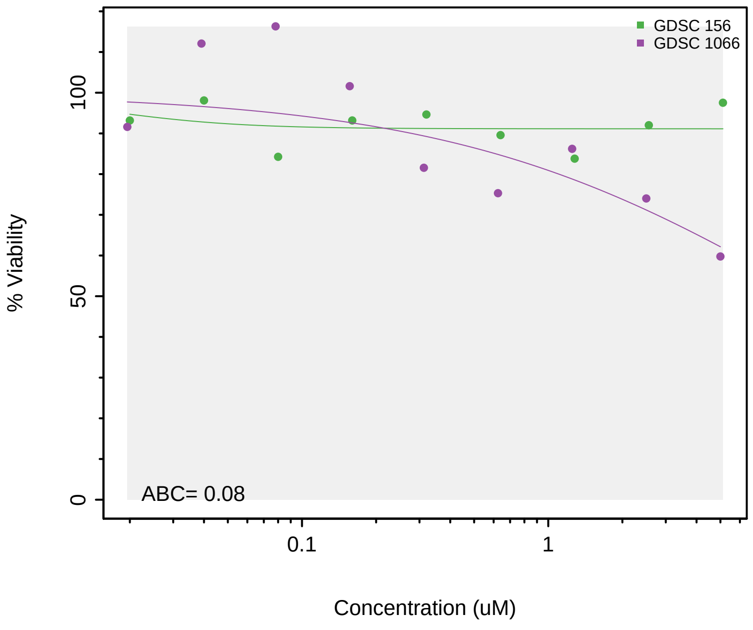

AZD6482:NCI−H524

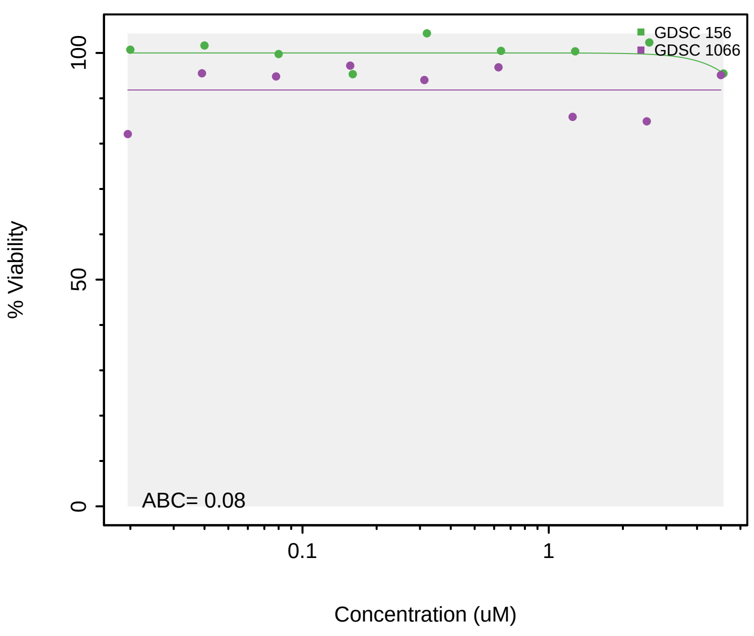

AZD6482:NMC−G1

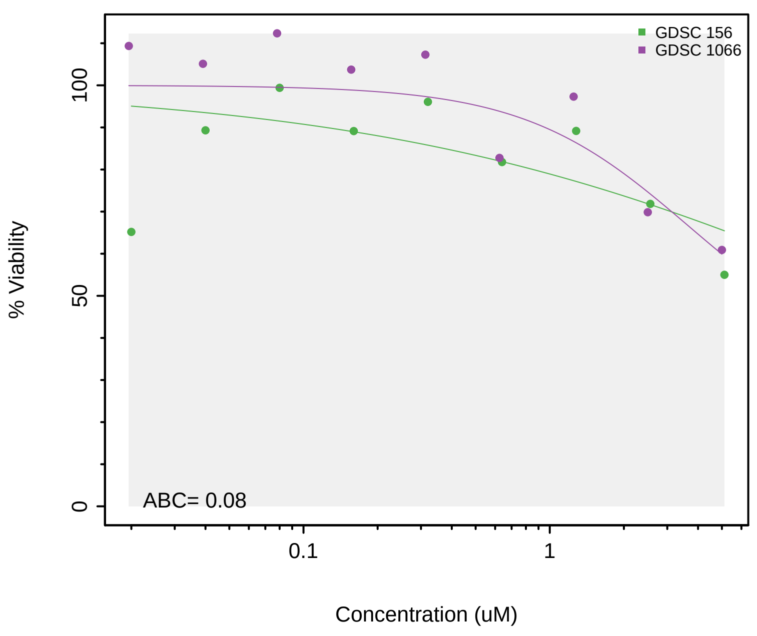

AZD6482:MES−SA

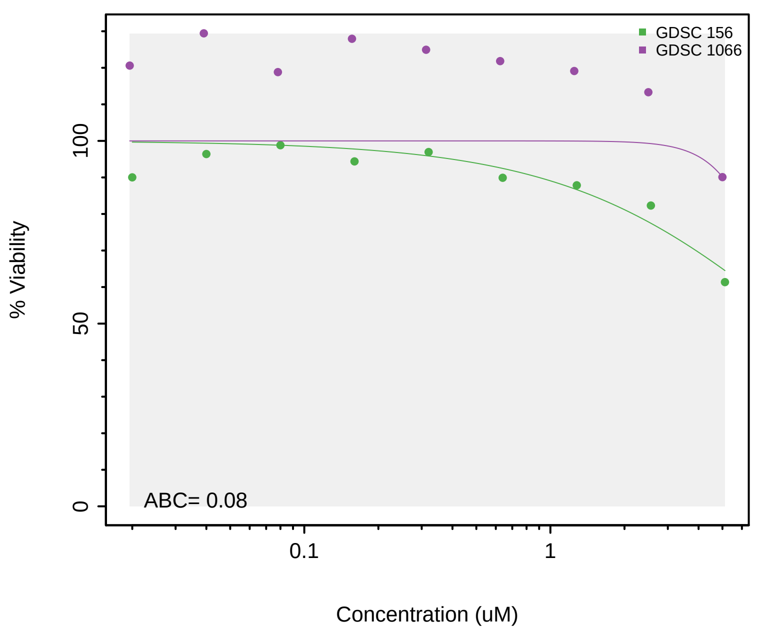

AZD6482:CAL−120

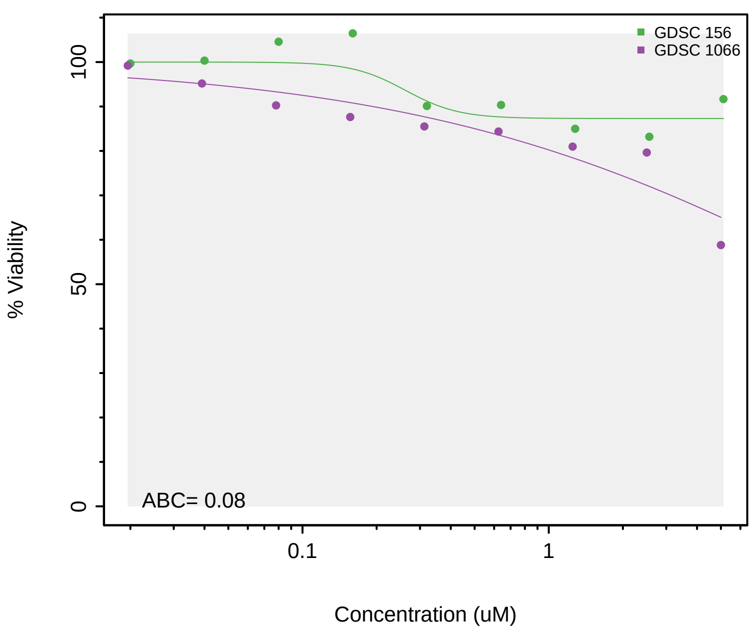

AZD6482:COLO−792

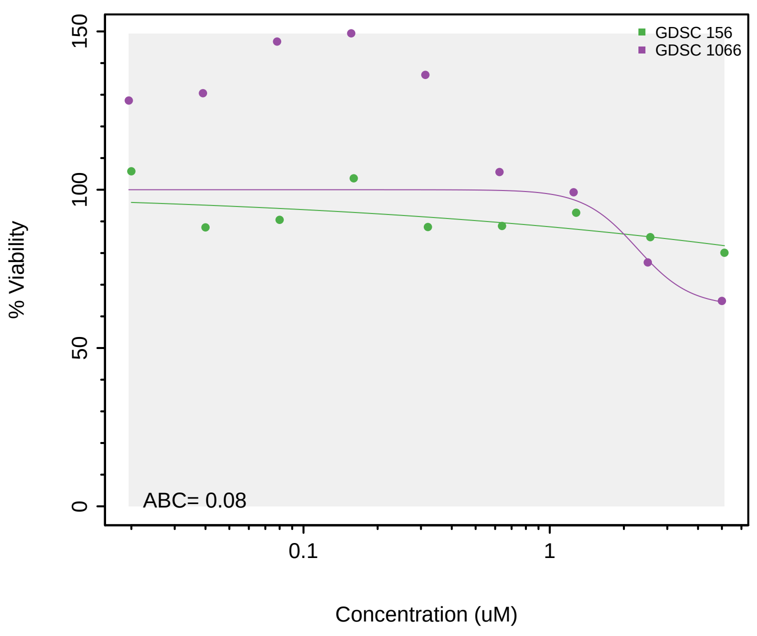

AZD6482:LS−123

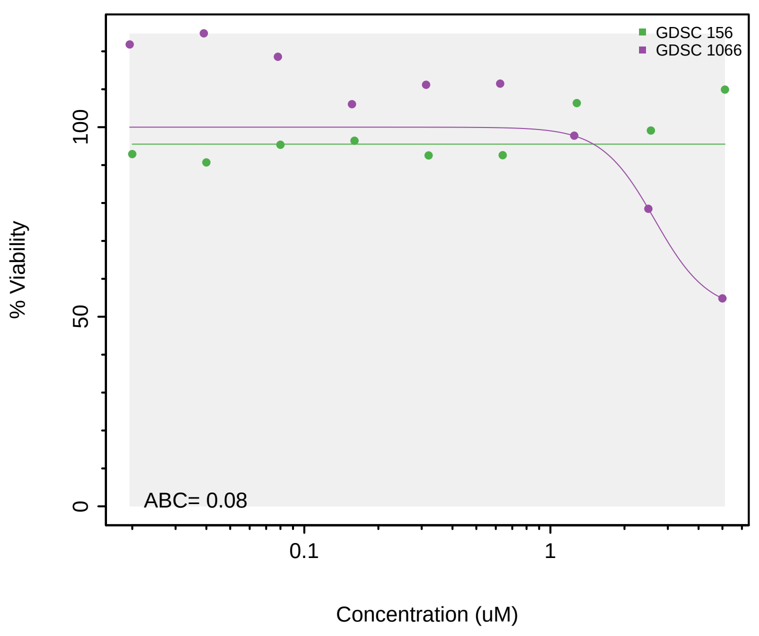

AZD6482:DoTc2−4510

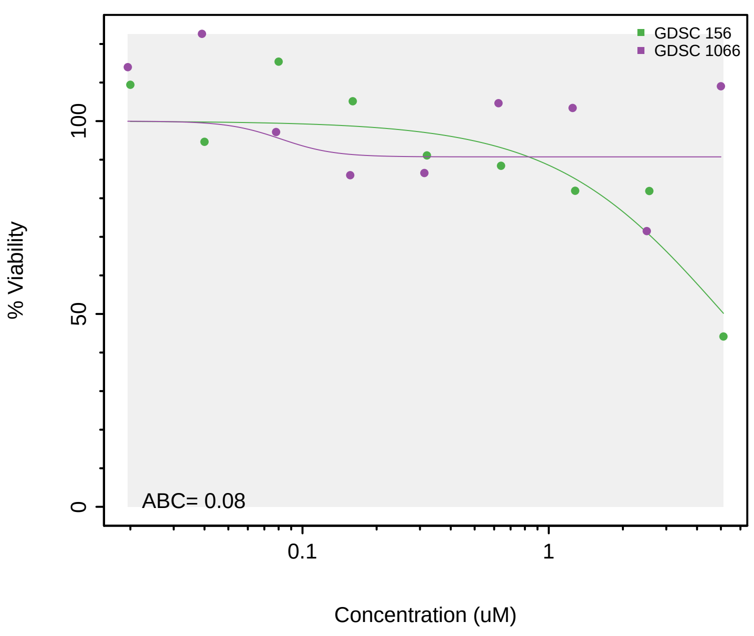

AZD6482:TK10

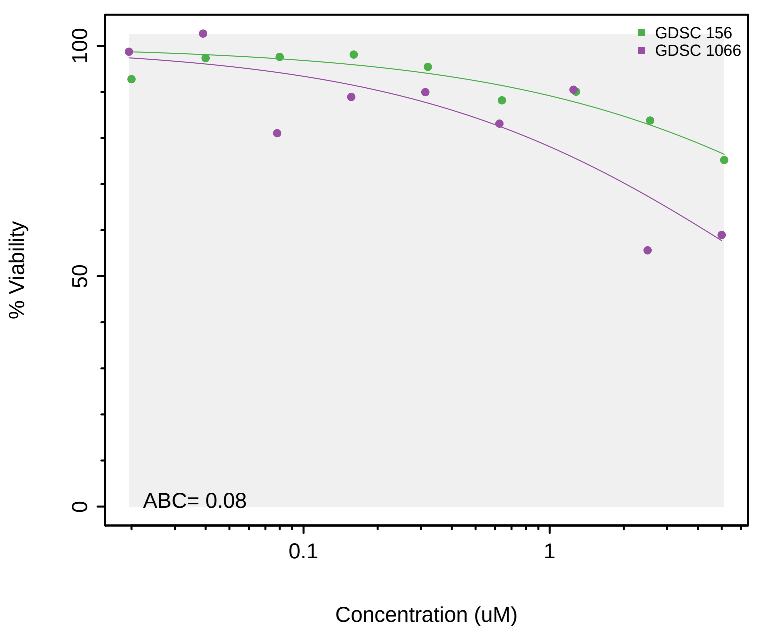

AZD6482:ETK−1

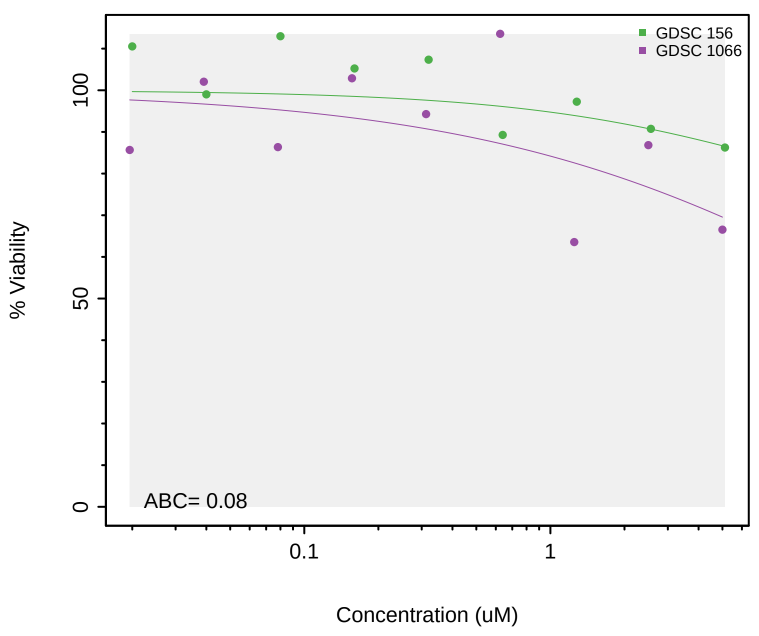

AZD6482:TE−6

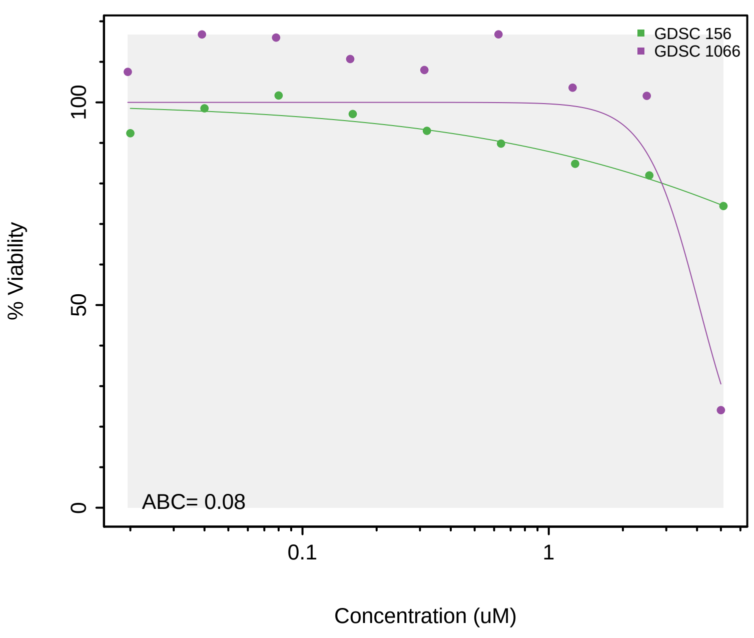

AZD6482:MEG−01

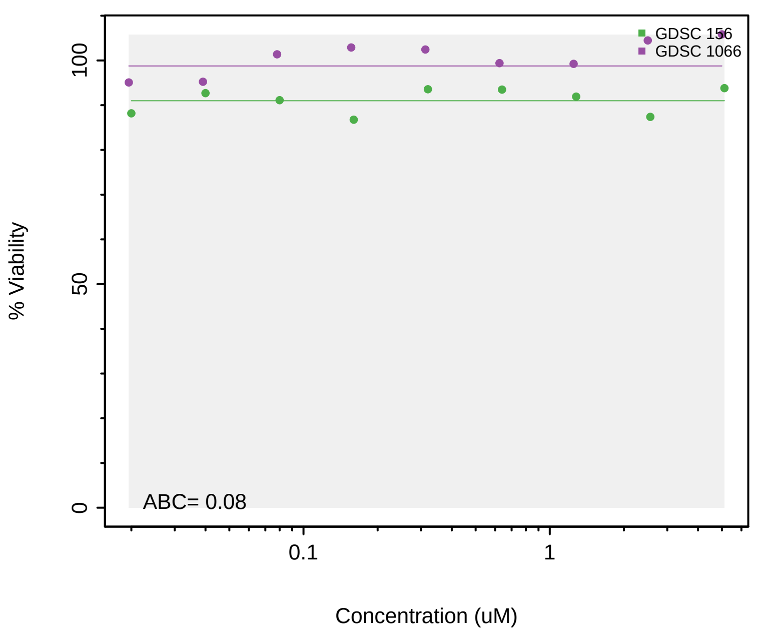

AZD6482:TCCSUP

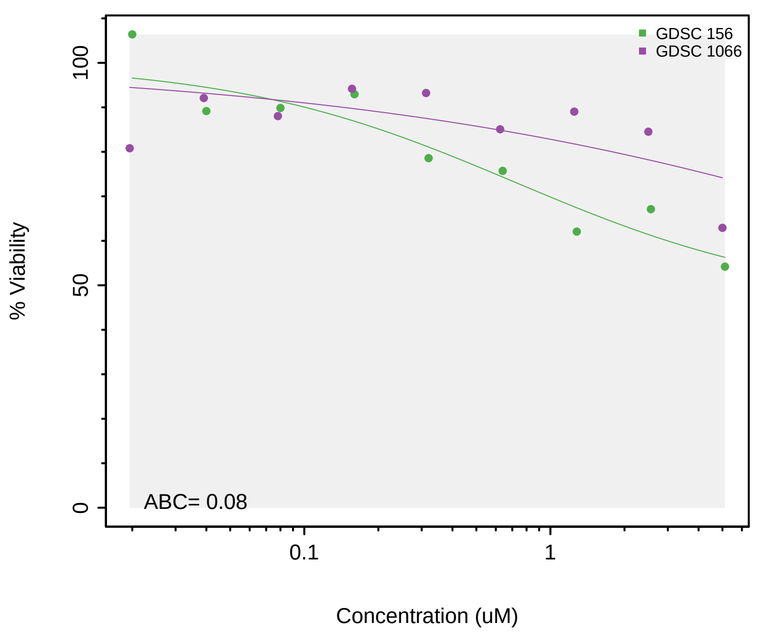

AZD6482:EW−7

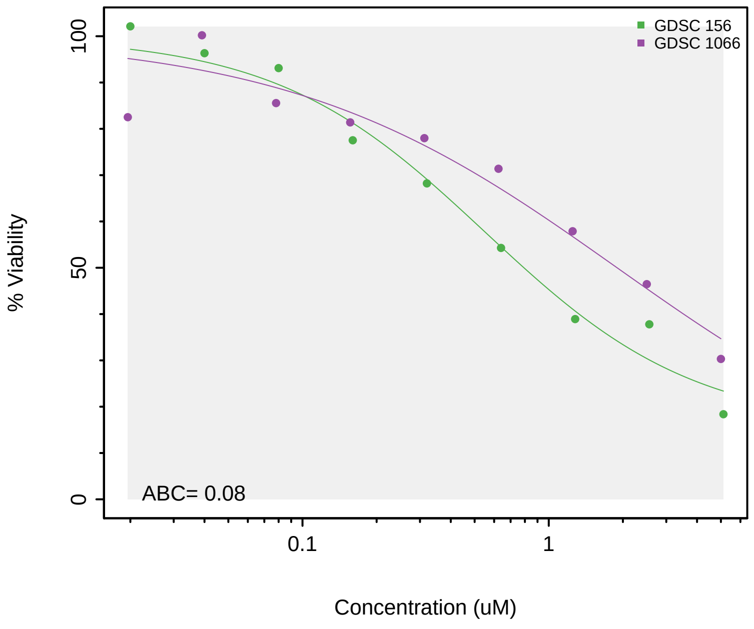

AZD6482:KYSE−520

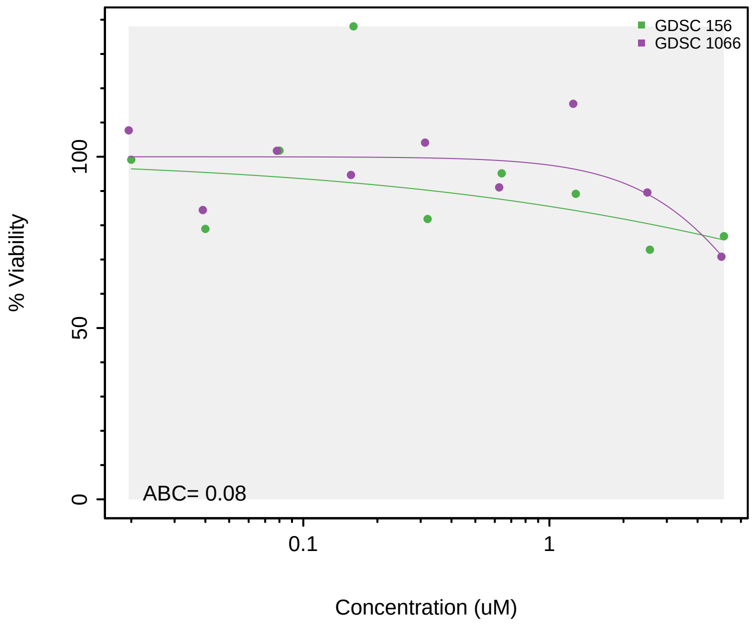

AZD6482:OPM−2

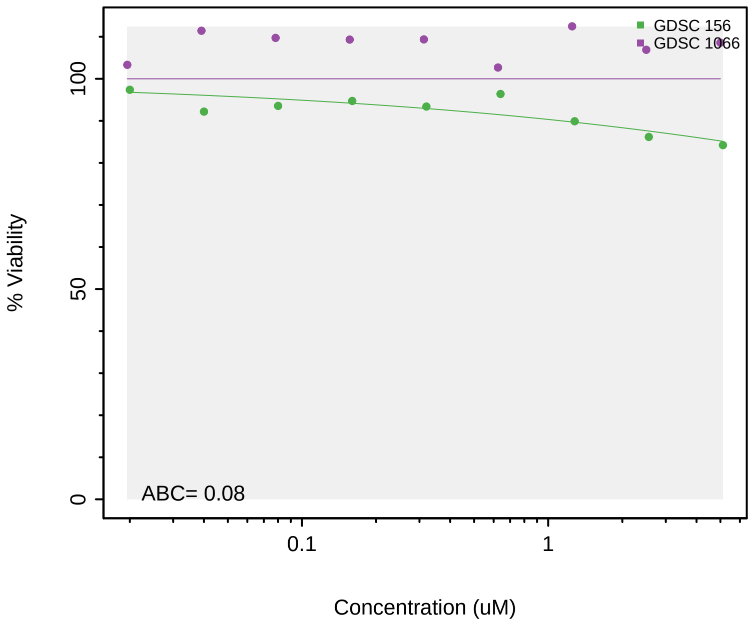

AZD6482:SNU−C2B

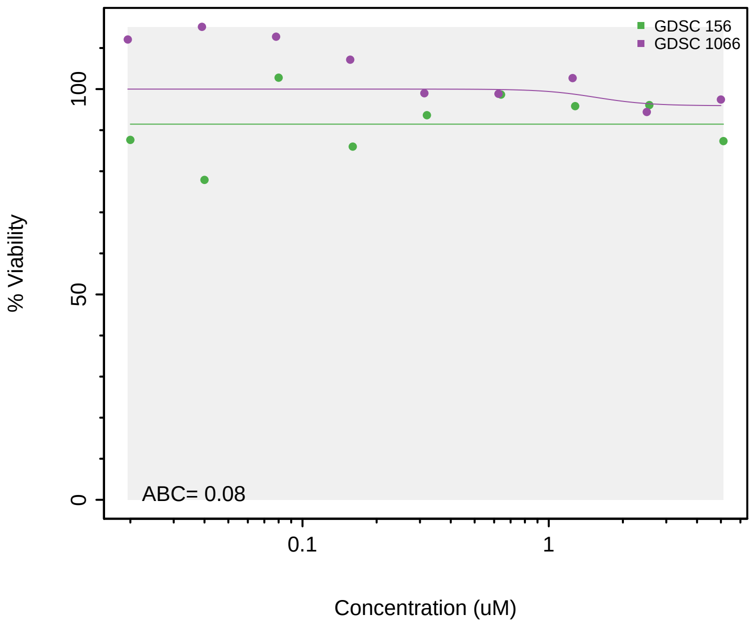

AZD6482:UACC−893

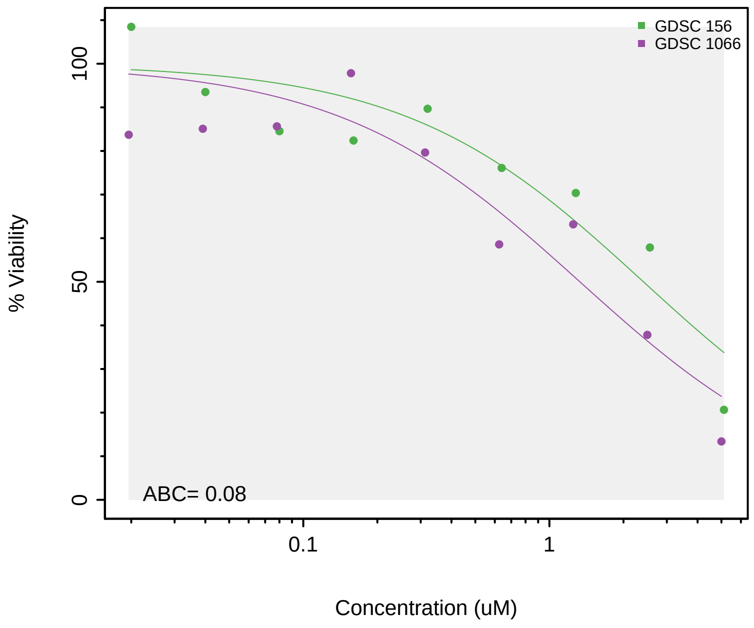

AZD6482:NCI−H1770

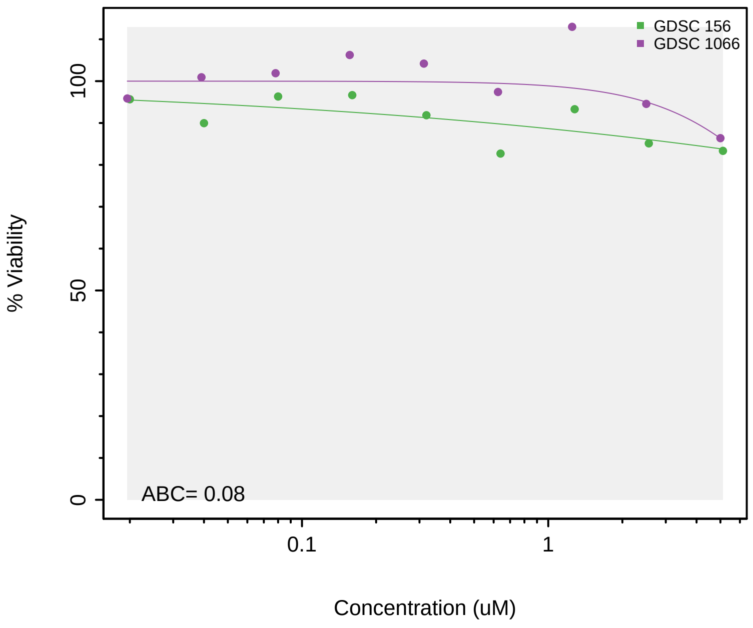

AZD6482:SF268

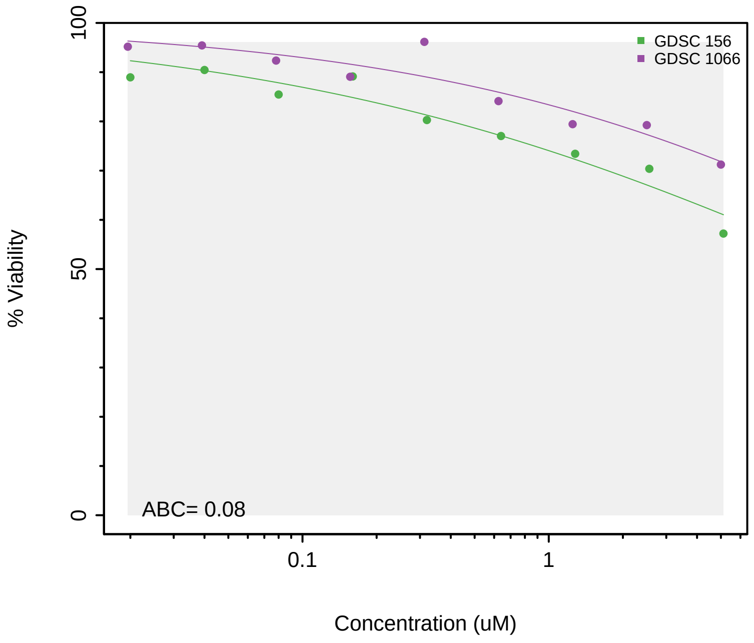

AZD6482:NCI−SNU−16

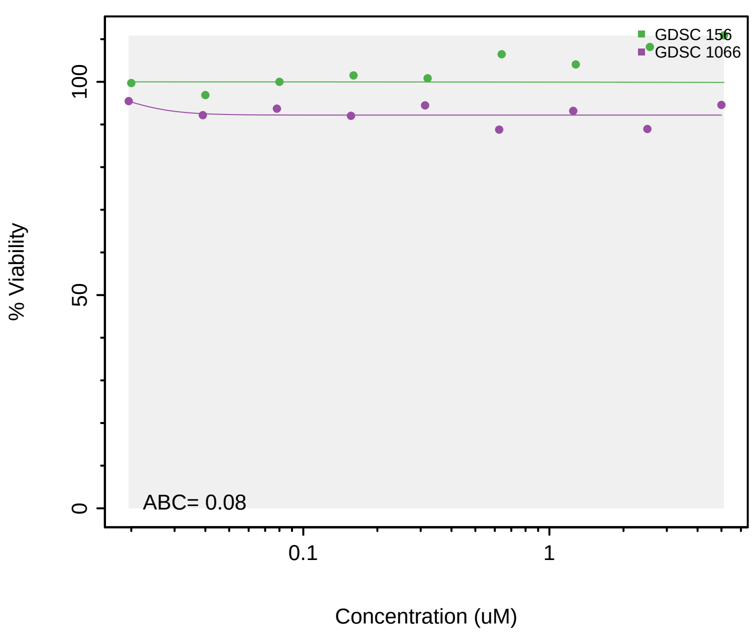

AZD6482:NCI−H650

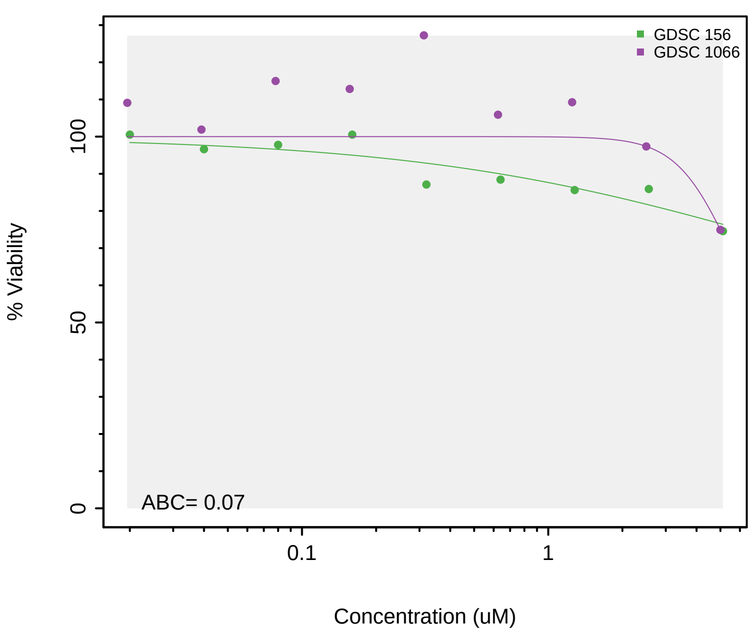

AZD6482:HLE

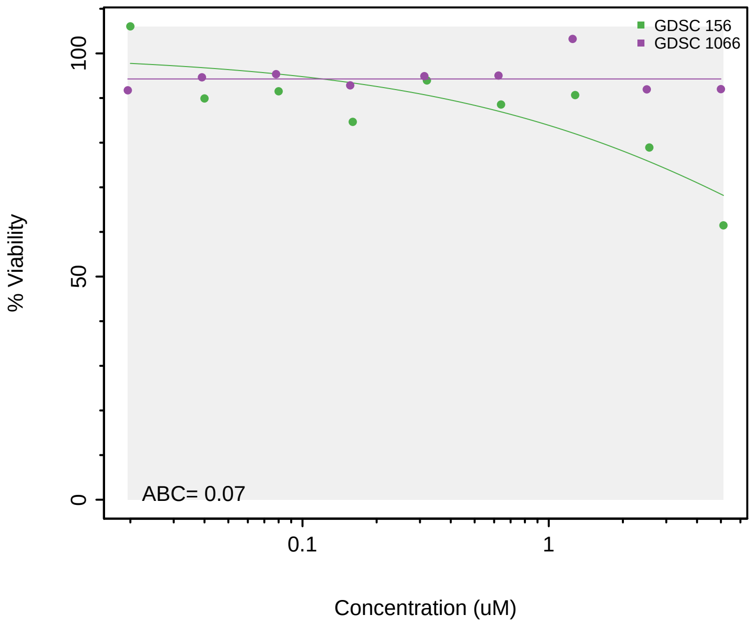

AZD6482:SW1710

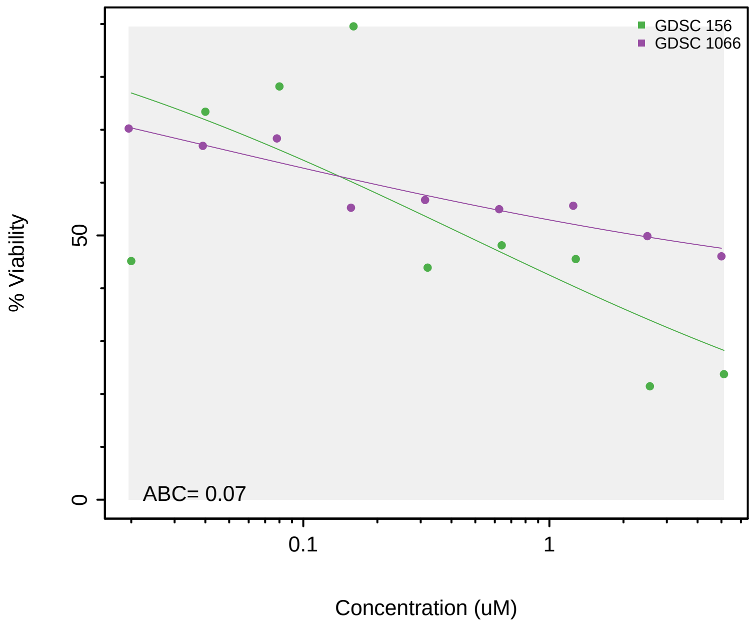

AZD6482:SK−LU−1

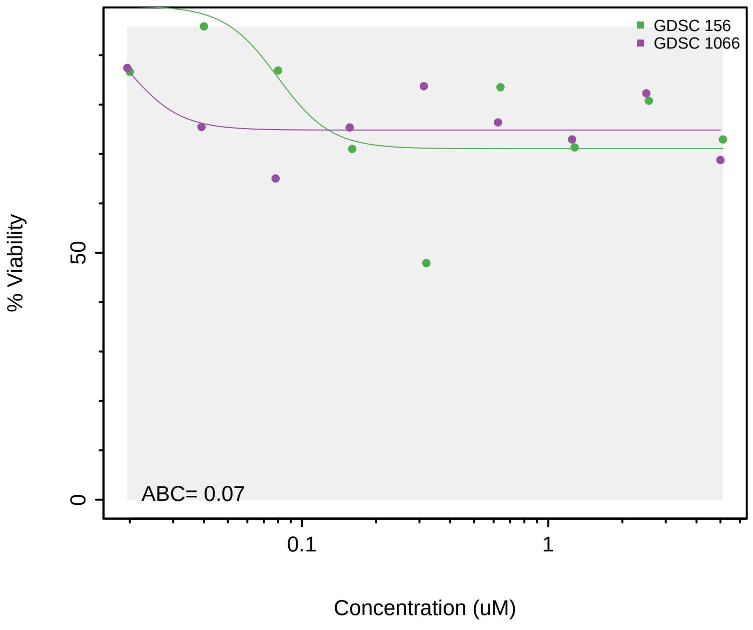

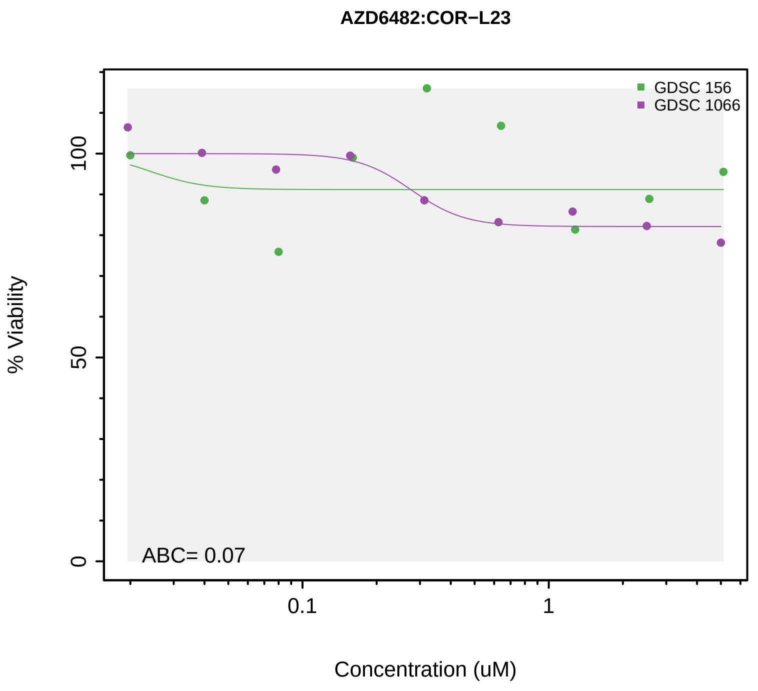
AZD6482:COR−L23
GDSC 156
GDSC 1066
ABC= 0.07
% Viability
Concentration (uM)

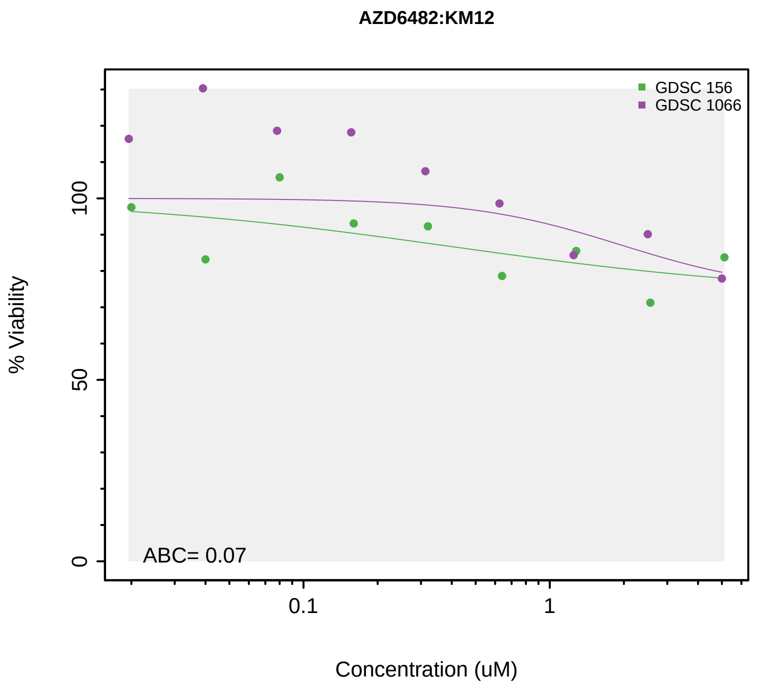
AZD6482:KM12
GDSC 156
GDSC 1066
ABC= 0.07
% Viability
Concentration (uM)

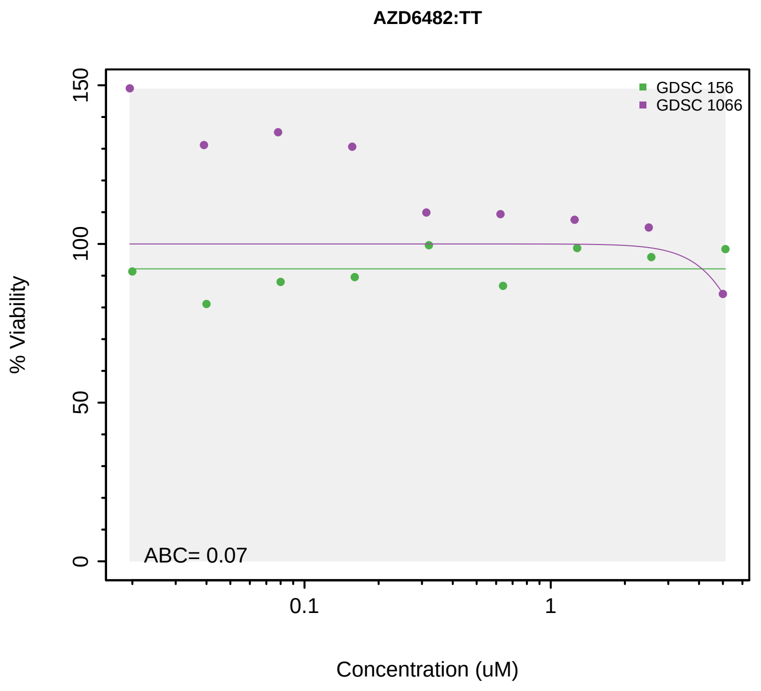
AZD6482:TT
GDSC 156
GDSC 1066
ABC= 0.07
% Viability
Concentration (uM)

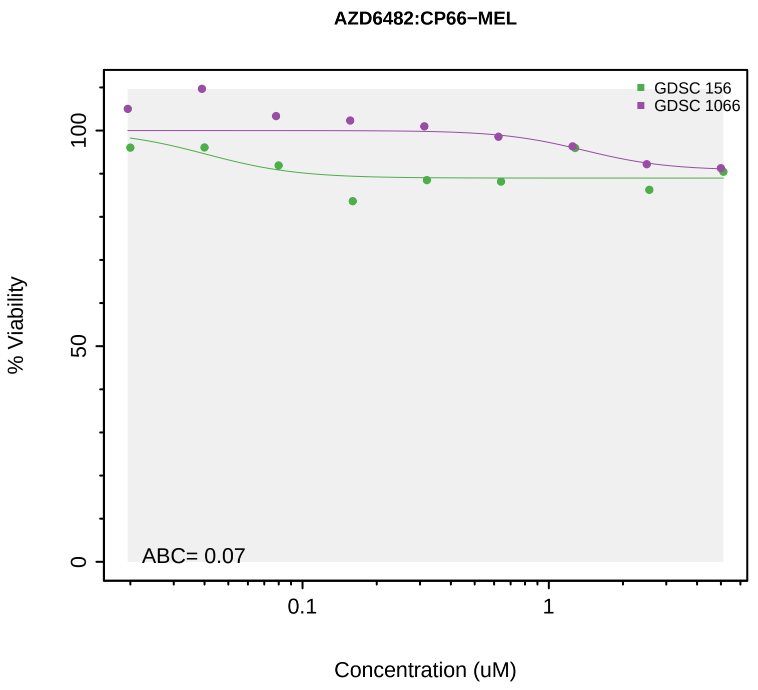
AZD6482:CP66−MEL
GDSC 156
GDSC 1066
ABC= 0.07
% Viability
Concentration (uM)

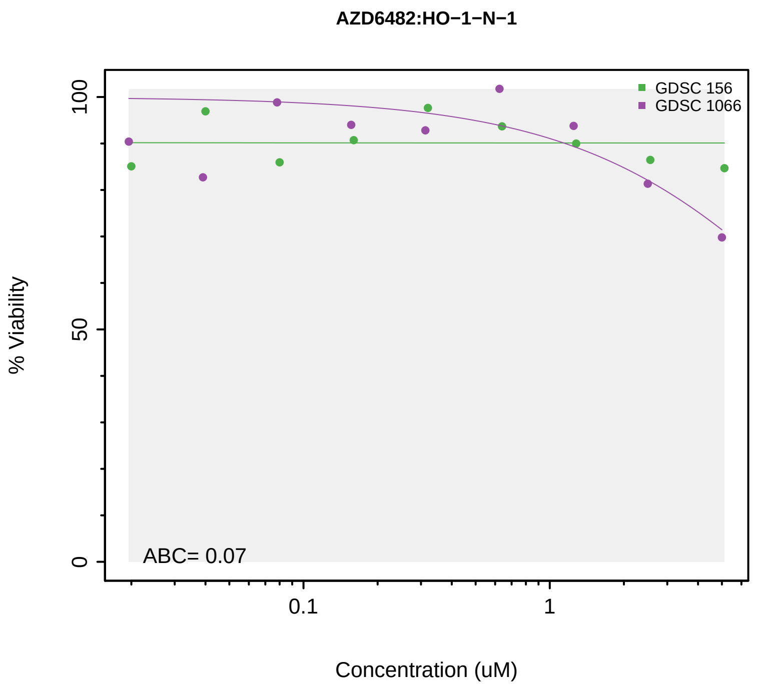
AZD6482:HO−1−N−1
GDSC 156
GDSC 1066
ABC= 0.07
% Viability
Concentration (uM)

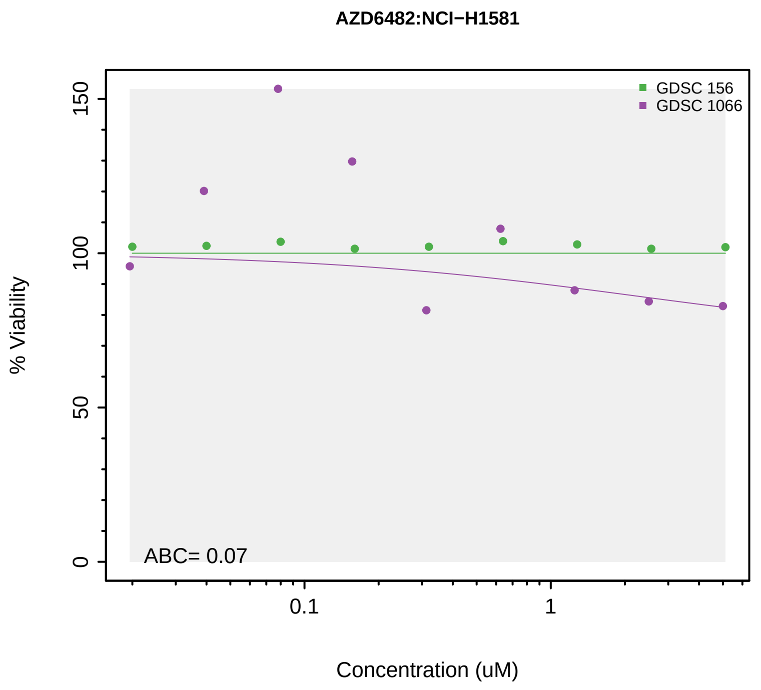
AZD6482:NCI−H1581
GDSC 156
GDSC 1066
ABC= 0.07
% Viability
Concentration (uM)

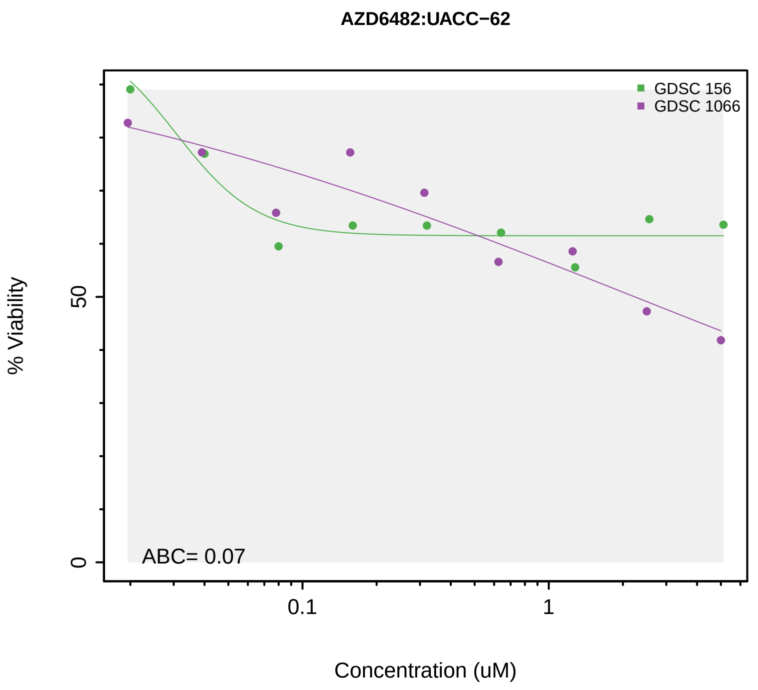
AZD6482:UACC−62
GDSC 156
GDSC 1066
ABC= 0.07
% Viability
Concentration (uM)

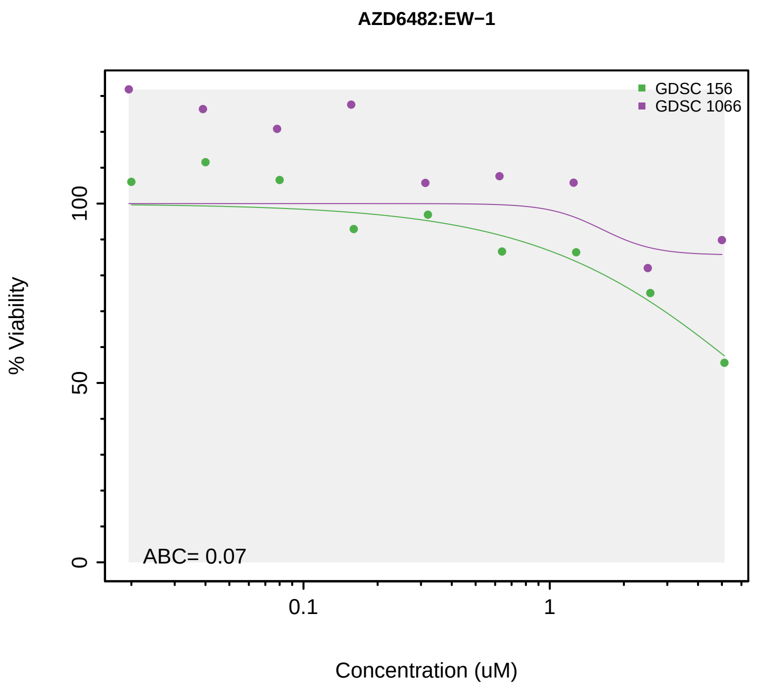
AZD6482:EW−1
GDSC 156
GDSC 1066
ABC= 0.07
% Viability
Concentration (uM)

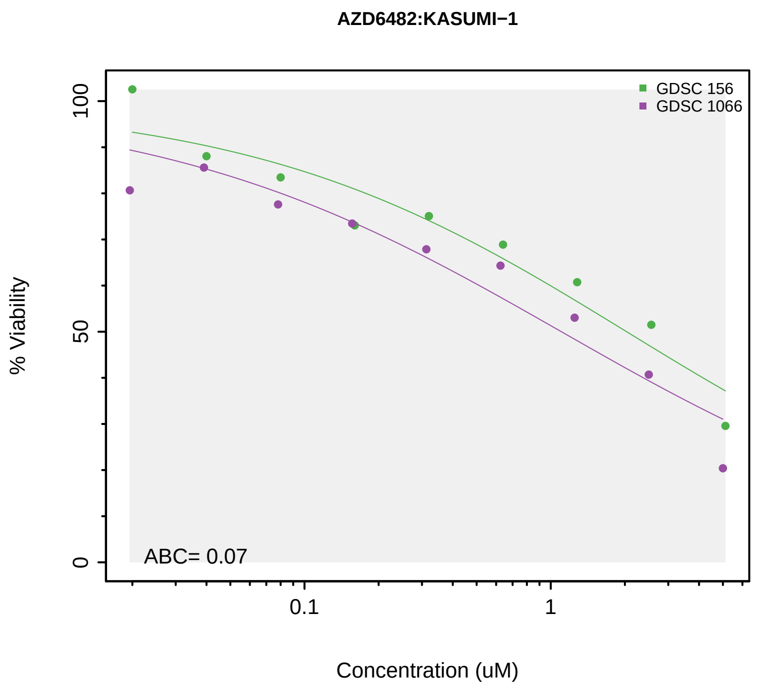
AZD6482:KASUMI−1
GDSC 156
GDSC 1066
ABC= 0.07
% Viability
Concentration (uM)

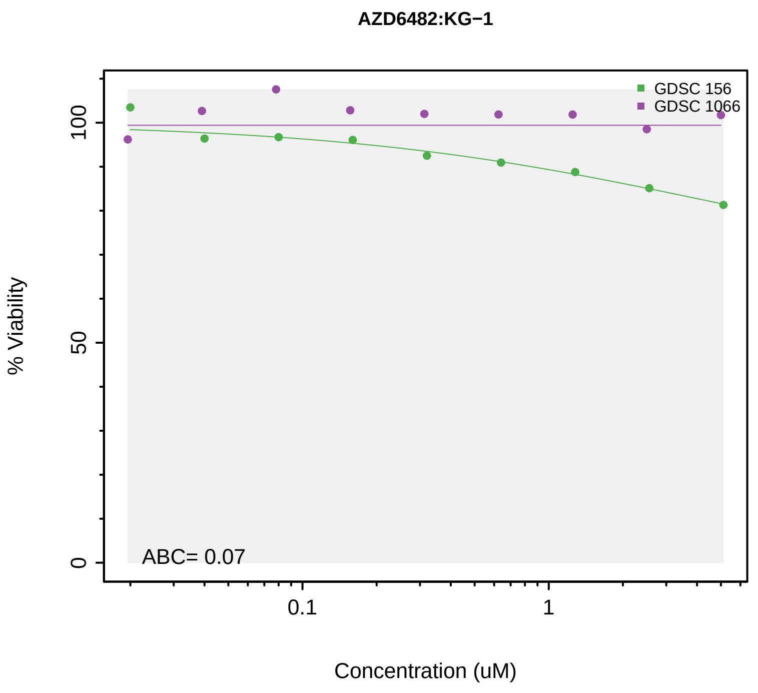
AZD6482:KG−1
GDSC 156
GDSC 1066
ABC= 0.07
% Viability
Concentration (uM)

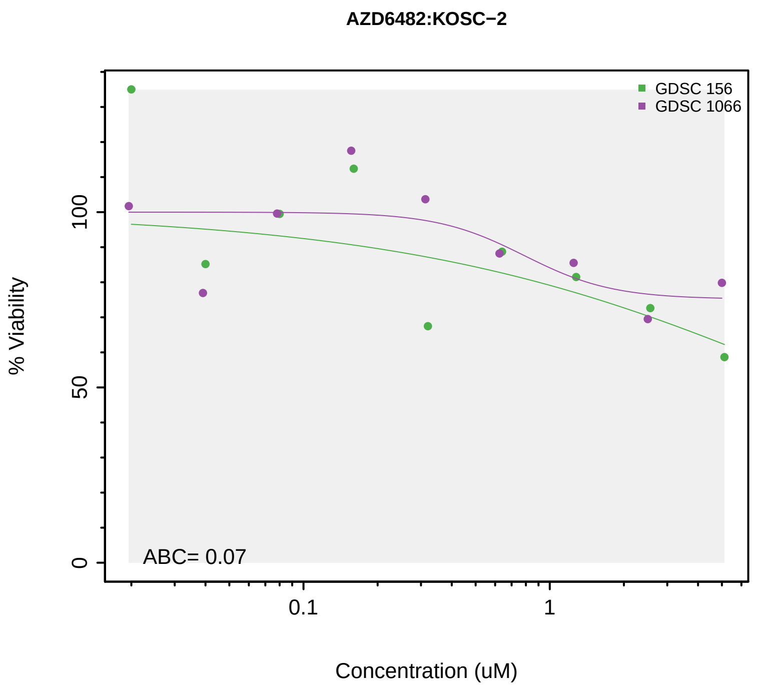
AZD6482:KOSC−2
GDSC 156
GDSC 1066
ABC= 0.07
% Viability
Concentration (uM)

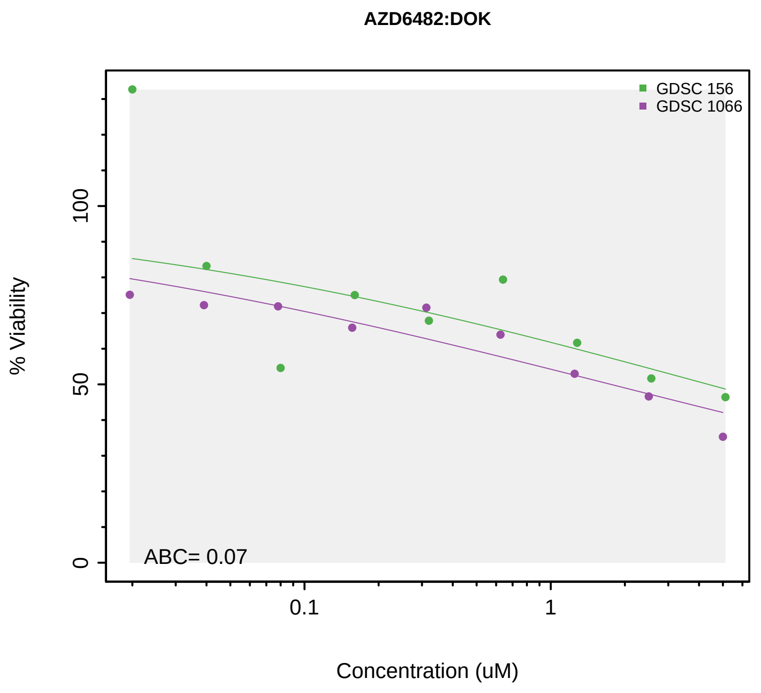
AZD6482:DOK
GDSC 156
GDSC 1066
ABC= 0.07
% Viability
Concentration (uM)

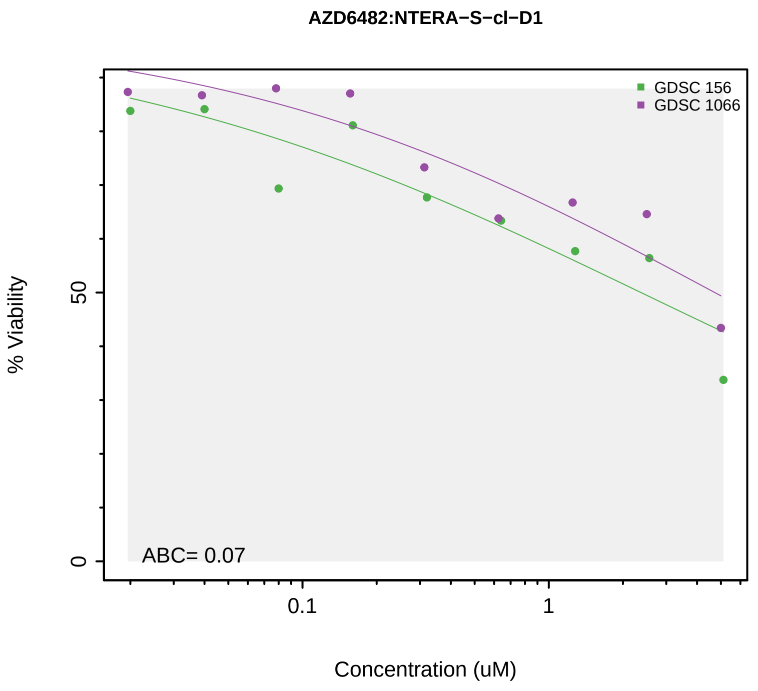
AZD6482:NTERA−S−cl−D1
GDSC 156
GDSC 1066
% Viability
ABC= 0.07
Concentration (uM)
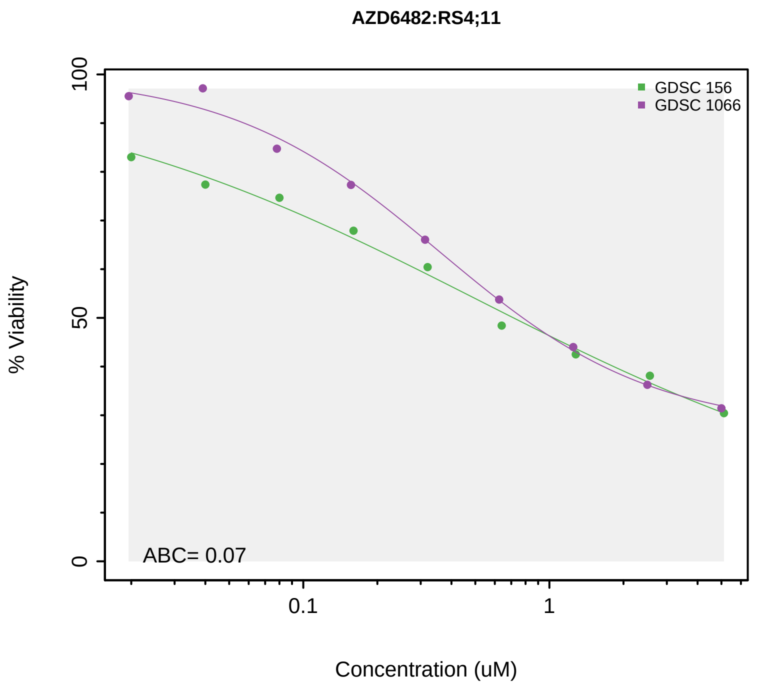
AZD6482:RS4;11
GDSC 156
GDSC 1066
% Viability
ABC= 0.07
Concentration (uM)
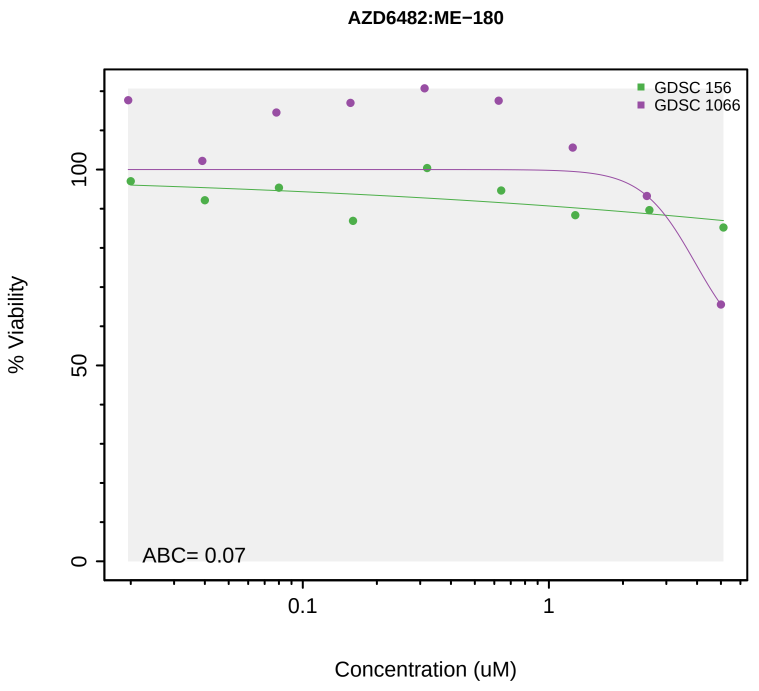
AZD6482:ME−180
GDSC 156
GDSC 1066
% Viability
ABC= 0.07
Concentration (uM)
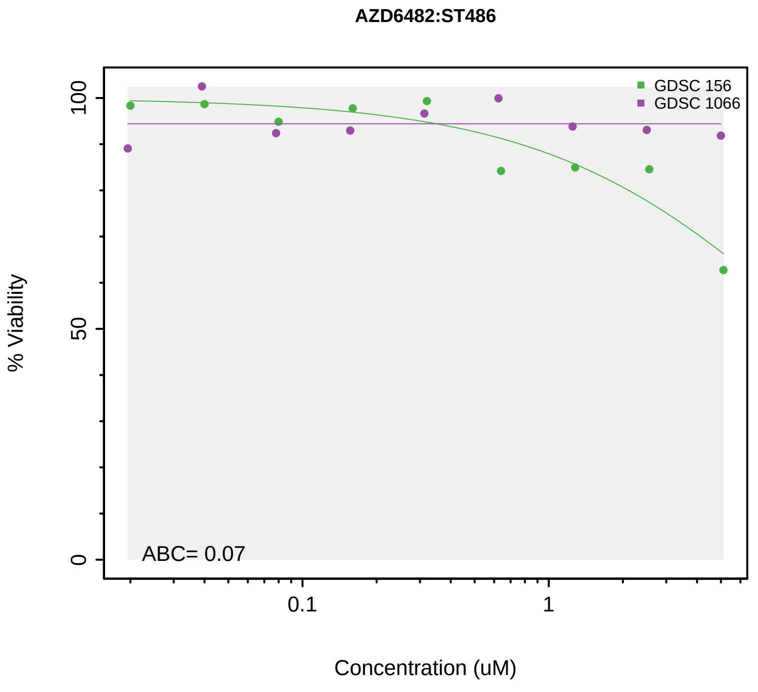
AZD6482:ST486
GDSC 156
GDSC 1066
% Viability
ABC= 0.07
Concentration (uM)
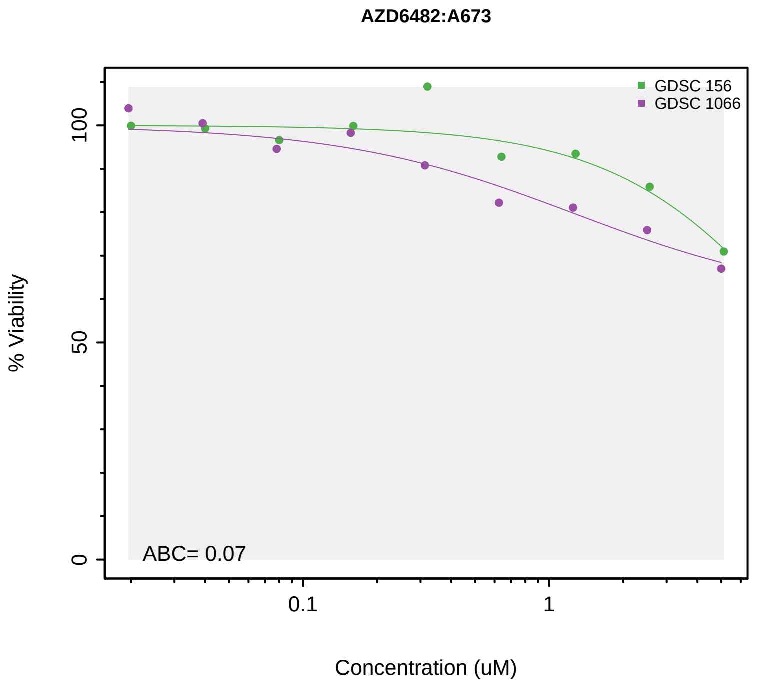
AZD6482:A673
GDSC 156
GDSC 1066
% Viability
ABC= 0.07
Concentration (uM)
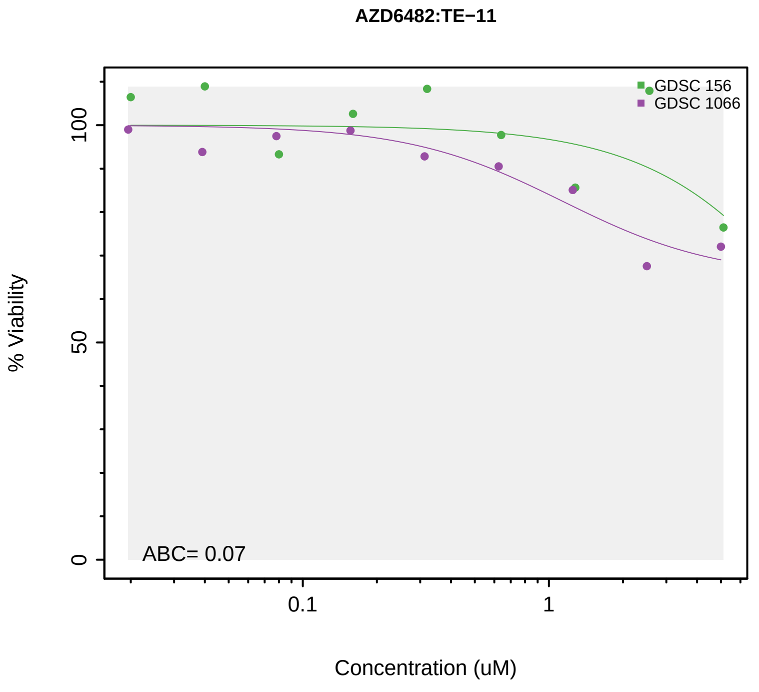
AZD6482:TE−11
GDSC 156
GDSC 1066
% Viability
ABC= 0.07
Concentration (uM)
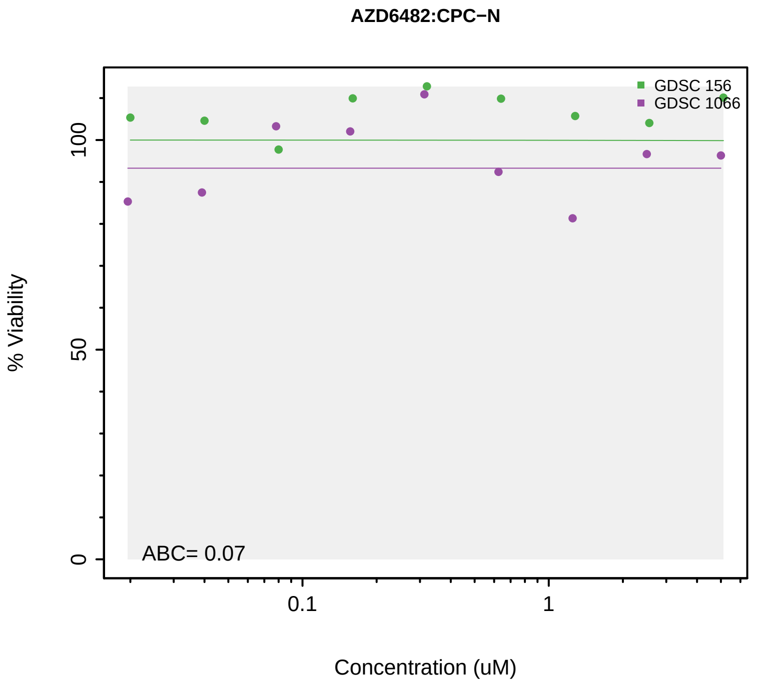
AZD6482:CPC−N
GDSC 156
GDSC 1066
% Viability
ABC= 0.07
Concentration (uM)
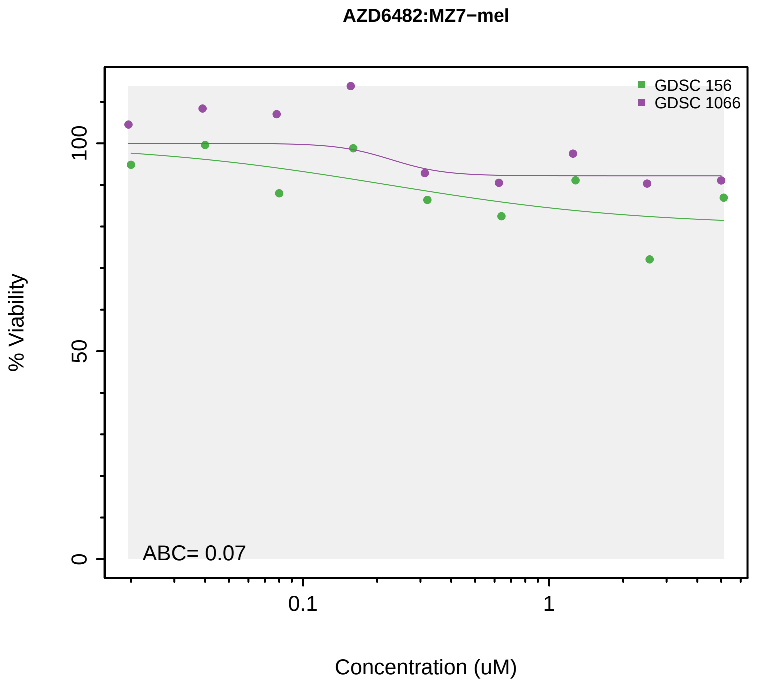
AZD6482:MZ7−mel
GDSC 156
GDSC 1066
% Viability
ABC= 0.07
Concentration (uM)
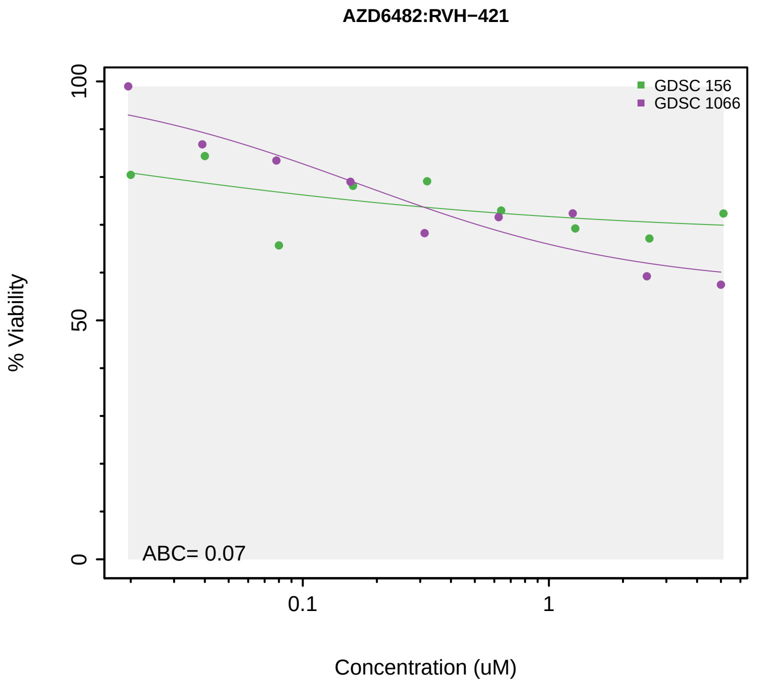
AZD6482:RVH−421
GDSC 156
GDSC 1066
% Viability
ABC= 0.07
Concentration (uM)
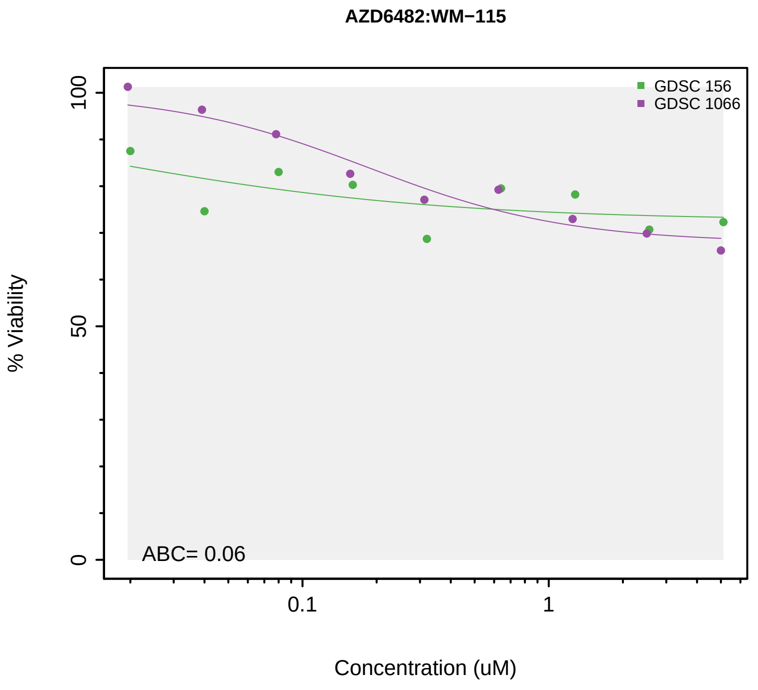
AZD6482:WM−115
GDSC 156
GDSC 1066
% Viability
ABC= 0.06
Concentration (uM)
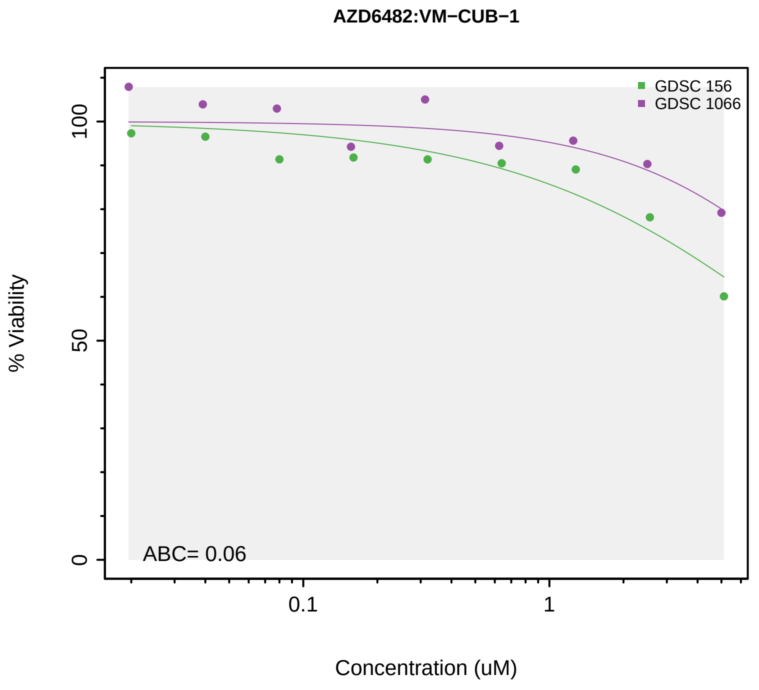
AZD6482:VM−CUB−1
GDSC 156
GDSC 1066
% Viability
ABC= 0.06
Concentration (uM)
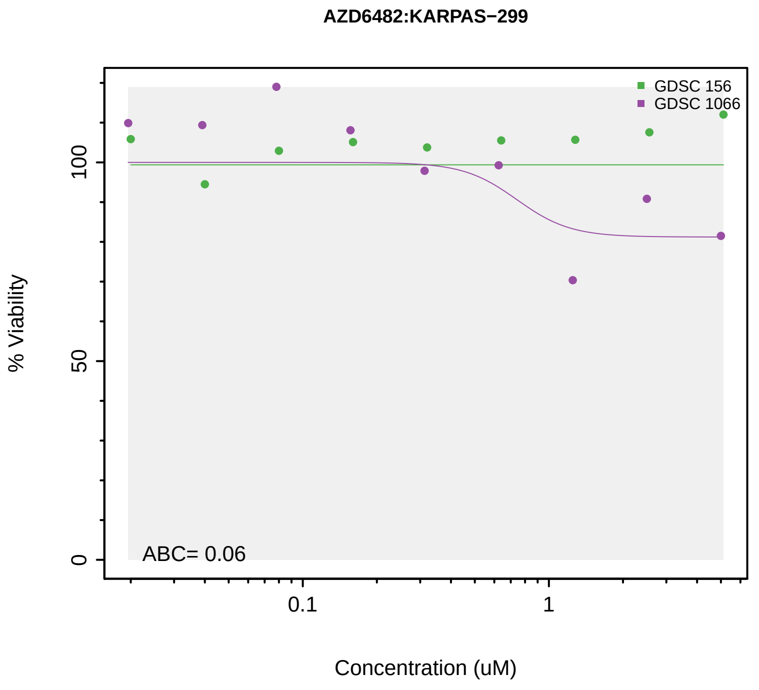
AZD6482:KARPAS−299
GDSC 156
GDSC 1066
% Viability
ABC= 0.06
Concentration (uM)

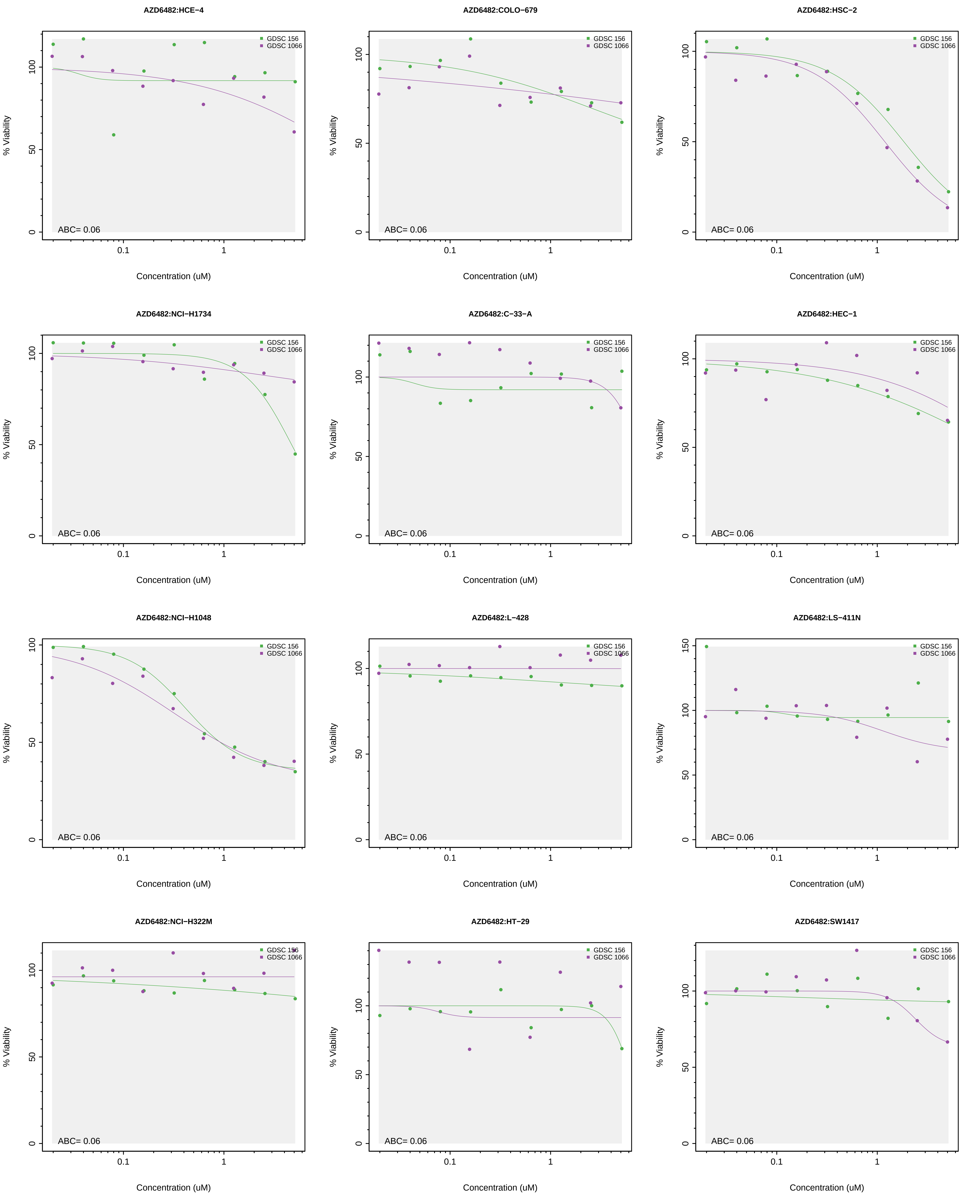
AZD6482:HCE−4
AZD6482:COLO−679
AZD6482:HSC−2
AZD6482:NCI−H1734
AZD6482:C−33−A
AZD6482:HEC−1
AZD6482:NCI−H1048
AZD6482:L−428
AZD6482:LS−411N
AZD6482:NCI−H322M
AZD6482:HT−29
AZD6482:SW1417
GDSC 156
GDSC 1066
ABC= 0.06
% Viability
Concentration (uM)
0
50
100
150
0.1
1

AZD6482:L−540

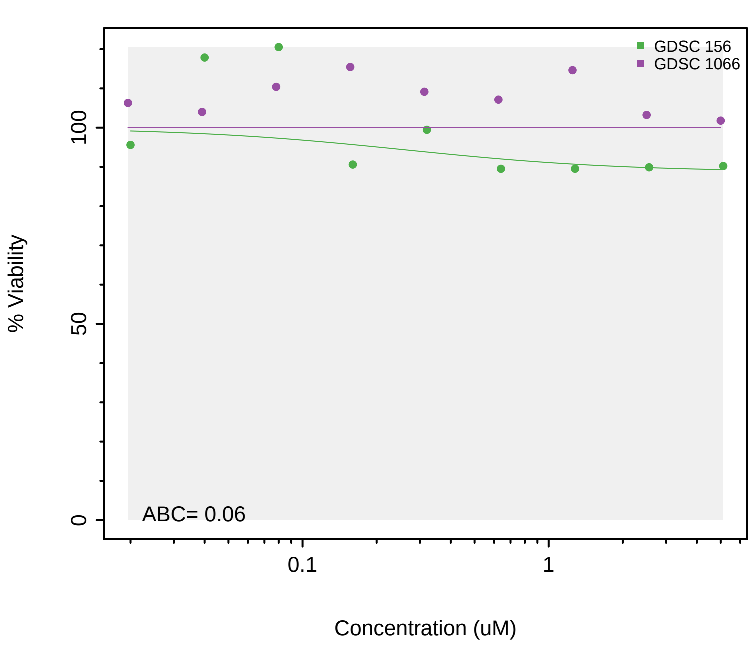

AZD6482:CAL−62

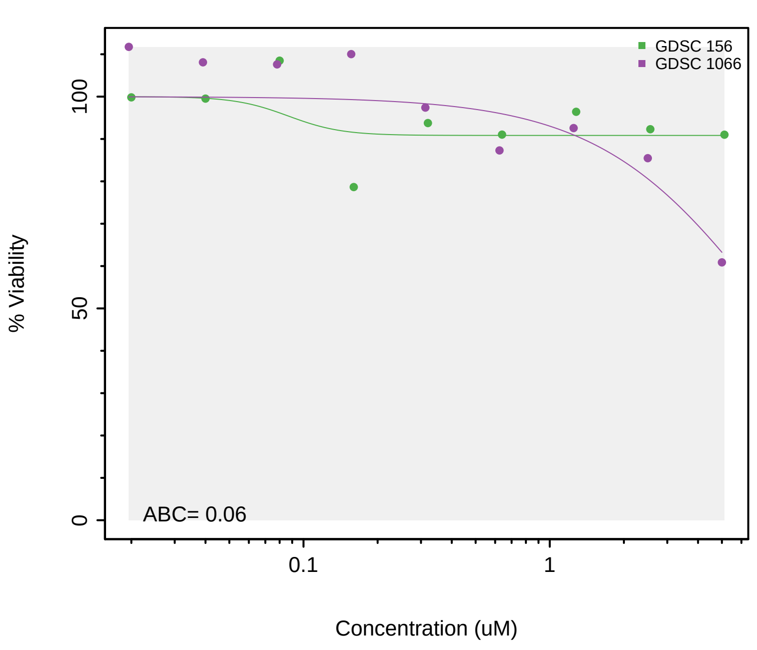

AZD6482:T−24

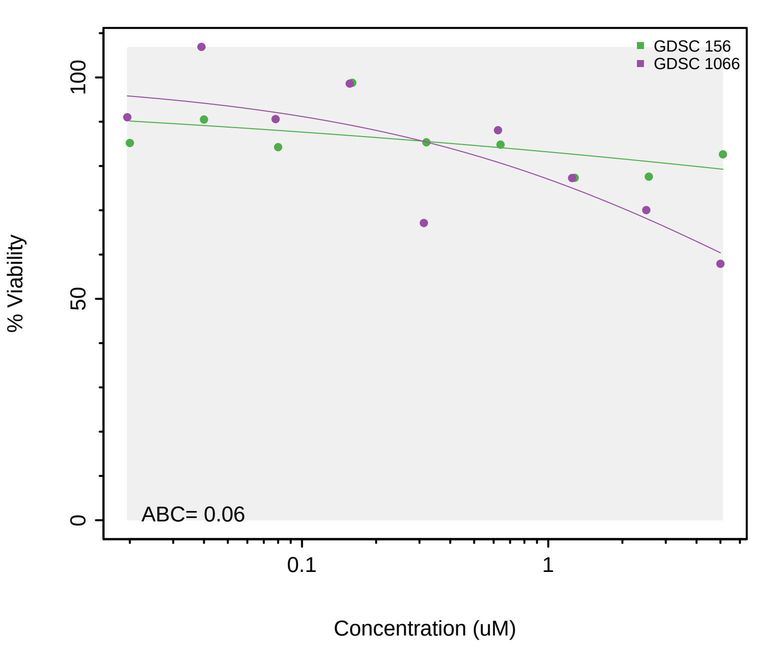

AZD6482:OMC−1

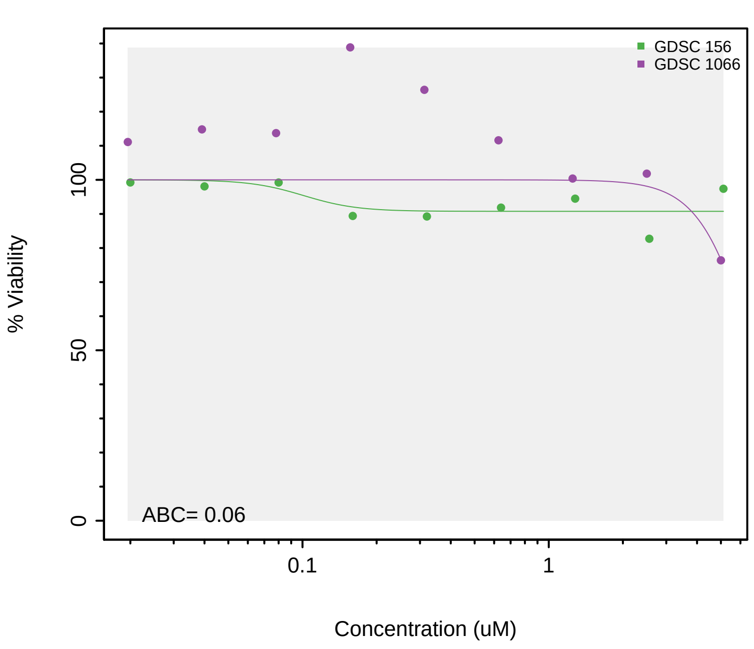

AZD6482:COR−L279

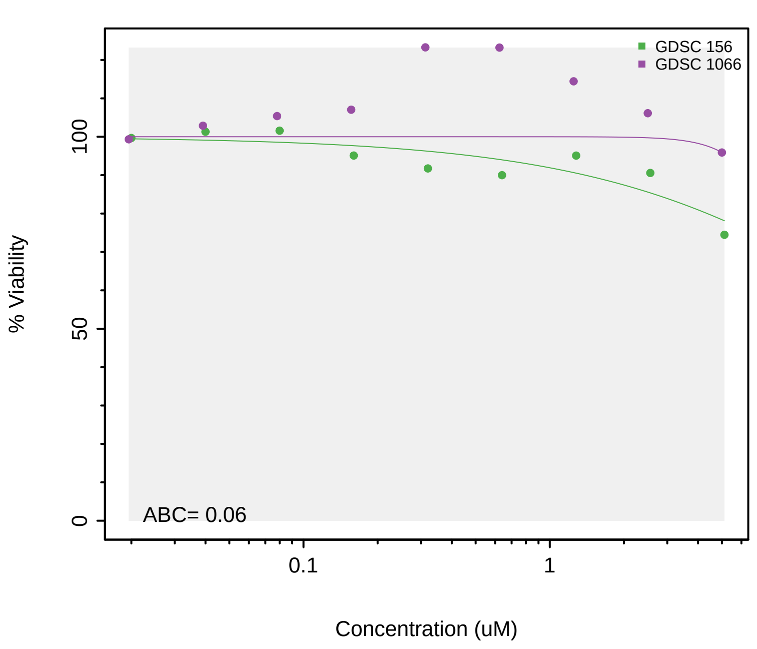

AZD6482:HuH−7

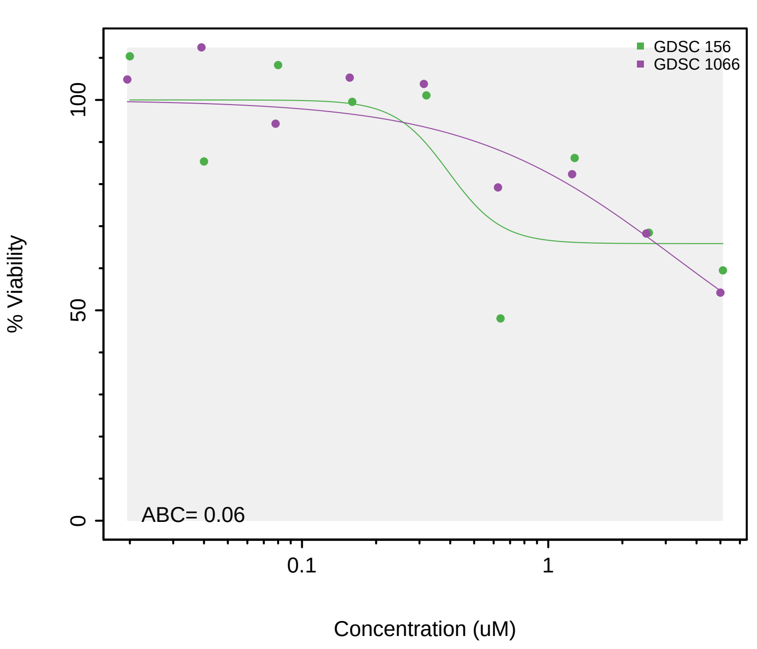

AZD6482:HCC1937

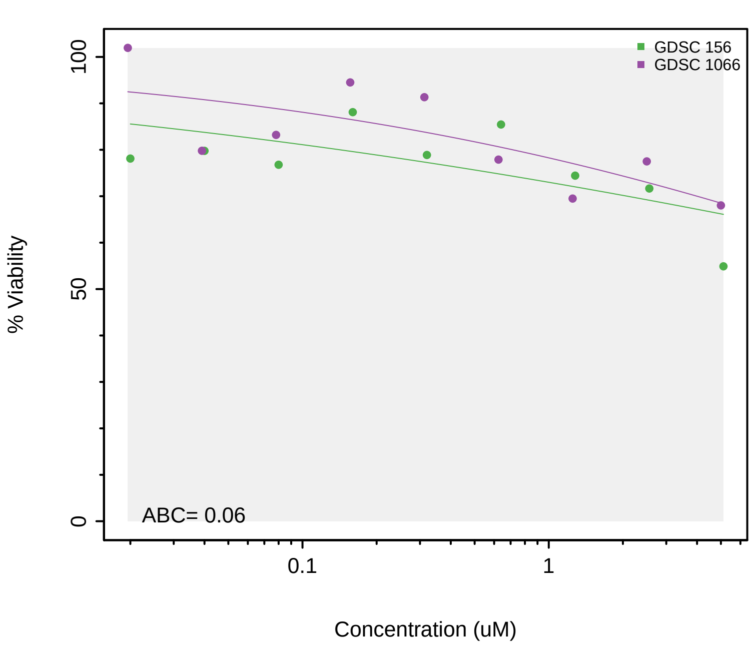

AZD6482:OVCAR−4

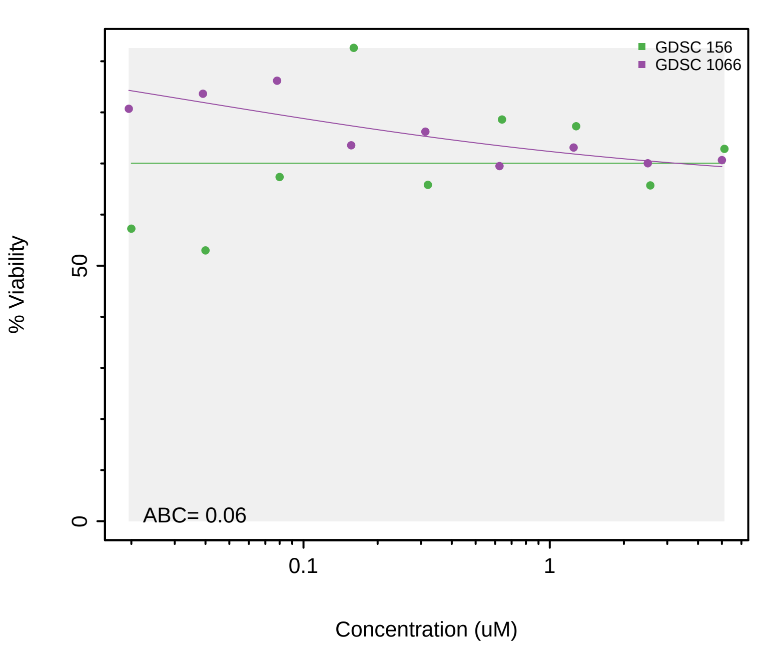

AZD6482:K−562

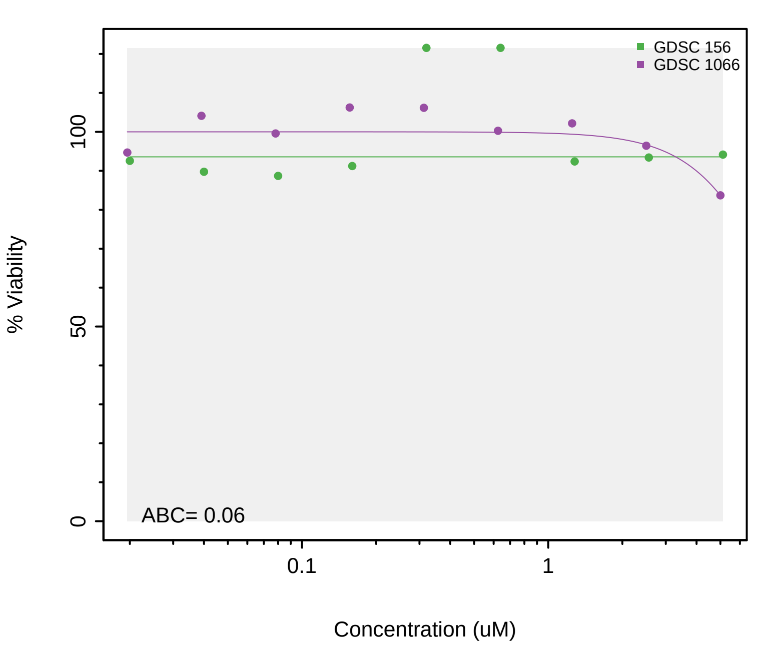

AZD6482:SK−HEP−1

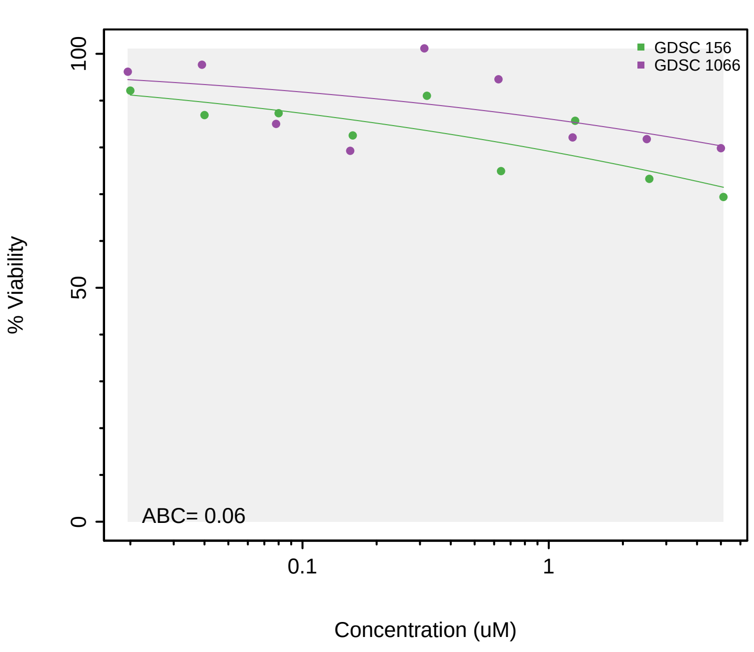

AZD6482:RD

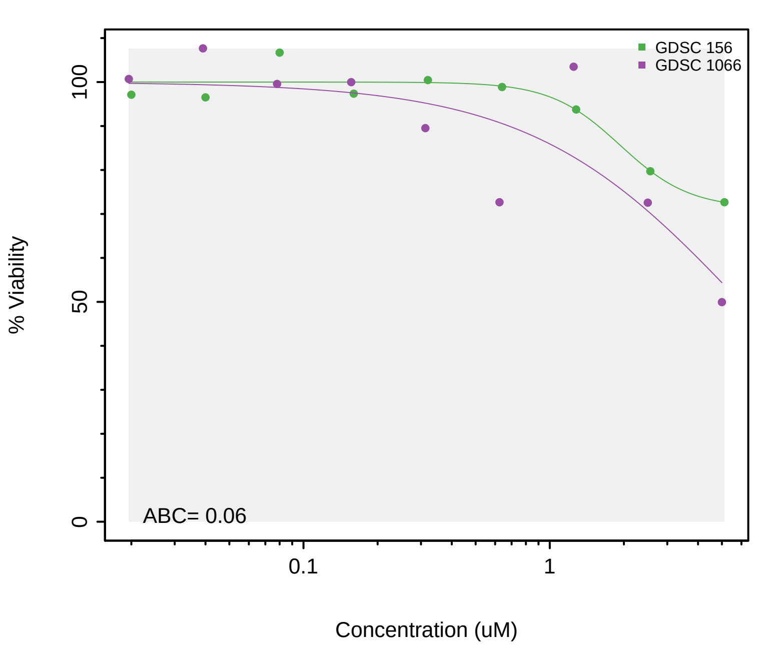

AZD6482:P30−OHK

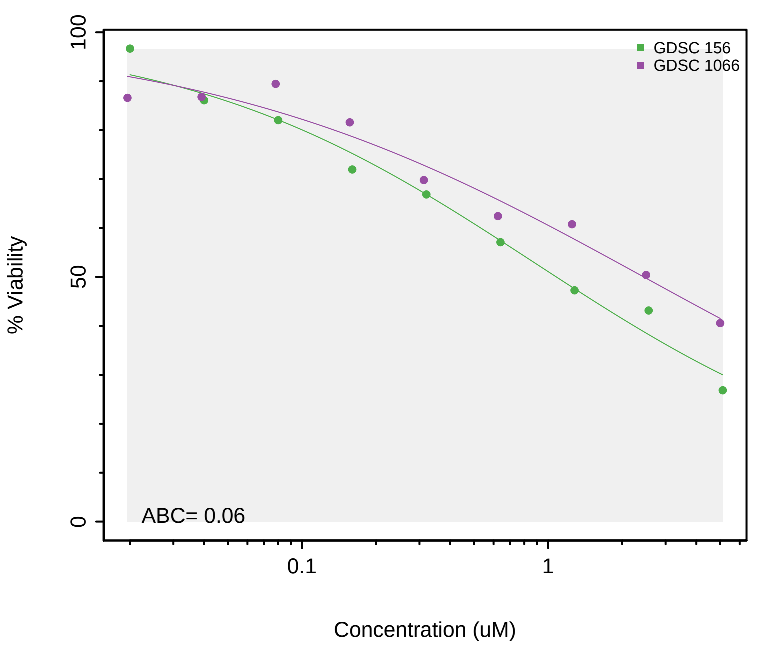

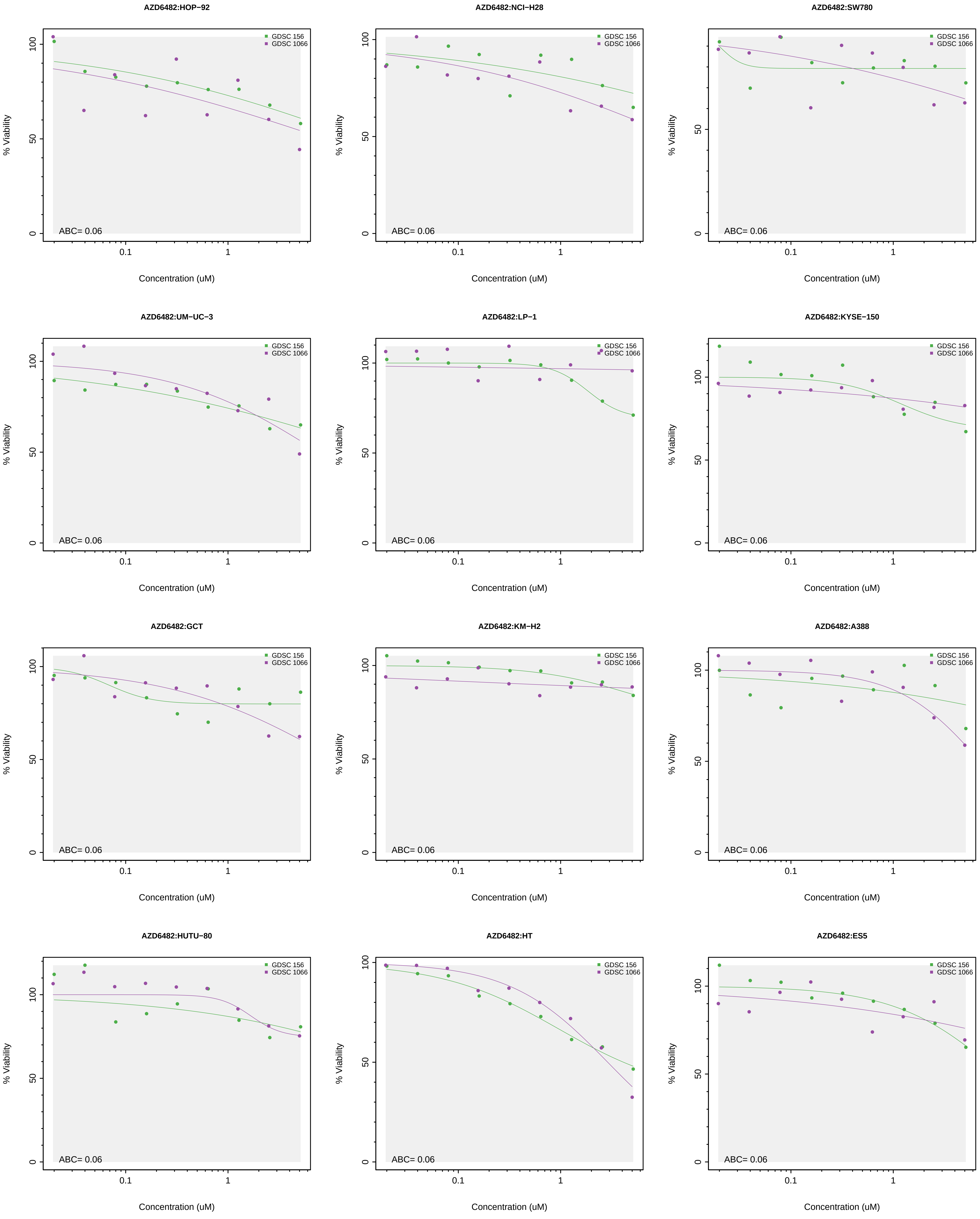
AZD6482:HOP−92
AZD6482:NCI−H28
AZD6482:SW780
AZD6482:UM−UC−3
AZD6482:LP−1
AZD6482:KYSE−150
AZD6482:GCT
AZD6482:KM−H2
AZD6482:A388
AZD6482:HUTU−80
AZD6482:HT
AZD6482:ES5
GDSC 156
GDSC 1066
ABC= 0.06
% Viability
Concentration (uM)

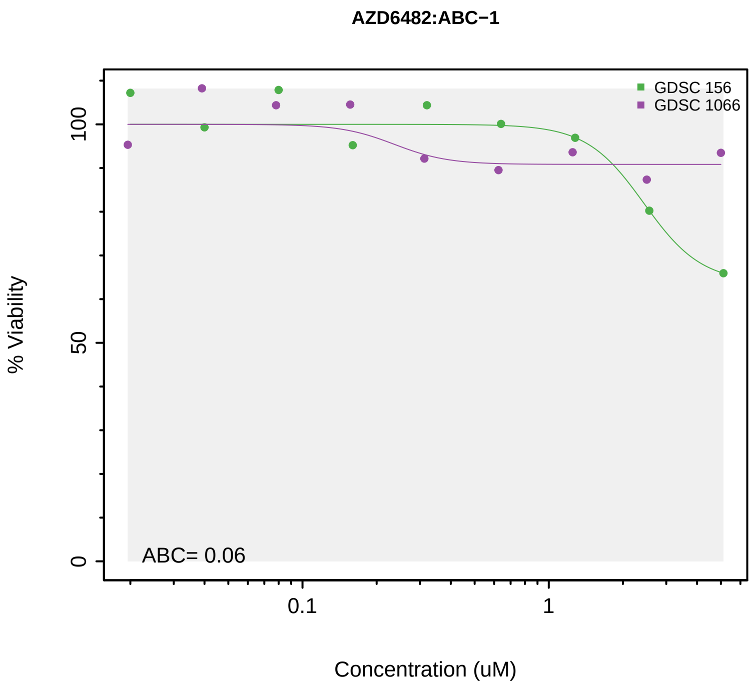
AZD6482:ABC−1
GDSC 156
GDSC 1066
ABC= 0.06
% Viability
0
50
100
0.1
1
Concentration (uM)

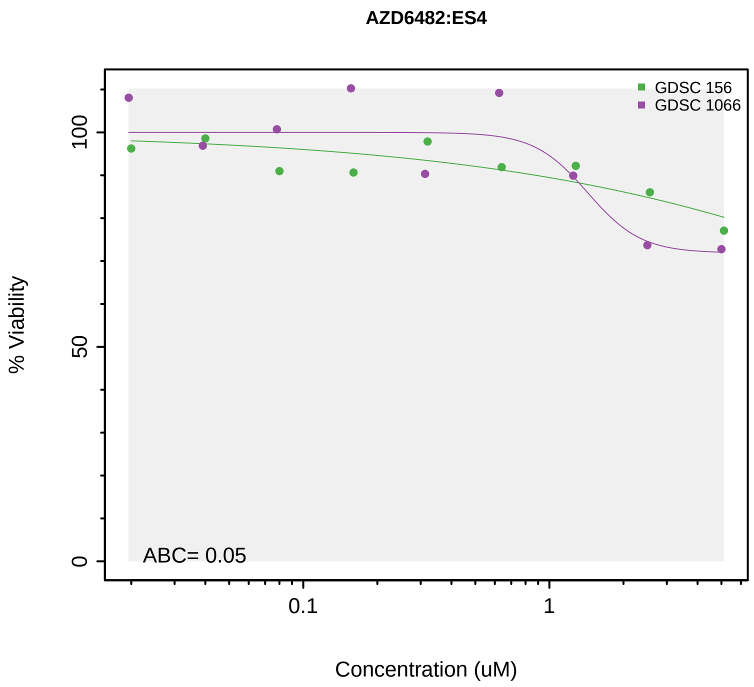
AZD6482:ES4
GDSC 156
GDSC 1066
ABC= 0.05
% Viability
0
50
100
0.1
1
Concentration (uM)

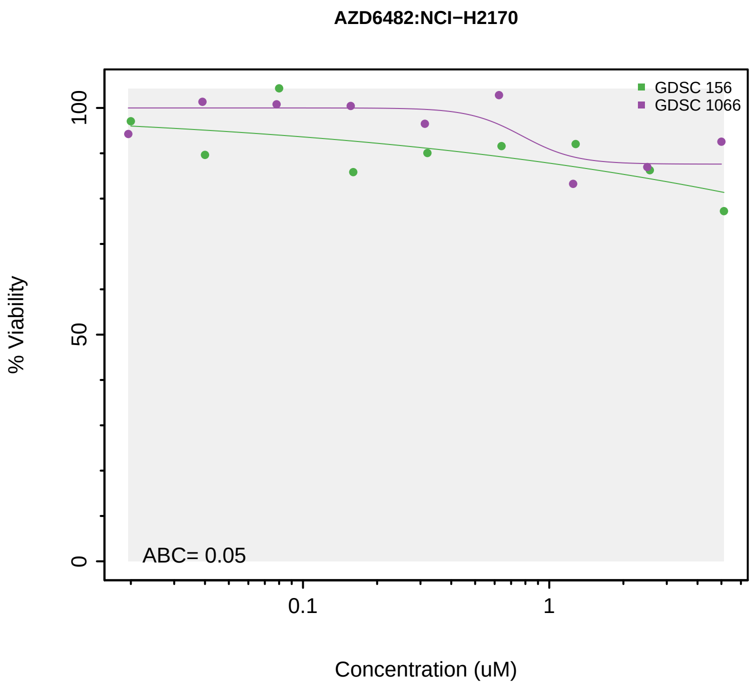
AZD6482:NCI−H2170
GDSC 156
GDSC 1066
ABC= 0.05
% Viability
0
50
100
0.1
1
Concentration (uM)

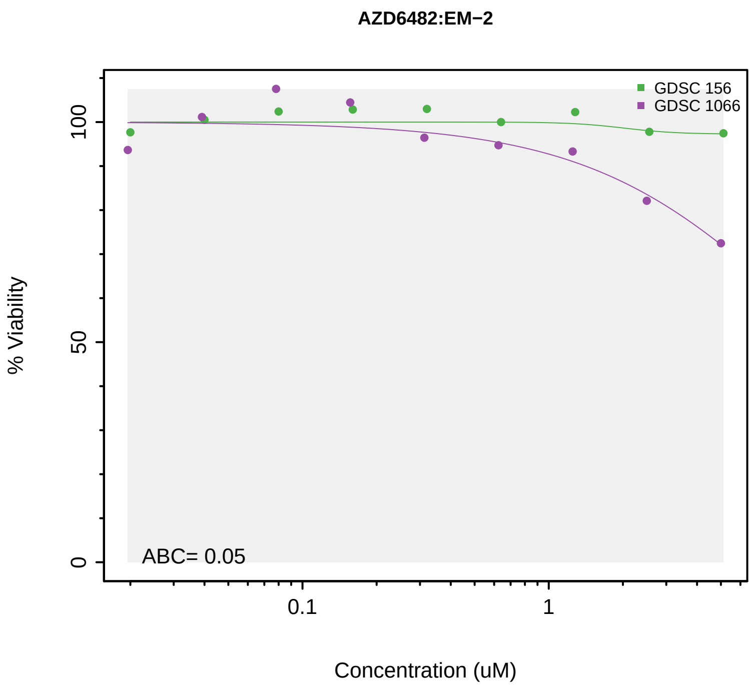
AZD6482:EM−2
GDSC 156
GDSC 1066
ABC= 0.05
% Viability
0
50
100
0.1
1
Concentration (uM)

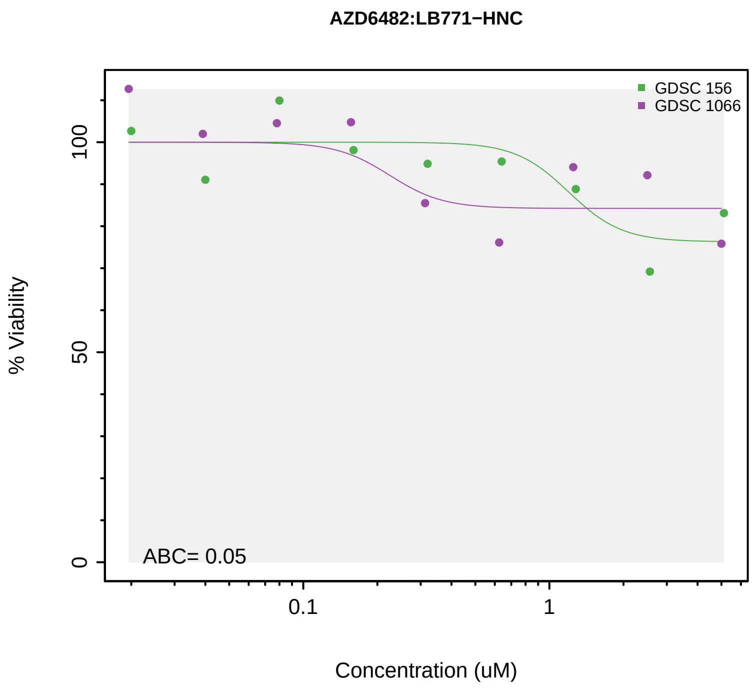
AZD6482:LB771−HNC
GDSC 156
GDSC 1066
ABC= 0.05
% Viability
0
50
100
0.1
1
Concentration (uM)

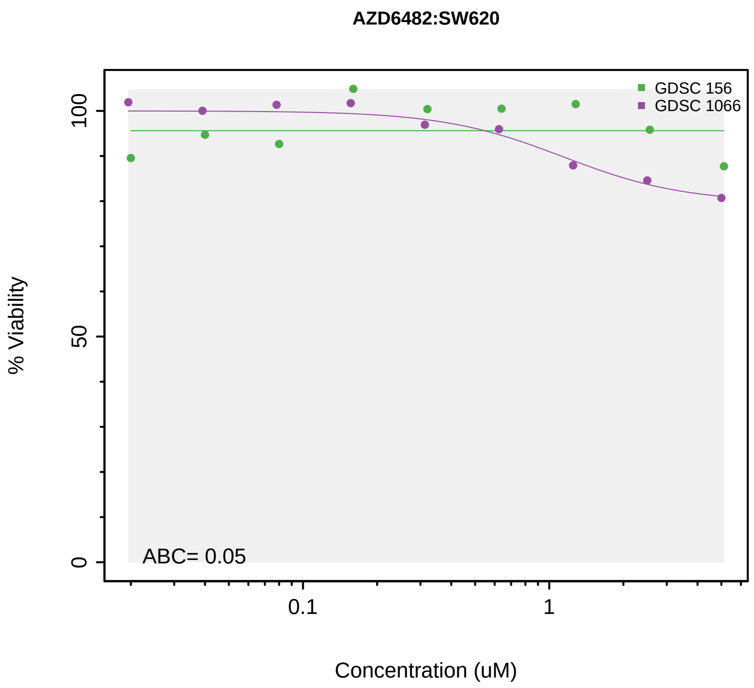
AZD6482:SW620
GDSC 156
GDSC 1066
ABC= 0.05
% Viability
0
50
100
0.1
1
Concentration (uM)

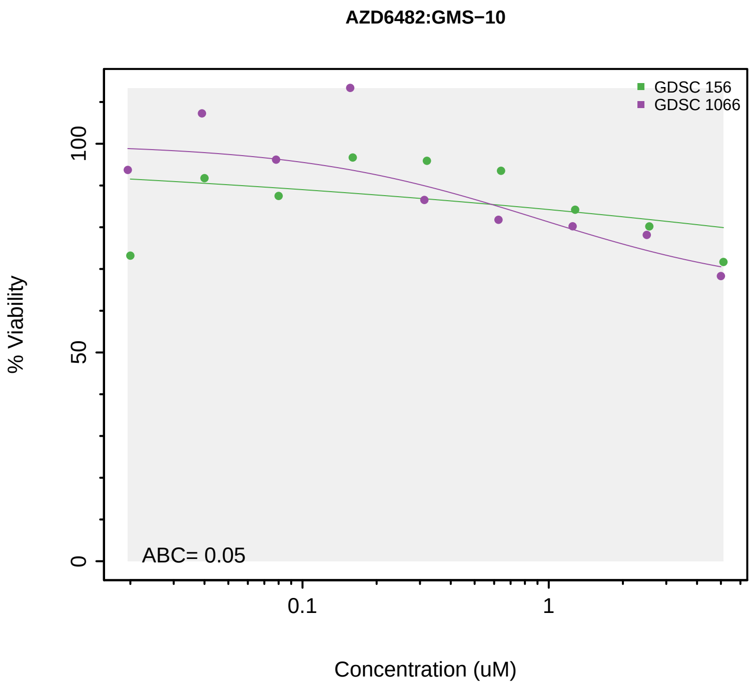
AZD6482:GMS−10
GDSC 156
GDSC 1066
ABC= 0.05
% Viability
0
50
100
0.1
1
Concentration (uM)

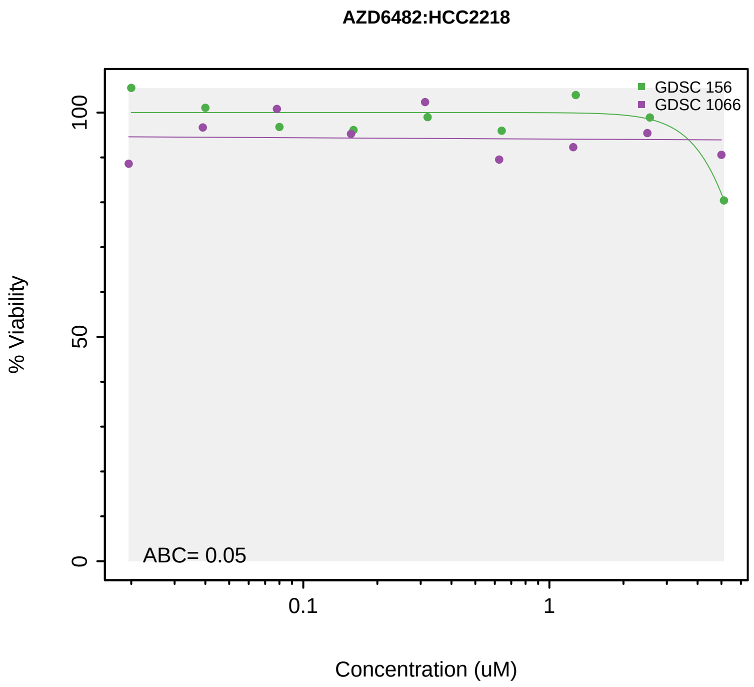
AZD6482:HCC2218
GDSC 156
GDSC 1066
ABC= 0.05
% Viability
0
50
100
0.1
1
Concentration (uM)

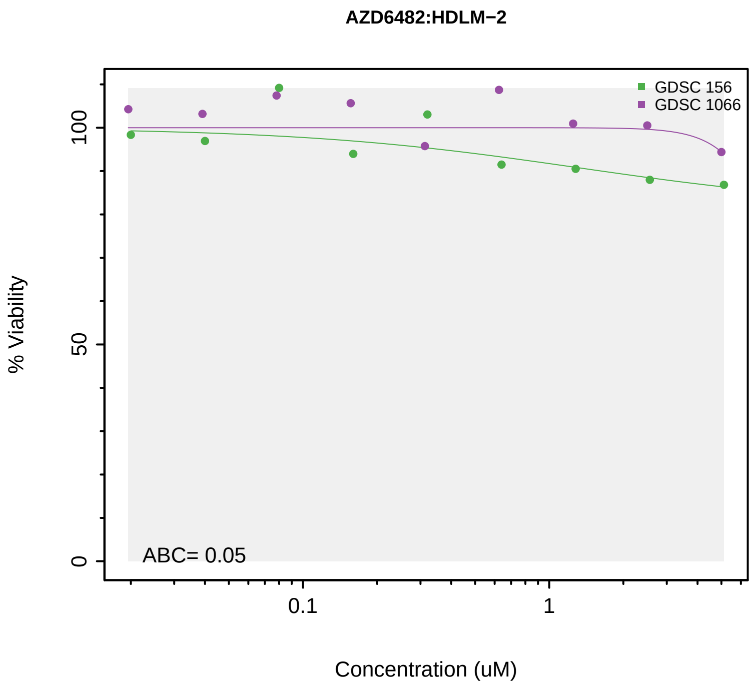
AZD6482:HDLM−2
GDSC 156
GDSC 1066
ABC= 0.05
% Viability
0
50
100
0.1
1
Concentration (uM)

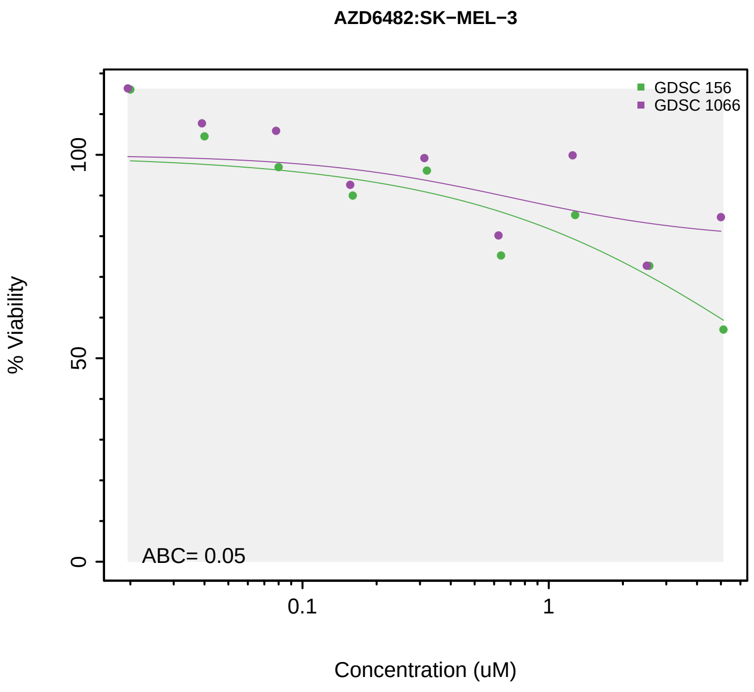
AZD6482:SK−MEL−3
GDSC 156
GDSC 1066
ABC= 0.05
% Viability
0
50
100
0.1
1
Concentration (uM)

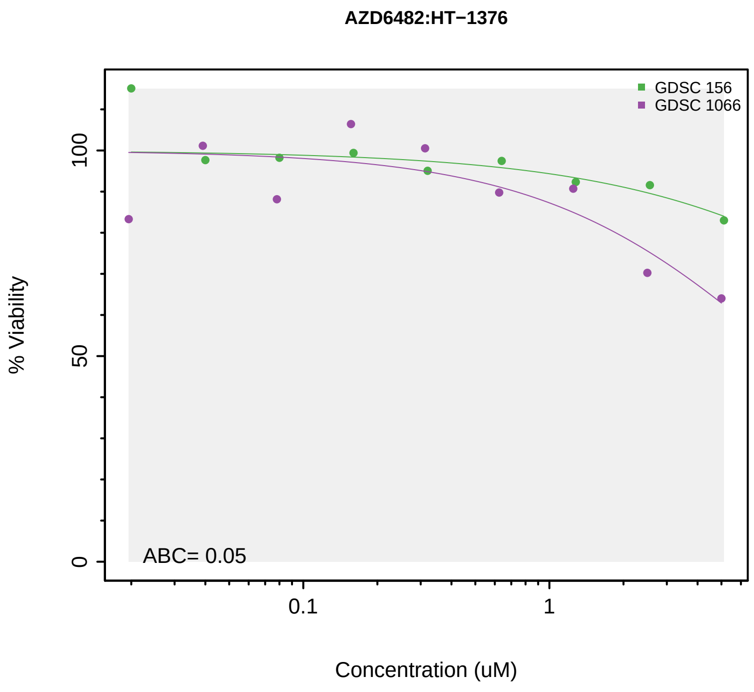
AZD6482:HT−1376
GDSC 156
GDSC 1066
ABC= 0.05
% Viability
0
50
100
0.1
1
Concentration (uM)

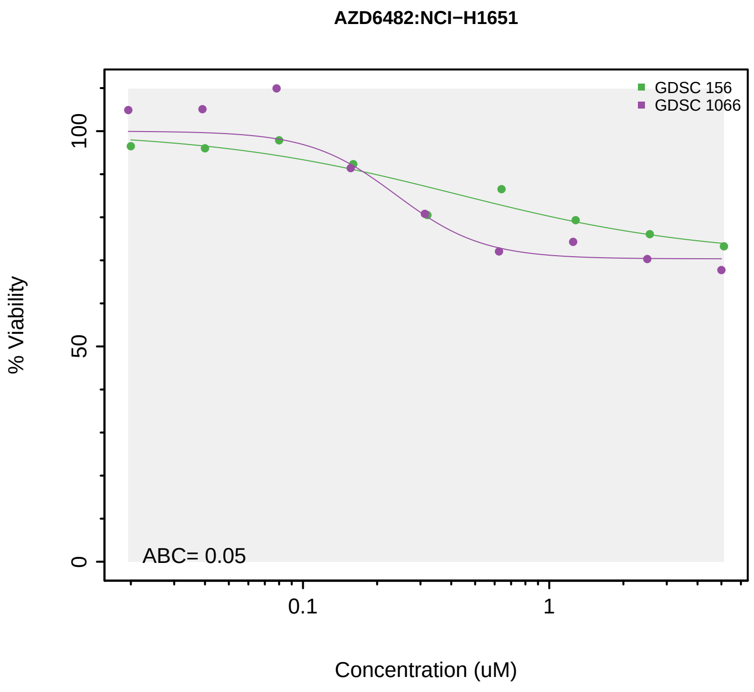
AZD6482:NCI−H1651
GDSC 156
GDSC 1066
ABC= 0.05
% Viability
0
50
100
0.1
1
Concentration (uM)

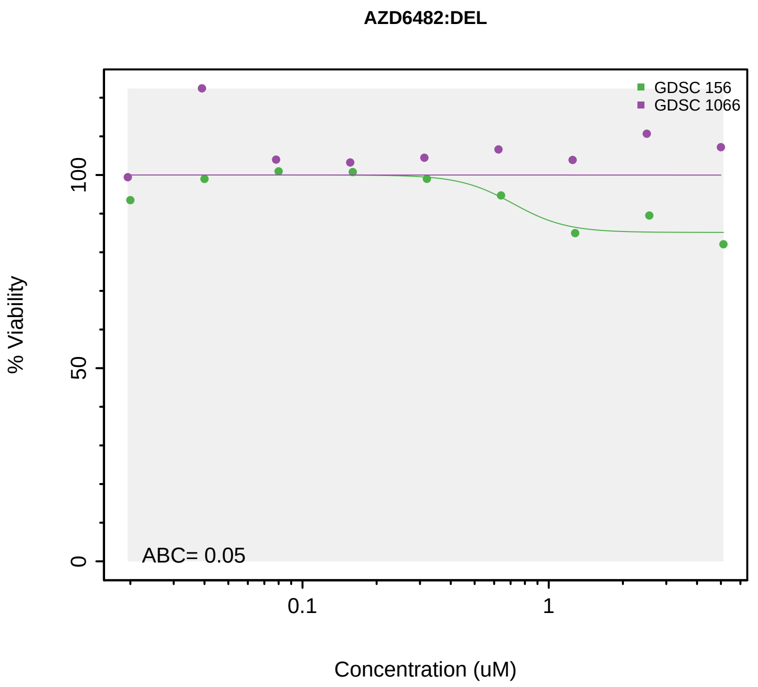
AZD6482:DEL
GDSC 156
GDSC 1066
% Viability
0
50
100
ABC= 0.05
0.1
1
Concentration (uM)

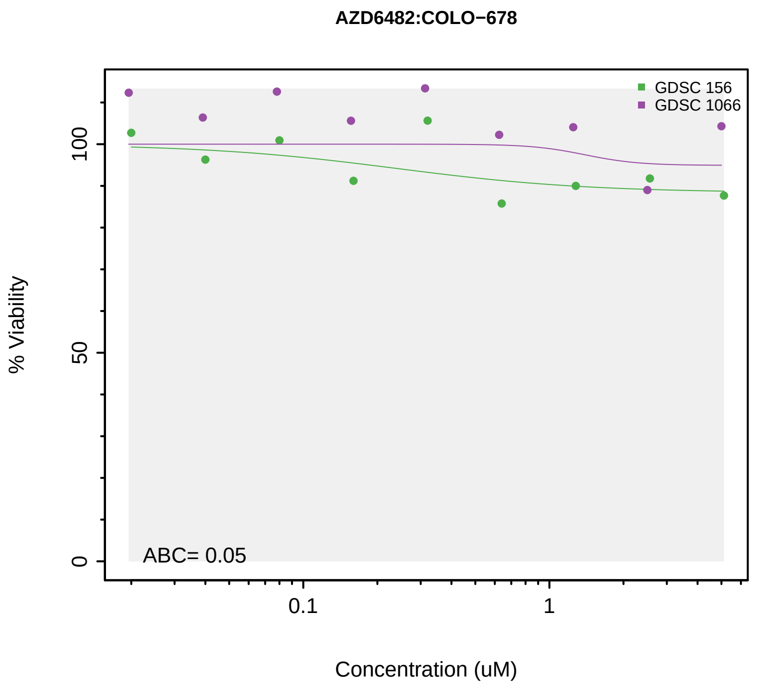
AZD6482:COLO−678
GDSC 156
GDSC 1066
% Viability
0
50
100
ABC= 0.05
0.1
1
Concentration (uM)

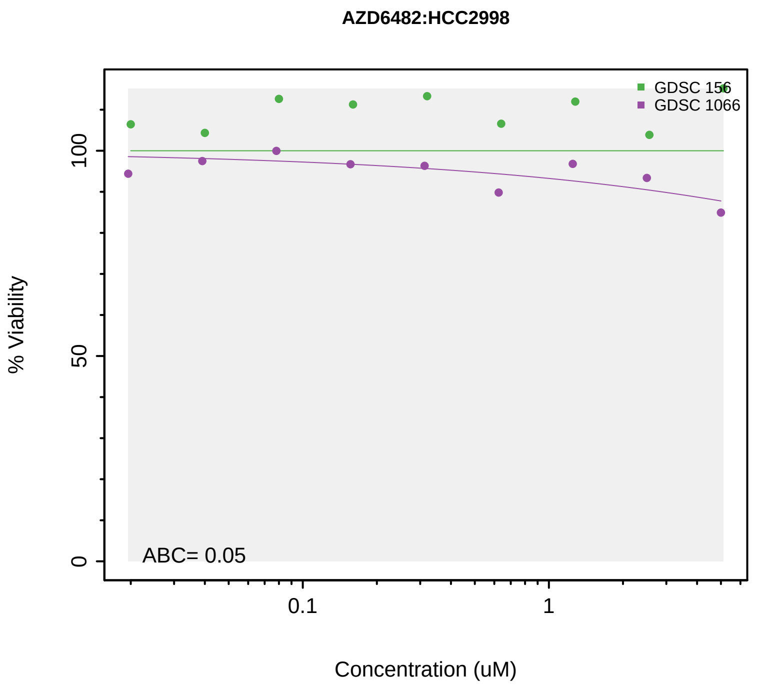
AZD6482:HCC2998
GDSC 156
GDSC 1066
% Viability
0
50
100
ABC= 0.05
0.1
1
Concentration (uM)

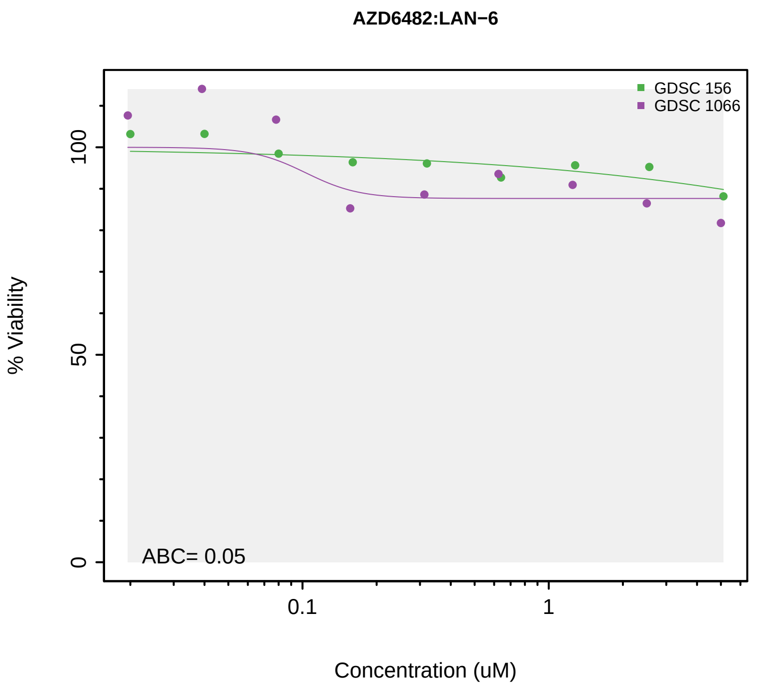
AZD6482:LAN−6
GDSC 156
GDSC 1066
% Viability
0
50
100
ABC= 0.05
0.1
1
Concentration (uM)

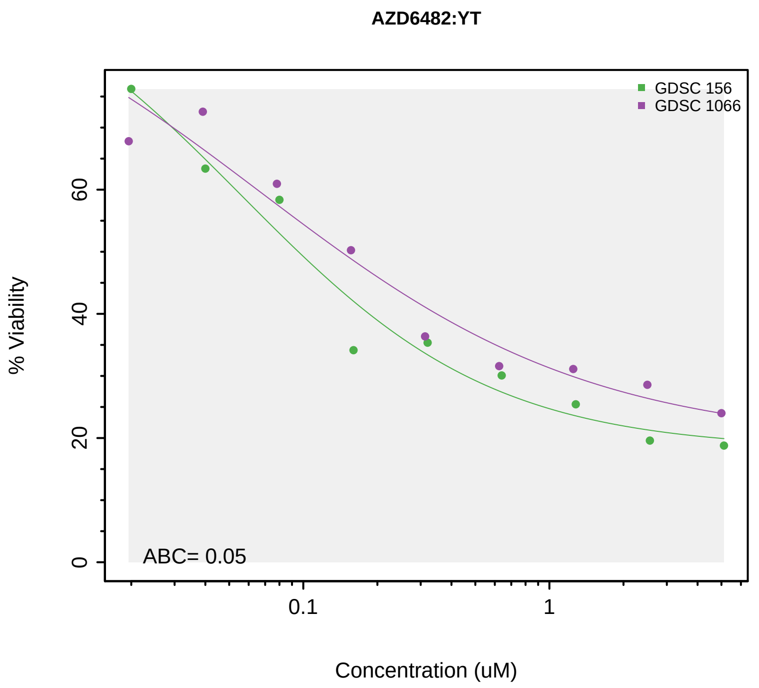
AZD6482:YT
GDSC 156
GDSC 1066
% Viability
0
20
40
60
ABC= 0.05
0.1
1
Concentration (uM)

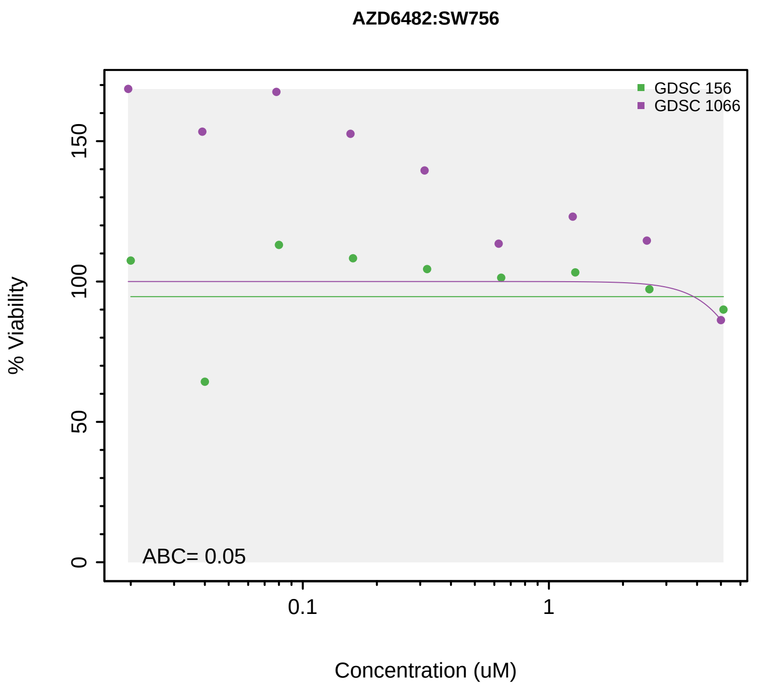
AZD6482:SW756
GDSC 156
GDSC 1066
% Viability
0
50
100
150
ABC= 0.05
0.1
1
Concentration (uM)

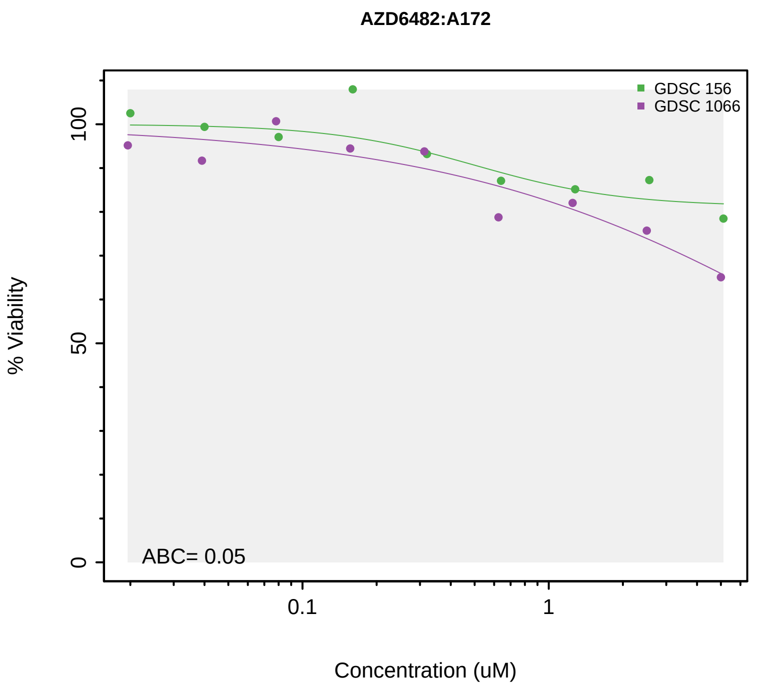
AZD6482:A172
GDSC 156
GDSC 1066
% Viability
0
50
100
ABC= 0.05
0.1
1
Concentration (uM)

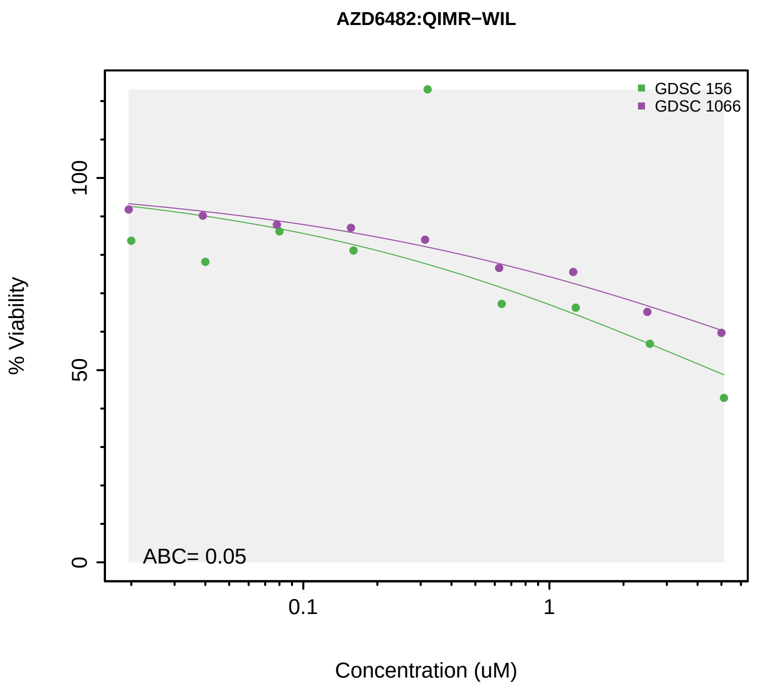
AZD6482:QIMR−WIL
GDSC 156
GDSC 1066
% Viability
0
50
100
ABC= 0.05
0.1
1
Concentration (uM)

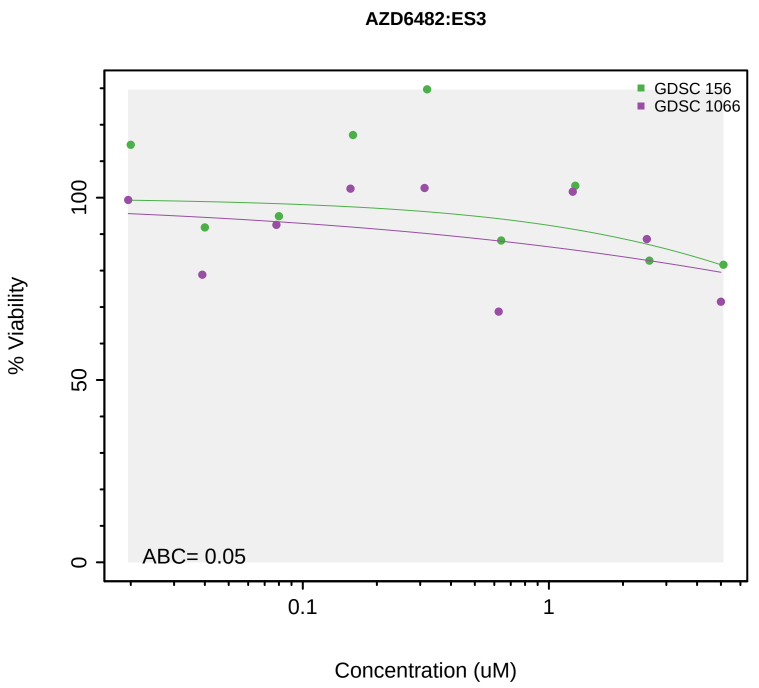
AZD6482:ES3
GDSC 156
GDSC 1066
% Viability
0
50
100
ABC= 0.05
0.1
1
Concentration (uM)

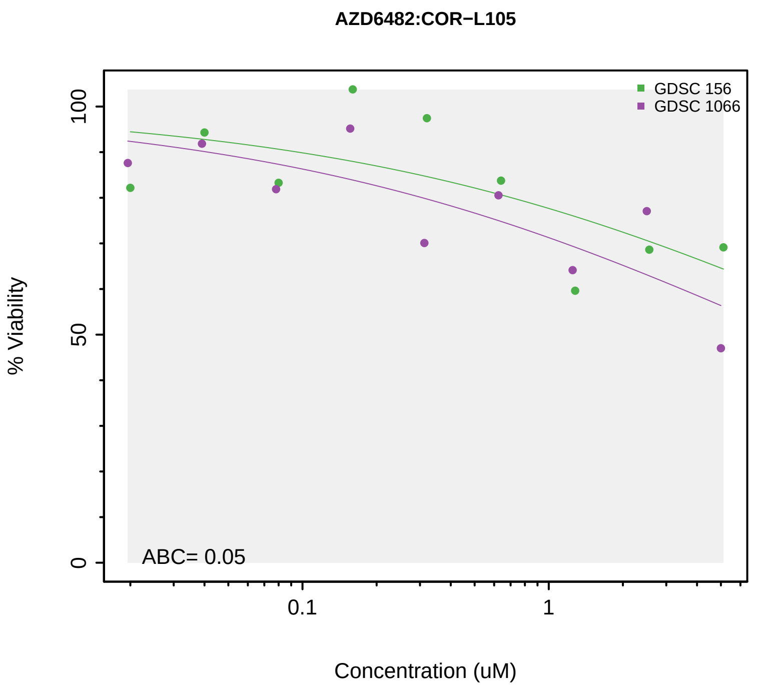
AZD6482:COR−L105
GDSC 156
GDSC 1066
% Viability
0
50
100
ABC= 0.05
0.1
1
Concentration (uM)

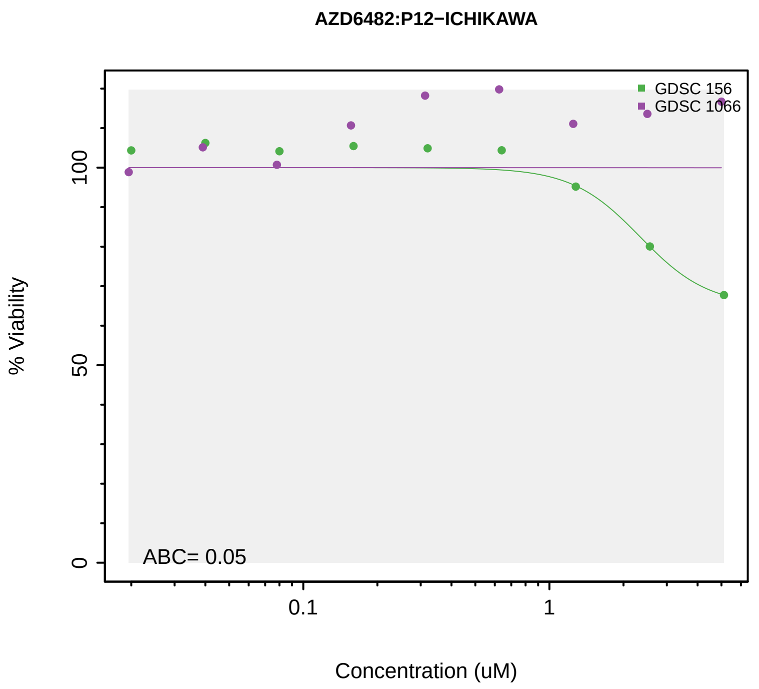
AZD6482:P12−ICHIKAWA
GDSC 156
GDSC 1066
% Viability
0
50
100
ABC= 0.05
0.1
1
Concentration (uM)

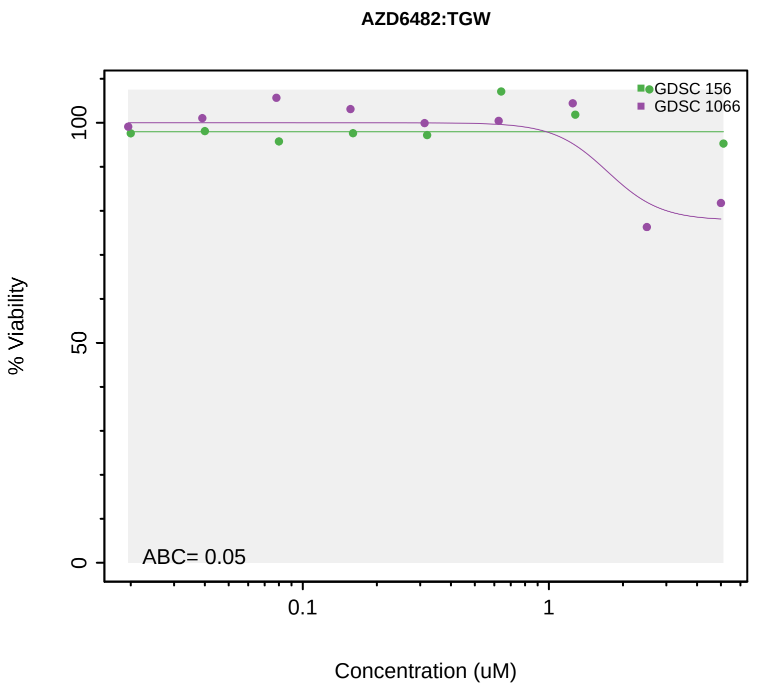
AZD6482:TGW
GDSC 156
GDSC 1066
% Viability
0
50
100
ABC= 0.05
0.1
1
Concentration (uM)

AZD6482:SIMA

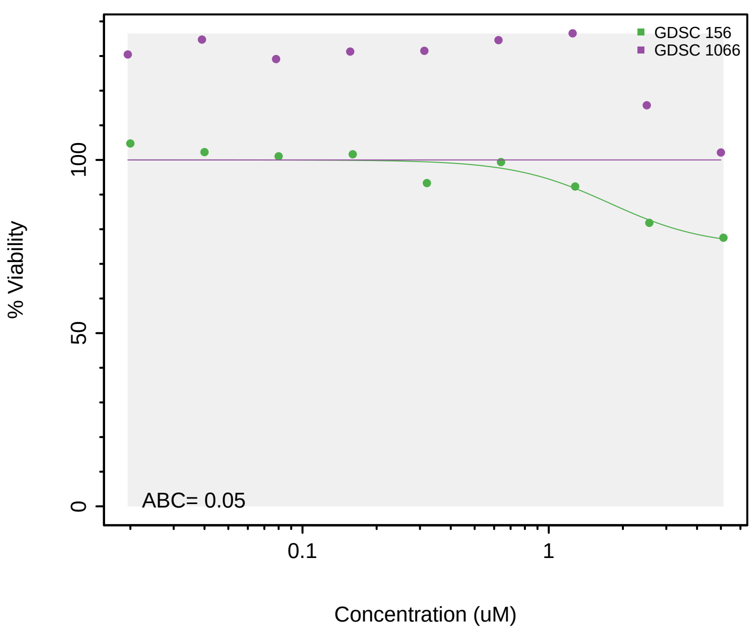

AZD6482:SW837

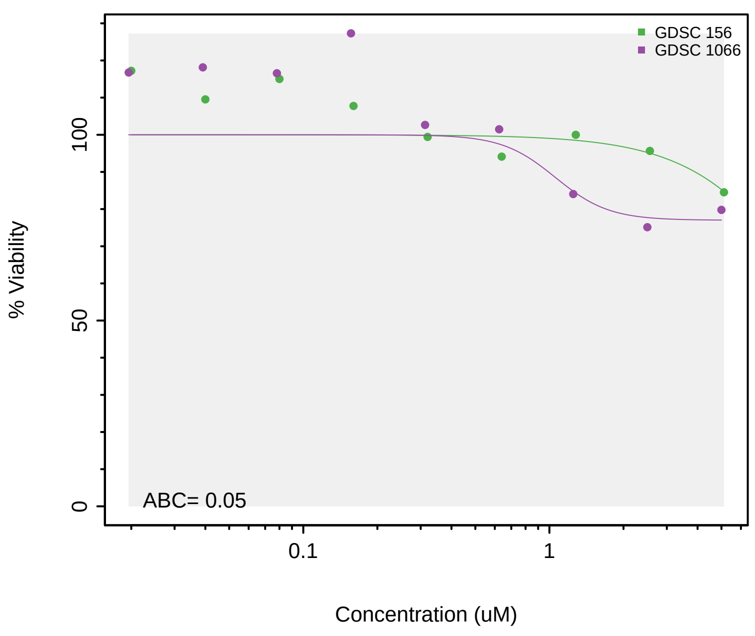

AZD6482:LOUCY

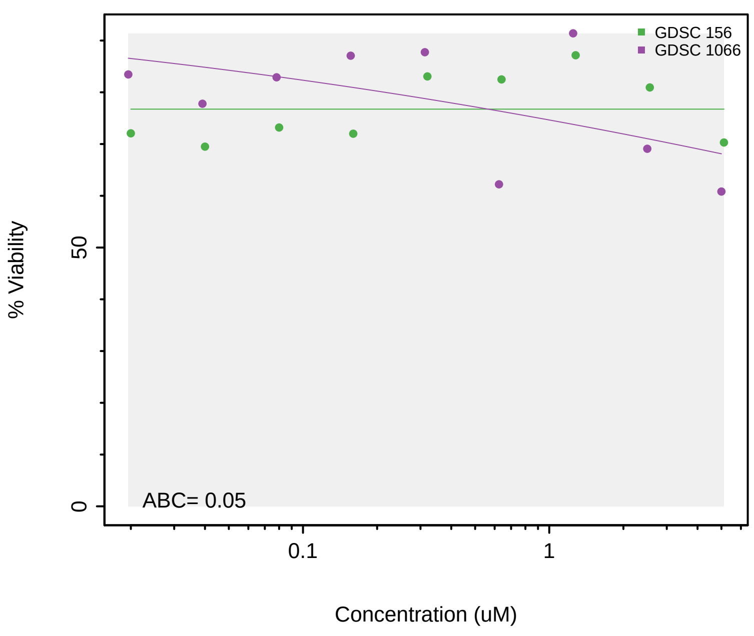

AZD6482:S−117

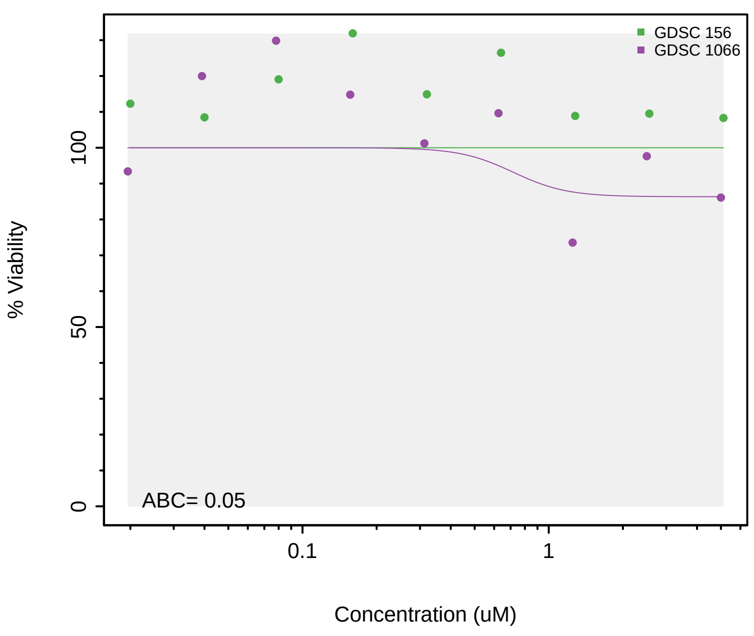

AZD6482:CCRF−CEM

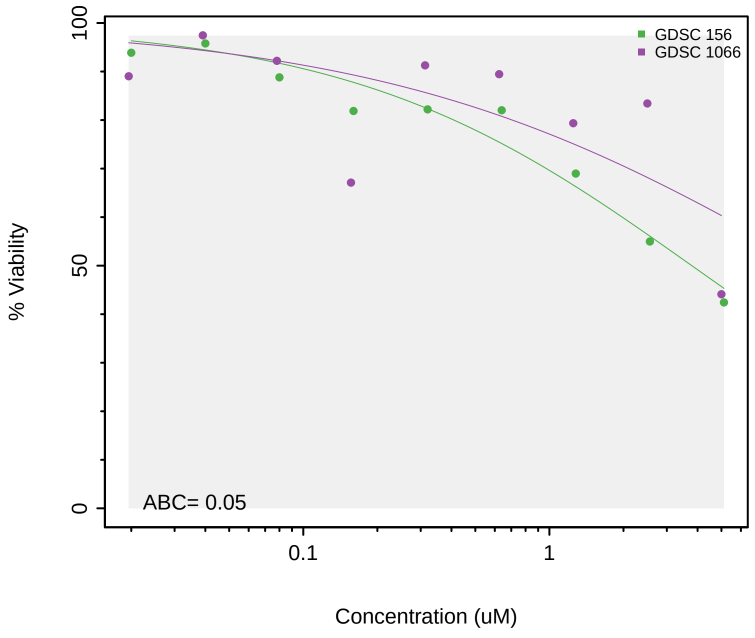

AZD6482:NB69

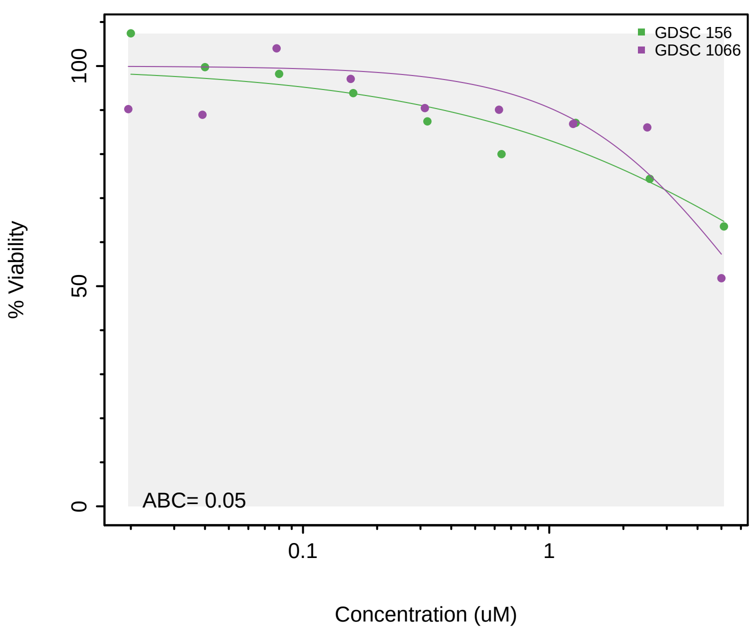

AZD6482:M059J

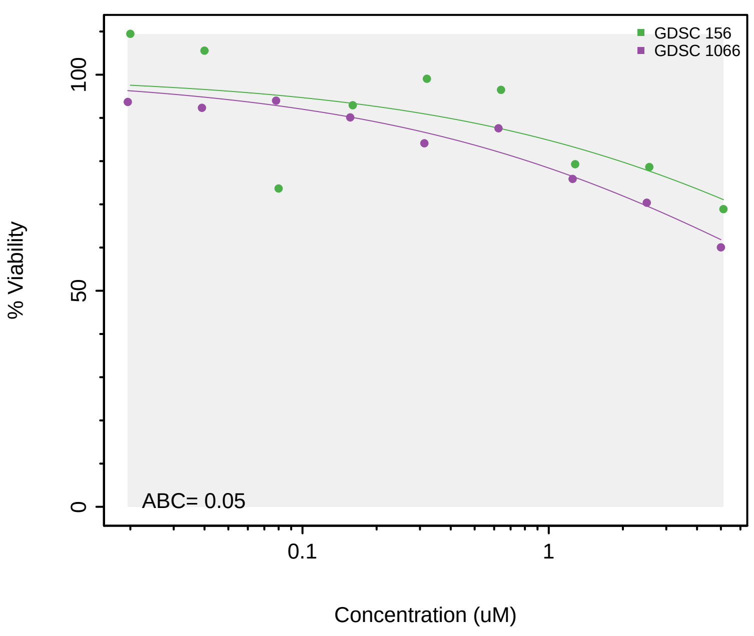

AZD6482:KYSE−410

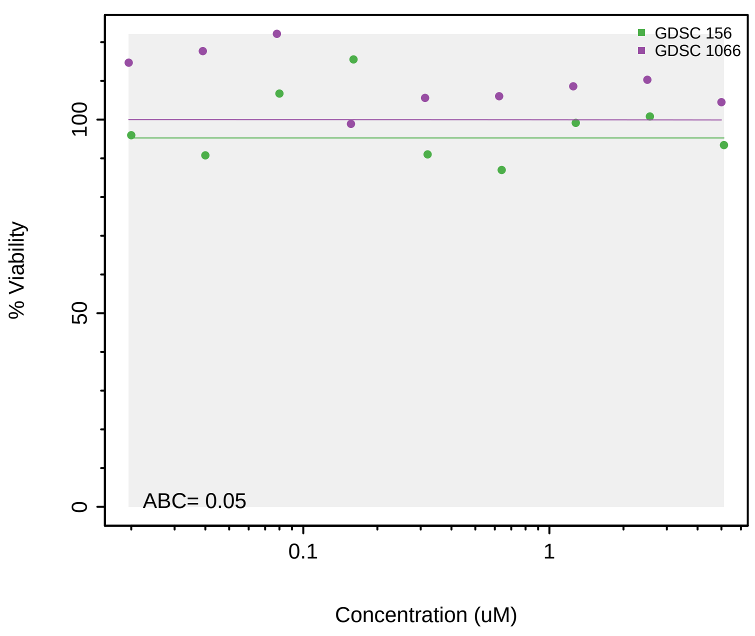

AZD6482:UMC−11

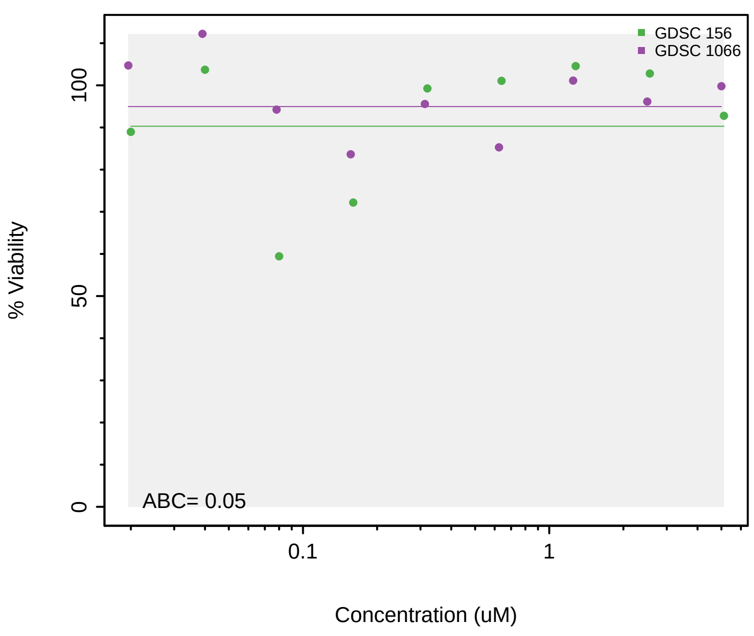

AZD6482:BEN

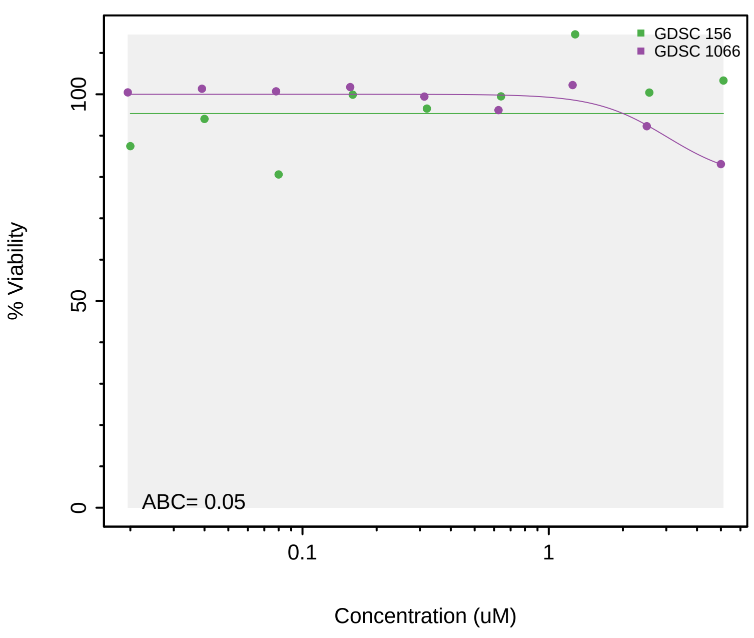

AZD6482:U−698−M

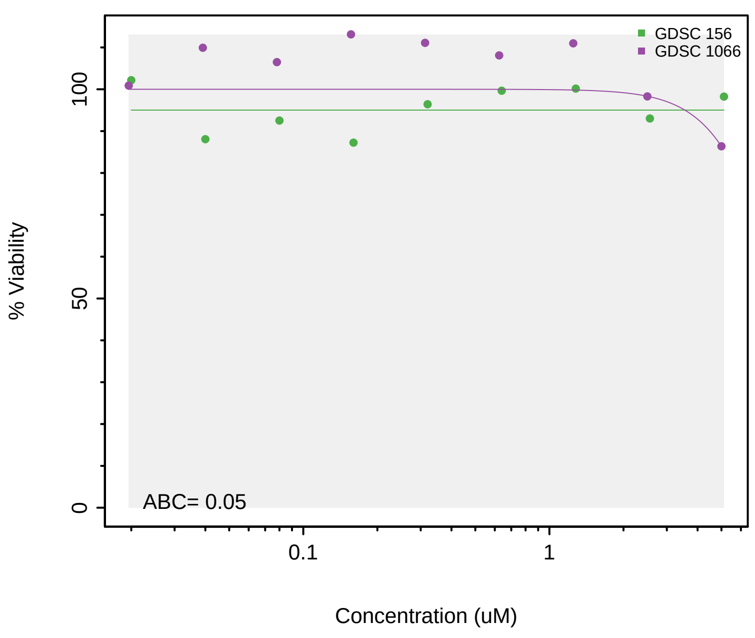

AZD6482:SCC−15

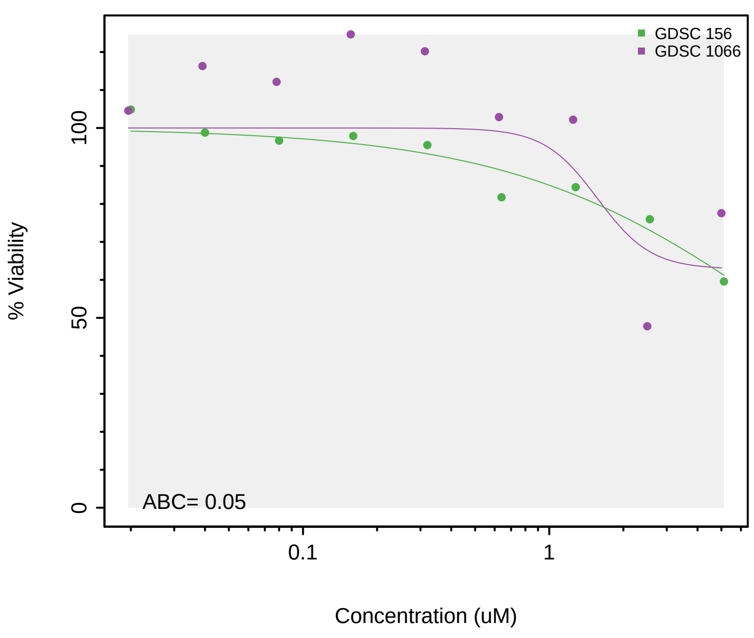

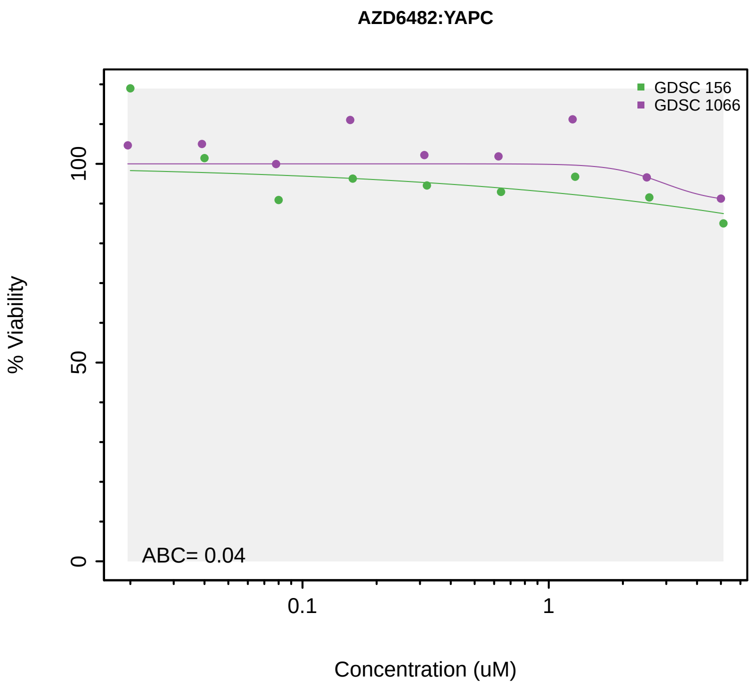
AZD6482:YAPC
GDSC 156
GDSC 1066
ABC= 0.04
% Viability
Concentration (uM)

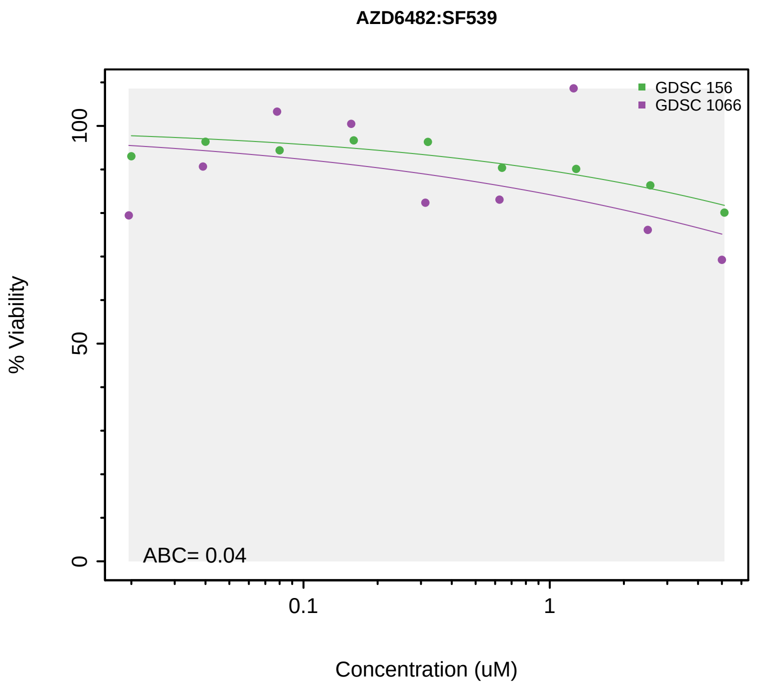
AZD6482:SF539
GDSC 156
GDSC 1066
ABC= 0.04
% Viability
Concentration (uM)

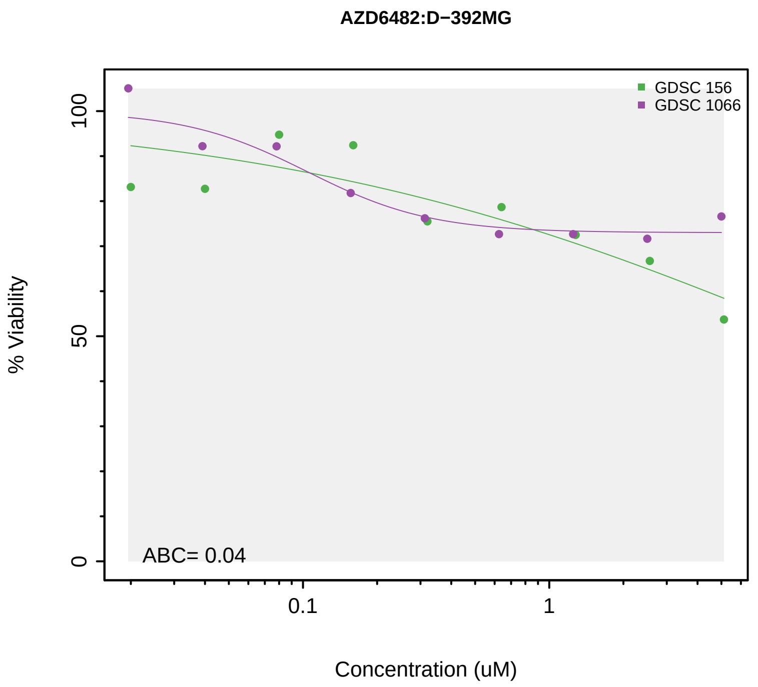
AZD6482:D−392MG
GDSC 156
GDSC 1066
ABC= 0.04
% Viability
Concentration (uM)

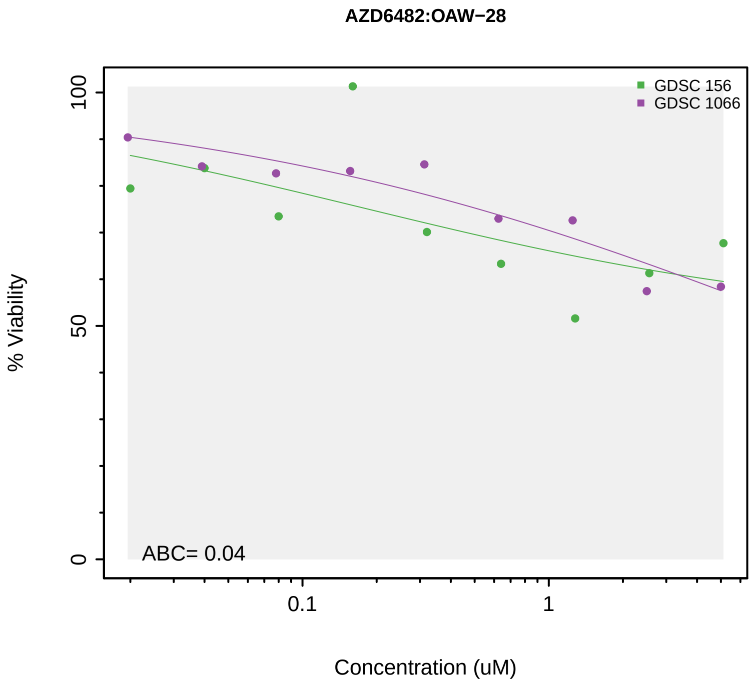
AZD6482:OAW−28
GDSC 156
GDSC 1066
ABC= 0.04
% Viability
Concentration (uM)

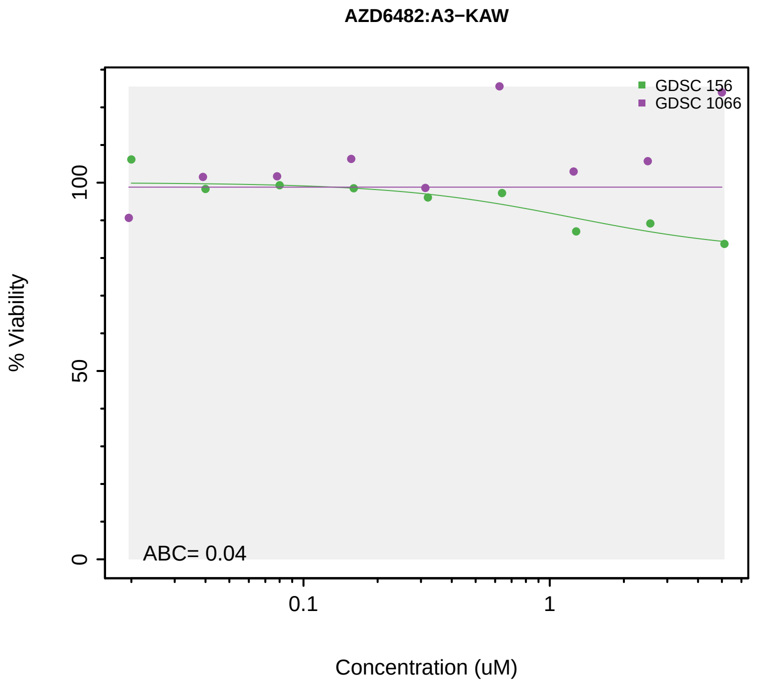
AZD6482:A3−KAW
GDSC 156
GDSC 1066
ABC= 0.04
% Viability
Concentration (uM)

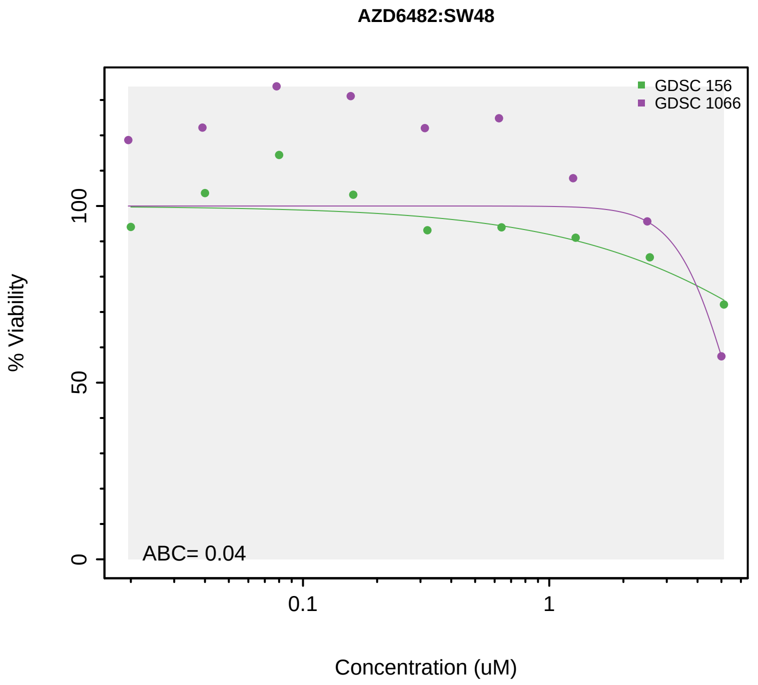
AZD6482:SW48
GDSC 156
GDSC 1066
ABC= 0.04
% Viability
Concentration (uM)

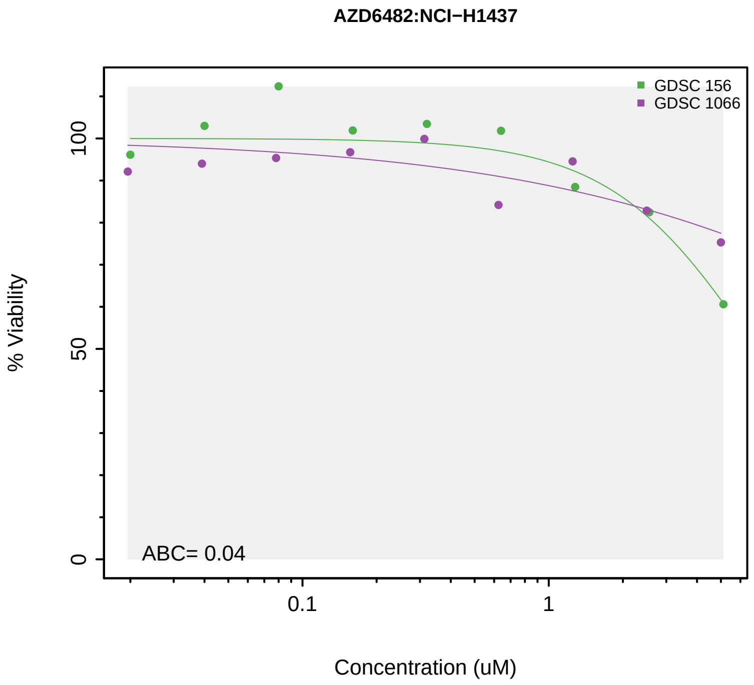
AZD6482:NCI−H1437
GDSC 156
GDSC 1066
ABC= 0.04
% Viability
Concentration (uM)

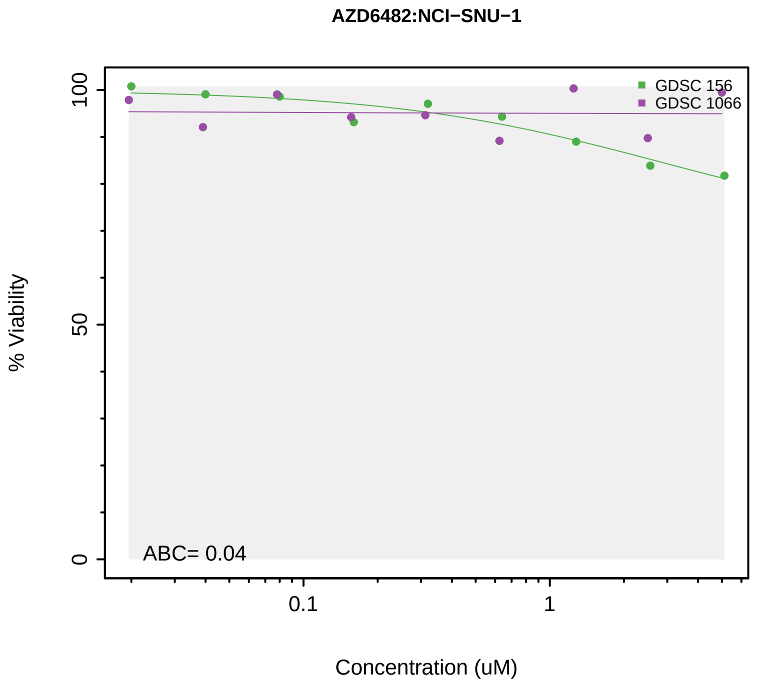
AZD6482:NCI−SNU−1
GDSC 156
GDSC 1066
ABC= 0.04
% Viability
Concentration (uM)

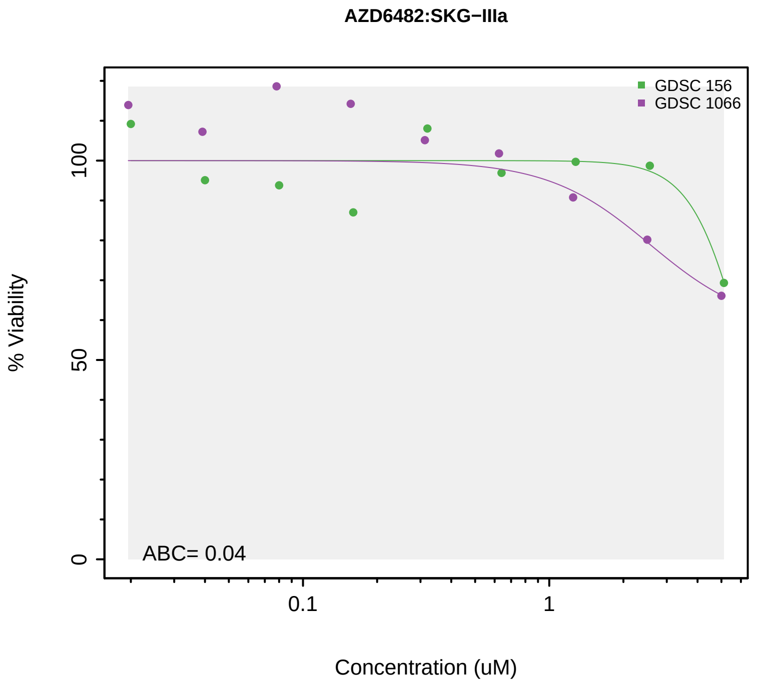
AZD6482:SKG−IIIa
GDSC 156
GDSC 1066
ABC= 0.04
% Viability
Concentration (uM)

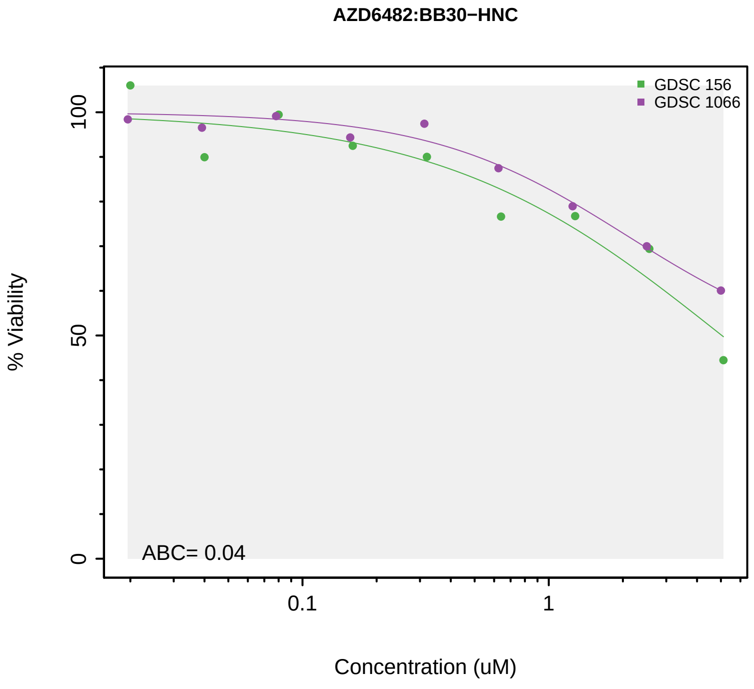
AZD6482:BB30−HNC
GDSC 156
GDSC 1066
ABC= 0.04
% Viability
Concentration (uM)

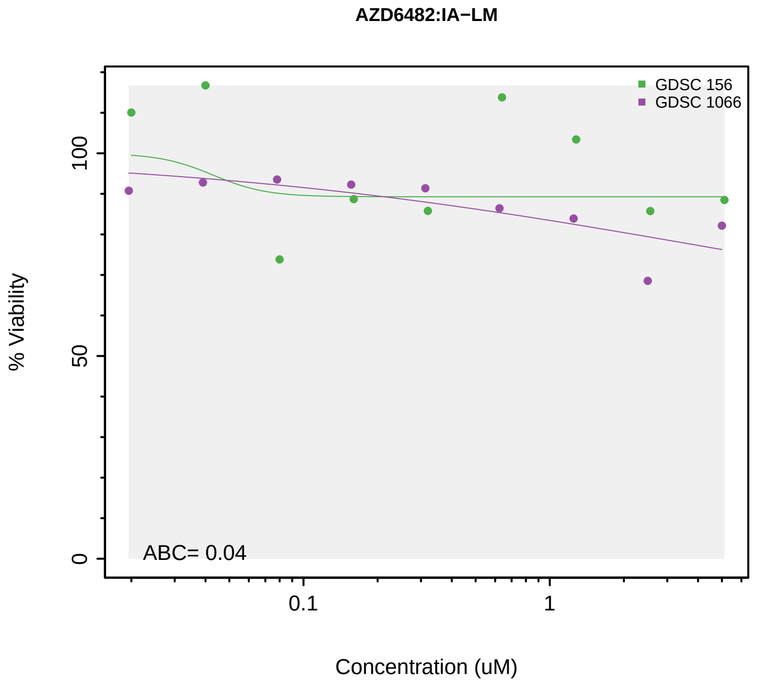
AZD6482:IA−LM
GDSC 156
GDSC 1066
ABC= 0.04
% Viability
Concentration (uM)

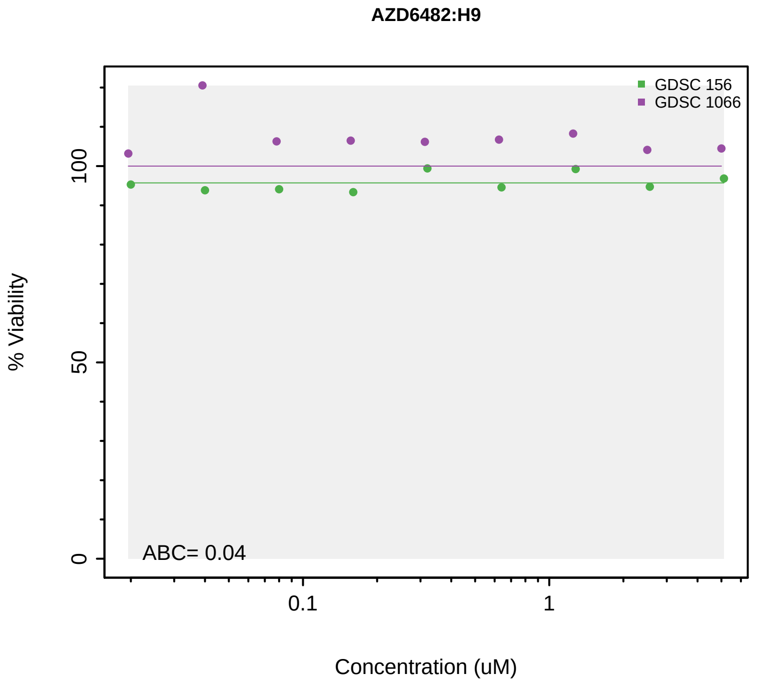
AZD6482:H9
GDSC 156
GDSC 1066
ABC= 0.04
% Viability
Concentration (uM)

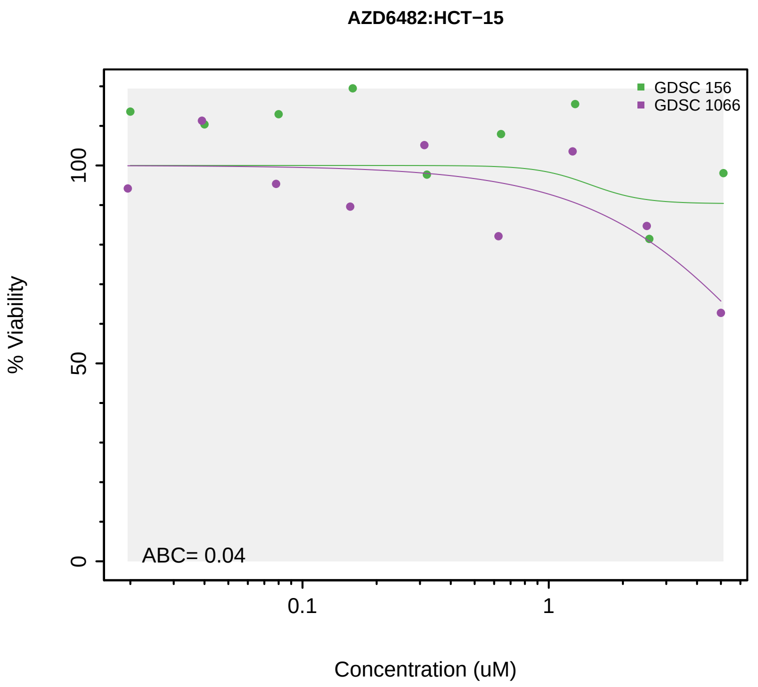
AZD6482:HCT−15
GDSC 156
GDSC 1066
% Viability
Concentration (uM)
ABC= 0.04

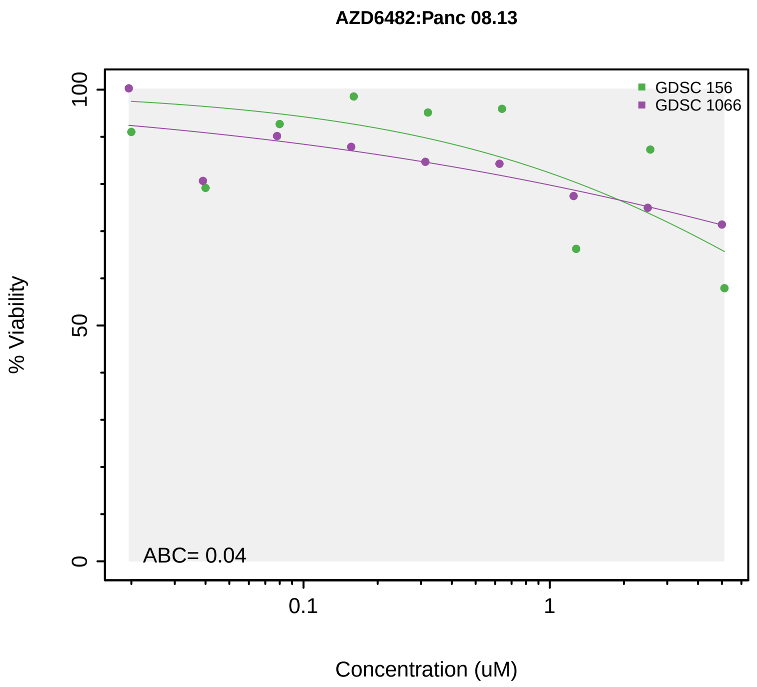
AZD6482:Panc 08.13
GDSC 156
GDSC 1066
% Viability
Concentration (uM)
ABC= 0.04

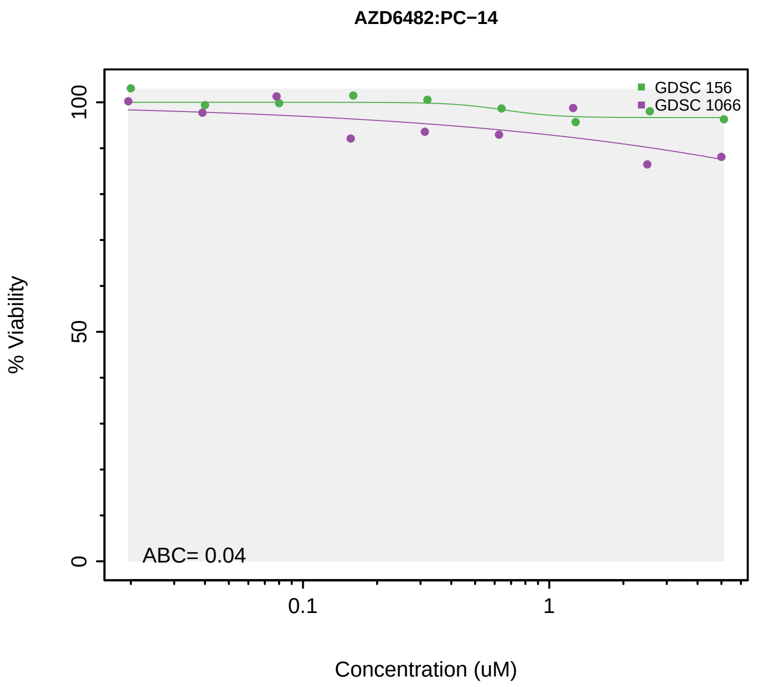
AZD6482:PC−14
GDSC 156
GDSC 1066
% Viability
Concentration (uM)
ABC= 0.04

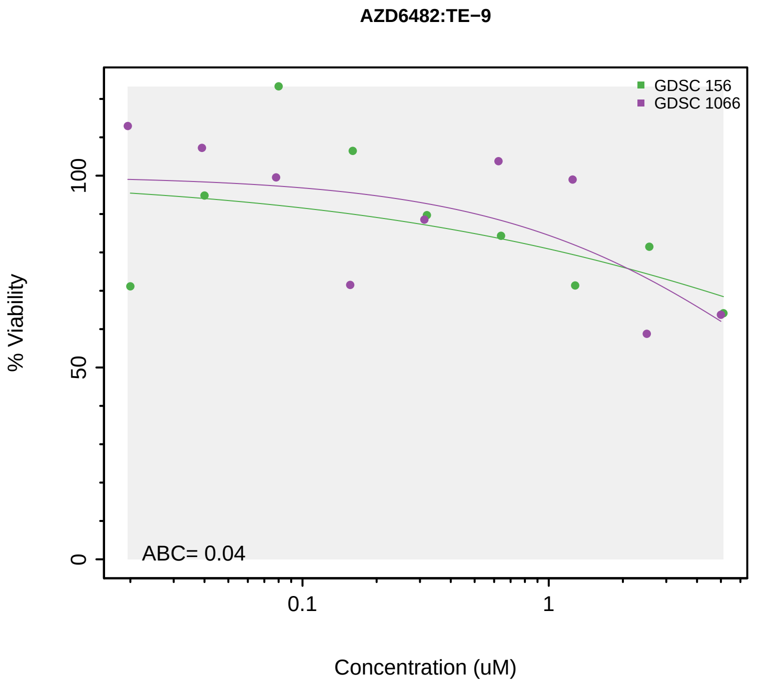
AZD6482:TE−9
GDSC 156
GDSC 1066
% Viability
Concentration (uM)
ABC= 0.04

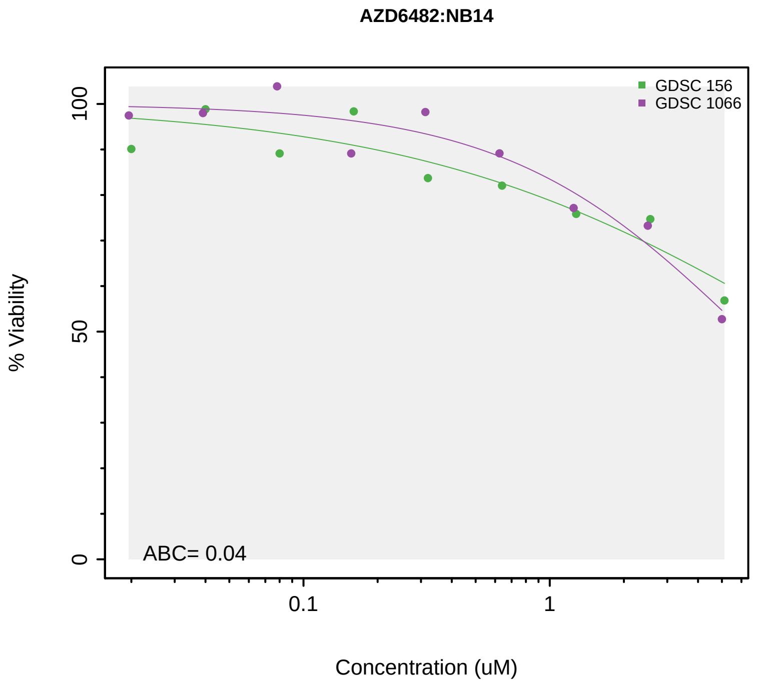
AZD6482:NB14
GDSC 156
GDSC 1066
% Viability
Concentration (uM)
ABC= 0.04

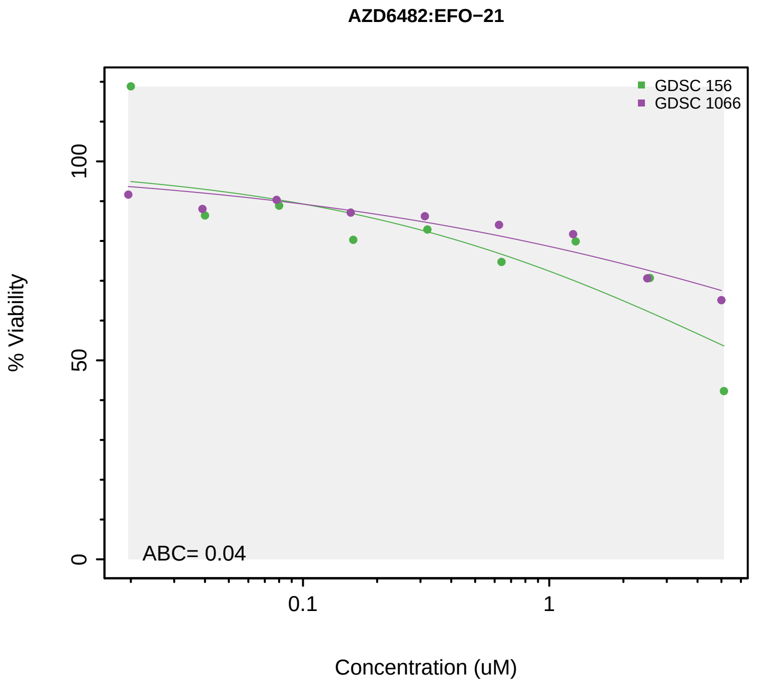
AZD6482:EFO−21
GDSC 156
GDSC 1066
% Viability
Concentration (uM)
ABC= 0.04

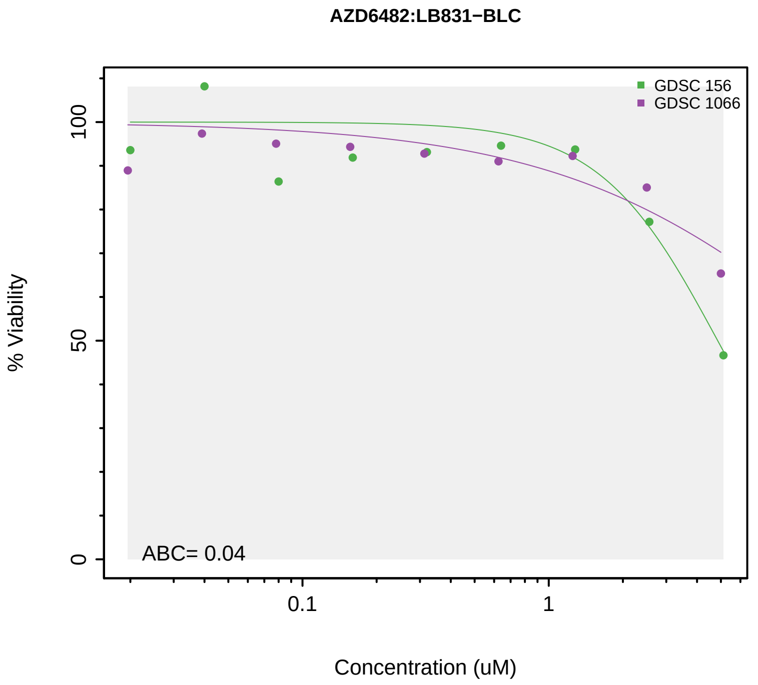
AZD6482:LB831−BLC
GDSC 156
GDSC 1066
% Viability
Concentration (uM)
ABC= 0.04

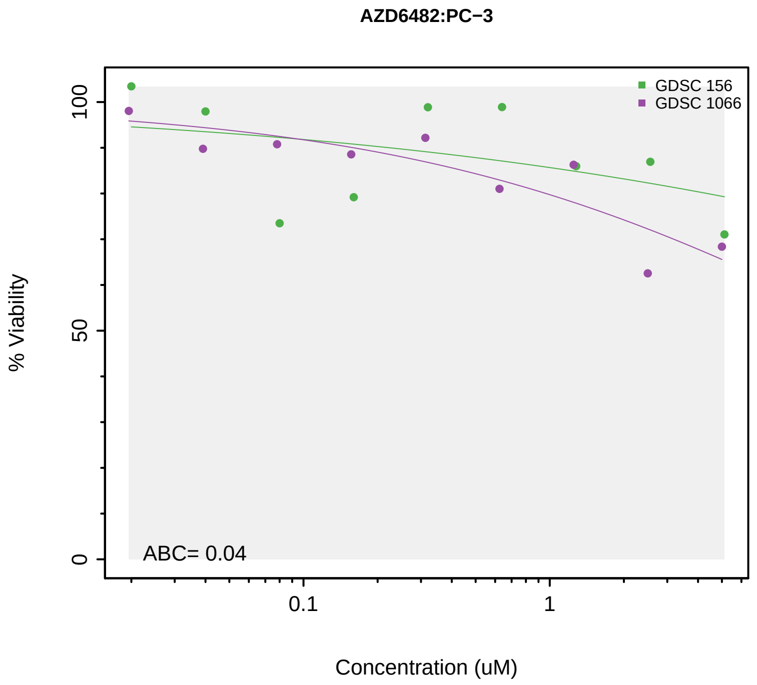
AZD6482:PC−3
GDSC 156
GDSC 1066
% Viability
Concentration (uM)
ABC= 0.04

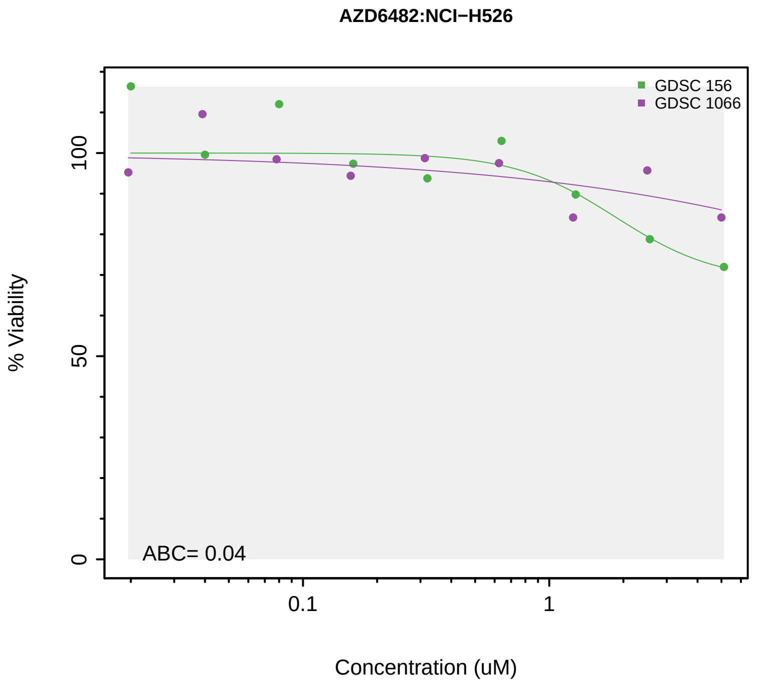
AZD6482:NCI−H526
GDSC 156
GDSC 1066
% Viability
Concentration (uM)
ABC= 0.04

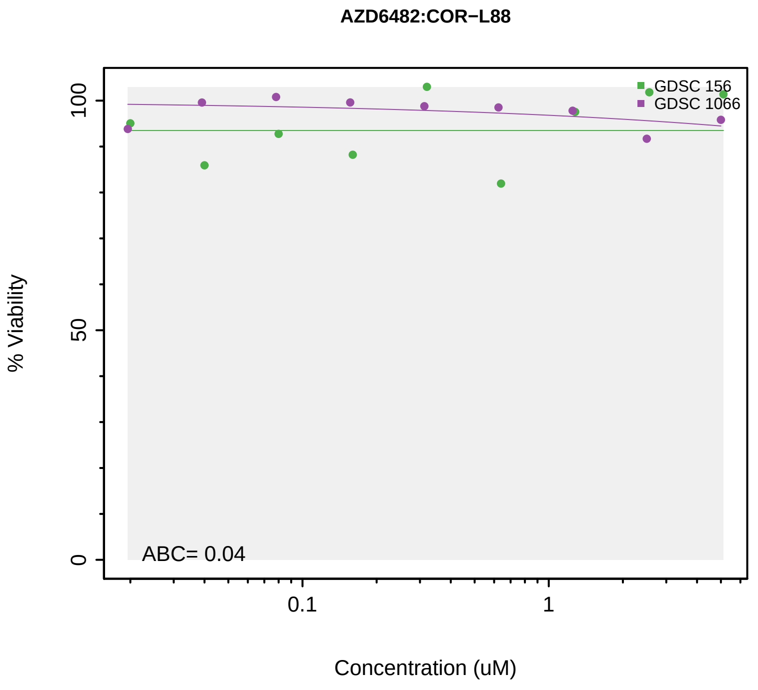
AZD6482:COR−L88
GDSC 156
GDSC 1066
% Viability
Concentration (uM)
ABC= 0.04

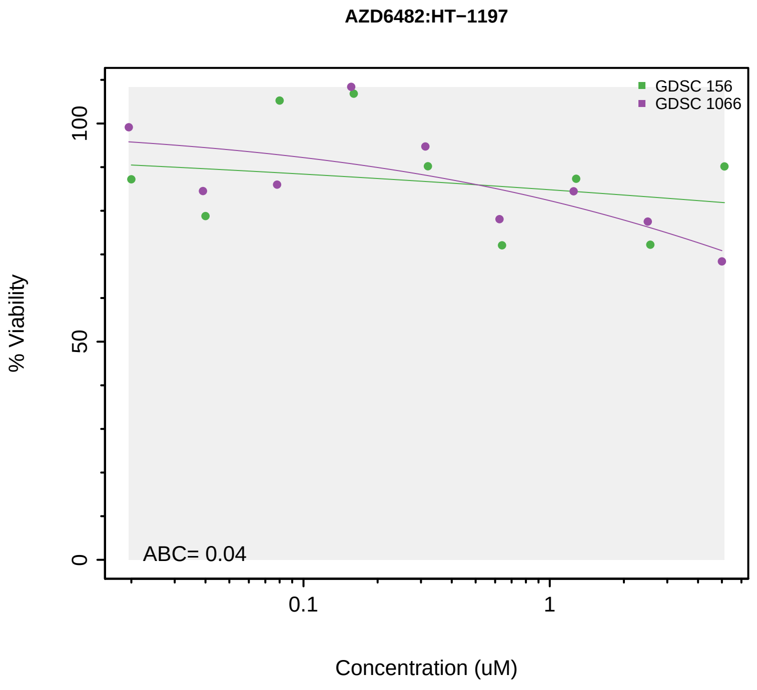
AZD6482:HT−1197
GDSC 156
GDSC 1066
% Viability
Concentration (uM)
ABC= 0.04

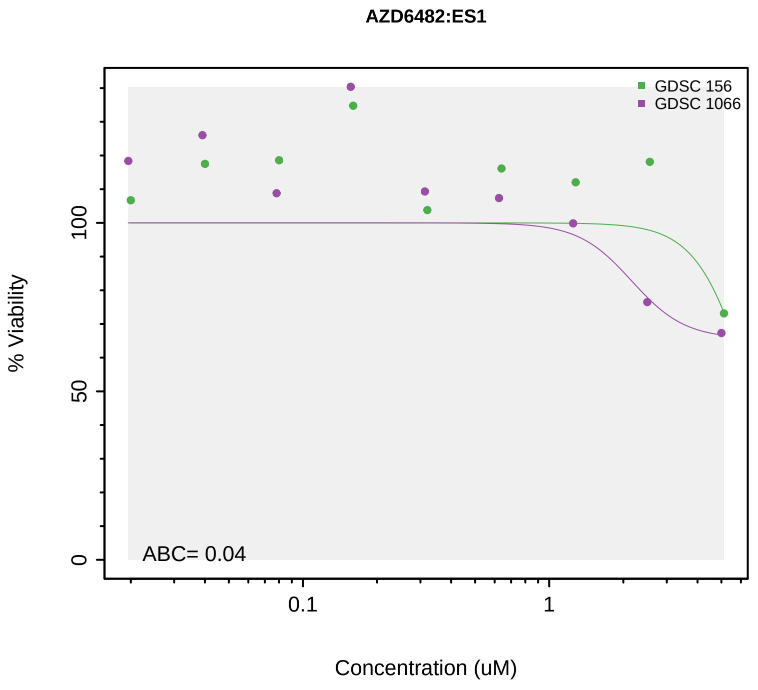
AZD6482:ES1
GDSC 156
GDSC 1066
% Viability
Concentration (uM)
ABC= 0.04

AZD6482:EPLC−272H

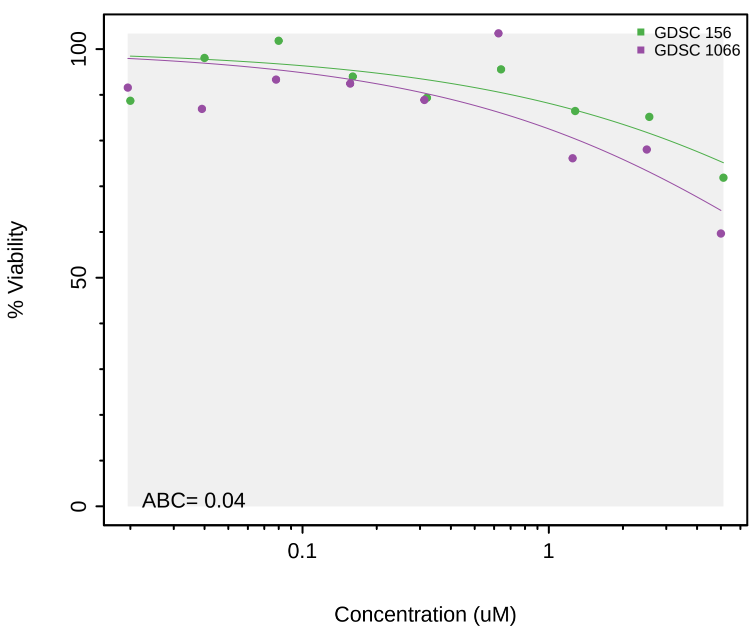

AZD6482:C2BBe1

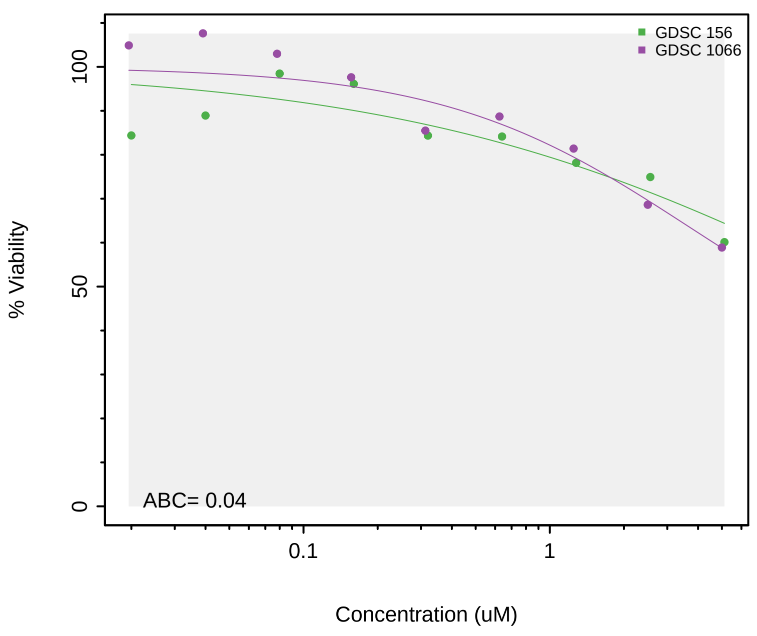

AZD6482:HSC−3

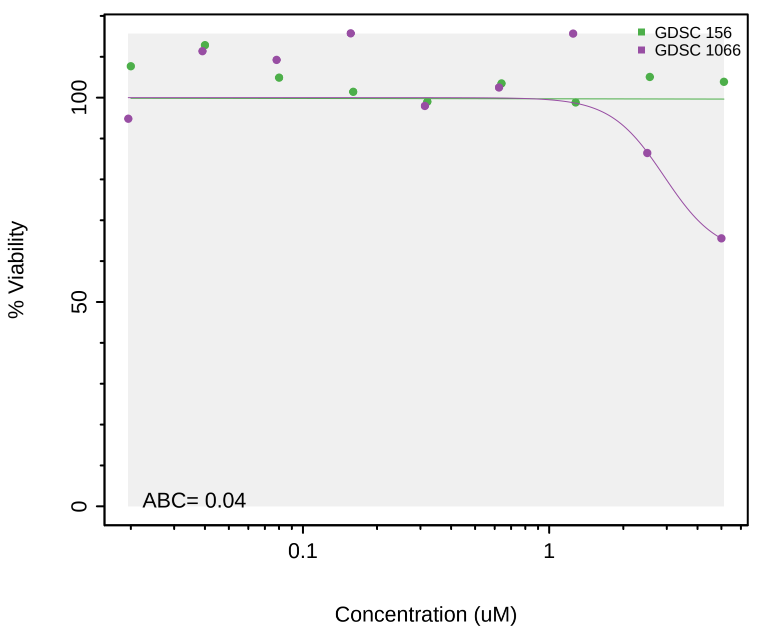

AZD6482:PFSK−1

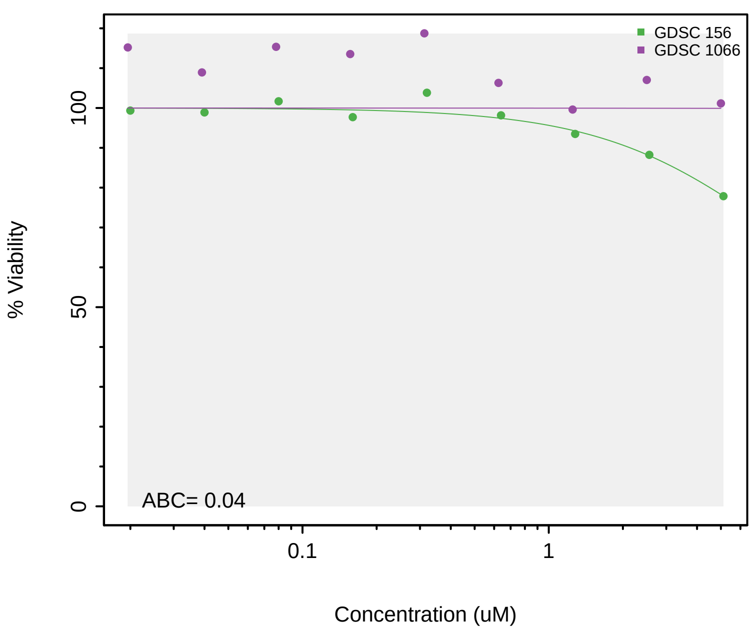

AZD6482:NCI−H1666

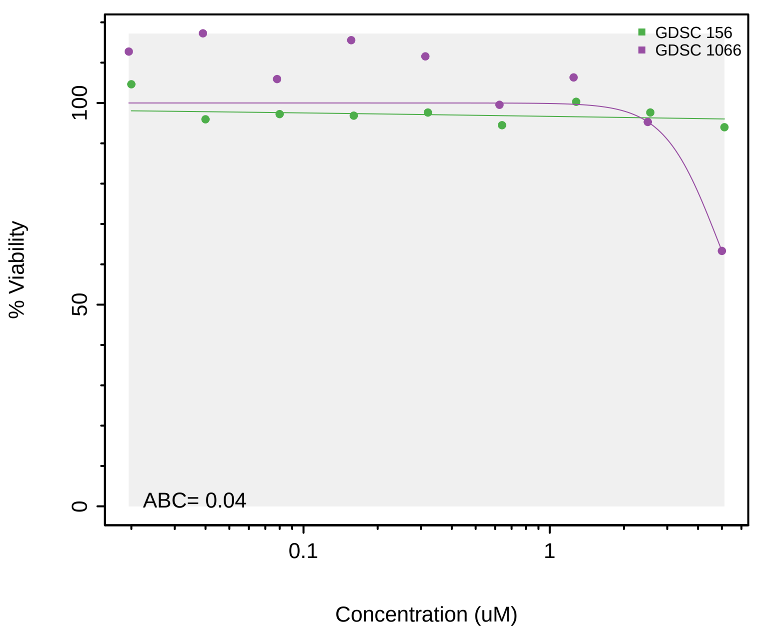

AZD6482:NCI−H1563

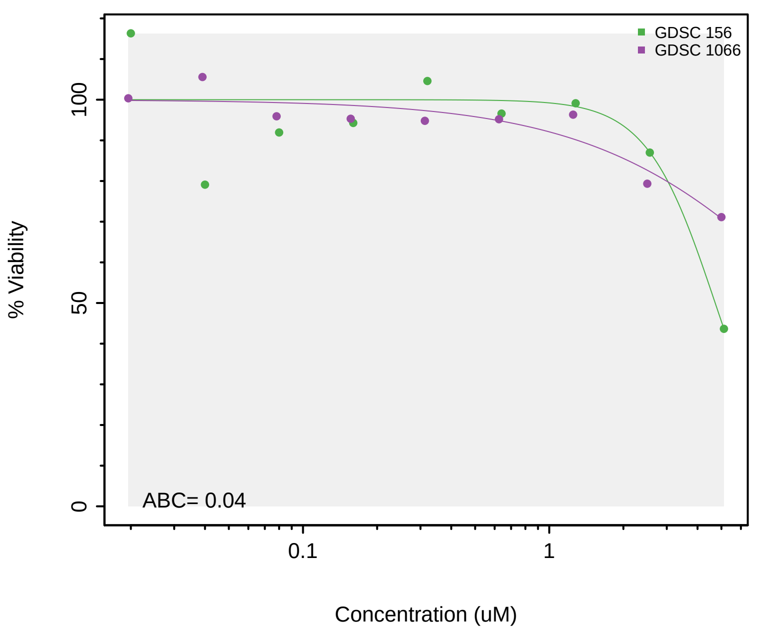

AZD6482:KYSE−70

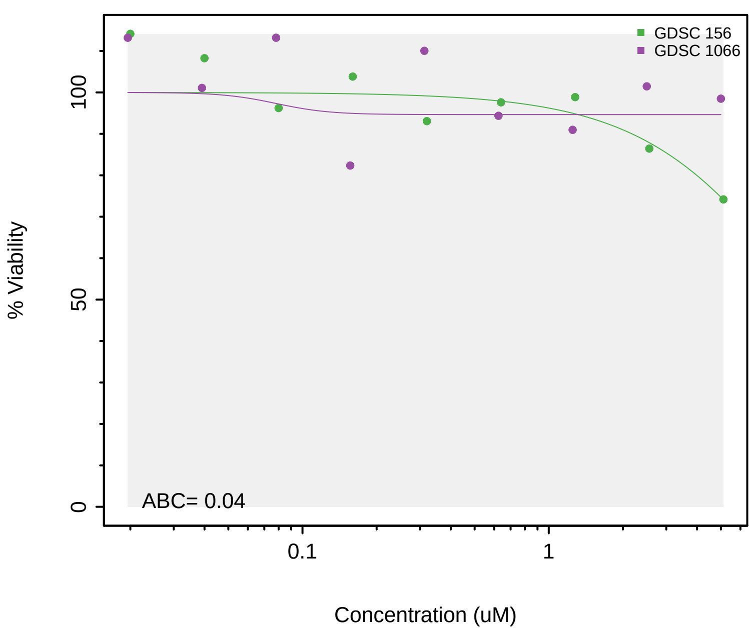

AZD6482:KYSE−140

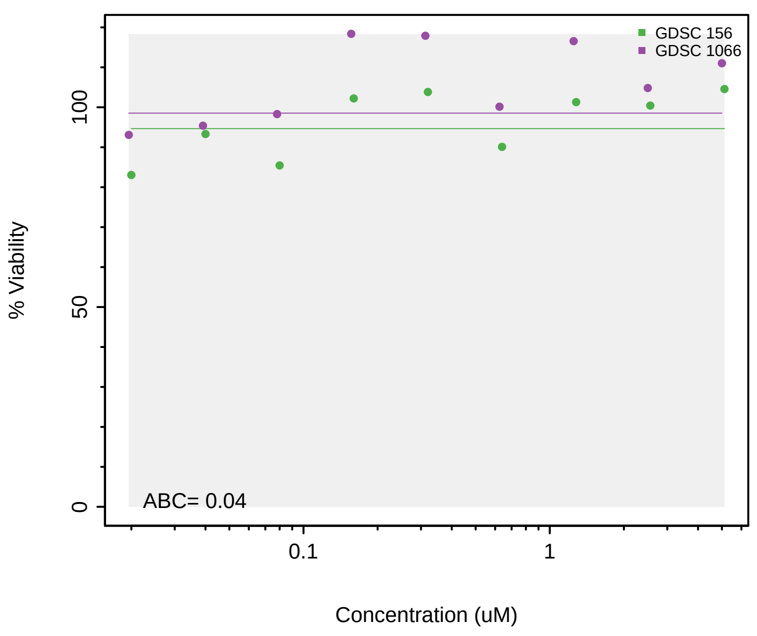

AZD6482:MIA−PaCa−2

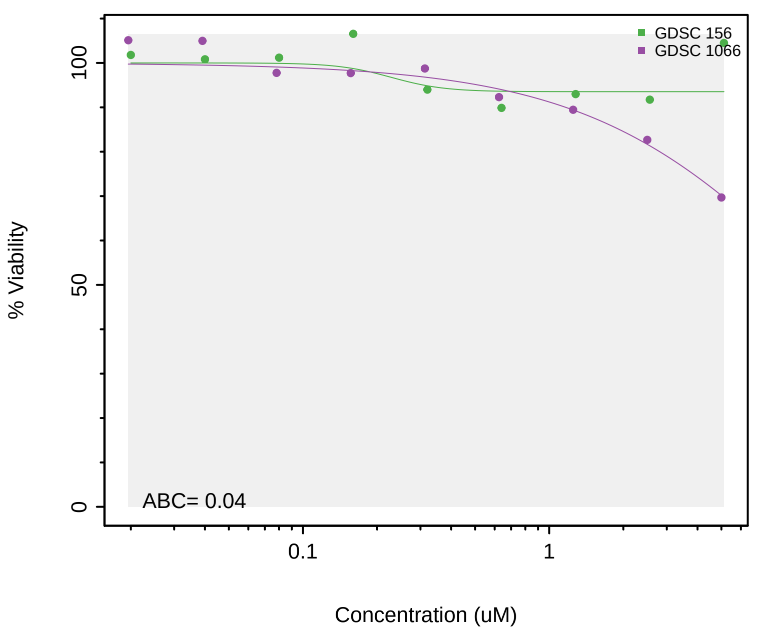

AZD6482:NCI−H2029

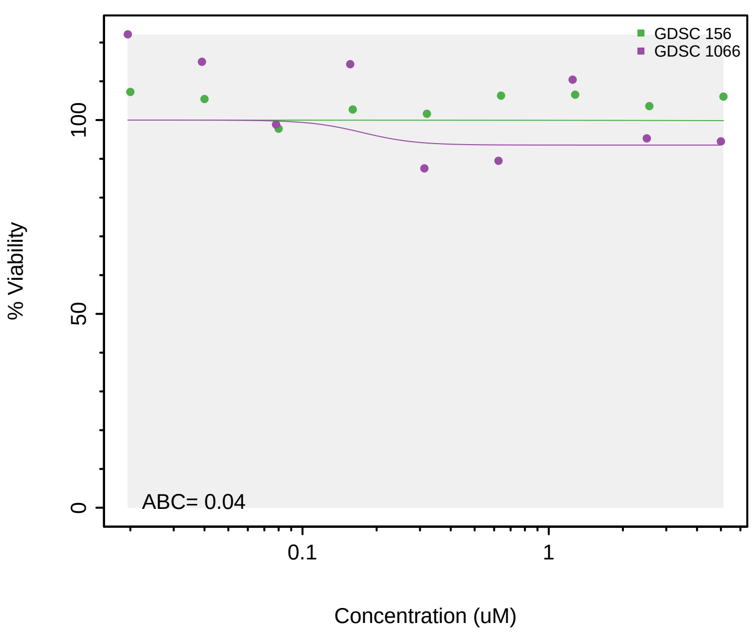

AZD6482:MMAC−SF

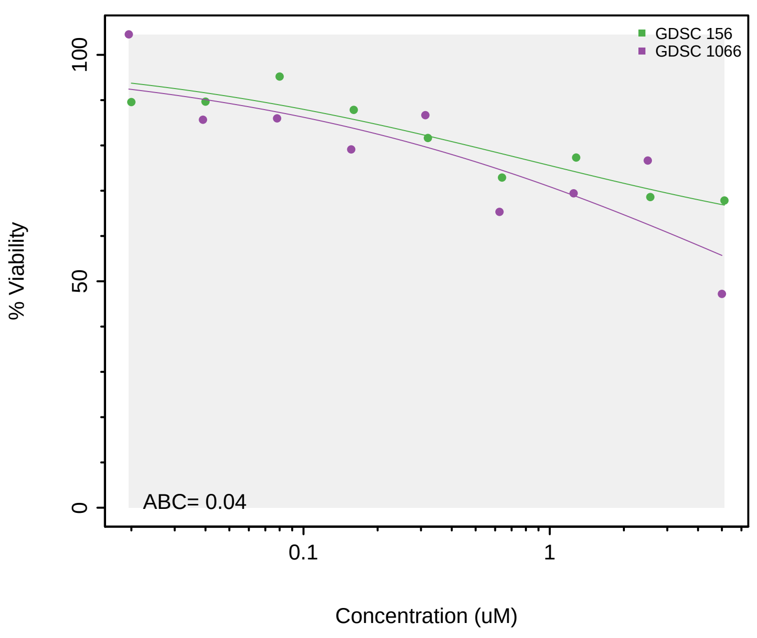

AZD6482:IST−SL1

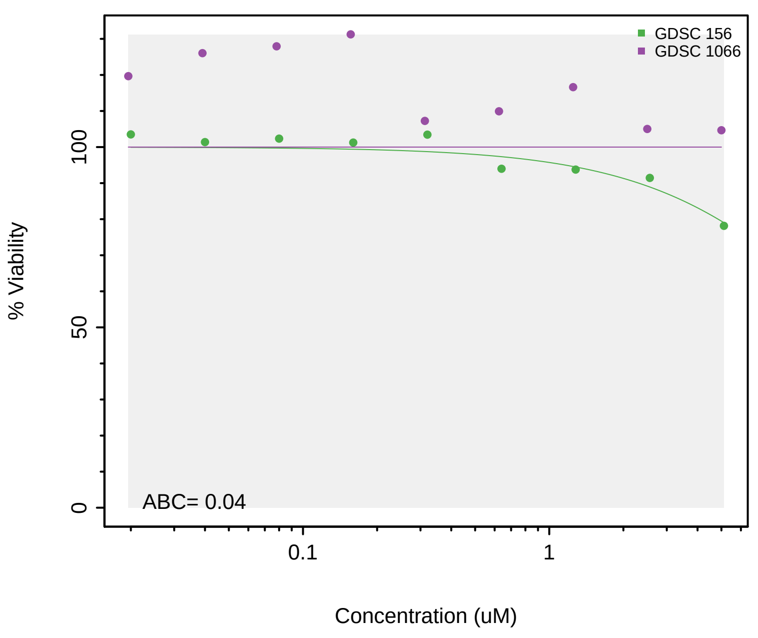

AZD6482:MS−1

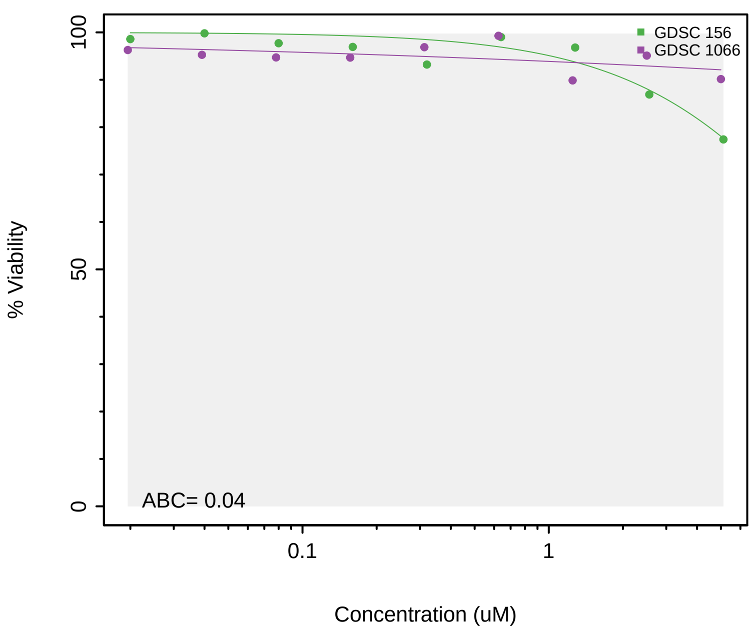

AZD6482:SK−N−AS

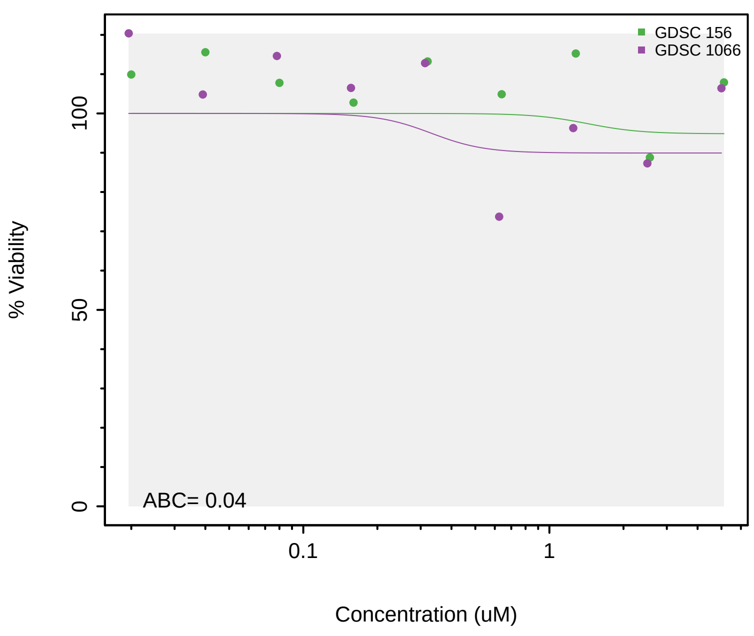

AZD6482:5637

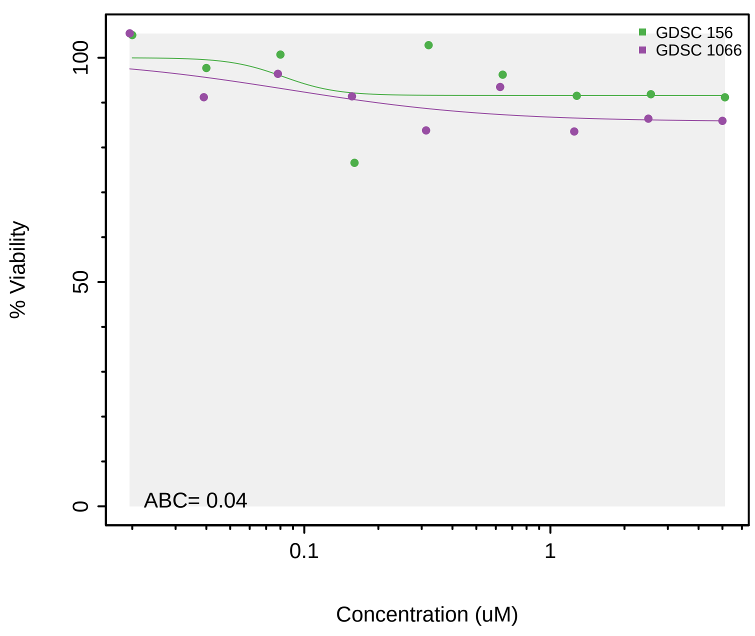

AZD6482:LCLC−97TM1

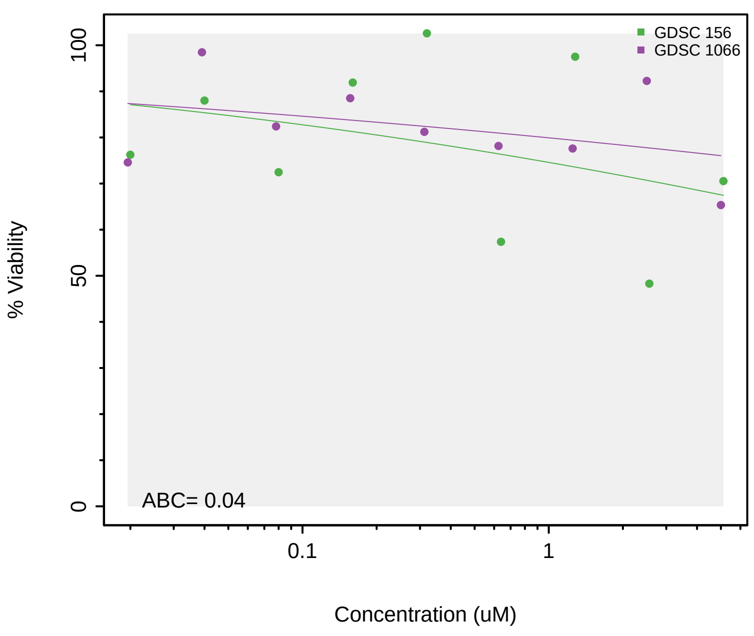

AZD6482:KARPAS−422

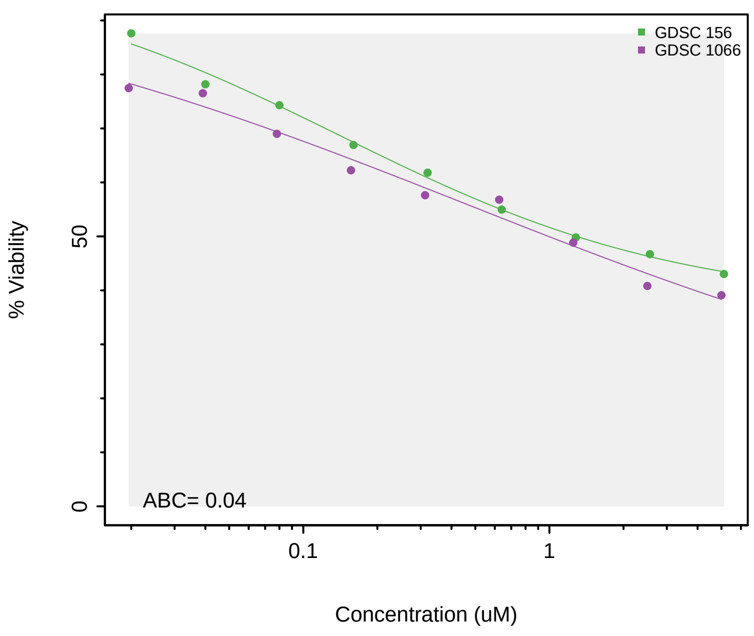

AZD6482:NCI−H1694

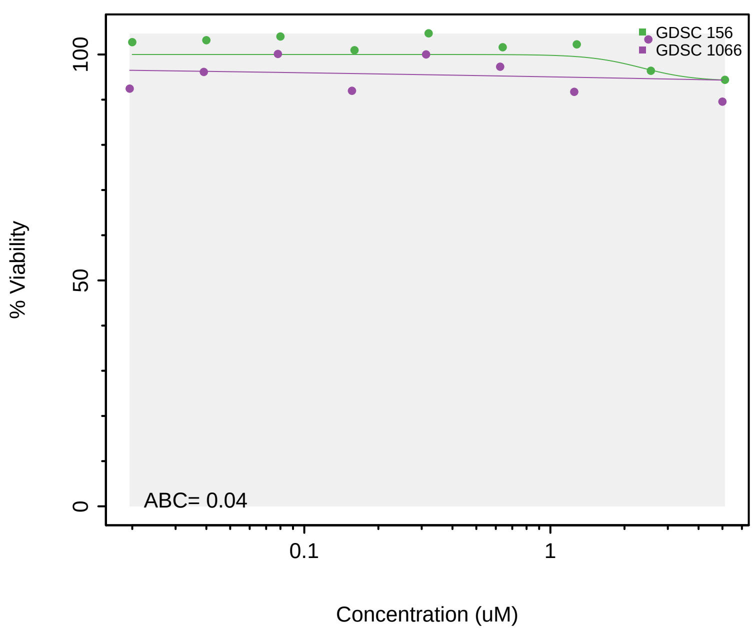

AZD6482:NCI−H596

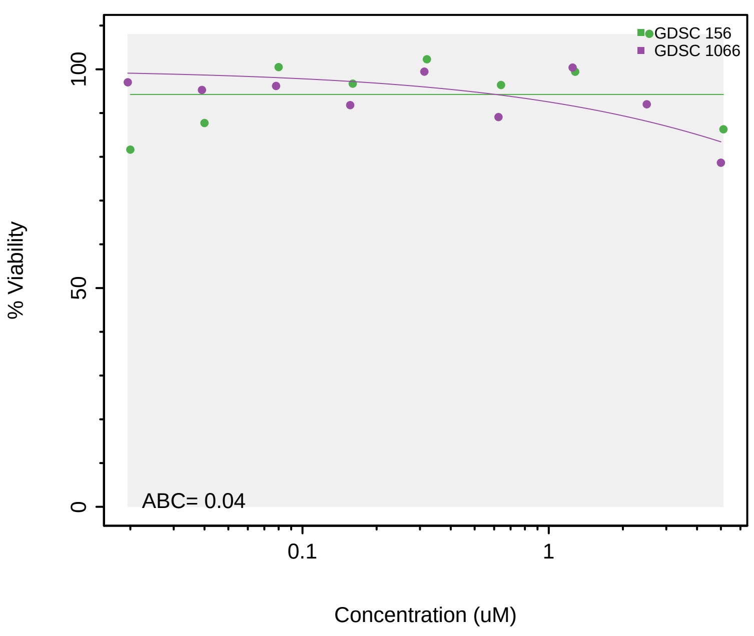

AZD6482:697

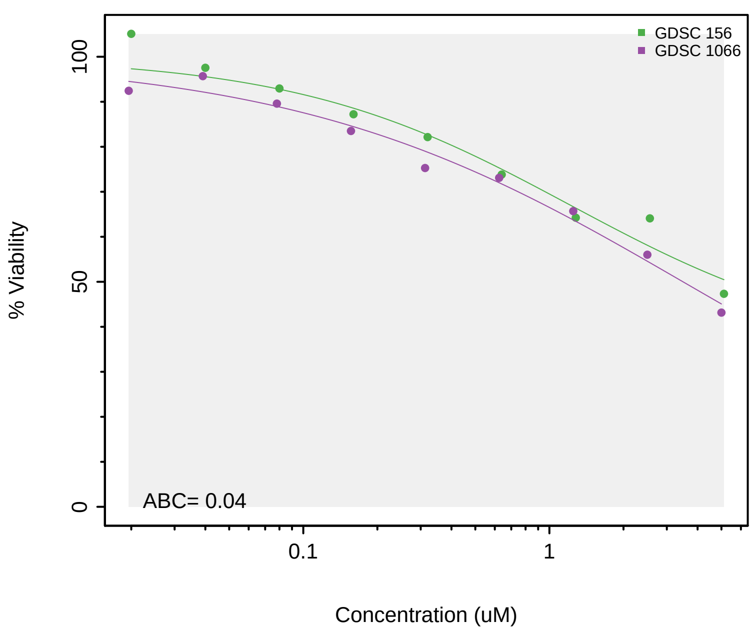

AZD6482:SK−MM−2

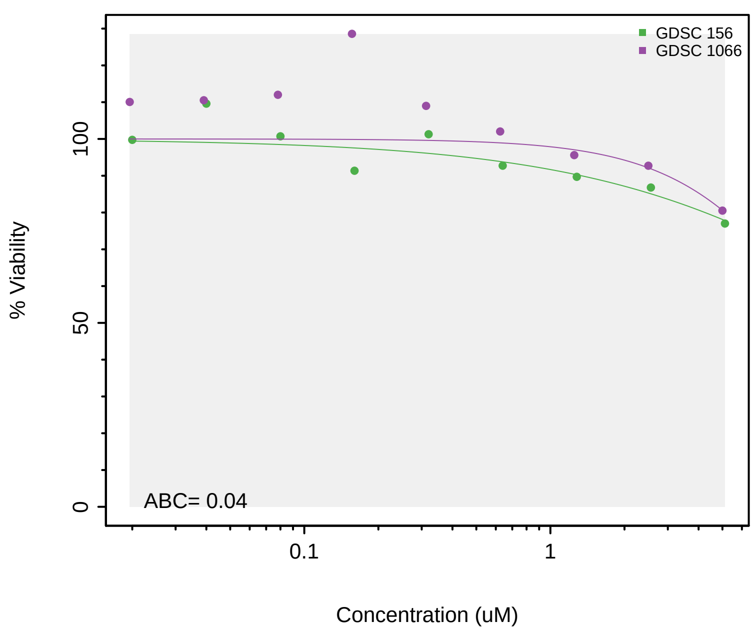

AZD6482:22RV1

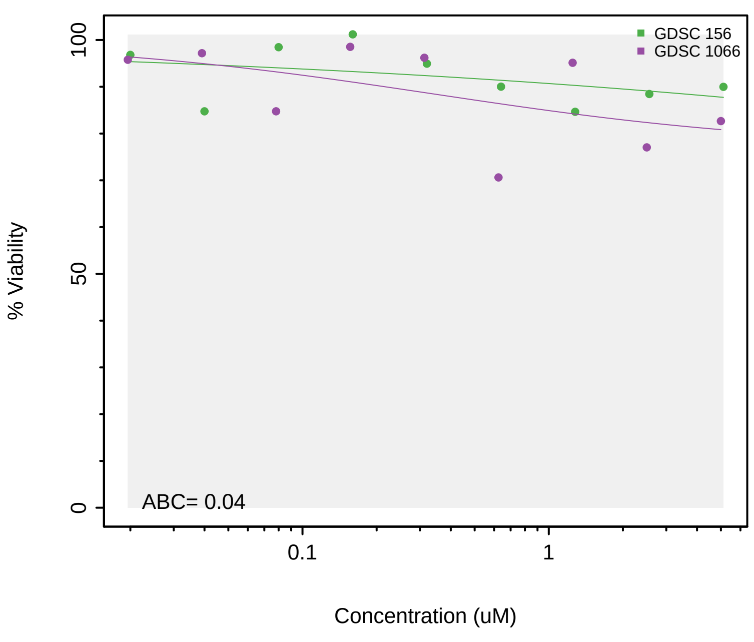

AZD6482:UACC−257

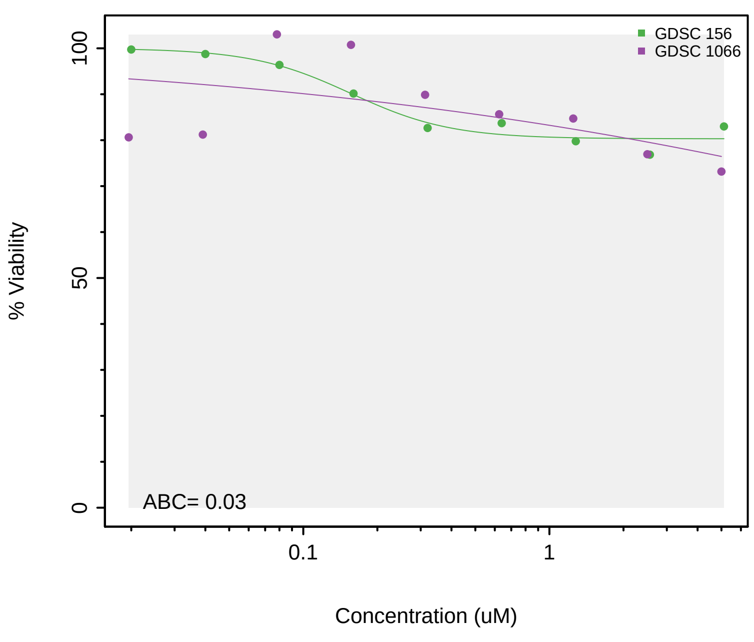

AZD6482:NBsusSR

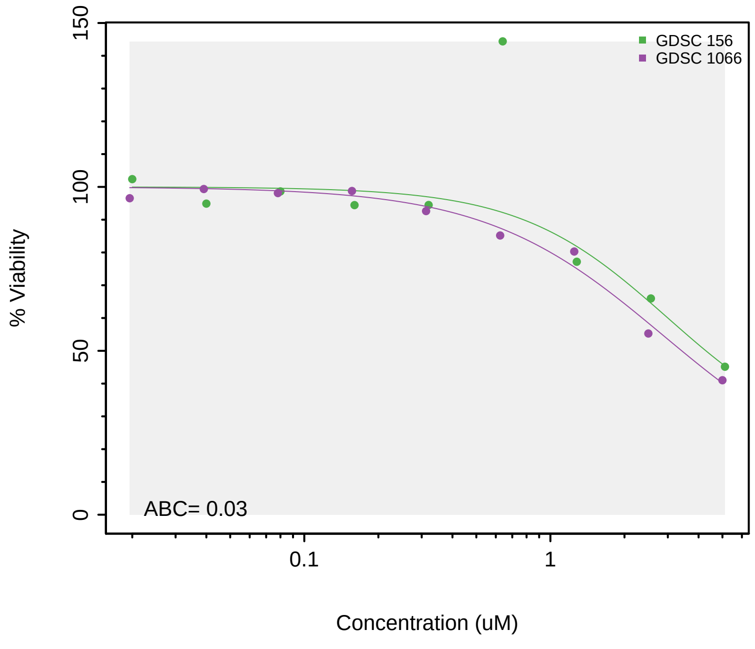

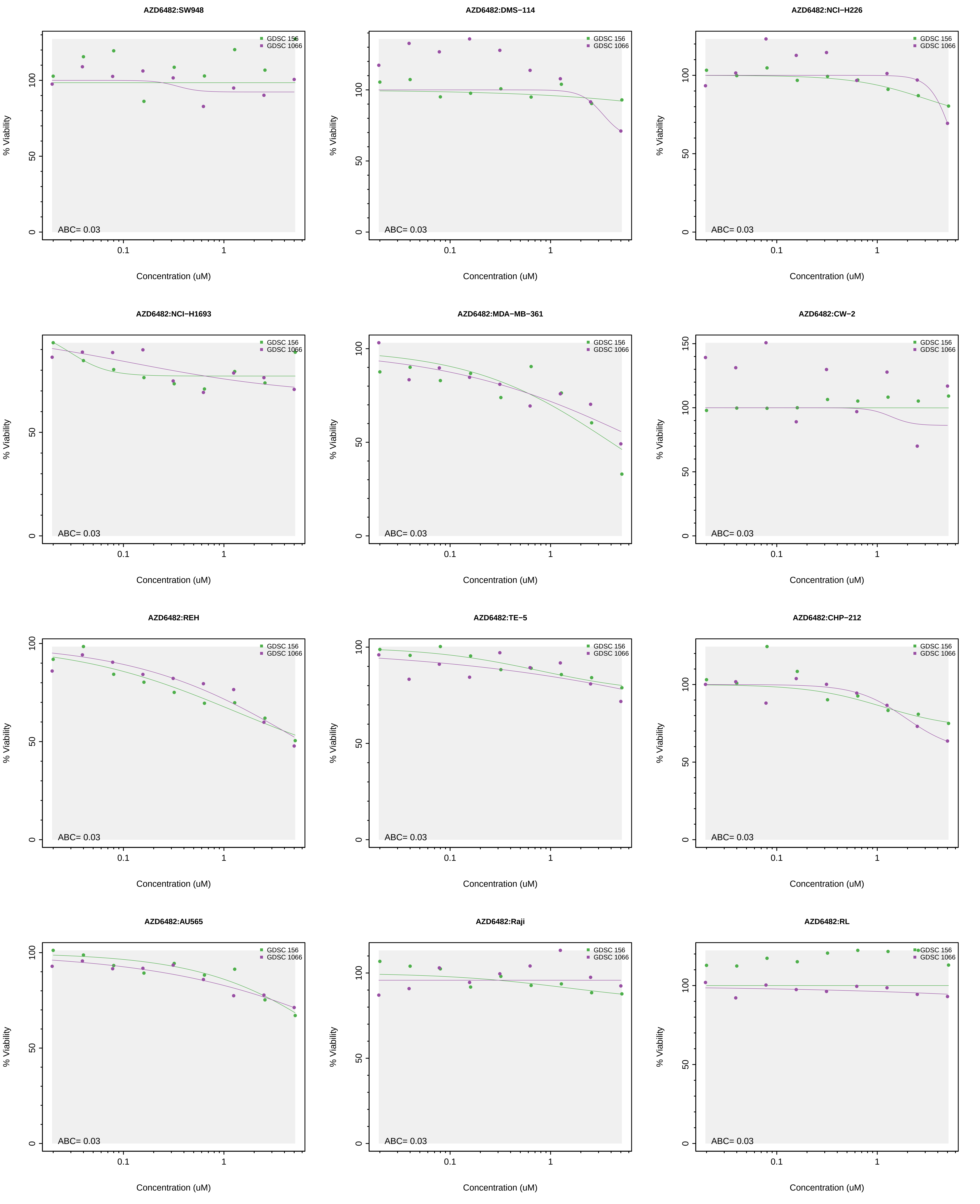
AZD6482:SW948
AZD6482:DMS−114
AZD6482:NCI−H226
AZD6482:NCI−H1693
AZD6482:MDA−MB−361
AZD6482:CW−2
AZD6482:REH
AZD6482:TE−5
AZD6482:CHP−212
AZD6482:AU565
AZD6482:Raji
AZD6482:RL
GDSC 156
GDSC 1066
ABC= 0.03
% Viability
Concentration (uM)

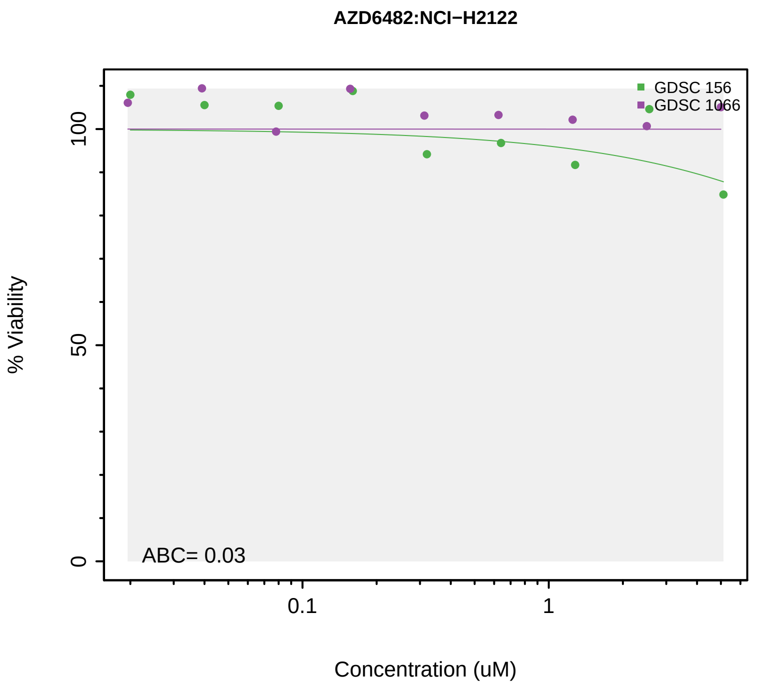
AZD6482:NCI−H2122
GDSC 156
GDSC 1066
ABC= 0.03
% Viability
Concentration (uM)

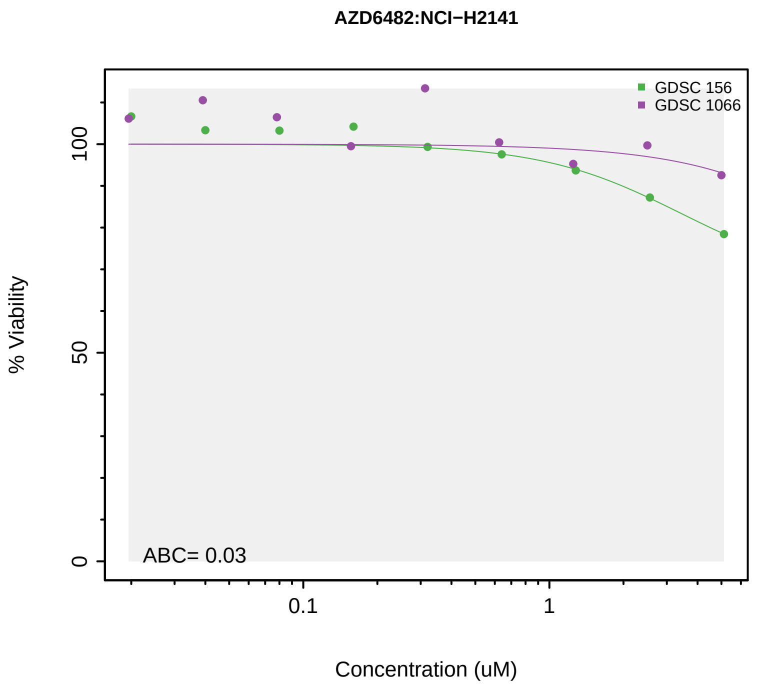
AZD6482:NCI−H2141
GDSC 156
GDSC 1066
ABC= 0.03
% Viability
Concentration (uM)

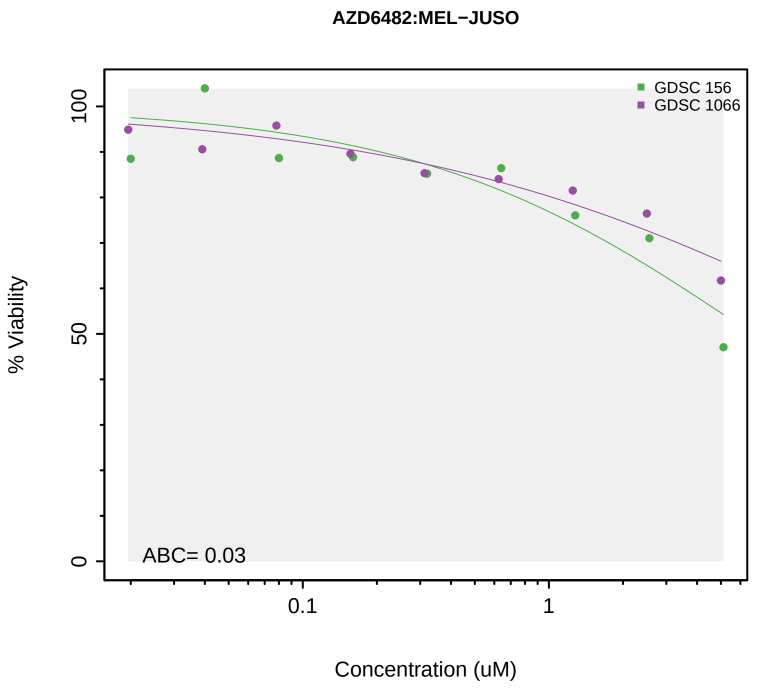
AZD6482:MEL−JUSO
GDSC 156
GDSC 1066
ABC= 0.03
% Viability
Concentration (uM)

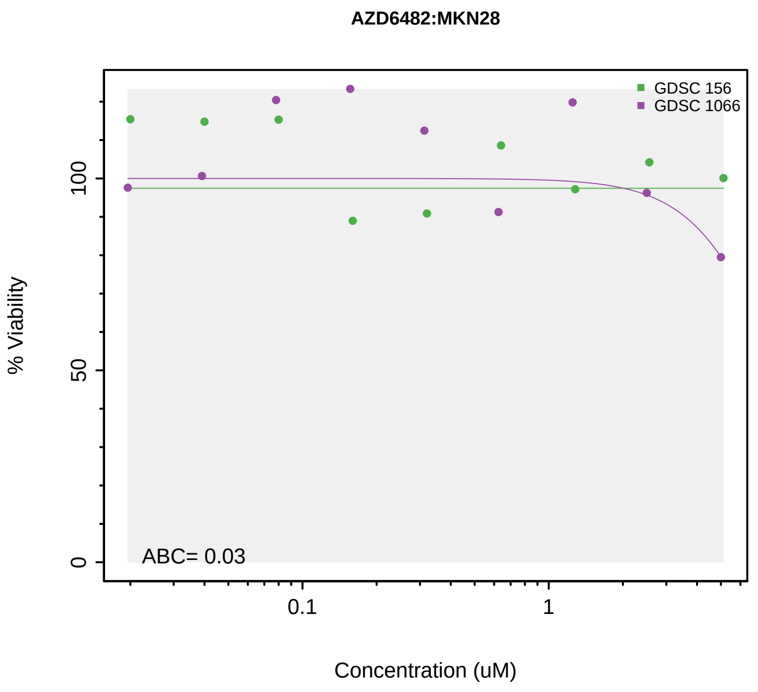
AZD6482:MKN28
GDSC 156
GDSC 1066
ABC= 0.03
% Viability
Concentration (uM)

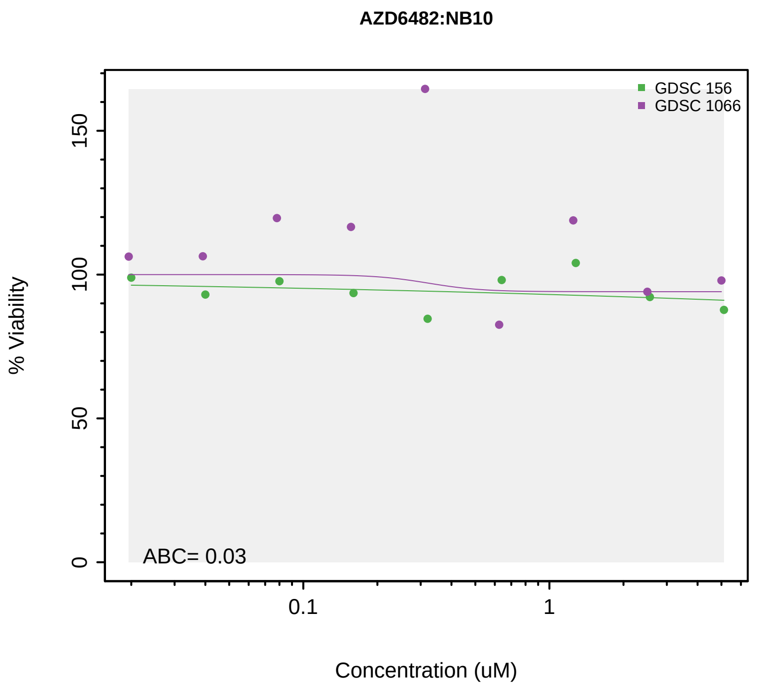
AZD6482:NB10
GDSC 156
GDSC 1066
ABC= 0.03
% Viability
Concentration (uM)

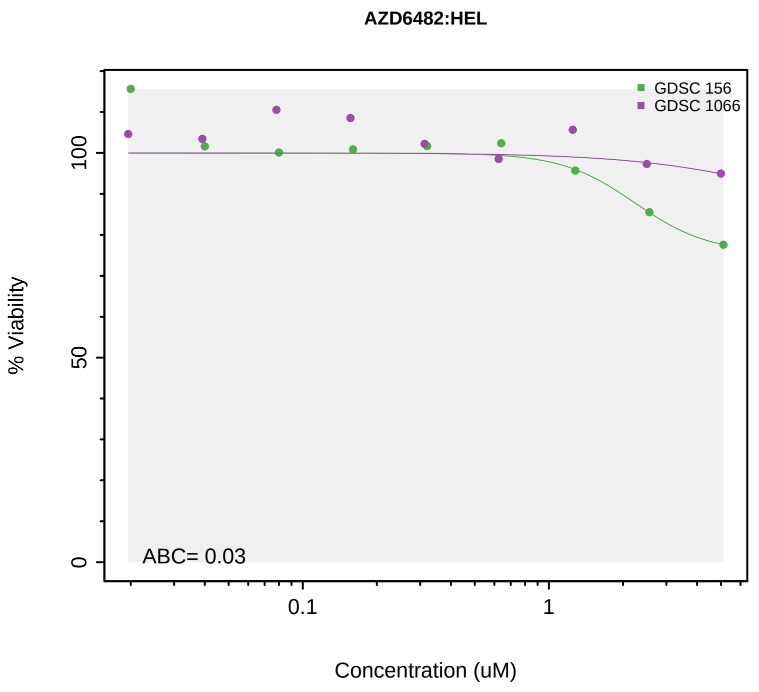
AZD6482:HEL
GDSC 156
GDSC 1066
ABC= 0.03
% Viability
Concentration (uM)

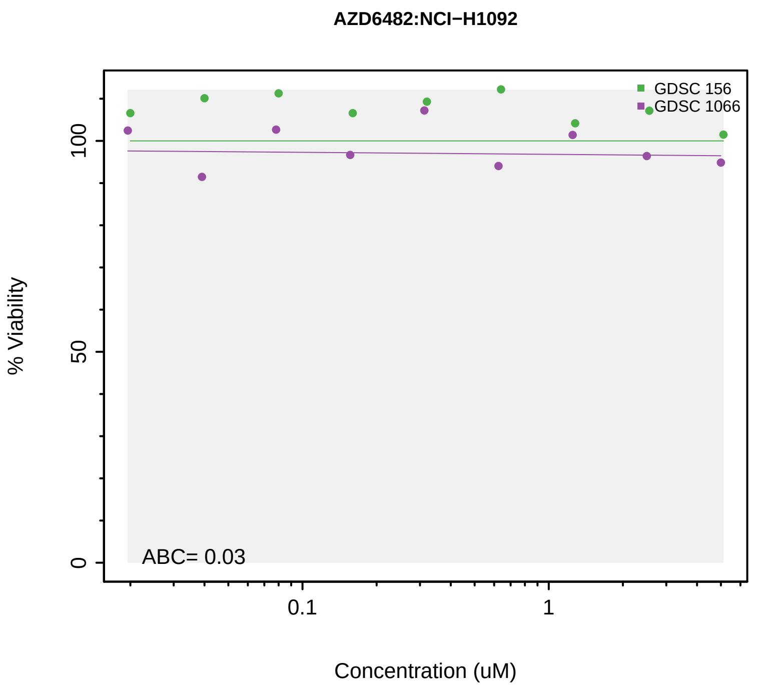
AZD6482:NCI−H1092
GDSC 156
GDSC 1066
ABC= 0.03
% Viability
Concentration (uM)

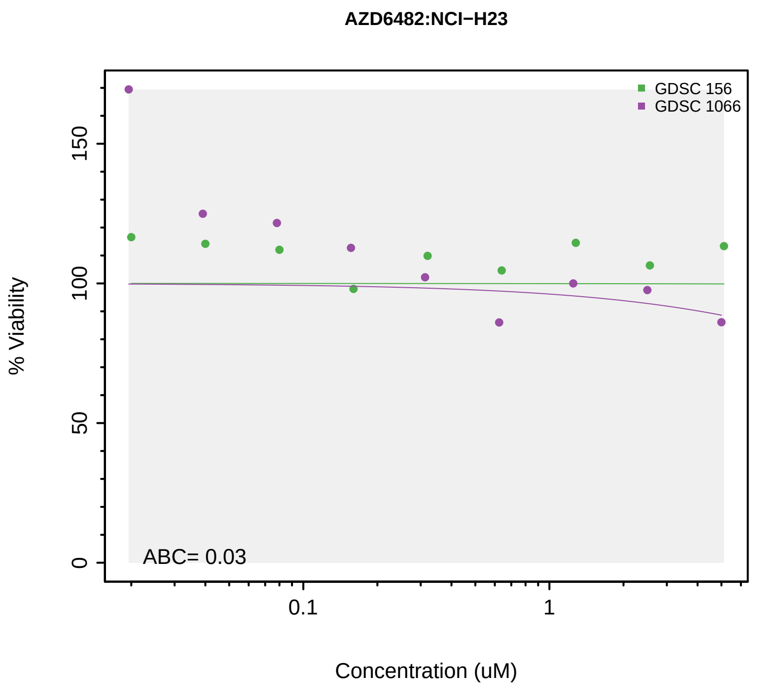
AZD6482:NCI−H23
GDSC 156
GDSC 1066
ABC= 0.03
% Viability
Concentration (uM)

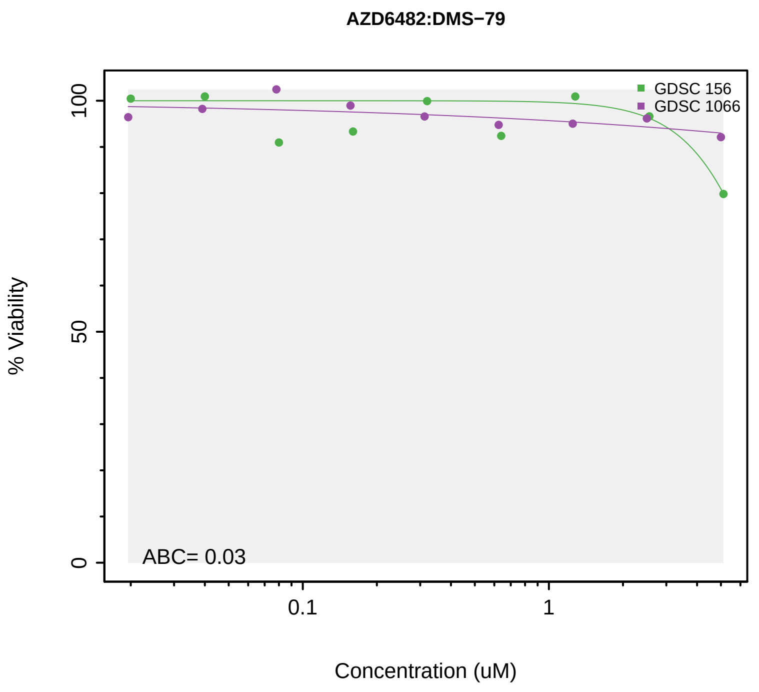
AZD6482:DMS−79
GDSC 156
GDSC 1066
ABC= 0.03
% Viability
Concentration (uM)

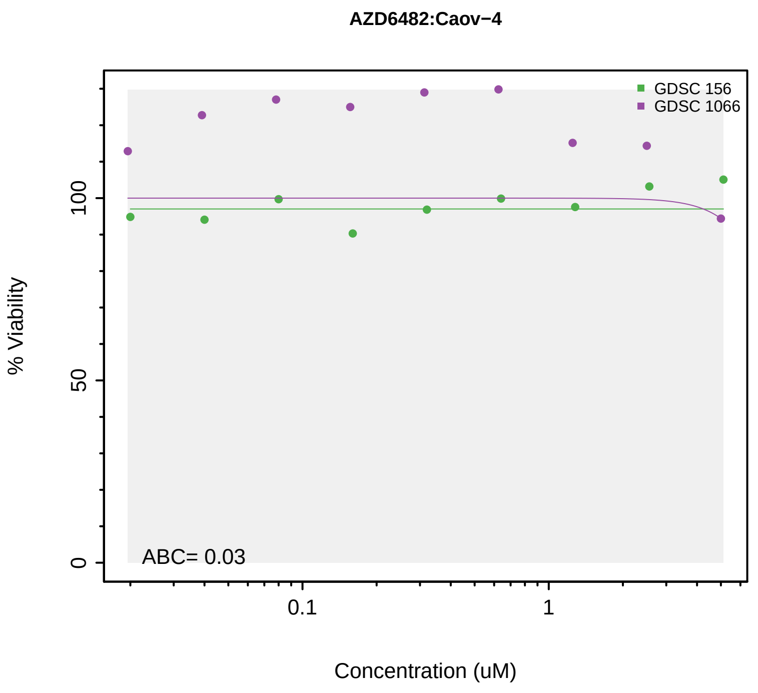
AZD6482:Caov−4
GDSC 156
GDSC 1066
ABC= 0.03
% Viability
Concentration (uM)

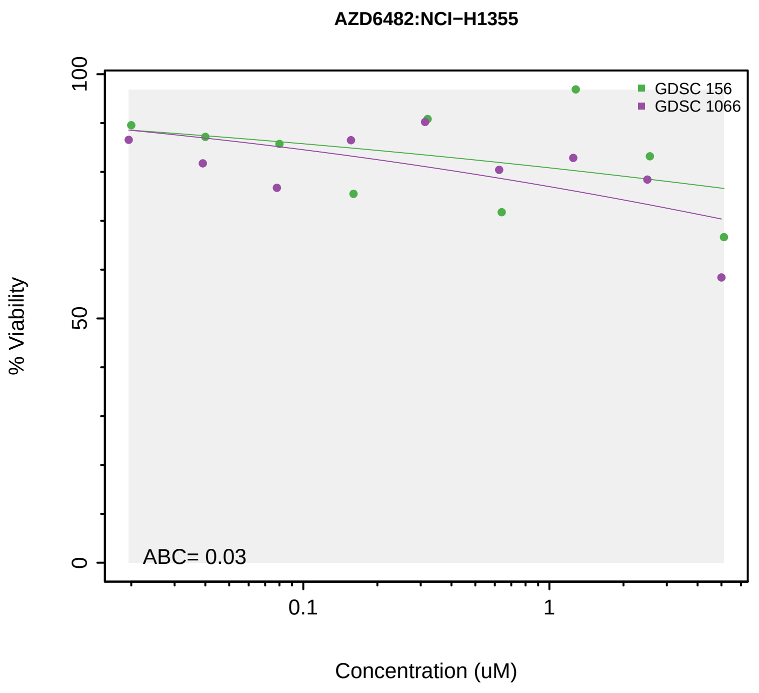
AZD6482:NCI−H1355
GDSC 156
GDSC 1066
ABC= 0.03
% Viability
Concentration (uM)

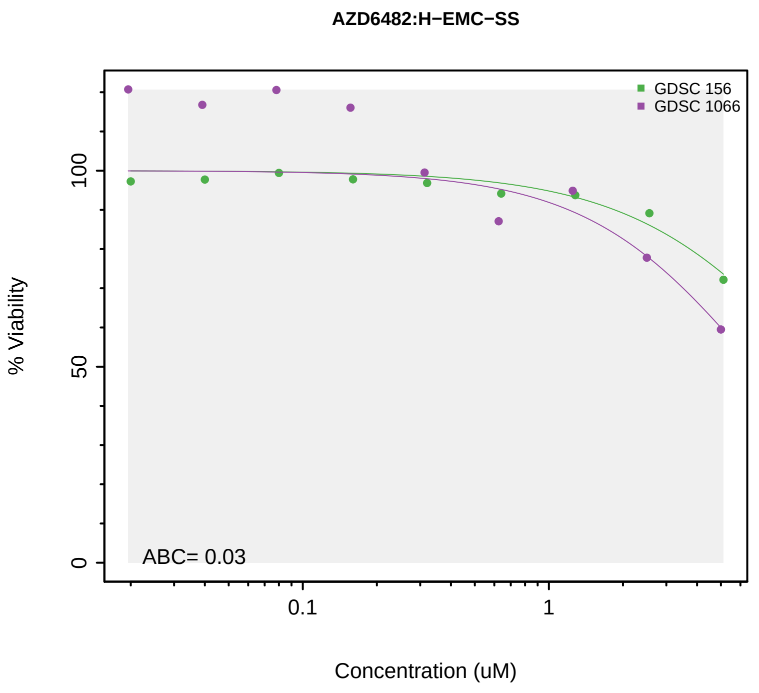
AZD6482:H−EMC−SS
GDSC 156
GDSC 1066
ABC= 0.03
% Viability
Concentration (uM)

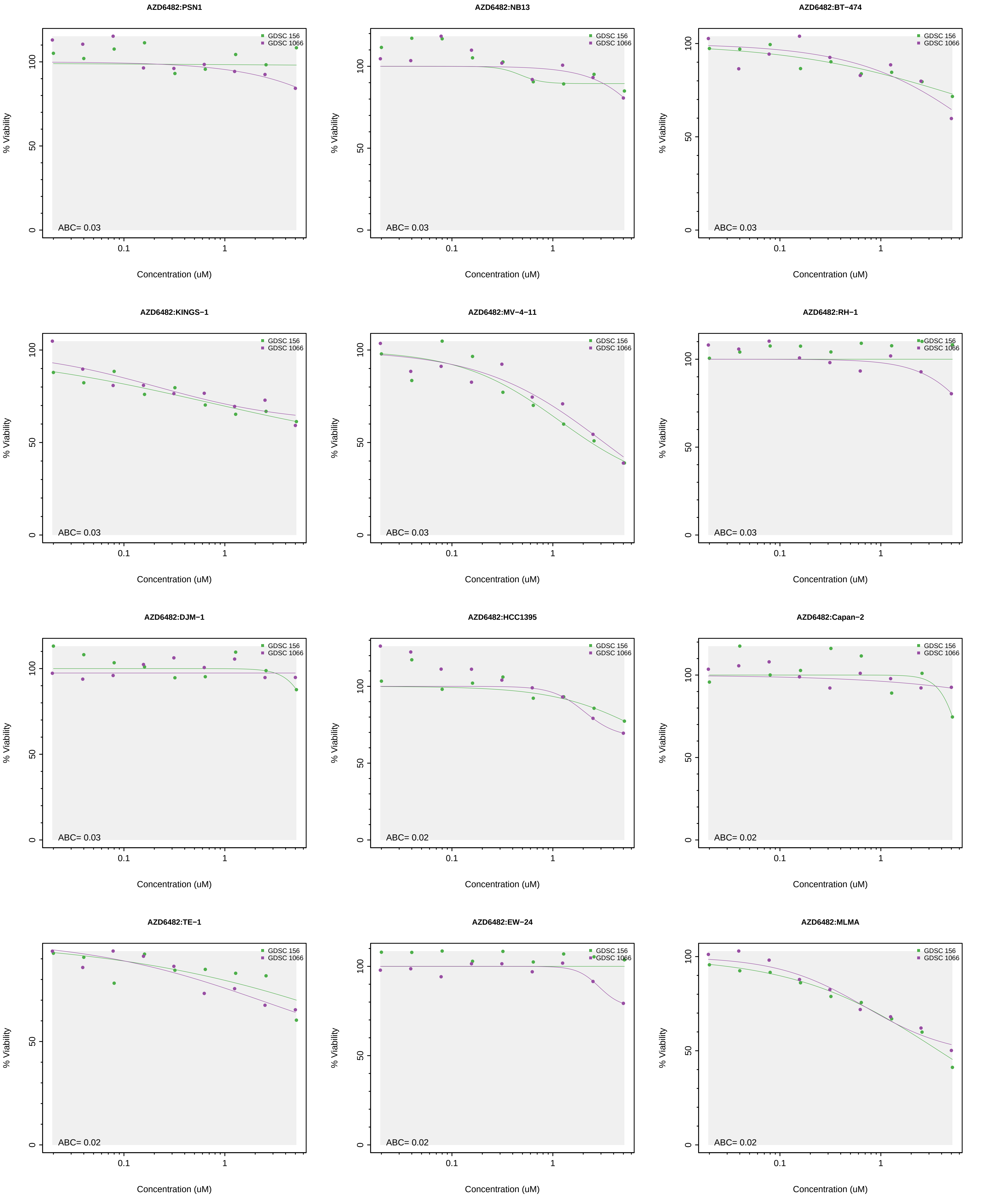
AZD6482:PSN1
ABC= 0.03
AZD6482:NB13
ABC= 0.03
AZD6482:BT−474
ABC= 0.03
AZD6482:KINGS−1
ABC= 0.03
AZD6482:MV−4−11
ABC= 0.03
AZD6482:RH−1
ABC= 0.03
AZD6482:DJM−1
ABC= 0.03
AZD6482:HCC1395
ABC= 0.02
AZD6482:Capan−2
ABC= 0.02
AZD6482:TE−1
ABC= 0.02
AZD6482:EW−24
ABC= 0.02
AZD6482:MLMA
ABC= 0.02
GDSC 156
GDSC 1066
% Viability
Concentration (uM)

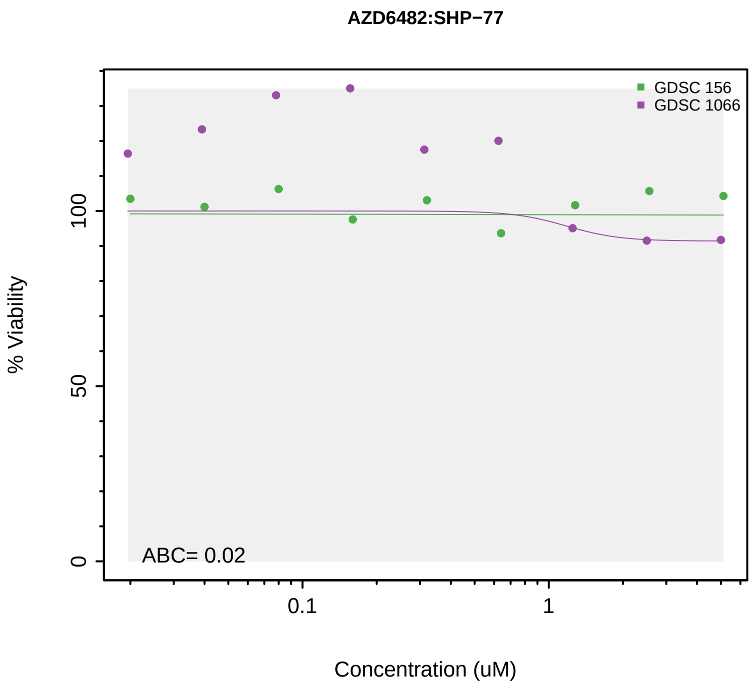
AZD6482:SHP−77
GDSC 156
GDSC 1066
% Viability
0
50
100
ABC= 0.02
0.1
1
Concentration (uM)

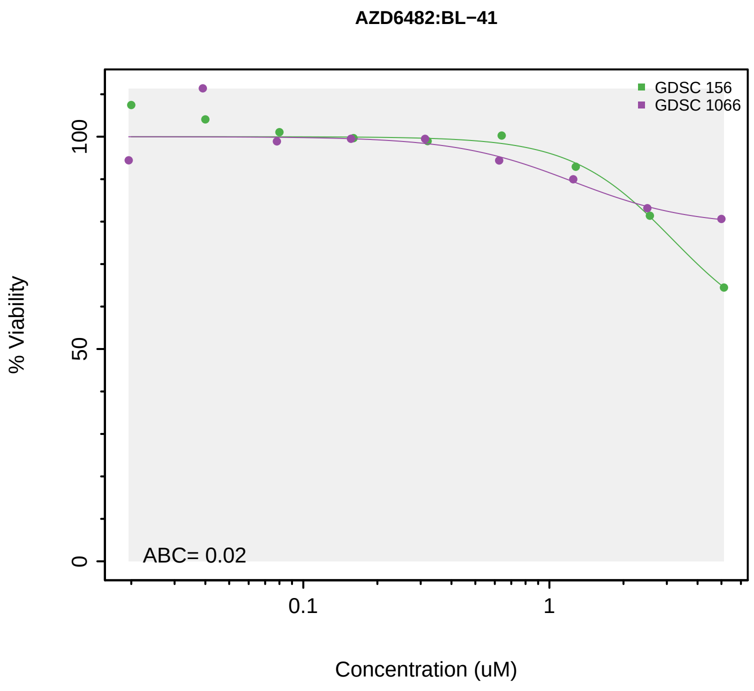
AZD6482:BL−41
GDSC 156
GDSC 1066
% Viability
0
50
100
ABC= 0.02
0.1
1
Concentration (uM)

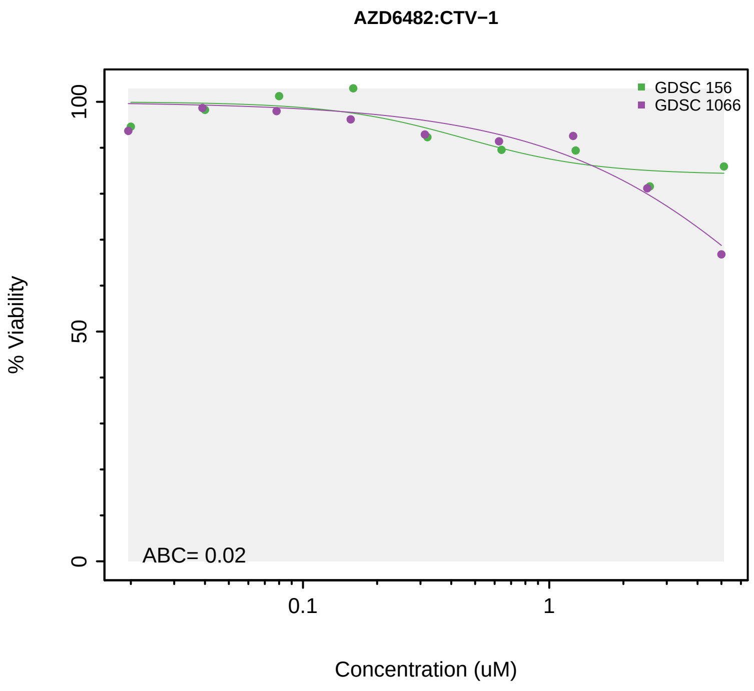
AZD6482:CTV−1
GDSC 156
GDSC 1066
% Viability
0
50
100
ABC= 0.02
0.1
1
Concentration (uM)

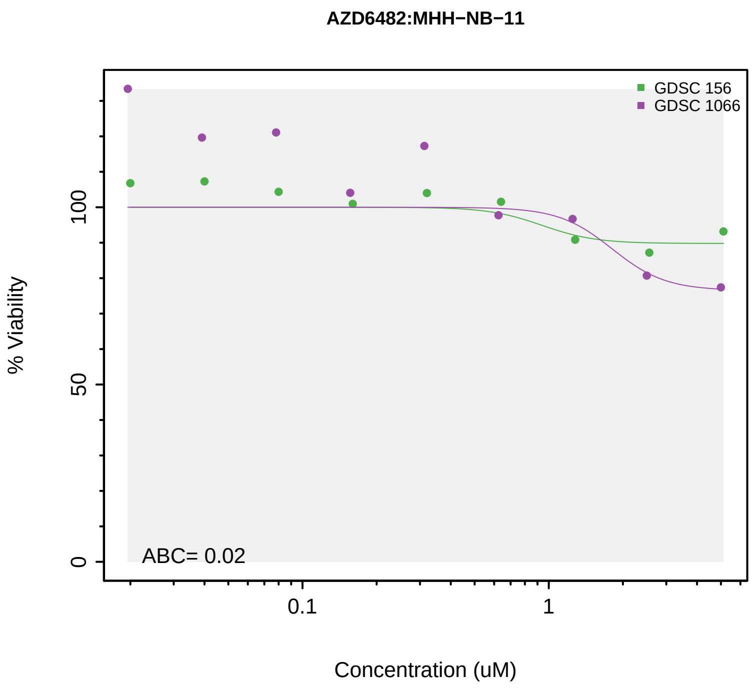
AZD6482:MHH−NB−11
GDSC 156
GDSC 1066
% Viability
0
50
100
ABC= 0.02
0.1
1
Concentration (uM)

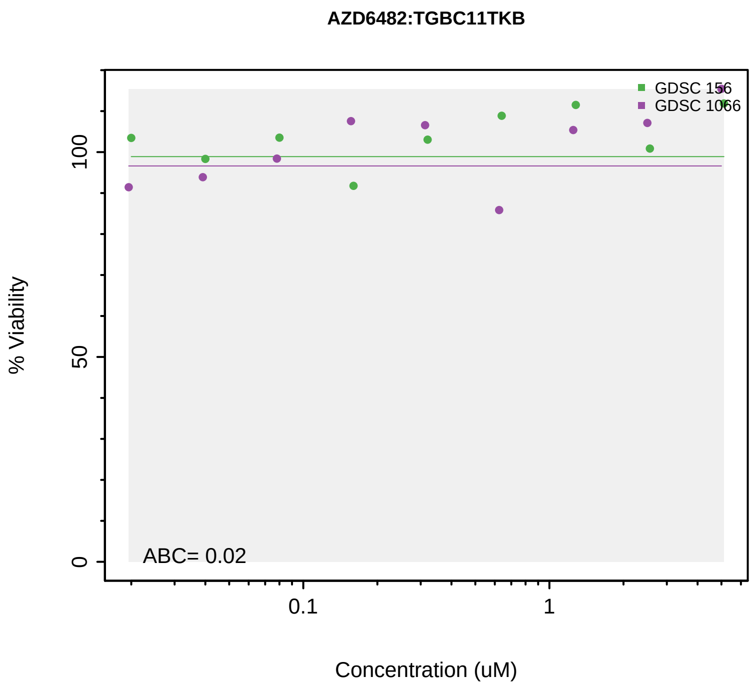
AZD6482:TGBC11TKB
GDSC 156
GDSC 1066
% Viability
0
50
100
ABC= 0.02
0.1
1
Concentration (uM)

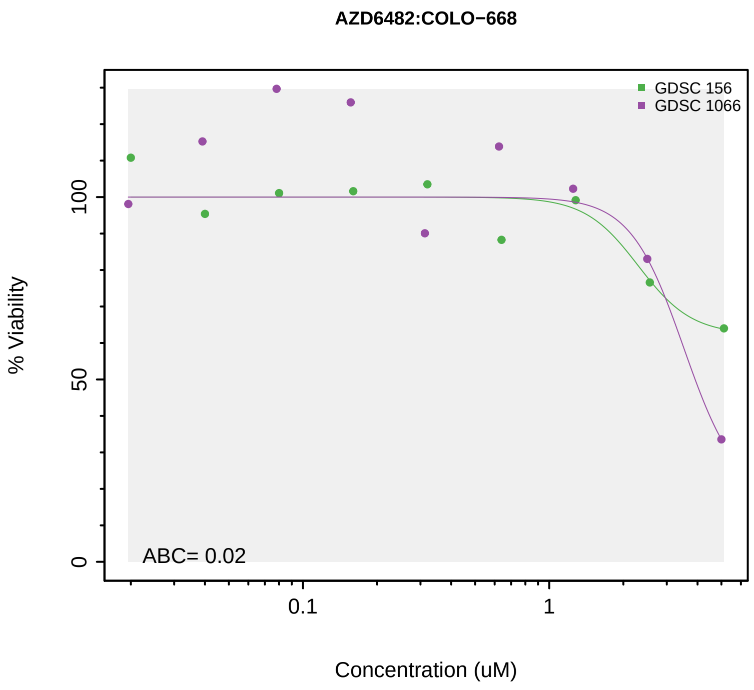
AZD6482:COLO−668
GDSC 156
GDSC 1066
% Viability
0
50
100
ABC= 0.02
0.1
1
Concentration (uM)

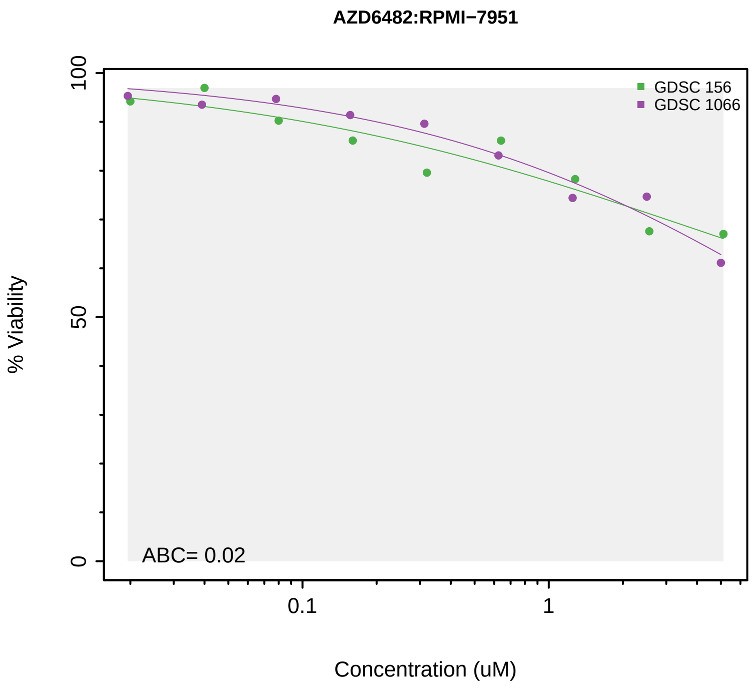
AZD6482:RPMI−7951
GDSC 156
GDSC 1066
% Viability
0
50
100
ABC= 0.02
0.1
1
Concentration (uM)

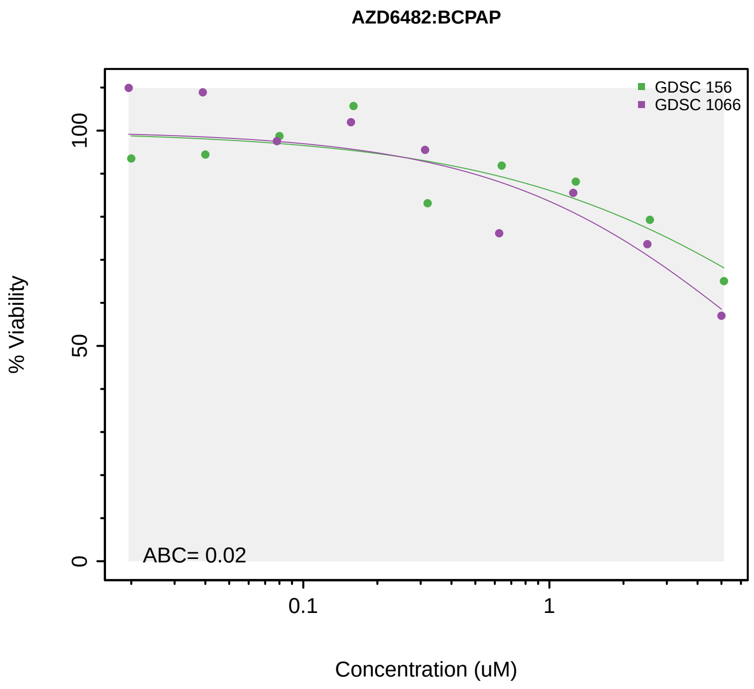
AZD6482:BCPAP
GDSC 156
GDSC 1066
% Viability
0
50
100
ABC= 0.02
0.1
1
Concentration (uM)

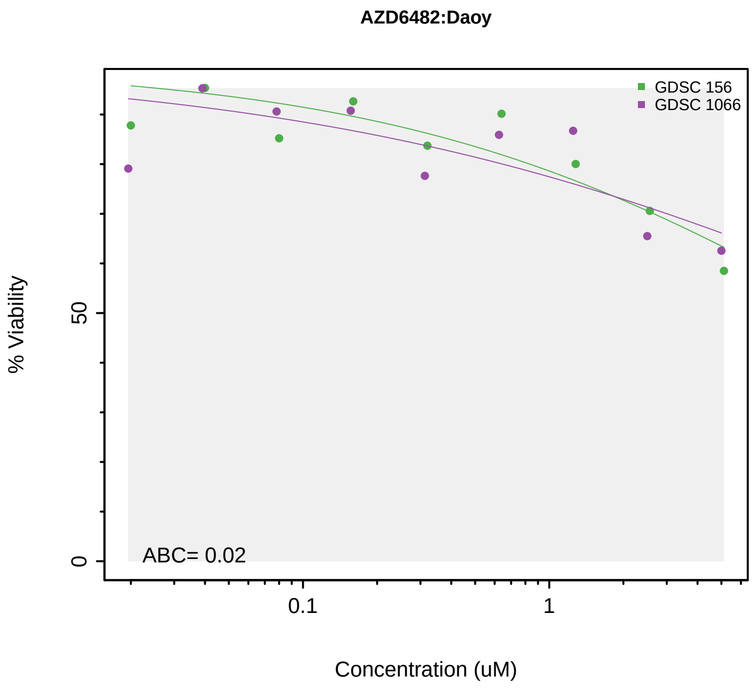
AZD6482:Daoy
GDSC 156
GDSC 1066
% Viability
0
50
ABC= 0.02
0.1
1
Concentration (uM)

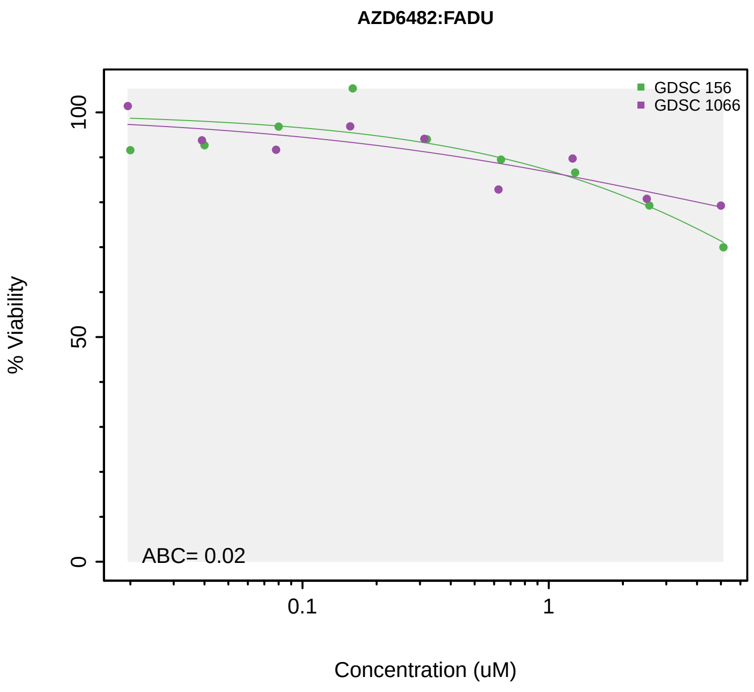
AZD6482:FADU
GDSC 156
GDSC 1066
% Viability
0
50
100
ABC= 0.02
0.1
1
Concentration (uM)

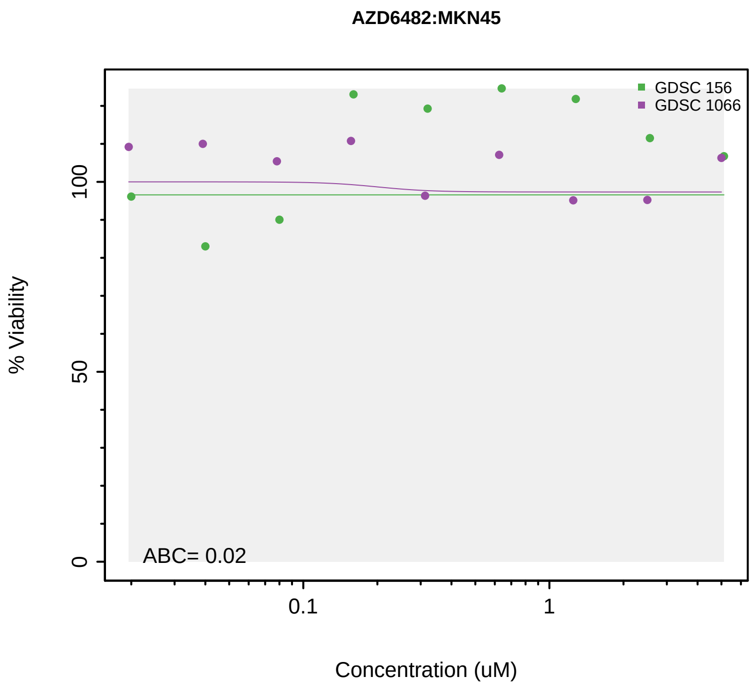
AZD6482:MKN45
GDSC 156
GDSC 1066
% Viability
0
50
100
ABC= 0.02
0.1
1
Concentration (uM)

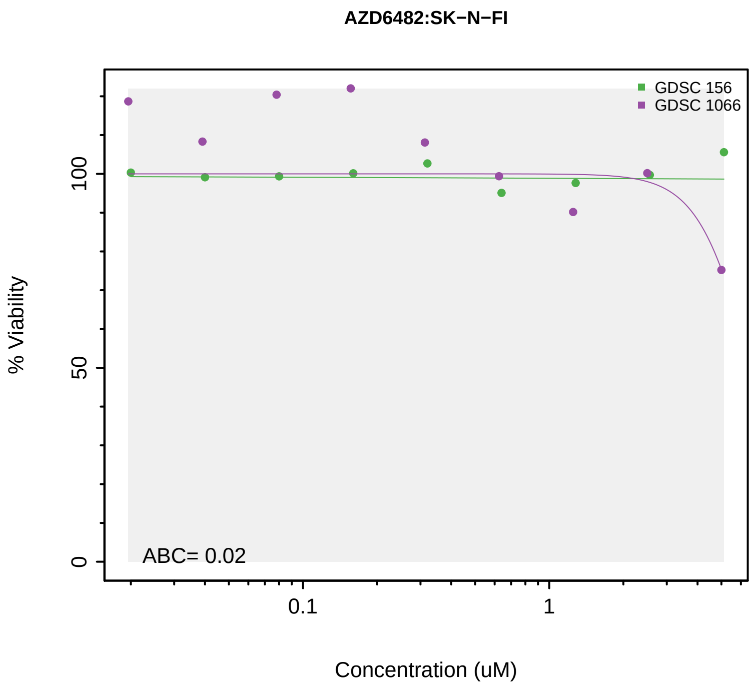
AZD6482:SK−N−FI
GDSC 156
GDSC 1066
% Viability
0
50
100
ABC= 0.02
0.1
1
Concentration (uM)

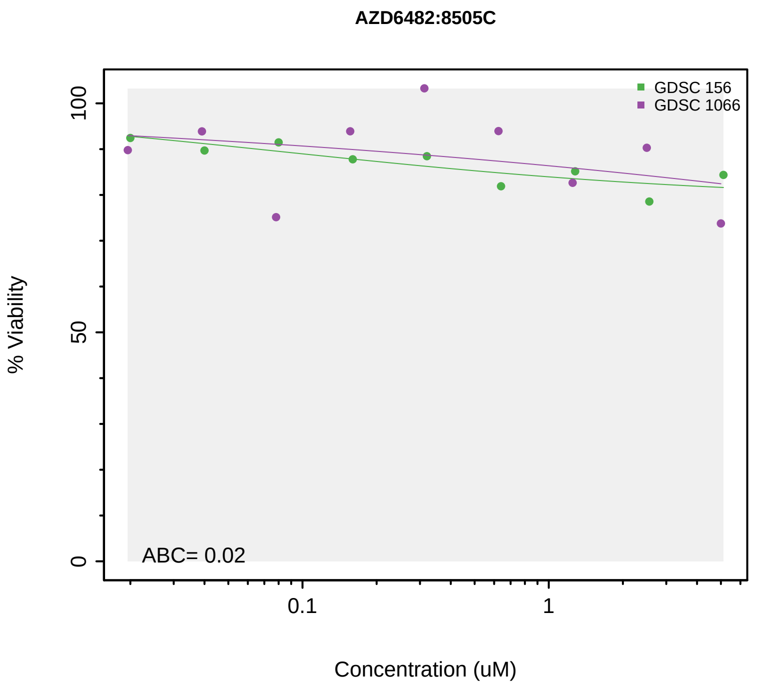
AZD6482:8505C
GDSC 156
GDSC 1066
ABC= 0.02
% Viability
Concentration (uM)

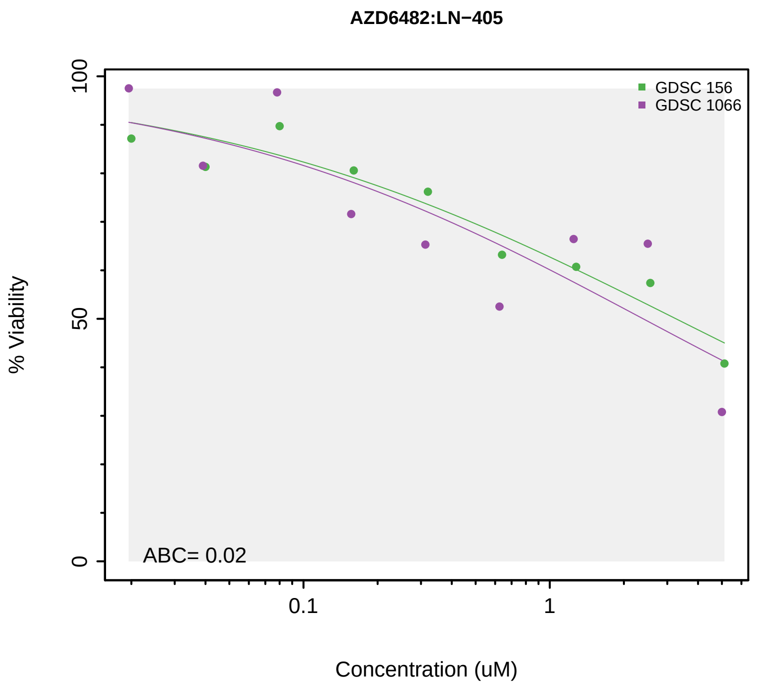
AZD6482:LN−405
GDSC 156
GDSC 1066
ABC= 0.02
% Viability
Concentration (uM)

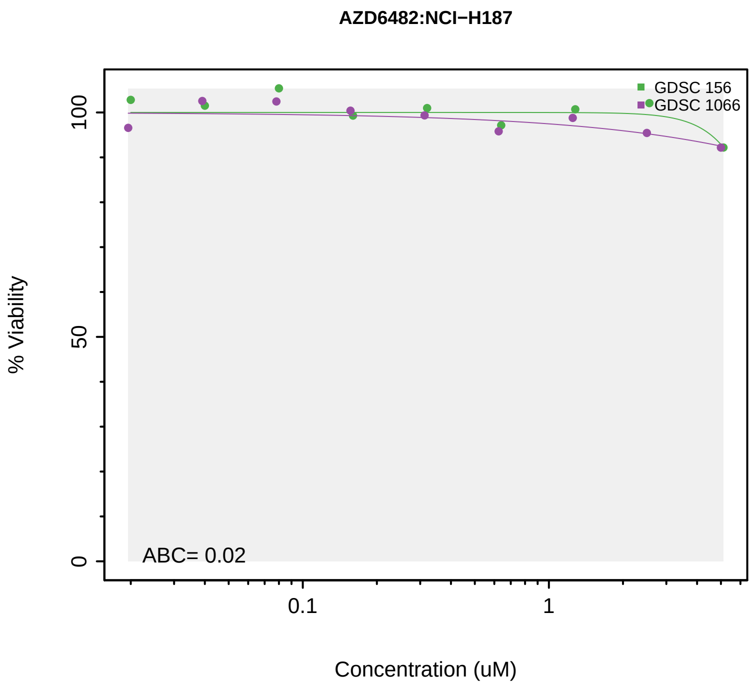
AZD6482:NCI−H187
GDSC 156
GDSC 1066
ABC= 0.02
% Viability
Concentration (uM)

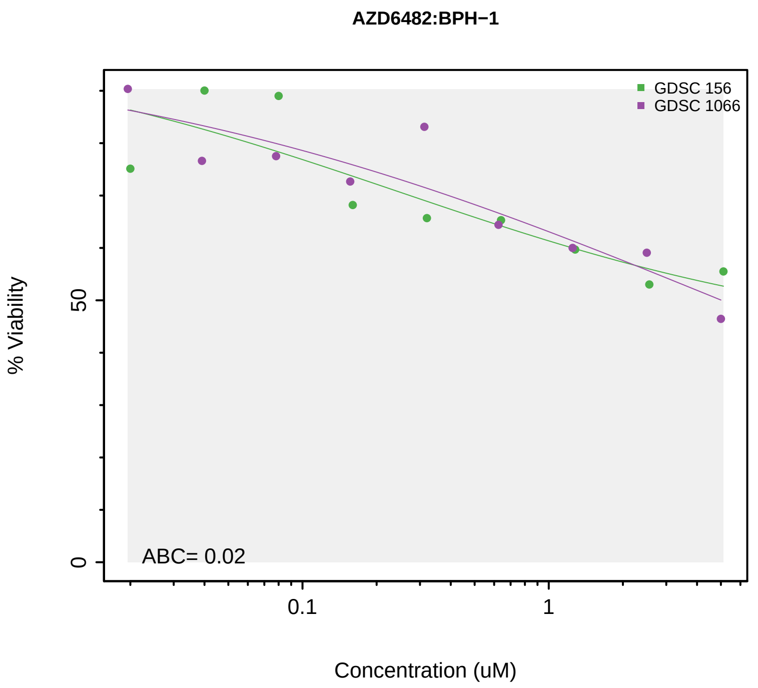
AZD6482:BPH−1
GDSC 156
GDSC 1066
ABC= 0.02
% Viability
Concentration (uM)

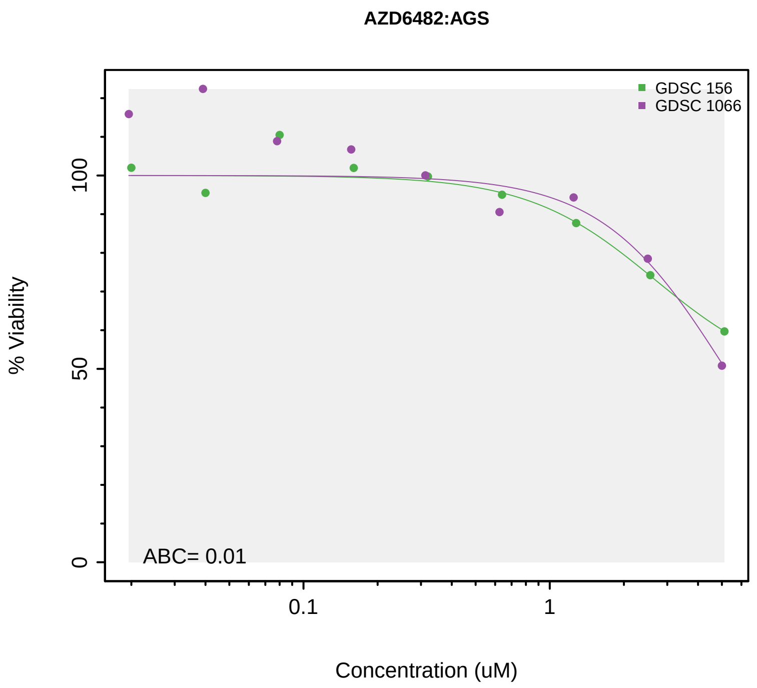
AZD6482:AGS
GDSC 156
GDSC 1066
ABC= 0.01
% Viability
Concentration (uM)

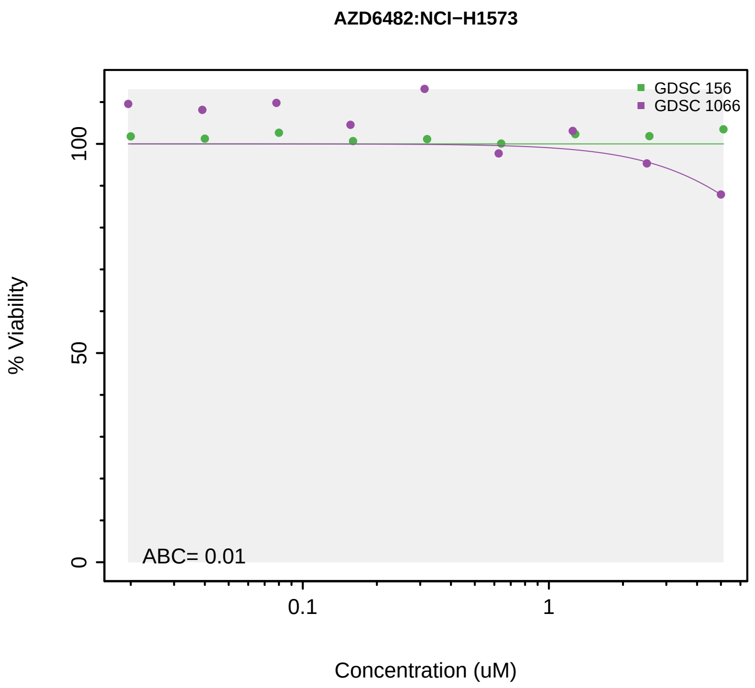
AZD6482:NCI−H1573
GDSC 156
GDSC 1066
ABC= 0.01
% Viability
Concentration (uM)

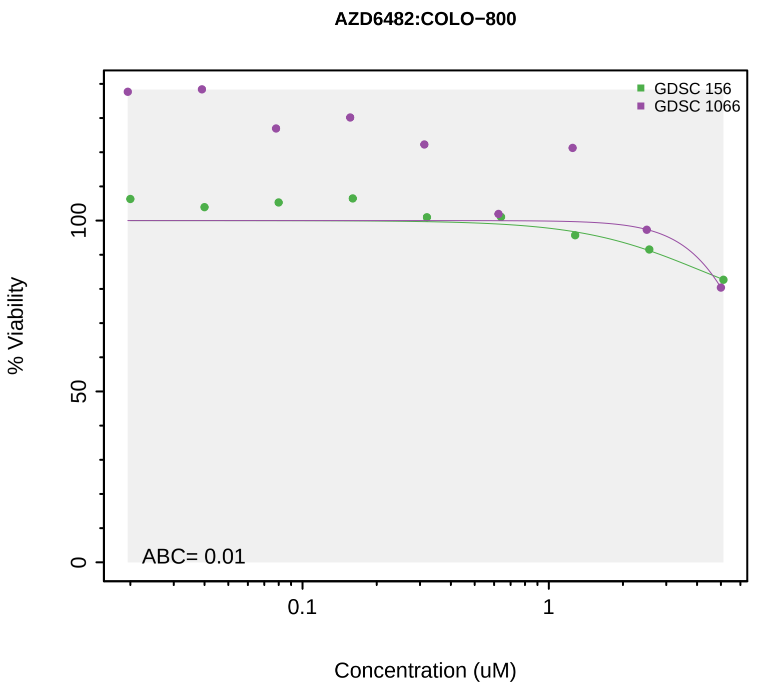
AZD6482:COLO−800
GDSC 156
GDSC 1066
ABC= 0.01
% Viability
Concentration (uM)

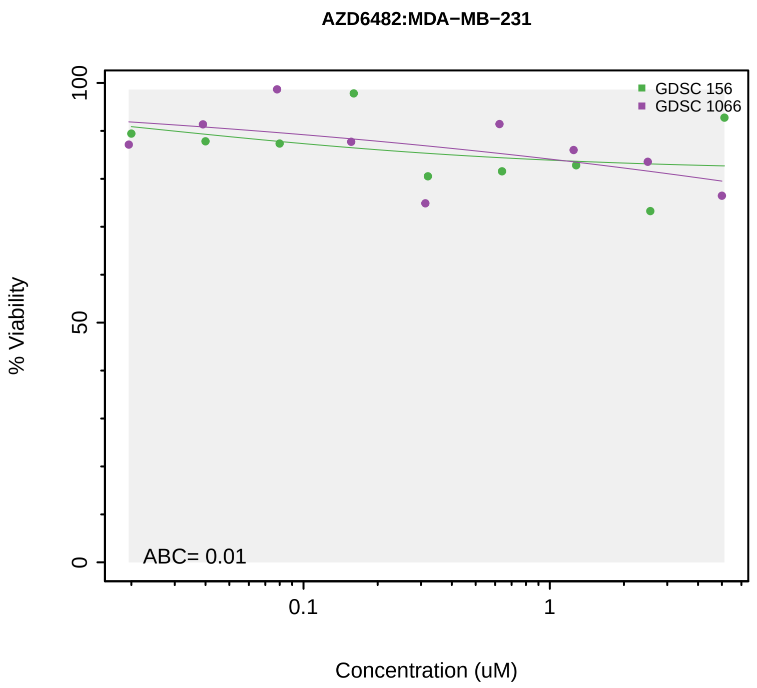
AZD6482:MDA−MB−231
GDSC 156
GDSC 1066
ABC= 0.01
% Viability
Concentration (uM)

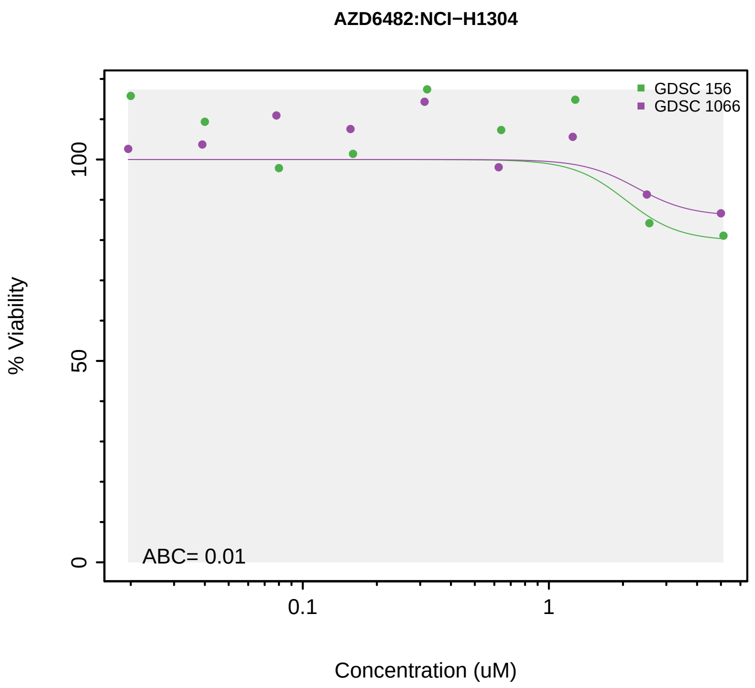
AZD6482:NCI−H1304
GDSC 156
GDSC 1066
ABC= 0.01
% Viability
Concentration (uM)

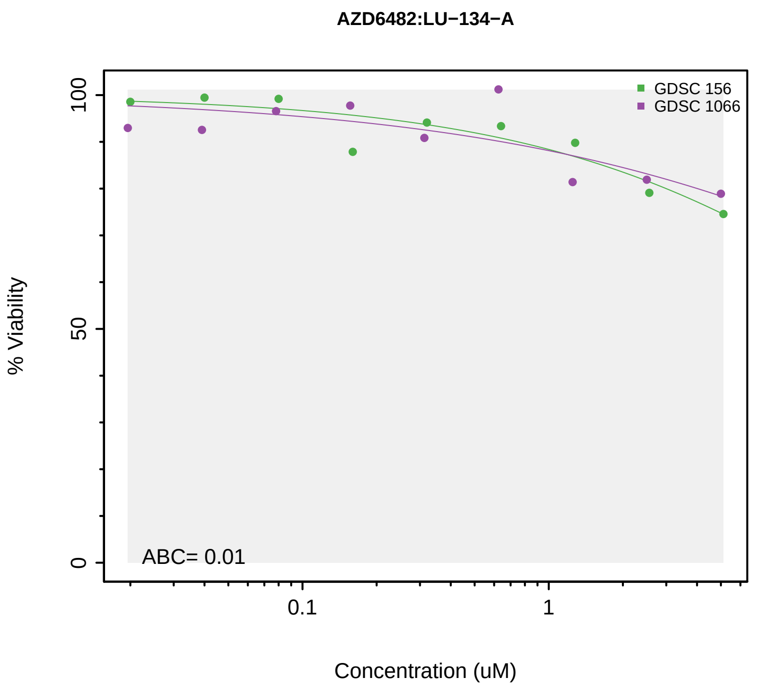
AZD6482:LU−134−A
GDSC 156
GDSC 1066
ABC= 0.01
% Viability
Concentration (uM)

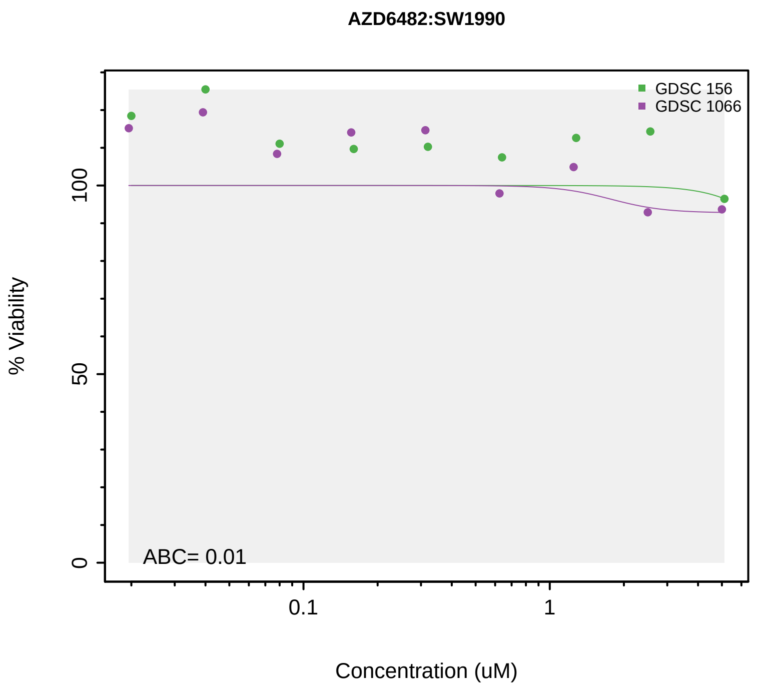
AZD6482:SW1990
GDSC 156
GDSC 1066
ABC= 0.01
% Viability
Concentration (uM)

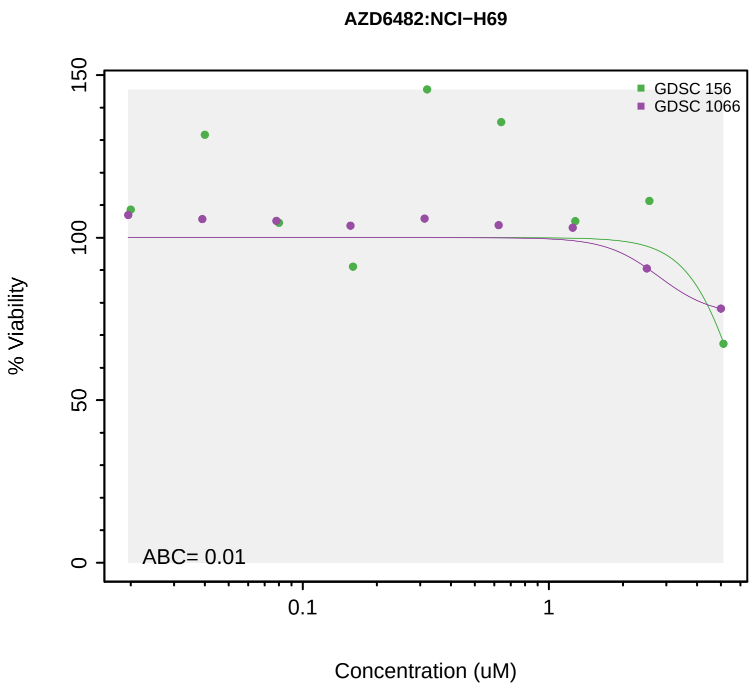
AZD6482:NCI−H69
GDSC 156
GDSC 1066
ABC= 0.01
% Viability
Concentration (uM)

AZD6482:NCI−H1703

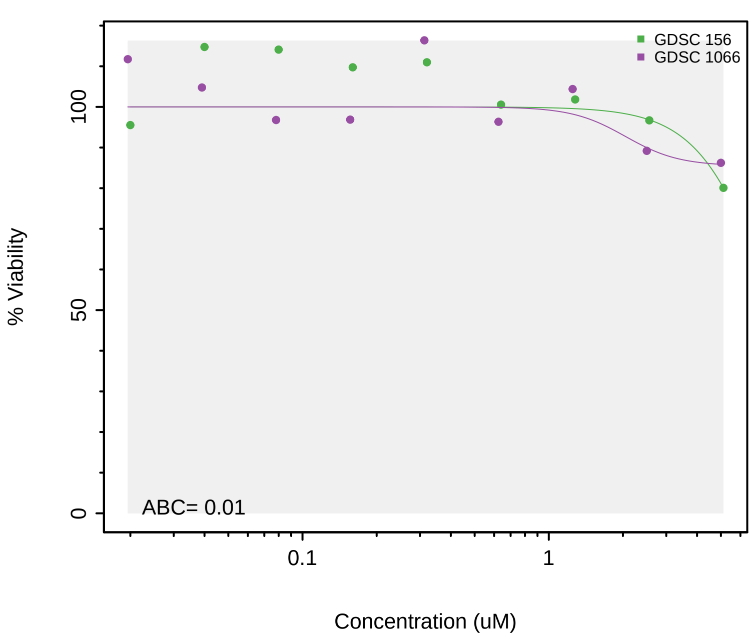

AZD6482:NCI−H1963

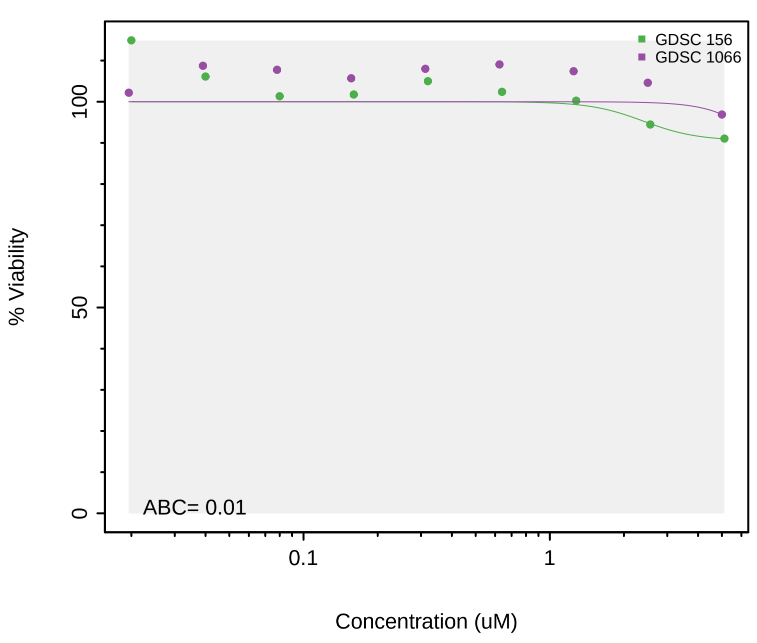

AZD6482:SBC−1

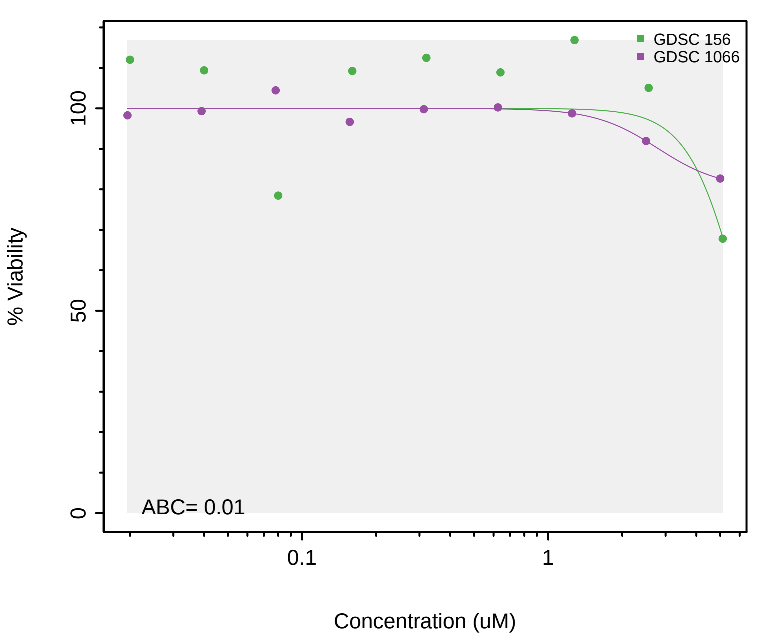

AZD6482:NCI−H2227

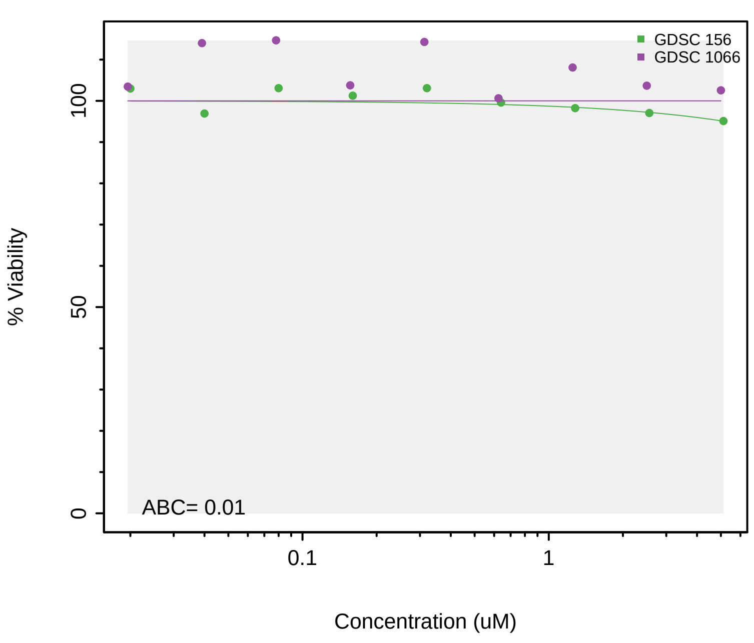

AZD6482:COLO−741

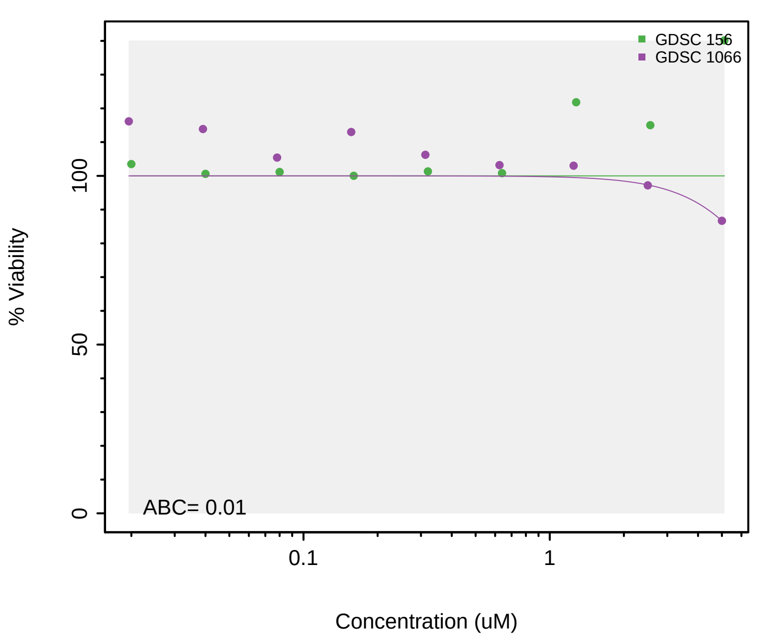

AZD6482:Saos−2

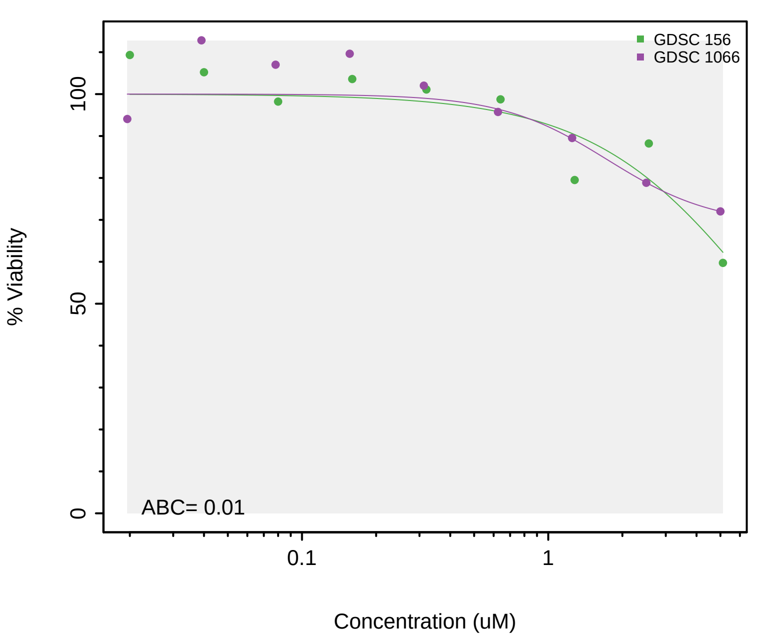

AZD6482:OCI−AML2

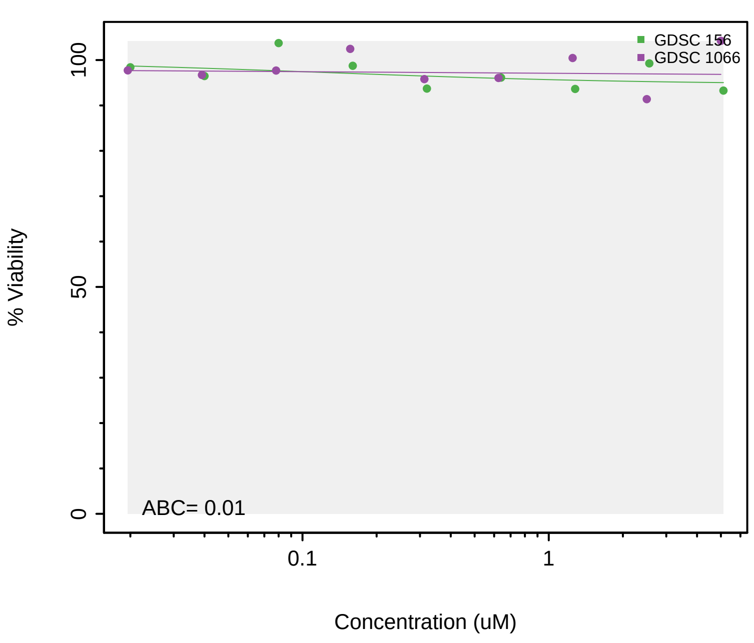

AZD6482:LU−135

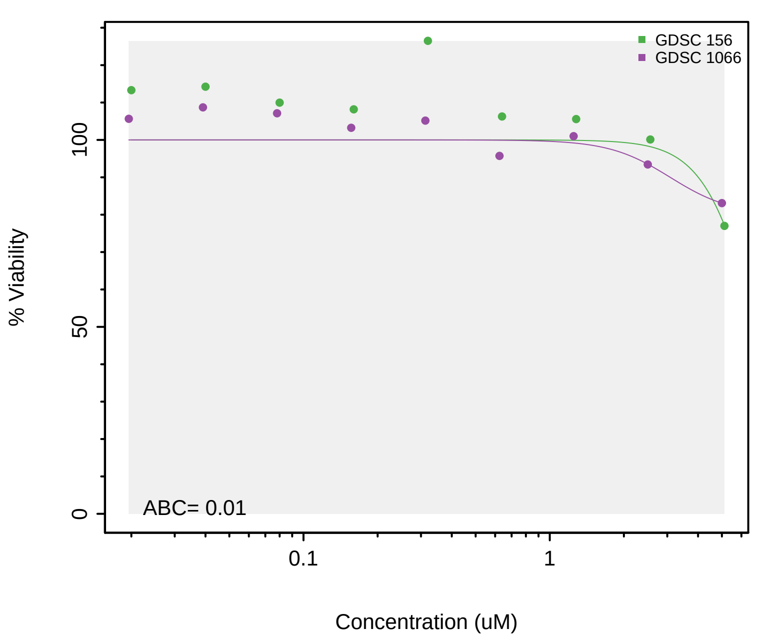

AZD6482:P31−FUJ

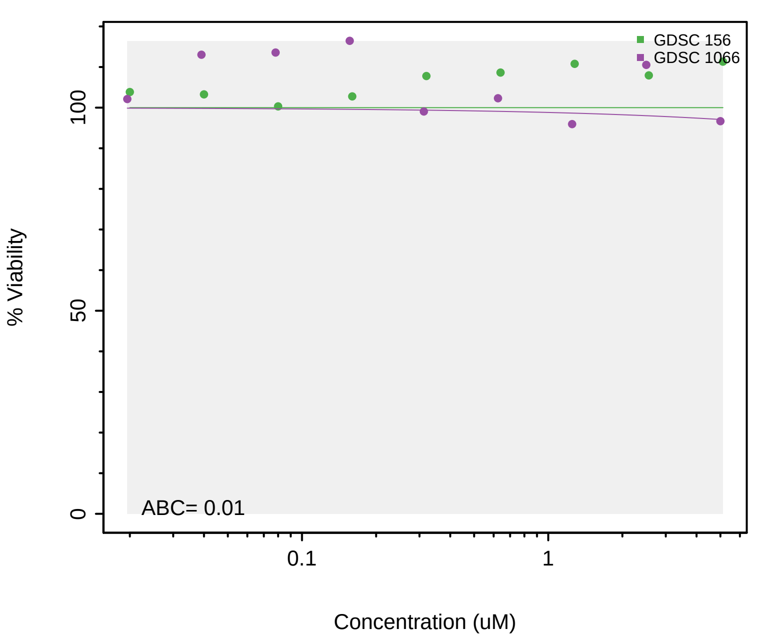

AZD6482:NCI−H720

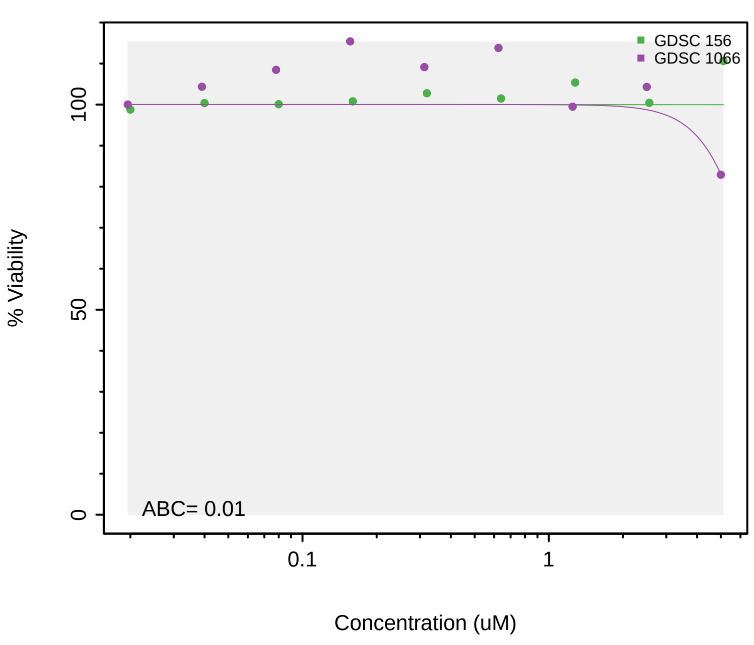

AZD6482:LU−99A

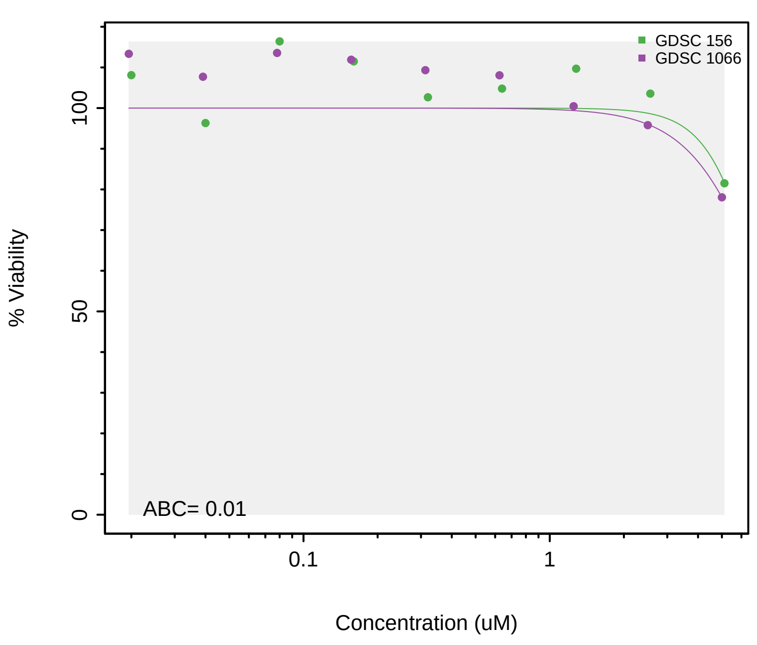

AZD6482:BOKU

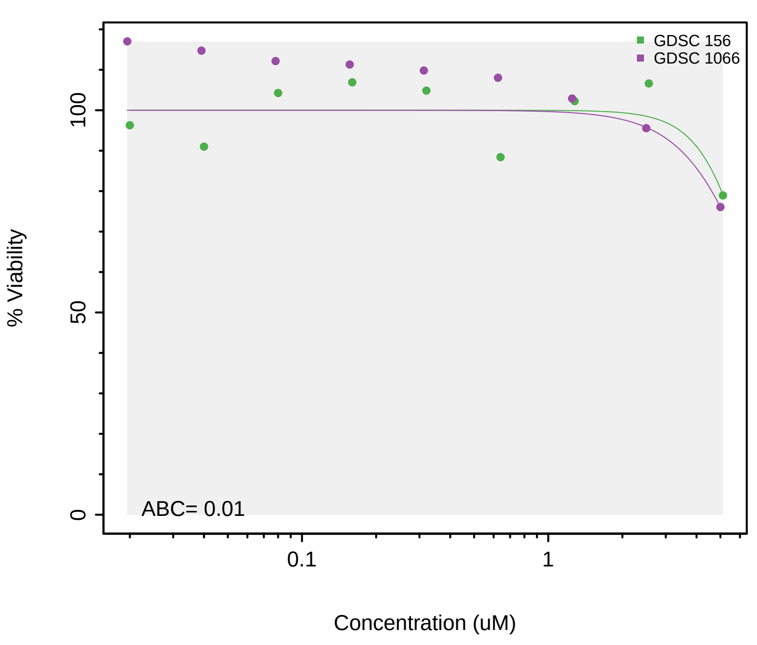

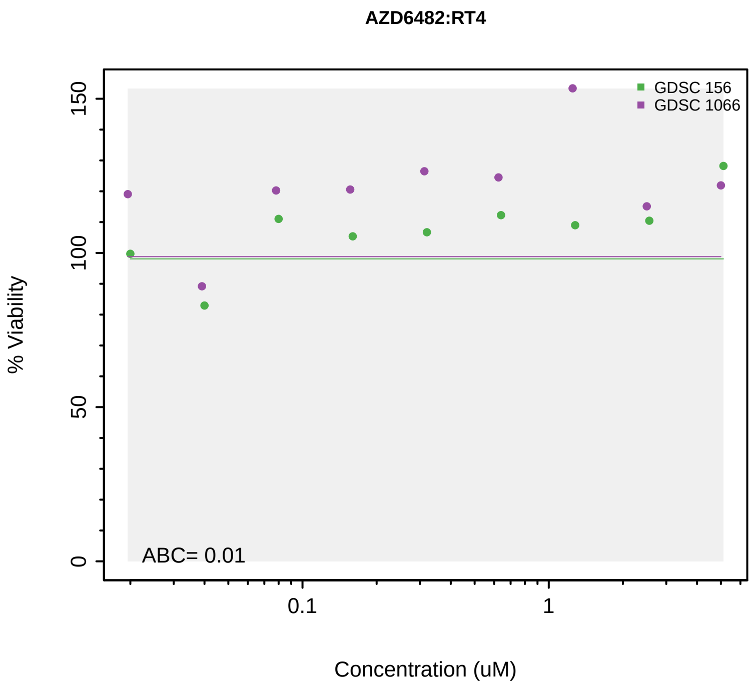
AZD6482:RT4
GDSC 156
GDSC 1066
ABC= 0.01
% Viability
Concentration (uM)

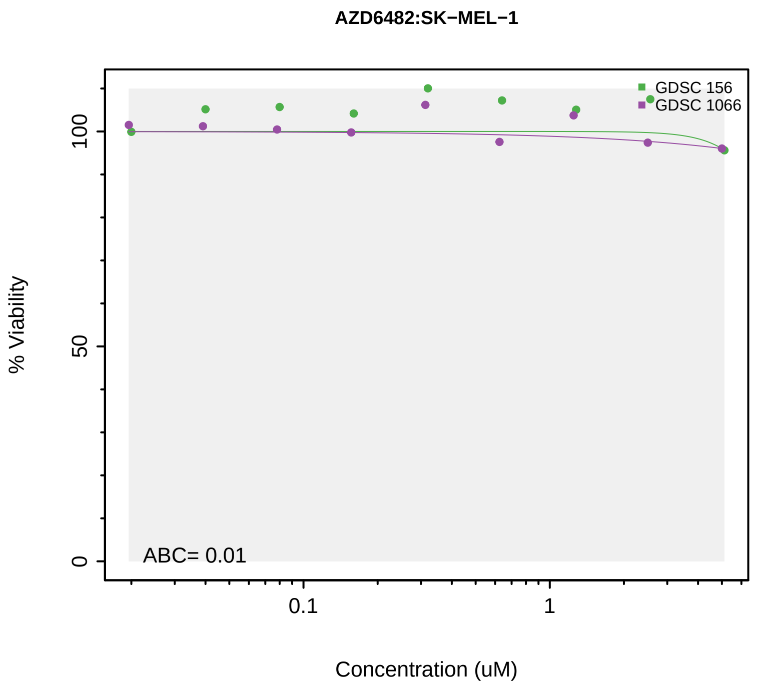
AZD6482:SK−MEL−1
GDSC 156
GDSC 1066
ABC= 0.01
% Viability
Concentration (uM)

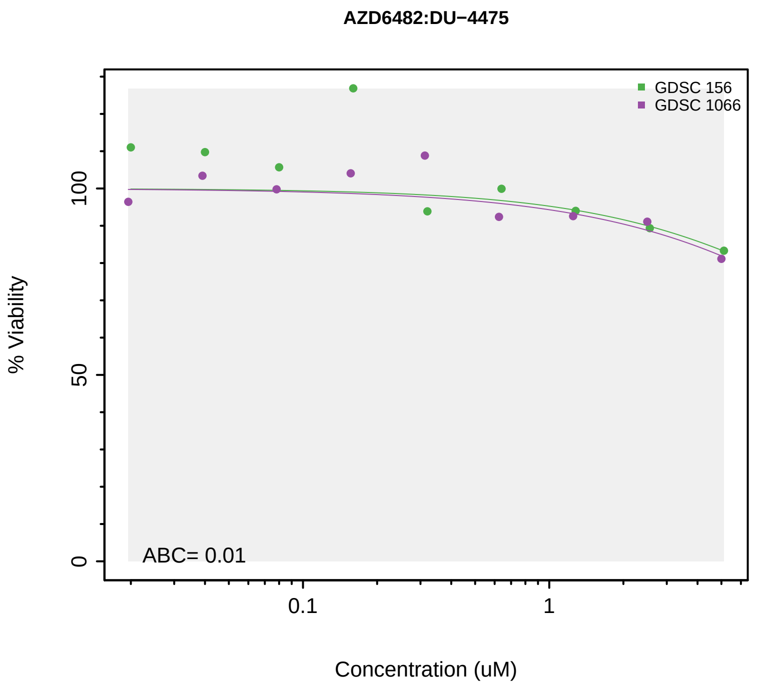
AZD6482:DU−4475
GDSC 156
GDSC 1066
ABC= 0.01
% Viability
Concentration (uM)

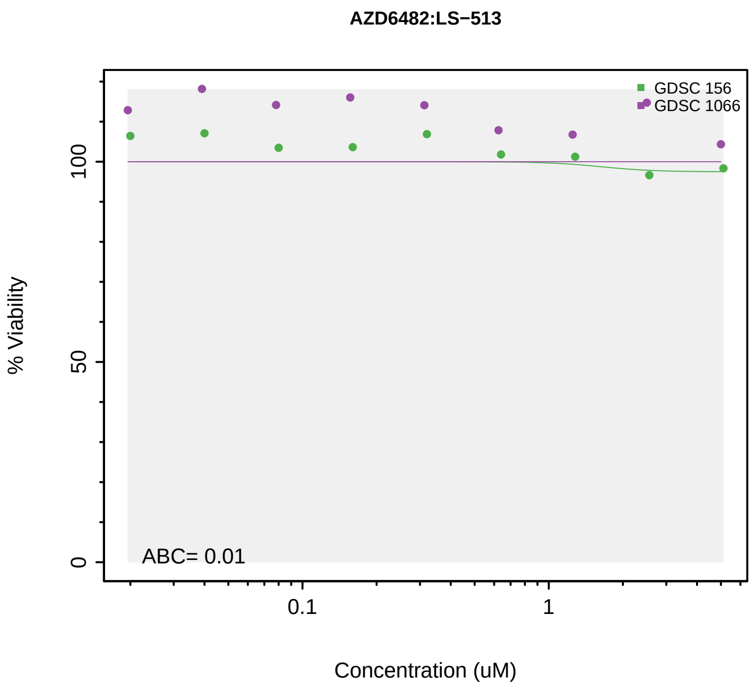
AZD6482:LS−513
GDSC 156
GDSC 1066
ABC= 0.01
% Viability
Concentration (uM)

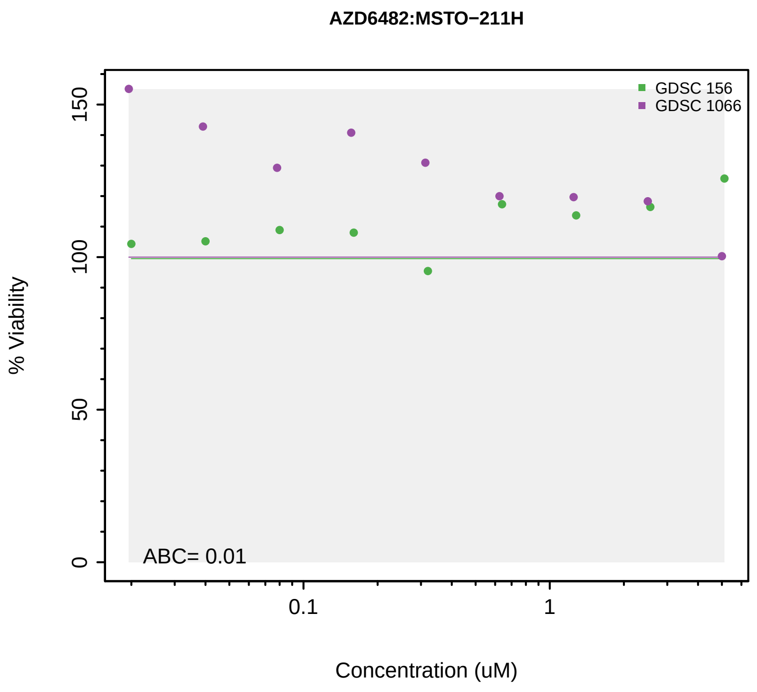
AZD6482:MSTO−211H
GDSC 156
GDSC 1066
ABC= 0.01
% Viability
Concentration (uM)

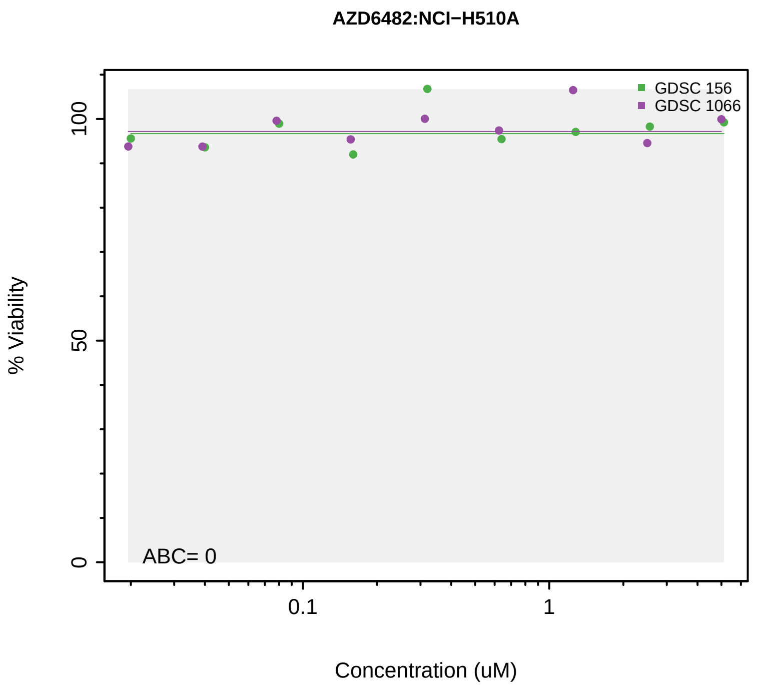
AZD6482:NCI−H510A
GDSC 156
GDSC 1066
ABC= 0
% Viability
Concentration (uM)

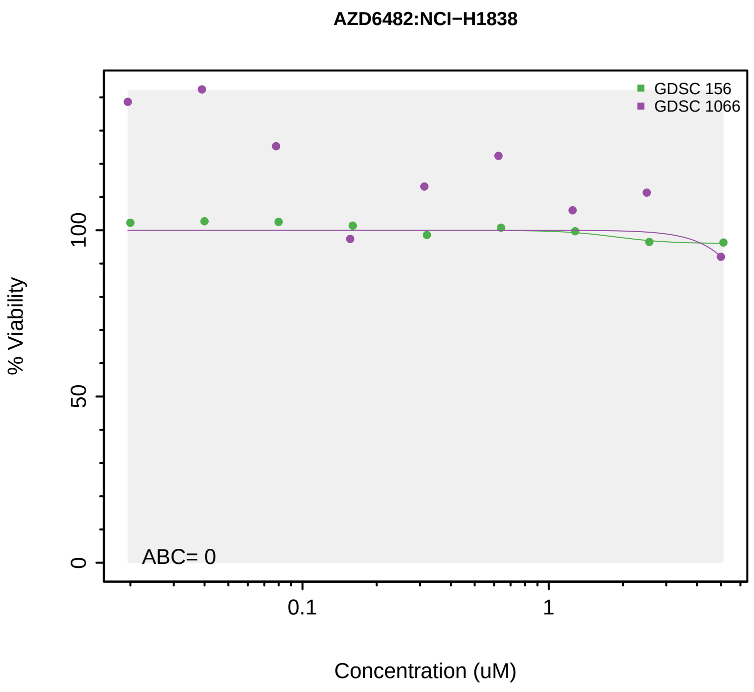
AZD6482:NCI−H1838
GDSC 156
GDSC 1066
ABC= 0
% Viability
Concentration (uM)

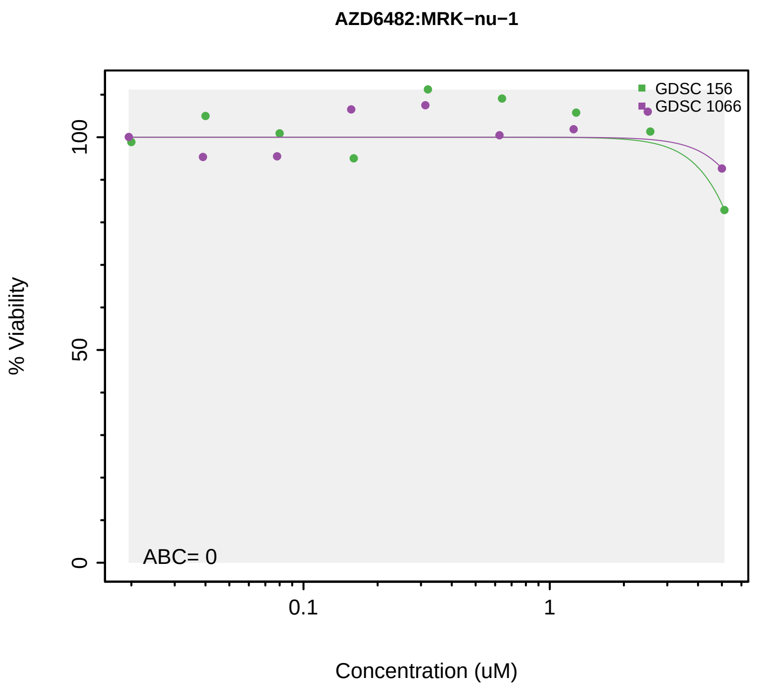
AZD6482:MRK−nu−1
GDSC 156
GDSC 1066
ABC= 0
% Viability
Concentration (uM)

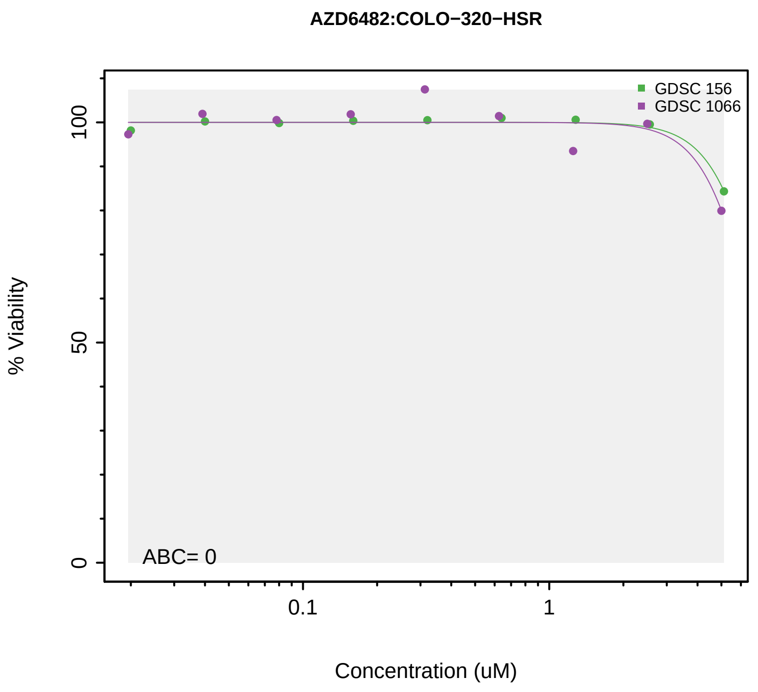
AZD6482:COLO−320−HSR
GDSC 156
GDSC 1066
ABC= 0
% Viability
Concentration (uM)

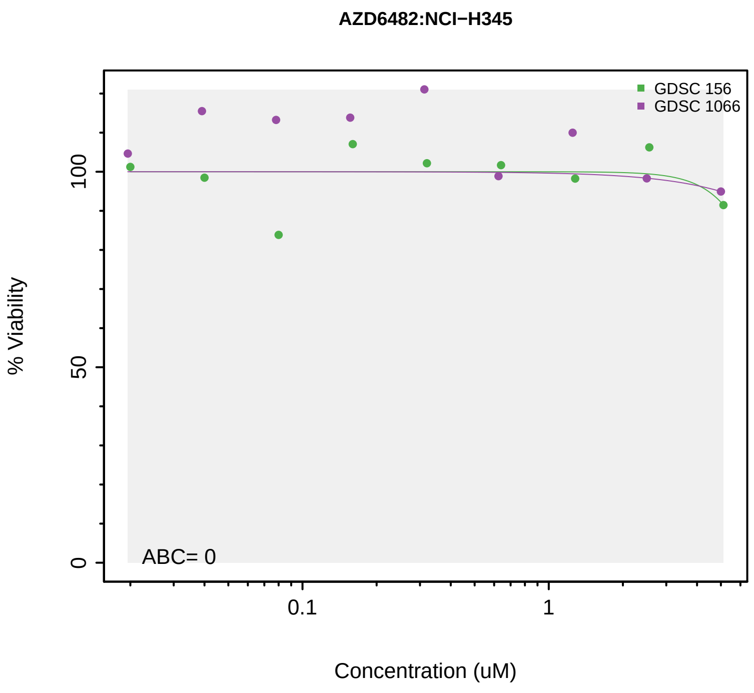
AZD6482:NCI−H345
GDSC 156
GDSC 1066
ABC= 0
% Viability
Concentration (uM)

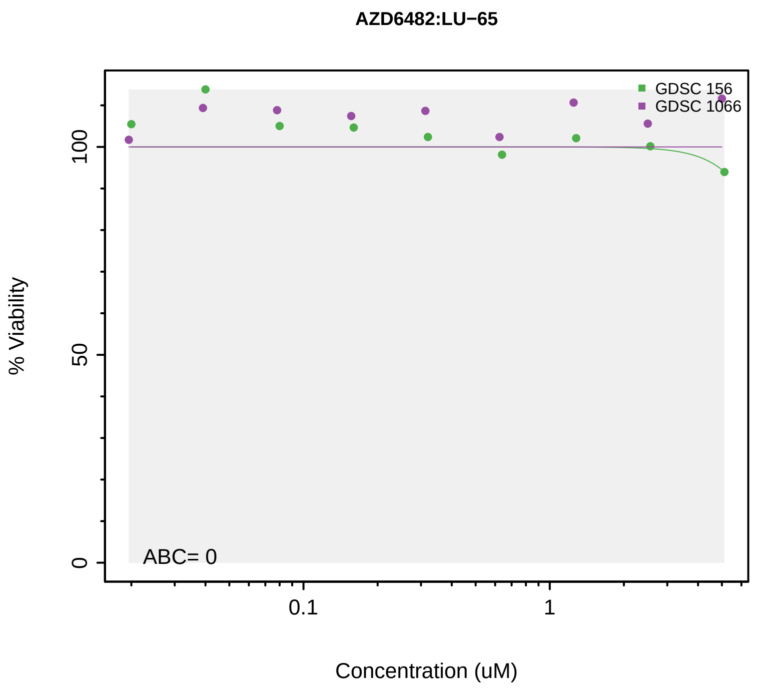
AZD6482:LU−65
GDSC 156
GDSC 1066
ABC= 0
% Viability
Concentration (uM)

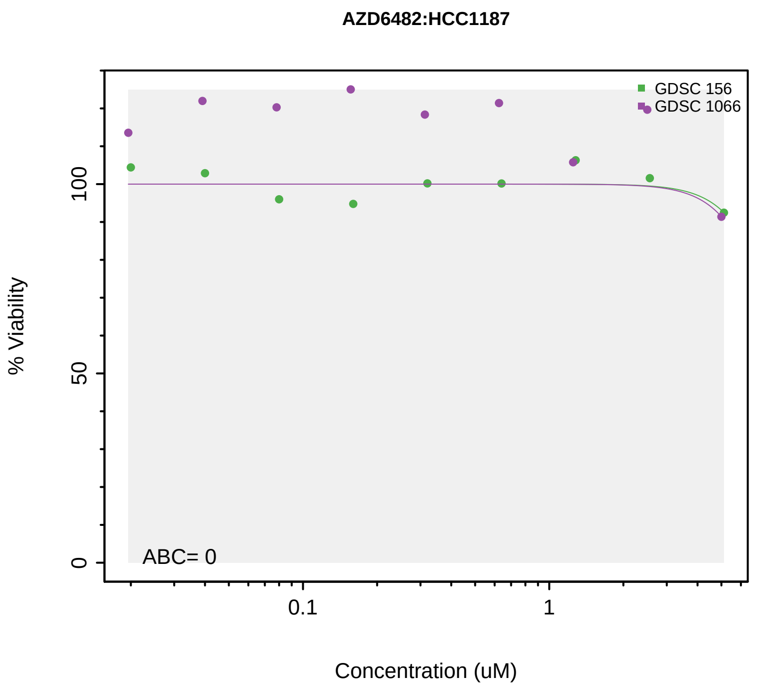
AZD6482:HCC1187
GDSC 156
GDSC 1066
ABC= 0
% Viability
Concentration (uM)

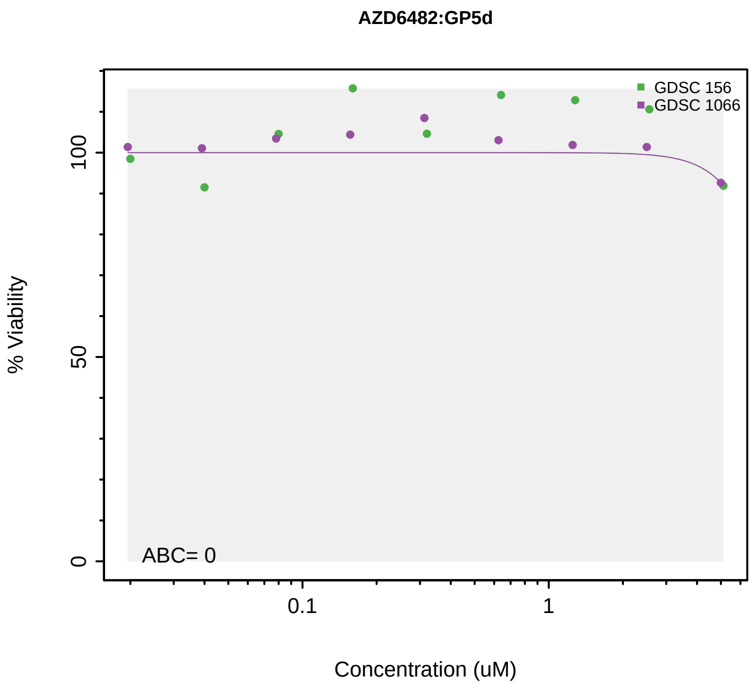
AZD6482:GP5d
GDSC 156
GDSC 1066
% Viability
100
50
0
ABC= 0
0.1
1
Concentration (uM)
