# Supplementary material for: Revisiting inconsistency in large pharmacogenomic studies
Source: F1000Res. 2017 Aug 11;5:2333. Originally published 2016 Sep 16. [Version 3] doi: 10.12688/f1000research.9611.3 (PMC5580432; doi:10.12688/f1000research.9611.3)
Supplement: Supplementary file 4 [file f1000research-5-13399-s0004.tgz › 0ab5d938-b220-4169-902a-c552a9942496.pdf]

Supplementary Methods

Full Reproducibility of the Analysis Results

We will describe how to fully reproduce the figures and tables reported in the main manuscript. We automated the analysis pipeline so that minimal manual interaction is required to reproduce our results. To do this, one must simply:

1. Set up the software environment
2. Run the R scripts
3. Generate the Supplementary Information

The code and associated files are publicly available on GitHub: https://github.com/bhklab/cdrug2.

Set up the software environment

We developed and tested our analysis pipeline using R running on Linux and Mac OSX platforms.

To mimic our software environment the following R packages should be installed.

- R version 3.3.1 (2016-06-21), `x86_64-apple-darwin13.4.0`
- Base packages: base, datasets, graphics, grDevices, grid, methods, parallel, stats, utils
- Other packages: Biobase 2.28.0, BiocGenerics 0.14.0, biomaRt 2.24.1, devtools 1.12.0, Formula 1.2-1, futile.logger 1.4.3, genefu 1.18.0, ggplot2 2.1.0, Hmisc 3.17-4, lattice 0.20-33, mclust 5.2, PharmacoGx 1.3.2, pROC 1.8, prodlim 1.5.7, psych 1.6.6, RColorBrewer 1.1-2, rJava 0.9-8, survcomp 1.18.0, survival 2.39-5, VennDiagram 1.6.17, xlsx 0.5.7, xlsxjars 0.6.1, xtable 1.8-2
- Loaded via a namespace (and not attached): acepack 1.3-3.3, amap 0.8-14, AnnotationDbi 1.30.1, BiocInstaller 1.18.5, bitops 1.0-6, bootstrap 2015.2, caTools 1.17.1, celestial 1.3, chron 2.3-47, cluster 2.0.4, colorspace 1.2-6, curl 1.2, data.table 1.9.6, DBI 0.5, digest 0.6.10, downloader 0.4, foreign 0.8-66, futile.options 1.0.0, gdata 2.17.0, GenomeInfoDb 1.4.3, git2r 0.15.0, gplots 3.0.1, gridExtra 2.2.1, gtable 0.2.0, gtools 3.5.0, httr 1.2.1, igraph 1.0.1, IRanges 2.2.9, KernSmooth 2.23-15, lambda.r 1.1.9, latticeExtra 0.6-28, lava 1.4.4, limma 3.24.15, lsa 0.73.1, magicaxis 2.0.0, magrittr 1.5, mapproj 1.2-4, maps 3.1.1, marray 1.46.0, MASS 7.3-45, Matrix 1.2-7, memoise 1.0.0, mnormt 1.5-4, munsell 0.4.3, nnet 7.3-12, piano 1.8.2, plotrix 3.6-3, plyr 1.8.4, R6 2.1.3, RANN 2.5, Rcpp 0.12.6, RCurl 1.95-4.8, relations 0.6-6, rmeta 2.16, rpart 4.1-10, RSQLite 1.0.0, S4Vectors 0.6.6, scales 0.4.0, sets 1.0-16, slam 0.1-37, sm 2.2-5.4, SnowballC 0.5.1, splines 3.3.1, stats4 3.3.1, SuppDists 1.1-9.2, survivalROC 1.0.3, tools 3.3.1, withr 1.0.2, XML 3.98-1.4

All these packages are available on CRAN[1] or Bioconductor[2] Run the following commands in a R session to install all the required packages:

```
source("http://bioconductor.org/biocLite.R")
biocLite(c("VennDiagram", "Hmisc", "xtable", "RColorBrewer", "pROC", "Biobase", "genefu"
"PharmacoGx", "xlsx"))
```

[1] http://cran.r-project.org
[2] http://www.bioconductor.org

Note that PharmacoGx requires that several packages are installed. However, all dependencies are available from CRAN or Bioconductor.

Once the packages are installed, clone the `cdrug2` GitHub repository (https://github.com/bhklab/cdrug2) This should create a directory on the file system containing the following files:

`cdrug2_analysis.R` Script generating all the figures and tables reported in the manuscript.

`cdrug2_foo.R` Additional functions implemented specifically for the analysis and results visualization.

All the files required to run the automated analysis pipeline are now in place. It is worth noting that 500 MB storage for downloading CGP and CCLE PSets which is done Automatically when user runs `cdrug2_analysis.R` script.

Run the R scripts

Open a terminal window and go to the `cdrug` directory. You can easily run the analysis pipeline either in batch mode or in a R session.
To run the full analysis pipeline in an R session, simply type the following command:

```
nbcore <- 4
#to allocate four CPU cores for instance.
source("code/cdrug2_analysis.R")
```

Key messages will be displayed to monitor the progress of the analysis.

The analysis pipeline was developed so that all intermediate analysis results are saved in the directories `data` and `saveres`. Therefore, in case of interruption, the pipeline will restart where it stopped.

Generate the Supplementary Information

After completion of the analysis pipeline a directory `output` will be created to contain all the intermediate results, tables and figures reported in the main manuscript and this Supplementary Information.

PharmacoGx: Structure of the PharmacoSet class

- @ `annotation`:
  - $ `name`: Acronym of the pharmacogenomic dataset.
  - $ `dateCreated`: When the object was created.
  - $ `sessionInfo`: Software environment used to create the object.
  - $ `call`: Set of parameters used to create the object.
- @ `datasetType`: Either 'sensitivity', 'perturbation', or 'both'
- @ `cell`: data frame annotating all cell lines investigated in the study.
- @ `drug`: data frame annotating all the drugs investigated in the study.
- @ `sensitivity`:
  - $ `n`: Number of experiments for each cell line treated with a given drug
  - $ `info`: Metadata for each pharmacological experiment.
  - $ `raw`: All cell viability measurements at each drug concentration from the drug dose-response curves.
  - $ `phenotype`: Drug sensitivity values summarizing each dose-response curve (IC_{50}, AUC, etc.)
- @ `perturbation`:

$ `n`: Number of experiments for each cell line perturbed by a given drug, for each molecular data type

$ `info`: 'The metadata for the perturbation experiments is available for each molecular type by calling the appropriate info function'

@ `molecularProfiles`: List of `ExpressionSet` objects containing the molecular profiles of the cell lines, such as mutations, gene expressions, or copy number variations.

SNP fingerprinting

The full pipeline to generate and compare cell line SNP fingerprints is provided below.

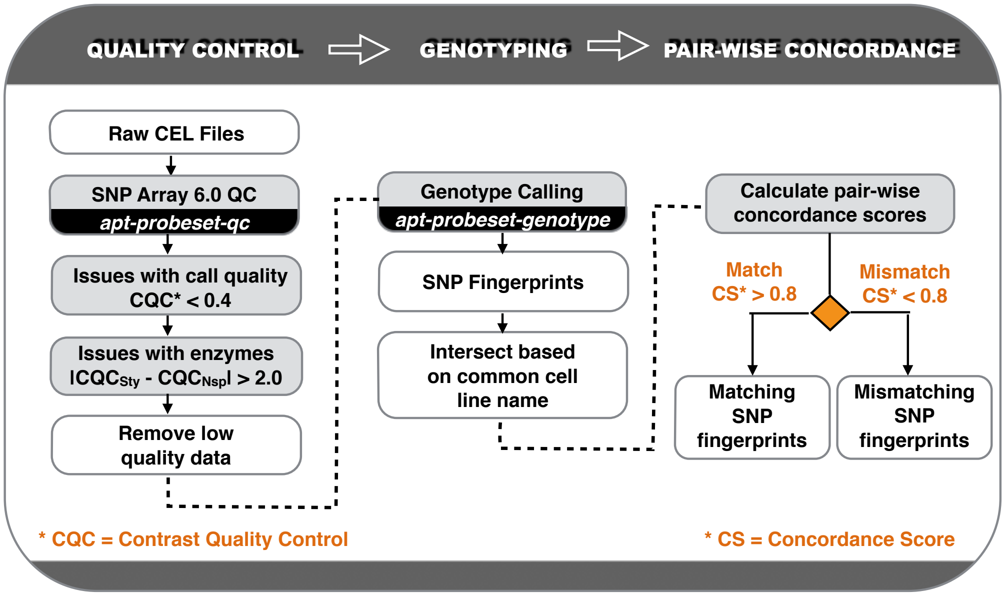

Affymetrix Power Tools (APT)

Quality control metrics were performed using the apt-probeset-qc program from the Affymetrix Power Tools (Version 1.16.1) suite of tools. Poor quality data was identified using the following criteria outlined via the Affymetrix White Pages [1]: (i) Contrast QC (CQC) sample values less than 0.4; (ii) the proportion of samples for a dataset that falls below 0.4 is greater than 10%; (iii) the mean CQC of all samples in a dataset is less than 1.7; and (iv) the absolute difference between the CQC of Nsp and Sty fragments is greater than 2 18,19. All raw CEL files that failed these metrics were removed from subsequent analysis. The apt-probeset-genotype program was used to call genotypes for the raw CEL files using the birdseed-v2 algorithm and default parameters 20. All remaining files were then intersected based on common cancer cell line names.

Pair-wise Concordance of SNP Fingerprints

Pairwise concordance scores between all unique SNP fingerprints were calculated using the formula given by Hong et al. [5]:

$$Conc_{i,j} = \frac{1}{N}\sum_{k=1}^{N} n_k = \begin{cases} 1 & \text{if } G_k^i = G_k^j \\ 0, & \text{if } G_k^i \neq G_k^j \end{cases}$$

where N is the total number of SNPs being compared (909,623 SNPs) and G is the genotype for SNP k in sample i or sample j. A concordance score greater than 0.80 was used to indicate consistent genetic identity.

Filtering drug dose-response curves

Assuming D_i and $\Delta(i, i+1)$ to be the viability of a cell in presence of ith drug concentration tested in a drug sensitivity experiment, and the difference of the cell viability measures in the presence of the $(i+1)^{st}$ and i^{th} drug concentrations, respectively. The trend of a drug-response curve is expected to be non-increasing. In order to have a non-noisy curve, one can assume that the $\Delta(i, i+1)$ must be less than a small positive threshold (ϵ) in a large fraction of the cases(ρ) (1). Since noise is unavoidable in the cell culturing and drug sensitivity experiments, it is expected to see some fluctuations in viability values. As a result, considering very small values for ϵ yields to identifying many non-noisy curves as noisy incorrectly. Clearly choosing the best value for ϵ and ρ is a trade-off between sensitivity and specificity. However, applying only equation (1) on the drug response curve will not filter all the noisy cases out. For instance, assume a dose response curve with monotonically increasing viability from 99% to 100% over ρ percent of successive drug concentrations; but viability falls monotonically to 20% over the last concentrations. Equation (1) does not detect this curve as noisy. Therefore, we have to add other constraints to filter out these types of noisy curves. These constraints require the sum of the $\Delta(i, i+1 \forall i$ to be less than ϵ (2) and the sum of the $\Delta(i, j) \forall i, j$ to be less than 2ϵ (3).

$$\Delta(i, j) = D_j - D_i$$

$$\frac{|\{i|\Delta(i, i+1) < \epsilon\}|}{|\{D_i\}| - 1} > \rho(1)$$

$$\sum_i \Delta(i, i+1) < \epsilon(2)$$

$$\sum_{j-i>=2} \Delta(i, j) < 2\epsilon(3)$$

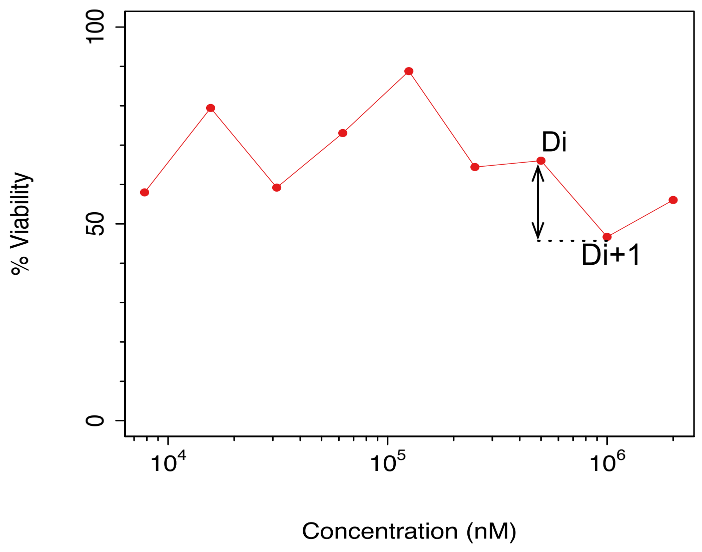

Fitting drug dose-response curves

The rate-limiting step in the killing of susceptible cancer cells by an anti-cancer drug is assumed to be the binding of a target receptor R to n molecules of a drug D, and the free drug molecules and unbound target are assumed to be in thermodynamic equilibrium with the bound drug-target complex:

$$R + nD \leftrightarrow RD_n$$

By the Law of Mass Action, this equilibrium is characterized by an equilibrium constant K satisfying the equation

$$K = \frac{[RD_n]}{[R][D]^n}$$

where brackets around a variable name denote the concentration of the molecule it represents. It then follows that the fraction f of receptors bound to ligands is given by

$$f = \frac{[RD_n]}{[R] + [RD_n]} = \frac{[D]^n}{[D]^n + K}$$

Since this binding is the rate-limiting step in the killing of cancer cells, the fraction $y(x)$ of susceptible cancer cells killed by a concentration x of the drug is approximately equal to the fraction of unbound receptors $1 - f$:

$$y(x) \approx 1 - f = \frac{1}{1 + (\frac{x}{\sqrt[n]{K}})^n}$$

We define $EC_{50} = \sqrt[n]{K}$ and interpret it as the concentration of the drug needed to have half of the target receptors bound at equilibrium. We also assume that a fraction E_∞ of the cancer cells are not susceptible to the drug at all. This leads to our final equation

$$y(x) = E_\infty + \frac{1 - E_\infty}{1 + (\frac{x}{EC_{50}})^{HS}}$$

where $y(x) = 0$ denotes death of all treated cells, $y(x) = 1$ denotes no effect of the drug dose, EC_{50} is the concentration at which $y(EC_{50}) = \frac{1}{2}$, and HS is a parameter describing the cooperativity of binding. $HS < 1$ denotes negative binding cooperativity, $HS = 1$ denotes noncooperative binding, and $HS > 1$ denotes positive binding cooperativity.

This is the basic mathematical structure that was posited to underlie the dose-response data observed in the study. Consequently, median cellular viability data from all datasets was fit by means of least-squares regression to equations of this type. To ensure robustness of the curve-fitting algorithms, bounds were placed on the values of each of these parameters. Drugs were assumed not to increase the fitness of malignant cells, so E_∞ was constrained to lie in the interval $[0, 1]$. Drugs were also assumed to have EC_{50} values within $[1\mathrm{pM}, 1\mathrm{M}]$, an interval containing the EC_{50} values reported by Barretina et al. [2]. Finally, we follow Fallahi et al. [3] in allowing HS to lie anywhere in $[0, 4]$.

Barretina et al. [2] fit dose-response data to one of three models. In most cases, their model of choice was identical to our own, with the addition of a maximum viability parameter E_0. Their dose response equation then became

$$y = E_\infty + \frac{E_0 - E_\infty}{1 + (\frac{x}{EC_{50}})^{HS}}$$

The inclusion of this parameter makes comparison of dose-response curves problematic. With its inclusion, the viability of the cell line in the absence of any drug becomes

$$y(0) = E_\infty + \frac{E_0 - E_\infty}{1 + (\frac{0}{EC_{50}})^{HS}} = E_\infty + E_0 - E_\infty = E_0$$

As a result, the viability measures of different drug-cell line combinations are normalized differently, and direct comparison of viability predictions from different dose-response curves is no longer appropriate. The IC_{50} values they reported, however, were simply the concentrations at which their fitted curves reached viability reduction of 50% of cellular viability. The end result was a reported IC_{50} value that assumed normalization of viability data to the negative control associated with a curve fitted assuming normalization of viability data to a reference level that was most consistent with the observed data. The IC_{50} values published in the paper's supplementary information thus represented viability reduction by a fraction that varied from cell line to cell line.

In GDSC [4], the following five-parameter model was used:

$$y = E_\infty + \frac{E_0 - E_\infty}{(1 + (\frac{x}{EC_{50}})^{HS})^S}$$

However, since the E_0 parameter is fixed by controls, their curve can be represented as

$$y = E_\infty + \frac{1 - E_\infty}{(E_\infty(1 + (\frac{x}{EC_{50}})^{HS})^S}$$

This parameter accounts for the presence of an antagonistic binding of the drug, and introduces asymmetry into the theoretical log dose-response curve. The extra parameter, known as the "Schild slope", allows the dose-response curve to be non-monotonic.

While this parameter is well-founded biologically, we chose not to use it in our own dose-response curves. As only medians of technical replicates are available for CCLE, using a 4-parameter model would have increased our susceptibility to overfitting noise in the sparse dose-response curves. Furthermore, we only rarely observed the non-monotonicity that necessitates the inclusion of a Schild slope parameter in a very small fraction of dose-response curves. For these reasons, we ultimately chose to use our simpler 3-parameter model to compare the dose-response curves from the GDSC and CCLE datasets.

Area between the drug dose-response curves (ABC)

The ABC is a function of two dose-response curves from CCLE and GDSC which come from the same drug-cell line combination. It is calculated by taking the unsigned area between the two curves over the intersection of the concentration range tested in the CCLE curve and that tested in the GDSC curve, and normalizing that area by the length of the intersection interval.

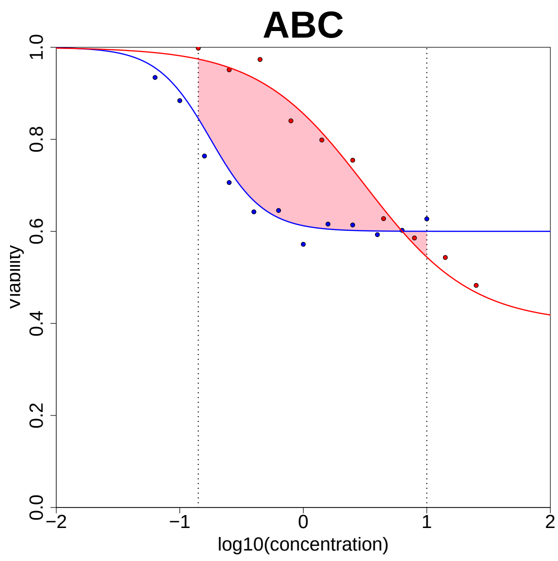

Consistency *across* vs. *between* cell lines

We assessed the concordance of the gene expression, mutation and drug sensitivity of CGP and CCLE studies *across* and *between* cell lines, as illustrated in the figure below. When data are compared across cell lines, we assess whether, for a given gene expression or drug, the cell line data were concordant (a gene is expressed at a similar level or similar response to a drug is observed in the same set of cell lines for instance; panel **A**). When data are compared between cell lines, we assessed whether, for a given cell line, the genomic and pharmacological profiles were concordant in the two studies (a given cell line harbours similar gene expression patterns or pharmacological responses for instance; panel **B**).

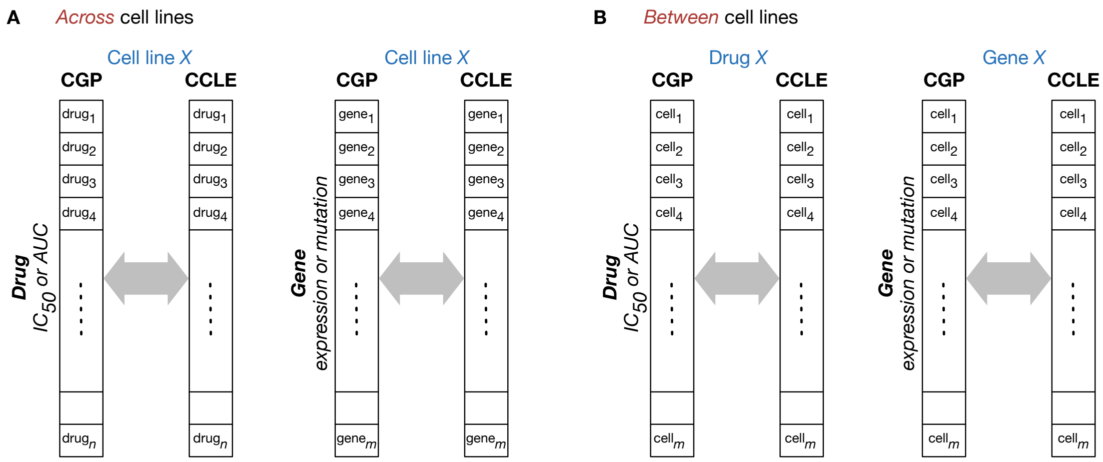

Acronyms

ABC	Area between the curves
AE	ArrayExpress by the European Bioinformatics Institute
AUC	Area under the dose response curve
AUC* or STAR	Area under the dose response curve calculated by considering only the common concentration range b
CCLE	The Cancer Cell Line Encyclopedia initiated by the Broad Institute of MIT and Harvard
CGHub	The Cancer Genomics Hub from the University of California Santa Cruz and the US National Cancer In
CGP	The Cancer Genome Project by the Wellcome Trust Sanger Institute
CMAP	Connectivity Map by the Broad Institute
COSMIC	Catalogue of Somatic Mutations in Cancer by the Wellcome Trust Sanger Institute
CRAMERV	Carmer's V
DXY	Somers' Dxy rank correlation
GDSC	The Cancer Genome Project initiated by the Wellcome Trust Sanger Institute
IC_{50}	Concentration at which the drug inhibited 50% of the maximum cellular growth
INFORM	Informedness
MCC	Matthews correlation coefficient
PCC	Pearson product-moment correlation coefficient
QC	Quality control
RMA	Robust multi-array normalization
SCC	Spearman rank correlation coefficient
SNP	Single nucleotide polymorphism

Definitions

Term	Drugs	Requirements	Sensitivity cut-off
No effect	sorafenib, erlotinib, and PHA–665752	at least five cells with AUC > 0.2 or IC_{50} < 1 μM	
Narrow effect	nilotinib, lapatinib, nutlin–3, PLX4720, crizotinib, PD–0332991, AZD0530, and TAE684	AUC MAD $\leq$ 0.13	
Broad effect	AZD6244, PD–0332901, 17-AAG and paclitaxel	AUC MAD > 0.13	
Targeted	sorafenib, erlotinib, PHA–665752, nilotinib, lapatinib, nutlin–3, PLX4720, crizotinib, PD–0332991, AZD0530, TAE684, AZD6244, PD–0332901 and 17-AAG		IC_{50} < 1 μM or AUC > 0.2
Cytotoxic	paclitaxel		IC_{50} < 10 μM or AUC > 0.4

References

[1] Affymetrix. Quality Control Assessment in Genotyping Console, 2008. URL http://media.affymetrix.com/support/technical/whitepapers/genotyping_console_cqc_whitepaper.pdf.

[2] Jordi Barretina, Giordano Caponigro, Nicolas Stransky, Kavitha Venkatesan, Adam A Margolin, Sungjoon Kim, Christopher J Wilson, Joseph Lehár, Gregory V Kryukov, Dmitriy Sonkin, Anupama Reddy, Manway Liu, Lauren Murray, Michael F Berger, John E Monahan, Paula Morais, Jodi Meltzer, Adam Korejwa, Judit Jané-Valbuena, Felipa A Mapa, Joseph Thibault, Eva Bric-Furlong, Pichai Raman, Aaron Shipway, Ingo H Engels, Jill Cheng, Guoying K Yu, Jianjun Yu, Peter Aspesi, Melanie de Silva, Kalpana Jagtap, Michael D Jones, Li Wang, Charles Hatton, Emanuele Palescandolo, Supriya Gupta, Scott Mahan, Carrie Sougnez, Robert C Onofrio, Ted Liefeld, Laura MacConaill, Wendy Winckler, Michael Reich, Nanxin Li, Jill P. Mesirov, Stacey B Gabriel, Gad Getz, Kristin Ardlie, Vivien Chan, Vic E Myer, Barbara L Weber, Jeff Porter, Markus Warmuth, Peter Finan, Jennifer L Harris, Matthew Meyerson, Todd R. Golub, Michael P Morrissey, William R Sellers, Robert Schlegel, and Levi A. Garraway. The Cancer Cell Line Encyclopedia enables predictive modelling of anticancer drug sensitivity. *Nature*, 483(7391):603–607, March 2012.

[3] Mohammad Fallahi-Sichani, Saman Honarnejad, Laura M Heiser, Joe W. Gray, and Peter K Sorger. Metrics other than potency reveal systematic variation in responses to cancer drugs. *Nature Chemical Biology*, September 2013.

[4] Mathew J Garnett, Elena J Edelman, Sonja J Heidorn, Chris D Greenman, Anahita Dastur, King Wai Lau, Patricia Greninger, I Richard Thompson, Xi Luo, Jorge Soares, Qingsong Liu, Francesco Iorio, Didier Surdez, Li Chen, Randy J Milano, Graham R Bignell, Ah T Tam, Helen Davies, Jesse A Stevenson, Syd Barthorpe, Stephen R Lutz, Fiona Kogera, Karl Lawrence, Anne McLaren-Douglas, Xeni Mitropoulos, Tatiana Mironenko, Helen Thi, Laura Richardson, Wenjun Zhou, Frances Jewitt, Tinghu Zhang, Patrick O'Brien, Jessica L Boisvert, Stacey Price, Wooyoung Hur, Wanjuan Yang, Xianming Deng, Adam Butler, Hwan Geun Choi, Jae Won Chang, Jose Baselga, Ivan Stamenkovic, Jeffrey A Engelman, Sreenath V Sharma, Olivier Delattre, Julio Saez-Rodriguez, Nathanael S Gray, Jeffrey Settleman, P Andrew Futreal, Daniel A Haber, Michael R Stratton, Sridhar Ramaswamy, Ultan McDermott, and Cyril H Benes. Systematic identification of genomic markers of drug sensitivity in cancer cells. *Nature*, 483(7391):570–575, March 2012.

[5] Huixiao Hong, Lei Xu, Jie Liu, Wendell D Jones, Zhenqiang Su, Baitang Ning, Roger Perkins, Weigong Ge, Kelci Miclaus, Li Zhang, Kyunghee Park, Bridgett Green, Tao Han, Hong Fang, Christophe G Lambert, Silvia C Vega, Simon M Lin, Nadereh Jafari, Wendy Czika, Russell D Wolfinger, Federico Goodsaid, Weida Tong, and Leming Shi. Technical Reproducibility of Genotyping SNP Arrays Used in Genome-Wide Association Studies. *PloS one*, 7(9):e44483, September 2012.
