# Supplementary material for: Revisiting inconsistency in large pharmacogenomic studies
Source: F1000Res. 2017 Aug 11;5:2333. Originally published 2016 Sep 16. [Version 3] doi: 10.12688/f1000research.9611.3 (PMC5580432; doi:10.12688/f1000research.9611.3)
Supplement: Supplementary file 5 [file f1000research-5-13399-s0003.tgz › d5bec513-43e7-44f6-88f3-f29a23f4b2db.pdf]

Supplementary Figures

A

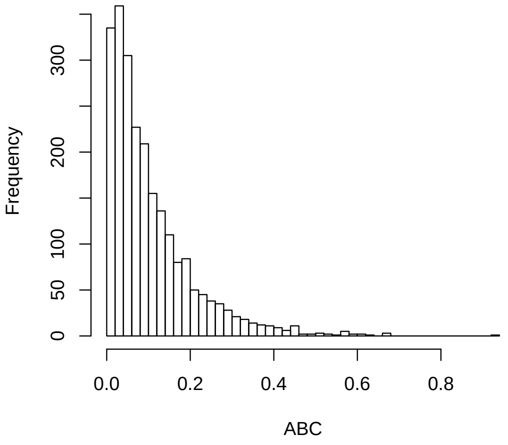

B

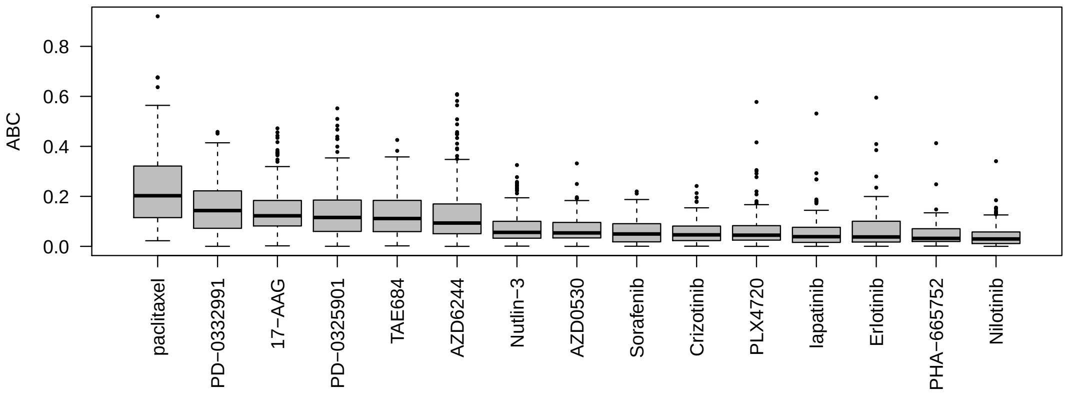

Supplementary Figure 1: (**A**) Histogram of ABC estimates for all common drug dose-response curves between GDSC and CCLE. (**B**) Boxes represent the median and inter quartile range of ABC for drug-cell line combinations screened in GDSC and CCLE.

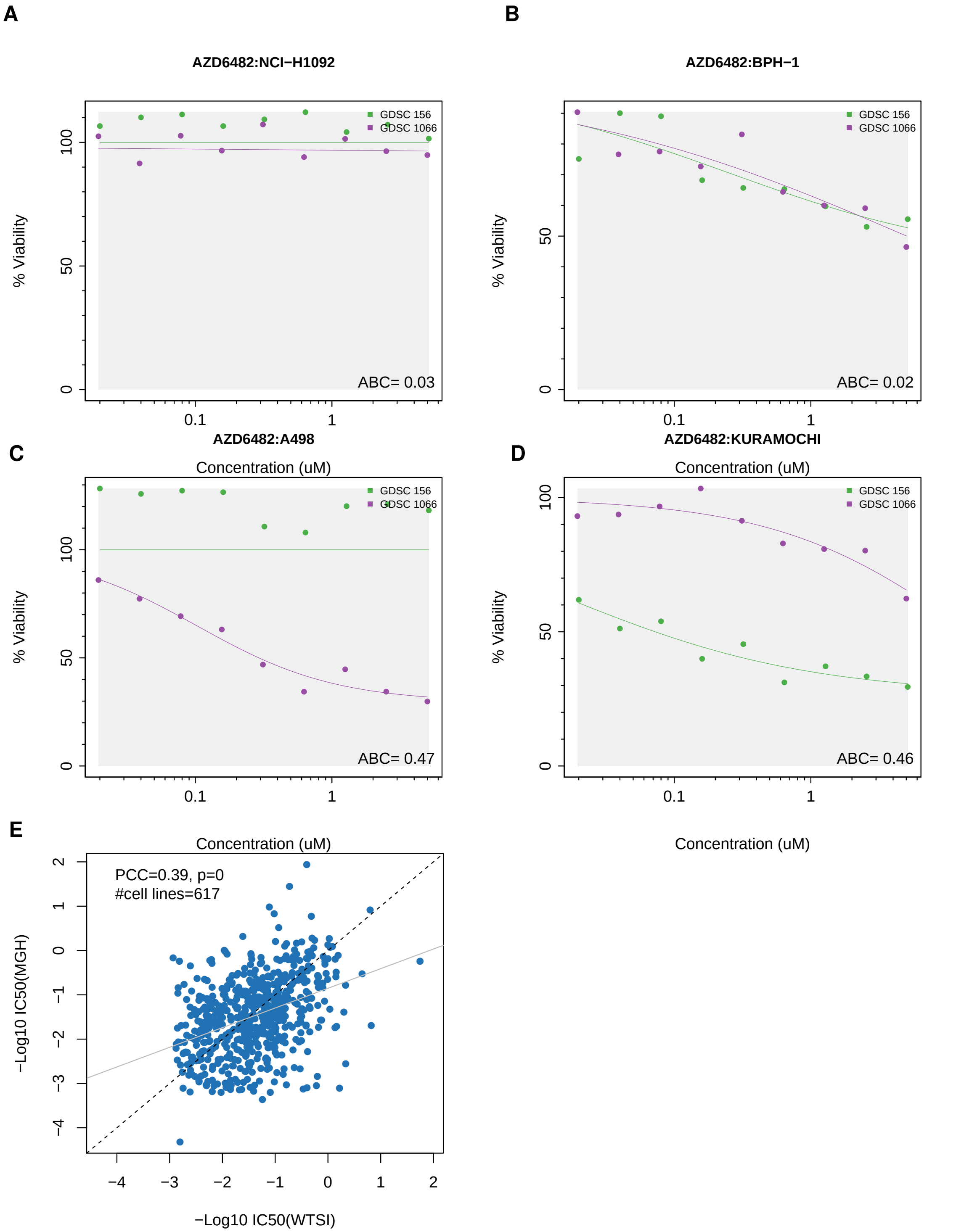

Supplementary Figure 2: Examples of (A,B) consistent and (C,D) inconsistent replicated experiments screening AZD6482 in GDSC. The grey area represents the common concentration range between studies. **(A)** NCI-H1092; **(B)** BPH-1; **(C)** A498; and **(D)** KURAMOCHI cell line treated with AZD6482. (**E**) Consistency of sensitivity profiles across replicated experiments of AZD6482 performed in different sites (MGH and WTSI).

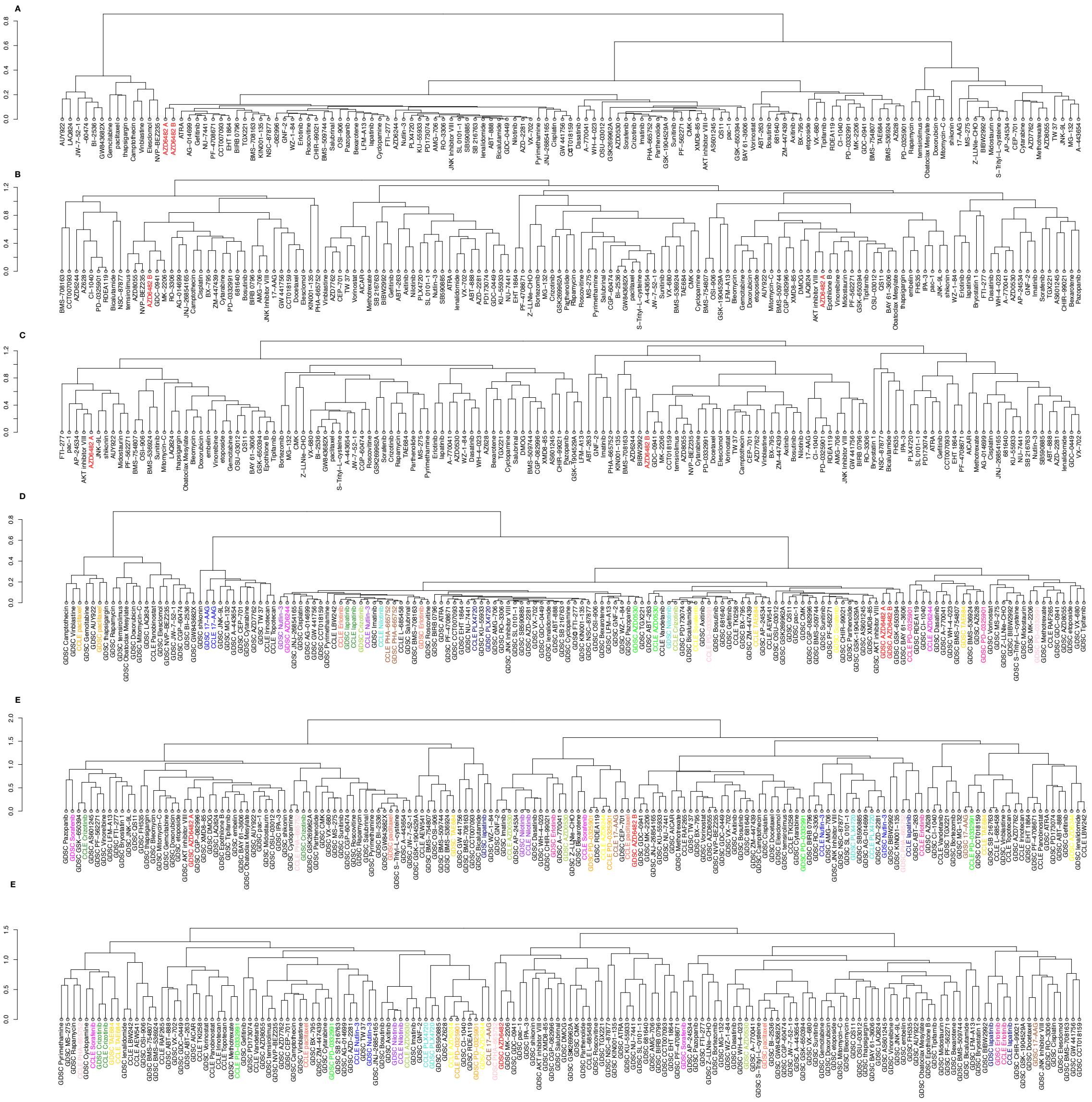

Supplementary Figure 3: Dendrogram of the clustering of all drugs in GDSC based on their (**A**) distance based on median ABC values (**B**) IC_{50}-based distance (**C**) AUC-based distance. Dendogram of the clustering of all drugs in CCLE and GDSC based on their (**D**) distance based on mean ABC values (**E**) IC_{50}-based distance (**F**) AUC-based distance. Distance based on IC_{50} and AUC used 1 minus Pearson correlation coefficient. Overlapping drugs are shown with the same colour.

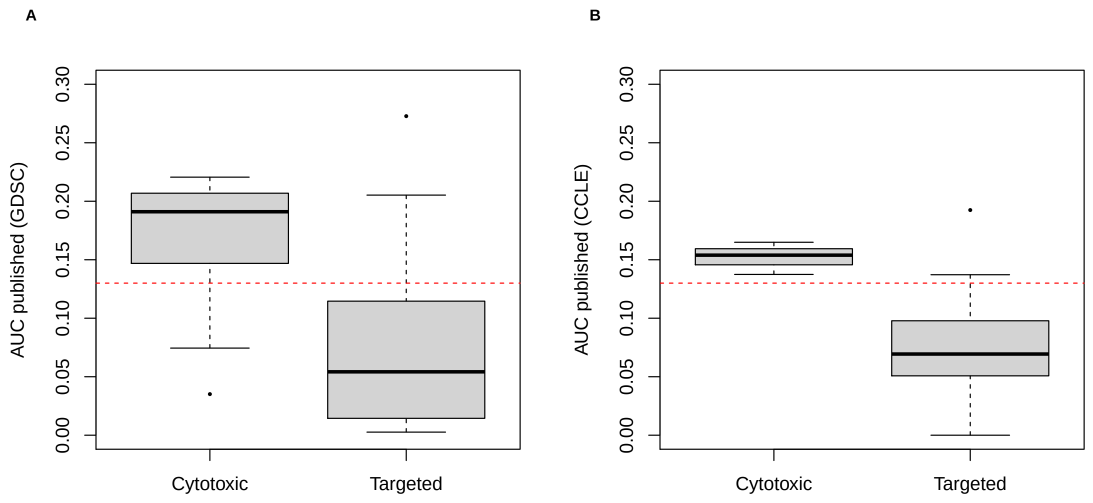

Supplementary Figure 4: Comparison of median absolute deviation (MAD) of published AUC values between cytotoxic and targeted drugs using all cell lines in (**A**) GDSC and (**B**) CCLE.

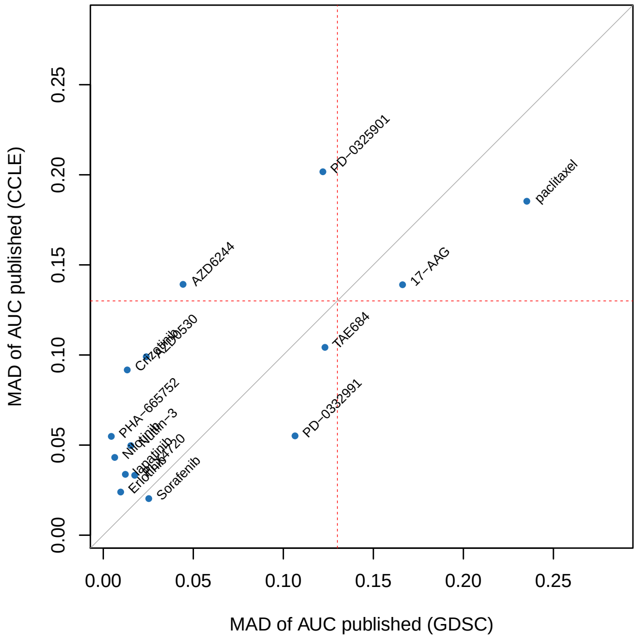

Supplementary Figure 5: Comparison of median absolute deviation (MAD) of published AUC values between drugs using common cell lines in (**A**) GDSC and (**B**) CCLE.

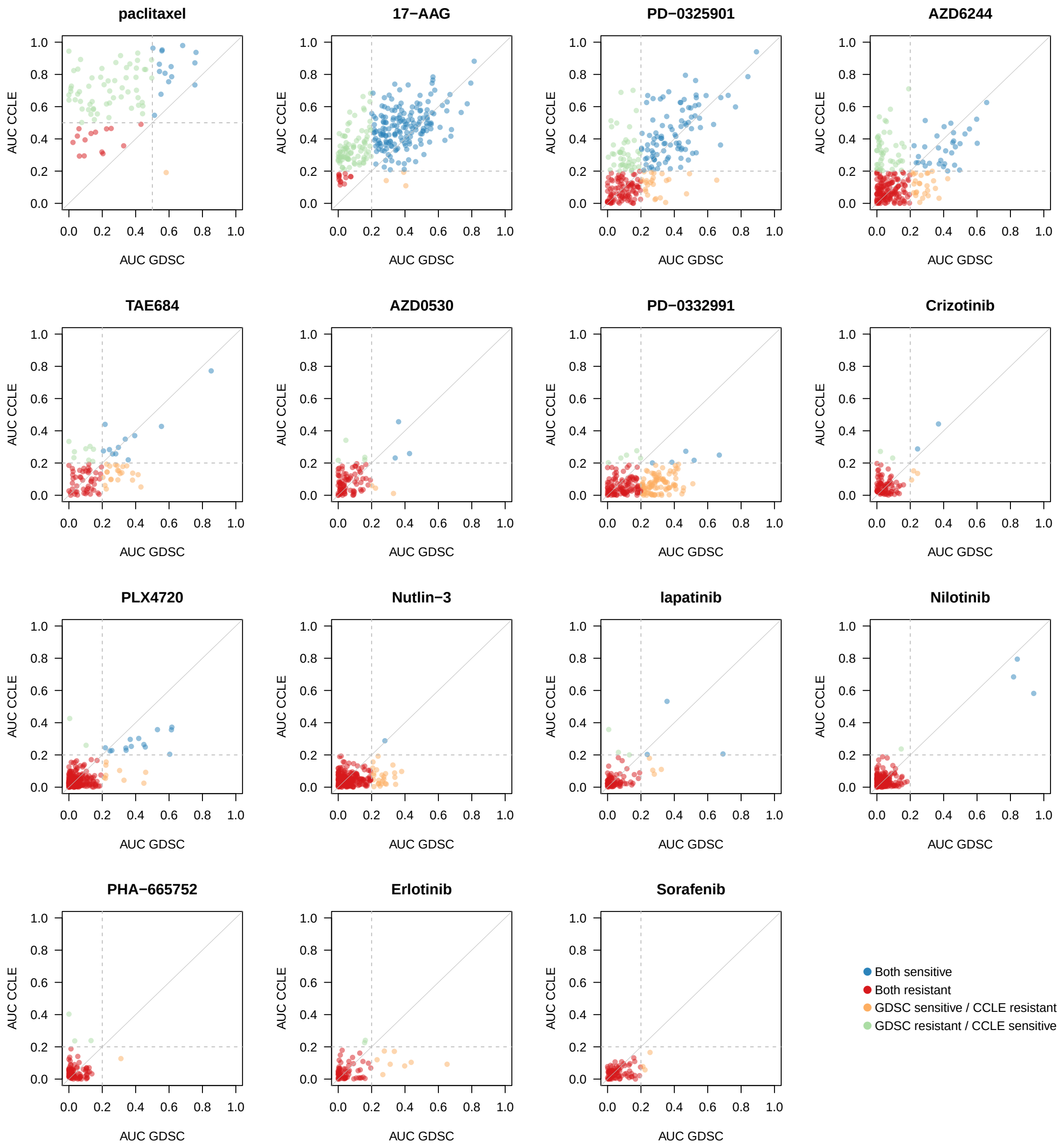

Supplementary Figure 6: Comparison of AUC values between GDSC and CCLE, as recomputed within *PharmacoGx*. Cell lines with AUC > 0.2 (AUC > 0.4 for paclitaxel) were considered as sensitive. In case of perfect consistency, all points would lie on the grey diagonal. The drugs are ranked based on their category: broad effect (AZD6244, PD-0325901, 17-AAG and paclitaxel), narrow effect (nilotinib, lapatinib, nutlin-3, PLX4720, crizotinib, PD-0332991, AZD0530, and TAE684) and no/little effect (sorafenib, erlotinib and PHA–665752).

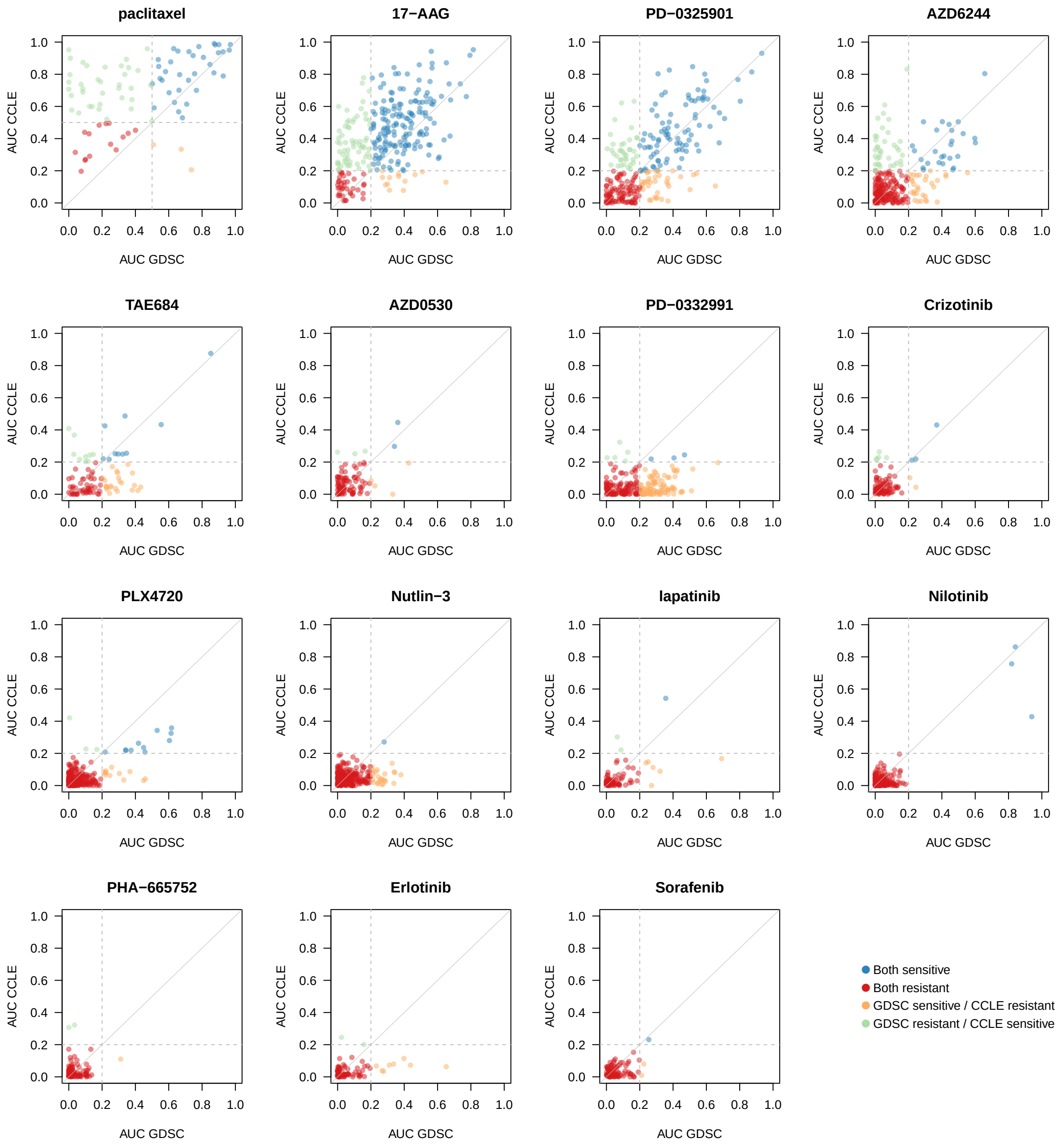

Supplementary Figure 7: Comparison of AUC* values between GDSC and CCLE, as recomputed within *PharmacoGx* using the concentration range common to GDSC and CCLE. Cell lines with AUC > 0.2 (AUC > 0.4 for paclitaxel) were considered as sensitive. In case of perfect consistency, all points would lie on the grey diagonal. The drugs are ranked based on their category: broad effect (AZD6244, PD-0325901, 17-AAG and paclitaxel), narrow effect (nilotinib, lapatinib, nutlin-3, PLX4720, crizotinib, PD-0332991, AZD0530, and TAE684) and no/little effect (sorafenib, erlotinib and PHA–665752).

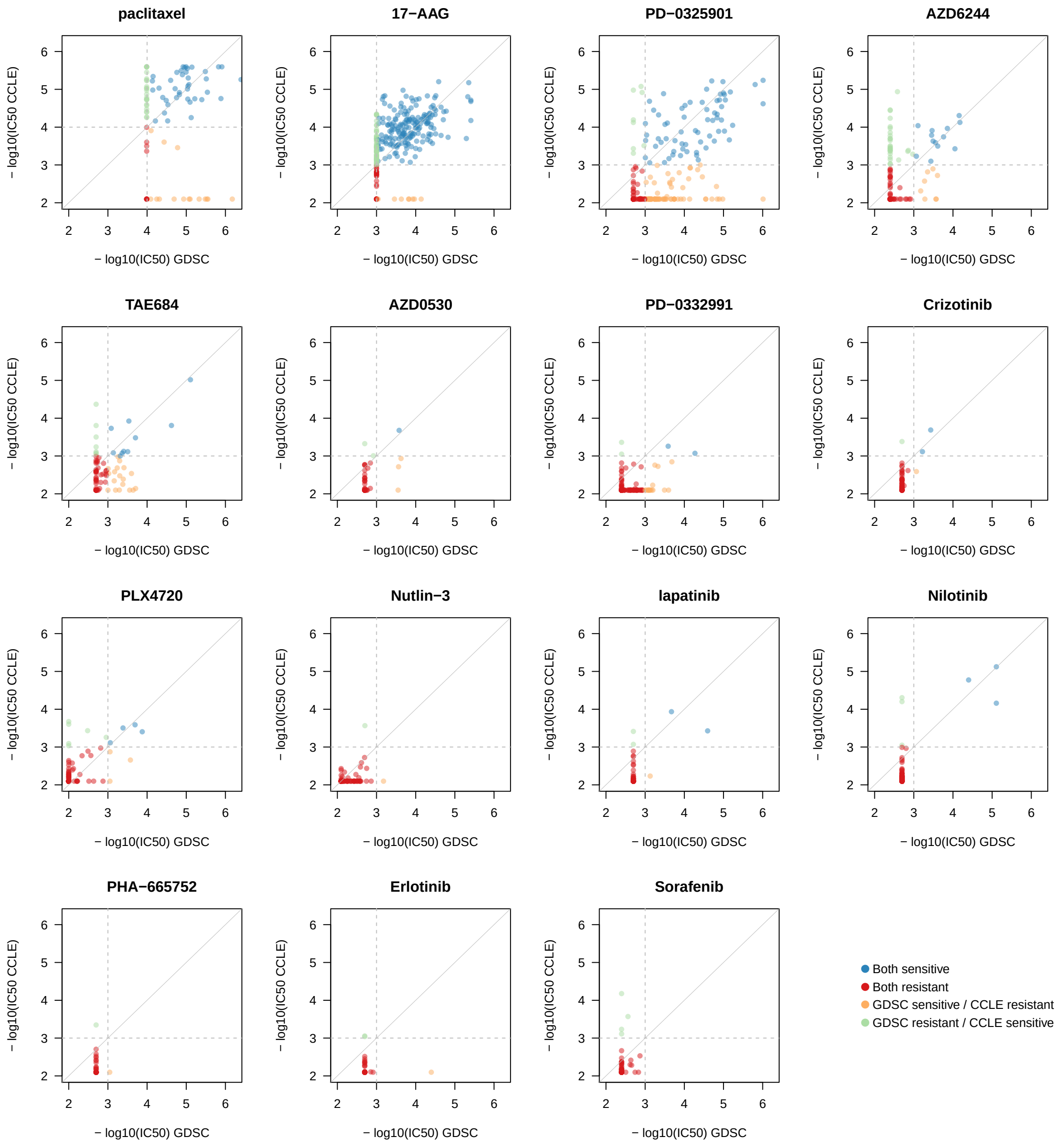

Supplementary Figure 8: Consistency of IC_{50} values between GDSC and CCLE, as published. Cell lines with $IC_{50} < 1\mu M$ ($IC_{50} < 10\mu M$ for paclitaxel) were considered as sensitive. In case of perfect consistency, all points would lie on the grey diagonal. The drugs are ranked based on their category: broad effect (AZD6244, PD-0325901, 17-AAG and paclitaxel), narrow effect (nilotinib, lapatinib, nutlin-3, PLX4720, crizotinib, PD-0332991, AZD0530, and TAE684) and no/little effect (sorafenib, erlotinib and PHA–665752).

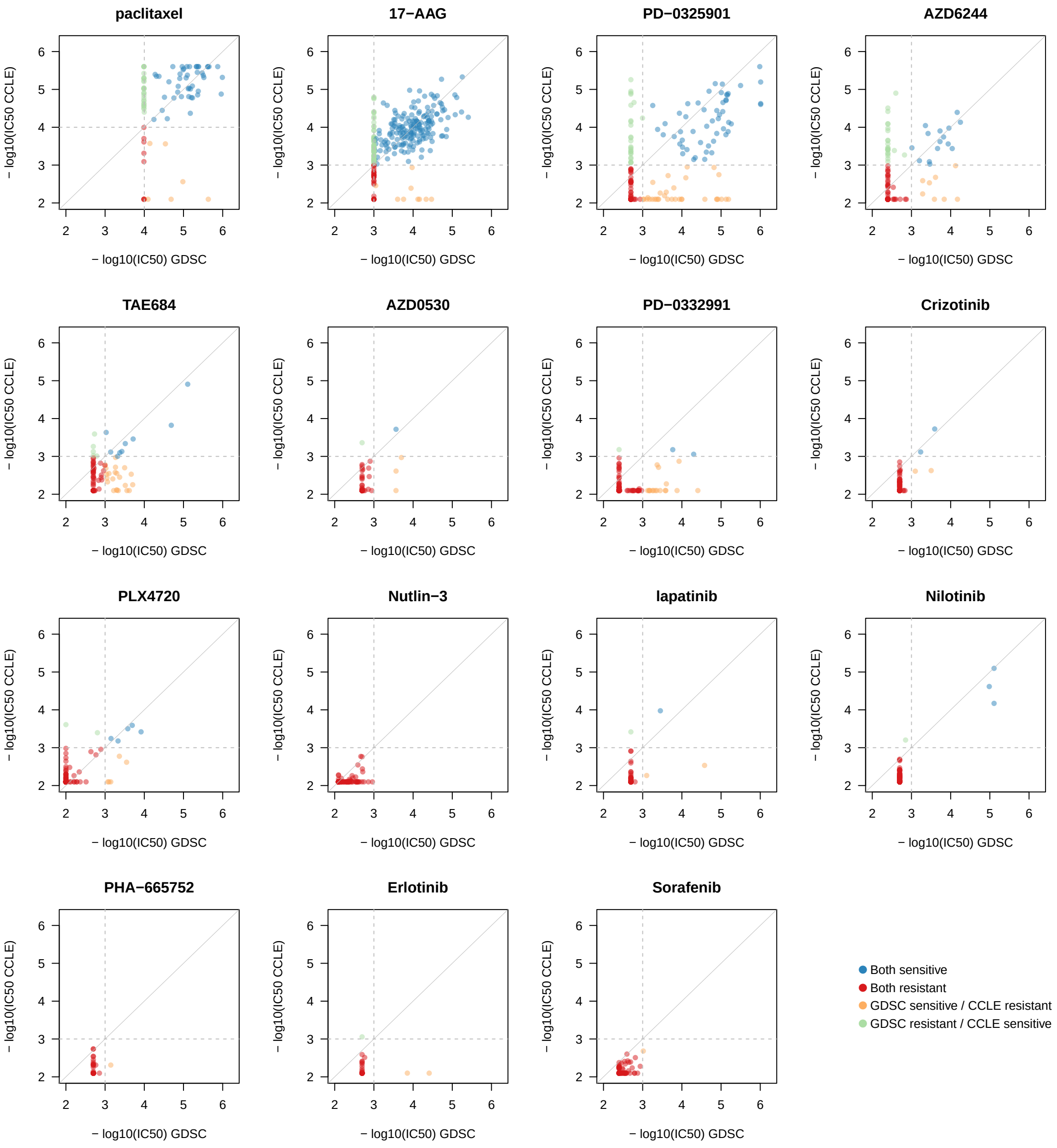

Supplementary Figure 9: Consistency of IC_{50} values between GDSC and CCLE, as recomputed within *PharmacoGx*. Cell lines with Cell lines with $IC_{50} < 1\mu M$ ($IC_{50} < 10\mu M$ for paclitaxel) were considered as sensitive. In case of perfect consistency, all points would lie on the grey diagonal. The drugs are ranked based on their category: broad effect (AZD6244, PD-0325901, 17-AAG and paclitaxel), narrow effect (nilotinib, lapatinib, nutlin-3, PLX4720, crizotinib, PD-0332991, AZD0530, and TAE684) and no/little effect (sorafenib, erlotinib and PHA–665752).

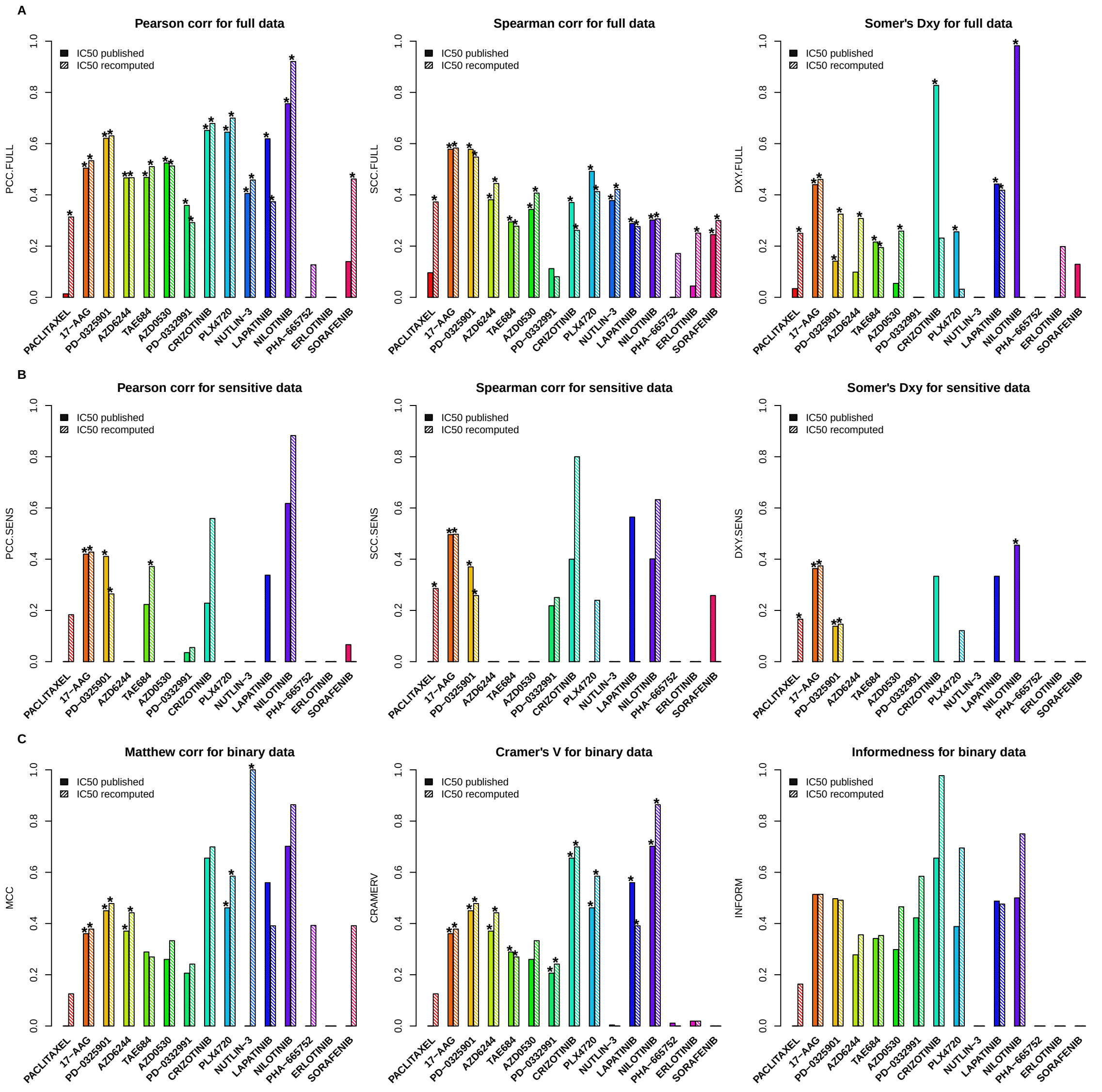

Supplementary Figure 10: Consistency of IC_{50} values between GDSC and CCLE, as published and recomputed within *PharmacoGx*.**(A)** Consistency assessed using the full set of cancer cell lines screened in both studies. **(B)** Consistency assessed using only sensitive cell lines ($IC_{50} < 1\mu M$ and $IC_{50} < 10\mu M$ for targeted and cytotoxic drugs, respectively). **(C)** Consistently assessed by discretizing the drug sensitivity data using the aforementioned cutoffs for IC_{50}. PCC: Pearson correlation coefficient; SCC: Spearman rank-based correlation coefficient; DXY: Somers' Dxy rank correlation; MCC: Matthews correlation coefficient; CRAMERV: Cramer's V statistic; INFORM: Informedness. The symbol '*' indicates whether the consistency is statistically significant ($p < 0.05$).

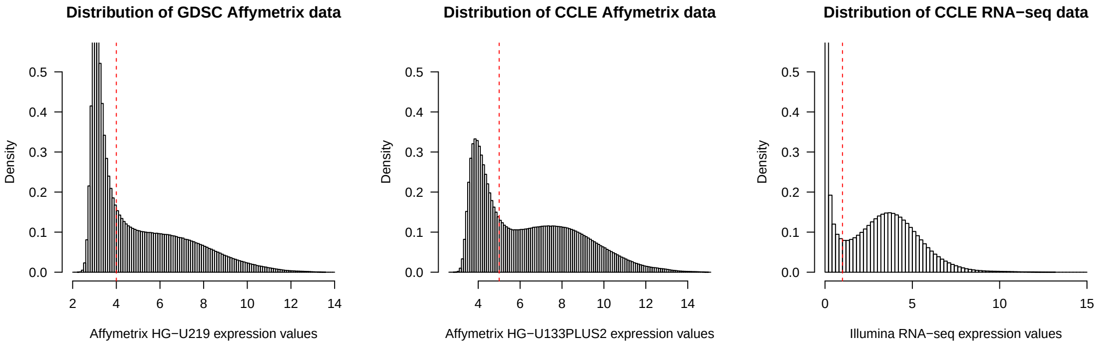

Supplementary Figure 11: Distribution of gene expression values and corresponding cutoffs for the microarray Affymetrix HG-U219 platform in GDSC (cutoff = 4), the microarray Affymetrix HG-U133PLUS2 platform in CCLE (cutoff = 5) and the new Illumina RNA-seq data in CCLE (cutoff = 1). To distinguish between lowly vs highly expressed genes, we fitted a mixture of two gaussians to each gene expression distribution and estimated the cutoffs as the 90% left interval of the distribution of the highly expressed genes.

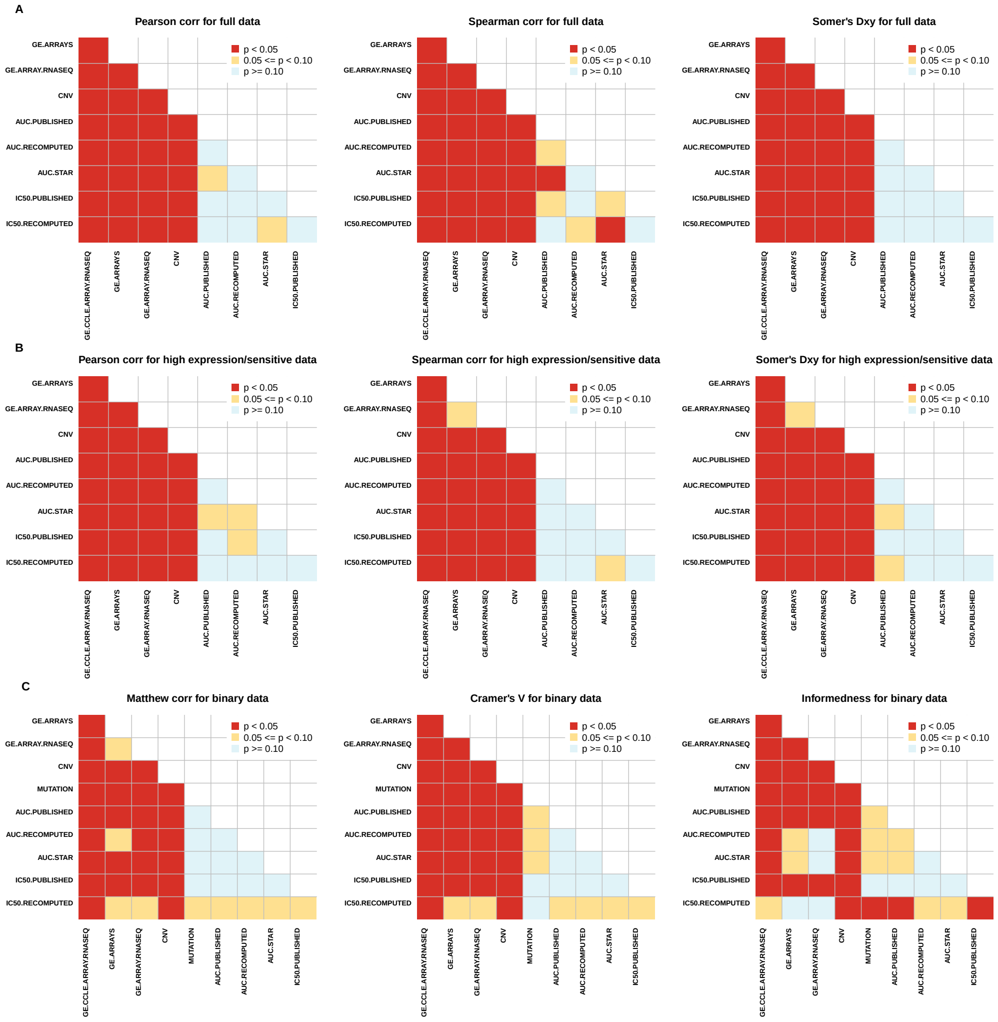

Supplementary Figure 12: Statistical test for difference in consistency for molecular and drug sensitivity data (**A**) for the full drug sensitivity data; (**B**) for the highly expressed genes and cell lines sensitive to the drugs (AUC > 0.2 / IC$_{50}$ $< 1\mu\mathrm{M}$ and AUC > 0.4 / IC$_{50}$ $< 10\mu\mathrm{M}$ for targeted and cytotoxic drugs, respectively); (**C**) for the binary gene expression and drug sensitivity calls. Each cell in the matrix represents the p-value (coded by colour) for a given pairwise comparison of consistency estimates. For instance, consistency of gene expression data is statistically significantly higher than consistency of drug sensitivity data. GE.CCLE.ARRAY.RNASEQ: Consistency between gene expression data generated using Affymetrix HG-U133PLUS2 microarray and Illumina RNA-seq platforms within CCLE; GE.ARRAYS: Consistency between gene expression data generated using Affymetrix HG-U133A and HG-U133PLUS2 microarray platforms in GDSC and CCLE, respectively; GE.ARRAY.RNASEQ: Consistency between gene expression data generated using Affymetrix HG-U133A microarray and Illumina RNA-seq platforms in GDSC and CCLE, respectively; CNV: Consistency of copy number variation data in CCLE and GDSC, respectively; MUTATION: Consistency of mutation profiles in CCLE and GDSC, respectively; AUC.PUBLISHED: Consistency of AUC values as published in GDSC and CCLE; AUC.PUBLISHED: Consistency of AUC values as published in GDSC and CCLE; AUC.RECOMPUTED: Consistency of AUC values in GDSC and CCLE as recomputed using *PharmacoGx*; AUC.STAR: Consistency of AUC values in GDSC and CCLE as recomputed from the common concentration range using *PharmacoGx* ; IC50.PUBLISHED: Consistency of IC$_{50}$ values as published in GDSC and CCLE; IC50.RECOMPUTED: Consistency of IC$_{50}$ values in GDSC and CCLE as recomputed using *PharmacoGx*.

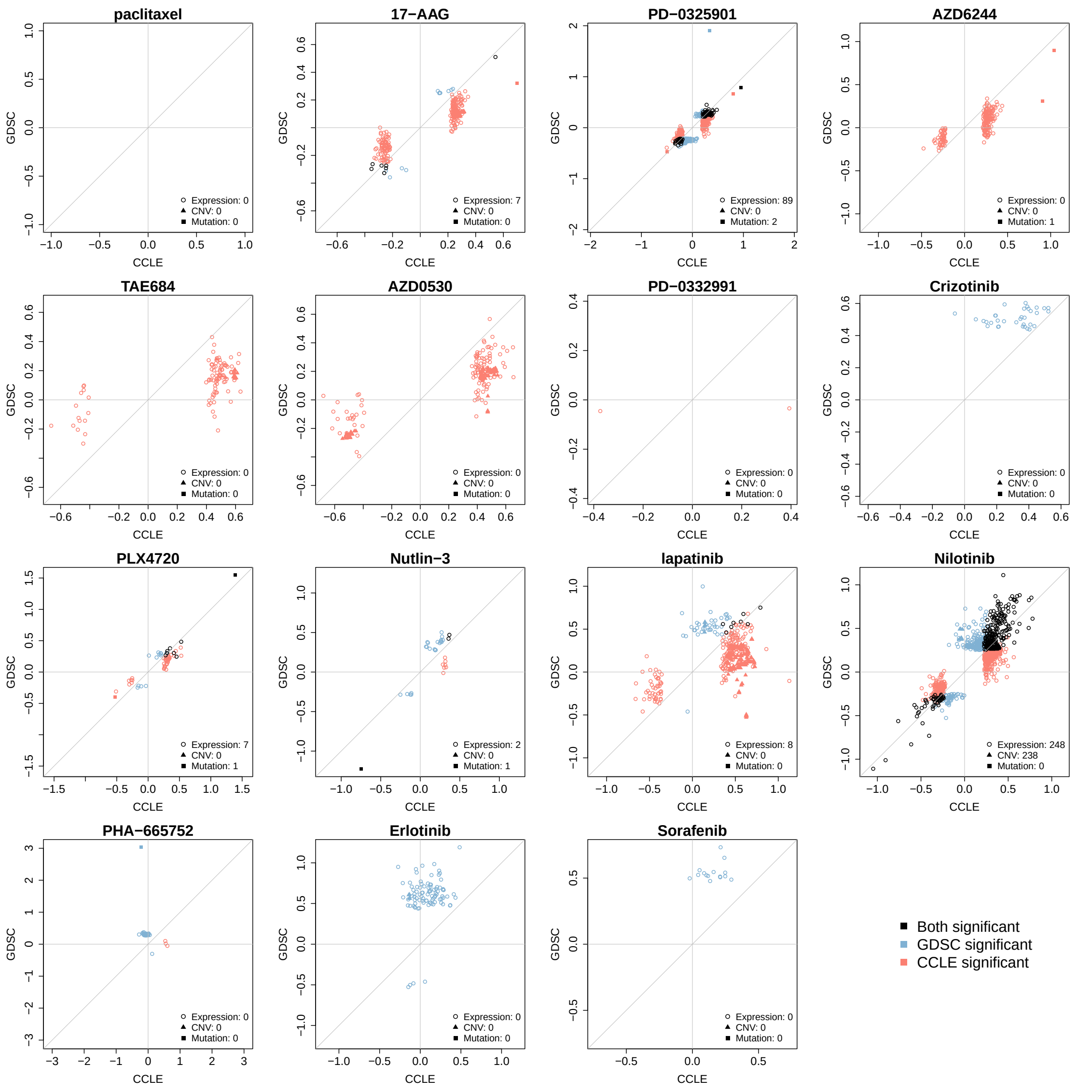

Supplementary Figure 13: Scatterplot representing the effect size of the significant gene-drug associations (FDR $< 5\%$) identified using continuous AUC and the common cell lines screened both in GDSC and CCLE. Gene-drug associations are identified using molecular profiles including gene expression, mutation and copy number variation data and continuous published AUC as input and output of a linear model, respectively. In case of perfect consistency, all points would lie on the grey diagonal.

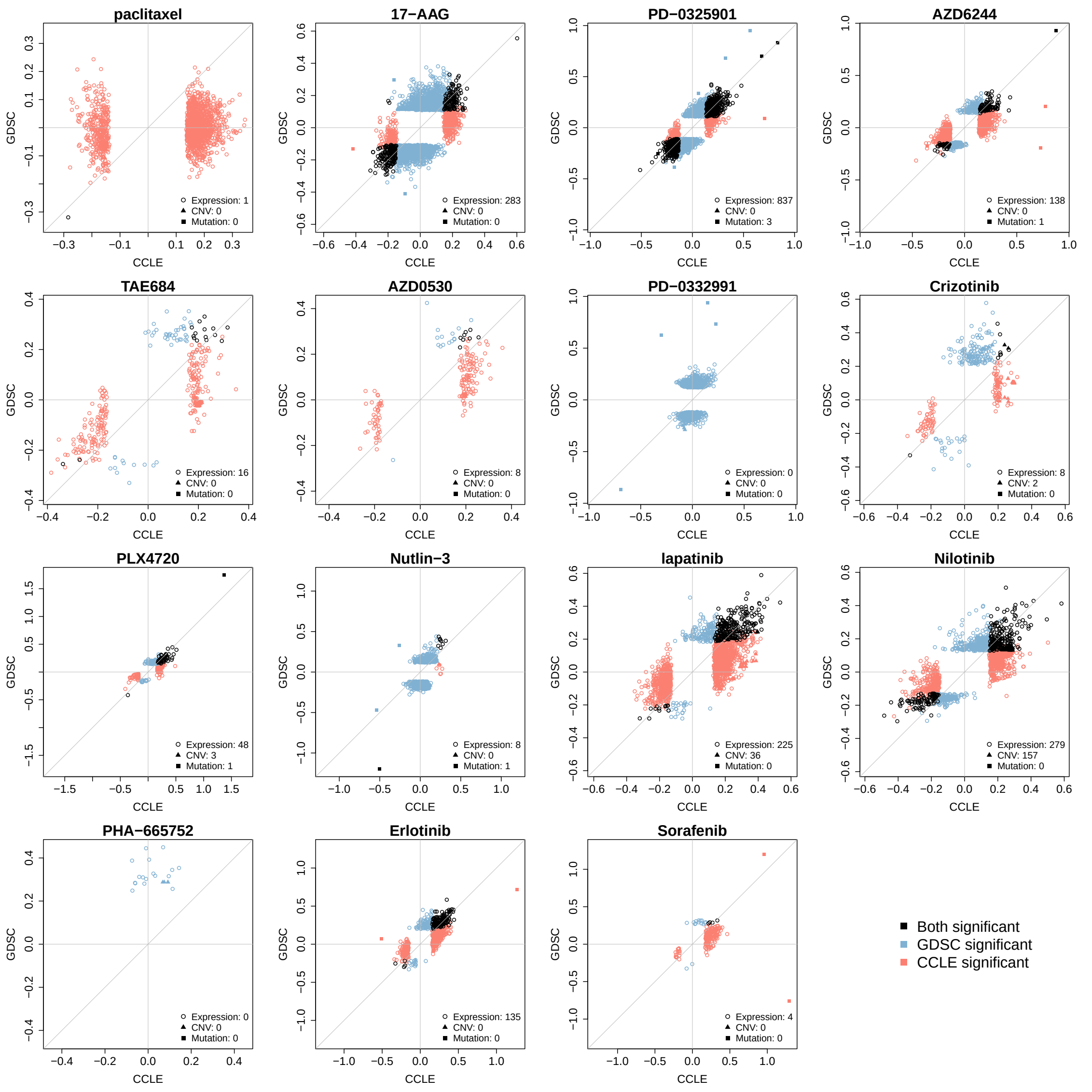

Supplementary Figure 14: Scatterplot representing the effect size of the significant gene-drug associations (FDR $< 5\%$) identified using continuous AUC and all cell lines screened in each study. Gene-drug associations are identified using molecular profiles including gene expression, mutation and copy number variation data and continuous published AUC as input and output of a linear model, respectively. In case of perfect consistency, all points would lie on the grey diagonal.

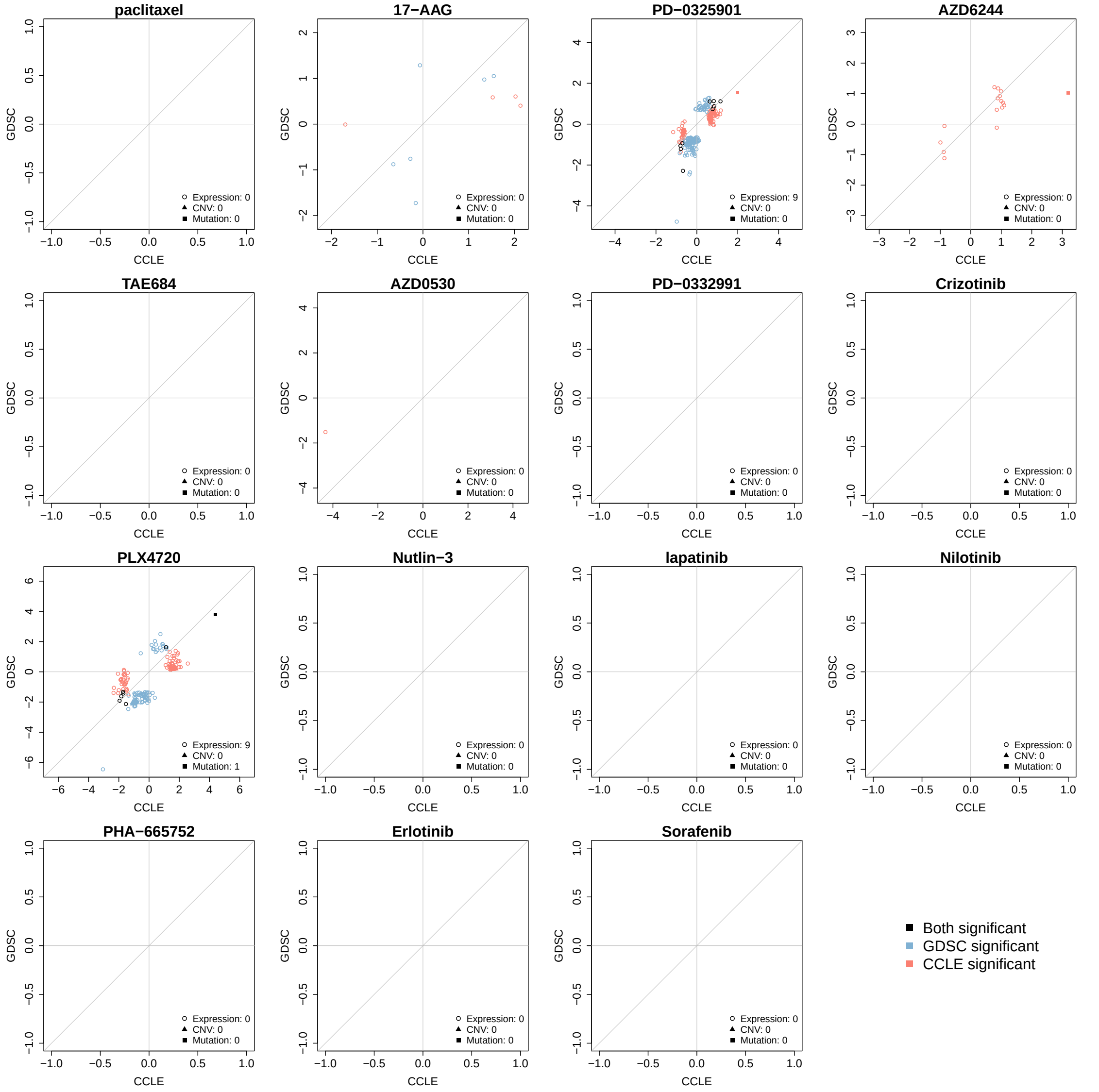

Supplementary Figure 15: Scatterplot representing the effect size of the significant gene-drug associations (FDR $< 5\%$) identified using discretized AUC and the common cell lines screened both in GDSC and CCLE. Gene-drug associations are identified using molecular profiles including gene expression, mutation and copy number variation data and discretized published AUC (AUC > 0.4 for paclitaxel, AUC > 0.2 for the other drugs) as input and output of a linear model, respectively. Note that the small number of cell lines classified as "sensitive" did not allow for finding enough significant gene-drug associations for the majority of the drugs. This is due to the lack of convergence of the logistic regression model when 3 or less cell lines are in one category.

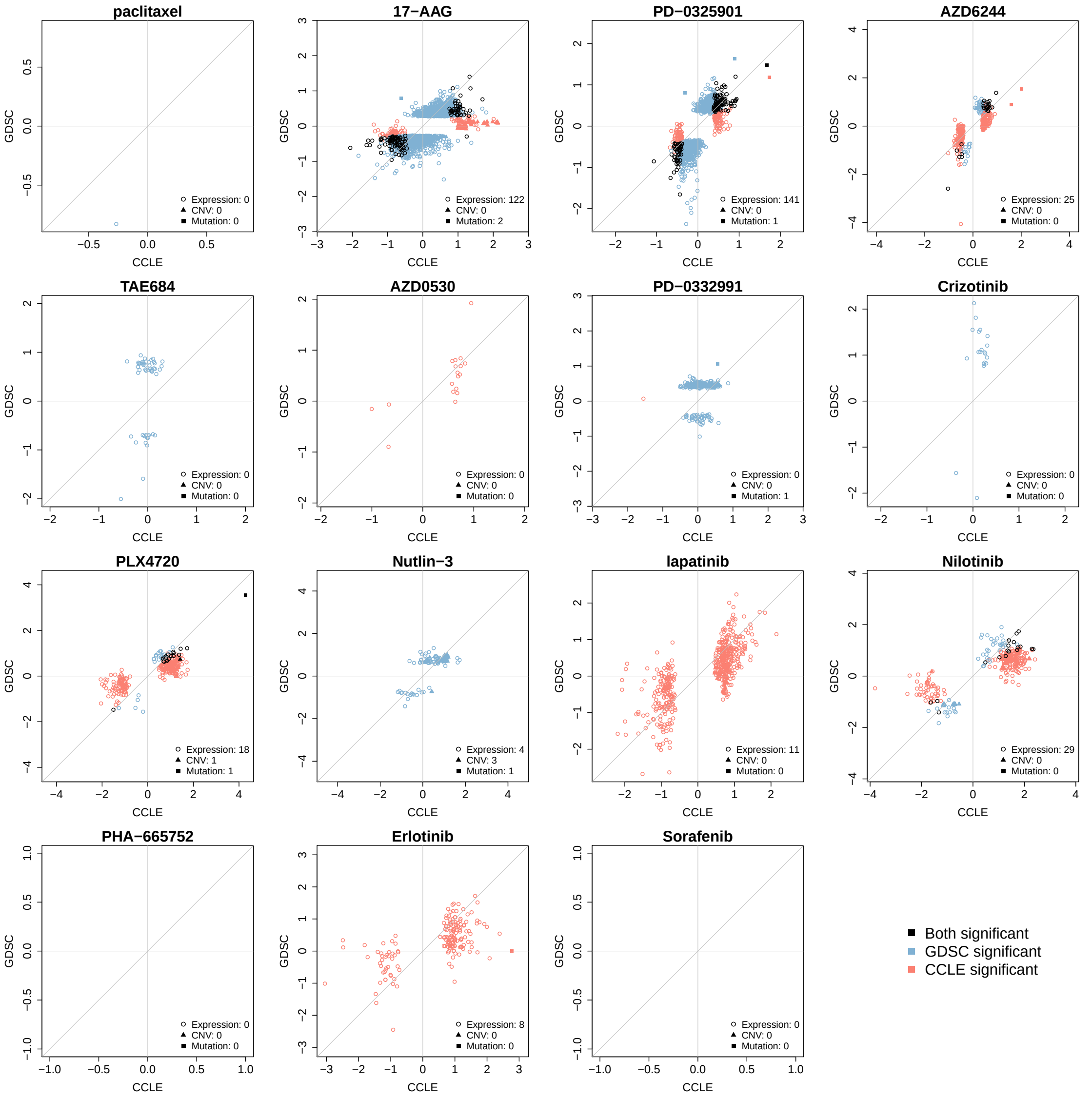

Supplementary Figure 16: Scatterplot representing the effect size of the significant gene-drug associations (FDR $< 5\%$) identified using discretized AUC and all cell lines screened in each study. Gene-drug associations are identified using molecular profiles including gene expression, mutation and copy number variation data and discretized published AUC (AUC > 0.4 for paclitaxel, AUC > 0.2 for the other drugs) as input and output of a linear model, respectively. Note that the small number of cell lines classified as "sensitive" did not allow for finding enough significant gene-drug associations for PHA-665752 and sorafenib. This is due to the lack of convergence of the logistic regression model when 3 or less cell lines are in one category.

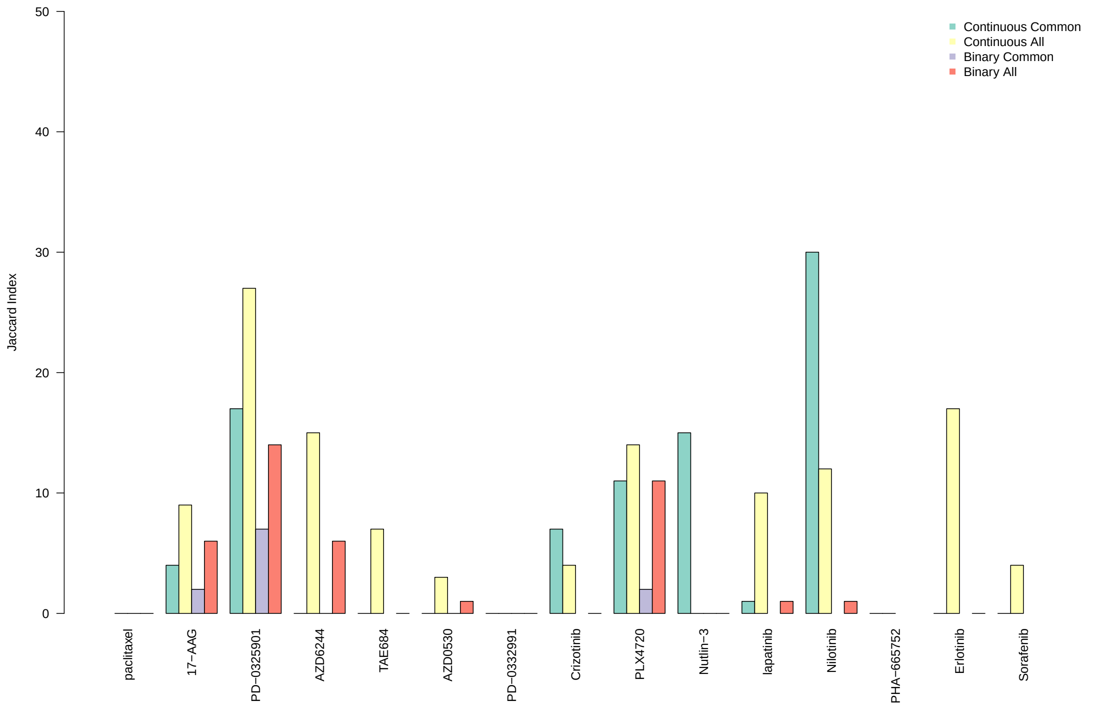

Supplementary Figure 17: Barplot representing the overlap, as estimated by the Jaccard index, between the gene-drug associations found in GDSC and CCLE. 'Continuous Common' refers to the associations identified using continuous published AUC values on the common cell lines in GDSC and CCLE; 'Continuous All' refers to the associations identified using continuous published AUC values on the entire panel of cell lines screened in each study; 'Binary Common' refers to the associations identified using the discretized (binary) published AUC values on the common cell lines in GDSC and CCLE; 'Binary All' refers to the associations identified using the discretized (binary) published AUC values on the entire panels of cell lines screened in each study

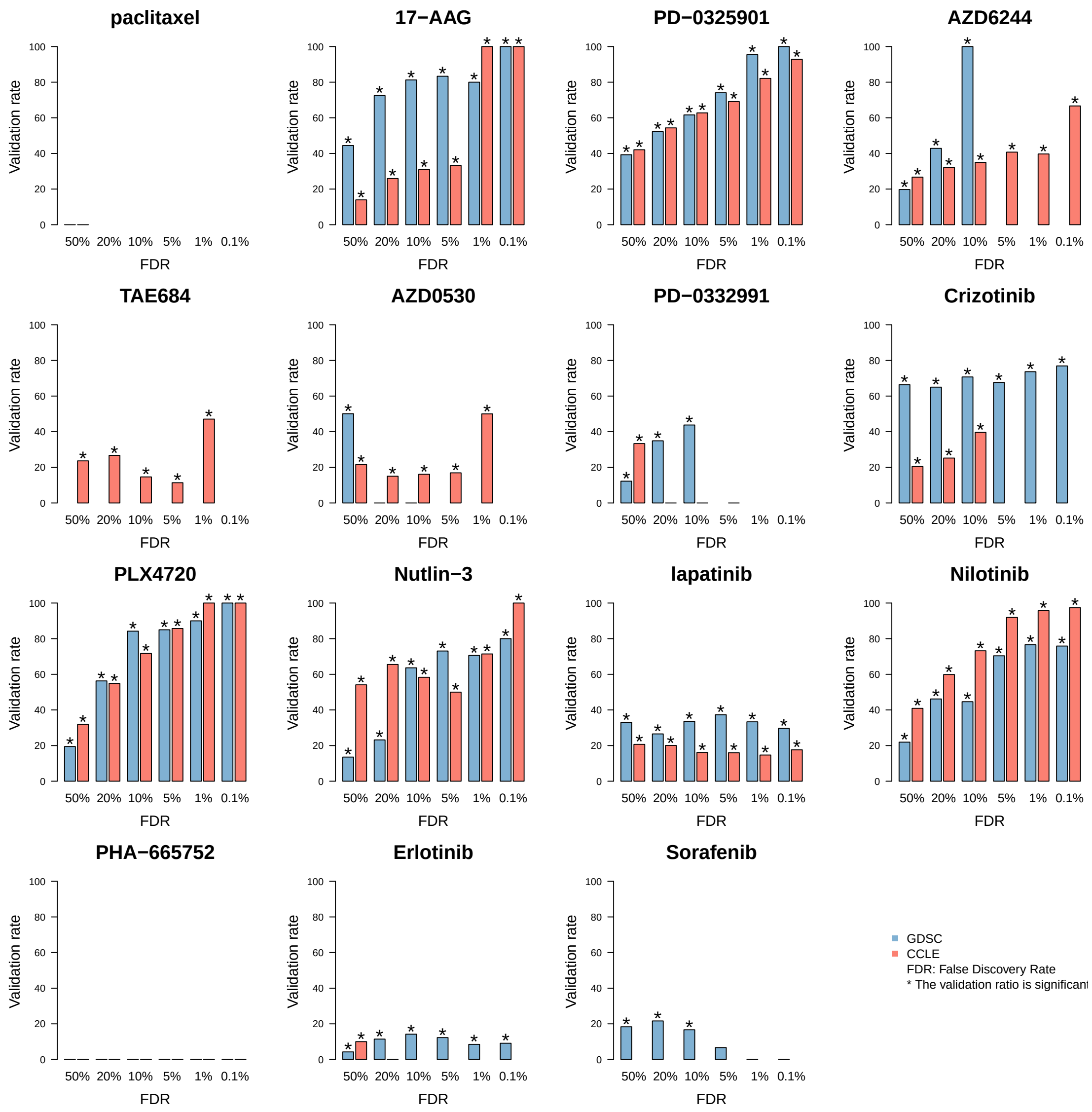

Supplementary Figure 18: Proportion of validated biomarkers with decreasing FDR using common cell lines screened both in GDSC and CCLE. Gene-drug associations are identified using molecular profiles including gene expression, mutation and copy number variation data and continuous published AUC as input and output of a linear mode, respectively. The symbol '*' represents the significance of the proportion of validated gene-drug associations, computed as the frequency of 1000 random subsets of markers of the same size having equal or greater validation rate compared to the observed rate.

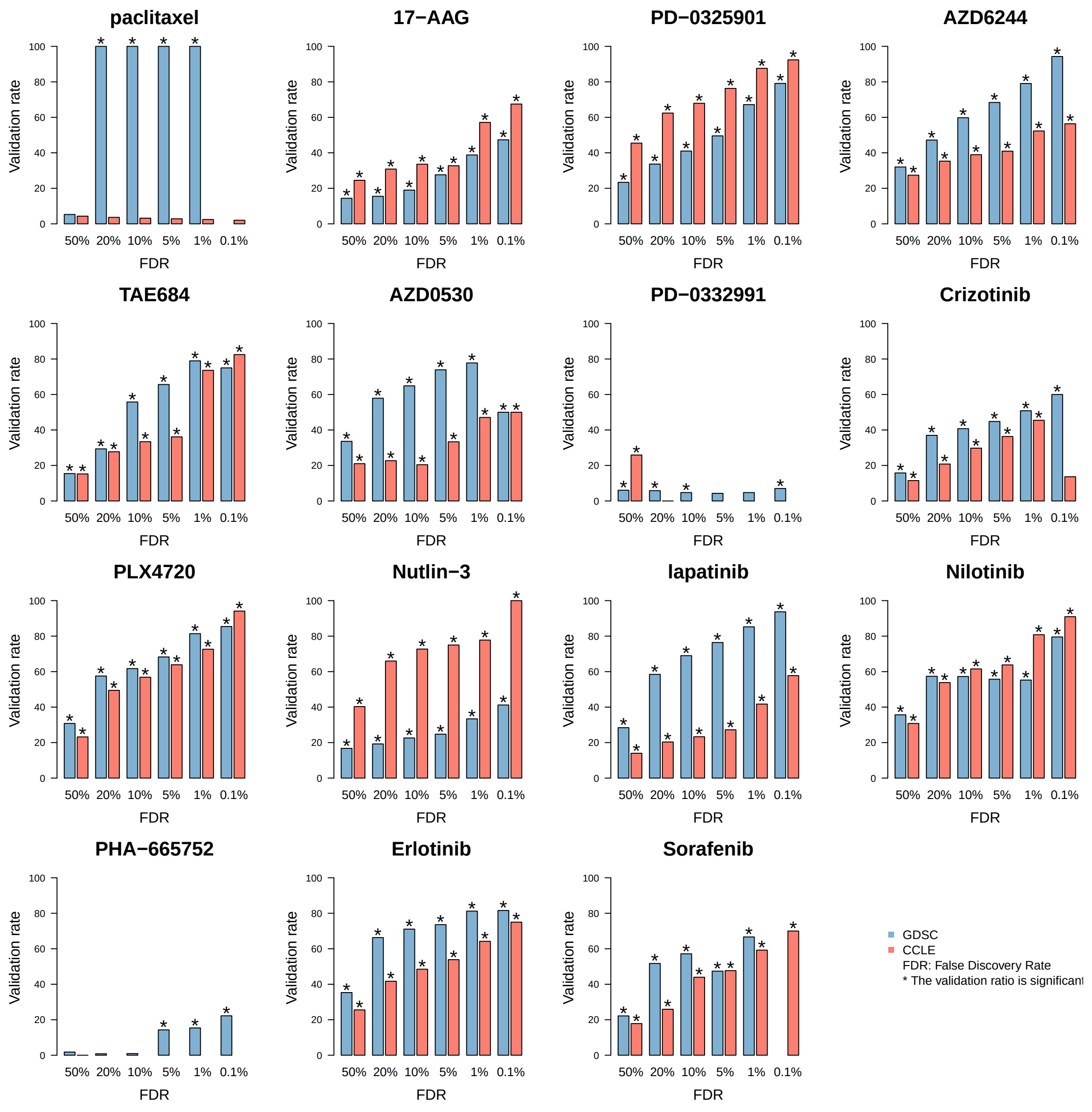

Supplementary Figure 19: Proportion of validated biomarkers with decreasing FDR using all cell lines in each study. Gene-drug associations are identified using molecular profiles including gene expression, mutation and copy number variation data and continuous published AUC as input and output of a linear mode, respectively. The symbol '*' represents the significance of the proportion of validated gene-drug associations, computed as the frequency of 1000 random subsets of markers of the same size having equal or greater validation rate compared to the observed rate.

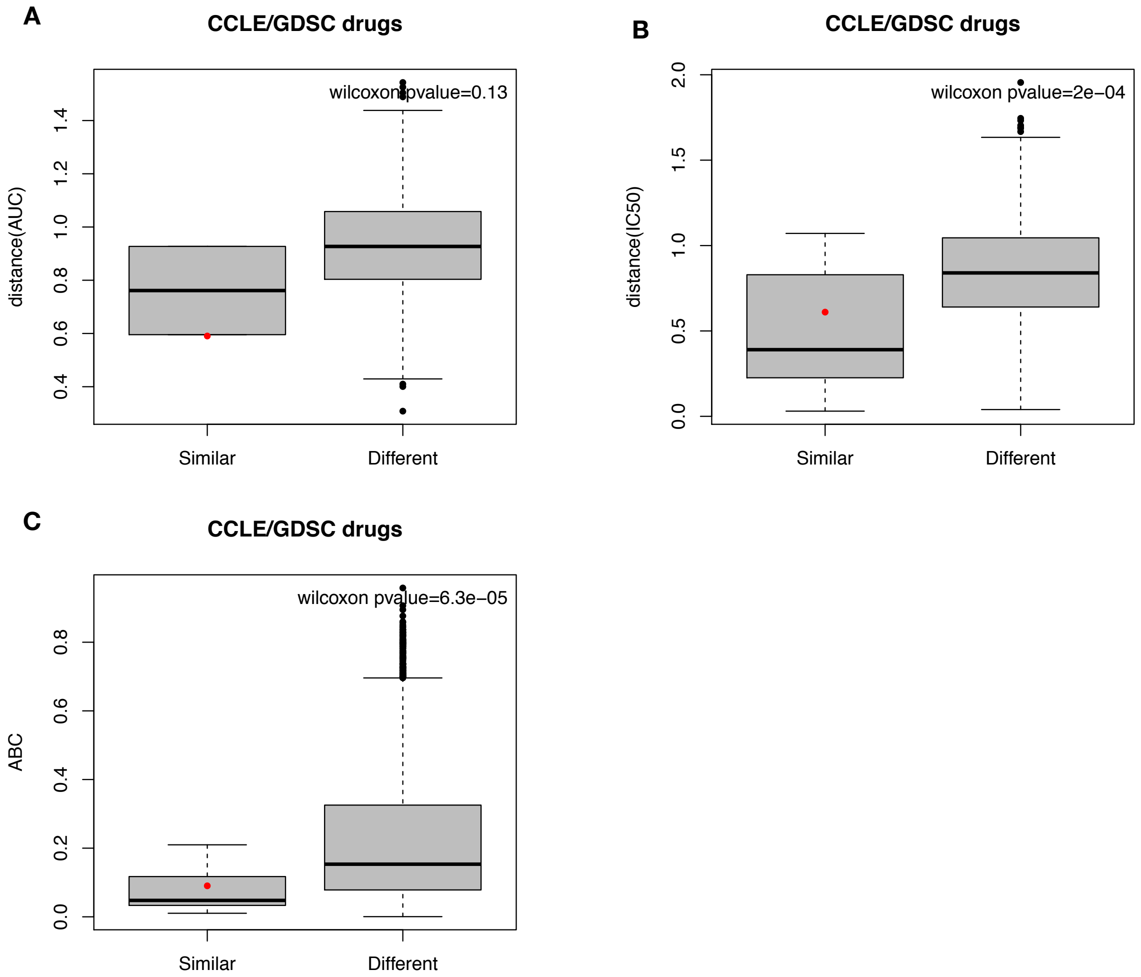

Supplementary Figure 20: Comparison of the distance between similar drugs versus different drugs using (**A**) distance based on 1-pearson correlation of published AUC; (**B**) distance based on 1-pearson correlation of published IC_{50}; and (**C**) Distance based on median ABC. The red point shows the distance between AZD6482 replicates in GDSC.

Supplementary Tables

	# GDSC	# CCLE	% GDSC	% CCLE	% Both
paclitaxel	0	0			
17-AAG	39	435	8	88	4
PD-0325901	455	811	30	53	17
AZD6244	0	418	0	100	0
TAE684	0	221	0	100	0
AZD0530	2	851	0	100	0
PD-0332991	19	3	86	14	0
Crizotinib	41	12	72	21	7
PLX4720	20	149	11	78	11
Nutlin-3	30	9	65	20	15
lapatinib	78	910	8	91	1
Nilotinib	1256	865	42	29	30
PHA-665752	39	7	85	15	0
Erlotinib	134	0	100	0	0
Sorafenib	31	1	97	3	0

Supplementary Table 1: Table reporting the total number of gene-drug associations identified using continuous published AUC and only the cell lines in common between GDSC and CCLE. The proportion of associations that are dataset-specific or reproducible across GDSC and CCLE are provided in the last three columns. The column '% Both' reports the overlap of gene-drug associations between the two studies, as computed using the Jaccard index.

	# GDSC	# CCLE	% GDSC	% CCLE	% Both
paclitaxel	1	2119	0	100	0
17-AAG	2950	978	68	23	9
PD-0325901	2847	738	58	15	27
AZD6244	603	1301	27	58	15
TAE684	97	318	22	71	7
AZD0530	31	314	9	88	3
PD-0332991	2635	0	100	0	0
Crizotinib	235	159	57	39	4
PLX4720	142	279	29	57	14
Nutlin-3	2609	9	99	0	0
lapatinib	291	2482	9	80	10
Nilotinib	1328	1975	35	53	12
PHA-665752	164	0	100	0	0
Erlotinib	174	757	15	67	17
Sorafenib	30	245	10	86	4

Supplementary Table 2: Table reporting the total number of gene-drug associations identified using continuous published AUC and all cell lines in GDSC and CCLE. The proportion of associations that are dataset-specific or reproducible across GDSC and CCLE are provided in the last three columns. The column '% Both' reports the overlap of gene-drug associations between the two studies, as computed using the Jaccard index
