# Supplementary material for: Revisiting inconsistency in large pharmacogenomic studies
Source: F1000Res. 2017 Aug 11;5:2333. Originally published 2016 Sep 16. [Version 3] doi: 10.12688/f1000research.9611.3 (PMC5580432; doi:10.12688/f1000research.9611.3)
Supplement: Peer review history

# Peer review: Referee response for version 3

**DOI:** 10.5256/f1000research.13399.r24961

**Published:** 2017-08-21

**Authors:** Michael T. Hallett

**Affiliations:** 1 Centre for Structural and Functional Genomics, Department of Biology, Concordia University, Montréal, QC, Canada

My concerns have been addressed, and the quality of the presentation is now more than sufficient to understand the details of their methodology.

 It is an important article, and the findings here contribute to (hopefully) a larger, quantitative discussion regarding pharmacogenomic studies. Differences (both technical and biological in nature) in methodologies between studies cause differences in results (eg which compounds are identified as affective). The methodological differences might be important: although they can "deflate" reproducibility, they also serve to probe a larger search space (that is, each assay may be exploring a subtly different part of a huge space of compounds with specific bioactivity). 

 Regardless, it is important to have the tools to have a quantitative discussion about whether two assays disagree due to "nuisances", or whether they disagree because they are fundamentally testing different hypothesis. This manuscript, and the software developed here, provide the community with important components to facilitate such discussions.

I have read this submission. I believe that I have an appropriate level of expertise to confirm that it is of an acceptable scientific standard.

# Peer review: Referee response for version 2

**DOI:** 10.5256/f1000research.13143.r24479

**Published:** 2017-07-26

**Authors:** Paul T. Spellman

**Affiliations:** 1 Department of Molecular and Medical Genetics, Oregon Health and Science University, Portland, OR, USA

"Our finding that molecular profiles are significantly more consistent than drug sensitivity data, indicates that the main barrier to biomarker development using these data is the unreliability in the reported response phenotypes for many drugs. For studies such as these to realize their full potential, additional work must be done to develop robust and reproducible experimental and analytical protocols so that the same compound, tested on the same set of cell lines by different groups, yields consistent and comparable results. Barring this, a predictive biomarker of response developed from one study is unlikely to be able to reliably validated on another, and consequently, is unlikely to be useful in predicting patient response."

 Again, I think the concern here is wrong for the same reasons I describe above. The main barrier is *not* the differences in quality between assays. The author's response to my initial concern shifts it to variability within an assay, but that is obvious -- if an assay doesn't work it can't be informative. Bad assays should be excluded. Period. The original premise of the work was that differences in results between assays was problematic but that is not the problem. Its that specific assays do not produce the high quality data necessary. 

 My further points remain. I agree it may be hard, but it is possible.

I have read this submission. I believe that I have an appropriate level of expertise to confirm that it is of an acceptable scientific standard, however I have significant reservations, as outlined above.

# Peer review: Referee response for version 1

**DOI:** 10.5256/f1000research.10354.r22599

**Published:** 2017-05-10

**Authors:** David G. Covell

**Affiliations:** 1 Screening Technologies Branch, Developmental Therapeutics Program, National Cancer Institute, Frederick, MD, USA

The paper under review, 'Revisiting inconsistency in large pharmacogenomics studies' by Zhaleh Safikhani, Petr Smirnov, Mark Freeman, Nehme El-Hachem, Adrian She, Quevedo Rene, Anna Goldenberg, Nicolai J. Birkbak, Christos Hatzis, Leming Shi, Andrew H. Beck, Hugo J.W.L. Aerts, John Quackenbush, Benjamin Haibe-Kains, reports an updated analysis of results from two previously published systematic drug screening projects[1,2]. As explained in their introductory material, this report is motivated in part by the expansion of data from these earlier studies, and as a means to document alternative data analysis strategies that have been proposed for improving the original publication[3].

 The authors address two highly important areas in basic and clinical research: data reproducibility and predictive (gene expression) biomarkers based on drug sensitivity data. The former issue represents a hallmark of basic science research; where results derived from different labs and measurement techniques serve to establish strong confidence in a proposed experimental protocol. The latter issue pertains best to highly confident (e.g. reproducible or consistent) experimental measurements; while data inconsistencies foreshadow a Pandora’s Box of alternatives in the search for the origins of these differences.

 With respect to the issue of data reproducibility, I find no fault in the new manuscript. All of the results reported in the original 2013 paper and current paper under review can be obtained from their Supplementary R code. With a bit of diligence and tenacity, sequentially stepping through their R-code will yield the reported figures and tables. Towards that end, the original R-code is tedious, but their addition of an open-source R-package, PharmacoGx, relieves much of the tedium. In fact, the authors must be applauded for making their analysis completely reproducible, a feat rarely achieved with biological results.

 Notwithstanding, the results remain largely the same; inconsistencies remain in the drug sensitivity profiles between the GCSC[2] and CCLE[1] groups. Data analysis based on alternative methods appear to be constructed around the arguments proposed in the pair of Brief Communications Arising from the original paper[4,5]. The general idea of this alternative data analysis is based on the limited role of weakly responsive tumor cells, and thus a failure to contribute to meaningful statistics. While segregating the data into three classes (drugs with no observed tumor cell activity, activity in a few tumor cells and activity in a large number of tumor cells) improves the statistics, the differences largely remain.

 Speculations about the differences between the two datasets focus, naturally, on each measurement platform. The current manuscript’s proposal of internal standardization may help identify the origin(s) of these differences, but this alone may not be sufficient. In this regard, I would recommend the Supplementary Information (all 47 pages) from the original article[3]. Specifically, Section 3, Comparison of experimental protocols, and the included Comparative table. The details of this section identify a number of platform differences that may underlie their measurement differences. Although not within the scope of the current article, a future study focused on these differences, combined with standardization, rather than looking for answers by segregating the data into three response classes, would be highly informative.

 An alternative speculation regarding data inconsistencies considers the ‘dated’ possibility that each tumor cell’s drug sensitivity and underlying phenotypic architecture (expression, mutation, snp, etc.) exemplifies a ‘snowflake’ phenomenon. Each tumor cell represents a unique circumstance, which can be modulated by any sort of environmental condition. Thus a drug response, even for the same tumor cell, may exhibit variation. Under these circumstances, the functional pathways, represented by groups of genes and their concordant expressions, become the focus, and derivation of pathway-based scoring schemes may significantly overcome inconsistencies between experimental groups. Clearly an appropriate pathway fitness scoring scheme has yet to be devised. 

 In summary, the analysis is sound, the results are clear, and the analyses of inconsistent data, as a means to obtain predictive biomarkers, remains a significant challenge.

I have read this submission. I believe that I have an appropriate level of expertise to confirm that it is of an acceptable scientific standard.

## References

[1] BarretinaJCaponigroGStranskyNVenkatesanKMargolinAAKimSWilsonCJLehárJKryukovGVSonkinDReddyALiuMMurrayLBergerMFMonahanJEMoraisPMeltzerJKorejwaAJané-ValbuenaJMapaFAThibaultJBric-FurlongERamanPShipwayAEngelsIHChengJYuGKYuJAspesiPde SilvaMJagtapKJonesMDWangLHattonCPalescandoloEGuptaSMahanSSougnezCOnofrioRCLiefeldTMacConaillLWincklerWReichMLiNMesirovJPGabrielSBGetzGArdlieKChanVMyerVEWeberBLPorterJWarmuthMFinanPHarrisJLMeyersonMGolubTRMorrisseyMPSellersWRSchlegelRGarrawayLA: The Cancer Cell Line Encyclopedia enables predictive modelling of anticancer drug sensitivity. *Nature*.2012;483(7391) : 10.1038/nature11003 603-7 10.1038/nature11003 22460905PMC3320027

[2] GarnettMJEdelmanEJHeidornSJGreenmanCDDasturALauKWGreningerPThompsonIRLuoXSoaresJLiuQIorioFSurdezDChenLMilanoRJBignellGRTamATDaviesHStevensonJABarthorpeSLutzSRKogeraFLawrenceKMcLaren-DouglasAMitropoulosXMironenkoTThiHRichardsonLZhouWJewittFZhangTO'BrienPBoisvertJLPriceSHurWYangWDengXButlerAChoiHGChangJWBaselgaJStamenkovicIEngelmanJASharmaSVDelattreOSaez-RodriguezJGrayNSSettlemanJFutrealPAHaberDAStrattonMRRamaswamySMcDermottUBenesCH: Systematic identification of genomic markers of drug sensitivity in cancer cells. *Nature*.2012;483(7391) : 10.1038/nature11005 570-5 10.1038/nature11005 22460902PMC3349233

[3] Haibe-KainsBEl-HachemNBirkbakNJJinACBeckAHAertsHJQuackenbushJ: Inconsistency in large pharmacogenomic studies. *Nature*.2013;504(7480) : 10.1038/nature12831 389-93 10.1038/nature12831 24284626PMC4237165

[4] BouhaddouMDiStefanoMSRieselEACarrascoEHolzapfelHYJonesDCSmithGRSternADSomaniSSThompsonTVBirtwistleMR: Drug response consistency in CCLE and CGP. *Nature*.2016;540(7631) : 10.1038/nature20580 E9-E10 10.1038/nature20580 PMC555488527905419

[5] SafikhaniZEl-HachemNSmirnovPFreemanMGoldenbergABirkbakNJBeckAHAertsHJQuackenbushJHaibe-KainsB: Safikhani et al. reply. *Nature*.2016;540(7631) : 10.1038/nature19839 E2-E4 10.1038/nature19839 27905430

# Response

**Published:** 2017-07-07

**Authors:** Benjamin Haibe-Kains

We thank Dr Covell for his constructive comments regarding our study. We are glad to hear that our PharmacoGx package is useful to reproduce our analysis results. The hope is that our package will enable other research groups to analyze and compare their own data with published large-scale pharmacogenomic datasets. 

We agree with the reviewer that more investigation is required to better assess the technical vs biological variations for each of the pharmacological assays. Then biological variations could be leveraged, at the pathway level as the reviewer suggested, to define more robust biomarkers.

# Peer review: Referee response for version 1

**DOI:** 10.5256/f1000research.10354.r17399

**Published:** 2016-12-21

**Authors:** Paul T. Spellman

**Affiliations:** 1 Department of Molecular and Medical Genetics, Oregon Health and Science University, Portland, OR, USA

Safikhani *et al.* have updated their previous analysis of two of the largest systematic drug screening projects linked to genomics data. The previous findings indicated that there is a lack of concordance between the two datasets that makes finding biomarkers of response difficult. Updating these studies with a wider array of methods in response to comments about the original article leaves largely the same result. Drug sensitivity profiles show significant variation between the two groups, likely due to differences in assay condition.

 Safikhani *et al.* follow this analysis up with a discussion on how to improve the situation and here I have some significant issues. The base argument is that the differences in platform are creating biases in the results and therefore the platforms need to be standardized. I think this is completely wrong. This makes sense if one platform were known to recapitulate *in vivo* response more accurately, but that is not true. We do not know if one platform is more physiologically relevant than another so the lack of standardization actually tells you something, it tells you when a predictor result is robust against biological context and is therefore more likely to work in new biological contexts. I would argue we need *more* variability in assays and platforms to broaden the scope of biological systems, not less.

 Similarly, the statement is made that there is a 75% validation rate for eight drugs but that "this does not suggest that one can use these studies to find new, reproducible gene-drug associations...", I actually think it does, but perhaps I am missing a subtlety.

 Finally, I think it is possible to set drug specific thresholds for each dataset. We have done this, I believe successfully, with datasets far smaller.

I have read this submission. I believe that I have an appropriate level of expertise to confirm that it is of an acceptable scientific standard, however I have significant reservations, as outlined above.

# Response

**Published:** 2017-07-12

**Authors:** Benjamin Haibe-Kains

*We thank the reviewer for his constructive comments. We agree with the reviewer that we need to update the discussion to reflect this important point. In the absence of “gold standard” screening platform, the best biomarkers are likely those that are robust to the use of different assays, as these assays assess different biological aspects of growth inhibition. However one must clearly distinguish between technical and biological variations. While biological variations might be interesting for biomarker discovery, assay variation must be kept as low as possible. Looking at the replicates performed for AZD6482 in GDSC, we found that drug sensitivity data lack consistency even when the same assay is used (see new Supplementary Figure 2E). In this setting, one cannot claim that the inconsistencies observed between GDSC and CCLE are solely due to differences in the type of assay used for drug screening. Even if we agree with the reviewer, we believe there is still work to be done to improve the robustness of each pharmacological assay. We have updated the discussion section of our manuscript accordingly.*

**Similarly, the statement is made that there is a 75% validation rate for eight drugs but that "this does not suggest that one can use these studies to find new, reproducible gene-drug associations...", I actually think it does, but perhaps I am missing a subtlety.**

*Although our new analysis revealed reasonable consistency for biomarker discovery for 8 out of 15 drugs, we could not get such a validation rate for the rest of the drugs where the biomarkers are only significant in one study but not the other.*

**Finally, I think it is possible to set drug specific thresholds for each dataset. We have done this, I believe successfully, with datasets far smaller.**

*Like this reviewer, we too tried to identify drug-specific cutoff that would allow us to binarize the drug sensitivity while optimising the consistency across datasets. Our best efforts were not successful though, except for Nilotinib (see Safikhani et al, Nature 2016; PMID: 27905430). Not to say that it is not feasible but we found it very challenging to define a cutoff within a dataset that would yield good concordance across datasets.*

# Peer review: Referee response for version 1

**DOI:** 10.5256/f1000research.10354.r16370

**Published:** 2016-11-03

**Authors:** Michael T. Hallett

**Affiliations:** 1 Centre for Structural and Functional Genomics, Department of Biology, Concordia University, Montréal, QC, Canada

This manuscript seeks to compare two large pharmacogenomics datasets (several hundred cancer cell lines screened against 15 common drugs) and evaluate their level of agreement via (1) the drug sensitivity values and (2) gene expression profiles of the cell lines. Broadly speaking, the value of these profiles is the discovery of (gene) biomarkers that could predict response of cells to the drugs. Previous efforts, including the authors' previous attempts, have had trouble with reproducibility. The authors have previously given harsh critiques regarding the reproducibility of the two datasets.

 This manuscript is very important, and it has the potential to dissect sources of both agreement and disagreement that can be amplified or minimized in the future respectively. The reviewer also has little doubt that there is in fact disagreement between these two datasets and, moreover, it is significant enough as to interfere with the discovery of biomarkers. The reviewer also agrees with the authors that this is important to point out and understand, and the "call to arms" in the Discussion (the best written part of the manuscript) should certainly be listened to.

 However, because this manuscript is very important , the cornerstones of the comparative analysis must be correct. The Supplemental Methods are near impossible to decipher and are littered with undefined terms, confusion in mathematical notation, poor equation formatting non-intuitive statements that do not assist the reader with understanding the numerous design choices (from throwing out poor quality data, to model fitting, to different measures of consistency). The Results section is not sufficient methodical to follow the argument of what does or does not represent ***statistically significant*** disagreement. Almost every paragraph until the conclusion presented serious challenges to this referee. They are included below.

 This is an important effort and the authors should return with an improved manuscript. Many of the co-authors are skilled mathematicians and they are strongly recommended to revisit every line of this manuscript to ensure correctness and to present with craftmanship. This is especially true in the Supplementary Methods that actually provide the "meat" of the methodology: this I believe must have been an oversight with this submission. 

 This manuscript needs to be published and I believe there are important lessons to be learnt here but there has to be a more focused, tighter argument to establish where there is disagreement and hypotheses as to why (in the Discussion) and what can be done about it. But the main issue here is that the basics of the paper are not solid, or at least they cannot be evaluated. The authors should be commended for the effort to be reproducible (in the sense they give the list of R packages used and their code) but that is only one aspect. The mathematics and statistics requires clarity and correctness. Terms such as "metric" should be used properly, and novel equations that are derived (e.g their "E" parameter) must be done so in a careful correct manner, with attempts made to justify these parameters (e.g \epsilon, \rho, 2*\epsilon, the $E$ parameter from the modified fit etc.

 I would be happy to view a revised version of the manuscript and I hope that my comments aid in this important project.

 pg5: What is “Dataset 1”? The link here doesn’t lead anywhere that I can tell.

 Figure 3. I’m not really sure what the value is in plotting the density functions for the mismatched and matched cell lines. First, wouldn’t one density function suffice with a threshold I guess? Second, do you really need it at all?

 In the Methods “Cell line identity….”, it is stated that 66 samples fell below threshold with a reference to the Supplementary methods. However I don’t see anything in the supplementary methods that discusses this. Moreover in the text, it seems that you threw 8 cases away. This is confusing.

 pg 5. I’m not sure what you mean by “remain stable or decrease monotonically”? 

 Do you just mean “monotonically non-increasing”?

 Please see comment regarding “Filtering of drug dose-response curves” form Supp Methods below. I think this really needs to be reworked, and I have to trust you guys here that you are doing the right thing.

 “as exemplified…” depicted?

 In Figure 4, is it possible to relate this back to the choice of \rho, \epsilon and 2 \cdot \epsilon from the Supp Methods, or perhaps integration a version of this figure (but annotated) into the Supp Methods. 

 In panel A, the grey area is a bit non-intuitive no? I would say that post 0.03, it’s looking pretty good, and it’s not measured in GDSC after that. However the first points are off.

 I don’t know what \epsilon or \rho are so it’s hard to relate what is depicted in Figure 4 back to your model.

 When I look through Supplemental Figure 1 (all the excluded comparisons), it seems like your criterion for excluding a comparison boils down cases where at least one of the curves has high variance, and the cutoff 2\epsilon I think  is a constant independent of the distribution of points for either curve. I don’t see in your equations how you encode that the sequence is monotonically non-increasing, or how “order” along the left to right sweep is incorporated.

**Supp Methods Filtering of drug dose-response curves**

 i +1^{st} -> (i+1)^{st}

 It seems like there is a formatting problem here. In my pdf there is something like I”A squiggle after “in some large fraction ??? of the cases (1).” I guess that’s supposed to be \rho right?

 I’m not sure I understand this sentence “Our quality control …” Are you saying that \Delta_{i, i+1} < \epsilon in some fraction \phi of the cases?

 equation #2 below:

 I also have never seen set notation such as \{ \Delta_{i,i+1} | \Delta_{i,i+1} < \epsilon \}.

 Are you trying to say “given all the \Deltas that are less than \epsilon?

 So the vertical | means cardinality here right?

 But then the denominator has the cardinality of a value, or do you mean absolute value?

 What is \rho? It’s undefined.

 “Unfortunately …”  The english is a bit rough - could be rephrased in terms of specificity and sensitive, I guess.

 I don’t understand the significance of the sentence “Consider, for instance, …” But in the main text, you say that it remains stable or decreases monotonically. Here it increases monotonically for many successive points, so this violates your model, no? 

 I think that this subsection wants simply to spell out mathematically the thresholds and also provide some rationale for the parameters. I think the text doesn’t really do a good job of establishing this rationale and needs work. Perhaps define the parameters precisely and then phrase the exposition in standard terms e.g. specific and sensitivity for different \epislon, etc. 

 Your equation (2) is inconsistent. In the text you specify \Sigma_{\forall i,j} \Delta_{i,j} but below your criterion seems to change to (the correct) $i < j$.  It is also sufficient to write \Sigma{ i < j } \Delta_{i,j} and avoid the double summation.

 You should probably define D_i in the text and not make the reader deduce it from the figure below

 The comments here w.r.t. the Supplementary Methods also apply to the associated subsection of the Methods. The mathematical correctness of some comments needs attention. For example, it is not quite correct to say that the “curve fitting would have yielded erroneous results”. The curve fitting is just that, curve fitting. It’s not really an error. Then in the Methods, you claim to use this equation but this is inconsistent with the discussion in the Supplemental Methods (where you have an equation with this undefined parameter $E$). 

 The least-squares method using a three-parameter sigmoid model. I understand the intuition for this, but when I look through Supplementary File 2, I think there are a lot cases where this is perhaps not the correct pattern to assume (e.g. straight lines). Moreover, there are some very strange fits, for example,

 AZD6244:G−361

 AZD6244:SK−MM−2

 PLX4720:MDA−MB−175−VII

 In some cases the curve is always above all of the observations. 

 Perhaps this is because the measurements of viability > 100%? In your model you have removed the $E_0$ from Barretina *et al.* for different reasons. 

**Supp Methods - Fitting of drug dose-response curves**

 I’m a bit lost with your choice of notation here. For example, you define y as an equation, not a function, and then write y(0) which I assume is supposed to be something like y(x). Ok but then you have y=0 and y = y(0) = 1. I’m not sure this is correct mathematically.  (I think you mean to say that y(x ) = … *where* y(0) =1. 

 “viability is reduced to half … concentration of the drug”… so the “Top”, E_\infinity … I find this a bit wordy.

 “The dose response equation now becomes …”

 So I deduce that E is the new parameter?!?!  Where has E_\infinity gone?!?! 

 (But then down below E is constrained to be in [0,1] and seems to related to the fitness of neoplastic cells.  I’m not sure I understand this.)

 There is no derivation of this formula whatsoever. In fact, I don’t see how this could be correct any longer. Couldn’t this be expressed as mixture of two cell types, and y would be then a sort of weighted average?

 I really don’t see how this was derived. This is a very central part of your paper (since the manuscript is measuring agreement) and therefore it needs to be bulletproof.

 Please define “extant drug”. Also HS is allowed to vary apparently but I don’t see where it is then optimized in your analysis later on. This is confusing.

**Consistency of Drug Sensitivity Data**

 Is the ABC method standard? Are there citations for this? You should probably define properly what you mean by the “insertion of the concentration range”. Elsewhere it seems that you are referring to this as “common concentration range” e.g. SFig 2

 More generally, isn’t this a sort of (non-statistical) version of the Kolmogorov-Smirnoff test?

 Figure 5A: Actually there are many such cases in your Supplementary Figures. Doesn’t this just mean that the range of concentrations are not sufficient in both datasets?

 pg 7 “We then computed the median …” I don’t understand what your distance metric is here. If I understand correctly, you could the ABC for each pair of drugs in the GDSC dataset. From that I can imagine a distance matrix D where D_ij i the ABC between drugs i and j. But you said you take the median ABC? median over what? cell lines? repeats? Whatever the case, are you sure it’s a distance metric?! Is it really true that distances derived from ABCs are metrics? I think this should be shown in the Supplemental Methods. Also as a minor comment, the caption in Supp Figure 3 says that you are using the mean ABC value but elsewhere it says median.

 p8. I am not sure what the significance of Supplementary Figure 3 is: are any of these clusters significant? Why are two drugs coloured red in panel A? On page 8 it is claimed that the samples split by hospital (MGH vs WTSI) but I don’t see how this is represented in Supplemental Figure 3. 

 pg 8. I have a very hard time estimating the significance of a statement like “…3 out of  15 common drugs clustered tightly”. I am not sure what tight means here. 

 When I look at Supp Figure 3 there has been no effort to annotate the clusters with their reproducibility e.g. pvclust or measure their significance in some other way. 

 When I look at the figure I think there appears to be a lot of co-clustering of the drugs, at least given that the median ABC across a diverse collection of cell lines might not be such a great “distance” measure. 

 pg 9. What do you mean by “highly targeted therapies”?

 pg 9. paragraph “Although the ABC values …”  I think the ABC is interesting but it takes a very prominent role in your paper when there are other standard techniques already like AUC and IC_50. Perhaps the manuscript should have comments about why have chosen this approach that is not standard. Also I think you would need to make precise what the differences are between how GDSC and CCLE computed the AUC and IC_50 that are different than how PharmacoGX does. This is a very central concept in your comparison so it would have to have a very solid definition and analysis. Supplemental Figure 4 suggests in a round-about way that the only difference in in the number of cell lines (figure caption). This is a bit confusing.

 Again, I am not sure what “Dataset 2” refers to. Perhaps the manuscript would benefit from the addition of some interpretation as to what you believe  Supp Figure 4 means.

 I don’t understand the definition of your three classes of drugs (no effect (AUC > 0.2); narrow effect AUC \leq 0.13 or broad affect AUC > 0.13). I don’t see how this definition clearly delineates between “no effect” and “narrow effect”. 

 The bottom paragraph of the first column is one sentence that spans 8.5 lines. It references 2 main figures of the paper and 5 supplementary figures. To be honest, this is very frustrating. I have gone through the Supplemental Methods very closely and I don’t see anywhere where the authors have distinguished between “recomputed AUC” and “AUC computed based on the common concentration range”. Then “IC_50 (figure figure) values” ?? 

 I’m not sure what to interpret re: Figure 7 for example. To me it looks like there is excellent agreement except for perhaps the first row. Only paclitaxel fits into this “cyotoxic drug’ category but for the life of me, I don’t see where this is defined. The authors just defined three types of drugs (no effect, narrow effect and broad effect) but that’s not what they are using here. I don’t understand this. To me it simply seems to be that at low AUCs there is high variance in the last 3 distributions of the first line (17-AAG PD-0325901 and AZD6244), but actually they look like they pretty well agree at higher AUCs. I’m not sure what that means 

 “and calculated the consistency of drug sensitivity data between studies using all common cases and only those that the data suggested were sensitive in at least on study.

 Maybe a table would help, especially if each of these different objects were properly defined in the Methods/Supp Methods. 

 “Given that no single metric can capture all forms of consistency, …” So you add three more. I don’t see the point here. Why these three? and how is something like pearson \rho applied here. What is the vector? I would guess that Supp Figures should show the distribution of correlations for all three distributions so that we can look the different moments of these distributions (e.g. skew). In Figure 8, there is a use of a * but how where these p-values estimated? Are these empirical estimations of the p-value?! 

 I am totally confused here. You say in Figure 8 that panel A is “full data”. But panel B is “sensitive cell lines”. Where is this defined? the parentheses beside this in the figure caption? But why did you introduce this “broad, narrow, no effect” definitions only to redefine something else here?

 I’m not sure I understand Supplemental Figure 11. Is this just all probe groups for the Affy arrays, or how were features chosen? 

 What is an “RNA-seq expression value”? how is this formed? rpm? Most importantly, I just don’t know what the message is here, and if there is any statistics

 to support that statement.

 I’m not sure I understand Figure 9 or what the take home message should be. I have a hard time understanding the labels along the x-axis in these figure. I just don’t really know statistically how one can conclude that gene expression is more “consistent” than the drug sensitivity values. There could be a million things going on in those arrays. There are so many more datapoint and you have literally a hundred thousand probes that probably don’t have an IQR > 1.5 on those arrays that “pump up” the correlation values, I would guess. What does this analysis mean?

I have read this submission. I believe that I have an appropriate level of expertise to state that I do not consider it to be of an acceptable scientific standard, for reasons outlined above.

# Response

**Published:** 2017-07-12

**Authors:** Benjamin Haibe-Kains

*We thank the reviewer for his constructive comments. We too believe it is important for the community to be aware of the challenges for biomarker discovery stemming from the lack of consistency across large-scale pharmacogenomic datasets. We have addressed most of the reviewer’s comments, as detailed below.*

**pg5: What is “Dataset 1”? The link here doesn’t lead anywhere that I can tell.**

*We have updated the manuscript to add a description of each “Dataset”.*

**Figure 3. I’m not really sure what the value is in plotting the density functions for the mismatched and matched cell lines. First, wouldn’t one density function suffice with a threshold I guess? Second, do you really need it at all?**

*The representation of both mismatched and matched cell line density functions serves to illustrates two main points: to give context to the concordance scores, and to show that the mismatched cell lines have a concordance score that is distinct and separate from the matched cell lines.  By representing only a single density function, a reader may not be able to appreciate the bimodal nature of matched/mismatched concordances, and that the distance between the two functions is large enough to allow for a robust classification scheme.*

**In the Methods “Cell line identity….”, it is stated that 66 samples fell below threshold with a reference to the Supplementary methods.** **However** **I don’t see anything in the supplementary methods that discusses this.** **Moreover** **in the text, it seems that you threw 8 cases away. This is confusing.**

*We* *analyzed* *the SNP array profiles of all 973 and 1190 CEL files available for GDSC and CCLE respectively. From these samples 66 and 5 with* *low quality* *SNP arrays in GDSC and CCLE have been removed from SNP fingerprinting pipeline. We continued the SNP fingerprinting pipeline with 503 cell lines with* *high quality* *SNP profiles available in common between CCLE and GDSC.*

*We compared the genotype concordance score for 503 out of 698 cell lines in common between CCLE and GDSC. We confirm that we removed 8 of 503 cell lines from analyses because their genotype concordance scores fell below the 0.8* *threshold* *, as well as within the range of genotype concordances for cell lines with discordant genotypes.  As such, we concluded that despite having the same annotations, these cell lines may have been contaminated or mislabelled in one of the two studies and further analysis on drug sensitivity cannot be compared between them. We have reported the quality scores and concordance scores in Dataset 1.*

**pg 5. I’m not sure what you mean by “remain stable or decrease monotonically”?**

**Do you just mean “monotonically non-increasing”?**

*That is correct. We changed the manuscript accordingly.*

**Please see comment regarding “Filtering of drug dose-response curves” form Supp Methods below. I think this really needs to be reworked, and I have to trust you guys here that you are doing the right thing.**

**“as exemplified…” depicted?**

*We agree and updated the manuscript accordingly.*

**In Figure 4, is it possible to relate this back to the choice of \rho, \epsilon and 2 \cdot \epsilon from the Supp Methods, or perhaps integration a version of this figure (but annotated) into the Supp** ****Methods.****

*Figure 4 legend is updated with the explanation of why these curves are identified as noisy.*

**In panel A, the grey area is a bit non-intuitive no? I would say that post 0.03, it’s looking pretty good, and it’s not measured in GDSC after that.** **However** **the first points are off.**

*In this* *panel* *the problematic curve is CCLE  While the curve is monotonically decreasing for just 62% of points (5 out of 8) it is expected to be for at least 80% (the value considered for the \rho parameter) of points. However, GDSC curve is consistently decreasing monotonically for all points except for one. So we think it is a good example of how the constraints we considered are useful in identifying noisy curves like CCLE curve in this panel.*

**I don’t know what \epsilon or \rho** **are** **so it’s hard to relate what is depicted in Figure 4 back to your model.**

*Figure 4 legend is updated with the default values of \epsilon and \rho which have been used to flag noisy curves in our study.*

**When I look through Supplemental Figure 1 (all the excluded comparisons), it seems like your criterion for excluding a comparison boils down cases where at least one of the curves has high variance, and the cutoff 2\epsilon I** **think  is** **a constant independent of the distribution of points for either curve. I don’t see in your equations how you encode that the sequence is monotonically non-increasing, or how “order” along the left to right sweep is incorporated.**

*By definition, we expect to see two types of drug response curves. When the cell is resistant to the drug it is expected that the viability fluctuates slightly around 100% and when the drug is sensitive a monotonically decreasing manner is expected to be seen. However, noise is unavoidable in these experiments. So we assumed that the viability of each point on the curve may get higher than the viability of its immediate prior at most with the size of \epsilon. To filter the largely noisy experiments and keep the slightly noisy ones at the same time, we consider this constraint to be true for the majority of points which is defined by \rho. Applying these simple constraints will result in omitting all the curves in which viability is increasing monotonically or if it is fluctuating largely.*

**Supp Methods Filtering of drug dose-response curves**

**i +1^{st} -> (i+1)^{st}**

*Thanks for pointing this out, we corrected this in the revised manuscript.*

**It seems like there is a formatting problem here. In my** **pdf** **there is something like I”A squiggle after “in some large fraction ??? of the cases (1).” I guess that’s supposed to be \rho right?**

*Thanks for pointing it out. You are right and we corrected this.*

**I’m not sure I understand this sentence “Our quality control …” Are you saying that \Delta_{i, i+1} < \epsilon in some fraction \phi of the cases?**

*You are correct and more explanation is presented in our previous comment.*

**equation #2 below:**

**I also have never seen set notation such as \{ \Delta_{i,i+1} | \Delta_{i,i+1} < \epsilon \}.**

**Are you trying to say “given all the \Deltas that are less than \epsilon?**

**So the vertical | means cardinality here right?**

**But then the denominator has the cardinality of a value, or do you mean absolute value?**

*Vertical bars are used as cardinality notation in both numerator and denominator of that equation. What is \rho? It’s undefined.*

**“Unfortunately …”  The** **english** **is a bit rough - could be rephrased in terms of specificity and sensitivity, I guess.**

*We updated the manuscript accordingly.*

**I don’t understand the significance of the sentence “Consider, for instance, …” But in the main text, you say that it remains stable or decreases monotonically. Here it increases monotonically for many successive points, so this violates your model, no?**

*Apply only equation (1) will not filter all the* *noisy* *cases and the curve explained in this sentence is one of those cases. Hence we also applied equations (2) and (3) to filter these remaining noisy curves. We clarified this in the revised manuscript.*

**I think that this subsection wants simply to spell out mathematically the thresholds and also provide some rationale for the parameters. I think the text doesn’t really do a good job of establishing this rationale and needs work. Perhaps define the parameters precisely and then phrase the exposition in standard terms e.g. specific and sensitivity for different \epislon, etc.**

*We thank the reviewer for his suggestion. We improved the clarity of our equations and clearly state the parameters we used. We agree that the selection of the parameter value is* *arbitrarily* *, although reasonable. It would be possible to create a set of manually curated curves as a gold standard set and tune our parameters accordingly. Although the idea is appealing, it would require a large set of curators as manual classification tends to be unstable too. Such an analysis is definitely of interest and we will pursue this in future studies.*

**Your equation (2) is inconsistent. In the text you specify \Sigma_{\forall i,j} \Delta_{i,j} but below your criterion seems to change to (the correct) $i < j$.  It is also sufficient to write \Sigma{ i < j } \Delta_{i,j} and avoid the double summation.**

*Corrected.*

**You should probably define D_i in the text and not make the reader deduce it from the figure below**

*Corrected.*

**The comments here w.r.t. the Supplementary Methods also apply to the associated subsection of the Methods. The mathematical correctness of some comments needs attention. For example, it is not quite correct to say that the “curve fitting would have yielded erroneous results”. The curve fitting is just that, curve fitting. It’s not really an error. Then in the Methods, you claim to use this equation but this is inconsistent with the discussion in the Supplemental Methods (where you have an equation with this undefined parameter $E$).**

*We agree with the reviewer and updated the manuscript accordingly.*

**The least-squares method using a three-parameter sigmoid model. I understand the intuition for this, but when I look through Supplementary File 2, I think there are a** **lot** **cases where this is perhaps not the correct pattern to assume (e.g. straight lines). Moreover, there are some very strange fits, for example,**

**AZD6244:G−361**

**AZD6244:SK−MM−2**

**PLX4720:MDA−MB−175−VII**

**In some** **cases** **the curve is always above all of the observations.**

**Perhaps this is** **because** **the measurements of viability > 100%? In your** **model** **you have removed the $E_0$ from Barretina et al. for different reasons.**

*Data points where the measured viability exceeds 100% always lie above their respective curves of best* *fit,* *since the functional form of the equation forces predicted viability to lie between 0 and 100% for all drug concentrations. The three-parameter sigmoid model’s intuition and flexibility* *makes* *it an attractive choice for the majority of cases, and for ease and uniformity of analysis, we felt it prudent to choose the same model to fit all curves, even if it may not have fit well in a few anomalous cases.*

**Supp Methods - Fitting of drug dose-response curves**

I’m a bit lost with your choice of notation here. For example, you define y as an equation, not a function, and then write y(0) which I assume is supposed to be something like y(x). Ok but then you have y=0 and y = y(0) = 1. I’m not sure this is correct mathematically.  (I think you mean to say that y(x ) = … *where* y(0) =1.

**“viability is reduced to half … concentration of the drug”… so the “Top”, E_\infinity … I find this a bit wordy.**

**“The dose response equation now becomes …”**

**So I deduce that E is the new parameter?!?!  Where has E_\infinity gone?!?!**

**(But then down below E is constrained to be in [0,1] and seems to related to the fitness of neoplastic cells.  I’m not sure I understand this.)**

**There is no derivation of this formula whatsoever. In fact, I don’t see how this could be correct any longer. Couldn’t this be expressed as** **mixture** **of two cell types, and y would be then a sort of weighted average?**

**I really don’t see how this was derived. This is a very central part of your paper (since the manuscript is measuring agreement) and therefore it needs to be bulletproof.**

**Please define “extant drug”.** **Also** **HS is allowed to vary apparently but I don’t see where it is then** **optimized** **in your analysis later on. This is confusing.**

*We thank the reviewer for his comments and we apologise for the lack of clarity of our Supplementary Methods. We have now rewritten this section to improve clarity and address all the reviewer’s comments.*

**Consistency of Drug Sensitivity Data**

**Is the ABC method standard? Are there citations for this? You should probably define properly what you mean by the “insertion of the concentration range”. Elsewhere it seems that you are referring to this as “common concentration range” e.g. SFig 2**

**More generally, isn’t this a sort of (non-statistical) version of the Kolmogorov-Smirnoff test?**

*We created and used the ABC method as a convenient and intuitive way of quantifying the agreement of analogous dose-response curves in different datasets over the intersection of the concentration ranges tested by them (henceforth referred to as their “common concentration range”). This method is inspired* *from* *two recent publications where the authors restricted the analysis to the common concentration range between datasets (Pozdeyev et al, Oncotarget 2016) and compared two curves directly (Yadav et al, Scientific Reports 2014).*

*While ABC does have some similarities to* *Kolmogoroff* *-Smirnoff, it evaluates the area between curves rather than the maximum vertical linear distance between them. Furthermore, it takes into account the behaviour of the fitted dose-response curves over their common concentration range only, rather than across their entire domains. Since the behaviour of fitted dose-response curves at concentrations far outside the concentration ranges over which they were fitted tends not to be robust to noise, we felt that ABC was a more appropriate test than Kolmogorov-Smirnoff for assessing accordance of dose-response curves in this study.*

*We updated the manuscript wit these references and clarifications.*

**Figure 5A: Actually there are many such cases in your Supplementary Figures. Doesn’t this just mean that the range of concentrations** **are** **not sufficient in both datasets?**

*Given the limited concentration range tested in* *high-throughput* *in vitro drug screening studies, such as GDSC nd CCLE, it is not possible to rule out that drug yielding no effect on cell viability could actually yield substantial effect at* *higher* *dose. However, these higher doses are likely to be clinically irrelevant.*

**pg 7 “We then computed the median …” I don’t understand what your distance metric is here. If I understand correctly, you could the ABC for each pair of drugs in the GDSC dataset. From** **that** **I can imagine a distance matrix D where D_ij** **i** **the ABC between drugs i and j. But you said you take the median ABC? median over what? cell lines? repeats? Whatever the case, are you sure it’s a distance metric?! Is it really true that distances derived from ABCs are metrics? I think this should be shown in the Supplemental Methods.**

*The ABC is computed for each cell lines and the median of ABC values was used as a measure of “distance” between two drugs.*

**Also as a minor comment, the caption in Supp Figure 3 says that you are using the mean ABC value but elsewhere it says median.**

*The caption has been corrected to read ‘median’ rather than ‘mean’.*

**p8. I am not sure what the significance of Supplementary Figure 3 is: are any of these clusters significant? Why are two drugs coloured red in panel A? On page 8 it is claimed that the samples split by** **hospital** **(MGH vs WTSI) but I don’t see how this is represented in Supplemental Figure 3.**

*As described in the manuscript, GDSC drug sensitivity experiments have been performed in two* *centers* *(MGH and MSTI) separately. The only drug has been tested by both centres is AZD6482 (the ones in red in panel A of Supplementary figure 3). The aim of that figure is to illustrate how well the biological replicates have been clustered together.* *However* *the other drugs are not expected to cluster according to their corresponding* *center* *thus there is not such a* *labeling* *in this figure.*

**pg 8. I have a very hard time estimating the significance of a statement like “…3 out of  15 common drugs clustered tightly”. I am not sure what tight means here.**

**When I look at Supp Figure 3 there has been no effort to annotate the clusters with their reproducibility e.g.** **pvclust** **or measure their significance in some other way.**

**When I look at the figure I think there appears to be a lot of co-clustering of the drugs, at least given that the median ABC across a diverse collection of cell lines might not be such a great “distance” measure.**

*We refer to the closest* *neighbor* *for each drug. We agree with the reviewer that our* *statement* *should be more quantitative.* *We therefore* *compared the ABC values between common drugs and different drugs and observed* *a significant differences* *(one-sided Wilcoxon test p-value = 0.004). We agree that median ABC might not be the best distance measure, this is why we updated Supplementary Figure 3 with other distance measures for completeness.*

**pg 9. What do you mean by “highly targeted therapies”?**

*It meant drugs for which there is* *a* *few* *sensitive cell lines in the CCLE and GDSC (narrow effect). However, we changed it to* *targeted* *to avoid any confusion.*

**pg 9. paragraph “Although the ABC values …”  I think the ABC is interesting but it takes a very prominent role in your paper when there are other standard techniques already like AUC and IC_50. Perhaps the manuscript should have comments about why have chosen this approach that is not standard.** **Also** **I think you would need to make precise what the differences are between how GDSC and CCLE computed the AUC and IC_50 that are different than how PharmacoGX does.**

*GDSC and CCLE fit a different family of curves to their dose-response data, as described in* *Haibe* *-Kains et al, Nature 2013. To eliminate this source of heterogeneity, we fitted the same three-parameter model for all the CCLE and GDSC curves, as implemented in PharmacoGx does. Once the curve is fitted, GDSC, CCLE, and PharmacoGx agree on how to calculate its AUC and IC_50.*

**This is a very central concept in your comparison so it would have to have a very solid definition and analysis. Supplemental Figure 4 suggests in a round-about way that the only difference** **in in** **the number of cell lines (figure caption). This is a bit confusing. Again, I am not sure what “Dataset 2” refers to. Perhaps the manuscript would benefit from the addition of some interpretation as to what you believe  Supp Figure 4 means.**

*We have updated the manuscript with a clear interpretation of Suppl Figure 4, which shows that drugs listed as targeted therapies exhibit less variation (as estimated by the median absolute deviation) in drug sensitivity (AUC) than cytotoxic therapies. Although expected, these results allowed us to define a cutoff fro MAD(AUC) to classify drug into broad vs narrow effect, as described in the manuscript.*

*We have also updated the manuscript to add a description of each “Dataset”, a denomination required by F1000Research formatting guidelines.*

**I don’t understand the definition of your three classes of drugs (no effect (AUC > 0.2); narrow effect AUC \leq 0.13 or broad affect AUC > 0.13). I don’t see how this definition clearly delineates between “no effect” and “narrow effect”.**

*We apologize for the confusion. We corrected the manuscript with the following definitions. Drugs with “no effect”: all AUC values < 0.2 (no sensitive cell lines); “narrow effect”: MAD(AUC) <= 0.13 (see Suppl Figure 4); “broad effect”: MAD(AUC) > 0.13 (see Supp Figure 4).*

**The bottom paragraph of the first column is one sentence that spans 8.5 lines. It references 2 main figures of the paper and 5 supplementary figures. To be honest, this is very frustrating. I have gone through the Supplemental Methods very closely and I don’t see anywhere where the authors have distinguished between “recomputed AUC” and “AUC computed based on the common concentration range”. Then “IC_50 (figure figure) values” ??**

*We have now clearly stated these definitions in the manuscript. AUC recomputed and AUC computed based on common concentration range both are computed by our PharmacoGx package by fitting the sigmoid model described in the Supplemental Methods. The only difference between these metrics is that the former is computed over the whole concentration range for each study while the former one is computed over the common concentration range between CCLE and GDSC. We updated the manuscript to reflect the fact that recomputed IC_50 values have been used in Supplementary Figure 8. Recomputed IC_50 values are inferred from the sigmoid model fitted to the data by the aim of PharmacoGx package.*

**I’m not sure what to interpret** **re:** **Figure 7 for example. To** **me** **it looks like there is excellent agreement except for perhaps the first row. Only paclitaxel fits into this “** **cyotoxic** **drug’ category but for the life of me, I don’t see where this is defined. The authors just defined three types of drugs (no effect, narrow effect and broad effect) but that’s not what they are using here. I don’t understand this. To** **me** **it simply seems to be that at low AUCs there is high variance in the last 3 distributions of the first line (17-AAG PD-0325901 and AZD6244), but** **actually** **they look like they pretty well agree at higher AUCs.**

*We agree with the reviewer that we have not been consistent in our definition of drugs with no, narrow and broad effect. This is now fixed in the updated manuscript. In Figure 7 (and all other figures) we have ordered the drugs by their “status” (no, narrow and broad effect). For the ease of interpretation, we also choose to* *color* *each AUC based on a standard cutoff for* *sensitivity* *of AUC > 0.2 (and therefore cell lines with AUC <= 0.2 are called “insensitive”). Although paclitaxel is the only drugs that* *is* *referred to as cytotoxic in the literature, we observed that 17-AAG, PD-0325901, and AZD6244 decreases cell viability for a large number of cell lines. As their MAD(AUC) > 0.13 (Supp Figure 4), we classified these drugs as “broad effect”. In this case, the consistency of drug sensitivity data (AUC) seems to be poor, with CCLE having much more sensitive cell lines than GDSC. Drugs with narrow effect (MAD(AUC) <= 0.13) (2 middle rows) yield better consistency for some drugs (e.g., crizotinib, PLX4720, lapatinib, lapatinib) but there are still cell lines with AUC > 0.2 (“sensitive”) that are far off the diagonal. The last row* *include* *all the drugs with “no effect”, i.e., the vast majority of cell lines yielded AUC <= 0.2, where no consistency is expected due to low* *signal / noise* *ratio.*

**I’m not sure what that means “and calculated the consistency of drug sensitivity data between studies using all common cases and only those that the data suggested were sensitive in at least on** **study** **.**

*The consistency of drug sensitivity data was performed twice. First using all the cell lines in common between the two studies. Second, using only the cell lines that are “sensitive” (AUC > 0.2) in at least one dataset. The second analysis aims to address a criticism we received from the community that only sensitive cell lines should be compared. We rephrased this part in the updated version of the manuscript.*

**Maybe a table would help, especially if each of these different objects** **were** **properly defined in the Methods/Supp Methods.**

*We enriched the “acronym table” in Supplementary Methods to add definitions of the additional objects and concepts used in our paper.*

**“Given that no single metric can capture all forms of consistency, …” So you add three more. I don’t see the point here. Why these three? and how is something like** **pearson** **\rho applied** **here.** What is the vector? I would guess that Supp Figures should show the distribution of correlations for all three distributions so that we can look the different moments of these distributions (e.g. skew). In Figure 8, there is a use of a * but how where these p-values estimated? Are these empirical estimations of the p-value?!

*In the absence of* *gold* *standard measure of consistency for drug sensitivity data, we decided to include other measures that could be used as* *alternative* *to Pearson and Spearman correlation already used in previous publications. The consistency measures are computed across cell lines. For each drug, a vector of drug sensitivity measurements (AUC’ IC_50,...) is extracted from GDSC and CCLE and then compared. P-values were computed analytically, as described in the updated Supplemental Methods. We updated the caption of Figure 8 to state these important points.*

**I am totally confused here. You say in Figure 8 that panel A is “full data”. But panel B is “sensitive cell lines”. Where is this defined? the parentheses beside this in the figure caption? But why did you introduce this “broad, narrow, no effect” definitions only to redefine something else here?**

*We* *apologise* *for the lack of definition and inconsistency. We have updated the figure to use a consistent classification of drugs and now clearly define the restriction to “sensitive data” (now renamed as “sensitive cell lines” for clarity).*

**I’m not sure I understand Supplemental Figure 11. Is this just all probe groups for the Affy arrays, or how were features chosen?**

**What is an “RNA-seq expression value”? how is this formed? rpm? Most importantly, I just don’t know what the message is here, and if there is any statistics**

**to support that statement.**

*Brainarray probe gene mapping* *cdf* *files have been used to quantify the expression value for each gene represented on the Affymetrix arrays. FPKM values for genes annotated by Gencode V.19 annotation were* *normalized* *by transforming them using log2(FPKM+1). The aim of Supplementary Figure 11 is to show the distribution of expression data for each platform and how well a mixture of 2* *gaussians* *could help define a cutoff to binarize the data. The caption has been updated to clearly state how the cutoffs have been determined.*

**I’m not sure I understand Figure 9 or what the take home message should be. I have a hard time understanding the labels along the x-axis in** **these figure** **. I just don’t really know statistically how one can conclude that gene expression is more “consistent” than the drug sensitivity values. There could be a million things going on in those arrays. There** **are** **so** **many** **more** **datapoint** **and you have literally a hundred thousand probes that probably don’t have an IQR > 1.5 on those arrays that “pump up” the correlation values, I would guess. What does this analysis mean?**

*We have now clearly defined the labels of the x-axis in the caption. We agree with the reviewer that* *sensitivity* *data and gene expression data have very different properties. However, as we looked at univariate biomarkers, one gene at a time, we sought to assess whether expression of each individual gene suffers from the same level of inconsistency than drug sensitivity data across cell lines. We have added a word of caution in the text to reflect on the limitation of this analysis.*
